# Supplementary material for: Jellyfish genomes reveal distinct homeobox gene clusters and conservation of small RNA processing
Source: Nat Commun. 2020 Jun 19;11:3051. doi: 10.1038/s41467-020-16801-9 (PMC7305137; doi:10.1038/s41467-020-16801-9)
Supplement: Supplementary file 1 — Supplementary Information [file 41467_2020_16801_MOESM1_ESM.pdf]

Supplementary Information

Jellyfish genomes reveal distinct homeobox gene clusters and conservation of small RNA processing

Nong et al

Supplementary Figures

Figure 1 - *Sanderia malayensis* and *Rhopilema esculentum* Hi-C information
Figure 2- Summary of ANTP-class homeobox gene arrangement in jellyfish genomes.
Figure 3 - Phylogenetic tree of HOXL subclass genes
Figure 4 - Phylogenetic tree of NKL subclass genes
Figure 5- Phylogenetic tree of all ANTP class genes
Figure 6- Phylogenetic tree of TALE class homeobox genes
Figure 7 - Sequences logos of ParaHox homeodomain sequences
Figure 8 - Syntenic blocks of Hox and ParaHox loci
Figure 9 - Syntenic blocks of other homeobox gene loci
Figure 10 - Phylogenetic tree of Argonaute/PIWI protein family.
Figure 11 - Hairpin sequence alignment and syntenic analyses of jellyfish microRNAs.
Figure 12 - Small RNA length distribution in different tissues of jellyfish.
Figure 13 - Phylogenetic trees and synteny analyses of ALDHs.

Supplementary Tables

Table 1 - *Sanderia malayensis* genome sequencing data information
Table 2 - *Rhopilema esculentum* genome sequencing data information
Table 3 - *R. esculentum* 21 pseudomolecules in the genome assembly
Table 4 - *S. malayensis* transcriptome sequencing data information
Table 5 - *R. esculentum* transcriptome sequencing data information
Table 6 - *A. aurelia* transcriptome sequencing data information
Table 7 - Species used in the phylogenomic analyses
Table 8 - Composition of transposable elements in the two jellyfish genomes
Table 9 - Repeat percentages and repeat compositions of sequenced cnidarians
Table 10 - Statistics of predicted gene models in the two jellyfish genomes
Table 11 - Comparison of assembled scyphozoan mitochondrial genomes
Table 12 - Genomic organisation of mitochondrial genes in *S. malayensis*
Table 13 - Genomic organization of mitochondrial genes in *R. esculentum*
Table 14 - Homeobox genes content in the two jellyfish genomes
Table 15 - *Sanderia* ANTP class homeobox genes and locations
Table 16 - *Rhopilema* ANTP class homeobox genes and locations
Table 17 - Summary of homeobox genes tree and synteny analyses
Table 18 - Fusion TALE-class homeobox gene in animals
Table 19 - Intron position of homeobox genes
Table 20 - ParaHox genes in cnidarians
Table 21 - Sequences of AGO-like and Piwi proteins
Table 22 - Sequences of conserved microRNAs

Table 23 - Graphical presentations in revealing the cases of microRNA arm switching
Table 24 - Sequences of ALDH proteins

Supplementary Discussion

1 - Heterozygosity
2 - Repeat content
3 - Gene model / Genome annotation
4 - Mitochondrial genomes
5 - Homeobox genes

Supplementary References

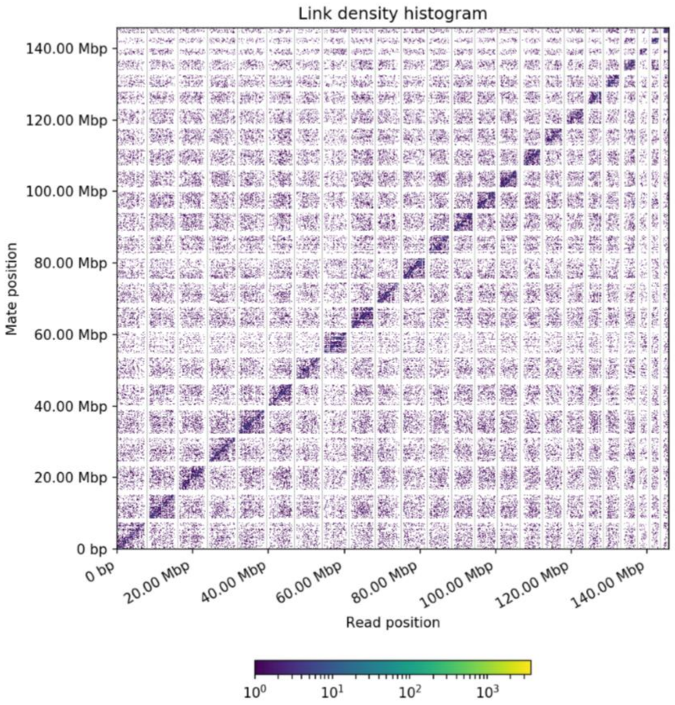

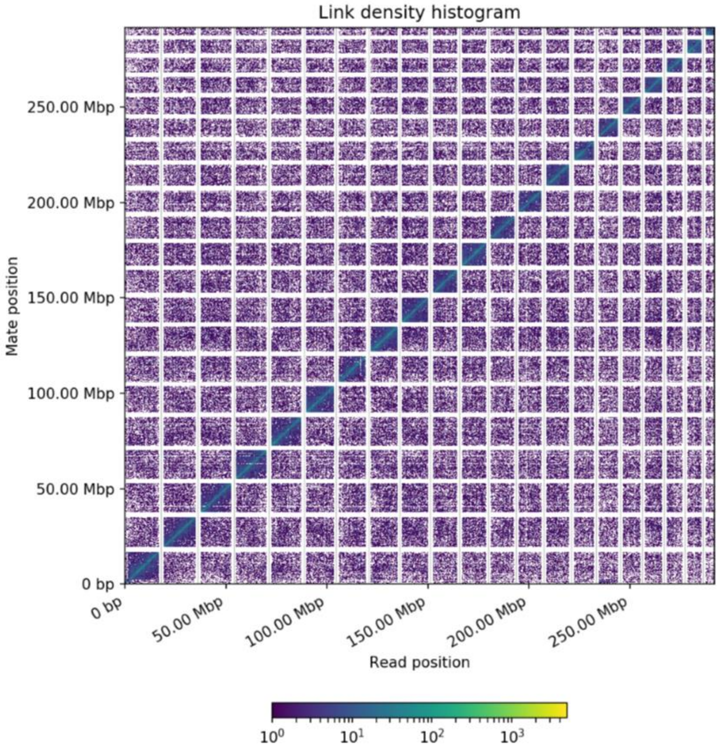

Supplementary Figure 1. *S. malayensis* (upper) and *R. esculentum* (lower) Hi-C information. The x- and y- axes give the mapping positions of the first and second read in the read pair respectively, grouped into bins. The colour of each square gives the number of read pairs within that bin. White vertical and black horizontal lines have been added to show the borders between scaffolds. Scaffolds less than 1 Mb are excluded.

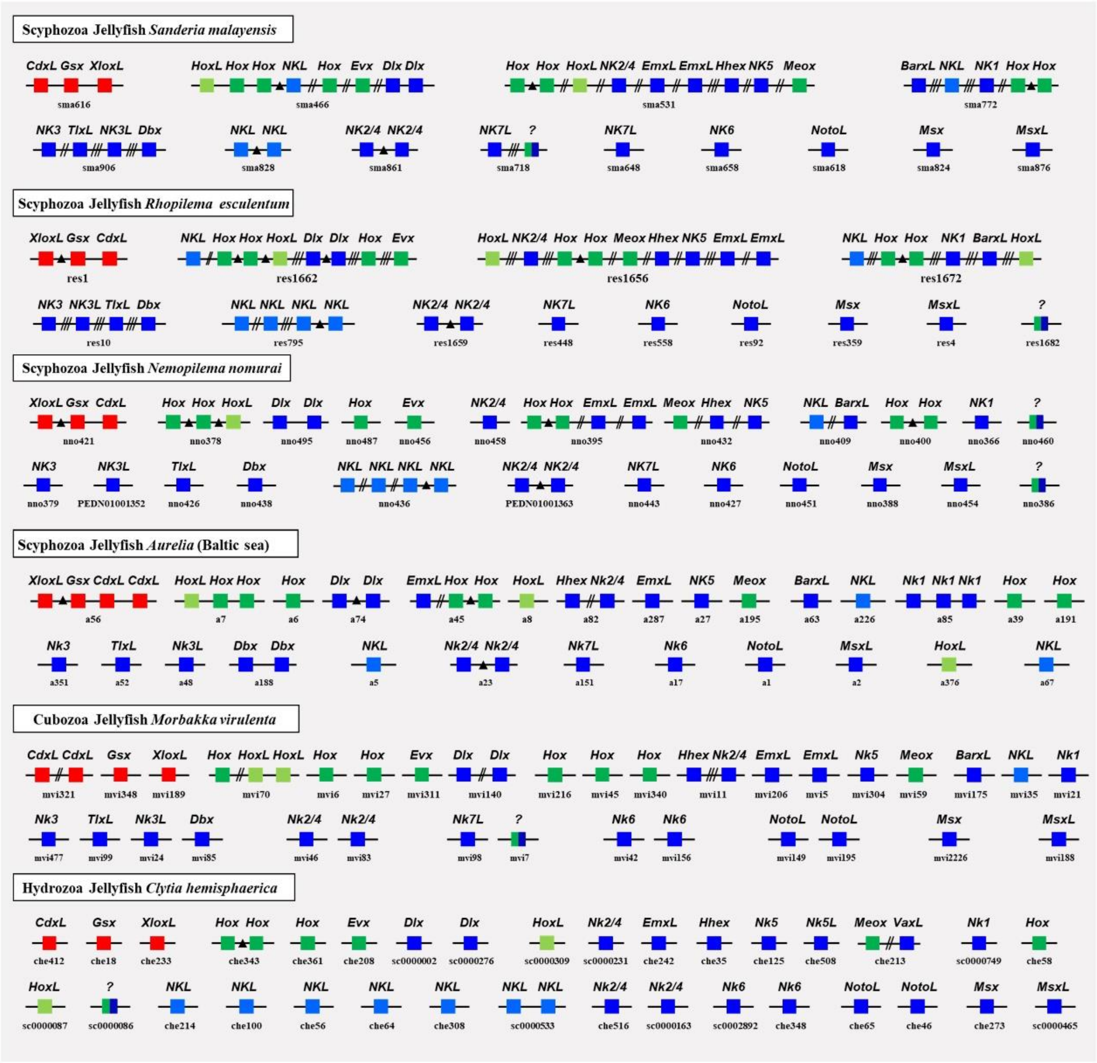

Supplementary Figure 2. Summary of ANTP-class homeobox gene arrangement in jellyfish genomes.

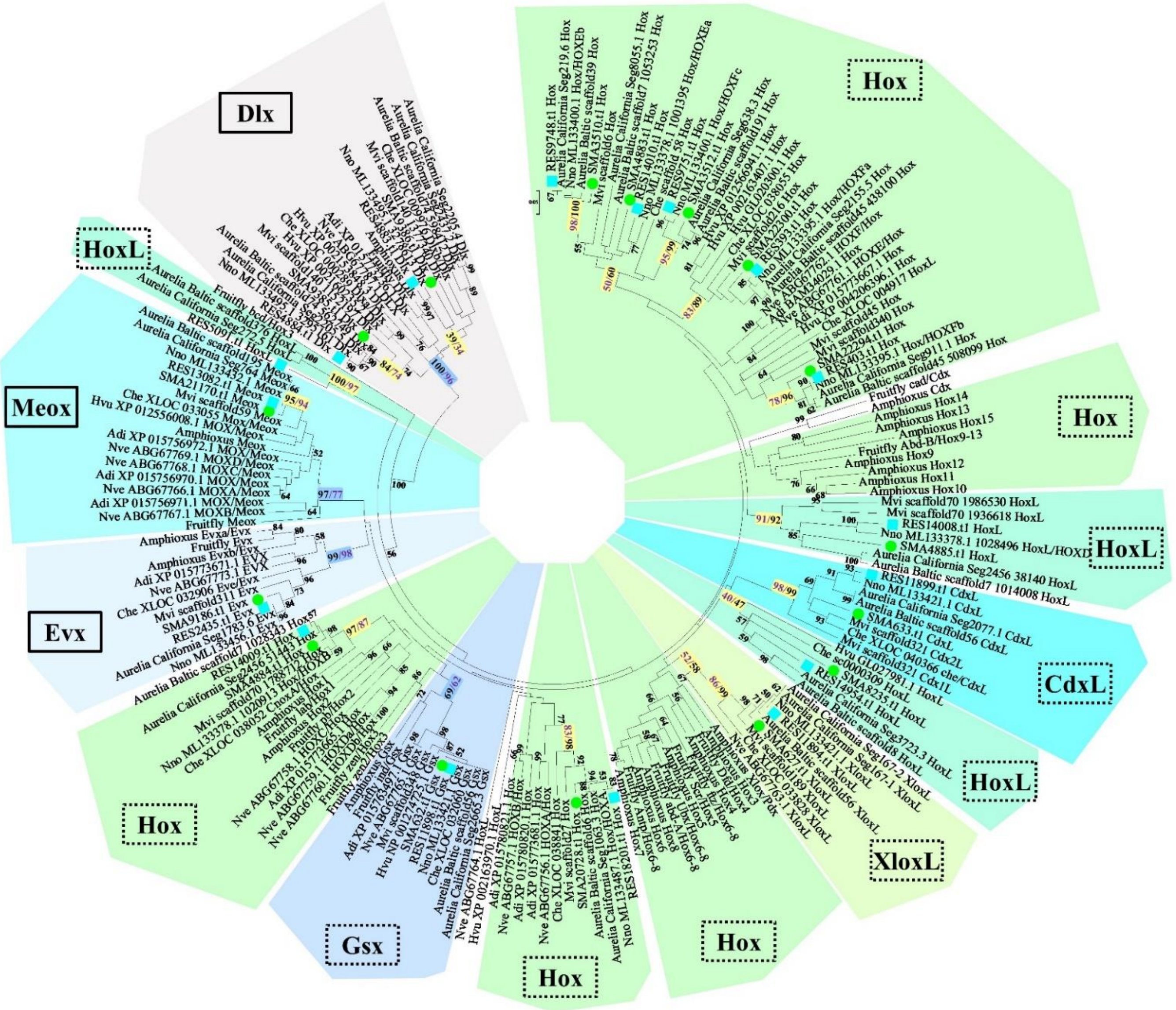

Supplementary Figure 3. Phylogenetic tree of HOXL subclass genes. The rooted phylogenetic tree was constructed with the LG (G) model using the Neighbour-joining (NJ) and Maximum Likelihood (ML) methods in MEGA7 (with 1000 replicates). Only bootstrap values larger than 50% are indicated for clarity (Black from NJ and purple from

ML). Genomic clustering of the three ParaHox genes in jellyfish supports their identity as orthologous to the ParaHox cluster of bilaterians, although whether the putative Pdx and Cdx genes are 1:1 orthologues of bilaterian is unclear. Nve, *Nematostella vectensis*; Adi, *Acropora digitifera*; Hvu*, Hydra vulgaris*; Che*, Clytia hemisphaerica*; Nno, *Nemopilema nomurai*; Mvi, *Morbakka virulenta*; *Aurelia*; Sma, *Sanderia malayensis*; Res*, Rhopilema esculentum*. Bilaterian homeobox genes from amphioxus (*Branchiostoma floridae*) and fruitfly (*Drosophila melanogaster*) are used as bilaterian represenatives.

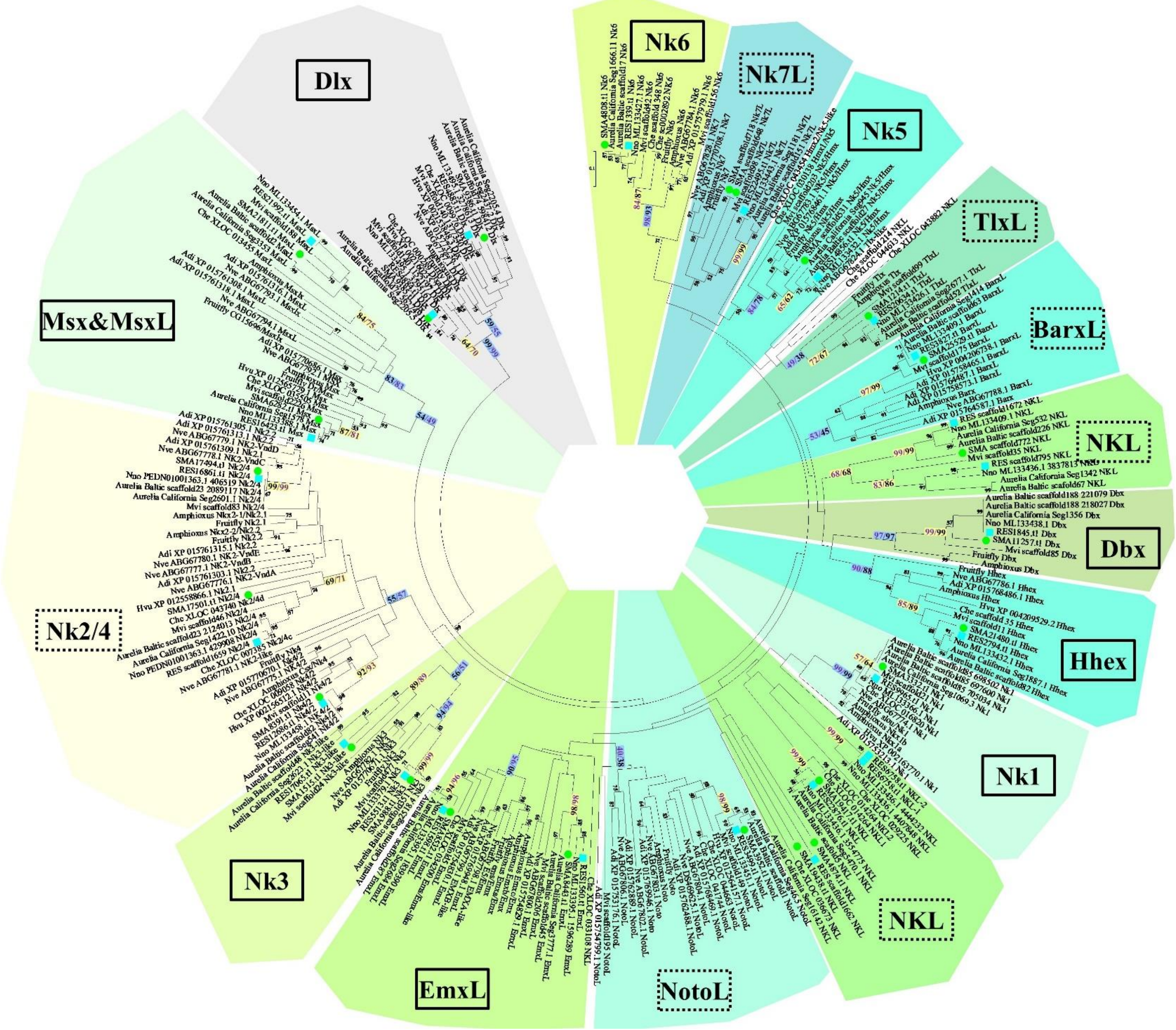
Nk6
Nk7L
Nk5
TlxL
BarxL
NKL
Dbx
Hhex
Nk1
NKL
NotoL
EmxL
Nk3
Nk2/4
Msx&MsxL
Dlx

Supplementary Figure 4. Phylogenetic tree of NKL subclass genes. The rooted phylogenetic tree was constructed with the LG (+G+I) model using the Neighbour-joining (NJ) and Maximum Likelihood (ML) methods in MEGA7 (with 1000 replicates). Only bootstrap values larger than 50% are indicated for clarity (Black from NJ and purple from ML). Nve, *Nematostella vectensis*; Adi, *Acropora digitifera*; Hvu, *Hydra vulgaris*; Che, *Clytia hemisphaerica*; Nno, *Nemopilema nomurai*; Mvi, *Morbakka virulenta*; *Aurelia* (2 strains), Aurelia_Baltic and Aurelia_California; Sma, *Sanderia malayensis*; Res, *Rhopilema esculentum*. Bilaterian homeobox genes from amphioxus (*Branchiostoma floridae*) and fruitfly (*Drosophila melanogaster*) are used as bilaterian represenatives.

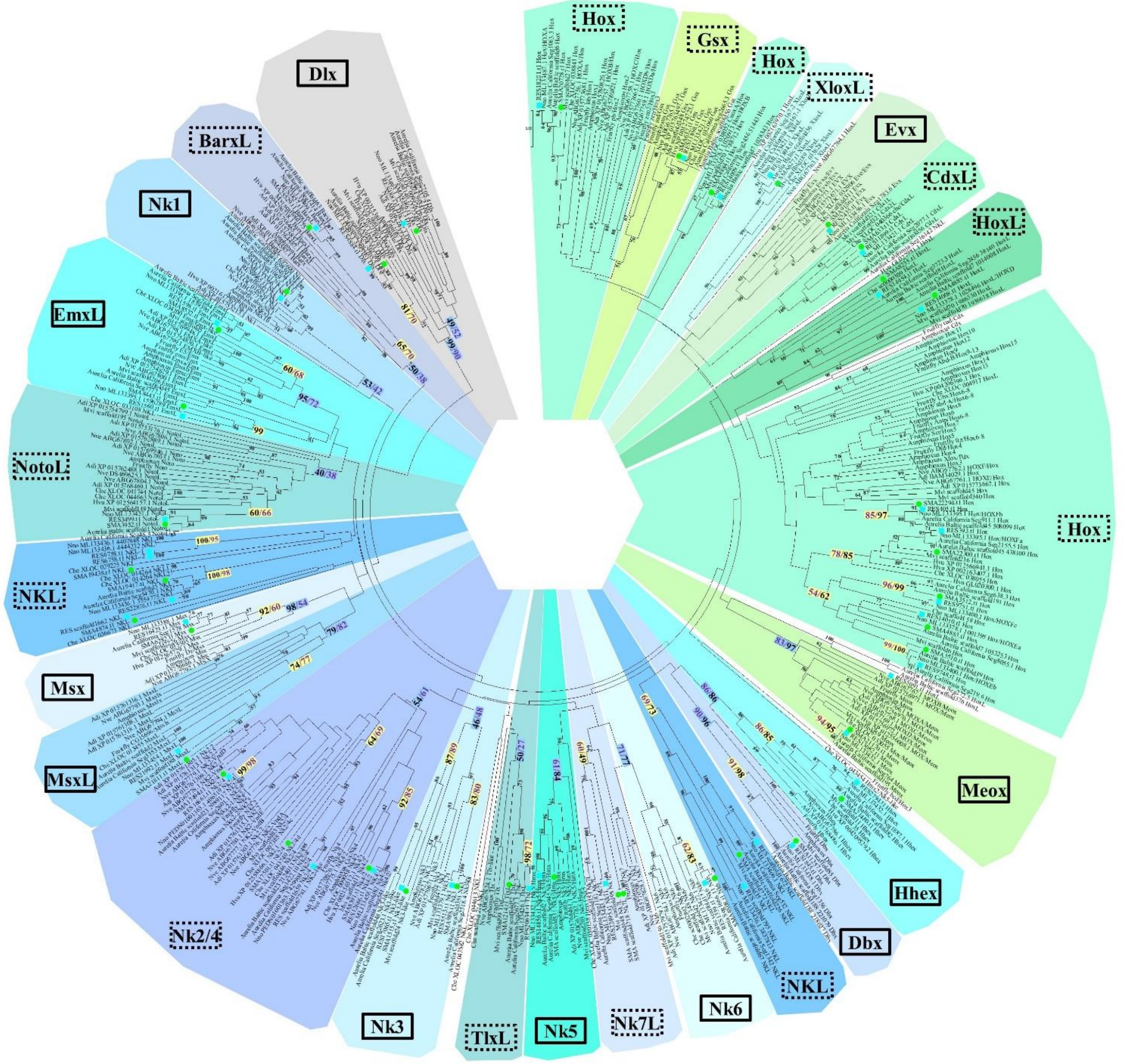
Dlx
BarxL
Nk1
EmxL
NotoL
NKL
Msx
MsxL
Nk2/4
Nk3
TlxL
Nk5
Nk7L
Nk6
NKL
Dbx
Hhex
Meox
Hox
HoxL
CdxL
Evx
XloxL
Hox
Gsx
Hox

Supplementary Figure 5. Phylogenetic tree of all ANTP class. The rooted phylogenetic tree was constructed with the LG (G) model using the Maximum Likelihood method in MEGA7 (with 1000 replicates). Only bootstrap values larger than 50% are indicated for clarity. Nve, *Nematostella vectensis*; Adi, *Acropora digitifera*; Hvu*, Hydra vulgaris*; Che*, Clytia hemisphaerica*; Nno, *Nemopilema nomurai*; Mvi, *Morbakka virulenta*; *Aurelia* (2 strains), Aurelia_Baltic and Aurelia_California; Sma, *Sanderia malayensis*; Res*, Rhopilema esculentum*. Bilaterian homeobox genes from Human *(Homo sapiens)*, amphioxus (*Branchiostoma floridae*) and fruitfly (*Drosophila melanogaster*) are used as bilaterian represenatives.

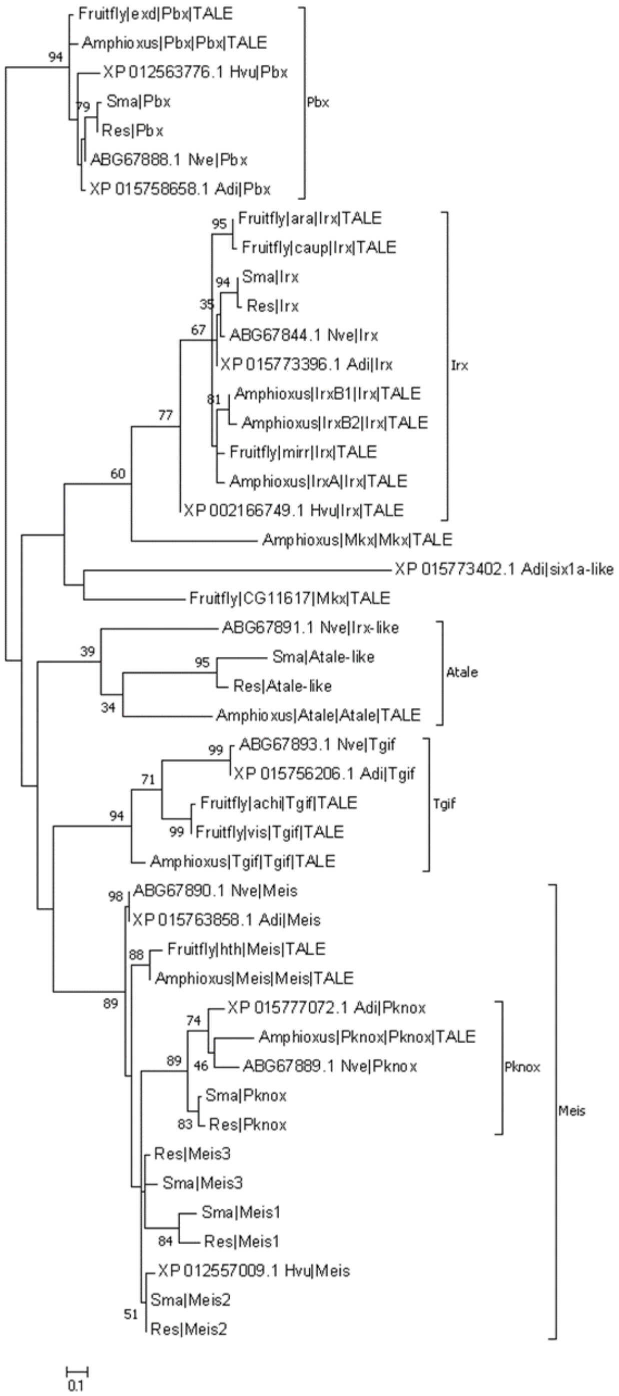

Supplementary Figure 6. Phylogenetic tree of TALE class homeobox genes. The phylogenetic tree was constructed with the LG model (G+I) using the Maximum Likelihood method in MEGA7. Bootstrap support values are based on 500 bootstraps and only values larger than 30 are indicated for clarity. Cnidarian homeobox genes from *Nematostella vectensis*, *Acropora digitifera*, *Hydra vulgaris*, *Sanderia malayensis* and *Rhopilema esculentum* are prefixed with Nve, Adi, Hvu, Sma and Res respectively. TALE class homeobox genes from amphioxus (*Branchiostoma floridae*) and fruitfly (*Drosophila melanogaster*) are used as bilaterian represenatives.

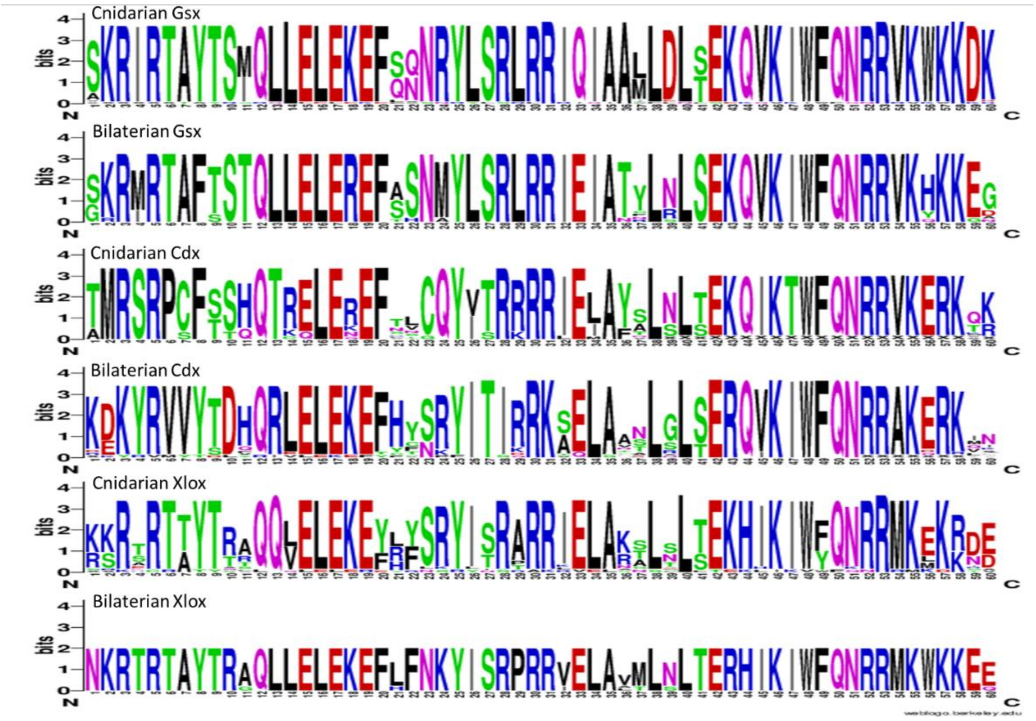

Supplementary Figure 7. Sequences logos of ParaHox homeodomain sequences from cnidarians and bilaterians (generated by WebLogo).

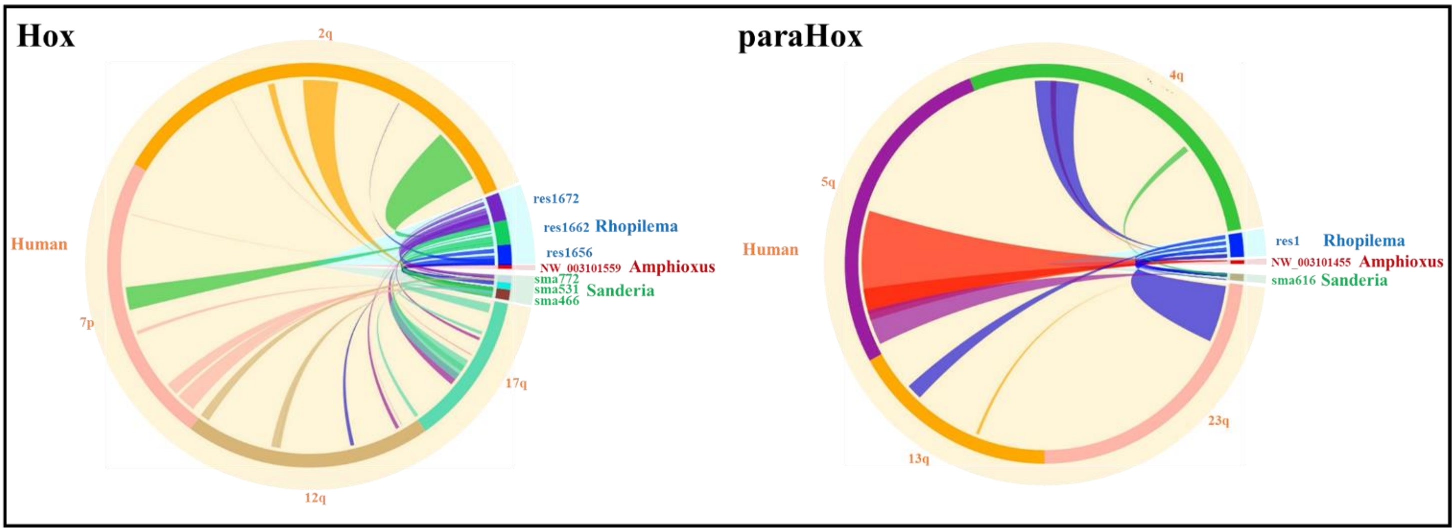

Supplementary Figure 8. Syntenic blocks between Hox and ParaHox loci of human *Homo sapiens*, amphioxus *Brachiostoma floridae*, and the two jellyfish genomes. The lines represent pairwise local alignments between scaffolds in respective genomes.

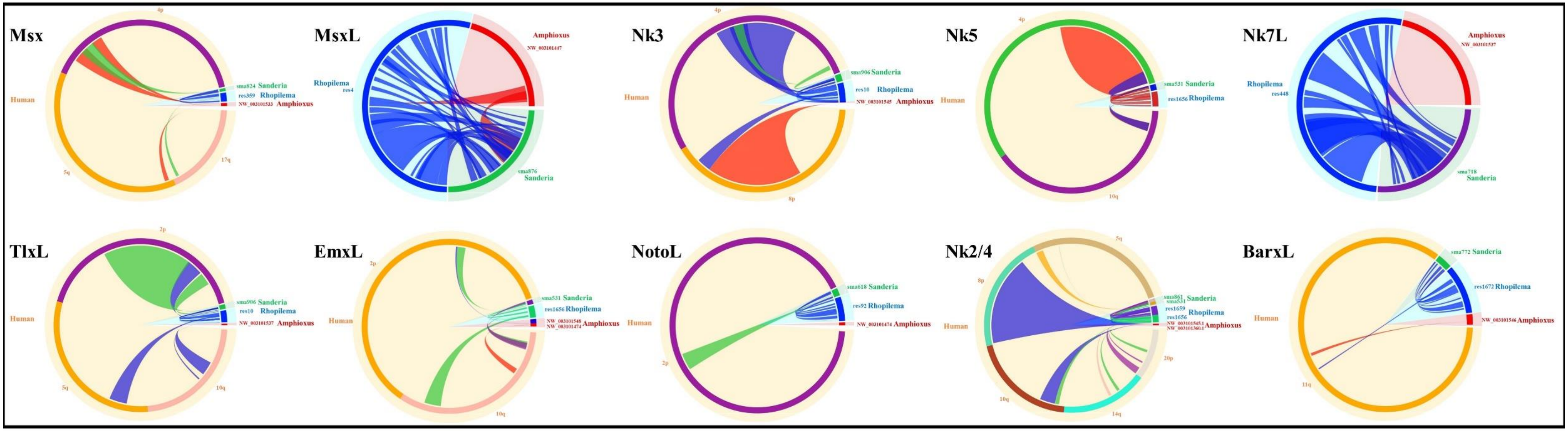

Supplementary Figure 9. Syntenic blocks between BarxL, EmxL, NK2/4, NotoL, Msx, MsxL, NK3, NK5, NK7L, and TlxL loci of human *Homo sapiens*, amphioxus *Brachiostoma floridae*, and the two jellyfish genomes. The lines represent pairwise local alignments between scaffolds in respective genomes.

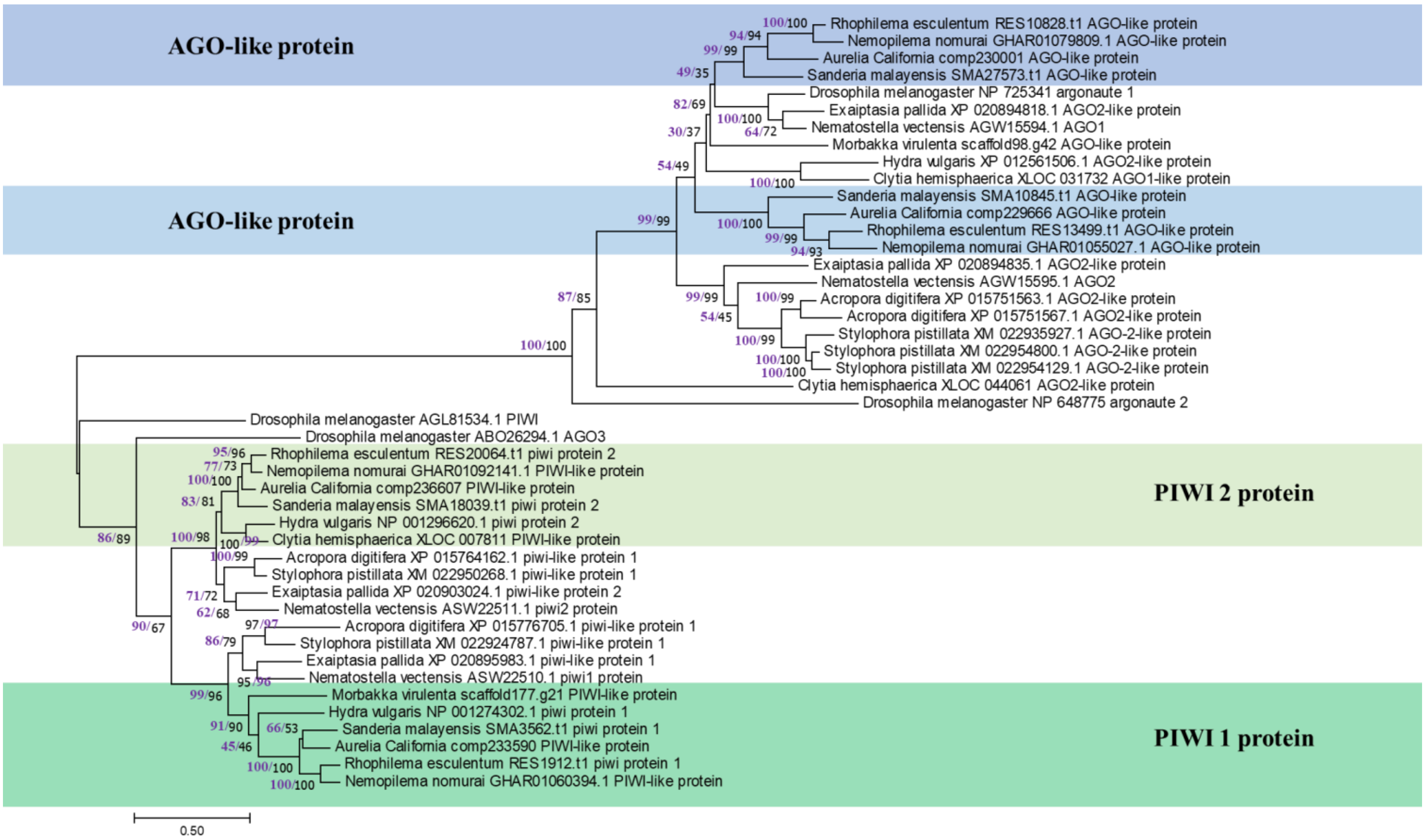

Supplementary Figure 10. Phylogenetic tree of Argonaute/PIWI protein family. Multiple alignments were performed using ClustalW and the rooted phylogenetic tree was constructed with the LG (G+I) model using the Maximum Likelihood method in MEGA7 (with 1000 replicates). Bootstrap values shown in purple and black are from neighbor-joining and maximum likelihood trees respectively.

A) miR-100

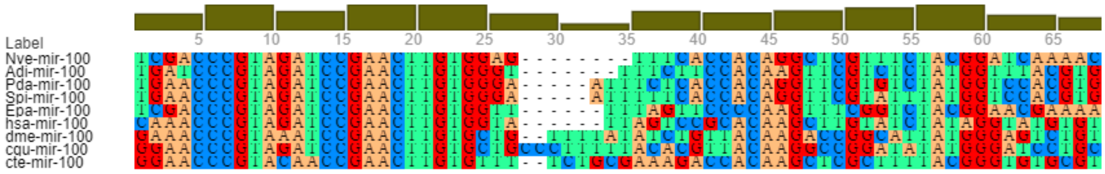

B) miR-2022

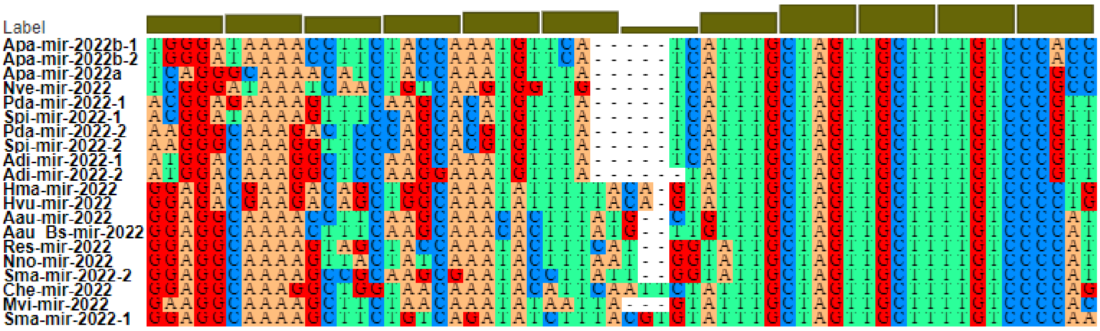

C) miR-2030

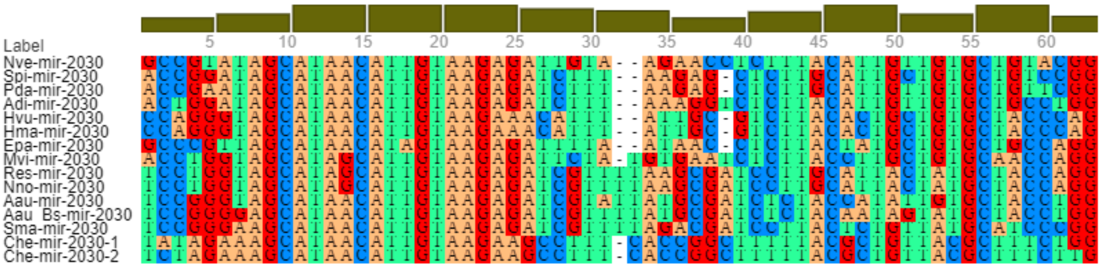

D) Six conserved scyphozoan novel microRNAs in all three jellyfish species

a.

One of these conserved scyphozoan jellyfish microRNAs also share high sequence similarity to one arm of the anthozoan miR-2023. Red arrow indicates scyphozoan specific changes in miR-2023-3p.

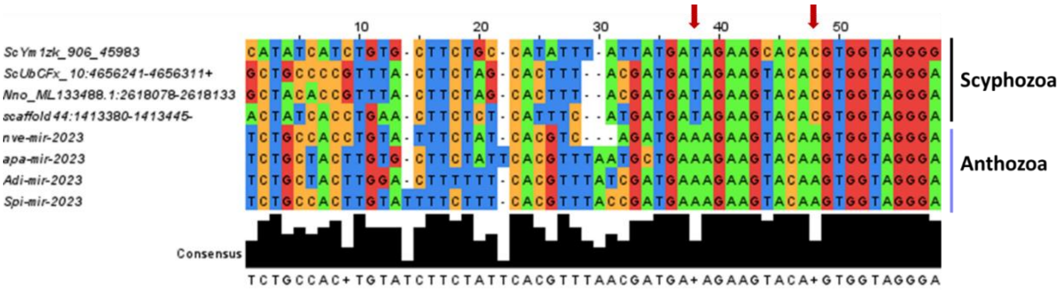

b.

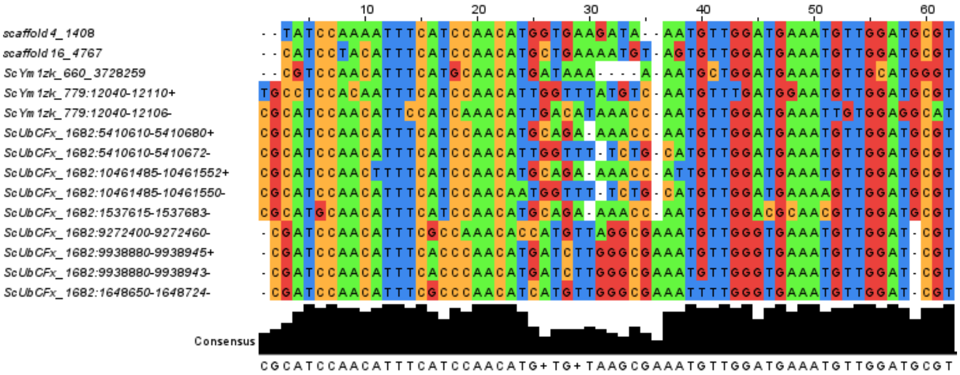

c.

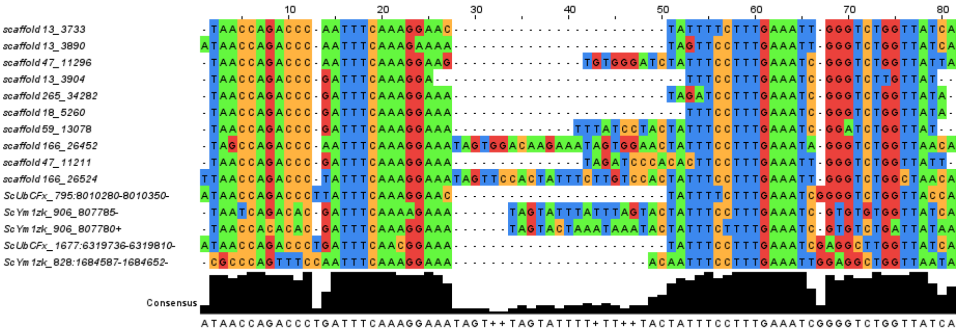

d.

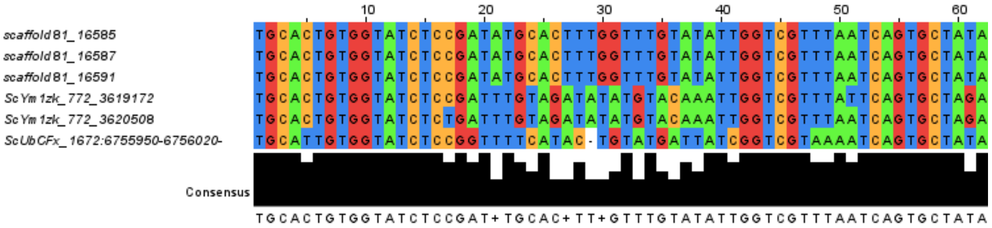

e.

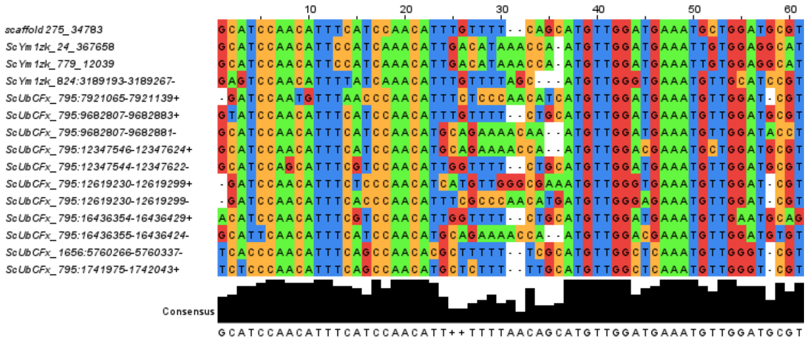

f.

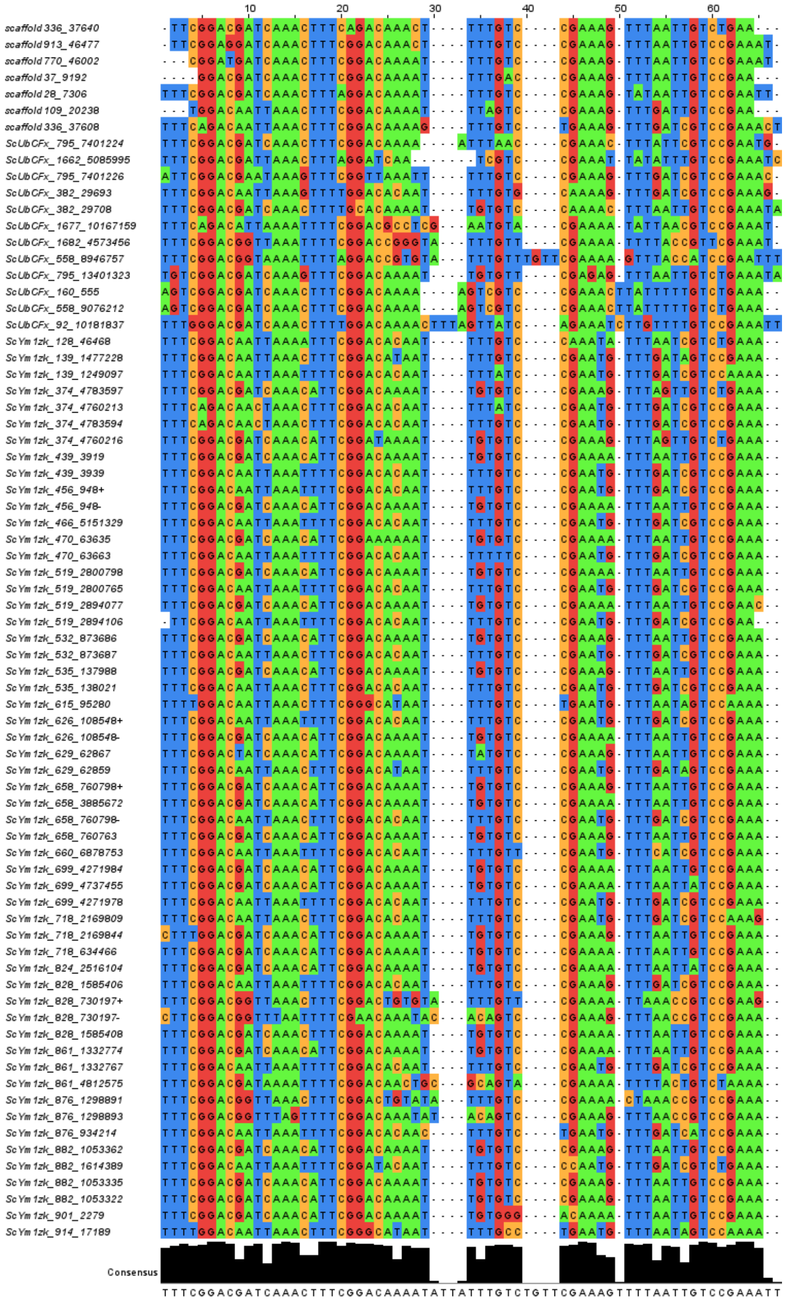

E) Four conserved miRNAs between jellyfish *Sanderia* and *Rhopilema*

a.

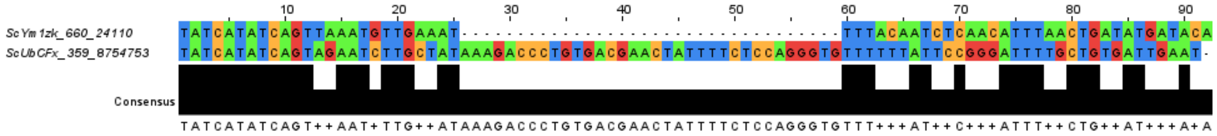

b.

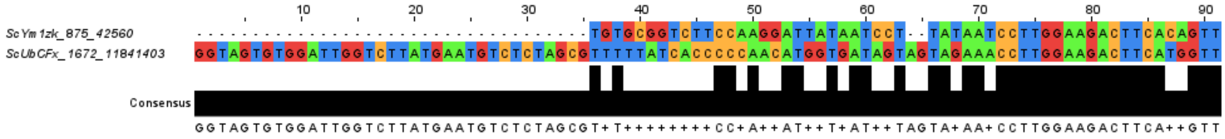

c.

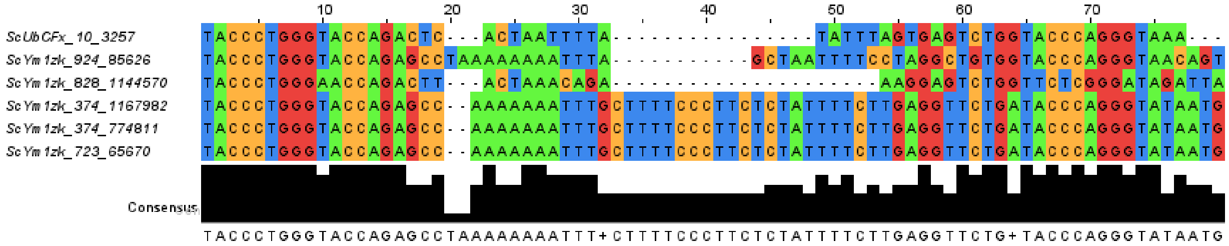

d.

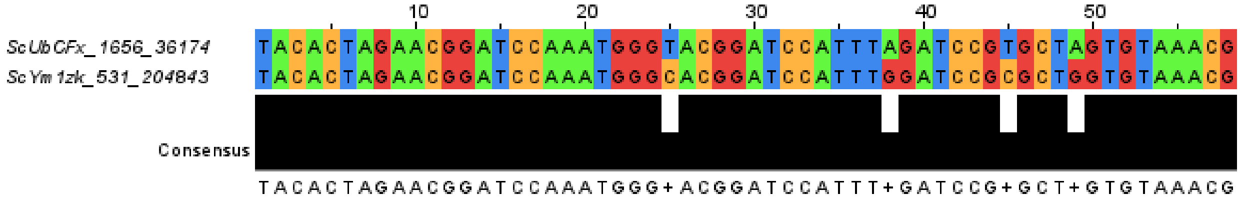

F) Twelve conserved microRNAs between jellyfish *Rhopilema* and *Aurelia*

a.

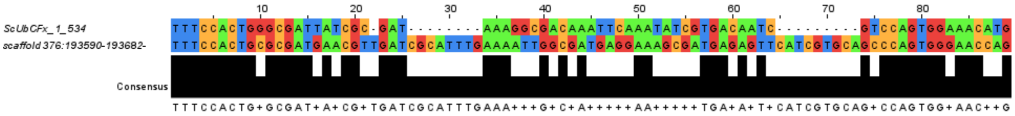

b.

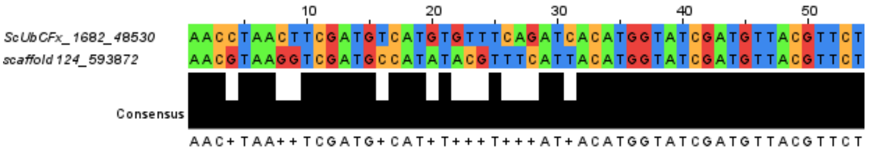

c.

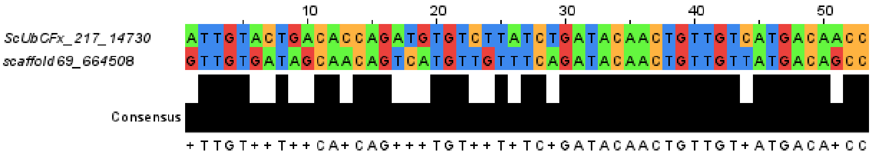

d.

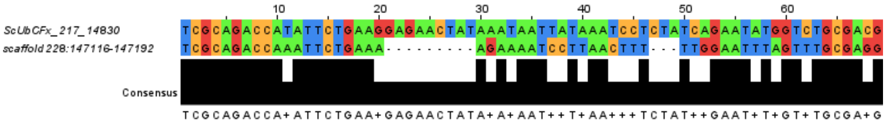

e.

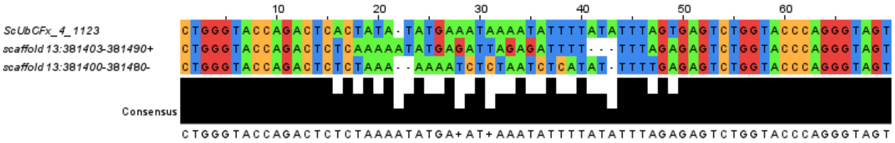

f.

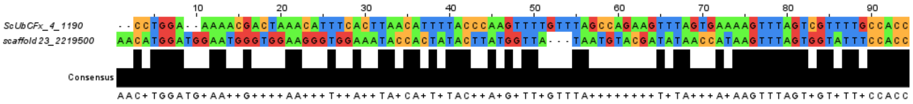

g.

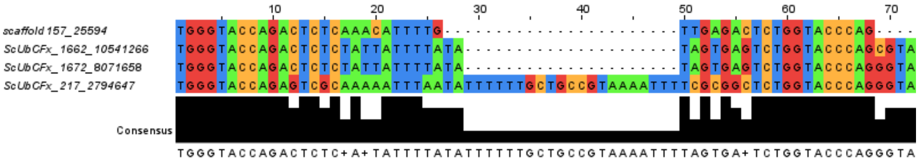

h.

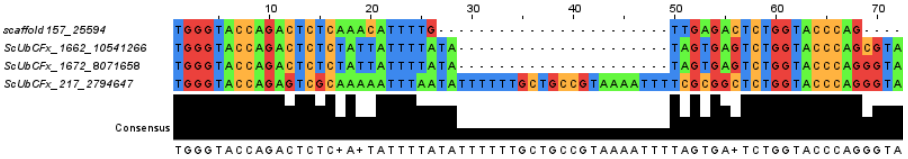

i.

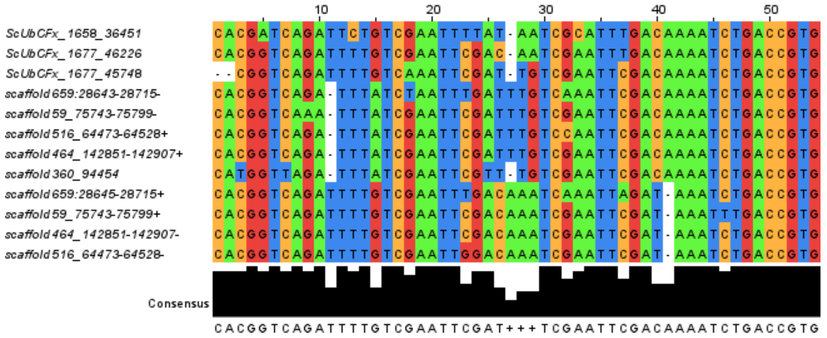
ScUbCFx_1658_36451
ScUbCFx_1677_46226
ScUbCFx_1677_45748
scaffold659:28643-28715-
scaffold59_75743-75799-
scaffold516_64473-64528+
scaffold464_142851-142907+
scaffold360_94454
scaffold659:28645-28715+
scaffold59_75743-75799+
scaffold464_142851-142907-
scaffold516_64473-64528-
Consensus
CACGGTCAGATTTTGTCGAATTCGAT+++TCGAATTCGACAAAATCTGACCGTG

j.

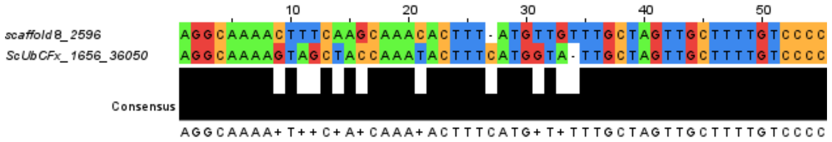
scaffold8_2596
ScUbCFx_1656_36050
Consensus
AGGCAAAA+T++C+A+CAAA+ACTTTCATG+T+TTTGCTAGTTGCTTTTGTCCCC

k.

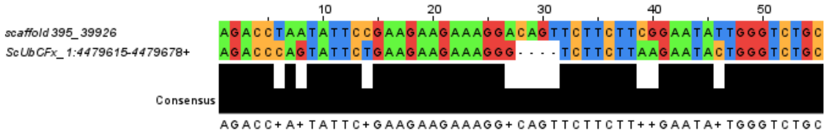
scaffold395_39926
ScUbCFx_1:4479615-4479678+
Consensus
AGACC+A+TATTC+GAAGAAGAAAGG+CAGTTCTTCTT++GAATA+TGGGTCTGC

l.

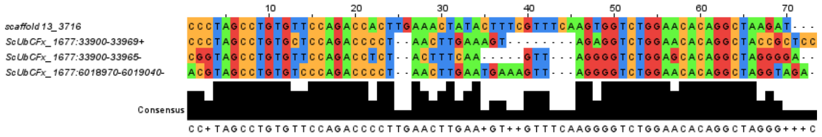
scaffold13_3716
ScUbCFx_1677:33900-33969+
ScUbCFx_1677:33900-33965-
ScUbCFx_1677:6018970-6019040-
Consensus
CC+TAGCCTGTGTTCCAGACCCCTTGAACTTGAA+GT++GTTTCAAGGGGTCTGGAACACAGGCTAGGG+++C

m.

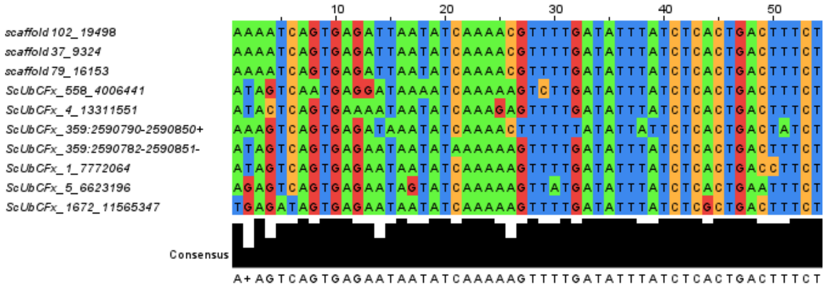
scaffold102_19498
scaffold37_9324
scaffold79_16153
ScUbCFx_558_4006441
ScUbCFx_4_13311551
ScUbCFx_359:2590790-2590850+
ScUbCFx_359:2590782-2590851-
ScUbCFx_1_7772064
ScUbCFx_5_6623196
ScUbCFx_1672_11565347
Consensus
A+AGTCAGTGAGAATAATATCAAAAAGTTTTGATATTTATCTCACTGACTTTCT

G) Two conserved miRNAs between *Aurelia* and *Sanderia*

a.

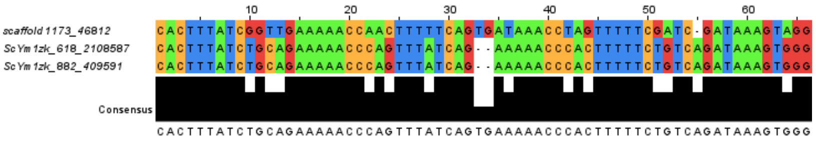

b.

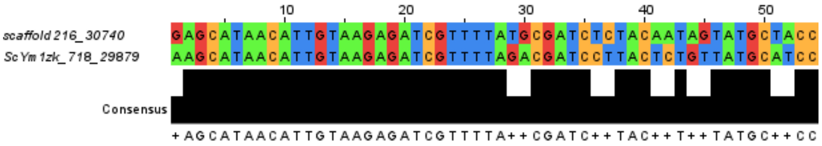

H)

conserved miRNA	Precursor location	speices
mir-2022	scaffold8	A. aurita_Baltic_sea
mir-2022	ScUbCFx_1656	R. esculentum
miR-2022	ScYm1zk_531	S. malayensis
mir-2030	scaffold216	A. aurita_Baltic_sea
mir-2030	ScUbCFx_448	R. esculentum
miR-2030	ScYm1zk_718	S. malayensis
Sma-Aau-Res_conserved-1	scaffold44	A. aurita_Baltic_sea
Sma-Aau-Res_conserved-1	ScUbCFx_10	R. esculentum
Sma-Aau-Res_conserved-1	ScYm1zk_906	S. malayensis
Sma-Aau-Res_conserved-2	scaffold16	A. aurita_Baltic_sea
Sma-Aau-Res_conserved-2	scaffold4	A. aurita_Baltic_sea
Sma-Aau-Res_conserved-2	ScUbCFx_1682	R. esculentum
Sma-Aau-Res_conserved-2	ScYm1zk_660	S. malayensis
Sma-Aau-Res_conserved-2	ScYm1zk_779	S. malayensis
Sma-Aau-Res_conserved-3	scaffold13	A. aurita_Baltic_sea
Sma-Aau-Res_conserved-3	scaffold166	A. aurita_Baltic_sea
Sma-Aau-Res_conserved-3	scaffold18	A. aurita_Baltic_sea
Sma-Aau-Res_conserved-3	scaffold265	A. aurita_Baltic_sea
Sma-Aau-Res_conserved-3	scaffold47	A. aurita_Baltic_sea
Sma-Aau-Res_conserved-3	scaffold59	A. aurita_Baltic_sea
Sma-Aau-Res_conserved-3	ScUbCFx_1677	R. esculentum
Sma-Aau-Res_conserved-3	ScUbCFx_795	R. esculentum
Sma-Aau-Res_conserved-3	ScYm1zk_828	S. malayensis
Sma-Aau-Res_conserved-3	ScYm1zk_906	S. malayensis
Sma-Aau-Res_conserved-4	scaffold275	A. aurita_Baltic_sea
Sma-Aau-Res_conserved-4	ScUbCFx_1656	R. esculentum
Sma-Aau-Res_conserved-4	ScUbCFx_795	R. esculentum
Sma-Aau-Res_conserved-4	ScYm1zk_24	S. malayensis
Sma-Aau-Res_conserved-4	ScYm1zk_779	S. malayensis
Sma-Aau-Res_conserved-4	ScYm1zk_824	S. malayensis
Sma-Aau-Res_conserved-5	scaffold81	A. aurita_Baltic_sea
Sma-Aau-Res_conserved-5	ScUbCFx_1672	R. esculentum
Sma-Aau-Res_conserved-5	ScYm1zk_772	S. malayensis
Sma-Aau-Res_conserved-6	scaffold109	A. aurita_Baltic_sea
Sma-Aau-Res_conserved-6	scaffold28	A. aurita_Baltic_sea
Sma-Aau-Res_conserved-6	scaffold336	A. aurita_Baltic_sea
Sma-Aau-Res_conserved-6	scaffold37	A. aurita_Baltic_sea
Sma-Aau-Res_conserved-6	scaffold770	A. aurita_Baltic_sea
Sma-Aau-Res_conserved-6	scaffold913	A. aurita_Baltic_sea
Sma-Aau-Res_conserved-6	ScUbCFx_160	R. esculentum
Sma-Aau-Res_conserved-6	ScUbCFx_1662	R. esculentum
Sma-Aau-Res_conserved-6	ScUbCFx_1677	R. esculentum
Sma-Aau-Res_conserved-6	ScUbCFx_1682	R. esculentum
Sma-Aau-Res_conserved-6	ScUbCFx_382	R. esculentum
Sma-Aau-Res_conserved-6	ScUbCFx_558	R. esculentum
Sma-Aau-Res_conserved-6	ScUbCFx_795	R. esculentum
Sma-Aau-Res_conserved-6	ScUbCFx_92	R. esculentum
Sma-Aau-Res_conserved-6	ScYm1zk_128	S. malayensis
Sma-Aau-Res_conserved-6	ScYm1zk_139	S. malayensis
Sma-Aau-Res_conserved-6	ScYm1zk_374	S. malayensis
Sma-Aau-Res_conserved-6	ScYm1zk_439	S. malayensis
Sma-Aau-Res_conserved-6	ScYm1zk_456	S. malayensis
Sma-Aau-Res_conserved-6	ScYm1zk_466	S. malayensis
Sma-Aau-Res_conserved-6	ScYm1zk_470	S. malayensis
Sma-Aau-Res_conserved-6	ScYm1zk_519	S. malayensis
Sma-Aau-Res_conserved-6	ScYm1zk_532	S. malayensis
Sma-Aau-Res_conserved-6	ScYm1zk_535	S. malayensis
Sma-Aau-Res_conserved-6	ScYm1zk_615	S. malayensis
Sma-Aau-Res_conserved-6	ScYm1zk_626	S. malayensis
Sma-Aau-Res_conserved-6	ScYm1zk_629	S. malayensis
Sma-Aau-Res_conserved-6	ScYm1zk_658	S. malayensis
Sma-Aau-Res_conserved-6	ScYm1zk_660	S. malayensis
Sma-Aau-Res_conserved-6	ScYm1zk_699	S. malayensis
Sma-Aau-Res_conserved-6	ScYm1zk_718	S. malayensis
Sma-Aau-Res_conserved-6	ScYm1zk_824	S. malayensis
Sma-Aau-Res_conserved-6	ScYm1zk_828	S. malayensis
Sma-Aau-Res_conserved-6	ScYm1zk_861	S. malayensis
Sma-Aau-Res_conserved-6	ScYm1zk_876	S. malayensis
Sma-Aau-Res_conserved-6	ScYm1zk_882	S. malayensis
Sma-Aau-Res_conserved-6	ScYm1zk_901	S. malayensis
Sma-Aau-Res_conserved-6	ScYm1zk_914	S. malayensis

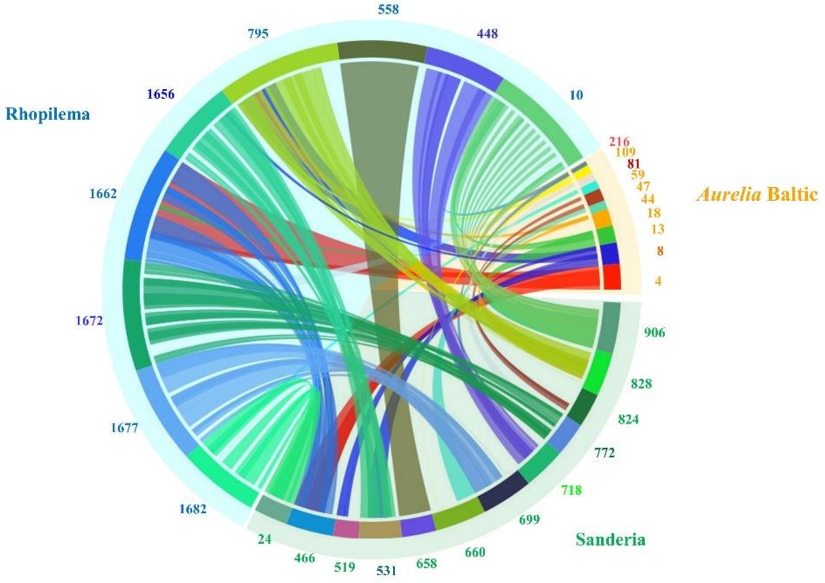

Supplementary Figure 11. Hairpin sequence alignment and syntenic analyses of jellyfish microRNAs. A) miR-100, B) miR-2022, C) miR-2030, D) Six conserved scyphozoan novel microRNAs in all three jellyfish species, E) Four conserved miRNAs between jellyfish *Sanderia* and *Rhopilema*, F) Twelve conserved microRNAs between jellyfish *Rhopilema* and *Aurelia*, G) Conserved miRNAs between *Aurelia* and *Sanderia*, H) Synteny analyses.

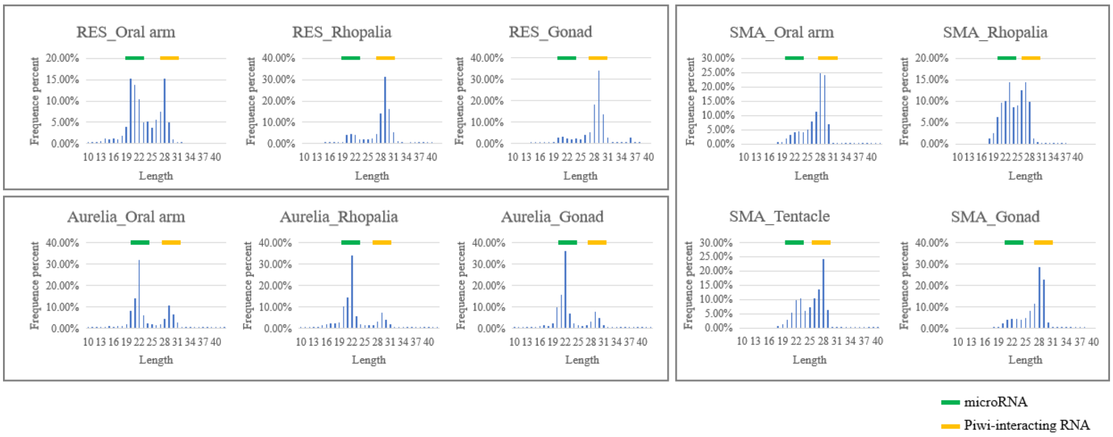

Supplementary Figure 12. Small RNA length distribution in different tissues of jellyfish. Abbreviations: *R. esculentum* (RES) , *S. malayensis* (SMA) and *Aurelia*.

A)

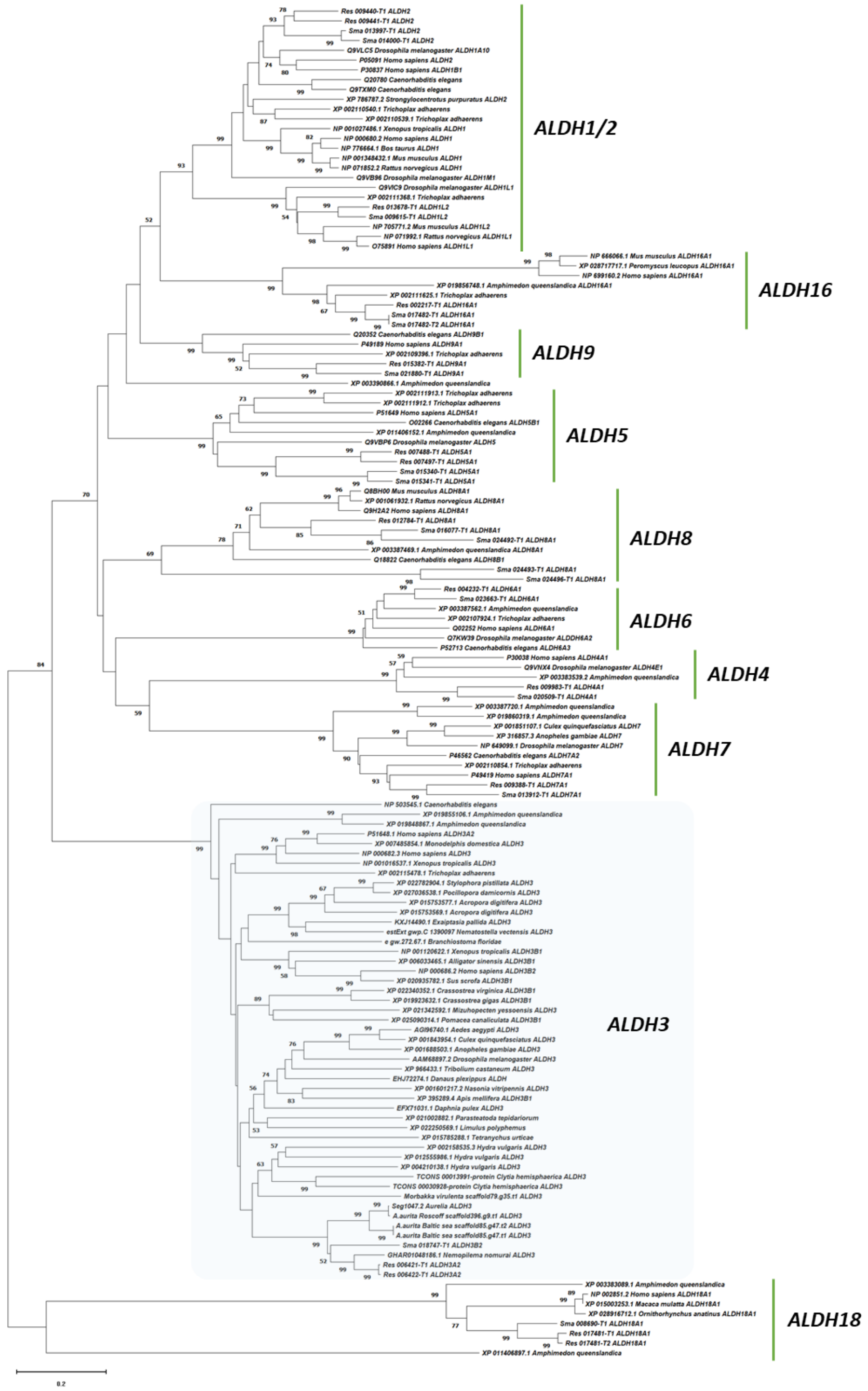
ALDH1/2
ALDH16
ALDH9
ALDH5
ALDH8
ALDH6
ALDH4
ALDH7
ALDH3
ALDH18

B)

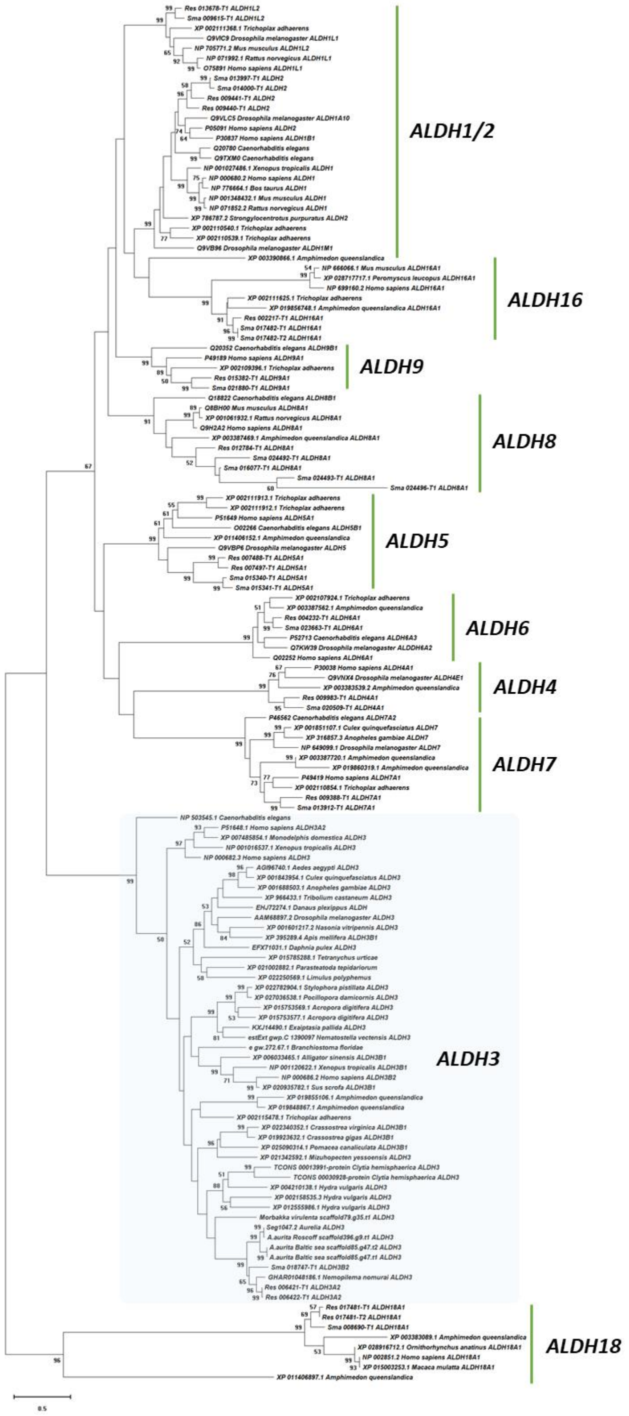
ALDH1/2
ALDH16
ALDH9
ALDH8
ALDH5
ALDH6
ALDH4
ALDH7
ALDH3
ALDH18

C)

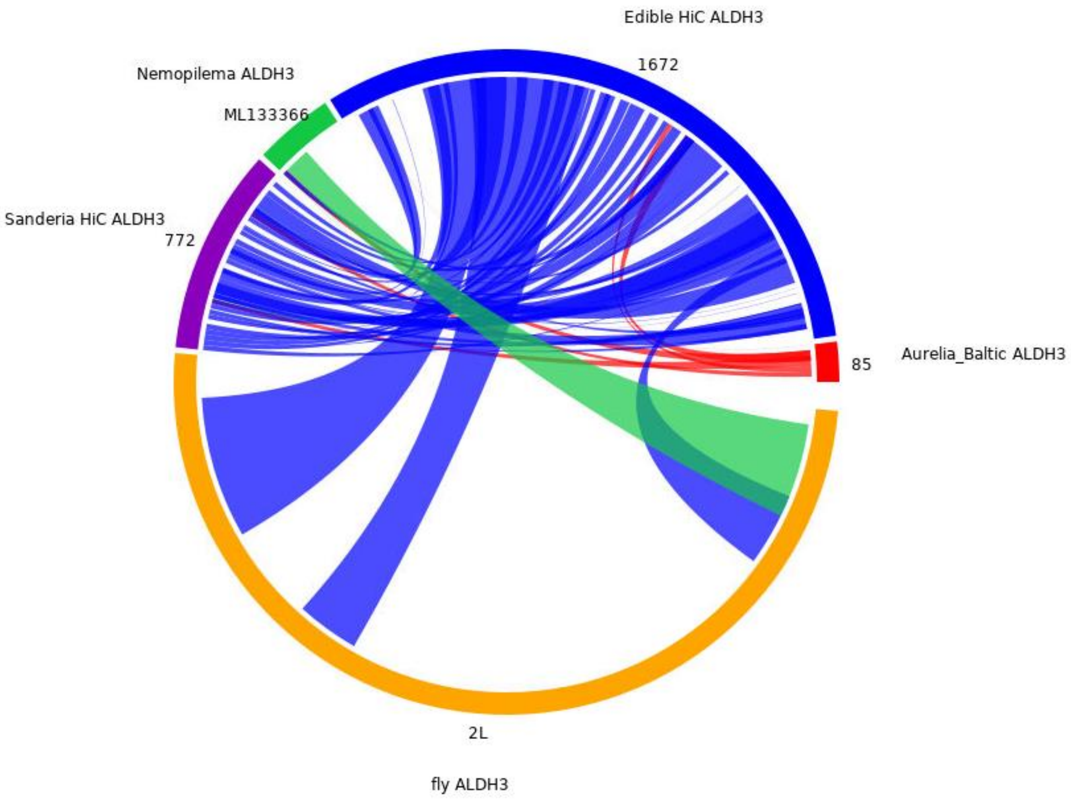

Supplementary Figure 13. Phylogenetic trees and synteny analyses of ALDHs. A) The Neighbor-Joining tree with 1000 bootstrap replicates; B) The Maximum Likelihood tree was constructed with the LG (G+I) model in MEGA7 (with 1000 bootstrap replicates).) Synteny of ALDH.

Supplementary Table 1. S*anderia malayensis* genome sequencing data information

Platform	Read length(bp)	Library size	Reads	Bases	Coverage*
HiSeq2500 125PE	125	200 bp	322,448,536	40,306,067,000	218.6
		500 bp	314,734,614	39,341,826,750	213.4
HiSeq4000 125PE		300 bp	290,161,232	36,270,154,000	196.7
		800 bp	173,845,904	21,730,738,000	117.9
HiSeq4000 50PE	49	5 kb	692,055,142	33,910,701,958	183.9
HiseqXten 150PE (Chromium WGS)	150	50 kb	181,100,754	27,346,213,854	148.3
PacBio RS II P6-C4	Average 8,428	20 kb	4,690,914	39,535,512,386	214.4
CHiCAGO	150	1-100kb	227,000,000	68,100,000,000	369.4
Hi-C		100-1000kb	219,000,000	65,700,000,000	356.3

***Estimated genome size = 184,371,355 bp**

Supplementary Table 2. *Rhopilema esculentum* genome sequencing data information

Platform	Read length(bp)	Library size	Reads	Bases	Coverage**
HiSeq4000 125PE	125	200 bp	204,508,418	25,563,552,250	99.6
		300 bp	211,057,000	26,382,125,000	102.8
HiSeq4000 125PE		500 bp	317,578,812	39,697,351,500	154.6
		800 bp	185,591,494	23,198,936,750	90.4
PacBio RS II P6-C4	Average 5,819	10 kb	1,381,093	8,036,359,407	31.3
CHiCAGO	150	1-100kb	153,000,000	45,900,000,000	178.8
Hi-C		100-1000kb	203,000,000	60,900,000,000	237.3

****Estimated genome size = 256,689,583 bp**

Supplementary Table 3. *R. esculentum* 21 pseudomolecules in the genome assembly.

Number	Length (bp)	Scaffold number	% of whole genome
**1**	16,759,797	ScUbCFx_795	6.53%
**2**	15,779,437	ScUbCFx_1662	6.15%
**3**	15,264,025	ScUbCFx_1	5.95%
**4**	15,034,379	ScUbCFx_1672	5.86%
**5**	14,788,415	ScUbCFx_10	5.76%
**6**	13,614,496	ScUbCFx_4	5.30%
**7**	13,390,862	ScUbCFx_217	5.22%
**8**	13,031,781	ScUbCFx_92	5.08%
**9**	12,930,448	ScUbCFx_1677	5.04%
**10**	11,943,657	ScUbCFx_558	4.65%
**11**	11,630,773	ScUbCFx_1659	4.53%
**12**	11,558,745	ScUbCFx_448	4.50%
**13**	11,157,820	ScUbCFx_1682	4.35%
**14**	11,089,206	ScUbCFx_1656	4.32%
**15**	9,803,757	ScUbCFx_359	3.82%
**16**	9,509,274	ScUbCFx_409	3.70%
**17**	8,633,203	ScUbCFx_5	3.36%
**18**	8,174,362	ScUbCFx_1658	3.18%
**19**	7,750,636	ScUbCFx_107	3.02%
**20**	6,928,765	ScUbCFx_979	2.70%
**21**	4,373,400	ScUbCFx_45	1.70%
			Total = 94.72%

Supplementary Table 4. *Sanderia malayensis* transcriptome sequencing data information

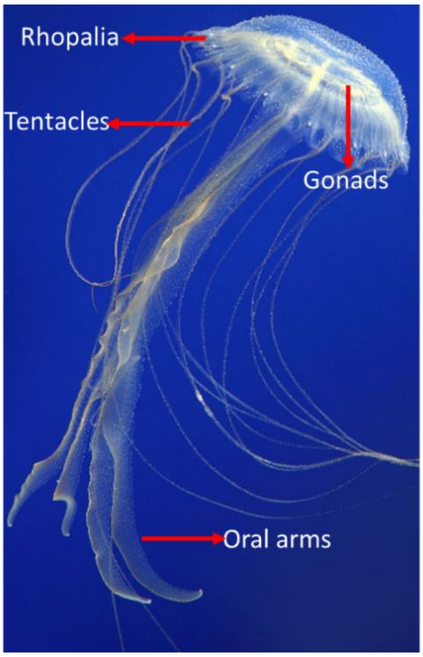

Type	Tissues	Platform	Reads	Bases	Accession
mRNA	Gonads (g2)	Hiseq4000 150PE	65,078,350	9,761,752,500	SRR8182683
	Oral arms (2B)		64,110,250	9,616,537,500	SRR8182684
	Tentacles (t6)		92,551,848	13,882,777,200	SRR8182681
	Rhopalia (B1)	Hiseq4000 100PE	57,372,996	5,737,299,600	SRR8182682
Small RNA	Gonads (g2)	Hiseq2000 50SE	25,404,258	677,082,507	SRR8181855
	Oral arms (2B)		28,809,031	774,332,717	SRR8181854
	Rhopalia (B1)	Hiseq2500 50SE	32,440,005	784,025,649	SRR8181853
	Tentacles (t6)		24,823,459	629,335,866	SRR8181852

Supplementary Table 5. *Rhopilema esculentum* transcriptome sequencing data information

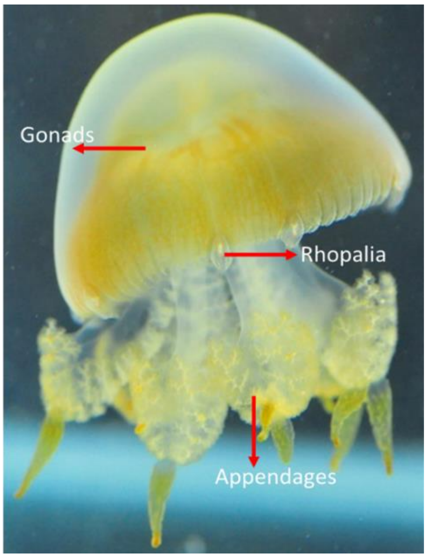

Type	Tissues	Platform	Reads	Bases	Accession
mRNA	Gonads (eg)	Hiseq4000 100PE	51,507,458	5,150,745,800	SRR8193750
	Appendages (ea)		60,987,726	6,098,772,600	SRR8193751
	Rhopalia (eb)		46,851,830	4,685,183,000	SRR8193752
Small RNA	Gonads (eg1)	Hiseq2500 50SE	26,663,434	732,091,624	SRR8195101
	Gonads (eg2)		25,893,533	738,808,278	SRR9590812
	Appendages (ea1)		28,239,931	691,365,149	SRR8195102
	Appendages (ea2)		13,715,456	280,965,327	SRR9590813
	Rhopalia (eb1)		23,942,602	674,594,689	SRR8195100
	Rhopalia (eb2)		19,454,355	525,275,296	SRR9590811

<u>Supplementary Table 6. *Aurelia* transcriptome sequencing data information</u>

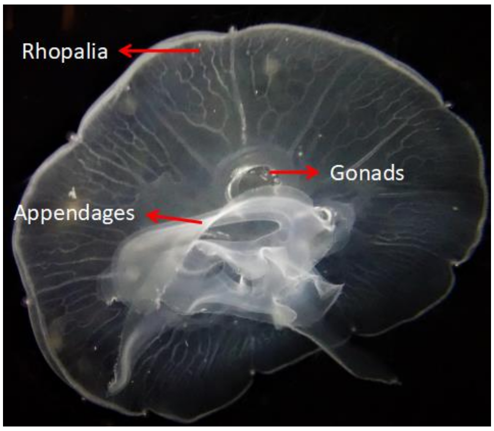

Type	Tissues	Platform	Reads	Bases	Accession
Small RNA	Gonads (G2)	Hiseq2500 50SE	18,987,518	435,376,288	SRR9657804
	Appendages (A2)		23,126,896	548,068,556	SRR9657805
	Rhopalia (B2)		18,546,845	418,657,215	SRR9657806

Supplementary Table 7. Species used in the phylogenomic analyses

Species	Link
Acropora digitifera	http://marinegenomics.oist.jp/coral/viewer/download?project_id=3
Amphimedon queenslandica	ftp://ftp.ensemblgenomes.org/pub/metazoa/release-35/fasta/amphimedon_queenslandica/pep/
Amplexidiscus fenestrafer	http://corallimorpharia.reefgenomics.org/
Aurelia sp.	https://davidadlergold.faculty.ucdavis.edu/jellyfish/
Branchiostoma floridae	http://genome.jgi.doe.gov/Brafl1/Brafl1.download.ftp.html
Clytia hemisphaerica	http://marimba.obs-vlfr.fr/downloads/
Danio rerio	ftp://ftp.ensembl.org/pub/release-89/fasta/danio_rerio/pep/
Discosoma sp.	http://corallimorpharia.reefgenomics.org/
Drosophila melanogaster	ftp://ftp.ensembl.org/pub/release-89/fasta/drosophila_melanogaster/pep/
Exaiptasia pallida	http://aiptasia.reefgenomics.org/download/
Homo sapiens	ftp://ftp.ensembl.org/pub/release-89/fasta/homo_sapiens/pep/
Hydra vulgaris	ftp://ftp.ncbi.nih.gov/genomes/Hydra_vulgaris/protein/
Lottia gigantea	http://genome.jgi.doe.gov/Lotgi1/Lotgi1.download.ftp.html
Monosiga brevicollis	http://genome.jgi.doe.gov/Monbr1/Monbr1.download.ftp.html
Nematostella vectensis	http://genome.jgi.doe.gov/Nemve1/Nemve1.download.ftp.html
Pleurobrachia bachei	http://neurobase.rc.ufl.edu/pleurobrachia/download
Pocillopora damicornis	http://pdam.reefgenomics.org/download/
Rhopilema esculentum	**This study**
Sanderia malayensis	**This study**
Stylophora pistillata	http://spis.reefgenomics.org/download/
Sycon ciliatum	http://datadryad.org/resource/doi:10.5061/dryad.tn0f3/3

Supplementary Table 8. Composition of transposable elements in the two jellyfish genomes

Species	*Sanderia malayensis*		*Rhopilema esculentum*	
**Repeat element**	**Count**	**bpMasked**	**Count**	**bpMasked**
**DNA Transposon**	**50576**	**23491092**	**54249**	**15094460**
Helitron	5098	7908426	2848	554950
TcMar	19798	3380123	19672	5114022
Polinton	1783	2710303	326	50909
Academ	5267	1689171	3179	928699
hAT	4375	1500837	12190	3732361
Zisupton	1391	869258	85	83679
Harbinger	2221	863338	3517	1228406
Kolobok	1615	863103	608	187551
ISL2EU	1755	813413	1248	629081
MuDR	1062	507873	4724	964967
Unknown	1337	403746	1897	303787
P	572	358351	726	391323
Ginger	1138	352267	674	108864
IS3EU	990	351452	258	28029
Transib	849	230751	/	/
EnSpm-CACTA	630	224233	1401	256497
Ginger1	305	188119	/	/
Sola3	45	115517	/	/
Ginger2	51	91772	/	/
Zator	167	41763	149	45892
Merlin	127	27276	69	12760
Sola2	/	/	511	330060
PiggyBac	/	/	167	142623
**LTR Retrotransposon**	**7608**	**6354107**	**6224**	**4185753**
Gypsy	3657	2901310	2489	2276879
BEL	1794	1688084	1306	1093373
DIRS	1149	951369	178	131167
Copia	876	763980	661	380630
Ngaro	132	49364	/	/
ERV1	/	/	96	31925
Unknown	/	/	1494	271779
**Non-LTR Retrotransposon**	**9890**	**4093680**	**31663**	**10349952**
Penelope	2899	864010	9657	2289450
Daphne	1546	687606	913	341752
CR1	1857	637788	3883	1060944
L2	518	329318	3581	937382
RTE	921	317020	1608	638766
L2A	623	307684	5042	1844408
L1-Tx1	242	197124	111	21372
CRE	356	193564	75	18173
Tad1	197	147855	/	/
R4	145	75477	365	307820
Crack	187	75457	2676	932927
RTEX	174	67436	921	320889
L1	37	52956	96	8399
Tx1	16	39484	220	298055
R1	31	36349	15	11541
Jockey	50	25905	/	/
I	30	21248	25	21374
Kiri	61	17399	347	75722
Hero	/	/	1587	995939
L2B	/	/	343	148313
Unknown	/	/	198	76726
**Low complexity**	**41549**	**3356522**	**33860**	**2310764**
Simple_repeat	36668	3068940	28862	2025350
Other low_complexity	4881	287582	4998	285414
**Satellite**	**1069**	**617784**	**261**	**237248**
**Unknown**	**109694**	**37370572**	**184615**	**44223298**
**Total**	**220386**	**75283757**	**310872**	**76401475**

Supplementary Table 9. Repeat percentages and repeat compositions of sequenced cnidarians.

Species	Repeat Category	DNA transposon			LTR Retrotransposon			Non-LTR Retrotransposon			Unclassified
Sanderia malayensis	Type	Helitron	TcMar	Polinton	Gypsy	BEL	DIRS	Penelope	Daphne	CR1	Unclassified
	Percentage	4.3%	1.9%	1.5%	1.6%	0.9%	0.5%	0.5%	0.4%	0.4%	20.4%
Rhopilema esculentum	Type	TcMar	hAT	Harbinger	Gypsy	BEL	Copia	Penelope	L2A	CR1	Unclassified
	Percentage	2.0%	1.5%	0.5%	0.9%	0.4%	0.2%	0.9%	0.7%	0.4%	17.3%
Exaiptasia pallida	Type	Polinton	EnSpm	Helitron	Gypsy	BEL	DIRS	CR1	Penelope	RTE	Unclassified
	Percentage	0.2%	0.2%	0.1%	0.5%	0.2%	0.2%	0.3%	0.3%	0.1%	14.0%
Nematostella vectensis	Type	Polinton	TcMar	hAT	Gypsy	DIRS	BEL	CR1	Penelope	RTE	Unclassified
	Percentage	3.0%	2.3%	2.1%	1.5%	0.4%	0.2%	1.0%	0.7%	0.4%	NA
Acropora digitifera	Type	hAT	TcMar	Kolobok	Gypsy	BEL	DIRS		NA		Unclassified
	Percentage	0.5%	0.4%	0.3%	1.4%	0.6%	0.5%		NA		NA
Hydra vulgaris	Type	hAT	TcMar	Transib	Gypsy	BEL	Copia	CR1	Penelope	L1	Unclassified
	Percentage	6.0%	4.2%	3.7%	2.1%	0.2%	0.1%	15.2%	2.2%	0.3%	15.6%
Stylophora pistillata	Type	Polinton	IS4	Sola	BEL	Gypsy	DIRS	CR1	Penelope	SINE	Unclassified
	Percentage	0.4%	0.2%	0.2%	0.3%	0.2%	0.2%	1.6%	1.4%	0.4%	17.0%
Aurelia aurita (Atlantic)	Type	hAT	TcMar	Kolobok	Gypsy	Copia	Ngaro	L2	CR1	Penelope	Unclassified
	Percentage	0.3%	0.1%	0.1%	0.2%	0.2%	0.1%	5.1%	1.5%	0.5%	32.0%
Aurelia aurita (Pacific)	Type	hAT	TcMar	PIF-ISL2EU	Gypsy	ERV	Copia	L2	CR1	L1	Unclassified
	Percentage	0.9%	0.8%	0.6%	2.2%	1.4%	1.0%	4.2%	1.4%	0.8%	19.3%
Morbakka virulenta	Type	MC-Chapaev	TcMar	hAT	Gypsy	Pao	Copia	RTE	Dong-R4	Penelope	Unclassified
	Percentage	1.3%	1.0%	0.9%	1.1%	0.3%	0.1%	9.2%	6.4%	1.9%	5.0%
Aurelia aurita	Type	Academ	Mariner/Tc	hAT	Gypsy	BEL	DIRS	CR1	L2A	Crack	Unclassified
	Percentage	0.3%	0.3%	0.2%	0.5%	0.3%	0.2%	4.3%	2.2%	1.1%	NA

Highlight: repeats are estimated using repeatmasker using the annotated repeat library provided by the original paper

Supplementary Table 10. Statistics of predicted gene models in the jellyfish genomes

Species	Assembled genome size(bp)	Number of Proteins	Sum of Amino Acids	Mean of Proteins	Sum of Exons(bp)	Mean of Exons	Sum of Introns(bp)	Mean of Introns	Number of gene loci	Sum of gene region(bp)	% of gene loci in genome	Average gene region
Sanderia malayensis	184,371,355	26,914	11,765,195	437	43,019,434	228	60,441,058	381	28526	93,435,282	50.68%	3,275
Rhopilema esculentum	256,689,583	18,923	9,636,271	509	36,712,848	237	149,561,064	1,099	17627	165137886	64.33%	9,368
Exaiptasia pallida	256,132,296	27,753	14,880,949	536	65,565,087	282	303,417,584	1,398	24491	157834213	61.62%	6,445
Nematostella vectensis	356,613,585	24,780	8,294,159	335	27,729,411	208	86,555,899	799	24773	114172274	32.02%	4,609
Acropora digitifera	447,497,157	33,878	15,729,665	464	76,330,869	296	574,341,465	2,560	32076	253648292	56.68%	7,908
Hydra vulgaris	852,170,992	21,990	10,036,274	456	33,744,933	222	6,221,150	3,800	22732	312113446	36.63%	13,730
Stylophora pistillata	400,120,318	33,252	18,275,238	550	86,199,075	296	513,138,303	1,905	27482	223994894	55.98%	8,151
Aurelia aurita (Atlantic)	376,952,359	38,007	17,244,864	454	100,279,977	369	343,391,322	1,466	28605	292225310	77.52%	10,216
Morbakka virulenta	951,575,644	28,983	14,362,356	496	73,580,670	351	660,896,111	3,656	24278	520633789	54.71%	21,445
Aurelia aurita	757,170,055	27,044	10,271,587	380	47,434,663	326	216,143,299	1,962	35273	263395770	34.79%	7,467

Supplementary Table 11. Comparison of assembled scyphozoan mitochondrial genomes

Species	*Aurelia aurita*	*Aurelia sp. nov.*	*Chrysaora quinquecirrha*	*Acromitus flagellatus*	*Chrysaora sp EK-2011; Cassiopea Andromeda; Cassiopea frondosa; Catostylus mosaicus; Cyanea capillata;Pelagia noctiluca; Rhizostoma Pulmo; Linuche unguiculata*	*Sanderia malayensis; Rhopilema esculentum*
Lengths (bp)	16937	16971	16775	14631	14415;15800;15949;14847;16202;15876; 752+5648;12129	17212; 17098
Reference	Shao et al., 2006	Hwang, Park, Won, & Lee, 2014	Hwang, Park, Won, Lee, et al., 2014	Huang, 2014	Kayal et al., 2012	This study

Supplementary Table 12. Genomic organisation of mitochondrial genes in *S. malayensis*

Gene	From	To	Strand	Size (bp)	Start	Stop
Telomere	1	477	/	477	/	/
polB	340	969	-	630	GTG	TAA
orf255	1250	1504	+	255	ATG	TAA
rrnL	1872	3400	-	1529	/	/
cox1	3658	5223	-	1566	ATG	TAA
cox2	5275	6042	+	768	ATG	TAG
tRNA-Trp(tca)	6049	6118	+	70	/	/
atp8	6124	6324	+	201	ATG	TAA
atp6	6324	7031	+	708	ATG	TAA
cox3	7031	7816	+	786	ATG	TAA
tRNA-Met(cat)	7822	7890	+	69	/	/
nad2	7896	9206	+	1311	ATG	TAA
nad5	9207	11021	+	1815	ATG	TAA
rrnS	11138	11990	+	853	/	/
nad6	12016	12558	+	543	ATG	TAA
nad3	12542	12904	+	363	GTG	TAA
nad4l	12895	13197	+	303	ATG	TAA
nad1	13197	14168	+	972	ATG	TAA
nad4	14168	15610	+	1443	ATG	TAA
cob	15615	16838	+	1224	ATG	TAA
Telomere	16736	17212	/	477	/	/

Supplementary Table 13. Genomic organization of mitochondrial genes in *R. esculentum*

Gene	From	To	Strand	Size (bp)	Start	Stop
Telomere	1	517	/	517	/	/
polB	430	1434	-	1005	ATG	TAA
tRNA-Asp(atc)	1447	1520	-	74	/	/
rrnL	1810	3247	-	1438	/	/
cox1	3521	5110	-	1590	ATG	TAA
tRNA-Asn(att)	5115	5187	+	73	/	/
cox2	5188	5937	+	750	ATG	TAA
tRNA-Trp(tca)	5942	6011	+	70	/	/
atp8	6018	6221	+	204	ATG	TAA
atp6	6225	6926	+	702	ATG	TAA
cox3	6927	7712	+	786	ATG	TAA
tRNA-Met(cat)	7721	7789	+	69	/	/
nad2	7794	9113	+	1320	ATG	TAA
nad5	9115	10932	+	1818	ATG	TAA
rrnS	10937	11864	+	928	/	/
nad6	11871	12416	+	546	ATG	TAG
nad3	12397	12756	+	360	GTG	TAA
nad4l	12747	13049	+	303	ATG	TAA
nad1	13049	14020	+	972	ATG	TAA
nad4	14026	15459	+	1434	ATG	TAA
cob	15463	16602	+	1140	ATG	TAG
Telomere	16583	17098	/	516	/	/

Supplementary Table 14. Homeobox genes content in the jellyfish genomes

Classes	Family	*Sanderia*	*Rhopilema*	*Nemopilema*	*Morbakka*	*Aurelia_* Baltic	*Clytia*
**ANTP**							
HOXL	CdxL	1	1	1	2	1	1
	Gsx	1	1	1	1	1	1
	XloxL	1	1	1	1	2	1
	Hox	7	7	7	6	7	4
	Hox-like	2	3	1	2	3	2
	Evx	1	1	1	1	0	1
	Meox	1	1	1	1	1	1
NKL	Dlx	2	2	2	2	2	2
	EmxL	2	2	2	2	2	1
	Hhex	1	1	1	1	1	1
	BarxL	1	1	1	1	1	0
	TlxL	1	1	1	1	1	0
	Dbx	1	1	1	1	2	0
	Msx	1	1	1	1	0	1
	MsxL	1	1	1	1	1	1
	NotoL	1	1	1	2	1	2
	Nk1	1	1	1	1	3	1
	Nk2/4	3	3	3	3	3	3
	Nk3	1	1	1	1	1	0
	NK3L	1	1	1	1	1	0
	Nk5/Hmx	1	1	1	1	1	2
	Nk6	1	1	1	2	1	2
	Nk7L	2	1	1	1	1	0
	Nk-like	4	6	5	1	3	8
Unclassified		1	1	2	1	0	1
	Total	40	42	40	38	40	36
**PRD**							
	Alx	1	1	2	1	1	0
	Arx	4	3	3	1	2	3
	Vsx-like	1	1	1	2	1	1
	Gsc	1	1	1	1	1	1
	Hbn	1	2	3	1	2	2
	Pax	1	1	1	1	2	0
	Pitx	1	1	1	1	1	1
	Otx	5	5	4	7	5	3
	Uncx	1	1	1	1	1	1
	Drgx-like	1	1	1	1	1	1
	Otp	1	1	1	1	1	1
	Dmbx-like	1	1	1	1	1	1
	Rax	1	1	1	2	1	0
Unclassified		12	10	11	4	7	3
	Total	32	30	32	25	27	18
**LIM**							
	Isl	1	1	1	1	1	1
	Lhx1/5	1	1	1	1	1	1
	Lhx2/9	1	1	1	1	1	1
	Lhx6/8	1	2	1	1	1	1
	Lmx	1	1	0	1	1	1
	Total	5	6	4	5	5	5
**POU**							
	Pou1	2	1	1	1	1	0
	Pou3	1	1	1	1	1	1
	Pou4	1	1	1	1	1	1
	Pou6-like	1	1	1	1	1	1
	Total	5	4	4	4	4	3
**SINE**							
	Six1/2	2	2	3	2	2	1
	Six3/6	1	1	1	2	1	1
	Six4/5	1	1	1	1	1	1
	Six-like	0	0	0	0	0	1
	Total	4	4	5	5	4	4
**TALE**							
	Irx	1	1	1	1	1	1
	Meis	3	3	3	1	2	1
	Pbx	1	1	1	1	1	1
	Pknox	1	1	1	1	1	1
	Atale-like	1	1	1	0	1	0
	Tale-like	0	0	0	0	0	1
	Total	7	7	7	4	6	5
**CERS**							
	Cers	1(partial)	1	1	1	1	1
**Other**							
	Total	7	4	2	1	3	5

Supplementary Table 15. *Sanderia* ANTP class homeobox genes and locations

gene ID	Sequences	Genomic location
>SMA633.t1 CdxL	TMRSRPCFTSHQTKQLENEFHSCQYITRRRRIEIAYALNLTEKQIKTWFQNRRVKERKVK	>ScYm1zk_616:3893016-3893195
>SMA632.t1 Gsx	SKRIRTAYTSIQLLELEKEFQNNRYLSRLRRIQIAAMLDLTEKQVKIWFQNRRVKWKKDK	>ScYm1zk_616:3904400-3904579
>SMA627.t1 XloxL	KKRTRTTYTRAQQVELEKEYHYSRYISRARRIELARALNLTEKHIKIWFQNRRMKEKRDD	>ScYm1zk_616:3921304-3921483
>SMA4885.t1 HoxL	TKQKQFKYTQKQLFELEKEFHTTPYLTGAKKVEISNKLNIEEKQVKIWFQNRRMRLKKET	>ScYm1zk_466:3135867-3136046
>SMA4884.t1 Hox	SKRFRTSFTTTQLTELEKEFHYNKYLTRRRRVELALGLNLTEKQVKVWFQNRRMKWKKQM	>ScYm1zk_466:3141964-3142143
>SMA4883.t1 Hox	CHRKRMTYTRHQTLELEKEFLYNQYLTKERRSQLSCSLVLSERQIKIWFQNRRMKSKKKE	>ScYm1zk_466:3146059-3146238
>SMA4874.t1 NKL	HRKPRTIFTKEQINSLEKRFSKKQYIGKKERRLMSKNMSLDEMKIKVWFQNRRAKVKRSK	>ScYm1zk_466:3178223-3178402
>SMA20728.t1 Hox	NNKKRVCFTQKQIVELEKEFHFNKYLTRARRVEISHSLRLSESQIKIWFQNRRMKYKREQ	>ScYm1zk_466:3895576-3895755
>SMA9186.t1 Evx	LHRTRTAFTREQLNKLEKEFSRENYISRARRCELATELSLPENTIKVWFQNRRMKCKRRR	>ScYm1zk_466:4316744-4316923
>SMA9385.t1 Dlx	TRKPRTIFNSFQLRELNRAFDRTHYLSLPERGELALALGLTQTQVKIWFQNRRSKYKKLL	>ScYm1zk_466:5160877-5161056
>SMA9386.t1 Dlx	KRKPRTIYSSHQLRELNKRFACAQYLALPDRAELATKLGLTQTQIKIWFQNKRSKLKKLI	>ScYm1zk_466:5172065-5172244
>SMA22300.t1 Hox	AKRKRMTYSRKQLLELEKEFHYNHFLKKERRTDLAKQLNLTERQIKIWFQNRRMKFKKEA	>ScYm1zk_531:357569-357748
>SMA22294.t1 Hox	KRRKRTAYTRKQLLELEKEFHFNHFLTRERRLQLATTLNLSERQIKIWFQNRRMKWKKRS	>ScYm1zk_531:414571-414750
>SMA8235.t1 HoxL	DKRKRTSYTKRQLIRLENEFQASNYITRAKRIELSLKMCLTEQQIKTWFQNRRMKMKKDK	>ScYm1zk_531:1137664-1137843
>SMA8391.t1 Nk2/4	KKKPRILFSQSQVTELEQRFKQQKYLNANEREQLASKLNLTATQIKIWFQNKRYKCKKQT	>ScYm1zk_531:1784024-1783893
>SMA8443.t1 EmxL	GRRIRTTFTRSQLKVLEKRFKSSHYIVGDERKQLAEELGLNESQVKIWFQNRRTKYKQDK	>ScYm1zk_531:2042468-2042647
>SMA8532.t1 EmxL	KKRHRTAFTPTQLLGLENSFEQNQYSVGEERKQLAAFLGLSETQIKVWFQNRRTKWKRIR	>ScYm1zk_531:2419502-2419681
>SMA21480.t1 Hhex	RKGGQVRFSHVQSVELERIFDHQKYISPQERKQLSLYLHLSERQVKTWFQNRRAKWRRVK	>ScYm1zk_531:3967874-3968002
>ScYm1zk_531:4104590-4104769 Nk5/Hmx	KKKTRTVFSRRQVYQLETAFDMKRYLSSSERASLANALKLSETQVKIWFQNRRNKWKRQI	>ScYm1zk_531:4104590-4104769
>SMA21170.t1 Meox	ARKERTAFTKQQIRDLESEFTKNNYLTRLRRYEIAVTLNLTERQVKVWFQNRRMKWKRVK	>ScYm1zk_531:4841865-4842005
>SMA25529.t1 BarxL	CRKPRTVFSELQLMVLNREFTEHKYLNTPQRTKLAERLGLNQTQVKTWFQNRRMKWKKET	>ScYm1zk_772:516161-516340
>ScYm1zk_772:2487695-2487876 NKL	LPSWRRKFSEGQTTNLEKVFTKQKYIAGKERAEIAGHLNLTTKQVKTWFQNRRTKWKKER	>ScYm1zk_772:2487695-2487876
>SMA3332.t1 Nk1	PRRIRTAFTYEQLVALENKFRSTRYLSVCERLNLALSLGLSETQVKIWFQNRRTKWKKQN	>ScYm1zk_772:3551682-3551861
>SMA3510.t1 Hox	RHRKRMTYSRNQILELEKEFLYSRYLTKERRKDLSETLHLTERQIKIWFQNRRTKSKKER	>ScYm1zk_772:4216913-4217092
>SMA3512.t1 Hox	PRQKRKTYTRHQTLELEKEFLFSQYLTKERRRELSQSLGLTERQIKIWFQNRRMKQRRTT	>ScYm1zk_772:4229706-4229885
>SMA988.t1 Nk3	KKRIRAAFSAGQVYELERIFDRQKYLSAPERAELSKALKLSEQQVKIWFQNRRYKTKRKV	>ScYm1zk_906:518446-518625
>SMA1214.t1 TlxL	RKKPRTSFTRSQISELEELFTEKKYLTSSERQRVAACLQLTDCQVKTWFQNRRTKWKRET	>ScYm1zk_906:1251747-1251926
>SMA1515.t1 Nk3L	RKRLRASFSHEQVVVLQKAFDKNKYLGSGERRELARKLGMTEQQVKIWFQNRRYKEKRKQ	>ScYm1zk_906:2340748-2340927
>SMA11257.t1 Dbx	VQQRRAVFSDYQRRQLEVEFLAHKYISKQERRSLANQLNLSESQVRIWFQNRRMKWRNSQ	>ScYm1zk_906:3351130-3351309
>SMA19438.t1 NKL	KRKSRTKFTSEQMRRLEESFARETYLTLKEVDRLADELNLEENCIRTWFQNRRTKMRRVD	>ScYm1zk_828:2707187-2707366
>SMA19417.t1 NKL	YPRERTTFTASQLKFLEELFRVKKYLALLERSKVATHLELSEKQVKTWFQNRRTKYRRQK	>ScYm1zk_828:2795053-2795232
>SMA17501.t1 Nk2/4	GKKRRTLFTTQQTWELEKAFSFQPYLSSPERDLLAERIHLSPDQIKIWFQNHRYKLRKYI	>ScYm1zk_861:2341212-2341391
>SMA17494.t1 Nk2/4	KRRRRILFTKAQTYELERRFMHQRYLSAPEREQLGRMINLSATQVKIWFQNHRYKYKRQK	>ScYm1zk_861:2363315-2363494
>ScYm1zk_718:1324-1503 Nk7L	RKKARTTFTGRQIYLLERVFFCSKYLSRLERQSIASQLNVTSTQVKTWFQNRRTKWKKDR	>ScYm1zk_718:1324-1503
>SMA2246.t1 ANTP	EKRKRTAYSNEKKDILERYFQKSIYATKSMVLEITKEVGLDKKKIDNWFRNRRSKLKKDE	>ScYm1zk_718:1087174-1087353
>ScYm1zk_648:17578-17757 Nk7L	RKKARTTFTGRQIYLLERVFFCSKYLSRLERQSIASQLNVTSTQVKTWFQNRRTKWKKDR	>ScYm1zk_648:17578-17757
>SMA4808.t1 Nk6	KKSSRPTFNGHQIYHLEKTFEQTKYLAGPERTRLAHYLAMTENQVKVWFQNRRTKWRKKT	>ScYm1zk_658:2571921-2572019
>SMA3952.t1 NotoL	SKRKRTIFTTEQLDRLEHEFQHQQYVVGQERKYLAVELGLNEIQVKVWFQNRRIKWRKQK	>ScYm1zk_618:1552613-1552792
>SMA6282.t1 Msx	NRKPRTPFSATQLLTLEQKFKRKQYLSIAERAELSENLNLSEQQVKIWFQNRRAKEKRIK	>ScYm1zk_824:2885898-2886077
>SMA21811.t1 MsxL	SRQPRVPFTPFQQATLENKFQLDHYLTSNAVSELSVVLNLPEQRIKIWFQNRRARERREG	>ScYm1zk_876:2979668-2979847

Supplementary Table 16. *Rhopilema* ANTP class homeobox genes and locations

gene ID	Sequences	Genomic location
>RES11894.t1 XloxL	KKRTRTTYTRAQQVELEKEYHYSRYISRARRIELARALSLTEKHIKIWFQNRRMKEKRDE	>ScUbCFx_1:14648587-14648766
>RES11898.t1 Gsx	SKRIRTAYTSIQLLELEKEFQNNRYLSRLRRIQIAAMLDLTEKQVKIWFQNRRVKWKKDK	>ScUbCFx_1:14696281-14696460
>RES11899.t1 CdxL	TMRSRPSFTSHQTRELEREFTNCQYISRRRRIEIAYSLNLSEKQIKTWFQNRRVKERKTR	>ScUbCFx_1:14718423-14718602
>ScUbCFx_1662:238698-238877 NKL	NRRPRTVFTMNQMFFLEEKFSFRPYVSKKERIEIAKRTNLDDNQIRVWFQNRRAKYNKIS	>ScUbCFx_1662:238698-238877
>RES14010.t1 Hox	RRRKRMTYTRSQTLELEKEFLYNQYLTKDRRSELSISLALSERQIKIWFQNRRMKCKKRT	>ScUbCFx_1662:412100-412279
>RES14009.t1 Hox	NKRFRTSFTTTQLTELEKEFHYNKYLTRRRRVELALGLNLTEKQVKVWFQNRRMKWKKQL	>ScUbCFx_1662:437366-437545
>RES14008.t1 HoxL	GSQSQFKYSQRQLFELEKEFHTLNYLTSTKRKELAQSLGLEEKQVKVWFQNRRMRQKKQR	>ScUbCFx_1662:454133-454312
>RES4883.t1 Dlx	RRKSRTIYNSFQLQELNTRFAKTQYLALPDRAELAAKLGLSQTQIKIWFQNKRSKLKKTL	>ScUbCFx_1662:4774662-4774841
>RES4884.t1 Dlx	QRKPRTIFNSFQLRELNRAFERTHYLSLPERGDLALALGLTQTQVKIWFQNRRSKYKKLL	>ScUbCFx_1662:4806048-4806227
>RES18201.t1 Hox	GNKKRVCFTQKQIVELEKEFHFNKYLTRARRVEISHALRLSESQIKIWFQNRRMKYKREQ	>ScUbCFx_1662:6126633-6126812
>RES2435.t1 Evx	LHRTRTAFTREQLNKLEKEFARENYISRARRCELAAELNLPENTIKVWFQNRRMKCKRRR	>ScUbCFx_1662:12102570-12102749
>RES14954.t1 HoxL	PKRRRTSYTRRQLLEMDKEFKANRYITRERRIELSMALGLTERQIKTWFQNRRMKQKKDL	>ScUbCFx_1656:1506758-1506937
>RES12686.t1 Nk2/4	KKKPRILFSQSQVTELEQRFKQQKYLNANEREQLANKLNLTPTQIKIWFQNKRYKCKKQS	>ScUbCFx_1656:2624081-2625918
>RES393.t1 Hox	AKRKRMTYSRKQLLELEKEFHYNHFVKKDRRAELAKMLNLTERQIKIWFQNRRMKFKKET	>ScUbCFx_1656:6772772-6772951
>RES403.t1 Hox	RRRKRTAYTRKQLVELEKEFHFNHFLTRERRLQLATSLNLSERQIKIWFQNRRMKWKKRG	>ScUbCFx_1656:6849527-6849706
>RES13082.t1 Meox	ARKERTAFTKQQIRDLENEFTKNNYLTRLRRYEIAVTLNLTERQVKVWFQNRRMKWKRVK	>ScUbCFx_1656:7278157-7277115
>RES2794.t1 Hhex	RKGGQVRFSHLQSVELERIFSIQKYISPQERKQLSNFLQLSERQVKTWFQNRRAKWRRIK	>ScUbCFx_1656:7609804-7610467
>RES14856.t1 Nk5/Hmx	RKKTRTVFSRRQVYQLESAFELKRYLSSSERASLASSLKLSETQVKIWFQNRRNKWKRQL	>ScUbCFx_1656:8409266-8409445
>RES1598.t1 EmxL	RKRHRTAFTPTQLLGLENAFEQSHYSVGEERKQLASFLGLSETQIKVWFQNRRTKWKRIR	>ScUbCFx_1656:9440075-9440254
>RES1560.t1 EmxL	GRRIRTTFTPSQLNVLEKRFKASHYIVGDERRELAQELRLNESQVKIWFQNRRTKYKQEK	>ScUbCFx_1656:9792688-9793536
>ScUbCFx_1672:1237496-1237675 NKL	LPSWRRKFTEGQTTNLEEIFLRQKYITGKERAEIANTLGLTTKQVKTWFQNRRTKWKREK	>ScUbCFx_1672:1237496-1237675
>RES9748.t1 Hox	RHRKRMTYSRNQILELEKEFLYSRYLTKERRKDLSDTLRLTERQIKIWFQNRRTKSKKER	>ScUbCFx_1672:4973019-4973198
>RES9751.t1 Hox	PRQKRKTYSRYQTLELEKEFLFNQYLTKERRRELSANLGLTERQIKIWFQNRRMKQRRTN	>ScUbCFx_1672:5014063-5014242
>RES9965.t1 Nk1	PRRIRTAFTYEQLVALENKFRTTRYLSVCERLNLALSLGLSETQVKIWFQNRRTKWKKQN	>ScUbCFx_1672:6045985-6046164
>RES3827.t1 BarxL	CRKPRTVFSELQLMVLNREFNEHKYLSTPQRTKLAERLGLNQTQVKTWFQNRRMKWKKET	>ScUbCFx_1672:7497344-7497523
>RES5091.t1 HoxL	SKRERTTFNKAQRCELEHQFQFSRYPSRFERAQLANFLGLTEFQVKVWFQNRRMKYKRWK	>ScUbCFx_1672:8861876-8862055
>RES5513.t1 Nk3	RKRIRAAFSAGQVYELERIFDRQKYLSAPERAELSKALKLSEQQVKIWFQNRRYKTKRKL	>ScUbCFx_10:930197-930376
>RES17065.t1 Nk3L	KKRMRASFSHDQVLALRKVFETKKYLGNTERTELAKQLNMTEQQIKIWFQNRRYKEKKQQ	>ScUbCFx_10:2893619-2893777
>RES20634.t1 TlxL	RKKPRTTFSRAQIAELEELFTEKKYLTSSERQKVASYLNLSDCQVKTWFQNRRTKWKRET	>ScUbCFx_10:6981483-6981662
>RES1845.t1 Dbx	MPPKRAVFSDYQRRQLEVEFLAHKYISKQERRLLAKRLELSESQVRIWFQNRRMKWRNRQ	>ScUbCFx_10:11328044-11328223
>RES22876.t1 NKL	YPRERTTFTAAQLKFLEELFRVKKYLTLIERSKVASHLELSEKQVKTWFQNRRTKYRRQK	>ScUbCFx_795:2980835-2981014
>ScUbCFx_795:3744831-3745010 NKL	SKVWRKKFTNLQRISLEESFKTQKYLSVKQRNKLAKEICLSSEQVKTWYQNRRTRWKRNQ	>ScUbCFx_795:3744831-3745010
>RES6758.t1 NKL-1	SKRLRTIFTAKQLQQLEDKFRESQYPNPQERYDMAEELGLSEKKVKTWFQNRRMKLKRDY	>ScUbCFx_795:15538399-15538578
>RES6758.t1 NKL-2	SKRLRTIFTAKQLQQLEDKFRESQYPNPQERYDMAEELGLSEKKVKTWFQNRRMKLKRDY	>ScUbCFx_795:15555764-15555943
>RES16861.t1 Nk2/4	KRRRRILFTKAQTYELERRFMHQRYLSAPEREQLGRMINLTATQVKIWFQNHRYKYKRQK	>ScUbCFx_1659:8130536-8130715
>ScUbCFx_1659:8155923-8156102 Nk2/4	GKKRRILFSRQQTWELERAFRYQPYLSSPERDMLARRIHLSPNQIKIWFQNHRYKLKKYV	>ScUbCFx_1659:8155923-8156102
>RES22495.t1 Nk7L	QKKSRTTFTGKQLYQLERKFSTAKYLSRIERQELAMNLNITHVQVKTWFQNRRTKWKKEL	>ScUbCFx_448:9073095-9073274
>RES1339.t1 Nk6	KKSSRPTFNGRQIYHLEKTFEQTKYLAGPERTRLAHYLAMTENQVKVWFQNRRTKWRKKT	>ScUbCFx_558:3778838-3778936&377839[illegible]
>RES3499.t1 NotoL	NKRKRTIFTAEQLDRLEQEFQQQQYVVGQERKYLAVELGLNEIQVKVWFQNRRIKWRKQK	>ScUbCFx_92:12776983-12777162
>RES16423.t1 Msx	NRKPRTPFSATQLLSLEQKFKRKQYLSIAERAELSENLSLSEQQVKIWFQNRRAKEKRLK	>ScUbCFx_359:2228699-2228878
>RES21992.t1 MsxL	SRQPRIPFTPFQQSNLESKFKKDHYLTPDAVKELSMLLDLPEQRIKIWFQNRRARERREN	>ScUbCFx_4:10610294-10610473
>RES15318.t1 ANTP	KKRIRTVITRRHKAKLELIFKKNQYQSRLDLAKIGRELGLPQHALKIWFQNRRAKFRKQN	>ScUbCFx_1682:1227718-1228111

Supplementary Table 17. Summary of homeobox gene tree and synteny analyses

Gene		HoxL tree	NKL tree	ANTP tree	Confidently assigned orthology by tree? B + C = between bilaterians and cnidarians C = only between cnidarians	Additional synteny analyses carried out to test orthology between bilaterians and cnidarians
Gsx		69/62 (bi), 98/- (c)	N/A	76/65 (bi), 99/74 (c)	C	Yes
XloxL		no(bi), 99/86 (c)	N/A	no(bi), 99/84 (c)	C	Yes
CdxL		no(bi), 99/98 (c)	N/A	no(bi), 100/93 (c)	C	Yes
Dlx		100/96 (bi)	99/99 (bi)	99/90(bi)	B+C, C	---
Meox		97/77(bi), 95/94(c)	N/A	97/83(bi), 95/94(c)	B+C, C	---
Evx		99/98(bi), 96/-(c)	N/A	99/93(bi), 97/82(c)	B+C, C	---
EmxL	SMA8532/RES1598		90/95(bi), 94/96(c)	95/72(bi), 60/68(c)	B+C, C	Yes
	SMA8443/RES1560		86/86(c)	99/-(c)	B+C	Yes
Hhex			88/90(bi), 89/85(c)	96/96(bi), 85/86(c)	B+C	---
BarxL			45/53(bi), 99/97(c)	50/38(bi), 65/70(c)	C	Yes
TlxL			38/49(bi), 67/72(c)	50/27(bi), 98/72(c)	C	Yes
Dbx			97/97(bi), 99/99(c)	96/90(bi), 98/91(c)	B+C, C	---
Msx			95/?(bi), 87/81(c)	98/54(bi), 92/60(c)	B+C, C	Yes
MsxL			83/83(bi), 84/75(c)	79/82(bi), 74/77(c)	B+C, C	Yes

<table>
<tr><th colspan="2">Gene</th><th>HoxL tree</th><th colspan="2">NKL tree</th><th colspan="2">ANTP tree</th><th>Confidently assigned orthology by tree?
B +C = between bilaterians and cnidarians
C = only between cnidarians</th><th>Additional synteny analyses carried out to test orthology between bilaterians and cnidarians</th></tr>
<tr><td colspan="2">NotoL</td><td></td><td colspan="2">38/40(bi), 99/98(c)</td><td colspan="2">40/38(bi), 60/66(c)</td><td>C</td><td>Yes</td></tr>
<tr><td colspan="2">Nk1</td><td></td><td colspan="2">99/99(bi), 64/57(c)</td><td colspan="2">99/-(bi)</td><td>B+C</td><td>---</td></tr>
<tr><td rowspan="3">Nk2/4</td><td>SMA17494/RES168 61 Nk2/4</td><td></td><td>99/99 (Noruma, Aurelia)</td><td rowspan="3">55/57(bi)</td><td>99/98 (Noruma, Aurelia)</td><td rowspan="3">54/61(bi)</td><td>C</td><td>Yes</td></tr>
<tr><td>SMA17501/RES sc1659 NK2/4</td><td></td><td>69/71(c)</td><td>64/69(c)</td><td></td><td>Yes</td></tr>
<tr><td>SMA8391/12686 Nk4/2</td><td></td><td>92/93(c)</td><td>92/85(c)</td><td>C</td><td>Yes</td></tr>
<tr><td colspan="2">Nk3</td><td></td><td>94/94(bi), 99/99(c)</td><td rowspan="2">56/51(bi)</td><td>97/-(bi), 83/80(c)</td><td rowspan="2">46/48(bi)</td><td>B+C, C</td><td>Yes</td></tr>
<tr><td colspan="2">Nk3L</td><td></td><td>89/89(c)</td><td>87/89(c)</td><td></td><td>Yes</td></tr>
<tr><td colspan="2">Nk5</td><td></td><td colspan="2">78/84(bi), 62/65(c)</td><td colspan="2">84/61(bi)</td><td>B+C</td><td>Yes</td></tr>
<tr><td colspan="2">Nk6</td><td></td><td colspan="2">93/98(bi), 87/84(c)</td><td colspan="2">77/71(bi), 83/62(c)</td><td>B+C</td><td>---</td></tr>
<tr><td colspan="2">Nk7L</td><td></td><td colspan="2">58/-(bi), 52/48(c)</td><td colspan="2">64/-(bi), 49/60(c)</td><td></td><td>Carried, but didn’t support</td></tr>
</table>

<table>
<tr><th>Gene</th><th colspan="2">HoxL tree</th><th>NKL tree</th><th colspan="2">ANTP tree</th><th>Confidently assigned orthology by tree?
B +C = between bilaterians and cnidarians
C = only between cnidarians</th><th>Additional synteny analyses carried out to test orthology between bilaterians and cnidarians</th></tr>
<tr><td>SMA20728/RES18201 Hox</td><td colspan="2">98/83(c)</td><td></td><td colspan="2">99/86(c)</td><td>C</td><td>Yes</td></tr>
<tr><td>SMA4884/RES14009 Hox</td><td colspan="2">97/87(c)</td><td></td><td colspan="2">99/85(c)</td><td>C</td><td>Yes</td></tr>
<tr><td>SMA22294/RES403 Hox</td><td colspan="2">96/78 (c)</td><td></td><td colspan="2">97/85(c)</td><td>C</td><td>Yes</td></tr>
<tr><td>SMA22300/RES393 Hox</td><td colspan="2">89/83(c)</td><td></td><td colspan="2">85/78(c)</td><td>C</td><td>Yes</td></tr>
<tr><td>RES9751/SMA3512 Hox</td><td>99/95(c)</td><td rowspan="3">60/50(c)</td><td></td><td>99/96(c)</td><td rowspan="3">62/54(c)</td><td>C</td><td>Yes</td></tr>
<tr><td>SMA4883/RES14010 Hox</td><td></td><td></td><td></td><td></td><td>Yes</td></tr>
<tr><td>SMA3510/RES9748 Hox</td><td>100/98(c)</td><td></td><td>100/99(c)</td><td>C</td><td>Yes</td></tr>
<tr><td>SMA8235/RES14954 HoxL</td><td colspan="2">47/40(c)</td><td></td><td colspan="2">61/-(c)</td><td></td><td>---</td></tr>
<tr><td>SMA4885/RES14008 HoxL</td><td colspan="2">92/91(c)</td><td></td><td colspan="2">89/87(c)</td><td></td><td>---</td></tr>
<tr><td>RES5091 HoxL</td><td colspan="2">100/97(with Aurelia)</td><td></td><td colspan="2">100/98(with Aurelia)</td><td>C</td><td>---</td></tr>
</table>

Supplementary Table 18. Fusion TALE-class homeobox gene in animals

Species	Phylum	Class	Accession
Nematostella vectensis	Cnidaria	Anthozoa	XP_001633591.1
Exaiptasia pallida	Cnidaria	Anthozoa	KXJ09686.1
Acropora digitifera	Cnidaria	Anthozoa	XP_015773402.1
Stylophora pistillata	Cnidaria	Anthozoa	XP_022778987.1; XP_022782439.1
Orbicella faveolata	Cnidaria	Anthozoa	XP_020619199.1
Craspedacusta sowerbyi	Cnidaria	Hydrozoa	AEP44002.1
Sanderia malayensis	Cnidaria	Scyphozoa	This study
Rhopilema esculentum	Cnidaria	Scyphozoa	This study
Branchiostoma floridae	Chordata	Leptocardii	XP_002590514.1
Branchiostoma belcheri	Chordata	Leptocardii	XP_019637243.1
Crassostrea gigas	Mollusca	Bivalvia	XP_019924140.1
Mizuhopecten yessoensis	Mollusca	Bivalvia	XP_021367327.1
Octopus bimaculoides	Mollusca	Cephalopoda	XP_014778251.1
Priapulus caudatus	Priapulida	Priapulimorpha	XP_014666768.1

Supplementary Table 19. Intron position of homeobox genes

Species	Phylum	Extended-Hox	NK	PRD	LIM	TALE
Nematostella vectensis	Cnidaria	No	Noto-like(i30)	i47	LMX(i30)	Meis/Pknox (i25-i51); other-TALE (i48)
Acropora digitifera		No	ANTP-divergent (i47); ANTP-divergent (i45)		LMX(i30)	Meis/Pknox (i25-i51); other-TALE (i48)
Hydra vulgaris		No	DLX (i35); NK2-like (i53); NK4 (i45); HX-like (i45); BARX-like (i45)		LMX(i30-i55); Lhx1/5-like (i39)	Meis/Pknox (i25-i51); other-TALE (i48)
Sanderia malayensis		MOX(i47)	NK6(i33); NK4(i45); HHEX(i45)		LMX(i30-i55)	Meis/Pknox (i25-i51); other-TALE (i48)
Rhopilema esculentum		MOX(i47)	NK6 (i33); NK3-like (I53); NK4 (i45); HHEX(i45); EMX-like (i45)		LMX(i30-i55)	Meis/Pknox (i25-i51); other-TALE (i48)
Trichoplax adhaerens	Placozoa	No	NK2 (i50); Dbx/Hlx (i50)		LMX(i30-i55)	Meis/Pknox (i25-i51); other-TALE (i48)
Amphimedon queenslandica	Polifera	No	No		No	TALE-divergent (i48)

Supplementary Table 20. ParaHox genes in cnidarians

Group	Life cycle/Stages	Class	Gsx	Xlox	Cdx	Evidence type	Cluster	Species
Most anthozoans	Polyp	Anthozoa	Yes	Yes	No	Genome	Yes	*Nematostella vectensis; Exaiptasia pallida; Porites lutea; Stylophora pistillata; Orbicella faveolata; Amplexidiscus fenestrafer; Discosoma sp.*
Acropora digitifera	Polyp	Anthozoa	Yes	No	No	Genome	/	*Acropora digitifera*
True jellyfish	Polyp and medusa	Scyphozoa	Yes	Yes	Yes	Genome and transcriptome	Yes	*Sanderia malayensis; Rhopilema esculentum; Aurelia aurita; Cyanea nozakii*
Stalked jellyfish	Attached medusa	Staurozoa	Yes	Yes	Yes	Transcriptome	NA	*Lucernariopsis campanulate (Calvadosia campanulate)*
Box jellyfish	Polyp and medusa	Cubozoa	Yes	Yes	Yes	Transcriptome	NA	*Alatina alata*
Hydra vulgaris	Polyp and no medusa	Hydrozoa	Yes	No	No	Genome	/	*Hydra vulgaris*
Liriope tetraphylla	Medusa, no polyp	Hydrozoa	Yes	Yes	Yes	Transcriptome	NA	*Liriope tetraphylla*
Hydrozoan jellyfish	Polyp and medusa	Hydrozoa	Yes	Yes	Yes	Transcriptome	NA	*Craspedacusta sowerbyi; Clytia hemisphaerica; Podocoryne carnea*
Ectopleura larynx	Polyp and attached, truncated medusa	Hydrozoa	Yes	Yes	Yes	Transcriptome	NA	*Ectopleura larynx*
Hydractinia echinata	Polyp but no feeding medusa	Hydrozoa	Yes	No	Yes	Genome	Yes	*Hydractinia echinata*
Siphonophores	Colonial organism composed of medusoid and polypoid zooids	Hydrozoa	Yes	Yes	Yes	Transcriptome	NA	*Nanomia bijuga; Agalma elegans*
Polypodium hydriforme	Endocellular parasite	Polypodiozoa	Partial	Yes	No	Genome and Transcriptome	NA	*Polypodium hydriforme*
Myxozoans	Obligate microscopic parasites	Myxozoa	No	No	No	Genome	/	*Sphaeromyxa zaharoni; Enteromyxum leei; Kudoa iwatai; Thelohanellus kitauei*

Supplementary Table 21. Sequences of AGO-like and Piwi proteins

>Sanderia malayensis SMA27573.t1 AGO-like protein

MSNNNGRGGEGREKKKRNKFNGRNNNGSINITLNNLSINGHGSSITQQKIKGQQVMLPTRSGFGKAGRKIQLSANFFEV
SFPDHEIYQYDVTLQPSSCPRQLNREIIEKMVAFYSDIFGEQRPVYDGRNAIFTIRPLTISPPKQEFLVKYNDEKESKDREFK
VTIKYVAHTSMLALHQVLRNQTSSMPHNTIQAMDVAMRHLPSLRYIPVGRSFFSPPDEPGCYQLGNGREVWFGYHQSL
RPSQWKMMINLDISSSAFYKTQPVLDLLCKIVQEDFKKKKRQLRDHERVKFLKEIKGLKIQITHLGKVKRKYRVTGVTL
LSTGCQTFKMENDGLVSDVIVASYFLERYNFKLKYPNLPCLQVGDPSKSVYLPIEVCEVVAGQRCLKKLNEKQSAEMIK
ATVTSASRRKKEIQSIARKADFSNDPYVKYFGIQVKPDMTVFDGRVIDSPVLQYGNELQTNKAQEIKPRNGVWNLQDKS
FRSSTAIDKWALVLFVQEKQCNYQAVKQFVNSLVEVGHGLNMPFAAQPLHCEYASRNANDVEGLFGTLIANHLDLQLIV
AVLSGKTPLYAEIKRLGDTLFGVPTQCIQMKTVLKAAKQSLANVCLKVNAKLGGINGIIAPRCKPKRIFDKPVVFLGADV
THPGPGDDTKPSIAAVVGSIDAHPMHYCTSVRVQKHRQEMIAELSSMVREILLAFYRSTRNKPSMIIMYRDGVSEGQFAQ
IVQHEVHAIRQACKSLEAGYEPGITFLVVQKRHHARFFAERQSDQVGKAGNIPPGTIVDRHICHPSEYDFYLCSHAGIQG
TSKPAHYHVLWDDNNITADELQQLTYMLCHTYVRCTRSVSIPAPAYYAHLAAFRARFHLMQKEGSIDSGSRSTPSVSSSE
HEIGISTMTDAVRPHVKLAQTLYFA

>Sanderia malayensis SMA10845.t1 AGO-like protein

MDKVNIAGTGESSSVTNEPGGQVKQRAPVPHLNGSEVTTSSRGASQAGDFSVNMSTETNKLADQVKKISINKTGSSAAS
TKPKLVGTQLLKRPNSGKMGRQIQLKANMIRLKIPEYGDIYHYDVVIVPDKCPSKTNQIVIKAMVDAYQTLFKDRPVFD
GKKNLFSKQPLPIGNKSQTFEVAVRDEGRDTDRSFKVTIKYASRVSLEALALGLKGRNEIPRETIHALEVVLRMAPSARYI
TVGRSFFSEPKPGTAIQLGGGCEMWYGSYQSIRPAQWGMLVNIDQAATAFHKSQLITDYLEELVRNCNLNEPLTDRDRV
NFEKKVRGFKVEFTYIVGQKRQKRIVGLSKESARNLTFERDDDGKKRKISVAQYFHEKYNIQLRYPNLQCLKAGSAEKTI
HLPMELCTIVPGQRCVKRLSESQTSVMIRKTATDALERKKNIESRVKQAEFDRDPHLKSFDMRIDNQFVSFEGRVLTPPKI
ALADKKVVMPRDGKWDMRGSKFYEGKKIDCWALLICVPYPKCQENTAVRFCDDLSRISSSLDMTINKRPAYVNYLKRD
MTVKQLFDDMLKNIPNVQLTVVVLDGTRYETVKSLADTYYGILTQCIKCKNVVKCNDQLLANLCMKINAKMAGINGV
FSSDIYRPRSVFERPVIFLGADVTHPSPGETRKPSIAAVVGSLDNHPMKYAARVRAQGPRKEIIEDLALMTKQLLLDFYRA
QKKSKPDKIIMYRDGVSEGQFSQVLEHELVAIRKACHDLDKDYQPGITFITVQKRHHVRLFADNIKDQVGKSKNIPAGTV
VDQAICHPTEFDFYLCSHNGIQGTSRPSHYRVLWDDSKFTADELQQLTYALCHNYARCTRSVSIPPPAYYAHHVAMRANY
HIQAMQLNNSDGSSTHSQSGSTNIDAVNDGLEAALKIHNNLLSKMYFI

>Sanderia malayensis SMA3562.t1 piwi protein 1

MTGRARGRSRGRSRAPQQQEAAPRPGAPPEEAPQQQVARGRGRTAPTPAQAAQPQAAQPQKPAADVAQVSQKLAATSI
TGAQREEQPPLSSSVLVTKPANIEQSTGTFGASVNLRANFFRLIKTPQFDGFFQYMVSFDPPIESRKFKSALLHDHAELLG
DVRAFDGMILYLPKKLPETETKLVSVTKRDQQQIEITVKYVNKFPFQSPTTLNLISVIFRRILKLMNMTQIGRSYFSKSRSI
ELEHIKLELWPGYETGIHTYDAGCLLNLDVSHKVMRTNTVLSILYDLYHQVGEKNFYNVATKKLVGEIVLTRYNNKTYKI
DDIAWEEHPTDSFPKRDGTSITYKEYYKGMYDYNPTDDKQPLLVSNPKKRDVRRGMTTPIKLLPEFCYITGISDEIRSNF
NIMKELSVHTRMAPQPRAQQLNNFMVELKRNDEANKKLTGWNIEFDSSLMNLKGRIFPPEKLSQREKSFSYKPEDADW
SRDMRGAALISTINLDDWALFFTRRDGGIAQDFYQTLCKVNGPMNINTRKPSMVELNDDRAETYIRSIKEQTTPSTQLVV
CIVPNNRKDRYDAIKKYCCVDKPVPSQVIVSRTLSKKQMLMSVSTKIGIQLNCKLGGEIWAVEIPVKNMMVVGYDVYH

DSLTKGKSIGGFVASTNQALTRYYSRITEQQTGIDIIDGLRTCMTSALRKYNEINNTLPDRIIIYRDGVGDGMLPMVYEHE

MPQVKKAIEAIAPGYNPKFAWIVVKKRINAKFFREGRDLANPIPGTVIDTEVTKPEWYDFYLVSQSVRQGTVSPTHYNVI

HDSSGLKPDHIQRLTYKLTHLYYNWQGTIRVPAPCQYAHKLAFLVGQSVHTEPAIALSERLYFL

>Sanderia malayensis SMA18039.t1 piwi protein 2

MSPPTQQLSSLSLDNEVAKATKRMEKGTEGTEIPLAVNYVPLKCKSNGFSQYAVSFSPDVESINMRRRMLNEHCDVVGK

TKSFDGGSLLLLNPLDSNVTILESTRITDGAKITITITFVKKLTPGDRQCTFLYNRIFRNIMRILGLCQIGRYYYSSKGQVSIP

QHKLELWPGYITSIEHYEGGLMLLADVSHRLLRMETTLQFMYDLYQKKPQNFQNNATKALVGSIVLTRYNNRTYRIDDI

AWDKNPQATFVAHNGEPMRYVDYYKTNYNQTLSDLEQPLLIHRPKPKKGSPAPVNEVICLIPELCSMTGLTDEIRSDFRV

MKDISAHTRVSPAQREESMLKFVSNVYSNPEATAELANWGLELDRAILQTRGRVLPNEKIIFGSKEIFSNREVDWSREIGR

ESVISGVNLNNWLVVFTKRDESKALEFYQTFAKVTTQMGFSVAQPNAVQLKDERTETYLQTIRQNLNPNVQIVVTIFPTS

RDDRYAAVKKLCCIESPVPSQCINARTIGQPNKLRSITQKIALQINCKLGGELWALEIPLRSVMIVGIDVYHDAGKGGRSIG

AFVASTNQQVTRWFSRVCFQKPGQELIDELKVIFAAALRKFHELNHDLPERIIVYRDGVGDGQLNVVSTYEVQQLSECFA

LFGPHYQPKMAVIVVQKRINTRIFAVAGNQKLDNPPPGTVLDHSVTRKDWFDFFLVSQHVRQGTVTPTHYVVVHDESGL

KADHLQRLSYKLTHLYYNWPGTVRVPAPCQYAHKLAYLVGCSLHQDPDLKLSDRLFFL

>Rhophilema esculentum RES10828.t1 AGO-like protein

MSDDGRGRGRGRGRKGKRSRKTEGLRPGPAMNPQFLSQQSQNDRPLATLGSTLQQVTKRTDKSLPKRPGFGNCGKKIT

LSANFFEVKYKDHDIYHYDVTIEPSKCPASLNRIAIERMVSVYPEVFGSHKPVFDGKKNMYTISPLDIGNGKKEFDITYLE

ENERERKLKVTLKWVALTSLSSLTEALRGNTNQIPYETMQAMDIALRHLPSLRYVPVGRSFFQPPDIKNAYQLGQGREIW

FGYHQSIRPAYWKTMINLDISSTTFYKSQPVTDFLNEVLDKVNINENKRPLGDHERIRFSKEVKGLKVEVTHLGNIKRKY

KVTGVTKESANEKTFTMEKDGANADVTVAAYFEERYKMKLRYPYLPCLQVGNPERGCFLPMEVCNIVSGQRCAKKLS

DRQTREMIKNTPKSASMRKEEIMKIVNKADFAKDPYLRYFGIEVSPQMVKVEGRILEPPTIEFGQMAQVLPRNGAWNLQ

DRAFHSAKKINVWVLLVLGSSQHCKQRVLMKFADEFVDVCSTAGMSVERKPTHFEYLRLGSTEVVGKQFERVIDTVLN

NNTSLDLIIVVLNGKNPCYELIKSLGDTYYGIPTQCVQYKNIEPLRRQTIVNLSLKVNTKLGGINSFIKRECKPKIFEKPVIV

LGADVTHPAPGDEQKPSIAAVVGSLDSYPMNYSTSIRVQKNRMEIIAELKDMIKELLNAFRNSTGHKPERIIMYRDGVSE

GQFQQVLLHEVKAIREACMMLEKDYEPGITFIVVQKRHHARFFPERHQDAVGTSGNIPPGTTVDSGICHPTEYDFYLCSH

AGIKGTSRPAHYHVLWDDNDLKPDELHNLTYMLCHTYVRCTRSVSIPAPAYYAHLAAFRARFHVDYQRDISAGSDQGG

SSSKKSISEAALKTLGDACRPHKKLSKTIYFA

>Rhophilema esculentum RES13499.t1 AGO-like protein

MENLNLQDNVKGLLKNEPNKLKNAQVQAKTVADSKEEKSSTGGAKKKATGDSTSTRTAIANLGSTSQGNRSSLETDNR

KLLARPGYGTVGRPIKLRANFLPMKIPTYGDIYHYDVSIVPEASRDINRAVIKKLEDNYGKVFNGEKPVFDGKKNIYTKK

RLPIENNGSEFTVKYHSESWRTEKEFKVTVKFASSVSIELLNQALAGKCEIPRETIQALELVLRMTPSARYTAIGRSFFSMP

TNPFDLGEGCELWYGTYKSIRPTQEGMMINIDLAATAFYKTQDVLDFLKEILQKNDIPRFLNDAERVRFSKSIKGLKVQL

NHIVGHKHQKRILGIAKESSEEIKFDLEQEGKKTRRISVAEYFKTTYNMVLRFPKLPCLKAGSDKKVVHLPMEVCTIVPG

QRCVKKLSEGQTAKMIRETAKNAKERQMIIECKVRDAEHATDPYLRHYEIEVEDKLVHVDGRVLNPPAIKHDERSALVT

PREGKWDMRGKGFFKGGNIDNWGLLICSSSRFLNESEARNFCNSLSRQAESLGMKMSPEPTQIVYYSERRKTLHQTLTD

LHEPCMKILLVILDGGLYAEIKYICDNDLGIQTQCIKGKNAKRPNPQLLANLCMKINAKVGGTNSYFEDNTGYKLYRNPK
VFGRPVIFLGADVTHPAPSETSRPSIAAVVGSLDKFATKYAARVDVQGHRKEIIANLQGMTKELLLEFKRATRSEPEKIIM
YRDGVSEGQFSQVLQHEVSAVQKACRSLHAEYKPLITFMTVQKRHHARLFPEDRRDEIGRSKNIPAGTVVDKTICHPTEF
DFYLCSHSGIQGTSRPAHYRVLWDDSRFTADELQTLTYSLCHNYARCTRSVSIPPPAYYAHHVAMRARYHVAAKEGSSQG
SSNSGSGSVGSASTSKSLQEAVTVHENLKSCMYFI

>Rhophilema esculentum RES1912.t1 piwi protein 1
MTGRARGRSRGRARAVQAEPAPVPGAPSSLEEAPQVPTQKVARGRGRAPTQAPPAATPQPSELKRLGEQVAEMKVSGG
ASAEERPYRGGDDGGLVTRPSNISETTGSFGRPISLKTNYFVLGTGSKFTGFFQYMVSYNPPIESRNLKFMLLKDHSELLG
TVRAFDGMILYLPKKLPDLETKLMSTRRSDGSSVEITIKFTNEFPFSSPRTIQLLNIIFRRILKIMEMQQIGRSFFSPKRNIDL
DQHRLQLWPGYNTSILRFDAGVLLNIDVSHKVMRLDTVLDILTELYSQVGESRFHNIATKKLVGETVLTRYNNKTYKIDD
IAWDKNLSHSFAKRDGSKITMRQYYKEAYDKDLTNDQQPLLVSNPKKRDIRRGQTEPILLVPELCYITGLSDDIRQNFQI
MKELAVHTRMGPEPRAKQLKAFMDELNRNAEASKQLTDWNLGFGNDLHSIKARNIPPETIAQKSKQFSYKAEDADWS
RDMRGANLISTINLNKWMILFSSRDKGIAQDFYQTLQKVNPPMGIEVNKPEMIMLKDDRADSYITAIREQKQPDTQLVL
CIVPNNRKDRYDAIKKYCCVDYAVPSQVVVSRTLSKKQMLMSVSTKIGIQLNCKLGGEVWAVDVPVKNMMVVGIDVY
HDSLTKGKSIGGFVASTNQSLTRYYSRITQQQTGLEFVDGLKTCMTAALRKFNEINKVLPDRIIVYRDGVGDGQIPMVYE
HEMPQIREAIAQINPGYNPKFAWVIVSKRINAKFFLEAGRNLANPPPGTIVDIDVTRSSEKYDFFLVSQSVRQGTVSPTHY
NIIYDTSGLKPDHIQRLTYKLTHLYYNWQGTIRVPAPCQYAHKLAFLVGQSVHKEPDLALADRLYYL

>Rhophilema esculentum RES20064.t1 piwi protein 2
MSGLGRGGRGALLLKALEKPVKLPGQPAPGQQQQQEQQPQEAKPTETDAAQKSLPVGRGISSQLMAQTASAASQPPPA
AESKPPVESLSKMTIQEPKITKKMEKGTAGAEVPVVANYIPIKATFGGAYQYAVSYSPDIESKSIRYQLLNEHRDVIGKTR
AFDGAVLFLPKKLDEDTKMLTSTRKTDGAEIRLTITFVKHLDPEDRQCMQLYNIMFRRVMRILEMSQVGRYYYNPNQPV
PIPQHKLELWPGYITAIEHYEGGLMLLADVSHRLLRTESVLQFMYAFYQKKPQNFQSEVTKKLVGNIVLTRYNNKTYRID
DIAWDKNPQNKFVFHSGEEMSYIDYYAKSYNKKIDDIDQPLLIHRPKPMKGQKAPANEVICLIPELCSMTGLTDEIRSDFR
VMKDISSHTRVSPGDREKSMKNFVSNIYSNEKALEELMNWGLELDRNLLQLQGRNLPAEKILLGSTSFTANREADWGR
EATRENVISAVNLNNWIVIYTKRDEAKAMDFCNTMGKVAPAMGIQVSQPMNVHLKDERTETYLKAIRDNLNPKVQMV
VTIFPTSRDDRYAAVKKLCCVESPVPSQCINARTISQANKLRSVTQKIALQINCKLGGELWALEIPMRSVMVVGIDVYHD
GGKGGRSIGGFVASTNKTFTRWYSRVCFQQPGQELIDGLKVCFTASLRKYHEVNHDLPERIIVYRDGVGDGQLHVVANY
EVQQFSDCFALFGESYKPKMAVIVVQKRINMRIFAATSQGGKLDNPPPGSVVDHTVTRKDWFDFFLVSQHVRQGTVSPT
HYVVVHDESGLKADHLQRLSYKLTHLYYNWPGTVRVPAPCQYAHKLAYLVGCSLHKDPALELSDRLFFL

Supplementary Table 22. Sequences of conserved microRNAs.

1) 8 miRNAs conserved in Aau, Res and Sma

	Species	ID	Sequences
mir-2022	Aau	scaffold8_2596	AGGCAAAACTTTCAAGCAAACACTTTATGT TGTTTGCTAGTTGCTTTTGTCCCC
	Res	ScUbCFx_1656_36050	AGGCAAAAGTAGCTACCAAATACTTTCATG GTATTGCTAGTTGCTTTTGTCCCC
	Sma	ScYm1zk_531_17221	AGGCAAAAGCTTCTGTCAGATATCTTTACG TGTATTTGCTAGTTGCTTTTGTCCCC
	Sma	ScYm1zk_531_17223	AGGCAAAAGCCGCAAGCGAATACCTTATT GGTATTGCTAGTTGCTTTTGTCCCC
mir-2030	Aau	scaffold216_30740	GAGCATAACATTGTAAGAGATCGTTTTATG CGATCTCTACAATAGTATGCTACC
	Aau	scaffold216_30742	GAGCATAACATTGTAAGAGATCGTTTTATG CGATCTCTACAATAGTATGCTACC
	Res	ScUbCFx_448_23551	TAGCATAGCATTGTAAGAGATCGTTTTAAG CGATCCTTGCATTACTATGCTACC
	Sma	ScYm1zk_718_29879	AAGCATAACATTGTAAGAGATCGTTTTAGA CGATCCTTACTCTGTTATGCATCC
1	Aau	scaffold44:1413380-1413445-	ACTATCACCTGAACTTCTCTCATTTCATGAT GATAGAAGTACACGTGGTAGGGA
	Res	ScUbCFx_10:4656241-4656311+	GCTGCCCCGTTTACTTCTAGCACTTTACGAT GATAGAAGTACACGTGGTAGGGA
	Sma	ScYm1zk_906_45983	CATATCATCTGTGCTTCTGCCATATTTATTA TGATAGAAGCACACGTGGTAGGGG
2	Aau	scaffold4_1408	TATCCAAAATTTCATCCAACATGGTGAAGA TAAATGTTGGATGAAATGTTGGATGCGT
	Aau	scaffold16_4767	CATCCTACATTTCATCCAACATGCTGAAAA TGTAGTGTTGGATGAAATGTTGGATGCGT
	Res	ScUbCFx_1682:5410610-5410680+	CGCATCCAACATTTCATCCAACATGCAGAA AACCAATGTTGGATGAAATGTTGGATGCGT
	Res	ScUbCFx_1682:10461485-10461552+	CGCATCCAACTTTTCATCCAACATGCAGAA AACCATTGTTGGATGAAATGTTGGATGCGT
	Res	ScUbCFx_1682:5410610-5410672-	CGCATCCAACATTTCATCCAACATTGGTTTT CTGCATGTTGGATGAAATGTTGGATGCGT
	Sma	ScYm1zk_660_3728259	CGTCCAACATTTCATGCAACATGATAAAAA ATGCTGGATGAAATGTTGCATGGGT

3	Aau	scaffold13_3733	TAACCAGACCCAATTTCAAAGGAACTATTTTCTTTGAAATTGGGTCTGGTTATCA
	Aau	scaffold13_3890	ATAACCAGACCCAATTTCAAAGAAAATAGTTCCTTTGAAATTGGGTCTGGTTATCA
	Aau	scaffold47_11296	TAACCAGACCCAATTTCAAAGGAAGTGTGGGATCTATTTCCTTTGAAATCGGGTCTGGTTATTA
	Aau	scaffold13_3904	TAACCAGACCCGATTTCAAAGGATTTCCTTTGAAATCGGGTCTTGTTAT
	Aau	scaffold265_34282	TAACCAGACCCGATTTCAAAGGAAATAGATCCTTTGAAATCGGGTCTGGTTATA
	Aau	scaffold18_5260	TAACCAGACCCGATTTCAAAGGAAATTTCCTTTGAAATCGGGTCTGGTTATA
	Aau	scaffold59_13078	TAACCAGACCCGATTTCAAAGGAAATTTATCCTACTATTTCCTTTGAAATCGGATCTGGTTAT
	Aau	scaffold166_26452	TAGCCAGACCCAATTTCAAAGGAAATAGTGGACAAGAAATAGTGGAACTATTTCCTTTGAAATAGGGTCTGGTTAACA
	Aau	scaffold47_11211	TAACCAGACCCGATTTCAAAGGAAATAGATCCCACACTTCCTTTGAAATTGGGTCTGGTTATT
	Aau	scaffold166_26524	TTAACCAGACCCTATTTCAAAGGAAATAGTTCCACTATTTCTTGTCCACTATTTCCTTTGAAATTGGGTCTGGCTAACA
	Res	ScUbCFx_795:8010280-8010350-	ATAACCAGACCCTTATTTCAAAGGAACTATTTTCTTTGAAATCGGGGTCTGGTTACCA
	Res	ScUbCFx_1677:6319736-6319810-	ATAACCAGACCCTGATTTCAACGGAAATATTTCCTTTGAAATCGAGGCTTGGTTATCA
	Sma	ScYm1zk_906_807785-	TAATCAGACACGATTTCAAAAGAAATAGTATTTATTTAGTACTATTTCCTTTGAAATCGTGTGTGGTTATCA
	Sma	ScYm1zk_828:1684587-1684652-	CGCCCAGTTTCCAATTTCAAAGGAAAACAATTTCCTTTGAAATTGGAGGCTGGTTAATA
4	Aau	scaffold275_34783	GCATCCAACATTTCATCCAACATTTGTTTTCAGCATGTTGGATGAAATGCTGGATGCGT
	Res	ScUbCFx_795:9682807-9682883+	GTATCCAACATTTCATCCAACATTTGTTTTCTGCATGTTGGATGAAATGTTGGATGCGT

	Res	ScUbCFx_795:9682807-9682881-	GCATCCAACATTTCATCCAACATGCAGAAA ACAAATGTTGGATGAAATGTTGGATACCT
	Res	ScUbCFx_795:12347544-12347622-	GCATCCAGCATTTCGTCCAACATTGGTTTTC TGCATGTTGGATGAAATGTTGGATGCGT
	Res	ScUbCFx_795:16436354-16436429+	ACATCCAACATTTCGTCCAACATTGGTTTTC TGCATGTTGGATGAAATGTTGAATGCAG
	Sma	ScYm1zk_24_367658	GCATCCAACATTCCATCAAACATTGACATA AACCAATGTTGGATGAAATTGTGGAGGCAT
	Sma	ScYm1zk_779_12039	GCATCCAACATTCCATCAAACATTGACATA AACCAATGTTGGATGAAATTGTGGAGGCAT
5	Aau	scaffold81_16585	TGCACTGTGGTATCTCCGATATGCACTTTG GTTTGTATATTGGTCGTTTAATCAGTGCTAT A
	Aau	scaffold81_16587	TGCACTGTGGTATCTCCGATATGCACTTTG GTTTGTATATTGGTCGTTTAATCAGTGCTAT A
	Aau	scaffold81_16591	TGCACTGTGGTATCTCCGATATGCACTTTG GTTTGTATATTGGTCGTTTAATCAGTGCTAT A
	Res	ScUbCFx_1672:6755950-6756020-	TGCATTGTGGTATCTCCGGTTTTCATACTGT ATGATTATCGGTCGTAAAATCAGTGCTATA
	Sma	ScYm1zk_772_3619172	TGCACTGTGGTATCTCCGATTTGTAGATAT ATGTACAAATTGGTCGTTTATTCAGTGCTA GA
	Sma	ScYm1zk_772_3620508	TGCACTGTGGTATCTCTGATTTGTAGATAT ATGTACAAATTGGTCGTTTAATCAGTGCTA GA
6	Aau	scaffold336_37640	TTCGGACGATCAAACTTTCAGACAAACTTT TGTCCGAAAGTTTAATTGTCTGAA
	Aau	scaffold913_46477	TTCGGAGGATCAAACTTTCGGACAAACTTT TGTCCGAAAGTTTAATTGTCCGAAAT
	Aau	scaffold770_46002	CGGATGATCAAACTTTCGGACAAAATTTTG TCCGAAAGTTTAATTGTCCGAAAT
	Aau	scaffold37_9192	GGACGATCAAACTTTCGGACAAAATTTTGA CCGAAAGTTTAATTGTCCGAA
	Aau	scaffold28_7306	TTTCGGACGATCAAACTTTAGGACAAAATT TTGTCCGAAAGTATAATTGTCCGAATT

Aau	scaffold109_20238	TGGACAATTAAACTTTCGGACAAAATTTAG TCCGAAAGTTTGATTGTCCGAA
Aau	scaffold336_37608	TTTCAGACAATTAAACTTTCGGACAAAAGT TTGTCTGAAAGTTTGATCGTCCGAAACT
Res	ScUbCFx_795_7401224	TTTCGGACGATCAAACTTTCGGACAAAAAT TTAACCGAAACTTTATTCGTCCGAATG
Res	ScUbCFx_1662_5085995	TTTCGGACGATTAAACTTTAGGATCAATCG TCCGAAATTATATTTGTCCGAAATC
Res	ScUbCFx_795_7401226	ATTCGGACGAATAAAGTTTCGGTTAAATTT TTGTCCGAAAGTTTGATCGTCCGAAAC
Res	ScUbCFx_382_29693	TTTCGGACAATTAAAGTTTTGGACACAATT TTGTGCAAAAGTTTGATCGTCCGAAAG
Res	ScUbCFx_382_29708	TTTCGGACGATCAAACTTTTGCACAAAATT GTGTCCAAAACTTTAATTGTCCGAAATA
Res	ScUbCFx_1677_10167159	TTTCAGACATTAAAATTTTCGGACGCCTCG AATGTACGAAAATATTAACGTCCGAAAT
Res	ScUbCFx_1682_4573456	TTTCGGACGGTTAAATTTTCGGACCGGGTA TTTGTTCGAAAATTTTACCGTTCGAAAT
Res	ScUbCFx_558_8946757	TTTCGGACGGTAAAATTTTAGGACCGTGTA TTTGTTTGTTCGAAAAGTTTACCATCCGAAT TT
Res	ScUbCFx_795_13401323	TGTCGGACGATCAAAGTTTCGGACAAAATT GTGTTCGAGAGTTTAATTGTCTGAAATA
Res	ScUbCFx_160_555	AGTCGGACGATCAAACTTTCGGACAAAAA GTCGTCCGAAACTTATTTTTGTCTGAAA
Res	ScUbCFx_558_9076212	AGTCGGACGATCAAACTTTCGGACAAAAA GTCGTCCGAAACTTATTTTTGTCTGAAA
Res	ScUbCFx_92_10181837	TTTGGGACGATCAAACTTTTGGACAAAACT TTAGTTATCAGAAATCTTGTTTTGTCCGAA ATT
Sma	ScYm1zk_128_46468	TTTCGGACAATTAAAATTTCGGACACAATT TTGTCCAAATATTTAATCGTCTGAAA
Sma	ScYm1zk_139_1477228	TTTCGGACAATTAAACTTTCGGACATAATT TTGTCCGAATGTTTGATAGTCCGAAA
Sma	ScYm1zk_139_1249097	TTTCGGACAATTAAATTTTCGGACACAATT TTATCCGAATGTTTGATCGTCCAAAA
Sma	ScYm1zk_374_4783597	TTTCGGACGATCAAACATTCGGACAAAATT GTGTCCGAAAGTTTAGTTGTCTGAAA

Sma	ScYm1zk_374_4760213	TTTCAGACAACTAAACTTTCGGACACAATT TTATCCGAATGTTTGATCGTCCGAAA
Sma	ScYm1zk_374_4783594	TTTCAGACAACTAAACTTTCGGACACAATT TTGTCCGAATGTTTGATCGTCCGAAA
Sma	ScYm1zk_374_4760216	TTTCGGACGATCAAACATTCGGATAAAATT GTGTCCGAAAGTTTAGTTGTCTGAAA
Sma	ScYm1zk_439_3919	TTTCGGACGATCAAACATTCGGACAAAATT GTGTCCGAAAATTTAATTGTCCGAAA
Sma	ScYm1zk_439_3939	TTTCGGACAATTAAATTTTCGGACACAATT TTGTCCGAATGTTTGATCGTCCGAAA
Sma	ScYm1zk_456_948+	TTTCGGACAATTAAATTTTCGGACACAATT TTGTCCGAATGTTTGATCGTCCGAAA
Sma	ScYm1zk_456_948-	TTTCGGACGATCAAACATTCGGACAAAATT GTGTCCGAAAATTTAATTGTCCGAAA
Sma	ScYm1zk_466_5151329	TTTCGGACAATTAAATTTTCGGACACAATT TTGTCCGAATGTTTGATCGTCCGAAA
Sma	ScYm1zk_470_63635	TTTCGGACGATCAAACATTCGGAAAAAATT GTGTCCGAAAATTTAATTGTCCGAAA
Sma	ScYm1zk_470_63663	TTTCGGACAATTAAATTTTCGGACACAATT TTTTCCGAATGTTTGATCGTCCGAAA
Sma	ScYm1zk_519_2800798	TTTCGGACGATCAAACATTCGGACAAAATT GTGTCCGAAAATTTAATTGTCCGAAA
Sma	ScYm1zk_519_2800765	TTTCGGACAATTAAATTTTCGGACACAATT TTGTCCGAATGTTTGATCGTCCGAAA
Sma	ScYm1zk_519_2894077	TTTCGGACGATCAAACATTCGGACAAAATT GTGTCCGAAAATTTAATTGTCCGAAC
Sma	ScYm1zk_519_2894106	TTCGGACAATTAAATTTTCGGACACAATTT TGTCCGAATGTTTGATCGTCCGAA
Sma	ScYm1zk_532_873686	TTTCGGACGATCAAACATTCGGACAAAATT GTGTCCGAAAGTTTAATTGTCCGAAA
Sma	ScYm1zk_532_873687	TTTCGGACAATTAAACTTTCGGACACAATT TTGTCCGAATGTTTGATCGTCCGAAA
Sma	ScYm1zk_535_137988	TTTCGGACGATCAAACATTCGGACAAAATT GTGTCCGAAAGTTTAATTGTCCGAAA
Sma	ScYm1zk_535_138021	TTTCGGACAATTAAACTTTCGGACACAATT TTGTCCGAATGTTTGATCGTCCGAAA
Sma	ScYm1zk_615_95280	TTTTGGACAATTAAACTTTCGGGCATAATT TTGTCTGAATGTTTAATAGTCCAAAA

Sma	ScYm1zk_626_108548+	TTTCGGACAATTAAATTTTCGGACACAATT TTGTCCGAATGTTTGATCGTCCGAAA
Sma	ScYm1zk_626_108548-	TTTCGGACGATCAAACATTCGGACAAAATT GTGTCCGAAAATTTAATTGTCCGAAA
Sma	ScYm1zk_629_62867	TTTCGGACTATCAAACATTCGGACAAAATT ATGTCCGAAAGTTTAATTGTCCGAAA
Sma	ScYm1zk_629_62859	TTTCGGACAATTAAACTTTCGGACATAATT TTGTCCGAATGTTTGATAGTCCGAAA
Sma	ScYm1zk_658_760798+	TTTCGGACGATCAAACATTCGGACAAAATT GTGTCCGAAAGTTTAATTGTCCGAAA
Sma	ScYm1zk_658_3885672	TTTCGGACGATCAAACATTCGGACAAAATT GTGTCCGAAAATTTAATTGTCCGAAA
Sma	ScYm1zk_658_760798-	TTTCGGACAATTAAACTTTCGGACACAATT TTGTCCGAATGTTTGATCGTCCGAAA
Sma	ScYm1zk_658_760763	TTTCGGACGATCAAACATTCGGACAAAATT GTGTCCGAAAGTTTAATTGTCCGAAA
Sma	ScYm1zk_660_6878753	TTTCGGACAATTAAATTTTCGGACACAATT TTGTTCGAATGTTTCATCGTCCGAAA
Sma	ScYm1zk_699_4271984	TTTCGGACGATCAAACATTCGGACAAAATT GTGTCCGAAAATTTAATTGTCCGAAA
Sma	ScYm1zk_699_4737455	TTTCGGACGATCAAACATTCGGACAAAATT GTGTCCGAAAATTTAATTATCCGAAA
Sma	ScYm1zk_699_4271978	TTTCGGACAATTAAATTTTCGGACACAATT TTGTCCGAATGTTTGATCGTCCGAAA
Sma	ScYm1zk_718_2169809	TTTCGGACAATTAAACTTTCGGACACAATT TTGTCCGAATGTTTGATCGTCCAAAG
Sma	ScYm1zk_718_2169844	CTTTGGACGATCAAACATTCGGACAAAATT GTGTCCGAAAGTTTAATTGTCCGAAA
Sma	ScYm1zk_718_634466	TTTCGGACGATCAAACATTCGGACAAAATT GTGTCCGAAAATTTAATTGTCCGAAA
Sma	ScYm1zk_824_2516104	TTTCGGACGATCAAACATTCGGACAAAATT GTGTCCGAAAATTTAATTATCCGAAA
Sma	ScYm1zk_828_1585406	TTTCGGACAATTAAATTTTCGGACACAATT TTGTCCGAAAGTTTGATCGTCCGAAA
Sma	ScYm1zk_828_730197+	TTTCGGACGGTTAAACTTTCGGACTGTGTA TTTGTTCGAAAATTAAACCGTCCGAAG
Sma	ScYm1zk_828_730197-	CTTCGGACGGTTTAATTTTCGAACAAATAC ACAGTCCGAAAGTTTAACCGTCCGAAA

Sma	ScYm1zk_828_1585408	TTTCGGACGATCAAACTTTCGGACAAAATT<br>GTGTCCGAAAATTTAATTGTCCGAAA
Sma	ScYm1zk_861_1332774	TTTCGGACGATCAAACATTCGGACAAAATT<br>GTGTCCGAAAATTTAATTGTCCGAAA
Sma	ScYm1zk_861_1332767	TTTCGGACAATTAAATTTTCGGACACAATT<br>TTGTCCGAATGTTTGATCGTCCGAAA
Sma	ScYm1zk_861_4812575	TTTCGGACGATAAAATTTTCGGACAACTGC<br>GCAGTACGAAAATTTTACTGTCTAAAA
Sma	ScYm1zk_876_1298891	TTTCGGACGGTTAAACTTTCGGACTGTATA<br>TTTGTCCGAAAACTAAACCGTCCGAAA
Sma	ScYm1zk_876_1298893	TTTCGGACGGTTTAGTTTTCGGACAAATAT<br>ACAGTCCGAAAGTTTAACCGTCCGAAA
Sma	ScYm1zk_876_934214	TTTCGGACAATTAAATTTTCGGACACAACT<br>TTGTCTGAATGTTTGATCATCCGAAA
Sma	ScYm1zk_882_1053362	TTTCGGACGATCAAACATTCGGACAAAATT<br>GTGTCCGAAAGTTTAATTGTCCGAAA
Sma	ScYm1zk_882_1614389	TTTCGGACAATTAAATTTTCGGATACAATT<br>TTGTCCCAATGTTTGATCGTCTGAAA
Sma	ScYm1zk_882_1053335	TTTCGGACGATCAAACATTCGGACAAAATT<br>GTGTCCGAAAGTTTAATTGTCCGAAA
Sma	ScYm1zk_882_1053322	TTTCGGACGATCAAACATTCGGACAAAATT<br>GTGTCCGAAAGTTTAATTGTCCGAAA
Sma	ScYm1zk_901_2279	TTTCGGACGATCAAACATTCGGACAAAATT<br>GTGGGACAAAATTTAATTGTCCGAAA
Sma	ScYm1zk_914_17189	TTTTGGACAATTAAACTTTCGGGCATAATT<br>TTGCCTGAATGTTTAATAGTCCAAAA

2) 4 miRNAs conserved in Sma and Res

	Species	ID	Sequences
1	Res	ScUbCFx_359_8754753	TATCATATCAGTAGAATCTTGCTATAAAGACC<br>CTGTGACGAACTATTTTCTCCAGGGTGTTTTTT<br>ATTCCGGGATTTTGCTGTGATTGAAT
	Sma	ScYm1zk_660_24110	TATCATATCAGTTAAATGTTGAAATTTTACAA<br>TCTCAACATTTAACTGATATGATACA
2	Res	ScUbCFx_1672_118414<br>03	GTCTTATGAATGTCTCTAGCGTTTTTATCACCC<br>CCAACATGGTGATAGTAGTAGAAACCTTGGA<br>AGACTTCATGGTT
	Sma	ScYm1zk_875_42560	TGTGCGGTCTTCCAAGGATTATAATCCTTATA

			ATCCTTGGAAGACTTCACAGTT
3	Res	ScUbCFx_10_3257	TACCCTGGGTACCAGACTCACTAATTTTATATTTAGTGAGTCTGGTACCCAGGGTAAA
	Sma	ScYm1zk_924_85626	TACCCTGGGTACCAGAGCCTAAAAAAAATTTAGCTAATTTTCCTAGGCTGTGGTACCCAGGGTAACAGT
	Sma	ScYm1zk_828_1144570	TACCCTGGGAACCAGACTTACTAAACAGAAAGGAGTCTGGTTCTCGGGATAGATTA
	Sma	ScYm1zk_374_1167982	TACCCTGGGTACCAGAGCCAAAAAAATTTGCTTTTCCCTTCTCTATTTTCTTGAGGTTCTGATACCCAGGGTATAATG
	Sma	ScYm1zk_374_774811	TACCCTGGGTACCAGAGCCAAAAAAATTTGCTTTTCCCTTCTCTATTTTCTTGAGGTTCTGATACCCAGGGTATAATG
	Sma	ScYm1zk_723_65670	TACCCTGGGTACCAGAGCCAAAAAAATTTGCTTTTCCCTTCTCTATTTTCTTGAGGTTCTGATACCCAGGGTATAATG
4	Res	ScUbCFx_1656_36174	TACACTAGAACGGATCCAAATGGGTACGGATCCATTTAGATCCGTGCTAGTGTAAACG
	Sma	ScYm1zk_531_204843	TACACTAGAACGGATCCAAATGGGCACGGATCCATTTGGATCCGCGCTGGTGTAAACG

3) 2 miRNAs conserved in Aau and Sma

	Species	ID	Sequences
1	Aau	scaffold1173_46812	CACTTTATCGGTTGAAAAACCAACTTTTTCAGTGATAAACCTAGTTTTTCGATCGATAAAGTAGG
	Sma	ScYm1zk_618_2108587	CACTTTATCTGCAGAAAAACCCAGTTTATCAGAAAAACCCACTTTTTCTGTCAGATAAAGTGGG
	Sma	ScYm1zk_882_409591	CACTTTATCTGCAGAAAAACCCAGTTTATCAGAAAAACCCACTTTTTCTGTCAGATAAAGTGGG
2	Aau	scaffold216_30740	GAGCATAACATTGTAAGAGATCGTTTTATGCGATCTCTACAATAGTATGCTACC
	Sma	ScYm1zk_718_29879	AAGCATAACATTGTAAGAGATCGTTTTAGACGATCCTTACTCTGTTATGCATCC

4) 12 miRNAs conserved in Res and Aau

	Species	ID	Sequences

1	Res	ScUbCFx_1_534	TTTCCACTGGGCGATTATCGCGATAAAGGCGA CAAATTCAAATATCGTGACAATCGTCCAGTGG AAACATG
	Aau	scaffold376:193590-193682-	TTTCCACTGCGCGATGAACGTTGATCGCATTT GAAAATTGGCGATGAGGAAAGCGATGAGAGT TCATCGTGCAGCCCAGTGGGAACCAG
2	Res	ScUbCFx_1682_48530	AACCTAACTTCGATGTCATGTGTTTCAGATCA CATGGTATCGATGTTACGTTCT
	Aau	scaffold124_593872	AACGTAAGGTCGATGCCATATACGTTTCATTA CATGGTATCGATGTTACGTTCT
3	Res	ScUbCFx_217_14730	ATTGTACTGACACCAGATGTGTCTTATCTGAT ACAACTGTTGTCATGACAACC
	Aau	scaffold69_664508	GTTGTGATAGCAACAGTCATGTTGTTTCAGAT ACAACTGTTGTTATGACAGCCAGCAC
4	Res	ScUbCFx_217_14830	TCGCAGACCATATTCTGAAGGAGAACTATAAA TAATTATAAATCCTCTATCAGAATATGGTCTG CGACG
	Aau	scaffold228:147116-147192	TCGCAGACCAAATTCTGAAAAGAAAATCCTTA ACTTTTTGGAATTTAGTTTGCGAGG
5	Res	ScUbCFx_4_1123	CTGGGTACCAGACTCACTATATATGAAATAAA ATATTTTATATTTAGTGAGTCTGGTACCCAGG GTAGT
	Aau	scaffold13:381403-381490+	CTGGGTACCAGACTCTCAAAAATATGAGATTA GAGATTTTTTTAGAGAGTCTGGTACCCAGGGT AGT
	Aau	scaffold13:381400-381480-	CTGGGTACCAGACTCTCTAAAAAAATCTCTAA TCTCATATTTTTGAGAGTCTGGTACCCAGGGT AGT
6	Res	ScUbCFx_4_1190	CCTGGAAAAACGACTAAACATTTCACTTAACA TTTTACCCAAGTTTTGTTTAGCCAGAAGTTTAG TGAAAAGTTTAGTCGTTTTGCCACC
	Aau	scaffold23_2219500	AACATGGATGGAATGGGTGGAAGGGTGGAAA TACCACTATACTTATGGTTATAATGTACGATA TAACCATAAGTTTAGTGGTATTTCCACC
7	Res	ScUbCFx_1658_36451	CACGATCAGATTCTGTCGAATTTTATAATCGC ATTTGACAAAATCTGACCGTG
	Res	ScUbCFx_1677_46226	CACGGTCAGATTTTGTCGAATTCGACAATCGA ATTTGACAAAATCTGACCGTG

	Res	ScUbCFx_1677_45748	CGGTCAGATTTTGTCAAATTCGATTGTCGAAT TCGACAAAATCTGACCGTG
	Aau	scaffold659:28643-28715-	CACGGTCAGATTTATCTAATTTGATTTGTCAA ATTCGACAAAATCTGACCGTG
	Aau	scaffold59_75743-75799-	CACGGTCAAATTTATCGAATTCGATTTGTCGA ATTCGACAAAATCTGACCGTG
	Aau	scaffold516_64473-64528+	CACGGTCAGATTTATCGAATTCGATTTGTCCA ATTCGACAAAATCTGACCGTG
	Aau	scaffold464_142851-142907+	CACGGTCAGATTTATCGAATTCGATTTGTCGA ATTCGACAAAATCTGACCGTG
	Aau	scaffold360_94454	CATGGTTAGATTTATCGAATTCGTTTGTCGAA TTCGACAAAATCTGACCGTG
	Aau	scaffold659:28645-28715+	CACGGTCAGATTTTGTCGAATTTGACAAATCA AATTAGATAAATCTGACCGTG
	Aau	scaffold59_75743-75799+	CACGGTCAGATTTTGTCGAATTCGACAAATCG AATTCGATAAATTTGACCGTG
	Aau	scaffold464_142851-142907-	CACGGTCAGATTTTGTCGAATTCGACAAATCG AATTCGATAAATCTGACCGTG
	Aau	scaffold516_64473-64528-	CACGGTCAGATTTTGTCGAATTGGACAAATCG AATTCGATAAATCTGACCGTG
8	Aau	scaffold157_25594	TGGGTACCAGACTCTCAAACATTTTGTTGAGA CTCTGGTACCCAG
	Res	ScUbCFx_1662_10541266	TGGGTACCAGACTCTCTATTATTTTATATAGTG AGTCTGGTACCCAGCGTA
	Res	ScUbCFx_1672_8071658	TGGGTACCAGACTCTCTATTATTTTATATAGTG AGTCTGGTACCCAGGGTA
	Res	ScUbCFx_217_2794647	TGGGTACCAGAGTCGCAAAAATTTAATATTTT TTGCTGCCGTAAAATTTTCGCGGCTCTGGTAC CCAGGGTA
9	Aau	scaffold8_2596	AGGCAAAACTTTCAAGCAAACACTTTATGTTG TTTGCTAGTTGCTTTTGTCCCC
	Res	ScUbCFx_1656_36050	AGGCAAAAGTAGCTACCAAATACTTTCATGGT ATTGCTAGTTGCTTTTGTCCCC
10	Aau	scaffold395_39926	AGACCTAATATTCCGAAGAAGAAAGGACAGT TCTTCTTCGGAATATTGGGTCTGC
	Res	ScUbCFx_1:4479615-4479678+	AGACCCAGTATTCTGAAGAAGAAAGGGTCTTC TTAAGAATACTGGGTCTGC
11	Aau	scaffold13_3716	CCCTAGCCTGTGTTCCAGACCACTTGAAACTA

			TACTTTCGTTTCAAGTGGTCTGGAACACAGGC TAAGAT
	Res	ScUbCFx_1677:33900-33969+	CCCTAGCCTGTGCTCCAGACCCCTAACTTGAA AGTAGAGGTCTGGAACACAGGCTACCGCTCC
	Res	ScUbCFx_1677:33900-33965-	CGGTAGCCTGTGTTCCAGACCTCTACTTTCAA GTTAGGGGTCTGGAGCACAGGCTAGGGGA
	Res	ScUbCFx_1677:6018970-6019040-	ACGTAGCCTGTGTCCCAGACCCCTAACTTGAA TGAAAGTTAGGGGTCTGGAACACAGGCTAGG TAGA
12	Aau	scaffold102_19498	AAAATCAGTGAGATTAATATCAAAACGTTTTG ATATTTATCTCACTGACTTTCT
	Aau	scaffold37_9324	AAAATCAGTGAGATTAATATCAAAACGTTTTG ATATTTATCTCACTGACTTTCT
	Aau	scaffold79_16153	AAAATCAGTGAGATTAATATCAAAACGTTTTG ATATTTATCTCACTGACTTTCT
	Res	ScUbCFx_558_4006441	ATAGTCAATGAGGATAAAATCAAAAAGTCTT GATATTTATCTCACTGACTTTCT
	Res	ScUbCFx_4_13311551	ATACTCAGTGAAAATAATATCAAAGAGTTTTG ATATTTATCTCACTGACTTTCT
	Res	ScUbCFx_359:2590782-2590851-	ATAGTCAGTGAGAATAATATAAAAAAGTTTTG ATATTTATCTCACTGACTTTCT
	Res	ScUbCFx_1_7772064	ATAGTCAGTGAGAATAATATCAAAAAGTTTTG ATATTTATCTCACTGACCTTCT
	Res	ScUbCFx_5_6623196	AGAGTCAGTGAGAATAGTATCAAAAAGTTAT GATATTTATCTCACTGAATTTCT
	Res	ScUbCFx_1672_11565347	TGAGATAGTGAGAATAATATCAAAAAGTTTTG ATATTTATCTCGCTGACTTTCT

Supplementary Table 23. Graphical presentations in revealing the cases of microRNA arm switching. The microRNA usage ratio was defined as 5p/(5p+3p) in the two genomes. Red box indicates 5p dominant (ratio>0.7), blue box represents 3p dominant (ratio<0.3), and yellow box means ratio in between (0.3-0.7). White box indicates no expression of both arms could be detected.

S. malayensis	**Gonads**	**Oral arm**	**Rhopalia**	**Tentacle**
ScYm1zk_139_2193				
ScYm1zk_191_2366				
ScYm1zk_198_2402				
ScYm1zk_320_3230				
ScYm1zk_320_3242				
ScYm1zk_361_3658				
ScYm1zk_361_3660				
ScYm1zk_361_3661				
ScYm1zk_361_3669				
ScYm1zk_361_3678				
ScYm1zk_361_3679				
ScYm1zk_361_3722				
ScYm1zk_361_3726				
ScYm1zk_374_7192				
ScYm1zk_397_10438				
ScYm1zk_397_10462				
ScYm1zk_406_10488				
ScYm1zk_434_10966				
ScYm1zk_454_11699				
ScYm1zk_454_11705				
ScYm1zk_454_11708				
ScYm1zk_454_11737				
ScYm1zk_454_11741				
ScYm1zk_454_11744				
ScYm1zk_454_11746				
ScYm1zk_475_13833				
ScYm1zk_480_14038				
ScYm1zk_480_14040				
ScYm1zk_480_14052				

ScYm1zk_494_14598				
ScYm1zk_495_14983				
ScYm1zk_531_17221				
ScYm1zk_531_17223				
ScYm1zk_531_17231				
ScYm1zk_531_17334				
ScYm1zk_531_17336				
ScYm1zk_531_17807				
ScYm1zk_531_17848				
ScYm1zk_532_18469				
ScYm1zk_540_19525				
ScYm1zk_564_19676				
ScYm1zk_612_19865				
ScYm1zk_612_19906				
ScYm1zk_616_20337				
ScYm1zk_659_24037				
ScYm1zk_659_24068				
ScYm1zk_660_24110				
ScYm1zk_660_24157				
ScYm1zk_660_24182				
ScYm1zk_660_24405				
ScYm1zk_660_24415				
ScYm1zk_660_24472				
ScYm1zk_660_24729				
ScYm1zk_660_24935				
ScYm1zk_660_25292				
ScYm1zk_696_26968				
ScYm1zk_696_26970				
ScYm1zk_715_28608				
ScYm1zk_715_28658				
ScYm1zk_718_28937				
ScYm1zk_718_29260				
ScYm1zk_718_29879				
ScYm1zk_729_31036				
ScYm1zk_729_32283				
ScYm1zk_742_33954				

ScYm1zk_744_34199				
ScYm1zk_744_34201				
ScYm1zk_744_34207				
ScYm1zk_744_34209				
ScYm1zk_744_34211				
ScYm1zk_758_34607				
ScYm1zk_758_34624				
ScYm1zk_772_35113				
ScYm1zk_785_36159				
ScYm1zk_810_36958				
ScYm1zk_810_36960				
ScYm1zk_810_36962				
ScYm1zk_810_36964				
ScYm1zk_810_36976				
ScYm1zk_810_36980				
ScYm1zk_810_36986				
ScYm1zk_810_36989				
ScYm1zk_828_39539				
ScYm1zk_843_40037				
ScYm1zk_863_41973				
ScYm1zk_865_42190				
ScYm1zk_875_42558				
ScYm1zk_875_42560				
ScYm1zk_875_42564				
ScYm1zk_876_43089				
ScYm1zk_882_44022				
ScYm1zk_882_44247				
ScYm1zk_882_44279				
ScYm1zk_882_44289				
ScYm1zk_882_44291				
ScYm1zk_882_44293				
ScYm1zk_882_44297				
ScYm1zk_882_44299				
ScYm1zk_882_44325				
ScYm1zk_906_45099				
ScYm1zk_906_45252				

ScYm1zk_906_45257				
ScYm1zk_906_45262				
ScYm1zk_906_45265				
ScYm1zk_906_45268				
ScYm1zk_906_45273				
ScYm1zk_906_45276				
ScYm1zk_906_45279				
ScYm1zk_906_45282				
ScYm1zk_906_45287				
ScYm1zk_906_45320				
ScYm1zk_906_45325				
ScYm1zk_906_45327				
ScYm1zk_906_45330				
ScYm1zk_906_45333				
ScYm1zk_906_45336				
ScYm1zk_906_45341				
ScYm1zk_906_45851				
ScYm1zk_906_45918				
ScYm1zk_906_45928				
ScYm1zk_906_45968				
ScYm1zk_906_45983				
ScYm1zk_924_48362				
ScYm1zk_926_48523				
ScYm1zk_936_48938				

R. esculentum	Appendages (ea1)	Appendages (ea2)	Gonads (eg1)	Gonads (eg2)	Rhopalia (eb1)	Rhopalia (eb2)
ScUbCFx_11_4192						
ScUbCFx_1656_35971						
ScUbCFx_1656_36050						
ScUbCFx_1656_36174						
ScUbCFx_1656_36176						
ScUbCFx_1658_36451						
ScUbCFx_1668_40329						
ScUbCFx_1672_41928						
ScUbCFx_1672_42972						
ScUbCFx_1673_44640						
ScUbCFx_1677_45748						
ScUbCFx_1677_46156						
ScUbCFx_1677_46251						
ScUbCFx_1682_48530						
ScUbCFx_217_14730						
ScUbCFx_217_14830						
ScUbCFx_217_17812						
ScUbCFx_217_17827						
ScUbCFx_217_18024						
ScUbCFx_359_19104						
ScUbCFx_359_19276						
ScUbCFx_384_20122						
ScUbCFx_4_1173						
ScUbCFx_4_1190						
ScUbCFx_4_1329						
ScUbCFx_4_1782						
ScUbCFx_4_1965						
ScUbCFx_4_1983						
ScUbCFx_409_21750						
ScUbCFx_409_21814						
ScUbCFx_448_23551						
ScUbCFx_448_23575						
ScUbCFx_45_5324						
ScUbCFx_558_24541						

ScUbCFx_65_7370						
ScUbCFx_65_7374						
ScUbCFx_65_7382						
ScUbCFx_65_7405						
ScUbCFx_795_28478						
ScUbCFx_879_33384						
ScUbCFx_979_34447						

A. aurelia	Arm	Bell	Gonad
scaffold109_20238			
scaffold13_3733			
scaffold13_3890			
scaffold16_4767			
scaffold166_26452			
scaffold166_26524			
scaffold176_27535			
scaffold204_29914			
scaffold265_34282			
scaffold3_906			
scaffold336_37608			
scaffold336_37640			
scaffold37_9192			
scaffold47_11296			
scaffold483_42298			
scaffold59_13078			
scaffold770_46002			
scaffold804_46141			
scaffold81_16585			
scaffold81_16587			
scaffold81_16591			
scaffold913_46477			
scaffold102_19498			
scaffold1173_46812			
scaffold12_3538			
scaffold124_22092			
scaffold13_3716			
scaffold13_3904			
scaffold130_22742			
scaffold131_22883			
scaffold134_23126			
scaffold14_4184			
scaffold156_25441			
scaffold157_25594			
scaffold17_4901			

scaffold17_4903			
scaffold172_27171			
scaffold18_5260			
scaffold189_28607			
scaffold196_29303			
scaffold216_30740			
scaffold216_30742			
scaffold275_34783			
scaffold28_7306			
**scaffold291_35518**			
scaffold3_1076			
scaffold355_38279			
scaffold360_38565			
scaffold368_38885			
scaffold37_9324			
scaffold395_39926			
scaffold4_1408			
scaffold411_40327			
scaffold437_41122			
scaffold445_41442			
scaffold47_11211			
scaffold5_1732			
scaffold54_12362			
scaffold550_43911			
scaffold635_45073			
scaffold64_13889			
scaffold69_14735			
scaffold73_15267			
scaffold79_16153			
scaffold8_2596			
scaffold8_2653			
scaffold8_2679			
scaffold84_16975			
scaffold92_18022			
scaffold93_18174			
scaffold96_18497			

Note:

	5p dominant
	3p dominant
	No preference
	No expression

Supplementary Table 24. Sequences of ALDH proteins

>Res_002217-T1 ALDH16A1

MASRRRTSATAKRDQERVTETMNIDVGAESGAPAAKLAKKETSKIGTIFNSLDYGPAPESAEIA
KRWIASHNGLLGHFIDGKWVKPEGRKRYDSFIPATGEKLSSTIQGEKEDLDQAVAAAKKAFKD
WSNLSPHKRARHIYSIARHVQKHIRLIAVLESLDNGKSIRETRDADTQIVARHLYHHAGWAELM
ETEMKGWSPIGVIGGIVPWNFPLMLLCWKVCPALAMGNTVVLKPASYTRLSALLFADICSEAG
LPPGVFNVVTGPGSFGSLLATHPDVDKVAFTGSTEVGMILRKLTAGSGKKLSLELGGKSPVVVF
DSADLDSAVEGIVQAIWFNQGQVCSAGSRLLVQETVAEQLIDKVKERMKHLRLGHSLDKCID
MGPIVDESQRKSIDEFVQHAKSEGAEVYQACASMPKTGCFYPPTLVTNVSTTSRIVMEEVFGP
VLTVMTFRTAKEAISLANNTRYGLGASVWSEKIGLAMEVACNIKAGTVWINNHNLFDAASGFG
GYKESGYGRDGGKEGLYEYVKPSWQKRARPNVESYDIKHFGPASSPPVLNANESLPANANSV
DPKVDRTHKLFVAGKQKRPDANYSRTILNKDGRIIGQVGDGNRKDIRDAVEAAAGALSGWSK
RTGHNKAQIIYYVAENLELRQSEIANRLQEEVGCDFEDAQKQVTASIQRLFHWAAYADKYGGS
VQETQQYGTVLRVHEPVGVLAIACPDESPLLSFISLLGPAIARGNTIVIVPSEKYPLSSMDVCQV
FETSDVPPGVVNVVTGSRDHLTKTLTEHQNVNAVWYFGSAEGSKFVEATSAFNIKRTWVNYG
ESRDWFDQQQGAGEEFLIRSVECKNVWIPMGHIYAN

>Res_004232-T1 ALDH6A1

MAKLLSGNRNGISKIACQAMRASLAARCYSPASSVPNTKLLIDGKFIESQTNEWIPLYNPATNE
VIGQVPKATQGEMEAAANAAAKAFPAWSNTSILARQQIMFKFQHLIKENMDKLAANVTLEQG
KTIPDAEGDVMRGLQVVEHACSVTSLQLGETLPSISRDMDTHSYRVPIGVCGGVCPFNFPAMIP
LWMFPLAIVCGNTYVMKPSEQDPGAAMMLAELAQQAGVPDGVLNVIHGAKESVDFICDNPTI
KAISFVGSDFVGQYIYERGSKNGKRVQSNMGAKNHGVVMPDANKEMTLNQLVGAAFGAAG
QRCMALSTAVFVGEAKEWIPELVERARKLKVNAGDQPGADLGPLISPAAKQRVLDLVQSGIDE
GAKIDLDGRGISVPGYEKGNFVGPTILSGVKPNMKCYTEEIFGPVLVILDTDTLDEAIEVVNSNP
YGNGTAIFTASGAVARKYRDSVDVGQIGINVPIPVPLPMFSFTGSRGSFRGDMNFYGKAGINFFT
QLKTVTSLWRAEDTLSKKAEVAFPKL

>Res_006421-T1 ALDH3A2

MAENIDYSDIVHHLRLSFNQGKIRPVEQRIQQLKQFLKMMTENKELFVEALHQDLRKPAFEAT
MMEYTVVVAELTKFIDNLPSWAAFQRTDADLMNKFNTCGIQYEPYGVALIIGAWNYPLLLLLQ
PLIGCIGAGNCAVLKPSELSPVTASLIESLVPRYLDEDCFRVIAGGKDETSALLRERFDYMFYTG
GHVVGQIVMQAAAKYLTPVTLELGGKSPCYVDGDCNFDITARRIVWGKYVNCGQTCLAPDYI
LCTKETEEKLTLSLKKALKEFYGENPKESKDLARIINERHFQRIRNLINQEKVVIGGDVDEDEK
YISPTVMTDVTSEDAVMKEEIFGPLLPIVKVISATEAVKFINKREKPLALYVFSKTQSIIDKFINET
SSGAVCVNDSIVQAAVPSLPFGGIGQSGMGAYHGKFSFESFSHRKACLIKKQNLEALNAIRYPP
YTDSNMKTACWFLMPSRNSKYMKFFFYFAVFGAVIYLLKSYNCLATVSKLWK

>Res_006422-T1 ALDH3A2

MAENIDYSDIVHHLRLSFNQGKIRPVEQRIQQLKQFLKMMTENKELFVEALHQDLRKPAFEATI
MEYTVVVAELTKFIDNLPSWAAFQRTDADLLNKFNTCGIQYEPYGVALIIGAWNYPLLLLLQPL
IGCIGAGNCAVLKPSELSPVTASLIESLVPRYLDEDCFRVIAGGKDETSALLRERFDYMFYTGGH
VVGQIVMQAAAKYLTPVTLELGGKSPCYVDGDCNFDITARRIIWGKYVNGGQTCLAPDYILCT
KETEEKLTLSLKKALKEFYGENPKESKDLARIINERHFQRIRNLINQEKVVIGGDVDEDEKYISP
TVMTDVTSEDAVMKEEIFGPLLPIVKVISATEAVKFINKREKPLALYVFSKTESIIDKFINETSSGA
VCVNDSIVQAAVPSLPFGGIGQSGMGAYHGKFSFECFSHRKACLIKKQNLEALNAIRYPPYTDS
NMKTACWFLMPSRNSNLTTAWQLFLDSGNE

>Res_007488-T1 ALDH5A1

MSKSLKAVSPILSCSKLLRSDPFINGTWVKCKKTFPVYSPSTGEHLIDVGDVEEEHVQLAIDAA
ANSLKDWSMLSGKERGALLITFYENVMIHKEPLAQIMSAECGRPIKKTRLEVDYGASFLQWFA
EESKRIYGDMIPAPSNSSRRFVLKRPVGVVGLITPWNFPLAMITRKLGAAMAAGCTSVIKPSEE
TPLTALALAAIAEESGIPAGVINVLTCSSESTPMVGDLLCDSQIVRKISFTGSTATGKLLLKKSASS
VKRMSLELGGNAPFIVFNSADIDLVVRAALSAKFRANGQTCIAATRFLIQDGVHQNFIQEMTKA
VKSLVVGDPFDQGTDVSSLINEKGMKKVETHVQDAISKGALPLVGCERHRLGPNFYKPSIIDGC
DETMLVMKEETFGPVISVMKFHSEEDAIRIANNSKYGLAGYVFSQDIAQIVRLSENIEVGMLGV
NDGAISSEMIPFGGIKESGIGREGSKYGIDEYLDLKFVSLGGLKSSL

>Res_007497-T1 ALDH5A1

MSKFSKAVSQILHGNKLTRSDPFINGTWMKCKKTFPVYSPSTGEHLIDVGDVEEENVQLAINA
AANSLKDWSMLSAKERGALLNMFHENVMSHREPLAQIMSAECGKTIKETKLEVDYGASFLQ
WFGEESKRIYGDLIPAANNKSRRFVLKRPVGVVGLITPWNFPLAMITRKLGAAMAAGCTSVIK
PSEETPLTALALAAIAEESGIPAGVVNVLTCSRESTPMVGDQLCDSQTVRKISFTGSTATGKLLL
KKSASNVKRLSLELGGNAPFIIFNSADISQVVNAALLAKFRGNGQTCIAANRFLVQEEIHPKFIE
EMKKAVNSLVVGDPFDQDTNVSSLINEAALKKVETHVQDAISKGAVPLVGCERHPLGTNFYKP
SIIDGCDETMLVMKEETFGPVISVMKFNSEEDAIGFANSSQNGLAGYVFSQNMDQVFRLSENIE
VGMLGINDSAISSEMIPFGGIKESGIGREGSKYGIDEYLDLKFVSLGGLK

>Res_009388-T1 ALDH7A1

MLQALNNGRHIRALGSLRSIHKALAKGLATDSSDQAYLINQPKYGFLKELGLAEENLGVFAGG
KWVGSGEWITSYFPGTGEAIAKVKQGTLIDYNNAVAEAKEASKIWREVPAPHRGEIIRQIGHAL
REKKTQLGKLISIEMGKIVPEGEGEVQEYIDICDFAVGLSRMLEGKALPSERPEHSLIEQWNPLG
TIGVITAFNFPVAVLGWNSAISLVCGNTQIWKGAPTTPLTSVATTRIMTEVLEANNYPGAICSMIC
GGADIGEAISKDERLNLVSFTGSTKVGHLVGNTVQNRFGRSLLELGGNNALIAMEDANLDLLIP
AVLFAAVGTAGQRCTTTRRLIVHDNIYDHVVEKLSKAYKQVKIGDPLDDGTLYGPLHSQQAVD
AYHKAVKDAQTQGGRLVCGGNRIDRPGFYVEPTIIADISHDASVVHTETFAPILYILKCSSFEQA

VEWNNEVKQGLSSSLFTKDPARIFKWMGASGSDCGIVNVNIPTSGAEIGGAFGGEKHTGGGRE
SGSDSWKQYMRRSTCTINYSKSLPLAQGIKFE

>Res_009440-T1 ALDH2
MPANTDSKVEIKFTQLFINNEFVNSVSGKVFPTINPASGEKICDVQEGDKADVDIAVKAAQQAF
KLGSKWRTMDASDRGLLLNRLADLIERDRKYLAELETLDNGKPYSDSYNIDLSLTIKCYRYYA
GWADKIHGKTIPTDGDFFNYTRREPVGVVGQIIPWNFPILMQAWKLGPALCCGNTVVMKLAE
QTPLTGLYVASLIKEAGFPPGVVNIISGYGPTAGAAISEHMDVDKVAFTGSTEIGKIIQQAAGKSN
LKNVTLELGGKSPNIIFADADIDEAVELSHFALFFNQGQCCCAGSRTFVEESVYDEFVKKSVSR
AQKRTVGNPMDSSTQQGPQIDKEQFDKILDLIESGKKQGAKLNCGGKRAGDKGFFIEPTVFSD
VTDDMRIAKEEIFGPVQQIIKFKDIDELIERANDTYYGLAAAVHTKDLNRALKVAHGIRAGTV
WVNCYDVLNAQSPFGGFKMSGSGRELGEYGLEQYTLVKSVIIKLDQKNA

>Res_009441-T1 ALDH2
MASVSSKILGCVRKVPSLSLSRFASQAAASRVISAPKVHFNQVFINNEFQDAVSGKVFPTVDPST
GDVICHVAEGDKADVDIAVKAAQEAFKLGSPWRTMDASGRGRLLNRLADLMERDKEYLAAL
ESLDNGKPFSDAYNADLSLSVNCLRYYAGWADKIHGKTIPVDGNFFNYTRREPIGVVGQIIPWN
FPLLMQAWKLGPALATGNVVVMKLAEQTPLTGLYVANLIREAGFPPGVVNIIPGYGPTAGNAIA
EHMFVDKVAFTGSTEVGKLIQQAAGRSNLKNVTLELGGKSPNIVFGDVNVDEAVELSHFALFF
NQGQCCCAGSRTFVEESIYDEFVAKSVERAKKRILGNPLDTSTTQGPQVDSEQMSKIIELIESGK
KEGASLQCGGGQVGDKGYYIAPTVFSDVSDDMRIASEEIFGPVQQIMKFKTADEVIERGNKTM
YGLAAAVQTNDLNKALKVAHGIRAGTVWVNCYDVLHAHSPFGGYKMSGTGRELGEYGLEQ
YSEVKSVIINVGQKNS

>Res_009983-T1 ALDH4A1
MALRLSGFGRFPSRISKVKCLRLLSSQQLPPNEPFTNFEDPIVRERLNEALARVNGQVEDIPIVIG
GQEFRTNDVKYQVSALIEKAIENSLSNRERWEKMPFSQRGPIFMRAAELMATKYRYDMLATT
MVGQAKTPIQADIDAVAESIDFLKFNHHYARDIYEGPALHQPKTVMNHVEYRGLEGFIAAISPF
NFTAIGVNLAGSPAMGGNVVLWKPSDTAMLSGWLVYKILRESGLPDGIIQFVPCDGPLFGDVIT
NSPDLAGISFTGSSKTFKNIWKSVGNGIENYKTFPRLVGECGGKNLHFIHESADVDTVVNGTILS
SFEFGGQKCSACSRLYVPDTLWPQIKEKLVNIMGQLKLGNPEEYSTYLSAVIDERSFDKISSYID
YARNSPQEEIVAGGKCDKSVGYFVEPTLIETKDPNSKLLNEEIFGPVLTAFVYPASEYKEYMKL
ASTTSPYGLTGSIFAKNRNVIEDACEIFKQSAGNFYINDKSTGSVVGQQPFGGARASGTNDKAG
MNTYMLKWLSPRSIKESLVHINNWRYPCMDS

>Res_012784-T1 ALDH8A1
MVVLQNFINGKFVDANDFIDSFEPSTGEVYAKVPASGPKDVELAIQAAENAKDGWAKCSRAK
RSFIMMKIADLLEARLEEFAKAESRDQGKPVWLAKTVDIPRAVHNFRFFSSAVLHFGNESSTQS

HLGALNYTVRSPVGVVGQITPWNLPLYLLTFKIAPAIAAGNCIVCKPSEFTSVTAWMLCGIFND
AGLPPGVVNMVFGNGKDTGAELVLNPAVRAISFTGSTLVGHYIQEKAAPLCKKLSLELGGKNA
AIVFDDANIEKAVSTCIRSAFSNQGEICLCTSRIFVQNKIYDEFTNRFVEQTRKIKVGPPEDDSSF
MGALVSKQHYEKVKYFISLAEEEGGKILCGEGKDEPLDLPEKNKNGYFVRPTVVVGLADNTK
CMQEEVFGPFVCIAPFDTEEEAVRRANGVNYGLCATVWSENGSRIHRVAPKLEAGTIWCNCWL
IRDLNMPFGGYKQSGLGREGYKDSLEFYTEVKTICVKIDEGS

>Res_013678-T1 ALDH1L2
MLSAARAFRQVIRGVSTSATSMGSLRIAVIGQSNFGAEVYKSLRTKGHEIVGVFTIPDVQGKAD
PLAQEAEKDGVAVFKFPRWQQKKVAIPEVVEKYKNVNAELNVMPFCSQFIPMDVINHPEKGSI
VYHPSILPRHRGASAINWTLMEGDSKGGFTIFYADDGLDTGPVLLQKVVDIDPNETVDTIYNRF
LYPEGIKGMVEAVELIANDCAPKVTQPKEGATYDKIWKKKQVAEIPWQRTGWELHNFIRGNDK
LPGAWSMINGEQVSFFGSSMYYKDLPRKATEVVIEGLEKPALVHKDGMIITGTDGQMINVKTL
QLGSGKMIPASKFGSTDDSQEELVLNDEEKELEKKIFKIWGGILNSSELEGSVDFFKAGAGSMD
VARLVEEVKSNCAVELENEEVYMNTVLSDFVRHTILRSRGFGKEEFKCDTIDLEVNNMTVKMP
YQLFIDGEMVNAKSGRTFETINPTDETVLASVALGGKDDVDDAVAAAKEAFYGDWGKMNAR
DRGALMYKLADLMDEHREELATIESLDSGAVYTLALKTHIGMSIDTLRYFAGWADKIQGGTIPI
NHARPNKNLCLTKKEPIGVCGLIVPWNYPLMMLAWKMGPLLATGNTVVLKPAQVTPMTALKF
AELAAKAGFPKGTINIVPGSGSVVGQRICDHPDIRKVGFTGSTPIGAGIMKSAAESNVKRVSLEL
GGKSPLIIFRDCDMEKAVRQGMYACMFNKGENCIAAGRLFIEESIHDEFVENVIAEIKKMKIGD
PLDRSTDHGPQNHKAHLESLLHYVEAGVKEGAKLVYGGKRVDRKGFFMEPAVFTDVTDDMFI
AKEESFGPVMIISKFRDNDVDGVLASANKTEYGLASGVFTNDMSKALRVADGLEAGTCFINTY
NKTDVAAPFGGFKQSGFGKDLGMEALNEYLKTKVITIEY

>Res_015382-T1 ALDH9A1
MLSYNFRMLRTPSNLCLSSASFVNTFVRAASFQAQLAELNFVNGRRSKPNDAGNCEVIDSIEPR
SGKTLGQFYLSGREEVSRAIDSAKIGLDIWRKISDFDKSRIMQNAAGILRERKADIVYLDAIDTG
RVISECEMDVDAAIESLEFYSSLLHVPFGQHIPFQDGSFAYTRREPLGICVGIGAWNFPFLNIAW
KVAPAISCGNAVVYKPSPFTPVSSVIFAEVLHEAGLPPGVLNVIQGGAETGSLLCRHEHVAKVSF
TGSGTTGTKIMQACAEGIKRVTLELGGKSPLVIFADAELENALNGALLANYLTQGQVCSNAAR
VYVQRPIFDEFLEKIVKKVNNITVGDPLKPSSQMGALISEEHLQKVLGYVDVAKKEGARILCGG
ERMRLDDPELQNGFYMSPCVMDNCNDQMTIVKEEHFGPVMSVLAFDTEEEAIARSNDTKYGL
AGGVFTKDLPRAHRFISNLQAGSCYINTYNMYPVQIPFGGYKQSGFGRENGLAVLDGYSQVKS
VYVENNDVSPPF

>Res_017481-T1 ALDH18A1
MLTKLAKLRSCTYLHRQTRLSPVKLFSRQLVLPGGPGPAVIHGLPSPQKKLPLSYRNQLKNAKR
VVVKLGSAVVTRDDECGLALGRLAAIVEQVSELQNAGKQMMVVTSGAVAYGKQRLRGEMS

MQQTLRQSLNYSRNGPTSHPKSSYIEPRACAAVGQGGLVSLYETMFNQYGISVAQVLVTKPDF
RDQYSRANLKTTLEELLKMHCIPIVNANDVVAPPPSQDVDLAGLTPRQQTVISLKDNDSLAALF
AVEMNADLLILLSDVDGIYTGPPDLETSRFIDVFRPGDIENIKFGGKSRVGLGGMESKVRAATW
ALENNCAVVIANGEFKDANIIKDIVNGRRIGTFFTLAEKIGASIEDQAAKAREGSRALQSLSPNQ
RAEIIYRLSELLLERQDDILSANQRDLDAARIAGNLPPPLMSRLALTNNKLKTLSDGLRKIADDS
FSNVGRVLKATRLASGLELNQITVPIGVLMVIFESRPDALPQVAALAIASANGLLLKGGKEAYH
SNHYLFSLVQEALSLHGAQGCPHASALISTRDEVSDLLQLENCIDLVIPRGSNELVRKIQAESKH
IPVLGHSEGICHVYVDKDADMEMALQIVIDSKCDYPAACNAMETILVHRELLRTSAFDQVLDE
LRNNNVIVHAGPRLAKALPFGPVPAKSLKVEYSSLECAMEIVDDVEDAIRHINEHGSAHTDAIV
TDCDRIAKRFIQGVDSACVFHNASTRFADGYRFGLGAEVGISTSRIHARGPVGVEGLLTTKWLL
RGDGHLVSQFGEDGKCKFVHEQLEVYPDSYNKRTASE

>Res_017481-T2 ALDH18A1
MNADLLILLSDVDGIYTGPPDLETSRFIDVFRPGDIENIKFGGKSRVGLGGMESKVRAATWALE
NNCAVVIANGEFKDANIIKDIVNGRRIGTFFTLAEKIGASIEDQAAKAREGSRALQSLSPNQRAE
IIYRLSELLLERQDDILSANQRDLDAARIAGNLPPPLMSRLALTNNKLKTLSDGLRKIADDSFSN
VGRVLKATRLASGLELNQITVPIGVLMVIFESRPDALPQVAALAIASANGLLLKGGKEAYHSNH
YLFSLVQEALSLHGAQGCPHASALISTRDEVSDLLQLENCIDLVIPRGSNELVRKIQAESKHIPVL
GHSEGICHVYVDKDADMEMALQIVIDSKCDYPAACNAMETILVHRELLRTSAFDQVLDELRN
NNVIVHAGPRLAKALPFGPVPAKSLKVEYSSLECAMEIVDDVEDAIRHINEHGSAHTDAIVTDC
DRIAKRFIQGVDSACVFHNASTRFADGYRFGLGAEVGISTSRIHARGPVGVEGLLTTKWLLRG
DGHLVSQFGEDGKCKFVHEQLEVYPDSYNKRTASE

>Sma_008690-T1 ALDH18A1
MALHYRNQLKNAKRIVIKLGSAVVTRDDECGVALGRLSAIVEQVSELQNSGKQMMVVTSGAV
AYGKQRLRGEMSMQQTLRQSLNYSKSGPSPNPKGTYIEPRACAAVGQGGLVSLYETMFSQYGI
SVAQVLVTKPDFRDAYSRANLKTTLEELLKMHCIPIVNANDTVAPPPSQDQDLTGLTPRQQTIIS
LKDNDSLAALFAVEMNADLLLLLSDVDGIYTGPPDQENSRLIDVFRPGDIENIKFGGKSRVGLG
GMESKVRAATWALENNCAVVIANGEFKETNLIKDIVDGRRCGTFFTLAEKVGPSVEDQAIKVK
RYFIVAAREGSGALQALTAQQRAEVIYRLSELLVEREQEILSANQRDLDAARVSGSLAAPLLSRL
ALSRSKIKTLSDGLKKIADDSFDNVGRLVKATRVAENLQLNQVTVPIGVLMVIFESRPDALPQV
AALAIATANGLLLKGGKEAYHSNNYLFSLVQEALSIHGPEGCPHAVALISTREGVSDLMQLENC
IDLVIPRGSSEMIRKIQSESKHIPVLGHSEGICHVYVDKDADLDMALRVVIDSKCDYPAACNAM
ETVLVHRELLRTSAFDQILDELRDNNVDVHAGPRLAKALPFGPVPAKTLKVEYGGLECAMEV
VDDVEDAIRHINVHGSSHTDSIITGNERTAWRFLQGVDSACVFHNASTRFADGYRFGLGAEVGI
STTRIHARGPVGVEGLLTTKWVLKGDGHTVSQFNEGGPCQYIHEPIEIETESRVPKTCALLFHN
LPNGVCKFYLEQLIYPLLLNHNSDLGEVAFGSPAPENEGRKMERLVDTGACAKSAKVNLLASA
YDLQSFGYFQRSSVKEFMGFTAKLLVERTQTGLRASVKEKEYFCHVFVRNDSLSGVVITDKDY

PSRVAFTFLGQLLDDFAIQVPALRWDSDGSDFVFNECETYLAKYQNPKEADPMMKVQTELDET
KIIMYNTIEKVLERGENLDDLVTKSEGLSTSSKAFYTTARKTNSCCGW

>Sma_009615-T1 ALDH1L2
MLSAGRLFKQVTRSVSTSASTMASLRVAVIGQSNFGAEVYKNLRLKGHEIVGVFTIPDVQGKA
DPLAQEAEKDGVQVFKFPKWQNKKVAIPDVLEKYKNVNAELNVMPFCSQFIPMEVIDCPEKG
SIVYHPSILPRHRGASAINWTLMEGDSVGGFTIFYADDGLDTGPILLQKTVDIDPNETVDTIYNR
FLYPEGIKGMVEAVELIAAGRAPKITQTEEGATYDKIWKKKSVAKIPFNKTGLELHNFIRGNDK
LPGAWAMIDGKQVTFFGSKMYRKAKLPRGTEVVIEGLEKPALVHKGGMILTGTDGQMVNVKS
LQLDDGRMIEAAKYGAADEVKEALVLTDDEKKIEKAIFNSWAGILNSQDISGDTDFFKAGAGS
MDVARLVEEVKECGVVLESEEVYMNTKYEDFVRFVILLSRGEGKEEFKCETIDVDVNKMTIR
MPYQLFIDGQFVDAESGRTFDTINPSDETVLAKVALGGKEDVDRAVEAAKDAFEEGEWGRMN
ARDRGALMYKLADLMEQHKEELATIESIDSGAVYTLAVKTHIGMSIDTIRYFAGWCDKILGSTI
PINHARPNKNLCYTKKEPIGVCGLIVPWNYPLMMLAWKMAPLLATGNTVVLKPAQVTPMTAL
KFAELAAKAGFPKGVINIVPGSGSVAGQAICDHPDIRKVGFTGSTPIGAGIMRSAAISNIKRVSLE
LGGKSPLVIFNDCDLEKAVRQGISACVFNKGENCIAAGRLFVEASIHDEFVANVITELKKMKIG
DPLDRSTDHGPQNHKAHLESLLHYIETGVNEGAKLVYGGKRLDRKGFFLEPAVFTDVTNDMFI
AKEESFGPVMIISKFANGDIEDCIESANRTEYGLASGVFTKDLSKALQVADRLDAGTCFINTYN
KTDVAAPFGGFKQSGFGKDLGMEALNEYLKTKVVTVEY

>Sma_013912-T1 ALDH7A1
MLQAASRSKLGNLLPCLVKQKFNMSSESAGKYLVSQPKYSFLKDLGLEEENLGVYDGTWSGS
GEVMTSYSPATGEPIAKAKQGTIDDYERVAQAAKEASKIWREVPAPHRGEIMRQIGHALREKIT
PLGKLVSLEMGKIAPEGIGEVQEFVDICDFTVGLSRMIDGKILPSERPNHALLEQWNPIGTIGIIT
AFNFPVAPFGWNASVSMVCGNTQVWKGAPSTPLTSIATTRIVADVLDANNFPGAISSMICGGA
DVGEAMANDERLNLVSFTGSTKIGHRVGNVVQNRFGRSLLELGGNNAIIAMEDANVNMLVPA
ALFAAVGTAGQRCTTTRRLILHENIHDEVVERLIKAYKQVRIGDPLDDGTLYGPLHSQAAVEQY
KAALIEAQAQGGKIECGGNVIDRPGYFVEPTIVTNVAHNAPIVQQETFVPILYVLKCQNLEQAIE
WNNEVKQGLSSSLFTQDPSKLFKWIGPSGSDCGIVNYNIPTSGAEIGGAFGGEKHTGGGRESGS
DSWKQYMRRSTCTINYGKELPLAQGIKFE

>Sma_013997-T1 ALDH2
MNNQVSKVISVTKKLTALNLSRCLSQVASENVIATPKVHFNQAFINNEFQDSVSGKTFPTINPST
GEVICQVSEGDKADVDIAVKAATEAFKRGSPWRKMDPSDRGILLNRLADLIERDREYLASLES
LDNGKPYSESFNADVPMAIKCLRYMAGWADKDHGKTIPVDGDYFSYTRHEPIGVIGQIIPWNF
PLVMQAWKLAPALAMGNTVVMKTAEQTPLSALYVADLVKEAGFPAGVVNIVPGYGPTAGAAI
AEHMGISKVAFTGSTEVGKLVQQAAGKSNLKNVTLELGGKSPNIVFPDVDIDEAVELSHFALFF
NQGQVCCAASRTFVHESIYDEFVEKSAQRAAQRKLGNPLDLSTNQGPQVSGEQFSRVMELIES

GKHEGATMHCGGEQAGDKGYFIKPTVFSNVTDNMRIATEEIFGPVQQIIKFSSIDEVIERANNTT
YGLAASVMTKDLNTALKVAHSVRAGSIWVNCYDVLPAQAPFGGYNMSGNGRELGEYGLRQY
SEVKSVIIKMDPVSVLCEPISQPEIRYTQEFCQWKNFSITLNPATGDKIVDVQEGDKADVDIAVK
AATEAFKLGSPWRRMDASDRGILLNRLADLIERDRDYLAQLETLDNGKTYADSYNIDLLLVVK
CYRYYAGWADKIHGKTIPTDGDFFNYTRLEPIGVVGQIIPWNFPLLMQAWKLGPALCCGNTIV
MKLAEQTPLTGLYVASLIKEAGFPAGVVNIIPGYGPTAGAAISEHMDIDKVAFTGSTEVGKLVQ
QAAGKSNLKNVTLELGGKSPNIVFADADLDEAVELSHFALFFNHGQCCCAGSRTFVEESIYDEF
VKKSVIRAQNRTVGNPMCKSTQQGPQIDDLQFNKILDLIESGKKEGAKLHCGGTQVGEKGYFI
APTVFSDVTDDMRIAKEEIFGPVQQILKFKDIDDVIERGNKTMYGLAASVMTKDLNKAFKCVV
WKTVNCYDVLNCQSPFGGFKMSGNGRELGEYGLQQYSEVKSVIIKLDQKNS

>Sma_014000-T1 ALDH2
MEEIFAIPVKYIESWLDLKENGKFKQYWVVLKTHYIFIFKDNNTHAREQYVGFLNIEVGETKCT
AKKPSKWYGDFFVEISKKKKFQFRVKFNEVMRSYKSNFYPSIKLLCGMGKWQPSDDILLRRW
LYAIDLVIRAEPPEPLPGDLDFKERTVFTPGPDKMKPRSQSVPVIPPIVRPPLNKSGSCDSTAISST
RATSLASSLRKISIQEADSETYDPIFIPPRDSERIKHHKIKMLPHGQWYFEGSEDKQFTNPTEAVQ
VLMKTTYAALYPQCIQSREDRASGDGEEDETQKFFPSEISSPGYDAAYESSFSPVTPSFGGHFPD
TSPDNTKRQRPTKSYTLPPSLTSPVSFDDHLPIQRNRAGSIGMNERHPTGVYENSNVVQRHRRS
NDSYVDMQLNIANNNETEDAPPPLPCKRRQSLRRQSDHDYVNLPVHGHLQVTYRVIFCSKGIL
IISFHNNTMNNQVSKVISVTKKLTALNLSRCLSQVASENVIATPKVHFNQAFINNEFQDSVSGKK
FPTINPSTGEVICQVSEGDKADVDIAVKAATEAFKRGSPWRRMDPSDRGILLNRLADLIERDRE
YLASLESLDNGKPYSESSNVDLPMAIKCLRYMAGWADKDHGKTMPVDGDYFSYTRHEPIGVV
GQIIPWTFPLVMQAWKLAPALAMGNTVVMKAATQTPLSALYVANLVKEAGFPAGVVNIVPGY
GSTAGTAITEHMGISKVAFTGSTEVGRLVQQAAGRSNLKNVTLELGGKSPNIVFPDVDIDEAVE
LSHFALFFNQGQVCCAASRTFVHESIYDEFVEKSAQRAAQRKLGNPLDLSTNQGPQVSGEQFS
RVMELIESGKRQGATMHCGGEQAGDKGYFIKPTVFSNVTDNMRIATEEVFGPVQQIMKFGNID
EVIERANNTTYGLAASVMTKDLNTALKVAHSVRAGCIWVNCYDVLSPQTPFGGYNMSGCGRE
LGEYGLQQYSEVKSSDIEDPTILLLICKGCIPCKLHAEGTPRQGQVYARVYMQLEIEQKHCANP
TPIITSYLCIGITMMQKYAHKPNGDGTNQQMLTEWQSITSN

>Sma_015340-T1 ALDH5A1
MSKLLKSAKQILSSNSLLRTKAYINGEWISLTKSFPVLSPATREVICEVTDADEDLTKSAIDAAKS
AFNKWSEVNCKERSLVLKEFCRHVVDHKEELALIMCAECGKTVAETRMEVNYGASFFEWFAE
EAKRTYGDILPEFGNSSMGLIIRQPVGVVGIITPWNFPLAMITRKLGAAMAVGCTTVIKPSEETP
LTALALAEIAEKAGIPQGVINVLPCSRLNTQAVGKLLCESSDVRKISFTGSTVVGKKLMMQAAN
DVKRVSMELGGNAPFVVFDSADINKVKKAVRGTKFRANGQTCISANRFIVQAGIHDRFVEEM
KILAEGLRIGDPFDEETELSSLIHEGAIEKIDLHIQDSVKKGAKAVAGCERHPLGPAYYKPSILVD
CTEDMLAMSCENFGPVIAITKFEAEDEAIRMMNNTQFGLAGYLFSEEMSQVVRVSKKMEVGM

VGANEGAISNEMVPFGGIKESGVGREGSKYGVDDYTELKYVCLGGL

>Sma_015341-T1 ALDH5A1

MSKLLKSAKQILSSNSLLRTKAYINGEWISLRRSFPVLSPATREVICEVTDADEDLTKSAIDAAKS
AFNKWSEVNCKERSLVLKEFFRRVMDHKEELSLLMSAECGKRIAETRMEVNYGASFFEWFAE
EAKRTYGDILPEFGNSSMGLITKQPVGVVGIITPWNSPLVTITRKLAAALAVGCTTVIKPSEETPL
TALALAEIAEKAGIPQGVINVLPCSRMSTPAVGKLICESSDVRKITFTGSTVVGKKLMMQAANN
VKRVSMELGGNAPFVVFDSADMNKVKEAVMGAKFKANGQMCTCANRFIVQAGIHDRFVEE
MKVLAEGLRIGDPFDEETELSSLIHEGAVEKIDLHIQDSLKKGAKVVAGCERHPLGPAYYKPSIL
VDCTEDMLAMSRENFGPVIAIKKFETEDEAIRMMNNTEFGLAGYVFSEEMSQAVRVSKKMEV
GLVGANEWAILSDMAPFGGIKESGVGKEGSKYGVDDYTQLKYVCLGGL

>Sma_016077-T1 ALDH8A1

MLLQLRSRCHKVFVFSLLLKSHMSSQRTWNPYFHSSTVASISSVVAPVITLVVTAAILPTVVTPT
PAIVSLVITYDVGQLDALAHYNTPGANINVFVPISSLFIRSLRILVPILLIRPLEKFMLKFPAVERA
MLKPLLQQQTTPGKEIGMTRYGVSAEIWSSTPVATRSSLMMKVADLLEANLDMFAKAESKDQ
GKPISLSRAVDIPRAIAPAIAAGNCVICKPSELTSVTAWMLCSIFNEAGIPPGVINMVFGSGAEVG
AEIVENPNICAISFTGSTGVGHYIQEKSAPFCKKLSLELGGKNSAIVFDDANLDACIQTCVRSSF
ANQGEICLCTSRIYVHRSIFDTFVKRFVEKTRSLKVGPPENDDVFMGALVSKDHLEKVKRYVQ
LAIEEGGTILCGEGKDELPDLPDANKQGYFMLPTAVMGLADDARCMQEEVFGPFVIKRVNNVT
YGLCSTLWTESGQRIHRVAPQLEVGTIWCNCWLVRDLNMPFGGFKQSGIGREGFKDSLEFYTE
VKTIYGASFTSPSGKQDKYETAGQDATFTFGYQVEALGSLQNIFCGYRGASPSTNIIAKVGTQP
SAITSNFRNRAQKGIDTTTQIGFKLLKVQLNDAGEFGCDMTYANNQIFSNYFVLKIYEAPRFGD
CLGPEGTKFIIREGQALNTTCAFYGNPQPTLTYQSSAPRDLKKRASTYSAITVQWLPPLDFGNA
PLISYQLEIEDSRPLHYSKNVTNSTLAGSTPSLEHTFDNLDAQVEYKISVVAYTDVGKGTPSGVE
IFKTVPYTPTVSGGRSSSNTGAIVGGVIGGLVFIVIIVAIVVWYRRREPAQKYPQSFVEEAVDYPA
AKSNMYSKVNKKKMPTSLEDESTSGYPDIIHQANADQRPHNPENQSLRADNPQDTSTSPSYES
SRKEYGYAVQANTIASTKEAPTALIDEEVYPIEVAKNDQRHHKKVYPQK

>Sma_017482-T1 ALDH16A1

MAGGKRGKSSASEAKSATLNEDGTPAKKQAKKELSKVATIFESLDYGPAPESGEIAKNWIASHG
DALGHFINGKWVKPEGRKKYDSYSPATGEKIASTIQGEAEDIDLAVAAAREAFKTWSKLSCHA
RARHLYSIARHVQKHMRLIAVLESLDNGKSIRETRDADVPIVVRHLYHHAGWAELMETEMKD
WSPVGVIGGIVPWNFPLMLLSWKVCPALAMGNTVVLKPASFTRLSALLFAEICAEAGLPPGVF
NVVTGPGSFGSKLATHPDVDKVAFTGSTEVGMILRRLTAGSGKKISLELGGKSPFVVFDSADLD
SAVEGVVQAIWFNQGQVCSAGSRLLVQETVAEKLIEKIKERMKHLRLGHSLDKCIDMGPIVDE
SQRKSIAKFVDDAKAEGAEVFQSYACMPSTGCFYPPTLVTNVSTTSNIVIEEVFGPVLAVLTFRT
AKEAISLANNTRYGLGASVWSENIGLAMEVACNIKAGSVWINNHNAFDAAAGIGGYKESGFG

RDGGKEGLYEYVKPSWQKTTKIEASELDTKSFGPASYPHVLSPNENPISNGHALEPKVDRTIKM
LVGGKQKRPDANYSRTILNVNGEIIGHVGDGNRKDIRDAVEAANGALAGWGKRTGHNKAQIL
YYIAENLELRLSEIAGRIKEETDVTMDAAEEQVNVAIRRLFHWAAYADKYGGNVQETQQYGT
VLRVHEPVGVIGIACPDDCPFLSFVSLLGPAIARGNTIVIIPSEKFPLSSMDLCQVFETSDVPAGVI
NVVTGSRDHLTKTLTEHQSISAMWYFGSTQGSKFVEEASAHNIKRTWVNYGASRDWFDEQQG
AGEEFLIRSVECKSVWIPMGHIFAN

>Sma_017482-T2 ALDH16A1
MAGGKRGKSSASEAKSATLNEDGTPAKKQAKKELSKVATIFESLDYGPAPESGEIAKNWIASHG
DALGHFINGKWVKPEGRKKYDSYSPATGEKIASTIQGEAEDIDLAVAAAREAFKTWSKLSCHA
RARHLYSIARHVQKHMRLIAVLESLDNGKSIRETRDADVPIVVRHLYHHAGWAELMETEMKD
WSPVGVIGGIVPWNFPLMLLSWKVCPALAMGNTVVLKPASFTRLSALLFAEICAEAGLPPGVF
NVVTGPGSFGSKLATHPDVDKVAFTGSTEVGMILRRLTAGSGKKISLELGGKSPFVVFDSADLD
SAVEGVVQAIWFNQGQVCSAGSRLLVQETVAEKLIEKIKERMKHLRLGHSLDKCIDMGPIVDE
SQRKSIAKFVDDAKAEGAEVFQSYACMPSTGCFYPPTLVTNVSTTSNIVIEEVFGPVLAVLTFRT
AKEAISLANNTRYGLGASVWSENIGLAMEVACNIKAGSVWINNHNAFDAAAGIGGYKESGFG
RDGGKEGLYEYVKPSWQKTTKIEASELDTKSFGPASYPHVLSPNENPISNGHALEPKVDRTIKM
LVGGKQKRPDANYSRTILNVNGEIIGHVGDGNRKDIRDAVEAANGALAGWGKRTGHNKAQIL
YYIAENLELRLSEIAGRIKEETDVTMDAAEEQVNVAIRRLFHWAAYADKYGGNVQVDQIYA

>Sma_018747-T1 ALDH3B2
MAEANIDYSDVVQQLRTTFRQGKTLPIWYRIGQLKQFLKMMTENKDLFVEALHQDLRKPKFE
STMMEYTVVIGELMKFIDNLPEWASFQKTEADLLNKLNTCGIKYEPYGVALIIGAWNYPLLLLL
QPFIGCLAAGNCAILKPSELSPATANLIESLLPRYLDEDCYKVIQGARDETTALLREKYDYIFYTG
GHVVGKIIMQAAAKYLTPVTLELGGKSPCYVDSDCHFDITARRIVWGKTEVKLVQSLKKALRE
FYGENPKDSGDLARIINNRHFERLMKLLDKEKIVIGGETDEAERYIAPTVMNRVTLDDAIMKEE
IFGPLLPIVNVANQSEAIKLIAEREKPLALYVFSNTKANIDTFLDKTSSGAVCVNDSIVQAAVPSIP
FGGVGQSGIGAYHGKFSFECFSHRKACLIKKQNLEPLNAIRYPPYTDSNLKTACWFLVPSRKSK
YLKYFFYVAVLGAVAYFCKYYDVCAQLKRFK

>Sma_020509-T1 ALDH4A1
MDYLQADKSTMSIINTAINYSMGVMGYHGMPPNEPATDFTDPRVREQLNEALSRVTSHIEDIPI
VIGGKDFRTAEHRYQVSPYDHKKRIAKYHYADKCPFFQNPFCCDLVSSTLIENAIENALINREK
WEKMPFDQRITTELLGPIFMRAAELMKTKYRYEMLACTMVGQAKTAIQADIDAVAESIDFLKF
NHYYAQEIYKGPKLHQCNDVMNHVMYRGLEGFVAAISPFNFTAIGVNLAGTPVMAGNVVVW
KPSDTAMLSAWLVYKIFMESGMPDGVVQFLPCDGPLFGDVMSKSPDLAGVSFTGSSKCYMTF
KHIWKTIGNNIDNYKTFPRLIGECGGKNFHFVHESADIGKYLLCTKNSVVNGTLRSAFEFSGQK
CSACSRLYVPDSLWPQIKSKLIQKMEQMKLGNPEEHSTFVSAVIDQVSFNKISGYIEHAKHSNE

VEIIMGGKCDDSTGYYVEPTLIETMDPKDKLMKEEIFGPVLTTYVYPAKHYKETMKMASETSP
YGLTGSIFSANRHVVEEACDVFRQSAGNFYINDKSTGSVVGQQPFGGARASGTNDKAGMNTY
ILKWMSARSVKETLTNLTEFKYKYMEE

>Sma_021880-T1 ALDH9A1
MAFRCLQGVLPIVKCELFPNYVFKQASKYTRRSVSTITAGDFEKLNFVDGKRCRPLEHGTCDV
GEDIEPRSGKVFGEFYYSNSNEVVRAVESAKQGHELWREMSEFQRSKIMQKAAEKLRERKSEI
ARIDSIDTGRVIWESEYDVEAAADCLDYYSGLIHTLVGQTLPSTSGSSFAYTRREPIGVCVGIGA
WNFPFLNIAWKISPAICCGNSVVYKPSPLAPVSSVLFAEILHEAGIPSGVLNVIQGGAEVGNLLC
KHDDVAKVSFTGSAETGTKVMQTCAEKIKNVTLELGGKSPLVIFADADIDNAVKGALMANYL
TQGEVCSNAARVFVEEPIFDEFLEKVVEKVKSIRVGDPLISDNQMGAVISEDHLQKVLAYVDGA
KQEGAEILHGGERLHPKEAAITDGFYMSPCVMTNCHDNMKIVKEEHFGPIMSLLKFNSEDEVI
RRANNTKYGLAGGAFTRDMPRAHRFIAKLQAGACYINNYNIYPVQLPFGGYKMSGIGRENGQ
AVLENYTQLKSVYIENQDVDYLFTFKTQILKHFIMNQTEAMIMVILLLFIISSINANAESNCPDG
HWFHSVGKKSTCCEYCSPGQYMLEPCNTVNRTKCAVCPAGTFTAGPNRFKKCLTCATCSLSHG
FKLKKKCTRVSNVECSCVPGYYIAMLHNLCKKCSTCKPGFEISKPCTADSNRECRSCSLGETFE
QKGKCIKCSQCKVKEIKQKNCTIFKDTVCEKFVPLTFPPTPTTRWKENDSGNTTKPIFISISVVFV
FVALCVFLCWIKKYGCFHRRADRHARTERLEANIASKPDSLNKREMVELLPKVPNTKTHLKNL
EVKDRHALAKLLNPNGCSNWESLAAAMKLSAIDIRNIERVASPTESLLFRYETSREFPTIEGLMQ
LFIDINRLDCFSHMRTALESVRMSEV

>Sma_023663-T1 ALDH6A1
MASNSLLQMCKSFAPHLSKAARPSYGVRCYSAGGSAPATKLFIGGKFIESKTNEYIPLYNPANN
EVIGQVPKATQEEMQAATDAAQQAFPSWSNMSVIARQQVMFKLQHLIKENMGRLAANITLEQ
GKTLADAEGDVLRGLQVTEHACSITSLQLGETMAGISKDMDTHSYRVPLGVCAGVTPFNFPA
MIPLWMFPLALVSGNTFVLKPSEQDPGAAMMLVQLAQDAGVPDGVLNVIHGAKESVDFICDN
PIIKAISFVGSDFVGQYIYERGSRNGKRVQSNMGAKNHGVVMPDANKEMTLNQLVGAAFGAA
GQRCMALSTAVFVGGAQDWIPELVERAKKLNVNAGDQPGADVGPLISPAAKQRVEDLIQSAID
EGASVDLDGRNVNVPGYKNGNFVGPTIISGVKPHMKCYTEEIFGPVLVILDTDSLEGAIDVVNK
NPYGNGTAIFTSSGAVARRYQHEVDCGQVGINVPIPVPLPMFSFTGSRGSFRGDMNFYGKAGIN
FYTQLKTVTSLWRMEDTESRKAEVSFPKL

>Sma_024492-T1 ALDH8A1
MIRVENFMNGKFIPASQFLDSFDPSTGKVHAQIPRSGKSDVEAAVAAADNARKGWSSTPVATRS
LLMMKVADLLEANLDMFAKAESKDQGKPISLSRAVDIPRAVHNFRFFATAILHFGNIAPAIAAG
NCVICKPSELTSVTAWMLCSIFNEAGIPPGVINMVFGSGAEVGAEIVENPNICAISFTGSTGVGH
YIQEKSAPFCKKLSLELGGKNSAIVFDDANLDACIQTCVRSSFANQGEICLCTSRIYVHRSIFDTF
IKRFVEKTRSLKVGPPENDVFMGALVSKDHLEKKQTGNQRENAHLEDSSNARRFSCRQQKTL

KQSFGKAKIHKNSTKRILILLHNVKQIGVFYSELGKKLKEFSDNGPNDVP

>Sma_024493-T1 ALDH8A1
MDDISTEHNRFADNVKRYVQLAIEEGGTILCGEGKDELPDLPDANKQVIKRVNNVTYGLCSTL
WTESGQRIHRVAPQLEVGTIWCNCWLVRDLNMPFGGFKQSGIGREGFKDSLEFYTEVKTICVK
TSY

>Sma_024496-T1 ALDH8A1
MDDISTEHNRFADNKVEQLLCGERGKDEIPDLPDANKQVILYIVAVIILFPGNKYYSTLKYVME
QLFGVQGYFMLPTAVMGLADDARCMQEEAGIIGCNCWLVRDLNMPFGGFKQSGIGREGFKDS
LEFYTEVKTICVKTSY

Supplementary Discussion

1. Heterozygosity

The estimated heterozygous rates are 0.97% for *S. malayensis* and 1.97% for *R. esculentum* respectively, which are higher than that of other cnidarians[1,2,3,4].

2. Repeat content

The estimated repeat percentages are 64% and 34% for *S. malayensis* and *R. esculentum* respectively. In contrast, the repeat percentages of sequenced anthozoans are all lower than 30%, while *H. vulgaris* has a high repeat percentage of 57%. The repeat content of *S. malayensis* is the highest among sequenced cnidarian genomes to date. In the repeat libraries constructed by RepeatModeler[5], there are 987 and 1495 repeat elements for *S. malayensis* and *R. esculentum* respectively. The repeat library of *S. malayensis* has an average size of 2,242 bp, which is higher than the 968 bp average size of the repeat library of *R. esculentum*. The longest repeat element is about 10 kb in the repeat library of *R. esculentum*, while 21 repeat elements in the repeat library of *S. malayensis* are longer than 10 kb.

Repeat compositions of the two jellyfish genomes were estimated by repeat-masking with respective repeat libraries. Based on genome-masking results, the percentages of various repeat elements were calculated. DNA transposons are the most abundant repeat category in both species, constituting 31.2% and 19.8% of masked repeats in the assemblies of *S. malayensis* and *R. esculentum* respectively. The three most abundant DNA transposons are Helitron, TcMar, and Polinton in *S. malayensis*, while TcMar, hAT, and Harbinger are three most abundant DNA transposons in *R. esculentum*. The Helitron alone constitutes about 10.5% of masked repeats in *S. malayensis*, while only 0.7% of masked repeats in *R. esculentum* are Helitrons. In terms of retrotransposons, *S. malayensis* has a higher percentage of long terminal repeat (LTR) retrotransposons than Non-LTR retrotransposons, while *R. esculentum* has more Non-LTR retrotransposons than LTR retrotransposons. LTR retrotransposons constitute 8.5% and 5.5% of masked repeats in *S. malayensis* and *R. esculentum* respectively, and the four most abundant LTR retrotransposons are Gypsy, BEL, DIRS, and Copia in both species. In *R. esculentum*, 13.6% of its masked repeats are Non-LTR retrotransposons, which constitute only 5.4% of masked repeats in *S. malayensis*.

3. Gene model / Genome annotation

28,526 and 17,627 gene models were predicted for *S. malayensis* and *R. esculentum* respectively, and the numbers of gene models are comparable to those of the other published cnidarian genomes shown in Table 1.3.3. The mean exon lengths were 250

bp for both jellyfishes, which are higher than most of the other published cnidarian genomes except *Exaiptasia pallida*. The mean intron lengths were 528 bp and 1,025 bp for *S. malayensis* and *R. esculentum* respectively, while mean deduced protein lengths were 448 aa and 476 aa respectively, comparable to other cnidarians.

Annotation statistics indicate that high-quality gene models had been achieved for both species. The predicted gene set of *S. malayensis* includes 94.1% Complete BUSCOs (2.9% Missing BUSCOs) with eukaryota_odb9 dataset and proteins mode and 91.4% Complete BUSCOs (4.9% Missing BUSCOs) with metazoa_odb9 dataset and proteins mode. The percentages of eukaryotic and metazoan BUSCO orthologues in the gene models of *R. esculentum* were 97.4% Complete BUSCOs (1.3% Missing BUSCOs) and 95.4% Complete BUSCOs (3.6% Missing BUSCOs) respectively. Considering the complete set of predicted proteins, 98.5% of gene models in *S. malayensis* and 96.9% of gene models in *R. esculentum* were apparently complete, with predicted start and stop codons. Around 72% gene models in *S. malayensis* and 81% of gene models in *R. esculentum* were supported by mRNA evidence, while 60.5% gene models in *S. malayensis* and 76.2% of gene models in *R. esculentum* were supported by homology evidence from comparison to the NCBI nr database. At least one protein domain predicted by InterProScan (version 5.26-65) was found in 86.4% and 92.4% of predicted proteins in *S. malayensis* and *R. esculentum* respectively.

4. Mitochondrial genomes

Both mitochondrial genomes had a complete set of all 13 protein-coding genes involved in energy production, 2 rRNAs (16S rRNA and 12S rRNA), 2 tRNAs for methionine and tryptophan, and a polB (polymerase domain of family B DNA polymerase) that shows similar gene order to other medusozoans[6]. Inverted terminal repeats or telomeres of 477 bp (GC content of 35%) in the *S. malayensis* mtDNA and 517 bp (GC content of 29%) in the *R. esculentum* mtDNA contain coding regions similarly to other linearlised mtDNA; gene conversion has been suggested as the mechanism for the expansion in telomeres of linear mitochondrial genomes[7].

5. Homeobox genes

100 and 98 homeobox genes were identified in the two jellyfish genomes, numbers comparable to other cnidarians[8,9]. Similar to other cnidarians, the two largest classes of homeobox genes in both jellyfish genomes are ANTP- and PRD- classes, which constitute more than 40% and 30% of total homeobox genes identified in the jellyfish genomes.

Seven TALE-class homeobox genes were identified in both jellyfish genomes,

including Meis, Pbx, Irx, and Pknox and Atale-like (the latter having highest identity in their homeodomains to the Atale gene of amphioxus, albeit this sequence identity being low, < 53%). The Atale gene was a novel TALE homeobox gene first identified in amphioxus and was thought to be cephalochordate-specific[10]. In the phylogenetic tree, these two Atale-like genes and a divergent TALE-class homeobox gene from *N. vectensis* form a clade with the Atale gene from amphioxus. Considering the high sequence divergence and relatively low support for this group of genes, we analysed other conserved domains in their protein sequences. Interestingly, all these proteins share a common feature of both a homeobox KN domain (Homeobox_KN) and N-terminal SD domain of SIX1 (SIX1_SD superfamily). Such a domain combination was also found in a six1a-like protein from *Acropora*, although its TALE homeobox domain did not group with those Atale-like genes in the tree. The presence of TALE class homeobox domain and SINE specific domain suggests that this protein could have evolved by a gene fusion event, or alternatively it could represent an ancestral state. Similar putative fusion proteins have been reported in the hydrozoan jellyfish *Craspedacusta sowerbyi* and suggested to result from the fusion of the TALE class homeobox gene Irx and the N-terminal SD domain of a SINE class homeobox gene[11].

Within the ANTP class of homeobox genes, most homeobox sequences do not have introns in jellyfish. We found an intron at homeodomain amino acid position 47 in the Mox genes of the two jellyfish species, absent in other cnidarians. Conserved introns at position 45 are also found in NK4 and Hhex genes of the two jellyfishes and the intron in NK4 is also conserved in *H. vulgaris*. Introns in NK6 at position 33 of the homeodomain were also conserved between the two jellyfishes.

For LIM class homeobox genes, introns were found in Lmx of cnidarians, but the intron patterns vary between anthozoans and medusozoans. Two conserved introns at positions 30 and 55 were found in the homeodomains of Lmx orthologues in medusozoans and proteins. These intron positions were also conserved in the Meis family protein ceh-25 of nematode *Caenorhabditis elegans*[12]. For Pbx and Irx family proteins, a single intron at about position 48 was identified.

Three-gene ParaHox cluster in both jellyfish

In bilaterians, the ParaHox cluster originally had three genes (*Gsx*, *Pdx* and *Cdx*), although Pdx has been secondarily lost in arthropods, and *Pdx* plus *Gsx* have been lost in the nematode *C. elegans*. In *N. vectensis*, Gsx and a potential Pdx/Cdx were found to be physically linked[8], and until the current study this was the most complete and best-characterized ParaHox cluster identified in cnidarians[13]. As explained below, we argue that the putative Pdx/Cdx is likely a Pdx orthologue, meaning that *N. vectensis*

has a *Gsx* plus *Pdx* ParaHox cluster.

In the two jellyfish species, we find three linked ParaHox genes, which phylogenetic analysis suggests are *Gsx*, *Pdx* and *Cdx*. Support values for some of these assignments are low, but the physical linkage is consistent with this being a 3-gene ParaHox gene cluster. Phylogenetic analysis alone does not definitely demonstrate that the three genes are each 1:1 orthologues of the three bilaterian ParaHox genes, and we cannot exclude the possibility that the putative *Pdx* and *Cdx* are derived by an independent duplication in cnidarian evolution. However, we suggest here that the three genes are orthologues of *Gsx*, *Pdx* and *Cdx*.

To further test this hypothesis, we analysed the distribution of ParaHox genes across cnidarians using available genomic and transcriptomic datasets. We find that most anthozoans only have orthologues of the two genes found in *N. vectensis,* which in phylogenetic analysis group with jellyfish putative *Gsx* and *Pdx* only. Coral *A. digitifera* has only *Gsx*. No ParaHox genes could be identified in the myxozoan genome. In another parasitic cnidarian, *P. hydriforme,* a homeobox similar to the jellyfish and *N. vectensis* putative *Pdx* gene and a partial *Gsx* were found in the genome.

In contrast, three ParaHox genes could be identified in the transcriptome of cubozoan *Alatina alata,* staurozoan *Lucernariopsis campanulate* and several scyphozoans. In hydrozoans, despite the presence of only Gsx in *H. vulgaris,* all three ParaHox genes were found in the transcriptomes of several hydrozoans. Intriguingly, these hydrozoans all contain a medusae stage or medusoid-like structure despite their diverse life cycles. For example, *Liriope tetraphylla* has no polyp stage[14], *Ectopleura larynx* has truncated medusae[15], and siphonophores are colonial organism composed of medusoid and polypoid zooids. In contrast, only Gsx and Cdx could be identified from the draft genome of *Hydractinia echinata,* which has no feeding medusa stage, while all three Parahox genes were identified from the transcriptome of hydrozoan jellyfish *Podocoryne carnea* belonging to the same family *Hydractiniidae*[16]. Thus, a complete set of three ParaHox genes was only identified in cnidarians with medusa stage or medusoid-like structures, indicating a correlation between the ParaHox cluster and the cnidarian body plans or life cycles.

Supplementary References

1. Bellis, E. S., Howe, D. K. & Denver, D. R. Genome-wide polymorphism and signatures of selection in the symbiotic sea anemone Aiptasia. *BMC Genomics* **17**, 160 (2016).
2. Chapman, J. A. et al. The dynamic genome of Hydra. *Nature* **464**, 592–596 (2010).
3. Putnam, N. H. et al. Sea anemone genome reveals ancestral eumetazoan gene repertoire and genomic organization. *Science* **317**, 86–94 (2007).
4. Shinzato, C., Mungpakdee, S., Arakaki, N. & Satoh, N. Genome-wide SNP analysis explains coral diversity and recovery in the Ryukyu Archipelago. *Sci. Rep.* **5**, 18211 (2015).
5. Smit, A. F. A. & Hubley, R. 2008-2017. RepeatModeler Open-1.0. Available from http://www.repeatmasker.org. (2017).
6. Kayal, E. et al. Evolution of linear mitochondrial genomes in medusozoan cnidarians. *Genome Biol. Evol.* **4**, 1-12 (2012).
7. Smith, D. R. & Keeling, P. J. Mitochondrial and plastid genome architecture: reoccurring themes, but significant differences at the extremes. *Proc. Natl. Acad. Sci.* **112**, 10177-10184 (2015).
8. Chourrout, D. et al. Minimal ProtoHox cluster inferred from bilaterian and cnidarian Hox complements. *Nature* **442**, 684–687 (2006).
9. Ryan, J. F. et al. The cnidarian-bilaterian ancestor possessed at least 56 homeoboxes: evidence from the starlet sea anemone, *Nematostella vectensis*. *Genome Biol*. **7**, R64 (2006).
10. Takatori, N. et al. Comprehensive survey and classification of homeobox genes in the genome of amphioxus, *Branchiostoma floridae*. *Dev. Genes Evol*. **218**, 579–90 (2008).
11. Hroudova, M. et al. Diversity, phylogeny and expression patterns of Pou and Six homeodomain transcription factors in hydrozoan jellyfish *Craspedacusta sowerbyi*. *PLoS One* **7**, e36420 (2012).
12. Bürglin, T. R. Analysis of TALE superclass homeobox genes MEIS, PBC, KNOX, Iroquois, TGIF reveals a novel domain conserved between plants and animals. *Nucleic Acids Res*. **25**, 4173–80 (1997).
13. Hui, J. H. L., Holland, P. W. H. & Ferrier, D. E. K. Do cnidarians have a ParaHox cluster? Analysis of synteny around a *Nematostella* homeobox gene cluster. *Evol. Dev*. **10**, 725–30 (2008).
14. Russell, F. S. *The Medusae of the British Isles* (Cambridge Univ. Press, Cambridge, 1953).
15. Nawrocki, A. M. & Cartwright, P. Expression of Wnt pathway genes in polyps and medusa-like structures of *Ectopleura larynx* (Cnidaria: Hydrozoa). *Evol. Dev.* **15**,

373–384 (2013).
16. Miglietta, M. P. & Cunningham, C. W. Evolution of life cycle, colony morphology, and host specificity in the family Hydractiniidae Hydrozoa, Cnidaria. *Evolution* **66**, 3876–901 2012.
