# Supplementary material for: Jellyfish genomes reveal distinct homeobox gene clusters and conservation of small RNA processing
Source: Nat Commun. 2020 Jun 19;11:3051. doi: 10.1038/s41467-020-16801-9 (PMC7305137; doi:10.1038/s41467-020-16801-9)
Supplement: Supplementary file 3 — Reporting Summary [file 41467_2020_16801_MOESM3_ESM.pdf]

nature research

Corresponding author(s): Jerome H.L. Hui, Peter W.H. Holland

Last updated by author(s): May 5, 2020

Reporting Summary

Nature Research wishes to improve the reproducibility of the work that we publish. This form provides structure for consistency and transparency in reporting. For further information on Nature Research policies, see Authors & Referees and the Editorial Policy Checklist.

Statistics

For all statistical analyses, confirm that the following items are present in the figure legend, table legend, main text, or Methods section.

n/a	Confirmed	
☐	☒	The exact sample size (*n*) for each experimental group/condition, given as a discrete number and unit of measurement
☐	☒	A statement on whether measurements were taken from distinct samples or whether the same sample was measured repeatedly
☒	☐	The statistical test(s) used AND whether they are one- or two-sided<br>*Only common tests should be described solely by name; describe more complex techniques in the Methods section.*
☒	☐	A description of all covariates tested
☒	☐	A description of any assumptions or corrections, such as tests of normality and adjustment for multiple comparisons
☒	☐	A full description of the statistical parameters including central tendency (e.g. means) or other basic estimates (e.g. regression coefficient) AND variation (e.g. standard deviation) or associated estimates of uncertainty (e.g. confidence intervals)
☒	☐	For null hypothesis testing, the test statistic (e.g. *F*, *t*, *r*) with confidence intervals, effect sizes, degrees of freedom and *P* value noted<br>*Give P values as exact values whenever suitable.*
☒	☐	For Bayesian analysis, information on the choice of priors and Markov chain Monte Carlo settings
☒	☐	For hierarchical and complex designs, identification of the appropriate level for tests and full reporting of outcomes
☒	☐	Estimates of effect sizes (e.g. Cohen's *d*, Pearson's *r*), indicating how they were calculated

Our web collection on statistics for biologists contains articles on many of the points above.

Software and code

Policy information about availability of computer code

Data collection	PacBio RS II P6-C4, Illumina, HiseqXten (Chromium WGS), CHiCAGO and Hi-C
Data analysis	FastQC, version 0.11.8, link https://www.bioinformatics.babraham.ac.uk/projects/fastqc/fastqc_v0.11.8.zip<br>minion, version 15-065, link http://wwwdev.ebi.ac.uk/enright-dev/kraken/reaper/src/reaper-15-065.tgz<br>cutadapt, version 1.10, link https://github.com/marcelm/cutadapt/tree/v1.10<br>DSK, version 2.1.0, link http://gatb-tools.gforge.inria.fr/versions/bin/dsk-2.1.0-Linux.tar.gz<br>Kraken, version 1.0, link https://github.com/DerrickWood/kraken<br>FALCON, version 0.7, link https://github.com/uwdata/falcon<br>pilon, version 1.20, link https://github.com/broadinstitute/pilon/releases/download/v1.20/pilon-1.20.jar<br>Supernova, version 2.1.0, link https://github.com/major/supernova/archive/v2.1.0.tar.gz<br>BUSCO, version 3, link https://gitlab.com/ezlab/busco<br>SOAPdenovo2, version 2.04-r240, link https://github.com/aquaskyline/SOAPdenovo2<br>MaSuRCA, version 3.2.1_01202017, link https://github.com/alekseyzimin/masurca<br>GapFiller, version 1-10, link https://github.com/dsarov/MGAP---Microbial-Genome-Assembler-Pipeline/tree/master/bin/GapFiller_v1-10_linux-x86_64<br>PBJelly, version 15.8.24, link https://sourceforge.net/p/pb-jelly/wiki/Home/<br>HaploMerger2, version 20161205, link https://github.com/mapleforest/HaploMerger2/releases/download/HaploMerger2_20161205/HaploMerger2_20161205.tar.gz<br>CAT, version Nov2016, link https://github.com/dutilh/CAT<br>assembly-stats, version 1.0.1, link https://github.com/rjchallis/assembly-stats<br>bwa, version 0.7.12-r1039, link https://sourceforge.net/projects/bio-bwa/files/bwa-0.7.12.tar.bz2/download<br>KAT, version 2.1.1, link https://github.com/TGAC/KAT<br>RepeatModeler, version 1.0.4, link http://www.repeatmasker.org/RMDownload.html<br>genometools, version 1.5.8, link http://genometools.org/pub/genometools-1.5.8.tar.gz<br>MITE_Hunter, version 11-2011, link http://target.iplantcollaborative.org/mite_hunter/MITE%20Hunter-11-2011.zip

TransposonPSI, version 08222010, link https://sourceforge.net/projects/transposonpsi/files/TransposonPSI_08222010.tgz/download
Trinity, version 2.4.0, link https://github.com/trinityrnaseq/trinityrnaseq/archive/Trinity-v2.4.0.tar.gz
TransDecoder, version 3.0.1, link https://github.com/TransDecoder/TransDecoder/archive/v3.0.1.tar.gz
AUGUSTUS, verion 3.3, link http://bioinf.uni-greifswald.de/augustus/binaries/old/augustus-3.3.tar.gz
Blast2Go, version 2.8, link https://www.blast2go.com/previous-versions
InterProScan, version 5.26-65.0, link https://github.com/ebi-pf-team/interproscan/wiki/Interproscan5_26_65_ReleaseNotes
Prokka, version 1.12-beta, link https://github.com/tseemann/prokka
MEGA, version 7, https://www.megasoftware.net/
OrthoMCL, version 2.0.9, http://orthomcl.org/common/downloads/software/v2.0/orthomclSoftware-v2.0.9.tar.gz
MUSCLE, version 3.8.31, link https://www.drive5.com/muscle/downloads.htm
trimAl, version 1.4, link https://github.com/scapella/trimal
RaxML, version 8.2.4, link https://github.com/stamatak/standard-RAxML
ProtTest, version 3.4, link https://github.com/ddarriba/prottest3
PAML, version 3.1, link http://abacus.gene.ucl.ac.uk/software/paml.html
SyMAP, version 4.2, link http://www.agcol.arizona.edu/software/symap/index.html
mirDeep2, version 2_0_0_8, link https://github.com/rajewsky-lab/mirdeep2
PASA, version 2.1.0, link https://github.com/PASApipeline/PASApipeline
blast+, version 2.2.31+, link https://ftp.ncbi.nlm.nih.gov/blast/executables/blast+/2.2.31/
funannotate, version 1.6.0, link https://funannotate.readthedocs.io/en/latest/

For manuscripts utilizing custom algorithms or software that are central to the research but not yet described in published literature, software must be made available to editors/reviewers. We strongly encourage code deposition in a community repository (e.g. GitHub). See the Nature Research guidelines for submitting code & software for further information.

Data

Policy information about availability of data

All manuscripts must include a data availability statement. This statement should provide the following information, where applicable:

- Accession codes, unique identifiers, or web links for publicly available datasets
- A list of figures that have associated raw data
- A description of any restrictions on data availability

All genome assemblies, raw reads of transcriptomic and small RNA sequencing are available in NCBI BioProject under accession number PRJNA505074. All the materials in this study are available upon request.

Field-specific reporting

Please select the one below that is the best fit for your research. If you are not sure, read the appropriate sections before making your selection.

- [x] Life sciences
- [ ] Behavioural & social sciences
- [ ] Ecological, evolutionary & environmental sciences

For a reference copy of the document with all sections, see nature.com/documents/nr-reporting-summary-flat.pdf

Life sciences study design

All studies must disclose on these points even when the disclosure is negative.

Sample size	Genomic DNA was extracted from single individuals from the amakusa jellyfish S. malayensis and edible jellyfish R. esculentum. mRNA and small RNA were extracted from a range of tissues of different individuals.
Data exclusions	No data were excluded from analysis.
Replication	Genomic and most of the transcriptomic sequencing were not replicated (except the sRNA data of edible jellyfish n=2).
Randomization	The jellyfish samples were collected and provided by the Ocean Park Hong Kong and a local supplier in Hong Kong.
Blinding	Blinding was not relevant for this study

Reporting for specific materials, systems and methods

We require information from authors about some types of materials, experimental systems and methods used in many studies. Here, indicate whether each material, system or method listed is relevant to your study. If you are not sure if a list item applies to your research, read the appropriate section before selecting a response.

Materials & experimental systems

n/a	Involved in the study
☒	☐ Antibodies
☒	☐ Eukaryotic cell lines
☒	☐ Palaeontology
☐	☒ Animals and other organisms
☒	☐ Human research participants
☒	☐ Clinical data

Methods

n/a	Involved in the study
☒	☐ ChIP-seq
☒	☐ Flow cytometry
☒	☐ MRI-based neuroimaging

Animals and other organisms

Policy information about studies involving animals; ARRIVE guidelines recommended for reporting animal research

Laboratory animals	This study did not involve laboratory animals.
Wild animals	Amakusa jellyfish S. malayensis used in this study included wild caught individuals obtained from a local supplier in Hong Kong or provided by the Ocean Park Hong Kong. Edible jellyfish R. esculentum used in this study were wild caught and provided by a local supplier in Hong Kong.
Field-collected samples	Medusae of both species were cultured in circulating artificial seawater (salinity 30 ppt) at room temperature at The Chinese University of Hong Kong. Individuals of S. malayensis were not fed for several days after arrival in the laboratory before extracting DNA or RNA. Individuals of R. esculentum were fed once per week with newly hatched Artemia, and were starved for at least two days before extracting DNA or RNA.
Ethics oversight	Not applicable.

Note that full information on the approval of the study protocol must also be provided in the manuscript.
