# Supplementary material for: Jellyfish genomes reveal distinct homeobox gene clusters and conservation of small RNA processing
Source: Nat Commun. 2020 Jun 19;11:3051. doi: 10.1038/s41467-020-16801-9 (PMC7305137; doi:10.1038/s41467-020-16801-9)
Supplement: Supplementary file 4 — Description of Additional Supplementary Files [file 41467_2020_16801_MOESM4_ESM.pdf]

Description of Additional Supplementary Files

File Name: Supplementary Data 1
Description: MicroRNA structures in *Sanderia malayensis*.

File Name: Supplementary Data 2
Description: MicroRNA structures in *Rhopilema esculentum*.

File Name: Supplementary Data 3
Description: MicroRNA structures in *Aurelia*.

File Name: Supplementary Data 4
Description: MicroRNA expression data and sequences of the three jellyfish.
