# Supplementary material for: Jellyfish genomes reveal distinct homeobox gene clusters and conservation of small RNA processing
Source: Nat Commun. 2020 Jun 19;11:3051. doi: 10.1038/s41467-020-16801-9 (PMC7305137; doi:10.1038/s41467-020-16801-9)
Supplement: Supplementary file 6 — Supplementary Data 2 [file 41467_2020_16801_MOESM6_ESM.pdf]

Provisional ID	:	ScUbCFx_1_534
Score total	:	1.6
Score for star read(s)	:	-1.3
Score for read counts	:	0
Score for mfe	:	1.9
Score for randfold	:	1.6
Score for cons. seed	:	-0.6
Total read count	:	2248
Mature read count	:	2167
Loop read count	:	2
Star read count	:	79

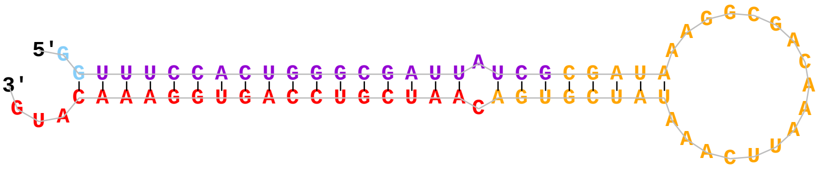

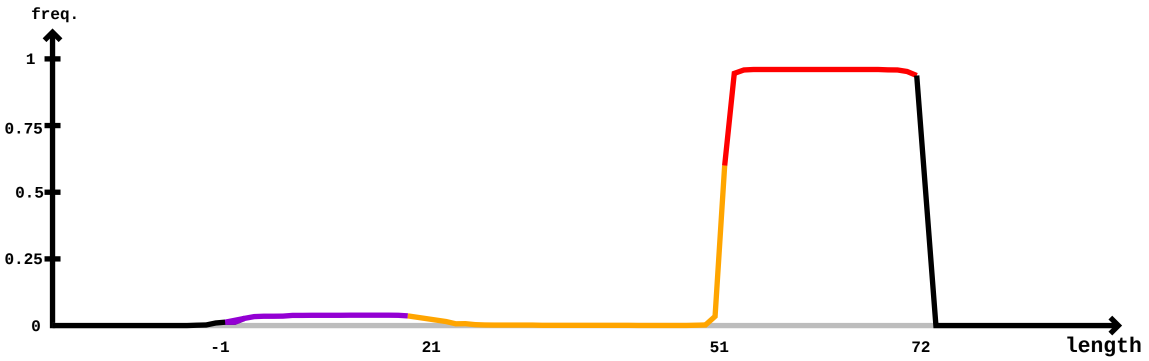

Star **Mature**

```
5'- aagauugcuaaagccugguuuccacugggcgauuaucgcgauaaaggcgacaaauucaaauaucgugacaaucguccaguggaaacaugauucaaucgugauuaucgcg  -3'   obs
    aagauugcuaaagccugguuuccacugggcgauuaucgcgauaaaggcgacaaauucaaauaucgugacaaucguccaguggaaacaugauucaaucgugauuaucgcg         exp
    ......((....))...(((((((((((((((((.((((((((.................)))))))).)))))))))))))))))..........((((.....))))  reads   mm   sample
    ...............uUguuuccacugggcgauuaucg.......................................................................  1       1    er2
    ..................uuuccacugggUgauuaucg.......................................................................  2       1    er2
    ..................uuuccacugggcgauuaucg.......................................................................  8       0    er2
    ..................uuuccacugggcgauuaucgcg.....................................................................  5       0    er2
    ...................uuccacugggcgauuaucgcg.....................................................................  5       0    er2
    ...................uuccacugggcgauuaucgcgaC...................................................................  5       1    er2
    .........................ugggcgauuaucgcgauaaaggcg............................................................  1       0    er2
    ..................................................................gaAaaucguccaguggaaac.......................  1       1    er2
    ...................................................................aAaaucguccaguggaaacaug....................  17      1    er2
    ...................................................................acaaucguccaguggaaacauA....................  2       1    er2
    ...................................................................acaaucguccaguggaaacauU....................  2       1    er2
    ....................................................................Aaaucguccaguggaaaca......................  1       1    er2
    ....................................................................caaucguccaguggaaacau.....................  4       0    er2
    ....................................................................caaucguccaguggaaacaug....................  171     0    er2
    ....................................................................Aaaucguccaguggaaacaug....................  78      1    er2
    ....................................................................caaucguccagAggaaacaug....................  1       1    er2
    ....................................................................caaucguccaguggaaacauA....................  54      1    er2
    ....................................................................caaucguccaguggaaacauU....................  3       1    er2
    ....................................................................caaucguccaAuggaaacaug....................  7       1    er2
    ....................................................................Naaucguccaguggaaacaug....................  1       1    er2
    .....................................................................aaucgGccaguggaaacau.....................  1       1    er2
    .....................................................................aaucguccaguggaaacauA....................  8       1    er2
    .....................................................................aaucgGccaguggaaacaug....................  107     1    er2
    .....................................................................aaucgAccaguggaaacaug....................  1       1    er2
    .....................................................................aaucguccaguggaaacaug....................  51      0    er2
    .....................................................................Naucguccaguggaaacaug....................  1       1    er2
    .....................................................................aaucguccaguggaaacauU....................  1       1    er2
    ......................................................................aucgGccaguggaaacaug....................  5       1    er2
    .......................................................................ucgGccaguggaaacaug....................  1       1    er2

    ...............uUguuuccacugggcgauu...........................................................................  1       1    ea1
    ...............ugguuuUcacugggcgauua..........................................................................  2       1    ea1
    ...............ugguuuUcacugggcgauuau.........................................................................  6       1    ea1
```

```
                  Star                                                Mature
aagauugcuaaagccugguuuccacugggcgauuaucgcgauaaaggcgacaaauucaaauaucgugacaaucguccaguggaaacaugauucaaucgugauuaucgcg
...............uUguuuccacugggcgauuauc........................................................................   1     1     ea1
................Uguuuccacugggcgauuauc........................................................................   5     1     ea1
..................uuuccacugggcgauuau.........................................................................   1     0     ea1
..................uuuccacugggcgauuauc........................................................................   1     0     ea1
..................uuuccacugggcgauuaucU.......................................................................   2     1     ea1
..................uuuccacugggcgauuaucg.......................................................................   5     0     ea1
..................Guuccacugggcgauuaucg.......................................................................   1     1     ea1
..................uuuccacugggcgauuaucA.......................................................................   1     1     ea1
..................Guuccacugggcgauuaucgcg.....................................................................   1     1     ea1
..................uuuccacugggcgauuaucgcg.....................................................................   2     0     ea1
...................uuccacugggcgauuauc........................................................................   1     0     ea1
...................uuccacugggcgauuaucgcg.....................................................................   2     0     ea1
....................uccacugggcgauuaucgcg.....................................................................   2     0     ea1
.......................acugggcgauuaucAcgaua..................................................................   1     1     ea1
.......................acugggcgauuaucgcgaua..................................................................   1     0     ea1
.......................acugggcCauuaucgcgaua..................................................................   1     1     ea1
.......................................Aauaaaggcgacaaauucaa..................................................   1     1     ea1
.................................................................ugacaaucguccaguggaaa........................   2     0     ea1
..................................................................gacaaucguccaguggaaaca......................   1     0     ea1
...................................................................acaaucguccaguggaaaca......................   3     0     ea1
...................................................................acaaucguccaguggaaacau.....................   2     0     ea1
...................................................................acaaucguccaguggaaacauA....................   4     1     ea1
...................................................................acaaucguccaguggaaacauU....................   1     1     ea1
....................................................................caaucgCccaguggaaaca......................   2     1     ea1
....................................................................caaucguccaguggaaaca......................   1     0     ea1
....................................................................caaucguccaguggaaacGu.....................   1     1     ea1
....................................................................caaucguccaguggaaacau.....................   15    0     ea1
....................................................................caaucguccagCggaaacaug....................   1     1     ea1
....................................................................Gaaucguccaguggaaacaug....................   1     1     ea1
....................................................................caaucguccaguggaGacaug....................   1     1     ea1
....................................................................cGaucguccaguggaaacaug....................   1     1     ea1
....................................................................caaucguccaguggaaacauU....................   18    1     ea1
....................................................................caaucguccaguggaaacauC....................   1     1     ea1
....................................................................caaucguccaguggUaacaug....................   1     1     ea1
....................................................................caaucguccaguggaaacaug....................   84    0     ea1
....................................................................caaucguccaguggaaacauA....................   109   1     ea1
....................................................................caaucguccaguAgaaacaug....................   1     1     ea1
.....................................................................aaucgGccaguggaaaca......................   1     1     ea1
.....................................................................aaucgGccaguggaaacau.....................   6     1     ea1
.....................................................................aaucguccaguggaaacauA....................   1     1     ea1
.....................................................................aaucgGccaguggaaacaug....................   79    1     ea1
.....................................................................aaucguccaguggaaacaug....................   1     0     ea1
......................................................................aucgGccaguggaaacaug....................   1     1     ea1

...................uuccacugggcgauuaucgcg.....................................................................   1     0     eg2
.........................................uaaaAgcgacaaauucaaaua...............................................   1     1     eg2
...................................................................acaaucguccaguggaaaca......................   2     0     eg2
....................................................................caaucguccaguggaaacauU....................   1     1     eg2
....................................................................caaucguccaguggaaacaug....................   18    0     eg2
....................................................................caaucguccaguggaaacauA....................   5     1     eg2
.....................................................................aaucgGccaguggaaacaug....................   2     1     eg2
.....................................................................aaucguccaguggaaacaug....................   4     0     eg2

...............ugguuuUcacugggcgauuau.........................................................................   3     1     ea2
..................uuuccacugggcgauuaucA.......................................................................   2     1     ea2
...................uuccacugggcgauuaucA.......................................................................   1     1     ea2
....................uccacugggcgauuaucgcg.....................................................................   1     0     ea2
.......................acugggcgauuaucAcgau...................................................................   3     1     ea2
.............................cgauuaucgcgauaaaggcg............................................................   1     0     ea2
..............................................................ucgugacaaucguccaguggaUa........................   1     1     ea2
...................................................................aAaaucguccaguggaaacaug....................   36    1     ea2
....................................................................caaucguccaguggaaacU......................   1     1     ea2
....................................................................caaucguccaguggaaacaug....................   445   0     ea2
....................................................................caaucguccaguggaaacauA....................   74    1     ea2
....................................................................Aaaucguccaguggaaacaug....................   82    1     ea2
....................................................................caaucguccaAuggaaacaug....................   12    1     ea2
....................................................................caaucguccaguggGaacaug....................   1     1     ea2
....................................................................Naaucguccaguggaaacaug....................   3     1     ea2
....................................................................caaucguccaguggaaacauU....................   16    1     ea2
....................................................................caaucguccaguggaaaAaug....................   1     1     ea2
```

Star **Mature**

```
aagauugcuaaagccugguuuccacugggcgauuaucgcgauaaaggcgacaaauucaaauaucgugacaaucguccaguggaaacaugauucaaucgugauuaucgcg
.....................................................................aaucguccaguggaaacauU....................    7      1      ea2
.....................................................................aaucguccaguggaaacaug....................    110    0      ea2
.....................................................................aaucguccaAuggaaacaug....................    5      1      ea2
.....................................................................aaucgGccaguggaaacaug....................    368    1      ea2
.....................................................................aaucguccaguggaaacauA....................    21     1      ea2
......................................................................aucgGccaguggaaacaug....................    23     1      ea2
.......................................................................ucgGccaguggaaacaug....................    3      1      ea2

.............Ucugguuuccacugggcgauua..........................................................................    3      1      er1
................Uguuuccacugggcgauuau.........................................................................    2      1      er1
.......................cacugggcgauuaucgcgauUa................................................................    1      1      er1
....................................................................caaucguccaguggaaacau.....................    1      0      er1
....................................................................caaucguccaguggaaacaug....................    4      0      er1
....................................................................caaucguccaguggaaacauA....................    1      1      er1
.....................................................................aaucgGccaguggaaacaug....................    2      1      er1

..............cuUguuuccacugggcgauuau.........................................................................    2      1      eg1
...............ugguuuUcacugggcgauuau.........................................................................    2      1      eg1
...............uUguuuccacugggcgauuauc........................................................................    1      1      eg1
..................uuuccacugggcgauuauc........................................................................    1      0      eg1
...................................................................acaaucguccaguggaaaca......................    1      0      eg1
...................................................................acaaucguccaguggaaacauU....................    1      1      eg1
...................................................................acaaucguccaguggaaacauA....................    1      1      eg1
...................................................................acaaucguccaguggaaacaug....................    1      0      eg1
....................................................................caaucguccaguggaaacau.....................    3      0      eg1
....................................................................caaucguccCguggaaacaug....................    1      1      eg1
....................................................................caaucguccaguggaaacauA....................    12     1      eg1
....................................................................caaAcguccaguggaaacaug....................    1      1      eg1
....................................................................caaucguccaguggaaacaug....................    30     0      eg1
....................................................................caaucguccaguggaaacauU....................    9      1      eg1
.....................................................................aaucgGccaguggaaacau.....................    1      1      eg1
.....................................................................aaucgGccaguggaaacaug....................    3      1      eg1
```

Provisional ID	:	ScUbCFx_4_1123
Score total	:	5.9
Score for star read(s)	:	-1.3
Score for read counts	:	0
Score for mfe	:	2.6
Score for randfold	:	1.6
Score for cons. seed	:	3
Total read count	:	2120
Mature read count	:	1537
Loop read count	:	0
Star read count	:	583

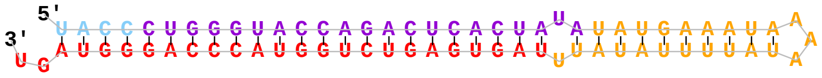

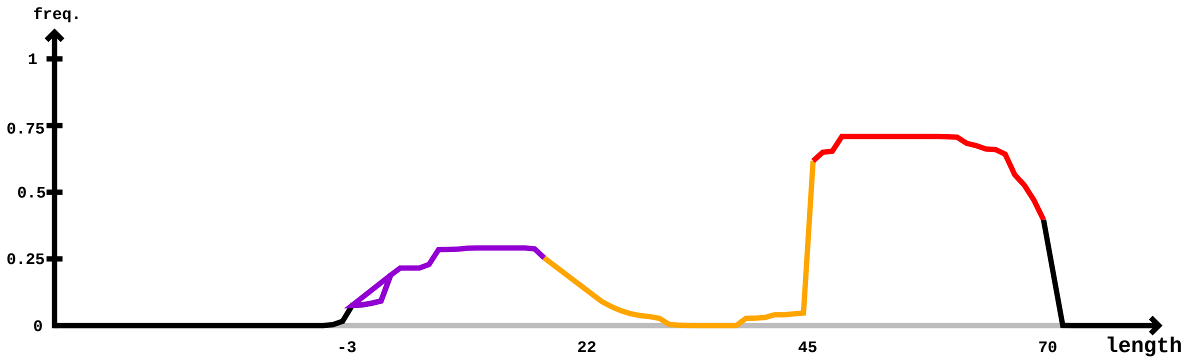

Star Mature

```
5'- agaaauacaaugggaagcccccucccucuacccuggguaccagacucacuauauaugaaauaaaauauuuuauauuuagugagucugguacccaggguagugucaggcuaaa   -3'   obs
    agaaauacaaugggaagcccccucccucuacccuggguaccagacucacuauauaugaaauaaaauauuuuauauuuagugagucugguacccaggguagugucaggcuaaa         exp
    ...........(((....)))(((..(.((((((((((((((((((((((((.((((((((((...)))))))))).))))))))))))))))))))))).)..))).....   reads   mm   sample
    ...........................cuacccuggguaccagacucU................................................................   6       1    eg2
    ...........................cuacccuggguaccagacucacu..............................................................   1       0    eg2
    ............................uacccugggCaccagacuca................................................................   1       1    eg2
    ............................uacccuggguaccagacucU................................................................   5       1    eg2
    ............................uacccuggguaccagacucC................................................................   1       1    eg2
    ............................uacccuggguaccagacuca................................................................   1       0    eg2
    ............................uacccugAguaccagacucacu..............................................................   1       1    eg2
    .............................acccuggguaccagacucU................................................................   2       1    eg2
    ...............................ccuggguaccagacucacuCu............................................................   2       1    eg2
    ................................cuggguaccagacucUcua.............................................................   2       1    eg2
    ................................cuggguaccagacucUcuau............................................................   6       1    eg2
    ................................cuCgguaccagacucacuaua...........................................................   1       1    eg2
    ................................cuggguaccagacucUcuaua...........................................................   15      1    eg2
    ................................Nuggguaccagacucacuaua...........................................................   1       1    eg2
    ................................cuggguaccagacucacuauaua.........................................................   2       0    eg2
    .................................uggguaccagacucUcuaua...........................................................   1       1    eg2
    .................................uggguacAagacucacuaua...........................................................   2       1    eg2
    ....................................guaccagacucacuauaua.........................................................   1       0    eg2
    ............................................................................uagugagucugguacccaggg...............   6       0    eg2
    .............................................................................agugagucugguaccUagg................   1       1    eg2
    .............................................................................agugagucugguacccaggg...............   1       0    eg2
    ...............................................................................ugagucugguGcccaggguag............   1       1    eg2
    ...............................................................................ugagucugguacccaggguaU............   1       1    eg2
    ...............................................................................ugagucugguacccaggguaUu...........   3       1    eg2

    ..........................uUuacccuggguaccagacuca................................................................   4       1    ea2
    ...........................cuacccuggguaccagacucC................................................................   2       1    ea2
    ...........................cuacccuggguaccagacuca................................................................   3       0    ea2
    ............................uacccuggguaccagacuc.................................................................   2       0    ea2
    ............................uacccuggguaccagacucC................................................................   4       1    ea2
    ............................uacccuggguaccagacucU................................................................   4       1    ea2
    ............................uacccuggguaccagacuca................................................................   5       0    ea2
    ............................uacccuUgguaccagacuca................................................................   1       1    ea2
```

Star **Mature**

agaaauacaaugggaagccccccucccucuacccuggguaccagacucacuauauaugaaauaaaaauauuuuauauuuagugagucugguacccaggguagugucaggcuaaa			
.............................uacccuggAuaccagacucacu...	3	1	ea2
...............................cccuggguaccagacucacA...	2	1	ea2
...............................cccuggAuaccagacucacua..	1	1	ea2
................................ccuggguaccagacucUcu...	1	1	ea2
.................................cuggguaccagacucacuau...	5	0	ea2
.................................cuggguaccagacucacuaua..	21	0	ea2
.................................cuggguaccagacucUcuaua..	1	1	ea2
.................................cuggguaccagacucacuCua..	1	1	ea2
.................................cuggguaccagacucacuauauU..	2	1	ea2
.................................cuggguaccagacucacuauaua..	6	0	ea2
..................................uggguaccagacucacuaua..	3	0	ea2
..................................uggguaccagacucacuCuaua..	1	1	ea2
..................................uggguaccagacucacuauaua..	6	0	ea2
.....................................guaccagacucacuauauaug..	2	0	ea2
......................................uaccagacucacuCuauau...	9	1	ea2
....uauauGagugagucugguaccc...................	1	1	ea2
....uuuagugagucugguacccU..................	3	1	ea2
....uagugagucugguaccca..................	2	0	ea2
....uagugagucugguacccag.................	4	0	ea2
....uagugagucugguacccagg................	1	0	ea2
....Nagugagucugguacccaggg...............	1	1	ea2
....uagugagucugguacccaggg...............	15	0	ea2
....uagugagucugguacccaggAua.............	1	1	ea2
....uagugagCcugguacccagggua.............	1	1	ea2
....uagugagucugguacccagggua.............	7	0	ea2
....uagugagucugguacccaggguG.............	13	1	ea2
....uagugagucugguacccagggCa.............	3	1	ea2
....uagugagucugguacccaggguaU............	4	1	ea2
....uagugagucugguacccaggguaUu...........	14	1	ea2
....uagugagucugguacccaggguaAu...........	6	1	ea2
....agugagucugguacccaggguaUu...........	4	1	ea2
....ugagucugguacccaggg...............	2	0	ea2
....ugagucugguacccaggguaU............	2	1	ea2
....ugagucugguacccaggguag............	2	0	ea2
....ugagucugguacccaggguaUu...........	27	1	ea2
...........................uUuacccuggguaccagacuca...	1	1	er2
............................cuacccuggguaccagacuca...	1	0	er2
............................cuacccuggguaccagacucacu...	3	0	er2
.............................uacccuUgguaccagacuca...	2	1	er2
.............................uacccuggguaccagacuca...	7	0	er2
.............................uacccuggguaccagacucaA..	2	1	er2
.............................uacccuggguaccagacucacu...	5	0	er2
.............................uacccuggguaccagacucacA...	1	1	er2
.............................uacccuggguaccagacucacuauaua..	1	0	er2
..............................acccuggguaccagacuca...	1	0	er2
...............................cccuggguaccagacucacuaCa..	1	1	er2
................................ccuggAuaccagacucacuaua..	2	1	er2
................................ccuggguaccagacucacuaua..	1	0	er2
................................ccuggguaccagacucacuaCa..	2	1	er2
................................ccCggguaccagacucacuauaua..	1	1	er2
.................................cuggguaccagacucacuau...	2	0	er2
.................................cuUgguaccagacucacuaua..	1	1	er2
.................................cugggGaccagacucacuaua..	1	1	er2
.................................cuggguaccagacucacuaua..	22	0	er2
.................................cuggguaccagacucacuCuau...	2	1	er2
.................................cuUgguaccagacucacuauaua..	1	1	er2
.................................cuggguaccagacucacuauaua..	9	0	er2
..................................uggguaccagacucacuaua..	10	0	er2
..................................uggguaccagacucacuauaua..	8	0	er2
..................................uUgguaccagacucacuauaua..	1	1	er2
......................................uaccagacucacuauauaug..	4	0	er2
....agacucacuauauauUaaau..	2	1	er2
....Nuagugagucugguacccaggg...............	1	1	er2
....uagugagucugguaccca..................	1	0	er2
....uagugagucugguacccU..................	3	1	er2
....uagugagucugguacccagg................	22	0	er2
....uagugagucugguacccaggg...............	108	0	er2
....Nagugagucugguacccagggu..............	1	1	er2
....uagugagucugguacccagggC..............	1	1	er2

Star Mature

```
agaaauacaaugggaagccccccucccucuacccuggguaccagacucacuauauaugaaauaaaauauuuuauauuuagugagucugguacccaggguagugucaggcuaaa
.............................................................................uagugagucugguacccagggu..............    71    0    er2
.............................................................................uagugagucugguacccaggguG.............    3     1    er2
.............................................................................uagugagucugguacccaggguU.............    21    1    er2
.............................................................................uagugagucugguacccaggAua.............    1     1    er2
.............................................................................Nagugagucugguacccagggua.............    1     1    er2
.............................................................................uagugagucugguacccagggua.............    34    0    er2
.............................................................................uagugagucugguacccaggguC.............    4     1    er2
.............................................................................uagugagucugguacccaggguaU............    124   1    er2
.............................................................................uagugagucugguacccaggguaUu...........    758   1    er2
.............................................................................uagugagucugguacccaggguaAu...........    3     1    er2
..............................................................................agugagucugguacccaggg...............    2     0    er2
..............................................................................agugagucugguacccagggu..............    4     0    er2
..............................................................................agugagucugguacccaggguC.............    2     1    er2
..............................................................................agugagucugguacccaggguU.............    2     1    er2
..............................................................................agugagucugguacccagggua.............    6     0    er2
..............................................................................agugagucugguacccaggguaU............    8     1    er2
..............................................................................agugagucugguacccaggguaUu...........    31    1    er2
...............................................................................gugagucugguacccaggg...............    2     0    er2
...............................................................................gugagucugguacccaggguaUu...........    1     1    er2
................................................................................ugagucugguacccaggguG.............    3     1    er2
................................................................................ugagucugguacccaggguag............    3     0    er2
................................................................................ugagucugguacccaggguaUu...........    28    1    er2

...........................ucuacccuggguaccagacuc.................................................................    1     0    ea1
...........................ucuacccuggguaccagacucU................................................................    1     1    ea1
...........................ucuacccuUgguaccagacucac...............................................................    1     1    ea1
............................cuacccuggguaccagacuA.................................................................    1     1    ea1
............................cuacccuAgguaccagacuca................................................................    2     1    ea1
............................cuacccuggguaccagacucU................................................................    2     1    ea1
............................cuacccuggguaccagacucGc...............................................................    2     1    ea1
............................cuacccuggguaccagacucaA...............................................................    1     1    ea1
............................cuaccUuggguaccagacucac...............................................................    1     1    ea1
............................cuacccuggguaccagacucUc...............................................................    1     1    ea1
............................cuacccuggguaccagacucacA..............................................................    1     1    ea1
............................cuacccuggguaccagacucGcuau............................................................    1     1    ea1
.............................uacccuggguaccagacuc.................................................................    3     0    ea1
.............................uacccuggguaccagacuca................................................................    4     0    ea1
.............................uacccuggguaccagacucC................................................................    6     1    ea1
.............................uacccuggguaccagacucU................................................................    12    1    ea1
.............................uacccuggguaccagacucUc...............................................................    7     1    ea1
.............................uacccuggguaccagacucac...............................................................    4     0    ea1
.............................uGcccuggguaccagacucac...............................................................    1     1    ea1
.............................uacccuggguaccagacucacu..............................................................    7     0    ea1
.............................uacccuAgguaccagacucacu..............................................................    3     1    ea1
.............................uacccuggguaccagacucUcu..............................................................    21    1    ea1
.............................uUcccuggguaccagacucacu..............................................................    1     1    ea1
.............................uacccuggguaccagacucacC..............................................................    2     1    ea1
.............................uacccuggguaccagacucUcua.............................................................    3     1    ea1
.............................uUcccuggguaccagacucacua.............................................................    1     1    ea1
.............................uacccuggguaccagacucUcuau............................................................    3     1    ea1
..............................Ucccuggguaccagacucacu..............................................................    1     1    ea1
...............................cccuggguaccagacucacu..............................................................    2     0    ea1
...............................cccuggguGccagacucacu..............................................................    1     1    ea1
...............................cccuggguaccagacucacua.............................................................    1     0    ea1
...............................cccuggguaccagacucacuau............................................................    1     0    ea1
...............................cccuggguaccagacucGcuau............................................................    1     1    ea1
...............................cccuggguaccagacucacuaCa...........................................................    1     1    ea1
................................ccuggguaccagacucacua.............................................................    2     0    ea1
................................ccuUgguaccagacucacuau............................................................    1     1    ea1
................................ccuggguaccagacucacuaCa...........................................................    2     1    ea1
................................ccuggguaccagacucacuauC...........................................................    1     1    ea1
................................Acuggguaccagacucacuaua...........................................................    1     1    ea1
................................ccuggguaccagacucacuaua...........................................................    1     0    ea1
.................................cuggguaccagacucUcu..............................................................    5     1    ea1
.................................cuggguaccagacucUcua.............................................................    2     1    ea1
.................................cuggguaccagacucacuau............................................................    7     0    ea1
.................................cuggguaccagacucUcuau............................................................    28    1    ea1
.................................cuggguaccagacucCcuaua...........................................................    1     1    ea1
.................................cuggguaccagacucUcuaua...........................................................    40    1    ea1
.................................cuggguaccagacucacuaua...........................................................    8     0    ea1
```

```
                                 Star                                        Mature
agaaauacaaugggaagccccccucccucuacccuggguaccagacucacuauauaugaaauaaaauauuuuauauuuagugagucugguacccaggguagugucaggcuaaa
.................................cuggguaccagacucacuauG...........................................................    2    1    ea1
.................................cuggguaccagacucacuauU...........................................................    1    1    ea1
.................................cuAgguaccagacucacuaua...........................................................    2    1    ea1
.................................cuggguaccagacucUcuauau..........................................................    1    1    ea1
.................................cuggguaccagacucacuauau..........................................................    1    0    ea1
.................................cuggguaccagacucacuauauau........................................................    1    0    ea1
..................................uggguaccagacucUcuau............................................................    2    1    ea1
..................................uggguaccagacucUcuaua...........................................................    15   1    ea1
..................................uggguaccagacucacuauau..........................................................    4    0    ea1
..................................uggguaccagacucacuaGau..........................................................    1    1    ea1
..................................uggguaccagacucacuaCaua.........................................................    1    1    ea1
..................................Gggguaccagacucacuauaua.........................................................    1    1    ea1
.....................................guaccagacucacuauau..........................................................    2    0    ea1
.....................................Auaccagacucacuauau..........................................................    3    1    ea1
.....................................guaccagacucGcuauau..........................................................    1    1    ea1
.....................................Auaccagacucacuauaua.........................................................    2    1    ea1
.....................................Auaccagacucacuauauau........................................................    3    1    ea1
.....................................guaccagacucacuauauaC........................................................    3    1    ea1
.....................................guaccagacucacuauauau........................................................    6    0    ea1
.....................................guaccagacucacuauauaug.......................................................    2    0    ea1
.....................................gCaccagacucacuauauaug.......................................................    1    1    ea1
......................................uaccagacucacuauaua.........................................................    1    0    ea1
......................................uaccagacucacuCuauau........................................................    1    1    ea1
......................................uaccagacucacuaAauau........................................................    2    1    ea1
......................................uaccagacucacuauauau........................................................    4    0    ea1
......................................uaccagacucGcuauauau........................................................    1    1    ea1
......................................uaccagacucacuauauaC........................................................    1    1    ea1
......................................uaccGgacucacuauauaug.......................................................    1    1    ea1
......................................uaccagacucacuauauaug.......................................................    11   0    ea1
......................................uacGagacucacuauauaug.......................................................    2    1    ea1
......................................uaccagacucacuauauauga......................................................    10   0    ea1
......................................uaccagacucacuauauaugU......................................................    1    1    ea1
......................................uaccagacucacuauUuauga......................................................    2    1    ea1
......................................uaccagacucacuCuauaugaa.....................................................    1    1    ea1
......................................uaccagacucacuauauaugaa.....................................................    6    0    ea1
......................................uaccagacucacuauauaugaaa....................................................    3    0    ea1
......................................Gaccagacucacuauauaugaaau...................................................    1    1    ea1
......................................uaccagacucacuauauaugaaau...................................................    17   0    ea1
......................................uaccagacucacuauauaugaaaC...................................................    2    1    ea1
......................................uaccagacucacuauauaugaaauU..................................................    1    1    ea1
......................................uaccagacucacuauauaugaaauUa.................................................    2    1    ea1
........................................cGagacucacuauauaug.......................................................    1    1    ea1
........................................ccagacucacuauauaugaaU....................................................    1    1    ea1
.........................................cagacucacuauauaugaaa....................................................    1    0    ea1
.........................................cGgacucacuauauaugaaa....................................................    1    1    ea1
.........................................cagacucacuauauaugaaau...................................................    1    0    ea1
.........................................cagacucacuauauaugaaaAa..................................................    1    1    ea1
.........................................cagacucacuauauaugaaauaaa................................................    1    0    ea1
......................................................................uuauauuuagugagucuggua......................    1    0    ea1
......................................................................Guauauuuagugagucuggua......................    1    1    ea1
......................................................................uuauauuuagugagucugguUc.....................    1    1    ea1
......................................................................uuauauGuagugagucugguacc....................    1    1    ea1
......................................................................uuauauuuagugagucugguacU....................    7    1    ea1
......................................................................Guauauuuagugagucugguacc....................    9    1    ea1
......................................................................uuauauuuagugagucugguacc....................    23   0    ea1
......................................................................uuauauuuGgugagucugguacc....................    1    1    ea1
......................................................................uuauauuuagugagucugguaccU...................    7    1    ea1
......................................................................uuauauuuagugagucugguaccA...................    3    1    ea1
.......................................................................uauauuuagugagucugguacc....................    1    0    ea1
........................................................................auauGuagugagucugguac.....................    2    1    ea1
........................................................................auaAuuagugagucugguacc....................    2    1    ea1
........................................................................auauGuagugagucugguaccc...................    1    1    ea1
........................................................................auaAuuagugagucugguaccca..................    1    1    ea1
.........................................................................uauGuagugagucugguacc....................    2    1    ea1
.........................................................................uauuuagugagucugguaccc...................    3    0    ea1
.........................................................................uauuuagugagucugguacccU..................    11   1    ea1
.........................................................................uauuuagugagucugguacccC..................    1    1    ea1
...........................................................................uuuagugagucugguacccC..................    2    1    ea1
...........................................................................uuuagugagucugguacccU..................    1    1    ea1
...........................................................................uGuagugagucugguacccaggg...............    1    1    ea1
```

```
                                   Star                                         Mature
agaaauacaaugggaagcccccucccucuacccuggguaccagacucacuauauaugaaauaaaaauauuuuauauuuagugagucugguacccaggguagugucaggcuaaa
...........................................................................uuuagugagucugguacccaggg...............   1   0   ea1
............................................................................Guagugagucugguacccaggg...............   5   1   ea1
............................................................................uuagugagucAgguacccagggu..............   1   1   ea1
.............................................................................uagugagucugguaccca..................   1   0   ea1
.............................................................................uagAgagucugguaccca..................   2   1   ea1
.............................................................................uagugagucugguaccUag.................   1   1   ea1
.............................................................................uagugagGcugguacccagg................   1   1   ea1
.............................................................................uagugagucugguaccUagg................   11  1   ea1
.............................................................................uagAgagucugguacccagg................   1   1   ea1
.............................................................................uagugagucugguacccagU................   1   1   ea1
.............................................................................uagugagucugguacccaggg...............   14  0   ea1
.............................................................................uagugagucugguacccaggA...............   2   1   ea1
.............................................................................uagugagucugguacccaggU...............   2   1   ea1
.............................................................................uUgugagucugguacccaggg...............   1   1   ea1
.............................................................................uagugagucugguaccUaggg...............   4   1   ea1
.............................................................................Gagugagucugguacccaggg...............   2   1   ea1
.............................................................................Gagugagucugguacccagggu..............   1   1   ea1
.............................................................................uagugagucugguacccagggu..............   2   0   ea1
.............................................................................uagugagucugguacccagggC..............   4   1   ea1
.............................................................................uagugagucugguacccaggguU.............   2   1   ea1
.............................................................................Gagugagucugguacccagggua.............   1   1   ea1
.............................................................................uagugagucugguacccagggua.............   6   0   ea1
.............................................................................uagugagucugguacccaggguag............   1   0   ea1
.............................................................................uagugagucugguacccaggguaUu...........   4   1   ea1
.............................................................................uagugagucugguacccaggguaguC..........   1   1   ea1
..............................................................................agugagucugguacccagg................   1   0   ea1
..............................................................................agugagucugguacccaggAu..............   1   1   ea1
..............................................................................agugagucugguacccagggC..............   1   1   ea1
..............................................................................agugagucugguacccagggua.............   3   0   ea1
..............................................................................agugagucugguacccaggguG.............   1   1   ea1
..............................................................................agugagucugguacccaAgguag............   1   1   ea1
..............................................................................agugagucugguacccaggguag............   2   0   ea1
..............................................................................agugagucugguacccaggguaUu...........   2   1   ea1
...............................................................................gugagucugguacccaggg...............   1   0   ea1
...............................................................................gugagucugguacccaggguU.............   3   1   ea1
...............................................................................gugagucugguacccagggua.............   1   0   ea1
...............................................................................gugagucugguGcccagggua.............   1   1   ea1
................................................................................ugagucugguacccagggA..............   1   1   ea1
................................................................................ugagucugguacccagggua.............   3   0   ea1
................................................................................ugagucugguacccaggguag............   3   0   ea1
................................................................................ugagucugguacccaAgguag............   1   1   ea1
................................................................................ugagucugguacccaggguaU............   10  1   ea1
................................................................................ugagucuggCacccaggguag............   1   1   ea1
................................................................................ugagucugguacccaggguagu...........   1   0   ea1
................................................................................ugagucugguacccaggguaUu...........   25  1   ea1
................................................................................ugagucugguacccaggguaguUu.........   1   1   ea1

...........................cuacccuggguaccagacuc..................................................................   1   0   eg1
............................uacccuggguaccagacucaA................................................................   1   1   eg1
............................uacccuggguaccagacucacu...............................................................   2   0   eg1
............................uacccuggguaccagacucUcu...............................................................   1   1   eg1
............................uacccugggAaccagacucacu...............................................................   2   1   eg1
............................uacccuggguaccagacucUcua..............................................................   2   1   eg1
............................uacccuggguaccagacucUcuau.............................................................   1   1   eg1
..............................cccuggguaccagacucacu...............................................................   1   0   eg1
..............................cccuggguaccagacucUcua..............................................................   1   1   eg1
..............................cccuggguaccagacucacuaC.............................................................   1   1   eg1
...............................ccuggguaccagacucacuau.............................................................   1   0   eg1
...............................ccuggguaccagacucacuauC............................................................   1   1   eg1
................................cuggguaccagacucUcu...............................................................   1   1   eg1
................................cuggguaccagacucacua..............................................................   1   0   eg1
................................cuCgguaccagacucacuau.............................................................   1   1   eg1
................................cuggguaccagacucacuau.............................................................   1   0   eg1
................................cuggguaccagacucUcuau.............................................................   2   1   eg1
................................cuggguaccagacucUcuaua............................................................   7   1   eg1
................................cuggguaccagacucacuaua............................................................   1   0   eg1
.................................uggguaccagacucUcuau.............................................................   1   1   eg1
.................................uggguaccagacucacuaua............................................................   2   0   eg1
.................................ugggAaccagacucacuauau...........................................................   1   1   eg1
....................................guaccagacucacuauauau.........................................................   2   0   eg1
```

Star Mature

```
agaaauacaaugggaagccccccucccucuacccuggguaccagacucacuauauaugaaauaaaauauuuuauauuuagugagucugguacccaggguagugucaggcuaaa
.....................................guaccagacucacuauauaugaaa....................................................   2   0   eg1
......................................uaccagacucacuauauaC........................................................   1   1   eg1
......................................uacGagacucacuauauaug.......................................................   2   1   eg1
......................................uaccagacucacuauauauga......................................................   2   0   eg1
......................................uaccagacucacuauauaugaa.....................................................   1   0   eg1
......................................uaccagacucacuauauaugaaa....................................................   3   0   eg1
......................................uaccagacucacuauauaugaaU....................................................   1   1   eg1
......................................uaccagacucacuauauaugaaau...................................................   2   0   eg1
......................................Gaccagacucacuauauaugaaau...................................................   1   1   eg1
......................................uaccagacucacuauauaugaaaC...................................................   2   1   eg1
.......................................accagacucacuauauaugaaa....................................................   1   0   eg1
.........................................cagacucacuauauaugaaau...................................................   1   0   eg1
.........................................cagacucacuauauaugaaaAa..................................................   2   1   eg1
......................................................................uuauauuuagugagucugguac.....................   1   0   eg1
......................................................................Guauauuuagugagucugguacc....................   1   1   eg1
......................................................................uuauauuuagugagucugguacc....................   1   0   eg1
.........................................................................uauuuagugagucugguacccU..................   1   1   eg1
.............................................................................uagugagucugguGcccaggg...............   1   1   eg1
.............................................................................uagugagucugguacccaggg...............   3   0   eg1
.............................................................................uagugagucugguacccagggCa.............   1   1   eg1
.............................................................................uagugagucugguacccaggguG.............   1   1   eg1
.............................................................................uagugagucugguacccagggua.............   2   0   eg1
..............................................................................agugagucugguacccaggg...............   1   0   eg1
..............................................................................agugagucugguacccagggua.............   1   0   eg1
................................................................................ugagCcugguacccagggu..............   1   1   eg1
................................................................................ugagucugguacccaggguaU............   2   1   eg1
................................................................................ugagucugguacccaggguag............   3   0   eg1
................................................................................ugagucugguacccaggguaUu...........   2   1   eg1

.............................uacccuAgguaccagacucacu..............................................................   1   1   er1
.............................uacccuggguacGagacucacu..............................................................   1   1   er1
.............................uacccuggguaccagacucUcu..............................................................   1   1   er1
.............................uacccuggguaccagacucUcua.............................................................   1   1   er1
................................ccuggguaccagacucacuauG...........................................................   1   1   er1
.................................cuggguaccagacucUcu..............................................................   1   1   er1
.................................cuggguaccagacucacuau............................................................   1   0   er1
.................................cuggguaccagacucacuaua...........................................................   1   0   er1
.................................cuggguaccagacucUcuaua...........................................................   5   1   er1
..................................uggguaccagacucUcuaua...........................................................   1   1   er1
..................................uggguaccagacucacuaua...........................................................   1   0   er1
.....................................Auaccagacucacuauau..........................................................   1   1   er1
......................................uaccagacucacuauauau........................................................   1   0   er1
......................................uaccagacucacuauauauga......................................................   1   0   er1
......................................uaccagacucacuauauaugaU.....................................................   1   1   er1
......................................Gaccagacucacuauauaugaaa....................................................   1   1   er1
......................................uGccagacucacuauauaugaaa....................................................   1   1   er1
......................................uaccagacucacuauauaugaaaC...................................................   2   1   er1
......................................Gaccagacucacuauauaugaaau...................................................   1   1   er1
......................................uaccagacucacuauauaCgaaau...................................................   1   1   er1
......................................uaccagacucacuauauaugaaau...................................................  18   0   er1
......................................uaccagacucacuauauaugaaaua..................................................   1   0   er1
......................................uaccagacucacuauauaugaaauU..................................................   2   1   er1
........................................ccagacucacuauauaugaaUu...................................................   1   1   er1
......................................................................uuauauuuagugagucugguacA....................   1   1   er1
......................................................................uuauauuuagugagucugguacc....................   2   0   er1
......................................................................Guauauuuagugagucugguacc....................   2   1   er1
.........................................................................uauuuagugagucugguaccA...................   3   1   er1
.........................................................................uauuuagugagucugguaccc...................   2   0   er1
.............................................................................uagugagucugguacccaggg...............   2   0   er1
.............................................................................uagugagucugguacccagggu..............   1   0   er1
.............................................................................uagugagucugguacccagggua.............   1   0   er1
................................................................................ugagucugguacccaggguaUu...........   1   1   er1
```

Provisional ID	:	ScUbCFx_4_1173
Score total	:	3831.4
Score for star read(s)	:	3.9
Score for read counts	:	3821.3
Score for mfe	:	1.6
Score for randfold	:	1.6
Score for cons. seed	:	3
Total read count	:	7507
Mature read count	:	7479
Loop read count	:	0
Star read count	:	28

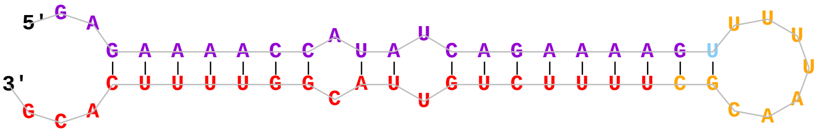

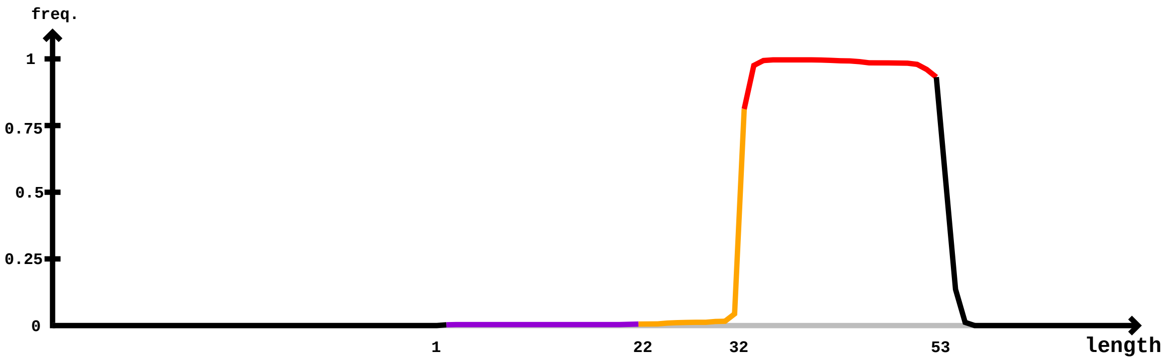


```
                                    Star                          Mature
5'- aggccccugccauucgaaaaaugcaacaucuaagugucgagaaaaccauaucagaaaaguuuuuaacgcuuuucuguuacgguuuucacgacacuugugaguaucuaaaa  -3'  obs
    aggccccugccauucgaaaaaugcaacaucuaagugucgagaaaaccauaucagaaaaguuuuuaacgcuuuucuguuacgguuuucacgacacuugugaguaucuaaaa       exp
    .(((.....))).............((.(((((((((((.(((((((.((.(((((((((.......))))))))).)).))))))).))))))))).))))........   reads   mm   sample
    ......................................gagaaaaccauaucagaaaagu..................................................   1       0    eg1
    ...........................................................uuuuuaacgcuuuucuguua...............................   1       0    eg1
    ..................................................................cgcuuuucuguuacgguuuu........................   1       0    eg1
    ..................................................................cgcuuuucuguuacgguuuuca......................   1       0    eg1
    ....................................................................cuuuucuguuacgguuuuca......................   1       0    eg1
    ....................................................................cuuuucuguuacgguuuucacg....................   1       0    eg1
    ....................................................................cuuuucuguuacgguuuucacgG...................   1       1    eg1
    .....................................................................uuuucuguuaUgguuuuca......................   1       1    eg1
    .....................................................................uuuucuguuacgguuuuca......................   3       0    eg1
    .....................................................................uuuucuguuacgguuuucaU.....................   1       1    eg1
    .....................................................................uuuucuguuacgguuuucac.....................   12      0    eg1
    .....................................................................uuuucuguuacgguuuucaG.....................   1       1    eg1
    .....................................................................uuuucuguuacgguuuucacA....................   15      1    eg1
    .....................................................................Guuucuguuacgguuuucacg....................   2       1    eg1
    .....................................................................uuuucuguuacgguCuucacg....................   1       1    eg1
    .....................................................................uuuucuguuacgguuuucacU....................   16      1    eg1
    .....................................................................uuuucugCuacgguuuucacg....................   1       1    eg1
    .....................................................................uuuucuguuacgguuuucacg....................   84      0    eg1
    .....................................................................uuuucuguuacgguuuucacgC...................   2       1    eg1
    .....................................................................uuuucuguuacgguuuucacgG...................   6       1    eg1
    .....................................................................uuuucuguuacgguuCucacga...................   1       1    eg1
    .....................................................................uuuucuguuacgguuuucacgU...................   42      1    eg1
    .....................................................................uuuucuguuacgguuuucacga...................   51      0    eg1
    .....................................................................uuuucuguuacgguuuCcacga...................   1       1    eg1
    .....................................................................uuuucuguuacgguuuucacgaA..................   21      1    eg1
    .....................................................................Cuuucuguuacgguuuucacgac..................   1       1    eg1
    .....................................................................uuuucuguuacgguuuucacgac..................   3       0    eg1
    .....................................................................uuuucuguuacgguuuucacgaU..................   9       1    eg1
    .....................................................................uuuucuguuacgguuuucacgaUa.................   5       1    eg1
    ......................................................................Guucuguuacgguuuucacg....................   1       1    eg1
    ......................................................................uuucuguuacgguuuucacA....................   1       1    eg1
    ......................................................................uuucuguuacgguuuucacU....................   1       1    eg1
    ......................................................................uuucuguuacgguuuucacg....................   3       0    eg1
```

Star **Mature**

```
aggcccccugccauucgaaaaaugcaacaucuaagugucgagaaaaccauaucagaaaaguuuuuaacgcuuuucuguuacgguuuucacgacacuugugaguaucuaaaa
.......................................................................uuucuguuacgguuuucacgG...................   1    1    eg1
.......................................................................uuucuguuacgguuuucacga...................   2    0    eg1
.......................................................................uuucuguuacgguuuucacgC...................   4    1    eg1
.......................................................................uuucuguuacgguuuucacgU...................   9    1    eg1
.......................................................................uuucuguuacgguuuucacgUc..................   1    1    eg1
.......................................................................uuucuguuacgguuuucacgaU..................   6    1    eg1
.......................................................................uuucuguuacgguuuucacgCc..................   1    1    eg1
.......................................................................uuucuguuacgguuuucacgaA..................   7    1    eg1
.......................................................................uuucuguuacgguuuucacgacC.................   1    1    eg1
.......................................................................uuucuguuacgguuuucacgaUa.................   1    1    eg1
........................................................................uucuguuacgguuuucacga...................   1    0    eg1
........................................................................uucuguuacgguuuucacgac..................   1    0    eg1
........................................................................uucuguuacgguuuucacgaA..................   1    1    eg1
........................................................................uucuguuacgguuuucacgaUa.................   1    1    eg1

.....................................................................cuuuucuguuacgguuuucacg....................   2    0    er1
.....................................................................cuuuucuguuacgguuuucacU....................   1    1    er1
......................................................................uuuucuguuacgguuuuca......................   2    0    er1
......................................................................uuuucuguuacgguuuucaU.....................   1    1    er1
......................................................................uuuucuguuacgguuuucac.....................   3    0    er1
......................................................................uuuucuguuacgguuuucacU....................   1    1    er1
......................................................................Cuuucuguuacgguuuucacg....................   1    1    er1
......................................................................Guuucuguuacgguuuucacg....................   2    1    er1
......................................................................uuuucuguuacgguuuucacA....................   2    1    er1
......................................................................uuuucuguuacgguuuucacC....................   1    1    er1
......................................................................uuuucuguuacggCuuucacg....................   1    1    er1
......................................................................uuuucuguuacgguuuucacg....................   45   0    er1
......................................................................uuuucuguuacgguuuucacgU...................   8    1    er1
......................................................................uuuucuguuacgguuuucacgC...................   2    1    er1
......................................................................uuuucuguuacgguuuucacUa...................   1    1    er1
......................................................................uuuucuguuacgguuuucacga...................   25   0    er1
......................................................................uuuucuguuacgguuuucacgac..................   1    0    er1
......................................................................uuuucuguuacgguuuucacgCc..................   1    1    er1
......................................................................uuuucuguuacgguuuucacgaA..................   11   1    er1
......................................................................uuuucuguuacgguuuucacgaU..................   5    1    er1
......................................................................uuuucuguuacgguuuucacgaUa.................   2    1    er1
.......................................................................uuucuguuacgguuuucacgU...................   2    1    er1
.......................................................................uuucuguuacgguuuucacga...................   2    0    er1
.......................................................................uuucuguuacgguuuucacgC...................   1    1    er1
.......................................................................uuucuguuacgguuuucacgaU..................   6    1    er1
.......................................................................uuucuguuacgguuuucacgac..................   2    0    er1
.......................................................................uuucuguuacgguuuucacgCc..................   1    1    er1
........................................................................uucuguuacgguuuucacgaA..................   1    1    er1

.......................................gagaaaaccauaucagaaaag...................................................   3    0    ea1
..........................................................aguuuuuaacgcuuuucuguA................................   1    1    ea1
............................................................uuuuuaacgcuuuucugu.................................   1    0    ea1
............................................................uuuuuaacgcuuuucuguA................................   1    1    ea1
............................................................uuuuuaacgcuuuucuguuA...............................   1    1    ea1
............................................................uuuuuaacgcuuuucuguuCcg.............................   1    1    ea1
.............................................................uuuuaacgcuuuucuguuac..............................   1    0    ea1
.............................................................uuuuaacgcuuuucuguuacA.............................   1    1    ea1
..............................................................uuuaacgcuuuucuguuacA.............................   1    1    ea1
..............................................................Guuaacgcuuuucuguuacgg............................   1    1    ea1
..............................................................uuuaacgcuuuucuguuacgguA..........................   1    1    ea1
...............................................................uuaacgcuuuucuguuac..............................   1    0    ea1
...............................................................uuaacgcuuuucuguuacgg............................   1    0    ea1
...............................................................uuaacgcuuuucuguuacgA............................   1    1    ea1
................................................................uaacgcuuuucuguuacgg............................   1    0    ea1
.................................................................aacgcuuuucuguuacgguuA.........................   1    1    ea1
...................................................................cgcuuuucuguuacgguuuu........................   1    0    ea1
...................................................................cgcuuuucuguuacgguuuuA.......................   1    1    ea1
.....................................................................cuuuucuguuacgguuuuc.......................   1    0    ea1
.....................................................................cuuuucuguuacgguuuucC......................   1    1    ea1
.....................................................................cuuuucuguuacgguuuuca......................   7    0    ea1
.....................................................................cuuuucuguuacgguuuucac.....................   1    0    ea1
.....................................................................cuuuucuguuacgguuuucacC....................   2    1    ea1
.....................................................................Guuuucuguuacgguuuucacg....................   1    1    ea1
.....................................................................cuuuucuguuacgguuuucacU....................   8    1    ea1
.....................................................................cuuuucuguuacgguuuucacg....................   37   0    ea1
```

```
                                        Star                        Mature
aggcccccugccauucgaaaaaugcaacaucuaagugucgagaaaaccauaucagaaaaguuuuuaacgcuuuucuguuacgguuuucacgacacuugugaguaucuaaaa
.....................................................................cuuuucuguuacgguuuucacA....................   7     1   ea1
.....................................................................cuuuucuguuacgguCuucacg....................   4     1   ea1
.....................................................................cuuuucuguuacgguuuucacgG...................   1     1   ea1
.....................................................................cuuuucuguuacgguuuucacgaA..................   2     1   ea1
.....................................................................cuuuucuguuacgguuuucacgaca.................   1     0   ea1
......................................................................uuuucuguuacgguuuuc.......................   14    0   ea1
......................................................................uuuucuguuacgguuCuc.......................   1     1   ea1
......................................................................uuuucuAuuacgguuuuca......................   1     1   ea1
......................................................................uuuucuguuacgguCuuca......................   1     1   ea1
......................................................................uuuucuguuacgguuuucG......................   1     1   ea1
......................................................................uCuucuguuacgguuuuca......................   1     1   ea1
......................................................................Guuucuguuacgguuuuca......................   3     1   ea1
......................................................................uuuucCguuacgguuuuca......................   1     1   ea1
......................................................................Cuuucuguuacgguuuuca......................   2     1   ea1
......................................................................uuuucuguuacgguuuuca......................   72    0   ea1
......................................................................uuuucuguuacgguuuCca......................   1     1   ea1
......................................................................uuuucuguuacgguuuucU......................   2     1   ea1
......................................................................uuuucuguuacgguuCuca......................   2     1   ea1
......................................................................Cuuucuguuacgguuuucac.....................   1     1   ea1
......................................................................uuuucuguuacgguuuucac.....................   96    0   ea1
......................................................................uuuucuguuacgguuuucaU.....................   9     1   ea1
......................................................................uuuucuguuacCguuuucac.....................   1     1   ea1
......................................................................Guuucuguuacgguuuucac.....................   4     1   ea1
......................................................................uuuucuguuacgguuuucaA.....................   1     1   ea1
......................................................................uCuucuguuacgguuuucacg....................   6     1   ea1
......................................................................uuuucuguuacgguuuCcacg....................   1     1   ea1
......................................................................uuuucuguCacgguuuucacg....................   1     1   ea1
......................................................................Auuucuguuacgguuuucacg....................   1     1   ea1
......................................................................uuuucuguuacgguuuucUcg....................   1     1   ea1
......................................................................Cuuucuguuacgguuuucacg....................   6     1   ea1
......................................................................uuuucuguuacgguuuucacC....................   14    1   ea1
......................................................................uuuucuguuacgguuuucacA....................   117   1   ea1
......................................................................uuuucuguuaUgguuuucacg....................   1     1   ea1
......................................................................uuuucuguuacgguuCucacg....................   4     1   ea1
......................................................................uuuCcuguuacgguuuucacg....................   3     1   ea1
......................................................................Guuucuguuacgguuuucacg....................   27    1   ea1
......................................................................uuuucugCuacgguuuucacg....................   2     1   ea1
......................................................................uuuucGguuacgguuuucacg....................   1     1   ea1
......................................................................uuuucAguuacgguuuucacg....................   1     1   ea1
......................................................................uuuucuguuacgguCuucacg....................   1     1   ea1
......................................................................uuuucCguuacgguuuucacg....................   5     1   ea1
......................................................................uuuucuguuacgguuuucacg....................   774   0   ea1
......................................................................uuuucuguuacgguuuucacU....................   127   1   ea1
......................................................................uuuucuguGacgguuuucacg....................   1     1   ea1
......................................................................uuuucuguuacggGuuucacg....................   2     1   ea1
......................................................................uuuucuguuacgguuuucGcg....................   3     1   ea1
......................................................................uuuucuguuacgguGuucacga...................   1     1   ea1
......................................................................uuuucuguuacgguuuucacCa...................   1     1   ea1
......................................................................uuuucuguuacgguuuucacgG...................   2     1   ea1
......................................................................uuuucuguuacggCuuucacga...................   1     1   ea1
......................................................................uuuCcuguuacgguuuucacga...................   1     1   ea1
......................................................................uuuucuguuacgguCuucacga...................   2     1   ea1
......................................................................uuuucuguuacgguuuucGcga...................   1     1   ea1
......................................................................uuuucuguuacgguuuucacgU...................   198   1   ea1
......................................................................uuuuUuguuacgguuuucacga...................   1     1   ea1
......................................................................uuuucuguuacgguuuucacgC...................   30    1   ea1
......................................................................uuuucuguuacgguuuucacUa...................   11    1   ea1
......................................................................uuuucuguuacgguuuucacga...................   177   0   ea1
......................................................................Guuucuguuacgguuuucacga...................   5     1   ea1
......................................................................Cuuucuguuacgguuuucacga...................   3     1   ea1
......................................................................uuuucuguuacgguuuucacgaU..................   22    1   ea1
......................................................................uuuucuguuacggCuuucacgac..................   2     1   ea1
......................................................................uuuucuguuacgguuuucacgaA..................   96    1   ea1
......................................................................uuuucuguuacgguuuucacgac..................   9     0   ea1
......................................................................uuuucuguuacgguuuucacgaGa.................   1     1   ea1
......................................................................uuuucuguuacgguuuucacgaUa.................   13    1   ea1
......................................................................uuuucuguuacgguuuucacgacU.................   3     1   ea1
.......................................................................Guucuguuacgguuuuca......................   1     1   ea1
.......................................................................uuucuguuacgguuuuca......................   2     0   ea1
.......................................................................uuucuguuacgguuuucac.....................   5     0   ea1
```

Star **Mature**

```
aggcccccugccauucgaaaaaugcaacaucuaagugucgagaaaaccauaucagaaaaguuuuuaacgcuuuucuguuacgguuuucacgacacuugugaguaucuaaaa
.......................................................................uuucuguuacgguuuucacU....................   1    1   ea1
.......................................................................uuucuguuacgguuuucacA....................   7    1   ea1
.......................................................................Guucuguuacgguuuucacg....................   4    1   ea1
.......................................................................uuucuguuacgguuuucacg....................   25   0   ea1
.......................................................................uuucuguuacgguuuucacgC...................   8    1   ea1
.......................................................................Guucuguuacgguuuucacga...................   1    1   ea1
.......................................................................uuucuguuacgguuuucacga...................   10   0   ea1
.......................................................................uuucuguuacgguuuucacgU...................   51   1   ea1
.......................................................................uuucuguuacgguuuucacgaA..................   5    1   ea1
.......................................................................uuucuguuacgguuuucacgaU..................   13   1   ea1
.......................................................................uuucuguuacgguuuucacgac..................   9    0   ea1
.......................................................................uuucuguuacgguuuucacgUc..................   5    1   ea1
.......................................................................uuucuguuacgguuuucacgCc..................   8    1   ea1
.......................................................................uuucuguuacgguuuucacgUca.................   1    1   ea1
.......................................................................uuucuguuacgguuuucacgacU.................   1    1   ea1
.......................................................................uuucuguuacgguuuucacgaUa.................   5    1   ea1
........................................................................uucuguuacgguuuucacga...................   2    0   ea1
........................................................................Gucuguuacgguuuucacga...................   1    1   ea1
........................................................................uucuguuacgguuuucacgU...................   1    1   ea1
........................................................................uucuguuacgguuuucacgaG..................   1    1   ea1
........................................................................uucuguuacgguuuucacgaUa.................   1    1   ea1
........................................................................uucuguuacgguuuucacgacU.................   1    1   ea1
.........................................................................ucuguuacgguuuucacgaA..................   3    1   ea1
.........................................................................ucuguuacgguuuucacgaAa.................   1    1   ea1

.......................................gagaaaaccauaucagaaaag...................................................   2    0   er2
.......................................gagaaaaccauaucagaaaagu..................................................   3    0   er2
..........................................................aguuuuuaacgcuuuucugu.................................   4    0   er2
............................................................uuuuuaacgcuuuucuguua...............................   1    0   er2
............................................................uuuuuaacgcuuuucuguuacC.............................   1    1   er2
.............................................................uuuuaacgcuuuucuguuacA.............................   1    1   er2
..............................................................uuuaacgcuuuucuguuacgg............................   2    0   er2
................................................................uaacgcuuuucuguuacggu...........................   1    0   er2
................................................................uaacgcuuuucuguuacgguuuA........................   2    1   er2
...................................................................cgcuuuucuguuacgguuuuA.......................   1    1   er2
.....................................................................cuuuucuguuacgguuuuca......................   1    0   er2
.....................................................................cuuuucuguuacgguuuucacg....................   16   0   er2
......................................................................uuuucuguuacgguuuucac.....................   4    0   er2
......................................................................uuuucuguuacgguuCucacg....................   1    1   er2
......................................................................Cuuucuguuacgguuuucacg....................   1    1   er2
......................................................................uuuucuguuacgguuuucacU....................   52   1   er2
......................................................................uuuucuguuacgguuuucacg....................   384  0   er2
......................................................................uuuucuguuacgguuuucacA....................   19   1   er2
......................................................................uuuucuguuacgguuuucacC....................   8    1   er2
......................................................................Nuuucuguuacgguuuucacg....................   1    1   er2
......................................................................uuuucuguuacgguuuucacgU...................   102  1   er2
......................................................................uuuucuguuacgguuuucacgC...................   6    1   er2
......................................................................uuuucuguuacgguuuucacUa...................   9    1   er2
......................................................................uuuucuguuacgguuuucacAa...................   1    1   er2
......................................................................uuuucuguuacgguuuucacga...................   35   0   er2
......................................................................uuuucuguuacgguuuucacgaU..................   39   1   er2
......................................................................uuuucuguuacgguuuucacgaA..................   82   1   er2
......................................................................uuuucuguuacgguuuucacgaG..................   1    1   er2
......................................................................uuuucuguuacgguuuucacgaUa.................   4    1   er2
.......................................................................uuucuguuacgguuuucac.....................   2    0   er2
.......................................................................uuucuguuacgguuuucacg....................   37   0   er2
.......................................................................uuucuguuacgguuuucacU....................   3    1   er2
.......................................................................uuucuguuacgguuuucacA....................   4    1   er2
.......................................................................uuucuguuacgguuuucacC....................   1    1   er2
.......................................................................uuucuguuacgguuuucacga...................   8    0   er2
.......................................................................uuucuguuacgguuuucacgU...................   83   1   er2
.......................................................................uuucuguuacgguuuucacgC...................   2    1   er2
.......................................................................uuucuguuacgguuuucacgaG..................   1    1   er2
.......................................................................uuucuguuacgguuuucacgUc..................   1    1   er2
.......................................................................uuucuguuacgguuuucacgaA..................   20   1   er2
.......................................................................uuucuguuacgguuuucacgaU..................   64   1   er2
.......................................................................uuucuguuacgguuuucacgCc..................   2    1   er2
.......................................................................uuucuguuacgguuuucacgac..................   3    0   er2
.......................................................................uuucuguuacgguuuucacgaUa.................   15   1   er2
........................................................................uucuguuacgguuuucacg....................   1    0   er2
```

Star **Mature**

```
aggcccccugccauucgaaaaaugcaacaucuaagugucgagaaaaccauaucagaaaaguuuuuaacgcuuuucuguuacgguuuucacgacacuugugaguaucuaaaa
........................................................................uucuguuacgguuuucacgU...................    9     1     er2
........................................................................uucuguuacgguuuucacgaA..................    8     1     er2
........................................................................uucuguuacgguuuucacgaU..................    9     1     er2
.........................................................................ucuguuacgguuuucacgaA..................    2     1     er2

.......................................gagaaaaccauaucagaaaag...................................................    12    0     ea2
........................................agaaaaccauaucagaaaag...................................................    5     0     ea2
........................................Ngaaaaccauaucagaaaag...................................................    1     1     ea2
..........................................................aguuuuuaacgcuuuucuguu................................    3     0     ea2
...........................................................guuuuuaacgcuuuucuguA................................    4     1     ea2
...........................................................guuuuuaacgcuuuucuguua...............................    3     0     ea2
............................................................uuuuuaacgcuuuucuguAa...............................    5     1     ea2
............................................................uuuuuaacgcuuuucuguua...............................    2     0     ea2
............................................................uuuuuaacgcuuuucuguuaA..............................    2     1     ea2
............................................................uuuuuaacgcuuuucuguuacU.............................    2     1     ea2
.............................................................uuuuaacgcuuuucuguuacA.............................    6     1     ea2
.............................................................uuuuaacgcuuuucuguuacg.............................    3     0     ea2
.............................................................uuuuaacgcuuuucuguuacgg............................    1     0     ea2
..............................................................uuuaacgcuuuucuguuacA.............................    1     1     ea2
..............................................................uuuaacgcuuuucuguuacg.............................    2     0     ea2
..............................................................uuuaacgcuuuucuguuacgg............................    17    0     ea2
...............................................................uuaacgcuuuucuguuacg.............................    1     0     ea2
...............................................................uuaacgcuuuucuguuacgg............................    7     0     ea2
................................................................uaacgcuuuucuguuacgg............................    3     0     ea2
.................................................................aacgcuuuucuguuacgguuA.........................    3     1     ea2
.................................................................aacgcuuuucuguuacgguuuucacg....................    1     0     ea2
...................................................................cgcuuuucuguuacgguuuuA.......................    8     1     ea2
...................................................................cgcuuuucuguuacgguuuuc.......................    1     0     ea2
...................................................................cgcuuuucuguuacgguuuuAa......................    4     1     ea2
...................................................................cgcuuuucuguuacgguuuuca......................    5     0     ea2
....................................................................gcuuuucuguuacgguuuuA.......................    6     1     ea2
.....................................................................Nuuuucuguuacgguuuuca......................    1     1     ea2
.....................................................................cuuuucuguuacUguuuuca......................    4     1     ea2
.....................................................................cuuuucuguuacgguuuuca......................    17    0     ea2
.....................................................................cuuuucuguuacgguuuucaA.....................    1     1     ea2
.....................................................................cuuuucuguuacgguuuucacA....................    2     1     ea2
.....................................................................cuuuucuguuacgguuuucacC....................    3     1     ea2
.....................................................................cuuuucuguuacggAuuucacg....................    1     1     ea2
.....................................................................cuuuucuguuacgguuuCcacg....................    1     1     ea2
.....................................................................cuuuucuguuacgguuuucacg....................    69    0     ea2
.....................................................................cuuuucuguuacgguuuucacU....................    17    1     ea2
......................................................................uuuucuguuacgguuuuca......................    9     0     ea2
......................................................................uuuucuguuacggGuuucac.....................    1     1     ea2
......................................................................uuuucuguuacgguuuucac.....................    60    0     ea2
......................................................................uuuucGguuacgguuuucac.....................    1     1     ea2
......................................................................uuuucuguuacgguuuucaUg....................    1     1     ea2
......................................................................uuuucuguuacgguuuucacg....................    1688  0     ea2
......................................................................uNuucuguuacgguuuucacg....................    1     1     ea2
......................................................................uuuucuAuuacgguuuucacg....................    3     1     ea2
......................................................................uuuCcuguuacgguuuucacg....................    2     1     ea2
......................................................................uuuucuguuacgguuuuAacg....................    1     1     ea2
......................................................................uuuGcuguuacgguuuucacg....................    1     1     ea2
......................................................................uuuucuguuacgguuCucacg....................    1     1     ea2
......................................................................uuuucuguuacgguuuucacA....................    92    1     ea2
......................................................................uuuucuguuacggGuuucacg....................    3     1     ea2
......................................................................uCuucuguuacgguuuucacg....................    1     1     ea2
......................................................................uuuucuguuacgguuuucacU....................    322   1     ea2
......................................................................Cuuucuguuacgguuuucacg....................    1     1     ea2
......................................................................uuuucuguuacgguGuucacg....................    1     1     ea2
......................................................................uuuucuguuacgguuuucacC....................    27    1     ea2
......................................................................uuuucuguGacgguuuucacg....................    1     1     ea2
......................................................................Nuuucuguuacgguuuucacg....................    3     1     ea2
......................................................................uuuucuguuacgguuuucacga...................    83    0     ea2
......................................................................uuuucuguuacgguuuucacgU...................    128   1     ea2
......................................................................uuuucuguuacgguuuucacgC...................    4     1     ea2
......................................................................uuuucuguuacgguuuucacUa...................    11    1     ea2
......................................................................uuuucuguuacgguuuucacgac..................    3     0     ea2
......................................................................uuuucuguuacgguuuucacgaA..................    176   1     ea2
......................................................................uuuucuguuacgguuuucacgaU..................    47    1     ea2
......................................................................uuuucuguuacgguuuucacgUc..................    6     1     ea2
```

```
                                       Star                          Mature
aggcccccugccauucgaaaaaugcaacaucuaagugucgagaaaaccauaucagaaaaguuuuuaacgcuuuucuguuacgguuuucacgacacuugugaguaucuaaaa
......................................................................uuuucuguuacgguuuucacgaUa.................    9     1    ea2
.......................................................................uuucuguuacgguuuucac.....................    12    0    ea2
.......................................................................uuucuguuacgguuuucaU.....................    1     1    ea2
.......................................................................uuucuguuacgguuuucacU....................    23    1    ea2
.......................................................................Nuucuguuacgguuuucacg....................    2     1    ea2
.......................................................................uuucuguuacgguuuucacA....................    19    1    ea2
.......................................................................uuucuguuacgguuuucacC....................    3     1    ea2
.......................................................................uuucuguuacggGuuucacg....................    2     1    ea2
.......................................................................uuucuguuacgguuuucacg....................    351   0    ea2
.......................................................................uuucuguuacgguuuucacgU...................    154   1    ea2
.......................................................................uuucuguuacgguuuucacga...................    14    0    ea2
.......................................................................uuucuguuacgguuuucacCa...................    3     1    ea2
.......................................................................uuucuguuacgguuuucacgC...................    18    1    ea2
.......................................................................uuucuguuacgguuuucacAa...................    3     1    ea2
.......................................................................uuucuguuacgguuuucacgaG..................    3     1    ea2
.......................................................................uuucuguuacgguuuucacgaA..................    65    1    ea2
.......................................................................uuucuguuacgguuuucacgUc..................    1     1    ea2
.......................................................................uuucuguuacgguuuucacgCc..................    4     1    ea2
.......................................................................uuucuguuacgguuuucacgaU..................    83    1    ea2
.......................................................................uuucuguuacgguuuucacgac..................    1     0    ea2
.......................................................................uuucuguuacgguuuucacgUca.................    2     1    ea2
.......................................................................uuucuguuacgguuuucacgaUa.................    10    1    ea2
........................................................................uucuguuacgguuuucac.....................    1     0    ea2
........................................................................uucuguuacgguuuucacg....................    20    0    ea2
........................................................................uucuguuacgguuuucacUa...................    2     1    ea2
........................................................................uucuguuacgguuuucacgU...................    15    1    ea2
........................................................................uucuguuacgguuuucacga...................    6     0    ea2
........................................................................uucuguuacgguuuucacgaG..................    2     1    ea2
........................................................................uucuguuacgguuuucacgaU..................    13    1    ea2
........................................................................uucuguuacgguuuucacgac..................    1     0    ea2
........................................................................uucuguuacgguuuucacgaA..................    36    1    ea2
........................................................................uucuguuacgguuuucacgaUa.................    6     1    ea2
.........................................................................ucuguuacgguuuucacg....................    5     0    ea2
.........................................................................ucuguuacgguuuucacga...................    6     0    ea2
.........................................................................ucuguuacgguuuucacgUca.................    2     1    ea2

.......................................gagaaaaccauaucagaaaaguA.................................................    1     1    eg2
......................................................................uuuucuguuacgguuuucacA....................    5     1    eg2
......................................................................uuuucuguuacgguuuucacU....................    19    1    eg2
......................................................................Nuuucuguuacgguuuucacg....................    2     1    eg2
......................................................................uuuucuguuacgguuuuUacg....................    3     1    eg2
......................................................................uuuucuguuacgguuuucacg....................    67    0    eg2
......................................................................uuuucuguuacgguuuucacga...................    11    0    eg2
......................................................................uuuucuguuacgguuuucacgU...................    2     1    eg2
......................................................................uuuucuguuacgguuuucacUa...................    4     1    eg2
......................................................................uuuucuguuacgguuuucacgaA..................    12    1    eg2
......................................................................uuuucuguuacgguuuucacgaU..................    3     1    eg2
......................................................................uuuucuguuacgguuuucacgaUa.................    4     1    eg2
.......................................................................uuucuguuacgguuuucacg....................    7     0    eg2
.......................................................................uuucuguuacgguuuucacgaA..................    2     1    eg2
.......................................................................uuucuguuacgguuuucacgaU..................    1     1    eg2
.......................................................................uuucuguuacgguuuucacgaUa.................    1     1    eg2
```

Provisional ID	:	ScUbCFx_4_1190
Score total	:	511.4
Score for star read(s)	:	3.9
Score for read counts	:	502.3
Score for mfe	:	0.5
Score for randfold	:	1.6
Score for cons. seed	:	3
Total read count	:	997
Mature read count	:	952
Loop read count	:	0
Star read count	:	45

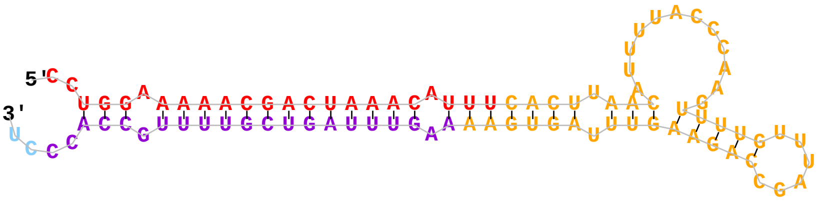

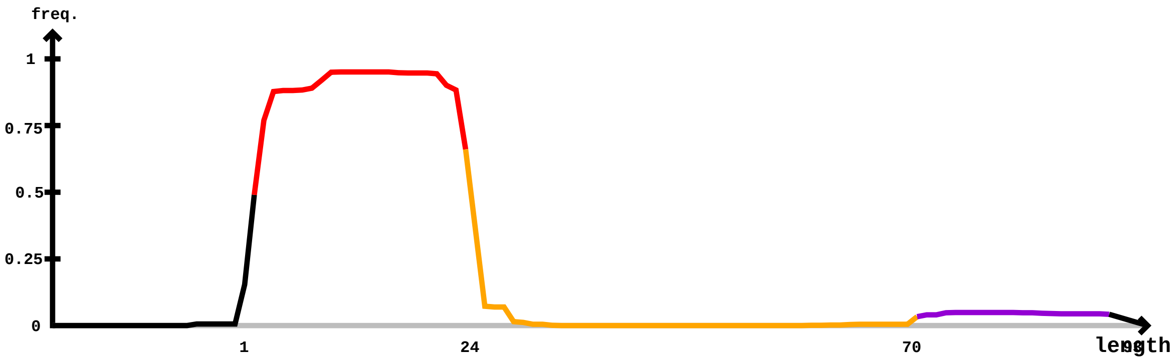


```
                                      Mature                                                        Star
5'- gaauucuuaguucuuaagcccuggaaaaacgacuaaacauuucacuuaacauuuuacccaaguuuuguuuagccagaaguuuagugaaaaguuuagucguuuugccacccug  -3'    obs
    gaauucuuaguucuuaagcccuggaaaaacgacuaaacauuucacuuaacauuuuacccaaguuuuguuuagccagaaguuuagugaaaaguuuagucguuuugccacccug          exp
    ((((.....))))........(((.((((((((((((.(((((((.(((..............(((((......)))))))).))))))).))))))))))))).))).....    reads    mm    sample
    ...................ccuggCaaaacgacuaaacauuu......................................................................    2        1     eg2
    ....................cuggCaaaacgacuaaacauu.......................................................................    3        1     eg2
    ....................cuggCaaaacgacuaaacauuu......................................................................    2        1     eg2
    ....................cuggaaaaacgacuaaacauuu......................................................................    3        0     eg2
    ....................cuggaaaaacgacuaaacauuuA.....................................................................    1        1     eg2
    .....................uggCaaaacgacuaaacauu.......................................................................    1        1     eg2
    .....................uggCaaaacgacuaaacauuu......................................................................    2        1     eg2
    .....................uggaaaaacgacuaaacauuu......................................................................    1        0     eg2
    .....................uggaaaaacgacuaaacauuuA.....................................................................    1        1     eg2
    ..........................aaacgacuaaacauuucacu..................................................................    2        0     eg2
    ...........................aacgacuaaacauuucacuua................................................................    2        0     eg2
    ...............................................................................uuuaguCaaaaguuuagucg.............    1        1     eg2
    ........................................................................................aaguuuagucguuuugUcacc...    2        1     eg2

    ..................cccuggCaaaacgacuaaacauuu......................................................................    6        1     ea2
    ...................ccuggCaaaacgacuaaacauu.......................................................................    2        1     ea2
    ...................ccuggCaaaacgacuaaacauuu......................................................................    48       1     ea2
    ....................cuggCaaaacgacuaaacauu.......................................................................    20       1     ea2
    ....................cuggaaaaacgacuaaacauu.......................................................................    2        0     ea2
    ....................cuggCaaaacgacuaaacauuu......................................................................    30       1     ea2
    ....................cuggaaaaacgacuaaacauuuA.....................................................................    1        1     ea2
    ....................cuggCaaaacgacuaaacauuuc.....................................................................    2        1     ea2
    .....................uggCaaaacgacuaaacauu.......................................................................    8        1     ea2
    .....................uggCaaaacgacuaaacauuu......................................................................    12       1     ea2
    .........................aaaacgacuaaacauuucacu..................................................................    1        0     ea2
    ..........................aaacgacuaaacUuuucacu..................................................................    7        1     ea2
    ..........................aaacgacuaaacauuucacu..................................................................    1        0     ea2
    ...........................aacgacuaaacUuuucacu..................................................................    2        1     ea2
    ...........................................................................................uuuagucguuuugccaccAu.    1        1     ea2

    ..................cGcuggaaaaacgacuaaaca.........................................................................    2        1     er2
    ..................cGcuggaaaaacgacuaaacauu.......................................................................    7        1     er2
    ..................cGcuggaaaaacgacuaaacauuu......................................................................    13       1     er2
```

Mature Star

```
gaauucuuaguucuuaagcccuggaaaaacgacuaaacauuucacuuaacauuuuacccaaguuuuguuuagccagaaguuuagugaaaaguuuagucguuuugccacccug
..................cGcuggaaaaacgacuaaacauuuca....................................................................   1   1   er2
...................ccuggCaaaacgacuaaacauu.......................................................................   1   1   er2
...................ccuggCaaaacgacuaaacauuu......................................................................   11  1   er2
...................Gcuggaaaaacgacuaaacauuu......................................................................   3   1   er2
....................cuggaaaaacgacuaaacauu.......................................................................   13  0   er2
....................cuggCaaaacgacuaaacauu.......................................................................   10  1   er2
....................cCggaaaaacgacuaaacauuu......................................................................   2   1   er2
....................cuggaaaaacgacuaaacauuu......................................................................   67  0   er2
....................cuggCaaaacgacuaaacauuu......................................................................   33  1   er2
....................cuggaaaaacgacuaaacauuuA.....................................................................   9   1   er2
.....................uggaaaaacgacuaaacUuu.......................................................................   1   1   er2
.....................uggCaaaacgacuaaacauu.......................................................................   3   1   er2
.....................uggCaaaacgacuaaacauuu......................................................................   4   1   er2
.....................uNgaaaaacgacuaaacauuu......................................................................   1   1   er2
.....................uggaaaaacgacuaaacauuu......................................................................   11  0   er2
.....................uggaaaaacgacuaaacauuuA.....................................................................   14  1   er2
......................ggaaaaacgacuaaacauuu......................................................................   4   0   er2
.........................aaaacgacuaaacUuuucacu..................................................................   1   1   er2
..........................aaacgacuaaacauuucacu..................................................................   2   0   er2
..........................aaacgacuaaacUuuucacu..................................................................   1   1   er2
...........................aacgacuaaacUuuucacu..................................................................   1   1   er2
...........................aacgacuaaacauuucacu..................................................................   2   0   er2
...........................aacgacuaaacauuucacuu.................................................................   1   0   er2
...........................aacgacuaaacauuucacuua................................................................   1   0   er2
...........................aacgacuaaacauuucacuuaaca.............................................................   1   0   er2
.................................................................................uagugaaaaguuuagucguG...........   2   1   er2
........................................................................................aaguuuagucguuuugccacc...   2   0   er2
........................................................................................aaguuuagucguuuugccacU...   1   1   er2
........................................................................................aaguuuagucguuuugccaccA..   1   1   er2

.............uuaagcccuggCaaaacgacu..............................................................................   2   1   ea1
.............uuaagcccuggCaaaacgacuaaac..........................................................................   2   1   ea1
..................cccuggCaaaacgacuaaaca.........................................................................   19  1   ea1
..................cccuggCaaaacgacuaaacau........................................................................   3   1   ea1
..................cccuggCaaaacgacuaaacauu.......................................................................   31  1   ea1
..................cccuggCaaaacgacuaaacauuu......................................................................   42  1   ea1
...................ccuggUaaaacgacuaaac..........................................................................   1   1   ea1
...................ccuggCaaaacgacuaaaca.........................................................................   15  1   ea1
...................ccuggCaaaacgacuaaacau........................................................................   8   1   ea1
...................ccuggCaaaacgacuaaacauu.......................................................................   53  1   ea1
...................ccuggCaaaacgacuaaacauuu......................................................................   103 1   ea1
...................ccuggCaaaacgacuaaacauuuc.....................................................................   2   1   ea1
...................ccuggCaaaacgacuaaacauuuca....................................................................   2   1   ea1
....................cuggUaaaacgacuaaacauu.......................................................................   1   1   ea1
....................cuggCaaaacgacuaaacauu.......................................................................   34  1   ea1
....................cuggaaaaacgacuaaacauuu......................................................................   1   0   ea1
....................cuggCaaaacgacuaaacauuu......................................................................   28  1   ea1
.....................uggCaaaacgacuaaacauu.......................................................................   5   1   ea1
.....................uggaaaaacgacuaaacUuu.......................................................................   4   1   ea1
.....................uggUaaaacgacuaaacauu.......................................................................   3   1   ea1
.....................uggaaaaacgGcuaaacauu.......................................................................   1   1   ea1
.....................uggUaaaacgacuaaacauuu......................................................................   1   1   ea1
.....................uggCaaaacgacuaaacauuu......................................................................   23  1   ea1
........................aaaaacgacuaaacUuuucacu..................................................................   2   1   ea1
.........................aaaacgacuaaacUuuucacu..................................................................   3   1   ea1
..........................aaacgacuaaacauuucacu..................................................................   1   0   ea1
..........................aaacgacuaaacUuuucacu..................................................................   13  1   ea1
...........................aacgacuaaacUuuucacu..................................................................   6   1   ea1
...........................aacgacuaaacauuucacC..................................................................   1   1   ea1
...........................aacgacuaaacauuucacu..................................................................   2   0   ea1
...........................aacgacuaaacUuuucacuu.................................................................   1   1   ea1
...........................aacgacuaaacauuucacuCa................................................................   1   1   ea1
..................................................................................agugaaaaguuuagucguuuC.........   1   1   ea1
........................................................................................aaguuuagucguuuugccaG....   1   1   ea1
........................................................................................aUguuuagucguuuugccacc...   1   1   ea1
........................................................................................aaguuuagucguuuugccacc...   7   0   ea1
........................................................................................aaguuuagucguuuugccaUc...   5   1   ea1
........................................................................................aaguuuagucguuuugUcacc...   5   1   ea1
........................................................................................aaguuuagucguuuugccacU...   1   1   ea1
........................................................................................aaguuuagucguuuugccaccU..   2   1   ea1
```

```
                   Mature                                                                 Star
gaauucuuaguucuuaagcccuggaaaaacgacuaaacauuucacuuaaacauuuuacccaaguuuuguuuagccagaaguuuagugaaaaguuuagucguuuugccacccug
..........................................................................................Uguuuagucguuuugccacc...   3    1    ea1
..........................................................................................Uguuuagucguuuugccacccu.   2    1    ea1
............................................................................................uuuagucguuuugccacA...   1    1    ea1
............................................................................................uuuagucguuuugccacc...   1    0    ea1
............................................................................................uuuagucguuuugccacccu.   1    0    ea1
............................................................................................uuuagucguuuugccaGccu.   2    1    ea1
............................................................................................uuuagucguuuugcAacccug   1    1    ea1
.............................................................................................uuagucguuuugccacccug   1    0    ea1

.............uuaagcccuggCaaaacgacu...............................................................................   1    1    er1
..................cccuggCaaaacgacuaaaca..........................................................................   3    1    er1
..................cccuggCaaaacgacuaaacau.........................................................................   1    1    er1
..................cccuggCaaaacgacuaaacauuu.......................................................................   2    1    er1
...................ccuggCaaaacgacuaaacauu........................................................................   5    1    er1
...................ccuggCaaaacgacuaaacauuu.......................................................................   5    1    er1
....................cuggaaaaacgacuaaacauuu.......................................................................   2    0    er1
....................cuggCaaaacgacuaaacauuu.......................................................................   5    1    er1
.....................uggCaaaacgacuaaacauu........................................................................   1    1    er1
.....................uggCaaaacgacuaaacauuu.......................................................................   2    1    er1
.....................uggaaaaacgacuaaacauuu.......................................................................   1    0    er1
..........................aaacgacuaaacUuuucacu...................................................................   1    1    er1
............................................................................................uuuagucguuuugccacccuA   1    1    er1

.............uuaagcccuggCaaaacgacua..............................................................................   1    1    eg1
..................cccuggCaaaacgacuaaaca..........................................................................   3    1    eg1
..................cccuggCaaaacgacuaaacau.........................................................................   1    1    eg1
..................cccuggCaaaacgacuaaacauu........................................................................   3    1    eg1
..................cccuggCaaaacgacuaaacauuu.......................................................................   14   1    eg1
...................ccuggCaaaacgacuaaaca..........................................................................   2    1    eg1
...................ccuggCaaaacgacuaaacau.........................................................................   5    1    eg1
...................ccuggUaaaacgacuaaacauu........................................................................   1    1    eg1
...................ccuggCaaaacgacuaaacauu........................................................................   10   1    eg1
...................ccuggCaaaacgacuaaacauuu.......................................................................   63   1    eg1
...................ccuggCaaaacgacuaaacauuuc......................................................................   1    1    eg1
....................cuggCaaaacgacuaaacauu........................................................................   2    1    eg1
....................cuggaaaaacgacuaaacauuC.......................................................................   2    1    eg1
....................cuggCaaaacgacuaaacauuu.......................................................................   11   1    eg1
....................cuggCaaaacgacuaaacauuuc......................................................................   1    1    eg1
.....................uggCaaaacgacuaaacauu........................................................................   1    1    eg1
.....................uggUaaaacgacuaaacauu........................................................................   1    1    eg1
.....................uggCaaaacgacuaaacauuu.......................................................................   8    1    eg1
.........................aaaacgacuaaacUuuucacu...................................................................   1    1    eg1
..........................aaacgacuaaacUuuucacu...................................................................   3    1    eg1
...........................aacgacuaaacUuuucacu...................................................................   3    1    eg1
...........................aacgacuaaacauuucacuC..................................................................   1    1    eg1
...........................aacgacuaaacauuucacuCa.................................................................   3    1    eg1
...........................aacgacuaaacauuucacuCaac...............................................................   3    1    eg1
............................acgacuaaacauuucacuCaac...............................................................   1    1    eg1
..............................................................................aguuuagugaaaaguuuagucguCu..........   1    1    eg1
.........................................................................................aaguuuagucguuuugUcacc...   1    1    eg1
..........................................................................................Uguuuagucguuuugccac....   1    1    eg1
..........................................................................................Uguuuagucguuuugccacc...   1    1    eg1
```

Provisional ID	:	ScUbCFx_4_1329
Score total	:	1250857
Score for star read(s)	:	3.9
Score for read counts	:	1250852
Score for mfe	:	0.1
Score for randfold	:	1.6
Score for cons. seed	:	-0.6
Total read count	:	2453504
Mature read count	:	2450768
Loop read count	:	0
Star read count	:	2736

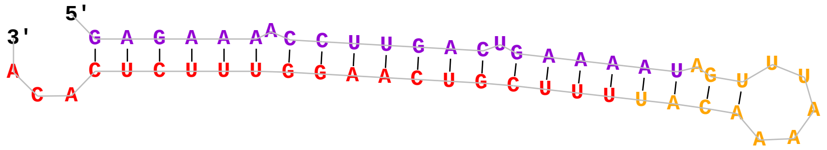

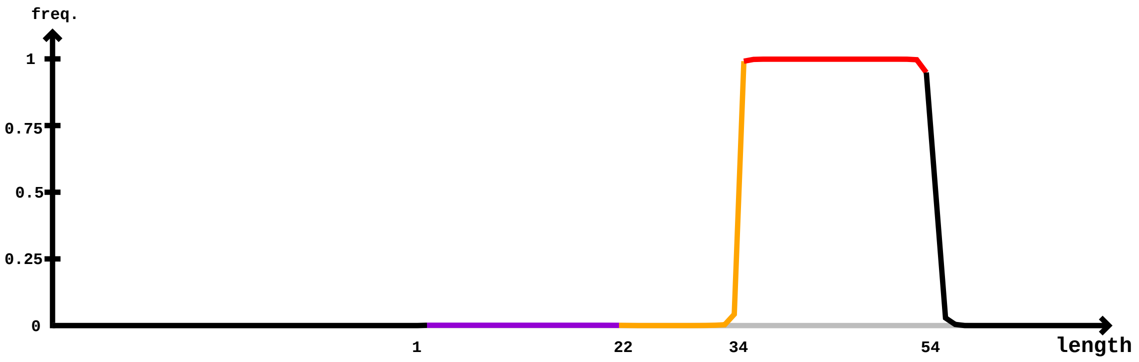

Star Mature

```
5'- aacagauaugauuaagaacagacaaaaaauaauucuugagaaaaccuugacugaaaauaguuuaaaacauuuucgucaagguuucucacaagaacuguuuaucucaaa  -3'  obs
    aacagauaugauuaagaacagacaaaaaauaauucuugagaaaaccuugacugaaaauaguuuaaaacauuuucgucaagguuucucacaagaacuguuuaucucaaa        exp
    ........(((....((((((..........((((((.((((((((((((.((((((.((.....)))))))))))))))))).)).))))))))))))...)))..   reads    mm     sample
    .....................................gagaaaaccuugacugaa.....................................................   3        0      eg1
    .....................................gagaaaaccuugacugaaU....................................................   1        1      eg1
    .....................................gagCaaaccuugacugaaa....................................................   1        1      eg1
    .....................................gagaaaaccuugacugaaa....................................................   13       0      eg1
    .....................................gagaaaaccuugacugaaaa...................................................   28       0      eg1
    .....................................gagaaaaccuugacugaaUa...................................................   1        1      eg1
    .....................................gagaaaaccuugacugaaaG...................................................   2        1      eg1
    .....................................gGgaaaaccuugacugaaaa...................................................   1        1      eg1
    .....................................gagaaaaccuugacugGaaa...................................................   1        1      eg1
    .....................................gagaaaaccuugGcugaaaa...................................................   1        1      eg1
    .....................................gagaaaaccuugacugaaaau..................................................   222      0      eg1
    .....................................gagaaaaccuuAacugaaaau..................................................   1        1      eg1
    .....................................gagaGaaccuugacugaaaau..................................................   1        1      eg1
    .....................................gGgaaaaccuugacugaaaau..................................................   3        1      eg1
    .....................................gagaaaaccuugacugaaaaG..................................................   4        1      eg1
    .....................................gagaaaCccuugacugaaaau..................................................   1        1      eg1
    .....................................gagaaaaccuugacugaaaaA..................................................   1        1      eg1
    .....................................gagaaaaccuugacugaaaCu..................................................   1        1      eg1
    .....................................gagaaaaccuugacAgaaaau..................................................   1        1      eg1
    .....................................gagaaaaccuugacugaaaaC..................................................   21       1      eg1
    .....................................gagaaaaccuCgacugaaaau..................................................   1        1      eg1
    .....................................Aagaaaaccuugacugaaaau..................................................   2        1      eg1
    .....................................gagaaGaccuugacugaaaau..................................................   1        1      eg1
    .....................................gagaaaaccuugaAugaaaau..................................................   1        1      eg1
    .....................................gagaaaaccuugacugaaaGu..................................................   1        1      eg1
    .....................................gagaaaaccuugacugaaaauG.................................................   1        1      eg1
    .....................................gagaaaaccuugacugaaaauU.................................................   12       1      eg1
    .....................................gagaaaaccuugacugaaaaua.................................................   11       0      eg1
    .....................................gagaaaaccuugacugaaaauC.................................................   1        1      eg1
    .....................................gagaaaaccuugacugaaaauag................................................   1        0      eg1
    .....................................gagaaaaccuugacugaaaauaguuua............................................   1        0      eg1
    ............................................................uuuaaaacauuuucgucaagg...........................   2        0      eg1
    ...............................................................aaaacauuuucgucaagguuucucacU.................   1        1      eg1
```

```
                                    Star                            Mature
aacagauaugauuaagaacagacaaaaaauaauucuugagaaaaccuugacugaaaauaguuuaaaacauuuucgucaagguuucucacaagaacuguuuaucucaaa
.................................................................aacauuuucgucaagguuucucaca..................    1    0    eg1
.................................................................aacauuuucgucaagguuucucacC..................    2    1    eg1
..................................................................acauuuucgucaagguuucuc.....................    4    0    eg1
..................................................................acauuuucgucaagguuucuca....................    2    0    eg1
..................................................................acauuuucgucaagguuucucac...................    1    0    eg1
..................................................................acauuuucgucaagguuucucacG..................    3    1    eg1
..................................................................acaAuuucgucaagguuucucaca..................    2    1    eg1
..................................................................acauuuucgucaagguuucucaca..................    10   0    eg1
..................................................................acauuuucgucaagguuucucacU..................    10   1    eg1
..................................................................acauuuucgucaagguuucucacaC.................    1    1    eg1
..................................................................acauuuucgucaagguuucucacaU.................    2    1    eg1
..................................................................acauuuucgucaagguuucucacUa.................    1    1    eg1
..................................................................acauuuucgucaagguuucucacaa.................    1    0    eg1
...................................................................cauuuucgucaagguuucuca....................    2    0    eg1
...................................................................cauuuucgucaagguuucucaU...................    2    1    eg1
...................................................................cauuuucgucaagguuucucac...................    2    0    eg1
...................................................................cauAuucgucaagguuucucaca..................    1    1    eg1
...................................................................cauuuucgucaagguuucucacG..................    1    1    eg1
...................................................................cauuuucgucaagguuucucacU..................    12   1    eg1
...................................................................cauuuucgucaagguuucucaca..................    11   0    eg1
...................................................................cauuuucgucaagguuucucaUa..................    1    1    eg1
...................................................................cauuuucgucaagguuucucacaU.................    1    1    eg1
....................................................................auuuucgucaagguuucuc.....................    1    0    eg1
....................................................................auuuucgucaagguuucucG....................    1    1    eg1
....................................................................auuuucgucaagguuucucU....................    1    1    eg1
....................................................................auuuucgucaagguuucuca....................    2    0    eg1
....................................................................auuuucgucaagguuucucaU...................    3    1    eg1
....................................................................auuuucgucaagguuucucac...................    30   0    eg1
....................................................................auuuucgucaagguuucucaca..................    66   0    eg1
....................................................................Uuuuucgucaagguuucucaca..................    2    1    eg1
....................................................................auuuucgucaagguuucucacU..................    13   1    eg1
....................................................................auuuucgucaagguuucucacG..................    3    1    eg1
....................................................................Guuuucgucaagguuucucaca..................    1    1    eg1
....................................................................Cuuuucgucaagguuucucaca..................    3    1    eg1
....................................................................Uuuuucgucaagguuucucacaa.................    1    1    eg1
....................................................................auuuucgucaagguuucucacaC.................    1    1    eg1
....................................................................auuuucgucaagguuucucacUa.................    2    1    eg1
....................................................................auuuucgucaGgguuucucacaa.................    2    1    eg1
....................................................................auuuucgucaagguuucucacaa.................    1    0    eg1
....................................................................auuuucgucaagguuucucacaU.................    2    1    eg1
....................................................................Cuuuucgucaagguuucucacaag................    1    1    eg1
.....................................................................uuuucgucaagguuucuG.....................    2    1    eg1
.....................................................................Cuuucgucaagguuucuc.....................    1    1    eg1
.....................................................................Guuucgucaagguuucuc.....................    2    1    eg1
.....................................................................uuuucgucaagguuucuU.....................    2    1    eg1
.....................................................................uuuucgucaagguuucuc.....................    13   0    eg1
.....................................................................uuuucgucaagguuucuca....................    4    0    eg1
.....................................................................uuuucgucaagguCucucac...................    1    1    eg1
.....................................................................uGuucgucaagguuucucac...................    1    1    eg1
.....................................................................uuuucgucaagguuucCcac...................    1    1    eg1
.....................................................................uuAucgucaagguuucucac...................    1    1    eg1
.....................................................................uuuucgucaagguuucAcac...................    1    1    eg1
.....................................................................uuuucgucaagguuucucac...................    216  0    eg1
.....................................................................uuuucgucaagguuucuUac...................    2    1    eg1
.....................................................................uuuucgucaagguuucucaU...................    20   1    eg1
.....................................................................uuuucgucaagguuucucUc...................    1    1    eg1
.....................................................................uuCucgucaagguuucucac...................    2    1    eg1
.....................................................................Guuucgucaagguuucucac...................    23   1    eg1
.....................................................................uuuucgucaagguuucucaA...................    4    1    eg1
.....................................................................uuuucgCcaagguuucucac...................    1    1    eg1
.....................................................................Cuuucgucaagguuucucac...................    12   1    eg1
.....................................................................uuuucgucaagguuucucUca..................    3    1    eg1
.....................................................................uuuucgucaagguuucucacU..................    270  1    eg1
.....................................................................uuuucgucUagguuucucaca..................    3    1    eg1
.....................................................................uuuucgucaagguuucucacC..................    35   1    eg1
.....................................................................uuuucgucGagguuucucaca..................    4    1    eg1
.....................................................................uuuucgucaaggGuucucaca..................    1    1    eg1
.....................................................................uuuCcgucaagguuucucaca..................    3    1    eg1
.....................................................................uuuucgCcaagguuucucaca..................    4    1    eg1
.....................................................................uAuucgucaagguuucucaca..................    1    1    eg1
```

Star Mature

```
aacagauaugauuaagaacagacaaaaaauaauucuugagaaaaccuugacugaaaauaguuuaaaacauuuucgucaagguuucucacaagaacuguuuaucucaaa
.....................................................................uuuuAgucaagguuucucaca..................	1	1	eg1
.....................................................................uuuucgucaagguuucucaUa..................	1	1	eg1
.....................................................................uCuucgucaagguuucucaca..................	8	1	eg1
.....................................................................Guuucgucaagguuucucaca..................	125	1	eg1
.....................................................................uuuuUgucaagguuucucaca..................	1	1	eg1
.....................................................................uuuucgucaaAguuucucaca..................	1	1	eg1
.....................................................................uuuucAucaagguuucucaca..................	2	1	eg1
.....................................................................uuuucguAaagguuucucaca..................	1	1	eg1
.....................................................................uuuucgucaagguuucCcaca..................	12	1	eg1
.....................................................................uuuucgucaagguuucucacG..................	112	1	eg1
.....................................................................uuuucgucaagguCucucaca..................	5	1	eg1
.....................................................................uuuucgucaGgguuucucaca..................	5	1	eg1
.....................................................................uuuucgucCagguuucucaca..................	1	1	eg1
.....................................................................uuuucgucaaUguuucucaca..................	2	1	eg1
.....................................................................uuuucgucaagguuCcucaca..................	1	1	eg1
.....................................................................uuuucgucaagguuucucaca..................	1748	0	eg1
.....................................................................uuuGcgucaagguuucucaca..................	1	1	eg1
.....................................................................uuuucgucaagguuucucGca..................	1	1	eg1
.....................................................................Cuuucgucaagguuucucaca..................	65	1	eg1
.....................................................................uuCucgucaagguuucucaca..................	1	1	eg1
.....................................................................uuuucgucaagguuuAucaca..................	2	1	eg1
.....................................................................uuuucgucaagguuucucacaC.................	37	1	eg1
.....................................................................uuuucgucaagguuucCcacaa.................	1	1	eg1
.....................................................................uuuuUgucaagguuucucacaa.................	1	1	eg1
.....................................................................uuuucgucaagguuucucacUa.................	16	1	eg1
.....................................................................uuuucgucaagguuucucacGa.................	1	1	eg1
.....................................................................Cuuucgucaagguuucucacaa.................	42	1	eg1
.....................................................................uuuucgucaagguuAcucacaa.................	1	1	eg1
.....................................................................uuuucgucaagguuucucacaU.................	326	1	eg1
.....................................................................uuuucgucaagguuucucacaG.................	38	1	eg1
.....................................................................uuuucgucaagguuucucacaa.................	306	0	eg1
.....................................................................uuuucgucGagguuucucacaa.................	3	1	eg1
.....................................................................Guuucgucaagguuucucacaa.................	9	1	eg1
.....................................................................uuuucgucaGgguuucucacaa.................	1	1	eg1
.....................................................................uuuucgucaagguuucucacCa.................	1	1	eg1
.....................................................................uuuucgCcaagguuucucacaa.................	1	1	eg1
.....................................................................uuuucgucaagguuucucGcaa.................	2	1	eg1
.....................................................................uuuucgucaagguuucucacaUg................	9	1	eg1
.....................................................................uuuucgucaagguuucucacaaC................	2	1	eg1
.....................................................................uuuucgucaagguuucucacaag................	8	0	eg1
.....................................................................uuuucgucaagguuucucacaaU................	64	1	eg1
.....................................................................uuuucgucaagguuucucacaaA................	4	1	eg1
.....................................................................uuuucgucaagguuucucacaaUa...............	8	1	eg1
......................................................................uuucgucaagguuucucG....................	3	1	eg1
......................................................................uuucgucaagguuucucU....................	4	1	eg1
......................................................................uuucgucaaggGuucuca....................	1	1	eg1
......................................................................uuucgucaagguuucGca....................	1	1	eg1
......................................................................uuucgucaagguuucuca....................	85	0	eg1
......................................................................Guucgucaagguuucuca....................	10	1	eg1
......................................................................uuucgucaagguuucuUa....................	1	1	eg1
......................................................................uuucgucaaAguuucuca....................	2	1	eg1
......................................................................uCucgucaagguuucuca....................	1	1	eg1
......................................................................uuucgucaagAuuucucac...................	2	1	eg1
......................................................................Guucgucaagguuucucac...................	459	1	eg1
......................................................................uuucgucaagguAucucac...................	1	1	eg1
......................................................................uuuUgucaagguuucucac...................	6	1	eg1
......................................................................uuucgucaaggAuucucac...................	2	1	eg1
......................................................................uuucgucaaUguuucucac...................	3	1	eg1
......................................................................uuucgucaagguuucAcac...................	8	1	eg1
......................................................................uuucgucaagguuucucGc...................	19	1	eg1
......................................................................uuucgucaCgguuucucac...................	1	1	eg1
......................................................................uuucgucaagguuucucUc...................	9	1	eg1
......................................................................uGucgucaagguuucucac...................	1	1	eg1
......................................................................Cuucgucaagguuucucac...................	24	1	eg1
......................................................................uuucguAaagguuucucac...................	2	1	eg1
......................................................................uuucgucaaggGuucucac...................	5	1	eg1
......................................................................uuucgucCagguuucucac...................	1	1	eg1
......................................................................uuucgucaagguuucucaA...................	65	1	eg1
......................................................................Auucgucaagguuucucac...................	4	1	eg1
......................................................................uuucgucaagguCucucac...................	7	1	eg1
```

```
                                     Star                             Mature
aacagauaugauuaagaacagacaaaaaauaauucuugagaaaaccuugacugaaaauaguuuaaaacauuuucgucaagguuucucacaagaacuguuuaucucaaa
......................................................................uCucgucaagguuucucac...................   14      1   eg1
......................................................................uuucAucaagguuucucac...................   8       1   eg1
......................................................................uuucgucaagguuucuUac...................   3       1   eg1
......................................................................uuucgucaaAguuucucac...................   6       1   eg1
......................................................................uuucgucaagguuucCcac...................   21      1   eg1
......................................................................uuucgucaGgguuucucac...................   15      1   eg1
......................................................................uuucgucaagguuucGcac...................   3       1   eg1
......................................................................uuucgucaagguuucucaG...................   17      1   eg1
......................................................................uuucgucaagguuCcucac...................   22      1   eg1
......................................................................uuucgucaagUuuucucac...................   1       1   eg1
......................................................................uuucgucaaggCuucucac...................   15      1   eg1
......................................................................uuucgucaagguuuUucac...................   4       1   eg1
......................................................................uuucgucaagguuAcucac...................   3       1   eg1
......................................................................uuucgucGagguuucucac...................   19      1   eg1
......................................................................uuucgGcaagguuucucac...................   1       1   eg1
......................................................................uuucgucaagguuucuAac...................   1       1   eg1
......................................................................uuucgCcaagguuucucac...................   21      1   eg1
......................................................................uuCcgucaagguuucucac...................   18      1   eg1
......................................................................uuucguUaagguuucucac...................   1       1   eg1
......................................................................uuucgucaUgguuucucac...................   2       1   eg1
......................................................................uuucgucaagguuucucac...................   5828    0   eg1
......................................................................uuucgucaagguuucucaU...................   671     1   eg1
......................................................................uAucgucaagguuucucac...................   2       1   eg1
......................................................................uuucgucUagguuucucac...................   1       1   eg1
......................................................................uuuAgucaagguuucucac...................   2       1   eg1
......................................................................uuucgAcaagguuucucac...................   4       1   eg1
......................................................................uuucgucaagguuucAcaca..................   27      1   eg1
......................................................................uuucgucaagguCucucaca..................   67      1   eg1
......................................................................uuucgucaagguuucuGaca..................   1       1   eg1
......................................................................uuucgucaaggCuucucaca..................   120     1   eg1
......................................................................uuucgucaaggGuucucaca..................   30      1   eg1
......................................................................uuucgucaaUguuucucaca..................   6       1   eg1
......................................................................uuucgucaUgguuucucaca..................   9       1   eg1
......................................................................uuCcgucaagguuucucaca..................   86      1   eg1
......................................................................uuucgucaagguAucucaca..................   2       1   eg1
......................................................................uuucgCcaagguuucucaca..................   124     1   eg1
......................................................................Guucgucaagguuucucaca..................   2036    1   eg1
......................................................................uuucgucaCgguuucucaca..................   7       1   eg1
......................................................................uuucAucaagguuucucaca..................   21      1   eg1
......................................................................uuucgucaagguuucucacU..................   7832    1   eg1
......................................................................uuucgucaagguuucucGca..................   122     1   eg1
......................................................................uAucgucaagguuucucaca..................   15      1   eg1
......................................................................uuuUgucaagguuucucaca..................   59      1   eg1
......................................................................uuucgucaaCguuucucaca..................   1       1   eg1
......................................................................uuucgucCagguuucucaca..................   6       1   eg1
......................................................................uuucgucaagguuucucacG..................   2139    1   eg1
......................................................................uuucgucaagguuucucaca..................   37447   0   eg1
......................................................................uuucgucUagguuucucaca..................   15      1   eg1
......................................................................uuucgucaagguuuAucaca..................   2       1   eg1
......................................................................uuucgucaagguuucuUaca..................   19      1   eg1
......................................................................uuucgucaaggAuucucaca..................   7       1   eg1
......................................................................uuucgucaagguuucucUca..................   16      1   eg1
......................................................................uuucguUaagguuucucaca..................   23      1   eg1
......................................................................uuuAgucaagguuucucaca..................   7       1   eg1
......................................................................uuucgucaagguuucucaAa..................   4       1   eg1
......................................................................uuucgucaagguGucucaca..................   3       1   eg1
......................................................................uuucgucaagguuCcucaca..................   112     1   eg1
......................................................................uuucgucaGgguuucucaca..................   115     1   eg1
......................................................................uuucgucGagguuucucaca..................   104     1   eg1
......................................................................uuucgucaagguuucucCca..................   5       1   eg1
......................................................................uuucgucaagUuuucucaca..................   6       1   eg1
......................................................................uGucgucaagguuucucaca..................   4       1   eg1
......................................................................uuGcgucaagguuucucaca..................   5       1   eg1
......................................................................uuucgGcaagguuucucaca..................   1       1   eg1
......................................................................uuucgucaagguuucuAaca..................   4       1   eg1
......................................................................uuucgucaagguuucucaUa..................   118     1   eg1
......................................................................uuucgucaagAuuucucaca..................   13      1   eg1
......................................................................Auucgucaagguuucucaca..................   14      1   eg1
......................................................................uuucgucaaAguuucucaca..................   25      1   eg1
......................................................................uuAcgucaagguuucucaca..................   11      1   eg1
```

Star Mature

```
aacagauaugauuaagaacagacaaaaaauaauucuugagaaaaccuugacugaaaauaguuuaaaacauuuucgucaagguuucucacaagaacuguuuaucucaaa
......................................................................uuucgucaagguuuUucaca..................	11	1	eg1
......................................................................uuucgucaagguuucucacC..................	823	1	eg1
......................................................................uuucUucaagguuucucaca..................	5	1	eg1
......................................................................uCucgucaagguuucucaca..................	105	1	eg1
......................................................................uuucgucaagguuucCcaca..................	148	1	eg1
......................................................................uuucgAcaagguuucucaca..................	12	1	eg1
......................................................................uuucguAaagguuucucaca..................	1	1	eg1
......................................................................uuuGgucaagguuucucaca..................	1	1	eg1
......................................................................uuucgucaagguuGcucaca..................	1	1	eg1
......................................................................uuucgucaagguuucGcaca..................	11	1	eg1
......................................................................Cuucgucaagguuucucaca..................	110	1	eg1
......................................................................uuucgucaagguuAcucaca..................	12	1	eg1
......................................................................uuuUgucaagguuucucacaa.................	20	1	eg1
......................................................................uuucgucaagguuucucacaa.................	27807	0	eg1
......................................................................uuucgucaagguuuGucacaa.................	1	1	eg1
......................................................................uuucAucaagguuucucacaa.................	12	1	eg1
......................................................................uuucgucaaggGuucucacaa.................	24	1	eg1
......................................................................uuucgucaagguuucucGcaa.................	78	1	eg1
......................................................................uuucgucaagguuucuGacaa.................	1	1	eg1
......................................................................uuCcgucaagguuucucacaa.................	67	1	eg1
......................................................................uuucguUaagguuucucacaa.................	9	1	eg1
......................................................................Guucgucaagguuucucacaa.................	1597	1	eg1
......................................................................uuucgucaagguuuUucacaa.................	14	1	eg1
......................................................................uuGcgucaagguuucucacaa.................	3	1	eg1
......................................................................uuucgucaagguuucucUcaa.................	18	1	eg1
......................................................................uuucgucaGgguuucucacaa.................	71	1	eg1
......................................................................uuucguAaagguuucucacaa.................	1	1	eg1
......................................................................uuucgucaagguuucucacaG.................	2195	1	eg1
......................................................................uuucgucaagguuucuUacaa.................	12	1	eg1
......................................................................uuucgucaUgguuucucacaa.................	5	1	eg1
......................................................................uuucgucCagguuucucacaa.................	4	1	eg1
......................................................................uuuAgucaagguuucucacaa.................	5	1	eg1
......................................................................uuAcgucaagguuucucacaa.................	4	1	eg1
......................................................................uuucgucaagguuucCcacaa.................	94	1	eg1
......................................................................uuuGgucaagguuucucacaa.................	2	1	eg1
......................................................................uuucgucaagguuAcucacaa.................	9	1	eg1
......................................................................uuucgucaagguCucucacaa.................	44	1	eg1
......................................................................uuucgucaagguuucucacCa.................	34	1	eg1
......................................................................uGucgucaagguuucucacaa.................	4	1	eg1
......................................................................uuucgucaaUguuucucacaa.................	4	1	eg1
......................................................................uuucgucaCgguuucucacaa.................	2	1	eg1
......................................................................uuucgucaagguuucucacUa.................	1072	1	eg1
......................................................................uuucgucaagUuuucucacaa.................	4	1	eg1
......................................................................uuucgucaagguuucuAacaa.................	8	1	eg1
......................................................................uuucgucaagguuGcucacaa.................	1	1	eg1
......................................................................Cuucgucaagguuucucacaa.................	111	1	eg1
......................................................................uuucgAcaagguuucucacaa.................	8	1	eg1
......................................................................uuucgucaagguuucAcacaa.................	20	1	eg1
......................................................................uuucgucaagguuucGcacaa.................	6	1	eg1
......................................................................uuucgucaagguuucucacGa.................	88	1	eg1
......................................................................uuucgucUagguuucucacaa.................	8	1	eg1
......................................................................uuucgCcaagguuucucacaa.................	76	1	eg1
......................................................................uuucgucaagAuuucucacaa.................	13	1	eg1
......................................................................uuucgucGagguuucucacaa.................	66	1	eg1
......................................................................uuucgucaagguAucucacaa.................	2	1	eg1
......................................................................uuucgucaagguuucucCcaa.................	2	1	eg1
......................................................................uuucgucaagguuucucacaU.................	24167	1	eg1
......................................................................uuucgucaaAguuucucacaa.................	12	1	eg1
......................................................................uCucgucaagguuucucacaa.................	72	1	eg1
......................................................................Auucgucaagguuucucacaa.................	22	1	eg1
......................................................................uuucgucaagguuucucaAaa.................	3	1	eg1
......................................................................uuucgucaaggCuucucacaa.................	95	1	eg1
......................................................................uuucgGcaagguuucucacaa.................	1	1	eg1
......................................................................uuucgucaagguuucucaUaa.................	23	1	eg1
......................................................................uuucgucaagguuCcucacaa.................	81	1	eg1
......................................................................uAucgucaagguuucucacaa.................	8	1	eg1
......................................................................uuucgucaagguuucucacaC.................	2559	1	eg1
......................................................................uuucgucaaggAuucucacaa.................	8	1	eg1
......................................................................uuucgucaagguuucucacUag................	5	1	eg1
......................................................................uuucgCcaagguuucucacaag................	1	1	eg1
```

```
                                     Star                             Mature
aacagauaugauuaagaacagacaaaaaauaauucuugagaaaaccuugacugaaaauaguuuaaaacauuuucgucaagguuucucacaagaacuguuuaucucaaa
......................................................................uuucgucaagguuucucacGag................    2     1     eg1
......................................................................uuucgucaagguuCcucacaag................    1     1     eg1
......................................................................Guucgucaagguuucucacaag................    11    1     eg1
......................................................................uuucgucaagguuucucacaCg................    18    1     eg1
......................................................................uuucgucGagguuucucacaag................    2     1     eg1
......................................................................uuucgucaagguuucucacaUg................    808   1     eg1
......................................................................uuucgucaagguuucucacaag................    291   0     eg1
......................................................................uuucgucaagguuucucacaaA................    466   1     eg1
......................................................................uuucgucaagguuucucacaaU................    3280  1     eg1
......................................................................uuucgucaagguuucucGcaag................    1     1     eg1
......................................................................uuucgucaagguuucucacCag................    1     1     eg1
......................................................................uuucgucaagguuucucacaaC................    294   1     eg1
......................................................................uuucgucaagguuucucacaaCa...............    19    1     eg1
......................................................................uuucgucaagguuucucacaagU...............    6     1     eg1
......................................................................uuucgucaagguuucucacaaAa...............    13    1     eg1
......................................................................uuucgucaagguuucucacaagC...............    5     1     eg1
......................................................................uuucgucaagguuucucacaagG...............    3     1     eg1
......................................................................uuucgucaagguuucucacaaga...............    1     0     eg1
......................................................................Guucgucaagguuucucacaaga...............    1     1     eg1
......................................................................uuucgucaagguuucucacaaUa...............    366   1     eg1
......................................................................uuucgucaagguuucucacaaAaa..............    2     1     eg1
......................................................................uuucgucaagguuucucacaaUaa..............    6     1     eg1
......................................................................uuucgucaagguuucucacaagaac.............    1     0     eg1
......................................................................uuucgucaagguuucucacaaUaac.............    1     1     eg1
......................................................................uuucgucaagguuucucacaGgaac.............    2     1     eg1
......................................................................Guucgucaagguuucucacaagaacu............    1     1     eg1
.......................................................................uucgucaagguuucucac...................    5     0     eg1
.......................................................................Gucgucaagguuucucac...................    1     1     eg1
.......................................................................Gucgucaagguuucucaca..................    2     1     eg1
.......................................................................uucgucaagguuucucaca..................    38    0     eg1
.......................................................................uucgucaagguuucucacU..................    4     1     eg1
.......................................................................uucgucaagguuucucacG..................    2     1     eg1
.......................................................................Gucgucaagguuucucacaa.................    2     1     eg1
.......................................................................uucgucaagguuucucacUa.................    1     1     eg1
.......................................................................uucgucaagguuucucacaC.................    2     1     eg1
.......................................................................uucgucaagguuucucacCa.................    1     1     eg1
.......................................................................uucgucaagguuucucacaa.................    48    0     eg1
.......................................................................uucgucaagguuucuUacaa.................    1     1     eg1
.......................................................................uucgucaagguuucucacaG.................    2     1     eg1
.......................................................................uuUgucaagguuucucacaa.................    4     1     eg1
.......................................................................uucgucaagguuucucacaU.................    36    1     eg1
.......................................................................uucgucaagguuucucacaaU................    14    1     eg1
.......................................................................uucgucaagguuucucacaag................    1     0     eg1
.......................................................................uucgucaagguuucucacaUg................    2     1     eg1
.......................................................................uucgucaagguuucucacaaA................    3     1     eg1
.......................................................................uucgucaagguuucucacaaC................    1     1     eg1
.......................................................................uucgucaagguuucucacaaUa...............    1     1     eg1
........................................................................ucgucaagguuucucacU..................    1     1     eg1
........................................................................Gcgucaagguuucucaca..................    2     1     eg1
........................................................................ucgucaagguuucucacaa.................    1     0     eg1
........................................................................ucgucaagguuucucacaaUa...............    1     1     eg1
......................................................................................cacaagaacuguuuaucuca..    1     0     eg1

.....................................gagaaaaccuugacugaaa....................................................    8     0     er1
.....................................gagaaaaccuugacugaaUa...................................................    1     1     er1
.....................................gagaaaaccuugacugaaaU...................................................    1     1     er1
.....................................gagaaaaccuugacugaaaa...................................................    17    0     er1
.....................................gagaaaaccuugGcugaaaau..................................................    1     1     er1
.....................................gagaaaaccuugacugaaaaA..................................................    2     1     er1
.....................................gagaaaaccuugacugaaaau..................................................    93    0     er1
.....................................gagGaaaccuugacugaaaau..................................................    1     1     er1
.....................................gagaaaaccuugacCgaaaau..................................................    1     1     er1
.....................................gagaaUaccuugacugaaaau..................................................    1     1     er1
.....................................gagaaaaccuugacugGaaau..................................................    1     1     er1
.....................................gagaaaaccuugacugaaaaC..................................................    9     1     er1
.....................................gagaaaaccuugacugaaaaua.................................................    1     0     er1
.....................................gagaaaaccuugacugaaaauU.................................................    9     1     er1
............................................................uuuaaaacauuuucgucaagg...........................    2     0     er1
.................................................................aaAauuuucgucaagguuucuc.....................    1     1     er1
.................................................................aacauuuucgucaagguuucucac...................    1     0     er1
```

```
                                    Star                           Mature
aacagauaugauuaagaacagacaaaaaauaauucuugagaaaaccuugacugaaaauaguuuaaaacauuuucgucaagguuucucacaagaacuguuuaucucaaa
.................................................................aacauuuucgucaagguuucucaca..................   1     0   er1
.................................................................aacauuuucgucaagguuucucacaa.................   2     0   er1
..................................................................acauuuucgucaagguuucucac...................   1     0   er1
..................................................................acauuCucgucaagguuucucaca..................   1     1   er1
..................................................................acauuuucgucaagguuucucaca..................   9     0   er1
..................................................................acauuuucgucaagguuucucacU..................   10    1   er1
..................................................................acauuuucgCcaagguuucucaca..................   1     1   er1
..................................................................acauuuucgucaagguuucucacC..................   1     1   er1
..................................................................acauuuucgucaagguuucucacaU.................   9     1   er1
..................................................................acauuuucgucaagguuucucacaa.................   1     0   er1
...................................................................cauuuucgucaagguuucuc.....................   1     0   er1
...................................................................cauuuucgucaagguuucuca....................   1     0   er1
...................................................................cauuuucgucaagguuucucac...................   3     0   er1
...................................................................cauuuucgucaagguuucucaca..................   11    0   er1
...................................................................cauuuucgucaagguuucucaUa..................   1     1   er1
...................................................................cauuuucgucaagguuucucacU..................   6     1   er1
....................................................................auuuucgucaagguuucuc.....................   1     0   er1
....................................................................auuuucgucaagguuucuca....................   1     0   er1
....................................................................auuuucgucaagguuucucaU...................   5     1   er1
....................................................................auuuucgucaagguuucucac...................   15    0   er1
....................................................................auuuAcgucaagguuucucac...................   1     1   er1
....................................................................auuuucgGcaagguuucucac...................   1     1   er1
....................................................................auuuucgucaagguuucucacC..................   5     1   er1
....................................................................auuuucgucaagguuucucacG..................   1     1   er1
....................................................................auuuucgucaagguuucucaca..................   85    0   er1
....................................................................auuuucgucaagguuucucacU..................   29    1   er1
....................................................................aCuuucgucaagguuucucaca..................   1     1   er1
....................................................................auuuucgucaGgguuucucaca..................   1     1   er1
....................................................................auuuucgucaagguuCcucaca..................   1     1   er1
....................................................................auuuucgucaagguuucCcaca..................   1     1   er1
....................................................................Cuuuucgucaagguuucucaca..................   2     1   er1
....................................................................Uuuuucgucaagguuucucaca..................   2     1   er1
....................................................................auuuucgucaagguuucucacaU.................   5     1   er1
....................................................................auuuucgucaagguuucucacaa.................   1     0   er1
.....................................................................Guuucgucaagguuucuc.....................   1     1   er1
.....................................................................uuuucgucaagguuucuU.....................   1     1   er1
.....................................................................uuuucgucaagguuucuc.....................   11    0   er1
.....................................................................uuuucgucaagguuucuca....................   2     0   er1
.....................................................................uuuucgucaagguuucucaU...................   19    1   er1
.....................................................................uuuucgucaaggAuucucac...................   1     1   er1
.....................................................................uuuucgucaagguuucucac...................   168   0   er1
.....................................................................Guuucgucaagguuucucac...................   15    1   er1
.....................................................................uuuucgucaagguuucucaA...................   2     1   er1
.....................................................................uuuucgucaagguuucCcac...................   1     1   er1
.....................................................................Cuuucgucaagguuucucac...................   9     1   er1
.....................................................................uuuucgucaGgguuucucac...................   2     1   er1
.....................................................................uuuucgucaagAuuucucaca..................   1     1   er1
.....................................................................uuuCcgucaagguuucucaca..................   2     1   er1
.....................................................................uuuucgucaagguuucucaUa..................   6     1   er1
.....................................................................uuuucgucaagguuucucaca..................   1788  0   er1
.....................................................................Guuucgucaagguuucucaca..................   96    1   er1
.....................................................................uuuucgucaaggGuucucaca..................   3     1   er1
.....................................................................uuGucgucaagguuucucaca..................   1     1   er1
.....................................................................uuuucgucaagguuucuUaca..................   3     1   er1
.....................................................................uuuucguUaagguuucucaca..................   1     1   er1
.....................................................................uuuucgucaagguCucucaca..................   3     1   er1
.....................................................................uuuucgucaGgguuucucaca..................   3     1   er1
.....................................................................uuuucgucaagguuucucCca..................   1     1   er1
.....................................................................uuuucgucaaAguuucucaca..................   3     1   er1
.....................................................................uuuucgCcaagguuucucaca..................   7     1   er1
.....................................................................uuuucgucaagguuucAcaca..................   2     1   er1
.....................................................................uuuAcgucaagguuucucaca..................   1     1   er1
.....................................................................uuuucgucaagguuuUucaca..................   1     1   er1
.....................................................................uuuucgucaagguuucucacU..................   180   1   er1
.....................................................................uAuucgucaagguuucucaca..................   2     1   er1
.....................................................................uuuucgucaaggCuucucaca..................   4     1   er1
.....................................................................uuuucgucaagguuucucacG..................   25    1   er1
.....................................................................uuuucgucaagguuucucGca..................   2     1   er1
.....................................................................uCuucgucaagguuucucaca..................   4     1   er1
.....................................................................uuuucgucaagguuucucacC..................   30    1   er1
```

Star Mature

```
aacagauaugauuaagaacagacaaaaaauaauucuugagaaaaccuugacugaaaauaguuuaaaacauuuucgucaagguuucucacaagaacuguuuaucucaaa
.....................................................................uuuucgucaagguuucCcaca.................. 3 1 er1
.....................................................................uuuucgucaagguuCcucaca.................. 4 1 er1
.....................................................................Cuuucgucaagguuucucaca.................. 27 1 er1
.....................................................................uuuuUgucaagguuucucaca.................. 1 1 er1
.....................................................................uuCucgucaagguuucucaca.................. 3 1 er1
.....................................................................uuuucgucGagguuucucaca.................. 2 1 er1
.....................................................................uuuucgucaagguuCcucacaa................. 2 1 er1
.....................................................................uuuucgucaagguuucucacUa................. 10 1 er1
.....................................................................uuuucgucGagguuucucacaa................. 2 1 er1
.....................................................................uuuucgCcaagguuucucacaa................. 1 1 er1
.....................................................................uuuucgucaagguuucucacaa................. 251 0 er1
.....................................................................uuuucgucaaggCuucucacaa................. 2 1 er1
.....................................................................uuuucgucaagguuucucacaU................. 297 1 er1
.....................................................................Cuuucgucaagguuucucacaa................. 25 1 er1
.....................................................................uuuucgucaagguuucCcacaa................. 2 1 er1
.....................................................................uuuucgucaagguuucucacaG................. 3 1 er1
.....................................................................uuuucgucaagguuucucacGa................. 1 1 er1
.....................................................................uuuucgucaagguuucucacaC................. 30 1 er1
.....................................................................Guuucgucaagguuucucacaa................. 9 1 er1
.....................................................................uuuucgucaagguuucucacaaU................ 35 1 er1
.....................................................................uuuucgucaagguuucucacaag................ 4 0 er1
.....................................................................uuuucgucaagguuucucacaaA................ 10 1 er1
.....................................................................uuuucgucaagguuucucacaaC................ 2 1 er1
.....................................................................uuuucgucaagguuucucacaUg................ 7 1 er1
.....................................................................uuuucgucaagguuucucacaaUa............... 3 1 er1
.....................................................................uuuucgucaagguuucucacaaUaa.............. 1 1 er1
......................................................................uuucgucaagguuucuca.................... 50 0 er1
......................................................................uuucgucaagguuucuGa.................... 1 1 er1
......................................................................uuucgucaagguuucucG.................... 2 1 er1
......................................................................uuucgucaGgguuucuca.................... 1 1 er1
......................................................................Guucgucaagguuucuca.................... 5 1 er1
......................................................................uuucgucaGgguuucucac................... 13 1 er1
......................................................................uuucgucaagguuucucaU................... 244 1 er1
......................................................................uuucguUaagguuucucac................... 1 1 er1
......................................................................uuucgucaagguuuUucac................... 3 1 er1
......................................................................Cuucgucaagguuucucac................... 10 1 er1
......................................................................uuucgucaagguGucucac................... 1 1 er1
......................................................................uuucgucaagguuCcucac................... 6 1 er1
......................................................................Auucgucaagguuucucac................... 1 1 er1
......................................................................Guucgucaagguuucucac................... 170 1 er1
......................................................................uuucgucaagguuucCcac................... 10 1 er1
......................................................................uuucgucaaAguuucucac................... 2 1 er1
......................................................................uuucgucaagguuucucac................... 2341 0 er1
......................................................................uuucgucaagguuucAcac................... 2 1 er1
......................................................................uuucgucaagguuucucaA................... 35 1 er1
......................................................................uuucgucGagguuucucac................... 3 1 er1
......................................................................uAucgucaagguuucucac................... 1 1 er1
......................................................................uuAcgucaagguuucucac................... 1 1 er1
......................................................................uuCcgucaagguuucucac................... 11 1 er1
......................................................................uuucgucaaggGuucucac................... 1 1 er1
......................................................................uuucgucaagguuucucUc................... 3 1 er1
......................................................................uuucgucaagguuucGcac................... 1 1 er1
......................................................................uuucgCcaagguuucucac................... 7 1 er1
......................................................................uuucgAcaagguuucucac................... 1 1 er1
......................................................................uuucAucaagguuucucac................... 4 1 er1
......................................................................uuucgucaagAuuucucac................... 1 1 er1
......................................................................uuucgucaaggCuucucac................... 8 1 er1
......................................................................uuuUgucaagguuucucac................... 3 1 er1
......................................................................uuucgucaagguuucuUac................... 1 1 er1
......................................................................uuucgucaagguuucucGc................... 6 1 er1
......................................................................uuucgucaagUuuucucac................... 1 1 er1
......................................................................uCucgucaagguuucucac................... 8 1 er1
......................................................................uuuAgucaagguuucucac................... 1 1 er1
......................................................................uuucgucaUgguuucucac................... 2 1 er1
......................................................................uuucgucaaggAuucucac................... 1 1 er1
......................................................................uuucgucaagguCucucac................... 5 1 er1
......................................................................uuucgucaaUguuucucac................... 1 1 er1
......................................................................uuucAucaagguuucucaca.................. 7 1 er1
......................................................................uuucgucaagguGucucaca.................. 1 1 er1
......................................................................uuucgucaagguuucucacU.................. 1792 1 er1
```

```
                                    Star                            Mature
aacagauaugauuaagaacagacaaaaaauaauucuugagaaaaccuugacugaaaauaguuuaaaacauuuucgucaagguuucucacaagaacuguuuaucucaaa
......................................................................uuucgucaagguuuUucaca..................   8       1   er1
......................................................................uuucgucaagguuuGucaca..................   1       1   er1
......................................................................uuucgucaaAguuucucaca..................   8       1   er1
......................................................................uAucgucaagguuucucaca..................   4       1   er1
......................................................................uuucgGcaagguuucucaca..................   1       1   er1
......................................................................uuucgucaaCguuucucaca..................   1       1   er1
......................................................................uuucgucaagguuucAcaca..................   4       1   er1
......................................................................uuucgucaagguuucucacG..................   142     1   er1
......................................................................uuuGgucaagguuucucaca..................   1       1   er1
......................................................................Guucgucaagguuucucaca..................   619     1   er1
......................................................................uuucgucaagUuuucucaca..................   1       1   er1
......................................................................uuucgucaagguuucucUca..................   4       1   er1
......................................................................uuucgucaagguuucuUaca..................   3       1   er1
......................................................................uuucgucaagguuAcucaca..................   2       1   er1
......................................................................uuucgucaUgguuucucaca..................   1       1   er1
......................................................................uuucgucaagguuucucGca..................   33      1   er1
......................................................................uuucgucaagguuucCcaca..................   38      1   er1
......................................................................uuucgucaagguuucGcaca..................   2       1   er1
......................................................................uuucgucCagguuucucaca..................   1       1   er1
......................................................................uuucgucaagguCucucaca..................   18      1   er1
......................................................................Cuucgucaagguuucucaca..................   47      1   er1
......................................................................uuucgAcaagguuucucaca..................   4       1   er1
......................................................................uCucgucaagguuucucaca..................   31      1   er1
......................................................................uuuAgucaagguuucucaca..................   2       1   er1
......................................................................uuucgucaaggGuucucaca..................   10      1   er1
......................................................................uuucgucaagAuuucucaca..................   4       1   er1
......................................................................uuucgucaagguuucucacC..................   185     1   er1
......................................................................uuucguAaagguuucucaca..................   2       1   er1
......................................................................uuucgucaagguuucuAaca..................   1       1   er1
......................................................................uGucgucaagguuucucaca..................   1       1   er1
......................................................................uuucgucUagguuucucaca..................   2       1   er1
......................................................................uuucguGaagguuucucaca..................   1       1   er1
......................................................................uuucgucaagguuucuCCca..................   4       1   er1
......................................................................uuucgucaagCuuucucaca..................   1       1   er1
......................................................................uuucgucaaggCuucucaca..................   35      1   er1
......................................................................Auucgucaagguuucucaca..................   8       1   er1
......................................................................uuucguUaagguuucucaca..................   2       1   er1
......................................................................uuucgucaaggAuucucaca..................   5       1   er1
......................................................................uuucgCcaagguuucucaca..................   38      1   er1
......................................................................uuucgucaagguuucucaUa..................   53      1   er1
......................................................................uuuUgucaagguuucucaca..................   11      1   er1
......................................................................uuCcgucaagguuucucaca..................   24      1   er1
......................................................................uuucgucaagguuucucaca..................   11581   0   er1
......................................................................uuucgucaCgguuucucaca..................   4       1   er1
......................................................................uuucgucGagguuucucaca..................   34      1   er1
......................................................................uuAcgucaagguuucucaca..................   4       1   er1
......................................................................uuucgucaagguuucucaAa..................   2       1   er1
......................................................................uuucgucaagguuCcucaca..................   40      1   er1
......................................................................uuucgucaGgguuucucaca..................   20      1   er1
......................................................................uuucgAcaagguuucucacaa.................   9       1   er1
......................................................................uuucgucaagguCucucacaa.................   26      1   er1
......................................................................uuucgucaagguuucucacaG.................   293     1   er1
......................................................................uGucgucaagguuucucacaa.................   2       1   er1
......................................................................uuucgucaagAuuucucacaa.................   8       1   er1
......................................................................uuucgucaagguuucAcacaa.................   16      1   er1
......................................................................uuucgucaUgguuucucacaa.................   4       1   er1
......................................................................uCucgucaagguuucucacaa.................   44      1   er1
......................................................................uuucgucaagguuucuCCcaa.................   4       1   er1
......................................................................uuucgucaagguuucucUcaa.................   14      1   er1
......................................................................uuucgucaagguuucucacGa.................   60      1   er1
......................................................................uuucgucaagguuuAucacaa.................   2       1   er1
......................................................................uuucgucCagguuucucacaa.................   3       1   er1
......................................................................Cuucgucaagguuucucacaa.................   71      1   er1
......................................................................uuucAucaagguuucucacaa.................   18      1   er1
......................................................................uAucgucaagguuucucacaa.................   7       1   er1
......................................................................uuucgucaaUguuucucacaa.................   2       1   er1
......................................................................uuucgCcaagguuucucacaa.................   55      1   er1
......................................................................uuGcgucaagguuucucacaa.................   3       1   er1
......................................................................uuucgucaagguuucuUacaa.................   2       1   er1
......................................................................uuuAgucaagguuucucacaa.................   4       1   er1
```

Star Mature

```
aacagauaugauuaagaacagacaaaaaauaauucuugagaaaaccuugacugaaaauaguuuaaaacauuucgucaagguuucucacaagaacuguuuaucucaaa
......................................................................uuucgucaCgguuucucacaa.................   3       1   er1
......................................................................uuuUgucaagguuucucacaa.................   20      1   er1
......................................................................uuucgucaagguuGcucacaa.................   3       1   er1
......................................................................uuucgucaagguGucucacaa.................   3       1   er1
......................................................................uuucgucaaAguuucucacaa.................   9       1   er1
......................................................................uuucgucaGgguuucucacaa.................   47      1   er1
......................................................................Guucgucaagguuucucacaa.................   1572    1   er1
......................................................................uuucgucaagguuucucacaC.................   1757    1   er1
......................................................................uuucgucaaggGuucucacaa.................   23      1   er1
......................................................................uuucgucUagguuucucacaa.................   3       1   er1
......................................................................uuucgucGagguuucucacaa.................   42      1   er1
......................................................................uuucgucaagguuucuAacaa.................   4       1   er1
......................................................................uuCcgucaagguuucucacaa.................   40      1   er1
......................................................................Auucgucaagguuucucacaa.................   12      1   er1
......................................................................uuucgucaagguuucCcacaa.................   52      1   er1
......................................................................uuucgucaagguuucucacaU.................   15493   1   er1
......................................................................uuucgucaagguuucucacaa.................   17703   0   er1
......................................................................uuAcgucaagguuucucacaa.................   9       1   er1
......................................................................uuucguAaagguuucucacaa.................   1       1   er1
......................................................................uuucgucaagguuuUucacaa.................   7       1   er1
......................................................................uuucgucaaggCuucucacaa.................   47      1   er1
......................................................................uuucgucaagguuucucGcaa.................   35      1   er1
......................................................................uuucgucaagguuucucacCa.................   10      1   er1
......................................................................uuucgucaagguuucucacUa.................   552     1   er1
......................................................................uuucgGcaagguuucucacaa.................   1       1   er1
......................................................................uuucguUaagguuucucacaa.................   7       1   er1
......................................................................uuucgucaagguuucGcacaa.................   4       1   er1
......................................................................uuucgucaagguuAcucacaa.................   8       1   er1
......................................................................uuucgucaagguuuGucacaa.................   1       1   er1
......................................................................uuucgucaagguuucucaAaa.................   1       1   er1
......................................................................uuucgucaaCguuucucacaa.................   2       1   er1
......................................................................uuucgucaagguuCcucacaa.................   51      1   er1
......................................................................uuucgucaagguuucucaUaa.................   16      1   er1
......................................................................uuucgucaaggAuucucacaa.................   8       1   er1
......................................................................uuucgucaagguuCcucacaag................   1       1   er1
......................................................................Guucgucaagguuucucacaag................   14      1   er1
......................................................................uuucgucaagguuucucacUag................   2       1   er1
......................................................................uuucgucaagguuucucacaaU................   1938    1   er1
......................................................................uuucgucaagguuucucacGag................   1       1   er1
......................................................................uuucgucaagguuucucacaUg................   326     1   er1
......................................................................uuucgucaagguuucucacaGg................   1       1   er1
......................................................................uuucgucaagguuucucacaag................   120     0   er1
......................................................................uuucgucaagguuucucacaaC................   193     1   er1
......................................................................Cuucgucaagguuucucacaag................   1       1   er1
......................................................................uuucgucaagguuucucacaCg................   2       1   er1
......................................................................uuucgucaagguuucucacaaA................   401     1   er1
......................................................................uuucgucaagguuucucacaagG...............   1       1   er1
......................................................................uuucgucaagguuucucacaagC...............   3       1   er1
......................................................................uuucgucaagguuucucacaaAa...............   6       1   er1
......................................................................uuucgucaagguuucucacaaCa...............   15      1   er1
......................................................................uuucgucaagguuucucacaaga...............   2       0   er1
......................................................................uuucgucaagguuucucacaaUa...............   411     1   er1
......................................................................uuucgucaagguuucucacaagU...............   3       1   er1
......................................................................uuucgucaagguuucucacaaUaa..............   12      1   er1
......................................................................uuucgucaagguuucucacaagCa..............   2       1   er1
......................................................................uuucgucaagguuucucacaaAaa..............   3       1   er1
......................................................................uuucgucaagguuucucacaGgaac.............   1       1   er1
......................................................................uuucgucaagguuucucacaagaUc.............   1       1   er1
......................................................................uuucgucaagguuucucacaagaac.............   1       0   er1
......................................................................uuucgucaagguuucucacaagaaU.............   1       1   er1
......................................................................uuucgucaagguuucucacaagaaG.............   1       1   er1
......................................................................uuucgucaagguuucucacaagaacu............   1       0   er1
......................................................................uuucgucaagguuucucacaUgaacu............   1       1   er1
......................................................................uuucgucaagguuucucacaagUacu............   1       1   er1
......................................................................uuucgucaagguuucucacaGgaacu............   1       1   er1
......................................................................uuucgucaagguuucucacaaCaacug...........   1       1   er1
.......................................................................uucgucaagguuucucac...................   2       0   er1
.......................................................................Gucgucaagguuucucaca..................   1       1   er1
.......................................................................uuUgucaagguuucucaca..................   2       1   er1
.......................................................................uucgucaagguuucucaca..................   6       0   er1
```

```
                                     Star                        Mature
aacagauaugauuaagaacagacaaaaaauaauucuugagaaaaccuugacugaaaauaguuuaaaacauuucgucaagguuucucacaagaacuguuuaucucaaa
.......................................................................uucgucaagguuucucacaC.................   2     1    er1
.......................................................................uucgCcaagguuucucacaa.................   1     1    er1
.......................................................................uucgucaagguuucucacaU.................   13    1    er1
.......................................................................Gucgucaagguuucucacaa.................   1     1    er1
.......................................................................uucgucaagguuucucacaa.................   16    0    er1
.......................................................................uucgucaagguuucucacaaU................   11    1    er1
.......................................................................uucgucaagguuucucacaaC................   1     1    er1
.......................................................................uucgucaagguuucucacaaA................   1     1    er1
.......................................................................uucgucaagguuucucacaaUa...............   2     1    er1
........................................................................Gcgucaagguuucucaca..................   1     1    er1
........................................................................ucgucaagguuucucaca..................   2     0    er1
........................................................................ucgucaagguuucucacaU.................   1     1    er1
........................................................................ucgucaagguuucCcacaa.................   1     1    er1
........................................................................Gcgucaagguuucucacaa.................   3     1    er1
........................................................................ucgucaagguuucucacaa.................   1     0    er1

.....................................gagaaaaccuugacugaaa....................................................   2     0    er2
.....................................gagaaaaccuugacugaaaa...................................................   14    0    er2
.....................................gagaaaaccuugacugaaaau..................................................   60    0    er2
.....................................gGgaaaaccuugacugaaaau..................................................   1     1    er2
.....................................Nagaaaaccuugacugaaaau..................................................   1     1    er2
.....................................gagaaaaccuugacugaaaauU.................................................   3     1    er2
..............................................uugacugaaaauaguuuaaaac........................................   8     0    er2
.................................................................aacauuuucgucaagguuucuc.....................   3     0    er2
.................................................................aacauuuucgucaagguuucuca....................   4     0    er2
.................................................................aacauuuucgucaagguuucucacU..................   20    1    er2
.................................................................aacauuuucgucaagguuucucaca..................   10    0    er2
.................................................................Cacauuuucgucaagguuucucaca..................   5     1    er2
..................................................................acauuuucgucaagguuucu......................   2     0    er2
..................................................................acauuuucgucaagguuucuc.....................   2     0    er2
..................................................................acauuuucgucaagguuucucU....................   1     1    er2
..................................................................acauuuucgucaagguuucuca....................   12    0    er2
..................................................................acauuuucgucaagguuucucac...................   5     0    er2
..................................................................acaAuuucgucaagguuucucaca..................   1     1    er2
..................................................................acauuuucgucaagguuucucacC..................   5     1    er2
..................................................................acauuuucgucaagguuucucacU..................   75    1    er2
..................................................................acauuuucgucaagguuucucaca..................   119   0    er2
..................................................................acauuuucgucaagguuucucacaa.................   8     0    er2
..................................................................acauuuucgucaagguuucucacUa.................   16    1    er2
..................................................................acauuuucgucaagguuucucacaU.................   12    1    er2
..................................................................acauuuucgucaagguuucucacaaA................   6     1    er2
...................................................................cauuuucgucaagguuucuca....................   17    0    er2
...................................................................cauuuucgucaagguuucucU....................   4     1    er2
...................................................................cauuuucgucaagguuucucac...................   9     0    er2
...................................................................cauuuucgucaagguuucucaU...................   1     1    er2
...................................................................Uauuuucgucaagguuucucaca..................   1     1    er2
...................................................................cauuuucgucaagguuucucacC..................   5     1    er2
...................................................................cauuuucgucaagguuucucaUa..................   3     1    er2
...................................................................cauuuucgucaagguuucucaca..................   472   0    er2
...................................................................cauuuucgucaagguuucucacG..................   3     1    er2
...................................................................cauuuucgucaagguuucucacU..................   257   1    er2
...................................................................caAuuucgucaagguuucucaca..................   1     1    er2
...................................................................Nauuuucgucaagguuucucaca..................   5     1    er2
...................................................................Aauuuucgucaagguuucucaca..................   2     1    er2
...................................................................cauuuucgucaagguuucucacaa.................   8     0    er2
...................................................................cauuuucgucaagguuucucacUa.................   3     1    er2
...................................................................cauuuucgucaagguuucucacaU.................   16    1    er2
....................................................................auuuucgucaagguuucuc.....................   7     0    er2
....................................................................auuuucgucaagguuucucU....................   8     1    er2
....................................................................auuuucgucaagguuucuca....................   18    0    er2
....................................................................auuuucgucaagguuucucac...................   31    0    er2
....................................................................auuuucgucaagguuucucaU...................   1     1    er2
....................................................................Cuuuucgucaagguuucucac...................   1     1    er2
....................................................................auuuucgucaagguuucucacG..................   1     1    er2
....................................................................Cuuuucgucaagguuucucaca..................   31    1    er2
....................................................................Nuuuucgucaagguuucucaca..................   1     1    er2
....................................................................auuuucgucaagguuucucacC..................   3     1    er2
....................................................................auuuucgucaagguuucucacU..................   222   1    er2
....................................................................Uuuuucgucaagguuucucaca..................   2     1    er2
....................................................................auuuucgucaagguuucucaca..................   569   0    er2
```

Star Mature

aacagauaugauuaagaacagacaaaaaauaauucuugagaaaaccuugacugaaaauaguuuaaaacauuuucgucaagguuucucacaagaacuguuuaucucaaa			
....auuuucgucGagguuucucaca..................	1	1	er2
....auuuucgucaagguuucucacaa.................	3	0	er2
....Cuuuucgucaagguuucucacaa.................	2	1	er2
....auuuucgucaagguuucucacaU.................	11	1	er2
....auuuucgucaagguuucucacUa.................	3	1	er2
....uuuucgucaagguuucucU....................	1	1	er2
....uuuucgucaagguuucuca....................	42	0	er2
....Cuuucgucaagguuucuca....................	1	1	er2
....uuuCcgucaagguuucuca....................	1	1	er2
....uuuucgucaagguuucucaU...................	11	1	er2
....uuuucgucaagguuucucac...................	282	0	er2
....Nuuucgucaagguuucucac...................	7	1	er2
....uuuucguUaagguuucucac...................	1	1	er2
....Cuuucgucaagguuucucac...................	6	1	er2
....uuCucgucaagguuucucaca..................	3	1	er2
....uuuucgucaaUguuucucaca..................	2	1	er2
....uuuucgucaaggGuucucaca..................	4	1	er2
....uuuucUucaagguuucucaca..................	1	1	er2
....uuuucgucaagguuucucacU..................	1576	1	er2
....uuuucgucaagguuucCcaca..................	5	1	er2
....uuuucguUaagguuucucaca..................	1	1	er2
....uuuucgAcaagguuucucaca..................	2	1	er2
....uuuuUgucaagguuucucaca..................	2	1	er2
....uuuucgucaagguuuUucaca..................	2	1	er2
....uuuucgucUagguuucucaca..................	1	1	er2
....uuuucgucaaggCuucucaca..................	2	1	er2
....uuuucgucaagguAucucaca..................	1	1	er2
....uuuucgucaagguuucuAaca..................	2	1	er2
....uuuucgucaagguuucucacG..................	7	1	er2
....uuuucgucGagguuucucaca..................	1	1	er2
....uuuucgucaagguuucuGaca..................	1	1	er2
....uuuucgucaaggAuucucaca..................	1	1	er2
....uuuucgucaagguCucucaca..................	4	1	er2
....Nuuucgucaagguuucucaca..................	103	1	er2
....Cuuucgucaagguuucucaca..................	382	1	er2
....uuuucgucaagguuucucacC..................	21	1	er2
....uuuucgucaagguuucucaca..................	10284	0	er2
....uuuAcgucaagguuucucaca..................	1	1	er2
....Guuucgucaagguuucucaca..................	8	1	er2
....uNuucgucaagguuucucaca..................	5	1	er2
....uuuucgucaagguuGcucaca..................	1	1	er2
....uuuucgucaaAguuucucaca..................	1	1	er2
....uuuucAucaagguuucucaca..................	1	1	er2
....uuuucgucaagguuucucaUa..................	2	1	er2
....uuuucgucaagguuucucGca..................	2	1	er2
....uuuucguAaagguuucucaca..................	3	1	er2
....uuuucgucaGgguuucucaca..................	3	1	er2
....uuuucgucaagguGucucaca..................	2	1	er2
....uCuucgucaagguuucucaca..................	2	1	er2
....uuuucgucaagguuucucacaa.................	591	0	er2
....Cuuucgucaagguuucucacaa.................	70	1	er2
....uuuucgucaagguuucucacaG.................	1	1	er2
....Nuuucgucaagguuucucacaa.................	1	1	er2
....uuuucgucaagguuucucacaU.................	473	1	er2
....uuuucgucaagguuucucacaC.................	4	1	er2
....uuuucgucaagguuucucacUa.................	34	1	er2
....uuuucgucaagguuucucacaUg................	16	1	er2
....uuuucgucaagguuucucacaaU................	84	1	er2
....uuuucgucaagguuucucacaag................	4	0	er2
....uuuucgucaagguuucucacaaA................	22	1	er2
....uuuucgucaagguuucucacaaAa...............	1	1	er2
....uuuucgucaagguuucucacaaCa...............	6	1	er2
....uuuucgucaagguuucucacaaUa...............	31	1	er2
....Cuucgucaagguuucuca....................	2	1	er2
....uuucgucaagguuucuca....................	532	0	er2
....uuucgucaagguuucucG....................	7	1	er2
....uuucgucaagguuucuAa....................	2	1	er2
....Nuucgucaagguuucuca....................	2	1	er2
....uuucgucaagguuucucU....................	19	1	er2
....Nuucgucaagguuucucac...................	17	1	er2

```
                                    Star                            Mature
aacagauaugauuaagaacagacaaaaaauaauucuugagaaaaccuugacugaaaauaguuuaaaacauuuucgucaagguuucucacaagaacuguuuaucucaaa
......................................................................uGucgucaagguuucucac...................   1       1   er2
......................................................................Auucgucaagguuucucac...................   2       1   er2
......................................................................uuucgucaaggCuucucac...................   1       1   er2
......................................................................uuucgucaagguuucucaU...................   83      1   er2
......................................................................uuucgucaagguuucucGc...................   2       1   er2
......................................................................uuGcgucaagguuucucac...................   1       1   er2
......................................................................uuucgucaaggGuucucac...................   3       1   er2
......................................................................uuucgucaagguuucucaA...................   14      1   er2
......................................................................uuucgucaagguuucucac...................   3189    0   er2
......................................................................Cuucgucaagguuucucac...................   5       1   er2
......................................................................uNucgucaagguuucucac...................   1       1   er2
......................................................................uuucgucaagguuuUucac...................   1       1   er2
......................................................................Guucgucaagguuucucac...................   3       1   er2
......................................................................uuucgucaagguuucCcac...................   2       1   er2
......................................................................uCucgucaagguuucucac...................   1       1   er2
......................................................................uuucgucaagguuCcucac...................   1       1   er2
......................................................................uuucgucaagguuucuGaca..................   2       1   er2
......................................................................uuucgucaagAuuucucaca..................   19      1   er2
......................................................................uuucgucaagguuAcucaca..................   4       1   er2
......................................................................uuucgucaaAguuucucaca..................   6       1   er2
......................................................................uuGcgucaagguuucucaca..................   11      1   er2
......................................................................uuucgucUagguuucucaca..................   2       1   er2
......................................................................uuucgucaagguGucucaca..................   16      1   er2
......................................................................uGucgucaagguuucucaca..................   28      1   er2
......................................................................uuucgucaagguuucucacG..................   159     1   er2
......................................................................uuucgucaCgguuucucaca..................   4       1   er2
......................................................................uuucgucaGgguuucucaca..................   58      1   er2
......................................................................uuucgucaagguuucGcaca..................   11      1   er2
......................................................................uuucgucaagguuucucacU..................   27882   1   er2
......................................................................uuucgucaaggGuucucaca..................   113     1   er2
......................................................................uuucgucaagguuCcucaca..................   52      1   er2
......................................................................uuucgucCagguuucucaca..................   2       1   er2
......................................................................uuuGgucaagguuucucaca..................   8       1   er2
......................................................................uuucgucaUgguuucucaca..................   8       1   er2
......................................................................uuucguGaagguuucucaca..................   6       1   er2
......................................................................uuucgucGagguuucucaca..................   36      1   er2
......................................................................uuucgucaagguuucAcaca..................   10      1   er2
......................................................................Cuucgucaagguuucucaca..................   105     1   er2
......................................................................uuAcgucaagguuucucaca..................   4       1   er2
......................................................................uuucguAaagguuucucaca..................   19      1   er2
......................................................................uuucgucaagguuucucGca..................   43      1   er2
......................................................................uuucgucaagguCucucaca..................   45      1   er2
......................................................................uAucgucaagguuucucaca..................   6       1   er2
......................................................................Auucgucaagguuucucaca..................   48      1   er2
......................................................................uuucgucaaggCuucucaca..................   60      1   er2
......................................................................uuucgCcaagguuucucaca..................   63      1   er2
......................................................................uuucgucaaUguuucucaca..................   13      1   er2
......................................................................uuucgAcaagguuucucaca..................   10      1   er2
......................................................................uuCcgucaagguuucucaca..................   37      1   er2
......................................................................uNucgucaagguuucucaca..................   78      1   er2
......................................................................uuucgucaagguuucucaca..................   193424  0   er2
......................................................................uuucgucaagguuucucCca..................   5       1   er2
......................................................................uuucgucaaCguuucucaca..................   1       1   er2
......................................................................uuucgucaagguuucucacC..................   233     1   er2
......................................................................uuucgucaagguuucucaUa..................   169     1   er2
......................................................................uuucUucaagguuucucaca..................   4       1   er2
......................................................................uuucgucaagguuucuUaca..................   19      1   er2
......................................................................uuucguUaagguuucucaca..................   15      1   er2
......................................................................uuucgucaaggAuucucaca..................   22      1   er2
......................................................................uuucgucaagguAucucaca..................   3       1   er2
......................................................................uuucgucaagguuuGucaca..................   1       1   er2
......................................................................uuucgucaagguuucucUca..................   10      1   er2
......................................................................uuuUgucaagguuucucaca..................   21      1   er2
......................................................................Guucgucaagguuucucaca..................   75      1   er2
......................................................................uuucgucaagguuuUucaca..................   13      1   er2
......................................................................uuucgucaagguuuAucaca..................   9       1   er2
......................................................................uuuAgucaagguuucucaca..................   16      1   er2
......................................................................uuucgucaagguuucucaAa..................   6       1   er2
......................................................................uuucgucaagguuGcucaca..................   15      1   er2
......................................................................uuucgucaagguuucCcaca..................   85      1   er2
```

Star **Mature**

```
aacagauaugauuaagaacagacaaaaaauaauucuugagaaaaccuugacugaaaauaguuuaaaacauuuucgucaagguuucucacaagaacuguuuaucucaaa
......................................................................uuucAucaagguuucucaca..................  29     1  er2
......................................................................uuucgucaagUuuucucaca..................  7      1  er2
......................................................................uuucgGcaagguuucucaca..................  43     1  er2
......................................................................Nuucgucaagguuucucaca..................  621    1  er2
......................................................................uCucgucaagguuucucaca..................  67     1  er2
......................................................................uuucgucaagguuucuAaca..................  5      1  er2
......................................................................uuucgucaagguuucucaGa..................  3      1  er2
......................................................................uCucgucaagguuucucacaa.................  14     1  er2
......................................................................uuucgucaagguuucucacGa.................  12     1  er2
......................................................................uuucguUaagguuucucacaa.................  1      1  er2
......................................................................uuucgucUagguuucucacaa.................  3      1  er2
......................................................................uuuAgucaagguuucucacaa.................  5      1  er2
......................................................................uuucgucaagguuucucacaC.................  202    1  er2
......................................................................uuucgucaagguuucucacaG.................  43     1  er2
......................................................................uuucgCcaagguuucucacaa.................  8      1  er2
......................................................................uuucgucaagguuucucacaa.................  44144  0  er2
......................................................................Guucgucaagguuucucacaa.................  12     1  er2
......................................................................uuucgucGagguuucucacaa.................  7      1  er2
......................................................................uuGcgucaagguuucucacaa.................  2      1  er2
......................................................................uuucgucaagguuucCcacaa.................  28     1  er2
......................................................................uuucgucaaggCuucucacaa.................  18     1  er2
......................................................................uuucgucCagguuucucacaa.................  1      1  er2
......................................................................uuucgucaagguuucucacCa.................  18     1  er2
......................................................................uuucgucaagguuucucaGaa.................  1      1  er2
......................................................................uuucAucaagguuucucacaa.................  5      1  er2
......................................................................uuucgucaagguuucuGacaa.................  3      1  er2
......................................................................uuucgucaagguuCcucacaa.................  4      1  er2
......................................................................uuucgucaagguuucucacaU.................  29833  1  er2
......................................................................uuucgGcaagguuucucacaa.................  10     1  er2
......................................................................uGucgucaagguuucucacaa.................  8      1  er2
......................................................................uuucgucaagAuuucucacaa.................  2      1  er2
......................................................................uuucgucaagguuucucaUaa.................  3      1  er2
......................................................................uuucgucaagguuucuUacaa.................  2      1  er2
......................................................................uuucguGaagguuucucacaa.................  1      1  er2
......................................................................uuucgucaagguuucuAacaa.................  7      1  er2
......................................................................uuucgucaagguuucAcacaa.................  2      1  er2
......................................................................uuucgucaagguuucGcacaa.................  1      1  er2
......................................................................uNucgucaagguuucucacaa.................  17     1  er2
......................................................................uuucgucaagguuuAucacaa.................  3      1  er2
......................................................................uuucgucaagguuuGucacaa.................  1      1  er2
......................................................................uuucgucaaCguuucucacaa.................  2      1  er2
......................................................................uuucgucaagguuuUucacaa.................  3      1  er2
......................................................................uuucgucaagguCucucacaa.................  8      1  er2
......................................................................uuucguAaagguuucucacaa.................  3      1  er2
......................................................................uuucgucaaggAuucucacaa.................  6      1  er2
......................................................................uuucgucaagguuucucaAaa.................  3      1  er2
......................................................................uuCcgucaagguuucucacaa.................  9      1  er2
......................................................................uuucgucaagguuucucUcaa.................  1      1  er2
......................................................................uuuUgucaagguuucucacaa.................  1      1  er2
......................................................................uuucgucaGgguuucucacaa.................  3      1  er2
......................................................................Cuucgucaagguuucucacaa.................  25     1  er2
......................................................................Auucgucaagguuucucacaa.................  11     1  er2
......................................................................uuAcgucaagguuucucacaa.................  1      1  er2
......................................................................uuucgucaagguuucucacUa.................  1463   1  er2
......................................................................uuucgucaaAguuucucacaa.................  1      1  er2
......................................................................uuucgucaaggGuucucacaa.................  28     1  er2
......................................................................uAucgucaagguuucucacaa.................  1      1  er2
......................................................................uuucgAcaagguuucucacaa.................  3      1  er2
......................................................................uuucgucaagguGucucacaa.................  2      1  er2
......................................................................uuucgucaagguuGcucacaa.................  4      1  er2
......................................................................uuucgucaagguuAcucacaa.................  1      1  er2
......................................................................Nuucgucaagguuucucacaa.................  147    1  er2
......................................................................uuucgucaagguuucucGcaa.................  10     1  er2
......................................................................uuucgGcaagguuucucacaag................  1      1  er2
......................................................................uuucgucaagguuucucacaaU................  5782   1  er2
......................................................................uuucgucaagguuucucacaaC................  115    1  er2
......................................................................uuucgucaagguuucucacaaA................  2498   1  er2
......................................................................Cuucgucaagguuucucacaag................  1      1  er2
......................................................................uuucgucaagguuucucacaag................  551    0  er2
......................................................................uuucgucaagguuucucacaUg................  965    1  er2
```

Star Mature

```
aacagauaugauuaagaacagacaaaaaauaauucuugagaaaaccuugacugaaaauaguuuaaaacauuuucgucaagguuucucacaagaacuguuuaucucaaa
......................................................................uuucgucaagguuucucacGag................   1      1    er2
......................................................................uuucgucaagguuucucacaGg................   1      1    er2
......................................................................Guucgucaagguuucucacaag................   1      1    er2
......................................................................uuucgucaagguuucucacaCg................   16     1    er2
......................................................................uuucgucaagguuucucacaaga...............   5      0    er2
......................................................................uuucgucaagguuucucacaaAa...............   62     1    er2
......................................................................uuucgucaagguuucucacaagU...............   1      1    er2
......................................................................uuucgucaagguuucucacaaUa...............   1691   1    er2
......................................................................uuucgucaagguuucucacaaCa...............   203    1    er2
......................................................................uuucgucaagguuucucacaaAaa..............   2      1    er2
......................................................................uuucgucaagguuucucacaUgaa..............   2      1    er2
......................................................................uuucgucaagguuucucacaaUaa..............   58     1    er2
.......................................................................uucgucaagguuucucaU...................   3      1    er2
.......................................................................Nucgucaagguuucucac...................   1      1    er2
.......................................................................uucgucaagguuucucac...................   35     0    er2
.......................................................................uucgucaagguuucucUc...................   2      1    er2
.......................................................................uucgucaaggGuucucac...................   1      1    er2
.......................................................................uucgucaaggGuucucaca..................   8      1    er2
.......................................................................uucguUaagguuucucaca..................   1      1    er2
.......................................................................uucgucaagguuucuUaca..................   1      1    er2
.......................................................................uuUgucaagguuucucaca..................   1      1    er2
.......................................................................uucgucaagguGucucaca..................   1      1    er2
.......................................................................uucgucaaggCuucucaca..................   3      1    er2
.......................................................................uucgucaagguCucucaca..................   1      1    er2
.......................................................................uucgucaagguuucuAaca..................   1      1    er2
.......................................................................uucgGcaagguuucucaca..................   1      1    er2
.......................................................................uucgucaaggAuucucaca..................   1      1    er2
.......................................................................uucgucaagguuucucaUa..................   4      1    er2
.......................................................................uucAucaagguuucucaca..................   2      1    er2
.......................................................................Cucgucaagguuucucaca..................   2      1    er2
.......................................................................uucgucaagguuucucacU..................   387    1    er2
.......................................................................uucgucCagguuucucaca..................   6      1    er2
.......................................................................uucgucaagguuucucGca..................   1      1    er2
.......................................................................uucgucaagguuucucacG..................   1      1    er2
.......................................................................Nucgucaagguuucucaca..................   8      1    er2
.......................................................................uucgucaGgguuucucaca..................   1      1    er2
.......................................................................uucgucaagguuucucaca..................   2458   0    er2
.......................................................................uucgucaagguuCcucaca..................   3      1    er2
.......................................................................uucgucaagguuucCcaca..................   1      1    er2
.......................................................................uucgucGagguuucucaca..................   1      1    er2
.......................................................................uucgucaagguCucucacaa.................   1      1    er2
.......................................................................uucgucaagguuucucacaC.................   2      1    er2
.......................................................................uucgucCagguuucucacaa.................   1      1    er2
.......................................................................uucgucGagguuucucacaa.................   1      1    er2
.......................................................................Cucgucaagguuucucacaa.................   1      1    er2
.......................................................................uucgucaagguuucucacGa.................   1      1    er2
.......................................................................uucgucaaggGuucucacaa.................   1      1    er2
.......................................................................uucgucaagguuucucacaa.................   625    0    er2
.......................................................................uucgucaagguuucucacaU.................   450    1    er2
.......................................................................Nucgucaagguuucucacaa.................   2      1    er2
.......................................................................uucgucaagguuucucacUa.................   17     1    er2
.......................................................................uucgucaagguuucucacaag................   15     0    er2
.......................................................................uucgucaagguuucucacaUg................   17     1    er2
.......................................................................uucgucaagguuucucacaaU................   121    1    er2
.......................................................................uucgucaagguuucucacaaA................   62     1    er2
.......................................................................uucgucaagguuucucacaaCa...............   3      1    er2
.......................................................................uucgucaagguuucucacaaUa...............   31     1    er2
........................................................................ucgucaagguuucucacU..................   34     1    er2
........................................................................ucgucaCgguuucucaca..................   1      1    er2
........................................................................Ncgucaagguuucucaca..................   1      1    er2
........................................................................Gcgucaagguuucucaca..................   1      1    er2
........................................................................ucgucaagguuucucaca..................   290    0    er2
........................................................................ucgucaagguuucucacUa.................   5      1    er2
........................................................................Ncgucaagguuucucacaa.................   1      1    er2
........................................................................ucgucaagguuucucacaU.................   34     1    er2
........................................................................ucgucaagguuucucacaa.................   68     0    er2
........................................................................ucgucaagguuucucacaaU................   8      1    er2
........................................................................ucgucaagguuucucacaaUa...............   7      1    er2

....................................Cgagaaaaccuugacugaaa....................................................   1      1    ea1
```

```
                                    Star                           Mature
aacagauaugauuaagaacagacaaaaaauaauucuugagaaaaccuugacugaaaauaguuuaaaacauuuucgucaagguuucucacaagaacuguuuaucucaaa
....................................Cgagaaaaccuugacugaaaa...................................................  1      1   ea1
....................................Cgagaaaaccuugacugaaaau..................................................  1      1   ea1
....................................ugagaaaaccuugacugaaaaC..................................................  1      1   ea1
.....................................gagaaaaccuugacugUa.....................................................  3      1   ea1
.....................................gaAaaaaccuugacugaa.....................................................  1      1   ea1
.....................................gagaaaaccuugacugaa.....................................................  59     0   ea1
.....................................gagaaaaccuugacugaaa....................................................  203    0   ea1
.....................................gagaaaaccuugacugaaU....................................................  6      1   ea1
.....................................gagaaaaccuugacugaUa....................................................  2      1   ea1
.....................................gagGaaaccuugacugaaa....................................................  6      1   ea1
.....................................gagaaaaGcuugacugaaa....................................................  1      1   ea1
.....................................gagaaaacAuugacugaaa....................................................  1      1   ea1
.....................................gagaaaaccuugacugaaG....................................................  5      1   ea1
.....................................gagaaGaccuugacugaaa....................................................  1      1   ea1
.....................................gagaaaaccuugacugaaaG...................................................  1      1   ea1
.....................................gagaaaaccuugacugaaGa...................................................  2      1   ea1
.....................................gagaaaaccuugacugaaUa...................................................  24     1   ea1
.....................................gGgaaaaccuugacugaaaa...................................................  1      1   ea1
.....................................gagaaaaccuugGcugaaaa...................................................  1      1   ea1
.....................................gagaaGaccuugacugaaaa...................................................  1      1   ea1
.....................................gUgaaaaccuugacugaaaa...................................................  1      1   ea1
.....................................gagaGaaccuugacugaaaa...................................................  1      1   ea1
.....................................gagaaaaccuugacugaaaU...................................................  4      1   ea1
.....................................gagaaaaccuugacugGaaa...................................................  1      1   ea1
.....................................gagaaaaccuugacugaaaC...................................................  3      1   ea1
.....................................Aagaaaaccuugacugaaaa...................................................  2      1   ea1
.....................................gagaaaaccGugacugaaaa...................................................  1      1   ea1
.....................................gagUaaaccuugacugaaaa...................................................  1      1   ea1
.....................................gagaaaaccuugacugaaaa...................................................  336    0   ea1
.....................................gagaaaaccuugacuAaaaa...................................................  2      1   ea1
.....................................gagGaaaccuugacugaaaa...................................................  3      1   ea1
.....................................gagaaaaccuugacugaaaUu..................................................  7      1   ea1
.....................................Aagaaaaccuugacugaaaau..................................................  1      1   ea1
.....................................gagaaaGccuugacugaaaau..................................................  4      1   ea1
.....................................gagGaaaccuugacugaaaau..................................................  4      1   ea1
.....................................gagaaaaccCugacugaaaau..................................................  3      1   ea1
.....................................gagaaGaccuugacugaaaau..................................................  8      1   ea1
.....................................gagaaaaccuugacugaaaaG..................................................  4      1   ea1
.....................................gagaaaacUuugacugaaaau..................................................  2      1   ea1
.....................................gGgaaaaccuugacugaaaau..................................................  1      1   ea1
.....................................gagaaaaccuugacugaaGau..................................................  3      1   ea1
.....................................gagaGaaccuugacugaaaau..................................................  3      1   ea1
.....................................gagaaaaccuugacugaaaau..................................................  1116   0   ea1
.....................................gUgaaaaccuugacugaaaau..................................................  1      1   ea1
.....................................gagaaaaccuugacugaUaau..................................................  3      1   ea1
.....................................gagaaaaccuuAacugaaaau..................................................  1      1   ea1
.....................................gaAaaaaccuugacugaaaau..................................................  1      1   ea1
.....................................gagaaaaccuugacugaaaCu..................................................  1      1   ea1
.....................................gagaaaaccuugacugaaUau..................................................  2      1   ea1
.....................................gagaaaaccuugacugGaaau..................................................  3      1   ea1
.....................................gagaaaaccuugacuAaaaau..................................................  4      1   ea1
.....................................gagaaaaccuugacugaGaau..................................................  3      1   ea1
.....................................gagaaaaccuugacugaaaaC..................................................  91     1   ea1
.....................................gagaaaaccuugacCgaaaau..................................................  1      1   ea1
.....................................gagCaaaccuugacugaaaau..................................................  1      1   ea1
.....................................gagaaaaccuugacugaaaaA..................................................  13     1   ea1
.....................................gagaaaaccuugaUugaaaaua.................................................  1      1   ea1
.....................................gagaaaaccuugacugaaaaua.................................................  28     0   ea1
.....................................gagaaaaccuugacugaaaaCa.................................................  2      1   ea1
.....................................gagaaaaccuugacugUaaaua.................................................  1      1   ea1
.....................................gagaaaaccuugacugaaaauU.................................................  44     1   ea1
.....................................gagGaaaccuugacugaaaaua.................................................  1      1   ea1
.....................................gagaaaaccuugacugaGaaua.................................................  1      1   ea1
.....................................gagaaaaccuugacugaaaauC.................................................  6      1   ea1
.....................................gagaaaaccuugacugaaaauag................................................  1      0   ea1
.....................................gagaaaaccuugacugaaaauaguuua............................................  1      0   ea1
.....................................gagaaaaccuugacugaaaauaguuuC............................................  1      1   ea1
...........................................accuugacugaaaauaguAua............................................  1      1   ea1
.........................................................uaguuuaaaacauuuucguc...............................  1      0   ea1
.........................................................uaguuuaaaacauuuucguca..............................  1      0   ea1
```

Star Mature

aacagauaugauuaagaacagacaaaaaauaauucuugagaaaaccuugacugaaaauaguuuaaaacauuuucgucaagguuucucacaagaacuguuuaucucaaa			
....Gaguuuaaaacauuuucguca..............................	1	1	ea1
....uuuaaaacauuuucgucaagA...........................	1	1	ea1
....Guuaaaacauuuucgucaagg...........................	1	1	ea1
....uuuaaaacaAuuucgucaagg...........................	1	1	ea1
....uuuaaaacauuuucgucaagg...........................	4	0	ea1
....uuuaaaacauuuucgucaagguA.........................	1	1	ea1
....Guaaaacauuuucgucaagg...........................	1	1	ea1
....uuaaaacauuuucgucaagg...........................	1	0	ea1
....aaacauuuucgucaagguuucucaca..................	1	0	ea1
....aacauuuucgucaagguuucA......................	1	1	ea1
....aacauuuucgucaagguuucucac...................	1	0	ea1
....Cacauuuucgucaagguuucucac...................	1	1	ea1
....aacauuuucgucaagguuCcucac...................	1	1	ea1
....aacauuuucgucaagguuucCcac...................	1	1	ea1
....aacauuuucgucaagguuucucacU..................	6	1	ea1
....aacauuuucgAcaagguuucucaca..................	1	1	ea1
....aacauuuucgucaagguuucucaca..................	11	0	ea1
....aacauuuucgucaagguuucucacC..................	1	1	ea1
....aacauuuucgucaagguuucucacaC.................	1	1	ea1
....aacauuuucgucaagguuucucacaU.................	2	1	ea1
....acauuuucgucaagguuu........................	1	0	ea1
....acauuuucgucaagguuucuU.....................	1	1	ea1
....acauuuucgucaagguuucuc.....................	7	0	ea1
....acauuuucgucaagguuucuca....................	14	0	ea1
....acauuCucgucaagguuucucac...................	1	1	ea1
....acauuuucgucaagguuucucaU...................	3	1	ea1
....acauuuucgucaagguuucucac...................	20	0	ea1
....Gcauuuucgucaagguuucucac...................	1	1	ea1
....acauuuucgucaagguuucucacU..................	56	1	ea1
....acauuuucgCcaagguuucucaca..................	3	1	ea1
....acauuuucgucaagguuucucacG..................	2	1	ea1
....Gcauuuucgucaagguuucucaca..................	2	1	ea1
....acauuuucgucaagguuucucaca..................	75	0	ea1
....acaCuuucgucaagguuucucaca..................	1	1	ea1
....acauuuucgucaagguuucucacC..................	6	1	ea1
....acauuuucgucaagguCucucaca..................	2	1	ea1
....acauuuucgucaagguuucucaUa..................	1	1	ea1
....acauuuucgucaagguuucucacaC.................	1	1	ea1
....acauuuucgucaagguuucucacUa.................	3	1	ea1
....acauuuucgucaagguuucucacaU.................	28	1	ea1
....acauuuucgucaagguuucucacaa.................	3	0	ea1
....acauuuucgucaagguuucucacaag................	1	0	ea1
....cauCuucgucaagguuuc.......................	1	1	ea1
....cauuuucgucaagguuucuc.....................	4	0	ea1
....cauuuucgucaagguuucucU....................	2	1	ea1
....cauuuucgucaagguuucuca....................	11	0	ea1
....cauuuucgCcaagguuucuca....................	2	1	ea1
....cauuuucgucaagguuucucac...................	11	0	ea1
....cauuuucgucaagguuucucaA...................	1	1	ea1
....cauuuucgucaagguuucucaU...................	2	1	ea1
....Gauuuucgucaagguuucucac...................	1	1	ea1
....caCuuucgucaagguuucucac...................	1	1	ea1
....cauuuucgucaaAguuucucaca..................	1	1	ea1
....cauuuucgucaagguuucucacC..................	8	1	ea1
....Gauuuucgucaagguuucucaca..................	1	1	ea1
....cauuuucgucaagguuuUucaca..................	2	1	ea1
....cauuuucgucaagguuucucaUa..................	1	1	ea1
....cauuuucgucaagguuucucacG..................	2	1	ea1
....cauuuucgucaagguuucCcaca..................	2	1	ea1
....Uauuuucgucaagguuucucaca..................	4	1	ea1
....cauuuucgucaagguuucucacU..................	82	1	ea1
....cauuuucgucaagguuucucaca..................	126	0	ea1
....cauuuucgucaagguuucucacaU.................	8	1	ea1
....cauuuucgucaagguuucucacaa.................	4	0	ea1
....cauuuucgucaagguuucucacaC.................	1	1	ea1
....cauuuucgucaagguuucucacUa.................	2	1	ea1
....cauuuucgucaagguuucucacaaU................	4	1	ea1
....auuuucgucaagguuucu......................	1	0	ea1
....auuuucgucaagguuucuc.....................	17	0	ea1
....Uuuuucgucaagguuucuc.....................	1	1	ea1

```
                                      Star                            Mature
aacagauaugauuaagaacagacaaaaaauaauucuugagaaaaccuugacugaaaauaguuuaaaacauuuucgucaagguuucucacaagaacuguuuaucucaaa
....................................................................auuuucgucGagguuucuc.....................   1     1   ea1
....................................................................Cuuuucgucaagguuucuc.....................   2     1   ea1
....................................................................auuuucgucaagguuucuU.....................   1     1   ea1
....................................................................auuuucgucaagguuucucC....................   7     1   ea1
....................................................................auuuucgucaagguuucuca....................   36    0   ea1
....................................................................auuuucgucaagguuucucU....................   1     1   ea1
....................................................................auuuucgucaagguuucucaA...................   10    1   ea1
....................................................................auuuucgucaagguuucucac...................   218   0   ea1
....................................................................auuuucgCcaagguuucucac...................   1     1   ea1
....................................................................auAuucgucaagguuucucac...................   1     1   ea1
....................................................................Guuuucgucaagguuucucac...................   7     1   ea1
....................................................................auCuucgucaagguuucucac...................   1     1   ea1
....................................................................auuuucgucaaggCuucucac...................   1     1   ea1
....................................................................auuuucgucaagguuucucaU...................   35    1   ea1
....................................................................auuCucgucaagguuucucac...................   1     1   ea1
....................................................................Uuuuucgucaagguuucucac...................   12    1   ea1
....................................................................auuuucgucaagguuucucGc...................   2     1   ea1
....................................................................Cuuuucgucaagguuucucac...................   4     1   ea1
....................................................................auuuucgucaagguuucuAac...................   1     1   ea1
....................................................................auuuucgucGagguuucucac...................   1     1   ea1
....................................................................auuuucgucaagguuucucaG...................   2     1   ea1
....................................................................auuuucgucaagguuucucacG..................   24    1   ea1
....................................................................auuuucgucaagguuucucGca..................   6     1   ea1
....................................................................auuuucgucaagguuCcucaca..................   1     1   ea1
....................................................................auuuucgCcaagguuucucaca..................   3     1   ea1
....................................................................auuuucgucaagguuucucaUa..................   1     1   ea1
....................................................................auuCucgucaagguuucucaca..................   8     1   ea1
....................................................................Cuuuucgucaagguuucucaca..................   74    1   ea1
....................................................................auuAucgucaagguuucucaca..................   1     1   ea1
....................................................................auuuucgucaagguAucucaca..................   1     1   ea1
....................................................................auuuucgucaagguuucucUca..................   1     1   ea1
....................................................................auuuucgucaGgguuucucaca..................   5     1   ea1
....................................................................auuuuUgucaagguuucucaca..................   1     1   ea1
....................................................................auuuucgucGagguuucucaca..................   8     1   ea1
....................................................................Uuuuucgucaagguuucucaca..................   34    1   ea1
....................................................................auuuucgucaagguuucucacU..................   276   1   ea1
....................................................................auCuucgucaagguuucucaca..................   4     1   ea1
....................................................................auuuucgucaagguCucucaca..................   1     1   ea1
....................................................................auuuucgucaagguuAcucaca..................   4     1   ea1
....................................................................auuuCcgucaagguuucucaca..................   2     1   ea1
....................................................................auuuucgucaagguuucucCca..................   1     1   ea1
....................................................................aGuuucgucaagguuucucaca..................   1     1   ea1
....................................................................auuuucguUaagguuucucaca..................   2     1   ea1
....................................................................auuuucgucaagguuucucacC..................   40    1   ea1
....................................................................auuuucgucaagguuucCcaca..................   7     1   ea1
....................................................................auuuucgucaagguuucucaca..................   1054  0   ea1
....................................................................auuuucgucaaggCuucucaca..................   3     1   ea1
....................................................................Guuuucgucaagguuucucaca..................   15    1   ea1
....................................................................auuuucgucaagguuucucacaG.................   1     1   ea1
....................................................................auuuucgucaagguuucucacCa.................   1     1   ea1
....................................................................Cuuuucgucaagguuucucacaa.................   4     1   ea1
....................................................................auuuucgucaagguuucucacaU.................   49    1   ea1
....................................................................auuuucgucaagguuucucacaa.................   6     0   ea1
....................................................................auuuucgucaagguuucucacaC.................   7     1   ea1
....................................................................auuuucgucaagguuucucacUa.................   4     1   ea1
....................................................................auuuucgucaagguuucucacaaA................   2     1   ea1
....................................................................auuuucgucaagguuucucacaaU................   4     1   ea1
....................................................................auuuucgucaagguuucucacaaC................   1     1   ea1
....................................................................auuuucgucaagguuucucacaUg................   1     1   ea1
.....................................................................uuuucguAaagguuucuc.....................   1     1   ea1
.....................................................................Cuuucgucaagguuucuc.....................   13    1   ea1
.....................................................................uuuucgucaagguuucuA.....................   8     1   ea1
.....................................................................uuuucgucaaggGuucuc.....................   1     1   ea1
.....................................................................Guuucgucaagguuucuc.....................   14    1   ea1
.....................................................................uuuucgucaagguuCcuc.....................   3     1   ea1
.....................................................................uuuucgucaagguuucuU.....................   18    1   ea1
.....................................................................uuuucgucaGgguuucuc.....................   2     1   ea1
.....................................................................uuuucgucaagguuucuc.....................   297   0   ea1
.....................................................................uuCucgucaagguuucuc.....................   6     1   ea1
.....................................................................uuuCcgucaagguuucuc.....................   1     1   ea1
```

```
                                     Star                            Mature
aacagauaugauuaagaacagacaaaaaauaauucuugagaaaaccuugacugaaaauaguuuaaaacauuuucgucaagguuucucacaagaacuguuuaucucaaa
.....................................................................uuuucgCcaagguuucuca....................  2      1   ea1
.....................................................................Guuucgucaagguuucuca....................  22     1   ea1
.....................................................................uuuucgucaagguuucuca....................  85     0   ea1
.....................................................................Cuuucgucaagguuucuca....................  3      1   ea1
.....................................................................uCuucgucaagguuucuca....................  1      1   ea1
.....................................................................uuuucgucaagguuucucG....................  2      1   ea1
.....................................................................uuuucgucaagguuucucC....................  1      1   ea1
.....................................................................uuuucgucaagguuucucU....................  6      1   ea1
.....................................................................uuuucgucaagguuucucGc...................  10     1   ea1
.....................................................................uuuucgucaagguuuAucac...................  1      1   ea1
.....................................................................uuuucgucaagguuucuUac...................  4      1   ea1
.....................................................................uuuucgucaaggCuucucac...................  6      1   ea1
.....................................................................uuuucgucaagguuuGucac...................  1      1   ea1
.....................................................................uuAucgucaagguuucucac...................  1      1   ea1
.....................................................................uAuucgucaagguuucucac...................  3      1   ea1
.....................................................................uuuucgucGagguuucucac...................  3      1   ea1
.....................................................................uuuucgucaagguCucucac...................  5      1   ea1
.....................................................................uuuuAgucaagguuucucac...................  1      1   ea1
.....................................................................uuuucgucaagguuucucaG...................  5      1   ea1
.....................................................................uuuucgucaagguuucucac...................  3028   0   ea1
.....................................................................uuuucgCcaagguuucucac...................  5      1   ea1
.....................................................................uuuucgucaagguuucCcac...................  11     1   ea1
.....................................................................uuuucgucaUgguuucucac...................  2      1   ea1
.....................................................................uuuucgucaagguuuUucac...................  1      1   ea1
.....................................................................uuuucgucaagguuCcucac...................  8      1   ea1
.....................................................................uuuucgucaagguuucucaU...................  375    1   ea1
.....................................................................uuuucgucaagguuAcucac...................  1      1   ea1
.....................................................................uuuCcgucaagguuucucac...................  2      1   ea1
.....................................................................uuuuUgucaagguuucucac...................  9      1   ea1
.....................................................................uuuucgucaagguuucucUc...................  4      1   ea1
.....................................................................uCuucgucaagguuucucac...................  12     1   ea1
.....................................................................uuuucgucaagguAucucac...................  1      1   ea1
.....................................................................uuuucgucaagguuucucaA...................  61     1   ea1
.....................................................................uuuucgucaGgguuucucac...................  4      1   ea1
.....................................................................uuuucgucaaggGuucucac...................  2      1   ea1
.....................................................................uuCucgucaagguuucucac...................  8      1   ea1
.....................................................................Cuuucgucaagguuucucac...................  129    1   ea1
.....................................................................uuuAcgucaagguuucucac...................  2      1   ea1
.....................................................................uuuucAucaagguuucucac...................  3      1   ea1
.....................................................................Guuucgucaagguuucucac...................  239    1   ea1
.....................................................................uuuucgucaagguuuUucaca..................  11     1   ea1
.....................................................................uuuucgucaagguuuGucaca..................  6      1   ea1
.....................................................................uGuucgucaagguuucucaca..................  5      1   ea1
.....................................................................uuuucCucaagguuucucaca..................  1      1   ea1
.....................................................................uuuucgucaagguuucuAaca..................  7      1   ea1
.....................................................................uuGucgucaagguuucucaca..................  2      1   ea1
.....................................................................Guuucgucaagguuucucaca..................  1479   1   ea1
.....................................................................uuuucgucaagUuuucucaca..................  5      1   ea1
.....................................................................uuuucgucaagguuucCcaca..................  114    1   ea1
.....................................................................uuuucgucaGgguuucucaca..................  78     1   ea1
.....................................................................uuuucgucaagguGucucaca..................  2      1   ea1
.....................................................................uuuucgucaaggGuucucaca..................  25     1   ea1
.....................................................................uuuucgucaagguuucAcaca..................  15     1   ea1
.....................................................................uuuucgucUagguuucucaca..................  7      1   ea1
.....................................................................uuAucgucaagguuucucaca..................  5      1   ea1
.....................................................................uuuucgucaUgguuucucaca..................  5      1   ea1
.....................................................................uuuucgucaagguuucuGaca..................  1      1   ea1
.....................................................................Cuuucgucaagguuucucaca..................  949    1   ea1
.....................................................................uuuucgucaagguuucucaUa..................  40     1   ea1
.....................................................................uuuucgCcaagguuucucaca..................  80     1   ea1
.....................................................................uuuucgucaagguuucucUca..................  18     1   ea1
.....................................................................uuuucgucaagguAucucaca..................  5      1   ea1
.....................................................................uuuucguUaagguuucucaca..................  12     1   ea1
.....................................................................uuuucgucaagguuucucaAa..................  1      1   ea1
.....................................................................uuuucgucaaggCuucucaca..................  105    1   ea1
.....................................................................uuuucgucaagguuucucaca..................  28327  0   ea1
.....................................................................uAuucgucaagguuucucaca..................  36     1   ea1
.....................................................................uuuucgucCagguuucucaca..................  4      1   ea1
.....................................................................uuuucgucaagguuucuCca..................  7      1   ea1
.....................................................................uuuucgAcaagguuucucaca..................  10     1   ea1
```

```
                                     Star                             Mature
aacagauaugauuaagaacagacaaaaaauaauucuugagaaaaccuugacugaaaauaguuuaaaacauuuucgucaagguuucucacaagaacuguuuaucucaaa
.....................................................................uuuucgucaagCuuucucaca..................    1       1       ea1
.....................................................................uuuucgucaagguuucucaGa..................    1       1       ea1
.....................................................................uCuucgucaagguuucucaca..................    106     1       ea1
.....................................................................uuuucgucaaggAuucucaca..................    4       1       ea1
.....................................................................uuuucgucaaUguuucucaca..................    3       1       ea1
.....................................................................uuuucgucaCgguuucucaca..................    4       1       ea1
.....................................................................uuuucgucaagguCucucaca..................    85      1       ea1
.....................................................................uuuucgucaagguuucuUaca..................    24      1       ea1
.....................................................................uuuuAgucaagguuucucaca..................    7       1       ea1
.....................................................................uuuucguAaagguuucucaca..................    3       1       ea1
.....................................................................uuuucgucaagguuucGcaca..................    2       1       ea1
.....................................................................uuuucgGcaagguuucucaca..................    4       1       ea1
.....................................................................uuuucguGaagguuucucaca..................    3       1       ea1
.....................................................................uuuucgucGagguuucucaca..................    70      1       ea1
.....................................................................uuuuUgucaagguuucucaca..................    28      1       ea1
.....................................................................uuuucgucaagguuuAucaca..................    4       1       ea1
.....................................................................uuuucgucaaAguuucucaca..................    22      1       ea1
.....................................................................uuuucgucaagguuucucacU..................    3265    1       ea1
.....................................................................uuuucgucaagguuCcucaca..................    85      1       ea1
.....................................................................uuCucgucaagguuucucaca..................    90      1       ea1
.....................................................................uuuGcgucaagguuucucaca..................    5       1       ea1
.....................................................................uuuCcgucaagguuucucaca..................    74      1       ea1
.....................................................................uuuucgucaagguuGcucaca..................    3       1       ea1
.....................................................................uuuucgucaagguuucucacG..................    423     1       ea1
.....................................................................uuuucgucaagguuucucGca..................    84      1       ea1
.....................................................................uuuucgucaagguuucucacC..................    397     1       ea1
.....................................................................uuuucgucaagguuAcucaca..................    15      1       ea1
.....................................................................uuuucAucaagguuucucaca..................    23      1       ea1
.....................................................................uuuAcgucaagguuucucaca..................    28      1       ea1
.....................................................................uuuucgucaagAuuucucaca..................    16      1       ea1
.....................................................................uuuucUucaagguuucucaca..................    1       1       ea1
.....................................................................uAuucgucaagguuucucacaa.................    1       1       ea1
.....................................................................uuuucgucaagguuucuUacaa.................    1       1       ea1
.....................................................................uuuucgucaagguuucucacaU.................    2847    1       ea1
.....................................................................uuuucguUaagguuucucacaa.................    2       1       ea1
.....................................................................uuuucgucaagguuucucUcaa.................    1       1       ea1
.....................................................................uuuCcgucaagguuucucacaa.................    3       1       ea1
.....................................................................uuuucgucaGgguuucucacaa.................    6       1       ea1
.....................................................................uuCucgucaagguuucucacaa.................    9       1       ea1
.....................................................................uuuucgucaagguuucucacCa.................    5       1       ea1
.....................................................................uuuucgucaagguuucucacaa.................    1936    0       ea1
.....................................................................Guuucgucaagguuucucacaa.................    79      1       ea1
.....................................................................uuuucgucaagAuuucucacaa.................    3       1       ea1
.....................................................................uuuucgucaaggCuucucacaa.................    9       1       ea1
.....................................................................uuuucgucaagguuucucacUa.................    199     1       ea1
.....................................................................uuuucgucaagguuuUucacaa.................    1       1       ea1
.....................................................................uuuucgucaagguuucucacaG.................    45      1       ea1
.....................................................................uuuucgucaagguCucucacaa.................    2       1       ea1
.....................................................................uuuuAgucaagguuucucacaa.................    1       1       ea1
.....................................................................Cuuucgucaagguuucucacaa.................    222     1       ea1
.....................................................................uuuucAucaagguuucucacaa.................    2       1       ea1
.....................................................................uuuucgucaaggAuucucacaa.................    1       1       ea1
.....................................................................uuuucgucGagguuucucacaa.................    6       1       ea1
.....................................................................uuuucgCcaagguuucucacaa.................    4       1       ea1
.....................................................................uuuucgucaagguuucucaUaa.................    1       1       ea1
.....................................................................uuuucgucaagguuucucGcaa.................    3       1       ea1
.....................................................................uuuucgucaagguuucuAacaa.................    2       1       ea1
.....................................................................uuuAcgucaagguuucucacaa.................    2       1       ea1
.....................................................................uuuucgucaagguuucAcacaa.................    1       1       ea1
.....................................................................uuuucgucaagguuCcucacaa.................    5       1       ea1
.....................................................................uuuucgucaagguuucCcacaa.................    12      1       ea1
.....................................................................uuuucgucaaAguuucucacaa.................    1       1       ea1
.....................................................................uuuucgucaagUuuucucacaa.................    1       1       ea1
.....................................................................uCuucgucaagguuucucacaa.................    5       1       ea1
.....................................................................uuuuUgucaagguuucucacaa.................    2       1       ea1
.....................................................................uuuucgucaagguuucucacaC.................    409     1       ea1
.....................................................................uuGucgucaagguuucucacaa.................    1       1       ea1
.....................................................................uuuucgucaagguuucucacGa.................    3       1       ea1
.....................................................................uuuucgucaagguuucucaAaa.................    3       1       ea1
.....................................................................uuuucgucaagguuGcucacaa.................    1       1       ea1
```

Star Mature			
aacagauaugauuaagaacagacaaaaaauaauucuugagaaaaccuugacugaaaauaguuuaaaacauuuucgucaagguuucucacaagaacuguuuaucucaaa			
....uuuucgucaagguuucucacaaU................	314	1	ea1
....uuuucgucaagguuucucacaUg................	70	1	ea1
....uuuucguUaagguuucucacaag................	1	1	ea1
....uuuucgucaagguuucucacaaC................	38	1	ea1
....uuuucgucaagguuucucacaag................	21	0	ea1
....uuuucgucaagguuucucacaaA................	52	1	ea1
....Cuuucgucaagguuucucacaag................	2	1	ea1
....uuuucgucaagguuucucacaaUa...............	65	1	ea1
....uuuucgucaagguuucucacaaga...............	1	0	ea1
....uuuucgucaagguuucucacaagG...............	1	1	ea1
....uuuucgucaagguuucucacaaCa...............	6	1	ea1
....uuuucgucaagguuucucacaUga...............	3	1	ea1
....uuuucgucaagguuucucacaaUaa..............	3	1	ea1
....uuucgucaagguuucuca....................	1303	0	ea1
....uuAcgucaagguuucuca....................	2	1	ea1
....uCucgucaagguuucuca....................	1	1	ea1
....uuucgucaaggCuucuca....................	11	1	ea1
....uuucgucaaCguuucuca....................	1	1	ea1
....uuCcgucaagguuucuca....................	5	1	ea1
....uuucAucaagguuucuca....................	1	1	ea1
....uuucgucGagguuucuca....................	6	1	ea1
....uuucgucaagguuucuAa....................	1	1	ea1
....uuuUgucaagguuucuca....................	2	1	ea1
....uuucgucaagguCucuca....................	4	1	ea1
....uuucgucaagguuucAca....................	7	1	ea1
....Cuucgucaagguuucuca....................	6	1	ea1
....uuucgucaagguuucuGa....................	1	1	ea1
....uuucgucaagguuAcuca....................	1	1	ea1
....uuucgucUagguuucuca....................	1	1	ea1
....uuucgucaagguuucucC....................	1	1	ea1
....uuucgucaagguuucucG....................	25	1	ea1
....uuucgucCagguuucuca....................	1	1	ea1
....uuucgucaagguuucuUa....................	1	1	ea1
....uuucgCcaagguuucuca....................	4	1	ea1
....Auucgucaagguuucuca....................	2	1	ea1
....Guucgucaagguuucuca....................	96	1	ea1
....uuucgucaagguuucucU....................	27	1	ea1
....uuucgucaagguuCcuca....................	3	1	ea1
....uuucgucaagguuucCca....................	4	1	ea1
....uuucgucaaggGuucuca....................	2	1	ea1
....uuucgucaGgguuucuca....................	8	1	ea1
....uuucgucaagguuGcucac...................	6	1	ea1
....uuucgucCagguuucucac...................	6	1	ea1
....uuucgucaagguuucucUc...................	94	1	ea1
....uuucgucaagguuucucaA...................	1592	1	ea1
....uuuAgucaagguuucucac...................	22	1	ea1
....uuucguUaagguuucucac...................	23	1	ea1
....uuucgAcaagguuucucac...................	27	1	ea1
....uuucgucaagguuucucGc...................	265	1	ea1
....Guucgucaagguuucucac...................	5367	1	ea1
....uuGcgucaagguuucucac...................	7	1	ea1
....uuucgucaagguuucuUac...................	32	1	ea1
....uuucgucaagguGucucac...................	6	1	ea1
....uuuGgucaagguuucucac...................	6	1	ea1
....uuucgucaagguuucucCc...................	20	1	ea1
....uuucgucaagguuuUucac...................	34	1	ea1
....uuuUgucaagguuucucac...................	75	1	ea1
....Auucgucaagguuucucac...................	49	1	ea1
....uuucgucaagAuuucucac...................	35	1	ea1
....uuucgucaagguuucuGac...................	2	1	ea1
....uuucgucaagguAucucac...................	6	1	ea1
....uuucgucaagguuucuAac...................	12	1	ea1
....uuucgucaagguuCcucac...................	248	1	ea1
....uuucCucaagguuucucac...................	9	1	ea1
....uuucgucaagguuucCcac...................	242	1	ea1
....uuucgGcaagguuucucac...................	8	1	ea1
....uuucgucaaggAuucucac...................	39	1	ea1
....uuucguAaagguuucucac...................	5	1	ea1
....uuucgucaCgguuucucac...................	6	1	ea1
....uuucgucaagguuucAcac...................	64	1	ea1

```
                                    Star                            Mature
aacagauaugauuaagaacagacaaaaaauaauucuugagaaaaccuugacugaaaauaguuuaaaacauuuucgucaagguuucucacaagaacuguuuaucucaaa
......................................................................uuCcgucaagguuucucac...................   174     1    ea1
......................................................................uuucAucaagguuucucac...................   51      1    ea1
......................................................................uuucgucaagguuucucaU...................   9964    1    ea1
......................................................................uCucgucaagguuucucac...................   225     1    ea1
......................................................................uuucgucaagguuuGucac...................   2       1    ea1
......................................................................uAucgucaagguuucucac...................   23      1    ea1
......................................................................uuucgucaagguuucucac...................   70861   0    ea1
......................................................................uuucgucaaggGuucucac...................   68      1    ea1
......................................................................uuucgucaagguuAcucac...................   37      1    ea1
......................................................................uuucgucaaggCuucucac...................   245     1    ea1
......................................................................uuucgucaaCguuucucac...................   2       1    ea1
......................................................................uuucgucaagUuuucucac...................   9       1    ea1
......................................................................uuucgucaGgguuucucac...................   186     1    ea1
......................................................................uuucgucaUgguuucucac...................   21      1    ea1
......................................................................Cuucgucaagguuucucac...................   297     1    ea1
......................................................................uuucgucUagguuucucac...................   25      1    ea1
......................................................................uGucgucaagguuucucac...................   9       1    ea1
......................................................................uuAcgucaagguuucucac...................   24      1    ea1
......................................................................uuucgCcaagguuucucac...................   228     1    ea1
......................................................................uuucgucaagguuucucaG...................   64      1    ea1
......................................................................uuucgucaaUguuucucac...................   6       1    ea1
......................................................................uuucgucGagguuucucac...................   156     1    ea1
......................................................................uuucgucaagguuuAucac...................   2       1    ea1
......................................................................uuucgucaagguCucucac...................   186     1    ea1
......................................................................uuucUucaagguuucucac...................   3       1    ea1
......................................................................uuucgucaagguuucGcac...................   17      1    ea1
......................................................................uuucgucaaAguuucucac...................   47      1    ea1
......................................................................uuucgucaagCuuucucac...................   2       1    ea1
......................................................................uuucgucaagguuucAcaca..................   476     1    ea1
......................................................................uuucgucaagguGucucaca..................   45      1    ea1
......................................................................uuucgucaagAuuucucaca..................   290     1    ea1
......................................................................uuucgCcaagguuucucaca..................   1732    1    ea1
......................................................................uuucgucUagguuucucaca..................   142     1    ea1
......................................................................uuucgucaagguAucucaca..................   60      1    ea1
......................................................................uuuUgucaagguuucucaca..................   598     1    ea1
......................................................................uuucgucaagguuucucUca..................   285     1    ea1
......................................................................uuucgucaagguuucucaGa..................   20      1    ea1
......................................................................uuucgucaagguuucucGca..................   1612    1    ea1
......................................................................uuucgucaaCguuucucaca..................   18      1    ea1
......................................................................uuucgucaagCuuucucaca..................   16      1    ea1
......................................................................uuucgucaagguuGcucaca..................   44      1    ea1
......................................................................uGucgucaagguuucucaca..................   58      1    ea1
......................................................................uuucguUaagguuucucaca..................   247     1    ea1
......................................................................Cuucgucaagguuucucaca..................   1982    1    ea1
......................................................................uuucgucaaggGuucucaca..................   519     1    ea1
......................................................................uuucgucaaggCuucucaca..................   1740    1    ea1
......................................................................uuucgucGagguuucucaca..................   1495    1    ea1
......................................................................uuucgucaCgguuucucaca..................   54      1    ea1
......................................................................uuGcgucaagguuucucaca..................   45      1    ea1
......................................................................uuucgucaaUguuucucaca..................   86      1    ea1
......................................................................uuucgucaagguuucuUaca..................   293     1    ea1
......................................................................uuuGgucaagguuucucaca..................   37      1    ea1
......................................................................uAucgucaagguuucucaca..................   178     1    ea1
......................................................................uCucgucaagguuucucaca..................   1626    1    ea1
......................................................................uuucgucaaggAuucucaca..................   213     1    ea1
......................................................................uuucgucaagguCucucaca..................   1178    1    ea1
......................................................................uuucgucaagguuuAucaca..................   61      1    ea1
......................................................................uuucgucaGgguuucucaca..................   1445    1    ea1
......................................................................uuucgucaagguuuUucaca..................   267     1    ea1
......................................................................uuucCucaagguuucucaca..................   26      1    ea1
......................................................................uuucgucaagguuucGcaca..................   150     1    ea1
......................................................................uuucgucaagguuucucacC..................   8373    1    ea1
......................................................................uuucAucaagguuucucaca..................   452     1    ea1
......................................................................Auucgucaagguuucucaca..................   393     1    ea1
......................................................................uuCcgucaagguuucucaca..................   1355    1    ea1
......................................................................uuucgucaagguuuGucaca..................   16      1    ea1
......................................................................uuucgucaagguuCcucaca..................   1903    1    ea1
......................................................................uuucgucaagUuuucucaca..................   69      1    ea1
......................................................................uuucguGaagguuucucaca..................   3       1    ea1
......................................................................uuucgucaagguuucucaUa..................   1436    1    ea1
```

```
                                       Star                            Mature
aacagauaugauuaagaacagacaaaaaauaauucuugagaaaaccuugacugaaaauaguuuaaaacauuuucgucaagguuucucacaagaacuguuuaucucaaa
......................................................................uuucguAaagguuucucaca..................    49      1    ea1
......................................................................uuucUucaagguuucucaca..................    25      1    ea1
......................................................................uuuAgucaagguuucucaca..................    150     1    ea1
......................................................................uuucgucaagguuucuAaca..................    88      1    ea1
......................................................................Guucgucaagguuucucaca..................    28377   1    ea1
......................................................................uuucgucaagguuAcucaca..................    197     1    ea1
......................................................................uuucgGcaagguuucucaca..................    55      1    ea1
......................................................................uuucgucaagguuucucacU..................    68863   1    ea1
......................................................................uuucgucaagguuucCcaca..................    2105    1    ea1
......................................................................uuucgucaagguuucucacG..................    7832    1    ea1
......................................................................uuucgucaaAguuucucaca..................    304     1    ea1
......................................................................uuucgucCagguuucucaca..................    66      1    ea1
......................................................................uuucgucaagguuucucCca..................    102     1    ea1
......................................................................uuucgAcaagguuucucaca..................    212     1    ea1
......................................................................uuucgucaagguuucuGaca..................    15      1    ea1
......................................................................uuucgucaUgguuucucaca..................    188     1    ea1
......................................................................uuucgucaagguuucucaAa..................    57      1    ea1
......................................................................uuAcgucaagguuucucaca..................    211     1    ea1
......................................................................uuucgucaagguuucucaca..................    549422  0    ea1
......................................................................uuucgucaagguuucucGcaa.................    461     1    ea1
......................................................................uuucgucaagguGucucacaa.................    21      1    ea1
......................................................................uuucgucaaAguuucucacaa.................    99      1    ea1
......................................................................uuucgucaagguuucucaUaa.................    124     1    ea1
......................................................................uuAcgucaagguuucucacaa.................    57      1    ea1
......................................................................uuucgucaagguuucucaGaa.................    9       1    ea1
......................................................................uuucgucaaCguuucucacaa.................    3       1    ea1
......................................................................uuucgucaagUuuucucacaa.................    11      1    ea1
......................................................................uuucguUaagguuucucacaa.................    71      1    ea1
......................................................................uuucgucaagguuucucacaa.................    163907  0    ea1
......................................................................uuuGgucaagguuucucacaa.................    13      1    ea1
......................................................................uuucgCcaagguuucucacaa.................    576     1    ea1
......................................................................uuucgucaagguuucucacCa.................    165     1    ea1
......................................................................uuucgucaagguuucucacaC.................    22665   1    ea1
......................................................................uuucgucaagguuuUucacaa.................    73      1    ea1
......................................................................uuucgucaaggCuucucacaa.................    549     1    ea1
......................................................................uuuUgucaagguuucucacaa.................    205     1    ea1
......................................................................Guucgucaagguuucucacaa.................    13401   1    ea1
......................................................................uuGcgucaagguuucucacaa.................    18      1    ea1
......................................................................uuucUucaagguuucucacaa.................    13      1    ea1
......................................................................uuucgucaagguuucucacaG.................    3064    1    ea1
......................................................................uuucgucaagCuuucucacaa.................    8       1    ea1
......................................................................uuucAucaagguuucucacaa.................    153     1    ea1
......................................................................uuucgucaagguuucAcacaa.................    138     1    ea1
......................................................................uuucgucaagguuucucacaU.................    175149  1    ea1
......................................................................Auucgucaagguuucucacaa.................    145     1    ea1
......................................................................uuucgAcaagguuucucacaa.................    54      1    ea1
......................................................................uCucgucaagguuucucacaa.................    478     1    ea1
......................................................................uuucgucGagguuucucacaa.................    406     1    ea1
......................................................................uuuAgucaagguuucucacaa.................    42      1    ea1
......................................................................Cuucgucaagguuucucacaa.................    599     1    ea1
......................................................................uuucguGaagguuucucacaa.................    2       1    ea1
......................................................................uuucgucaagguuucuUacaa.................    86      1    ea1
......................................................................uuucgucaCgguuucucacaa.................    23      1    ea1
......................................................................uuucgucaagguAucucacaa.................    14      1    ea1
......................................................................uuucgucUagguuucucacaa.................    50      1    ea1
......................................................................uuucgucaagguuuGucacaa.................    3       1    ea1
......................................................................uuucgucaagguuucucacUa.................    9581    1    ea1
......................................................................uuucgucaagguuucuAacaa.................    31      1    ea1
......................................................................uuucgucaagguuuAucacaa.................    17      1    ea1
......................................................................uuucgucaUgguuucucacaa.................    56      1    ea1
......................................................................uuucgucaagguuucCcacaa.................    659     1    ea1
......................................................................uuucgucaagguuucuGacaa.................    4       1    ea1
......................................................................uuucgucaagguuucucCcaa.................    22      1    ea1
......................................................................uGucgucaagguuucucacaa.................    20      1    ea1
......................................................................uuucgucaagguuucGcacaa.................    34      1    ea1
......................................................................uuucgucaagguuGcucacaa.................    16      1    ea1
......................................................................uuCcgucaagguuucucacaa.................    393     1    ea1
......................................................................uuucgucaagguuucucaAaa.................    19      1    ea1
......................................................................uuucCucaagguuucucacaa.................    9       1    ea1
......................................................................uuucgucaagguCucucacaa.................    355     1    ea1
```

```
                                     Star                             Mature
aacagauaugauuaagaacagacaaaaaauaauucuugagaaaaccuugacugaaaauaguuuaaaacauuuucgucaagguuucucacaagaacuguuuaucucaaa
......................................................................uuucgucaagguuucucacGa.................   548     1    ea1
......................................................................uuucguAaagguuucucacaa.................   16      1    ea1
......................................................................uuucgucCagguuucucacaa.................   19      1    ea1
......................................................................uuucgucaaggGuucucacaa.................   141     1    ea1
......................................................................uuucgucaagAuuucucacaa.................   87      1    ea1
......................................................................uuucgucaGgguuucucacaa.................   447     1    ea1
......................................................................uuucgGcaagguuucucacaa.................   23      1    ea1
......................................................................uuucgucaaUguuucucacaa.................   19      1    ea1
......................................................................uuucgucaagguuucucUcaa.................   91      1    ea1
......................................................................uuucgucaagguuCcucacaa.................   553     1    ea1
......................................................................uAucgucaagguuucucacaa.................   66      1    ea1
......................................................................uuucgucaaggAuucucacaa.................   62      1    ea1
......................................................................uuucgucaagguuAcucacaa.................   63      1    ea1
......................................................................uuucUucaagguuucucacaag................   2       1    ea1
......................................................................uAucgucaagguuucucacaag................   1       1    ea1
......................................................................uuCcgucaagguuucucacaag................   2       1    ea1
......................................................................uuucgucaagguuucucacaaA................   3514    1    ea1
......................................................................uuucgucaagguuucucacaaC................   2206    1    ea1
......................................................................uuucgucaagguuucucacaag................   1172    0    ea1
......................................................................uuucgucaagguuucucacaUg................   3936    1    ea1
......................................................................uuucgucaagCuuucucacaag................   1       1    ea1
......................................................................uuucgucaaggGuucucacaag................   2       1    ea1
......................................................................uuuUgucaagguuucucacaag................   2       1    ea1
......................................................................uuucgucaaggCuucucacaag................   5       1    ea1
......................................................................uuucgucGagguuucucacaag................   5       1    ea1
......................................................................uuucgCcaagguuucucacaag................   2       1    ea1
......................................................................uuAcgucaagguuucucacaag................   1       1    ea1
......................................................................uuucgucaagguuucucaUaag................   1       1    ea1
......................................................................uCucgucaagguuucucacaag................   4       1    ea1
......................................................................Guucgucaagguuucucacaag................   74      1    ea1
......................................................................uuucgucaagguuucucacUag................   12      1    ea1
......................................................................uuuAgucaagguuucucacaag................   1       1    ea1
......................................................................uuucgucaagguuuAucacaag................   1       1    ea1
......................................................................uuucgucaagguCucucacaag................   4       1    ea1
......................................................................uuucgucaagguuucAcacaag................   1       1    ea1
......................................................................uuucgucaagguuucucacCag................   2       1    ea1
......................................................................uuucgucaagguuucCcacaag................   7       1    ea1
......................................................................Auucgucaagguuucucacaag................   1       1    ea1
......................................................................uuucgucaagguuucucGcaag................   3       1    ea1
......................................................................uuucgucaagguuucucacaGg................   7       1    ea1
......................................................................uuucgucaagguuCcucacaag................   1       1    ea1
......................................................................uuucgucaagguuucucacGag................   2       1    ea1
......................................................................uuucgucaagguuucucacaCg................   47      1    ea1
......................................................................uuucgucaagguuucucacaaU................   19273   1    ea1
......................................................................Cuucgucaagguuucucacaag................   8       1    ea1
......................................................................uuucgucaagguuuUucacaag................   2       1    ea1
......................................................................uuucgucaagguuucuUacaag................   1       1    ea1
......................................................................uuucgucaagguuucucacaaUa...............   5372    1    ea1
......................................................................uuucgucaagguuucucacaagG...............   8       1    ea1
......................................................................uuucgucaagguuucucacaUga...............   13      1    ea1
......................................................................uuucgucaagguuucucacaaCa...............   161     1    ea1
......................................................................uuucgucaagguuucucacaagC...............   13      1    ea1
......................................................................uuucgucaagguuucucacaagU...............   16      1    ea1
......................................................................uuucgucaagguuucucacaaAa...............   50      1    ea1
......................................................................uuucgucaagguuucucacaaga...............   14      0    ea1
......................................................................uuucgucaagguuucucacaagaC..............   1       1    ea1
......................................................................uuucgucaagguuucucacaagCa..............   2       1    ea1
......................................................................uuucgucaagguuucucacaaCaa..............   2       1    ea1
......................................................................uuucgucaagguuucucacaaUaa..............   69      1    ea1
......................................................................uuucgucaagguuucucacaagGa..............   2       1    ea1
......................................................................uuucgucaagguuucucacaagUa..............   6       1    ea1
......................................................................uuucgucaagguuucucacaaAaa..............   3       1    ea1
......................................................................uuucgucaagguuucucacaaUaac.............   2       1    ea1
......................................................................uuucgucaagguuucucacaGgaac.............   5       1    ea1
......................................................................uuucgucaagguuucucacaagaac.............   4       0    ea1
......................................................................uuucgucaagguuucucacaagaaA.............   2       1    ea1
......................................................................uuucgucaagguuucucacaagaaG.............   1       1    ea1
......................................................................uuucgucaagguuucucacaagaUc.............   4       1    ea1
......................................................................uuucgucaagguuucucacaagaacG............   3       1    ea1
......................................................................uuucgucaagguuucucacaGgaacu............   1       1    ea1
```

Star Mature

aacagauaugauuaagaacagacaaaaaauaauucuugagaaaaccuugacugaaaauaguuuaaaacauuuucgucaagguuucucacaagaacuguuuaucucaaa			
....uuucgucaagguuucucacaUgaacu............	1	1	ea1
....uuucgucaagguuucucacaagaacA............	5	1	ea1
....uuucgucaagguuucucacaagaCcu............	3	1	ea1
....uuucgucaagguuucucacaagaacu............	6	0	ea1
....uuucgucaagguuucucacaagaacug...........	3	0	ea1
....uuucgucaagguuucucacaagaacuA...........	1	1	ea1
....uuucgucaagguuucucacaagaCcug...........	1	1	ea1
....uucgucaaggGuucucac...................	2	1	ea1
....uucgucaagguuucucGc...................	2	1	ea1
....uucgucaagguuucucaU...................	10	1	ea1
....Gucgucaagguuucucac...................	4	1	ea1
....uucgucaagguuucucac...................	90	0	ea1
....uucgucaagguuucucaA...................	2	1	ea1
....uuUgucaagguuucucac...................	6	1	ea1
....uucgCcaagguuucucaca..................	2	1	ea1
....uucgucaagguuucucGca..................	1	1	ea1
....uCcgucaagguuucucaca..................	2	1	ea1
....Gucgucaagguuucucaca..................	27	1	ea1
....uucgucaagguuucucaca..................	564	0	ea1
....uucgucaGgguuucucaca..................	1	1	ea1
....uucgucaagguuucucacG..................	7	1	ea1
....uucgucaagguuucCcaca..................	2	1	ea1
....Aucgucaagguuucucaca..................	1	1	ea1
....uucgucaagguCucucaca..................	1	1	ea1
....uucUucaagguuucucaca..................	1	1	ea1
....uucgucaagguuCcucaca..................	3	1	ea1
....uucgucGagguuucucaca..................	2	1	ea1
....uucgucaagguuucucacU..................	54	1	ea1
....uucgucaagguuucucaUa..................	1	1	ea1
....uucgucaagguuucucacC..................	10	1	ea1
....Cucgucaagguuucucaca..................	1	1	ea1
....uuUgucaagguuucucaca..................	38	1	ea1
....uucgucaaggCuucucaca..................	4	1	ea1
....uucgucaaggAuucucaca..................	1	1	ea1
....uucgucaagguuucucacaC.................	50	1	ea1
....uucgucaagguuucucacGa.................	1	1	ea1
....Gucgucaagguuucucacaa.................	21	1	ea1
....uucgucaagguuucucacaU.................	318	1	ea1
....uucgucaagguuCcucacaa.................	1	1	ea1
....uucgucaaCguuucucacaa.................	2	1	ea1
....Cucgucaagguuucucacaa.................	7	1	ea1
....uuUgucaagguuucucacaa.................	11	1	ea1
....uCcgucaagguuucucacaa.................	2	1	ea1
....uucgucaagguuucucGcaa.................	1	1	ea1
....uucgucaagguuucucacUa.................	23	1	ea1
....uucgucaagguuucucacaa.................	345	0	ea1
....uucgucaagguuucucacaG.................	4	1	ea1
....uucgCcaagguuucucacaa.................	1	1	ea1
....uucgucaGgguuucucacaa.................	3	1	ea1
....uucgucGagguuucucacaa.................	3	1	ea1
....uucgucaagguuucucacaaA................	17	1	ea1
....uucgucaagguuucucacaaC................	8	1	ea1
....uucgucaagguuucucacaag................	5	0	ea1
....Gucgucaagguuucucacaag................	1	1	ea1
....uucgucaagguuucucacaaU................	71	1	ea1
....uucgucaagguuucucacaUg................	12	1	ea1
....uucgucaagguuucucacaaCa...............	1	1	ea1
....uucgucaagguuucucacaaUa...............	19	1	ea1
....uucgucaagguuucucacaaUaa..............	2	1	ea1
....ucgucaagguuucucacC..................	1	1	ea1
....ucgucaagguuucucaca..................	27	0	ea1
....Gcgucaagguuucucaca..................	7	1	ea1
....ucgucaagguuucucacU..................	8	1	ea1
....ucgucaagguuucucacaU.................	22	1	ea1
....Gcgucaagguuucucacaa.................	9	1	ea1
....ucgucaagguuucucCcaa.................	1	1	ea1
....ucgucaagguuucucacaC.................	3	1	ea1
....ucgucaagguuucucacaa.................	9	0	ea1
....ucgucaagguuucucacaaU................	5	1	ea1
....ucgucaagguuucucacaaUa...............	2	1	ea1

```
                                     Star                               Mature
aacagauaugauuaagaacagacaaaaaauaauucuugagaaaaccuugacugaaaauaguuuaaaacauuuucgucaagguuucucacaagaacuguuuaucucaaa
.........................................................................cgucaagguuucucacaa.................   3    0   ea1
.........................................................................cgucaagguuucucacaag................   1    0   ea1

.....................................gagaaaaccuugacugUa.....................................................   1    1   eg2
.....................................gagaaaaccuugacugaaa....................................................   1    0   eg2
.....................................gagaaaaccuugacugaaaa...................................................   5    0   eg2
.....................................gagaaaaccuugacugaaaau..................................................   16   0   eg2
.....................................gagaaaaccuugacugaaaauU.................................................   2    1   eg2
.....................................gagaaaaccuugacugaaaaua.................................................   1    0   eg2
.............................................................uuaaaacauuuucgucaagg...........................   1    0   eg2
.................................................................aacauuuucgucaagguuucucaca..................   2    0   eg2
..................................................................acauuuucgucaagguuucuca....................   2    0   eg2
..................................................................acauuuucgucaagguuucucaca..................   3    0   eg2
..................................................................acauuuucgucaagguuucucacUa................   2    1   eg2
...................................................................cauuuucgucaagguuucucac...................   1    0   eg2
...................................................................caGuuucgucaagguuucucaca..................   1    1   eg2
...................................................................cauuuucgucaagguuucucaca..................   11   0   eg2
....................................................................auuuucgucaagguuucucaca..................   26   0   eg2
....................................................................Cuuuucgucaagguuucucaca..................   4    1   eg2
....................................................................auuuucgucaagguuucucacU..................   6    1   eg2
....................................................................auNuucgucaagguuucucaca..................   1    1   eg2
....................................................................auuuucgucaagguuucucacUa.................   2    1   eg2
.....................................................................uuuucgucaagguuucuc.....................   2    0   eg2
.....................................................................uuuucgucaagguuucuca....................   10   0   eg2
.....................................................................uuuucgucaagguuucucac...................   13   0   eg2
.....................................................................uuuucgucaaggGuucucac...................   1    1   eg2
.....................................................................uuuucgucaagguuucucacU..................   114  1   eg2
.....................................................................uuuucgucaagguuucucGca..................   2    1   eg2
.....................................................................Cuuucgucaagguuucucaca..................   41   1   eg2
.....................................................................uuuucgucaaggGuucucaca..................   1    1   eg2
.....................................................................uuuucgucaagguuucucaca..................   772  0   eg2
.....................................................................uuuucgucaagguuucucacG..................   2    1   eg2
.....................................................................Nuuucgucaagguuucucaca..................   21   1   eg2
.....................................................................uuuucgucaagguuucucacC..................   3    1   eg2
.....................................................................Nuuucgucaagguuucucacaa.................   1    1   eg2
.....................................................................Guuucgucaagguuucucacaa.................   1    1   eg2
.....................................................................uuuucgucaagguuucucacaa.................   40   0   eg2
.....................................................................uuuucgucaagguuucucacaU.................   55   1   eg2
.....................................................................Cuuucgucaagguuucucacaa.................   9    1   eg2
.....................................................................uuuucgucaagguuucucacaG.................   1    1   eg2
.....................................................................uuuucgucaagguuucucacUa.................   4    1   eg2
.....................................................................uuuucgucaagguuucucacaUg................   4    1   eg2
.....................................................................uuuucgucaagguuucucacaaA................   5    1   eg2
.....................................................................uuuucgucaagguuucucacaaU................   2    1   eg2
.....................................................................uuuucgucaagguuucucacaaAa...............   1    1   eg2
.....................................................................uuuucgucaagguuucucacaaUa...............   4    1   eg2
......................................................................Guucgucaagguuucuca....................   2    1   eg2
......................................................................uuucgucaaggGuucuca....................   1    1   eg2
......................................................................uuucgucaagguuucuca....................   40   0   eg2
......................................................................uuucgucaagguuucucU....................   2    1   eg2
......................................................................uuucgucaagguuucucUc...................   1    1   eg2
......................................................................uuucgucaaggCuucucac...................   1    1   eg2
......................................................................uuucgucaagguCucucac...................   1    1   eg2
......................................................................uuucgucaagguuucucac...................   191  0   eg2
......................................................................uuucgucaagguuucucaG...................   1    1   eg2
......................................................................uuucgucaagguuucucaU...................   4    1   eg2
......................................................................Nuucgucaagguuucucac...................   1    1   eg2
......................................................................uuucgucaagguuucucUca..................   2    1   eg2
......................................................................uuucgucaagguuNcucaca..................   1    1   eg2
......................................................................uuucgucaagguAucucaca..................   1    1   eg2
......................................................................uuuUgucaagguuucucaca..................   3    1   eg2
......................................................................uuucgucaagguuucucaUa..................   80   1   eg2
......................................................................uuGcgucaagguuucucaca..................   2    1   eg2
......................................................................uuucgucGagguuucucaca..................   1    1   eg2
......................................................................uuCcgucaagguuucucaca..................   2    1   eg2
......................................................................uuucgucaagguuucuUaca..................   1    1   eg2
......................................................................uuucgucaagguuucucaGa..................   10   1   eg2
......................................................................uAucgucaagguuucucaca..................   1    1   eg2
......................................................................uCucgucaagguuucucaca..................   4    1   eg2
......................................................................Nuucgucaagguuucucaca..................   125  1   eg2
```

```
                                                                      Star                      Mature
aacagauaugauuaagaacagacaaaaaauaauucuugagaaaaccuugacugaaaauaguuuaaaacauuuucgucaagguuucucacaagaacuguuuaucucaaa
......................................................................uuucgucaagguCucucaca..................  9      1   eg2
......................................................................uuucgucaagguuucucacG..................  10     1   eg2
......................................................................uuucgucaagguuucucCca..................  2      1   eg2
......................................................................uuucgucaagguGucucaca..................  8      1   eg2
......................................................................uuucgucaaUguuucucaca..................  6      1   eg2
......................................................................uuucgGcaagguuucucaca..................  2      1   eg2
......................................................................uuucgucaagguuucucacC..................  27     1   eg2
......................................................................uuucgucaagguuucucGca..................  5      1   eg2
......................................................................uuucgucaagguuGcucaca..................  1      1   eg2
......................................................................uuucgucaagCuuucucaca..................  1      1   eg2
......................................................................uuucgucaagguuucucaca..................  18541  0   eg2
......................................................................uuucgucaaggCuucucaca..................  9      1   eg2
......................................................................uuucgucaagguuucGcaca..................  4      1   eg2
......................................................................uuNcgucaagguuucucaca..................  2      1   eg2
......................................................................uuucUucaagguuucucaca..................  2      1   eg2
......................................................................uuucgucaagguuuGucaca..................  3      1   eg2
......................................................................uuuGgucaagguuucucaca..................  1      1   eg2
......................................................................uuucgucaGgguuucucaca..................  4      1   eg2
......................................................................uGucgucaagguuucucaca..................  4      1   eg2
......................................................................uuucguAaagguuucucaca..................  1      1   eg2
......................................................................uuucgucaagguuCcucaca..................  4      1   eg2
......................................................................uuucgucaagguuucucacU..................  3501   1   eg2
......................................................................uuucgucaaggGuucucaca..................  20     1   eg2
......................................................................uuucgucaagAuuucucaca..................  5      1   eg2
......................................................................uuucgucaagguuucCcaca..................  4      1   eg2
......................................................................uuuNgucaagguuucucaca..................  1      1   eg2
......................................................................Cuucgucaagguuucucaca..................  8      1   eg2
......................................................................uuucgucaagUuuucucaca..................  10     1   eg2
......................................................................uuucCucaagguuucucaca..................  1      1   eg2
......................................................................uuucguUaagguuucucaca..................  2      1   eg2
......................................................................uuucguGaagguuucucaca..................  1      1   eg2
......................................................................uuucgucaaggAuucucaca..................  4      1   eg2
......................................................................uuucgucaagguuuAucaca..................  4      1   eg2
......................................................................Guucgucaagguuucucaca..................  22     1   eg2
......................................................................uuucgucaagguuuUucaca..................  1      1   eg2
......................................................................uuucgucaagguuucAcaca..................  2      1   eg2
......................................................................uuucAucaagguuucucaca..................  4      1   eg2
......................................................................uuucgucaagguuAcucaca..................  2      1   eg2
......................................................................uuucgCcaagguuucucaca..................  4      1   eg2
......................................................................uuucgucaagguuucucacaC.................  20     1   eg2
......................................................................uuucgucaagguGucucacaa.................  3      1   eg2
......................................................................Cuucgucaagguuucucacaa.................  1      1   eg2
......................................................................uuucgucaagguuucucaUaa.................  3      1   eg2
......................................................................uuucgucaagguuucucacaU.................  2922   1   eg2
......................................................................uuucgucaaggCuucucacaa.................  3      1   eg2
......................................................................Auucgucaagguuucucacaa.................  3      1   eg2
......................................................................uuucgucaagguuucucGcaa.................  1      1   eg2
......................................................................uuucguAaagguuucucacaa.................  1      1   eg2
......................................................................Guucgucaagguuucucacaa.................  1      1   eg2
......................................................................uuucgucaagUuuucucacaa.................  1      1   eg2
......................................................................uuucUucaagguuucucacaa.................  1      1   eg2
......................................................................uuucgucaagguuucucaGaa.................  3      1   eg2
......................................................................uuucgucaagguuucucacCa.................  7      1   eg2
......................................................................uuuAgucaagguuucucacaa.................  1      1   eg2
......................................................................uuucgucaagguuucucacaa.................  3530   0   eg2
......................................................................uuucgucGagguuucucacaa.................  1      1   eg2
......................................................................uuucgucaagguuucucacUa.................  316    1   eg2
......................................................................uuucgucaagguuCcucacaa.................  1      1   eg2
......................................................................uuucgucaagguuucGcacaa.................  1      1   eg2
......................................................................uuucguUaagguuucucacaa.................  1      1   eg2
......................................................................uuucgucaagguuucucacGa.................  1      1   eg2
......................................................................uuucgucaagguuucucacaG.................  5      1   eg2
......................................................................Nuucgucaagguuucucacaa.................  20     1   eg2
......................................................................uuucgucaagguuucucacaaC................  4      1   eg2
......................................................................uuucgucaagguuucucacaCg................  2      1   eg2
......................................................................uuucgucaagguuucucacaag................  31     0   eg2
......................................................................uuucgucaagguuucucacaUg................  157    1   eg2
......................................................................uuucgucaagguuucucacaaA................  241    1   eg2
......................................................................uuucgucaagguuucucacaaU................  375    1   eg2
......................................................................uuucgucaagguuucucacaUga...............  1      1   eg2
```

```
                                     Star                            Mature
aacagauaugauuaagaacagacaaaaaauaauucuugagaaaaccuugacugaaaauaguuuaaaacauuuucgucaagguuucucacaagaacuguuuaucucaaa
......................................................................uuucgucaagguuucucacaaUa...............   128   1   eg2
......................................................................uuucgucaagguuucucacaaCa...............   20    1   eg2
......................................................................uuucgucaagguuucucacaaAa...............   1     1   eg2
......................................................................uuucgucaagguuucucacaaAaa..............   1     1   eg2
......................................................................uuucgucaagguuucucacaaUaa..............   3     1   eg2
.......................................................................uucgucaagguuucucac...................   2     0   eg2
.......................................................................uucgucaagguuucucaca..................   207   0   eg2
.......................................................................uucCucaagguuucucaca..................   1     1   eg2
.......................................................................uucgGcaagguuucucaca..................   1     1   eg2
.......................................................................uucgucaaggGuucucaca..................   22    1   eg2
.......................................................................Nucgucaagguuucucaca..................   3     1   eg2
.......................................................................uucgucaagguuucucacU..................   33    1   eg2
.......................................................................Nucgucaagguuucucacaa.................   1     1   eg2
.......................................................................uucgucaaggGuucucacaa.................   2     1   eg2
.......................................................................uucgucaagguuucucacaU.................   62    1   eg2
.......................................................................uucgucaagguuucucacaa.................   44    0   eg2
.......................................................................uucgucaagguuucucacUa.................   1     1   eg2
.......................................................................uucgucaagguuucucacaUg................   2     1   eg2
.......................................................................uucgucaagguuucucacaaA................   5     1   eg2
.......................................................................uucgucaagguuucucacaaU................   5     1   eg2
.......................................................................uucgucaagguuucucacaaUa...............   1     1   eg2
........................................................................ucgucaagguuucucacU..................   5     1   eg2
........................................................................ucgucaaggGuucucaca..................   1     1   eg2
........................................................................ucgucaagguuucucaca..................   29    0   eg2
........................................................................ucgucaagguuucucacaU.................   4     1   eg2
........................................................................ucgucaagguuucucacaa.................   6     0   eg2

.....auaugauuaagaacagaca....................................................................................   3     0   ea2
.....................................gagaaaaccuugacugUa.....................................................   2     1   ea2
.....................................gagaaaaccuugacugaa.....................................................   5     0   ea2
.....................................gagaaaaccuugacugaaa....................................................   7     0   ea2
.....................................gagaaaaccuugacugaaaa...................................................   24    0   ea2
.....................................gagaaaaccuugacugaaaau..................................................   55    0   ea2
.....................................gagaaaaccuugacugaaaaua.................................................   1     0   ea2
.....................................gagaaaaccuugacugaaaauU.................................................   6     1   ea2
.........................................................uaguuuaaaacauuuucguca..............................   3     0   ea2
............................................................uuuaaaacauuuucgucaagg...........................   1     0   ea2
.................................................................Cacauuuucgucaagguuucucaca..................   2     1   ea2
.................................................................aacauuuucgucaagguuucucacU..................   3     1   ea2
.................................................................aacauuuucgucaagguuucucaca..................   13    0   ea2
..................................................................acauuuucgucaagguuucu......................   1     0   ea2
..................................................................acauuuucgucaagguuucuc.....................   15    0   ea2
..................................................................acauuuucgucaagguuucuca....................   9     0   ea2
..................................................................acauuuucgucaagguuucucU....................   3     1   ea2
..................................................................acauuuucgucaagguuucCca....................   1     1   ea2
..................................................................acauuuucgucaagguuucucac...................   7     0   ea2
..................................................................acauuuucgucaaggGuucucaca..................   1     1   ea2
..................................................................acauuuucgucaagguuucucaUa..................   1     1   ea2
..................................................................acauuuucgucaagguuucucaca..................   110   0   ea2
..................................................................acauuCucgucaagguuucucaca..................   1     1   ea2
..................................................................acauuuucgucaagguuucucacC..................   1     1   ea2
..................................................................acauuuucgucaagguuucucacU..................   44    1   ea2
..................................................................acauuuucgucaagguuucucacaa.................   1     0   ea2
..................................................................acauuuucgucaagguuucucacaU.................   2     1   ea2
...................................................................cauuuucgucaagguuucu......................   15    0   ea2
...................................................................cauuuucgucaagguuucuc.....................   7     0   ea2
...................................................................cauuuucgucaGgguuucuc.....................   1     1   ea2
...................................................................cauuuucgucaagguuuGuca....................   1     1   ea2
...................................................................cauuuucgucaagguuucucU....................   1     1   ea2
...................................................................cauuuucgucaagguuucuca....................   47    0   ea2
...................................................................cauuuucgucaagguuucucaU...................   1     1   ea2
...................................................................cauuuucgucaagguuucucac...................   13    0   ea2
...................................................................cauuuucgucaagguuucucaA...................   1     1   ea2
...................................................................cauuuucgucaagguuucucacC..................   3     1   ea2
...................................................................Nauuuucgucaagguuucucaca..................   2     1   ea2
...................................................................cauuuucgucaagguuucucacU..................   241   1   ea2
...................................................................cauAuucgucaagguuucucaca..................   1     1   ea2
...................................................................cauuuucgucaagguuucucacG..................   10    1   ea2
...................................................................cauuuucgucaaggGuucucaca..................   1     1   ea2
...................................................................cauuuucguUaagguuucucaca..................   4     1   ea2
```

Star Mature

aacagauaugauuaagaacagacaaaaaauaauucuugagaaaaccuugacugaaaauaguuuaaaacauuuucgucaagguuucucacaagaacuguuuaucucaaa			
....cauuuucgucaagguuucucaUa..................	5	1	ea2
....cauuuucgucaagguuucucaca..................	419	0	ea2
....cauuuucgucaagguuucucacUa.................	4	1	ea2
....cauuuucgucaagguuucucacaa.................	1	0	ea2
....cauuuucgucaagguuucucacaU.................	1	1	ea2
....auuuucgucaagguuucuc.....................	12	0	ea2
....auuuucgucaagguuucucU....................	3	1	ea2
....Cuuuucgucaagguuucuca....................	3	1	ea2
....auuuucgucaagguuucuca....................	40	0	ea2
....auuuucgucaagguuucucC....................	5	1	ea2
....auuuucgucaagguuucucac...................	50	0	ea2
....auuuucgucaagguuucucaU...................	2	1	ea2
....auuuucgucaagguuucCcaca..................	2	1	ea2
....auuuucgucaGgguuucucaca..................	1	1	ea2
....Cuuuucgucaagguuucucaca..................	58	1	ea2
....auuuucgucGagguuucucaca..................	1	1	ea2
....auuuucgucaagguuucucacU..................	322	1	ea2
....auuuucgucaagguuucucacG..................	7	1	ea2
....Uuuuucgucaagguuucucaca..................	2	1	ea2
....auuCucgucaagguuucucaca..................	1	1	ea2
....auuuucgucaagguuucucaca..................	800	0	ea2
....auuuucgucaagguuucucacC..................	9	1	ea2
....Nuuuucgucaagguuucucaca..................	2	1	ea2
....auuuucgucaagguuucucaUa..................	2	1	ea2
....auuuucgCcaagguuucucaca..................	1	1	ea2
....auuuucgucaagguuucucacaa.................	3	0	ea2
....Cuuuucgucaagguuucucacaa.................	3	1	ea2
....auuuucgucaagguuucucacUa.................	2	1	ea2
....uuuucgucaagguuucuAa....................	7	1	ea2
....Nuuucgucaagguuucuca....................	2	1	ea2
....uuuucgucaagguuucuca....................	75	0	ea2
....uuuucgucaagguuucucU....................	4	1	ea2
....uuuucgucaagguuucucaU...................	6	1	ea2
....Nuuucgucaagguuucucac...................	6	1	ea2
....uuuucgucaagguuucucCc...................	1	1	ea2
....Cuuucgucaagguuucucac...................	4	1	ea2
....uuuucgucaaggGuucucac...................	1	1	ea2
....uuuucgucaagguuucucac...................	566	0	ea2
....uuuucgucaagguGucucac...................	1	1	ea2
....uuuucgucCagguuucucaca..................	1	1	ea2
....uuuGcgucaagguuucucaca..................	1	1	ea2
....uuuucgucaagguAucucaca..................	1	1	ea2
....uuuucgucaaggCuucucaca..................	7	1	ea2
....uuuucgucaGgguuucucaca..................	9	1	ea2
....uuuucgucGagguuucucaca..................	4	1	ea2
....uuuuGgucaagguuucucaca..................	1	1	ea2
....uuuucguUaagguuucucaca..................	3	1	ea2
....uuuucgucaaAguuucucaca..................	1	1	ea2
....uuuucgucaagguuCcucaca..................	5	1	ea2
....uuuucgucUagguuucucaca..................	2	1	ea2
....uuuucgucaaggGuucucaca..................	22	1	ea2
....Guuucgucaagguuucucaca..................	13	1	ea2
....uuuucguAaagguuucucaca..................	2	1	ea2
....uuuucgucaagguuucGcaca..................	3	1	ea2
....uuuucgucaagguuucucacG..................	24	1	ea2
....uuuucgucaagguCucucaca..................	5	1	ea2
....uGuucgucaagguuucucaca..................	1	1	ea2
....uuuucgucaaggAuucucaca..................	2	1	ea2
....uuuucgucaagguuuAucaca..................	2	1	ea2
....uuuucgGcaagguuucucaca..................	3	1	ea2
....uCuucgucaagguuucucaca..................	10	1	ea2
....uuuucgucaagguuucAcaca..................	3	1	ea2
....uuuucgucaagguGucucaca..................	4	1	ea2
....uuAucgucaagguuucucaca..................	1	1	ea2
....uAuucgucaagguuucucaca..................	1	1	ea2
....uuuucgCcaagguuucucaca..................	14	1	ea2
....uuCucgucaagguuucucaca..................	4	1	ea2
....uuuucgucaagguuucucaGa..................	1	1	ea2
....uNuucgucaagguuucucaca..................	12	1	ea2
....uuGucgucaagguuucucaca..................	2	1	ea2

```
                                      Star                           Mature
aacagauaugauuaagaacagacaaaaaauaauucuugagaaaaccuugacugaaaauaguuuaaaacauuuucgucaagguuucucacaagaacuguuuaucucaaa
.....................................................................uuuucgucaagguuucucacU..................    4889    1    ea2
.....................................................................uuuucgucaagguuAcucaca..................    1       1    ea2
.....................................................................uuuucgucaagguuucCcaca..................    8       1    ea2
.....................................................................uuuucgAcaagguuucucaca..................    3       1    ea2
.....................................................................uuuAcgucaagguuucucaca..................    2       1    ea2
.....................................................................uuuucgucaagguuucucaUa..................    8       1    ea2
.....................................................................uuuucgucaagguuucucaAa..................    2       1    ea2
.....................................................................uuuucAucaagguuucucaca..................    7       1    ea2
.....................................................................uuuCcgucaagguuucucaca..................    2       1    ea2
.....................................................................uuuucgucaaUguuucucaca..................    2       1    ea2
.....................................................................Nuuucgucaagguuucucaca..................    119     1    ea2
.....................................................................uuuucgucaagguuucucGca..................    6       1    ea2
.....................................................................uuuuAgucaagguuucucaca..................    4       1    ea2
.....................................................................uuuucgucaagguuucucacC..................    84      1    ea2
.....................................................................Cuuucgucaagguuucucaca..................    510     1    ea2
.....................................................................uuuucgucaagguuucuUaca..................    1       1    ea2
.....................................................................uuuucgucaagguuucucaca..................    19686   0    ea2
.....................................................................uuuucgucaagguuucucCca..................    4       1    ea2
.....................................................................uuuucgucaagguuucucacUa.................    118     1    ea2
.....................................................................uuuucgucaagguuucucacaa.................    399     0    ea2
.....................................................................Guuucgucaagguuucucacaa.................    1       1    ea2
.....................................................................uuuucgucaagguuucucacaC.................    6       1    ea2
.....................................................................Cuuucgucaagguuucucacaa.................    57      1    ea2
.....................................................................uuuucgucaagguuucucacaU.................    583     1    ea2
.....................................................................uuuucgucaagguuucucacCa.................    1       1    ea2
.....................................................................uuuucgucaagguuucucacaaU................    61      1    ea2
.....................................................................Cuuucgucaagguuucucacaag................    1       1    ea2
.....................................................................uuuucgucaagguuucucacaaA................    21      1    ea2
.....................................................................uuuucgucaagguuucucacaag................    5       0    ea2
.....................................................................uuuucgucaagguuucucacaUg................    9       1    ea2
.....................................................................uuuucgucaagguuucucacaaUa...............    8       1    ea2
......................................................................uuucgGcaagguuucuca....................    1       1    ea2
......................................................................Nuucgucaagguuucuca....................    9       1    ea2
......................................................................uuucgucaagguuucucU....................    20      1    ea2
......................................................................Auucgucaagguuucuca....................    4       1    ea2
......................................................................Cuucgucaagguuucuca....................    1       1    ea2
......................................................................uuucgucaGgguuucuca....................    1       1    ea2
......................................................................uuucgucaagguuucuca....................    1785    0    ea2
......................................................................Guucgucaagguuucuca....................    2       1    ea2
......................................................................uNucgucaagguuucuca....................    1       1    ea2
......................................................................uuucgucaagAuuucuca....................    1       1    ea2
......................................................................uuucgucaagguuucuAa....................    12      1    ea2
......................................................................uuucgucaagguuucucG....................    21      1    ea2
......................................................................uuucgucaagguuucucac...................    6122    0    ea2
......................................................................uuucgucaagguuucucaU...................    145     1    ea2
......................................................................Cuucgucaagguuucucac...................    6       1    ea2
......................................................................uuucgucaagguuucucaA...................    14      1    ea2
......................................................................uNucgucaagguuucucac...................    2       1    ea2
......................................................................uuucgAcaagguuucucac...................    1       1    ea2
......................................................................uuucgucaaggAuucucac...................    4       1    ea2
......................................................................uuucgucaaggCuucucac...................    2       1    ea2
......................................................................Guucgucaagguuucucac...................    5       1    ea2
......................................................................uuucAucaagguuucucac...................    1       1    ea2
......................................................................uuucUucaagguuucucac...................    1       1    ea2
......................................................................Nuucgucaagguuucucac...................    32      1    ea2
......................................................................uuucgucaagguuucucUc...................    2       1    ea2
......................................................................uuucgCcaagguuucucac...................    1       1    ea2
......................................................................uuucgucaagguuucucaG...................    2       1    ea2
......................................................................uuucgucaagguuucuUac...................    1       1    ea2
......................................................................uuucgucaagguuucucGc...................    3       1    ea2
......................................................................uuuUgucaagguuucucac...................    2       1    ea2
......................................................................uuucgucaaCguuucucac...................    1       1    ea2
......................................................................uuucgucaagguGucucac...................    1       1    ea2
......................................................................uuucgucGagguuucucac...................    3       1    ea2
......................................................................uuucgucaaggGuucucac...................    7       1    ea2
......................................................................uuucgucaGgguuucucac...................    1       1    ea2
......................................................................uuucgucaagguuucGcac...................    1       1    ea2
......................................................................uuucgGcaagguuucucac...................    1       1    ea2
......................................................................uuucgucaagguuucCcac...................    2       1    ea2
......................................................................uuucgucaagguuCcucac...................    1       1    ea2
```

Star Mature

```
aacagauaugauuaagaacagacaaaaaauaauucuugagaaaaccuugacugaaaauaguuuaaaacauuuucgucaagguuucucacaagaacuguuuaucucaaa
......................................................................uuucgucaagguuucucCc...................	3	1	ea2
......................................................................uGucgucaagguuucucac...................	2	1	ea2
......................................................................Auucgucaagguuucucac...................	5	1	ea2
......................................................................uuucgucaagguuucucaAa..................	39	1	ea2
......................................................................uuucgucaagguGucucaca..................	45	1	ea2
......................................................................uuucgucaagguuucucUca..................	16	1	ea2
......................................................................Auucgucaagguuucucaca..................	107	1	ea2
......................................................................uuuGgucaagguuucucaca..................	7	1	ea2
......................................................................uuGcgucaagguuucucaca..................	39	1	ea2
......................................................................Guucgucaagguuucucaca..................	172	1	ea2
......................................................................uuucgAcaagguuucucaca..................	49	1	ea2
......................................................................uNucgucaagguuucucaca..................	171	1	ea2
......................................................................uuucgucaagguuucucacG..................	365	1	ea2
......................................................................uuucgucaagguuucCcaca..................	165	1	ea2
......................................................................uuuAgucaagguuucucaca..................	45	1	ea2
......................................................................uuucgucaaUguuucucaca..................	27	1	ea2
......................................................................uuucgucaagguuGcucaca..................	41	1	ea2
......................................................................uCucgucaagguuucucaca..................	167	1	ea2
......................................................................uuucAucaagguuucucaca..................	69	1	ea2
......................................................................uuucgucaaCguuucucaca..................	2	1	ea2
......................................................................uuAcgucaagguuucucaca..................	9	1	ea2
......................................................................uuucgucaagguuucucacC..................	962	1	ea2
......................................................................uuucgucaaAguuucucaca..................	16	1	ea2
......................................................................uuucgucaagguuucucaca..................	378763	0	ea2
......................................................................uuucgucaagguuuGucaca..................	7	1	ea2
......................................................................uuucgucaagguuucucGca..................	84	1	ea2
......................................................................uuucgucaGgguuucucaca..................	114	1	ea2
......................................................................uuucgucaagguuuUucaca..................	13	1	ea2
......................................................................uuucgucaagUuuucucaca..................	16	1	ea2
......................................................................uuucgGcaagguuucucaca..................	211	1	ea2
......................................................................uAucgucaagguuucucaca..................	11	1	ea2
......................................................................uuucgucaagguCucucaca..................	90	1	ea2
......................................................................uuucUucaagguuucucaca..................	24	1	ea2
......................................................................uuucgucaagguuAcucaca..................	15	1	ea2
......................................................................uuucgucGagguuucucaca..................	98	1	ea2
......................................................................uuucguGaagguuucucaca..................	6	1	ea2
......................................................................uuucgucaagguuucucaUa..................	340	1	ea2
......................................................................uuucgucaagAuuucucaca..................	29	1	ea2
......................................................................uuucgCcaagguuucucaca..................	153	1	ea2
......................................................................uuucgucaCgguuucucaca..................	3	1	ea2
......................................................................uuucgucaagCuuucucaca..................	7	1	ea2
......................................................................uuucgucaagguuucuAaca..................	30	1	ea2
......................................................................uuucgucaagguuucGcaca..................	22	1	ea2
......................................................................uuucgucaagguuCcucaca..................	106	1	ea2
......................................................................uuucgucaagguuuAucaca..................	26	1	ea2
......................................................................uuucgucaagguuucuGaca..................	4	1	ea2
......................................................................uuucgucaagguuucucaGa..................	7	1	ea2
......................................................................uuucgucaagguuucucCca..................	8	1	ea2
......................................................................uuuUgucaagguuucucaca..................	44	1	ea2
......................................................................uuucgucaagguAucucaca..................	14	1	ea2
......................................................................uuucgucaaggGuucucaca..................	354	1	ea2
......................................................................uuucgucaagguuucAcaca..................	19	1	ea2
......................................................................uuucgucaaggAuucucaca..................	102	1	ea2
......................................................................uuucgucaUgguuucucaca..................	11	1	ea2
......................................................................uuucgucCagguuucucaca..................	2	1	ea2
......................................................................uuCcgucaagguuucucaca..................	57	1	ea2
......................................................................uuucgucaagguuucuUaca..................	29	1	ea2
......................................................................uuucguAaagguuucucaca..................	28	1	ea2
......................................................................Nuucgucaagguuucucaca..................	1382	1	ea2
......................................................................uuucgucUagguuucucaca..................	9	1	ea2
......................................................................Cuucgucaagguuucucaca..................	219	1	ea2
......................................................................uuucguUaagguuucucaca..................	31	1	ea2
......................................................................uuucgucaaggCuucucaca..................	191	1	ea2
......................................................................uGucgucaagguuucucaca..................	30	1	ea2
......................................................................uuucgucaagguuucucacU..................	88032	1	ea2
......................................................................uuuCucaagguuucucaca..................	7	1	ea2
......................................................................Nuucgucaagguuucucacaa.................	170	1	ea2
......................................................................uuucgucaagguuucucacaG.................	72	1	ea2
......................................................................Guucgucaagguuucucacaa.................	55	1	ea2
......................................................................uAucgucaagguuucucacaa.................	4	1	ea2
```

```
                                                                   Star                          Mature
aacagauaugauuaagaacagacaaaaaauaauucuugagaaaaccuugacugaaaauaguuuaaaacauuuucgucaagguuucucacaagaacuguuuaucucaaa
......................................................................uuucgucaagAuuucucacaa.................   1       1   ea2
......................................................................uuucgucaagguuucucaGaa.................   1       1   ea2
......................................................................uuucgucaagguuucAcacaa.................   2       1   ea2
......................................................................uuucgucaagguuucucacGa.................   12      1   ea2
......................................................................uuucgucaGgguuucucacaa.................   5       1   ea2
......................................................................uuucgucaagguuucuUacaa.................   4       1   ea2
......................................................................uuucgAcaagguuucucacaa.................   4       1   ea2
......................................................................uuucgucaagguuuAucacaa.................   4       1   ea2
......................................................................uuucgucaaggGuucucacaa.................   49      1   ea2
......................................................................uuucgucaagguuucuGacaa.................   1       1   ea2
......................................................................uuucgucaagguuucGcacaa.................   1       1   ea2
......................................................................uuucgucaagguuucucacaU.................   39023   1   ea2
......................................................................uuucgucaagguuucucacCa.................   141     1   ea2
......................................................................uNucgucaagguuucucacaa.................   15      1   ea2
......................................................................uuuAgucaagguuucucacaa.................   6       1   ea2
......................................................................uuucgucaaAguuucucacaa.................   1       1   ea2
......................................................................uuucgucaagguuucucCcaa.................   2       1   ea2
......................................................................uuucUucaagguuucucacaa.................   2       1   ea2
......................................................................uuucgucaagguuuUucacaa.................   1       1   ea2
......................................................................uuucguAaagguuucucacaa.................   5       1   ea2
......................................................................uuucgucUagguuucucacaa.................   1       1   ea2
......................................................................uuAcgucaagguuucucacaa.................   3       1   ea2
......................................................................Cuucgucaagguuucucacaa.................   52      1   ea2
......................................................................uuucgucaagguuucucGcaa.................   8       1   ea2
......................................................................uuucgucaagUuuucucacaa.................   1       1   ea2
......................................................................uuucgucaagguuucucacUa.................   4062    1   ea2
......................................................................uuucgucaaggCuucucacaa.................   14      1   ea2
......................................................................Auucgucaagguuucucacaa.................   30      1   ea2
......................................................................uuucgucGagguuucucacaa.................   10      1   ea2
......................................................................uCucgucaagguuucucacaa.................   18      1   ea2
......................................................................uuucgucaUgguuucucacaa.................   2       1   ea2
......................................................................uGucgucaagguuucucacaa.................   9       1   ea2
......................................................................uuucAucaagguuucucacaa.................   14      1   ea2
......................................................................uuucgucaagguuucucaAaa.................   6       1   ea2
......................................................................uuucgCcaagguuucucacaa.................   13      1   ea2
......................................................................uuucgucaagguuucucUcaa.................   3       1   ea2
......................................................................uuucgucaagguuucCcacaa.................   25      1   ea2
......................................................................uuucgucaagguuCcucacaa.................   22      1   ea2
......................................................................uuucgucaagguuucucaUaa.................   12      1   ea2
......................................................................uuucgucaagguuucucacaa.................   48600   0   ea2
......................................................................uuucgucaagguuucucacaC.................   358     1   ea2
......................................................................uuucgucaagCuuucucacaa.................   1       1   ea2
......................................................................uuucgucaCgguuucucacaa.................   1       1   ea2
......................................................................uuucgucaaUguuucucacaa.................   1       1   ea2
......................................................................uuucgucaagguCucucacaa.................   14      1   ea2
......................................................................uuucguGaagguuucucacaa.................   2       1   ea2
......................................................................uuucguUaagguuucucacaa.................   3       1   ea2
......................................................................uuucgucaagguuGcucacaa.................   5       1   ea2
......................................................................uuuGgucaagguuucucacaa.................   1       1   ea2
......................................................................uuucgucaagguuucuAacaa.................   6       1   ea2
......................................................................uuuUgucaagguuucucacaa.................   7       1   ea2
......................................................................uuucgucaagguuuGucacaa.................   4       1   ea2
......................................................................uuucgGcaagguuucucacaa.................   14      1   ea2
......................................................................uuCcgucaagguuucucacaa.................   5       1   ea2
......................................................................uuucgucaagguGucucacaa.................   7       1   ea2
......................................................................uuGcgucaagguuucucacaa.................   7       1   ea2
......................................................................uuucgucaaggAuucucacaa.................   12      1   ea2
......................................................................uuucgucaagguuAcucacaa.................   1       1   ea2
......................................................................uuucgGcaagguuucucacaag................   1       1   ea2
......................................................................uuucgucaagguuGcucacaag................   1       1   ea2
......................................................................uuucgucaagguuCcucacaag................   1       1   ea2
......................................................................uuucgucaagguCucucacaag................   1       1   ea2
......................................................................uuucgucaagguuucucCcaag................   1       1   ea2
......................................................................uuucgucaagguuucucacaaU................   4220    1   ea2
......................................................................Auucgucaagguuucucacaag................   1       1   ea2
......................................................................uuucgucaagguuucucacUag................   6       1   ea2
......................................................................uuucgucaagguuucucacaCg................   40      1   ea2
......................................................................Nuucgucaagguuucucacaag................   2       1   ea2
......................................................................uuucgucaagguuucucacaUg................   1771    1   ea2
......................................................................uuucgucaagguuucucacaaA................   2235    1   ea2
```

Star Mature

aacagauaugauuaagaacagacaaaaaauaauucuugagaaaaccuugacugaaaauaguuuaaaacauuuucgucaagguuucucacaagaacuguuuaucucaaa			
....uuucgucaagguuucucacaaC................	60	1	ea2
....uuucgucaagguuucucacaag................	421	0	ea2
....uuucgucaagguuucucacaaAa...............	19	1	ea2
....uuucgucaagguuucucacaaCa...............	228	1	ea2
....uuucgucaagguuucucacaaUa...............	1436	1	ea2
....uuucgucaagguuucucacaUga...............	6	1	ea2
....uuucgucaagguuucucacaagCa..............	3	1	ea2
....uuucgucaagguuucucacaUgaa..............	1	1	ea2
....uuucgucaagguuucucacaaUaa..............	24	1	ea2
....uuucgucaagguuucucacaagaacA............	1	1	ea2
....uucgucaagguuucucac...................	72	0	ea2
....uucgucaagguuucucaU...................	3	1	ea2
....Nucgucaagguuucucac...................	2	1	ea2
....uucgucaaggCuucucaca..................	1	1	ea2
....uucgucaagguuGcucaca..................	2	1	ea2
....uucgucaagguuucucaAa..................	3	1	ea2
....uucgucGagguuucucaca..................	1	1	ea2
....uucgucaagguuucucacC..................	5	1	ea2
....uuAgucaagguuucucaca..................	1	1	ea2
....uucgucaagguuucucaGa..................	1	1	ea2
....uucgucaagguuucCcaca..................	3	1	ea2
....uNcgucaagguuucucaca..................	2	1	ea2
....uucgucaagguGucucaca..................	1	1	ea2
....uCcgucaagguuucucaca..................	1	1	ea2
....uuUgucaagguuucucaca..................	4	1	ea2
....uucAucaagguuucucaca..................	1	1	ea2
....uucgGcaagguuucucaca..................	4	1	ea2
....uucgucaagguuucucaUa..................	11	1	ea2
....uucgucaaggGuucucaca..................	24	1	ea2
....uucgucaGgguuucucaca..................	1	1	ea2
....uucgucaagUuuucucaca..................	1	1	ea2
....uucgucaagguuucucCca..................	2	1	ea2
....uucgucaagguuucucacG..................	4	1	ea2
....Nucgucaagguuucucaca..................	18	1	ea2
....uucgucaUgguuucucaca..................	2	1	ea2
....uucgucaagAuuucucaca..................	3	1	ea2
....uucgucCagguuucucaca..................	21	1	ea2
....uucgucaaggAuucucaca..................	2	1	ea2
....uucgucaagguuucucacU..................	1296	1	ea2
....uucgucaagguuCcucaca..................	2	1	ea2
....uucgucaagguuucuAaca..................	1	1	ea2
....uucgucaagguuucucaca..................	6486	0	ea2
....uucgucaagguuucucGca..................	1	1	ea2
....uucgucaagguuucucacaU.................	738	1	ea2
....uucgucaagguuucucacCa.................	5	1	ea2
....uucgucaagguuucucacaa.................	796	0	ea2
....uucgCcaagguuucucacaa.................	4	1	ea2
....uucgucaagguuucucacaG.................	3	1	ea2
....uGcgucaagguuucucacaa.................	1	1	ea2
....uucgucaagguuucucacUa.................	65	1	ea2
....uucgucCagguuucucacaa.................	2	1	ea2
....uucgGcaagguuucucacaa.................	1	1	ea2
....uucgucaaggGuucucacaa.................	6	1	ea2
....uucgucaaUguuucucacaa.................	1	1	ea2
....uucgucaagUuuucucacaa.................	1	1	ea2
....uucgucaagguuucucacaC.................	13	1	ea2
....uucgucaagguuucuUacaa.................	1	1	ea2
....uucgucaagguuucCcacaa.................	1	1	ea2
....uucgucaagguuucucacaaA................	55	1	ea2
....uucgucaagguuucucacaUg................	21	1	ea2
....uucgucaagguuucucacaaU................	90	1	ea2
....uucgucaagguuucucacaag................	10	0	ea2
....uucgucaagguuucucacGag................	1	1	ea2
....uucgucaagguuucucacaaUa...............	28	1	ea2
....ucgucaagguuCcucaca..................	1	1	ea2
....Ncgucaagguuucucaca..................	3	1	ea2
....ucgucaagguuucucacU..................	236	1	ea2
....Acgucaagguuucucaca..................	1	1	ea2
....ucgucaaggGuucucaca..................	1	1	ea2
....ucgucaagguuucucaca..................	1000	0	ea2

```
                                    Star                            Mature
aacagauaugauuaagaacagacaaaaaauaauucuugagaaaaccuugacugaaaauaguuuaaaacauuuucgucaagguuucucacaagaacuguuuaucucaaa
........................................................................ucgucaagguuucucacaU.................   151   1   ea2
........................................................................ucgucaagguuucucacaC.................   7     1   ea2
........................................................................Ncgucaagguuucucacaa.................   1     1   ea2
........................................................................ucgucaagguCucucacaa.................   1     1   ea2
........................................................................ucgucaagguuucucacUa.................   27    1   ea2
........................................................................ucgucaagguuucucacaa.................   148   0   ea2
........................................................................ucgucaagguuucucacaag................   6     0   ea2
........................................................................ucgucaagguuucucacaUg................   4     1   ea2
........................................................................ucgucaagguuucucacaaU................   12    1   ea2
........................................................................ucgucaagguuucucacaaA................   7     1   ea2
........................................................................ucgucaagguuucucacaaCa...............   1     1   ea2
```

Provisional ID : ScUbCFx_4_1782
Score total : 1.4
Score for star read(s) : -1.3
Score for read counts : 0
Score for mfe : 1.7
Score for randfold : 1.6
Score for cons. seed : -0.6
Total read count : 6563
Mature read count : 6545
Loop read count : 0
Star read count : 18

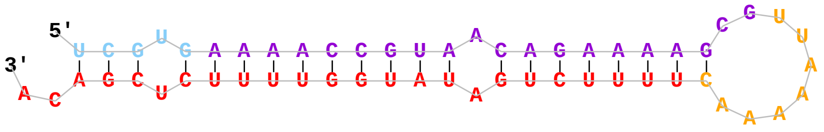

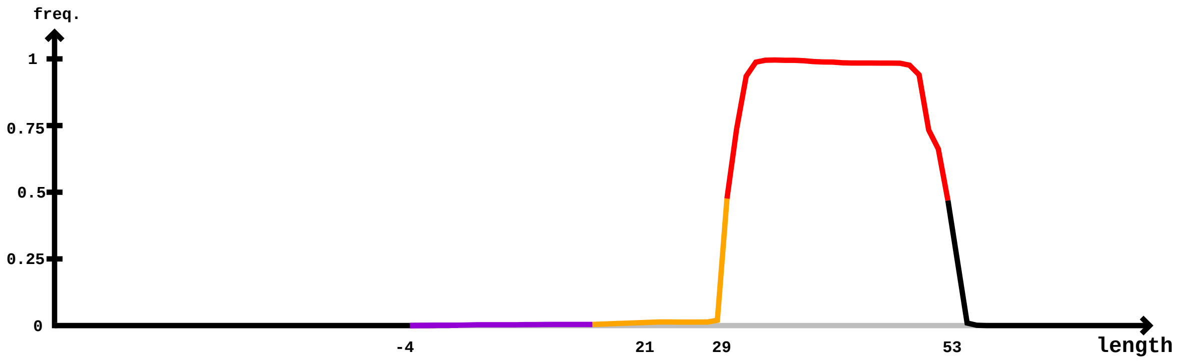

Star Mature

```
5'- cucugaauaucuuuccuuuuagauacucacaagugucgugaaaaccguaacagaaaagcguuaaaaacuuuucugauaugguuuucucgacacuuagauguugcauuuuucg -3'  obs
    cucugaauaucuuuccuuuuagauacucacaagugucgugaaaaccguaacagaaaagcguuaaaaacuuuucugauaugguuuucucgacacuuagauguugcauuuuucg       exp
    ...(((.(((((.........))))).))).((((((((.(((((((((.((((((((.........)))))))).))))))))).)))))))))..................  reads  mm  sample
    ....................................cgugaaaaccguaacagaaU........................................................  1  1  ea1
    ......................................ugaaaaccguaacagaaaag......................................................  1  0  ea1
    .......................................................aagcguuaaaaacuuuucuga....................................  1  0  ea1
    ........................................................agcguuaaaaacuuuucuga....................................  1  0  ea1
    ........................................................Ugcguuaaaaacuuuucuga....................................  1  1  ea1
    ........................................................aAcguuaaaaacuuuucuga....................................  1  1  ea1
    ........................................................agcguuaaaaacuuuucugau...................................  1  0  ea1
    ........................................................agcguuaaaaacuuuucugaA...................................  1  1  ea1
    .........................................................gcguuaaaaacuuuucugaua..................................  2  0  ea1
    ..........................................................cguuaaaaacuuuucugau...................................  1  0  ea1
    ..........................................................cguuaaaaacuuuucugauG..................................  1  1  ea1
    ..........................................................cguuaaaaacuuuucugaua..................................  3  0  ea1
    ..........................................................cguuaaaaacuuuucugaCa..................................  1  1  ea1
    ..........................................................cguuaaaaacuuuucugauaugg...............................  1  0  ea1
    ...........................................................guuaaaaacuuuucugauauA................................  1  1  ea1
    ............................................................uuaaaaacuuuucugauau.................................  2  0  ea1
    ............................................................uuaaaaGcuuuucugauaug................................  1  1  ea1
    ............................................................uuaaaaacuuuucugauauA................................  2  1  ea1
    ............................................................uuaaaaacuuuucugauaug................................  6  0  ea1
    .............................................................uaaaaacuuuucugauaugg...............................  1  0  ea1
    .............................................................uaaaaacuuuucugauaugguuA............................  1  1  ea1
    ...............................................................aaaaGuuuucugauaugguuu............................  1  1  ea1
    .................................................................aacuuuucugauaugguuuuc..........................  1  0  ea1
    ..................................................................acuuuucugauaugguuuucu.........................  1  0  ea1
    ...................................................................cuuuucugauaugguuuucA.........................  1  1  ea1
    ...................................................................cuuuucugauaugguuuucu.........................  2  0  ea1
    ...................................................................cuuuucugauaugguuuucC.........................  1  1  ea1
    ...................................................................cuuuucugauaugguuuucuc........................  1  0  ea1
    ...................................................................cuuuucugauaugguuuucucU.......................  1  1  ea1
    ...................................................................cuuuucugauaugguuuucucg.......................  1  0  ea1
    ...................................................................cuuuucugauaugguuuucucA.......................  1  1  ea1
    ...................................................................cuuuucugauaugguuuucucAa......................  4  1  ea1
    ....................................................................uuuucugauaugguuuucu.........................  3  0  ea1
```

Star Mature

```
cucugaauaucuuuccuuuuagauacucacaagugucgugaaaaccguaacagaaaagcguuaaaaacuuuucugauaugguuuucucgacacuuagauguugcauuuuucg
....................................................................uuuucugauaugguuuucC.........................   1     1   ea1
....................................................................Guuucugauaugguuuucuc........................   4     1   ea1
....................................................................uuuucugauaugguuuucuA........................   2     1   ea1
....................................................................uuuucugauaugguuuucuc........................   31    0   ea1
....................................................................uuuucugauaugguuuucuG........................   1     1   ea1
....................................................................uuuucugauaugguuCucucg.......................   1     1   ea1
....................................................................uuuucugUuaugguuuucucg.......................   1     1   ea1
....................................................................uuuucugauaugguuuucucg.......................   136   0   ea1
....................................................................Guuucugauaugguuuucucg.......................   12    1   ea1
....................................................................uuuucugauaugguuuucucA.......................   36    1   ea1
....................................................................uuuucugauaugguCuucucg.......................   1     1   ea1
....................................................................Cuuucugauaugguuuucucg.......................   1     1   ea1
....................................................................uuuucugauaugguuuucucU.......................   20    1   ea1
....................................................................uuuucugauaCgguuuucucg.......................   1     1   ea1
....................................................................uuuucugauaugguuuucucC.......................   17    1   ea1
....................................................................uuuucugauaugguuuucucAa......................   1     1   ea1
....................................................................uuuucugauaugguuuCcucga......................   1     1   ea1
....................................................................uuuucugauaugguCuucucga......................   6     1   ea1
....................................................................uuuucugauaugguuuuUucga......................   1     1   ea1
....................................................................uuuucugauaugguuuucucga......................   151   0   ea1
....................................................................uuCucugauaugguuuucucga......................   1     1   ea1
....................................................................uuuucugauaugguuuucucgC......................   8     1   ea1
....................................................................Guuucugauaugguuuucucga......................   17    1   ea1
....................................................................uuuucugauaugguuuucucCa......................   3     1   ea1
....................................................................uuuucugauauggCuuucucga......................   1     1   ea1
....................................................................uuuucugauaugguuuucucgU......................   18    1   ea1
....................................................................uuuucugauaugguuuucucgG......................   2     1   ea1
....................................................................uuuucugaCaugguuuucucga......................   1     1   ea1
....................................................................uuuucugauaugguuuucuUga......................   1     1   ea1
....................................................................uuuucugauauggGuuucucga......................   1     1   ea1
....................................................................uuuucugauaugguuuucucgGc.....................   5     1   ea1
....................................................................Guuucugauaugguuuucucgac.....................   14    1   ea1
....................................................................uuuucugauaugguuuucucgac.....................   224   0   ea1
....................................................................uuuucugauaCgguuuucucgac.....................   1     1   ea1
....................................................................Cuuucugauaugguuuucucgac.....................   1     1   ea1
....................................................................uuuucugauaugguuuucucgaA.....................   35    1   ea1
....................................................................uuuucugauaugguuuucucgaG.....................   1     1   ea1
....................................................................uuuucugauaugguuuCcucgac.....................   1     1   ea1
....................................................................uuuucugauaugguuuucucAac.....................   2     1   ea1
....................................................................uuuucugauaugguuuucucgaU.....................   63    1   ea1
....................................................................uuuucugauaugguuuucCcgac.....................   1     1   ea1
....................................................................uuuucugauaugguuuucucgCc.....................   1     1   ea1
....................................................................uuuCcugauaugguuuucucgac.....................   1     1   ea1
....................................................................uuuucugauaugguuuucucgacU....................   117   1   ea1
....................................................................uuuucugauauggGuuucucgaca....................   1     1   ea1
....................................................................uuuucugauaugguuuCcucgaca....................   2     1   ea1
....................................................................uuuucugauaugguCuucucgaca....................   4     1   ea1
....................................................................uCuucugauaugguuuucucgaca....................   3     1   ea1
....................................................................uuuucugauaugAuuuucucgaca....................   1     1   ea1
....................................................................uuuucugauGugguuuucucgaca....................   1     1   ea1
....................................................................uuuucugauaugguAuucucgaca....................   1     1   ea1
....................................................................Guuucugauaugguuuucucgaca....................   45    1   ea1
....................................................................uuuucugauaugguuuucucgaca....................   552   0   ea1
....................................................................uuuucugauaugguuuucCcgaca....................   2     1   ea1
....................................................................uuuucugauaugguuuucucAaca....................   3     1   ea1
....................................................................uuuucugauaugguuuucucgacG....................   22    1   ea1
....................................................................uuuucugGuaugguuuucucgaca....................   1     1   ea1
....................................................................uuuucugauaugguuuucucgGca....................   2     1   ea1
....................................................................uuuucugauaugUuuuucucgaca....................   1     1   ea1
....................................................................uuuucugauaugguuuucucgaAa....................   1     1   ea1
....................................................................uuuucCgauaugguuuucucgaca....................   1     1   ea1
....................................................................uuuucugauaugguuuucuUgaca....................   2     1   ea1
....................................................................uuuucugauaugguuuucucgacC....................   22    1   ea1
....................................................................uuuucugauaugguuuucucgaUa....................   28    1   ea1
....................................................................Cuuucugauaugguuuucucgaca....................   4     1   ea1
....................................................................uuuucugauaugguuuucucgacUc...................   3     1   ea1
....................................................................uuuucugauaugguuuucucgacaA...................   9     1   ea1
....................................................................Guuucugauaugguuuucucgacac...................   1     1   ea1
....................................................................uuuucugauaugguuuucucgacaU...................   11    1   ea1
....................................................................uuuucugauaugguuuucucgacCc...................   6     1   ea1
```

```
                                            Star                 Mature
cucugaauaucuuuccuuuuagauacucacaagugucgugaaaaccguaacagaaaagcguuaaaaacuuuucugauaugguuuucucgacacuuagauguugcauuuuucg
....................................................................uuuucugauaugguuuucucgacaUu.................. 5 1 ea1
....................................................................uuuucugauaugguuuucucgacacA.................. 2 1 ea1
....................................................................uuuucugauaugguuuucucgacaUuu................. 1 1 ea1
....................................................................uuuucugauaugguuuucucgacaAuu................. 1 1 ea1
.....................................................................uuucugauaugguuuucu......................... 1 0 ea1
.....................................................................uuucugauaugguuuucuc........................ 23 0 ea1
.....................................................................uuucugauaugguuuucuU........................ 3 1 ea1
.....................................................................Guucugauaugguuuucuc........................ 1 1 ea1
.....................................................................uuucugauaugAuuuucuc........................ 1 1 ea1
.....................................................................Guucugauaugguuuucucg....................... 4 1 ea1
.....................................................................uuucugauaugguuuucucU....................... 10 1 ea1
.....................................................................uuucugauaugguuuucucC....................... 9 1 ea1
.....................................................................uuucugauaugguuuucucg....................... 33 0 ea1
.....................................................................uuucugauaugguuuucCcg....................... 1 1 ea1
.....................................................................uuucugauaugguuuucucA....................... 23 1 ea1
.....................................................................uuCcugauaugguuuucucga...................... 1 1 ea1
.....................................................................uuucugauaugguuuucucgC...................... 2 1 ea1
.....................................................................uuucugauaugguuuucucUa...................... 1 1 ea1
.....................................................................uuucugauaugguuCucucga...................... 1 1 ea1
.....................................................................uuucugauaugguuuucucga...................... 9 0 ea1
.....................................................................Guucugauaugguuuucucga...................... 1 1 ea1
.....................................................................uuucugauaugguuuucucAa...................... 3 1 ea1
.....................................................................uuucugauaugguuuucucgaA..................... 5 1 ea1
.....................................................................uuucugauaugguuuucucAac..................... 6 1 ea1
.....................................................................uuucugauaugguuuucucgac..................... 63 0 ea1
.....................................................................uuucugauaugguuuucucgaU..................... 3 1 ea1
.....................................................................uuucugauaugguuuucucgGc..................... 1 1 ea1
.....................................................................uCucugauaugguuuucucgac..................... 1 1 ea1
.....................................................................Guucugauaugguuuucucgac..................... 10 1 ea1
.....................................................................uuucugauaGgguuuucucgaca.................... 1 1 ea1
.....................................................................uuucugauaugguuuucucgaca.................... 174 0 ea1
.....................................................................Guucugauaugguuuucucgaca.................... 16 1 ea1
.....................................................................uuucugauaugguuuucucAaca.................... 3 1 ea1
.....................................................................uuucugauaugguuuucucgacU.................... 111 1 ea1
.....................................................................uuucAgauaugguuuucucgaca.................... 1 1 ea1
.....................................................................uuucugauaugguuuucCcgaca.................... 1 1 ea1
.....................................................................uuucugauaugguuuucucgacC.................... 11 1 ea1
.....................................................................uuucCgauaugguuuucucgaca.................... 2 1 ea1
.....................................................................uuucugauGugguuuucucgaca.................... 1 1 ea1
.....................................................................uuucugauaugguuuucucgacG.................... 5 1 ea1
.....................................................................uuucugauauggCuuucucgaca.................... 1 1 ea1
.....................................................................uuucugauaugguuuCcucgaca.................... 3 1 ea1
.....................................................................uuucugauaugguuuucucgCca.................... 1 1 ea1
.....................................................................uuucugauaugguuuuAucgaca.................... 1 1 ea1
.....................................................................uuucugauaugguuuucucgacac................... 2 0 ea1
.....................................................................uuucugauaugguuuucucgacUc................... 1 1 ea1
.....................................................................uuucugauaugguuuucucgacaA................... 2 1 ea1
.....................................................................uuucugauaugguuuucucgacaU................... 4 1 ea1
.....................................................................uuucugauaugguuuucucgacaAu.................. 3 1 ea1
.....................................................................uuucugauaugguuuucucgacaUu.................. 2 1 ea1
.....................................................................uuucugauaugguuuucucgacacu.................. 1 0 ea1
......................................................................uucugauagguAuucuc......................... 1 1 ea1
......................................................................uucugauaugguuuucuU........................ 1 1 ea1
......................................................................uucugauaugguuuucuc........................ 9 0 ea1
......................................................................uucugauauggCuuucuc........................ 1 1 ea1
......................................................................uucugauaugguuuucuA........................ 1 1 ea1
......................................................................uucugauaugguuuucucg....................... 12 0 ea1
......................................................................Gucugauaugguuuucucg....................... 1 1 ea1
......................................................................uucugauaugguuuucucA....................... 6 1 ea1
......................................................................uucugauaugguuuucucUa...................... 1 1 ea1
......................................................................uucugauaugguuuucucAa...................... 4 1 ea1
......................................................................uucugauaugguuuucucga...................... 5 0 ea1
......................................................................uucugauaugguuuucucgaA..................... 1 1 ea1
......................................................................Gucugauaugguuuucucgac..................... 3 1 ea1
......................................................................uucugauaugguuuucuCac..................... 1 1 ea1
......................................................................uucugauaugguuuucucgac..................... 71 0 ea1
......................................................................uucugauaugguuuucucAac..................... 7 1 ea1
......................................................................uucugauagguCuucucgac..................... 1 1 ea1
......................................................................uCcugauaugguuuucucgac..................... 1 1 ea1
......................................................................uucugauaugguuuucucgaU..................... 13 1 ea1
```

```
                                        Star                       Mature
cucugaauaucuuuccuuuuagauacucacaagugucgugaaaaccguaacagaaaagcguuaaaaacuuuucugauaugguuuucucgacacuuagauguugcauuuucg
......................................................................uucugauaugguuuucucAaca...................    3     1     ea1
......................................................................uucugauagguCuucucgaca....................    1     1     ea1
......................................................................uucugauGugguuuucucgaca...................    1     1     ea1
......................................................................uucugauaugguuuucucgacC...................    11    1     ea1
......................................................................uucugauauggGuuucucgaca...................    2     1     ea1
......................................................................Gucugauaugguuuucucgaca...................    24    1     ea1
......................................................................uucugauaugguuuucucgaUa...................    2     1     ea1
......................................................................uucugauaugguuuucucgacG...................    4     1     ea1
......................................................................uucugauaugguuuucucgGca...................    1     1     ea1
......................................................................uCcugauaugguuuucucgaca...................    3     1     ea1
......................................................................uucugauaugguuuCcucgaca...................    1     1     ea1
......................................................................uucugGuaugguuuucucgaca...................    2     1     ea1
......................................................................uucugauaugguuuucucgacU...................    97    1     ea1
......................................................................uucugauaugguuuucucgaca...................    193   0     ea1
......................................................................uucugauaugguuuucucgacaU..................    14    1     ea1
......................................................................uucugauaugguuuucucgacaA..................    1     1     ea1
......................................................................uucugauaugguuuucucgacUc..................    1     1     ea1
......................................................................uucugauaugguuuucucgacac..................    5     0     ea1
......................................................................uucugauaugguuuucucgacCc..................    1     1     ea1
......................................................................uucugGuaugguuuucucgacacu.................    1     1     ea1
......................................................................uucugauaugguuuucucgacaUu.................    2     1     ea1
......................................................................Gucugauaugguuuucucgacacu.................    1     1     ea1
......................................................................uucugauaugguuuucucgacacC.................    2     1     ea1
......................................................................uucugauaugguuuucucgacacA.................    6     1     ea1
......................................................................uucugauaugguuuucucgacacG.................    1     1     ea1
......................................................................uucugauaugguuuucucgacacu.................    5     0     ea1
......................................................................uucugauaugguuuucucgacaAu.................    2     1     ea1
......................................................................uucugauaugguuuucucgacacuA................    3     1     ea1
.......................................................................ucugauaugguuuucucA......................    1     1     ea1
.......................................................................ucugauaugguuuucucg......................    3     0     ea1
.......................................................................ucugauaugguuuucucgU.....................    1     1     ea1
.......................................................................ucugauaugguuuucucgaA....................    1     1     ea1
.......................................................................ucugauaugguuuucucgac....................    16    0     ea1
.......................................................................ucugauaugguuuucucgaU....................    3     1     ea1
.......................................................................ucugauaugguuuucucgaG....................    1     1     ea1
.......................................................................Gcugauaugguuuucucgac....................    1     1     ea1
.......................................................................ucugauaugguuuucucAac....................    6     1     ea1
.......................................................................ucugauaugguuuucucgacG...................    2     1     ea1
.......................................................................ucugauGugguuuucucgaca...................    1     1     ea1
.......................................................................ucugauaugguuuucucgacU...................    11    1     ea1
.......................................................................ucugauaugguuuucucAaca...................    6     1     ea1
.......................................................................ucugauaugguuuucucgGca...................    1     1     ea1
.......................................................................ucugGuaugguuuucucgaca...................    1     1     ea1
.......................................................................ucugauaugguuuucucgaca...................    64    0     ea1
.......................................................................Gcugauaugguuuucucgaca...................    7     1     ea1
.......................................................................ucugauaugguuuucucgacC...................    3     1     ea1
.......................................................................Ccugauaugguuuucucgaca...................    1     1     ea1
.......................................................................ucugauaugguuuucucgacaA..................    1     1     ea1
........................................................................cugauaugguuuucucgaU....................    2     1     ea1
........................................................................cugauaugguuuucucgac....................    2     0     ea1
........................................................................Gugauaugguuuucucgaca...................    1     1     ea1
........................................................................cugauaugguuuucucgaca...................    11    0     ea1
........................................................................cugauaugguuuucucgacaU..................    1     1     ea1
.........................................................................ugauaugguuuucucgacacA.................    1     1     ea1

........................................aaaaccguaacagaaaagcg...................................................    3     0     er2
..........................................aaccguaacagaaaagcguuaaaaac...........................................    2     0     er2
...............................................uaacagaaaagcAuuaaaaac...........................................    2     1     er2
........................................................agcguuaaaaacuuuucuga...................................    1     0     er2
........................................................agcguuaaaaacuuuucugaAa.................................    1     1     er2
..........................................................cguuaaaaacuuuucugaua.................................    2     0     er2
............................................................uuaaaaacuuuucugauauA...............................    3     1     er2
.............................................................uaaaaacuuuucugauaugg..............................    1     0     er2
...................................................................cuuuucugauaugguuuucu........................    7     0     er2
...................................................................cuuuucugauaugguuuucucC......................    1     1     er2
...................................................................cuuuucugauaugguuuucucAa.....................    2     1     er2
....................................................................uuuucugauaugguuuucu........................    2     0     er2
....................................................................uuuucugauaugguuuucuc.......................    16    0     er2
....................................................................uuuucugauaugguuuucucg......................    30    0     er2
....................................................................uuuucugauaugguuuucucC......................    20    1     er2
```

```
                                        Star                        Mature
cucugaauaucuuuccuuuuagauacucacaagugucgugaaaaccguaacagaaaagcguuaaaaacuuuucugauaugguuuucucgacacuuagauguugcauuuuucg
....................................................................uuuucugauaugguuuucucA.......................    20   1   er2
....................................................................uuuucugauaugguuuucucU.......................    16   1   er2
....................................................................Cuuucugauaugguuuucucg.......................    2    1   er2
....................................................................uuuucugauaugguuuucucga......................    38   0   er2
....................................................................uuuucugauaugguuuucucgac.....................    77   0   er2
....................................................................uuuucugauaugguuuucucAac.....................    14   1   er2
....................................................................uuuucugauaugguuuucucgaU.....................    10   1   er2
....................................................................uuuucugauaugguuuucucgCc.....................    2    1   er2
....................................................................uuuucugauaugguuuucucgaA.....................    6    1   er2
....................................................................uGuucugauaugguuuucucgaca....................    1    1   er2
....................................................................uuuucugauaugguuuucucgacU....................    65   1   er2
....................................................................uuuucugauaugguuuucucgacC....................    10   1   er2
....................................................................uuuucugauauggGuuucucgaca....................    1    1   er2
....................................................................uuuucugauaugguuuucucgaUa....................    4    1   er2
....................................................................uuuucugauaugguuuucucgaca....................    94   0   er2
.....................................................................uuucugauaugguuuucuc........................    4    0   er2
.....................................................................uuucugauaugguuuucuG........................    2    1   er2
.....................................................................uuucugauaugguuuAcucg.......................    2    1   er2
.....................................................................uuucugauaugguuuucucg.......................    20   0   er2
.....................................................................uuucugauaugguuuucucA.......................    41   1   er2
.....................................................................uuucugauaugguuuucucC.......................    9    1   er2
.....................................................................uuucugauaugguuuucucU.......................    12   1   er2
.....................................................................uuucugauaugguuuucucga......................    3    0   er2
.....................................................................uuucugauaugguuuucucAa......................    4    1   er2
.....................................................................uuucugauaugguuuucucCa......................    1    1   er2
.....................................................................uuucugauaugguuuucucAac.....................    18   1   er2
.....................................................................uuucugauaugguuuucucgCc.....................    1    1   er2
.....................................................................uuucugauaugguuuucucgaA.....................    1    1   er2
.....................................................................uuucugauaugguuuucucgaU.....................    3    1   er2
.....................................................................uuucugauaugguuuucucgac.....................    30   0   er2
.....................................................................uuucugGuaugguuuucucgac.....................    1    1   er2
.....................................................................uNucugauaugguuuucucgac.....................    1    1   er2
.....................................................................uuucugauaugguuCucucgaca....................    2    1   er2
.....................................................................uuucugauaugguuuucucAaca....................    2    1   er2
.....................................................................uuucugauaugguuuucucgaca....................    65   0   er2
.....................................................................uuucugauaugguuuucucgacC....................    28   1   er2
.....................................................................uuucugauaugguuuucucgCca....................    6    1   er2
.....................................................................uuucugauaugguuuucucgacU....................    145  1   er2
.....................................................................uuucugauaugguuuucucgacacu..................    2    0   er2
......................................................................uucugauaugguuuucucA.......................    11   1   er2
......................................................................uucugauaugguuuucucAa......................    2    1   er2
......................................................................uucugauaugguuuucucga......................    4    0   er2
......................................................................uucugauaugguuuucucUa......................    10   1   er2
......................................................................uucugauaugguuuucucgac.....................    7    0   er2
......................................................................uucugauaugguuuucucAac.....................    16   1   er2
......................................................................uucugauaugguuuucucgacG....................    1    1   er2
......................................................................uucugauaugguuuucucgacU....................    31   1   er2
......................................................................uucugauaugguuuucucgacC....................    9    1   er2
......................................................................uucugauaugguuuucucAaca....................    5    1   er2
......................................................................uucugauaugguuuucucgaca....................    66   0   er2
......................................................................uucugauaugguuuucucgacaAu..................    1    1   er2
......................................................................uucugauaugguuuucucgacacA..................    2    1   er2
.......................................................................ucugauaugguuuucucg.......................    1    0   er2
.......................................................................Ncugauaugguuuucucg.......................    1    1   er2
.......................................................................ucugauaugguuuucucA.......................    5    1   er2
.......................................................................ucugauaugguuuucucga......................    4    0   er2
.......................................................................ucugauaugguuuucucAa......................    3    1   er2
.......................................................................ucugauaugguuuucucAac.....................    7    1   er2
.......................................................................ucugauaugguuuucucgac.....................    12   0   er2
.......................................................................ucugauaugguuuucucgCc.....................    2    1   er2
.......................................................................Ncugauaugguuuucucgaca....................    1    1   er2
.......................................................................ucugauaugguuuucucgacU....................    6    1   er2
.......................................................................ucugauaugguuuucucgaca....................    18   0   er2
........................................................................cugauaugguuuucucAac.....................    5    1   er2
........................................................................cugauaugguuuucucgaca....................    3    0   er2
.........................................................................ugauaugguuuucucgacaAu..................    3    1   er2

........................................aaaaccguaaGagaaaagcg....................................................    3    1   ea2
.........................................aaaccguaacagaaaagcg....................................................    4    0   ea2
..........................................aaccguaacagaaaagcAuua.................................................    4    1   ea2
```

```
                                               Star                        Mature
cucugaauaucuuuccuuuuagauacucacaagugucgugaaaaccguaacagaaaagcguuaaaaacuuuucugauaugguuuucucgacacuuagauguugcauuuuucg
...............................................uaacagaaaagcguuaaGaacuu..........................................   2     1    ea2
.................................................acGgaaaagcguuaaaaacu...........................................   2     1    ea2
..................................................cGgaaaagcguuaaaaacuuu.........................................   1     1    ea2
.......................................................aagcguuaaGaacuuuucuga....................................   3     1    ea2
.......................................................aGgcguuaaaaacuuuucuga....................................   3     1    ea2
........................................................agcguuaaaaacuuuucu......................................   7     0    ea2
........................................................agcguuaaaaacuuuucugaA...................................   10    1    ea2
.........................................................gcguuaaaaacuuuucugaA...................................   3     1    ea2
..........................................................cguuaaaaacuuuucugau...................................   4     0    ea2
............................................................uuaaaaacuuuucugauaug................................   1     0    ea2
.............................................................uaaaGacuuuucugauaugg...............................   1     1    ea2
...................................................................cuuuucugauaugguuuuc..........................   2     0    ea2
...................................................................cuuuucugauaugguuuucu.........................   20    0    ea2
...................................................................cuuuucugauaugguuuucuc........................   1     0    ea2
....................................................................uuuucugauaugguuuucu.........................   9     0    ea2
....................................................................uuuucugauaugguuuucuc........................   49    0    ea2
....................................................................Guuucugauaugguuuucuc........................   1     1    ea2
....................................................................Nuuucugauaugguuuucuc........................   1     1    ea2
....................................................................uuuucugauaugguuuucuG........................   10    1    ea2
....................................................................uuuucugauaugguuuucucU.......................   19    1    ea2
....................................................................uuuucugauaugguuuucucg.......................   39    0    ea2
....................................................................uuuucugauaugguuuucucA.......................   97    1    ea2
....................................................................uuuucugauaugguAuucucg.......................   2     1    ea2
....................................................................uuuucugauaugguuuCcucg.......................   1     1    ea2
....................................................................uuuucugauauAguuuucucg.......................   5     1    ea2
....................................................................uuuucugauaugguuuucucC.......................   21    1    ea2
....................................................................uuuucugauaugguuuAcucg.......................   1     1    ea2
....................................................................Cuuucugauaugguuuucucga......................   1     1    ea2
....................................................................uuuucugauaugguuuucucga......................   7     0    ea2
....................................................................uuuucugauaugguuuucucAac.....................   8     1    ea2
....................................................................uuuucugauaugguuuucucgaA.....................   8     1    ea2
....................................................................uuuucugauaugguuuucucgac.....................   10    0    ea2
....................................................................Nuuucugauaugguuuucucgaca....................   1     1    ea2
....................................................................uuuucugauaugguuuucucgaUa....................   6     1    ea2
....................................................................uuuucugauaugguuuucucgacU....................   2     1    ea2
....................................................................uuuucugauaugguuuucucgaca....................   20    0    ea2
....................................................................uuuucugauaugguuuucucAaca....................   7     1    ea2
.....................................................................uuucugauaugguuuucuc........................   42    0    ea2
.....................................................................uuucugauaugguuuucCc........................   1     1    ea2
.....................................................................uuucugauaugguuuAcucg.......................   4     1    ea2
.....................................................................uuucugauaugguuCucucg.......................   6     1    ea2
.....................................................................uuucugauaugguuuucucA.......................   189   1    ea2
.....................................................................uuucugauaugguuuucucg.......................   73    0    ea2
.....................................................................Guucugauaugguuuucucg.......................   1     1    ea2
.....................................................................uuucugauaugguuuucucU.......................   50    1    ea2
.....................................................................uuucugauaugguuuucucC.......................   25    1    ea2
.....................................................................uuucugauaugguuuucucgU......................   1     1    ea2
.....................................................................uuucugauaugguuuucucCa......................   3     1    ea2
.....................................................................uuucugauaugguuuucucAa......................   3     1    ea2
.....................................................................uuucugauaugguuuucucga......................   12    0    ea2
.....................................................................uuucugauaugguuuucucAac.....................   62    1    ea2
.....................................................................uuucugauaugguuuucucgaA.....................   14    1    ea2
.....................................................................uuucugauaugguuuucucgac.....................   16    0    ea2
.....................................................................uuucugauaugguuuucucgaU.....................   4     1    ea2
.....................................................................uuucugauaugguuuucucgCc.....................   8     1    ea2
.....................................................................uuucugauaugguuuucucUac.....................   1     1    ea2
.....................................................................uuucugauaugguuuucucAaca....................   17    1    ea2
.....................................................................uuucugauaugguuuucucgacU....................   9     1    ea2
.....................................................................uuucugauaugguuuucucgaUa....................   2     1    ea2
.....................................................................uuucugauaugguuuucucgaca....................   45    0    ea2
.....................................................................uuucugauaugguuuucucgacaU...................   1     1    ea2
.....................................................................uuucugauaugguuuucucgacaA...................   1     1    ea2
......................................................................Nucugauaugguuuucuc........................   1     1    ea2
......................................................................uucugauaugguuuucuc........................   24    0    ea2
......................................................................uucugauaugguuuucucU.......................   26    1    ea2
......................................................................uucugauaugguuuucucC.......................   9     1    ea2
......................................................................uucugauaugguAuucucg.......................   3     1    ea2
......................................................................uucugauaugguuuucucA.......................   128   1    ea2
......................................................................uucugauaugguuuucucg.......................   39    0    ea2
......................................................................uucugauaugguuuucucCa......................   1     1    ea2
```

Star Mature

```
cucugaauaucuuuccuuuuagauacucacaagugucgugaaaaccguaacagaaaagcguuaaaaaacuuuucugauaugguuuucucgacacuuagauguugcauuuuucg
.......................................................................uucugauaugguuuucucUa......................  1  1  ea2
.......................................................................uucugauaugguuuucucAa......................  2  1  ea2
.......................................................................uucugauaugguuuucucga......................  22  0  ea2
.......................................................................uucugauaugguuuucucgU......................  4  1  ea2
.......................................................................uucugauaugguuuucucgaA.....................  1  1  ea2
.......................................................................uucugauaugguuuucucgCc.....................  1  1  ea2
.......................................................................uucugauaugguuuucucAac.....................  54  1  ea2
.......................................................................uucugauaugguuuucucgaU.....................  10  1  ea2
.......................................................................uucugauaugguuuucucgac.....................  14  0  ea2
.......................................................................uucugauaugguuuucucgaca....................  38  0  ea2
.......................................................................uucugauaugguuuucucgaAa....................  2  1  ea2
.......................................................................uucugauaugguuuucucAaca....................  18  1  ea2
.......................................................................uucugauaugguuuucucgaUa....................  7  1  ea2
.......................................................................uucugauaugguuuucucgacU....................  7  1  ea2
.......................................................................uuAugauaugguuuucucgaca....................  1  1  ea2
.......................................................................uucugauaugguuuucucgacaA...................  2  1  ea2
.......................................................................uucugauaugguuuucucgacaU...................  3  1  ea2
.......................................................................uucugauaugguuuucucgacacA..................  1  1  ea2
........................................................................ucugauaugguuuucucg.......................  4  0  ea2
........................................................................ucugauaugguuuucucU.......................  2  1  ea2
........................................................................ucugauaugguuuucucA.......................  12  1  ea2
........................................................................ucugauaugguuuucucAa......................  5  1  ea2
........................................................................ucugauaugguuuucucgaA.....................  5  1  ea2
........................................................................ucugauaugguuuucucgac.....................  5  0  ea2
........................................................................ucugauaugguuuucucAac.....................  63  1  ea2
........................................................................ucugauaugguuuucucgaca....................  15  0  ea2
........................................................................ucugauaugguuuucucgaUa....................  2  1  ea2
........................................................................ucugauaugguuuucucAaca....................  13  1  ea2
.........................................................................cugauaugguuuucucAac.....................  3  1  ea2
.........................................................................cugauaugguuuucucAaca....................  6  1  ea2
.........................................................................cugauaugguuuucCcgaca....................  1  1  ea2
.........................................................................cugauaugguuuucucgaca....................  8  0  ea2

......................................ugaaaaccguaacagaaaagcA.....................................................  1  1  eg2
.................................................acagaaaagcguuaaaaaacu...........................................  1  0  eg2
............................................................uuaaaaaacuuuucugauaug................................  1  0  eg2
.....................................................................uuuucugauaugguuuucucU.......................  7  1  eg2
.....................................................................uuuucugauaugguuuucucg.......................  7  0  eg2
.....................................................................uuuucugauaugguuuucucA.......................  1  1  eg2
.....................................................................uuuucugauaugguuuucuGg.......................  1  1  eg2
.....................................................................uuuucugauaugguuuucucUa......................  2  1  eg2
.....................................................................uuuucugauaugguuuucucga......................  6  0  eg2
.....................................................................uuuucugauaugguuuucucgaA.....................  1  1  eg2
.....................................................................uuuucugauaugguuuucucgac.....................  1  0  eg2
.....................................................................uuuucugauaugguuuucucAac.....................  1  1  eg2
.....................................................................uuuucugauaugguuuucucgaca....................  18  0  eg2
.....................................................................uuuucugauaugguuuucucgacaA...................  2  1  eg2
......................................................................uuucugauaugguuuucucU.......................  3  1  eg2
......................................................................uuucugauaugguuuucucgac.....................  2  0  eg2
......................................................................uuucugauaugguuuucucAac.....................  1  1  eg2
......................................................................uuucugauaugguuuucucgaca....................  9  0  eg2
......................................................................uuucugauaugguuuucucgacC...................  1  1  eg2
.......................................................................uucugauaugguuuucucCa......................  1  1  eg2
.......................................................................uucugauaugguuuucucgaU.....................  1  1  eg2
.......................................................................uucugauaugguuuucucAac.....................  4  1  eg2
.......................................................................uucugauaugguuuucucgac.....................  2  0  eg2
.......................................................................uucugauaugguuuucucgaca....................  18  0  eg2
.......................................................................uucugauaugguuuucucgacaA...................  1  1  eg2
........................................................................ucugauaugguuuucucgaA.....................  1  1  eg2
........................................................................ucugauaugguuuucucAac.....................  1  1  eg2
........................................................................ucugauaugguuuucucgaca....................  4  0  eg2
........................................................................ucugauaugguuuucucgacaA...................  1  1  eg2
.........................................................................cugauaugguuuucucgaca....................  1  0  eg2
.........................................................................cugauaugguuuucucgacaA...................  1  1  eg2

..........................................................cguuaaaaaacuuuucugau...................................  1  0  eg1
..........................................................cguuaaaaaacuuuucugauaA.................................  1  1  eg1
............................................................uuaaaaaacuuuucugauaug................................  2  0  eg1
............................................................uuaaaaaacuuuucugauaugA...............................  2  1  eg1
.....................................................................uuuucugauaugguuuucuc........................  3  0  eg1
```

Star Mature

```
cucugaauaucuuuccuuuuagauacucacaagugucgugaaaaccguaacagaaaagcguuaaaaacuuuucugauaugguuuucucgacacuuagauguugcauuuuucg
....................................................................uuuucugauaugguuuucucA.......................  8   1  eg1
....................................................................uuuucugauaugguuuucucg.......................  13  0  eg1
....................................................................uuuucugauaugguuCucucg.......................  1   1  eg1
....................................................................uuuucugauaCgguuuucucg.......................  1   1  eg1
....................................................................uuuucugauaugguuuucucU.......................  1   1  eg1
....................................................................Cuuucugauaugguuuucucg.......................  1   1  eg1
....................................................................uuuucugauaugguuuucucUa......................  2   1  eg1
....................................................................uuuucugauaugguuuucucgG......................  1   1  eg1
....................................................................uuuucugauaugguuuucucgU......................  1   1  eg1
....................................................................uuuucugauaugguuuucucga......................  20  0  eg1
....................................................................uuuucugauaugguuuucucgac.....................  20  0  eg1
....................................................................uuuucugauaugguuuucucgaA.....................  5   1  eg1
....................................................................uuuucAgauaugguuuucucgac.....................  1   1  eg1
....................................................................Guuucugauaugguuuucucgac.....................  1   1  eg1
....................................................................uuuucugauaugguuCucucgac.....................  1   1  eg1
....................................................................uuuucugauaugguuuucucAac.....................  1   1  eg1
....................................................................uuuucugauaugguuuucucgaU.....................  11  1  eg1
....................................................................Cuuucugauaugguuuucucgaca....................  2   1  eg1
....................................................................Guuucugauaugguuuucucgaca....................  6   1  eg1
....................................................................uuuucugauaugguuuucucgacC....................  2   1  eg1
....................................................................uuuucugauaugguuuucucgaca....................  40  0  eg1
....................................................................uuuucugauaugguuuucucgacG....................  8   1  eg1
....................................................................uuuucugauaugguuuucucgaUa....................  2   1  eg1
....................................................................uuuCcugauaugguuuucucgaca....................  1   1  eg1
....................................................................uuuucugauaugguuuucucgacU....................  16  1  eg1
....................................................................uuuucugauaugguuuucucgacaU...................  1   1  eg1
....................................................................uuuucugauaugguuuucucgacaA...................  1   1  eg1
....................................................................uuuucugauaugguuuucucgacUc...................  1   1  eg1
.....................................................................uuucugauaugguuuucuc........................  1   0  eg1
.....................................................................Cuucugauaugguuuucuc........................  1   1  eg1
.....................................................................uuucugauaugguuuucucg.......................  5   0  eg1
.....................................................................Guucugauaugguuuucucg.......................  1   1  eg1
.....................................................................uuucugauaugguuuucucA.......................  6   1  eg1
.....................................................................uuucugauaugguuuucucU.......................  2   1  eg1
.....................................................................Guucugauaugguuuucucga......................  1   1  eg1
.....................................................................uuucugauaugguuuucucga......................  1   0  eg1
.....................................................................uuucugauaugguuuucucAac.....................  1   1  eg1
.....................................................................uuucugauaugguuuucucgCc.....................  1   1  eg1
.....................................................................uuucugUuaugguuuucucgac.....................  1   1  eg1
.....................................................................uuucugauaugguuuucucgac.....................  10  0  eg1
.....................................................................Cuucugauaugguuuucucgaca....................  1   1  eg1
.....................................................................Guucugauaugguuuucucgaca....................  1   1  eg1
.....................................................................uuucugauaugguuuucucgacC....................  1   1  eg1
.....................................................................uuucugauaugguuuucucgacU....................  17  1  eg1
.....................................................................uuucugauaugguuuucucgacG....................  2   1  eg1
.....................................................................uuucugauaugguuuucucgaca....................  14  0  eg1
.....................................................................uuucugauaugguuuucucgacUc...................  1   1  eg1
......................................................................uucugauaugguuuucuc........................  1   0  eg1
......................................................................uucugauaugguuuucucAa......................  1   1  eg1
......................................................................uucugauaugguuuucucgU......................  1   1  eg1
......................................................................uucugauaugguuuucucga......................  1   0  eg1
......................................................................uucugauaugguuuucucgac.....................  2   0  eg1
......................................................................Gucugauaugguuuucucgac.....................  1   1  eg1
......................................................................uucugauaugguuuucuUgac.....................  1   1  eg1
......................................................................uucugauaugguuuucucgaU.....................  2   1  eg1
......................................................................uucugauaugguuuucucgacG....................  2   1  eg1
......................................................................uucugauaugguuuucucgacC....................  3   1  eg1
......................................................................uucugauaugguuuucucgacU....................  24  1  eg1
......................................................................Gucugauaugguuuucucgaca....................  5   1  eg1
......................................................................uucugauaugguuuucucgaca....................  21  0  eg1
......................................................................uucugauaugguuuucucgacaA...................  1   1  eg1
......................................................................uucugauaugguuuucucgacUc...................  1   1  eg1
......................................................................uucugauaugguuuucucgacaU...................  3   1  eg1
......................................................................uucugauaugguuuucucgacacA..................  1   1  eg1
......................................................................uucugauaugguuuucucgacaUu..................  1   1  eg1
.......................................................................ucugauaugguuuucucgaG.....................  1   1  eg1
.......................................................................ucugauaugguuuucucgac.....................  2   0  eg1
.......................................................................ucugauaugguuuucucgaUa....................  1   1  eg1
.......................................................................ucugauaugguuuucucgaca....................  3   0  eg1
.......................................................................ucugauaugguuuucucAaca....................  2   1  eg1
```

```
                                        Star                        Mature
cucugaauaucuuuccuuuuagauacucacaagugucgugaaaaccguaacagaaaagcguuaaaaacuuuucugauaugguuuucucgacacuuagauguugcauuuuucg
.......................................................................ucugauaugguuuucucgacG....................	1	1	eg1
.........................................................................ugauaugguuuucucgacacA..................	1	1	eg1

......................................ugaaaaccguaacagaaaagc.....................................................	1	0	er1
....................................................................uuuucugauaugguuuucu.........................	1	0	er1
....................................................................uuuucugauaugguuuucuc........................	2	0	er1
....................................................................uuuCcugauaugguuuucucg.......................	2	1	er1
....................................................................uuuucugauaugguuuucucg.......................	30	0	er1
....................................................................uuuucugauaugguuuucucU.......................	1	1	er1
....................................................................uuuucugauaugguuuucucA.......................	3	1	er1
....................................................................uuuucugauaugguuuucucgC......................	3	1	er1
....................................................................uuuucugauaugguuuucucga......................	45	0	er1
....................................................................uuuucugauaugguuuucucgU......................	2	1	er1
....................................................................uuuucugauaugguGuucucga......................	1	1	er1
....................................................................Guuucugauaugguuuucucga......................	1	1	er1
....................................................................uuuucugauauggCuuucucga......................	1	1	er1
....................................................................uuuucugauaugguuuucucgaU.....................	26	1	er1
....................................................................uuuucugauaugguuuCcucgac.....................	3	1	er1
....................................................................uuuucugauauggGuuucucgac.....................	1	1	er1
....................................................................uuuucugauaugguuuucucgCc.....................	1	1	er1
....................................................................uuuucugauaugguuuucucgac.....................	53	0	er1
....................................................................uuuucugauaugguuuucucgaA.....................	5	1	er1
....................................................................Guuucugauaugguuuucucgac.....................	4	1	er1
....................................................................uuuucugauaugguuuucucgaG.....................	1	1	er1
....................................................................uuuucugauauggCuuucucgaca....................	1	1	er1
....................................................................uuuucugauaugguuuucucgaca....................	72	0	er1
....................................................................uuuucugauaugguuuucucgacU....................	67	1	er1
....................................................................uuuucugauaugguuuucucgaUa....................	6	1	er1
....................................................................uuuucugauaugguuuucucgacC....................	23	1	er1
....................................................................Guuucugauaugguuuucucgaca....................	8	1	er1
....................................................................uuuucugaAaugguuuucucgaca....................	2	1	er1
....................................................................uuuucugauaugguuuucucgacG....................	3	1	er1
....................................................................uuuCcugauaugguuuucucgaca....................	1	1	er1
....................................................................uuuucugauaAgguuuucucgaca....................	1	1	er1
....................................................................uuuucugauaugguuuucucgaGa....................	1	1	er1
....................................................................uuuucugauaugguuuucucgacCc...................	2	1	er1
....................................................................uuuucugauaugguuuucucgacUc...................	2	1	er1
....................................................................uuuucugauaugguuuucucgacaU...................	1	1	er1
....................................................................uuuucugauaugguuuucucgacacA..................	1	1	er1
....................................................................uuuucugauaugguuuucucgacaUu..................	1	1	er1
....................................................................uuuucugauaugguuuucucgacacC..................	1	1	er1
.....................................................................Guucugauaugguuuucucg.......................	1	1	er1
.....................................................................uuucugauaugguuuucucA.......................	1	1	er1
.....................................................................uuucugauaugguuuucucg.......................	2	0	er1
.....................................................................uuucugauaugguuuucucga......................	2	0	er1
.....................................................................uuucugGuaugguuuucucgac.....................	1	1	er1
.....................................................................uuucugauaugguuuucucgac.....................	13	0	er1
.....................................................................uuucugauaugguuuucucgaU.....................	4	1	er1
.....................................................................Guucugauaugguuuucucgac.....................	1	1	er1
.....................................................................uuucugauaugguuuucucgacC....................	2	1	er1
.....................................................................uuucugauaugguuuucucgacU....................	30	1	er1
.....................................................................Guucugauaugguuuucucgaca....................	2	1	er1
.....................................................................uuucugauaugguuuucucgacG....................	1	1	er1
.....................................................................uuucugauaugguuuucucgaca....................	25	0	er1
.....................................................................uuucugauaugguuuucucgacCc...................	1	1	er1
.....................................................................uuucugauaugguuuucucgacUc...................	3	1	er1
.....................................................................uuucugauaugguuuucucgacaU...................	1	1	er1
.....................................................................uuucugauaugguuuucucgacaUu..................	1	1	er1
.....................................................................uuucugauaugguuuucucgacaAu..................	2	1	er1
.....................................................................uuucugauaugguuuucucgacaAuu.................	1	1	er1
......................................................................uucugauaugguuuucucUa......................	1	1	er1
......................................................................uucugauaugguuuucucAac.....................	3	1	er1
......................................................................uucugauauggGuuucucgac.....................	1	1	er1
......................................................................uucugauaugguuuucucgac.....................	15	0	er1
......................................................................uucugauaugguuuucucgaU.....................	2	1	er1
......................................................................uucugauaugguuuucucgaca....................	43	0	er1
......................................................................Gucugauaugguuuucucgaca....................	7	1	er1
......................................................................uucugauaugguuuucucgacC....................	8	1	er1
......................................................................uucugauaugguuuucucgacU....................	20	1	er1
......................................................................uucugauaugguuuucucgacG....................	1	1	er1
```

Star **Mature**

```
cucugaauaucuuuccuuuuagauacucacaagugucgugaaaaccguaacagaaaagcguuaaaaacuuuucugauaugguuuucucgacacuuagauguugcauuuuucg
......................................................................uucugauaugguuuucucgacaU...................    2    1    er1
......................................................................uucugauaugguuuucucgacaA...................    1    1    er1
......................................................................uucugauaugguuuucucgacUc...................    1    1    er1
......................................................................uucugauaugguuuucucgacac...................    1    0    er1
......................................................................uucugauaugguuuucucgacacu..................    2    0    er1
......................................................................uucugauaugguCuucucgacacu..................    2    1    er1
.......................................................................ucugauaugguuuucucgac.....................    2    0    er1
.......................................................................ucugauaugguuuucucgaU.....................    3    1    er1
.......................................................................ucugauaugguuuucucgacG....................    1    1    er1
.......................................................................ucugauaugguuuucucgacU....................    2    1    er1
.......................................................................ucugauaugguuuucucgaca....................    8    0    er1
.......................................................................ucugauaugguuuucucgacUc...................    1    1    er1
........................................................................cugauaugguuuucucgaca....................    3    0    er1
........................................................................cugauaugguuuucucgacaAuu.................    1    1    er1
```

Provisional ID	:	ScUbCFx_4_1965
Score total	:	3.8
Score for star read(s)	:	-1.3
Score for read counts	:	0
Score for mfe	:	0.5
Score for randfold	:	1.6
Score for cons. seed	:	3
Total read count	:	1561
Mature read count	:	1560
Loop read count	:	1
Star read count	:	0

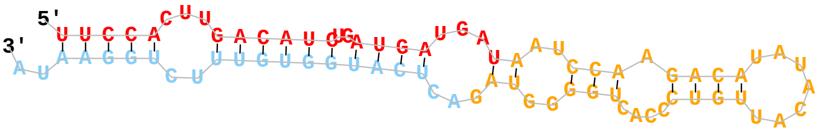

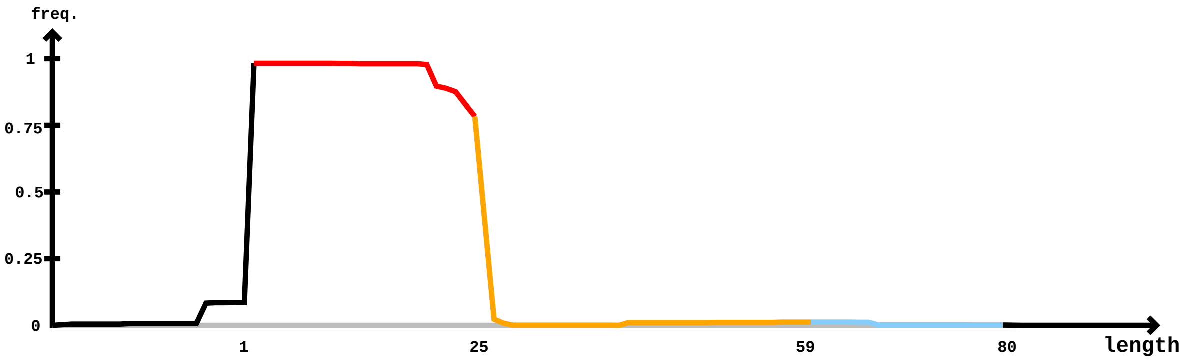


```
                    Mature                                                       Star
5'- aaggauuuuugcaauauuguuccacuugacaucugaugaugauaauccaagacauauacauugucccacugggguagacucaugguguuucuggaauagagcagaggccaaag   -3'    exp
    ..((.(((((((....((((((((...((((((..((((...((..(((.((((.......))))....)))..))...))))))))))..)))))))).))))))))).... reads      mm       sample
    ......uuuugcaauauuguuccacuugac...................................................................................  1          0        eg1
    .............uauuguuccacuugacaucugaug............................................................................  5          0        eg1
    .............uauuguuccacuugacaucugauga...........................................................................  1          0        eg1
    .............uauuguuccacuugacaucugaugaug.........................................................................  1          0        eg1
    ...................uuccacuugacaucugaCg...........................................................................  1          1        eg1
    ...................uuccacuugacaucugaug...........................................................................  4          0        eg1
    ...................uuccacuugacaucugauga..........................................................................  2          0        eg1
    ...................uuccacuugacaucugaugau.........................................................................  9          0        eg1
    ...................uuccacuugacaucCgaugaug........................................................................  3          1        eg1
    ...................uuccacuugacaucugaugaug........................................................................  8          0        eg1
    ...................uuccacuugacaucugaugauA........................................................................  1          1        eg1
    ...................uuccacuugacaucugaugaCg........................................................................  1          1        eg1
    ...................Guccacuugacaucugaugaug........................................................................  1          1        eg1
    ...................uuccacuugacaucugaugaCga.......................................................................  1          1        eg1
    ...................uuccacuugacaucugaugaugG.......................................................................  1          1        eg1
    ...................uuccacuugacaucugaugauga.......................................................................  15         0        eg1
    ...................Guccacuugacaucugaugauga.......................................................................  2          1        eg1
    ...................uuccacCugacaucugaugaugau......................................................................  1          1        eg1
    ...................uuccacuugacaucugaugaCgau......................................................................  9          1        eg1
    ...................uuccacuugacaucugUugaugau......................................................................  3          1        eg1
    ...................uuccacuugacaucugaugaugaC......................................................................  22         1        eg1
    ...................uuccacuugUcaucugaugaugau......................................................................  1          1        eg1
    ...................Guccacuugacaucugaugaugau......................................................................  14         1        eg1
    ...................uuccacuugacaucugaugaugau......................................................................  208        0        eg1
    ...................uuccGcuugacaucugaugaugau......................................................................  1          1        eg1
    ...................uCccacuugacaucugaugaugau......................................................................  1          1        eg1
    ...................uuccacuugacaucugaugaugaG......................................................................  1          1        eg1
    ...................uucAacuugacaucugaugaugau......................................................................  1          1        eg1
    ...................uuccacuugGcaucugaugaugau......................................................................  1          1        eg1
    ...................uuccacuugacaucugaugaugaA......................................................................  1          1        eg1
    ...................uuccacuugacaucugaugaCgaua.....................................................................  1          1        eg1
    ...................uuccacuugacaucugUugaugaua.....................................................................  1          1        eg1
    ...................uuccacuugacaucugaugaugaua.....................................................................  31         0        eg1
    ...................uuccacuugacaucugaugaugauU.....................................................................  2          1        eg1
```

Mature / Star			
aaggauuuuugcaauauuguuccacuugacaucugaugaugauaauccaagacauauacauugucccacugggguagacucaugguguuucuggaauagagcagaggccaaag			
...................uuccacuugacaucugaugaugauG...	9	1	eg1
...................uuccacuugacaucugGugaugaua...	1	1	eg1
...................Guccacuugacaucugaugaugaua...	3	1	eg1
...................uuccacuugGcaucugaugaugaua...	1	1	eg1
...................uuccacuugacaucugaugaugauaa..	10	0	eg1
...................uuccacuugacaucugaugaugauaau...	4	0	eg1
....ugauUauccaagacauau..	1	1	eg1
....cauugucccacugggguagacucaug.............................	7	0	eg1
....uagacucaugguguuucuggaauag..............	1	0	eg1
......uuuugcaauauuguuccacuug...	1	0	er1
..............Gauuguuccacuugacaucugaug...	1	1	er1
..............uauuguuccacuugacaucugaug...	9	0	er1
...................uuccacuugacaucugau..	1	0	er1
...................uuccacuugacaucugaug...	1	0	er1
...................uuccacuugacaucugauUa..	1	1	er1
...................uuccacuugacaucugaugau...	4	0	er1
...................uuccacuugacaucugaugaCg..	2	1	er1
...................uuccacuugacaucugaugauA..	1	1	er1
...................uuccacuugacaucugaugaug..	4	0	er1
...................uuccacuugacaucugaCgaug..	2	1	er1
...................Cuccacuugacaucugaugaug..	1	1	er1
...................uuccacuugacaucugaugauga...	13	0	er1
...................uuccacuugaUaucugaugauga...	1	1	er1
...................uuccacuugacaucugaugaugG...	1	1	er1
...................uuccGcuugacaucugaugaugau..	1	1	er1
...................uCccacuugacaucugaugaugau..	1	1	er1
...................uuccacuugGcaucugaugaugau..	2	1	er1
...................uuccacuugacaucugaugaugaG..	2	1	er1
...................uuccacuugacaucugaugaugau..	104	0	er1
...................Guccacuugacaucugaugaugau..	13	1	er1
...................uuccacuugacaucuUaugaugau..	1	1	er1
...................uuccacuugacaucugaugaCgau..	4	1	er1
...................uuccacuugacauUugaugaugau..	1	1	er1
...................uuccacuugacaucCgaugaugau..	2	1	er1
...................uuccacuugacaucugaugaugaC..	13	1	er1
...................uuccacuugacaucugaugaugGu..	1	1	er1
...................uuccacuugacaucugaugaugauG...	5	1	er1
...................Guccacuugacaucugaugaugaua...	1	1	er1
...................uuccacuugacGucugaugaugaua...	2	1	er1
...................uuccacuugacaucugaugaugaua...	17	0	er1
...................uuccacuugacaucugaugaugGua...	1	1	er1
...................uuccacuugacaucugaugaCgaua...	2	1	er1
...................uuccacuugacaucugaugaugaCa...	1	1	er1
...................uuccacuugacaucugaugaugauaau...	2	0	er1
..............uauuguuccacuugacaucugaug...	11	0	er2
..............uauuguuccacuugacaucugauA...	1	1	er2
..............uauuguuccacuugacaucugaugauA..	3	1	er2
...................uuccacuugacaucugaugaugau..	18	0	er2
...................uuccacuugacaucugaugaugaua...	9	0	er2
....cauugucccacugggguagacucaug.............................	1	0	er2
aaggauuuuugcaauauug..	1	0	ea1
..............uauuguuccacuugacaucugau..	2	0	ea1
..............uauuguuccacuugacaucugauA...	5	1	ea1
..............uauuguuccacuugacaucugaug...	28	0	ea1
..............Gauuguuccacuugacaucugaug...	2	1	ea1
..............uauuguuccacuugacaucugaugauA..	1	1	ea1
..............uauuguuccacuugacaucugaugaug..	8	0	ea1
.................uguuccacuugacaucugaugaugaC..	1	1	ea1
...................uuccacuugacaucugau..	2	0	ea1
...................Guccacuugacaucugaug...	1	1	ea1
...................uuccacuugacaucugauA...	1	1	ea1
...................uuccacuugacaucugaug...	11	0	ea1
...................uuccacuugacaucugauUa..	1	1	ea1
...................uuccacuugacaucugaugU..	1	1	ea1
...................uuccacuugacaucugauga..	4	0	ea1
...................uuccacuugacaucugaugau...	4	0	ea1
...................uuccacuugacaucugaugaC...	3	1	ea1

```
                   Mature                                                  Star
aaggauuuuugcaauauuguuccacuugacaucugaugaugauaauccaagacauauacauugucccacugggguagacucaugguguuucuggaauagagcagaggccaaag
...................uuccacuugGcaucugaugau.........................................................................    2     1     ea1
...................uuccacuugacaucugaugauA........................................................................    3     1     ea1
...................Guccacuugacaucugaugaug........................................................................    1     1     ea1
...................uGccacuugacaucugaugaug........................................................................    1     1     ea1
...................uuccacuugacaucugaugaug........................................................................    26    0     ea1
...................Auccacuugacaucugaugaug........................................................................    1     1     ea1
...................uuccacuugacaucugaugaCg........................................................................    11    1     ea1
...................uuccacuGgacaucugaugauga.......................................................................    2     1     ea1
...................uuccacuugacaucugaugaCga.......................................................................    8     1     ea1
...................uuccacuugacaucugaugauga.......................................................................    32    0     ea1
...................uuccacuugacaCcugaugauga.......................................................................    1     1     ea1
...................uuccacuugacGucugaugaugau......................................................................    2     1     ea1
...................uuccacuugacaucugaugGugau......................................................................    3     1     ea1
...................uuccacuugacaucCgaugaugau......................................................................    5     1     ea1
...................uuccacuCgacaucugaugaugau......................................................................    1     1     ea1
...................uuccacCugacaucugaugaugau......................................................................    3     1     ea1
...................uuccacuugacaucugaugaugau......................................................................    491   0     ea1
...................Guccacuugacaucugaugaugau......................................................................    29    1     ea1
...................uuccacuugacaucugaugaugUu......................................................................    2     1     ea1
...................uuccacuugacaucugaugaCgau......................................................................    11    1     ea1
...................uuccacuugaUaucugaugaugau......................................................................    1     1     ea1
...................Cuccacuugacaucugaugaugau......................................................................    1     1     ea1
...................uuccacuugacaucugaugaugaA......................................................................    3     1     ea1
...................uuccacuugacaucugaCgaugau......................................................................    4     1     ea1
...................uuccacuugacaucugGugaugau......................................................................    1     1     ea1
...................uuccacuugacaucugaugaugaC......................................................................    66    1     ea1
...................uuccacuugGcaucugaugaugau......................................................................    1     1     ea1
...................uAccacuugacaucugaugaugau......................................................................    1     1     ea1
...................uuccaUuugacaucugaugaugau......................................................................    1     1     ea1
...................uuccacuugacaCcugaugaugau......................................................................    5     1     ea1
...................uuccacuugacaucugaugaugaG......................................................................    6     1     ea1
...................uucUacuugacaucugaugaugau......................................................................    2     1     ea1
...................uuccacuugGcaucugaugaugaua.....................................................................    1     1     ea1
...................uuccacuugacaucugaugaCgaua.....................................................................    3     1     ea1
...................Cuccacuugacaucugaugaugaua.....................................................................    2     1     ea1
...................Guccacuugacaucugaugaugaua.....................................................................    9     1     ea1
...................uuccacuugacaucugaugaugaua.....................................................................    86    0     ea1
...................uuccacuugacaucugaugaugauG.....................................................................    15    1     ea1
...................uuccacuugacaCcugaugaugaua.....................................................................    1     1     ea1
...................uuccacuugUcaucugaugaugaua.....................................................................    2     1     ea1
...................uuccacuugacaucugaugaugauU.....................................................................    4     1     ea1
...................Guccacuugacaucugaugaugauaa....................................................................    2     1     ea1
...................uuccacuugacaucugaugaugauaa....................................................................    14    0     ea1
...................uuccacuugacaucugaugaugauaau...................................................................    6     0     ea1
...................uuccacuugacaucugaugaCgauaau...................................................................    1     1     ea1
.........................................................cauugucccacugggguagacuAaug..............................    2     1     ea1
.........................................................cauugucccacugggguagacucaug..............................    4     0     ea1
..........................................................................uagacucaugguguuucuggaauag..............    1     0     ea1

......uuuugcaauauuguuccacuugac...................................................................................    1     0     eg2
..............uauuguuccacuugacaucugauA...........................................................................    3     1     eg2
..............uauuguuccacuugacaucugaug...........................................................................    11    0     eg2
..............uauuguuccacuugacaucugauga..........................................................................    2     0     eg2
...................uuccacuugacaucugaugaCg........................................................................    1     1     eg2
...................uuccacuugacaucugaugauga.......................................................................    3     0     eg2
...................uuccacuugacaucugaugaugau......................................................................    23    0     eg2
...................uuccacuugacaucugaugaugaua.....................................................................    10    0     eg2
.........................................................cauugucccacugggguagacuca................................    1     0     eg2
.........................................................cauugucccacugggguagacucaug..............................    3     0     eg2
...................................................................acugggguagacucaugguguuucugg...................    1     0     eg2

aaggauuuuugcaauauug..............................................................................................    7     0     ea2
..............uauuguuccacuugacaucugaug...........................................................................    37    0     ea2
..............Nauuguuccacuugacaucugaug...........................................................................    2     1     ea2
...............auuguuccacuugacaucugaug...........................................................................    3     0     ea2
...................uuccacuugacaucugaug...........................................................................    3     0     ea2
...................uuccacuugacaucugauAa..........................................................................    3     1     ea2
...................uuccacuugacaucugaugaugau......................................................................    1     0     ea2
...................uuccacuugacaucugaugaugaua.....................................................................    3     0     ea2
```

Provisional ID : ScUbCFx_4_1983
Score total : 3.8
Score for star read(s) : -1.3
Score for read counts : 0
Score for mfe : 0.5
Score for randfold : 1.6
Score for cons. seed : 3
Total read count : 1561
Mature read count : 1560
Loop read count : 1
Star read count : 0

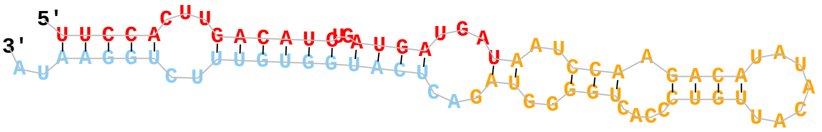

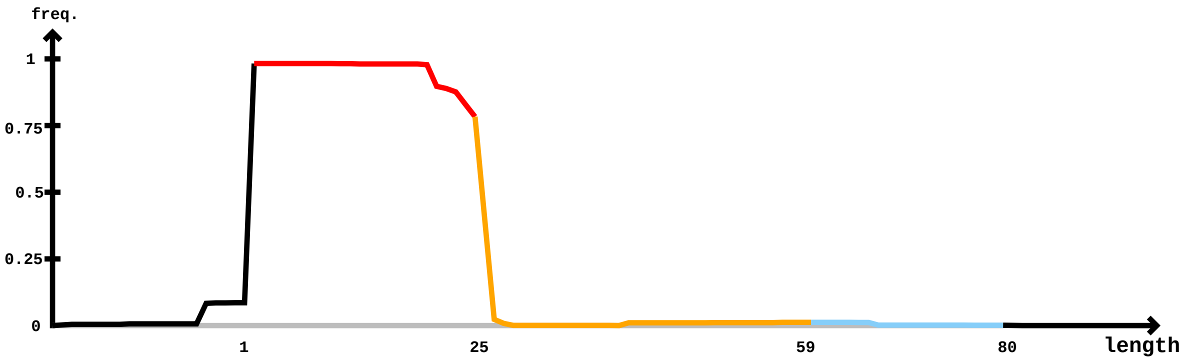

Mature Star

```
5'- aaggauuuuugcaauauuguuccacuugacaucugaugaugauaauccaagacauauacauugucccacugggguagacucaugguguuucuggaauagagcagaggccaaag   -3'    exp
    ..((.(((((((....((((((((...((((((..((((...((..(((.((((.......))))....)))..))...))))))))))..)))))))).)))))))))....   reads    mm    sample
    ..............uauuguuccacuugacaucugaug...........................................................................   11       0     er2
    ..............uauuguuccacuugacaucugauA...........................................................................   1        1     er2
    ..............uauuguuccacuugacaucugaugauA........................................................................   3        1     er2
    ...................uuccacuugacaucugaugaugau......................................................................   18       0     er2
    ...................uuccacuugacaucugaugaugaua.....................................................................   9        0     er2
    ..........................................................cauugucccacugggguagacucaug.............................   1        0     er2

    aaggauuuuugcaauauug..............................................................................................   1        0     ea1
    ..............uauuguuccacuugacaucugau............................................................................   2        0     ea1
    ..............uauuguuccacuugacaucugauA...........................................................................   5        1     ea1
    ..............uauuguuccacuugacaucugaug...........................................................................   28       0     ea1
    ..............Gauuguuccacuugacaucugaug...........................................................................   2        1     ea1
    ..............uauuguuccacuugacaucugaugauA........................................................................   1        1     ea1
    ..............uauuguuccacuugacaucugaugaug........................................................................   8        0     ea1
    .................uguuccacuugacaucugaugaugaC......................................................................   1        1     ea1
    ...................uuccacuugacaucugau............................................................................   2        0     ea1
    ...................Guccacuugacaucugaug...........................................................................   1        1     ea1
    ...................uuccacuugacaucugauA...........................................................................   1        1     ea1
    ...................uuccacuugacaucugaug...........................................................................   11       0     ea1
    ...................uuccacuugacaucugauUa..........................................................................   1        1     ea1
    ...................uuccacuugacaucugaugU..........................................................................   1        1     ea1
    ...................uuccacuugacaucugauga..........................................................................   4        0     ea1
    ...................uuccacuugacaucugaugaC.........................................................................   3        1     ea1
    ...................uuccacuugacaucugaugau.........................................................................   4        0     ea1
    ...................uuccacuugGcaucugaugau.........................................................................   2        1     ea1
    ...................Guccacuugacaucugaugaug........................................................................   1        1     ea1
    ...................uGccacuugacaucugaugaug........................................................................   1        1     ea1
    ...................uuccacuugacaucugaugaug........................................................................   26       0     ea1
    ...................Auccacuugacaucugaugaug........................................................................   1        1     ea1
    ...................uuccacuugacaucugaugauA........................................................................   3        1     ea1
    ...................uuccacuugacaucugaugaCg........................................................................   11       1     ea1
    ...................uuccacuugacaCcugaugauga.......................................................................   1        1     ea1
    ...................uuccacuugacaucugaugaCga.......................................................................   8        1     ea1
    ...................uuccacuugacaucugaugauga.......................................................................   32       0     ea1
```

```
                   Mature                                                   Star
aaggauuuuugcaauauuguuccacuugacaucugaugaugauaauccaagacauauacauugucccacugggguagacucaugguguuucuggaauagagcagaggccaaag
...................uuccacuGgacaucugaugauga.......................................................................   2     1    ea1
...................uuccacuugacaucugaugaugUu......................................................................   2     1    ea1
...................uuccacuugacGucugaugaugau......................................................................   2     1    ea1
...................uuccacuugaUaucugaugaugau......................................................................   1     1    ea1
...................uuccacuugacaucugaCgaugau......................................................................   4     1    ea1
...................uuccacuugacaCcugaugaugau......................................................................   5     1    ea1
...................uuccacuugacaucCgaugaugau......................................................................   5     1    ea1
...................uuccacuugacaucugaugaugaC......................................................................   66    1    ea1
...................Guccacuugacaucugaugaugau......................................................................   29    1    ea1
...................Cuccacuugacaucugaugaugau......................................................................   1     1    ea1
...................uucUacuugacaucugaugaugau......................................................................   2     1    ea1
...................uuccacuugacaucugaugaugaG......................................................................   6     1    ea1
...................uuccaUuugacaucugaugaugau......................................................................   1     1    ea1
...................uuccacuugacaucugGugaugau......................................................................   1     1    ea1
...................uuccacuugacaucugaugGugau......................................................................   3     1    ea1
...................uuccacuugGcaucugaugaugau......................................................................   1     1    ea1
...................uuccacuCgacaucugaugaugau......................................................................   1     1    ea1
...................uuccacuugacaucugaugaugau......................................................................   491   0    ea1
...................uuccacCugacaucugaugaugau......................................................................   3     1    ea1
...................uuccacuugacaucugaugaCgau......................................................................   11    1    ea1
...................uuccacuugacaucugaugaugaA......................................................................   3     1    ea1
...................uAccacuugacaucugaugaugau......................................................................   1     1    ea1
...................uuccacuugacaucugaugaugauG.....................................................................   15    1    ea1
...................uuccacuugacaCcugaugaugaua.....................................................................   1     1    ea1
...................Cuccacuugacaucugaugaugaua.....................................................................   2     1    ea1
...................uuccacuugUcaucugaugaugaua.....................................................................   2     1    ea1
...................Guccacuugacaucugaugaugaua.....................................................................   9     1    ea1
...................uuccacuugacaucugaugaugaua.....................................................................   86    0    ea1
...................uuccacuugacaucugaugaugauU.....................................................................   4     1    ea1
...................uuccacuugacaucugaugaCgaua.....................................................................   3     1    ea1
...................uuccacuugGcaucugaugaugaua.....................................................................   1     1    ea1
...................Guccacuugacaucugaugaugauaa....................................................................   2     1    ea1
...................uuccacuugacaucugaugaugauaa....................................................................   14    0    ea1
...................uuccacuugacaucugaugaCgauaau...................................................................   1     1    ea1
...................uuccacuugacaucugaugaugauaau...................................................................   6     0    ea1
..........................................................cauugucccacugggguagacucaug.............................   4     0    ea1
..........................................................cauugucccacugggguagacuAaug.............................   2     1    ea1
..........................................................................uagacucaugguguuucuggaauag..............   1     0    ea1

......uuuugcaauauuguuccacuugac...................................................................................   1     0    eg2
..............uauuguuccacuugacaucugaug...........................................................................   11    0    eg2
..............uauuguuccacuugacaucugauA...........................................................................   3     1    eg2
..............uauuguuccacuugacaucugauga..........................................................................   2     0    eg2
...................uuccacuugacaucugaugaCg........................................................................   1     1    eg2
...................uuccacuugacaucugaugauga.......................................................................   3     0    eg2
...................uuccacuugacaucugaugaugau......................................................................   23    0    eg2
...................uuccacuugacaucugaugaugaua.....................................................................   10    0    eg2
..........................................................cauugucccacugggguagacuca...............................   1     0    eg2
..........................................................cauugucccacugggguagacucaug.............................   3     0    eg2
...................................................................acugggguagacucaugguguuucugg...................   1     0    eg2

aaggauuuuugcaauauug..............................................................................................   7     0    ea2
..............Nauuguuccacuugacaucugaug...........................................................................   2     1    ea2
..............uauuguuccacuugacaucugaug...........................................................................   37    0    ea2
...............auuguuccacuugacaucugaug...........................................................................   3     0    ea2
...................uuccacuugacaucugaug...........................................................................   3     0    ea2
...................uuccacuugacaucugauAa..........................................................................   3     1    ea2
...................uuccacuugacaucugaugaugau......................................................................   1     0    ea2
...................uuccacuugacaucugaugaugaua.....................................................................   3     0    ea2

......uuuugcaauauuguuccacuugac...................................................................................   1     0    eg1
..............uauuguuccacuugacaucugaug...........................................................................   5     0    eg1
..............uauuguuccacuugacaucugauga..........................................................................   1     0    eg1
..............uauuguuccacuugacaucugaugaug........................................................................   1     0    eg1
...................uuccacuugacaucugaug...........................................................................   4     0    eg1
...................uuccacuugacaucugaCg...........................................................................   1     1    eg1
...................uuccacuugacaucugauga..........................................................................   2     0    eg1
...................uuccacuugacaucugaugau.........................................................................   9     0    eg1
...................Guccacuugacaucugaugaug........................................................................   1     1    eg1
...................uuccacuugacaucCgaugaug........................................................................   3     1    eg1
```

Mature   Star

```
aaggauuuuugcaauauuguuccacuugacaucugaugaugauaauccaagacauauacauugucccacugggguagacucaugguguuucuggaauagagcagaggccaaag
...................uuccacuugacaucugaugaCg........................................................................    1    1    eg1
...................uuccacuugacaucugaugaug........................................................................    8    0    eg1
...................uuccacuugacaucugaugauA........................................................................    1    1    eg1
...................uuccacuugacaucugaugaCga.......................................................................    1    1    eg1
...................Guccacuugacaucugaugauga.......................................................................    2    1    eg1
...................uuccacuugacaucugaugaugG.......................................................................    1    1    eg1
...................uuccacuugacaucugaugauga.......................................................................    15   0    eg1
...................uuccacuugacaucugaugaugaC......................................................................    22   1    eg1
...................uuccacuugUcaucugaugaugau......................................................................    1    1    eg1
...................uuccGcuugacaucugaugaugau......................................................................    1    1    eg1
...................uuccacuugGcaucugaugaugau......................................................................    1    1    eg1
...................uuccacCugacaucugaugaugau......................................................................    1    1    eg1
...................uuccacuugacaucugaugaugaA......................................................................    1    1    eg1
...................Guccacuugacaucugaugaugau......................................................................    14   1    eg1
...................uuccacuugacaucugaugaugau......................................................................    208  0    eg1
...................uCccacuugacaucugaugaugau......................................................................    1    1    eg1
...................uuccacuugacaucugaugaCgau......................................................................    9    1    eg1
...................uuccacuugacaucugUugaugau......................................................................    3    1    eg1
...................uuccacuugacaucugaugaugaG......................................................................    1    1    eg1
...................uucAacuugacaucugaugaugau......................................................................    1    1    eg1
...................uuccacuugacaucugaugaCgaua.....................................................................    1    1    eg1
...................uuccacuugacaucugaugaugaua.....................................................................    31   0    eg1
...................uuccacuugacaucugGugaugaua.....................................................................    1    1    eg1
...................Guccacuugacaucugaugaugaua.....................................................................    3    1    eg1
...................uuccacuugacaucugUugaugaua.....................................................................    1    1    eg1
...................uuccacuugGcaucugaugaugaua.....................................................................    1    1    eg1
...................uuccacuugacaucugaugaugauU.....................................................................    2    1    eg1
...................uuccacuugacaucugaugaugauG.....................................................................    9    1    eg1
...................uuccacuugacaucugaugaugauaa....................................................................    10   0    eg1
...................uuccacuugacaucugaugaugauaaau..................................................................    4    0    eg1
.......................................ugauUauccaagacauau........................................................    1    1    eg1
..........................................................cauugucccacugggguagacucaug.............................    7    0    eg1
..........................................................................uagacucaugguguuucuggaauag..............    1    0    eg1

......uuuugcaauauuguuccacuug.....................................................................................    1    0    er1
..............Gauuguuccacuugacaucugaug...........................................................................    1    1    er1
..............uauuguuccacuugacaucugaug...........................................................................    9    0    er1
...................uuccacuugacaucugau............................................................................    1    0    er1
...................uuccacuugacaucugaug...........................................................................    1    0    er1
...................uuccacuugacaucugauUa..........................................................................    1    1    er1
...................uuccacuugacaucugaugau.........................................................................    4    0    er1
...................Cuccacuugacaucugaugaug........................................................................    1    1    er1
...................uuccacuugacaucugaCgaug........................................................................    2    1    er1
...................uuccacuugacaucugaugauA........................................................................    1    1    er1
...................uuccacuugacaucugaugaug........................................................................    4    0    er1
...................uuccacuugacaucugaugaCg........................................................................    2    1    er1
...................uuccacuugacaucugaugauga.......................................................................    13   0    er1
...................uuccacuugacaucugaugaugG.......................................................................    1    1    er1
...................uuccacuugaUaucugaugauga.......................................................................    1    1    er1
...................uuccacuugacauUugaugaugau......................................................................    1    1    er1
...................uuccacuugacaucugaugaugaC......................................................................    13   1    er1
...................Guccacuugacaucugaugaugau......................................................................    13   1    er1
...................uuccacuugacaucugaugaugau......................................................................    104  0    er1
...................uCccacuugacaucugaugaugau......................................................................    1    1    er1
...................uuccGcuugacaucugaugaugau......................................................................    1    1    er1
...................uuccacuugGcaucugaugaugau......................................................................    2    1    er1
...................uuccacuugacaucugaugaugaG......................................................................    2    1    er1
...................uuccacuugacaucuUaugaugau......................................................................    1    1    er1
...................uuccacuugacaucugaugaCgau......................................................................    4    1    er1
...................uuccacuugacaucCgaugaugau......................................................................    2    1    er1
...................uuccacuugacaucugaugaugGu......................................................................    1    1    er1
...................uuccacuugacaucugaugaugaCa.....................................................................    1    1    er1
...................uuccacuugacaucugaugaugGua.....................................................................    1    1    er1
...................uuccacuugacaucugaugaugaua.....................................................................    17   0    er1
...................uuccacuugacGucugaugaugaua.....................................................................    2    1    er1
...................uuccacuugacaucugaugaugauG.....................................................................    5    1    er1
...................Guccacuugacaucugaugaugaua.....................................................................    1    1    er1
...................uuccacuugacaucugaugaCgaua.....................................................................    2    1    er1
...................uuccacuugacaucugaugaugauaaau..................................................................    2    0    er1
```

Provisional ID : ScUbCFx_10_3257
Score total : 734.7
Score for star read(s) : 3.9
Score for read counts : 724.1
Score for mfe : 2.1
Score for randfold : 1.6
Score for cons. seed : 3
Total read count : 1432
Mature read count : 1253
Loop read count : 0
Star read count : 179

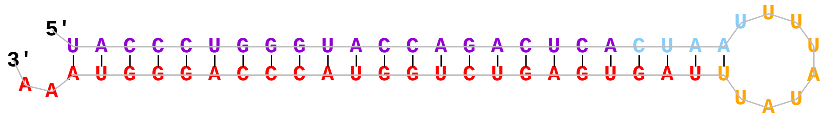

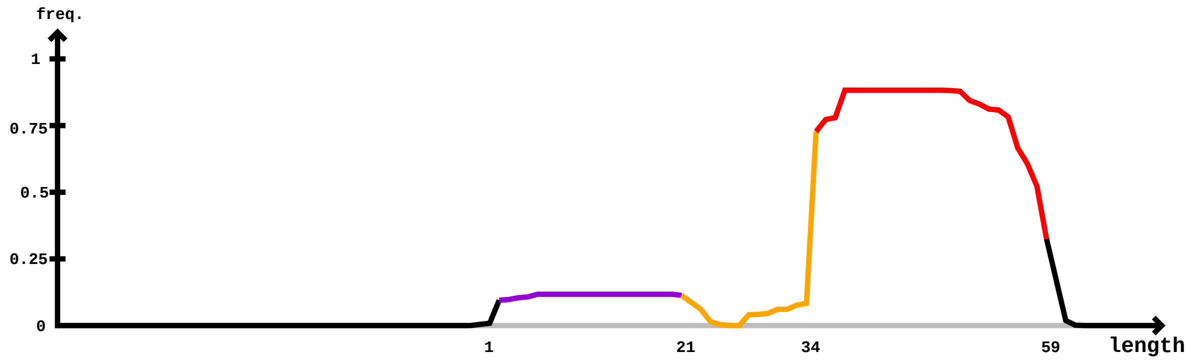

Star Mature

```
5'- gcucaaauuuccuaaacaauuuaacaaaguagcauaaauauccuauacccuggguaccagacucacuaauuuuauauuuagugagucugguacccaggguaaauauccuaaaug  -3'   obs
    gcucaaauuuccuaaacaauuuaacaaaguagcauaaauauccuauacccuggguaccagacucacuaauuuuauauuuagugagucugguacccaggguaaauauccuaaaug         exp
    .............................................((((((((((((((((((((((((........))))))))))))))))))))))))).............   reads   mm   sample
    .............................................uacccuggguaccagacucU.................................................   5       1    eg2
    .............................................uacccugggCaccagacuca.................................................   1       1    eg2
    .............................................uacccuggguaccagacuca.................................................   1       0    eg2
    .............................................uacccuggguaccagacucC.................................................   1       1    eg2
    .............................................uacccugAguaccagacucacu...............................................   1       1    eg2
    ..............................................acccuggguaccagacucU.................................................   2       1    eg2
    .................................................cuggguaccagacucUcua..............................................   2       1    eg2
    ............................................................................uAuagugagucugguacccaggguaaa...........   1       1    eg2
    ..............................................................................uagugagucugguacccaggg...............   6       0    eg2
    ..............................................................................uagugaguGugguacccaggguaaa...........   1       1    eg2
    ...............................................................................agugagucugguaccUagg................   1       1    eg2
    ...............................................................................agugagucugguacccaggg...............   1       0    eg2
    ................................................................................gAgagucugguacccagggua.............   1       1    eg2
    .................................................................................ugagucugguacccagggua.............   1       0    eg2
    .................................................................................ugagucugguacccaggguaU............   1       1    eg2
    .................................................................................ugagucugguacccaggguaUa...........   5       1    eg2

    ...........................................uUuacccuggguaccagacuca.................................................   4       1    ea2
    ............................................auacccuggguaccagacuca.................................................   2       0    ea2
    .............................................uacccuggguaccagacuc..................................................   2       0    ea2
    .............................................uacccuUgguaccagacuca.................................................   1       1    ea2
    .............................................uacccuggguaccagacucU.................................................   4       1    ea2
    .............................................uacccuggguaccagacucC.................................................   4       1    ea2
    .............................................uacccuggguaccagacuca.................................................   5       0    ea2
    .............................................uacccuggAuaccagacucacu...............................................   3       1    ea2
    ...............................................cccuggguaccagacucacA...............................................   2       1    ea2
    ...............................................cccuggAuaccagacucacua..............................................   1       1    ea2
    ................................................ccuggguaccagacucUcu...............................................   1       1    ea2
    ................................................ccuggguaccagacucacuUa.............................................   2       1    ea2
    ........................................................................uauauGuagugagucugguaccc...................   1       1    ea2
    ............................................................................uuuagugagucugguacccU..................   3       1    ea2
    ............................................................................uAuagugagucugguacccaggguaa............   1       1    ea2
    ..............................................................................uagugagucugguaccca..................   2       0    ea2
```

Star **Mature**

gcucaaauuuccuaaacaauuuaacaaaguagcauaauauccuauacccuggguaccagacucacuaauuuuauauuuagugagucugguacccaggguaaauauccuaaaug			
....uagugagucugguacccag.................	4	0	ea2
....uagugagucugguacccagg................	1	0	ea2
....Nagugagucugguacccaggg...............	1	1	ea2
....uagugagucugguacccaggg...............	15	0	ea2
....uagugagucugguacccaggAua.............	1	1	ea2
....uagugagucugguacccaggguG.............	13	1	ea2
....uagugagCcugguacccagggua.............	1	1	ea2
....uagugagucugguacccagggua.............	7	0	ea2
....uagugagucugguacccagggCa.............	3	1	ea2
....uagugagucugguacccaggguaU............	4	1	ea2
....uagugagucugguacccaggguUa............	4	1	ea2
....uagugagucugguacccaggguaa............	9	0	ea2
....Gagugagucugguacccaggguaa............	1	1	ea2
....uagugagucugguacccaggguaaC...........	2	1	ea2
....uagugagucugguacccaggguaaU...........	6	1	ea2
....uagugagucugguacccaggguaUa...........	39	1	ea2
....uagugagucugguacccaggguaaG...........	4	1	ea2
....uagugagucugguacccaggguaaUu..........	1	1	ea2
....uagugagucugguacccaggguaaaua.........	1	0	ea2
....agugagucugguacccaggguaa............	1	0	ea2
....agugagucugguacccaggguUa............	3	1	ea2
....agugagucugguacccaggguaUa...........	2	1	ea2
....ugagucugguacccaggg...............	2	0	ea2
....ugagucugguacccaggguaU............	2	1	ea2
....ugagucugguacccagggAaaa...........	3	1	ea2
....ugagucugguacccaggguaUa...........	14	1	ea2
....ugagucugguacccaggguaaCu..........	3	1	ea2
....ugagucugguacccaggguaaUua.........	5	1	ea2
....uUuacccuggguaccagacuca...	1	1	er2
....uacccuggguaccagacuca...	7	0	er2
....uacccuUgguaccagacuca...	2	1	er2
....uacccuggguaccagacucaA..	2	1	er2
....uacccuggguaccagacucacu...	5	0	er2
....uacccuggguaccagacucacA...	1	1	er2
....acccuggguaccagacuca...	1	0	er2
....uAuagugagucugguacccaggguaa............	2	1	er2
....Nuagugagucugguacccaggg...............	1	1	er2
....uagugagucugguacccU..................	3	1	er2
....uagugagucugguaccca..................	1	0	er2
....uagugagucugguacccagg................	22	0	er2
....uagugagucugguacccaggg...............	108	0	er2
....uagugagucugguacccagggu..............	71	0	er2
....Nagugagucugguacccagggu..............	1	1	er2
....uagugagucugguacccagggC..............	1	1	er2
....Nagugagucugguacccagggua.............	1	1	er2
....uagugagucugguacccaggguU.............	21	1	er2
....uagugagucugguacccaggguG.............	3	1	er2
....uagugagucugguacccagggua.............	34	0	er2
....uagugagucugguacccaggAua.............	1	1	er2
....uagugagucugguacccaggguC.............	4	1	er2
....Nagugagucugguacccaggguaa............	1	1	er2
....uagugagucugguacccaggguUa............	69	1	er2
....uagugagucugguacccaggguaU............	124	1	er2
....uagugagucugguacccaggguaa............	19	0	er2
....uagugagucugguacccaggguaUa...........	282	1	er2
....uagugagucugguacccaggguaaG...........	1	1	er2
....uagugagucugguacccaggguUaa...........	2	1	er2
....uagugagucugguacccaggguaaU...........	3	1	er2
....uagugagucugguacccaggguaaC...........	4	1	er2
....uagugagucugguacccaggguaaa...........	4	0	er2
....uagugagucugguacccaggguaUau..........	1	1	er2
....uagugagucugguacccaggguaaUu..........	1	1	er2
....uagugagucugguacccaggguaCaua.........	1	1	er2
....agugagucugguacccaggg...............	2	0	er2
....agugagucugguacccagggu..............	4	0	er2
....agugagucugguacccaggguU.............	2	1	er2
....agugagucugguacccagggua.............	6	0	er2
....agugagucugguacccaggguC.............	2	1	er2
....agugagucugguacccaggguUa............	3	1	er2

```
                                            Star                              Mature
gcucaaauuuccuaaacaauuuaacaaaguagcauaauauccuauacccuggguaccagacucacuaauuuuauauuuagugagucugguacccaggguaaauauccuaaaug
..............................................................................agugagucugguacccaggguaa............    7    0    er2
..............................................................................agugagucugguacccaggguaU............    8    1    er2
..............................................................................agugagucugguacccaggguUaa...........    2    1    er2
..............................................................................agugagucugguacccaggguaaa...........    1    0    er2
..............................................................................agugagucugguacccaggguaUa...........    7    1    er2
...............................................................................gugagucugguacccaggg...............    2    0    er2
...............................................................................gugagucugguacccaggguaaUua.........    1    1    er2
................................................................................ugagucugguacccaggguG.............    3    1    er2
................................................................................ugagucugguaccAaggguaa............    1    1    er2
................................................................................ugagucugguacccaggguaa............    2    0    er2
................................................................................ugagucugguacccaggguaUa...........    21   1    er2
................................................................................ugagucugguacccaggguaaCu..........    8    1    er2
................................................................................ugagucugguacccaggguaaUu..........    5    1    er2
................................................................................ugagucugguacccaggguaaau..........    3    0    er2
................................................................................ugagucugguacccaggguaaUua.........    2    1    er2
................................................................................ugagucugguacccaggguaaCua.........    6    1    er2
................................................................................ugagucugguacccaggguaaaua.........    1    0    er2

..........................................uauacccuggguaccagacuc..................................................    1    0    ea1
..........................................uauacccuggguaccagacucU.................................................    1    1    ea1
...........................................auacccuggguaccagacucC.................................................    1    1    ea1
...........................................auacccuggguaccagacucacA...............................................    2    1    ea1
............................................uacccuggguaccagacuc..................................................    3    0    ea1
............................................uacccuggguaccagacucU.................................................    12   1    ea1
............................................uacccuggguaccagacuca.................................................    4    0    ea1
............................................uacccuggguaccagacucC.................................................    6    1    ea1
............................................uGcccuggguaccagacucac................................................    1    1    ea1
............................................uacccuggguaccagacucac................................................    4    0    ea1
............................................uacccuggguaccagacucUc................................................    7    1    ea1
............................................uacccuggguaccagacucUcu...............................................    21   1    ea1
............................................uUcccuggguaccagacucacu...............................................    1    1    ea1
............................................uacccuggguaccagacucacu...............................................    7    0    ea1
............................................uacccuggguaccagacucacC...............................................    2    1    ea1
............................................uacccuAgguaccagacucacu...............................................    3    1    ea1
............................................uUcccuggguaccagacucacua..............................................    1    1    ea1
............................................uacccuggguaccagacucUcua..............................................    3    1    ea1
.............................................Ucccuggguaccagacucacu...............................................    1    1    ea1
..............................................cccuggguaccagacucacu...............................................    2    0    ea1
..............................................cccugggugccagacucacu...............................................    1    1    ea1
..............................................cccuggguaccagacucacua..............................................    1    0    ea1
...............................................ccuggguaccagacucacua..............................................    2    0    ea1
................................................cuggguaccagacucUcu...............................................    5    1    ea1
................................................cuggguaccagacucUcua..............................................    2    1    ea1
................................................cuggguaccagacucUcuaa.............................................    1    1    ea1
................................................cuggguaccagacucacuaa.............................................    1    0    ea1
................................................cuggguaccagacucacuaUu............................................    1    1    ea1
......................................................................Guauauuuagugagucuggua......................    1    1    ea1
......................................................................uuauauuuagugagucuggua......................    1    0    ea1
......................................................................uuauauuuagugagucugguUc.....................    1    1    ea1
......................................................................uuauauGuagugagucugguacc....................    1    1    ea1
......................................................................Guauauuuagugagucugguacc....................    9    1    ea1
......................................................................uuauauuuagugagucugguacc....................    23   0    ea1
......................................................................uuauauuuGgugagucugguacc....................    1    1    ea1
......................................................................uuauauuuagugagucugguacU....................    7    1    ea1
......................................................................uuauauuuagugagucugguaccU...................    7    1    ea1
......................................................................uuauauuuagugagucugguaccA...................    3    1    ea1
.......................................................................uauauuuagugagucugguacc....................    1    0    ea1
........................................................................auauGuagugagucugguac.....................    2    1    ea1
........................................................................auaAuuagugagucugguacc....................    2    1    ea1
........................................................................auauGuagugagucugguaccc...................    1    1    ea1
........................................................................auaAuuagugagucugguaccca..................    1    1    ea1
.........................................................................uauGuagugagucugguacc....................    2    1    ea1
.........................................................................uauuuagugagucugguaccc...................    3    0    ea1
.........................................................................uauuuagugagucugguacccU..................    11   1    ea1
.........................................................................uauuuagugagucugguacccC..................    1    1    ea1
...........................................................................uuuagugagucugguacccC..................    2    1    ea1
...........................................................................uuuagugagucugguacccU..................    1    1    ea1
...........................................................................uGuagugagucugguacccaggg...............    1    1    ea1
...........................................................................uuuagugagucugguacccaggg...............    1    0    ea1
...........................................................................uAuagugagucugguacccaggguaa............    8    1    ea1
```

Star Mature

```
gcucaaauuuccuaaacaauuuaacaaaguagcauaauauccuauacccuggguaccagacucacuaauuuuauauuuagugagucugguacccaggguaaauauccuaaaug
...........................................................................uAuagugagucugguacccaggguaaa...........  3   1   ea1
............................................................................Guagugagucugguacccaggg...............  5   1   ea1
............................................................................uuagugagucAgguacccagggu..............  1   1   ea1
............................................................................Auagugagucugguacccaggguaa............  3   1   ea1
.............................................................................uagAgagucugguaccca..................  2   1   ea1
.............................................................................uagugagucugguaccca..................  1   0   ea1
.............................................................................uagugagucugguaccUag.................  1   1   ea1
.............................................................................uagAgagucugguacccagg................  1   1   ea1
.............................................................................uagugagGcugguacccagg................  1   1   ea1
.............................................................................uagugagucugguacccagU................  1   1   ea1
.............................................................................uagugagucugguaccUagg................  11  1   ea1
.............................................................................uagugagucugguacccaggg...............  14  0   ea1
.............................................................................uagugagucugguacccaggU...............  2   1   ea1
.............................................................................Gagugagucugguacccaggg...............  2   1   ea1
.............................................................................uagugagucugguaccUaggg...............  4   1   ea1
.............................................................................uagugagucugguacccaggA...............  2   1   ea1
.............................................................................uUgugagucugguacccaggg...............  1   1   ea1
.............................................................................Gagugagucugguacccagggu..............  1   1   ea1
.............................................................................uagugagucugguacccagggC..............  4   1   ea1
.............................................................................uagugagucugguacccagggu..............  2   0   ea1
.............................................................................uagugagucugguacccagggua.............  6   0   ea1
.............................................................................Gagugagucugguacccagggua.............  1   1   ea1
.............................................................................uagugagucugguacccaggguU.............  2   1   ea1
.............................................................................Gagugagucugguacccaggguaa............  2   1   ea1
..............................................................................agugagucugguacccagg................  1   0   ea1
..............................................................................agugagucugguacccaggAu..............  1   1   ea1
..............................................................................agugagucugguacccagggC..............  1   1   ea1
..............................................................................agugagucugguacccaggguG.............  1   1   ea1
..............................................................................agugagucugguacccagggua.............  3   0   ea1
..............................................................................agugagucugguacccaggguaa............  1   0   ea1
..............................................................................agAgagucugguacccaggguaa............  2   1   ea1
..............................................................................agugagucugguacccaggguUa............  1   1   ea1
..............................................................................agugagucugguacccaggguaUa...........  2   1   ea1
...............................................................................gugagucugguacccaggg...............  1   0   ea1
...............................................................................gugagucugguGcccagggua.............  1   1   ea1
...............................................................................gugagucugguacccaggguU.............  3   1   ea1
...............................................................................gugagucugguacccagggua.............  1   0   ea1
...............................................................................gugagucugguacccaggguaUa...........  1   1   ea1
................................................................................ugagucugguacccagggA..............  1   1   ea1
................................................................................ugagucugguacccagggua.............  3   0   ea1
................................................................................ugagucugguacccaggguaU............  10  1   ea1
................................................................................ugagucugguacccaggguaa............  2   0   ea1
................................................................................ugagucugguacccaggguUa............  1   1   ea1
................................................................................ugagucugguacccaggguaaa...........  5   0   ea1
................................................................................ugagucugguacccaggguaUa...........  16  1   ea1
................................................................................ugagucugguacccaggguaaCu..........  4   1   ea1
................................................................................ugagucugguacccaggguaaUu..........  4   1   ea1
................................................................................ugagucugguacccaggguaaau..........  1   0   ea1
................................................................................ugagucugguacccaggguaaaua.........  2   0   ea1
................................................................................Ggagucugguacccaggguaaaua.........  1   1   ea1
................................................................................ugagucugguacccaggguaaUua.........  5   1   ea1
................................................................................ugagucugguacccaggguaCauau........  1   1   ea1

...........................................auacccuggguaccagacucacA...............................................  1   1   eg1
............................................uacccuggguaccagacucaA................................................  1   1   eg1
............................................uacccuggguaccagacucUcu...............................................  1   1   eg1
............................................uacccugggAaccagacucacu...............................................  2   1   eg1
............................................uacccuggguaccagacucacu...............................................  2   0   eg1
............................................uacccuggguaccagacucUcua..............................................  2   1   eg1
..............................................cccuggguaccagacucacu...............................................  1   0   eg1
..............................................cccuggguaccagacucUcua..............................................  1   1   eg1
..............................................cccuggguaccagacucacuaC.............................................  1   1   eg1
................................................cuggguaccagacucUcu...............................................  1   1   eg1
................................................cuggguaccagacucacua..............................................  1   0   eg1
......................................................................uuauauuuagugagucugguac.....................  1   0   eg1
......................................................................uuauauuuagugagucugguacc....................  1   0   eg1
......................................................................Guauauuuagugagucugguacc....................  1   1   eg1
.........................................................................uauuuagugagucugguacccU..................  1   1   eg1
...........................................................................uAuagugagucugguacccaggguaa............  1   1   eg1
.............................................................................uagugagucugguGcccaggg...............  1   1   eg1
```

```
                                             Star                           Mature
gcucaaauuuccuaaacaauuuaacaaaguagcauaaauauccuauacccuggguaccagacucacuaauuuuauauuuagugagucugguacccaggguaaauauccuaaaug
..............................................................................uagugagucugguacccaggg...............   3   0   eg1
..............................................................................uagugagucugguacccaggguG.............   1   1   eg1
..............................................................................uagugagucugguacccagggCa.............   1   1   eg1
..............................................................................uagugagucugguacccagggua.............   2   0   eg1
...............................................................................agugagucugguacccaggg...............   1   0   eg1
...............................................................................agugagucugguacccagggua.............   1   0   eg1
.................................................................................ugagCcugguacccagggu..............   1   1   eg1
.................................................................................ugagucugguacccaggguaU............   2   1   eg1
.................................................................................ugagucugguacccaggguaUa...........   2   1   eg1
.................................................................................ugagucugguacccaggguaaUu..........   1   1   eg1
.................................................................................ugagucugguacccaggguaaUua.........   1   1   eg1

.............................................uacccuggguacGagacucacu...............................................   1   1   er1
.............................................uacccuAgguaccagacucacu...............................................   1   1   er1
.............................................uacccuggguaccagacucUcu...............................................   1   1   er1
.............................................uacccuggguaccagacucUcua..............................................   1   1   er1
.................................................cuggguaccagacucUcu...............................................   1   1   er1
.......................................................................uuauauuuagugagucugguacA....................   1   1   er1
.......................................................................Guauauuuagugagucugguacc....................   2   1   er1
.......................................................................uuauauuuagugagucugguacc....................   2   0   er1
..........................................................................uauuuagugagucugguaccc...................   2   0   er1
..........................................................................uauuuagugagucugguaccA...................   3   1   er1
..............................................................................uagugagucugguacccaggg...............   2   0   er1
..............................................................................uagugagucugguacccagggu..............   1   0   er1
..............................................................................uagugagucugguacccagggua.............   1   0   er1
...............................................................................agugagucugguacccaggguaa............   2   0   er1
.................................................................................ugagucugguacccaggguaa............   2   0   er1
.................................................................................ugagucugguacccaggguaUa...........   1   1   er1
.................................................................................ugagucugguacccaggguaaCu..........   1   1   er1
.................................................................................ugagucugguacccaggguaUaua.........   1   1   er1
.................................................................................ugagucugguacccaggguaaUuau........   1   1   er1
```

Provisional ID	:	ScUbCFx_10_3327
Score total	:	1842.7
Score for star read(s)	:	3.9
Score for read counts	:	1835.5
Score for mfe	:	2.3
Score for randfold	:	1.6
Score for cons. seed	:	-0.6
Total read count	:	3612
Mature read count	:	1611
Loop read count	:	0
Star read count	:	2001

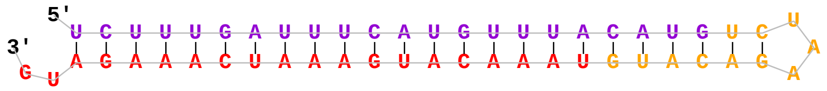

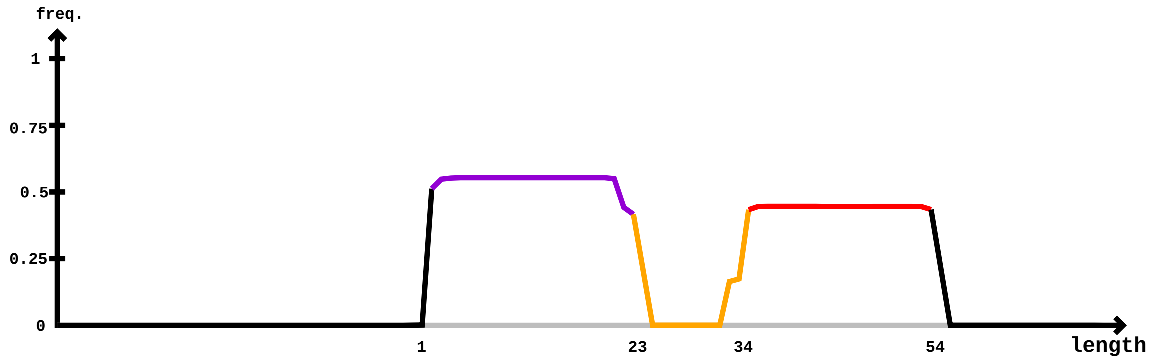

Star **Mature**

```
5'- guauggauggguuguuggaaacucaacagaagcaucaucuuugauuucauguuuacaugucuaagacauguaaacaugaaaucaaagaugaugucuugcuguuuaaauc   -3'   obs
    guauggauggguuguuggaaacucaacagaagcaucaucuuugauuucauguuuacaugucuaagacauguaaacaugaaaucaaagaugaugucuugcuguuuaaauc         exp
    .......((((((......))))))((((((((((((((((((((((((((((((((((((...))))))))))))))))))))))))))))).))).)))).......    reads   mm    sample
    .....................................ucuuugauuucauguuuacau...................................................    1       0     eg1
    .....................................ucuuugauuucauguuuacaug..................................................    3       0     eg1
    ....................................................................uguaaacaugaaaucaaagau....................    3       0     eg1
    ....................................................................uguaaacaugaaaucaaagauA...................    3       1     eg1
    ....................................................................uguaaacaugaaaucaaagaug...................    6       0     eg1
    ......................................................................uaaacaugaaaucaaagauA...................    1       1     eg1

    .....................................ucuuugauuucauguuuacau...................................................    2       0     er1
    .....................................ucuuugauuucauguuuacauA..................................................    1       1     er1
    .....................................Gcuuugauuucauguuuacaug..................................................    1       1     er1
    .....................................ucuuugauuucauguuuacaug..................................................    14      0     er1
    ......................................cuuugauuucauguuuacaug..................................................    1       0     er1
    ....................................................................uguaaacaugaaaucaaagau....................    5       0     er1
    ....................................................................uguaaacaugaaaucaaagaug...................    25      0     er1
    ....................................................................uguaaacaugaaaucaaagauA...................    5       1     er1
    ....................................................................uguaaacaugaaaucaaagaCg...................    1       1     er1
    ....................................................................uguaaacaCgaaaucaaagaug...................    1       1     er1
    ......................................................................uaaacaugaaaucaaagauA...................    1       1     er1
    ......................................................................uaaacaugaGaucaaagaug...................    4       1     er1
    ......................................................................uaaacaugaaaucaaagaug...................    2       0     er1
    ......................................................................uaaacaugaaaucaaagauCa..................    1       1     er1

    ...................................Aaucuuugauuucauguuuacaug..................................................    2       1     er2
    ....................................aucuuugauuucauguuuacauA..................................................    2       1     er2
    .....................................ucuuugauuucauguuuac.....................................................    13      0     er2
    .....................................ucuuugauuucGuguuuaca....................................................    5       1     er2
    .....................................ucuuugaCuucauguuuaca....................................................    1       1     er2
    .....................................ucuuugauuucauguuuaca....................................................    337     0     er2
    .....................................ucuuugauuucauguuuacU....................................................    1       1     er2
    .....................................Ncuuugauuucauguuuaca....................................................    3       1     er2
    .....................................ucuuugauuucauguuuacau...................................................    33      0     er2
    .....................................ucuuugauuucauguuuacaA...................................................    35      1     er2
    .....................................ucuuugauuucauguuuacauA..................................................    526     1     er2
```

Star Mature

guauggauggguuguuggaaacucaacagaagcaucaucuuugauuucauguuuacaugucuaagacauguaaacaugaaaucaaagaugaugucuugcuguuuaaauc			
.....................................ucuuugauuucauguuuacauC..	2	1	er2
.....................................ucuuugauuucGuguuuacaug..	7	1	er2
.....................................ucuuugauuucauguuuacaug..	622	0	er2
.....................................ucuuugauuucauguuuaUaug..	1	1	er2
.....................................Ncuuugauuucauguuuacaug..	3	1	er2
.....................................ucuuugauuucauguuuacauU..	33	1	er2
.....................................ucuuugauuucauguuuacaugu...	4	0	er2
.....................................ucuuugauuucauguuuacaugA...	1	1	er2
......................................cuuugauuucauguuuaca..	14	0	er2
......................................cuuugauuucauguuuacau...	1	0	er2
......................................cuuugauuucauguuuacauA..	36	1	er2
......................................cuuugauuucauguuuacaug..	57	0	er2
......................................cuuugauuuAauguuuacaug..	2	1	er2
....uuugauuucauguuuaca..	1	0	er2
....uuugauuucauguuuacaug..	11	0	er2
....uuugauuucauguuuacauA..	3	1	er2
....uugauuucauguuuacauA..	5	1	er2
....ugucuaagacauguaaacaug...............................	2	0	er2
....uguaaacaugaaaucaaagau....................	4	0	er2
....uguaaacaugaaaucaaagauA...................	41	1	er2
....Nguaaacaugaaaucaaagaug...................	3	1	er2
....uguaGacaugaaaucaaagaug...................	1	1	er2
....uAuaaacaugaaaucaaagaug...................	1	1	er2
....uguGaacaugaaaucaaagaug...................	3	1	er2
....uguaaacaugaaaucaaagaug...................	393	0	er2
....guaaacaugaaaucaaagauA...................	5	1	er2
....guaaacaugaaaucaaagaug...................	29	0	er2
....uaaacaugaaaucaaagau....................	12	0	er2
....uaGacaugaaaucaaagaug...................	1	1	er2
....uaaacaugaaaucaaagauA...................	69	1	er2
....Naaacaugaaaucaaagaug...................	4	1	er2
....uaaacaugaaaucaaagauC...................	2	1	er2
....uaaacaugaaaucGaagaug...................	1	1	er2
....uaaacaugaGaucaaagaug...................	2	1	er2
....Gaaacaugaaaucaaagaug...................	1	1	er2
....uaaacaugaaaucaaagaug...................	767	0	er2
....uaaacaugaaaucaaagauAa..................	1	1	er2
....aaacaugaaaucaaagauA...................	8	1	er2
....aaacaugaaaucaaagaug...................	26	0	er2
....aaacaugaaaucaaagauAa..................	2	1	er2
.....................................ucuuugauuucauguuuaca..	2	0	ea1
.....................................ucuuugauuucauguuuacau...	4	0	ea1
.....................................ucuuugauuucauguuuacaA...	1	1	ea1
.....................................ucuuugauuucauguuuacauU..	1	1	ea1
.....................................Gcuuugauuucauguuuacaug..	3	1	ea1
.....................................ucuuugauuucauguuuacauA..	15	1	ea1
.....................................ucuuugauuucauguuuacaug..	16	0	ea1
......................................cuuugauuucauguuuacaug..	4	0	ea1
......................................cCuugauuucauguuuacaug..	2	1	ea1
....uguaaacaugaaaucaaaga.....................	3	0	ea1
....uguGaacaugaaaucaaagau....................	1	1	ea1
....uguaaacaugaaaucaaagaA....................	1	1	ea1
....uguaaacaugaaaucaaagau....................	6	0	ea1
....Gguaaacaugaaaucaaagaug...................	1	1	ea1
....uguaaacaugaaaucaaagaCg...................	1	1	ea1
....uguaaacaugaaaucaaagauU...................	1	1	ea1
....uguaaacaugaaaucaaagaug...................	29	0	ea1
....uguaaacaugaaaucaaagauA...................	41	1	ea1
....uguaaacaugaaaucaaagauAa..................	1	1	ea1
....Gaaacaugaaaucaaagau....................	1	1	ea1
....uaaacaugaaaucaaagau....................	3	0	ea1
....uaaacaugaaaucaaagaug...................	6	0	ea1
....uaaacaugaaaucaaagauA...................	14	1	ea1
....uaaacaugaaaucaaagauAa..................	1	1	ea1
....aaagaugaugucuugcuguuuaUa..	1	1	ea1
.....................................ucuuugauuucauguuuaca..	1	0	eg2
.....................................ucuuugauuucauguuuacaug..	1	0	eg2
....uguaaacaugaaaucaaagauA...................	1	1	eg2

```
                                     Star                            Mature
guauggauggguuguuggaaacucaacagaagcaucaucuuugauuucauguuuacaugucuaagacauguaaacaugaaaucaaagaugaugucuugcuguuuaaauc
......................................................................uaaacaugaaaucaaagaug...................    2     0     eg2

.....................................ucuuugauuucauguuuaca....................................................    25    0     ea2
.....................................ucuuugauuucauguuuacau...................................................    5     0     ea2
.....................................ucuuugauuucauguuuacaA...................................................    12    1     ea2
.....................................ucuuugauuucauguuuacauA..................................................    77    1     ea2
.....................................ucuuugauuucauguuuacaug..................................................    36    0     ea2
......................................cuuugauuucauguuuaca....................................................    1     0     ea2
......................................cuuugauuucauguuuacauA..................................................    6     1     ea2
......................................cuuugauuucauguuuacaug..................................................    5     0     ea2
....................................................................uguaaacaugaaaucaaagauA...................    3     1     ea2
....................................................................uguaaacaugaaaucaaagaug...................    7     0     ea2
.....................................................................guaaacaugaaaucaaagaug...................    2     0     ea2
......................................................................uaaacaugaaaucaaagauA...................    7     1     ea2
......................................................................uaaacaugaaaucaaagaug...................    35    0     ea2
.......................................................................aaacaugaaaucaaagau....................    4     0     ea2
.......................................................................aaacaugaaaucaaagaug...................    3     0     ea2
.......................................................................aaacaugaaUucaaagaug...................    1     1     ea2
........................................................................aacaugaaaucaaagaug...................    2     0     ea2
```

Provisional ID	:	ScUbCFx_10_3858
Score total	:	1842.7
Score for star read(s)	:	3.9
Score for read counts	:	1835.5
Score for mfe	:	2.3
Score for randfold	:	1.6
Score for cons. seed	:	-0.6
Total read count	:	3612
Mature read count	:	1611
Loop read count	:	0
Star read count	:	2001

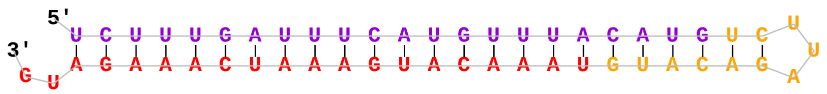

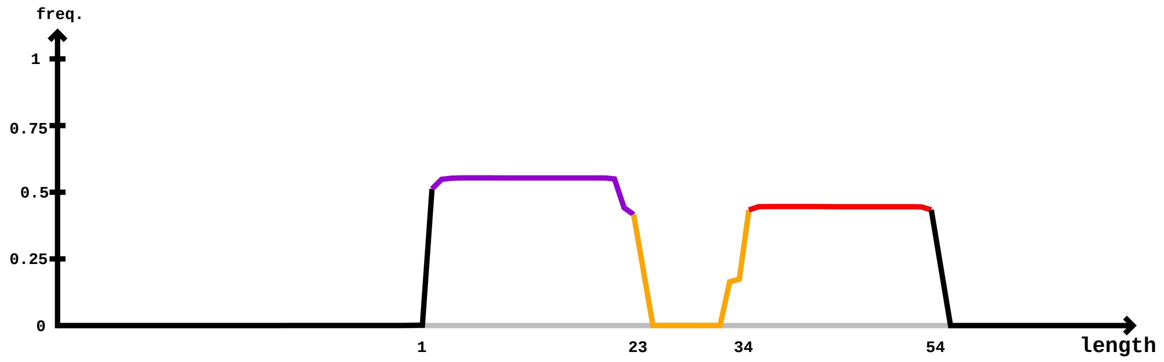

Star **Mature**

```
5'- cacaaauuuugucauagauuuaaacagcaagacaucaucuuugauuucauguuuacaugucuuagacauguaaacaugaaaucaaagaugaugcuucuguugaguuucca  -3'  obs
    cacaaauuuugucauagauuuaaacagcaagacaucaucuuugauuucauguuuacaugucuuagacauguaaacaugaaaucaaagaugaugcuucuguugaguuucca       exp
    ...............(((((((.(((.(((.((((((((((((((((((((((((((((((...)))))))))))))))))))))))))))))))))))))))))))...  reads  mm  sample
    .....................................ucuuugauuucauguuuaca.....................................................  1      0   eg2
    .....................................ucuuugauuucauguuuacaug...................................................  1      0   eg2
    ....................................................................uguaaacaugaaaucaaagauA....................  1      1   eg2
    ......................................................................uaaacaugaaaucaaagaug....................  2      0   eg2

    .....................................ucuuugauuucauguuuaca.....................................................  25     0   ea2
    .....................................ucuuugauuucauguuuacaA....................................................  12     1   ea2
    .....................................ucuuugauuucauguuuacau....................................................  5      0   ea2
    .....................................ucuuugauuucauguuuacauA...................................................  77     1   ea2
    .....................................ucuuugauuucauguuuacaug...................................................  36     0   ea2
    ......................................cuuugauuucauguuuaca.....................................................  1      0   ea2
    ......................................cuuugauuucauguuuacauA...................................................  6      1   ea2
    ......................................cuuugauuucauguuuacaug...................................................  5      0   ea2
    ....................................................................uguaaacaugaaaucaaagauA....................  3      1   ea2
    ....................................................................uguaaacaugaaaucaaagaug....................  7      0   ea2
    .....................................................................guaaacaugaaaucaaagaug....................  2      0   ea2
    ......................................................................uaaacaugaaaucaaagauA....................  7      1   ea2
    ......................................................................uaaacaugaaaucaaagaug....................  35     0   ea2
    .......................................................................aaacaugaaaucaaagau.....................  4      0   ea2
    .......................................................................aaacaugaaaucaaagaug....................  3      0   ea2
    .......................................................................aaacaugaaUucaaagaug....................  1      1   ea2
    ........................................................................aacaugaaaucaaagaug....................  2      0   ea2

    ...................................Aaucuuugauuucauguuuacaug...................................................  2      1   er2
    ....................................aucuuugauuucauguuuacauA...................................................  2      1   er2
    .....................................ucuuugauuucauguuuac......................................................  13     0   er2
    .....................................ucuuugauuucauguuuacU.....................................................  1      1   er2
    .....................................ucuuugaCuucauguuuaca.....................................................  1      1   er2
    .....................................Ncuuugauuucauguuuaca.....................................................  3      1   er2
    .....................................ucuuugauuucauguuuaca.....................................................  337    0   er2
    .....................................ucuuugauuucGuguuuaca.....................................................  5      1   er2
    .....................................ucuuugauuucauguuuacaA....................................................  35     1   er2
    .....................................ucuuugauuucauguuuacau....................................................  33     0   er2
```

Star **Mature**

```
cacaaauuuugucauagauuuaaacagcaagacaucaucuuugauuucauguuuacaugucuuagacauguaaacaugaaaucaaagaugaugcuucuguugaguuucca
.....................................ucuuugauuucauguuuacauU...................................................    33    1    er2
.....................................Ncuuugauuucauguuuacaug...................................................    3     1    er2
.....................................ucuuugauuucauguuuacauA...................................................    526   1    er2
.....................................ucuuugauuucauguuuaUaug...................................................    1     1    er2
.....................................ucuuugauuucGuguuuacaug...................................................    7     1    er2
.....................................ucuuugauuucauguuuacaug...................................................    622   0    er2
.....................................ucuuugauuucauguuuacauC...................................................    2     1    er2
.....................................ucuuugauuucauguuuacaugu..................................................    4     0    er2
.....................................ucuuugauuucauguuuacaugA..................................................    1     1    er2
......................................cuuugauuucauguuuaca.....................................................    14    0    er2
......................................cuuugauuucauguuuacau....................................................    1     0    er2
......................................cuuugauuuAauguuuacaug...................................................    2     1    er2
......................................cuuugauuucauguuuacaug...................................................    57    0    er2
......................................cuuugauuucauguuuacauA...................................................    36    1    er2
.......................................uuugauuucauguuuaca.....................................................    1     0    er2
.......................................uuugauuucauguuuacaug...................................................    11    0    er2
.......................................uuugauuucauguuuacauA...................................................    3     1    er2
........................................uugauuucauguuuacauA...................................................    5     1    er2
....................................................................uguaaacaugaaaucaaagau.....................    4     0    er2
....................................................................uguaaacaugaaaucaaagaug....................    393   0    er2
....................................................................uAuaaacaugaaaucaaagaug....................    1     1    er2
....................................................................uguaaacaugaaaucaaagauA....................    41    1    er2
....................................................................Nguaaacaugaaaucaaagaug....................    3     1    er2
....................................................................uguGaacaugaaaucaaagaug....................    3     1    er2
....................................................................uguaGacaugaaaucaaagaug....................    1     1    er2
.....................................................................guaaacaugaaaucaaagaug....................    29    0    er2
.....................................................................guaaacaugaaaucaaagauA....................    5     1    er2
......................................................................uaaacaugaaaucaaagau.....................    12    0    er2
......................................................................uaaacaugaaaucGaagaug....................    1     1    er2
......................................................................uaaacaugaaaucaaagauC....................    2     1    er2
......................................................................Gaaacaugaaaucaaagaug....................    1     1    er2
......................................................................uaaacaugaaaucaaagauA....................    69    1    er2
......................................................................Naaacaugaaaucaaagaug....................    4     1    er2
......................................................................uaaacaugaaaucaaagaug....................    767   0    er2
......................................................................uaGacaugaaaucaaagaug....................    1     1    er2
......................................................................uaaacaugaGaucaaagaug....................    2     1    er2
......................................................................uaaacaugaaaucaaagauAa...................    1     1    er2
.......................................................................aaacaugaaaucaaagaug....................    26    0    er2
.......................................................................aaacaugaaaucaaagauA....................    8     1    er2
.......................................................................aaacaugaaaucaaagauAa...................    2     1    er2

.....................................ucuuugauuucauguuuaca.....................................................    2     0    ea1
.....................................ucuuugauuucauguuuacau....................................................    4     0    ea1
.....................................ucuuugauuucauguuuacaA....................................................    1     1    ea1
.....................................ucuuugauuucauguuuacauU...................................................    1     1    ea1
.....................................ucuuugauuucauguuuacaug...................................................    16    0    ea1
.....................................Gcuuugauuucauguuuacaug...................................................    3     1    ea1
.....................................ucuuugauuucauguuuacauA...................................................    15    1    ea1
......................................cCuugauuucauguuuacaug...................................................    2     1    ea1
......................................cuuugauuucauguuuacaug...................................................    4     0    ea1
.........................................................ugucuuagacauguaaacaug................................    1     0    ea1
....................................................................uguaaacaugaaaucaaaga......................    3     0    ea1
....................................................................uguaaacaugaaaucaaagaA.....................    1     1    ea1
....................................................................uguaaacaugaaaucaaagau.....................    6     0    ea1
....................................................................uguGaacaugaaaucaaagau.....................    1     1    ea1
....................................................................Gguaaacaugaaaucaaagaug....................    1     1    ea1
....................................................................uguaaacaugaaaucaaagauU....................    1     1    ea1
....................................................................uguaaacaugaaaucaaagauA....................    41    1    ea1
....................................................................uguaaacaugaaaucaaagaug....................    29    0    ea1
....................................................................uguaaacaugaaaucaaagaCg....................    1     1    ea1
....................................................................uguaaacaugaaaucaaagauAa...................    1     1    ea1
......................................................................uaaacaugaaaucaaagau.....................    3     0    ea1
......................................................................Gaaacaugaaaucaaagau.....................    1     1    ea1
......................................................................uaaacaugaaaucaaagauA....................    14    1    ea1
......................................................................uaaacaugaaaucaaagaug....................    6     0    ea1
......................................................................uaaacaugaaaucaaagauAa...................    1     1    ea1

.....................................ucuuugauuucauguuuacau....................................................    1     0    eg1
.....................................ucuuugauuucauguuuacaug...................................................    3     0    eg1
....................................................................uguaaacaugaaaucaaagau.....................    3     0    eg1
```

```
                                     Star                             Mature
cacaaauuuugucauagauuuaaacagcaagacaucaucuuugauuucauguuuacaugucuuagacauguaaacaugaaaucaaagaugaugcuucuguugaguuucca
....................................................................uguaaacaugaaaucaaagauA....................    3    1    eg1
....................................................................uguaaacaugaaaucaaagaug....................    6    0    eg1
......................................................................uaaacaugaaaucaaagauA....................    1    1    eg1

......................aacagcaagacaucaucuuuga..................................................................    1    0    er1
.....................................ucuuugauuucauguuuacau....................................................    2    0    er1
.....................................ucuuugauuucauguuuacauA...................................................    1    1    er1
.....................................Gcuuugauuucauguuuacaug...................................................    1    1    er1
.....................................ucuuugauuucauguuuacaug...................................................    14   0    er1
......................................cuuugauuucauguuuacaug...................................................    1    0    er1
...........................................................Gcuuagacauguaaacauga...............................    1    1    er1
....................................................................uguaaacaugaaaucaaagau.....................    5    0    er1
....................................................................uguaaacaugaaaucaaagaCg....................    1    1    er1
....................................................................uguaaacaCgaaaucaaagaug....................    1    1    er1
....................................................................uguaaacaugaaaucaaagauA....................    5    1    er1
....................................................................uguaaacaugaaaucaaagaug....................    25   0    er1
......................................................................uaaacaugaaaucaaagaug....................    2    0    er1
......................................................................uaaacaugaaaucaaagauA....................    1    1    er1
......................................................................uaaacaugaGaucaaagaug....................    4    1    er1
......................................................................uaaacaugaaaucaaagauCa...................    1    1    er1
```

Provisional ID	:	ScUbCFx_11_4192
Score total	:	1932.9
Score for star read(s)	:	3.9
Score for read counts	:	1925.7
Score for mfe	:	-1.2
Score for randfold	:	1.6
Score for cons. seed	:	3
Total read count	:	3789
Mature read count	:	3770
Loop read count	:	0
Star read count	:	19

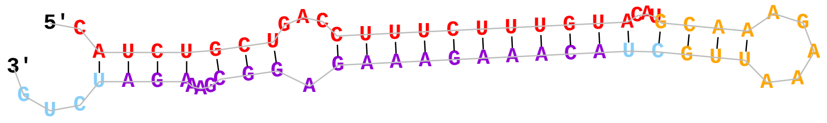

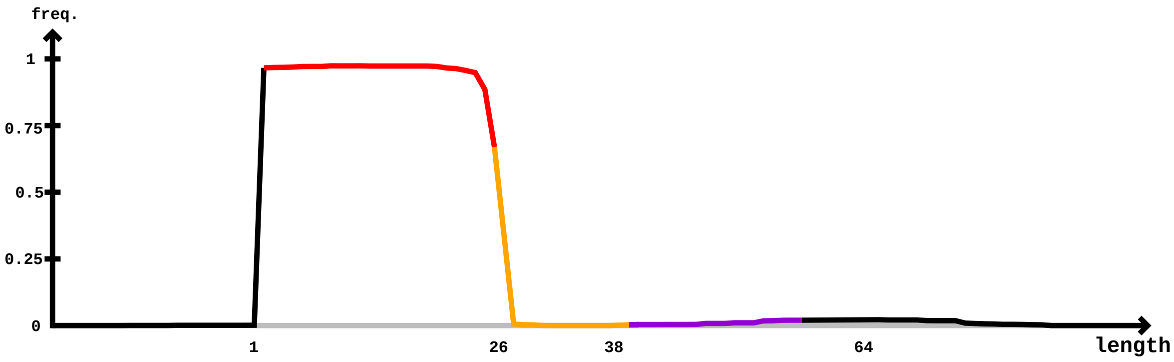


```
                         Mature                                        Star
5'- agacgaaaagucaugucaaacaucugcugaccuuucuuuguacaugcaaagaaauugcuacaaagaaagaggcgaaagaucuguguauugaauaauaauuguguauucaaaa  -3'   obs
    agacgaaaagucaugucaaacaucugcugaccuuucuuuguacaugcaaagaaauugcuacaaagaaagaggcgaaagaucuguguauugaauaauaauuguguauucaaaa        exp
    .(((.....)))....((...(((((((...(((((((((((...((((.....))))))))))))))).)))...))))...))..(((((((.........)))))))..   reads   mm   sample
    ....................caucugcugaccuuucuu..........................................................................   1       0    er1
    ....................caucugcugaccuuucuuu.........................................................................   5       0    er1
    ....................caucugcugaccAuucuuug........................................................................   2       1    er1
    ....................caucugcugaccuuucuuugu.......................................................................   4       0    er1
    ....................caucugcugaccAuucuuugu.......................................................................   1       1    er1
    ....................caCcugcugaccuuucuuugua......................................................................   1       1    er1
    ....................caucugcugaccuuucuuugua......................................................................   2       0    er1
    ....................Gaucugcugaccuuucuuuguac.....................................................................   1       1    er1
    ....................caucugcugaccuuucuuuguac.....................................................................   22      0    er1
    ....................caucugcugaccuuucuuuguaU.....................................................................   1       1    er1
    ....................caucugcugaccGuucuuuguac.....................................................................   1       1    er1
    ....................caucGgcugaccuuucuuuguac.....................................................................   1       1    er1
    ....................caucugcugaccAuucuuuguac.....................................................................   2       1    er1
    ....................caucugcCgaccuuucuuuguaca....................................................................   1       1    er1
    ....................caucugcugaccAuucuuuguaca....................................................................   4       1    er1
    ....................caucugcugaccuuucuuuguacG....................................................................   6       1    er1
    ....................caucugcugaccuuucuuuguacU....................................................................   1       1    er1
    ....................Gaucugcugaccuuucuuuguaca....................................................................   3       1    er1
    ....................caucugcugaccCuucuuuguaca....................................................................   1       1    er1
    ....................caucugcugaccuuucuuugCaca....................................................................   2       1    er1
    ....................caucugcuAaccuuucuuuguaca....................................................................   1       1    er1
    ....................caucugcugaccuuucuuuguaca....................................................................   110     0    er1
    ....................caucugcugaccuuucuCuguaca....................................................................   1       1    er1
    ....................caucugcugaccCuucuuuguacau...................................................................   1       1    er1
    ....................caucugcugaccuuucuuuguacau...................................................................   53      0    er1
    ....................caucugcugaccAuucuuuguacau...................................................................   3       1    er1
    ....................cGucugcugaccuuucuuuguacau...................................................................   1       1    er1
    ....................caucugcugaccuuucuuuguacaC...................................................................   449     1    er1
    ....................caucugUugaccuuucuuuguacau...................................................................   1       1    er1
    ....................caucugcugaccuuucuuuguacaA...................................................................   6       1    er1
    ....................caucugcugaccuuucuuuguacaugc.................................................................   1       0    er1
    ......................ucugcugaccuuucuuuguaUaugcaa...............................................................   1       1    er1
    ........................cugcugaccuuucuuuguacau..................................................................   1       0    er1
```

Mature **Star**

```
agacgaaaagucaugucaaacaucugcugaccuuucuuuguacaugcaaagaaauugcuacaaagaaagaggcgaaagaucuguguauugaauaauaauuguguauucaaaa
.............................................................................gaucuguguauugaauaauU...............  1  1  er1
.............................................................................gaucuguguauugaauaauaauugC..........  1  1  er1

....................caucugcugaccuuucuuu.........................................................................  2  0  eg1
....................cGucugcugaccuuucuuu.........................................................................  1  1  eg1
....................caucugcugaccuuucuuC.........................................................................  1  1  eg1
....................caucugcugaccuuucuuug........................................................................  2  0  eg1
....................caucugcugaccuuucuuugu.......................................................................  6  0  eg1
....................caucugcugaccuuucuuugG.......................................................................  3  1  eg1
....................Gaucugcugaccuuucuuugua......................................................................  1  1  eg1
....................caucugcugaccuuucuuugua......................................................................  9  0  eg1
....................caucugcugaccuuucuuuguG......................................................................  1  1  eg1
....................caucugcugaccuuucuuuguaU.....................................................................  10  1  eg1
....................Gaucugcugaccuuucuuuguac.....................................................................  2  1  eg1
....................caucugcugaccAuucuuuguac.....................................................................  3  1  eg1
....................caucugcugaccuuucuuuguac.....................................................................  41  0  eg1
....................caucugcugaccuuucuuuguaA.....................................................................  1  1  eg1
....................Gaucugcugaccuuucuuuguaca....................................................................  1  1  eg1
....................caucCgcugaccuuucuuuguaca....................................................................  1  1  eg1
....................cUucugcugaccuuucuuuguaca....................................................................  1  1  eg1
....................caucugcugaccuuucuuuguaca....................................................................  192  0  eg1
....................caucugcugaccuuucuuCguaca....................................................................  1  1  eg1
....................caucugcugaccuuucuuuguacU....................................................................  2  1  eg1
....................caucugcugaccAuucuuuguaca....................................................................  12  1  eg1
....................caucugcuAaccuuucuuuguaca....................................................................  1  1  eg1
....................caucugcugaccuuucuuGguaca....................................................................  1  1  eg1
....................caucugcugaccuuucuuuguacG....................................................................  12  1  eg1
....................caucugcugaccuuucuuuguacau...................................................................  56  0  eg1
....................Gaucugcugaccuuucuuuguacau...................................................................  1  1  eg1
....................caucugcugaccuuucuuuguaUau...................................................................  1  1  eg1
....................caucugcugaccuuucuuuguacaA...................................................................  11  1  eg1
....................caucugcugaccuuucuuuguacaC...................................................................  554  1  eg1
....................caucugcugaccAuucuuuguacau...................................................................  3  1  eg1
....................caucugcugaccuuucuuuguacaCg..................................................................  2  1  eg1
....................caucugcugaccuuucuuuguacaugc.................................................................  2  0  eg1
.....................aucugcugaccuuucuuuguacaC...................................................................  1  1  eg1
.....................aucugcugaccuuucuuuguacaCg..................................................................  1  1  eg1
.......................cugcugaccuuucuuuguacaC...................................................................  1  1  eg1
.........................................................cuacaaagaaagaggcgaaagaucug.............................  2  0  eg1
.........................................................cuacaaagaaagaggcgUaagaucugu............................  1  1  eg1
..................................................................aagaggcgaaagaucuAuguauu.......................  3  1  eg1
..................................................................aagaggcgaaagaucuAuguauugaau...................  1  1  eg1
.....................................................................aggcgUaagaucuguguauugaau...................  3  1  eg1
.........................................................................gaaagaucuAuguauugaauaau................  1  1  eg1
.........................................................................gaaagaucuguguauugaauaauU...............  1  1  eg1
.............................................................................gaucuguguauugaauaau................  1  0  eg1
.............................................................................gaucuguguauugaauaauaauu............  2  0  eg1
.............................................................................gaucCguguauugaauaauaauu............  1  1  eg1
.............................................................................gaucuguguauugaauaauaauug...........  1  0  eg1
.............................................................................gaucuguguauugaauaauaauugu..........  4  0  eg1

........................ugcugaccAuucuuuguaca....................................................................  5  1  ea2
.........................gcugaccAuucuuuguaca....................................................................  1  1  ea2
...........................ugaccuuucuuuguacaugca................................................................  1  0  ea2
...........................ugaccuuucuuuguacaugcaa...............................................................  5  0  ea2
..........................................................uacaaagaaagaggcgaaag..................................  1  0  ea2
...........................................................acaaagaaagaggcgaaaga.................................  5  0  ea2
..................................................................aagaggcgaaagaucugug...........................  3  0  ea2
........................................................................cgaaagaucuguguauugaau...................  1  0  ea2
........................................................................cgaaagaucuAuguauugaau...................  1  1  ea2
........................................................................cgaaagaucuUuguauugaau...................  14  1  ea2
........................................................................cgaaagaucuUuguauugaaua..................  1  1  ea2
..........................................................................aaagaucuUuguauugaaua..................  1  1  ea2
..........................................................................aaagaucuguguauugaaua..................  2  0  ea2

......aaagucaugucaaacaucugcA....................................................................................  1  1  eg2
........................ugcugaccAuucuuuguaca....................................................................  1  1  eg2
...........................ugaccuuucuuuguacaugcaa...............................................................  1  0  eg2
...........................ugaccuuucuuuguacaugcaaa..............................................................  1  0  eg2
........................................................................cgaaagaucuUuguauugaau...................  4  1  eg2
```

Mature / Star

agacgaaaagucaugucaaacaucugcugaccuuucuuuguacaugcaaagaaauugcuacaaagaaagaggcgaaagaucuguguauugaauaauaauuguguauucaaaa			
....cgaaagaucuguguauugaau...................	1	0	eg2
....aaagaucuguguauugaaua..................	2	0	eg2
...........caugucaaacaucugcugaA...	3	1	ea1
...................Ccaucugcugaccuuucuuuguacau...	1	1	ea1
....................caucugcugaccuuucuu..	5	0	ea1
....................caucugcugaccuuucuuu...	7	0	ea1
....................caucugcugaccAuucuuu...	2	1	ea1
....................caucugcugaccuuucuuC...	1	1	ea1
....................caucCgcugaccuuucuuu...	3	1	ea1
....................caucugcugaccuuucuuug..	5	0	ea1
....................caucugcugaccuuucuuugC...	1	1	ea1
....................caucugcugaccuuucuuugu...	12	0	ea1
....................caucugcugaccuuucAuugua..	1	1	ea1
....................caucugcugaccuuucuuugua..	16	0	ea1
....................caucCgcugaccuuucuuugua..	1	1	ea1
....................caucugcuAaccuuucuuuguac...	1	1	ea1
....................caucugcugaccuuucuuuguac...	118	0	ea1
....................caucugcugaccuuucuuuguaA...	5	1	ea1
....................caucugcugaccuuucuuuguaU...	17	1	ea1
....................Gaucugcugaccuuucuuuguac...	2	1	ea1
....................caucugcugaccuuucuCuguac...	1	1	ea1
....................caucugAugaccuuucuuuguac...	1	1	ea1
....................caucugcugaccuuucuuCguac...	1	1	ea1
....................caucugcugaccAuucuuuguac...	11	1	ea1
....................caucugcugaccuuucuuugCaca..	3	1	ea1
....................caucugcugaccuuucuCuguaca..	1	1	ea1
....................caucugcCgaccuuucuuuguaca..	4	1	ea1
....................caucugcuAaccuuucuuuguaca..	1	1	ea1
....................cauGugcugaccuuucuuuguaca..	1	1	ea1
....................caucugcugaccAuucuuuguaca..	27	1	ea1
....................cauUugcugaccuuucuuuguaca..	1	1	ea1
....................caucugcugaccuuucuuuguacG..	5	1	ea1
....................caucugcugaccCuucuuuguaca..	4	1	ea1
....................caucugcugaccuuucuuuguaUa..	3	1	ea1
....................caucugcugaccuuucuuuguGca..	1	1	ea1
....................caucugcugaccuuucAuuguaca..	1	1	ea1
....................caucugcugaccuuucuuuguaca..	408	0	ea1
....................caucugcugGccuuucuuuguaca..	1	1	ea1
....................caucugcugaccuuucuuuguaGa..	1	1	ea1
....................caucugcugaccuuucuuuguacU..	1	1	ea1
....................caucugcugaccuuucuuuguUca..	2	1	ea1
....................caCcugcugaccuuucuuuguaca..	2	1	ea1
....................Gaucugcugaccuuucuuuguaca..	4	1	ea1
....................cGucugcugaccuuucuuuguaca..	1	1	ea1
....................caucugcuAaccuuucuuuguacau...	1	1	ea1
....................caucugcugaccuuCcuuuguacau...	1	1	ea1
....................Uaucugcugaccuuucuuuguacau...	1	1	ea1
....................caucugcugaccuuucuuuguacaG...	3	1	ea1
....................caucugcugaccuuucuuuguacaC...	1207	1	ea1
....................caCcugcugaccuuucuuuguacau...	1	1	ea1
....................caucugcugaccuuucuuuguaGau...	1	1	ea1
....................caucugcugaccAuucuuuguacau...	7	1	ea1
....................caucugcugaccuCucuuuguacau...	2	1	ea1
....................caucugcugaccuuucuuuguacaA...	34	1	ea1
....................caucugcugGccuuucuuuguacau...	2	1	ea1
....................caucAgcugaccuuucuuuguacau...	1	1	ea1
....................caucugcugaccuuucuuuguGcau...	1	1	ea1
....................Gaucugcugaccuuucuuuguacau...	2	1	ea1
....................caucugcugaccuuucuCuguacau...	1	1	ea1
....................caucugcugaccuuucuuuguacau...	152	0	ea1
....................caucugcugaccuuucuuuguacaCg..	2	1	ea1
....................caucugcugaccuuucuuuguacauA..	1	1	ea1
....................caucugcugaccuuucuuuguaUaugc...	1	1	ea1
....................caucugcugaccuCucuuuguacaugc...	1	1	ea1
....................caucugcugaccuuucuuuguacaugU...	1	1	ea1
....................caucugcugaccuuucuuuguacaugc...	7	0	ea1
.....................aucugcugaccuuucuuuguaca..	1	0	ea1
.....................aucugcugaccuuucuuuguacaC...	2	1	ea1
......................ucugcugaccAuucuuuguac...	1	1	ea1

Mature Star

```
agacgaaaagucaugucaaacaucugcugaccuuucuuuguacaugcaaagaaauugcuacaaagaaagaggcgaaagaucuguguauugaauaauaauuguguauucaaaa
.......................cugcugaccuuucuuuguaca....................................................................    2    0    ea1
........................ugcugaccuuucuuuguacG....................................................................    1    1    ea1
.........................................................cuacaaagaaagaggcgaaagauc...............................    1    0    ea1
.........................................................cuacaaagaaagaggcgUaagaucug.............................    2    1    ea1
..............................................................aagaaagaggcgaaagaucugu............................    1    0    ea1
.................................................................aaagaggcgaaagaucuAuguauu.......................    1    1    ea1
..................................................................aagaggcgUaagaucuguguauu.......................    5    1    ea1
..................................................................aagaggcgUaagaucuguguauug......................    1    1    ea1
..................................................................aagaggcgUaagaucuguguauugaau...................    1    1    ea1
.....................................................................aggcgaaagaucuAuguauugaau...................    4    1    ea1
.....................................................................aggcgaaagaucuAuguauugaauaa.................    2    1    ea1
..........................................................................aaagaucuguguauugaauaa.................    1    0    ea1
...........................................................................aagaucuguguauugaauaauaau.............    1    0    ea1
.............................................................................gaucuguguauugaauaauU...............    3    1    ea1
.............................................................................gaucuguguauugaauaauaau.............    1    0    ea1
.............................................................................gaucuguguauugaauaauaauu............    1    0    ea1
.............................................................................gaucuguguauugaauaauaauug...........    1    0    ea1
.............................................................................gaucuguguauugaauaauaauugu..........    4    0    ea1
.............................................................................gaucuguguauugaauaauaauuAu..........    1    1    ea1

........................ugcugaccAuucuuuguaca....................................................................    1    1    er2
...........................ugaccuuucuuuguacaugcaa...............................................................    1    0    er2
..............................ccuuucuuuguacaugcaa...............................................................    1    0    er2
..........................................................uacaaagaaagaggcgaaaA.................................    3    1    er2
........................................................................cgaaagaucuguguauugaau...................    2    0    er2
........................................................................cgaaagaucuAuguauugaau...................    1    1    er2
........................................................................cgaaagaucuUuguauugaau...................    1    1    er2
........................................................................cgaaagaucuAuguauugaauaa.................    2    1    er2
..........................................................................aaagaucuAuguauugaau...................    1    1    er2
```

Provisional ID	:	ScUbCFx_45_5324
Score total	:	705.6
Score for star read(s)	:	3.9
Score for read counts	:	694.5
Score for mfe	:	2.6
Score for randfold	:	1.6
Score for cons. seed	:	3
Total read count	:	1374
Mature read count	:	557
Loop read count	:	0
Star read count	:	817

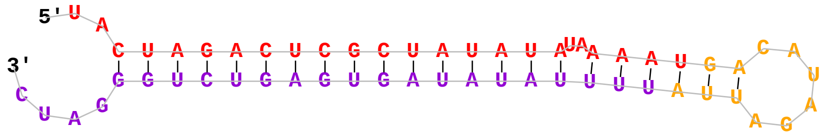

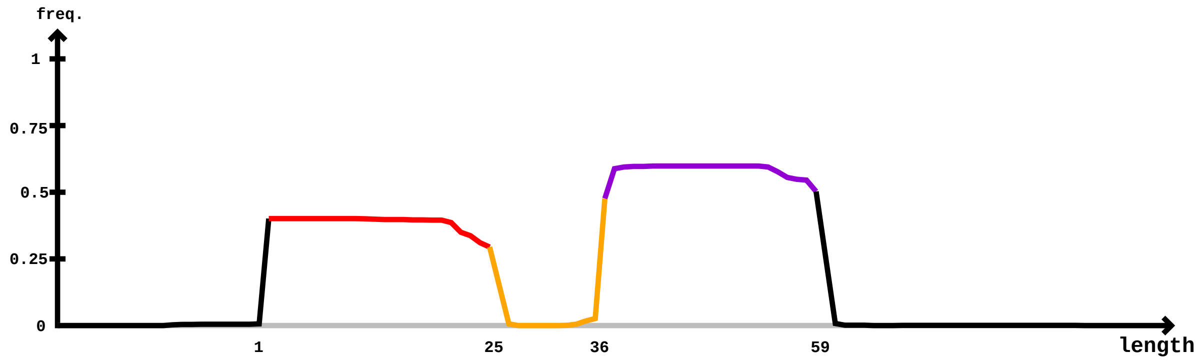

Mature **Star**

```
5'- agccauugggcuacccugggguacuagacucgcuauauauaaaaugacauagauuauuuuauauagugagucugggauccaggguaccauugggcuagcaggggcuauagccccu   -3'   obs
    agccauugggcuacccugggguacuagacucgcuauauauaaaaugacauagauuauuuuauauagugagucugggauccaggguaccauugggcuagcaggggcuauagccccu         exp
    ((((....((.((((((((((.((((((((((((((((..((((((......)))))))))))))))))))))).))))))))))))....))))...((((((....))))))   reads   mm   sample
    ...........uacccuggguacuagacucC...................................................................................   2       1    ea2
    .....................................................uauuuuauauagugagucugg........................................   1       0    ea2

    ..........cuacccuggguacuagacuc....................................................................................   1       0    ea1
    ..........cuacccuggguacCagacucgc..................................................................................   2       1    ea1
    ..........cuacccuggguacCagacucgcuau...............................................................................   1       1    ea1
    .............cccuggguacCagacucgcuau...............................................................................   1       1    ea1
    ...................guacCagacucgcuauau.............................................................................   1       1    ea1
    ...................guacuagacucgcuGuauaua..........................................................................   1       1    ea1
    ....................uacCagacucgcuauauau...........................................................................   1       1    ea1
    ....................uacuagacucgcuauauau...........................................................................   7       0    ea1
    ....................uacuagacucgcuauauaA...........................................................................   2       1    ea1
    ....................uacuagacucgcuauauaC...........................................................................   2       1    ea1
    ....................uacCagacucgcuauauaua..........................................................................   1       1    ea1
    ....................Gacuagacucgcuauauaua..........................................................................   2       1    ea1
    ....................uacuagacucgcuauauaua..........................................................................   36      0    ea1
    ....................uacuagacucgcuauauaAa..........................................................................   1       1    ea1
    ....................uacuagacucgcuauauauC..........................................................................   1       1    ea1
    ....................uacuagacucgcuauauauaU.........................................................................   1       1    ea1
    ....................Gacuagacucgcuauauauaa.........................................................................   1       1    ea1
    ....................uacuagacucgcuauauauaa.........................................................................   12      0    ea1
    ....................uacuagacucgcuauauauUa.........................................................................   1       1    ea1
    ....................uacuagacucgcuauauauaaa........................................................................   17      0    ea1
    ....................Gacuagacucgcuauauauaaa........................................................................   1       1    ea1
    ....................uacuagacucgcuauauauaaU........................................................................   8       1    ea1
    ....................uacuagacucgcuauauauaaG........................................................................   1       1    ea1
    ....................uacuagacucgcuauauauaaC........................................................................   1       1    ea1
    ....................uacCagacucgcuauauauaaa........................................................................   1       1    ea1
    ....................Gacuagacucgcuauauauaaaa.......................................................................   1       1    ea1
    ....................uacuagacucgcuauauauaaaa.......................................................................   3       0    ea1
    ....................uacuagacucgcuauauauGaaa.......................................................................   1       1    ea1
    ....................uacuagacucgcuauauauaaaU.......................................................................   10      1    ea1
    ....................uacuagacucgcuauauauaaUa.......................................................................   1       1    ea1
```

Mature / Star			
agccauugggcuacccuggguacuagacucgcuauauauaaaaugacauagauuauuuuauauagugagucugggauccaggguaccauugggcuagcaggggcuauagccccu			
uacuagacucgcuauauauaGaau	1	1	ea1
uacuagacucgcuauauauaaaaA	1	1	ea1
uacuagacAcgcuauauauaaaau	1	1	ea1
uacuagacucgcuauauauaaGau	1	1	ea1
uacuagacucgcuauauauaaaaC	18	1	ea1
uacuagacucgcuauauauaaaau	160	0	ea1
uacuagacucgcuauGuauaaaau	1	1	ea1
uacuagacucgcuauauauaaaUu	2	1	ea1
Gacuagacucgcuauauauaaaau	14	1	ea1
uacuagacucgcuauauauaaaCu	1	1	ea1
uacCagacucgcuauauauaaaau	1	1	ea1
uacuagacucgcuauauauaaaGu	2	1	ea1
uacuagacucgcuauauauaaaaug	14	0	ea1
uacuagacucgcuauauauaaaauC	1	1	ea1
uacuagacAcgcuauauauaaaaug	1	1	ea1
uacuagacucgcuauauauaaaaCg	2	1	ea1
Gacuagacucgcuauauauaaaaug	2	1	ea1
uacuagacucgcuauauauaaaauU	26	1	ea1
uacuGgacucgcuauauauaaaaug	1	1	ea1
uacuagacucgcuauauauaaaauA	26	1	ea1
uacuagacucgcuauauauaaaauUa	4	1	ea1
uacuagacucgcuauauauaaaaugU	1	1	ea1
uacuagacucgcuauauauaaaauAa	3	1	ea1
auuauuuuauauagugagucugU	1	1	ea1
uuauuuuauauagugagucug	2	0	ea1
uuauuuuauauagugagucugg	2	0	ea1
Guauuuuauauagugagucugg	1	1	ea1
uauuuuauauaCugagucu	1	1	ea1
uauuuuauauagugagucug	1	0	ea1
uauuuuauauaCugagucugg	2	1	ea1
uauuuuauauagugagucugg	1	0	ea1
uUuuuuauauagugagucugg	1	1	ea1
uauuuuauauagugagucugA	1	1	ea1
uauuuuauauagugagucugU	2	1	ea1
auuuuauauaCugagucu	1	1	ea1
auuuuauauagugagucu	2	0	ea1
auuuuauauagugagucug	2	0	ea1
auuuuauauagugagAcugg	1	1	ea1
auuuuauauagugagucugg	4	0	ea1
Cuuuuauauagugagucugggau	1	1	ea1
uuuuauauagugagucug	13	0	ea1
Guuuauauagugagucug	3	1	ea1
uuuuauauagugagucuA	1	1	ea1
uuuuauauagugagucugg	2	0	ea1
Guuuauauagugagucuggg	1	1	ea1
uuuuauauagugagucuggg	5	0	ea1
uuuuauauagugagucuggA	1	1	ea1
uuuuauauagugagucuggga	3	0	ea1
uuuuauauagugagucugggau	26	0	ea1
uuuuauaCagugagucugggau	1	1	ea1
uuuuauauagugagucuggUau	1	1	ea1
uuuuauauagugagucugggaA	4	1	ea1
uuuuauauagugagucugggaC	2	1	ea1
Guuuauauagugagucugggau	3	1	ea1
uuuuauauagugagucugggaG	1	1	ea1
Guuuauauagugagucugggauc	10	1	ea1
uuuuauauagugagucugggauc	116	0	ea1
uuuuaCauagugagucugggauc	1	1	ea1
uuuuauauagugagucCgggauc	1	1	ea1
uuuuauauagugagucugggauG	2	1	ea1
uuuuGuauagugagucugggauc	1	1	ea1
uuuuauauagugagucugggauA	78	1	ea1
uuuuauUuagugagucugggauc	1	1	ea1
uuuuauauagugGgucugggauc	2	1	ea1
uuuuauauagugagucugggUuc	2	1	ea1
uuuuauauagugagucugggauU	44	1	ea1
uuuuauauagugagucugggGuc	1	1	ea1
uuuuauauagugagucugggauUc	1	1	ea1
uuuuauauagugagucugggaucU	35	1	ea1
uuuuauauagugagucugggaucA	130	1	ea1

```
                    Mature                            Star
agccauugggcuacccugggauacuagacucgcuauauauaaaaugacauagauuauuuuauauagugagucugggauccagguaccauugggcuagcaggggcuauagccccu
.......................................................uuuuauauagugagucugggaucc..................................  5   0   ea1
.......................................................uuuuauauagugagucugggauAc..................................  1   1   ea1
.......................................................uuuuauauagugagucugggaucG..................................  4   1   ea1
.......................................................uuuuauauagugagucugggaucUa.................................  3   1   ea1
.......................................................uuuuauauagugagucugggaucAa.................................  2   1   ea1
........................................................uuuauauagugagucugg.......................................  1   0   ea1
........................................................uuuauauagugagucuggg......................................  1   0   ea1
........................................................uuuauauagugagucuggga.....................................  1   0   ea1
........................................................uuuauauagugagucuggUau....................................  2   1   ea1
........................................................Guuauauagugagucugggau....................................  2   1   ea1
........................................................uuuauauagugagucugggauA...................................  8   1   ea1
........................................................uuuauauagugagucugggUuc...................................  2   1   ea1
........................................................Guuauauagugagucugggauc...................................  3   1   ea1
........................................................uuuauauagugagucugggauU...................................  7   1   ea1
........................................................uuuauauagugagucugggauc...................................  26  0   ea1
........................................................uuuauauagugagucugggaucG..................................  2   1   ea1
........................................................Guuauauagugagucugggaucc..................................  1   1   ea1
........................................................uuuauauagugagucugggaucc..................................  4   0   ea1
........................................................uuuauauagugagucugggaucA..................................  52  1   ea1
........................................................uuuauauagugagucugggaucU..................................  15  1   ea1
........................................................uuuauauagugagucugggaucAa.................................  3   1   ea1
........................................................uuuauauagugagucugggaucUa.................................  1   1   ea1
.........................................................uuauauagugagucugggauA...................................  1   1   ea1
.........................................................uuauauagugagucuggUaucc..................................  2   1   ea1
.........................................................uuauauagugagucugggaucA..................................  3   1   ea1
..........................................................uauauagugagucuggUaucc..................................  2   1   ea1
..........................................................uauauagugagucugggaucA..................................  1   1   ea1
............................................................uauagugagucugggaCccaggg..............................  1   1   ea1
............................................................uauagugagucuggUauccaggg..............................  1   1   ea1
......................................................................................cauugggcuagcaggggcu........  1   0   ea1

.....................................................uauuuuauauagugagucugU.......................................  3   1   er2

....................uacuagacucgcuauauau..........................................................................  1   0   eg1
....................Gacuagacucgcuauauaua.........................................................................  1   1   eg1
....................uacuagacucgcuauauaua.........................................................................  4   0   eg1
....................Gacuagacucgcuauauauaa........................................................................  1   1   eg1
....................uacuagacucgcuauauauaa........................................................................  2   0   eg1
....................uacuagacucgcuauauauaaa.......................................................................  6   0   eg1
....................Gacuagacucgcuauauauaaaa......................................................................  1   1   eg1
....................uacuagacucgcuauauauaaaa......................................................................  1   0   eg1
....................uacuagacucgcuauauauaaaU......................................................................  1   1   eg1
....................uacCagacucgcuauauauaaaau.....................................................................  1   1   eg1
....................Gacuagacucgcuauauauaaaau.....................................................................  1   1   eg1
....................uacuagacucgcuauauauaaaGu.....................................................................  1   1   eg1
....................uacuagacucgcuauauauaaaau.....................................................................  52  0   eg1
....................uacuagacucgcuauauauaaaaC.....................................................................  4   1   eg1
....................uacuagacucgcuauauauaaaauA....................................................................  4   1   eg1
....................uacuagacucgcuauauauaaaaug....................................................................  12  0   eg1
....................uacuagacucgcuauauauaaaauU....................................................................  12  1   eg1
....................................................uuauuuuauauagugagucugg.......................................  1   0   eg1
.....................................................uauuuuauauagugagucugU.......................................  1   1   eg1
.....................................................uauuuuauauagugagucugg.......................................  3   0   eg1
......................................................Uuuuuauauagugagucu.........................................  1   1   eg1
......................................................auuuuauauagugagucugg.......................................  1   0   eg1
.......................................................Guuuauauagugagucug........................................  1   1   eg1
.......................................................uuuuauauagugagucug........................................  2   0   eg1
.......................................................uuuuauauagugagucugg.......................................  1   0   eg1
.......................................................uuuuauauagugagucuggg......................................  2   0   eg1
.......................................................uuuuauauagugGgucugggau....................................  1   1   eg1
.......................................................Guuuauauagugagucugggau....................................  1   1   eg1
.......................................................uuuuauauagugagucugggau....................................  9   0   eg1
.......................................................uuuuauauagugagucugggaA....................................  1   1   eg1
.......................................................uuuuauauagugagucugggauA...................................  4   1   eg1
.......................................................uuuuauauagugagucugggUuc...................................  1   1   eg1
.......................................................uuuuauauagugagucugggauU...................................  1   1   eg1
.......................................................uuuuauauagugagucugggauG...................................  1   1   eg1
.......................................................Guuuauauagugagucugggauc...................................  3   1   eg1
.......................................................uuuuauauagugagucugggauc...................................  15  0   eg1
.......................................................uuuuauauagugagucugggaucU..................................  10  1   eg1
```

```
                    Mature                            Star
agccauugggcuacccuggg
uacuagacucgcuauauauaaaaugacauagauuauuuuauauagugagucugggauccaggguaccauugggcuagcaggggcuauagccccu
```

```
agccauugggcuacccugggUACUAGACUCGCUAUAUAUAAAAUgacauagauuaUUUUAUAUAGUGAGUCUGGGAUCcaggguaccauugggcuagcaggggcuauagccccu
.......................................................uuuuauauagugagucugggaucA...................................  27  1  eg1
.......................................................uuuuauauagugagucugggaucc...................................  1   0  eg1
.......................................................uuuuauauagugagucugggaucG...................................  3   1  eg1
........................................................uuuauauagugagucugggauc....................................  3   0  eg1
........................................................uuuauauagugagucugggaucc...................................  2   0  eg1
........................................................uuuauauagugagucugggaucG...................................  1   1  eg1
........................................................uuuauauagugagucugggaucA...................................  7   1  eg1
........................................................Guuauauagugagucugggaucc...................................  1   1  eg1
.........................................................uuauauagugagucugggaucA...................................  2   1  eg1

....................uacuagacucgcuauauaua..........................................................................  3   0  er1
....................uacCagacucgcuauauaua..........................................................................  1   1  er1
....................uacuagacucgcuauauauaaa........................................................................  2   0  er1
....................uacuagacucgcuauauauaaaa.......................................................................  4   0  er1
....................uacuagacucgcuauauauaaaaG......................................................................  1   1  er1
....................uacuagacucgcuauauauaaaau......................................................................  20  0  er1
....................uacuagacucgcuauauauaaaaC......................................................................  2   1  er1
....................Gacuagacucgcuauauauaaaau......................................................................  2   1  er1
....................uacuagacucgcuauaAauaaaaug.....................................................................  2   1  er1
....................uacuagacucgcuauauauaaaauA.....................................................................  3   1  er1
....................uacuagacucgcuauauauaaaauU.....................................................................  6   1  er1
....................Gacuagacucgcuauauauaaaaug.....................................................................  1   1  er1
....................uacuagacucgcuauauauaaaaug.....................................................................  5   0  er1
....................uacuagacucgcuauauauaaaauAa....................................................................  1   1  er1
.......................................................uuuuauauagugagucugggau......................................  4   0  er1
.......................................................Guuuauauagugagucugggau......................................  1   1  er1
.......................................................Guuuauauagugagucugggauc.....................................  4   1  er1
.......................................................uuuuauauagugagucugggauU.....................................  1   1  er1
.......................................................uuuuauaCagugagucugggauc....................................  1   1  er1
.......................................................uuuuauauagugagucugggauc....................................  15  0  er1
.......................................................uuuuauauagugagucugggauA....................................  2   1  er1
.......................................................uuuuauauagugagucugggaucU...................................  3   1  er1
.......................................................uuuuauauagugagucugggaucc...................................  1   0  er1
.......................................................uuuuauauagugagucugggaucA...................................  10  1  er1
........................................................uuuauauagugagucugggauc....................................  3   0  er1
........................................................uuuauauagugagucugggaucU...................................  6   1  er1
........................................................uuuauauagugagucugggaucA...................................  3   1  er1
.........................................................uuauauagugagucugggaucA...................................  1   1  er1
```

Provisional ID : ScUbCFx_45_5919
Score total : 5.5
Score for star read(s) : -1.3
Score for read counts : 0
Score for mfe : 2.2
Score for randfold : 1.6
Score for cons. seed : 3
Total read count : 12473
Mature read count : 11963
Loop read count : 26
Star read count : 484

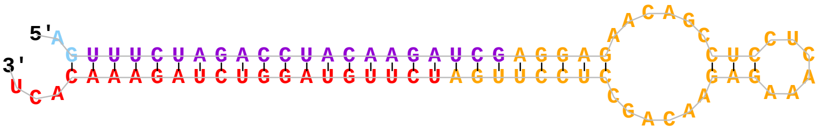

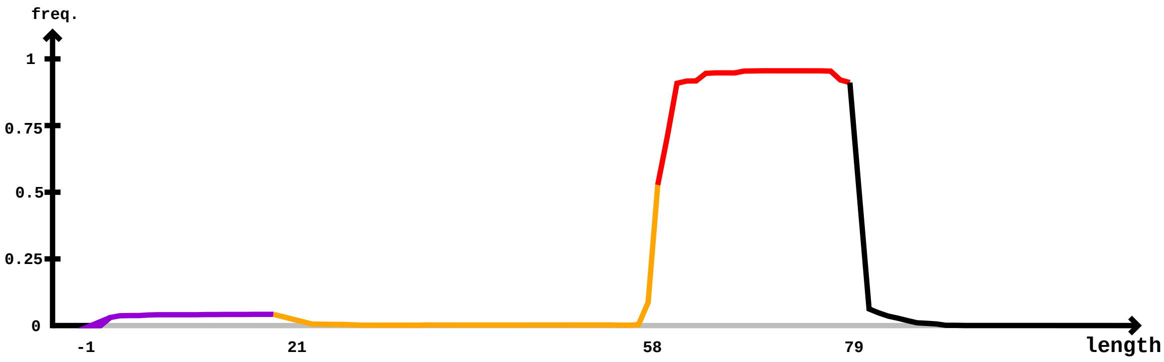

Star **Mature**

```
5'- acaguuucuagaccuacaagaucgaggagaacagccuccucaaagagaacagccuccuugaucuuguaggucuagaaacacuguugaaacaagugugauucugccaucggu  -3'  obs
    acaguuucuagaccuacaagaucgaggagaacagccuccucaaagagaacagccuccuugaucuuguaggucuagaaacacuguugaaacaagugugauucugccaucggu       exp
    ...((((((((((((((((((((((((((......(((......)))......))))))))))))))))))))))))))...((.(((.((....)).))).)).......  reads  mm  sample
    ....uuucuagaccuacaagaucg.......................................................................................  2  0  eg2
    ......ucuagaccuacaagaucgagg....................................................................................  2  0  eg2
    ..............uacaagaucgaggagaacagcA...........................................................................  2  1  eg2
    ..............uacaagaucgaggagaacagccuA.........................................................................  1  1  eg2
    ..............uacaagaucgaggagaacagccuc.........................................................................  2  0  eg2
    ................caagaucgaggagaacagccuA.........................................................................  2  1  eg2
    ...........................................................gaucuuguaggucuagaaa.................................  1  0  eg2
    ...........................................................gCucuuguaggucuagaaac................................  1  1  eg2
    ...........................................................Aaucuuguaggucuagaaaca...............................  4  1  eg2
    ............................................................aucuuguaggucuagaaaca...............................  16  0  eg2
    ............................................................aucuuguaggucuagaaacacA.............................  12  1  eg2
    ............................................................aucuuguaggucuagaaacacu.............................  8  0  eg2
    ............................................................aucCuguaggucuagaaacacu.............................  5  1  eg2
    ............................................................aucuuguaggucGagaaacacu.............................  2  1  eg2
    ............................................................aucuuguaggucuagaaacacuA............................  1  1  eg2
    .............................................................ucuuguaggucuagaaaca...............................  5  0  eg2
    .............................................................ucuuguaggucuagaaacaA..............................  1  1  eg2
    .............................................................ucuuguagguUuagaaacacu.............................  2  1  eg2
    .............................................................ucuuguagAucuagaaacacu.............................  9  1  eg2
    .............................................................ucuuguaggucuagGaacacu.............................  1  1  eg2
    .............................................................ucuuguaggucuagaaacGcu.............................  1  1  eg2
    .............................................................ucuuguaggucuagaaGcacu.............................  3  1  eg2
    .............................................................ucuuguaggucGagaaacacu.............................  1  1  eg2
    .............................................................ucuuguaggucuagaaacaUu.............................  1  1  eg2
    .............................................................Ncuuguaggucuagaaacacu.............................  1  1  eg2
    .............................................................Gcuuguaggucuagaaacacu.............................  2  1  eg2
    .............................................................ucuuguaggucuagaaacacC.............................  3  1  eg2
    .............................................................ucuuguaggucuagaaacacu.............................  170  0  eg2
    .............................................................ucuuguaggucuagaaacacA.............................  82  1  eg2
    .............................................................ucCuguaggucuagaaacacu.............................  1  1  eg2
    .............................................................ucuuguaggucuagaaacacug............................  2  0  eg2
    .............................................................ucuuguaggucGagaaacacug............................  1  1  eg2
    .............................................................ucuuguaggucuagaaacacuA............................  5  1  eg2
```

Star **Mature**

```
acaguuucuagaccuacaagaucgaggagaacagccuccucaaagagaacagccuccuugaucuuguaggucuagaaacacuguugaaacaagugugauucugccaucggu
.............................................................ucuuguaggucuagaaacacuU............................  5    1  eg2
.............................................................ucuuguaggucuagaaacacuUu...........................  1    1  eg2
.............................................................ucuuguaggucuagaaacacugu...........................  1    0  eg2
.............................................................ucuuguaggucuagaaacacuAu...........................  1    1  eg2
.............................................................ucuuguaggucuagaaacacuUuu..........................  1    1  eg2
..............................................................cuuguaggucuagaaaca...............................  1    0  eg2
..............................................................cuuguaggucuagaaacacu.............................  24   0  eg2
..............................................................cuuguaggucuagaaacacA.............................  1    1  eg2
..............................................................Uuuguaggucuagaaacacu.............................  1    1  eg2
..............................................................cuuguaggucuagaaGcacu.............................  1    1  eg2
..............................................................cuuguaggucuagaaacaUu.............................  1    1  eg2
..............................................................cuuguaggucGagaaacacu.............................  3    1  eg2
..............................................................cuuguaggucGagaaacacug............................  1    1  eg2
..............................................................cuuguaggucuagaaacacuA............................  3    1  eg2
..............................................................cuuguaggucuagaaacacug............................  18   0  eg2
...............................................................uuguaggucuagaaacacu.............................  15   0  eg2
...............................................................uuguaggucuagaaGcacu.............................  1    1  eg2
...............................................................uuguaggucuagaaacacC.............................  1    1  eg2
...............................................................uuguaggucuagaaacGcug............................  1    1  eg2
...............................................................uuguaggucGagaaacacug............................  2    1  eg2
...............................................................uuguaggucuagaaacacuA............................  2    1  eg2
...............................................................uuguaggucuagaaacacug............................  22   0  eg2
...............................................................uuguaggucuagaaacacugu...........................  7    0  eg2
...............................................................uuguaggAcuagaaacacugu...........................  1    1  eg2
...............................................................uuguaggucuagaaacacuUu...........................  3    1  eg2
...............................................................uuguaggucGagaaacacugu...........................  6    1  eg2
...............................................................uuguaggucuagaaacacuUuu..........................  1    1  eg2
...............................................................uuguaggucuagaaacacuguuC.........................  1    1  eg2
...............................................................uuguaggucuagaaacacuguuUa........................  3    1  eg2
................................................................uguaggucuagaaacacugu...........................  1    0  eg2
................................................................uguaggucuagaaacacuguuUa........................  2    1  eg2
.................................................................guaggucuagaaacacuguuCa........................  1    1  eg2
.................................................................guaggucuagaaacacuguuUa........................  1    1  eg2
..................................................................uaggucuagaaacacuguu..........................  1    0  eg2
..................................................................uaggucuagaaacacuguuU.........................  4    1  eg2
..................................................................uaggucuagaaacGcuguug.........................  1    1  eg2
..................................................................uaggucuagaaGcacuguug.........................  1    1  eg2
..................................................................uaggucuagaaacacuguug.........................  2    0  eg2
..................................................................uaggucuGgaaacacuguug.........................  1    1  eg2
..................................................................uaggucuagaaacacuguuCa........................  2    1  eg2
..................................................................uaggucuagaaacacuguugG........................  2    1  eg2
..................................................................uaggucuagaaacacuguuga........................  4    0  eg2
..................................................................uaggucuagaaacacuguuUa........................  19   1  eg2
..................................................................uaggucuGgaaacacuguugaa.......................  3    1  eg2
..................................................................uaggucuagaaacacuguuUaa.......................  3    1  eg2
..................................................................uaggucuagaaacacuguugaa.......................  6    0  eg2
..................................................................uaggucuagaaacacuguugaaa......................  2    0  eg2
..................................................................uaggucuagaaacacuguugaaU......................  2    1  eg2
......................................................................ucuagaaacacuguugaaac.....................  2    0  eg2
......................................................................ucuagaaacacuguugaaaca....................  3    0  eg2
......................................................................ucuagaaacacuguugaaacU....................  2    1  eg2
.......................................................................cuagaaacacuguugaaaca....................  1    0  eg2

...guuucuCgaccuacaagaucga.......................................................................................  2    1  ea2
....uuucuagaccuacaagaucU.......................................................................................  1    1  ea2
....uuucuCgaccuacaagaucg.......................................................................................  300  1  ea2
....uuucuagaccuacaagaucg.......................................................................................  7    0  ea2
....uuucuagaccuacaagaucgaA.....................................................................................  5    1  ea2
.....uucuCgaccuacaagaucg.......................................................................................  59   1  ea2
.....uucuGgaccuacaagaucg.......................................................................................  3    1  ea2
.....uucuagaccuacaagaucga......................................................................................  1    0  ea2
.....uucuagaccuacaagUucga......................................................................................  5    1  ea2
......ucuCgaccuacaagaucg.......................................................................................  3    1  ea2
......ucuagaccuacaagaucg.......................................................................................  1    0  ea2
........uagaccuacaagUucgaggag..................................................................................  4    1  ea2
........uagaccuacaagaucgaggaA..................................................................................  3    1  ea2
........uagaccuacaagaucgaggaUa.................................................................................  9    1  ea2
........uagaccuacaagaucgaggaCa.................................................................................  1    1  ea2
.........Cgaccuacaagaucgaggag..................................................................................  3    1  ea2
.............uacaagaucgaggagaacagccu...........................................................................  3    0  ea2
```

Star Mature

```
acaguuucuagaccuacaagaucgaggagaacagccuccucaaagagaacagccuccuugaucuuguaggucuagaaacacuguugaaacaagugugauucugccaucggu
................caagaucgaggagaacagccu..........................................................................	1	0	ea2
...................gaucgaggagaacagccuc.........................................................................	5	0	ea2
.....................................ccucaaagagaacagccucc......................................................	1	0	ea2
.....................................ccucaGagagaacagccuccu.....................................................	2	1	ea2
.....................................ccucaaagagaacagccuccuA....................................................	1	1	ea2
......................................cucaaagagaacagccucc......................................................	3	0	ea2
................................................acagccuccuugaucuuguaA..........................................	2	1	ea2
....................................................ccuccuugaucuuguaggucA......................................	2	1	ea2
...........................................................gaucuuguaggucuagaa..................................	1	0	ea2
...........................................................gaucuuguaggucuagaaac................................	1	0	ea2
...........................................................gaucuuguaggucuagaaaca...............................	3	0	ea2
...........................................................gaucuuguaggucuagaaCca...............................	3	1	ea2
............................................................aucuuguaggucuagaaac................................	1	0	ea2
............................................................aucuuguaggucuagaaaA................................	2	1	ea2
............................................................aucuuguaggucGagaaaca...............................	1	1	ea2
............................................................aucuuguaggucuagaaCca...............................	1	1	ea2
............................................................aucuuguaggucuagaaaca...............................	177	0	ea2
............................................................Nucuuguaggucuagaaaca...............................	3	1	ea2
............................................................aucuuAuaggucuagaaaca...............................	2	1	ea2
............................................................aucuuguaggucuagaaacC...............................	2	1	ea2
............................................................aucuuguaggucuaAaaaca...............................	3	1	ea2
............................................................aucuuguaggucuagaaacaA..............................	5	1	ea2
............................................................aucuuguaggucuagaaacaU..............................	2	1	ea2
............................................................aucuuguaggucCagaaacac..............................	1	1	ea2
............................................................aucuuguaggucuagaaacac..............................	2	0	ea2
............................................................aucuuguaggucuagaaacacA.............................	173	1	ea2
............................................................aucuuguaggucGagaaacacu.............................	13	1	ea2
............................................................Cucuuguaggucuagaaacacu.............................	2	1	ea2
............................................................aucuuguaggucuagaaacaAu.............................	3	1	ea2
............................................................aucuuguaggucuagaaacacu.............................	109	0	ea2
............................................................aNcuuguaggucuagaaacacu.............................	1	1	ea2
............................................................aucuuguaggucuagaaacacC.............................	3	1	ea2
............................................................aucuuguaggucuagaaacacuU............................	3	1	ea2
............................................................aucuuguaggucuagaaacacuA............................	1	1	ea2
............................................................aucuuguaggucuagaaacacuUu...........................	3	1	ea2
.............................................................ucuuguaggucuagaaaca...............................	37	0	ea2
.............................................................ucuuguaggucGagaaacac..............................	6	1	ea2
.............................................................ucuugGaggucuagaaacac..............................	1	1	ea2
.............................................................ucuuguaggucuagaaacaG..............................	6	1	ea2
.............................................................ucuuguaggucuagaaacac..............................	28	0	ea2
.............................................................ucuuguaggucuagaaacaA..............................	1	1	ea2
.............................................................ucuuguaggucuagGaacacu.............................	12	1	ea2
.............................................................ucuuguaggucuagaaGcacu.............................	16	1	ea2
.............................................................uNuuguaggucuagaaacacu.............................	1	1	ea2
.............................................................ucuuAuaggucuagaaacacu.............................	7	1	ea2
.............................................................ucuuguaggucuagaaacacG.............................	25	1	ea2
.............................................................ucuuguagAucuagaaacacu.............................	2	1	ea2
.............................................................ucuuguaggucuagaaacGcu.............................	11	1	ea2
.............................................................ucuuguaggucuagaaacaAu.............................	3	1	ea2
.............................................................ucuuguaggucGagaaacacu.............................	165	1	ea2
.............................................................Gcuuguaggucuagaaacacu.............................	1	1	ea2
.............................................................ucuuguaggucuagaaacaUu.............................	2	1	ea2
.............................................................ucuuguaggucuUgaaacacu.............................	4	1	ea2
.............................................................ucuuUuaggucuagaaacacu.............................	1	1	ea2
.............................................................ucuuguaggucuagaaacacC.............................	5	1	ea2
.............................................................Ccuuguaggucuagaaacacu.............................	1	1	ea2
.............................................................ucuGguaggucuagaaacacu.............................	1	1	ea2
.............................................................Ncuuguaggucuagaaacacu.............................	7	1	ea2
.............................................................ucCuguaggucuagaaacacu.............................	3	1	ea2
.............................................................Acuuguaggucuagaaacacu.............................	1	1	ea2
.............................................................ucuuguaggucuagaaacacA.............................	1179	1	ea2
.............................................................ucGuguaggucuagaaacacu.............................	2	1	ea2
.............................................................ucuuguaggucCagaaacacu.............................	1	1	ea2
.............................................................ucuuguaggucuagaaacacu.............................	1552	0	ea2
.............................................................ucuuguaggucuagaaacacuA............................	54	1	ea2
.............................................................ucuuguaggucuagaaacacuU............................	13	1	ea2
.............................................................ucuuguaggucGagaaacacug............................	10	1	ea2
.............................................................ucuuguaggucuagaaacacuUu...........................	1	1	ea2
..............................................................cuuguaggucuagaaaca...............................	1	0	ea2
..............................................................cuuguaggucuagaaacac..............................	4	0	ea2
```

Star **Mature**

acaguuucuagaccuacaagaucgaggagaacagccuccucaaagagaacagccuccuugaucuuguaggucuagaaacacuguugaaacaagugugauucugccaucggu			
....cuuguaggucuagGaacac..............................	4	1	ea2
....cuuguaggucuagaaacaA..............................	2	1	ea2
....cuuguaggucGagaaacac..............................	3	1	ea2
....Nuuguaggucuagaaacacu.............................	1	1	ea2
....cuuguaggucuagaaacaUu.............................	2	1	ea2
....cuuguaggucGagaaacacu.............................	270	1	ea2
....cuuguaggucuagaaacacC.............................	1	1	ea2
....cuuguaggCcuagaaacacu.............................	1	1	ea2
....cuuguaggucuagaaacGcu.............................	4	1	ea2
....cuuguaggucuagaaaUacu.............................	1	1	ea2
....cuugGaggucuagaaacacu.............................	1	1	ea2
....cuuguaggucuagaaacacu.............................	873	0	ea2
....cuAguaggucuagaaacacu.............................	1	1	ea2
....cuuAuaggucuagaaacacu.............................	4	1	ea2
....cuuguaggucuagGaacacu.............................	3	1	ea2
....cuuguaggucuagaaacaAu.............................	1	1	ea2
....cuuguaggucuagaaacacA.............................	74	1	ea2
....cuuguaggucuagaaGcacu.............................	9	1	ea2
....cuuguaggucuagaaacacug............................	98	0	ea2
....cuuguaggucuagaaacacuU............................	13	1	ea2
....cuuguaggucuagaaGcacug............................	1	1	ea2
....cuuguaggucuagaaacacuA............................	41	1	ea2
....cuuguaggucGagaaacacug............................	66	1	ea2
....cuuguaggucuagaaacacuUu...........................	3	1	ea2
....cuuguaggucuagaaaUacugu...........................	1	1	ea2
....uuguaggucuagaaacac..............................	1	0	ea2
....uuAuaggucuagaaacacu.............................	1	1	ea2
....uuguaUgucuagaaacacu.............................	1	1	ea2
....uuguaggucuagGaacacu.............................	2	1	ea2
....uuguaggucuagaaacGcu.............................	10	1	ea2
....uuguaggucuagaaGcacu.............................	16	1	ea2
....Nuguaggucuagaaacacu.............................	2	1	ea2
....uuguaggCcuagaaacacu.............................	2	1	ea2
....uuguaggucuagaaacacu.............................	1060	0	ea2
....uuguaggAcuagaaacacu.............................	1	1	ea2
....uuguaggucGagaaacacu.............................	224	1	ea2
....uAguaggucuagaaacacu.............................	3	1	ea2
....uuguaggGcuagaaacacu.............................	1	1	ea2
....uuguaggucuagUaacacu.............................	1	1	ea2
....uuguaggucuagaaacacA.............................	19	1	ea2
....uuguaggucGagaaacacug............................	230	1	ea2
....uuguaggucuagaaacacGg............................	1	1	ea2
....uuguaggucuagaaacacuU............................	2	1	ea2
....uuguaggucuagaaacacuA............................	16	1	ea2
....uuguaggucuagGaacacug............................	3	1	ea2
....uuguaggucuagaaacacug............................	54	0	ea2
....uuguaCgucuagaaacacug............................	1	1	ea2
....uuguaggucGagaaacacugu...........................	17	1	ea2
....uuguaggucuagaaacacuUu...........................	1	1	ea2
....uuguaggucuagaaGcacugu...........................	3	1	ea2
....uuguaggucuagaaacacugu...........................	14	0	ea2
....uuAuaggucuagaaacacugu...........................	3	1	ea2
....uuguaggucGagaaacacuguu..........................	2	1	ea2
....uuguaggucuagaaacacuguu..........................	3	0	ea2
....uuguaggucuagaaacacuguuU.........................	1	1	ea2
....uuguaggucuagaaacacuguuUa........................	4	1	ea2
....uguaggucGagaaacacu.............................	2	1	ea2
....uguaggucuagaaacacu.............................	4	0	ea2
....uguaggucuagaaacacugu...........................	3	0	ea2
....uguaggucuagaaacGcugu...........................	1	1	ea2
....uguaggucuagaaacacuguu..........................	4	0	ea2
....uguagAucuagaaacacuguu..........................	6	1	ea2
....uguaggucuagaaacacuguuUa........................	2	1	ea2
....uaggucuagGaacacuguu..........................	3	1	ea2
....uaggucuagaaacacuguu..........................	32	0	ea2
....uaggucGagaaacacuguu..........................	3	1	ea2
....uGggucuagaaacacuguug.........................	3	1	ea2
....uaggucuagaaacacuguuU.........................	12	1	ea2
....uaggucuagaaacGcuguug.........................	7	1	ea2
....uaggucuagaaacacuguug.........................	10	0	ea2

Star **Mature**

```
acaguuucuagaccuacaagaucgaggagaacagccuccucaaagagaacagccuccuugaucuuguaggucuagaaacacuguugaaacaagugugauucugccaucggu
..................................................................uaggucuagaaGcacuguug.........................	6	1	ea2
..................................................................uaggucuagaaacacugAug.........................	1	1	ea2
..................................................................uaggucuagaaacacuguugG........................	13	1	ea2
..................................................................uaggucuagaaacacuguuCa........................	5	1	ea2
..................................................................uaggucuagaaacacuguuga........................	1	0	ea2
..................................................................uaggucuagaaacacuguuUa........................	11	1	ea2
..................................................................uaggucuagaaacacuguugU........................	3	1	ea2
..................................................................uaggucuagaaacacuguugUa.......................	5	1	ea2
..................................................................Naggucuagaaacacuguugaa.......................	1	1	ea2
..................................................................uaggucuagaaacacuguugaU.......................	1	1	ea2
..................................................................uaggucuagaaGcacuguugaa.......................	2	1	ea2
..................................................................uaggucuagaaacacuguuUaa.......................	8	1	ea2
..................................................................uaggucuGgaaacacuguugaa.......................	1	1	ea2
..................................................................uaggucuagaaacacuguugaa.......................	22	0	ea2
..................................................................uaggucuagaaacacuguugaUa......................	1	1	ea2
..................................................................uaggucuagaaacacuguugaaa......................	2	0	ea2
...................................................................aggucGagaaacacuguu..........................	1	1	ea2
...................................................................aggucuagaaacacuguu..........................	10	0	ea2
...................................................................aggucuagaaacGcuguugaa.......................	3	1	ea2
......................................................................ucuagaaacacuguugaaac.....................	20	0	ea2
......................................................................ucGagaaacacuguugaaaca....................	6	1	ea2
......................................................................ucuGgaaacacuguugaaaca....................	1	1	ea2
......................................................................ucuagaaacacuguugaaaca....................	19	0	ea2

....uuucuCgaccuacaagaucg.......................................................................................	49	1	er2
....uuucuagaccuacaagaucg.......................................................................................	1	0	er2
....uuucuagaccuacaagaucga......................................................................................	1	0	er2
.....uucuCgaccuacaagaucg.......................................................................................	10	1	er2
.....uucuagaccuacaagaucgaA.....................................................................................	1	1	er2
......ucuagaccuacaagaucgagg....................................................................................	1	0	er2
........uagaccuacaagaucgaggaUa.................................................................................	2	1	er2
........uagaccuacaagaucgaggagG.................................................................................	1	1	er2
............ccuacaagaucgaggagaaca..............................................................................	1	0	er2
................caagaucgaggagaacagcc...........................................................................	2	0	er2
.....................................ccucaaagagaacagccucc......................................................	1	0	er2
.....................................ccucaaagagaacagccuccuuA...................................................	7	1	er2
...........................................................gaucuuguaggucuagaaCca...............................	1	1	er2
...........................................................Aaucuuguaggucuagaaaca...............................	3	1	er2
...........................................................gaucuuguaggucuagaaacacA.............................	5	1	er2
............................................................aucuuguaggucGagaaaca...............................	1	1	er2
............................................................aucuuguaggucuagaaaca...............................	64	0	er2
............................................................aucuuguaggCcuagaaaca...............................	3	1	er2
............................................................aucuuguaggucuaAaaaca...............................	11	1	er2
............................................................aucuuguaggucuagaaacac..............................	5	0	er2
............................................................aucuuguaggucuaAaaacac..............................	1	1	er2
............................................................aucuuguaggucuagaaacaU..............................	3	1	er2
............................................................aucuuguaggucuagaaacaA..............................	7	1	er2
............................................................aucuuguaggucuagaaacacC.............................	5	1	er2
............................................................aucuuguaggucuagaaacacA.............................	101	1	er2
............................................................aucuuguaggucuagaaacacu.............................	79	0	er2
............................................................aucuuguaggucGagaaacacu.............................	11	1	er2
............................................................aucuuguaggucuagaaacGcu.............................	1	1	er2
............................................................aucuuguaggucuaAaaacacu.............................	16	1	er2
............................................................aucuuguaggucuagaaacacuU............................	4	1	er2
............................................................aucuuguaggucuagaaacacuA............................	6	1	er2
.............................................................ucuuguaggucuagaaac................................	3	0	er2
.............................................................ucuuguaggucuagaaaca...............................	28	0	er2
.............................................................ucuuguaggucuagaaacac..............................	17	0	er2
.............................................................ucuuguaggucGagaaacacu.............................	81	1	er2
.............................................................ucuuguaggucuagaaGcacu.............................	9	1	er2
.............................................................Ccuuguaggucuagaaacacu.............................	3	1	er2
.............................................................ucuuguaggucuagaaacacG.............................	2	1	er2
.............................................................ucuuguaggucuagaaacacu.............................	805	0	er2
.............................................................ucuuguaggucuUgaaacacu.............................	2	1	er2
.............................................................ucuuguaggucuagaaacGcu.............................	1	1	er2
.............................................................ucuuAuaggucuagaaacacu.............................	2	1	er2
.............................................................ucuuguagAucuagaaacacu.............................	4	1	er2
.............................................................ucuuguaggucuagGaacacu.............................	7	1	er2
.............................................................ucuuguaggucuagaaacacA.............................	517	1	er2
.............................................................ucuuguaggucuagaaacaAu.............................	5	1	er2
```

Star **Mature**

acaguuucuagaccuacaagaucgaggagaacagccuccucaaagagaacagccuccuugaucuuguaggucuagaaacacuguugaaacaagugugauucugccaucggu

....ucuuguaggucuagaaacacC.............................	6	1	er2
....ucuugGaggucuagaaacacu.............................	1	1	er2
....ucuuguaggucuagaaacacuU............................	4	1	er2
....ucuuguaggucuagaaacacug............................	5	0	er2
....ucuuguaggucGagaaacacug............................	3	1	er2
....ucuuguaggucuagaaacacuA............................	38	1	er2
....ucuuguaggucuagaaacacuUu...........................	4	1	er2
....cuuguaggucuagaaacac..............................	1	0	er2
....cuuguaggucuagaaacacA.............................	15	1	er2
....cCuguaggucuagaaacacu.............................	1	1	er2
....Nuuguaggucuagaaacacu.............................	1	1	er2
....cuuguaggucGagaaacacu.............................	118	1	er2
....cuuguaggucuUgaaacacu.............................	2	1	er2
....cuuguaggucuagaaacacC.............................	5	1	er2
....cuuguaggucuagaaacacu.............................	300	0	er2
....cuuguaggucuagaaGcacu.............................	1	1	er2
....cuuUuaggucuagaaacacu.............................	1	1	er2
....cuuguaggucuagaaacacuA............................	20	1	er2
....cuuguaggucGagaaacacug............................	46	1	er2
....cuuguaggucuagGaacacug............................	3	1	er2
....cuuguaggucuagaaacacug............................	36	0	er2
....cuuguaggucuagaaacacuU............................	12	1	er2
....cuuguaggucuagaaGcacug............................	1	1	er2
....cuuguaggucuagaaacacuUu...........................	2	1	er2
....uuguaggucuagaaacaA..............................	1	1	er2
....uuguaggucuagaaGcacu.............................	3	1	er2
....uuguaggucuagaaacacu.............................	309	0	er2
....uuguaggucuagaaacacA.............................	1	1	er2
....uuguaggucuagaaacacC.............................	1	1	er2
....uuguaggucGagaaacacu.............................	66	1	er2
....uuguaggucGagaaacacug............................	85	1	er2
....uuguaggucuagaaacacug............................	40	0	er2
....uuguaggucuagaaacacuA............................	2	1	er2
....uuguaggucuagaaacacugu...........................	13	0	er2
....uuguaggucuagGaacacugu...........................	1	1	er2
....uuguaggucGagaaacacugu...........................	10	1	er2
....uuguaggucGagaaacacuguu..........................	9	1	er2
....uuguaggucuagaaacacuguU..........................	1	1	er2
....uuguaggucuagaaacacuguUa.........................	6	1	er2
....uguaggucuagGaacacu.............................	2	1	er2
....uguaggucGagaaacacu.............................	3	1	er2
....uguaggucGagaaacacug............................	1	1	er2
....uguaggucuagaaacacugu...........................	4	0	er2
....uguaggucGagaaacacugu...........................	2	1	er2
....uguaggucuagaaacacuguu..........................	4	0	er2
....uguaggucuagaaacacuguuU.........................	3	1	er2
....uguaggucuagaaacacuguuUaa.......................	1	1	er2
....guaggucuagaaacacuguu..........................	1	0	er2
....uaggucGagaaacacuguu..........................	3	1	er2
....uaggucuagaaacacuguu..........................	12	0	er2
....uCggucuagaaacacuguu..........................	1	1	er2
....uaggucGagaaacacuguug.........................	1	1	er2
....uaggucuagaaacacuguug.........................	6	0	er2
....uaggucuagaaacacuguuU.........................	9	1	er2
....uaggucuagaaacacuguuCa........................	3	1	er2
....uaggucuagaaacGcuguuga........................	1	1	er2
....uaggucuagaaacacuguuga........................	2	0	er2
....uaggucuagaaacacuguuUa........................	6	1	er2
....uaggucuagaaacacuguugG........................	4	1	er2
....uaggucuagaaacacuguuUaa.......................	25	1	er2
....uaggucuagaaacacuguugaa.......................	16	0	er2
....uaggucuagaaacacuguugCa.......................	3	1	er2
....uGggucuagaaacacuguugaa.......................	1	1	er2
....uaggucuagaaacacuguugUa.......................	2	1	er2
....uaggucuagaaacacuguugaUa......................	4	1	er2
....uaggucuagaaacacuguugaaa......................	13	0	er2
....aggucuagaaacacuguuU.........................	1	1	er2
....aggucuagaaacacuguuUa........................	2	1	er2
....aggucuagaaacacuguuCa........................	1	1	er2
....aggucuagaaacacuguuUaa.......................	1	1	er2

```
    Star                                                    Mature
acaguuucuagaccuacaagaucgaggagaacagccuccucaaagagaacagccuccuugaucuuguaggucuagaaacacuguugaaacaagugugauucugccaucggu
...................................................................aggucuagaaacacuguugaa.......................  2   0   er2
......................................................................ucuagaaacacuguugaaac.....................  4   0   er2
......................................................................ucGagaaacacuguugaaac.....................  2   1   er2
......................................................................ucGagaaacacuguugaaaca....................  2   1   er2
......................................................................ucuagaaacacuguugGaaca....................  3   1   er2
......................................................................ucuagaaacacuguugaaaca....................  18  0   er2
......................................................................ucuagaaacacuguGgaaaca....................  1   1   er2
.......................................................................cuagaaacacuguugaaaca....................  5   0   er2
........................................................................uGgaaacacuguugaaacaag..................  3   1   er2

....Guucuagaccuacaagauc........................................................................................  2   1   ea1
....uuucuagaccuacaagaucg.......................................................................................  6   0   ea1
....uuucuagaccuacaagaucga......................................................................................  2   0   ea1
....uuucuGgaccuacaagaucga......................................................................................  1   1   ea1
....uuucuagaccuacaagaucgag.....................................................................................  1   0   ea1
....uuucuagaccuacaagauUgag.....................................................................................  1   1   ea1
.....uucuGgaccuacaagaucg.......................................................................................  1   1   ea1
.....uucuagaccuacaagaucga......................................................................................  1   0   ea1
.....uucuagaccuacGagaucga......................................................................................  1   1   ea1
.....uucuagaccuacaagaucgaU.....................................................................................  1   1   ea1
......ucuGgaccuacaagaucga......................................................................................  2   1   ea1
........Gagaccuacaagaucgagga...................................................................................  1   1   ea1
........uagaccuacaagaucgaggaA..................................................................................  1   1   ea1
........uagaccuacaagaucgaggaCa.................................................................................  1   1   ea1
........uagaccuacaagaucgaggaUaa................................................................................  1   1   ea1
.........agaccuacaagaucgagg....................................................................................  2   0   ea1
.........agaccuacaagaucgaggag..................................................................................  1   0   ea1
.........agaccuacaagaucgaggaA..................................................................................  1   1   ea1
.........agaccuacaagaucgaggaCa.................................................................................  2   1   ea1
.....................................ccucaaagagaacagccucc......................................................  1   0   ea1
.....................................ccucaaagGgaacagccucc......................................................  2   1   ea1
.....................................ccucaaagagaacagccuccu.....................................................  2   0   ea1
......................................cucaaagagaacagccuccuu....................................................  2   0   ea1
......................................cucaaagagaacagccuccuuU...................................................  1   1   ea1
.......................................ucaaagagaacagccuccuu....................................................  1   0   ea1
...........................................................gaucuuguaggucuagaaaca...............................  4   0   ea1
...........................................................Aaucuuguaggucuagaaaca...............................  2   1   ea1
...........................................................Aaucuuguaggucuagaaacac..............................  3   1   ea1
............................................................aucuuguaggucuagaaac................................  2   0   ea1
............................................................aucuuguaggucuagaaaca...............................  28  0   ea1
............................................................aucuuguaggucuagaaacC...............................  2   1   ea1
............................................................aucuuguaggucuagaaaUa...............................  1   1   ea1
............................................................aucuuguaggucuagaaacG...............................  2   1   ea1
............................................................aucuuguaggucuagaaacaA..............................  1   1   ea1
............................................................aucuuguaggucuagaaacac..............................  7   0   ea1
............................................................aucuuguaggucuagaaacacu.............................  29  0   ea1
............................................................aucuuguaggucuagaaacaUu.............................  1   1   ea1
............................................................aucuuguaggucuagaaacacC.............................  9   1   ea1
............................................................aucuuguaggucuagaaacacG.............................  1   1   ea1
............................................................aucuuguaggucuagaaacacA.............................  59  1   ea1
............................................................aucuuguaggucuagaaacacuU............................  2   1   ea1
............................................................aucuuguaggucuagaaacacuA............................  4   1   ea1
............................................................aucuuguaggucuagaaacacuUu...........................  1   1   ea1
.............................................................ucuuguaggucuagaaac................................  1   0   ea1
.............................................................ucuuguaggucuagaaaca...............................  4   0   ea1
.............................................................ucuuguaggucuagaaacG...............................  1   1   ea1
.............................................................Ccuuguaggucuagaaacac..............................  1   1   ea1
.............................................................ucuuguaggucGagaaacac..............................  1   1   ea1
.............................................................ucuuguaggucuagaaacac..............................  6   0   ea1
.............................................................ucuugCaggucuagaaacacu.............................  1   1   ea1
.............................................................uUuuguaggucuagaaacacu.............................  2   1   ea1
.............................................................Gcuuguaggucuagaaacacu.............................  16  1   ea1
.............................................................ucuuguaggucuagGaacacu.............................  1   1   ea1
.............................................................ucuuguaggucCagaaacacu.............................  1   1   ea1
.............................................................Ccuuguaggucuagaaacacu.............................  1   1   ea1
.............................................................ucCuguaggucuagaaacacu.............................  2   1   ea1
.............................................................ucuuguaggucuagaaacacC.............................  27  1   ea1
.............................................................ucuuAuaggucuagaaacacu.............................  2   1   ea1
.............................................................ucuuguaggucuagaaGcacu.............................  14  1   ea1
.............................................................ucuuguaggucuUgaaacacu.............................  1   1   ea1
```

```
 Star                                                     Mature
acaguuucuagaccuacaagaucgaggagaacagccuccucaaagagaacagccuccuugaucuuguaggucuagaaacacuguugaaacaagugugauucugccaucggu
.............................................................ucuuguaggucuagaaacacu.............................    200    0    ea1
.............................................................ucuuguaggCcuagaaacacu.............................    2      1    ea1
.............................................................ucuuguaggucuagaaacacA.............................    160    1    ea1
.............................................................ucuuguaggucGagaaacacu.............................    1      1    ea1
.............................................................ucuuguaggucuagaaacGcu.............................    2      1    ea1
.............................................................ucuuguaggucuagaaacacG.............................    8      1    ea1
.............................................................ucuuguaggucuagGaacacug............................    1      1    ea1
.............................................................ucuuguaggucuagaaacacug............................    2      0    ea1
.............................................................ucuuguaggucuagaaacacuU............................    5      1    ea1
.............................................................ucuuguaggucuagaaacacuA............................    10     1    ea1
.............................................................ucuuguaggucuagaaacacuC............................    1      1    ea1
.............................................................ucuuguaggucuagaaacacuUu...........................    1      1    ea1
.............................................................ucuuguaggucuagaaacacuUuu..........................    1      1    ea1
..............................................................cuuguaggucuagaaacac..............................    2      0    ea1
..............................................................cuuguaggucuagaaacacu.............................    70     0    ea1
..............................................................cuuguaggucuagaaacGcu.............................    1      1    ea1
..............................................................cuuguaggucuagaaacacG.............................    1      1    ea1
..............................................................cuuguaggCcuagaaacacu.............................    1      1    ea1
..............................................................cuuguaggucuagaaacacA.............................    6      1    ea1
..............................................................cuuguaggucuagaaacacC.............................    8      1    ea1
..............................................................cuuguaggucuaAaaacacu.............................    2      1    ea1
..............................................................cuuguaggucuagaaacacCg............................    1      1    ea1
..............................................................cuuguaggucuagaaacacug............................    48     0    ea1
..............................................................cuuguaggucuagaaacacuA............................    6      1    ea1
..............................................................cuuguaggucuagaaacGcug............................    2      1    ea1
..............................................................cuuguaggucuagaaGcacug............................    2      1    ea1
..............................................................cuuguaggucuaAaaacacug............................    3      1    ea1
..............................................................Guuguaggucuagaaacacug............................    2      1    ea1
..............................................................cuuguaggucuagaaacacuU............................    1      1    ea1
..............................................................cuuguaggucuagaaacacuC............................    1      1    ea1
..............................................................cuuguaggucuagaaacacuUu...........................    3      1    ea1
..............................................................cuuguaggucuagaaacacugC...........................    1      1    ea1
..............................................................cuuguaggucuagaaacacuguuC.........................    1      1    ea1
..............................................................cuuguaggucuagaaacacuguug.........................    2      0    ea1
..............................................................cuuguaggucuagaaacacuguuU.........................    1      1    ea1
...............................................................uuguaggucuagaaacacA.............................    2      1    ea1
...............................................................uuguaggucuaAaaacacu.............................    2      1    ea1
...............................................................uuguaggucuagaaacacu.............................    63     0    ea1
...............................................................uuguaggucuagaaGcacu.............................    1      1    ea1
...............................................................Guguaggucuagaaacacu.............................    6      1    ea1
...............................................................uuguaggucuagaaacacC.............................    6      1    ea1
...............................................................uuguaggucuagaaacGcug............................    1      1    ea1
...............................................................uuguaggucuagaUacacug............................    1      1    ea1
...............................................................Guguaggucuagaaacacug............................    10     1    ea1
...............................................................uuguaggucuaAaaacacug............................    2      1    ea1
...............................................................uuguaggucGagaaacacug............................    3      1    ea1
...............................................................uuguaggucuagaaacacuA............................    6      1    ea1
...............................................................uuguaggucuagaaacacug............................    44     0    ea1
...............................................................uuguaggucuagaaacacugu...........................    18     0    ea1
...............................................................uuguaggucuagaaacGcugu...........................    2      1    ea1
...............................................................uuguaggucuagaaacacugA...........................    2      1    ea1
...............................................................uuguaggucuagaaacacugC...........................    1      1    ea1
...............................................................Guguaggucuagaaacacugu...........................    9      1    ea1
...............................................................uuguaggucuagaaacacuguu..........................    3      0    ea1
...............................................................Guguaggucuagaaacacuguu..........................    2      1    ea1
...............................................................uuguaggucuagaaacacuguuU.........................    1      1    ea1
...............................................................uuguaggucuagaaacacuguuC.........................    1      1    ea1
...............................................................uuguaggucuagaaacacuguuUa........................    2      1    ea1
................................................................uguaggucuagaaacGcugu...........................    3      1    ea1
................................................................uguaggucGagaaacacugu...........................    2      1    ea1
................................................................uguaggucuagGaacacugu...........................    1      1    ea1
................................................................Gguaggucuagaaacacugu...........................    4      1    ea1
................................................................uguaggucuagaaacacugu...........................    9      0    ea1
................................................................uguaggucuagaaacacugC...........................    1      1    ea1
................................................................Gguaggucuagaaacacuguu..........................    2      1    ea1
................................................................uguaggucuaAaaacacuguu..........................    1      1    ea1
................................................................uAuaggucuagaaacacuguu..........................    1      1    ea1
................................................................uguaggucuagaaacacuguu..........................    6      0    ea1
................................................................uguagAucuagaaacacuguu..........................    1      1    ea1
................................................................uguaggucuagaaacacuguuU.........................    1      1    ea1
```

Star **Mature**

```
acaguuucuagaccuacaagaucgaggagaacagccuccucaaagagaacagccuccuugaucuuguaggucuagaaacacuguugaaacaagugugauucugccaucggu
................................................................uguaggucuagaaacacuguuUa........................ 1 1 ea1
.................................................................guaggucuagaaacacuguu.......................... 1 0 ea1
.................................................................guaggucuagaaacacuguuU......................... 1 1 ea1
..................................................................uaggucuagaaacacuguC.......................... 1 1 ea1
..................................................................uaggucuaAaaacacuguu.......................... 1 1 ea1
..................................................................uaggucuagaaacacuguu.......................... 13 0 ea1
..................................................................uaggucuagaaacGcuguug......................... 1 1 ea1
..................................................................uGggucuagaaacacuguug......................... 2 1 ea1
..................................................................uaggucuagaaacacuguug......................... 2 0 ea1
..................................................................uaggucuagaaGcacuguug......................... 1 1 ea1
..................................................................uaggucuagaaacacuguuU......................... 3 1 ea1
..................................................................uaggucuGgaaacacuguuga........................ 1 1 ea1
..................................................................uaggucuagaaacacuguugG........................ 1 1 ea1
..................................................................uaggucuagaaacacuguuUa........................ 4 1 ea1
..................................................................uGggucuagaaacacuguuga........................ 2 1 ea1
..................................................................uGggucuagaaacacuguugaa....................... 1 1 ea1
..................................................................uaggucuagaaacacuguugaC....................... 1 1 ea1
...................................................................aggucuagaaacacuguC.......................... 1 1 ea1
......................................................................ucuGgaaacacuguugaaaca.................... 1 1 ea1
......................................................................ucuagaaacacuguugaaaca.................... 1 0 ea1
......................................................................ucuagaaacacuguugGaaca.................... 2 1 ea1
........................................................................uagaaacacuguugGaacaag.................. 2 1 ea1
........................................................................uagaaacacuguugaaacGag.................. 3 1 ea1
.................................................................................uguugaaacaagugugauucA......... 1 1 ea1
........................................................................................acaagugugauucugccaucC.. 1 1 ea1

....uuucuagaccuacaagCucgagg.................................................................................... 1 1 er1
.....uucuagaccuacaagaucga...................................................................................... 1 0 er1
.....uucuagaccuacaagaucgaU..................................................................................... 1 1 er1
.....uuUuagaccuacaagaucgagg.................................................................................... 1 1 er1
........uGgaccuacaagaucgagga................................................................................... 1 1 er1
........uagaccuacaagaucgaggaAa................................................................................. 1 1 er1
..............uacaagaucgaggagaacagccuccG....................................................................... 1 1 er1
............................................................aucuuguaggucuaAaaaca............................... 2 1 er1
............................................................aucuuguaggucuagaaacac.............................. 1 0 er1
............................................................aucuuguaggucuagaaacacA............................. 5 1 er1
............................................................aucuuguaggucuagaaacacu............................. 4 0 er1
............................................................aucuuguaggucuagaaacacuA............................ 2 1 er1
............................................................aucuuguaggucuagaaacacuUu........................... 1 1 er1
.............................................................ucuuguaggucuagaaacacA............................. 11 1 er1
.............................................................ucuuguaggucuagaaacacG............................. 1 1 er1
.............................................................ucuuguaggucuagaaacacu............................. 17 0 er1
.............................................................Gcuuguaggucuagaaacacu............................. 4 1 er1
.............................................................ucuuguaggucuagaaacacug............................ 1 0 er1
.............................................................ucuuguaggucuagaaacacugC........................... 1 1 er1
.............................................................ucuuguaggucGagaaacacuguu.......................... 1 1 er1
.............................................................ucuuguaggucuagaaacacuguu.......................... 1 0 er1
..............................................................cuuguaggucuagaaacacu............................. 7 0 er1
..............................................................Guuguaggucuagaaacacu............................. 1 1 er1
..............................................................cuuguaggucuaAaaacacug............................ 1 1 er1
..............................................................cuuguaggucuagaaacacug............................ 4 0 er1
..............................................................cuuguaggucuagaaacacuA............................ 1 1 er1
...............................................................uuguaggucuagaaacacu............................. 1 0 er1
...............................................................uuguaggucuagaaacacug............................ 1 0 er1
...............................................................Guguaggucuagaaacacugu........................... 1 1 er1
...............................................................uuguaggucuaAaaacacuguu.......................... 1 1 er1
................................................................uguaggucuagaaacacu............................. 1 0 er1
................................................................Gguaggucuagaaacacu............................. 1 1 er1
................................................................uguaggucuagaaacUcuguu.......................... 1 1 er1
................................................................uguaggucuagaaacacuguu.......................... 7 0 er1
................................................................uguaggucuagaaacacugCu.......................... 1 1 er1
................................................................Gguaggucuagaaacacuguu.......................... 1 1 er1
................................................................uguaggucuagaaacacuguuU......................... 1 1 er1
................................................................uguaggucuagaaacacuguuUa........................ 1 1 er1
.................................................................guaggucuagaaacacuguuU......................... 1 1 er1
..................................................................uaggucuagaaacacuguuU......................... 1 1 er1

....uuucuagaccuacaagau......................................................................................... 2 0 eg1
........uagaccuacaagaucgaggaCa................................................................................. 1 1 eg1
........uagaccuacaagaucgaggaUa................................................................................. 1 1 eg1
```

Star **Mature**

```
acaguuucuagaccuacaagaucgaggagaacagccuccucaaagagaacagccuccuugaucuuguaggucuagaaacacuguugaaacaagugugauucugccaucggu
.........agaccuacaagaucgagg....................................................................................	1	0	eg1
.........agaccuacaagaucgaggaCa.................................................................................	1	1	eg1
.....................................ccucaaagagaacagccucc......................................................	1	0	eg1
.....................................ccucaaagagaacagccuccuu....................................................	1	0	eg1
............................................................aucuuguaggucuaAaaac................................	2	1	eg1
............................................................aucuuguaggucuagaaaca...............................	5	0	eg1
............................................................aucuuguaggucuaAaaacacu.............................	1	1	eg1
............................................................aucuuguaggucuagaaacacA.............................	9	1	eg1
............................................................aucuuguaggucuagaaacacC.............................	1	1	eg1
............................................................aucuuguaggucuagaaacacG.............................	4	1	eg1
............................................................aucuuguaggCcuagaaacacu.............................	1	1	eg1
............................................................aucuuguaggucuagaaacacu.............................	12	0	eg1
............................................................aucuuguaggucuagaaacacuA............................	1	1	eg1
.............................................................ucuuguaggucuagaaaca...............................	2	0	eg1
.............................................................Gcuuguaggucuagaaacac..............................	1	1	eg1
.............................................................ucuuguaggucuagaaacaA..............................	1	1	eg1
.............................................................ucuCguaggucuagaaacacu.............................	1	1	eg1
.............................................................ucuuguaggucuagaaacacA.............................	31	1	eg1
.............................................................Gcuuguaggucuagaaacacu.............................	3	1	eg1
.............................................................ucuuguaggucuagaaacacu.............................	44	0	eg1
.............................................................ucuuguaggucuagaaacacC.............................	5	1	eg1
.............................................................ucuuguaAgucuagaaacacu.............................	1	1	eg1
.............................................................ucuuguaggCcuagaaacacu.............................	1	1	eg1
.............................................................ucuuguaggucuagaaacacG.............................	3	1	eg1
.............................................................ucuuguaggucuagaaacacuU............................	1	1	eg1
.............................................................ucuuguaggucuagaaacacuA............................	4	1	eg1
.............................................................ucuuguaggucuagaaacacug............................	2	0	eg1
.............................................................ucuuguaggucuagaaacacuguu..........................	4	0	eg1
..............................................................cuuguaggucuagaaacacC.............................	2	1	eg1
..............................................................cuuguaggucuagaaacacG.............................	1	1	eg1
..............................................................cuuguaggucuagaaacacu.............................	14	0	eg1
..............................................................cuuguaggucuUgaaacacu.............................	2	1	eg1
..............................................................cuuguaggucuagaaacacuU............................	3	1	eg1
..............................................................cuuguaggucuagaaGcacug............................	2	1	eg1
..............................................................cuuguaggucuagGaacacug............................	1	1	eg1
..............................................................cuuguaggucuagaaacacug............................	10	0	eg1
..............................................................cuuguaggucuagaaacacuA............................	4	1	eg1
..............................................................cuuguaggucuagaaacacugu...........................	1	0	eg1
..............................................................cuuguaggucuagaaacacuguC..........................	1	1	eg1
...............................................................uuguaggucuagaaacac..............................	1	0	eg1
...............................................................uuguaggucuagaaacacC.............................	1	1	eg1
...............................................................uuguaggucuagaaacacu.............................	3	0	eg1
...............................................................Guguaggucuagaaacacug............................	5	1	eg1
...............................................................uuguaggucGagaaacacug............................	2	1	eg1
...............................................................uugCaggucuagaaacacug............................	2	1	eg1
...............................................................uuguaggucuagaaacCcug............................	1	1	eg1
...............................................................uuguaggucuagaaacacuA............................	1	1	eg1
...............................................................uuguaggucuagaaacacug............................	10	0	eg1
...............................................................Guguaggucuagaaacacugu...........................	1	1	eg1
...............................................................uuguaggucuagaaacacugC...........................	1	1	eg1
...............................................................uuguaggucuagaaacacugu...........................	7	0	eg1
...............................................................uuguaggucuagaaacGcugu...........................	1	1	eg1
...............................................................uuguaggucuagaaacacuUu...........................	1	1	eg1
...............................................................Guguaggucuagaaacacuguu..........................	1	1	eg1
...............................................................uuguaggucuagaaacacuguG..........................	1	1	eg1
...............................................................uuguaggucuagaaacacuguug.........................	1	0	eg1
................................................................uguaggucuagaaacacugG...........................	1	1	eg1
................................................................uguaggucuagaaacacugu...........................	6	0	eg1
................................................................Gguaggucuagaaacacugu...........................	1	1	eg1
................................................................Gguaggucuagaaacacuguu..........................	1	1	eg1
................................................................uguaggucuaAaaacacuguu..........................	1	1	eg1
................................................................uguaggucuagaaacacuguu..........................	1	0	eg1
................................................................uguaggucuagaaacacuguuU.........................	1	1	eg1
................................................................uguaggucuagaaacacuguuUa........................	2	1	eg1
.................................................................Auaggucuagaaacacuguu..........................	1	1	eg1
.................................................................guaggucuagaaacacuguu..........................	1	0	eg1
..................................................................uaggucuagaGacacuguug.........................	1	1	eg1
..................................................................uaggucuagaaacacuguuU.........................	5	1	eg1
..................................................................uaggucuagaaacacuguug.........................	1	0	eg1
..................................................................uaggucuagaaacacuguuga........................	1	0	eg1
```

Star Mature

```
acaguuucuagaccuacaagaucgaggagaacagccuccucaaagagaacagccuccuugaucuuguaggucuagaaacacuguugaaacaagugugauucugccaucggu
..................................................................uGggucuagaaacacuguuga........................	1	1	eg1
......................................................................ucuagaaacGcuguugaaac.....................	1	1	eg1
......................................................................ucuagaaacacuguugGaac.....................	1	1	eg1
......................................................................ucuagaaacacuguugaaacU....................	1	1	eg1
...................................................................................uugaaacaagugugauucug........	1	0	eg1
```

Provisional ID	:	ScUbCFx_65_7370
Score total	:	21354.1
Score for star read(s)	:	3.9
Score for read counts	:	21345
Score for mfe	:	0.6
Score for randfold	:	1.6
Score for cons. seed	:	3
Total read count	:	41879
Mature read count	:	41754
Loop read count	:	0
Star read count	:	125

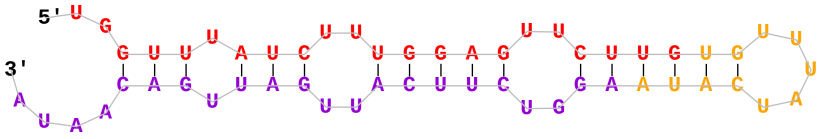

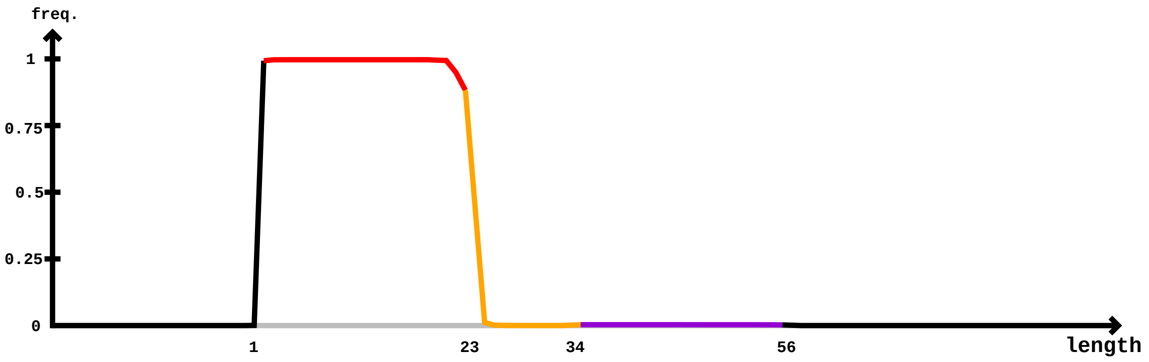

Mature **Star**

```
5'- agucauuuuaauauaccucaugguuuaucuuuggaguucuuguguuuaucauaaggucuucauugauugacaauaagguauauaauggacuuucagauuugacagcuua   -3'   obs
    agucauuuuaauauaccucaugguuuaucuuuggaguucuuguguuuaucauaaggucuucauugauugacaauaagguauauaauggacuuucagauuugacagcuua         exp
    .((((.....((((((((.((.(((.(((..(((((..((((((.....))))))..)))))..))).))).)).))))))))..(((....)))....))))......   reads   mm   sample
    ...................Cugguuuaucuuuggaguucuug...................................................................   2       1    eg1
    ....................ugguuuaucuuuggaguC.......................................................................   1       1    eg1
    ....................ugguuuaucuuuggaguG.......................................................................   1       1    eg1
    ....................ugguuuaucuuuggaguu.......................................................................   9       0    eg1
    ....................Ggguuuaucuuuggaguu.......................................................................   3       1    eg1
    ....................ugguuuaucuuuggaguuc......................................................................   1       0    eg1
    ....................ugguuuaucuuuggaguuU......................................................................   1       1    eg1
    ....................ugguuuaucuuuggagCucu.....................................................................   1       1    eg1
    ....................ugguuuaucuuuggaguucG.....................................................................   18      1    eg1
    ....................Cgguuuaucuuuggaguucu.....................................................................   1       1    eg1
    ....................ugguuuaucuuuggaguucu.....................................................................   190     0    eg1
    ....................ugguuuaucuuuggGguucu.....................................................................   2       1    eg1
    ....................Ggguuuaucuuuggaguucu.....................................................................   17      1    eg1
    ....................ugguuuaucCuuggaguucu.....................................................................   3       1    eg1
    ....................ugguuuaucuuuggaguuUu.....................................................................   1       1    eg1
    ....................uggCuuaucuuuggaguucu.....................................................................   1       1    eg1
    ....................ugguuuaucuuCggaguucu.....................................................................   1       1    eg1
    ....................ugguuuaucuuuggaguucA.....................................................................   10      1    eg1
    ....................ugguuuaucuuuggaguucC.....................................................................   21      1    eg1
    ....................ugguuuGucuuuggaguucuu....................................................................   1       1    eg1
    ....................ugguuuaucuCuggaguucuu....................................................................   1       1    eg1
    ....................uAguuuaucuuuggaguucuu....................................................................   1       1    eg1
    ....................ugguuuaucuuuggaguucuu....................................................................   248     0    eg1
    ....................ugguuuaucuuuggaguuUuu....................................................................   1       1    eg1
    ....................ugguuuaucuuuggaguucuC....................................................................   32      1    eg1
    ....................ugguCuaucuuuggaguucuu....................................................................   1       1    eg1
    ....................Agguuuaucuuuggaguucuu....................................................................   1       1    eg1
    ....................uggCuuaucuuuggaguucuu....................................................................   3       1    eg1
    ....................Ggguuuaucuuuggaguucuu....................................................................   55      1    eg1
    ....................ugguuuaucAuuggaguucuu....................................................................   1       1    eg1
    ....................ugguuuauUuuuggaguucuu....................................................................   1       1    eg1
    ....................ugguuuaucuuuggGguucuu....................................................................   1       1    eg1
    ....................ugguuuaucuuuggaguucuG....................................................................   6       1    eg1
```

Mature **Star**

```
agucauuuuaauauaccucaugguuuaucuuuggaguucuuguguuuaucauaaggucuucauugauugacaauaagguauauaauggacuucagauuugacagcuua
....................ugguuuaucuuuggaguCcuu...................................................................   2     1   eg1
....................ugguuuaucuuuggaguucuA...................................................................   5     1   eg1
....................uggGuuaucuuuggaguucuug..................................................................   7     1   eg1
....................ugguuuaucuuuggaguucuGg..................................................................   1     1   eg1
....................ugguuuGucuuuggaguucuug..................................................................   5     1   eg1
....................uAguuuaucuuuggaguucuug..................................................................   1     1   eg1
....................ugguAuaucuuuggaguucuug..................................................................   2     1   eg1
....................ugguuCaucuuuggaguucuug..................................................................   4     1   eg1
....................ugguuuaucuuAggaguucuug..................................................................   2     1   eg1
....................ugguuuaucCuuggaguucuug..................................................................   16    1   eg1
....................ugguuuaucuAuggaguucuug..................................................................   1     1   eg1
....................ugguuuaucuuuggCguucuug..................................................................   1     1   eg1
....................ugguuuaucuuuggaguCcuug..................................................................   10    1   eg1
....................ugAuuuaucuuuggaguucuug..................................................................   2     1   eg1
....................ugguuuaucuCuggaguucuug..................................................................   1     1   eg1
....................ugguuuaucuuuggaguucuuC..................................................................   9     1   eg1
....................ugguuuaucuuGggaguucuug..................................................................   1     1   eg1
....................ugguuuaucuuuggaguucuCg..................................................................   40    1   eg1
....................ugguuuaucuuuggaguuUuug..................................................................   3     1   eg1
....................Agguuuaucuuuggaguucuug..................................................................   2     1   eg1
....................uggCuuaucuuuggaguucuug..................................................................   8     1   eg1
....................ugguuuaucuuuggagCucuug..................................................................   7     1   eg1
....................ugguuuaucuuuggaguucCug..................................................................   6     1   eg1
....................ugUuuuaucuuuggaguucuug..................................................................   1     1   eg1
....................ugguuuaucuuuggUguucuug..................................................................   2     1   eg1
....................ugguuuaucuuuggGguucuug..................................................................   9     1   eg1
....................ugguuuaucuuuggaguucuAg..................................................................   1     1   eg1
....................Cgguuuaucuuuggaguucuug..................................................................   9     1   eg1
....................ugguuuAcuuuggaguucuug...................................................................   2     1   eg1
....................ugguuuaCcuuuggaguucuug..................................................................   9     1   eg1
....................ugguuuaucuuuggaAuucuug..................................................................   2     1   eg1
....................ugguuuaucuuCggaguucuug..................................................................   7     1   eg1
....................ugguuuaucuuugAaguucuug..................................................................   2     1   eg1
....................ugguuuaucuuuggaguucuuA..................................................................   370   1   eg1
....................ugguCuaucuuuggaguucuug..................................................................   7     1   eg1
....................ugguuuauUuuuggaguucuug..................................................................   1     1   eg1
....................ugguuuaucuuuggaguucuuU..................................................................   113   1   eg1
....................ugguuuaucuuuggaguucuug..................................................................   3203  0   eg1
....................Ggguuuaucuuuggaguucuug..................................................................   535   1   eg1
....................Cgguuuaucuuuggaguucuugu.................................................................   1     1   eg1
....................Ggguuuaucuuuggaguucuugu.................................................................   17    1   eg1
....................ugguuuaucuuuggaguucuugu.................................................................   66    0   eg1
....................ugguuuaucuuuggaguucuuAu.................................................................   7     1   eg1
....................ugguuuaucuuuggaguucuugA.................................................................   10    1   eg1
....................ugguuuaucCuuggaguucuugu.................................................................   1     1   eg1
....................ugguuuaucuuuggaguucuugG.................................................................   9     1   eg1
....................ugguuuaucuuuggaguucuuUu.................................................................   12    1   eg1
....................ugguCuaucuuuggaguucuugu.................................................................   1     1   eg1
....................ugguuuaucuuuggaguucuugC.................................................................   11    1   eg1
....................ugguuuaucuuuggaguucuCgu.................................................................   1     1   eg1
....................ugguuuaucuuuggaguucuugug................................................................   1     0   eg1
....................ugguuuaucuuuggaguucuuguU................................................................   6     1   eg1
....................Ggguuuaucuuuggaguucuugug................................................................   2     1   eg1
....................ugguuuaucuuuggaguucuuguC................................................................   2     1   eg1
....................ugguuuaucuuuggaguucuuguA................................................................   39    1   eg1
....................ugguuuaucuuuggaguucuuguUu...............................................................   2     1   eg1
....................ugguuuaucuuuggaguucuuguAu...............................................................   8     1   eg1
....................Ggguuuaucuuuggaguucuuguguuu.............................................................   1     1   eg1
....................ugguuuaucuuuggaguucuuguguuu.............................................................   1     0   eg1
.....................ggGuuaucuuuggaguucuug..................................................................   1     1   eg1
.....................gguuuaucuuuggaguucuug..................................................................   3     0   eg1
........................................uguguuuaucauaaggucuuc...............................................   1     0   eg1
....................................................aaggucuucauugauugacaau..................................   3     0   eg1
....................................................Gaggucuucauugauugacaau..................................   1     1   eg1
....................................................aaggucuucauugauugacaauU.................................   2     1   eg1
....................................................aaggucuucauugauugacaauUa................................   2     1   eg1
.....................................................aggucuucauugauugacaau..................................   1     0   eg1
.....................................................aggucuucauugauugacaaua.................................   1     0   eg1
.....................................................aggucuucauugauugacaauU.................................   2     1   eg1
.....................................................Gggucuucauugauugacaaua.................................   1     1   eg1
```

Mature **Star**

```
agucauuuuaauauaccucaugguuuaucuuuggaguucuuguguuuaucauaaggucuucauugauugacaauaagguauauaauggacuuucagauuugacagcuua
....................................................aggucuucauugauugacaauUa..................................  1     1   eg1

...................Cugguuuaucuuuggaguucuug...................................................................  2     1   er1
....................ugguuuaucuuuggaguu.......................................................................  3     0   er1
....................ugguuuaucuuuggaguC.......................................................................  2     1   er1
....................ugguuuaucuuuggaguuc......................................................................  4     0   er1
....................ugguuuaucCuuggaguuc......................................................................  1     1   er1
....................ugguuuaucuuuggaguucC.....................................................................  12    1   er1
....................ugguuuaucuuuggaguucA.....................................................................  1     1   er1
....................ugguuuaucGuuggaguucu.....................................................................  1     1   er1
....................ugguuuaucuuuggaguucu.....................................................................  109   0   er1
....................Ggguuuaucuuuggaguucu.....................................................................  19    1   er1
....................ugguuuaucuuuggaguucuC....................................................................  9     1   er1
....................ugguuuaCcuuuggaguucuu....................................................................  1     1   er1
....................ugguAuaucuuuggaguucuu....................................................................  1     1   er1
....................ugguuuaucuuuggaguucuG....................................................................  1     1   er1
....................ugAuuuaucuuuggaguucuu....................................................................  2     1   er1
....................ugguuuaucuuuggGguucuu....................................................................  1     1   er1
....................Ggguuuaucuuuggaguucuu....................................................................  35    1   er1
....................ugguuuaucuuuggaguucuu....................................................................  166   0   er1
....................ugguCuaucuuuggaguucuu....................................................................  3     1   er1
....................ugguuuaucuuuAgaguucuu....................................................................  1     1   er1
....................ugguuuaucuuuggaAuucuu....................................................................  1     1   er1
....................ugguGuaucuuuggaguucuug...................................................................  1     1   er1
....................ugguuuaucuuugUaguucuug...................................................................  1     1   er1
....................ugguuAaucuuuggaguucuug...................................................................  1     1   er1
....................ugguuuaucuuuggaguuUuug...................................................................  1     1   er1
....................ugguuuaucuuuggaguCcuug...................................................................  11    1   er1
....................ugguuuaucuuuggaAuucuug...................................................................  3     1   er1
....................ugguuuaucuuuggaguucuCg...................................................................  4     1   er1
....................ugguuuaucuuugAaguucuug...................................................................  1     1   er1
....................ugUuuuaucuuuggaguucuug...................................................................  2     1   er1
....................ugguuuaucuuuggaguAcuug...................................................................  1     1   er1
....................Cgguuuaucuuuggaguucuug...................................................................  3     1   er1
....................ugguuuaucCuuggaguucuug...................................................................  8     1   er1
....................ugguuuaucuuCggaguucuug...................................................................  7     1   er1
....................ugguuuaucuuuggaguGcuug...................................................................  1     1   er1
....................ugguCuaucuuuggaguucuug...................................................................  12    1   er1
....................ugAuuuaucuuuggaguucuug...................................................................  1     1   er1
....................ugguuuaucuuuggaguucAug...................................................................  2     1   er1
....................ugguuuaucuuuggaguucuuA...................................................................  261   1   er1
....................uggGuuaucuuuggaguucuug...................................................................  2     1   er1
....................Agguuuaucuuuggaguucuug...................................................................  2     1   er1
....................Ggguuuaucuuuggaguucuug...................................................................  400   1   er1
....................ugguuuGucuuuggaguucuug...................................................................  5     1   er1
....................ugguuuaucuuuggaguucuGg...................................................................  1     1   er1
....................ugguuCaucuuuggaguucuug...................................................................  3     1   er1
....................ugguuuaucuuuggaguucuug...................................................................  2379  0   er1
....................ugguuuaucuuuggaguucuuC...................................................................  18    1   er1
....................ugguuuaucuuuggGguucuug...................................................................  11    1   er1
....................ugguuuaCcuuuggaguucuug...................................................................  2     1   er1
....................ugguuuaucuuuggUguucuug...................................................................  4     1   er1
....................ugguuuaucuuuggagGucuug...................................................................  3     1   er1
....................ugguuuaucuCuggaguucuug...................................................................  4     1   er1
....................ugguuuaucuuuggaguucCug...................................................................  1     1   er1
....................uggCuuaucuuuggaguucuug...................................................................  5     1   er1
....................uggAuuaucuuuggaguucuug...................................................................  2     1   er1
....................ugguuuaucAuuggaguucuug...................................................................  2     1   er1
....................ugguuuauUuuggaguucuug....................................................................  3     1   er1
....................ugguuuaucuuuggCguucuug...................................................................  1     1   er1
....................ugguuuaucuuuggagCucuug...................................................................  1     1   er1
....................ugguuuaGcuuuggaguucuug...................................................................  1     1   er1
....................ugguuuaucuuuggaguucuuU...................................................................  73    1   er1
....................Ggguuuaucuuuggaguucuugu..................................................................  8     1   er1
....................Cgguuuaucuuuggaguucuugu..................................................................  1     1   er1
....................ugguuuaucuuuggaguucuugC..................................................................  8     1   er1
....................ugguuuaucuuuggaguCcuugu..................................................................  2     1   er1
....................ugguuuaucuuuggaguucuuAu..................................................................  3     1   er1
....................ugguuuaucuuuggagCucuugu..................................................................  1     1   er1
....................ugguuuaucuuuggaguucuugA..................................................................  11    1   er1
```

Mature Star

```
agucauuuuaauauaccucaugguuuaucuuuggaguucuuguguuuaucauaaggucuucauugauugacaauaagguauauaauggacuuucagauuugacagcuua
....................ugguuuaucuuuggaguucuugu..................................................................  54    0  er1
....................ugguuuaucuuuggaguucuuUu..................................................................  7     1  er1
....................ugguCuaucuuuggaguucuugu..................................................................  1     1  er1
....................Ggguuuaucuuuggaguucuugug.................................................................  1     1  er1
....................ugguuuaucuuuggaguucuuguU.................................................................  4     1  er1
....................ugguuuaucuuuggaguucuugug.................................................................  1     0  er1
....................ugguuuaucuuuggaguucuuguA.................................................................  29    1  er1
....................ugguuuaucuuuggaguucuuguAu................................................................  4     1  er1
....................Ggguuuaucuuuggaguucuuguguuu..............................................................  1     1  er1
....................ugguuuaucuuuggaguucuuguguuu..............................................................  3     0  er1
.....................gguuuaucuuuggaguucuug...................................................................  3     0  er1
....................................................aaggucuucauugauugacaauU..................................  2     1  er1
....................................................aaggucuucauugauugacaaua..................................  2     0  er1
....................................................aaggucuucauugauugacaauUa.................................  1     1  er1
.....................................................aggucuucauugauugacaaua..................................  1     0  er1
.....................................................aggucuucauugauugacaauU..................................  1     1  er1
.....................................................aggucuucauugauugacaauUa.................................  3     1  er1

....................ugguuuaucuuuggaguucu.....................................................................  7     0  er2
....................ugguuuaucuuuggaguucuu....................................................................  46    0  er2
....................ugguuuaucuuuggaguucuA....................................................................  5     1  er2
....................ugguCuaucuuuggaguucuug...................................................................  1     1  er2
....................ugguuuaucuuuggagCucuug...................................................................  1     1  er2
....................ugguuuaucuuuggaguucuug...................................................................  768   0  er2
....................Ngguuuaucuuuggaguucuug...................................................................  2     1  er2
....................uggGuuaucuuuggaguucuug...................................................................  2     1  er2
....................ugguuuaucuuuggaguucuuA...................................................................  118   1  er2
....................Cgguuuaucuuuggaguucuug...................................................................  1     1  er2
....................ugguuuaucuuuggGguucuug...................................................................  1     1  er2
....................ugguuuaucuuuggaguucuuU...................................................................  31    1  er2
....................ugguuuaucuuuggaguCcuug...................................................................  1     1  er2
....................ugguAuaucuuuggaguucuug...................................................................  1     1  er2
....................ugguuuaucuuuggaguucuuUu..................................................................  3     1  er2
....................ugguuuaucuuuggaguucuugu..................................................................  16    0  er2
....................ugguuuaucuuuggaguucuuAu..................................................................  9     1  er2
....................ugguuuauUuuuggaguucuugu..................................................................  2     1  er2
....................ugguuuaucuuuggaguucuuguA.................................................................  8     1  er2
.....................gguuuaucuuuggaguucuu....................................................................  3     0  er2
.....................gguuuaucuuuggaguucuug...................................................................  48    0  er2
.....................gguuuaucuuuggaguucuuA...................................................................  6     1  er2
.....................Uguuuaucuuuggaguucuug...................................................................  1     1  er2
.....................gguuuaucuuuggaguucuugu..................................................................  1     0  er2
.....................gguuuaucuuuggaguucuuUu..................................................................  1     1  er2
....................................................aaggucuucauugauugacaau...................................  7     0  er2
....................................................aaggucuucauugauugacaauU..................................  3     1  er2
.....................................................aggucuucauugauugacaaua..................................  7     0  er2
.....................................................aggucuucauugauugacaauU..................................  10    1  er2
.....................................................aggucuucauugauugacaauUa.................................  8     1  er2

...................Cugguuuaucuuuggaguucuu....................................................................  2     1  ea1
...................Cugguuuaucuuuggaguucuug...................................................................  13    1  ea1
....................Ggguuuaucuuuggaguu.......................................................................  8     1  ea1
....................ugguuuaucuuuggaguA.......................................................................  1     1  ea1
....................ugguuuaucuuuggaguu.......................................................................  34    0  ea1
....................ugguuuaucuuuggaguC.......................................................................  3     1  ea1
....................uggCuuaucuuuggaguu.......................................................................  1     1  ea1
....................ugguuuaucuuuggaguuA......................................................................  1     1  ea1
....................Ggguuuaucuuuggaguuc......................................................................  10    1  ea1
....................ugguuuGucuuuggaguuc......................................................................  1     1  ea1
....................ugguuuaucuuuggaguuc......................................................................  27    0  ea1
....................ugguuuaucuuuggagGuc......................................................................  1     1  ea1
....................ugguuuaucuuuggaguuU......................................................................  2     1  ea1
....................ugguuuaucuuuggaguucG.....................................................................  17    1  ea1
....................ugguuuaucuuuAgaguucu.....................................................................  3     1  ea1
....................ugguuuaucCuuggaguucu.....................................................................  2     1  ea1
....................ugguuuaucuuAggaguucu.....................................................................  2     1  ea1
....................ugguuuaucuuuggaguucA.....................................................................  73    1  ea1
....................Cgguuuaucuuuggaguucu.....................................................................  3     1  ea1
....................ugguuuaucuuuggaguucC.....................................................................  126   1  ea1
....................ugguuuaucuuuggaguucu.....................................................................  1028  0  ea1
```

Mature **Star**

```
agucauuuuaauauaccucaugguuuaucuuuggaguucuuguguuuaucauaaggucuucauugauugacaauaagguauauaauggacuuucagauuugacagcuua
....................ugguuuaCcuuuggaguucu.....................................................................  3      1  ea1
....................ugguuuaucuCuggaguucu.....................................................................  3      1  ea1
....................ugguuCaucuuuggaguucu.....................................................................  1      1  ea1
....................ugguuuaucuuuggGguucu.....................................................................  5      1  ea1
....................ugguCuaucuuuggaguucu.....................................................................  3      1  ea1
....................ugguuuaucuuuggaguCcu.....................................................................  2      1  ea1
....................uggGuuaucuuuggaguucu.....................................................................  1      1  ea1
....................ugguAuaucuuuggaguucu.....................................................................  1      1  ea1
....................Ggguuuaucuuuggaguucu.....................................................................  152    1  ea1
....................uggCuuaucuuuggaguucu.....................................................................  5      1  ea1
....................Agguuuaucuuuggaguucu.....................................................................  1      1  ea1
....................ugguuuaucuuuggagCucu.....................................................................  3      1  ea1
....................ugguuuaucuuCggaguucu.....................................................................  3      1  ea1
....................ugguuuaucuAuggaguucu.....................................................................  2      1  ea1
....................ugguuuaCcuuuggaguucuu....................................................................  6      1  ea1
....................uCguuuaucuuuggaguucuu....................................................................  1      1  ea1
....................ugguuuaucuuuggaguucuu....................................................................  1635   0  ea1
....................Ggguuuaucuuuggaguucuu....................................................................  240    1  ea1
....................uggGuuaucuuuggaguucuu....................................................................  3      1  ea1
....................Cgguuuaucuuuggaguucuu....................................................................  3      1  ea1
....................uggAuuaucuuuggaguucuu....................................................................  1      1  ea1
....................ugguuuaucuuuggaguucuA....................................................................  38     1  ea1
....................ugguuuaucuuuggaguuUuu....................................................................  2      1  ea1
....................ugguuuGucuuuggaguucuu....................................................................  1      1  ea1
....................ugguuuaucuuuggaguCcuu....................................................................  4      1  ea1
....................ugguuuaucCuuggaguucuu....................................................................  6      1  ea1
....................ugguuuaucuuuggagCucuu....................................................................  1      1  ea1
....................ugguuCaucuuuggaguucuu....................................................................  4      1  ea1
....................ugguuuaucuuCggaguucuu....................................................................  4      1  ea1
....................ugguuuCucuuuggaguucuu....................................................................  1      1  ea1
....................ugguuuaucuuuggGguucuu....................................................................  6      1  ea1
....................uUguuuaucuuuggaguucuu....................................................................  3      1  ea1
....................ugguuuaucuuuggaguucuG....................................................................  10     1  ea1
....................uAguuuaucuuuggaguucuu....................................................................  2      1  ea1
....................ugguuuaucuuuggagAucuu....................................................................  1      1  ea1
....................ugguuuaucuuAggaguucuu....................................................................  1      1  ea1
....................ugguuuaucuuuggaguGcuu....................................................................  1      1  ea1
....................ugguuuaucuuugAaguucuu....................................................................  1      1  ea1
....................ugguuuaucuuuggaguucAu....................................................................  1      1  ea1
....................ugguuuaucuuuAgaguucuu....................................................................  1      1  ea1
....................ugguuuaucuuuggaguucCu....................................................................  4      1  ea1
....................ugguuuauUuuuggaguucuu....................................................................  1      1  ea1
....................uggCuuaucuuuggaguucuu....................................................................  7      1  ea1
....................ugguuuaucuCuggaguucuu....................................................................  3      1  ea1
....................ugguuuaucAuuggaguucuu....................................................................  1      1  ea1
....................ugguuuaucuuuggaguucGu....................................................................  1      1  ea1
....................ugguCuaucuuuggaguucuu....................................................................  5      1  ea1
....................ugguuuaucuAuggaguucuu....................................................................  6      1  ea1
....................ugguAuaucuuuggaguucuu....................................................................  1      1  ea1
....................ugguuuaucuuuggaguucuC....................................................................  147    1  ea1
....................ugguuuauGuuuggaguucuug...................................................................  1      1  ea1
....................ugguuuaucuuuggaguuGuug...................................................................  3      1  ea1
....................ugguuGaucuuuggaguucuug...................................................................  4      1  ea1
....................ugguuuaucuuuggUguucuug...................................................................  11     1  ea1
....................ugguuuaucuuAggaguucuug...................................................................  13     1  ea1
....................ugguuuaucuuuggaguGcuug...................................................................  2      1  ea1
....................ugguuuaCcuuuggaguucuug...................................................................  40     1  ea1
....................ugguGuaucuuuggaguucuug...................................................................  1      1  ea1
....................uggAuuaucuuuggaguucuug...................................................................  5      1  ea1
....................uggGuuaucuuuggaguucuug...................................................................  9      1  ea1
....................ugguuuaucuuuggaguucuuC...................................................................  78     1  ea1
....................uggCuuaucuuuggaguucuug...................................................................  74     1  ea1
....................uAguuuaucuuuggaguucuug...................................................................  10     1  ea1
....................ugguuuaucuuGggaguucuug...................................................................  8      1  ea1
....................ugguuCaucuuuggaguucuug...................................................................  41     1  ea1
....................ugAuuuaucuuuggaguucuug...................................................................  3      1  ea1
....................ugguuuaucuuuggaguucuug...................................................................  18752  0  ea1
....................ugguuuaucuuuggaguucuAg...................................................................  3      1  ea1
....................ugguuuaucuuuggaguucuCg...................................................................  51     1  ea1
....................ugguuuaucuuuggaguucGug...................................................................  3      1  ea1
```

```
                    Mature                          Star
agucauuuuaauauaccucaugguuuaucuuuggaguucuuguguuuaucauaaggucuucauugauugacaauaagguauauaauggacuuucagauuugacagcuua
....................ugguuuaucuuuggaUuucuug...................................................................   5      1    ea1
....................ugguuuaGcuuuggaguucuug...................................................................   1      1    ea1
....................ugguuuaucuuuggaguucAug...................................................................   6      1    ea1
....................ugguuuaucAuuggaguucuug...................................................................   10     1    ea1
....................uUguuuaucuuuggaguucuug...................................................................   5      1    ea1
....................ugguuuaucuuuUgaguucuug...................................................................   5      1    ea1
....................ugguuuaucuCuggaguucuug...................................................................   39     1    ea1
....................ugguuuaucuuuggaCuucuug...................................................................   2      1    ea1
....................Ggguuuaucuuuggaguucuug...................................................................   2914   1    ea1
....................ugguuuaucuuuggaguucCug...................................................................   31     1    ea1
....................ugguuuauUuuuggaguucuug...................................................................   8      1    ea1
....................ugguuuaucuuugAaguucuug...................................................................   8      1    ea1
....................Aggguuuaucuuuggaguucuug..................................................................   17     1    ea1
....................ugguuuauAuuuggaguucuug...................................................................   2      1    ea1
....................ugguuuaucuuuggaAuucuug...................................................................   8      1    ea1
....................ugguuuaucuuuggGguucuug...................................................................   47     1    ea1
....................ugguuAaucuuuggaguucuug...................................................................   4      1    ea1
....................ugguuuaucuuuAgaguucuug...................................................................   12     1    ea1
....................ugguuuaucuuuggaguAcuug...................................................................   5      1    ea1
....................ugguuuaucuuCggaguucuug...................................................................   71     1    ea1
....................ugguuuaucuuuggagCucuug...................................................................   55     1    ea1
....................ugguuuaucCuuggaguucuug...................................................................   52     1    ea1
....................Cgguuuaucuuuggaguucuug...................................................................   42     1    ea1
....................ugguCuaucuuuggaguucuug...................................................................   57     1    ea1
....................ugguuuaucuAuggaguucuug...................................................................   7      1    ea1
....................ugguAuaucuuuggaguucuug...................................................................   8      1    ea1
....................ugguuuaucuuuggagAucuug...................................................................   4      1    ea1
....................ugguuuaucuuuggaguucuuU...................................................................   551    1    ea1
....................ugguuuaucuuuggaguuAuug...................................................................   3      1    ea1
....................ugguuuaucuuuggaguucuuA...................................................................   2962   1    ea1
....................ugguuuaucuuuggaguucuGg...................................................................   5      1    ea1
....................ugUuuuaucuuuggaguucuug...................................................................   5      1    ea1
....................ugguuuaucuuuggaguCcuug...................................................................   65     1    ea1
....................ugguuuaucuuugUaguucuug...................................................................   1      1    ea1
....................ugguuuaucuuuggCguucuug...................................................................   2      1    ea1
....................ugguuuGucuuuggaguucuug...................................................................   21     1    ea1
....................ugguuuaucuuuggaguuUuug...................................................................   8      1    ea1
....................Ggguuuaucuuuggaguucuugu..................................................................   55     1    ea1
....................ugguuuaucuuGggaguucuugu..................................................................   1      1    ea1
....................ugguuuaucuuuggaguucCugu..................................................................   1      1    ea1
....................uCguuuaucuuuggaguucuugu..................................................................   1      1    ea1
....................ugguuuaucuuuggaguucuugG..................................................................   7      1    ea1
....................ugguuuaucuuuggaguucuuUu..................................................................   49     1    ea1
....................ugguCuaucuuuggaguucuugu..................................................................   1      1    ea1
....................ugUuuuaucuuuggaguucuugu..................................................................   2      1    ea1
....................uggAuuaucuuuggaguucuugu..................................................................   3      1    ea1
....................ugguAuaucuuuggaguucuugu..................................................................   1      1    ea1
....................ugguuuaCcuuuggaguucuugu..................................................................   2      1    ea1
....................ugguuuaucuuuggaguucuugA..................................................................   64     1    ea1
....................ugguuuaucuuuggaguucuuAu..................................................................   52     1    ea1
....................ugguuuaucCuuggaguucuugu..................................................................   2      1    ea1
....................uggCuuaucuuuggaguucuugu..................................................................   2      1    ea1
....................ugguuuaucuuuggaguucuugu..................................................................   331    0    ea1
....................ugguuuaucuuuggaUuucuugu..................................................................   1      1    ea1
....................ugguuuaucuuuggaAuucuugu..................................................................   1      1    ea1
....................ugguuuaucuuuggGguucuugu..................................................................   2      1    ea1
....................ugguuuaucuuuggaguucuugC..................................................................   39     1    ea1
....................ugguuCaucuuuggaguucuugu..................................................................   1      1    ea1
....................ugguuuaucuuuggaguucuuCu..................................................................   2      1    ea1
....................ugguuuaucuuuggaguucuuguC.................................................................   8      1    ea1
....................ugguuuaucuuuggaguucuuAug.................................................................   1      1    ea1
....................ugguuuaucuuuggaguucuugug.................................................................   14     0    ea1
....................Ggguuuaucuuuggaguucuugug.................................................................   1      1    ea1
....................ugguuuaucuuuggaguucuuguA.................................................................   272    1    ea1
....................ugguuuaucuuuggaguucuuguU.................................................................   24     1    ea1
....................ugguuuaucuuuggaguucuugugG................................................................   1      1    ea1
....................ugguuuaucuuuggaguucuuguUu................................................................   5      1    ea1
....................ugguuuaucuuuggaguucuuguAu................................................................   20     1    ea1
....................Ggguuuaucuuuggaguucuugugu................................................................   1      1    ea1
....................ugguuuaucuuuggaguucuugugu................................................................   2      0    ea1
```

Mature **Star**

agucauuuuaauauaccucaugguuuaucuuuggaguucuuguguuuaucauaaggucuucauugauugacaauaagguauauaauggacuuucagauuugacagcuua			
....................ugguuuaucuuuggaguucuuguguu...	2	0	ea1
....................ugguuuaucuuuggaguucuuguAuu...	2	1	ea1
....................ugguuuaucuuuggaguucuuguguuu..	3	0	ea1
....................ugguuuaCcuuuggaguucuuguguuu..	1	1	ea1
....................Ggguuuaucuuuggaguucuuguguuu..	1	1	ea1
.....................gguuuaucuuuggaguucuug...	14	0	ea1
.....................gguuuaucuuuggaguucuuA...	7	1	ea1
.....................ggGuuaucuuuggaguucuug...	2	1	ea1
.....................Uguuuaucuuuggaguucuug...	1	1	ea1
......................guuuaucuuuggaguucuug...	3	0	ea1
......................guuuaucuuuggaguucuugug...	2	0	ea1
......................Auuuaucuuuggaguucuugug...	1	1	ea1
..............................uuggaguucuuguguuuauca..	1	0	ea1
....uguuuaucauaaggucuucauA...	1	1	ea1
....aaggucuucauugauugaca.....................................	1	0	ea1
....aaggucuucauugauugacaau...................................	1	0	ea1
....Gaggucuucauugauugacaaua..................................	1	1	ea1
....aaggucuucauugauugacaauU..................................	6	1	ea1
....aaggucuucauugauugacaaua..................................	9	0	ea1
....aaggucuucauugauugacaauUa.................................	7	1	ea1
....aggucuucauugauugacaau...................................	1	0	ea1
....aggucuucauugauugacaauU..................................	1	1	ea1
....aggucuucauugauugacaauC..................................	1	1	ea1
....aggucuucauugauugacaaua..................................	3	0	ea1
....aggucuucauugauugacaauUa.................................	4	1	ea1
...................Cugguuuaucuuuggaguucuug...	1	1	eg2
....................ugguuuaucuuuggaguucuu..	4	0	eg2
....................ugguuuaucuuuggaguucuuA...	5	1	eg2
....................ugguuuaucuuuggagGucuug...	1	1	eg2
....................Ngguuuaucuuuggaguucuug...	1	1	eg2
....................ugguuuaucuuuggaguucuug...	107	0	eg2
....................ugguuuaucuuuggaguucuuU...	3	1	eg2
....................ugguuuaucuuuggaguucuuguA...	1	1	eg2
.....................gguuuaucuuuggaguucuuU...	2	1	eg2
.....................ggGuuaucuuuggaguucuug...	1	1	eg2
.....................gguuuaucuuuggaguucuug...	3	0	eg2
....aaggucuCcauugauugacaau...................................	1	1	eg2
....aggucuucauugauugacaauU..................................	1	1	eg2
....aggucuucauugauugacaaua..................................	2	0	eg2
....aggucuucauugauugacaauUa.................................	3	1	eg2
....................ugguuuaucuuuggaguucu...	4	0	ea2
....................ugguuuaucuuuggaguucAu..	1	1	ea2
....................ugguuuaucuuuggaguucuA..	3	1	ea2
....................ugguuuaucuuuggaguucuu..	31	0	ea2
....................ugguCuaucuuuggaguucuug...	4	1	ea2
....................ugguuuaucuuuggaguucuuA...	70	1	ea2
....................ugguuuaucuuuggaguucuuU...	8	1	ea2
....................ugguuuaucuuuggGguucuug...	1	1	ea2
....................uAguuuaucuuuggaguucuug...	2	1	ea2
....................ugguuuaucuuuggaguucuug...	466	0	ea2
....................Ngguuuaucuuuggaguucuug...	3	1	ea2
....................ugguuuaucuuuggaguucuuAu..	1	1	ea2
....................ugguuuaucuuuggaguucuugA..	1	1	ea2
....................ugguuuaucuuuggaguucuuUu..	2	1	ea2
....................ugguuuaucuuuggaguucuuguA...	12	1	ea2
....................ugguuuaucuuuggaguucuugug...	3	0	ea2
.....................gguuuaucuuuggaguucuu..	1	0	ea2
.....................gguuuaucuuuggaguucuug...	38	0	ea2
.....................gguuuaucuuuggaguucuuU...	3	1	ea2
.....................gguuuaucuuuggaguucuuA...	8	1	ea2
......................guuuaucuuuggaguucuuU...	7	1	ea2
....uuuaucauaaggucuucauuga...	5	0	ea2
....uuaucauaaggucuucauuga...	1	0	ea2
....aaggucuucauugauugacaau...................................	2	0	ea2
....aaggucuucauugauugacaauG..................................	1	1	ea2
....aaggucuucauugauugacaauaa.................................	2	0	ea2
....aggucuucauugauugaca.....................................	5	0	ea2
....aggucuucauugauugacaau...................................	2	0	ea2

```
                    Mature                                   Star
agucauuuuaauauaccucaugguuuaucuuuggaguucuuguguuuaucauaaggucuucauugauugacaauaagguauauaauggacuuucagauuugacagcuua
.....................................................aggucuucauugauugacaauU..................................    7    1    ea2
.....................................................aggucuucauugauugacaaua..................................    5    0    ea2
```

Provisional ID	:	ScUbCFx_65_7374
Score total	:	21354.1
Score for star read(s)	:	3.9
Score for read counts	:	21345
Score for mfe	:	0.6
Score for randfold	:	1.6
Score for cons. seed	:	3
Total read count	:	41879
Mature read count	:	41754
Loop read count	:	0
Star read count	:	125

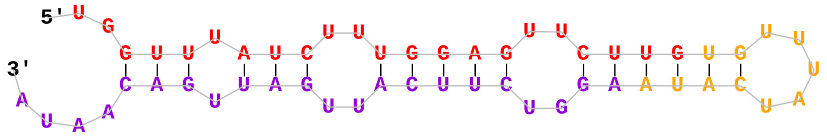

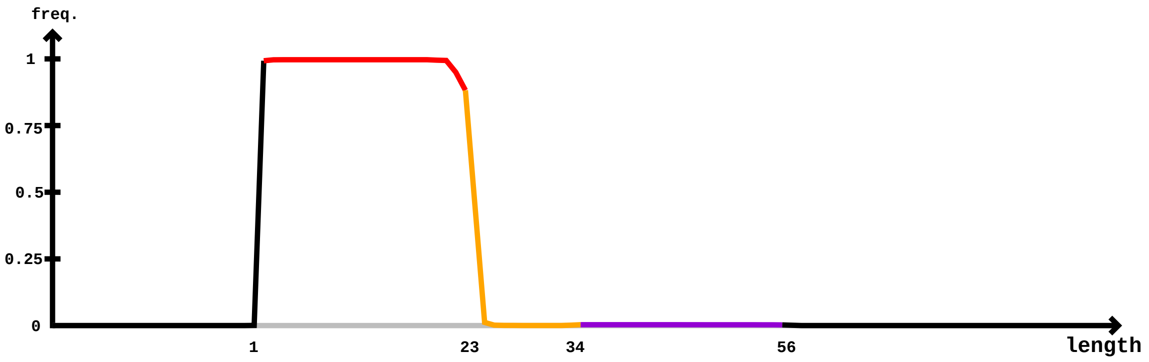

Mature **Star**

5'- agucauuuuaauauaccucaugguuuaucuuuggaguucuuguguuuaucauaaggucuucauugauugacaauaagguauauaauggacuuucagauuugacagcuua -3'	obs		
agucauuuuaauauaccucaugguuuaucuuuggaguucuuguguuuaucauaaggucuucauugauugacaauaagguauauaauggacuuucagauuugacagcuua	exp		
.((((.....((((((((.((.(((.(((..(((((..((((((.....))))))..))))).))).))).)).))))))))..(((....)))....))))......	reads	mm	sample
...................Cugguuuaucuuuggaguucuu..	2	1	ea1
...................Cugguuuaucuuuggaguucuug...	13	1	ea1
....................ugguuuaucuuuggaguA...	1	1	ea1
....................ugguuuaucuuuggaguu...	34	0	ea1
....................Ggguuuaucuuuggaguu...	8	1	ea1
....................uggCuuaucuuuggaguu...	1	1	ea1
....................ugguuuaucuuuggaguC...	3	1	ea1
....................ugguuuGucuuuggaguuc..	1	1	ea1
....................Ggguuuaucuuuggaguuc..	10	1	ea1
....................ugguuuaucuuuggaguuc..	27	0	ea1
....................ugguuuaucuuuggagGuc..	1	1	ea1
....................ugguuuaucuuuggaguuA..	1	1	ea1
....................ugguuuaucuuuggaguuU..	2	1	ea1
....................ugguuuaucuuuggGguucu...	5	1	ea1
....................Agguuuaucuuuggaguucu...	1	1	ea1
....................Cgguuuaucuuuggaguucu...	3	1	ea1
....................ugguuuaCcuuuggaguucu...	3	1	ea1
....................ugguAuaucuuuggaguucu...	1	1	ea1
....................ugguuuaucuuuggaguucC...	126	1	ea1
....................ugguuuaucuuuggaguucu...	1028	0	ea1
....................ugguuuaucuCuggaguucu...	3	1	ea1
....................ugguuuaucuuuggaguCcu...	2	1	ea1
....................uggCuuaucuuuggaguucu...	5	1	ea1
....................ugguuuaucuuAggaguucu...	2	1	ea1
....................ugguuuaucuuuggagCucu...	3	1	ea1
....................ugguuuaucuAuggaguucu...	2	1	ea1
....................uggGuuaucuuuggaguucu...	1	1	ea1
....................ugguCuaucuuuggaguucu...	3	1	ea1
....................ugguuCaucuuuggaguucu...	1	1	ea1
....................ugguuuaucuuCggaguucu...	3	1	ea1
....................ugguuuaucuuuggaguucA..	73	1	ea1
....................ugguuuaucuuuggaguucG..	17	1	ea1
....................ugguuuaucCuuggaguucu...	2	1	ea1

Mature   Star

agucauuuuaauauaccucaugguuuaucuuuggaguucuuguguuuaucauaaggucuucauugauugacaauaagguauauaauggacuuucagauuugacagcuua			
....................ugguuuaucuuuAgaguucu...	3	1	ea1
....................Ggguuuaucuuuggaguucu...	152	1	ea1
....................uUguuuaucuuuggaguucuu..	3	1	ea1
....................ugguuuaucuuuggaguucAu..	1	1	ea1
....................ugguuuaucuCuggaguucuu..	3	1	ea1
....................ugguuuaucCuuggaguucuu..	6	1	ea1
....................ugguuuCucuuuggaguucuu..	1	1	ea1
....................ugguuuaucuuuggaguuUuu..	2	1	ea1
....................ugguAuaucuuuggaguucuu..	1	1	ea1
....................ugguuuaucuuuggaguucuC..	147	1	ea1
....................ugguuuaucuuuggaguucCu..	4	1	ea1
....................uCguuuaucuuuggaguucuu..	1	1	ea1
....................Cgguuuaucuuuggaguucuu..	3	1	ea1
....................ugguuuaucuuuggagAucuu..	1	1	ea1
....................Ggguuuaucuuuggaguucuu..	240	1	ea1
....................ugguuuaucuuugAaguucuu..	1	1	ea1
....................ugguuuaucuuuAgaguucuu..	1	1	ea1
....................ugguuuauUuuggaguucuu..	1	1	ea1
....................ugguuuaCcuuuggaguucuu..	6	1	ea1
....................ugguuuaucuuuggaguCcuu..	4	1	ea1
....................ugguuuaucuuuggaguucuu..	1635	0	ea1
....................ugguuuaucuuuggaguucuA..	38	1	ea1
....................ugguuuaucuuAggaguucuu..	1	1	ea1
....................uggCuuaucuuuggaguucuu..	7	1	ea1
....................uggGuuaucuuuggaguucuu..	3	1	ea1
....................ugguuuaucuuuggaguucuG..	10	1	ea1
....................ugguuuaucAuuggaguucuu..	1	1	ea1
....................ugguuuaucuAuggaguucuu..	6	1	ea1
....................uggAuuaucuuuggaguucuu..	1	1	ea1
....................ugguuuaucuuuggGguucuu..	6	1	ea1
....................ugguuuaucuuCggaguucuu..	4	1	ea1
....................uAguuuaucuuuggaguucuu..	2	1	ea1
....................ugguuuGucuuuggaguucuu..	1	1	ea1
....................ugguuCaucuuuggaguucuu..	4	1	ea1
....................ugguuuaucuuuggagCucuu..	1	1	ea1
....................ugguCuaucuuuggaguucuu..	5	1	ea1
....................ugguuuaucuuuggaguGcuu..	1	1	ea1
....................ugguuuaucuuuggaguucGu..	1	1	ea1
....................Ggguuuaucuuuggaguucuug...	2914	1	ea1
....................ugguuuaucuuuggagAucuug...	4	1	ea1
....................ugguuuaucuuGggaguucuug...	8	1	ea1
....................ugguuuaucuuuggaguucAug...	6	1	ea1
....................ugguGuaucuuuggaguucuug...	1	1	ea1
....................ugguuuaucuuuggaCuucuug...	2	1	ea1
....................ugguuuaucuuuAgaguucuug...	12	1	ea1
....................ugguuuaucuuuggaguucuug...	18752	0	ea1
....................ugguuuaucuuuggCguucuug...	2	1	ea1
....................ugguuAaucuuuggaguucuug...	4	1	ea1
....................ugguuuauUuuggaguucuug...	8	1	ea1
....................ugguuuaucuuuggaguucCug...	31	1	ea1
....................ugguuuaucuuuggaguucuuU...	551	1	ea1
....................Agguuuaucuuuggaguucuug...	17	1	ea1
....................ugguuuaucuuuggaguuAuug...	3	1	ea1
....................ugguuuaucuuugUaguucuug...	1	1	ea1
....................ugguuGaucuuuggaguucuug...	4	1	ea1
....................ugguuuaucuuuggaguucuCg...	51	1	ea1
....................uAguuuaucuuuggaguucuug...	10	1	ea1
....................ugguuuaucuuuggaguucuAg...	3	1	ea1
....................ugguuuaucuuuggaUuucuug...	5	1	ea1
....................ugguuuaCcuuuggaguucuug...	40	1	ea1
....................ugguuuaucuCuggaguucuug...	39	1	ea1
....................ugguuuaucuuuggaguCcuug...	65	1	ea1
....................ugguuuaucuuuUgaguucuug...	5	1	ea1
....................ugguuuaucuuuggaguucuGg...	5	1	ea1
....................ugguuuaucuuuggaguucuuC...	78	1	ea1
....................ugguuuaucCuuggaguucuug...	52	1	ea1
....................ugguuuaucuuuggaguuUuug...	8	1	ea1
....................ugguuuaucuuugAaguucuug...	8	1	ea1
....................uggAuuaucuuuggaguucuug...	5	1	ea1
....................ugguuuaucAuuggaguucuug...	10	1	ea1

```
                    Mature                Star
agucauuuuaauauaccucaugguuuaucuuuggaguucuuguguuuaucauaaggucuucauugauugacaauaagguauauaauggacuuucagauuugacagcuua
....................Cgguuuaucuuuggaguucuug...................................................................   42     1    ea1
....................ugguuuaGcuuuggaguucuug...................................................................   1      1    ea1
....................ugguuuaucuAuggaguucuug...................................................................   7      1    ea1
....................ugguuuauGuuuggaguucuug...................................................................   1      1    ea1
....................uUguuuaucuuuggaguucuug...................................................................   5      1    ea1
....................uggGuuaucuuuggaguucuug...................................................................   9      1    ea1
....................ugguuCaucuuuggaguucuug...................................................................   41     1    ea1
....................ugguuuaucuuuggGguucuug...................................................................   47     1    ea1
....................uggCuuaucuuuggaguucuug...................................................................   74     1    ea1
....................ugguuuGucuuuggaguucuug...................................................................   21     1    ea1
....................ugguCuaucuuuggaguucuug...................................................................   57     1    ea1
....................ugguuuaucuuuggaAuucuug...................................................................   8      1    ea1
....................ugguuuauAuuuggaguucuug...................................................................   2      1    ea1
....................ugguuuaucuuuggUguucuug...................................................................   11     1    ea1
....................ugguuuaucuuCggaguucuug...................................................................   71     1    ea1
....................ugguuuaucuuuggaguAcuug...................................................................   5      1    ea1
....................ugUuuuaucuuuggaguucuug...................................................................   5      1    ea1
....................ugguuuaucuuAggaguucuug...................................................................   13     1    ea1
....................ugAuuuaucuuuggaguucuug...................................................................   3      1    ea1
....................ugguuuaucuuuggaguuGuug...................................................................   3      1    ea1
....................ugguuuaucuuuggagCucuug...................................................................   55     1    ea1
....................ugguuuaucuuuggaguucuuA...................................................................   2962   1    ea1
....................ugguuuaucuuuggaguGcuug...................................................................   2      1    ea1
....................ugguuuaucuuuggaguucGug...................................................................   3      1    ea1
....................ugguAuaucuuuggaguucuug...................................................................   8      1    ea1
....................ugguuuaucuuGggaguucuugu..................................................................   1      1    ea1
....................ugguuuaucuuuggaguucuuAu..................................................................   52     1    ea1
....................uggCuuaucuuuggaguucuugu..................................................................   2      1    ea1
....................ugguuuaucuuuggaguucuugG..................................................................   7      1    ea1
....................ugguCuaucuuuggaguucuugu..................................................................   1      1    ea1
....................ugguuuaucCuuggaguucuugu..................................................................   2      1    ea1
....................ugguuuaucuuuggaguucuugu..................................................................   331    0    ea1
....................ugguuuaucuuuggaguucuugC..................................................................   39     1    ea1
....................ugguuuaucuuuggaguucCugu..................................................................   1      1    ea1
....................ugguuuaucuuuggaguucuugA..................................................................   64     1    ea1
....................ugguuuaucuuuggaUuucuugu..................................................................   1      1    ea1
....................ugguuuaucuuuggGguucuugu..................................................................   2      1    ea1
....................uCguuuaucuuuggaguucuugu..................................................................   1      1    ea1
....................ugguuuaucuuuggaguucuuUu..................................................................   49     1    ea1
....................ugguAuaucuuuggaguucuugu..................................................................   1      1    ea1
....................ugguuuaucuuuggaguucuuCu..................................................................   2      1    ea1
....................ugUuuuaucuuuggaguucuugu..................................................................   2      1    ea1
....................ugguuuaucuuuggaAuucuugu..................................................................   1      1    ea1
....................uggAuuaucuuuggaguucuugu..................................................................   3      1    ea1
....................ugguuCaucuuuggaguucuugu..................................................................   1      1    ea1
....................Ggguuuaucuuuggaguucuugu..................................................................   55     1    ea1
....................ugguuuaCcuuuggaguucuugu..................................................................   2      1    ea1
....................ugguuuaucuuuggaguucuuguU.................................................................   24     1    ea1
....................ugguuuaucuuuggaguucuuguA.................................................................   272    1    ea1
....................ugguuuaucuuuggaguucuugug.................................................................   14     0    ea1
....................ugguuuaucuuuggaguucuuguC.................................................................   8      1    ea1
....................ugguuuaucuuuggaguucuuAug.................................................................   1      1    ea1
....................Ggguuuaucuuuggaguucuugug.................................................................   1      1    ea1
....................ugguuuaucuuuggaguucuugugu................................................................   2      0    ea1
....................Ggguuuaucuuuggaguucuugugu................................................................   1      1    ea1
....................ugguuuaucuuuggaguucuuguUu................................................................   5      1    ea1
....................ugguuuaucuuuggaguucuugugG................................................................   1      1    ea1
....................ugguuuaucuuuggaguucuuguAu................................................................   20     1    ea1
....................ugguuuaucuuuggaguucuuguAuu...............................................................   2      1    ea1
....................ugguuuaucuuuggaguucuuguguu...............................................................   2      0    ea1
....................ugguuuaCcuuuggaguucuuguguuu..............................................................   1      1    ea1
....................ugguuuaucuuuggaguucuuguguuu..............................................................   3      0    ea1
....................Ggguuuaucuuuggaguucuuguguuu..............................................................   1      1    ea1
.....................gguuuaucuuuggaguucuug...................................................................   14     0    ea1
.....................Uguuuaucuuuggaguucuug...................................................................   1      1    ea1
.....................gguuuaucuuuggaguucuuA...................................................................   7      1    ea1
.....................ggGuuaucuuuggaguucuug...................................................................   2      1    ea1
......................guuuaucuuuggaguucuug...................................................................   3      0    ea1
......................guuuaucuuuggaguucuugug.................................................................   2      0    ea1
......................Auuuaucuuuggaguucuugug.................................................................   1      1    ea1
```

```
                    Mature                Star
agucauuuuaauauaccucaugguuuaucuuuggaguucuuguguuuaucauaaggucuucauugauugacaauaagguauauaauggacuucagauuugacagcuua
..............................uuggaguucuuguguuuauca.........................................................   1    0    ea1
..........................................uguuuaucauaaggucuucauA............................................   1    1    ea1
....................................................aaggucuucauugauugaca....................................   1    0    ea1
....................................................aaggucuucauugauugacaau..................................   1    0    ea1
....................................................aaggucuucauugauugacaaua.................................   9    0    ea1
....................................................Gaggucuucauugauugacaaua.................................   1    1    ea1
....................................................aaggucuucauugauugacaauU.................................   6    1    ea1
....................................................aaggucuucauugauugacaauUa................................   7    1    ea1
.....................................................aggucuucauugauugacaau..................................   1    0    ea1
.....................................................aggucuucauugauugacaaua.................................   3    0    ea1
.....................................................aggucuucauugauugacaauU.................................   1    1    ea1
.....................................................aggucuucauugauugacaauC.................................   1    1    ea1
.....................................................aggucuucauugauugacaauUa................................   4    1    ea1

....................ugguuuaucuuuggaguucu....................................................................   7    0    er2
....................ugguuuaucuuuggaguucuu...................................................................   46   0    er2
....................ugguuuaucuuuggaguucuA...................................................................   5    1    er2
....................uggGuuaucuuuggaguucuug..................................................................   2    1    er2
....................Ngguuuaucuuuggaguucuug..................................................................   2    1    er2
....................Cgguuuaucuuuggaguucuug..................................................................   1    1    er2
....................ugguAuaucuuuggaguucuug..................................................................   1    1    er2
....................ugguCuaucuuuggaguucuug..................................................................   1    1    er2
....................ugguuuaucuuuggGguucuug..................................................................   1    1    er2
....................ugguuuaucuuuggagCucuug..................................................................   1    1    er2
....................ugguuuaucuuuggaguucuuU..................................................................   31   1    er2
....................ugguuuaucuuuggaguucuuA..................................................................   118  1    er2
....................ugguuuaucuuuggaguucuug..................................................................   768  0    er2
....................ugguuuaucuuuggaguCcuug..................................................................   1    1    er2
....................ugguuuaucuuuggaguucuugu.................................................................   16   0    er2
....................ugguuuauUuuuggaguucuugu.................................................................   2    1    er2
....................ugguuuaucuuuggaguucuuUu.................................................................   3    1    er2
....................ugguuuaucuuuggaguucuuAu.................................................................   9    1    er2
....................ugguuuaucuuuggaguucuuguA................................................................   8    1    er2
.....................gguuuaucuuuggaguucuu...................................................................   3    0    er2
.....................Uguuuaucuuuggaguucuug..................................................................   1    1    er2
.....................gguuuaucuuuggaguucuuA..................................................................   6    1    er2
.....................gguuuaucuuuggaguucuug..................................................................   48   0    er2
.....................gguuuaucuuuggaguucuugu.................................................................   1    0    er2
.....................gguuuaucuuuggaguucuuUu.................................................................   1    1    er2
....................................................aaggucuucauugauugacaau..................................   7    0    er2
....................................................aaggucuucauugauugacaauU.................................   3    1    er2
.....................................................aggucuucauugauugacaaua.................................   7    0    er2
.....................................................aggucuucauugauugacaauU.................................   10   1    er2
.....................................................aggucuucauugauugacaauUa................................   8    1    er2

....................ugguuuaucuuuggaguucu....................................................................   4    0    ea2
....................ugguuuaucuuuggaguucuu...................................................................   31   0    ea2
....................ugguuuaucuuuggaguucuA...................................................................   3    1    ea2
....................ugguuuaucuuuggaguucAu...................................................................   1    1    ea2
....................ugguCuaucuuuggaguucuug..................................................................   4    1    ea2
....................uAguuuaucuuuggaguucuug..................................................................   2    1    ea2
....................Ngguuuaucuuuggaguucuug..................................................................   3    1    ea2
....................ugguuuaucuuuggaguucuug..................................................................   466  0    ea2
....................ugguuuaucuuuggaguucuuU..................................................................   8    1    ea2
....................ugguuuaucuuuggGguucuug..................................................................   1    1    ea2
....................ugguuuaucuuuggaguucuuA..................................................................   70   1    ea2
....................ugguuuaucuuuggaguucuuAu.................................................................   1    1    ea2
....................ugguuuaucuuuggaguucuugA.................................................................   1    1    ea2
....................ugguuuaucuuuggaguucuuUu.................................................................   2    1    ea2
....................ugguuuaucuuuggaguucuuguA................................................................   12   1    ea2
....................ugguuuaucuuuggaguucuugug................................................................   3    0    ea2
.....................gguuuaucuuuggaguucuu...................................................................   1    0    ea2
.....................gguuuaucuuuggaguucuuU..................................................................   3    1    ea2
.....................gguuuaucuuuggaguucuug..................................................................   38   0    ea2
.....................gguuuaucuuuggaguucuuA..................................................................   8    1    ea2
......................guuuaucuuuggaguucuuU..................................................................   7    1    ea2
............................................uuuaucauaaggucuucauuga..........................................   5    0    ea2
.............................................uuaucauaaggucuucauuga..........................................   1    0    ea2
....................................................aaggucuucauugauugacaau..................................   2    0    ea2
....................................................aaggucuucauugauugacaauG.................................   1    1    ea2
```

```
                    Mature                Star
agucauuuuaauauaccucaugguuuaucuuuggaguucuuguguuuaucauaaggucuucauugauugacaauaagguauauaauggacuuucagauuugacagcuua
....................................................aaggucuucauugauugacaauaa.................................   2     0     ea2
.....................................................aggucuucauugauugaca.....................................   5     0     ea2
.....................................................aggucuucauugauugacaau...................................   2     0     ea2
.....................................................aggucuucauugauugacaaua..................................   5     0     ea2
.....................................................aggucuucauugauugacaauU..................................   7     1     ea2

...................Cugguuuaucuuuggaguucuug...................................................................   1     1     eg2
....................ugguuuaucuuuggaguucuu....................................................................   4     0     eg2
....................ugguuuaucuuuggaguucuuU...................................................................   3     1     eg2
....................Ngguuuaucuuuggaguucuug...................................................................   1     1     eg2
....................ugguuuaucuuuggagGucuug...................................................................   1     1     eg2
....................ugguuuaucuuuggaguucuug...................................................................   107   0     eg2
....................ugguuuaucuuuggaguucuuA...................................................................   5     1     eg2
....................ugguuuaucuuuggaguucuuguA.................................................................   1     1     eg2
.....................gguuuaucuuuggaguucuuU...................................................................   2     1     eg2
.....................gguuuaucuuuggaguucuug...................................................................   3     0     eg2
.....................ggGuuaucuuuggaguucuug...................................................................   1     1     eg2
....................................................aaggucuCcauugauugacaau...................................   1     1     eg2
.....................................................aggucuucauugauugacaaua..................................   2     0     eg2
.....................................................aggucuucauugauugacaauU..................................   1     1     eg2
.....................................................aggucuucauugauugacaauUa.................................   3     1     eg2

...................Cugguuuaucuuuggaguucuug...................................................................   2     1     eg1
....................ugguuuaucuuuggaguC.......................................................................   1     1     eg1
....................ugguuuaucuuuggaguG.......................................................................   1     1     eg1
....................Ggguuuaucuuuggaguu.......................................................................   3     1     eg1
....................ugguuuaucuuuggaguu.......................................................................   9     0     eg1
....................ugguuuaucuuuggaguuc......................................................................   1     0     eg1
....................ugguuuaucuuuggaguuU......................................................................   1     1     eg1
....................ugguuuaucuuCggaguucu.....................................................................   1     1     eg1
....................ugguuuaucuuuggaguucG.....................................................................   18    1     eg1
....................Cgguuuaucuuuggaguucu.....................................................................   1     1     eg1
....................ugguuuaucuuuggaguucA.....................................................................   10    1     eg1
....................ugguuuaucuuuggGguucu.....................................................................   2     1     eg1
....................ugguuuaucuuuggaguucu.....................................................................   190   0     eg1
....................ugguuuaucuuuggaguucC.....................................................................   21    1     eg1
....................uggCuuaucuuuggaguucu.....................................................................   1     1     eg1
....................ugguuuaucCuuggaguucu.....................................................................   3     1     eg1
....................ugguuuaucuuuggaguuUu.....................................................................   1     1     eg1
....................Ggguuuaucuuuggaguucu.....................................................................   17    1     eg1
....................ugguuuaucuuuggagCucu.....................................................................   1     1     eg1
....................ugguuuaucuCuggaguucuu....................................................................   1     1     eg1
....................ugguuuaucuuuggGguucuu....................................................................   1     1     eg1
....................uAguuuaucuuuggaguucuu....................................................................   1     1     eg1
....................ugguuuaucuuuggaguucuC....................................................................   32    1     eg1
....................ugguuuaucuuuggaguuUuu....................................................................   1     1     eg1
....................ugguuuauUuuuggaguucuu....................................................................   1     1     eg1
....................uggCuuaucuuuggaguucuu....................................................................   3     1     eg1
....................ugguCuaucuuuggaguucuu....................................................................   1     1     eg1
....................Agguuuaucuuuggaguucuu....................................................................   1     1     eg1
....................ugguuuaucuuuggaguucuG....................................................................   6     1     eg1
....................ugguuuaucuuuggaguucuu....................................................................   248   0     eg1
....................ugguuuaucuuuggaguucuA....................................................................   5     1     eg1
....................ugguuuaucAuuggaguucuu....................................................................   1     1     eg1
....................Ggguuuaucuuuggaguucuu....................................................................   55    1     eg1
....................ugguuuaucuuuggaguCcuu....................................................................   2     1     eg1
....................ugguuuGucuuuggaguucuu....................................................................   1     1     eg1
....................ugguuuGucuuuggaguucuug...................................................................   5     1     eg1
....................ugguuuaucuuCggaguucuug...................................................................   7     1     eg1
....................ugguuuaucuCuggaguucuug...................................................................   1     1     eg1
....................uggGuuaucuuuggaguucuug...................................................................   7     1     eg1
....................ugguuuaucuuugAaguucuug...................................................................   2     1     eg1
....................ugguuuaucuuuggaguuUuug...................................................................   3     1     eg1
....................ugguuuaucuuuggaguucuAg...................................................................   1     1     eg1
....................Agguuuaucuuuggaguucuug...................................................................   2     1     eg1
....................uggCuuaucuuuggaguucuug...................................................................   8     1     eg1
....................ugguuuaucuuuggaguCcuug...................................................................   10    1     eg1
....................ugAuuuaucuuuggaguucuug...................................................................   2     1     eg1
....................ugguuuaucuuAggaguucuug...................................................................   2     1     eg1
....................ugguuuaucuuuggCguucuug...................................................................   1     1     eg1
```

Mature **Star**

```
agucauuuuaauauaccucaugguuuaucuuuggaguucuuguguuuaucauaaggucuucauugauugacaauaagguauauaauggacuuucagauuugacagcuua
....................ugguuuaucuuuggaguucuuC...................................................................   9      1   eg1
....................ugguuuaucuuuggaguucuCg...................................................................   40     1   eg1
....................ugguuuaucuuuggaguucCug...................................................................   6      1   eg1
....................ugguuuaCcuuuggaguucuug...................................................................   9      1   eg1
....................ugguuuaucuuGggaguucuug...................................................................   1      1   eg1
....................ugguuCaucuuuggaguucuug...................................................................   4      1   eg1
....................uAguuuaucuuuggaguucuug...................................................................   1      1   eg1
....................Ggguuuaucuuuggaguucuug...................................................................   535    1   eg1
....................ugguuuaucCuuggaguucuug...................................................................   16     1   eg1
....................ugguCuaucuuuggaguucuug...................................................................   7      1   eg1
....................ugguuuaucuuuggaguucuug...................................................................   3203   0   eg1
....................ugguuuaucuuuggagCucuug...................................................................   7      1   eg1
....................ugguuuaAcuuuggaguucuug...................................................................   2      1   eg1
....................ugguuuaucuuuggaguucuuU...................................................................   113    1   eg1
....................ugguuuaucuuuggaguucuGg...................................................................   1      1   eg1
....................ugguuuauUuuuggaguucuug...................................................................   1      1   eg1
....................ugguuuaucuAuggaguucuug...................................................................   1      1   eg1
....................ugguAuaucuuuggaguucuug...................................................................   2      1   eg1
....................ugUuuuaucuuuggaguucuug...................................................................   1      1   eg1
....................ugguuuaucuuuggUguucuug...................................................................   2      1   eg1
....................ugguuuaucuuuggaAuucuug...................................................................   2      1   eg1
....................Cgguuuaucuuuggaguucuug...................................................................   9      1   eg1
....................ugguuuaucuuuggGguucuug...................................................................   9      1   eg1
....................ugguuuaucuuuggaguucuuA...................................................................   370    1   eg1
....................ugguCuaucuuuggaguucuugu..................................................................   1      1   eg1
....................ugguuuaucuuuggaguucuuAu..................................................................   7      1   eg1
....................Ggguuuaucuuuggaguucuugu..................................................................   17     1   eg1
....................ugguuuaucuuuggaguucuuUu..................................................................   12     1   eg1
....................ugguuuaucuuuggaguucuugG..................................................................   9      1   eg1
....................ugguuuaucuuuggaguucuCgu..................................................................   1      1   eg1
....................Cgguuuaucuuuggaguucuugu..................................................................   1      1   eg1
....................ugguuuaucuuuggaguucuugC..................................................................   11     1   eg1
....................ugguuuaucuuuggaguucuugA..................................................................   10     1   eg1
....................ugguuuaucuuuggaguucuugu..................................................................   66     0   eg1
....................ugguuuaucCuuggaguucuugu..................................................................   1      1   eg1
....................ugguuuaucuuuggaguucuuguC.................................................................   2      1   eg1
....................Ggguuuaucuuuggaguucuugug.................................................................   2      1   eg1
....................ugguuuaucuuuggaguucuuguA.................................................................   39     1   eg1
....................ugguuuaucuuuggaguucuuguU.................................................................   6      1   eg1
....................ugguuuaucuuuggaguucuugug.................................................................   1      0   eg1
....................ugguuuaucuuuggaguucuuguUu................................................................   2      1   eg1
....................ugguuuaucuuuggaguucuuguAu................................................................   8      1   eg1
....................Ggguuuaucuuuggaguucuuguguuu..............................................................   1      1   eg1
....................ugguuuaucuuuggaguucuuguguuu..............................................................   1      0   eg1
.....................ggGuuaucuuuggaguucuug...................................................................   1      1   eg1
.....................gguuuaucuuuggaguucuug...................................................................   3      0   eg1
........................................uguguuuaucauaaggucuuc................................................   1      0   eg1
....................................................aaggucuucauugauugacaau...................................   3      0   eg1
....................................................Gaggucuucauugauugacaau...................................   1      1   eg1
....................................................aaggucuucauugauugacaauU..................................   2      1   eg1
....................................................aaggucuucauugauugacaauUa.................................   2      1   eg1
.....................................................aggucuucauugauugacaau...................................   1      0   eg1
.....................................................aggucuucauugauugacaauU..................................   2      1   eg1
.....................................................aggucuucauugauugacaaua..................................   1      0   eg1
.....................................................Gggucuucauugauugacaaua..................................   1      1   eg1
.....................................................aggucuucauugauugacaauUa.................................   1      1   eg1

...................Cugguuuaucuuuggaguucuug...................................................................   2      1   er1
....................ugguuuaucuuuggaguu.......................................................................   3      0   er1
....................ugguuuaucuuuggaguC.......................................................................   2      1   er1
....................ugguuuaucuuuggaguuc......................................................................   4      0   er1
....................ugguuuaucCuuggaguuc......................................................................   1      1   er1
....................ugguuuaucGuuggaguucu.....................................................................   1      1   er1
....................Ggguuuaucuuuggaguucu.....................................................................   19     1   er1
....................ugguuuaucuuuggaguucA.....................................................................   1      1   er1
....................ugguuuaucuuuggaguucC.....................................................................   12     1   er1
....................ugguuuaucuuuggaguucu.....................................................................   109    0   er1
....................ugguuuaucuuuggaguucuC....................................................................   9      1   er1
....................ugguCuaucuuuggaguucuu....................................................................   3      1   er1
....................ugguuuaucuuuAgaguucuu....................................................................   1      1   er1
```

```
                    Mature                           Star
agucauuuuaauauaccucaugguuuaucuuuggaguucuuguguuuaucauaaggucuucauugauugacaauaagguauauaauggacuuucagauuugacagcuua
....................ugguuuaCcuuuggaguucuu....................................................................    1      1    er1
....................ugAuuuaucuuuggaguucuu....................................................................    2      1    er1
....................ugguuuaucuuuggaguucuu....................................................................    166    0    er1
....................ugguuuaucuuuggaAuucuu....................................................................    1      1    er1
....................ugguAuaucuuuggaguucuu....................................................................    1      1    er1
....................ugguuuaucuuuggaguucuG....................................................................    1      1    er1
....................ugguuuaucuuuggGguucuu....................................................................    1      1    er1
....................Ggguuuaucuuuggaguucuu....................................................................    35     1    er1
....................uggCuuaucuuuggaguucuug...................................................................    5      1    er1
....................ugguuuaucuuuggCguucuug...................................................................    1      1    er1
....................ugguuuaucuuuggaguucAug...................................................................    2      1    er1
....................ugguuuaucuuuggaguGcuug...................................................................    1      1    er1
....................ugguuuaucuCuggaguucuug...................................................................    4      1    er1
....................ugguuuaucuuugAaguucuug...................................................................    1      1    er1
....................ugguuuaucuuuggaguucuuU...................................................................    73     1    er1
....................ugguuuauUuuuggaguucuug...................................................................    3      1    er1
....................ugguuuGucuuuggaguucuug...................................................................    5      1    er1
....................ugguuuaucuuuggaguucuuA...................................................................    261    1    er1
....................ugAuuuaucuuuggaguucuug...................................................................    1      1    er1
....................ugguuuaucuuuggagCucuug...................................................................    1      1    er1
....................Ggguuuaucuuuggaguucuug...................................................................    400    1    er1
....................ugguuuaucuuuggGguucuug...................................................................    11     1    er1
....................ugguuCaucuuuggaguucuug...................................................................    3      1    er1
....................ugguuuaucAuuggaguucuug...................................................................    2      1    er1
....................uggAuuaucuuuggaguucuug...................................................................    2      1    er1
....................ugguuAaucuuuggaguucuug...................................................................    1      1    er1
....................ugguuuaGcuuuggaguucuug...................................................................    1      1    er1
....................ugguuuaucuuCggaguucuug...................................................................    7      1    er1
....................ugguuuaucuuuggaguCcuug...................................................................    11     1    er1
....................ugguuuaucuuuggaAuucuug...................................................................    3      1    er1
....................Agguuuaucuuuggaguucuug...................................................................    2      1    er1
....................ugguCuaucuuuggaguucuug...................................................................    12     1    er1
....................ugguuuaucuuuggaguAcuug...................................................................    1      1    er1
....................ugguuuaucuuuggaguucCug...................................................................    1      1    er1
....................ugguuuaucuuuggaguuUuug...................................................................    1      1    er1
....................ugguuuaucuuuggaguucuGg...................................................................    1      1    er1
....................ugguuuaucuuuggagGucuug...................................................................    3      1    er1
....................uggGuuaucuuuggaguucuug...................................................................    2      1    er1
....................ugguGuaucuuuggaguucuug...................................................................    1      1    er1
....................ugguuuaucCuuggaguucuug...................................................................    8      1    er1
....................ugguuuaCcuuuggaguucuug...................................................................    2      1    er1
....................Cgguuuaucuuuggaguucuug...................................................................    3      1    er1
....................ugguuuaucuuuggUguucuug...................................................................    4      1    er1
....................ugguuuaucuuugUaguucuug...................................................................    1      1    er1
....................ugguuuaucuuuggaguucuCg...................................................................    4      1    er1
....................ugguuuaucuuuggaguucuuC...................................................................    18     1    er1
....................ugguuuaucuuuggaguucuug...................................................................    2379   0    er1
....................ugUuuuaucuuuggaguucuug...................................................................    2      1    er1
....................ugguuuaucuuuggaguCcuugu..................................................................    2      1    er1
....................Cgguuuaucuuuggaguucuugu..................................................................    1      1    er1
....................ugguuuaucuuuggaguucuuAu..................................................................    3      1    er1
....................ugguuuaucuuuggagCucuugu..................................................................    1      1    er1
....................ugguuuaucuuuggaguucuugC..................................................................    8      1    er1
....................ugguuuaucuuuggaguucuugA..................................................................    11     1    er1
....................ugguuuaucuuuggaguucuuUu..................................................................    7      1    er1
....................ugguCuaucuuuggaguucuugu..................................................................    1      1    er1
....................Ggguuuaucuuuggaguucuugu..................................................................    8      1    er1
....................ugguuuaucuuuggaguucuugu..................................................................    54     0    er1
....................ugguuuaucuuuggaguucuuguU.................................................................    4      1    er1
....................ugguuuaucuuuggaguucuugug.................................................................    1      0    er1
....................Ggguuuaucuuuggaguucuugug.................................................................    1      1    er1
....................ugguuuaucuuuggaguucuuguA.................................................................    29     1    er1
....................ugguuuaucuuuggaguucuuguAu................................................................    4      1    er1
....................ugguuuaucuuuggaguucuuguguuu..............................................................    3      0    er1
....................Ggguuuaucuuuggaguucuuguguuu..............................................................    1      1    er1
.....................gguuuaucuuuggaguucuug...................................................................    3      0    er1
....................................................aaggucuucauugauugacaauU..................................    2      1    er1
....................................................aaggucuucauugauugacaaua..................................    2      0    er1
....................................................aaggucuucauugauugacaauUa.................................    1      1    er1
.....................................................aggucuucauugauugacaaua..................................    1      0    er1
```

```
                    Mature                          Star
agucauuuuaauauaccucaugguuuaucuuuggaguucuuguguuuaucauaaggucuucauugauugacaauaagguauauaauggacuuucagauuugacagcuua
.....................................................aggucuucauugauugacaauU..................................    1    1    er1
.....................................................aggucuucauugauugacaauUa.................................    3    1    er1
```

Provisional ID	:	ScUbCFx_65_7382
Score total	:	21354.1
Score for star read(s)	:	3.9
Score for read counts	:	21345
Score for mfe	:	0.6
Score for randfold	:	1.6
Score for cons. seed	:	3
Total read count	:	41879
Mature read count	:	41754
Loop read count	:	0
Star read count	:	125

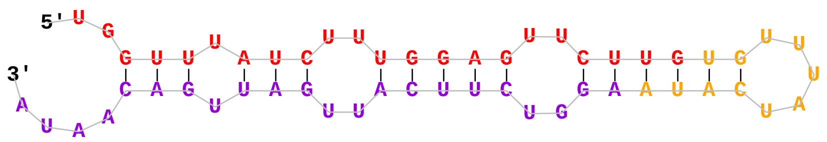

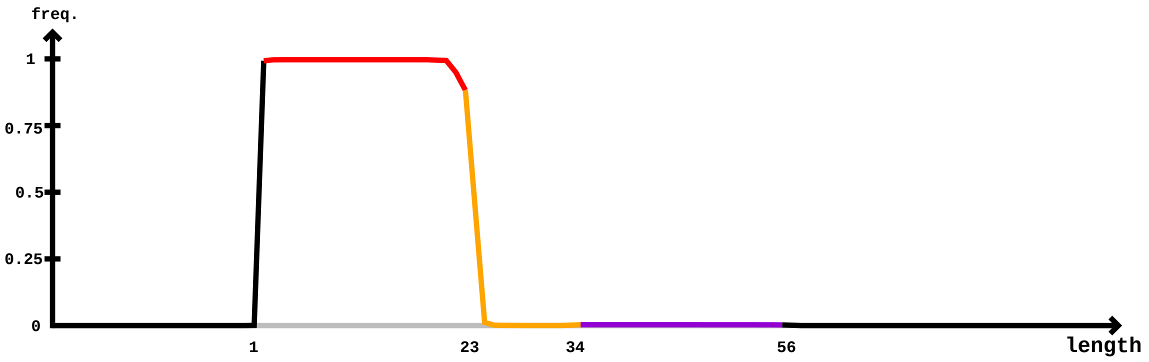


```
                        Mature                  Star
5'- agucauuuuaauauaccucaugguuuaucuuuggaguucuuguguuuaucauaaggucuucauugauugacaauaagguauauaauggaauuucagauuugacagcuua  -3'   obs
    agucauuuuaauauaccucaugguuuaucuuuggaguucuuguguuuaucauaaggucuucauugauugacaauaagguauauaauggaauuucagauuugacagcuua         exp
    .((((.....((((((((.((.(((.(((..(((((..((((((.....))))))..))))).))).))).)).))))))))..(((....)))....)))).....    reads    mm    sample
    ...................Cugguuuaucuuuggaguucuug...................................................................    2        1     eg1
    ....................Ggguuuaucuuuggaguu.......................................................................    3        1     eg1
    ....................ugguuuaucuuuggaguG.......................................................................    1        1     eg1
    ....................ugguuuaucuuuggaguC.......................................................................    1        1     eg1
    ....................ugguuuaucuuuggaguu.......................................................................    9        0     eg1
    ....................ugguuuaucuuuggaguuU......................................................................    1        1     eg1
    ....................ugguuuaucuuuggaguuc......................................................................    1        0     eg1
    ....................uggCuuaucuuuggaguucu.....................................................................    1        1     eg1
    ....................ugguuuaucuuCggaguucu.....................................................................    1        1     eg1
    ....................ugguuuaucuuuggaguucA.....................................................................    10       1     eg1
    ....................ugguuuaucuuuggaguucG.....................................................................    18       1     eg1
    ....................Ggguuuaucuuuggaguucu.....................................................................    17       1     eg1
    ....................Cgguuuaucuuuggaguucu.....................................................................    1        1     eg1
    ....................ugguuuaucuuuggaguucC.....................................................................    21       1     eg1
    ....................ugguuuaucuuuggagCucu.....................................................................    1        1     eg1
    ....................ugguuuaucCuuggaguucu.....................................................................    3        1     eg1
    ....................ugguuuaucuuuggaguuUu.....................................................................    1        1     eg1
    ....................ugguuuaucuuuggaguucu.....................................................................    190      0     eg1
    ....................ugguuuaucuuuggGguucu.....................................................................    2        1     eg1
    ....................uggCuuaucuuuggaguucuu....................................................................    3        1     eg1
    ....................ugguuuauUuuuggaguucuu....................................................................    1        1     eg1
    ....................ugguCuaucuuuggaguucuu....................................................................    1        1     eg1
    ....................ugguuuaucuuuggaguucuA....................................................................    5        1     eg1
    ....................ugguuuaucAuuggaguucuu....................................................................    1        1     eg1
    ....................ugguuuaucuuuggaguucuu....................................................................    248      0     eg1
    ....................ugguuuaucuCuggaguucuu....................................................................    1        1     eg1
    ....................ugguuuGucuuuggaguucuu....................................................................    1        1     eg1
    ....................ugguuuaucuuuggaguucuG....................................................................    6        1     eg1
    ....................ugguuuaucuuuggaguCcuu....................................................................    2        1     eg1
    ....................ugguuuaucuuuggaguuUuu....................................................................    1        1     eg1
    ....................ugguuuaucuuuggGguucuu....................................................................    1        1     eg1
    ....................Ggguuuaucuuuggaguucuu....................................................................    55       1     eg1
    ....................ugguuuaucuuuggaguucuC....................................................................    32       1     eg1
```

```
                    Mature                Star
agucauuuuaauauaccucauggguuuaucuuuggaguucuuguguuuaucauaaggucuucauugauugacaauaagguauauaauggaauuucagauuugacagcuua
....................uAguuuaucuuuggaguucuu....................................................................    1      1      eg1
....................Agguuuaucuuuggaguucuu....................................................................    1      1      eg1
....................ugguuuGucuuuggaguucuug...................................................................    5      1      eg1
....................ugguuuaCcuuuggaguucuug...................................................................    9      1      eg1
....................Cgguuuaucuuuggaguucuug...................................................................    9      1      eg1
....................uggCuuaucuuuggaguucuug...................................................................    8      1      eg1
....................ugguuuaAcuuuggaguucuug...................................................................    2      1      eg1
....................ugguuuaucuuuggaguucuAg...................................................................    1      1      eg1
....................ugguuuaucuuuggaAuucuug...................................................................    2      1      eg1
....................ugguuuaucuuugAaguucuug...................................................................    2      1      eg1
....................ugguuuauUuuuggaguucuug...................................................................    1      1      eg1
....................ugguuuaucuuuggaguucuug...................................................................    3203   0      eg1
....................ugguuCaucuuuggaguucuug...................................................................    4      1      eg1
....................ugguuuaucuuuggaguucCug...................................................................    6      1      eg1
....................ugguuuaucuuuggaguucuuC...................................................................    9      1      eg1
....................ugguuuaucuuGggaguucuug...................................................................    1      1      eg1
....................ugAuuuaucuuuggaguucuug...................................................................    2      1      eg1
....................Agguuuaucuuuggaguucuug...................................................................    2      1      eg1
....................ugguuuaucuuuggagCucuug...................................................................    7      1      eg1
....................ugguuuaucCuuggaguucuug...................................................................    16     1      eg1
....................ugguuuaucuuuggaguucuuA...................................................................    370    1      eg1
....................ugguuuaucuuuggaguucuuU...................................................................    113    1      eg1
....................ugguCuaucuuuggaguucuug...................................................................    7      1      eg1
....................ugUuuuaucuuuggaguucuug...................................................................    1      1      eg1
....................ugguuuaucuuuggaguucuGg...................................................................    1      1      eg1
....................ugguuuaucuCuggaguucuug...................................................................    1      1      eg1
....................ugguuuaucuuuggUguucuug...................................................................    2      1      eg1
....................ugguuuaucuuuggaguCcuug...................................................................    10     1      eg1
....................ugguuuaucuuuggCguucuug...................................................................    1      1      eg1
....................ugguuuaucuuAggaguucuug...................................................................    2      1      eg1
....................ugguuuaucuuCggaguucuug...................................................................    7      1      eg1
....................ugguuuaucuuuggGguucuug...................................................................    9      1      eg1
....................ugguuuaucuAuggaguucuug...................................................................    1      1      eg1
....................Ggguuuaucuuuggaguucuug...................................................................    535    1      eg1
....................ugguuuaucuuuggaguuUuug...................................................................    3      1      eg1
....................uAguuuaucuuuggaguucuug...................................................................    1      1      eg1
....................uggGuuaucuuuggaguucuug...................................................................    7      1      eg1
....................ugguuuaucuuuggaguucuCg...................................................................    40     1      eg1
....................ugguAuaucuuuggaguucuug...................................................................    2      1      eg1
....................ugguuuaucuuuggaguucuugC..................................................................    11     1      eg1
....................ugguuuaucuuuggaguucuugu..................................................................    66     0      eg1
....................ugguuuaucuuuggaguucuugG..................................................................    9      1      eg1
....................ugguuuaucuuuggaguucuuAu..................................................................    7      1      eg1
....................ugguuuaucuuuggaguucuuUu..................................................................    12     1      eg1
....................ugguuuaucuuuggaguucuugA..................................................................    10     1      eg1
....................ugguuuaucuuuggaguucuCgu..................................................................    1      1      eg1
....................Cgguuuaucuuuggaguucuugu..................................................................    1      1      eg1
....................ugguuuaucCuuggaguucuugu..................................................................    1      1      eg1
....................Ggguuuaucuuuggaguucuugu..................................................................    17     1      eg1
....................ugguCuaucuuuggaguucuugu..................................................................    1      1      eg1
....................ugguuuaucuuuggaguucuuguU.................................................................    6      1      eg1
....................ugguuuaucuuuggaguucuugug.................................................................    1      0      eg1
....................ugguuuaucuuuggaguucuuguA.................................................................    39     1      eg1
....................Ggguuuaucuuuggaguucuugug.................................................................    2      1      eg1
....................ugguuuaucuuuggaguucuuguC.................................................................    2      1      eg1
....................ugguuuaucuuuggaguucuuguAu................................................................    8      1      eg1
....................ugguuuaucuuuggaguucuuguUu................................................................    2      1      eg1
....................Ggguuuaucuuuggaguucuuguguuu..............................................................    1      1      eg1
....................ugguuuaucuuuggaguucuuguguuu..............................................................    1      0      eg1
.....................ggGuuaucuuuggaguucuug...................................................................    1      1      eg1
.....................gguuuaucuuuggaguucuug...................................................................    3      0      eg1
........................................uguguuuaucauaaggucuuc................................................    1      0      eg1
....................................................Gaggucuucauugauugacaau...................................    1      1      eg1
....................................................aaggucuucauugauugacaau...................................    3      0      eg1
....................................................aaggucuucauugauugacaauU..................................    2      1      eg1
....................................................aaggucuucauugauugacaauUa.................................    2      1      eg1
.....................................................aggucuucauugauugacaau...................................    1      0      eg1
.....................................................aggucuucauugauugacaauU..................................    2      1      eg1
.....................................................aggucuucauugauugacaaua..................................    1      0      eg1
.....................................................Gggucuucauugauugacaaua..................................    1      1      eg1
```

```
                    Mature                Star
agucauuuuaauauaccucaugguuuaucuuuggaguucuuguguuuaucauaaggucuucauugauugacaauaagguauauaauggaauuucagauuugacagcuua
.....................................................aggucuucauugauugacaauUa.................................      1     1     eg1

...................Cugguuuaucuuuggaguucuug...................................................................      2     1     er1
....................ugguuuaucuuuggaguC.......................................................................      2     1     er1
....................ugguuuaucuuuggaguu.......................................................................      3     0     er1
....................ugguuuaucCuuggaguuc......................................................................      1     1     er1
....................ugguuuaucuuuggaguuc......................................................................      4     0     er1
....................ugguuuaucuuuggaguucC.....................................................................      12    1     er1
....................ugguuuaucuuuggaguucA.....................................................................      1     1     er1
....................ugguuuaucGuuggaguucu.....................................................................      1     1     er1
....................Ggguuuaucuuuggaguucu.....................................................................      19    1     er1
....................ugguuuaucuuuggaguucu.....................................................................      109   0     er1
....................ugguuuaucuuuggaguucuG....................................................................      1     1     er1
....................Ggguuuaucuuuggaguucuu....................................................................      35    1     er1
....................ugguuuaucuuuggaguucuu....................................................................      166   0     er1
....................ugguuuaucuuuggaAuucuu....................................................................      1     1     er1
....................ugguuuaucuuuggGguucuu....................................................................      1     1     er1
....................ugguuuaCcuuuggaguucuu....................................................................      1     1     er1
....................ugguuuaucuuuggaguucuC....................................................................      9     1     er1
....................ugguAuaucuuuggaguucuu....................................................................      1     1     er1
....................ugAuuuaucuuuggaguucuu....................................................................      2     1     er1
....................ugguuuaucuuuAgaguucuu....................................................................      1     1     er1
....................ugguCuaucuuuggaguucuu....................................................................      3     1     er1
....................ugguuuGucuuuggaguucuug...................................................................      5     1     er1
....................ugguuuaucuuuggCguucuug...................................................................      1     1     er1
....................ugguuuaucAuuggaguucuug...................................................................      2     1     er1
....................ugguuuaucuuuggaAuucuug...................................................................      3     1     er1
....................ugguuuaucuuuggaguucuug...................................................................      2379  0     er1
....................ugguuuaucuuuggGguucuug...................................................................      11    1     er1
....................Cgguuuaucuuuggaguucuug...................................................................      3     1     er1
....................ugguuuaucuuuggaguucuCg...................................................................      4     1     er1
....................ugguuuaCcuuuggaguucuug...................................................................      2     1     er1
....................ugguuAaucuuuggaguucuug...................................................................      1     1     er1
....................ugguuuaucuuuggaguCcuug...................................................................      11    1     er1
....................ugUuuuaucuuuggaguucuug...................................................................      2     1     er1
....................Ggguuuaucuuuggaguucuug...................................................................      400   1     er1
....................ugguCuaucuuuggaguucuug...................................................................      12    1     er1
....................ugguuCaucuuuggaguucuug...................................................................      3     1     er1
....................ugguuuaucuuuggaguucAug...................................................................      2     1     er1
....................ugguuuaucuuuggaguucuuU...................................................................      73    1     er1
....................ugguuuaucuuuggUguucuug...................................................................      4     1     er1
....................ugguuuauUuuggaguucuug....................................................................      3     1     er1
....................ugguuuaucuuuggaguuUuug...................................................................      1     1     er1
....................ugguuuaucuuuggaguucCug...................................................................      1     1     er1
....................ugguGuaucuuuggaguucuug...................................................................      1     1     er1
....................ugguuuaucuuuggagGucuug...................................................................      3     1     er1
....................ugguuuaucuCuggaguucuug...................................................................      4     1     er1
....................ugguuuaucuuugAaguucuug...................................................................      1     1     er1
....................ugguuuaucuuuggaguAcuug...................................................................      1     1     er1
....................uggCuuaucuuuggaguucuug...................................................................      5     1     er1
....................ugguuuaucuuuggaguucuuA...................................................................      261   1     er1
....................ugguuuaucuuCggaguucuug...................................................................      7     1     er1
....................uggAuuaucuuuggaguucuug...................................................................      2     1     er1
....................ugguuuaucCuuggaguucuug...................................................................      8     1     er1
....................ugguuuaucuuugUaguucuug...................................................................      1     1     er1
....................Agguuuaucuuuggaguucuug...................................................................      2     1     er1
....................ugguuuaucuuuggaguucuGg...................................................................      1     1     er1
....................ugguuuaucuuuggaguucuuC...................................................................      18    1     er1
....................ugguuuaucuuuggagCucuug...................................................................      1     1     er1
....................ugguuuaGcuuuggaguucuug...................................................................      1     1     er1
....................uggGuuaucuuuggaguucuug...................................................................      2     1     er1
....................ugAuuuaucuuuggaguucuug...................................................................      1     1     er1
....................ugguuuaucuuuggaguGcuug...................................................................      1     1     er1
....................ugguuuaucuuuggaguucuuAu..................................................................      3     1     er1
....................ugguuuaucuuuggaguucuuUu..................................................................      7     1     er1
....................Ggguuuaucuuuggaguucuugu..................................................................      8     1     er1
....................ugguuuaucuuuggaguucuugu..................................................................      54    0     er1
....................ugguCuaucuuuggaguucuugu..................................................................      1     1     er1
....................Cgguuuaucuuuggaguucuugu..................................................................      1     1     er1
....................ugguuuaucuuuggaguucuugC..................................................................      8     1     er1
```

Mature Star

```
agucauuuuaauauaccucaugguuuaucuuuggaguucuuguguuuaucauaaggucuucauugauugacaauaagguauauaauggaauuucagauuugacagcuua
....................ugguuuaucuuuggaguucuugA..................................................................    11    1    er1
....................ugguuuaucuuuggagCucuugu..................................................................    1     1    er1
....................ugguuuaucuuuggaguCcuugu..................................................................    2     1    er1
....................ugguuuaucuuuggaguucuugug.................................................................    1     0    er1
....................ugguuuaucuuuggaguucuuguA.................................................................    29    1    er1
....................Ggguuuaucuuuggaguucuugug.................................................................    1     1    er1
....................ugguuuaucuuuggaguucuuguU.................................................................    4     1    er1
....................ugguuuaucuuuggaguucuuguAu................................................................    4     1    er1
....................Ggguuuaucuuuggaguucuuguguuu..............................................................    1     1    er1
....................ugguuuaucuuuggaguucuuguguuu..............................................................    3     0    er1
.....................gguuuaucuuuggaguucuug...................................................................    3     0    er1
....................................................aaggucuucauugauugacaauU..................................    2     1    er1
....................................................aaggucuucauugauugacaaua..................................    2     0    er1
....................................................aaggucuucauugauugacaauUa.................................    1     1    er1
.....................................................aggucuucauugauugacaaua..................................    1     0    er1
.....................................................aggucuucauugauugacaauU..................................    1     1    er1
.....................................................aggucuucauugauugacaauUa.................................    3     1    er1

....................ugguuuaucuuuggaguucu.....................................................................    7     0    er2
....................ugguuuaucuuuggaguucuA....................................................................    5     1    er2
....................ugguuuaucuuuggaguucuu....................................................................    46    0    er2
....................ugguuuaucuuuggaguucuuU...................................................................    31    1    er2
....................ugguuuaucuuuggaguucuuA...................................................................    118   1    er2
....................ugguuuaucuuuggaguCcuug...................................................................    1     1    er2
....................Ngguuuaucuuuggaguucuug...................................................................    2     1    er2
....................ugguuuaucuuuggGguucuug...................................................................    1     1    er2
....................Cgguuuaucuuuggaguucuug...................................................................    1     1    er2
....................ugguAuaucuuuggaguucuug...................................................................    1     1    er2
....................ugguCuaucuuuggaguucuug...................................................................    1     1    er2
....................ugguuuaucuuuggagCucuug...................................................................    1     1    er2
....................ugguuuaucuuuggaguucuug...................................................................    768   0    er2
....................uggGuuaucuuuggaguucuug...................................................................    2     1    er2
....................ugguuuaucuuuggaguucuuUu..................................................................    3     1    er2
....................ugguuuaucuuuggaguucuugu..................................................................    16    0    er2
....................ugguuuauUuuuggaguucuugu..................................................................    2     1    er2
....................ugguuuaucuuuggaguucuuAu..................................................................    9     1    er2
....................ugguuuaucuuuggaguucuuguA.................................................................    8     1    er2
.....................gguuuaucuuuggaguucuu....................................................................    3     0    er2
.....................gguuuaucuuuggaguucuuA...................................................................    6     1    er2
.....................gguuuaucuuuggaguucuug...................................................................    48    0    er2
.....................Uguuuaucuuuggaguucuug...................................................................    1     1    er2
.....................gguuuaucuuuggaguucuuUu..................................................................    1     1    er2
.....................gguuuaucuuuggaguucuugu..................................................................    1     0    er2
....................................................aaggucuucauugauugacaau...................................    7     0    er2
....................................................aaggucuucauugauugacaauU..................................    3     1    er2
.....................................................aggucuucauugauugacaauU..................................    10    1    er2
.....................................................aggucuucauugauugacaaua..................................    7     0    er2
.....................................................aggucuucauugauugacaauUa.................................    8     1    er2

...................Cugguuuaucuuuggaguucuu....................................................................    2     1    ea1
...................Cugguuuaucuuuggaguucuug...................................................................    13    1    ea1
....................ugguuuaucuuuggaguu.......................................................................    34    0    ea1
....................ugguuuaucuuuggaguA.......................................................................    1     1    ea1
....................Ggguuuaucuuuggaguu.......................................................................    8     1    ea1
....................ugguuuaucuuuggaguC.......................................................................    3     1    ea1
....................uggCuuaucuuuggaguu.......................................................................    1     1    ea1
....................Ggguuuaucuuuggaguuc......................................................................    10    1    ea1
....................ugguuuaucuuuggaguuA......................................................................    1     1    ea1
....................ugguuuaucuuuggaguuc......................................................................    27    0    ea1
....................ugguuuaucuuuggagGuc......................................................................    1     1    ea1
....................ugguuuGucuuuggaguuc......................................................................    1     1    ea1
....................ugguuuaucuuuggaguuU......................................................................    2     1    ea1
....................ugguuuaucuuuggaguCcu.....................................................................    2     1    ea1
....................ugguuuaucuCuggaguucu.....................................................................    3     1    ea1
....................Agguuuaucuuuggaguucu.....................................................................    1     1    ea1
....................ugguuuaucuuuAgaguucu.....................................................................    3     1    ea1
....................ugguuuaucuuuggaguucC.....................................................................    126   1    ea1
....................Cgguuuaucuuuggaguucu.....................................................................    3     1    ea1
....................ugguuuaucuuuggGguucu.....................................................................    5     1    ea1
....................ugguAuaucuuuggaguucu.....................................................................    1     1    ea1
```

```
                    Mature                           Star
agucauuuuaauauaccucaugguuuaucuuuggaguucuuguguuuaucauaaggucuucauugauugacaauaagguauauaauggaauuucagauuugacagcuua
....................uggCuuaucuuuggaguucu.....................................................................  5     1  ea1
....................ugguuuaucuAuggaguucu.....................................................................  2     1  ea1
....................Ggguuuaucuuuggaguucu.....................................................................  152   1  ea1
....................ugguCuaucuuuggaguucu.....................................................................  3     1  ea1
....................ugguuCaucuuuggaguucu.....................................................................  1     1  ea1
....................ugguuuaucCuuggaguucu.....................................................................  2     1  ea1
....................ugguuuaucuuuggaguucu.....................................................................  1028  0  ea1
....................uggGuuaucuuuggaguucu.....................................................................  1     1  ea1
....................ugguuuaCcuuuggaguucu.....................................................................  3     1  ea1
....................ugguuuaucuuCggaguucu.....................................................................  3     1  ea1
....................ugguuuaucuuuggaguucA.....................................................................  73    1  ea1
....................ugguuuaucuuuggagCucu.....................................................................  3     1  ea1
....................ugguuuaucuuAggaguucu.....................................................................  2     1  ea1
....................ugguuuaucuuuggaguucG.....................................................................  17    1  ea1
....................ugguuuaucuuuggagAucuu....................................................................  1     1  ea1
....................ugguuuaucuuuggaguucuu....................................................................  1635  0  ea1
....................ugguAuaucuuuggaguucuu....................................................................  1     1  ea1
....................Cgguuuaucuuuggaguucuu....................................................................  3     1  ea1
....................ugguuuaucuAuggaguucuu....................................................................  6     1  ea1
....................ugguuuaucuuuggaguucuG....................................................................  10    1  ea1
....................uggGuuaucuuuggaguucuu....................................................................  3     1  ea1
....................ugguuuaucCuuggaguucuu....................................................................  6     1  ea1
....................ugguuuaucuuCggaguucuu....................................................................  4     1  ea1
....................ugguuuaucuuuggaguucAu....................................................................  1     1  ea1
....................ugguuuGucuuuggaguucuu....................................................................  1     1  ea1
....................ugguuuaucuuuggaguucuC....................................................................  147   1  ea1
....................ugguuuCucuuuggaguucuu....................................................................  1     1  ea1
....................ugguuuaucuuuggagCucuu....................................................................  1     1  ea1
....................ugguuuaucuuAggaguucuu....................................................................  1     1  ea1
....................ugguuuaucAuuggaguucuu....................................................................  1     1  ea1
....................ugguuuaucuuuggaguGcuu....................................................................  1     1  ea1
....................uggCuuaucuuuggaguucuu....................................................................  7     1  ea1
....................ugguuuaucuuuggGguucuu....................................................................  6     1  ea1
....................ugguuuaucuuuggaguucCu....................................................................  4     1  ea1
....................ugguuuaucuuugAaguucuu....................................................................  1     1  ea1
....................uCguuuaucuuuggaguucuu....................................................................  1     1  ea1
....................ugguCuaucuuuggaguucuu....................................................................  5     1  ea1
....................ugguuuaucuuuggaguuUuu....................................................................  2     1  ea1
....................ugguuuaucuuuggaguucGu....................................................................  1     1  ea1
....................uAguuuaucuuuggaguucuu....................................................................  2     1  ea1
....................ugguuuaucuuuggaguucuA....................................................................  38    1  ea1
....................ugguuCaucuuuggaguucuu....................................................................  4     1  ea1
....................ugguuuaCcuuuggaguucuu....................................................................  6     1  ea1
....................ugguuuaucuuuggaguCcuu....................................................................  4     1  ea1
....................uUguuuaucuuuggaguucuu....................................................................  3     1  ea1
....................ugguuuauUuuuggaguucuu....................................................................  1     1  ea1
....................ugguuuaucuuuAgaguucuu....................................................................  1     1  ea1
....................uggAuuaucuuuggaguucuu....................................................................  1     1  ea1
....................Ggguuuaucuuuggaguucuu....................................................................  240   1  ea1
....................ugguuuaucuCuggaguucuu....................................................................  3     1  ea1
....................Agguuuaucuuuggaguucuug...................................................................  17    1  ea1
....................ugguuuaucuuuggaguucAug...................................................................  6     1  ea1
....................ugUuuuaucuuuggaguucuug...................................................................  5     1  ea1
....................uUguuuaucuuuggaguucuug...................................................................  5     1  ea1
....................ugguuuaucuuuggGguucuug...................................................................  47    1  ea1
....................ugguuuaucuuuggaguucGug...................................................................  3     1  ea1
....................ugguuuaucuuuggUguucuug...................................................................  11    1  ea1
....................ugguuuaucuuuggaguAcuug...................................................................  5     1  ea1
....................ugguuCaucuuuggaguucuug...................................................................  41    1  ea1
....................ugguuuaucuuuggaUuucuug...................................................................  5     1  ea1
....................ugguuuaucuuuggaguGcuug...................................................................  2     1  ea1
....................ugguAuaucuuuggaguucuug...................................................................  8     1  ea1
....................ugguuuaucuuuggaguuAuug...................................................................  3     1  ea1
....................ugguuuaucuuuggCguucuug...................................................................  2     1  ea1
....................ugguuuaucuuuggaguucuCg...................................................................  51    1  ea1
....................ugguCuaucuuuggaguucuug...................................................................  57    1  ea1
....................ugguuuaucuuuggaguucuAg...................................................................  3     1  ea1
....................ugguuuaucuuugAaguucuug...................................................................  8     1  ea1
....................ugguuuaucuuuggaguucuGg...................................................................  5     1  ea1
....................ugguuGaucuuuggaguucuug...................................................................  4     1  ea1
```

```
                    Mature                  Star
agucauuuuaauauaccucaugguuuaucuuuggaguucuuguguuuaucauaaggucuucauugauugacaauaagguauauaauggaauuucagauuugacagcuua
....................ugguuuauGuuuggaguucuug...................................................................	1	1	ea1
....................ugguuuaucuuuggaguuGuug...................................................................	3	1	ea1
....................ugguuuauAuuuggaguucuug...................................................................	2	1	ea1
....................ugguuAaucuuuggaguucuug...................................................................	4	1	ea1
....................ugguuuaucuAuggaguucuug...................................................................	7	1	ea1
....................ugguuuaucuuGggaguucuug...................................................................	8	1	ea1
....................Ggguuuaucuuuggaguucuug...................................................................	2914	1	ea1
....................ugguuuaucuuuggaCuucuug...................................................................	2	1	ea1
....................ugguuuaucuCuggaguucuug...................................................................	39	1	ea1
....................uggGuuaucuuuggaguucuug...................................................................	9	1	ea1
....................ugguuuaucuuuggaguucuuC...................................................................	78	1	ea1
....................ugguuuaGcuuuggaguucuug...................................................................	1	1	ea1
....................ugguuuaucuuuggaguucuuA...................................................................	2962	1	ea1
....................ugguuuaucCuuggaguucuug...................................................................	52	1	ea1
....................ugguuuaucuuuggaguuUuug...................................................................	8	1	ea1
....................ugguuuaucuuCggaguucuug...................................................................	71	1	ea1
....................ugguuuaucuuuggagCucuug...................................................................	55	1	ea1
....................uAguuuaucuuuggaguucuug...................................................................	10	1	ea1
....................ugguuuaCcuuuggaguucuug...................................................................	40	1	ea1
....................ugguuuaucuuuggaguucuuU...................................................................	551	1	ea1
....................ugguuuGucuuuggaguucuug...................................................................	21	1	ea1
....................ugguGuaucuuuggaguucuug...................................................................	1	1	ea1
....................ugguuuaucuuuggaguucuug...................................................................	18752	0	ea1
....................ugguuuaucuuuUgaguucuug...................................................................	5	1	ea1
....................uggAuuaucuuuggaguucuug...................................................................	5	1	ea1
....................Cgguuuaucuuuggaguucuug...................................................................	42	1	ea1
....................uggCuuaucuuuggaguucuug...................................................................	74	1	ea1
....................ugguuuaucuuuggaguucCug...................................................................	31	1	ea1
....................ugAuuuaucuuuggaguucuug...................................................................	3	1	ea1
....................ugguuuaucuuuggaAuucuug...................................................................	8	1	ea1
....................ugguuuauUuuuggaguucuug...................................................................	8	1	ea1
....................ugguuuaucuuuggaguCcuug...................................................................	65	1	ea1
....................ugguuuaucuuuAgaguucuug...................................................................	12	1	ea1
....................ugguuuaucAuuggaguucuug...................................................................	10	1	ea1
....................ugguuuaucuuugUaguucuug...................................................................	1	1	ea1
....................ugguuuaucuuAggaguucuug...................................................................	13	1	ea1
....................ugguuuaucuuuggagAucuug...................................................................	4	1	ea1
....................ugguuuaucuuuggaUuucuugu..................................................................	1	1	ea1
....................ugguuuaucuuuggaguucuuCu..................................................................	2	1	ea1
....................ugguuuaucuuuggaguucuuUu..................................................................	49	1	ea1
....................ugguuuaucuuuggaguucuuAu..................................................................	52	1	ea1
....................ugguuCaucuuuggaguucuugu..................................................................	1	1	ea1
....................ugguuuaucuuuggaguucuugu..................................................................	331	0	ea1
....................ugguuuaucCuuggaguucuugu..................................................................	2	1	ea1
....................Ggguuuaucuuuggaguucuugu..................................................................	55	1	ea1
....................ugguAuaucuuuggaguucuugu..................................................................	1	1	ea1
....................ugguuuaucuuuggaguucuugA..................................................................	64	1	ea1
....................ugguuuaucuuuggGguucuugu..................................................................	2	1	ea1
....................ugguuuaucuuuggaAuucuugu..................................................................	1	1	ea1
....................ugUuuuaucuuuggaguucuugu..................................................................	2	1	ea1
....................ugguuuaucuuuggaguucCugu..................................................................	1	1	ea1
....................ugguuuaucuuGggaguucuugu..................................................................	1	1	ea1
....................ugguuuaucuuuggaguucuugG..................................................................	7	1	ea1
....................ugguuuaCcuuuggaguucuugu..................................................................	2	1	ea1
....................uggCuuaucuuuggaguucuugu..................................................................	2	1	ea1
....................uggAuuaucuuuggaguucuugu..................................................................	3	1	ea1
....................ugguuuaucuuuggaguucuugC..................................................................	39	1	ea1
....................ugguCuaucuuuggaguucuugu..................................................................	1	1	ea1
....................uCguuuaucuuuggaguucuugu..................................................................	1	1	ea1
....................ugguuuaucuuuggaguucuuAug.................................................................	1	1	ea1
....................ugguuuaucuuuggaguucuuguU.................................................................	24	1	ea1
....................ugguuuaucuuuggaguucuugug.................................................................	14	0	ea1
....................ugguuuaucuuuggaguucuuguC.................................................................	8	1	ea1
....................ugguuuaucuuuggaguucuuguA.................................................................	272	1	ea1
....................Ggguuuaucuuuggaguucuugug.................................................................	1	1	ea1
....................ugguuuaucuuuggaguucuugugG................................................................	1	1	ea1
....................ugguuuaucuuuggaguucuuguUu................................................................	5	1	ea1
....................ugguuuaucuuuggaguucuugugu................................................................	2	0	ea1
....................ugguuuaucuuuggaguucuuguAu................................................................	20	1	ea1
....................Ggguuuaucuuuggaguucuugugu................................................................	1	1	ea1
```

```
                    Mature                           Star
agucauuuuaauauaccucaugguuuaucuuuggaguucuuguguuuaucauaaggucuucauugauugacaauaagguauauaauggaauuucagauuugacagcuua
....................ugguuuaucuuuggaguucuuguAuu...............................................................   2     1    ea1
....................ugguuuaucuuuggaguucuuguguu...............................................................   2     0    ea1
....................ugguuuaCcuuuggaguucuuguguuu..............................................................   1     1    ea1
....................Ggguuuaucuuuggaguucuuguguuu..............................................................   1     1    ea1
....................ugguuuaucuuuggaguucuuguguuu..............................................................   3     0    ea1
.....................ggGuuaucuuuggaguucuug...................................................................   2     1    ea1
.....................gguuuaucuuuggaguucuuA...................................................................   7     1    ea1
.....................Uguuuaucuuuggaguucuug...................................................................   1     1    ea1
.....................gguuuaucuuuggaguucuug...................................................................   14    0    ea1
......................guuuaucuuuggaguucuug...................................................................   3     0    ea1
......................guuuaucuuuggaguucuugug.................................................................   2     0    ea1
......................Auuuaucuuuggaguucuugug.................................................................   1     1    ea1
..............................uuggaguucuuguguuuauca..........................................................   1     0    ea1
..........................................uguuuaucauaaggucuucauA.............................................   1     1    ea1
....................................................aaggucuucauugauugaca.....................................   1     0    ea1
....................................................aaggucuucauugauugacaau...................................   1     0    ea1
....................................................aaggucuucauugauugacaauU..................................   6     1    ea1
....................................................Gaggucuucauugauugacaaua..................................   1     1    ea1
....................................................aaggucuucauugauugacaaua..................................   9     0    ea1
....................................................aaggucuucauugauugacaauUa.................................   7     1    ea1
.....................................................aggucuucauugauugacaau...................................   1     0    ea1
.....................................................aggucuucauugauugacaaua..................................   3     0    ea1
.....................................................aggucuucauugauugacaauU..................................   1     1    ea1
.....................................................aggucuucauugauugacaauC..................................   1     1    ea1
.....................................................aggucuucauugauugacaauUa.................................   4     1    ea1

...................Cugguuuaucuuuggaguucuug...................................................................   1     1    eg2
....................ugguuuaucuuuggaguucuu....................................................................   4     0    eg2
....................ugguuuaucuuuggagGucuug...................................................................   1     1    eg2
....................ugguuuaucuuuggaguucuug...................................................................   107   0    eg2
....................ugguuuaucuuuggaguucuuU...................................................................   3     1    eg2
....................Ngguuuaucuuuggaguucuug...................................................................   1     1    eg2
....................ugguuuaucuuuggaguucuuA...................................................................   5     1    eg2
....................ugguuuaucuuuggaguucuuguA.................................................................   1     1    eg2
.....................ggGuuaucuuuggaguucuug...................................................................   1     1    eg2
.....................gguuuaucuuuggaguucuug...................................................................   3     0    eg2
.....................gguuuaucuuuggaguucuuU...................................................................   2     1    eg2
....................................................aaggucuCcauugauugacaau...................................   1     1    eg2
.....................................................aggucuucauugauugacaaua..................................   2     0    eg2
.....................................................aggucuucauugauugacaauU..................................   1     1    eg2
.....................................................aggucuucauugauugacaauUa.................................   3     1    eg2

....................ugguuuaucuuuggaguucu.....................................................................   4     0    ea2
....................ugguuuaucuuuggaguucuu....................................................................   31    0    ea2
....................ugguuuaucuuuggaguucAu....................................................................   1     1    ea2
....................ugguuuaucuuuggaguucuA....................................................................   3     1    ea2
....................Ngguuuaucuuuggaguucuug...................................................................   3     1    ea2
....................ugguuuaucuuuggaguucuuU...................................................................   8     1    ea2
....................ugguCuaucuuuggaguucuug...................................................................   4     1    ea2
....................ugguuuaucuuuggaguucuug...................................................................   466   0    ea2
....................ugguuuaucuuuggaguucuuA...................................................................   70    1    ea2
....................uAguuuaucuuuggaguucuug...................................................................   2     1    ea2
....................ugguuuaucuuuggGguucuug...................................................................   1     1    ea2
....................ugguuuaucuuuggaguucuuUu..................................................................   2     1    ea2
....................ugguuuaucuuuggaguucuuAu..................................................................   1     1    ea2
....................ugguuuaucuuuggaguucuugA..................................................................   1     1    ea2
....................ugguuuaucuuuggaguucuuguA.................................................................   12    1    ea2
....................ugguuuaucuuuggaguucuugug.................................................................   3     0    ea2
.....................gguuuaucuuuggaguucuu....................................................................   1     0    ea2
.....................gguuuaucuuuggaguucuug...................................................................   38    0    ea2
.....................gguuuaucuuuggaguucuuU...................................................................   3     1    ea2
.....................gguuuaucuuuggaguucuuA...................................................................   8     1    ea2
......................guuuaucuuuggaguucuuU...................................................................   7     1    ea2
............................................uuuaucauaaggucuucauuga...........................................   5     0    ea2
.............................................uuaucauaaggucuucauuga...........................................   1     0    ea2
....................................................aaggucuucauugauugacaau...................................   2     0    ea2
....................................................aaggucuucauugauugacaauG..................................   1     1    ea2
....................................................aaggucuucauugauugacaauaa.................................   2     0    ea2
.....................................................aggucuucauugauugaca.....................................   5     0    ea2
.....................................................aggucuucauugauugacaau...................................   2     0    ea2
```

Mature Star

```
agucauuuuaauauaccucaugguuuaucuuuggaguucuuguguuuaucauaaggucuucauugauugacaauaagguauauaauggaauuucagauuugacagcuua
.....................................................aggucuucauugauugacaaua..................................    5    0    ea2
.....................................................aggucuucauugauugacaauU..................................    7    1    ea2
```

Provisional ID : ScUbCFx_65_7405
Score total : 21354
Score for star read(s) : 3.9
Score for read counts : 21345
Score for mfe : 0.5
Score for randfold : 1.6
Score for cons. seed : 3
Total read count : 41879
Mature read count : 41754
Loop read count : 0
Star read count : 125

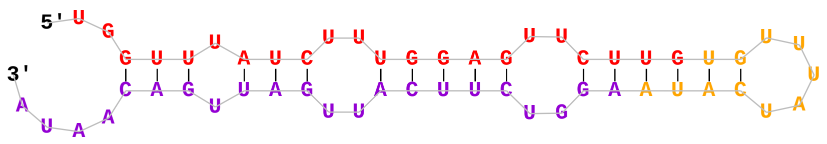

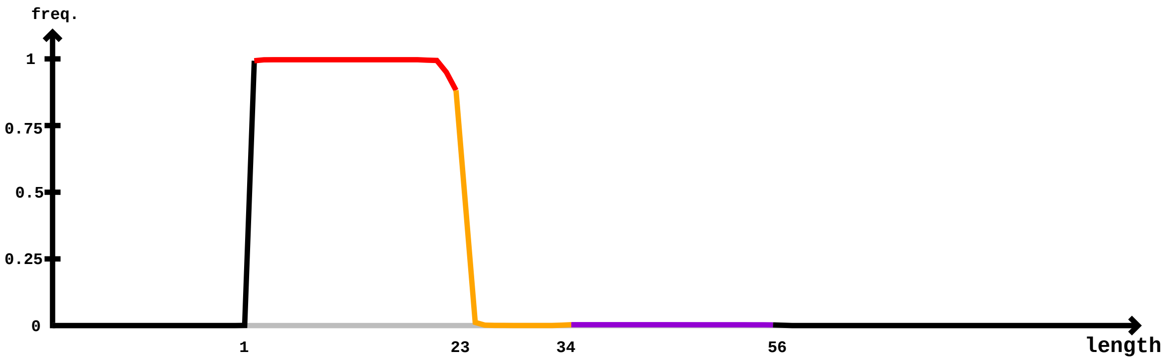

Mature Star

```
5'- gucauuuuaauauaccucaugguuuaucuuuggaguucuuguguuuaucauaaggucuucauugauugacaauaagguauauaauggacuuucagauuugacagcuuagaa  -3'   obs
    gucauuuuaauauaccucaugguuuaucuuuggaguucuuguguuuaucauaaggucuucauugauugacaauaagguauauaauggacuuucagauuugacagcuuagaa         exp
    ((((.....((((((((.((.(((.(((..(((((..((((((.....))))))..)))))..))).))).)).))))))))..(((....)))....)))).........   reads   mm   sample
    ..................Cugguuuaucuuuggaguucuug......................................................................   2   1   eg1
    ...................Ggguuuaucuuuggaguu..........................................................................   3   1   eg1
    ...................ugguuuaucuuuggaguG..........................................................................   1   1   eg1
    ...................ugguuuaucuuuggaguu..........................................................................   9   0   eg1
    ...................ugguuuaucuuuggaguC..........................................................................   1   1   eg1
    ...................ugguuuaucuuuggaguuU.........................................................................   1   1   eg1
    ...................ugguuuaucuuuggaguuc.........................................................................   1   0   eg1
    ...................Ggguuuaucuuuggaguucu........................................................................   17   1   eg1
    ...................ugguuuaucuuuggagCucu........................................................................   1   1   eg1
    ...................ugguuuaucuuuggGguucu........................................................................   2   1   eg1
    ...................ugguuuaucuuuggaguucG........................................................................   18   1   eg1
    ...................ugguuuaucuuuggaguucu........................................................................   190   0   eg1
    ...................ugguuuaucuuuggaguucA........................................................................   10   1   eg1
    ...................uggCuuaucuuuggaguucu........................................................................   1   1   eg1
    ...................Cgguuuaucuuuggaguucu........................................................................   1   1   eg1
    ...................ugguuuaucuuCggaguucu........................................................................   1   1   eg1
    ...................ugguuuaucuuuggaguuUu........................................................................   1   1   eg1
    ...................ugguuuaucuuuggaguucC........................................................................   21   1   eg1
    ...................ugguuuaucCuuggaguucu........................................................................   3   1   eg1
    ...................ugguuuaucuCuggaguucuu.......................................................................   1   1   eg1
    ...................ugguuuauUuuuggaguucuu.......................................................................   1   1   eg1
    ...................ugguuuaucuuuggaguCcuu.......................................................................   2   1   eg1
    ...................ugguuuaucuuuggaguucuA.......................................................................   5   1   eg1
    ...................ugguuuaucuuuggaguucuu.......................................................................   248   0   eg1
    ...................ugguuuaucAuuggaguucuu.......................................................................   1   1   eg1
    ...................uAguuuaucuuuggaguucuu.......................................................................   1   1   eg1
    ...................ugguCuaucuuuggaguucuu.......................................................................   1   1   eg1
    ...................ugguuuaucuuuggGguucuu.......................................................................   1   1   eg1
    ...................ugguuuGucuuuggaguucuu.......................................................................   1   1   eg1
    ...................Agguuuaucuuuggaguucuu.......................................................................   1   1   eg1
    ...................ugguuuaucuuuggaguucuG.......................................................................   6   1   eg1
    ...................ugguuuaucuuuggaguuUuu.......................................................................   1   1   eg1
    ...................ugguuuaucuuuggaguucuC.......................................................................   32   1   eg1
```

```
                   Mature                           Star
gucauuuuaauauaccucaugguuuaucuuuggaguucuuguguuuaucauaaggucuucauugauugacaauaagguauauaaauggacuuucagauuugacagcuuagaa
...................uggCuuaucuuuggaguucuu........................................................................ 3    1 eg1
...................Ggguuuaucuuuggaguucuu........................................................................ 55   1 eg1
...................ugguuuaucuuuggaguucuGg....................................................................... 1    1 eg1
...................ugguuuaucuuuggGguucuug....................................................................... 9    1 eg1
...................ugguuuaucuuuggaguucuCg....................................................................... 40   1 eg1
...................ugguuuaucuuuggUguucuug....................................................................... 2    1 eg1
...................ugguuuaucuuuggaguucCug....................................................................... 6    1 eg1
...................ugUuuuaucuuuggaguucuug....................................................................... 1    1 eg1
...................uAguuuaucuuuggaguucuug....................................................................... 1    1 eg1
...................ugguuuaCcuuuggaguucuug....................................................................... 9    1 eg1
...................ugguuuGucuuuggaguucuug....................................................................... 5    1 eg1
...................ugguuuaucuuGggaguucuug....................................................................... 1    1 eg1
...................ugguuuaucuuuggaAuucuug....................................................................... 2    1 eg1
...................ugAuuuaucuuuggaguucuug....................................................................... 2    1 eg1
...................ugguAuaucuuuggaguucuug....................................................................... 2    1 eg1
...................ugguCuaucuuuggaguucuug....................................................................... 7    1 eg1
...................ugguuuaucuuAggaguucuug....................................................................... 2    1 eg1
...................ugguuuaucuuuGAaguucuug....................................................................... 2    1 eg1
...................uggCuuaucuuuggaguucuug....................................................................... 8    1 eg1
...................ugguuuaucuuuggCguucuug....................................................................... 1    1 eg1
...................ugguuuaucCuuggaguucuug....................................................................... 16   1 eg1
...................ugguuuaucuCuggaguucuug....................................................................... 1    1 eg1
...................ugguuuaucuuuggaguucuAg....................................................................... 1    1 eg1
...................ugguuuaucuAuggaguucuug....................................................................... 1    1 eg1
...................ugguuuauUuuuggaguucuug....................................................................... 1    1 eg1
...................ugguuuaucuuuggaguCcuug....................................................................... 10   1 eg1
...................uggGuuaucuuuggaguucuug....................................................................... 7    1 eg1
...................ugguuuaucuuuggaguuUuug....................................................................... 3    1 eg1
...................ugguuuaucuuuggaguucuuC....................................................................... 9    1 eg1
...................ugguuuaucuuuggagCucuug....................................................................... 7    1 eg1
...................Agguuuaucuuuggaguucuug....................................................................... 2    1 eg1
...................ugguuCaucuuuggaguucuug....................................................................... 4    1 eg1
...................ugguuuaucuuuggaguucuuA....................................................................... 370  1 eg1
...................ugguuuaucuuuggaguucuuU....................................................................... 113  1 eg1
...................ugguuuaucuuuggaguucuug....................................................................... 3203 0 eg1
...................Ggguuuaucuuuggaguucuug....................................................................... 535  1 eg1
...................ugguuuaucuuCggaguucuug....................................................................... 7    1 eg1
...................Cgguuuaucuuuggaguucuug....................................................................... 9    1 eg1
...................ugguuuaAcuuuggaguucuug....................................................................... 2    1 eg1
...................ugguuuaucuuuggaguucuuUu...................................................................... 12   1 eg1
...................ugguCuaucuuuggaguucuugu...................................................................... 1    1 eg1
...................Cgguuuaucuuuggaguucuugu...................................................................... 1    1 eg1
...................Ggguuuaucuuuggaguucuugu...................................................................... 17   1 eg1
...................ugguuuaucuuuggaguucuugC...................................................................... 11   1 eg1
...................ugguuuaucuuuggaguucuugG...................................................................... 9    1 eg1
...................ugguuuaucuuuggaguucuugu...................................................................... 66   0 eg1
...................ugguuuaucuuuggaguucuCgu...................................................................... 1    1 eg1
...................ugguuuaucuuuggaguucuugA...................................................................... 10   1 eg1
...................ugguuuaucuuuggaguucuuAu...................................................................... 7    1 eg1
...................ugguuuaucCuuggaguucuugu...................................................................... 1    1 eg1
...................ugguuuaucuuuggaguucuuguC..................................................................... 2    1 eg1
...................Ggguuuaucuuuggaguucuugug..................................................................... 2    1 eg1
...................ugguuuaucuuuggaguucuuguU..................................................................... 6    1 eg1
...................ugguuuaucuuuggaguucuuguA..................................................................... 39   1 eg1
...................ugguuuaucuuuggaguucuugug..................................................................... 1    0 eg1
...................ugguuuaucuuuggaguucuuguAu.................................................................... 8    1 eg1
...................ugguuuaucuuuggaguucuuguUu.................................................................... 2    1 eg1
...................ugguuuaucuuuggaguucuuguguuu.................................................................. 1    0 eg1
...................Ggguuuaucuuuggaguucuuguguuu.................................................................. 1    1 eg1
....................gguuuaucuuuggaguucuug....................................................................... 3    0 eg1
....................ggGuuaucuuuggaguucuug....................................................................... 1    1 eg1
.......................................uguguuuaucauaaggucuuc.................................................... 1    0 eg1
...................................................aaggucuucauugauugacaau....................................... 3    0 eg1
...................................................Gaggucuucauugauugacaau....................................... 1    1 eg1
...................................................aaggucuucauugauugacaauU...................................... 2    1 eg1
...................................................aaggucuucauugauugacaauUa..................................... 2    1 eg1
....................................................aggucuucauugauugacaau....................................... 1    0 eg1
....................................................Gggucuucauugauugacaaua...................................... 1    1 eg1
....................................................aggucuucauugauugacaauU...................................... 2    1 eg1
....................................................aggucuucauugauugacaaua...................................... 1    0 eg1
```

```
                   Mature                Star
gucauuuuaauauaccucaugguuuaucuuuggaguucuuguguuuaucauaaggucuucauugauugacaauaagguauauaauggacuuucagauuugacagcuuagaa
....................................................aggucuucauugauugacaauUa....................................    1     1     eg1

..................Cugguuuaucuuuggaguucuug......................................................................    2     1     er1
...................ugguuuaucuuuggaguC..........................................................................    2     1     er1
...................ugguuuaucuuuggaguu..........................................................................    3     0     er1
...................ugguuuaucuuuggaguuc.........................................................................    4     0     er1
...................ugguuuaucCuuggaguuc.........................................................................    1     1     er1
...................ugguuuaucuuuggaguucA........................................................................    1     1     er1
...................ugguuuaucGuuggaguucu........................................................................    1     1     er1
...................Ggguuuaucuuuggaguucu........................................................................    19    1     er1
...................ugguuuaucuuuggaguucC........................................................................    12    1     er1
...................ugguuuaucuuuggaguucu........................................................................    109   0     er1
...................ugguuuaucuuuggaguucuC.......................................................................    9     1     er1
...................ugguuuaCcuuuggaguucuu.......................................................................    1     1     er1
...................ugguuuaucuuuggaAuucuu.......................................................................    1     1     er1
...................ugguuuaucuuuggaguucuu.......................................................................    166   0     er1
...................ugguCuaucuuuggaguucuu.......................................................................    3     1     er1
...................ugAuuuaucuuuggaguucuu.......................................................................    2     1     er1
...................ugguuuaucuuuAgaguucuu.......................................................................    1     1     er1
...................ugguAuaucuuuggaguucuu.......................................................................    1     1     er1
...................ugguuuaucuuuggGguucuu.......................................................................    1     1     er1
...................ugguuuaucuuuggaguucuG.......................................................................    1     1     er1
...................Ggguuuaucuuuggaguucuu.......................................................................    35    1     er1
...................ugguuAaucuuuggaguucuug......................................................................    1     1     er1
...................ugguuuaucuuuggUguucuug......................................................................    4     1     er1
...................ugguuuaCcuuuggaguucuug......................................................................    2     1     er1
...................ugguuuaucuuuggaguucuuC......................................................................    18    1     er1
...................ugguuuaucuuuggaguAcuug......................................................................    1     1     er1
...................ugguuuaucuuuggCguucuug......................................................................    1     1     er1
...................Ggguuuaucuuuggaguucuug......................................................................    400   1     er1
...................ugguuuaucuuuggaguucuug......................................................................    2379  0     er1
...................ugguuuaucuuuggaguucuGg......................................................................    1     1     er1
...................Agguuuaucuuuggaguucuug......................................................................    2     1     er1
...................uggCuuaucuuuggaguucuug......................................................................    5     1     er1
...................ugguuuaucAuuggaguucuug......................................................................    2     1     er1
...................ugguuuaucuuuggagCucuug......................................................................    1     1     er1
...................ugguuuauUuuuggaguucuug......................................................................    3     1     er1
...................ugguuuGucuuuggaguucuug......................................................................    5     1     er1
...................ugguuuaGcuuuggaguucuug......................................................................    1     1     er1
...................ugguuuaucuuuggaguucuuA......................................................................    261   1     er1
...................uggGuuaucuuuggaguucuug......................................................................    2     1     er1
...................ugguuuaucuuugUaguucuug......................................................................    1     1     er1
...................ugguuuaucuuuggaguuUuug......................................................................    1     1     er1
...................uggAuuaucuuuggaguucuug......................................................................    2     1     er1
...................ugguuuaucCuuggaguucuug......................................................................    8     1     er1
...................ugguuuaucuuugAaguucuug......................................................................    1     1     er1
...................ugguuuaucuuCggaguucuug......................................................................    7     1     er1
...................ugguuuaucuuuggaguucuuU......................................................................    73    1     er1
...................ugguuuaucuuuggaguucuCg......................................................................    4     1     er1
...................ugguuuaucuuuggaguCcuug......................................................................    11    1     er1
...................ugguuuaucuuuggaguGcuug......................................................................    1     1     er1
...................ugguuuaucuuuggGguucuug......................................................................    11    1     er1
...................ugguGuaucuuuggaguucuug......................................................................    1     1     er1
...................ugguuuaucuuuggaAuucuug......................................................................    3     1     er1
...................ugUuuuaucuuuggaguucuug......................................................................    2     1     er1
...................ugAuuuaucuuuggaguucuug......................................................................    1     1     er1
...................ugguuuaucuuuggagGucuug......................................................................    3     1     er1
...................ugguuuaucuCuggaguucuug......................................................................    4     1     er1
...................ugguuuaucuuuggaguucAug......................................................................    2     1     er1
...................ugguCuaucuuuggaguucuug......................................................................    12    1     er1
...................ugguuCaucuuuggaguucuug......................................................................    3     1     er1
...................ugguuuaucuuuggaguucCug......................................................................    1     1     er1
...................Cgguuuaucuuuggaguucuug......................................................................    3     1     er1
...................ugguuuaucuuuggaguCcuugu.....................................................................    2     1     er1
...................ugguuuaucuuuggaguucuugA.....................................................................    11    1     er1
...................ugguCuaucuuuggaguucuugu.....................................................................    1     1     er1
...................ugguuuaucuuuggaguucuugu.....................................................................    54    0     er1
...................ugguuuaucuuuggaguucuuAu.....................................................................    3     1     er1
...................ugguuuaucuuuggaguucuuUu.....................................................................    7     1     er1
...................ugguuuaucuuuggaguucuugC.....................................................................    8     1     er1
```

```
                   Mature                           Star
gucauuuuaauauaccucaugguuuaucuuuggaguucuuguguuuaucauaaggucuucauugauugacaauaagguauauaauggacuuucagauuugacagcuuagaa
...................Ggguuuaucuuuggaguucuugu.....................................................................  8    1  er1
...................ugguuuaucuuuggagCucuugu.....................................................................  1    1  er1
...................Cgguuuaucuuuggaguucuugu.....................................................................  1    1  er1
...................ugguuuaucuuuggaguucuugug....................................................................  1    0  er1
...................ugguuuaucuuuggaguucuuguU....................................................................  4    1  er1
...................ugguuuaucuuuggaguucuuguA....................................................................  29   1  er1
...................Ggguuuaucuuuggaguucuugug....................................................................  1    1  er1
...................ugguuuaucuuuggaguucuuguAu...................................................................  4    1  er1
...................ugguuuaucuuuggaguucuuguguuu.................................................................  3    0  er1
...................Ggguuuaucuuuggaguucuuguguuu.................................................................  1    1  er1
....................gguuuaucuuuggaguucuug......................................................................  3    0  er1
...................................................aaggucuucauugauugacaaua.....................................  2    0  er1
...................................................aaggucuucauugauugacaauU.....................................  2    1  er1
...................................................aaggucuucauugauugacaauUa....................................  1    1  er1
....................................................aggucuucauugauugacaaua.....................................  1    0  er1
....................................................aggucuucauugauugacaauU.....................................  1    1  er1
....................................................aggucuucauugauugacaauUa....................................  3    1  er1

..................Cugguuuaucuuuggaguucuug......................................................................  1    1  eg2
...................ugguuuaucuuuggaguucuu.......................................................................  4    0  eg2
...................ugguuuaucuuuggaguucuuU......................................................................  3    1  eg2
...................Ngguuuaucuuuggaguucuug......................................................................  1    1  eg2
...................ugguuuaucuuuggaguucuug......................................................................  107  0  eg2
...................ugguuuaucuuuggagGucuug......................................................................  1    1  eg2
...................ugguuuaucuuuggaguucuuA......................................................................  5    1  eg2
...................ugguuuaucuuuggaguucuuguA....................................................................  1    1  eg2
....................gguuuaucuuuggaguucuuU......................................................................  2    1  eg2
....................gguuuaucuuuggaguucuug......................................................................  3    0  eg2
....................ggGuuaucuuuggaguucuug......................................................................  1    1  eg2
...................................................aaggucuCcauugauugacaau......................................  1    1  eg2
....................................................aggucuucauugauugacaauU.....................................  1    1  eg2
....................................................aggucuucauugauugacaaua.....................................  2    0  eg2
....................................................aggucuucauugauugacaauUa....................................  3    1  eg2

...................ugguuuaucuuuggaguucu........................................................................  4    0  ea2
...................ugguuuaucuuuggaguucAu.......................................................................  1    1  ea2
...................ugguuuaucuuuggaguucuA.......................................................................  3    1  ea2
...................ugguuuaucuuuggaguucuu.......................................................................  31   0  ea2
...................ugguuuaucuuuggaguucuuA......................................................................  70   1  ea2
...................ugguuuaucuuuggGguucuug......................................................................  1    1  ea2
...................Ngguuuaucuuuggaguucuug......................................................................  3    1  ea2
...................uAguuuaucuuuggaguucuug......................................................................  2    1  ea2
...................ugguuuaucuuuggaguucuug......................................................................  466  0  ea2
...................ugguuuaucuuuggaguucuuU......................................................................  8    1  ea2
...................ugguCuaucuuuggaguucuug......................................................................  4    1  ea2
...................ugguuuaucuuuggaguucuuAu.....................................................................  1    1  ea2
...................ugguuuaucuuuggaguucuuUu.....................................................................  2    1  ea2
...................ugguuuaucuuuggaguucuugA.....................................................................  1    1  ea2
...................ugguuuaucuuuggaguucuuguA....................................................................  12   1  ea2
...................ugguuuaucuuuggaguucuugug....................................................................  3    0  ea2
....................gguuuaucuuuggaguucuu.......................................................................  1    0  ea2
....................gguuuaucuuuggaguucuuA......................................................................  8    1  ea2
....................gguuuaucuuuggaguucuuU......................................................................  3    1  ea2
....................gguuuaucuuuggaguucuug......................................................................  38   0  ea2
.....................guuuaucuuuggaguucuuU......................................................................  7    1  ea2
...........................................uuuaucauaaggucuucauuga..............................................  5    0  ea2
............................................uuaucauaaggucuucauuga..............................................  1    0  ea2
...................................................aaggucuucauugauugacaau......................................  2    0  ea2
...................................................aaggucuucauugauugacaauG.....................................  1    1  ea2
...................................................aaggucuucauugauugacaauaa....................................  2    0  ea2
....................................................aggucuucauugauugaca........................................  5    0  ea2
....................................................aggucuucauugauugacaau......................................  2    0  ea2
....................................................aggucuucauugauugacaauU.....................................  7    1  ea2
....................................................aggucuucauugauugacaaua.....................................  5    0  ea2

...................ugguuuaucuuuggaguucu........................................................................  7    0  er2
...................ugguuuaucuuuggaguucuA.......................................................................  5    1  er2
...................ugguuuaucuuuggaguucuu.......................................................................  46   0  er2
...................ugguuuaucuuuggaguucuuU......................................................................  31   1  er2
...................Ngguuuaucuuuggaguucuug......................................................................  2    1  er2
```

```
                   Mature                            Star
gucauuuuaauauaccucaugguuuaucuuuggaguucuuguguuuaucauaaggucuucauugauugacaauaagguauauaauggacuuucagauuugacagcuuagaa
...................Cgguuuaucuuuggaguucuug......................................................................    1     1    er2
...................uggGuuaucuuuggaguucuug......................................................................    2     1    er2
...................ugguuuaucuuuggagCucuug......................................................................    1     1    er2
...................ugguuuaucuuuggaguucuug......................................................................    768   0    er2
...................ugguuuaucuuuggGguucuug......................................................................    1     1    er2
...................ugguuuaucuuuggaguucuuA......................................................................    118   1    er2
...................ugguCuaucuuuggaguucuug......................................................................    1     1    er2
...................ugguuuaucuuuggaguCcuug......................................................................    1     1    er2
...................ugguAuaucuuuggaguucuug......................................................................    1     1    er2
...................ugguuuaucuuuggaguucuugu.....................................................................    16    0    er2
...................ugguuuaucuuuggaguucuuAu.....................................................................    9     1    er2
...................ugguuuauUuuuggaguucuugu.....................................................................    2     1    er2
...................ugguuuaucuuuggaguucuuUu.....................................................................    3     1    er2
...................ugguuuaucuuuggaguucuuguA....................................................................    8     1    er2
....................gguuuaucuuuggaguucuu.......................................................................    3     0    er2
....................Uguuuaucuuuggaguucuug......................................................................    1     1    er2
....................gguuuaucuuuggaguucuuA......................................................................    6     1    er2
....................gguuuaucuuuggaguucuug......................................................................    48    0    er2
....................gguuuaucuuuggaguucuugu.....................................................................    1     0    er2
....................gguuuaucuuuggaguucuuUu.....................................................................    1     1    er2
...................................................aaggucuucauugauugacaau......................................    7     0    er2
...................................................aaggucuucauugauugacaauU.....................................    3     1    er2
....................................................aggucuucauugauugacaauU.....................................    10    1    er2
....................................................aggucuucauugauugacaaua.....................................    7     0    er2
....................................................aggucuucauugauugacaauUa....................................    8     1    er2

..................Cugguuuaucuuuggaguucuu.......................................................................    2     1    ea1
..................Cugguuuaucuuuggaguucuug......................................................................    13    1    ea1
...................ugguuuaucuuuggaguu..........................................................................    34    0    ea1
...................ugguuuaucuuuggaguA..........................................................................    1     1    ea1
...................ugguuuaucuuuggaguC..........................................................................    3     1    ea1
...................Ggguuuaucuuuggaguu..........................................................................    8     1    ea1
...................uggCuuaucuuuggaguu..........................................................................    1     1    ea1
...................ugguuuaucuuuggagGuc.........................................................................    1     1    ea1
...................ugguuuaucuuuggaguuc.........................................................................    27    0    ea1
...................ugguuuaucuuuggaguuA.........................................................................    1     1    ea1
...................ugguuuGucuuuggaguuc.........................................................................    1     1    ea1
...................Ggguuuaucuuuggaguuc.........................................................................    10    1    ea1
...................ugguuuaucuuuggaguuU.........................................................................    2     1    ea1
...................ugguuuaucuuAggaguucu........................................................................    2     1    ea1
...................ugguuuaucuuCggaguucu........................................................................    3     1    ea1
...................uggGuuaucuuuggaguucu........................................................................    1     1    ea1
...................ugguuuaucCuuggaguucu........................................................................    2     1    ea1
...................Agguuuaucuuuggaguucu........................................................................    1     1    ea1
...................ugguCuaucuuuggaguucu........................................................................    3     1    ea1
...................ugguuuaucuuuAgaguucu........................................................................    3     1    ea1
...................ugguuuaucuAuggaguucu........................................................................    2     1    ea1
...................uggCuuaucuuuggaguucu........................................................................    5     1    ea1
...................ugguuuaucuuuggaguCcu........................................................................    2     1    ea1
...................Cgguuuaucuuuggaguucu........................................................................    3     1    ea1
...................Ggguuuaucuuuggaguucu........................................................................    152   1    ea1
...................ugguuuaucuuuggaguucG........................................................................    17    1    ea1
...................ugguuuaucuuuggGguucu........................................................................    5     1    ea1
...................ugguuuaucuuuggagCucu........................................................................    3     1    ea1
...................ugguuCaucuuuggaguucu........................................................................    1     1    ea1
...................ugguuuaCcuuuggaguucu........................................................................    3     1    ea1
...................ugguAuaucuuuggaguucu........................................................................    1     1    ea1
...................ugguuuaucuCuggaguucu........................................................................    3     1    ea1
...................ugguuuaucuuuggaguucC........................................................................    126   1    ea1
...................ugguuuaucuuuggaguucA........................................................................    73    1    ea1
...................ugguuuaucuuuggaguucu........................................................................    1028  0    ea1
...................uUguuuaucuuuggaguucuu.......................................................................    3     1    ea1
...................ugguuuaucuuuAgaguucuu.......................................................................    1     1    ea1
...................ugguuuaucuCuggaguucuu.......................................................................    3     1    ea1
...................ugguuuaucuuuggaguucuA.......................................................................    38    1    ea1
...................uggAuuaucuuuggaguucuu.......................................................................    1     1    ea1
...................uggCuuaucuuuggaguucuu.......................................................................    7     1    ea1
...................uggGuuaucuuuggaguucuu.......................................................................    3     1    ea1
...................ugguuuaucuuuggaguucuG.......................................................................    10    1    ea1
...................ugguuuaucuuAggaguucuu.......................................................................    1     1    ea1
```

```
                   Mature                          Star
gucauuuuaauauaccucaugguuuaucuuuggaguucuuguguuuaucauaaggucuucauugauugacaauaagguauauaauggacuuucagauuugacagcuuagaa
...................ugguuuaucuuuggagCucuu.......................................................................   1      1    ea1
...................ugguuuaucuuuggaguucCu.......................................................................   4      1    ea1
...................ugguuuaucuAuggaguucuu.......................................................................   6      1    ea1
...................ugguuuaucuuuggaguGcuu.......................................................................   1      1    ea1
...................Ggguuuaucuuuggaguucuu.......................................................................   240    1    ea1
...................ugguAuaucuuuggaguucuu.......................................................................   1      1    ea1
...................uAguuuaucuuuggaguucuu.......................................................................   2      1    ea1
...................ugguuuaCcuuuggaguucuu.......................................................................   6      1    ea1
...................Cgguuuaucuuuggaguucuu.......................................................................   3      1    ea1
...................ugguuuaucuuCggaguucuu.......................................................................   4      1    ea1
...................ugguuuaucAuuggaguucuu.......................................................................   1      1    ea1
...................ugguuuCucuuuggaguucuu.......................................................................   1      1    ea1
...................ugguuuGucuuuggaguucuu.......................................................................   1      1    ea1
...................ugguCuaucuuuggaguucuu.......................................................................   5      1    ea1
...................ugguuuauUuuuggaguucuu.......................................................................   1      1    ea1
...................ugguuuaucuuuggaguucuC.......................................................................   147    1    ea1
...................uCguuuaucuuuggaguucuu.......................................................................   1      1    ea1
...................ugguuuaucuuuggaguucuu.......................................................................   1635   0    ea1
...................ugguuuaucuuuggaguucAu.......................................................................   1      1    ea1
...................ugguuuaucuuuggGguucuu.......................................................................   6      1    ea1
...................ugguuuaucuuuggagAucuu.......................................................................   1      1    ea1
...................ugguuuaucuuuggaguucGu.......................................................................   1      1    ea1
...................ugguuCaucuuuggaguucuu.......................................................................   4      1    ea1
...................ugguuuaucuuuggaguuUuu.......................................................................   2      1    ea1
...................ugguuuaucuuugAaguucuu.......................................................................   1      1    ea1
...................ugguuuaucuuuggaguCcuu.......................................................................   4      1    ea1
...................ugguuuaucCuuggaguucuu.......................................................................   6      1    ea1
...................ugguuuaucCuuggaguucuug......................................................................   52     1    ea1
...................ugguuuaucuuuggagAucuug......................................................................   4      1    ea1
...................ugguuuaucuuCggaguucuug......................................................................   71     1    ea1
...................ugguuuaucuuuggagCucuug......................................................................   55     1    ea1
...................ugguuuaucuuuggaguuAuug......................................................................   3      1    ea1
...................ugguuuaucuuuggaguucAug......................................................................   6      1    ea1
...................ugguuuaucuuuggaUuucuug......................................................................   5      1    ea1
...................ugguuuaucuuuggaAuucuug......................................................................   8      1    ea1
...................ugUuuuaucuuuggaguucuug......................................................................   5      1    ea1
...................ugguuuauAuuuggaguucuug......................................................................   2      1    ea1
...................ugguuAaucuuuggaguucuug......................................................................   4      1    ea1
...................ugguCuaucuuuggaguucuug......................................................................   57     1    ea1
...................ugguuuauUuuuggaguucuug......................................................................   8      1    ea1
...................ugguuuaucuAuggaguucuug......................................................................   7      1    ea1
...................ugguuuaucuuuggCguucuug......................................................................   2      1    ea1
...................ugguuuGucuuuggaguucuug......................................................................   21     1    ea1
...................ugguuuaucuCuggaguucuug......................................................................   39     1    ea1
...................ugguuGaucuuuggaguucuug......................................................................   4      1    ea1
...................ugguuuaucuuuggaguGcuug......................................................................   2      1    ea1
...................ugguuuaucuuuggaguucuuU......................................................................   551    1    ea1
...................ugguuuaucuuugUaguucuug......................................................................   1      1    ea1
...................ugguuuaucuuuggaguucuGg......................................................................   5      1    ea1
...................ugguuuauGuuuggaguucuug......................................................................   1      1    ea1
...................Ggguuuaucuuuggaguucuug......................................................................   2914   1    ea1
...................ugguAuaucuuuggaguucuug......................................................................   8      1    ea1
...................ugguuuaucuuuUgaguucuug......................................................................   5      1    ea1
...................uUguuuaucuuuggaguucuug......................................................................   5      1    ea1
...................ugguuuaucuuuggaguucuug......................................................................   18752  0    ea1
...................Agguuuaucuuuggaguucuug......................................................................   17     1    ea1
...................ugguuCaucuuuggaguucuug......................................................................   41     1    ea1
...................ugguuuaucuuGggaguucuug......................................................................   8      1    ea1
...................ugguuuaucuuuggUguucuug......................................................................   11     1    ea1
...................uggCuuaucuuuggaguucuug......................................................................   74     1    ea1
...................ugguuuaCcuuuggaguucuug......................................................................   40     1    ea1
...................ugguuuaucuuugAaguucuug......................................................................   8      1    ea1
...................ugguuuaucuuuggaguCcuug......................................................................   65     1    ea1
...................ugguuuaucuuuggaguAcuug......................................................................   5      1    ea1
...................ugguuuaucuuAggaguucuug......................................................................   13     1    ea1
...................ugguuuaucuuuAgaguucuug......................................................................   12     1    ea1
...................uAguuuaucuuuggaguucuug......................................................................   10     1    ea1
...................ugguuuaucuuuggaguuGuug......................................................................   3      1    ea1
...................Cgguuuaucuuuggaguucuug......................................................................   42     1    ea1
...................ugAuuuaucuuuggaguucuug......................................................................   3      1    ea1
```

Mature / Star			
gucauuuuaauauaccucaugguuuaucuuuggaguucuuguguuuaucauaaggucuucauugauugacaauaagguauauaauggacuuucagauuugacagcuuagaa			
...................ugguuuaucAuuggaguucuug..	10	1	ea1
...................ugguuuaucuuuggaguucuuA..	2962	1	ea1
...................ugguuuaucuuuggaCuucuug..	2	1	ea1
...................ugguuuaucuuuggaguucCug..	31	1	ea1
...................ugguuuaucuuuggaguucGug..	3	1	ea1
...................ugguuuaucuuuggaguucuuC..	78	1	ea1
...................ugguuuaGcuuuggaguucuug..	1	1	ea1
...................ugguuuaucuuuggaguuUuug..	8	1	ea1
...................ugguGuaucuuuggaguucuug..	1	1	ea1
...................ugguuuaucuuuggGguucuug..	47	1	ea1
...................ugguuuaucuuuggaguucuAg..	3	1	ea1
...................uggAuuaucuuuggaguucuug..	5	1	ea1
...................ugguuuaucuuuggaguucuCg..	51	1	ea1
...................uggGuuaucuuuggaguucuug..	9	1	ea1
...................ugUuuuaucuuuggaguucuugu...	2	1	ea1
...................ugguuuaCcuuuggaguucuugu...	2	1	ea1
...................ugguuuaucuuuggaguucuuAu...	52	1	ea1
...................ugguuuaucuuuggaguucuugA...	64	1	ea1
...................ugguuuaucuuuggaguucuugu...	331	0	ea1
...................uCguuuaucuuuggaguucuugu...	1	1	ea1
...................Ggguuuaucuuuggaguucuugu...	55	1	ea1
...................ugguAuaucuuuggaguucuugu...	1	1	ea1
...................ugguCuaucuuuggaguucuugu...	1	1	ea1
...................ugguuuaucCuuggaguucuugu...	2	1	ea1
...................ugguuuaucuuuggaAuucuugu...	1	1	ea1
...................uggAuuaucuuuggaguucuugu...	3	1	ea1
...................ugguuuaucuuuggaguucCugu...	1	1	ea1
...................ugguuuaucuuuggaguucuuCu...	2	1	ea1
...................ugguuuaucuuuggaguucuuUu...	49	1	ea1
...................ugguuuaucuuuggaUuucuugu...	1	1	ea1
...................ugguuuaucuuGggaguucuugu...	1	1	ea1
...................ugguuuaucuuuggaguucuugC...	39	1	ea1
...................ugguuCaucuuuggaguucuugu...	1	1	ea1
...................ugguuuaucuuuggGguucuugu...	2	1	ea1
...................ugguuuaucuuuggaguucuugG...	7	1	ea1
...................uggCuuaucuuuggaguucuugu...	2	1	ea1
...................ugguuuaucuuuggaguucuuguC..	8	1	ea1
...................Ggguuuaucuuuggaguucuugug..	1	1	ea1
...................ugguuuaucuuuggaguucuuguA..	272	1	ea1
...................ugguuuaucuuuggaguucuugug..	14	0	ea1
...................ugguuuaucuuuggaguucuuguU..	24	1	ea1
...................ugguuuaucuuuggaguucuuAug..	1	1	ea1
...................ugguuuaucuuuggaguucuuguUu...	5	1	ea1
...................Ggguuuaucuuuggaguucuugugu...	1	1	ea1
...................ugguuuaucuuuggaguucuugugu...	2	0	ea1
...................ugguuuaucuuuggaguucuuguAu...	20	1	ea1
...................ugguuuaucuuuggaguucuugugG...	1	1	ea1
...................ugguuuaucuuuggaguucuuguguu..	2	0	ea1
...................ugguuuaucuuuggaguucuuguAuu..	2	1	ea1
...................ugguuuaCcuuuggaguucuuguguuu...	1	1	ea1
...................Ggguuuaucuuuggaguucuuguguuu...	1	1	ea1
...................ugguuuaucuuuggaguucuuguguuu...	3	0	ea1
....................Uguuuaucuuuggaguucuug..	1	1	ea1
....................ggGuuaucuuuggaguucuug..	2	1	ea1
....................gguuuaucuuuggaguucuuA..	7	1	ea1
....................gguuuaucuuuggaguucuug..	14	0	ea1
.....................guuuaucuuuggaguucuug..	3	0	ea1
.....................guuuaucuuuggaguucuugug..	2	0	ea1
.....................Auuuaucuuuggaguucuugug..	1	1	ea1
.............................uuggaguucuuguguuuauca...	1	0	ea1
....uguuuaucauaaggucuucauA..	1	1	ea1
....aaggucuucauugauugaca..	1	0	ea1
....aaggucuucauugauugacaau......................................	1	0	ea1
....Gaggucuucauugauugacaaua.....................................	1	1	ea1
....aaggucuucauugauugacaaua.....................................	9	0	ea1
....aaggucuucauugauugacaauU.....................................	6	1	ea1
....aaggucuucauugauugacaauUa....................................	7	1	ea1
....aggucuucauugauugacaau......................................	1	0	ea1
....aggucuucauugauugacaauC.....................................	1	1	ea1
....aggucuucauugauugacaaua.....................................	3	0	ea1

```
                    Mature                          Star
gucauuuuaauauaccucaugguuuaucuuuggaguucuuguguuuaucauaaggucuucauugauugacaauaagguauauaauggacuuucagauuugacagcuuagaa
....................................................aggucuucauugauugacaauU......................................     1      1      ea1
....................................................aggucuucauugauugacaauUa.....................................     4      1      ea1
```

Provisional ID	:	ScUbCFx_107_10289
Score total	:	1303.5
Score for star read(s)	:	3.9
Score for read counts	:	1292.5
Score for mfe	:	2.5
Score for randfold	:	1.6
Score for cons. seed	:	3
Total read count	:	2547
Mature read count	:	2377
Loop read count	:	0
Star read count	:	170

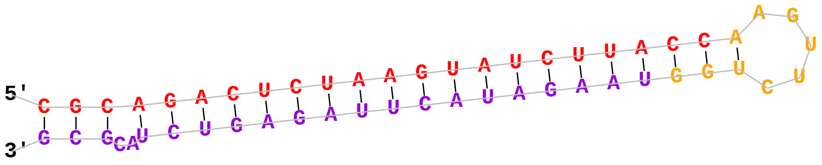

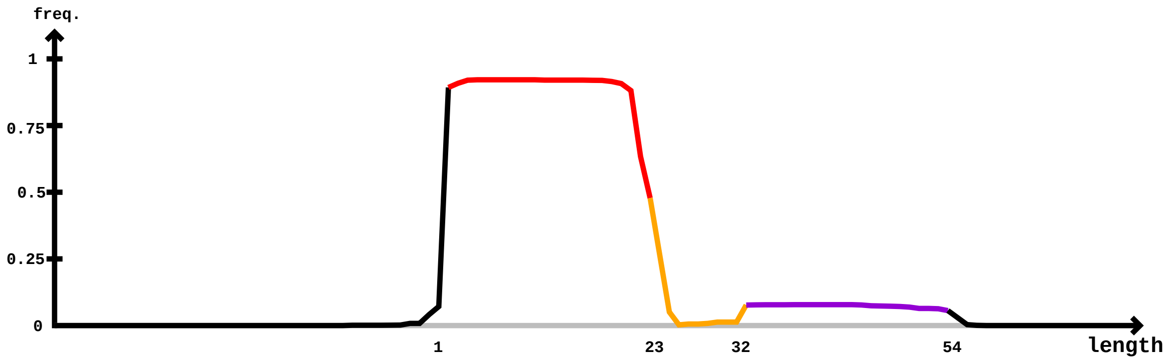

Mature **Star**

```
5'- uauuuacuaacuaaggaaacaccucaagacuagccugcgcgcagacucuaaguaucuuaccaaguucugguaagauacuuagagucuacgcgcaggcuacucauaacuggu  -3'   obs
    uauuuacuaacuaaggaaacaccucaagacuagccugcgcgcagacucuaaguaucuuaccaaguucugguaagauacuuagagucuacgcgcaggcuacucauaacuggu         exp
    .....((((.....(....).......((.(((((((((((.((((((((((((((((((((.....)))))))))))))))))))).))))))))))).)).....))))   reads   mm   sample
    ...................................ugcgcgcagacucuaaguauc.......................................................   1       0    eg1
    ...................................ugcgcgcagacucuaaguUucu......................................................   1       1    eg1
    .....................................cgcgcagacucuaaguaucuua....................................................   1       0    eg1
    .....................................cgcgcagacucuaaguaucuuacc..................................................   1       0    eg1
    ......................................gcgcagacucGaaguaucuua....................................................   2       1    eg1
    ......................................gcgcagacucuaaguaucuuU....................................................   1       1    eg1
    ......................................gcgcagacucuaaguaucuuac...................................................   1       0    eg1
    .......................................cgcagacucuaaguaucuu.....................................................   1       0    eg1
    .......................................cgcagacucuaaguaucuuG....................................................   1       1    eg1
    .......................................cgcagacucuaaguaucuua....................................................   4       0    eg1
    .......................................cgcagacucAaaguaucuua....................................................   1       1    eg1
    .......................................cgcagacucuaaguaucuuU....................................................   3       1    eg1
    .......................................cgcagacucuaaguaucuuaU...................................................   1       1    eg1
    .......................................Ggcagacucuaaguaucuuac...................................................   2       1    eg1
    .......................................cgcagacucuaaguaGcuuac...................................................   1       1    eg1
    .......................................cgcagacucuaaguaucuuac...................................................   16      0    eg1
    .......................................cgcagacucuaaguaucuuacU..................................................   7       1    eg1
    .......................................cgcagacucuaaguaAcuuacc..................................................   1       1    eg1
    .......................................cgcagacucuaaguaucuuacc..................................................   10      0    eg1
    .......................................cgcagacucuaaguaucuuacA..................................................   8       1    eg1
    .......................................cgcagacucuaagCaucuuacc..................................................   1       1    eg1
    .......................................cgcagacucuaGguaucuuacc..................................................   1       1    eg1
    .......................................cgcagacucuaaguUucuuacc..................................................   1       1    eg1
    .......................................cgcagacucuaaguaucuuaccC.................................................   2       1    eg1
    .......................................cgcagacucuaaguaucuuacca.................................................   2       0    eg1
    .......................................cgcagacucuaaguaucuuacUa.................................................   1       1    eg1
    .......................................cgcGgacucuaaguaucuuaccaa................................................   4       1    eg1
    .......................................cgcagacucuaaguaucuuaccaU................................................   1       1    eg1
    .........................................cagacucuaaguaucuuacc..................................................   1       0    eg1
    ................................................................uucugguaagauacuuagagu..........................   2       0    eg1
    ......................................................................uaagauacuuagagucuacgcC...................   2       1    eg1
    ......................................................................uaagauacuuagagucuacgcU...................   1       1    eg1
    ......................................................................uaagauacuuagagucuacgcg...................   7       0    eg1
```

```
                                       Mature          Star
uauuuacuaacuaaggaaacaccucaagacuagccugcgcgcagacucuaaguaucuuaccaaguucugguaagauacuuagagucuacgcgcaggcuacucauaacuggu
......................................................................uaagGuacuuagagucuacgcg...................  1   1   eg1
......................................................................uaagaAacuuagagucuacgcg...................  1   1   eg1
......................................................................uaagauacuuagagucuacgcgUa.................  2   1   eg1

.....................................cgcgcagacucuaaguaucu......................................................  1   0   er1
.....................................Ugcgcagacucuaaguaucu......................................................  2   1   er1
.....................................cgcgcagacucuaaguaucuuU....................................................  2   1   er1
.....................................cgcgcagacucuaaguUucuua....................................................  1   1   er1
......................................gcgcagacucuaaguaucuuC....................................................  1   1   er1
......................................gcgcagacucuaaguaucuuU....................................................  1   1   er1
......................................gcgcagacucuaaguaucuuac...................................................  1   0   er1
......................................gcgcagacucuaaguaucuuacU..................................................  2   1   er1
......................................gcgcagacucuaaguaucuuaccC.................................................  1   1   er1
.......................................cgcagacucuaaguaucuua....................................................  3   0   er1
.......................................cgcagacucuaaguaucuuU....................................................  1   1   er1
.......................................cgcagacucuaaguaucuuac...................................................  21  0   er1
.......................................Ggcagacucuaaguaucuuac...................................................  1   1   er1
.......................................cgcagacucuaagCaucuuac...................................................  1   1   er1
.......................................cgcagacucuaaguaucuuacA..................................................  11  1   er1
.......................................cgcagacucuaaguaucuuacc..................................................  4   0   er1
.......................................cgcagacucuaaguaCcuuacc..................................................  1   1   er1
.......................................cgcagacucuaaguaucuuacU..................................................  12  1   er1
.......................................cgcagacucuaaguaucuuacUa.................................................  3   1   er1
.......................................cgcagacucuaaguaucuuaccCa................................................  1   1   er1
.......................................cgcGgacucuaaguaucuuaccaa................................................  6   1   er1
......................................................................uaagaAacuuagagucuacgcg...................  1   1   er1
......................................................................uaagauacuuagagucuacgcg...................  1   0   er1
......................................................................uaagauacuuagagucuacgcA...................  1   1   er1
......................................................................uaagauacuuagagucuacgcgUa.................  1   1   er1

.............................cuagccugcgcgcagacucu..............................................................  3   0   ea1
.................................ccugcgcAcagacucuaagua.........................................................  1   1   ea1
...................................ugcgcgcagacucCaaguauc.......................................................  1   1   ea1
...................................ugcgcgcagacucuaaguauU.......................................................  1   1   ea1
...................................ugcgcgcagacucuaaguUuc.......................................................  1   1   ea1
...................................ugcgcgcagacucuaaguauc.......................................................  7   0   ea1
...................................ugcgcgcagacucuaaguaucu......................................................  2   0   ea1
...................................ugcgcgcagacucuaaguaucA......................................................  1   1   ea1
.....................................cgcgcagacucCaaguaucu......................................................  2   1   ea1
.....................................cgcgcagacucuaaguaucu......................................................  5   0   ea1
.....................................Ugcgcagacucuaaguaucu......................................................  2   1   ea1
.....................................Ugcgcagacucuaaguaucuu.....................................................  3   1   ea1
.....................................cgcgcagacucuaaguaucuA.....................................................  1   1   ea1
.....................................cgcgcagacucuaaguaucuu.....................................................  5   0   ea1
.....................................cgcgcagacucuaaguGucuu.....................................................  3   1   ea1
.....................................Ggcgcagacucuaaguaucuu.....................................................  2   1   ea1
.....................................cgcgcagacucuaaguaucuC.....................................................  1   1   ea1
.....................................cgcgcagacucuaaguaucuuC....................................................  1   1   ea1
.....................................cgcgcagacucuaaguaucuua....................................................  4   0   ea1
.....................................Ugcgcagacucuaaguaucuua....................................................  3   1   ea1
.....................................cgcgcagacucuaaguaucuuU....................................................  2   1   ea1
.....................................cgcgcagacucuaaguaucuuac...................................................  9   0   ea1
.....................................cgcgcagacucuaaguaucuuacU..................................................  4   1   ea1
.....................................Ugcgcagacucuaaguaucuuacc..................................................  2   1   ea1
.....................................cgcgcagacucuaaguaucuuacA..................................................  5   1   ea1
.....................................cgcgcagacucuaaguaucuuacc..................................................  5   0   ea1
.....................................cgcgcagacucuaaAuaucuuacc..................................................  2   1   ea1
.....................................cgcgcagacucuaaguaucuuaccU.................................................  2   1   ea1
.....................................cgcgcagacucuaaguaucuuaccC.................................................  1   1   ea1
.....................................cgcgcagacucuaaguaucuuacca.................................................  1   0   ea1
......................................gcgcagacucGaaguaucu......................................................  1   1   ea1
......................................gcgcagacucGaaguaucuu.....................................................  5   1   ea1
......................................gcgcagacucuaaguaucuu.....................................................  1   0   ea1
......................................gcgcagacucuaaguaucuC.....................................................  1   1   ea1
......................................gcgcagacucuaaguaucuua....................................................  5   0   ea1
......................................gcgcagacucuaCguaucuua....................................................  1   1   ea1
......................................gcgcagacucuaaguaucuuU....................................................  11  1   ea1
......................................gcgcagacucGaaguaucuua....................................................  7   1   ea1
......................................gcgcagacucuaaguaucuuac...................................................  1   0   ea1
......................................gcgcagacucuaaguaucuuCc...................................................  1   1   ea1
```

Mature Star

```
uauuuacuaacuaaggaaacaccucaagacuagccugcgcgcagacucuaaguaucuuaccaaguucugguaagauacuuagagucuacgcgcaggcuacucauaacuggu
.....................................gcgcagacucuaaguaucuuacc...................................................	1	0	ea1
.......................................cgcagacucuaaguaucCu.....................................................	1	1	ea1
.......................................cgcagacucuaaguaucuu.....................................................	6	0	ea1
.......................................cgcagacucGaaguaucuu.....................................................	1	1	ea1
.......................................cgcagacucuaaguaucuC.....................................................	4	1	ea1
.......................................cgcagacucuaaguaucuua....................................................	85	0	ea1
.......................................cgcUgacucuaaguaucuua....................................................	1	1	ea1
.......................................cgcagacucuaaguaucuuG....................................................	1	1	ea1
.......................................cgcagacucuaaguUucuua....................................................	3	1	ea1
.......................................Ggcagacucuaaguaucuua....................................................	1	1	ea1
.......................................cgcagacucuaaguaucuuC....................................................	3	1	ea1
.......................................cgcagacucuaaguaucuuU....................................................	21	1	ea1
.......................................cgcagacucuaaguauAuuac...................................................	1	1	ea1
.......................................Ggcagacucuaaguaucuuac...................................................	1	1	ea1
.......................................cgcagGcucuaaguaucuuac...................................................	1	1	ea1
.......................................cgcagacucuaaguaucuuaU...................................................	15	1	ea1
.......................................cgcagacucuaGguaucuuac...................................................	1	1	ea1
.......................................cgcagacucuaaguaCcuuac...................................................	2	1	ea1
.......................................cgcagacucuGaguaucuuac...................................................	1	1	ea1
.......................................cgcagacucuaaguaucuuac...................................................	223	0	ea1
.......................................cgcagacucuaaguGucuuac...................................................	1	1	ea1
.......................................cgcagacCcuaaguaucuuac...................................................	3	1	ea1
.......................................cgcagacucCaaguaucuuac...................................................	1	1	ea1
.......................................cgcagacucuaaguaucuuaA...................................................	6	1	ea1
.......................................Ggcagacucuaaguaucuuacc..................................................	4	1	ea1
.......................................cgcagacucCaaguaucuuacc..................................................	1	1	ea1
.......................................cgcagacucuGaguaucuuacc..................................................	2	1	ea1
.......................................cgcagacucuaaguaucuuacU..................................................	134	1	ea1
.......................................cgcagacucuaaguaucuuacA..................................................	191	1	ea1
.......................................cgcagGcucuaaguaucuuacc..................................................	2	1	ea1
.......................................cgcagacucuaaguauUuuacc..................................................	1	1	ea1
.......................................cgcagacucuaaguUucuuacc..................................................	4	1	ea1
.......................................cgcagacucuaaguaucuGacc..................................................	1	1	ea1
.......................................cgcagacucuaGguaucuuacc..................................................	1	1	ea1
.......................................cgcagacucuaaguaAcuuacc..................................................	1	1	ea1
.......................................Agcagacucuaaguaucuuacc..................................................	1	1	ea1
.......................................cgcagacCcuaaguaucuuacc..................................................	2	1	ea1
.......................................cgcagacucuaaguaucuuacc..................................................	439	0	ea1
.......................................cgcagacucuaaguaCcuuacc..................................................	1	1	ea1
.......................................cgcagacucuaaguGucuuacc..................................................	1	1	ea1
.......................................cgcagacucuaaguUucuuacca.................................................	1	1	ea1
.......................................cgcagacucuaaguaucuuacAa.................................................	1	1	ea1
.......................................cgcagacucuaaguaucuuacca.................................................	39	0	ea1
.......................................cgcagacucuaaguaucuuaccU.................................................	13	1	ea1
.......................................cgcagacucuaaguaucCuacca.................................................	1	1	ea1
.......................................cgcagacucuaaguaucuuaccC.................................................	11	1	ea1
.......................................cgcagacucuaaguaucuuacUa.................................................	10	1	ea1
.......................................cgcagacucuaGguaucuuacca.................................................	2	1	ea1
.......................................cgcGgacucuaaguaucuuaccaa................................................	60	1	ea1
.......................................cgcagacucuaaguaucuuaccaa................................................	2	0	ea1
.......................................cgcagacucuaaguaucuuaccUa................................................	2	1	ea1
.......................................cgcagacucuaaguaucuuacUaa................................................	6	1	ea1
.......................................cgUagacucuaaguaucuuaccaa................................................	1	1	ea1
.......................................cgcagacucuaaguaucuuaccCa................................................	2	1	ea1
.......................................cgcagacucuaaguaucuuaccaU................................................	7	1	ea1
.......................................cgcagacucuaaguaucuuaccaGg...............................................	2	1	ea1
........................................gcagacucuaaguaucuuacU..................................................	2	1	ea1
.........................................cagacucuaaguaucuuacc..................................................	2	0	ea1
.........................................cagacucuaaguaucuuacU..................................................	4	1	ea1
.........................................cagacucGaaguaucuuacc..................................................	3	1	ea1
.........................................cagacucuaaguaucuuaUc..................................................	1	1	ea1
.........................................cagacucuaaguaucuuacca.................................................	1	0	ea1
.........................................cagacucuaaguaucuuacUa.................................................	2	1	ea1
.........................................cagacucuaGguaucuuacca.................................................	1	1	ea1
.........................................cGgacucuaaguaucuuaccaa................................................	1	1	ea1
..........................................agacucuaaguaucuuacAa.................................................	1	1	ea1
..........................................agacucAaaguaucuuacca.................................................	1	1	ea1
..........................................agacucuaaguaucuuaccaag...............................................	2	0	ea1
.............................................................aaguucugguaagauacuuag.............................	1	0	ea1
...............................................................uucugguaagaAcuuaga..............................	8	1	ea1
```

Mature **Star**

```
uauuuacuaacuaaggaaacaccucaagacuagccugcgcgcagacucuaaguaucuuaccaaguucugguaagauacuuagagucuacgcgcaggcuacucauaacuggu
................................................................uucugguaagaAcuuagag............................  2  1  ea1
..................................................................cugguaagaAcuuagaguc..........................  1  1  ea1
..................................................................cugguaagaAcuuagagucu.........................  1  1  ea1
..................................................................cugguaagaAcuuagagucua........................  1  1  ea1
...................................................................ugguaagauacuuagaguA.........................  1  1  ea1
...................................................................ugguaagaAcuuagaguc..........................  1  1  ea1
...................................................................ugguaagauacuuagagucu........................  1  0  ea1
...................................................................ugguaagaAcuuagagucu.........................  1  1  ea1
...................................................................ugguaagauacuuagagucA........................  1  1  ea1
...................................................................ugguaagaAcuuagagucua........................  6  1  ea1
......................................................................uaagaAcuuagagucua........................  3  1  ea1
......................................................................uaagaAcuuagagucuacg......................  1  1  ea1
......................................................................uaagauacuuagagucuacg.....................  2  0  ea1
......................................................................uaagauacuuagagucuacAc....................  1  1  ea1
......................................................................uaagauacuuagagucuacgA....................  3  1  ea1
......................................................................uaagauacuuagagucuacgc....................  13  0  ea1
......................................................................uaagauacuuagagucuacgG....................  1  1  ea1
......................................................................uaagauacuuagGgucuacgcg...................  1  1  ea1
......................................................................uaagauacuuagagucuacgcC...................  2  1  ea1
......................................................................uGagauacuuagagucuacgcg...................  2  1  ea1
......................................................................uaagauacuuagagucAacgcg...................  1  1  ea1
......................................................................uaagauacuuagagucuacgcg...................  77  0  ea1
......................................................................uaagauacuuagagucuacUcg...................  4  1  ea1
......................................................................uaagaAcuuagagucuacgcg....................  6  1  ea1
......................................................................uaagauacuuagagucuacgcU...................  3  1  ea1
......................................................................uaagaCacuuagagucuacgcg...................  1  1  ea1
......................................................................uaagauacuuagagucuacgcA...................  11  1  ea1
......................................................................uaagauacuuagagucuacgcgc..................  2  0  ea1
......................................................................uaagauacuuagagucuCcgcgc..................  1  1  ea1
......................................................................uaagauacuuagagucuacgcgU..................  5  1  ea1
......................................................................uaagauacuuagagCcuacgcgc..................  1  1  ea1
......................................................................uaagauacuuagagucuacgcgcC.................  1  1  ea1
......................................................................uaagauacuuagagucuacgcgUa.................  2  1  ea1
.......................................................................Gagauacuuagagucuacgcg...................  1  1  ea1
.......................................................................aagauacuuagagucuacgcgcaU................  1  1  ea1
........................................................................agauacuuCgagucuacgcgca.................  1  1  ea1
...........................................................................uacuuagagucuacgcgcaC................  1  1  ea1

..................................cugcgUgcagacucuaagua.........................................................  1  1  er2
...................................ugcgcgcagacucuaaguau........................................................  1  0  er2
.....................................cgcgcagacucuaaguaucu......................................................  1  0  er2
.....................................cgcgcagacucuaaguaucuuG....................................................  3  1  er2
......................................gcgcagacucuaaguaucuA.....................................................  1  1  er2
......................................gcgcagacucuaaguaucuuU....................................................  2  1  er2
......................................gcgcagacucuaaguaucuua....................................................  2  0  er2
.......................................cgcagacucuaaguaucuu.....................................................  10  0  er2
.......................................cgcagacucCaaguaucuua....................................................  1  1  er2
.......................................cgcagacucuaaguaucuuU....................................................  17  1  er2
.......................................cgcagacucuaaguUucuua....................................................  2  1  er2
.......................................cgcagacucuaaguaucuua....................................................  109  0  er2
.......................................Ngcagacucuaaguaucuua....................................................  1  1  er2
.......................................cgcagacucuaaguaCcuua....................................................  1  1  er2
.......................................cgcagacucuaaguaucuuaA...................................................  7  1  er2
.......................................cgcagacucuaaguaucuuaU...................................................  9  1  er2
.......................................cgcagacucuaaguaucuuac...................................................  40  0  er2
.......................................cgcagacucuaaguaucuuacA..................................................  20  1  er2
.......................................cgcagacucuaaguaucuuacc..................................................  17  0  er2
.......................................cgcagacucuaaguaucuuacU..................................................  15  1  er2
.......................................cgcagacucuaaguaucuuacca.................................................  1  0  er2
.......................................cgcGgacucuaaguaucuuaccaa................................................  16  1  er2
........................................gcagacucuaaguaucuua....................................................  3  0  er2
........................................gcagacucuaaguaucuuac...................................................  1  0  er2
........................................gcagacucuaaguaucuuacA..................................................  1  1  er2
.........................................cagacucuaaguaucuuacU..................................................  2  1  er2
.........................................cagacucuaagGaucuuacc..................................................  1  1  er2
.........................................cagacucuaaguaucuuaAcaa................................................  4  1  er2
...................................................................ugguaagaAcuuagagucu.........................  2  1  er2

.....................................cgcgcagacucuaaguaucA......................................................  2  1  ea2
.....................................cgcgcagacucuaaguaucuA.....................................................  3  1  ea2
```

Mature Star

```
uauuuacuaacuaaggaaacaccucaagacuagccugcgcgcagacucuaaguaucuuaccaaguucugguaagauacuuagagucuacgcgcaggcuacucauaacuggu
.....................................cgcgcagacucuaaguaucuu.....................................................   1   0   ea2
.....................................cgcgcagacucuaaguUucuua....................................................   4   1   ea2
......................................gcgcagacucuaaguaucuu.....................................................   6   0   ea2
......................................gcgcagacucuaaguaucuua....................................................   5   0   ea2
......................................gcgcagacucuaaguaucuuU...................................................   11   1   ea2
......................................gcgcagacucuaaguaucuuCc..................................................   2   1   ea2
......................................gcgcagacucuaaguaucuuacc..................................................   4   0   ea2
.......................................cgcagacucuaaguaucuu.....................................................   12   0   ea2
.......................................cgcagacucuUaguaucuua....................................................   1   1   ea2
.......................................cgcagacucuaaguaucuuG....................................................   5   1   ea2
.......................................Ngcagacucuaaguaucuua....................................................   1   1   ea2
.......................................cgcagacucuaaguaucuuU....................................................   51   1   ea2
.......................................cgcagacucuaaguaucuua....................................................   182   0   ea2
.......................................cAcagacucuaaguaucuua....................................................   1   1   ea2
.......................................cAcagacucuaaguaucuuac...................................................   1   1   ea2
.......................................cgcagacucuaaguaucuuaA...................................................   4   1   ea2
.......................................cgcagacucuaaguaucuuac...................................................   16   0   ea2
.......................................Ngcagacucuaaguaucuuacc..................................................   1   1   ea2
.......................................cgcagacucuaaguaucuuacA..................................................   24   1   ea2
.......................................cgcagacucuaaguaucuuacc..................................................   24   0   ea2
.......................................cgcagacucuaaguaucuuacU..................................................   13   1   ea2
.......................................cgcagacucuaaguaucuuaccU.................................................   1   1   ea2
.......................................cgcagacucuaaguaucuuacca.................................................   2   0   ea2
.......................................cgcagacucuaaguaucuuacUaa................................................   1   1   ea2
.......................................cgcGgacucuaaguaucuuaccaa................................................   7   1   ea2
........................................gcagacucuaaguaucuuU....................................................   3   1   ea2
........................................Ucagacucuaaguaucuua....................................................   1   1   ea2
........................................gcagacucuaaguaucuua....................................................   22   0   ea2
........................................gcagacucuaaguaucuuac...................................................   5   0   ea2
........................................gcagacucuaaguaucuuacA..................................................   2   1   ea2
........................................gcGgacucuaaguaucuuaccaa................................................   1   1   ea2
.........................................cagacucuaaguaucuua....................................................   3   0   ea2
.........................................cagacucuaaguaucuuacc..................................................   3   0   ea2
.........................................cagacuAuaaguaucuuacc..................................................   1   1   ea2
..............................................................aguucugguaagauacuuag.............................   2   0   ea2
..................................................................cugguaagaAcuuagagucua........................   3   1   ea2
......................................................................uaagauacuuagagucuacgcg...................   3   0   ea2

.......................................cgcagacucuaaguaucuuU....................................................   14   1   eg2
.......................................cgcagacucuaaguaucuua....................................................   27   0   eg2
.......................................cgcagacucuaaguaucuuaU...................................................   1   1   eg2
.......................................cgcagacucuaaguaucuuaA...................................................   5   1   eg2
.......................................cgcagacucuaaguaucuuac...................................................   3   0   eg2
.......................................cgcagacucuaaguaucuuacc..................................................   1   0   eg2
.......................................cgcagacucuaaguaucuuacU..................................................   4   1   eg2
.......................................cgcagacucuaaguaucuuacA..................................................   1   1   eg2
.......................................cgcagacucuaaguaucuuacca.................................................   2   0   eg2
.......................................cgcagacucuaaguaucuuacUaa................................................   2   1   eg2
.......................................cgcagacucuaaguaucuuaccaa................................................   1   0   eg2
......................................................................uaagauacuuagagucuacgcg...................   1   0   eg2
```

Provisional ID	:	ScUbCFx_107_10607
Score total	:	6988.8
Score for star read(s)	:	3.9
Score for read counts	:	6977.6
Score for mfe	:	2.7
Score for randfold	:	1.6
Score for cons. seed	:	3
Total read count	:	13698
Mature read count	:	13570
Loop read count	:	0
Star read count	:	128

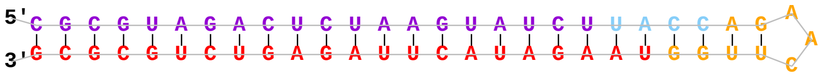

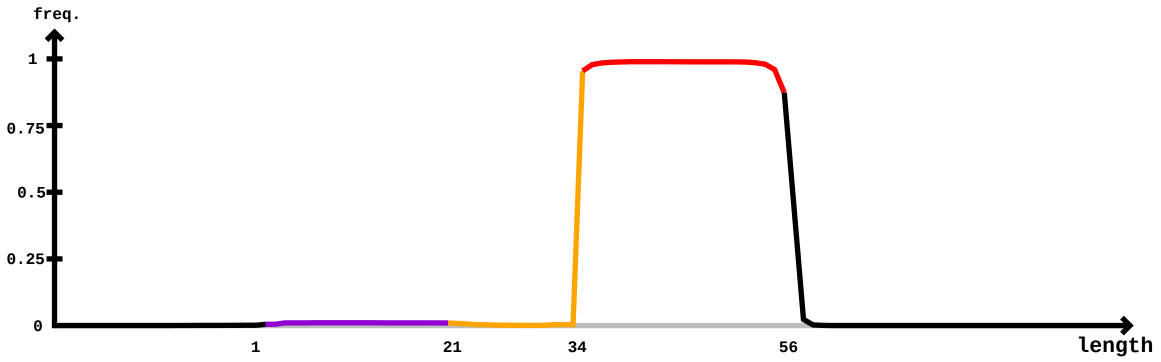

Star Mature

```
5'- uaccaguuaugagaguagccugcgcguagacucuaaguaucuuaccagaacuugguaagauacuuagagucugcgcgcaggcuagucuugagguguuuccuuaguuaguaaa   -3'    obs
    uaccaguuaugagaguagccugcgcguagacucuaaguaucuuaccagaacuugguaagauacuuagagucugcgcgcaggcuagucuugagguguuuccuuaguuaguaaa          exp
    (((.......((.((((((((((((((((((((((((((((((((((...)))))))))))))))))))))))))))))))))).)).(((((.....)))))....)))..   reads     mm     sample
    ......................cgcguagacucuaaguaucu......................................................................   2         0      eg1
    ........................cguagacucuaaguaucuuacc..................................................................   1         0      eg1
    ........................cguagacucuaaguaucuuacU..................................................................   1         1      eg1
    ..............................................agaacuugguaagauacuu...............................................   1         0      eg1
    ....................................................ugguaagaAacuuagagucugc......................................   1         1      eg1
    .......................................................uaagaAacuuagagucugc......................................   2         1      eg1
    .......................................................uaagaAacuuagagucugcg.....................................   19        1      eg1
    .......................................................uaagauacuuagagucugcg.....................................   1         0      eg1
    .......................................................uaagauacuuagagucugcCc....................................   1         1      eg1
    .......................................................uaagauacuuagagucugcgA....................................   1         1      eg1
    .......................................................uaagauacuuagagucugcgc....................................   1         0      eg1
    .......................................................uaagauacuuagagucugcgU....................................   1         1      eg1
    .......................................................uaagaAacuuagagucugcgc....................................   44        1      eg1
    .......................................................uaagauacuuagagucugcgcA...................................   9         1      eg1
    .......................................................uaagaAacuuagagucugcgcg...................................   502       1      eg1
    .......................................................uaagauacuuagagucugcgcg...................................   11        0      eg1
    .......................................................uaagauacuuagagucugcgcU...................................   1         1      eg1
    .......................................................uGagauacuuagagucugcgcg...................................   1         1      eg1
    .......................................................uaagauacuuagagucugcgcgc..................................   1         0      eg1
    .......................................................uaagauacuuagagucugcgcgU..................................   5         1      eg1
    .......................................................uaagaAacuuagagucugcgcgc..................................   9         1      eg1
    .......................................................uaagauacuuagagucugcgcAc..................................   2         1      eg1
    .......................................................uaagauacuuagagucugcgcUca.................................   1         1      eg1
    .......................................................uaagauacuuagagucugcgcgcC.................................   1         1      eg1
    .......................................................uaagaAacuuagagucugcgcgca.................................   1         1      eg1
    ........................................................aagaAacuuagagucugcgcg...................................   2         1      eg1
    ........................................................aagauacuuagagucugcgcg...................................   2         0      eg1
    ........................................................aagauacuuagagucugcgcgc..................................   2         0      eg1
    ........................................................aagaAacuuagagucugcgcgc..................................   1         1      eg1
    ........................................................aagauacuuagagucugcgcgcC.................................   2         1      eg1
    ........................................................aagauacuuagagucugcgcgcU.................................   13        1      eg1
    ........................................................aagauacuuagagucugcgcgcaU................................   3         1      eg1
    ........................................................aagauacuuagagucugcgcgcUg................................   3         1      eg1
```

```
                    Star                            Mature
uaccaguuaugaguagccugcgcguagacucuaaguaucuuaccagaacugguaagauacuuagagucugcgcgcaggcuagucuugagguguuuccuuaguuaguaaa
......................................................agaAacuuagagucugcgcg...................................   1    1    eg1
......................................................agauacuuagagucugcgcgcU.................................   1    1    eg1
.......................................................gaAacuuagagucugcgc....................................   1    1    eg1
.......................................................gauacuuagagucugcgcgc..................................   1    0    eg1
.......................................................gauacuuagagucugcgcgcU.................................   1    1    eg1
.......................................................gauacuuagagucugcgcgcC.................................   2    1    eg1
.........................................................uacuuagagucugcgcgcagU...............................   2    1    eg1
.........................................................uacuuagagucugcgcgcagg...............................   1    0    eg1

...........aguagccugcgcguagacucu.............................................................................   2    0    er1
.............uagccugcgcguagacucua............................................................................   1    0    er1
................ccugcgcguagacucuaagua........................................................................   1    0    er1
.................cugcgcguagacucuaaguauc......................................................................   1    0    er1
..................ugcgcguagacucuaaguauc......................................................................   1    0    er1
..................ugcgcguagacucuaaguaucA.....................................................................   2    1    er1
....................cgcguagacucuaaguaucu.....................................................................   5    0    er1
......................cguagacucuaaguaucuuacc.................................................................   3    0    er1
......................cguGgacucuaaguaucuuacca................................................................   1    1    er1
......................cguagacucuaaguaucuuaccag...............................................................   1    0    er1
............................................agaacugguaagauacuuag.............................................   1    0    er1
.................................................ugguaagaAacuuagagucug.......................................   1    1    er1
....................................................uaagaAacuuagagucug.......................................   1    1    er1
....................................................uaagaAacuuagagucugc......................................   6    1    er1
....................................................uaagaAacuuagagucugcg.....................................   14   1    er1
....................................................uaagauacuuagagucugcgc....................................   1    0    er1
....................................................uaagauacuuagagucugcgG....................................   1    1    er1
....................................................uaagaAacuuagagucugcgc....................................   75   1    er1
....................................................uaagauacuuagagucugcgA....................................   1    1    er1
....................................................uaagauacuuagagucugcgcg...................................   17   0    er1
....................................................uaagauacuuagagucugcgcA...................................   3    1    er1
....................................................uaagaGacuuagagucugcgcg...................................   2    1    er1
....................................................uaagaAacuuagagucugcgcg...................................   764  1    er1
....................................................uaagauacuuagagucugcgcU...................................   3    1    er1
....................................................uaagaAacuuagagucugcgcgc..................................   25   1    er1
....................................................uaagauacuuagagucugcgcgG..................................   1    1    er1
....................................................uaagauacuuagagucugcgcgU..................................   12   1    er1
....................................................uaagaAacuuagagucugcgcgca.................................   5    1    er1
....................................................uaagauacuuagagucugcgcgUa.................................   3    1    er1
....................................................uaagauacuuagagucugcgcUca.................................   1    1    er1
....................................................uaagauacuuagagucugcgcgca.................................   1    0    er1
.....................................................aagauacuuagagucugcgcA...................................   1    1    er1
.....................................................aagaAacuuagagucugcgcg...................................   1    1    er1
.....................................................aagauacuuagagucugcgcg...................................   1    0    er1
.....................................................aagauacuuagagucugcgcgc..................................   2    0    er1
.....................................................aagauacuuagagucugcgcgG..................................   1    1    er1
.....................................................aagauacuuagagucugcgcgU..................................   1    1    er1
.....................................................aagauacuuagagucugcgcgca.................................   4    0    er1
.....................................................aagauacuuagagucugcgcgcC.................................   1    1    er1
.....................................................aagauacuuagagucugcgcgcU.................................   3    1    er1
.....................................................aagauacuuagagucugcgcgcaUg...............................   1    1    er1
......................................................agauacuuagagucugcgcgcU.................................   1    1    er1
.......................................................gauacuuagagucugcgcgcU.................................   3    1    er1
........................................................auacuuagagucugcgcgcag................................   1    0    er1
.........................................................uacuuagagucugcgcgc..................................   2    0    er1
.........................................................uacuuagGgucugcgcgcagg...............................   1    1    er1

....................cgcguagacucuaaguaucu.....................................................................   9    0    er2
......................cguagacucuaaguaucuua...................................................................   6    0    er2
......................cguagacucuaaguaucuuU...................................................................   5    1    er2
......................cguagacucuaaguaucuuac..................................................................   1    0    er2
......................cguagacucuaaguaucuuacc.................................................................   5    0    er2
...........................................cagaacugguaagauacuuC..............................................   2    1    er2
................................................uugguaagauacuuagagucA........................................   3    1    er2
.................................................ugguaagaAacuuagagucu........................................   2    1    er2
....................................................uaagaAacuuagagucugcg.....................................   1    1    er2
....................................................uaagaAacuuagagucugcgc....................................   1    1    er2
....................................................uaagaAacuuagagucugcgcg...................................   355  1    er2
....................................................uaagauacuuagagucugcgcg...................................   9    0    er2
....................................................uaagauacuuagagucugcgcgU..................................   1    1    er2
....................................................uaagauacuuagagucugcgcgUa.................................   4    1    er2
```

Star **Mature**

uaccaguuaugaguagccugcgcguagacucuaaguaucuuaccagaacuugguaagauacuuagagucugcgcgcaggcuagucuugagguguuuccuuaguuaguaaa			
....aagaAacuuagagucugcg.....................................	1	1	er2
....aagaAacuuagagucugcgcg...................................	21	1	er2
....aagauacuuagagucugcgcg...................................	7	0	er2
....aagauacuuagagucugcgcgU..................................	6	1	er2
....aagauacuuagagucugcgcgca.................................	1	0	er2
....aagauacuuagagucugcgcgcU.................................	3	1	er2
....aagauacuuagagucugcgcgUa.................................	2	1	er2
....agaAacuuagagucugcgcg...................................	3	1	er2
....agauacuuagagucugcgcA...................................	2	1	er2
....agauacuuagagucugcgcgcU.................................	2	1	er2
....auacuuagagucugcgcgca.................................	1	0	er2
....uacuuagagucugcgcgcagC...............................	1	1	er2
...........aguagccugcgcguagacucu..	2	0	ea1
..............agccugcgcguagacucu..	1	0	ea1
.................cugcgcguagacucuaaguauc...	1	0	ea1
..................ugcgcguagacucuaaguau..	1	0	ea1
..................ugcgcguagacucuaaguaucA..	2	1	ea1
....................cgcguagacucuaaguaucA..	1	1	ea1
....................cgcguagacucuaaguaucu..	15	0	ea1
....................cgcguagacucuaaguaucC..	1	1	ea1
....................cgcguagacucuaaguaucuu...	2	0	ea1
....................cgcguagacucuaaguaucuua..	1	0	ea1
....................cgcguagacucuaaguaucuuacA..	1	1	ea1
......................cguagacucuaaguaucuuU..	2	1	ea1
......................cguagacucuaaguaucuua..	5	0	ea1
......................cguagacucuaaguaucuuac...	1	0	ea1
......................cguagacucuaaguaucuuacc..	5	0	ea1
......................cguGgacucuaaguaucuuacc..	4	1	ea1
......................cguagacucuaaguaucuuacU..	3	1	ea1
......................cguagacucuaaguaucuuaccaA..	1	1	ea1
.........................agacucuaaguaucuuacAa...	1	1	ea1
.........................agacucAaaguaucuuacca...	1	1	ea1
.........................agacucCaaguaucuuaccaga...	2	1	ea1
.........................agacucAaaguaucuuaccaga...	1	1	ea1
.........................agacucuaaguaucuuaccaUa...	2	1	ea1
..........................gacucuaaguUucuuaccag..	1	1	ea1
...................................uaucuuaccagaacuuggAa...	1	1	ea1
....ccagaacuugguaagauacA..	1	1	ea1
....cGagaacuugguaagauacuu...	1	1	ea1
....agaacuugguaagauacuua..	1	0	ea1
....ugguaagauacuuagaguA...	1	1	ea1
....ugguaagaAacuuagaguc...	1	1	ea1
....ugguaagauacuuagagucA..	1	1	ea1
....ugguaagaAacuuagagucu..	1	1	ea1
....ugguaagauacuuagagucu..	1	0	ea1
....ugguaagaAacuuagagucug.......................................	18	1	ea1
....ugguaagauacuuagagucug.......................................	1	0	ea1
....ugguaagauacuuGgagucug.......................................	2	1	ea1
....ugguaagauacuuGgagucugc......................................	1	1	ea1
....ugguaagaAacuuagagucugc......................................	1	1	ea1
....ugguaagaAacuuagagucugcgcg...................................	1	1	ea1
....gguaagaAacuuagagucug.......................................	3	1	ea1
....guaagaAacuuagagucugc......................................	1	1	ea1
....uaagaAacuuagagucug.......................................	8	1	ea1
....uaagauacuuagagucugU......................................	2	1	ea1
....uaagaAacuuagagucugc......................................	56	1	ea1
....uaagauacuuagagucugcg.....................................	4	0	ea1
....uaagauacuuGgagucugcg.....................................	1	1	ea1
....uaagaAacuuagagucugcg.....................................	234	1	ea1
....uaagauacuuagagucugcgc....................................	38	0	ea1
....uaagaGacuuagagucugcgc....................................	5	1	ea1
....uaagauacuuagagucugcgA....................................	5	1	ea1
....uaagauacuuGgagucugcgc....................................	1	1	ea1
....uaagauacuuagagucugcgU....................................	12	1	ea1
....Gaagauacuuagagucugcgc....................................	1	1	ea1
....uaagaAacuuagagucugcgc....................................	983	1	ea1
....uaagauacuuagagucugcgG....................................	3	1	ea1
....uaagauacuuagCgucugcgc....................................	1	1	ea1
....uaagauacuuagaAucugcgcg...................................	1	1	ea1

```
                    Star                            Mature
uaccaguuaugaguagccugcgcguagacucuaaguaucuuaccagaacuugguaagauacuuagagucugcgcgcaggcuagucuugagguguuuccuuaguuaguaaa
.....................................................uaagauacuuagagucugcgcg...................................   85     0    ea1
.....................................................uaagauacuuagagucugcgcC...................................   8      1    ea1
.....................................................Gaagauacuuagagucugcgcg...................................   4      1    ea1
.....................................................uaagauacuuagagucugUgcg...................................   1      1    ea1
.....................................................uaagauacuuagagucugcgcU...................................   29     1    ea1
.....................................................uaagaAacuuagagucugcgcg...................................   8641   1    ea1
.....................................................uaagauacuuagagucugcgcA...................................   69     1    ea1
.....................................................uaagaGacuuagagucugcgcg...................................   23     1    ea1
.....................................................uaagauacuuGgagucugcgcg...................................   7      1    ea1
.....................................................uaagauacuuagagucugcgcAc..................................   3      1    ea1
.....................................................uaagaAacuuagagucugcgcgc..................................   303    1    ea1
.....................................................uaagauacuuagagucugcgcgU..................................   16     1    ea1
.....................................................uaagauacuuagagucuCcgcgc..................................   1      1    ea1
.....................................................uaagauacuuagagucugcgcgG..................................   1      1    ea1
.....................................................Gaagauacuuagagucugcgcgc..................................   1      1    ea1
.....................................................uaagauacuuagagucugcgcgcU.................................   3      1    ea1
.....................................................uaagaAacuuagagucugcgcgca.................................   68     1    ea1
.....................................................uaagaGacuuagagucugcgcgca.................................   1      1    ea1
.....................................................uaagauacuuagagucugcgcgca.................................   1      0    ea1
.....................................................uaagauacuuagagucugcgcAca.................................   3      1    ea1
.....................................................uaagauacuuagagucugcgcgUa.................................   17     1    ea1
.....................................................uaagaAacuuagagucugcgcgcag................................   2      1    ea1
.....................................................uaagauacuuagagucugcgcgUag................................   1      1    ea1
......................................................aagauacuuagagucugc......................................   1      0    ea1
......................................................aagauacuuagagucugcC.....................................   2      1    ea1
......................................................aagauacuuagagucugcg.....................................   1      0    ea1
......................................................aagauacuuagagucugcgc....................................   2      0    ea1
......................................................aagaAacuuagagucugcgc....................................   2      1    ea1
......................................................aagauacuuagagucugcgcU...................................   1      1    ea1
......................................................aagauacuuagagucugcgcA...................................   5      1    ea1
......................................................aagauacuuagagucugcgcg...................................   12     0    ea1
......................................................aagaAacuuagagucugcgcg...................................   18     1    ea1
......................................................aagauacuuagagucugcgcgG..................................   3      1    ea1
......................................................aagauacuuagagucugcgcgU..................................   4      1    ea1
......................................................aagauacuuagagucugcgcAc..................................   1      1    ea1
......................................................aagauacuuagagucugcgcgc..................................   10     0    ea1
......................................................aagaAacuuagagucugcgcgc..................................   9      1    ea1
......................................................aagauacuuagagucugcgcgcU.................................   45     1    ea1
......................................................aagauacuuagagucugcgcgcC.................................   5      1    ea1
......................................................aagaGacuuagagucugcgcgca.................................   1      1    ea1
......................................................aagauacuuagagucugcgcgcG.................................   1      1    ea1
......................................................aagauacuuagagucugcgcgca.................................   15     0    ea1
......................................................aagaAacuuagagucugcgcgca.................................   4      1    ea1
......................................................aagauacuuagagucugcgcgcUg................................   3      1    ea1
......................................................aagauacuuagagucugcgcgcaU................................   5      1    ea1
......................................................aagauacuuagagucugcgcgcaUg...............................   1      1    ea1
.......................................................agaAacuuagagucugcgc....................................   2      1    ea1
.......................................................agauacuuagagucugcgc....................................   2      0    ea1
.......................................................agauacuuagagucugcgcU...................................   1      1    ea1
.......................................................agaAacuuagagucugcgcg...................................   50     1    ea1
.......................................................agauacuuagagucugcgcg...................................   1      0    ea1
.......................................................agaAacuuagagucugcgcgc..................................   4      1    ea1
.......................................................agauacuuagagucugcgcgU..................................   1      1    ea1
.......................................................agauacuuagagucugcgcgc..................................   1      0    ea1
.......................................................agaAacuuagagucugcgcgca.................................   1      1    ea1
.......................................................agauacuuagagucugcgcgcC.................................   1      1    ea1
.......................................................agauacuuagagucugcgcgcU.................................   6      1    ea1
.......................................................agauacuuagagucugcgcgUa.................................   1      1    ea1
.......................................................agauacuuagagucugcgcgca.................................   1      0    ea1
.......................................................agauacuuagagucugcgcgcaU................................   1      1    ea1
........................................................gauacuuagagucugcgcgcU.................................   15     1    ea1
........................................................gauacuuagagucugcgcgca.................................   4      0    ea1
........................................................gauacuuagagucugcgcgcC.................................   7      1    ea1
.........................................................auacuuagagucugcgcgcC.................................   2      1    ea1
.........................................................auacuuagagucugcgcgAa.................................   1      1    ea1
.........................................................auacuuagagucugcgcgca.................................   4      0    ea1
.........................................................auacuuagagucugcgcgcU.................................   3      1    ea1
.........................................................aAacuuagagucugcgcgca.................................   3      1    ea1
.........................................................auacuuagagucugcgcgcaU................................   1      1    ea1
.........................................................aAacuuagagucugcgcgcag...............................   1      1    ea1
```

```
                    Star                             Mature
uaccaguuaugaguagccugcgcguagacucuaaguaucuuaccagaacuugguaagauacuuagagucugcgcgcaggcuagucuugagguguuuccuuaguuaguaaa
..........................................................uacuuGgagucugcgcgc..................................  1    1    ea1
..........................................................uacuuagagucugcgcgcU.................................  1    1    ea1
..........................................................uacuuagagucugcgcgcaU................................  1    1    ea1
..........................................................uacuuGgagucugcgcgcagg...............................  1    1    ea1
..........................................................uacuuagagucugcgcgcagg...............................  1    0    ea1

.............uagccugcgcguagacucua.............................................................................  2    0    eg2
......................cguGgacucuaaguaucuua....................................................................  1    1    eg2
......................Nguagacucuaaguaucuua....................................................................  1    1    eg2
......................cguGgacucuaaguaucuuacca.................................................................  3    1    eg2
.....................................................uaagauacuuagagucugcg.....................................  1    0    eg2
.....................................................uaagauacuuagagucugcgcA...................................  2    1    eg2
.....................................................uaagauacuuagagucugcgcg...................................  3    0    eg2
.....................................................uaagauacuuagagucugcgcgUa.................................  1    1    eg2
......................................................aagauacuuagagucugcgcgc..................................  1    0    eg2

....................cgcguagacucuaaguaucu......................................................................  7    0    ea2
....................cgcguagacucuaaguaucuA.....................................................................  3    1    ea2
....................cgcguagacucuaaguaucuua....................................................................  2    0    ea2
....................cgcguagacucuaaguaucuuacA..................................................................  1    1    ea2
......................cguagacucuaaguaucuu.....................................................................  2    0    ea2
......................cguagacucuaaguaucuua....................................................................  17   0    ea2
......................cguGgacucuaaguaucuuacca.................................................................  1    1    ea2
.............................................gaacuugguaagauacuuag.............................................  1    0    ea2
..................................................ugguaagaAacuuagagucug.......................................  3    1    ea2
.....................................................uaagaAacuuagagucugcg.....................................  6    1    ea2
.....................................................uaagauacuuagagucugcgcg...................................  10   0    ea2
.....................................................uaagaAacuuagagucugcgcg...................................  497  1    ea2
.....................................................uaagaGacuuagagucugcgcg...................................  1    1    ea2
.....................................................uaagauacuuagagucugcgcgUa.................................  2    1    ea2
......................................................aagauacuuagagucugcgc....................................  4    0    ea2
......................................................aagauacuuagagucugUgc....................................  1    1    ea2
......................................................aagauacuuagagucugcgcg...................................  9    0    ea2
......................................................aagauacuuagagucugUgcg...................................  2    1    ea2
......................................................aagaAacuuagagucugcgcg...................................  65   1    ea2
......................................................aagauacuuagagucugcgcgcU.................................  6    1    ea2
......................................................aagauacuuagagucugcgcgca.................................  4    0    ea2
.......................................................agaAacuuagagucugcgcg...................................  1    1    ea2
.......................................................agauacuuagagucugcgcCc..................................  1    1    ea2
........................................................gaAacuuagagucugcgcg...................................  3    1    ea2
```

Provisional ID	:	ScUbCFx_217_14663
Score total	:	62.7
Score for star read(s)	:	3.9
Score for read counts	:	55.7
Score for mfe	:	2.1
Score for randfold	:	1.6
Score for cons. seed	:	-0.6
Total read count	:	121
Mature read count	:	93
Loop read count	:	0
Star read count	:	28

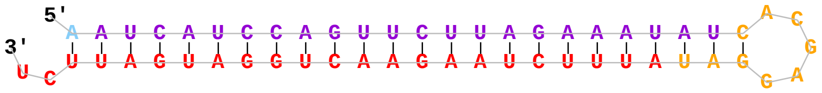

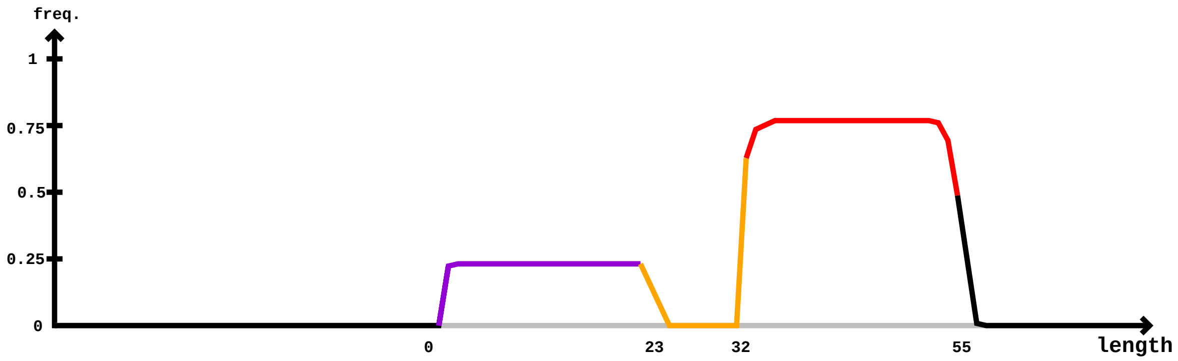


```
                                       Star                          Mature
5'- uuuauaauacauuucuuuauaagaauauucagguucagaaucauccaguucuuagaaauaucacgaggauauuucuaagaacuggaugauucucagccacaaauugcaaauu   -3'   obs
    uuuauaauacauuucuuuauaagaauauucagguucagaaucauccaguucuuagaaauaucacgaggauauuucuaagaacuggaugauucucagccacaaauugcaaauu         exp
    (((((((.........)))))))........((((.((((((((((((((((((((((((((.....))))))))))))))))))))))))))).))))..............   reads   mm   sample
    ......................................................................Cuuucuaagaacuggaugauuc....................   1       1    eg1
    ......................................................................auuucuaagaacuggaugauuc....................   10      0    eg1
    ......................................................................auuucuaagaacuggaugauuA....................   1       1    eg1
    ......................................................................auuucuaagaacuggaugauucu...................   4       0    eg1
    ......................................................................auuucuaagaacuggaugauucA...................   1       1    eg1
    ......................................................................auuucuaagaacuggaugauucG...................   1       1    eg1
    ......................................................................auuucuaagaacuggaugauucC...................   4       1    eg1
    .........................................................................ucuaagaacuggaugauucCca.................   1       1    eg1

    .......................................aucauccaguucuuagaaauau...................................................   1       0    er1
    ......................................................................auuucuaagaacuggaugau......................   1       0    er1
    ......................................................................auuucuaagaacuggaugauuc....................   1       0    er1
    ......................................................................auuucuaagaacuggaugauucA...................   2       1    er1
    .......................................................................uuucuaagaacuggaugauucC...................   1       1    er1

    .......................................aucauccaguucuuagaaaua....................................................   1       0    ea1
    .......................................aucauccaguucuuagaaauaC...................................................   2       1    ea1
    .......................................aucauccaguucuuagaaauau...................................................   5       0    ea1
    .......................................aucauccaguucuuagaaauCu...................................................   1       1    ea1
    .......................................aucauccaguucuuagaaauauA..................................................   1       1    ea1
    ......................................................................auuucuaagaacuggaugauu.....................   3       0    ea1
    ......................................................................auuucuaagaacuggaugauuc....................   8       0    ea1
    ......................................................................auuucuaagaacuggaugauucu...................   6       0    ea1
    ......................................................................auuucuaagaacuggaugauucC...................   2       1    ea1
    ......................................................................auuucuaagaacuggaugauucA...................   13      1    ea1
    ......................................................................auuucuaagaacuggaugauucuA..................   1       1    ea1
    .......................................................................Auucuaagaacuggaugauu.....................   2       1    ea1
    .......................................................................uuucuaagaacuggaugauucC...................   1       1    ea1

    .......................................aucauccaguucuuagaaauau...................................................   9       0    er2
    .......................................aucauccaguucuuagaaauauA..................................................   3       1    er2
    ......................................................................auuucuaagaacuggaugauuc....................   4       0    er2
    ......................................................................auuucuaagaacuggaugauucu...................   2       0    er2
```

Star **Mature**

```
uuuauaauacauuucuuuauaagaauauucagguucagaaucauccaguucuuagaaauaucacgaggauauuucuaagaacuggaugauucucagccacaaauugcaaauu
......................................................................auuucuaagaacuggaugauucC...................   1   1   er2
......................................................................auuucuaagaacuggaugauucA...................   5   1   er2
.......................................................................uuucuaagaacuggaugauucC...................   2   1   er2
........................................................................uucuaagaacuggaugauucC...................   2   1   er2
.........................................................................ucuaagaacuggaugauucC...................   1   1   er2

.......................................aucauccaguucuuagaaauau...................................................   1   0   ea2
.......................................aucauccaguucuuagaaauauA..................................................   3   1   ea2
........................................ucauccaguucuuagaaaua....................................................   1   0   ea2
.......................................................................uuucuaagaacuggaugauG.....................   3   1   ea2
.......................................................................uuucuaagaacuggaugauucA...................   3   1   ea2

......................................................................auuucuaagaacuggaugauucu...................   3   0   eg2
......................................................................auuucuaagaacuggaugauucA...................   2   1   eg2
.......................................................................uuucuaagaacuggaugauucu...................   1   0   eg2
```

Provisional ID	:	ScUbCFx_217_14730
Score total	:	608.2
Score for star read(s)	:	3.9
Score for read counts	:	597.6
Score for mfe	:	2.1
Score for randfold	:	1.6
Score for cons. seed	:	3
Total read count	:	1184
Mature read count	:	1150
Loop read count	:	0
Star read count	:	34

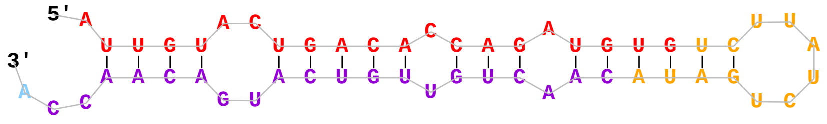

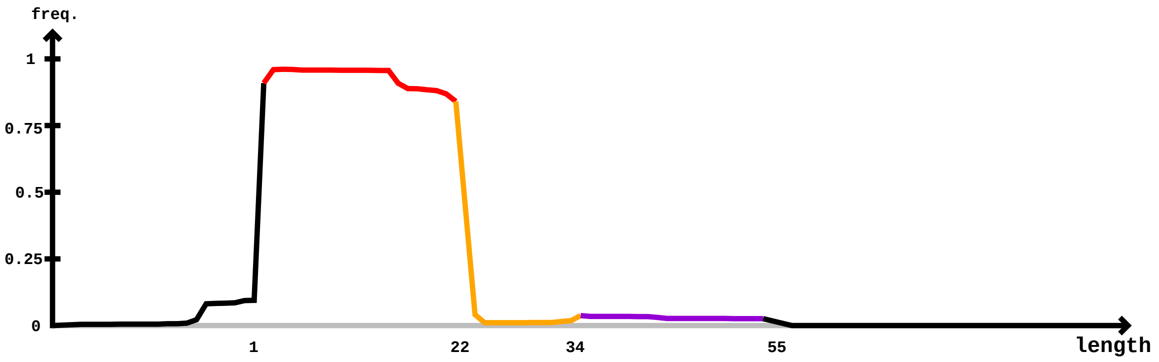

Mature **Star**

```
5'- uggccaaggaacauuugcugauuguacugacaccagaugugucuuaucugauacaacuguugucaugacaaccagcaguuccuuaggugaccugauggaaauuucaggaa  -3'   obs
    uggccaaggaacauuugcugauuguacugacaccagaugugucuuaucugauacaacuguugucaugacaaccagcaguuccuuaggugaccugauggaaauuucaggaa        exp
    ..((((((((((...(((((.((((..(((((.(((.((((((......)))))).))).)))))..)))).))))))))))))).)))..(((((........))))).. reads  mm  sample
    .............uuugcugauuguacugacacc............................................................................ 1      0   eg2
    ..............uugcugauuguacugacacc............................................................................ 6      0   eg2
    .................cugauuguacugacaccaga......................................................................... 2      0   eg2
    ....................auuguacugacaccagaug....................................................................... 1      0   eg2
    ....................auuguacugacaccagaugug..................................................................... 41     0   eg2
    ....................auuguaUugacaccagaugug..................................................................... 1      1   eg2
    .....................................................caacuguugucaugacaacca.................................... 1      0   eg2

    .............uuugcugauuguacugacacca........................................................................... 5      0   ea2
    ..............uugUugauuguacugacacc............................................................................ 1      1   ea2
    ..............uugcugauuguacugacacc............................................................................ 13     0   ea2
    ..............uugcugauuguacugacacca........................................................................... 9      0   ea2
    ..................ugauuguacugacaccagaug....................................................................... 2      0   ea2
    ..................ugauuguacugacaccagaugug..................................................................... 4      0   ea2
    ..................ugauuguacugacaccagaugAg..................................................................... 1      1   ea2
    ...................Cauuguacugacaccagaugug..................................................................... 1      1   ea2
    ....................auuguacugacaccagaugug..................................................................... 270    0   ea2
    ....................Nuuguacugacaccagaugug..................................................................... 1      1   ea2
    ....................auuguacugacaccagauguA..................................................................... 1      1   ea2
    ....................auuguacugGcaccagaugug..................................................................... 1      1   ea2
    ....................auuguaUugacaccagaugug..................................................................... 1      1   ea2
    ....................auuguacugacaccagaugugu.................................................................... 16     0   ea2
    ....................auuguacugacaccagauguguA................................................................... 1      1   ea2
    .....................uuguacugacaccagaugug..................................................................... 38     0   ea2
    .....................uuguacugacaccagauguA..................................................................... 1      1   ea2
    ..................................agaugugucuuaucugauaA........................................................ 4      1   ea2

    .ggccaaggaacauuugcugauug...................................................................................... 2      0   er2
    .....aaggaacauuugcugauuguacu.................................................................................. 1      0   er2
    ..........acauuugcugauuguacugaca.............................................................................. 1      0   er2
    ..............uugcugauuguacugacacA............................................................................ 2      1   er2
    ..............uugcugauuguacugacacc............................................................................ 8      0   er2
    ..............uugcugauuguacugacacca........................................................................... 3      0   er2
```

```
                    Mature                Star
uggccaaggaacauuugcugauuguacugacaccagaugugucuuaucugauacaacuguugucaugacaaccagcaguuccuuaggugaccugauggaaauuucaggaa
...............ugcugauuguacugacacc............................................................................    1     0    er2
...................Cauuguacugacaccagaugug.....................................................................    1     1    er2
....................auuguacugacaccagaugug.....................................................................    155   0    er2
....................aGuguacugacaccagaugugu....................................................................    1     1    er2
....................auuguacugacaccagaugugu....................................................................    14    0    er2
.....................uuguacugacaccagaugu......................................................................    1     0    er2
.....................Nuguacugacaccagaugug.....................................................................    2     1    er2
.....................uuguacugacacUagaugug.....................................................................    1     1    er2
.....................uuguacugacaccagaugug.....................................................................    20    0    er2
......................uguacugacaccagaugug.....................................................................    2     0    er2
....................................................acaacuguugucaugacaacc.....................................    3     0    er2

uggccaaggaacauuugcu...........................................................................................    2     0    ea1
uggccaaggaacauuugcugauuA......................................................................................    1     1    ea1
............auuugcugauuguacugacacc............................................................................    1     0    ea1
............auuugcugauuguacugacacca...........................................................................    1     0    ea1
.............uuugcugauuguacugacacc............................................................................    7     0    ea1
.............Guugcugauuguacugacaccaga.........................................................................    1     1    ea1
..............uugcugauuguacugacacA............................................................................    1     1    ea1
..............uugcugauuguacugacacc............................................................................    12    0    ea1
..............Gugcugauuguacugacacc............................................................................    1     1    ea1
..............uugcugauuAuacugacacc............................................................................    1     1    ea1
..............uugcugauuguacugacacU............................................................................    6     1    ea1
..............uugcugauuguacugacaccC...........................................................................    1     1    ea1
..............uugcugauuguacugacacca...........................................................................    5     0    ea1
..............uugcugauuguacugacaccaC..........................................................................    1     1    ea1
..............uugcugauuguacugacaccaUau........................................................................    1     1    ea1
..............uugcugauuguacugacaccagauA.......................................................................    1     1    ea1
..............uugcugauuguacugacaccagaugug.....................................................................    3     0    ea1
...............ugcugauuguacugacaccaUa.........................................................................    1     1    ea1
..................ugauuguacugacaccaga.........................................................................    1     0    ea1
..................ugauuguacugacaccagauA.......................................................................    1     1    ea1
..................Ggauuguacugacaccagaugu......................................................................    1     1    ea1
..................ugauuguacugacaccagaugug.....................................................................    1     0    ea1
...................Cauuguacugacaccagaugugu....................................................................    1     1    ea1
....................auuguacugacaccagau........................................................................    3     0    ea1
....................auuguacugacaccagauA.......................................................................    1     1    ea1
....................Cuuguacugacaccagaug.......................................................................    1     1    ea1
....................auuguacugacaccagaug.......................................................................    8     0    ea1
....................Guuguacugacaccagaug.......................................................................    1     1    ea1
....................auCguacugacaccagaug.......................................................................    1     1    ea1
....................auuguacugacaccagaugu......................................................................    23    0    ea1
....................auAguacugacaccagaugu......................................................................    1     1    ea1
....................auuguacugacaccagaugA......................................................................    1     1    ea1
....................Guuguacugacaccagaugu......................................................................    1     1    ea1
....................auuguacuAacaccagaugu......................................................................    1     1    ea1
....................auuguacugacaccagaugC......................................................................    1     1    ea1
....................auuguacugacaccagauguA.....................................................................    45    1    ea1
....................auuguacugacaccagauAug.....................................................................    1     1    ea1
....................auugCacugacaccagaugug.....................................................................    1     1    ea1
....................auuguacugacGccagaugug.....................................................................    1     1    ea1
....................auuguaUugacaccagaugug.....................................................................    1     1    ea1
....................auuguacugacaccagaugCg.....................................................................    1     1    ea1
....................auuguacugacaccUgaugug.....................................................................    1     1    ea1
....................auuguaAugacaccagaugug.....................................................................    1     1    ea1
....................auuguacugacaccagauguU.....................................................................    10    1    ea1
....................Guuguacugacaccagaugug.....................................................................    7     1    ea1
....................auuguacugacUccagaugug.....................................................................    1     1    ea1
....................auuAuacugacaccagaugug.....................................................................    1     1    ea1
....................auuguacugacaccagaCgug.....................................................................    2     1    ea1
....................auCguacugacaccagaugug.....................................................................    1     1    ea1
....................auuguacugacaccagaugug.....................................................................    287   0    ea1
....................auuguGcugacaccagaugug.....................................................................    1     1    ea1
....................auuguacCgacaccagaugug.....................................................................    1     1    ea1
....................auuguacugacaccagaugugC....................................................................    4     1    ea1
....................auuguacugacaccagaugugu....................................................................    20    0    ea1
....................auuguacugacaccagauguguc...................................................................    1     0    ea1
....................auuguacugacaccagauguguU...................................................................    2     1    ea1
....................auuguacugacaccagauguguA...................................................................    28    1    ea1
.....................uuguacugacaccagaugug.....................................................................    2     0    ea1
```

Mature Star

```
uggccaaggaacauuugcugauuguacugacaccagaugugucuuaucugauacaacuguugucaugacaaccagcaguuccuuaggugaccugauggaaauuucaggaa
.....................uuguacugacaccagaugugugA..................................................................   1   1   ea1
.......................................ugucuuaucugauacaacug...................................................   1   0   ea1
.......................................ugucuuaucugauacaacuguA.................................................   1   1   ea1
.........................................ucuuaucugauacaacuguA.................................................   1   1   ea1
.........................................ucuuaucugauacaacuguug................................................   1   0   ea1
..........................................cuuaucugauacaacuguu.................................................   1   0   ea1
..........................................cuuaucugauacaacuguug................................................   4   0   ea1
...........................................uuaucugGuacaacuguu.................................................   1   1   ea1
................................................ugauacaacuguugucaugac.........................................   1   0   ea1
...................................................uacaacuguugucaugacaacc.....................................   3   0   ea1
...................................................uacaacuguugucaugacaacU.....................................   2   1   ea1
....................................................acaacuguugucaugacaacc.....................................   1   0   ea1
.....................................................caacuguugucaugacaacU.....................................   5   1   ea1
.....................................................caacuguugucaugacaacc.....................................   9   0   ea1
.....................................................caacuguugucaugacaacA.....................................   1   1   ea1
.....................................................caacuguugucaugaUaacc.....................................   1   1   ea1
.....................................................caacuguugucaugacaaccU....................................   3   1   ea1
.....................................................caacuguugucaugacaacca....................................   2   0   ea1
.....................................................caacuguugucaugacaacUa....................................   1   1   ea1

............auuugcugauuguacugacacU............................................................................   1   1   eg1
.............uuugcugauuguacugacacU............................................................................   1   1   eg1
..............uugcugauuguacugacaccU...........................................................................   1   1   eg1
....................Guuguacugacaccagaugu......................................................................   1   1   eg1
....................auugCacugacaccagaugu......................................................................   1   1   eg1
....................auuguacugacaccagaugu......................................................................   4   0   eg1
....................Guuguacugacaccagaugug.....................................................................   2   1   eg1
....................auuguacugacaccagaugCg.....................................................................   2   1   eg1
....................auuguacugacaccagauguA.....................................................................   6   1   eg1
....................auuguacugacaccagaugug.....................................................................   33  0   eg1
....................auuguacugacaccagaugugu....................................................................   3   0   eg1
....................auuguacugacaccagauguguG...................................................................   1   1   eg1
....................auuguacugacaccagauguguA...................................................................   5   1   eg1
....................auuguacugacaccagauguguc...................................................................   1   0   eg1
.....................................................caacuguugucaugacaacca....................................   1   0   eg1
.....................................................caacuguugucaugacaaccaU...................................   1   1   eg1

uggccaaggaacauuugcugauu.......................................................................................   1   0   er1
..........acauuugcugauuguacugacacU............................................................................   1   1   er1
.............uuugcugauuguacugacacc............................................................................   2   0   er1
..............uugcugauuguacugacacc............................................................................   1   0   er1
..............uugcugauuguacugacacca...........................................................................   1   0   er1
................gcugauuguacugacaccagauA.......................................................................   1   1   er1
....................auuguacugacaccagaugu......................................................................   1   0   er1
....................auuguacugacaccagauguU.....................................................................   3   1   er1
....................auuguacugacaccagauguA.....................................................................   4   1   er1
....................auuguacugacaccagaugug.....................................................................   33  0   er1
....................aCuguacugacaccagaugug.....................................................................   1   1   er1
....................auuguacugacaccagaugugu....................................................................   1   0   er1
....................auuguacugacaccagauguguA...................................................................   1   1   er1
```

Provisional ID	:	ScUbCFx_217_14830
Score total	:	9774.3
Score for star read(s)	:	3.9
Score for read counts	:	9763.3
Score for mfe	:	2.5
Score for randfold	:	1.6
Score for cons. seed	:	3
Total read count	:	19162
Mature read count	:	19068
Loop read count	:	0
Star read count	:	94

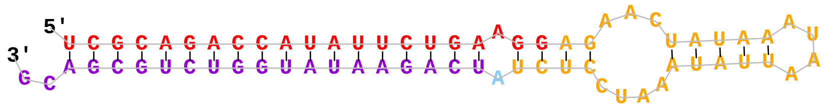

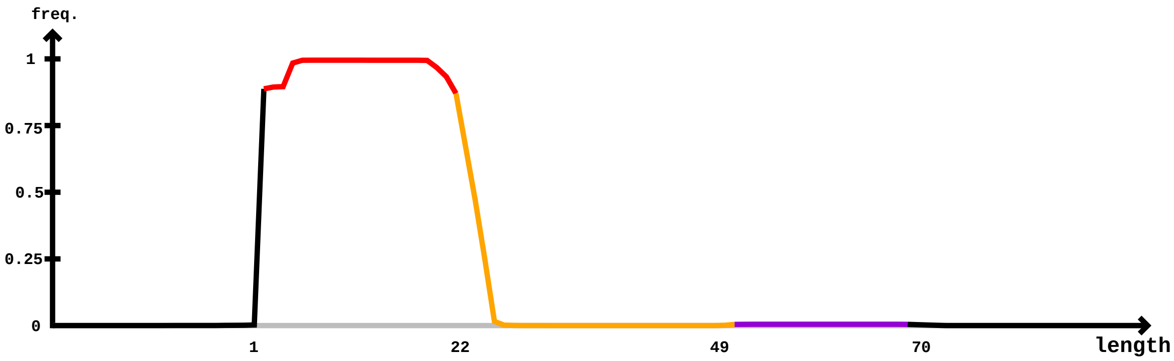

Mature **Star**

```
5'- aacuugaaugcuagccugcgucgcagaccauauucugaaggagaacuauaaaauaauuauaaauccucuaucagaauauggucugcgacgcaggcuacuugaaugccagggag -3'   obs
    aacuugaaugcuagccugcgucgcagaccauauucugaaggagaacuauaaaauaauuauaaauccucuaucagaauauggucugcgacgcaggcuacuugaaugccagggag       exp
    ..((((.((..(((((((((((((((((((((((((((.((((...(((((....)))))....)))).))))))))))))))))))))))))))).....))..))))...   reads   mm   sample
    ..................cgucgcagaccauUuucuga...........................................................................   3       1    er1
    ....................ucgcagaccauauucuga...........................................................................   19      0    er1
    ....................ucgcagGccauauucuga...........................................................................   1       1    er1
    ....................Gcgcagaccauauucuga...........................................................................   3       1    er1
    ....................ucgcGgaccauauucugaa..........................................................................   1       1    er1
    ....................Ccgcagaccauauucugaa..........................................................................   1       1    er1
    ....................ucgcagaccauauucugaa..........................................................................   29      0    er1
    ....................Gcgcagaccauauucugaa..........................................................................   4       1    er1
    ....................Gcgcagaccauauucugaag.........................................................................   8       1    er1
    ....................ucgcagaccauauucugaag.........................................................................   52      0    er1
    ....................ucgcagaccauauucugaaU.........................................................................   1       1    er1
    ....................Ccgcagaccauauucugaag.........................................................................   1       1    er1
    ....................ucgcagaccauauucugaaA.........................................................................   3       1    er1
    ....................ucgcagaccauUuucugaag.........................................................................   1       1    er1
    ....................ucgcagaccauauucuCaag.........................................................................   1       1    er1
    ....................Gcgcagaccauauucugaagg........................................................................   42      1    er1
    ....................ucgcagaccauauucugaagA........................................................................   28      1    er1
    ....................ucgcagaccauauucugaaAg........................................................................   2       1    er1
    ....................ucgcagaccauauAcugaagg........................................................................   1       1    er1
    ....................ucgcagaccauauucugaagC........................................................................   1       1    er1
    ....................ucgcagaccauauucugaUgg........................................................................   1       1    er1
    ....................ucgcagaccauauucugaGgg........................................................................   1       1    er1
    ....................ucgcagaccauauucugaagg........................................................................   359     0    er1
    ....................ucgcagaccaGauucugaagg........................................................................   1       1    er1
    ....................ucgcagaccauauucugaagU........................................................................   3       1    er1
    ....................ucgcagaUcauauucugaagg........................................................................   1       1    er1
    ....................ucAcagaccauauucugaagg........................................................................   3       1    er1
    ....................ucgcGgaccauauucugaagg........................................................................   2       1    er1
    ....................ucgcagaccauauucugUagg........................................................................   1       1    er1
    ....................Ccgcagaccauauucugaagg........................................................................   1       1    er1
    ....................ucgcagaccauauucuCaagg........................................................................   4       1    er1
    ....................ucgcagaccauauCcugaagg........................................................................   1       1    er1
    ....................Gcgcagaccauauucugaagga.......................................................................   22      1    er1
```

```
                    Mature                                               Star
aacuugaaugcuagccugcgucgcagaccauauucugaaggagaacuauaaaauaauuauaaauccucuaucagaauauggucugcgacgcaggcuacuugaaugccagggag
....................ucgcGgaccauauucugaagga.......................................................................    1      1    er1
....................ucgcagaccauauucugaaggG.......................................................................    2      1    er1
....................ucgcagaccaCauucugaagga.......................................................................    2      1    er1
....................ucgUagaccauauucugaagga.......................................................................    1      1    er1
....................ucgcagaccauauucugaagga.......................................................................    113    0    er1
....................ucgcagaccauauucugaGgga.......................................................................    1      1    er1
....................ucgcagaccauauucuCaagga.......................................................................    8      1    er1
....................ucgcagaccauauucugaaggU.......................................................................    3      1    er1
....................ucgcagaccauauucuUaagga.......................................................................    1      1    er1
....................ucgcagaAcauauucugaaggag......................................................................    1      1    er1
....................ucgcagaccauauucCgaaggag......................................................................    1      1    er1
....................ucgcagaccGuauucugaaggag......................................................................    1      1    er1
....................ucgcagUccauauucugaaggag......................................................................    1      1    er1
....................ucgcGgaccauauucugaaggag......................................................................    3      1    er1
....................ucgcagaccaCauucugaaggag......................................................................    1      1    er1
....................ucgcagaccauauucugaaggag......................................................................    345    0    er1
....................Ccgcagaccauauucugaaggag......................................................................    1      1    er1
....................ucgcagaccauauucugaaggaU......................................................................    32     1    er1
....................ucgcagaccauauucAgaaggag......................................................................    1      1    er1
....................ucgcagaccauauucugaaUgag......................................................................    1      1    er1
....................ucgcagaccauauucugaaggaC......................................................................    3      1    er1
....................ucgcagaccUuauucugaaggag......................................................................    1      1    er1
....................ucgcUgaccauauucugaaggag......................................................................    1      1    er1
....................ucgcagaccauauucugaaggGg......................................................................    1      1    er1
....................ucgcagaccauauucuCaaggag......................................................................    41     1    er1
....................ucgcagaccauauucugaUggag......................................................................    3      1    er1
....................Gcgcagaccauauucugaaggag......................................................................    54     1    er1
....................ucgcagacUauauucugaaggag......................................................................    1      1    er1
....................ucgcagaccauauucugaaggagC.....................................................................    10     1    er1
....................ucgcagaccauauucugaaggagG.....................................................................    1      1    er1
....................ucgcagaccauauucugaaggaUa.....................................................................    132    1    er1
....................ucgcagaccauauucugaaggaCa.....................................................................    37     1    er1
....................Gcgcagaccauauucugaaggaga.....................................................................    5      1    er1
....................ucgcagaccauauucugaaggaga.....................................................................    34     0    er1
....................ucgcagaccauauucugaaggagU.....................................................................    108    1    er1
....................ucgcagaccauauucugaaggagUa....................................................................    17     1    er1
....................ucgcagaccauauucugaaggaUaa....................................................................    8      1    er1
....................ucgcagaccauauucugaaggagaG....................................................................    1      1    er1
....................ucgcagaccauauucugaaggagaa....................................................................    1      0    er1
....................ucgcagaccauauucugaaggaCaa....................................................................    1      1    er1
....................ucgcagaccauauucugaaggagUac...................................................................    1      1    er1
......................gcagaccauauucugaaggaUa.....................................................................    1      1    er1
.......................cagaccauauucugaagU........................................................................    1      1    er1
.......................cagaccauauucugaagA........................................................................    1      1    er1
.......................cagaccauUuucugaagg........................................................................    2      1    er1
.......................cagaccauUuucugaagga.......................................................................    7      1    er1
.......................cagaccauUuucugaaggag......................................................................    1      1    er1
.......................cagaccauauucugaaggag......................................................................    18     0    er1
.......................cagaccauauucugaaggaU......................................................................    2      1    er1
.......................cagaccauauucugaaggaC......................................................................    3      1    er1
.......................cagaccauauucugaaggagU.....................................................................    10     1    er1
.......................cagaccauauucugaaggaUa.....................................................................    4      1    er1
.......................cagaccauauucugaaggagC.....................................................................    5      1    er1
.......................cagaccauauucugaaggaCa.....................................................................    12     1    er1
.......................cagaccauauucugaaggaga.....................................................................    1      0    er1
.......................cagaccauauucugaaggagUa....................................................................    8      1    er1
.......................cagaccauauucugaaggagaa....................................................................    2      0    er1
.......................cagaccauauucugaaggaCaa....................................................................    1      1    er1
........................agaccauauucugaaggagU.....................................................................    2      1    er1

...........uagccugcgucAcagaccau..................................................................................    1      1    eg1
...................Uucgcagaccauauucugaagg........................................................................    1      1    eg1
....................Ccgcagaccauauucuga...........................................................................    1      1    eg1
....................ucgcagaccauauucuga...........................................................................    46     0    eg1
....................ucgcagaccauUuucuga...........................................................................    1      1    eg1
....................Gcgcagaccauauucuga...........................................................................    8      1    eg1
....................ucgcagaccauauucugG...........................................................................    3      1    eg1
....................ucgcGgaccauauucuga...........................................................................    1      1    eg1
....................ucgcagaccauauucuCa...........................................................................    1      1    eg1
....................ucgcagaccauauucugaG..........................................................................    3      1    eg1
```

Mature **Star**

aacuugaaugcuagccugcgucgcagaccauauucugaaggagaacuauaaauaauuauaaauccucuaucagaauauggucugcgacgcaggcuacuugaaugccagggag			
....................ucgUagaccauauucugaa...	1	1	eg1
....................ucgcagaccauUuucugaa...	1	1	eg1
....................Gcgcagaccauauucugaa...	9	1	eg1
....................ucgcagaccauauucugaa...	78	0	eg1
....................ucgcGgaccauauucugaag..	3	1	eg1
....................Ccgcagaccauauucugaag..	1	1	eg1
....................ucgcagaccauauucugaag..	123	0	eg1
....................ucgcagaccauauucugaaU..	1	1	eg1
....................ucgcagaccauauucugaaA..	7	1	eg1
....................Gcgcagaccauauucugaag..	13	1	eg1
....................ucgcagaccaCauucugaag..	1	1	eg1
....................ucgcagaccauaCucugaagg...	4	1	eg1
....................ucgcUgaccauauucugaagg...	1	1	eg1
....................ucgcagaccauauucuCaagg...	6	1	eg1
....................ucgcagaccauauucuUaagg...	1	1	eg1
....................ucgcagaccauauucugaagA...	60	1	eg1
....................ucgcagaccauauucAgaagg...	1	1	eg1
....................ucgcagaccaCauucugaagg...	4	1	eg1
....................Ccgcagaccauauucugaagg...	3	1	eg1
....................ucgcagaccauauucugaaCg...	6	1	eg1
....................ucgcagaccauauucugaagg...	765	0	eg1
....................ucgcagaccauauucGgaagg...	1	1	eg1
....................ucgcagaccauauucugaGgg...	2	1	eg1
....................ucgcagaccauGuucugaagg...	2	1	eg1
....................ucgcagacUauauucugaagg...	2	1	eg1
....................ucgcagaccauUuucugaagg...	3	1	eg1
....................ucgcGgaccauauucugaagg...	3	1	eg1
....................ucgcagaccauauucugaagC...	1	1	eg1
....................ucgcagaccGuauucugaagg...	3	1	eg1
....................ucgcagaccauauucugaUgg...	1	1	eg1
....................ucgcagaccauauucCgaagg...	1	1	eg1
....................ucgcagaccauauuUugaagg...	2	1	eg1
....................ucgcagaccauauucugaagU...	2	1	eg1
....................Gcgcagaccauauucugaagg...	84	1	eg1
....................ucgcagGccauauucugaagg...	2	1	eg1
....................ucgcagaccaCauucugaagga..	1	1	eg1
....................Gcgcagaccauauucugaagga..	25	1	eg1
....................ucgcagaccauauucAgaagga..	1	1	eg1
....................ucgcagaccauauucugaaggU..	4	1	eg1
....................ucgcagaccauauucuCaagga..	8	1	eg1
....................ucgcagaccauauucugaagga..	222	0	eg1
....................ucgcaAaccauauucugaagga..	1	1	eg1
....................ucgcagGccauauucugaagga..	1	1	eg1
....................ucgcagaccauauucugaGgga..	3	1	eg1
....................ucgUagaccauauucugaagga..	1	1	eg1
....................ucgcagaccGuauucugaagga..	1	1	eg1
....................ucgcGgaccauauucugaagga..	2	1	eg1
....................ucgcagaccauauucugaagUa..	1	1	eg1
....................ucgcagaccauauucugaaggG..	7	1	eg1
....................ucgcagaccauauucugGagga..	2	1	eg1
....................ucgcagaccauauucuCaaggag...	18	1	eg1
....................ucgcagaccauauucugaaggag...	466	0	eg1
....................ucgcagaccauUuucugaaggag...	1	1	eg1
....................ucgcagaccauauucugaaggaU...	53	1	eg1
....................ucgcagacUauauucugaaggag...	1	1	eg1
....................ucgcagaccauauucCgaaggag...	2	1	eg1
....................ucgcagaccaCauucugaaggag...	2	1	eg1
....................ucgcagaccauauucugaGggag...	1	1	eg1
....................ucgcagaccauauucugaaggUg...	1	1	eg1
....................ucAcagaccauauucugaaggag...	1	1	eg1
....................ucgcagaccauauCcugaaggag...	1	1	eg1
....................ucgGagaccauauucugaaggag...	1	1	eg1
....................Gcgcagaccauauucugaaggag...	61	1	eg1
....................ucgcagaccauaCucugaaggag...	4	1	eg1
....................ucgcagaccGuauucugaaggag...	1	1	eg1
....................ucgcagaccauauucugaaUgag...	1	1	eg1
....................Ccgcagaccauauucugaaggag...	3	1	eg1
....................ucgcGgaccauauucugaaggag...	1	1	eg1
....................ucgcagaccauauucugaaggaC...	6	1	eg1
....................ucgcagaccauauucugaaggaCa..	27	1	eg1

```
                    Mature                                              Star
aacuugaaugcuagccugcgucgcagaccauauucugaaggagaacuauaaauaauuauaaauccucuaucagaauauggucugcgacgcaggcuacuugaaugccagggag
....................ucgcagaccauauucugaaggagU....................................................................   119   1   eg1
....................ucgcagaccauauucugaaggaUa....................................................................   211   1   eg1
....................ucgcagaccauauucugaaggagC....................................................................   16    1   eg1
....................Gcgcagaccauauucugaaggaga....................................................................   4     1   eg1
....................ucgcagaccauauucugaaggaga....................................................................   18    0   eg1
....................ucgcagaccauauucugaaggagG....................................................................   9     1   eg1
....................ucgcagaccauauucugaaggaCaa...................................................................   1     1   eg1
....................ucgcagaccauauucugaaggaUaa...................................................................   5     1   eg1
....................ucgcagaccauauucugaaggagCa...................................................................   1     1   eg1
....................ucgcagaccauauucugaaggagUa...................................................................   20    1   eg1
....................ucgcagaccauauucugaaggagaU...................................................................   1     1   eg1
....................ucgcagaccauauucugaaggagUac..................................................................   1     1   eg1
....................ucgcagGccauauucugaaggagaac..................................................................   1     1   eg1
.....................cgcagaccauauucugaagg.......................................................................   2     0   eg1
......................gcagaccauauucugaagg.......................................................................   4     0   eg1
......................gcagaccauauucugaaggaga....................................................................   1     0   eg1
.......................cagaccauauucugaagg.......................................................................   7     0   eg1
.......................cagaccauUuucugaagg.......................................................................   2     1   eg1
.......................cagaccauCuucugaagga......................................................................   1     1   eg1
.......................cagaccauUuucugaagga......................................................................   12    1   eg1
.......................cagaccauUuucugaaggag.....................................................................   4     1   eg1
.......................cagaccauauucugaaggag.....................................................................   27    0   eg1
.......................cagaccauauucugaaggGg.....................................................................   1     1   eg1
.......................cagaccauauucugaaggaU.....................................................................   1     1   eg1
.......................cagUccauauucugaaggag.....................................................................   1     1   eg1
.......................cagaccauauucugaGggag.....................................................................   2     1   eg1
.......................cagaccauUuucugaaggaga....................................................................   1     1   eg1
.......................cagaccauauucugaaggaCa....................................................................   13    1   eg1
.......................cagaccauauucugaaggaUa....................................................................   9     1   eg1
.......................cagaccauauucugaaggaga....................................................................   1     0   eg1
.......................cagaccauauucugaaggagU....................................................................   11    1   eg1
.......................cagaccauauucugaaggaCaa...................................................................   1     1   eg1
.......................cagaccauauucugaaggagCa...................................................................   1     1   eg1
.......................cagaccauauucugaaggagUa...................................................................   4     1   eg1
.......................cagaccauauucugaaggagaA..................................................................   1     1   eg1
........................agaccauaCucugaaggag.....................................................................   1     1   eg1
........................agaccauauucugaaggag.....................................................................   2     0   eg1
........................agaccauauucugaaggaCa....................................................................   1     1   eg1
........................agaccauauucugaaggagCa...................................................................   1     1   eg1
........................agaccauauucugaaggagaa...................................................................   1     0   eg1
.....................................................................ucagaaAauggucugcgacg.......................   1     1   eg1
.....................................................................ucagaaAauggucugcgacgca.....................   3     1   eg1
......................................................................cagaaAauggucugcgacgca.....................   1     1   eg1

................ugcgucgcagaccauauucug...........................................................................   3     0   ea2
................Agcgucgcagaccauauucug...........................................................................   2     1   ea2
.................gUgucgcagaccauauucug...........................................................................   4     1   ea2
...................Cucgcagaccauauucugaag........................................................................   3     1   ea2
...................Uucgcagaccauauucugaag........................................................................   3     1   ea2
....................ucgcagaccauauucuga..........................................................................   18    0   ea2
....................ucgcagaccauauucugaa.........................................................................   23    0   ea2
....................ucgcagaccauUuucugaa.........................................................................   2     1   ea2
....................ucgcagaccauUuucugaag........................................................................   2     1   ea2
....................ucgcagaccauauucugaGg........................................................................   1     1   ea2
....................ucgcagaccauauucugaaA........................................................................   17    1   ea2
....................ucgcagGccauauucugaag........................................................................   1     1   ea2
....................ucgcagaccauauucugaag........................................................................   183   0   ea2
....................ucgcagCccauauucugaagg.......................................................................   1     1   ea2
....................Ncgcagaccauauucugaagg.......................................................................   3     1   ea2
....................ucgUagaccauauucugaagg.......................................................................   3     1   ea2
....................ucgcagaccauUuucugaagg.......................................................................   2     1   ea2
....................ucgcagaccauauucugaagg.......................................................................   494   0   ea2
....................ucAcagaccauauucugaagg.......................................................................   1     1   ea2
....................ucgcagaccauauucugaagA.......................................................................   2     1   ea2
....................ucgcagGccauauucugaagg.......................................................................   1     1   ea2
....................uNgcagaccauauucugaagg.......................................................................   1     1   ea2
....................Ncgcagaccauauucugaagga......................................................................   1     1   ea2
....................ucgcagaccauauucugaagga......................................................................   89    0   ea2
....................ucgUagaccauauucugaaggag.....................................................................   6     1   ea2
....................ucgcagaccauauucugaaggag.....................................................................   409   0   ea2
```

```
                    Mature                                                Star
aacuugaaugcuagccugcgucgcagaccauauucugaaggagaacuauaaaauaauuauaaauccucuaucagaauauggucugcgacgcaggcuacuugaaugccagggag
....................ucgcagaccauUuucugaaggag......................................................................    2     1    ea2
....................ucgcagaccauauucugaaggaU......................................................................    12    1    ea2
....................ucgcagaccauauucugaaggaC......................................................................    5     1    ea2
....................Ncgcagaccauauucugaaggag......................................................................    2     1    ea2
....................ucgcagaccauauucugaaggUg......................................................................    1     1    ea2
....................uNgcagaccauauucugaaggag......................................................................    2     1    ea2
....................ucgcagaccauauucugaaggagU.....................................................................    57    1    ea2
....................ucgcagaccauauucugaaggaUa.....................................................................    62    1    ea2
....................ucgcagaccauauucugaaggaga.....................................................................    34    0    ea2
....................ucgcagaccauauucugaaggagC.....................................................................    6     1    ea2
....................ucgcagaccauauucugaaggaCa.....................................................................    161   1    ea2
....................ucgcagaccauauucugaaggagaa....................................................................    1     0    ea2
....................ucgcagaccauauucugaaggaUaa....................................................................    1     1    ea2
.....................cgcagaccauauucugaag.........................................................................    3     0    ea2
.....................cgcagaccauauucugaagg........................................................................    20    0    ea2
.....................cgcagaccauauucugaaggag......................................................................    20    0    ea2
.....................cgcagaccauauucugaaggagU.....................................................................    2     1    ea2
.....................cgcagaccauauucugaaggaCa.....................................................................    2     1    ea2
.....................cgcagaccauauucugaaggaga.....................................................................    2     0    ea2
.....................cgcagaccauauucugaaggaUa.....................................................................    6     1    ea2
......................gcagaccauauucugaaggag......................................................................    3     0    ea2
.......................cagaccauauucugaagg........................................................................    4     0    ea2
.......................cagaccauauucugaaggG.......................................................................    14    1    ea2
.......................cagaccauauucugaaggaU......................................................................    2     1    ea2
.......................cagaccauauucugaaggCg......................................................................    3     1    ea2
.......................cagaccauauucugaaggag......................................................................    35    0    ea2
.......................cagaccauauucugaaggagU.....................................................................    16    1    ea2
.......................cagaccauauucugaaggaUa.....................................................................    27    1    ea2
.......................cagaccauauucugaaggaCa.....................................................................    352   1    ea2
.......................cagaccauauucugaaggagCa....................................................................    1     1    ea2
.......................cagaccauauucugaaggagUa....................................................................    2     1    ea2
.......................cagaccauauucugaaggaCaa....................................................................    3     1    ea2
.......................cagaccauauucugaaggagaaA...................................................................    3     1    ea2
........................agaccauauucugaaggag......................................................................    6     0    ea2
........................agaccauauucugaaggaCa.....................................................................    62    1    ea2
........................agaccauauucugaaggaUa.....................................................................    9     1    ea2
........................agaccauauucugaaggagU.....................................................................    2     1    ea2
........................agaccauauucugaaggagaa....................................................................    2     0    ea2
........................agaccauauucugaaggagUa....................................................................    1     1    ea2
.....................................................................Uucagaauauggucugcga.........................    3     1    ea2
.....................................................................Uucagaauauggucugcgacg.......................    4     1    ea2
......................................................................ucagaauauggucugcgacg.......................    11    0    ea2
......................................................................ucagaauauggucugcgacgUa.....................    5     1    ea2

...........uagccugcgucAcagaccau..................................................................................    1     1    er2
................ugcgucgcagaccauauucug............................................................................    2     0    er2
...................Cucgcagaccauauucugaaggaga.....................................................................    2     1    er2
....................ucgcagaccauauuUuga...........................................................................    2     1    er2
....................ucgcagaccauauucuga...........................................................................    10    0    er2
....................ucgcagaccauUuucugaa..........................................................................    1     1    er2
....................ucgcagaccauauucugaa..........................................................................    10    0    er2
....................ucgcagaccauauucugaU..........................................................................    1     1    er2
....................ucgcagaccauauucugaag.........................................................................    97    0    er2
....................ucgcagaccauauucuCaag.........................................................................    1     1    er2
....................ucgcagaccauauucugaaA.........................................................................    9     1    er2
....................ucgcagaccauUuucugaagg........................................................................    1     1    er2
....................ucgcagaccauauucugaagg........................................................................    461   0    er2
....................Ncgcagaccauauucugaagg........................................................................    2     1    er2
....................ucgcaAaccauauucugaagg........................................................................    1     1    er2
....................Ccgcagaccauauucugaagg........................................................................    1     1    er2
....................ucAcagaccauauucugaagg........................................................................    4     1    er2
....................ucgcagaccauauucugaaggU.......................................................................    5     1    er2
....................ucgcagaccauauucugaagga.......................................................................    89    0    er2
....................ucgcagaccauauucugaaggC.......................................................................    1     1    er2
....................ucUcagaccauauucugaaggag......................................................................    1     1    er2
....................ucAcagaccauauucugaaggag......................................................................    9     1    er2
....................ucgUagaccauauucugaaggag......................................................................    1     1    er2
....................ucgcagaccauUuucugaaggag......................................................................    2     1    er2
....................ucgcagaccauauucugaaggaU......................................................................    47    1    er2
....................Ncgcagaccauauucugaaggag......................................................................    2     1    er2
```

Mature / Star

```
aacuugaaugcuagccugcgucgcagaccauauucugaaggagaacuauaaauaauuauaaauccucuaucagaauauggucugcgacgcaggcuacuugaaugccagggag
....................ucgcagaccauauucGgaaggag.....................................................................	1	1	er2
....................ucgcagaccauauucugaaggag.....................................................................	554	0	er2
....................ucgcagaccauauucugaaggaC.....................................................................	4	1	er2
....................ucgcagaccauauucugaaggagU....................................................................	153	1	er2
....................ucgcagaccauauucugaaggaga....................................................................	34	0	er2
....................ucgcagaccauauucugaaggaCa....................................................................	253	1	er2
....................ucgcagaccauauucugaaggagC....................................................................	8	1	er2
....................ucgcagaccauauucugaaggaUa....................................................................	223	1	er2
....................ucgcagaccauauucugaaggagG....................................................................	1	1	er2
....................ucgcagaccauauucugaaggagUa...................................................................	2	1	er2
....................ucgcagaccauauucugaaggaUaa...................................................................	8	1	er2
.....................cgcagaccauauucugaag........................................................................	7	0	er2
.....................cgcagaccauauucugaagg.......................................................................	11	0	er2
.....................cgcagaccauauucugaagUa......................................................................	1	1	er2
.....................cgcagaccauauucugaagga......................................................................	3	0	er2
.....................cgcagaccauauucugaaggag.....................................................................	3	0	er2
.....................cgcagaccauauucugaaggaU.....................................................................	7	1	er2
.....................cgcagaccauauucugaaggagU....................................................................	15	1	er2
.....................cgcagaccauauucugaaggaCa....................................................................	11	1	er2
.....................cgcagaccauauucugaaggaUa....................................................................	11	1	er2
......................gcagaccauauucugaag........................................................................	1	0	er2
......................gcagaccauauucugaagg.......................................................................	1	0	er2
......................gcagaccauauucugaaggaUa....................................................................	4	1	er2
.......................cagaccauauucugaagg.......................................................................	4	0	er2
.......................cagaccauauucugaagga......................................................................	5	0	er2
.......................cagaccauauucugaaggag.....................................................................	24	0	er2
.......................cagaccauauucugaaggaU.....................................................................	1	1	er2
.......................cagaccauauucugaagCag.....................................................................	1	1	er2
.......................cagaccauauucugaaggagU....................................................................	24	1	er2
.......................cagaccauauucugaaggaCa....................................................................	256	1	er2
.......................cagaccauauucugaaggaUa....................................................................	28	1	er2
.......................cagaccauauucugaaggagC....................................................................	1	1	er2
.......................cagaccauauucugaaggaCaa...................................................................	8	1	er2
.......................cagaccauauucugaaggagUa...................................................................	8	1	er2
.......................cagaccauauucugaaggaUaa...................................................................	1	1	er2
.......................cagaccauauucugaaggagCa...................................................................	5	1	er2
........................agaccauauucugaagga......................................................................	4	0	er2
........................agaccauauucugaaggag.....................................................................	1	0	er2
........................agaccauauucugaaggaU.....................................................................	2	1	er2
........................agaccauauucugaaggGg.....................................................................	7	1	er2
........................agaccauauucugaaggagC....................................................................	3	1	er2
........................agaccauauucugaaggaUa....................................................................	4	1	er2
........................agaccauauucugaaggaCa....................................................................	34	1	er2
........................agaccauauucugaaggagU....................................................................	1	1	er2
........................agaccauauucugaaggagCa...................................................................	1	1	er2
........................agaccauauucugaaggaUaa...................................................................	1	1	er2
........................agaccauauucugaaggagaa...................................................................	1	0	er2
........................agaccauauucugaaggagUa...................................................................	4	1	er2
........................agaccauauucugaaggaCaa...................................................................	6	1	er2
........................agaccauauucugaaggagaaA..................................................................	3	1	er2
........................agaccauauucugaaggagaaAu.................................................................	1	1	er2
.........................gaccauauucugaaggag.....................................................................	1	0	er2
.........................gaccauauucugaaggagU....................................................................	3	1	er2
.........................gaccauauucugaaggaCa....................................................................	1	1	er2
....................................................................Uucagaauauggucugcgac........................	2	1	er2
....................................................................Uucagaauauggucugcgacg.......................	9	1	er2
.....................................................................ucagaauauggucugcgacg.......................	15	0	er2
.....................................................................ucagaauauggucugcgacgc......................	2	0	er2
.....................................................................ucagaauauggucugcgacgU......................	1	1	er2
.....................................................................ucagaauauggucugcgacgUa.....................	3	1	er2
......................................................................cagaauauggucugcgacg.......................	1	0	er2
......................................................................cagaauauggucugcgacgcU.....................	1	1	er2
.......................................................................agaauauggucugcgacgcU.....................	4	1	er2

..........cuagccugcgucAcagaccau.................................................................................	1	1	ea1
...........uagccugcgucAcagaccau.................................................................................	1	1	ea1
................ugcgucgcagaccauauucug...........................................................................	1	0	ea1
................ugcgucgcagaccauUuucug...........................................................................	1	1	ea1
..................cgucgcagaccauUuucug...........................................................................	1	1	ea1
..................cgucgcagaccauUuucuga..........................................................................	1	1	ea1
```

Mature   Star

```
aacuugaaugcuagccugcgucgcagaccauauucugaaggagaacuauaaaauaauuauaaaauccucuaucagaauauggucugcgacgcaggcuacuugaaugccagggag
..................cUucgcagaccauauucugaa...........................................................................    1     1    ea1
...................Uucgcagaccauauucugaa...........................................................................    1     1    ea1
...................Uucgcagaccauauucugaagg.........................................................................    2     1    ea1
...................Cucgcagaccauauucugaagg.........................................................................    1     1    ea1
...................Cucgcagaccauauucugaagga........................................................................    4     1    ea1
...................Cucgcagaccauauucugaaggag.......................................................................    2     1    ea1
....................ucgcagacUauauucuga............................................................................    1     1    ea1
....................ucgcagaccauauucuCa............................................................................    2     1    ea1
....................ucgcagaccauaCucuga............................................................................    1     1    ea1
....................ucAcagaccauauucuga............................................................................    1     1    ea1
....................ucgcagaccauUuucuga............................................................................    1     1    ea1
....................ucgcagaccauauucuAa............................................................................    2     1    ea1
....................ucgcagaccauGuucuga............................................................................    1     1    ea1
....................Gcgcagaccauauucuga............................................................................    39    1    ea1
....................ucgcagaccauauucuga............................................................................    331   0    ea1
....................ucgcagaccauauucugG............................................................................    2     1    ea1
....................ucgcagaccauauucugU............................................................................    8     1    ea1
....................ucgcagaccGuauucuga............................................................................    1     1    ea1
....................ucgcagaccauauucCga............................................................................    2     1    ea1
....................ucgcGgaccauauucuga............................................................................    2     1    ea1
....................ucgcagUccauauucuga............................................................................    1     1    ea1
....................ucgcagaccauauucCgaa...........................................................................    2     1    ea1
....................ucCcagaccauauucugaa...........................................................................    1     1    ea1
....................ucgcagaccauauucugaa...........................................................................    408   0    ea1
....................ucgcagaccauauCcugaa...........................................................................    1     1    ea1
....................ucgcagaccauauucugGa...........................................................................    5     1    ea1
....................Ccgcagaccauauucugaa...........................................................................    1     1    ea1
....................ucgcagaccauauucugaU...........................................................................    2     1    ea1
....................Gcgcagaccauauucugaa...........................................................................    60    1    ea1
....................ucgcagaccGuauucugaa...........................................................................    3     1    ea1
....................ucgcagGccauauucugaa...........................................................................    3     1    ea1
....................ucgcagaccUuauucugaa...........................................................................    1     1    ea1
....................ucgcagaccauauucugaG...........................................................................    5     1    ea1
....................ucgcagaccauUuucugaa...........................................................................    2     1    ea1
....................ucgcGgaccauauucugaa...........................................................................    2     1    ea1
....................ucgcagaccGuauucugaag..........................................................................    1     1    ea1
....................ucgcagaccauaCucugaag..........................................................................    1     1    ea1
....................ucgcagaccauauucuCaag..........................................................................    8     1    ea1
....................ucgcagaccauauucugaGg..........................................................................    3     1    ea1
....................ucgcagaccaCauucugaag..........................................................................    3     1    ea1
....................ucgcGgaccauauucugaag..........................................................................    2     1    ea1
....................Gcgcagaccauauucugaag..........................................................................    65    1    ea1
....................ucgcCgaccauauucugaag..........................................................................    1     1    ea1
....................Acgcagaccauauucugaag..........................................................................    1     1    ea1
....................ucgcagaccauauucugaag..........................................................................    518   0    ea1
....................ucgcagaccauauCcugaag..........................................................................    1     1    ea1
....................ucgcagaccauauucugGag..........................................................................    2     1    ea1
....................ucgcagaccauauuAugaag..........................................................................    1     1    ea1
....................ucgAagaccauauucugaag..........................................................................    1     1    ea1
....................ucgcagaccauauucugUag..........................................................................    1     1    ea1
....................Ccgcagaccauauucugaag..........................................................................    1     1    ea1
....................ucgcagGccauauucugaag..........................................................................    1     1    ea1
....................ucgcagaccauauucugaaA..........................................................................    46    1    ea1
....................ucgcagaccauauucugaaU..........................................................................    3     1    ea1
....................ucgcagaccauaCucugaagg.........................................................................    3     1    ea1
....................ucgcGgaccauauucugaagg.........................................................................    11    1    ea1
....................ucgcagaccauauucugaagC.........................................................................    17    1    ea1
....................ucCcagaccauauucugaagg.........................................................................    1     1    ea1
....................ucAcagaccauauucugaagg.........................................................................    4     1    ea1
....................Acgcagaccauauucugaagg.........................................................................    2     1    ea1
....................ucgUagaccauauucugaagg.........................................................................    3     1    ea1
....................ucgcUgaccauauucugaagg.........................................................................    2     1    ea1
....................ucgcagaccauauuAugaagg.........................................................................    1     1    ea1
....................ucgcagaccauauucugaaCg.........................................................................    1     1    ea1
....................ucgcagaccauauucugaagg.........................................................................    2742  0    ea1
....................ucgcagaccauauuUugaagg.........................................................................    3     1    ea1
....................Gcgcagaccauauucugaagg.........................................................................    359   1    ea1
....................ucgcagaccauauucCgaagg.........................................................................    6     1    ea1
....................ucgcagaccauauucuCaagg.........................................................................    24    1    ea1
....................ucgcagaccauUuucugaagg.........................................................................    8     1    ea1
```

```
                    Mature                                            Star
aacuugaaugcuagccugcgucgcagaccauauucugaaggagaacuauaaauaauuauaaauccucuaucagaauauggucugcgacgcaggcuacuugaaugccagggag
....................ucgcagaccauauucugaUgg.......................................................................   2     1   ea1
....................ucgcagaccauaGucugaagg.......................................................................   1     1   ea1
....................ucgcagaccauauucGgaagg.......................................................................   1     1   ea1
....................ucgcagaccauauucugGagg.......................................................................   13    1   ea1
....................ucgcagaccauauucugaagA.......................................................................   233   1   ea1
....................ucgcagaccCuauucugaagg.......................................................................   1     1   ea1
....................ucgcaUaccauauucugaagg.......................................................................   1     1   ea1
....................ucgcagaccauauucAgaagg.......................................................................   2     1   ea1
....................ucgcagaccauauucugaGgg.......................................................................   12    1   ea1
....................ucgcagaccaAauucugaagg.......................................................................   2     1   ea1
....................ucgcagaccauauucugaaAg.......................................................................   2     1   ea1
....................ucgcagaccauauucugCagg.......................................................................   1     1   ea1
....................ucgcagaUcauauucugaagg.......................................................................   1     1   ea1
....................ucgcagaccUuauucugaagg.......................................................................   4     1   ea1
....................Ccgcagaccauauucugaagg.......................................................................   8     1   ea1
....................ucgcagaccauauCcugaagg.......................................................................   5     1   ea1
....................ucgcaAaccauauucugaagg.......................................................................   4     1   ea1
....................ucgcagacAauauucugaagg.......................................................................   1     1   ea1
....................ucgcagaccGuauucugaagg.......................................................................   3     1   ea1
....................ucgcagaccaCauucugaagg.......................................................................   3     1   ea1
....................ucgAagaccauauucugaagg.......................................................................   1     1   ea1
....................ucgcagaccauauucugaagU.......................................................................   17    1   ea1
....................ucgcagaccauauucugUagg.......................................................................   2     1   ea1
....................ucgcagaccauaAucugaagg.......................................................................   2     1   ea1
....................ucgcagaccauGuucugaagg.......................................................................   5     1   ea1
....................ucgcagGccauauucugaagg.......................................................................   8     1   ea1
....................ucgcagaccauauucugaUgga......................................................................   1     1   ea1
....................ucgcagaccauauucAgaagga......................................................................   1     1   ea1
....................uGgcagaccauauucugaagga......................................................................   2     1   ea1
....................ucgcagaccauauucugaagUa......................................................................   3     1   ea1
....................ucgcagaccauauucuCaagga......................................................................   43    1   ea1
....................Gcgcagaccauauucugaagga......................................................................   76    1   ea1
....................ucgcagaccauaCucugaagga......................................................................   1     1   ea1
....................ucgcagaccauauucugGagga......................................................................   1     1   ea1
....................ucgcCgaccauauucugaagga......................................................................   1     1   ea1
....................Ccgcagaccauauucugaagga......................................................................   4     1   ea1
....................ucgcagaUcauauucugaagga......................................................................   2     1   ea1
....................ucgcagaccauUuucugaagga......................................................................   1     1   ea1
....................ucgcagaAcauauucugaagga......................................................................   1     1   ea1
....................ucgcagaccGuauucugaagga......................................................................   1     1   ea1
....................ucgcagaccauauucugaagga......................................................................   652   0   ea1
....................ucgcagaccauauucCgaagga......................................................................   1     1   ea1
....................ucgcagaccauauucugaGgga......................................................................   3     1   ea1
....................ucgcagaccaCauucugaagga......................................................................   1     1   ea1
....................ucgcagaccauauucugaaggG......................................................................   7     1   ea1
....................ucgcagaccauauucugaaggU......................................................................   14    1   ea1
....................ucgcGgaccauauucugaagga......................................................................   3     1   ea1
....................ucgcagaccCuauucugaagga......................................................................   1     1   ea1
....................ucgcagaccauauucugaagAa......................................................................   1     1   ea1
....................ucgcagGccauauucugaagga......................................................................   1     1   ea1
....................ucgAagaccauauucugaagga......................................................................   1     1   ea1
....................ucgcagaccauGuucugaagga......................................................................   1     1   ea1
....................ucgcagaccauauCcugaagga......................................................................   1     1   ea1
....................ucgcaAaccauauucugaagga......................................................................   2     1   ea1
....................Acgcagaccauauucugaaggag.....................................................................   5     1   ea1
....................ucgcagaccauaCucugaaggag.....................................................................   1     1   ea1
....................ucgcagaccauauucuCaaggag.....................................................................   131   1   ea1
....................ucgcagaccauauucuAaaggag.....................................................................   1     1   ea1
....................ucgcagaccauauucugaaggGg.....................................................................   5     1   ea1
....................ucgcagaccauauucugaagAag.....................................................................   1     1   ea1
....................ucgcagGccauauucugaaggag.....................................................................   5     1   ea1
....................ucgcagaccauauucugGaggag.....................................................................   3     1   ea1
....................ucgcagaccGuauucugaaggag.....................................................................   2     1   ea1
....................ucgcagaccauauucugaaggUg.....................................................................   2     1   ea1
....................ucgUagaccauauucugaaggag.....................................................................   1     1   ea1
....................ucgcagaccauauCcugaaggag.....................................................................   3     1   ea1
....................Gcgcagaccauauucugaaggag.....................................................................   125   1   ea1
....................ucAcagaccauauucugaaggag.....................................................................   1     1   ea1
....................ucgcagaccauauucugaGggag.....................................................................   2     1   ea1
....................ucgcagaccauauucugaaggaC.....................................................................   42    1   ea1
```

Mature **Star**

aacuugaaugcuagccugcgucgcagaccauauucugaaggagaacuauaaaauaauuauaaauccucuaucagaauauggucugcgacgcaggcuacuugaaugccagggag			
....................ucgAagaccauauucugaaggag..	2	1	ea1
....................ucgcagaccaAauucugaaggag..	1	1	ea1
....................ucgcagaccauauuUugaaggag..	1	1	ea1
....................ucgcagaccauUuucugaaggag..	1	1	ea1
....................ucgcagUccauauucugaaggag..	1	1	ea1
....................ucgcGgaccauauucugaaggag..	11	1	ea1
....................ucgcagaccauauucugaaggag..	1153	0	ea1
....................ucgcagacAauauucugaaggag..	1	1	ea1
....................ucgcagaAcauauucugaaggag..	1	1	ea1
....................ucgcagaccauauucCgaaggag..	5	1	ea1
....................ucgcaAaccauauucugaaggag..	2	1	ea1
....................ucgcagaUcauauucugaaggag..	1	1	ea1
....................ucgcagaccauauucugaaggaU..	175	1	ea1
....................ucgcagaccGuauucugaaggaga...	1	1	ea1
....................ucgcagaccauauucugaaggaCa...	196	1	ea1
....................ucgcagaccauauucugaaggaUa...	873	1	ea1
....................ucgcagaccauauucugaaggagU...	290	1	ea1
....................ucgcagaccauaCucugaaggaga...	1	1	ea1
....................ucgcagaccauauucugaagCaga...	1	1	ea1
....................ucgcagaccauauucugaaggagG...	7	1	ea1
....................Ccgcagaccauauucugaaggaga...	1	1	ea1
....................ucgcagaccauauucuCaaggaga...	1	1	ea1
....................ucgcagaccauauucugaaggagC...	63	1	ea1
....................ucgcagaccauauucugaaggaga...	101	0	ea1
....................Gcgcagaccauauucugaaggaga...	9	1	ea1
....................ucgcagaccauauucugaaggagUa..	43	1	ea1
....................ucgcagaccauauucugaaggagaa..	2	0	ea1
....................ucgcagaccauauucugaaggagCa..	1	1	ea1
....................ucgcagaccauauucugaaggaUaa..	18	1	ea1
....................ucgcagaccauauucugaaggagaU..	2	1	ea1
....................ucgcagaccauauucugaaggagGa..	1	1	ea1
....................ucgcagaccauauucugaaggaCaa..	1	1	ea1
....................ucgcagaccauauucugaaggagaG..	1	1	ea1
....................ucgcagaccauauucugaaggagaCc...	1	1	ea1
....................ucgcagaccauauucugaaggagaaA...	1	1	ea1
....................ucgcagaccauauucugaaggagaacA..	1	1	ea1
....................ucgcagaccauauucugaaggagUacu..	1	1	ea1
....................ucgcagaccauauucugaaggagaaUu..	2	1	ea1
.....................cgcagaccauauucugaa..	1	0	ea1
.....................cgcagaccauauucugaagga...	2	0	ea1
.....................cgcagaccauauucugaaggaUa...	1	1	ea1
.....................cgcagaccauauucugaaggagU...	1	1	ea1
......................gcagaccauUuucugaagg..	1	1	ea1
......................gcagaccauauucugaagg..	2	0	ea1
......................gcagaccauauucuCaagga...	1	1	ea1
......................gcagaccauUuucugaagga...	1	1	ea1
......................gcagaccauauucugaaggag..	2	0	ea1
......................gcagaccauauucugaaggaga...	1	0	ea1
......................gcagaccauauucugaaggaUa...	2	1	ea1
.......................cagaccauauucugaagA..	4	1	ea1
.......................cagaccauauucugaagg..	47	0	ea1
.......................cagaccauUuucugaagg..	20	1	ea1
.......................cagGccauauucugaagg..	1	1	ea1
.......................Gagaccauauucugaagg..	1	1	ea1
.......................cagaUcauauucugaagg..	2	1	ea1
.......................cagaccGuauucugaagga...	1	1	ea1
.......................cagaccauUuucugaagga...	83	1	ea1
.......................cagaccauCuucugaagga...	1	1	ea1
.......................cagaccauauuUugaagga...	1	1	ea1
.......................cagaccauauucugaagga...	28	0	ea1
.......................cagUccauauucugaaggag..	1	1	ea1
.......................cagaccauauucugaGggag..	2	1	ea1
.......................cagaccauauucugaaggag..	156	0	ea1
.......................cagaccauauucugaaggGg..	1	1	ea1
.......................cagaccauUuucugaaggag..	14	1	ea1
.......................cagaccauGuucugaaggag..	1	1	ea1
.......................cagaccauauucugaaggaU..	4	1	ea1
.......................cagaUcauauucugaaggag..	2	1	ea1
.......................cagaccauauucugaaggaC..	7	1	ea1
.......................cagaccauauucuCaaggag..	1	1	ea1

Mature **Star**

```
aacuugaaugcuagccugcgucgcagaccauauucugaaggagaacuauaaaauaauuauaaauccucuaucagaauauggucugcgacgcaggcuacuugaaugccagggag
.......................cagaccauauuUugaaggag......................................................................	1	1	ea1
.......................Gagaccauauucugaaggag......................................................................	2	1	ea1
.......................cagaccauauucCgaaggag......................................................................	3	1	ea1
.......................cGgaccauauucugaaggag......................................................................	1	1	ea1
.......................cagaccauauucugCaggaga.....................................................................	1	1	ea1
.......................Uagaccauauucugaaggaga.....................................................................	1	1	ea1
.......................cagaccauauucugaaggagG.....................................................................	3	1	ea1
.......................cagaccauauucugaaggaCa.....................................................................	104	1	ea1
.......................cagaccauauucugaaggaga.....................................................................	9	0	ea1
.......................cagaccauauucugaaggagU.....................................................................	58	1	ea1
.......................cagaccauauucugaaggaUa.....................................................................	38	1	ea1
.......................cagaccauauucugaaggagC.....................................................................	18	1	ea1
.......................cagaccauUuucugaaggaga.....................................................................	5	1	ea1
.......................cagaccauauucugaaggaUaa....................................................................	1	1	ea1
.......................cagaccauauucugaaggagUa....................................................................	45	1	ea1
.......................cagaccauauucugaaggagCa....................................................................	13	1	ea1
.......................cagaccauauucugaaggaCaa....................................................................	1	1	ea1
.......................cagaccauauucugaaggagaa....................................................................	4	0	ea1
.......................Gagaccauauucugaaggagaac...................................................................	1	1	ea1
.......................cagaccauauucugaaggagaaA...................................................................	4	1	ea1
.......................cagaccauauucugaaggagaCcu..................................................................	1	1	ea1
........................agaccauUuucugaagga.......................................................................	1	1	ea1
........................agaccauauucugaagga.......................................................................	3	0	ea1
........................agaccauUuucugaaggag......................................................................	1	1	ea1
........................agaccauauucugaaggag......................................................................	6	0	ea1
........................agaccauauucugaaggaCa.....................................................................	8	1	ea1
........................agaccauauucugaaggagC.....................................................................	4	1	ea1
........................agaccauauucugaaggaga.....................................................................	5	0	ea1
........................agaccauauucugaaggagU.....................................................................	1	1	ea1
........................agaccauauucugaaggagaC....................................................................	2	1	ea1
........................agaccauauucugaaggagaa....................................................................	1	0	ea1
........................agaccauUuucugaaggagaa....................................................................	1	1	ea1
........................agaccauauucugaaggagaaA...................................................................	3	1	ea1
........................agaccauauucugaaggagaac...................................................................	1	0	ea1
........................agaccauauucugaaggagaacu..................................................................	1	0	ea1
........................agaccauauucugaaggagaacuaua...............................................................	1	0	ea1
.........................gaccauauucugaaggagaacA..................................................................	1	1	ea1
.....................................................................Uucagaauauggucugcga.........................	2	1	ea1
......................................................................ucagaaAauggucugcga.........................	2	1	ea1
......................................................................ucagaauauggucugcgacg.......................	2	0	ea1
......................................................................ucagaaAauggucugcgacg.......................	14	1	ea1
......................................................................uGagaauauggucugcgacg.......................	2	1	ea1
......................................................................ucagaaCauggucugcgacg.......................	1	1	ea1
......................................................................ucagaaAauggucugcgacgca.....................	2	1	ea1
.......................................................................cagaauauggucugcgaA........................	3	1	ea1
```

Provisional ID : ScUbCFx_217_17812
Score total : 239.7
Score for star read(s) : 3.9
Score for read counts : 230.6
Score for mfe : 0.6
Score for randfold : 1.6
Score for cons. seed : 3
Total read count : 464
Mature read count : 457
Loop read count : 0
Star read count : 7

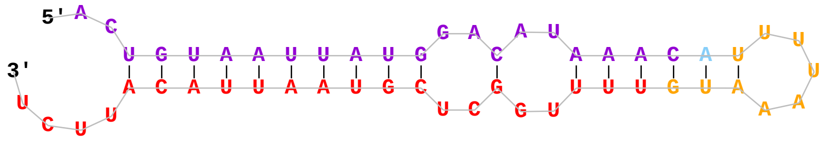

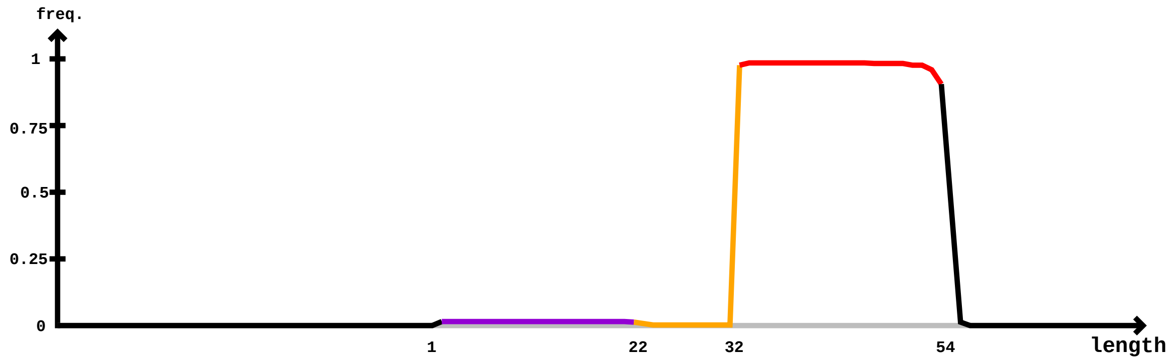

Star Mature

```
5'- aggaugaugauaacaaguggaaacauguuugugaucagacuguaauuauggacauaaacauuuuaaauguuuuggcucguaauuacauucuaaucauuauauuuucacuua  -3'   obs
    aggaugaugauaacaaguggaaacauguuugugaucagacuguaauuauggacauaaacauuuuaaauguuuuggcucguaauuacauucuaaucauuauauuuucacuua        exp
    ..............(((((((((.(((...(((((.(((.((((((((((..(..((((((.....))))))..)..)))))))))).))).)))))))).))))))))).   reads   mm   sample
    .....................................................................uuuuggcucguaauuacauA.....................   1       1    eg2
    .....................................................................uuuuggcucguaauuacauucA...................   7       1    eg2
    .....................................................................uuuuggcucguaauuacauucu...................   8       0    eg2
    .....................................................................uuuuggcucguaauuacauucua..................   6       0    eg2

    .....................................................................uuuuggcucguaauuacauuc....................   1       0    ea2
    .....................................................................uuuuggcucguaauuacauucu...................   61      0    ea2
    .....................................................................uuuuggcucguaauuacauucA...................   13      1    ea2
    .....................................................................uuuuggcucguaauuacauucua..................   11      0    ea2

    .....................................................................uuuuggcucguaauuacauuc....................   2       0    er2
    .....................................................................uuuuggcucguaauuacauucu...................   30      0    er2
    .....................................................................uuuuggcucguaauuacauucA...................   4       1    er2
    .....................................................................uuuuggcucguaauuacauucua..................   6       0    er2
    .....................................................................uuuuggcucguaauuacauucCa..................   1       1    er2

    ......................................acuguaauuauggacauaaaU....................................................   1       1    ea1
    ......................................acuguaauuauggacauaaac....................................................   1       0    ea1
    .....................................................................uuuuggcucguaauuaca........................   1       0    ea1
    .....................................................................uuuuggcucguaauuacauA.....................   2       1    ea1
    .....................................................................uuuuggcucguaauuacauu.....................   4       0    ea1
    .....................................................................uuuuggcucguaauuacauuc....................   11      0    ea1
    .....................................................................uuuuggcucguaauuacauuU....................   1       1    ea1
    .....................................................................uuuuggcucgCaauuacauuc....................   1       1    ea1
    .....................................................................Cuuuggcucguaauuacauucu...................   1       1    ea1
    .....................................................................uuuuggcuUguaauuacauucu...................   1       1    ea1
    .....................................................................uuuuggcucguaauuacauucC...................   13      1    ea1
    .....................................................................Guuuggcucguaauuacauucu...................   13      1    ea1
    .....................................................................uuuCggcucguaauuacauucu...................   1       1    ea1
    .....................................................................uuCuggcucguaauuacauucu...................   1       1    ea1
    .....................................................................uuuuggcucguaauuacauucA...................   18      1    ea1
    .....................................................................uuuuggcucguaauuacauucu...................   79      0    ea1
    .....................................................................uuuuggcucguaauuacauucua..................   13      0    ea1
```

```
                                      Star                          Mature
aggaugaugauaacaaguggaaacauguuugugaucagacuguaauuauggacauaaacauuuuaaauguuuuggcucguaauuacauucuaaucauuauauuuucacuua
.....................................................................uuuuggcucguaauuacauucuU...................   1   1   ea1
.....................................................................Guuuggcucguaauuacauucua...................   4   1   ea1
.....................................................................uuuuggcucguaauuacauucuaU..................   2   1   ea1
.....................................................................uuuuggcucguaauuacauucuaa..................   1   0   ea1
.....................................................................uuuuggcucguaauuacauucuUa..................   2   1   ea1
......................................................................uuuggcucguaauuacauucu....................   3   0   ea1

......................................acuguaauuauggacauaaa.....................................................   1   0   eg1
......................................acuguaauuauggacauaaac....................................................   1   0   eg1
............................................................uuuuaaauguuuuggcucguaUu............................   1   1   eg1
.....................................................................uuuuggcucguaauuaca........................   2   0   eg1
.....................................................................uuuuggcucguaauuacauuU.....................   1   1   eg1
.....................................................................uuuuggcucguaauuacauuc.....................   5   0   eg1
.....................................................................Guuuggcucguaauuacauuc.....................   2   1   eg1
.....................................................................uuuuggcucguaauuacauucu....................  55   0   eg1
.....................................................................Guuuggcucguaauuacauucu....................   2   1   eg1
.....................................................................uuuuggcucguaauuacauucC....................   5   1   eg1
.....................................................................uuuuggcucguaauuacauucA....................   6   1   eg1
.....................................................................uuuuggcucguaauuacauucuU...................   2   1   eg1
.....................................................................uuuuggcucguaauuacauucua...................   6   0   eg1
.....................................................................uuuuggcucguaauuacauucuUa..................   1   1   eg1
......................................................................uuuggcucguaauuacauucua...................   1   0   eg1

......................................acuguaauuauggacauaaac....................................................   2   0   er1
......................................acuguaauuauggacauaaaca...................................................   1   0   er1
.....................................................................uuuuggcucguaauuacauu......................   1   0   er1
.....................................................................uuuuggcucguaauuacauuc.....................   1   0   er1
.....................................................................uuuuggcucguaauuacauucA....................   5   1   er1
.....................................................................Guuuggcucguaauuacauucu....................   5   1   er1
.....................................................................uuuuggcucguaauuacauucC....................   4   1   er1
.....................................................................uuuuggcucguaauuacauucu....................  27   0   er1
.....................................................................uuuuggcucguaauuacauucua...................   2   0   er1
```

Provisional ID	:	ScUbCFx_217_17827
Score total	:	5472.4
Score for star read(s)	:	3.9
Score for read counts	:	5461.9
Score for mfe	:	2
Score for randfold	:	1.6
Score for cons. seed	:	3
Total read count	:	10725
Mature read count	:	9505
Loop read count	:	2
Star read count	:	1218

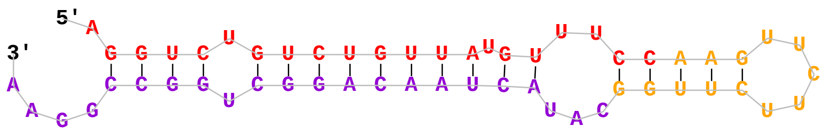

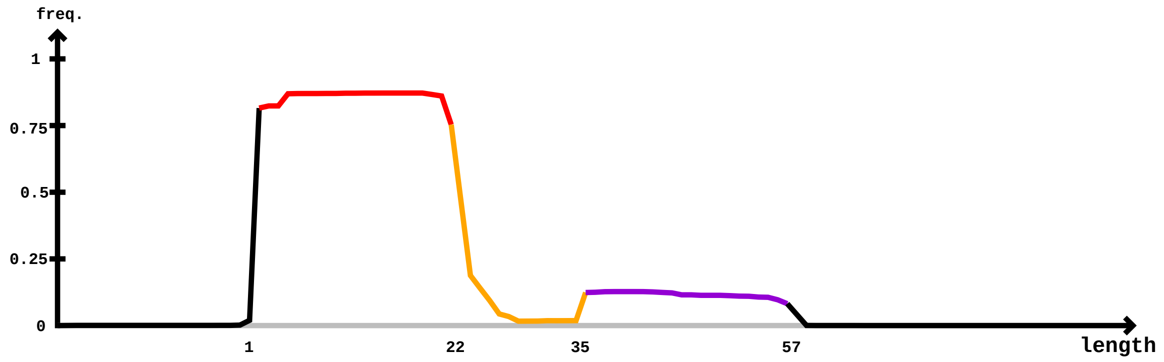

Mature **Star**

```
5'- cugccauggacugcccuccaggucugucuguuauguuuccaaguucuucuuggcauacuaacaggcuggccggaaggguauucauguuggcaauucaauggaaauccuaa   -3'   obs
    cugccauggacugcccuccaggucugucuguuauguuuccaaguucuucuuggcauacuaacaggcuggccggaaggguauucauguuggcaauucaauggaaauccuaa         exp
    .(((((..((.((((((((.((((.((((((((.((..(((((.....)))))...)))))))))).)))))).)))))).))....)))))).......((....))...   reads   mm   sample
    ..................caggucugucuguuauguuuc.......................................................................   2       0    er1
    ..................caggucugCcuguuauguuuc.......................................................................   2       1    er1
    ..................caggucugucuguuauguuucA......................................................................   1       1    er1
    ..................caggucugucuguuauguuucc......................................................................   1       0    er1
    ..................caggucugucuguuauguuucU......................................................................   1       1    er1
    ...................aggucugucuguuauguu.........................................................................   1       0    er1
    ...................aggucCgucuguuauguuuc.......................................................................   1       1    er1
    ...................aggucugucuguuauguuAc.......................................................................   1       1    er1
    ...................aggucugucugCuauguuuc.......................................................................   1       1    er1
    ...................Gggucugucuguuauguuuc.......................................................................   3       1    er1
    ...................aggucugucuguuauguuuU.......................................................................   2       1    er1
    ...................aggucugucuguuGuguuuc.......................................................................   1       1    er1
    ...................aggucugCcuguuauguuuc.......................................................................   1       1    er1
    ...................aggucugucuguuauguuuc.......................................................................   23      0    er1
    ...................aggucugucuguuauguuucc......................................................................   157     0    er1
    ...................agUucugucuguuauguuucc......................................................................   1       1    er1
    ...................aggucugucCguuauguuucc......................................................................   1       1    er1
    ...................aggucugucuguuGuguuucc......................................................................   1       1    er1
    ...................aggucugucuguuaugCuucc......................................................................   1       1    er1
    ...................aggucCgucuguuauguuucc......................................................................   1       1    er1
    ...................aggucugucuguuauguuucA......................................................................   21      1    er1
    ...................Gggucugucuguuauguuucc......................................................................   3       1    er1
    ...................agAucugucuguuauguuucc......................................................................   1       1    er1
    ...................aggucugucuguuauguuucU......................................................................   18      1    er1
    ...................aggucugucuguuauguuuccU.....................................................................   3       1    er1
    ...................aggucugucuguuauguuuccC.....................................................................   5       1    er1
    ...................aggucugucuguuauguuucca.....................................................................   20      0    er1
    ...................aggucugucuguuauguuuccaC....................................................................   5       1    er1
    ...................aggucugucuguuauguuuccaa....................................................................   3       0    er1
    ...................aggucugucuguuauguuuccaU....................................................................   13      1    er1
    ...................aggucugucuguuauguuuccCa....................................................................   4       1    er1
    ...................aggucugucuguuauguuuccaag...................................................................   6       0    er1
    ...................aggucugucuguuauguuuccaaU...................................................................   19      1    er1
```

Mature **Star**

cugccauggacugcccuccaggucugucuguuauguuuccaaguucuucuuggcauacuaacaggcuggccggaaggguauucauguuggcaauucaauggaaauccuaa			
...................aggucugucuguuauguuuccaaC...	5	1	er1
...................aggucugucuguuauguuuccaaAu..	1	1	er1
...................aggucugucuguuauguuuccaagu..	16	0	er1
...................aggucCgucuguuauguuuccaagu..	2	1	er1
...................aggucugucuguuauguuuccaagA..	1	1	er1
...................aggucugucuguuauguuuccaaUu..	21	1	er1
...................Gggucugucuguuauguuuccaagu..	1	1	er1
...................aggucugucuguuauAuuuccaagu..	1	1	er1
...................aggucugucuguuauguuuccaagC..	2	1	er1
...................aggucugucuguuauguGuccaagu..	1	1	er1
...................aggucugucuguuauguuuccaaguu...	10	0	er1
...................aggucugucuguuauguuuccaaguuU..	1	1	er1
...................aggucugucuguuauguuuccaaguuA..	1	1	er1
...................aggucugucuguuauguuuccaaguuc..	14	0	er1
......................ucugucuguuauguuuccaC..	2	1	er1
......................ucugucuguuauguuuccaaC...	5	1	er1
......................ucugucuguuauguuuccaagC..	3	1	er1
......................ucugucuguuauguuuccaagu..	1	0	er1
............................uguuauguuuccaaguucuuU...	1	1	er1
....aaguucuucuuggcauacua..	1	0	er1
....Gucuucuuggcauacuaaca...	1	1	er1
....cuucuuggcauacuaacagg...	2	0	er1
....uucuuggcauacuaacaggcug..	2	0	er1
....ucuuggcauacuaacaggcuggcA.......................................	1	1	er1
....uuggcauacuaacaggcuggc..	1	0	er1
....cauacuaacaggcuggcU.......................................	1	1	er1
....cauacuaacaggcuggcc.......................................	1	0	er1
....cauacuaacaggcuggccgg.....................................	4	0	er1
....cauacuaacaggcuggccgU.....................................	1	1	er1
....cauacuaacaggcuggccgga....................................	8	0	er1
....cauacuaacaggcuggccggU....................................	1	1	er1
....cauacuaacaggcuggccggaC...................................	8	1	er1
....cauacuaacaggcuggccggaU...................................	72	1	er1
....cauacuaacaggcuggccggUa...................................	5	1	er1
....cauacuaacaggcuggccggaa...................................	6	0	er1
....cGuacuaacaggcuggccggaa...................................	1	1	er1
....cauacuaacaggcuggccggaG...................................	1	1	er1
....cauacuaacaggcuggccggaaA..................................	4	1	er1
....cauacuaacaggcuggccggaaC..................................	1	1	er1
....cauacuaacaggcuggccggaaU..................................	4	1	er1
....cauacuaacaggcuggccggaUg..................................	3	1	er1
....uacuaacaggcuggccggaa...................................	1	0	er1
cugccauggacugcccucU...	2	1	eg1
..................caggucugucuguuauguuuc...	3	0	eg1
...................aggucugucuguuauguG...	2	1	eg1
...................aggucugucuguuauguuu..	2	0	eg1
...................Gggucugucuguuauguuuc...	2	1	eg1
...................aggucugucuguuauguuCc...	2	1	eg1
...................aggucugucuguuauguuuA...	2	1	eg1
...................aggucugucuguuauguuuU...	5	1	eg1
...................aggucugucuAuuauguuuc...	1	1	eg1
...................aggucugucuguuauguuuc...	67	0	eg1
...................aggucugucuguuaugCuucc..	1	1	eg1
...................agguUugucuguuauguuucc..	1	1	eg1
...................aggucugucuguuauguuucU..	32	1	eg1
...................Gggucugucuguuauguuucc..	5	1	eg1
...................aggucugucuguuauguuucA..	47	1	eg1
...................aggucugucugCuauguuucc..	1	1	eg1
...................aggucugucuguuauguuCcc..	2	1	eg1
...................aggucugucuguuGuguuucc..	2	1	eg1
...................aggucugucuguuauguuGcc..	1	1	eg1
...................aggucugucuguuauguuucc..	348	0	eg1
...................aggucugucuguuauguuucG..	2	1	eg1
...................aAgucugucuguuauguuucc..	1	1	eg1
...................aggCcugucuguuauguuucc..	1	1	eg1
...................aggucugucuguuauguuucca...	68	0	eg1
...................aggucugucuguuauguuuccG...	2	1	eg1
...................aggucugucuguuauguuuccU...	5	1	eg1
...................aggucugucuguuauguuuccC...	12	1	eg1

```
                   Mature              Star
cugccauggacugcccuccaggucugucuguuauguuuccaaguucuucuuggcauacuaacaggcuggccggaaggguauucauguuggcaauucaauggaaauccuaa
...................aggucugucuguuauguuAcca.....................................................................    1    1    eg1
...................aggucugucuguuauguuucAa.....................................................................    1    1    eg1
...................aggucugucuguuauguuuccCa....................................................................    4    1    eg1
...................aggucugucuguuauguuuccaa....................................................................    12   0    eg1
...................aggucugucuguuauguuuccaG....................................................................    7    1    eg1
...................aggucugucuguuauguuuccaC....................................................................    12   1    eg1
...................aggucugucuguuauguuuccaU....................................................................    24   1    eg1
...................aggucugucuguuauguuuccaaU...................................................................    13   1    eg1
...................aggucugucuguuauguuuccaag...................................................................    10   0    eg1
...................aggucugucuguuauguuuccaaC...................................................................    2    1    eg1
...................aggucugucuguuauguuuccaaUu..................................................................    19   1    eg1
...................aggucugucuguuauguuuccaagA..................................................................    1    1    eg1
...................aggucugucuguuauguuuccaagG..................................................................    1    1    eg1
...................aggucugucuguuauguuuccaagu..................................................................    27   0    eg1
...................aggucugucuguuauguuuccaagC..................................................................    5    1    eg1
...................Gggucugucuguuauguuuccaagu..................................................................    2    1    eg1
...................aggucugucuguuauguuuccaaguG.................................................................    2    1    eg1
...................aggucugucuguuauguuuccaaguu.................................................................    23   0    eg1
...................aggucugucuguuauguuuccaaUuu.................................................................    1    1    eg1
...................aggucugucuguuauguuuccaaguC.................................................................    5    1    eg1
...................aggucugucuguuauguuuccaaguuU................................................................    1    1    eg1
...................aggucugucuguuaCguuuccaaguuc................................................................    1    1    eg1
...................aggucugucuguuaugCuuccaaguuc................................................................    1    1    eg1
...................aggucugucuguuauguuuccaaguuA................................................................    1    1    eg1
...................aggucugucuguuauguuuccaaguuc................................................................    19   0    eg1
...................Gggucugucuguuauguuuccaaguuc................................................................    3    1    eg1
......................ucugucuguuauguuuccaC....................................................................    10   1    eg1
......................ucugucuguuauguuuccaU....................................................................    2    1    eg1
......................ucugucuguuauguuuccCa....................................................................    1    1    eg1
......................ucugucuguuauguuuccaag...................................................................    1    0    eg1
......................ucugucuguuauguuuccaaU...................................................................    1    1    eg1
......................ucugucuguuauguuuccaaC...................................................................    9    1    eg1
......................ucugucuguuauguuuccaagC..................................................................    5    1    eg1
......................Gcugucuguuauguuuccaagu..................................................................    1    1    eg1
......................ucugucuguuauguuuccaagu..................................................................    2    0    eg1
......................ucugucuguuauguuuccaaguu.................................................................    1    0    eg1
......................ucugucuguuauguuuccaaguuc................................................................    1    0    eg1
......................ucugucuguuauguuuccaaguuA................................................................    1    1    eg1
........................................Gaguucuucuuggcauacua..................................................    1    1    eg1
........................................aaguucuucuuggcauacua..................................................    1    0    eg1
.........................................aguucuucuuggcauacuaCc................................................    1    1    eg1
...........................................uucuucuuggcauacuaaca...............................................    5    0    eg1
.............................................cuucuuggcauacuaacaggcuA..........................................    3    1    eg1
.................................................uuggcauacuaacaggcuggcU.......................................    1    1    eg1
....................................................gcauacuaacaggcuggccgga....................................    1    0    eg1
.....................................................cauacuaacaggcuggcc.......................................    1    0    eg1
.....................................................cauacuaacaggcuAgccgg.....................................    1    1    eg1
.....................................................cauacuaacaggcuggccgg.....................................    6    0    eg1
.....................................................cauacuaacaggcuggccggC....................................    2    1    eg1
.....................................................cauacuaacaggcuggccggU....................................    1    1    eg1
.....................................................cauacuaacaggcuggccgga....................................    3    0    eg1
.....................................................cauacuaacaggcuggccggaa...................................    3    0    eg1
.....................................................cauacuaacaggcuggccggaU...................................    26   1    eg1
.....................................................cauacuaacaggcuggccggaG...................................    3    1    eg1
.....................................................cauacuaacaggcuggccggaC...................................    4    1    eg1
.....................................................cauacuaacaggcuggccggaUg..................................    7    1    eg1
.....................................................cauacuaacaggcuggccggaaU..................................    6    1    eg1
.....................................................cauacuaacaggcuggccggaaA..................................    2    1    eg1
.....................................................cauacuaacaggcuggccggaagU.................................    1    1    eg1
.......................................................uacuaacaggcuggccggaU...................................    1    1    eg1
.......................................................uacuaacaggcuggccggaaA..................................    1    1    eg1
........................................................acuaacaggcuggccggaU...................................    1    1    eg1

...................aggucugucuguuauguu.........................................................................    1    0    eg2
...................aggucugucuguuauguuA........................................................................    2    1    eg2
...................aggucugucuguuauguuuc.......................................................................    5    0    eg2
...................aggucugucuguuauguuucc......................................................................    7    0    eg2
...................aggucugucuguuauguuucca.....................................................................    1    0    eg2
...................aggucugucuguuauguuuccaU....................................................................    2    1    eg2
...................aggucugucuguuauguuuccCa....................................................................    1    1    eg2
```

Mature **Star**

cugccauggacugcccuccaggucugucuguuauguuuccaaguucuucuuggcauacuaacaggcuggccggaaggguauucauguuggcaauucaauggaaauccuaa

...................aggucugucuguuauguuuccaaU...	1	1	eg2
...................aggucugucuguuauguuuccaag...	4	0	eg2
...................aggucugucuguuauguuuccaagu..	1	0	eg2
...................aggucugucuguuauguuuccaaUu..	5	1	eg2
...................aggucugucuguuauguuuccaaguu...	3	0	eg2
...................aggucugucuguuauguuuccaaguuc..	1	0	eg2
......................ucugucuguuauguuuccaC..	3	1	eg2
cugccauggacugcccucc...	2	0	ea2
.................ccaggucugucuguuauguuucc..	1	0	ea2
..................caggGcugucuguuauguuuc...	1	1	ea2
..................caggucugucuguuauguuuc...	15	0	ea2
..................caggucugucuguuauguuucA..	17	1	ea2
..................caggucugucuguuauguuucc..	19	0	ea2
..................caggucugucuguuauguuucca...	2	0	ea2
..................caggucugucuguuauguuuccaC..	1	1	ea2
..................caggucugucuguuauguuuccaag...	1	0	ea2
...................aggucugucuguuauguA...	6	1	ea2
...................aggucugucuguuauguu...	4	0	ea2
...................aggucugucuguuauguuu..	12	0	ea2
...................aggucugucCguuauguuuc...	1	1	ea2
...................aggucGgucuguuauguuuc...	1	1	ea2
...................aggucugucAguuauguuuc...	1	1	ea2
...................aggucugucuguuauguuuc...	153	0	ea2
...................aggucugucuguuauguuucA..	217	1	ea2
...................aggucugucuguuauguuucU..	1	1	ea2
...................Uggucugucuguuauguuucc..	1	1	ea2
...................aggucugucugGuauguuucc..	1	1	ea2
...................aggucugucuguuauguuucc..	804	0	ea2
...................aUgucugucuguuauguuucc..	1	1	ea2
...................Nggucugucuguuauguuucc..	5	1	ea2
...................aggucugucuguuauguuuccU...	7	1	ea2
...................aggucugucuguuauguuuAca...	9	1	ea2
...................aggCcugucuguuauguuucca...	1	1	ea2
...................aggucugucuguuauguuuccC...	11	1	ea2
...................aggucugucuguuauguuucca...	79	0	ea2
...................aggucugucuguuauguuuccaU..	5	1	ea2
...................aggucugucuguuauguuuccaC..	10	1	ea2
...................aggucugucuguuauguuuccaa..	3	0	ea2
...................aggucugucuguuauguuuccCa..	3	1	ea2
...................aggucugucuguuauguuuccaaC...	1	1	ea2
...................aggucugucuguuauguuuccaag...	12	0	ea2
...................aggucugucuguuauguuuccaaU...	19	1	ea2
...................aggucugucuguuauguuuccaaUu..	2	1	ea2
...................aggucugucuguuauguuuccaagu..	5	0	ea2
...................aggucugucuguuauguuuccaaAu..	6	1	ea2
...................aggucugucuguuauguuuccaaguu...	2	0	ea2
...................aggucugucuguuauguuuccaaguuA..	1	1	ea2
....................Ugucugucuguuauguuuc..	1	1	ea2
....................ggucugucuguuauguuuc..	2	0	ea2
....................ggucugucuguuauguuucA...	18	1	ea2
....................ggucugucuguuauguuucc...	30	0	ea2
......................ucugucuguuauguuucc..	4	0	ea2
......................ucugucuguuauguuucca...	10	0	ea2
......................ucugGcuguuauguuucca...	1	1	ea2
......................ucugucuguuauguuuccaC..	25	1	ea2
......................ucugucuguuauguuuccaa..	2	0	ea2
......................ucugucuguuauguuuccaag...	9	0	ea2
......................ucugucuguuauguuuccaaU...	9	1	ea2
......................ucugucuguuauguuuccaaC...	30	1	ea2
......................ucugucuguuauguuuccaaCu..	1	1	ea2
......................ucugucuguuauguuuccaagC..	2	1	ea2
......................ucugucuguuauguuuccaaguuA..	5	1	ea2
.......................cugucuguuauguuuccCa..	1	1	ea2
.......................cugucuguuauguuuccaa..	3	0	ea2
.......................cugucuguuauguuuccaaC...	2	1	ea2
........................ugucuguuauguuuccaC..	2	1	ea2
..........................ucuguuauguuuccaaguA...	3	1	ea2
............................uguuauguuuccaaguucuucA..	3	1	ea2
..............................uuauguuuccaaguucuA..	4	1	ea2

```
                   Mature                           Star
cugccauggacugcccuccaggucugucuguuauguuuccaaguucuucuuggcauacuaacaggcuggccggaagguauucauguuggcaauucaauggaaauccuaa
........................................aaguucuucuuggcauacua.................................................   1     0    ea2
........................................aaguucuucuuggcauacuaa................................................   3     0    ea2
.........................................aguucuucuuggcauacuCa................................................   1     1    ea2
.........................................aguucuucuuggcauacuaCc...............................................   1     1    ea2
.........................................aguucuucuuggcauacuaaca..............................................   1     0    ea2
..........................................guucuucuuggcauacuaa................................................   4     0    ea2
..........................................guucuucuuggcauacuaaca..............................................   1     0    ea2
...........................................uucuucuuggcauacuaac...............................................   3     0    ea2
...........................................uucuucuuggcauacuaaca..............................................   19    0    ea2
...........................................uucuucuuggcauacuaacU..............................................   1     1    ea2
.............................................cuucuuggcauacuaacagg............................................   2     0    ea2
..............................................uucuuggcauacuaacaggcugg........................................   4     0    ea2
.................................................uuggcauacuaacaggcug.........................................   2     0    ea2
.................................................uuggcauacuaacaggcugg........................................   6     0    ea2
..................................................uggcauacuaacaggcug.........................................   3     0    ea2
.....................................................cauacuaacaggcuggccg.....................................   1     0    ea2
.....................................................cauacuaacaggcuggccggU...................................   4     1    ea2
.....................................................cauacuaacaggcuggccgga...................................   3     0    ea2
.....................................................cauacuaacaggcuggccggaU..................................   1     1    ea2
.....................................................cauacuaacaggcuggccggaaA.................................   6     1    ea2
......................................................auacuaacaggcuggccg.....................................   2     0    ea2
......................................................auacuaacaggcuggccggC...................................   1     1    ea2
.......................................................uacuaacaggcuggccgg....................................   3     0    ea2
.......................................................uacuaacaggcuggccggU...................................   1     1    ea2

...............cuccaggucugucuguuauguu........................................................................   1     0    er2
.................ccaggucugucuguuauguA........................................................................   2     1    er2
..................caggucugucuguuauguuuc......................................................................   30    0    er2
..................caggucugucuguuauguuuA......................................................................   5     1    er2
..................caggucugucuguuauguuCc......................................................................   1     1    er2
..................cGggucugucuguuauguuucc.....................................................................   1     1    er2
..................caggucugucuguuauguuucA.....................................................................   18    1    er2
..................caggucugucuguuauguuucc.....................................................................   27    0    er2
..................caggucugucuguuauguuucca....................................................................   5     0    er2
..................caggucugucuguuauguuuccaaU..................................................................   5     1    er2
...................aggucugucuguuauguC........................................................................   1     1    er2
...................aggucugucuguuauguu........................................................................   11    0    er2
...................aggucugucuguuauguuu.......................................................................   1     0    er2
...................aggucugucuguuauguuCc......................................................................   2     1    er2
...................aggucugucuguuauguuuA......................................................................   6     1    er2
...................aggucugucuguuauguuuc......................................................................   162   0    er2
...................aggucugucuguuauguuucA.....................................................................   142   1    er2
...................aggucugucuguuauguuucc.....................................................................   466   0    er2
...................aggucGgucuguuauguuucc.....................................................................   1     1    er2
...................aggucugucuguuauguuCcc.....................................................................   1     1    er2
...................Nggucugucuguuauguuucc.....................................................................   5     1    er2
...................aggucugucuguuauguuuAc.....................................................................   1     1    er2
...................aggCcugucuguuauguuucc.....................................................................   1     1    er2
...................aggucugucuguuauguuucU.....................................................................   4     1    er2
...................aggucugucuguuGuguuucca....................................................................   1     1    er2
...................aggucugucuguuauguuuccC....................................................................   13    1    er2
...................aggucugucuguuauguuucAa....................................................................   4     1    er2
...................aggucugucuguuauguuucca....................................................................   75    0    er2
...................aggucugucuguuauguuuccaa...................................................................   3     0    er2
...................aggucugucuguuauguuucUaa...................................................................   2     1    er2
...................aggucugucuguuauguuuccaC...................................................................   7     1    er2
...................aggucugucuguuauguuuccCa...................................................................   3     1    er2
...................aggucugucuguuauguuuccaU...................................................................   30    1    er2
...................aggucugucuguuauguuuccaaU..................................................................   77    1    er2
...................aggucugucuguuauguuuccaag..................................................................   15    0    er2
...................aggucugucuguuauguuuccaaUu.................................................................   35    1    er2
...................aggucugucuguuauguuuccaagu.................................................................   30    0    er2
...................aggucugucuguuauguuuccaaguu................................................................   11    0    er2
...................aggucugucuguuauguuuccaaguA................................................................   1     1    er2
...................aggucugucuguuauguuuccaaguuc...............................................................   10    0    er2
...................aggucugucuguuauguuuccaaguCc...............................................................   2     1    er2
....................ggucugucuguuauguuuc......................................................................   11    0    er2
....................ggucugucuguuauguuucc.....................................................................   11    0    er2
....................ggucugucuguuauguuucA.....................................................................   8     1    er2
....................ggucugucuguuauguuuccaU...................................................................   2     1    er2
```

```
                   Mature             Star
cugccauggacugcccuccaggucugucuguuauguuuccaaguucuucuuggcauacuaacaggcuggccggaaggguauucauguuggcaauucaauggaaauccuaa
....................ggucugucuguuauguuuccaaU...................................................................   2   1   er2
....................ggucugucuguuauguuuccaagu..................................................................   1   0   er2
....................ggucugucuguuauguuuccaaguu.................................................................   2   0   er2
......................ucugucuguuauguuucc......................................................................   1   0   er2
......................ucugucuguuauguuucca.....................................................................   2   0   er2
......................ucugucuguuauguuuccaC....................................................................   7   1   er2
......................ucugucuguuauguuuccaaU...................................................................   7   1   er2
......................ucugucuguuauguuuccaag...................................................................   6   0   er2
......................ucugucuguuauguuuccaaC...................................................................   8   1   er2
......................ucugucuguuauguuuccaagC..................................................................   2   1   er2
......................ucugucuguuauguuuccaaCu..................................................................   5   1   er2
......................ucugucuguuauguuuccaaguA.................................................................   1   1   er2
......................ucugucuguuauguuuccaaguuA................................................................   6   1   er2
.......................cugucuguuauguuuccaaC...................................................................   2   1   er2
............................uguuauguuuccaaguucuucA............................................................   1   1   er2
........................................aaguucuucuuggcauacua..................................................   2   0   er2
.........................................aguucuucuuggcauacuaa.................................................   10  0   er2
.........................................aguucuucuuggcauacuaCc................................................   2   1   er2
.........................................aguucuucuuggcauacuaaca...............................................   3   0   er2
...........................................uucuucuuggcauacuaac................................................   1   0   er2
...........................................uucuucuuggcauacuaaca...............................................   8   0   er2
............................................ucuucuuggcauacuaacagg.............................................   3   0   er2
.............................................cuucuuggcauacuaacagg.............................................   1   0   er2
.................................................uuggcauacuaacaggcugg.........................................   1   0   er2
.................................................uuggcauacuaacaggcuggccgg.....................................   1   0   er2
.....................................................cauacuaacaggcuggccgg.....................................   3   0   er2
.....................................................cauacuaacaggcuggccggU....................................   6   1   er2
.....................................................cauacuaacaggcuggccggC....................................   2   1   er2
.....................................................cauacuaacaggcuggccgga....................................   1   0   er2
.....................................................cauacuaacaggcuggccgUa....................................   2   1   er2
.....................................................cauacuaacaggcuggccgUaa...................................   1   1   er2
.....................................................cauacuaacaggcuggccggaU...................................   4   1   er2
.....................................................cauacuaacaggcuggccggaG...................................   1   1   er2
.....................................................cauacuaacaggcuggccggaa...................................   2   0   er2
.....................................................cauacuaacaggcuggccggaaA..................................   5   1   er2
.....................................................cauacuaacaggcuggccggaUg..................................   3   1   er2
......................................................auacuaacaggcuggccggaaA..................................   1   1   er2

cugccauggacugcccucc...........................................................................................   2   0   ea1
cugccauggacugcccucU...........................................................................................   1   1   ea1
.................ccaggucugucuguuauguuuc.......................................................................   5   0   ea1
.................Gcaggucugucuguuauguuuc.......................................................................   1   1   ea1
.................ccaggucugucuguuauguuuU.......................................................................   1   1   ea1
.................ccaggucugucuguuauguuucc......................................................................   1   0   ea1
.................Ucaggucugucuguuauguuucc......................................................................   1   1   ea1
..................caggucugucuguuauguuuU.......................................................................   1   1   ea1
..................Gaggucugucuguuauguuuc.......................................................................   1   1   ea1
..................caggucugucuguuauguuuc.......................................................................   11  0   ea1
..................caggucugucuguuauguCucc......................................................................   1   1   ea1
..................caggucugucuguuauguuucU......................................................................   1   1   ea1
..................caggucugucuguuauguuucA......................................................................   3   1   ea1
..................caggucugucuguuauguuucc......................................................................   13  0   ea1
..................caggucugucuguuauguuucca.....................................................................   8   0   ea1
..................caggucugucuguuauguuuccaU....................................................................   1   1   ea1
..................caggucugucuguuauguuuccaagu..................................................................   2   0   ea1
...................Gggucugucuguuauguu.........................................................................   2   1   ea1
...................aggucugucuguuauguA.........................................................................   1   1   ea1
...................aggucugucuguuauguG.........................................................................   1   1   ea1
...................aggucugucuguuauguu.........................................................................   21  0   ea1
...................aggucugGcuguuauguu.........................................................................   1   1   ea1
...................aggucugucuguuauguC.........................................................................   5   1   ea1
...................aggucugucuguuauguuA........................................................................   2   1   ea1
...................Gggucugucuguuauguuu........................................................................   1   1   ea1
...................aggucCgucuguuauguuu........................................................................   1   1   ea1
...................aggucugucuguuauguuu........................................................................   23  0   ea1
...................aggucugucuguuauguuC........................................................................   15  1   ea1
...................aggucugucuguuauguAu........................................................................   4   1   ea1
...................Uggucugucuguuauguuuc.......................................................................   2   1   ea1
...................aggucugucuguuauguuCc.......................................................................   2   1   ea1
...................Gggucugucuguuauguuuc.......................................................................   27  1   ea1
```

```
                  Mature             Star
cugccauggacugcccuccaggucugucuguuauguuuccaaguucuucuuggcauacuaacaggcuggccggaaggguauucauguuggcaauucaauggaaauccuaa
...................Cggucugucuguuauguuuc.......................................................................   1     1   ea1
...................aggucugucugCuauguuuc.......................................................................   4     1   ea1
...................aggucugucuguuaugCuuc.......................................................................   2     1   ea1
...................aggucugucuguuauguuuA.......................................................................   38    1   ea1
...................aggucugCcuguuauguuuc.......................................................................   3     1   ea1
...................aggucugucCguuauguuuc.......................................................................   1     1   ea1
...................aggucugucuguuauguuuU.......................................................................   25    1   ea1
...................aggucugucAguuauguuuc.......................................................................   1     1   ea1
...................aggucugucuguCauguuuc.......................................................................   1     1   ea1
...................aggucugucuguuauguuuc.......................................................................   546   0   ea1
...................aggucugucuguuGuguuuc.......................................................................   2     1   ea1
...................aggucuguUuguuauguuuc.......................................................................   1     1   ea1
...................aggCcugucuguuauguuuc.......................................................................   2     1   ea1
...................aggucugucuguuauguuAc.......................................................................   2     1   ea1
...................aggucCgucuguuauguuuc.......................................................................   1     1   ea1
...................aggucugucuguuauguuuG.......................................................................   1     1   ea1
...................aggucugucuguuauguuuAc......................................................................   3     1   ea1
...................aggucugucuguuGuguuucc......................................................................   2     1   ea1
...................aggucAgucuguuauguuucc......................................................................   2     1   ea1
...................aggCcugucuguuauguuucc......................................................................   3     1   ea1
...................aggGcugucuguuauguuucc......................................................................   1     1   ea1
...................aggucugucuAuuauguuucc......................................................................   1     1   ea1
...................aggucuguUuguuauguuucc......................................................................   4     1   ea1
...................aggucugucuguuauguuucA......................................................................   503   1   ea1
...................aggucugucuguuaugCuucc......................................................................   4     1   ea1
...................aggucugucuguuauguuucG......................................................................   13    1   ea1
...................aAgucugucuguuauguuucc......................................................................   1     1   ea1
...................aUgucugucuguuauguuucc......................................................................   3     1   ea1
...................aggucugucuguuauguuAcc......................................................................   2     1   ea1
...................Cggucugucuguuauguuucc......................................................................   2     1   ea1
...................Gggucugucuguuauguuucc......................................................................   56    1   ea1
...................aggucugucuguuaugGuucc......................................................................   5     1   ea1
...................aggucGgucuguuauguuucc......................................................................   1     1   ea1
...................aggucugucGguuauguuucc......................................................................   4     1   ea1
...................aggucugucuguuaCguuucc......................................................................   6     1   ea1
...................aggucuAucuguuauguuucc......................................................................   1     1   ea1
...................aggucugucuguuauguuuUc......................................................................   3     1   ea1
...................aggucugucAguuauguuucc......................................................................   2     1   ea1
...................aggucugucuguuauguuucU......................................................................   433   1   ea1
...................aggucugucugCuauguuucc......................................................................   6     1   ea1
...................aggucCgucuguuauguuucc......................................................................   9     1   ea1
...................aggucugucuguuauguuucc......................................................................   1905  0   ea1
...................aggAcugucuguuauguuucc......................................................................   3     1   ea1
...................aggucugCcuguuauguuucc......................................................................   4     1   ea1
...................aggucugucuguCauguuucc......................................................................   1     1   ea1
...................aggucugucCguuauguuucc......................................................................   3     1   ea1
...................aggucugucuguuauguCucc......................................................................   5     1   ea1
...................agguUugucuguuauguuucc......................................................................   1     1   ea1
...................aggucugucuguuauguuCcc......................................................................   6     1   ea1
...................aggucugucuguAauguuucc......................................................................   3     1   ea1
...................aggucugucuguuauguuucUa.....................................................................   3     1   ea1
...................aggucugucuguuauguuuAca.....................................................................   2     1   ea1
...................aggCcugucuguuauguuucca.....................................................................   1     1   ea1
...................Gggucugucuguuauguuucca.....................................................................   14    1   ea1
...................aggucugCcuguuauguuucca.....................................................................   1     1   ea1
...................aggucugucuguuauguuuUca.....................................................................   1     1   ea1
...................aggucugucuguuGuguuucca.....................................................................   1     1   ea1
...................aggucugucuguuauguuucca.....................................................................   345   0   ea1
...................aggucugucuguuaCguuucca.....................................................................   1     1   ea1
...................aggucugucuguuauguuucAa.....................................................................   3     1   ea1
...................aggucugucuguuaAguuucca.....................................................................   1     1   ea1
...................aggucugucuguuauguuuccU.....................................................................   25    1   ea1
...................aggucugucCguuauguuucca.....................................................................   1     1   ea1
...................aggucCgucuguuauguuucca.....................................................................   1     1   ea1
...................aggucugucuguCauguuucca.....................................................................   1     1   ea1
...................aggucugucuguuauguuuccG.....................................................................   3     1   ea1
...................aggucugucuguuauguuuccC.....................................................................   88    1   ea1
...................aggucugucuguuauguuuccaC....................................................................   41    1   ea1
...................aggucugucuguuauguuuccaU....................................................................   120   1   ea1
...................aggucugucuguuauguuuccCa....................................................................   17    1   ea1
```

```
                   Mature                           Star
cugccauggacugcccuccaggucugucuguuauguuuccaaguucuucuuggcauacuaacaggcuggccggaaggguauucauguuggcaauucaauggaaauccuaa
...................aggucugucuguuauguuuccaa....................................................................   36   0   ea1
...................aggucAgucuguuauguuuccaa....................................................................   2    1   ea1
...................aggucugucuguuauguuuccaG....................................................................   2    1   ea1
...................aggucugucuguuauguuuccaaU...................................................................   131  1   ea1
...................aggucugucuguuauguCuccaag...................................................................   1    1   ea1
...................aggucugucuguuauguuuccaaC...................................................................   24   1   ea1
...................aggucugucuguuauguuuccaUg...................................................................   2    1   ea1
...................agAucugucuguuauguuuccaag...................................................................   1    1   ea1
...................aggucugucuguuauguuuccaaA...................................................................   7    1   ea1
...................aggucugucuguuaugCuuccaag...................................................................   2    1   ea1
...................Gggucugucuguuauguuuccaag...................................................................   3    1   ea1
...................aggucugucuguuauguuuccaag...................................................................   71   0   ea1
...................aggucCgucuguuauguuuccaag...................................................................   1    1   ea1
...................aggucugucugCuauguuuccaag...................................................................   1    1   ea1
...................aggucugucuguuauguuuccaagC..................................................................   26   1   ea1
...................Gggucugucuguuauguuuccaagu..................................................................   5    1   ea1
...................aggucugucuguuauAuuuccaagu..................................................................   1    1   ea1
...................aggucugucuguuaCguuuccaagu..................................................................   1    1   ea1
...................aggucugucuguuauguuuccaGgu..................................................................   2    1   ea1
...................aggucuAucuguuauguuuccaagu..................................................................   1    1   ea1
...................aggucugucuguuauguuuccUagu..................................................................   1    1   ea1
...................aggucugucuguuauguuuccaaUu..................................................................   99   1   ea1
...................aggucugucuguuauguuucUaagu..................................................................   1    1   ea1
...................aggucugucuguuauguuuccaaAu..................................................................   2    1   ea1
...................aggucugucuguuauguuuccaagu..................................................................   122  0   ea1
...................aggucugucuguuGuguuuccaagu..................................................................   1    1   ea1
...................aggCcugucuguuauguuuccaagu..................................................................   1    1   ea1
...................aggucugucuguuauguuCccaagu..................................................................   3    1   ea1
...................aggucugCcuguuauguuuccaagu..................................................................   1    1   ea1
...................aggucugucuguuauguuuccaaguu.................................................................   34   0   ea1
...................aggucugucuguuauguuuccaGguu.................................................................   1    1   ea1
...................aggucAgucuguuauguuuccaaguu.................................................................   2    1   ea1
...................aggucugucuguuauguuuccaaUuu.................................................................   4    1   ea1
...................Gggucugucuguuauguuuccaaguu.................................................................   1    1   ea1
...................aggCcugucuguuauguuuccaaguu.................................................................   1    1   ea1
...................aggucugucuguuauguuuccaaguA.................................................................   1    1   ea1
...................aggucugucuguuauguuuccaaguG.................................................................   1    1   ea1
...................aggucugucuguuauguuuccaaguC.................................................................   4    1   ea1
...................aggucugucuguuauguuuccaaguuU................................................................   7    1   ea1
...................aggucugucuguuauguuCccaaguuc................................................................   3    1   ea1
...................Gggucugucuguuauguuuccaaguuc................................................................   4    1   ea1
...................aggucugucuguuauguuuccaGguuc................................................................   1    1   ea1
...................aggucugucCguuauguuuccaaguuc................................................................   1    1   ea1
...................aggucugucugCuauguuuccaaguuc................................................................   1    1   ea1
...................aggucugucuguuauguCuccaaguuc................................................................   2    1   ea1
...................aggucugucuguuauguuuccaaguuA................................................................   1    1   ea1
...................aggucCgucuguuauguuuccaaguuc................................................................   1    1   ea1
...................aggucugucuguuauguuuccaaguGc................................................................   1    1   ea1
...................aggucugucuguuauguuuccaaguuc................................................................   103  0   ea1
....................ggucugucuguuauguuuccaU....................................................................   1    1   ea1
......................ucugucuguuauguuucU......................................................................   1    1   ea1
......................ucugucuguuauguuucA......................................................................   1    1   ea1
......................ucugucuguuauguuucc......................................................................   5    0   ea1
......................Gcugucuguuauguuucc......................................................................   3    1   ea1
......................Gcugucuguuauguuucca.....................................................................   4    1   ea1
......................ucugucuguuauguuucca.....................................................................   16   0   ea1
......................ucugucuguuauguAucca.....................................................................   2    1   ea1
......................ucugucuguuauguuuccC.....................................................................   1    1   ea1
......................ucugucuguuauguuuccaU....................................................................   18   1   ea1
......................ucugucuguuauguuuccaG....................................................................   1    1   ea1
......................ucugucuguuauguuuccaC....................................................................   77   1   ea1
......................ucugucuguuauguuuccaa....................................................................   3    0   ea1
......................ucugucuguuauguuuccaaU...................................................................   11   1   ea1
......................Gcugucuguuauguuuccaag...................................................................   5    1   ea1
......................ucugucuguuauguuuccaaA...................................................................   5    1   ea1
......................ucugucuguuauguuuccaag...................................................................   20   0   ea1
......................ucugucuguuauguuuccaaC...................................................................   33   1   ea1
......................ucugCcuguuauguuuccaagu..................................................................   1    1   ea1
......................ucCgucuguuauguuuccaagu..................................................................   1    1   ea1
......................ucugucuguuauguuuccaaCu..................................................................   13   1   ea1
```

Mature   Star

```
cugccauggacugcccuccaggucugucuguuauguuuccaaguucuucuuggcauacuaacaggcuggccggaaggguauucauguuggcaauucaauggaaauccuaa
......................ucugucuguuauguuuccaaUu..................................................................    3    1    ea1
......................ucugucuguuauguuCccaagu..................................................................    1    1    ea1
......................Ccugucuguuauguuuccaagu..................................................................    1    1    ea1
......................ucugucuguuauguuuccaagu..................................................................    8    0    ea1
......................ucugucuguuauguuuccaagA..................................................................    2    1    ea1
......................ucugucuguuauguuuccaagC..................................................................    60   1    ea1
......................Gcugucuguuauguuuccaagu..................................................................    2    1    ea1
......................ucugucuguuauguuuccaaguu.................................................................    6    0    ea1
......................ucugucuguuauguuuccaaguC.................................................................    1    1    ea1
......................ucugucuguuauguuuccaaguA.................................................................    4    1    ea1
......................Gcugucuguuauguuuccaaguu.................................................................    2    1    ea1
......................ucugucuguuauguuuccaaguCc................................................................    1    1    ea1
......................Gcugucuguuauguuuccaaguuc................................................................    1    1    ea1
......................ucugucuguuauguuuccaaguuA................................................................    2    1    ea1
......................ucugucuguuauguuuccaaguuc................................................................    1    0    ea1
.......................cugucuguuauguuucca.....................................................................    1    0    ea1
............................uguuauguuuccaaguucuuc.............................................................    3    0    ea1
............................uguuauguuuccaaguucuGc.............................................................    1    1    ea1
............................uguuauguuuccaaguucuucu............................................................    1    0    ea1
..............................uuauguuuccaaguucuucu............................................................    1    0    ea1
................................auguuuccaaguucuucuAg..........................................................    1    1    ea1
.......................................caaguucuucuuggcauacuaacagg.............................................    1    0    ea1
........................................aaguucuucuuggcauacu...................................................    2    0    ea1
........................................Gaguucuucuuggcauacua..................................................    1    1    ea1
........................................aaguucuucuuggcauacua..................................................    5    0    ea1
........................................aaguucuucuuggcauacuaa.................................................    3    0    ea1
........................................aaguucuucuuggcauacuUa.................................................    1    1    ea1
........................................aaguucuucuuggcauacuaaca...............................................    6    0    ea1
.........................................aguucuucuuggcauacuaa.................................................    1    0    ea1
.........................................aguucuucuuggcauacuaUc................................................    1    1    ea1
.........................................aguucuucuuggcauacuaac................................................    1    0    ea1
.........................................aguucuucuuggcauacuaCca...............................................    2    1    ea1
..........................................guucuucuuggcauacuaac................................................    1    0    ea1
...........................................uucuucuuggcauacuaac................................................    8    0    ea1
...........................................uucuucuuggcauacuaaca...............................................    29   0    ea1
...........................................uucCucuuggcauacuaaca...............................................    1    1    ea1
...........................................Gucuucuuggcauacuaaca...............................................    4    1    ea1
...........................................uucuucuuggcauacuaacaggc............................................    1    0    ea1
............................................ucuucuuggcauacuaacagg.............................................    2    0    ea1
.............................................cuucuuggcauacuaacagA.............................................    1    1    ea1
.............................................cuucuuggcauacuaacagg.............................................    8    0    ea1
..............................................uucuuggcauacuaacaggcug..........................................    1    0    ea1
...............................................ucuuggcauacuaacaggcug..........................................    1    0    ea1
................................................cuuggcauacuaacaggcug..........................................    2    0    ea1
................................................cuuggcauacuaacaggcugC.........................................    1    1    ea1
................................................cuuggcauacuaacaggcugU.........................................    1    1    ea1
................................................cuuggcauacuaacaggcuggcA.......................................    2    1    ea1
.................................................uuggcauacuaacaggcugg.........................................    6    0    ea1
.................................................uuggcauacuaacaggcuggc........................................    2    0    ea1
.................................................uuggcauacuaacaggcuggA........................................    1    1    ea1
..................................................uggcauacuaacaggcuggU........................................    1    1    ea1
..................................................uggcauacuaacaggcuggcG.......................................    1    1    ea1
...................................................ggcauacuaacaggcuggcG.......................................    1    1    ea1
....................................................gcauacuaacaggcuggccgg.....................................    1    0    ea1
....................................................gcauacuaacaggcuggccgga....................................    2    0    ea1
.....................................................cauacuaacaggcuggcU.......................................    8    1    ea1
.....................................................cauacuaacUggcuggcc.......................................    2    1    ea1
.....................................................cauacuaacaggcuggcA.......................................    2    1    ea1
.....................................................cauacuaacaggcuggcc.......................................    13   0    ea1
.....................................................cauacuaacaggcuggccA......................................    1    1    ea1
.....................................................cauacuaacaggcuggccg......................................    6    0    ea1
.....................................................cauacuaacaggcuggccgg.....................................    66   0    ea1
.....................................................cauaUuaacaggcuggccgg.....................................    1    1    ea1
.....................................................cGuacuaacaggcuggccgg.....................................    1    1    ea1
.....................................................cauacuaacaggcuggccgU.....................................    3    1    ea1
.....................................................cauacuaacaggcuggccgA.....................................    6    1    ea1
.....................................................cauacuaacaggcuggccgC.....................................    1    1    ea1
.....................................................caCacuaacaggcuggccgga....................................    1    1    ea1
.....................................................cauacuaacaggcuggccggC....................................    7    1    ea1
.....................................................cauacuaGcaggcuggccgga....................................    1    1    ea1
```

Mature Star

```
cugccauggacugcccuccaggucugucuguuauguuuccaaguucuucuuggcauacuaacaggcuggccggaaggguauucauguuggcaauucaauggaaauccuaa
.....................................................Gauacuaacaggcuggccgga....................................  2    1  ea1
.....................................................cauacuaacaggcuggccgga....................................  86   0  ea1
.....................................................cauacuaacaggcCggccgga....................................  1    1  ea1
.....................................................cauacuaacaggcuggccggU....................................  28   1  ea1
.....................................................cauacuaacaggcuggccggaU...................................  507  1  ea1
.....................................................cauacuaacaggcuggccggaG...................................  9    1  ea1
.....................................................cauacuaacaggcuggccgUaa...................................  1    1  ea1
.....................................................cauacuaacaggcuggccggaC...................................  41   1  ea1
.....................................................cauacuaacaggcuggccggaa...................................  34   0  ea1
.....................................................cauacuaacaggcuggccggCa...................................  2    1  ea1
.....................................................cauacuaacaggcuggccggUa...................................  16   1  ea1
.....................................................cauacuaacaggcuggccggaag..................................  2    0  ea1
.....................................................cauacuaacaggcuggccggaaC..................................  4    1  ea1
.....................................................cauacuaacaggcuggccggaUg..................................  12   1  ea1
.....................................................cauacuaacaggcuggccggaaU..................................  32   1  ea1
.....................................................cauacuaacaggcuggccggaaA..................................  44   1  ea1
.....................................................cauacuaacaggcuggccggaaUg.................................  2    1  ea1
.....................................................cauacuaacaggcuggccggaaAg.................................  1    1  ea1
......................................................auacuaacaggcuggccgg.....................................  5    0  ea1
......................................................auacuaacaggcuggccggaU...................................  1    1  ea1
......................................................auacuaacaggcuggccggaa...................................  1    0  ea1
.......................................................uacuaacaggcuggccgg.....................................  1    0  ea1
.......................................................uacuaacaggcuggccggaa...................................  1    0  ea1
.......................................................uacuaacaggcuggccggaC...................................  2    1  ea1
.......................................................uacuaacaggcuggccggaU...................................  7    1  ea1
.......................................................Gacuaacaggcuggccggaa...................................  1    1  ea1
.......................................................uacuaacaggcuggccggaag..................................  1    0  ea1
.......................................................uacuaacaggcuggccggaaA..................................  1    1  ea1
.......................................................uacuaacaggcuggccggaUg..................................  1    1  ea1
.......................................................uacuaacaggcuggccggaaU..................................  1    1  ea1
........................................................acuaacaggcuggccggaU...................................  4    1  ea1
.............................................................caggcuggccggaaggguauu............................  1    0  ea1
```

Provisional ID	:	ScUbCFx_217_18024
Score total	:	4.4
Score for star read(s)	:	-1.3
Score for read counts	:	0
Score for mfe	:	1.1
Score for randfold	:	1.6
Score for cons. seed	:	3
Total read count	:	315
Mature read count	:	241
Loop read count	:	0
Star read count	:	74

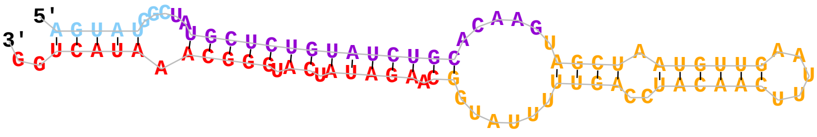

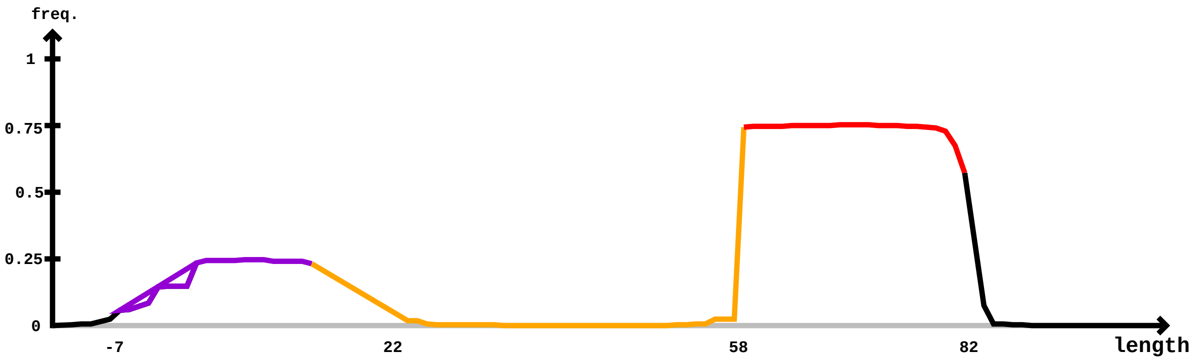

Star Mature

```
5'- agaacaguauggcuaugcucuguaucugcacaaguagcuaauguugaauuucaacauccaguuuuuauggcaagauaucaugggcaaauacuggcaauggcuugagaaagucag  -3'   obs
    agaacaguauggcuaugcucuguaucugcacaaguagcuaauguugaauuucaacauccaguuuuuauggcaagauaucaugggcaaauacuggcaauggcuugagaaagucag         exp
    ....((((((.....((((((((((((((......((((.((((((.....))))))..))))......)).))))).)).))))).))))))....((((((....)))))).  reads   mm   sample
    .........uggcuaugcucuguaucugc.....................................................................................  2       0    er1
    .........uggcuaugcucuguaucugca....................................................................................  1       0    er1
    ..............augcucuguaucugcacaagA...............................................................................  1       1    er1
    ...............................................................uuuauggcaagauaucauggg..............................  1       0    er1
    ...................................................................Gggcaagauaucaugggcaaauacug.....................  1       1    er1
    ......................................................................caagauaucaugggcaaauacuC.....................  1       1    er1
    ......................................................................caagauaucaugggcaaauacu......................  2       0    er1
    ......................................................................caagauaucaugggcaaauacug.....................  4       0    er1
    ......................................................................caagauaucaugggcaaauacugg....................  18      0    er1
    ......................................................................caagauaucaugggcaaauaUugg....................  1       1    er1
    ......................................................................caagauaucaugggcaaauacuggA...................  1       1    er1
    ......................................................................caagauaucaugggcaaauacuggc...................  7       0    er1
    ......................................................................caagauaucaugggcaaauacuggca..................  2       0    er1

    .....aguauggcuaugcucuguaucugc.....................................................................................  1       0    eg1
    .....aguauggcuaugcucuguaucugcaU...................................................................................  1       1    eg1
    .........uggcuaugcucuguaucugU.....................................................................................  1       1    eg1
    .........uggcuaugcucuguaucugc.....................................................................................  1       0    eg1
    .........uggcuaugcucuguaucugca....................................................................................  1       0    eg1
    .........uggcuaugcucuguaucugcaA...................................................................................  1       1    eg1
    .............uaugcucuguaucugcaca..................................................................................  1       0    eg1
    .............uaugcucuguaucugcacaag................................................................................  5       0    eg1
    .............uaugcucuguaucugcacaaguaA.............................................................................  4       1    eg1
    ..............augcucuguaucugcacaagu...............................................................................  1       0    eg1
    ..............augcucuguaucugcacaaguagU............................................................................  1       1    eg1
    ...................................................................uggcaagauaucaugggcaaauacu......................  1       0    eg1
    ...................................................................uggcaagauaucaugggcaaauacug.....................  1       0    eg1
    ......................................................................caagauaucaugggcaaau.........................  1       0    eg1
    ......................................................................caagauaucaugggcaaauacu......................  4       0    eg1
    ......................................................................caagauaucaugggcaaauacuC.....................  1       1    eg1
    ......................................................................caagauaucaugggcaaauacug.....................  2       0    eg1
    ......................................................................Gaagauaucaugggcaaauacugg....................  1       1    eg1
    ......................................................................caagauaucaugggcaaauacuCg....................  1       1    eg1
```

Star Mature

```
agaacaguauggcuaugcucuguaucugcacaaguagcuaauguugaauuucaacauccaguuuuauggcaagauaucaugggcaaauacuggcaauggcuugagaaagucag
.....................................................................caagauaucaugggcaaauacugg....................  27  0  eg1
.....................................................................caagauGucaugggcaaauacugg....................  2  1  eg1
.....................................................................caagaAaucaugggcaaauacugg....................  1  1  eg1
.....................................................................caagauaucaugggcaaauacuggc...................  18  0  eg1
.....................................................................caagauaucaugggcaaauacuggca..................  6  0  eg1
.....................................................................caagauaucaugggcaaauacuggcG..................  3  1  eg1

.gaacaguauggcuaugcucu............................................................................................  1  0  eg2
...acaguauggcuaugcucuguaA........................................................................................  3  1  eg2
....caguauggcuaugcucuguauA.......................................................................................  3  1  eg2
.....aguauggcuaugcucuguaucu......................................................................................  1  0  eg2
.....aguauggcuaugcCcuguaucug.....................................................................................  1  1  eg2
.....aguauggcuaugcucuguaucugc....................................................................................  6  0  eg2
......guauggcuaugcucuguaucA......................................................................................  1  1  eg2
........auggcuaugcucuguaucug.....................................................................................  1  0  eg2
........auggcuGugcucuguaucugca...................................................................................  1  1  eg2
.........uggcuaugcucuguaucugca...................................................................................  2  0  eg2
..........ggcuaCgcucuguaucugca...................................................................................  1  1  eg2
.............uaugcucuguaucugcacaa................................................................................  1  0  eg2
.............uaugcucuguaucugcacaag...............................................................................  1  0  eg2
.............uaugcucuguaucugcacaaU...............................................................................  1  1  eg2
.............uaugcucuguaucugcacaaAu..............................................................................  1  1  eg2
................................................................uauggcaagauaucaugggcaa...........................  1  0  eg2
.....................................................................caagauaucaugggcaaauacug.....................  1  0  eg2
.....................................................................caagauaucaugggcaaauacugg....................  7  0  eg2
......................................................................aagauaucaugggcaaauaUuggc...................  1  1  eg2
..........................................................................uaucaugggcaaauacuggcaau................  1  0  eg2

.............uaugcucuguaucugcacaag...............................................................................  7  0  ea2

.............uaugcucuguaucugcacaG................................................................................  2  1  er2

aUaacaguauggcuaugcucu............................................................................................  1  1  ea1
.....aguauggcuaugcucuguaucugc....................................................................................  1  0  ea1
.......uauggcuaugcucuguaucugcaca.................................................................................  3  0  ea1
.......uauggcuaugcucuguaucugcacaag...............................................................................  1  0  ea1
........Guggcuaugcucuguaucug.....................................................................................  1  1  ea1
........auggcuaugcucuguaucug.....................................................................................  1  0  ea1
.........uggcuaugcucuguaucug.....................................................................................  1  0  ea1
.........Gggcuaugcucuguaucugc....................................................................................  1  1  ea1
.........uggcuaugcucuguaucugc....................................................................................  4  0  ea1
.........uggcuaugcucuguaucugca...................................................................................  5  0  ea1
.............uaugcucuguaucugcaca.................................................................................  1  0  ea1
.............uaugcucuguaucugcacaaU...............................................................................  1  1  ea1
.............uaugcucuguaucugcacaag...............................................................................  4  0  ea1
..................Gcuguaucugcacaaguagcuaaugu.....................................................................  1  1  ea1
..................................................................uggcaagauaucaugggcaaauac.......................  1  0  ea1
..................................................................uggcaagauaucaugggcaaauacu......................  1  0  ea1
..................................................................uggcaagauaucaugggcaaauacug.....................  1  0  ea1
.....................................................................caagauaucaugggcaaaua........................  1  0  ea1
.....................................................................caagauaucaugggcaaauac.......................  3  0  ea1
.....................................................................caagauaucaugggcaaauacG......................  1  1  ea1
.....................................................................caagauaucaugggcaaauacC......................  2  1  ea1
.....................................................................caagauaucaugggcaaauacu......................  5  0  ea1
.....................................................................caagauaucaugggcaaauacCg.....................  1  1  ea1
.....................................................................caagauaucaugggcaaauacuA.....................  2  1  ea1
.....................................................................caagauaucaugggcaaauacug.....................  21  0  ea1
.....................................................................caagauaucaugggcaaauacugA....................  4  1  ea1
.....................................................................caagauaucaugggcaaauacugg....................  57  0  ea1
.....................................................................caagauaucGugggcaaauacugg....................  1  1  ea1
.....................................................................caagauaucaugggcaaauaUugg....................  1  1  ea1
.....................................................................cGagauaucaugggcaaauacugg....................  1  1  ea1
.....................................................................caagauaucaugggcaaauacuggA...................  2  1  ea1
.....................................................................Gaagauaucaugggcaaauacuggc...................  1  1  ea1
.....................................................................caagauaucaugggcaaauacuggU...................  2  1  ea1
.....................................................................caagauaucaugggcaaauacuggc...................  11  0  ea1
.....................................................................caagauaucaugggcaaauacuggca..................  9  0  ea1
.....................................................................Aaagauaucaugggcaaauacuggca..................  1  1  ea1
.....................................................................Uaagauaucaugggcaaauacuggca..................  1  1  ea1
.....................................................................caagauaucGugggcaaauacuggca..................  1  1  ea1
```

```
               Star                                       Mature
agaacaguauggcuaugcucuguaucugcacaaguagcuaauguugaauuucaacauccaguuuuuauggcaagauaucaugggcaaauacuggcaauggcuugagaaagucag
................................................................................ugggcaaauacuggcaaugC..............	1	1	ea1
```

Provisional ID	:	ScUbCFx_359_19104
Score total	:	303.5
Score for star read(s)	:	3.9
Score for read counts	:	293.8
Score for mfe	:	1.2
Score for randfold	:	1.6
Score for cons. seed	:	3
Total read count	:	588
Mature read count	:	572
Loop read count	:	0
Star read count	:	16

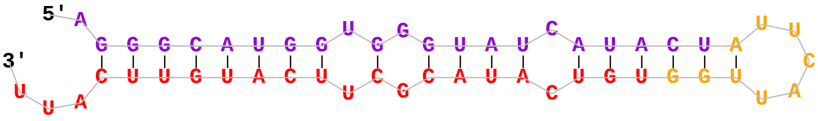

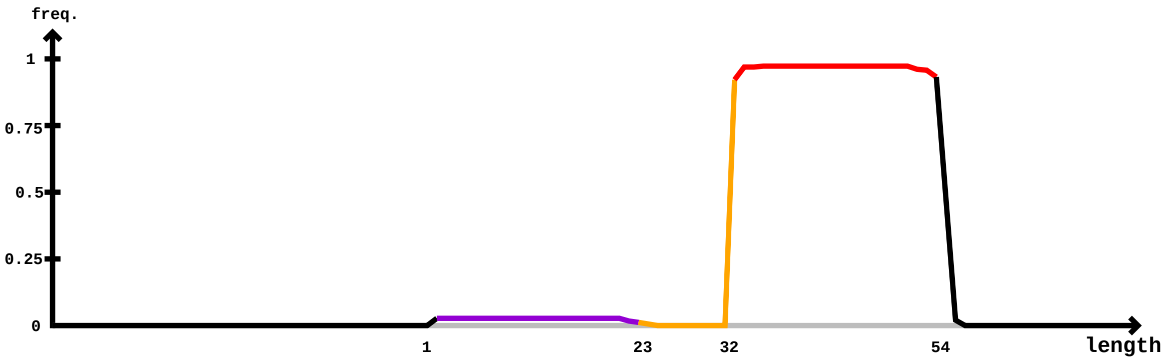

Star　　　　　　Mature

```
5'- aaauuuguucaagaauauaaaagauuuacauccaguucagggcaugguggguaucauacuauucauuggugucauacgcuucauguucauugaacuugauguuuuuucuua  -3'  obs
    aaauuuguucaagaauauaaaagauuuacauccaguucagggcaugguggguaucauacuauucauuggugucauacgcuucauguucauugaacuugauguuuuuucuua       exp
    ....................((((...(((((.((((((((((((((.(.((((.((((((.....)))))).)))).).))))))))..)))))).)))))....)))).   reads  mm  sample
    .....................................................................ugucauacgcuucauguucauu....................   37     0   er2
    .....................................................................Ngucauacgcuucauguucauu....................   1      1   er2
    .....................................................................ugucauacgcuucauguucauA....................   5      1   er2
    .....................................................................ugucauacgcuucauguucauuA...................   23     1   er2
    .....................................................................ugucauacgcuucauguucauuUa..................   5      1   er2
    ......................................................................gucauacgcuucauguucauu....................   1      0   er2

    ......................................agggcaugguggguaucaua.....................................................   3      0   ea1
    ......................................agggcaugguggguaucauac....................................................   1      0   ea1
    ......................................agggcaugguggguaucauacu...................................................   3      0   ea1
    .....................................................................ugucauacgcuucauguuU.......................   2      1   ea1
    .....................................................................ugucauacgcuucauguuc.......................   5      0   ea1
    .....................................................................ugucauacgcuucauguuca......................   2      0   ea1
    .....................................................................ugucauacgcuucauguucaG.....................   1      1   ea1
    .....................................................................ugucauacgcuucauguucau.....................   7      0   ea1
    .....................................................................ugucauacgcuucauguucauG....................   1      1   ea1
    .....................................................................ugucauacgcuucauguucauu....................   49     0   ea1
    .....................................................................ugucauacgcuucauguucauC....................   3      1   ea1
    .....................................................................ugucauGcgcuucauguucauu....................   2      1   ea1
    .....................................................................ugucauacgcuucauguucauuA...................   4      1   ea1
    .....................................................................ugucauacgcuucauguucauuU...................   1      1   ea1
    .....................................................................ugucauacgcuucauguucauug...................   4      0   ea1

    ......................................agggcaugguggguaucaua.....................................................   3      0   eg2
    ......................................agggcaugguggguaucauac....................................................   1      0   eg2
    ......................................agggcaugguggguaucauacu...................................................   1      0   eg2
    .....................................................................ugucauacgcuucauguucau.....................   1      0   eg2
    .....................................................................ugucauacgcuucauguucauC....................   1      1   eg2
    .....................................................................ugucauacgcuucauguucauA....................   14     1   eg2
    .....................................................................ugucauacgcuucauguucauu....................   135    0   eg2
    .....................................................................ugucauacgcuucauguucauuA...................   66     1   eg2
    .....................................................................ugucauacgcuucauguucauug...................   5      0   eg2
    .....................................................................ugucauacgcuucauguucauuAa..................   7      1   eg2
```

```
                                      Star                      Mature
aaauuuguucaagaauauaaaagauuuacauccaguucagggcaugguggguaucauacuauucauuggugucauacgcuucauguucauugaacuugauguuuuuucuua
......................................................................gucauacgcuucauguucauA....................   1    1   eg2
......................................................................gucauacgcuucauguucauu....................   14   0   eg2
........................................................................cauacgcuucauguucauuA...................   2    1   eg2

.....................................................................ugucauacgcuucauguucau.....................   1    0   ea2
.....................................................................ugucauacgcuucauguucauu....................   38   0   ea2
.....................................................................ugucauacgcuucauguucauA....................   5    1   ea2
.....................................................................ugucauacgcuucauguucUuu....................   3    1   ea2
.....................................................................ugucauacgcuucauguucauC....................   1    1   ea2
.....................................................................ugucauacgcuucauguucauuA...................   20   1   ea2
......................................................................gucauacgcuucauguucauu....................   10   0   ea2
......................................................................gucauacgcuucauguucauuA...................   2    1   ea2

......................................agggcaugguggguaucauac....................................................   1    0   er1
.....................................................................ugucauacgcuucauguucau.....................   1    0   er1
.....................................................................Ggucauacgcuucauguucauu....................   1    1   er1
.....................................................................ugucauacgcuucCuguucauu....................   1    1   er1
.....................................................................ugucUuacgcuucauguucauu....................   1    1   er1
.....................................................................ugucauacgcuucauguucauC....................   2    1   er1
.....................................................................ugucauacgcuucauguucauu....................   17   0   er1
.....................................................................ugucauacgcuucauguucauuA...................   3    1   er1
.....................................................................ugucauacgcuucauguucauuU...................   1    1   er1

......................................agggcaugguggguaucauacu...................................................   3    0   eg1
.....................................................................ugucauacgcuucauguucau.....................   1    0   eg1
.....................................................................ugucauacgcuucauguucaG.....................   1    1   eg1
.....................................................................ugucauacgcuucauguucaC.....................   2    1   eg1
.....................................................................ugucauacgcuucauguucauA....................   2    1   eg1
.....................................................................ugucauacgcuucauguucauG....................   1    1   eg1
.....................................................................Ggucauacgcuucauguucauu....................   2    1   eg1
.....................................................................ugucauacAcuucauguucauu....................   2    1   eg1
.....................................................................ugucauacgcuucauguucUuu....................   1    1   eg1
.....................................................................ugucauacgcuucauguucauu....................   45   0   eg1
.....................................................................ugucauacgcuucauguucauC....................   6    1   eg1
.....................................................................ugucauacgcuucauguucauuA...................   1    1   eg1
.....................................................................ugucauacgcuucauguucauug...................   2    0   eg1
```

Provisional ID	:	ScUbCFx_359_19276
Score total	:	1558
Score for star read(s)	:	3.9
Score for read counts	:	1546.9
Score for mfe	:	2.6
Score for randfold	:	1.6
Score for cons. seed	:	3
Total read count	:	3046
Mature read count	:	2485
Loop read count	:	0
Star read count	:	561

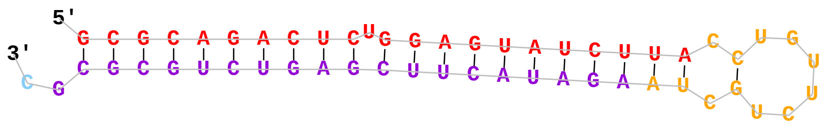

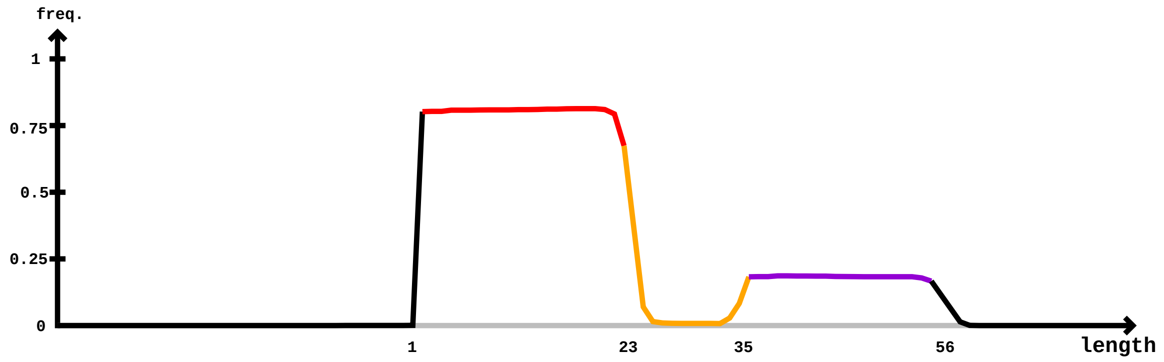


```
                                       Mature                           Star
5'- aacuuuaccugagaagggccuguuuaccuggccugcgcgcagacucuggaguaucuuaccuguucugcuaagauacuucgagucugcgcgcaggcuguguuuaccaggcc  -3'   obs
    aacuuuaccugagaagggccuguuuaccuggccugcgcgcagacucuggaguaucuuaccuguucugcuaagauacuucgagucugcgcgcaggcuguguuuaccaggcc         exp
    ..(((.....)))...(((((((..((.((((((((((((((((((.(((((((((((.(......).))))))))))))))))))))))))))))).))..).))))))  reads     mm     sample
    ....................................gAgcagacucuggaguauc.......................................................  1         1      eg1
    ....................................gAgcagacucuggaguaucu......................................................  2         1      eg1
    ....................................gAgcagacucuggaguaucuu.....................................................  29        1      eg1
    ....................................gAgcagacucuggaguaucuua....................................................  134       1      eg1
    ....................................gAgcagacucuggaguaucuuac...................................................  1         1      eg1
    ....................................gAgcagacucuggaguaucuuacc..................................................  18        1      eg1
    ....................................gAgcagacucuggaguaucuuaccu.................................................  1         1      eg1
    .................................................aguaucuuaccuguucugAu.........................................  2         1      eg1
    ........................................................uaccuguucugAuaagauacu.................................  1         1      eg1
    ....................................................................uaagauacuucgaAucugcgc.....................  1         1      eg1
    ....................................................................uaagauacuucgagucugcgcg....................  1         0      eg1
    ....................................................................uaagauacuucgagucugcgcgU...................  2         1      eg1
    ....................................................................uaagauacuucgagucugcgcgUa..................  1         1      eg1
    .....................................................................aagauacuucgagucugcgU.....................  1         1      eg1
    .....................................................................aagauacuucgagucugcgc.....................  1         0      eg1
    .....................................................................aagauacuucgagucugcgcg....................  14        0      eg1
    .....................................................................aagauacuucgagucugcgcA....................  1         1      eg1
    .....................................................................aagauacuucgagucugcgcU....................  1         1      eg1
    .....................................................................aagauGcuucgagucugcgcg....................  1         1      eg1
    .....................................................................aagauacuucgagucugcgcgUa..................  1         1      eg1
    .....................................................................aagauacuucgagucugcgcgcaC.................  1         1      eg1
    ......................................................................agauacuucgagucugcgc.....................  2         0      eg1
    ......................................................................agauacuucgagucugcgcg....................  13        0      eg1
    ......................................................................agauacuucgagucugcgcU....................  3         1      eg1
    ......................................................................agauacuucgagucugcgcA....................  2         1      eg1
    ......................................................................agauacuucgagucugcgcgU...................  3         1      eg1
    ......................................................................agauacuucgagucugcgcgc...................  1         0      eg1
    ......................................................................Ggauacuucgagucugcgcgc...................  1         1      eg1
    ......................................................................agauacuucgagucugcgcgca..................  3         0      eg1
    ......................................................................agauacuucgagucugcgcgcG..................  1         1      eg1
    ......................................................................agauacuucgagucugcgcgUa..................  1         1      eg1
    ......................................................................agauacuucgagucugcgcgcC..................  2         1      eg1
    ......................................................................agauacuucgagucugcgcgcU..................  9         1      eg1
```

Mature Star

```
aacuuuaccugagaagggccuguuuaccuggccugcgcgcagacucuggaguaucuuaccuguucugcuaagauacuucgagucugcgcgcaggcuguguuuaccaggcc
......................................................................agauacuucgagucugcgcgcaU.................	1	1	eg1
......................................................................agauacuucgagucugcgcgcaC.................	1	1	eg1
......................................................................agauacuucgagucugcgcgcaUg................	1	1	eg1
.......................................................................gauacuucgagucugcgcgcC..................	4	1	eg1

....................................gAgcagacucuggaguaucu......................................................	6	1	er1
....................................gAgcagacucuggaguaucuu.....................................................	23	1	er1
....................................gGgcagacucuggaguaucuua....................................................	1	1	er1
....................................gAgcagacucuggaguaucuua....................................................	130	1	er1
....................................gAgcagacucuggaguaucuuac...................................................	2	1	er1
....................................gAgcagacucuggaguaucuuacc..................................................	19	1	er1
....................................gAgcagacucuggaguaucuuaccu.................................................	2	1	er1
....................................................................uaagauacuucgagucugcgA.....................	1	1	er1
....................................................................uaagauacuucgagucugcgcg....................	1	0	er1
....................................................................uaagauacuucgagucugcgcgca..................	1	0	er1
.....................................................................aagauacuucgagucugcg......................	2	0	er1
.....................................................................aagauacuucgagucugcgc.....................	1	0	er1
.....................................................................aagauacuucgagucugcgcC....................	1	1	er1
.....................................................................aagauacuucgagucugcgcU....................	2	1	er1
.....................................................................aagauacuucgagucugcgcg....................	2	0	er1
.....................................................................aagauacuucgagucugcgcgcU..................	4	1	er1
.....................................................................Gagauacuucgagucugcgcgca..................	1	1	er1
......................................................................agauacuucgagucugcgcC....................	1	1	er1
......................................................................agauacuucgagucugcgcA....................	1	1	er1
......................................................................agauacuucgagucugcgcg....................	2	0	er1
......................................................................agauacuucgagucugcgcgU...................	2	1	er1
......................................................................agauacuucgagucugcgcgc...................	3	0	er1
......................................................................agauacuucgagucugcgcgca..................	2	0	er1
......................................................................agauacuucgagucugcgcgcU..................	2	1	er1
......................................................................agauacuucgagucugcgcgcUg.................	1	1	er1
......................................................................agauacuucgagucugcgcgcaU.................	1	1	er1
......................................................................agauacuucgagucugcgcgcaC.................	1	1	er1
......................................................................agauacuucgagucugcgcgcaA.................	3	1	er1

............................uggccugcgUgcagacucug..............................................................	1	1	ea1
...................................cgAgcagacucuggaguaucuua....................................................	1	1	ea1
....................................gAgcagacucuggaguauc.......................................................	11	1	ea1
....................................gAgcagacucuggaguaucu......................................................	44	1	ea1
....................................gAgcagacucuggaguaucuu.....................................................	311	1	ea1
....................................gAgcagacucuggaguaucuua....................................................	1536	1	ea1
....................................gcgcagacucuggaguaucuuC....................................................	1	1	ea1
....................................gGgcagacucuggaguaucuua....................................................	6	1	ea1
....................................gUgcagacucuggaguaucuua....................................................	1	1	ea1
....................................gcgcagacucuggaguaucuua....................................................	1	0	ea1
....................................gAgcagacucuggaguaucuuac...................................................	25	1	ea1
....................................gAgcagacucuggaguaucuuacc..................................................	132	1	ea1
....................................gAgcagacucuggaguaucuuaccu.................................................	6	1	ea1
.....................................Agcagacucuggaguaucuuacc..................................................	1	1	ea1
.......................................cagacucuggaguaucuC.....................................................	3	1	ea1
.......................................cagacucuggaguaucuuacU..................................................	1	1	ea1
.......................................cagacucuggaguaucuuacc..................................................	1	0	ea1
.......................................cagacucuggaguaucuuaccC.................................................	2	1	ea1
.......................................cagacucuggaguaucuuaccu.................................................	4	0	ea1
.......................................Gagacucuggaguaucuuaccu.................................................	1	1	ea1
.......................................cagacucuggaguaucuuaccuA................................................	1	1	ea1
.......................................cagacucuggaguaucuuaccuU................................................	1	1	ea1
..........................................acucuggaguaucuuacAug................................................	2	1	ea1
...........................................cucuggaguaucuuaccugu...............................................	1	0	ea1
..............................................uggaguaucuuGccuguucug...........................................	3	1	ea1
................................................gaguaucuuaccuguucugAua........................................	1	1	ea1
.................................................aguaucuuaccuguucugAu.........................................	1	1	ea1
.................................................aguaucuuaccuguucugAuaa.......................................	1	1	ea1
...................................................uaucuuaccuguucugAuaa.......................................	2	1	ea1
....................................................aucuuaccuguucugAua........................................	1	1	ea1
.......................................................uuaccuguucugAuaagaua...................................	2	1	ea1
..........................................................ccuguucugAuaagauacuuc...............................	2	1	ea1
...........................................................cuguucugAuaagauacuuc...............................	1	1	ea1
............................................................uguucugAuaagauacuucg..............................	1	1	ea1
............................................................uguucugAuaagauacuucga.............................	1	1	ea1
...................................................................cuaagaAacuucgagucugcgcg....................	2	1	ea1
```

Mature **Star**

```
aacuuuaccugagaagggccuguuuaccuggccugcgcgcagacucuggaguaucuuaccuguucugcuaagauacuucgagucugcgcgcaggcuguguuuaccaggcc
....................................................................uaagauacuucgagucugcA......................  2  1  ea1
....................................................................uaagauacuucgagucugcg......................  1  0  ea1
....................................................................uaagauacuuGgagucugcg......................  1  1  ea1
....................................................................uaagauacuucgagucugcgc.....................  1  0  ea1
....................................................................uaagauacuuGgagucugcgc.....................  1  1  ea1
....................................................................uaagauacuuGgagucugcgcg....................  7  1  ea1
....................................................................uaagauacuucgagucugcgcU....................  2  1  ea1
....................................................................uaagauacuucgagucugcgcg....................  6  0  ea1
....................................................................uaagauacuucgagucugcgcA....................  2  1  ea1
....................................................................uaagauacuucgagucugcgcgU...................  1  1  ea1
....................................................................uaagauacuucgagucugcgcgc...................  5  0  ea1
....................................................................uaagauacuucgagucugcgcgcU..................  19  1  ea1
....................................................................uaagauacuucgagucugcgcgUa..................  1  1  ea1
....................................................................uaagauacuucgagucugcgcgcC..................  3  1  ea1
....................................................................uaagauacuucgagucugcgcgca..................  5  0  ea1
.....................................................................aagauacuucgagucugcg......................  1  0  ea1
.....................................................................aagauacuucgagucugcgc.....................  19  0  ea1
.....................................................................aagauacuucgagucugcgU.....................  1  1  ea1
.....................................................................aagauacuucgagucugcgcA....................  16  1  ea1
.....................................................................aagauacuucgagucugcgcU....................  3  1  ea1
.....................................................................Gagauacuucgagucugcgcg....................  1  1  ea1
.....................................................................aagauacuucgagucugcgcC....................  2  1  ea1
.....................................................................aagauacuucgagucugcgcg....................  53  0  ea1
.....................................................................aagauacuucgagucugcgcgU...................  7  1  ea1
.....................................................................aagauacuucgagucugcgcgc...................  2  0  ea1
.....................................................................aagauacuucgagucugcgcgUa..................  3  1  ea1
.....................................................................aagauacuucgagucugcgcgca..................  15  0  ea1
.....................................................................aagauacuucgagucugcgcgcU..................  5  1  ea1
.....................................................................aagauacuucgagucugcgcgcaU.................  4  1  ea1
.....................................................................aagauacuucgagucugcgcgcaA.................  4  1  ea1
.....................................................................aagauacuucgagucugcgcgcaC.................  1  1  ea1
......................................................................Ggauacuucgagucugcg......................  1  1  ea1
......................................................................agauacuucgagucugcg......................  5  0  ea1
......................................................................agauacuucgagucugcA......................  1  1  ea1
......................................................................agauacuucgagucugcgc.....................  7  0  ea1
......................................................................agauacuucgagucugcgU.....................  1  1  ea1
......................................................................agGuacuucgagucugcgc.....................  1  1  ea1
......................................................................agauacuucgGgucugcgcg....................  1  1  ea1
......................................................................agGuacuucgagucugcgcg....................  1  1  ea1
......................................................................agauacuucgaAucugcgcg....................  1  1  ea1
......................................................................agauacuucgagucugcgcA....................  18  1  ea1
......................................................................agauacuucgagucugcgcU....................  3  1  ea1
......................................................................agauacuucgagucugAgcg....................  2  1  ea1
......................................................................Ggauacuucgagucugcgcg....................  1  1  ea1
......................................................................agauacuucgagucugcgcg....................  93  0  ea1
......................................................................agaCacuucgagucugcgcg....................  1  1  ea1
......................................................................agauacuucgagucCgcgcg....................  1  1  ea1
......................................................................Ggauacuucgagucugcgcgc...................  2  1  ea1
......................................................................agauacuucgagucugcgcgU...................  11  1  ea1
......................................................................agauacuucgagucugcgcgc...................  21  0  ea1
......................................................................agauacuCcgagucugcgcgc...................  2  1  ea1
......................................................................agauacuucgagucugcgcgcU..................  15  1  ea1
......................................................................agauacuucgagucugcgcgcG..................  1  1  ea1
......................................................................agauacuucgagucugcgcgcC..................  2  1  ea1
......................................................................agauacuucgagucugcgcgUa..................  1  1  ea1
......................................................................agauacuucgagucuAcgcgca..................  1  1  ea1
......................................................................agauacuucgagucugcgcgca..................  30  0  ea1
......................................................................Ggauacuucgagucugcgcgca..................  1  1  ea1
......................................................................agauacuucgGgucugcgcgca..................  1  1  ea1
......................................................................agauacuucgagucugcgcgcag.................  1  0  ea1
......................................................................agauacuucgagucugcgcgcaA.................  4  1  ea1
......................................................................agauacuucgagucugcgcgcaU.................  12  1  ea1
......................................................................agauacuucgagucugcgcgcaC.................  1  1  ea1
.......................................................................gauacuucgagucugcgcA....................  1  1  ea1
.......................................................................gauacuucgagucugcgcgG...................  1  1  ea1
.......................................................................gauacuucgagucugcgcAca..................  1  1  ea1
.........................................................................uacuuGgagucugcgcgc...................  1  1  ea1
.........................................................................uacuucgagucugcgcgc...................  2  0  ea1
.........................................................................uacuucgagucugcgcgcaC.................  2  1  ea1
.........................................................................uacuucgagucugcgcgcaU.................  3  1  ea1
```

Mature Star

```
aacuuuaccugagaagggccuguuuaccuggccugcgcgcagacucuggaguaucuuaccuguucugcuaagauacuucgagucugcgcgcaggcuguguuuaccaggcc
.........................................................................uacuuGgagucugcgcgcagg................    1    1    ea1

....................................gAgcagacucuggaguaucuu.....................................................    1    1    er2
............................................................uguucugcuaagauacuucgag............................    1    0    er2

....................................gAgcagacucuggaguaucuua....................................................    17   1    ea2
......................................................................agauacuucgagucugcgcg....................    2    0    ea2

....................................gAgcagacucuggaguaucuua....................................................    3    1    eg2
.....................................Agcagacucuggaguaucuua....................................................    2    1    eg2
................................................gaguaucuuaccGguucug...........................................    1    1    eg2
................................................gaguaucuuaccuguucugA..........................................    1    1    eg2
...................................................uaucuuaccuguucugAuaa.......................................    2    1    eg2
...........................................................cuguucugAuaagauacuuc...............................    1    1    eg2
```

Provisional ID	:	ScUbCFx_384_20122
Score total	:	63.3
Score for star read(s)	:	3.9
Score for read counts	:	53.1
Score for mfe	:	1.7
Score for randfold	:	1.6
Score for cons. seed	:	3
Total read count	:	116
Mature read count	:	112
Loop read count	:	0
Star read count	:	4

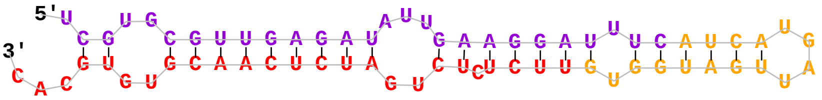

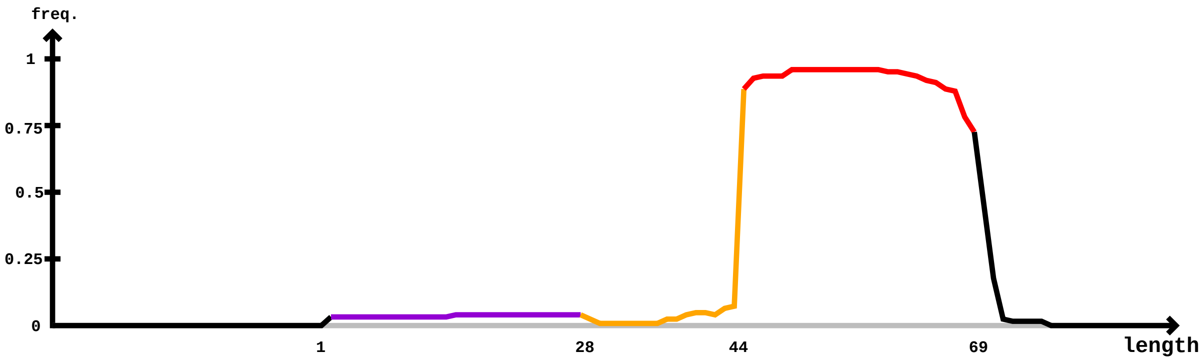

Star Mature

```
5'- gaacacugcauggugugguccaauguaucgugcguugagauauugaaggauuucaucaugauugauggug uucucucugaucucaacgugugcacagucgcuuccuuuguggaug   -3'   obs
    gaacacugcauggugugguccaauguaucgugcguugagauauugaaggauuucaucaugauugauggugu ucucucugaucucaacgugugcacagucgcuuccuuuguggaug         exp
    .....(..((.((.((((.....(((..((..(((((((((...(((((((.((((((....)))))).))))).))..)))))))))..)).))).))))..))..))..)...   reads   mm   sample
    ...........................ucgugcguugagauauugaaggauuuc.............................................................   1   0   eg1
    ........................................uauugaaggauuucaucaugauugaug................................................   1   0   eg1
    ..............................................................ugauggugu ucucucugaucucaacgu..........................   2   0   eg1
    ................................................................auggugu ucucucugaucucaacgugu........................   1   0   eg1
    .................................................................ugguguucucucugaucucaacgugu........................   1   0   eg1
    ....................................................................uguucucucugaucucaacgugugU......................   1   1   eg1
    ....................................................................uguucucucugaucucaacgugugcac....................   1   0   eg1
    .....................................................................guucucucugaucucaac............................   1   0   eg1
    ......................................................................uucucucugaucucaacgCg.........................   1   1   eg1
    ......................................................................uucucucugaucucaacgugu........................   1   0   eg1
    ......................................................................Gucucucugaucucaacgugugc......................   1   1   eg1
    ......................................................................uucucucugaucucaacgugugc......................   6   0   eg1
    ......................................................................uucucucugaucucaacgugugU......................   1   1   eg1
    ......................................................................Gucucucugaucucaacgugugca.....................   2   1   eg1
    ......................................................................uucucucugaucucaacgugugca.....................   1   0   eg1
    ......................................................................uucucucugaucucaacgugugcaU....................   3   1   eg1
    ......................................................................Gucucucugaucucaacgugugcac....................   2   1   eg1
    ......................................................................uucucucugaucucaacgugugcac....................   19  0   eg1
    ......................................................................uucucucugaucucaacgugugcaca...................   7   0   eg1
    ......................................................................uucucucugaucucaacgugugcacG...................   1   1   eg1
    ......................................................................Gucucucugaucucaacgugugcaca...................   1   1   eg1
    ......................................................................uucuUucugaucucaacgugugcacag..................   1   1   eg1
    ......................................................................uucucucugaucucaacgugugcacag..................   11  0   eg1
    .......................................................................ucucucugaucucaacgugugc......................   1   0   eg1
    .......................................................................ucucucugaucucaacgugugcacag..................   1   0   eg1
    ...........................................................................ucugaucucaacgugugcac....................   1   0   eg1

    ...........................ucgugcguugagauauugaaggauuuc.............................................................   3   0   er1
    .......................................................................ucucucugaucucaacgugugcacagu.................   1   0   er1
    ...........................................................................ucugaucucaacgugugcacagucgcu.............   2   0   er1

    ................................................................aCgguguuucucucugaucuca..............................   1   1   ea1
    ....................................................................uguucucucugaucucaacgugugcac....................   1   0   ea1
```

```
                          Star                                          Mature
gaacacugcauggugugguccaauguaucgugcguugagauauugaaggauuucaucaugauugauggugUUCUCUCUGAUCUCAACGUGUGCACagucgcuucccuuuguggaug
......................................................................uucucucugaucucaacg............................   1    0    ea1
......................................................................uucucucugaucucaacgugug........................   1    0    ea1
......................................................................uucucucugaucucaacgugugc.......................   2    0    ea1
......................................................................uucucucugaucucaacgugugca......................   3    0    ea1
......................................................................uucucucugaucucaacgugugcac.....................   18   0    ea1
......................................................................uucucucugaucucaacgugugAac.....................   2    1    ea1
......................................................................Gucucucugaucucaacgugugcac.....................   1    1    ea1
......................................................................Gucucucugaucucaacgugugcaca....................   1    1    ea1
......................................................................uucucucugaucucaacgugugcaca....................   6    0    ea1
......................................................................uucucucugaucucaacgugugcacG....................   2    1    ea1
......................................................................uucucucugaucucaacgugugcacag...................   6    0    ea1
.......................................................................ucucucugaucucaacgugugcac.....................   2    0    ea1
........................................................................cucucugaucucaacgugugca......................   1    0    ea1
```

Provisional ID	:	ScUbCFx_409_21750
Score total	:	4.2
Score for star read(s)	:	-1.3
Score for read counts	:	0
Score for mfe	:	0.9
Score for randfold	:	1.6
Score for cons. seed	:	3
Total read count	:	304
Mature read count	:	221
Loop read count	:	9
Star read count	:	74

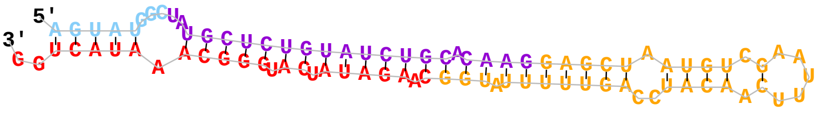

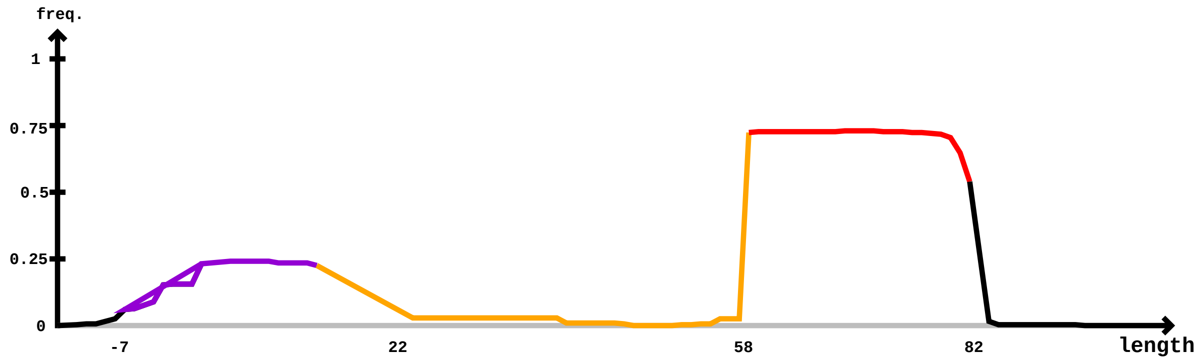


```
                 Star                                                     Mature
5'- agaacaguauggcuaugcucuguaucugcacaaggagcuaaugucgaauuucaacauccaguuuuuauggcaagauaucaugggcaaauacuggccauggcuugaggaagucag  -3'    obs
    agaacaguauggcuaugcucuguaucugcacaaggagcuaaugucgaauuucaacauccaguuuuuauggcaagauaucaugggcaaauacuggccauggcuugaggaagucag          exp
    .(..((((((.....(((((((((((((.((((((((((.((((.(.....).))))..))))))).)))).))))).)).))))).))))))..).((((((....)))))).   reads   mm   sample
    .....aguauggcuaugcucuguaucugc.....................................................................................   1       0    eg1
    .....aguauggcuaugcucuguaucugcaU...................................................................................   1       1    eg1
    .........uggcuaugcucuguaucugc.....................................................................................   1       0    eg1
    .........uggcuaugcucuguaucugU.....................................................................................   1       1    eg1
    .........uggcuaugcucuguaucugca....................................................................................   1       0    eg1
    .........uggcuaugcucuguaucugcaA...................................................................................   1       1    eg1
    .............uaugcucuguaucugcaca..................................................................................   1       0    eg1
    .............uaugcucuguaucugcacaag................................................................................   5       0    eg1
    ...............................aaggagcuaaugucgaauuucaacau.........................................................   1       0    eg1
    ...................................................................uggcaagauaucaugggcaaauacu......................   1       0    eg1
    ...................................................................uggcaagauaucaugggcaaauacug.....................   1       0    eg1
    ......................................................................caagauaucaugggcaaau.........................   1       0    eg1
    ......................................................................caagauaucaugggcaaauacu......................   4       0    eg1
    ......................................................................caagauaucaugggcaaauacC......................   1       1    eg1
    ......................................................................caagauaucaugggcaaauacug.....................   2       0    eg1
    ......................................................................caagauGucaugggcaaauacugg....................   2       1    eg1
    ......................................................................Gaagauaucaugggcaaauacugg....................   1       1    eg1
    ......................................................................caagauaucaugggcaaauacugg....................   27      0    eg1
    ......................................................................caagauaucaugggcaaauacuCg....................   1       1    eg1
    ......................................................................caagaAaucaugggcaaauacugg....................   1       1    eg1
    ......................................................................caagauaucaugggcaaauacuggc...................   18      0    eg1
    ......................................................................caagauaucaugggcaaauacuggcG..................   3       1    eg1
    ................................................................................ugggcaaauacuggccauggcuuga.........   1       0    eg1

    .........uggcuaugcucuguaucugc.....................................................................................   2       0    er1
    .........uggcuaugcucuguaucugca....................................................................................   1       0    er1
    ..............augcucuguaucugcacaagA...............................................................................   1       1    er1
    ...............................................................uuuauggcaagauaucauggg..............................   1       0    er1
    ...................................................................Gggcaagauaucaugggcaaauacug.....................   1       1    er1
    ......................................................................caagauaucaugggcaaauacC......................   1       1    er1
    ......................................................................caagauaucaugggcaaauacu......................   2       0    er1
    ......................................................................caagauaucaugggcaaauacug.....................   4       0    er1
    ......................................................................caagauaucaugggcaaauacugg....................   18      0    er1
```

Star　　　　　　　　　　　　　　Mature

```
agaacaguauggcuaugcucuguaucugcacaaggagcuaaugucgaauuucaacauccaguuuuuauggcaagauaucaugggcaaauacuggccauggcuugaggaagucag
......................................................................caagauaucaugggcaaauaUugg....................    1    1    er1
......................................................................caagauaucaugggcaaauacuggc...................    7    0    er1
......................................................................caagauaucaugggcaaauacuggA...................    1    1    er1

.............uaugcucuguaucugcacaG.................................................................................    2    1    er2

aUaacaguauggcuaugcucu.............................................................................................    1    1    ea1
.....aguauggcuaugcucuguaucugc.....................................................................................    1    0    ea1
.......uauggcuaugcucuguaucugcaca..................................................................................    3    0    ea1
.......uauggcuaugcucuguaucugcacaag................................................................................    1    0    ea1
........Guggcuaugcucuguaucug......................................................................................    1    1    ea1
........auggcuaugcucuguaucug......................................................................................    1    0    ea1
.........uggcuaugcucuguaucug......................................................................................    1    0    ea1
.........uggcuaugcucuguaucugc.....................................................................................    4    0    ea1
.........Gggcuaugcucuguaucugc.....................................................................................    1    1    ea1
.........uggcuaugcucuguaucugca....................................................................................    5    0    ea1
.............uaugcucuguaucugcaca..................................................................................    1    0    ea1
.............uaugcucuguaucugcacaag................................................................................    4    0    ea1
.............uaugcucuguaucugcacaaU................................................................................    1    1    ea1
...............................aaggagcuaaugucgaauuucaacauc........................................................    1    0    ea1
...............................aaggagcuaaugucgaauuucaacauU........................................................    1    1    ea1
...................................................................uggcaagauaucaugggcaaauac.......................    1    0    ea1
...................................................................uggcaagauaucaugggcaaauacu......................    1    0    ea1
...................................................................uggcaagauaucaugggcaaauacug.....................    1    0    ea1
......................................................................caagauaucaugggcaaaua........................    1    0    ea1
......................................................................caagauaucaugggcaaauac.......................    3    0    ea1
......................................................................caagauaucaugggcaaauacG......................    1    1    ea1
......................................................................caagauaucaugggcaaauacu......................    5    0    ea1
......................................................................caagauaucaugggcaaauacC......................    2    1    ea1
......................................................................caagauaucaugggcaaauacuA.....................    2    1    ea1
......................................................................caagauaucaugggcaaauacCg.....................    1    1    ea1
......................................................................caagauaucaugggcaaauacug.....................    21   0    ea1
......................................................................caagauaucaugggcaaauacugA....................    4    1    ea1
......................................................................cGagauaucaugggcaaauacugg....................    1    1    ea1
......................................................................caagauaucaugggcaaauaUugg....................    1    1    ea1
......................................................................caagauaucGugggcaaauacugg....................    1    1    ea1
......................................................................caagauaucaugggcaaauacugg....................    57   0    ea1
......................................................................caagauaucaugggcaaauacuggA...................    2    1    ea1
......................................................................caagauaucaugggcaaauacuggc...................    11   0    ea1
......................................................................Gaagauaucaugggcaaauacuggc...................    1    1    ea1
......................................................................caagauaucaugggcaaauacuggU...................    2    1    ea1
......................................................................caagGuaucaugggcaaauacuggcc..................    1    1    ea1

.gaacaguauggcuaugcucu.............................................................................................    1    0    eg2
...acaguauggcuaugcucuguaA.........................................................................................    3    1    eg2
....caguauggcuaugcucuguauA........................................................................................    3    1    eg2
.....aguauggcuaugcucuguaucu.......................................................................................    1    0    eg2
.....aguauggcuaugcCcuguaucug......................................................................................    1    1    eg2
.....aguauggcuaugcucuguaucugc.....................................................................................    6    0    eg2
......guauggcuaugcucuguaucA.......................................................................................    1    1    eg2
........auggcuaugcucuguaucug......................................................................................    1    0    eg2
........auggcuGugcucuguaucugca....................................................................................    1    1    eg2
.........uggcuaugcucuguaucugca....................................................................................    2    0    eg2
..........ggcuaCgcucuguaucugca....................................................................................    1    1    eg2
.............uaugcucuguaucugcacaa.................................................................................    1    0    eg2
.............uaugcucuguaucugcacaag................................................................................    1    0    eg2
.............uaugcucuguaucugcacaaU................................................................................    1    1    eg2
...............ugcucuguaucugcacaaAg...............................................................................    1    1    eg2
.................................................................uauggcaagauaucaugggcaa...........................    1    0    eg2
......................................................................caagauaucaugggcaaauacug.....................    1    0    eg2
......................................................................caagauaucaugggcaaauacugg....................    7    0    eg2
.......................................................................aagauaucaugggcaaauaUuggc...................    1    1    eg2

.............uaugcucuguaucugcacaag................................................................................    7    0    ea2
................gcucuguaucugcacaagg...............................................................................    1    0    ea2
...............................aaggagcuaaugucgaauuu...............................................................    6    0    ea2
```

Provisional ID	:	ScUbCFx_409_21814
Score total	:	4.3
Score for star read(s)	:	-1.3
Score for read counts	:	0
Score for mfe	:	1
Score for randfold	:	1.6
Score for cons. seed	:	3
Total read count	:	300
Mature read count	:	221
Loop read count	:	5
Star read count	:	74

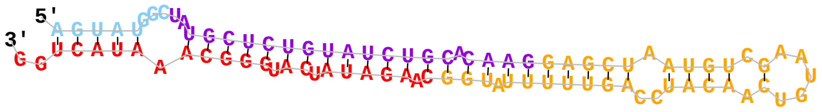

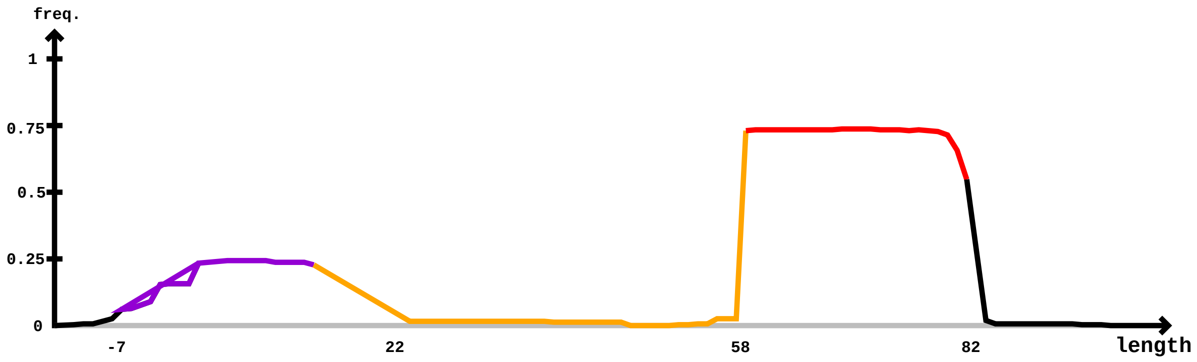


```
                  Star                                              Mature
5'- agaacaguauggcuaugcucuguaucugcacaaggagcuaaugucgaaugucaacauccaguuuuuauggcaagauaucaugggcaaauacuggccauggcuugagaaagucag   -3'   obs
    agaacaguauggcuaugcucuguaucugcacaaggagcuaaugucgaaugucaacauccaguuuuuauggcaagauaucaugggcaaauacuggccauggcuugagaaagucag         exp
    .(..((((((.....((((((((((((((.(((((((((.((((.(.....).))))..))))))).)))).))))).)).))))).))))))..).((((((....)))))).   reads   mm   sample
    .gaacaguauggcuaugcucu.............................................................................................   1   0   eg2
    ...acaguauggcuaugcucuguaA.........................................................................................   3   1   eg2
    ....caguauggcuaugcucuguauA........................................................................................   3   1   eg2
    .....aguauggcuaugcucuguaucu.......................................................................................   1   0   eg2
    .....aguauggcuaugcCcuguaucug......................................................................................   1   1   eg2
    .....aguauggcuaugcucuguaucugc.....................................................................................   6   0   eg2
    ......guauggcuaugcucuguaucA.......................................................................................   1   1   eg2
    ........auggcuaugcucuguaucug......................................................................................   1   0   eg2
    ........auggcuGugcucuguaucugca....................................................................................   1   1   eg2
    .........uggcuaugcucuguaucugca....................................................................................   2   0   eg2
    ..........ggcuaCgcucuguaucugca....................................................................................   1   1   eg2
    .............uaugcucuguaucugcacaa.................................................................................   1   0   eg2
    .............uaugcucuguaucugcacaaU................................................................................   1   1   eg2
    .............uaugcucuguaucugcacaag................................................................................   1   0   eg2
    ...............ugcucuguaucugcacaaAg...............................................................................   1   1   eg2
    ..............................caaggagcuaaugucgaaug................................................................   1   0   eg2
    .................................................................uauggcaagauaucaugggcaa...........................   1   0   eg2
    ......................................................................caagauaucaugggcaaauacug.....................   1   0   eg2
    ......................................................................caagauaucaugggcaaauacugg....................   7   0   eg2
    .......................................................................aagauaucaugggcaaauaUuggc...................   1   1   eg2

    .............uaugcucuguaucugcacaag................................................................................   7   0   ea2
    ................gcucuguaucugcacaagg...............................................................................   1   0   ea2

    .............uaugcucuguaucugcacaG.................................................................................   2   1   er2

    aUaacaguauggcuaugcucu.............................................................................................   1   1   ea1
    .....aguauggcuaugcucuguaucugc.....................................................................................   1   0   ea1
    .......uauggcuaugcucuguaucugcaca..................................................................................   3   0   ea1
    .......uauggcuaugcucuguaucugcacaag................................................................................   1   0   ea1
    ........auggcuaugcucuguaucug......................................................................................   1   0   ea1
    ........Guggcuaugcucuguaucug......................................................................................   1   1   ea1
    .........uggcuaugcucuguaucug......................................................................................   1   0   ea1
```

```
              Star                          Mature
agaacaguauggcuaugcucuguaucugcacaaggagcuaaugucgaaugucaacauccaguuuuuauggcaagauaucaugggcaaauacuggccauggcuugagaaagucag
.........uggcuaugcucuguaucugc.....................................................................................	4	0	ea1
.........Gggcuaugcucuguaucugc.....................................................................................	1	1	ea1
.........uggcuaugcucuguaucugca....................................................................................	5	0	ea1
.............uaugcucuguaucugcaca..................................................................................	1	0	ea1
.............uaugcucuguaucugcacaaU................................................................................	1	1	ea1
.............uaugcucuguaucugcacaag................................................................................	4	0	ea1
...............................aaggagcuaaugucgaaugucaacauc........................................................	1	0	ea1
...............................aaggagcuaaugucgaaugucaacauA........................................................	3	1	ea1
...................................................................uggcaagauaucaugggcaaauac.......................	1	0	ea1
...................................................................uggcaagauaucaugggcaaauacu......................	1	0	ea1
...................................................................uggcaagauaucaugggcaaauacug.....................	1	0	ea1
......................................................................caagauaucaugggcaaaua........................	1	0	ea1
......................................................................caagauaucaugggcaaauac.......................	3	0	ea1
......................................................................caagauaucaugggcaaauacG......................	1	1	ea1
......................................................................caagauaucaugggcaaauacC......................	2	1	ea1
......................................................................caagauaucaugggcaaauacu......................	5	0	ea1
......................................................................caagauaucaugggcaaauacCg.....................	1	1	ea1
......................................................................caagauaucaugggcaaauacug.....................	21	0	ea1
......................................................................caagauaucaugggcaaauacuA.....................	2	1	ea1
......................................................................caagauaucaugggcaaauacugA....................	4	1	ea1
......................................................................caagauaucGugggcaaauacugg....................	1	1	ea1
......................................................................cGagauaucaugggcaaauacugg....................	1	1	ea1
......................................................................caagauaucaugggcaaauaUugg....................	1	1	ea1
......................................................................caagauaucaugggcaaauacugg....................	57	0	ea1
......................................................................caagauaucaugggcaaauacuggU...................	2	1	ea1
......................................................................Gaagauaucaugggcaaauacuggc...................	1	1	ea1
......................................................................caagauaucaugggcaaauacuggc...................	11	0	ea1
......................................................................caagauaucaugggcaaauacuggA...................	2	1	ea1
......................................................................caagGuaucaugggcaaauacuggcc..................	1	1	ea1
........................................................................................uacuggccauggcuCgagaa......	1	1	ea1

.....aguauggcuaugcucuguaucugc.....................................................................................	1	0	eg1
.....aguauggcuaugcucuguaucugcaU...................................................................................	1	1	eg1
.........uggcuaugcucuguaucugU.....................................................................................	1	1	eg1
.........uggcuaugcucuguaucugc.....................................................................................	1	0	eg1
.........uggcuaugcucuguaucugca....................................................................................	1	0	eg1
.........uggcuaugcucuguaucugcaA...................................................................................	1	1	eg1
.............uaugcucuguaucugcaca..................................................................................	1	0	eg1
.............uaugcucuguaucugcacaag................................................................................	5	0	eg1
...................................................................uggcaagauaucaugggcaaauacu......................	1	0	eg1
...................................................................uggcaagauaucaugggcaaauacug.....................	1	0	eg1
......................................................................caagauaucaugggcaaau.........................	1	0	eg1
......................................................................caagauaucaugggcaaauacC......................	1	1	eg1
......................................................................caagauaucaugggcaaauacu......................	4	0	eg1
......................................................................caagauaucaugggcaaauacug.....................	2	0	eg1
......................................................................caagauaucaugggcaaauacuCg....................	1	1	eg1
......................................................................caagauGucaugggcaaauacugg....................	2	1	eg1
......................................................................Gaagauaucaugggcaaauacugg....................	1	1	eg1
......................................................................caagaAaucaugggcaaauacugg....................	1	1	eg1
......................................................................caagauaucaugggcaaauacugg....................	27	0	eg1
......................................................................caagauaucaugggcaaauacuggc...................	18	0	eg1
......................................................................caagauaucaugggcaaauacuggcG..................	3	1	eg1
................................................................................ugggcaaauacuggccauggcuuga.........	1	0	eg1

.........uggcuaugcucuguaucugc.....................................................................................	2	0	er1
.........uggcuaugcucuguaucugca....................................................................................	1	0	er1
..............augcucuguaucugcacaagA...............................................................................	1	1	er1
...............................................................uuuauggcaagauaucauggg..............................	1	0	er1
...................................................................Gggcaagauaucaugggcaaauacug.....................	1	1	er1
......................................................................caagauaucaugggcaaauacC......................	1	1	er1
......................................................................caagauaucaugggcaaauacu......................	2	0	er1
......................................................................caagauaucaugggcaaauacug.....................	4	0	er1
......................................................................caagauaucaugggcaaauaUugg....................	1	1	er1
......................................................................caagauaucaugggcaaauacugg....................	18	0	er1
......................................................................caagauaucaugggcaaauacuggc...................	7	0	er1
......................................................................caagauaucaugggcaaauacuggA...................	1	1	er1
```

Provisional ID	:	ScUbCFx_448_23551
Score total	:	32615
Score for star read(s)	:	3.9
Score for read counts	:	32608
Score for mfe	:	2.1
Score for randfold	:	1.6
Score for cons. seed	:	-0.6
Total read count	:	63971
Mature read count	:	63951
Loop read count	:	0
Star read count	:	20

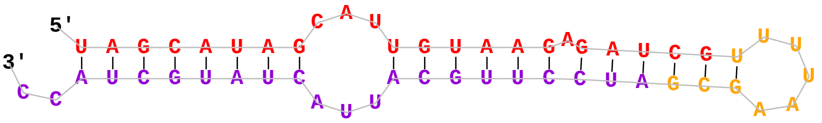

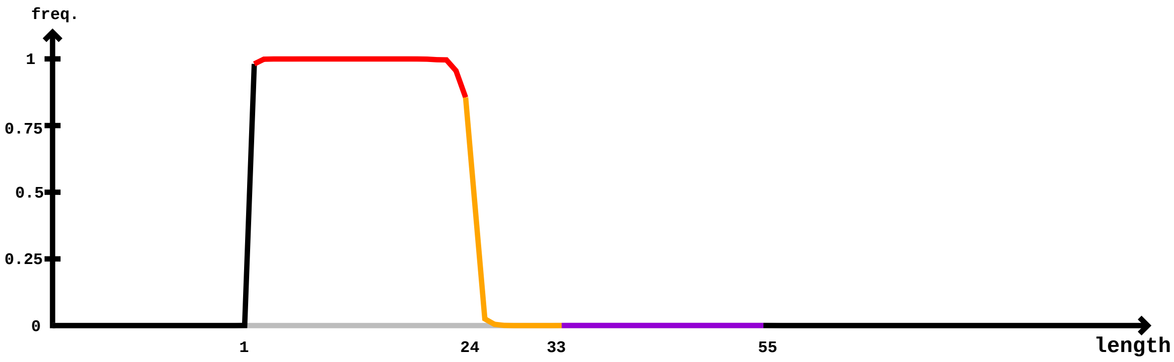

Mature Star

```
5'- gaccaauuccaauuccugguagcauagcauuguaagagaucguuuuaagcgauccuugcauuacuaugcuaccagguauuguaccgagguuggagguagaaacgaggcaaag  -3'   obs
    gaccaauuccaauuccugguagcauagcauuguaagagaucguuuuaagcgauccuugcauuacuaugcuaccagguauuguaccgagguuggagguagaaacgaggcaaag         exp
    .(((.....((((.(((((((((((((...((((((.((((((.....))))))))))))...))))))))))))).))))..(((....))).)))...............   reads    mm    sample
    ...................uagcauagcauuguaagag..........................................................................   7        0     eg1
    ...................Gagcauagcauuguaagaga.........................................................................   1        1     eg1
    ...................uagcauagcauuguaagaga.........................................................................   1        0     eg1
    ...................uagcauagcauuguaagagau........................................................................   50       0     eg1
    ...................uagcauagcauuguaagagaA........................................................................   5        1     eg1
    ...................uagcauagcauuguaagagaC........................................................................   3        1     eg1
    ...................Gagcauagcauuguaagagau........................................................................   3        1     eg1
    ...................uagcauagcauCguaagagauc.......................................................................   1        1     eg1
    ...................uagcauagcauuguaagGgauc.......................................................................   2        1     eg1
    ...................uagcauCgcauuguaagagauc.......................................................................   1        1     eg1
    ...................uagUauagcauuguaagagauc.......................................................................   1        1     eg1
    ...................uagcauagcauuguaagagauc.......................................................................   157      0     eg1
    ...................Gagcauagcauuguaagagauc.......................................................................   10       1     eg1
    ...................uagcauagcauuguaagagauA.......................................................................   8        1     eg1
    ...................uagcauagcaCuguaagagauc.......................................................................   2        1     eg1
    ...................uagcauagcauuguaagUgauc.......................................................................   1        1     eg1
    ...................uagcauagcauuguaagagauU.......................................................................   5        1     eg1
    ...................uagAauagcauuguaagagaucg......................................................................   1        1     eg1
    ...................uagcauagcaCuguaagagaucg......................................................................   4        1     eg1
    ...................uagcauagcGuuguaagagaucg......................................................................   4        1     eg1
    ...................uagcauagcauuguaagagaAcg......................................................................   1        1     eg1
    ...................uagUauagcauuguaagagaucg......................................................................   1        1     eg1
    ...................uaAcauagcauuguaagagaucg......................................................................   3        1     eg1
    ...................uagcGuagcauuguaagagaucg......................................................................   3        1     eg1
    ...................uagcauGgcauuguaagagaucg......................................................................   4        1     eg1
    ...................uagcauagcauuAuaagagaucg......................................................................   1        1     eg1
    ...................uagcauagcauuguaagagaCcg......................................................................   6        1     eg1
    ...................uagcauagcauuguaagagaucA......................................................................   251      1     eg1
    ...................uagcauagcauuguaagagauAg......................................................................   1        1     eg1
    ...................uagcauagcauuguaagagaucg......................................................................   1390     0     eg1
    ...................uagcauagcauuguaagagaucC......................................................................   10       1     eg1
    ...................uagcauagcauuguaGgagaucg......................................................................   6        1     eg1
    ...................uGgcauagcauuguaagagaucg......................................................................   3        1     eg1
```

Mature Star

```
gaccaauuccaauuccugguagcauagcauuguaagagaucguuuuaagcgauccuugcauuacuaugcuaccagguauuguaccgagguuggagguagaaacgaggcaaag
...................uagcauagcauCguaagagaucg......................................................................    3     1     eg1
...................uagcauagcauuguaagGgaucg......................................................................    5     1     eg1
...................uagcauagcauuguaagagauUg......................................................................    2     1     eg1
...................uUgcauagcauuguaagagaucg......................................................................    2     1     eg1
...................uagcauagcauuguGagagaucg......................................................................    4     1     eg1
...................Cagcauagcauuguaagagaucg......................................................................    5     1     eg1
...................uagcauagcauuguaagagaucU......................................................................    45    1     eg1
...................Gagcauagcauuguaagagaucg......................................................................    102   1     eg1
...................uagcaCagcauuguaagagaucg......................................................................    2     1     eg1
...................uagcauagcauuguaUgagaucg......................................................................    1     1     eg1
...................uagcauagcauuguaagUgaucg......................................................................    2     1     eg1
...................uagcauagcauuguaagagGucg......................................................................    4     1     eg1
...................uagcauagcauuguaagagUucg......................................................................    1     1     eg1
...................Gagcauagcauuguaagagaucgu.....................................................................    2     1     eg1
...................uagcauagcauAguaagagaucgu.....................................................................    1     1     eg1
...................uagcauagcauuguaagagaucUu.....................................................................    1     1     eg1
...................uagcauagcauuguaagagaucgG.....................................................................    1     1     eg1
...................uagcauagcauuguaagagaucgC.....................................................................    3     1     eg1
...................uagcauagcauuguaagagaucgu.....................................................................    25    0     eg1
...................uagcauagcauuguaagagaucgA.....................................................................    12    1     eg1
...................uagcauagcauuguaagagaucAuu....................................................................    1     1     eg1
...................uagcauagcauuguaagagaucguu....................................................................    19    0     eg1
...................uagcauagcauuguaagagaucgAu....................................................................    3     1     eg1
...................uagcauagcauuguaagagaucguC....................................................................    5     1     eg1
...................uagcauagcGuuguaagagaucguu....................................................................    1     1     eg1
...................uagcauagcauuguaagagaucguA....................................................................    28    1     eg1
...................uagcauagcauuguaagagaucgAuu...................................................................    2     1     eg1
...................uagcauagcauuguaagagaucguuu...................................................................    5     0     eg1
...................Gagcauagcauuguaagagaucguuu...................................................................    2     1     eg1
...................uagcauagcauuguaagagaucguCu...................................................................    1     1     eg1
...................uagcauagcauuguaagagaucguAu...................................................................    3     1     eg1
...................uagcauagcauuguaagagaucguAuu..................................................................    1     1     eg1
...................Gagcauagcauuguaagagaucguuuu..................................................................    1     1     eg1
...................uagcauagcauuguaagagaucguuuA..................................................................    3     1     eg1
....................agcauagcauuguaagagaucg......................................................................    3     0     eg1

...................uagcauagcauuguaagag..........................................................................    4     0     er1
...................Gagcauagcauuguaagag..........................................................................    1     1     er1
...................uagcauagcauuguaagaga.........................................................................    3     0     er1
...................uagcauagcauuguaagagau........................................................................    28    0     er1
...................Gagcauagcauuguaagagau........................................................................    1     1     er1
...................uagcauagcauuguGagagauc.......................................................................    1     1     er1
...................Cagcauagcauuguaagagauc.......................................................................    1     1     er1
...................uagcauagcauuguaagagauA.......................................................................    7     1     er1
...................uagcauagcauuguaagagGuc.......................................................................    1     1     er1
...................Gagcauagcauuguaagagauc.......................................................................    13    1     er1
...................uagcauagcauuguaagGgauc.......................................................................    1     1     er1
...................uagcauagcauuguaagagaCc.......................................................................    3     1     er1
...................uagcauagcauuguaagagauU.......................................................................    3     1     er1
...................uagcauagcauuguaagagUuc.......................................................................    1     1     er1
...................uagcauGgcauuguaagagauc.......................................................................    1     1     er1
...................uagcGuagcauuguaagagauc.......................................................................    3     1     er1
...................uagcauagcauuguaagagauc.......................................................................    106   0     er1
...................uagcauagcauuguUagagaucg......................................................................    1     1     er1
...................uagcauagcauuguaagagaucU......................................................................    28    1     er1
...................uagcaCagcauuguaagagaucg......................................................................    1     1     er1
...................Cagcauagcauuguaagagaucg......................................................................    1     1     er1
...................uagcGuagcauuguaagagaucg......................................................................    4     1     er1
...................uagcauagcauuguaagagGucg......................................................................    7     1     er1
...................Gagcauagcauuguaagagaucg......................................................................    98    1     er1
...................uagcauagcauuguaagGgaucg......................................................................    4     1     er1
...................uagcauagcauuguaGgagaucg......................................................................    1     1     er1
...................uagcauagcauCguaagagaucg......................................................................    3     1     er1
...................uagcauagcaCuguaagagaucg......................................................................    2     1     er1
...................uagcauagcCuuguaagagaucg......................................................................    1     1     er1
...................uagcauagcauuguaagagaucg......................................................................    879   0     er1
...................uagcauagcauuguGagagaucg......................................................................    3     1     er1
...................uagcauGgcauuguaagagaucg......................................................................    1     1     er1
...................uagcauagcauuguaagagaucC......................................................................    10    1     er1
...................uaAcauagcauuguaagagaucg......................................................................    4     1     er1
```

```
                   Mature                          Star
gaccaauuccaauuccugguagcauagcauuguaagagaucguuuuaagcgauccuugcauuacuaugcuaccagguauuguaccgagguuggagguagaaacgaggcaaag
...................uagcauagcauuguaagagaucA......................................................................   115    1   er1
...................uagcauagcauuAuaagagaucg......................................................................   1      1   er1
...................uagcauagcGuuguaagagaucg......................................................................   3      1   er1
...................uagcauagcauuguaagagauUg......................................................................   1      1   er1
...................uagcauagUauuguaagagaucg......................................................................   2      1   er1
...................uagcauagcauugCaagagaucg......................................................................   1      1   er1
...................uagcauUgcauuguaagagaucg......................................................................   1      1   er1
...................uagcauagcauAguaagagaucg......................................................................   1      1   er1
...................uagcauagcauuguaaAagaucg......................................................................   1      1   er1
...................uagcauagcauuguaagagUucg......................................................................   1      1   er1
...................uagcUuagcauuguaagagaucg......................................................................   1      1   er1
...................uGgcauagcauuguaagagaucg......................................................................   2      1   er1
...................uagcauagcauuguaagagaucgC.....................................................................   3      1   er1
...................Gagcauagcauuguaagagaucgu.....................................................................   2      1   er1
...................uagcauagcauuguaagagaucgu.....................................................................   16     0   er1
...................uagcauagcauuguaagagaucUu.....................................................................   4      1   er1
...................uagcauagcauuguaagagaucgA.....................................................................   6      1   er1
...................uagcauagcauuguaagagaucgAu....................................................................   4      1   er1
...................uagcauagcauuguaagagaucguA....................................................................   9      1   er1
...................uagcauagcauuguaagagaucguC....................................................................   1      1   er1
...................uagcauagcauuguaagagaucguu....................................................................   24     0   er1
...................uagcauagcGuuguaagagaucguu....................................................................   2      1   er1
...................uagcauagcauuguaagagaucgAuu...................................................................   1      1   er1
...................uagcauagcauuguaagagaucguuA...................................................................   3      1   er1
...................uagcauagcauuguaagagaucguAu...................................................................   6      1   er1
...................uagcauagcauuguaagagaucguuu...................................................................   1      0   er1
...................uagcauagcauuguaagagaucguuC...................................................................   1      1   er1
...................uagcauagcauuguaagagaucguuuu..................................................................   1      0   er1
...................uagcauagcauuguaagagaucguuuA..................................................................   1      1   er1

..................Cuagcauagcauuguaagagaucg......................................................................   1      1   er2
...................uagcauagcauuguaagag..........................................................................   3      0   er2
...................uagcauagcauuguaagaga.........................................................................   4      0   er2
...................uagcauagcauuguaagagau........................................................................   37     0   er2
...................uagcauagcauuguaagagauc.......................................................................   445    0   er2
...................uagcauagcauuguaagagauA.......................................................................   133    1   er2
...................Nagcauagcauuguaagagauc.......................................................................   3      1   er2
...................uCgcauagcauuguaagagauc.......................................................................   1      1   er2
...................uagcauagcauuguaagagauG.......................................................................   1      1   er2
...................uagcauagcauuguGagagaucg......................................................................   1      1   er2
...................uagcauagcauuguaagGgaucg......................................................................   1      1   er2
...................uGgcauagcauuguaagagaucg......................................................................   1      1   er2
...................uagcauagcaCuguaagagaucg......................................................................   1      1   er2
...................Nagcauagcauuguaagagaucg......................................................................   7      1   er2
...................uagcGuagcauuguaagagaucg......................................................................   1      1   er2
...................uagcauGgcauuguaagagaucg......................................................................   1      1   er2
...................uagcauagcauuguaagagaGcg......................................................................   1      1   er2
...................uagcauagcauuguaagagaucU......................................................................   1475   1   er2
...................uagcauagcauuguaagagaAcg......................................................................   1      1   er2
...................uagcauagcauuguaagagauAg......................................................................   1      1   er2
...................uagcauagcauuguaGgagaucg......................................................................   2      1   er2
...................uagcauagcauuguaagagaCcg......................................................................   3      1   er2
...................uagcauagcUuuguaagagaucg......................................................................   1      1   er2
...................uagcauagcauuguaagagaucg......................................................................   1810   0   er2
...................uagcauagcauuguaagagaucC......................................................................   6      1   er2
...................uagcauagcauuguaagagaucA......................................................................   403    1   er2
...................uagcauagcGuuguaagagaucg......................................................................   2      1   er2
...................uagcauagcauuguaagagaucAu.....................................................................   14     1   er2
...................uagcauagcauuguaagagaucgA.....................................................................   79     1   er2
...................uagcauagcauuguaagagaucUu.....................................................................   11     1   er2
...................uagcauagcauuguaagagaucgu.....................................................................   4      0   er2
...................uagcauagcauuguaagagaucgG.....................................................................   2      1   er2
...................uagcauagcauuguaagagaucguu....................................................................   5      0   er2
...................uagcauagcauuguaagagaucgAu....................................................................   40     1   er2
...................uagcauagcauuguaagagaucguA....................................................................   17     1   er2
...................uagcauagcauuguaagagaucAuu....................................................................   12     1   er2
...................uagcauagcauuguaagagaucUuu....................................................................   2      1   er2
...................uagcauagcauuguaagagaucguuu...................................................................   2      0   er2
...................uagcauagcauuguaagagaucgAuu...................................................................   13     1   er2
...................uagcauagcauuguaagagaucguuAu..................................................................   3      1   er2
```

```
                   Mature                         Star
gaccaauuccaauuccugguagcauagcauuguaagagaucguuuuaagcgauccuugcauuacuaugcuaccagguauuguaccgagguuggagguagaaacgaggcaaag
....................agcauagcauuguaagaga.........................................................................  3     0  er2
....................agcauagcauuguaagagau........................................................................  3     0  er2
....................agcauagcauuguaagagauA.......................................................................  8     1  er2
....................agcauagcauuguaagagauc.......................................................................  56    0  er2
....................agAauagcauuguaagagauc.......................................................................  1     1  er2
....................agcauagcauuguaagagaucg......................................................................  385   0  er2
....................agcauagcauuguaagagaucC......................................................................  1     1  er2
....................agcauagcauuguaagagaAcg......................................................................  1     1  er2
....................agcGuagcauuguaagagaucg......................................................................  1     1  er2
....................agcauagcauuguaagagaucA......................................................................  30    1  er2
....................agcauagcauuguaagagaucU......................................................................  86    1  er2
....................agcauagAauuguaagagaucg......................................................................  1     1  er2
....................Ngcauagcauuguaagagaucg......................................................................  2     1  er2
....................agcauagcauuguaagagGucg......................................................................  1     1  er2
....................agcauagcauuguaagagaucgA.....................................................................  3     1  er2
....................agcauagcauuguaagagaucAu.....................................................................  4     1  er2
....................agcauagcauuguaagagaucAuu....................................................................  1     1  er2
....................agcauagcauuguaagagaucguA....................................................................  5     1  er2
....................agcauagcauuguaagagaucgAu....................................................................  10    1  er2
....................agcauagcauuguaagagaucguAu...................................................................  1     1  er2
.....................gcauagcauuguaagagauc.......................................................................  3     0  er2
.....................gcauagcauuguaagagaucg......................................................................  16    0  er2
.....................gcauagcauuguaagagaucU......................................................................  7     1  er2
.....................gcauagcauuguaagagaucgAu....................................................................  3     1  er2
...................................................auccuugcauuacuaugcuacc.......................................  4     0  er2
....................................................uccuugcauuacuaugcuacca......................................  3     0  er2
....................................................uccuugcauuacuaugcuaccU......................................  3     1  er2

..................Cuagcauagcauuguaag............................................................................  3     1  ea1
..................Cuagcauagcauuguaagagau........................................................................  3     1  ea1
..................Cuagcauagcauuguaagagauc.......................................................................  3     1  ea1
..................Uuagcauagcauuguaagagaucg......................................................................  1     1  ea1
..................Cuagcauagcauuguaagagaucg......................................................................  11    1  ea1
...................uagcauagcauugCaaga...........................................................................  1     1  ea1
...................uagcauagcauuguaaga...........................................................................  32    0  ea1
...................Gagcauagcauuguaaga...........................................................................  7     1  ea1
...................Gagcauagcauuguaagag..........................................................................  15    1  ea1
...................uagcauagcauuguaagag..........................................................................  90    0  ea1
...................uagcauagcauuguGagag..........................................................................  2     1  ea1
...................uagcauagcauuguaagaA..........................................................................  8     1  ea1
...................Gagcauagcauuguaagaga.........................................................................  3     1  ea1
...................uagcauagcauuguaagaga.........................................................................  24    0  ea1
...................uagcauagcauugCaagagau........................................................................  1     1  ea1
...................uagcauagcauuguaagagaC........................................................................  221   1  ea1
...................uaAcauagcauuguaagagau........................................................................  4     1  ea1
...................uagUauagcauuguaagagau........................................................................  2     1  ea1
...................uagcauagcauCguaagagau........................................................................  4     1  ea1
...................uagcauagcauuguaagagUu........................................................................  5     1  ea1
...................uagcauagcaCuguaagagau........................................................................  8     1  ea1
...................uagcauagcauuguUagagau........................................................................  1     1  ea1
...................uagcauagcauuguaagagau........................................................................  1827  0  ea1
...................uagcauagcauuguaagGgau........................................................................  1     1  ea1
...................uagcauagcauuguaGgagau........................................................................  3     1  ea1
...................uagcauagUauuguaagagau........................................................................  1     1  ea1
...................uagcauagcauuAuaagagau........................................................................  4     1  ea1
...................uagcauagcauuguaUgagau........................................................................  3     1  ea1
...................uagcauagcauAguaagagau........................................................................  1     1  ea1
...................Gagcauagcauuguaagagau........................................................................  237   1  ea1
...................uagcUuagcauuguaagagau........................................................................  4     1  ea1
...................uagcauagcauuguaagagaG........................................................................  36    1  ea1
...................uagcauGgcauuguaagagau........................................................................  7     1  ea1
...................uagcauagcauuguGagagau........................................................................  5     1  ea1
...................Cagcauagcauuguaagagau........................................................................  3     1  ea1
...................uagcauaAcauuguaagagau........................................................................  1     1  ea1
...................Aagcauagcauuguaagagau........................................................................  2     1  ea1
...................uagcauagcauuguaagagaA........................................................................  37    1  ea1
...................uUgcauagcauuguaagagau........................................................................  1     1  ea1
...................uagcaCagcauuguaagagau........................................................................  6     1  ea1
...................uGgcauagcauuguaagagau........................................................................  8     1  ea1
...................uagcauagcGuuguaagagau........................................................................  6     1  ea1
```

```
                  Mature              Star
gaccaauuccaauuccugguagcauagcauuguaagagaucguuuuaagcgauccuugcauuacuaugcuaccagguauuguaccgagguuggagguagaaacgaggcaaag
...................uagcauagcauuguaagagGu........................................................................ 6 1 ea1
...................uagcauagcauGguaagagau........................................................................ 1 1 ea1
...................uagcGuagcauuguaagagau........................................................................ 4 1 ea1
...................uagcauagcaAuguaagagauc....................................................................... 1 1 ea1
...................uagcCuagcauuguaagagauc....................................................................... 2 1 ea1
...................Cagcauagcauuguaagagauc....................................................................... 12 1 ea1
...................uagcauagcauuguaagagGuc....................................................................... 11 1 ea1
...................uagcauagcauGguaagagauc....................................................................... 2 1 ea1
...................Gagcauagcauuguaagagauc....................................................................... 442 1 ea1
...................uagcauagcauuguaagagaCc....................................................................... 6 1 ea1
...................uagcauagcGuuguaagagauc....................................................................... 5 1 ea1
...................uagcauagcauuguaagagaGc....................................................................... 6 1 ea1
...................uagcauagcCuuguaagagauc....................................................................... 2 1 ea1
...................uagcauagcauuguaUgagauc....................................................................... 2 1 ea1
...................uagcauagcauuguaagagUuc....................................................................... 23 1 ea1
...................uagcauagcauuguaCgagauc....................................................................... 3 1 ea1
...................uaAcauagcauuguaagagauc....................................................................... 4 1 ea1
...................uagcauagcauuguaagagauU....................................................................... 267 1 ea1
...................uagcauagcauuguaagagauc....................................................................... 3754 0 ea1
...................uagcauagcauuguGagagauc....................................................................... 3 1 ea1
...................uagcauagcauuguaagGgauc....................................................................... 8 1 ea1
...................uagcauaAcauuguaagagauc....................................................................... 3 1 ea1
...................uagcauagcauAguaagagauc....................................................................... 1 1 ea1
...................uagcauagcauuguaagagauA....................................................................... 248 1 ea1
...................uagcauagcauuguaGgagauc....................................................................... 11 1 ea1
...................uagcauagcauCguaagagauc....................................................................... 9 1 ea1
...................uagcauagcauugGaagagauc....................................................................... 1 1 ea1
...................uagUauagcauuguaagagauc....................................................................... 5 1 ea1
...................uagcauagcauuguaagagaAc....................................................................... 1 1 ea1
...................Aagcauagcauuguaagagauc....................................................................... 8 1 ea1
...................uagcauagcUuuguaagagauc....................................................................... 1 1 ea1
...................uagcauUgcauuguaagagauc....................................................................... 4 1 ea1
...................uagcauagcauuguaaAagauc....................................................................... 4 1 ea1
...................uagcauagcaGuguaagagauc....................................................................... 2 1 ea1
...................uagcauagcauugCaagagauc....................................................................... 4 1 ea1
...................uagcauagcauuguUagagauc....................................................................... 2 1 ea1
...................uagcUuagcauuguaagagauc....................................................................... 6 1 ea1
...................uagcaCagcauuguaagagauc....................................................................... 5 1 ea1
...................uGgcauagcauuguaagagauc....................................................................... 13 1 ea1
...................uagcauagcaCuguaagagauc....................................................................... 12 1 ea1
...................uagcauagcauuguaagCgauc....................................................................... 1 1 ea1
...................uagcauGgcauuguaagagauc....................................................................... 4 1 ea1
...................uagcauagcauuAuaagagauc....................................................................... 3 1 ea1
...................uagcGuagcauuguaagagauc....................................................................... 14 1 ea1
...................uagcauagcauuguaagagauG....................................................................... 2 1 ea1
...................uUgcauagcauuguaagagauc....................................................................... 1 1 ea1
...................uagcauagcauuguaagagauGg...................................................................... 1 1 ea1
...................uagcauagcauuguGagagaucg...................................................................... 67 1 ea1
...................uagcauagcauuguUagagaucg...................................................................... 16 1 ea1
...................uagcauagcauuguCagagaucg...................................................................... 1 1 ea1
...................uagcauagcauugGaagagaucg...................................................................... 3 1 ea1
...................uagUauagcauuguaagagaucg...................................................................... 30 1 ea1
...................uCgcauagcauuguaagagaucg...................................................................... 1 1 ea1
...................uagcauagcaAuguaagagaucg...................................................................... 7 1 ea1
...................uagcauagGauuguaagagaucg...................................................................... 2 1 ea1
...................uagcauagcGuuguaagagaucg...................................................................... 82 1 ea1
...................uagcauagcauuguaagagaucA...................................................................... 4675 1 ea1
...................uagcauagcauuguaagagaucg...................................................................... 30092 0 ea1
...................uagcauagcaCuguaagagaucg...................................................................... 64 1 ea1
...................uagcCuagcauuguaagagaucg...................................................................... 3 1 ea1
...................uagcGuagcauuguaagagaucg...................................................................... 82 1 ea1
...................uagcaAagcauuguaagagaucg...................................................................... 16 1 ea1
...................uagcauagcauuguaUgagaucg...................................................................... 17 1 ea1
...................uagcauagcauuguaaAagaucg...................................................................... 15 1 ea1
...................uagcauagcauuguaagagaAcg...................................................................... 7 1 ea1
...................uagcauagcauuguaagagaCcg...................................................................... 68 1 ea1
...................uagcaCagcauuguaagagaucg...................................................................... 96 1 ea1
...................Gagcauagcauuguaagagaucg...................................................................... 3044 1 ea1
...................uaAcauagcauuguaagagaucg...................................................................... 18 1 ea1
...................uagcauagcauugAaagagaucg...................................................................... 6 1 ea1
```

Mature Star

```
gaccaauuccaauuccugguagcauagcauuguaagagaucguuuuaagcgauccuugcauuacuaugcuaccagguauuguaccgagguuggagguagaaacgaggcaaag
...................uagcauagcauGguaagagaucg......................................................................  3     1  ea1
...................uagcauagcauuguaagagUucg......................................................................  10    1  ea1
...................uagcauagcauuguaagCgaucg......................................................................  6     1  ea1
...................uagcauagcauuguaagaCaucg......................................................................  5     1  ea1
...................uUgcauagcauuguaagagaucg......................................................................  15    1  ea1
...................uagcauagcauuguaagagaucC......................................................................  330   1  ea1
...................uagcauagcauuguaagagGucg......................................................................  96    1  ea1
...................uagcauagcauuguaagaAaucg......................................................................  12    1  ea1
...................uagcauCgcauuguaagagaucg......................................................................  14    1  ea1
...................uaCcauagcauuguaagagaucg......................................................................  1     1  ea1
...................uagcauagcauuguaagUgaucg......................................................................  8     1  ea1
...................uagcauagcauuguaagGgaucg......................................................................  88    1  ea1
...................uagcauagUauuguaagagaucg......................................................................  22    1  ea1
...................uagcauagcauuguaagagaucU......................................................................  1498  1  ea1
...................Aagcauagcauuguaagagaucg......................................................................  29    1  ea1
...................uGgcauagcauuguaagagaucg......................................................................  82    1  ea1
...................Cagcauagcauuguaagagaucg......................................................................  97    1  ea1
...................uagcauagcauuguaGgagaucg......................................................................  93    1  ea1
...................uagcauagcauuguaagagauUg......................................................................  29    1  ea1
...................uagcauUgcauuguaagagaucg......................................................................  9     1  ea1
...................uagcUuagcauuguaagagaucg......................................................................  20    1  ea1
...................uagcauaAcauuguaagagaucg......................................................................  10    1  ea1
...................uagcauagcauuguaagagauAg......................................................................  7     1  ea1
...................uagcauagcauCguaagagaucg......................................................................  85    1  ea1
...................uagcauaCcauuguaagagaucg......................................................................  1     1  ea1
...................uagcauagcauugCaagagaucg......................................................................  58    1  ea1
...................uagAauagcauuguaagagaucg......................................................................  7     1  ea1
...................uagcauagcauuUuaagagaucg......................................................................  3     1  ea1
...................uagcauGgcauuguaagagaucg......................................................................  63    1  ea1
...................uagcauagcauAguaagagaucg......................................................................  11    1  ea1
...................uaUcauagcauuguaagagaucg......................................................................  6     1  ea1
...................uagcauagcauuguaCgagaucg......................................................................  12    1  ea1
...................uagcauagcaGuguaagagaucg......................................................................  3     1  ea1
...................uagGauagcauuguaagagaucg......................................................................  2     1  ea1
...................uagcauaUcauuguaagagaucg......................................................................  6     1  ea1
...................uagcauagcauuguaagaUaucg......................................................................  1     1  ea1
...................uagcauagcUuuguaagagaucg......................................................................  8     1  ea1
...................uagcauagcCuuguaagagaucg......................................................................  3     1  ea1
...................uagcauagcauuguaaUagaucg......................................................................  2     1  ea1
...................uagcauagcauuAuaagagaucg......................................................................  16    1  ea1
...................uagcauagcauuguaagagaGcg......................................................................  4     1  ea1
...................uagcauagcauuCuaagagaucg......................................................................  3     1  ea1
...................uagcauagAauuguaagagaucg......................................................................  1     1  ea1
...................uGgcauagcauuguaagagaucgu.....................................................................  3     1  ea1
...................uagcauagcauuguaagagaucgA.....................................................................  347   1  ea1
...................uagcaCagcauuguaagagaucgu.....................................................................  1     1  ea1
...................uagcauagcauuguaagagaucUu.....................................................................  20    1  ea1
...................uagcauagcauugAaagagaucgu.....................................................................  1     1  ea1
...................uagcauagcauuguaagagaucgu.....................................................................  815   0  ea1
...................uagcUuagcauuguaagagaucgu.....................................................................  1     1  ea1
...................uagcauagcauuguGagagaucgu.....................................................................  4     1  ea1
...................uagcauagcaCuguaagagaucgu.....................................................................  1     1  ea1
...................uagcauagUauuguaagagaucgu.....................................................................  1     1  ea1
...................uagcauagAauuguaagagaucgu.....................................................................  1     1  ea1
...................uagcauagcauuguaagagaucAu.....................................................................  24    1  ea1
...................uagcauagcauuguaagagaucgC.....................................................................  90    1  ea1
...................uagcauagcauuguaUgagaucgu.....................................................................  1     1  ea1
...................uagcauUgcauuguaagagaucgu.....................................................................  1     1  ea1
...................uagcauagcauuguaagagaucgG.....................................................................  25    1  ea1
...................uagcauagcauuguaagagGucgu.....................................................................  6     1  ea1
...................uagcauGgcauuguaagagaucgu.....................................................................  2     1  ea1
...................uaAcauagcauuguaagagaucgu.....................................................................  1     1  ea1
...................uagcauagcauuguaagagaCcgu.....................................................................  1     1  ea1
...................uagcauagcGuuguaagagaucgu.....................................................................  2     1  ea1
...................uCgcauagcauuguaagagaucgu.....................................................................  1     1  ea1
...................Gagcauagcauuguaagagaucgu.....................................................................  112   1  ea1
...................uagcauagcauAguaagagaucgu.....................................................................  1     1  ea1
...................uagcauagcaAuguaagagaucgu.....................................................................  1     1  ea1
...................uagcauagcauCguaagagaucgu.....................................................................  3     1  ea1
...................uagcauagcauuguaGgagaucgu.....................................................................  1     1  ea1
```

```
                   Mature                          Star
gaccaauuccaauuccugguagcauagcauuguaagagaucguuuuaagcgauccuugcauuacuaugcuaccagguauuguaccgagguuggagguagaaacgaggcaaag
...................uagcauagcauuAuaagagaucgu.....................................................................    1      1    ea1
...................Cagcauagcauuguaagagaucgu.....................................................................    3      1    ea1
...................uagcauagcauuguaagGgaucgu.....................................................................    2      1    ea1
...................uagcauagcauuguaagagaucgCu....................................................................    6      1    ea1
...................uagcauagcauuguaagagaucguC....................................................................    74     1    ea1
...................uagcauagcauuguaagGgaucguu....................................................................    3      1    ea1
...................uagcauagcaCuguaagagaucguu....................................................................    2      1    ea1
...................Gagcauagcauuguaagagaucguu....................................................................    47     1    ea1
...................uagcauagcauuguaagagaucguA....................................................................    401    1    ea1
...................Cagcauagcauuguaagagaucguu....................................................................    3      1    ea1
...................uagcauagcauuguGagagaucguu....................................................................    1      1    ea1
...................uagcauagcauuguCagagaucguu....................................................................    1      1    ea1
...................uagcauagcauuguaagagaucguu....................................................................    461    0    ea1
...................uagcCuagcauuguaagagaucguu....................................................................    1      1    ea1
...................uagcauagcauuguaagagaucCuu....................................................................    1      1    ea1
...................uagcauagcUuuguaagagaucguu....................................................................    1      1    ea1
...................uagcauagcauuguaagaAaucguu....................................................................    1      1    ea1
...................uagcauagcauuguaagagGucguu....................................................................    1      1    ea1
...................uagcaCagcauuguaagagaucguu....................................................................    3      1    ea1
...................uagcGuagcauuguaagagaucguu....................................................................    2      1    ea1
...................uagcauagcauuguaagagUucguu....................................................................    1      1    ea1
...................uagcauUgcauuguaagagaucguu....................................................................    1      1    ea1
...................uagcauagcauuguaagagaucAuu....................................................................    10     1    ea1
...................uagcauagcauuguaagagauUguu....................................................................    2      1    ea1
...................uagcauagcauuguaagagaucUuu....................................................................    2      1    ea1
...................uagcauagcauuguaagagaucguG....................................................................    6      1    ea1
...................uagcauagcauuguaagagaucgAu....................................................................    54     1    ea1
...................uagcauagUauuguaagagaucguu....................................................................    1      1    ea1
...................uagcauagcauuguaagagaucguuG...................................................................    3      1    ea1
...................Gagcauagcauuguaagagaucguuu...................................................................    1      1    ea1
...................uagcauagcauuguaagagaucgAuu...................................................................    18     1    ea1
...................uagcauagcauuguaagagaucguCu...................................................................    1      1    ea1
...................uagcauagcauuguaagagaucAuuu...................................................................    3      1    ea1
...................uagcauagcauuguaagagaCcguuu...................................................................    1      1    ea1
...................uagcauagcauuguaagagaucguAu...................................................................    127    1    ea1
...................uagcauagcauuguaagagaucguuC...................................................................    8      1    ea1
...................Cagcauagcauuguaagagaucguuu...................................................................    1      1    ea1
...................uagcauagcauuguaagagaucguuu...................................................................    37     0    ea1
...................uagcauagcauuguaagagaucguuA...................................................................    17     1    ea1
...................uagcauagcauuguaagagaucguuuA..................................................................    5      1    ea1
...................uagcauagcauuguaagagaucguuuu..................................................................    4      0    ea1
...................uagcauagcauuguGagagaucguuuu..................................................................    1      1    ea1
...................uagcauagcauuguaagagaucguAuu..................................................................    14     1    ea1
....................agcauagcauuguaagagGu........................................................................    1      1    ea1
....................agcauagcauuguaagagauc.......................................................................    3      0    ea1
....................agcauagcauuguaagagaucg......................................................................    5      0    ea1
....................agcauagcauuguaagagaucA......................................................................    2      1    ea1
....................Cgcauagcauuguaagagaucg......................................................................    1      1    ea1
.....................gcauagcauuguaagagauc.......................................................................    1      0    ea1
.....................gcauagcauuguaagagaucg......................................................................    5      0    ea1
.....................gcauagcauuguaagagaucA......................................................................    2      1    ea1
......................cauagcauuguaagagaucg......................................................................    1      0    ea1
.......................auagcauuguaagagaucg......................................................................    3      0    ea1
.......................Guagcauuguaagagaucg......................................................................    1      1    ea1
........................uagcauuguaagagaucg......................................................................    2      0    ea1
.............................uuguaagagaucguuuuaagcA.............................................................    1      1    ea1
...................................................auccuugcauuacuaugcuacU.......................................    2      1    ea1

...................uagcauagcauuguaagagauc.......................................................................    2      0    eg2
...................uagcauagcauuguaagagauA.......................................................................    5      1    eg2
...................uagcauagcaAuguaagagaucg......................................................................    1      1    eg2
...................Aagcauagcauuguaagagaucg......................................................................    1      1    eg2
...................Nagcauagcauuguaagagaucg......................................................................    4      1    eg2
...................uagcauagcauuguaagagaucA......................................................................    53     1    eg2
...................uagcGuagcauuguaagagaucg......................................................................    1      1    eg2
...................uagcauagcauuguaGgagaucg......................................................................    1      1    eg2
...................uagcauagcauuguaagagaucg......................................................................    455    0    eg2
...................uagcauagcauuguaagagaucU......................................................................    24     1    eg2
...................uagcauagcauuguaagagauGg......................................................................    1      1    eg2
...................uagcauagcauuguaagagaucAu.....................................................................    2      1    eg2
```

Mature Star

```
gaccaauuccaauuccugguagcauagcauuguaagagaucguuuuaagcgauccuugcauuacuaugcuaccagguauuguaccgagguuggagguagaaacgaggcaaag
....................agcauagcauuguaagagaucg......................................................................    18     0    eg2
....................agcauagcauuguaagagaucA......................................................................    4      1    eg2
.....................gcauagcauuguaagagaucg......................................................................    1      0    eg2
...................................................auccuugcauuacuaugcuaccU......................................    1      1    eg2

...................uagcauagcauuguaagag..........................................................................    3      0    ea2
...................uagcauagcauuguaagaga.........................................................................    9      0    ea2
...................Nagcauagcauuguaagagau........................................................................    1      1    ea2
...................uagcauagcauuguaagagaA........................................................................    1      1    ea2
...................uagcauagcauuguaagagau........................................................................    19     0    ea2
...................uagcGuagcauuguaagagauc.......................................................................    1      1    ea2
...................uagcauagcauuguaagagauA.......................................................................    78     1    ea2
...................uagcauaUcauuguaagagauc.......................................................................    1      1    ea2
...................uagcauagcauGguaagagauc.......................................................................    1      1    ea2
...................uNgcauagcauuguaagagauc.......................................................................    1      1    ea2
...................uagcauagcauuguaagaAauc.......................................................................    1      1    ea2
...................uagcauagcauuguaagagGuc.......................................................................    1      1    ea2
...................uagcauagcauugGaagagauc.......................................................................    1      1    ea2
...................uagcauagcauuguaagagauc.......................................................................    331    0    ea2
...................uagcauagcauuguaagagaucU......................................................................    453    1    ea2
...................uagcauagcauuguaagagaucC......................................................................    1      1    ea2
...................uagcauagGauuguaagagaucg......................................................................    1      1    ea2
...................uagcauagcauuguaagagaucA......................................................................    211    1    ea2
...................Nagcauagcauuguaagagaucg......................................................................    10     1    ea2
...................uagcauagcauGguaagagaucg......................................................................    1      1    ea2
...................uNgcauagcauuguaagagaucg......................................................................    3      1    ea2
...................uagcauagcauuguaaUagaucg......................................................................    1      1    ea2
...................uaAcauagcauuguaagagaucg......................................................................    1      1    ea2
...................uagcauagcauuguaagagauAg......................................................................    3      1    ea2
...................uagcauagcauuguaagGgaucg......................................................................    1      1    ea2
...................uagcauagcauuguaagagaucg......................................................................    1221   0    ea2
...................uagcauagcauuguaagagaucAu.....................................................................    6      1    ea2
...................uagcauagcauuguaagagaucgu.....................................................................    4      0    ea2
...................uagcauagcauuguaagagaucgA.....................................................................    15     1    ea2
...................uagcauagcauuguaagagaucgAu....................................................................    6      1    ea2
...................uagcauagcauuguaagagaucguu....................................................................    10     0    ea2
...................uagcauagcauuguaagagaucguA....................................................................    4      1    ea2
...................uagcauagcauuguaagagaucguG....................................................................    2      1    ea2
...................uagcauagcauuguaagagaucguAu...................................................................    4      1    ea2
...................uagcauagcauuguaagagaucgAuu...................................................................    3      1    ea2
...................uagcauagcauuguaagagaucguAuu..................................................................    1      1    ea2
....................agcauagcauuguaagagauA.......................................................................    6      1    ea2
....................agcauagcauuguaagagauc.......................................................................    72     0    ea2
....................agcauagcauuguaagagaCc.......................................................................    1      1    ea2
....................agcGuagcauuguaagagaucg......................................................................    2      1    ea2
....................agcauagcauuguaagagaAcg......................................................................    1      1    ea2
....................agcauagcauuguaagagaucA......................................................................    19     1    ea2
....................agcauagcauuguaagagaucU......................................................................    25     1    ea2
....................agcauagcauuguaagagaucg......................................................................    362    0    ea2
....................agcauagcauuguaagagaucgA.....................................................................    1      1    ea2
....................agcauagcauuguaagagaucAu.....................................................................    1      1    ea2
....................agcauagcauuguaagagaucgAu....................................................................    3      1    ea2
.....................gcauagcauuguaagagauc.......................................................................    2      0    ea2
.....................gcauagcauuguaagagaucg......................................................................    9      0    ea2
.....................gcauagcauuguaagagaucA......................................................................    2      1    ea2
...................................................auccuugcauuacuaugcuacc.......................................    4      0    ea2
....................................................uccuugcauuacuaugcuaccU......................................    3      1    ea2
```

Provisional ID : ScUbCFx_448_23575
Score total : 21354.3
Score for star read(s) : 3.9
Score for read counts : 21345
Score for mfe : 0.9
Score for randfold : 1.6
Score for cons. seed : 3
Total read count : 41879
Mature read count : 41754
Loop read count : 0
Star read count : 125

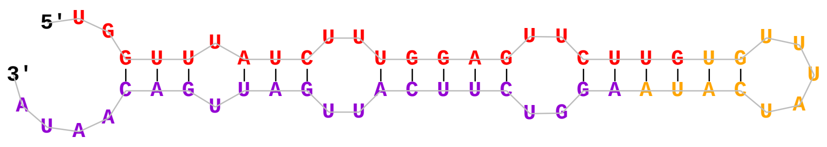

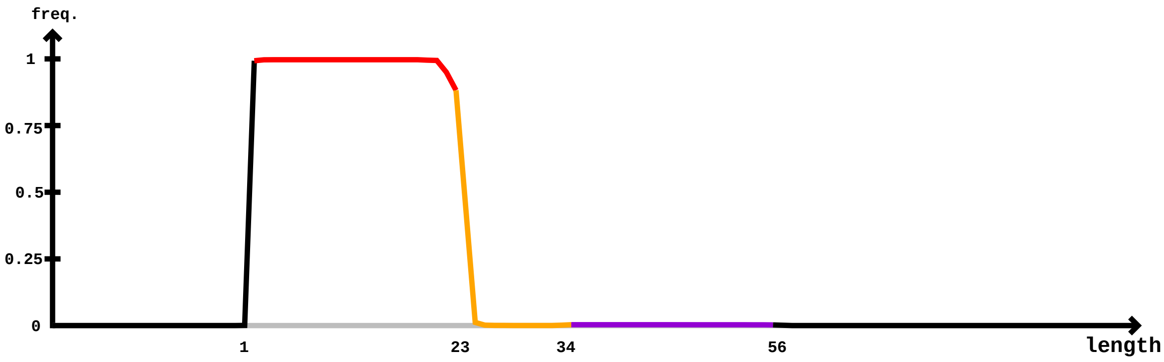

Mature Star

```
5'- gucauuuugauauaccucaugguuuaucuuuggaguucuuguguuuaucauaaggucuucauugauugacaauaagguauauaauggacuuucagauuugacagcuuagaa -3' obs
    gucauuuugauauaccucaugguuuaucuuuggaguucuuguguuuaucauaaggucuucauugauugacaauaagguauauaauggacuuucagauuugacagcuuagaa      exp
    ((((.(((((((((((.((.(((.(((..(((((..((((((.....))))))..)))))..))).))).)).)))))))..........)))))..))))........      reads  mm  sample
    ..................Cugguuuaucuuuggaguucuug......................................................................      1      1   eg2
    ...................ugguuuaucuuuggaguucuu.......................................................................      4      0   eg2
    ...................ugguuuaucuuuggaguucuuA......................................................................      5      1   eg2
    ...................Ngguuuaucuuuggaguucuug......................................................................      1      1   eg2
    ...................ugguuuaucuuuggaguucuuU......................................................................      3      1   eg2
    ...................ugguuuaucuuuggagGucuug......................................................................      1      1   eg2
    ...................ugguuuaucuuuggaguucuug......................................................................      107    0   eg2
    ...................ugguuuaucuuuggaguucuuguA....................................................................      1      1   eg2
    ....................ggGuuaucuuuggaguucuug......................................................................      1      1   eg2
    ....................gguuuaucuuuggaguucuuU......................................................................      2      1   eg2
    ....................gguuuaucuuuggaguucuug......................................................................      3      0   eg2
    ...................................................aaggucuCcauugauugacaau......................................      1      1   eg2
    ....................................................aggucuucauugauugacaaua.....................................      2      0   eg2
    ....................................................aggucuucauugauugacaauU.....................................      1      1   eg2
    ....................................................aggucuucauugauugacaauUa....................................      3      1   eg2

    ...................ugguuuaucuuuggaguucu........................................................................      4      0   ea2
    ...................ugguuuaucuuuggaguucuu.......................................................................      31     0   ea2
    ...................ugguuuaucuuuggaguucuA.......................................................................      3      1   ea2
    ...................ugguuuaucuuuggaguucAu.......................................................................      1      1   ea2
    ...................ugguuuaucuuuggaguucuuA......................................................................      70     1   ea2
    ...................ugguCuaucuuuggaguucuug......................................................................      4      1   ea2
    ...................Ngguuuaucuuuggaguucuug......................................................................      3      1   ea2
    ...................ugguuuaucuuuggaguucuuU......................................................................      8      1   ea2
    ...................ugguuuaucuuuggGguucuug......................................................................      1      1   ea2
    ...................ugguuuaucuuuggaguucuug......................................................................      466    0   ea2
    ...................uAguuuaucuuuggaguucuug......................................................................      2      1   ea2
    ...................ugguuuaucuuuggaguucuuAu.....................................................................      1      1   ea2
    ...................ugguuuaucuuuggaguucuugA.....................................................................      1      1   ea2
    ...................ugguuuaucuuuggaguucuuUu.....................................................................      2      1   ea2
    ...................ugguuuaucuuuggaguucuuguA....................................................................      12     1   ea2
    ...................ugguuuaucuuuggaguucuugug....................................................................      3      0   ea2
    ....................gguuuaucuuuggaguucuu.......................................................................      1      0   ea2
```

Mature   Star

```
gucauuuugauauaccucaugguuuaucuuuggaguucuuguguuuaucauaaggucuucauugauugacaauaagguauauaauggacuuucagauuugacagcuuagaa
....................gguuuaucuuuggaguucuuA......................................................................   8     1   ea2
....................gguuuaucuuuggaguucuug......................................................................   38    0   ea2
....................gguuuaucuuuggaguucuuU......................................................................   3     1   ea2
.....................guuuaucuuuggaguucuuU......................................................................   7     1   ea2
...........................................uuuaucauaaggucuucauuga..............................................   5     0   ea2
............................................uuaucauaaggucuucauuga..............................................   1     0   ea2
...................................................aaggucuucauugauugacaau......................................   2     0   ea2
...................................................aaggucuucauugauugacaauG.....................................   1     1   ea2
...................................................aaggucuucauugauugacaauaa....................................   2     0   ea2
....................................................aggucuucauugauugaca........................................   5     0   ea2
....................................................aggucuucauugauugacaau......................................   2     0   ea2
....................................................aggucuucauugauugacaauU.....................................   7     1   ea2
....................................................aggucuucauugauugacaaua.....................................   5     0   ea2

...................ugguuuaucuuuggaguucu........................................................................   7     0   er2
...................ugguuuaucuuuggaguucuu.......................................................................   46    0   er2
...................ugguuuaucuuuggaguucuA.......................................................................   5     1   er2
...................uggGuuaucuuuggaguucuug......................................................................   2     1   er2
...................ugguuuaucuuuggaguucuug......................................................................   768   0   er2
...................ugguCuaucuuuggaguucuug......................................................................   1     1   er2
...................Ngguuuaucuuuggaguucuug......................................................................   2     1   er2
...................ugguuuaucuuuggagCucuug......................................................................   1     1   er2
...................Cgguuuaucuuuggaguucuug......................................................................   1     1   er2
...................ugguuuaucuuuggGguucuug......................................................................   1     1   er2
...................ugguuuaucuuuggaguucuuU......................................................................   31    1   er2
...................ugguuuaucuuuggaguucuuA......................................................................   118   1   er2
...................ugguuuaucuuuggaguCcuug......................................................................   1     1   er2
...................ugguAuaucuuuggaguucuug......................................................................   1     1   er2
...................ugguuuaucuuuggaguucuugu.....................................................................   16    0   er2
...................ugguuuaucuuuggaguucuuAu.....................................................................   9     1   er2
...................ugguuuauUuuuggaguucuugu.....................................................................   2     1   er2
...................ugguuuaucuuuggaguucuuUu.....................................................................   3     1   er2
...................ugguuuaucuuuggaguucuuguA....................................................................   8     1   er2
....................gguuuaucuuuggaguucuu.......................................................................   3     0   er2
....................gguuuaucuuuggaguucuuA......................................................................   6     1   er2
....................Uguuuaucuuuggaguucuug......................................................................   1     1   er2
....................gguuuaucuuuggaguucuug......................................................................   48    0   er2
....................gguuuaucuuuggaguucuuUu.....................................................................   1     1   er2
....................gguuuaucuuuggaguucuugu.....................................................................   1     0   er2
...................................................aaggucuucauugauugacaau......................................   7     0   er2
...................................................aaggucuucauugauugacaauU.....................................   3     1   er2
....................................................aggucuucauugauugacaauU.....................................   10    1   er2
....................................................aggucuucauugauugacaaua.....................................   7     0   er2
....................................................aggucuucauugauugacaauUa....................................   8     1   er2

..................Cugguuuaucuuuggaguucuu.......................................................................   2     1   ea1
..................Cugguuuaucuuuggaguucuug......................................................................   13    1   ea1
...................Ggguuuaucuuuggaguu..........................................................................   8     1   ea1
...................ugguuuaucuuuggaguu..........................................................................   34    0   ea1
...................ugguuuaucuuuggaguA..........................................................................   1     1   ea1
...................uggCuuaucuuuggaguu..........................................................................   1     1   ea1
...................ugguuuaucuuuggaguC..........................................................................   3     1   ea1
...................ugguuuaucuuuggaguuc.........................................................................   27    0   ea1
...................ugguuuaucuuuggaguuA.........................................................................   1     1   ea1
...................ugguuuGucuuuggaguuc.........................................................................   1     1   ea1
...................ugguuuaucuuuggaguuU.........................................................................   2     1   ea1
...................ugguuuaucuuuggagGuc.........................................................................   1     1   ea1
...................Ggguuuaucuuuggaguuc.........................................................................   10    1   ea1
...................Ggguuuaucuuuggaguucu........................................................................   152   1   ea1
...................ugguuuaucuAuggaguucu........................................................................   2     1   ea1
...................Agguuuaucuuuggaguucu........................................................................   1     1   ea1
...................ugguuuaucuuuggaguucu........................................................................   1028  0   ea1
...................ugguuuaucuuuggaguucC........................................................................   126   1   ea1
...................ugguuuaucuuuggaguucA........................................................................   73    1   ea1
...................ugguuuaucuuuggGguucu........................................................................   5     1   ea1
...................ugguCuaucuuuggaguucu........................................................................   3     1   ea1
...................ugguuuaucuuuggaguucG........................................................................   17    1   ea1
...................ugguuuaucuCuggaguucu........................................................................   3     1   ea1
...................ugguuCaucuuuggaguucu........................................................................   1     1   ea1
...................Cgguuuaucuuuggaguucu........................................................................   3     1   ea1
```

```
                   Mature                          Star
gucauuuugauauaccucaugguuuaucuuuggaguucuuguguuuaucauaaggucuucauugauugacaauaagguauauaaggacuuucagauuugacagcuuagaa
...................ugguAuaucuuuggaguucu.......................................................................   1     1   ea1
...................ugguuuaucuuCggaguucu.......................................................................   3     1   ea1
...................uggGuuaucuuuggaguucu.......................................................................   1     1   ea1
...................uggCuuaucuuuggaguucu.......................................................................   5     1   ea1
...................ugguuuaucuuAggaguucu.......................................................................   2     1   ea1
...................ugguuuaucuuuggaguCcu.......................................................................   2     1   ea1
...................ugguuuaucCuuggaguucu.......................................................................   2     1   ea1
...................ugguuuaucuuuggagCucu.......................................................................   3     1   ea1
...................ugguuuaCcuuuggaguucu.......................................................................   3     1   ea1
...................ugguuuaucuuuAgaguucu.......................................................................   3     1   ea1
...................uggGuuaucuuuggaguucuu......................................................................   3     1   ea1
...................ugguuuaucCuuggaguucuu......................................................................   6     1   ea1
...................Ggguuuaucuuuggaguucuu......................................................................   240   1   ea1
...................ugguuuaucuuuggaguucGu......................................................................   1     1   ea1
...................ugguuuaCcuuuggaguucuu......................................................................   6     1   ea1
...................ugguuuaucuuuggaguucAu......................................................................   1     1   ea1
...................ugguuuaucuuuggaguucuC......................................................................   147   1   ea1
...................ugguuuCucuuuggaguucuu......................................................................   1     1   ea1
...................ugguuuaucuuuggaguGcuu......................................................................   1     1   ea1
...................ugguCuaucuuuggaguucuu......................................................................   5     1   ea1
...................uggCuuaucuuuggaguucuu......................................................................   7     1   ea1
...................uAguuuaucuuuggaguucuu......................................................................   2     1   ea1
...................ugguAuaucuuuggaguucuu......................................................................   1     1   ea1
...................ugguuuaucuuuggaguucCu......................................................................   4     1   ea1
...................ugguuuaucuuuggaguucuG......................................................................   10    1   ea1
...................ugguuuGucuuuggaguucuu......................................................................   1     1   ea1
...................uCguuuaucuuuggaguucuu......................................................................   1     1   ea1
...................ugguuuauUuuuggaguucuu......................................................................   1     1   ea1
...................ugguuuaucuuuggaguucuA......................................................................   38    1   ea1
...................ugguuuaucuuuggGguucuu......................................................................   6     1   ea1
...................ugguuuaucuCuggaguucuu......................................................................   3     1   ea1
...................ugguuuaucuuCggaguucuu......................................................................   4     1   ea1
...................ugguuuaucAuuggaguucuu......................................................................   1     1   ea1
...................ugguuuaucuuuggagCucuu......................................................................   1     1   ea1
...................uUguuuaucuuuggaguucuu......................................................................   3     1   ea1
...................ugguuuaucuuuAgaguucuu......................................................................   1     1   ea1
...................ugguuuaucuuuggaguCcuu......................................................................   4     1   ea1
...................ugguuuaucuAuggaguucuu......................................................................   6     1   ea1
...................ugguuuaucuuugAaguucuu......................................................................   1     1   ea1
...................Cgguuuaucuuuggaguucuu......................................................................   3     1   ea1
...................ugguuuaucuuuggaguucuu......................................................................   1635  0   ea1
...................uggAuuaucuuuggaguucuu......................................................................   1     1   ea1
...................ugguuuaucuuuggaguuUuu......................................................................   2     1   ea1
...................ugguuuaucuuAggaguucuu......................................................................   1     1   ea1
...................ugguuCaucuuuggaguucuu......................................................................   4     1   ea1
...................ugguuuaucuuuggagAucuu......................................................................   1     1   ea1
...................ugguuuGucuuuggaguucuug.....................................................................   21    1   ea1
...................ugguuuaucuuuggaUuucuug.....................................................................   5     1   ea1
...................ugguuuaucuuuggagAucuug.....................................................................   4     1   ea1
...................ugguuuauAuuuggaguucuug.....................................................................   2     1   ea1
...................ugguuCaucuuuggaguucuug.....................................................................   41    1   ea1
...................ugguuuaucuuuggGguucuug.....................................................................   47    1   ea1
...................ugguuuaucuuuggaCuucuug.....................................................................   2     1   ea1
...................uggGuuaucuuuggaguucuug.....................................................................   9     1   ea1
...................uUguuuaucuuuggaguucuug.....................................................................   5     1   ea1
...................ugguCuaucuuuggaguucuug.....................................................................   57    1   ea1
...................ugguuuaucuuAggaguucuug.....................................................................   13    1   ea1
...................ugguuGaucuuuggaguucuug.....................................................................   4     1   ea1
...................ugguGuaucuuuggaguucuug.....................................................................   1     1   ea1
...................ugUuuuaucuuuggaguucuug.....................................................................   5     1   ea1
...................ugguuuaucuuuggaguCcuug.....................................................................   65    1   ea1
...................ugguuuaucuuGggaguucuug.....................................................................   8     1   ea1
...................ugguuuaucuuuggaguuUuug.....................................................................   8     1   ea1
...................ugguAuaucuuuggaguucuug.....................................................................   8     1   ea1
...................ugguuuaucuuCggaguucuug.....................................................................   71    1   ea1
...................ugguuuaucuuuggaguucuCg.....................................................................   51    1   ea1
...................ugguuuaucuuuggaguAcuug.....................................................................   5     1   ea1
...................ugguuuaucuuuAgaguucuug.....................................................................   12    1   ea1
...................ugguuuauGuuuggaguucuug.....................................................................   1     1   ea1
...................ugguuuaucuuuggaguucuAg.....................................................................   3     1   ea1
```

Mature Star

```
gucauuuugauauaccucaugguuuaucuuuggaguucuuguguuuaucauaaggucuucauugauugacaauaagguauauaaauggacuuucagauuugacagcuuagaa
...................ugguuuaucuuuggaguucuuU.......................................................................  551    1    ea1
...................ugguuuaGcuuuggaguucuug.......................................................................  1      1    ea1
...................ugguuuaucuuuggCguucuug.......................................................................  2      1    ea1
...................ugguuuauUuuuggaguucuug.......................................................................  8      1    ea1
...................ugguuuaucuAuggaguucuug.......................................................................  7      1    ea1
...................ugguuuaucuuuggaguucuuA.......................................................................  2962   1    ea1
...................ugguuuaucuuuggaAuucuug.......................................................................  8      1    ea1
...................Ggguuuaucuuuggaguucuug.......................................................................  2914   1    ea1
...................ugguuuaucuuuggaguGcuug.......................................................................  2      1    ea1
...................ugguuuaucuuuggaguuGuug.......................................................................  3      1    ea1
...................ugguuuaucCuuggaguucuug.......................................................................  52     1    ea1
...................ugguuuaucuuuggaguucuGg.......................................................................  5      1    ea1
...................ugguuuaCcuuuggaguucuug.......................................................................  40     1    ea1
...................ugguuuaucuuuggagCucuug.......................................................................  55     1    ea1
...................ugguuuaucuuuggUguucuug.......................................................................  11     1    ea1
...................Agguuuaucuuuggaguucuug.......................................................................  17     1    ea1
...................uggAuuaucuuuggaguucuug.......................................................................  5      1    ea1
...................ugguuuaucuuuggaguucGug.......................................................................  3      1    ea1
...................ugguuuaucAuuggaguucuug.......................................................................  10     1    ea1
...................ugguuuaucuuuggaguuAuug.......................................................................  3      1    ea1
...................ugguuuaucuuuggaguucAug.......................................................................  6      1    ea1
...................ugguuuaucuuugUaguucuug.......................................................................  1      1    ea1
...................Cgguuuaucuuuggaguucuug.......................................................................  42     1    ea1
...................uAguuuaucuuuggaguucuug.......................................................................  10     1    ea1
...................ugguuuaucuuuggaguucuug.......................................................................  18752  0    ea1
...................ugguuuaucuuugAaguucuug.......................................................................  8      1    ea1
...................ugguuuaucuuuggaguucCug.......................................................................  31     1    ea1
...................uggCuuaucuuuggaguucuug.......................................................................  74     1    ea1
...................ugguuuaucuuUgaguucuug.......................................................................  5      1    ea1
...................ugguuAaucuuuggaguucuug.......................................................................  4      1    ea1
...................ugguuuaucuuuggaguucuuC.......................................................................  78     1    ea1
...................ugguuuaucuCuggaguucuug.......................................................................  39     1    ea1
...................ugAuuuaucuuuggaguucuug.......................................................................  3      1    ea1
...................ugguuuaucuuuggaguucuugu......................................................................  331    0    ea1
...................ugguuCaucuuuggaguucuugu......................................................................  1      1    ea1
...................ugguuuaCcuuuggaguucuugu......................................................................  2      1    ea1
...................ugguuuaucuuuggaUuucuugu......................................................................  1      1    ea1
...................ugguAuaucuuuggaguucuugu......................................................................  1      1    ea1
...................ugguuuaucuuuggaguucuugC......................................................................  39     1    ea1
...................ugUuuuaucuuuggaguucuugu......................................................................  2      1    ea1
...................Ggguuuaucuuuggaguucuugu......................................................................  55     1    ea1
...................ugguuuaucCuuggaguucuugu......................................................................  2      1    ea1
...................ugguCuaucuuuggaguucuugu......................................................................  1      1    ea1
...................uggAuuaucuuuggaguucuugu......................................................................  3      1    ea1
...................ugguuuaucuuuggaguucuuUu......................................................................  49     1    ea1
...................ugguuuaucuuuggaguucCugu......................................................................  1      1    ea1
...................uggCuuaucuuuggaguucuugu......................................................................  2      1    ea1
...................ugguuuaucuuGggaguucuugu......................................................................  1      1    ea1
...................ugguuuaucuuuggaguucuuCu......................................................................  2      1    ea1
...................ugguuuaucuuuggaguucuugG......................................................................  7      1    ea1
...................uCguuuaucuuuggaguucuugu......................................................................  1      1    ea1
...................ugguuuaucuuuggGguucuugu......................................................................  2      1    ea1
...................ugguuuaucuuuggaguucuuAu......................................................................  52     1    ea1
...................ugguuuaucuuuggaguucuugA......................................................................  64     1    ea1
...................ugguuuaucuuuggaAuucuugu......................................................................  1      1    ea1
...................ugguuuaucuuuggaguucuuguC.....................................................................  8      1    ea1
...................ugguuuaucuuuggaguucuuAug.....................................................................  1      1    ea1
...................Ggguuuaucuuuggaguucuugug.....................................................................  1      1    ea1
...................ugguuuaucuuuggaguucuuguU.....................................................................  24     1    ea1
...................ugguuuaucuuuggaguucuugug.....................................................................  14     0    ea1
...................ugguuuaucuuuggaguucuuguA.....................................................................  272    1    ea1
...................ugguuuaucuuuggaguucuugugu....................................................................  2      0    ea1
...................ugguuuaucuuuggaguucuugugG....................................................................  1      1    ea1
...................ugguuuaucuuuggaguucuuguUu....................................................................  5      1    ea1
...................ugguuuaucuuuggaguucuuguAu....................................................................  20     1    ea1
...................Ggguuuaucuuuggaguucuugugu....................................................................  1      1    ea1
...................ugguuuaucuuuggaguucuuguguu...................................................................  2      0    ea1
...................ugguuuaucuuuggaguucuuguAuu...................................................................  2      1    ea1
...................ugguuuaucuuuggaguucuuguguuu..................................................................  3      0    ea1
...................Ggguuuaucuuuggaguucuuguguuu..................................................................  1      1    ea1
```

Mature **Star**

```
gucauuuugauauaccucaugguuuaucuuuggaguucuuguguuuaucauaaggucuucauugauugacaauaagguauauaauggacuuucagauuugacagcuuagaa
...................ugguuuaCcuuuggaguucuuguguuu.................................................................    1    1    ea1
....................ggGuuaucuuuggaguucuug......................................................................    2    1    ea1
....................gguuuaucuuuggaguucuug......................................................................    14   0    ea1
....................Uguuuaucuuuggaguucuug......................................................................    1    1    ea1
....................gguuuaucuuuggaguucuuA......................................................................    7    1    ea1
.....................guuuaucuuuggaguucuug......................................................................    3    0    ea1
.....................guuuaucuuuggaguucuugug....................................................................    2    0    ea1
.....................Auuuaucuuuggaguucuugug....................................................................    1    1    ea1
.............................uuggaguucuuguguuuauca.............................................................    1    0    ea1
.........................................uguuuaucauaaggucuucauA................................................    1    1    ea1
...................................................aaggucuucauugauugaca........................................    1    0    ea1
...................................................aaggucuucauugauugacaau......................................    1    0    ea1
...................................................aaggucuucauugauugacaaua.....................................    9    0    ea1
...................................................aaggucuucauugauugacaauU.....................................    6    1    ea1
...................................................Gaggucuucauugauugacaaua.....................................    1    1    ea1
...................................................aaggucuucauugauugacaauUa....................................    7    1    ea1
....................................................aggucuucauugauugacaau......................................    1    0    ea1
....................................................aggucuucauugauugacaauU.....................................    1    1    ea1
....................................................aggucuucauugauugacaauC.....................................    1    1    ea1
....................................................aggucuucauugauugacaaua.....................................    3    0    ea1
....................................................aggucuucauugauugacaauUa....................................    4    1    ea1

..................Cugguuuaucuuuggaguucuug......................................................................    2    1    er1
...................ugguuuaucuuuggaguu..........................................................................    3    0    er1
...................ugguuuaucuuuggaguC..........................................................................    2    1    er1
...................ugguuuaucCuuggaguuc.........................................................................    1    1    er1
...................ugguuuaucuuuggaguuc.........................................................................    4    0    er1
...................ugguuuaucuuuggaguucC........................................................................    12   1    er1
...................ugguuuaucGuuggaguucu........................................................................    1    1    er1
...................ugguuuaucuuuggaguucA........................................................................    1    1    er1
...................Ggguuuaucuuuggaguucu........................................................................    19   1    er1
...................ugguuuaucuuuggaguucu........................................................................    109  0    er1
...................ugAuuuaucuuuggaguucuu.......................................................................    2    1    er1
...................ugguCuaucuuuggaguucuu.......................................................................    3    1    er1
...................ugguuuaCcuuuggaguucuu.......................................................................    1    1    er1
...................ugguuuaucuuuggGguucuu.......................................................................    1    1    er1
...................Ggguuuaucuuuggaguucuu.......................................................................    35   1    er1
...................ugguuuaucuuuggaguucuC.......................................................................    9    1    er1
...................ugguuuaucuuuggaAuucuu.......................................................................    1    1    er1
...................ugguAuaucuuuggaguucuu.......................................................................    1    1    er1
...................ugguuuaucuuuggaguucuG.......................................................................    1    1    er1
...................ugguuuaucuuuAgaguucuu.......................................................................    1    1    er1
...................ugguuuaucuuuggaguucuu.......................................................................    166  0    er1
...................ugguuuaucuuuggaguuUuug......................................................................    1    1    er1
...................ugguuuaucuuuggagGucuug......................................................................    3    1    er1
...................ugguuuaucuCuggaguucuug......................................................................    4    1    er1
...................ugguuuaGcuuuggaguucuug......................................................................    1    1    er1
...................ugguuuaucuuuggaguucuGg......................................................................    1    1    er1
...................ugguuuaucuuuggGguucuug......................................................................    11   1    er1
...................ugguuuaucuuuggaguucuuA......................................................................    261  1    er1
...................ugguuuaucuuuggaguucAug......................................................................    2    1    er1
...................ugguuuaucuuuggCguucuug......................................................................    1    1    er1
...................ugguuuaucuuuggaAuucuug......................................................................    3    1    er1
...................ugguuuaucuuCggaguucuug......................................................................    7    1    er1
...................ugguGuaucuuuggaguucuug......................................................................    1    1    er1
...................uggCuuaucuuuggaguucuug......................................................................    5    1    er1
...................Ggguuuaucuuuggaguucuug......................................................................    400  1    er1
...................ugUuuuaucuuuggaguucuug......................................................................    2    1    er1
...................ugguuuauUuuuggaguucuug......................................................................    3    1    er1
...................ugguuuaucCuuggaguucuug......................................................................    8    1    er1
...................ugguuuaucuuuggaguucuuU......................................................................    73   1    er1
...................ugguuAaucuuuggaguucuug......................................................................    1    1    er1
...................ugguuuaucuuuggaguucuuC......................................................................    18   1    er1
...................ugguuuaucuuuggaguucuCg......................................................................    4    1    er1
...................ugAuuuaucuuuggaguucuug......................................................................    1    1    er1
...................ugguuuaCcuuuggaguucuug......................................................................    2    1    er1
...................ugguuuaucuuuggUguucuug......................................................................    4    1    er1
...................Cgguuuaucuuuggaguucuug......................................................................    3    1    er1
...................ugguuuaucuuuggaguucCug......................................................................    1    1    er1
...................ugguuuaucuuuggaguCcuug......................................................................    11   1    er1
```

```
                  Mature                Star
gucauuuugauauaccucaugguuuaucuuuggaguucuuguguuuaucauaaggucuucauugauugacaauaagguauauaauggacuuucagauuugacagcuuagaa
...................ugguuCaucuuuggaguucuug......................................................................   3     1   er1
...................ugguuuaucuuugAaguucuug......................................................................   1     1   er1
...................uggAuuaucuuuggaguucuug......................................................................   2     1   er1
...................ugguuuaucAuuggaguucuug......................................................................   2     1   er1
...................ugguuuaucuuugUaguucuug......................................................................   1     1   er1
...................ugguuuGucuuuggaguucuug......................................................................   5     1   er1
...................ugguCuaucuuuggaguucuug......................................................................   12    1   er1
...................uggGuuaucuuuggaguucuug......................................................................   2     1   er1
...................Agguuuaucuuuggaguucuug......................................................................   2     1   er1
...................ugguuuaucuuuggaguucuug......................................................................   2379  0   er1
...................ugguuuaucuuuggaguGcuug......................................................................   1     1   er1
...................ugguuuaucuuuggagCucuug......................................................................   1     1   er1
...................ugguuuaucuuuggaguAcuug......................................................................   1     1   er1
...................ugguuuaucuuuggaguucuuUu.....................................................................   7     1   er1
...................ugguuuaucuuuggaguucuuAu.....................................................................   3     1   er1
...................Cgguuuaucuuuggaguucuugu.....................................................................   1     1   er1
...................ugguuuaucuuuggaguucuugC.....................................................................   8     1   er1
...................ugguCuaucuuuggaguucuugu.....................................................................   1     1   er1
...................ugguuuaucuuuggagCucuugu.....................................................................   1     1   er1
...................ugguuuaucuuuggaguucuugA.....................................................................   11    1   er1
...................ugguuuaucuuuggaguCcuugu.....................................................................   2     1   er1
...................Ggguuuaucuuuggaguucuugu.....................................................................   8     1   er1
...................ugguuuaucuuuggaguucuugu.....................................................................   54    0   er1
...................ugguuuaucuuuggaguucuugug....................................................................   1     0   er1
...................ugguuuaucuuuggaguucuuguU....................................................................   4     1   er1
...................ugguuuaucuuuggaguucuuguA....................................................................   29    1   er1
...................Ggguuuaucuuuggaguucuugug....................................................................   1     1   er1
...................ugguuuaucuuuggaguucuuguAu...................................................................   4     1   er1
...................ugguuuaucuuuggaguucuuguguuu.................................................................   3     0   er1
...................Ggguuuaucuuuggaguucuuguguuu.................................................................   1     1   er1
....................gguuuaucuuuggaguucuug......................................................................   3     0   er1
........................................aaggucuucauugauugacaauU................................................   2     1   er1
........................................aaggucuucauugauugacaaua................................................   2     0   er1
........................................aaggucuucauugauugacaauUa...............................................   1     1   er1
.........................................aggucuucauugauugacaauU................................................   1     1   er1
.........................................aggucuucauugauugacaaua................................................   1     0   er1
.........................................aggucuucauugauugacaauUa...............................................   3     1   er1

..................Cugguuuaucuuuggaguucuug......................................................................   2     1   eg1
...................ugguuuaucuuuggaguG..........................................................................   1     1   eg1
...................ugguuuaucuuuggaguC..........................................................................   1     1   eg1
...................ugguuuaucuuuggaguu..........................................................................   9     0   eg1
...................Ggguuuaucuuuggaguu..........................................................................   3     1   eg1
...................ugguuuaucuuuggaguuc.........................................................................   1     0   eg1
...................ugguuuaucuuuggaguuU.........................................................................   1     1   eg1
...................ugguuuaucuuCggaguucu........................................................................   1     1   eg1
...................ugguuuaucuuuggaguucA........................................................................   10    1   eg1
...................ugguuuaucuuuggagCucu........................................................................   1     1   eg1
...................ugguuuaucuuuggaguuUu........................................................................   1     1   eg1
...................Ggguuuaucuuuggaguucu........................................................................   17    1   eg1
...................ugguuuaucuuuggaguucC........................................................................   21    1   eg1
...................ugguuuaucuuuggaguucu........................................................................   190   0   eg1
...................ugguuuaucCuuggaguucu........................................................................   3     1   eg1
...................uggCuuaucuuuggaguucu........................................................................   1     1   eg1
...................ugguuuaucuuuggGguucu........................................................................   2     1   eg1
...................Cgguuuaucuuuggaguucu........................................................................   1     1   eg1
...................ugguuuaucuuuggaguucG........................................................................   18    1   eg1
...................ugguuuaucAuuggaguucuu.......................................................................   1     1   eg1
...................uAguuuaucuuuggaguucuu.......................................................................   1     1   eg1
...................Agguuuaucuuuggaguucuu.......................................................................   1     1   eg1
...................ugguuuaucuuuggaguCcuu.......................................................................   2     1   eg1
...................ugguuuaucuuuggaguucuu.......................................................................   248   0   eg1
...................ugguuuaucuCuggaguucuu.......................................................................   1     1   eg1
...................uggCuuaucuuuggaguucuu.......................................................................   3     1   eg1
...................ugguuuaucuuuggaguucuG.......................................................................   6     1   eg1
...................ugguuuauUuuuggaguucuu.......................................................................   1     1   eg1
...................ugguuuaucuuuggaguucuC.......................................................................   32    1   eg1
...................ugguuuGucuuuggaguucuu.......................................................................   1     1   eg1
...................ugguuuaucuuuggGguucuu.......................................................................   1     1   eg1
...................ugguuuaucuuuggaguucuA.......................................................................   5     1   eg1
```

```
                   Mature                          Star
gucauuuugauauaccucaugguuuaucuuuggaguucuuguguuuaucauaaggucuucauugauugacaauaagguauauaaauggacuuucagauuugacagcuuagaa
...................ugguuuaucuuuggaguuUuu........................................................................  1     1  eg1
...................Ggguuuaucuuuggaguucuu........................................................................  55    1  eg1
...................ugguCuaucuuuggaguucuu........................................................................  1     1  eg1
...................ugguuuaucuuuggUguucuug.......................................................................  2     1  eg1
...................ugguuuaucuuuggagCucuug.......................................................................  7     1  eg1
...................ugguuuaucCuuggaguucuug.......................................................................  16    1  eg1
...................ugguuuaucuuuggaguucuuA.......................................................................  370   1  eg1
...................ugguuCaucuuuggaguucuug.......................................................................  4     1  eg1
...................ugguuuaucuuuggCguucuug.......................................................................  1     1  eg1
...................ugUuuuaucuuuggaguucuug.......................................................................  1     1  eg1
...................Agguuuaucuuuggaguucuug.......................................................................  2     1  eg1
...................ugguuuaucuCuggaguucuug.......................................................................  1     1  eg1
...................ugguuuaucuuuggaguucuuC.......................................................................  9     1  eg1
...................Cgguuuaucuuuggaguucuug.......................................................................  9     1  eg1
...................ugguuuaCcuuuggaguucuug.......................................................................  9     1  eg1
...................Ggguuuaucuuuggaguucuug.......................................................................  535   1  eg1
...................ugguuuaucuuuggaguucCug.......................................................................  6     1  eg1
...................ugguCuaucuuuggaguucuug.......................................................................  7     1  eg1
...................ugguuuaucuuuggaguucuuU.......................................................................  113   1  eg1
...................ugguuuaucuuuggaguuUuug.......................................................................  3     1  eg1
...................ugguuuaucuuuggaguucuCg.......................................................................  40    1  eg1
...................ugguuuaucuuuggaguucuAg.......................................................................  1     1  eg1
...................uggCuuaucuuuggaguucuug.......................................................................  8     1  eg1
...................uAguuuaucuuuggaguucuug.......................................................................  1     1  eg1
...................ugguuuaucuuuggaguCcuug.......................................................................  10    1  eg1
...................ugguuuaucuuAggaguucuug.......................................................................  2     1  eg1
...................ugguuuaucuuGggaguucuug.......................................................................  1     1  eg1
...................ugguuuaucuuuggGguucuug.......................................................................  9     1  eg1
...................ugguuuGucuuuggaguucuug.......................................................................  5     1  eg1
...................ugguuuauUuuuggaguucuug.......................................................................  1     1  eg1
...................ugguuuaucuAuggaguucuug.......................................................................  1     1  eg1
...................ugguuuaAcuuuggaguucuug.......................................................................  2     1  eg1
...................ugguuuaucuuuggaguucuug.......................................................................  3203  0  eg1
...................uggGuuaucuuuggaguucuug.......................................................................  7     1  eg1
...................ugguuuaucuuugAaguucuug.......................................................................  2     1  eg1
...................ugguuuaucuuCggaguucuug.......................................................................  7     1  eg1
...................ugguAuaucuuuggaguucuug.......................................................................  2     1  eg1
...................ugguuuaucuuuggaguucuGg.......................................................................  1     1  eg1
...................ugguuuaucuuuggaAuucuug.......................................................................  2     1  eg1
...................ugAuuuaucuuuggaguucuug.......................................................................  2     1  eg1
...................ugguuuaucuuuggaguucuugA......................................................................  10    1  eg1
...................ugguuuaucuuuggaguucuugC......................................................................  11    1  eg1
...................ugguuuaucCuuggaguucuugu......................................................................  1     1  eg1
...................ugguuuaucuuuggaguucuuUu......................................................................  12    1  eg1
...................ugguuuaucuuuggaguucuCgu......................................................................  1     1  eg1
...................Ggguuuaucuuuggaguucuugu......................................................................  17    1  eg1
...................Cgguuuaucuuuggaguucuugu......................................................................  1     1  eg1
...................ugguuuaucuuuggaguucuuAu......................................................................  7     1  eg1
...................ugguuuaucuuuggaguucuugG......................................................................  9     1  eg1
...................ugguuuaucuuuggaguucuugu......................................................................  66    0  eg1
...................ugguCuaucuuuggaguucuugu......................................................................  1     1  eg1
...................ugguuuaucuuuggaguucuugug.....................................................................  1     0  eg1
...................ugguuuaucuuuggaguucuuguA.....................................................................  39    1  eg1
...................ugguuuaucuuuggaguucuuguU.....................................................................  6     1  eg1
...................ugguuuaucuuuggaguucuuguC.....................................................................  2     1  eg1
...................Ggguuuaucuuuggaguucuugug.....................................................................  2     1  eg1
...................ugguuuaucuuuggaguucuuguUu....................................................................  2     1  eg1
...................ugguuuaucuuuggaguucuuguAu....................................................................  8     1  eg1
...................Ggguuuaucuuuggaguucuuguguuu..................................................................  1     1  eg1
...................ugguuuaucuuuggaguucuuguguuu..................................................................  1     0  eg1
....................ggGuuaucuuuggaguucuug.......................................................................  1     1  eg1
....................gguuuaucuuuggaguucuug.......................................................................  3     0  eg1
.......................................uguguuuaucauaaggucuuc....................................................  1     0  eg1
...................................................aaggucuucauugauugacaau.......................................  3     0  eg1
...................................................Gaggucuucauugauugacaau.......................................  1     1  eg1
...................................................aaggucuucauugauugacaauU......................................  2     1  eg1
...................................................aaggucuucauugauugacaauUa.....................................  2     1  eg1
....................................................aggucuucauugauugacaau.......................................  1     0  eg1
....................................................aggucuucauugauugacaaua......................................  1     0  eg1
....................................................aggucuucauugauugacaauU......................................  2     1  eg1
```

```
                   Mature                          Star
gucauuuugauauaccucaugguuuaucuuuggaguucuuguguuuaucauaaggucuucauugauugacaauaagguauauaauggacuuucagauuugacagcuuagaa
....................................................Gggucuucauugauugacaaua.....................................    1    1    eg1
....................................................aggucuucauugauugacaauUa....................................    1    1    eg1
```

Provisional ID	:	ScUbCFx_558_24078
Score total	:	4654.9
Score for star read(s)	:	3.9
Score for read counts	:	4648.2
Score for mfe	:	1.8
Score for randfold	:	1.6
Score for cons. seed	:	-0.6
Total read count	:	9129
Mature read count	:	2621
Loop read count	:	0
Star read count	:	6508

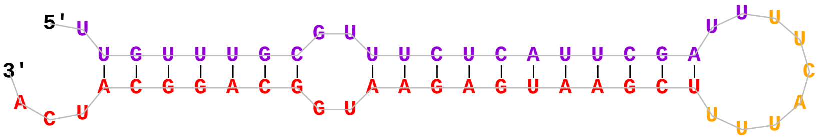

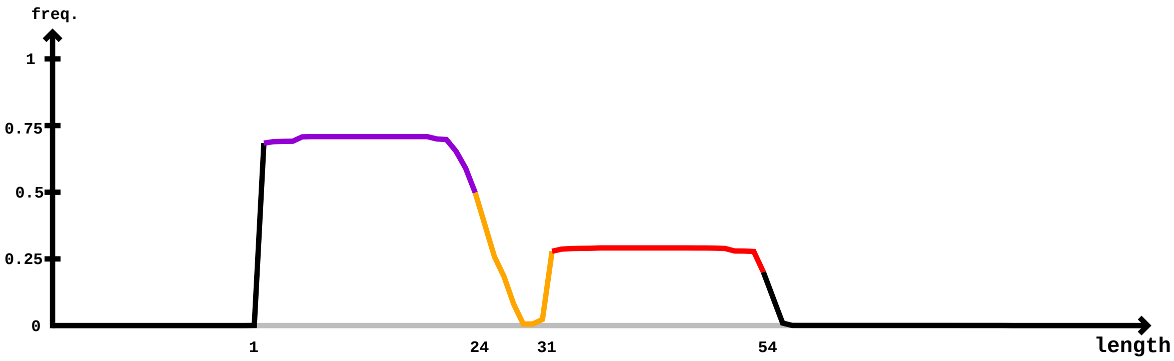

Star **Mature**

```
5'- cauacaguguaucggcaauguuguuugcguuucucauucgauuuucauuuucgaaugagaauggcaggcaucauugcugauugaccuaaaaagagacagaagaauuucaccg   -3'    obs
    cauacaguguaucggcaauguuguuugcguuucucauucgauuuucauuuucgaaugagaauggcaggcaucauugcugauugaccuaaaaagagacagaagaauuucaccg          exp
    ......((..(((((((((.(((((((..((((((((((.........)))))))))))..))))))).))))))))))..)).......((((........))))....   reads      mm        sample
    ....................uuguuugcguuucucauu..........................................................................   3          0         eg1
    ....................uuguuugcguuucucauuc.........................................................................   1          0         eg1
    ....................Guguuugcguuucucauucg........................................................................   1          1         eg1
    ....................uuguuugcguuucucauucg........................................................................   4          0         eg1
    ....................uuguuugcguuucucauucA........................................................................   1          1         eg1
    ....................uuguuugcguuCcucauucga.......................................................................   1          1         eg1
    ....................uuguuugcguuucucauucga.......................................................................   5          0         eg1
    ....................Guguuugcguuucucauucgau......................................................................   1          1         eg1
    ....................uuguuugcguuucucauucgaA......................................................................   7          1         eg1
    ....................uuguuugcguuucucauucgau......................................................................   20         0         eg1
    ....................uuguuugcguuucucauuGgau......................................................................   1          1         eg1
    ....................Guguuugcguuucucauucgauu.....................................................................   2          1         eg1
    ....................uuguuugcguuucucGuucgauu.....................................................................   1          1         eg1
    ....................uuguuugcguuucucauucgauA.....................................................................   12         1         eg1
    ....................uuguuugcguuucucauucgaAu.....................................................................   1          1         eg1
    ....................uuguuugcguuucucauucgauu.....................................................................   5          0         eg1
    ....................uuguuugcguuucucauucgauC.....................................................................   1          1         eg1
    ....................uuguuugcguuucucauucgauuu....................................................................   5          0         eg1
    ....................uuguuugcguuucucauucgauuG....................................................................   3          1         eg1
    ....................uuguuugcguuucucauucgauuA....................................................................   9          1         eg1
    ....................uuguuugcguuucucauucgauuuA...................................................................   4          1         eg1
    ....................uuguuugcguuucucauucgauuuu...................................................................   2          0         eg1
    ....................uuguuugcguuucucauucgauuuG...................................................................   3          1         eg1
    ....................uuguuugcguuucucauucgauuAu...................................................................   2          1         eg1
    ....................uuguuugcguuucucauucgauuCu...................................................................   1          1         eg1
    ....................uuguuugcguuucucauucgauuuuA..................................................................   9          1         eg1
    ....................uuguuugcguuucucauucgauuuuc..................................................................   2          0         eg1
    ....................uuguuugcguuucucauucgauuuuU..................................................................   3          1         eg1
    ....................Guguuugcguuucucauucgauuuuc..................................................................   1          1         eg1
    ....................uuguuugcguuucucauucgauuuuAa.................................................................   2          1         eg1
    ....................uuguCugcguuucucauucgauuuuca.................................................................   1          1         eg1
    ....................uuguuugcguuucucauucgauuuuca.................................................................   2          0         eg1
    .......................uugcguuucucauucgauuuu....................................................................   3          0         eg1
```

Star Mature

```
cauacaguguaucggcaauguuguuugcguuucucauucgauuuucauuuucgaaugagaauggcaggcaucauugcugauugaccuaaaaagagacagaagaauuucaccg
........................uugcguuucucauucgauuuuA..................................................................  2    1    eg1
........................uugcguuucucauucgauuuuc..................................................................  5    0    eg1
.................................................uucgaaugagaauggcaggc...........................................  1    0    eg1
.................................................uucgaaugagaauggcaggcauA........................................  1    1    eg1
.................................................uucgaaugagaauggcaggcauc........................................  8    0    eg1
.................................................uucgaaugagaauggcaggcaucU.......................................  6    1    eg1
.................................................uucgaaugagaauggcaggcauca.......................................  4    0    eg1
.................................................Gucgaaugagaauggcaggcauca.......................................  1    1    eg1
..................................................Gcgaaugagaauggcaggc...........................................  1    1    eg1
..................................................ucgaaugagaauggcaggc...........................................  3    0    eg1
..................................................Gcgaaugagaauggcaggcau.........................................  3    1    eg1
..................................................ucgaaugagaauggcaggcauc........................................  25   0    eg1
..................................................Gcgaaugagaauggcaggcauc........................................  7    1    eg1
..................................................ucgaaugagaauggcaggcaCc........................................  1    1    eg1
..................................................ucgaaCgagaauggcaggcauc........................................  1    1    eg1
..................................................ucgaaugagaauggcaggcauU........................................  1    1    eg1
..................................................ucgaaugagaauggcaggcauca.......................................  20   0    eg1
..................................................ucgaaugagaauggcaggcaucG.......................................  3    1    eg1
..................................................Gcgaaugagaauggcaggcauca.......................................  10   1    eg1
..................................................ucgaaugagaauggcaggcaucC.......................................  9    1    eg1
..................................................ucgaaugagaauggcaggcaucU.......................................  51   1    eg1
..................................................ucgaaugagaauggcaggcaucUu......................................  1    1    eg1
..................................................Gcgaaugagaauggcaggcaucauu.....................................  1    1    eg1
..................................................ucgaaugagaauggcaggcaucauu.....................................  1    0    eg1
...................................................cgaaugagaauggcaggc...........................................  1    0    eg1
...................................................cgaaugagaauggcaggcauc........................................  2    0    eg1
...................................................cgaaugagaauggcaggcaucU.......................................  1    1    eg1
....................................................gaaugagaauggcaggcaucU.......................................  1    1    eg1

....................uuguuugcguuucucauuc.........................................................................  1    0    er1
....................uuguuugcguuucucauucg........................................................................  5    0    er1
....................Guguuugcguuucucauucg........................................................................  1    1    er1
....................uuguuugcguuucucauucga.......................................................................  18   0    er1
....................uuguuugcguuucucauucgU.......................................................................  2    1    er1
....................uuguuugcguuucucauucgG.......................................................................  1    1    er1
....................Guguuugcguuucucauucga.......................................................................  5    1    er1
....................uuguuugcguuucucauucgau......................................................................  16   0    er1
....................uuguuugcguuucucauucgaC......................................................................  3    1    er1
....................Guguuugcguuucucauucgau......................................................................  4    1    er1
....................uuguuugcguuucucaCucgau......................................................................  1    1    er1
....................uuguuugcguuucucauucgaA......................................................................  8    1    er1
....................uuguuugcguuucucauucgauA.....................................................................  19   1    er1
....................uuguuugcguuucucauucgaAu.....................................................................  2    1    er1
....................uuguuugcguuucucauucgaCu.....................................................................  1    1    er1
....................uuguuugcguuucucauucgauu.....................................................................  4    0    er1
....................uuguuugcguuucucauucgauC.....................................................................  1    1    er1
....................uuguuugcguuucucauucgauuG....................................................................  1    1    er1
....................uuguuugcguuucucauucgauuu....................................................................  12   0    er1
....................uuguuugcguuucucauucgauuC....................................................................  1    1    er1
....................uuguuugcguuucucauucgauuA....................................................................  7    1    er1
....................uuguuugcguuucucauucgauuuu...................................................................  10   0    er1
....................uuguuugcguuucucauucgauuuA...................................................................  8    1    er1
....................uuguuugcguuucucauucgauuuC...................................................................  1    1    er1
....................uuguuugcguuucucauucgauuuuA..................................................................  17   1    er1
....................Guguuugcguuucucauucgauuuuc..................................................................  4    1    er1
....................uuguuugcguuucucauucgauuuuG..................................................................  1    1    er1
....................uuguCugcguuucucauucgauuuuc..................................................................  2    1    er1
....................uuguuugcguuucucauucgauuuuU..................................................................  10   1    er1
....................uuguuugcguuucucauucgauuuuc..................................................................  9    0    er1
....................uuguuugcguuucucauucgauuuucC.................................................................  1    1    er1
....................uuguuugcguuucucauucgauuuuUa.................................................................  1    1    er1
....................uuguuugcguuucCcauucgauuuuca.................................................................  1    1    er1
....................uuguuugcguuucucauucgauuuuAa.................................................................  11   1    er1
....................Guguuugcguuucucauucgauuuuca.................................................................  1    1    er1
....................uuguuugcguuucucauucgauuuucU.................................................................  1    1    er1
.......................uugcguuucucauucgauuuu....................................................................  1    0    er1
.......................uugcguuucucauucgauuuuc...................................................................  1    0    er1
.......................uugcguuucucauucgauuuuU...................................................................  1    1    er1
........................uugcguuucucauucgauuuu...................................................................  1    0    er1
........................uugcguuucucauucgauuuuA..................................................................  4    1    er1
```

Star **Mature**

```
cauacaguguaucggcaauguuguuugcguuucucauucgauuuucauuuucgaaugagaauggcaggcaucauugcugauugaccuaaaaagagacagaagaauuucaccg
........................uugcguuucucauucgauuuuc..................................................................	1	0	er1
........................uugcguuucucauucgauuuuca.................................................................	1	0	er1
........................uugcguuucucauucgauuuuAa.................................................................	1	1	er1
..............................................auuuucgaaugagaauggcaggca..........................................	1	0	er1
..............................................auuuucgaaugagaauggcaggcauc........................................	1	0	er1
...............................................uuuucgaaugagaauggcaggcauc........................................	1	0	er1
...............................................uuuucgaaugagaauggcaggcGuca.......................................	1	1	er1
...............................................uuuucgaaugagaauggcaggcaucU.......................................	1	1	er1
.................................................Gucgaaugagaauggcaggcauc........................................	1	1	er1
.................................................uucgaaugagaauggcaggcauc........................................	2	0	er1
..................................................ucgaaugagaauggcaggc...........................................	2	0	er1
..................................................ucgaaugagaauggcaggcau.........................................	1	0	er1
..................................................ucgaaugagaauggcaggcauc........................................	17	0	er1
..................................................ucgaaugagaaugAcaggcauc........................................	1	1	er1
..................................................Gcgaaugagaauggcaggcauc........................................	6	1	er1
..................................................ucgaaugagaauggcaggcaucC.......................................	3	1	er1
..................................................Gcgaaugagaauggcaggcauca.......................................	8	1	er1
..................................................ucgaaugagaauggcaggcauca.......................................	15	0	er1
..................................................ucgaaugagaauggcaggcaucU.......................................	38	1	er1
..................................................ucgaaugagaauggcaggcaucUu......................................	2	1	er1
..................................................ucgaaugagaauggcaggcaucauA.....................................	3	1	er1
..................................................ucgaaugagaauggcaggcaucauu.....................................	1	0	er1
...................................................cgaaugagaauggcaggcaucU.......................................	2	1	er1
.......................................................ugagaauggcaggcaucauA.....................................	1	1	er1

....................uuguuugcguuucucauu..........................................................................	31	0	ea1
....................uuguuugcguuucucGuu..........................................................................	2	1	ea1
....................uuguuugcguuucucauA..........................................................................	1	1	ea1
....................uuguuugcguuucucUuu..........................................................................	1	1	ea1
....................Guguuugcguuucucauu..........................................................................	3	1	ea1
....................uuguuugcguuucucauuc.........................................................................	1	0	ea1
....................Guguuugcguuucucauuc.........................................................................	2	1	ea1
....................uuguuugcguuucucauucg........................................................................	37	0	ea1
....................Guguuugcguuucucauucg........................................................................	6	1	ea1
....................uuguuugcguuucucauucA........................................................................	6	1	ea1
....................Guguuugcguuucucauucga.......................................................................	7	1	ea1
....................uuguuugcguuucucauucgU.......................................................................	1	1	ea1
....................uuguCugcguuucucauucga.......................................................................	1	1	ea1
....................uuguuugcguuucucauucgG.......................................................................	1	1	ea1
....................Cuguuugcguuucucauucga.......................................................................	1	1	ea1
....................uuguuugcguuucucauucga.......................................................................	47	0	ea1
....................uuguuCgcguuucucauucga.......................................................................	1	1	ea1
....................uuguuugcguuucucauucgau......................................................................	58	0	ea1
....................uugAuugcguuucucauucgau......................................................................	1	1	ea1
....................Guguuugcguuucucauucgau......................................................................	11	1	ea1
....................uuguuugcguuucucauucgaA......................................................................	17	1	ea1
....................uuguuugcguuucucauucgaG......................................................................	2	1	ea1
....................uuguuugcguuucucauucgaC......................................................................	10	1	ea1
....................Guguuugcguuucucauucgauu.....................................................................	2	1	ea1
....................uuguuugcguuucucauucgauA.....................................................................	74	1	ea1
....................uuguuugcguuucucauucgauu.....................................................................	37	0	ea1
....................uuguuugcguuucucauucgauG.....................................................................	6	1	ea1
....................uuguuugcguuucucauucgauC.....................................................................	9	1	ea1
....................uuguuugcguuucucauucgaCu.....................................................................	4	1	ea1
....................uuguuugcguuucucauucgaAu.....................................................................	17	1	ea1
....................uuguuugcguuucucauucgauuu....................................................................	19	0	ea1
....................uuguuugcguuucucauucgauuC....................................................................	2	1	ea1
....................uuguuugcguuucucauucgauAu....................................................................	1	1	ea1
....................uuguuugcguuucucauucgauuA....................................................................	43	1	ea1
....................uuguuugcguuucucauucgaAuu....................................................................	2	1	ea1
....................uuguuugcguuucucauucgauuG....................................................................	24	1	ea1
....................Guguuugcguuucucauucgauuu....................................................................	3	1	ea1
....................uuguuugcguuucucauucgauuuu...................................................................	16	0	ea1
....................uuguuugcguuucucauucgauuuG...................................................................	11	1	ea1
....................uuguuugcguuucucauucgauuuA...................................................................	39	1	ea1
....................uuguuugcguuucucauucgauuAu...................................................................	7	1	ea1
....................uuguuugcguCucucauucgauuuu...................................................................	1	1	ea1
....................Guguuugcguuucucauucgauuuu...................................................................	2	1	ea1
....................uuguuugcguuucucauucgauuuuG..................................................................	3	1	ea1
....................uuguuugcguuucucauucgauuuuA..................................................................	63	1	ea1
```

Star Mature

cauacaguguaucggcaauguuguuugcguuucucauucgauuuucauuuucgaaugagaauggcaggcaucauugcugauugaccuaaaaagagacagaagaauuucaccg			
....................uuguuugcguuucucauucgauuuuU..	7	1	ea1
....................uuguuugcguuucucauucgauuAuc..	1	1	ea1
....................uuguuugcguuucucauucgauuuuc..	11	0	ea1
....................uuguuugcguuucucauucgauuuucU...	4	1	ea1
....................uuguuugcguuucucauucgauuuuAa...	13	1	ea1
....................uuguuugcguuucucauucgauuuCca...	1	1	ea1
....................uuguuugcguuucucauucgauuuuca...	12	0	ea1
....................Guguuugcguuucucauucgauuuuca...	1	1	ea1
....................uuguuugcguuucucauucgauuuuUa...	4	1	ea1
.......................uuugcguuucucauucgauuuuA..	1	1	ea1
........................uugcguuucucauucgauuuA...	1	1	ea1
........................uugcgGuucucauucgauuuu...	1	1	ea1
........................uugcguuucucauucgauuuu...	9	0	ea1
........................uugcgCuucucauucgauuuu...	1	1	ea1
........................Gugcguuucucauucgauuuu...	1	1	ea1
........................uugcguuucucauucgauuuuU..	1	1	ea1
........................uugcguuucucauucgauuuuA..	19	1	ea1
........................uugcguuucucauuUgauuuuca...	1	1	ea1
........................uugcguuucucauucgauuuuca...	4	0	ea1
........................uugcguuucucauucgauuuuAa...	8	1	ea1
........................uugcguuucucauucgauuCuca...	1	1	ea1
........................uugcguuucucauucgauuuCca...	1	1	ea1
........................uugcguuucucauucgauuuuUa...	1	1	ea1
........................Gugcguuucucauucgauuuuca...	1	1	ea1
.........................ugcguuucucauucgauuuu...	1	0	ea1
.........................ugcguuucucauucgauuuuA..	1	1	ea1
....auuuucgaaugagaauggcagU..	1	1	ea1
....auuuucgaaugagaauggcaggcauc..	2	0	ea1
....auuuucgaaugagaauggcaggcauca.......................................	2	0	ea1
....auuuucgaaugagaauggcaggcaucU.......................................	1	1	ea1
....uuuucgaaugagaauggU...	1	1	ea1
....uuuucgaaugagaauggc...	1	0	ea1
....uuuucgaaugagaauggUag...	1	1	ea1
....uuuucgaaugagaauggcaggcauc..	10	0	ea1
....uuuucgaaugagaauggcaggcauG..	2	1	ea1
....uuuucgaaugagaauggcaggcauA..	1	1	ea1
....uuuucgaaugagaauggcaggCuca..	1	1	ea1
....uuuucgaaugagaauggcaggcaucU.......................................	3	1	ea1
....uucgaaugagaauggcag...	1	0	ea1
....Gucgaaugagaauggcag...	1	1	ea1
....uucgaaugagaauggcaggc...	3	0	ea1
....uucgaaugagaauggcaggcau...	2	0	ea1
....uucgaaugagaauggcaggcaC...	1	1	ea1
....Gucgaaugagaauggcaggcauc..	2	1	ea1
....uucgaaugagaauggcaggcauc..	67	0	ea1
....uucgaaugagaauggcaggcauU..	5	1	ea1
....uucgaaugagaauggcaggcauA..	6	1	ea1
....Cucgaaugagaauggcaggcauc..	2	1	ea1
....uucgaaugagaauggcaggcaCc..	1	1	ea1
....uucgaaugagaauggcaggcaAca.......................................	1	1	ea1
....uucgaaugagaauggcaggcaucU.......................................	8	1	ea1
....uucgaaugagaauggcaggcauca.......................................	13	0	ea1
....uucgaaugagaauggcaggcaucC.......................................	1	1	ea1
....uucgaaugagaauggcaggcaucaAu.....................................	1	1	ea1
....uucgaaugagaauggcaggcaucauA.....................................	5	1	ea1
....ucgaaugagaauggcagg..	1	0	ea1
....Gcgaaugagaauggcagg..	2	1	ea1
....ucgaaugagaauggcagA..	1	1	ea1
....ucgaaugagaauggcaggU...	8	1	ea1
....ucgaaugagaauggcaggA...	2	1	ea1
....ucgaaugUgaauggcaggc...	1	1	ea1
....ucgaaugagaauggcaggc...	58	0	ea1
....Gcgaaugagaauggcaggc...	5	1	ea1
....ucgaaugagaauggcaggca..	1	0	ea1
....ucgaaugagaauggcaggcaA...	1	1	ea1
....Gcgaaugagaauggcaggcau...	3	1	ea1
....ucgaaugagaauggcaggcau...	3	0	ea1
....Ccgaaugagaauggcaggcauc..	2	1	ea1
....ucgaaugagaaugCcaggcauc..	2	1	ea1
....ucgaaCgagaauggcaggcauc..	1	1	ea1

```
                    Star                          Mature
cauacaguguaucggcaauguuguuugcguuucucauucgauuuucauuuucgaaugagaauggcaggcaucauugcugauugaccuaaaaagagacagaagaauuucaccg
..................................................Gcgaaugagaauggcaggcauc.........................................   41   1   ea1
..................................................ucgaaugagaauggcaggcauU........................................   33   1   ea1
..................................................ucgaaugagaauggcaggcaCc........................................   4    1   ea1
..................................................ucgaaugagaGuggcaggcauc........................................   1    1   ea1
..................................................ucgaaugagaauAgcaggcauc........................................   1    1   ea1
..................................................ucgaaugagaauggcaggUauc........................................   1    1   ea1
..................................................ucgaaugagaaCggcaggcauc........................................   2    1   ea1
..................................................ucgaaugagaauggcaAgcauc........................................   1    1   ea1
..................................................ucgaaugagaauggcGggcauc........................................   1    1   ea1
..................................................ucgaaugagaauggcaggcCuc........................................   1    1   ea1
..................................................ucgaaugagaauggcaggcauA........................................   19   1   ea1
..................................................ucgaaugagaauggcaggcGuc........................................   1    1   ea1
..................................................ucgaaugagaauggcaggcauc........................................   296  0   ea1
..................................................ucgGaugagaauggcaggcauc........................................   1    1   ea1
..................................................ucgaaugagaauggcaggcaucU.......................................   600  1   ea1
..................................................ucgaaugagaauggcaggcauUa.......................................   1    1   ea1
..................................................ucgaaugagaauggcaggUauca.......................................   2    1   ea1
..................................................ucgaaugagaauggcaggcaucC.......................................   87   1   ea1
..................................................ucgaaCgagaauggcaggcauca.......................................   1    1   ea1
..................................................ucUaaugagaauggcaggcauca.......................................   1    1   ea1
..................................................ucgaGugagaauggcaggcauca.......................................   2    1   ea1
..................................................ucgaaugGgaauggcaggcauca.......................................   1    1   ea1
..................................................ucgaaugagaauggcaggcauca.......................................   172  0   ea1
..................................................ucgaaugagaauAgcaggcauca.......................................   1    1   ea1
..................................................uUgaaugagaauggcaggcauca.......................................   1    1   ea1
..................................................ucgaaugagaauggcaggcaucG.......................................   4    1   ea1
..................................................ucgaaugagaaugUcaggcauca.......................................   1    1   ea1
..................................................ucgaaugagaauggcaggAauca.......................................   1    1   ea1
..................................................Ccgaaugagaauggcaggcauca.......................................   1    1   ea1
..................................................Gcgaaugagaauggcaggcauca.......................................   75   1   ea1
..................................................ucgGaugagaauggcaggcauca.......................................   2    1   ea1
..................................................ucgaaugagaauggcaggcaucaA......................................   1    1   ea1
..................................................ucgaaugagaauggcaggcaucau......................................   2    0   ea1
..................................................ucgaaugagaauggcaggcaucUu......................................   15   1   ea1
..................................................ucgaaugagaauggcaggcaucauC.....................................   1    1   ea1
..................................................ucgaaugagaauggcaggcaucaAu.....................................   1    1   ea1
..................................................ucgaaugagaauggcaggcaucauA.....................................   9    1   ea1
..................................................ucgaaugagaauggcaggcaucUuu.....................................   16   1   ea1
...................................................cgaaugagaauggcaggc...........................................   2    0   ea1
...................................................cgaaugagaauggcaggcauU........................................   1    1   ea1
...................................................cgaaugagaauggcaggcauc........................................   5    0   ea1
...................................................cgaaugagaauggcaggcaucC.......................................   2    1   ea1
...................................................cgaaugagaauggcaggcauca.......................................   8    0   ea1
...................................................cgaaugagaauggcaggcaucU.......................................   15   1   ea1
...................................................cgaaugagaauggcaggcaucUu......................................   2    1   ea1
....................................................gaaugagaauggcaggcauc........................................   1    0   ea1
....................................................gaaugagaauggcaggcauca.......................................   1    0   ea1
....................................................gaaugagaauggcaggcaucC.......................................   1    1   ea1
....................................................gaaugagaauggcaggcaucUu......................................   2    1   ea1

...................Cuuguuugcguuucucauucgau......................................................................   1    1   er2
...................guugGuugcguuucucauucgauu.....................................................................   1    1   er2
...................Cuuguuugcguuucucauucgauuuuc..................................................................   2    1   er2
....................uuguuugcguuucucauA..........................................................................   12   1   er2
....................uuguuugcguuucucauu..........................................................................   4    0   er2
....................uuguuugcguuucucauG..........................................................................   1    1   er2
....................uuguuugcguuucucauuA.........................................................................   5    1   er2
....................uuguuugcguuucucauuc.........................................................................   3    0   er2
....................uuguuugcguuucucauucA........................................................................   9    1   er2
....................uuguuugcguuucucauucg........................................................................   87   0   er2
....................uuguuugcguuucucauucga.......................................................................   159  0   er2
....................uuguuugcguuucucGuucga.......................................................................   1    1   er2
....................uuguuugUguuucucauucga.......................................................................   2    1   er2
....................uuguuugcguuucucauucgau......................................................................   172  0   er2
....................uuguuugcguuucucauucgaA......................................................................   60   1   er2
....................uuguuugcguuucucauucgaG......................................................................   4    1   er2
....................uuguuugcguuucucauucgauu.....................................................................   124  0   er2
....................uuguuugcguuucucauucgauA.....................................................................   332  1   er2
....................uuguuugcguuucucauucgauG.....................................................................   23   1   er2
....................uuguuugcguuucucauucgaAu.....................................................................   10   1   er2
```

```
                    Star                         Mature
cauacaguguaucggcaauguuguuugcguuucucauucgauuuucauuuucgaaugagaauggcaggcaucauugcugauugaccuaaaaagagacagaagaauuucaccg
....................uuguuugcguuucucauucgauAu.................................................................... 3    1    er2
....................uuguuugcguuucucauucgaAuu.................................................................... 3    1    er2
....................uuguuugcguuucucauucgauuu.................................................................... 41   0    er2
....................uuguuugcguuucucauucgauuA.................................................................... 157  1    er2
....................uuguuugcguuucucauucgauuG.................................................................... 23   1    er2
....................uuguuugcguuucucauucgauuuA................................................................... 180  1    er2
....................uuguuugcguuucucauucgauuuu................................................................... 61   0    er2
....................uuguuugcguuucucauucgauuAu................................................................... 20   1    er2
....................uuguuugcguuucucauucgauuuC................................................................... 2    1    er2
....................uuguuugcguuucucauucgauuuG................................................................... 15   1    er2
....................Nuguuugcguuucucauucgauuuu................................................................... 1    1    er2
....................uuguuugcguuucucauucgauAuu................................................................... 3    1    er2
....................uuguuugcguuucucauucgauuuuA.................................................................. 355  1    er2
....................uuguuugcguuucucauucgauuuAc.................................................................. 2    1    er2
....................uuguuugcguuucucauucgauuuuc.................................................................. 59   0    er2
....................uuguuCgcguuucucauucgauuuuc.................................................................. 1    1    er2
....................uuguuugcguuucucauucgauuuuG.................................................................. 6    1    er2
....................Guguuugcguuucucauucgauuuuc.................................................................. 1    1    er2
....................uuguuugcguuucucauucgauuuCc.................................................................. 1    1    er2
....................uuguuugcguuucucauucgauuuuU.................................................................. 51   1    er2
....................uuguuugcguuucucauucgauuuuca................................................................. 201  0    er2
....................uuguuugcguuucGcauucgauuuuca................................................................. 1    1    er2
....................uuguuugcguuucucauucgauuuCca................................................................. 6    1    er2
....................uuguuugcguuucucauucgauuuuUa................................................................. 45   1    er2
....................Nuguuugcguuucucauucgauuuuca................................................................. 2    1    er2
....................uuguuugcguuucucauucgauuuAca................................................................. 3    1    er2
....................uuguuugcguuucucauucgauuuuAa................................................................. 158  1    er2
....................uuguuugcguuucucauucgauuuucU................................................................. 6    1    er2
....................uuguuugcguuucucauucgauuuucG................................................................. 2    1    er2
.....................uguuugcguuucucauucga........................................................................ 5    0    er2
.....................uguuugcguuucucauucgaA....................................................................... 1    1    er2
.....................uguuugcguuucucauucgauA...................................................................... 3    1    er2
.....................uguuugcguuucucauucgauu...................................................................... 3    0    er2
.....................uguuugcguuucucauucgauuG..................................................................... 2    1    er2
.....................uguuugcguuucucauucgauuA..................................................................... 1    1    er2
.....................uguuugcguuucucauucgauuuu.................................................................... 1    0    er2
.....................uguuugcguuucucauucgauuuuG................................................................... 1    1    er2
.....................uguuugcguuucucauucgauuuuA................................................................... 2    1    er2
.....................uguuugcguuucucauucgauuuuU................................................................... 2    1    er2
.....................uguuugcguuucucauucgauuuuUa.................................................................. 1    1    er2
.....................uguuugcguuucucauucgauuuuAa.................................................................. 2    1    er2
.....................uguuugcguuucucauucgauuuuca.................................................................. 2    0    er2
......................guuugcguuucucauucg........................................................................ 1    0    er2
......................guuugcguuucucauucga....................................................................... 2    0    er2
........................uugcguuucucauucgauuA.................................................................... 1    1    er2
........................uugcguuucucauucgauuu.................................................................... 5    0    er2
........................uugcguuucucauucgauuuA................................................................... 17   1    er2
........................uugcguuucucauucgauuuuc.................................................................. 7    0    er2
........................uugcguuucucauucgauuuuca................................................................. 8    0    er2
........................uugcguuucucauucgauuuuAa................................................................. 6    1    er2
.........................ugcguuucucauucgauuuA................................................................... 1    1    er2
.........................ugcguuucucauucgauuuAa.................................................................. 1    1    er2
...............................................uuuucgaaugagaauggcaggcauc........................................ 7    0    er2
...............................................uuuucgaaugagaauggcaggcauca....................................... 3    0    er2
...............................................uuuucgaaugagaauggcaggcaucU....................................... 4    1    er2
................................................uuucgaaugagaauggcaggcaucauA..................................... 1    1    er2
.................................................uucgaaugagaauggcaggcauc........................................ 2    0    er2
.................................................uucgaaugagaauggcaggcauca....................................... 1    0    er2
..................................................ucgaaugagaauggcaggcauc........................................ 34   0    er2
..................................................ucgaaugagaauggcaggcauU........................................ 4    1    er2
..................................................ucgaaugagaauggcaggcauA........................................ 2    1    er2
..................................................ucgaaugagaauggcaggcauca....................................... 19   0    er2
..................................................ucgaaugagaauggcaggcaucU....................................... 119  1    er2
..................................................ucgaaugagaauggcaggcaucC....................................... 16   1    er2
..................................................ucgaaugagaauggcaggcaucUu...................................... 6    1    er2
..................................................ucgaaugagaauggcaggcaucau...................................... 6    0    er2
..................................................ucgaaugagaauggcaggcaucauA..................................... 3    1    er2
..................................................ucgaaugagaauggcaggcaucauG..................................... 1    1    er2
..................................................ucgaaugagaauggcaggcaucUuu..................................... 9    1    er2
..................................................ucgaaugagaauggcaggcaucauu..................................... 4    0    er2
```

Star Mature

```
cauacaguguaucggcaauguuguuugcguuucucauucgauuuucauuuucgaaugagaauggcaggcaucauugcugauugaccuaaaaagagacagaagaauuucaccg
..................................................ucgaaugagaauggcaggcaucauuA....................................    1    1    er2
...................................................cgaaugagaauggcaggcauc........................................    4    0    er2
...................................................cgaaugagaauggcaggcauU........................................    2    1    er2
...................................................cgaaugagaauggcaggcaucU.......................................    7    1    er2
...................................................cgaaugagaauggcaggcauca.......................................    2    0    er2
....................................................gaaugagaauggcaggcaucUu......................................    1    1    er2
....................................................gaaugagaauggcaggcaucauA.....................................    3    1    er2
......................................................augagaauggcaggcaucU.......................................    1    1    er2
.......................................................ugagaauggcaggcaucauu.....................................    1    0    er2
.........................................................................uugcugauugaccuaaaaagagaca..............    1    0    er2

....................uuguuugcguuucucauA..........................................................................    23   1    ea2
....................uuguuugcguuucucauuA.........................................................................    5    1    ea2
....................Guguuugcguuucucauucg........................................................................    1    1    ea2
....................Nuguuugcguuucucauucg........................................................................    1    1    ea2
....................uuguuugcguuucucauucA........................................................................    16   1    ea2
....................uuguuugcguuucucauucg........................................................................    193  0    ea2
....................uuguuugcguuucucauucgU.......................................................................    1    1    ea2
....................Cuguuugcguuucucauucga.......................................................................    1    1    ea2
....................uuguuugcguuucucauucga.......................................................................    217  0    ea2
....................uuguuugcguuucucauucCa.......................................................................    2    1    ea2
....................uuguuugcguuucucauucgau......................................................................    179  0    ea2
....................uuguuugcguuucucauucgaA......................................................................    81   1    ea2
....................uuguuugcguuucucauucgaC......................................................................    4    1    ea2
....................uuguuugcguuucucauucgaG......................................................................    14   1    ea2
....................uuguuugcguuucucauucgauC.....................................................................    1    1    ea2
....................uuguuugcguuucucauucgaAu.....................................................................    4    1    ea2
....................uuguuugcguuucucauucgauu.....................................................................    106  0    ea2
....................uuguuugcguuucucauucgauA.....................................................................    366  1    ea2
....................uuguuugcguuucucauucgauG.....................................................................    31   1    ea2
....................uuguuugcguuucucauucgaCu.....................................................................    2    1    ea2
....................uuguuugcguuucucauucgauAu....................................................................    2    1    ea2
....................uuguuugcguuucucauucgauuA....................................................................    66   1    ea2
....................uuguuugcguuucucauucgauuG....................................................................    20   1    ea2
....................uuguuugcguuucucauucgauuu....................................................................    31   0    ea2
....................uuguuugcguCucucauucgauuu....................................................................    1    1    ea2
....................uuguuugcguuucucauucgauuuG...................................................................    4    1    ea2
....................uuguuugcguuucucauucgauuAu...................................................................    15   1    ea2
....................uuguuugcguuucucauucgauAuu...................................................................    3    1    ea2
....................uuguuugcguuucucauucgauuuA...................................................................    50   1    ea2
....................uuguuugcguuucucauucgauuuu...................................................................    12   0    ea2
....................uuguuugcguuucucauucgauuuuU..................................................................    4    1    ea2
....................uuguuugcguuucucauucgauuuuG..................................................................    2    1    ea2
....................uuguuugcguuucucauucgauuuuc..................................................................    22   0    ea2
....................uuguuugcguuucucauucgauuuuA..................................................................    83   1    ea2
....................uuguuugcguuucucauucgauuuAca.................................................................    3    1    ea2
....................uuguuugcguuucucauucgauuuuUa.................................................................    5    1    ea2
....................uuguuugcguuucucauucgauuuuAa.................................................................    35   1    ea2
....................uuguuugcguuucucauucgauuuuca.................................................................    50   0    ea2
.....................uguuugcguuucucauucg........................................................................    1    0    ea2
.....................uguuugcguuucucauucga.......................................................................    4    0    ea2
.....................uguuugcguuucucauucgau......................................................................    4    0    ea2
.....................uguuugcguuucucauucgaA......................................................................    2    1    ea2
.....................uguuugcguuucucauucgauu.....................................................................    2    0    ea2
.....................uguuugcguuucucauucgauA.....................................................................    2    1    ea2
.....................uguuugcguuucucauucgauuG....................................................................    1    1    ea2
.....................uguuugcguuucucauucgauuuA...................................................................    1    1    ea2
......................guuugcguuucucauucgauA.....................................................................    3    1    ea2
........................uugcguuucucauucgauuu....................................................................    2    0    ea2
........................uugcguuucucauucgauuuA...................................................................    7    1    ea2
........................uugcguuucucauucgauuuuA..................................................................    7    1    ea2
........................uugcguuucucauucgauuuuc..................................................................    3    0    ea2
........................uugcguuucucauucgauuuuAa.................................................................    7    1    ea2
........................uugcguuucucauucgauuuuca.................................................................    11   0    ea2
........................uugcguuucucauucgauuuucaA................................................................    1    1    ea2
.........................ugcguuucucauucgauuuuA..................................................................    3    1    ea2
..............................................auuucgaaugagaauggcaggcaucU........................................    2    1    ea2
...............................................uuuucgaaugagaauggcag.............................................    2    0    ea2
...............................................uuuucgaaugagCauggcaggcauc........................................    1    1    ea2
...............................................uuuucgaaugagaauggcaggcauc........................................    2    0    ea2
```

Star Mature

```
cauacaguguaucggcaauguuguuugcguuucucauucgauuuucauuuucgaaugagaauggcaggcaucauugcugauugaccuaaaaagagacagaagaauuucaccg
...............................................uuuucgaaugagaauggcaggcauca.......................................  1    0    ea2
.................................................uucgaaugagaauggcaggcauc........................................  2    0    ea2
.................................................uucgaaugagaauggcaggcaucU.......................................  1    1    ea2
.................................................uucgaaugagaauggcaggcauca.......................................  3    0    ea2
.................................................uucgaaugagaauggcaggcGucau......................................  1    1    ea2
..................................................ucgaaugagaauggcagg............................................  6    0    ea2
..................................................ucgaaugagaauggcaggcauc........................................  39   0    ea2
..................................................ucgaaugagaauggcaggcauA........................................  5    1    ea2
..................................................ucgaaugagaauggcaggcauU........................................  3    1    ea2
..................................................ucgaaugagaauggcaggcauG........................................  4    1    ea2
..................................................ucgaaugagaauggcaggcaucU.......................................  124  1    ea2
..................................................ucgaaugagaauggcaggcaAca.......................................  6    1    ea2
..................................................Gcgaaugagaauggcaggcauca.......................................  1    1    ea2
..................................................ucgaaugagaauggcaggcaucC.......................................  6    1    ea2
..................................................ucgaaugagaauggcaggcauca.......................................  43   0    ea2
..................................................ucgaaugagaauggcaggcaucUu......................................  3    1    ea2
..................................................ucgaaugagaauggcaggcaucauu.....................................  10   0    ea2
...................................................cgaaugagaauggcaggcauc........................................  2    0    ea2
...................................................cgaaugagaauggcaggcauca.......................................  2    0    ea2
...................................................cgaaugagaauggcaggcaucU.......................................  6    1    ea2
....................................................gaaugagaauggcaggcaucUu......................................  1    1    ea2
.....................................................aaugagaauggcaggcauca.......................................  6    0    ea2
......................................................augagaauggcaggcaucauA.....................................  1    1    ea2
.......................................................ugagaauggcaggcaucUu......................................  3    1    ea2
.......................................................ugagaauggcaggcaucauA.....................................  3    1    ea2
.......................................................ugagaauggcaggcaucauu.....................................  2    0    ea2
.........................................................................uugcugauugaccuaaaaag...................  1    0    ea2

....................uuguuugcguuucucauuU.........................................................................  1    1    eg2
....................Nuguuugcguuucucauucg........................................................................  1    1    eg2
....................uuguuugcguuucucauucg........................................................................  30   0    eg2
....................uuguuugcguGucucauucg........................................................................  1    1    eg2
....................uuguuugcguuucCcauucga.......................................................................  2    1    eg2
....................uuguuugcguuucucauucga.......................................................................  94   0    eg2
....................uuguuugcguuucucauuUga.......................................................................  1    1    eg2
....................uuguuugcguuucucauucgaC......................................................................  1    1    eg2
....................uuguuugcguGucucauucgau......................................................................  1    1    eg2
....................uuguuugcguuucucauucgaA......................................................................  53   1    eg2
....................uuguuugcguuucucauucgau......................................................................  102  0    eg2
....................uuguuugcguuucucauucgaG......................................................................  2    1    eg2
....................uuguGugcguuucucauucgau......................................................................  1    1    eg2
....................uuguuugcguuucucauucgaAu.....................................................................  4    1    eg2
....................uuguuugcguuucucauucgaCu.....................................................................  1    1    eg2
....................uuguuugcguuucucauucgauu.....................................................................  58   0    eg2
....................uuguuugcguuucucauucgauG.....................................................................  3    1    eg2
....................uuguuugcguuucucauucgauA.....................................................................  280  1    eg2
....................uuguuugUguuucucauucgauu.....................................................................  1    1    eg2
....................uuguuugcguuucucauucgauuu....................................................................  42   0    eg2
....................uuguuugcguuucucauucgauuG....................................................................  10   1    eg2
....................uuguuugcguuucucauucgauCu....................................................................  1    1    eg2
....................uuguuugcguuucucauucgauAu....................................................................  1    1    eg2
....................uuguuugcguuucucauucgauuA....................................................................  102  1    eg2
....................uuguuugcguuucucauucgauuAu...................................................................  2    1    eg2
....................uuguuugcguuucucauucgauuuu...................................................................  16   0    eg2
....................uuguuugcguuucucauucgauuuG...................................................................  13   1    eg2
....................uuguuugcguuucucauucgauuuA...................................................................  150  1    eg2
....................uuguuugcguuucucauucgauuuuU..................................................................  2    1    eg2
....................uuguuugcguuucucauucgauuuuA..................................................................  133  1    eg2
....................uuguuugcguuucucauucgauuuuc..................................................................  4    0    eg2
....................uuguuugcguuucucauucgauuuuG..................................................................  3    1    eg2
....................uuguuugcguuucucauucgauuuuAa.................................................................  43   1    eg2
....................uuguuugcguuucucauucgauAuuca.................................................................  1    1    eg2
....................uuguuugcguuucucauucgauuuucU.................................................................  2    1    eg2
....................uuguuugcguuucucauucgauuuuUa.................................................................  4    1    eg2
....................uuguuugcguuucucauucgauuuuca.................................................................  43   0    eg2
....................uuguuugcguuucucauuUgauuuuca.................................................................  1    1    eg2
....................uuguuugcguuucucauucgauuuucG.................................................................  3    1    eg2
.....................uguuugcguuucucauucga.......................................................................  2    0    eg2
.....................uguuugcguuucucauucgau......................................................................  1    0    eg2
.....................uguuugcguuucucauucgauA.....................................................................  2    1    eg2
```

Star **Mature**

```
cauacaguguaucggcaaug[uuguuugcguuucucauucgauu]uucauuu[ucgaaugagaauggcaggcauca]uugcugauugaccuaaaaagagacagaagaauuucaccg
```

Alignment	Count		
.....................uguuugcguuucucauucgauuA..	1	1	eg2
.....................uguuugcguuucucauucgauuuAa..	2	1	eg2
......................guuugcguuucucauucgau..	3	0	eg2
......................guuugcguuucucauucgauuA..	1	1	eg2
........................uugcguuucucauucgauuuuca...	1	0	eg2
........................uugcguuucucauucgauuuuAa...	1	1	eg2
....uucgaaugagaauggcaggcauc..	1	0	eg2
....uucgaaugagaauggcaggcauca.......................................	4	0	eg2
....Ncgaaugagaauggcaggcau...	1	1	eg2
....ucgaaugagaauggcaggcauc..	7	0	eg2
....ucgaaugagaauggcaggcauA..	1	1	eg2
....ucgaaugagaauggcaggcaucC.......................................	9	1	eg2
....ucgaaugagaauggcaggcauca.......................................	27	0	eg2
....ucgaaugagaauggcaggcaucU.......................................	100	1	eg2
....ucgaaugagaauggcaggcaucau......................................	1	0	eg2
....cgaaugagaauggcaggcauca.......................................	2	0	eg2
....cgaaugagaauggcaggcaucU.......................................	5	1	eg2
....cgaaugagaauggcaggcaucC.......................................	1	1	eg2
....gaaugagaauggcaggcaucU.......................................	2	1	eg2
....gaaugagaauggcaggcaucau......................................	1	0	eg2
....gaaugagaauggcaggcaucaA......................................	1	1	eg2
....gaaugagaauggcaggcaucUu......................................	1	1	eg2
....gaaugagaauggcaggcaucauA.....................................	2	1	eg2
....augagaauggcaggcaucU.......................................	3	1	eg2
....ugagaauggcaggcaucauA.....................................	1	1	eg2
....uugcugauugaccuaaaaagagaca..............	2	0	eg2

Provisional ID	:	ScUbCFx_558_24541
Score total	:	72.3
Score for star read(s)	:	3.9
Score for read counts	:	61.8
Score for mfe	:	2
Score for randfold	:	1.6
Score for cons. seed	:	3
Total read count	:	133
Mature read count	:	117
Loop read count	:	0
Star read count	:	16

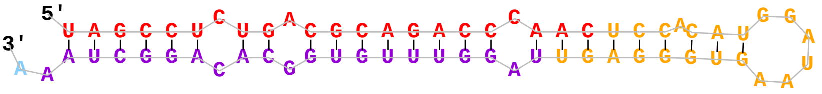

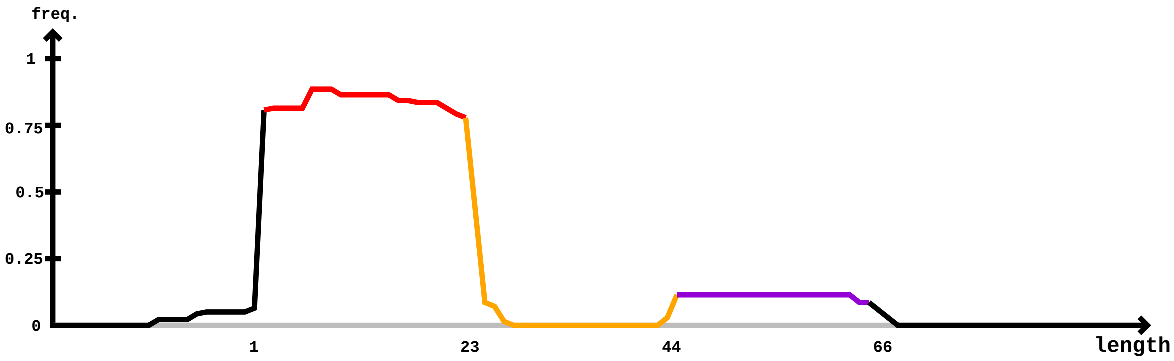

Mature Star

```
5'- gauggcagauggaaugacuguagccucugacgcagacccaacuccacauggauaaguggggaguuagguuuguggcacaggcuaaauucauugcagcaucaaucguuuuuaag  -3'   obs
    gauggcagauggaaugacuguagccucugacgcagacccaacuccacauggauaaguggggaguuagguuuguggcacaggcuaaauucauugcagcaucaaucguuuuuaag        exp
    ((((.....((.(((((...((((((.((.((((((((.((((((.(((......))))))))).)))))))).)).))))))...))))).)).)))).............   reads   mm   sample
    ....................uagccucugacgcagaccU..........................................................................   1       1    ea1
    ....................uagccucugacgcagaccca.........................................................................   2       0    ea1
    ....................uagccucugacgcagacccaa........................................................................   2       0    ea1
    ....................Gagccucugacgcagacccaac.......................................................................   1       1    ea1
    ....................uagccucugacgcagacccaac.......................................................................   23      0    ea1
    ....................uagccCcugacgcagacccaac.......................................................................   1       1    ea1
    ....................uagccucugacgcagacccaaU.......................................................................   2       1    ea1
    ....................uagccucugacgcagacccaacG......................................................................   1       1    ea1
    ....................uagccucugacgcagacccaacA......................................................................   8       1    ea1
    ....................uagccucugacgcagacccaaUu......................................................................   1       1    ea1
    ....................uagccucugacgcagacccaacC......................................................................   6       1    ea1
    ....................uagccucugacgcagacccaacu......................................................................   19      0    ea1
    ....................Gagccucugacgcagacccaacu......................................................................   1       1    ea1
    ....................uagccucugacgcagacccaacuU.....................................................................   1       1    ea1
    ................................................................uGgguuuguggcacaggcuaa............................   4       1    ea1

    .............augacuguagccucugacgca...............................................................................   3       0    er2
    ..............ugacuguagccucugacgcaUa.............................................................................   1       1    er2
    ................................................................uagguuuguggcacaggcuaa............................   1       0    er2
    ................................................................uagguuuguggcacaggcuaaa...........................   1       0    er2
    ................................................................uagguuuguggcacaggcuaaau..........................   4       0    er2

    .........uggaaugacuguagcUucu.....................................................................................   3       1    ea2
    .........................ucugacgcagacccaacucA....................................................................   3       1    ea2
    .........................ucugacgcagacccaacucc....................................................................   5       0    ea2
    .........................ucugacgcagacccaacucca...................................................................   2       0    ea2
    ...............................................................uuagguuuguggcacaggcG..............................   4       1    ea2
    ................................................................uagguuuguggcacaggcuaaU...........................   1       1    ea2

    ....................uagccucugacgcagacccaac.......................................................................   4       0    er1
    ....................uagccucugacgcagacccaacA......................................................................   1       1    er1
    ....................uagccucugacgcagacccaacu......................................................................   2       0    er1
    .....................agccucugacgcagacccaac.......................................................................   1       0    er1
```

Mature **Star**

```
gauggcagauggaaugacuguagccucugacgcagacccaacuccacauggauaagugggaguuagguuuguggcacaggcuaaauucauugcagcaucaaucguuuuuaag
...............................................................uGgguuuguggcacaggcuaa............................    1    1    er1

...................guagccucugacgcagacccaacu.....................................................................    1    0    eg1
...................Uuagccucugacgcagacccaacu.....................................................................    1    1    eg1
....................uagccucugacgcagaccc.........................................................................    2    0    eg1
....................uagccucugacgcagaccca........................................................................    1    0    eg1
....................uagccucugacgcagacccaac......................................................................    15   0    eg1
....................uagccucugacgcagacccaacC.....................................................................    1    1    eg1
....................uagccucugacgcagacccaacA.....................................................................    3    1    eg1
....................uagccucugacgcagacccaacu.....................................................................    5    0    eg1
....................uagccucugacgcagacccaacuU....................................................................    1    1    eg1
```

Provisional ID	:	ScUbCFx_558_24648
Score total	:	89.2
Score for star read(s)	:	3.9
Score for read counts	:	78.1
Score for mfe	:	2.6
Score for randfold	:	1.6
Score for cons. seed	:	3
Total read count	:	165
Mature read count	:	164
Loop read count	:	0
Star read count	:	1

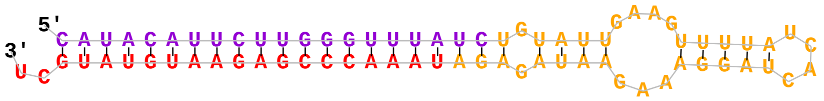

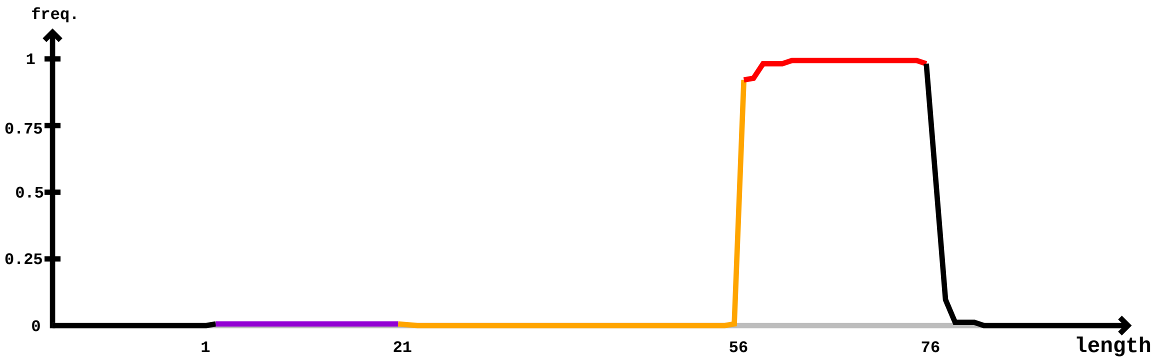

Star **Mature**

```
5'- uguucuagccgccagcauacauucuuggguuuaucuguauugaaguuuuaucacuaggaaagaauagagauaaacccgagaauguaugcuggcggcuggugggaccauugg   -3'   obs
    uguucuagccgccagcauacauucuuggguuuaucuguauugaaguuuuaucacuaggaaagaauagagauaaacccgagaauguaugcuggcggcuggugggaccauugg         exp
    .(((((((((((((((((((((((((((((((((((.((((....(((((....)))))...)))).))))))))))))))))))))))))))))))))..)))......   reads   mm   sample
    .....................................................................auaaacccgagaauguaugcuA...................   1   1   ea1
    ......................................................................uaaacccgagaauguaugc.....................   1   0   ea1
    ......................................................................uaaacccgagaauguaugU.....................   1   1   ea1
    ......................................................................Gaaacccgagaauguaugcu....................   6   1   ea1
    ......................................................................uaaacccgagaauguaugcA....................   2   1   ea1
    ......................................................................uaaacccgagaauguaugcC....................   3   1   ea1
    ......................................................................uaaacccgagaauguaugcu....................   14   0   ea1
    ......................................................................uaaacccgagaauguaugcuC...................   1   1   ea1
    ......................................................................uaaacccgagaauguaugcuA...................   18   1   ea1
    ......................................................................uaaacccgagaauguaugcuU...................   1   1   ea1
    ......................................................................uaaacccgagaauguaugcuAg..................   1   1   ea1
    ........................................................................aacccgagaauguaugcugg..................   3   0   ea1
    ...........................................................................cAgagaauguaugcuggcgg...............   2   1   ea1

    ......................................................................uaaacccgagaauguaugcA....................   1   1   er2
    ......................................................................uaaacccgagaauguaugcu....................   45   0   er2
    ......................................................................uaaacccgagaauguaugAu....................   1   1   er2
    ......................................................................uaaacccgagaauguaugcuC...................   2   1   er2
    ......................................................................uaaacccgagaauguaugcuU...................   8   1   er2
    ......................................................................uaaacccgagaauguaugcuA...................   5   1   er2
    ......................................................................uaaacccgagaauguaugcugg..................   2   0   er2
    .......................................................................aaacccgagaauguaugcu....................   1   0   er2

    ......................................................................uaaacccgagaauguaugcu....................   10   0   ea2
    ......................................................................uaaacccgagaauguaugcuC...................   3   1   ea2
    ......................................................................uaaacccgagaauguaugcuUg..................   2   1   ea2
    ........................................................................aacccgagaauguaugcugg..................   4   0   ea2

    ........................................................................aacccgagaauguaugcugg..................   1   0   eg2

    ......................................................................uaaacccgagaauguaugcu....................   3   0   eg1
    ......................................................................Gaaacccgagaauguaugcu....................   2   1   eg1
    ......................................................................uaaacccgagaauguaugcuA...................   3   1   eg1
```

Star Mature

```
uguucuagccgccagcauacauucuuggguuuaucuguauugaaguuuuaucacuaggaaagaauagagauaaacccgagaauguaugcuggcggcugguggaccauugg
......................................................................uaaacccgagaauguaugcuU...................    1    1    eg1
........................................................................aUcccgagaauguaugcugg..................    1    1    eg1

...............cauacauucuuggguuuauc...........................................................................    1    0    er1
......................................................................Gaaacccgagaauguaugcu....................    5    1    er1
......................................................................uaaacccgagaauguaugcC....................    2    1    er1
......................................................................uaaacccgagaauguaugcu....................    3    0    er1
......................................................................uaaacccgagaauguaugcuA...................    4    1    er1
......................................................................uaaacccgagaauguaugcuU...................    1    1    er1
```

Provisional ID	:	ScUbCFx_558_24778
Score total	:	4.1
Score for star read(s)	:	-1.3
Score for read counts	:	0
Score for mfe	:	0.8
Score for randfold	:	1.6
Score for cons. seed	:	3
Total read count	:	3465
Mature read count	:	3198
Loop read count	:	0
Star read count	:	267

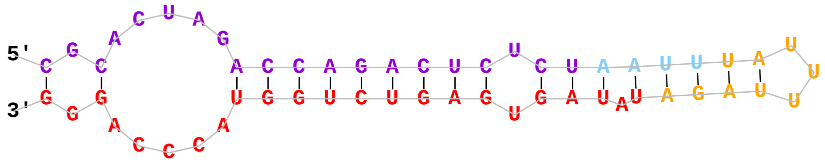

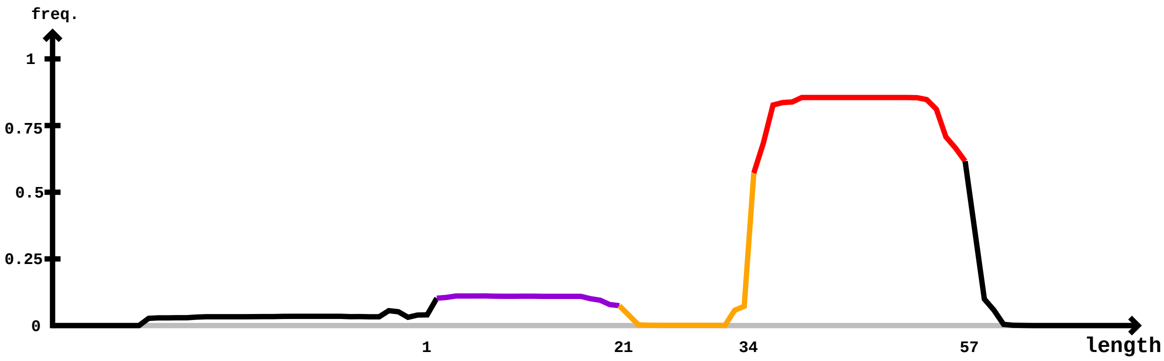


```
                                           Star                      Mature
5'- ugauugguuaauaucagcacaccagaacgagaauggggcgcacuagaccagacucucuaauuuauuuuagauauagugagucugguacccaggguacccaggcuaaaagca  -3'  obs
    ugauugguuaauaucagcacaccagaacgagaauggggcgcacuagaccagacucucuaauuuauuuuagauauagugagucugguacccaggguacccaggcuaaaagca       exp
    ...(((((............)))))....((..((((((.(.....(((((((((.((((((((...))))).))).))))))))).....).)).))))..)).......  reads  mm  sample
    ........uaauaucagcacaccagaacgag................................................................................  1      0   er1
    ........Gaauaucagcacaccagaacgaga...............................................................................  1      1   er1
    ........uaauaucagcacaccagaacgagaau.............................................................................  2      0   er1
    ........uaCuaucagcacaccagaacgagaaug............................................................................  1      1   er1
    ........Gaauaucagcacaccagaacgagaaug............................................................................  1      1   er1
    ........uaauaucagcacaccagaacgagaaug............................................................................  16     0   er1
    ...................................gggcgcacuagaccagacucA.......................................................  1      1   er1
    ......................................cgcacuagaccagacucucua....................................................  1      0   er1
    .....................................................................gauauagugagucugguacA......................  1      1   er1
    .....................................................................gauauagugagucugguaccc.....................  3      0   er1
    .....................................................................gauauagugagucugguaccca....................  3      0   er1
    .....................................................................gauauagugagucugguacccU....................  3      1   er1
    .......................................................................Gauagugagucugguaccc.....................  1      1   er1
    .......................................................................uauagugagucugguaccc.....................  1      0   er1
    .......................................................................uauagugagucugguacccU....................  2      1   er1
    .......................................................................uauagugagucugguaccca....................  1      0   er1
    .......................................................................uauagugagucugguacccC....................  1      1   er1
    .......................................................................uauagugagucugguacccag...................  3      0   er1
    .......................................................................uauagugagucugguacccagg..................  2      0   er1
    .......................................................................uauagugagucugguacccaggA.................  2      1   er1
    .......................................................................uauagugagucugguacccGggg.................  1      1   er1
    .......................................................................uauagugagucugguacccaggg.................  27     0   er1
    .......................................................................uauagugagucugguacccaggU.................  1      1   er1
    .......................................................................Gauagugagucugguacccaggg.................  4      1   er1
    .......................................................................uauagugagucugguacccagggAa...............  3      1   er1
    ........................................................................auagugagucugguacccaggg.................  10     0   er1
    .........................................................................uagugagucugguacccaggg.................  2      0   er1
    .........................................................................uagugagucugguacccagggu................  1      0   er1
    .........................................................................uagugagucugguacccagggua...............  1      0   er1

    ........uaauaucagcacaccagaacg..................................................................................  4      0   eg1
    ........uaauaucagcacaccagaacgagU...............................................................................  1      1   eg1
    ........uaauaCcagcacaccagaacgagaau.............................................................................  1      1   eg1
```

Star Mature

ugauugguuaauaucagcacaccagaacgagaauggggcgcacuagaccagacucucuaauuuauuuuagauauagugagucugguacccaggguacccaggcuaaaagca			
........uaauaucagcGcaccagaacgagaau...	1	1	eg1
........uaauaucagcacaccagaacgagaau...	8	0	eg1
........Gaauaucagcacaccagaacgagaaug..	2	1	eg1
........uaauaucagcacaccagaacgagaauA..	2	1	eg1
........uaauaucUgcacaccagaacgagaaug..	1	1	eg1
........uaauaucagcacGccagaacgagaaug..	1	1	eg1
........uaauaucagcacaccagaacgagaaug..	20	0	eg1
.............ucagcacaccagaacgagaaug..	1	0	eg1
..............cagcacaUcagaacgagaaug..	1	1	eg1
..............cagcacaccagaacgagaaugA...	1	1	eg1
...................caUcagaacgagaauggggcgcacu...	1	1	eg1
......................cagaacgagaauggggcgcacu...	1	0	eg1
.................................uggggcgcacuagaccagacu...	2	0	eg1
.................................uggggcgcacuagaccGgacu...	1	1	eg1
.................................Gggggcgcacuagaccagacucu...	1	1	eg1
.................................uggggcgcacuagaccagacuAu...	1	1	eg1
...................................gggcgcacuagaccagacu...	1	0	eg1
...................................gggcgcacuagaccagacuA..	1	1	eg1
....................................ggcgcacuagaccagacucu...	3	0	eg1
....................................ggcgcacuagaccagacucG...	1	1	eg1
....................................ggcgcacuagaccagacucuc..	3	0	eg1
....................................ggcgcacuagaccagacucucA...	2	1	eg1
....................................ggcgcacuagaccagacucucu...	1	0	eg1
....................................ggcgcacuagaccagacucucuUau..	1	1	eg1
......................................cgcacuagaccUgacucucu...	1	1	eg1
......................................cgcacuagaccagacucucG...	1	1	eg1
......................................cgcacuagaccagacucucu...	24	0	eg1
......................................cgcacuagaccagUcucucu...	1	1	eg1
......................................cgcacuagaccagacucucC...	1	1	eg1
......................................cgcacuagaccagacucucua..	1	0	eg1
......................................cgcacuagaccagacucucuUa...	1	1	eg1
....cacuagaccagacucucC...	1	1	eg1
....uagaccagacucucuaUuuuauuuu...	1	1	eg1
....gauauagugagucugguaU.......................	1	1	eg1
....gauauagugagucugguaccc.....................	2	0	eg1
....gauauagugagucugguaccca....................	1	0	eg1
....gauauagugagucugguacccU....................	6	1	eg1
....gauauagugagucugguacccC....................	1	1	eg1
....gauauagugagucugguacccG....................	1	1	eg1
....gauauagugagucugguacccagggu................	2	0	eg1
....auauagugagucugguaccca....................	1	0	eg1
....uauaguAagucugguaccc.....................	1	1	eg1
....uauagugagucugguacccU....................	2	1	eg1
....uauagugagucugguaccca....................	1	0	eg1
....uauagugagucugguaccAa....................	1	1	eg1
....uauagugagucugguacccag...................	2	0	eg1
....uauagugagucugguacAcag...................	1	1	eg1
....Gauagugagucugguacccagg..................	1	1	eg1
....uauagugagucugguacccagg..................	2	0	eg1
....uauagugagucugguacccaggU.................	1	1	eg1
....Gauagugagucugguacccaggg.................	7	1	eg1
....uauagugagucugguacccaggg.................	21	0	eg1
....uaGagugagucugguacccaggg.................	1	1	eg1
....uauaguAagucugguacccaggg.................	1	1	eg1
....uauagugagucugguacccaggA.................	4	1	eg1
....uauagugagucugguacccagggu................	1	0	eg1
....uauagugagucugguacccagggua...............	1	0	eg1
....uauagugagucugguacccaggguaA..............	1	1	eg1
....uauagugagucugguacccaggguaU..............	1	1	eg1
....auagugagucugguacccaggg.................	9	0	eg1
....uagugagucugguacccaggg.................	3	0	eg1
....uagugagucugguGcccaggg.................	1	1	eg1
....uagugagucugguacccagggCa...............	1	1	eg1
....uagugagucugguacccaggguG...............	1	1	eg1
....uagugagucugguacccagggua...............	2	0	eg1
....agugagucugguacccaggg.................	1	0	eg1
....agugagucugguacccagggua...............	1	0	eg1
....ugagCcugguacccagggu................	1	1	eg1
....ugagucugguacccaggguaU..............	2	1	eg1
....ugagucugguacccaggguaUc.............	1	1	eg1

Star Mature

ugauugguuaauaucagcacaccagaacgagaauggggcgcacuagaccagacucucuaauuuauuuuagauauagugagucugguacccaggguacccaggcuaaaagca

........uaauaucagcacGccagaacg..	1	1	ea1
........uaauaucagcacaccagaacgagaau...	2	0	ea1
........uaauaucagcacaccagaacgagaauA..	3	1	ea1
........Gaauaucagcacaccagaacgagaaug..	1	1	ea1
........uaauaucagcacaccagaacgagaaug..	18	0	ea1
.............uAagcacaccagaacgagaaug..	1	1	ea1
.............ucagcacaccagaacgagaaug..	2	0	ea1
.............ucagcacaccagaacgagaauggggc..	1	0	ea1
..............cagcacaccagaacgagaauA..	1	1	ea1
..............cagcacaccagaacgagaaug..	1	0	ea1
....................aUcagaacgagaauggggcgcacuaga..	1	1	ea1
......................cagaacgagaauggggcgcacu...	1	0	ea1
......................cagaacgagaauggggcgcacA...	1	1	ea1
.......................Cgaacgagaauggggcgcacua..	1	1	ea1
..............................gaauggggcAcacuagacc..	1	1	ea1
..............................gaauggggcgcacuagacc..	1	0	ea1
................................auggggcgcacuagaccagacA...	2	1	ea1
.................................uggggcgcacuagaccagacA...	3	1	ea1
.................................uggggcgcacuagaccagacu...	8	0	ea1
.................................Gggggcgcacuagaccagacu...	1	1	ea1
.................................uggggcgcacuagaccagacuA..	9	1	ea1
.................................uggggcgcacuagaccagacuAu...	9	1	ea1
...................................gggcgcacuagaAcagacucu...	1	1	ea1
...................................gggcgcacuagaccagacucu...	6	0	ea1
....................................ggcgcacuagaccagGcucu...	1	1	ea1
....................................ggcgcacuagaccagacucC...	1	1	ea1
....................................ggcgcacuagaccagacucu...	11	0	ea1
....................................ggcgcacuagaccagacucuc..	4	0	ea1
....................................ggcgcacuagaccagacucucu...	1	0	ea1
....................................ggcgcacuagaccagacucucA...	4	1	ea1
....................................ggcgcacuagaccagacucucua..	1	0	ea1
.....................................gcgcacuagaccagacucucu...	1	0	ea1
.....................................gcgcacuagaccagacucucC...	1	1	ea1
......................................cgcacuagaccagacucuc..	3	0	ea1
......................................Ggcacuagaccagacucucu...	1	1	ea1
......................................cgcacuGgaccagacucucu...	1	1	ea1
......................................cgcacuagGccagacucucu...	2	1	ea1
......................................cgcacuagaccagacCcucu...	1	1	ea1
......................................cgcacuagaccagacucucG...	1	1	ea1
......................................cgcacuagaccagacGcucu...	1	1	ea1
......................................cgcacuagaccagacucAcu...	1	1	ea1
......................................cgcacuagaccGgacucucu...	1	1	ea1
......................................cgcacuagaccagacucucC...	6	1	ea1
......................................Ugcacuagaccagacucucu...	1	1	ea1
......................................cgcacuagaAcagacucucu...	1	1	ea1
......................................cAcacuagaccagacucucu...	3	1	ea1
......................................cgcacuagaccagacucucu...	97	0	ea1
......................................cgcacuagaccagacucucA...	4	1	ea1
......................................cgcacuagaccagGcucucu...	1	1	ea1
......................................cgcacuagaccagacucucua..	5	0	ea1
......................................cgcacuagaccagacucucuU..	4	1	ea1
......................................cgcacuagaccagacucucuaau..	1	0	ea1
....cacuagaccagacucucua..	1	0	ea1
....cacuagaccagacucucuaC...	2	1	ea1
....ccagacucucuaUuuuauuu..	1	1	ea1
....ccagacucucuaUuuuauuua...	1	1	ea1
....aCauauagugagucugguaccc.....................	1	1	ea1
....Cauauagugagucugguac.......................	1	1	ea1
....gaGauagugagucugguacc......................	2	1	ea1
....gauauagugagucugguacU......................	2	1	ea1
....gauauagugagucugguacA......................	4	1	ea1
....gauauagugagucugguacG......................	1	1	ea1
....gauauagugagucugguacc......................	8	0	ea1
....gauauagugagucCgguaccc.....................	1	1	ea1
....gauauagugagucugguaccA.....................	8	1	ea1
....gauauagugGgucugguaccc.....................	1	1	ea1
....gauauagugagucugguaccU.....................	14	1	ea1
....gauauagugagucugguaccc.....................	54	0	ea1
....Cauauagugagucugguaccca....................	3	1	ea1

Star Mature

ugauugguuaauaucagcacaccagaacgagaauggggcgcacuagaccagacucucuaauuuauuuuagauauagugagucugguacccaggguacccaggcuaaaagca			
....gauauagugagucugguacccU....................	20	1	ea1
....gauauagugagucugguaccca....................	20	0	ea1
....gauauagugagucugguacccC....................	46	1	ea1
....gauauagugagucugguacAca....................	1	1	ea1
....gauauagugagucugguacccG....................	1	1	ea1
....gauauagugagucugguacccaAg..................	1	1	ea1
....auauagugGgucugguac.......................	1	1	ea1
....auauagugagucugguacc......................	2	0	ea1
....auauagugagucugguacA......................	1	1	ea1
....auauagugagucugguaccU.....................	1	1	ea1
....auauagugagucugguaccc.....................	1	0	ea1
....auauagugagucugguacccU....................	1	1	ea1
....auauagugagucugguacAca....................	3	1	ea1
....auauagugagucugguacccC....................	6	1	ea1
....Cuauagugagucugguacccaggg.................	1	1	ea1
....uauagugagucugguacc......................	3	0	ea1
....uauagugagucugguacA......................	1	1	ea1
....uauagugagucugguaccA.....................	3	1	ea1
....uauagugagGcugguaccc.....................	1	1	ea1
....uauagugagucugguaccU.....................	9	1	ea1
....uauagCgagucugguaccc.....................	2	1	ea1
....Gauagugagucugguaccc.....................	3	1	ea1
....uauagugagucugguaccc.....................	19	0	ea1
....uauagCgagucugguaccca....................	2	1	ea1
....uauagugagCcugguaccca....................	1	1	ea1
....uauagAgagucugguaccca....................	1	1	ea1
....uauagugagucugguacccU....................	21	1	ea1
....uauagugagucugguacccG....................	1	1	ea1
....Cauagugagucugguaccca....................	1	1	ea1
....uauagugagucugguacccC....................	28	1	ea1
....uaGagugagucugguaccca....................	1	1	ea1
....uauagugagucugguaccca....................	16	0	ea1
....Gauagugagucugguaccca....................	2	1	ea1
....uauagugagucugguacccaA...................	2	1	ea1
....uauagugagucugguacccaU...................	2	1	ea1
....uauagugagucugguacccaC...................	1	1	ea1
....Gauagugagucugguacccag...................	8	1	ea1
....uaGagugagucugguacccag...................	1	1	ea1
....uauagugagucugguacccag...................	46	0	ea1
....Cauagugagucugguacccag...................	1	1	ea1
....uauagugagucugguacccagA..................	7	1	ea1
....uauagugaAucugguacccagg..................	1	1	ea1
....Gauagugagucugguacccagg..................	16	1	ea1
....uauagugagucugguacccagg..................	59	0	ea1
....uauagugagucugguacccGgg..................	1	1	ea1
....uauagugagucugguacccagC..................	1	1	ea1
....uauagugagucugguacccagU..................	4	1	ea1
....uauagugagucugguacccaggU.................	6	1	ea1
....uauagugagucugguacccaggg.................	725	0	ea1
....uauGgugagucugguacccaggg.................	2	1	ea1
....uaGagugagucugguacccaggg.................	3	1	ea1
....uauagugagucuggCacccaggg.................	1	1	ea1
....uGuagugagucugguacccaggg.................	1	1	ea1
....uauagugagucugguaccUaggg.................	1	1	ea1
....uauagugGgucugguacccaggg.................	5	1	ea1
....Gauagugagucugguacccaggg.................	179	1	ea1
....uauagugagucugguaAccaggg.................	1	1	ea1
....Cauagugagucugguacccaggg.................	6	1	ea1
....uauagugagucugguacccagUg.................	1	1	ea1
....uaCagugagucugguacccaggg.................	1	1	ea1
....uauUgugagucugguacccaggg.................	1	1	ea1
....uauagugaAucugguacccaggg.................	1	1	ea1
....uauagugagucuggGacccaggg.................	1	1	ea1
....uauagugagucugguGcccaggg.................	4	1	ea1
....uauagugagucugguacccaggC.................	5	1	ea1
....uauagugagucugguaUccaggg.................	1	1	ea1
....uauagugagucCgguacccaggg.................	2	1	ea1
....uauagugagucugguacccaggA................	51	1	ea1
....uauCgugagucugguacccaggg.................	1	1	ea1
....uauagugCgucugguacccaggg.................	3	1	ea1

Star **Mature**

```
ugauugguuaauaucagcacaccagaacgagaaugggg cgcacuagaccagacucucu aauuuauuuuaga uauagugagucugguacccaggg uacccaggcuaaaagca
```

```
ugauugguuaauaucagcacaccagaacgagaauggggcgcacuagaccagacucucuaauuuauuuuagauauagugagucugguacccaggguacccaggcuaaaagca
.......................................................................uauagugagucugguacccagggu................ 6    0  ea1
.......................................................................uauGgugagucugguacccagggu................ 1    1  ea1
.......................................................................uauagugagucugguacccagggA................ 6    1  ea1
.......................................................................uauagugagucugguacccagggAa............... 2    1  ea1
.......................................................................uauagugagucugguacccagggCa............... 2    1  ea1
.......................................................................uauagugagucugguacccagggua............... 2    0  ea1
.......................................................................uauagugagucugguacccaggguaA.............. 8    1  ea1
.......................................................................uauagugagucugguacccaggguac.............. 1    0  ea1
.......................................................................uauagugagucugguacccaggguaU.............. 1    1  ea1
........................................................................auagugagucugguaccc..................... 1    0  ea1
........................................................................auagugagucugguaccU..................... 1    1  ea1
........................................................................auagugagucugguacccag................... 5    0  ea1
........................................................................auagugagucugguacccagg.................. 4    0  ea1
........................................................................Guagugagucugguacccaggg................. 5    1  ea1
........................................................................aCagugagucugguacccaggg................. 1    1  ea1
........................................................................auagugagucugguGcccaggg................. 1    1  ea1
........................................................................auagugagucugguacccaggU................. 4    1  ea1
........................................................................auagugagucugguacccaggA................. 8    1  ea1
........................................................................auagugagucugguacccaggg................. 222  0  ea1
........................................................................auagugagucugguacccaggC................. 3    1  ea1
........................................................................auagugCgucugguacccaggg................. 1    1  ea1
........................................................................auagugagucuggCacccaggg................. 1    1  ea1
........................................................................auagugUgucugguacccaggg................. 1    1  ea1
........................................................................auagCgagucugguacccaggg................. 1    1  ea1
........................................................................auagugagucugguacccagggu................ 5    0  ea1
........................................................................auagugagucugguacccagggC................ 1    1  ea1
........................................................................auagugagucugguacccagggA................ 2    1  ea1
........................................................................auagugagucugguacccaggguU............... 3    1  ea1
........................................................................auagugagucugguacccagggua............... 4    0  ea1
........................................................................auagugagucugguacccagggCa............... 2    1  ea1
........................................................................auagugagucugguacccaggguG............... 1    1  ea1
........................................................................auagugagucugguacccagggAa............... 1    1  ea1
........................................................................auagugagucugguacccaggguaA.............. 3    1  ea1
........................................................................auagugagucugguacccaggguaU.............. 1    1  ea1
.........................................................................uagAgagucugguaccca.................... 2    1  ea1
.........................................................................uagugagucugguaccca.................... 1    0  ea1
.........................................................................uagugagucugguaccUag................... 1    1  ea1
.........................................................................uagugagGcugguacccagg.................. 1    1  ea1
.........................................................................uagugagucugguaccUagg.................. 11   1  ea1
.........................................................................uagAgagucugguacccagg.................. 1    1  ea1
.........................................................................uagugagucugguacccagU.................. 1    1  ea1
.........................................................................Gagugagucugguacccaggg................. 2    1  ea1
.........................................................................uagugagucugguacccaggg................. 14   0  ea1
.........................................................................uagugagucugguacccaggU................. 2    1  ea1
.........................................................................uagugagucugguaccUaggg................. 4    1  ea1
.........................................................................uagugagucugguacccaggA................. 2    1  ea1
.........................................................................uUgugagucugguacccaggg................. 1    1  ea1
.........................................................................uagugagucugguacccagggC................ 4    1  ea1
.........................................................................uagugagucugguacccagggu................ 2    0  ea1
.........................................................................Gagugagucugguacccagggu................ 1    1  ea1
.........................................................................uagugagucugguacccaggguU............... 2    1  ea1
.........................................................................Gagugagucugguacccagggua............... 1    1  ea1
.........................................................................uagugagucugguacccagggua............... 6    0  ea1
..........................................................................agugagucugguacccagg.................. 1    0  ea1
..........................................................................agugagucugguacccaggAu................ 1    1  ea1
..........................................................................agugagucugguacccagggC................ 1    1  ea1
..........................................................................agugagucugguacccaggguG............... 1    1  ea1
..........................................................................agugagucugguacccagggua............... 3    0  ea1
...........................................................................gugagucugguacccaggg................. 1    0  ea1
...........................................................................gugagucugguGcccagggua............... 1    1  ea1
...........................................................................gugagucugguacccagggua............... 1    0  ea1
...........................................................................gugagucugguacccaggguU............... 3    1  ea1
............................................................................ugagucugguacccagggA................ 1    1  ea1
............................................................................ugagucugguacccagggua............... 3    0  ea1
............................................................................ugagucugguacccaggguUc.............. 1    1  ea1
............................................................................Ggagucugguacccaggguac.............. 1    1  ea1
............................................................................ugagucugguacccaggguaU.............. 10   1  ea1
............................................................................ugagucugguacccaggguac.............. 9    0  ea1
............................................................................ugagucugguacccaggguacc............. 1    0  ea1
............................................................................ugagucugguacccaggguaUc............. 3    1  ea1
```

Star **Mature**

```
ugauugguuaauaucagcacaccagaacgagaauggggcgcacuagaccagacucucuaauuuauuuuagauauagugagucugguacccaggguacccaggcuaaaagca
............................................................................ugagucugguacccaggguaAcc............	2	1	ea1
............................................................................ugagucugguacccaggguaGcca...........	2	1	ea1

..............cagcacaccagaacgagaaCgg...........................................................................	1	1	er2
.................................uggggcgcacuagaccagacuG........................................................	1	1	er2
.................................uggggcgcacuagaccagacuA........................................................	5	1	er2
.................................uggggcgcacuagaccagacuAu.......................................................	15	1	er2
.................................uggggcgcacuagaccagacucu.......................................................	1	0	er2
......................................cgcacuagaccagacucucu.....................................................	21	0	er2
......................................cgcacuagaccagacucucG.....................................................	1	1	er2
......................................cAcacuagaccagacucucu.....................................................	3	1	er2
......................................cgcacuagaccagacucucua....................................................	3	0	er2
.......................................gcacuagaccagacucucua....................................................	2	0	er2
......................................................................auauagugagucugguaccc.....................	3	0	er2
......................................................................auauagugagucugguacccU....................	10	1	er2
......................................................................auauagugagucugguacccC....................	4	1	er2
......................................................................auauagugagucugguacccaU...................	1	1	er2
.......................................................................uauagugagucugguaccca....................	6	0	er2
.......................................................................uauagugagucugguacccU....................	27	1	er2
.......................................................................uauagugagucugguacccC....................	15	1	er2
.......................................................................uauagugagucugguacccag...................	15	0	er2
.......................................................................Nauagugagucugguacccag...................	1	1	er2
.......................................................................Cauagugagucugguacccag...................	2	1	er2
.......................................................................uauagugagucugguacccagg..................	7	0	er2
.......................................................................uauagugagucugguacccagU..................	5	1	er2
.......................................................................uaGagugagucugguacccagg..................	3	1	er2
.......................................................................uauagugagucugguacccaggg.................	47	0	er2
.......................................................................Nauagugagucugguacccaggg.................	1	1	er2
.......................................................................uauagugagucugguacccaggguaA..............	2	1	er2
........................................................................auagugagucugguacccag...................	1	0	er2
........................................................................auagugagucugguacccagg..................	2	0	er2
........................................................................auagugagucugguacccaggU.................	1	1	er2
........................................................................Nuagugagucugguacccaggg.................	1	1	er2
........................................................................auagugagucugguacccaggg.................	9	0	er2
........................................................................auagugagucugguacccagggC................	4	1	er2
.........................................................................uagugagucugguaccca....................	1	0	er2
.........................................................................uagugagucugguacccU....................	3	1	er2
.........................................................................uagugagucugguacccagg..................	22	0	er2
.........................................................................uagugagucugguacccaggg.................	108	0	er2
.........................................................................uagugagucugguacccagggC................	1	1	er2
.........................................................................Nagugagucugguacccagggu................	1	1	er2
.........................................................................uagugagucugguacccagggu................	71	0	er2
.........................................................................uagugagucugguacccaggguC...............	4	1	er2
.........................................................................Nagugagucugguacccagggua...............	1	1	er2
.........................................................................uagugagucugguacccaggguG...............	3	1	er2
.........................................................................uagugagucugguacccaggAua...............	1	1	er2
.........................................................................uagugagucugguacccagggua...............	34	0	er2
.........................................................................uagugagucugguacccaggguU...............	21	1	er2
.........................................................................uagugagucugguacccaggguaU..............	124	1	er2
.........................................................................uagugagucugguacccaggguac..............	1	0	er2
.........................................................................uagugagucugguacccaggguaAc.............	4	1	er2
..........................................................................agugagucugguacccaggg.................	2	0	er2
..........................................................................agugagucugguacccagggu................	4	0	er2
..........................................................................agugagucugguacccaggguU...............	2	1	er2
..........................................................................agugagucugguacccagggua...............	6	0	er2
..........................................................................agugagucugguacccaggguC...............	2	1	er2
..........................................................................agugagucugguacccaggguaU..............	8	1	er2
...........................................................................gugagucugguacccaggg.................	2	0	er2
............................................................................ugagucugguacccaggguG...............	3	1	er2
............................................................................ugagucugguacccaggguac..............	8	0	er2

........uaauaucagcacaccagaacga.................................................................................	1	0	ea2
........uaauaucagcacaccagaacgagaaug............................................................................	3	0	ea2
.............ucagcacaccagaacgag................................................................................	2	0	ea2
.................................uggggcgcacuagaccagacA.........................................................	3	1	ea2
.................................uggggcgcacuagaccagacu.........................................................	7	0	ea2
.................................uggggcgcacuagaccagacuG........................................................	3	1	ea2
.................................uggggcgcacuagaccagacuAu.......................................................	8	1	ea2
....................................ggcgcacuagaccagacucuc......................................................	6	0	ea2
......................................cgcacuagaccagacucucu.....................................................	28	0	ea2
```

```
                                      Star                             Mature
ugauugguuaauaucagcacaccagaacgagaauggggcgcacuagaccagacucucuaauuuauuuuagauauagugagucugguacccaggguacccaggcuaaaagca
......................................cAcacuagaccagacucucu.....................................................   3   1   ea2
......................................cgcacuagaccagacucucA.....................................................   1   1   ea2
......................................cgcacuagaccagacucCcu.....................................................   1   1   ea2
......................................cgcacuagaccagacucucua....................................................   2   0   ea2
.......................................gcacuagaccagacucucu.....................................................   8   0   ea2
.......................................gcacuagaccagacucucua....................................................   2   0   ea2
........................................cacuagaccagacucucu.....................................................   16  0   ea2
.....................................................................Cauauagugagucugguacc......................   1   1   ea2
......................................................................auauagugagucugguaccca....................   4   0   ea2
......................................................................Nuauagugagucugguaccca....................   1   1   ea2
......................................................................auauagugagucugguacccC....................   2   1   ea2
......................................................................auauagugagucugguacccU....................   11  1   ea2
......................................................................aGauagugagucugguacccagg..................   1   1   ea2
.......................................................................uauagugagucugguaccc.....................   3   0   ea2
.......................................................................uauagugagucugguacccC....................   15  1   ea2
.......................................................................uauagugagucugguaccca....................   17  0   ea2
.......................................................................uauagugagucugguacccU....................   46  1   ea2
.......................................................................uaGagugagucugguaccca....................   1   1   ea2
.......................................................................Nauagugagucugguacccag...................   1   1   ea2
.......................................................................uaGagugagucugguacccag...................   6   1   ea2
.......................................................................uauagugagucugguacccag...................   49  0   ea2
.......................................................................uauagugagucugguacccagU..................   3   1   ea2
.......................................................................uauagugUgucugguacccagg..................   2   1   ea2
.......................................................................uauagugagucugguacccagg..................   11  0   ea2
.......................................................................uauagugagucugguacccagA..................   1   1   ea2
.......................................................................uauagGgagucugguacccaggg.................   1   1   ea2
.......................................................................uauagugagucugguacccaggg.................   138 0   ea2
.......................................................................Nauagugagucugguacccaggg.................   1   1   ea2
.......................................................................uauagugagucugguacccagggua...............   4   0   ea2
.......................................................................uauagugagucugguacccaggguaU..............   1   1   ea2
.......................................................................uauagugagucugguacccaggguaA..............   1   1   ea2
........................................................................auagugagucugguacccU....................   3   1   ea2
........................................................................aGagugagucugguaccca....................   2   1   ea2
........................................................................auagugagucugguacccag...................   1   0   ea2
........................................................................auagugagucugguacccagg..................   8   0   ea2
........................................................................auagugagucugguacccaggg.................   83  0   ea2
........................................................................auagugagucugguacccagggCa...............   2   1   ea2
.........................................................................uagugagucugguaccca....................   2   0   ea2
.........................................................................uagugagucugguacccag...................   4   0   ea2
.........................................................................uagugagucugguacccagg..................   1   0   ea2
.........................................................................uagugagucugguacccaggg.................   15  0   ea2
.........................................................................Nagugagucugguacccaggg.................   1   1   ea2
.........................................................................uagugagucugguacccagggCa...............   3   1   ea2
.........................................................................uagugagucugguacccaggAua...............   1   1   ea2
.........................................................................uagugagucugguacccagggua...............   7   0   ea2
.........................................................................uagugagucugguacccaggguG...............   13  1   ea2
.........................................................................uagugagCcugguacccagggua...............   1   1   ea2
.........................................................................uagugagucugguacccaggguaU..............   4   1   ea2
.........................................................................uagugagucugguacccaggguaAc.............   2   1   ea2
............................................................................ugagucugguacccaggg.................   2   0   ea2
............................................................................ugagucugguacccaggguaU..............   2   1   ea2
............................................................................ugagucugguacccaggguac..............   5   0   ea2
............................................................................ugagucugguaccGaggguac..............   2   1   ea2
............................................................................ugagucugguacccaggguaUc.............   2   1   ea2

........uaauaucagcacaccagaacgagaau.............................................................................   1   0   eg2
........uaauaucagcacaccagaacgagaaug............................................................................   8   0   eg2
.........aauaucagcacaccagaacgagaaug............................................................................   1   0   eg2
.........aauaucagcacaccagaacgagaaugg...........................................................................   6   0   eg2
...........uaucagcacaccagaacgagaaug............................................................................   2   0   eg2
.............ucagcacaccagaacgagaaug............................................................................   1   0   eg2
.............ucagcacaccagaacgagaauggggc........................................................................   1   0   eg2
.................................uggggcgcacuagaccagacA.........................................................   5   1   eg2
.................................uggggcgcacuagaccagacuA........................................................   2   1   eg2
.................................uggggcgcacuagaccagacuAu.......................................................   1   1   eg2
......................................cgcacuagaccagacucucu.....................................................   3   0   eg2
......................................cgcacuagaccagacucucua....................................................   1   0   eg2
......................................cgcacuagaccagacucucuUa...................................................   1   1   eg2
.......................................gcacuagaccagacucucu.....................................................   1   0   eg2
......................................................................auauagugagucugguacccU....................   2   1   eg2
```

```
                                      Star                            Mature
ugauugguuaauaucagcacaccagaacgagaaugggcgcacuagaccagacucucuaauuuauuuuagauauagugagucugguacccaggguacccaggcuaaaagca
......................................................................uauagugagucugguaccca....................   6   0   eg2
......................................................................uauagugagucugguacccC....................   2   1   eg2
......................................................................uauagugagucugguacccU....................   9   1   eg2
......................................................................uauagugagucugguacccag...................   2   0   eg2
......................................................................uauagugagucugguUcccagg..................   2   1   eg2
......................................................................uauagugagucugguacccagg..................   2   0   eg2
......................................................................uauagugagucugguacccaggg.................  19   0   eg2
.......................................................................auagugagucugguacccaggg.................   6   0   eg2
........................................................................uagugagucugguacccaggg.................   6   0   eg2
.........................................................................agugagucugguaccUagg..................   1   1   eg2
.........................................................................agugagucugguacccaggg.................   1   0   eg2
...........................................................................ugagucugguacccaggguac..............   1   0   eg2
...........................................................................ugagucugguacccagggua U..............   1   1   eg2
```

Provisional ID	:	ScUbCFx_795_26213
Score total	:	1.7
Score for star read(s)	:	-1.3
Score for read counts	:	0
Score for mfe	:	-1.5
Score for randfold	:	1.6
Score for cons. seed	:	3
Total read count	:	811
Mature read count	:	807
Loop read count	:	0
Star read count	:	4

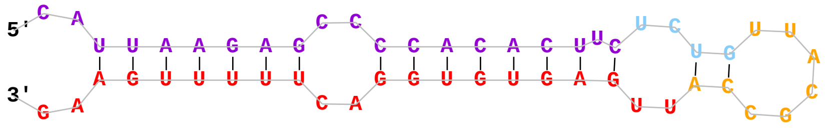

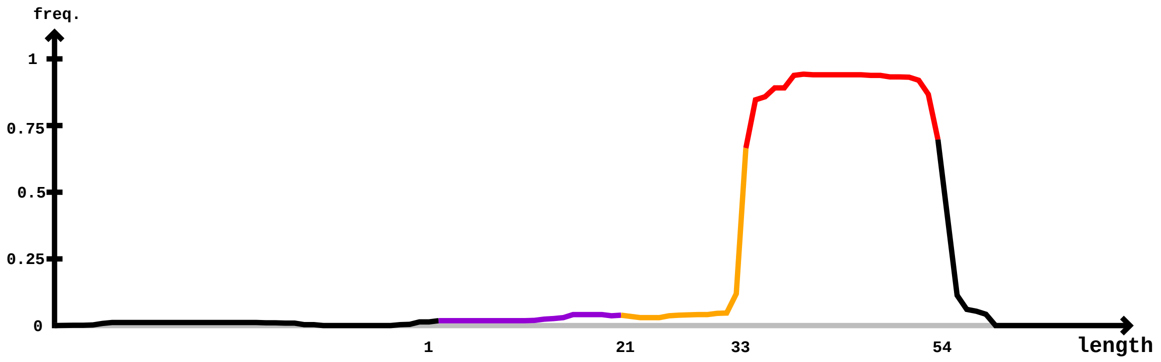

Star **Mature**

```
5'- aacuugacucaaauuuaguguacccaaaauacuacuuucauuaagagccccacacuucucuguuacgccauugaguguggacuuuuugaagagaucucaaaaguucaaga  -3'   obs
    aacuugacucaaauuuaguguacccaaaauacuacuuucauuaagagccccacacuucucuguuacgccauugaguguggacuuuuugaagagaucucaaaaguucaaga         exp
    ..(((((((......((((((.......)))))).((((.(((((((..(((((((.(..((......))..))))))))..))))))).)))).......)).))))).    reads     mm      sample
    .................................................ccacacuucucuguuacgccU........................................    1         1       er1
    ...............................................................uacgccauugaguguggacuuuuuga.....................    1         0       er1
    ....................................................................Aauugaguguggacuuuuugaag...................    1         1       er1
    .......................................................................Ggaguguggacuuuuugaag...................    1         1       er1
    .......................................................................ugaguguggacuuuuugaag...................    1         0       er1
    .........................................................................aguguggacuuuuugaGgag.................    2         1       er1
    ...........................................................................uguggacuuuuugaagagauc..............    1         0       er1

    .................................................ccacacuucucuguuacgcca........................................    1         0       eg1
    .................................................ccacacuucucuguuacgccau.......................................    1         0       eg1
    ..................................................cacacuucucuguuacgccauu......................................    1         0       eg1
    ....................................................cacuucucuguuacgccauu......................................    2         0       eg1
    ........................................................uUucuguuacgccauugagug.................................    1         1       eg1
    ..............................................................Guacgccauugaguguggacu...........................    1         1       eg1
    ..............................................................uuacgccauugaguguggacu...........................    1         0       eg1
    ..............................................................uuacgccauugaguguggacuuuuC.......................    1         1       eg1
    .....................................................................auugaguguggacuuuuuga.....................    1         0       eg1
    .....................................................................auugaguguggacuuuuugaa....................    1         0       eg1
    ......................................................................uugaguguggacuuuuuga.....................    1         0       eg1
    ......................................................................Gugaguguggacuuuuugaa....................    2         1       eg1
    .......................................................................ugaguguggacuuuuuga.....................    2         0       eg1
    .......................................................................ugaguguggacuuuuugaag...................    2         0       eg1
    .......................................................................Ggaguguggacuuuuugaag...................    1         1       eg1
    ...........................................................................uguggacuuuuugaagagauc..............    2         0       eg1
    ...........................................................................uguggacuuuuugaagagauA..............    1         1       eg1

    ...uugacucaaauuuaguguacA......................................................................................    1         1       ea1
    ................................................cccacacuucucuguuacgcc.........................................    1         0       ea1
    .................................................ccacacuucucuguuacgcca........................................    1         0       ea1
    ..................................................cacacuucucuguuacgccau.......................................    1         0       ea1
    ...................................................acacuucucuguuacgccau.......................................    1         0       ea1
    ...................................................acacuucucuguuacgccauu......................................    1         0       ea1
    ...................................................acacuucucuguuacgccauuUa....................................    1         1       ea1
```

```
                                       Star                   Mature
aacuugacucaaauuuaguguacccaaaauacuacuuucauuaagagccccacacuucucuguuacgccauugaguguggacuuuuugaagagaucucaaaaguucaaga
....................................................cacuucucuguuacgccau.......................................   1   0   ea1
....................................................cacuucucuguuacgccauu......................................   3   0   ea1
....................................................cacuuUucuguuacgccauu......................................   1   1   ea1
....................................................cacuucucuguuacgccauuUa....................................   3   1   ea1
......................................................cuucucuguuacgccauuga....................................   1   0   ea1
......................................................cuuUucuguuacgccauuga....................................   1   1   ea1
..............................................................uuacgccauugaguguggacuAu.........................   3   1   ea1
.....................................................................auugaguguggacuuuuuga.....................   4   0   ea1
.....................................................................auugaguguggacuuuuugaaA...................   1   1   ea1
......................................................................uugaguguggacuuuuug......................   3   0   ea1
......................................................................Gugaguguggacuuuuuga.....................   2   1   ea1
......................................................................uugaguguggacuuuuuAa.....................   1   1   ea1
......................................................................uugaguguggacuuuuuga.....................   5   0   ea1
......................................................................uugaguguggacuuuuugaa....................   5   0   ea1
......................................................................uugaguguggacuuuuugaaA...................   1   1   ea1
......................................................................Gugaguguggacuuuuugaag...................   1   1   ea1
......................................................................uugaguguggacuuuuugaag...................   2   0   ea1
......................................................................uugaguguggacuuuuugaaga..................   1   0   ea1
......................................................................uugaguguggacuuuuugaagagauc..............   2   0   ea1
.......................................................................ugaguguggacuuuuugaa....................   1   0   ea1
.......................................................................ugaguguggacuuuuugaag...................   1   0   ea1
.......................................................................Ggaguguggacuuuuugaag...................   1   1   ea1
.........................................................................aguguggacuuuuugaagag.................   1   0   ea1
...........................................................................uguggacuuuuugaagagauc..............   3   0   ea1
...........................................................................uguggacuuuuugaagagauA..............   2   1   ea1

aacuugacucaaauuuagug..........................................................................................   1   0   er2
...uugacucaaauuuaguguacA......................................................................................   3   1   er2
....................................uucauuaagagccccacacu......................................................   2   0   er2
....................................uucauuaagagccccacacuuA....................................................   3   1   er2
.....................................................................auugaguguggacuuuuuga.....................   1   0   er2
.....................................................................auugaguguggacuuuuugaa....................   6   0   er2
.....................................................................auugaguguggacuuuuugaaA...................   6   1   er2
.....................................................................auugaguguggacuuuuugaag...................   2   0   er2
.....................................................................auugaguguggacuuuuugaaAa..................   2   1   er2
......................................................................uugaguguggacuuuuuga.....................   9   0   er2
......................................................................uugaguguggacuuuuugaa....................   31  0   er2
......................................................................uugaguguggGcuuuuugaa....................   1   1   er2
......................................................................uugaguguggacuuuuugaag...................   22  0   er2
......................................................................uugaguguggacuuuuugaaA...................   55  1   er2
......................................................................uugaguguggacuuuuugaaAa..................   18  1   er2
......................................................................uugaguguggacuuuuugaagag.................   1   0   er2
.......................................................................ugaguguggacuuuuugaa....................   2   0   er2
.......................................................................ugagGguggacuuuuugaag...................   1   1   er2
.......................................................................ugaguguggacuuuuugaaA...................   7   1   er2
.......................................................................ugaguguggacuuuuugaag...................   39  0   er2
.......................................................................ugaguguggacuuuuugaaga..................   5   0   er2
.......................................................................ugaguguggacuuuuugaaUa..................   4   1   er2
.......................................................................ugaguguggacuuuuugaaAa..................   1   1   er2
.......................................................................ugaguguggacuuuuugaagaAa................   3   1   er2
........................................................................gaguguggacuuuuugaa....................   1   0   er2
........................................................................gaguguggacuuuuugaaA...................   1   1   er2
........................................................................gaguguggacuuuuugaag...................   1   0   er2
........................................................................gaguguggacuuuuugaaga..................   2   0   er2
........................................................................gaguguggacuuuuugaaUa..................   1   1   er2
........................................................................gaguguggacuuuuugaagaCa................   2   1   er2
.........................................................................aguguggacuuuuugaag...................   1   0   er2
.........................................................................aguguggacuuuuugaaAa..................   1   1   er2
.........................................................................aguguggacuuuuugaagag.................   7   0   er2
...........................................................................uguggacuuuuugaagaAa................   1   1   er2
.............................................................................uggacuuuuugaagagauc..............   1   0   er2

..cuugacucaaauuuagugua........................................................................................   1   0   ea2
....ugacucaaauuuaguguaccca....................................................................................   3   0   ea2
..................................cuuucauuaagagccccaca........................................................   3   0   ea2
....................................uucauuaagagccccacacu......................................................   3   0   ea2
......................................cauuaagagccccacacuuU....................................................   4   1   ea2
.........................................................Uucuguuacgccauugagug.................................   2   1   ea2
...................................................................ccauugaguguggacuuuuug......................   1   0   ea2
...................................................................ccauugaguguggacuuuuuA......................   1   1   ea2
```

Star **Mature**

aacuugacucaaauuuaguguacccaaaauacuacuuucauuaagagccccacacuucucuguuacgccauugaguguggacuuuuugaagagaucucaaaaguucaaga			
....auugaguguggacuuuuug......................	3	0	ea2
....auugaguguggacuuuuugaa....................	7	0	ea2
....auugaguguggacuuuuugaaA...................	5	1	ea2
....auugaguguggacuuuuugaag...................	8	0	ea2
....auugaguguggacuuuuugaaUa..................	1	1	ea2
....auugaguguggacuuuuugaaAa..................	1	1	ea2
....uugaguguggacuuuuuga.....................	2	0	ea2
....uugaguguggacuuuuugaa....................	57	0	ea2
....Nugaguguggacuuuuugaa....................	1	1	ea2
....uugaguguggacuuuuugaaA...................	99	1	ea2
....uugaguguggacuuuuugaag...................	25	0	ea2
....uugaguguggacuuuuugaaUa..................	2	1	ea2
....uugaguguggacuuuuugaaAa..................	24	1	ea2
....uugaguguggacuCuuugaagag.................	1	1	ea2
....uugaguguggacuuuuugaagag.................	10	0	ea2
....Nugaguguggacuuuuugaagag.................	1	1	ea2
....ugaguguggacuuuuuga.....................	6	0	ea2
....ugaguguggacuuuuugaag...................	46	0	ea2
....ugaguguggacuuuuugaaA...................	12	1	ea2
....ugagAguggacuuuuugaag...................	4	1	ea2
....Ngaguguggacuuuuugaag...................	1	1	ea2
....ugaguguggacuuuuugaagC..................	1	1	ea2
....ugaguguggacuuuuugaaAa..................	4	1	ea2
....gaguguggacuuuuugaag...................	3	0	ea2
....gaAuguggacuuuuugaaga..................	1	1	ea2
....gaguguggacuuuuugaaAa..................	3	1	ea2
....aguguggacuuuuugaaA...................	2	1	ea2
....aguguggacuuuuugaagag.................	11	0	ea2
....guguggacuuuuugaagag.................	7	0	ea2
....uguggacuuuuugaagagaA...............	6	1	ea2
....uguggacuuuuugaagagauc..............	6	0	ea2
....uguggacuuuuugaagagauA..............	11	1	ea2
....guggacuuuuugaagagauc..............	4	0	ea2
...uugacucaaauuuaguguacc..	1	0	eg2
...................................uuucauuaagagccccacacuuU..	1	1	eg2
....cuucucuguuacgccauuga....................................	1	0	eg2
....uacgccauugaguguggacuuuuuga.....................	1	0	eg2
....acgccauugaguguggacuuu.........................	1	0	eg2
....cgccauugaguguggacuuu.........................	1	0	eg2
....ccauugaguguggacuuuuug......................	2	0	eg2
....auugaguguggacuuuuuga.....................	1	0	eg2
....auugaguguggacuuuuugaa....................	3	0	eg2
....auugaguguggacuuuuugaaA...................	8	1	eg2
....auugaguguggacuuuuugaaAa..................	3	1	eg2
....uugaguguggacuuuuuga.....................	8	0	eg2
....uugaguguggacuuuuugaa....................	30	0	eg2
....uugaguguggacuuuuugaag...................	7	0	eg2
....uugaguguggacuuuuugaaA...................	23	1	eg2
....uugaguguggacuuuuugaaAa..................	14	1	eg2
....uugaguguggacuuuuugaaUa..................	8	1	eg2
....uugaguguggacuuuuugaaCa..................	4	1	eg2
....uugaguguggacuuuuugaagag.................	1	0	eg2
....ugaguguggacuuuuuga.....................	1	0	eg2
....ugaguguggacuuuuugaag...................	11	0	eg2
....ugaguguggacuuuuugaaA...................	3	1	eg2
....ugaguguggacuuuuugaaga..................	1	0	eg2
....gaguguggacuuuuugaag...................	2	0	eg2
....Naguguggacuuuuugaag...................	1	1	eg2
....aguguggacuuuuugaagag.................	4	0	eg2
....uguggacuuuuugaagagaA...............	4	1	eg2
....uguggacuuuuugaagagauA..............	4	1	eg2

Provisional ID	:	ScUbCFx_795_27849
Score total	:	2.2
Score for star read(s)	:	-1.3
Score for read counts	:	0
Score for mfe	:	2.5
Score for randfold	:	1.6
Score for cons. seed	:	-0.6
Total read count	:	457
Mature read count	:	413
Loop read count	:	26
Star read count	:	18

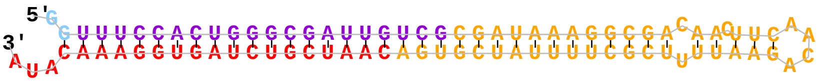

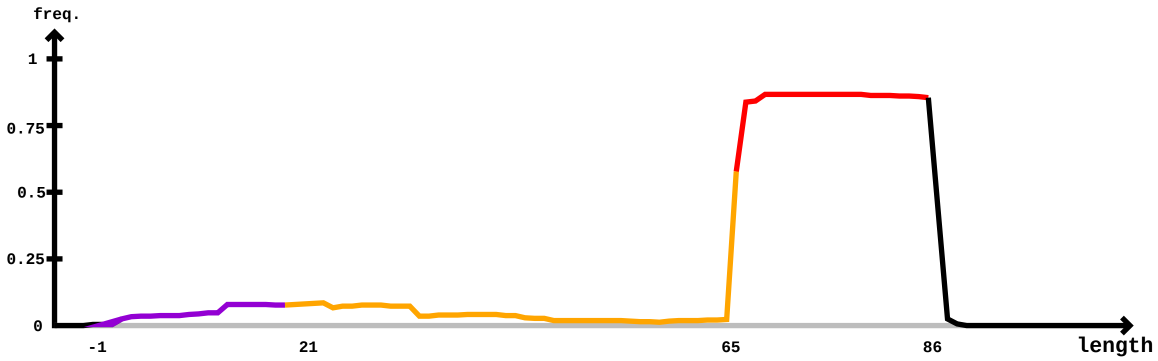


```
          Star                                                            Mature
5'- ccugguuuccacugggcgauugucgcgauaaaggcgacaacuucaacagaauuuucgcuuuuaucgugacaaucgucuaguggaaacauaauuuaaucgugauugucgcg   -3'    obs
    ccugguuuccacugggcgauugucgcgauaaaggcgacaacuucaacagaauuuucgcuuuuaucgugacaaucgucuaguggaaacauaauuuaaucgugauugucgcg          exp
    ....(((((((((((((((((((((((((((((((((.((.(((....))))).)))))))))))))))))))))))))))))))))..........((((.....))))    reads    mm    sample
    ..ugguuuccacugggcgauugu.......................................................................................    1    0    er1
    ..............................................................aucgugacaaucgucuaguggaUa........................    1    1    er1
    .....................................................................caaucgucCaguggaaacaua....................    1    1    er1

    .........................cgauaaCggcgacaacuuca.................................................................    1    1    eg1
    ............................uaaaggcgacaacuucaacaga............................................................    2    0    eg1
    ....................................................................acaaucgucCaguggaaacaua....................    1    1    eg1
    .....................................................................caaucgucCaguggaaacaua....................    12    1    eg1
    .....................................................................caaucgucuaguggaaacauU....................    1    1    eg1
    ......................................................................aaucgucuaguggaaacaua....................    1    0    eg1

    .....uuuccacugggcgauugucgcA...................................................................................    3    1    ea2
    .............gggcgauugucgcgauaaaggcg..........................................................................    1    0    ea2
    ................cgauugucgcgauaaaggcg..........................................................................    15    0    ea2
    ..........................gauaaaggcgacaacuucaac...............................................................    4    0    ea2
    ..........................gauaaaggcgacaacuucaaU...............................................................    5    1    ea2
    ......................................aacuucaacagaCuuuucgc....................................................    1    1    ea2
    ......................................aacuucaacagaCuuuucgcu...................................................    1    1    ea2
    ...............................................agaauuuucgcuuuuaucgug..........................................    6    0    ea2
    ..............................................................aucgugacaaucgucuagugg...........................    1    0    ea2
    .....................................................................caaucgucCaguggaaacaua....................    74    1    ea2
    .....................................................................caaucgucuaguggaaacauU....................    3    1    ea2
    .....................................................................caaucgucuaguggaaacaua....................    6    0    ea2
    ......................................................................aaucgucuaguggaaacau.....................    1    0    ea2
    ......................................................................aaucgucCaguggaaacaua....................    21    1    ea2
    ......................................................................aaucgucuaguggaaacauU....................    2    1    ea2
    ......................................................................aaucgucuaguggaaacaua....................    45    0    ea2
    ......................................................................aaucgucuaguggaaacauaU...................    7    1    ea2
    ......................................................................aaucgucuaguggaaacauUa...................    8    1    ea2
    ........................................................................ucgucuaguggaaacauaau..................    9    0    ea2

    .....................................................................caaucgucCaguggaaacaua....................    5    1    eg2
    .......................................................................aucgucuaguggaaacauaU...................    1    1    eg2
```

Star **Mature**

ccugguuuccacugggcgauugucgcgauaaaggcgacaacuucaacagaauuuucgcuuuuaucgugacaaucgucuaguggaaacauaauuuaaucgugauugucgcg

..uUguuuccacugggcgauu...	1	1	ea1
.....Guuccacugggcgauugucg...	1	1	ea1
.....uuuccacugggcgauugucA...	1	1	ea1
.....uuuccacugggcgauugucg...	4	0	ea1
.....uuuccacugggcgauugucgcg...	1	0	ea1
......uuccacugggcgauugucgcg...	4	0	ea1
.......uccacugggcgauugucgcg...	1	0	ea1
.........cacugggcgauugucgcg...	1	0	ea1
............ugggcgauugucgcgauaaaA...	2	1	ea1
..............ggcgauugucgcgauaaaggcA..	1	1	ea1
...........................auaaaggcgacaacuucaac...	1	0	ea1
............................uaaaggcgacaacuucaaca..	1	0	ea1
..............................aaggcgacaacuucaacagU..	2	1	ea1
....uucaacagaCuuucgcuuu..	1	1	ea1
....ugacaaucgucuaguggaaacC......................	1	1	ea1
....acaaucgucCaguggaaacaua....................	4	1	ea1
....caaucgucuaguggaaacaua....................	1	0	ea1
....caaucgucCaguggaaacaua....................	109	1	ea1
....aaucgucCaguggaaacaua....................	1	1	ea1
....aaucgucuaguggaaacauaC...................	1	1	ea1
..............gAcgauugucgcgauaaaggcg..	1	1	er2
.......................cgcgauaaaggcgacaacuuca...	1	0	er2
....ucgugacaaucgucuagugg...........................	1	0	er2
....acaaucgucCaguggaaacaua....................	2	1	er2
....caaucgucCaguggaaacaua....................	54	1	er2
....caaucgucuaguggaaacauUa...................	1	1	er2
....aaucgucuaguggaaacau.....................	1	0	er2
....aaucgucCaguggaaacaua....................	8	1	er2
....aaucgucuaguggaaacaua....................	19	0	er2
....Naucgucuaguggaaacaua....................	1	1	er2
....aaucgucuaguggaaacauU....................	2	1	er2
....aaucgucuaguggaaacauUa...................	1	1	er2
....aaucgucuaguggaaacauaU...................	6	1	er2
....aucgucuaguggaaacauaa...................	1	0	er2
....ucgucuaguggaaacauaauu.................	3	0	er2

Provisional ID : ScUbCFx_795_28339
Score total : 2.2
Score for star read(s) : -1.3
Score for read counts : 0
Score for mfe : 2.5
Score for randfold : 1.6
Score for cons. seed : -0.6
Total read count : 457
Mature read count : 413
Loop read count : 26
Star read count : 18

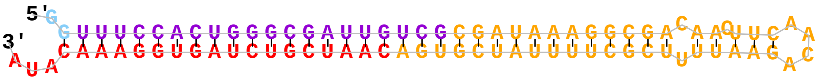

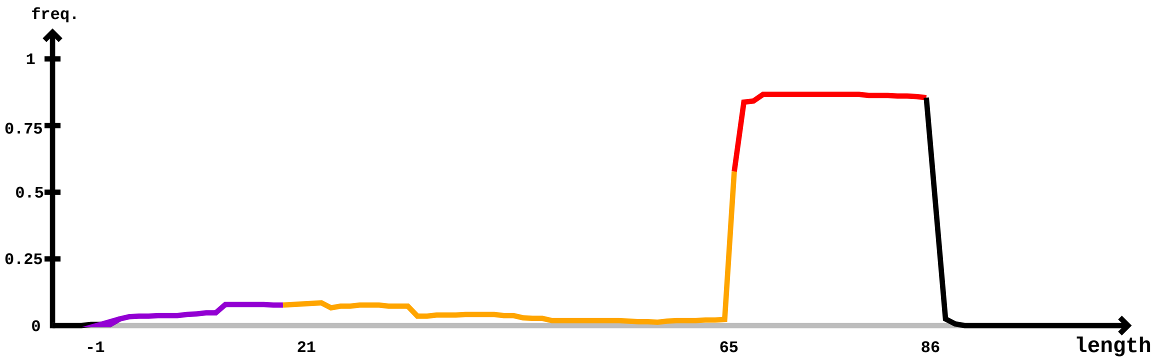

Star Mature

```
5'- ccugguuuccacugggcgauugucgcgauaaaggcgacaacuucaacagaauuuucgcuuuuaucgugacaaucgucuaguggaaacauaauuuaaucgugauuaucgcg  -3'  obs
    ccugguuuccacugggcgauugucgcgauaaaggcgacaacuucaacagaauuuucgcuuuuaucgugacaaucgucuaguggaaacauaauuuaaucgugauuaucgcg       exp
    ....(((((((((((((((((((((((((((((((((.((.(((....))))).)))))))))))))))))))))))))))))))))..........((((.....))))   reads  mm  sample
    ..ugguuuccacugggcgauugu.......................................................................................   1      0   er1
    ..............................................................aucgugacaaucgucuaguggaUa........................   1      1   er1
    .....................................................................caaucgucCaguggaaacaua....................   1      1   er1

    .........................cgauaaCggcgacaacuuca.................................................................   1      1   eg1
    ............................uaaaggcgacaacuucaacaga............................................................   2      0   eg1
    ....................................................................acaaucgucCaguggaaacaua....................   1      1   eg1
    .....................................................................caaucgucuaguggaaacauU....................   1      1   eg1
    .....................................................................caaucgucCaguggaaacaua....................   12     1   eg1
    ......................................................................aaucgucuaguggaaacaua....................   1      0   eg1

    .....................................................................caaucgucCaguggaaacaua....................   5      1   eg2
    .......................................................................aucgucuaguggaaacauaU...................   1      1   eg2

    .....uuuccacugggcgauugucgcA...................................................................................   3      1   ea2
    .............gggcgauugucgcgauaaaggcg..........................................................................   1      0   ea2
    ................cgauugucgcgauaaaggcg..........................................................................   15     0   ea2
    ..........................gauaaaggcgacaacuucaac...............................................................   4      0   ea2
    ..........................gauaaaggcgacaacuucaaU...............................................................   5      1   ea2
    ......................................aacuucaacagaCuuuucgc....................................................   1      1   ea2
    ......................................aacuucaacagaCuuuucgcu...................................................   1      1   ea2
    ...............................................agaauuuucgcuuuuaucgug..........................................   6      0   ea2
    ..............................................................aucgugacaaucgucuagugg...........................   1      0   ea2
    .....................................................................caaucgucuaguggaaacaua....................   6      0   ea2
    .....................................................................caaucgucCaguggaaacaua....................   74     1   ea2
    .....................................................................caaucgucuaguggaaacauU....................   3      1   ea2
    ......................................................................aaucgucuaguggaaacau.....................   1      0   ea2
    ......................................................................aaucgucCaguggaaacaua....................   21     1   ea2
    ......................................................................aaucgucuaguggaaacaua....................   45     0   ea2
    ......................................................................aaucgucuaguggaaacauU....................   2      1   ea2
    ......................................................................aaucgucuaguggaaacauUa...................   8      1   ea2
    ......................................................................aaucgucuaguggaaacauaU...................   7      1   ea2
    ........................................................................ucgucuaguggaaacauaau..................   9      0   ea2
```

Star **Mature**

```
ccugguuuccacugggcgauugucgcgauaaaggcgacaacuucaacagaauuuucgcuuuuaucgugacaaucgucuaguggaaacauaauuuaaucgugauuaucgcg

..............gAcgauugucgcgauaaaggcg..........................................................................   1   1   er2
.......................cgcgauaaaggcgacaacuuca.................................................................   1   0   er2
...............................................................ucgugacaaucgucuagugg...........................   1   0   er2
....................................................................acaaucgucCaguggaaacaua....................   2   1   er2
.....................................................................caaucgucCaguggaaacaua....................   54  1   er2
.....................................................................caaucgucuaguggaaacauUa...................   1   1   er2
......................................................................aaucgucuaguggaaacau.....................   1   0   er2
......................................................................aaucgucuaguggaaacauU....................   2   1   er2
......................................................................Naucgucuaguggaaacaua....................   1   1   er2
......................................................................aaucgucuaguggaaacaua....................   19  0   er2
......................................................................aaucgucCaguggaaacaua....................   8   1   er2
......................................................................aaucgucuaguggaaacauUa...................   1   1   er2
......................................................................aaucgucuaguggaaacauaU...................   6   1   er2
.......................................................................aucgucuaguggaaacauaa...................   1   0   er2
........................................................................ucgucuaguggaaacauaauu.................   3   0   er2

..uUguuuccacugggcgauu.........................................................................................   1   1   ea1
.....Guuccacugggcgauugucg.....................................................................................   1   1   ea1
.....uuuccacugggcgauugucg.....................................................................................   4   0   ea1
.....uuuccacugggcgauugucA.....................................................................................   1   1   ea1
.....uuuccacugggcgauugucgcg...................................................................................   1   0   ea1
......uuccacugggcgauugucgcg...................................................................................   4   0   ea1
.......uccacugggcgauugucgcg...................................................................................   1   0   ea1
.........cacugggcgauugucgcg...................................................................................   1   0   ea1
............ugggcgauugucgcgauaaaA.............................................................................   2   1   ea1
..............ggcgauugucgcgauaaaggcA..........................................................................   1   1   ea1
...........................auaaaggcgacaacuucaac...............................................................   1   0   ea1
............................uaaaggcgacaacuucaaca..............................................................   1   0   ea1
..............................aaggcgacaacuucaacagU............................................................   2   1   ea1
.........................................uucaacagaCuuuucgcuuu.................................................   1   1   ea1
..................................................................ugacaaucgucuaguggaaacC......................   1   1   ea1
....................................................................acaaucgucCaguggaaacaua....................   4   1   ea1
.....................................................................caaucgucuaguggaaacaua....................   1   0   ea1
.....................................................................caaucgucCaguggaaacaua....................   109 1   ea1
......................................................................aaucgucCaguggaaacaua....................   1   1   ea1
......................................................................aaucgucuaguggaaacauaC...................   1   1   ea1
```

Provisional ID	:	ScUbCFx_795_28478
Score total	:	5783.3
Score for star read(s)	:	3.9
Score for read counts	:	5775.9
Score for mfe	:	2.5
Score for randfold	:	1.6
Score for cons. seed	:	-0.6
Total read count	:	11341
Mature read count	:	11305
Loop read count	:	0
Star read count	:	36

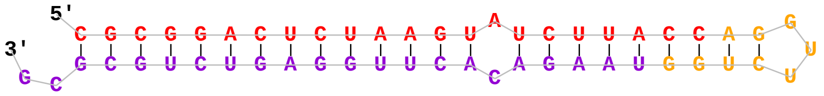

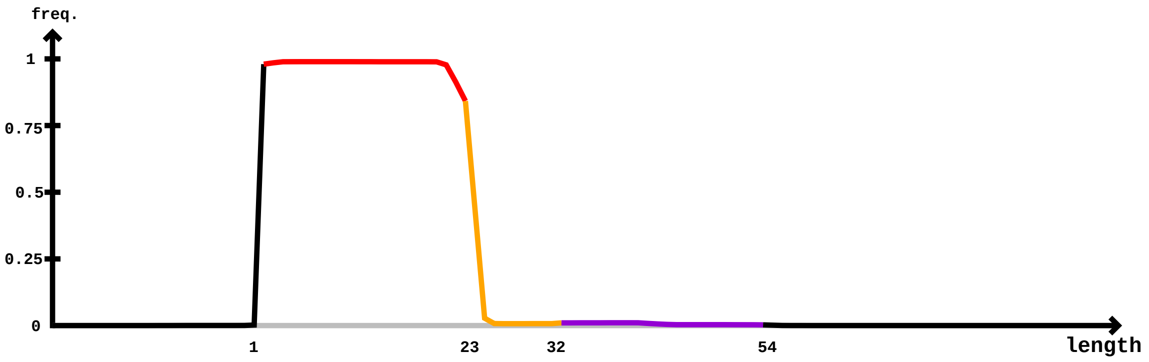

Mature Star

```
5'- cauaguuuuguuagccugcgcgcggacucuaaguaucuuaccagguucugguaagacacuuggagucugcgcgcaggccaaguuuuguaggaugcaaauugagcgcagu -3' obs
    cauaguuuuguuagccugcgcgcggacucuaaguaucuuaccagguucugguaagacacuuggagucugcgcgcaggccaaguuuuguaggaugcaaauugagcgcagu      exp
    ....((((.....(((((((((((((((((((((.(((((((((...))))))))).))))))))))))))))))))).....(((((.....)))))..)))).....   reads  mm  sample
    ....................cgUggacucuaaguaucuua.....................................................................   1      1   eg2
    ....................cgUggacucuaaguaucuuacca..................................................................   3      1   eg2

    ....................cgcggGcucuaaguaucuua.....................................................................   1      1   ea2
    ....................cgcggacucuaaguaucuua.....................................................................   29     0   ea2
    ....................cgcggacucuaaguaucuuU.....................................................................   6      1   ea2
    ....................cgcggacucuaaguaucuuac....................................................................   35     0   ea2
    ....................cgcggacucuaaguaucuuaA....................................................................   8      1   ea2
    ....................cgcggacucuaaguaucuuaU....................................................................   2      1   ea2
    ....................Ngcggacucuaaguaucuuacc...................................................................   2      1   ea2
    ....................cgcggacucuaagAaucuuacc...................................................................   1      1   ea2
    ....................cgcggacucuaaguaucuuacA...................................................................   34     1   ea2
    ....................cgcggacucuaaguaucuuacG...................................................................   2      1   ea2
    ....................cgcggacucuaaguaucuuacU...................................................................   4      1   ea2
    ....................cgcggacucuaaguaucuuacc...................................................................   271    0   ea2
    ....................cAcggacucuaaguaucuuacc...................................................................   3      1   ea2
    ....................cgcggacucuGaguaucuuacca..................................................................   1      1   ea2
    ....................cgcggacucuaaguaucuuaccG..................................................................   2      1   ea2
    ....................cgcggacucuaaguaucuuacca..................................................................   70     0   ea2
    ....................cgUggacucuaaguaucuuacca..................................................................   1      1   ea2
    ....................cgcggacucuaaguaucuuaccU..................................................................   2      1   ea2
    ....................cgcggacucuaaguaucuuaccaU.................................................................   9      1   ea2
    ....................cgcggacucuaaguaucuuaccaA.................................................................   7      1   ea2
    .....................gcggacucuaaguaucuuacc...................................................................   27     0   ea2
    .....................gcggacucuaaguaucuuacA...................................................................   2      1   ea2
    .....................gcggacucuaaguaucuuacca..................................................................   3      0   ea2
    .....................gcggacucuaaguaucuuaccaA.................................................................   1      1   ea2
    ......................cggacucuaaguaucuuaA....................................................................   4      1   ea2
    ........................................ccagguucugguaagacacu.................................................   8      0   ea2
    ........................................ccagguucugguaagaAacu.................................................   7      1   ea2
    .........................................cagguucugguaagaAacuu................................................   4      1   ea2
    .........................................cagguucugguaagacacuuA...............................................   2      1   ea2
    ..........................................agguucugguaagacacuuA...............................................   7      1   ea2
```

```
                    Mature                         Star
cauaguuuuguuagccugcgcgcggacucuaaguaucuuaccagguucugguaagacacuuggagucugcgcgcaggccaaguuuuguaggaugcaaauugagcgcagu
..........................................agguucugguaagacacuuAg..............................................   3     1    ea2
...........................................gguucugguaagacacuuAg..............................................   2     1    ea2

....................cgcggacucuaaguaucuua.....................................................................   8     0    er2
....................cgcggacucuaaguaucuuU.....................................................................   3     1    er2
....................cAcggacucuaaguaucuua.....................................................................   1     1    er2
....................cgcggacucuaaguaucuuac....................................................................   16    0    er2
....................cgcggacucuaaguaucuuaA....................................................................   4     1    er2
....................cgcggacucuaaguaucuuaU....................................................................   1     1    er2
....................cgcggacucuaaguaucuuacG...................................................................   3     1    er2
....................cgcggacucuaaguaucuuacA...................................................................   13    1    er2
....................cgcggacucuaaguaucuuacc...................................................................   219   0    er2
....................cgcggacucuaaguaucuuacU...................................................................   6     1    er2
....................cgcggacucuaaguaucGuacc...................................................................   1     1    er2
....................cgcggacucuaaguaucuuacca..................................................................   66    0    er2
....................cgcggacucuaaguaucuuacAa..................................................................   2     1    er2
....................Ngcggacucuaaguaucuuacca..................................................................   1     1    er2
....................cgcggacucuaaguaucuuaccag.................................................................   2     0    er2
....................cgcggacucuaaguaucuuaccaA.................................................................   16    1    er2
.....................gcggacucuaaguaucuua.....................................................................   1     0    er2
.....................gcggacucuaaguaucuuaA....................................................................   6     1    er2
.....................gcggacucuaaguaucuuacc...................................................................   8     0    er2
.....................gcggacucuaaguaucuuacAa..................................................................   2     1    er2
.....................gcggacucuaaguaucuuacca..................................................................   1     0    er2
........................................ccagguucugguaagacacu.................................................   8     0    er2
........................................ccagguucugguaagacacuu................................................   1     0    er2
.........................................cagguucugguaagacacuu................................................   2     0    er2
.........................................cagguucugguaagacacuuA...............................................   9     1    er2
..........................................agguucugguaagacacuuA...............................................   1     1    er2
..........................................agguucugguaagacacuuAg..............................................   6     1    er2

.........guuagccugcgcgcAgacucu...............................................................................   1     1    ea1
...........uagccugcgcgcggacucuaU.............................................................................   1     1    ea1
............agccugcgcgcggacucuaa.............................................................................   1     0    ea1
................ugcgcgcggacucuaaguauc........................................................................   1     0    ea1
..................cgcgcggacucuaaguaucuA......................................................................   1     1    ea1
...................gcgcggacucuaaguaucuuU.....................................................................   4     1    ea1
...................gcgcggacucuaaguaucuuac....................................................................   3     0    ea1
...................gcgcggacucuaaguaucuuUc....................................................................   1     1    ea1
...................gcgcggacucuaaguaucuuacc...................................................................   3     0    ea1
...................gcgcggacucuaaguaucuuacU...................................................................   2     1    ea1
...................Ccgcggacucuaaguaucuuacc...................................................................   1     1    ea1
...................gcgcggacucuaaguaucuuacAa..................................................................   1     1    ea1
....................cgcggacucuaaguaucu.......................................................................   3     0    ea1
....................cgcggacucuaaguaucuG......................................................................   1     1    ea1
....................cgcggaUucuaaguaucu.......................................................................   1     1    ea1
....................cgcggacCcuaaguaucu.......................................................................   1     1    ea1
....................cgcggacucuaaguaucuu......................................................................   113   0    ea1
....................cgcggacucuaaguaucuA......................................................................   5     1    ea1
....................cgcggacucuaaguauUuu......................................................................   1     1    ea1
....................cgAggacucuaaguaucuu......................................................................   1     1    ea1
....................Agcggacucuaaguaucuua.....................................................................   1     1    ea1
....................cgcggacucuaaguaCcuua.....................................................................   2     1    ea1
....................Ggcggacucuaaguaucuua.....................................................................   6     1    ea1
....................cgcggacucuaagCaucuua.....................................................................   2     1    ea1
....................cgcggacucuaaguaucCua.....................................................................   1     1    ea1
....................cgcggacucuaaguaucuuG.....................................................................   3     1    ea1
....................cgcggacucuaaguaucuuC.....................................................................   12    1    ea1
....................cgcUgacucuaaguaucuua.....................................................................   1     1    ea1
....................cgcggacCcuaaguaucuua.....................................................................   2     1    ea1
....................cgcggacucuaaguaucuAa.....................................................................   2     1    ea1
....................cgcggacucCaaguaucuua.....................................................................   2     1    ea1
....................cgcggacucAaaguaucuua.....................................................................   1     1    ea1
....................cgcggacucuaaguGucuua.....................................................................   2     1    ea1
....................cgcggacucuaaguaucuua.....................................................................   478   0    ea1
....................cgcggacuUuaaguaucuua.....................................................................   2     1    ea1
....................cgcggacucuaaguaucGua.....................................................................   2     1    ea1
....................cgcggacucuaaguauUuua.....................................................................   1     1    ea1
....................Ugcggacucuaaguaucuua.....................................................................   1     1    ea1
....................cgcggacucuGaguaucuua.....................................................................   1     1    ea1
```

```
                    Mature                        Star
cauaguuuuguuagccugcgcgcggacucuaaguaucuuaccagguucugguaagacacuuggagucugcgcgcaggccaaguuuuguaggaugcaaauugagcgcagu
....................cgcggacucuaaguaucuuU.....................................................................	155	1	ea1
....................Ggcggacucuaaguaucuuac....................................................................	6	1	ea1
....................cgcggacucuaaguaucuuaU....................................................................	70	1	ea1
....................cgcggacucuGaguaucuuac....................................................................	2	1	ea1
....................cgcgAacucuaaguaucuuac....................................................................	1	1	ea1
....................Agcggacucuaaguaucuuac....................................................................	1	1	ea1
....................cgcggacucuaaguaucuuac....................................................................	577	0	ea1
....................cgcggacucuaGguaucuuac....................................................................	6	1	ea1
....................cAcggacucuaaguaucuuac....................................................................	2	1	ea1
....................cgcggacucuaaguaucuuUc....................................................................	2	1	ea1
....................cgcggGcucuaaguaucuuac....................................................................	2	1	ea1
....................cgcggacucuaaguaucuuGc....................................................................	2	1	ea1
....................cgcggacucuaaguaucuuCc....................................................................	1	1	ea1
....................cgcggacucuaaguauUuuac....................................................................	1	1	ea1
....................cgcggacucuaUguaucuuac....................................................................	2	1	ea1
....................cgcggacucuaaguaucuuaA....................................................................	24	1	ea1
....................cgcggacucuaaguaucuuacc...................................................................	4129	0	ea1
....................cgUggacucuaaguaucuuacc...................................................................	4	1	ea1
....................cgcggacucuaGguaucuuacc...................................................................	3	1	ea1
....................cgcggGcucuaaguaucuuacc...................................................................	11	1	ea1
....................cgcggacucuGaguaucuuacc...................................................................	14	1	ea1
....................cgcggacucuaaguaCcuuacc...................................................................	6	1	ea1
....................cgcggacucuaaguauUuuacc...................................................................	2	1	ea1
....................cgcggacAcuaaguaucuuacc...................................................................	1	1	ea1
....................cgcggacucuaaguaucAuacc...................................................................	2	1	ea1
....................cgcgCacucuaaguaucuuacc...................................................................	1	1	ea1
....................cgcggacucuaUguaucuuacc...................................................................	2	1	ea1
....................cCcggacucuaaguaucuuacc...................................................................	2	1	ea1
....................cgcggacucAaaguaucuuacc...................................................................	1	1	ea1
....................cgcggacucCaaguaucuuacc...................................................................	7	1	ea1
....................cgcggacucuaaAuaucuuacc...................................................................	3	1	ea1
....................cgcggacucuaagAaucuuacc...................................................................	1	1	ea1
....................cgcggacucuaaguaucuuacU...................................................................	848	1	ea1
....................cgcggaUucuaaguaucuuacc...................................................................	2	1	ea1
....................cAcggacucuaaguaucuuacc...................................................................	4	1	ea1
....................Agcggacucuaaguaucuuacc...................................................................	1	1	ea1
....................Ggcggacucuaaguaucuuacc...................................................................	75	1	ea1
....................cgcggacucuaagCaucuuacc...................................................................	5	1	ea1
....................cgcggacCcuaaguaucuuacc...................................................................	18	1	ea1
....................cgcggacucuUaguaucuuacc...................................................................	2	1	ea1
....................cgcggacucuaaguGucuuacc...................................................................	3	1	ea1
....................cgcggacucuaaguaAcuuacc...................................................................	1	1	ea1
....................cgcggacucuaaguaucuuacG...................................................................	15	1	ea1
....................Ugcggacucuaaguaucuuacc...................................................................	5	1	ea1
....................cgcggacucuaaguaucuuacA...................................................................	707	1	ea1
....................cgcggacucuaaguaGcuuacc...................................................................	3	1	ea1
....................cgcggacucuaaguaucuuGcc...................................................................	2	1	ea1
....................cgcgAacucuaaguaucuuacc...................................................................	3	1	ea1
....................cgcggacucGaaguaucuuacc...................................................................	3	1	ea1
....................cgcggacucuaaguaucuuaUc...................................................................	3	1	ea1
....................cgcggacucuaaguaucCuacc...................................................................	12	1	ea1
....................cgcggacucuCaguaucuuacc...................................................................	1	1	ea1
....................cgcggacucuaaguaucuuacAa..................................................................	15	1	ea1
....................cgcggacucuaaguaucuAacca..................................................................	1	1	ea1
....................cgcggacucuaaguauUuuacca..................................................................	2	1	ea1
....................cgcggacucuaUguaucuuacca..................................................................	3	1	ea1
....................cgcggacucuaaguaucuuaccC..................................................................	118	1	ea1
....................cgcggacCcuaaguaucuuacca..................................................................	3	1	ea1
....................cgcggacucCaaguaucuuacca..................................................................	2	1	ea1
....................cgcggacucuaGguaucuuacca..................................................................	7	1	ea1
....................cgAggacucuaaguaucuuacca..................................................................	1	1	ea1
....................cgcggacucuaaAuaucuuacca..................................................................	1	1	ea1
....................cgcgAacucuaaguaucuuacca..................................................................	1	1	ea1
....................cgcggacucuaagCaucuuacca..................................................................	3	1	ea1
....................cgcggaUucuaaguaucuuacca..................................................................	1	1	ea1
....................cgcggacucuaaguaCcuuacca..................................................................	3	1	ea1
....................cgcggacucuaaguaucuuGcca..................................................................	2	1	ea1
....................cgcggacucuaaguaucuuacca..................................................................	1442	0	ea1
....................cgcggacucuaaguaucuuacUa..................................................................	9	1	ea1
....................cgcggacucuaaguaucCuacca..................................................................	6	1	ea1
```

Mature Star

```
cauaguuuuguuagccugcgcgcggacucuaaguaucuuaccagguucugguaagacacuuggagucugcgcgcaggccaaguuuuguaggaugcaaauugagcgcagu
....................cgcggacucuaaguaucuuaccG..................................................................	27	1	ea1
....................cgcgUacucuaaguaucuuacca..................................................................	1	1	ea1
....................cgcggacucuGaguaucuuacca..................................................................	2	1	ea1
....................Ggcggacucuaaguaucuuacca..................................................................	30	1	ea1
....................cgcggacuUuaaguaucuuacca..................................................................	1	1	ea1
....................cgcggacucuaaguauAuuacca..................................................................	1	1	ea1
....................cgcggGcucuaaguaucuuacca..................................................................	4	1	ea1
....................Ugcggacucuaaguaucuuacca..................................................................	1	1	ea1
....................cgcggacucuaaguaucAuacca..................................................................	2	1	ea1
....................cgcggacucuaaguGucuuacca..................................................................	7	1	ea1
....................cgcggacucuaaguaAcuuacca..................................................................	1	1	ea1
....................cgcggacucuaaguaucuuaccU..................................................................	100	1	ea1
....................cgcggacucuaaguGucuuaccag.................................................................	1	1	ea1
....................cgcggacucuaaguaucCuaccag.................................................................	1	1	ea1
....................cgcggacucuaaguaucuuaccag.................................................................	11	0	ea1
....................cgcggacucuaaguaucuuaccaU.................................................................	67	1	ea1
....................cAcggacucuaaguaucuuaccag.................................................................	1	1	ea1
....................cgcggacucuaaguaucuuaccaC.................................................................	12	1	ea1
....................cgcggacucuaaguaucuuaccaA.................................................................	60	1	ea1
....................cgcggacucuaaguaucuuaccagg................................................................	3	0	ea1
....................cgcAgacucuaaguaucuuaccagg................................................................	2	1	ea1
.....................gcggacucuaaguaucuuacc...................................................................	3	0	ea1
.....................Ccggacucuaaguaucuuacca..................................................................	1	1	ea1
.....................gcggacucuaaguaucuuacca..................................................................	2	0	ea1
......................cggacucuaaguaucuuac....................................................................	2	0	ea1
......................cggacucuaaguaucuuacU...................................................................	5	1	ea1
......................cggacucuaaguaucuuacc...................................................................	7	0	ea1
......................cggacucuaaguaucuuacca..................................................................	6	0	ea1
......................cggacucuaaguaucuuaccC..................................................................	1	1	ea1
......................Gggacucuaaguaucuuacca..................................................................	1	1	ea1
......................cggacucuaaguaucuuaccU..................................................................	2	1	ea1
......................cggacucuaaguaucuuaUca..................................................................	1	1	ea1
......................cggacucuaaguaucuuaccaA.................................................................	1	1	ea1
......................cggacucCaaguaucuuaccag.................................................................	2	1	ea1
......................cggacucuaaguaucuuaccag.................................................................	8	0	ea1
......................cggacucuaaguaucuuaccaC.................................................................	2	1	ea1
......................cggacucuaaguaucuuaccagg................................................................	1	0	ea1
.......................ggacucuaaguaucuuaccag.................................................................	2	0	ea1
........................gacucuaaguUucuuaccag.................................................................	1	1	ea1
........................................ccagguucugguaagaAacu.................................................	2	1	ea1
........................................ccagguucugguaagaAacuu................................................	3	1	ea1
.........................................cagguucugguaagaAac..................................................	1	1	ea1
.........................................cagguucugguaagaAacuu................................................	4	1	ea1
..........................................agguucugguaagaAacuu................................................	6	1	ea1
..........................................agguucugguaagaAacuug...............................................	1	1	ea1
................................................ugguaagaUacuuggagucug........................................	2	1	ea1
................................................ugguaagaUacuuggagucugc.......................................	1	1	ea1
...................................................uaagaAacuuggagucugcg......................................	1	1	ea1
...................................................uaagaUacuuggagucugcg......................................	1	1	ea1
...................................................uaagaAacuuggagucugcgc.....................................	2	1	ea1
...................................................uaagaUacuuggagucugcgc.....................................	1	1	ea1
...................................................uaagaAacuuggagucugcgcg....................................	12	1	ea1
...................................................uaagaUacuuggagucugcgcg....................................	7	1	ea1
...................................................uaagaAacuuggagucugcgcgc...................................	1	1	ea1
...................................................uaagaAacuuggagucugcgcgca..................................	1	1	ea1
.....................................................agacacuuCgagucugcgcg....................................	1	1	ea1
.....................................................agaAacuuggagucugcgcg....................................	1	1	ea1
........................................................Uacuuggagucugcgcgc...................................	1	1	ea1
........................................................Uacuuggagucugcgcgcagg................................	1	1	ea1

...................Ccgcggacucuaaguaucuuacc...................................................................	2	1	er1
...................gcgcggacucuaaguaucuuacc...................................................................	1	0	er1
....................cgcggacucuaaguaucuu......................................................................	3	0	er1
....................cgcggacucuaaguaucuua.....................................................................	18	0	er1
....................cgcggacucuaaguaucuuac....................................................................	20	0	er1
....................cgcggacucuaaguaucCuac....................................................................	1	1	er1
....................cgcggacucuaaguaucCuacc...................................................................	4	1	er1
....................cgcggacucuaaguaucuuGcc...................................................................	1	1	er1
....................cgcggacucuaaguaucuuacU...................................................................	40	1	er1
....................cgcggacucuaagCaucuuacc...................................................................	1	1	er1
```

```
                    Mature                        Star
cauaguuuuguuagccugcgcgcggacucuaaguaucuuaccagguucugguaagacacuuggagucugcgcgcaggccaaguuuuguaggaugcaaauugagcgcagu
....................Ggcggacucuaaguaucuuacc...................................................................   2     1    er1
....................cgcggacucuaaguaucuuacc...................................................................   277   0    er1
....................cgcggacucuaaguaucuuacA...................................................................   38    1    er1
....................cgcggacuUuaaguaucuuacc...................................................................   2     1    er1
....................cgcggacucuaaguaCcuuacc...................................................................   1     1    er1
....................cAcggacucuaaguaucuuacc...................................................................   1     1    er1
....................Ugcggacucuaaguaucuuacc...................................................................   1     1    er1
....................cgcggacucuaaguaucuuaccC..................................................................   4     1    er1
....................cgUggacucuaaguaucuuacca..................................................................   1     1    er1
....................cgcggacucuaaUuaucuuacca..................................................................   1     1    er1
....................cgcggacucuaaguaucuuaccU..................................................................   4     1    er1
....................Ggcggacucuaaguaucuuacca..................................................................   2     1    er1
....................cgcggacucuaaguaucuuaccG..................................................................   2     1    er1
....................cgcggacucuaaguaucuuacca..................................................................   88    0    er1
....................cgcggacucuaaguaCcuuacca..................................................................   1     1    er1
....................cgcggacucuaaguaucuuaccaA.................................................................   6     1    er1
....................cgcggacucuaaguaucuuaccag.................................................................   1     0    er1
....................cgcggacucuaaguaucuuaccaU.................................................................   7     1    er1
......................cggacucuaaguaucuuac....................................................................   1     0    er1
...................................................uaagaAacuuggagucugcgc.....................................   1     1    er1
...................................................uaagaAacuuggagucugcgcg....................................   2     1    er1
...................................................uaagaAacuuggagucugcgcgca..................................   1     1    er1

....................cgcggacucuaaguaucCu......................................................................   2     1    eg1
....................cgcggacucuaaguaucuuG.....................................................................   1     1    eg1
....................cgcggacucuaaguaucuuU.....................................................................   2     1    eg1
....................cgcggacucuaaguaucuua.....................................................................   18    0    eg1
....................cgcggacucuaaguaucuuC.....................................................................   1     1    eg1
....................cgcggacucuaaguaucuuac....................................................................   26    0    eg1
....................cgcggacucuaaguaucuuaA....................................................................   1     1    eg1
....................cgcggacucuaaguaucuuaU....................................................................   4     1    eg1
....................cgcggacucuaaguaucuuacU...................................................................   30    1    eg1
....................cgcggacucuaaguaucuuacA...................................................................   33    1    eg1
....................cgcggacucuaaguaucuuacc...................................................................   226   0    eg1
....................cgcggacucuaaguaucuuaUc...................................................................   1     1    eg1
....................Ggcggacucuaaguaucuuacc...................................................................   3     1    eg1
....................cgcggacucuaUguaucuuacc...................................................................   1     1    eg1
....................cgcggacucuaaguaucuuacG...................................................................   2     1    eg1
....................cgcggacucuGaguaucuuacca..................................................................   1     1    eg1
....................cgcggacucuaaguaucuuaccU..................................................................   6     1    eg1
....................cgcggacucuaaguaucuuaccC..................................................................   6     1    eg1
....................cgcggacucuaaguaucuuaccG..................................................................   4     1    eg1
....................cgcggacucuaaguaucuuacca..................................................................   61    0    eg1
....................cgcggacucuaaguaucuuaccaU.................................................................   4     1    eg1
....................cgcggacucuaaguaucuuaccaC.................................................................   1     1    eg1
....................cgcggacucuaaguaucuuaccaA.................................................................   4     1    eg1
......................cggacucuaaguaucuuacc...................................................................   1     0    eg1
......................cggacucuaaguaucuuaccag.................................................................   1     0    eg1
........................................ccagguucugguaagaAacu.................................................   1     1    eg1
.........................................cagguucugguaagaAacuu................................................   3     1    eg1
...................................................uaagaAacuuggagucugcgcg....................................   2     1    eg1
```

Provisional ID	: ScUbCFx_795_28571
Score total	: 1.2
Score for star read(s)	: -1.3
Score for read counts	: 0
Score for mfe	: 1.5
Score for randfold	: 1.6
Score for cons. seed	: -0.6
Total read count	: 2792
Mature read count	: 2792
Loop read count	: 0
Star read count	: 0

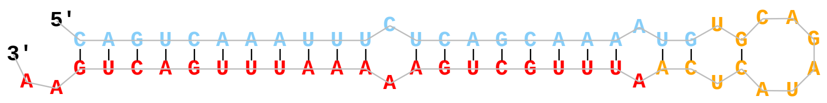

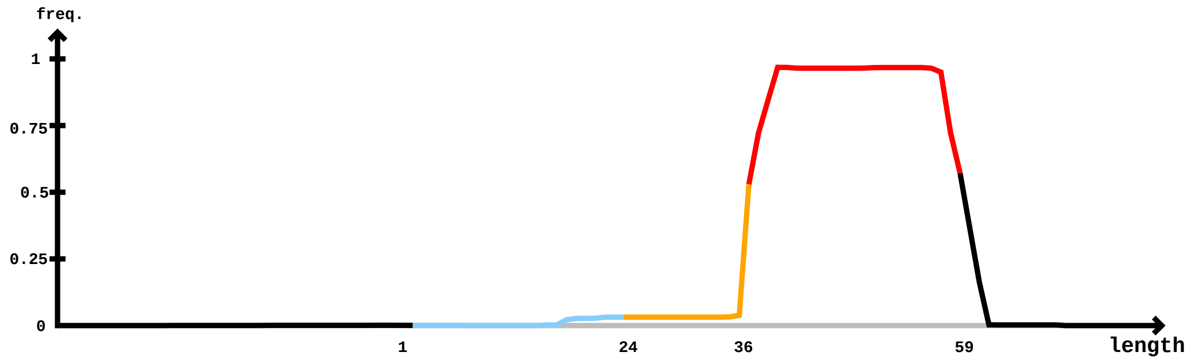

Star Mature

5'- uuacuuaauuugauucagcaacagcaguuacucuucagucaaauuucucagcaaaaugugcagauacucaauuugcugaaaaauuugacugaaaguaauguguaaccuuugc -3'	exp		
.................((((..(((((((((.(((((((((((((.((((((((.((.(......).)).)))))))).))))))))))))).)))))).))).....))))	reads	mm	sample
....................acagcaguuacucuucaguca..	1	0	ea1
...............................ucuucaguUaaauuucucagca..	1	1	ea1
....aaaaugugcagauacucaauu..	1	0	ea1
....aaaaugugcagauacucaauuA.......................................	1	1	ea1
....aauuugcugaaaaauuugacuA......................	1	1	ea1
....aauuugcugaaaaauuugacug......................	5	0	ea1
....auuugcugaGaaauuugac........................	1	1	ea1
....auuugcugaaaaauuugac........................	4	0	ea1
....auuugcugaaaaauuugacC.......................	4	1	ea1
....auuugcugaaaaauuugacu.......................	22	0	ea1
....auuugcugaGaaauuugacu.......................	1	1	ea1
....Guuugcugaaaaauuugacu.......................	1	1	ea1
....auuugcugaaaaauuugacA.......................	2	1	ea1
....auuugcugaaaaauuugacuA......................	8	1	ea1
....auuugcugaaaaauuugGcug......................	2	1	ea1
....auuugcugaaaaauuugacuU......................	5	1	ea1
....auuugcugGaaaauuugacug......................	1	1	ea1
....auuugcugaGaaauuugacug......................	1	1	ea1
....auuugcugaaaaauuGgacug......................	1	1	ea1
....Guuugcugaaaaauuugacug......................	4	1	ea1
....auuugcugaaaaauCugacug......................	2	1	ea1
....auuugcugaaaaauuugacug......................	154	0	ea1
....auuugcugaaaaauuugacGg......................	3	1	ea1
....auuugcugaaGaauuugacug......................	1	1	ea1
....auuugcugaaaaauuugacCg......................	4	1	ea1
....auuugcugaaaaauuugGcuga.....................	1	1	ea1
....auuugcugaaaaauuugacuAa.....................	3	1	ea1
....auuugcugaGaaauuugacuga.....................	2	1	ea1
....Cuuugcugaaaaauuugacuga.....................	1	1	ea1
....auuugcugaaaaauuGgacuga.....................	1	1	ea1
....auuugcugaUaaauuugacuga.....................	2	1	ea1
....Guuugcugaaaaauuugacuga.....................	9	1	ea1
....auuugcugaaaaauCugacuga.....................	2	1	ea1
....auuugcugaaaaaCuugacuga.....................	1	1	ea1

Star Mature

uuacuuaauuugauucagcaacagcaguuacucuucagucaaauuucucagcaaaaugugcagauacucaauuugcugaaaaauuugacugaaaaguaauguguaaccuuugc			
....auuugcCgaaaaauuugacuga.....................	1	1	ea1
....auuugcugaaaaauuugacugC.....................	3	1	ea1
....auuCgcugaaaaauuugacuga.....................	1	1	ea1
....auuugcugaaaaauuugacuga.....................	168	0	ea1
....auuugcugaaGaauuugacuga.....................	1	1	ea1
....auuugcugaaaaauuugacugG.....................	4	1	ea1
....auuugcugaaaaauuugacugU.....................	2	1	ea1
....auuugcugaaaaauuCgacugaa....................	2	1	ea1
....Guuugcugaaaaauuugacugaa....................	7	1	ea1
....auuugcugaaaaauuugacugaC....................	5	1	ea1
....auuugcugaGaaauuugacugaa....................	1	1	ea1
....auuugcugGaaaauuugacugaa....................	2	1	ea1
....auuugcugUaaaauuugacugaa....................	1	1	ea1
....aCuugcugaaaaauuugacugaa....................	1	1	ea1
....auuugcugaaaaauuugacugUa....................	1	1	ea1
....auuugcugaaaaauuugacugaG....................	2	1	ea1
....auuugcugaaaaauuugacugaa....................	260	0	ea1
....auuugcugaaaaauuugacugaU....................	7	1	ea1
....auuugcugaaaUauuugacugaa....................	1	1	ea1
....auuugcAgaaaaauuugacugaa....................	1	1	ea1
....auuugcugaaaGauuugacugaa....................	1	1	ea1
....auuugcugaaaaGuuugacugaa....................	2	1	ea1
....auuugcugaaaaauuugacugaUa...................	21	1	ea1
....Guuugcugaaaaauuugacugaaa...................	1	1	ea1
....auuugcugaaaaauuugacugaaG...................	5	1	ea1
....auuugcugaaaaauuugacugaaU...................	93	1	ea1
....auuugcugaaaaauuugacugaaC...................	17	1	ea1
....auuugcugaaaaauuugacugaaa...................	47	0	ea1
....auuugcugaaaaauuugacugGaa...................	1	1	ea1
....auuugcugaGaaauuugacugaaa...................	1	1	ea1
....auuugcugaaaaauuugacugaCa...................	41	1	ea1
....auuugcugaaaaauuugacugaaaa..................	3	0	ea1
....auuugcugaaaaauuugacugaaUa..................	85	1	ea1
....auuugcugaaaaauuugacugaUaa..................	1	1	ea1
....auuugcugaaaaauuugacugaaCa..................	2	1	ea1
....uuugcugaaaaauuugac........................	1	0	ea1
....uuugcugaaaaauuugacu.......................	1	0	ea1
....uuugcugaaaaaAuugacug......................	3	1	ea1
....uuugcugaaaaauuugacug......................	3	0	ea1
....uuugcugaaaaauuugGcug......................	1	1	ea1
....Guugcugaaaaauuugacug......................	2	1	ea1
....uuugcugaaaaaAuugacuga.....................	1	1	ea1
....uuugcugaaaaauuugacugU.....................	2	1	ea1
....uuugcugaaaaauuugacuAa.....................	1	1	ea1
....uuugcuAaaaaauuugacuga.....................	1	1	ea1
....uuugcugaaaaauuugacuga.....................	9	0	ea1
....Guugcugaaaaauuugacuga.....................	3	1	ea1
....uuugcugaaGaauuugacugaa....................	1	1	ea1
....uuugcuAaaaaauuugacugaa....................	2	1	ea1
....Guugcugaaaaauuugacugaa....................	5	1	ea1
....uuugcugaaaaauuugacugaa....................	19	0	ea1
....uuugcugaaaaauuugacugaU....................	1	1	ea1
....uuugcugaaaaauuugacugaG....................	2	1	ea1
....uuugcugaaaaauuugacugaaC...................	4	1	ea1
....uuugcugaaaaauuugacugaaa...................	12	0	ea1
....uuugcugaaaaauuugacugaCa...................	3	1	ea1
....uuugcugaaaaauuugacugaaG...................	1	1	ea1
....uuugcugaaaaauuugacugaUa...................	3	1	ea1
....Auugcugaaaaauuugacugaaa...................	1	1	ea1
....uuugcugaaaaauuugacugaaU...................	18	1	ea1
....uuugcugaaaaauuugacugaaaa..................	1	0	ea1
....uuugcugaaaaauuugacugaaUa..................	58	1	ea1
....uuugcugaaaaauuugacugaaaG..................	1	1	ea1
....uuugcugaaaaauuugacugaaCa..................	15	1	ea1
....uugcugaaaaauuugacu.......................	1	0	ea1
....uugcugaaaaauuugacug......................	3	0	ea1
....uugcugaaaaauuugacuA......................	1	1	ea1
....Gugcugaaaaauuugacuga.....................	3	1	ea1
....uugcugaaaaauuugacuAa.....................	1	1	ea1
....uugcugaaaaauuugacuga.....................	12	0	ea1

Star Mature

```
uuacuuaauuugauucagcaacagcaguuacucuucagucaaauuucucagcaaaaugugcagauacucaauuugcugaaaaauuugacugaaaaguaauguguaaccuuugc
........................................................................uugcugaaaaauCugacugaa....................  1  1  ea1
........................................................................uugcugaaaaauuugacugGa....................  1  1  ea1
........................................................................uugcugGaaaauuugacugaa....................  2  1  ea1
........................................................................Gugcugaaaaauuugacugaa....................  2  1  ea1
........................................................................uugcugaaaaauuugacugaa....................  21  0  ea1
........................................................................uugcugaaaaauuugacugaaa...................  24  0  ea1
........................................................................uugcugaaaaauuugacugaCa...................  9  1  ea1
........................................................................uCgcugaaaaauuugacugaaa...................  1  1  ea1
........................................................................uugcugaaaaauuugacugaaU...................  16  1  ea1
........................................................................uugcugaaGaauuugacugaaa...................  2  1  ea1
........................................................................Gugcugaaaaauuugacugaaa...................  1  1  ea1
........................................................................uugcugaaaaauuugacugaaC...................  10  1  ea1
........................................................................uugcugaaaaauuugacugaUa...................  8  1  ea1
........................................................................uugcugaaaaauuugacugaaaa..................  4  0  ea1
........................................................................uugcugaaaaauuugacugaaUa..................  50  1  ea1
........................................................................uugcugaaaaGuuugacugaaaa..................  1  1  ea1
........................................................................uugcugaaaaauuugacugaaCa..................  25  1  ea1
........................................................................uugcugaaaaauuugacugaUaa..................  1  1  ea1
........................................................................uugcugaaaaauuugacugaaaU..................  2  1  ea1
........................................................................uugcugaaaaauuugacugaaUag.................  1  1  ea1
.........................................................................ugcugaaaaauuugacug......................  15  0  ea1
.........................................................................ugcugaaaaauuugacuA......................  4  1  ea1
.........................................................................Ggcugaaaaauuugacug......................  2  1  ea1
.........................................................................ugcugaaaaaGuugacuga.....................  1  1  ea1
.........................................................................Ggcugaaaaauuugacuga.....................  2  1  ea1
.........................................................................ugcugaaaaauuugacuga.....................  25  0  ea1
.........................................................................ugcAgaaaaauuugacugaa....................  1  1  ea1
.........................................................................Ggcugaaaaauuugacugaa....................  9  1  ea1
.........................................................................ugcugaaaGauuugacugaa....................  1  1  ea1
.........................................................................ugcugaaaaauuugGcugaa....................  2  1  ea1
.........................................................................ugcugaaaaauuugaAugaa....................  1  1  ea1
.........................................................................ugcCgaaaaauuugacugaa....................  1  1  ea1
.........................................................................ugcugaaaaauuugacugaa....................  81  0  ea1
.........................................................................ugcugaaaaauuugacugUa....................  2  1  ea1
.........................................................................ugcugaaaaaCuugacugaa....................  1  1  ea1
.........................................................................ugcugaaaaauuugacugaaa...................  33  0  ea1
.........................................................................ugcugaaaaauuugacugaaC...................  1  1  ea1
.........................................................................ugcugaaaaauuugacugaCa...................  3  1  ea1
.........................................................................ugcugaaaaauuugacugaUa...................  9  1  ea1
.........................................................................ugcugaaaaaCuugacugaaa...................  1  1  ea1
.........................................................................Ggcugaaaaauuugacugaaa...................  4  1  ea1
.........................................................................ugcugaaaaauuugacugaaU...................  11  1  ea1
.........................................................................ugcugaaaaauuugacugaaCa..................  17  1  ea1
.........................................................................ugcugaaaaauuugacugaaUa..................  62  1  ea1
.........................................................................ugcugaaaaauuugacugaCaa..................  1  1  ea1
...........................................................................cugaaaaauuugacugaaa...................  1  0  ea1
...........................................................................cugaaaaauuugacugaaCa..................  2  1  ea1
...........................................................................cugaaaaauuugacugaaUa..................  2  1  ea1
...................................................................................uuugacugaaaGguaaugug..........  1  1  ea1

.................................................agcaaaaugugcagauacuca...........................................  2  0  er2
...................................................caaaaugugcagauacuca...........................................  4  0  er2
...................................................caaaaugugcagauacucaa..........................................  16  0  er2
...................................................caaaaugugcagauacucaaC.........................................  8  1  er2
...................................................caaaauAugcagauacucaau.........................................  3  1  er2
...................................................caaaaugugcagauacucaauuA.......................................  1  1  er2
....................................................aaaaugugcagauacucaU..........................................  2  1  er2
....................................................aaaauAugcagauacucaau.........................................  1  1  er2
....................................................aaaaugugcagauacucaau.........................................  1  0  er2
.....................................................aaaugugcagauacucaauu........................................  1  0  er2
.......................................................augugcagauacucaauuug......................................  4  0  er2
....................................................................caauuugcugaaaaauuugacug......................  2  0  er2
.....................................................................aauuugcugaaaaauuugacug......................  8  0  er2
......................................................................auuugcugaaaaauuugacug......................  40  0  er2
......................................................................auuugcugaaaaauuugaGug......................  2  1  er2
......................................................................auuugcugaaaaauuugacuAa.....................  3  1  er2
......................................................................auuugcugaaaaauuugacugC.....................  2  1  er2
......................................................................auuugcugaaaaauuugacuga.....................  15  0  er2
......................................................................auuugcugaaaaauuugacugaa....................  5  0  er2
......................................................................auuugcugaaaaauuugacugaCa...................  6  1  er2
```

```
                                                   Star                                Mature
uuacuuaauuugauucagcaacagcaguuacucuucagucaaauuucucagcaaaaugugcagauacucaauuugcugaaaaauuugacugaaaaguaauguguaaccuuugc
......................................................................auuugcugaaaaauuugacugaaUa..................    3     1    er2
.......................................................................uuugcugaaaaaAuugacug......................    5     1    er2
.......................................................................uuugcugaaaaauuugacug......................    85    0    er2
.......................................................................uuugcugaaaaauuugacugU.....................    6     1    er2
.......................................................................uuugcugaaaaauuugacuAa.....................    8     1    er2
.......................................................................uuugcugaaaaauuugacuga.....................    9     0    er2
.......................................................................uuugcugaaaaauuugacugaG....................    4     1    er2
.......................................................................uuugcugaaaaauuugacugaC....................    2     1    er2
.......................................................................uuugcugaaaaauuugacugaa....................    1     0    er2
.......................................................................uuugcugaaaaauuugacugaaU...................    7     1    er2
.......................................................................uuugcugaaaaauuugacugaCa...................    3     1    er2
.......................................................................uuugcugaaaaauuugacugaaCa..................    1     1    er2
.......................................................................uuugcugaaaaauuugacugaaUa..................    2     1    er2
........................................................................uugcugaaaaauuugacuA......................    2     1    er2
........................................................................uugcugaaaaauuugacug......................    6     0    er2
........................................................................Nugcugaaaaauuugacuga.....................    1     1    er2
........................................................................uugcugaaaaauuugacuAa.....................    4     1    er2
........................................................................uugcugaaaaauuugacuga.....................    5     0    er2
........................................................................uugcugaaaaauuugacugaa....................    5     0    er2
........................................................................uugcugaaaaauuugacugUa....................    2     1    er2
........................................................................uugcugaaaaauuugacugaC....................    2     1    er2
........................................................................uugcugaaaaauuugacuAaa....................    2     1    er2
........................................................................uugcugaaaaauuugacugaaU...................    1     1    er2
........................................................................uugcugaaaaauuugacugaCa...................    1     1    er2
........................................................................uugcugaaaaauuugacugaaUa..................    2     1    er2
.........................................................................ugcugaaaaauuugacug......................    9     0    er2
.........................................................................ugcugaaaaauuugacugaa....................    1     0    er2
.........................................................................ugcugaaaaauuugacugaaUa..................    4     1    er2
.........................................................................ugcugaaaaauuugacugaaCa..................    5     1    er2
..........................................................................gcugaaaaauuugacugaaCa..................    2     1    er2

.................................................agcaaaaugugcagauacuc............................................    3     0    ea2
...................................................caaaaugugcagauacuca...........................................    7     0    ea2
...................................................caaaaugugcagauacucaa..........................................    6     0    ea2
...................................................caaaaugugcagauacucaau.........................................    6     0    ea2
...................................................caaaaugugcagauacucaaA.........................................    2     1    ea2
....................................................aaaaugugcagauacucaau.........................................    5     0    ea2
....................................................aaaaugugcagauacucaauuA.......................................    1     1    ea2
.....................................................aaaugugcagauacucaau.........................................    1     0    ea2
.......................................................augugcagauacucaauuug......................................    8     0    ea2
....................................................................caauuugcugaaaaauAugacug......................    1     1    ea2
.....................................................................aauuugcugaaaaauuugacu.......................    1     0    ea2
.....................................................................aauuugcugaaaaauuugacug......................    2     0    ea2
......................................................................auuugcugaaaaauuugacug......................    8     0    ea2
......................................................................auuugcugaaaaauuugacugU.....................    1     1    ea2
......................................................................auuugcugaaaaauuugacuga.....................    4     0    ea2
......................................................................auuugcugaaaaauuugacugaG....................    5     1    ea2
......................................................................auuugcugaaaaauuugacugaa....................    3     0    ea2
......................................................................auuugcugaaaaauuugacugaCa...................    6     1    ea2
......................................................................auuugcugaaaaauuugacugaaa...................    1     0    ea2
.......................................................................uuugcugaaaaauuugacGg......................    1     1    ea2
.......................................................................uuugcugaaaaaGuugacug......................    2     1    ea2
.......................................................................uuugcugaaaaauGugacug......................    1     1    ea2
.......................................................................uuugcugaaaaauuugacug......................    158   0    ea2
.......................................................................uuugcugaaGaauuugacug......................    6     1    ea2
.......................................................................uuugcugaaaaauuugacuga.....................    9     0    ea2
.......................................................................uuugcugaaaaauuugacugG.....................    2     1    ea2
.......................................................................uuugcugaaaaauuugacuAa.....................    3     1    ea2
.......................................................................uuugcugaaaaauuugacugaa....................    5     0    ea2
.......................................................................uuugcugaaaaauuugacugaG....................    1     1    ea2
.......................................................................uuugcugaaaaauuugacugaaU...................    2     1    ea2
.......................................................................uuugcugaaaaauuugacugaaUa..................    3     1    ea2
.......................................................................uuugcugaaaaauuugacugaaCa..................    6     1    ea2
........................................................................uugcugaaaaauuugacuA......................    3     1    ea2
........................................................................uugcugaaaaauuugacug......................    24    0    ea2
........................................................................Nugcugaaaaauuugacug......................    1     1    ea2
........................................................................uugcugaaaaauuugacugG.....................    10    1    ea2
........................................................................uugcugaaaaauuugacuAa.....................    6     1    ea2
........................................................................Nugcugaaaaauuugacuga.....................    1     1    ea2
........................................................................uugcugaaaaauuugacuga.....................    19    0    ea2
```

Star   Mature

```
uuacuuaauuugauucagcaacagcaguuacucuucagucaaauuucucagcaaaaugugcagauacucaauuugcugaaaaauuugacugaaaaguaauguguaaccuuugc
........................................................................uugcugaaaaauuugacugaCa...................    3    1    ea2
........................................................................uugcugaaaaauuugacugaaa...................    1    0    ea2
........................................................................uugcugaaaaauuugacugaaUa..................    1    1    ea2
.........................................................................ugcugaaaaauuugacugaG....................    2    1    ea2
.........................................................................ugcugaaaaauuugacugaa....................    1    0    ea2
.........................................................................ugcugaaaaauuugacugaUa...................    3    1    ea2
.........................................................................ugcugaaaaauuugacugaaCa..................    4    1    ea2
.........................................................................ugcugaaaaauuugacugaaUa..................    1    1    ea2

.................................................agcaaaaugugcagauacucUa..........................................    1    1    eg2
...................................................caGaaugugcagauacucaa..........................................    1    1    eg2
...................................................caaaaugugcagauacucaau.........................................    2    0    eg2
.....................................................aaaaugugcagauacucaau........................................    1    0    eg2
.....................................................aaaaugugcagauacucaauA.......................................    1    1    eg2
......................................................aaaugugcagauacucaauuug.....................................    1    0    eg2
.....................................................................aauuugcugaaaaauuugacug......................    1    0    eg2
......................................................................auuugcugaaaaauuugacug......................    11    0    eg2
......................................................................auuugcugaaaaauuugacuA......................    1    1    eg2
......................................................................auuugcugaaaaauuugacuga.....................    2    0    eg2
......................................................................auuugcugaaaaauuugacugUa....................    2    1    eg2
......................................................................auuugcugaaaaauuugacugaa....................    1    0    eg2
......................................................................auuugcugaaaaauuugacugaCa...................    1    1    eg2
......................................................................auuugcugaaaGauuugacugaaa...................    1    1    eg2
......................................................................auuugcugaaaaauuugacugaaa...................    1    0    eg2
......................................................................auuugcugaaaaauuugacugaaU...................    3    1    eg2
......................................................................auuugcugaaaaauuugacugaaCa..................    2    1    eg2
.......................................................................uuugcugaaaaauuugacug......................    14    0    eg2
.......................................................................uuugcugaaaaauuugacugC.....................    1    1    eg2
.......................................................................uuugcugaaaaauuugacuga.....................    1    0    eg2
.......................................................................uuugcugaaaaauuugacugU.....................    1    1    eg2
.......................................................................Nuugcugaaaaauuugacugaa....................    1    1    eg2
.......................................................................uuugcugaaaaauuugacugUa....................    1    1    eg2
.......................................................................uuugcugaaaaauuugacugaa....................    3    0    eg2
.......................................................................uuugcugaaaaauuugacugUaa...................    1    1    eg2
.......................................................................uuugcugaaaaauuugacugaaUa..................    4    1    eg2
........................................................................uugcugaaGaauuugacu.......................    1    1    eg2
........................................................................uugcugaaaaauuugacug......................    1    0    eg2
........................................................................uugcugaaaaauuugacuga.....................    2    0    eg2
........................................................................uugcugaaaaauuugacugaG....................    1    1    eg2
........................................................................uugcugaaaaauuugacugaa....................    3    0    eg2
........................................................................uugcugaaaaauuugacugaCa...................    1    1    eg2
........................................................................uugcugaaaaauuugacugaaa...................    5    0    eg2
........................................................................uugcugaaaaauuugacugaaU...................    2    1    eg2
........................................................................uugcugaaaaauuugacugaaCa..................    2    1    eg2
.........................................................................ugcugaaaaauuugacugaC....................    1    1    eg2
.........................................................................ugcugaaaaauuugacugaaU...................    1    1    eg2
.........................................................................ugcugaaaaauuugacugaaUa..................    1    1    eg2
...................................................................................uuugacugaaaaguaaugug..........    4    0    eg2
....................................................................................uugacugaaaaguaaugug..........    1    0    eg2

..........ugauuUagcaacagcaguuacucu...............................................................................    1    1    er1
.....................................................................Cauuugcugaaaaauuugacuga.....................    1    1    er1
......................................................................auuugcugaaaaauuugac........................    1    0    er1
......................................................................auuugcugaaaaauuugacA.......................    1    1    er1
......................................................................auuugcugaaaaauuugacC.......................    1    1    er1
......................................................................auuugcugaaaaauuugacu.......................    4    0    er1
......................................................................auuugcugaaaaauuugacuA......................    3    1    er1
......................................................................auuugUugaaaaauuugacug......................    1    1    er1
......................................................................Guuugcugaaaaauuugacug......................    1    1    er1
......................................................................auuugcugaaaaauuugacuU......................    1    1    er1
......................................................................auuugcugaaaaauuugacug......................    10    0    er1
......................................................................Guuugcugaaaaauuugacuga.....................    1    1    er1
......................................................................auuugcugaaaaauuugacugU.....................    1    1    er1
......................................................................auuugcugaaaaauuugacuga.....................    28    0    er1
......................................................................Guuugcugaaaaauuugacugaa....................    1    1    er1
......................................................................auuugcugaaaaauuugacugaa....................    34    0    er1
......................................................................auuugcugaaaaauuugacugaU....................    4    1    er1
......................................................................auuugcugaaaaauuugacugaC....................    3    1    er1
......................................................................Uuuugcugaaaaauuugacugaaa...................    2    1    er1
......................................................................auuugcugaaaaauuugacugaUa...................    5    1    er1
```

```
                                                   Star                              Mature
uuacuuaauuugauucagcaacagcaguuacucuucagucaaauuucucagcaaaaugugcagauacucaauuugcugaaaaauuugacugaaaaguaauguguaaccuuugc
......................................................................auuugcugaaaaauuugacugaaU...................   25   1   er1
......................................................................auuugcugaaaaauuugacugaaa...................   6   0   er1
......................................................................auuugcugaaaaauuugacugaaC...................   4   1   er1
......................................................................auuugcugaaaaauuugacugaCa...................   3   1   er1
......................................................................auuugcugaaaaauuugacugUaaa..................   1   1   er1
......................................................................auuugcugaaaaauuugacugaaUa..................   13   1   er1
......................................................................auuugcugaaaaauuugacugaCaa..................   1   1   er1
......................................................................auuugcugaaaaauuugacugaUaa..................   1   1   er1
.......................................................................uuugcugaaaaauuugacuga.....................   1   0   er1
.......................................................................uuugcugaaaaauuugacugaaC...................   1   1   er1
.......................................................................uuugcugaaaaauuugacugaCa...................   1   1   er1
.......................................................................uuugcugaaaaauuugacugaaU...................   5   1   er1
.......................................................................uuugcugaaaaauuugacugaaa...................   3   0   er1
.......................................................................uuugcugaaaaauuugacugaaUa..................   10   1   er1
.......................................................................uuugcugaaaaauuugacugaaCa..................   2   1   er1
........................................................................uugcugaaaaauuugacuAa.....................   1   1   er1
........................................................................uugcugaaaaauuugacuga.....................   4   0   er1
........................................................................Gugcugaaaaauuugacugaa....................   1   1   er1
........................................................................uugcugaaaaauuugacugaa....................   3   0   er1
........................................................................uugcugaaaaauuugacugaCa...................   4   1   er1
........................................................................uugcugaaaaauuugacugaaa...................   5   0   er1
........................................................................uugcugaaaaauuugacugaaU...................   8   1   er1
........................................................................uugcugaaaaauuugacugaaUa..................   11   1   er1
........................................................................uugcugaaaaauuugacugaaCa..................   5   1   er1
.........................................................................ugcugaaaaauuugacugaa....................   3   0   er1
.........................................................................ugcugaaaaauuugacugaaa...................   1   0   er1
.........................................................................ugcugaaaaauuugacugaaU...................   6   1   er1
.........................................................................ugcugaGaaauuugacugaaa...................   1   1   er1
.........................................................................ugcugaaaaauuugacugaaUa..................   8   1   er1

.....................................................................aauuugcugaaaaauuugacug......................   1   0   eg1
......................................................................auuugcugaaaaauuugacu.......................   2   0   eg1
......................................................................auuugcugGaaaauuugacug......................   1   1   eg1
......................................................................auuugcugaaaaauuugacug......................   16   0   eg1
......................................................................aCuugcugaaaaauuugacug......................   1   1   eg1
......................................................................auuugcugaaaaauuugacugG.....................   1   1   eg1
......................................................................auuugcCgaaaaauuugacuga.....................   1   1   eg1
......................................................................auuugcugaaaaauuugacugU.....................   1   1   eg1
......................................................................auuugcugaaaaauuugacuga.....................   17   0   eg1
......................................................................Guuugcugaaaaauuugacuga.....................   3   1   eg1
......................................................................auuugcugaaaaauuugacugaG....................   1   1   eg1
......................................................................auuugcugaaaaauuugacugaU....................   3   1   eg1
......................................................................auuugcugaaaaauuugacugaa....................   22   0   eg1
......................................................................auuugcugaaaaauuugacugaaG...................   3   1   eg1
......................................................................auuugcugaaaaauuugacugaaC...................   1   1   eg1
......................................................................auuugcugaaaaauuugacugaaU...................   22   1   eg1
......................................................................auuugcugaaaaauuugacugaCa...................   8   1   eg1
......................................................................auuugcugaaaaauuugacugaUa...................   1   1   eg1
......................................................................auuugcugaaaaauuugacugaCaa..................   1   1   eg1
......................................................................auuugcugaaaaauuugacugaaUa..................   10   1   eg1
.......................................................................uuugcugaaaaauuugacu.......................   1   0   eg1
.......................................................................uuugcugaaaaauuugacug......................   1   0   eg1
.......................................................................uuugcugaaaaauuugacuga.....................   2   0   eg1
.......................................................................Guugcugaaaaauuugacuga.....................   1   1   eg1
.......................................................................uuugcugaaaaaAuugacugaa....................   2   1   eg1
.......................................................................uuugcugaaaaauuugacugaaU...................   3   1   eg1
.......................................................................uuugcugaaaaauuugacugaaa...................   1   0   eg1
.......................................................................uuugcugaaaaauuugacugaCa...................   1   1   eg1
.......................................................................uuugcugaaaaauuugacugaaG...................   1   1   eg1
.......................................................................uuugcugaaaaauuugacugaaUa..................   8   1   eg1
.......................................................................uuugcugaaaaauuugacugaaCa..................   3   1   eg1
........................................................................uugcugaaaaauuugacuga.....................   1   0   eg1
........................................................................uugcugaaaaauuugacugaC....................   1   1   eg1
........................................................................uugcugaaaaauuugacugaa....................   3   0   eg1
........................................................................uugcugaaaaauuugacugaaU...................   5   1   eg1
........................................................................uugcugaaaaauuugacugaaa...................   1   0   eg1
........................................................................uugcugaaaaauuugacugaaG...................   1   1   eg1
........................................................................uugcugaaaaauuugacugaCa...................   1   1   eg1
........................................................................uugcugaaaaauuugacugaaC...................   1   1   eg1
........................................................................uugcugaaaaauuugacugaaUa..................   8   1   eg1
```

Star Mature

```
uuacuuaauuugauucagcaacagcaguuacucuucagucaaauuucucagcaaaaugugcagauacucaauuugcugaaaaauuugacugaaaaguaauguguaaccuuugc
........................................................................uugcugaaaaauuugacugaaCa..................	1	1	eg1
.........................................................................Ggcugaaaaauuugacugaa....................	1	1	eg1
.........................................................................ugcugaaaaauuugacugaG....................	2	1	eg1
.........................................................................ugcugaaaaauuugacugaa....................	1	0	eg1
.........................................................................ugcugaaaaauuugacugaaa...................	1	0	eg1
.........................................................................ugcugaaaaauuugacugaaU...................	2	1	eg1
.........................................................................ugcugaaaaauuugacugaaC...................	3	1	eg1
.........................................................................ugcugaaaaauuugacugaUa...................	1	1	eg1
.........................................................................ugcugaaaaauuugacugaaUa..................	3	1	eg1
.........................................................................ugcugaaaaauuugacugaaCa..................	2	1	eg1
..................................................................................auuugacugaaaaguaaugug..........	1	0	eg1
```

Provisional ID	:	ScUbCFx_879_33384
Score total	:	4.1
Score for star read(s)	:	-1.3
Score for read counts	:	0
Score for mfe	:	0.8
Score for randfold	:	1.6
Score for cons. seed	:	3
Total read count	:	1560
Mature read count	:	1560
Loop read count	:	0
Star read count	:	0

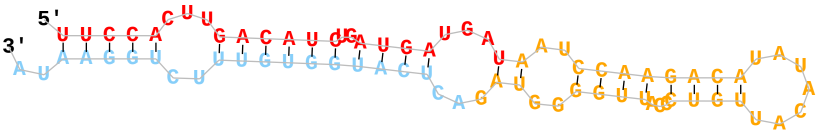

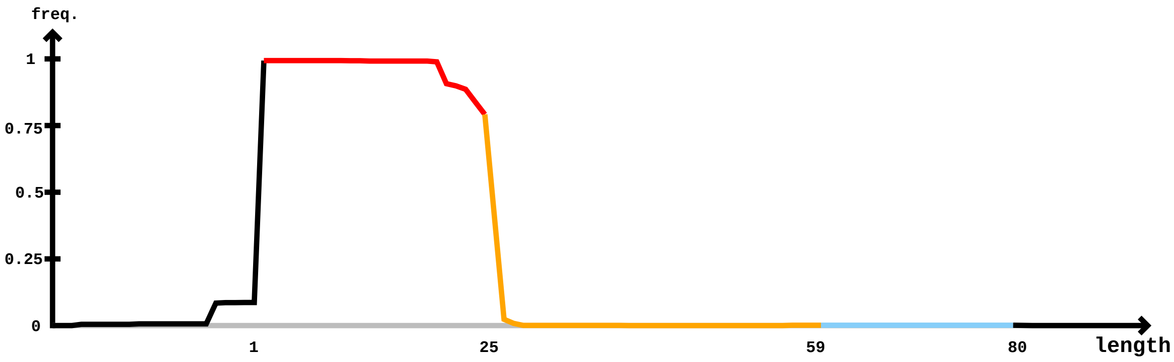

Mature **Star**

```
5'- aaaggauuuuugcaauauuguuccacuugacaucugaugaugauaauccaagacauauacauugucccauugggguagacucauggguguuucuggaauagagcagaggccaa  -3'   exp
    ...((.(((((((....((((((((...((((((..((((...((..((((((((.......))))...))))..))...))))))))))..)))))))).))))))))..   reads   mm   sample
    .......uuuugcaauauuguuccacuug...................................................................................   1       0    er1
    ..............uauuguuccacuugacaucugaug..........................................................................   9       0    er1
    ..............Gauuguuccacuugacaucugaug..........................................................................   1       1    er1
    ....................uuccacuugacaucugau..........................................................................   1       0    er1
    ....................uuccacuugacaucugaug.........................................................................   1       0    er1
    ....................uuccacuugacaucugauUa........................................................................   1       1    er1
    ....................uuccacuugacaucugaugau.......................................................................   4       0    er1
    ....................Cuccacuugacaucugaugaug......................................................................   1       1    er1
    ....................uuccacuugacaucugaugaug......................................................................   4       0    er1
    ....................uuccacuugacaucugaCgaug......................................................................   2       1    er1
    ....................uuccacuugacaucugaugauA......................................................................   1       1    er1
    ....................uuccacuugacaucugaugaCg......................................................................   2       1    er1
    ....................uuccacuugaUaucugaugauga.....................................................................   1       1    er1
    ....................uuccacuugacaucugaugaugG.....................................................................   1       1    er1
    ....................uuccacuugacaucugaugauga.....................................................................   13      0    er1
    ....................uuccacuugacaucugaugaugaG....................................................................   2       1    er1
    ....................uuccacuugacaucugaugaugaC....................................................................   13      1    er1
    ....................Guccacuugacaucugaugaugau....................................................................   13      1    er1
    ....................uuccacuugacaucugaugaugGu....................................................................   1       1    er1
    ....................uuccacuugacaucCgaugaugau....................................................................   2       1    er1
    ....................uuccacuugacaucuUaugaugau....................................................................   1       1    er1
    ....................uuccacuugacauUugaugaugau....................................................................   1       1    er1
    ....................uuccacuugacaucugaugaugau....................................................................   104     0    er1
    ....................uuccacuugacaucugaugaCgau....................................................................   4       1    er1
    ....................uuccacuugGcaucugaugaugau....................................................................   2       1    er1
    ....................uuccGcuugacaucugaugaugau....................................................................   1       1    er1
    ....................uCccacuugacaucugaugaugau....................................................................   1       1    er1
    ....................Guccacuugacaucugaugaugaua...................................................................   1       1    er1
    ....................uuccacuugacaucugaugaugauG...................................................................   5       1    er1
    ....................uuccacuugacaucugaugaugaua...................................................................   17      0    er1
    ....................uuccacuugacaucugaugaugaCa...................................................................   1       1    er1
    ....................uuccacuugacGucugaugaugaua...................................................................   2       1    er1
    ....................uuccacuugacaucugaugaCgaua...................................................................   2       1    er1
    ....................uuccacuugacaucugaugaugGua...................................................................   1       1    er1
```

```
                    Mature                                                     Star
aaaggauuuuugcaauauuguuccacuugacaucugaugaugauaauccaagacauauacauugucccauuggggguagacucaugguguuucuggaauagagcagaggccaa
....................uuccacuugacaucugaugaugauaau..................................................................   2    0   er1

.......uuuugcaauauuguuccacuugac..................................................................................   1    0   eg1
...............uauuguuccacuugacaucugaug..........................................................................   5    0   eg1
...............uauuguuccacuugacaucugauga.........................................................................   1    0   eg1
...............uauuguuccacuugacaucugaugaug.......................................................................   1    0   eg1
....................uuccacuugacaucugaug..........................................................................   4    0   eg1
....................uuccacuugacaucugaCg..........................................................................   1    1   eg1
....................uuccacuugacaucugauga.........................................................................   2    0   eg1
....................uuccacuugacaucugaugau........................................................................   9    0   eg1
....................uuccacuugacaucugaugaug.......................................................................   8    0   eg1
....................uuccacuugacaucCgaugaug.......................................................................   3    1   eg1
....................uuccacuugacaucugaugauA.......................................................................   1    1   eg1
....................Guccacuugacaucugaugaug.......................................................................   1    1   eg1
....................uuccacuugacaucugaugaCg.......................................................................   1    1   eg1
....................uuccacuugacaucugaugaugG......................................................................   1    1   eg1
....................uuccacuugacaucugaugaCga......................................................................   1    1   eg1
....................uuccacuugacaucugaugauga......................................................................   15   0   eg1
....................Guccacuugacaucugaugauga......................................................................   2    1   eg1
....................uucAacuugacaucugaugaugau.....................................................................   1    1   eg1
....................uuccacuugacaucugaugaugaG.....................................................................   1    1   eg1
....................uuccacuugacaucugaugaugau.....................................................................   208  0   eg1
....................uuccacuugacaucugUugaugau.....................................................................   3    1   eg1
....................uuccacCugacaucugaugaugau.....................................................................   1    1   eg1
....................uuccacuugacaucugaugaugaC.....................................................................   22   1   eg1
....................uuccacuugUcaucugaugaugau.....................................................................   1    1   eg1
....................Guccacuugacaucugaugaugau.....................................................................   14   1   eg1
....................uuccacuugacaucugaugaugaA.....................................................................   1    1   eg1
....................uCccacuugacaucugaugaugau.....................................................................   1    1   eg1
....................uuccGcuugacaucugaugaugau.....................................................................   1    1   eg1
....................uuccacuugacaucugaugaCgau.....................................................................   9    1   eg1
....................uuccacuugGcaucugaugaugau.....................................................................   1    1   eg1
....................Guccacuugacaucugaugaugaua....................................................................   3    1   eg1
....................uuccacuugacaucugaugaugauG....................................................................   9    1   eg1
....................uuccacuugGcaucugaugaugaua....................................................................   1    1   eg1
....................uuccacuugacaucugaugaCgaua....................................................................   1    1   eg1
....................uuccacuugacaucugUugaugaua....................................................................   1    1   eg1
....................uuccacuugacaucugaugaugaua....................................................................   31   0   eg1
....................uuccacuugacaucugGugaugaua....................................................................   1    1   eg1
....................uuccacuugacaucugaugaugauU....................................................................   2    1   eg1
....................uuccacuugacaucugaugaugauaa...................................................................   10   0   eg1
....................uuccacuugacaucugaugaugauaau..................................................................   4    0   eg1
........................................ugauUauccaagacauau.......................................................   1    1   eg1
............................................................................uagacucaugguguuucuggaauag............   1    0   eg1

.aaggauuuuugcaauauug.............................................................................................   7    0   ea2
...............Nauuguuccacuugacaucugaug..........................................................................   2    1   ea2
...............uauuguuccacuugacaucugaug..........................................................................   37   0   ea2
................auuguuccacuugacaucugaug..........................................................................   3    0   ea2
....................uuccacuugacaucugaug..........................................................................   3    0   ea2
....................uuccacuugacaucugauAa.........................................................................   3    1   ea2
....................uuccacuugacaucugaugaugau.....................................................................   1    0   ea2
....................uuccacuugacaucugaugaugaua....................................................................   3    0   ea2

.......uuuugcaauauuguuccacuugac..................................................................................   1    0   eg2
...............uauuguuccacuugacaucugauA..........................................................................   3    1   eg2
...............uauuguuccacuugacaucugaug..........................................................................   11   0   eg2
...............uauuguuccacuugacaucugauga.........................................................................   2    0   eg2
....................uuccacuugacaucugaugaCg.......................................................................   1    1   eg2
....................uuccacuugacaucugaugauga......................................................................   3    0   eg2
....................uuccacuugacaucugaugaugau.....................................................................   23   0   eg2
....................uuccacuugacaucugaugaugaua....................................................................   10   0   eg2

.aaggauuuuugcaauauug.............................................................................................   1    0   ea1
...............uauuguuccacuugacaucugau...........................................................................   2    0   ea1
...............Gauuguuccacuugacaucugaug..........................................................................   2    1   ea1
...............uauuguuccacuugacaucugauA..........................................................................   5    1   ea1
...............uauuguuccacuugacaucugaug..........................................................................   28   0   ea1
...............uauuguuccacuugacaucugaugauA.......................................................................   1    1   ea1
...............uauuguuccacuugacaucugaugaug.......................................................................   8    0   ea1
```

Mature Star

```
aaaggauuuuugcaauauuguuccacuugacaucugaugaugauaauccaagacauauacauugucccauuggggguagacucaugguguuucuggaauagagcagaggccaa
..................uguuccacuugacaucugaugaugaC.....................................................................   1     1    ea1
....................uuccacuugacaucugau...........................................................................   2     0    ea1
....................Guccacuugacaucugaug..........................................................................   1     1    ea1
....................uuccacuugacaucugauA..........................................................................   1     1    ea1
....................uuccacuugacaucugaug..........................................................................   11    0    ea1
....................uuccacuugacaucugauUa.........................................................................   1     1    ea1
....................uuccacuugacaucugauga.........................................................................   4     0    ea1
....................uuccacuugacaucugaugU.........................................................................   1     1    ea1
....................uuccacuugacaucugaugaC........................................................................   3     1    ea1
....................uuccacuugacaucugaugau........................................................................   4     0    ea1
....................uuccacuugGcaucugaugau........................................................................   2     1    ea1
....................uuccacuugacaucugaugaug.......................................................................   26    0    ea1
....................Auccacuugacaucugaugaug.......................................................................   1     1    ea1
....................uuccacuugacaucugaugauA.......................................................................   3     1    ea1
....................uuccacuugacaucugaugaCg.......................................................................   11    1    ea1
....................Guccacuugacaucugaugaug.......................................................................   1     1    ea1
....................uGccacuugacaucugaugaug.......................................................................   1     1    ea1
....................uuccacuugacaucugaugauga......................................................................   32    0    ea1
....................uuccacuugacaucugaugaCga......................................................................   8     1    ea1
....................uuccacuGgacaucugaugauga......................................................................   2     1    ea1
....................uuccacuugacaCcugaugauga......................................................................   1     1    ea1
....................uuccacuugacaucugaugaCgau.....................................................................   11    1    ea1
....................Guccacuugacaucugaugaugau.....................................................................   29    1    ea1
....................uuccacuCgacaucugaugaugau.....................................................................   1     1    ea1
....................uuccacuugacaCcugaugaugau.....................................................................   5     1    ea1
....................uuccacuugacaucugaugaugau.....................................................................   491   0    ea1
....................uuccaUuugacaucugaugaugau.....................................................................   1     1    ea1
....................uuccacCugacaucugaugaugau.....................................................................   3     1    ea1
....................uAccacuugacaucugaugaugau.....................................................................   1     1    ea1
....................uuccacuugacaucugaCgaugau.....................................................................   4     1    ea1
....................uuccacuugaUaucugaugaugau.....................................................................   1     1    ea1
....................uuccacuugacaucugaugaugaA.....................................................................   3     1    ea1
....................uuccacuugGcaucugaugaugau.....................................................................   1     1    ea1
....................uuccacuugacaucugGugaugau.....................................................................   1     1    ea1
....................uuccacuugacaucCgaugaugau.....................................................................   5     1    ea1
....................uuccacuugacaucugaugaugUu.....................................................................   2     1    ea1
....................uucUacuugacaucugaugaugau.....................................................................   2     1    ea1
....................uuccacuugacaucugaugGugau.....................................................................   3     1    ea1
....................uuccacuugacaucugaugaugaC.....................................................................   66    1    ea1
....................uuccacuugacaucugaugaugaG.....................................................................   6     1    ea1
....................Cuccacuugacaucugaugaugau.....................................................................   1     1    ea1
....................uuccacuugacGucugaugaugau.....................................................................   2     1    ea1
....................uuccacuugGcaucugaugaugaua....................................................................   1     1    ea1
....................Guccacuugacaucugaugaugaua....................................................................   9     1    ea1
....................uuccacuugacaucugaugaugaua....................................................................   86    0    ea1
....................uuccacuugacaucugaugaugauG....................................................................   15    1    ea1
....................uuccacuugacaucugaugaCgaua....................................................................   3     1    ea1
....................Cuccacuugacaucugaugaugaua....................................................................   2     1    ea1
....................uuccacuugacaucugaugaugauU....................................................................   4     1    ea1
....................uuccacuugacaCcugaugaugaua....................................................................   1     1    ea1
....................uuccacuugUcaucugaugaugaua....................................................................   2     1    ea1
....................uuccacuugacaucugaugaugauaa...................................................................   14    0    ea1
....................Guccacuugacaucugaugaugauaa...................................................................   2     1    ea1
....................uuccacuugacaucugaugaCgauaau..................................................................   1     1    ea1
....................uuccacuugacaucugaugaugauaau..................................................................   6     0    ea1
............................................................................uagacucaugguguuucuggaauag............   1     0    ea1

...............uauuguuccacuugacaucugauA..........................................................................   1     1    er2
...............uauuguuccacuugacaucugaug..........................................................................   11    0    er2
...............uauuguuccacuugacaucugaugauA.......................................................................   3     1    er2
....................uuccacuugacaucugaugaugau.....................................................................   18    0    er2
....................uuccacuugacaucugaugaugaua....................................................................   9     0    er2
```

Provisional ID	:	ScUbCFx_979_34011
Score total	:	969.9
Score for star read(s)	:	3.9
Score for read counts	:	959.1
Score for mfe	:	2.3
Score for randfold	:	1.6
Score for cons. seed	:	3
Total read count	:	1893
Mature read count	:	1883
Loop read count	:	0
Star read count	:	10

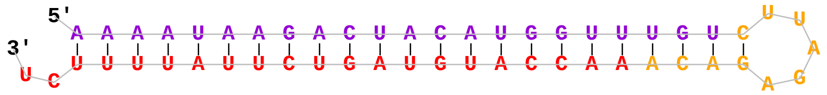

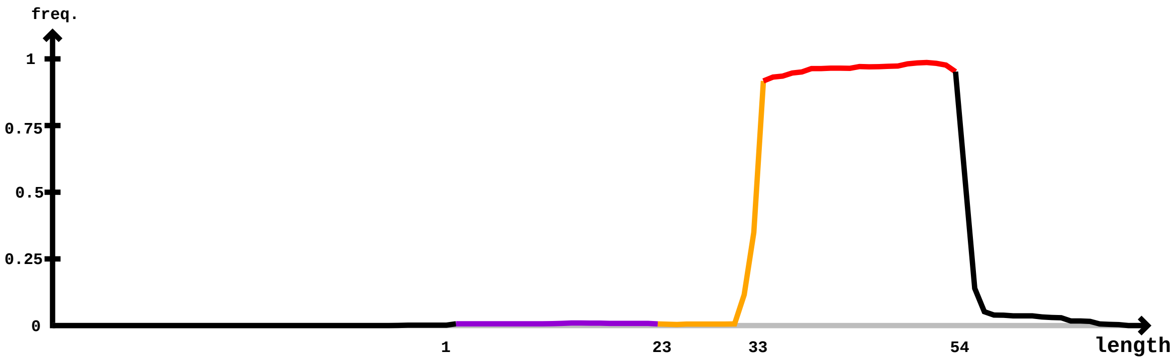

Star **Mature**

```
5'- uugaggaaaguuuuuugaagauuugaagaaagaguugaagaaaauaaagacuacaugguuugucuuagagacaaaccauguagucuuauuuucuucaacucuaugauaaauau   -3'    obs
    uugaggaaaguuuuuugaagauuugaagaaagaguugaagaaaauaaagacuacaugguuugucuuagagacaaaccauguagucuuauuuucuucaacucuaugauaaauau          exp
    ..........(((((..(....)..)))))((((((((((((((((((((((((((((((((((.....))))))))))))))))))))))))))))))))).......... .   reads   mm   sample
    ..................................uugaagaaaauaaagacuaca..........................................................   1       0    er1
    ........................................aaaauaaagacuacaugguuug...................................................   1       0    er1
    .......................................................................caaaccauguagucuuauuuucu...................   24      0    er1
    .......................................................................caaaccaCguagucuuauuuucu...................   1       1    er1
    .......................................................................caaaccauguagucuuauuuucC...................   2       1    er1
    .......................................................................caaaccauguagucuuauuuucA...................   1       1    er1
    .......................................................................caaaccauguagucuuauuuucuuA.................   1       1    er1
    ........................................................................aaaccauguagucuuauuuu.....................   1       0    er1
    ........................................................................aaaccauguagucuuauuuucu...................   27      0    er1
    ........................................................................aaaccauguagucuuauuuucC...................   2       1    er1
    ........................................................................aaaccUuguagucuuauuuucu...................   1       1    er1
    ........................................................................aaaccauguagucuuauuuucG...................   1       1    er1
    ........................................................................aaaccauguagucuuauCuucu...................   1       1    er1
    ........................................................................aaaccauguagucuuauuuucA...................   4       1    er1
    ........................................................................aaaccauguagucuuauuuucuA..................   3       1    er1
    ........................................................................aaaccauguagucuuauuuucuu..................   1       0    er1
    ........................................................................aaaccauguagucuuauuuucuuA.................   1       1    er1
    .........................................................................aaUcauguagucuuauuuuc....................   2       1    er1
    .........................................................................aaccauguagucuuauuuuc....................   2       0    er1
    .........................................................................aaccUuguagucuuauuuucu...................   1       1    er1
    .........................................................................aaccaugCagucuuauuuucu...................   1       1    er1
    .........................................................................aaUcauguagucuuauuuucu...................   1       1    er1
    .........................................................................aaccauguagucuuauuuucu...................   32      0    er1
    .........................................................................aaccauguagucuuauuuucA...................   2       1    er1
    .........................................................................aaccauguagucuuauuuucC...................   21      1    er1
    .........................................................................aaccauguagucuuauuuucuu..................   1       0    er1
    .........................................................................aaccauguagucuuauuuucuC..................   2       1    er1
    .........................................................................aaccauguagucuuauuuucuA..................   5       1    er1
    .........................................................................aaccauguagucuuauuuucuuU.................   2       1    er1
    .........................................................................aaccauguagucuuauuuucuuA.................   10      1    er1
    .........................................................................aaccauguagucuuauuuucuuUa................   4       1    er1
    ..............................................................................uguagucuuaCuucuucaac...............   1       1    er1
    ..............................................................................uguagucuuaCuucuucaacucuaug.........   7       1    er1
```

```
                                        Star                        Mature
uugaggaaaguuuuuugaagauuugaagaaagaguugaagaaaauaagacuacaugguuugucuuagagacaaaccauguagucuuauuuucuucaacucuaugauaaauau
..................................................................................ucuuauuuucuucaacucuau.........    1     0     er1
......................................................................................auuuucuucaacucuaugau......    1     0     er1
.........................................................................................uucuucaacucuaugauaCa...    2     1     er1

........................................aaaauaagacuacaugguuugu..................................................    1     0     eg1
..................................................uacaugguuugucuuagaAac.........................................    1     1     eg1
................................................................uagagacaaaccauguaguA............................    1     1     eg1
.....................................................................acaaaccauguagucuuauuuucA...................    1     1     eg1
......................................................................caaaccaugCagucuuauuuucu...................    1     1     eg1
......................................................................caaaccauguagucuuauuuuUu...................    1     1     eg1
......................................................................caaaccauguagucuuGuuuucu...................    1     1     eg1
......................................................................caaaccauguagucuuauuuucC...................    2     1     eg1
......................................................................caaaccauguagucuuauuuucA...................    4     1     eg1
......................................................................caaaccauguagucuuauuuucu...................    21    0     eg1
......................................................................caaaccauguagucuuauuuucuA..................    2     1     eg1
.......................................................................aaaccauguagucuuauuuu.....................    1     0     eg1
.......................................................................aaaccauguagucuuauuuuc....................    1     0     eg1
.......................................................................aaaccauguagucuuauuuucA...................    3     1     eg1
.......................................................................aaaccauguagucuuauuuucC...................    16    1     eg1
.......................................................................aaaccauguagucuuauuuucu...................    63    0     eg1
.......................................................................aaaccauguagucuuauuuucG...................    2     1     eg1
.......................................................................Gaaccauguagucuuauuuucu...................    1     1     eg1
.......................................................................aaaccauguagucuuauuuucuC..................    1     1     eg1
.......................................................................aaaccauguagucuuauuuucuA..................    6     1     eg1
........................................................................aaUcauguagucuuauuuuc....................    1     1     eg1
........................................................................aaccauguagucuuauuuuG....................    2     1     eg1
........................................................................aaccauguagucuuauuuucu...................    101   0     eg1
........................................................................aaccauguagucuuaAuuucu...................    1     1     eg1
........................................................................aaccauguagucuuauuuucC...................    19    1     eg1
........................................................................Gaccauguagucuuauuuucu...................    2     1     eg1
........................................................................aaccauguagucuuauuuucA...................    2     1     eg1
........................................................................aaccauguagCcuuauuuucu...................    1     1     eg1
........................................................................aaccauguagucuuauuuucG...................    4     1     eg1
........................................................................aaccGuguagucuuauuuucu...................    1     1     eg1
........................................................................Uaccauguagucuuauuuucuu..................    1     1     eg1
........................................................................aaccauguagucuuauuuucuA..................    12    1     eg1
........................................................................aaccauguagucuuauuuucuu..................    14    0     eg1
........................................................................aaccauguagucuuauuuucuC..................    9     1     eg1
........................................................................aaccauguagucuuauuuucCu..................    2     1     eg1
........................................................................aaccauguagucuuauuuucuuc.................    1     0     eg1
........................................................................aaccauguagucuuauuuucuuU.................    5     1     eg1
........................................................................aaccauguagucuuauuuucuuA.................    12    1     eg1
........................................................................aaccauguagucuuauuuucuuUa................    6     1     eg1
.........................................................................accauguagucuuauuuucC...................    2     1     eg1
..........................................................................ccauguagucuuauuuucuuU.................    1     1     eg1
...........................................................................Gauguagucuuauuuucuuc.................    1     1     eg1
.............................................................................uguagucCuauuuucuucaacucu...........    1     1     eg1
.............................................................................uguagucuuaCuuucuucaacucu...........    1     1     eg1
.............................................................................Gguagucuuauuuucuucaacucuau.........    1     1     eg1
.............................................................................uguagucuuaCuuucuucaacucuaug........    2     1     eg1
..................................................................................ucuuauuuucuucaacucua..........    2     0     eg1
....................................................................................uuauuuucuucaacucuaug........    1     0     eg1
.......................................................................................uuuucuucaacucuaugaua.....    2     0     eg1

........................................aaaauaagacuacaugguuug...................................................    1     0     eg2
.......................................................................aaaccauguagucuuauuuuc....................    1     0     eg2
.......................................................................aaaccauguagucuuauuuucu...................    1     0     eg2
........................................................................aaccauguagucuuaCuuucu...................    1     1     eg2
........................................................................aaccauguagucuuauuuucA...................    1     1     eg2
........................................................................aaccauguagucuuauuuucuA..................    4     1     eg2
........................................................................aaccauguagucuuauuuucuuA.................    1     1     eg2
.........................................................................accauguagucuuauuuucA...................    1     1     eg2
.........................................................................accauguagucuuauuuucu...................    1     0     eg2
.........................................................................accauguagucuuauuuucuuA.................    1     1     eg2
...........................................................................cauguagucuuauuuucuuA.................    2     1     eg2
...........................................................................cauguagucuuauuuucuuUa................    1     1     eg2
............................................................................auguagucuuauuuucuuca................    2     0     eg2
.....................................................................................uauuuucuucaacucuaugauaUau..    2     1     eg2
.......................................................................................uuuucuucaacucuaugauaCau..    1     1     eg2

........................................aaaauaagacuacaugguuug...................................................    3     0     ea2
```

```
                                        Star                            Mature
uugaggaaaguuuuuugaagauuugaagaaagaguugaagaaaauaagacuacaugguuugucuuagagacaaaccauguagucuuauuuucuucaacucuaugauaaauau
........................................aaaauaagacuacaugguuugu..................................................   3   0   ea2
...................................................acauggAuugucuuagagaca........................................   2   1   ea2
......................................................................caaaccauguagucuuauCu......................   3   1   ea2
........................................................................aaccauguagucuuauuuucA...................   9   1   ea2
........................................................................aaccauguagucuuauuuucu...................   7   0   ea2
........................................................................aaccauguagucuuauuuucuA..................   7   1   ea2
........................................................................aaccauguagucuuauuuucuu..................   6   0   ea2
........................................................................aaccauguagucuuauuuucuuA.................   13  1   ea2
.........................................................................accauguagucuuauuuucA...................   5   1   ea2
.........................................................................accauguagucuuauuuucC...................   2   1   ea2
.........................................................................accauguagucuuauuuucuA..................   2   1   ea2
.........................................................................accauguagucuuauuuucuuA.................   1   1   ea2
..........................................................................ccauguagucuuauuuucuuA.................   4   1   ea2
...........................................................................cauguagucuuauuuucuuA.................   3   1   ea2
...........................................................................cauguagucuuauuuucuuUa................   2   1   ea2
............................................................................auguagucuuauuuucuuA.................   6   1   ea2
.............................................................................uguagucuuaCuucuucaacucuaug.........   1   1   ea2
...............................................................................uagucuuauuuucuucaacucuauA........   1   1   ea2
..................................................................................ucuuauuuucuucaacucuaug........   5   0   ea2
.......................................................................................uuuucuucaacucuaugaua.....   2   0   ea2

...................................ugaagaaaauaagacuacaug........................................................   1   0   er2
....................................................caugguuugucuuagaAaca........................................   2   1   er2
......................................................................caaaccauguagucuuauuuucA...................   2   1   er2
......................................................................caaaccauguagucuuauuuucu...................   5   0   er2
......................................................................caaaccauguagucuuauuuucuA..................   1   1   er2
.......................................................................Naaccauguagucuuauuuucu...................   1   1   er2
.......................................................................aaaccauguagucuuauuuucu...................   8   0   er2
.......................................................................aaaccauguagucuuauuuucuA..................   9   1   er2
........................................................................aaccauguagucuuauuuucC...................   1   1   er2
........................................................................aaccauguagucuuauuuucu...................   19  0   er2
........................................................................aaccauguagucuuauuuucuA..................   10  1   er2
........................................................................aaccauguagucuuauuuucuuA.................   11  1   er2
........................................................................aaccauguagucuuauuuucuuU.................   1   1   er2
.........................................................................accauguagucuuauuuucu...................   1   0   er2
.........................................................................accauguagucuuauuuucA...................   1   1   er2
.........................................................................accauguagucuuauuuucuuA.................   3   1   er2
.........................................................................accauguagucuuauuuucuuUa................   1   1   er2
...............................................................................uGgucuuauuuucuucaacucu...........   1   1   er2
..................................................................................ucuuauuuucuucaacucuauA........   5   1   er2
.......................................................................................uuuucuucaacucuaugaua.....   3   0   er2
.......................................................................................Cuuucuucaacucuaugauaa....   1   1   er2
........................................................................................uuucuucaacucuaAgaua.....   1   1   er2
........................................................................................uuucuucaacucuaugaua.....   6   0   er2

...................................Ggaagaaaauaagacuacaug........................................................   1   1   ea1
........................................aaaauaagacuacaugguuugu..................................................   1   0   ea1
....................................................caugguuugucuuagagaAa........................................   1   1   ea1
.............................................................ucuuagagacaaaccauguaA..............................   1   1   ea1
..............................................................cuuagagacaaaccaugua...............................   1   0   ea1
................................................................uagagacaaaccauguagu.............................   2   0   ea1
......................................................................caaaccauguagucuuauuA......................   3   1   ea1
......................................................................Gaaaccauguagucuuauuu......................   1   1   ea1
......................................................................caaaccauguagucuuauuuA.....................   1   1   ea1
......................................................................caaaccauguagucuuauuuu.....................   1   0   ea1
......................................................................caaaccauguagucuuauuuC.....................   1   1   ea1
......................................................................caaaccauguagucuuauuuuc....................   4   0   ea1
......................................................................caaaccauguagucuuauuuuA....................   2   1   ea1
......................................................................caaaccauguagucuuauuuucu...................   74  0   ea1
......................................................................caaaccauguagucuuaCuuucu...................   1   1   ea1
......................................................................caaGccauguagucuuauuuucu...................   2   1   ea1
......................................................................caaaccauAuagucuuauuuucu...................   1   1   ea1
......................................................................caaaccauguagucuuauuuucC...................   21  1   ea1
......................................................................caaaccauguagucuCauuuucu...................   1   1   ea1
......................................................................Gaaaccauguagucuuauuuucu...................   2   1   ea1
......................................................................caaaccauguagucuuauuuuUu...................   1   1   ea1
......................................................................caaaccauguagucuuauuuucA...................   17  1   ea1
......................................................................caaaccauguagucuuauuuucG...................   2   1   ea1
......................................................................caaaccauguagucuuauuuucuA..................   10  1   ea1
.......................................................................aaaccauguagucuuauu.......................   1   0   ea1
```

```
                                                          Star                           Mature
uugaggaaaguuuuuugaagauuugaagaaagaguugaagaaaauaaagacuacaugguuugucuuagagacaaaccauguagucuuauuuucuucaacucuaugauaaauau
........................................................................aaaccauguagucuuauuC......................	1	1	ea1
........................................................................aaaccauguagucuuauuuuU....................	1	1	ea1
........................................................................aaaccauguagucuuauuuuc....................	11	0	ea1
........................................................................aaaccauguagucuuauuuucA...................	21	1	ea1
........................................................................aaaccauguagucuuauuuucC...................	43	1	ea1
........................................................................aGaccauguagucuuauuuucu...................	2	1	ea1
........................................................................aaaccauguagucuuauuuucG...................	1	1	ea1
........................................................................aaaccauguagucuuaCuuucu...................	1	1	ea1
........................................................................aaaccauguagucuuaAuuucu...................	1	1	ea1
........................................................................aaaccauguaAucuuauuuucu...................	1	1	ea1
........................................................................aaaccaugCagucuuauuuucu...................	1	1	ea1
........................................................................aaaccaCguagucuuauuuucu...................	1	1	ea1
........................................................................aaaccauguagucuuauCuucu...................	1	1	ea1
........................................................................aaacAauguagucuuauuuucu...................	1	1	ea1
........................................................................aaaccauguagucuuauuuucu...................	181	0	ea1
........................................................................aaaccUuguagucuuauuuucu...................	1	1	ea1
........................................................................Gaaccauguagucuuauuuucu...................	5	1	ea1
........................................................................aaaccGuguagucuuauuuucu...................	1	1	ea1
........................................................................aaaccauguagucuuauuuucCu..................	1	1	ea1
........................................................................aaaccauguagucuuauuuucuA..................	28	1	ea1
........................................................................aaaccauguagucuuauuuucuG..................	1	1	ea1
........................................................................aaaccauguagucuuauuuucuuU.................	1	1	ea1
.........................................................................aaccauguagucuuauuuu.....................	8	0	ea1
.........................................................................aaccauguagucuuauuuuA....................	2	1	ea1
.........................................................................aaccauguagucCuauuuuc....................	1	1	ea1
.........................................................................aaccauguagucuuauuuuU....................	3	1	ea1
.........................................................................aaccauguagucuuauuuuc....................	13	0	ea1
.........................................................................Gaccauguagucuuauuuuc....................	1	1	ea1
.........................................................................aaccauguagucuuauuuuG....................	2	1	ea1
.........................................................................aaccauguagucuuauuuucA...................	38	1	ea1
.........................................................................aaccaugCagucuuauuuucu...................	5	1	ea1
.........................................................................aaccaCguagucuuauuuucu...................	2	1	ea1
.........................................................................aaccauguagucuuauuCucu...................	1	1	ea1
.........................................................................aaccauguGgucuuauuuucu...................	1	1	ea1
.........................................................................aaccauguaguAuuauuuucu...................	1	1	ea1
.........................................................................aaccauguagucuuauCuucu...................	3	1	ea1
.........................................................................aaccauguagucuCauuuucu...................	2	1	ea1
.........................................................................aaccauguagucuuauuuucC...................	106	1	ea1
.........................................................................Gaccauguagucuuauuuucu...................	7	1	ea1
.........................................................................aaccauguagucuuauuuucG...................	2	1	ea1
.........................................................................aaccauguagucuuauuuucu...................	344	0	ea1
.........................................................................aaccauguagucuuaCuuucu...................	1	1	ea1
.........................................................................aaccauguagucuuauuuuUu...................	1	1	ea1
.........................................................................aaccauguagucCuauuuucu...................	1	1	ea1
.........................................................................Gaccauguagucuuauuuucuu..................	1	1	ea1
.........................................................................aGccauguagucuuauuuucuu..................	3	1	ea1
.........................................................................aaccauguagucuuauuuucCu..................	9	1	ea1
.........................................................................aaccauguagucuuauuuucuu..................	21	0	ea1
.........................................................................aaccauguagucuuauuuucuC..................	11	1	ea1
.........................................................................aaccauguagucuuauuuucuA..................	78	1	ea1
.........................................................................aaccauguagucuuauuuucuuU.................	11	1	ea1
.........................................................................aaccauguagucuuauuuucuuA.................	64	1	ea1
.........................................................................aaccauguagucuuauuuucuuc.................	5	0	ea1
.........................................................................aaccauguagucuuauuuucuuca................	1	0	ea1
.........................................................................aaccauguagucuuauuuucuuUa................	8	1	ea1
..........................................................................aUcauguagucuuauuuucu...................	1	1	ea1
..........................................................................accauguagucuuauuuucu...................	3	0	ea1
..........................................................................accauguagucuuauuuucC...................	4	1	ea1
...........................................................................ccauguagucuuauuuucuu..................	1	0	ea1
...........................................................................ccauguagucuuauuuucuuA.................	1	1	ea1
............................................................................cauguagucuuauuuCcu...................	1	1	ea1
............................................................................cauguagucuuauuuucu...................	2	0	ea1
............................................................................cauguagucuuauuuucuuc.................	4	0	ea1
............................................................................cauguagucuGauuuucuuc.................	5	1	ea1
............................................................................cauguagucuuauuuucuuU.................	2	1	ea1
..............................................................................uguagucuGauuuucuucaa...............	1	1	ea1
..............................................................................uguagucuGauuuucuucaac..............	4	1	ea1
..............................................................................uguagucuuauuuucuucaacucu...........	1	0	ea1
..............................................................................uguagucuuauCuucuucaacucu...........	3	1	ea1
..............................................................................uguagucuuaCuuucuucaacucua..........	2	1	ea1
```

```
                                        Star                            Mature
uugaggaaaguuuuuugaagauuugaagaaagaguugaagaaaauaagacuacaugguuugucuuagagacaaaccauguagucuuauuuucuucaacucuaugauaaauau
...............................................................................uagucuuauuuucuucaacucu...........  1  0  ea1
..................................................................................ucuuauuuucuucaacucuaug........  1  0  ea1
....................................................................................Guauuuucuucaacucuaug........  1  1  ea1
.....................................................................................uaCuuucuucaacucuaugauaa....  1  1  ea1
......................................................................................auuCucuucaacucuaugaua.....  1  1  ea1
.......................................................................................uuuucuucaacucuaugau......  2  0  ea1
.......................................................................................uuuucuucaacucuaugaua.....  2  0  ea1
.......................................................................................uuuucCucaacucuaugaua.....  2  1  ea1
.......................................................................................uuuucuucaacucuaugauaa....  1  0  ea1
.........................................................................................uucuucaacucuaugauaaau..  2  0  ea1
..........................................................................................ucuucaacucuaugauaCau..  1  1  ea1
..........................................................................................ucuucaacucuaugauaaau..  1  0  ea1
```

Provisional ID	:	ScUbCFx_979_34362
Score total	:	971.9
Score for star read(s)	:	3.9
Score for read counts	:	961.1
Score for mfe	:	2.3
Score for randfold	:	1.6
Score for cons. seed	:	3
Total read count	:	1897
Mature read count	:	1883
Loop read count	:	0
Star read count	:	14

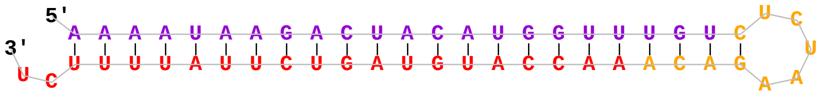

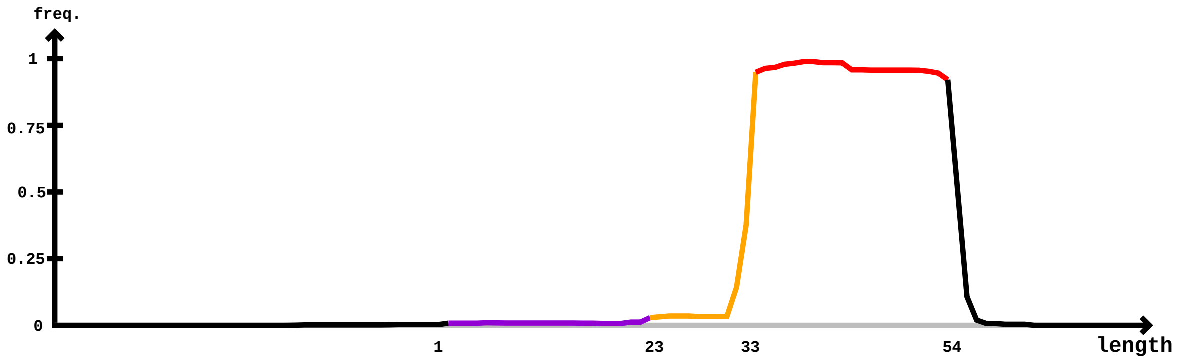

Star **Mature**

```
5'- auaaaaauacauauaaauauauuuaucauagaguugaagaaaauaagacuacaugguuugucucuaagacaaaccauguagucuuauuuucuucaacucuuucuucaaaucu  -3'   obs
    auaaaaauacauauaaauauauuuaucauagaguugaagaaaauaagacuacaugguuugucucuaagacaaaccauguagucuuauuuucuucaacucuuucuucaaaucu         exp
    .............................(((((((((((((((((((((((((((((((((.....)))))))))))))))))))))))))))))))))............   reads   mm   sample
    .......................................aaaauaagacuacaugguuug....................................................   3       0    ea2
    .......................................aaaauaagacuacaugguuugu...................................................   3       0    ea2
    ............................................aagacuacaugguuuAucucu...............................................   1       1    ea2
    ..........................................................ugucucuaagacaaaccaug..................................   10      0    ea2
    ............................................................ucucuaagacaaaccaugua................................   1       0    ea2
    ............................................................ucucuaagacaaaccauguag...............................   1       0    ea2
    .............................................................cucuaagacaaaccauguag...............................   1       0    ea2
    .....................................................................caaaccauguagucuuauCu.......................   3       1    ea2
    .......................................................................aaccauguagucuuauuuucA....................   9       1    ea2
    .......................................................................aaccauguagucuuauuuucu....................   7       0    ea2
    .......................................................................aaccauguagucuuauuuucuu...................   6       0    ea2
    .......................................................................aaccauguagucuuauuuucuA...................   7       1    ea2
    .......................................................................aaccauguagucuuauuuucuuA..................   13      1    ea2
    ........................................................................accauguagucuuauuuucA....................   5       1    ea2
    ........................................................................accauguagucuuauuuucC....................   2       1    ea2
    ........................................................................accauguagucuuauuuucuA...................   2       1    ea2
    ........................................................................accauguagucuuauuuucuuA..................   1       1    ea2
    .........................................................................ccauguagucuuauuuucuuA..................   4       1    ea2
    ..........................................................................cauguagucuuauuuucuuA..................   3       1    ea2
    ..........................................................................cauguagucuuauuuucuuUa.................   2       1    ea2
    ...........................................................................auguagucuuauuuucuuA..................   6       1    ea2

    .......................................aaaauaagacuacaugguuug....................................................   1       0    eg2
    ......................................................................aaaccauguagucuuauuuuc.....................   1       0    eg2
    ......................................................................aaaccauguagucuuauuuucu....................   1       0    eg2
    .......................................................................aaccauguagucuuaCuuucu....................   1       1    eg2
    .......................................................................aaccauguagucuuauuuucA....................   1       1    eg2
    .......................................................................aaccauguagucuuauuuucuA...................   4       1    eg2
    .......................................................................aaccauguagucuuauuuucuuA..................   1       1    eg2
    ........................................................................accauguagucuuauuuucA....................   1       1    eg2
    ........................................................................accauguagucuuauuuucu....................   1       0    eg2
    ........................................................................accauguagucuuauuuucuuA..................   1       1    eg2
    ..........................................................................cauguagucuuauuuucuuA..................   2       1    eg2
```

```
                                            Star                          Mature
auaaaaauacauauaaaauauauuuaucauagaguugaagaaaauaagacuacaugguuugucucuaagacaaaccauguagucuuauuuucuucaacucuuucuucaaaucu
...........................................................................cauguagucuuauuuucuuUa.................   1    1    eg2
............................................................................auguagucuuauuuucuuca.................   2    0    eg2

........................uaucauagaguugGagaaaaua...................................................................   1    1    ea1
.........................aucauagaguugaagaaaGu....................................................................   2    1    ea1
...................................Ggaagaaaauaagacuacaug.........................................................   1    1    ea1
........................................aaaauaagacuacaugguuugu...................................................   1    0    ea1
............................................uaagacuacaugguuuAucucu...............................................   1    1    ea1
.............................................................ucucuaagacaaaccauguaA...............................   1    1    ea1
.............................................................ucucuaagacaaacUauguag...............................   2    1    ea1
.............................................................ucucuaagacaaaccauguag...............................   23   0    ea1
..............................................................cucuaagacaaaccauguag...............................   10   0    ea1
..............................................................cucuaagacaaaccauguaA...............................   2    1    ea1
...............................................................ucuaagacaaaccauguaguA.............................   2    1    ea1
......................................................................Gaaaccauguagucuuauuu.......................   1    1    ea1
......................................................................caaaccauguagucuuauuA.......................   3    1    ea1
......................................................................caaaccauguagucuuauuuC......................   1    1    ea1
......................................................................caaaccauguagucuuauuuA......................   1    1    ea1
......................................................................caaaccauguagucuuauuuu......................   1    0    ea1
......................................................................caaaccauguagucuuauuuuc.....................   4    0    ea1
......................................................................caaaccauguagucuuauuuuA.....................   2    1    ea1
......................................................................caaaccauAuagucuuauuuucu....................   1    1    ea1
......................................................................caaaccauguagucuuauuuucG....................   2    1    ea1
......................................................................Gaaaccauguagucuuauuuucu....................   2    1    ea1
......................................................................caaaccauguagucuuauuuucu....................   74   0    ea1
......................................................................caaaccauguagucuCauuuucu....................   1    1    ea1
......................................................................caaaccauguagucuuauuuucA....................   17   1    ea1
......................................................................caaaccauguagucuuauuuuUu....................   1    1    ea1
......................................................................caaaccauguagucuuaCuuucu....................   1    1    ea1
......................................................................caaaccauguagucuuauuuucC....................   21   1    ea1
......................................................................caaGccauguagucuuauuuucu....................   2    1    ea1
......................................................................caaaccauguagucuuauuuucuA...................   10   1    ea1
.......................................................................aaaccauguagucuuauu........................   1    0    ea1
.......................................................................aaaccauguagucuuauuC.......................   1    1    ea1
.......................................................................aaaccauguagucuuauuuuU.....................   1    1    ea1
.......................................................................aaaccauguagucuuauuuuc.....................   11   0    ea1
.......................................................................aaaccauguaAucuuauuuucu....................   1    1    ea1
.......................................................................aaaccauguagucuuauuuucC....................   43   1    ea1
.......................................................................aaacAauguagucuuauuuucu....................   1    1    ea1
.......................................................................aaaccaugCagucuuauuuucu....................   1    1    ea1
.......................................................................aaaccauguagucuuauuuucG....................   1    1    ea1
.......................................................................aaaccauguagucuuauuuucu....................   181  0    ea1
.......................................................................aaaccauguagucuuauCuucu....................   1    1    ea1
.......................................................................aaaccauguagucuuaAuuucu....................   1    1    ea1
.......................................................................aaaccauguagucuuaCuuucu....................   1    1    ea1
.......................................................................Gaaccauguagucuuauuuucu....................   5    1    ea1
.......................................................................aGaccauguagucuuauuuucu....................   2    1    ea1
.......................................................................aaaccUuguagucuuauuuucu....................   1    1    ea1
.......................................................................aaaccauguagucuuauuuucA....................   21   1    ea1
.......................................................................aaaccaCguagucuuauuuucu....................   1    1    ea1
.......................................................................aaaccGuguagucuuauuuucu....................   1    1    ea1
.......................................................................aaaccauguagucuuauuuucuG...................   1    1    ea1
.......................................................................aaaccauguagucuuauuuucCu...................   1    1    ea1
.......................................................................aaaccauguagucuuauuuucuA...................   28   1    ea1
.......................................................................aaaccauguagucuuauuuucuuU..................   1    1    ea1
........................................................................aaccauguagucuuauuuu......................   8    0    ea1
........................................................................Gaccauguagucuuauuuuc.....................   1    1    ea1
........................................................................aaccauguagucuuauuuuc.....................   13   0    ea1
........................................................................aaccauguagucuuauuuuA.....................   2    1    ea1
........................................................................aaccauguagucCuauuuuc.....................   1    1    ea1
........................................................................aaccauguagucuuauuuuG.....................   2    1    ea1
........................................................................aaccauguagucuuauuuuU.....................   3    1    ea1
........................................................................aaccauguagucuuauuuucC....................   106  1    ea1
........................................................................aaccauguaguAuuauuuucu....................   1    1    ea1
........................................................................aaccaCguagucuuauuuucu....................   2    1    ea1
........................................................................aaccauguagucuuaCuuucu....................   1    1    ea1
........................................................................Gaccauguagucuuauuuucu....................   7    1    ea1
........................................................................aaccauguagucuCauuuucu....................   2    1    ea1
........................................................................aaccauguagucuuauuuucG....................   2    1    ea1
........................................................................aaccauguGgucuuauuuucu....................   1    1    ea1
```

```
                                        Star                            Mature
auaaaaauacauauaaaauauauuuaucauagaguugaagaaaauaagacuacaugguuugucucuaagacaaaccauguagucuuauuuucuucaacucuuucuucaaaucu
........................................................................aaccauguagucuuauuCucu....................    1    1    ea1
........................................................................aaccauguagucCuauuuucu....................    1    1    ea1
........................................................................aaccauguagucuuauCuucu....................    3    1    ea1
........................................................................aaccauguagucuuauuuuUu....................    1    1    ea1
........................................................................aaccaugCagucuuauuuucu....................    5    1    ea1
........................................................................aaccauguagucuuauuuucA....................    38   1    ea1
........................................................................aaccauguagucuuauuuucu....................    344  0    ea1
........................................................................aGccauguagucuuauuuucuu...................    3    1    ea1
........................................................................aaccauguagucuuauuuucuu...................    21   0    ea1
........................................................................aaccauguagucuuauuuucuC...................    11   1    ea1
........................................................................aaccauguagucuuauuuucCu...................    9    1    ea1
........................................................................Gaccauguagucuuauuuucuu...................    1    1    ea1
........................................................................aaccauguagucuuauuuucuA...................    78   1    ea1
........................................................................aaccauguagucuuauuuucuuA..................    64   1    ea1
........................................................................aaccauguagucuuauuuucuuU..................    11   1    ea1
........................................................................aaccauguagucuuauuuucuuc..................    5    0    ea1
........................................................................aaccauguagucuuauuuucuuUa.................    8    1    ea1
........................................................................aaccauguagucuuauuuucuuca.................    1    0    ea1
.........................................................................aUcauguagucuuauuuucu....................    1    1    ea1
.........................................................................accauguagucuuauuuucu....................    3    0    ea1
.........................................................................accauguagucuuauuuucC....................    4    1    ea1
..........................................................................ccauguagucuuauuuucuu...................    1    0    ea1
..........................................................................ccauguagucuuauuuucuuA..................    1    1    ea1
...........................................................................cauguagucuuauuuCcu....................    1    1    ea1
...........................................................................cauguagucuuauuuucu....................    2    0    ea1
...........................................................................cauguagucuuauuuucuuc..................    4    0    ea1
...........................................................................cauguagucuGauuuucuuc..................    5    1    ea1
...........................................................................cauguagucuuauuuucuuU..................    2    1    ea1
.............................................................................uguagucuGauuuucuucaa................    1    1    ea1
.............................................................................uguagucuGauuuucuucaac...............    4    1    ea1
.............................................................................uguagucuuauCuucuucaacucu............    3    1    ea1
.............................................................................uguagucuuauuuucuucaacucu............    1    0    ea1
...............................................................................uagucuuauuuucuucaacucu............    1    0    ea1

...................................ugaagaaaauaagacuacaug.........................................................    1    0    er2
............................................uaagacuacaugguuuAucucu...............................................    2    1    er2
.............................................................ucucuaagacaaaccauguag...............................    3    0    er2
......................................................................caaaccauguagucuuauuuucA....................    2    1    er2
......................................................................caaaccauguagucuuauuuucu....................    5    0    er2
......................................................................caaaccauguagucuuauuuucuA...................    1    1    er2
.......................................................................aaaccauguagucuuauuuucu....................    8    0    er2
.......................................................................Naaccauguagucuuauuuucu....................    1    1    er2
.......................................................................aaaccauguagucuuauuuucuA...................    9    1    er2
........................................................................aaccauguagucuuauuuucu....................    19   0    er2
........................................................................aaccauguagucuuauuuucC....................    1    1    er2
........................................................................aaccauguagucuuauuuucuA...................    10   1    er2
........................................................................aaccauguagucuuauuuucuuU..................    1    1    er2
........................................................................aaccauguagucuuauuuucuuA..................    11   1    er2
.........................................................................accauguagucuuauuuucu....................    1    0    er2
.........................................................................accauguagucuuauuuucA....................    1    1    er2
.........................................................................accauguagucuuauuuucuuA..................    3    1    er2
.........................................................................accauguagucuuauuuucuuUa.................    1    1    er2
...............................................................................uGgucuuauuuucuucaacucu............    1    1    er2

..................................uugaagaaaauaagacuaca...........................................................    1    0    er1
........................................aaaauaagacuacaugguuug....................................................    1    0    er1
.............................................................ucucuaagacaaaccauguag...............................    2    0    er1
.............................................................ucucuaagacaaaccauguaA...............................    1    1    er1
.............................................................ucucuaagacaaaccaCguag...............................    1    1    er1
......................................................................caaaccauguagucuuauuuucA....................    1    1    er1
......................................................................caaaccaCguagucuuauuuucu....................    1    1    er1
......................................................................caaaccauguagucuuauuuucC....................    2    1    er1
......................................................................caaaccauguagucuuauuuucu....................    24   0    er1
......................................................................caaaccauguagucuuauuuucuuA..................    1    1    er1
.......................................................................aaaccauguagucuuauuuu......................    1    0    er1
.......................................................................aaaccauguagucuuauuuucG....................    1    1    er1
.......................................................................aaaccUuguagucuuauuuucu....................    1    1    er1
.......................................................................aaaccauguagucuuauCuucu....................    1    1    er1
.......................................................................aaaccauguagucuuauuuucu....................    27   0    er1
.......................................................................aaaccauguagucuuauuuucC....................    2    1    er1
```

Star Mature

auaaaaauacauauaaaauauauuuaucauagaguugaagaaaauaagacuacaugguuugucucuaagacaaaccauguagucuuauuuucuucaacucuuucuucaaaucu			
....aaaccauguagucuuauuuucA....................	4	1	er1
....aaaccauguagucuuauuuucuu...................	1	0	er1
....aaaccauguagucuuauuuucuA...................	3	1	er1
....aaaccauguagucuuauuuucuuA..................	1	1	er1
....aaUcauguagucuuauuuuc.....................	2	1	er1
....aaccauguagucuuauuuuc.....................	2	0	er1
....aaccauguagucuuauuuucC....................	21	1	er1
....aaccauguagucuuauuuucu....................	32	0	er1
....aaUcauguagucuuauuuucu....................	1	1	er1
....aaccUuguagucuuauuuucu....................	1	1	er1
....aaccauguagucuuauuuucA....................	2	1	er1
....aaccaugCagucuuauuuucu....................	1	1	er1
....aaccauguagucuuauuuucuu...................	1	0	er1
....aaccauguagucuuauuuucuC...................	2	1	er1
....aaccauguagucuuauuuucuA...................	5	1	er1
....aaccauguagucuuauuuucuuA..................	10	1	er1
....aaccauguagucuuauuuucuuU..................	2	1	er1
....aaccauguagucuuauuuucuuUa.................	4	1	er1
....uguagucuuaCuuucuucaac...............	1	1	er1
....aaaauaagacuacaugguuugu...	1	0	eg1
....ucucuaagacaaaccauguag...............................	3	0	eg1
....cucuaagacaaaccauguag...............................	2	0	eg1
....acaaaccauguagucuuauuuucA....................	1	1	eg1
....caaaccauguagucuuauuuucC....................	2	1	eg1
....caaaccauguagucuuauuuucu....................	21	0	eg1
....caaaccauguagucuuauuuuUu....................	1	1	eg1
....caaaccaugCagucuuauuuucu....................	1	1	eg1
....caaaccauguagucuuauuuucA....................	4	1	eg1
....caaaccauguagucuuGuuuucu....................	1	1	eg1
....caaaccauguagucuuauuuucuA...................	2	1	eg1
....aaaccauguagucuuauuuu......................	1	0	eg1
....aaaccauguagucuuauuuuc.....................	1	0	eg1
....aaaccauguagucuuauuuucA....................	3	1	eg1
....aaaccauguagucuuauuuucC....................	16	1	eg1
....Gaaccauguagucuuauuuucu....................	1	1	eg1
....aaaccauguagucuuauuuucG....................	2	1	eg1
....aaaccauguagucuuauuuucu....................	63	0	eg1
....aaaccauguagucuuauuuucuA...................	6	1	eg1
....aaaccauguagucuuauuuucuC...................	1	1	eg1
....aaUcauguagucuuauuuuc.....................	1	1	eg1
....aaccauguagucuuauuuuG.....................	2	1	eg1
....aaccauguagucuuauuuucC....................	19	1	eg1
....aaccGuguagucuuauuuucu....................	1	1	eg1
....aaccauguagucuuauuuucA....................	2	1	eg1
....aaccauguagucuuauuuucu....................	101	0	eg1
....aaccauguagCcuuauuuucu....................	1	1	eg1
....aaccauguagucuuaAuuucu....................	1	1	eg1
....Gaccauguagucuuauuuucu....................	2	1	eg1
....aaccauguagucuuauuuucG....................	4	1	eg1
....aaccauguagucuuauuuucuA...................	12	1	eg1
....aaccauguagucuuauuuucuC...................	9	1	eg1
....Uaccauguagucuuauuuucuu...................	1	1	eg1
....aaccauguagucuuauuuucuu...................	14	0	eg1
....aaccauguagucuuauuuucCu...................	2	1	eg1
....aaccauguagucuuauuuucuuA..................	12	1	eg1
....aaccauguagucuuauuuucuuU..................	5	1	eg1
....aaccauguagucuuauuuucuuc..................	1	0	eg1
....aaccauguagucuuauuuucuuUa.................	6	1	eg1
....accauguagucuuauuuucC....................	2	1	eg1
....ccauguagucuuauuuucuuU..................	1	1	eg1
....Gauguagucuuauuuucuuc..................	1	1	eg1
....uguagucuuaCuuucuucaacucu............	1	1	eg1
....uguagucCuauuuucuucaacucu............	1	1	eg1

Provisional ID	:	ScUbCFx_979_34447
Score total	:	57.3
Score for star read(s)	:	3.9
Score for read counts	:	50.6
Score for mfe	:	1.8
Score for randfold	:	1.6
Score for cons. seed	:	-0.6
Total read count	:	111
Mature read count	:	94
Loop read count	:	0
Star read count	:	17

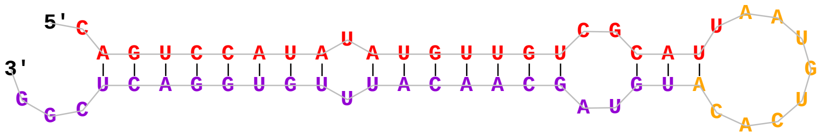

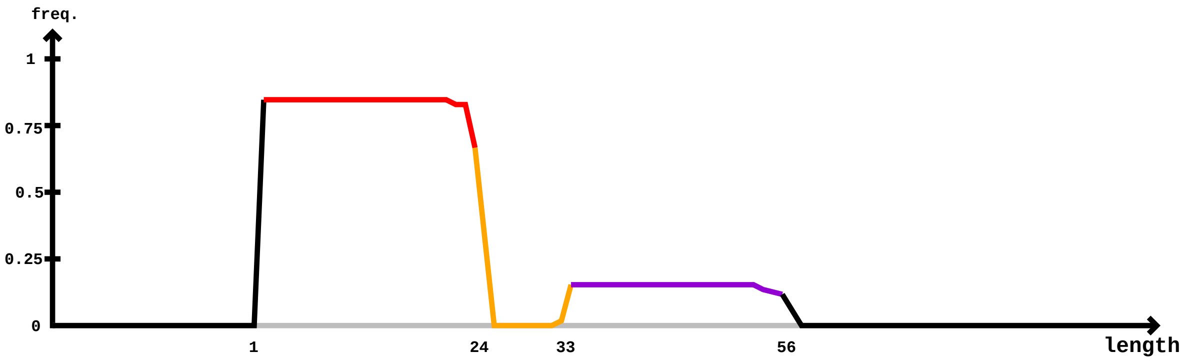

Mature Star

```
5'- guaucaaguagacugugccccaguccauauauguugucgcauuaaugucacauguagcaacauuuguggacucgggcaaaggcuaauaucaagacgauagucaaccuauaagu  -3'  obs
    guaucaaguagacugugccccaguccauauauguugucgcauuaaugucacauguagcaacauuuguggacucgggcaaaggcuaauaucaagacgauagucaaccuauaagu       exp
    ........(((.((.(((((.((((((((.(((((((..(((.........)))..))))))).)))))))).))))).)).)))........(((....)))...........  reads  mm  sample
    ....................caguccauauauguugucgcauu......................................................................  1      0   eg1

    ....................caguccauauauguugucgcauu......................................................................  7      0   er1
    ....................caguccauauauguugucgcauuU.....................................................................  1      1   er1

    ....................caguccauauauguugucgc.........................................................................  2      0   ea1
    ....................caguccauauauguugucgcaC.......................................................................  1      1   ea1
    ....................caguccauauauguugucgcau.......................................................................  3      0   ea1
    ....................caguccauauaugCugucgcau.......................................................................  2      1   ea1
    ....................caguccauauauguugucgcauA......................................................................  1      1   ea1
    ....................cagCccauauauguugucgcauu......................................................................  2      1   ea1
    ....................caguccauauauguugucgcauu......................................................................  4      0   ea1
    ....................caguccauauauguugucgcauC......................................................................  1      1   ea1
    ....................caguccaCauauguugucgcauu......................................................................  1      1   ea1
    ....................caguccauauauguugucgcauuC.....................................................................  1      1   ea1
    ....................caguccaCauauguugucgcauua.....................................................................  1      1   ea1
    ....................caguccauauauguugucgcauuU.....................................................................  1      1   ea1
    ...................................................auguagcaacauuuguggacu.........................................  1      0   ea1
    ....................................................uguGgcaacauuuguggacu.........................................  1      1   ea1
    ....................................................uguGgcaacauuuguggacuc........................................  1      1   ea1
    ....................................................uguGgcaacauuuguggacucg.......................................  1      1   ea1
    ....................................................uguGgcaacauuuguggacucgg......................................  2      1   ea1
    ....................................................uguagcaacauuuguggacucgg......................................  2      0   ea1

    ....................caguccauauauguugucgcau.......................................................................  10     0   er2
    ....................caguccauauauguugucgcauu......................................................................  20     0   er2
    ....................caguccauauauguugucgcauua.....................................................................  1      0   er2
    ....................caguccauauauguugucgcauuU.....................................................................  17     1   er2
    ....................................................uguGgcaacauuuguggacucgg......................................  3      1   er2

    ....................caguccauauauguugucgcau.......................................................................  2      0   ea2
    ....................caguccauauauguugucgcauu......................................................................  7      0   ea2
    ....................................................uguGgcaacauuuguggacucgg......................................  1      1   ea2
```

Mature **Star**

guaucaaguagacugugcccaguccauauauguugucgcauuaaugucacauguagcaacauuuguggacucgggcaaaggcuaauaucaagacgauagucaaccuauaagu

....................caguccauauauguugucgcauu..	4	0	eg2
....................caguccauauauguugucgcauuU...	4	1	eg2
....auguGgcaacauuuguggacucgg......................................	1	1	eg2
....uguGgcaacauuuguggacucgg......................................	3	1	eg2
....uguagcaacauuuguggacucgg......................................	1	0	eg2

Provisional ID : ScUbCFx_1656_35971
Score total : 1451295.7
Score for star read(s) : 3.9
Score for read counts : 1451289.2
Score for mfe : 1.5
Score for randfold : 1.6
Score for cons. seed : -0.6
Total read count : 2846653
Mature read count : 2846262
Loop read count : 0
Star read count : 391

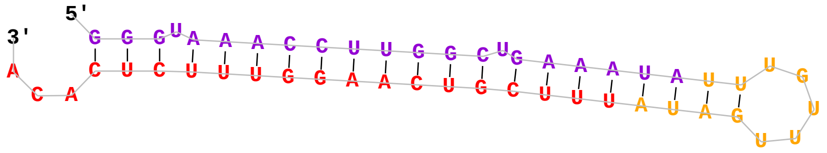

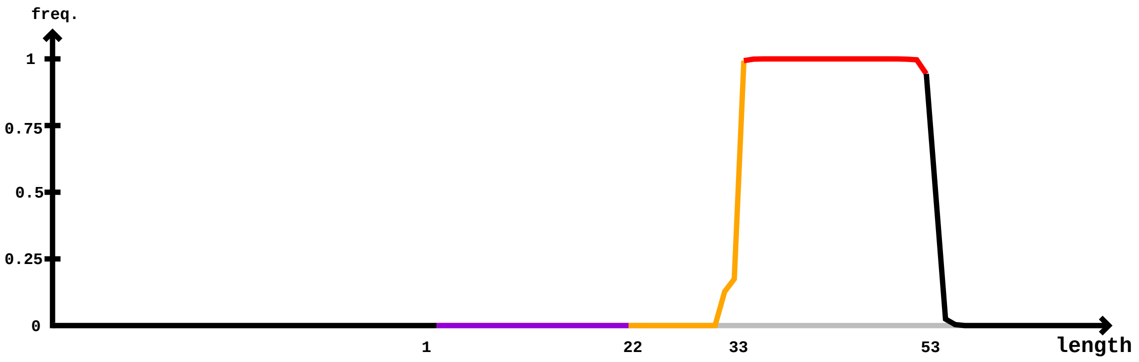

Star Mature

```
5'- augaaaaaccaaagaaguaaaacguuuucuuuucucuugggguaaaccuuggcugaaauauuuguuugauauuucgucaagguuucucacaagagaaauggcuucgguc -3' obs
    augaaaaaccaaagaaguaaaacguuuucuuuucucuuggguaaaccuuggcugaaauauuuguuugauauuucgucaagguuucucacaagagaaauggcuucgguc     exp
    ..........(((((((........)))))))((((((((..((((((((((.((((((((.....))))))))))))))))))..).)))))))...(((....)))    reads  mm  sample
    ................................................uggcugaaauauuuguuugau.......................................    1      0   ea2
    .................................................................ugauCuuucgucaagguuucucaca..................    1      1   ea2
    ...................................................................auauuucgucaagguuucuca....................    1      0   ea2
    ...................................................................Cuauuucgucaagguuucucac...................    1      1   ea2
    ...................................................................Cuauuucgucaagguuucucaca..................    53     1   ea2
    ...................................................................auauuucgucaagguuucucacU.................    4      1   ea2
    ...................................................................aAauuucgucaagguuucucaca..................    1      1   ea2
    ....................................................................uauuucgucaagguuucA......................    1      1   ea2
    ....................................................................uauuucgucaagguuucuc.....................    5      0   ea2
    ....................................................................Nauuucgucaagguuucuca....................    1      1   ea2
    ....................................................................uauuGcgucaagguuucuca....................    1      1   ea2
    ....................................................................uauuucgucaagguuucuca....................    165    0   ea2
    ....................................................................uauuucgucaagguuucucU....................    3      1   ea2
    ....................................................................uaGuucgucaagguuucuca....................    1      1   ea2
    ....................................................................uauuucgucaagguuucucG....................    2      1   ea2
    ....................................................................uauuucgucaagguuucucC....................    1      1   ea2
    ....................................................................uauuucgucaaggGuucucac...................    1      1   ea2
    ....................................................................Cauuucgucaagguuucucac...................    3      1   ea2
    ....................................................................uauuucgucaaggCuucucac...................    2      1   ea2
    ....................................................................uauuucgucaagguCucucac...................    1      1   ea2
    ....................................................................uauuucgucaagguuucucac...................    813    0   ea2
    ....................................................................uauuucgucaagguuucucaU...................    27     1   ea2
    ....................................................................uauuucgucaagguuucucaA...................    1      1   ea2
    ....................................................................uauuucgucaagguuucCcac...................    1      1   ea2
    ....................................................................uaGuucgucaagguuucucac...................    1      1   ea2
    ....................................................................uauuucgucGagguuucucac...................    1      1   ea2
    ....................................................................Nauuucgucaagguuucucac...................    5      1   ea2
    ....................................................................uauuucgucaagguGucucaca..................    4      1   ea2
    ....................................................................uauuuUgucaagguuucucaca..................    1      1   ea2
    ....................................................................uauuucgucaagguuucucacU.................    13583  1   ea2
    ....................................................................Gauuucgucaagguuucucaca..................    10     1   ea2
    ....................................................................uauuucgCcaagguuucucaca..................    4      1   ea2
    ....................................................................Nauuucgucaagguuucucaca..................    81     1   ea2
```

Star Mature

augaaaaaccaaagaaguaaaacguuuucuuuucucuuggguaaaccuuggcugaaauauuuguuugauauuucgucaagguuucucacaagagaaauggcuucgguc			
....uauuucgucaagguCucucaca..................	13	1	ea2
....uauuGcgucaagguuucucaca..................	8	1	ea2
....uauuucgucaagguuucAcaca..................	1	1	ea2
....uauuucgucaCgguuucucaca..................	1	1	ea2
....uauuucgucaagguuucuUaca..................	2	1	ea2
....uauuucgucaagguuucucaca..................	18478	0	ea2
....uauuucgucaagguuucGcaca..................	5	1	ea2
....uauuucguUaagguuucucaca..................	2	1	ea2
....uaCuucgucaagguuucucaca..................	4	1	ea2
....uauuucgucaagguuucucaUa..................	63	1	ea2
....uauuucAucaagguuucucaca..................	3	1	ea2
....uCuuucgucaagguuucucaca..................	1	1	ea2
....uauuucgucaGgguuucucaca..................	4	1	ea2
....uUuuucgucaagguuucucaca..................	2	1	ea2
....uauuucgucaaUguuucucaca..................	1	1	ea2
....uauuucgucCagguuucucaca..................	1	1	ea2
....uauuucgucaagAuuucucaca..................	1	1	ea2
....uauuucgucGagguuucucaca..................	4	1	ea2
....Cauuucgucaagguuucucaca..................	38	1	ea2
....uauuuAgucaagguuucucaca..................	4	1	ea2
....uauCucgucaagguuucucaca..................	4	1	ea2
....uNuuucgucaagguuucucaca..................	10	1	ea2
....uauuucgucaagguuucucacG..................	79	1	ea2
....uauuucgucaaggCuucucaca..................	10	1	ea2
....uauuAcgucaagguuucucaca..................	2	1	ea2
....uauuucgucaagUuuucucaca..................	1	1	ea2
....uauuucgucaagguuucuAaca..................	2	1	ea2
....uauuucgucaagguuucucGca..................	5	1	ea2
....uaAuucgucaagguuucucaca..................	1	1	ea2
....uauuucgucaagguuucucaAa..................	1	1	ea2
....uauGucgucaagguuucucaca..................	5	1	ea2
....uauuucgucaagguuucCcaca..................	14	1	ea2
....uauuucguAaagguuucucaca..................	3	1	ea2
....uauuCcgucaagguuucucaca..................	2	1	ea2
....uauuucgucaagguuuUucaca..................	3	1	ea2
....uauuucgucaaggGuucucaca..................	29	1	ea2
....uGuuucgucaagguuucucaca..................	4	1	ea2
....uauuucgucaagguuuGucaca..................	1	1	ea2
....uaGuucgucaagguuucucaca..................	2	1	ea2
....uauuucgucaaggAuucucaca..................	4	1	ea2
....uauuucgucaagguuCcucaca..................	1	1	ea2
....uauuucgucaagguuucucacC..................	237	1	ea2
....uauuucgucaagguuAcucaca..................	2	1	ea2
....uauuucgucaagguuucuGaca..................	1	1	ea2
....uauuucgucaagguAucucaca..................	1	1	ea2
....uauuucgucaagguuucuCca..................	1	1	ea2
....uauuucgucaagguuucucacaU.................	62	1	ea2
....uauuucgucaagguuucucacUa.................	227	1	ea2
....uauuucgucaagguuucucacaa.................	6	0	ea2
....uauuucgucaagguuucucacCa.................	4	1	ea2
....uauuucgucaagguuucucacaaU................	3	1	ea2
....uauuucgucaagguuucucacaUg................	2	1	ea2
....auuucgucaagguuucuc.....................	3	0	ea2
....auuucgucaaggGuucuca....................	2	1	ea2
....auuucgucaagguuCcuca....................	1	1	ea2
....auuucgucaagguuucuca....................	125	0	ea2
....auuucgucaaggCuucuca....................	1	1	ea2
....aCuucgucaagguuucuca....................	1	1	ea2
....auuucgucaagguuucucU....................	1	1	ea2
....Nuuucgucaagguuucuca....................	2	1	ea2
....auuucUucaagguuucucac...................	1	1	ea2
....Nuuucgucaagguuucucac...................	6	1	ea2
....auuucgucaagguuucucac...................	643	0	ea2
....auuucgucaagguuucCcac...................	1	1	ea2
....auuucgucaagAuuucucac...................	1	1	ea2
....Cuuucgucaagguuucucac...................	4	1	ea2
....auuucgucaagguuucucGc...................	1	1	ea2
....auuucgucaagguuucucaU...................	36	1	ea2
....auuucgucaagguuucucaA...................	1	1	ea2
....auuucgucaaggCuucucac...................	1	1	ea2

Star **Mature**

augaaaaaccaaagaaguaaaacguuuucuuuucucuuggguaaaccuuggcugaaauauuuguuugauauuucgucaagguuucucacaagagaaauggcuucgguc			
....auuucgucaagguuuAucac...................	2	1	ea2
....auuucgucaagguuucucCc...................	1	1	ea2
....auuucgucaagguGucucac...................	1	1	ea2
....aCuucgucaagguuucucac...................	1	1	ea2
....auuucgucaaggGuucucac...................	5	1	ea2
....auuucgucaaUguuucucac...................	1	1	ea2
....auuucguUaagguuucucaca..................	2	1	ea2
....auCucgucaagguuucucaca..................	13	1	ea2
....auuucAucaagguuucucaca..................	1	1	ea2
....auuucgucaagUuuucucaca..................	2	1	ea2
....auuucgucaagguuCcucaca..................	4	1	ea2
....aGuucgucaagguuucucaca..................	3	1	ea2
....auuucgucaagguuucucGca..................	4	1	ea2
....auuucgucaagguuucGcaca..................	1	1	ea2
....auuucgucaaggAuucucaca..................	4	1	ea2
....auuucgucaagguGucucaca..................	4	1	ea2
....auuucgCcaagguuucucaca..................	2	1	ea2
....auuucgucaaggGuucucaca..................	75	1	ea2
....auuucgucaagAuuucucaca..................	5	1	ea2
....auuucgucaagguAucucaca..................	1	1	ea2
....auuucgucaagguuucuUaca..................	1	1	ea2
....auuucgucaaggCuucucaca..................	9	1	ea2
....auuucgucaagguuucucacC.................	252	1	ea2
....auuucgucaagguuucAcaca..................	1	1	ea2
....auAucgucaagguuucucaca..................	1	1	ea2
....aAuucgucaagguuucucaca..................	4	1	ea2
....auuucgucaaAguuucucaca..................	3	1	ea2
....auuucgucaagguuucCcaca..................	14	1	ea2
....auuucgucaagguuuUucaca..................	5	1	ea2
....auuucgucaagguuucucaca..................	18314	0	ea2
....auuucgucaGgguuucucaca..................	5	1	ea2
....auuuAgucaagguuucucaca..................	1	1	ea2
....Cuuucgucaagguuucucaca..................	510	1	ea2
....auGucgucaagguuucucaca..................	1	1	ea2
....auuucgucaagguuucucCca..................	1	1	ea2
....auuucgucCagguuucucaca..................	2	1	ea2
....auuucgucaagguuucucUca..................	2	1	ea2
....auuucgucaagguCucucaca..................	3	1	ea2
....auuucgucaagguuucucacU.................	11562	1	ea2
....auuuUgucaagguuucucaca..................	2	1	ea2
....aNuucgucaagguuucucaca..................	4	1	ea2
....auuucgucaagguuucucacG.................	59	1	ea2
....auuucgucaagguuucucaUa..................	53	1	ea2
....auuucUucaagguuucucaca..................	2	1	ea2
....auuucgGcaagguuucucaca..................	15	1	ea2
....Nuuucgucaagguuucucaca..................	119	1	ea2
....auuucgucaCgguuucucaca..................	2	1	ea2
....auuucgucGagguuucucaca..................	5	1	ea2
....Guuucgucaagguuucucaca..................	13	1	ea2
....auuucgucaagguuucuAaca..................	1	1	ea2
....auuucgucUagguuucucaca..................	2	1	ea2
....aCuucgucaagguuucucaca..................	8	1	ea2
....auCcgucaagguuucucaca..................	5	1	ea2
....auuAcgucaagguuucucaca..................	2	1	ea2
....auuucgucaagguuuAucaca..................	1	1	ea2
....auuucCucaagguuucucaca..................	2	1	ea2
....auuucgucaagguuucucacaa.................	24	0	ea2
....auuucgucaagguuucucacaG.................	1	1	ea2
....auuucgucaagguuucucacaU.................	95	1	ea2
....auuucgucaagguuucucacCa.................	6	1	ea2
....auuucgucaagguCucucacaa.................	1	1	ea2
....auuucgucaagguuucucacUa.................	167	1	ea2
....Guuucgucaagguuucucacaa.................	1	1	ea2
....Cuuucgucaagguuucucacaa.................	57	1	ea2
....auuucgucaagguuucucacaaA................	2	1	ea2
....Cuuucgucaagguuucucacaag................	1	1	ea2
....auuucgucaagguuucucacaaU................	2	1	ea2
....auuucgucaagguuucucacaUg................	2	1	ea2
....auuucgucaagguuucucacaag................	4	0	ea2
....uuucgucaagguuucuAa....................	12	1	ea2

Star Mature

```
augaaaaaccaaagaaguaaaacguuuucuuuucucuuggguaaaccuuggcugaaauauuuguuugauauuucgucaagguuucucacaagagaaauggcuucgguc
......................................................................uuucgucaagguuucucG....................	21	1	ea2
......................................................................Guucgucaagguuucuca....................	2	1	ea2
......................................................................uuucgucaagguuucucU....................	20	1	ea2
......................................................................Auucgucaagguuucuca....................	4	1	ea2
......................................................................uNucgucaagguuucuca....................	1	1	ea2
......................................................................Cuucgucaagguuucuca....................	1	1	ea2
......................................................................uuucgucaGgguuucuca....................	1	1	ea2
......................................................................uuucgucaagguuucuca....................	1785	0	ea2
......................................................................uuucgGcaagguuucuca....................	1	1	ea2
......................................................................uuucgucaagAuuucuca....................	1	1	ea2
......................................................................Nuucgucaagguuucuca....................	9	1	ea2
......................................................................uuucgucaaggAuucucac...................	4	1	ea2
......................................................................Guucgucaagguuucucac...................	5	1	ea2
......................................................................uuucgucaagguGucucac...................	1	1	ea2
......................................................................uNucgucaagguuucucac...................	2	1	ea2
......................................................................uuucgucaGgguuucucac...................	1	1	ea2
......................................................................uuucAucaagguuucucac...................	1	1	ea2
......................................................................uuucgucaagguuucGcac...................	1	1	ea2
......................................................................uGucgucaagguuucucac...................	2	1	ea2
......................................................................uuucgucGagguuucucac...................	3	1	ea2
......................................................................uuucgGcaagguuucucac...................	1	1	ea2
......................................................................uuucgucaaggGuucucac...................	7	1	ea2
......................................................................uuucUucaagguuucucac...................	1	1	ea2
......................................................................Auucgucaagguuucucac...................	5	1	ea2
......................................................................uuucgucaagguuucucUc...................	2	1	ea2
......................................................................uuucgucaagguuucucac...................	6122	0	ea2
......................................................................uuucgucaaggCuucucac...................	2	1	ea2
......................................................................uuucgucaaCguuucucac...................	1	1	ea2
......................................................................uuuUgucaagguuucucac...................	2	1	ea2
......................................................................uuucgucaagguuucuUac...................	1	1	ea2
......................................................................uuucgucaagguuucucaG...................	2	1	ea2
......................................................................Cuucgucaagguuucucac...................	6	1	ea2
......................................................................uuucgucaagguuucucaA...................	14	1	ea2
......................................................................uuucgucaagguuCcucac...................	1	1	ea2
......................................................................uuucgucaagguuucucCc...................	3	1	ea2
......................................................................uuucgucaagguuucucaU...................	145	1	ea2
......................................................................Nuucgucaagguuucucac...................	32	1	ea2
......................................................................uuucgucaagguuucCcac...................	2	1	ea2
......................................................................uuucgAcaagguuucucac...................	1	1	ea2
......................................................................uuucgCcaagguuucucac...................	1	1	ea2
......................................................................uuucgucaagguuucucGc...................	3	1	ea2
......................................................................uuucgucaagguuucucacG..................	365	1	ea2
......................................................................uuucgucaagguuucucUca..................	16	1	ea2
......................................................................uuucgucaagguuGcucaca..................	41	1	ea2
......................................................................uuucgucaaUguuucucaca..................	27	1	ea2
......................................................................uuucgucaaggAuucucaca..................	102	1	ea2
......................................................................uuucgucUagguuucucaca..................	9	1	ea2
......................................................................uuucgucaagguuucucGca..................	84	1	ea2
......................................................................uuucgCcaagguuucucaca..................	153	1	ea2
......................................................................uuucgucaaggGuucucaca..................	354	1	ea2
......................................................................uuucgucaagguuucucacC..................	962	1	ea2
......................................................................uuucguUaagguuucucaca..................	31	1	ea2
......................................................................uuuUgucaagguuucucaca..................	44	1	ea2
......................................................................uuucgucaagCuuucucaca..................	7	1	ea2
......................................................................uuucgucaagUuuucucaca..................	16	1	ea2
......................................................................uuucgucaagguuucucacU..................	88032	1	ea2
......................................................................uuucgucaagguuucCcaca..................	165	1	ea2
......................................................................uuucgucaaCguuucucaca..................	2	1	ea2
......................................................................uuucgucaCgguuucucaca..................	3	1	ea2
......................................................................uuucgucaagguuucucaca..................	378763	0	ea2
......................................................................uuucgAcaagguuucucaca..................	49	1	ea2
......................................................................uCucgucaagguuucucaca..................	167	1	ea2
......................................................................uuucgucCagguuucucaca..................	2	1	ea2
......................................................................Auucgucaagguuucucaca..................	107	1	ea2
......................................................................uGucgucaagguuucucaca..................	30	1	ea2
......................................................................uuucgucaagguuucGcaca..................	22	1	ea2
......................................................................uuucgucaagguuuAucaca..................	26	1	ea2
......................................................................uuucguAaagguuucucaca..................	28	1	ea2
......................................................................uuucgucaagguuAcucaca..................	15	1	ea2
......................................................................uuAcgucaagguuucucaca..................	9	1	ea2
```

Star **Mature**

```
augaaaaaccaaagaaguaaaacguuuucuuuucucuuggguaaaccuuggcugaaauauuuguuugauauuucgucaagguuucucacaagagaaauggcuucgguc
......................................................................uuucgucaagAuuucucaca..................	29	1	ea2
......................................................................uuucgucaagguuucucaGa..................	7	1	ea2
......................................................................uuucgucaagguCucucaca..................	90	1	ea2
......................................................................uuucgucaagguuuUucaca..................	13	1	ea2
......................................................................uuucgucaagguuuGucaca..................	7	1	ea2
......................................................................uuucgucaagguuucucaUa..................	340	1	ea2
......................................................................uuucgucaagguuCcucaca..................	106	1	ea2
......................................................................uuucguGaagguuucucaca..................	6	1	ea2
......................................................................uAucgucaagguuucucaca..................	11	1	ea2
......................................................................uuuAgucaagguuucucaca..................	45	1	ea2
......................................................................uuucgucaagguuucucaAa..................	39	1	ea2
......................................................................uuuGgucaagguuucucaca..................	7	1	ea2
......................................................................uuGcgucaagguuucucaca..................	39	1	ea2
......................................................................uuucgucaagguuucuUaca..................	29	1	ea2
......................................................................uuucgucaagguAucucaca..................	14	1	ea2
......................................................................uuucUucaagguuucucaca..................	24	1	ea2
......................................................................uuucgucaUgguuucucaca..................	11	1	ea2
......................................................................Cuucgucaagguuucucaca..................	219	1	ea2
......................................................................uuucgGcaagguuucucaca..................	211	1	ea2
......................................................................Nuucgucaagguuucucaca..................	1382	1	ea2
......................................................................uuCcgucaagguuucucaca..................	57	1	ea2
......................................................................uuucgucaagguuucuGaca..................	4	1	ea2
......................................................................uuucgucaagguuucuAaca..................	30	1	ea2
......................................................................uuucgucGagguuucucaca..................	98	1	ea2
......................................................................uuucgucaagguuucAcaca..................	19	1	ea2
......................................................................uuucAucaagguuucucaca..................	69	1	ea2
......................................................................uuucgucaaggCuucucaca..................	191	1	ea2
......................................................................uuucgucaagguGucucaca..................	45	1	ea2
......................................................................Guucgucaagguuucucaca..................	172	1	ea2
......................................................................uNucgucaagguuucucaca..................	171	1	ea2
......................................................................uuucCucaagguuucucaca..................	7	1	ea2
......................................................................uuucgucaGgguuucucaca..................	114	1	ea2
......................................................................uuucgucaagguuucucCca..................	8	1	ea2
......................................................................uuucgucaaAguuucucaca..................	16	1	ea2
......................................................................uuucgucaagguuucucacUa.................	4062	1	ea2
......................................................................uuucAucaagguuucucacaa.................	14	1	ea2
......................................................................uuucgucaagguuucucaAaa.................	6	1	ea2
......................................................................uuucgucaagguCucucacaa.................	14	1	ea2
......................................................................uuucgucaagguuucCcacaa.................	25	1	ea2
......................................................................uuucgucaagguuucuGacaa.................	1	1	ea2
......................................................................uuucgGcaagguuucucacaa.................	14	1	ea2
......................................................................uuucgucaagCuuucucacaa.................	1	1	ea2
......................................................................uAucgucaagguuucucacaa.................	4	1	ea2
......................................................................uuucgucaagUuuucucacaa.................	1	1	ea2
......................................................................uuucgucaagguuucucCcaa.................	2	1	ea2
......................................................................uuucguAaagguuucucacaa.................	5	1	ea2
......................................................................uGucgucaagguuucucacaa.................	9	1	ea2
......................................................................uuucgucGagguuucucacaa.................	10	1	ea2
......................................................................uuuAgucaagguuucucacaa.................	6	1	ea2
......................................................................uuucgucaagguuucucacaa.................	48600	0	ea2
......................................................................uuucguUaagguuucucacaa.................	3	1	ea2
......................................................................uuCcgucaagguuucucacaa.................	5	1	ea2
......................................................................uuucgucaagguuucucUcaa.................	3	1	ea2
......................................................................uuucgucaagguuucucacCa.................	141	1	ea2
......................................................................uuucgAcaagguuucucacaa.................	4	1	ea2
......................................................................uCucgucaagguuucucacaa.................	18	1	ea2
......................................................................uuucgucaagguuucucaUaa.................	12	1	ea2
......................................................................uuucgucaaAguuucucacaa.................	1	1	ea2
......................................................................uuucgCcaagguuucucacaa.................	13	1	ea2
......................................................................Guucgucaagguuucucacaa.................	55	1	ea2
......................................................................uuucgucaagguuucucacGa.................	12	1	ea2
......................................................................uuucgucaagguuucucacaU.................	39023	1	ea2
......................................................................uuucgucaagguuAcucacaa.................	1	1	ea2
......................................................................Nuucgucaagguuucucacaa.................	170	1	ea2
......................................................................uuucgucaaUguuucucacaa.................	1	1	ea2
......................................................................uuucgucaagguuuGucacaa.................	4	1	ea2
......................................................................uuucgucaagguuuUucacaa.................	1	1	ea2
......................................................................uuGcgucaagguuucucacaa.................	7	1	ea2
......................................................................uuucgucaCgguuucucacaa.................	1	1	ea2
......................................................................uuucgucaagguGucucacaa.................	7	1	ea2
```

Star Mature

augaaaaaccaaagaaguaaaacguuuucuuuucucuuggguaaaccuuggcugaaauauuuguuugauauuucgucaagguuucucacaagagaaauggcuucgguc			
....uuucguGaagguuucucacaa.................	2	1	ea2
....uuucgucaUgguuucucacaa.................	2	1	ea2
....uNucgucaagguuucucacaa.................	15	1	ea2
....uuucgucaaggGuucucacaa.................	49	1	ea2
....uuucUucaagguuucucacaa.................	2	1	ea2
....uuucgucaagguuucuAacaa.................	6	1	ea2
....Auucgucaagguuucucacaa.................	30	1	ea2
....uuucgucaagguuuAucacaa.................	4	1	ea2
....uuucgucaagguuCcucacaa.................	22	1	ea2
....uuuUgucaagguuucucacaa.................	7	1	ea2
....uuAcgucaagguuucucacaa.................	3	1	ea2
....uuucgucaagguuucuUacaa.................	4	1	ea2
....uuucgucaagguuucAcacaa.................	2	1	ea2
....uuucgucUagguuucucacaa.................	1	1	ea2
....uuucgucaagguuucucGcaa.................	8	1	ea2
....uuucgucaaggAuucucacaa.................	12	1	ea2
....uuuGgucaagguuucucacaa.................	1	1	ea2
....uuucgucaagguuucGcacaa.................	1	1	ea2
....Cuucgucaagguuucucacaa.................	52	1	ea2
....uuucgucaagguuucucaGaa.................	1	1	ea2
....uuucgucaaggCuucucacaa.................	14	1	ea2
....uuucgucaagguuGcucacaa.................	5	1	ea2
....uuucgucaagguuucucacaC.................	358	1	ea2
....uuucgucaagguuucucacaG.................	72	1	ea2
....uuucgucaagAuuucucacaa.................	1	1	ea2
....uuucgucaGgguuucucacaa.................	5	1	ea2
....uuucgucaagguuucucacaCg................	40	1	ea2
....uuucgucaagguuucucCcaag................	1	1	ea2
....uuucgGcaagguuucucacaag................	1	1	ea2
....uuucgucaagguuucucacaag................	421	0	ea2
....uuucgucaagguuucucacaaC................	60	1	ea2
....Nuucgucaagguuucucacaag................	2	1	ea2
....uuucgucaagguCucucacaag................	1	1	ea2
....uuucgucaagguuucucacaUg................	1771	1	ea2
....uuucgucaagguuCcucacaag................	1	1	ea2
....uuucgucaagguuucucacaaA................	2235	1	ea2
....Auucgucaagguuucucacaag................	1	1	ea2
....uuucgucaagguuucucacaaU................	4220	1	ea2
....uuucgucaagguuGcucacaag................	1	1	ea2
....uuucgucaagguuucucacUag................	6	1	ea2
....uuucgucaagguuucucacaaCa...............	228	1	ea2
....uuucgucaagguuucucacaUga...............	6	1	ea2
....uuucgucaagguuucucacaaAa...............	19	1	ea2
....uuucgucaagguuucucacaaUa...............	1436	1	ea2
....uuucgucaagguuucucacaaUag..............	2	1	ea2
....uuucgucaagguuucucacaaAag..............	4	1	ea2
....Nucgucaagguuucucac...................	2	1	ea2
....uucgucaagguuucucac...................	72	0	ea2
....uucgucaagguuucucaU...................	3	1	ea2
....uucgucaagguuucCcaca..................	3	1	ea2
....uucgucaagguuucucacC..................	5	1	ea2
....uuAgucaagguuucucaca..................	1	1	ea2
....uNcgucaagguuucucaca..................	2	1	ea2
....uucgucaaggCuucucaca..................	1	1	ea2
....uucgucaagguuucucGca..................	1	1	ea2
....uucgucaagguuucucaAa..................	3	1	ea2
....Nucgucaagguuucucaca..................	18	1	ea2
....uucgGcaagguuucucaca..................	4	1	ea2
....uucAucaagguuucucaca..................	1	1	ea2
....uucgucaagguuucucacU..................	1296	1	ea2
....uucgucaagguuGcucaca..................	2	1	ea2
....uucgucaagguuucucacG..................	4	1	ea2
....uucgucaagguuCcucaca..................	2	1	ea2
....uucgucaagUuuucucaca..................	1	1	ea2
....uucgucaUgguuucucaca..................	2	1	ea2
....uucgucaagguGucucaca..................	1	1	ea2
....uucgucaagAuuucucaca..................	3	1	ea2
....uucgucaaggGuucucaca..................	24	1	ea2
....uucgucaaggAuucucaca..................	2	1	ea2
....uuUgucaagguuucucaca..................	4	1	ea2

```
                                      Star                            Mature
augaaaaaccaaagaaguaaaacguuuucuuuucucuuggguaaaccuuggcugaaauauuuguuugauauuucgucaagguuucucacaagagaaauggcuucgguc
.......................................................................uucgucaagguuucucaca..................   6486   0   ea2
.......................................................................uucgucGagguuucucaca..................   1      1   ea2
.......................................................................uucgucaagguuucucaUa..................   11     1   ea2
.......................................................................uucgucaagguuucuAaca..................   1      1   ea2
.......................................................................uucgucaagguuucucaGa..................   1      1   ea2
.......................................................................uucgucCagguuucucaca..................   21     1   ea2
.......................................................................uCcgucaagguuucucaca..................   1      1   ea2
.......................................................................uucgucaagguuucucCca..................   2      1   ea2
.......................................................................uucgucaGgguuucucaca..................   1      1   ea2
.......................................................................uucgucCagguuucucacaa.................   2      1   ea2
.......................................................................uucgucaagguuucCcacaa.................   1      1   ea2
.......................................................................uucgucaagguuucucacaG.................   3      1   ea2
.......................................................................uucgucaagguuucucacaC.................   13     1   ea2
.......................................................................uGcgucaagguuucucacaa.................   1      1   ea2
.......................................................................uucgucaagUuuucucacaa.................   1      1   ea2
.......................................................................uucgGcaagguuucucacaa.................   1      1   ea2
.......................................................................uucgucaagguuucucacUa.................   65     1   ea2
.......................................................................uucgucaagguuucucacCa.................   5      1   ea2
.......................................................................uucgucaagguuucucacaU.................   738    1   ea2
.......................................................................uucgCcaagguuucucacaa.................   4      1   ea2
.......................................................................uucgucaaUguuucucacaa.................   1      1   ea2
.......................................................................uucgucaaggGuucucacaa.................   6      1   ea2
.......................................................................uucgucaagguuucucacaa.................   796    0   ea2
.......................................................................uucgucaagguuucuUacaa.................   1      1   ea2
.......................................................................uucgucaagguuucucacaUg................   21     1   ea2
.......................................................................uucgucaagguuucucacaaU................   90     1   ea2
.......................................................................uucgucaagguuucucacaaA................   55     1   ea2
.......................................................................uucgucaagguuucucacGag................   1      1   ea2
.......................................................................uucgucaagguuucucacaag................   10     0   ea2
.......................................................................uucgucaagguuucucacaaUa...............   28     1   ea2
........................................................................Ncgucaagguuucucaca..................   3      1   ea2
........................................................................ucgucaaggGuucucaca..................   1      1   ea2
........................................................................ucgucaagguuucucacU..................   236    1   ea2
........................................................................ucgucaagguuCcucaca..................   1      1   ea2
........................................................................Acgucaagguuucucaca..................   1      1   ea2
........................................................................ucgucaagguuucucaca..................   1000   0   ea2
........................................................................ucgucaagguuucucacaU.................   151    1   ea2
........................................................................ucgucaagguCucucacaa.................   1      1   ea2
........................................................................ucgucaagguuucucacUa.................   27     1   ea2
........................................................................Ncgucaagguuucucacaa.................   1      1   ea2
........................................................................ucgucaagguuucucacaa.................   148    0   ea2
........................................................................ucgucaagguuucucacaC.................   7      1   ea2
........................................................................ucgucaagguuucucacaaU................   12     1   ea2
........................................................................ucgucaagguuucucacaag................   6      0   ea2
........................................................................ucgucaagguuucucacaaA................   7      1   ea2
........................................................................ucgucaagguuucucacaUg................   4      1   ea2
........................................................................ucgucaagguuucucacaaCa...............   1      1   ea2

...................................................................auauuucgucaagguuucucU....................   1      1   eg2
...................................................................Cuauuucgucaagguuucucaca..................   11     1   eg2
...................................................................aAauuucgucaagguuucucaca..................   1      1   eg2
....................................................................uauuucgucaagguuucucG....................   1      1   eg2
....................................................................uauuucgucaagguuucuca....................   13     0   eg2
....................................................................uauuucgucaagguuucucU....................   3      1   eg2
....................................................................uauuucgucaaUguuucuca....................   1      1   eg2
....................................................................uauuucgucaagguuucGcac...................   1      1   eg2
....................................................................uauuucgucaagguuucucac...................   88     0   eg2
....................................................................uauuucgucaagguuucucaU...................   9      1   eg2
....................................................................uauuucgucaagguCucucac...................   1      1   eg2
....................................................................uauuucgucaagguuucCcaca..................   5      1   eg2
....................................................................uauuucgucaagguuGcucaca..................   1      1   eg2
....................................................................uauuucgucaagUuuucucaca..................   1      1   eg2
....................................................................Nauuucgucaagguuucucaca..................   25     1   eg2
....................................................................uauuucgucaagguuucucaca..................   4137   0   eg2
....................................................................uauuucgGcaagguuucucaca..................   3      1   eg2
....................................................................uauuucgucaagguuucucGca..................   1      1   eg2
....................................................................uauuucgucaagguuucAcaca..................   1      1   eg2
....................................................................uauuucgucGagguuucucaca..................   1      1   eg2
....................................................................uauuucgucaagguCucucaca..................   1      1   eg2
....................................................................uauGucgucaagguuucucaca..................   3      1   eg2
```

Star Mature

```
augaaaaaccaaagaaguaaaacguuuucuuuucucuuggguaaaccuuggcugaaauauuuguuugauauuucgucaagguuucucacaagagaaauggcuucgguc
....................................................................uauuucgucaaggCuucucaca..................   1     1   eg2
....................................................................uauuucgucaagguuucuAaca..................   5     1   eg2
....................................................................Gauuucgucaagguuucucaca..................   2     1   eg2
....................................................................uauuucgucaagguuucucacG..................   14    1   eg2
....................................................................uauCucgucaagguuucucaca..................   1     1   eg2
....................................................................uauuucgucaagguGucucaca..................   2     1   eg2
....................................................................uauuucgucaagguuucuGaca..................   1     1   eg2
....................................................................uauuucgucaagguuucucacU..................   1634  1   eg2
....................................................................uauuucgucaaUguuucucaca..................   3     1   eg2
....................................................................uauuCcgucaagguuucucaca..................   2     1   eg2
....................................................................uauuucgCcaagguuucucaca..................   1     1   eg2
....................................................................uauuucAucaagguuucucaca..................   1     1   eg2
....................................................................uauuucgucaaggGuucucaca..................   3     1   eg2
....................................................................uauuGcgucaagguuucucaca..................   1     1   eg2
....................................................................uauuucgucaagguuucucaUa..................   15    1   eg2
....................................................................uauuucgucaagguuuGucaca..................   2     1   eg2
....................................................................uauuucgucaagguuucucacC..................   25    1   eg2
....................................................................uauuucgucaagguuucucacUa.................   80    1   eg2
....................................................................uauuucgucaagguuucucacCa.................   2     1   eg2
....................................................................uauuucgucaagguuucucacaU.................   30    1   eg2
....................................................................uauuucgucaagguuucucacaa.................   12    0   eg2
....................................................................uauuucgucaagguuucucaUaa.................   1     1   eg2
....................................................................uauuucgucaagguuucucacaUg................   2     1   eg2
....................................................................uauuucgucaagguuucucacaaA................   4     1   eg2
.....................................................................auuucgucaagguuucuca....................   5     0   eg2
.....................................................................auuucgucaaggGuucucac...................   1     1   eg2
.....................................................................auuucgucaagguuucucaU...................   1     1   eg2
.....................................................................auuucgucaagguuucucac...................   40    0   eg2
.....................................................................auuucgucaagguuucucaA...................   1     1   eg2
.....................................................................auuNcgucaagguuucucaca..................   1     1   eg2
.....................................................................auuucgGcaagguuucucaca..................   1     1   eg2
.....................................................................auuucgucaagguuucucacC..................   23    1   eg2
.....................................................................auuucgucaagguuucucUca..................   1     1   eg2
.....................................................................auNucgucaagguuucucaca..................   1     1   eg2
.....................................................................auuucgucaagUuuucucaca..................   3     1   eg2
.....................................................................auuucAucaagguuucucaca..................   1     1   eg2
.....................................................................auuucgucGagguuucucaca..................   1     1   eg2
.....................................................................auuucgucaagguuucucacG..................   9     1   eg2
.....................................................................auuGcgucaagguuucucaca..................   1     1   eg2
.....................................................................auuucgucaaUguuucucaca..................   1     1   eg2
.....................................................................Nuuucgucaagguuucucaca..................   21    1   eg2
.....................................................................auuucgucaagguuucucGca..................   4     1   eg2
.....................................................................auuucgucaagguuucCcaca..................   3     1   eg2
.....................................................................auuucgucaagguuucucaUa..................   12    1   eg2
.....................................................................auCucgucaagguuucucaca..................   2     1   eg2
.....................................................................auuucgucaagguuucGcaca..................   1     1   eg2
.....................................................................auuucgucaagguuucucacU..................   794   1   eg2
.....................................................................Cuuucgucaagguuucucaca..................   41    1   eg2
.....................................................................auuucguUaagguuucucaca..................   1     1   eg2
.....................................................................auuucgucaaggCuucucaca..................   1     1   eg2
.....................................................................auuucgucaagguuucucaca..................   2080  0   eg2
.....................................................................auuucgucaaggGuucucaca..................   42    1   eg2
.....................................................................auuucgucaagguuucucacaU.................   26    1   eg2
.....................................................................auuucgucaagguuucucacaa.................   9     0   eg2
.....................................................................Nuuucgucaagguuucucacaa.................   1     1   eg2
.....................................................................Guuucgucaagguuucucacaa.................   1     1   eg2
.....................................................................auuucgucaagguuucucacUa.................   41    1   eg2
.....................................................................auuucgucaagguuucucacCa.................   1     1   eg2
.....................................................................Cuuucgucaagguuucucacaa.................   9     1   eg2
.....................................................................auuucgucaagguuucucacaaU................   2     1   eg2
......................................................................uuucgucaagguuucucU....................   2     1   eg2
......................................................................uuucgucaaggGuucuca....................   1     1   eg2
......................................................................Guucgucaagguuucuca....................   2     1   eg2
......................................................................uuucgucaagguuucuca....................   40    0   eg2
......................................................................uuucgucaagguuucucUc...................   1     1   eg2
......................................................................uuucgucaagguCucucac...................   1     1   eg2
......................................................................uuucgucaaggCuucucac...................   1     1   eg2
......................................................................Nuucgucaagguuucucac...................   1     1   eg2
......................................................................uuucgucaagguuucucaG...................   1     1   eg2
......................................................................uuucgucaagguuucucac...................   191   0   eg2
```

```
                                                        Star                              Mature
augaaaaaccaaagaaguaaaacguuuucuuuucucuuggguaaaccuuggcugaaauauuuguuugauauuucgucaagguuucucacaagagaaauggcuucgguc
......................................................................uuucgucaagguuucucaU...................   4       1       eg2
......................................................................uGucgucaagguuucucaca..................   4       1       eg2
......................................................................uuucgCcaagguuucucaca..................   4       1       eg2
......................................................................uuucguGaagguuucucaca..................   1       1       eg2
......................................................................uuucgucaaggGuucucaca..................   20      1       eg2
......................................................................Cuucgucaagguuucucaca..................   8       1       eg2
......................................................................uuucgucaagguuucucaca..................   18541   0       eg2
......................................................................uuucguAaagguuucucaca..................   1       1       eg2
......................................................................uuucgucaagguuucAcaca..................   2       1       eg2
......................................................................uuNcgucaagguuucucaca..................   2       1       eg2
......................................................................uuCcgucaagguuucucaca..................   2       1       eg2
......................................................................uuucgucaagguuucucacU..................   3501    1       eg2
......................................................................uuucAucaagguuucucaca..................   4       1       eg2
......................................................................uuGcgucaagguuucucaca..................   2       1       eg2
......................................................................uuucgucGagguuucucaca..................   1       1       eg2
......................................................................uuucgucaagguuAcucaca..................   2       1       eg2
......................................................................uuucgucaagguuucucGca..................   5       1       eg2
......................................................................uCucgucaagguuucucaca..................   4       1       eg2
......................................................................uuucgucaagguuGcucaca..................   1       1       eg2
......................................................................uuucgucaagguuucCcaca..................   4       1       eg2
......................................................................uuucgucaaUguuucucaca..................   6       1       eg2
......................................................................uuucgucaaggCuucucaca..................   9       1       eg2
......................................................................uuucgucaagguGucucaca..................   8       1       eg2
......................................................................uuuNgucaagguuucucaca..................   1       1       eg2
......................................................................uuucgucaagCuuucucaca..................   1       1       eg2
......................................................................uuucgucaagguuNcucaca..................   1       1       eg2
......................................................................uuucgucaagAuuucucaca..................   5       1       eg2
......................................................................uuucgucaagguuucucCca..................   2       1       eg2
......................................................................uuucgucaagguuucucacG..................   10      1       eg2
......................................................................uuucgucaagguuucucaUa..................   80      1       eg2
......................................................................uuucgucaagguuuGucaca..................   3       1       eg2
......................................................................uuuUgucaagguuucucaca..................   3       1       eg2
......................................................................uuucCucaagguuucucaca..................   1       1       eg2
......................................................................Nuucgucaagguuucucaca..................   125     1       eg2
......................................................................uAucgucaagguuucucaca..................   1       1       eg2
......................................................................uuucgucaagUuuucucaca..................   10      1       eg2
......................................................................uuucgucaagguAucucaca..................   1       1       eg2
......................................................................uuucguUaagguuucucaca..................   2       1       eg2
......................................................................uuuGgucaagguuucucaca..................   1       1       eg2
......................................................................uuucgucaagguCucucaca..................   9       1       eg2
......................................................................Guucgucaagguuucucaca..................   22      1       eg2
......................................................................uuucgucaagguuucucaGa..................   10      1       eg2
......................................................................uuucUucaagguuucucaca..................   2       1       eg2
......................................................................uuucgucaGgguuucucaca..................   4       1       eg2
......................................................................uuucgucaagguuuAucaca..................   4       1       eg2
......................................................................uuucgucaagguuucucUca..................   2       1       eg2
......................................................................uuucgucaagguuucGcaca..................   4       1       eg2
......................................................................uuucgucaagguuuUucaca..................   1       1       eg2
......................................................................uuucgucaagguuucuUaca..................   1       1       eg2
......................................................................uuucgGcaagguuucucaca..................   2       1       eg2
......................................................................uuucgucaaggAuucucaca..................   4       1       eg2
......................................................................uuucgucaagguuucucacC..................   27      1       eg2
......................................................................uuucgucaagguuCcucaca..................   4       1       eg2
......................................................................uuucgucaagguuucucacaG.................   5       1       eg2
......................................................................uuucgucaagUuuucucacaa.................   1       1       eg2
......................................................................uuucguAaagguuucucacaa.................   1       1       eg2
......................................................................uuucguUaagguuucucacaa.................   1       1       eg2
......................................................................uuucgucaagguuucucGcaa.................   1       1       eg2
......................................................................uuucgucaagguuucucacaU.................   2922    1       eg2
......................................................................Cuucgucaagguuucucacaa.................   1       1       eg2
......................................................................uuucgucaagguuucucacCa.................   7       1       eg2
......................................................................Auucgucaagguuucucacaa.................   3       1       eg2
......................................................................uuucgucaagguuucGcacaa.................   1       1       eg2
......................................................................uuucgucaagguuucucacUa.................   316     1       eg2
......................................................................uuucgucaagguuucucaUaa.................   3       1       eg2
......................................................................uuucgucaagguuCcucacaa.................   1       1       eg2
......................................................................Nuucgucaagguuucucacaa.................   20      1       eg2
......................................................................uuucgucaagguuucucaGaa.................   3       1       eg2
......................................................................uuucgucaaggCuucucacaa.................   3       1       eg2
......................................................................uuucgucGagguuucucacaa.................   1       1       eg2
```

Star Mature

```
augaaaaaccaaagaaguaaaacguuuucuuuucucuuggguaaaccuuggcugaaauauuuguuugauauuucgucaagguuucucacaagagaaauggcuucgguc
......................................................................Guucgucaagguuucucacaa.................  1     1   eg2
......................................................................uuucgucaagguuucucacGa.................  1     1   eg2
......................................................................uuucgucaagguuucucacaC.................  20    1   eg2
......................................................................uuucgucaagguuucucacaa.................  3530  0   eg2
......................................................................uuucUucaagguuucucacaa.................  1     1   eg2
......................................................................uuuAgucaagguuucucacaa.................  1     1   eg2
......................................................................uuucgucaagguGucucacaa.................  3     1   eg2
......................................................................uuucgucaagguuucucacaaA................  241   1   eg2
......................................................................uuucgucaagguuucucacaaU................  375   1   eg2
......................................................................uuucgucaagguuucucacaag................  31    0   eg2
......................................................................uuucgucaagguuucucacaCg................  2     1   eg2
......................................................................uuucgucaagguuucucacaUg................  157   1   eg2
......................................................................uuucgucaagguuucucacaaC................  4     1   eg2
......................................................................uuucgucaagguuucucacaUga...............  1     1   eg2
......................................................................uuucgucaagguuucucacaaCa...............  20    1   eg2
......................................................................uuucgucaagguuucucacaaAa...............  1     1   eg2
......................................................................uuucgucaagguuucucacaaUa...............  128   1   eg2
......................................................................uuucgucaagguuucucacaaAag..............  1     1   eg2
.......................................................................uucgucaagguuucucac...................  2     0   eg2
.......................................................................uucgucaaggGuucucaca..................  22    1   eg2
.......................................................................uucgGcaagguuucucaca..................  1     1   eg2
.......................................................................uucgucaagguuucucaca..................  207   0   eg2
.......................................................................Nucgucaagguuucucaca..................  3     1   eg2
.......................................................................uucgucaagguuucucacU..................  33    1   eg2
.......................................................................uucCucaagguuucucaca..................  1     1   eg2
.......................................................................Nucgucaagguuucucacaa.................  1     1   eg2
.......................................................................uucgucaagguuucucacaa.................  44    0   eg2
.......................................................................uucgucaagguuucucacaU.................  62    1   eg2
.......................................................................uucgucaaggGuucucacaa.................  2     1   eg2
.......................................................................uucgucaagguuucucacUa.................  1     1   eg2
.......................................................................uucgucaagguuucucacaaA................  5     1   eg2
.......................................................................uucgucaagguuucucacaaU................  5     1   eg2
.......................................................................uucgucaagguuucucacaUg................  2     1   eg2
.......................................................................uucgucaagguuucucacaaUa...............  1     1   eg2
........................................................................ucgucaagguuucucacU..................  5     1   eg2
........................................................................ucgucaaggGuucucaca..................  1     1   eg2
........................................................................ucgucaagguuucucaca..................  29    0   eg2
........................................................................ucgucaagguuucucacaU.................  4     1   eg2
........................................................................ucgucaagguuucucacaa.................  6     0   eg2

......................................ggguaaaccuuggcugaa....................................................  1     0   ea1
......................................ggguaaaccuuggcugaaa...................................................  17    0   ea1
......................................ggguaaaAcuuggcugaaa...................................................  1     1   ea1
......................................ggguaaaccuuggcCgaaau..................................................  1     1   ea1
......................................ggguaaaccuuggcugaaaA..................................................  1     1   ea1
......................................ggguaaaccuuggcugaaau..................................................  72    0   ea1
......................................ggguaaaccuuggcugaaaC..................................................  4     1   ea1
......................................ggguaaaccuuggcugaaauC.................................................  2     1   ea1
......................................ggguaaGccuuggcugaaaua.................................................  1     1   ea1
......................................ggguaaaccuuggcugaaaAa.................................................  2     1   ea1
......................................ggguaaaccuGggcugaaaua.................................................  1     1   ea1
......................................gggCaaaccuuggcugaaaua.................................................  2     1   ea1
......................................ggguaaaccuuggcCgaaaua.................................................  1     1   ea1
......................................ggguaaaccuuggcugaGaua.................................................  1     1   ea1
......................................ggguaaaUcuuggcugaaaua.................................................  1     1   ea1
......................................ggguaaaccuuggcugaaaua.................................................  140   0   ea1
......................................ggguaaaccuuggcugaaGua.................................................  1     1   ea1
......................................ggguaaaccuuggcugaaauU.................................................  8     1   ea1
......................................gggCaaaccuuggcugaaauau................................................  1     1   ea1
......................................ggguaaaccuuggcugaaauau................................................  6     0   ea1
......................................ggguaaaccuuggcugaaauaC................................................  1     1   ea1
......................................ggguaaaccuuggcugaaauauu...............................................  22    0   ea1
......................................ggguaaGccuuggcugaaauauu...............................................  2     1   ea1
......................................ggguaaaccuuggcugaaauauC...............................................  5     1   ea1
......................................ggguaaaccCuggcugaaauauu...............................................  1     1   ea1
......................................ggguaaaccuuggcugaaauauuu..............................................  1     0   ea1
.........................................uaaaccuuggcugaaauauuu..............................................  1     0   ea1
..........................................aaaccuuggcugaaauauuuguA...........................................  1     1   ea1
...........................................aaccuuggcugaaauauuuguA...........................................  1     1   ea1
...........................................aaccuuggcugaaauauuuguuA..........................................  1     1   ea1
```

Star Mature

augaaaaaccaaagaaguaaaacguuuucuuuucucuuggguaaaccuuggcugaaauauuuguuugauauuucgucaagguuucucacaagagaaauggcuucgguc			
....cuuggcugaaauauuuguuA..	2	1	ea1
....cuuggcugaaauauuuguuug...	1	0	ea1
....uuggcugaaauauuuguuug...	3	0	ea1
....uuggcugaaauauuuguuuga..	2	0	ea1
....uuggcugGaauauuuguuugaua......................................	2	1	ea1
....uuggcugaaauauuuguuugaua......................................	1	0	ea1
....uggcugaaauauuuguuugaua......................................	1	0	ea1
....uauuuguuugauauuucguc...............................	2	0	ea1
....auuuguuugauauuucguca..............................	1	0	ea1
....uuugauauuucgucaagguuucucacU..................	2	1	ea1
....uugauauuucgucaagguuucucacU..................	1	1	ea1
....ugauauuucgucaagguuuc.......................	2	0	ea1
....ugauauuucgucaagguuucucacC..................	1	1	ea1
....ugauauuucgucaagguuucucacU..................	1	1	ea1
....ugauauuucgucaagguuucucaUa..................	1	1	ea1
....gauCuuucgucaagguuucucac...................	1	1	ea1
....Aauauuucgucaagguuucucaca..................	1	1	ea1
....gauauuucgucaagguuucucaca..................	1	0	ea1
....gauCuuucgucaagguuucucaca..................	2	1	ea1
....Cuauuucgucaagguuucuc.....................	5	1	ea1
....auauuucgucaagguuucuc.....................	1	0	ea1
....auauuucgucaagguuucuca....................	3	0	ea1
....Cuauuucgucaagguuucuca....................	1	1	ea1
....auauuucgucaagguuucucU....................	2	1	ea1
....auauuucgucaagguuucucac...................	1	0	ea1
....Cuauuucgucaagguuucucac...................	27	1	ea1
....Uuauuucgucaagguuucucac...................	1	1	ea1
....auauuucgucaagguuucucacU..................	8	1	ea1
....Cuauuucgucaagguuucucaca..................	177	1	ea1
....auUuuucgucaagguuucucaca..................	1	1	ea1
....aAauuucgucaagguuucucaca..................	1	1	ea1
....auauuucgucaagguuucucacC..................	1	1	ea1
....auauuucgucaagguuucucaca..................	1	0	ea1
....auCuuucgucaagguuucucacaa.................	1	1	ea1
....aAauuucgucaagguuucucacaa.................	1	1	ea1
....uauuucgucaagguuucC......................	1	1	ea1
....uauuucgucaagguuucA......................	2	1	ea1
....uauuucgucaagguuucu......................	39	0	ea1
....uGuuucgucaagguuucu......................	1	1	ea1
....Gauuucgucaagguuucu......................	3	1	ea1
....uUuuucgucaagguuucuc.....................	1	1	ea1
....uauuucgucaagguuucCc.....................	2	1	ea1
....uauuucguUaagguuucuc.....................	2	1	ea1
....uauuucgCcaagguuucuc.....................	6	1	ea1
....uauuucgucaaUguuucuc.....................	1	1	ea1
....uauuuUgucaagguuucuc.....................	1	1	ea1
....uauCucgucaagguuucuc.....................	4	1	ea1
....uauuucguAaagguuucuc.....................	1	1	ea1
....uauuucgucaagguuuUuc.....................	1	1	ea1
....uauuucgucaagguuucuU.....................	128	1	ea1
....Cauuucgucaagguuucuc.....................	4	1	ea1
....uauuucAucaagguuucuc.....................	2	1	ea1
....uauuucgucaGgguuucuc.....................	3	1	ea1
....uauuucgucaaggCuucuc.....................	2	1	ea1
....uauuucgucaagguuCcuc.....................	6	1	ea1
....uauuucgucaagguuucuA.....................	58	1	ea1
....uGuuucgucaagguuucuc.....................	3	1	ea1
....uauuucgucGagguuucuc.....................	5	1	ea1
....uauuAcgucaagguuucuc.....................	1	1	ea1
....uauuucUucaagguuucuc.....................	1	1	ea1
....uauuucgucaagguuucGc.....................	2	1	ea1
....uauuucgucaagguuucuc.....................	1462	0	ea1
....Gauuucgucaagguuucuc.....................	107	1	ea1
....uaGuucgucaagguuucuc.....................	2	1	ea1
....uauuucgucaaggGuucuc.....................	1	1	ea1
....uaCuucgucaagguuucuc.....................	3	1	ea1
....uauuucgucaagguuucucU....................	29	1	ea1
....uauuucgucaagguuucucG....................	12	1	ea1
....uGuuucgucaagguuucuca....................	4	1	ea1
....uauuucgucaUgguuucuca....................	2	1	ea1

Star **Mature**

augaaaaaccaaagaaguaaaacguuuucuuuucucuugggguaaaccuuggcugaaauauuuguuugauauuucgucaagguuucucacaagagaaauggcuucgguc

....uaGuucgucaagguuucuca....................	1	1	ea1
....uauuucgucaagguuucCca....................	3	1	ea1
....uaCuucgucaagguuucuca....................	4	1	ea1
....uCuuucgucaagguuucuca....................	1	1	ea1
....uauuucgucaagguuucuca....................	918	0	ea1
....uauuucgucaagguuucuUa....................	1	1	ea1
....uauuucgucaagAuuucuca....................	1	1	ea1
....uauuuUgucaagguuucuca....................	1	1	ea1
....uauuucgucaagguCucuca....................	3	1	ea1
....Cauuucgucaagguuucuca....................	6	1	ea1
....Gauuucgucaagguuucuca....................	78	1	ea1
....uauuucgucaagguuCcuca....................	3	1	ea1
....uauuucgCcaagguuucuca....................	3	1	ea1
....uauuCcgucaagguuucuca....................	2	1	ea1
....uauuucgucaaggCuucuca....................	4	1	ea1
....uauuucgucaaggGuucuca....................	2	1	ea1
....uauCucgucaagguuucuca....................	2	1	ea1
....uauuucgAcaagguuucuca....................	1	1	ea1
....uauuucgucaagguuucAca....................	2	1	ea1
....uauuucgucaagguuucucC....................	10	1	ea1
....uauuucgucGagguuucuca....................	1	1	ea1
....uauuucgucaaAguuucucac...................	10	1	ea1
....uauuucgucaagguuucucaA...................	399	1	ea1
....uaAuucgucaagguuucucac...................	4	1	ea1
....uauuucgucaagguAucucac...................	1	1	ea1
....uauuucgucaagguuucucaU...................	3033	1	ea1
....uaGuucgucaagguuucucac...................	4	1	ea1
....uauuucgucaagguuucGcac...................	1	1	ea1
....uauuucgucCagguuucucac...................	3	1	ea1
....uCuuucgucaagguuucucac...................	8	1	ea1
....uauuucCucaagguuucucac...................	1	1	ea1
....uauuucgucaGgguuucucac...................	53	1	ea1
....uauuucgucaaggCuucucac...................	43	1	ea1
....uauuucguUaagguuucucac...................	7	1	ea1
....uGuuucgucaagguuucucac...................	31	1	ea1
....uauuucgucaagguuucuUac...................	5	1	ea1
....uauuucgucaagguuucucCc...................	6	1	ea1
....uauuucgucaagguuucucUc...................	30	1	ea1
....uauuucgucGagguuucucac...................	54	1	ea1
....uauuucgucaaggAuucucac...................	9	1	ea1
....uauuucgucaagguuucuAac...................	4	1	ea1
....uauuGcgucaagguuucucac...................	1	1	ea1
....uauuucgucaagguuucucac...................	17014	0	ea1
....uauuucgucaagguuuUucac...................	6	1	ea1
....Cauuucgucaagguuucucac...................	44	1	ea1
....uauuucgucaagguuCcucac...................	55	1	ea1
....uauuucgucaaUguuucucac...................	3	1	ea1
....uauuuAgucaagguuucucac...................	10	1	ea1
....uauuucgucaagguuucucaG...................	18	1	ea1
....uauuucgucaUgguuucucac...................	7	1	ea1
....uauGucgucaagguuucucac...................	3	1	ea1
....uauuucgucaagUuuucucac...................	3	1	ea1
....uauuucAucaagguuucucac...................	9	1	ea1
....uauuucgAcaagguuucucac...................	7	1	ea1
....uauuucgucaagguuucucGc...................	48	1	ea1
....uauuucgucaagCuuucucac...................	1	1	ea1
....uauuucgucaagAuuucucac...................	8	1	ea1
....uaCuucgucaagguuucucac...................	60	1	ea1
....uauuucgucaagguuAcucac...................	11	1	ea1
....Gauuucgucaagguuucucac...................	1047	1	ea1
....uauuCcgucaagguuucucac...................	34	1	ea1
....uauuAcgucaagguuucucac...................	11	1	ea1
....uauuucgucaagguuGcucac...................	1	1	ea1
....uauuucgucaagguuucCcac...................	63	1	ea1
....uauuucgucaagguuucAcac...................	17	1	ea1
....uauuucguAaagguuucucac...................	1	1	ea1
....uauAucgucaagguuucucac...................	6	1	ea1
....uauuucgucUagguuucucac...................	7	1	ea1
....uauuucgucaaggGuucucac...................	22	1	ea1
....uauuuUgucaagguuucucac...................	23	1	ea1

Star Mature

```
augaaaaaccaaagaaguaaaacguuuucuuuucucuuggguaaaccuuggcugaaauauuuguuugauauuucgucaagguuucucacaagagaaauggcuucgguc
...................................................................uauuucgucaCgguuucucac....................	4	1	ea1
...................................................................uauuucgCcaagguuucucac....................	54	1	ea1
...................................................................uUuuucgucaagguuucucac....................	12	1	ea1
...................................................................uauCucgucaagguuucucac....................	47	1	ea1
...................................................................uauuucgucaagguGucucac....................	4	1	ea1
...................................................................uauuucgucaagguCucucac....................	31	1	ea1
...................................................................uauuucgucaagguGucucaca...................	12	1	ea1
...................................................................uauuucgucaagguuucucUca...................	57	1	ea1
...................................................................uauuucgucaagguuucucacU...................	67526	1	ea1
...................................................................uauuucgucaagguuuUucaca...................	66	1	ea1
...................................................................uauuucgucaagguuucucCca...................	18	1	ea1
...................................................................uauuucgucaaggGuucucaca...................	135	1	ea1
...................................................................uauuucgucaagguuucucaGa...................	3	1	ea1
...................................................................uauuuUgucaagguuucucaca...................	88	1	ea1
...................................................................uauuucgucaagguuucucacG...................	2376	1	ea1
...................................................................uaGuucgucaagguuucucaca...................	16	1	ea1
...................................................................uauuCcgucaagguuucucaca...................	338	1	ea1
...................................................................uauuucgucaaggCuucucaca...................	374	1	ea1
...................................................................Cauuucgucaagguuucucaca...................	276	1	ea1
...................................................................uauuuGgucaagguuucucaca...................	1	1	ea1
...................................................................uauAucgucaagguuucucaca...................	43	1	ea1
...................................................................uauuucguAaagguuucucaca...................	10	1	ea1
...................................................................uauuucgucaaggAuucucaca...................	58	1	ea1
...................................................................uauuucguUaagguuucucaca...................	50	1	ea1
...................................................................uauuucgGcaagguuucucaca...................	21	1	ea1
...................................................................uauuucgucaagguuCcucaca...................	425	1	ea1
...................................................................uauuucgucaagguuGcucaca...................	23	1	ea1
...................................................................uCuuucgucaagguuucucaca...................	34	1	ea1
...................................................................uauuGcgucaagguuucucaca...................	6	1	ea1
...................................................................uauuucgucaagguuucGcaca...................	13	1	ea1
...................................................................uauuucAucaagguuucucaca...................	91	1	ea1
...................................................................uauuucgucCagguuucucaca...................	16	1	ea1
...................................................................uauuucgAcaagguuucucaca...................	50	1	ea1
...................................................................uauuucgucaagguuAcucaca...................	86	1	ea1
...................................................................uaCuucgucaagguuucucaca...................	321	1	ea1
...................................................................uauuucgucUagguuucucaca...................	42	1	ea1
...................................................................uaAuucgucaagguuucucaca...................	21	1	ea1
...................................................................uauuucgucaagguuucucGca...................	323	1	ea1
...................................................................uauuucgucaagguCucucaca...................	291	1	ea1
...................................................................uauuucgucaagguuucuUaca...................	52	1	ea1
...................................................................uauuucgucaagUuuucucaca...................	10	1	ea1
...................................................................uauuucgucGagguuucucaca...................	311	1	ea1
...................................................................uGuuucgucaagguuucucaca...................	242	1	ea1
...................................................................uauuucgucaagguAucucaca...................	11	1	ea1
...................................................................uauuucgucaagguuuGucaca...................	7	1	ea1
...................................................................uauuAgucaagguuucucaca...................	31	1	ea1
...................................................................uauuucgucaaUguuucucaca...................	24	1	ea1
...................................................................uauGucgucaagguuucucaca...................	11	1	ea1
...................................................................uauuucCucaagguuucucaca...................	5	1	ea1
...................................................................uauuucgucaaAguuucucaca...................	70	1	ea1
...................................................................uauuucgucaagguuucucaca...................	115532	0	ea1
...................................................................uauuucgucaagAuuucucaca...................	74	1	ea1
...................................................................Gauuucgucaagguuucucaca...................	7098	1	ea1
...................................................................uauuucUucaagguuucucaca...................	2	1	ea1
...................................................................uUuuucgucaagguuucucaca...................	34	1	ea1
...................................................................uauuucgucaagguuucAcaca...................	81	1	ea1
...................................................................uauuucgucaagguuucCcaca...................	506	1	ea1
...................................................................uauAcgucaagguuucucaca...................	88	1	ea1
...................................................................uauuucgucaCgguuucucaca...................	12	1	ea1
...................................................................uauuucgucaaCguuucucaca...................	4	1	ea1
...................................................................uauuucgucaagguuucuGaca...................	3	1	ea1
...................................................................uauuucgucaGgguuucucaca...................	350	1	ea1
...................................................................uauuucgucaagCuuucucaca...................	4	1	ea1
...................................................................uauuucgucaagguuucucaAa...................	10	1	ea1
...................................................................uauuucgCcaagguuucucaca...................	388	1	ea1
...................................................................uauuucgucaagguuucucacC...................	8731	1	ea1
...................................................................uauuucgucaagguuucuAaca...................	11	1	ea1
...................................................................uauuucgucaUgguuucucaca...................	48	1	ea1
...................................................................uauCucgucaagguuucucaca...................	383	1	ea1
...................................................................uauuucgucaagguuuAucaca...................	17	1	ea1
```

Star Mature

augaaaaaccaaagaaguaaaacguuuucuuuucucuuggguaaaccuuggcugaaauauuuguuugauauuucgucaagguuucucacaagagaaauggcuucgguc			
....uauuucgucaagguuucucaUa..................	824	1	ea1
....uauuucgucaagguuucucacGa.................	1	1	ea1
....uauuucgucaagguuCcucacaa.................	2	1	ea1
....uauuucgucaagguuucCcacaa.................	1	1	ea1
....uauuucgucaagguuucucacUa.................	2319	1	ea1
....uauuucgucaagguuucucCcaa.................	1	1	ea1
....uauuucgucaagguuucucGcaa.................	1	1	ea1
....uauuucgucaaggCuucucacaa.................	1	1	ea1
....uauuucgucaagguuucucUcaa.................	2	1	ea1
....Cauuucgucaagguuucucacaa.................	2	1	ea1
....Gauuucgucaagguuucucacaa.................	21	1	ea1
....uauuuUgucaagguuucucacaa.................	2	1	ea1
....uauuucgucaagguuucucacaG.................	30	1	ea1
....uCuuucgucaagguuucucacaa.................	1	1	ea1
....uauuucgucaagguuucucacaU.................	1669	1	ea1
....uauuucgucaagguuucucaUaa.................	6	1	ea1
....uauuucgucaaggGuucucacaa.................	2	1	ea1
....uauuucgucaagguuucGcacaa.................	1	1	ea1
....uauuucgucaagguuucucacaa.................	270	0	ea1
....uauuucgucaagguuucucacaC.................	234	1	ea1
....uauuucgucaagguuucucacCa.................	37	1	ea1
....uauuucgucaagguuucucacaGg................	1	1	ea1
....uauuucgucaagguuucucacaaU................	21	1	ea1
....uauuucgucaagguuucucacaaA................	2	1	ea1
....uauuucgucaagguuucucacaUg................	42	1	ea1
....uauuucgucaagguuucucacaCg................	2	1	ea1
....uauuucgucaagguuucucacaag................	2	0	ea1
....uauuucgucaagguuucucacUag................	5	1	ea1
....uauuucgucaagguuucucacaaAa...............	6	1	ea1
....uauuucgucaagguuucucacaaUa...............	3	1	ea1
....Guuucgucaagguuucuc.....................	14	1	ea1
....auuCcgucaagguuucuc.....................	4	1	ea1
....auuucgucaagguuucuc.....................	602	0	ea1
....auuucgucaagguuucCc.....................	3	1	ea1
....auuucgucaagguuCcuc.....................	2	1	ea1
....auuucgucaaggCuucuc.....................	2	1	ea1
....Cuuucgucaagguuucuc.....................	13	1	ea1
....auuucgucaagguuucuA.....................	13	1	ea1
....auuucgucGagguuucuc.....................	3	1	ea1
....auuucgucaGgguuucuc.....................	5	1	ea1
....auuGcgucaagguuucuc.....................	1	1	ea1
....aCuucgucaagguuucuc.....................	1	1	ea1
....auuuUgucaagguuucuc.....................	2	1	ea1
....auuucgucUagguuucuc.....................	1	1	ea1
....auuucgCcaagguuucuc.....................	1	1	ea1
....auuucgucaagguCucuc.....................	1	1	ea1
....auuucgucaagAuuucuc.....................	1	1	ea1
....auuucgucaaUguuucuc.....................	2	1	ea1
....auuucgucaagguuucuU.....................	44	1	ea1
....auuucgucaagguuucCca....................	2	1	ea1
....auuucgucaagguuucucC....................	4	1	ea1
....auuucgucaagAuuucuca....................	4	1	ea1
....Guuucgucaagguuucuca....................	22	1	ea1
....Cuuucgucaagguuucuca....................	3	1	ea1
....auuucgucaagguuucucG....................	2	1	ea1
....auuucgucaagguCucuca....................	2	1	ea1
....auuucgucaagguuucucU....................	7	1	ea1
....auuucgucaaggCuucuca....................	1	1	ea1
....auuucgucaagguuucuca....................	204	0	ea1
....auuucgCcaagguuucuca....................	1	1	ea1
....auuucAucaagguuucuca....................	1	1	ea1
....aCuucgucaagguuucuca....................	1	1	ea1
....auuucgucaagguuucucGc...................	26	1	ea1
....auuucguGaagguuucucac...................	1	1	ea1
....auuucgucaaAguuucucac...................	6	1	ea1
....auuucgucaagguCucucac...................	15	1	ea1
....auuucgucaaggGuucucac...................	5	1	ea1
....auuucgucaagguuucucaA...................	130	1	ea1
....auuucgucaagguuuAucac...................	1	1	ea1
....auuucgucaagguuucucac...................	5783	0	ea1

```
                                         Star                              Mature
augaaaaaccaaagaaguaaaacguuuucuuuucucuugggguaaaccuuggcugaaauauuuguuugauauuucgucaagguuucucacaagagaaauggcuucgguc
.....................................................................auuucgucaagguuuUucac...................   4      1     ea1
.....................................................................auuucguAaagguuucucac...................   1      1     ea1
.....................................................................Cuuucgucaagguuucucac...................   129    1     ea1
.....................................................................Guuucgucaagguuucucac...................   239    1     ea1
.....................................................................auuucgucaagguuAcucac...................   2      1     ea1
.....................................................................auuucgucaagguuucAcac...................   6      1     ea1
.....................................................................auuucgucaagAuuucucac...................   2      1     ea1
.....................................................................auuucAucaagguuucucac...................   7      1     ea1
.....................................................................auuucgucUagguuucucac...................   3      1     ea1
.....................................................................auuuUgucaagguuucucac...................   8      1     ea1
.....................................................................auuucgucaagguuucCcac...................   21     1     ea1
.....................................................................auuAcgucaagguuucucac...................   4      1     ea1
.....................................................................auuucgucaagguuCcucac...................   17     1     ea1
.....................................................................auGucgucaagguuucucac...................   1      1     ea1
.....................................................................auuucgucaagguuucucCc...................   4      1     ea1
.....................................................................auuucgucaagguuucucaU...................   920    1     ea1
.....................................................................auuucgucaagguuucucUc...................   8      1     ea1
.....................................................................auuucgucaagguuuGucac...................   3      1     ea1
.....................................................................auCucgucaagguuucucac...................   11     1     ea1
.....................................................................auuucgAcaagguuucucac...................   5      1     ea1
.....................................................................auuucgucaCgguuucucac...................   1      1     ea1
.....................................................................auuuAgucaagguuucucac...................   1      1     ea1
.....................................................................auuucgCcaagguuucucac...................   11     1     ea1
.....................................................................auuucgucaagguuucucaG...................   3      1     ea1
.....................................................................auuucgucGagguuucucac...................   23     1     ea1
.....................................................................auuCcgucaagguuucucac...................   17     1     ea1
.....................................................................auAucgucaagguuucucac...................   5      1     ea1
.....................................................................auuucgucaaggCuucucac...................   19     1     ea1
.....................................................................aCuucgucaagguuucucac...................   24     1     ea1
.....................................................................auuucguUaagguuucucac...................   1      1     ea1
.....................................................................auuucgucaGgguuucucac...................   16     1     ea1
.....................................................................auuucgucaUgguuucucac...................   4      1     ea1
.....................................................................aGuucgucaagguuucucac...................   2      1     ea1
.....................................................................auuucCucaagguuucucaca..................   2      1     ea1
.....................................................................auuucgucGagguuucucaca..................   102    1     ea1
.....................................................................auuucgucaagguuucucUca..................   16     1     ea1
.....................................................................auuuAgucaagguuucucaca..................   8      1     ea1
.....................................................................auuucgucaagguuucucacC..................   1361   1     ea1
.....................................................................aGuucgucaagguuucucaca..................   6      1     ea1
.....................................................................auuucgucaagguuuUucaca..................   19     1     ea1
.....................................................................auuucgucaaUguuucucaca..................   3      1     ea1
.....................................................................auuucgucaagguuGcucaca..................   2      1     ea1
.....................................................................auuucgucaagguuCcucaca..................   112    1     ea1
.....................................................................auuucgCcaagguuucucaca..................   74     1     ea1
.....................................................................auuucgucaaggAuucucaca..................   11     1     ea1
.....................................................................Guuucgucaagguuucucaca..................   1479   1     ea1
.....................................................................auuucgucaagguuucucacG..................   534    1     ea1
.....................................................................auuucgucaagguuucucaAa..................   3      1     ea1
.....................................................................auuucgAcaagguuucucaca..................   11     1     ea1
.....................................................................auuucgucaaAguuucucaca..................   10     1     ea1
.....................................................................auuucgucaaggCuucucaca..................   112    1     ea1
.....................................................................auuucgucaUgguuucucaca..................   11     1     ea1
.....................................................................auuucgucaagguCucucaca..................   79     1     ea1
.....................................................................auuucgucaagUuuucucaca..................   4      1     ea1
.....................................................................auuucguAaagguuucucaca..................   6      1     ea1
.....................................................................aCuucgucaagguuucucaca..................   88     1     ea1
.....................................................................auuucgucaagguuucucCca..................   4      1     ea1
.....................................................................auuucgucaaggGuucucaca..................   16     1     ea1
.....................................................................auuucgucaagguuucGcaca..................   3      1     ea1
.....................................................................auuucgucaagguuucucaUa..................   223    1     ea1
.....................................................................auuucguUaagguuucucaca..................   25     1     ea1
.....................................................................auuucgucUagguuucucaca..................   8      1     ea1
.....................................................................auuucgucaagAuuucucaca..................   11     1     ea1
.....................................................................auAucgucaagguuucucaca..................   8      1     ea1
.....................................................................auuucgucaCgguuucucaca..................   7      1     ea1
.....................................................................auuucgucaagguuAcucaca..................   13     1     ea1
.....................................................................auuucgucaagguAucucaca..................   1      1     ea1
.....................................................................auuGcgucaagguuucucaca..................   3      1     ea1
.....................................................................auuucgucaagguuucucGca..................   103    1     ea1
.....................................................................auuuUgucaagguuucucaca..................   41     1     ea1
```

Star **Mature**

```
augaaaaaccaaagaaguaaaacguuuucuuuucucuugggguaaaccuuggcugaaauauuuguuugauauuucgucaagguuucucacaagagaaauggcuucgguc
.....................................................................aAuucgucaagguuucucaca..................	4	1	ea1
.....................................................................auuucgucaagguuuGucaca..................	2	1	ea1
.....................................................................auuucAucaagguuucucaca..................	27	1	ea1
.....................................................................auuucgucaagguuucucacU..................	10509	1	ea1
.....................................................................auCucgucaagguuucucaca..................	76	1	ea1
.....................................................................auuucgucaagguuuAucaca..................	6	1	ea1
.....................................................................auuucgucaagguuucuAaca..................	4	1	ea1
.....................................................................auuucgucaagguuucAcaca..................	25	1	ea1
.....................................................................auuucgucCagguuucucaca..................	4	1	ea1
.....................................................................auuAcgucaagguuucucaca..................	13	1	ea1
.....................................................................auGucgucaagguuucucaca..................	3	1	ea1
.....................................................................auuucgucaagguuucucaca..................	31022	0	ea1
.....................................................................auuucgucaGgguuucucaca..................	86	1	ea1
.....................................................................auuucgucaagCuuucucaca..................	2	1	ea1
.....................................................................Cuuucgucaagguuucucaca..................	949	1	ea1
.....................................................................auuucgucaagguuucCcaca..................	109	1	ea1
.....................................................................auuucgucaagguuucuUaca..................	13	1	ea1
.....................................................................auuCcgucaagguuucucaca..................	87	1	ea1
.....................................................................auuucgucaagguGucucaca..................	3	1	ea1
.....................................................................auuucgucaagguuucucacGa.................	4	1	ea1
.....................................................................Guuucgucaagguuucucacaa.................	79	1	ea1
.....................................................................auuucgucaagguuucucaUaa.................	2	1	ea1
.....................................................................auuucgucaagguuGcucacaa.................	1	1	ea1
.....................................................................auuucgucaagguuucucacaG.................	15	1	ea1
.....................................................................auuucgucaagguuucucacaU.................	2142	1	ea1
.....................................................................auuucgCcaagguuucucacaa.................	1	1	ea1
.....................................................................auuucgucaaggGuucucacaa.................	1	1	ea1
.....................................................................auuucgucaaggCuucucacaa.................	5	1	ea1
.....................................................................auuucgucaagguuucucacCa.................	8	1	ea1
.....................................................................auuucgucaagguuucucacaC.................	235	1	ea1
.....................................................................aAuucgucaagguuucucacaa.................	1	1	ea1
.....................................................................Cuuucgucaagguuucucacaa.................	222	1	ea1
.....................................................................auCucgucaagguuucucacaa.................	2	1	ea1
.....................................................................auuucgucaagguuucuUacaa.................	1	1	ea1
.....................................................................auuCcgucaagguuucucacaa.................	1	1	ea1
.....................................................................auuucgucaagguuucucacaa.................	387	0	ea1
.....................................................................auuucgucaagguuucucUcaa.................	1	1	ea1
.....................................................................auuucgucaagguuucucacUa.................	433	1	ea1
.....................................................................auuucgGcaagguuucucacaa.................	1	1	ea1
.....................................................................auuucgucaagguuucucacaaU................	112	1	ea1
.....................................................................auuucgucaagguuucucacaaC................	18	1	ea1
.....................................................................auuucgucaagguuucucacaag................	8	0	ea1
.....................................................................auuucgucaagguuucCcacaag................	1	1	ea1
.....................................................................auuucgucaagguuucucacaUg................	48	1	ea1
.....................................................................Cuuucgucaagguuucucacaag................	2	1	ea1
.....................................................................auuucgucaagguuucucacaaA................	22	1	ea1
.....................................................................auuucgucaagguuucucacaaCa...............	1	1	ea1
.....................................................................auuucgucaagguuucucacaaAa...............	3	1	ea1
.....................................................................auuucgucaagguuucucacaaUa...............	17	1	ea1
......................................................................uuucgucaagguuucAca....................	7	1	ea1
......................................................................uuucAucaagguuucuca....................	1	1	ea1
......................................................................uuucgucaagguuucucU....................	27	1	ea1
......................................................................uuucgucaagguuucuUa....................	1	1	ea1
......................................................................uuucgucaagguuucuGa....................	1	1	ea1
......................................................................uuucgucaagguuucCca....................	4	1	ea1
......................................................................Cuucgucaagguuucuca....................	6	1	ea1
......................................................................uuucgucaagguuCcuca....................	3	1	ea1
......................................................................uuucgucaagguuAcuca....................	1	1	ea1
......................................................................uuucgucaaggCuucuca....................	11	1	ea1
......................................................................uCucgucaagguuucuca....................	1	1	ea1
......................................................................uuucgucaagguuucuAa....................	1	1	ea1
......................................................................uuucgucaGgguuucuca....................	8	1	ea1
......................................................................uuuUgucaagguuucuca....................	2	1	ea1
......................................................................uuucgucaagguCucuca....................	4	1	ea1
......................................................................Guucgucaagguuucuca....................	96	1	ea1
......................................................................uuCcgucaagguuucuca....................	5	1	ea1
......................................................................uuucgCcaagguuucuca....................	4	1	ea1
......................................................................uuucgucaagguuucucC....................	1	1	ea1
......................................................................uuAcgucaagguuucuca....................	2	1	ea1
......................................................................uuucgucaagguuucucG....................	25	1	ea1
```

```
                                            Star                          Mature
augaaaaaccaaagaaguaaaacguuuucuuuucucuuggguaaaccuuggcugaaauauuuguuugauauuucgucaagguuucucacaagagaaauggcuucgguc
......................................................................uuucgucaagguuucuca....................   1303    0    ea1
......................................................................uuucgucaaCguuucuca....................   1       1    ea1
......................................................................Auucgucaagguuucuca....................   2       1    ea1
......................................................................uuucgucUagguuucuca....................   1       1    ea1
......................................................................uuucgucCagguuucuca....................   1       1    ea1
......................................................................uuucgucGagguuucuca....................   6       1    ea1
......................................................................uuucgucaaggGuucuca....................   2       1    ea1
......................................................................uuucgucaaCguuucucac...................   2       1    ea1
......................................................................uuucgucaagguuucCcac...................   242     1    ea1
......................................................................uuucgucaagguuucuGac...................   2       1    ea1
......................................................................uuuGgucaagguuucucac...................   6       1    ea1
......................................................................uuucgucaCgguuucucac...................   6       1    ea1
......................................................................uuucgucaagguuucucGc...................   265     1    ea1
......................................................................uuucgucaagUuuucucac...................   9       1    ea1
......................................................................uuucgucaagAuuucucac...................   35      1    ea1
......................................................................uuucgucaagguCucucac...................   186     1    ea1
......................................................................uCucgucaagguuucucac...................   225     1    ea1
......................................................................uuucCucaagguuucucac...................   9       1    ea1
......................................................................uuucgCcaagguuucucac...................   228     1    ea1
......................................................................uuucguUaagguuucucac...................   23      1    ea1
......................................................................uuucgucaaAguuucucac...................   47      1    ea1
......................................................................Auucgucaagguuucucac...................   49      1    ea1
......................................................................uuucgucaaggGuucucac...................   68      1    ea1
......................................................................uuucgucaGgguuucucac...................   186     1    ea1
......................................................................uuucguAaagguuucucac...................   5       1    ea1
......................................................................uuucgucaagguuGcucac...................   6       1    ea1
......................................................................uuucgucGagguuucucac...................   156     1    ea1
......................................................................uuucgucaagguuucuAac...................   12      1    ea1
......................................................................uuucgAcaagguuucucac...................   27      1    ea1
......................................................................uuucgucaagguuucGcac...................   17      1    ea1
......................................................................uuucgucaagguuucuUac...................   32      1    ea1
......................................................................uuucgucaagguuuUucac...................   34      1    ea1
......................................................................uGucgucaagguuucucac...................   9       1    ea1
......................................................................uuuAgucaagguuucucac...................   22      1    ea1
......................................................................uuucgucaUgguuucucac...................   21      1    ea1
......................................................................uuucgucaagguuuGucac...................   2       1    ea1
......................................................................uuucgucaagguuAcucac...................   37      1    ea1
......................................................................uuucgGcaagguuucucac...................   8       1    ea1
......................................................................uuuUgucaagguuucucac...................   75      1    ea1
......................................................................uuucgucUagguuucucac...................   25      1    ea1
......................................................................uuucUucaagguuucucac...................   3       1    ea1
......................................................................uuucAucaagguuucucac...................   51      1    ea1
......................................................................uuucgucaagguGucucac...................   6       1    ea1
......................................................................uuucgucaaggCuucucac...................   245     1    ea1
......................................................................uuucgucaaggAuucucac...................   39      1    ea1
......................................................................uuucgucaagguuucAcac...................   64      1    ea1
......................................................................uuGcgucaagguuucucac...................   7       1    ea1
......................................................................uuucgucaagCuuucucac...................   2       1    ea1
......................................................................uuucgucaagguuucucac...................   70861   0    ea1
......................................................................uuAcgucaagguuucucac...................   24      1    ea1
......................................................................uuucgucaagguuuAucac...................   2       1    ea1
......................................................................uuucgucaagguuucucUc...................   94      1    ea1
......................................................................uuucgucaagguAucucac...................   6       1    ea1
......................................................................Cuucgucaagguuucucac...................   297     1    ea1
......................................................................uuucgucaagguuucucaU...................   9964    1    ea1
......................................................................uuucgucaaUguuucucac...................   6       1    ea1
......................................................................uuucgucaagguuucucCc...................   20      1    ea1
......................................................................uuCcgucaagguuucucac...................   174     1    ea1
......................................................................uuucgucaagguuCcucac...................   248     1    ea1
......................................................................uuucgucaagguuucucaA...................   1592    1    ea1
......................................................................Guucgucaagguuucucac...................   5367    1    ea1
......................................................................uuucgucaagguuucucaG...................   64      1    ea1
......................................................................uAucgucaagguuucucac...................   23      1    ea1
......................................................................uuucgucCagguuucucac...................   6       1    ea1
......................................................................uuucgucaagAuuucucaca..................   290     1    ea1
......................................................................uuucgucUagguuucucaca..................   142     1    ea1
......................................................................uuucgucaagguuucucacC..................   8373    1    ea1
......................................................................uuucgucaaAguuucucaca..................   304     1    ea1
......................................................................uuucgucaagguuucuCca..................   102     1    ea1
......................................................................uuucgucaagguuAcucaca..................   197     1    ea1
```

```
                                     Star                                Mature
augaaaaaccaaagaaguaaaacguuuucuuuucucuuggguaaaccuuggcugaaauauuuguuugauauuucgucaagguuucucacaagagaaauggcuucgguc
......................................................................uuucgucaaggGuucucaca..................   519      1   ea1
......................................................................uuucAucaagguuucucaca..................   452      1   ea1
......................................................................uuucgucaagguuucuUaca..................   293      1   ea1
......................................................................uuucguGaagguuucucaca..................   3        1   ea1
......................................................................uuucgucaUgguuucucaca..................   188      1   ea1
......................................................................uuucUucaagguuucucaca..................   25       1   ea1
......................................................................uuucgucaagguuucucaGa..................   20       1   ea1
......................................................................uuucgAcaagguuucucaca..................   212      1   ea1
......................................................................uuucgucaagguuucucacG..................   7832     1   ea1
......................................................................uuucgucaaCguuucucaca..................   18       1   ea1
......................................................................uuucgucaCgguuucucaca..................   54       1   ea1
......................................................................uuucgCcaagguuucucaca..................   1732     1   ea1
......................................................................uuucgucaagguCucucaca..................   1178     1   ea1
......................................................................uuucgucaaggCuucucaca..................   1740     1   ea1
......................................................................uuucgucaagguuGcucaca..................   44       1   ea1
......................................................................uuucgGcaagguuucucaca..................   55       1   ea1
......................................................................uuucgucaagguuCcucaca..................   1903     1   ea1
......................................................................uuucgucaGgguuucucaca..................   1445     1   ea1
......................................................................uuuUgucaagguuucucaca..................   598      1   ea1
......................................................................uuucgucaagguuucGcaca..................   150      1   ea1
......................................................................uuucgucaagguuucuGaca..................   15       1   ea1
......................................................................uuucgucaagCuuucucaca..................   16       1   ea1
......................................................................uuucgucaagguuuUucaca..................   267      1   ea1
......................................................................Auucgucaagguuucucaca..................   393      1   ea1
......................................................................uuucgucaagguuucucGca..................   1612     1   ea1
......................................................................uuucgucaaUguuucucaca..................   86       1   ea1
......................................................................uuucgucaagguuucucUca..................   285      1   ea1
```
[truncated: 1,236,763 more chars]
