# Supplementary material for: Jellyfish genomes reveal distinct homeobox gene clusters and conservation of small RNA processing
Source: Nat Commun. 2020 Jun 19;11:3051. doi: 10.1038/s41467-020-16801-9 (PMC7305137; doi:10.1038/s41467-020-16801-9)
Supplement: Supplementary file 7 — Supplementary Data 3 [file 41467_2020_16801_MOESM7_ESM.pdf]

Provisional ID	:	scaffold3_906
Score total	:	5.1
Score for star read(s)	:	-1.3
Score for read counts	:	0
Score for mfe	:	1.8
Score for randfold	:	1.6
Score for cons. seed	:	3
Total read count	:	269
Mature read count	:	141
Loop read count	:	0
Star read count	:	128

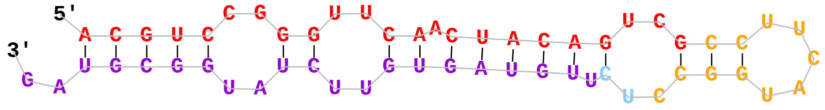

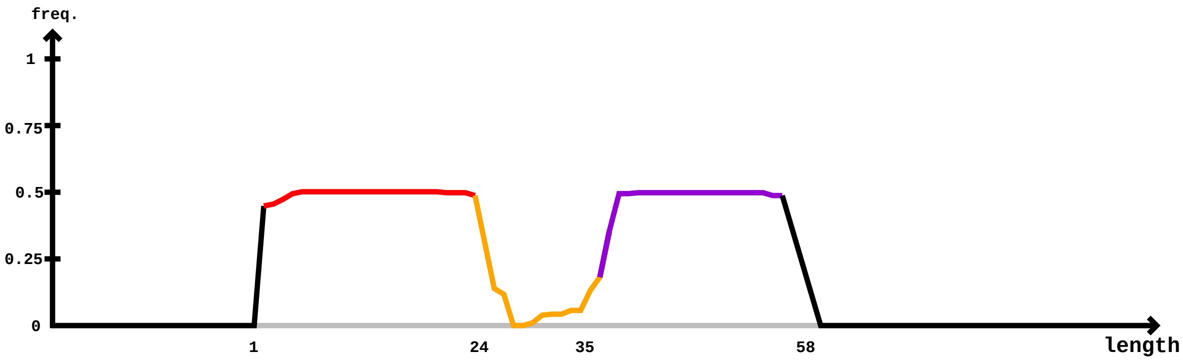

Mature Star

```
5'- ucgagauuucgaaggcccccacguccggguucaacuacagucgccuucauggccucuuguaguguucuauggcguaggggcuugagaaauaauuguucgucuacuugcagaac  -3'   obs
    ucgagauuucgaaggcccccacguccggguucaacuacagucgccuucauggccucuuguaguguucuauggcguaggggcuugagaaauaauuguucgucuacuugcagaac         exp
    .....(((((..(((((((.(((((..((..((.((((((..(((.....)))..).)))))))..))..))))).)))))))..)))))....((((((......)).))))   reads   mm   sample
    ....................acguccggguucaacuacaguc.......................................................................   1       0    0B2
    ....................acguccggguucaacuacagucg......................................................................   50      0    0B2
    ....................acguccggguucaacuacagucgccu...................................................................   5       0    0B2
    ......................guccggguucaacuacagucg......................................................................   5       0    0B2
    .......................uccggguucaacuacagucg......................................................................   3       0    0B2
    ........................ccggguucaacuacagucgcA....................................................................   2       1    0B2
    ....................................................ccucuuguaguguGcuauggcgu......................................   1       1    0B2
    ......................................................ucuuguaguguGcuauggcgua.....................................   1       1    0B2
    ......................................................ucuuguaguguGcuauggcguag....................................   6       1    0B2
    .......................................................cuuguaguguGcuauggcgua.....................................   1       1    0B2
    ........................................................uuguaguguGcuauggcgu......................................   6       1    0B2
    ........................................................uuguaguguGcuauggcguag....................................   4       1    0B2
    .........................................................uguaguguGcuauggcgua.....................................   2       1    0B2
    .........................................................uguaguguGcuauggcguag....................................   10      1    0B2

    ....................acguccggguucaacuacagucg......................................................................   19      0    0G2
    .................................................uggccucuuguaguguGcuauggcgu......................................   4       1    0G2
    .................................................uggccucuuguaguguGcuauggcgua.....................................   1       1    0G2
    ....................................................ccucuuguaguguGcuauggcgu......................................   3       1    0G2
    ......................................................ucuuguaguguGcuauggcguag....................................   8       1    0G2
    .......................................................cuuguaguguGcuauggcgua.....................................   2       1    0G2
    .......................................................cuuguaguguGcuauggcguag....................................   1       1    0G2
    ........................................................uuguaguguGcuauggcgu......................................   2       1    0G2
    ........................................................uuguaguguGcuauggcgua.....................................   2       1    0G2
    ........................................................uuguaguguGcuauggcguag....................................   12      1    0G2
    .........................................................uguaguguGcuauggcgua.....................................   6       1    0G2
    .........................................................uguaguguGcuauggcguag....................................   15      1    0G2

    ....................acguccggguucaacuacU..........................................................................   1       1    0A2
    ....................acguccggguucaacuacagucg......................................................................   18      0    0A2
    ....................acgCccggguucaacuacagucgcc....................................................................   1       1    0A2
    ....................acguccggguucaacuacagucgcc....................................................................   3       0    0A2
    ....................acguccggguucaacuacagucgccu...................................................................   28      0    0A2
```

Mature Star

```
ucgagauuucgaaggcccccacguccggguucaacuacagucgccuucauggccucuuguaguguucuauggcguaggggcuugagaaauaauuguucgucuacuugcagaac
.....................cguccggguucaacuacaguc.......................................................................   2   0   0A2
.......................uccggguucaacuacagucg......................................................................   3   0   0A2
................................................auggccucuuguaguguGcuauggcgu......................................   3   1   0A2
.................................................uggccucuuguaguguGcuauggcgu......................................   3   1   0A2
..................................................ggccucuuguaguguGcuauggcguag....................................   1   1   0A2
......................................................ucuuguaguguGcuauggcgua.....................................   3   1   0A2
......................................................ucuuguaguguGcuauggcguag....................................   3   1   0A2
.......................................................cuuguaguguGcuauggc........................................   3   1   0A2
.......................................................cuuguaguguGcuauggcgua.....................................   2   1   0A2
.......................................................cuuguaguguGcuauggcguag....................................   5   1   0A2
........................................................uuguaguguGcuauggcgu......................................   5   1   0A2
........................................................uuguaguguGcuauggcgua.....................................   4   1   0A2
........................................................uuguaguguGcuauggcguag....................................  14   1   0A2
.........................................................uguaguguGcuauggcgua.....................................   4   1   0A2
.........................................................uguaguguGcuauggcguag....................................   2   1   0A2
...........................................................uaguguGcuauggcguag....................................   1   1   0A2
```

Provisional ID	:	scaffold3_1076
Score total	:	5.9
Score for star read(s)	:	-1.3
Score for read counts	:	0
Score for mfe	:	2.6
Score for randfold	:	1.6
Score for cons. seed	:	3
Total read count	:	823
Mature read count	:	712
Loop read count	:	0
Star read count	:	111

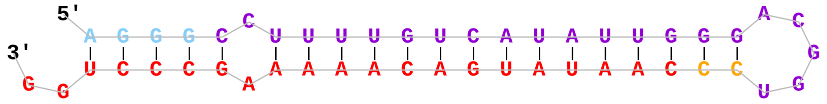

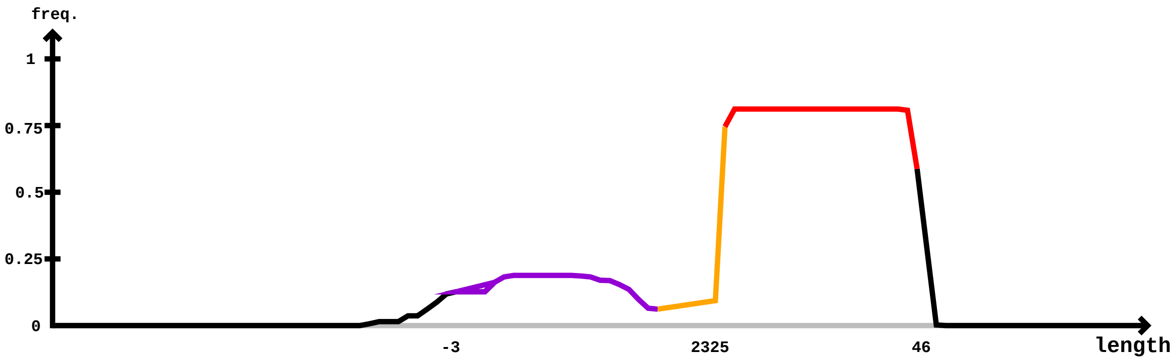

Star Mature

```
5'- gcuacgugauaaacuaauauauaugaucaaccucguccccagggccuuuugucauauugggacgguucccaauaugacaaaaagcccuggugacgagguugauauaugauacu   -3'   obs
    gcuacgugauaaacuaauauauaugaucaaccucguccccagggccuuuugucauauugggacgguucccaauaugacaaaaagcccuggugacgagguugauauaugauacu         exp
    ...................((((((.(((((((((((.(((((((.(((((((((((((((.....))))))))))))))).))))))).))))))))))))))))).....   reads   mm   sample
    ................................ucguccccaggUccuuuuguca..........................................................   3       1    0B2
    ...................................uccccagggccuuuuguG...........................................................   2       1    0B2
    ...................................uccccagggccuuuuguGau.........................................................   5       1    0B2
    ...................................uccccagggccuuuuguGauau.......................................................   1       1    0B2
    ...................................uccccagggccuuuuguGauauu......................................................   2       1    0B2
    .....................................cccagggccuuuugucauauug.....................................................   7       0    0B2
    .....................................cccagggccuCuugucauauug.....................................................   1       1    0B2
    .....................................cccagggccuCuugucauauugg....................................................   4       1    0B2
    ......................................ccagggccuuuugucauauu......................................................   4       0    0B2
    ......................................ccagggccuuuugucGuauug.....................................................   4       1    0B2
    ......................................ccagggccuuuugucauauug.....................................................   1       0    0B2
    .......................................cagggccuuuugucauauug.....................................................   1       0    0B2
    ........................................agggccuuuugucauauu......................................................   1       0    0B2
    ........................................agggccuuuugucauauug.....................................................   3       0    0B2
    ............................................ccuuuugucauGuugggacgg...............................................   6       1    0B2
    ............................................ccuuuugucauauugggacgg...............................................   2       0    0B2
    ............................................ccuuuugucauGuugggacggu..............................................   5       1    0B2
    ............................................ccuuuugucauauugggacggu..............................................   7       0    0B2
    .............................................cuuuugucauauugggacg................................................   5       0    0B2
    .............................................cuuuugucauauuUggacggu..............................................   1       1    0B2
    .............................................cuuuugucauauugggacggu..............................................   2       0    0B2
    ...................................................................ccaauaugacaaaaagcccu.........................   3       0    0B2
    ...................................................................ccaauaugacaaaaagcccug........................   19      0    0B2
    ...................................................................ccaauaugacaaaaagcccugU.......................   3       1    0B2
    ...................................................................ccaauaugacaaaaagcccugg.......................   2       0    0B2
    ...................................................................Ncaauaugacaaaaagcccugg.......................   1       1    0B2
    ...................................................................ccaauaugacaaaaagcccugguU.....................   2       1    0B2
    ....................................................................caauaugacaaaaagcccu.........................   1       0    0B2
    ....................................................................caauaugacaaaaagcccug........................   30      0    0B2
    ....................................................................caauaugacaaaaagUccug........................   1       1    0B2
    ....................................................................caauaugacaaaaagcccugU.......................   1       1    0B2
    ....................................................................caauaugacaaaaagcccCgg.......................   1       1    0B2
    ....................................................................Naauaugacaaaaagcccugg.......................   1       1    0B2
```

```
                                            Star                  Mature
gcuacgugauaaacuaauauauaugaucaaccucguccccagggccuuuugucauauugggacgguccccaauaugacaaaaagcccuggugacgagguugauauaugauacu
....................................................................caauauUacaaaaagcccugg.......................    1     1    0B2
....................................................................caauaugacaaaaagcccugg.......................    99    0    0B2
....................................................................caauaugacaaaaagcccuggG......................    21    1    0B2
.....................................................................aauaugacaaaaagcccug........................    3     0    0B2
.....................................................................aauaugacaaaaagcccugg.......................    4     0    0B2

...................................uccccagggccuuuuguGaua........................................................    1     1    0G2
...................................ucccGagggccuuuugucauau.......................................................    4     1    0G2
.....................................cccagggccuCuugucauaug......................................................    1     1    0G2
......................................ccagggccuuuugucauauug.....................................................    2     0    0G2
......................................ccagggccuuuugucauauugg....................................................    3     0    0G2
.......................................cagggccuuuugucauauA......................................................    4     1    0G2
.......................................cagggccuuuugucauauug.....................................................    5     0    0G2
.......................................cagggccuuuugucauauugg....................................................    9     0    0G2
.......................................cagggccuuuugucauauuggg...................................................    2     0    0G2
........................................agggccuuuugucauauugg....................................................    2     0    0G2
............................................ccuuuugucauauugggacggu..............................................    1     0    0G2
.............................................cuuuugucauauUggacgg................................................    5     1    0G2
..............................................uuuugucauauugggacA................................................    1     1    0G2
..............................................uuuugucauauugggacgg...............................................    3     0    0G2
...................................................................ccaauaugacaaaaagcccug........................    9     0    0G2
...................................................................ccaauaugacaaaaagcccugU.......................    11    1    0G2
...................................................................ccaauaugacaaaaagcccugg.......................    7     0    0G2
....................................................................caaGugacaaaaagcccug.........................    1     1    0G2
....................................................................caauaugacaaaaagcccug........................    46    0    0G2
....................................................................caauGugacaaaaagcccug........................    8     1    0G2
....................................................................caauaugacaaaaagcccGgg.......................    1     1    0G2
....................................................................caauaugacaaaaagcccugg.......................    111   0    0G2
....................................................................caauaugacaaaaagcccugU.......................    6     1    0G2
....................................................................caauGugacaaaaagcccugg.......................    13    1    0G2
....................................................................caauaugacaaaaagcccuggG......................    42    1    0G2
.....................................................................aauaugacaaaaagcccug........................    6     0    0G2
.....................................................................aauaugacaaaaagcccugg.......................    13    0    0G2

...............................cucgucccUagggccuuuugucau.........................................................    6     1    0A2
................................ucguccccagggccuuuugucauUu.......................................................    4     1    0A2
...................................uccGcagggccuuuugucauau.......................................................    4     1    0A2
.....................................cccagggccuCuugucauauu......................................................    3     1    0A2
.....................................cccagggccuuuugucauaug......................................................    1     0    0A2
.....................................cccagggccuCuugucauauugg....................................................    5     1    0A2
......................................ccagggccuuuugucauauu......................................................    2     0    0A2
......................................ccagggccuuuugucauauuA.....................................................    1     1    0A2
......................................ccagggccuuuugucauauug.....................................................    6     0    0A2
.......................................cagggccGuuugucauauugg....................................................    1     1    0A2
.......................................cagggccuuuugucauauugg....................................................    5     0    0A2
........................................agggccuuuugucauauuggg...................................................    1     0    0A2
............................................ccuuuugucauGuugggacg................................................    1     1    0A2
............................................ccuuuugucauauugggacggu..............................................    9     0    0A2
.............................................cuuuugucauauugggacg................................................    2     0    0A2
.............................................cuuuugucauauugggacggu..............................................    3     0    0A2
..............................................uuuugucauauugggacggu..............................................    1     0    0A2
...................................................................ccaauaugacaaaaagcccug........................    23    0    0A2
...................................................................ccaauaugacaaaaagcccugU.......................    2     1    0A2
....................................................................caauaugacaaaaagcccug........................    37    0    0A2
....................................................................caauaugacaaaaagcccGg........................    1     1    0A2
....................................................................caauaugacaaaaagcccugg.......................    109   0    0A2
....................................................................caauaugacaaaGagcccugg.......................    1     1    0A2
....................................................................caauaugacaaaaagcccugU.......................    8     1    0A2
....................................................................caauaugacaaaaagccGugg.......................    1     1    0A2
....................................................................caauaugacaaaaagcccuggG......................    31    1    0A2
.....................................................................aauaugacaaaaagcccug........................    9     0    0A2
.....................................................................aauaugacaaaaagcccugg.......................    20    0    0A2
.....................................................................aauaugacaaaaagcccuggG......................    3     1    0A2
```

Provisional ID	:	scaffold4_1408
Score total	:	348.3
Score for star read(s)	:	3.9
Score for read counts	:	341.2
Score for mfe	:	2.2
Score for randfold	:	1.6
Score for cons. seed	:	-0.6
Total read count	:	681
Mature read count	:	680
Loop read count	:	0
Star read count	:	1

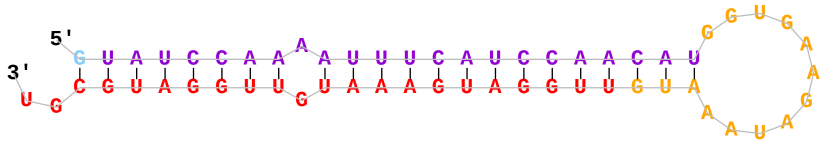

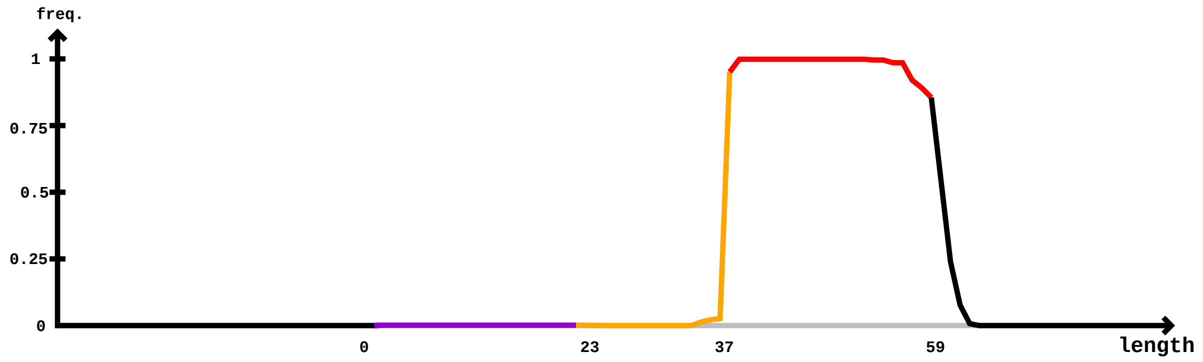


```
                                 Star                            Mature
5'- auguuggaugacccaacauggguugucaaacguauccaaaauuucauccaacauggugaagauaaauguuggaugaaauguuggaugcguuacaccggggugcugucaacacaa   -3'    obs
    auguuggaugacccaacauggguugucaaacguauccaaaauuucauccaacauggugaagauaaauguuggaugaaauguuggaugcguuacaccggggugcugucaacacaa          exp
    .(((((((..((((.....))))..)).(((((((((((.((((((((((((((...........)))))))))))))).))))))))))).(((....)))....)))))...   reads   mm   sample
    ..................................................................uguuggaugaaauguuggauA...........................   4       1    0B2
    ..................................................................uguuggaugaaauAuuggaug...........................   1       1    0B2
    ...................................................................guuggaugaaauguuggauA...........................   2       1    0B2
    ....................................................................uuggaugaaauguuggauA...........................   2       1    0B2
    ....................................................................uuggaugaaauAuuggaugc..........................   1       1    0B2
    ....................................................................uuggaugaaauguuggaugc..........................   1       0    0B2
    ....................................................................uuggaugaaauguuggaugcg.........................   5       0    0B2
    ....................................................................uuggaugaaauguuggauAcgu........................   1       1    0B2
    ....................................................................uuggaugaaauguuggaugUgu........................   1       1    0B2
    ....................................................................uuggaugaaauUuuggaugcgu........................   16      1    0B2
    ....................................................................uuggaugaaauguuggaugcgu........................   79      0    0B2
    ....................................................................uuggaugaaauguuggaugcgA........................   2       1    0B2
    ....................................................................uuggaugaaauUuuggaugcguu.......................   11      1    0B2
    ....................................................................uuggaugaaauguuggaugcguu.......................   52      0    0B2
    ....................................................................uuggaugaaauguuggaGgcguu.......................   1       1    0B2
    ....................................................................uuAgaugaaauguuggaugcguu.......................   6       1    0B2
    ....................................................................uuggaugaaauguuggaugcAuu.......................   3       1    0B2
    ....................................................................uuggaugaaauguuggaugcguua......................   7       0    0B2
    ....................................................................uuggaugaaauguuggaugcgAua......................   3       1    0B2
    ....................................................................uuggaugaaauUuuggaugcguua......................   3       1    0B2
    ....................................................................uuggaugaaauguuggaugcguAa......................   6       1    0B2
    ....................................................................uuggaugaaauguuggaugcguuaA.....................   8       1    0B2
    ....................................................................uuggaugaaauguuggaugcguuaU.....................   5       1    0B2
    .....................................................................uggaugaaauguuggauA...........................   3       1    0B2
    .....................................................................uggaugaaauguuggaugcgu........................   2       0    0B2
    .....................................................................uggaugaaauUuuggaugcgu........................   3       1    0B2
    .....................................................................uggaugaaauguuggaugcgAu.......................   1       1    0B2
    .....................................................................uggaugaaauguuggaugcguu.......................   5       0    0B2

    ................................Cauccaaaauuucauccaacau............................................................   1       1    0G2
    ....................................................................uuggaugaaauAuuggaug...........................   3       1    0G2
    ....................................................................uuggaugaaauguuggauA...........................   9       1    0G2
    ....................................................................uuggaugaaauguuggaugc..........................   7       0    0G2
```

Star Mature

auguuggaugacccaacauggguugucaaacguauccaaaauuucauccaacauggugaagauaaaauguuggaugaaauguuggaugcguuacaccggggugcugucaacacaa			
....uuggaugaaauguuggaugcg.........................	8	0	0G2
....uuggaugaaauUuuggaugcgu........................	11	1	0G2
....uuAgaugaaauguuggaugcgu........................	1	1	0G2
....uuggaugaaauguuggaugcCu........................	3	1	0G2
....uuggaugaaauguuggauAcgu........................	6	1	0G2
....uuggaugaaauguuggaugcgu........................	18	0	0G2
....uuggaugaaauUuuggaugcguu.......................	4	1	0G2
....uuggaugaaaCguuggaugcguu.......................	1	1	0G2
....uuggaugaaauguuggaugcguu.......................	42	0	0G2
....uuggaugaaauguuggaugcguua......................	27	0	0G2
....Nuggaugaaauguuggaugcguua......................	1	1	0G2
....uuggaugaaauUuuggaugcguua......................	3	1	0G2
....uuggaugaaauguuggaugcguAa......................	7	1	0G2
....uuggaugaaauguuggaugcguuaU.....................	1	1	0G2
....uuggaugaaauguuggaugcguuaA.....................	5	1	0G2
....uuggaugaaauguuggaugcguuaUa....................	3	1	0G2
....uggaCgaaauguuggaug...........................	2	1	0G2
....uggaugaaauguuggaugc..........................	4	0	0G2
....uggaugaaauguuggaugcguu.......................	3	0	0G2
....uggaugaaauguuggaugcguua......................	1	0	0G2

....auguuggaugaaauguug...............................	2	0	0A2
....auguuggaugaaauAuugga.............................	7	1	0A2
....uguuggaugaaauguuggaugcA.........................	1	1	0A2
....guuggaugaaauguuggaugc..........................	1	0	0A2
....uuggaugaaauguuggaug...........................	3	0	0A2
....uuggaugaaauguuggauA...........................	16	1	0A2
....uuggaugaaauguuggaugc..........................	6	0	0A2
....uuggaugaaauguuggaugcg.........................	10	0	0A2
....uuggaugaaauguuggauUcg.........................	1	1	0A2
....uuAgaugaaauguuggaugcgu........................	3	1	0A2
....uuggaugaaauguuggauAcgu........................	8	1	0A2
....Nuggaugaaauguuggaugcgu........................	1	1	0A2
....uuggaugaaauguuggaugcgu........................	44	0	0A2
....uuggaugaaauguuggaugcCu........................	2	1	0A2
....uuggaugaaauUuuggaugcgu........................	15	1	0A2
....uuggaugaaauguuggaugcguu.......................	65	0	0A2
....uuggaugaaauguuggaugcgAu.......................	2	1	0A2
....uuggaugaaauguuggaugUguu.......................	2	1	0A2
....uuggaugaaauUuuggaugcguu.......................	7	1	0A2
....uuggaugaaauUuuggaugcguua......................	8	1	0A2
....uuggaugaaauguuggauAcguua......................	6	1	0A2
....uuggaugaaauguuggaugcguuC......................	6	1	0A2
....uuggaugaaauguuggaugcguua......................	20	0	0A2
....uuggaugaaauCuuggaugcguua......................	3	1	0A2
....uuggaugaaauguuggaugcguAa......................	6	1	0A2
....uuggaugaaauguuggaugcguuaA.....................	16	1	0A2
....uuggaugaaauguuggaugcguuaU.....................	13	1	0A2
....uuggaugaaauguuggaugcguuaUa....................	2	1	0A2
....uggaugaaauguuggaugcguu.......................	4	0	0A2
....uggaugaaauguuggaugcguua......................	5	0	0A2

Provisional ID	:	scaffold5_1732
Score total	:	262.7
Score for star read(s)	:	3.9
Score for read counts	:	255.5
Score for mfe	:	2.3
Score for randfold	:	1.6
Score for cons. seed	:	-0.6
Total read count	:	513
Mature read count	:	508
Loop read count	:	0
Star read count	:	5

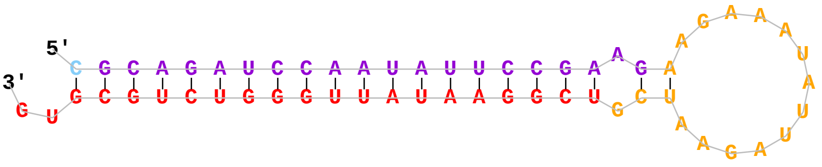

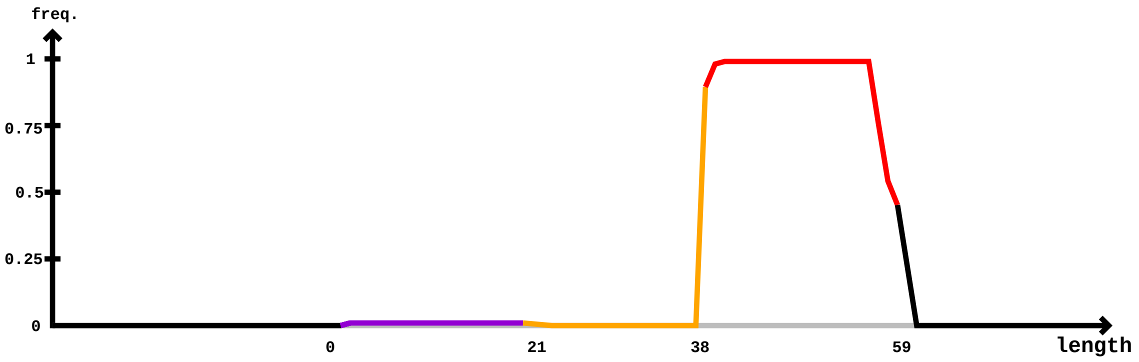


```
                               Star                                  Mature
5'- acgacggcaacagguauagccuccugcgcgcagauccaauauuccgaagaagaaauauuagaaucgucggaauauugggucugcgugcaggcuacaacagguauugca  -3'  obs
    acgacggcaacagguauagccuccugcgcgcagauccaauauuccgaagaagaaauauuagaaucgucggaauauugggucugcgugcaggcuacaacagguauugca       exp
    .....(....)..(((..(((((((((((((((((((((((((((((.((.............)).))))))))))))))))))))))))).......))))..))).  reads  mm  sample
    .............................gcagauccaauauucUgaag...........................................................  5      1   0G2
    ..................................................................ucggGauauugggucugc........................  1      1   0G2
    ..................................................................ucggaauauugggucugc........................  56     0   0G2
    ..................................................................ucggaauauugggucugcg.......................  15     0   0G2
    ..................................................................ucggaauauugggucugcA.......................  2      1   0G2
    ..................................................................ucggaauauugggucugcgu......................  12     0   0G2
    ..................................................................ucggaauauugggucugcguC.....................  3      1   0G2
    ..................................................................ucggaauauugggucugcguA.....................  13     1   0G2
    ..................................................................ucggaauauugggucugcguU.....................  45     1   0G2
    ...................................................................cggaauauugggucugcA.......................  7      1   0G2
    ...................................................................cggaauauugggucugcguU.....................  11     1   0G2
    ....................................................................ggaauauugggucugcgu......................  1      0   0G2

    ..................................................................ucggaauauugggucugc........................  39     0   0B2
    ..................................................................Ncggaauauugggucugc........................  1      1   0B2
    ..................................................................ucggaauaGugggucugc........................  1      1   0B2
    ..................................................................ucggaauauugggucugcA.......................  3      1   0B2
    ..................................................................ucggaauauugggucugcg.......................  47     0   0B2
    ..................................................................ucggaauauugggucugcgu......................  6      0   0B2
    ..................................................................ucggaauauugggucugcUu......................  3      1   0B2
    ..................................................................Ncggaauauugggucugcgu......................  1      1   0B2
    ..................................................................ucggaauauugggucugcguU.....................  66     1   0B2
    ..................................................................ucggaauauugggucugcguA.....................  10     1   0B2
    ..................................................................ucggaauauugggucugcguC.....................  2      1   0B2
    ..................................................................ucggaauauugggucugcgug.....................  1      0   0B2
    ...................................................................cggaauauugggucugcg.......................  2      0   0B2
    ...................................................................cggaauauugggucugcguU.....................  12     1   0B2
    ....................................................................ggaauauugggucugcguU.....................  1      1   0B2

    ..................................................................ucAgaauauugggucugc........................  1      1   0A2
    ..................................................................ucggaauauugggucugc........................  19     0   0A2
    ..................................................................ucggaauauugggucugcg.......................  27     0   0A2
    ..................................................................ucggaauauugggucugcA.......................  6      1   0A2
```

Star Mature

```
acgacggcaacagguauagccuccugcgcgcagauccaauauuccgaagaagaaauauuagaaucgucggaauauugggucugcgugcaggcuacaacagguauugca
..................................................................ucggaauauugggucugcgu......................	20	0	0A2
..................................................................ucggaauauugggucugcguU.....................	46	1	0A2
..................................................................ucggaauauugggucugcguA.....................	13	1	0A2
...................................................................cggaauauugggucugcA.......................	3	1	0A2
...................................................................cggaauauugggucugcguU.....................	9	1	0A2
....................................................................ggaauauugggucugcgu......................	3	0	0A2
```

Provisional ID	:	scaffold8_2596
Score total	:	2393.7
Score for star read(s)	:	3.9
Score for read counts	:	2384.1
Score for mfe	:	1.1
Score for randfold	:	1.6
Score for cons. seed	:	3
Total read count	:	4688
Mature read count	:	4679
Loop read count	:	0
Star read count	:	9

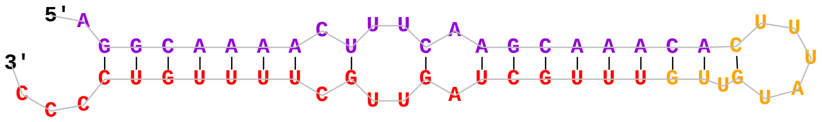

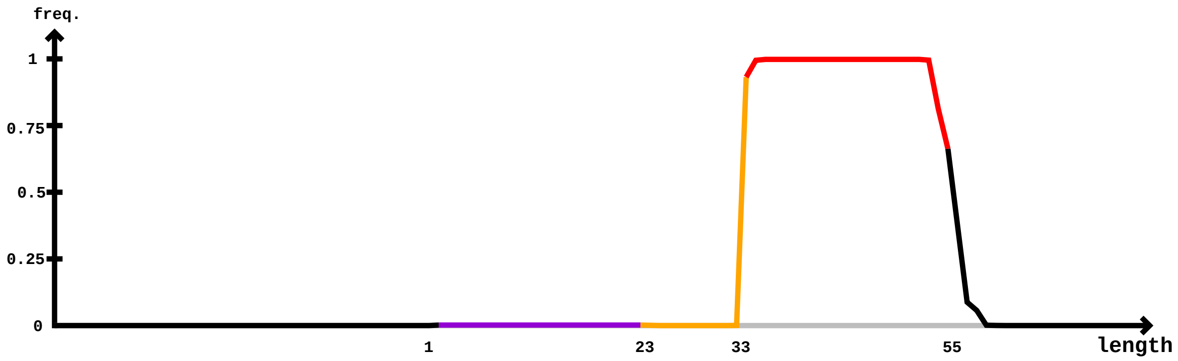

Star   Mature

```
5'- caaaauucgcaguaacuccuuagacacuuucugagcggaggcaaaacuuucaagcaaacacuuuauguuguuugcuaguugcuuuuguccccauucuggugacaggaguugu  -3'   obs
    caaaauucgcaguaacuccuuagacacuuucugagcggaggcaaaacuuucaagcaaacacuuuauguuguuugcuaguugcuuuuguccccauucuggugacaggaguugu         exp
    ............((((((((....((((....(((.((.(((((((.(..(.(((((((((.....).)))))))).)..).))))))).)).))).)))).)))))))).   reads   mm   sample
    ......................................................................uuugcuaguugcuuuugucc......................   78      0    0G2
    ......................................................................uuugcuaguugcuuuugucA......................   1       1    0G2
    ......................................................................Nuugcuaguugcuuuugucc......................   1       1    0G2
    ......................................................................uuugcuaguugcuuuuguccA.....................   5       1    0G2
    ......................................................................uuugcuaguugcuuuuguccc.....................   23      0    0G2
    ......................................................................uuugcuaguugcuuuuguccU.....................   3       1    0G2
    ......................................................................uuugcuaguugcCuuuguccc.....................   2       1    0G2
    ......................................................................uuugcuaguugcuuuugucccU....................   6       1    0G2
    ......................................................................uuugcuaguugcuuuugucccc....................   61      0    0G2
    ......................................................................uuugcuaguugcuuuuguAccc....................   1       1    0G2
    ......................................................................uuugcuaguugcuuuuguccccU...................   44      1    0G2
    ......................................................................uuugcuaguugcuuuugucccca...................   9       0    0G2
    ......................................................................uuugcuaguugcuuuuguccccUu..................   9       1    0G2
    ......................................................................uuugcuaguugcuuuuguccccauu.................   1       0    0G2
    ......................................................................uuugcuaguugcuuuuguccccUuu.................   22      1    0G2
    .......................................................................Nugcuaguugcuuuugucc......................   1       1    0G2
    .......................................................................uugcuaguugcuuuugucc......................   2       0    0G2
    .......................................................................uugcuaguugcuuuuguccc.....................   1       0    0G2
    .......................................................................uugcuaguugcuuuugucccc....................   5       0    0G2
    .......................................................................uugcuaguugcuuuuguccccU...................   2       1    0G2
    ........................................................................ugcuaguugcuuuugucccc....................   1       0    0G2
    ........................................................................ugcuaguugcuuuuguccccU...................   2       1    0G2

    ......................................aggcaaaacCuucaagcaaaca....................................................   7       1    0B2
    .....................................................................Cuuugcuaguugcuuuugucccc....................   2       1    0B2
    ......................................................................uuugcuaguugcuuuuguc.......................   11      0    0B2
    ......................................................................uuugcuaguuAcuuuugucc......................   2       1    0B2
    ......................................................................Nuugcuaguugcuuuugucc......................   1       1    0B2
    ......................................................................uuugcuaguugcuuuugucU......................   1       1    0B2
    ......................................................................uuugcuaguugcuuuugucA......................   1       1    0B2
    ......................................................................uuugcuaguugcuuuugucc......................   490     0    0B2
    ......................................................................uCugcuaguugcuuuugucc......................   1       1    0B2
    ......................................................................uuugcuaUuugcuuuuguccc.....................   1       1    0B2
```

```
                                      Star                  Mature
caaaauucgcaguaacuccuuagacacuuucugagcggaggcaaaacuuucaagcaaacacuuuauguuguuugcuaguugcuuuugucccc auucuggugacaggaguugu
......................................................................uuugcuaguugcuuuuguccU.....................   12     1   0B2
......................................................................uuugcuaguGgcuuuuguccc.....................   1      1   0B2
......................................................................Nuugcuaguugcuuuuguccc.....................   5      1   0B2
......................................................................uuugcuaguuAcuuuuguccc.....................   1      1   0B2
......................................................................uuugcuaguugcuuuuguccA.....................   4      1   0B2
......................................................................Guugcuaguugcuuuuguccc.....................   1      1   0B2
......................................................................uuuUcuaguugcuuuuguccc.....................   1      1   0B2
......................................................................uuugcuaguugcuuuuguccc.....................   413    0   0B2
......................................................................uuugcuaguugcuCuuguccc.....................   1      1   0B2
......................................................................uuugcuaguugcuuuugucccU....................   48     1   0B2
......................................................................uuugcuaguugcuuuGgucccc....................   1      1   0B2
......................................................................uuugcuaguugcuuuugucccA....................   3      1   0B2
......................................................................uuugAuaguugcuuuugucccc....................   1      1   0B2
......................................................................uuugUuaguugcuuuugucccc....................   1      1   0B2
......................................................................Nuugcuaguugcuuuugucccc....................   4      1   0B2
......................................................................uuugcuaguugcuuuugucccc....................   1005   0   0B2
......................................................................uuugcuaguuAcuuuugucccc....................   1      1   0B2
......................................................................uuugcuaguugcuuuugucccG....................   1      1   0B2
......................................................................uuugcuaguugcuuuugucGcc....................   1      1   0B2
......................................................................uuugcuGguugcuuuugucccc....................   2      1   0B2
......................................................................uuugcuaguugcuuuugCcccc....................   3      1   0B2
......................................................................uuugcuaguugcuuuugucccca...................   17     0   0B2
......................................................................uuugcuaguugcuuuuguccccU...................   490    1   0B2
......................................................................uuugcuaguugcuuuuguccccC...................   3      1   0B2
......................................................................uuugcuaguugcuuuuguccccG...................   2      1   0B2
......................................................................uuugcuaguugcuuuuguccccUu..................   87     1   0B2
......................................................................uuugcuaguugcuuuuguccccUauu................   5      1   0B2
......................................................................uuugcuaguugcuuuuguccccUuu.................   125    1   0B2
......................................................................uuugcuaguugcuuuuguccccauu.................   6      0   0B2
......................................................................uuugcuaguugcuuuuguccccUuucu...............   2      1   0B2
.......................................................................Nugcuaguugcuuuugucc......................   1      1   0B2
.......................................................................uugcuaguugcuuuugucc......................   39     0   0B2
.......................................................................uugcuaguugcuuuuguccc.....................   24     0   0B2
.......................................................................uuAcuaguugcuuuugucccc....................   1      1   0B2
.......................................................................uugcuaguugcuuuugucccU....................   8      1   0B2
.......................................................................uugcuaguugcuuuugucccc....................   97     0   0B2
.......................................................................uugcuaguugcuuuuguccccU...................   44     1   0B2
.......................................................................uugcuaguugcuuuugucccca...................   2      0   0B2
.......................................................................uugcuaguugcuuuuguccccUu..................   11     1   0B2
.......................................................................uugcuaguugcuuuuguccccUuu.................   5      1   0B2
........................................................................ugcuaguugcuuuugucc......................   3      0   0B2
........................................................................ugcuaguugcuuuuguccc.....................   5      0   0B2
........................................................................ugcuaguugcuuuuguccccUuu.................   3      1   0B2

......................................aggcaaaacCuucaagcaaaca....................................................   2      1   0A2
......................................................................uuugcuaguugcuuuuguc.......................   2      0   0A2
......................................................................Cuugcuaguugcuuuugucc......................   3      1   0A2
......................................................................Nuugcuaguugcuuuugucc......................   2      1   0A2
......................................................................uuugcuaguugAuuuugucc......................   1      1   0A2
......................................................................uuugcuaguugcuuuuguUc......................   1      1   0A2
......................................................................uuugcGaguugcuuuugucc......................   1      1   0A2
......................................................................uuugcuaguugcuuuugucc......................   217    0   0A2
......................................................................uuuUcuaguugcuuuugucc......................   1      1   0A2
......................................................................uuugcuaguugcuuuuguccc.....................   173    0   0A2
......................................................................uuugcuaguugcuGuuguccc.....................   1      1   0A2
......................................................................uuCgcuaguugcuuuuguccc.....................   1      1   0A2
......................................................................uuugcuaguugcuuuuguccA.....................   5      1   0A2
......................................................................uuugcuaguugcuuuuUuccc.....................   1      1   0A2
......................................................................uuugcuUguugcuuuugucccc....................   1      1   0A2
......................................................................uuugcuaguugcuuuuguUccc....................   2      1   0A2
......................................................................uuugcuaguugcuuuugucccA....................   2      1   0A2
......................................................................uuugcGaguugcuuuugucccc....................   2      1   0A2
......................................................................uuugcuaguugcGuuugucccc....................   1      1   0A2
......................................................................uuugcuaguugGuuuugucccc....................   1      1   0A2
......................................................................uNugcuaguugcuuuugucccc....................   1      1   0A2
......................................................................uuugAuaguugcuuuugucccc....................   1      1   0A2
......................................................................uuugcuaguugcuuuugucccc....................   408    0   0A2
......................................................................uuugcuaguugcuuuugucccU....................   36     1   0A2
......................................................................uuugcuaguugcuuuugucGcc....................   1      1   0A2
......................................................................Nuugcuaguugcuuuugucccc....................   1      1   0A2
```

Star **Mature**

```
caaaauucgcaguaacuccuuagacacuuucugagcggaggcaaaacuuucaagcaaacacuuuauguuguuugcuaguugcuuuuguccccauucuggugacaggaguugu
......................................................................uuugcuaguugcuuuuguccccU....................    325    1    0A2
......................................................................uuugcuaguugcuuuuguccccC....................    2      1    0A2
......................................................................uuugcuaguugcuuuugucccca....................    18     0    0A2
......................................................................uuugcuCguugcuuuugucccca....................    1      1    0A2
......................................................................uuugcuaguugcuuuuguccccUu...................    38     1    0A2
......................................................................uuugcuaguugcuuuuguccccCuu..................    4      1    0A2
......................................................................uuugcuaguugcuuuuguccccauu..................    6      0    0A2
......................................................................uuugcuaguugcuuuuguccccUuu..................    77     1    0A2
......................................................................uuugcuaguugcuuuuguccccauA..................    4      1    0A2
......................................................................uuugcuaguugcuuuuguccccauuU.................    4      1    0A2
.......................................................................uugcuaguugcuuuugucc.......................    8      0    0A2
.......................................................................uugcuaguugcuuuuUucc.......................    1      1    0A2
.......................................................................uugcuaguugcuuuuguccA......................    1      1    0A2
.......................................................................uugcuaguugcuCuuguccc......................    1      1    0A2
.......................................................................uugcuaguugcuuuuguccc......................    12     0    0A2
.......................................................................uugcuagGugcuuuuguccc......................    1      1    0A2
.......................................................................uugcuaguugcuuuuguUcc......................    1      1    0A2
.......................................................................uugcuaguugcuuuugGcccc.....................    1      1    0A2
.......................................................................uugcuaguugcuuuugucccc.....................    12     0    0A2
.......................................................................uugcuaguugcuuuuguccccU....................    12     1    0A2
.......................................................................uugcuaguugcuuuuguccccUuu..................    2      1    0A2
........................................................................ugcuaguugcuuuugucc.......................    2      0    0A2
```

Provisional ID	:	scaffold8_2653
Score total	:	503.7
Score for star read(s)	:	3.9
Score for read counts	:	492.6
Score for mfe	:	2.6
Score for randfold	:	1.6
Score for cons. seed	:	3
Total read count	:	978
Mature read count	:	976
Loop read count	:	0
Star read count	:	2

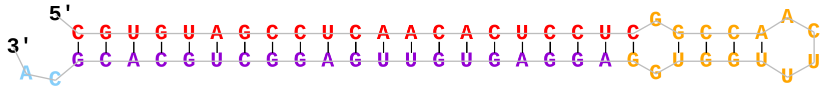

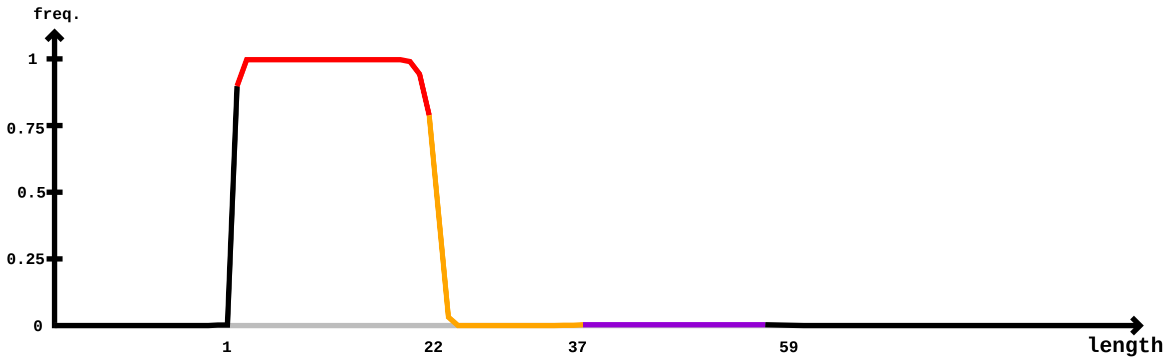

Mature **Star**

```
5'- ucaaaaugaguagcaugcguguagccucaacacuccucggccaacuuugguggaggaguguugaggcugcacgcaggcuaaaaaugacccuuuucuugucguaaugucauc  -3'   obs
    ucaaaaugaguagcaugcguguagccucaacacuccucggccaacuuugguggaggaguguugaggcugcacgcaggcuaaaaaugacccuuuucuugucguaaugucauc        exp
    .....((((.((((.(((((((((((((((((((((((.((((....)))).))))))))))))))))))))))).)))).)...(((((.........)))))....)))).   reads   mm   sample
    ...............ugcguguagccucaacacuccu..........................................................................   2       0    0B2
    .................cguguagccucaacGcucc...........................................................................   2       1    0B2
    .................cguguagccucaacacuccu..........................................................................   33      0    0B2
    .................cguguagccucaacacuccC..........................................................................   2       1    0B2
    .................cguguagccucaacacuccuU.........................................................................   8       1    0B2
    .................cguguagccucaacacuccuc.........................................................................   152     0    0B2
    .................cguguagccucaacacuccucC........................................................................   10      1    0B2
    .................cguguagccucaacacuccucg........................................................................   135     0    0B2
    .................Nguguagccucaacacuccucg........................................................................   1       1    0B2
    .................cguguagccucaacacuccucgU.......................................................................   11      1    0B2
    ..................guguagccucaacacuccu..........................................................................   4       0    0B2
    ..................guguagccucaacacuccuU.........................................................................   2       1    0B2
    ..................guguagccucaacacuccuc.........................................................................   19      0    0B2
    ..................guguagccucaacacuccucg........................................................................   7       0    0B2
    ...................................................Cgaggaguguugaggcugcacg......................................   1       1    0B2

    .................cguguagccucaacacucA...........................................................................   1       1    0G2
    .................cguguagccucaacacucc...........................................................................   23      0    0G2
    .................cguguagccucaacacuccu..........................................................................   68      0    0G2
    .................cguguagccGcaacacuccuc.........................................................................   1       1    0G2
    .................cguguagccucaacacuccuc.........................................................................   104     0    0G2
    .................cguguagccucaacacuccuU.........................................................................   13      1    0G2
    .................cguguagccucaacacuccuA.........................................................................   4       1    0G2
    .................cguguagccucaacacuccucg........................................................................   67      0    0G2
    .................cguguagccucaacacuccucC........................................................................   4       1    0G2
    .................cguguagccucaacacuccucgA.......................................................................   5       1    0G2
    .................cguguagccucaacacuccucgU.......................................................................   11      1    0G2
    ..................Nuguagccucaacacuccuc.........................................................................   1       1    0G2
    ..................guguagccucaacacuccuc.........................................................................   20      0    0G2
    ..................guguagccucaacacuccucg........................................................................   3       0    0G2

    .................cguguagccucaacacuc............................................................................   7       0    0A2
    .................cguguagccucaacacucU...........................................................................   3       1    0A2
```

Mature Star

```
ucaaaaugaguagcaugcguguagccucaacacuccucggccaacuuugguggaggaguguugaggcugcacgcaggcuaaaaaugacccuuuucuugucguaaugucauc
.................cguguagccucaacacucc...........................................................................	7	0	0A2
.................cguguagccucaacacucA...........................................................................	6	1	0A2
.................cguguagccucaacacuccu..........................................................................	24	0	0A2
.................cguguagccucaacacuccA..........................................................................	8	1	0A2
.................cguguagccucaacacuccuc.........................................................................	73	0	0A2
.................cguguagccucaacacuccuA.........................................................................	2	1	0A2
.................cguguagccucaacacuccuU.........................................................................	5	1	0A2
.................cguguagccucaacacuccucg........................................................................	82	0	0A2
.................cguguagccucaacacuccucC........................................................................	1	1	0A2
.................cguguagccucaacacuccucgU.......................................................................	3	1	0A2
.................cguguagccucaacacuccucgA.......................................................................	1	1	0A2
..................guguagccucaacacucA...........................................................................	4	1	0A2
..................guguagccucaacacuccu..........................................................................	10	0	0A2
..................guguagccucaacacuccuc.........................................................................	17	0	0A2
..................guguagccucaacacuccucg........................................................................	10	0	0A2
.....................................................aggaguguugaggcugcacU......................................	2	1	0A2
```

Provisional ID	:	scaffold8_2679
Score total	:	5.4
Score for star read(s)	:	-1.3
Score for read counts	:	0
Score for mfe	:	2.1
Score for randfold	:	1.6
Score for cons. seed	:	3
Total read count	:	1041
Mature read count	:	1041
Loop read count	:	0
Star read count	:	0

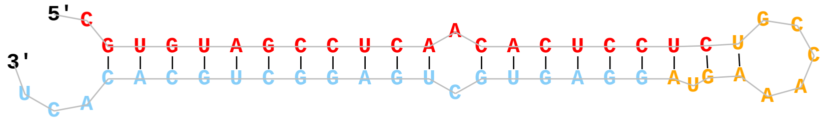

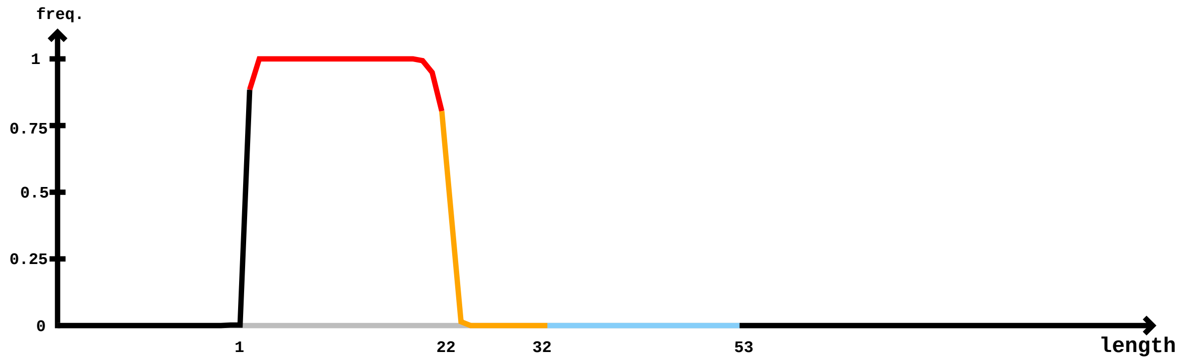

Mature **Star**

```
5'- uacauaauggguagccugcguguagccucaacacuccucugccaaaguaggagugcugaggcugcacacugcagaauggguggguaugcuaccuggaggcaaaaacguguuu   -3'    exp
    ............((((..((((((((((.((((((((.....)).)))))).)))))))))).........((((((.....))))))..)))).................. reads   mm   sample
    ..................cguguagccucaacacuc............................................................................ 7       0    0A2
    ..................cguguagccucaacacucc........................................................................... 7       0    0A2
    ..................cguguagccucaacacucA........................................................................... 6       1    0A2
    ..................cguguagccucaacacucU........................................................................... 3       1    0A2
    ..................cguguagccucaacacuccA.......................................................................... 8       1    0A2
    ..................cguguagccucaacacuccu.......................................................................... 24      0    0A2
    ..................cguguagccucaacacuccuc......................................................................... 73      0    0A2
    ..................cguguagccucaacacuccuU......................................................................... 5       1    0A2
    ..................cguguagccucaacacuccuA......................................................................... 2       1    0A2
    ..................Nguguagccucaacacuccucu........................................................................ 1       1    0A2
    ..................cguguagccucaacacuccucu........................................................................ 88      0    0A2
    ..................cguguagccucaacacuccucC........................................................................ 1       1    0A2
    ..................cguguagccucaacacuccucuU....................................................................... 8       1    0A2
    ..................cguguagccucaacacuccucuA....................................................................... 2       1    0A2
    ...................guguagccucaacacucA........................................................................... 4       1    0A2
    ...................guguagccucaacacuccu.......................................................................... 10      0    0A2
    ...................guguagccucaacacuccuc......................................................................... 17      0    0A2
    ...................guguagccucaacacuccucu........................................................................ 9       0    0A2

    ...............ugcguguagccucaacacuccu........................................................................... 2       0    0B2
    ..................cguguagccucaacGcucc........................................................................... 2       1    0B2
    ..................cguguagccucaacacuccC.......................................................................... 2       1    0B2
    ..................cguguagccucaacacuccu.......................................................................... 33      0    0B2
    ..................cguguagccucaacacuccuU......................................................................... 8       1    0B2
    ..................cguguagccucaacacuccuc......................................................................... 152     0    0B2
    ..................cguguagccucaacGcuccucu........................................................................ 2       1    0B2
    ..................cguguagccucaacacuccucu........................................................................ 151     0    0B2
    ..................Nguguagccucaacacuccucu........................................................................ 1       1    0B2
    ..................cguguagccucaacacuccucC........................................................................ 10      1    0B2
    ..................cguguagccucaacacuAcucu........................................................................ 1       1    0B2
    ..................cguguagAcucaacacuccucu........................................................................ 1       1    0B2
    ..................cguguagccucaacacuccucuU....................................................................... 2       1    0B2
    ...................guguagccucaacacuccu.......................................................................... 4       0    0B2
    ...................guguagccucaacacuccuc......................................................................... 19      0    0B2
```

Mature Star

```
uacauaauggguagccugcguguagccucaacacuccucugccaaaguaggagugcugaggcugcacacugcagaauggguggguaugcuaccuggaggcaaaaacguguuu
...................guguagccucaacacuccuU.........................................................................  2    1  0B2
...................guguagccucaacacuccucu........................................................................  28   0  0B2

..................cguguagccucaacacucc...........................................................................  23   0  0G2
..................cguguagccucaacacucA...........................................................................  1    1  0G2
..................cguguagccucaacacuccu..........................................................................  68   0  0G2
..................cguguagccucaacacuccuc.........................................................................  104  0  0G2
..................cguguagccucaacacuccuU.........................................................................  13   1  0G2
..................cguguagccucaacacuccuA.........................................................................  4    1  0G2
..................cguguagccGcaacacuccuc.........................................................................  1    1  0G2
..................cguguagccucaacacuccuUu........................................................................  10   1  0G2
..................cguguagccucaacacuccucC........................................................................  4    1  0G2
..................cguguagccucaacacuccucu........................................................................  88   0  0G2
..................cguguagccucaacacuccucuU.......................................................................  3    1  0G2
...................Nuguagccucaacacuccuc.........................................................................  1    1  0G2
...................guguagccucaacacuccuc.........................................................................  20   0  0G2
...................guguagccucaacacuccuUu........................................................................  6    1  0G2
```

Provisional ID	:	scaffold12_3538
Score total	:	3.8
Score for star read(s)	:	-1.3
Score for read counts	:	0
Score for mfe	:	0.5
Score for randfold	:	1.6
Score for cons. seed	:	3
Total read count	:	331
Mature read count	:	331
Loop read count	:	0
Star read count	:	0

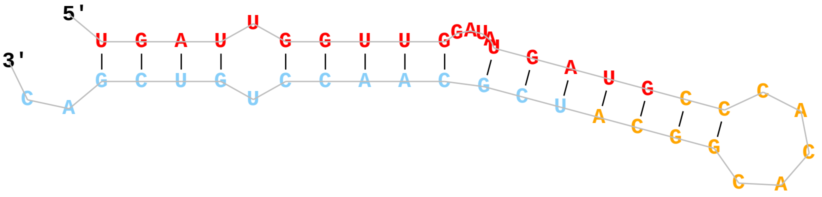

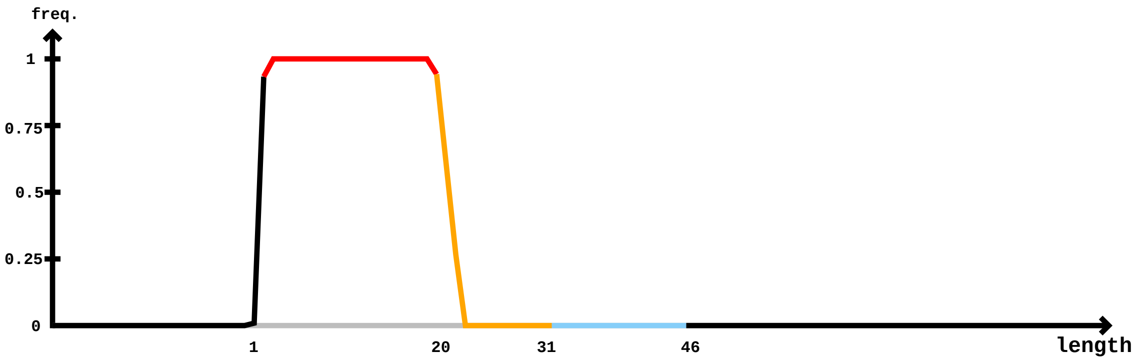

Mature **Star**

```
5'- uuguagggagaacuuuugguugauugguuggauaugaugcccacacggcaucgcaaccugucgacaaacuuuuuuuaaccauugacuaugcuuuagcaauaggaucug   -3'   exp
    ......((((((..((((.(((((.(((((....(((((((.....))))))))))))).)))))))))..))))))..((((((.(((.....))))))).)).....   reads   mm   sample
    ...................Gugauugguuggauaugaug.....................................................................   1   1   0B2
    ....................ugGuugguuggauaugau......................................................................   2   1   0B2
    ....................ugauugguuggauaugau......................................................................   15   0   0B2
    ....................ugGuugguuggauaugaug.....................................................................   6   1   0B2
    ....................ugauugguuggauaugauA.....................................................................   10   1   0B2
    ....................ugauugguuggauaugGug.....................................................................   8   1   0B2
    ....................ugauugguuggauaugaug.....................................................................   24   0   0B2
    ....................ugauugguuggauaugaugG....................................................................   7   1   0B2
    ....................ugauugguuggauaugaugGc...................................................................   37   1   0B2
    ....................ugauugguuAgauaugaugcc...................................................................   2   1   0B2
    .....................gauugguuggauaugaug.....................................................................   3   0   0B2
    .....................gauugguuggauaugaugG....................................................................   4   1   0B2

    ....................ugauugguuggauaugau......................................................................   1   0   0G2
    ....................ugauugguuggauaugauA.....................................................................   8   1   0G2
    ....................Ngauugguuggauaugaug.....................................................................   2   1   0G2
    ....................ugauugguuggGuaugaug.....................................................................   4   1   0G2
    ....................ugGuugguuggauaugaug.....................................................................   5   1   0G2
    ....................ugauugguuggauaugaug.....................................................................   44   0   0G2
    ....................ugauugguuggauaugaugG....................................................................   6   1   0G2
    ....................ugauugguuggauaugaugGc...................................................................   19   1   0G2
    .....................gauugguuggauaugaugGc...................................................................   2   1   0G2

    ...................Gugauugguuggauaugaug.....................................................................   2   1   0A2
    ....................ugauugguuggauaugGu......................................................................   1   1   0A2
    ....................ugaCugguuggauaugaug.....................................................................   1   1   0A2
    ....................ugGuugguuggauaugaug.....................................................................   8   1   0A2
    ....................ugauugguuggauaugGug.....................................................................   3   1   0A2
    ....................ugauugguuggauaugaug.....................................................................   50   0   0A2
    ....................ugauugguuggauaugauA.....................................................................   2   1   0A2
    ....................ugauugAuuggauaugaug.....................................................................   2   1   0A2
    ....................ugauugguuggauaugaugA....................................................................   2   1   0A2
    ....................ugauugguuggauaugaugG....................................................................   9   1   0A2
    ....................ugauugguuggauaugaugGc...................................................................   28   1   0A2
```

Mature   Star

```
uuguagggagaacuuuugguugauugguuggauaugaugcccacacggcaucgcaaccugucgacaaacuuuuuuuaaccauugacuaugcuuuagcaauaggaucug
.....................gauugguuggauaugaug.....................................................................    13    0    0A2
```

Provisional ID : scaffold13_3716
Score total : 5.6
Score for star read(s) : -1.3
Score for read counts : 0
Score for mfe : 2.3
Score for randfold : 1.6
Score for cons. seed : 3
Total read count : 2409
Mature read count : 2409
Loop read count : 0
Star read count : 0

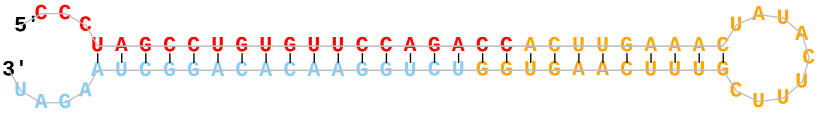

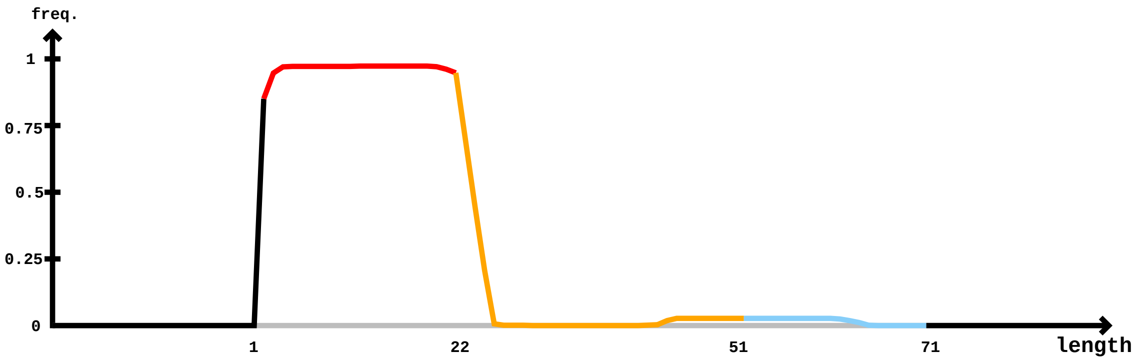

Mature Star

```
5'- ugcuuauauaagcuuagauucccuagccuguguuccagaccacuugaaacuauacuuucguuucaagugguucuggaacacaggcuaagauucccuuuaauaucagaaa -3'   exp
    .((((....))))...((.((..(((((((((((((((((((((((((((.........))))))))))))))))))))))))))).)).))................   reads   mm   sample
    ....................cccuagccuguguuccag......................................................................   3       0    0A2
    ....................cccuagccuguguuccaU......................................................................   1       1    0A2
    ....................cccuagccuguguuccaga.....................................................................   6       0    0A2
    ....................cccuagccuguguuccagac....................................................................   7       0    0A2
    ....................cccGagccuguguuccagacc...................................................................   1       1    0A2
    ....................cccuagccuguguuccagacU...................................................................   4       1    0A2
    ....................cccuagccuguguuccagacc...................................................................   230     0    0A2
    ....................cccuagccuguguuccaUacc...................................................................   3       1    0A2
    ....................cccuagccuguguuccagacA...................................................................   36      1    0A2
    ....................cccuagccuguguuccagaccU..................................................................   6       1    0A2
    ....................cccuagccuguguuccagacca..................................................................   54      0    0A2
    ....................cccuagccuguguuccagaccaA.................................................................   35      1    0A2
    ....................cccuagccuguguuccagaccaU.................................................................   176     1    0A2
    ....................cccuagccuguguuccagaccac.................................................................   26      0    0A2
    ....................cccuagccuguguuccagaccaAu................................................................   8       1    0A2
    ....................cccuagccuguguuccagaccacu................................................................   33      0    0A2
    ....................cccuagccuguguuccagaccacA................................................................   37      1    0A2
    ....................cccuagccuguguuccagaccaUu................................................................   116     1    0A2
    ....................cccuagccuguguuccagaccaUuu...............................................................   3       1    0A2
    .....................ccuagccuguguuccaga.....................................................................   1       0    0A2
    .....................ccuagccuguguuccagacc...................................................................   7       0    0A2
    .....................ccuagccuguguuccagacA...................................................................   5       1    0A2
    .....................ccuagccuguguuccagacca..................................................................   13      0    0A2
    .....................ccuagccuguguuccagaccU..................................................................   1       1    0A2
    .....................ccuagccuguguuccagaccaU.................................................................   20      1    0A2
    .....................ccuagccuguguuccagaccac.................................................................   8       0    0A2
    .....................ccuagccuguguuccagaccaA.................................................................   1       1    0A2
    .....................ccuagccuguguuccagaccaUu................................................................   11      1    0A2
    .....................ccuagccuguguuccagaccacA................................................................   4       1    0A2
    .....................ccuagccuguguuccagaccacu................................................................   37      0    0A2
    .....................ccuagccuguguuccagaccacuA...............................................................   2       1    0A2
    ......................cuagccuguguuccagacc...................................................................   1       0    0A2
    ......................cuagccuguguuccagaccac.................................................................   1       0    0A2
    ......................cuagccuguguuccagaccaU.................................................................   2       1    0A2
```

Mature Star

ugcuuauauaagcuuagauucccuagccuguguuccagaccacuugaaacuauacuuucguuucaaguggucuggaacacaggcuaagauucccuuuaauaucagaaa			
......................cuagccuguguuccagaccacu..	20	0	0A2
......................cuagccuguguuccagaccaUu..	2	1	0A2
......................cuagccuguguucAagaccacu..	1	1	0A2
......................cuagccGguguuccagaccacu..	1	1	0A2
.......................uagccuguguuccagaccacu..	4	0	0A2
....Ncaaguggucuggaacaca...........................	1	1	0A2
....ucaaguggucuggaacaca...........................	4	0	0A2
....ucaaguggucuggaacacag..........................	1	0	0A2
....ucaaguggucuggaacacagg.........................	7	0	0A2
....caaguggucuggaacacagg.........................	7	0	0A2
....................cccuagccuguguuccaA..	2	1	0G2
....................cccuagccuguguuccaga...	4	0	0G2
....................cccuagccuguguuUcagac..	2	1	0G2
....................cccuagccuguguuccagac..	6	0	0G2
....................cccuagccuguguuccagacU...	3	1	0G2
....................cccuagccuguguuccagacA...	36	1	0G2
....................cccuagccuguguuccagacc...	105	0	0G2
....................cccuagccuguguuccagacca..	35	0	0G2
....................cccuagccuguguuccagaccU..	10	1	0G2
....................cccuagccuguguuccagaccaA...	13	1	0G2
....................cccuagccuguguuccagaccaU...	86	1	0G2
....................cccuagccuguguuccagaccac...	3	0	0G2
....................cccuagccuguguuccagaccaG...	2	1	0G2
....................cccuagccuguguuccagaccaUu..	35	1	0G2
....................cccuagccuguguuccagaccacA..	4	1	0G2
....................cccuagccuguguuccagaccacu..	19	0	0G2
....................cccuagccuAuguuccagaccacu..	2	1	0G2
....................cccuagccuguguuccagaccaUuu...	4	1	0G2
.....................ccuagccuguguuccagacc...	4	0	0G2
.....................ccuagccuguguuccagacca..	11	0	0G2
.....................ccuagccuguguuccagaccaU...	12	1	0G2
.....................ccuagccuguguuccagaccaUu..	10	1	0G2
.....................ccuagccuguguuccagaccacu..	5	0	0G2
......................cuagccuguguuccagacc...	1	0	0G2
......................cuagccuguguuccagaccaU...	3	1	0G2
......................cuagccuguguuccagaccacu..	2	0	0G2
......................cuagccuguguuccagaccacuu...	4	0	0G2
....uAucaaguggucuggaacaca...........................	1	1	0G2
....uNucaaguggucuggaacaca...........................	1	1	0G2
....uucaaguggucuggaacaAag..........................	3	1	0G2
....ucaaguggucuggaacaca...........................	2	0	0G2
....ucaaguggucuggaacacag..........................	9	0	0G2
....ucaaUuggucuggaacacag..........................	5	1	0G2
....................cccuagccuguguuccaga...	11	0	0B2
....................cccuagccuguguuccagac..	19	0	0B2
....................cccuagccuguguGccagacc...	1	1	0B2
....................cccuagccuguguuccagacA...	17	1	0B2
....................cccuagccuguguuccagacU...	4	1	0B2
....................cccuagccuguguuccagacc...	493	0	0B2
....................Nccuagccuguguuccagacc...	4	1	0B2
....................cccuagAcuguguuccagacc...	1	1	0B2
....................cccuagccuguguuccagacca..	88	0	0B2
....................cccuagccuguguCccagacca..	1	1	0B2
....................cccuagccuguguuccagaccU..	10	1	0B2
....................Nccuagccuguguuccagaccac...	1	1	0B2
....................cccuagccuguguuccagaccaU...	149	1	0B2
....................cccuagccuguguuccagaccac...	13	0	0B2
....................cccuagccuguguuccagaccaA...	28	1	0B2
....................cccuagccuguguuccagaccacu..	31	0	0B2
....................cccuagccuguguuccagaccaUu..	59	1	0B2
....................cccuagccuguguuccagaccacA..	22	1	0B2
....................cccuagccuguguuccagaccacC..	1	1	0B2
.....................ccuagccuguguuccaga...	1	0	0B2
.....................ccuagccugCguuccagacc...	2	1	0B2
.....................ccuagccuguguuccagacc...	42	0	0B2
.....................ccuagccuguguuccagaUc...	1	1	0B2
.....................ccuagccugugAuccagacca..	1	1	0B2
.....................ccuagccuguguuccagacca..	8	0	0B2

Mature **Star**

```
ugcuuauauaagcuuagauucccuagccuguguuccagaccacuugaaacuauacuuucguuucaaguggucuggaacacaggcuaagauucccuuuaauaucagaaa
.....................ccuagccuguguuccagaccac.................................................................    5    0    0B2
.....................ccuagccuguguuccagaccaU.................................................................    9    1    0B2
.....................ccuagccuguguuccagaccaUu................................................................    10   1    0B2
.....................ccuagccuguguuccagaccacu................................................................    5    0    0B2
.....................ccuagccuguguuccagaccacA................................................................    2    1    0B2
......................cuagccuguguuccagacc...................................................................    4    0    0B2
......................cuagccuguguuccagaccacu................................................................    16   0    0B2
..............................uguuccagaccacuugaG............................................................    3    1    0B2
............................................................uuucaaguggucuggaacaAa...........................    1    1    0B2
............................................................uAucaaguggucuggaacaca...........................    1    1    0B2
.............................................................Nucaaguggucuggaacaca...........................    1    1    0B2
..............................................................Ncaaguggucuggaacac............................    1    1    0B2
..............................................................ucaaguggucuggaacac............................    5    0    0B2
..............................................................ucaaguggucuggaacacaggc........................    3    0    0B2
...............................................................caaguggucuggaaAaca...........................    2    1    0B2
...............................................................caaguggucuggaacaca...........................    1    0    0B2
...............................................................caaguggucuggaacacagg.........................    11   0    0B2
```

Provisional ID	:	scaffold13_3733
Score total	:	6116.4
Score for star read(s)	:	3.9
Score for read counts	:	6105.3
Score for mfe	:	2.6
Score for randfold	:	1.6
Score for cons. seed	:	3
Total read count	:	11987
Mature read count	:	11923
Loop read count	:	0
Star read count	:	64

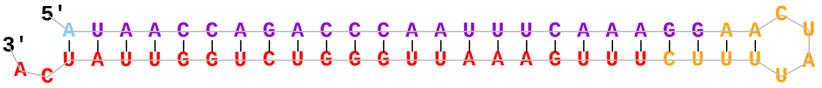

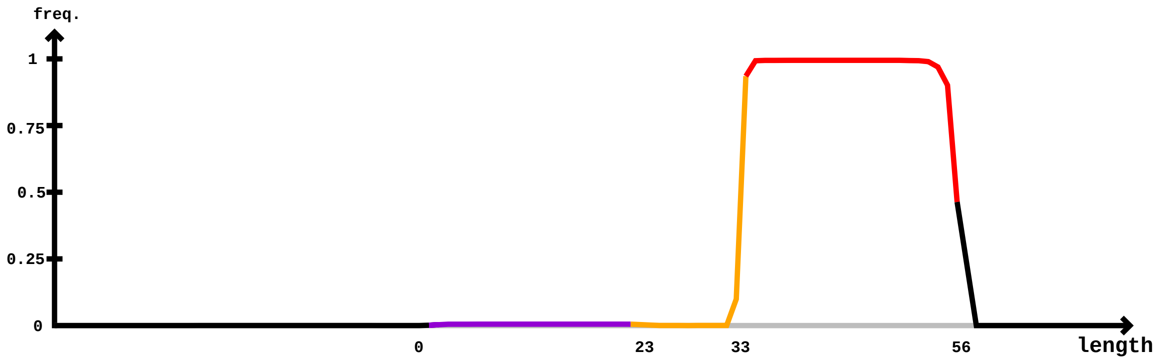

Star **Mature**

```
5'- ccucaaccauagguggaucuucacuuaagguagccugauaaccagacccaauuucaaaggaacuauuuucuuugaaauugggucugguuaucaggcuaacuuaagguggc  -3'   obs
    ccucaaccauagguggaucuucacuuaagguagccugauaaccagacccaauuucaaaggaacuauuuucuuugaaauugggucugguuaucaggcuaacuuaagguggc          exp
    ......(((....)))....((((((((.((((((((((((((((((((((((((((((((((....)))))))))))))))))))))))))))))))).))))).)))).    reads     mm       sample
    .............uggaucuucacuuaagguG..............................................................................    1         1        0A2
    .....................................Uuaaccagacccaauuucaaag...................................................    3         1        0A2
    .....................................Cuaaccagacccaauuucaaagg..................................................    2         1        0A2
    .....................................Uuaaccagacccaauuucaaagg..................................................    2         1        0A2
    ......................................uaaccagacccaauuucaaagg..................................................    5         0        0A2
    ......................................uaaccagacccaauuucaaaAg..................................................    2         1        0A2
    ......................................uaaccagacccaauuucaaaAga.................................................    2         1        0A2
    .......................................aaccagacccaauuucaaaAg..................................................    3         1        0A2
    .......................................aaccagacccaauuucaaagg..................................................    7         0        0A2
    .......................................aaccagacccaauuucaaaAga.................................................    3         1        0A2
    .................................................................uuuCcuuugaaauugggucugguuau...................    5         1        0A2
    .....................................................................cuuugaaauugggucugU.......................    3         1        0A2
    .....................................................................cuuugaaauugggucugg.......................    7         0        0A2
    .....................................................................cuuugaaauugggucugguu.....................    15        0        0A2
    .....................................................................cuuugaaauGggucugguua.....................    1         1        0A2
    .....................................................................cuuugaaauugggucugguAa....................    2         1        0A2
    .....................................................................cuuugaaauugggucugguua....................    60        0        0A2
    .....................................................................Uuuugaaauugggucugguua....................    23        1        0A2
    .....................................................................cuuugaaauugggucugguuau...................    18        0        0A2
    .....................................................................Uuuugaaauugggucugguuau...................    33        1        0A2
    .....................................................................cuuugaaauugggucugguuaA...................    153       1        0A2
    .....................................................................cuuugaaauugggucugguuUu...................    2         1        0A2
    .....................................................................Uuuugaaauugggucugguuauc..................    6         1        0A2
    .....................................................................cuuugaaauugggucugguuauA..................    24        1        0A2
    .....................................................................cuuugaaauugggucugguuaAc..................    6         1        0A2
    .....................................................................cuuugaaauugggucugguuauAa.................    3         1        0A2
    .....................................................................cuuugaaauugggucugguuaAca.................    12        1        0A2
    .....................................................................cuuugaaauugggucugguuaucU.................    4         1        0A2
    ......................................................................uuugaaauuAggucugguua....................    2         1        0A2
    ......................................................................uuGgaaauugggucugguuau...................    1         1        0A2
    ......................................................................uuugaaauugggucugguuaA...................    27        1        0A2
    ......................................................................uuugaaauugggucugguuau...................    4         0        0A2
    ......................................................................uuugaaauugggucugguAau...................    4         1        0A2
```

Star Mature

```
ccucaaccauagguggaucuucacuuaagguagccugauaaccagacccaauuucaaaggaacuauuuucuuugaaauugggucugguuaucaggcuaacuuaagguggc
......................................................................uuugaaauugUgucugguuauc..................  8     1   0A2
......................................................................uuugaaauugggucugguuauc..................  18    0   0A2
......................................................................uuugaaauGgggucugguuauc..................  1     1   0A2
......................................................................uuugaaauugggucugguuaAc..................  1259  1   0A2
......................................................................uuugaaauugggucugguuauA..................  6     1   0A2
......................................................................uuugaaauugggucugguuauca.................  19    0   0A2
......................................................................uuugaaauAgggucugguuauca.................  1     1   0A2
......................................................................uuugaaauugggucugguuaAca.................  1353  1   0A2
......................................................................uuugaaauugggucugguuaucU.................  10    1   0A2
......................................................................uuugaaauugUgucugguuauca.................  7     1   0A2
.......................................................................uugaaauugggucugguuauc..................  32    0   0A2
.......................................................................uugaaauugggucugguuaAc..................  57    1   0A2
.......................................................................uugaaauugggucugguuauca.................  13    0   0A2
.......................................................................uugaaauugggucugguuaucU.................  29    1   0A2
.......................................................................uugaaauugggucugguuaAca.................  67    1   0A2
........................................................................ugaaauugggucugguuaucU.................  1     1   0A2
........................................................................ugaaauugggucugguuaAca.................  1     1   0A2
........................................................................ugaaauugggucuUguuauca.................  3     1   0A2
..........................................................................aaauugggucugguuaAca.................  2     1   0A2

.....................................Uuaaccagacccaauuucaaag...................................................  1     1   0B2
.....................................Uuaaccagacccaauuucaaagg..................................................  2     1   0B2
......................................uaaccagacccaauuucaaagg..................................................  4     0   0B2
......................................uaaccagacccaauuucaaaAga.................................................  1     1   0B2
.......................................aaccagacccaauuucaaagg..................................................  2     0   0B2
.......................................aaccagacccaauuucaaagga.................................................  2     0   0B2
..........................................cagacccaauuucaaaggaaUu..............................................  3     1   0B2
.....................................................................cuuugaaauugggucugguA.....................  1     1   0B2
.....................................................................cuuugaaauugggucugguu.....................  27    0   0B2
.....................................................................cuuugaaauugggucugguuU....................  8     1   0B2
.....................................................................cuuugaaauugggucugguua....................  49    0   0B2
.....................................................................Uuuugaaauugggucugguua....................  11    1   0B2
.....................................................................cuuugaaauugggucugguuUu...................  1     1   0B2
.....................................................................cuuugaaauugggucugguuaA...................  162   1   0B2
.....................................................................cuuugaaauugggucuggCuau...................  2     1   0B2
.....................................................................Uuuugaaauugggucugguuau...................  34    1   0B2
.....................................................................cuuugaaauugggucugguuau...................  35    0   0B2
.....................................................................cuuugaaauugggucugguuaAc..................  27    1   0B2
.....................................................................cuuugaaauugggucugguuauA..................  8     1   0B2
.....................................................................cuuugaaauugggucugguuaucU.................  1     1   0B2
.....................................................................Uuuugaaauugggucugguuauca.................  4     1   0B2
.....................................................................cuuugaaauugggucugguuaAca.................  17    1   0B2
......................................................................uuugaaauugggucugguuau...................  4     0   0B2
......................................................................uuugaaauugggucugguuaA...................  19    1   0B2
......................................................................uuugaaauugggucugguuaAc..................  2229  1   0B2
......................................................................uuugaaauugggucugguuauc..................  18    0   0B2
......................................................................uuugaaauugUgucugguuauc..................  7     1   0B2
......................................................................uuugaaauugUgucugguuauca.................  8     1   0B2
......................................................................uuugaaauugggucugguuaAca.................  1223  1   0B2
......................................................................uuugaaauugggucugguuauca.................  13    0   0B2
......................................................................uuugaaauugggucugguuaucU.................  13    1   0B2
.......................................................................uugaaauugggucugguuauc..................  28    0   0B2
.......................................................................uugaaauugggucugguuaAc..................  92    1   0B2
.......................................................................uugaaauugggucugguuaucU.................  31    1   0B2
.......................................................................uugaaauugggucugguuaAca.................  82    1   0B2
.......................................................................uugaaauugggucugguuauca.................  33    0   0B2
.......................................................................uugGaauugggucugguuauca.................  6     1   0B2
........................................................................ugaaauugggucugguuaAc..................  7     1   0B2
........................................................................ugaaauugggucugguuaucU.................  1     1   0B2

.....................................Uuaaccagacccaauuucaaag...................................................  5     1   0G2
......................................uaaccGgacccaauuucaaagg..................................................  1     1   0G2
......................................uaaccagacccaauuucaaagg..................................................  8     0   0G2
.......................................aaccagacccaauuucaaa....................................................  2     0   0G2
.......................................aaccagacccaauuucaaagg..................................................  4     0   0G2
.....................................................................Uuuugaaauugggucuggu......................  6     1   0G2
.....................................................................cuuugaaauugggucugguuU....................  1     1   0G2
.....................................................................cuuugaaauugggucAgguua....................  1     1   0G2
.....................................................................cuuugaaauugggucugguua....................  70    0   0G2
.....................................................................Uuuugaaauugggucugguua....................  9     1   0G2
```

Star Mature

```
ccucaaccauagguggaucuucacuuaagguagccugauaaccagacccaauuucaaaggaacuauuuucuuugaaauugggucugguuaucaggcuaacuuaagguggc
.....................................................................cuuugaaauugggucuggCuau...................     9     1     0G2
.....................................................................cuuugaaauugggucugguuau...................     22    0     0G2
.....................................................................cuuugaaauugggucugguuaA...................     191   1     0G2
.....................................................................Uuuugaaauugggucugguuau...................     65    1     0G2
.....................................................................cuuugaaauugggucugguuauA..................     25    1     0G2
.....................................................................Uuuugaaauugggucugguuauc..................     2     1     0G2
.....................................................................cuuugaaauugggucugguuaAc..................     5     1     0G2
.....................................................................cuuugaaauugggucugguuaAca.................     14    1     0G2
.....................................................................cuuugaaauugggucugguuaucU.................     7     1     0G2
......................................................................uuugaaauugggucugguua....................     4     0     0G2
......................................................................uuugaaauugggucugguuaA...................     22    1     0G2
......................................................................uuugaaauugUgucugguuauc..................     1     1     0G2
......................................................................uuugaaauugggucugguuaAc..................     1323  1     0G2
......................................................................uuugaaauugggucugguuauc..................     6     0     0G2
......................................................................uuugaaauugggucugguuauA..................     8     1     0G2
......................................................................uuugaaauugggucugguuaAca.................     2385  1     0G2
......................................................................uuugaaauugggucugguuaucU.................     14    1     0G2
......................................................................uuugaaauGggucugguuauca.................     1     1     0G2
......................................................................uuugaaauugggucugguuauca.................     7     0     0G2
.......................................................................uugaaauugggucugguuaA...................     4     1     0G2
.......................................................................uugaaauugggucugguuauA..................     4     1     0G2
.......................................................................uugaaauugggucugguuaAc..................     38    1     0G2
.......................................................................uugaaauugggucugguuauc..................     7     0     0G2
.......................................................................uugaaauugggucuUguuauca.................     2     1     0G2
.......................................................................uugaaauGggucugguuauca.................     1     1     0G2
.......................................................................uugaaauugggucugguuauca.................     34    0     0G2
.......................................................................uugaaauugggucugguuaAca.................     96    1     0G2
.......................................................................uugaaauugggucugguuaucU.................     35    1     0G2
.......................................................................uugaaauugUgucugguuauca.................     1     1     0G2
.......................................................................uugaaauAgggucugguuauca.................     1     1     0G2
........................................................................ugaaauugggucugguuaAc..................     2     1     0G2
........................................................................ugaaauugggucugguuauca.................     1     0     0G2
...........................................................................aauugggucugguuaucaU................     1     1     0G2
```

Provisional ID : scaffold13_3890
Score total : 5.7
Score for star read(s) : -1.3
Score for read counts : 0
Score for mfe : 2.4
Score for randfold : 1.6
Score for cons. seed : 3
Total read count : 11945
Mature read count : 11925
Loop read count : 0
Star read count : 20

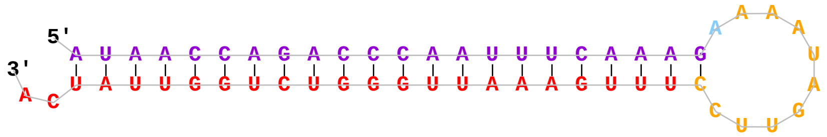

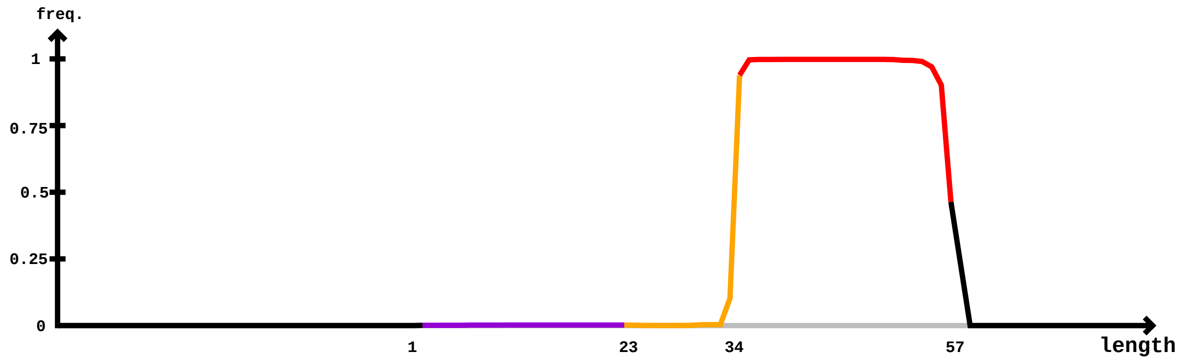

Star Mature

```
5'- aauauuuuuaguagcaagccaccuuaaguuagccugauaaccagacccaauuucaaagaaaauaguuccuuugaaauugggucugguuaucaggcuaccuuaagugaagauc   -3'    obs
    aauauuuuuaguagcaagccaccuuaaguuagccugauaaccagacccaauuucaaagaaaauaguuccuuugaaauugggucugguuaucaggcuaccuuaagugaagauc          exp
    ...................(((.(((((.(((((((((((((((((((((((((((((..........))))))))))))))))))))))))))))).))))))))......    reads   mm   sample
    ....................................Uuaaccagacccaauuucaaag......................................................    3       1    0A2
    ................................................................Nuuccuuugaaauugggucug...........................    1       1    0A2
    ................................................................Uuuccuuugaaauugggucugguuau......................    5       1    0A2
    .................................................................uuccuuugaaauugggucugg..........................    7       0    0A2
    ....................................................................cUuugaaauugggucugguuau......................    2       1    0A2
    ....................................................................cuuugaaauugggucugU..........................    3       1    0A2
    ....................................................................cuuugaaauugggucugg..........................    7       0    0A2
    ....................................................................cuuugaaauugggucugguu........................    15      0    0A2
    ....................................................................Uuuugaaauugggucugguua.......................    23      1    0A2
    ....................................................................cuuugaaauugggucugguua.......................    60      0    0A2
    ....................................................................cuuugaaauGgggucugguua.......................    1       1    0A2
    ....................................................................cuuugaaauugggucugguAa.......................    2       1    0A2
    ....................................................................cuuugaaauugggucugguuUu......................    2       1    0A2
    ....................................................................Uuuugaaauugggucugguuau......................    33      1    0A2
    ....................................................................cuuugaaauugggucugguuaA......................    153     1    0A2
    ....................................................................cuuugaaauugggucugguuau......................    18      0    0A2
    ....................................................................cuuugaaauugggucugguuauA.....................    24      1    0A2
    ....................................................................Uuuugaaauugggucugguuauc.....................    6       1    0A2
    ....................................................................cuuugaaauugggucugguuaAc.....................    6       1    0A2
    ....................................................................cuuugaaauugggucugguuauAa....................    3       1    0A2
    ....................................................................cuuugaaauugggucugguuaAca....................    12      1    0A2
    ....................................................................cuuugaaauugggucugguuaucU....................    4       1    0A2
    .....................................................................uuugaaauuAggucugguua.......................    2       1    0A2
    .....................................................................uuugaaauugggucugguuau......................    4       0    0A2
    .....................................................................uuugaaauugggucugguAau......................    4       1    0A2
    .....................................................................uuugaaauugggucugguuaA......................    27      1    0A2
    .....................................................................uuGgaaauugggucugguuau......................    1       1    0A2
    .....................................................................uuugaaauugggucugguuauc.....................    18      0    0A2
    .....................................................................uuugaaauGgggucugguuauc.....................    1       1    0A2
    .....................................................................uuugaaauugggucugguuaAc.....................    1259    1    0A2
    .....................................................................uuugaaauugUgucugguuauc.....................    8       1    0A2
    .....................................................................uuugaaauugggucugguuauA.....................    6       1    0A2
    .....................................................................uuugaaauugggucugguuauca....................    19      0    0A2
```

```
                                    Star                            Mature
aauauuuuuaguagcaagccaccuuaaguuagccugauaaccagacccaauuucaaagaaaauaguuccuuugaaauugggucugguuaucaggcuaccuuaagugaagauc
.....................................................................uuugaaauugggucugguuaAca....................    1353    1    0A2
.....................................................................uuugaaauAgggucugguuauca....................    1       1    0A2
.....................................................................uuugaaauugUgucugguuauca....................    7       1    0A2
.....................................................................uuugaaauugggucugguuaucU....................    10      1    0A2
......................................................................uugaaauugggucugguuaAc.....................    57      1    0A2
......................................................................uugaaauugggucugguuauc.....................    32      0    0A2
......................................................................uugaaauugggucugguuaucU....................    29      1    0A2
......................................................................uugaaauugggucugguuauca....................    13      0    0A2
......................................................................uugaaauugggucugguuaAca....................    67      1    0A2
.......................................................................ugaaauugggucuUguuauca....................    3       1    0A2
.......................................................................ugaaauugggucugguuaAca....................    1       1    0A2
.......................................................................ugaaauugggucugguuaucU....................    1       1    0A2
.........................................................................aaauugggucugguuaAca....................    2       1    0A2

....................................Uuaaccagacccaauuucaaag......................................................    5       1    0G2
......................................aaccagacccaauuucaaa.......................................................    2       0    0G2
.........................................cagacccaauuCcaaagaa....................................................    3       1    0G2
................................................................guuccuuugaaauugggucug...........................    1       0    0G2
.................................................................uuccuuugaaauugggucugC..........................    3       1    0G2
.................................................................uuccuuugaaauugggucugU..........................    2       1    0G2
.................................................................uuccuuugaaauugggucugguA........................    2       1    0G2
....................................................................Uuugaaauugggucuggu..........................    6       1    0G2
....................................................................cuugaaauugggucAgguua........................    1       1    0G2
....................................................................cuugaaauugggucugguua........................    70      0    0G2
....................................................................Uuugaaauugggucugguua........................    9       1    0G2
....................................................................cuugaaauugggucugguuU........................    1       1    0G2
....................................................................cuugaaauugggucugguuaA.......................    191     1    0G2
....................................................................cuugaaauugggucugguuau.......................    22      0    0G2
....................................................................cuugaaauugggucuggCuau.......................    9       1    0G2
....................................................................Uuugaaauugggucugguuau.......................    65      1    0G2
....................................................................Uuugaaauugggucugguuauc......................    2       1    0G2
....................................................................cuugaaauugggucugguuaAc......................    5       1    0G2
....................................................................cuugaaauugggucugguuauA......................    25      1    0G2
....................................................................cuugaaauugggucugguuaAca.....................    14      1    0G2
....................................................................cuugaaauugggucugguuaucU.....................    7       1    0G2
.....................................................................uuugaaauugggucugguua.......................    4       0    0G2
.....................................................................uuugaaauugggucugguuaA......................    22      1    0G2
.....................................................................uuugaaauugggucugguuauA.....................    8       1    0G2
.....................................................................uuugaaauugUgucugguuauc.....................    1       1    0G2
.....................................................................uuugaaauugggucugguuauc.....................    6       0    0G2
.....................................................................uuugaaauugggucugguuaAc.....................    1323    1    0G2
.....................................................................uuugaaauugggucugguuaucU....................    14      1    0G2
.....................................................................uuugaaauugggucugguuaAca....................    2385    1    0G2
.....................................................................uuugaaauugggucugguuauca....................    7       0    0G2
.....................................................................uuugaaauGgggucugguuauca....................    1       1    0G2
......................................................................uugaaauugggucugguuaA......................    4       1    0G2
......................................................................uugaaauugggucugguuauc.....................    7       0    0G2
......................................................................uugaaauugggucugguuauA.....................    4       1    0G2
......................................................................uugaaauugggucugguuaAc.....................    38      1    0G2
......................................................................uugaaauugggucugguuauca....................    34      0    0G2
......................................................................uugaaauGgggucugguuauca....................    1       1    0G2
......................................................................uugaaauAgggucugguuauca....................    1       1    0G2
......................................................................uugaaauugggucuUguuauca....................    2       1    0G2
......................................................................uugaaauugUgucugguuauca....................    1       1    0G2
......................................................................uugaaauugggucugguuaAca....................    96      1    0G2
......................................................................uugaaauugggucugguuaucU....................    35      1    0G2
.......................................................................ugaaauugggucugguuaAc.....................    2       1    0G2
.......................................................................ugaaauugggucugguuauca....................    1       0    0G2
..........................................................................aauugggucugguuaucaU...................    1       1    0G2

....................................Uuaaccagacccaauuucaaag......................................................    1       1    0B2
.........................................cagacccaauuCcaaaga.....................................................    6       1    0B2
.............................................cccGauuucaaagaaaauag...............................................    3       1    0B2
.................................................................uuccuuugaaauugggucug...........................    6       0    0B2
.................................................................uuccuuugaaauugggucugg..........................    8       0    0B2
.................................................................uuccuuugaaauugggucugguA........................    2       1    0B2
..................................................................uccuuugaaauugggucugU..........................    2       1    0B2
....................................................................cuugaaauugggucugguu.........................    27      0    0B2
....................................................................cuugaaauugggucugguA.........................    1       1    0B2
....................................................................Uuugaaauugggucugguua........................    11      1    0B2
```

Star Mature

```
aauauuuuuaguagcaagccaccuuaaguuagccugauaaccagacccaauuucaaagaaaauaguuccuuugaaauugggucugguuaucaggcuaccuuaagugaagauc
....................................................................cuuugaaauugggucugguua.......................  49    0  0B2
....................................................................cuuugaaauugggucugguuU.......................  8     1  0B2
....................................................................cuuugaaauugggucuggCuau......................  2     1  0B2
....................................................................cuuugaaauugggucugguuau......................  35    0  0B2
....................................................................Uuuugaaauugggucugguuau......................  34    1  0B2
....................................................................cuuugaaauugggucugguuaA......................  162   1  0B2
....................................................................cuuugaaauugggucugguuUu......................  1     1  0B2
....................................................................cuuugaaauugggucugguuaAc.....................  27    1  0B2
....................................................................cuuugaaauugggucugguuauA.....................  8     1  0B2
....................................................................cuuugaaauugggucugguuaucU....................  1     1  0B2
....................................................................Uuuugaaauugggucugguuauca....................  4     1  0B2
....................................................................cuuugaaauugggucugguuaAca....................  17    1  0B2
.....................................................................uuugaaauugggucugguuaA......................  19    1  0B2
.....................................................................uuugaaauugggucugguuau......................  4     0  0B2
.....................................................................uuugaaauugggucugguuauc.....................  18    0  0B2
.....................................................................uuugaaauugggucugguuaAc.....................  2229  1  0B2
.....................................................................uuugaaauugUgucugguuauc.....................  7     1  0B2
.....................................................................uuugaaauugggucugguuaAca....................  1223  1  0B2
.....................................................................uuugaaauugggucugguuaucU....................  13    1  0B2
.....................................................................uuugaaauugggucugguuauca....................  13    0  0B2
.....................................................................uuugaaauugUgucugguuauca....................  8     1  0B2
......................................................................uugaaauugggucugguuauc.....................  28    0  0B2
......................................................................uugaaauugggucugguuaAc.....................  92    1  0B2
......................................................................uugaaauugggucugguuaAca....................  82    1  0B2
......................................................................uugaaauugggucugguuaucU....................  31    1  0B2
......................................................................uugaaauugggucugguuauca....................  33    0  0B2
......................................................................uugGaauugggucugguuauca....................  6     1  0B2
.......................................................................ugaaauugggucugguuaAc.....................  7     1  0B2
.......................................................................ugaaauugggucugguuaucU....................  1     1  0B2
```

Provisional ID	:	scaffold13_3904
Score total	:	480.4
Score for star read(s)	:	3.9
Score for read counts	:	469.7
Score for mfe	:	2.3
Score for randfold	:	1.6
Score for cons. seed	:	3
Total read count	:	933
Mature read count	:	506
Loop read count	:	0
Star read count	:	427

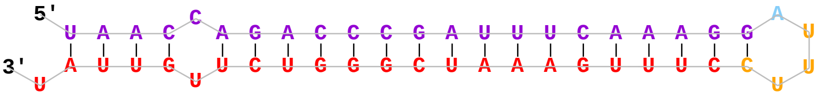

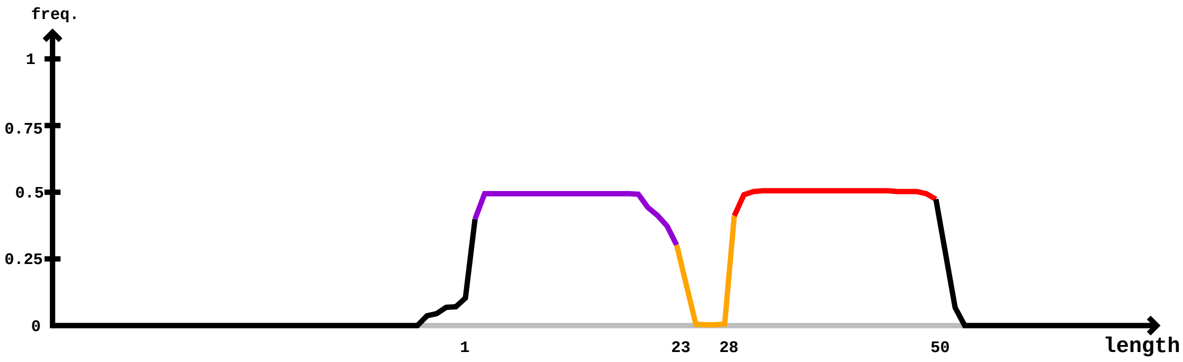

Star **Mature**

```
5'- uccuccugaguuuccuaucuugcaguugggguguauaaccugauaaccagacccgauuucaaaggauuuccuuugaaaucgggucuuguuaucaggcuauuggguguagggcuc  -3'   obs
    uccuccugaguuuccuaucuugcaguugggguguauaaccugauaaccagacccgauuucaaaggauuuccuuugaaaucgggucuuguuaucaggcuauuggguguagggcuc         exp
    ...(((((.....((((...((((......))))...(((((((((.(((((((((((((((((....))))))))))))))))).)))))))))....))))..)))))...    reads   mm    sample
    ......................................cAugauaaccagacccgauuuca.....................................................    20      1     0A2
    .......................................cugauaaccaCacccgauuuca.....................................................    1       1     0A2
    .......................................Augauaaccagacccgauuuca.....................................................    7       1     0A2
    ........................................ugauaaccagaAccgauuucaa....................................................    10      1     0A2
    ........................................ugauaaccagacccgauuucaa....................................................    2       0     0A2
    ........................................ugauaaccagaAccgauuucaaa...................................................    5       1     0A2
    ..........................................auaaccagacccgauuucaa....................................................    4       0     0A2
    ..........................................auaaccagaAccgauuucaaa...................................................    6       1     0A2
    ..........................................auaaccagacccgauuucaaa...................................................    1       0     0A2
    ...........................................uaaccagaAccgauuucaaa...................................................    1       1     0A2
    ...........................................Caaccagacccgauuucaaa...................................................    3       1     0A2
    ...........................................uaaccagacccgauuucaaa...................................................    1       0     0A2
    ...........................................Caaccagacccgauuucaaag..................................................    10      1     0A2
    ...........................................uaaccagacccgauuucaaag..................................................    8       0     0A2
    ...........................................Caaccagacccgauuucaaagg.................................................    99      1     0A2
    ...........................................uaGccagacccgauuucaaagg.................................................    4       1     0A2
    ...........................................uaaccagacccgauuucaaaAg.................................................    4       1     0A2
    ...........................................Caaccagacccgauuucaaagga................................................    4       1     0A2
    ............................................aaccagacccgauuucaa....................................................    8       0     0A2
    ............................................aaccagacccgauuucaaag..................................................    3       0     0A2
    ............................................aaccagacccgauuucaaaA..................................................    2       1     0A2
    ............................................aaccagacccgauuucaaagg.................................................    3       0     0A2
    ............................................aaccagacccgauuucaaaAga................................................    23      1     0A2
    .....................................................................cUuuugaaaucgggucuuguuau......................    3       1     0A2
    ......................................................................Uuuugaaaucgggucuuguu........................    2       1     0A2
    ......................................................................Uuuugaaaucgggucuuguua.......................    2       1     0A2
    ......................................................................Uuuugaaaucgggucuuguuau......................    127     1     0A2
    .......................................................................uuugaaaucgggucuuguuau......................    6       0     0A2
    .......................................................................uuugaaaucgggucuuguuauc.....................    3       0     0A2
    .......................................................................uuugaaaucgggucuuguuaAca....................    3       1     0A2
    .......................................................................uuugaaaucgggucuuguuauca....................    16      0     0A2
    ........................................................................uugaaaucgggucuuguuauca....................    2       0     0A2
    ........................................................................uGgaaaucgggucuuguuauca....................    1       1     0A2
```

Star **Mature**

uccuccugaguuuccuaucuugcaguuggguguauaaccugauaaccagacccgauuucaaaggauuuccuuugaaaucgggucuuguuaucaggcuauuggguguagggcuc			
....ugaaauUgggucuuguuauca....................	3	1	0A2
.....................................cAugauaaccagacccgauuuc..	2	1	0G2
.....................................cAugauaaccagacccgauuuca...	15	1	0G2
....ugauaaccagacccgauuucaa..	3	0	0G2
....ugauaaccagaAccgauuucaaa...	2	1	0G2
....gauaaccagacccgauuuca...	2	0	0G2
....auaaccagacccgauuuca...	2	0	0G2
....auaaccagacccgauuucaa..	1	0	0G2
....auaaccagacccgauuucaaa...	2	0	0G2
....Nuaaccagacccgauuucaaa...	1	1	0G2
....uaaccGgacccgauuucaaa...	4	1	0G2
....Caaccagacccgauuucaaag..	2	1	0G2
....uaaccagacccgauuucaaaAg...	1	1	0G2
....Caaccagacccgauuucaaagg...	10	1	0G2
....uaaccagacccgauuucaaagg...	7	0	0G2
....aaccagacccgauuucaaag..	9	0	0G2
....aaccagacccgauuucaaaAga..	1	1	0G2
....Nuuugaaaucgggucuuguuau......................	1	1	0G2
....Uuuugaaaucgggucuuguuau......................	27	1	0G2
....Uuuugaaaucgggucuuguuauca....................	1	1	0G2
....uuugaaaucgggucuAguuauca....................	1	1	0G2
....uugaaaucgggucuuguuauca....................	7	0	0G2
....uugaaauUgggucuuguuauca....................	2	1	0G2
....ugauaaccagaAccgauuucaa..	2	1	0B2
....auaaccagacccgauuucaaa...	11	0	0B2
....aCaaccagacccgauuucaaag..	5	1	0B2
....Caaccagacccgauuuca...	3	1	0B2
....Caaccagacccgauuucaaa...	3	1	0B2
....Caaccagacccgauuucaaag..	26	1	0B2
....Caaccagacccgauuucaaagg...	98	1	0B2
....uaaccagacccgauuucaaaAg...	2	1	0B2
....uaGccagacccgauuucaaagg...	1	1	0B2
....uaaccagacccgauuucaaagga..	7	0	0B2
....aaccagacccgauuucaaag..	7	0	0B2
....aaccagacccgauuucaaagg...	32	0	0B2
....aaccGgacccgauuucaaagg...	5	1	0B2
....aaccagacGcgauuucaaagg...	1	1	0B2
....aaccagacccgaCuucaaaggau...	2	1	0B2
....uuuccuuugaaauUgggucuu...........................	3	1	0B2
....Uuuugaaaucgggucuuguu........................	6	1	0B2
....Uuuugaaaucgggucuuguua.......................	19	1	0B2
....Nuuugaaaucgggucuuguuau......................	1	1	0B2
....Uuuugaaaucgggucuuguuau......................	217	1	0B2
....cuuugaaaucgggucuuguuau......................	1	0	0B2
....Uuuugaaaucgggucuuguuauca....................	4	1	0B2
....uuugaaaucgggucuuguuau......................	8	0	0B2
....uuugaaaucgggucuuguuauA.....................	3	1	0B2
....uuugaaaucgggucuuguuaAc.....................	2	1	0B2
....uuugaaaucgggucuuguuauc.....................	10	0	0B2
....uuugaaaucgggucuuguuauUa....................	3	1	0B2
....uuugaaaucgggucuuguuauca....................	25	0	0B2

Provisional ID	:	scaffold14_4184
Score total	:	152334.1
Score for star read(s)	:	3.9
Score for read counts	:	152326.2
Score for mfe	:	-0.5
Score for randfold	:	1.6
Score for cons. seed	:	3
Total read count	:	298793
Mature read count	:	297196
Loop read count	:	0
Star read count	:	1597

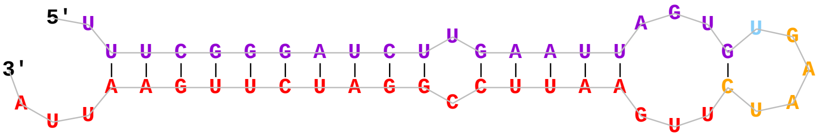

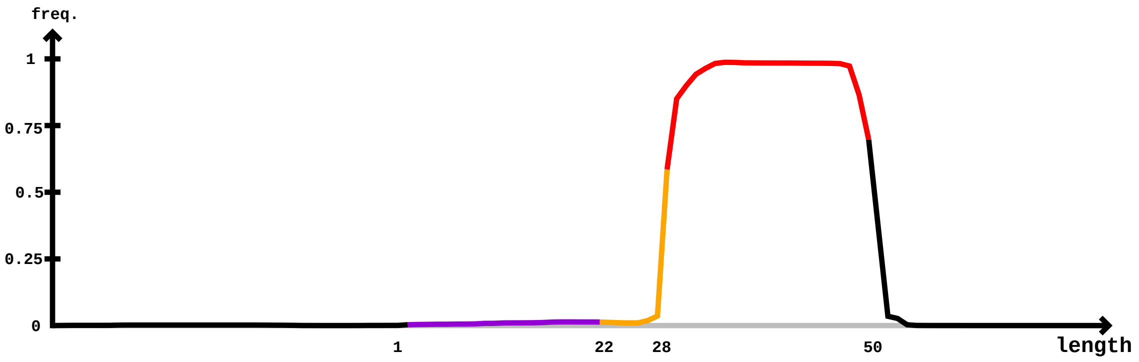


```
                                   Star                       Mature
5'- uugauuucuggaucuugaauuaguuugaaucuugauuucgggaucuugaauuagugugaaucuugaauuccggaucuugaauuaguaugaaucuugaauucuagaucu   -3'   obs
    uugauuucuggaucuugaauuaguuugaaucuugauuucgggaucuugaauuagugugaaucuugaauuccggaucuugaauuaguaugaaucuugaauucuagaucu         exp
    .........((((((.(((((.....((.((.((..((((((((((.(((((...(.....)...))))).))))))))))....)).)).))...))))).))))))   reads   mm   sample
    uugaGuucuggaucuugaau........................................................................................   17      1    0A2
    uugaGuucuggaucuugaauu.......................................................................................   20      1    0A2
    uugaGuucuggaucuugaauua......................................................................................   10      1    0A2
    .ugaGuucuggaucuugaau........................................................................................   1       1    0A2
    .ugaGuucuggaucuugaauua......................................................................................   6       1    0A2
    ....Cuucuggaucuugaauua......................................................................................   2       1    0A2
    ....uuucuggaucuAgaauuagu....................................................................................   1       1    0A2
    .....uucuggaucuugaauuag.....................................................................................   37      0    0A2
    .....uucuggaucuAgaauuagu....................................................................................   40      1    0A2
    .....uucuggaucuugaauuagGu...................................................................................   2       1    0A2
    .....uucCggaucuugaauuaguu...................................................................................   6       1    0A2
    .....uucCggaucuugaauuaguuu..................................................................................   3       1    0A2
    ......ucuggaucuAgaauuagu....................................................................................   8       1    0A2
    .............................ucuugauuucgggaucuugaau.........................................................   5       0    0A2
    ..............................cuugauuucgggaucuGgaauua........................................................   5       1    0A2
    ...............................uugauuucgggaucuugaauu........................................................   15      0    0A2
    ...............................uugauuucgggaucuugaauua.......................................................   9       0    0A2
    ...............................uugauuucgggaucuugaauuag......................................................   5       0    0A2
    ................................ugauuucgggaucuugaauu........................................................   5       0    0A2
    ................................ugauuucgggaucuugaauua.......................................................   9       0    0A2
    ..................................auuucggCaucuugaauuag......................................................   1       1    0A2
    ..................................auuucgggaucuGgaauuag......................................................   1       1    0A2
    ..................................auuucgggaucuugaauuag......................................................   9       0    0A2
    ..................................auuucgggaucuugaauuagug....................................................   4       0    0A2
    ...................................uuucgggaucuugaauua.......................................................   5       0    0A2
    ...................................uuucgggaucuugaauuagu.....................................................   11      0    0A2
    ...................................uuucgggaucuugGauuagug....................................................   1       1    0A2
    ...................................Nuucgggaucuugaauuagug....................................................   1       1    0A2
    ...................................uGucgggaucuugaauuagug....................................................   1       1    0A2
    ...................................uuucgggaucuGgaauuagug....................................................   22      1    0A2
    ...................................uuucgggaucuugaauuagug....................................................   141     0    0A2
    ...................................uuucgggaucuugaauuGgug....................................................   4       1    0A2
    ...................................uuucgggaucuugaauuagugug..................................................   22      0    0A2
```

```
                                   Star                        Mature
uugauuucuggaucuugaauuaguuugaaucuugauuucgggaucuugaauuagugugugaaucuugaauuccggaucuugaauuaguaugaaucuugaauucuagaucu
...................................Nuucgggaucuugaauuagugug....................................................  1   1   0A2
....................................uucgggaucuugaauuag........................................................  1   0   0A2
....................................uucgggaucuugaauuagG.......................................................  6   1   0A2
....................................uucgggaucuugaauuGgug......................................................  5   1   0A2
....................................uucgggaucuugaauuagug......................................................  33  0   0A2
....................................uucgggaucuugaauuagGg......................................................  3   1   0A2
....................................uucgggaucuGgaauuagug......................................................  11  1   0A2
....................................uucgggaucuugaauuagugug....................................................  8   0   0A2
....................................uucgggaucuugaauuaguguAa...................................................  5   1   0A2
.....................................ucgggaucuugaauuagug......................................................  10  0   0A2
.....................................ucgggaucuGgaauuagug......................................................  6   1   0A2
.....................................ucgggaucuugaauuagGg......................................................  1   1   0A2
.....................................ucgggaucuGgaauuagugug....................................................  1   1   0A2
.....................................ucgggaucuugaauuagugug....................................................  1   0   0A2
......................................cgggaucuugaauuagug......................................................  12  0   0A2
......................................cgggaucuugaauuagGgu.....................................................  1   1   0A2
......................................cgggaucuugaGuuagugug....................................................  1   1   0A2
......................................cgggaucuugaauuagugug....................................................  24  0   0A2
......................................cgggaucuugaauuagugugaa..................................................  10  0   0A2
......................................cgggauGuugaauuagugugaa..................................................  1   1   0A2
......................................cgggaucuugaauuagugugaaA.................................................  5   1   0A2
......................................cgggaucuugaauuagugugaauA................................................  7   1   0A2
......................................cgggaucuugaauuagugugaaucA...............................................  8   1   0A2
.......................................Nggaucuugaauuagugug....................................................  1   1   0A2
.......................................Nggaucuugaauuagugugaa..................................................  1   1   0A2
........................................ggaucuugaauuagugug....................................................  10  0   0A2
........................................ggaucuGgaauuagugug....................................................  6   1   0A2
........................................ggaucuugaauuaguguAa...................................................  3   1   0A2
........................................ggaucuGgaauuagugugaa..................................................  4   1   0A2
........................................ggaucuugaauuaguguAaa..................................................  3   1   0A2
........................................ggaucuugaauuagugugaa..................................................  10  0   0A2
........................................ggaucuugaauuagugugaaA.................................................  6   1   0A2
.........................................gaucuugaauuagugugaauc................................................  2   0   0A2
.........................................gaucuGgaauuagugugaauc................................................  6   1   0A2
.........................................gaucuGgaauuagugugaaucu...............................................  2   1   0A2
..........................................aucuGgaauuagugugaauc................................................  3   1   0A2
..........................................aucuGgaauuagugugaaucu...............................................  34  1   0A2
..........................................aucuugaauuagugugaaucu...............................................  2   0   0A2
...........................................ucuGgaauuagugugaauc................................................  7   1   0A2
...........................................ucuugaauuagugugaaucu...............................................  90  0   0A2
...........................................ucuGgaauuagugugaaucu...............................................  71  1   0A2
...........................................ucuugaauuagugugaaucuu..............................................  1   0   0A2
...........................................ucuGgaauuagugugaaucuu..............................................  9   1   0A2
............................................cuGgaauuagugugaaucu...............................................  12  1   0A2
............................................cuugaauuagugugaaucu...............................................  3   0   0A2
............................................cuGgaauuagugugaaucuu..............................................  6   1   0A2
.............................................uugaauuagugugaaucuu..............................................  7   0   0A2
.............................................uugaauuagugugaaucuug.............................................  29  0   0A2
.............................................Nugaauuagugugaaucuuga............................................  2   1   0A2
.............................................uugaauuagugugaaucuuga............................................  77  0   0A2
.............................................uNgaauuagugugaaucuuga............................................  1   1   0A2
.............................................uugaaCuagugugaaucuuga............................................  1   1   0A2
.............................................uugaauCagugugaaucuuga............................................  1   1   0A2
.............................................uugaauuagugugaaucuugaa...........................................  12  0   0A2
.............................................uugaauuagugugaaucuugaau..........................................  5   0   0A2
.............................................uugaauuagugugaaucuugaauA.........................................  5   1   0A2
..............................................ugaauuagugugaaucuuga............................................  1   0   0A2
..............................................Cgaauuagugugaaucuugaa...........................................  1   1   0A2
..............................................ugaauuagugugaaucuugaa...........................................  1   0   0A2
..............................................ugaauuagugugaaucuugaauuAcg......................................  9   1   0A2
...............................................gaauuagugugaaucuug.............................................  2   0   0A2
...............................................Caauuagugugaaucuugaa...........................................  1   1   0A2
...............................................gaauuagugugaaucuugaa...........................................  13  0   0A2
................................................aauuagugugaaucuugaau..........................................  4   0   0A2
................................................aauuagugugaaucuugaauA.........................................  30  1   0A2
................................................aGuuagugugaaucuugaauu.........................................  2   1   0A2
................................................aauuagugugaaucuugaauu.........................................  9   0   0A2
................................................aauuagugugaaucuugaauuc........................................  1   0   0A2
.................................................auuagugugaaucuugaauu.........................................  28  0   0A2
.................................................auuagugugaaucuugaauA.........................................  3   1   0A2
```

Star **Mature**

```
uugauuucuggaucuugaauuaguuugaaucuugauuucgggaucuugaauuagugugaaucuugaauuccggaucuugaauuaguaugaaucuugaauucuagaucu
.................................................auuagugugaaucuugaauuc......................................  24  0  0A2
.................................................Cuuagugugaaucuugaauuc......................................  1  1  0A2
..................................................uuagugugaaucuugaauu.......................................  14  0  0A2
..................................................uuagugugaaucuugaauuc......................................  66  0  0A2
..................................................uuagugugaaucuugaauucc.....................................  1  0  0A2
..................................................uuagugugaGucuugaauuccgga..................................  3  1  0A2
..................................................uuagugugaaucuugaauuUcggauc................................  5  1  0A2
..................................................uuagugugaaucuugaaCuccggaucu...............................  3  1  0A2
..................................................uuagugugaaucuugaauuUcggaucu...............................  4  1  0A2
..................................................uuagugugaaucuugaauuccggaucA...............................  4  1  0A2
...................................................uagugugaaucuugaauu.......................................  2  0  0A2
...................................................uagugugaaucuugaauuc......................................  12  0  0A2
...................................................uagugugaGucuugaauucc.....................................  2  1  0A2
...................................................uaguguUaaucuugaauucc.....................................  1  1  0A2
...................................................uagugugaaucuugaauucc.....................................  21  0  0A2
...................................................uaguguCaaucuugaauuccg....................................  4  1  0A2
...................................................uagugugaaucuugaaCuccg....................................  5  1  0A2
...................................................uagugugaaucuugaauuUcgg...................................  1  1  0A2
...................................................uagugugaaucuugaauuccggauc................................  3  0  0A2
...................................................uagugugaaucuugaauuccggaucuA..............................  1  1  0A2
....................................................agugugaaucuugaauuc......................................  1  0  0A2
....................................................agugugaaucuugaauucc.....................................  1  0  0A2
....................................................aguguCaaucuugaauucc.....................................  1  1  0A2
....................................................agugugaGucuugaauuccgga..................................  2  1  0A2
......................................................ugugaaucuugaaCuccgga..................................  2  1  0A2
......................................................ugugUaucuugaauuccgga..................................  2  1  0A2
........................................................ugaaucuugaauuccggau.................................  3  0  0A2
........................................................ugaaucuugaauuccggauA................................  12  1  0A2
........................................................ugaaucuugaauuccggauc................................  3  0  0A2
........................................................ugaGucuugaauuccggaucu...............................  2  1  0A2
........................................................ugaCucuugaauuccggaucuug.............................  1  1  0A2
........................................................ugaaucuugaauuccggaucuAgaa...........................  11  1  0A2
........................................................ugaaucuugaauuccggaucuugaau..........................  3  0  0A2
........................................................ugaaucuugaauuccggaucuAgaau..........................  3  1  0A2
........................................................ugaaucuugaauuccggaucuAgaauu.........................  1  1  0A2
........................................................ugaaucuugaauuccCgaucuugaauu.........................  1  1  0A2
........................................................ugaaucuugaauuccggaucuugaauu.........................  19  0  0A2
..........................................................aaucuugaauuccggaucuA..............................  1  1  0A2
..........................................................aaucuugaauuccggaucuAg.............................  2  1  0A2
..........................................................aaucuugaauuccggaucuug.............................  11  0  0A2
..........................................................aaucuugaauuccggaucuAgaauua........................  3  1  0A2
..........................................................aaucuugaauuccggaucuAgaauuag.......................  1  1  0A2
...........................................................aucuugaauuccggaucuu..............................  6  0  0A2
...........................................................aucuugaauuccggaucuug.............................  3  0  0A2
...........................................................aucGugaauuccggaucuug.............................  3  1  0A2
...........................................................aucuugaauuccggaucuugaauAa........................  2  1  0A2
...........................................................aucuugaauuccggaucuugUauua........................  3  1  0A2
...........................................................aucuugaauuccggaucuAgaauuagu......................  2  1  0A2
............................................................ucuugaauuccggaucuA..............................  1  1  0A2
............................................................ucuugaauuccggaucuug.............................  6  0  0A2
............................................................ucuugaauuccggaucuuga............................  7  0  0A2
............................................................ucuugaauuccggaucuAga............................  40  1  0A2
............................................................ucGugaauuccggaucuugaa...........................  26  1  0A2
............................................................Ccuugaauuccggaucuugaa...........................  5  1  0A2
............................................................ucuugaauuccggaucuCgaa...........................  1  1  0A2
............................................................ucuugaauuccggaucuAgaa...........................  55  1  0A2
............................................................ucuugaauuccggaucuugaa...........................  22  0  0A2
............................................................ucuugaauuccggaucuAgaau..........................  12  1  0A2
............................................................ucuugaauuccggaucuugaau..........................  184  0  0A2
............................................................ucGugaauuccggaucuugaau..........................  34  1  0A2
............................................................Ccuugaauuccggaucuugaau..........................  1  1  0A2
............................................................uUuugaauuccggaucuugaauu.........................  1  1  0A2
............................................................ucuugaauuccggaucuugaauu.........................  23  0  0A2
............................................................ucGugaauuccggaucuugaauu.........................  3  1  0A2
............................................................uGuugaauuccggaucuugaauu.........................  7  1  0A2
............................................................ucuugaauuccggaucuAgaauua........................  22  1  0A2
............................................................ucuugaauuccggaucuugaauAa........................  232  1  0A2
............................................................ucuugaauuccggaucuugaauua........................  113  0  0A2
............................................................ucuugaauuccggaucuugaaCua........................  1  1  0A2
............................................................uUuugaauuccggaucuugaauua........................  3  1  0A2
```

Star **Mature**

```
uugauuucuggaucuugaauuaguuugaaucuugauuucgggaucuugaauuagugugaaucuugaauuccggaucuugaauuaguaugaaucuugaauucuagaucu
............................................................ucuugaauuccggaucuCgaauua........................ 1 1 0A2
............................................................uGuugaauuccggaucuugaauua........................ 2 1 0A2
............................................................ucuugaGuuccggaucuugaauua........................ 1 1 0A2
............................................................ucuugaauuccggaucuugaauuag....................... 24 0 0A2
............................................................ucuugaauuccggaucuAgaauuag....................... 19 1 0A2
............................................................ucuugaauuccggaucuAgaauuagu...................... 121 1 0A2
............................................................ucuugaauuccggaucuugaauuaUua..................... 10 1 0A2
............................................................ucuugaauuccggaucuugaauuagua..................... 11 0 0A2
.............................................................cuugaauuccggaucuAga............................ 17 1 0A2
.............................................................cuugaauuccggaucuAgaa........................... 291 1 0A2
.............................................................Nuugaauuccggaucuugaa........................... 1 1 0A2
.............................................................cGugaauuccggaucuugaa........................... 2 1 0A2
.............................................................cuugaauuccggaucuugaa........................... 95 0 0A2
.............................................................cuugaGuuccggaucuugaa........................... 1 1 0A2
.............................................................cuugaauuccggaucuugaau.......................... 152 0 0A2
.............................................................cuugaauuccggaucuAgaau.......................... 42 1 0A2
.............................................................cGugaauuccggaucuugaau.......................... 29 1 0A2
.............................................................cuugaaGuccggaucuugaauu......................... 1 1 0A2
.............................................................cuugaauuccggaucuAgaauu......................... 57 1 0A2
.............................................................cGugaauuccggaucuugaauu......................... 6 1 0A2
.............................................................cuugaauuccggaucuugaauu......................... 229 0 0A2
.............................................................cuugaauuccggaucuugaauAa........................ 134 1 0A2
.............................................................cuugaGuuccggaucuugaauua........................ 1 1 0A2
.............................................................Nuugaauuccggaucuugaauua........................ 1 1 0A2
.............................................................cuugaauuccggaGcuugaauua........................ 1 1 0A2
.............................................................cuugaauuccggaucuugaauCa........................ 24 1 0A2
.............................................................cuugaauuccggaucuugGauua........................ 3 1 0A2
.............................................................cuugaaGuccggaucuugaauua........................ 2 1 0A2
.............................................................cGugaauuccggaucuugaauua........................ 5 1 0A2
.............................................................cuugaauuccggCucuugaauua........................ 1 1 0A2
.............................................................cuugaauuccggaucuAgaauua........................ 122 1 0A2
.............................................................cuugaauuccggaucuugaauua........................ 580 0 0A2
.............................................................cuugaauuccggaucuugaaCuag....................... 2 1 0A2
.............................................................cuugaauuccggaucuAgaauuag....................... 1 1 0A2
.............................................................cuugaauuccggaucuugaauuag....................... 6 0 0A2
.............................................................cuugaauuccggaucuAgaauuagu...................... 3 1 0A2
.............................................................cuugaauuccggaucuugaauuaAua..................... 3 1 0A2
.............................................................cuugaauuccggaucuugaauuaUua..................... 7 1 0A2
.............................................................cuugaauuccggaucuAgaauuaguau.................... 1 1 0A2
..............................................................uugaauuccggaucuAga............................ 6 1 0A2
..............................................................uugaauuccggaucuAgaa........................... 192 1 0A2
..............................................................uugaauuccggaucuugaa........................... 49 0 0A2
..............................................................Gugaauuccggaucuugaau.......................... 18 1 0A2
..............................................................uugaauuccggauAuugaau.......................... 2 1 0A2
..............................................................uugaGuuccggaucuugaau.......................... 81 1 0A2
..............................................................Nugaauuccggaucuugaau.......................... 15 1 0A2
..............................................................uugaauuccggaucuCgaau.......................... 2 1 0A2
..............................................................uugaauuccggaucuugaau.......................... 7322 0 0A2
..............................................................uugaauuccggaucuugaaC.......................... 2 1 0A2
..............................................................uugaauuccggaucuGgaau.......................... 4 1 0A2
..............................................................uugaauuccggaucuugaaG.......................... 1 1 0A2
..............................................................uugaauuAcggaucuugaau.......................... 3 1 0A2
..............................................................uugaauuccggaucuAgaau.......................... 1577 1 0A2
..............................................................Augaauuccggaucuugaau.......................... 2 1 0A2
..............................................................Cugaauuccggaucuugaau.......................... 3 1 0A2
..............................................................uugaauuccggaucuugUau.......................... 1 1 0A2
..............................................................uugaaGuccggaucuugaau.......................... 8 1 0A2
..............................................................uNgaauuccggaucuugaau.......................... 3 1 0A2
..............................................................uugaauAccggaucuugaau.......................... 5 1 0A2
..............................................................uugaauuccggaucCugaau.......................... 3 1 0A2
..............................................................uugaauuccggaCcuugaau.......................... 4 1 0A2
..............................................................uugaauuccggaGcuugaau.......................... 7 1 0A2
..............................................................uugaauuccggGucuugaau.......................... 13 1 0A2
..............................................................uugaauuccggCucuugaau.......................... 1 1 0A2
..............................................................uugaauuccggaucAugaau.......................... 1 1 0A2
..............................................................uugaauuccggaucuugaauu......................... 9162 0 0A2
..............................................................uugaauuccggaCcuugaauu......................... 4 1 0A2
..............................................................uugaGuuccggaucuugaauu......................... 155 1 0A2
..............................................................Augaauuccggaucuugaauu......................... 1 1 0A2
..............................................................uugaauuccggCucuugaauu......................... 3 1 0A2
```

Star Mature

```
uugauuucuggaucuugaauuaguuugaaucuugauuucgggaucuugaauuagugugaaucuugaauuccggaucuugaauuaguaugaaucuugaauucuagaucu
..............................................................uugaauuccggauGuugaauu.........................   1       1   0A2
..............................................................uugaauuccggaucuAgaauu.........................   2314    1   0A2
..............................................................uugaauuccggaucuugaaGu.........................   1       1   0A2
..............................................................Gugaauuccggaucuugaauu.........................   7       1   0A2
..............................................................Cugaauuccggaucuugaauu.........................   1       1   0A2
..............................................................uugaauuccggaucuGgaauu.........................   3       1   0A2
..............................................................uugaauuccggaucCugaauu.........................   2       1   0A2
..............................................................uugaauAccggaucuugaauu.........................   7       1   0A2
..............................................................uugaauuccggaucuugaaCu.........................   5       1   0A2
..............................................................uugaaGuccggaucuugaauu.........................   1       1   0A2
..............................................................uugaauuccggaucuugGauu.........................   3       1   0A2
..............................................................uugaauuAcggaucuugaauu.........................   5       1   0A2
..............................................................uugaauuccggGucuugaauu.........................   3       1   0A2
..............................................................uNgaauuccggaucuugaauu.........................   1       1   0A2
..............................................................Nugaauuccggaucuugaauu.........................   28      1   0A2
..............................................................uGgaauuccggaucuugaauu.........................   5       1   0A2
..............................................................uugaauuccggaucAugaauu.........................   3       1   0A2
..............................................................uugaauuccggaucuCgaauu.........................   23      1   0A2
..............................................................uugaauuccggauAuugaauu.........................   1       1   0A2
..............................................................uuNaauuccggaucuugaauu.........................   1       1   0A2
..............................................................uugaauuccggaucuuUaauu.........................   7       1   0A2
..............................................................uugaauuccggaGcuugaauu.........................   9       1   0A2
..............................................................uugaauuAcggaucuugaauua........................   20      1   0A2
..............................................................uugaauuccggaucuugGauua........................   20      1   0A2
..............................................................Augaauuccggaucuugaauua........................   9       1   0A2
..............................................................uugaauuccggaucuGgaauua........................   4       1   0A2
..............................................................uuNaauuccggaucuugaauua........................   2       1   0A2
..............................................................uugaauuccggaucuugCauua........................   1       1   0A2
..............................................................uugaaGuccggaucuugaauua........................   9       1   0A2
..............................................................Cugaauuccggaucuugaauua........................   16      1   0A2
..............................................................uAgaauuccggaucuugaauua........................   4       1   0A2
..............................................................uugaauuccgCaucuugaauua........................   1       1   0A2
..............................................................uugaauuccggaucuugUauua........................   1       1   0A2
..............................................................uugaGuuccggaucuugaauua........................   625     1   0A2
..............................................................uugaauuGcggaucuugaauua........................   3       1   0A2
..............................................................uugaauuccggCucuugaauua........................   6       1   0A2
..............................................................uugaauuccggaucuugaaCua........................   47      1   0A2
..............................................................Nugaauuccggaucuugaauua........................   49      1   0A2
..............................................................uNgaauuccggaucuugaauua........................   5       1   0A2
..............................................................uugaauuccggauGuugaauua........................   2       1   0A2
..............................................................uugaauuccggaucAugaauua........................   3       1   0A2
..............................................................uugaauuccggaGcuugaauua........................   19      1   0A2
..............................................................uugaauAccggaucuugaauua........................   10      1   0A2
..............................................................Gugaauuccggaucuugaauua........................   37      1   0A2
..............................................................uugaauuccggaucuugaauua........................   23495   0   0A2
..............................................................uugaauuccggaucuuUaauua........................   15      1   0A2
..............................................................uugaauuccggauAuugaauua........................   11      1   0A2
..............................................................uugaauuccggaucuugaauCa........................   1979    1   0A2
..............................................................uugaauuccCgaucuugaauua........................   1       1   0A2
..............................................................uugaauuccggaucuugaaGua........................   4       1   0A2
..............................................................uugaauuccggaucuCgaauua........................   11      1   0A2
..............................................................uugaauuccggGucuugaauua........................   5       1   0A2
..............................................................uugaauuccggaucuugaauAa........................   3722    1   0A2
..............................................................uGgaauuccggaucuugaauua........................   7       1   0A2
..............................................................uugaauuccggaucCugaauua........................   4       1   0A2
..............................................................uugaauuccggaCcuugaauua........................   7       1   0A2
..............................................................uugaauuccggaucuAgaauua........................   5193    1   0A2
..............................................................uugaauuccggaucuugaaCuag.......................   20      1   0A2
..............................................................uugaauuccggaucuugaauAag.......................   38      1   0A2
..............................................................uugaauuccggaucuuUaauuag.......................   2       1   0A2
..............................................................uugaauuccggaucuugaauuag.......................   284     0   0A2
..............................................................uugaauuccggaucuugaaGuag.......................   2       1   0A2
..............................................................uugaauuccggaucuGgaauuag.......................   1       1   0A2
..............................................................uugaGuuccggaucuugaauuag.......................   13      1   0A2
..............................................................uugaauuccggaucuugaauCag.......................   4       1   0A2
..............................................................uugaauuccggaucuAgaauuag.......................   1270    1   0A2
..............................................................uugaauuccggaucuugaauuagA......................   7       1   0A2
..............................................................uugaauuccggaucuugaauuaCu......................   6       1   0A2
..............................................................uugaauuccggaucuAgaauuagu......................   51      1   0A2
..............................................................uugaauuccggaucuugaauAagu......................   4       1   0A2
```

Star Mature

uugauuucuggaucuugaauuaguuugaaucuugauuucgggaucuugaauuagugugaaucuugaauuccggaucuugaauuaguaugaaucuugaauucuagaucu			
....uugaauuccggaucuAgaauuagua.....................	5	1	0A2
....uugaauuccggaucuugaauuaUua.....................	112	1	0A2
....uugaauuccggaucuugaauuaAua.....................	13	1	0A2
....uugaauuccggaucuugaauuagua.....................	4	0	0A2
....uugaauuccggaucuugaauuaguaA....................	3	1	0A2
....uugaauuccggaucuugaauuaUuau....................	2	1	0A2
....uugaauuccggaucuugaauuaAuau....................	3	1	0A2
....uugaauuccggaucuAgaauuaguau....................	4	1	0A2
....ugaauuccggaucuAgaa...........................	49	1	0A2
....ugaauuccggaucuugaaG..........................	1	1	0A2
....ugaauuccggGucuugaau..........................	3	1	0A2
....Agaauuccggaucuugaau..........................	1	1	0A2
....ugaGuuccggaucuugaau..........................	6	1	0A2
....ugaauuccggaucuAgaau..........................	86	1	0A2
....ugaauuccggaGcuugaau..........................	1	1	0A2
....ugaauuccggaucuugaau..........................	317	0	0A2
....ugaauuccggaucuugaaCu.........................	7	1	0A2
....ugaauuccggaucuugaaGu.........................	1	1	0A2
....uNaauuccggaucuugaauu.........................	2	1	0A2
....ugaauuccgCaucuugaauu.........................	1	1	0A2
....ugaauuccggaucuugGauu.........................	1	1	0A2
....Ggaauuccggaucuugaauu.........................	2	1	0A2
....ugaaGuccggaucuugaauu.........................	4	1	0A2
....ugaauuccggaucCugaauu.........................	1	1	0A2
....ugaauuccggaGcuugaauu.........................	3	1	0A2
....ugaauuccggaucuCgaauu.........................	3	1	0A2
....ugaauuccggGucuugaauu.........................	2	1	0A2
....ugaauuAcggaucuugaauu.........................	2	1	0A2
....ugaauuccggaucuAgaauu.........................	751	1	0A2
....ugaauuccggaucuugaauu.........................	2250	0	0A2
....ugaGuuccggaucuugaauu.........................	43	1	0A2
....Ngaauuccggaucuugaauu.........................	3	1	0A2
....ugaauuccggaucuuUaauu.........................	2	1	0A2
....ugaCuuccggaucuugaauu.........................	1	1	0A2
....ugaauuccggaCcuugaauu.........................	1	1	0A2
....ugaauuccggaucuugaaCua........................	20	1	0A2
....Ggaauuccggaucuugaauua........................	4	1	0A2
....ugaGuuccggaucuugaauua........................	256	1	0A2
....ugaauuccggaucCugaauua........................	3	1	0A2
....Ngaauuccggaucuugaauua........................	26	1	0A2
....ugaauuccggCucuugaauua........................	8	1	0A2
....ugaauuccggaucuugaauua........................	13165	0	0A2
....ugaauuccggaucuugaaGua........................	2	1	0A2
....ugaauuGcggaucuugaauua........................	11	1	0A2
....ugaauuccggaGcuugaauua........................	8	1	0A2
....ugaauuccggaucuGgaauua........................	2	1	0A2
....ugaauuccggaucuugCauua........................	2	1	0A2
....ugaauuccggaCcuugaauua........................	6	1	0A2
....ugaauuccggaucuCgaauua........................	6	1	0A2
....uNaauuccggaucuugaauua........................	3	1	0A2
....ugaauuccggaucuugGauua........................	10	1	0A2
....ugaaGuccggaucuugaauua........................	21	1	0A2
....ugaauuccggauAuugaauua........................	6	1	0A2
....ugaauuAcggaucuugaauua........................	11	1	0A2
....ugaauuccggaucuugaauCa........................	317	1	0A2
....ugaauuccggaucuAgaauua........................	4768	1	0A2
....ugaauuccggGucuugaauua........................	10	1	0A2
....Agaauuccggaucuugaauua........................	6	1	0A2
....ugaauuccggaucuugaauAa........................	271	1	0A2
....ugaauuccggaucuuUaauua........................	11	1	0A2
....ugaauuccggaucuugaaGuag.......................	7	1	0A2
....ugaauuccggGucuugaauuag.......................	1	1	0A2
....ugaauuccggaucuugaauCag.......................	12	1	0A2
....ugaaGuccggaucuugaauuag.......................	2	1	0A2
....ugaauuccggaucCugaauuag.......................	2	1	0A2
....ugaauuccggaucuugaauuag.......................	947	0	0A2
....ugaauuccggaucuAgaauuag.......................	3791	1	0A2
....ugaGuuccggaucuugaauuag.......................	18	1	0A2
....ugaauuccggaucuugaauAag.......................	10	1	0A2
....Agaauuccggaucuugaauuag.......................	1	1	0A2

```
                                   Star                Mature
uugauuucuggaucuugaauuaguuugaaucuugauuucgggaucuugaauuagugugaaucuugaauuccggaucuugaauuaguaugaaucuugaauucuagaucu
...............................................................ugaauuccggaucuugaauuagu......................	2	0	0A2
...............................................................ugaauuccggaucuugaauuagC......................	4	1	0A2
...............................................................ugaauuccggaucuAgaauuagu......................	196	1	0A2
...............................................................ugaauuccggaucuugaauuaUua.....................	46	1	0A2
...............................................................Ngaauuccggaucuugaauuagua.....................	1	1	0A2
...............................................................ugaauuccggaucuAgaauuagua.....................	82	1	0A2
...............................................................ugaauuccggaucuugaauuaAua.....................	8	1	0A2
...............................................................ugaauuccggaucuugaauuaguaA....................	2	1	0A2
...............................................................ugaauuccggaucuAgaauuaguau....................	23	1	0A2
................................................................gaauuccggaucuugaau..........................	21	0	0A2
................................................................gaauuccggaucuAgaau..........................	7	1	0A2
................................................................gaauuccggGucuugaauu.........................	1	1	0A2
................................................................gaauuccggaucuugaauu.........................	107	0	0A2
................................................................gaauuccggaucuAgaauu.........................	46	1	0A2
................................................................gaGuuccggaucuugaauu.........................	2	1	0A2
................................................................gaauuccggaucuuUaauua........................	1	1	0A2
................................................................gaaGuccggaucuugaauua........................	1	1	0A2
................................................................gaauuccggaucuugaauua........................	835	0	0A2
................................................................gaauuccggaCcuugaauua........................	3	1	0A2
................................................................gaauuccggaucuugaauAa........................	3	1	0A2
................................................................gaauuccggaucuAgaauua........................	463	1	0A2
................................................................gaGuuccggaucuugaauua........................	23	1	0A2
................................................................gaauuccggaucuugaaCua........................	2	1	0A2
................................................................Naauuccggaucuugaauua........................	2	1	0A2
................................................................gaauuccggaucuugaaGua........................	1	1	0A2
................................................................gaauuccggaucuugaauCa........................	84	1	0A2
................................................................gaauuccggaucuugGauua........................	1	1	0A2
................................................................gaauuccggaucuAgaauuag.......................	2687	1	0A2
................................................................gaauuccggaCcuugaauuag.......................	1	1	0A2
................................................................gaauuccggaucuugaauuag.......................	543	0	0A2
................................................................gaauuccggauAuugaauuag.......................	1	1	0A2
................................................................Naauuccggaucuugaauuag.......................	2	1	0A2
................................................................gaauuccggGucuugaauuag.......................	1	1	0A2
................................................................gaauuccggaucuugaauuagu......................	16	0	0A2
................................................................gaauuccggaucuAgaauuagu......................	94	1	0A2
................................................................gaauuccggaucuAgaauuagua.....................	5	1	0A2
................................................................gaauuccggaucuAgaauuaguau....................	4	1	0A2
.................................................................aauuccggaucuugaauu.........................	10	0	0A2
.................................................................aauuccggaucuugaauAa........................	16	1	0A2
.................................................................aauuccggaucuugaauCa........................	17	1	0A2
.................................................................aauuccggCucuugaauua........................	1	1	0A2
.................................................................aauuccggaucuugaauua........................	526	0	0A2
.................................................................aauuAcggaucuugaauua........................	1	1	0A2
.................................................................Nauuccggaucuugaauua........................	1	1	0A2
.................................................................aGuuccggaucuugaauua........................	7	1	0A2
.................................................................aauuGcggaucuugaauua........................	1	1	0A2
.................................................................aNuuccggaucuugaauuag.......................	1	1	0A2
.................................................................aauuccggaucuugaaCuag.......................	1	1	0A2
.................................................................aauuccggaGcuugaauuag.......................	1	1	0A2
.................................................................aauuccggaCcuugaauuag.......................	1	1	0A2
.................................................................aauuccCgaucuugaauuag.......................	4	1	0A2
.................................................................aauuccggaucuGgaauuag.......................	2	1	0A2
.................................................................aauuccggaucuCgaauuag.......................	3	1	0A2
.................................................................aauuccggCucuugaauuag.......................	2	1	0A2
.................................................................aauuccggaucuugaaGuag.......................	1	1	0A2
.................................................................aauuccggaucuugaauuag.......................	3749	0	0A2
.................................................................aauuccggaucuugaauAag.......................	1	1	0A2
.................................................................aCuuccggaucuugaauuag.......................	2	1	0A2
.................................................................aGuuccggaucuugaauuag.......................	69	1	0A2
.................................................................aauuAcggaucuugaauuag.......................	1	1	0A2
.................................................................aauuccggaucAugaauuag.......................	6	1	0A2
.................................................................aauuccggGucuugaauuag.......................	1	1	0A2
.................................................................aauuccggaucuuUaauuag.......................	6	1	0A2
.................................................................Nauuccggaucuugaauuag.......................	12	1	0A2
.................................................................aauuccggaucuugGauuag.......................	6	1	0A2
.................................................................aauuccggaucuugaauCag.......................	15	1	0A2
.................................................................aauuccggaucuugCauuag.......................	1	1	0A2
.................................................................aauuccggaucuugaauuagA......................	19	1	0A2
.................................................................aauuccggaucuugaauuagu......................	13	0	0A2
.................................................................aauuccggaucuugaauuaguU.....................	1	1	0A2
```

Star **Mature**

```
uugauuucuggaucuugaauuaguuugaaucuugauuucgggaucuugaauuagugugaaucuugaauuccggaucuugaauuaguaugaaucuugaauucuagaucu
.................................................................aauuccggaucuugaauuagua.....................   12     0   0A2
.................................................................aauuccggaucuugaauuaAua.....................   1      1   0A2
.................................................................aauuccggaucuugaauuagAa.....................   3      1   0A2
.................................................................aauuccggaucuAgaauuagua.....................   65     1   0A2
.................................................................aauuccggaucuugaauuaUua.....................   18     1   0A2
.................................................................aauuccggaucuugaauuaguaA....................   8      1   0A2
..................................................................auuccggaucuugaauua........................   183    0   0A2
..................................................................Guuccggaucuugaauua........................   4      1   0A2
..................................................................auuccggaucuugaauCa........................   10     1   0A2
..................................................................Nuuccggaucuugaauua........................   2      1   0A2
..................................................................auuccggaucuugaauAa........................   4      1   0A2
..................................................................auuccggaucuugaaCua........................   2      1   0A2
..................................................................auuccggaucuugaauCag.......................   9      1   0A2
..................................................................auuccggaucuuUaauuag.......................   3      1   0A2
..................................................................auuccgCaucuugaauuag.......................   1      1   0A2
..................................................................Guuccggaucuugaauuag.......................   12     1   0A2
..................................................................auuccggaucuugaauAag.......................   3      1   0A2
..................................................................Nuuccggaucuugaauuag.......................   4      1   0A2
..................................................................auuccggaucAugaauuag.......................   12     1   0A2
..................................................................auuccggaucuCgaauuag.......................   2      1   0A2
..................................................................auuccggaGcuugaauuag.......................   2      1   0A2
..................................................................auuccggaucuGgaauuag.......................   2      1   0A2
..................................................................auuccggaucuugaauuag.......................   1042   0   0A2
..................................................................Cuuccggaucuugaauuag.......................   5      1   0A2
..................................................................auuccggGucuugaauuag.......................   2      1   0A2
..................................................................auuccggCucuugaauuag.......................   2      1   0A2
..................................................................auuccggaucuugaauuUgu......................   1      1   0A2
..................................................................auuAcggaucuugaauuagu......................   1      1   0A2
..................................................................auuUcggaucuugaauuagu......................   1      1   0A2
..................................................................auuccggaucuugaauuagu......................   186    0   0A2
..................................................................auuccggaucuugaauuagC......................   4      1   0A2
..................................................................auuccggaucuGgaauuagu......................   3      1   0A2
..................................................................auuccggaucuugaauuagA......................   12     1   0A2
..................................................................auuccggaucAugaauuagua.....................   3      1   0A2
..................................................................auuccggaucuugaauuaUua.....................   43     1   0A2
..................................................................auuccggUucuugaauuagua.....................   1      1   0A2
..................................................................auuccggaucuugaauuagua.....................   93     0   0A2
..................................................................auuccggaGcuugaauuagua.....................   1      1   0A2
..................................................................auuccggaucuAgaauuagua.....................   868    1   0A2
..................................................................auuccggaucuugaauuaAua.....................   3      1   0A2
..................................................................auuccggaucuugaauuaguU.....................   6      1   0A2
..................................................................auuccggaucuAgaauuaguau....................   24     1   0A2
..................................................................auuccggaucuugaauuaguaA....................   93     1   0A2
..................................................................auuccggaucuugaauuaguauUa..................   9      1   0A2
...................................................................uuccggaucuCgaauuag.......................   1      1   0A2
...................................................................Nuccggaucuugaauuag.......................   2      1   0A2
...................................................................uuccggaucuugaauuag.......................   979    0   0A2
...................................................................uuccggaCcuugaauuag.......................   1      1   0A2
...................................................................uuccggaucuugaauCag.......................   29     1   0A2
...................................................................uuccggGucuugaauuag.......................   1      1   0A2
...................................................................uuccggCucuugaauuag.......................   2      1   0A2
...................................................................uuccggaucuugGauuag.......................   3      1   0A2
...................................................................uuccggaucAugaauuag.......................   3      1   0A2
...................................................................uuccggauAuugaauuag.......................   1      1   0A2
...................................................................uuccggaucuugaauuagu......................   81     0   0A2
...................................................................uuccggaucuGgaauuagu......................   40     1   0A2
...................................................................uuccggaucuugaauuagA......................   11     1   0A2
...................................................................uuccggaucuugaauuaguU.....................   6      1   0A2
...................................................................uuccggUucuugaauuagua.....................   4      1   0A2
...................................................................uuccggaucuugGauuagua.....................   4      1   0A2
...................................................................uuccggaucuGgaauuagua.....................   9      1   0A2
...................................................................uuccggaucuugaauuaUua.....................   15     1   0A2
...................................................................uuccggaucuugaauAagua.....................   1      1   0A2
...................................................................uuccggaucuAgaauuagua.....................   958    1   0A2
...................................................................uuccggaucuugaauuaAua.....................   1      1   0A2
...................................................................uuccggaucuugaauuagAa.....................   2      1   0A2
...................................................................uuccggaucuugaauuagua.....................   61     0   0A2
...................................................................uuccggaucuugaauuaguCu....................   3      1   0A2
...................................................................uuccggaucuugaauuaguau....................   3      0   0A2
...................................................................uuccggaucuugaauuaguUu....................   3      1   0A2
```

Star Mature

uugauuucuggaucuugaauuaguuugaaucuugauuucgggaucuugaauuagugugaaucuugaauuccggaucuugaauuaguaugaaucuugaauucuagaucu			
....uuccggaucuAgaauuaguau....................	47	1	0A2
....uuccggaucuugaauuaAuau....................	2	1	0A2
....uuccggaucuugaauuaguaA....................	51	1	0A2
....uuccggaucuugaauuaguaAg...................	14	1	0A2
....uuccggaucuAgaauuaguaug...................	3	1	0A2
....uuccggaucuugaauuaguauA...................	2	1	0A2
....uuccggaucuAgaauuaguauga..................	5	1	0A2
....uuccggaucuAgaauuaguaugaaucu..............	2	1	0A2
....uccggaucuGgaauuagu......................	11	1	0A2
....uccggaucuugaauuagA......................	1	1	0A2
....uccggaucuugaauuagu......................	9	0	0A2
....uccggaucuugaauuagAa.....................	6	1	0A2
....uccggaucuugaauuagua.....................	11	0	0A2
....uccggaucuAgaauuagua.....................	427	1	0A2
....uccggaucuGgaauuagua.....................	5	1	0A2
....uccggaucuugaauuaguaA....................	28	1	0A2
....uccggaucuugaauuaguau....................	1	0	0A2
....uccggaucuAgaauuaguau....................	21	1	0A2
....uccggaucuugaauuaguaAg...................	1	1	0A2
....uccggaucuAgaauuaguaugaaucuu.............	2	1	0A2
....ccggaucuAgaauuagua.....................	37	1	0A2
....ccggaucuugaauuagua.....................	3	0	0A2
....ccggaucuugaauuaguaA....................	22	1	0A2
....ccggaucuAgaauuaguaug...................	1	1	0A2
....ccggaucuugaauuaguaug...................	1	0	0A2
....cggaucuAgaauuaguau....................	7	1	0A2
....cggaucuugaauuaguaA....................	6	1	0A2
....cggaucuAgaauuaguaug...................	1	1	0A2
....cggaucuugaauuaguauUa..................	5	1	0A2
....ucuAgaauuaguaugaaucu..............	3	1	0A2
uugaGuucuggaucuugaau..	24	1	0G2
uugaGuucuggaucuugaauu...	43	1	0G2
uugaGuucuggaucuugaauua..	41	1	0G2
.ugaGuucuggaucuugaauu...	8	1	0G2
..gaGuucuggaucuugaauua..	3	1	0G2
....Guucuggaucuugaauuag...	13	1	0G2
.....uucuggaucuugaauuag...	24	0	0G2
.....uucuggaucuugaauuagA..	2	1	0G2
.....uucuggaucuAgaauuagu..	72	1	0G2
.....uucuggaucuugaauuagu..	2	0	0G2
.....uucuggaucuGgaauuagu..	2	1	0G2
.....uucuggaucuugaauuaUuu...	4	1	0G2
.....uucCggaucuugaauuaguu...	9	1	0G2
......ucuggaucuAgaauuagu..	13	1	0G2
......ucuggaucuugaauuagGu...	1	1	0G2
......ucuggaucuAgaauuaguuu..	3	1	0G2
......ucuggaucuugaauuaguuuA...	3	1	0G2
............ucuAgaauuaguuugaaucu..	5	1	0G2
...............................uugauuucgggaucuugaauA..	7	1	0G2
...............................uugauuucgggaucuugaauua...	16	0	0G2
...............................uugauuucgggaucuGgaauua...	4	1	0G2
...............................uugauuucgggaucuugaauAa...	3	1	0G2
................................ugauuucgggaucuGgaauu..	5	1	0G2
................................ugauuucgggaucuugaauua...	3	0	0G2
.................................gauuucgggaucuugaauuag..	5	0	0G2
..................................auuucgggaucuugaauuag..	5	0	0G2
...................................uuucgggaucuugaauuagu...	26	0	0G2
...................................Nuucgggaucuugaauuagu...	1	1	0G2
...................................uuucgggaucuGgaauuagu...	15	1	0G2
...................................uuucgggaucuugaauuagug..	198	0	0G2
...................................Cuucgggaucuugaauuagug..	1	1	0G2
...................................uuucgggaucuugaauuGgug..	33	1	0G2
...................................uuucgggaucuGgaauuagug..	22	1	0G2
...................................uuucgggaucuugaGuuagug..	3	1	0G2
...................................uuucgggaucuugaauuagugug..	46	0	0G2
...................................uuucgggaucuCgaauuagugug..	1	1	0G2
...................................uuucgggaucuGgaauuagugug..	5	1	0G2
....................................uucgggaucuugaauuagG...	10	1	0G2
....................................uucgggaucuugaauuGgug..	2	1	0G2

Star **Mature**

```
uugauuucuggaucuugaauuaguuugaaucuugauuucgggaucuugaauuagugugaaucuugaauuccggaucuugaauuaguaugaaucuugaauucuagaucu
.....................................uucgggaucuGgaauuagug....................................................	10	1	0G2
.....................................uucgggaucuugaauuagGg....................................................	6	1	0G2
.....................................uucgggaucuugaauuagug....................................................	55	0	0G2
.....................................uucgggaucuugaauuagugug..................................................	1	0	0G2
.....................................uucgggaucuugaauuagugugaaucA.............................................	4	1	0G2
......................................ucgggaucuugaauuagG.....................................................	4	1	0G2
......................................ucgggaucuGgaauuagug....................................................	6	1	0G2
......................................ucgggaucuugaauuagug....................................................	12	0	0G2
......................................ucgggaucuugaauuaguguU..................................................	6	1	0G2
......................................ucgggaucuGgaauuagugug..................................................	8	1	0G2
......................................ucgggaucuugaauuagugug..................................................	23	0	0G2
.......................................cgggaucuugaauuagGg....................................................	5	1	0G2
.......................................cgggaucuugaauuagug....................................................	13	0	0G2
.......................................cgggaucuugaauuagugug..................................................	6	0	0G2
.......................................cgggaucuugaauuagGgug..................................................	2	1	0G2
.......................................cgggaucuugaauuagugugaa................................................	6	0	0G2
.......................................cgggaucuugaauuGgugugaa................................................	4	1	0G2
.......................................cgggaucuugaauuagugugaaA...............................................	7	1	0G2
.......................................cgggaucuugaauuagugugaauA..............................................	5	1	0G2
.......................................cgggaucuugaauuagugugaaucA.............................................	2	1	0G2
........................................Nggaucuugaauuagugug..................................................	1	1	0G2
........................................gggaucuugaauuagugugaa................................................	3	0	0G2
........................................Nggaucuugaauuagugugaa................................................	1	1	0G2
........................................Nggaucuugaauuagugugaau...............................................	1	1	0G2
.........................................ggaucuugaauuagugug..................................................	19	0	0G2
.........................................ggaucuGgaauuagugug..................................................	22	1	0G2
.........................................ggaucuugaauuaguguA..................................................	1	1	0G2
.........................................ggaucuugaauuaguguAa.................................................	4	1	0G2
.........................................ggaucuugaauuaguguAaa................................................	4	1	0G2
.........................................ggaucuugaauuagugugaau...............................................	2	0	0G2
.........................................ggaucuugaauuagugugaaA...............................................	1	1	0G2
.........................................ggaucuugaauuagugugaauA..............................................	7	1	0G2
...........................................aucuGgaauuagugugaauc..............................................	10	1	0G2
...........................................aucuGgaauuagugugaaucu.............................................	76	1	0G2
...........................................aucuugaauuagugugaaucu.............................................	5	0	0G2
...........................................aucuGgaauuagugugaaucuu............................................	2	1	0G2
............................................ucuGgaauuagugugaauc..............................................	6	1	0G2
............................................ucuugaauuagugugaaucu.............................................	89	0	0G2
............................................ucuugaGuuagugugaaucu.............................................	13	1	0G2
............................................ucuGgaauuagugugaaucu.............................................	121	1	0G2
............................................ucuugaauuagugugaaucuA............................................	2	1	0G2
............................................ucuGgaauuagugugaaucuu............................................	10	1	0G2
............................................ucuGgaauuagugugaaucuug...........................................	5	1	0G2
.............................................cuGgaauuagugugaaucuu............................................	7	1	0G2
.............................................cuugaauuagugugaaucuuga..........................................	1	0	0G2
..............................................uugaauuagugugaaucuug...........................................	59	0	0G2
..............................................uuAaauuagugugaaucuug...........................................	4	1	0G2
..............................................uugaGuuagugugaaucuuga..........................................	2	1	0G2
..............................................uugaauuagugugaaucuuga..........................................	133	0	0G2
..............................................Nugaauuagugugaaucuugaa.........................................	1	1	0G2
..............................................uugaauuagugugaaucuugaa.........................................	8	0	0G2
..............................................uugaauuagugugaaucuugaau........................................	1	0	0G2
..............................................uugaauuagugugaaucuugaauA.......................................	4	1	0G2
...............................................ugaauuagugugaaucuug...........................................	5	0	0G2
...............................................ugaauuagugugaaucuuga..........................................	25	0	0G2
...............................................ugaauuagugugaaucuugaa.........................................	1	0	0G2
...............................................ugaauuagugugaaucuugaau........................................	1	0	0G2
................................................gaauuagugugaaucuuga..........................................	7	0	0G2
................................................gaauuagugugaaucuugaauA.......................................	5	1	0G2
.................................................aauuagugugaaucuugaau........................................	12	0	0G2
.................................................aauuagugugaaucuugaauA.......................................	27	1	0G2
..................................................auuagugugaaucuugaauA.......................................	9	1	0G2
..................................................auuagugugaaucuugaauu.......................................	34	0	0G2
..................................................Cuuagugugaaucuugaauuc......................................	1	1	0G2
..................................................auuagugugaaucuugaauuc......................................	61	0	0G2
..................................................auGagugugaaucuugaauuc......................................	1	1	0G2
...................................................uuaguguAaaucuugaauu.......................................	1	1	0G2
...................................................uuagugugaaucuugaauu.......................................	12	0	0G2
...................................................uuagugugaaucuugaauuc......................................	76	0	0G2
...................................................uuagugugUaucuugaauucc.....................................	5	1	0G2
```

Star **Mature**

```
uugauuucuggaucuugaauuaguuugaaucuugauuucgggaucuugaauuagugugaaucuugaauuccggaucuugaauuaguaugaaucuugaauucuagaucu
..................................................uuagugugaaucuugaauucc.....................................	7	0	0G2
..................................................uuagugugaaucuugaauuUcg....................................	1	1	0G2
..................................................uuagugugaaucuugaauuccA....................................	6	1	0G2
..................................................uuagugugaaucuugaauuUcgg...................................	4	1	0G2
..................................................uuagugugaaucuugaauuUcggau.................................	1	1	0G2
..................................................uuagugugaaucuugaauuccggau.................................	1	0	0G2
..................................................uuagugugaaucuugaauuccggauc................................	7	0	0G2
..................................................uuagugugaaucuugaauuUcggauc................................	3	1	0G2
..................................................uuagugugaaucuugaaCuccggaucu...............................	6	1	0G2
..................................................uuagugugaaucuugaauuccggaucA...............................	3	1	0G2
..................................................uuagugugaaucuugaauuccggaucu...............................	10	0	0G2
..................................................uuagugugaaucuugaUuuccggaucu...............................	3	1	0G2
..................................................uuagugugaaucuugaauuUcggaucu...............................	10	1	0G2
...................................................uagugugaaucuugaauu.......................................	2	0	0G2
...................................................uagugugaaucuugaauuc......................................	24	0	0G2
...................................................uagugugaaucCugaauuc......................................	1	1	0G2
...................................................uagugugUaucuugaauucc.....................................	4	1	0G2
...................................................uagugugaaucuugaauucc.....................................	16	0	0G2
...................................................uagugugCaucuugaauucc.....................................	6	1	0G2
...................................................uagugugaGucuugaauucc.....................................	2	1	0G2
...................................................uagugugaaucuGgaauucc.....................................	4	1	0G2
...................................................uaguguCaaucuugaauuccg....................................	6	1	0G2
...................................................uagugugaaucuugaauuccgg...................................	9	0	0G2
...................................................uagugugaaucuugaauuUcgg...................................	5	1	0G2
...................................................uagugugaaucuugaauuccggauU................................	5	1	0G2
...................................................uagugugaaucCugaauuccggauc................................	1	1	0G2
...................................................uagugugaaucuugaauuccggaucA...............................	4	1	0G2
....................................................agugugaaucuugaauuc......................................	3	0	0G2
....................................................agugugaGucuugaauuccg....................................	4	1	0G2
....................................................aguguCaaucuugaauuccg....................................	6	1	0G2
....................................................agugugaaucuugaaCuccgg...................................	2	1	0G2
.....................................................gugugaaucuugaauuccggaucuAga............................	6	1	0G2
......................................................ugugaaucuugaaCuccgg...................................	7	1	0G2
......................................................ugugaaucuugaauuccggauA................................	2	1	0G2
......................................................ugugaaucuugaauuccggaucuAga............................	3	1	0G2
.......................................................Nugaaucuugaauuccggau.................................	1	1	0G2
.......................................................gugaaucuugaauuccggaucuugaau..........................	3	0	0G2
........................................................ugaaucuugaauuccggauc................................	3	0	0G2
........................................................ugaCucuugaauuccggauc................................	4	1	0G2
........................................................ugaaucuugaauuccggauA................................	10	1	0G2
........................................................ugaGucuugaauuccggauc................................	3	1	0G2
........................................................ugaaucuugaauuccggaucu...............................	20	0	0G2
........................................................ugaaucuugaauuccggaucA...............................	1	1	0G2
........................................................ugaaucuugaauuccCgaucuugaauu.........................	3	1	0G2
........................................................ugaaucuugaauuccggaucuugaauu.........................	22	0	0G2
........................................................ugaaucuugaauuccggaucuAgaauu.........................	3	1	0G2
.........................................................gaaucuugaauuccggauc................................	6	0	0G2
.........................................................gaaucuugaauuccggaucuAgaauua........................	2	1	0G2
..........................................................aaucuugaauuccggaucu...............................	5	0	0G2
..........................................................aaucuugaauuccggaucA...............................	5	1	0G2
..........................................................aaucuugaauuccggaucuA..............................	5	1	0G2
..........................................................aaucuugaauuccggaucuAg.............................	18	1	0G2
..........................................................aGucuugaauuccggaucuug.............................	10	1	0G2
..........................................................aaucuugaauuccggaucuugUa...........................	5	1	0G2
..........................................................aaucuugaauuccggaucuAgaauuag.......................	5	1	0G2
...........................................................aucuugaauuccggaucuug.............................	3	0	0G2
...........................................................aucuugaauuccggaucuAga............................	2	1	0G2
...........................................................aucuugaauuccggaucuugaau..........................	8	0	0G2
...........................................................aucuugaauuccggaucuugaauua........................	13	0	0G2
...........................................................aucuugaauuccggaucuugaauuag.......................	1	0	0G2
...........................................................aucuugaauuccggaucuAgaauuagu......................	9	1	0G2
............................................................ucuugaauuccggaucuA..............................	2	1	0G2
............................................................ucuugaauuccggaucuug.............................	1	0	0G2
............................................................ucuugaauuccggaucuuga............................	19	0	0G2
............................................................ucuugaauuccggaucuAga............................	51	1	0G2
............................................................ucuugaauuccggaucuugaa...........................	26	0	0G2
............................................................ucGugaauuccggaucuugaa...........................	16	1	0G2
............................................................ucuugaauuccggaucuAgaa...........................	74	1	0G2
............................................................ucuugaauuccggaucuugaau..........................	227	0	0G2
............................................................Ccuugaauuccggaucuugaau..........................	1	1	0G2
```

Star Mature

uugauuucuggaucuugaauuaguuugaaucuugauuucgggaucuugaauuagugugaaucuugaauuccggaucuugaauuaguaugaaucuugaauucuagaucu

....ucuugaauuccggaucuugaaG..........................	3	1	0G2
....ucuugaauuccggaucuAgaau..........................	10	1	0G2
....ucGugaauuccggaucuugaau..........................	47	1	0G2
....ucuugaauuAcggaucuugaau..........................	1	1	0G2
....ucuugaauuccggaCcuugaau..........................	1	1	0G2
....ucuugaaGuccggaucuugaauu.........................	10	1	0G2
....ucuugaauuccggaucuugaauu.........................	52	0	0G2
....uGuugaauuccggaucuugaauu.........................	4	1	0G2
....uUuugaauuccggaucuugaauu.........................	11	1	0G2
....ucuugaauuccggaucuugaauAa........................	165	1	0G2
....ucuugaauuccggaucuGgaauua........................	3	1	0G2
....ucuugaauuccggaucuAgaauua........................	7	1	0G2
....uUuugaauuccggaucuugaauua........................	6	1	0G2
....ucuugaauuccggaucuugaauua........................	100	0	0G2
....ucuugaauuccggaucuAgaauuag.......................	7	1	0G2
....ucuugaauuccggaucuugaauAag.......................	3	1	0G2
....ucuugaauuccggaucuugaauuag.......................	12	0	0G2
....ucuugaauuccggaucuAgaauuagu......................	123	1	0G2
....cuugaauuccggaucuug.............................	7	0	0G2
....cuugaauuccggaucuAga............................	54	1	0G2
....cuugaauuccggaucuuga............................	2	0	0G2
....Nuugaauuccggaucuugaa...........................	2	1	0G2
....cGugaauuccggaucuugaa...........................	1	1	0G2
....cuugaauuccggaucuugaa...........................	96	0	0G2
....cuugaauuccggaucuAgaa...........................	363	1	0G2
....cuugaauuccggaucuugaau..........................	227	0	0G2
....cGugaauuccggaucuugaau..........................	15	1	0G2
....Nuugaauuccggaucuugaau..........................	2	1	0G2
....cuugaauuccggaucuAgaau..........................	21	1	0G2
....cNugaauuccggaucuugaauu.........................	1	1	0G2
....cuugaauuccggaucuAgaauu.........................	74	1	0G2
....cAugaauuccggaucuugaauu.........................	1	1	0G2
....cuugaauuccggaucuugaauu.........................	226	0	0G2
....cuugaauuccggaucuugaauCa........................	35	1	0G2
....cuugaauuccggaucuugaaCua........................	5	1	0G2
....cuugaauuccggaucuAgaauua........................	160	1	0G2
....Nuugaauuccggaucuugaauua........................	1	1	0G2
....cuugaauuccggaucuugaaGua........................	1	1	0G2
....cuugaauuccggaucuugaauAa........................	68	1	0G2
....cuugaauuccggaucuugaauua........................	516	0	0G2
....cuugaauuccggaucuugaauuag.......................	4	0	0G2
....cuugaauuccggaucuAgaauuag.......................	2	1	0G2
....cuugaauuccggaucuAgaauuagu......................	5	1	0G2
....uugaauuccggaucuAga............................	26	1	0G2
....uugaauuccggaucuAgaa...........................	238	1	0G2
....uugaauuccggaucuugaa...........................	103	0	0G2
....uugaauuccggaCcuugaau..........................	1	1	0G2
....uugaauuccggauAuugaau..........................	5	1	0G2
....uugaauuccggaucuGgaau..........................	3	1	0G2
....uugaauuccggaucuugaaC..........................	3	1	0G2
....uugaauuAcggaucuugaau..........................	3	1	0G2
....uGgaauuccggaucuugaau..........................	2	1	0G2
....Augaauuccggaucuugaau..........................	6	1	0G2
....uAgaauuccggaucuugaau..........................	2	1	0G2
....uugaaGuccggaucuugaau..........................	1	1	0G2
....Gugaauuccggaucuugaau..........................	12	1	0G2
....uugaauuccggaGcuugaau..........................	2	1	0G2
....uugaGuuccggaucuugaau..........................	57	1	0G2
....uugaauuccggaucuugaaG..........................	7	1	0G2
....uugaauuccggaucuugaau..........................	7306	0	0G2
....uugaauuccggaucCugaau..........................	1	1	0G2
....Nugaauuccggaucuugaau..........................	28	1	0G2
....uNgaauuccggaucuugaau..........................	6	1	0G2
....uugaauuccggGucuugaau..........................	7	1	0G2
....Cugaauuccggaucuugaau..........................	2	1	0G2
....uugaauuccggaucuAgaau..........................	1498	1	0G2
....uugaauuccggCucuugaau..........................	1	1	0G2
....uugaauuccggaGcuugaauu.........................	2	1	0G2
....Cugaauuccggaucuugaauu.........................	3	1	0G2
....uugaauuccggaucuugaaGu.........................	4	1	0G2

Star Mature

```
uugauuucuggaucuugaauuaguuugaaucuugauuucgggaucuugaauuagugugaaucuugaauuccggaucuugaauuaguaugaaucuugaauucuagaucu
..............................................................uugaauuAcggaucuugaauu.........................	2	1	0G2
..............................................................Augaauuccggaucuugaauu.........................	2	1	0G2
..............................................................uugaauuccggaucCugaauu.........................	2	1	0G2
..............................................................Nugaauuccggaucuugaauu.........................	30	1	0G2
..............................................................uugaauuccggaucuugaauu.........................	10633	0	0G2
..............................................................uugaauuccggaucuugGauu.........................	7	1	0G2
..............................................................uugaauuccggaCcuugaauu.........................	5	1	0G2
..............................................................uugaauuccggaucuGgaauu.........................	3	1	0G2
..............................................................uugaauuccggaucuuUaauu.........................	1	1	0G2
..............................................................uugaauuccggCucuugaauu.........................	4	1	0G2
..............................................................uugaauuccggaucuAgaauu.........................	2620	1	0G2
..............................................................Gugaauuccggaucuugaauu.........................	5	1	0G2
..............................................................uugaaGuccggaucuugaauu.........................	2	1	0G2
..............................................................uugaauuccggGucuugaauu.........................	4	1	0G2
..............................................................uNgaauuccggaucuugaauu.........................	4	1	0G2
..............................................................uugaauuccggaucuCgaauu.........................	5	1	0G2
..............................................................uugaGuuccggaucuugaauu.........................	130	1	0G2
..............................................................uugaauuccggaucuugaaCu.........................	19	1	0G2
..............................................................uGgaauuccggaucuugaauu.........................	1	1	0G2
..............................................................uugaauuccggauAuugaauua........................	7	1	0G2
..............................................................uugaauuccggaCcuugaauua........................	4	1	0G2
..............................................................uugaauuccggaucAugaauua........................	1	1	0G2
..............................................................uugaauuccggauGuugaauua........................	3	1	0G2
..............................................................uugaauuccggaucCugaauua........................	9	1	0G2
..............................................................uugaauuccggaucuGgaauua........................	6	1	0G2
..............................................................uNgaauuccggaucuugaauua........................	12	1	0G2
..............................................................uugaauuccggaucuugCauua........................	1	1	0G2
..............................................................uugaauuccCgaucuugaauua........................	8	1	0G2
..............................................................uugaauuccggaucuugaauua........................	29090	0	0G2
..............................................................uugaaGuccggaucuugaauua........................	18	1	0G2
..............................................................Nugaauuccggaucuugaauua........................	82	1	0G2
..............................................................Augaauuccggaucuugaauua........................	7	1	0G2
..............................................................uAgaauuccggaucuugaauua........................	2	1	0G2
..............................................................uugaauuccggaucuugGauua........................	37	1	0G2
..............................................................uugaauuccggaucuugaaCua........................	32	1	0G2
..............................................................uugaGuuccggaucuugaauua........................	639	1	0G2
..............................................................uugaauuccggGucuugaauua........................	8	1	0G2
..............................................................uugaCuuccggaucuugaauua........................	1	1	0G2
..............................................................uugaauuccggaucuAgaauua........................	6712	1	0G2
..............................................................uugaauuccggaGcuugaauua........................	7	1	0G2
..............................................................uugaauuccggaucuugaaGua........................	3	1	0G2
..............................................................uugaauuccggaucuugaauAa........................	2680	1	0G2
..............................................................uugaauuGcggaucuugaauua........................	1	1	0G2
..............................................................uugaauuccggaucuuUaauua........................	3	1	0G2
..............................................................uugaauAccggaucuugaauua........................	11	1	0G2
..............................................................Gugaauuccggaucuugaauua........................	12	1	0G2
..............................................................Cugaauuccggaucuugaauua........................	17	1	0G2
..............................................................uugaauuccggaucuCgaauua........................	5	1	0G2
..............................................................uGgaauuccggaucuugaauua........................	1	1	0G2
..............................................................uugaauuccggaucuugaauCa........................	2426	1	0G2
..............................................................uugaauuccgCaucuugaauua........................	1	1	0G2
..............................................................uugaauuAcggaucuugaauua........................	14	1	0G2
..............................................................uAgaauuccggaucuugaauuag.......................	3	1	0G2
..............................................................uugaauuccggaucuugaauCag.......................	8	1	0G2
..............................................................uugaauuccggaucuugaaCuag.......................	15	1	0G2
..............................................................uugaGuuccggaucuugaauuag.......................	7	1	0G2
..............................................................uNgaauuccggaucuugaauuag.......................	1	1	0G2
..............................................................uugaauuccggaucuAgaauuag.......................	1313	1	0G2
..............................................................uugaauuccggaucuGgaauuag.......................	1	1	0G2
..............................................................uugaauuccggaucuugaauAag.......................	9	1	0G2
..............................................................Cugaauuccggaucuugaauuag.......................	1	1	0G2
..............................................................Nugaauuccggaucuugaauuag.......................	1	1	0G2
..............................................................uugaaGuccggaucuugaauuag.......................	7	1	0G2
..............................................................uugaauuccggaucuCgaauuag.......................	4	1	0G2
..............................................................uugaauuccggaucuugaauuag.......................	303	0	0G2
..............................................................uugaauuccggaucuugGauuag.......................	1	1	0G2
..............................................................uugaauuccggaucuAgaauuagu......................	32	1	0G2
..............................................................uugaauuccggaucuugaauuagA......................	3	1	0G2
..............................................................uugaauuccggaucuugaauuaAua.....................	15	1	0G2
..............................................................uugaauuccggaucuugaauuaUua.....................	77	1	0G2
```

Star Mature

uugauuucuggaucuugaauuaguuugaaucuugauuucgggaucuugaauuagugugaaucuugaauuccggaucuugaauuaguaugaaucuugaauucuagaucu			
....uugaauuccggaucuAgaauuagua.....................	5	1	0G2
....uugaauuccggaucuugaauuaguaA....................	3	1	0G2
....uugaauuccggaucuAgaauuaguau....................	5	1	0G2
....uugaauuccggaucuugaauuaUuau....................	1	1	0G2
....ugaauuccggaucuAgaa...........................	66	1	0G2
....ugaauuccggaucuugaa...........................	2	0	0G2
....ugaauuccggaucuGgaa...........................	1	1	0G2
....ugaauuAcggaucuugaau..........................	1	1	0G2
....ugaauuccggaucuugaau..........................	240	0	0G2
....ugaauuccggaucuAgaau..........................	68	1	0G2
....uNaauuccggaucuugaauu.........................	1	1	0G2
....ugaauuAcggaucuugaauu.........................	1	1	0G2
....ugaauuccggaucuugaaCu.........................	4	1	0G2
....ugaauuccggaucuugaauu.........................	2509	0	0G2
....ugaGuuccggaucuugaauu.........................	32	1	0G2
....Ngaauuccggaucuugaauu.........................	7	1	0G2
....Agaauuccggaucuugaauu.........................	7	1	0G2
....ugaaGuccggaucuugaauu.........................	1	1	0G2
....ugaauuccggaucuCgaauu.........................	1	1	0G2
....ugaauuccggCucuugaauu.........................	1	1	0G2
....ugaauuccggaucuAgaauu.........................	850	1	0G2
....ugaauuccggaCcuugaauua........................	4	1	0G2
....ugaauuccggaucuCgaauua........................	10	1	0G2
....ugaGuuccggaucuugaauua........................	306	1	0G2
....ugaauuccggCucuugaauua........................	3	1	0G2
....ugaauuccggaucCugaauua........................	3	1	0G2
....ugaauuccggaucuugaauua........................	15838	0	0G2
....ugaauuccggaucuuUaauua........................	5	1	0G2
....ugaauuccggaucuugUauua........................	2	1	0G2
....ugaauuccCgaucuugaauua........................	1	1	0G2
....ugaauuccggaucuugaauAa........................	147	1	0G2
....ugaauuccggauAuugaauua........................	5	1	0G2
....ugaauuccggaucuugGauua........................	21	1	0G2
....ugaauuAcggaucuugaauua........................	16	1	0G2
....Agaauuccggaucuugaauua........................	3	1	0G2
....ugaauuccggGucuugaauua........................	1	1	0G2
....ugaauuccggaGcuugaauua........................	7	1	0G2
....Ggaauuccggaucuugaauua........................	7	1	0G2
....ugaauuccggaucuAgaauua........................	6001	1	0G2
....ugaauuccggaucuugaaCua........................	18	1	0G2
....ugaauuccggaucuugaauCa........................	474	1	0G2
....ugaaGuccggaucuugaauua........................	26	1	0G2
....uNaauuccggaucuugaauua........................	11	1	0G2
....ugaauuccggaucAugaauua........................	3	1	0G2
....ugaauuccggaucuGgaauua........................	1	1	0G2
....Ngaauuccggaucuugaauua........................	53	1	0G2
....ugaauuccggaucuGgaauuag.......................	2	1	0G2
....Agaauuccggaucuugaauuag.......................	1	1	0G2
....ugaauuccggaucuAgaauuag.......................	4535	1	0G2
....ugaauuccggaucuugaauCag.......................	8	1	0G2
....ugaGuuccggaucuugaauuag.......................	12	1	0G2
....ugaauuccggGucuugaauuag.......................	1	1	0G2
....ugaauuccggaucuugaauAag.......................	1	1	0G2
....ugaauuccggaucuugGauuag.......................	1	1	0G2
....ugaauuccggaGcuugaauuag.......................	1	1	0G2
....ugaauuccggaucuugaauuag.......................	1046	0	0G2
....Ngaauuccggaucuugaauuag.......................	7	1	0G2
....ugaauuccggaucuugaauuagA......................	2	1	0G2
....ugaauuccggaucuAgaauuagu......................	235	1	0G2
....ugaauuccggaucuugaauuagu......................	2	0	0G2
....ugaGuuccggaucuugaauuagua.....................	4	1	0G2
....ugaauuccggaucuAgaauuagua.....................	70	1	0G2
....ugaauuccggaucuugaauuaguU.....................	2	1	0G2
....ugaauuccggaucuugaauuaAua.....................	6	1	0G2
....ugaauuccggaucuugaauuagua.....................	9	0	0G2
....ugaauuccggaucuugaauuaUua.....................	33	1	0G2
....ugaauuccggaucuAgaauuaguau....................	6	1	0G2
....ugaauuccggaucuugaauuaguaA....................	3	1	0G2
....gaauuccggaucuAgaau..........................	15	1	0G2
....gaauuccggaucuugaau..........................	10	0	0G2

```
                                   Star                          Mature
uugauuucuggaucuugaauuaguuugaaucuugauuucgggaucuugaauuagugugugaaucuugaauuccggaucuugaauuaguaugaaucuugaauucuagaucu
..................................................................gaauuccggaucuAgaauu.........................   35     1     0G2
..................................................................gaauuccggaucuCgaauu.........................   2      1     0G2
..................................................................gaauuccggaucuugaauu.........................   143    0     0G2
..................................................................gaauuccggaucuuUaauu.........................   1      1     0G2
..................................................................gaauuccggauAuugaauu.........................   1      1     0G2
..................................................................gaGuuccggaucuugaauua........................   13     1     0G2
..................................................................gaauuccggaucuugaauCa........................   103    1     0G2
..................................................................gNauuccggaucuugaauua........................   1      1     0G2
..................................................................gaauuccggaucCugaauua........................   1      1     0G2
..................................................................gaauuccggaucuAgaauua........................   607    1     0G2
..................................................................Naauuccggaucuugaauua........................   5      1     0G2
..................................................................gaauuccggaucuuUaauua........................   1      1     0G2
..................................................................gaauuccggauAuugaauua........................   1      1     0G2
..................................................................gaauuccggaucuugaauAa........................   4      1     0G2
..................................................................gaauuccggaucuugaauua........................   1083   0     0G2
..................................................................gaauuccggaucuugaaCua........................   4      1     0G2
..................................................................gaauuccggaGcuugaauua........................   1      1     0G2
..................................................................gaauuccggaucuugaauuag.......................   561    0     0G2
..................................................................gaGuuccggaucuugaauuag.......................   6      1     0G2
..................................................................gaauuccggaucuAgaauuag.......................   3229   1     0G2
..................................................................gaauuccggaucuugaauuagA......................   8      1     0G2
..................................................................gaauuccggaucuAgaauuagu......................   76     1     0G2
..................................................................gaauuccggaucuAgaauuagua.....................   11     1     0G2
...................................................................aauuccggaucuugaauu.........................   4      0     0G2
...................................................................aauuccggaucuugaauCa........................   19     1     0G2
...................................................................aNuuccggaucuugaauua........................   1      1     0G2
...................................................................aauuccggaucuugaauua........................   496    0     0G2
...................................................................aauuccggaucuugaauAa........................   11     1     0G2
...................................................................aauuccggaucuuUaauuag.......................   9      1     0G2
...................................................................aauuccggaucuugaauCag.......................   20     1     0G2
...................................................................aauuccggaucAugaauuag.......................   2      1     0G2
...................................................................Nauuccggaucuugaauuag.......................   6      1     0G2
...................................................................aauuccggaGcuugaauuag.......................   1      1     0G2
...................................................................aauuccggaucuCgaauuag.......................   15     1     0G2
...................................................................aauAccggaucuugaauuag.......................   1      1     0G2
...................................................................aauuccggaucuGgaauuag.......................   6      1     0G2
...................................................................aauuccggaucuugaaCuag.......................   2      1     0G2
...................................................................aGuuccggaucuugaauuag.......................   56     1     0G2
...................................................................aauuccggGucuugaauuag.......................   7      1     0G2
...................................................................aauuccggaucuugaauuag.......................   4093   0     0G2
...................................................................aauuccggauAuugaauuag.......................   2      1     0G2
...................................................................aNuuccggaucuugaauuag.......................   1      1     0G2
...................................................................aauuccggaCcuugaauuag.......................   2      1     0G2
...................................................................aauuccggaucuugaauuagA......................   4      1     0G2
...................................................................aauuccggaucuugaauCagu......................   4      1     0G2
...................................................................aauuccggaucuugaauuagu......................   20     0     0G2
...................................................................Gauuccggaucuugaauuagua.....................   8      1     0G2
...................................................................aauuccggaucuugaauuagua.....................   6      0     0G2
...................................................................aauuccggaucuugaauuaUua.....................   3      1     0G2
...................................................................aauuccggaucuugaauuagAa.....................   2      1     0G2
...................................................................aauuccggaucuAgaauuagua.....................   104    1     0G2
...................................................................aauuccggaucuugaauuaguaA....................   7      1     0G2
....................................................................auuccggaucuugaauCa........................   12     1     0G2
....................................................................auuccggaucuGgaauua........................   1      1     0G2
....................................................................auuccggaucuugaauAa........................   10     1     0G2
....................................................................auuccggaGcuugaauua........................   1      1     0G2
....................................................................Guuccggaucuugaauua........................   4      1     0G2
....................................................................auuccggaucuugaauua........................   179    0     0G2
....................................................................aNuccggaucuugaauua........................   1      1     0G2
....................................................................auuccggaucAugaauuag.......................   14     1     0G2
....................................................................auuccggaucuugaauCag.......................   11     1     0G2
....................................................................auuccggaucuugaauuag.......................   1187   0     0G2
....................................................................Guuccggaucuugaauuag.......................   2      1     0G2
....................................................................auuccggauAuugaauuag.......................   1      1     0G2
....................................................................auuAcggaucuugaauuag.......................   1      1     0G2
....................................................................Nuuccggaucuugaauuag.......................   2      1     0G2
....................................................................auuccggaucuGgaauuag.......................   12     1     0G2
....................................................................auuccggaucuGgaauuagu......................   10     1     0G2
....................................................................auuccggaucuugaauuagA......................   45     1     0G2
....................................................................auuUcggaucuugaauuagu......................   3      1     0G2
```

```
                                                   Star                     Mature
uugauuucuggaucuugaauuaguuugaaucuugauuucgggaucuugaauuagugugugaaucuugaauuccggaucuugaauuaguaugaaucuugaauucuagaucu
....................................................................auuccggaucuugaauuagu......................    158    0    0G2
....................................................................auuccggaucuAgaauuagua.....................    932    1    0G2
....................................................................auuccggaucuGgaauuagua.....................    3      1    0G2
....................................................................Cuuccggaucuugaauuagua.....................    3      1    0G2
....................................................................auuccggaucuugaauuaUua.....................    65     1    0G2
....................................................................auuccggaucuugaauuagAa.....................    1      1    0G2
....................................................................auuccggaucuugaauuaAua.....................    14     1    0G2
....................................................................auuccggaucuugaauuagua.....................    102    0    0G2
....................................................................auuccggaucuugaauuaguaA....................    44     1    0G2
....................................................................auuccggaucuAgaauuaguau....................    30     1    0G2
....................................................................auuccggaucuugaauuaguauAa..................    1      1    0G2
.....................................................................uuccggaucuugaauCag.......................    5      1    0G2
.....................................................................uuccggaucuuUaauuag.......................    4      1    0G2
.....................................................................uuccggGucuugaauuag.......................    4      1    0G2
.....................................................................uuccggaucuugaauuag.......................    933    0    0G2
.....................................................................Nuccggaucuugaauuag.......................    3      1    0G2
.....................................................................uuccggaucuGgaauuag.......................    4      1    0G2
.....................................................................uuccggaucuugGauuag.......................    1      1    0G2
.....................................................................uuccggaucuugaauuagu......................    80     0    0G2
.....................................................................uuccggaucuGgaauuagu......................    58     1    0G2
.....................................................................uuccggaucuugaauuagA......................    3      1    0G2
.....................................................................uuccggaucuugaauuagua.....................    38     0    0G2
.....................................................................uuccggaucuugaauuaUua.....................    22     1    0G2
.....................................................................uuccggaucuugaauuaguU.....................    9      1    0G2
.....................................................................uuccggaucuAgaauuagua.....................    966    1    0G2
.....................................................................uuccggaucuGgaauuagua.....................    2      1    0G2
.....................................................................uuccggaucuugaauuagAa.....................    8      1    0G2
.....................................................................uuccggaucuugaauuaAua.....................    1      1    0G2
.....................................................................uuccggaucuAgaauuaguau....................    18     1    0G2
.....................................................................uuccggaucuugaauuaguaA....................    99     1    0G2
.....................................................................uuccggaucuAgaauuaguaug...................    4      1    0G2
......................................................................uccggaucuugaauuagA......................    3      1    0G2
......................................................................uccggaucuugaauuagu......................    15     0    0G2
......................................................................uccggaucuGgaauuagu......................    5      1    0G2
......................................................................Nccggaucuugaauuagua.....................    1      1    0G2
......................................................................uccggaucuugaauuagua.....................    15     0    0G2
......................................................................uccggaucuAgaauuagua.....................    450    1    0G2
......................................................................uccggaucuAgaauuaguau....................    33     1    0G2
......................................................................uccggaucuugaauuaguaA....................    7      1    0G2
......................................................................uccggaucuugaauuaguauA...................    2      1    0G2
......................................................................uccggaucuugaauuaguaAg...................    2      1    0G2
......................................................................uccggaucuAgaauuaguaug...................    7      1    0G2
.......................................................................ccggaucuAgaauuagua.....................    15     1    0G2
.......................................................................ccggaucuugaauuagAa.....................    1      1    0G2
.......................................................................ccggaucuugaauuaguaA....................    30     1    0G2
.......................................................................ccggaucuAgaauuaguau....................    2      1    0G2
........................................................................cggaucuugaauuaguaA....................    11     1    0G2
........................................................................cggaucuGgaauuaguaug...................    1      1    0G2
........................................................................cggaucuAgaauuaguaug...................    1      1    0G2
........................................................................cggaucuAgaauuaguauga..................    3      1    0G2
.............................................................................cuAgaauuaguaugaaucu..............    3      1    0G2

uugaGuucuggaucuugaau..........................................................................................    21     1    0B2
uugaGuucuggaucuugaauu.........................................................................................    17     1    0B2
uugaGuucuggaucuugaauua........................................................................................    6      1    0B2
uugaGuucuggaucuugaauuag.......................................................................................    2      1    0B2
..gaGuucuggaucuugaauu.........................................................................................    2      1    0B2
....Guucuggaucuugaauuag.......................................................................................    1      1    0B2
.....uucuggaucuuAaauuag.......................................................................................    2      1    0B2
.....uucuggaucuugaauuag.......................................................................................    22     0    0B2
.....uucuggaucuugaauuagA......................................................................................    1      1    0B2
.....uucuggaucuAgaauuagu......................................................................................    54     1    0B2
.....uucCggaucuugaauuaguu.....................................................................................    1      1    0B2
.....uucuggaucuugaauuaguu.....................................................................................    3      0    0B2
......ucuggaucuugaauuagu......................................................................................    1      0    0B2
......ucuggaucuAgaauuagu......................................................................................    12     1    0B2
......ucuggaucuugaauuaguuA....................................................................................    2      1    0B2
......ucuggaucuAgaauuaguuu....................................................................................    3      1    0B2
......ucCggaucuugaauuaguuu....................................................................................    1      1    0B2
.........ggaucuAgaauuaguuuga..................................................................................    2      1    0B2
```

Star **Mature**

```
uugauuucuggaucuugaauuaguuugaaucuugauuucgggaucuugaauuagugugaaucuugaauuccggaucuugaauuaguaugaaucuugaauucuagaucu
.........................ugaaucuugauuucgggauc...............................................................  1    0  0B2
.........................ugaGucuugauuucgggauc...............................................................  1    1  0B2
............................aucuugauuucgggaucuu.............................................................  1    0  0B2
..............................cuuAauuucgggaucuugaauu........................................................  2    1  0B2
...............................uugauuucgggaucuugaau.........................................................  2    0  0B2
...............................uugauuucgggaucuGgaauu........................................................  4    1  0B2
...............................uugauuucgggaucuugaauu........................................................  9    0  0B2
...............................uugauuucgggaucuugaauA........................................................  2    1  0B2
...............................uugauuucgggaucuugaauua.......................................................  11   0  0B2
................................ugauuucgggaucuugaau.........................................................  1    0  0B2
................................ugauuucgggaucuugaauuag......................................................  6    0  0B2
.................................gauuucgggaucuugaauua.......................................................  6    0  0B2
..................................auuucgggaucuugaauuag......................................................  2    0  0B2
..................................Cuuucgggaucuugaauuagug....................................................  4    1  0B2
..................................auuucgggaucuugaauuagug....................................................  3    0  0B2
...................................uuucgggaucuGgaauuagu.....................................................  7    1  0B2
...................................uuucgggaucuugaauuagu.....................................................  19   0  0B2
...................................Nuucgggaucuugaauuagu.....................................................  1    1  0B2
...................................uuucgggaucuGgaauuagug....................................................  35   1  0B2
...................................uuucgggaucuugaauuGgug....................................................  12   1  0B2
...................................uuucgggaucuugaGuuagug....................................................  3    1  0B2
...................................uuucgggaucuugaauuagug....................................................  145  0  0B2
...................................uuucgggaucuugaauuagugug..................................................  10   0  0B2
...................................uuucgggaucuugaauuaguguga.................................................  9    0  0B2
...................................uuucgggaucuugaaCuaguguga.................................................  1    1  0B2
...................................uuucgggaucuugaauuaguguAa.................................................  2    1  0B2
...................................uuucgggaucuugaauuagugugaa................................................  3    0  0B2
....................................uucgggaucuGgaauuagu.....................................................  1    1  0B2
....................................uucgggaucuugaauuagG.....................................................  2    1  0B2
....................................uucgggaucuugaauuagu.....................................................  8    0  0B2
....................................uucgggaucuugaauuagug....................................................  48   0  0B2
....................................uucgggaucuugaauuGgug....................................................  5    1  0B2
....................................uucgggaucuugaauuagGg....................................................  16   1  0B2
....................................uucgggaucuugaauuaguguga.................................................  1    0  0B2
....................................uucgggaucuugaauuagugugaaucu.............................................  1    0  0B2
.....................................ucgggaucuugaauuagug....................................................  6    0  0B2
.....................................ucgggaucuGgaauuagug....................................................  2    1  0B2
.....................................ucgggaucuugaauuagGg....................................................  6    1  0B2
.....................................ucgggaucuugaauuGgug....................................................  1    1  0B2
.....................................ucgggaucuugaauuagugu...................................................  3    0  0B2
.....................................ucgggaucuugaauuagugug..................................................  7    0  0B2
.....................................ucgggaucuugaauuagGgug..................................................  3    1  0B2
.....................................ucgggaucuugaauuagugugaauUuu............................................  2    1  0B2
......................................cgggaucuugaauuagug....................................................  13   0  0B2
......................................cgggaucuugaauuagGg....................................................  7    1  0B2
......................................Ngggaucuugaauuagug....................................................  1    1  0B2
......................................cgggaucuugaauuaguguA..................................................  3    1  0B2
......................................cgggaucuGgaauuagugug..................................................  3    1  0B2
......................................cgggaucuugaauuagugug..................................................  14   0  0B2
......................................cgggaucuugaauuagGgug..................................................  2    1  0B2
......................................cgggaucuugaauuaguguga.................................................  2    0  0B2
......................................cgggaucuugaauuagugugaa................................................  11   0  0B2
......................................cgggaucuugaauuagugugaaA...............................................  5    1  0B2
......................................cgggaucuugaauuagugugaaucA.............................................  1    1  0B2
.......................................Nggaucuugaauuagugug..................................................  3    1  0B2
.......................................Nggaucuugaauuagugugaa................................................  1    1  0B2
.......................................Nggaucuugaauuagugugaau...............................................  1    1  0B2
........................................ggaucuGgaauuagugug..................................................  7    1  0B2
........................................ggaucuugaauuaguguA..................................................  2    1  0B2
........................................ggaucuugaauuagugug..................................................  10   0  0B2
........................................ggaucuugaauuaguguga.................................................  8    0  0B2
........................................ggaucuugaGuuaguguga.................................................  1    1  0B2
........................................Ngaucuugaauuagugugaa................................................  1    1  0B2
........................................ggaucuugaauuagugugaU................................................  6    1  0B2
........................................ggaucuugaauuagugugaa................................................  21   0  0B2
........................................ggaucuugaGuuagugugaa................................................  3    1  0B2
........................................ggaucuGgaauuagugugaa................................................  11   1  0B2
........................................ggaucuugaauuagugugaaA...............................................  1    1  0B2
........................................ggaucuugaauuagugugaau...............................................  6    0  0B2
..........................................aucuGgaauuagugugaauc..............................................  11   1  0B2
```

Star Mature

uugauuucuggaucuugaauuaguuugaaucuugauuucgggaucuugaauuagugugaaucuugaauuccggaucuugaauuaguaugaaucuugaauucuagaucu			
....aucuugaauuagugugaaucu...	6	0	0B2
....aucuGgaauuagugugaaucu...	6	1	0B2
....aucuugaauuagugugaaucuug...	4	0	0B2
....ucuCgaauuagugugaaucu...	1	1	0B2
....ucuugaauuagugugaaucu...	71	0	0B2
....Ncuugaauuagugugaaucu...	2	1	0B2
....ucuugaauCagugugaaucu...	1	1	0B2
....ucuGgaauuagugugaaucu...	95	1	0B2
....ucuugaauuagugugaaucuu..	5	0	0B2
....ucuGgaauuagugugaaucuu..	4	1	0B2
....ucuugaauuagugugaaucuug...	9	0	0B2
....ucuGgaauuagugugaaucuug...	5	1	0B2
....cuGgaauuagugugaaucu...	1	1	0B2
....cuugaauuagugugaaucu...	5	0	0B2
....cuugaauuagugugaaucuu..	1	0	0B2
....cuGgaauuagugugaaucuu..	4	1	0B2
....cuGgaauuagugugaaucuug...	7	1	0B2
....cuGgaauuagugugaaucuuga..	2	1	0B2
....uugaauuagugugaaucuu..	9	0	0B2
....uugaauuagugugaaucuug...	23	0	0B2
....uugaauuagugugaGucuuga..	4	1	0B2
....uugaauuagugugaaucuuga..	36	0	0B2
....uugaaCuagugugaaucuuga..	1	1	0B2
....uugaauuagugugaaucuugaa...	20	0	0B2
....Nugaauuagugugaaucuugaa...	1	1	0B2
....Gugaauuagugugaaucuugaa...	1	1	0B2
....uugaauuagugugaaucuugaauu.......................................	4	0	0B2
....ugaauuagugugaGucuuga..	1	1	0B2
....ugaauuagugugaaucuuga..	7	0	0B2
....Ngaauuagugugaaucuugaa...	1	1	0B2
....ugaauuagugugaaucuugaa...	1	0	0B2
....ugaauuagugugaaucuugaauA.......................................	2	1	0B2
....gaauuagugugaaucuugaauA.......................................	6	1	0B2
....aauuagugugaaucuugaau..	8	0	0B2
....aauuagugugaaucuugaauA.......................................	10	1	0B2
....aauuagugugaaucuugaauu.......................................	6	0	0B2
....aauuagugugaaucCugaauuc......................................	1	1	0B2
....auuagugugaaucuugaauu.......................................	41	0	0B2
....auuagugugaaucuugaauuc......................................	38	0	0B2
....auuagugugaaucuugaauucc.....................................	3	0	0B2
....uuagugugaaucuugaauu.......................................	22	0	0B2
....uuagugugaaucuugaGuuc......................................	3	1	0B2
....uuagugugaaucuCgaauuc......................................	2	1	0B2
....uuagugugaaucuugaaCuc......................................	1	1	0B2
....uuagugugaaucuugaauuc......................................	191	0	0B2
....uuagugugaaucuugaauuU......................................	5	1	0B2
....Nuagugugaaucuugaauuc......................................	1	1	0B2
....uuagugugUaucuugaauucc.....................................	2	1	0B2
....uuagugugaaucuGgaauucc.....................................	5	1	0B2
....uuagugugaaucuugaauucc.....................................	2	0	0B2
....uuagugugaaucuugaauuccg....................................	1	0	0B2
....uuagugugaaucuugaaCuccg....................................	1	1	0B2
....uuagugugaaucuugaauuGcg....................................	10	1	0B2
....uuagugugaaucuugaaCuccgga..................................	3	1	0B2
....uuagugugaaucuugaauuGcggauc................................	3	1	0B2
....uuagugugaaucuugaauuccggauc................................	5	0	0B2
....uuagugugaaucuugaauCccggauc................................	2	1	0B2
....uuagugugaaucCugaauuccggauc................................	4	1	0B2
....uuagugugaaucuugaauuUcggauc................................	8	1	0B2
....uuagugugaaucuugaauuccggaucu...............................	3	0	0B2
....uuagugugaaucuugaauuccggaucA...............................	2	1	0B2
....uuagugugaaucuugaauuUcggaucu...............................	2	1	0B2
....uuagugugaaucuugaauCccggaucu...............................	5	1	0B2
....uuagugugaaucuugaauuGcggaucu...............................	1	1	0B2
....uuagugugaaucuugaaCuccggaucu...............................	2	1	0B2
....uagugugaaucuugaauu.......................................	7	0	0B2
....uagugugaaucuugaauuc......................................	24	0	0B2
....uagugugaaucuugaauucc.....................................	2	0	0B2
....uaguguCaaucuugaauuccg....................................	3	1	0B2
....uagugugaaucuugaaCuccg....................................	2	1	0B2

Star Mature

```
uugauuucuggaucuugaauuaguuugaaucuugauuucgggaucuugaauuagugugaaucuugaauuccggaucuugaauuaguaugaaucuugaauucuagaucu
...................................................uagugugaaucuugaauuUcgg...................................	3	1	0B2
...................................................uagugugaaucuugaauuccggauc................................	4	0	0B2
...................................................uagugugaaucuugaauuUcggauc................................	2	1	0B2
...................................................uagugugaaucuugaUuuccggauc................................	2	1	0B2
...................................................uagugugaaucuugaauuccggaucA...............................	2	1	0B2
....................................................agugugaaucuugaauuc......................................	6	0	0B2
....................................................agugugaaucuugaaCucc.....................................	2	1	0B2
....................................................agugugaaucuugaaCuccg....................................	9	1	0B2
....................................................agugugaaucuugaauuUcgg...................................	3	1	0B2
....................................................agugugaaucuugaaCuccgga..................................	5	1	0B2
.....................................................gugugaaucuugaauuccgg...................................	1	0	0B2
......................................................uguAaaucuugaauuccgga..................................	5	1	0B2
......................................................ugugaaAcuugaauuccgga..................................	2	1	0B2
......................................................ugugaaucuugaauuccggau.................................	5	0	0B2
......................................................ugugaaucuugaauuccggauA................................	5	1	0B2
......................................................ugugaaucuugaauuccggauc................................	4	0	0B2
........................................................ugaaucuugaauuccggauc................................	5	0	0B2
........................................................ugaaucuugaauuccggaucA...............................	3	1	0B2
........................................................ugaaucuugaauuccggaucuAgaau..........................	6	1	0B2
........................................................ugaaucuugaauuccggaucuugaau..........................	3	0	0B2
........................................................ugaaucuugaauuccggaucuugaauu.........................	3	0	0B2
........................................................ugaaucuugaauuccggaucuAgaauu.........................	7	1	0B2
.........................................................gaaucuugaauuccggaucuugaauu.........................	1	0	0B2
.........................................................gaaucuugaauuccggaucuAgaauua........................	3	1	0B2
..........................................................aaucuugaauuccggaucuAg.............................	6	1	0B2
..........................................................aaucuugaauuccggaucuug.............................	2	0	0B2
...........................................................aucuugaauuccggaucu...............................	4	0	0B2
...........................................................aucuugaauuccggaucuA..............................	2	1	0B2
...........................................................aucuugaauuccggaucuu..............................	6	0	0B2
...........................................................aucuugaauuccggaucuuga............................	5	0	0B2
...........................................................aucGugaauuccggaucuuga............................	7	1	0B2
...........................................................Cucuugaauuccggaucuugaa...........................	2	1	0B2
...........................................................aucuugaauuccggaucuugaau..........................	11	0	0B2
...........................................................aucuugaauuccggaucuugaauua........................	2	0	0B2
............................................................ucuugaauuccggaucuA..............................	1	1	0B2
............................................................ucuugaauuccggaucuu..............................	1	0	0B2
............................................................ucuugaauuccggaucuug.............................	5	0	0B2
............................................................ucuugaauuccggaucuAg.............................	11	1	0B2
............................................................ucuugaauuccggaucuuga............................	15	0	0B2
............................................................ucuugaauuccggaucuAga............................	44	1	0B2
............................................................ucuugaauuccggaucuugaa...........................	20	0	0B2
............................................................ucGugaauuccggaucuugaa...........................	26	1	0B2
............................................................ucuugaauuccggaucuAgaa...........................	44	1	0B2
............................................................ucuugaauuccggaucuGgaa...........................	4	1	0B2
............................................................ucuugaauuccggaucuugaaG..........................	5	1	0B2
............................................................ucuugaauuccggaucuugaau..........................	262	0	0B2
............................................................Ncuugaauuccggaucuugaau..........................	3	1	0B2
............................................................ucuugaauuccggaucuCgaau..........................	2	1	0B2
............................................................ucuugaauuccggaucuAgaau..........................	16	1	0B2
............................................................Ccuugaauuccggaucuugaau..........................	1	1	0B2
............................................................ucGugaauuccggaucuugaau..........................	18	1	0B2
............................................................Ncuugaauuccggaucuugaauu.........................	1	1	0B2
............................................................uUuugaauuccggaucuugaauu.........................	3	1	0B2
............................................................ucuugaauuccggaucuAgaauu.........................	10	1	0B2
............................................................ucuugaauuccggaucuugaauu.........................	42	0	0B2
............................................................uGuugaauuccggaucuugaauu.........................	7	1	0B2
............................................................ucuugaauuccggaucuAgaauua........................	8	1	0B2
............................................................ucuugaauuccggaucuugaauAa........................	66	1	0B2
............................................................uGuugaauuccggaucuugaauua........................	6	1	0B2
............................................................ucuugaauuccggaucuugaauua........................	63	0	0B2
............................................................uUuugaauuccggaucuugaauua........................	9	1	0B2
............................................................ucuugaauuccggaucuugaauCa........................	2	1	0B2
............................................................ucuugaauuccggaucuugaauuag.......................	1	0	0B2
............................................................ucuugaauuccggaucuAgaauuag.......................	11	1	0B2
............................................................ucuugaauuccggaucuugaauAag.......................	3	1	0B2
............................................................ucuugaauuccggaucuAgaauuagu......................	84	1	0B2
............................................................ucuugaauuccggaucuugaauuagu......................	4	0	0B2
............................................................ucuugaaCuccggaucuugaauuagu......................	1	1	0B2
............................................................ucuugaauuccggaucuugaauuaUua.....................	2	1	0B2
.............................................................cuugaauuccggaucuug.............................	2	0	0B2
```

```
                                   Star                      Mature
uugauuucuggaucuugaauuaguuugaaucuugauuucgggaucuugaauuagugugaaucuugaauuccggaucuugaauuaguaugaaucuugaauucuagaucu
.............................................................cuugaauuccggaucuAg.............................   1      1    0B2
.............................................................cuugaauuccggaucuuga............................   5      0    0B2
.............................................................cuugaauuccggaucuAga............................   11     1    0B2
.............................................................cuugaauuccggaucuugaa...........................   90     0    0B2
.............................................................Nuugaauuccggaucuugaa...........................   1      1    0B2
.............................................................cGugaauuccggaucuugaa...........................   4      1    0B2
.............................................................cuugaauuccggaucuAgaa...........................   366    1    0B2
.............................................................Nuugaauuccggaucuugaau..........................   1      1    0B2
.............................................................cuugaauuccggaucuugaaC..........................   1      1    0B2
.............................................................cGugaauuccggaucuugaau..........................   28     1    0B2
.............................................................cuugaauuccggaucuugaau..........................   204    0    0B2
.............................................................cuugaauuccggaucuAgaau..........................   15     1    0B2
.............................................................cuugaauuccggaGcuugaau..........................   1      1    0B2
.............................................................Nuugaauuccggaucuugaauu.........................   1      1    0B2
.............................................................cuugaauuccggaucuAgaauu.........................   74     1    0B2
.............................................................cuugaauuccggaucuugaauu.........................   234    0    0B2
.............................................................cuugaauuccggaucuugaauAa........................   38     1    0B2
.............................................................cuugaauuccggaucuAgaauua........................   37     1    0B2
.............................................................cuugaauuccggaucuugaauCa........................   27     1    0B2
.............................................................cuugaauuccggaucuugaauua........................   239    0    0B2
.............................................................cuugaauuccggauGuugaauua........................   7      1    0B2
.............................................................cuugaauuccggaucuugGauua........................   3      1    0B2
.............................................................Guugaauuccggaucuugaauua........................   1      1    0B2
.............................................................Nuugaauuccggaucuugaauua........................   2      1    0B2
.............................................................cuugaauuccggaucuugaauuag.......................   2      0    0B2
.............................................................cuugaauuccggaucuAgaauuag.......................   8      1    0B2
.............................................................cuugaauuccggaucuAgaauuagu......................   4      1    0B2
.............................................................cuugaauuccggaucuugaauuagu......................   3      0    0B2
.............................................................cuugaauuccggaucuugaauuaUua.....................   7      1    0B2
.............................................................cuugaauuccggaucuugaauuaAua.....................   1      1    0B2
..............................................................uugaauuccggaucuAga............................   14     1    0B2
..............................................................uugaauuccggCucuugaa...........................   1      1    0B2
..............................................................uugaauuccggaucuugaa...........................   53     0    0B2
..............................................................uugaauuccggaucuAgaa...........................   284    1    0B2
..............................................................uugaauuccggaucuGgaa...........................   1      1    0B2
..............................................................uugaauuccgCaucuugaau..........................   4      1    0B2
..............................................................uGgaauuccggaucuugaau..........................   1      1    0B2
..............................................................uugaauuccggaucuCgaau..........................   2      1    0B2
..............................................................uugaauuccggaucuAgaau..........................   1941   1    0B2
..............................................................uugaauuccggaGcuugaau..........................   4      1    0B2
..............................................................uugaGuuccggaucuugaau..........................   91     1    0B2
..............................................................Nugaauuccggaucuugaau..........................   73     1    0B2
..............................................................uugaauuccggaCcuugaau..........................   3      1    0B2
..............................................................uugaauuccggauAuugaau..........................   1      1    0B2
..............................................................uugaCuuccggaucuugaau..........................   1      1    0B2
..............................................................Gugaauuccggaucuugaau..........................   7      1    0B2
..............................................................uugaauuccggaucuugaaC..........................   10     1    0B2
..............................................................uugaauuccggaucuGgaau..........................   9      1    0B2
..............................................................Cugaauuccggaucuugaau..........................   8      1    0B2
..............................................................uugaauuccggCucuugaau..........................   1      1    0B2
..............................................................uugaauuccggGucuugaau..........................   8      1    0B2
..............................................................uugaauuccggaucAugaau..........................   4      1    0B2
..............................................................uugaauuccggaucuugaau..........................   9489   0    0B2
..............................................................uugaauuccggaucuugaaG..........................   1      1    0B2
..............................................................uugaauuccggaucuAgaauu.........................   2752   1    0B2
..............................................................uugaaGuccggaucuugaauu.........................   4      1    0B2
..............................................................uugaauuccggaCcuugaauu.........................   2      1    0B2
..............................................................Cugaauuccggaucuugaauu.........................   2      1    0B2
..............................................................uugaauuccggCucuugaauu.........................   2      1    0B2
..............................................................uugaauuccggaucuugaauu.........................   9659   0    0B2
..............................................................Gugaauuccggaucuugaauu.........................   9      1    0B2
..............................................................uugaauuccggaucuugaaCu.........................   33     1    0B2
..............................................................uugaauuccggauAuugaauu.........................   1      1    0B2
..............................................................uugaauuccggauNuugaauu.........................   1      1    0B2
..............................................................uugaauuccggaGcuugaauu.........................   3      1    0B2
..............................................................uugaauAccggaucuugaauu.........................   1      1    0B2
..............................................................uugaauuccggaucuGgaauu.........................   2      1    0B2
..............................................................Nugaauuccggaucuugaauu.........................   77     1    0B2
..............................................................uugaauuccggaucuugaaGu.........................   3      1    0B2
..............................................................uugaGuuccggaucuugaau..........................   166    1    0B2
```

Star **Mature**

uugauuucuggaucuugaauuaguuugaaucuugauuucgggaucuugaauuagugugaaucuugaauuccggaucuugaauuaguaugaaucuugaauucuagaucu			
....Augaauuccggaucuugaauu.........................	3	1	0B2
....uugaauuccggaucuCgaauu.........................	9	1	0B2
....uugaauuccggaucCugaauu.........................	4	1	0B2
....uugaauuAcggaucuugaauu.........................	3	1	0B2
....uugaauuccggaucuugGauu.........................	6	1	0B2
....uugaauuccggGucuugaauu.........................	1	1	0B2
....uugaGuuccggaucuugaauua........................	263	1	0B2
....uugaauuccggaucuugaaCua........................	16	1	0B2
....uugaauuccggaucuugaauua........................	10885	0	0B2
....Augaauuccggaucuugaauua........................	3	1	0B2
....uugaauuccggaucuugUauua........................	1	1	0B2
....uugaauNccggaucuugaauua........................	1	1	0B2
....uugaauuccggaucuCgaauua........................	15	1	0B2
....uugaauuccggGucuugaauua........................	2	1	0B2
....Cugaauuccggaucuugaauua........................	9	1	0B2
....uugaauuccggaucuugaauAa........................	819	1	0B2
....uugaauuccggaGcuugaauua........................	4	1	0B2
....uugaauuccggauAuugaauua........................	1	1	0B2
....uGgaauuccggaucuugaauua........................	2	1	0B2
....uugaauuccggaucuuUaauua........................	1	1	0B2
....Nugaauuccggaucuugaauua........................	91	1	0B2
....uugaauuccggNucuugaauua........................	1	1	0B2
....uugaauuccggaucuGgaauua........................	2	1	0B2
....uugaCuuccggaucuugaauua........................	2	1	0B2
....uugaauuGcggaucuugaauua........................	2	1	0B2
....uugaauuccggCucuugaauua........................	9	1	0B2
....uugaauuccggaCcuugaauua........................	3	1	0B2
....uugaauuccggaucuugGauua........................	18	1	0B2
....uugaauuccggaucuugaauCa........................	934	1	0B2
....uugaauuAcggaucuugaauua........................	1	1	0B2
....uugaaGuccggaucuugaauua........................	12	1	0B2
....uugaauuccggaucuAgaauua........................	2593	1	0B2
....Gugaauuccggaucuugaauua........................	4	1	0B2
....uugaauuccggaucuAgaauuag.......................	757	1	0B2
....uugaauuccggaucuugaauAag.......................	4	1	0B2
....uugaGuuccggaucuugaauuag.......................	14	1	0B2
....Cugaauuccggaucuugaauuag.......................	9	1	0B2
....uugaaGuccggaucuugaauuag.......................	2	1	0B2
....uugaauuccCgaucuugaauuag.......................	1	1	0B2
....Nugaauuccggaucuugaauuag.......................	5	1	0B2
....uugaauuccggauGuugaauuag.......................	1	1	0B2
....uugaauuccggaucuugaaCuag.......................	4	1	0B2
....uugaauuccggaucuugaauuag.......................	243	0	0B2
....uugaauuccggaucuAgaauuagu......................	16	1	0B2
....uugaauuccggaucuugaauuagu......................	3	0	0B2
....uugaauuccggaucuugaauuagA......................	3	1	0B2
....uugaauuccggaucuugaUuuagu......................	5	1	0B2
....Nugaauuccggaucuugaauuagua.....................	1	1	0B2
....uugaauuccggaucuAgaauuagua.....................	2	1	0B2
....uugaauuccggaucuugaauuagua.....................	1	0	0B2
....uugaauuccggaucuugaauuaUua.....................	12	1	0B2
....uugaauuccggaucuAgaauuaguau....................	2	1	0B2
....ugaauuccggaucuAgaa...........................	70	1	0B2
....ugaauuccggaucuugaa...........................	5	0	0B2
....ugaGuuccggaucuugaau..........................	7	1	0B2
....ugaauuccggaucuAgaau..........................	149	1	0B2
....ugaauuccggaucuugaau..........................	498	0	0B2
....ugaauuccggaGcuugaau..........................	2	1	0B2
....Ngaauuccggaucuugaau..........................	2	1	0B2
....ugaauuccggaucCugaauu.........................	2	1	0B2
....ugaGuuccggaucuugaauu.........................	66	1	0B2
....ugaauuccggaucuuUaauu.........................	1	1	0B2
....ugaauuccggaucuugaaGu.........................	1	1	0B2
....ugaauuccggGucuugaauu.........................	2	1	0B2
....ugaauuccggaucuAgaauu.........................	1400	1	0B2
....ugaauuccggaucuCgaauu.........................	2	1	0B2
....ugaauuccggaGcuugaauu.........................	1	1	0B2
....ugaauuccggaucuugaauu.........................	3055	0	0B2
....ugaauuccggauAuugaauu.........................	1	1	0B2
....Ngaauuccggaucuugaauu.........................	14	1	0B2

```
                                                Star                      Mature
uugauuucuggaucuugaauuaguuugaaucuugauuucgggaucuugaauuagugugaaucuugaauuccggaucuugaauuaguaugaaucuugaauucuagaucu
...............................................................ugaaGuccggaucuugaauu.........................    5      1    0B2
...............................................................ugaauuccggaucuGgaauua........................    1      1    0B2
...............................................................ugaGuuccggaucuugaauua........................    146    1    0B2
...............................................................ugaaGuccggaucuugaauua........................    22     1    0B2
...............................................................ugaauuccgCaucuugaauua........................    1      1    0B2
...............................................................ugaauuccggGucuugaauua........................    1      1    0B2
...............................................................ugaauuccggaGcuugaauua........................    5      1    0B2
...............................................................ugaauuccggaucuugaauAa........................    58     1    0B2
...............................................................ugaauuccggaucuugGauua........................    10     1    0B2
...............................................................ugaauuccggaucuugaaCua........................    6      1    0B2
...............................................................Ngaauuccggaucuugaauua........................    56     1    0B2
...............................................................ugaauuccCgaucuugaauua........................    1      1    0B2
...............................................................ugaauuccggaucuugaauCa........................    280    1    0B2
...............................................................ugaauuccggaucAugaauua........................    4      1    0B2
...............................................................ugaauuccggaucuugaauua........................    7913   0    0B2
...............................................................ugaauuccggauAuugaauua........................    4      1    0B2
...............................................................ugaauuccggaucCugaauua........................    3      1    0B2
...............................................................ugaauuccggaCcuugaauua........................    2      1    0B2
...............................................................ugaauuccggaucuCgaauua........................    3      1    0B2
...............................................................ugaauuAcggaucuugaauua........................    1      1    0B2
...............................................................ugaauuccggaucuAgaauua........................    3149   1    0B2
...............................................................ugaauuccggaucuugUauua........................    1      1    0B2
...............................................................uNaauuccggaucuugaauua........................    1      1    0B2
...............................................................Ggaauuccggaucuugaauua........................    4      1    0B2
...............................................................ugaauuccggaucuugaauAag.......................    9      1    0B2
...............................................................ugaauuccggaucuAgaauuag.......................    2559   1    0B2
...............................................................ugaauuccggGucuugaauuag.......................    1      1    0B2
...............................................................Ngaauuccggaucuugaauuag.......................    6      1    0B2
...............................................................ugaGuuccggaucuugaauuag.......................    13     1    0B2
...............................................................ugaauuccggaucuugGauuag.......................    1      1    0B2
...............................................................ugaauuccggaucuugaauuag.......................    725    0    0B2
...............................................................ugaauuccggaucuugaauCag.......................    13     1    0B2
...............................................................ugaauuccggaucuGgaauuag.......................    1      1    0B2
...............................................................ugaauuccggaucuugaauuagu......................    1      0    0B2
...............................................................ugaauuccggaucuAgaauuagu......................    170    1    0B2
...............................................................ugaauuccggaucuugaauuagA......................    1      1    0B2
...............................................................ugaauuccggaucuugaauCagu......................    3      1    0B2
...............................................................ugaauuccggaucuAgaauuagua.....................    21     1    0B2
...............................................................ugaauuccggaucuugaauuagua.....................    9      0    0B2
...............................................................ugaauuccggaucuugaauuaUua.....................    10     1    0B2
...............................................................ugaauuccggaucuugaauuaguaA....................    1      1    0B2
...............................................................ugaauuccggaucuAgaauuaguau....................    4      1    0B2
................................................................gaauuccggaucuAgaau..........................    1      1    0B2
................................................................gaauuccggaucuugaau..........................    21     0    0B2
................................................................gaauuccggaucuAgaauu.........................    86     1    0B2
................................................................gaauuccggaucuugaauu.........................    131    0    0B2
................................................................gaGuuccggaucuugaauua........................    7      1    0B2
................................................................gaauuccggaucuugCauua........................    1      1    0B2
................................................................gaauuccggaucuugaauAa........................    2      1    0B2
................................................................gaauuccggaucuAgaauua........................    431    1    0B2
................................................................gaauuccggaucuugaauua........................    687    0    0B2
................................................................gaauuccggaucAugaauua........................    2      1    0B2
................................................................gaauuccggaucuugaauCa........................    59     1    0B2
................................................................gaaGuccggaucuugaauua........................    3      1    0B2
................................................................Naauuccggaucuugaauua........................    10     1    0B2
................................................................gaauuccggaucuCgaauuag.......................    1      1    0B2
................................................................gaauuccggaucuugaaCuag.......................    3      1    0B2
................................................................gaauuccggaucuugaauuag.......................    492    0    0B2
................................................................gaGuuccggaucuugaauuag.......................    1      1    0B2
................................................................gaauuccggaucuugaauCag.......................    3      1    0B2
................................................................Naauuccggaucuugaauuag.......................    5      1    0B2
................................................................gaauuccggaucuAgaauuag.......................    2009   1    0B2
................................................................gaauuccggaucCugaauuag.......................    1      1    0B2
................................................................gaauuccggaucuugaauuagA......................    1      1    0B2
................................................................gaauuccggaucuAgaauuagu......................    50     1    0B2
................................................................gaauuccggaucuugaauuaUua.....................    1      1    0B2
................................................................gaauuccggaucuAgaauuagua.....................    7      1    0B2
.................................................................aauuccggaucuugaauu.........................    7      0    0B2
.................................................................aCuuccggaucuugaauua........................    1      1    0B2
.................................................................aauuccggaucuugaauua........................    313    0    0B2
```

Star Mature

```
uugauuucuggaucuugaauuaguuugaaucuugauuucgggaucuugaauuagugugugaaucuugaauuccggaucuugaauuaguaugaaucuugaauucuagaucu
..................................................................aauuccggaucuCgaauua.........................   1      1   0B2
..................................................................aauuccggaucuugaauAa.........................   4      1   0B2
..................................................................aauuccggaCcuugaauua.........................   1      1   0B2
..................................................................aauuccggaucuugaauCa.........................   8      1   0B2
..................................................................aauuccggaucuugaaGuag........................   1      1   0B2
..................................................................aauAccggaucuugaauuag........................   2      1   0B2
..................................................................Nauuccggaucuugaauuag........................   12     1   0B2
..................................................................aauuccggaucuuUaauuag........................   7      1   0B2
..................................................................aauuccggaucuugaauuag........................   3015   0   0B2
..................................................................aauuccggaucuugaauCag........................   13     1   0B2
..................................................................aGuuccggaucuugaauuag........................   35     1   0B2
..................................................................aauuccggaucuCgaauuag........................   2      1   0B2
..................................................................aauuccggaucuugaauAag........................   1      1   0B2
..................................................................aauuccggaGcuugaauuag........................   1      1   0B2
..................................................................aauuccggaucuugGauuag........................   11     1   0B2
..................................................................aauuccggaucAugaauuag........................   1      1   0B2
..................................................................aauuccggaCcuugaauuag........................   1      1   0B2
..................................................................aaGuccggaucuugaauuag........................   7      1   0B2
..................................................................aauuccggaucuugaauuagu.......................   3      0   0B2
..................................................................aauuccggaucuugaauuagA.......................   9      1   0B2
..................................................................aauuccggaucuugaauuaUua......................   7      1   0B2
..................................................................aauuccggaucuAgaauuagua......................   47     1   0B2
..................................................................aauuccggaucuAgaauuaguau.....................   1      1   0B2
..................................................................aGuuccggaucuugaauuaguau.....................   2      1   0B2
...................................................................auuccggaucuugaauCa.........................   4      1   0B2
...................................................................Nuuccggaucuugaauua.........................   2      1   0B2
...................................................................auuccggaGcuugaauua.........................   1      1   0B2
...................................................................auuccggaucuugaauua.........................   110    0   0B2
...................................................................auuccggaucuugaauAa.........................   1      1   0B2
...................................................................auuccggGucuugaauuag........................   1      1   0B2
...................................................................Nuuccggaucuugaauuag........................   3      1   0B2
...................................................................auuccggaucuugaauCag........................   8      1   0B2
...................................................................auuccggauAuugaauuag........................   1      1   0B2
...................................................................auuccggaucAugaauuag........................   5      1   0B2
...................................................................auuccggaucuugaauuag........................   701    0   0B2
...................................................................auuccggaGcuugaauuag........................   2      1   0B2
...................................................................Guuccggaucuugaauuag........................   9      1   0B2
...................................................................aCuccggaucuugaauuagu.......................   1      1   0B2
...................................................................auuccggaucuugaauuagu.......................   131    0   0B2
...................................................................auuccggaucuugaauuagA.......................   44     1   0B2
...................................................................auuccggaucuGgaauuagu.......................   9      1   0B2
...................................................................auuccggaucuugaGuuagu.......................   1      1   0B2
...................................................................auuccggaucuugaauuaUua......................   12     1   0B2
...................................................................auuccggaucuGgaauuagua......................   2      1   0B2
...................................................................auuccggUucuugaauuagua......................   6      1   0B2
...................................................................auuccggaucuAgaauuagua......................   366    1   0B2
...................................................................Nuuccggaucuugaauuagua......................   1      1   0B2
...................................................................auuccggaucuugaauuaguU......................   3      1   0B2
...................................................................auuccggaucuugaauuagua......................   29     0   0B2
...................................................................auuccggaucuugaauuaguaA.....................   17     1   0B2
...................................................................auuccggaucuAgaauuaguau.....................   14     1   0B2
...................................................................auuccggaucuAgaauuaguaug....................   4      1   0B2
....................................................................uuccggaucuugGauuag........................   1      1   0B2
....................................................................uuccggaGcuugaauuag........................   1      1   0B2
....................................................................Nuccggaucuugaauuag........................   6      1   0B2
....................................................................uuccggaucuGgaauuag........................   2      1   0B2
....................................................................uuccggaucuugaauuag........................   503    0   0B2
....................................................................uuccggaucuugaauCag........................   5      1   0B2
....................................................................uuccggaucuugaauuagA.......................   12     1   0B2
....................................................................uuccggaucuugaauuagu.......................   79     0   0B2
....................................................................Nuccggaucuugaauuagu.......................   1      1   0B2
....................................................................uuccggaucuGgaauuagu.......................   17     1   0B2
....................................................................Nuccggaucuugaauuagua......................   1      1   0B2
....................................................................uuccggaucuugaauuagua......................   24     0   0B2
....................................................................uuccggaucuugaauuaguU......................   1      1   0B2
....................................................................uuccggaucuGgaauuagua......................   2      1   0B2
....................................................................uuccgUaucuugaauuagua......................   1      1   0B2
....................................................................uuccggaucuAgaauuagua......................   370    1   0B2
....................................................................uuccggaucuugaauuGgua......................   4      1   0B2
....................................................................uuccggaucuugaauuaUua......................   5      1   0B2
```

```
                                   Star                      Mature
uugauuucuggaucuugaauuaguuugaaucuugauuucgggaucuugaauuagugugaaucuugaauuccggaucuugaauuaguaugaaucuugaauucuagaucu
...................................................................uuccggaucuugaaGuagua.....................   1     1    0B2
...................................................................uuccggaucuugaauuaguaA....................   6     1    0B2
...................................................................uuccggUucuugaauuaguau....................   4     1    0B2
...................................................................uuccggaucuAgaauuaguau....................   21    1    0B2
...................................................................uuccggaucuAgaauuaguaug...................   2     1    0B2
...................................................................uuccggaucuAgaauuaguauga..................   11    1    0B2
...................................................................uuccggaucuAgaauuaguaugaauc...............   5     1    0B2
....................................................................uccggaucuugaauuagu......................   5     0    0B2
....................................................................uccggaucuGgaauuagu......................   15    1    0B2
....................................................................uccggaucuugaauuagua.....................   16    0    0B2
....................................................................uccggaucuAgaauuagua.....................   188   1    0B2
....................................................................uccggaucuugaauuaguUu....................   1     1    0B2
....................................................................uccggaucuugaauuaguau....................   2     0    0B2
....................................................................uccggaucuugaauuaguaA....................   2     1    0B2
....................................................................uccggaucuAgaauuaguau....................   13    1    0B2
....................................................................uccggaucuAgaauuaguaug...................   2     1    0B2
....................................................................uccggaucuugaauuaguaAg...................   1     1    0B2
.....................................................................ccggaucuAgaauuagua.....................   38    1    0B2
.....................................................................ccggUucuugaauuagua.....................   1     1    0B2
.....................................................................ccggaucuugaauuaguaA....................   14    1    0B2
.....................................................................ccggaucuAgaauuaguaug...................   2     1    0B2
......................................................................cggaucuAgaauuaguau....................   1     1    0B2
......................................................................cggaucuugaauuaguaA....................   22    1    0B2
......................................................................cggaucuugaauuaguNug...................   1     1    0B2
......................................................................cggaucuAgaauuaguauga..................   5     1    0B2
......................................................................cggaucuugaauuaguaugaa.................   1     0    0B2
..........................................................................ucuAgaauuaguaugaaucu..............   1     1    0B2
```

Provisional ID	: scaffold16_4767
Score total	: 539.7
Score for star read(s)	: 3.9
Score for read counts	: 532.4
Score for mfe	: 2.4
Score for randfold	: 1.6
Score for cons. seed	: -0.6
Total read count	: 1056
Mature read count	: 950
Loop read count	: 0
Star read count	: 106

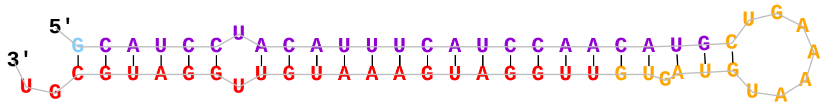

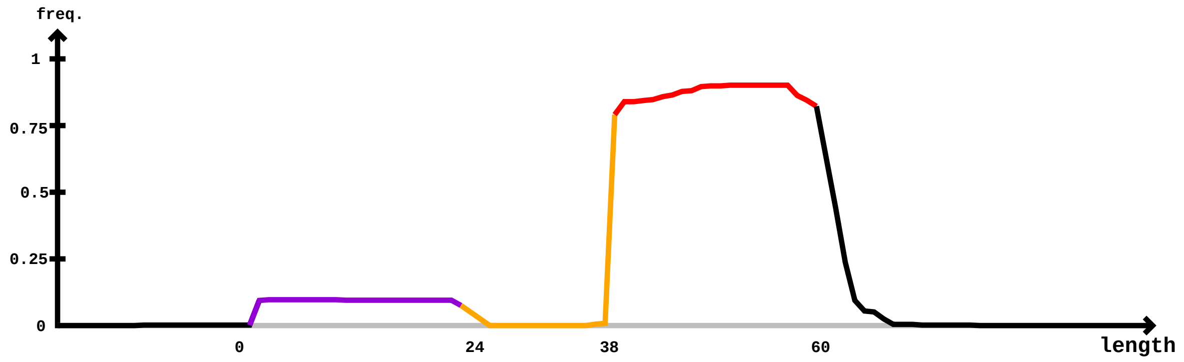

Star Mature

```
5'- guucagccccggucaaacgcauccuacauuucauccaacaugcugaaaauguaguguuggaugaaauguuggaugcguuugaccacccauguugugucauccaacauuugca   -3'   obs
    guucagccccggucaaacgcauccuacauuucauccaacaugcugaaaauguaguguuggaugaaauguuggaugcguuugaccacccauguugugucauccaacauuugca         exp
    .....((...((((((((((((((.((((((((((((((((((.......))).))))))))))))))).))))))))))))))....((((((.(....)))))))..)).   reads   mm   sample
    .......cccggucaaacgcauccAaca....................................................................................   2       1    0G2
    ...................cauccuacauuucauccaaca........................................................................   8       0    0G2
    ...................cauccuacauuucauccaacau.......................................................................   14      0    0G2
    ...................cauccuacauuucauccaacauU......................................................................   12      1    0G2
    ...................cauccuacauuucauccaacauA......................................................................   14      1    0G2
    ........................................................uuggaugaaauguuggauA.....................................   9       1    0G2
    ........................................................uuggaugaaauAuuggaug.....................................   3       1    0G2
    ........................................................uuggaugaaauguuggaugc....................................   7       0    0G2
    ........................................................uuggaugaaauguuggaugcg...................................   8       0    0G2
    ........................................................uuggaugaaauguuggaugcCu..................................   3       1    0G2
    ........................................................uuAgaugaaauguuggaugcgu..................................   1       1    0G2
    ........................................................uuggaugaaauUuuggaugcgu..................................   11      1    0G2
    ........................................................uuggaugaaauguuggauAcgu..................................   6       1    0G2
    ........................................................uuggaugaaauguuggaugcgu..................................   18      0    0G2
    ........................................................uuggaugaaauUuuggaugcguu.................................   4       1    0G2
    ........................................................uuggaugaaaCguuggaugcguu.................................   1       1    0G2
    ........................................................uuggaugaaauguuggaugcguu.................................   42      0    0G2
    ........................................................uuggGugaaauguuggaugcguuu................................   1       1    0G2
    ........................................................uuggaugaaauguuggaugcguuu................................   71      0    0G2
    ........................................................uuggaugaaauguuggaugcguAu................................   6       1    0G2
    ........................................................uuggaugaaauguuggaugcguuuA...............................   38      1    0G2
    ........................................................uuggaugaaauguuggaugcguuuU...............................   14      1    0G2
    ........................................................uuggaugaaauguuggaugcguuuUa..............................   16      1    0G2
    .........................................................uggaCgaaauguuggaug.....................................   2       1    0G2
    .........................................................uggaugaaauguuggaugc....................................   4       0    0G2
    .........................................................uggaugaaauguuggaugcguu.................................   3       0    0G2
    .........................................................uggaugaaauguuggaugcguuuA...............................   1       1    0G2
    .........................................................uggaugaaauguuggaugcguuuUa..............................   3       1    0G2
    .........................................................uggaugaaauguuggaugcguuugacc............................   1       0    0G2
    ...........................................................gaugaaauguuggaugcguuuUa..............................   5       1    0G2
    .............................................................ugaaGuguuggaugcguuuga..............................   3       1    0G2
    .................................................................auguuggaugcguuugacca...........................   12      0    0G2

    ...................cauccuacauuucauccaaca........................................................................   5       0    0B2
```

```
                   Star                                    Mature
guucagccccggucaaacgcauccuacauuucauccaacaugcugaaaauguaguguuggaugaaauguuggaugcguuugaccacccauguugugucauccaacauuugca
...................cauccuacauuucauccaacau.......................................................................  10  0  0B2
...................cauccuacauuucauccaacauA......................................................................  8   1  0B2
....................auccuacauuucauccaaca........................................................................  3   0  0B2
......................................................uguuggaugaaauAuuggaug.....................................  1   1  0B2
......................................................uguuggaugaaauguuggauA......................................  4   1  0B2
.......................................................guuggaugaaauguuggauA......................................  2   1  0B2
........................................................uuggaugaaauguuggauA......................................  2   1  0B2
........................................................uuggaugaaauAuuggaugc....................................  1   1  0B2
........................................................uuggaugaaauguuggaugc....................................  1   0  0B2
........................................................uuggaugaaauguuggaugcg...................................  5   0  0B2
........................................................uuggaugaaauUuuggaugcgu..................................  16  1  0B2
........................................................uuggaugaaauguuggaugcgu..................................  79  0  0B2
........................................................uuggaugaaauguuggaugcgA..................................  2   1  0B2
........................................................uuggaugaaauguuggaugUgu..................................  1   1  0B2
........................................................uuggaugaaauguuggauAcgu..................................  1   1  0B2
........................................................uuAgaugaaauguuggaugcguu.................................  6   1  0B2
........................................................uuggaugaaauguuggaugcguu.................................  52  0  0B2
........................................................uuggaugaaauUuuggaugcguu.................................  11  1  0B2
........................................................uuggaugaaauguuggaugcAuu.................................  3   1  0B2
........................................................uuggaugaaauguuggaGgcguu.................................  1   1  0B2
........................................................uuggaugaaauguuggaugcguAu................................  4   1  0B2
........................................................uuggaugaaauguuggaugcguuu................................  78  0  0B2
........................................................uuggaugaaauUuuggaugcguuu................................  2   1  0B2
........................................................Nuggaugaaauguuggaugcguuu................................  1   1  0B2
........................................................uuggaugaaauguuggaugcguuuU...............................  25  1  0B2
........................................................uuggaugaaauguuggaugcguuuA...............................  24  1  0B2
........................................................uuggaugaaauguuggaugcguuuUa..............................  8   1  0B2
.........................................................uggaugaaauguuggauA......................................  3   1  0B2
.........................................................uggaugaaauguuggaugcgu..................................  2   0  0B2
.........................................................uggaugaaauUuuggaugcgu..................................  3   1  0B2
.........................................................uggaugaaauguuggaugcguu.................................  5   0  0B2
.........................................................uggaugaaauguuggaugcgAu.................................  1   1  0B2
.........................................................uAgaugaaauguuggaugcguuu................................  2   1  0B2
.........................................................uggaugaaauguuggaugcguuu................................  3   0  0B2
............................................................augaaauguuggaugcguuug...............................  4   0  0B2
.............................................................ugaaauguuggaugcguuugacU............................  2   1  0B2
.............................................................ugaaauguuggaugcguuugacA............................  1   1  0B2
..............................................................gaaauguuggaugcguuuA...............................  1   1  0B2
..............................................................gaaGuguuggaugcguuuga..............................  3   1  0B2
...............................................................aUauguuggaugcguuugac.............................  4   1  0B2
...............................................................aaauguuggaugcguuugacA............................  10  1  0B2
.................................................................auguuggaugcguuugacca...........................  3   0  0B2
....................................................................uuggaugcguuugaccaAcc........................  3   1  0B2

...................cauccuacauuucaCccaaca........................................................................  1   1  0A2
...................cauccuacauuucauccaaca........................................................................  5   0  0A2
...................cauccuacauuucauccaacau.......................................................................  10  0  0A2
...................cauccuacauuucauccaacauA......................................................................  16  1  0A2
......................................................uguuggaugaaauguuggaugcA...................................  1   1  0A2
.......................................................guuggaugaaauguuggaugc....................................  1   0  0A2
........................................................uuggaugaaauguuggaug.....................................  3   0  0A2
........................................................uuggaugaaauguuggauA......................................  16  1  0A2
........................................................uuggaugaaauguuggaugc....................................  6   0  0A2
........................................................uuggaugaaauguuggauUcg...................................  1   1  0A2
........................................................uuggaugaaauguuggaugcg...................................  10  0  0A2
........................................................uuggaugaaauguuggaugcCu..................................  2   1  0A2
........................................................Nuggaugaaauguuggaugcgu..................................  1   1  0A2
........................................................uuAgaugaaauguuggaugcgu..................................  3   1  0A2
........................................................uuggaugaaauUuuggaugcgu..................................  15  1  0A2
........................................................uuggaugaaauguuggaugcgu..................................  44  0  0A2
........................................................uuggaugaaauguuggauAcgu..................................  8   1  0A2
........................................................uuggaugaaauguuggaugUguu.................................  2   1  0A2
........................................................uuggaugaaauguuggaugcgAu.................................  2   1  0A2
........................................................uuggaugaaauUuuggaugcguu.................................  7   1  0A2
........................................................uuggaugaaauguuggaugcguu.................................  65  0  0A2
........................................................uuggaugaaauUuuggaugcguuu................................  2   1  0A2
........................................................uuggaugaaauguuggaugcguuC................................  6   1  0A2
........................................................uuggaugaaauguuggaugcguuu................................  40  0  0A2
........................................................uuggaugaaauguuggaugcguAu................................  8   1  0A2
........................................................uuggaugaaauguuggaugcguuuU...............................  36  1  0A2
```

Star Mature

```
guucagccccggucaaacgcauccuacauuucauccaacaugcugaaaauguaguguuggaugaaauguuggaugcguuugaccacccauguugugucauccaacauuugca
........................................................uuggaugaaauguuggaugcguuuA...............................   14   1   0A2
.........................................................uggaugaaauguuggaugcguu.................................   4    0   0A2
.........................................................uggaugaaauguuggaugcguuu................................   3    0   0A2
.........................................................uggaugaaauguuggaugcguuuA...............................   3    1   0A2
.........................................................uggaugaaauguuggaugcguuuUa..............................   1    1   0A2
.........................................................uggaugaaauguuggaugcguuugacc............................   10   0   0A2
............................................................ugaaauguuggaugcguuuga...............................   5    0   0A2
............................................................ugaaauguuggaugcguuugacA.............................   1    1   0A2
.............................................................gaaauguuggaugcguuugacA.............................   3    1   0A2
..............................................................aaauguuggaugcguuugacA.............................   1    1   0A2
................................................................Uauguuggaugcguuugacca...........................   3    1   0A2
.................................................................auguuggaugcguuugacca...........................   2    0   0A2
..................................................................uguuggaugcguuugacca...........................   3    0   0A2
...........................................................................cguuugaccaAccauguug..................   2    1   0A2
```

Provisional ID	:	scaffold17_4901
Score total	:	3.4
Score for star read(s)	:	-1.3
Score for read counts	:	0
Score for mfe	:	0.1
Score for randfold	:	1.6
Score for cons. seed	:	3
Total read count	:	1190
Mature read count	:	1190
Loop read count	:	0
Star read count	:	0

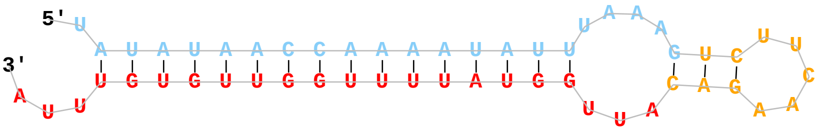

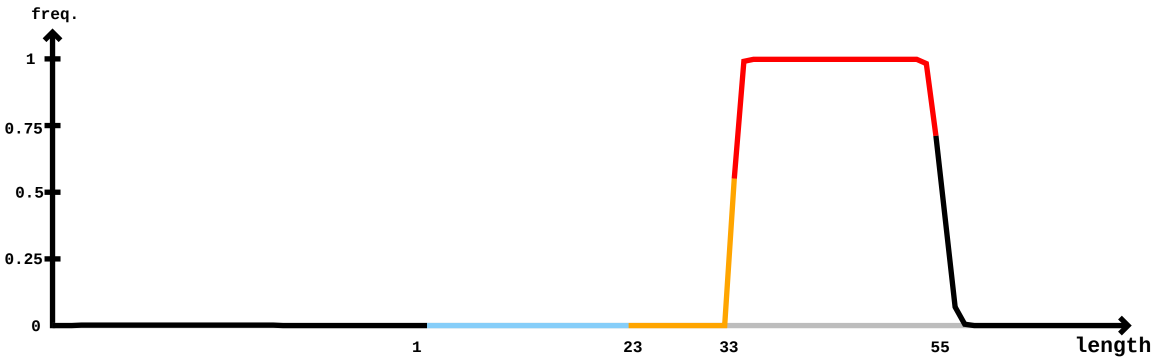

Star Mature

```
5'- auucggaugcugaucgcugaucauacacuugaacauauauauaaccaaaauauuuaaagucuucaagacauugguauuuugguuguguuuaugcucaaagauauaaaugu   -3'    exp
    ..........(((((...)))))).....((((.((((.((((((((((((((((....(((.....)))...)))))))))))))))).)))).))))............   reads    mm    sample
    .....................................................................Cuugguauuuugguuguguu.....................   4        1     0A2
    .....................................................................Cuugguauuuugguuguguuu....................   83       1     0A2
    .....................................................................Cuugguauuuugguuguguuua...................   150      1     0A2
    .....................................................................Cuugguauuuugguuguguuuau..................   12       1     0A2
    ......................................................................uugAuauuuugguuguguuu....................   23       1     0A2
    ......................................................................uugguauuuugguuguguuu....................   26       0     0A2
    ......................................................................uugguauuuugguuguguuua...................   93       0     0A2
    ......................................................................uugAuauuuugguuguguuua...................   40       1     0A2
    ......................................................................uugguauuuugguuguguuuU...................   3        1     0A2
    ......................................................................uugguauuuuggGuguguuua...................   1        1     0A2
    ......................................................................uugAuauuuugguuguguuuau..................   4        1     0A2
    ......................................................................uugguauuuugguuguguuuUu..................   4        1     0A2
    ......................................................................uugguauuuugguuguguuuauA.................   21       1     0A2
    ......................................................................uugguauuuugguuguguuuauC.................   2        1     0A2
    ......................................................................uugguauuuugguuguguuuauU.................   5        1     0A2
    .......................................................................ugguauuuugguuguguuua...................   1        0     0A2

    .uucggaugcugauUgcugauc........................................................................................   2        1     0B2
    .....................................................................Cuugguauuuugguuguguu.....................   7        1     0B2
    .....................................................................Cuugguauuuugguuguguuu....................   86       1     0B2
    .....................................................................Nuugguauuuugguuguguuu....................   1        1     0B2
    .....................................................................Cuugguauuuugguuguguuua...................   51       1     0B2
    .....................................................................Cuugguauuuugguuguguuuau..................   22       1     0B2
    ......................................................................uugguauuuugguuguguuu....................   9        0     0B2
    ......................................................................uugAuauuuugguuguguuu....................   5        1     0B2
    ......................................................................uugAuauuuugguuguguuua...................   15       1     0B2
    ......................................................................uugguauuuugguuguguuuU...................   5        1     0B2
    ......................................................................uugguauuuugguuguguuua...................   68       0     0B2
    ......................................................................uugguauuuugguuguguuuaA..................   2        1     0B2
    ......................................................................uugguauuuugguuguguuuau..................   13       0     0B2
    ......................................................................uugguauuuugguuguguuuauU.................   14       1     0B2
    ......................................................................uugguauuuugguuguguuuauA.................   6        1     0B2
    ......................................................................uugguauuuugguuguguuuauUc................   6        1     0B2
    .......................................................................ugguauuuugguuguguuua...................   1        0     0B2
```

```
                                    Star                          Mature
auucggaugcugaucgcugaucauacacuugaacauauauauaaccaaaauauuuaaagucuucaagacauugguauuuugguuguguuuaugcucaaagauauaaaugu
.......................................................................ugguauuuuUguuguguuua...................   1     1    0B2

.....................................................................Cuugguauuuugguugugu......................   8     1    0G2
.....................................................................Cuugguauuuugguuguguu.....................   60    1    0G2
.....................................................................Cuugguauuuugguuguguuua...................   158   1    0G2
.....................................................................Cuugguauuuugguuguguuuau..................   15    1    0G2
......................................................................uugguauuuugguuguguu.....................   21    0    0G2
......................................................................uugAuauuuugguuguguu.....................   6     1    0G2
......................................................................uugguauuuuggCuguguu.....................   1     1    0G2
......................................................................uugAuauuuugguuguguuua...................   19    1    0G2
......................................................................uugguauuuugguuguguuuU...................   5     1    0G2
......................................................................uugguauuuugguuguguuua...................   44    0    0G2
......................................................................uugguauuuugguuguguuuau..................   32    0    0G2
......................................................................uugAuauuuugguuguguuuau..................   2     1    0G2
......................................................................uugguauuuugguuguguuuauU.................   2     1    0G2
......................................................................uugguauuuugguuguguuuauA.................   27    1    0G2
.......................................................................ugguauuuugguuguguu.....................   2     0    0G2
.......................................................................ugguauuuugguuguguuua...................   3     0    0G2
.......................................................................uggGauuuugguuguguuua...................   1     1    0G2
```

Provisional ID	:	scaffold17_4903
Score total	:	2.4
Score for star read(s)	:	-1.3
Score for read counts	:	0
Score for mfe	:	-0.8
Score for randfold	:	1.6
Score for cons. seed	:	3
Total read count	:	1190
Mature read count	:	1190
Loop read count	:	0
Star read count	:	0

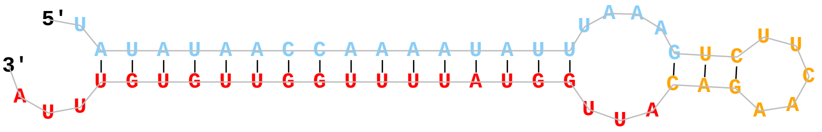

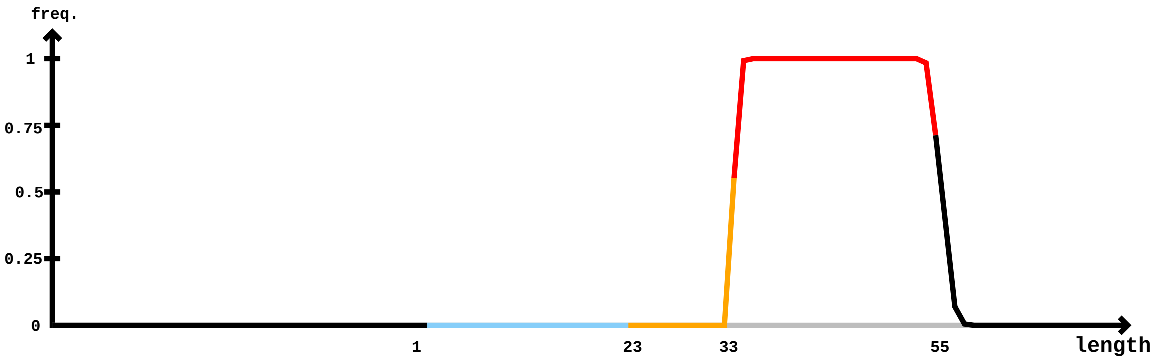

Star Mature

```
5'- auucagaugccgcucuccgaucauacacuugaacauauauauaaccaaaauauuuaaagucuucaagacauugguauuuugguuguguuuaugcucaaagauauaaaugu  -3'   exp
    ............................((((.((((.((((((((((((((((....(((.....)))...)))))))))))))))).)))).))))............   reads   mm   sample
    .....................................................................Cuugguauuuugguuguguu.....................   4       1    0A2
    .....................................................................Cuugguauuuugguuguguuu....................   83      1    0A2
    .....................................................................Cuugguauuuugguuguguuua...................   150     1    0A2
    .....................................................................Cuugguauuuugguuguguuuau..................   12      1    0A2
    ......................................................................uugguauuuugguuguguuu....................   26      0    0A2
    ......................................................................uugAuauuuugguuguguuu....................   23      1    0A2
    ......................................................................uugguauuuugguuguguuua...................   93      0    0A2
    ......................................................................uugguauuuuggGuguguuua...................   1       1    0A2
    ......................................................................uugguauuuugguuguguuuU...................   3       1    0A2
    ......................................................................uugAuauuuugguuguguuua...................   40      1    0A2
    ......................................................................uugAuauuuugguuguguuuau..................   4       1    0A2
    ......................................................................uugguauuuugguuguguuuUu..................   4       1    0A2
    ......................................................................uugguauuuugguuguguuuauC.................   2       1    0A2
    ......................................................................uugguauuuugguuguguuuauU.................   5       1    0A2
    ......................................................................uugguauuuugguuguguuuauA.................   21      1    0A2
    .......................................................................ugguauuuugguuguguuua...................   1       0    0A2

    .....................................................................Cuugguauuuugguuguguu.....................   8       1    0G2
    .....................................................................Cuugguauuuugguuguguuu....................   60      1    0G2
    .....................................................................Cuugguauuuugguuguguuua...................   158     1    0G2
    .....................................................................Cuugguauuuugguuguguuuau..................   15      1    0G2
    ......................................................................uugguauuuuggCuguguuu....................   1       1    0G2
    ......................................................................uugguauuuugguuguguuu....................   21      0    0G2
    ......................................................................uugAuauuuugguuguguuu....................   6       1    0G2
    ......................................................................uugguauuuugguuguguuua...................   44      0    0G2
    ......................................................................uugAuauuuugguuguguuua...................   19      1    0G2
    ......................................................................uugguauuuugguuguguuuU...................   5       1    0G2
    ......................................................................uugAuauuuugguuguguuuau..................   2       1    0G2
    ......................................................................uugguauuuugguuguguuuau..................   32      0    0G2
    ......................................................................uugguauuuugguuguguuuauA.................   27      1    0G2
    ......................................................................uugguauuuugguuguguuuauU.................   2       1    0G2
    .......................................................................ugguauuuugguuguguuu....................   2       0    0G2
    .......................................................................ugguauuuugguuguguuua...................   3       0    0G2
    .......................................................................uggGauuuugguuguguuua...................   1       1    0G2
```

```
                                     Star                            Mature
auucagaugccgcucuccgaucauacacuugaacauauauauaaccaaaauauuuaaagucuucaagacauugguauuuugguuguguuuaugcucaaagauauaaaugu

.....................................................................Cuugguauuuugguuguguu.....................  7   1  0B2
.....................................................................Cuugguauuuugguuguguuu....................  86  1  0B2
.....................................................................Nuugguauuuugguuguguuu....................  1   1  0B2
.....................................................................Cuugguauuuugguuguguuua...................  51  1  0B2
.....................................................................Cuugguauuuugguuguguuuau..................  22  1  0B2
......................................................................uugguauuuugguuguguuu....................  9   0  0B2
......................................................................uugAuauuuugguuguguuu....................  5   1  0B2
......................................................................uugguauuuugguuguguuuU...................  5   1  0B2
......................................................................uugguauuuugguuguguuua...................  68  0  0B2
......................................................................uugAuauuuugguuguguuua...................  15  1  0B2
......................................................................uugguauuuugguuguguuuaA..................  2   1  0B2
......................................................................uugguauuuugguuguguuuau..................  13  0  0B2
......................................................................uugguauuuugguuguguuuauU.................  14  1  0B2
......................................................................uugguauuuugguuguguuuauA.................  6   1  0B2
......................................................................uugguauuuugguuguguuuauUc................  6   1  0B2
.......................................................................ugguauuuuUguuguguuua...................  1   1  0B2
.......................................................................ugguauuuugguuguguuua...................  1   0  0B2
```

Provisional ID	:	scaffold18_5260
Score total	:	3085.8
Score for star read(s)	:	3.9
Score for read counts	:	3074.9
Score for mfe	:	2.4
Score for randfold	:	1.6
Score for cons. seed	:	3
Total read count	:	6043
Mature read count	:	5614
Loop read count	:	0
Star read count	:	429

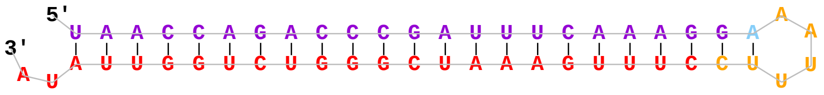

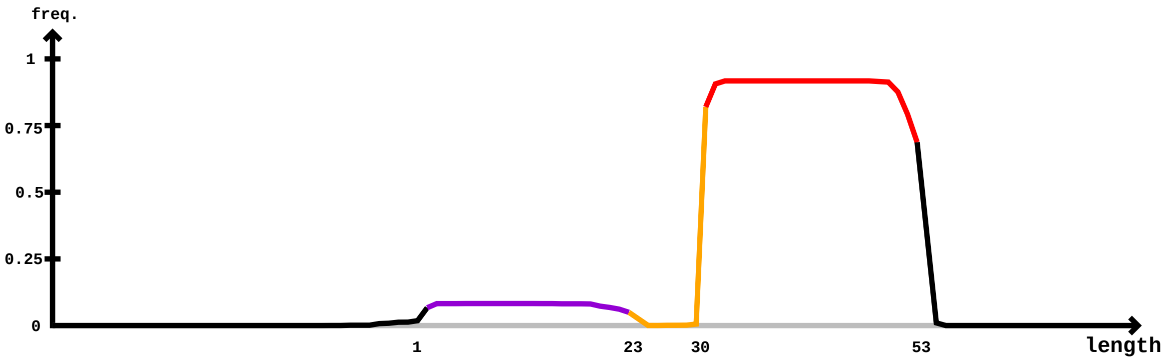


```
                                                 Star                        Mature
5'- aauaugaacgcgaaaaacguucauuauacuagccugauaaccagacccgauuucaaaggaaauuuccuuugaaaucgggucugguuauaaggcuaucauuauacgugaaca   -3'   obs
    aauaugaacgcgaaaaacguucauuauacuagccugauaaccagacccgauuucaaaggaaauuuccuuugaaaucgggucugguuauaaggcuaucauuauacgugaaca         exp
    ..................((((((((((.((((((.((((((((((((((((((((((((....)))))))))))))))))))))))).))))))....)))).)))))).     reads    mm    sample
    ...........................acuagccugauaaccagacccU..............................................................     1        1     0B2
    .............................uagccugauaacUagacccgau............................................................     5        1     0B2
    ..................................ugauaaccagaAccgauuucaa.......................................................     2        1     0B2
    ....................................auaaccagacccgauuucaaa......................................................     11       0     0B2
    ....................................aCaaccagacccgauuucaaag.....................................................     5        1     0B2
    .....................................Caaccagacccgauuuca........................................................     3        1     0B2
    .....................................Caaccagacccgauuucaaa......................................................     3        1     0B2
    .....................................Caaccagacccgauuucaaag.....................................................     26       1     0B2
    .....................................uaaccagacccgauuucaaaAg....................................................     2        1     0B2
    .....................................Caaccagacccgauuucaaagg....................................................     98       1     0B2
    .....................................uaGccagacccgauuucaaagg....................................................     1        1     0B2
    .....................................uaaccagacccgauuucaaagga...................................................     7        0     0B2
    ......................................aaccagacccgauuucaaag.....................................................     7        0     0B2
    ......................................aaccGgacccgauuucaaagg....................................................     5        1     0B2
    ......................................aaccagacccgauuucaaagg....................................................     32       0     0B2
    ......................................aaccagacGcgauuucaaagg....................................................     1        1     0B2
    ..............................................................uuuccuuuUaaaucgggucugg...........................     1        1     0B2
    .................................................................ccuuugaaaucgggucugguu.........................     1        0     0B2
    ..................................................................cuuugaaaucgggucugg...........................     6        0     0B2
    ..................................................................cuuugaaaucgggucuggu..........................     6        0     0B2
    ..................................................................cuuugaaaucgggucugguu.........................     72       0     0B2
    ..................................................................cuuugaaaucgggucugguC.........................     1        1     0B2
    ..................................................................Nuuugaaaucgggucugguua........................     2        1     0B2
    ..................................................................cuuugaaaucgggucugguua........................     101      0     0B2
    ..................................................................cuuugaaaucgggucugguuG........................     7        1     0B2
    ..................................................................cuuugaaaucgggucugguuC........................     5        1     0B2
    ..................................................................cuuugaaaCcgggucugguuau.......................     1        1     0B2
    ..................................................................cuuugaaaucgggucugguuGu.......................     14       1     0B2
    ..................................................................cuuugaaaucgggucugguuUu.......................     14       1     0B2
    ..................................................................cuuugaaaucgggucuggGuau.......................     1        1     0B2
    ..................................................................cuuuAaaaucgggucugguuau.......................     2        1     0B2
    ..................................................................cuuugaaaucgggucugguuau.......................     83       0     0B2
    ..................................................................cAuugaaaucgggucugguuau.......................     1        1     0B2
```

Star **Mature**

```
aauaugaacgcgaaaaacguucauuauacuagccugauaaccagacccgauuucaaaggaaauuuccuuugaaaucgggucugguuauaaggcuaucauuauacgugaaca
..................................................................Nuuugaaaucgggucugguuau.......................	2	1	0B2
..................................................................cuuugaaaGcgggucugguuaua......................	1	1	0B2
..................................................................Uuuugaaaucgggucugguuaua......................	32	1	0B2
..................................................................cuuugaaaucgggucuggCuaua......................	1	1	0B2
..................................................................cuuugaaaucgggucugguuaAa......................	273	1	0B2
..................................................................cuuugaaaucgggucugguuauG......................	2	1	0B2
..................................................................cuuugaaaucgggucCgguuaua......................	1	1	0B2
..................................................................cuuugaaaucgggucugguuGua......................	7	1	0B2
..................................................................Nuuugaaaucgggucugguuaua......................	3	1	0B2
..................................................................cuuugaaaucgggucugguuaCa......................	17	1	0B2
..................................................................cuuugaaaucgggucugguuUua......................	68	1	0B2
..................................................................cuuugaaauUgggucugguuaua......................	8	1	0B2
..................................................................cuuugaaaucgggucugguuaua......................	348	0	0B2
..................................................................cuuugaaaucgggucugguuaAaa.....................	35	1	0B2
..................................................................cuuugaaaucgggucugguuauaG.....................	1	1	0B2
..................................................................cuuugaaaucgggucugguuauaU.....................	16	1	0B2
..................................................................cuuugaaaucgggucugguuaCaa.....................	14	1	0B2
..................................................................cuuugaaaucgggucugguuauaC.....................	8	1	0B2
..................................................................cuuugaaaucgggucugguuauaa.....................	18	0	0B2
..................................................................Nuuugaaaucgggucugguuauaa.....................	1	1	0B2
..................................................................cuuugaaaucgggucugguuauaaU....................	3	1	0B2
..................................................................cuuugaaaucgggucugguuauaaA....................	1	1	0B2
...................................................................uuugaaaucgggucugguua........................	17	0	0B2
...................................................................uuugaaaucgggucugguuau.......................	23	0	0B2
...................................................................uuugaaaucgggucugguuGu.......................	3	1	0B2
...................................................................uuugaaaucgggucugguuaua......................	27	0	0B2
...................................................................uuugaaaucgggucugguuUua......................	6	1	0B2
...................................................................uuugaaaucggAucugguuaua......................	5	1	0B2
...................................................................uuugaaaucgggucugguuGua......................	1	1	0B2
...................................................................Nuugaaaucgggucugguuaua......................	1	1	0B2
...................................................................uuugaaaucgggucugguuaAa......................	26	1	0B2
...................................................................uuugaaaucgggucuUguuaua......................	3	1	0B2
...................................................................uuugaaaucgggucugguuUuaa.....................	3	1	0B2
...................................................................uuugaaaucgggucugguuauaaA....................	2	1	0B2
...................................................................uuugaaaucgggucugguuauaaC....................	1	1	0B2
....................................................................uugaaaucgggucugguA.........................	6	1	0B2

................................cAugauaaccagacccgauuuc.........................................................	2	1	0G2
................................cAugauaaccagacccgauuuca........................................................	15	1	0G2
..................................ugauaaccagacccgauuucaa.......................................................	3	0	0G2
..................................ugauaaccagaAccgauuucaaa......................................................	2	1	0G2
...................................gauaaccagacccgauuuca........................................................	2	0	0G2
....................................auaaccagacccgauuuca........................................................	2	0	0G2
....................................auaaccagacccgauuucaa.......................................................	1	0	0G2
....................................auaaccagacccgauuucaaa......................................................	2	0	0G2
....................................Nuaaccagacccgauuucaaa......................................................	1	1	0G2
.....................................uaaccGgacccgauuucaaa......................................................	4	1	0G2
.....................................Caaccagacccgauuucaaag.....................................................	2	1	0G2
.....................................uaaccagacccgauuucaaagg....................................................	7	0	0G2
.....................................Caaccagacccgauuucaaagg....................................................	10	1	0G2
.....................................uaaccagacccgauuucaaaAg....................................................	1	1	0G2
......................................aaccagacccgauuucaaag.....................................................	9	0	0G2
......................................aaccagacccgauuucaaaAga...................................................	1	1	0G2
.................................................................ccuuugaaaucgggucugguua........................	3	0	0G2
.................................................................cUuugaaaucgggucugguuaua.......................	8	1	0G2
..................................................................cuuugaaaucgggucugg...........................	6	0	0G2
..................................................................cuuugaaGucgggucuggu..........................	1	1	0G2
..................................................................cuuugaaaucgggucuggu..........................	4	0	0G2
..................................................................cuuugaaaucgggucugguu.........................	67	0	0G2
..................................................................cuuugaaaucgggucugguuG........................	3	1	0G2
..................................................................cuuugaaaucgggucugguua........................	111	0	0G2
..................................................................Nuuugaaaucgggucugguua........................	1	1	0G2
..................................................................cuuugaaaucgggucugguuUu.......................	12	1	0G2
..................................................................cuuugaaaucgggucugguuau.......................	163	0	0G2
..................................................................cuuugaaaucgggucugguuGu.......................	27	1	0G2
..................................................................Nuuugaaaucgggucugguuau.......................	1	1	0G2
..................................................................cAuugaaaucgggucugguuau.......................	1	1	0G2
..................................................................cuuugaaaucgggucugguuaG.......................	6	1	0G2
..................................................................cuuugaaaucgggucugguuaCa......................	6	1	0G2
..................................................................cuuugaaaucgggucugguuaAa......................	327	1	0G2
```

Star Mature

```
aauaugaacgcgaaaaacguucauuauacuagccugauaaccagacccgauuucaaaggaaauuuccuuugaaaucgggucugguuauaaggcuaucauuauacgugaaca
..................................................................cuuugaaauUgggucugguuaua......................  25   1  0G2
..................................................................Uuuugaaaucgggucugguuaua......................  79   1  0G2
..................................................................cuCugaaaucgggucugguuaua......................  3    1  0G2
..................................................................cuuugaaaucgggucugguuUua......................  57   1  0G2
..................................................................cNuugaaaucgggucugguuaua......................  1    1  0G2
..................................................................cuuugaaaucgggucugguuaua......................  423  0  0G2
..................................................................cuuugaaaucgggucugguuaCaa.....................  9    1  0G2
..................................................................cuuugaaaucgggucugguuaAaa.....................  37   1  0G2
..................................................................Uuuugaaaucgggucugguuauaa.....................  7    1  0G2
..................................................................cuuugaaaucgggucugguuauaU.....................  13   1  0G2
..................................................................cuuugaaaucgggucugguuauaC.....................  1    1  0G2
..................................................................cuuugaaaucgggucugguuauaG.....................  2    1  0G2
..................................................................cuuugaaaucgggucugguuGuaa.....................  2    1  0G2
..................................................................cuuugaaaucgggucugguuUuaa.....................  4    1  0G2
..................................................................cuuugaaaucgggucugguuauaa.....................  80   0  0G2
..................................................................cuuugaaaucgggucugguuauaaA....................  14   1  0G2
..................................................................cuuugaaaucgggucugguuauaGg....................  4    1  0G2
...................................................................uuugaaaucgggucugguua........................  23   0  0G2
...................................................................uuugaaaucgggucugguuG........................  7    1  0G2
...................................................................uuugaaaucgggucugguuau.......................  2    0  0G2
...................................................................uuugaaaucgggucugguuUua......................  12   1  0G2
...................................................................Cuugaaaucgggucugguuaua......................  2    1  0G2
...................................................................uuugaaaucgggucugguuaua......................  52   0  0G2
...................................................................uuugaaauUgggucugguuaua......................  8    1  0G2
...................................................................uuugaaaucgggucugguuaCa......................  4    1  0G2
...................................................................uuugaaaucgggucugguuaAa......................  24   1  0G2
...................................................................uuugaaaucggAucugguuaua......................  13   1  0G2
...................................................................uuugaaaucgggucugguuauaaU....................  1    1  0G2
....................................................................uugaaaucgggucugguua........................  2    0  0G2
....................................................................uugaaaucgggGcugguua........................  1    1  0G2
....................................................................uugaaaucgggucugguuau.......................  2    0  0G2
....................................................................uugaaaCcgggucugguuau.......................  1    1  0G2
....................................................................uugaaaucgggucugguuaua......................  19   0  0G2
....................................................................uugaGaucgggucugguuaua......................  2    1  0G2
....................................................................uugaaauUgggucugguuaua......................  4    1  0G2
....................................................................uugaaaucgggucugguuauaG.....................  1    1  0G2

.............................uagccugauaaccagacccgauuuA.........................................................  2    1  0A2
................................cAugauaaccagacccgauuuca........................................................  20   1  0A2
.................................cugauaaccaCacccgauuuca........................................................  1    1  0A2
.................................Augauaaccagacccgauuuca........................................................  7    1  0A2
..................................ugauaaccagacccgauuucaa.......................................................  2    0  0A2
..................................ugauaaccagaAccgauuucaa.......................................................  10   1  0A2
..................................ugauaaccagaAccgauuucaaa......................................................  5    1  0A2
....................................auaaccagacccgauuucaa.......................................................  4    0  0A2
....................................auaaccagacccgauuucaaa......................................................  1    0  0A2
....................................auaaccagaAccgauuucaaa......................................................  6    1  0A2
.....................................Caaccagacccgauuucaaa......................................................  3    1  0A2
.....................................uaaccagaAccgauuucaaa......................................................  1    1  0A2
.....................................uaaccagacccgauuucaaa......................................................  1    0  0A2
.....................................uaaccagacccgauuucaaag.....................................................  8    0  0A2
.....................................Caaccagacccgauuucaaag.....................................................  10   1  0A2
.....................................uaGccagacccgauuucaaagg....................................................  4    1  0A2
.....................................Caaccagacccgauuucaaagg....................................................  99   1  0A2
.....................................uaaccagacccgauuucaaaAg....................................................  4    1  0A2
.....................................Caaccagacccgauuucaaagga...................................................  4    1  0A2
......................................aaccagacccgauuucaa.......................................................  8    0  0A2
......................................aaccagacccgauuucaaaA.....................................................  2    1  0A2
......................................aaccagacccgauuucaaag.....................................................  3    0  0A2
......................................aaccagacccgauuucaaagg....................................................  3    0  0A2
......................................aaccagacccgauuucaaaAga...................................................  23   1  0A2
.........................................cagacccgauuucaaaAgaa..................................................  1    1  0A2
.........................................cagacccgauuucaaaAgaaa.................................................  1    1  0A2
..............................................................uuuccuuugaaauUgggucugguuau.......................  5    1  0A2
...............................................................uucUuuugaaaucgggucugguua........................  1    1  0A2
................................................................ucUuuugaaaucgggucugguua........................  1    1  0A2
................................................................ucUuuugaaaucgggucugguuau.......................  1    1  0A2
.................................................................ccuuugaaaucgggucugguua........................  2    0  0A2
.................................................................ccuuugaaaucgggucugUuua........................  6    1  0A2
.................................................................cUuuugaaaucgggucugguuaua......................  1    1  0A2
```

Star **Mature**

aauaugaacgcgaaaaacguucauuauacuagccugauaaccagacccgauuucaaaggaaauuuccuuugaaaucgggucugguuauaaggcuaucauuauacgugaaca			
....cUuuugaaaucgggucugguuauaa.....................	4	1	0A2
....cuuugaaaucgggucugg...........................	1	0	0A2
....cuuugaaaucgggucuggu..........................	2	0	0A2
....cuuugaaaucgggucugguu.........................	68	0	0A2
....cuuugaaaucgggucugguuG........................	10	1	0A2
....cuuugaaaucgggucugguua........................	186	0	0A2
....cuuugaaaucgggucugguuC........................	5	1	0A2
....cuuugaaauGgggucugguua........................	1	1	0A2
....Nuuugaaaucgggucugguua........................	1	1	0A2
....cuuugaGaucgggucugguua........................	1	1	0A2
....cuuugaaaucggguUugguuau.......................	1	1	0A2
....cuuugaaaucgggucugguuaC.......................	7	1	0A2
....cuuugaaaucgggucugguuGu.......................	40	1	0A2
....cuuugaaaucgggCcugguuau.......................	1	1	0A2
....cuuugaaaucgggucugguuUu.......................	33	1	0A2
....cuuugaGaucgggucugguuau.......................	1	1	0A2
....Nuuugaaaucgggucugguuau.......................	1	1	0A2
....cuuugaCaucgggucugguuau.......................	1	1	0A2
....cuuugaaaucgggucugguuau.......................	150	0	0A2
....cuuugaaaucgggucugguuUua......................	161	1	0A2
....cuuugaaauUgggucugguuaua......................	24	1	0A2
....cuuugaCaucgggucugguuaua......................	1	1	0A2
....cuuugaaaucgggCcugguuaua......................	1	1	0A2
....cuuugaaaucAggucugguuaua......................	1	1	0A2
....cuuugaGaucgggucugguuaua......................	3	1	0A2
....cuuugaaaucgggucuAguuaua......................	2	1	0A2
....cuuugaaaucgggucugguuaua......................	840	0	0A2
....cuuugaaaucgggucugguuaAa......................	341	1	0A2
....cuuugaaaucgggucugguuaCa......................	31	1	0A2
....Uuuugaaaucgggucugguuaua......................	53	1	0A2
....cuuugaaaucgggGcugguuaua......................	1	1	0A2
....cuuugaaaucgggucugUuuaua......................	1	1	0A2
....cuuugaaaGcgggucugguuaua......................	1	1	0A2
....cuuugaaaucggUucugguuaua......................	1	1	0A2
....cuuugaaaucgggucugguuGua......................	2	1	0A2
....cuuugaaaucggAucugguuaua......................	1	1	0A2
....Nuuugaaaucgggucugguuaua......................	4	1	0A2
....cuuugaaaucgggucugguuUuaa.....................	8	1	0A2
....Uuuugaaaucgggucugguuauaa.....................	4	1	0A2
....cCuugaaaucgggucugguuauaa.....................	5	1	0A2
....cuuugaaaucgggucugguuaCaa.....................	16	1	0A2
....Nuuugaaaucgggucugguuauaa.....................	1	1	0A2
....cuuugaaaucgggucugguuauaG.....................	11	1	0A2
....cuuugaaaucgggucugguuauaa.....................	131	0	0A2
....cuuugaaauUgggucugguuauaa.....................	3	1	0A2
....cuuugaaaucgggucugguuGuaa.....................	2	1	0A2
....cuuugaaaucgggucuggGuauaa.....................	1	1	0A2
....cuuugaaaucUggucugguuauaa.....................	1	1	0A2
....cuuugaaaucgggucugguuaAaa.....................	98	1	0A2
....cuuugaaaucgggucugguuauaU.....................	25	1	0A2
....cuuugaaaucgggucugguuauaaU....................	10	1	0A2
....cuuugaaaucgggucugguuauaaA....................	21	1	0A2
....uuugaaaucgggGcugguu.........................	1	1	0A2
....uuugaaaucgggucugguu.........................	11	0	0A2
....uuugaaaucgggucugguua........................	13	0	0A2
....uuuAaaaucgggucugguuau.......................	6	1	0A2
....uuugaaaucgggGcugguuau.......................	1	1	0A2
....uuugaaaucgggucugguuau.......................	35	0	0A2
....uuugaaaucgggucugguGau.......................	1	1	0A2
....uuugaaaucUggucugguuau.......................	1	1	0A2
....uuugaaaucgggucugguuUu.......................	1	1	0A2
....uuugaaaucgggucugguuaua......................	65	0	0A2
....uuugaaaucggAucugguuaua......................	19	1	0A2
....uuuAaaaucgggucugguuaua......................	1	1	0A2
....uuugaaauUgggucugguuaua......................	6	1	0A2
....uuugaaaucgggucugguuaAa......................	33	1	0A2
....uuugaaaucgggucugguuUua......................	46	1	0A2
....uuugaaaucggAucugguuauaa.....................	3	1	0A2
....uuugaaaucgggucugguuauaU.....................	3	1	0A2
....uuugaaaucgggucugguuauaa.....................	1	0	0A2

```
                                    Star                           Mature
aauaugaacgcgaaaaacguucauuauacuagccugauaaccagacccgauuucaaaggaaauuuccuuugaaaucgggucugguuauaaggcuaucauuauacgugaaca
...................................................................uuugaaaucgggucugguuaAaa.....................    10    1    0A2
...................................................................uuugaaaucgggucugguuauaaA....................    5     1    0A2
....................................................................uugaaaucggAucugguuaua......................    2     1    0A2
....................................................................uugaaaucggguGugguuaua......................    1     1    0A2
....................................................................uugaaaucgggucugguuaua......................    29    0    0A2
```

Provisional ID	:	scaffold28_7306
Score total	:	15094.3
Score for star read(s)	:	3.9
Score for read counts	:	15087.9
Score for mfe	:	1.5
Score for randfold	:	1.6
Score for cons. seed	:	-0.6
Total read count	:	29606
Mature read count	:	29602
Loop read count	:	0
Star read count	:	4

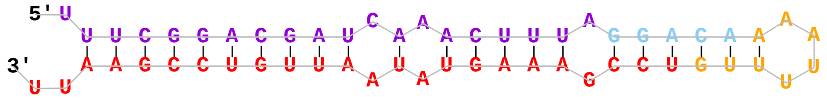

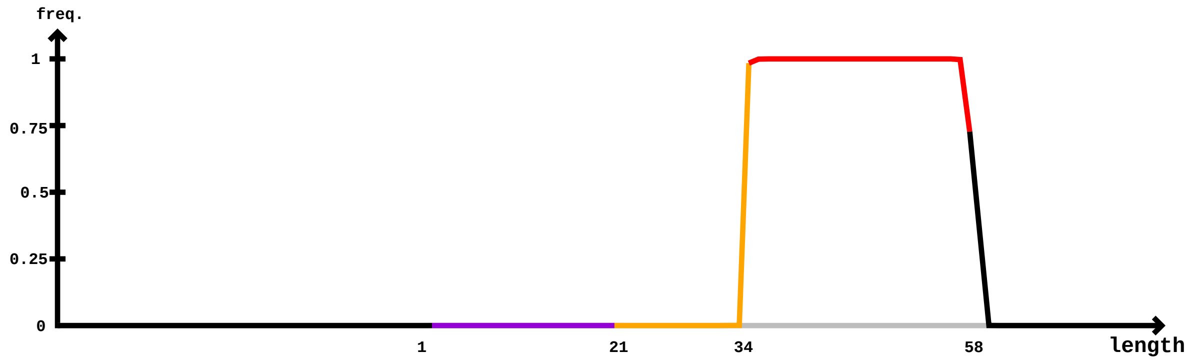

Star **Mature**

```
5'- aagggacaguaaucaaaauuacaguaaaaccgaaaaguuucggacgaucaaacuuuaggacaaaauuuuguccgaaaguauaauuguccgaauucuggugauguccgaaaguu   -3'   obs
    aagggacaguaaucaaaauuacaguaaaaccgaaaaguuucggacgaucaaacuuuaggacaaaauuuuguccgaaaguauaauuguccgaauucuggugauguccgaaaguu          exp
    ...((((((((((....)))))......((((((....((((((((((.(.(((((.((((((....)))))).))))).).))))))))))))).)))..)))))....... reads   mm   sample
    .....................................uuucggacgaucaaacuuuagg...................................................... 1       0    0G2
    ......................................................................uccgaaaguauaauuguccgaa..................... 25      0    0G2
    ......................................................................uccgaaaguauaauuguUcgaa..................... 1       1    0G2
    ......................................................................uccgaaaguauaauuguccgaU..................... 10      1    0G2
    ......................................................................uccgaaaguauaauuguccgaau.................... 12      0    0G2
    ......................................................................uccgaaaguUuaauuguccgaau.................... 2444    1    0G2
    ......................................................................uccgaaaguUuaauuguccgaauu................... 6481    1    0G2
    ......................................................................uccgaaaguauaauuguccgaauu................... 4       0    0G2
    ......................................................................uccgaaaguauaauuguccgaauA................... 3       1    0G2
    ......................................................................uccgaaaguCuaauuguccgaauu................... 2       1    0G2
    ......................................................................uccgaaaguUuaauuguccgaauuc.................. 7       1    0G2
    ......................................................................uccgaaaguUuaauuguccgaauucu................. 5       1    0G2
    .......................................................................ccgaaaguUuaauuguccgaau.................... 107     1    0G2
    .......................................................................ccgaaaguauaauuguccgaau.................... 8       0    0G2
    .......................................................................ccgaaaguauaauuguccgaauA................... 1       1    0G2
    .......................................................................ccgaaaguUuaauuguccgaauu................... 40      1    0G2
    ........................................................................cgaaaguUuaauuguccgaau.................... 6       1    0G2

    ......................................................................uccgaaaguGuaauuguccgaa..................... 1       1    0B2
    ......................................................................uccgaaaguNuaauuguccgaa..................... 1       1    0B2
    ......................................................................uccgaaaguauaauuguccgaa..................... 12      0    0B2
    ......................................................................uccgaaaguauaauuguccgaau.................... 27      0    0B2
    ......................................................................uccgaaaguauaauuguccgaaC.................... 1       1    0B2
    ......................................................................uccgaaaguUuaauuguccgaau.................... 2550    1    0B2
    ......................................................................Nccgaaaguauaauuguccgaau.................... 1       1    0B2
    ......................................................................uccgaaaguGuaauuguccgaau.................... 3       1    0B2
    ......................................................................uccgaaaguCuaauuguccgaau.................... 6       1    0B2
    ......................................................................uccgaaaguauaauuguccgaauu................... 11      0    0B2
    ......................................................................uccgaaaguNuaauuguccgaauu................... 1       1    0B2
    ......................................................................uccgaaaguauaauuguccgaauA................... 9       1    0B2
    ......................................................................uccgaaaguUuaauuguccgaauu................... 6612    1    0B2
    ......................................................................uccgaaaguUuaauuguccgaauuc.................. 14      1    0B2
    ......................................................................uccgaaaguUuaauuguccgaauucu................. 3       1    0B2
```

```
                                     Star                           Mature
aagggacaguaaucaaaauuacaguaaaaccgaaaaguuucggacgaucaaacuuuaggacaaaauuuuguccgaaaguauaauuguccgaauucuggugauguccgaaaguu
.......................................................................ccgaaaguauaauuguccgaau....................    1     0    0B2
.......................................................................ccgaaaguUuaauuguccgaau....................    107   1    0B2
.......................................................................ccgaaaguauaauuguccgaauA...................    2     1    0B2
.......................................................................ccgaaaguUuaauuguccgaauu...................    56    1    0B2
.......................................................................ccgaaaguCuaauuguccgaauu...................    1     1    0B2
........................................................................cgaaaguUuaauuguccgaau....................    4     1    0B2
........................................................................cgaaaguUuaauuguccgaauu...................    8     1    0B2

.....................................uuucggaGgaucaaacuuua........................................................    3     1    0A2
....................................................................uguccgaaaguUuaauuguccgaauu...................    11    1    0A2
......................................................................uccgaaaguCuaauuguccgaa.....................    1     1    0A2
......................................................................uccgaaaguauaauuguccgaa.....................    21    0    0A2
......................................................................uccgaaaguGuaauuguccgaa.....................    1     1    0A2
......................................................................Cccgaaaguauaauuguccgaa.....................    1     1    0A2
......................................................................uccgaaaguGuaauuguccgaau....................    1     1    0A2
......................................................................uccgaaaguUuaauuguccgaau....................    2617  1    0A2
......................................................................uccgaaaguauaauuguccgaau....................    32    0    0A2
......................................................................uccgaaaguGuaauuguccgaauu...................    2     1    0A2
......................................................................uccgaaaguauaauuguccgaauA...................    1     1    0A2
......................................................................uccgaaaguUuaauuguccgaauu...................    8190  1    0A2
......................................................................uccgaaaguUuaauuguccgaauuc..................    8     1    0A2
.......................................................................ccgaaaguauaauuguccgaau....................    5     0    0A2
.......................................................................ccgaaaguUuaauuguccgaau....................    68    1    0A2
.......................................................................ccgaaaguUuaauuguccgaauu...................    48    1    0A2
........................................................................cgaaaguUuaauuguccgaau....................    8     1    0A2
```

Provisional ID	:	scaffold37_9192
Score total	:	3294.8
Score for star read(s)	:	3.9
Score for read counts	:	3288.5
Score for mfe	:	1.4
Score for randfold	:	1.6
Score for cons. seed	:	-0.6
Total read count	:	6462
Mature read count	:	5150
Loop read count	:	0
Star read count	:	1312

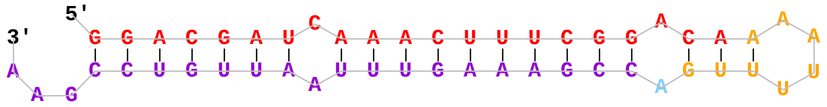

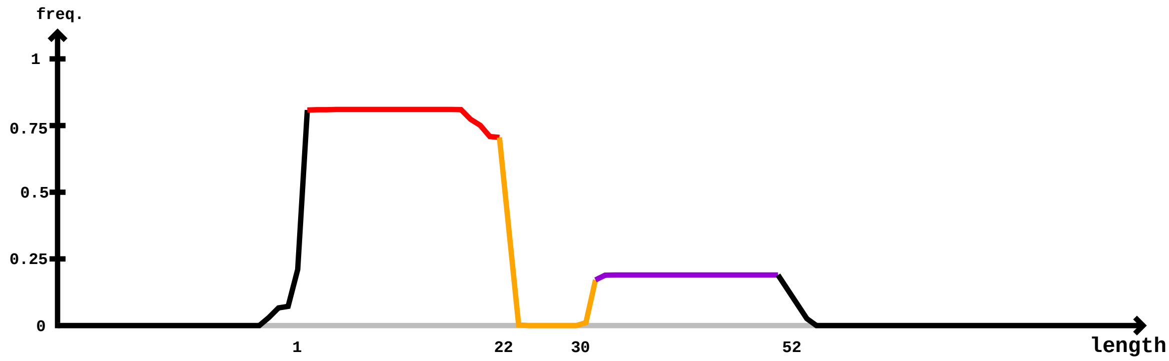


```
                                    Mature                        Star
5'- guuacauuaaaaccgaaaagauucggacgaucaaacuuucggacaaaauuuugaccgaaaguuuaauuguccgaaaucuugugauguccgaaaguuuuaaauuucauugug  -3'   obs
    guuacauuaaaaccgaaaagauucggacgaucaaacuuucggacaaaauuuugaccgaaaguuuaauuguccgaaaucuugugauguccgaaaguuuuaaauuucauugug        exp
    ...((((((........((((((((((((((.((((((((((.(((....))).)))))))))).)))))))).)))))).))))))..((((........))))......   reads   mm   sample
    ....................auucggacgaucaaacuuucg......................................................................   46      0    0B2
    ....................Nuucggacgaucaaacuuucg......................................................................   2       1    0B2
    ....................Nuucggacgaucaaacuuucgg.....................................................................   5       1    0B2
    ....................auucggacgaucaaacuuucgg.....................................................................   26      0    0B2
    ....................Nuucggacgaucaaacuuucgga....................................................................   2       1    0B2
    ....................auucggacgaucaaacuuucgga....................................................................   8       0    0B2
    .....................uucggacgaucaaacuuucg......................................................................   42      0    0B2
    .....................uucggacgaucGaacuuucg......................................................................   5       1    0B2
    .....................Nucggacgaucaaacuuucg......................................................................   1       1    0B2
    .....................Nucggacgaucaaacuuucgg.....................................................................   1       1    0B2
    .....................uucggacgaucaaacuuucgg.....................................................................   38      0    0B2
    .....................uucggacgaucaaacuuucggaca..................................................................   3       0    0B2
    ......................Ccggacgaucaaacuuucggaca..................................................................   7       1    0B2
    ......................ucggacAaucaaacuuucggaca..................................................................   7       1    0B2
    .......................cggacgaGcaaacuuucgga....................................................................   1       1    0B2
    .......................cggGcgaucaaacuuucgga....................................................................   14      1    0B2
    .......................Aggacgaucaaacuuucgga....................................................................   1       1    0B2
    .......................cggaAgaucaaacuuucgga....................................................................   3       1    0B2
    .......................cgUacgaucaaacuuucgga....................................................................   2       1    0B2
    .......................Nggacgaucaaacuuucgga....................................................................   49      1    0B2
    .......................cggacgGucaaacuuucgga....................................................................   2       1    0B2
    .......................cggacUaucaaacuuucgga....................................................................   15      1    0B2
    .......................cggacgCucaaacuuucggac...................................................................   1       1    0B2
    .......................cggacUaucaaacuuucggac...................................................................   7       1    0B2
    .......................Nggacgaucaaacuuucggac...................................................................   1       1    0B2
    .......................cggGcgaucaaacuuucggaca..................................................................   118     1    0B2
    .......................cggacgauGaaacuuucggaca..................................................................   3       1    0B2
    .......................cggacgaCcaaacuuucggaca..................................................................   57      1    0B2
    .......................cggacgGucaaacuuucggaca..................................................................   1       1    0B2
    .......................cggacgaGcaaacuuucggaca..................................................................   1       1    0B2
    .......................cggacUaucaaacuuucggaca..................................................................   33      1    0B2
    .......................Nggacgaucaaacuuucggaca..................................................................   31      1    0B2
    .......................cggacgCucaaacuuucggaca..................................................................   2       1    0B2
```

Mature Star

```
guuacauuaaaaccgaaaagauucggacgaucaaacuuucggacaaaauuuugaccgaaaguuuaauuguccgaaaucuugugauguccgaaaguuuuaaauuucauugug
.......................cgUacgaucaaacuuucggaca..................................................................   1      1   0B2
.......................cggacAaucaaacuuucggaca..................................................................   20     1   0B2
.......................cggacAaucaaacuuucggacaa.................................................................   4      1   0B2
.......................cggGcgaucaaacuuucggacaa.................................................................   3      1   0B2
.......................cggacUaucaaacuuucggacaa.................................................................   4      1   0B2
.......................Nggacgaucaaacuuucggacaa.................................................................   1      1   0B2
.......................Nggacgaucaaacuuucggacaaa................................................................   1      1   0B2
........................ggCcgaucaaacuuucgga....................................................................   1      1   0B2
........................ggacgaAcaaacuuucggac...................................................................   1      1   0B2
........................ggGcgaucaaacuuucggaca..................................................................   7      1   0B2
........................ggacAaucaaacuuucggaca..................................................................   1613   1   0B2
........................ggacUaucaaacuuucggaca..................................................................   113    1   0B2
........................ggacAaucaaacuuucggacaa.................................................................   1      1   0B2
........................ggacAaucaaacuuucggacaaa................................................................   4      1   0B2
.........................gacgaucaaacuuucggaca..................................................................   5      0   0B2
.........................gacgaucaaacuuucggacaaU................................................................   3      1   0B2
...........................cgaucaaacuuucggacGaa................................................................   7      1   0B2
.....................................................accgaaaguuuGauuguccgaa....................................   1      1   0B2
.....................................................Nccgaaaguuuaauuguccgaa....................................   20     1   0B2
.....................................................Cccgaaaguuuaauuguccgaa....................................   6      1   0B2
.....................................................Nccgaaaguuuaauuguccgaaa...................................   1      1   0B2
......................................................ccgaaaguuuaauGguccgaa....................................   1      1   0B2
......................................................ccgaaaguuuaauuguccgaa....................................   173    0   0B2
......................................................ccgaaaguuuaauuguccgaC....................................   1      1   0B2
......................................................cGgaaaguuuaauuguccgaa....................................   1      1   0B2
......................................................Ncgaaaguuuaauuguccgaa....................................   1      1   0B2
......................................................ccgaaaguuuaauuguccgaaU...................................   107    1   0B2
......................................................ccgaaaguuuaauuguccgaUa...................................   10     1   0B2
......................................................ccgaaaguuuaauuguccgaaC...................................   26     1   0B2
......................................................ccgaaaguuuaauuguccgaCa...................................   2      1   0B2
......................................................ccgaaaguuuaauuUuccgaaa...................................   1      1   0B2
......................................................ccgaaaguuuaauuguccgaaa...................................   20     0   0B2
......................................................ccgaaaguuuaauuguccgaaUu..................................   56     1   0B2
......................................................ccgaaaguuuaauuguccgaaaG..................................   1      1   0B2
......................................................ccgaaaguuuaauuguccgaaCu..................................   8      1   0B2
.......................................................cgaaaguuuaauuguccgaa....................................   29     0   0B2
.......................................................cgaaaguuuaauuguccgaaU...................................   4      1   0B2
.......................................................cgaaaguuuaauuguccgaaC...................................   2      1   0B2
.......................................................cgaaaguuuaauuguccgaaUu..................................   8      1   0B2
........................................................gaaaguuuaauuguccgaUa...................................   2      1   0B2

....................auucggacgaucaaacuuucg......................................................................   30     0   0G2
....................Nuucggacgaucaaacuuucg......................................................................   1      1   0G2
....................auucggacgaucaaacuuucgg.....................................................................   13     0   0G2
....................auucggacgaucaaacuuucggaA...................................................................   3      1   0G2
.....................uucggacgaucaaacuuucg......................................................................   48     0   0G2
.....................uucggacgaucaaaGuuucg......................................................................   2      1   0G2
.....................uucggacgaucaaacuuucgg.....................................................................   17     0   0G2
.....................uucggacgaucGaacuuucgga....................................................................   2      1   0G2
.....................uucggacgaucaaacuuucggaAa..................................................................   1      1   0G2
......................Ccggacgaucaaacuuucgga....................................................................   4      1   0G2
......................ucggacAaucaaacuuucggaca..................................................................   9      1   0G2
.......................cggGcgaucaaacuuucgg.....................................................................   3      1   0G2
.......................cggGcgaucaaacuuucgga....................................................................   8      1   0G2
.......................cggacgGucaaacuuucgga....................................................................   6      1   0G2
.......................cggacUaucaaacuuucgga....................................................................   19     1   0G2
.......................cggacgCucaaacuuucgga....................................................................   3      1   0G2
.......................Aggacgaucaaacuuucgga....................................................................   3      1   0G2
.......................Gggacgaucaaacuuucgga....................................................................   1      1   0G2
.......................cgAacgaucaaacuuucgga....................................................................   1      1   0G2
.......................Nggacgaucaaacuuucgga....................................................................   31     1   0G2
.......................cgUacgaucaaacuuucgga....................................................................   2      1   0G2
.......................cggaAgaucaaacuuucgga....................................................................   1      1   0G2
.......................cggacgaGcaaacuuucggac...................................................................   1      1   0G2
.......................cggacUaucaaacuuucggac...................................................................   4      1   0G2
.......................cggacUaucaaacuuucggaca..................................................................   57     1   0G2
.......................cggacgGucaaacuuucggaca..................................................................   2      1   0G2
.......................cggacgaGcaaacuuucggaca..................................................................   2      1   0G2
.......................cggacAaucaaacuuucggaca..................................................................   13     1   0G2
.......................cggacgauAaaacuuucggaca..................................................................   1      1   0G2
```

```
                       Mature                Star
guuacauuaaaaccgaaaagauucggacgaucaaacuuucggacaaaauuuugaccgaaaguuuaauuguccgaaaucuugugauguccgaaaguuuuaaauuucauugug
.......................Aggacgaucaaacuuucggaca..................................................................  1    1  0G2
.......................Nggacgaucaaacuuucggaca..................................................................  7    1  0G2
.......................cggacgauGaaacuuucggaca..................................................................  1    1  0G2
.......................cggacgaCcaaacuuucggaca..................................................................  46   1  0G2
.......................cggacgUucaaacuuucggaca..................................................................  2    1  0G2
.......................cggCcgaucaaacuuucggaca..................................................................  1    1  0G2
.......................cggGcgaucaaacuuucggaca..................................................................  23   1  0G2
.......................Nggacgaucaaacuuucggacaa.................................................................  2    1  0G2
.......................cggacUaucaaacuuucggacaa.................................................................  7    1  0G2
.......................cggGcgaucaaacuuucggacaa.................................................................  2    1  0G2
........................ggacgGucaaacuuucgga....................................................................  1    1  0G2
........................ggacUaucaaacuuucgga....................................................................  10   1  0G2
........................gNacgaucaaacuuucgga....................................................................  1    1  0G2
........................ggGcgaucaaacuuucgga....................................................................  1    1  0G2
........................ggacAaucaaacuuucggaca..................................................................  984  1  0G2
........................gNacgaucaaacuuucggaca..................................................................  2    1  0G2
........................ggacUaucaaacuuucggaca..................................................................  100  1  0G2
........................ggGcgaucaaacuuucggaca..................................................................  3    1  0G2
........................ggacUaucaaacuuucggacaa.................................................................  2    1  0G2
.....................................................Gccgaaaguuuaauuguccgaa....................................  2    1  0G2
.....................................................Cccgaaaguuuaauuguccgaa....................................  8    1  0G2
.....................................................Nccgaaaguuuaauuguccgaa....................................  14   1  0G2
.....................................................accgaaaguuuaauuguccgaaU...................................  1    1  0G2
.....................................................accgaaaguuuaauuguccgaaa...................................  1    0  0G2
.....................................................Gccgaaaguuuaauuguccgaaa...................................  1    1  0G2
.....................................................Nccgaaaguuuaauuguccgaaa...................................  1    1  0G2
.....................................................accgaaaguuuaauuguccgaaUu..................................  1    1  0G2
......................................................ccgaaaguuuaauuguccgaa....................................  215  0  0G2
......................................................Ncgaaaguuuaauuguccgaa....................................  1    1  0G2
......................................................ccgaaaguuuaauuguccgaUa...................................  6    1  0G2
......................................................ccgaaaguuuaauuguccgaaa...................................  25   0  0G2
......................................................ccgaaaguuuaauuguccgaCa...................................  4    1  0G2
......................................................ccgaaaguuuaauuguccgaaU...................................  107  1  0G2
......................................................ccgaaaguuuaauuguccgaaC...................................  3    1  0G2
......................................................ccgaaaguuuaauuguccgaaau..................................  1    0  0G2
......................................................ccgaaaguuuaauuguccgaaUu..................................  40   1  0G2
.......................................................cgaaaguuuaauuguccgaa....................................  28   0  0G2
.......................................................cgaaaguuuaauuguccgaaU...................................  6    1  0G2
.......................................................cgaaaguuuaauuguccgaUa...................................  8    1  0G2
.......................................................cgaaaguuuaauuguccgaaaG..................................  10   1  0G2
........................................................gaaaguuuaauuguccgaaaG..................................  3    1  0G2

....................auucggacgaucaaacuuucg......................................................................  24   0  0A2
....................Guucggacgaucaaacuuucg......................................................................  2    1  0A2
....................auucggacgaucaaacuuucgg.....................................................................  30   0  0A2
....................Nuucggacgaucaaacuuucgg.....................................................................  1    1  0A2
....................auucggacgaucaaacuuucgga....................................................................  13   0  0A2
....................auucggacgaucaaacuuucggaUa..................................................................  4    1  0A2
.....................uucggacgaucaaacuuuc.......................................................................  5    0  0A2
.....................uucggacgaucaaacuuucg......................................................................  48   0  0A2
.....................uucggacgaucaaacuuucgg.....................................................................  21   0  0A2
.....................uucggacgaucaaacuuucgga....................................................................  10   0  0A2
.....................uucggacgaucaaacuuucggaU...................................................................  2    1  0A2
.....................uucggacgaucaaacuuucggaUa..................................................................  2    1  0A2
......................Ccggacgaucaaacuuucgga....................................................................  5    1  0A2
......................Ncggacgaucaaacuuucgga....................................................................  1    1  0A2
......................ucggacAaucaaacuuucggaca..................................................................  7    1  0A2
.......................cggGcgaucaaacuuucg......................................................................  1    1  0A2
.......................cgUacgaucaaacuuucgga....................................................................  2    1  0A2
.......................Nggacgaucaaacuuucgga....................................................................  19   1  0A2
.......................cggacgaGcaaacuuucgga....................................................................  6    1  0A2
.......................cggacgGucaaacuuucgga....................................................................  7    1  0A2
.......................cggacgauGaaacuuucgga....................................................................  4    1  0A2
.......................Aggacgaucaaacuuucgga....................................................................  1    1  0A2
.......................cggacUaucaaacuuucgga....................................................................  21   1  0A2
.......................cggacgUucaaacuuucgga....................................................................  1    1  0A2
.......................cggGcgaucaaacuuucgga....................................................................  3    1  0A2
.......................cggacgCucaaacuuucgga....................................................................  5    1  0A2
.......................cggacUaucaaacuuucggac...................................................................  2    1  0A2
.......................cggUcgaucaaacuuucggaca..................................................................  1    1  0A2
```

Mature Star

```
guuacauuaaaaccgaaaagauucggacgaucaaacuuucggacaaaauuuugaccgaaaguuuaauuguccgaaaucuugugauguccgaaaguuuaaauuucauugug
.......................cgUacgaucaaacuuucggaca.................................................................   2   1   0A2
.......................cggacgauAaaacuuucggaca.................................................................   3   1   0A2
.......................cggCcgaucaaacuuucggaca.................................................................   1   1   0A2
.......................Nggacgaucaaacuuucggaca.................................................................   13   1   0A2
.......................cggacgaGcaaacuuucggaca.................................................................   4   1   0A2
.......................cggacgCucaaacuuucggaca.................................................................   4   1   0A2
.......................cggacgaAcaaacuuucggaca.................................................................   1   1   0A2
.......................cggacgaCcaaacuuucggaca.................................................................   33   1   0A2
.......................cggacgGucaaacuuucggaca.................................................................   8   1   0A2
.......................cgAacgaucaaacuuucggaca.................................................................   1   1   0A2
.......................cggacAaucaaacuuucggaca.................................................................   19   1   0A2
.......................cggacgUucaaacuuucggaca.................................................................   1   1   0A2
.......................cggacUaucaaacuuucggaca.................................................................   60   1   0A2
.......................cgNacgaucaaacuuucggaca.................................................................   1   1   0A2
.......................cggGcgaucaaacuuucggaca.................................................................   78   1   0A2
.......................cgCacgaucaaacuuucggaca.................................................................   1   1   0A2
.......................cggaAgaucaaacuuucggaca.................................................................   1   1   0A2
.......................Gggacgaucaaacuuucggaca.................................................................   1   1   0A2
.......................cggacgauGaaacuuucggacaa................................................................   2   1   0A2
.......................cggacgGucaaacuuucggacaa................................................................   1   1   0A2
.......................cggacgaCcaaacuuucggacaa................................................................   1   1   0A2
.......................cggacgaGcaaacuuucggacaa................................................................   2   1   0A2
.......................cggacUaucaaacuuucggacaa................................................................   3   1   0A2
.......................cggacgUucaaacuuucggacaa................................................................   1   1   0A2
.......................cggCcgaucaaacuuucggacaa................................................................   1   1   0A2
.......................Nggacgaucaaacuuucggacaa................................................................   2   1   0A2
.......................cggGcgaucaaacuuucggacaa................................................................   12   1   0A2
........................ggacUaucaaacuuucgga...................................................................   1   1   0A2
........................ggacgaGcaaacuuucggaca.................................................................   2   1   0A2
........................ggacgaCcaaacuuucggaca.................................................................   1   1   0A2
........................ggacUaucaaacuuucggaca.................................................................   76   1   0A2
........................ggGcgaucaaacuuucggaca.................................................................   3   1   0A2
........................ggacAaucaaacuuucggaca.................................................................   1204   1   0A2
........................ggacgauAaaacuuucggaca.................................................................   1   1   0A2
........................ggacgaAcaaacuuucggaca.................................................................   1   1   0A2
........................ggacgCucaaacuuucggacaa................................................................   1   1   0A2
........................ggacAaucaaacuuucggacaa................................................................   3   1   0A2
........................ggacAaucaaacuuucggacaaa...............................................................   2   1   0A2
.....................................................Cccgaaaguuuaauuguccgaa...................................   2   1   0A2
.....................................................Gccgaaaguuuaauuguccgaa...................................   4   1   0A2
.....................................................Nccgaaaguuuaauuguccgaa...................................   10   1   0A2
.....................................................accgaaaguuuaauuguccgaaUu.................................   2   1   0A2
......................................................ccgaaaguuuaauuguccgaa...................................   141   0   0A2
......................................................ccgaaaguuuaauugucAgaa...................................   3   1   0A2
......................................................ccgaaaguuuaauuguccgaaC..................................   19   1   0A2
......................................................ccgaaaguuuaauuguccgaaU..................................   68   1   0A2
......................................................ccgaaaguuuaauuguccgaaa..................................   12   0   0A2
......................................................ccgaaaguuuaauuguccgaUa..................................   7   1   0A2
......................................................ccgaaaguuuaauuguccgaaUu.................................   48   1   0A2
......................................................ccgaaaguuuaauuguccgaaCu.................................   2   1   0A2
.......................................................cgaaaguuuaauuguccgaa...................................   16   0   0A2
.......................................................cgaaaguuuaauuguccgaaC..................................   1   1   0A2
.......................................................cgaaaguuuaauuguccgaaU..................................   8   1   0A2
.......................................................cgaaaguuuaauuguccgaaa..................................   2   0   0A2
```

Provisional ID	:	scaffold37_9324
Score total	:	198
Score for star read(s)	:	3.9
Score for read counts	:	191.3
Score for mfe	:	1.8
Score for randfold	:	1.6
Score for cons. seed	:	-0.6
Total read count	:	387
Mature read count	:	381
Loop read count	:	0
Star read count	:	6

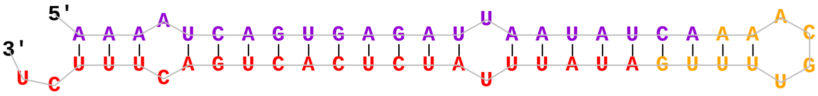

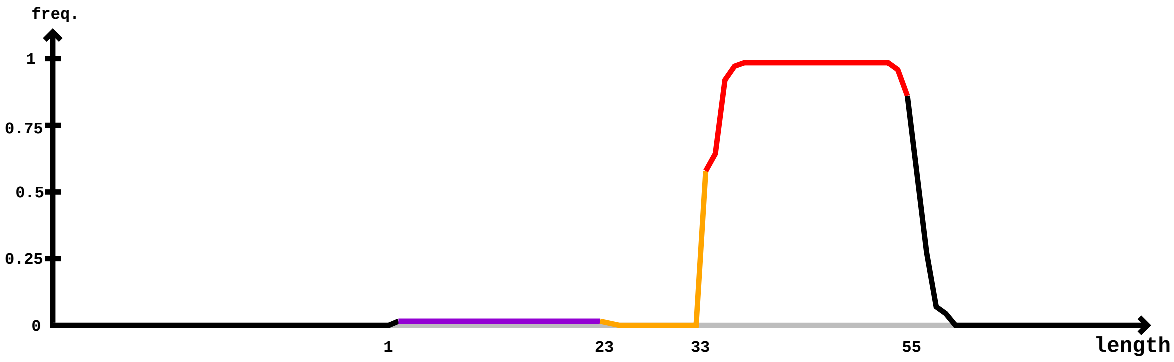


```
                                        Star                                 Mature
5'- ccaaaauuugucucacuuggcaauuuucagugagaaaaucagugagauuaauaucaaaacguuuugauauuuaucucacugacuuucucacuuaauaaaggaagccgcuaau   -3'   obs
    ccaaaauuugucucacuuggcaauuuucagugagaaaaucagugagauuaauaucaaaacguuuugauauuuaucucacugacuuucucacuuaauaaaggaagccgcuaau         exp
    ((.....(((((......))))).....(((((((((.((((((((((.(((((((((....))))))))).)))))))))).))))))))).......))...........   reads   mm   sample
    ..................................aaaaucagugagaAuaauauca........................................................   6       1    0A2
    ..................................................................auauuuaucucacugacuuuc.........................   10      0    0A2
    ..................................................................auauuuaucucacugacuuucu........................   52      0    0A2
    ..................................................................auauuuaucucacugacuuucA........................   5       1    0A2
    ..................................................................auauuuaucucacugacuuucuU.......................   6       1    0A2
    ..................................................................auauuuaucucacugacuuucuA.......................   15      1    0A2
    ...................................................................uauuuaucucacugacuuucu........................   4       0    0A2
    ....................................................................auuuaucucacugacuuuc.........................   2       0    0A2
    ....................................................................Guuuaucucacugacuuucuca......................   4       1    0A2
    ....................................................................auuuaucucacugacuuucuca......................   27      0    0A2
    ....................................................................auuuaucucacugacuuucucaUu....................   2       1    0A2
    .....................................................................uuuaucucacugacuuucuc.......................   2       0    0A2
    .....................................................................uuuaucucacugacuuucuca......................   7       0    0A2
    .....................................................................uuuaucucacugacuuucucaUu....................   4       1    0A2
    ......................................................................uuaucucacugacuuucuca......................   5       0    0A2

    ..................................................................auauuuaucucacugacuuuc.........................   12      0    0B2
    ..................................................................auauuuaucucacugacuuucA........................   2       1    0B2
    ..................................................................auauuuaucucacugacuuucu........................   36      0    0B2
    ..................................................................auauuuaucucacugacuuucuU.......................   4       1    0B2
    ...................................................................uauuuaucucacugacuuucu........................   4       0    0B2
    ...................................................................uauuuaucucacugacuuucuc.......................   4       0    0B2
    ....................................................................Nuuuaucucacugacuuucuc.......................   1       1    0B2
    ....................................................................auuuaucucacugacuuucuc.......................   18      0    0B2
    ....................................................................auuuaucucacugacuuucuca......................   12      0    0B2
    ....................................................................auuuaucucacugacuuucucaA.....................   1       1    0B2
    ....................................................................auuuaucucacugacuuucucaAu....................   7       1    0B2
    .....................................................................uuuaucucacugacuuucucaUu....................   4       1    0B2

    ..................................................................auauuuaucucacugacuuu..........................   5       0    0G2
    ..................................................................auauuuaucucacugacuuuc.........................   14      0    0G2
    ..................................................................auauuuaucucacugacuuucu........................   49      0    0G2
    ..................................................................auauuuaucucacugacuuucA........................   8       1    0G2
```

```
                                  Star                              Mature
ccaaaauuugucucacuuggcaauuuucagugagaaaaucagugagauuaauaucaaaacguuuugauauuuaucucacugacuuucucacuuaauaaaggaagccgcuaau
..................................................................auauuuaucucacugacuuucuA.......................    6    1    0G2
...................................................................uauuuaucucacugacuuu..........................    5    0    0G2
...................................................................uauuuaucucacugacuuucu........................    8    0    0G2
....................................................................auuuaucCcacugacuuucuc.......................    1    1    0G2
....................................................................auuuaucucacugacuuucuc.......................    2    0    0G2
....................................................................auuuaucucacugacuuucucU......................    2    1    0G2
....................................................................auuuaucucacugacuuucuUa......................    1    1    0G2
....................................................................auuuaucucacuCacuuucuca......................    1    1    0G2
....................................................................auuuaucucacugacuuucuca......................    17   0    0G2
....................................................................auuuaucucacugacuuucucaA.....................    9    1    0G2
.....................................................................uuuaucucacugacuuucuca......................    3    0    0G2
```

Provisional ID	:	scaffold47_11211
Score total	:	1768.9
Score for star read(s)	:	3.9
Score for read counts	:	1758
Score for mfe	:	2.4
Score for randfold	:	1.6
Score for cons. seed	:	3
Total read count	:	3460
Mature read count	:	3030
Loop read count	:	0
Star read count	:	430

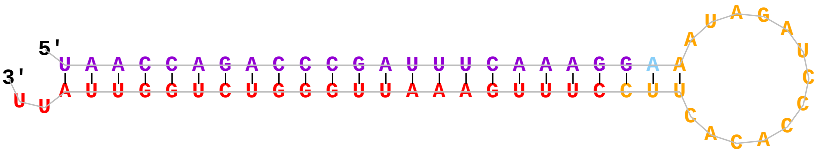

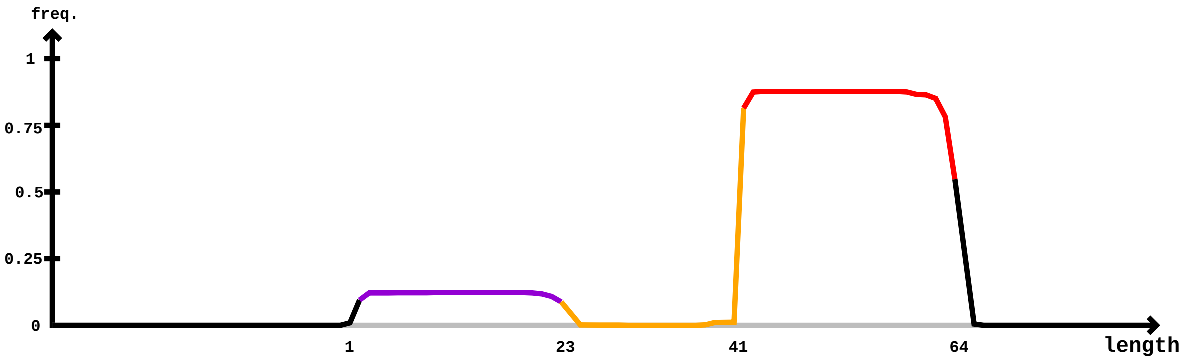

Star Mature

```
5'- uuuuacgaagccgugccauagauagccuaauaaccagacccgauuucaaaggaaauagaucccacacuuccuuugaaauugggucugguuauuaggcuaugccauagacuuua   -3'   obs
    uuuuacgaagccgugccauagauagccuaauaaccagacccgauuucaaaggaaauagaucccacacuuccuuugaaauugggucugguuauuaggcuaugccauagacuuua         exp
    ......(((((..((.((....((((((((((((((((((((((((((((((((.............)))))))))))))))))))))))))))))))))).))..).)))).    reads   mm   sample
    .............................auaaccagacccgauuuca.................................................................    2       0    0G2
    .............................auaaccagacccgauuucaa................................................................    1       0    0G2
    .............................auaaccagacccgauuucaaa...............................................................    2       0    0G2
    .............................Nuaaccagacccgauuucaaa...............................................................    1       1    0G2
    ..............................uaaccGgacccgauuucaaa...............................................................    4       1    0G2
    ..............................Caaccagacccgauuucaaag..............................................................    2       1    0G2
    ..............................uaaccagacccgauuucaaagg.............................................................    7       0    0G2
    ..............................Caaccagacccgauuucaaagg.............................................................    10      1    0G2
    ..............................uaaccagacccgauuucaaaAg.............................................................    1       1    0G2
    ...............................aaccagacccgauuucaaag..............................................................    9       0    0G2
    ...............................aaccagacccgauuucaaaAga............................................................    1       1    0G2
    ...................................................................uuccuuugaaauugggucugU.........................    2       1    0G2
    ...................................................................uuccuuugaaauugggucugC.........................    3       1    0G2
    ...................................................................uuccuuugaaauugggucuggA........................    2       1    0G2
    ......................................................................Uuugaaauugggucuggu.........................    6       1    0G2
    ......................................................................cuuugaaauugggucugguua......................    70      0    0G2
    ......................................................................cuuugaaauugggucAgguua......................    1       1    0G2
    ......................................................................cuuugaaauugggucugguuU......................    1       1    0G2
    ......................................................................Uuuugaaauugggucugguua......................    9       1    0G2
    ......................................................................cuuugaaauugggucugguuaA.....................    191     1    0G2
    ......................................................................cuuugaaauugggucugguuau.....................    22      0    0G2
    ......................................................................cuuugaaauugggucuggCuau.....................    9       1    0G2
    ......................................................................Uuuugaaauugggucugguuau.....................    65      1    0G2
    ......................................................................Uuuugaaauugggucugguuauu....................    36      1    0G2
    ......................................................................cuuugaaauugggucugguuUuu....................    5       1    0G2
    ......................................................................cuuugaaauugggucugguuauu....................    19      0    0G2
    ......................................................................cuuugaaauugggucugguuaAu....................    341     1    0G2
    ......................................................................cuuugaaauugggucugguuauA....................    25      1    0G2
    ......................................................................cuuugaaauugggucugguuauua...................    25      0    0G2
    ......................................................................Uuuugaaauugggucugguuauua...................    5       1    0G2
    ......................................................................cuuugaaauugggucugguuaAua...................    112     1    0G2
    ......................................................................cuuugaaauugggucugguuauuC...................    9       1    0G2
    ......................................................................Nuuugaaauugggucugguuauua...................    2       1    0G2
```

Star Mature

```
uuuuacgaagccgugccauagauagccuaauaaccagacccgauuucaaaggaaauagaucccacacuuccuuugaaauugggucugguuauuaggcuaugccauagacuuua
......................................................................cuuugaaauugggucugguuauuaA..................   1    1   0G2
......................................................................cuuugaaauugggucugguuauuaU..................   4    1   0G2
.......................................................................uuugaaauugggucugguua......................   4    0   0G2
.......................................................................uuugaaauugggucugguuaA.....................   22   1   0G2
.......................................................................uuugaaauugggucugguuauA....................   8    1   0G2
.......................................................................uuugaaauugggucugguuaAu....................   12   1   0G2
.......................................................................uuugaaauugggucugguuauua...................   4    0   0G2
.......................................................................uuugaaauugggucugguuaAua...................   12   1   0G2
........................................................................uugaaauugggucugguuaA.....................   4    1   0G2
........................................................................uugaaauugggucugguuauA....................   4    1   0G2

.............................auaaccagacccgauuucaaa...............................................................   11   0   0B2
.............................aCaaccagacccgauuucaaag..............................................................   5    1   0B2
..............................Caaccagacccgauuuca.................................................................   3    1   0B2
..............................Caaccagacccgauuucaaa...............................................................   3    1   0B2
..............................Caaccagacccgauuucaaag..............................................................   26   1   0B2
..............................uaGccagacccgauuucaaagg.............................................................   1    1   0B2
..............................Caaccagacccgauuucaaagg.............................................................   98   1   0B2
..............................uaaccagacccgauuucaaaAg.............................................................   2    1   0B2
..............................uaaccagacccgauuucaaagga............................................................   7    0   0B2
...............................aaccagacccgauuucaaag..............................................................   7    0   0B2
...............................aaccagacccgauuucaaagg.............................................................   32   0   0B2
...............................aaccGgacccgauuucaaagg.............................................................   5    1   0B2
...............................aaccagacGcgauuucaaagg.............................................................   1    1   0B2
......................................cccgauuucaaagAaaauag.......................................................   3    1   0B2
...................................................................uuccuuugaaauugggucug..........................   6    0   0B2
...................................................................uuccuuugaaauugggucugg.........................   8    0   0B2
...................................................................uuccuuugaaauugggucugguA.......................   2    1   0B2
....................................................................uccuuugaaauugggucugU.........................   2    1   0B2
......................................................................cuuugaaauugggucugguA.......................   1    1   0B2
......................................................................cuuugaaauugggucugguu.......................   27   0   0B2
......................................................................Uuuugaaauugggucugguua......................   11   1   0B2
......................................................................cuuugaaauugggucugguuU......................   8    1   0B2
......................................................................cuuugaaauugggucugguua......................   49   0   0B2
......................................................................cuuugaaauugggucuggCuau.....................   2    1   0B2
......................................................................cuuugaaauugggucugguuUu.....................   1    1   0B2
......................................................................cuuugaaauugggucugguuaA.....................   162  1   0B2
......................................................................Uuuugaaauugggucugguuau.....................   34   1   0B2
......................................................................cuuugaaauugggucugguuau.....................   35   0   0B2
......................................................................cuuugaaauugggucugguuauA....................   8    1   0B2
......................................................................cuuugaaauugggucCgguuauu....................   1    1   0B2
......................................................................Uuuugaaauugggucugguuauu....................   37   1   0B2
......................................................................cuuugaaauugggucuggCuauu....................   2    1   0B2
......................................................................cuuugaaauugggucugguuauu....................   16   0   0B2
......................................................................cuuugaaauugggucugguuaAu....................   456  1   0B2
......................................................................Uuuugaaauugggucugguuauua...................   1    1   0B2
......................................................................cuuugaaauugggucugguuaAua...................   46   1   0B2
......................................................................cuuugaaauugggucugguuauua...................   4    0   0B2
......................................................................cuuugaaauugggucugguuauuU...................   11   1   0B2
......................................................................cuuugaaauugggucugguuauuC...................   9    1   0B2
......................................................................cuuugaaauugggucugguuauuaC..................   6    1   0B2
.......................................................................uuugaaauugggucugguuaA.....................   19   1   0B2
.......................................................................uuugaaauugggucugguuau.....................   4    0   0B2
.......................................................................uuugaaauugggucugguuaAu....................   16   1   0B2
.......................................................................uuugaaauugggucugguuauu....................   11   0   0B2
.......................................................................uuugaaauugggucugguuaAua...................   1    1   0B2

.............................auaaccagacccgauuucaa................................................................   4    0   0A2
.............................auaaccagacccgauuucaaa...............................................................   1    0   0A2
.............................auaaccagaAccgauuucaaa...............................................................   6    1   0A2
..............................uaaccagacccgauuucaaa...............................................................   1    0   0A2
..............................Caaccagacccgauuucaaa...............................................................   3    1   0A2
..............................uaaccagaAccgauuucaaa...............................................................   1    1   0A2
..............................uaaccagacccgauuucaaag..............................................................   8    0   0A2
..............................Caaccagacccgauuucaaag..............................................................   10   1   0A2
..............................uaaccagacccgauuucaaaAg.............................................................   4    1   0A2
..............................uaGccagacccgauuucaaagg.............................................................   4    1   0A2
..............................Caaccagacccgauuucaaagg.............................................................   99   1   0A2
..............................Caaccagacccgauuucaaagga............................................................   4    1   0A2
...............................aaccagacccgauuucaa................................................................   8    0   0A2
```

```
                              Star                                    Mature
uuuuacgaagccgugccauagauagccuaauaaccagacccgauuucaaaggaaauagaucccacacuuccuuugaaauugggucugguuauuaggcuaugccauagacuuua
...............................aaccagacccgauuucaaag..............................................................    3     0    0A2
...............................aaccagacccgauuucaaaA..............................................................    2     1    0A2
...............................aaccagacccgauuucaaagg.............................................................    3     0    0A2
...............................aaccagacccgauuucaaaAga............................................................    23    1    0A2
..................................cagacccgauuucaaaAgaa...........................................................    1     1    0A2
..................................cagacccgauuucaaaAgaaa..........................................................    1     1    0A2
..................................................................Nuuccuuugaaauugggucug..........................    1     1    0A2
..................................................................Uuuccuuugaaauugggucugguuau.....................    5     1    0A2
...................................................................uuccuuugaaauugggucugg.........................    7     0    0A2
...................................................................uuccuuugaaauugggucugguuUuu....................    1     1    0A2
.....................................................................cUuuugaaauugggucugguuau.....................    2     1    0A2
......................................................................cuuugaaauugggucugg.........................    7     0    0A2
......................................................................cuuugaaauugggucugU.........................    3     1    0A2
......................................................................cuuugaaauugggucugguu.......................    15    0    0A2
......................................................................cuuugaaauGgggucugguua......................    1     1    0A2
......................................................................cuuugaaauugggucugguua......................    60    0    0A2
......................................................................Uuuugaaauugggucugguua......................    23    1    0A2
......................................................................cuuugaaauugggucugguAa......................    2     1    0A2
......................................................................cuuugaaauugggucugguuau.....................    18    0    0A2
......................................................................cuuugaaauugggucugguuUu.....................    2     1    0A2
......................................................................cuuugaaauugggucugguuaA.....................    153   1    0A2
......................................................................Uuuugaaauugggucugguuau.....................    33    1    0A2
......................................................................cuuugaaauugggucugguuaAu....................    396   1    0A2
......................................................................Uuuugaaauugggucugguuauu....................    17    1    0A2
......................................................................cuuugaaauugggucugguuUuu....................    6     1    0A2
......................................................................cuuugaaauugUgucugguuauu....................    1     1    0A2
......................................................................cuuugaaauugggucugguuauu....................    10    0    0A2
......................................................................cuuugaaauugggucugguuauA....................    24    1    0A2
......................................................................cuuugaaauugggucuggCuauu....................    9     1    0A2
......................................................................cuuugaaauugggucugguuauuU...................    10    1    0A2
......................................................................cuuugaaauugggucugguuUuua...................    1     1    0A2
......................................................................cuuugaaauugggucugguuauua...................    24    0    0A2
......................................................................cuuugaaauugggucugguuaAua...................    88    1    0A2
......................................................................cuuugaaauugggucugguuauuC...................    7     1    0A2
......................................................................cuuugaaauugggucugguuauAa...................    3     1    0A2
......................................................................Uuuugaaauugggucugguuauua...................    4     1    0A2
......................................................................cuuugaaauugggucugguuaAuag..................    4     1    0A2
.......................................................................uuugaaauuAggucugguua......................    2     1    0A2
.......................................................................uuugaaauugggucugguAau.....................    4     1    0A2
.......................................................................uuugaaauugggucugguuau.....................    4     0    0A2
.......................................................................uuGgaaauugggucugguuau.....................    1     1    0A2
.......................................................................uuugaaauugggucugguuaA.....................    27    1    0A2
.......................................................................uuugaaauugggucugguuauA....................    6     1    0A2
.......................................................................uuugaaauugggucugguuaAu....................    45    1    0A2
.......................................................................uuugaaauugggucuggCuauu....................    4     1    0A2
.......................................................................uuugaaauugggucugguuaAua...................    4     1    0A2
.......................................................................uuugaaauugggucugguuauuaU..................    2     1    0A2
```

Provisional ID : scaffold47_11296
Score total : 3956.3
Score for star read(s) : 3.9
Score for read counts : 3945.2
Score for mfe : 2.6
Score for randfold : 1.6
Score for cons. seed : 3
Total read count : 7750
Mature read count : 7686
Loop read count : 0
Star read count : 64

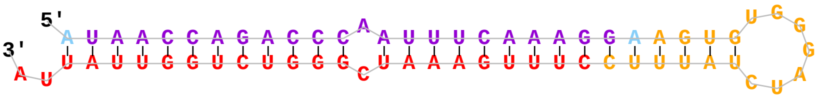

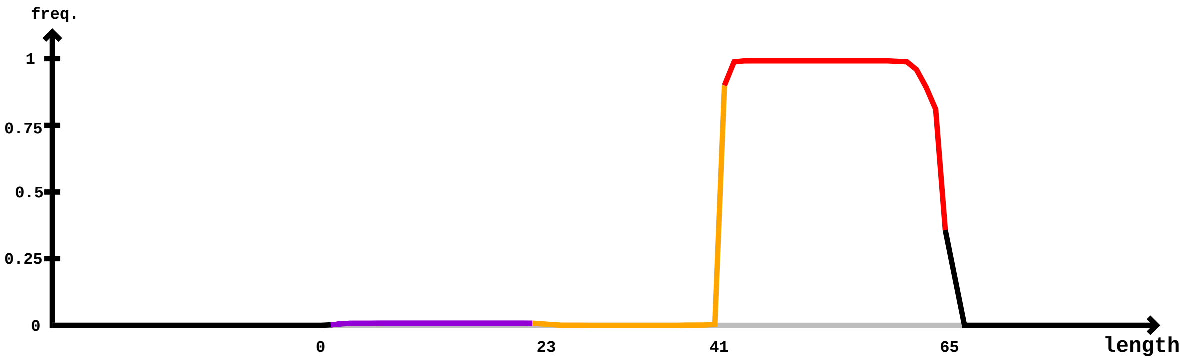

Star Mature

```
5'- cucgaguaaagucuauggcauagccuaauaaccagacccaauuucaaaggaagugugggaucuauuuccuuugaaaucgggucugguuauuaggcuaucuauggcacggcuuc  -3'  obs
    cucgaguaaagucuauggcauagccuaauaaccagacccaauuucaaaggaagugugggaucuauuuccuuugaaaucgggucugguuauuaggcuaucuauggcacggcuuc        exp
    ...((((...(((((((((.(((((((((((((((((((((.(((((((((((((.......))))))))))))))).)))))))))))))))))))))))))).)).)))).   reads   mm   sample
    ...........................Uuaaccagacccaauuucaaag................................................................   3       1    0A2
    ...........................Cuaaccagacccaauuucaaagg...............................................................   2       1    0A2
    ...........................Uuaaccagacccaauuucaaagg...............................................................   2       1    0A2
    ............................uaaccagacccaauuucaaaAg...............................................................   2       1    0A2
    ............................uaaccagacccaauuucaaagg...............................................................   5       0    0A2
    ............................uaaccagacccaauuucaaaAga..............................................................   2       1    0A2
    .............................aaccagacccaauuucaaagg...............................................................   7       0    0A2
    .............................aaccagacccaauuucaaaAg...............................................................   3       1    0A2
    .............................aaccagacccaauuucaaaAga..............................................................   3       1    0A2
    ................................................................uuuccuuugaaauUgggucugguuau.......................   5       1    0A2
    .................................................................uucUuuugaaaucgggucugguua........................   1       1    0A2
    ..................................................................ucUuuugaaaucgggucugguua........................   1       1    0A2
    ..................................................................ucUuuugaaaucgggucugguuau.......................   1       1    0A2
    ...................................................................ccuuugaaaucgggucugUuua........................   6       1    0A2
    ...................................................................ccuuugaaaucgggucugguua........................   2       0    0A2
    ...................................................................Ucuuugaaaucgggucugguuauu......................   1       1    0A2
    ...................................................................ccuuugaaaucgggucugguuaAua.....................   1       1    0A2
    ....................................................................cuuugaaaucgggucugg...........................   1       0    0A2
    ....................................................................cuuugaaaucgggucuggu..........................   2       0    0A2
    ....................................................................cuuugaaaucgggucugguu.........................   68      0    0A2
    ....................................................................cuuugaaauGggucugguua........................   1       1    0A2
    ....................................................................cuuugaaaucgggucugguuG........................   10      1    0A2
    ....................................................................Nuuugaaaucgggucugguua........................   1       1    0A2
    ....................................................................cuuugaGaucgggucugguua........................   1       1    0A2
    ....................................................................cuuugaaaucgggucugguuC........................   5       1    0A2
    ....................................................................cuuugaaaucgggucugguua........................   186     0    0A2
    ....................................................................cuuugaGaucgggucugguuau.......................   1       1    0A2
    ....................................................................cuuugaCaucgggucugguuau.......................   1       1    0A2
    ....................................................................cuuugaaaucgggucugguuUu.......................   33      1    0A2
    ....................................................................cuuugaaaucgggucugguuau.......................   150     0    0A2
    ....................................................................cuuugaaaucgggucugguuaC.......................   7       1    0A2
    ....................................................................cuuugaaaucgggCcugguuau.......................   1       1    0A2
    ....................................................................cuuugaaaucggguUugguuau.......................   1       1    0A2
```

```
                            Star                                       Mature
cucgaguaaagucuauggcauagccuaauaaccagacccaauuucaaaggaagugugggaucuauuuccuuugaaaucgggucugguuauuaggcuaucuauggcacggcuuc
....................................................................Nuuugaaaucgggucugguuau.......................    1      1      0A2
....................................................................cuuugaaaucgggucugguuGu.......................    40     1      0A2
....................................................................cuuugaaaCcgggucugguuauu......................    1      1      0A2
....................................................................cuuugaaaucggguGugguuauu......................    1      1      0A2
....................................................................cCuugaaaucgggucugguuauu......................    1      1      0A2
....................................................................Uuuugaaaucgggucugguuauu......................    35     1      0A2
....................................................................cuuugaaaucgggucugguuUuu......................    111    1      0A2
....................................................................cuuugaaaucgggucugguuauu......................    323    0      0A2
....................................................................cuuugaaaucgggucugUuuauu......................    7      1      0A2
....................................................................Nuuugaaaucgggucugguuauu......................    1      1      0A2
....................................................................cuuugaaaucgggucugguuaAu......................    576    1      0A2
....................................................................cuuugaaaucgggucuggGuauu......................    1      1      0A2
....................................................................cuuugaaaucgggucugguuauuU.....................    58     1      0A2
....................................................................cuuugaaaucgggucugguuaAua.....................    918    1      0A2
....................................................................cuuugaaaucgggucugguuauua.....................    92     0      0A2
....................................................................cuuugaaaucgggucugguuUuua.....................    27     1      0A2
....................................................................Uuuugaaaucgggucugguuauua.....................    10     1      0A2
....................................................................cuuugaaaucgggucugguuauuC.....................    8      1      0A2
....................................................................cuuugaaaucgggucugguuauuaU....................    2      1      0A2
....................................................................cuuugaaaucgggucugguuaAuag....................    5      1      0A2
....................................................................cuuugaaaucgggucugguuauuaA....................    31     1      0A2
.....................................................................uuugaaaucgggGcugguu.........................    1      1      0A2
.....................................................................uuugaaaucgggucugguu.........................    11     0      0A2
.....................................................................uuugaaaucgggucugguua........................    13     0      0A2
.....................................................................uuugaaaucgggucugguuau.......................    35     0      0A2
.....................................................................uuugaaaucgggGcugguuau.......................    1      1      0A2
.....................................................................uuugaaaucgggucugguuUu.......................    1      1      0A2
.....................................................................uuugaaaucgggucugguGau.......................    1      1      0A2
.....................................................................uuuAaaaucgggucugguuau.......................    6      1      0A2
.....................................................................uuugaaaucUggucugguuau.......................    1      1      0A2
.....................................................................uuugaaaucgggucugguuauu......................    45     0      0A2
.....................................................................uuugaaaucgggucugguuaAu......................    33     1      0A2
.....................................................................uuugaaaucggAucugguuauu......................    9      1      0A2
.....................................................................uuugaaaucgggucugguuUuu......................    13     1      0A2
.....................................................................uuugaaaucgggucugguuauuC.....................    3      1      0A2
.....................................................................uuugaaaucgggucugguuauuU.....................    12     1      0A2
.....................................................................uuugaaaucgggGcugguuauua.....................    1      1      0A2
.....................................................................uuugaaaucgggucugguuaGua.....................    1      1      0A2
.....................................................................uuugaaaucgggucugguuaAua.....................    90     1      0A2
.....................................................................uuugaaaucgggucugguuauua.....................    5      0      0A2
.....................................................................uuugaaaucgggucugguuauuag....................    2      0      0A2
......................................................................uugaaaucgggucugguuauu......................    2      0      0A2
......................................................................uugaaaucgggucugguuaAua.....................    1      1      0A2

...........................Uuaaccagacccaauuucaaag................................................................    5      1      0G2
............................uaaccagacccaauuucaaagg...............................................................    8      0      0G2
............................uaaccGgacccaauuucaaagg...............................................................    1      1      0G2
.............................aaccagacccaauuucaaa.................................................................    2      0      0G2
.............................aaccagacccaauuucaaagg...............................................................    4      0      0G2
...................................................................ccuuugaaaucgggucugguua........................    3      0      0G2
...................................................................cUuuugaaaucgggucugguuauu......................    5      1      0G2
....................................................................cuuugaaaucgggucugg...........................    6      0      0G2
....................................................................cuuugaaaucgggucuggu..........................    4      0      0G2
....................................................................cuuugaaGucgggucuggu..........................    1      1      0G2
....................................................................cuuugaaaucgggucugguu.........................    67     0      0G2
....................................................................Nuuugaaaucgggucugguua........................    1      1      0G2
....................................................................cuuugaaaucgggucugguua........................    111    0      0G2
....................................................................cuuugaaaucgggucugguuG........................    3      1      0G2
....................................................................Nuuugaaaucgggucugguuau.......................    1      1      0G2
....................................................................cuuugaaaucgggucugguuGu.......................    27     1      0G2
....................................................................cuuugaaaucgggucugguuau.......................    163    0      0G2
....................................................................cAuugaaaucgggucugguuau.......................    1      1      0G2
....................................................................cuuugaaaucgggucugguuUu.......................    12     1      0G2
....................................................................cuuugaaaucgggucugguuaG.......................    6      1      0G2
....................................................................cuuugaaaucgggucugguuaAu......................    494    1      0G2
....................................................................cuuugaaaucgggucugguuUuu......................    118    1      0G2
....................................................................Uuuugaaaucgggucugguuauu......................    24     1      0G2
....................................................................Nuuugaaaucgggucugguuauu......................    2      1      0G2
....................................................................cuuugaaaucgggucuggCuauu......................    1      1      0G2
....................................................................cuuugaaaucgggAcugguuauu......................    1      1      0G2
```

```
                            Star                                      Mature
cucgaguaaagucuauggcauagccuaauaaccagacccaauuucaaaggaagugugggaucuauuuccuuugaaaucgggucugguuauuaggcuaucuauggcacggcuuc
....................................................................cuuugaaaucgggucugguuauu......................    341   0   0G2
....................................................................cuuugaaaucgggucugguuGuu......................    8     1   0G2
....................................................................cuuugaaaucgggCcugguuauu......................    1     1   0G2
....................................................................cuuugaaaucgggucugguuauua.....................    53    0   0G2
....................................................................cuuugaGaucgggucugguuauua.....................    1     1   0G2
....................................................................cuuugaaaucgggucugguuGuua.....................    4     1   0G2
....................................................................Uuuugaaaucgggucugguuauua.....................    5     1   0G2
....................................................................cuuugaaaucgggucugguuauuC.....................    12    1   0G2
....................................................................cuuugaaaucgggucugguuaAua.....................    734   1   0G2
....................................................................cuuugaaaucgggucugguuauuU.....................    31    1   0G2
....................................................................cuuugaUaucgggucugguuauua.....................    1     1   0G2
....................................................................cuuugaaaucgggucugguuUuua.....................    31    1   0G2
....................................................................cuuugaaaucgggucugguuauuaA....................    11    1   0G2
.....................................................................uuugaaaucgggucugguua........................    23    0   0G2
.....................................................................uuugaaaucgggucugguuG........................    7     1   0G2
.....................................................................uuugaaaucgggucugguuau.......................    2     0   0G2
.....................................................................uuugGaaucgggucugguuau.......................    1     1   0G2
.....................................................................uuugaaaucgggucugguuauu......................    36    0   0G2
.....................................................................uuugaaaucgggucugguuUuu......................    6     1   0G2
.....................................................................uuugaaaGcgggucugguuauu......................    2     1   0G2
.....................................................................uuugaaaucgggucugguuaAu......................    62    1   0G2
.....................................................................Nuugaaaucgggucugguuauu......................    2     1   0G2
.....................................................................uuugaaaucgggucugguuauua.....................    2     0   0G2
.....................................................................uuugaaaucgggucugguuUuua.....................    3     1   0G2
.....................................................................uuugaaaucgggucugguuaAua.....................    51    1   0G2
.....................................................................uuugaaaucgggucugguuauuU.....................    6     1   0G2
......................................................................uugaaaucgggGcugguua........................    1     1   0G2
......................................................................uugaaaucgggucugguua........................    2     0   0G2
......................................................................uugaaaucgggucugguuau.......................    2     0   0G2
......................................................................uugaaaCcgggucugguuau.......................    1     1   0G2
......................................................................uugaaaucgggucugguuauu......................    4     0   0G2
......................................................................uugaaaucgggucugguuUuu......................    6     1   0G2

...........................Uuaaccagacccaauuucaaag................................................................    1     1   0B2
...........................Uuaaccagacccaauuucaaagg...............................................................    2     1   0B2
............................uaaccagacccaauuucaaagg...............................................................    4     0   0B2
............................uaaccagacccaauuucaaaAga..............................................................    1     1   0B2
.............................aaccagacccaauuucaaagg...............................................................    2     0   0B2
.............................aaccagacccaauuucaaagga..............................................................    2     0   0B2
................................cagacccaauuucaaaggaaUu...........................................................    3     1   0B2
.................................................................uuccuuuUaaaucgggucugg...........................    1     1   0B2
...................................................................ccuuugaaaucgggucugguu.........................    1     0   0B2
....................................................................cuuugaaaucgggucugg...........................    6     0   0B2
....................................................................cuuugaaaucgggucuggu..........................    6     0   0B2
....................................................................cuuugaaaucgggucugguu.........................    72    0   0B2
....................................................................cuuugaaaucgggucugguC.........................    1     1   0B2
....................................................................cuuugaaaucgggucugguuC........................    5     1   0B2
....................................................................Nuugaaaucgggucugguua.........................    2     1   0B2
....................................................................cuuugaaaucgggucugguuG........................    7     1   0B2
....................................................................cuuugaaaucgggucugguua........................    101   0   0B2
....................................................................cuuuAaaucgggucugguuau........................    2     1   0B2
....................................................................cAuugaaaucgggucugguuau.......................    1     1   0B2
....................................................................cuuugaaaucgggucugguuUu.......................    14    1   0B2
....................................................................cuuugaaaucgggucuggGuau.......................    1     1   0B2
....................................................................cuuugaaaucgggucugguuGu.......................    14    1   0B2
....................................................................Nuugaaaucgggucugguuau........................    2     1   0B2
....................................................................cuuugaaaucgggucugguuau.......................    83    0   0B2
....................................................................cuuugaaaCcgggucugguuau.......................    1     1   0B2
....................................................................cuuugaaaucggAucugguuauu......................    3     1   0B2
....................................................................cuuugaaaucgggucugguuauu......................    399   0   0B2
....................................................................cCuugaaaucgggucugguuauu......................    4     1   0B2
....................................................................cuuugaaaucgggucugguuGuu......................    4     1   0B2
....................................................................cuuugaaaucgggucugguuaAu.......................    532   1   0B2
....................................................................Nuugaaaucgggucugguuauu.......................    7     1   0B2
....................................................................cuuugaaaucgggucugguuUuu......................    120   1   0B2
....................................................................Uuugaaaucgggucugguuauu.......................    40    1   0B2
....................................................................cuuugaaaucgggucugguuauG......................    2     1   0B2
....................................................................cuuUaaaucgggucugguuauu.......................    1     1   0B2
....................................................................Nuugaaaucgggucugguuauua......................    1     1   0B2
....................................................................cuuugaaaucgggucugguuauua.....................    46    0   0B2
```

Star Mature

cucgaguaaagucuauggcauagccuaauaaccagacccaauuucaaaggaagugugggaucuauuuccuuugaaaucgggucugguuauuaggcuaucuauggcacggcuuc			
....cuuugaaaucgggucugguuaAua.....................	355	1	0B2
....cuuugaaaucgggucugguuaCua.....................	1	1	0B2
....cuuugaaaucgggucugguuauuU.....................	75	1	0B2
....cuuugaaaucgggucugguuauuC.....................	11	1	0B2
....cuuugaGaucgggucugguuauua.....................	1	1	0B2
....cuuugaaaucgggucugguuUuua.....................	13	1	0B2
....Uuuugaaaucgggucugguuauua.....................	4	1	0B2
....cuuugaaaucgggucugguuauuaU....................	1	1	0B2
....cuuugaaaucgggucugguuauuaA....................	18	1	0B2
....uuugaaaucgggucugguua........................	17	0	0B2
....uuugaaaucgggucugguuau.......................	23	0	0B2
....uuugaaaucgggucugguuGu.......................	3	1	0B2
....uuugaaaucgggucugguuaAu......................	67	1	0B2
....uuugaaaucggAucugguuauu......................	4	1	0B2
....uuugaaaucgggucugguuauu......................	47	0	0B2
....uuugaaaucgggucuUguuauua.....................	3	1	0B2
....uuugaaaucgggucugguuauuC.....................	1	1	0B2
....uuugaaaucgggucugguuaAua.....................	28	1	0B2
....uuugaaaucgggucugguuauuU.....................	3	1	0B2
....uuuAaaaucgggucugguuauua.....................	1	1	0B2
....uugaaaucgggucugguA.........................	6	1	0B2
....uugaaaucgggucugguuUuu......................	4	1	0B2
....ugaaaucgggucugguuauu......................	2	0	0B2

Provisional ID	:	scaffold54_12362
Score total	:	2.2
Score for star read(s)	:	-1.3
Score for read counts	:	0
Score for mfe	:	2.5
Score for randfold	:	1.6
Score for cons. seed	:	-0.6
Total read count	:	3203
Mature read count	:	3203
Loop read count	:	0
Star read count	:	0

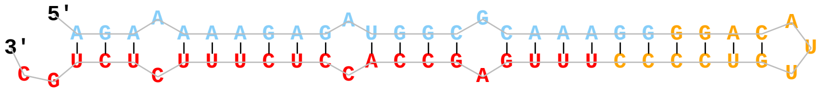

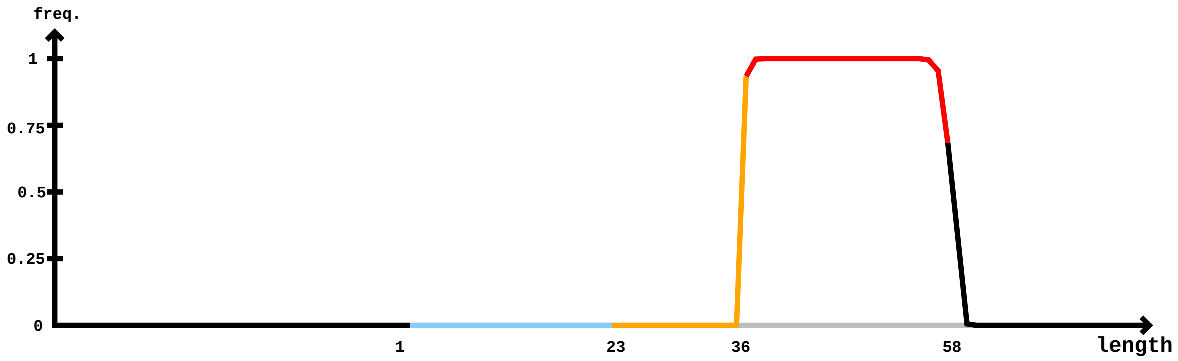

Star Mature

```
5'- auagugacacaaacaacgaacaauuugaagcuggcagaaaaagagauggcgcaaaggggacauuguccccuuugagccaccucuuucucugccagcuucaagcguggaaaag   -3'    exp
    ....................((.(((((((((((((((.((((((.((((.((((((((((...)))))))))).)))).)))))).)))))))))))))))..))......     reads    mm    sample
    ......................................................................uuugagccaccucuuucucu......................     17       0     0B2
    ......................................................................uuugagccaccucuuucucuU.....................     7        1     0B2
    ......................................................................uuugagccaccucGuucucug.....................     1        1     0B2
    ......................................................................uuugagccaccucuuucucug.....................     275      0     0B2
    ......................................................................Nuugagccaccucuuucucug.....................     1        1     0B2
    ......................................................................uuugagccaccucuuucucuA.....................     2        1     0B2
    ......................................................................uuugagccaccucuuucucGg.....................     1        1     0B2
    ......................................................................Guugagccaccucuuucucug.....................     1        1     0B2
    ......................................................................uuugGgccaccucuuucucug.....................     7        1     0B2
    ......................................................................uuugagccaccucuuucucugc....................     533      0     0B2
    ......................................................................uuugagccaccucuuucucugU....................     29       1     0B2
    ......................................................................uNugagccaccucuuucucugc....................     1        1     0B2
    ......................................................................uuugagUcaccucuuucucugc....................     1        1     0B2
    ......................................................................uuugagccaccucuuucucCgc....................     1        1     0B2
    ......................................................................uuugGgccaccucuuucucugc....................     2        1     0B2
    ......................................................................Cuugagccaccucuuucucugc....................     1        1     0B2
    ......................................................................Nuugagccaccucuuucucugc....................     4        1     0B2
    ......................................................................uuCgagccaccucuuucucugc....................     1        1     0B2
    ......................................................................uuugagccaccucuuucucugcA...................     97       1     0B2
    ......................................................................uuugagccaccucuuucucugcU...................     7        1     0B2
    ......................................................................uuugagccaccucuuucucugcUa..................     2        1     0B2
    ......................................................................uuugagccaccucuuucucugcAa..................     7        1     0B2
    .......................................................................uugagccaccucuuucucug.....................     18       0     0B2
    .......................................................................uugagccaccucuuucucugc....................     40       0     0B2
    .......................................................................uugagccaccucuuucucugcA...................     7        1     0B2
    .......................................................................uugagccaccucuuucucugcU...................     3        1     0B2
    ........................................................................ugagccaccucuuucucugc....................     3        0     0B2
    ........................................................................ugagccaccucuuucucugU....................     3        1     0B2

    ......................................................................uuugagccaccucuuucuc.......................     8        0     0G2
    ......................................................................uuugagccaccucuuucucu......................     51       0     0G2
    ......................................................................uuugagccaccucuuucucA......................     30       1     0G2
    ......................................................................uuugagccaccucuuucucuU.....................     16       1     0G2
    ......................................................................uuugagccaccucuCucucug.....................     1        1     0G2
```

Star Mature

```
auagugacacaaacaacgaacaauuugaagcuggcagaaaaagagauggcgcaaaggggacauugucccuuugagccaccucuuucucugccagcuucaagcguggaaaag
......................................................................uuugagccaccucuuucGcug.....................	1	1	0G2
......................................................................uuugagccaccucuuucucug.....................	229	0	0G2
......................................................................uuugagccaccucuuucucuA.....................	2	1	0G2
......................................................................uuugGgccaccucuuucucug.....................	4	1	0G2
......................................................................uNugagccaccucuuucucugc....................	1	1	0G2
......................................................................uuugagccaccucuuucucugc....................	428	0	0G2
......................................................................uuugagUcaccucuuucucugc....................	2	1	0G2
......................................................................uuugGgccaccucuuucucugc....................	7	1	0G2
......................................................................uuugagccaccucuuucucugU....................	24	1	0G2
......................................................................uuugagccaccucuuucucuAc....................	1	1	0G2
......................................................................uuugagccaccucuuucucugcA...................	107	1	0G2
......................................................................uuugagccaccucuuucucugcU...................	15	1	0G2
......................................................................uuugagccaccucuuucucugcAa..................	6	1	0G2
.......................................................................uugagccaccucuuucucu......................	3	0	0G2
.......................................................................uugagccaccucuuucucug.....................	18	0	0G2
.......................................................................uugagccaccucuuucucugU....................	5	1	0G2
.......................................................................uugagccaccucuuucucugc....................	35	0	0G2
.......................................................................uugagccaccucuuucucugcc...................	6	0	0G2
.......................................................................uugagccaccucuuucucugcA...................	5	1	0G2
.......................................................................uugagccaccucuuucucugcAa..................	1	1	0G2

......................................................................uuugagccaccucuuucuA.......................	7	1	0A2
......................................................................uuugagccaccucuuucucu......................	22	0	0A2
......................................................................uuugagccaccucuuucucC......................	1	1	0A2
......................................................................uuugagccaccucuuucucA......................	6	1	0A2
......................................................................uuugagccaccucuuucucug.....................	248	0	0A2
......................................................................uuugGgccaccucuuucucug.....................	5	1	0A2
......................................................................uuugagccaccucuuucucuA.....................	9	1	0A2
......................................................................Nuugagccaccucuuucucug.....................	1	1	0A2
......................................................................uuugagccaccucuuucucugU....................	63	1	0A2
......................................................................uuugagccaccucuuucucugc....................	444	0	0A2
......................................................................Guugagccaccucuuucucugc....................	1	1	0A2
......................................................................uuugagccGccucuuucucugc....................	9	1	0A2
......................................................................uuugagAcaccucuuucucugc....................	1	1	0A2
......................................................................uuugagUcaccucuuucucugc....................	5	1	0A2
......................................................................uuNgagccaccucuuucucugc....................	1	1	0A2
......................................................................Nuugagccaccucuuucucugc....................	1	1	0A2
......................................................................uuugagccaccucuuucucugcA...................	229	1	0A2
......................................................................uuugagccaccucuuucucugcc...................	1	0	0A2
......................................................................uuugagccaccucuuucucugcU...................	5	1	0A2
......................................................................uuugagccaccucuuucucugcAa..................	1	1	0A2
.......................................................................uugagccaccucuuucucA......................	1	1	0A2
.......................................................................uugagccaccucuuucucug.....................	16	0	0A2
.......................................................................uugagccaccucuuucucugc....................	23	0	0A2
.......................................................................uugagccaccucuuucucugU....................	2	1	0A2
.......................................................................uugagccaccucuuucucugcA...................	23	1	0A2
```

Provisional ID	:	scaffold59_13078
Score total	:	745.5
Score for star read(s)	:	3.9
Score for read counts	:	734.8
Score for mfe	:	2.2
Score for randfold	:	1.6
Score for cons. seed	:	3
Total read count	:	1453
Mature read count	:	1058
Loop read count	:	0
Star read count	:	395

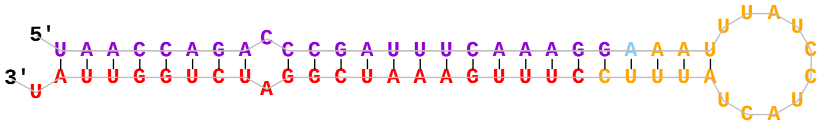

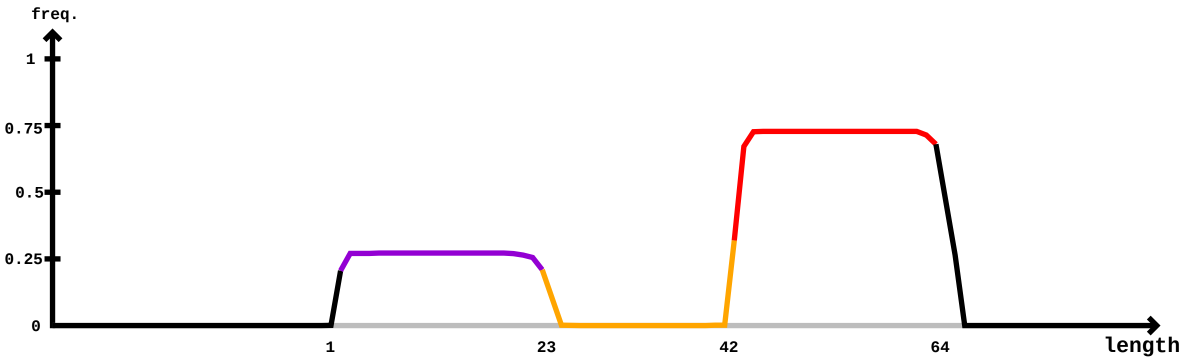

Star Mature

```
5'- guucaguuucuugggugcacaauauuaguaaccagacccgauuucaaaggaaauuuauccuacuauuuccuuugaaaucggaucugguuaucaggcuagccuugcauucaagu  -3'  obs
    guucaguuucuugggugcacaauauuaguaaccagacccgauuucaaaggaaauuuauccuacuauuuccuuugaaaucggaucugguuaucaggcuagccuugcauucaagu       exp
    .........((((((((((........(((((((((.((((((((((((((((...........)))))))))))))))).))))))))).(((....))))))))))))).  reads  mm  sample
    ...........................Caaccagacccgauuuca...................................................................  3  1  0B2
    ...........................Caaccagacccgauuucaaa.................................................................  3  1  0B2
    ...........................Caaccagacccgauuucaaag................................................................  26  1  0B2
    ...........................uaaccagacccgauuucaaaAg...............................................................  2  1  0B2
    ...........................uaGccagacccgauuucaaagg...............................................................  1  1  0B2
    ...........................Caaccagacccgauuucaaagg...............................................................  98  1  0B2
    ...........................uaaccagacccgauuucaaagga..............................................................  7  0  0B2
    ............................aaccagacccgauuucaaag................................................................  7  0  0B2
    ............................aaccGgacccgauuucaaagg...............................................................  5  1  0B2
    ............................aaccagacccgauuucaaagg...............................................................  32  0  0B2
    ............................aaccagacGcgauuucaaagg...............................................................  1  1  0B2
    .................................................................ucUuuugaaaucggaucugguua........................  2  1  0B2
    ...................................................................Uuuugaaaucggaucugguu.........................  11  1  0B2
    ...................................................................Uuuugaaaucggaucugguua........................  12  1  0B2
    ...................................................................Nuuugaaaucggaucugguua........................  1  1  0B2
    ...................................................................Guuugaaaucggaucugguuau.......................  1  1  0B2
    ...................................................................cuuugaaaucggaucugguuau.......................  1  0  0B2
    ...................................................................Uuuugaaaucggaucugguuau.......................  63  1  0B2
    ...................................................................Uuuugaaaucggaucugguuauc......................  7  1  0B2
    ...................................................................cuuugaaaucggaucugguuauU......................  3  1  0B2
    ...................................................................Uuuugaaaucggaucugguuauca.....................  4  1  0B2
    ....................................................................uuugaaaucggaucugguu.........................  4  0  0B2
    ....................................................................uuugaaaucggaucugguuau.......................  15  0  0B2
    ....................................................................uuugaaaucggaucugguuauA......................  5  1  0B2
    ....................................................................uuugaaaucggaucugguuauc......................  39  0  0B2
    ....................................................................uuugaaaucggaucugguuauU......................  4  1  0B2
    ....................................................................uuugaaaucggaucugguuaucU.....................  3  1  0B2
    ....................................................................uuugGaaucggaucugguuauca.....................  3  1  0B2
    ....................................................................uuugaGaucggaucugguuauca.....................  3  1  0B2
    ....................................................................uuugaaaucggaucugguuaAca.....................  1  1  0B2
    ....................................................................uuugaaaucggaucugguuaucG.....................  2  1  0B2
    ....................................................................uuugaaaucggaucugguuauca.....................  77  0  0B2
    .....................................................................Nugaaaucggaucugguuauc......................  1  1  0B2
```

Star **Mature**

guucaguuucuugggugcacaauauuaguaaccagacccgauuucaaaggaaauuuauccuacuauuuccuuugaaaucggaucugguuaucaggcuagccuugcauucaagu

....uugaaaucggaucugguuauc.....................	16	0	0B2
....uugaaaucggaucugguuauca....................	13	0	0B2
....ugaaaucggaucugguuaucU....................	1	1	0B2
...........................Nuaaccagacccgauuucaaa...	1	1	0G2
............................uaaccGgacccgauuucaaa...	4	1	0G2
............................Caaccagacccgauuucaaag..	2	1	0G2
............................uaaccagacccgauuucaaagg...	7	0	0G2
............................uaaccagacccgauuucaaaAg...	1	1	0G2
............................Caaccagacccgauuucaaagg...	10	1	0G2
.............................aaccagacccgauuucaaag..	9	0	0G2
.............................aaccagacccgauuucaaaAga..	1	1	0G2
....Uuuugaaaucggaucugguua.......................	16	1	0G2
....Uuuugaaaucggaucugguuau......................	145	1	0G2
....Uuuugaaaucggaucugguuauc.....................	4	1	0G2
....Uuuugaaaucggaucugguuauca....................	13	1	0G2
....uuugaaaucggaucugguu........................	2	0	0G2
....Guugaaaucggaucugguuauc.....................	1	1	0G2
....uuugaaaucggaucugAuuauc.....................	1	1	0G2
....uuugaaaucggaucugguuauA.....................	13	1	0G2
....uuugaaaucggaucugguuauc.....................	43	0	0G2
....uuugaaaucggaucugguuauca....................	96	0	0G2
....uuugaaaucggaucugguuaAca....................	4	1	0G2
....uNugaaaucggaucugguuauca....................	1	1	0G2
....uuugaaaucgAaucugguuauca....................	1	1	0G2
....uuugaaaucggaucugguuaucU....................	8	1	0G2
....uuugaGaucggaucugguuauca....................	2	1	0G2
....uugaaaucggaucugguuauc.....................	6	0	0G2
....uugaGaucggaucugguuauca....................	2	1	0G2
....uugaaaucggaucugguuauca....................	11	0	0G2
....uugaaaucggaucugguuaAca....................	1	1	0G2
....uugaaaucggCucugguuauca....................	1	1	0G2
............................Caaccagacccgauuucaaa...	3	1	0A2
............................uaaccagaAccgauuucaaa...	1	1	0A2
............................uaaccagacccgauuucaaa...	1	0	0A2
............................Caaccagacccgauuucaaag..	10	1	0A2
............................uaaccagacccgauuucaaag..	8	0	0A2
............................uaGccagacccgauuucaaagg...	4	1	0A2
............................uaaccagacccgauuucaaaAg...	4	1	0A2
............................Caaccagacccgauuucaaagg...	99	1	0A2
............................Caaccagacccgauuucaaagga..	4	1	0A2
.............................aaccagacccgauuucaa..	8	0	0A2
.............................aaccagacccgauuucaaag..	3	0	0A2
.............................aaccagacccgauuucaaaA..	2	1	0A2
.............................aaccagacccgauuucaaagg...	3	0	0A2
.............................aaccagacccgauuucaaaAga..	23	1	0A2
................................cagacccgauuucaaaAgaa...	1	1	0A2
................................cagacccgauuucaaaAgaaa..	1	1	0A2
....Uuuugaaaucggaucugguu........................	3	1	0A2
....Uuuugaaaucggaucugguua.......................	15	1	0A2
....Uuuugaaaucggaucugguuau......................	158	1	0A2
....Nuuugaaaucggaucugguuau......................	1	1	0A2
....Uuuugaaaucggaucugguuauc.....................	1	1	0A2
....cuuugaaaucggaucugguuauA.....................	1	1	0A2
....Uuuugaaaucggaucugguuauca....................	2	1	0A2
....uuugaaaucggaucugguua.......................	4	0	0A2
....uuugaaaucggaucugguuau......................	11	0	0A2
....uuugGaaucggaucugguuauc.....................	2	1	0A2
....uuugaaaucggaucugguuauU.....................	9	1	0A2
....uuugaaaucggaucugguuauA.....................	19	1	0A2
....uuugaaaucggaucugguuauc.....................	25	0	0A2
....uuugaaaucggaucugguuauca....................	105	0	0A2
....uuugaaaucggaucugguuaucU....................	5	1	0A2
....uuugaaaucggaucugUuuauca....................	1	1	0A2
....uuugaaaucggaucugguuauAa....................	3	1	0A2
....uugaaaucggaucugguuauA.....................	2	1	0A2
....uugaaaucggaucugguuauc.....................	6	0	0A2
....uugaaaCcggaucugguuauc.....................	1	1	0A2
....Nugaaaucggaucugguuauca....................	1	1	0A2

```
                            Star                                    Mature
guucaguuucuugggugcacaauauuaguaaccagacccgauuucaaaggaaauuuauccuacuauuuccuuugaaaucggaucugguuaucaggcuagccuugcauucaagu
.......................................................................uugaaaucggaucugguuauca....................    19    0    0A2
........................................................................ugaaaucggaucugguuauca....................    1     0    0A2
```

Provisional ID	:	scaffold64_13889
Score total	:	6
Score for star read(s)	:	-1.3
Score for read counts	:	0
Score for mfe	:	2.7
Score for randfold	:	1.6
Score for cons. seed	:	3
Total read count	:	152
Mature read count	:	152
Loop read count	:	0
Star read count	:	0

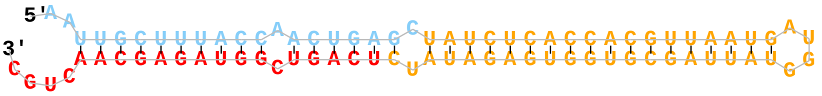

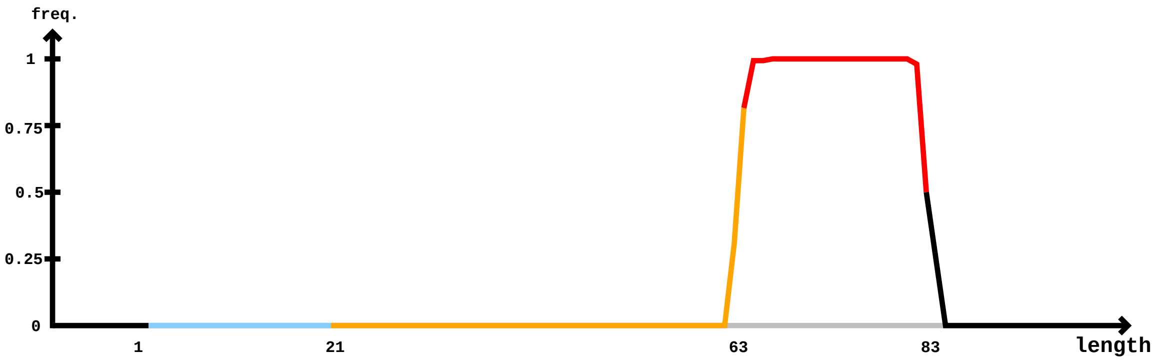

Star   Mature

```
5'- aucuaggcaauugcuuuaccaacugagcuaucucaccacguuaaugaugguauuagcguggugagauaucucagucgguagagcaacugccuagauaggaggagguccca   -3'    exp
    ((((((((.((((((((((.((((((.(((((((((((((((((((....))))))))))))))))))).)))))).)))))))))).)))))))).(((.....)))..    reads      mm       sample
    .....................................................................cucagucgguagagcaacug.....................    17         0        0B2
    .....................................................................cucagucgguGgagcaacug.....................    6          1        0B2
    ......................................................................ucagucgguagagcaacug.....................    1          0        0B2
    ......................................................................ucagucgguagagcaacugc....................    25         0        0B2
    ......................................................................ucagucgguagagcaacugcc...................    10         0        0B2
    ......................................................................ucagucgguagagcaacugcU...................    3          1        0B2
    .......................................................................cagucgguagagcaacug.....................    17         0        0B2
    .......................................................................cGgucgguagagcaacugc....................    2          1        0B2
    .......................................................................Nagucgguagagcaacugcc...................    1          1        0B2
    .......................................................................cagucgguagagcaacugcc...................    4          0        0B2
    .........................................................................gucgguagagcaacugAc...................    1          1        0B2

    .....................................................................cucagucgguagagcaacug.....................    15         0        0G2
    ......................................................................ucagucgguagagcaGcu......................    1          1        0G2
    ......................................................................ucagucgguagagcaacu......................    2          0        0G2
    ......................................................................ucagucgguagagcaacug.....................    5          0        0G2
    ......................................................................ucagucgguagagcaacugc....................    4          0        0G2
    ......................................................................ucagucgguagagcaacugcc...................    13         0        0G2
    .......................................................................cagucgguagagcaaAug.....................    3          1        0G2

    .....................................................................cucagucgguagagcaacug.....................    9          0        0A2
    ......................................................................ucagucgguagagcaacugc....................    8          0        0A2
    ......................................................................ucagucgguagagcaacugcA...................    2          1        0A2
    ......................................................................ucagucgguagagcaacugcc...................    2          0        0A2
    ......................................................................ucGgucgguagagcaacugcc...................    1          1        0A2
```

Provisional ID	:	scaffold69_14735
Score total	:	3.5
Score for star read(s)	:	-1.3
Score for read counts	:	0
Score for mfe	:	0.2
Score for randfold	:	1.6
Score for cons. seed	:	3
Total read count	:	223
Mature read count	:	208
Loop read count	:	15
Star read count	:	0

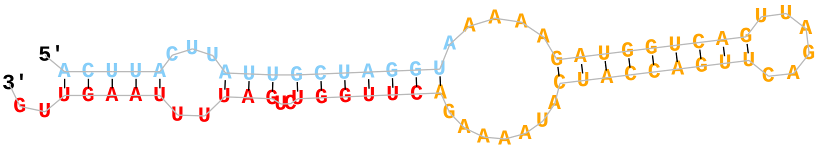

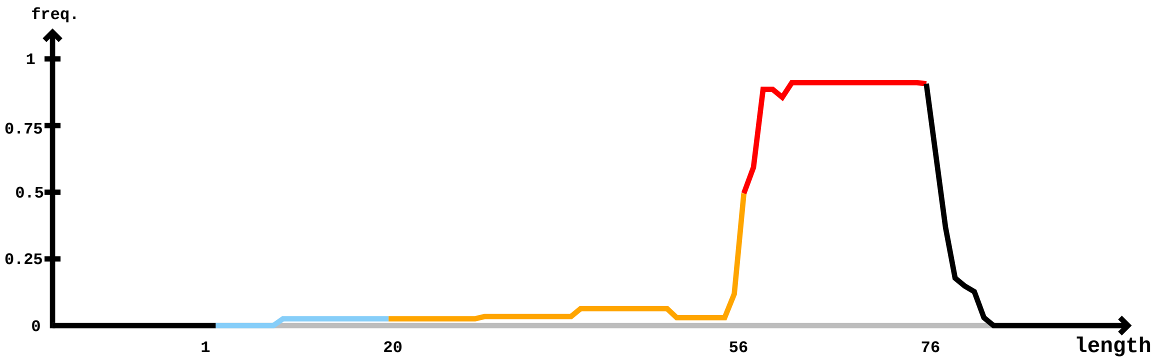

Star   Mature

```
5'- agauuuguugccuuuacuuacuuauugcuagguaaaaagauggucaguuagacuugaccaucauaaaagacuuggucugauuuuaaguuguaauagauuuguuuuuuaug   -3'    exp
    ((((((((((....(((((...((((((((((.....((((((((((......))))))))))......."))))))..)))..))))).))))))))))..........    reads    mm    sample
    ......................uauugcuaCguaaaaagaugg...................................................................    3        1     0B2
    ...........................................ucaguCagacuugaccauca...............................................    8        1     0B2
    ......................................................................cuuggucugauuuuaaguug....................    17       0     0B2
    ........................................................................uggucugauuuuaaguugua..................    3        0     0B2
    ...........................................................................ucugauuuuaaguuguaaua...............    3        0     0B2

    ......................uauugcuaCguaaaaagaugg...................................................................    3        1     0G2
    .....................................................uugaccaucauaaaagacuug....................................    2        0     0G2
    .....................................................................acuuggucugauuuuaaguug....................    9        0     0G2
    ......................................................................cuuggucugauuuuaaguug....................    24       0     0G2
    .......................................................................uuggucugauuuuaaguugu...................    12       0     0G2
    .......................................................................uuggucugauuuuaaguugua..................    6        0     0G2
    ........................................................................uggucugauuuuaaguugua..................    20       0     0G2
    ........................................................................uggucugauuuuaaguuguaa.................    2        0     0G2
    ........................................................................uggucugauuuuaaguuguaau................    5        0     0G2
    ........................................................................uggucugauuuuaaguuguaaua...............    4        0     0G2
    ...........................................................................ucugauuuuaaguuguaaua...............    5        0     0G2

    .....................................................uugaccaucauaaaagacuug....................................    5        0     0A2
    .....................................................................acuuggucugauuuuaaguug....................    12       0     0A2
    ......................................................................cuuggucugauuuuaaguu.....................    1        0     0A2
    ......................................................................cGuggucugauuuuaaguug....................    1        1     0A2
    ......................................................................cuuggucugauuuuaaguuA....................    3        1     0A2
    ......................................................................cuuggucugauuuuaaguug....................    43       0     0A2
    .......................................................................uuggucugauuuuaaguugu...................    4        0     0A2
    .......................................................................uuggucugauuuuaaguugua..................    1        0     0A2
    ........................................................................uggucugauuuuaaguug....................    1        0     0A2
    ........................................................................uggucugauuuuaaNuug....................    1        1     0A2
    ........................................................................uggucugauuuuaaguugua..................    15       0     0A2
    ........................................................................uggucugauuuuaaguuguaa.................    5        0     0A2
    ........................................................................uggucugauuuuaaguuguaaua...............    6        0     0A2
    ........................................................................uggucugauuuuaaguuguaauU...............    5        1     0A2
    ........................................................................uggucugauuuuaaguuguaauaA..............    2        1     0A2
    ...........................................................................ucugauuuuaaguuguaauaA..............    5        1     0A2
```

Star Mature

agauuuguugccuuuacuuacuuauugcuagguaaaaagaugguucaguuagacuugaccaucauaaaaagacuuggucugauuuuaaguuguaauagauuuguuuuuuaug

Provisional ID	:	scaffold73_15267
Score total	:	0.6
Score for star read(s)	:	-1.3
Score for read counts	:	0
Score for mfe	:	0.9
Score for randfold	:	1.6
Score for cons. seed	:	-0.6
Total read count	:	416399
Mature read count	:	416399
Loop read count	:	0
Star read count	:	0

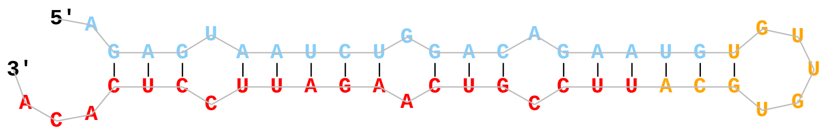

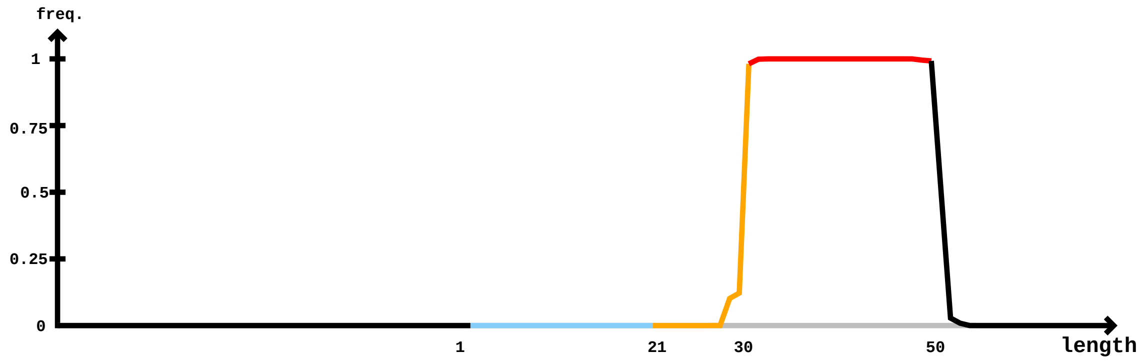

Star Mature

```
5'- uuuuaucacugaauugcaauagcguauacuugcauuucuuaagaguaaucuggacagaaugugguugugcauuccgucaagauuccucacaagaacugcaguguuaaac  -3'    exp
    .((((.(((((...(((((((.....)).)))))).(((((..(((.(((((.(((.((((((.....)))))).))).))))).)))..)))))...))))).)))).   reads   mm    sample
    .............................................................uguuguAcauuccgucaagauuccuca....................   4       1     0A2
    ................................................................uguAcauuccgucaagauuccu......................   3       1     0A2
    ................................................................uguAcauuccgucaagauuccuca....................   2       1     0A2
    ................................................................uguAcauuccgucaagauuccucaca..................   1       1     0A2
    ..................................................................uAcauuccgucaagauuccuca....................   3       1     0A2
    ..................................................................uAcauuccgucaagauuccucaca..................   7       1     0A2
    ...................................................................Ccauuccgucaagauuccucaca..................   8       1     0A2
    ....................................................................cauuccgucaagauuccu......................   1       0     0A2
    ....................................................................cauuccgucaagauuccuc.....................   23      0     0A2
    ....................................................................cauuccgucaagauuccuU.....................   1       1     0A2
    ....................................................................cauuccgucaagauuccucC....................   1       1     0A2
    ....................................................................cauuccgucaagauuccucG....................   1       1     0A2
    ....................................................................Nauuccgucaagauuccuca....................   1       1     0A2
    ....................................................................cauuccgucaagauuccuca....................   296     0     0A2
    ....................................................................cauuccgucaagauuccucU....................   55      1     0A2
    ....................................................................cauuccgucaagauGccuca....................   1       1     0A2
    ....................................................................cauuccgucaagauuccucaA...................   2       1     0A2
    ....................................................................cauuccUucaagauuccucac...................   1       1     0A2
    ....................................................................cauuccgucaagauuccucUc...................   4       1     0A2
    ....................................................................cauuccgucaagauuccucac...................   146     0     0A2
    ....................................................................cauuccgucaagauuAcucaca..................   2       1     0A2
    ....................................................................cauuccgCcaagauuccucaca..................   3       1     0A2
    ....................................................................cauucGgucaagauuccucaca..................   1       1     0A2
    ....................................................................cauuccgucGagauuccucaca..................   3       1     0A2
    ....................................................................cauuccgucaagauuccAcaca..................   1       1     0A2
    ....................................................................Uauuccgucaagauuccucaca..................   6       1     0A2
    ....................................................................cauuccgucaagauuccucUca..................   6       1     0A2
    ....................................................................cNuuccgucaagauuccucaca..................   2       1     0A2
    ....................................................................cauuccgucaagauuccucacG..................   12      1     0A2
    ....................................................................cauuccgAcaagauuccucaca..................   1       1     0A2
    ....................................................................cauuccgucaagGuuccucaca..................   3       1     0A2
    ....................................................................cauuccgucaagauAccucaca..................   1       1     0A2
    ....................................................................cauuccguUaagauuccucaca..................   2       1     0A2
    ....................................................................cauuccAucaagauuccucaca..................   9       1     0A2
```

Star **Mature**

```
uuuuaucacugaauugcaauagcguauacuugcauuucuuaagaguaaucuggacagaaugcuguugugcauuccgucaagauuccucacaagaacugcaguguuaaac
....................................................................cauuccgucaagauuccucacU..................   19315   1   0A2
....................................................................cauuccgucaagauuccucaAa..................   2       1   0A2
....................................................................cauuccgucaagauuccucGca..................   2       1   0A2
....................................................................cauuccgucaagaCuccucaca..................   2       1   0A2
....................................................................caGuccgucaagauuccucaca..................   1       1   0A2
....................................................................cauuccguAaagauuccucaca..................   1       1   0A2
....................................................................cauuccgucaaCauuccucaca..................   1       1   0A2
....................................................................caCuccgucaagauuccucaca..................   1       1   0A2
....................................................................Gauuccgucaagauuccucaca..................   2       1   0A2
....................................................................cauuGcgucaagauuccucaca..................   1       1   0A2
....................................................................cauuccgucaagauuccuAaca..................   1       1   0A2
....................................................................cauuccgucaagauucAucaca..................   2       1   0A2
....................................................................Nauuccgucaagauuccucaca..................   21      1   0A2
....................................................................cauuccgucaagauuUcucaca..................   4       1   0A2
....................................................................cauuccgucaagauuccucaca..................   7121    0   0A2
....................................................................cauuccgGcaagauuccucaca..................   1       1   0A2
....................................................................cauuccgucaagauucGucaca..................   1       1   0A2
....................................................................cauuccgucaagauuccCcaca..................   4       1   0A2
....................................................................cauuccUucaagauuccucaca..................   1       1   0A2
....................................................................Aauuccgucaagauuccucaca..................   2       1   0A2
....................................................................cauuccgucaGgauuccucaca..................   19      1   0A2
....................................................................cauuUcgucaagauuccucaca..................   1       1   0A2
....................................................................cauuccgucaagauuccucacC..................   35      1   0A2
....................................................................cauuccgucaagauCccucaca..................   2       1   0A2
....................................................................cauuccgucaagauuccucacaU.................   84      1   0A2
....................................................................cauuccgucaagauuccucacUa.................   518     1   0A2
....................................................................cauuccgucaagauuccucacCa.................   6       1   0A2
....................................................................cauuccgucaagauuccucacaa.................   19      0   0A2
....................................................................cauuccgucaagauuccucacaaU................   16      1   0A2
....................................................................cauuccgucaagauuccucacaaA................   5       1   0A2
....................................................................cauuccgucaagauuccucacaaUa...............   17      1   0A2
.....................................................................auuccgucaagauuccuA.....................   1       1   0A2
.....................................................................auuccgucaagauuccuc.....................   10      0   0A2
.....................................................................Nuuccgucaagauuccuca....................   1       1   0A2
.....................................................................auuccgucaagauuccuca....................   59      0   0A2
.....................................................................auuccgucaagauuccucU....................   12      1   0A2
.....................................................................auuccgucaagaCuccuca....................   1       1   0A2
.....................................................................auuccgucaagauuccucac...................   36      0   0A2
.....................................................................auuccgucaagauucAucac...................   1       1   0A2
.....................................................................Nuuccgucaagauuccucac...................   2       1   0A2
.....................................................................auuccgucaagauuccucUc...................   3       1   0A2
.....................................................................auuccgGcaagauuccucaca..................   2       1   0A2
.....................................................................auuccgucaagauuccuUaca..................   1       1   0A2
.....................................................................auuccgucCagauuccucaca..................   1       1   0A2
.....................................................................Guuccgucaagauuccucaca..................   1       1   0A2
.....................................................................auuccgucaagauuccucacU..................   3198    1   0A2
.....................................................................aNuccgucaagauuccucaca..................   1       1   0A2
.....................................................................Cuuccgucaagauuccucaca..................   40      1   0A2
.....................................................................auuccAucaagauuccucaca..................   1       1   0A2
.....................................................................auuccgucaagauuccucacG..................   3       1   0A2
.....................................................................auuccgucaagauuccucaca..................   1369    0   0A2
.....................................................................Nuuccgucaagauuccucaca..................   1       1   0A2
.....................................................................auuccgAcaagauuccucaca..................   1       1   0A2
.....................................................................auuccgucaagauuccucacC..................   2       1   0A2
.....................................................................auuAcgucaagauuccucaca..................   1       1   0A2
.....................................................................auucUgucaagauuccucaca..................   1       1   0A2
.....................................................................auuccgucaagauuccucacUa.................   65      1   0A2
.....................................................................Cuuccgucaagauuccucacaa.................   6       1   0A2
.....................................................................auuccgucaagauuccucacaa.................   23      0   0A2
.....................................................................auuccgucaagauuccucacaU.................   24      1   0A2
.....................................................................auuccgucCagauuccucacaa.................   1       1   0A2
.....................................................................auuccgucaagauuccucacaaU................   26      1   0A2
.....................................................................auuccgucaagauuccucacaaA................   5       1   0A2
.....................................................................auuccgucaagauuccucacaaUa...............   19      1   0A2
.....................................................................auuccgucaagauuccucacaaUaa..............   2       1   0A2
......................................................................uuccgucaagauuccuca....................   432     0   0A2
......................................................................uuccgucaGgauuccuca....................   5       1   0A2
......................................................................uuccguAaagauuccuca....................   1       1   0A2
......................................................................uuccgucGagauuccuca....................   1       1   0A2
......................................................................Nuccgucaagauuccuca....................   1       1   0A2
```

Star Mature

uuuuaucacugaauugcaauagcguauacuugcauuucuuaagaguaaucuggacagaauguguugugcauuccgucaagauuccucacaagaacugcaguguuaaac			
....Cuccgucaagauuccuca....................	1	1	0A2
....uuccgucaagauuccucU....................	199	1	0A2
....uuccguUaagauuccuca....................	1	1	0A2
....uuccguGaagauuccucac...................	2	1	0A2
....uuccgucaagauuccucaG...................	1	1	0A2
....Nuccgucaagauuccucac...................	2	1	0A2
....uuccgucaagauuccucac...................	439	0	0A2
....uuccgucaagauuccucUc...................	1	1	0A2
....uuccgGcaagauuccucac...................	1	1	0A2
....uuccgucaagauuccucGca..................	7	1	0A2
....uuccgucaagauuccucacU..................	119027	1	0A2
....uuccgucaaAauuccucaca..................	3	1	0A2
....uuccgucaagauCccucaca..................	4	1	0A2
....uuccgucaagauuccucaGa..................	1	1	0A2
....uuccgucaagauuccucaAa..................	6	1	0A2
....uuccgucaagUuuccucaca..................	1	1	0A2
....uNccgucaagauuccucaca..................	6	1	0A2
....uuccgucaagauuccuAaca..................	4	1	0A2
....Guccgucaagauuccucaca..................	11	1	0A2
....uuccgucaagauucAucaca..................	7	1	0A2
....uuccgGcaagauuccucaca..................	35	1	0A2
....uuccCucaagauuccucaca..................	1	1	0A2
....uuccgucaagGuuccucaca..................	3	1	0A2
....uuccguAaagauuccucaca..................	19	1	0A2
....uuccgucaagauuccucacC..................	152	1	0A2
....uuccgucaagauuccAcaca..................	3	1	0A2
....uuccgucUagauuccucaca..................	4	1	0A2
....uucUgucaagauuccucaca..................	4	1	0A2
....uuccgucaaUauuccucaca..................	8	1	0A2
....uGccgucaagauuccucaca..................	2	1	0A2
....Cuccgucaagauuccucaca..................	11	1	0A2
....uuccgucaagauuccuGaca..................	1	1	0A2
....uuccUucaagauuccucaca..................	4	1	0A2
....uuccguGaagauuccucaca..................	40	1	0A2
....uuccgucaGgauuccucaca..................	76	1	0A2
....uucGgucaagauuccucaca..................	3	1	0A2
....uuccgucaagauuccucacG..................	32	1	0A2
....Auccgucaagauuccucaca..................	7	1	0A2
....uuccgCcaagauuccucaca..................	12	1	0A2
....uuccgucaagauuAcucaca..................	6	1	0A2
....uuccgucaagaCuccucaca..................	14	1	0A2
....uuccgucGagauuccucaca..................	11	1	0A2
....uuccgucaagauAccucaca..................	2	1	0A2
....uuUcgucaagauuccucaca..................	10	1	0A2
....uuccguUaagauuccucaca..................	5	1	0A2
....uCccgucaagauuccucaca..................	14	1	0A2
....uuccgucaagCuuccucaca..................	1	1	0A2
....Nuccgucaagauuccucaca..................	75	1	0A2
....uuccgucaagauuccuUaca..................	6	1	0A2
....uuccgucaagaGuccucaca..................	6	1	0A2
....uuccgAcaagauuccucaca..................	2	1	0A2
....uuccgucaagauuccucaca..................	35257	0	0A2
....uuccAucaagauuccucaca..................	8	1	0A2
....uuccgucaagaAuccucaca..................	2	1	0A2
....uuccgucaagauuccCcaca..................	8	1	0A2
....uucAgucaagauuccucaca..................	6	1	0A2
....uAccgucaagauuccucaca..................	2	1	0A2
....uuccgucaagauuUcucaca..................	6	1	0A2
....uuccgucaagauGccucaca..................	8	1	0A2
....uuccgucaagauuccucUca..................	2	1	0A2
....uuccgucaagauuccGcaca..................	5	1	0A2
....uuccgucaagauucGucaca..................	1	1	0A2
....uuAcgucaagauuccucaca..................	7	1	0A2
....uuUcgucaagauuccucacaa.................	1	1	0A2
....uuccAucaagauuccucacaa.................	1	1	0A2
....uuccgucaagauuccucacaG.................	4	1	0A2
....uuccgucaagauuccucacCa.................	33	1	0A2
....uuccgucaagauuccucacUa.................	1774	1	0A2
....uuccgucaagauuccuUacaa.................	1	1	0A2
....uuccgucaagauuccucacaa.................	2943	0	0A2

Star Mature

```
uuuuaucacugaauugcaauagcguauacuugcauuucuuaagaguaaucuggacagaauguguugugcauuccgucaagauuccucacaagaacugcaguguuaaac
......................................................................uuccguGaagauuccucacaa.................  4     1  0A2
......................................................................uuccgucaaAauuccucacaa.................  1     1  0A2
......................................................................uuccgucaagauuccucacGa.................  1     1  0A2
......................................................................uuAcgucaagauuccucacaa.................  3     1  0A2
......................................................................uuccgucaagauuccucacaC.................  1     1  0A2
......................................................................uuccgucaagauuAcucacaa.................  2     1  0A2
......................................................................Cuccgucaagauuccucacaa.................  1     1  0A2
......................................................................uuccgCcaagauuccucacaa.................  1     1  0A2
......................................................................uuccgucUagauuccucacaa.................  1     1  0A2
......................................................................uNccgucaagauuccucacaa.................  1     1  0A2
......................................................................uuccguUaagauuccucacaa.................  1     1  0A2
......................................................................Nuccgucaagauuccucacaa.................  4     1  0A2
......................................................................uuccgucaagUuuccucacaa.................  1     1  0A2
......................................................................uuccgucaagauuccuAacaa.................  1     1  0A2
......................................................................uuccgucaagaCuccucacaa.................  2     1  0A2
......................................................................uuccgucaagauuccucacaU.................  361   1  0A2
......................................................................Guccgucaagauuccucacaa.................  1     1  0A2
......................................................................uuccgucaagauuccucaAaa.................  2     1  0A2
......................................................................uuccgucaagauuccucacaaA................  152   1  0A2
......................................................................uuccgucaagauuccucacaaU................  4006  1  0A2
......................................................................uuccgucaagauuccucacCag................  1     1  0A2
......................................................................uuccgucaagauuccucacaag................  80    0  0A2
......................................................................uuccgucaagauuccucacaaC................  7     1  0A2
......................................................................uuccgucaagauuccucacaaAa...............  10    1  0A2
......................................................................uuccgucaagauuccucacaaCa...............  11    1  0A2
......................................................................uuccgucaagauuccucacaaUa...............  2268  1  0A2
......................................................................uuccgucaagauuccucacaaUaa..............  11    1  0A2
......................................................................uuccgucaagauuccucacaagaaA.............  3     1  0A2
.......................................................................uccgGcaagauuccucaca..................  1     1  0A2
.......................................................................uccgucGagauuccucaca..................  1     1  0A2
.......................................................................uccgucCagauuccucaca..................  1     1  0A2
.......................................................................uccgucaagauAccucaca..................  2     1  0A2
.......................................................................uccgAcaagauuccucaca..................  1     1  0A2
.......................................................................uccgucaagauuccuAaca..................  1     1  0A2
.......................................................................Gccgucaagauuccucaca..................  1     1  0A2
.......................................................................uccgucaagauuccucacU..................  2134  1  0A2
.......................................................................uccgucaagauuccucacG..................  2     1  0A2
.......................................................................uAcgucaagauuccucaca..................  1     1  0A2
.......................................................................uccgucaagauuccucaca..................  858   0  0A2
.......................................................................uccgCcaagauuccucaca..................  2     1  0A2
.......................................................................uNcgucaagauuccucaca..................  1     1  0A2
.......................................................................uccUucaagauuccucaca..................  1     1  0A2
.......................................................................uccgucaagauuccucacC..................  5     1  0A2
.......................................................................Nccgucaagauuccucaca..................  2     1  0A2
.......................................................................uccgucaagauuccucacUa.................  40    1  0A2
.......................................................................uccgucaagauuccucacaa.................  93    0  0A2
.......................................................................uccgucaagauuccucacCa.................  2     1  0A2
.......................................................................uccgucaagauuccucacaU.................  8     1  0A2
.......................................................................ucAgucaagauuccucacaa.................  1     1  0A2
.......................................................................uccgucaagauuccucacaaA................  3     1  0A2
.......................................................................uccgucaagauuccucacaaU................  109   1  0A2
.......................................................................uccgucaagauuccucacaaCa...............  2     1  0A2
.......................................................................uccgucaagauuccucacaaga...............  12    0  0A2
.......................................................................uccgucaagauuccucacaaUa...............  77    1  0A2
.......................................................................uccgucaagauuccucacaagaaA.............  6     1  0A2
........................................................................ccgucaagauuccucaca..................  55    0  0A2
........................................................................ccgucaagauuccucacU..................  136   1  0A2
........................................................................ccgucaagaCuccucaca..................  1     1  0A2
........................................................................ccgucaagauuccucacUa.................  7     1  0A2
........................................................................ccgucaagauuccucacaaU................  6     1  0A2
.........................................................................cgucaagauuccucacaaU................  3     1  0A2

................................................................uguAcauuccgucaagauuccucaca..................  2     1  0B2
....................................................................cauuccgucaagauuccuc.....................  2     0  0B2
....................................................................Nauuccgucaagauuccuca....................  1     1  0B2
....................................................................cauuccgucaagauuccucU....................  17    1  0B2
....................................................................cauuccgucaagauuccuca....................  59    0  0B2
....................................................................cauuccgucaagauuccucUc...................  5     1  0B2
....................................................................cauuccgCcaagauuccucac...................  1     1  0B2
....................................................................cauuccgucaagauuccucac...................  79    0  0B2
```

```
                                                           Star                          Mature
uuuuaucacugaauugcaauagcguauacuugcauuucuuaagaguaaucuggacagaauguguugugcauuccgucaagauuccucacaagaacugcaguguuaaac
...................................................................cauuccgucaagauuccucaA....................	10	1	0B2
...................................................................cauuccgucaagauuccucUca...................	1	1	0B2
...................................................................cauuccUucaagauuccucaca...................	1	1	0B2
...................................................................cauCccgucaagauuccucaca...................	1	1	0B2
...................................................................cauuccgucGagauuccucaca...................	1	1	0B2
...................................................................cauuccCucaagauuccucaca...................	1	1	0B2
...................................................................cauuccgucaagauuccucaca...................	1701	0	0B2
...................................................................Uauuccgucaagauuccucaca...................	1	1	0B2
...................................................................Nauuccgucaagauuccucaca...................	14	1	0B2
...................................................................cauuccgucaagauuccucaGa...................	1	1	0B2
...................................................................cauuccgucaagauuccucacG...................	1	1	0B2
...................................................................cauuccgGcaagauuccucaca...................	1	1	0B2
...................................................................cauuccgucaagauuccCcaca...................	3	1	0B2
...................................................................cauuccgucaagauuccucacC...................	18	1	0B2
...................................................................Gauuccgucaagauuccucaca...................	1	1	0B2
...................................................................cauuccgucaagauuccucCca...................	1	1	0B2
...................................................................cauuccgucaagauuccucaAa...................	3	1	0B2
...................................................................cauuccgucaagauuccucacU...................	6265	1	0B2
...................................................................cauuccgucaagauuccucacaU..................	11	1	0B2
...................................................................cauuccgucaagauuccucacCa..................	1	1	0B2
...................................................................cauuccgucaagauuccucacaa..................	3	0	0B2
...................................................................cauuccgucaagauuccucacUa..................	89	1	0B2
...................................................................cauuccgucaagauuccucacaaU.................	1	1	0B2
...................................................................cauuccgucaagauuccucacaaUa................	1	1	0B2
....................................................................auuccgucaagauuccucU.....................	16	1	0B2
....................................................................auuccgucaagauuccuca.....................	16	0	0B2
....................................................................Nuuccgucaagauuccucac....................	1	1	0B2
....................................................................auuccgucaagauuccucac....................	29	0	0B2
....................................................................Cuuccgucaagauuccucaca...................	30	1	0B2
....................................................................auuccgucaagauuccucaca...................	484	0	0B2
....................................................................auuccgucaagauuccucacC...................	10	1	0B2
....................................................................auuccgucaagauuccucacG...................	1	1	0B2
....................................................................auuAcgucaagauuccucaca...................	1	1	0B2
....................................................................auuccgucaCgauuccucaca...................	1	1	0B2
....................................................................Nuuccgucaagauuccucaca...................	3	1	0B2
....................................................................auuccgucaagauuccucacU...................	1423	1	0B2
....................................................................auuccgucaagauCccucaca...................	1	1	0B2
....................................................................auuccgucaagauuccCcaca...................	1	1	0B2
....................................................................auuccgucaagauuccucacUa..................	21	1	0B2
....................................................................auuccgucaagauuccucacaa..................	4	0	0B2
....................................................................Cuuccgucaagauuccucacaa..................	3	1	0B2
....................................................................auuccgucaagauuccucacaaU.................	15	1	0B2
....................................................................auuccgucaagauuccucacaaUa................	3	1	0B2
.....................................................................uuccgucaagauuccucU.....................	124	1	0B2
.....................................................................uuccgucaagauuccuca.....................	237	0	0B2
.....................................................................Nuccgucaagauuccucac....................	3	1	0B2
.....................................................................uuccgucaagauuccucA.....................	1	1	0B2
.....................................................................uuccgucaagauuccucGc....................	1	1	0B2
.....................................................................uuccgucaagaCuccucac....................	3	1	0B2
.....................................................................uuccAucaagauuccucac....................	1	1	0B2
.....................................................................uuccgucaagauuccucac....................	346	0	0B2
.....................................................................uuccgucaagauuccucUc....................	2	1	0B2
.....................................................................uuccgucaaCauuccucaca...................	1	1	0B2
.....................................................................uuccgucaagauuccucaca...................	23202	0	0B2
.....................................................................uuccgucaagauuccucacU...................	82955	1	0B2
.....................................................................uuccguUaagauuccucaca...................	2	1	0B2
.....................................................................uucUgucaagauuccucaca...................	11	1	0B2
.....................................................................uuccgGcaagauuccucaca...................	16	1	0B2
.....................................................................uuccgucaagauucAucaca...................	1	1	0B2
.....................................................................uuccgucaagaCuccucaca...................	5	1	0B2
.....................................................................uuccgucaagGuuccucaca...................	4	1	0B2
.....................................................................uuccgucaagauuccucGca...................	1	1	0B2
.....................................................................uuccUucaagauuccucaca...................	4	1	0B2
.....................................................................Cuccgucaagauuccucaca...................	7	1	0B2
.....................................................................uuccgucaagauuccGcaca...................	1	1	0B2
.....................................................................uuAcgucaagauuccucaca...................	5	1	0B2
.....................................................................uuccAucaagauuccucaca...................	10	1	0B2
.....................................................................uuccgucaGgauuccucaca...................	36	1	0B2
.....................................................................Guccgucaagauuccucaca...................	3	1	0B2
.....................................................................uuccgAcaagauuccucaca...................	1	1	0B2
```

Star Mature

```
uuuuaucacugaauugcaauagcguauacuugcauuucuuaagaguaaucuggacagaauguguugugcauuccgucaagauuccucacaagaacugcaguguuaaac
......................................................................uuccguAaagauuccucaca..................   7      1   0B2
......................................................................uuccgucaagauuccucUca..................   6      1   0B2
......................................................................uuccgucaCgauuccucaca..................   1      1   0B2
......................................................................uGccgucaagauuccucaca..................   2      1   0B2
......................................................................uuUcgucaagauuccucaca..................   1      1   0B2
......................................................................uuccgucaagauuccucCca..................   2      1   0B2
......................................................................uuccgucaagauuccucacC..................   154    1   0B2
......................................................................uuccgucaagauuccucaAa..................   14     1   0B2
......................................................................uuccgucaagauuccucaGa..................   1      1   0B2
......................................................................uuccgucaagauuccCcaca..................   23     1   0B2
......................................................................Auccgucaagauuccucaca..................   1      1   0B2
......................................................................uucAgucaagauuccucaca..................   4      1   0B2
......................................................................uuccgucaagauuccucacG..................   35     1   0B2
......................................................................uuccgucaagauuccAcaca..................   1      1   0B2
......................................................................uCccgucaagauuccucaca..................   5      1   0B2
......................................................................uuccgucGagauuccucaca..................   5      1   0B2
......................................................................Nuccgucaagauuccucaca..................   180    1   0B2
......................................................................uNccgucaagauuccucaca..................   1      1   0B2
......................................................................uuccgucaagauCccucaca..................   4      1   0B2
......................................................................uuccgucCagauuccucaca..................   2      1   0B2
......................................................................uuccgucaagauucGucaca..................   1      1   0B2
......................................................................uuccgucaaUauuccucaca..................   5      1   0B2
......................................................................uuccgucaagaGuccucaca..................   2      1   0B2
......................................................................uuccgucaagauuccuGaca..................   2      1   0B2
......................................................................uuccgucaagauGccucaca..................   7      1   0B2
......................................................................uAccgucaagauuccucaca..................   1      1   0B2
......................................................................uuccgucaaAauuccucaca..................   1      1   0B2
......................................................................uuccCucaagauuccucaca..................   1      1   0B2
......................................................................uuccgCcaagauuccucaca..................   4      1   0B2
......................................................................uuccgucaagauuccuUaca..................   1      1   0B2
......................................................................Cuccgucaagauuccucacaa.................   4      1   0B2
......................................................................uuccgucaagauuccucacCa.................   13     1   0B2
......................................................................uuccgucaagauuccucacaa.................   2069   0   0B2
......................................................................uuccgGcaagauuccucacaa.................   2      1   0B2
......................................................................uuccgCcaagauuccucacaa.................   1      1   0B2
......................................................................uuccgucaagauuccucacaC.................   1      1   0B2
......................................................................uuccgucaagauuccCcacaa.................   1      1   0B2
......................................................................uuccgucaGgauuccucacaa.................   1      1   0B2
......................................................................uuccgucaagauuAcucacaa.................   1      1   0B2
......................................................................Nuccgucaagauuccucacaa.................   20     1   0B2
......................................................................uuccgucaagauuccucacGa.................   8      1   0B2
......................................................................uuccgucaagauuccucacUa.................   702    1   0B2
......................................................................uuccgucaagaGuccucacaa.................   1      1   0B2
......................................................................uuccgucaagauuccucacaU.................   259    1   0B2
......................................................................uuccgucaagauuccucacaG.................   17     1   0B2
......................................................................uuccgucaagauuccucacaaA................   69     1   0B2
......................................................................uuccgucaagauuccucacaaU................   1710   1   0B2
......................................................................uuccgucaagauuccucacaag................   61     0   0B2
......................................................................uuccgucaagauuccucacaUg................   4      1   0B2
......................................................................uuccgucaagauucGucacaag................   1      1   0B2
......................................................................uuccgucaagauuccucacaaUa...............   364    1   0B2
......................................................................uuccgucaagauuccucacaaCa...............   9      1   0B2
......................................................................uuccgucaagauuccucacaaUaa..............   1      1   0B2
.......................................................................uccgAcaagauuccucaca..................   1      1   0B2
.......................................................................uccgucaagauuccucacC..................   5      1   0B2
.......................................................................uccgucaagauuccGcaca..................   1      1   0B2
.......................................................................Gccgucaagauuccucaca..................   1      1   0B2
.......................................................................uccgucaagauuccucacU..................   1472   1   0B2
.......................................................................uccgGcaagauuccucaca..................   1      1   0B2
.......................................................................uccgucaagauuccucaca..................   518    0   0B2
.......................................................................uccgucaagauuccCcaca..................   3      1   0B2
.......................................................................Nccgucaagauuccucaca..................   5      1   0B2
.......................................................................uccgucaagauuccucacaa.................   43     0   0B2
.......................................................................uccgucaagauuccucacUa.................   7      1   0B2
.......................................................................uccgucaagauuccucacaaA................   1      1   0B2
.......................................................................uccgucaagauuccucacaaU................   37     1   0B2
.......................................................................uccgucaagauuccucacaag................   3      0   0B2
.......................................................................uccgucaagauuccucacaaga...............   9      0   0B2
.......................................................................uccgucaagauuccucacaaUa...............   8      1   0B2
........................................................................ccgucaagauuccucacU..................   121    1   0B2
```

Star Mature

```
uuuuaucacugaauugcaauagcguauacuugcauuucuuaagaguaaucuggacagaauguguugugcauuccgucaagauuccucacaagaacugcaguguuaaac
........................................................................ccgucaagauuccucacC..................   1      1   0B2
........................................................................ccgucaagauuccucaca..................   24     0   0B2
........................................................................ccgucaagauuccucacaU.................   6      1   0B2
........................................................................ccgucaagauuccucacUa.................   2      1   0B2
........................................................................ccgucaagauuccucacaaU................   5      1   0B2
........................................................................ccgucaagauuccucacaaga...............   4      0   0B2

...............................................................uuguAcauuccgucaagauuccucac...................   7      1   0G2
..................................................................uAcauuccgucaagauuccucaca..................   3      1   0G2
....................................................................cauuccgucaagauuccuc.....................   5      0   0G2
....................................................................cauuccgucaagauuccucU....................   25     1   0G2
....................................................................cauuccgCcaagauuccuca....................   1      1   0G2
....................................................................Nauuccgucaagauuccuca....................   1      1   0G2
....................................................................cauuccgucaagauuccuca....................   73     0   0G2
....................................................................cauuccgucaagauuccucaA...................   1      1   0G2
....................................................................cauuccgucaagauuccucac...................   37     0   0G2
....................................................................Nauuccgucaagauuccucac...................   1      1   0G2
....................................................................cauuccgucaagauuccucacU..................   4263   1   0G2
....................................................................caGuccgucaagauuccucaca..................   1      1   0G2
....................................................................caCuccgucaagauuccucaca..................   1      1   0G2
....................................................................cauuccgucaagauuccGcaca..................   1      1   0G2
....................................................................Aauuccgucaagauuccucaca..................   12     1   0G2
....................................................................cauuccgucaagauuccucaca..................   1616   0   0G2
....................................................................cauuccgGcaagauuccucaca..................   1      1   0G2
....................................................................cauuccgucaGgauuccucaca..................   4      1   0G2
....................................................................cauuccgucaagauuccucacC..................   15     1   0G2
....................................................................cauuccgucaagauuccAcaca..................   1      1   0G2
....................................................................Nauuccgucaagauuccucaca..................   8      1   0G2
....................................................................cauuccgucaagauuccucacG..................   1      1   0G2
....................................................................cauuccgucaagaAuccucaca..................   1      1   0G2
....................................................................cauuccgCcaagauuccucaca..................   1      1   0G2
....................................................................cauCccgucaagauuccucaca..................   1      1   0G2
....................................................................Uauuccgucaagauuccucaca..................   1      1   0G2
....................................................................cauuccgucaagauuccucacaU.................   30     1   0G2
....................................................................cauuccgucaagauuccucacaa.................   6      0   0G2
....................................................................cauuccgucaagauuccucacUa.................   78     1   0G2
....................................................................cauuccgucaagauuccucacCa.................   1      1   0G2
....................................................................cauuccgucaagauuccucacaaA................   1      1   0G2
....................................................................cauuccgucaagauuccucacaaU................   4      1   0G2
....................................................................cauuccgucaagauuccucacaaUa...............   7      1   0G2
.....................................................................auuccgucaagauuccuca....................   13     0   0G2
.....................................................................auuccgucaagauuccucac...................   9      0   0G2
.....................................................................auuccgucaagauuccucaca..................   388    0   0G2
.....................................................................auuccgucaagauuccucacC..................   1      1   0G2
.....................................................................Cuuccgucaagauuccucaca..................   34     1   0G2
.....................................................................auuccgucaagauuccucacU..................   998    1   0G2
.....................................................................auuccgucaagauuccucacaU.................   3      1   0G2
.....................................................................auuccgucaagauuccucacUa.................   14     1   0G2
.....................................................................auuccgucaagauuccucacaaA................   2      1   0G2
.....................................................................auuccgucaagauuccucacaaU................   19     1   0G2
......................................................................uuccgucaagauuccuca....................   145    0   0G2
......................................................................uuccgGcaagauuccuca....................   1      1   0G2
......................................................................uuccgucaagauuccucU....................   52     1   0G2
......................................................................Nuccgucaagauuccuca....................   1      1   0G2
......................................................................uuAcgucaagauuccuca....................   1      1   0G2
......................................................................uNccgucaagauuccucac...................   2      1   0G2
......................................................................uuccgucaagauuccucUc...................   5      1   0G2
......................................................................uuccgucaagauuccucac...................   124    0   0G2
......................................................................Guccgucaagauuccucac...................   1      1   0G2
......................................................................uuccgGcaagauuccucac...................   1      1   0G2
......................................................................uuccgucaagauuccAcaca..................   1      1   0G2
......................................................................uAccgucaagauuccucaca..................   1      1   0G2
......................................................................uuccgucaagauuccuGaca..................   1      1   0G2
......................................................................uuccgucaagauuUcucaca..................   4      1   0G2
......................................................................uuccgCcaagauuccucaca..................   9      1   0G2
......................................................................uuccAucaagauuccucaca..................   9      1   0G2
......................................................................uuccgucaagauuccCcaca..................   14     1   0G2
......................................................................uuccgucaagauCccucaca..................   6      1   0G2
......................................................................uuccgucaagauuccucaAa..................   2      1   0G2
......................................................................uuccgucaagauuccucacU..................   51925  1   0G2
```

Star Mature

```
uuuuaucacugaauugcaauagcguauacuugcauuucuuaagaguaaucuggacagaauguguugugcauuccgucaagauuccucacaagaacugcaguguuaaac
......................................................................uNccgucaagauuccucaca..................   19      1   0G2
......................................................................uuccgucaagauucAucaca..................   1       1   0G2
......................................................................uuccgucaaAauuccucaca..................   2       1   0G2
......................................................................uuccgucaagauuccucCca..................   1       1   0G2
......................................................................uuccgucaagauuccucacG..................   23      1   0G2
......................................................................uuccgucaagauAccucaca..................   1       1   0G2
......................................................................uuccUucaagauuccucaca..................   1       1   0G2
......................................................................uuccguUaagauuccucaca..................   3       1   0G2
......................................................................Auccgucaagauuccucaca..................   8       1   0G2
......................................................................Cuccgucaagauuccucaca..................   5       1   0G2
......................................................................uucUgucaagauuccucaca..................   1       1   0G2
......................................................................uuccgucaagaGuccucaca..................   2       1   0G2
......................................................................uuccgucaagGuuccucaca..................   7       1   0G2
......................................................................uuccgucaagauuccuUaca..................   7       1   0G2
......................................................................uuccgucaagauuccucGca..................   5       1   0G2
......................................................................uucGgucaagauuccucaca..................   3       1   0G2
......................................................................uuccguAaagauuccucaca..................   6       1   0G2
......................................................................uuccgucaagauuAcucaca..................   1       1   0G2
......................................................................uCccgucaagauuccucaca..................   7       1   0G2
......................................................................uuccgucaGgauuccucaca..................   55      1   0G2
......................................................................uuccgucaagauuccucaca..................   19778   0   0G2
......................................................................uucAgucaagauuccucaca..................   7       1   0G2
......................................................................uuccgucaagaCuccucaca..................   6       1   0G2
......................................................................uuccgucaagUuuccucaca..................   1       1   0G2
......................................................................uuccgGcaagauuccucaca..................   8       1   0G2
......................................................................uuAcgucaagauuccucaca..................   3       1   0G2
......................................................................uuccgucaagauuccucacC..................   67      1   0G2
......................................................................uuccgucGagauuccucaca..................   5       1   0G2
......................................................................uuUcgucaagauuccucaca..................   6       1   0G2
......................................................................uuccgucaagauucGucaca..................   1       1   0G2
......................................................................Nuccgucaagauuccucaca..................   63      1   0G2
......................................................................uuccgucaagauuccuAaca..................   1       1   0G2
......................................................................uuccgAcaagauuccucaca..................   5       1   0G2
......................................................................Guccgucaagauuccucaca..................   11      1   0G2
......................................................................uuccgucaagauGccucacaa.................   1       1   0G2
......................................................................uuccgucaagaCuccucacaa.................   2       1   0G2
......................................................................uuccgucaagauuccucacaU.................   226     1   0G2
......................................................................uuccgucaagauuccucacaG.................   5       1   0G2
......................................................................uuccgucaagauuccucacaa.................   1648    0   0G2
......................................................................uuccgucaagauuccucacUa.................   643     1   0G2
......................................................................uuccgucaagauuccucacGa.................   2       1   0G2
......................................................................Nuccgucaagauuccucacaa.................   6       1   0G2
......................................................................uuccgucaagauuccGcacaa.................   1       1   0G2
......................................................................uuccgCcaagauuccucacaa.................   1       1   0G2
......................................................................uuUcgucaagauuccucacaa.................   1       1   0G2
......................................................................uuccgucaagauuccucacCa.................   1       1   0G2
......................................................................uucAgucaagauuccucacaa.................   1       1   0G2
......................................................................uuccgAcaagauuccucacaa.................   1       1   0G2
......................................................................uuccgucaagauuccucacaaA................   72      1   0G2
......................................................................uuccgucaagauuccucacaag................   57      0   0G2
......................................................................uuccgucaagauuccucacaaU................   1527    1   0G2
......................................................................uuccgucaagauuccucacaaC................   2       1   0G2
......................................................................uuccgucaagauuccucacaaCa...............   5       1   0G2
......................................................................uuccgucaagauuccucacaaUa...............   798     1   0G2
......................................................................uuccgucaagauuccucacaaUaa..............   7       1   0G2
.......................................................................uUcgucaagauuccucaca..................   1       1   0G2
.......................................................................uccAucaagauuccucaca..................   1       1   0G2
.......................................................................Cccgucaagauuccucaca..................   1       1   0G2
.......................................................................uccgucaagauuccuUaca..................   1       1   0G2
.......................................................................uAcgucaagauuccucaca..................   1       1   0G2
.......................................................................uccgucaagauuccucaca..................   509     0   0G2
.......................................................................uccgucaagauuccucacU..................   1083    1   0G2
.......................................................................Gccgucaagauuccucaca..................   1       1   0G2
.......................................................................uccgAcaagauuccucaca..................   1       1   0G2
.......................................................................uccgucaagauuccucacaa.................   28      0   0G2
.......................................................................uccgucaagauuccucacUa.................   7       1   0G2
.......................................................................uccgucaagauuccucacaG.................   3       1   0G2
.......................................................................uccgucaagauuccucacaU.................   3       1   0G2
.......................................................................uccgucaagauuccucacaaU................   63      1   0G2
.......................................................................uccgucaagauuccucacaaUa...............   17      1   0G2
```

```
                                        Star                         Mature
uuuuaucacugaauugcaauagcguauacuugcauuucuuaagaguaaucuggacagaauguguugugcauuccgucaagauuccucacaagaacugcaguguuaaac
.......................................................................uccgucaagauuccucacaaga...............   6    0    0G2
.......................................................................uccgucaagauuccucacaagaU..............   1    1    0G2
........................................................................ccgucaagauuccucacU..................   87   1    0G2
........................................................................ccgucaagauuccucaca..................   23   0    0G2
........................................................................ccgucaagauuccucacaU.................   10   1    0G2
```

Provisional ID : scaffold79_16153
Score total : 198.4
Score for star read(s) : 3.9
Score for read counts : 191.3
Score for mfe : 2.1
Score for randfold : 1.6
Score for cons. seed : -0.6
Total read count : 387
Mature read count : 381
Loop read count : 0
Star read count : 6

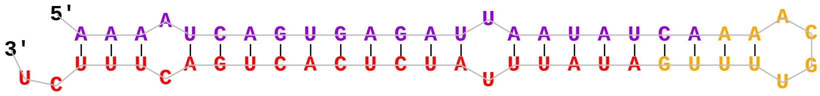

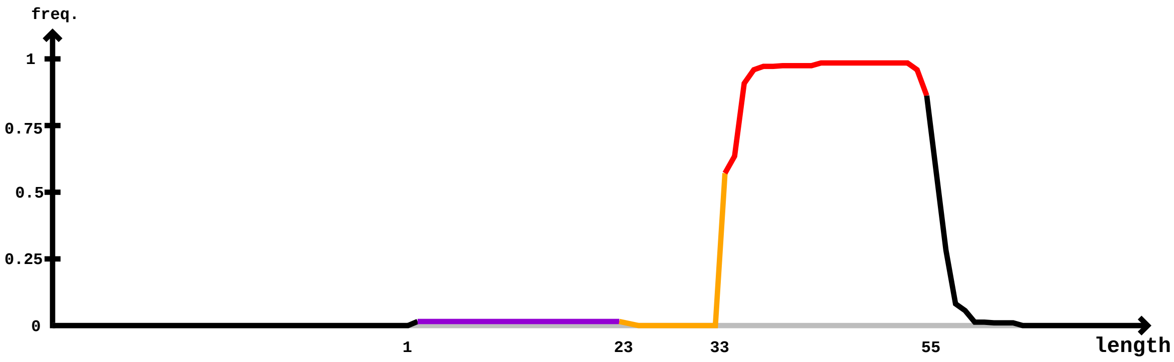


```
                                    Star                          Mature
5'- gccaaaaaauuuucucacuggccaauuuucagugagaaaaucagugagauuaauaucaaaacguuuugauauuuaucucacugacuuucucacugaacagaggaggccgcua  -3'   obs
    gccaaaaaauuuucucacuggccaauuuucagugagaaaaucagugagauuaauaucaaaacguuuugauauuuaucucacugacuuucucacugaacagaggaggccgcua         exp
    ...................((((..((((((((((((((.((((((((((.(((((((((....))))))))).)))))))))).)))))))))))...)))..))))....   reads   mm   sample
    ....................................aaaaucagugagaAuaauauca......................................................   6       1    0A2
    ....................................................................auauuuaucucacugacuuuc.......................   10      0    0A2
    ....................................................................auauuuaucucacugacuuucu......................   52      0    0A2
    ....................................................................auauuuaucucacugacuuucA......................   5       1    0A2
    ....................................................................auauuuaucucacugacuuucuA.....................   15      1    0A2
    ....................................................................auauuuaucucacugacuuucuU.....................   6       1    0A2
    .....................................................................uauuuaucucacugacuuucu......................   4       0    0A2
    ......................................................................auuuaucucacugacuuuc.......................   2       0    0A2
    ......................................................................auuuaucucacugacuuucuca....................   27      0    0A2
    ......................................................................Guuuaucucacugacuuucuca....................   4       1    0A2
    ......................................................................auuuaucucacugacuuucucaUu..................   2       1    0A2
    .......................................................................uuuaucucacugacuuucuc.....................   2       0    0A2
    .......................................................................uuuaucucacugacuuucuca....................   7       0    0A2
    .......................................................................uuuaucucacugacuuucucaUu..................   4       1    0A2
    ........................................................................uuaucucacugacuuucuca....................   5       0    0A2
    ..........................................................................auUucacugacuuucucacuga................   1       1    0A2

    ....................................................................auauuuaucucacugacuuuc.......................   12      0    0B2
    ....................................................................auauuuaucucacugacuuucu......................   36      0    0B2
    ....................................................................auauuuaucucacugacuuucA......................   2       1    0B2
    ....................................................................auauuuaucucacugacuuucuU.....................   4       1    0B2
    .....................................................................uauuuaucucacugacuuucu......................   4       0    0B2
    .....................................................................uauuuaucucacugacuuucuc.....................   4       0    0B2
    ......................................................................auuuaucucacugacuuucuc.....................   18      0    0B2
    ......................................................................Nuuuaucucacugacuuucuc.....................   1       1    0B2
    ......................................................................auuuaucucacugacuuucuca....................   12      0    0B2
    ......................................................................auuuaucucacugacuuucucaA...................   1       1    0B2
    ......................................................................auuuaucucacugacuuucucaAu..................   7       1    0B2
    .......................................................................uuuaucucacugacuuucucaUu..................   4       1    0B2

    ....................................................................auauuuaucucacugacuuu........................   5       0    0G2
    ....................................................................auauuuaucucacugacuuuc.......................   14      0    0G2
    ....................................................................auauuuaucucacugacuuucu......................   49      0    0G2
```

Star **Mature**

gccaaaaaauuuucucacuggccaauuuucagugagaaaaucagugagauuaauaucaaaacguuuugauauuuaucucacugacuuucucacugaacagaggaggccgcua			
....auauuuaucucacugacuuucA......................	8	1	0G2
....auauuuaucucacugacuuucuA.....................	6	1	0G2
....uauuuaucucacugacuuu........................	5	0	0G2
....uauuuaucucacugacuuucu......................	8	0	0G2
....auuuaucucacugacuuucuc.....................	2	0	0G2
....auuuaucCcacugacuuucuc.....................	1	1	0G2
....auuuaucucacugacuuucuUa....................	1	1	0G2
....auuuaucucacugacuuucuca....................	17	0	0G2
....auuuaucucacugacuuucucU....................	2	1	0G2
....auuuaucucacuCacuuucuca....................	1	1	0G2
....auuuaucucacugacuuucucaA...................	9	1	0G2
....uuuaucucacugacuuucuca....................	3	0	0G2
....cacugacuuucucacugaGca.............	4	1	0G2

Provisional ID	:	scaffold81_16585
Score total	:	227.9
Score for star read(s)	:	3.9
Score for read counts	:	222.4
Score for mfe	:	0.6
Score for randfold	:	1.6
Score for cons. seed	:	-0.6
Total read count	:	448
Mature read count	:	440
Loop read count	:	0
Star read count	:	8

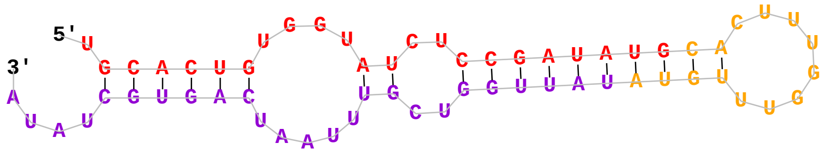

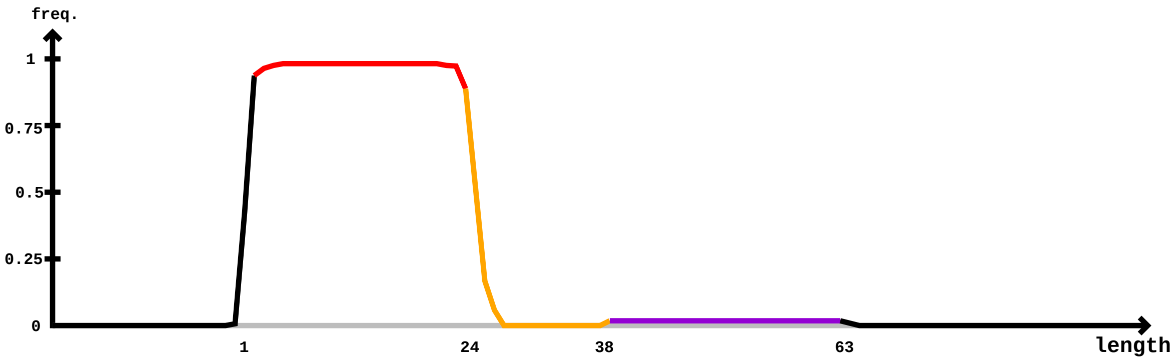

Mature **Star**

Sequence		reads	mm	sample
5'- caucagccucuuuugagccugcacugugguaucuccgauaugcacuuugguuuguauauuggucguuuaaucagugcuauaucaacuuagauacugagcaucguuagaaaau	-3'	obs		
caucagccucuuuugagccugcacugugguaucuccgauaugcacuuugguuuguauauuggucguuuaaucagugcuauaucaacuuagauacugagcaucguuagaaaau		exp		
..((((..(((.((((....((((((....((..((((((((((........))))))))))..)).....))))))....))))...)))..))))...............		reads	mm	sample
.................ccugcacugugguaucuccgauaugca..		3	0	0A2
..................Nugcacugugguaucuccgauau...		2	1	0A2
..................cugcacugugguaucuccgauauU..		2	1	0A2
..................cugcacugugguaucuccgauaug..		8	0	0A2
..................cugcacugugguaucCccgauaugc...		1	1	0A2
..................cugcacugugguaucuccgauaugc...		61	0	0A2
..................cugcacugugguaucuccgauaugca..		11	0	0A2
..................cugcacugugguaucuccgauaugcU..		1	1	0A2
..................cugcacugugguaucuccgauaugcac...		10	0	0A2
...................ugcacugugguaucuccgau...		2	0	0A2
...................ugcacugugguaucuccgaua..		1	0	0A2
...................ugcacugugguaucuccgauau...		14	0	0A2
...................ugcacugugguaucuccgauauA..		81	1	0A2
...................ugcacugugguaucuccgauauU..		11	1	0A2
...................ugcacuAugguaucuccgauaug..		1	1	0A2
...................ugcacugugguaucuccgauaug..		22	0	0A2
...................ugcacugugguaucuccgauaugc...		6	0	0A2
...................ugcacugugguaucuccgauaugcac...		2	0	0A2
....................gcacugugguaucuccgauau...		4	0	0A2
....................gcacugugguaucuccgauaug..		1	0	0A2
.....................cacugugguaucuccgauaugc...		3	0	0A2
......................acugugguaucuccgauaugc...		3	0	0A2
..................Nugcacugugguaucuccgau...		1	1	0B2
..................cugcacugugguaucuccgauau...		3	0	0B2
..................cugcacugugguaucuccgauaug..		2	0	0B2
..................cugcacugugguaucuccgauaugc...		13	0	0B2
..................cugcacugugguaucuccgauaugca..		15	0	0B2
..................cugcacugugguaucuccgauaugcac...		4	0	0B2
...................ugcacugugguaucuccgauaA...		1	1	0B2
...................ugcacugugguaucuccgauau...		11	0	0B2
...................ugcacugugguaucuccgauaug..		12	0	0B2
...................ugcacugugguaucuccgauauA..		10	1	0B2

Mature Star

```
caucagccucuuuugagccugcacugugguaucuccgauaugcacuuugguuuguauauuggucguuuaaucagugcuauaucaacuuagauacugagcaucguuagaaaau
...................ugcacugugguaucuccgauaugc.....................................................................  8   0   0B2
...................ugcacugugguaucuccgauaugca....................................................................  7   0   0B2

..................cugcacugugguaucuccgauauA......................................................................  6   1   0G2
..................cugcacugugguaucuccgauaug......................................................................  6   0   0G2
..................cugcacugugguaucuccgauaugc.....................................................................  24  0   0G2
..................cugcacugugguaucuccgauaugca....................................................................  12  0   0G2
..................cugcacugugguaucuccgauaugcac..................................................................  6   0   0G2
...................ugcacugugguaucuccgauau.......................................................................  3   0   0G2
...................ugcacugugguaucuccgauauU......................................................................  2   1   0G2
...................ugcacugugguaucuccgauaug......................................................................  2   0   0G2
...................ugcacugugguaucuccgauauA......................................................................  25  1   0G2
...................ugcacugugguaucuccgauaugc.....................................................................  4   0   0G2
...................ugcacugugguaucuccgauaugcac...................................................................  4   0   0G2
....................gcacugugguaucuccgauauA......................................................................  7   1   0G2
.....................cacugugguaucuccgauaugc.....................................................................  2   0   0G2
........................................................uauuggucguuuaaucagugcuaua...............................  8   0   0G2
```

Provisional ID	:	scaffold81_16587
Score total	:	227.9
Score for star read(s)	:	3.9
Score for read counts	:	222.4
Score for mfe	:	0.6
Score for randfold	:	1.6
Score for cons. seed	:	-0.6
Total read count	:	448
Mature read count	:	440
Loop read count	:	0
Star read count	:	8

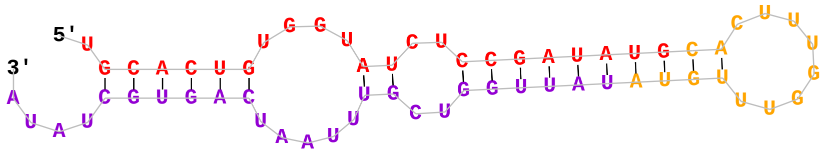

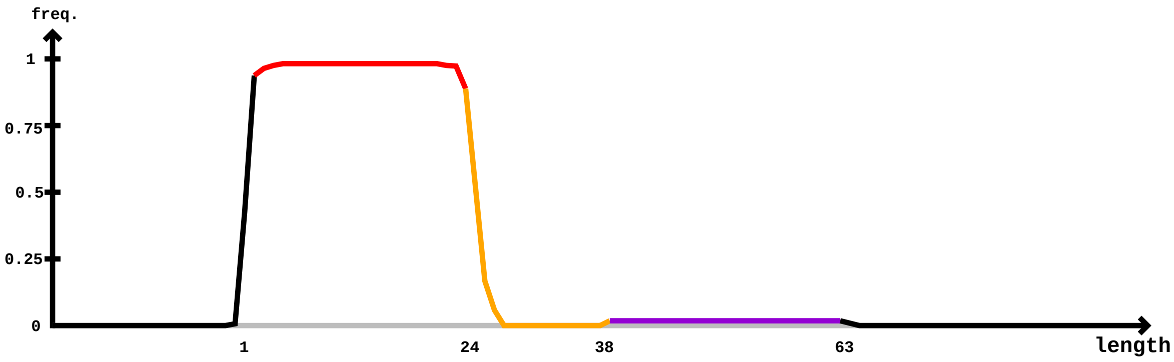

Mature Star

```
5'- caucagccucuuuugagccugcacugugguaucuccgauaugcacuuugguuuguauauuggucguuuaaucagugcuauaucaacuuagauacugagcaucguuagaaaau  -3'   obs
    caucagccucuuuugagccugcacugugguaucuccgauaugcacuuugguuuguauauuggucguuuaaucagugcuauaucaacuuagauacugagcaucguuagaaaau        exp
    ..((((..(((.((((....((((((....((..((((((((((........))))))))))..)).....))))))....))))...)))..))))...............  reads   mm    sample
    .................ccugcacugugguaucuccgauaugca....................................................................  3       0     0A2
    ..................Nugcacugugguaucuccgauau.......................................................................  2       1     0A2
    ..................cugcacugugguaucuccgauauU......................................................................  2       1     0A2
    ..................cugcacugugguaucuccgauaug......................................................................  8       0     0A2
    ..................cugcacugugguaucCccgauaugc.....................................................................  1       1     0A2
    ..................cugcacugugguaucuccgauaugc.....................................................................  61      0     0A2
    ..................cugcacugugguaucuccgauaugcU....................................................................  1       1     0A2
    ..................cugcacugugguaucuccgauaugca....................................................................  11      0     0A2
    ..................cugcacugugguaucuccgauaugcac...................................................................  10      0     0A2
    ...................ugcacugugguaucuccgau.........................................................................  2       0     0A2
    ...................ugcacugugguaucuccgaua........................................................................  1       0     0A2
    ...................ugcacugugguaucuccgauau.......................................................................  14      0     0A2
    ...................ugcacuAugguaucuccgauaug......................................................................  1       1     0A2
    ...................ugcacugugguaucuccgauaug......................................................................  22      0     0A2
    ...................ugcacugugguaucuccgauauA......................................................................  81      1     0A2
    ...................ugcacugugguaucuccgauauU......................................................................  11      1     0A2
    ...................ugcacugugguaucuccgauaugc.....................................................................  6       0     0A2
    ...................ugcacugugguaucuccgauaugcac...................................................................  2       0     0A2
    ....................gcacugugguaucuccgauau.......................................................................  4       0     0A2
    ....................gcacugugguaucuccgauaug......................................................................  1       0     0A2
    .....................cacugugguaucuccgauaugc.....................................................................  3       0     0A2
    ......................acugugguaucuccgauaugc.....................................................................  3       0     0A2

    ..................Nugcacugugguaucuccgau.........................................................................  1       1     0B2
    ..................cugcacugugguaucuccgauau.......................................................................  3       0     0B2
    ..................cugcacugugguaucuccgauaug......................................................................  2       0     0B2
    ..................cugcacugugguaucuccgauaugc.....................................................................  13      0     0B2
    ..................cugcacugugguaucuccgauaugca....................................................................  15      0     0B2
    ..................cugcacugugguaucuccgauaugcac...................................................................  4       0     0B2
    ...................ugcacugugguaucuccgauau.......................................................................  11      0     0B2
    ...................ugcacugugguaucuccgauaA.......................................................................  1       1     0B2
    ...................ugcacugugguaucuccgauaug......................................................................  12      0     0B2
    ...................ugcacugugguaucuccgauauA......................................................................  10      1     0B2
```

Mature Star

caucagccucuuuugagccugcacugugguaucuccgauaugcacuuugguuuguauauuggucguuuaaucagugcuauaucaacuuagauacugagcaucguuagaaaau			
...................ugcacugugguaucuccgauaugc...	8	0	0B2
...................ugcacugugguaucuccgauaugca..	7	0	0B2
..................cugcacugugguaucuccgauauA..	6	1	0G2
..................cugcacugugguaucuccgauaug..	6	0	0G2
..................cugcacugugguaucuccgauaugc...	24	0	0G2
..................cugcacugugguaucuccgauaugca..	12	0	0G2
..................cugcacugugguaucuccgauaugcac...	6	0	0G2
...................ugcacugugguaucuccgauau...	3	0	0G2
...................ugcacugugguaucuccgauaug..	2	0	0G2
...................ugcacugugguaucuccgauauA..	25	1	0G2
...................ugcacugugguaucuccgauauU..	2	1	0G2
...................ugcacugugguaucuccgauaugc...	4	0	0G2
...................ugcacugugguaucuccgauaugcac...	4	0	0G2
....................gcacugugguaucuccgauauA..	7	1	0G2
.....................cacugugguaucuccgauaugc...	2	0	0G2
....uauuggucguuuaaucagugcuaua...............................	8	0	0G2

Provisional ID	:	scaffold81_16591
Score total	:	239
Score for star read(s)	:	3.9
Score for read counts	:	235.7
Score for mfe	:	-1.4
Score for randfold	:	1.6
Score for cons. seed	:	-0.6
Total read count	:	474
Mature read count	:	466
Loop read count	:	0
Star read count	:	8

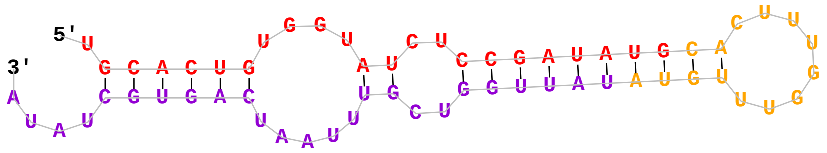

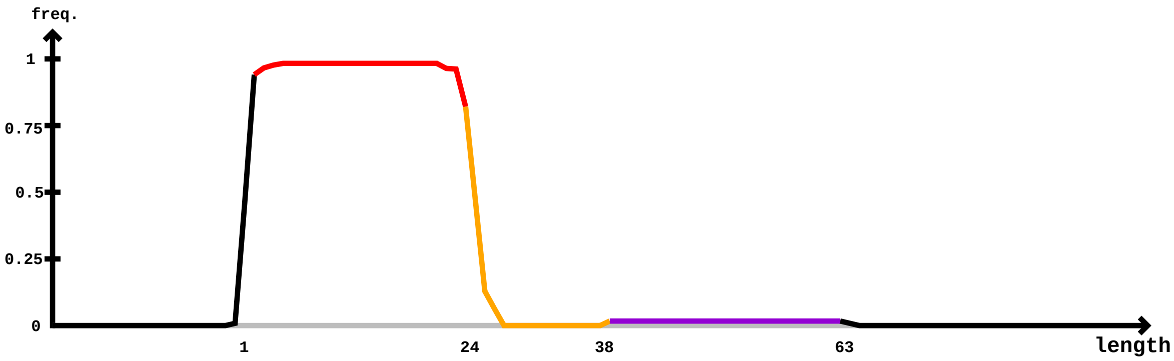

Mature Star

```
5'- gucuuuauaaaaaaucguuugcacugugguaucuccgauaugcacuuugguuuguauauuggucguuuaaucagugcuauaucaacuuauauuucuuuggagaauauuuuga  -3'  obs
    gucuuuauaaaaaaucguuugcacugugguaucuccgauaugcacuuugguuuguauauuggucguuuaaucagugcuauaucaacuuauauuucuuuggagaauauuuuga       exp
    ...................((((((....((..((((((((((........))))))))))..)).....))))))....((((..(((.((((....)))))))..))))   reads   mm   sample
    ..................uugcacugugguaucuccgauau.......................................................................   12   0   0A2
    ..................Nugcacugugguaucuccgauau.......................................................................   2    1   0A2
    ..................uugcacugugguaucuccgauauA......................................................................   10   1   0A2
    ..................uugcacugugguaucuccgauaug......................................................................   5    0   0A2
    ..................uugcacugugguauUuccgauaug......................................................................   4    1   0A2
    ..................uugcacugugguaucuccgauaugc.....................................................................   59   0   0A2
    ..................uugcacugCgguaucuccgauaugc.....................................................................   1    1   0A2
    ..................uugcacugugguaucuccgauaugA.....................................................................   3    1   0A2
    ..................uugcacugugguaucuccgauaugGac...................................................................   1    1   0A2
    ..................uugcacugugguaucuccgauaugcac...................................................................   15   0   0A2
    ...................ugcacugugguaucuccgau.........................................................................   2    0   0A2
    ...................ugcacugugguaucuccgaua........................................................................   1    0   0A2
    ...................ugcacugugguaucuccgauau.......................................................................   14   0   0A2
    ...................ugcacuAugguaucuccgauaug......................................................................   1    1   0A2
    ...................ugcacugugguaucuccgauaug......................................................................   22   0   0A2
    ...................ugcacugugguaucuccgauauA......................................................................   81   1   0A2
    ...................ugcacugugguaucuccgauauU......................................................................   11   1   0A2
    ...................ugcacugugguaucuccgauaugc.....................................................................   6    0   0A2
    ...................ugcacugugguaucuccgauaugcac...................................................................   2    0   0A2
    ....................gcacugugguaucuccgauau.......................................................................   4    0   0A2
    ....................gcacugugguaucuccgauaug......................................................................   1    0   0A2
    .....................cacugugguaucuccgauaugc.....................................................................   3    0   0A2
    ......................acugugguaucuccgauaugc.....................................................................   3    0   0A2

    .................uuugcacugugguaucuccgauaugca....................................................................   1    0   0B2
    ..................uugcacugugguaucuccgau.........................................................................   5    0   0B2
    ..................Nugcacugugguaucuccgau.........................................................................   1    1   0B2
    ..................uugcacuguUguaucuccgau.........................................................................   1    1   0B2
    ..................uugcacugugguaucuccgauau.......................................................................   12   0   0B2
    ..................uugcacugugguaucuccgauauA......................................................................   2    1   0B2
    ..................uugcacugugguaucuccgauaug......................................................................   2    0   0B2
    ..................uugcacugugguaucuccgauaugc.....................................................................   17   0   0B2
    ..................uugcacugugguaucuccgauaugca....................................................................   6    0   0B2
```

Mature **Star**

```
gucuuuauaaaaaaucguuugcacugugguaucuccgauaugcacuuugguuuguauauuggucguuuaaucagugcuauaucaacuuauauuucuuuggagaauauuuuga
..................uugcacugugguaucuccgauaugcac...................................................................   8   0   0B2
...................ugcacugugguaucuccgauaA.......................................................................   1   1   0B2
...................ugcacugugguaucuccgauau.......................................................................  11   0   0B2
...................ugcacugugguaucuccgauaug......................................................................  12   0   0B2
...................ugcacugugguaucuccgauauA......................................................................  10   1   0B2
...................ugcacugugguaucuccgauaugc.....................................................................   8   0   0B2
...................ugcacugugguaucuccgauaugca....................................................................   7   0   0B2

.................uuugcacugugguaucuccgauaugca....................................................................   3   0   0G2
..................uugcacugugguaucuccgauau.......................................................................   8   0   0G2
..................uugcacugugguaucuccgauauA......................................................................   1   1   0G2
..................uugcacugugguaucuccgauaug......................................................................   5   0   0G2
..................uugcacugugguaucuccgauaugc.....................................................................  19   0   0G2
..................uugcacugugguaucuccgauaugca....................................................................  14   0   0G2
...................ugcacugugguaucuccgauau.......................................................................   3   0   0G2
...................ugcacugugguaucuccgauauU......................................................................   2   1   0G2
...................ugcacugugguaucuccgauaug......................................................................   2   0   0G2
...................ugcacugugguaucuccgauauA......................................................................  25   1   0G2
...................ugcacugugguaucuccgauaugc.....................................................................   4   0   0G2
...................ugcacugugguaucuccgauaugcac...................................................................   4   0   0G2
....................gcacugugguaucuccgauauA......................................................................   7   1   0G2
.....................cacugugguaucuccgauaugc.....................................................................   2   0   0G2
........................................................uauuggucguuuaaucagugcuaua...............................   8   0   0G2
```

Provisional ID	:	scaffold84_16975
Score total	:	25704
Score for star read(s)	:	3.9
Score for read counts	:	25695.3
Score for mfe	:	0.2
Score for randfold	:	1.6
Score for cons. seed	:	3
Total read count	:	50412
Mature read count	:	48826
Loop read count	:	0
Star read count	:	1586

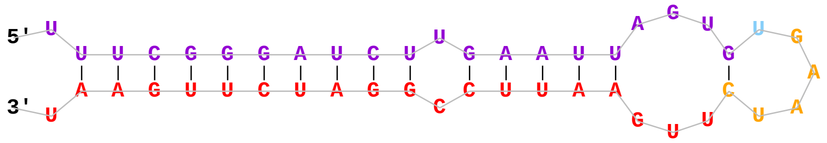

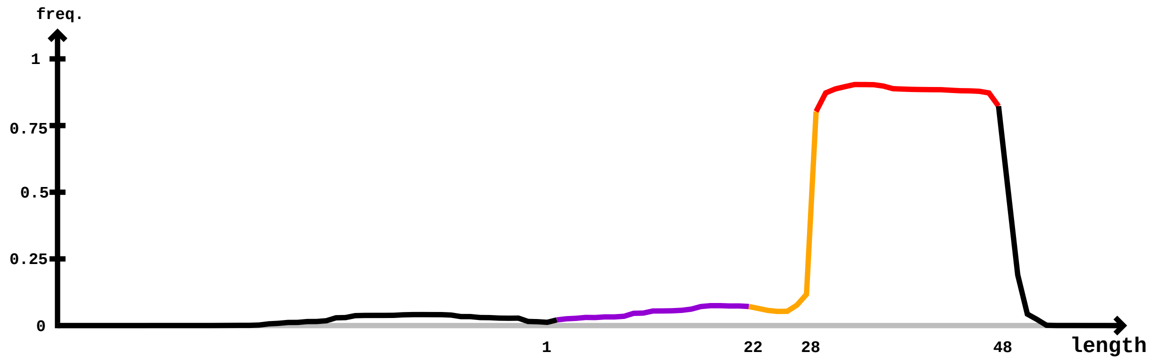

Star Mature

```
5'- cuuagaucuugaaucuugaauucgggaucuugaauuagugugaaucuugauuucgggaucuugaauuagugugaaucuugaauuccggaucuugaaucuuggaucuuga   -3'   obs
    cuuagaucuugaaucuugaauucgggaucuugaauuagugugaaucuugauuucgggaucuugaauuagugugaaucuugaauuccggaucuugaaucuuggaucuuga         exp
    ...((((((((((.....)))))))))))..........((.((..((((.((((((((.(((((...(.....)...))))).)))))))))))..)).))....   reads   mm   sample
    ..uGgaucuugaaucuugaauuc......................................................................................   1   1   0B2
    ................ugaauucgggUucuugaauua........................................................................   5   1   0B2
    .................gaauucgggaucuAgaauuag.......................................................................   4   1   0B2
    ...................Nuucgggaucuugaauuagu......................................................................   2   1   0B2
    ...................auucgggaucuugaauuagG......................................................................   6   1   0B2
    ...................auucgggaucuAgaauuagug.....................................................................   4   1   0B2
    ....................uucgggaucuugaauuagu......................................................................   16   0   0B2
    ....................uucgggaucuugaauuagG......................................................................   4   1   0B2
    ....................uucgggaucuGgaauuagu......................................................................   2   1   0B2
    ....................uucgggaucuugaauuagGg.....................................................................   32   1   0B2
    ....................uucgggaucuugaauuagug.....................................................................   96   0   0B2
    ....................uucgggaucuugaauuGgug.....................................................................   10   1   0B2
    ....................uucgggaucuugaauuaguguga..................................................................   2   0   0B2
    ....................uucgggaucuugaauuagugugaaucu..............................................................   2   0   0B2
    .....................ucgggaucuugaauuagug.....................................................................   12   0   0B2
    .....................ucgggaucuugaauuagGg.....................................................................   12   1   0B2
    .....................ucgggaucuugaauuGgug.....................................................................   2   1   0B2
    .....................ucgggaucuGgaauuagug.....................................................................   4   1   0B2
    .....................ucgggaucuugaauuagugu....................................................................   6   0   0B2
    .....................ucgggaucuugaauuagGgug...................................................................   6   1   0B2
    .....................ucgggaucuugaauuagugug...................................................................   14   0   0B2
    .....................ucgggaucuugaauuagugugaauUuu.............................................................   4   1   0B2
    ......................Ngggaucuugaauuagug.....................................................................   2   1   0B2
    ......................cgggaucuugaauuagug.....................................................................   26   0   0B2
    ......................cgggaucuugaauuagGg.....................................................................   14   1   0B2
    ......................cgggaucuugaauuagugug...................................................................   28   0   0B2
    ......................cgggaucuGgaauuagugug...................................................................   6   1   0B2
    ......................cgggaucuugaauuagGgug...................................................................   4   1   0B2
    ......................cgggaucuugaauuaguguA...................................................................   6   1   0B2
    ......................cgggaucuugaauuaguguga..................................................................   4   0   0B2
    ......................cgggaucuugaauuagugugaa.................................................................   22   0   0B2
    ......................cgggaucuugaauuagugugaaA................................................................   10   1   0B2
    ......................cgggaucuugaauuagugugaaucA..............................................................   2   1   0B2
```

Star **Mature**

cuuagaucuugaaucuugaauucgggaucuugaauuagugugaaucuugauuucgggaucuugaauuagugugaaucuugaauuccggaucuugaaucuuggaucuuga			
.......................Nggaucuugaauuagugug...	6	1	0B2
.......................Nggaucuugaauuagugugaa...	2	1	0B2
.......................Nggaucuugaauuagugugaau..	2	1	0B2
........................ggaucuugaauuaguguA...	4	1	0B2
........................ggaucuGgaauuagugug...	14	1	0B2
........................ggaucuugaauuagugug...	20	0	0B2
........................ggaucuugaauuaguguga..	16	0	0B2
........................ggaucuugaGuuaguguga..	2	1	0B2
........................ggaucuugaauuagugugaU...	12	1	0B2
........................Ngaucuugaauuagugugaa...	2	1	0B2
........................ggaucuGgaauuagugugaa...	22	1	0B2
........................ggaucuugaauuagugugaa...	42	0	0B2
........................ggaucuugaGuuagugugaa...	6	1	0B2
........................ggaucuugaauuagugugaau..	12	0	0B2
........................ggaucuugaauuagugugaaA..	2	1	0B2
..........................aucuGgaauuagugugaauc...	22	1	0B2
..........................aucuGgaauuagugugaaucu..	12	1	0B2
..........................aucuugaauuagugugaaucu..	12	0	0B2
..........................aucuugaauuagugugaaucuug..	8	0	0B2
...........................ucuGgaauuagugugaaucu..	190	1	0B2
...........................ucuCgaauuagugugaaucu..	2	1	0B2
...........................ucuugaauCagugugaaucu..	2	1	0B2
...........................ucuugaauuagugugaaucu..	142	0	0B2
...........................Ncuugaauuagugugaaucu..	4	1	0B2
...........................ucuGgaauuagugugaaucuu...	8	1	0B2
...........................ucuugaauuagugugaaucuu...	10	0	0B2
...........................ucuugaauuagugugaaucuug..	18	0	0B2
...........................ucuGgaauuagugugaaucuug..	10	1	0B2
............................cuGgaauuagugugaaucu..	2	1	0B2
............................cuugaauuagugugaaucu..	10	0	0B2
............................cuGgaauuagugugaaucuu...	8	1	0B2
............................cuugaauuagugugaaucuu...	2	0	0B2
............................cuGgaauuagugugaaucuug..	14	1	0B2
............................cuGgaauuagugugaaucuuga...	4	1	0B2
.............................uugaauuagugugaaucuu...	18	0	0B2
.............................uugaauuagugugaaucuug..	46	0	0B2
.............................uugaauuagugugaaucuuga...	72	0	0B2
.............................uugaauuagugugaGucuuga...	8	1	0B2
.............................uugaaCuagugugaaucuuga...	2	1	0B2
.............................uugaauuagugugaaucuugau..	3	0	0B2
..............................ugaauuagugugaaucuuga...	14	0	0B2
..............................ugaauuagugugaGucuuga...	2	1	0B2
..............................ugaauuagugugaaucuugau..	2	0	0B2
.................................auuagugugaaucuugauuucggg..	1	0	0B2
..................................uuagugugaaucuugaGuuc...	6	1	0B2
..................................uuagugugaaucuugauuuc...	4	0	0B2
..................................uuagugugaaucuugauuucg..	3	0	0B2
..................................uuagugugaaucuugauuucggg..	5	0	0B2
..................................uuagugugaaucuugauuucgggauc...	7	0	0B2
..................................uuagugugaaucuugauuucgggaucu..	14	0	0B2
...................................uagugugaaucuugauuucg..	5	0	0B2
...................................uagugugaaucuugauuucggg..	6	0	0B2
...................................uagugugaaucuugauuucgggaA..	1	1	0B2
...................................uagugugaaucuugauuucCggauc...	2	1	0B2
...................................uagugugaaucuugauuucgggaucu..	1	0	0B2
....ugaaucuugauuucgggauc...	1	0	0B2
....ugaGucuugauuucgggauc...	1	1	0B2
....aucuugauuucgggaucuu...	1	0	0B2
....cuuAauuucgggaucuugaauu..	2	1	0B2
....uugauuucgggaucuugaau...	2	0	0B2
....uugauuucgggaucuGgaauu..	4	1	0B2
....uugauuucgggaucuugaauA..	2	1	0B2
....uugauuucgggaucuugaauu..	9	0	0B2
....uugauuucgggaucuugaauua...	11	0	0B2
....ugauuucgggaucuugaau...	1	0	0B2
....ugauuucgggaucuugaauuag..	6	0	0B2
....gauuucgggaucuugaauua...	6	0	0B2
....auuucgggaucuugaauuag..	2	0	0B2
....auuucgggaucuugaauuagug......................................	3	0	0B2
....Cuuucgggaucuugaauuagug......................................	4	1	0B2

```
                                                  Star                      Mature
cuuagaucuugaaucuugaauucgggaucuugaauuagugugaaucuugauuucgggaucuugaauuagugugugaaucuugaauuccggaucuugaaucuuggaucuuga
..................................................uuucgggaucuGgaauuagu.........................................   7    1   0B2
..................................................uuucgggaucuugaauuagu.........................................   19   0   0B2
..................................................Nuucgggaucuugaauuagu.........................................   2    1   0B2
..................................................uuucgggaucuugaauuGgug........................................   12   1   0B2
..................................................uuucgggaucuugaauuagug........................................   145  0   0B2
..................................................uuucgggaucuugaGuuagug........................................   3    1   0B2
..................................................uuucgggaucuGgaauuagug........................................   35   1   0B2
..................................................uuucgggaucuugaauuagugug......................................   10   0   0B2
..................................................uuucgggaucuugaaCuaguguga.....................................   1    1   0B2
..................................................uuucgggaucuugaauuaguguAa.....................................   2    1   0B2
..................................................uuucgggaucuugaauuaguguga.....................................   9    0   0B2
..................................................uuucgggaucuugaauuagugugaa....................................   3    0   0B2
...................................................uucgggaucuugaauuagG.........................................   4    1   0B2
...................................................uucgggaucuGgaauuagu.........................................   2    1   0B2
...................................................uucgggaucuugaauuagu.........................................   16   0   0B2
...................................................uucgggaucuugaauuagug........................................   96   0   0B2
...................................................uucgggaucuugaauuagGg........................................   32   1   0B2
...................................................uucgggaucuugaauuGgug........................................   10   1   0B2
...................................................uucgggaucuugaauuaguguga.....................................   2    0   0B2
...................................................uucgggaucuugaauuagugugaaucu.................................   2    0   0B2
....................................................ucgggaucuGgaauuagug........................................   4    1   0B2
....................................................ucgggaucuugaauuagGg........................................   12   1   0B2
....................................................ucgggaucuugaauuagug........................................   12   0   0B2
....................................................ucgggaucuugaauuGgug........................................   2    1   0B2
....................................................ucgggaucuugaauuagugu.......................................   6    0   0B2
....................................................ucgggaucuugaauuagGgug......................................   6    1   0B2
....................................................ucgggaucuugaauuagugug......................................   14   0   0B2
....................................................ucgggaucuugaauuagugugaauUuu...............................   4    1   0B2
.....................................................cgggaucuugaauuagGg........................................   14   1   0B2
.....................................................cgggaucuugaauuagug........................................   26   0   0B2
.....................................................Ngggaucuugaauuagug........................................   2    1   0B2
.....................................................cgggaucuugaauuagugug......................................   28   0   0B2
.....................................................cgggaucuugaauuagGgug......................................   4    1   0B2
.....................................................cgggaucuGgaauuagugug......................................   6    1   0B2
.....................................................cgggaucuugaauuaguguA......................................   6    1   0B2
.....................................................cgggaucuugaauuaguguga.....................................   4    0   0B2
.....................................................cgggaucuugaauuagugugaa....................................   22   0   0B2
.....................................................cgggaucuugaauuagugugaaA...................................   10   1   0B2
.....................................................cgggaucuugaauuagugugaaucA.................................   2    1   0B2
......................................................Nggaucuugaauuagugug......................................   6    1   0B2
......................................................Nggaucuugaauuagugugaa....................................   2    1   0B2
......................................................Nggaucuugaauuagugugaau...................................   2    1   0B2
.......................................................ggaucuugaauuagugug......................................   20   0   0B2
.......................................................ggaucuugaauuaguguA......................................   4    1   0B2
.......................................................ggaucuGgaauuagugug......................................   14   1   0B2
.......................................................ggaucuugaGuuaguguga.....................................   2    1   0B2
.......................................................ggaucuugaauuaguguga.....................................   16   0   0B2
.......................................................Ngaucuugaauuagugugaa....................................   2    1   0B2
.......................................................ggaucuugaauuagugugaa....................................   42   0   0B2
.......................................................ggaucuGgaauuagugugaa....................................   22   1   0B2
.......................................................ggaucuugaauuagugugaU....................................   12   1   0B2
.......................................................ggaucuugaGuuagugugaa....................................   6    1   0B2
.......................................................ggaucuugaauuagugugaaA...................................   2    1   0B2
.......................................................ggaucuugaauuagugugaau...................................   12   0   0B2
.........................................................aucuGgaauuagugugaauc..................................   22   1   0B2
.........................................................aucuugaauuagugugaaucu.................................   12   0   0B2
.........................................................aucuGgaauuagugugaaucu.................................   12   1   0B2
.........................................................aucuugaauuagugugaaucuug...............................   8    0   0B2
..........................................................ucuGgaauuagugugaaucu.................................   190  1   0B2
..........................................................Ncuugaauuagugugaaucu.................................   4    1   0B2
..........................................................ucuugaauCagugugaaucu.................................   2    1   0B2
..........................................................ucuugaauuagugugaaucu.................................   142  0   0B2
..........................................................ucuCgaauuagugugaaucu.................................   2    1   0B2
..........................................................ucuugaauuagugugaaucuu................................   10   0   0B2
..........................................................ucuGgaauuagugugaaucuu................................   8    1   0B2
..........................................................ucuGgaauuagugugaaucuug...............................   10   1   0B2
..........................................................ucuugaauuagugugaaucuug...............................   18   0   0B2
...........................................................cuugaauuagugugaaucu.................................   10   0   0B2
...........................................................cuGgaauuagugugaaucu.................................   2    1   0B2
...........................................................cuGgaauuagugugaaucuu................................   8    1   0B2
```

Star **Mature**

cuuagaucuugaaucuugaauucgggaucuugaauuagugugaaucuugauuucgggaucuugaauuagugugaaucuugaauuccggaucuugaaucuuggaucuuga			
....cuugaauuagugugaaucuu..............................	2	0	0B2
....cuGgaauuagugugaaucuug.............................	14	1	0B2
....cuGgaauuagugugaaucuuga............................	4	1	0B2
....uugaauuagugugaaucuu..............................	18	0	0B2
....uugaauuagugugaaucuug.............................	46	0	0B2
....uugaauuagugugaaucuuga............................	72	0	0B2
....uugaaCuagugugaaucuuga............................	2	1	0B2
....uugaauuagugugaGucuuga............................	8	1	0B2
....uugaauuagugugaaucuugaa...........................	20	0	0B2
....Nugaauuagugugaaucuugaa...........................	1	1	0B2
....Gugaauuagugugaaucuugaa...........................	1	1	0B2
....uugaauuagugugaaucuugaauu.........................	4	0	0B2
....ugaauuagugugaaucuuga............................	14	0	0B2
....ugaauuagugugaGucuuga............................	2	1	0B2
....Ngaauuagugugaaucuugaa...........................	1	1	0B2
....ugaauuagugugaaucuugaa...........................	1	0	0B2
....ugaauuagugugaaucuugaauA.........................	2	1	0B2
....gaauuagugugaaucuugaauA.........................	6	1	0B2
....aauuagugugaaucuugaau..........................	8	0	0B2
....aauuagugugaaucuugaauA.........................	10	1	0B2
....aauuagugugaaucuugaauu.........................	6	0	0B2
....aauuagugugaaucCugaauuc........................	1	1	0B2
....auuagugugaaucuugaauu.........................	41	0	0B2
....auuagugugaaucuugaauuc........................	38	0	0B2
....auuagugugaaucuugaauucc.......................	3	0	0B2
....uuagugugaaucuugaauu.........................	22	0	0B2
....uuagugugaaucuugaauuU........................	5	1	0B2
....uuagugugaaucuCgaauuc........................	2	1	0B2
....Nuagugugaaucuugaauuc........................	1	1	0B2
....uuagugugaaucuugaaCuc........................	1	1	0B2
....uuagugugaaucuugaauuc........................	191	0	0B2
....uuagugugaaucuugaGuuc........................	6	1	0B2
....uuagugugaaucuugaauucc.......................	2	0	0B2
....uuagugugUaucuugaauucc.......................	2	1	0B2
....uuagugugaaucuGgaauucc.......................	5	1	0B2
....uuagugugaaucuugaauuGcg......................	10	1	0B2
....uuagugugaaucuugaaCuccg......................	1	1	0B2
....uuagugugaaucuugaauuccg......................	1	0	0B2
....uuagugugaaucuugaaCuccgga....................	3	1	0B2
....uuagugugaaucuugaauuUcggauc..................	8	1	0B2
....uuagugugaaucCugaauuccggauc..................	4	1	0B2
....uuagugugaaucuugaauuccggauc..................	5	0	0B2
....uuagugugaaucuugaauCccggauc..................	2	1	0B2
....uuagugugaaucuugaauuGcggauc..................	3	1	0B2
....uuagugugaaucuugaauuUcggaucu.................	2	1	0B2
....uuagugugaaucuugaauuccggaucA.................	2	1	0B2
....uuagugugaaucuugaauuccggaucu.................	3	0	0B2
....uuagugugaaucuugaauCccggaucu.................	5	1	0B2
....uuagugugaaucuugaaCuccggaucu.................	2	1	0B2
....uuagugugaaucuugaauuGcggaucu.................	1	1	0B2
....uagugugaaucuugaauu.........................	7	0	0B2
....uagugugaaucuugaauuc........................	24	0	0B2
....uagugugaaucuugaauucc.......................	2	0	0B2
....uaguguCaaucuugaauuccg......................	3	1	0B2
....uagugugaaucuugaaCuccg......................	2	1	0B2
....uagugugaaucuugaauuUcgg.....................	3	1	0B2
....uagugugaaucuugaUuuccggauc..................	2	1	0B2
....uagugugaaucuugaauuccggauc..................	4	0	0B2
....uagugugaaucuugaauuUcggauc..................	2	1	0B2
....uagugugaaucuugaauuccggaucA.................	2	1	0B2
....agugugaaucuugaauuc........................	6	0	0B2
....agugugaaucuugaaCucc.......................	2	1	0B2
....agugugaaucuugaaCuccg......................	9	1	0B2
....agugugaaucuugaauuUcgg.....................	3	1	0B2
....agugugaaucuugaaCuccgga....................	5	1	0B2
....gugugaaucuugaauuccgg.....................	1	0	0B2
....uguAaaucuugaauuccgga....................	5	1	0B2
....ugugaaAcuugaauuccgga....................	2	1	0B2
....ugugaaucuugaauuccggau...................	5	0	0B2
....ugugaaucuugaauuccggauc..................	4	0	0B2

Star Mature

```
cuuagaucuugaaucuugaauucgggaucuugaauuagugugaaucuugauuucgggaucuugaauuagugugaaucuugaauuccggaucuugaaucuuggaucuuga
.....................................................................ugugaaucuugaauuccggauA..................	5	1	0B2
.......................................................................ugaaucuugaauuccggauc..................	5	0	0B2
.......................................................................ugaaucuugaauuccggaucA.................	3	1	0B2
.......................................................................ugaaucuugaauuccggaucuAgaau............	6	1	0B2
.......................................................................ugaaucuugaauuccggaucuugaau............	3	0	0B2
.......................................................................ugaaucuugaauuccggaucuugaauc...........	3	0	0B2
.......................................................................ugaaucuugaauucUggaucuugaauc...........	2	1	0B2
.......................................................................ugaaucuugaauuccggaucuAgaauc...........	2	1	0B2
.........................................................................aaucuugaauuccggaucuug...............	2	0	0B2
.........................................................................aaucuugaauuccggaucuAg...............	6	1	0B2
..........................................................................aucuugaauuccggaucu.................	4	0	0B2
..........................................................................aucuugaauuccggaucuu................	6	0	0B2
..........................................................................aucuugaauuccggaucuA................	2	1	0B2
..........................................................................aucuugaauuccggaucuuga..............	5	0	0B2
..........................................................................aucGugaauuccggaucuuga..............	7	1	0B2
..........................................................................Cucuugaauuccggaucuugaa.............	2	1	0B2
..........................................................................aucuugaauuccggaucuugaau............	11	0	0B2
..........................................................................aucuugaauuccggaucuugaauUuu.........	1	1	0B2
...........................................................................ucuugaauuccggaucuu................	1	0	0B2
...........................................................................ucuugaauuccggaucuA................	1	1	0B2
...........................................................................ucuugaauuccggaucuAg...............	11	1	0B2
...........................................................................ucuugaauuccggaucuug...............	5	0	0B2
...........................................................................ucuugaauuccggaucuuga..............	15	0	0B2
...........................................................................ucuugaauuccggaucuAga..............	44	1	0B2
...........................................................................ucuugaauuccggaucuugaa.............	20	0	0B2
...........................................................................ucuugaauuccggaucuAgaa.............	44	1	0B2
...........................................................................ucGugaauuccggaucuugaa.............	26	1	0B2
...........................................................................ucuugaauuccggaucuGgaa.............	4	1	0B2
...........................................................................ucuugaauuccggaucuugaau............	262	0	0B2
...........................................................................ucGugaauuccggaucuugaau............	18	1	0B2
...........................................................................ucuugaauuccggaucuugaaG............	5	1	0B2
...........................................................................Ncuugaauuccggaucuugaau............	3	1	0B2
...........................................................................ucuugaauuccggaucuAgaau............	16	1	0B2
...........................................................................Ccuugaauuccggaucuugaau............	1	1	0B2
...........................................................................ucuugaauuccggaucuCgaau............	2	1	0B2
...........................................................................ucuugaauuccggaucuugaauc...........	1	0	0B2
...........................................................................ucuugaauuccggaucuugaaucu..........	4	0	0B2
...........................................................................ucuugaauucUggaucuugaaucu..........	4	1	0B2
...........................................................................ucuugaauuccggaucuugaaucA..........	2	1	0B2
...........................................................................ucuugaauuccggaucuugaaucuuCg.......	3	1	0B2
............................................................................cuugaauuccggaucuug...............	2	0	0B2
............................................................................cuugaauuccggaucuAg...............	1	1	0B2
............................................................................cuugaauuccggaucuAga..............	11	1	0B2
............................................................................cuugaauuccggaucuuga..............	5	0	0B2
............................................................................cuugaauuccggaucuAgaa.............	366	1	0B2
............................................................................cGugaauuccggaucuugaa.............	4	1	0B2
............................................................................cuugaauuccggaucuugaa.............	90	0	0B2
............................................................................Nuugaauuccggaucuugaa.............	1	1	0B2
............................................................................cuugaauuccggaucuugaau............	204	0	0B2
............................................................................cuugaauuccggaucuAgaau............	15	1	0B2
............................................................................cuugaauuccggaGcuugaau............	1	1	0B2
............................................................................cGugaauuccggaucuugaau............	28	1	0B2
............................................................................Nuugaauuccggaucuugaau............	1	1	0B2
............................................................................cuugaauuccggaucuugaaC............	1	1	0B2
............................................................................cuugaauuccggaucuugaauc...........	22	0	0B2
............................................................................cuugaauuccggaucuugaaucA..........	27	1	0B2
............................................................................cuugaauuccggaucuugaaucu..........	2	0	0B2
............................................................................cuugaauuccggaucuugaaucuA.........	2	1	0B2
............................................................................cuugaauuccggaucuugaauUuu.........	4	1	0B2
.............................................................................uugaauuccggaucuAga..............	14	1	0B2
.............................................................................uugaauuccggaucuAgaa.............	284	1	0B2
.............................................................................uugaauuccggaucuGgaa.............	1	1	0B2
.............................................................................uugaauuccggaucuugaa.............	53	0	0B2
.............................................................................uugaauuccggCucuugaa.............	1	1	0B2
.............................................................................uugaauuccggaucuAgaau............	1941	1	0B2
.............................................................................uugaCuuccggaucuugaau............	1	1	0B2
.............................................................................Nugaauuccggaucuugaau............	73	1	0B2
.............................................................................uugaauuccggaucuCgaau............	2	1	0B2
.............................................................................uugaauuccggaucuGgaau............	9	1	0B2
.............................................................................Gugaauuccggaucuugaau............	7	1	0B2
```

```
                                                                            Star                     Mature
cuuagaucuugaaucuugaauucgggaucuugaauuagugugaaucuugauuucgggaucuugaauuagugugaaucuugaauuccggaucuugaaucuuggaucuuga
.............................................................................uugaauuccggaucuugaau............   9489   0   0B2
.............................................................................uugaauuccgCaucuugaau............   4      1   0B2
.............................................................................uugaauuccggCucuugaau............   1      1   0B2
.............................................................................uugaauuccggaGcuugaau............   4      1   0B2
.............................................................................uugaauuccggaucuugaaC............   10     1   0B2
.............................................................................uugaauuccggaucAugaau............   4      1   0B2
.............................................................................uugaauuccggauAuugaau............   1      1   0B2
.............................................................................Cugaauuccggaucuugaau............   8      1   0B2
.............................................................................uGgaauuccggaucuugaau............   1      1   0B2
.............................................................................uugaGuuccggaucuugaau............   91     1   0B2
.............................................................................uugaauuccggGucuugaau............   8      1   0B2
.............................................................................uugaauuccggaCcuugaau............   3      1   0B2
.............................................................................uugaauuccggaucuugaaG............   1      1   0B2
.............................................................................uugaauuccgUaucuugaauc...........   1      1   0B2
.............................................................................uugaauuccggaucuugaaCc...........   8      1   0B2
.............................................................................uugaauuccggaucuugaauc...........   690    0   0B2
.............................................................................uugaauuccggaucuugaGuc...........   1      1   0B2
.............................................................................uugaauuccggaucuAgaauc...........   8      1   0B2
.............................................................................Nugaauuccggaucuugaauc...........   5      1   0B2
.............................................................................uugaauucUggaucuugaauc...........   4      1   0B2
.............................................................................uugaauuUcggaucuugaauc...........   3      1   0B2
.............................................................................uugaauuccggaucuugaaucA..........   934    1   0B2
.............................................................................uugaauuccggaucuugaaucu..........   49     0   0B2
.............................................................................uugaauuccggaucuugaGucu..........   3      1   0B2
.............................................................................uugaauuccggaucuugaaucAu.........   15     1   0B2
.............................................................................uugaauuccggaucuugaaucuA.........   3      1   0B2
.............................................................................uugaauuccggaucuugaauUuu.........   36     1   0B2
.............................................................................uugaauuccggaucuugaaucuu.........   1      0   0B2
.............................................................................uugaauuccggaucuugaaucuuA........   4      1   0B2
.............................................................................uugaauuccggaucuugaaucuuU........   3      1   0B2
..............................................................................ugaauuccggaucuAgaa.............   70     1   0B2
..............................................................................ugaauuccggaucuugaa.............   5      0   0B2
..............................................................................ugaauuccggaGcuugaau............   2      1   0B2
..............................................................................ugaauuccggaucuAgaau............   149    1   0B2
..............................................................................Ngaauuccggaucuugaau............   2      1   0B2
..............................................................................ugaauuccggaucuugaau............   498    0   0B2
..............................................................................ugaGuuccggaucuugaau............   7      1   0B2
..............................................................................uUaauuccggaucuugaauc...........   1      1   0B2
..............................................................................ugaGuuccggaucuugaauc...........   6      1   0B2
..............................................................................ugaauuccggaucuugaauc...........   248    0   0B2
..............................................................................ugaauuccggaGcuugaauc...........   1      1   0B2
..............................................................................ugaauuUcggaucuugaauc...........   1      1   0B2
..............................................................................Ngaauuccggaucuugaauc...........   1      1   0B2
..............................................................................Ngaauuccggaucuugaaucu..........   2      1   0B2
..............................................................................ugaGuuccggaucuugaaucu..........   2      1   0B2
..............................................................................ugaauuccggaucuugaGucu..........   2      1   0B2
..............................................................................ugaauuccggaucuugaaucu..........   308    0   0B2
..............................................................................ugaauuccggaucuugaCucu..........   2      1   0B2
..............................................................................ugaauuccggaucuugaaucA..........   280    1   0B2
..............................................................................Cgaauuccggaucuugaaucu..........   1      1   0B2
..............................................................................ugaauCccggaucuugaaucu..........   2      1   0B2
..............................................................................ugaauuccggauUuugaaucu..........   2      1   0B2
..............................................................................ugaauuccggaucuugaaucAu.........   8      1   0B2
..............................................................................ugaauuccggaucuugaaucuu.........   7      0   0B2
..............................................................................ugaauuccggaucuugaauUuu.........   15     1   0B2
..............................................................................ugaauuccggaucuugaaucuA.........   70     1   0B2
..............................................................................ugaauuccggaucuugaaucuuA........   12     1   0B2
..............................................................................ugaauuccggaucuugaaucuuU........   3      1   0B2
...............................................................................gaauuccggaucuugaau............   21     0   0B2
...............................................................................gaauuccggaucuAgaau............   1      1   0B2
...............................................................................gaauuccggaucuugaauc...........   23     0   0B2
...............................................................................gaauuccggaucuugaaucA..........   59     1   0B2
...............................................................................gaauuccggaucuGgaaucu..........   1      1   0B2
...............................................................................gaauuccggaucuugaaucu..........   92     0   0B2
...............................................................................Naauuccggaucuugaaucu..........   1      1   0B2
...............................................................................gaauuccggaucuAgaaucu..........   5      1   0B2
...............................................................................gaauuccggaucuugaaucuu.........   7      0   0B2
...............................................................................gaauuccggaucuugaGucuu.........   1      1   0B2
...............................................................................gaauuccggaucuugaauUuu.........   4      1   0B2
...............................................................................gaauuccggaucuugaaucuA.........   36     1   0B2
```

Star **Mature**

cuuagaucuugaaucuugaauucgggaucuugaauuagugugaaucuugauuucgggaucuugaauuagugugaaucuugaauuccggaucuugaaucuuggaucuuga			
....gaauuccggaucuugaaucuuA........	10	1	0B2
....gaauuccggaucuugaaucuuU........	12	1	0B2
....aauuccggaucuugaauc...........	3	0	0B2
....aauuccggaucuugaaucu..........	6	0	0B2
....aauuccggaucuugaaucA..........	8	1	0B2
....aauuccggaucuugaaucuA.........	3	1	0B2
....aauuccggaucuugaauUuu.........	28	1	0B2
....aauuccggaucuugaaucuu.........	39	0	0B2
....aauuccggaucuugaaCcuug........	2	1	0B2
....aauuccggaucuugaGucuug........	5	1	0B2
....aauuccggaucuugaaucuuC........	3	1	0B2
....aauuccggaucuugaaucuuU........	17	1	0B2
....aauuccggaucuugaaucuuA........	31	1	0B2
....aauuccggaucuugaauUuug........	4	1	0B2
....aauuccggaucuugaaucuug........	11	0	0B2
....auuccggaucuugaaucA..........	4	1	0B2
....auuccggaucuugaaucu..........	2	0	0B2
....auuccggaucuugaaucuA.........	1	1	0B2
....auuccggaucuugaauUuu.........	7	1	0B2
....auuccggaucuugaUucuug........	1	1	0B2
....auuccggaucuugaaucuuA........	22	1	0B2
....auuccggaucuugaaucuug........	114	0	0B2
....auuccggaucuugaaucGug........	8	1	0B2
....auuccggaucuugaaucuuU........	3	1	0B2
....Guuccggaucuugaaucuug........	4	1	0B2
....Nuuccggaucuugaaucuug........	3	1	0B2
....auuccggaucuugaauUuug........	12	1	0B2
....auuccggUucuugaaucuug........	5	1	0B2
....auuccggaucuugaauUuugg.......	3	1	0B2
....auuccggaucuugaaucuuUg.......	3	1	0B2
....uuccggaucuugaauUuu.........	2	1	0B2
....uuccggaucuugaaucuug........	5	0	0B2
....uuccggaucuugaaucuuA........	3	1	0B2
....uuccggaucuugaauUuug........	2	1	0B2
....uuccggaucuugaaucGug........	2	1	0B2
....aucuugaaucuuggaucuu..	1	0	0B2
........uugaaucuugaauucgggauA..	2	1	0G2
...............uugaauucgggaucuugaauA...	1	1	0G2
...............uugaauucgggaucuAgaauu...	4	1	0G2
...............uugaauucgggaucuugaauAa..	3	1	0G2
................ugaauucgggaucuAgaauua..	3	1	0G2
................ugaauucgggaucuugaauua..	1	0	0G2
...................Nuucgggaucuugaauuagu..	2	1	0G2
...................aucgggaucuugaauuagG...	9	1	0G2
...................aucgggaucuAgaauuagu...	26	1	0G2
...................aucgggaucuAgaauuagug..	10	1	0G2
...................Cuucgggaucuugaauuagug...	2	1	0G2
....................uucgggaucuugaauuagG..	20	1	0G2
....................uucgggaucuugaauuGgug...	4	1	0G2
....................uucgggaucuugaauuagGg...	12	1	0G2
....................uucgggaucuugaauuagug...	110	0	0G2
....................uucgggaucuGgaauuagug...	20	1	0G2
....................uucgggaucuugaauuagugug...	2	0	0G2
....................uucgggaucuugaauuagugugaaucA..	8	1	0G2
.....................ucgggaucuugaauuagG..	8	1	0G2
.....................ucgggaucuGgaauuagug...	12	1	0G2
.....................ucgggaucuugaauuagug...	24	0	0G2
.....................ucgggaucuugaauuagugug...	46	0	0G2
.....................ucgggaucuugaauuaguguU...	12	1	0G2
.....................ucgggaucuGgaauuagugug...	16	1	0G2
......................cgggaucuugaauuagGg...	10	1	0G2
......................cgggaucuugaauuagug...	26	0	0G2
......................cgggaucuugaauuagGgug...	4	1	0G2
......................cgggaucuugaauuagugug...	12	0	0G2
......................cgggaucuugaauuagugugaa...	12	0	0G2
......................cgggaucuugaauuGgugugaa...	8	1	0G2
......................cgggaucuugaauuagugugaaA..	14	1	0G2
......................cgggaucuugaauuagugugaauA...	10	1	0G2
......................cgggaucuugaauuagugugaaucA..	4	1	0G2

Star Mature

cuuagaucuugaaucuugaauucgggaucuugaauuagugugaaucuugauuucgggaucuugaauuagugugaaucuugaauuccggaucuugaaucuuggaucuuga			
.......................Nggaucuugaauuagugug...	2	1	0G2
.......................Nggaucuugaauuagugugaa...	2	1	0G2
.......................gggaucuugaauuagugugaa...	6	0	0G2
.......................Nggaucuugaauuagugugaau..	2	1	0G2
........................ggaucuugaauuaguguA...	2	1	0G2
........................ggaucuugaauuagugug...	38	0	0G2
........................ggaucuGgaauuagugug...	44	1	0G2
........................ggaucuugaauuaguguAa..	8	1	0G2
........................ggaucuugaauuaguguAaa...	8	1	0G2
........................ggaucuugaauuagugugaaA..	2	1	0G2
........................ggaucuugaauuagugugaau..	4	0	0G2
........................ggaucuugaauuagugugaauA...	14	1	0G2
..........................aucuGgaauuagugugaauc...	20	1	0G2
..........................aucuugaauuagugugaaucu..	10	0	0G2
..........................aucuGgaauuagugugaaucu..	152	1	0G2
..........................aucuGgaauuagugugaaucuu...	4	1	0G2
...........................ucuGgaauuagugugaauc...	12	1	0G2
...........................ucuGgaauuagugugaaucu..	242	1	0G2
...........................ucuugaGuuagugugaaucu..	26	1	0G2
...........................ucuugaauuagugugaaucu..	178	0	0G2
...........................ucuugaauuagugugaaucuA...	4	1	0G2
...........................ucuGgaauuagugugaaucuu...	20	1	0G2
...........................ucuGgaauuagugugaaucuug..	10	1	0G2
............................cuGgaauuagugugaaucuu...	14	1	0G2
............................cuugaauuagugugaaucuuga...	2	0	0G2
.............................uugaauuagugugaaucuug..	118	0	0G2
.............................uuAaauuagugugaaucuug..	8	1	0G2
.............................uugaauuagugugaaucuuga...	266	0	0G2
.............................uugaGuuagugugaaucuuga...	4	1	0G2
.............................uGgaauuagugugaaucuugau..	5	1	0G2
..............................ugaauuagugugaaucuug..	10	0	0G2
..............................ugaauuagugugaaucuuga...	50	0	0G2
...............................gaauuagugugaaucuuga...	14	0	0G2
.................................auuagugugaaucuugauuucg..	1	0	0G2
..................................uuagugugaaucuugauuuc...	15	0	0G2
..................................uuagugugaaucuugauuucg..	5	0	0G2
..................................uuagugugaaucuugauuucgggaucu..	17	0	0G2
..................................uuagugugaaucuugauuucCggaucu..	3	1	0G2
..................................uuagugugaaucuugauuuGgggaucu..	1	1	0G2
...................................uagugugaaucuCgauuucg..	2	1	0G2
...................................uagugugaaucuugauuucg..	6	0	0G2
...................................uagugugaaucuCgauuucgg...	5	1	0G2
...................................uagugugaaucuugauuucggg..	2	0	0G2
....................................agugugaaucuugauuucg..	7	0	0G2
....................................agugugaaucuugauuucgg...	4	0	0G2
.....................................Cugugaaucuugauuucgggaucuug..	11	1	0G2
....uugauuucgggaucuugaauA..	7	1	0G2
....uugauuucgggaucuugaauAa...	3	1	0G2
....uugauuucgggaucuGgaauua...	4	1	0G2
....uugauuucgggaucuugaauua...	16	0	0G2
....ugauuucgggaucuGgaauu..	5	1	0G2
....ugauuucgggaucuugaauua...	3	0	0G2
....gauuucgggaucuugaauuag..	5	0	0G2
....auuucgggaucuugaauuag..	5	0	0G2
....Nuucgggaucuugaauuagu...	2	1	0G2
....uuucgggaucuGgaauuagu...	15	1	0G2
....uuucgggaucuugaauuagu...	26	0	0G2
....uuucgggaucuugaGuuagug..	3	1	0G2
....uuucgggaucuGgaauuagug..	22	1	0G2
....uuucgggaucuugaauuagug..	198	0	0G2
....uuucgggaucuugaauuGgug..	33	1	0G2
....Cuucgggaucuugaauuagug..	2	1	0G2
....uuucgggaucuGgaauuagugug......................................	5	1	0G2
....uuucgggaucuugaauuagugug......................................	46	0	0G2
....uuucgggaucuCgaauuagugug......................................	1	1	0G2
....uucgggaucuugaauuagG...	20	1	0G2
....uucgggaucuGgaauuagug..	20	1	0G2
....uucgggaucuugaauuGgug..	4	1	0G2
....uucgggaucuugaauuagug..	110	0	0G2
....uucgggaucuugaauuagGg..	12	1	0G2

Star **Mature**

cuuagaucuugaaucuugaauucgggaucuugaauuagugugaaucuugauuucgggaucuugaauuagugugaaucuugaauuccggaucuugaaucuuggaucuuga			
....uucgggaucuugaauuagugug....................................	2	0	0G2
....uucgggaucuugaauuagugugaaucA...............................	8	1	0G2
....ucgggaucuugaauuagG.......................................	8	1	0G2
....ucgggaucuGgaauuagug......................................	12	1	0G2
....ucgggaucuugaauuagug......................................	24	0	0G2
....ucgggaucuugaauuagugug....................................	46	0	0G2
....ucgggaucuGgaauuagugug....................................	16	1	0G2
....ucgggaucuugaauuaguguU....................................	12	1	0G2
....cgggaucuugaauuagug......................................	26	0	0G2
....cgggaucuugaauuagGg......................................	10	1	0G2
....cgggaucuugaauuagGgug....................................	4	1	0G2
....cgggaucuugaauuagugug....................................	12	0	0G2
....cgggaucuugaauuGgugugaa..................................	8	1	0G2
....cgggaucuugaauuagugugaa..................................	12	0	0G2
....cgggaucuugaauuagugugaaA.................................	14	1	0G2
....cgggaucuugaauuagugugaauA................................	10	1	0G2
....cgggaucuugaauuagugugaaucA...............................	4	1	0G2
....Nggaucuugaauuagugug....................................	2	1	0G2
....gggaucuugaauuagugugaa..................................	6	0	0G2
....Nggaucuugaauuagugugaa..................................	2	1	0G2
....Nggaucuugaauuagugugaau.................................	2	1	0G2
....ggaucuugaauuagugug....................................	38	0	0G2
....ggaucuugaauuaguguA....................................	2	1	0G2
....ggaucuGgaauuagugug....................................	44	1	0G2
....ggaucuugaauuaguguAa...................................	8	1	0G2
....ggaucuugaauuaguguAaa..................................	8	1	0G2
....ggaucuugaauuagugugaau.................................	4	0	0G2
....ggaucuugaauuagugugaaA.................................	2	1	0G2
....ggaucuugaauuagugugaauA................................	14	1	0G2
....aucuGgaauuagugugaauc................................	20	1	0G2
....aucuGgaauuagugugaaucu...............................	152	1	0G2
....aucuugaauuagugugaaucu...............................	10	0	0G2
....aucuGgaauuagugugaaucuu..............................	4	1	0G2
....ucuGgaauuagugugaauc................................	12	1	0G2
....ucuGgaauuagugugaaucu...............................	242	1	0G2
....ucuugaGuuagugugaaucu...............................	26	1	0G2
....ucuugaauuagugugaaucu...............................	178	0	0G2
....ucuGgaauuagugugaaucuu..............................	20	1	0G2
....ucuugaauuagugugaaucuA..............................	4	1	0G2
....ucuGgaauuagugugaaucuug.............................	10	1	0G2
....cuGgaauuagugugaaucuu..............................	14	1	0G2
....cuugaauuagugugaaucuuga............................	2	0	0G2
....uugaauuagugugaaucuug.............................	118	0	0G2
....uuAaauuagugugaaucuug.............................	8	1	0G2
....uugaGuuagugugaaucuuga............................	4	1	0G2
....uugaauuagugugaaucuuga............................	266	0	0G2
....Nugaauuagugugaaucuugaa...........................	1	1	0G2
....uugaauuagugugaaucuugaa...........................	8	0	0G2
....uugaauuagugugaaucuugaau..........................	1	0	0G2
....uugaauuagugugaaucuugaauA.........................	4	1	0G2
....ugaauuagugugaaucuug.............................	10	0	0G2
....ugaauuagugugaaucuuga............................	50	0	0G2
....ugaauuagugugaaucuugaa...........................	1	0	0G2
....ugaauuagugugaaucuugaau..........................	1	0	0G2
....gaauuagugugaaucuuga............................	14	0	0G2
....gaauuagugugaaucuugaauA........................	5	1	0G2
....aauuagugugaaucuugaau..........................	12	0	0G2
....aauuagugugaaucuugaauA.........................	27	1	0G2
....auuagugugaaucuugaauu.........................	34	0	0G2
....auuagugugaaucuugaauA.........................	9	1	0G2
....auGagugugaaucuugaauuc........................	1	1	0G2
....auuagugugaaucuugaauuc........................	61	0	0G2
....Cuuagugugaaucuugaauuc........................	1	1	0G2
....uuagugugaaucuugaauu.........................	12	0	0G2
....uuaguguAaaucuugaauu.........................	1	1	0G2
....uuagugugaaucuugaauuc........................	76	0	0G2
....uuagugugaaucuugaauucc.......................	7	0	0G2
....uuagugugUaucuugaauucc.......................	5	1	0G2
....uuagugugaaucuugaauuUcg......................	1	1	0G2
....uuagugugaaucuugaauuccA......................	6	1	0G2

Star **Mature**

cuuagaucuugaaucuugaauucgggaucuugaauuagugugaaucuugauuucgggaucuugaauuagugugugaaucuugaauuccggaucuugaaucuuggaucuuga			
....uuagugugaaucuugaauuUcgg......................	4	1	0G2
....uuagugugaaucuugaauuccggau....................	1	0	0G2
....uuagugugaaucuugaauuUcggau....................	1	1	0G2
....uuagugugaaucuugaauuccggauc...................	7	0	0G2
....uuagugugaaucuugaauuUcggauc...................	3	1	0G2
....uuagugugaaucuugaauuccggaucA..................	3	1	0G2
....uuagugugaaucuugaauuccggaucu..................	10	0	0G2
....uuagugugaaucuugaaCuccggaucu..................	6	1	0G2
....uuagugugaaucuugaUuuccggaucu..................	3	1	0G2
....uuagugugaaucuugaauuUcggaucu..................	10	1	0G2
....uagugugaaucuugaauu..........................	2	0	0G2
....uagugugaaucuugaauuc.........................	24	0	0G2
....uagugugaaucCugaauuc.........................	1	1	0G2
....uagugugaaucuugaauucc........................	16	0	0G2
....uagugugUaucuugaauucc........................	4	1	0G2
....uagugugaGucuugaauucc........................	2	1	0G2
....uagugugaaucuGgaauucc........................	4	1	0G2
....uagugugCaucuugaauucc........................	6	1	0G2
....uaguguCaaucuugaauuccg.......................	6	1	0G2
....uagugugaaucuugaauuccgg......................	9	0	0G2
....uagugugaaucuugaauuUcgg......................	5	1	0G2
....uagugugaaucuugaauuccggauU...................	5	1	0G2
....uagugugaaucCugaauuccggauc...................	1	1	0G2
....uagugugaaucuugaauuccggaucA..................	4	1	0G2
....agugugaaucuugaauuc.........................	3	0	0G2
....aguguCaaucuugaauuccg.......................	6	1	0G2
....agugugaGucuugaauuccg.......................	4	1	0G2
....agugugaaucuugaaCuccgg......................	2	1	0G2
....gugugaaucuugaauuccggaucuAga...............	6	1	0G2
....ugugaaucuugaaCuccgg......................	7	1	0G2
....ugugaaucuugaauuccggauA...................	2	1	0G2
....ugugaaucuugaauuccggaucuAga...............	3	1	0G2
....Nugaaucuugaauuccggau....................	1	1	0G2
....gugaaucuugaauuccggaucuugaau.............	3	0	0G2
....ugaCucuugaauuccggauc...................	4	1	0G2
....ugaaucuugaauuccggauc...................	3	0	0G2
....ugaGucuugaauuccggauc...................	3	1	0G2
....ugaaucuugaauuccggauA...................	10	1	0G2
....ugaaucuugaauuccggaucA..................	1	1	0G2
....ugaaucuugaauuccggaucu..................	20	0	0G2
....gaaucuugaauuccggauc...................	6	0	0G2
....aaucuugaauuccggaucA..................	5	1	0G2
....aaucuugaauuccggaucu..................	5	0	0G2
....aaucuugaauuccggaucuA.................	5	1	0G2
....aaucuugaauuccggaucuAg................	18	1	0G2
....aGucuugaauuccggaucuug................	10	1	0G2
....aaucuugaauuccggaucuugUa..............	5	1	0G2
....aucuugaauuccggaucuug................	3	0	0G2
....aucuugaauuccggaucuAga...............	2	1	0G2
....aucuugaauuccggaucuugaau.............	8	0	0G2
....ucuugaauuccggaucuA.................	2	1	0G2
....ucuugaauuccggaucuug................	1	0	0G2
....ucuugaauuccggaucuAga...............	51	1	0G2
....ucuugaauuccggaucuuga...............	19	0	0G2
....ucuugaauuccggaucuAgaa..............	74	1	0G2
....ucuugaauuccggaucuugaa..............	26	0	0G2
....ucGugaauuccggaucuugaa..............	16	1	0G2
....ucGugaauuccggaucuugaau.............	47	1	0G2
....ucuugaauuccggaucuAgaau.............	10	1	0G2
....Ccuugaauuccggaucuugaau.............	1	1	0G2
....ucuugaauuAcggaucuugaau.............	1	1	0G2
....ucuugaauuccggaucuugaau.............	227	0	0G2
....ucuugaauuccggaucuugaaG.............	3	1	0G2
....ucuugaauuccggaCcuugaau.............	1	1	0G2
....ucuugaauuccggaucuugaauUuu..........	2	1	0G2
....ucuugaauuccggaucuugaaucuA..........	3	1	0G2
....ucuugaauuccggaucuugaaucuuA.........	3	1	0G2
....ucuugaauuccggaucuugaaucuuCg........	1	1	0G2
....cuugaauuccggaucuug................	7	0	0G2
....cuugaauuccggaucuAga...............	54	1	0G2

Star Mature

cuuagaucuugaaucuugaauucgggaucuugaauuagugugaaucuugauuucgggaucuugaauuagugugaaucuugaauuccggaucuugaaucuuggaucuuga

....cuugaauuccggaucuuga..............	2	0	0G2
....Nuugaauuccggaucuugaa.............	2	1	0G2
....cGugaauuccggaucuugaa.............	1	1	0G2
....cuugaauuccggaucuAgaa.............	363	1	0G2
....cuugaauuccggaucuugaa.............	96	0	0G2
....Nuugaauuccggaucuugaau............	2	1	0G2
....cGugaauuccggaucuugaau............	15	1	0G2
....cuugaauuccggaucuAgaau............	21	1	0G2
....cuugaauuccggaucuugaau............	227	0	0G2
....cuugaauuccggaucuugaauc...........	2	0	0G2
....cuugaauuccggaucuugaaucA..........	35	1	0G2
....uugaauuccggaucuAga..............	26	1	0G2
....uugaauuccggaucuAgaa.............	238	1	0G2
....uugaauuccggaucuugaa.............	103	0	0G2
....Augaauuccggaucuugaau............	6	1	0G2
....uugaauuccggaucuGgaau............	3	1	0G2
....Gugaauuccggaucuugaau............	12	1	0G2
....uGgaauuccggaucuugaau............	2	1	0G2
....uugaauuccggaGcuugaau............	2	1	0G2
....uugaaGuccggaucuugaau............	1	1	0G2
....uugaauuccggaucCugaau............	1	1	0G2
....uugaauuccggaucuugaaG............	7	1	0G2
....Cugaauuccggaucuugaau............	2	1	0G2
....uugaauuccggauAuugaau............	5	1	0G2
....uugaauuccggaucuugaaC............	3	1	0G2
....uugaauuccggGucuugaau............	7	1	0G2
....uugaauuccggCucuugaau............	1	1	0G2
....uAgaauuccggaucuugaau............	2	1	0G2
....Nugaauuccggaucuugaau............	28	1	0G2
....uugaauuccggaCcuugaau............	1	1	0G2
....uugaauuccggaucuAgaau............	1498	1	0G2
....uugaauuccggaucuugaau............	7306	0	0G2
....uugaGuuccggaucuugaau............	57	1	0G2
....uNgaauuccggaucuugaau............	6	1	0G2
....uugaauuAcggaucuugaau............	3	1	0G2
....Nugaauuccggaucuugaauc...........	2	1	0G2
....uugaauCccggaucuugaauc...........	1	1	0G2
....uugaauuccggaucuugaauc...........	578	0	0G2
....uugaauuccggaucuAgaauc...........	5	1	0G2
....uugaauuccggaucuugaGuc...........	5	1	0G2
....uugaauuccggGucuugaauc...........	1	1	0G2
....uCgaauuccggaucuugaauc...........	1	1	0G2
....uugaauuccggaucuugaaGc...........	1	1	0G2
....uugaauuccggaucuGgaauc...........	1	1	0G2
....Augaauuccggaucuugaauc...........	1	1	0G2
....uugaGuuccggaucuugaauc...........	1	1	0G2
....uugaauuccggaucuugaaucA..........	2426	1	0G2
....uugaauuccggaucuugaaucu..........	77	0	0G2
....uugaauuccggaucuugaauGu..........	1	1	0G2
....uugaauuccggaucuAgaaucu..........	5	1	0G2
....uuCaauuccggaucuugaaucu..........	2	1	0G2
....uugaauuccggaucuugaaucG..........	1	1	0G2
....uugaauuccggaucuugaaucuu.........	8	0	0G2
....uugaauuccggaucuugaaucAu.........	37	1	0G2
....uugaauuccggaucuugaaucuA.........	11	1	0G2
....uugaauuccggaucuugaauUuu.........	15	1	0G2
....uugaauuccggaucuugaaucuuA........	1	1	0G2
....ugaauuccggaucuAgaa.............	66	1	0G2
....ugaauuccggaucuGgaa.............	1	1	0G2
....ugaauuccggaucuugaa.............	2	0	0G2
....ugaauuAcggaucuugaau............	1	1	0G2
....ugaauuccggaucuugaau............	240	0	0G2
....ugaauuccggaucuAgaau............	68	1	0G2
....ugaauuccggaucuugaauc...........	116	0	0G2
....ugaauuUcggaucuugaauc...........	1	1	0G2
....Ngaauuccggaucuugaauc...........	1	1	0G2
....ugaauuccggaAcuugaauc...........	1	1	0G2
....ugaauuccggaucuugaaucu..........	301	0	0G2
....ugaGuuccggaucuugaaucu..........	4	1	0G2
....ugaauuccggaucuugaaucA..........	474	1	0G2

```
                                                          Star                    Mature
cuuagaucuugaaucuugaauucgggaucuugaauuagugugaaucuugauuucgggaucuugaauuagugugaaucuugaauuccggaucuugaaucuuggaucuuga
..............................................................................ugaauuccggaucuugaaucuA.........   148   1   0G2
..............................................................................ugaauuccggaucuugaaucAu.........   4     1   0G2
..............................................................................ugaauuccggaucuugaaucuu.........   14    0   0G2
..............................................................................ugaauuccggaucuugaauUuu.........   20    1   0G2
..............................................................................ugaauuccggaucuugaaucuuA........   29    1   0G2
..............................................................................ugaauuccggaucuugaaucuuU........   21    1   0G2
..............................................................................ugaauuccggaucuugaaucuug........   3     0   0G2
...............................................................................gaauuccggaucuugaau............   10    0   0G2
...............................................................................gaauuccggaucuAgaau............   15    1   0G2
...............................................................................gaauuccggaucuugaauc...........   8     0   0G2
...............................................................................gaauuccggaucuugaaucu..........   105   0   0G2
...............................................................................gaauuccggaucuugaaucG..........   1     1   0G2
...............................................................................gaauuccggaucuugaaucA..........   103   1   0G2
...............................................................................gaauuccggaucuugaaucuA.........   51    1   0G2
...............................................................................gaauuccggaucuugaauUuu.........   8     1   0G2
...............................................................................gaauuccggaucuugaaucuuA........   4     1   0G2
...............................................................................gaauuccggaucuugaauUuug........   2     1   0G2
...............................................................................gaauuccggaucuugaaucuuU........   17    1   0G2
................................................................................aauuccggaucuugaaucA..........   19    1   0G2
................................................................................aauuccggaucuugaGucuu.........   10    1   0G2
................................................................................aauuccggaucuugaaucuu.........   46    0   0G2
................................................................................aauuccggaucuAgaaucuu.........   6     1   0G2
................................................................................aauuccggaucuugaauUuu.........   24    1   0G2
................................................................................aauuccggaucuugaaucAu.........   2     1   0G2
................................................................................aauuccggUucuugaaucuug........   6     1   0G2
................................................................................aauuccggaucuugaaucuug........   60    0   0G2
................................................................................aauuccggaucuugaaCcuug........   8     1   0G2
................................................................................aauuccggaucuugaaucuuA........   66    1   0G2
................................................................................aauuccggaucuugaauUuug........   28    1   0G2
.................................................................................auuccggaucuugaaucu..........   9     0   0G2
.................................................................................auuccggaucuugaaucA..........   12    1   0G2
.................................................................................auuccggaucuugaaucuA.........   4     1   0G2
.................................................................................auuccggaucuugaaucAu.........   1     1   0G2
.................................................................................auuccggaucuugaauUuu.........   2     1   0G2
.................................................................................auuccggaucuugaaucuu.........   24    0   0G2
.................................................................................Uuuccggaucuugaaucuug........   2     1   0G2
.................................................................................auuccggaucuugaaucuug........   112   0   0G2
.................................................................................auuccggaucuugaaucuuA........   53    1   0G2
.................................................................................auuccggaucuugaaucuuU........   25    1   0G2
.................................................................................auuccggaucuugaaCcuug........   7     1   0G2
.................................................................................auuccggaucuugaauUuug........   16    1   0G2
.................................................................................auuccggaucuGgaaucuug........   1     1   0G2
.................................................................................auuccggGucuugaaucuug........   4     1   0G2
.................................................................................auuccggaucuugaaucGug........   1     1   0G2
.................................................................................auuccggaucuugaaucuuUg.......   3     1   0G2
.................................................................................auuccggaucuugaaucuuCg.......   3     1   0G2
..................................................................................uuccggaucuugaaucuuA........   5     1   0G2
..................................................................................uuccggaucuugaaucGug........   11    1   0G2
..................................................................................uuccggaucuugaaucuug........   3     0   0G2
..................................................................................uuccggaucuugaaucuuCg.......   7     1   0G2

.............ucuugaauucgggaucuugaauAa........................................................................   1     1   0A2
...............uugaauucgggaucuugaauG.........................................................................   1     1   0A2
...............uugaauucgggaucuugaauA.........................................................................   2     1   0A2
...............uugaauucgggaucuugaauua........................................................................   2     0   0A2
................ugaauucgggaucuugaauu.........................................................................   1     0   0A2
................ugaauucgggaucuugaauua........................................................................   2     0   0A2
.................gaauucgggaucuugaauua........................................................................   1     0   0A2
.................gaauucgggaucuAgaauua........................................................................   1     1   0A2
.................gaauucgggaucuugaauuag.......................................................................   1     0   0A2
.................gaauucgggaucuAgaauuag.......................................................................   1     1   0A2
...................auucgggaucuAgaauuagu......................................................................   9     1   0A2
...................auucgggaucuAgaauuagug.....................................................................   7     1   0A2
...................Nuucgggaucuugaauuagug.....................................................................   2     1   0A2
...................auucgggaucuugaauuagug.....................................................................   1     0   0A2
...................Nuucgggaucuugaauuagugug...................................................................   2     1   0A2
....................uucgggaucuugaauuag.......................................................................   2     0   0A2
....................uucgggaucuugaauuagG......................................................................   12    1   0A2
....................uucgggaucuGgaauuagug.....................................................................   22    1   0A2
....................uucgggaucuugaauuagug.....................................................................   66    0   0A2
```

Star **Mature**

```
cuuagaucuugaaucuugaauucgggaucuugaauuagugugaaucuugauuucgggaucuugaauuagugugaaucuugaauuccggaucuugaaucuuggaucuuga
....................uucgggaucuugaauuGgug.....................................................................   10   1   0A2
....................uucgggaucuugaauuagGg.....................................................................   6    1   0A2
....................uucgggaucuugaauuagugug...................................................................   16   0   0A2
....................uucgggaucuugaauuaguguAa..................................................................   10   1   0A2
.....................ucgggaucuugaauuagug.....................................................................   20   0   0A2
.....................ucgggaucuGgaauuagug.....................................................................   12   1   0A2
.....................ucgggaucuugaauuagGg.....................................................................   2    1   0A2
.....................ucgggaucuGgaauuagugug...................................................................   2    1   0A2
.....................ucgggaucuugaauuagugug...................................................................   2    0   0A2
......................cgggaucuugaauuagug.....................................................................   24   0   0A2
......................cgggaucuugaauuagGgu....................................................................   2    1   0A2
......................cgggaucuugaGuuagugug...................................................................   2    1   0A2
......................cgggaucuugaauuagugug...................................................................   48   0   0A2
......................cgggauGuugaauuagugugaa.................................................................   2    1   0A2
......................cgggaucuugaauuagugugaa.................................................................   20   0   0A2
......................cgggaucuugaauuagugugaaA................................................................   10   1   0A2
......................cgggaucuugaauuagugugaauA...............................................................   14   1   0A2
......................cgggaucuugaauuagugugaaucA..............................................................   16   1   0A2
.......................Nggaucuugaauuagugug...................................................................   2    1   0A2
.......................Nggaucuugaauuagugugaa.................................................................   2    1   0A2
........................ggaucuugaauuagugug...................................................................   20   0   0A2
........................ggaucuGgaauuagugug...................................................................   12   1   0A2
........................ggaucuugaauuaguguAa..................................................................   6    1   0A2
........................ggaucuGgaauuagugugaa.................................................................   8    1   0A2
........................ggaucuugaauuaguguAaa.................................................................   6    1   0A2
........................ggaucuugaauuagugugaa.................................................................   20   0   0A2
........................ggaucuugaauuagugugaaA................................................................   12   1   0A2
.........................gaucuGgaauuagugugaauc...............................................................   12   1   0A2
.........................gaucuugaauuagugugaauc...............................................................   4    0   0A2
.........................gaucuGgaauuagugugaaucu..............................................................   4    1   0A2
..........................aucuGgaauuagugugaauc...............................................................   6    1   0A2
..........................aucuGgaauuagugugaaucu..............................................................   68   1   0A2
..........................aucuugaauuagugugaaucu..............................................................   4    0   0A2
...........................ucuGgaauuagugugaauc...............................................................   14   1   0A2
...........................ucuugaauuagugugaaucu..............................................................   180  0   0A2
...........................ucuGgaauuagugugaaucu..............................................................   142  1   0A2
...........................ucuugaauuagugugaaucuu.............................................................   2    0   0A2
...........................ucuGgaauuagugugaaucuu.............................................................   18   1   0A2
............................cuugaauuagugugaaucu..............................................................   6    0   0A2
............................cuGgaauuagugugaaucu..............................................................   24   1   0A2
............................cuGgaauuagugugaaucuu.............................................................   12   1   0A2
.............................uugaauuagugugaaucuu.............................................................   14   0   0A2
.............................uugaauuagugugaaucuug............................................................   58   0   0A2
.............................uugaaCuagugugaaucuuga...........................................................   2    1   0A2
.............................uugaauCagugugaaucuuga...........................................................   2    1   0A2
.............................uugaauuagugugaaucuuga...........................................................   154  0   0A2
.............................Nugaauuagugugaaucuuga...........................................................   4    1   0A2
.............................uNgaauuagugugaaucuuga...........................................................   2    1   0A2
.............................uugaauuagugugaaucuugauu.........................................................   6    0   0A2
..............................ugaauuagugugaaucuuga...........................................................   2    0   0A2
...............................gaauuagugugaaucuug............................................................   4    0   0A2
.................................auuagugugaaucuCgauuuc.......................................................   7    1   0A2
.................................auuagugugaaucuugauuucg......................................................   5    0   0A2
.................................auuagugugaaucuCgauuucg......................................................   2    1   0A2
..................................uuagugugaaucuugauuuc.......................................................   7    0   0A2
..................................uuagugugaaucuugauuucg......................................................   4    0   0A2
..................................uuagugugaaucuugauuucgg.....................................................   4    0   0A2
..................................uuagugugaaucuugauuucgggauc.................................................   3    0   0A2
..................................uuagugugaaucuugauuucgggaucu................................................   9    0   0A2
...................................uagugugaaucuugauuucg......................................................   7    0   0A2
...................................uagugugaaucuugauuucggg....................................................   9    0   0A2
............................................ucuugauuucgggaucuugaau...........................................   5    0   0A2
.............................................cuugauuucgggaucuGgaauua.........................................   5    1   0A2
..............................................uugauuucgggaucuugaau...........................................   15   0   0A2
..............................................uugauuucgggaucuugaauua.........................................   9    0   0A2
..............................................uugauuucgggaucuugaauuag........................................   5    0   0A2
...............................................ugauuucgggaucuugaau...........................................   5    0   0A2
...............................................ugauuucgggaucuugaauua.........................................   9    0   0A2
.................................................auuucggCaucuugaauuag........................................   1    1   0A2
.................................................auuucgggaucuugaauuag........................................   9    0   0A2
```

Star **Mature**

```
cuuagaucuugaaucuugaauucgggaucuugaauuagugugaaucuugauuucgggaucuugaauuagugugaaucuugaauuccggaucuugaaucuuggaucuuga
.................................................auuucgggaucuGgaauuag........................................ 1 1 0A2
.................................................auuucgggaucuugaauuagug...................................... 4 0 0A2
..................................................uuucgggaucuugaauua......................................... 5 0 0A2
..................................................uuucgggaucuugaauuagu....................................... 11 0 0A2
..................................................uGucgggaucuugaauuagug...................................... 1 1 0A2
..................................................uuucgggaucuugGauuagug...................................... 1 1 0A2
..................................................uuucgggaucuGgaauuagug...................................... 22 1 0A2
..................................................uuucgggaucuugaauuGgug...................................... 4 1 0A2
..................................................Nuucgggaucuugaauuagug...................................... 2 1 0A2
..................................................uuucgggaucuugaauuagug...................................... 141 0 0A2
..................................................Nuucgggaucuugaauuagugug.................................... 2 1 0A2
..................................................uuucgggaucuugaauuagugug.................................... 22 0 0A2
...................................................uucgggaucuugaauuag........................................ 2 0 0A2
...................................................uucgggaucuugaauuagG....................................... 12 1 0A2
...................................................uucgggaucuugaauuagGg...................................... 6 1 0A2
...................................................uucgggaucuugaauuagug...................................... 66 0 0A2
...................................................uucgggaucuGgaauuagug...................................... 22 1 0A2
...................................................uucgggaucuugaauuGgug...................................... 10 1 0A2
...................................................uucgggaucuugaauuagugug.................................... 16 0 0A2
...................................................uucgggaucuugaauuaguguAa................................... 10 1 0A2
....................................................ucgggaucuugaauuagGg...................................... 2 1 0A2
....................................................ucgggaucuGgaauuagug...................................... 12 1 0A2
....................................................ucgggaucuugaauuagug...................................... 20 0 0A2
....................................................ucgggaucuGgaauuagugug.................................... 2 1 0A2
....................................................ucgggaucuugaauuagugug.................................... 2 0 0A2
.....................................................cgggaucuugaauuagug...................................... 24 0 0A2
.....................................................cgggaucuugaauuagGgu..................................... 2 1 0A2
.....................................................cgggaucuugaGuuagugug.................................... 2 1 0A2
.....................................................cgggaucuugaauuagugug.................................... 48 0 0A2
.....................................................cgggaucuugaauuagugugaa.................................. 20 0 0A2
.....................................................cgggauGuugaauuagugugaa.................................. 2 1 0A2
.....................................................cgggaucuugaauuagugugaaA................................. 10 1 0A2
.....................................................cgggaucuugaauuagugugaauA................................ 14 1 0A2
.....................................................cgggaucuugaauuagugugaaucA............................... 16 1 0A2
......................................................Nggaucuugaauuagugug.................................... 2 1 0A2
......................................................Nggaucuugaauuagugugaa.................................. 2 1 0A2
.......................................................ggaucuGgaauuagugug.................................... 12 1 0A2
.......................................................ggaucuugaauuagugug.................................... 20 0 0A2
.......................................................ggaucuugaauuaguguAa................................... 6 1 0A2
.......................................................ggaucuGgaauuagugugaa.................................. 8 1 0A2
.......................................................ggaucuugaauuagugugaa.................................. 20 0 0A2
.......................................................ggaucuugaauuaguguAaa.................................. 6 1 0A2
.......................................................ggaucuugaauuagugugaaA................................. 12 1 0A2
........................................................gaucuGgaauuagugugaauc................................ 12 1 0A2
........................................................gaucuugaauuagugugaauc................................ 4 0 0A2
........................................................gaucuGgaauuagugugaaucu............................... 4 1 0A2
.........................................................aucuGgaauuagugugaauc................................ 6 1 0A2
.........................................................aucuGgaauuagugugaaucu............................... 68 1 0A2
.........................................................aucuugaauuagugugaaucu............................... 4 0 0A2
..........................................................ucuGgaauuagugugaauc................................ 14 1 0A2
..........................................................ucuugaauuagugugaaucu............................... 180 0 0A2
..........................................................ucuGgaauuagugugaaucu............................... 142 1 0A2
..........................................................ucuugaauuagugugaaucuu.............................. 2 0 0A2
..........................................................ucuGgaauuagugugaaucuu.............................. 18 1 0A2
...........................................................cuugaauuagugugaaucu............................... 6 0 0A2
...........................................................cuGgaauuagugugaaucu............................... 24 1 0A2
...........................................................cuGgaauuagugugaaucuu.............................. 12 1 0A2
............................................................uugaauuagugugaaucuu.............................. 14 0 0A2
............................................................uugaauuagugugaaucuug............................. 58 0 0A2
............................................................uugaauCagugugaaucuuga............................ 2 1 0A2
............................................................uNgaauuagugugaaucuuga............................ 2 1 0A2
............................................................uugaauuagugugaaucuuga............................ 154 0 0A2
............................................................Nugaauuagugugaaucuuga............................ 4 1 0A2
............................................................uugaaCuagugugaaucuuga............................ 2 1 0A2
............................................................uugaauuagugugaaucuugaa........................... 12 0 0A2
............................................................uugaauuagugugaaucuugaau.......................... 5 0 0A2
............................................................uugaauuagugugaaucuugaauA......................... 5 1 0A2
.............................................................ugaauuagugugaaucuuga............................ 2 0 0A2
.............................................................Cgaauuagugugaaucuugaa........................... 1 1 0A2
.............................................................ugaauuagugugaaucuugaa........................... 1 0 0A2
```

Star **Mature**

cuuagaucuugaaucuugaauucgggaucuugaauuagugugaaucuugauuucgggaucuugaauuagugugaaucuugaauuccggaucuugaaucuuggaucuuga			
....ugaauuagugugaaucuugaauuAcg......................	9	1	0A2
....gaauuagugugaaucuug.............................	4	0	0A2
....Caauuagugugaaucuugaa...........................	1	1	0A2
....gaauuagugugaaucuugaa...........................	13	0	0A2
....aauuagugugaaucuugaau..........................	4	0	0A2
....aauuagugugaaucuugaauu.........................	9	0	0A2
....aauuagugugaaucuugaauA.........................	30	1	0A2
....aGuuagugugaaucuugaauu.........................	2	1	0A2
....aauuagugugaaucuugaauuc........................	1	0	0A2
....auuagugugaaucuugaauu.........................	28	0	0A2
....auuagugugaaucuugaauA.........................	3	1	0A2
....Cuuagugugaaucuugaauuc........................	1	1	0A2
....auuagugugaaucuugaauuc........................	24	0	0A2
....uuagugugaaucuugaauu.........................	14	0	0A2
....uuagugugaaucuugaauuc........................	66	0	0A2
....uuagugugaaucuugaauucc.......................	1	0	0A2
....uuagugugaGucuugaauuccgga....................	3	1	0A2
....uuagugugaaucuugaauuUcggauc..................	5	1	0A2
....uuagugugaaucuugaaCuccggaucu.................	3	1	0A2
....uuagugugaaucuugaauuccggaucA.................	4	1	0A2
....uuagugugaaucuugaauuUcggaucu.................	4	1	0A2
....uagugugaaucuugaauu.........................	2	0	0A2
....uagugugaaucuugaauuc........................	12	0	0A2
....uagugugaGucuugaauucc.......................	2	1	0A2
....uaguguUaaucuugaauucc.......................	1	1	0A2
....uagugugaaucuugaauucc.......................	21	0	0A2
....uagugugaaucuugaaCuccg......................	5	1	0A2
....uaguguCaaucuugaauuccg......................	4	1	0A2
....uagugugaaucuugaauuUcgg.....................	1	1	0A2
....uagugugaaucuugaauuccggauc..................	3	0	0A2
....uagugugaaucuugaauuccggaucuA................	1	1	0A2
....agugugaaucuugaauuc........................	1	0	0A2
....agugugaaucuugaauucc.......................	1	0	0A2
....aguguCaaucuugaauucc.......................	1	1	0A2
....agugugaGucuugaauuccgga....................	2	1	0A2
....ugugaaucuugaaCuccgga....................	2	1	0A2
....ugugUaucuugaauuccgga....................	2	1	0A2
....ugaaucuugaauuccggau...................	3	0	0A2
....ugaaucuugaauuccggauA..................	12	1	0A2
....ugaaucuugaauuccggauc..................	3	0	0A2
....ugaGucuugaauuccggaucu.................	2	1	0A2
....ugaCucuugaauuccggaucuug...............	1	1	0A2
....ugaaucuugaauuccggaucuAgaa.............	11	1	0A2
....ugaaucuugaauuccggaucuugaau............	3	0	0A2
....ugaaucuugaauuccggaucuAgaau............	3	1	0A2
....aaucuugaauuccggaucuA................	1	1	0A2
....aaucuugaauuccggaucuug...............	11	0	0A2
....aaucuugaauuccggaucuAg...............	2	1	0A2
....aucuugaauuccggaucuu................	6	0	0A2
....aucuugaauuccggaucuug...............	3	0	0A2
....aucGugaauuccggaucuug...............	3	1	0A2
....ucuugaauuccggaucuA................	1	1	0A2
....ucuugaauuccggaucuug...............	6	0	0A2
....ucuugaauuccggaucuuga..............	7	0	0A2
....ucuugaauuccggaucuAga..............	40	1	0A2
....ucGugaauuccggaucuugaa.............	26	1	0A2
....ucuugaauuccggaucuCgaa.............	1	1	0A2
....ucuugaauuccggaucuAgaa.............	55	1	0A2
....ucuugaauuccggaucuugaa.............	22	0	0A2
....Ccuugaauuccggaucuugaa.............	5	1	0A2
....ucGugaauuccggaucuugaau............	34	1	0A2
....Ccuugaauuccggaucuugaau............	1	1	0A2
....ucuugaauuccggaucuugaau............	184	0	0A2
....ucuugaauuccggaucuAgaau............	12	1	0A2
....ucuugaauuccggaucuugaauUuu.........	2	1	0A2
....ucuugaauuccggaucuugaaucuuCg.......	11	1	0A2
....ucuugaauuccggaucuugaaucuuUg.......	5	1	0A2
....cuugaauuccggaucuAga..............	17	1	0A2
....cGugaauuccggaucuugaa.............	2	1	0A2
....cuugaauuccggaucuugaa.............	95	0	0A2

Star Mature

cuuagaucuugaaucuugaauucgggaucuugaauuagugugaaucuugauuucgggaucuugaauuagugugaaucuugaauuccggaucuugaaucuuggaucuuga			
....cuugaauuccggaucuAgaa..............	291	1	0A2
....Nuugaauuccggaucuugaa..............	1	1	0A2
....cuugaGuuccggaucuugaa..............	1	1	0A2
....cuugaauuccggaucuugaau.............	152	0	0A2
....cGugaauuccggaucuugaau.............	29	1	0A2
....cuugaauuccggaucuAgaau.............	42	1	0A2
....cuugaauuccggaucuugaauc............	4	0	0A2
....cuugaauuccggaucuugaaucA...........	24	1	0A2
....cuugaauuccggaucuugaaucu...........	7	0	0A2
....cuugaauuccggaucuugaauUuu..........	3	1	0A2
....uugaauuccggaucuAga...............	6	1	0A2
....uugaauuccggaucuAgaa..............	192	1	0A2
....uugaauuccggaucuugaa..............	49	0	0A2
....uugaauuccggaucuugaaC.............	2	1	0A2
....uugaauuccggaucuAgaau.............	1577	1	0A2
....uugaauuccggGucuugaau.............	13	1	0A2
....Cugaauuccggaucuugaau.............	3	1	0A2
....uugaauuccggaucCugaau.............	3	1	0A2
....uugaauuccggaucuGgaau.............	4	1	0A2
....Gugaauuccggaucuugaau.............	18	1	0A2
....uugaauuAcggaucuugaau.............	3	1	0A2
....uugaauuccggauAuugaau.............	2	1	0A2
....Augaauuccggaucuugaau.............	2	1	0A2
....uugaauAccggaucuugaau.............	5	1	0A2
....uugaGuuccggaucuugaau.............	81	1	0A2
....uugaauuccggaucuugaaG.............	1	1	0A2
....uugaauuccggaucuugUau.............	1	1	0A2
....uugaauuccggaCcuugaau.............	4	1	0A2
....Nugaauuccggaucuugaau.............	15	1	0A2
....uugaauuccggaucuCgaau.............	2	1	0A2
....uugaauuccggCucuugaau.............	1	1	0A2
....uugaaGuccggaucuugaau.............	8	1	0A2
....uNgaauuccggaucuugaau.............	3	1	0A2
....uugaauuccggaucuugaau.............	7322	0	0A2
....uugaauuccggaucAugaau.............	1	1	0A2
....uugaauuccggaGcuugaau.............	7	1	0A2
....uugaauuccggaucuugaGuc............	1	1	0A2
....uugaauuccggaucuugaaGc............	1	1	0A2
....uugaauuccggaucuugaaCc............	3	1	0A2
....uugaGuuccggaucuugaauc............	5	1	0A2
....uGgaauuccggaucuugaauc............	1	1	0A2
....uugaaCuccggaucuugaauc............	1	1	0A2
....uugaauuccggaucuAgaauc............	11	1	0A2
....uugaauGccggaucuugaauc............	1	1	0A2
....Cugaauuccggaucuugaauc............	2	1	0A2
....uugaauuAcggaucuugaauc............	1	1	0A2
....uugaauuccggCucuugaauc............	1	1	0A2
....uugaauuccggaucuugaauc............	499	0	0A2
....uugaauuccggaucuAgaaucu...........	5	1	0A2
....uugaauuccggaucuugaaucA...........	1979	1	0A2
....uugaauuccggaucuugaaCcu...........	2	1	0A2
....uugaauuccggaucuugaaucu...........	28	0	0A2
....uugaauuccggaucuugaauUuu..........	29	1	0A2
....uugaauuccggaucuugaaucuA..........	4	1	0A2
....uugaauuccggaucuugaaucuu..........	4	0	0A2
....uugaauuccggaucuugaaucAu..........	28	1	0A2
....uugaauuccggaucuugaaucuuA.........	13	1	0A2
....ugaauuccggaucuAgaa..............	49	1	0A2
....ugaauuccggGucuugaau.............	3	1	0A2
....ugaauuccggaucuAgaau.............	86	1	0A2
....ugaauuccggaGcuugaau.............	1	1	0A2
....ugaauuccggaucuugaau.............	317	0	0A2
....ugaGuuccggaucuugaau.............	6	1	0A2
....ugaauuccggaucuugaaG.............	1	1	0A2
....Agaauuccggaucuugaau.............	1	1	0A2
....ugaauuccggaucuAgaauc............	2	1	0A2
....ugaauuccggaucuugaauc............	161	0	0A2
....ugaGuuccggaucuugaaucu...........	2	1	0A2
....ugaauucGggaucuugaaucu...........	1	1	0A2
....ugaauuccggaucuugaaucA...........	317	1	0A2

```
                                                         Star                       Mature
cuuagaucuugaaucuugaauucgggaucuugaauuagugugaaucuugauuucgggaucuugaauuagugugaaucuugaauuccggaucuugaaucuuggaucuuga
..............................................................................ugaauuccggaucuugaaucu..........   245   0   0A2
..............................................................................ugaauuccggaucuugaauUuu.........   12    1   0A2
..............................................................................ugaauuccggaucuugaaucuu.........   6     0   0A2
..............................................................................ugaauuccggaucuugaaucuA.........   125   1   0A2
..............................................................................ugaauuccggaucuugaaucAu.........   21    1   0A2
..............................................................................ugaauuccggaucuugaauUuug........   4     1   0A2
..............................................................................ugaauuccggaucuugaaucuuA........   8     1   0A2
..............................................................................ugaauuccggaucuugaaucuuU........   6     1   0A2
..............................................................................ugaauuccggaucuugaaucuug........   5     0   0A2
..............................................................................ugaauuccggaucuugaaucAug........   1     1   0A2
...............................................................................gaauuccggaucuAgaau............   7     1   0A2
...............................................................................gaauuccggaucuugaau............   21    0   0A2
...............................................................................gaauuccggaucuugaauc...........   15    0   0A2
...............................................................................gaauucUggaucuugaauc...........   1     1   0A2
...............................................................................gaauuccggaucuugaaucu..........   61    0   0A2
...............................................................................gaauuccggaucuugaaucA..........   84    1   0A2
...............................................................................gaauuccggaucuugaaucG..........   3     1   0A2
...............................................................................gaauuccggaucuugaaucuA.........   52    1   0A2
...............................................................................gaauuccggaucuugaaucAu.........   7     1   0A2
...............................................................................gaauuccggaucuugaaucuu.........   13    0   0A2
...............................................................................gaauuccggaucuugaauUuu.........   3     1   0A2
...............................................................................gaauuccggaucuugaaucuuU........   23    1   0A2
...............................................................................gaauuccggaucuugaaucuuA........   10    1   0A2
................................................................................aauuccggaucuugaaucu..........   6     0   0A2
................................................................................aauuccggaucuugaaucA..........   17    1   0A2
................................................................................Nauuccggaucuugaaucuu.........   1     1   0A2
................................................................................aauuccggaucuugaauUuu.........   21    1   0A2
................................................................................aauuccggaucuugaaucuu.........   19    0   0A2
................................................................................aauuccggaucuugaaucuA.........   9     1   0A2
................................................................................aauuccggaucuugaaucuuU........   6     1   0A2
................................................................................aauuccggaucuugaaucuuA........   25    1   0A2
................................................................................aauuccggaucuugaaCcuug........   6     1   0A2
................................................................................aauuccggaucuugaaucuug........   65    0   0A2
................................................................................aauuccggaucuugaGucuug........   2     1   0A2
................................................................................aauuccggaucuugaauUuug........   9     1   0A2
.................................................................................auuccggaucuugaaucA..........   10    1   0A2
.................................................................................auuccggaucuugaauUuu.........   4     1   0A2
.................................................................................auuccggaucuugaaCcuu.........   2     1   0A2
.................................................................................auuccggaucuugaaucuu.........   2     0   0A2
.................................................................................auuccggGucuugaaucuu.........   4     1   0A2
.................................................................................auuccggaucuugaaucGu.........   3     1   0A2
.................................................................................auuccggaucuugaaucuA.........   8     1   0A2
.................................................................................auuccggaucuugaauUuug........   33    1   0A2
.................................................................................auuccggaucuugaaucuug........   111   0   0A2
.................................................................................auuccggaucuugaaucuuA........   79    1   0A2
.................................................................................auuccggaucuugaaucuuU........   4     1   0A2
.................................................................................auuccggaucuugaaucGug........   4     1   0A2
.................................................................................auuccggaucuugaaCcuug........   2     1   0A2
.................................................................................Cuuccggaucuugaaucuug........   1     1   0A2
.................................................................................auuccggaucuugaGucuug........   3     1   0A2
.................................................................................auuccgUaucuugaaucuug........   3     1   0A2
.................................................................................auuccggaucuugaauUuugg.......   5     1   0A2
..................................................................................uuccggaucuugaauUuug........   2     1   0A2
..................................................................................uuccggaucuugaaucuug........   5     0   0A2
..................................................................................uuccggaucuugaaucuuA........   9     1   0A2
..................................................................................uuccggaucuugaaucuuUg.......   3     1   0A2
...................................................................................uccggaucuugaaucuuCgauc....   2     1   0A2
.....................................................................................cggaucuugaaucuuggaucA...   1     1   0A2
........................................................................................aucuugaaucuuggaucuuA.   5     1   0A2
```

Provisional ID	:	scaffold92_18022
Score total	:	0
Score for star read(s)	:	-1.3
Score for read counts	:	0
Score for mfe	:	0.2
Score for randfold	:	1.6
Score for cons. seed	:	-0.6
Total read count	:	412739
Mature read count	:	412739
Loop read count	:	0
Star read count	:	0

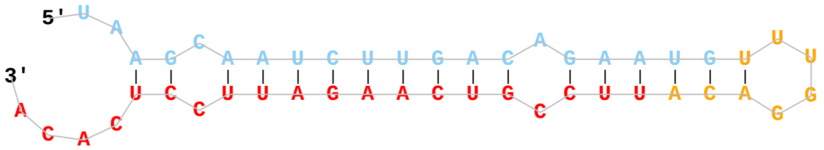

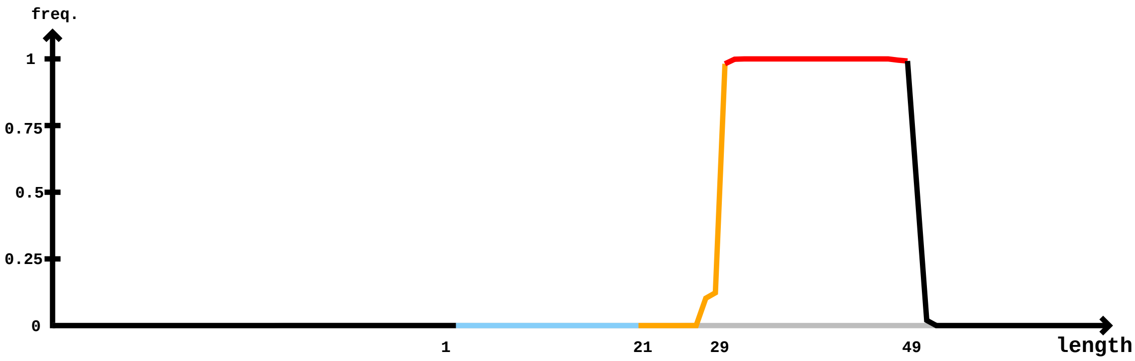

Star Mature

```
5'- ggauucaaagucgcgauucaaaucaaaacugugcuucuuauaagcaaucuugacagaauguuuggacauuccgucaagauuccucacaaggacugcaggaaagggaag   -3'    exp
    ...(((....((..(((....)))....((((..(((((.(.((.(((((((((.((((((....)))))).))))))))).)).).)))))..))))))....))).   reads   mm    sample
    ......................ucaaaacugugcuucuuauC..................................................................   26      1     0B2
    ......................ucaaaacugugcuucuuauCa.................................................................   3       1     0B2
    .......................caaaacugugcuucuuauCa.................................................................   5       1     0B2
    ..............................................................ugUacauuccgucaagauuccucaca....................   2       1     0B2
    .................................................................acauuccgucaagauuccucacU....................   3       1     0B2
    ..................................................................cauuccgucaagauuccuc.......................   2       0     0B2
    ..................................................................Nauuccgucaagauuccuca......................   1       1     0B2
    ..................................................................cauuccgucaagauuccuca......................   59      0     0B2
    ..................................................................cauuccgucaagauuccucU......................   17      1     0B2
    ..................................................................cauuccgucaagauuccucUc.....................   5       1     0B2
    ..................................................................cauuccgucaagauuccucac.....................   79      0     0B2
    ..................................................................cauuccgCcaagauuccucac.....................   1       1     0B2
    ..................................................................cauuccgucaagauuccucaA.....................   10      1     0B2
    ..................................................................cauuccgucaagauuccucCca....................   1       1     0B2
    ..................................................................cauuccCucaagauuccucaca....................   1       1     0B2
    ..................................................................cauuccgGcaagauuccucaca....................   1       1     0B2
    ..................................................................cauuccgucaagauuccucacG....................   1       1     0B2
    ..................................................................cauuccgucGagauuccucaca....................   1       1     0B2
    ..................................................................cauuccgucaagauuccucUca....................   1       1     0B2
    ..................................................................Nauuccgucaagauuccucaca....................   14      1     0B2
    ..................................................................cauuccgucaagauuccucacC....................   18      1     0B2
    ..................................................................Gauuccgucaagauuccucaca....................   1       1     0B2
    ..................................................................cauuccgucaagauuccCcaca....................   3       1     0B2
    ..................................................................cauuccgucaagauuccucaGa....................   1       1     0B2
    ..................................................................Uauuccgucaagauuccucaca....................   1       1     0B2
    ..................................................................cauuccUucaagauuccucaca....................   1       1     0B2
    ..................................................................cauuccgucaagauuccucaAa....................   3       1     0B2
    ..................................................................cauuccgucaagauuccucaca....................   1701    0     0B2
    ..................................................................cauuccgucaagauuccucacU....................   6265    1     0B2
    ..................................................................cauCccgucaagauuccucaca....................   1       1     0B2
    ..................................................................cauuccgucaagauuccucacUa...................   89      1     0B2
    ..................................................................cauuccgucaagauuccucacaU...................   11      1     0B2
    ..................................................................cauuccgucaagauuccucacCa...................   1       1     0B2
    ..................................................................cauuccgucaagauuccucacaa...................   3       0     0B2
```

```
                                        Star                          Mature
ggauucaaagucgcgauucaaaucaaaacugugcuucuuauaagcaaucuugacagaauguuuggacauuccgucaagauuccucacaaggacugcaggaaagggaag
..................................................................cauuccgucaagauuccucacaaU..................  1      1  0B2
...................................................................auuccgucaagauuccuca......................  16     0  0B2
...................................................................auuccgucaagauuccucU......................  16     1  0B2
...................................................................auuccgucaagauuccucac.....................  29     0  0B2
...................................................................Nuuccgucaagauuccucac.....................  1      1  0B2
...................................................................auuAcgucaagauuccucaca....................  1      1  0B2
...................................................................auuccgucaagauuccucacU....................  1423   1  0B2
...................................................................auuccgucaagauuccucacG....................  1      1  0B2
...................................................................auuccgucaagauuccucaca....................  484    0  0B2
...................................................................auuccgucaagauuccCcaca....................  1      1  0B2
...................................................................Cuuccgucaagauuccucaca....................  30     1  0B2
...................................................................auuccgucaCgauuccucaca....................  1      1  0B2
...................................................................auuccgucaagauuccucacC....................  10     1  0B2
...................................................................Nuuccgucaagauuccucaca....................  3      1  0B2
...................................................................auuccgucaagauCccucaca....................  1      1  0B2
...................................................................Cuuccgucaagauuccucacaa...................  3      1  0B2
...................................................................auuccgucaagauuccucacaa...................  4      0  0B2
...................................................................auuccgucaagauuccucacUa...................  21     1  0B2
...................................................................auuccgucaagauuccucacaaU..................  15     1  0B2
...................................................................auuccgucaagauuccucacaaAg.................  1      1  0B2
....................................................................uuccgucaagauuccuca......................  237    0  0B2
....................................................................uuccgucaagauuccucU......................  124    1  0B2
....................................................................uuccAucaagauuccucac.....................  1      1  0B2
....................................................................uuccgucaagauuccucac.....................  346    0  0B2
....................................................................uuccgucaagauuccucUc.....................  2      1  0B2
....................................................................uuccgucaagauuccucaA.....................  1      1  0B2
....................................................................Nuccgucaagauuccucac.....................  3      1  0B2
....................................................................uuccgucaagaCuccucac.....................  3      1  0B2
....................................................................uuccgucaagauuccucGc.....................  1      1  0B2
....................................................................uuccUucaagauuccucaca....................  4      1  0B2
....................................................................uuccgucaagauuccucCca....................  2      1  0B2
....................................................................uuccgucaagauuccuGaca....................  2      1  0B2
....................................................................uuccgucaagauuccCcaca....................  23     1  0B2
....................................................................uuccgucaagauucGucaca....................  1      1  0B2
....................................................................uuccgucaagaGuccucaca....................  2      1  0B2
....................................................................uuccgucaagaCuccucaca....................  5      1  0B2
....................................................................uuccgucaagauuccGcaca....................  1      1  0B2
....................................................................uuccgucaagauuccucUca....................  6      1  0B2
....................................................................uuccgucaagauuccucaAa....................  14     1  0B2
....................................................................uuccAucaagauuccucaca....................  10     1  0B2
....................................................................uCccgucaagauuccucaca....................  5      1  0B2
....................................................................uuUcgucaagauuccucaca....................  1      1  0B2
....................................................................Nuccgucaagauuccucaca....................  180    1  0B2
....................................................................uuccgucaagauuccAcaca....................  1      1  0B2
....................................................................uuccgAcaagauuccucaca....................  1      1  0B2
....................................................................uuccgucCagauuccucaca....................  2      1  0B2
....................................................................uuccgucaagauucAucaca....................  1      1  0B2
....................................................................uNccgucaagauuccucaca....................  1      1  0B2
....................................................................uuccgGcaagauuccucaca....................  16     1  0B2
....................................................................uuccgucaagauuccucaGa....................  1      1  0B2
....................................................................Auccgucaagauuccucaca....................  1      1  0B2
....................................................................uuccgucaagauuccucacU....................  82955  1  0B2
....................................................................uuccgucGagauuccucaca....................  5      1  0B2
....................................................................uuccgucaagauuccucaca....................  23202  0  0B2
....................................................................uuccgucaagauCccucaca....................  4      1  0B2
....................................................................uAccgucaagauuccucaca....................  1      1  0B2
....................................................................uuccgucaagauGccucaca....................  7      1  0B2
....................................................................uuccgucaagauuccucGca....................  1      1  0B2
....................................................................uuccCucaagauuccucaca....................  1      1  0B2
....................................................................uucAgucaagauuccucaca....................  4      1  0B2
....................................................................uuAcgucaagauuccucaca....................  5      1  0B2
....................................................................uuccgucaagGuuccucaca....................  4      1  0B2
....................................................................uuccgucaaUauuccucaca....................  5      1  0B2
....................................................................uuccgucaCgauuccucaca....................  1      1  0B2
....................................................................uuccgucaaAauuccucaca....................  1      1  0B2
....................................................................uucUgucaagauuccucaca....................  11     1  0B2
....................................................................uuccgucaagauuccuUaca....................  1      1  0B2
....................................................................uuccgucaGgauuccucaca....................  36     1  0B2
....................................................................uuccguAaagauuccucaca....................  7      1  0B2
....................................................................uuccgucaaCauuccucaca....................  1      1  0B2
```

Star Mature

```
ggauucaaagucgcgauucaaaucaaaacugugcuucuuauaagcaaucuugacagaauguuuggacauuccgucaagauuccucacaaggacugcaggaaagggaag
....................................................................uGccgucaagauuccucaca....................    2       1    0B2
....................................................................uuccgCcaagauuccucaca....................    4       1    0B2
....................................................................uuccguUaagauuccucaca....................    2       1    0B2
....................................................................Cuccgucaagauuccucaca....................    7       1    0B2
....................................................................Guccgucaagauuccucaca....................    3       1    0B2
....................................................................uuccgucaagauuccucacC....................    154     1    0B2
....................................................................uuccgucaagauuccucacG....................    35      1    0B2
....................................................................Nuccgucaagauuccucacaa...................    20      1    0B2
....................................................................uuccgucaagauuccucacaa...................    2069    0    0B2
....................................................................uuccgucaGgauuccucacaa...................    1       1    0B2
....................................................................uuccgucaagauuAcucacaa...................    1       1    0B2
....................................................................uuccgucaagauuccucacUa...................    702     1    0B2
....................................................................Cuccgucaagauuccucacaa...................    4       1    0B2
....................................................................uuccgucaagauuccucacGa...................    8       1    0B2
....................................................................uuccgucaagauuccucacaC...................    1       1    0B2
....................................................................uuccgucaagauuccucacCa...................    13      1    0B2
....................................................................uuccgucaagauuccucacaU...................    259     1    0B2
....................................................................uuccgucaagauuccucacaG...................    17      1    0B2
....................................................................uuccgCcaagauuccucacaa...................    1       1    0B2
....................................................................uuccgucaagaGuccucacaa...................    1       1    0B2
....................................................................uuccgucaagauuccCcacaa...................    1       1    0B2
....................................................................uuccgGcaagauuccucacaa...................    2       1    0B2
....................................................................uuccgucaagauucGucacaag..................    1       1    0B2
....................................................................uuccgucaagauuccucacaag..................    61      0    0B2
....................................................................uuccgucaagauuccucacaaU..................    1710    1    0B2
....................................................................uuccgucaagauuccucacaaA..................    69      1    0B2
....................................................................uuccgucaagauuccucacaUg..................    4       1    0B2
....................................................................uuccgucaagauuccucacaaAg.................    3       1    0B2
.....................................................................uccgucaagauuccucacU....................    1472    1    0B2
.....................................................................Nccgucaagauuccucaca....................    5       1    0B2
.....................................................................Gccgucaagauuccucaca....................    1       1    0B2
.....................................................................uccgAcaagauuccucaca....................    1       1    0B2
.....................................................................uccgucaagauuccucacC....................    5       1    0B2
.....................................................................uccgGcaagauuccucaca....................    1       1    0B2
.....................................................................uccgucaagauuccucaca....................    518     0    0B2
.....................................................................uccgucaagauuccGcaca....................    1       1    0B2
.....................................................................uccgucaagauuccCcaca....................    3       1    0B2
.....................................................................uccgucaagauuccucacUa...................    7       1    0B2
.....................................................................uccgucaagauuccucacaa...................    43      0    0B2
.....................................................................uccgucaagauuccucacaag..................    3       0    0B2
.....................................................................uccgucaagauuccucacaaA..................    1       1    0B2
.....................................................................uccgucaagauuccucacaaU..................    37      1    0B2
......................................................................ccgucaagauuccucacU....................    121     1    0B2
......................................................................ccgucaagauuccucaca....................    24      0    0B2
......................................................................ccgucaagauuccucacC....................    1       1    0B2
......................................................................ccgucaagauuccucacaU...................    6       1    0B2
......................................................................ccgucaagauuccucacUa...................    2       1    0B2
......................................................................ccgucaagauuccucacaaU..................    5       1    0B2

......................ucaaaacugugcuucuuauC..................................................................    7       1    0G2
......................ucaaaacugugcuucuuauCa.................................................................    5       1    0G2
.......................caaaacugugcuucuuauC..................................................................    1       1    0G2
.............................................................uugUacauuccgucaagauuccucac.....................    7       1    0G2
................................................................Uacauuccgucaagauuccucaca....................    3       1    0G2
..................................................................cauuccgucaagauuccuc.......................    5       0    0G2
..................................................................cauuccgucaagauuccucU......................    25      1    0G2
..................................................................cauuccgCcaagauuccuca......................    1       1    0G2
..................................................................cauuccgucaagauuccuca......................    73      0    0G2
..................................................................Nauuccgucaagauuccuca......................    1       1    0G2
..................................................................Nauuccgucaagauuccucac.....................    1       1    0G2
..................................................................cauuccgucaagauuccucaA.....................    1       1    0G2
..................................................................cauuccgucaagauuccucac.....................    37      0    0G2
..................................................................cauCccgucaagauuccucaca....................    1       1    0G2
..................................................................cauuccgucaagauuccucacC....................    15      1    0G2
..................................................................cauuccgucaGgauuccucaca....................    4       1    0G2
..................................................................cauuccgucaagauuccucacG....................    1       1    0G2
..................................................................cauuccgGcaagauuccucaca....................    1       1    0G2
..................................................................caCuccgucaagauuccucaca....................    1       1    0G2
..................................................................cauuccgucaagaAuccucaca....................    1       1    0G2
..................................................................cauuccgCcaagauuccucaca....................    1       1    0G2
```

Star Mature

ggauucaaagucgcgauucaaaucaaaacugugcuucuuauaagcaaucuugacagaauguuuggacauuccgucaagauuccucacaaggacugcaggaaagggaag

....cauuccgucaagauuccucacU....................	4263	1	0G2
....Uauuccgucaagauuccucaca....................	1	1	0G2
....Aauuccgucaagauuccucaca....................	12	1	0G2
....Nauuccgucaagauuccucaca....................	8	1	0G2
....cauuccgucaagauuccAcaca....................	1	1	0G2
....cauuccgucaagauuccucaca....................	1616	0	0G2
....caGuccgucaagauuccucaca....................	1	1	0G2
....cauuccgucaagauuccGcaca....................	1	1	0G2
....cauuccgucaagauuccucacaa...................	6	0	0G2
....cauuccgucaagauuccucacUa...................	78	1	0G2
....cauuccgucaagauuccucacCa...................	1	1	0G2
....cauuccgucaagauuccucacaU...................	30	1	0G2
....cauuccgucaagauuccucacaaU..................	4	1	0G2
....cauuccgucaagauuccucacaaA..................	1	1	0G2
....auuccgucaagauuccuca......................	13	0	0G2
....auuccgucaagauuccucac.....................	9	0	0G2
....auuccgucaagauuccucacC....................	1	1	0G2
....auuccgucaagauuccucaca....................	388	0	0G2
....Cuuccgucaagauuccucaca....................	34	1	0G2
....auuccgucaagauuccucacU....................	998	1	0G2
....auuccgucaagauuccucacaU...................	3	1	0G2
....auuccgucaagauuccucacUa...................	14	1	0G2
....auuccgucaagauuccucacaaA..................	2	1	0G2
....auuccgucaagauuccucacaaU..................	19	1	0G2
....uuAcgucaagauuccuca......................	1	1	0G2
....uuccgucaagauuccucU......................	52	1	0G2
....uuccgGcaagauuccuca......................	1	1	0G2
....uuccgucaagauuccuca......................	145	0	0G2
....Nuccgucaagauuccuca......................	1	1	0G2
....Guccgucaagauuccucac.....................	1	1	0G2
....uNccgucaagauuccucac.....................	2	1	0G2
....uuccgGcaagauuccucac.....................	1	1	0G2
....uuccgucaagauuccucac.....................	124	0	0G2
....uuccgucaagauuccucUc.....................	5	1	0G2
....uuccAucaagauuccucaca....................	9	1	0G2
....Nuccgucaagauuccucaca....................	63	1	0G2
....uuccgucaagauCccucaca....................	6	1	0G2
....uuccguAaagauuccucaca....................	6	1	0G2
....uuccgucaaAauuccucaca....................	2	1	0G2
....uuccgucaagauuccucGca....................	5	1	0G2
....uuccgucaagauuAcucaca....................	1	1	0G2
....uuccgGcaagauuccucaca....................	8	1	0G2
....uuccUucaagauuccucaca....................	1	1	0G2
....uuccgucaagauuccucacU....................	51925	1	0G2
....uuccgucaagaCuccucaca....................	6	1	0G2
....uuccgucaagauuccucaca....................	19778	0	0G2
....uNccgucaagauuccucaca....................	19	1	0G2
....uuccgucaagaGuccucaca....................	2	1	0G2
....uuccgucaGgauuccucaca....................	55	1	0G2
....uuccgucaagauAccucaca....................	1	1	0G2
....Guccgucaagauuccucaca....................	11	1	0G2
....uuccgucaagauuccucCca....................	1	1	0G2
....Auccgucaagauuccucaca....................	8	1	0G2
....uuccgucaagGuuccucaca....................	7	1	0G2
....uuccgucaagauuccuUaca....................	7	1	0G2
....uuccgucaagauuccAcaca....................	1	1	0G2
....uuccguUaagauuccucaca....................	3	1	0G2
....uuccgucaagauuccuGaca....................	1	1	0G2
....uuccgucGagauuccucaca....................	5	1	0G2
....uuccgucaagauucGucaca....................	1	1	0G2
....uuccgucaagauucAucaca....................	1	1	0G2
....uCccgucaagauuccucaca....................	7	1	0G2
....uuccgucaagauuccucaAa....................	2	1	0G2
....uucGgucaagauuccucaca....................	3	1	0G2
....uuccgucaagauuccucacC....................	67	1	0G2
....uucUgucaagauuccucaca....................	1	1	0G2
....uuccgucaagauuccuAaca....................	1	1	0G2
....uuccgucaagUuuccucaca....................	1	1	0G2
....uuccgCcaagauuccucaca....................	9	1	0G2
....uuccgucaagauuccucacG....................	23	1	0G2

Star **Mature**

```
ggauucaaagucgcgauucaaaucaaaacugugcuucuuauaagcaaucuugacagaauguuuggacauuccgucaagauuccucacaaggacugcaggaaagggaag
....................................................................uAccgucaagauuccucaca....................   1     1   0G2
....................................................................uuccgucaagauuccCcaca....................   14    1   0G2
....................................................................Cuccgucaagauuccucaca....................   5     1   0G2
....................................................................uuccgAcaagauuccucaca....................   5     1   0G2
....................................................................uuUcgucaagauuccucaca....................   6     1   0G2
....................................................................uuAcgucaagauuccucaca....................   3     1   0G2
....................................................................uuccgucaagauuUcucaca....................   4     1   0G2
....................................................................uucAgucaagauuccucaca....................   7     1   0G2
....................................................................Nuccgucaagauuccucacaa...................   6     1   0G2
....................................................................uuUcgucaagauuccucacaa...................   1     1   0G2
....................................................................uuccgucaagauuccucacUa...................   643   1   0G2
....................................................................uucAgucaagauuccucacaa...................   1     1   0G2
....................................................................uuccgucaagauuccucacaU...................   226   1   0G2
....................................................................uuccgucaagauuccucacaG...................   5     1   0G2
....................................................................uuccgucaagauGccucacaa...................   1     1   0G2
....................................................................uuccgucaagauuccucacaa...................   1648  0   0G2
....................................................................uuccgucaagauuccucacGa...................   2     1   0G2
....................................................................uuccgAcaagauuccucacaa...................   1     1   0G2
....................................................................uuccgucaagaCuccucacaa...................   2     1   0G2
....................................................................uuccgucaagauuccGcacaa...................   1     1   0G2
....................................................................uuccgCcaagauuccucacaa...................   1     1   0G2
....................................................................uuccgucaagauuccucacCa...................   1     1   0G2
....................................................................uuccgucaagauuccucacaaC..................   2     1   0G2
....................................................................uuccgucaagauuccucacaag..................   57    0   0G2
....................................................................uuccgucaagauuccucacaaA..................   72    1   0G2
....................................................................uuccgucaagauuccucacaaU..................   1527  1   0G2
....................................................................uuccgucaagauuccucacaaUg.................   2     1   0G2
.....................................................................uAcgucaagauuccucaca....................   1     1   0G2
.....................................................................uccAucaagauuccucaca....................   1     1   0G2
.....................................................................uUcgucaagauuccucaca....................   1     1   0G2
.....................................................................Cccgucaagauuccucaca....................   1     1   0G2
.....................................................................uccgucaagauuccucaca....................   509   0   0G2
.....................................................................uccgucaagauuccuUaca....................   1     1   0G2
.....................................................................uccgAcaagauuccucaca....................   1     1   0G2
.....................................................................Gccgucaagauuccucaca....................   1     1   0G2
.....................................................................uccgucaagauuccucacU....................   1083  1   0G2
.....................................................................uccgucaagauuccucacaG...................   3     1   0G2
.....................................................................uccgucaagauuccucacaU...................   3     1   0G2
.....................................................................uccgucaagauuccucacUa...................   7     1   0G2
.....................................................................uccgucaagauuccucacaa...................   28    0   0G2
.....................................................................uccgucaagauuccucacaaU..................   63    1   0G2
......................................................................ccgucaagauuccucacU....................   87    1   0G2
......................................................................ccgucaagauuccucaca....................   23    0   0G2
......................................................................ccgucaagauuccucacaU...................   10    1   0G2

......................ucaaaacugugcuucuuauC..................................................................   1     1   0A2
..............................................................ugUacauuccgucaagauuccu........................   3     1   0A2
..............................................................ugUacauuccgucaagauuccuca......................   2     1   0A2
..............................................................ugUacauuccgucaagauuccucaca....................   1     1   0A2
................................................................Uacauuccgucaagauuccuca......................   3     1   0A2
................................................................Uacauuccgucaagauuccucaca....................   7     1   0A2
.................................................................acauuccgucaagauuccuca......................   2     0   0A2
.................................................................Ccauuccgucaagauuccucaca....................   8     1   0A2
..................................................................cauuccgucaagauuccu........................   1     0   0A2
..................................................................cauuccgucaagauuccuU.......................   1     1   0A2
..................................................................cauuccgucaagauuccuc.......................   23    0   0A2
..................................................................cauuccgucaagauuccucC......................   1     1   0A2
..................................................................cauuccgucaagauuccucG......................   1     1   0A2
..................................................................cauuccgucaagauuccuca......................   296   0   0A2
..................................................................cauuccgucaagauuccucU......................   55    1   0A2
..................................................................Nauuccgucaagauuccuca......................   1     1   0A2
..................................................................cauuccgucaagauGccuca......................   1     1   0A2
..................................................................cauuccgucaagauuccucac.....................   146   0   0A2
..................................................................cauuccgucaagauuccucUc.....................   4     1   0A2
..................................................................cauuccUucaagauuccucac.....................   1     1   0A2
..................................................................cauuccgucaagauuccucaA.....................   2     1   0A2
..................................................................cauuccgucaagauAccucaca....................   1     1   0A2
..................................................................cauucGgucaagauuccucaca....................   1     1   0A2
..................................................................cauuccgCcaagauuccucaca....................   3     1   0A2
..................................................................cauuccgucaaCauuccucaca....................   1     1   0A2
```

Star Mature

ggauucaaagucgcgauucaaaucaaaacugugcuucuuauaagcaaucuugacagaauguuuggacauuccgucaagauuccucacaaggacugcaggaaagggaag

....cauuccgucaagauuccucUca....................	6	1	0A2
....cauuccgucaagauuccucacC....................	35	1	0A2
....cauuccgucaagauuccucaca....................	7121	0	0A2
....cauuccgucaagauuccucGca....................	2	1	0A2
....caGuccgucaagauuccucaca....................	1	1	0A2
....cauuccgucGagauuccucaca....................	3	1	0A2
....cauuccgucaagauuccucacG....................	12	1	0A2
....cNuuccgucaagauuccucaca....................	2	1	0A2
....caCuccgucaagauuccucaca....................	1	1	0A2
....cauuccAucaagauuccucaca....................	9	1	0A2
....cauuccgucaagauuccuAaca....................	1	1	0A2
....cauuccgucaagauuccucaAa....................	2	1	0A2
....cauuccgucaagauucGucaca....................	1	1	0A2
....cauuccgucaagauuccucacU....................	19315	1	0A2
....cauuccUucaagauuccucaca....................	1	1	0A2
....Uauuccgucaagauuccucaca....................	6	1	0A2
....cauuccgucaagaCuccucaca....................	2	1	0A2
....cauuccgucaagauCccucaca....................	2	1	0A2
....cauuccgGcaagauuccucaca....................	1	1	0A2
....cauuccgucaagauuccAcaca....................	1	1	0A2
....cauuccgucaagauucAucaca....................	2	1	0A2
....cauuccguUaagauuccucaca....................	2	1	0A2
....cauuccgAcaagauuccucaca....................	1	1	0A2
....cauuccgucaagauuAcucaca....................	2	1	0A2
....cauuUcgucaagauuccucaca....................	1	1	0A2
....cauuGcgucaagauuccucaca....................	1	1	0A2
....Aauuccgucaagauuccucaca....................	2	1	0A2
....cauuccgucaagauuUcucaca....................	4	1	0A2
....cauuccgucaagauuccCcaca....................	4	1	0A2
....cauuccguAaagauuccucaca....................	1	1	0A2
....cauuccgucaGgauuccucaca....................	19	1	0A2
....Gauuccgucaagauuccucaca....................	2	1	0A2
....cauuccgucaagGuuccucaca....................	3	1	0A2
....Nauuccgucaagauuccucaca....................	21	1	0A2
....cauuccgucaagauuccucacaU...................	84	1	0A2
....cauuccgucaagauuccucacUa...................	518	1	0A2
....cauuccgucaagauuccucacaa...................	19	0	0A2
....cauuccgucaagauuccucacCa...................	6	1	0A2
....cauuccgucaagauuccucacaaU..................	16	1	0A2
....cauuccgucaagauuccucacaaA..................	5	1	0A2
....auuccgucaagauuccuA.......................	1	1	0A2
....auuccgucaagauuccuc.......................	10	0	0A2
....Nuuccgucaagauuccuca......................	1	1	0A2
....auuccgucaagaCuccuca......................	1	1	0A2
....auuccgucaagauuccucU......................	12	1	0A2
....auuccgucaagauuccuca......................	59	0	0A2
....Nuuccgucaagauuccucac.....................	2	1	0A2
....auuccgucaagauuccucac.....................	36	0	0A2
....auuccgucaagauucAucac.....................	1	1	0A2
....auuccgucaagauuccucUc.....................	3	1	0A2
....auuccgucaagauuccucacG....................	3	1	0A2
....auuAcgucaagauuccucaca....................	1	1	0A2
....auuccgucaagauuccucaca....................	1369	0	0A2
....auuccgucaagauuccuUaca....................	1	1	0A2
....Guuccgucaagauuccucaca....................	1	1	0A2
....auuccgucCagauuccucaca....................	1	1	0A2
....aNuccgucaagauuccucaca....................	1	1	0A2
....auuccgucaagauuccucacC....................	2	1	0A2
....Cuuccgucaagauuccucaca....................	40	1	0A2
....auuccgGcaagauuccucaca....................	2	1	0A2
....auucUgucaagauuccucaca....................	1	1	0A2
....auuccAucaagauuccucaca....................	1	1	0A2
....Nuuccgucaagauuccucaca....................	1	1	0A2
....auuccgucaagauuccucacU....................	3198	1	0A2
....auuccgAcaagauuccucaca....................	1	1	0A2
....auuccgucaagauuccucacaU...................	24	1	0A2
....auuccgucaagauuccucacUa...................	65	1	0A2
....Cuuccgucaagauuccucacaa...................	6	1	0A2
....auuccgucaagauuccucacaa...................	23	0	0A2
....auuccgucCagauuccucacaa...................	1	1	0A2

```
                                         Star                Mature
ggauucaaagucgcgauucaaaucaaaacugugcuucuuauaagcaaucuugacagaauguuuggacauuccgucaagauuccucacaaggacugcaggaaagggaag
...................................................................auuccgucaagauuccucacaaU..................  26      1  0A2
...................................................................auuccgucaagauuccucacaaA..................  5       1  0A2
....................................................................uuccguUaagauuccuca......................  1       1  0A2
....................................................................uuccguAaagauuccuca......................  1       1  0A2
....................................................................uuccgucaagauuccuca......................  432     0  0A2
....................................................................uuccgucGagauuccuca......................  1       1  0A2
....................................................................Cuccgucaagauuccuca......................  1       1  0A2
....................................................................Nuccgucaagauuccuca......................  1       1  0A2
....................................................................uuccgucaGgauuccuca......................  5       1  0A2
....................................................................uuccgucaagauuccucU......................  199     1  0A2
....................................................................Nuccgucaagauuccucac.....................  2       1  0A2
....................................................................uuccgucaagauuccucUc.....................  1       1  0A2
....................................................................uuccgucaagauuccucaG.....................  1       1  0A2
....................................................................uuccguGaagauuccucac.....................  2       1  0A2
....................................................................uuccgGcaagauuccucac.....................  1       1  0A2
....................................................................uuccgucaagauuccucac.....................  439     0  0A2
....................................................................uuccgucaagauGccucaca....................  8       1  0A2
....................................................................uuccgAcaagauuccucaca....................  2       1  0A2
....................................................................uuccgucaagaCuccucaca....................  14      1  0A2
....................................................................uuccguAaagauuccucaca....................  19      1  0A2
....................................................................uuccgucaagauuccuUaca....................  6       1  0A2
....................................................................uuccgucaagauuccuAaca....................  4       1  0A2
....................................................................uuUcgucaagauuccucaca....................  10      1  0A2
....................................................................uuccgucaagauuUcucaca....................  6       1  0A2
....................................................................uuccgucaagauuccucGca....................  7       1  0A2
....................................................................Auccgucaagauuccucaca....................  7       1  0A2
....................................................................uuccgucaagCuuccucaca....................  1       1  0A2
....................................................................uuccUucaagauuccucaca....................  4       1  0A2
....................................................................uuccgucaagauuccucacC....................  152     1  0A2
....................................................................uuccAucaagauuccucaca....................  8       1  0A2
....................................................................uuccgucaagUuuccucaca....................  1       1  0A2
....................................................................uuccgucGagauuccucaca....................  11      1  0A2
....................................................................uuccgucaaUauuccucaca....................  8       1  0A2
....................................................................uuccgucaagauuccucUca....................  2       1  0A2
....................................................................Cuccgucaagauuccucaca....................  11      1  0A2
....................................................................uuccguUaagauuccucaca....................  5       1  0A2
....................................................................uuccgucaagaGuccucaca....................  6       1  0A2
....................................................................uuccgucaagauucGucaca....................  1       1  0A2
....................................................................uuccgucaagauuccuGaca....................  1       1  0A2
....................................................................uuccgucaagauuccCcaca....................  8       1  0A2
....................................................................uuAcgucaagauuccucaca....................  7       1  0A2
....................................................................uuccgucaagauuccGcaca....................  5       1  0A2
....................................................................uuccgucaagauuccucaGa....................  1       1  0A2
....................................................................uucGgucaagauuccucaca....................  3       1  0A2
....................................................................uucUgucaagauuccucaca....................  4       1  0A2
....................................................................uuccgucaagauAccucaca....................  2       1  0A2
....................................................................uNccgucaagauuccucaca....................  6       1  0A2
....................................................................uuccgucaagauCccucaca....................  4       1  0A2
....................................................................uuccgGcaagauuccucaca....................  35      1  0A2
....................................................................uuccgucaagauuccAcaca....................  3       1  0A2
....................................................................uuccgucaagauucAucaca....................  7       1  0A2
....................................................................Guccgucaagauuccucaca....................  11      1  0A2
....................................................................uuccguGaagauuccucaca....................  40      1  0A2
....................................................................uuccgucaagauuAcucaca....................  6       1  0A2
....................................................................uGccgucaagauuccucaca....................  2       1  0A2
....................................................................uCccgucaagauuccucaca....................  14      1  0A2
....................................................................uuccgucaGgauuccucaca....................  76      1  0A2
....................................................................uuccgucaagaAuccucaca....................  2       1  0A2
....................................................................uAccgucaagauuccucaca....................  2       1  0A2
....................................................................Nuccgucaagauuccucaca....................  75      1  0A2
....................................................................uuccgucaagauuccucacU....................  119027  1  0A2
....................................................................uuccCucaagauuccucaca....................  1       1  0A2
....................................................................uuccgucaagauuccucacG....................  32      1  0A2
....................................................................uuccgucaaAauuccucaca....................  3       1  0A2
....................................................................uuccgucUagauuccucaca....................  4       1  0A2
....................................................................uuccgucaagauuccucaAa....................  6       1  0A2
....................................................................uuccgucaagauuccucaca....................  35257   0  0A2
....................................................................uuccgucaagGuuccucaca....................  3       1  0A2
....................................................................uuccgCcaagauuccucaca....................  12      1  0A2
....................................................................uucAgucaagauuccucaca....................  6       1  0A2
```

Star Mature

ggauucaaagucgcgauucaaaucaaaacugugcuucuuauaagcaaucuugacagaauguuuggacauuccgucaagauuccucacaaggacugcaggaaagggaag			
....uuccguGaagauuccucacaa...................	4	1	0A2
....uuccgucaagauuccucacaa...................	2943	0	0A2
....uuccguUaagauuccucacaa...................	1	1	0A2
....Nuccgucaagauuccucacaa...................	4	1	0A2
....Cuccgucaagauuccucacaa...................	1	1	0A2
....uuccgucaagauuccucacGa...................	1	1	0A2
....uuccgucaagauuccucacUa...................	1774	1	0A2
....uuccgucaagaCuccucacaa...................	2	1	0A2
....uuccAucaagauuccucacaa...................	1	1	0A2
....uuccgucaagauuccucaAaa...................	2	1	0A2
....uuccgucaagauuccucacaC...................	1	1	0A2
....uuccgucaagauuccuUacaa...................	1	1	0A2
....uuAcgucaagauuccucacaa...................	3	1	0A2
....uuccgucaagUuuccucacaa...................	1	1	0A2
....uuccgucaagauuccucacaU...................	361	1	0A2
....uuccgCcaagauuccucacaa...................	1	1	0A2
....uuccgucaagauuccucacCa...................	33	1	0A2
....uNccgucaagauuccucacaa...................	1	1	0A2
....uuccgucaagauuccuAacaa...................	1	1	0A2
....uuccgucaagauuccucacaG...................	4	1	0A2
....uuccgucaaAauuccucacaa...................	1	1	0A2
....uuccgucaagauuAcucacaa...................	2	1	0A2
....uuccgucUagauuccucacaa...................	1	1	0A2
....uuUcgucaagauuccucacaa...................	1	1	0A2
....Guccgucaagauuccucacaa...................	1	1	0A2
....uuccgucaagauuccucacaag..................	80	0	0A2
....uuccgucaagauuccucacaaA..................	152	1	0A2
....uuccgucaagauuccucacCag..................	1	1	0A2
....uuccgucaagauuccucacaaU..................	4006	1	0A2
....uuccgucaagauuccucacaaC..................	7	1	0A2
....uuccgucaagauuccucacaaUg.................	11	1	0A2
....uuccgucaagauuccucacaaAga................	1	1	0A2
....uccUucaagauuccucaca....................	1	1	0A2
....uccgucaagauuccucacU....................	2134	1	0A2
....Gccgucaagauuccucaca....................	1	1	0A2
....uAcgucaagauuccucaca....................	1	1	0A2
....uNcgucaagauuccucaca....................	1	1	0A2
....uccgucaagauAccucaca....................	2	1	0A2
....uccgCcaagauuccucaca....................	2	1	0A2
....uccgucaagauuccucaca....................	858	0	0A2
....uccgucCagauuccucaca....................	1	1	0A2
....uccgGcaagauuccucaca....................	1	1	0A2
....uccgucaagauuccucacG....................	2	1	0A2
....uccgucaagauuccucacC....................	5	1	0A2
....uccgAcaagauuccucaca....................	1	1	0A2
....uccgucGagauuccucaca....................	1	1	0A2
....uccgucaagauuccuAaca....................	1	1	0A2
....Nccgucaagauuccucaca....................	2	1	0A2
....uccgucaagauuccucacUa...................	40	1	0A2
....uccgucaagauuccucacaU...................	8	1	0A2
....ucAgucaagauuccucacaa...................	1	1	0A2
....uccgucaagauuccucacCa...................	2	1	0A2
....uccgucaagauuccucacaa...................	93	0	0A2
....uccgucaagauuccucacaaU..................	109	1	0A2
....uccgucaagauuccucacaaA..................	3	1	0A2
....ccgucaagaCuccucaca....................	1	1	0A2
....ccgucaagauuccucacU....................	136	1	0A2
....ccgucaagauuccucaca....................	55	0	0A2
....ccgucaagauuccucacUa...................	7	1	0A2
....ccgucaagauuccucacaaU..................	6	1	0A2
....cgucaagauuccucacaaU..................	3	1	0A2

Provisional ID	:	scaffold93_18174
Score total	:	0.9
Score for star read(s)	:	-1.3
Score for read counts	:	0
Score for mfe	:	1.2
Score for randfold	:	1.6
Score for cons. seed	:	-0.6
Total read count	:	2551
Mature read count	:	2551
Loop read count	:	0
Star read count	:	0

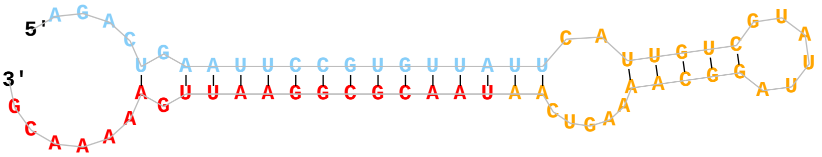

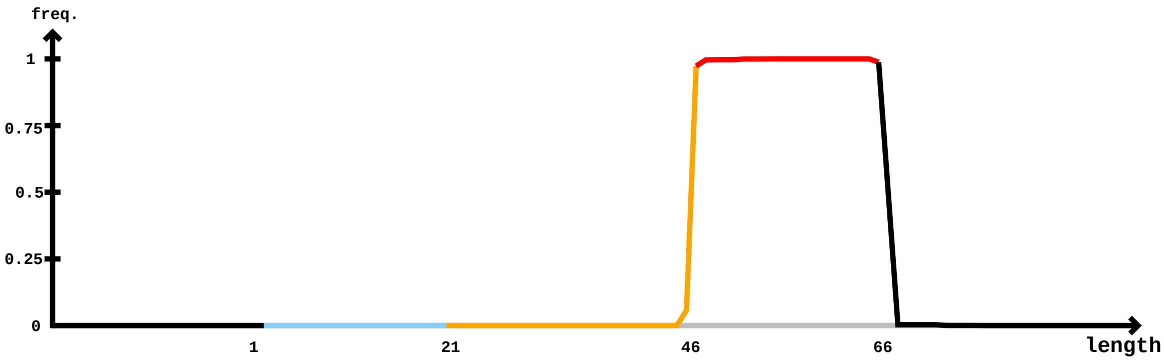

Star Mature

```
5'- cggaacaggaauuuucugcgagacugaauuccguguuauucauugucguauuaggcaaaagucaauaacgcggaauugaaaaacguggaaaauuuuuuuagcacugaaaau   -3'    exp
    (((...((((((((((..((....(.((((((((((((((..(((((......))))).....)))))))))))))).)....))..))))))))))......))).....    reads    mm    sample
    ................................................................auaacgcggaauugaaaaac...........................    4    0    0A2
    ................................................................auaacgcggaauugaaaaacA..........................    2    1    0A2
    ................................................................auaGcgcggaauugaaaaacg..........................    1    1    0A2
    ................................................................auaacgcgCaauugaaaaacg..........................    1    1    0A2
    ................................................................aCaacgcggaauugaaaaacg..........................    3    1    0A2
    ................................................................auGacgcggaauugaaaaacg..........................    3    1    0A2
    ................................................................Guaacgcggaauugaaaaacg..........................    2    1    0A2
    ................................................................auaacgcggaauugaaaaacg..........................    21    0    0A2
    .................................................................Caacgcggaauugaaaaac...........................    3    1    0A2
    .................................................................uaacgcggaauugaaaaac...........................    7    0    0A2
    .................................................................uaacgcggaauugaaaaUcg..........................    3    1    0A2
    .................................................................uaacgAggaauugaaaaacg..........................    1    1    0A2
    .................................................................uaacgcggaauugaaUaacg..........................    77    1    0A2
    .................................................................uaacgcggaauugaGaaacg..........................    3    1    0A2
    .................................................................uaaGgcggaauugaaaaacg..........................    4    1    0A2
    .................................................................uaacgcAgaauugaaaaacg..........................    1    1    0A2
    .................................................................uGacgcggaauugaaaaacg..........................    24    1    0A2
    .................................................................uaacgcggaauugaaaaacg..........................    574    0    0A2
    .................................................................uaacgcggaauugaaaGacg..........................    1    1    0A2
    .................................................................uaGcgcggaauugaaaaacg..........................    65    1    0A2
    .................................................................Caacgcggaauugaaaaacg..........................    6    1    0A2
    .................................................................uaacgcggaauugaaaaacU..........................    2    1    0A2
    .................................................................uaacgcggaauugaaaaacA..........................    27    1    0A2
    .................................................................uaacgcggaauugaaaaacgA.........................    4    1    0A2
    ..................................................................aacgcggaauugaaaaacg..........................    11    0    0A2
    ...................................................................acgcggaauugaaaaacg..........................    2    0    0A2

    ................................................................auaacgcggaauugaaaaac...........................    12    0    0B2
    ................................................................auaacgcggaauugaaaaUc...........................    2    1    0B2
    ................................................................aCaacgcggaauugaaaaacg..........................    1    1    0B2
    ................................................................auaacgcggaauugaaaaacg..........................    31    0    0B2
    ................................................................auaacgcggaauugaaaaacA..........................    8    1    0B2
    ................................................................auGacgcggaauugaaaaacg..........................    2    1    0B2
    .................................................................Naacgcggaauugaaaaacg..........................    3    1    0B2
```

```
                    Star                                         Mature
cggaacaggaauuuucugcgagacugaauuccguguuauucauugucguauuaggcaaaagucaauaacgcggaauugaaaaacguggaaaauuuuuuuagcacugaaaau
.................................................................Caacgcggaauugaaaaacg..........................    24     1     0B2
.................................................................uaacgcggaauugaaaaUcg..........................    5      1     0B2
.................................................................uaacgAggaauugaaaaacg..........................    2      1     0B2
.................................................................uaGcgcggaauugaaaaacg..........................    67     1     0B2
.................................................................uaacgcggaauugaaaaacg..........................    523    0     0B2
.................................................................uGacgcggaauugaaaaacg..........................    32     1     0B2
.................................................................uaacgcggaauugaaaaacA..........................    31     1     0B2
.................................................................uaacgcggaauugaaUaacg..........................    46     1     0B2
.................................................................uaacgcggaauugaaaaacgA.........................    22     1     0B2
..................................................................aacgcggaauugaaaaacg..........................    27     0     0B2
..................................................................aGcgcggaauugaaaaacg..........................    2      1     0B2
..................................................................aacgcggaauugaaaaacgA.........................    8      1     0B2
......................................................................cggaauugaaaaacguggaUa....................    1      1     0B2
......................................................................cggaauugaaaaacguggaaa....................    6      0     0B2
.........................................................................aauugaaaaacguggaaaUuuu................    1      1     0B2

................................................................auaacgcggaauugaaaaacg..........................    20     0     0G2
................................................................auGacgcggaauugaaaaacg..........................    3      1     0G2
................................................................auaacgcggaauugaaaaacA..........................    29     1     0G2
................................................................auaacgcggaauugaaUaacg..........................    2      1     0G2
.................................................................uaacgcggaauugaaaaac...........................    4      0     0G2
.................................................................uGacgcggaauugaaaaacg..........................    34     1     0G2
.................................................................Gaacgcggaauugaaaaacg..........................    1      1     0G2
.................................................................Aaacgcggaauugaaaaacg..........................    1      1     0G2
.................................................................Caacgcggaauugaaaaacg..........................    28     1     0G2
.................................................................uaacgcggaauugaaUaacg..........................    64     1     0G2
.................................................................uaaAgcggaauugaaaaacg..........................    1      1     0G2
.................................................................uaacgcggaauugaaaaacg..........................    580    0     0G2
.................................................................uaGcgcggaauugaaaaacg..........................    29     1     0G2
.................................................................uaacgcAgaauugaaaaacg..........................    6      1     0G2
.................................................................Naacgcggaauugaaaaacg..........................    1      1     0G2
.................................................................uaacgcggaauugaaaaacA..........................    34     1     0G2
.................................................................uaacgcggaauugaaaaacgA.........................    3      1     0G2
..................................................................aacgcggaauugaaaaacg..........................    3      0     0G2
..................................................................aGcgcggaauugaaaaacg..........................    3      1     0G2
..................................................................aacgcggaauugaaaaacgA.........................    5      1     0G2
```

Provisional ID	:	scaffold96_18497
Score total	:	5.2
Score for star read(s)	:	-1.3
Score for read counts	:	0
Score for mfe	:	1.9
Score for randfold	:	1.6
Score for cons. seed	:	3
Total read count	:	1370
Mature read count	:	1352
Loop read count	:	18
Star read count	:	0

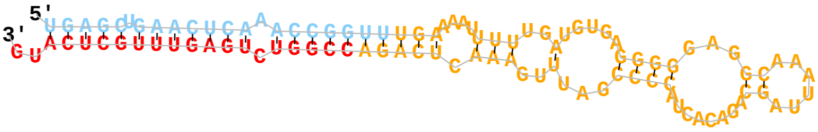

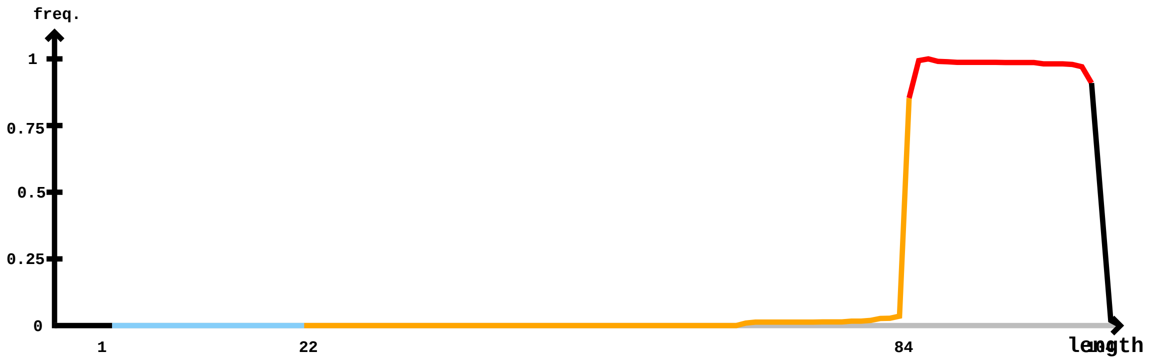

Star   Mature

```
5'- uaagugagcugaacucaaaccgguuugaaaauuuuugaugugagggggaggcaaauuagcagacacuaccccgauuugaaacucagaccggucugaguuugcucaugug   -3'    exp
    ...((((((.(((((((.((((((((((....(((..(.....((((...((......))........))))...)..))).)))))))))).)))))))))))))...   reads    mm    sample
    ......................................................................ccgauuugaGacucagaccg...................   2        1     0G2
    ......................................................................ccgauuugaaacucagaccA...................   6        1     0G2
    .......................................................................................ccggucugaguuugcucau...   16       0     0G2
    .......................................................................................ccggucugaguuugcucauA..   9        1     0G2
    .......................................................................................ccggucugaguuugcucauU..   56       1     0G2
    .......................................................................................ccggucugaguuugcucaug..   133      0     0G2
    .......................................................................................ccggucugaguuugcucauAu.   12       1     0G2
    .......................................................................................ccggucugaguuugcucauUu.   94       1     0G2
    .......................................................................................ccggucugaguuugcucaugu.   26       0     0G2
    .......................................................................................ccggucugaguuugcucauCu.   1        1     0G2
    ........................................................................................cggucugaguuugcucauU..   3        1     0G2
    ........................................................................................cggucugaguuugcucaug..   18       0     0G2
    ........................................................................................cggucugaguuugcucauA..   5        1     0G2
    ........................................................................................cggucugaguuugcucaugu.   1        0     0G2
    ........................................................................................cggucugaguuugcucauUu.   12       1     0G2
    ........................................................................................cggucugaguuugcucauguA   5        1     0G2
    .........................................................................................ggucugaguuugcucauA..   4        1     0G2
    .........................................................................................ggucugaguuugcucauU..   1        1     0G2

    .......................................................................cgauuugaaacucagaccAg..................   2        1     0B2
    .......................................................................cgauuugaaacucagaccggu.................   3        0     0B2
    ...................................................................................cagaccggucugaguuugcuca....   1        0     0B2
    ....................................................................................agaccggucugaguuugcuA.....   3        1     0B2
    ......................................................................................Cccggucugaguuugcucaug..   6        1     0B2
    .......................................................................................ccggucugaguuugcucau...   25       0     0B2
    .......................................................................................ccggucugaguuugcucauU..   83       1     0B2
    .......................................................................................ccggucuAaguuugcucaug..   1        1     0B2
    .......................................................................................Ncggucugaguuugcucaug..   1        1     0B2
    .......................................................................................ccggucugaguuugcucaug..   172      0     0B2
    .......................................................................................ccggucGgaguuugcucaug..   1        1     0B2
    .......................................................................................ccggucugaguuugcucauA..   11       1     0B2
    .......................................................................................cAggucugaguuugcucaug..   1        1     0B2
    .......................................................................................ccggucugaguuugcucauUu.   39       1     0B2
    .......................................................................................ccggucugaguuugcucauCu.   5        1     0B2
```

Star **Mature**

```
uaagugagcugaacucaaaccgguuugaaaauuuuugaugugagggggaggcaaauuagcagacacuaccccgauuugaaacucagaccggucugaguuugcucaugug
.......................................................................................ccggucugaguGugcucaugu.    1     1    0B2
.......................................................................................ccggucugaguuugcucaugu.    12    0    0B2
.......................................................................................ccggucugaguuugcucauguA    5     1    0B2
........................................................................................cggucugaguuugcucau...    5     0    0B2
........................................................................................cggucugaguuugcucaug..    16    0    0B2
........................................................................................cggucugaguuugcucauU..    12    1    0B2
........................................................................................cggucugaguuugcucauAu.    5     1    0B2
........................................................................................cggucugaguuugcucaugu.    3     0    0B2
........................................................................................cggucugaguuugcucauUu.    21    1    0B2
........................................................................................cggucugaguuugcucauguA    1     1    0B2
.........................................................................................ggucugaguuugcucauUu.    3     1    0B2

......................................................................ccgauuugaaacucagaccA...................    5     1    0A2
..............................................................................aaacucGgaccggucugag............    1     1    0A2
.................................................................................cucagaccggucugaguuuC........    4     1    0A2
...................................................................................cagaccggucugaguuug........    3     0    0A2
....................................................................................agaccggucugaguuugcuca....    6     0    0A2
....................................................................................agaccggucugaguuugcucauA..    1     1    0A2
.....................................................................................gaccggucugaguuugcuca....    1     0    0A2
......................................................................................accggucugaguuCgcucau...    5     1    0A2
.......................................................................................ccggucugaguuugcuca....    4     0    0A2
.......................................................................................ccggucugaguuugcucau...    25    0    0A2
.......................................................................................ccggucugaguuuAcucaug..    2     1    0A2
.......................................................................................ccggucugaguuugcucauU..    72    1    0A2
.......................................................................................ccggucugaguuugcCcaug..    1     1    0A2
.......................................................................................ccggucugaguuugcucaug..    152   0    0A2
.......................................................................................ccggucugaguuugcucauA..    23    1    0A2
.......................................................................................ccggucugaguuugcucauUu.    107   1    0A2
.......................................................................................ccggucugaguuugcucaugu.    33    0    0A2
.......................................................................................ccggucugaguuugcucauguU    1     1    0A2
.......................................................................................ccggucugaguuugcucauguA    12    1    0A2
........................................................................................cggucugaguuugcucau...    4     0    0A2
........................................................................................cggucugagCuugcucau...    5     1    0A2
........................................................................................cggucugaguuugcucauA..    1     1    0A2
........................................................................................cggucugaguuugcucaug..    10    0    0A2
........................................................................................cggucugaguuugcucauU..    30    1    0A2
........................................................................................cggucugaguuugcucauUu.    27    1    0A2
........................................................................................cggucugaguuugcucaugu.    8     0    0A2
........................................................................................cggucugaguuugcucauguA    3     1    0A2
.........................................................................................ggucugaguuugcucauUu.    1     1    0A2
```

Provisional ID	:	scaffold102_19498
Score total	:	197.9
Score for star read(s)	:	3.9
Score for read counts	:	191.3
Score for mfe	:	1.6
Score for randfold	:	1.6
Score for cons. seed	:	-0.6
Total read count	:	387
Mature read count	:	381
Loop read count	:	0
Star read count	:	6

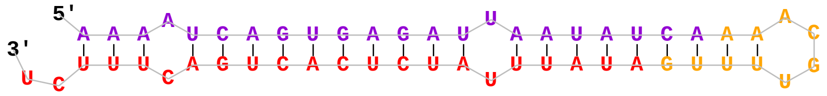

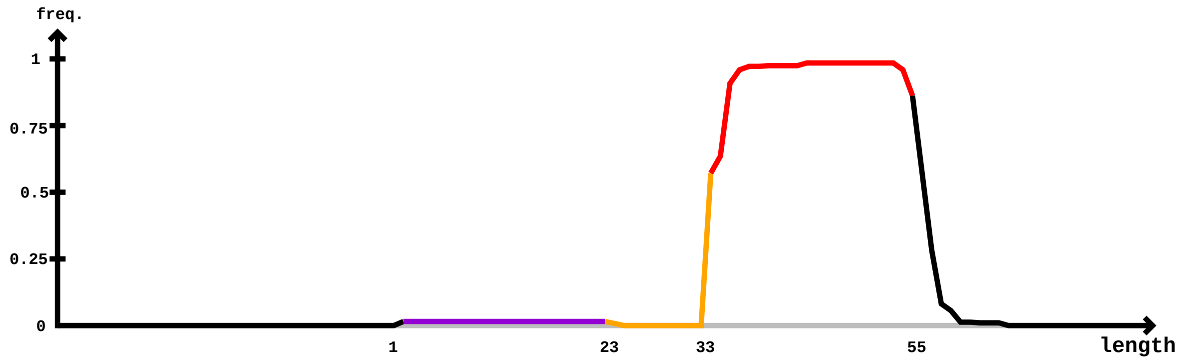


```
                                                   Star                           Mature
5'- ucucaaaauuucccacuggccaauuuucagugaaaaaaucagugagauuaauaucaaaacguuuugauauuuaucucacugacuuucucacugaacaaaggaagccgcuaau  -3'   obs
    ucucaaaauuucccacuggccaauuuucagugaaaaaaucagugagauuaauaucaaaacguuuugauauuuaucucacugacuuucucacugaacaaaggaagccgcuaau         exp
    .................(((.....((((((((.(((.((((((((((.(((((((((....))))))))).)))))))))).))).))))))))........)))......   reads   mm   sample
    ..................................aaaaucagugagaAuaauauca........................................................   6       1    0A2
    ..................................................................auauuuaucucacugacuuuc.........................   10      0    0A2
    ..................................................................auauuuaucucacugacuuucA........................   5       1    0A2
    ..................................................................auauuuaucucacugacuuucu........................   52      0    0A2
    ..................................................................auauuuaucucacugacuuucuA.......................   15      1    0A2
    ..................................................................auauuuaucucacugacuuucuU.......................   6       1    0A2
    ...................................................................uauuuaucucacugacuuucu........................   4       0    0A2
    ....................................................................auuuaucucacugacuuuc.........................   2       0    0A2
    ....................................................................Guuuaucucacugacuuucuca.......................   4       1    0A2
    ....................................................................auuuaucucacugacuuucuca......................   27      0    0A2
    ....................................................................auuuaucucacugacuuucucaUu....................   2       1    0A2
    .....................................................................uuuaucucacugacuuucuc.......................   2       0    0A2
    .....................................................................uuuaucucacugacuuucuca......................   7       0    0A2
    .....................................................................uuuaucucacugacuuucucaUu....................   4       1    0A2
    ......................................................................uuaucucacugacuuucuca......................   5       0    0A2
    ........................................................................auUucacugacuuucucacuga..................   1       1    0A2

    ..................................................................auauuuaucucacugacuuu..........................   5       0    0G2
    ..................................................................auauuuaucucacugacuuuc.........................   14      0    0G2
    ..................................................................auauuuaucucacugacuuucu........................   49      0    0G2
    ..................................................................auauuuaucucacugacuuucA........................   8       1    0G2
    ..................................................................auauuuaucucacugacuuucuA.......................   6       1    0G2
    ...................................................................uauuuaucucacugacuuu..........................   5       0    0G2
    ...................................................................uauuuaucucacugacuuucu........................   8       0    0G2
    ....................................................................auuuaucucacugacuuucuc.......................   2       0    0G2
    ....................................................................auuuaucCcacugacuuucuc.......................   1       1    0G2
    ....................................................................auuuaucucacugacuuucuca......................   17      0    0G2
    ....................................................................auuuaucucacugacuuucuUa......................   1       1    0G2
    ....................................................................auuuaucucacugacuuucucU......................   2       1    0G2
    ....................................................................auuuaucucacuCacuuucuca......................   1       1    0G2
    ....................................................................auuuaucucacugacuuucucaA.....................   9       1    0G2
    .....................................................................uuuaucucacugacuuucuca......................   3       0    0G2
    ............................................................................cacugacuuucucacugaGca...............   4       1    0G2
```

```
                                 Star                           Mature
ucucaaaauuucccacuggccaauuuucagugaaaaaaucagugagauuaauaucaaaacguuuugauauuuaucucacugacuuucucacugaacaaaggaagccgcuaau

..................................................................auauuuaucucacugacuuuc.........................   12   0   0B2
..................................................................auauuuaucucacugacuuucA........................   2    1   0B2
..................................................................auauuuaucucacugacuuucu........................   36   0   0B2
..................................................................auauuuaucucacugacuuucuU.......................   4    1   0B2
...................................................................uauuuaucucacugacuuucu........................   4    0   0B2
...................................................................uauuuaucucacugacuuucuc.......................   4    0   0B2
....................................................................Nuuuaucucacugacuuucuc.......................   1    1   0B2
....................................................................auuuaucucacugacuuucuc.......................   18   0   0B2
....................................................................auuuaucucacugacuuucuca......................   12   0   0B2
....................................................................auuuaucucacugacuuucucaA.....................   1    1   0B2
....................................................................auuuaucucacugacuuucucaAu....................   7    1   0B2
.....................................................................uuuaucucacugacuuucucaUu....................   4    1   0B2
```

Provisional ID : scaffold109_20238
Score total : 29094
Score for star read(s) : 3.9
Score for read counts : 29087.2
Score for mfe : 1.9
Score for randfold : 1.6
Score for cons. seed : -0.6
Total read count : 57065
Mature read count : 47293
Loop read count : 0
Star read count : 9772

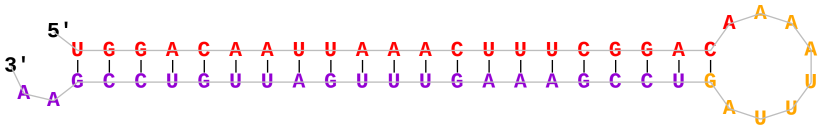

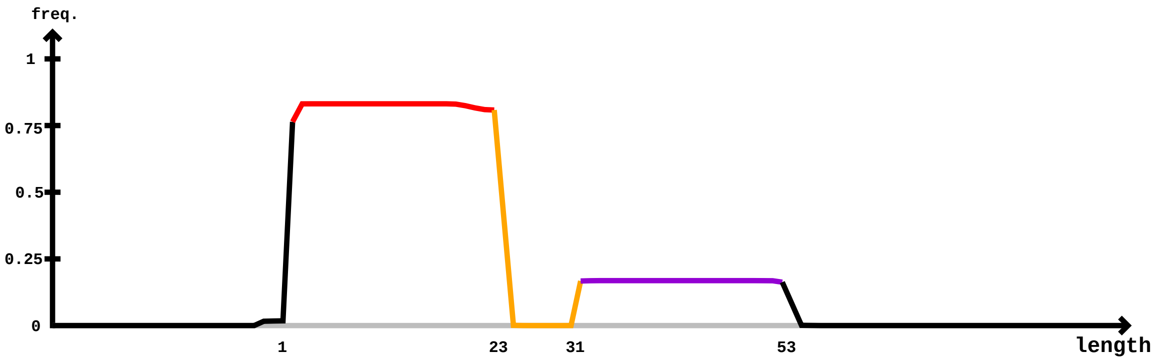

Mature Star

```
5'- aacuuucggacaaaaccagauuuuggacaauuaaacuuucggacaaaauuuaguccgaaaguuugauuguccgaaacuuuucgguuuuaauguaaguaauaauaaauuac  -3'   obs
    aacuuucggacaaaaccagauuuuggacaauuaaacuuucggacaaaauuuaguccgaaaguuugauuguccgaaacuuuucgguuuuaauguaaguaauaauaaauuac         exp
    .((((.((...(((((((((((((((((((((((((((((((((........))))))))))))))))))))))))..))).))))))..)).))))............   reads   mm   sample
    ....................uuuuggacaauuaaacuuuc.....................................................................   13      0    0A2
    ....................uuuuggacaauuaaacuuucA....................................................................   17      1    0A2
    ....................uuuAggacaauuaaacuuucg....................................................................   2       1    0A2
    ....................uuuuggacaauuaaacuuucg....................................................................   8       0    0A2
    ....................uuuuggacCauuaaacuuucgg...................................................................   12      1    0A2
    ....................uuuuggGcaauuaaacuuucgg...................................................................   1       1    0A2
    ....................uuuuggacaauuaaacuuucgg...................................................................   63      0    0A2
    ....................uuuuggacaauuaaacuuucgga..................................................................   8       0    0A2
    ....................uuuuggacaauuaaacuuucggU..................................................................   5       1    0A2
    ....................uuuuggacaauuaaacuuucggaUa................................................................   5       1    0A2
    .....................uuuggacaauuaaacuuucgga..................................................................   1       0    0A2
    ......................uuggacaauuaaacuuucggaca................................................................   2       0    0A2
    ......................uuggacaauCaaacuuucggaca................................................................   2       1    0A2
    .......................uggacaauCaaacuuucg....................................................................   6       1    0A2
    .......................uggacaauCaaacuuucgg...................................................................   9       1    0A2
    .......................uggacaauCaaacuuucgga..................................................................   106     1    0A2
    .......................uggacaauCaaacuuucggac.................................................................   20      1    0A2
    .......................uggacUauuaaacuuucggaca................................................................   32      1    0A2
    .......................uggacCauuaaacuuucggaca................................................................   51      1    0A2
    .......................uggacaauCaaacuuucggaca................................................................   13832   1    0A2
    .......................uggacaauuaaacuuucggaca................................................................   1       0    0A2
    .......................uggacaauAaaacuuucggaca................................................................   3       1    0A2
    .......................Nggacaauuaaacuuucggaca................................................................   1       1    0A2
    .......................uggacaauGaaacuuucggaca................................................................   1       1    0A2
    .......................uggacaauCaaacuuucggacaa...............................................................   53      1    0A2
    .......................uggacaauCaaacuuucggacaaa..............................................................   22      1    0A2
    ........................ggacaauuaaacuuucggG..................................................................   3       1    0A2
    ........................ggacaauuaaacuuucgga..................................................................   10      0    0A2
    ........................ggacaauuaaacuuucggaca................................................................   9       0    0A2
    ........................Ugacaauuaaacuuucggaca................................................................   2       1    0A2
    ........................ggacCauuaaacuuucggaca................................................................   7       1    0A2
    ........................ggacaauuaaacuuucAgaca................................................................   8       1    0A2
    ........................ggacaauCaaacuuucggaca................................................................   1204    1    0A2
```

Mature **Star**

```
aacuuucggacaaaaccagauuuuggacaauuaaacuuucggacaaaauuuaguccgaaaguuugauuguccgaaacuuucgguuuuaauguaaguaauaauaaauuac
........................ggacaauCaaacuuucggacaa...............................................................    3     1   0A2
........................ggacaauCaaacuuucggacaaa..............................................................    2     1   0A2
.........................gacaauuaaacuuucggaca................................................................    3     0   0A2
.....................................................uccgaaaguuugauuguUc.....................................    4     1   0A2
.....................................................uccgaaaguuugauuguUcga...................................    27    1   0A2
.....................................................uccgaaaguuugauuguccga...................................    30    0   0A2
.....................................................uccgaaaguuugauCguccga...................................    4     1   0A2
.....................................................Nccgaaaguuugauuguccga...................................    1     1   0A2
.....................................................uccgaGaguuugauuguccgaa..................................    64    1   0A2
.....................................................uccgaaaguuugaGuguccgaa..................................    1     1   0A2
.....................................................uccgaaaguuugauuguccgaa..................................    1235  0   0A2
.....................................................uccCaaaguuugauuguccgaa..................................    1     1   0A2
.....................................................uccgaaaguuugauuguUcgaa..................................    955   1   0A2
.....................................................uccgaaaguuugGuuguccgaa..................................    2     1   0A2
.....................................................uGcgaaaguuugauuguccgaa..................................    1     1   0A2
.....................................................uccgaaaguuugauugucUgaa..................................    1     1   0A2
.....................................................uccgaaaguCugauuguccgaa..................................    1     1   0A2
.....................................................uccgaaaguuugauGguccgaa..................................    1     1   0A2
.....................................................Nccgaaaguuugauuguccgaa..................................    5     1   0A2
.....................................................uccgaaaguuugauCguccgaa..................................    2     1   0A2
.....................................................Gccgaaaguuugauuguccgaa..................................    1     1   0A2
.....................................................uccgaaaguuCgauuguccgaa..................................    1     1   0A2
.....................................................uccgaaCguuugauuguccgaa..................................    1     1   0A2
.....................................................uccgaaaguuugauuguccAaa..................................    6     1   0A2
.....................................................uccgaaaguuugauugCccgaa..................................    1     1   0A2
.....................................................uccgaaaguuugauuguccgaU..................................    4     1   0A2
.....................................................uccgaaCguuugauuguccgaaa.................................    2     1   0A2
.....................................................uccgaaaguuugauuguccgaaG.................................    2     1   0A2
.....................................................uccgaaaguuugauuguccgaUa.................................    11    1   0A2
.....................................................uccgaaaguuugauuguccgaaU.................................    281   1   0A2
.....................................................uccgaaaguuugauuguccgaaa.................................    103   0   0A2
.....................................................uccgaaaguuugauuguUcgaaa.................................    53    1   0A2
.....................................................uccgaaaguuugauCguccgaaa.................................    12    1   0A2
.....................................................uccgaGaguuugauuguccgaaa.................................    3     1   0A2
.....................................................uccgaaaguuugauuguccgaaUc................................    5     1   0A2
.....................................................uccgaaaguuugauuguccgaaaA................................    11    1   0A2
.....................................................uccgaaaguuugauuguccgaaaAu...............................    7     1   0A2
.....................................................uccgaaaguuugauuguccgaaaUu...............................    1     1   0A2
......................................................ccgaaaguuugauuguUcgaa..................................    9     1   0A2
......................................................ccgaaaguuugauugucAgaaa.................................    1     1   0A2
......................................................ccgaaaguuugauuguccgaaa.................................    3     0   0A2
.......................................................cgaaaguuugauuguccgaa..................................    11    0   0A2
.......................................................cgaGaguuugauuguccgaa..................................    2     1   0A2
.......................................................cgaaaguuugauuguUcgaa..................................    1     1   0A2
.......................................................cgaaaguuugauCguccgaaa.................................    5     1   0A2

....................uuuuggacaauuaaacuuuc.....................................................................    43    0   0G2
....................uuuuggacaauCaaacuuucg....................................................................    3     1   0G2
....................uuuuggacaauuaaacuuucA....................................................................    229   1   0G2
....................uuuuggacaauuaaacuuucg....................................................................    34    0   0G2
....................uuuuggacaauuaaacuuuGg....................................................................    3     1   0G2
....................uuuuggacGauuaaacuuucg....................................................................    2     1   0G2
....................uuuuggacaauuaaacuuucgA...................................................................    2     1   0G2
....................Guuuggacaauuaaacuuucgg...................................................................    1     1   0G2
....................uuuuggacaauCaaacuuucgg...................................................................    9     1   0G2
....................uuuuggacaauuaaacuuucgg...................................................................    276   0   0G2
....................uuuuggacaauuaaacuuucggU..................................................................    12    1   0G2
....................uuuuggacaauuaaacuuucgga..................................................................    65    0   0G2
....................uuuuggacaauuaaacuuucggaUa................................................................    13    1   0G2
.....................uuuggacaauuaaacuuuc.....................................................................    3     0   0G2
.....................uuuggacaauuaaacuuucA....................................................................    4     1   0G2
.....................uuuggacaauuaaacuuucg....................................................................    4     0   0G2
.....................uuuggacaauuaaacuuucgg...................................................................    23    0   0G2
.....................uuuggacaauuaaacuuucgga..................................................................    3     0   0G2
.....................uuuggacaauCaaacuuucgga..................................................................    1     1   0G2
.....................uuuggacaauuaaacuuucggaUa................................................................    1     1   0G2
......................uuggacaauuaaacuuucg....................................................................    2     0   0G2
......................uuggacaauCaaacuuucggaca................................................................    5     1   0G2
......................uuggacaauuaaacuuucggacUa...............................................................    7     1   0G2
.......................uggacaauCaaacuuucg....................................................................    5     1   0G2
```

```
                       Mature                      Star
aacuuucggacaaaaccagauuuuggacaauuaaacuuucggacaaaauuuaguccgaaaguuugauuguccgaaacuuucgguuuuaauguaaguaauaauaaauuac
.......................uggacaauCaaacuuucgga..................................................................   75      1   0G2
.......................uggacaauCaaacuuucggac.................................................................   16      1   0G2
.......................uggacCauuaaacuuucggaca................................................................   19      1   0G2
.......................uggacUauuaaacuuucggaca................................................................   18      1   0G2
.......................uggacaauAaaacuuucggaca................................................................   3       1   0G2
.......................uggacaauuaaacuuucggaca................................................................   8       0   0G2
.......................uggacaauCaaacuuucggaca................................................................   10793   1   0G2
.......................uggacaauCaaacuuucggacaa...............................................................   71      1   0G2
.......................uggacaauCaaacuuucggacaaa..............................................................   2       1   0G2
........................ggacaauAaaacuuucggaca................................................................   1       1   0G2
........................ggacaauCaaacuuucggaca................................................................   984     1   0G2
........................ggacUauuaaacuuucggaca................................................................   4       1   0G2
........................ggacaauuaaacuuucAgaca................................................................   8       1   0G2
........................ggacaauuaaacuuucAgacaa...............................................................   9       1   0G2
.........................gacaauuaaacuuucggacaa...............................................................   8       0   0G2
...........................caauuaaacuuucggacaaaa.............................................................   1       0   0G2
...................................................Uguccgaaaguuugauuguccg....................................   1       1   0G2
....................................................guccgaaaguuugauCguccga...................................   1       1   0G2
....................................................Cuccgaaaguuugauuguccgaa..................................   5       1   0G2
.....................................................uccgaaaguuugauuguUc.....................................   8       1   0G2
.....................................................uccgaaaguuugauuguUcg....................................   2       1   0G2
.....................................................uccgaaaguuugauuguccA....................................   9       1   0G2
.....................................................uccgaaaguuugauuguccga...................................   46      0   0G2
.....................................................uccgaaaguuugauuguUcga...................................   78      1   0G2
.....................................................uccgaaaguuugauuguccgU...................................   1       1   0G2
.....................................................Nccgaaaguuugauuguccgaa..................................   4       1   0G2
.....................................................uccgaaagCuugauuguccgaa..................................   2       1   0G2
.....................................................uccgaaaguuugauuguccAaa..................................   1       1   0G2
.....................................................uAcgaaaguuugauuguccgaa..................................   3       1   0G2
.....................................................uccgaaagGuugauuguccgaa..................................   1       1   0G2
.....................................................uccgaaaguuugauuguccgGa..................................   1       1   0G2
.....................................................uccgaaaguuugauuguccgaU..................................   14      1   0G2
.....................................................uccgaGaguuugauuguccgaa..................................   79      1   0G2
.....................................................Cccgaaaguuugauuguccgaa..................................   2       1   0G2
.....................................................ucUgaaaguuugauuguccgaa..................................   3       1   0G2
.....................................................uccgaaaguuugauuguUcgaa..................................   2286    1   0G2
.....................................................uccgaaaguuugauuguccgaa..................................   1052    0   0G2
.....................................................uccgaaaguCugauuguccgaa..................................   2       1   0G2
.....................................................uccgaaaguuugauuguccgaaC.................................   7       1   0G2
.....................................................uccgaGaguuugauuguccgaaa.................................   7       1   0G2
.....................................................uccgaaaguuugauuguccgaUa.................................   17      1   0G2
.....................................................uccgaaaguuugauuguccgaaa.................................   26      0   0G2
.....................................................uccgaaaguuugauuguccgaaG.................................   1       1   0G2
.....................................................uccgaaaguuugauuguccgaaU.................................   300     1   0G2
.....................................................uccgaaaguuugauuguUcgaaa.................................   84      1   0G2
.....................................................uccgaaaguuugauuguccgaaaA................................   9       1   0G2
.....................................................uccgaaaguuugauuguccgaaaAu...............................   4       1   0G2
.....................................................uccgaaaguuugauuguccgaaaUu...............................   8       1   0G2
......................................................ccgaaaguuugauuguccgaa..................................   6       0   0G2
......................................................ccgaaaguuugauuguUcgaa..................................   2       1   0G2
......................................................ccgaaaguuugauuguccgaaa.................................   5       0   0G2
......................................................ccgaaaguuugauuguccgaaU.................................   7       1   0G2
......................................................ccgaGaguuugauuguccgaaa.................................   4       1   0G2
.......................................................cgaaaguuugauuguUcga...................................   1       1   0G2

....................Nuuuggacaauuaaacuuu......................................................................   1       1   0B2
....................uuuuggacaauuaaacuuu......................................................................   3       0   0B2
....................uuuuggacaauuaaacuuucA....................................................................   7       1   0B2
....................uuuuggacCauuaaacuuucg....................................................................   11      1   0B2
....................uuuuggacaauuaaacuuucg....................................................................   11      0   0B2
....................uuuuggacaauuaaacuuucgg...................................................................   56      0   0B2
....................Nuuuggacaauuaaacuuucgg...................................................................   1       1   0B2
....................uuGuggacaauuaaacuuucgg...................................................................   1       1   0B2
....................uuuuggacaauCaaacuuucgga..................................................................   1       1   0B2
....................uuuuggacCauuaaacuuucgga..................................................................   1       1   0B2
....................uuuuggacaauuaaacuuucgga..................................................................   9       0   0B2
....................uuuuggacaauuaaacuuucggU..................................................................   8       1   0B2
....................uuuuggacCauuaaacuuucggaca................................................................   3       1   0B2
.....................uuuggacaauuaaacuuucgg...................................................................   11      0   0B2
......................uuggacaauuaaacuuucggaca................................................................   8       0   0B2
```

```
                      Mature              Star
aacuuucggacaaaaccagauuuuggacaauuaaacuuucggacaaaauuuaguccgaaaguuugauuguccgaaacuuucgguuuuaauguaaguaauaauaaauuac
......................uuggacaauCaaacuuucggaca................................................................    11      1    0B2
.......................uggacaauCaaacuuucgg...................................................................    16      1    0B2
.......................uggacaauCaaacuuucgga..................................................................    62      1    0B2
.......................uggacaauCaaacuuucggac.................................................................    54      1    0B2
.......................uggacCauuaaacuuucggac.................................................................    2       1    0B2
.......................uggacaauAaaacuuucggaca................................................................    4       1    0B2
.......................uggacaauGaaacuuucggaca................................................................    1       1    0B2
.......................uggacaauCaaacuuucggaca................................................................    17840   1    0B2
.......................uggacaauuaaacuuucggaca................................................................    11      0    0B2
.......................uggacUauuaaacuuucggaca................................................................    9       1    0B2
.......................uggacCauuaaacuuucggaca................................................................    53      1    0B2
.......................Aggacaauuaaacuuucggaca................................................................    1       1    0B2
.......................uggacUauuaaacuuucggacaa...............................................................    4       1    0B2
.......................uggacaauCaaacuuucggacaa...............................................................    69      1    0B2
.......................uggacaauCaaacuuucggacaaa..............................................................    12      1    0B2
.......................uggacaauCaaacuuucggacaaaa.............................................................    3       1    0B2
........................ggacaauuaaacuuucggG..................................................................    1       1    0B2
........................ggacUauuaaacuuucggaca................................................................    4       1    0B2
........................ggacaauuaaacuuucAgaca................................................................    8       1    0B2
........................ggacCauuaaacuuucggaca................................................................    11      1    0B2
........................ggacaauuaaacuuucggaca................................................................    5       0    0B2
........................ggacaauCaaacuuucggaca................................................................    1613    1    0B2
........................Ngacaauuaaacuuucggaca................................................................    1       1    0B2
........................ggacaauCaaacuuucggacaa...............................................................    1       1    0B2
........................ggacaauuaaacuuucggacaaU..............................................................    2       1    0B2
........................ggacaauCaaacuuucggacaaa..............................................................    4       1    0B2
........................Ugacaauuaaacuuucggacaaa..............................................................    1       1    0B2
.....................................................uccgaaaguuugauuguccA.....................................    4       1    0B2
.....................................................uccgaaaguuugauuguUcg.....................................    2       1    0B2
.....................................................uccgaaaguuugauugAccga....................................    1       1    0B2
.....................................................uccgaaaguuugauuguccga....................................    46      0    0B2
.....................................................uccgaaagGuugauuguccga....................................    1       1    0B2
.....................................................uccgaaaguuugauuguccgU....................................    5       1    0B2
.....................................................uccgaaaguuugauuguUcga....................................    59      1    0B2
.....................................................Cccgaaaguuugauuguccga....................................    1       1    0B2
.....................................................uccgaaaguuugaCuguccgaa...................................    1       1    0B2
.....................................................Accgaaaguuugauuguccgaa...................................    1       1    0B2
.....................................................uccgaaaguuugauuguUcgaa...................................    1123    1    0B2
.....................................................Nccgaaaguuugauuguccgaa...................................    6       1    0B2
.....................................................uccgaaaguuugGuuguccgaa...................................    2       1    0B2
.....................................................uccgaaaguuugauuguccgaa...................................    1040    0    0B2
.....................................................uccgaaaguuugauCguccgaa...................................    8       1    0B2
.....................................................uccgaGaguuugauuguccgaa...................................    46      1    0B2
.....................................................uccgaaaguuugauuguccgaU...................................    14      1    0B2
.....................................................uccCaaaguuugauuguccgaa...................................    2       1    0B2
.....................................................uccgaaaguuugauugCccgaa...................................    1       1    0B2
.....................................................uccgaaaguuugauuguUcgaaa..................................    14      1    0B2
.....................................................uccgaGaguuugauuguccgaaa..................................    6       1    0B2
.....................................................uccgaaaguuugauuguccgaaC..................................    5       1    0B2
.....................................................uccgaaaguuugauuguccgaaG..................................    4       1    0B2
.....................................................uccgaaaguuugauuguccgaaa..................................    42      0    0B2
.....................................................uccgaaaguuugauugucAgaaa..................................    1       1    0B2
.....................................................uccgaaaguuugauuguccgaUa..................................    8       1    0B2
.....................................................uccgaaaguuugauuguccgaaU..................................    339     1    0B2
.....................................................uccgaaaguuugauCguccgaaa..................................    1       1    0B2
.....................................................uccgaaaguuugauuguccgaaUc.................................    4       1    0B2
.....................................................uccgaaaguuugauuguccgaaaAu................................    2       1    0B2
......................................................ccgaaaguuugauuguccgaa...................................    12      0    0B2
......................................................ccgaaaguuugauuguccgaaU..................................    8       1    0B2
......................................................ccgaaaguuugauuguccgaaaUu................................    1       1    0B2
.......................................................cgaaaguuugauuguccgaa...................................    1       0    0B2
```

Provisional ID	:	scaffold124_22092
Score total	:	34554.3
Score for star read(s)	:	3.9
Score for read counts	:	34543.8
Score for mfe	:	2
Score for randfold	:	1.6
Score for cons. seed	:	3
Total read count	:	67768
Mature read count	:	67682
Loop read count	:	0
Star read count	:	86

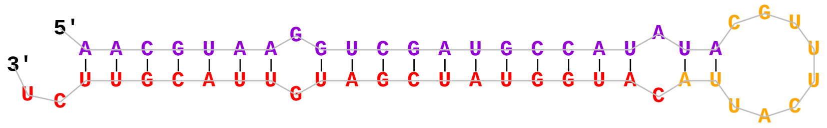

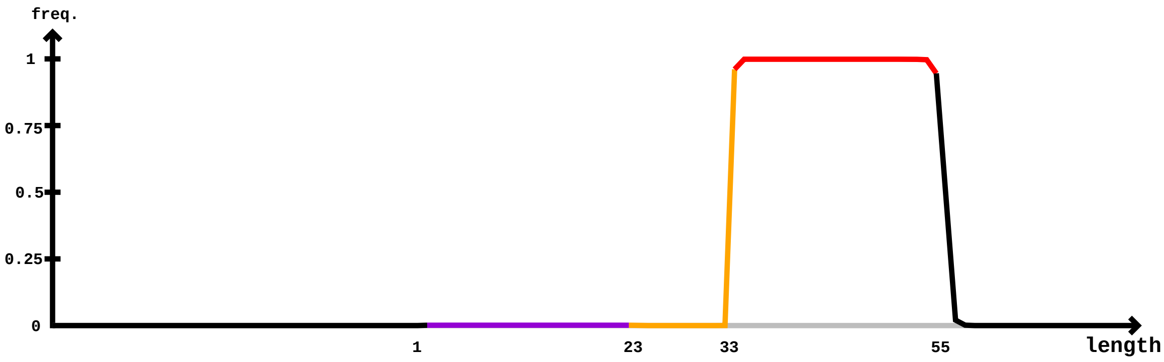

Star **Mature**

```
5'- gagcugguacuugcugggaaaggcauagcaauugaagaacguaaggucgaugccauauacguuucauuacaugguaucgauguuacguucuuuggaugcauaguuugagaa  -3'   obs
    gagcugguacuugcugggaaaggcauagcaauugaagaacguaaggucgaugccauauacguuucauuacaugguaucgauguuacguucuuuggaugcauaguuugagaa         exp
    ((((((.....((((......))))..(((.(..((((((((((.(((((((((((.((........)).))))))))))).))))))))))..).))).)))))).....   reads   mm   sample
    .....................................aacgCaaggucgaugccauau.....................................................   3       1    0B2
    .....................................aacgCaaggucgaugccauaua....................................................   31      1    0B2
    ....................................................................Ccaugguaucgauguuacguucu....................   6       1    0B2
    .....................................................................caugguaucgauguuacg........................   2       0    0B2
    .....................................................................caugguaucgauguuacgu.......................   7       0    0B2
    .....................................................................caugguaucgauguuacguu......................   12      0    0B2
    .....................................................................caugguaucgauguuacguuU.....................   8       1    0B2
    .....................................................................caugguaucgauguuGcguuc.....................   2       1    0B2
    .....................................................................caugguaucgaAguuacguuc.....................   1       1    0B2
    .....................................................................cauggGaucgauguuacguuc.....................   1       1    0B2
    .....................................................................Naugguaucgauguuacguuc.....................   6       1    0B2
    .....................................................................caugguaucgauguuacguuc.....................   941     0    0B2
    .....................................................................caugguaucgauguuacAuuc.....................   1       1    0B2
    .....................................................................cGugguaucgauguuacguuc.....................   7       1    0B2
    .....................................................................caugguaucgauguuaAguuc.....................   1       1    0B2
    .....................................................................caGgguaucgauguuacguuc.....................   1       1    0B2
    .....................................................................caugguaucgauguuacguGc.....................   1       1    0B2
    .....................................................................caugguaucgauguuacguuA.....................   1       1    0B2
    .....................................................................caugguaucgaCguuacguuc.....................   2       1    0B2
    .....................................................................caugguauUgauguuacguuc.....................   5       1    0B2
    .....................................................................caugguaucgaugGuacguucu....................   4       1    0B2
    .....................................................................caugguaucgauguuacgGucu....................   3       1    0B2
    .....................................................................cauggGaucgauguuacguucu....................   4       1    0B2
    .....................................................................cCugguaucgauguuacguucu....................   1       1    0B2
    .....................................................................Naugguaucgauguuacguucu....................   83      1    0B2
    .....................................................................cGugguaucgauguuacguucu....................   1       1    0B2
    .....................................................................caGgguaucgauguuacguucu....................   2       1    0B2
    .....................................................................cauAguaucgauguuacguucu....................   1       1    0B2
    .....................................................................caugguaucgauguuacguucC....................   27      1    0B2
    .....................................................................caugguaucgaugAuacguucu....................   1       1    0B2
    .....................................................................caugguaGcgauguuacguucu....................   3       1    0B2
    .....................................................................caugguaucgauguuacgCucu....................   8       1    0B2
    .....................................................................caugguaAcgauguuacguucu....................   1       1    0B2
```

Star Mature

```
gagcugguacuugcugggaaaggcauagcaauugaagaacguaaggucgaugccauauacguuucauuacaugguaucgauguuacguucuuuggaugcauaguuugagaa
.....................................................................Aaugguaucgauguuacguucu....................    1      1    0B2
.....................................................................caugguaucgauguuacguucG....................    6      1    0B2
.....................................................................cauggCaucgauguuacguucu....................    3      1    0B2
.....................................................................caugguaucgauguuacguCcu....................    2      1    0B2
.....................................................................caugguaCcgauguuacguucu....................    1      1    0B2
.....................................................................caugguaucgaAguuacguucu....................    1      1    0B2
.....................................................................cauUguaucgauguuacguucu....................    3      1    0B2
.....................................................................caugguaucgGuguuacguucu....................    3      1    0B2
.....................................................................caugguaucgauguuacguuGu....................    1      1    0B2
.....................................................................caugguaucgauguuacguucu....................    12967  0    0B2
.....................................................................caugguaucgauguuacAuucu....................    2      1    0B2
.....................................................................caugguaucgauguuacguucA....................    944    1    0B2
.....................................................................caugguaucgaugCuacguucu....................    1      1    0B2
.....................................................................caugguaucgauguuacUuucu....................    2      1    0B2
.....................................................................caCgguaucgauguuacguucu....................    4      1    0B2
.....................................................................caugguaucgauguuacguuAu....................    2      1    0B2
.....................................................................caugguaucgauguCacguucu....................    1      1    0B2
.....................................................................Uaugguaucgauguuacguucu....................    10     1    0B2
.....................................................................caugguaucgauguuGcguucu....................    42     1    0B2
.....................................................................caugguaucgaGguuacguucu....................    5      1    0B2
.....................................................................caugguaucgaCguuacguucu....................    1      1    0B2
.....................................................................caugguaucgauguuaUguucu....................    1      1    0B2
.....................................................................caugguaucgauguGacguucu....................    1      1    0B2
.....................................................................caugguGucgauguuacguucu....................    6      1    0B2
.....................................................................caugguauUgauguuacguucu....................    8      1    0B2
.....................................................................caugguaucgauUuuacguucu....................    1      1    0B2
.....................................................................caugguaucgauguuacguGcu....................    2      1    0B2
.....................................................................Naugguaucgauguuacguucuu...................    3      1    0B2
.....................................................................caugguaucgauguuacguucuC...................    60     1    0B2
.....................................................................caugguaucgauguuacgGucuu...................    1      1    0B2
.....................................................................caugguaucgauguuacguucuu...................    297    0    0B2
.....................................................................caugguaucgauguuacguucAu...................    10     1    0B2
.....................................................................caugguaucgauguuacguucuG...................    7      1    0B2
.....................................................................caugguaucgauguuacguucuA...................    896    1    0B2
.....................................................................caugguaucgauguuacgGucuuu..................    1      1    0B2
.....................................................................caugguaucgauguuacguucuAu..................    58     1    0B2
.....................................................................cauggGaucgauguuacguucuuu..................    1      1    0B2
.....................................................................caugguaucgauguuacguucuuA..................    24     1    0B2
.....................................................................caugguaucgauguuacguucAuu..................    4      1    0B2
.....................................................................caugguaucgauguuacguucuuu..................    160    0    0B2
.....................................................................caugguaucgauguuacguucuCu..................    5      1    0B2
.....................................................................caugguaucgauguuacguucuuC..................    6      1    0B2
.....................................................................caugguaucgauguuaNguucuuu..................    1      1    0B2
.....................................................................caugguaucgauguuacguucuuuU.................    14     1    0B2
.....................................................................caugguaucgauguuacguucuuuA.................    9      1    0B2
.....................................................................caugguaucgauguuacguucuuuC.................    4      1    0B2
......................................................................augguaucgauguuacguu......................    1      0    0B2
......................................................................augguaucgauguuacguG......................    1      1    0B2
......................................................................augguaucgauguuacguuc.....................    26     0    0B2
......................................................................augguaucgauguuacguucu....................    513    0    0B2
......................................................................augguaucgaugGuacguucu....................    1      1    0B2
......................................................................auAguaucgauguuacguucu....................    1      1    0B2
......................................................................auggGaucgauguuacguucu....................    1      1    0B2
......................................................................augguaucgauguuacguucA....................    36     1    0B2
......................................................................augguaucgauguuGcguucu....................    10     1    0B2
......................................................................Nugguaucgauguuacguucu....................    9      1    0B2
......................................................................aGgguaucgauguuacguucu....................    1      1    0B2
......................................................................augguaucgauguuacguucuA...................    37     1    0B2
......................................................................augguaucgauguuacguucuu...................    13     0    0B2
......................................................................augguaucgauguuacguucuuu..................    2      0    0B2
......................................................................augguaucgauguuacguucuAu..................    2      1    0B2

.....................................aacgCaaggucgaugccauaua....................................................    22     1    0G2
.......................................cgCaaggucgaugccauaua....................................................    3      1    0G2
.....................................................................caugguaucgauguuacg........................    1      0    0G2
.....................................................................caugguaucgauguuacA........................    13     1    0G2
.....................................................................caugguaucgauguuacgu.......................    4      0    0G2
.....................................................................caugguaucgauguuacguu......................    19     0    0G2
.....................................................................caugguaucgauguuacgCu......................    1      1    0G2
.....................................................................caugguaucgauguuacguA......................    5      1    0G2
```

Star **Mature**

```
gagcugguacuugcugggaaaggcauagcaauugaagaacguaaggucgaugccauauacguuucauuacaugguaucgauguuacguucuuuggaugcauaguuugagaa
.....................................................................caugguauUgauguuacguuc.....................    3      1    0G2
.....................................................................caugguUucgauguuacguuc.....................    1      1    0G2
.....................................................................cauggGaucgauguuacguuc.....................    1      1    0G2
.....................................................................caugguaucgauguuacguuA.....................    20     1    0G2
.....................................................................caugguaucgauguuacgCuc.....................    1      1    0G2
.....................................................................caugguaucgauguuGcguuc.....................    8      1    0G2
.....................................................................caugguaucgauguuacguuc.....................    1264   0    0G2
.....................................................................Naugguaucgauguuacguuc.....................    3      1    0G2
.....................................................................caugguaucgaugGuacguuc.....................    1      1    0G2
.....................................................................caugguaucgauguuacguuU.....................    4      1    0G2
.....................................................................caugguaucgauguuacguCcu....................    2      1    0G2
.....................................................................caugguaucgauguuaUguucu....................    3      1    0G2
.....................................................................caugguGucgauguuacguucu....................    3      1    0G2
.....................................................................caugguaucgauguuGcguucu....................    43     1    0G2
.....................................................................caugguaucgauCuuacguucu....................    4      1    0G2
.....................................................................Naugguaucgauguuacguucu....................    39     1    0G2
.....................................................................caugguaucgaAguuacguucu....................    1      1    0G2
.....................................................................caugguaucgaugCuacguucu....................    5      1    0G2
.....................................................................caugguaucgauguuacguucC....................    21     1    0G2
.....................................................................caugguaucgauguuacguucu....................    19111  0    0G2
.....................................................................caugguaucgauguuacguuAu....................    11     1    0G2
.....................................................................caugguaucgauguuaGguucu....................    1      1    0G2
.....................................................................caugguCucgauguuacguucu....................    2      1    0G2
.....................................................................cGugguaucgauguuacguucu....................    2      1    0G2
.....................................................................caugguaucgauAuuacguucu....................    2      1    0G2
.....................................................................cauggGaucgauguuacguucu....................    6      1    0G2
.....................................................................caugguaucgauguuacguucA....................    2876   1    0G2
.....................................................................cUugguaucgauguuacguucu....................    1      1    0G2
.....................................................................caugguaucgauguuaAguucu....................    2      1    0G2
.....................................................................caugguaucgauguuacgCucu....................    5      1    0G2
.....................................................................caugguaucgauguCacguucu....................    3      1    0G2
.....................................................................caugguaCcgauguuacguucu....................    4      1    0G2
.....................................................................caugguaucgaCguuacguucu....................    8      1    0G2
.....................................................................cauUguaucgauguuacguucu....................    1      1    0G2
.....................................................................cNugguaucgauguuacguucu....................    11     1    0G2
.....................................................................Uaugguaucgauguuacguucu....................    6      1    0G2
.....................................................................caugguaucgauguuacgGucu....................    4      1    0G2
.....................................................................caugguaucUauguuacguucu....................    2      1    0G2
.....................................................................caugguaucgauguuacguAcu....................    2      1    0G2
.....................................................................caugguaucgaugAuacguucu....................    1      1    0G2
.....................................................................cauggCaucgauguuacguucu....................    3      1    0G2
.....................................................................caAgguaucgauguuacguucu....................    1      1    0G2
.....................................................................caugguaucgauguGacguucu....................    1      1    0G2
.....................................................................caugguaucgCuguuacguucu....................    1      1    0G2
.....................................................................caugguaucgauguuacguuUu....................    4      1    0G2
.....................................................................caugguauUgauguuacguucu....................    10     1    0G2
.....................................................................caugUuaucgauguuacguucu....................    1      1    0G2
.....................................................................caugguaucgauguuCcguucu....................    1      1    0G2
.....................................................................caugAuaucgauguuacguucu....................    1      1    0G2
.....................................................................caugguauAgauguuacguucu....................    6      1    0G2
.....................................................................caugguaucgauguuacguucG....................    31     1    0G2
.....................................................................caugguaucgGuguuacguucu....................    3      1    0G2
.....................................................................caugguaucgauguuacAuucu....................    3      1    0G2
.....................................................................caCgguaucgauguuacguucu....................    3      1    0G2
.....................................................................caugguaucgaGguuacguucu....................    8      1    0G2
.....................................................................Aaugguaucgauguuacguucu....................    5      1    0G2
.....................................................................caugguaucgauguuacguGcu....................    5      1    0G2
.....................................................................caugguUucgauguuacguucu....................    2      1    0G2
.....................................................................caugguaucgaugGuacguucu....................    6      1    0G2
.....................................................................caugCuaucgauguuacguucu....................    1      1    0G2
.....................................................................caCgguaucgauguuacguucuu...................    3      1    0G2
.....................................................................caugguaucgauguuacguucuG...................    7      1    0G2
.....................................................................caugguaucgauguuacgCucuu...................    3      1    0G2
.....................................................................caugguaucgauguuacgGucuu...................    1      1    0G2
.....................................................................caugguaucgauguuacguucuu...................    457    0    0G2
.....................................................................Naugguaucgauguuacguucuu...................    4      1    0G2
.....................................................................caugguaucgauguuacguucuA...................    2822   1    0G2
.....................................................................caugguaucgauguuacguucAu...................    22     1    0G2
.....................................................................caugguaucgauguuacguucuC...................    27     1    0G2
.....................................................................caugguaucgauguuacguucuAu..................    214    1    0G2
```

```
                                    Star                          Mature
gagcugguacuugcugggaaaggcauagcaauugaagaacguaaggucgaugccauauacguuucauuacaugguaucgauguuacguucuuuggaugcauaguuugagaa
.....................................................................caugguaucgauguuacguucAuu..................    23     1     0G2
.....................................................................caugCuaucgauguuacguucuuu..................    1      1     0G2
.....................................................................Naugguaucgauguuacguucuuu..................    2      1     0G2
.....................................................................caugguaucgGuguuacguucuuu..................    1      1     0G2
.....................................................................caugguaucgauguuGcguucuuu..................    4      1     0G2
.....................................................................caugguaucgauguuacguucuuu..................    159    0     0G2
.....................................................................caugguaucgauguuacguucuCu..................    3      1     0G2
.....................................................................caugguaucgauAuuacguucuuu..................    1      1     0G2
.....................................................................caugguaucgauguuacguucuuA..................    117    1     0G2
.....................................................................caugguaucgauguuacguucuuC..................    11     1     0G2
.....................................................................caugguaucgauguuacguucuuuA.................    13     1     0G2
.....................................................................caugguaucgauguuacguucuuAg.................    2      1     0G2
.....................................................................caugguaucgauguuacguucuuuU.................    14     1     0G2
......................................................................augguaucgauguuacguu......................    2      0     0G2
......................................................................Nugguaucgauguuacguuc.....................    1      1     0G2
......................................................................augguaucgauguuacguuc.....................    80     0     0G2
......................................................................augguaucgauguuacguGcu....................    1      1     0G2
......................................................................augguaucgauguuacguuAu....................    2      1     0G2
......................................................................augguaucgauguuacguucG....................    1      1     0G2
......................................................................Nugguaucgauguuacguucu....................    1      1     0G2
......................................................................augguaucgaCguuacguucu....................    1      1     0G2
......................................................................aGgguaucgauguuacguucu....................    1      1     0G2
......................................................................augguaucgaugGuacguucu....................    1      1     0G2
......................................................................augguaucgauguuacgAucu....................    1      1     0G2
......................................................................augguGucgauguuacguucu....................    1      1     0G2
......................................................................augguaCcgauguuacguucu....................    1      1     0G2
......................................................................augguaucgauguuGcguucu....................    1      1     0G2
......................................................................augguaucgauguuacguucA....................    180    1     0G2
......................................................................augguaucgauguuUcguucu....................    1      1     0G2
......................................................................augguaucgauguuacgCucu....................    1      1     0G2
......................................................................augguaucgauguuacguucu....................    680    0     0G2
......................................................................augguaucgauguuacguucuC...................    5      1     0G2
......................................................................augguaucgauguuacguucuA...................    147    1     0G2
......................................................................augguaucgauguuacguucuu...................    19     0     0G2
......................................................................augguaucgauguuacguucuuu..................    10     0     0G2
......................................................................augguaucgauguuacguucuuA..................    10     1     0G2
......................................................................augguaucgauguuacguucuAu..................    7      1     0G2

.....................................aacgCaaggucgaugccauaua....................................................    27     1     0A2
...................................................................Aacaugguaucgauguuacguucu....................    3      1     0A2
....................................................................Ccaugguaucgauguuacguucu....................    4      1     0A2
.....................................................................caugguaucgauguuacgu.......................    2      0     0A2
.....................................................................Naugguaucgauguuacguu......................    2      1     0A2
.....................................................................caugguaucgauguuacguu......................    31     0     0A2
.....................................................................caugguaucgauguuacguA......................    27     1     0A2
.....................................................................Aaugguaucgauguuacguuc.....................    2      1     0A2
.....................................................................cauggAaucgauguuacguuc.....................    1      1     0A2
.....................................................................caugguauAgauguuacguuc.....................    1      1     0A2
.....................................................................caugguaucgaugCuacguuc.....................    1      1     0A2
.....................................................................caugguaucgauguuacgCuc.....................    1      1     0A2
.....................................................................caGgguaucgauguuacguuc.....................    1      1     0A2
.....................................................................Naugguaucgauguuacguuc.....................    1      1     0A2
.....................................................................caugguaucgauguuacguuc.....................    998    0     0A2
.....................................................................caugguaucgauguuacgGuc.....................    2      1     0A2
.....................................................................cNugguaucgauguuacguuc.....................    1      1     0A2
.....................................................................caugguaucgauguuacguuA.....................    15     1     0A2
.....................................................................caugguaucgaCguuacguuc.....................    3      1     0A2
.....................................................................caugguGucgauguuacguuc.....................    1      1     0A2
.....................................................................caugguauUgauguuacguuc.....................    2      1     0A2
.....................................................................caugguaCcgauguuacguucu....................    1      1     0A2
.....................................................................caugguaucgaugGuacguucu....................    2      1     0A2
.....................................................................cGugguaucgauguuacguucu....................    8      1     0A2
.....................................................................caugguaucgauguuacUuucu....................    2      1     0A2
.....................................................................caugguaucgauguNacguucu....................    1      1     0A2
.....................................................................caGgguaucgauguuacguucu....................    2      1     0A2
.....................................................................caugguaucCauguuacguucu....................    2      1     0A2
.....................................................................caugguaucgCuguuacguucu....................    2      1     0A2
.....................................................................caugguaucgauguuacguucG....................    6      1     0A2
.....................................................................caugguauAgauguuacguucu....................    2      1     0A2
.....................................................................cCugguaucgauguuacguucu....................    2      1     0A2
```

Star **Mature**

gagcugguacuugcugggaaaggcauagcaauugaagaacguaaggucgaugccauauacguuucauuacaugguaucgauguuacguucuuuggaugcauaguuugagaa			
....caugguaucgauguuaUguucu....................	5	1	0A2
....caugguaucgauguuGcguucu....................	48	1	0A2
....cNugguaucgauguuacguucu....................	3	1	0A2
....caugguaucgauguuacguCcu....................	6	1	0A2
....caugguaucgauguCacguucu....................	1	1	0A2
....caugUuaucgauguuacguucu....................	1	1	0A2
....Naugguaucgauguuacguucu....................	34	1	0A2
....caugguauUgauguuacguucu....................	2	1	0A2
....caugguaucgaCguuacguucu....................	3	1	0A2
....caugguaucgauguuaAguucu....................	2	1	0A2
....cauggGaucgauguuacguucu....................	9	1	0A2
....caugguUucgauguuacguucu....................	3	1	0A2
....caugguaucgauguuacguucA....................	2052	1	0A2
....cauUguaucgauguuacguucu....................	3	1	0A2
....caugguaucgauUuuacguucu....................	3	1	0A2
....Uaugguaucgauguuacguucu....................	3	1	0A2
....caugguaucgauguuacgCucu....................	6	1	0A2
....cauCguaucgauguuacguucu....................	2	1	0A2
....caugguCucgauguuacguucu....................	5	1	0A2
....caugguaAcgauguuacguucu....................	2	1	0A2
....caugguaucgauguuacguucC....................	21	1	0A2
....caugguaucgauguuacguucu....................	14568	0	0A2
....caugguaucgaugCuacguucu....................	1	1	0A2
....caugguaucgauguuacAuucu....................	4	1	0A2
....caugguaucgauguuacguuGu....................	1	1	0A2
....caugguGucgauguuacguucu....................	13	1	0A2
....Aaugguaucgauguuacguucu....................	9	1	0A2
....caugguaucgauguuacguuAu....................	6	1	0A2
....caugguaucgaugAuacguucu....................	1	1	0A2
....caugguaucgauguuacgGucu....................	5	1	0A2
....caugguaucgaGguuacguucu....................	2	1	0A2
....caugguaucgauguuacguGcu....................	3	1	0A2
....caCgguaucgauguuacguucu....................	3	1	0A2
....caugguaGcgauguuacguucu....................	4	1	0A2
....Gaugguaucgauguuacguucu....................	1	1	0A2
....caugguaucgGuguuacguucu....................	1	1	0A2
....cauggCaucgauguuacguucu....................	9	1	0A2
....caugguaucUauguuacguucu....................	3	1	0A2
....caugguaucgauguuacgGucuu...................	2	1	0A2
....caugguaucgauguuacguucuC...................	27	1	0A2
....caugguaucgauguuacguucuA...................	2034	1	0A2
....caugguaucgauguuacguucuG...................	3	1	0A2
....caugguaucgauguuacguucAu...................	13	1	0A2
....caugguaucgauguuacguucuu...................	301	0	0A2
....caugguGucgauguuacguucuu...................	1	1	0A2
....caugguauUgauguuacguucuu...................	1	1	0A2
....caugguaucgGuguuacguucuu...................	1	1	0A2
....caugguaucgauguuacguucuCu..................	8	1	0A2
....caugguaucgauguuacguucuuC..................	14	1	0A2
....caugguaucgauguuacguucAuu..................	21	1	0A2
....caugguaucgauguuacguucuuu..................	195	0	0A2
....caugguaucgauguuacguucuuA..................	57	1	0A2
....caugguaucgauguuacguucuGu..................	9	1	0A2
....caugUuaucgauguuacguucuuu..................	1	1	0A2
....caugguaucgauguuacguucuAu..................	138	1	0A2
....caugguaucgauguuacguucuuuU.................	18	1	0A2
....caugguaucgauguuacguucuuuA.................	30	1	0A2
....augguaucgauguuacguuA.....................	4	1	0A2
....Nugguaucgauguuacguuc.....................	2	1	0A2
....augguaucgauguuacguuc.....................	36	0	0A2
....auggua Gcgauguuacguucu....................	1	1	0A2
....augguaucgauguuacguucA....................	101	1	0A2
....aNgguaucgauguuacguucu....................	1	1	0A2
....Nugguaucgauguuacguucu....................	3	1	0A2
....augguaucgauguuacguucu....................	467	0	0A2
....auggGaucgauguuacguucu....................	1	1	0A2
....augguaucgauguuacguucuC...................	2	1	0A2
....augguaucgauguuacguucuA...................	122	1	0A2
....augguaucgauguuacguucuAu..................	8	1	0A2
....augguaucgauguuacguucuuu..................	6	0	0A2

Star Mature

```
gagcugguacuugcugggaaaggcauagcaauugaagaacguaaggucgaugccauauacguuucauuacaugguaucgauguuacguucuuuggaugcauaguuugagaa
......................................................................augguaucgauguuacguucuuA..................    4    1    0A2
```

Provisional ID	:	scaffold130_22742
Score total	:	5.3
Score for star read(s)	:	-1.3
Score for read counts	:	0
Score for mfe	:	2
Score for randfold	:	1.6
Score for cons. seed	:	3
Total read count	:	352
Mature read count	:	352
Loop read count	:	0
Star read count	:	0

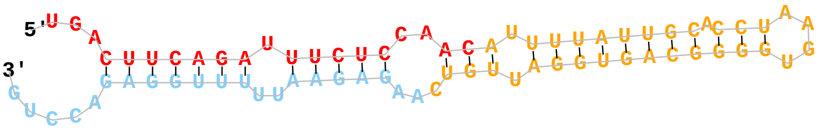

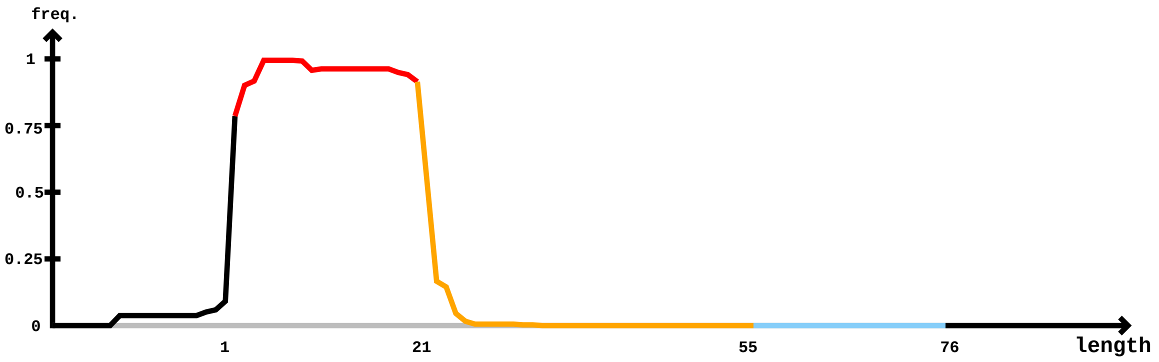

Mature **Star**

```
5'- agauccagggaucugauugacuucagauuucuccaacauuuuauugcaccuaaguggggcaguggauugucaagagaauuuuuggagaccugaaggucucuggauugaau  -3'   exp
    .((((((((((((......(((((((.(((((..(((.((((((((.(((....))))))))))).)))...)))))..)))))))......))))))))))))....  reads    mm    sample
    .....cagggaucugaGugacuuca.....................................................................................  2        1     0A2
    ................Gugacuucagauuucuccaac.........................................................................  11       1     0A2
    .................ugacuucGgauuucuccaa..........................................................................  2        1     0A2
    .................ugacuucagauuucuccaa..........................................................................  2        0     0A2
    .................ugacuucGgauuucuccaac.........................................................................  2        1     0A2
    .................ugacuucagauuucuccaaU.........................................................................  5        1     0A2
    .................ugacuucagauCucuccaac.........................................................................  3        1     0A2
    .................ugacGucagauuucuccaac.........................................................................  1        1     0A2
    .................ugacuucagauuucuccaac.........................................................................  75       0     0A2
    .................ugacuucagauuucuccaaUa........................................................................  2        1     0A2
    .................ugacuucagauuucuccaaca........................................................................  15       0     0A2
    .................ugacuucagauuucuccaaAa........................................................................  3        1     0A2
    .................ugacuucagauuucuccaacaAu......................................................................  2        1     0A2
    .................ugacuucagauuucuccaacauuuA....................................................................  4        1     0A2
    ..................gacuucagauCucuccaac.........................................................................  3        1     0A2
    ..................gacuucagauuucuccaac.........................................................................  11       0     0A2
    ...................acuucagauuucuccaacauu......................................................................  5        0     0A2
    ....................cuucGgauuucuccaacauu......................................................................  9        1     0A2
    ....................cuucagauuucuccaacauu......................................................................  7        0     0A2
    ....................cuucagauuucuccaacauuu.....................................................................  3        0     0A2
    ..........................auuucuccaacauuuuauugcGc.............................................................  1        1     0A2

    ...............aGugacuucagauuucucca...........................................................................  3        1     0G2
    .................ugacuucagauuucuccaac.........................................................................  30       0     0G2
    .................ugacuucagauuAcuccaac.........................................................................  3        1     0G2
    .................ugacuucagauuucuccaaU.........................................................................  3        1     0G2
    .................ugacuucagauuucuccaacU........................................................................  1        1     0G2
    .................ugacuucagauuucuccaaca........................................................................  22       0     0G2
    .................ugacuucagauuucuccaaAa........................................................................  8        1     0G2
    .................ugacuucagauuucuccaacaA.......................................................................  8        1     0G2
    .................ugacuucagauuucuccaacauu......................................................................  8        0     0G2
    ..................gacuucGgauuucuccaa..........................................................................  3        1     0G2
    ..................gacuucagauuucuccaac.........................................................................  12       0     0G2
    ..................gacuucagauuucuccaaca........................................................................  5        0     0G2
```

```
                Mature                                            Star
agauccagggaucugauugacuucagauuucuccaacauuuuauugcaccuaaguggggcaguggauugucaagagaauuuuuggagaccugaaggucucuggauugaau
...................acuucagauuucuccaacauA......................................................................    1    1    0G2

.....cagggaucugaGugacuuc......................................................................................    1    1    0B2
.....cagggaucugaGugacuuca.....................................................................................    11   1    0B2
..............gaGugacuucagauuucucc............................................................................    5    1    0B2
................Nugacuucagauuucuccaa..........................................................................    1    1    0B2
.................ugacuucagauuucuccaa..........................................................................    2    0    0B2
.................ugacuucagauuucuccaac.........................................................................    50   0    0B2
.................ugacuucGgauuucuccaac.........................................................................    1    1    0B2
.................ugacuucagauuucuccaaUa........................................................................    4    1    0B2
.................ugacuucagauuucuccaacaAu......................................................................    3    1    0B2
..................gacuucagauuucuccaacU........................................................................    1    1    0B2
..................gacuucagauuucuccaaca........................................................................    8    0    0B2
....................cuucGgauuucuccaacauu.....................................................................    2    1    0B2
....................cuucagauuucuccaacauuu.....................................................................    8    0    0B2
..........................auuucuccaacauuuAauugc...............................................................    1    1    0B2
```

Provisional ID	:	scaffold131_22883
Score total	:	0.7
Score for star read(s)	:	-1.3
Score for read counts	:	0
Score for mfe	:	1
Score for randfold	:	1.6
Score for cons. seed	:	-0.6
Total read count	:	8617
Mature read count	:	8617
Loop read count	:	0
Star read count	:	0

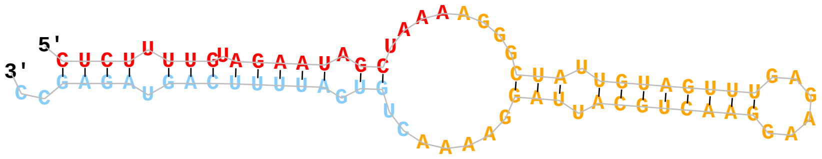

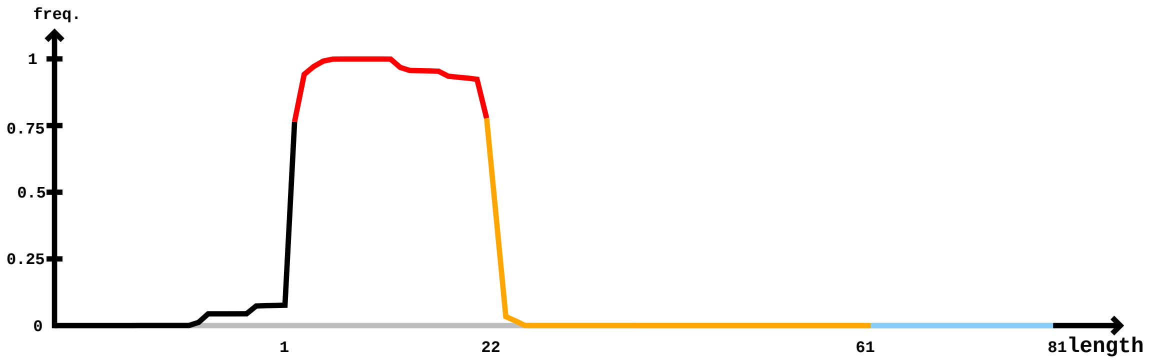

Mature   Star

```
5'- aucgaaauaucguucgauucgggcucuuuuguagaauagcuaaaagggcuauuguaguuugagaaggaacugcauuaggaaaacugugauuuucaguagagcccuuaaa  -3'    exp
    ((((((......))))))..(((((((.(((.(((((.((........(((.((((((((......)))))))).))).......)).)))))))).))))))).....    reads     mm     sample
    .............ucgauCcgggcucuuuuguag...........................................................................    41        1      0G2
    ..............cgauCcgggcucuuuuguag...........................................................................    29        1      0G2
    ..............cgauCcgggcucuuuuguaga..........................................................................    30        1      0G2
    ...................cgggcucuuuuguagaaAag......................................................................    1         1      0G2
    ...................cgggcucuuuuguagaauag......................................................................    46        0      0G2
    ...................cgggcucuuuuguagaauGg......................................................................    4         1      0G2
    ...................cgggcucuuuuguagaauaA......................................................................    2         1      0G2
    ...................cggAcucuuuuguagaauag......................................................................    1         1      0G2
    ...................cgggcucuuuuguagaauagc.....................................................................    2         0      0G2
    ...................cgggcucuuuuguagGauagc.....................................................................    1         1      0G2
    ...................cgggcucuuuuguagaauGgc.....................................................................    1         1      0G2
    ...................cgggcucuuuuguagaauagcu....................................................................    1         0      0G2
    ...................cgggcucuuuuguagaauagcA....................................................................    12        1      0G2
    ...................cgggcucuuuuguagaauGgcua...................................................................    1         1      0G2
    ...................cgggcucuuuuguagaauagcAaa..................................................................    16        1      0G2
    ....................gggcucuuuuguagaauag......................................................................    4         0      0G2
    ....................ggAcucuuuuguagaauag......................................................................    1         1      0G2
    ......................Ccucuuuuguagaauagcuaaa.................................................................    9         1      0G2
    .......................cucuuuuguagaauagcua...................................................................    16        0      0G2
    .......................cucuuuuguagaauagcCaa..................................................................    1         1      0G2
    .......................cuAuuuuguagaauagcuaa..................................................................    1         1      0G2
    .......................Nucuuuuguagaauagcuaa..................................................................    2         1      0G2
    .......................cucuuuuguagaaCagcuaa..................................................................    2         1      0G2
    .......................cucuuGuguagaauagcuaa..................................................................    2         1      0G2
    .......................cucuuuuguagaauagcuaa..................................................................    414       0      0G2
    .......................cucuuuuguagaauagcuaU..................................................................    4         1      0G2
    .......................cucuuuuguagaauagcGaa..................................................................    1         1      0G2
    .......................cucuuuuguagGauagcuaa..................................................................    7         1      0G2
    .......................cucuuuuguagaGuagcuaa..................................................................    9         1      0G2
    .......................cucuuuuguagGauagcuaaa.................................................................    1         1      0G2
    .......................cucuuuuguaAaauagcuaaa.................................................................    1         1      0G2
    .......................cucuuuuguagaauGgcuaaa.................................................................    42        1      0G2
    .......................cucuuGuguagaauagcuaaa.................................................................    1         1      0G2
    .......................cucuuuuguagaauagcuaaa.................................................................    1700      0      0G2
```

Mature	Star		
aucgaaauaucguucgauucgggcucuuuuguagaauagcuaaaagggcuauuguaguuugagaaggaacugcauuaggaaaacugugauuuucaguagagcccuuaaa			
.......................cucuuuuguagaauaAcuaaa...	5	1	0G2
.......................cucuuuuguagaauagcuaaG...	12	1	0G2
.......................cNcuuuuguagaauagcuaaa...	1	1	0G2
.......................cucuuCuguagaauagcuaaa...	1	1	0G2
.......................cucCuuuguagaauagcuaaa...	1	1	0G2
.......................cucuuuuguagaauagcuaaU...	1	1	0G2
.......................cucuuuuguagaauagcuaUa...	7	1	0G2
.......................cAcuuuuguagaauagcuaaa...	6	1	0G2
.......................cucuuuuguagaaCagcuaaa...	11	1	0G2
.......................Nucuuuuguagaauagcuaaa...	4	1	0G2
.......................cucuuuuguagaauagcuaaaa..	74	0	0G2
.......................Nucuuuuguagaauagcuaaaa..	1	1	0G2
.......................cucuuuuguagaauagcuaaCa..	11	1	0G2
.......................cAcuuuuguagaauagcuaaaa..	2	1	0G2
.......................cucuuuuguagaauagcuaaaaA...	14	1	0G2
.......................cucuuuuguagaauagcuaaaag...	6	0	0G2
........................ucuuuuguagaauGgcuaa..	7	1	0G2
........................ucuuuuguagaauagcuaa..	42	0	0G2
........................Ncuuuuguagaauagcuaaa...	1	1	0G2
........................ucuGuuguagaauagcuaaa...	1	1	0G2
........................ucuuuuguagaauagcuaaU...	1	1	0G2
........................ucuuuuguagaauGgcuaaa...	2	1	0G2
........................ucuuuuguagGauagcuaaa...	7	1	0G2
........................ucuuuuguagaauagcuaaa...	407	0	0G2
........................ucuuuuguagaauagcAaaa...	1	1	0G2
........................ucuuuuguagaauagcCaaa...	2	1	0G2
........................ucuuAuguagaauagcuaaa...	1	1	0G2
........................ucuCuuguagaauagcuaaa...	7	1	0G2
........................Ncuuuuguagaauagcuaaaa..	1	1	0G2
........................ucuuCuguagaauagcuaaaa..	1	1	0G2
........................ucuuuuguagaauagcuaaUa..	1	1	0G2
........................ucuuuuguagaauagcuaaaa..	141	0	0G2
........................ucCuuuguagaauagcuaaaa..	4	1	0G2
........................ucuuuuguagaauGgcuaaaa..	7	1	0G2
........................ucuuuuguagaauagcuaaaaA...	9	1	0G2
........................ucuuuuguagaauagcuaaaag...	1	0	0G2
.........................cuuuuguagaauGgcuaaa...	13	1	0G2
.........................cuuuuguagaauagcuaaa...	23	0	0G2
.........................cuuuuguagaauagcuaaaa..	51	0	0G2
.........................cuuuuguagGauagcuaaaa..	2	1	0G2
.........................cuuuuguagaauGgcuaaaa..	4	1	0G2
.........................cuuuuguagaauagcuaaaaA...	11	1	0G2
..........................uuuuguagaauagcuaaa...	2	0	0G2
..........................uuuuguagaauagcuaaaa..	12	0	0G2
..........................uuuuguagaauagcuaaaaU...	5	1	0G2
..........................uuuuguagaauagcuaaaag...	8	0	0G2
..........................uuuuguagaauagcuaaaCgg..	6	1	0G2
..........................uuuuguagaauagcuaaCagg..	1	1	0G2
..........................uuuuguagaauagcuaaaagg..	17	0	0G2
...........................uuuguagaauagcuaaaCgg..	7	1	0G2
...........................uuuguagaauagcuaaaagg..	10	0	0G2
.............ucgauCcgggcucuuuugua..	1	1	0B2
.............ucgauCcgggcucuuuuguag...	27	1	0B2
.............ucgauCcgggcucuuuuguaga..	2	1	0B2
..............cgauCcgggcucuuuuguag...	72	1	0B2
..............cgauCcgggcucuuuuguaga..	26	1	0B2
..............cgauCcgggcucuuuuguagaa...	7	1	0B2
..................Ncgggcucuuuuguagaau..	1	1	0B2
...................cgggcucuuuuguagGaua...	5	1	0B2
...................cgggcucuuuuguagaauag..	46	0	0B2
...................cgggcucuuuuguGgaauag..	6	1	0B2
...................cgggcucuuuuguagaauagc...	8	0	0B2
...................cgggcucuuuuguagaauGgc...	3	1	0B2
...................cgggcucuuuuguagaauagcu..	1	0	0B2
...................cgggcucuuuuguagaauagcA..	2	1	0B2
...................cgggcucuuuuguagaauagcua...	1	0	0B2
...................cgggcucuuuuguagaauagcAaa..	2	1	0B2
....................Nggcucuuuuguagaauag..	1	1	0B2
....................gggcucuuuuguagaauag..	2	0	0B2

Mature Star

aucgaaauaucguucgauucgggcucuuuuguagaauagcuaaaagggcuauuguaguuugagaaggaacugcauuaggaaaacugugauuuucaguagagcccuuaaa			
....................gggcucuuuuguagaauagc...	2	0	0B2
.....................ggcucuuuuguagaauag..	2	0	0B2
.......................cucuuuuguagaauagcua...	17	0	0B2
.......................cucuuuuguagaauagcuaa..	243	0	0B2
.......................cucuuuuguagaauGgcuaa..	4	1	0B2
.......................cucuuuuguagGauagcuaa..	8	1	0B2
.......................cAcuuuuguagaauagcuaa..	3	1	0B2
.......................Nucuuuuguagaauagcuaa..	2	1	0B2
.......................cucuuGuguagaauagcuaa..	1	1	0B2
.......................cucuuuuguagaauGgcuaaa...	18	1	0B2
.......................cucCuuuguagaauagcuaaa...	2	1	0B2
.......................cucuuuuguagaauagcuaUa...	14	1	0B2
.......................cucuuuuguagGauagcuaaa...	7	1	0B2
.......................Nucuuuuguagaauagcuaaa...	3	1	0B2
.......................cucuuuuguagaauagcuaaG...	8	1	0B2
.......................Aucuuuuguagaauagcuaaa...	1	1	0B2
.......................cucuuuuguagaauagcCaaa...	9	1	0B2
.......................cucuuuuguagaGuagcuaaa...	4	1	0B2
.......................cucuuuuguagaauagcuaaU...	14	1	0B2
.......................cuGuuuuguagaauagcuaaa...	1	1	0B2
.......................cucuuuGguagaauagcuaaa...	1	1	0B2
.......................cucuuuuguagaauagcuaaa...	935	0	0B2
.......................cuAuuuuguagaauagcuaaa...	2	1	0B2
.......................cucuuuuguagaauGgcuaaaa..	1	1	0B2
.......................cucuuuuguagaauagcuaaaa..	40	0	0B2
.......................cucuuuuguagaauagcuaaaU..	3	1	0B2
.......................cucuuuuguagaauagcuaaaaA...	1	1	0B2
........................ucuuuuguagaauagcuaa..	16	0	0B2
........................ucuuCuguagaauagcuaaa...	1	1	0B2
........................Acuuuuguagaauagcuaaa...	2	1	0B2
........................ucuuuGguagaauagcuaaa...	1	1	0B2
........................ucuuuuguagaauagcuaaa...	278	0	0B2
........................Ncuuuuguagaauagcuaaa...	2	1	0B2
........................ucuuuuguagaauGgcuaaa...	2	1	0B2
........................ucuuuuguagaauagcuaaUa..	1	1	0B2
........................ucuuuuguagaaGagcuaaaa..	1	1	0B2
........................ucuuuuguagaauagcuaaaa..	65	0	0B2
........................Ncuuuuguagaauagcuaaaa..	1	1	0B2
.........................cuuuuguagaauagcuaaa...	24	0	0B2
.........................cuuuuguagaauagcuaaaa..	28	0	0B2
..........................uuuuguagaauagcuaaa...	3	0	0B2
..........................uuuuguagaauagcuaaaa..	7	0	0B2
..........................uuuuguagaGuagcuaaaag...	2	1	0B2
..........................uuuuguagaauagcuaaaCg...	5	1	0B2
..........................uuuuguagaauagcuaaaag...	9	0	0B2
..........................uuuuguagaauagcuaaaaA...	3	1	0B2
..........................uuuuguagaauagcuaaaagU..	3	1	0B2
..........................uuuuguagaauagcuaaaCgg..	6	1	0B2
..........................uuuuguagaauagcuaaaaUg..	1	1	0B2
..........................uuuuguagaauagcuaaaagg..	28	0	0B2
...........................uuuguagaauagcuaaaagU..	1	1	0B2
...........................uuuguagaauagcuaaaagg..	4	0	0B2
...........................uuuguagaauagcuaaaCgg..	7	1	0B2
...........................uuuguagaGuagcuaaaagg..	5	1	0B2
............................uuguagaauagcuaaaagU..	3	1	0B2
.......uaucguucgauucgggcucA..	3	1	0A2
.............ucgauCcgggcucuuuugua..	1	1	0A2
.............ucgauCcgggcucuuuuguag...	33	1	0A2
.............ucgauCcgggcucuuuuguaga..	3	1	0A2
..............cgauCcgggcucuuuuguag...	89	1	0A2
..............cgauCcgggcucuuuuguaga..	46	1	0A2
.................uucgggcucuuuuguagaaua...	1	0	0A2
...................cgggcucuuuuguagaaA..	5	1	0A2
...................cgggcucuuuuguagaaAa...	2	1	0A2
...................cgggcucuuuuguGgaaua...	4	1	0A2
...................cgggcucuuCuguagaaua...	2	1	0A2
...................cgggcucuuuuguGgaauag..	5	1	0A2
...................cgggcucuuuuguagaauGg..	1	1	0A2
...................cgggcucuuuuguagaauag..	47	0	0A2

Mature   Star

```
aucgaaauaucguucgauucgggcucuuuuguagaauagcuaaaaagggcuauuguaguuugagaaggaacugcauuaggaaaacugugauuuucaguagagcccuuaaa
...................cgggcucuuuuguagaauagc......................................................................	17	0	0A2
...................cNggcucuuuuguagaauagc......................................................................	1	1	0A2
...................cgggcucuuuuguagaauagcu.....................................................................	6	0	0A2
...................cgggcucuuuuguagaauagcA.....................................................................	9	1	0A2
...................cgggcucuuuuguagaauagcua....................................................................	2	0	0A2
...................cgggcucuuuuguagaauagcAa....................................................................	4	1	0A2
...................cgggcucuuuuguagaauagcAaa...................................................................	5	1	0A2
....................Cggcucuuuuguagaauag.......................................................................	1	1	0A2
....................gggcucuuuuguagaauag.......................................................................	2	0	0A2
.....................ggcucuuuuguagaauaA.......................................................................	3	1	0A2
.......................cucuuuuguagaauagcua....................................................................	2	0	0A2
.......................cucuuuGguagaauagcuaa...................................................................	5	1	0A2
.......................cucuuuuguagaauagcGaa...................................................................	5	1	0A2
.......................cuAuuuuguagaauagcuaa...................................................................	1	1	0A2
.......................cucuuuuguagaauGgcuaa...................................................................	12	1	0A2
.......................cucuuuuguagaaCagcuaa...................................................................	3	1	0A2
.......................cucuuuuguagaauagcuaa...................................................................	486	0	0A2
.......................cucuuuuguagaauagcAaa...................................................................	9	1	0A2
.......................cucuGuuguagaauagcuaa...................................................................	1	1	0A2
.......................cucuuuAguagaauagcuaa...................................................................	1	1	0A2
.......................Nucuuuuguagaauagcuaa...................................................................	1	1	0A2
.......................cucuuuuguagGauagcuaa...................................................................	2	1	0A2
.......................cucuuuuguagaauagcuaU...................................................................	2	1	0A2
.......................cucuuuuguagaauagcuaaU..................................................................	9	1	0A2
.......................cucuuuuguagaauagcuaaG..................................................................	3	1	0A2
.......................cucuGuuguagaauagcuaaa..................................................................	2	1	0A2
.......................cucuuuuguagaauagcuaaa..................................................................	1884	0	0A2
.......................cucuuuuguagaauagUuaaa..................................................................	1	1	0A2
.......................Nucuuuuguagaauagcuaaa..................................................................	6	1	0A2
.......................cucuuuugAagaauagcuaaa..................................................................	1	1	0A2
.......................cucuuuGguagaauagcuaaa..................................................................	5	1	0A2
.......................cucuAuuguagaauagcuaaa..................................................................	1	1	0A2
.......................cucuuuuguagaauaAcuaaa..................................................................	2	1	0A2
.......................cucuuuuguagaauagcuaUa..................................................................	3	1	0A2
.......................cucuCuuguagaauagcuaaa..................................................................	1	1	0A2
.......................cucuuuuguaUaauagcuaaa..................................................................	1	1	0A2
.......................cucuuCuguagaauagcuaaa..................................................................	2	1	0A2
.......................cucuuGuguagaauagcuaaa..................................................................	1	1	0A2
.......................cAcuuuuguagaauagcuaaa..................................................................	10	1	0A2
.......................cucuuuuguagGauagcuaaa..................................................................	3	1	0A2
.......................cucuuuugCagaauagcuaaa..................................................................	1	1	0A2
.......................cucuuuuguagaauGgcuaaa..................................................................	34	1	0A2
.......................cucCuuuguagaauagcuaaa..................................................................	2	1	0A2
.......................cucuuuAguagaauagcuaaa..................................................................	3	1	0A2
.......................Aucuuuuguagaauagcuaaa..................................................................	1	1	0A2
.......................cucuuuCguagaauagcuaaa..................................................................	2	1	0A2
.......................cucuuuuguagaGuagcuaaa..................................................................	2	1	0A2
.......................cucuuuuguagaauagcuaaUa.................................................................	2	1	0A2
.......................cucuuuGguagaauagcuaaaa.................................................................	2	1	0A2
.......................cucuuuuguagaauagcuaaaU.................................................................	4	1	0A2
.......................cucuuuuguagaauagcuaaaa.................................................................	172	0	0A2
.......................cucuuuuguagaauGgcuaaaa.................................................................	4	1	0A2
.......................cuGuuuuguagaauagcuaaaa.................................................................	2	1	0A2
.......................cucuuCuguagaauagcuaaaa.................................................................	1	1	0A2
.......................cucuuuuguagaauGgcuaaaag................................................................	2	1	0A2
.......................cucuuuuguagaauagcuaaaaA................................................................	5	1	0A2
........................ucGuuuguagaauagcuaa...................................................................	1	1	0A2
........................ucuuuuguagaauagcuaa...................................................................	40	0	0A2
........................ucuuuuguagGauagcuaaa..................................................................	1	1	0A2
........................ucuuuuguagaauagcuaaa..................................................................	411	0	0A2
........................ucuGuuguagaauagcuaaa..................................................................	1	1	0A2
........................Ncuuuuguagaauagcuaaa..................................................................	2	1	0A2
........................uGuuuuguagaauagcuaaa..................................................................	1	1	0A2
........................ucuuuGguagaauagcuaaa..................................................................	2	1	0A2
........................ucuuuuguagaauGgcuaaa..................................................................	15	1	0A2
........................ucuuuuguagaGuagcuaaa..................................................................	3	1	0A2
........................ucuuuugGagaauagcuaaa..................................................................	1	1	0A2
........................ucuuuuguagaauGgcuaaaa.................................................................	8	1	0A2
........................ucuuuuguagaGuagcuaaaa.................................................................	1	1	0A2
........................ucuuuuguagaauagcuaaaa.................................................................	133	0	0A2
```

```
                        Mature                                                        Star
aucgaaauaucguucgauucgggcucuuuuguagaauagcuaaaagggcuauuguaguuugagaaggaacugcauuaggaaaacugugauuuucaguagagcccuuaaa
........................ucuuuuguagGauagcuaaaa................................................................   5    1    0A2
........................ucuuuuguagaauagcuaaaag...............................................................   3    0    0A2
........................ucuuuuguagaauagcuaaaaA...............................................................   13   1    0A2
........................ucuuuuguagaauagcuaaaaU...............................................................   2    1    0A2
.........................cuuuuguagaauagcuaC..................................................................   1    1    0A2
.........................cuuuuguagaauagcuaaa.................................................................   36   0    0A2
.........................cuuuuguagaauagcuaaaa................................................................   53   0    0A2
.........................cuuuuguagaauagcuaaCa................................................................   6    1    0A2
.........................cuuuuguagaauGgcuaaaa................................................................   9    1    0A2
.........................cuuuuguagaauagcuaaaaA...............................................................   9    1    0A2
.........................cuuuuguagaauagcuaaaaU...............................................................   6    1    0A2
..........................uuuuguagGauagcuaaa.................................................................   1    1    0A2
..........................uuuuguagaauagcuaaa.................................................................   1    0    0A2
..........................uuuuguagaauagcuaaaa................................................................   7    0    0A2
..........................uuGuguagaauagcuaaaag...............................................................   2    1    0A2
..........................uuuuguagaauagcuaaaag...............................................................   19   0    0A2
..........................uuuuguagaauagcuaaaaA...............................................................   6    1    0A2
..........................uuuuguagaauagcuaaaCg...............................................................   1    1    0A2
..........................uuuuguagaauagcuaaaCgg..............................................................   2    1    0A2
..........................uuuuguagaauagcuaaaaUg..............................................................   1    1    0A2
..........................uuuuguagaauagcuaaaagg..............................................................   32   0    0A2
...........................uuuguagaauagcuaaaa................................................................   2    0    0A2
...........................uuuguagaauagcuaaaag...............................................................   4    0    0A2
...........................uuuguagaauagcuaaaaU...............................................................   2    1    0A2
...........................uuuguagaauagcuaaCagg..............................................................   5    1    0A2
...........................uuuguagaauagcuaaaagg..............................................................   13   0    0A2
...........................uuuguagaauagcuaaaCgg..............................................................   10   1    0A2
.......................................cuaaaaggUcuauuguaguu..................................................   2    1    0A2
```

Provisional ID	:	scaffold134_23126
Score total	:	1.8
Score for star read(s)	:	-1.3
Score for read counts	:	0
Score for mfe	:	2.1
Score for randfold	:	1.6
Score for cons. seed	:	-0.6
Total read count	:	6156
Mature read count	:	6156
Loop read count	:	0
Star read count	:	0

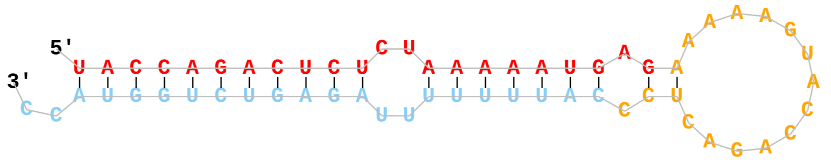

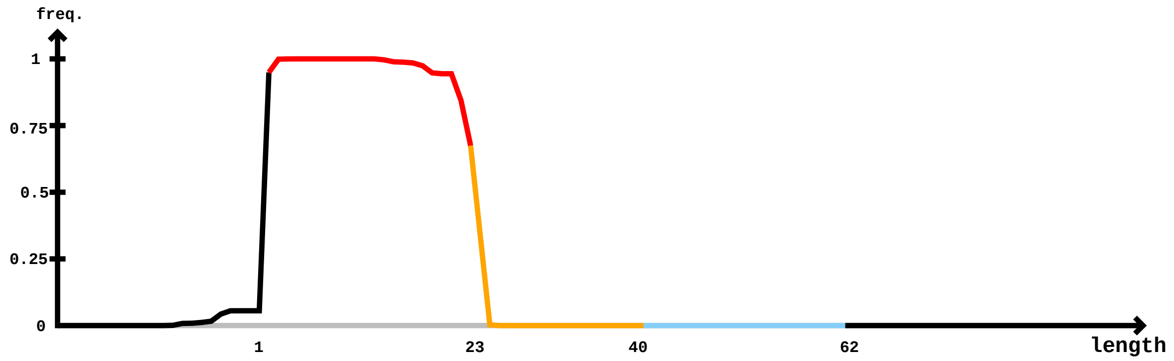

Mature **Star**

```
5'- guguaaaucgguacccugggUACCAGACUCUCUAAAAAAUGAGaaaaaguaccagacucccauuuuuuuagagucugguacccagguaguaaaucggcgacucuguucuga -3'    exp
    .........(.((((((((((((((((((..(((((((.((............)).)))))))..)))))))))))))))))))).)...((((.(((...)))))))    reads    mm    sample
    ...........uacccuggguaccagacucuc...............................................................................    6    0    0G2
    ...........uacccuggguaccagCcucucu..............................................................................    5    1    0G2
    ...........uacccuggguaccagacucucu..............................................................................    3    0    0G2
    ...........uacccuggguaccGgacucucu..............................................................................    4    1    0G2
    .............cccuggguaccagacucucuaaa...........................................................................    2    0    0G2
    ..............ccuggguaccagacucucu..............................................................................    2    0    0G2
    ..............ccuAgguaccagacucucua.............................................................................    6    1    0G2
    ..............ccuggguaccagacucucuaUa...........................................................................    2    1    0G2
    ..............ccuggguaccagacucucuaaCa..........................................................................    1    1    0G2
    ...............cuggguaccagacucucuaa............................................................................    7    0    0G2
    ...............cuggguaccagacucucuaaU...........................................................................    6    1    0G2
    ...............cuggguaccagacucucuaaa...........................................................................    2    0    0G2
    ...............cuggguaccagacucucuaaC...........................................................................    5    1    0G2
    ...............cuggguaccagacucucuaaCa..........................................................................    16    1    0G2
    ...............cuggguaccagacucucuaaUa..........................................................................    1    1    0G2
    ................uggguaccagacucucuaaU...........................................................................    8    1    0G2
    ................uggguaccagacucucuaaCa..........................................................................    25    1    0G2
    ....................uaccagacucucuaaaaaaug......................................................................    71    0    0G2
    ....................uaccagacucucuaaaaaGug......................................................................    36    1    0G2
    ....................uaccagacucucuaaaaaauga.....................................................................    202    0    0G2
    ....................uaccaAacucucuaaaaaauga.....................................................................    1    1    0G2
    ....................uaccGgacucucuaaaaaauga.....................................................................    15    1    0G2
    ....................uaAcagacucucuaaaaaauga.....................................................................    2    1    0G2
    ....................uaccagacucAcuaaaaaauga.....................................................................    1    1    0G2
    ....................uaccagacucucuaaaaaaugaA....................................................................    684    1    0G2
    ....................uaccagacucucuaaaaaaugaU....................................................................    1    1    0G2
    ....................uaccagacucucuaaaaaaugaAa...................................................................    23    1    0G2
    .....................accagacucucuaaaaaaug......................................................................    1    0    0G2
    .....................accagacucucuaaaaaauga.....................................................................    11    0    0G2
    .....................accagacucucuaaaaaaugaA....................................................................    38    1    0G2
    .....................accagacucucuaaaaaaugaAa...................................................................    3    1    0G2
    ......................ccagacucucuaaaaaaugaA....................................................................    4    1    0G2

    ...........uacUcuggguaccagacucuc...............................................................................    3    1    0B2
```

```
                    Mature                                      Star
guguaaaucgguacccugggg uaccagacucucuaaaaaugagaaaaaguaccagacucccauuuuuuuagagucugguacccagguaguaaaucggcgacucuguucuga
```

```
guguaaaucgguacccuggguaccagacucucuaaaaaugagaaaaaguaccagacucccauuuuuuuagagucugguacccagguaguaaaucggcgacucuguucuga
...........uacccuggguaccagacucuc..............................................................................    3     0    0B2
...........uacccuggguaccagCcucucu.............................................................................    13    1    0B2
...........uacccuggguaccagacucucu.............................................................................    1     0    0B2
...........uacccuggguaccagacucuAu.............................................................................    1     1    0B2
............acccuAgguaccagacucuc..............................................................................    5     1    0B2
.............cccuggguaccagacucucu.............................................................................    4     0    0B2
.............cccuggguaccagacucucuaU...........................................................................    1     1    0B2
.............cccuggguaccagacucucuaC...........................................................................    3     1    0B2
.............cccuggguaccagacucucuaaU..........................................................................    2     1    0B2
..............ccuAgguaccagacucucu.............................................................................    2     1    0B2
..............ccuggguaccagacucucuaaU..........................................................................    1     1    0B2
...............cuggguaccagacucucuaaC..........................................................................    15    1    0B2
...............cuggguaccagacucucuaaU..........................................................................    6     1    0B2
...............cuggguaccagacucucuaaUa.........................................................................    3     1    0B2
...............cuggguaccagacucucuaaCa.........................................................................    38    1    0B2
...............cuggguaccagacucucuaaUaa........................................................................    2     1    0B2
................uggAuaccagacucucuaa...........................................................................    1     1    0B2
................uggguaccagacucucuaaC..........................................................................    5     1    0B2
................uggAuaccagacucucuaaa..........................................................................    8     1    0B2
................uggguaccagacucucuaaCa.........................................................................    10    1    0B2
.................ggguaccagacucucuaaCaau.......................................................................    1     1    0B2
....................Naccagacucucuaaaaaug......................................................................    1     1    0B2
....................uaccagacucucuaaaaGug......................................................................    57    1    0B2
....................uaccagacucucuaaaaaug......................................................................    100   0    0B2
....................uaccaUacucucuaaaaaug......................................................................    1     1    0B2
....................uaccagaUucucuaaaaauga.....................................................................    1     1    0B2
....................uaccGgacucucuaaaaauga.....................................................................    14    1    0B2
....................uaccagacucucuaaaaGuga.....................................................................    9     1    0B2
....................uaccagacucCcuaaaaauga.....................................................................    1     1    0B2
....................Naccagacucucuaaaaauga.....................................................................    1     1    0B2
....................uaccagacucucuaaaaauga.....................................................................    345   0    0B2
....................uaccagacucucuaaaaaugaU....................................................................    1     1    0B2
....................uaccagacucucuaaaaaugag....................................................................    1     0    0B2
....................uaccagacucucuaaaaaugaA....................................................................    1427  1    0B2
....................uaccagacucucuaaaaaugaAa...................................................................    15    1    0B2
.....................Nccagacucucuaaaaaug......................................................................    1     1    0B2
.....................accagacucucuaaaaaug......................................................................    6     0    0B2
.....................accagacucucuaaaaGug......................................................................    11    1    0B2
.....................accagacucucuaaaaauga.....................................................................    29    0    0B2
.....................aAcagacucucuaaaaauga.....................................................................    1     1    0B2
.....................acAagacucucuaaaaauga.....................................................................    1     1    0B2
.....................accagacucucuaaaaaugaA....................................................................    82    1    0B2

..........guacccuggguaccagCcucuc..............................................................................    6     1    0A2
...........uacccuggguaGcagacucuc..............................................................................    1     1    0A2
...........uacccuggguaccagCcucucu.............................................................................    3     1    0A2
...........uacccuggguaccagacucucu.............................................................................    3     0    0A2
.............cccuAgguaccagacucucu.............................................................................    1     1    0A2
.............cccuggguaccagacucucuaU...........................................................................    4     1    0A2
.............cccuAgguaccagacucucuaa...........................................................................    1     1    0A2
.............cccuggguaccagacucucuaaU..........................................................................    1     1    0A2
..............ccuAgguaccagacucucu.............................................................................    3     1    0A2
..............ccuggguacAagacucucua............................................................................    1     1    0A2
..............ccuggguaccagacucucua............................................................................    1     0    0A2
..............ccuggguaccagacucucuaaa..........................................................................    2     0    0A2
..............ccuggguaccagacucucuaaUa.........................................................................    1     1    0A2
..............ccuggguaccagacucucuaaCaa........................................................................    8     1    0A2
...............cuggguaccagacucucu.............................................................................    3     0    0A2
...............cuggguaccagacucucuaa...........................................................................    2     0    0A2
...............cuggguaccagacucucuaaCa.........................................................................    43    1    0A2
...............cuggguaccagacucucuaaUa.........................................................................    18    1    0A2
...............cuggguaccagacucucuaaCaa........................................................................    8     1    0A2
................uggAuaccagacucucuaaa..........................................................................    4     1    0A2
................uggguaccagacucucuaaCa.........................................................................    15    1    0A2
................uggguaccagacucucuaaUa.........................................................................    4     1    0A2
....................uaccagacucucuaUaaa........................................................................    2     1    0A2
....................uaccGgacucucuaaaaaug......................................................................    8     1    0A2
....................Naccagacucucuaaaaaug......................................................................    1     1    0A2
....................uaccagacucucuaaaaaGg......................................................................    1     1    0A2
....................uaccagacucucuaaaaauA......................................................................    3     1    0A2
```

Mature Star

guguaaaucgguacccuggguaccagacucucuaaaaaugagaaaaaguaccagacucccauuuuuuuagagucugguacccagguaguaaaucggcgacucuguucuga			
....................uaccagacucucuaGaaaug..	2	1	0A2
....................uaccagacucucuaaaaGug..	60	1	0A2
....................uaccagacucucuaaaaaug..	274	0	0A2
....................uaUcagacucucuaaaaaug..	1	1	0A2
....................uNccagacucucuaaaaauga...	1	1	0A2
....................uaccagaUucucuaaaaauga...	1	1	0A2
....................uaccGgacucucuaaaaauga...	14	1	0A2
....................uaccagacucucGaaaaauga...	1	1	0A2
....................uaccagacucucuaaaaauga...	434	0	0A2
....................Naccagacucucuaaaaauga...	1	1	0A2
....................uaccagacucucuaaaaaugaU..	1	1	0A2
....................uaccagacucucuaaaaaugaA..	1937	1	0A2
....................uaccagacucucuaaaaaugaAa...	57	1	0A2
....................uaccagacucucuaaaaaugaAaa..	16	1	0A2
.....................accagacucucuaaaaaug..	15	0	0A2
.....................accagacucGcuaaaaaug..	1	1	0A2
.....................accagacucucuaaaaauga...	19	0	0A2
.....................accagacucucuaaaaaugaA..	94	1	0A2
.....................accagacucucuaaaaaugaAa...	8	1	0A2
.....................accagacucucuaaaaaugaAaa..	3	1	0A2
......................ccagacucucuaaaaaug..	1	0	0A2
......................ccagacucucuaaaaauga...	1	0	0A2
.......................cagacucucuaaaaaugaA..	1	1	0A2

Provisional ID	:	scaffold156_25441
Score total	:	0.2
Score for star read(s)	:	-1.3
Score for read counts	:	0
Score for mfe	:	-3
Score for randfold	:	1.6
Score for cons. seed	:	3
Total read count	:	101
Mature read count	:	101
Loop read count	:	0
Star read count	:	0

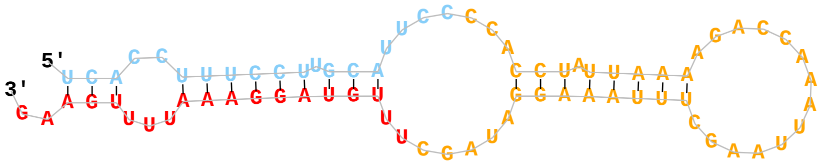

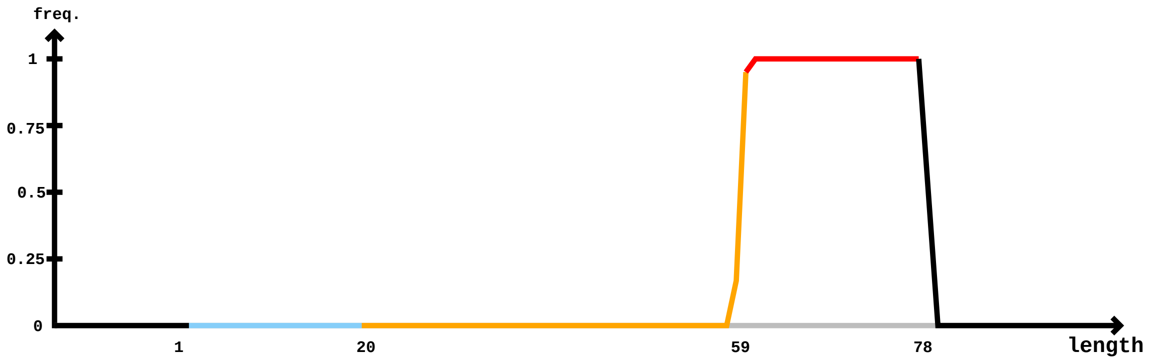

Star **Mature**

```
5'- auuucgacagcuucaccuuuccuugcauuccccaccuauuaaaagaccaaauuaagcuuuaaaggauagcuuuguaggaaauuuugaagucugucucaguauuagagcc   -3'    exp
    .....((((((((((..((((((.(((.......(((.(((((..............))))))))).......)))))))))...))))).)))))..............    reads      mm          sample
    .....................................................................Uuuuguaggaaauuuugaag....................    11         1           0A2
    ......................................................................uuuguaggaaauuuugaag....................    6          0           0A2
    ......................................................................uuuguaggaaauuuugaaA....................    1          1           0A2
    ......................................................................uuuguGggaaauuuugaag....................    5          1           0A2
    ......................................................................uuuguaggaaauuuugUag....................    5          1           0A2
    ......................................................................uuuguaggaaauuuugaagG...................    13         1           0A2
    ......................................................................uuuguaggaaauuuugaagA...................    2          1           0A2
    .......................................................................uuguaggaaauuuugUag....................    1          1           0A2

    ......................................................................uuuguaggaaauuuugCag....................    2          1           0B2
    ......................................................................uuuguaggaaauuuugaagG...................    2          1           0B2

    .....................................................................Uuuuguaggaaauuuugaag....................    6          1           0G2
    ......................................................................uuuguaggaaauuuugaag....................    26         0           0G2
    ......................................................................uuuguaggaaauuuugUag....................    5          1           0G2
    ......................................................................uuuguaggaaauuuugaagG...................    12         1           0G2
    .......................................................................uuguaggaaauuuugaag....................    4          0           0G2
```

Provisional ID	:	scaffold157_25594
Score total	:	837.3
Score for star read(s)	:	3.9
Score for read counts	:	830.6
Score for mfe	:	1.7
Score for randfold	:	1.6
Score for cons. seed	:	-0.6
Total read count	:	1641
Mature read count	:	1157
Loop read count	:	0
Star read count	:	484

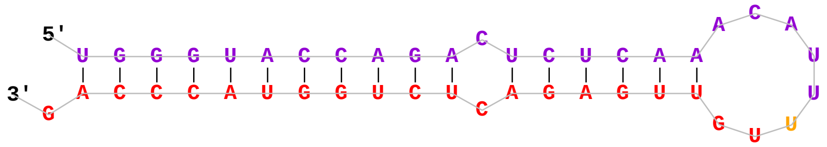

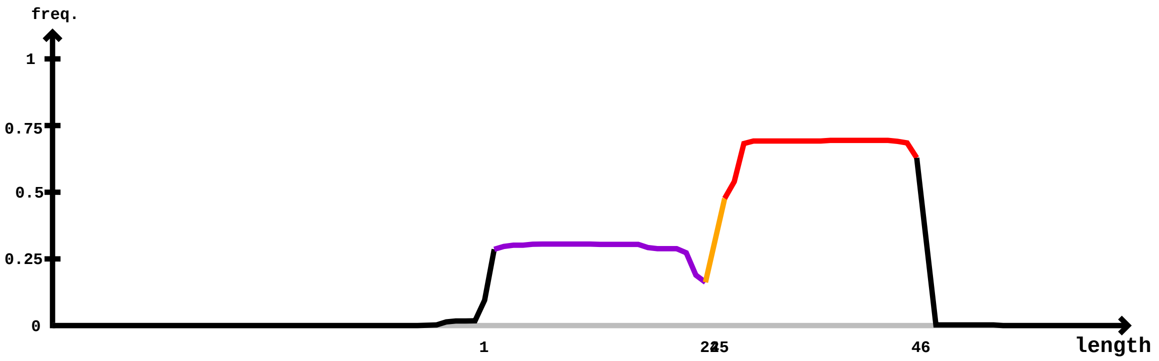

Star Mature

```
5'- uuuuauauucaucuauuuauuucugcucuuuacuccuccuacccuggguaccagacucucaaacauuuuguugagacucugguacccaggguauacuccuccguacaaga  -3'  obs
    uuuuauauucaucuauuuauuucugcucuuuacuccuccuacccuggguaccagacucucaaacauuuuguugagacucugguacccaggguauacuccuccguacaaga       exp
    ..........................(((((((......((((((((((((((((.((((((........)))))).)))))))))))))))).........))).))))  reads  mm  sample
    .......................................uacccuggguaccagacucuc..................................................  3      0   0B2
    .......................................uacUcuggguaccagacucuc..................................................  3      1   0B2
    .......................................uacccugggAaccagacucuca.................................................  6      1   0B2
    ........................................acccuAgguaccagacucuc..................................................  5      1   0B2
    ...........................................cuggguaccagacucucUaac..............................................  15     1   0B2
    ...........................................cuggguaccagacucucUaaca.............................................  38     1   0B2
    ...........................................cuggguaccagacucucUaacau............................................  6      1   0B2
    ............................................uggguaccagacucucUaac..............................................  5      1   0B2
    ............................................uggguaccagacucucUaaca.............................................  10     1   0B2
    ............................................uggguaccagacucucUaacau............................................  26     1   0B2
    ............................................uggguaccagacucucUaacauu...........................................  56     1   0B2
    ............................................uggguaccagacucucUaacauuu..........................................  14     1   0B2
    .............................................ggguaccagacucucUaacauu...........................................  6      1   0B2
    .............................................ggguaccagacucucUaacauuu..........................................  2      1   0B2
    .................................................................uuuuCuugagacucugguacc........................  6      1   0B2
    ..................................................................uuuCuugagacucugguaccc.......................  6      1   0B2
    ...................................................................uuCuugagacucugguaccca......................  1      1   0B2
    ...................................................................uuCuugagacucugguacccag.....................  22     1   0B2
    ....................................................................uCuugagacucugguaccca......................  16     1   0B2
    ....................................................................uCuugagacucugguacccag.....................  151    1   0B2
    ....................................................................uCuugagacucugguacccagg....................  5      1   0B2
    .....................................................................Nuugagacucugguacccag.....................  1      1   0B2
    .....................................................................Cuugagacucugguacccag.....................  18     1   0B2
    .....................................................................Cuugagacucugguacccagg....................  10     1   0B2
    ......................................................................uugagacucugguacccag.....................  12     0   0B2
    ......................................................................uugagacucugguacccagg....................  30     0   0B2
    .......................................................................ugagacucugguacccag.....................  1      0   0B2
    .......................................................................ugagacucugguacccCg.....................  1      1   0B2
    .......................................................................ugagacucugguacccagg....................  3      0   0B2
    ...............................................................................ugguacccaggguaGacu.............  4      1   0B2

    ......................................cuacccuggguaccagacucuU..................................................  2      1   0G2
    .......................................uacccuggguaccagacucuc..................................................  6      0   0G2
```

Star Mature

```
uuuuauauucaucuauuuauuucugcucuuuacuccuccuacccuggguaccagacucucaaacauuuuguugagacucugguacccaggguauacuccuccguacaaga
..........................................ccuggguaccagacucucUaaca.............................................   1     1    0G2
...........................................cuggguaccagacucucCaac..............................................   1     1    0G2
...........................................cuggguaccagacucucUaac..............................................   5     1    0G2
...........................................cuggguaccagacucucUaaca.............................................   16    1    0G2
............................................uggguaccagacucucUaaca.............................................   25    1    0G2
............................................uggguaccagacucucUaacau............................................   6     1    0G2
............................................uggguaccagacucucUaacauu...........................................   49    1    0G2
............................................uggguaccagacucucUaacauuu..........................................   9     1    0G2
.............................................ggguaccagacucucUaacauu...........................................   3     1    0G2
..............................................gguaccagacucucUaacauuu..........................................   2     1    0G2
..............................................gguaccagacucucUaacauuuu.........................................   6     1    0G2
..................................................................uuuCuugagacucugguaccc.......................   3     1    0G2
...................................................................uuCuugagacucugguacccag.....................   20    1    0G2
....................................................................uCuugagacucugguaccca......................   20    1    0G2
....................................................................uCuugagacucugguacccag.....................   142   1    0G2
.....................................................................Cuugagacucugguaccca......................   2     1    0G2
.....................................................................Cuugagacucugguacccag.....................   24    1    0G2
......................................................................uugagacucugguacccag.....................   48    0    0G2
......................................................................uugagacucugguacccagg....................   38    0    0G2
......................................................................Augagacucugguacccagg....................   1     1    0G2
......................................................................uugagacCcugguacccagg....................   1     1    0G2
.......................................................................ugagGcucugguacccag.....................   1     1    0G2
.......................................................................ugagacucugguacccag.....................   1     0    0G2

.....................................ccuacccuggguaccaga.......................................................   2     0    0A2
.......................................uacccuggguaGcagacucuc..................................................   1     1    0A2
........................................acccuggCuaccagacucuca.................................................   1     1    0A2
...........................................cuggguaccagacucucUaaca.............................................   43    1    0A2
...........................................cuggguaccagacucucUaacau............................................   6     1    0A2
............................................uggguaccagacucucUaaca.............................................   15    1    0A2
............................................uggguaccagacucucUaacau............................................   9     1    0A2
............................................uggguaccagacucucUaacauu...........................................   86    1    0A2
............................................uggguaccagacucucUaacauuu..........................................   11    1    0A2
.............................................ggguaccagacucucUaacau............................................   5     1    0A2
.............................................ggguaccagacucucUaacauu...........................................   2     1    0A2
................................................uaccagacucucUaacau............................................   1     1    0A2
................................................uaccagacucucUaacauu...........................................   2     1    0A2
................................................uaccagacucucUaacauuu..........................................   3     1    0A2
.................................................accagacucucUaacauuuu.........................................   1     1    0A2
.................................................................uuuuCuugagacucugguaccc.......................   1     1    0A2
..................................................................uuuCuugagacucugguaccca......................   5     1    0A2
...................................................................uuCuugagacucugguaccca......................   9     1    0A2
...................................................................uuCuugagacucugguacccag.....................   12    1    0A2
....................................................................uCuugagacucugguaccca......................   25    1    0A2
....................................................................uCuugagacucugguacccag.....................   344   1    0A2
....................................................................uCuugagacucugguacccagg....................   7     1    0A2
.....................................................................Cuugagacucugguaccca......................   14    1    0A2
.....................................................................Cuugagacucugguacccag.....................   38    1    0A2
.....................................................................Cuugagacucugguacccagg....................   6     1    0A2
......................................................................uugagacucugguaccca......................   2     0    0A2
......................................................................uugagacucuCguacccag.....................   1     1    0A2
......................................................................uugagacucugguacccaA.....................   1     1    0A2
......................................................................uugagacucugguacccag.....................   46    0    0A2
......................................................................uugagacucugguacccagg....................   59    0    0A2
......................................................................NugagacucugguacccagG....................   1     1    0A2
.......................................................................ugagacucugguacccag.....................   4     0    0A2
.......................................................................ugagacucugguacccagg....................   5     0    0A2
```

Provisional ID	:	scaffold166_26452
Score total	:	5962.3
Score for star read(s)	:	3.9
Score for read counts	:	5951.3
Score for mfe	:	2.5
Score for randfold	:	1.6
Score for cons. seed	:	3
Total read count	:	11685
Mature read count	:	10902
Loop read count	:	0
Star read count	:	783

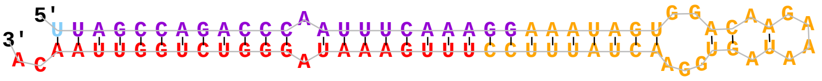

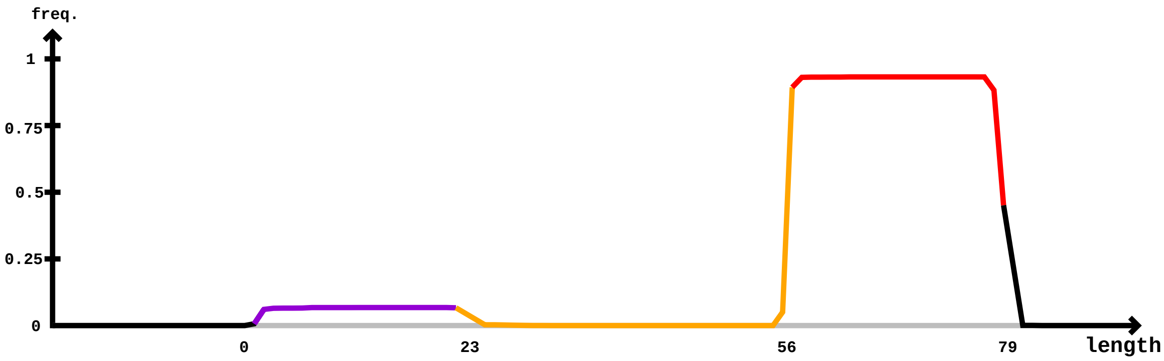

Star Mature

```
5'- auccuguuagcauauccuguuagccagacccaauuucaaaggaaauaguggacaagaaauaguggaacuauuuccuuugaaauagggucugguuaacaggcuaggaugcac  -3'   obs
    auccuguuagcauauccuguuagccagacccaauuucaaaggaaauaguggacaagaaauaguggaacuauuuccuuugaaauagggucugguuaacaggcuaggaugcac          exp
    .........((((..((((((((((((((((.(((((((((((((((((..((.(....).))...))))))))))))))))).)))))))))))))))).....))))..   reads   mm   sample
    ...................Cuagccagacccaauuucaaa.......................................................................   1       1    0A2
    ...................uuagccagacccaauuucaaa.......................................................................   8       0    0A2
    ...................uuagccagacccaauuucaaag......................................................................   13      0    0A2
    ...................uuaAccagacccaauuucaaag......................................................................   3       1    0A2
    ...................uuagccagacccaauuucaaagg.....................................................................   2       0    0A2
    ...................uuaAccagacccaauuucaaagg.....................................................................   2       1    0A2
    ....................uagccagacccaauuuca.........................................................................   1       0    0A2
    ....................uagccagacccaauuucaaa.......................................................................   2       0    0A2
    ....................uagccagacccaauuucaaag......................................................................   24      0    0A2
    ....................uagccaUacccaauuucaaagg.....................................................................   1       1    0A2
    ....................uagccagacccaauuucaaagg.....................................................................   239     0    0A2
    ....................uagccagacccGauuucaaagg.....................................................................   4       1    0A2
    ....................uagccagacUcaauuucaaagg.....................................................................   1       1    0A2
    ....................uagUcagacccaauuucaaagg.....................................................................   2       1    0A2
    ....................uagccagacccaauuucaaagga....................................................................   1       0    0A2
    .....................agccagacccaauuucaaag......................................................................   5       0    0A2
    .....................agccagacccaauuucaaagg.....................................................................   17      0    0A2
    .....................Ggccagacccaauuucaaagg.....................................................................   2       1    0A2
    .....................agccagacccaauuucaaagga....................................................................   6       0    0A2
    ......................gccagacccaauuucaaagga....................................................................   3       0    0A2
    ..........................................................................cuuugaaauGgggucugguua................   1       1    0A2
    ..........................................................................cuuugaaauUgggucugguuaa...............   153     1    0A2
    ..........................................................................cuuugaaauUgggucugguuaac..............   6       1    0A2
    ..........................................................................cuuugaaauUgggucugguuaaca.............   12      1    0A2
    ...........................................................................uuugaaauUgggucugguuaa...............   27      1    0A2
    ...........................................................................uuugaaauUgggucugguuaac..............   1259    1    0A2
    ...........................................................................uuugaaauGgggucugguuaac..............   1       1    0A2
    ...........................................................................uuugaaauagggucugguuaaca.............   9       0    0A2
    ...........................................................................uuugaaauGgggucugguuaaca.............   2       1    0A2
    ...........................................................................uuugaaauUgggucugguuaaca.............   1353    1    0A2
    ...........................................................................uuugaaauagggucugguuaUca.............   1       1    0A2
    ............................................................................uugaaauUgggucugguuaac..............   57      1    0A2
    ............................................................................uugaaauUgggucugguuaaca.............   67      1    0A2
```

```
                    Star                                                   Mature
auccuguuagcauauccuguuagccagacccaauuucaaaggaaauaguggacaagaaauaguggaacuauuuccuuugaaauagggucugguuaacaggcuaggaugcac
.............................................................................ugaaauUgggucugguuaaca.............    1      1     0A2
...............................................................................aaauUgggucugguuaaca.............    2      1     0A2

...................uuaAccagacccaauuucaaag......................................................................    5      1     0G2
...................uuagccagacccaauuucaaag......................................................................    21     0     0G2
...................uuagccagacccaauuucaaagg.....................................................................    4      0     0G2
....................uagccagacccaauuucaaGg......................................................................    4      1     0G2
....................uagccagacccaauuucaaag......................................................................    11     0     0G2
....................uagccagaAccaauuucaaagg.....................................................................    1      1     0G2
....................uagccagacccaauuucaaagg.....................................................................    158    0     0G2
....................uagUcagacccaauuucaaagg.....................................................................    1      1     0G2
....................uagccagacccaauuucaaagga....................................................................    3      0     0G2
....................uagccaAacccaauuucaaagga....................................................................    1      1     0G2
....................uagccagacccaauuucaaaggaaa..................................................................    8      0     0G2
.....................Ggccagacccaauuucaaagga.....................................................................    4      1     0G2
.....................agccagacccaauuucaaagga.....................................................................    6      0     0G2
......................gccagacccaauuucaaagg.....................................................................    3      0     0G2
.........................agacccaauuucaaAgaaau..................................................................    7      1     0G2
.........................agacccaauuucaaAgaaaua.................................................................    8      1     0G2
.........................agacccaauuucaaAgaaauag................................................................    6      1     0G2
..........................................................................cuuugaaauUgggucugguuaa...............    191    1     0G2
..........................................................................cuuugaaauGgggucugguuaa...............    1      1     0G2
..........................................................................cuuugaaauUgggucugguuaac..............    5      1     0G2
..........................................................................cuuugaaauUgggucugguuaaca.............    14     1     0G2
...........................................................................uuugaaauUgggucugguuaa...............    22     1     0G2
...........................................................................uuugaaauUgggucugguuaac..............    1323   1     0G2
...........................................................................uuugaaauGgggucugguuaaca.............    1      1     0G2
...........................................................................uuugaaauUgggucugguuaaca.............    2385   1     0G2
............................................................................uugaaauUgggucugguuaa...............    4      1     0G2
............................................................................uugaaauUgggucugguuaac..............    38     1     0G2
............................................................................uugaaauGgggucugguuaaca.............    1      1     0G2
............................................................................uugaaauagggucugguuaUca.............    1      1     0G2
............................................................................uugaaauUgggucugguuaaca.............    96     1     0G2
.............................................................................ugaaauUgggucugguuaac..............    2      1     0G2

...................uuaAccagacccaauuucaaag......................................................................    1      1     0B2
...................uuagccagacccaauuucaaag......................................................................    8      0     0B2
...................uuagccagacccaauuucaaagg.....................................................................    8      0     0B2
...................uuaAccagacccaauuucaaagg.....................................................................    2      1     0B2
...................Nuagccagacccaauuucaaagg.....................................................................    1      1     0B2
....................uagccagacccaauuucaaag......................................................................    3      0     0B2
....................uagccagacccaauuucaGag......................................................................    3      1     0B2
....................uGgccagacccaauuucaaagg.....................................................................    2      1     0B2
....................uagccagacccGauuucaaagg.....................................................................    1      1     0B2
....................uagccagacccaauuucaaagg.....................................................................    153    0     0B2
....................Nagccagacccaauuucaaagg.....................................................................    2      1     0B2
....................uagccagacccaauuucaaagga....................................................................    4      0     0B2
.....................agccagacccaauuucaaagg.....................................................................    6      0     0B2
.....................agccagacccaauuucaaagga....................................................................    4      0     0B2
........................cagacccaauuucaaaggaaUu.................................................................    3      1     0B2
..............................caauuucaaaggaCauagug.............................................................    2      1     0B2
..........................................................................cuuugaaauUgggucugguuaa...............    162    1     0B2
..........................................................................cuuugaaauUgggucugguuaac..............    27     1     0B2
..........................................................................cuuugaaauUgggucugguuaaca.............    17     1     0B2
...........................................................................uuugaaauagggucugguuaa...............    1      0     0B2
...........................................................................uuugaaauUgggucugguuaa...............    19     1     0B2
...........................................................................uuugaaauGgggucugguuaac..............    1      1     0B2
...........................................................................uuugaaauUgggucugguuaac..............    2229   1     0B2
...........................................................................uuugaaauUgggucugguuaaca.............    1223   1     0B2
............................................................................uugaaauUgggucugguuaac..............    92     1     0B2
............................................................................uugaaauUgggucugguuaaca.............    82     1     0B2
.............................................................................ugaaauUgggucugguuaac..............    7      1     0B2
.................................................................................auagggucugguuaacaUgc..........    7      1     0B2
```

Provisional ID	:	scaffold166_26524
Score total	:	5816.3
Score for star read(s)	:	3.9
Score for read counts	:	5805.5
Score for mfe	:	2.3
Score for randfold	:	1.6
Score for cons. seed	:	3
Total read count	:	11399
Mature read count	:	11386
Loop read count	:	0
Star read count	:	13

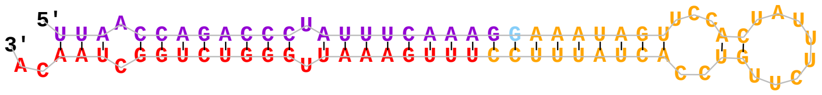

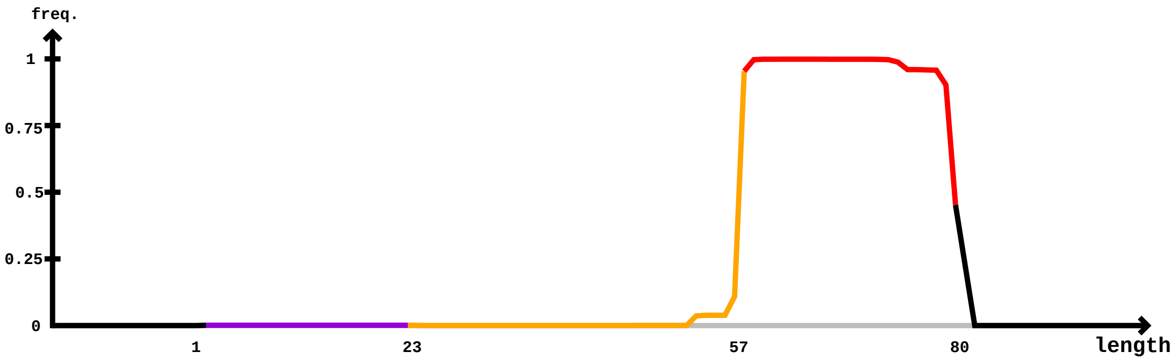

Star Mature

```
5'- ugcauccuagccuguuaaccagacccuauuucaaaggaaauaguuccacuauuucuuguccacuauuuccuuugaaauugggucuggcuaacaggauaugcuaacaggauau   -3'   obs
    ugcauccuagccuguuaaccagacccuauuucaaaggaaauaguuccacuauuucuuguccacuauuuccuuugaaauugggucuggcuaacaggauaugcuaacaggauau         exp
    .((((.....(((((((.((((((((.(((((((((((((((((...((........))..))))))))))))))))).)))))))).)))))))..))))...........   reads   mm   sample
    ..............uuaaccagacccAauuucaaag............................................................................   3       1    0A2
    ..............uuaaccagacccAauuucaaagg...........................................................................   2       1    0A2
    .................................................................Nuuccuuugaaauugggucug..........................   1       1    0A2
    .................................................................uuuccuuugaaauugggucug..........................   33      0    0A2
    .................................................................uuuccuuugaaauugggucugg.........................   47      0    0A2
    .................................................................uuuccuuugaGauugggucugg.........................   4       1    0A2
    .................................................................uuuccuuugaaauugggucugU.........................   57      1    0A2
    ..................................................................uuccuuugaaauugggucugg.........................   7       0    0A2
    .....................................................................cuuugaaauugggucugg.........................   7       0    0A2
    .....................................................................cuuugaaauugggucugU.........................   3       1    0A2
    .....................................................................cuuugaaauugggucuggcu.......................   9       0    0A2
    .....................................................................cuuugaaauugggucuggcua......................   2       0    0A2
    .....................................................................cuuugaaauugggucuggcuU......................   4       1    0A2
    .....................................................................cuuugaaauugggucuggUuaa.....................   153     1    0A2
    .....................................................................cuuugaaauugggucuggcuaa.....................   7       0    0A2
    .....................................................................cuuugaaauCgggucuggcuaa.....................   3       1    0A2
    .....................................................................cuuugaaauugggucuggcuCa.....................   5       1    0A2
    .....................................................................cuuugaaauugggucuggUuaac....................   6       1    0A2
    .....................................................................cuuugaaauugggucuggcuaaU....................   44      1    0A2
    .....................................................................cuuugaaauugggucuggcuaaUa...................   11      1    0A2
    .....................................................................cuuugaaauugggucuggUuaaca...................   12      1    0A2
    ......................................................................uuugaaauugggucuggUuaa.....................   27      1    0A2
    ......................................................................uuugaaauugggucuggcuaa.....................   12      0    0A2
    ......................................................................uuugaaauugggucuggcuCa.....................   6       1    0A2
    ......................................................................uuugaGauugggucuggcuaac....................   2       1    0A2
    ......................................................................uuugaaauugggucuggcuaaU....................   12      1    0A2
    ......................................................................uuugaaauugggucuggUuaac....................   1259    1    0A2
    ......................................................................uuugaaauugggucuggcuaac....................   14      0    0A2
    ......................................................................uuugaaauCgggucuggcuaaca...................   2       1    0A2
    ......................................................................uuugaaauugggucuggcuaaca...................   14      0    0A2
    ......................................................................uuugaaauugggucuggUuaaca...................   1353    1    0A2
    ......................................................................uuugaaauugggucuggcuaaUa...................   1       1    0A2
    ......................................................................uuugaaauugggucuggcuaacaA..................   2       1    0A2
```

Star Mature

```
ugcauccuagccuguuaaccagacccuauuucaaaggaaauaguuccacuauuucuuguccacuauuuccuuugaaauugggucuggcuaacaggauaugcuaacaggauau
.......................................................................uugaaauugggucuggcuaac....................	13	0	0A2
.......................................................................uugaaauugggucuggcuaaU....................	1	1	0A2
.......................................................................uugaaauugggucuggUuaac....................	57	1	0A2
.......................................................................uugaGauugggucuggcuaaca...................	9	1	0A2
.......................................................................uugaaauugggucuggUuaaca...................	67	1	0A2
.......................................................................uugaaauugggucuggcuaaca...................	14	0	0A2
........................................................................ugaaauugggucuggUuaaca...................	1	1	0A2
..........................................................................aaauugggucuggUuaaca...................	2	1	0A2

..............uuaaccagacccAauuucaaag............................................................................	5	1	0G2
.................................................................uuuccuuugaaauuggguc............................	6	0	0G2
.................................................................uuuccuuugaaauugggucu...........................	3	0	0G2
.................................................................uuuccuuugaaauugggucuA..........................	2	1	0G2
.................................................................uuuccuuugaaauugggucug..........................	26	0	0G2
.................................................................uuuccuuugaaauugggucugU.........................	34	1	0G2
.................................................................uuuccuuugaaauugggucugg.........................	36	0	0G2
.................................................................uuuccuuugaaauugggucugA.........................	6	1	0G2
..................................................................uuccuuugaaauugggucugC.........................	3	1	0G2
..................................................................uuccuuugaaauugggucugU.........................	2	1	0G2
.....................................................................cuuugaaauugggucuggGuaa.....................	1	1	0G2
.....................................................................cuuugaaauugggucuggcuaU.....................	9	1	0G2
.....................................................................cuuugaaauugggucuggcuaa.....................	6	0	0G2
.....................................................................cuuugaaauugggucuggUuaa.....................	191	1	0G2
.....................................................................cuuugaaauugggucuggcuaaG....................	6	1	0G2
.....................................................................cuuugaaauugggucuggcuaaU....................	50	1	0G2
.....................................................................cuuugaaauugggucuggUuaac....................	5	1	0G2
.....................................................................cuuugaaauugggucuggUuaaca...................	14	1	0G2
......................................................................uuugaaauugggucuggcuaa.....................	7	0	0G2
......................................................................uuugaaauugggucuggcuGa.....................	1	1	0G2
......................................................................uuugaaauugggucuggUuaa.....................	22	1	0G2
......................................................................uuugaaauugggucuggcuaac....................	16	0	0G2
......................................................................uuugaaauugggucuggcuaaU....................	9	1	0G2
......................................................................uuugaaauugggucuggUuaac....................	1323	1	0G2
......................................................................uuugaaauugggucuggUuaaca...................	2385	1	0G2
......................................................................uuugaaauugggucuggcuaaca...................	13	0	0G2
......................................................................uuugaaauugggucuggcuaacU..................	5	1	0G2
......................................................................uuugaaGuugggucuggcuaaca...................	6	1	0G2
.......................................................................uugaaauugggucuggUuaa.....................	4	1	0G2
.......................................................................uugaaauugggucuggUuaac....................	38	1	0G2
.......................................................................uugaaauugggucuggcuaac....................	15	0	0G2
.......................................................................uugaaauugggucuggUuaaca...................	96	1	0G2
.......................................................................Cugaaauugggucuggcuaaca...................	1	1	0G2
.......................................................................uugaaauugggucuggGuaaca...................	1	1	0G2
.......................................................................uugaaauugggucuggcuaaca...................	11	0	0G2
........................................................................ugaaauugggucuggcuaac....................	3	0	0G2
........................................................................ugaaauugggucuggUuaac....................	2	1	0G2
........................................................................ugaaaAugggucuggcuaac....................	1	1	0G2
........................................................................ugaaauugggucuggcuaaca...................	4	0	0G2

..............uuaaccagacccAauuucaaag............................................................................	1	1	0B2
..............uuaaccagacccAauuucaaagg...........................................................................	2	1	0B2
...........................................................ccacuGuuuccuuugaaauu.................................	4	1	0B2
.................................................................uuuccuuugaaauugggucu...........................	8	0	0B2
.................................................................uuuccuuugaaauugggucuU..........................	3	1	0B2
.................................................................uuuccuuugaaauugggAcug..........................	1	1	0B2
.................................................................uuuccuuugaaauugggucug..........................	37	0	0B2
.................................................................uuuccuuugaaauugggucugg.........................	89	0	0B2
.................................................................uuuccuuugaaauugggucugU.........................	22	1	0B2
.................................................................uuuccuuugGaauugggucugg.........................	5	1	0B2
.................................................................uuuccuuugaaauugggucugUcu.......................	3	1	0B2
.................................................................uuuccuuugaaauugggucuggAu.......................	2	1	0B2
.................................................................uuuccuuugaaauugggucuggAua......................	1	1	0B2
..................................................................uuccuuugaaauugggucug..........................	6	0	0B2
..................................................................uuccuuugaaauugggucugg.........................	8	0	0B2
...................................................................uccuuugaaauugggucugU.........................	2	1	0B2
.....................................................................cuuugaaauugggucuggc........................	2	0	0B2
.....................................................................Uuuugaaauugggucuggcua.......................	1	1	0B2
.....................................................................cuuugaaauugggucuggcuU......................	2	1	0B2
.....................................................................cuuugaaauugggucuggUuaa.....................	162	1	0B2
.....................................................................cuuugaaauugggucuggcuaU.....................	2	1	0B2
```

Star **Mature**

```
ugcauccuagccuguuaaccagacccuauuucaaaggaaauaguuccacuauuucuuguccacuauuuccuuugaaauugggucuggcuaacaggauaugcuaacaggauau
.....................................................................Nuuugaaauugggucuggcuaa.....................	1	1	0B2
.....................................................................cuuugaaauugggucuggcuaa.....................	13	0	0B2
.....................................................................cuuugaaauugggucuggcuaaU....................	53	1	0B2
.....................................................................cuuugaaauugggucuggcuaaG....................	3	1	0B2
.....................................................................cuuugaaauugggucuggUuaac....................	27	1	0B2
.....................................................................cuuugaaauugggucuggcuaacU...................	1	1	0B2
.....................................................................cuuugaaauugggucuggUuaaca...................	17	1	0B2
.....................................................................cuuugaaauugggucuggcuaaUa...................	2	1	0B2
......................................................................uuugaaauugggucuggcuaa.....................	3	0	0B2
......................................................................uuugaaauugggucuggUuaa.....................	19	1	0B2
......................................................................Nuugaaauugggucuggcuaac....................	1	1	0B2
......................................................................uuugaaauugggucuggUuaac....................	2229	1	0B2
......................................................................uuugGaauugggucuggcuaac....................	1	1	0B2
......................................................................uuugaaauugggucuggGuaac....................	2	1	0B2
......................................................................uuugaaauugggucuggcuaaU....................	7	1	0B2
......................................................................uuugaaauugggucuggcuaac....................	32	0	0B2
......................................................................uuugaaauugggucuggcuaacU...................	6	1	0B2
......................................................................uuugaaauuAggucuggcuaaca...................	1	1	0B2
......................................................................uuugaaauugggucuggUuaaca...................	1223	1	0B2
......................................................................uuugaaauugggucuggcuaaca...................	3	0	0B2
.......................................................................uugaaauugggucuggcuaac....................	11	0	0B2
.......................................................................uugaaauugggucuggUuaac....................	92	1	0B2
.......................................................................uugaaauugggucuggUuaaca...................	82	1	0B2
........................................................................ugaaauugggucuggGuaac....................	1	1	0B2
........................................................................ugaaauugggucuggUuaac....................	7	1	0B2
........................................................................ugaaauugggucuggcuaaca...................	1	0	0B2
```

Provisional ID	:	scaffold172_27171
Score total	:	4.7
Score for star read(s)	:	-1.3
Score for read counts	:	0
Score for mfe	:	1.4
Score for randfold	:	1.6
Score for cons. seed	:	3
Total read count	:	1935
Mature read count	:	1934
Loop read count	:	0
Star read count	:	1

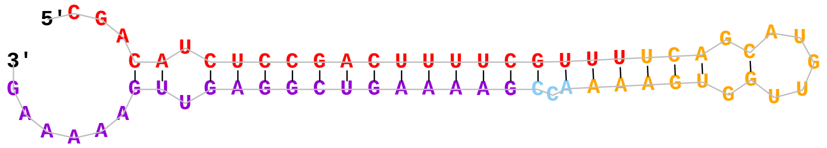

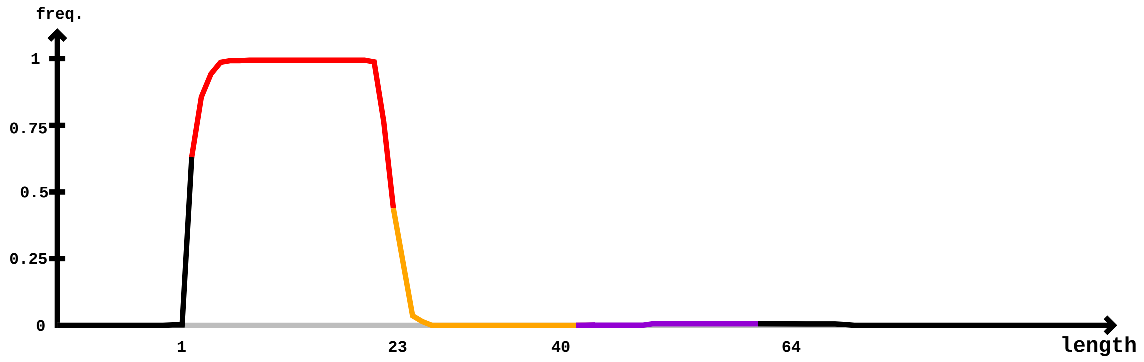

Mature **Star**

```
5'- aaaucccugccacgacaucuccgacuuuucguuuucagcauguuggugaaaaccgaaaagucggaguugaaaaaguuugaguccuugcuccaagaucuccgagaucuc   -3'   obs
    aaaucccugccacgacaucuccgacuuuucguuuucagcauguuggugaaaaccgaaaagucggaguugaaaaaguuugaguccuugcuccaagaucuccgagaucuc         exp
    ..............((.((((((((((((((((((.(.....).)))))).))))))))))))).)).........((((....))))..(((((.....))))).   reads   mm   sample
    ..........cacgacaucuccgacuuuucg.............................................................................   3       0    0B2
    ............cAacaucuccgacuuuucgu............................................................................   77      1    0B2
    ............cgacaucuccgacuuuucAu............................................................................   2       1    0B2
    ............cgacaucuccgacuuuucgu............................................................................   54      0    0B2
    ............Ngacaucuccgacuuuucguu...........................................................................   2       1    0B2
    ............Ugacaucuccgacuuuucguu...........................................................................   1       1    0B2
    ............cAacaucuccgacuuuucguu...........................................................................   41      1    0B2
    ............cgaUaucuccgacuuuucguu...........................................................................   2       1    0B2
    ............cgacaucuccgGcuuuucguu...........................................................................   2       1    0B2
    ............cgacaucuccgacuuuucguA...........................................................................   20      1    0B2
    ............cgacaucuccgacuuuucguu...........................................................................   95      0    0B2
    ............cgacaucuccgacuuCucguuu..........................................................................   7       1    0B2
    ............Ugacaucuccgacuuuucguuu..........................................................................   2       1    0B2
    ............cgacaucuccgacuuuucguuu..........................................................................   25      0    0B2
    ............Ngacaucuccgacuuuucguuu..........................................................................   1       1    0B2
    ............cgacaucuccgacuuuucguuA..........................................................................   69      1    0B2
    ............cAacaucuccgacuuuucguuu..........................................................................   3       1    0B2
    ............cgacaucuccgacuuuucguuuu.........................................................................   11      0    0B2
    ............cgacaucuccgacuuuucguuAu.........................................................................   5       1    0B2
    ............cgacaucuccgacuuuucguuuuU........................................................................   2       1    0B2
    .............gacaucuccgacuuuucgu............................................................................   12      0    0B2
    .............Aacaucuccgacuuuucgu............................................................................   9       1    0B2
    .............gacauAuccgacuuuucguu...........................................................................   1       1    0B2
    .............Nacaucuccgacuuuucguu...........................................................................   1       1    0B2
    .............Aacaucuccgacuuuucguu...........................................................................   32      1    0B2
    .............gacaucuccgacuuuucguu...........................................................................   31      0    0B2
    .............gacaucuccgacuuuucguuA..........................................................................   24      1    0B2
    .............Aacaucuccgacuuuucguuu..........................................................................   21      1    0B2
    .............gacaucuccgacuuuucguuu..........................................................................   21      0    0B2
    .............gacaucuccgacuuuucguuAu.........................................................................   4       1    0B2
    ..............acaucuccgacuuuucguuu..........................................................................   16      0    0B2
    ..............Ncaucuccgacuuuucguuu..........................................................................   1       1    0B2
    ..............acaucuccgacuuuucguuA..........................................................................   10      1    0B2
```

Mature Star

```
aaaucccugccacgacaucuccgacuuuucguuuucagcauguugggugaaaaccgaaaagucggaguugaaaaaguugaguccuugcuccaagaucuccgagaucuc
..............acaucuccgacuuuucgGuu..........................................................................   1     1     0B2
..............acaucuUcgacuuuucguuuu.........................................................................   5     1     0B2
..............acaucuccgacuuuucguuuu.........................................................................   9     0     0B2
...............caucuccgacuuuucguuu..........................................................................   11    0     0B2
...............caucuccgacuGuucguuuu.........................................................................   1     1     0B2
...............caucuccgacuuuucguuuu.........................................................................   13    0     0B2
...............caucuccgacuuuucguuuuA........................................................................   4     1     0B2
...............caucuccgacuuuucguuuuAa.......................................................................   2     1     0B2
..................cuccgacuuuucguuuucC.......................................................................   4     1     0B2

............cAacaucuccgacuuuucg.............................................................................   2     1     0G2
............cgacaucuccgacuuuucgu............................................................................   43    0     0G2
............cgacaucuccgGcuuuucgu............................................................................   2     1     0G2
............cAacaucuccgacuuuucgu............................................................................   64    1     0G2
............cgacaucuccgacuuuucguu...........................................................................   26    0     0G2
............cgacaucuccgacuuuucguA...........................................................................   15    1     0G2
............cAacaucuccgacuuuucguu...........................................................................   37    1     0G2
............cgacaucuccgacuuuucAuu...........................................................................   6     1     0G2
............cgacaucuccgacuuuucCuu...........................................................................   3     1     0G2
............cgacaucuccgacuuuucgGu...........................................................................   1     1     0G2
............cgacaucuccgacuuuucguuu..........................................................................   2     0     0G2
............cgacaucuccgacuuuuGguuu..........................................................................   1     1     0G2
............cgacaucuccgacuuuucguuA..........................................................................   116   1     0G2
............cgacaucuccgacuuuucguuAu.........................................................................   1     1     0G2
............cgacaucuccgacuuuucguuuA.........................................................................   2     1     0G2
.............Aacaucuccgacuuuucg.............................................................................   3     1     0G2
.............gacaucuccgacuuuucgu............................................................................   13    0     0G2
.............Aacaucuccgacuuuucgu............................................................................   22    1     0G2
.............Aacaucuccgacuuuucguu...........................................................................   32    1     0G2
.............gacaucuccgacuuuucguu...........................................................................   42    0     0G2
.............gacaucuccgacuuuucguuu..........................................................................   24    0     0G2
.............Aacaucuccgacuuuucguuu..........................................................................   11    1     0G2
.............gacaucuccgacuuuucguuA..........................................................................   15    1     0G2
.............gacaucuccgacuuuucguuuUa........................................................................   3     1     0G2
..............acaucuccgacuuuucguA...........................................................................   2     1     0G2
..............acaucuccgacuuuucguu...........................................................................   6     0     0G2
..............acaucuccgacuuuucguuu..........................................................................   29    0     0G2
..............acaucuccgacuuuucguuA..........................................................................   18    1     0G2
..............acaucuccgGcuuuucguuu..........................................................................   2     1     0G2
..............acaucuccgacuuuucguuuG.........................................................................   4     1     0G2
..............acaucuccgacuuuucguuuu.........................................................................   4     0     0G2
..............acaucuccgacuuuucguuuuUa.......................................................................   9     1     0G2
...............caucuccgacuuuucguuA..........................................................................   1     1     0G2
...............caucuccgacuuuucguuuu.........................................................................   10    0     0G2
...............caucuccgacuuuuUguuuu.........................................................................   5     1     0G2
...............caucuccgacuuuucguuuG.........................................................................   4     1     0G2
...............caucuccgacuuuucguuuuAa.......................................................................   4     1     0G2
................aucuccgacuuuucguuuuA........................................................................   5     1     0G2
................aucuccgacuuuucguuuuAa.......................................................................   5     1     0G2

............Ugacaucuccgacuuuucg.............................................................................   1     1     0A2
............cAacaucuccgacuuuucg.............................................................................   3     1     0A2
............cgacaucuccgacuuuucgu............................................................................   72    0     0A2
............cAacaucuccgacuuuucgu............................................................................   60    1     0A2
............Agacaucuccgacuuuucguu...........................................................................   1     1     0A2
............cgacaucuccgacuuuucguu...........................................................................   93    0     0A2
............cgacaucCccgacuuuucguu...........................................................................   1     1     0A2
............cAacaucuccgacuuuucguu...........................................................................   32    1     0A2
............cgacaucuccgacuuuucguA...........................................................................   20    1     0A2
............cgacaucuccgacuuuucUuu...........................................................................   1     1     0A2
............cgacGucuccgacuuuucguu...........................................................................   3     1     0A2
............Ugacaucuccgacuuuucguu...........................................................................   16    1     0A2
............cAacaucuccgacuuuucguuu..........................................................................   10    1     0A2
............cgacaucuccgacuuuucguuu..........................................................................   15    0     0A2
............cgacaucuccgacuuuucguuA..........................................................................   123   1     0A2
............Ugacaucuccgacuuuucguuu..........................................................................   7     1     0A2
............Ngacaucuccgacuuuucguuuu.........................................................................   1     1     0A2
............Ugacaucuccgacuuuucguuuu.........................................................................   5     1     0A2
............cgacaucuccgacuuuucguuAu.........................................................................   9     1     0A2
............cgacaucuccgacuuuucguAuu.........................................................................   3     1     0A2
```

Mature Star

```
aaaucccugccacgacaucuccgacuuuucguuuucagcauguuggugaaaaccgaaaagucggaguugaaaaaguuugaguccuugcuccaagaucuccgagaucuc
............cgacaucuccgacuuuucguuuu.........................................................................   3    0    0A2
............cgacaucuccgacuuuucguuuuA........................................................................   3    1    0A2
.............gacaucuccgacuuuucA.............................................................................   1    1    0A2
.............Aacaucuccgacuuuucgu............................................................................   3    1    0A2
.............gacaucuccgacuuuucgu............................................................................   4    0    0A2
.............Uacaucuccgacuuuucguu...........................................................................   3    1    0A2
.............gacaucuccgacuuuucguA...........................................................................   2    1    0A2
.............Aacaucuccgacuuuucguu...........................................................................   17   1    0A2
.............gaAaucuccgacuuuucguu...........................................................................   1    1    0A2
.............gacaucuccgacuuuucguu...........................................................................   35   0    0A2
.............gacaucuccgacuuuucguuA..........................................................................   11   1    0A2
.............gacaucuccgacuuuucguuu..........................................................................   18   0    0A2
.............Aacaucuccgacuuuucguuu..........................................................................   13   1    0A2
.............gacaucuccgacuuuucguuuu.........................................................................   1    0    0A2
.............gacaucuccgacuuuucguuuuA........................................................................   7    1    0A2
.............gacaucuccgacuuuucguuuuAa.......................................................................   1    1    0A2
..............acaucuccgacuuuucguu...........................................................................   7    0    0A2
..............acaucuccgacuuuucguuA..........................................................................   20   1    0A2
..............acaucuccgacuuuucgGuu..........................................................................   1    1    0A2
..............acaucuccgacuuuucguuu..........................................................................   16   0    0A2
..............acaucuccgacuuuucguuuuA........................................................................   8    1    0A2
...............caucuccgacuuuucguuu..........................................................................   2    0    0A2
...............caucuccgacuuuucguuuu.........................................................................   14   0    0A2
...............caucuccgacuCuucguuuu.........................................................................   3    1    0A2
...............caucuccgacuuuucguuuuA........................................................................   11   1    0A2
................Gucuccgacuuuucguuuu.........................................................................   2    1    0A2
......................................................gaaaagucggagAugaaaaag.................................   1    1    0A2
............................................................ucggagAugaaaaaguuuga............................   4    1    0A2
............................................................ucggagAugaaaaaguuugag...........................   6    1    0A2
```

Provisional ID	:	scaffold176_27535
Score total	:	2.2
Score for star read(s)	:	-1.3
Score for read counts	:	0
Score for mfe	:	-1
Score for randfold	:	1.6
Score for cons. seed	:	3
Total read count	:	300453
Mature read count	:	299327
Loop read count	:	0
Star read count	:	1126

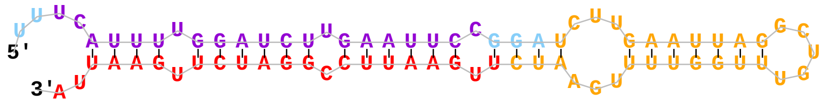

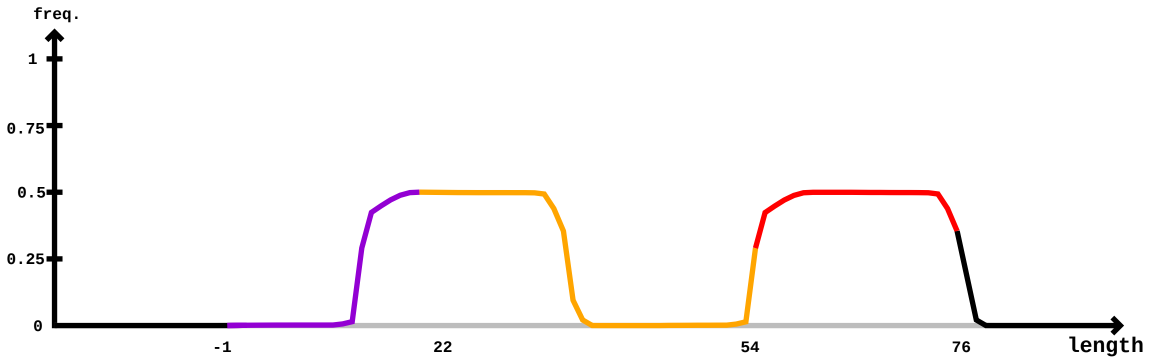

Star **Mature**

```
5'- guuucuuauuagugugguucauuuuggaucuugaauuccggaucuugaauuaggcuguuugguuuugaaucuugaauuccggaucuugaauuaggcuguuugauuuuga   -3'   obs
    guuucuuauuagugugguuucauuuuggaucuugaauuccggaucuugaauuaggcuguuugguuuugaaucuugaauuccggaucuugaauuaggcuguuugauuuuga         exp
    ...((....((((.((.((((....((((((.((((((.((((...(((((((.....)))))))...)))).)))))).))))))))))).)).))))...))......   reads   mm   sample
    ..............uguuucauuGuggaucuuga..........................................................................   2   1   0B2
    ..............uguGucauuuuggaucuugaa.........................................................................   6   1   0B2
    ................uuucauuCuggaucuugaau........................................................................   2   1   0B2
    ................uuucauuCuggaucuugaauu.......................................................................   7   1   0B2
    ................uuucauuuuggaucuugaauA.......................................................................   4   1   0B2
    ................uuucauuGuggaucuugaauu.......................................................................   1   1   0B2
    ................uuucauuCuggaucuugaauuc......................................................................   18   1   0B2
    ................uuucauuCuggaucuugaauucc.....................................................................   10   1   0B2
    .................uucauuCuggaucuugaauu.......................................................................   7   1   0B2
    .................uucauuGuggaucuugaauu.......................................................................   4   1   0B2
    .................uucauuCuggaucuugaauuc......................................................................   48   1   0B2
    .................uucauuGuggaucuugaauuc......................................................................   1   1   0B2
    .................uucGuuuuggaucuugaauucc.....................................................................   2   1   0B2
    .................uucauuCuggaucuugaauucc.....................................................................   11   1   0B2
    ..................ucauuCuggaucuugaauuc......................................................................   44   1   0B2
    ..................ucauuuuggaucuugaauuA......................................................................   3   1   0B2
    ..................ucauuCuggaucuugaauucc.....................................................................   49   1   0B2
    ..................ucauuuuggaucuugaauucA.....................................................................   19   1   0B2
    ..................ucauuuuggaucuugaauucU.....................................................................   2   1   0B2
    ...................cauuCuggaucuugaauuc......................................................................   9   1   0B2
    ...................cGuuuuggaucuugaauucc.....................................................................   2   1   0B2
    ...................cauuCuggaucuugaauucc.....................................................................   41   1   0B2
    ...................cauuuuggaucuugaauucA.....................................................................   2   1   0B2
    ...................cauuCuggaucuugaauuccg....................................................................   2   1   0B2
    ...................cauuuuggaucuugaauuccg....................................................................   1   0   0B2
    ....................auuCuggaucuugaauucc.....................................................................   25   1   0B2
    ....................aCuuuggaucuugaauuccg....................................................................   5   1   0B2
    ....................auuuuggaucuugaauucUg....................................................................   2   1   0B2
    ....................auuCuggaucuugaauuccg....................................................................   8   1   0B2
    ....................auuuuggGucuugaauuccg....................................................................   4   1   0B2
    ....................auuuuggaucuugaauuccg....................................................................   2   0   0B2
    ....................auuuuggaucuugaauuccgg...................................................................   5   0   0B2
    .....................uuuuggaucuugaauuccg....................................................................   1   0   0B2
```

```
                  Star                                                   Mature
guuucuuauuaguguguuucauuuuggaucuugaauuccggaucuugaauuaggcuguuugguuuugaaucuugaauuccggaucuugaauuaggcuguuugauuuuga
.....................uuuuggaucuugaauucUg.....................................................................	3	1	0B2
.....................uuCuggaucuugaauuccg.....................................................................	5	1	0B2
.....................uuuuggGucuugaauuccg.....................................................................	3	1	0B2
.....................uuuuggGucuugaauuccgga...................................................................	17	1	0B2
.....................uuuuggaucuugaauuccgga...................................................................	4	0	0B2
.....................uuuuggaucuugaauucUgga...................................................................	1	1	0B2
......................uuuggUucuugaauuccgg....................................................................	3	1	0B2
......................uuuggGucuugaauuccgga...................................................................	1	1	0B2
......................uuuggaucuugaauuccgga...................................................................	1	0	0B2
.......................uuggGucuugaauuccgga...................................................................	2	1	0B2
.......................uuggGucuugaauuccggau..................................................................	2	1	0B2
.......................uuggGucuugaauuccggauc.................................................................	2	1	0B2
.......................uuggaucuugaauuccggaucA................................................................	3	1	0B2
........................uggaucuugaauuUcgga...................................................................	1	1	0B2
........................uggaucuugaauuccCgaucuugaauu..........................................................	1	1	0B2
...........................aucuugaauuccggaucu................................................................	8	0	0B2
...........................aucuugaauuccggaucuA...............................................................	4	1	0B2
...........................aucuugaauuccggaucuu...............................................................	12	0	0B2
...........................aucuugaauuccggaucuuga.............................................................	10	0	0B2
...........................aucGugaauuccggaucuuga.............................................................	14	1	0B2
...........................Cucuugaauuccggaucuugaa............................................................	4	1	0B2
...........................aucuugaauuccggaucuugaau...........................................................	22	0	0B2
...........................aucuugaauuccggaucuugaauua.........................................................	4	0	0B2
............................ucuugaauuccggaucuA...............................................................	2	1	0B2
............................ucuugaauuccggaucuu...............................................................	2	0	0B2
............................ucuugaauuccggaucuug..............................................................	10	0	0B2
............................ucuugaauuccggaucuAg..............................................................	22	1	0B2
............................ucuugaauuccggaucuuga.............................................................	30	0	0B2
............................ucuugaauuccggaucuAga.............................................................	88	1	0B2
............................ucGugaauuccggaucuugaa............................................................	52	1	0B2
............................ucuugaauuccggaucuugaa............................................................	40	0	0B2
............................ucuugaauuccggaucuGgaa............................................................	8	1	0B2
............................ucuugaauuccggaucuAgaa............................................................	88	1	0B2
............................Ccuugaauuccggaucuugaau...........................................................	2	1	0B2
............................ucuugaauuccggaucuCgaau...........................................................	4	1	0B2
............................Ncuugaauuccggaucuugaau...........................................................	6	1	0B2
............................ucGugaauuccggaucuugaau...........................................................	36	1	0B2
............................ucuugaauuccggaucuugaaG...........................................................	10	1	0B2
............................ucuugaauuccggaucuugaau...........................................................	524	0	0B2
............................ucuugaauuccggaucuAgaau...........................................................	32	1	0B2
............................ucuugaauuccggaucuAgaauu..........................................................	20	1	0B2
............................uGuugaauuccggaucuugaauu..........................................................	14	1	0B2
............................uUuugaauuccggaucuugaauu..........................................................	6	1	0B2
............................Ncuugaauuccggaucuugaauu..........................................................	2	1	0B2
............................ucuugaauuccggaucuugaauu..........................................................	84	0	0B2
............................ucuugaauuccggaucuugaauua.........................................................	126	0	0B2
............................ucuugaauuccggaucuugaauCa.........................................................	4	1	0B2
............................ucuugaauuccggaucuugaauAa.........................................................	132	1	0B2
............................ucuugaauuccggaucuAgaauua.........................................................	16	1	0B2
............................uUuugaauuccggaucuugaauua.........................................................	18	1	0B2
............................uGuugaauuccggaucuugaauua.........................................................	12	1	0B2
............................ucuugaauuccggaucuugaauAag........................................................	6	1	0B2
............................ucuugaauuccggaucuAgaauuag........................................................	22	1	0B2
............................ucuugaauuccggaucuugaauuag........................................................	2	0	0B2
............................ucuugaauuccggaucuugaauuagg.......................................................	4	0	0B2
............................ucuugaauuccggaucuugaaCuagg.......................................................	4	1	0B2
.............................cuugaauuccggaucuug..............................................................	4	0	0B2
.............................cuugaauuccggaucuAg..............................................................	2	1	0B2
.............................cuugaauuccggaucuuga.............................................................	10	0	0B2
.............................cuugaauuccggaucuAga.............................................................	22	1	0B2
.............................cGugaauuccggaucuugaa............................................................	8	1	0B2
.............................cuugaauuccggaucuAgaa............................................................	732	1	0B2
.............................cuugaauuccggaucuugaa............................................................	180	0	0B2
.............................Nuugaauuccggaucuugaa............................................................	2	1	0B2
.............................cuugaauuccggaucuAgaau...........................................................	30	1	0B2
.............................Nuugaauuccggaucuugaau...........................................................	2	1	0B2
.............................cGugaauuccggaucuugaau...........................................................	56	1	0B2
.............................cuugaauuccggaGcuugaau...........................................................	2	1	0B2
.............................cuugaauuccggaucuugaau...........................................................	408	0	0B2
.............................cuugaauuccggaucuugaaC...........................................................	2	1	0B2
```

```
                    Star                                                    Mature
guuucuuauuaguguguuucauuuuggaucuugaauuccggaucuugaauuaggcuguuugguuuugaaucuugaauuccggaucuugaauuaggcuguuugauuuuga
.............................cuugaauuccggaucuAgaauu..........................................................  148     1     0B2
.............................Nuugaauuccggaucuugaauu..........................................................  2       1     0B2
.............................cuugaauuccggaucuugaauu..........................................................  468     0     0B2
.............................cuugaauuccggaucuugaauua.........................................................  478     0     0B2
.............................Nuugaauuccggaucuugaauua.........................................................  4       1     0B2
.............................Guugaauuccggaucuugaauua.........................................................  2       1     0B2
.............................cuugaauuccggaucuugaauCa.........................................................  54      1     0B2
.............................cuugaauuccggauGuugaauua.........................................................  14      1     0B2
.............................cuugaauuccggaucuugaauAa.........................................................  76      1     0B2
.............................cuugaauuccggaucuAgaauua.........................................................  74      1     0B2
.............................cuugaauuccggaucuugGauua.........................................................  6       1     0B2
.............................cuugaauuccggaucuugaauuag........................................................  4       0     0B2
.............................cuugaauuccggaucuAgaauuag........................................................  16      1     0B2
.............................cuugaauuccggaucuugaauuagg.......................................................  2       0     0B2
..............................uugaauuccggaucuAga.............................................................  28      1     0B2
..............................uugaauuccggaucuAgaa............................................................  568     1     0B2
..............................uugaauuccggaucuGgaa............................................................  2       1     0B2
..............................uugaauuccggaucuugaa............................................................  106     0     0B2
..............................uugaauuccggCucuugaa............................................................  2       1     0B2
..............................Gugaauuccggaucuugaau...........................................................  14      1     0B2
..............................uugaGuuccggaucuugaau...........................................................  182     1     0B2
..............................uugaauuccggCucuugaau...........................................................  2       1     0B2
..............................uugaauuccgCaucuugaau...........................................................  8       1     0B2
..............................uugaauuccggaGcuugaau...........................................................  8       1     0B2
..............................uugaauuccggaucuugaaG...........................................................  2       1     0B2
..............................Cugaauuccggaucuugaau...........................................................  16      1     0B2
..............................uugaauuccggGucuugaau...........................................................  16      1     0B2
..............................uugaauuccggaCcuugaau...........................................................  6       1     0B2
..............................uugaauuccggaucAugaau...........................................................  8       1     0B2
..............................uugaauuccggaucuugaaC...........................................................  20      1     0B2
..............................uugaauuccggauAuugaau...........................................................  2       1     0B2
..............................uugaauuccggaucuCgaau...........................................................  4       1     0B2
..............................uugaauuccggaucuAgaau...........................................................  3882    1     0B2
..............................uugaCuuccggaucuugaau...........................................................  2       1     0B2
..............................uugaauuccggaucuugaau...........................................................  18978   0     0B2
..............................Nugaauuccggaucuugaau...........................................................  146     1     0B2
..............................uugaauuccggaucuGgaau...........................................................  18      1     0B2
..............................uGgaauuccggaucuugaau...........................................................  2       1     0B2
..............................uugaauuccggaucuugaaCu..........................................................  66      1     0B2
..............................uugaauuccggaucuCgaauu..........................................................  18      1     0B2
..............................uugaGuuccggaucuugaauu..........................................................  332     1     0B2
..............................uugaauuAcggaucuugaauu..........................................................  6       1     0B2
..............................uugaauuccggauAuugaauu..........................................................  2       1     0B2
..............................uugaaGuccggaucuugaauu..........................................................  8       1     0B2
..............................uugaauuccggaucuAgaauu..........................................................  5504    1     0B2
..............................uugaauuccggaCcuugaauu..........................................................  4       1     0B2
..............................Nugaauuccggaucuugaauu..........................................................  154     1     0B2
..............................Augaauuccggaucuugaauu..........................................................  6       1     0B2
..............................uugaauuccggaucuugaaGu..........................................................  6       1     0B2
..............................uugaauuccggauNuugaauu..........................................................  2       1     0B2
..............................Gugaauuccggaucuugaauu..........................................................  18      1     0B2
..............................uugaauuccggaucuugGauu..........................................................  12      1     0B2
..............................uugaauAccggaucuugaauu..........................................................  2       1     0B2
..............................uugaauuccggaucuGgaauu..........................................................  4       1     0B2
..............................uugaauuccggaGcuugaauu..........................................................  6       1     0B2
..............................uugaauuccggCucuugaauu..........................................................  4       1     0B2
..............................Cugaauuccggaucuugaauu..........................................................  4       1     0B2
..............................uugaauuccggaucCugaauu..........................................................  8       1     0B2
..............................uugaauuccggaucuugaauu..........................................................  19318   0     0B2
..............................uugaauuccggGucuugaauu..........................................................  2       1     0B2
..............................uugaauuccggaucuugaauCa.........................................................  1868    1     0B2
..............................uugaauuccggaucuugaaCua.........................................................  32      1     0B2
..............................uugaauNccggaucuugaauua.........................................................  2       1     0B2
..............................uugaauuccggaucuuUaauua.........................................................  2       1     0B2
..............................uugaauuccggaGcuugaauua.........................................................  8       1     0B2
..............................uugaauuccggGucuugaauua.........................................................  4       1     0B2
..............................uugaauuccggauAuugaauua.........................................................  2       1     0B2
..............................uugaauuGcggaucuugaauua.........................................................  4       1     0B2
..............................uugaauuccggaucuGgaauua.........................................................  4       1     0B2
..............................uugaauuAcggaucuugaauua.........................................................  2       1     0B2
```

Star **Mature**

guuucuuauuaguguguuucauuuuggaucuugaauuccggaucuugaauuaggcuguuugguuuugaaucuugaauuccggaucuugaauuaggcuguuugauuuuga			
..............................uugaauuccggaucuugaauua...	21770	0	0B2
..............................uugaauuccggaucuCgaauua...	30	1	0B2
..............................uugaCuuccggaucuugaauua...	4	1	0B2
..............................uugaauuccggCucuugaauua...	18	1	0B2
..............................uugaaGuccggaucuugaauua...	24	1	0B2
..............................uugaauuccggNucuugaauua...	2	1	0B2
..............................uugaauuccggaucuugUauua...	2	1	0B2
..............................uugaauuccggaucuugaauAa...	1638	1	0B2
..............................uugaGuuccggaucuugaauua...	526	1	0B2
..............................uugaauuccggaCcuugaauua...	6	1	0B2
..............................Nugaauuccggaucuugaauua...	182	1	0B2
..............................uugaauuccggaucuugGauua...	36	1	0B2
..............................Augaauuccggaucuugaauua...	6	1	0B2
..............................uGgaauuccggaucuugaauua...	4	1	0B2
..............................Cugaauuccggaucuugaauua...	18	1	0B2
..............................uugaauuccggaucuAgaauua...	5186	1	0B2
..............................Gugaauuccggaucuugaauua...	8	1	0B2
..............................uugaGuuccggaucuugaauuag..	28	1	0B2
..............................uugaauuccggaucuAgaauuag..	1514	1	0B2
..............................uugaauuccggaucuugaauAag..	8	1	0B2
..............................Cugaauuccggaucuugaauuag..	18	1	0B2
..............................Nugaauuccggaucuugaauuag..	10	1	0B2
..............................uugaauuccggauGuugaauuag..	2	1	0B2
..............................uugaaGuccggaucuugaauuag..	4	1	0B2
..............................uugaauuccggaucuugaaCuag..	8	1	0B2
..............................uugaauuccCgaucuugaauuag..	2	1	0B2
..............................uugaauuccggaucuugaauuag..	486	0	0B2
..............................uugaauuccggaucuugaaCuagg...	4	1	0B2
..............................uugaGuuccggaucuugaauuagg...	6	1	0B2
..............................uugaauuccggaucuugaauuagA...	6	1	0B2
...............................ugaauuccggaucuugaa..	10	0	0B2
...............................ugaauuccggaucuAgaa..	140	1	0B2
...............................ugaauuccggaucuugaau...	996	0	0B2
...............................ugaauuccggaucuAgaau...	298	1	0B2
...............................ugaauuccggaGcuugaau...	4	1	0B2
...............................ugaGuuccggaucuugaau...	14	1	0B2
...............................Ngaauuccggaucuugaau...	4	1	0B2
...............................ugaauuccggaucuuUaauu..	2	1	0B2
...............................ugaauuccggaucuugaauu..	6110	0	0B2
...............................ugaauuccggaGcuugaauu..	2	1	0B2
...............................ugaauuccggaucuAgaauu..	2800	1	0B2
...............................ugaauuccggaucuCgaauu..	4	1	0B2
...............................ugaauuccggGucuugaauu..	4	1	0B2
...............................ugaaGuccggaucuugaauu..	10	1	0B2
...............................Ngaauuccggaucuugaauu..	28	1	0B2
...............................ugaauuccggauAuugaauu..	2	1	0B2
...............................ugaauuccggaucuugaaGu..	2	1	0B2
...............................ugaGuuccggaucuugaauu..	132	1	0B2
...............................ugaauuccggaucCugaauu..	4	1	0B2
...............................ugaaGuccggaucuugaauua...	44	1	0B2
...............................ugaauuccggaucuugaauAa...	116	1	0B2
...............................uNaauuccggaucuugaauua...	2	1	0B2
...............................Ggaauuccggaucuugaauua...	8	1	0B2
...............................ugaauuccggaucAugaauua...	8	1	0B2
...............................ugaauuccggaGcuugaauua...	10	1	0B2
...............................ugaauuccggaucuugaaCua...	12	1	0B2
...............................ugaauuccggaucuAgaauua...	6298	1	0B2
...............................ugaauuccggaucuGgaauua...	2	1	0B2
...............................ugaauuccggGucuugaauua...	2	1	0B2
...............................ugaauuccggaucCugaauua...	6	1	0B2
...............................ugaGuuccggaucuugaauua...	292	1	0B2
...............................Ngaauuccggaucuugaauua...	112	1	0B2
...............................ugaauuccggauAuugaauua...	8	1	0B2
...............................ugaauuAcggaucuugaauua...	2	1	0B2
...............................ugaauuccggaucuugGauua...	20	1	0B2
...............................ugaauuccgCaucuugaauua...	2	1	0B2
...............................ugaauuccggaucuugaauCa...	560	1	0B2
...............................ugaauuccCgaucuugaauua...	2	1	0B2
...............................ugaauuccggaucuCgaauua...	6	1	0B2
...............................ugaauuccggaCcuugaauua...	4	1	0B2

```
                                   Star                                                     Mature
guuucuuauuaguguguuucauuuuggaucuugaauuccggaucuugaauuaggcuguuugguuuugaaucuugaauuccggaucuugaauuaggcuguuugauuuuga
...............................ugaauuccggaucuugaauua.........................................................    15826    0    0B2
...............................ugaauuccggaucuugUauua.........................................................    2        1    0B2
...............................ugaauuccggaucuugGauuag........................................................    2        1    0B2
...............................ugaauuccggaucuAgaauuag........................................................    5118     1    0B2
...............................ugaauuccggaucuugaauAag........................................................    18       1    0B2
...............................Ngaauuccggaucuugaauuag........................................................    12       1    0B2
...............................ugaauuccggaucuugaauuag........................................................    1450     0    0B2
...............................ugaauuccggaucuugaauCag........................................................    26       1    0B2
...............................ugaGuuccggaucuugaauuag........................................................    26       1    0B2
...............................ugaauuccggGucuugaauuag........................................................    2        1    0B2
...............................ugaauuccggaucuGgaauuag........................................................    2        1    0B2
...............................ugaauuccggaucuugaaCuagg.......................................................    2        1    0B2
...............................ugaauuccggaucuugaauuaUg.......................................................    2        1    0B2
...............................ugaaGuccggaucuugaauuagg.......................................................    2        1    0B2
...............................ugaauuccggaucuugaauuagg.......................................................    122      0    0B2
...............................ugaauuccggaucuugaauuagA.......................................................    2        1    0B2
................................gaauuccggaucuAgaau...........................................................    2        1    0B2
................................gaauuccggaucuugaau...........................................................    42       0    0B2
................................gaauuccggaucuAgaauu..........................................................    172      1    0B2
................................gaauuccggaucuugaauu..........................................................    262      0    0B2
................................gaauuccggaucuugaauCa.........................................................    118      1    0B2
................................gaauuccggaucuugaauAa.........................................................    4        1    0B2
................................gaauuccggaucuugCauua.........................................................    2        1    0B2
................................gaauuccggaucuAgaauua.........................................................    862      1    0B2
................................gaGuuccggaucuugaauua.........................................................    14       1    0B2
................................gaauuccggaucAugaauua.........................................................    4        1    0B2
................................Naauuccggaucuugaauua.........................................................    20       1    0B2
................................gaauuccggaucuugaauua.........................................................    1374     0    0B2
................................gaaGuccggaucuugaauua.........................................................    6        1    0B2
................................gaauuccggaucuugaauCag........................................................    6        1    0B2
................................gaauuccggaucuugaauuag........................................................    984      0    0B2
................................gaGuuccggaucuugaauuag........................................................    2        1    0B2
................................gaauuccggaucuugaaCuag........................................................    6        1    0B2
................................gaauuccggaucCugaauuag........................................................    2        1    0B2
................................gaauuccggaucuAgaauuag........................................................    4018     1    0B2
................................Naauuccggaucuugaauuag........................................................    10       1    0B2
................................gaauuccggaucuCgaauuag........................................................    2        1    0B2
................................gaauuccggaucuugaauGagg.......................................................    2        1    0B2
................................gaauuccggaucuugaauuagA.......................................................    2        1    0B2
................................gaauuccggaucuugaauuagg.......................................................    22       0    0B2
................................Aaauuccggaucuugaauuagg.......................................................    16       1    0B2
.................................aauuccggaucuugaauu..........................................................    14       0    0B2
.................................aauuccggaucuugaauua.........................................................    626      0    0B2
.................................aauuccggaCcuugaauua.........................................................    2        1    0B2
.................................aauuccggaucuugaauCa.........................................................    16       1    0B2
.................................aauuccggaucuugaauAa.........................................................    8        1    0B2
.................................aauuccggaucuCgaauua.........................................................    2        1    0B2
.................................aCuuccggaucuugaauua.........................................................    2        1    0B2
.................................aauuccggaucuuUaauuag........................................................    14       1    0B2
.................................aauuccggaucuugaauuag........................................................    6030     0    0B2
.................................aGuuccggaucuugaauuag........................................................    70       1    0B2
.................................aauuccggaucuugaauAag........................................................    2        1    0B2
.................................aauuccggaucuCgaauuag........................................................    4        1    0B2
.................................aauuccggaucuugGauuag........................................................    22       1    0B2
.................................aauuccggaucAugaauuag........................................................    2        1    0B2
.................................aauAccggaucuugaauuag........................................................    4        1    0B2
.................................aaGuccggaucuugaauuag........................................................    14       1    0B2
.................................aauuccggaucuugaaGuag........................................................    2        1    0B2
.................................aauuccggaCcuugaauuag........................................................    2        1    0B2
.................................Nauuccggaucuugaauuag........................................................    24       1    0B2
.................................aauuccggaucuugaauCag........................................................    26       1    0B2
.................................aauuccggaGcuugaauuag........................................................    2        1    0B2
.................................aauuccggaucuugGauuagg.......................................................    2        1    0B2
.................................Nauuccggaucuugaauuagg.......................................................    4        1    0B2
.................................aauuccggaucuCgaauuagg.......................................................    2        1    0B2
.................................aauuccggaucuugaauuaUg.......................................................    8        1    0B2
.................................aauuccggaucuAgaauuagg.......................................................    20       1    0B2
.................................aauuccggaucuugaauuagg.......................................................    486      0    0B2
.................................aauuccggaCcuugaauuagg.......................................................    2        1    0B2
.................................aauuccggaucuugaauuagA.......................................................    18       1    0B2
```

Star	Mature			

guuucuuauuagugugu**ucauuuuggaucuugaauucc**ggaucuugaauuaggcuguuugguuuugaauc**uugaauuccggaucuugaauua**ggcuguuugauuuuga			
.................................auuccggaucuugaauCa...	8	1	0B2
.................................Nuuccggaucuugaauua...	4	1	0B2
.................................auuccggaucuugaauua...	220	0	0B2
.................................auuccggaGcuugaauua...	2	1	0B2
.................................auuccggaucuugaauAa...	2	1	0B2
.................................auuccggaucAugaauuag..	10	1	0B2
.................................Guuccggaucuugaauuag..	18	1	0B2
.................................Nuuccggaucuugaauuag..	6	1	0B2
.................................auuccggauAuugaauuag..	2	1	0B2
.................................auuccggGucuugaauuag..	2	1	0B2
.................................auuccggaucuugaauuag..	1402	0	0B2
.................................auuccggaucuugaauCag..	16	1	0B2
.................................auuccggaGcuugaauuag..	4	1	0B2
.................................Guuccggaucuugaauuagg...	66	1	0B2
.................................auuccggaucuugaaCuagg...	2	1	0B2
.................................auuccggaucAugaauuagg...	56	1	0B2
.................................auuccggaucuAgaauuagg...	38	1	0B2
.................................auuccggGucuugaauuagg...	12	1	0B2
.................................auuccggaucuugaauAagg...	2	1	0B2
.................................auuccggaucuugaauuagA...	88	1	0B2
.................................auuccggaucuugaaGuagg...	2	1	0B2
.................................auuccggaGcuugaauuagg...	4	1	0B2
.................................auuccggaucuugaauuaUg...	18	1	0B2
.................................auuccggaucuCgaauuagg...	6	1	0B2
.................................auucGggaucuugaauuagg...	12	1	0B2
.................................auuccggCucuugaauuagg...	2	1	0B2
.................................auuccggaucuugGauuagg...	4	1	0B2
.................................auuccggaucuugaauuagg...	3760	0	0B2
.................................auuccgCaucuugaauuagg...	2	1	0B2
.................................Nuuccggaucuugaauuagg...	30	1	0B2
.................................auuccggaucuugaauGagg...	4	1	0B2
.................................auuccggaCcuugaauuagg...	2	1	0B2
.................................auuccggaucuugaauuaggcA...	10	1	0B2
..................................uuccggaucuGgaauuag..	4	1	0B2
..................................uuccggaucuugGauuag..	2	1	0B2
..................................Nuccggaucuugaauuag..	12	1	0B2
..................................uuccggaucuugaauCag..	10	1	0B2
..................................uuccggaGcuugaauuag..	2	1	0B2
..................................uuccggaucuugaauuag..	1006	0	0B2
..................................uuccggaucuGgaauuagg...	8	1	0B2
..................................uuccggaucAugaauuagg...	56	1	0B2
..................................uuAcggaucuugaauuagg...	4	1	0B2
..................................uuccggaucuugaauuagg...	1688	0	0B2
..................................uuccggaucuuUaauuagg...	4	1	0B2
..................................uucAggaucuugaauuagg...	16	1	0B2
..................................uuccggaGcuugaauuagg...	2	1	0B2
..................................uuccggaucuugGauuagg...	4	1	0B2
..................................uucGggaucuugaauuagg...	4	1	0B2
..................................uuccggaucuugaaCuagg...	10	1	0B2
..................................uuccggaucuugaauuagA...	24	1	0B2
..................................uuccggauAuugaauuagg...	2	1	0B2
..................................uuccggaucuAgaauuagg...	16	1	0B2
..................................Nuccggaucuugaauuagg...	8	1	0B2
..................................uuccggaucuugaauuaggcA...	4	1	0B2
...................................uccggaucuugaauuagg...	384	0	0B2
...................................uccggaucAugaauuagg...	2	1	0B2
...................................uccggaucuAgaauuagg...	8	1	0B2
...................................uccggaCcuugaauuagg...	6	1	0B2
...................................Nccggaucuugaauuagg...	2	1	0B2
...................................uccggGucuugaauuagg...	2	1	0B2
.....................................cggaucuugaauuaggcuA..	6	1	0B2
....uuuugaCucuugaauuccg............................	4	1	0B2
....uuuugaCucuugaauuccgg...........................	9	1	0B2
....uuuuAaaucuugaauuccgg...........................	2	1	0B2
....uuuugaaucuugaauuccgg...........................	37	0	0B2
....uuuugaaucuugaauuccgga..........................	23	0	0B2
....uuuugaCucuugaauuccgga..........................	10	1	0B2
....uuuugaGucuugaauuccgga..........................	7	1	0B2
....Nuuugaaucuugaauuccgga..........................	1	1	0B2
....uuuugaaucGugaauuccgga..........................	2	1	0B2

```
                Star                                                  Mature
guuucuuauuaguguguuucauuuuggaucuugaauuccggaucuugaauuaggcuguuugguuuugaaucuugaauuccggaucuugaauuaggcuguuugauuuuga
..............................................................uuuugaaucuugaauuccggaucA.......................   4     1    0B2
..............................................................uuuugaaucuugaauuccggaucuuU.....................   2     1    0B2
...............................................................uuugaGucuugaauuccgga..........................   3     1    0B2
...............................................................uuugaaucuugaauuccgga..........................   13    0    0B2
...............................................................uuugaCucuugaauuccgga..........................   1     1    0B2
................................................................uugaaucuugaauuccgga..........................   2     0    0B2
................................................................uugaGucuugaauuccggau.........................   3     1    0B2
................................................................uugaaucuugaauuccggau.........................   4     0    0B2
................................................................uugaaucuugaauuccggauA........................   5     1    0B2
................................................................uugaaucuugaauuccggauc........................   17    0    0B2
................................................................uugaaucuugaauuccggaucA.......................   7     1    0B2
................................................................uugaGucuugaauuccggaucu.......................   3     1    0B2
................................................................uugaaucuugaauuccggaucuugaau..................   8     0    0B2
.................................................................ugaaucuugaauuccggauc........................   5     0    0B2
.................................................................ugaaucuugaauuccggaucA.......................   3     1    0B2
.................................................................ugaaucuugaauuccggaucuugaau..................   3     0    0B2
.................................................................ugaaucuugaauuccggaucuAgaau..................   6     1    0B2
.................................................................ugaaucuugaauuccggaucuugaauu.................   3     0    0B2
.................................................................ugaaucuugaauuccggaucuAgaauu.................   7     1    0B2
..................................................................gaaucuugaauuccggaucuugaauu.................   1     0    0B2
..................................................................gaaucuugaauuccggaucuAgaauua................   3     1    0B2
...................................................................aaucuugaauuccggaucuAg.....................   6     1    0B2
...................................................................aaucuugaauuccggaucuug.....................   2     0    0B2
....................................................................aucuugaauuccggaucu.......................   8     0    0B2
....................................................................aucuugaauuccggaucuu......................   12    0    0B2
....................................................................aucuugaauuccggaucuA......................   4     1    0B2
....................................................................aucuugaauuccggaucuuga....................   10    0    0B2
....................................................................aucGugaauuccggaucuuga....................   14    1    0B2
....................................................................Cucuugaauuccggaucuugaa...................   4     1    0B2
....................................................................aucuugaauuccggaucuugaau..................   22    0    0B2
....................................................................aucuugaauuccggaucuugaauua................   4     0    0B2
.....................................................................ucuugaauuccggaucuu......................   2     0    0B2
.....................................................................ucuugaauuccggaucuA......................   2     1    0B2
.....................................................................ucuugaauuccggaucuug.....................   10    0    0B2
.....................................................................ucuugaauuccggaucuAg.....................   22    1    0B2
.....................................................................ucuugaauuccggaucuuga....................   30    0    0B2
.....................................................................ucuugaauuccggaucuAga....................   88    1    0B2
.....................................................................ucuugaauuccggaucuGgaa...................   8     1    0B2
.....................................................................ucuugaauuccggaucuAgaa...................   88    1    0B2
.....................................................................ucuugaauuccggaucuugaa...................   40    0    0B2
.....................................................................ucGugaauuccggaucuugaa...................   52    1    0B2
.....................................................................ucuugaauuccggaucuugaaG..................   10    1    0B2
.....................................................................ucuugaauuccggaucuCgaau..................   4     1    0B2
.....................................................................ucuugaauuccggaucuAgaau..................   32    1    0B2
.....................................................................Ccuugaauuccggaucuugaau..................   2     1    0B2
.....................................................................Ncuugaauuccggaucuugaau..................   6     1    0B2
.....................................................................ucuugaauuccggaucuugaau..................   524   0    0B2
.....................................................................ucGugaauuccggaucuugaau..................   36    1    0B2
.....................................................................ucuugaauuccggaucuugaauu.................   84    0    0B2
.....................................................................ucuugaauuccggaucuAgaauu.................   20    1    0B2
.....................................................................uUuugaauuccggaucuugaauu.................   6     1    0B2
.....................................................................uGuugaauuccggaucuugaauu.................   14    1    0B2
.....................................................................Ncuugaauuccggaucuugaauu.................   2     1    0B2
.....................................................................uUuugaauuccggaucuugaauua................   18    1    0B2
.....................................................................ucuugaauuccggaucuugaauAa................   132   1    0B2
.....................................................................uGuugaauuccggaucuugaauua................   12    1    0B2
.....................................................................ucuugaauuccggaucuAgaauua................   16    1    0B2
.....................................................................ucuugaauuccggaucuugaauua................   126   0    0B2
.....................................................................ucuugaauuccggaucuugaauCa................   4     1    0B2
.....................................................................ucuugaauuccggaucuAgaauuag...............   22    1    0B2
.....................................................................ucuugaauuccggaucuugaauAag...............   6     1    0B2
.....................................................................ucuugaauuccggaucuugaauuag...............   2     0    0B2
.....................................................................ucuugaauuccggaucuugaauuagg..............   4     0    0B2
.....................................................................ucuugaauuccggaucuugaaCuagg..............   4     1    0B2
......................................................................cuugaauuccggaucuug.....................   4     0    0B2
......................................................................cuugaauuccggaucuAg.....................   2     1    0B2
......................................................................cuugaauuccggaucuuga....................   10    0    0B2
......................................................................cuugaauuccggaucuAga....................   22    1    0B2
......................................................................cGugaauuccggaucuugaa...................   8     1    0B2
......................................................................cuugaauuccggaucuAgaa...................   732   1    0B2
```

Star / Mature			
guuucuuauuaguguguuucauuuuggaucuugaauuccggaucuugaauuaggcuguuugguuuugaaucuugaauuccggaucuugaauuaggcuguuugauuuuga			
....cuugaauuccggaucuugaa...................	180	0	0B2
....Nuugaauuccggaucuugaa...................	2	1	0B2
....cuugaauuccggaucuAgaau..................	30	1	0B2
....Nuugaauuccggaucuugaau..................	2	1	0B2
....cuugaauuccggaGcuugaau..................	2	1	0B2
....cuugaauuccggaucuugaau..................	408	0	0B2
....cuugaauuccggaucuugaaC..................	2	1	0B2
....cGugaauuccggaucuugaau..................	56	1	0B2
....cuugaauuccggaucuAgaauu.................	148	1	0B2
....cuugaauuccggaucuugaauu.................	468	0	0B2
....Nuugaauuccggaucuugaauu.................	2	1	0B2
....cuugaauuccggauGuugaauua................	14	1	0B2
....cuugaauuccggaucuugGauua................	6	1	0B2
....cuugaauuccggaucuugaauCa................	54	1	0B2
....cuugaauuccggaucuAgaauua................	74	1	0B2
....Nuugaauuccggaucuugaauua................	4	1	0B2
....cuugaauuccggaucuugaauAa................	76	1	0B2
....cuugaauuccggaucuugaauua................	478	0	0B2
....Guugaauuccggaucuugaauua................	2	1	0B2
....cuugaauuccggaucuAgaauuag...............	16	1	0B2
....cuugaauuccggaucuugaauuag...............	4	0	0B2
....cuugaauuccggaucuugaauuagg..............	2	0	0B2
....uugaauuccggaucuAga....................	28	1	0B2
....uugaauuccggCucuugaa...................	2	1	0B2
....uugaauuccggaucuAgaa...................	568	1	0B2
....uugaauuccggaucuGgaa...................	2	1	0B2
....uugaauuccggaucuugaa...................	106	0	0B2
....Cugaauuccggaucuugaau..................	16	1	0B2
....uugaauuccgCaucuugaau..................	8	1	0B2
....uugaCuuccggaucuugaau..................	2	1	0B2
....uugaGuuccggaucuugaau..................	182	1	0B2
....uugaauuccggaucuugaaG..................	2	1	0B2
....Nugaauuccggaucuugaau..................	146	1	0B2
....uugaauuccggaucuCgaau..................	4	1	0B2
....uugaauuccggGucuugaau..................	16	1	0B2
....uugaauuccggauAuugaau..................	2	1	0B2
....uGgaauuccggaucuugaau..................	2	1	0B2
....uugaauuccggaucAugaau..................	8	1	0B2
....Gugaauuccggaucuugaau..................	14	1	0B2
....uugaauuccggaucuGgaau..................	18	1	0B2
....uugaauuccggaCcuugaau..................	6	1	0B2
....uugaauuccggaucuugaau..................	18978	0	0B2
....uugaauuccggCucuugaau..................	2	1	0B2
....uugaauuccggaucuugaaC..................	20	1	0B2
....uugaauuccggaGcuugaau..................	8	1	0B2
....uugaauuccggaucuAgaau..................	3882	1	0B2
....uugaauuccggaucCugaauu.................	8	1	0B2
....uugaauuccggaucuugaaGu.................	6	1	0B2
....uugaauuccggGucuugaauu.................	2	1	0B2
....uugaauuccggauNuugaauu.................	2	1	0B2
....uugaauuccggaucuugGauu.................	12	1	0B2
....Cugaauuccggaucuugaauu.................	4	1	0B2
....uugaauuccggaCcuugaauu.................	4	1	0B2
....uugaauuccggaucuCgaauu.................	18	1	0B2
....uugaGuuccggaucuugaauu.................	332	1	0B2
....uugaauuccggaucuugaaCu.................	66	1	0B2
....uugaaGuccggaucuugaauu.................	8	1	0B2
....uugaauuccggaucuGgaauu.................	4	1	0B2
....Gugaauuccggaucuugaauu.................	18	1	0B2
....uugaauuccggaucuugaauu.................	19318	0	0B2
....uugaauuccggauAuugaauu.................	2	1	0B2
....uugaauuccggaGcuugaauu.................	6	1	0B2
....uugaauuccggCucuugaauu.................	4	1	0B2
....Nugaauuccggaucuugaauu.................	154	1	0B2
....uugaauuccggaucuAgaauu.................	5504	1	0B2
....uugaauuAcggaucuugaauu.................	6	1	0B2
....uugaauAccggaucuugaauu.................	2	1	0B2
....Augaauuccggaucuugaauu.................	6	1	0B2
....uugaauuccggaucuAgaauua................	5186	1	0B2
....uugaauuccggaucuugaauAa................	1638	1	0B2

Star **Mature**

guuucuuauuagugugguuucauuuuggaucuugaauuccggaucuugaauuaggcuguuugguuuugaaucuugaauuccggaucuugaauuaggcuguuugauuuuga			
....uugaauuccggaucuCgaauua................	30	1	0B2
....Nugaauuccggaucuugaauua................	182	1	0B2
....uugaCuuccggaucuugaauua................	4	1	0B2
....uugaauuccggaGcuugaauua................	8	1	0B2
....uugaauuccggNucuugaauua................	2	1	0B2
....uGgaauuccggaucuugaauua................	4	1	0B2
....uugaGuuccggaucuugaauua................	526	1	0B2
....uugaauuccggauAuugaauua................	2	1	0B2
....uugaauuccggaucuugaauCa................	1868	1	0B2
....uugaauuccggaCcuugaauua................	6	1	0B2
....uugaauuccggaucuugaaCua................	32	1	0B2
....uugaauuccggaucuugaauua................	21770	0	0B2
....uugaauuccggaucuugGauua................	36	1	0B2
....uugaauuGcggaucuugaauua................	4	1	0B2
....Augaauuccggaucuugaauua................	6	1	0B2
....uugaauNccggaucuugaauua................	2	1	0B2
....uugaauuccggaucuuUaauua................	2	1	0B2
....uugaauuccggCucuugaauua................	18	1	0B2
....Cugaauuccggaucuugaauua................	18	1	0B2
....uugaauuccggaucuGgaauua................	4	1	0B2
....uugaaGuccggaucuugaauua................	24	1	0B2
....Gugaauuccggaucuugaauua................	8	1	0B2
....uugaauuccggaucuugUauua................	2	1	0B2
....uugaauuAcggaucuugaauua................	2	1	0B2
....uugaauuccggGucuugaauua................	4	1	0B2
....uugaauuccggaucuugaauAag...............	8	1	0B2
....Cugaauuccggaucuugaauuag...............	18	1	0B2
....Nugaauuccggaucuugaauuag...............	10	1	0B2
....uugaauuccggaucuugaauuag...............	486	0	0B2
....uugaaGuccggaucuugaauuag...............	4	1	0B2
....uugaauuccggauGuugaauuag...............	2	1	0B2
....uugaGuuccggaucuugaauuag...............	28	1	0B2
....uugaauuccggaucuugaaCuag...............	8	1	0B2
....uugaauuccCgaucuugaauuag...............	2	1	0B2
....uugaauuccggaucuAgaauuag...............	1514	1	0B2
....uugaGuuccggaucuugaauuagg..............	6	1	0B2
....uugaauuccggaucuugaaCuagg..............	4	1	0B2
....uugaauuccggaucuugaauuagA..............	6	1	0B2
....ugaauuccggaucuugaa...................	10	0	0B2
....ugaauuccggaucuAgaa...................	140	1	0B2
....ugaauuccggaGcuugaau..................	4	1	0B2
....ugaauuccggaucuAgaau..................	298	1	0B2
....Ngaauuccggaucuugaau..................	4	1	0B2
....ugaGuuccggaucuugaau..................	14	1	0B2
....ugaauuccggaucuugaau..................	996	0	0B2
....ugaauuccggaucuAgaauu.................	2800	1	0B2
....ugaauuccggaGcuugaauu.................	2	1	0B2
....Ngaauuccggaucuugaauu.................	28	1	0B2
....ugaauuccggaucuugaauu.................	6110	0	0B2
....ugaauuccggaucCugaauu.................	4	1	0B2
....ugaauuccggGucuugaauu.................	4	1	0B2
....ugaauuccggaucuuUaauu.................	2	1	0B2
....ugaGuuccggaucuugaauu.................	132	1	0B2
....ugaauuccggaucuCgaauu.................	4	1	0B2
....ugaauuccggaucuugaaGu.................	2	1	0B2
....ugaauuccggauAuugaauu.................	2	1	0B2
....ugaaGuccggaucuugaauu.................	10	1	0B2
....ugaauuccggaucuugUauua................	2	1	0B2
....ugaauuccggaucuugaauCa................	560	1	0B2
....ugaauuccggaucuugaauua................	15826	0	0B2
....ugaauuAcggaucuugaauua................	2	1	0B2
....ugaauuccggGucuugaauua................	2	1	0B2
....ugaauuccggaGcuugaauua................	10	1	0B2
....ugaauuccggaucuugGauua................	20	1	0B2
....ugaauuccggaucCugaauua................	6	1	0B2
....ugaauuccggaucAugaauua................	8	1	0B2
....ugaGuuccggaucuugaauua................	292	1	0B2
....ugaauuccggauAuugaauua................	8	1	0B2
....uNaauuccggaucuugaauua................	2	1	0B2
....ugaauuccggaucuAgaauua................	6298	1	0B2

Star Mature

```
guuucuuauuaguguguuucauuuuggaucuugaauuccggaucuugaauuaggcuguuugguuuugaaucuugaauuccggaucuugaauuaggcuguuugauuuuga
........................................................................ugaauuccggaucuCgaauua................	6	1	0B2
........................................................................ugaauuccggaCcuugaauua................	4	1	0B2
........................................................................ugaauuccggaucuugaaCua................	12	1	0B2
........................................................................ugaauuccggaucuGgaauua................	2	1	0B2
........................................................................ugaauuccCgaucuugaauua................	2	1	0B2
........................................................................ugaauuccggaucuugaauAa................	116	1	0B2
........................................................................Ggaauuccggaucuugaauua................	8	1	0B2
........................................................................ugaauuccgCaucuugaauua................	2	1	0B2
........................................................................Ngaauuccggaucuugaauua................	112	1	0B2
........................................................................ugaaGuccggaucuugaauua................	44	1	0B2
........................................................................Ngaauuccggaucuugaauuag...............	12	1	0B2
........................................................................ugaauuccggaucuugGauuag...............	2	1	0B2
........................................................................ugaGuuccggaucuugaauuag...............	26	1	0B2
........................................................................ugaauuccggaucuugaauuag...............	1450	0	0B2
........................................................................ugaauuccggGucuugaauuag...............	2	1	0B2
........................................................................ugaauuccggaucuugaauCag...............	26	1	0B2
........................................................................ugaauuccggaucuGgaauuag...............	2	1	0B2
........................................................................ugaauuccggaucuAgaauuag...............	5118	1	0B2
........................................................................ugaauuccggaucuugaauAag...............	18	1	0B2
........................................................................ugaaGuccggaucuugaauuagg..............	2	1	0B2
........................................................................ugaauuccggaucuugaauuagA..............	2	1	0B2
........................................................................ugaauuccggaucuugaaCuagg..............	2	1	0B2
........................................................................ugaauuccggaucuugaauuaUg..............	2	1	0B2
........................................................................ugaauuccggaucuugaauuagg..............	122	0	0B2
.........................................................................gaauuccggaucuAgaau..................	2	1	0B2
.........................................................................gaauuccggaucuugaau..................	42	0	0B2
.........................................................................gaauuccggaucuAgaauu.................	172	1	0B2
.........................................................................gaauuccggaucuugaauu.................	262	0	0B2
.........................................................................gaauuccggaucuugaauua................	1374	0	0B2
.........................................................................gaGuuccggaucuugaauua................	14	1	0B2
.........................................................................gaauuccggaucuugaauCa................	118	1	0B2
.........................................................................gaaGuccggaucuugaauua................	6	1	0B2
.........................................................................gaauuccggaucuugaauAa................	4	1	0B2
.........................................................................Naauuccggaucuugaauua................	20	1	0B2
.........................................................................gaauuccggaucuAgaauua................	862	1	0B2
.........................................................................gaauuccggaucAugaauua................	4	1	0B2
.........................................................................gaauuccggaucuugCauua................	2	1	0B2
.........................................................................gaauuccggaucuAgaauuag...............	4018	1	0B2
.........................................................................gaGuuccggaucuugaauuag...............	2	1	0B2
.........................................................................gaauuccggaucuugaauCag...............	6	1	0B2
.........................................................................gaauuccggaucuugaaCuag...............	6	1	0B2
.........................................................................gaauuccggaucuugaauuag...............	984	0	0B2
.........................................................................gaauuccggaucuCgaauuag...............	2	1	0B2
.........................................................................gaauuccggaucCugaauuag...............	2	1	0B2
.........................................................................Naauuccggaucuugaauuag...............	10	1	0B2
.........................................................................Aaauuccggaucuugaauuagg..............	16	1	0B2
.........................................................................gaauuccggaucuugaauuagg..............	22	0	0B2
.........................................................................gaauuccggaucuugaauGagg..............	2	1	0B2
.........................................................................gaauuccggaucuugaauuagA..............	2	1	0B2
..........................................................................aauuccggaucuugaauu.................	14	0	0B2
..........................................................................aauuccggaucuCgaauua................	2	1	0B2
..........................................................................aCuuccggaucuugaauua................	2	1	0B2
..........................................................................aauuccggaucuugaauCa................	16	1	0B2
..........................................................................aauuccggaucuugaauua................	626	0	0B2
..........................................................................aauuccggaucuugaauAa................	8	1	0B2
..........................................................................aauuccggaCcuugaauua................	2	1	0B2
..........................................................................Nauuccggaucuugaauuag...............	24	1	0B2
..........................................................................aGuuccggaucuugaauuag...............	70	1	0B2
..........................................................................aauuccggaCcuugaauuag...............	2	1	0B2
..........................................................................aauuccggaucuugGauuag...............	22	1	0B2
..........................................................................aauuccggaucuuUaauuag...............	14	1	0B2
..........................................................................aauuccggaucuugaauuag...............	6030	0	0B2
..........................................................................aaGuccggaucuugaauuag...............	14	1	0B2
..........................................................................aauuccggaucuCgaauuag...............	4	1	0B2
..........................................................................aauuccggaucAugaauuag...............	2	1	0B2
..........................................................................aauuccggaGcuugaauuag...............	2	1	0B2
..........................................................................aauuccggaucuugaaGuag...............	2	1	0B2
..........................................................................aauAccggaucuugaauuag...............	4	1	0B2
..........................................................................aauuccggaucuugaauCag...............	26	1	0B2
..........................................................................aauuccggaucuugaauAag...............	2	1	0B2
```

Star **Mature**

```
guuucuuauuaguguguuucauuuuggaucuugaauuccggaucuugaauuaggcuguuugguuuugaaucuugaauuccggaucuugaauuaggcuguuugauuuuga
..........................................................................Nauuccggaucuugaauuagg..............   4     1   0B2
..........................................................................aauuccggaucuugGauuagg..............   2     1   0B2
..........................................................................aauuccggaCcuugaauuagg..............   2     1   0B2
..........................................................................aauuccggaucuugaauuaUg..............   8     1   0B2
..........................................................................aauuccggaucuugaauuagg..............   486   0   0B2
..........................................................................aauuccggaucuCgaauuagg..............   2     1   0B2
..........................................................................aauuccggaucuugaauuagA..............   18    1   0B2
..........................................................................aauuccggaucuAgaauuagg..............   20    1   0B2
...........................................................................auuccggaGcuugaauua................   2     1   0B2
...........................................................................Nuuccggaucuugaauua................   4     1   0B2
...........................................................................auuccggaucuugaauua................   220   0   0B2
...........................................................................auuccggaucuugaauAa................   2     1   0B2
...........................................................................auuccggaucuugaauCa................   8     1   0B2
...........................................................................auuccggaucAugaauuag...............   10    1   0B2
...........................................................................auuccggaucuugaauCag...............   16    1   0B2
...........................................................................auuccggaucuugaauuag...............   1402  0   0B2
...........................................................................auuccggGucuugaauuag...............   2     1   0B2
...........................................................................auuccggauAuugaauuag...............   2     1   0B2
...........................................................................Guuccggaucuugaauuag...............   18    1   0B2
...........................................................................Nuuccggaucuugaauuag...............   6     1   0B2
...........................................................................auuccggaGcuugaauuag...............   4     1   0B2
...........................................................................auuccggaGcuugaauuagg..............   4     1   0B2
...........................................................................auuccggaucuugGauuagg..............   4     1   0B2
...........................................................................auuccggaucuugaaGuagg..............   2     1   0B2
...........................................................................auuccggaCcuugaauuagg..............   2     1   0B2
...........................................................................auuccggGucuugaauuagg..............   12    1   0B2
...........................................................................auuccggCucuugaauuagg..............   2     1   0B2
...........................................................................auuccggaucAugaauuagg..............   56    1   0B2
...........................................................................auucGggaucuugaauuagg..............   12    1   0B2
...........................................................................Nuuccggaucuugaauuagg..............   30    1   0B2
...........................................................................auuccggaucuCgaauuagg..............   6     1   0B2
...........................................................................auuccgCaucuugaauuagg..............   2     1   0B2
...........................................................................Guuccggaucuugaauuagg..............   66    1   0B2
...........................................................................auuccggaucuugaauuaUg..............   18    1   0B2
...........................................................................auuccggaucuugaauuagg..............   3760  0   0B2
...........................................................................auuccggaucuugaaCuagg..............   2     1   0B2
...........................................................................auuccggaucuAgaauuagg..............   38    1   0B2
...........................................................................auuccggaucuugaauAagg..............   2     1   0B2
...........................................................................auuccggaucuugaauGagg..............   4     1   0B2
...........................................................................auuccggaucuugaauuagA..............   88    1   0B2
...........................................................................auuccggaucuugaauuaggcA............   10    1   0B2
............................................................................uuccggaucuGgaauuag...............   4     1   0B2
............................................................................uuccggaucuugaauCag...............   10    1   0B2
............................................................................uuccggaucuugaauuag...............   1006  0   0B2
............................................................................uuccggaGcuugaauuag...............   2     1   0B2
............................................................................Nuccggaucuugaauuag...............   12    1   0B2
............................................................................uuccggaucuugGauuag...............   2     1   0B2
............................................................................uuccggaucuugaauuagg..............   1688  0   0B2
............................................................................uuccggaucuGgaauuagg..............   8     1   0B2
............................................................................uuccggaucuugGauuagg..............   4     1   0B2
............................................................................Nuccggaucuugaauuagg..............   8     1   0B2
............................................................................uuccggaucuAgaauuagg..............   16    1   0B2
............................................................................uuccggaucAugaauuagg..............   56    1   0B2
............................................................................uuccggaGcuugaauuagg..............   2     1   0B2
............................................................................uuccggaucuugaauuagA..............   24    1   0B2
............................................................................uucAggaucuugaauuagg..............   16    1   0B2
............................................................................uuccggauAuugaauuagg..............   2     1   0B2
............................................................................uuccggaucuugaaCuagg..............   10    1   0B2
............................................................................uuccggaucuuUaauuagg..............   4     1   0B2
............................................................................uucGggaucuugaauuagg..............   4     1   0B2
............................................................................uuAcggaucuugaauuagg..............   4     1   0B2
............................................................................uuccggaucuugaauuaggcA............   4     1   0B2
.............................................................................uccggaucAugaauuagg..............   2     1   0B2
.............................................................................uccggaucuugaauuagg..............   384   0   0B2
.............................................................................Nccggaucuugaauuagg..............   2     1   0B2
.............................................................................uccggaCcuugaauuagg..............   6     1   0B2
.............................................................................uccggaucuAgaauuagg..............   8     1   0B2
.............................................................................uccggGucuugaauuagg..............   2     1   0B2
...............................................................................cggaucuugaauuaggcuA...........   6     1   0B2

.............guguuucauuuuggaucuug............................................................................   5     0   0G2
```

```
                    Star                                                      Mature
guuucuuauuaguguguuucauuuuggaucuugaauuccggaucuugaauuaggcuguuugguuuugaaucuugaauuccggaucuugaauuaggcuguuugauuuuga
................uuucauuCuggaucuugaauu........................................................................  5    1  0G2
................uuucauuCuggaucuugaauuc.......................................................................  2    1  0G2
................uuucauuCuggaucuugaauucc......................................................................  5    1  0G2
.................uucauuuuggGucuugaauu........................................................................  6    1  0G2
.................uucauuCuggaucuugaauuc.......................................................................  56   1  0G2
.................uucauuuuggaucuugaauucA......................................................................  8    1  0G2
.................uucauuCuggaucuugaauucc......................................................................  5    1  0G2
..................ucauuGuggaucuugaauu........................................................................  4    1  0G2
..................ucauuCuggaucuugaauuc.......................................................................  14   1  0G2
..................ucauuuuggaucuugaauuA.......................................................................  8    1  0G2
..................ucauuuuggaucuugaauucA......................................................................  19   1  0G2
..................ucauuuuggaucuugaauucc......................................................................  4    0  0G2
..................ucauuCuggaucuugaauucc......................................................................  39   1  0G2
..................ucauuuuggaucuugaauuccg.....................................................................  2    0  0G2
..................ucauuCuggaucuugaauuccg.....................................................................  9    1  0G2
...................cauuCuggaucuugaauuc.......................................................................  11   1  0G2
...................cauuCuggaucuugaauucc......................................................................  57   1  0G2
...................cauuuuggaucuugaauucA......................................................................  21   1  0G2
...................cGuuuuggaucuugaauuccg.....................................................................  1    1  0G2
...................cauuuuggaucuugaauuccgga...................................................................  5    0  0G2
....................auuCuggaucuugaauucc......................................................................  7    1  0G2
....................auuuuggaucuugaauuccg.....................................................................  7    0  0G2
....................auuCuggaucuugaauuccg.....................................................................  13   1  0G2
....................auuuuggaucuugaauucUg.....................................................................  3    1  0G2
.....................uuCuggaucuugaauucc......................................................................  6    1  0G2
.....................uuuuggGucuugaauuccg.....................................................................  2    1  0G2
.....................Cuuuggaucuugaauuccgg....................................................................  2    1  0G2
.....................uuuuggGucuugaauuccgg....................................................................  1    1  0G2
.....................uuuuggaucuugaauuccgga...................................................................  10   0  0G2
.....................uuuuggGucuugaauuccgga...................................................................  23   1  0G2
......................uuuggGucuugaauuccgga...................................................................  4    1  0G2
.......................uuggGucuugaauuccgg....................................................................  5    1  0G2
.......................uuggaucuugaauuccgga...................................................................  8    0  0G2
.......................uuggaucuugaauuccggau..................................................................  1    0  0G2
.......................uuggaucuugaauuccggauA.................................................................  1    1  0G2
.......................uuggGucuugaauuccggauc.................................................................  11   1  0G2
.......................uuggaucuugaauuccggauc.................................................................  5    0  0G2
..........................gGucuugaauuccggaucuu...............................................................  3    1  0G2
...........................aucuugaauuccggaucuug..............................................................  6    0  0G2
...........................aucuugaauuccggaucuAga.............................................................  4    1  0G2
...........................aucuugaauuccggaucuugaau...........................................................  16   0  0G2
...........................aucuugaauuccggaucuugaauua.........................................................  26   0  0G2
...........................aucuugaauuccggaucuugaauuag........................................................  2    0  0G2
............................ucuugaauuccggaucuA...............................................................  4    1  0G2
............................ucuugaauuccggaucuug..............................................................  2    0  0G2
............................ucuugaauuccggaucuAga.............................................................  102  1  0G2
............................ucuugaauuccggaucuuga.............................................................  38   0  0G2
............................ucGugaauuccggaucuugaa............................................................  32   1  0G2
............................ucuugaauuccggaucuAgaa............................................................  148  1  0G2
............................ucuugaauuccggaucuugaa............................................................  52   0  0G2
............................ucuugaauuAcggaucuugaau...........................................................  2    1  0G2
............................Ccuugaauuccggaucuugaau...........................................................  2    1  0G2
............................ucuugaauuccggaucuugaau...........................................................  454  0  0G2
............................ucuugaauuccggaucuAgaau...........................................................  20   1  0G2
............................ucuugaauuccggaCcuugaau...........................................................  2    1  0G2
............................ucGugaauuccggaucuugaau...........................................................  94   1  0G2
............................ucuugaauuccggaucuugaaG...........................................................  6    1  0G2
............................ucuugaaGuccggaucuugaauu..........................................................  20   1  0G2
............................uUuugaauuccggaucuugaauu..........................................................  22   1  0G2
............................ucuugaauuccggaucuugaauu..........................................................  104  0  0G2
............................uGuugaauuccggaucuugaauu..........................................................  8    1  0G2
............................ucuugaauuccggaucuGgaauua.........................................................  6    1  0G2
............................ucuugaauuccggaucuAgaauua.........................................................  14   1  0G2
............................ucuugaauuccggaucuugaauua.........................................................  200  0  0G2
............................ucuugaauuccggaucuugaauAa.........................................................  330  1  0G2
............................uUuugaauuccggaucuugaauua.........................................................  12   1  0G2
............................ucuugaauuccggaucuugaauuag........................................................  24   0  0G2
............................ucuugaauuccggaucuugaauAag........................................................  6    1  0G2
............................ucuugaauuccggaucuAgaauuag........................................................  14   1  0G2
............................ucuugaauuccggaucuugaauuagg.......................................................  20   0  0G2
```

Star Mature

```
guuucuuauuagugugguuucauuuuggaucuugaauuccggaucuugaauuaggcuguuugguuuugaaucuugaauuccggaucuugaauuaggcuguuugauuuuga
.............................cuugaauuccggaucuug..............................................................  14     0  0G2
.............................cuugaauuccggaucuAga.............................................................  108    1  0G2
.............................cuugaauuccggaucuuga.............................................................  4      0  0G2
.............................Nuugaauuccggaucuugaa............................................................  4      1  0G2
.............................cuugaauuccggaucuugaa............................................................  192    0  0G2
.............................cGugaauuccggaucuugaa............................................................  2      1  0G2
.............................cuugaauuccggaucuAgaa............................................................  726    1  0G2
.............................cuugaauuccggaucuugaau...........................................................  454    0  0G2
.............................cGugaauuccggaucuugaau...........................................................  30     1  0G2
.............................Nuugaauuccggaucuugaau...........................................................  4      1  0G2
.............................cuugaauuccggaucuAgaau...........................................................  42     1  0G2
.............................cuugaauuccggaucuugaauu..........................................................  452    0  0G2
.............................cuugaauuccggaucuAgaauu..........................................................  148    1  0G2
.............................cNugaauuccggaucuugaauu..........................................................  2      1  0G2
.............................cAugaauuccggaucuugaauu..........................................................  2      1  0G2
.............................cuugaauuccggaucuugaauAa.........................................................  136    1  0G2
.............................cuugaauuccggaucuAgaauua.........................................................  320    1  0G2
.............................Nuugaauuccggaucuugaauua.........................................................  2      1  0G2
.............................cuugaauuccggaucuugaaGua.........................................................  2      1  0G2
.............................cuugaauuccggaucuugaauua.........................................................  1032   0  0G2
.............................cuugaauuccggaucuugaauCa.........................................................  70     1  0G2
.............................cuugaauuccggaucuugaaCua.........................................................  10     1  0G2
.............................cuugaauuccggaucuugaauuag........................................................  8      0  0G2
.............................cuugaauuccggaucuAgaauuag........................................................  4      1  0G2
..............................uugaauuccggaucuAga.............................................................  52     1  0G2
..............................uugaauuccggaucuAgaa............................................................  476    1  0G2
..............................uugaauuccggaucuugaa............................................................  206    0  0G2
..............................uugaauuccggaGcuugaau...........................................................  4      1  0G2
..............................uNgaauuccggaucuugaau...........................................................  12     1  0G2
..............................uugaauuAcggaucuugaau...........................................................  6      1  0G2
..............................uugaauuccggaucuugaaG...........................................................  14     1  0G2
..............................uugaauuccggaucuugaaC...........................................................  6      1  0G2
..............................uugaauuccggaucuugaau...........................................................  14612  0  0G2
..............................uGgaauuccggaucuugaau...........................................................  4      1  0G2
..............................Augaauuccggaucuugaau...........................................................  12     1  0G2
..............................uugaauuccggCucuugaau...........................................................  2      1  0G2
..............................uugaaGuccggaucuugaau...........................................................  2      1  0G2
..............................uugaauuccggaucuAgaau...........................................................  2996   1  0G2
..............................Nugaauuccggaucuugaau...........................................................  56     1  0G2
..............................uugaGuuccggaucuugaau...........................................................  114    1  0G2
..............................uugaauuccggaucuGgaau...........................................................  6      1  0G2
..............................Gugaauuccggaucuugaau...........................................................  24     1  0G2
..............................uugaauuccggaCcuugaau...........................................................  2      1  0G2
..............................uAgaauuccggaucuugaau...........................................................  4      1  0G2
..............................uugaauuccggaucCugaau...........................................................  2      1  0G2
..............................uugaauuccggGucuugaau...........................................................  14     1  0G2
..............................Cugaauuccggaucuugaau...........................................................  4      1  0G2
..............................uugaauuccggauAuugaau...........................................................  10     1  0G2
..............................Cugaauuccggaucuugaauu..........................................................  6      1  0G2
..............................uugaauuccggaucuugGauu..........................................................  14     1  0G2
..............................Augaauuccggaucuugaauu..........................................................  4      1  0G2
..............................uugaauuccggaCcuugaauu..........................................................  10     1  0G2
..............................uugaauuccggaucuugaaCu..........................................................  38     1  0G2
..............................uNgaauuccggaucuugaauu..........................................................  8      1  0G2
..............................uugaauuccggaucuGgaauu..........................................................  6      1  0G2
..............................uugaauuccggaucuuUaauu..........................................................  2      1  0G2
..............................uugaGuuccggaucuugaauu..........................................................  260    1  0G2
..............................uGgaauuccggaucuugaauu..........................................................  2      1  0G2
..............................uugaauuccggaucCugaauu..........................................................  4      1  0G2
..............................uugaauuccggCucuugaauu..........................................................  8      1  0G2
..............................uugaauuccggaGcuugaauu..........................................................  4      1  0G2
..............................uugaauuccggaucuAgaauu..........................................................  5240   1  0G2
..............................uugaauuccggaucuugaaGu..........................................................  8      1  0G2
..............................Nugaauuccggaucuugaauu..........................................................  60     1  0G2
..............................uugaauuccggGucuugaauu..........................................................  8      1  0G2
..............................Gugaauuccggaucuugaauu..........................................................  10     1  0G2
..............................uugaauuAcggaucuugaauu..........................................................  4      1  0G2
..............................uugaauuccggaucuCgaauu..........................................................  10     1  0G2
..............................uugaauuccggaucuugaauu..........................................................  21266  0  0G2
..............................uugaaGuccggaucuugaauu..........................................................  4      1  0G2
```

Star Mature

```
guuucuuauuaguguguuucauuuuggaucuugaauuccggaucuugaauuaggcuguuugguuuugaaucuugaauuccggaucuugaauuaggcuguuugauuuuga
..............................uugaauuccggaucuugGauua.........................................................   74      1   0G2
..............................uugaauuccggaucuugaauAa.........................................................   5360    1   0G2
..............................uugaauuccggauGuugaauua.........................................................   6       1   0G2
..............................Gugaauuccggaucuugaauua.........................................................   24      1   0G2
..............................uugaauAccggaucuugaauua.........................................................   22      1   0G2
..............................uugaauuccCgaucuugaauua.........................................................   16      1   0G2
..............................uugaauuccggGucuugaauua.........................................................   16      1   0G2
..............................uugaaGuccggaucuugaauua.........................................................   36      1   0G2
..............................uugaauuccggaucuCgaauua.........................................................   10      1   0G2
..............................uugaCuuccggaucuugaauua.........................................................   2       1   0G2
..............................uugaauuccgCaucuugaauua.........................................................   2       1   0G2
..............................uugaauuccggaCcuugaauua.........................................................   8       1   0G2
..............................uugaauuAcggaucuugaauua.........................................................   28      1   0G2
..............................uugaauuccggaucuugaauCa.........................................................   4852    1   0G2
..............................Cugaauuccggaucuugaauua.........................................................   34      1   0G2
..............................uugaauuccggaucuugaauua.........................................................   58180   0   0G2
..............................uAgaauuccggaucuugaauua.........................................................   4       1   0G2
..............................uugaauuccggaucuugaaGua.........................................................   6       1   0G2
..............................uugaauuccggauAuugaauua.........................................................   14      1   0G2
..............................Nugaauuccggaucuugaauua.........................................................   164     1   0G2
..............................uugaauuccggaucAugaauua.........................................................   2       1   0G2
..............................uugaauuccggaucuugCauua.........................................................   2       1   0G2
..............................uugaauuccggaucuugaaCua.........................................................   64      1   0G2
..............................uugaauuccggaGcuugaauua.........................................................   14      1   0G2
..............................Augaauuccggaucuugaauua.........................................................   14      1   0G2
..............................uugaauuccggaucuuUaauua.........................................................   6       1   0G2
..............................uugaGuuccggaucuugaauua.........................................................   1278    1   0G2
..............................uNgaauuccggaucuugaauua.........................................................   24      1   0G2
..............................uGgaauuccggaucuugaauua.........................................................   2       1   0G2
..............................uugaauuccggaucuGgaauua.........................................................   12      1   0G2
..............................uugaauuccggaucuAgaauua.........................................................   13424   1   0G2
..............................uugaauuGcggaucuugaauua.........................................................   2       1   0G2
..............................uugaauuccggaucCugaauua.........................................................   18      1   0G2
..............................uNgaauuccggaucuugaauuag........................................................   2       1   0G2
..............................uugaauuccggaucuugaauAag........................................................   18      1   0G2
..............................uugaauuccggaucuAgaauuag........................................................   2626    1   0G2
..............................uugaaGuccggaucuugaauuag........................................................   14      1   0G2
..............................Cugaauuccggaucuugaauuag........................................................   2       1   0G2
..............................uugaauuccggaucuCgaauuag........................................................   8       1   0G2
..............................uugaauuccggaucuugaauCag........................................................   16      1   0G2
..............................uugaauuccggaucuugaaCuag........................................................   30      1   0G2
..............................uugaGuuccggaucuugaauuag........................................................   14      1   0G2
..............................Nugaauuccggaucuugaauuag........................................................   2       1   0G2
..............................uugaauuccggaucuugaauuag........................................................   606     0   0G2
..............................uugaauuccggaucuugGauuag........................................................   2       1   0G2
..............................uAgaauuccggaucuugaauuag........................................................   6       1   0G2
..............................uugaauuccggaucuGgaauuag........................................................   2       1   0G2
..............................uugaauuccggaucuugaauuaUg.......................................................   2       1   0G2
..............................uugaauuccggaucuugaauuagA.......................................................   6       1   0G2
..............................uugaauuccggaucAugaauuagg.......................................................   22      1   0G2
..............................uugaauuccggaucuugaauuagg.......................................................   2       0   0G2
..............................uugaauuccggaucuugaauuaggcuA....................................................   2       1   0G2
...............................ugaauuccggaucuugaa............................................................   4       0   0G2
...............................ugaauuccggaucuAgaa............................................................   132     1   0G2
...............................ugaauuccggaucuGgaa............................................................   2       1   0G2
...............................ugaauuccggaucuugaau...........................................................   480     0   0G2
...............................ugaauuAcggaucuugaau...........................................................   2       1   0G2
...............................ugaauuccggaucuAgaau...........................................................   136     1   0G2
...............................Agaauuccggaucuugaauu..........................................................   14      1   0G2
...............................ugaauuAcggaucuugaauu..........................................................   2       1   0G2
...............................uNaauuccggaucuugaauu..........................................................   2       1   0G2
...............................ugaauuccggaucuugaauu..........................................................   5018    0   0G2
...............................ugaGuuccggaucuugaauu..........................................................   64      1   0G2
...............................ugaauuccggaucuugaaCu..........................................................   8       1   0G2
...............................ugaaGuccggaucuugaauu..........................................................   2       1   0G2
...............................ugaauuccggCucuugaauu..........................................................   2       1   0G2
...............................Ngaauuccggaucuugaauu..........................................................   14      1   0G2
...............................ugaauuccggaucuCgaauu..........................................................   2       1   0G2
...............................ugaauuccggaucuAgaauu..........................................................   1700    1   0G2
...............................ugaauuccCgaucuugaauua.........................................................   2       1   0G2
```

Star **Mature**

```
guuucuuauuaguguguuucauuuuggaucuugaauuccggaucuugaauuaggcuguuugguuuugaaucuugaauuccggaucuugaauuaggcuguuugauuuuga
...............................Ggaauuccggaucuugaauua.........................................................   14      1   0G2
...............................ugaauuccggaCcuugaauua.........................................................   8       1   0G2
...............................ugaauuccggCucuugaauua.........................................................   6       1   0G2
...............................ugaaGuccggaucuugaauua.........................................................   52      1   0G2
...............................ugaauuccggauAuugaauua.........................................................   10      1   0G2
...............................ugaauuccggaucAugaauua.........................................................   6       1   0G2
...............................ugaauuccggaucuugGauua.........................................................   42      1   0G2
...............................ugaauuccggaGcuugaauua.........................................................   14      1   0G2
...............................ugaauuccggaucuuUaauua.........................................................   10      1   0G2
...............................ugaauuccggaucuugaaCua.........................................................   36      1   0G2
...............................ugaauuccggaucuugUauua.........................................................   4       1   0G2
...............................ugaauuccggaucuGgaauua.........................................................   2       1   0G2
...............................ugaauuccggaucCugaauua.........................................................   6       1   0G2
...............................ugaauuccggGucuugaauua.........................................................   2       1   0G2
...............................ugaauuccggaucuugaauua.........................................................   31676   0   0G2
...............................ugaauuccggaucuugaauAa.........................................................   294     1   0G2
...............................ugaauuAcggaucuugaauua.........................................................   32      1   0G2
...............................ugaauuccggaucuCgaauua.........................................................   20      1   0G2
...............................uNaauuccggaucuugaauua.........................................................   22      1   0G2
...............................Ngaauuccggaucuugaauua.........................................................   106     1   0G2
...............................ugaGuuccggaucuugaauua.........................................................   612     1   0G2
...............................Agaauuccggaucuugaauua.........................................................   6       1   0G2
...............................ugaauuccggaucuugaauCa.........................................................   948     1   0G2
...............................ugaauuccggaucuAgaauua.........................................................   12002   1   0G2
...............................ugaauuccggaGcuugaauuag........................................................   2       1   0G2
...............................ugaauuccggaucuGgaauuag........................................................   4       1   0G2
...............................ugaauuccggGucuugaauuag........................................................   2       1   0G2
...............................ugaauuccggaucuugaauuag........................................................   2092    0   0G2
...............................ugaGuuccggaucuugaauuag........................................................   24      1   0G2
...............................ugaauuccggaucuugaauCag........................................................   16      1   0G2
...............................Agaauuccggaucuugaauuag........................................................   2       1   0G2
...............................Ngaauuccggaucuugaauuag........................................................   14      1   0G2
...............................ugaauuccggaucuAgaauuag........................................................   9070    1   0G2
...............................ugaauuccggaucuugaauAag........................................................   2       1   0G2
...............................ugaauuccggaucuugGauuag........................................................   2       1   0G2
...............................ugaauuccggaucuugaauuagg.......................................................   146     0   0G2
...............................ugaauuccggaucuugaauuagA.......................................................   4       1   0G2
...............................ugaaGuccggaucuugaauuagg.......................................................   10      1   0G2
................................gaauuccggaucuAgaau...........................................................   30      1   0G2
................................gaauuccggaucuugaau...........................................................   20      0   0G2
................................gaauuccggaucuuUaauu..........................................................   2       1   0G2
................................gaauuccggaucuugaauu..........................................................   286     0   0G2
................................gaauuccggaucuCgaauu..........................................................   4       1   0G2
................................gaauuccggaucuAgaauu..........................................................   70      1   0G2
................................gaauuccggauAuugaauu..........................................................   2       1   0G2
................................gaauuccggaGcuugaauua.........................................................   2       1   0G2
................................gaauuccggaucCugaauua.........................................................   2       1   0G2
................................gaauuccggaucuugaauAa.........................................................   8       1   0G2
................................gaauuccggaucuuUaauua.........................................................   2       1   0G2
................................gaauuccggaucuugaauua.........................................................   2166    0   0G2
................................gNauuccggaucuugaauua.........................................................   2       1   0G2
................................gaauuccggauAuugaauua.........................................................   2       1   0G2
................................gaauuccggaucuugaauCa.........................................................   206     1   0G2
................................gaauuccggaucuugaaCua.........................................................   8       1   0G2
................................gaauuccggaucuAgaauua.........................................................   1214    1   0G2
................................Naauuccggaucuugaauua.........................................................   10      1   0G2
................................gaGuuccggaucuugaauua.........................................................   26      1   0G2
................................gaGuuccggaucuugaauuag........................................................   12      1   0G2
................................gaauuccggaucuAgaauuag........................................................   6458    1   0G2
................................gaauuccggaucuugaauuag........................................................   1122    0   0G2
................................gaauuccggaucuAgaauuagg.......................................................   6       1   0G2
................................gaauuccggaucuugaauuagA.......................................................   16      1   0G2
................................gaauuccggaucuugaauuagg.......................................................   10      0   0G2
.................................aauuccggaucuugaauu..........................................................   8       0   0G2
.................................aauuccggaucuugaauAa.........................................................   22      1   0G2
.................................aauuccggaucuugaauua.........................................................   992     0   0G2
.................................aauuccggaucuugaauCa.........................................................   38      1   0G2
.................................aNuuccggaucuugaauua.........................................................   2       1   0G2
.................................aauAccggaucuugaauuag........................................................   2       1   0G2
.................................aauuccggaucuuUaauuag........................................................   18      1   0G2
```

Star Mature

```
guuucuuauuaguguguuucauuuuggaucuugaauuccggaucuugaauuaggcuguuugguuuugaaucuugaauuccggaucuugaauuaggcuguuugauuuuga
.................................Nauuccggaucuugaauuag........................................................ 12    1  0G2
.................................aauuccggaucuGgaauuag........................................................ 12    1  0G2
.................................aauuccggaucuugaauuag........................................................ 8186  0  0G2
.................................aauuccggaucAugaauuag........................................................ 4     1  0G2
.................................aauuccggaucuCgaauuag........................................................ 30    1  0G2
.................................aauuccggauAuugaauuag........................................................ 4     1  0G2
.................................aauuccggaucuugaaCuag........................................................ 4     1  0G2
.................................aGuuccggaucuugaauuag........................................................ 112   1  0G2
.................................aauuccggaGcuugaauuag........................................................ 2     1  0G2
.................................aauuccggaCcuugaauuag........................................................ 4     1  0G2
.................................aauuccggGucuugaauuag........................................................ 14    1  0G2
.................................aNuuccggaucuugaauuag........................................................ 2     1  0G2
.................................aauuccggaucuugaauCag........................................................ 40    1  0G2
.................................aauuccggaucuugaauuagg....................................................... 558   0  0G2
.................................aauuccggaucuugaauuaUg....................................................... 2     1  0G2
.................................aauuccggaucuAgaauuagg....................................................... 14    1  0G2
.................................aGuuccggaucuugaauuagg....................................................... 14    1  0G2
.................................Nauuccggaucuugaauuagg....................................................... 2     1  0G2
.................................aauuccggaucuugaauuagA....................................................... 8     1  0G2
.................................aauuccggaucAugaauuagg....................................................... 8     1  0G2
.................................aNuuccggaucuugaauuagg....................................................... 2     1  0G2
..................................auuccggaucuugaauAa......................................................... 20    1  0G2
..................................auuccggaucuugaauCa......................................................... 24    1  0G2
..................................Guuccggaucuugaauua......................................................... 8     1  0G2
..................................auuccggaGcuugaauua......................................................... 2     1  0G2
..................................auuccggaucuugaauua......................................................... 358   0  0G2
..................................aNuccggaucuugaauua......................................................... 2     1  0G2
..................................auuccggaucuGgaauua......................................................... 2     1  0G2
..................................Nuuccggaucuugaauuag........................................................ 4     1  0G2
..................................auuccggaucAugaauuag........................................................ 28    1  0G2
..................................auuccggaucuugaauCag........................................................ 22    1  0G2
..................................auuccggaucuugaauuag........................................................ 2374  0  0G2
..................................Guuccggaucuugaauuag........................................................ 4     1  0G2
..................................auuccggauAuugaauuag........................................................ 2     1  0G2
..................................auuccggaucuGgaauuag........................................................ 24    1  0G2
..................................auuAcggaucuugaauuag........................................................ 2     1  0G2
..................................auuccggaucuAgaauuagg....................................................... 38    1  0G2
..................................auuccggGucuugaauuagg....................................................... 2     1  0G2
..................................auuccggaucAugaauuagg....................................................... 118   1  0G2
..................................auuccggaucuGgaauuagg....................................................... 4     1  0G2
..................................auuAcggaucuugaauuagg....................................................... 6     1  0G2
..................................Nuuccggaucuugaauuagg....................................................... 16    1  0G2
..................................aNuccggaucuugaauuagg....................................................... 2     1  0G2
..................................auuccggaucuugGauuagg....................................................... 2     1  0G2
..................................auuccggaucuugaaGuagg....................................................... 2     1  0G2
..................................auucGggaucuugaauuagg....................................................... 18    1  0G2
..................................auuccggaucuugaauuagA....................................................... 90    1  0G2
..................................auuccggCucuugaauuagg....................................................... 18    1  0G2
..................................auuccggaucuugaauuaUg....................................................... 26    1  0G2
..................................auuccggaucuugaauCagg....................................................... 2     1  0G2
..................................auuccggaucuugaaCuagg....................................................... 32    1  0G2
..................................auuccggaucuCgaauuagg....................................................... 18    1  0G2
..................................Guuccggaucuugaauuagg....................................................... 64    1  0G2
..................................auuccggaucuugaauuagg....................................................... 4754  0  0G2
..................................auuccggaCcuugaauuagg....................................................... 2     1  0G2
...................................uuccggaucuugGauuag........................................................ 2     1  0G2
...................................Nuccggaucuugaauuag........................................................ 6     1  0G2
...................................uuccggaucuugaauCag........................................................ 10    1  0G2
...................................uuccggGucuugaauuag........................................................ 8     1  0G2
...................................uuccggaucuugaauuag........................................................ 1866  0  0G2
...................................uuccggaucuuUaauuag........................................................ 8     1  0G2
...................................uuccggaucuGgaauuag........................................................ 8     1  0G2
...................................uuccggauGuugaauuagg....................................................... 6     1  0G2
...................................uuccggaucuugaaCuagg....................................................... 26    1  0G2
...................................uuccggaucuugaauuagA....................................................... 6     1  0G2
...................................uuccggaucuGgaauuagg....................................................... 8     1  0G2
...................................uuccggaucuugaauuagg....................................................... 2582  0  0G2
...................................uuccggaucuAgaauuagg....................................................... 22    1  0G2
...................................uuccggaucuugaauuaUg....................................................... 8     1  0G2
...................................Nuccggaucuugaauuagg....................................................... 8     1  0G2
```

```
                  Star                                              Mature
guuucuuauuaguguguuucauuuuggaucuugaauuccggaucuugaauuaggcuguuugguuuugaaucuugaauuccggaucuugaauuaggcuguuugauuuuga
...................................uuccggaCcuugaauuagg.......................................................    4     1    0G2
...................................uuccggaucAugaauuagg.......................................................    28    1    0G2
...................................uucGggaucuugaauuagg.......................................................    20    1    0G2
...................................uuccggGucuugaauuagg.......................................................    4     1    0G2
...................................uNccggaucuugaauuagg.......................................................    4     1    0G2
...................................uuccggCucuugaauuagg.......................................................    2     1    0G2
....................................uccggaucuGgaauuagg.......................................................    6     1    0G2
....................................uccggGucuugaauuagg.......................................................    2     1    0G2
....................................Nccggaucuugaauuagg.......................................................    6     1    0G2
....................................uccggaucuCgaauuagg.......................................................    20    1    0G2
....................................uccggaucuugaauuagA.......................................................    6     1    0G2
....................................uccggaucuugaauuaUg.......................................................    2     1    0G2
....................................uccggaucuugaauuagg.......................................................    888   0    0G2
....................................uccggaucuAgaauuagg.......................................................    10    1    0G2
....................................ucGggaucuugaauuagg.......................................................    8     1    0G2
....................................uccggaucAugaauuagg.......................................................    10    1    0G2
.....................................ccggaucuugaauuaggcuA....................................................    6     1    0G2
..............................................................uuuugaGucuugaauuccgg...........................    3     1    0G2
..............................................................uuuugaaucGugaauuccgg...........................    4     1    0G2
..............................................................uuuugaaucuugaauuccgg...........................    62    0    0G2
..............................................................uuCugaaucuugaauuccgg...........................    11    1    0G2
..............................................................uuuuAaaucuugaauuccgg...........................    3     1    0G2
..............................................................uuuugaCucuugaauuccgg...........................    3     1    0G2
..............................................................uNuugaaucuugaauuccgga..........................    1     1    0G2
..............................................................uuuugaCucuugaauuccgga..........................    10    1    0G2
..............................................................Nuuugaaucuugaauuccgga..........................    1     1    0G2
..............................................................uuuugaaucuugaauuccgga..........................    23    0    0G2
..............................................................uuuugaaucuugaauuccggauA........................    18    1    0G2
...............................................................uuugaaucuugaauuccgg...........................    1     0    0G2
...............................................................uuugaaucuugaauuccgga..........................    2     0    0G2
...............................................................uuugaaucuugaauuccggauA........................    14    1    0G2
...............................................................uuugaaucuugaauuccggauc........................    5     0    0G2
................................................................uugaaucuugaauuccggau.........................    2     0    0G2
................................................................Nugaaucuugaauuccggau.........................    1     1    0G2
................................................................uugaaucuugaauuccggauA........................    27    1    0G2
................................................................uugaaucGugaauuccggauc........................    1     1    0G2
................................................................uugaaucuugaauuccggaucA.......................    12    1    0G2
................................................................uugaaucuugaauuccggaucuugaau..................    13    0    0G2
.................................................................ugaaucuugaauuccggauc........................    3     0    0G2
.................................................................ugaCucuugaauuccggauc........................    4     1    0G2
.................................................................ugaGucuugaauuccggauc........................    3     1    0G2
.................................................................ugaaucuugaauuccggauA........................    10    1    0G2
.................................................................ugaaucuugaauuccggaucA.......................    1     1    0G2
.................................................................ugaaucuugaauuccggaucu.......................    20    0    0G2
.................................................................ugaaucuugaauuccggaucuAgaauu.................    3     1    0G2
.................................................................ugaaucuugaauuccggaucuugaauu.................    22    0    0G2
.................................................................ugaaucuugaauuccCgaucuugaauu.................    3     1    0G2
..................................................................gaaucuugaauuccggauc........................    6     0    0G2
..................................................................gaaucuugaauuccggaucuAgaauua................    2     1    0G2
...................................................................aaucuugaauuccggaucA.......................    5     1    0G2
...................................................................aaucuugaauuccggaucu.......................    5     0    0G2
...................................................................aaucuugaauuccggaucuA......................    5     1    0G2
...................................................................aaucuugaauuccggaucuAg.....................    18    1    0G2
...................................................................aGucuugaauuccggaucuug.....................    10    1    0G2
...................................................................aaucuugaauuccggaucuugUa...................    5     1    0G2
...................................................................aaucuugaauuccggaucuAgaauuag...............    5     1    0G2
....................................................................aucuugaauuccggaucuug.....................    6     0    0G2
....................................................................aucuugaauuccggaucuAga....................    4     1    0G2
....................................................................aucuugaauuccggaucuugaau..................    16    0    0G2
....................................................................aucuugaauuccggaucuugaauua................    26    0    0G2
....................................................................aucuugaauuccggaucuugaauuag...............    2     0    0G2
.....................................................................ucuugaauuccggaucuA......................    4     1    0G2
.....................................................................ucuugaauuccggaucuug.....................    2     0    0G2
.....................................................................ucuugaauuccggaucuAga....................    102   1    0G2
.....................................................................ucuugaauuccggaucuuga....................    38    0    0G2
.....................................................................ucuugaauuccggaucuAgaa...................    148   1    0G2
.....................................................................ucuugaauuccggaucuugaa...................    52    0    0G2
.....................................................................ucGugaauuccggaucuugaa...................    32    1    0G2
.....................................................................Ccuugaauuccggaucuugaau..................    2     1    0G2
.....................................................................ucuugaauuccggaucuugaaG..................    6     1    0G2
```

Star **Mature**

guuucuuauuaguguguuucauuuuggaucuugaauuccggaucuugaauuaggcuguuugguuuugaaucuugaauuccggaucuugaauuaggcuguuugauuuuga			
....ucuugaauuAcggaucuugaau..................	2	1	0G2
....ucuugaauuccggaucuAgaau..................	20	1	0G2
....ucuugaauuccggaCcuugaau..................	2	1	0G2
....ucGugaauuccggaucuugaau..................	94	1	0G2
....ucuugaauuccggaucuugaau..................	454	0	0G2
....uGuugaauuccggaucuugaauu.................	8	1	0G2
....ucuugaauuccggaucuugaauu.................	104	0	0G2
....ucuugaaGuccggaucuugaauu.................	20	1	0G2
....uUuugaauuccggaucuugaauu.................	22	1	0G2
....ucuugaauuccggaucuGgaauua................	6	1	0G2
....ucuugaauuccggaucuugaauAa................	330	1	0G2
....ucuugaauuccggaucuAgaauua................	14	1	0G2
....ucuugaauuccggaucuugaauua................	200	0	0G2
....uUuugaauuccggaucuugaauua................	12	1	0G2
....ucuugaauuccggaucuugaauAag...............	6	1	0G2
....ucuugaauuccggaucuAgaauuag...............	14	1	0G2
....ucuugaauuccggaucuugaauuag...............	24	0	0G2
....ucuugaauuccggaucuugaauuagg..............	20	0	0G2
....cuugaauuccggaucuug.....................	14	0	0G2
....cuugaauuccggaucuAga....................	108	1	0G2
....cuugaauuccggaucuuga....................	4	0	0G2
....Nuugaauuccggaucuugaa...................	4	1	0G2
....cuugaauuccggaucuAgaa...................	726	1	0G2
....cGugaauuccggaucuugaa...................	2	1	0G2
....cuugaauuccggaucuugaa...................	192	0	0G2
....cuugaauuccggaucuAgaau..................	42	1	0G2
....cuugaauuccggaucuugaau..................	454	0	0G2
....Nuugaauuccggaucuugaau..................	4	1	0G2
....cGugaauuccggaucuugaau..................	30	1	0G2
....cAugaauuccggaucuugaauu.................	2	1	0G2
....cuugaauuccggaucuugaauu.................	452	0	0G2
....cNugaauuccggaucuugaauu.................	2	1	0G2
....cuugaauuccggaucuAgaauu.................	148	1	0G2
....cuugaauuccggaucuAgaauua................	320	1	0G2
....cuugaauuccggaucuugaauAa................	136	1	0G2
....cuugaauuccggaucuugaaGua................	2	1	0G2
....cuugaauuccggaucuugaaCua................	10	1	0G2
....cuugaauuccggaucuugaauua................	1032	0	0G2
....Nuugaauuccggaucuugaauua................	2	1	0G2
....cuugaauuccggaucuugaauCa................	70	1	0G2
....cuugaauuccggaucuAgaauuag...............	4	1	0G2
....cuugaauuccggaucuugaauuag...............	8	0	0G2
....uugaauuccggaucuAga....................	52	1	0G2
....uugaauuccggaucuAgaa...................	476	1	0G2
....uugaauuccggaucuugaa...................	206	0	0G2
....Augaauuccggaucuugaau..................	12	1	0G2
....Gugaauuccggaucuugaau..................	24	1	0G2
....uugaauuccggaCcuugaau..................	2	1	0G2
....Cugaauuccggaucuugaau..................	4	1	0G2
....uugaauuccggaucuugaaC..................	6	1	0G2
....Nugaauuccggaucuugaau..................	56	1	0G2
....uugaauuccggCucuugaau..................	2	1	0G2
....uugaauuccggGucuugaau..................	14	1	0G2
....uNgaauuccggaucuugaau..................	12	1	0G2
....uugaauuccggaucuAgaau..................	2996	1	0G2
....uugaaGuccggaucuugaau..................	2	1	0G2
....uGgaauuccggaucuugaau..................	4	1	0G2
....uugaGuuccggaucuugaau..................	114	1	0G2
....uugaauuccggaGcuugaau..................	4	1	0G2
....uugaauuccggauAuugaau..................	10	1	0G2
....uugaauuccggaucuugaau..................	14612	0	0G2
....uugaauuccggaucuugaaG..................	14	1	0G2
....uugaauuccggaucuGgaau..................	6	1	0G2
....uugaauuAcggaucuugaau..................	6	1	0G2
....uugaauuccggaucCugaau..................	2	1	0G2
....uAgaauuccggaucuugaau..................	4	1	0G2
....uugaauuccggaucuugaaGu.................	8	1	0G2
....uugaauuccggaucuugaauu.................	21266	0	0G2
....uugaauuccggaucuAgaauu.................	5240	1	0G2
....uugaGuuccggaucuugaauu.................	260	1	0G2

Star **Mature**

```
guuucuuauuaguguguuucauuuuggaucuugaauuccggaucuugaauuaggcuguuugguuuugaaucuugaauuccggaucuugaauuaggcuguuugauuuuga
.......................................................................uugaauuccggaucuuUaauu.................	2	1	0G2
.......................................................................uugaauuAcggaucuugaauu.................	4	1	0G2
.......................................................................uugaauuccggaucuugaaCu.................	38	1	0G2
.......................................................................uugaauuccggaucCugaauu.................	4	1	0G2
.......................................................................Nugaauuccggaucuugaauu.................	60	1	0G2
.......................................................................uugaauuccggGucuugaauu.................	8	1	0G2
.......................................................................Gugaauuccggaucuugaauu.................	10	1	0G2
.......................................................................Augaauuccggaucuugaauu.................	4	1	0G2
.......................................................................uNgaauuccggaucuugaauu.................	8	1	0G2
.......................................................................Cugaauuccggaucuugaauu.................	6	1	0G2
.......................................................................uugaauuccggaGcuugaauu.................	4	1	0G2
.......................................................................uugaauuccggCucuugaauu.................	8	1	0G2
.......................................................................uugaauuccggaCcuugaauu.................	10	1	0G2
.......................................................................uugaauuccggaucuugGauu.................	14	1	0G2
.......................................................................uugaaGuccggaucuugaauu.................	4	1	0G2
.......................................................................uugaauuccggaucuCgaauu.................	10	1	0G2
.......................................................................uugaauuccggaucuGgaauu.................	6	1	0G2
.......................................................................uGgaauuccggaucuugaauu.................	2	1	0G2
.......................................................................Nugaauuccggaucuugaauua................	164	1	0G2
.......................................................................uugaCuuccggaucuugaauua................	2	1	0G2
.......................................................................uugaauuccggaucAugaauua................	2	1	0G2
.......................................................................uugaauuccggaucuAgaauua................	13424	1	0G2
.......................................................................uugaauuccggaucuugaauCa................	4852	1	0G2
.......................................................................uGgaauuccggaucuugaauua................	2	1	0G2
.......................................................................uugaauuccggaucuugGauua................	74	1	0G2
.......................................................................Augaauuccggaucuugaauua................	14	1	0G2
.......................................................................uugaauuccggaucuugCauua................	2	1	0G2
.......................................................................uugaauuccggauGuugaauua................	6	1	0G2
.......................................................................uugaaGuccggaucuugaauua................	36	1	0G2
.......................................................................uugaGuuccggaucuugaauua................	1278	1	0G2
.......................................................................uugaauuccggaGcuugaauua................	14	1	0G2
.......................................................................uugaauAccggaucuugaauua................	22	1	0G2
.......................................................................uugaauuccggaucuugaauua................	58180	0	0G2
.......................................................................uugaauuccggaucuCgaauua................	10	1	0G2
.......................................................................uugaauuccggaucuugaaGua................	6	1	0G2
.......................................................................Cugaauuccggaucuugaauua................	34	1	0G2
.......................................................................uugaauuccggauAuugaauua................	14	1	0G2
.......................................................................uugaauuccggGucuugaauua................	16	1	0G2
.......................................................................Gugaauuccggaucuugaauua................	24	1	0G2
.......................................................................uugaauuccggaCcuugaauua................	8	1	0G2
.......................................................................uugaauuccggaucuGgaauua................	12	1	0G2
.......................................................................uugaauuccggaucuuUaauua................	6	1	0G2
.......................................................................uugaauuccggaucuugaaCua................	64	1	0G2
.......................................................................uugaauuccgCaucuugaauua................	2	1	0G2
.......................................................................uAgaauuccggaucuugaauua................	4	1	0G2
.......................................................................uugaauuccCgaucuugaauua................	16	1	0G2
.......................................................................uugaauuccggaucuugaauAa................	5360	1	0G2
.......................................................................uugaauuGcggaucuugaauua................	2	1	0G2
.......................................................................uugaauuAcggaucuugaauua................	28	1	0G2
.......................................................................uNgaauuccggaucuugaauua................	24	1	0G2
.......................................................................uugaauuccggaucCugaauua................	18	1	0G2
.......................................................................uugaGuuccggaucuugaauuag...............	14	1	0G2
.......................................................................Nugaauuccggaucuugaauuag...............	2	1	0G2
.......................................................................uugaauuccggaucuugGauuag...............	2	1	0G2
.......................................................................uugaauuccggaucuGgaauuag...............	2	1	0G2
.......................................................................uugaauuccggaucuugaauCag...............	16	1	0G2
.......................................................................uugaauuccggaucuAgaauuag...............	2626	1	0G2
.......................................................................uugaauuccggaucuugaauAag...............	18	1	0G2
.......................................................................uNgaauuccggaucuugaauuag...............	2	1	0G2
.......................................................................uAgaauuccggaucuugaauuag...............	6	1	0G2
.......................................................................uugaaGuccggaucuugaauuag...............	14	1	0G2
.......................................................................uugaauuccggaucuCgaauuag...............	8	1	0G2
.......................................................................uugaauuccggaucuugaaCuag...............	30	1	0G2
.......................................................................Cugaauuccggaucuugaauuag...............	2	1	0G2
.......................................................................uugaauuccggaucuugaauuag...............	606	0	0G2
.......................................................................uugaauuccggaucAugaauuagg..............	22	1	0G2
.......................................................................uugaauuccggaucuugaauuagg..............	2	0	0G2
.......................................................................uugaauuccggaucuugaauuaUg..............	2	1	0G2
.......................................................................uugaauuccggaucuugaauuagA..............	6	1	0G2
.......................................................................uugaauuccggaucuugaauuaggcuA...........	2	1	0G2
```

Star **Mature**

guuucuuauuaguguguuucauuuuggaucuugaauuccggaucuugaauuaggcuguuugguuuugaaucuugaauuccggaucuugaauuaggcuguuugauuuuga

....ugaauuccggaucuGgaa...................	2	1	0G2
....ugaauuccggaucuAgaa...................	132	1	0G2
....ugaauuccggaucuugaa...................	4	0	0G2
....ugaauuccggaucuAgaau..................	136	1	0G2
....ugaauuAcggaucuugaau..................	2	1	0G2
....ugaauuccggaucuugaau..................	480	0	0G2
....Agaauuccggaucuugaauu.................	14	1	0G2
....Ngaauuccggaucuugaauu.................	14	1	0G2
....ugaauuAcggaucuugaauu.................	2	1	0G2
....ugaauuccggCucuugaauu.................	2	1	0G2
....ugaaGuccggaucuugaauu.................	2	1	0G2
....ugaGuuccggaucuugaauu.................	64	1	0G2
....ugaauuccggaucuAgaauu.................	1700	1	0G2
....ugaauuccggaucuugaaCu.................	8	1	0G2
....uNaauuccggaucuugaauu.................	2	1	0G2
....ugaauuccggaucuugaauu.................	5018	0	0G2
....ugaauuccggaucuCgaauu.................	2	1	0G2
....ugaauuccggaucuugaauua................	31676	0	0G2
....ugaGuuccggaucuugaauua................	612	1	0G2
....ugaauuccCgaucuugaauua................	2	1	0G2
....ugaauuccggaucCugaauua................	6	1	0G2
....ugaauuccggaucuGgaauua................	2	1	0G2
....ugaauuccggaucAugaauua................	6	1	0G2
....ugaauuccggaucuuUaauua................	10	1	0G2
....ugaauuccggaucuAgaauua................	12002	1	0G2
....ugaauuccggaucuugaauAa................	294	1	0G2
....ugaauuccggaucuugUauua................	4	1	0G2
....ugaauuccggaCcuugaauua................	8	1	0G2
....ugaauuccggaucuugGauua................	42	1	0G2
....ugaauuAcggaucuugaauua................	32	1	0G2
....ugaauuccggCucuugaauua................	6	1	0G2
....ugaauuccggaucuugaaCua................	36	1	0G2
....Ggaauuccggaucuugaauua................	14	1	0G2
....ugaauuccggaGcuugaauua................	14	1	0G2
....ugaauuccggaucuCgaauua................	20	1	0G2
....Agaauuccggaucuugaauua................	6	1	0G2
....Ngaauuccggaucuugaauua................	106	1	0G2
....ugaauuccggaucuugaauCa................	948	1	0G2
....ugaaGuccggaucuugaauua................	52	1	0G2
....uNaauuccggaucuugaauua................	22	1	0G2
....ugaauuccggauAuugaauua................	10	1	0G2
....ugaauuccggGucuugaauua................	2	1	0G2
....Ngaauuccggaucuugaauuag...............	14	1	0G2
....ugaauuccggaucuugGauuag...............	2	1	0G2
....ugaauuccggaGcuugaauuag...............	2	1	0G2
....Agaauuccggaucuugaauuag...............	2	1	0G2
....ugaauuccggGucuugaauuag...............	2	1	0G2
....ugaauuccggaucuugaauAag...............	2	1	0G2
....ugaauuccggaucuugaauCag...............	16	1	0G2
....ugaauuccggaucuGgaauuag...............	4	1	0G2
....ugaauuccggaucuAgaauuag...............	9070	1	0G2
....ugaauuccggaucuugaauuag...............	2092	0	0G2
....ugaGuuccggaucuugaauuag...............	24	1	0G2
....ugaaGuccggaucuugaauuagg..............	10	1	0G2
....ugaauuccggaucuugaauuagA..............	4	1	0G2
....ugaauuccggaucuugaauuagg..............	146	0	0G2
....gaauuccggaucuugaau..................	20	0	0G2
....gaauuccggaucuAgaau..................	30	1	0G2
....gaauuccggaucuuUaau..................	2	1	0G2
....gaauuccggauAuugaau..................	2	1	0G2
....gaauuccggaucuAgaauu.................	70	1	0G2
....gaauuccggaucuugaauu.................	286	0	0G2
....gaauuccggaucuCgaauu.................	4	1	0G2
....gNauuccggaucuugaauua................	2	1	0G2
....gaauuccggaucuuUaauua................	2	1	0G2
....gaauuccggauAuugaauua................	2	1	0G2
....gaauuccggaucuugaaCua................	8	1	0G2
....gaauuccggaucuugaauAa................	8	1	0G2
....gaauuccggaucCugaauua................	2	1	0G2
....gaauuccggaGcuugaauua................	2	1	0G2

Star Mature

guuucuuauuaguguguuucauuuuggaucuugaauuccggaucuugaauuaggcuguuugguuuugaaucuugaauuccggaucuugaauuaggcuguuugauuuuga			
....gaauuccggaucuugaauua................	2166	0	0G2
....Naauuccggaucuugaauua................	10	1	0G2
....gaauuccggaucuugaauCa................	206	1	0G2
....gaauuccggaucuAgaauua................	1214	1	0G2
....gaGuuccggaucuugaauua................	26	1	0G2
....gaGuuccggaucuugaauuag...............	12	1	0G2
....gaauuccggaucuAgaauuag...............	6458	1	0G2
....gaauuccggaucuugaauuag...............	1122	0	0G2
....gaauuccggaucuAgaauuagg..............	6	1	0G2
....gaauuccggaucuugaauuagg..............	10	0	0G2
....gaauuccggaucuugaauuagA..............	16	1	0G2
....aauuccggaucuugaauu.................	8	0	0G2
....aNuuccggaucuugaauua................	2	1	0G2
....aauuccggaucuugaauua................	992	0	0G2
....aauuccggaucuugaauAa................	22	1	0G2
....aauuccggaucuugaauCa................	38	1	0G2
....aauuccggaCcuugaauuag...............	4	1	0G2
....aauuccggaucuugaaCuag...............	4	1	0G2
....aauuccggaucuuUaauuag...............	18	1	0G2
....aauuccggaucuCgaauuag...............	30	1	0G2
....aauuccggaucuugaauuag...............	8186	0	0G2
....aGuuccggaucuugaauuag...............	112	1	0G2
....aauuccggGucuugaauuag...............	14	1	0G2
....aauuccggaucuGgaauuag...............	12	1	0G2
....aauAccggaucuugaauuag...............	2	1	0G2
....aauuccggaGcuugaauuag...............	2	1	0G2
....aauuccggauAuugaauuag...............	4	1	0G2
....Nauuccggaucuugaauuag...............	12	1	0G2
....aNuuccggaucuugaauuag...............	2	1	0G2
....aauuccggaucAugaauuag...............	4	1	0G2
....aauuccggaucuugaauCag...............	40	1	0G2
....aauuccggaucuAgaauuagg..............	14	1	0G2
....aauuccggaucuugaauuagg..............	558	0	0G2
....aGuuccggaucuugaauuagg..............	14	1	0G2
....aauuccggaucAugaauuagg..............	8	1	0G2
....aauuccggaucuugaauuagA..............	8	1	0G2
....Nauuccggaucuugaauuagg..............	2	1	0G2
....aNuuccggaucuugaauuagg..............	2	1	0G2
....aauuccggaucuugaauuaUg..............	2	1	0G2
....auuccggaucuGgaauua................	2	1	0G2
....auuccggaucuugaauCa................	24	1	0G2
....aNuccggaucuugaauua................	2	1	0G2
....auuccggaucuugaauua................	358	0	0G2
....auuccggaucuugaauAa................	20	1	0G2
....auuccggaGcuugaauua................	2	1	0G2
....Guuccggaucuugaauua................	8	1	0G2
....auuccggaucuGgaauuag...............	24	1	0G2
....auuccggaucAugaauuag...............	28	1	0G2
....auuccggauAuugaauuag...............	2	1	0G2
....Guuccggaucuugaauuag...............	4	1	0G2
....auuccggaucuugaauuag...............	2374	0	0G2
....auuAcggaucuugaauuag...............	2	1	0G2
....Nuuccggaucuugaauuag...............	4	1	0G2
....auuccggaucuugaauCag...............	22	1	0G2
....auuccggaucuugaauuagg..............	4754	0	0G2
....auuccggaucuugaauCagg..............	2	1	0G2
....auuccggaucuugaaGuagg..............	2	1	0G2
....auuccggaucuGgaauuagg..............	4	1	0G2
....auuccggaCcuugaauuagg..............	2	1	0G2
....Nuuccggaucuugaauuagg..............	16	1	0G2
....auuccggaucuugaauuaUg..............	26	1	0G2
....auuAcggaucuugaauuagg..............	6	1	0G2
....auuccggCucuugaauuagg..............	18	1	0G2
....auuccggaucuugGauuagg..............	2	1	0G2
....Guuccggaucuugaauuagg..............	64	1	0G2
....auuccggGucuugaauuagg..............	2	1	0G2
....auuccggaucAugaauuagg..............	118	1	0G2
....aNuccggaucuugaauuagg..............	2	1	0G2
....auuccggaucuAgaauuagg..............	38	1	0G2
....auuccggaucuCgaauuagg..............	18	1	0G2

Star Mature

guuucuuauuaguguguuucauuuuggaucuugaauuccggaucuugaauuaggcuguuugguuuugaaucuugaauuccggaucuugaauuaggcuguuugauuuuga			
....auucGggaucuugaauuagg..............	18	1	0G2
....auuccggaucuugaaCuagg..............	32	1	0G2
....auuccggaucuugaauuagA..............	90	1	0G2
....uuccggaucuugGauuag...............	2	1	0G2
....uuccggaucuugaauCag...............	10	1	0G2
....uuccggaucuugaauuag...............	1866	0	0G2
....uuccggaucuGgaauuag...............	8	1	0G2
....uuccggaucuuUaauuag...............	8	1	0G2
....Nuccggaucuugaauuag...............	6	1	0G2
....uuccggGucuugaauuag...............	8	1	0G2
....uuccggaucuugaauuagA..............	6	1	0G2
....uucGggaucuugaauuagg..............	20	1	0G2
....uuccggaucAugaauuagg..............	28	1	0G2
....uuccggauGuugaauuagg..............	6	1	0G2
....uuccggaucuugaaCuagg..............	26	1	0G2
....uNccggaucuugaauuagg..............	4	1	0G2
....uuccggGucuugaauuagg..............	4	1	0G2
....uuccggaucuugaauuaUg..............	8	1	0G2
....uuccggaCcuugaauuagg..............	4	1	0G2
....uuccggaucuAgaauuagg..............	22	1	0G2
....uuccggaucuugaauuagg..............	2582	0	0G2
....uuccggCucuugaauuagg..............	2	1	0G2
....uuccggaucuGgaauuagg..............	8	1	0G2
....Nuccggaucuugaauuagg..............	8	1	0G2
....Nccggaucuugaauuagg..............	6	1	0G2
....ucGggaucuugaauuagg..............	8	1	0G2
....uccggaucAugaauuagg..............	10	1	0G2
....uccggaucuugaauuagg..............	888	0	0G2
....uccggGucuugaauuagg..............	2	1	0G2
....uccggaucuugaauuagA..............	6	1	0G2
....uccggaucuugaauuaUg..............	2	1	0G2
....uccggaucuAgaauuagg..............	10	1	0G2
....uccggaucuGgaauuagg..............	6	1	0G2
....uccggaucuCgaauuagg..............	20	1	0G2
....ccggaucuugaauuaggcuA...........	6	1	0G2
..............uguuucauuCuggaucuugaa..	2	1	0A2
................uuucauuCuggaucuugaauu..	1	1	0A2
................uuucauuCuggaucuugaauuc...	11	1	0A2
................uuucGuuuuggaucuugaauuc...	7	1	0A2
.................uucauuuuggaucuugaauuA...	2	1	0A2
.................uucauuCuggaucuugaauuc...	29	1	0A2
.................uucauuCuggaucuugaauucc..	14	1	0A2
.................uucauuuuggaucuugaauucA..	4	1	0A2
..................ucauuGuggaucuugaauuc...	1	1	0A2
..................ucauuCuggaucuugaauuc...	30	1	0A2
..................ucauuuuggaucuugaauucG..	3	1	0A2
..................ucauuCuggaucuugaauucc..	65	1	0A2
..................ucauuuuggaucuugaauucA..	23	1	0A2
..................ucauuuuggaucuCgaauuccg...	4	1	0A2
..................ucauuuuggaucuugaauuccg...	7	0	0A2
...................cauuCuggaucuugaauuc...	6	1	0A2
...................cauuuuggaucuugaauucA..	3	1	0A2
...................cauuuuggaucuugaauucc..	1	0	0A2
...................cauuCuggaucuugaauucc..	23	1	0A2
...................cauuuuggGucuugaauucc..	3	1	0A2
....................auuCuggaucuugaauucc..	17	1	0A2
....................auuuuggaucuugaauucA..	2	1	0A2
....................auuCuggaucuugaauuccg...	4	1	0A2
....................auuuuggaucuugaauuccg...	2	0	0A2
....................auuuuggGucuugaauuccg...	3	1	0A2
....................aCuuuggaucuugaauuccg...	2	1	0A2
....................auuuugUaucuugaauuccgg..	1	1	0A2
.....................uuuuggGucuugaauuccg...	13	1	0A2
.....................uuuuggaucuugaauuccg...	5	0	0A2
.....................uuuuggaucuugaauucUg...	3	1	0A2
.....................uuuuggGucuugaauuccgg..	2	1	0A2
.....................uuuuggaucuugaGuuccgg..	1	1	0A2
.....................uuuuggGucuugaauuccgga...	20	1	0A2
.....................uuuuggaucuugaauuccgga...	15	0	0A2

Star **Mature**

```
guuucuuauuaguguguuucauuuuggaucuugaauuccggaucuugaauuaggcuguuugguuuugaaucuugaauuccggaucuugaauuaggcuguuugauuuuga
......................uuuggGucuugaauuccgga...................................................................  2  1  0A2
.......................uuggaucuugaauuccggaucA................................................................  3  1  0A2
........................uggGucuugaauuccggauc.................................................................  2  1  0A2
...........................aucuugaauuccggaucuu...............................................................  12  0  0A2
...........................aucGugaauuccggaucuug..............................................................  6  1  0A2
...........................aucuugaauuccggaucuug..............................................................  6  0  0A2
...........................aucuugaauuccggaucuugUauua.........................................................  6  1  0A2
...........................aucuugaauuccggaucuugaauAa.........................................................  4  1  0A2
...........................aucuugaauuccggaucuugaauuagg.......................................................  14  0  0A2
............................ucuugaauuccggaucA................................................................  2  1  0A2
............................ucuugaauuccggaucuug..............................................................  12  0  0A2
............................ucuugaauuccggaucuuga.............................................................  14  0  0A2
............................ucuugaauuccggaucuAga.............................................................  80  1  0A2
............................ucuugaauuccggaucuCgaa............................................................  2  1  0A2
............................ucGugaauuccggaucuugaa............................................................  52  1  0A2
............................ucuugaauuccggaucuugaa............................................................  44  0  0A2
............................Ccuugaauuccggaucuugaa............................................................  10  1  0A2
............................ucuugaauuccggaucuAgaa............................................................  110  1  0A2
............................ucGugaauuccggaucuugaau...........................................................  68  1  0A2
............................Ccuugaauuccggaucuugaau...........................................................  2  1  0A2
............................ucuugaauuccggaucuugaau...........................................................  368  0  0A2
............................ucuugaauuccggaucuAgaau...........................................................  24  1  0A2
............................ucGugaauuccggaucuugaauu..........................................................  6  1  0A2
............................uGuugaauuccggaucuugaauu..........................................................  14  1  0A2
............................ucuugaauuccggaucuugaauu..........................................................  46  0  0A2
............................uUuugaauuccggaucuugaauu..........................................................  2  1  0A2
............................ucuugaauuccggaucuugaauua.........................................................  226  0  0A2
............................uUuugaauuccggaucuugaauua.........................................................  6  1  0A2
............................ucuugaauuccggaucuugaauAa.........................................................  464  1  0A2
............................ucuugaGuuccggaucuugaauua.........................................................  2  1  0A2
............................ucuugaauuccggaucuCgaauua.........................................................  2  1  0A2
............................ucuugaauuccggaucuAgaauua.........................................................  44  1  0A2
............................uGuugaauuccggaucuugaauua.........................................................  4  1  0A2
............................ucuugaauuccggaucuugaaCua.........................................................  2  1  0A2
............................ucuugaauuccggaucuAgaauuag........................................................  38  1  0A2
............................ucuugaauuccggaucuugaauuag........................................................  48  0  0A2
............................ucuugaauuccggaucuugaauuagg.......................................................  36  0  0A2
............................ucuugaauuccggaucuugaaCuagg.......................................................  14  1  0A2
............................ucuugaauuccggaucuugaauuaggc......................................................  18  0  0A2
.............................cuugaauuccggaucuAga.............................................................  34  1  0A2
.............................cuugaauuccggaucuAgaa............................................................  582  1  0A2
.............................cuugaGuuccggaucuugaa............................................................  2  1  0A2
.............................cuugaauuccggaucuugaa............................................................  190  0  0A2
.............................Nuugaauuccggaucuugaa............................................................  2  1  0A2
.............................cGugaauuccggaucuugaa............................................................  4  1  0A2
.............................cuugaauuccggaucuAgaau...........................................................  84  1  0A2
.............................cuugaauuccggaucuugaau...........................................................  304  0  0A2
.............................cGugaauuccggaucuugaau...........................................................  58  1  0A2
.............................cuugaauuccggaucuAgaauu..........................................................  114  1  0A2
.............................cuugaauuccggaucuugaauu..........................................................  458  0  0A2
.............................cGugaauuccggaucuugaauu..........................................................  12  1  0A2
.............................cuugaaGuccggaucuugaauu..........................................................  2  1  0A2
.............................cuugaauuccggaucuugaauAa.........................................................  268  1  0A2
.............................cuugaauuccggaucuugaauCa.........................................................  48  1  0A2
.............................cuugaauuccggaucuugGauua.........................................................  6  1  0A2
.............................cuugaauuccggaucuAgaauua.........................................................  244  1  0A2
.............................cuugaGuuccggaucuugaauua.........................................................  2  1  0A2
.............................cuugaauuccggaucuugaauua.........................................................  1160  0  0A2
.............................cuugaaGuccggaucuugaauua.........................................................  4  1  0A2
.............................cuugaauuccggCucuugaauua.........................................................  2  1  0A2
.............................cGugaauuccggaucuugaauua.........................................................  10  1  0A2
.............................cuugaauuccggaGcuugaauua.........................................................  2  1  0A2
.............................Nuugaauuccggaucuugaauua.........................................................  2  1  0A2
.............................cuugaauuccggaucuAgaauuag........................................................  2  1  0A2
.............................cuugaauuccggaucuugaauuag........................................................  12  0  0A2
.............................cuugaauuccggaucuugaaCuag........................................................  4  1  0A2
.............................cuugaauuccggaucuugaauuagg.......................................................  6  0  0A2
..............................uugaauuccggaucuAga.............................................................  12  1  0A2
..............................uugaauuccggaucuugaa............................................................  98  0  0A2
..............................uugaauuccggaucuAgaa............................................................  384  1  0A2
```

Star	Mature			
guuucuuauuaguguguuucauuuuggaucuugaauuccggaucuugaauuaggcuguuugguuuugaaucuugaauuccggaucuugaauuaggcuguuugauuuuga				
..............................uugaauuccggaGcuugaau...		14	1	0A2
..............................uugaauuccggaucuCgaau...		4	1	0A2
..............................Nugaauuccggaucuugaau...		30	1	0A2
..............................uNgaauuccggaucuugaau...		6	1	0A2
..............................uugaGuuccggaucuugaau...		162	1	0A2
..............................uugaauuccggaucuAgaau...		3154	1	0A2
..............................Augaauuccggaucuugaau...		4	1	0A2
..............................Gugaauuccggaucuugaau...		36	1	0A2
..............................uugaauuccggaucuugaaG...		2	1	0A2
..............................uugaauuccggaucuugaaC...		4	1	0A2
..............................uugaauuccggGucuugaau...		26	1	0A2
..............................uugaauuccggaucuugaau...		14644	0	0A2
..............................uugaauuccggaucCugaau...		6	1	0A2
..............................uugaauuAcggaucuugaau...		6	1	0A2
..............................uugaauuccggauAuugaau...		4	1	0A2
..............................uugaauuccggaucuGgaau...		8	1	0A2
..............................uugaaGuccggaucuugaau...		16	1	0A2
..............................uugaauuccggaucuugUau...		2	1	0A2
..............................uugaauuccggaucAugaau...		2	1	0A2
..............................uugaauuccggaCcuugaau...		8	1	0A2
..............................uugaauuccggCucuugaau...		2	1	0A2
..............................Cugaauuccggaucuugaau...		6	1	0A2
..............................uugaauAccggaucuugaau...		10	1	0A2
..............................uugaGuuccggaucuugaauu..		310	1	0A2
..............................uugaauuccggaucCugaauu..		4	1	0A2
..............................uugaauAccggaucuugaauu..		14	1	0A2
..............................uugaauuccggaucuGgaauu..		6	1	0A2
..............................uugaauuccggauAuugaauu..		2	1	0A2
..............................uugaauuccggGucuugaauu..		6	1	0A2
..............................uugaauuccggaucuuUaauu..		14	1	0A2
..............................uuNaauuccggaucuugaauu..		2	1	0A2
..............................uugaauuccggaCcuugaauu..		8	1	0A2
..............................uugaauuccggaucuugaauu..		18324	0	0A2
..............................uugaauuccggaGcuugaauu..		18	1	0A2
..............................uugaauuccggaucuugaaCu..		10	1	0A2
..............................uugaauuccggaucAugaauu..		6	1	0A2
..............................Augaauuccggaucuugaauu..		2	1	0A2
..............................uGgaauuccggaucuugaauu..		10	1	0A2
..............................uugaauuccggaucuugaaGu..		2	1	0A2
..............................uugaauuccggCucuugaauu..		6	1	0A2
..............................Nugaauuccggaucuugaauu..		56	1	0A2
..............................uugaauuAcggaucuugaauu..		10	1	0A2
..............................uugaaGuccggaucuugaauu..		2	1	0A2
..............................Gugaauuccggaucuugaauu..		14	1	0A2
..............................uugaauuccggauGuugaauu..		2	1	0A2
..............................uugaauuccggaucuCgaauu..		46	1	0A2
..............................uugaauuccggaucuugGauu..		6	1	0A2
..............................uugaauuccggaucuAgaauu..		4628	1	0A2
..............................Cugaauuccggaucuugaauu..		2	1	0A2
..............................uNgaauuccggaucuugaauu..		2	1	0A2
..............................uugaauuccggaCcuugaauua...		14	1	0A2
..............................uugaauuccggaucuuUaauua...		30	1	0A2
..............................Nugaauuccggaucuugaauua...		98	1	0A2
..............................uugaaGuccggaucuugaauua...		18	1	0A2
..............................uugaauuccggaucuAgaauua...		10386	1	0A2
..............................uugaauuccggaucuugaaCua...		94	1	0A2
..............................uugaauuccggaucuugaaGua...		8	1	0A2
..............................uugaauuGcggaucuugaauua...		6	1	0A2
..............................uNgaauuccggaucuugaauua...		10	1	0A2
..............................uugaauuccggaucuugaauCa...		3958	1	0A2
..............................uugaauuccggauGuugaauua...		4	1	0A2
..............................uugaauuccggaucuugGauua...		40	1	0A2
..............................uugaauuccggaucuugCauua...		2	1	0A2
..............................Augaauuccggaucuugaauua...		18	1	0A2
..............................uugaauuccggauAuugaauua...		22	1	0A2
..............................uugaauuccggaucuugUauua...		2	1	0A2
..............................uugaauuccggaucCugaauua...		8	1	0A2
..............................Gugaauuccggaucuugaauua...		74	1	0A2
..............................uugaauuccggGucuugaauua...		10	1	0A2
..............................uugaauuccggaucuGgaauua...		8	1	0A2

Star   Mature

```
guuucuuauuaguguguuucauuuuggaucuugaauuccggaucuugaauuaggcuguuugguuuugaaucuugaauuccggaucuugaauuaggcuguuugauuuuga
..............................uugaauuccggaGcuugaauua.........................................................    38      1    0A2
..............................uugaauuccCgaucuugaauua.........................................................    2       1    0A2
..............................uugaauuccggaucAugaauua.........................................................    6       1    0A2
..............................Cugaauuccggaucuugaauua.........................................................    32      1    0A2
..............................uugaauuccggCucuugaauua.........................................................    12      1    0A2
..............................uugaauuccggaucuugaauua.........................................................    46990   0    0A2
..............................uAgaauuccggaucuugaauua.........................................................    8       1    0A2
..............................uGgaauuccggaucuugaauua.........................................................    14      1    0A2
..............................uugaauuccgCaucuugaauua.........................................................    2       1    0A2
..............................uuNaauuccggaucuugaauua.........................................................    4       1    0A2
..............................uugaauuAcggaucuugaauua.........................................................    40      1    0A2
..............................uugaauuccggaucuCgaauua.........................................................    22      1    0A2
..............................uugaauuccggaucuugaauAa.........................................................    7444    1    0A2
..............................uugaGuuccggaucuugaauua.........................................................    1250    1    0A2
..............................uugaauAccggaucuugaauua.........................................................    20      1    0A2
..............................uugaauuccggaucuGgaauuag........................................................    2       1    0A2
..............................uugaGuuccggaucuugaauuag........................................................    26      1    0A2
..............................uugaauuccggaucuAgaauuag........................................................    2540    1    0A2
..............................uugaauuccggaucuugaaGuag........................................................    4       1    0A2
..............................uugaauuccggaucuugaauuag........................................................    568     0    0A2
..............................uugaauuccggaucuugaauCag........................................................    8       1    0A2
..............................uugaauuccggaucuugaaCuag........................................................    40      1    0A2
..............................uugaauuccggaucuuUaauuag........................................................    4       1    0A2
..............................uugaauuccggaucuugaauAag........................................................    76      1    0A2
..............................uugaauuccggaucuugaauuagg.......................................................    22      0    0A2
..............................uugaauuccggaucuugaauuagA.......................................................    14      1    0A2
...............................ugaauuccggaucuAgaa............................................................    98      1    0A2
...............................ugaauuccggaucuugaaG...........................................................    2       1    0A2
...............................Agaauuccggaucuugaau...........................................................    2       1    0A2
...............................ugaauuccggaucuugaau...........................................................    634     0    0A2
...............................ugaauuccggGucuugaau...........................................................    6       1    0A2
...............................ugaauuccggaucuAgaau...........................................................    172     1    0A2
...............................ugaauuccggaGcuugaau...........................................................    2       1    0A2
...............................ugaGuuccggaucuugaau...........................................................    12      1    0A2
...............................uNaauuccggaucuugaauu..........................................................    4       1    0A2
...............................ugaauuccggaucuugaaCu..........................................................    14      1    0A2
...............................ugaauuccggaucCugaauu..........................................................    2       1    0A2
...............................ugaauuccggaucuuUaauu..........................................................    4       1    0A2
...............................ugaauuccggaucuugaauu..........................................................    4500    0    0A2
...............................ugaauuccggaCcuugaauu..........................................................    2       1    0A2
...............................ugaauuAcggaucuugaauu..........................................................    4       1    0A2
...............................Ngaauuccggaucuugaauu..........................................................    6       1    0A2
...............................ugaauuccggaucuCgaauu..........................................................    6       1    0A2
...............................ugaGuuccggaucuugaauu..........................................................    86      1    0A2
...............................ugaauuccggGucuugaauu..........................................................    4       1    0A2
...............................ugaaGuccggaucuugaauu..........................................................    8       1    0A2
...............................ugaauuccggaGcuugaauu..........................................................    6       1    0A2
...............................ugaauuccggaucuugGauu..........................................................    2       1    0A2
...............................ugaCuuccggaucuugaauu..........................................................    2       1    0A2
...............................ugaauuccggaucuAgaauu..........................................................    1502    1    0A2
...............................ugaauuccgCaucuugaauu..........................................................    2       1    0A2
...............................ugaauuccggaucuugaaGu..........................................................    2       1    0A2
...............................Ggaauuccggaucuugaauu..........................................................    4       1    0A2
...............................ugaauuccggaucuGgaauua.........................................................    4       1    0A2
...............................ugaauuccggGucuugaauua.........................................................    20      1    0A2
...............................ugaauuccggaCcuugaauua.........................................................    12      1    0A2
...............................Ggaauuccggaucuugaauua.........................................................    8       1    0A2
...............................ugaauuccggaucuugaauAa.........................................................    542     1    0A2
...............................ugaauuccggaucuAgaauua.........................................................    9536    1    0A2
...............................uNaauuccggaucuugaauua.........................................................    6       1    0A2
...............................ugaauuccggaucuugGauua.........................................................    20      1    0A2
...............................Ngaauuccggaucuugaauua.........................................................    52      1    0A2
...............................ugaGuuccggaucuugaauua.........................................................    512     1    0A2
...............................ugaauuccggaucuugaaGua.........................................................    4       1    0A2
...............................ugaauuccggaucCugaauua.........................................................    6       1    0A2
...............................ugaaGuccggaucuugaauua.........................................................    42      1    0A2
...............................ugaauuccggaucuuUaauua.........................................................    22      1    0A2
...............................ugaauuGcggaucuugaauua.........................................................    22      1    0A2
...............................ugaauuccggaucuugaauua.........................................................    26330   0    0A2
...............................ugaauuccggaucuugaauCa.........................................................    634     1    0A2
```

Star **Mature**

```
guuucuuauuaguguguuucauuuuggaucuugaauuccggaucuugaauuaggcuguuugguuuugaaucuugaauuccggaucuugaauuaggcuguuugauuuuga
...............................Agaauuccggaucuugaauua.........................................................  12    1  0A2
...............................ugaauuccggauAuugaauua.........................................................  12    1  0A2
...............................ugaauuccggCucuugaauua.........................................................  16    1  0A2
...............................ugaauuccggaucuugCauua.........................................................  4     1  0A2
...............................ugaauuccggaGcuugaauua.........................................................  16    1  0A2
...............................ugaauuccggaucuugaaCua.........................................................  40    1  0A2
...............................ugaauuccggaucuCgaauua.........................................................  12    1  0A2
...............................ugaauuAcggaucuugaauua.........................................................  22    1  0A2
...............................ugaauuccggaucuAgaauuag........................................................  7582  1  0A2
...............................ugaaGuccggaucuugaauuag........................................................  4     1  0A2
...............................ugaauuccggaucCugaauuag........................................................  4     1  0A2
...............................ugaGuuccggaucuugaauuag........................................................  36    1  0A2
...............................ugaauuccggaucuugaauuag........................................................  1894  0  0A2
...............................ugaauuccggaucuugaauCag........................................................  24    1  0A2
...............................ugaauuccggGucuugaauuag........................................................  2     1  0A2
...............................ugaauuccggaucuugaaGuag........................................................  14    1  0A2
...............................ugaauuccggaucuugaauAag........................................................  20    1  0A2
...............................Agaauuccggaucuugaauuag........................................................  2     1  0A2
...............................ugaauuccggaucuugaauuagg.......................................................  58    0  0A2
...............................ugaauuccggaucuugaauuagC.......................................................  8     1  0A2
...............................ugaauuAcggaucuugaauuagg.......................................................  4     1  0A2
...............................ugaauuccggauAuugaauuagg.......................................................  2     1  0A2
...............................ugaauuccggaucuAgaauuagg.......................................................  8     1  0A2
...............................ugaauuccggaucuugaaCuagg.......................................................  4     1  0A2
................................gaauuccggaucuAgaau...........................................................  14    1  0A2
................................gaauuccggaucuugaau...........................................................  42    0  0A2
................................gaauuccggaucuugaauu..........................................................  214   0  0A2
................................gaauuccggaucuAgaauu..........................................................  92    1  0A2
................................gaGuuccggaucuugaauu..........................................................  4     1  0A2
................................gaauuccggGucuugaauu..........................................................  2     1  0A2
................................gaauuccggaucuugGauua.........................................................  2     1  0A2
................................gaauuccggaucuuUaauua.........................................................  2     1  0A2
................................gaauuccggaucuugaauAa.........................................................  6     1  0A2
................................gaauuccggaucuugaauCa.........................................................  168   1  0A2
................................gaauuccggaucuugaaCua.........................................................  4     1  0A2
................................gaauuccggaucuugaaGua.........................................................  2     1  0A2
................................Naauuccggaucuugaauua.........................................................  4     1  0A2
................................gaauuccggaCcuugaauua.........................................................  6     1  0A2
................................gaGuuccggaucuugaauua.........................................................  46    1  0A2
................................gaauuccggaucuAgaauua.........................................................  926   1  0A2
................................gaaGuccggaucuugaauua.........................................................  2     1  0A2
................................gaauuccggaucuugaauua.........................................................  1670  0  0A2
................................gaauuccggauAuugaauuag........................................................  2     1  0A2
................................gaauuccggaucuAgaauuag........................................................  5374  1  0A2
................................Naauuccggaucuugaauuag........................................................  4     1  0A2
................................gaauuccggGucuugaauuag........................................................  2     1  0A2
................................gaauuccggaCcuugaauuag........................................................  2     1  0A2
................................gaauuccggaucuugaauuag........................................................  1086  0  0A2
................................gaauuccggaucuAgaauuagg.......................................................  10    1  0A2
................................gaauuccggaucuugaauuagg.......................................................  48    0  0A2
.................................aauuccggaucuugaauu..........................................................  20    0  0A2
.................................Nauuccggaucuugaauua.........................................................  2     1  0A2
.................................aauuccggaucuugaauCa.........................................................  34    1  0A2
.................................aauuccggaucuugaauua.........................................................  1052  0  0A2
.................................aGuuccggaucuugaauua.........................................................  14    1  0A2
.................................aauuccggaucuugaauAa.........................................................  32    1  0A2
.................................aauuccggCucuugaauua.........................................................  2     1  0A2
.................................aauuAcggaucuugaauua.........................................................  2     1  0A2
.................................aauuGcggaucuugaauua.........................................................  2     1  0A2
.................................aNuuccggaucuugaauuag........................................................  2     1  0A2
.................................aauuccggaucuugaaCuag........................................................  2     1  0A2
.................................aauuccggaucuGgaauuag........................................................  4     1  0A2
.................................aauuccggaucuuUaauuag........................................................  12    1  0A2
.................................aauuccggaucuugGauuag........................................................  12    1  0A2
.................................aauuccggaucuugaaGuag........................................................  2     1  0A2
.................................aauuAcggaucuugaauuag........................................................  2     1  0A2
.................................aauuccggGucuugaauuag........................................................  2     1  0A2
.................................aauuccggaucuCgaauuag........................................................  6     1  0A2
.................................aauuccggaucuugCauuag........................................................  2     1  0A2
.................................aauuccggaucuugaauAag........................................................  2     1  0A2
```

Star **Mature**

guuucuuauuaguguguuucauuuuggaucuugaauuccggaucuugaauuaggcuguuugguuuugaaucuugaauuccggaucuugaauuaggcuguuugauuuuga			
.................................Nauuccggaucuugaauuag..	24	1	0A2
.................................aGuuccggaucuugaauuag..	138	1	0A2
.................................aauuccggaucuugaauuag..	7498	0	0A2
.................................aauuccggaucuugaauCag..	30	1	0A2
.................................aCuuccggaucuugaauuag..	4	1	0A2
.................................aauuccggaucAugaauuag..	12	1	0A2
.................................aauuccCgaucuugaauuag..	8	1	0A2
.................................aauuccggCucuugaauuag..	4	1	0A2
.................................aauuccggaCcuugaauuag..	2	1	0A2
.................................aauuccggaGcuugaauuag..	2	1	0A2
.................................aauuccggaucuCgaauuagg...	6	1	0A2
.................................aauuccggaucuugaauuagg...	540	0	0A2
.................................Nauuccggaucuugaauuagg...	2	1	0A2
.................................aauuccggaucuugaaCuagg...	2	1	0A2
.................................aauuccggaucuAgaauuagg...	22	1	0A2
.................................aauuccggaucuugaauuagA...	38	1	0A2
.................................aauuccggaucuGgaauuagg...	2	1	0A2
.................................aauuccggGucuugaauuagg...	2	1	0A2
..................................Guuccggaucuugaauua...	8	1	0A2
..................................Nuuccggaucuugaauua...	4	1	0A2
..................................auuccggaucuugaaCua...	4	1	0A2
..................................auuccggaucuugaauAa...	8	1	0A2
..................................auuccggaucuugaauCa...	20	1	0A2
..................................auuccggaucuugaauua...	366	0	0A2
..................................auuccggaucAugaauuag..	24	1	0A2
..................................auuccggaGcuugaauuag..	4	1	0A2
..................................Guuccggaucuugaauuag..	24	1	0A2
..................................auuccggaucuugaauAag..	6	1	0A2
..................................auuccggaucuugaauuag..	2084	0	0A2
..................................auuccggaucuCgaauuag..	4	1	0A2
..................................auuccggaucuugaauCag..	18	1	0A2
..................................auuccgCaucuugaauuag..	2	1	0A2
..................................auuccggCucuugaauuag..	4	1	0A2
..................................auuccggaucuuUaauuag..	6	1	0A2
..................................auuccggGucuugaauuag..	4	1	0A2
..................................Cuuccggaucuugaauuag..	10	1	0A2
..................................auuccggaucuGgaauuag..	4	1	0A2
..................................Nuuccggaucuugaauuag..	8	1	0A2
..................................auuccggGucuugaauuagg...	2	1	0A2
..................................auuccggaucuugaauuaUg...	28	1	0A2
..................................auuccggaucuugaauuagg...	4064	0	0A2
..................................auuccggaucuugaauuagC...	8	1	0A2
..................................Guuccggaucuugaauuagg...	82	1	0A2
..................................auuccggaucuCgaauuagg...	6	1	0A2
..................................auuccggaucuGgaauuagg...	2	1	0A2
..................................Cuuccggaucuugaauuagg...	8	1	0A2
..................................auuccggaucuugGauuagg...	6	1	0A2
..................................auuccggaucuugaaCuagg...	8	1	0A2
..................................Nuuccggaucuugaauuagg...	8	1	0A2
..................................auuccggaucAugaauuagg...	44	1	0A2
..................................auuccggaucuAgaauuagg...	58	1	0A2
..................................auuccggaucuugaauuCgg...	2	1	0A2
..................................auuccggaucuugaauuagA...	24	1	0A2
..................................auuccggaucuugaauGagg...	4	1	0A2
..................................auuAcggaucuugaauuagg...	16	1	0A2
..................................auuccggaucuuUaauuagg...	6	1	0A2
...................................uuccggaucuugaauCag..	58	1	0A2
...................................uuccggaucuugaauuag..	1958	0	0A2
...................................uuccggGucuugaauuag..	2	1	0A2
...................................uuccggCucuugaauuag..	4	1	0A2
...................................uuccggauAuugaauuag..	2	1	0A2
...................................uuccggaucuCgaauuag..	2	1	0A2
...................................uuccggaCcuugaauuag..	2	1	0A2
...................................uuccggaucAugaauuag..	6	1	0A2
...................................uuccggaucuugGauuag..	6	1	0A2
...................................Nuccggaucuugaauuag..	4	1	0A2
...................................uuccggaucuugaaGuagg...	2	1	0A2
...................................Nuccggaucuugaauuagg...	4	1	0A2
...................................uuccggaucAugaauuagg...	42	1	0A2
...................................uuccggaucuAgaauuagg...	20	1	0A2

Star Mature

```
guuucuuauuaguguguuucauuuuggaucuugaauuccggaucuugaauuaggcuguuugguuuugaaucuugaauuccggaucuugaauuaggcuguuugauuuuga
...................................uuccggaucuGgaauuagg.......................................................	8	1	0A2
...................................uuccggaGcuugaauuagg.......................................................	4	1	0A2
...................................uuccggaucuugaauuagA.......................................................	22	1	0A2
...................................uuccggaucuugaauuaUg.......................................................	4	1	0A2
...................................uuccggGucuugaauuagg.......................................................	6	1	0A2
...................................uuccggaucuuUaauuagg.......................................................	4	1	0A2
...................................uucGggaucuugaauuagg.......................................................	12	1	0A2
...................................uuccggCucuugaauuagg.......................................................	2	1	0A2
...................................uuccggaucuugaaCuagg.......................................................	8	1	0A2
...................................uuccggaucuugGauuagg.......................................................	2	1	0A2
...................................uuccggaucuugaauuagg.......................................................	2276	0	0A2
...................................uuAcggaucuugaauuagg.......................................................	2	1	0A2
...................................uuccggaucuugCauuagg.......................................................	4	1	0A2
...................................uuccggaucuugaauuagUcu.....................................................	6	1	0A2
...................................uuccggaucuugaauuaggcuC....................................................	8	1	0A2
....................................uccggaucAugaauuagg.......................................................	10	1	0A2
....................................uccggaucuugaauuagg.......................................................	632	0	0A2
....................................uccggaucuugaauuagA.......................................................	2	1	0A2
....................................uccggaucuAgaauuagg.......................................................	6	1	0A2
....................................uAcggaucuugaauuagg.......................................................	4	1	0A2
....................................uccggaucuGgaauuagg.......................................................	6	1	0A2
....................................uccggaCcuugaauuagg.......................................................	2	1	0A2
....................................Nccggaucuugaauuagg.......................................................	4	1	0A2
....................................uccggaucuugaauuaggcuA....................................................	2	1	0A2
.............................................................Cuuuugaaucuugaauuccgga..........................	2	1	0A2
..............................................................uuuugaauUuugaauuccgg...........................	5	1	0A2
..............................................................uuuugaaucuugaauuccgg...........................	71	0	0A2
..............................................................uuuugaaucuugaauuccggG..........................	4	1	0A2
..............................................................uuuugaaucuugaauuccgga..........................	14	0	0A2
..............................................................uuuugaGucuugaauuccgga..........................	9	1	0A2
..............................................................uuuugaaucGugaauuccgga..........................	5	1	0A2
..............................................................uuuugaaucuugaauuccggau.........................	7	0	0A2
..............................................................uuuugaaucuugaauuccggaG.........................	1	1	0A2
...............................................................uuugaaucuugaauuccgg...........................	7	0	0A2
...............................................................uuugaCucuugaauuccgga..........................	3	1	0A2
...............................................................uuugaaucuugaauuccgga..........................	7	0	0A2
................................................................uugaaucuugaauuccggauA........................	14	1	0A2
................................................................uugaGucuugaauuccggauc........................	3	1	0A2
................................................................uugaaucuugaauuccggauc........................	3	0	0A2
................................................................uugaaucuugaauuccggaucA.......................	7	1	0A2
................................................................uugaaucuugaauuccggaucuugaau..................	21	0	0A2
.................................................................ugaaucuugaauuccggau.........................	3	0	0A2
.................................................................ugaaucuugaauuccggauc........................	3	0	0A2
.................................................................ugaaucuugaauuccggauA........................	12	1	0A2
.................................................................ugaGucuugaauuccggaucu.......................	2	1	0A2
.................................................................ugaCucuugaauuccggaucuug.....................	1	1	0A2
.................................................................ugaaucuugaauuccggaucuAgaa...................	11	1	0A2
.................................................................ugaaucuugaauuccggaucuugaau..................	3	0	0A2
.................................................................ugaaucuugaauuccggaucuAgaau..................	3	1	0A2
.................................................................ugaaucuugaauuccggaucuugaauu.................	19	0	0A2
.................................................................ugaaucuugaauuccCgaucuugaauu.................	1	1	0A2
.................................................................ugaaucuugaauuccggaucuAgaauu.................	1	1	0A2
...................................................................aaucuugaauuccggaucuA......................	1	1	0A2
...................................................................aaucuugaauuccggaucuug.....................	11	0	0A2
...................................................................aaucuugaauuccggaucuAg.....................	2	1	0A2
...................................................................aaucuugaauuccggaucuAgaauua................	3	1	0A2
...................................................................aaucuugaauuccggaucuAgaauuag...............	1	1	0A2
....................................................................aucuugaauuccggaucuu......................	12	0	0A2
....................................................................aucGugaauuccggaucuug.....................	6	1	0A2
....................................................................aucuugaauuccggaucuug.....................	6	0	0A2
....................................................................aucuugaauuccggaucuugUauua................	6	1	0A2
....................................................................aucuugaauuccggaucuugaauAa................	4	1	0A2
....................................................................aucuugaauuccggaucuugaauuagg..............	14	0	0A2
.....................................................................ucuugaauuccggaucuA......................	2	1	0A2
.....................................................................ucuugaauuccggaucuug.....................	12	0	0A2
.....................................................................ucuugaauuccggaucuuga....................	14	0	0A2
.....................................................................ucuugaauuccggaucuAga....................	80	1	0A2
.....................................................................ucuugaauuccggaucuCgaa...................	2	1	0A2
.....................................................................Ccuugaauuccggaucuugaa...................	10	1	0A2
.....................................................................ucGugaauuccggaucuugaa...................	52	1	0A2
```

Star **Mature**

guuucuuauuaguguguuucauuuuggaucuugaauuccggaucuugaauuaggcuguuugguuuugaaucuugaauuccggaucuugaauuaggcuguuugauuuuga			
....ucuugaauuccggaucuugaa...................	44	0	0A2
....ucuugaauuccggaucuAgaa...................	110	1	0A2
....ucuugaauuccggaucuAgaau..................	24	1	0A2
....Ccuugaauuccggaucuugaau..................	2	1	0A2
....ucGugaauuccggaucuugaau..................	68	1	0A2
....ucuugaauuccggaucuugaau..................	368	0	0A2
....ucGugaauuccggaucuugaauu.................	6	1	0A2
....uUuugaauuccggaucuugaauu.................	2	1	0A2
....ucuugaauuccggaucuugaauu.................	46	0	0A2
....uGuugaauuccggaucuugaauu.................	14	1	0A2
....ucuugaGuuccggaucuugaauua................	2	1	0A2
....ucuugaauuccggaucuugaauAa................	464	1	0A2
....uGuugaauuccggaucuugaauua................	4	1	0A2
....ucuugaauuccggaucuCgaauua................	2	1	0A2
....ucuugaauuccggaucuugaaCua................	2	1	0A2
....ucuugaauuccggaucuAgaauua................	44	1	0A2
....uUuugaauuccggaucuugaauua................	6	1	0A2
....ucuugaauuccggaucuugaauua................	226	0	0A2
....ucuugaauuccggaucuAgaauuag...............	38	1	0A2
....ucuugaauuccggaucuugaauuag...............	48	0	0A2
....ucuugaauuccggaucuugaaCuagg..............	14	1	0A2
....ucuugaauuccggaucuugaauuagg..............	36	0	0A2
....ucuugaauuccggaucuugaauuaggc.............	18	0	0A2
....cuugaauuccggaucuAga....................	34	1	0A2
....cGugaauuccggaucuugaa...................	4	1	0A2
....cuugaauuccggaucuAgaa...................	582	1	0A2
....cuugaGuuccggaucuugaa...................	2	1	0A2
....Nuugaauuccggaucuugaa...................	2	1	0A2
....cuugaauuccggaucuugaa...................	190	0	0A2
....cGugaauuccggaucuugaau..................	58	1	0A2
....cuugaauuccggaucuAgaau..................	84	1	0A2
....cuugaauuccggaucuugaau..................	304	0	0A2
....cuugaaGuccggaucuugaauu.................	2	1	0A2
....cuugaauuccggaucuugaauu.................	458	0	0A2
....cGugaauuccggaucuugaauu.................	12	1	0A2
....cuugaauuccggaucuAgaauu.................	114	1	0A2
....cuugaauuccggCucuugaauua................	2	1	0A2
....cuugaauuccggaucuugGauua................	6	1	0A2
....cuugaGuuccggaucuugaauua................	2	1	0A2
....cuugaauuccggaGcuugaauua................	2	1	0A2
....cuugaauuccggaucuugaauua................	1160	0	0A2
....Nuugaauuccggaucuugaauua................	2	1	0A2
....cGugaauuccggaucuugaauua................	10	1	0A2
....cuugaauuccggaucuAgaauua................	244	1	0A2
....cuugaaGuccggaucuugaauua................	4	1	0A2
....cuugaauuccggaucuugaauAa................	268	1	0A2
....cuugaauuccggaucuugaauCa................	48	1	0A2
....cuugaauuccggaucuugaauuag...............	12	0	0A2
....cuugaauuccggaucuugaaCuag...............	4	1	0A2
....cuugaauuccggaucuAgaauuag...............	2	1	0A2
....cuugaauuccggaucuugaauuagg..............	6	0	0A2
....uugaauuccggaucuAga....................	12	1	0A2
....uugaauuccggaucuAgaa...................	384	1	0A2
....uugaauuccggaucuugaa...................	98	0	0A2
....Gugaauuccggaucuugaau..................	36	1	0A2
....uugaauuccggaucuugaaC..................	4	1	0A2
....Nugaauuccggaucuugaau..................	30	1	0A2
....uugaauuccggaucuAgaau..................	3154	1	0A2
....Augaauuccggaucuugaau..................	4	1	0A2
....uugaauuccggaGcuugaau..................	14	1	0A2
....uugaauAccggaucuugaau..................	10	1	0A2
....uugaauuccggaucuGgaau..................	8	1	0A2
....uugaauuccggaucuugUau..................	2	1	0A2
....uugaauuccggaucuugaau..................	14644	0	0A2
....uugaauuAcggaucuugaau..................	6	1	0A2
....uugaauuccggauAuugaau..................	4	1	0A2
....uNgaauuccggaucuugaau..................	6	1	0A2
....uugaaGuccggaucuugaau..................	16	1	0A2
....uugaauuccggaucCugaau..................	6	1	0A2
....uugaauuccggaucuCgaau..................	4	1	0A2

```
                    Star                                                 Mature
guuucuuauuaguguguuucauuuuggaucuugaauuccggaucuugaauuaggcuguuugguuuugaaucuugaauuccggaucuugaauuaggcuguuugauuuuga
.......................................................................uugaauuccggaucAugaau..................  2      1  0A2
.......................................................................Cugaauuccggaucuugaau..................  6      1  0A2
.......................................................................uugaauuccggCucuugaau..................  2      1  0A2
.......................................................................uugaauuccggGucuugaau..................  26     1  0A2
.......................................................................uugaGuuccggaucuugaau..................  162    1  0A2
.......................................................................uugaauuccggaucuugaaG..................  2      1  0A2
.......................................................................uugaauuccggaCcuugaau..................  8      1  0A2
.......................................................................uugaaGuccggaucuugaauu.................  2      1  0A2
.......................................................................uugaauuccggaucuugaaGu.................  2      1  0A2
.......................................................................uugaauuccggaGcuugaauu.................  18     1  0A2
.......................................................................uugaauuccggaucuugaauu.................  18324  0  0A2
.......................................................................uugaGuuccggaucuugaauu.................  310    1  0A2
.......................................................................uGgaauuccggaucuugaauu.................  10     1  0A2
.......................................................................Nugaauuccggaucuugaauu.................  56     1  0A2
.......................................................................uugaauuccggaucCugaauu.................  4      1  0A2
.......................................................................uugaauuccggaCcuugaauu.................  8      1  0A2
.......................................................................uugaauuccggaucuAgaauu.................  4628   1  0A2
.......................................................................uugaauuccggaucuuUaauu.................  14     1  0A2
.......................................................................uugaauuAcggaucuugaauu.................  10     1  0A2
.......................................................................uugaauuccggCucuugaauu.................  6      1  0A2
.......................................................................Gugaauuccggaucuugaauu.................  14     1  0A2
.......................................................................uugaauuccggGucuugaauu.................  6      1  0A2
.......................................................................uugaauuccggaucuugaaCu.................  10     1  0A2
.......................................................................uugaauuccggauGuugaauu.................  2      1  0A2
.......................................................................Augaauuccggaucuugaauu.................  2      1  0A2
.......................................................................uNgaauuccggaucuugaauu.................  2      1  0A2
.......................................................................Cugaauuccggaucuugaauu.................  2      1  0A2
.......................................................................uugaauuccggaucuGgaauu.................  6      1  0A2
.......................................................................uugaauuccggaucuugGauu.................  6      1  0A2
.......................................................................uugaauuccggaucAugaauu.................  6      1  0A2
.......................................................................uuNaauuccggaucuugaauu.................  2      1  0A2
.......................................................................uugaauuccggauAuugaauu.................  2      1  0A2
.......................................................................uugaauuccggaucuCgaauu.................  46     1  0A2
.......................................................................uugaauAccggaucuugaauu.................  14     1  0A2
.......................................................................uugaauuccggaucuugaaCua................  94     1  0A2
.......................................................................uugaauuccggaucuugaauAa................  7444   1  0A2
.......................................................................uugaauuccgCaucuugaauua................  2      1  0A2
.......................................................................uugaauuccggaucuugCauua................  2      1  0A2
.......................................................................uugaauuccggaucuuUaauua................  30     1  0A2
.......................................................................uugaauAccggaucuugaauua................  20     1  0A2
.......................................................................uugaauuccggaucCugaauua................  8      1  0A2
.......................................................................Augaauuccggaucuugaauua................  18     1  0A2
.......................................................................uugaauuGcggaucuugaauua................  6      1  0A2
.......................................................................Nugaauuccggaucuugaauua................  98     1  0A2
.......................................................................Gugaauuccggaucuugaauua................  74     1  0A2
.......................................................................uugaauuccggaucuugUauua................  2      1  0A2
.......................................................................Cugaauuccggaucuugaauua................  32     1  0A2
.......................................................................uugaauuccggauAuugaauua................  22     1  0A2
.......................................................................uNgaauuccggaucuugaauua................  10     1  0A2
.......................................................................uugaauuccggaucuCgaauua................  22     1  0A2
.......................................................................uugaauuccggaucuAgaauua................  10386  1  0A2
.......................................................................uugaauuccggaucuugaaGua................  8      1  0A2
.......................................................................uugaauuAcggaucuugaauua................  40     1  0A2
.......................................................................uugaauuccggaucuugGauua................  40     1  0A2
.......................................................................uugaauuccggCucuugaauua................  12     1  0A2
.......................................................................uugaauuccggaCcuugaauua................  14     1  0A2
.......................................................................uGgaauuccggaucuugaauua................  14     1  0A2
.......................................................................uuNaauuccggaucuugaauua................  4      1  0A2
.......................................................................uugaauuccggaucuugaauua................  46990  0  0A2
.......................................................................uugaauuccggauGuugaauua................  4      1  0A2
.......................................................................uugaauuccggaGcuugaauua................  38     1  0A2
.......................................................................uugaaGuccggaucuugaauua................  18     1  0A2
.......................................................................uugaauuccCgaucuugaauua................  2      1  0A2
.......................................................................uugaauuccggaucuGgaauua................  8      1  0A2
.......................................................................uugaauuccggaucuugaauCa................  3958   1  0A2
.......................................................................uugaauuccggaucAugaauua................  6      1  0A2
.......................................................................uugaGuuccggaucuugaauua................  1250   1  0A2
.......................................................................uAgaauuccggaucuugaauua................  8      1  0A2
.......................................................................uugaauuccggGucuugaauua................  10     1  0A2
.......................................................................uugaauuccggaucuAgaauuag...............  2540   1  0A2
```

```
                         Star                                                      Mature
guuucuuauuaguguguuucauuuuggaucuugaauuccggaucuugaauuaggcuguuugguuuugaaucuugaauuccggaucuugaauuaggcuguuugauuuuga
.......................................................................uugaauuccggaucuugaaGuag...............    4        1    0A2
.......................................................................uugaauuccggaucuugaauAag...............    76       1    0A2
.......................................................................uugaauuccggaucuuUaauuag...............    4        1    0A2
.......................................................................uugaauuccggaucuugaauCag...............    8        1    0A2
.......................................................................uugaauuccggaucuGgaauuag...............    2        1    0A2
.......................................................................uugaauuccggaucuugaauuag...............    568      0    0A2
.......................................................................uugaauuccggaucuugaaCuag...............    40       1    0A2
.......................................................................uugaGuuccggaucuugaauuag...............    26       1    0A2
.......................................................................uugaauuccggaucuugaauuagA..............    14       1    0A2
.......................................................................uugaauuccggaucuugaauuagg..............    22       0    0A2
........................................................................ugaauuccggaucuAgaa...................    98       1    0A2
........................................................................ugaauuccggGucuugaau..................    6        1    0A2
........................................................................ugaauuccggaucuAgaau..................    172      1    0A2
........................................................................Agaauuccggaucuugaau..................    2        1    0A2
........................................................................ugaauuccggaucuugaaG..................    2        1    0A2
........................................................................ugaauuccggaucuugaau..................    634      0    0A2
........................................................................ugaGuuccggaucuugaau..................    12       1    0A2
........................................................................ugaauuccggaGcuugaau..................    2        1    0A2
........................................................................ugaauuccggaucuuUaauu.................    4        1    0A2
........................................................................ugaauuccgCaucuugaauu.................    2        1    0A2
........................................................................ugaauuccggGucuugaauu.................    4        1    0A2
........................................................................ugaauuccggaucuAgaauu.................    1502     1    0A2
........................................................................ugaauuccggaucCugaauu.................    2        1    0A2
........................................................................ugaauuccggaucuugaaGu.................    2        1    0A2
........................................................................ugaauuAcggaucuugaauu.................    4        1    0A2
........................................................................ugaCuuccggaucuugaauu.................    2        1    0A2
........................................................................ugaGuuccggaucuugaauu.................    86       1    0A2
........................................................................ugaauuccggaucuCgaauu.................    6        1    0A2
........................................................................ugaauuccggaucuugaaCu.................    14       1    0A2
........................................................................uNaauuccggaucuugaauu.................    4        1    0A2
........................................................................ugaauuccggaucuugaauu.................    4500     0    0A2
........................................................................ugaauuccggaCcuugaauu.................    2        1    0A2
........................................................................ugaauuccggaGcuugaauu.................    6        1    0A2
........................................................................Ggaauuccggaucuugaauu.................    4        1    0A2
........................................................................ugaauuccggaucuugGauu.................    2        1    0A2
........................................................................Ngaauuccggaucuugaauu.................    6        1    0A2
........................................................................ugaaGuccggaucuugaauu.................    8        1    0A2
........................................................................ugaauuccggaucuAgaauua................    9536     1    0A2
........................................................................ugaauuccggaucuugaauAa................    542      1    0A2
........................................................................ugaauuccggaucuugaaGua................    4        1    0A2
........................................................................ugaauuccggaucuCgaauua................    12       1    0A2
........................................................................ugaauuccggaucuugaauua................    26330    0    0A2
........................................................................ugaauuccggauAuugaauua................    12       1    0A2
........................................................................ugaauuccggaucuugaaCua................    40       1    0A2
........................................................................ugaauuccggaucuGgaauua................    4        1    0A2
........................................................................Ngaauuccggaucuugaauua................    52       1    0A2
........................................................................ugaauuccggGucuugaauua................    20       1    0A2
........................................................................ugaauuAcggaucuugaauua................    22       1    0A2
........................................................................ugaauuGcggaucuugaauua................    22       1    0A2
........................................................................uNaauuccggaucuugaauua................    6        1    0A2
........................................................................ugaaGuccggaucuugaauua................    42       1    0A2
........................................................................ugaauuccggaGcuugaauua................    16       1    0A2
........................................................................ugaGuuccggaucuugaauua................    512      1    0A2
........................................................................Agaauuccggaucuugaauua................    12       1    0A2
........................................................................Ggaauuccggaucuugaauua................    8        1    0A2
........................................................................ugaauuccggaCcuugaauua................    12       1    0A2
........................................................................ugaauuccggaucuugCauua................    4        1    0A2
........................................................................ugaauuccggaucCugaauua................    6        1    0A2
........................................................................ugaauuccggCucuugaauua................    16       1    0A2
........................................................................ugaauuccggaucuugaauCa................    634      1    0A2
........................................................................ugaauuccggaucuuUaauua................    22       1    0A2
........................................................................ugaauuccggaucuugGauua................    20       1    0A2
........................................................................ugaauuccggaucuugaauuag...............    1894     0    0A2
........................................................................Agaauuccggaucuugaauuag...............    2        1    0A2
........................................................................ugaauuccggaucuugaaGuag...............    14       1    0A2
........................................................................ugaGuuccggaucuugaauuag...............    36       1    0A2
........................................................................ugaauuccggGucuugaauuag...............    2        1    0A2
........................................................................ugaauuccggaucuugaauAag...............    20       1    0A2
........................................................................ugaauuccggaucuugaauCag...............    24       1    0A2
........................................................................ugaauuccggaucCugaauuag...............    4        1    0A2
```

Star Mature

guuucuuauuaguguguuucauuuuggaucuugaauuccggaucuugaauuaggcuguuugguuuugaaucuugaauuccggaucuugaauuaggcuguuugauuuuga			
....ugaauuccggaucuAgaauuag...............	7582	1	0A2
....ugaaGuccggaucuugaauuag...............	4	1	0A2
....ugaauuccggaucuugaauuagg..............	58	0	0A2
....ugaauuccggauAuugaauuagg..............	2	1	0A2
....ugaauuAcggaucuugaauuagg..............	4	1	0A2
....ugaauuccggaucuugaaCuagg..............	4	1	0A2
....ugaauuccggaucuugaauuagC..............	8	1	0A2
....ugaauuccggaucuAgaauuagg..............	8	1	0A2
....gaauuccggaucuAgaau..................	14	1	0A2
....gaauuccggaucuugaau..................	42	0	0A2
....gaauuccggGucuugaauu.................	2	1	0A2
....gaGuuccggaucuugaauu.................	4	1	0A2
....gaauuccggaucuugaauu.................	214	0	0A2
....gaauuccggaucuAgaauu.................	92	1	0A2
....gaauuccggaucuugaauCa................	168	1	0A2
....gaaGuccggaucuugaauua................	2	1	0A2
....gaauuccggaucuugGauua................	2	1	0A2
....Naauuccggaucuugaauua................	4	1	0A2
....gaauuccggaCcuugaauua................	6	1	0A2
....gaauuccggaucuuUaauua................	2	1	0A2
....gaauuccggaucuAgaauua................	926	1	0A2
....gaGuuccggaucuugaauua................	46	1	0A2
....gaauuccggaucuugaauAa................	6	1	0A2
....gaauuccggaucuugaauua................	1670	0	0A2
....gaauuccggaucuugaaGua................	2	1	0A2
....gaauuccggaucuugaaCua................	4	1	0A2
....Naauuccggaucuugaauuag...............	4	1	0A2
....gaauuccggGucuugaauuag...............	2	1	0A2
....gaauuccggauAuugaauuag...............	2	1	0A2
....gaauuccggaCcuugaauuag...............	2	1	0A2
....gaauuccggaucuugaauuag...............	1086	0	0A2
....gaauuccggaucuAgaauuag...............	5374	1	0A2
....gaauuccggaucuAgaauuagg..............	10	1	0A2
....gaauuccggaucuugaauuagg..............	48	0	0A2
....aauuccggaucuugaauu.................	20	0	0A2
....aauuccggaucuugaauua................	1052	0	0A2
....aauuccggaucuugaauCa................	34	1	0A2
....aauuccggCucuugaauua................	2	1	0A2
....aGuuccggaucuugaauua................	14	1	0A2
....Nauuccggaucuugaauua................	2	1	0A2
....aauuAcggaucuugaauua................	2	1	0A2
....aauuGcggaucuugaauua................	2	1	0A2
....aauuccggaucuugaauAa................	32	1	0A2
....aauuccggGucuugaauuag...............	2	1	0A2
....aauuccggaucuugaauuag...............	7498	0	0A2
....Nauuccggaucuugaauuag...............	24	1	0A2
....aauuAcggaucuugaauuag...............	2	1	0A2
....aCuuccggaucuugaauuag...............	4	1	0A2
....aauuccggaucuugaaCuag...............	2	1	0A2
....aauuccggaucuCgaauuag...............	6	1	0A2
....aauuccggaucuuUaauuag...............	12	1	0A2
....aauuccggaucuGgaauuag...............	4	1	0A2
....aauuccggaCcuugaauuag...............	2	1	0A2
....aauuccggaucuugaauCag...............	30	1	0A2
....aauuccggaucuugGauuag...............	12	1	0A2
....aauuccggCucuugaauuag...............	4	1	0A2
....aauuccggaGcuugaauuag...............	2	1	0A2
....aauuccggaucuugaauAag...............	2	1	0A2
....aNuuccggaucuugaauuag...............	2	1	0A2
....aauuccggaucuugaaGuag...............	2	1	0A2
....aauuccCgaucuugaauuag...............	8	1	0A2
....aGuuccggaucuugaauuag...............	138	1	0A2
....aauuccggaucAugaauuag...............	12	1	0A2
....aauuccggaucuugCauuag...............	2	1	0A2
....Nauuccggaucuugaauuagg..............	2	1	0A2
....aauuccggaucuugaauuagg..............	540	0	0A2
....aauuccggaucuGgaauuagg..............	2	1	0A2
....aauuccggaucuAgaauuagg..............	22	1	0A2
....aauuccggaucuCgaauuagg..............	6	1	0A2
....aauuccggaucuugaauuagA..............	38	1	0A2

```
                   Star                                                Mature
guuucuuauuaguguguuucauuuuggaucuugaauuccggaucuugaauuaggcuguuugguuuugaaucuugaauuccggaucuugaauuaggcuguuugauuuuga
..........................................................................aauuccggGucuugaauuagg..............    2     1    0A2
..........................................................................aauuccggaucuugaaCuagg..............    2     1    0A2
...........................................................................Guuccggaucuugaauua................    8     1    0A2
...........................................................................auuccggaucuugaauCa................    20    1    0A2
...........................................................................Nuuccggaucuugaauua................    4     1    0A2
...........................................................................auuccggaucuugaauua................    366   0    0A2
...........................................................................auuccggaucuugaauAa................    8     1    0A2
...........................................................................auuccggaucuugaaCua................    4     1    0A2
...........................................................................auuccgCaucuugaauuag...............    2     1    0A2
...........................................................................Nuuccggaucuugaauuag...............    8     1    0A2
...........................................................................auuccggaucuGgaauuag...............    4     1    0A2
...........................................................................Guuccggaucuugaauuag...............    24    1    0A2
...........................................................................Cuuccggaucuugaauuag...............    10    1    0A2
...........................................................................auuccggCucuugaauuag...............    4     1    0A2
...........................................................................auuccggaucuugaauCag...............    18    1    0A2
...........................................................................auuccggGucuugaauuag...............    4     1    0A2
...........................................................................auuccggaucuugaauAag...............    6     1    0A2
...........................................................................auuccggaucuuUaauuag...............    6     1    0A2
...........................................................................auuccggaGcuugaauuag...............    4     1    0A2
...........................................................................auuccggaucuCgaauuag...............    4     1    0A2
...........................................................................auuccggaucAugaauuag...............    24    1    0A2
...........................................................................auuccggaucuugaauuag...............    2084  0    0A2
...........................................................................auuccggaucuugGauuagg..............    6     1    0A2
...........................................................................auuccggaucuugaauuaUg..............    28    1    0A2
...........................................................................auuccggaucuugaauuCgg..............    2     1    0A2
...........................................................................auuccggaucuugaauGagg..............    4     1    0A2
...........................................................................auuAcggaucuugaauuagg..............    16    1    0A2
...........................................................................auuccggaucuAgaauuagg..............    58    1    0A2
...........................................................................Guuccggaucuugaauuagg..............    82    1    0A2
...........................................................................auuccggaucuugaaCuagg..............    8     1    0A2
...........................................................................Nuuccggaucuugaauuagg..............    8     1    0A2
...........................................................................auuccggGucuugaauuagg..............    2     1    0A2
...........................................................................auuccggaucuuUaauuagg..............    6     1    0A2
...........................................................................auuccggaucuugaauuagA..............    24    1    0A2
...........................................................................auuccggaucuCgaauuagg..............    6     1    0A2
...........................................................................Cuuccggaucuugaauuagg..............    8     1    0A2
...........................................................................auuccggaucuugaauuagg..............    4064  0    0A2
...........................................................................auuccggaucAugaauuagg..............    44    1    0A2
...........................................................................auuccggaucuGgaauuagg..............    2     1    0A2
...........................................................................auuccggaucuugaauuagC..............    8     1    0A2
............................................................................uuccggCucuugaauuag...............    4     1    0A2
............................................................................uuccggaucuCgaauuag...............    2     1    0A2
............................................................................uuccggaucuugGauuag...............    6     1    0A2
............................................................................uuccggaucuugaauuag...............    1958  0    0A2
............................................................................uuccggaCcuugaauuag...............    2     1    0A2
............................................................................uuccggauAuugaauuag...............    2     1    0A2
............................................................................uuccggaucuugaauCag...............    58    1    0A2
............................................................................uuccggaucAugaauuag...............    6     1    0A2
............................................................................Nuccggaucuugaauuag...............    4     1    0A2
............................................................................uuccggGucuugaauuag...............    2     1    0A2
............................................................................uuAcggaucuugaauuagg..............    2     1    0A2
............................................................................uuccggaucAugaauuagg..............    42    1    0A2
............................................................................uuccggaucuugaaCuagg..............    8     1    0A2
............................................................................Nuccggaucuugaauuagg..............    4     1    0A2
............................................................................uuccggaGcuugaauuagg..............    4     1    0A2
............................................................................uuccggaucuugaauuagA..............    22    1    0A2
............................................................................uuccggaucuugCauuagg..............    4     1    0A2
............................................................................uuccggaucuuUaauuagg..............    4     1    0A2
............................................................................uuccggCucuugaauuagg..............    2     1    0A2
............................................................................uucGggaucuugaauuagg..............    12    1    0A2
............................................................................uuccggaucuugaaGuagg..............    2     1    0A2
............................................................................uuccggaucuugaauuaUg..............    4     1    0A2
............................................................................uuccggaucuugaauuagg..............    2276  0    0A2
............................................................................uuccggaucuGgaauuagg..............    8     1    0A2
............................................................................uuccggGucuugaauuagg..............    6     1    0A2
............................................................................uuccggaucuugGauuagg..............    2     1    0A2
............................................................................uuccggaucuAgaauuagg..............    20    1    0A2
............................................................................uuccggaucuugaauuagUcu............    6     1    0A2
............................................................................uuccggaucuugaauuaggcuC...........    8     1    0A2
.............................................................................uccggaucuAgaauuagg..............    6     1    0A2
```

Star Mature

```
guuucuuauuaguguguuucauuuuggaucuugaauuccggaucuugaauuaggcuguuugguuuugaaucuugaauuccggaucuugaauuaggcuguuugauuuuga
.............................................................................Nccggaucuugaauuagg..............    4     1    0A2
.............................................................................uccggaucuGgaauuagg..............    6     1    0A2
.............................................................................uccggaucAugaauuagg..............    10    1    0A2
.............................................................................uAcggaucuugaauuagg..............    4     1    0A2
.............................................................................uccggaucuugaauuagg..............    632   0    0A2
.............................................................................uccggaucuugaauuagA..............    2     1    0A2
.............................................................................uccggaCcuugaauuagg..............    2     1    0A2
.............................................................................uccggaucuugaauuaggcuA...........    2     1    0A2
```

Provisional ID	:	scaffold189_28607
Score total	:	2.9
Score for star read(s)	:	-1.3
Score for read counts	:	0
Score for mfe	:	-0.3
Score for randfold	:	1.6
Score for cons. seed	:	3
Total read count	:	158
Mature read count	:	158
Loop read count	:	0
Star read count	:	0

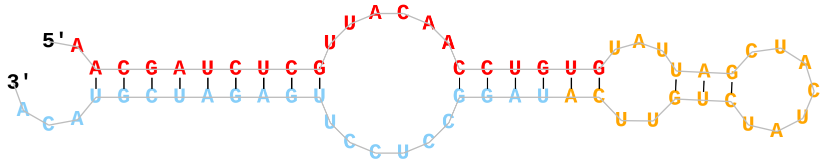

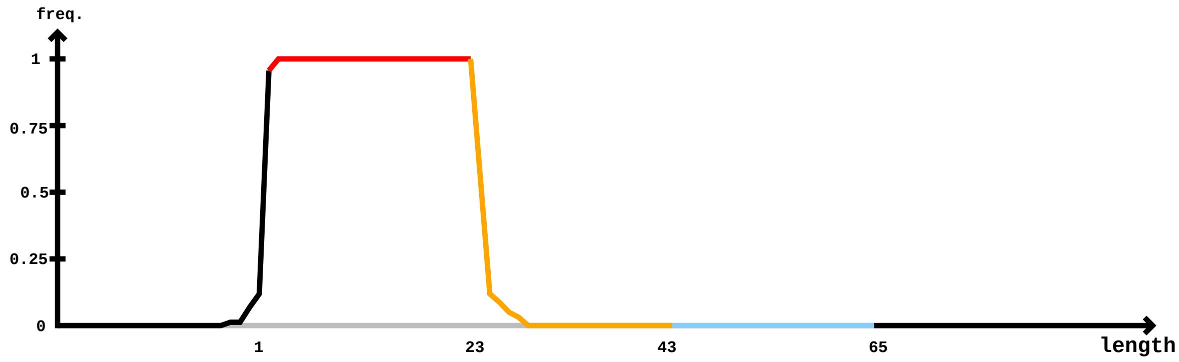

Mature Star

```
5'- uaugcuguuugcccaaauuaaacgaucucguuacaaccugugauauuagcuacuaucuguucauaggccuccuugagaucguacaauauucggacuuuuuagcauuccgugaa   -3'    exp
    .((((((...(.((.(((...(((((((((......((((((...(((.......)))..))))))......))))))))).....))).)).)....))))))........    reads    mm    sample
    ..................uaaacgaucucguuacaaccuguguau...................................................................    2        0     0A2
    ...................aaacgaucucguuacaaccugugua....................................................................    5        0     0A2
    ....................aGcgaucucguuacaaccugug......................................................................    2        1     0A2
    ....................aacgaucucguuacaaccugug......................................................................    57       0     0A2
    .....................acgaucucguuacaaccugug......................................................................    2        0     0A2
    .....................acgaucucguuacaaccuguguauAa.................................................................    5        1     0A2

    ................auuaaacgaucucguuacaaccugug......................................................................    2        0     0B2
    ..................uaaacgaucucguuacaaccugugu.....................................................................    7        0     0B2
    ....................aacgaucucguuacaaccugug......................................................................    31       0     0B2
    ....................Nacgaucucguuacaaccugug......................................................................    1        1     0B2

    ...................aaacgaucucguuacaaccuguguauA..................................................................    3        1     0G2
    ....................aacgaucucguuacaaccugug......................................................................    39       0     0G2
    ....................aacgaucucguuacaaccuguguau...................................................................    3        0     0G2
    ....................aacgaucucguuacaacAuguguau...................................................................    1        1     0G2
```

Provisional ID	: scaffold196_29303
Score total	: 4.8
Score for star read(s)	: -1.3
Score for read counts	: 0
Score for mfe	: 1.5
Score for randfold	: 1.6
Score for cons. seed	: 3
Total read count	: 599
Mature read count	: 599
Loop read count	: 0
Star read count	: 0

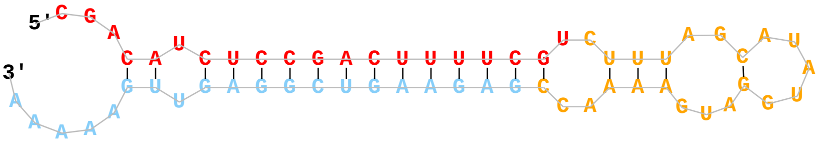

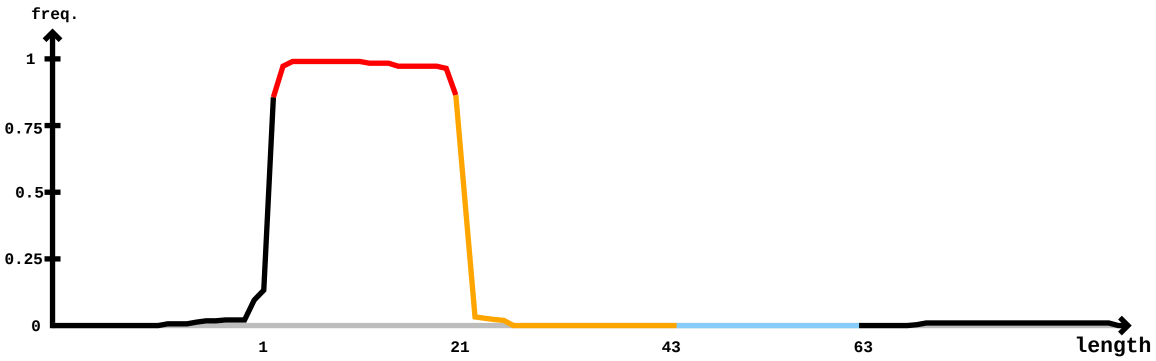

Mature Star

5'- gaagggagaaaaucccugccccgacaucuccgacuuuucgucuuuagcauauggaugaaaaccgagaagucggaguugaaaaaguucgagucuuugcuccgacaucucca -3' exp

....(((((...............((.(((((((((((((..(((..(.....)...)))..))))))))))))).)).....(((.((((....)))).))).))))).	reads	mm	sample
..........aaucccugcccGgacaucucc...	4	1	0B2
.............cccugcccGgacaucuccgac..	4	1	0B2
..............ccugcccGgacaucuccgac..	3	1	0B2
...................cccgacaucuccgacuuuuc...	4	0	0B2
...................cccgacaucuccgacuuuucA..	1	1	0B2
...................cccgacaucucUgacuuuucg..	1	1	0B2
...................cccgacaucuccgacuuuucgu...	1	0	0B2
....................ccgacaucuccgacuuuuc...	1	0	0B2
....................ccgacaucuccgacuuuucg..	7	0	0B2
....................ccAacaucuccgacuuuucg..	2	1	0B2
....................ccAacaucuccgacuuuucgu...	1	1	0B2
.....................cgacaucuccgacuuuucgu...	54	0	0B2
.....................cgacaucuccgacuuuucAu...	2	1	0B2
.....................cAacaucuccgacuuuucgu...	77	1	0B2
.....................cgacaucuccgacuuuucguA..	20	1	0B2
.....................cgacaucuccgacuuuucguc..	4	0	0B2
.....................cgacaucuccgacuuuucguUuuu...	2	1	0B2
......................gacaucuccgacuuuucgu...	12	0	0B2
......................Aacaucuccgacuuuucgu...	9	1	0B2
................ugccccgacaucuccgacuuuucgA...	2	1	0G2
...................cccgacaucuccgacuuuucg..	13	0	0G2
...................cccgacaucuccgacuuuucA..	2	1	0G2
...................Nccgacaucuccgacuuuucg..	1	1	0G2
...................ccGgacaucuccgacuuuucgu...	1	1	0G2
....................ccgacaucuccgacuuuucA..	1	1	0G2
....................ccgacaucuccgacuuuucgu...	1	0	0G2
.....................cAacaucuccgacuuuucg..	2	1	0G2
.....................cAacaucuccgacuuuucgu...	64	1	0G2
.....................cgacaucuccgGcuuuucgu...	2	1	0G2
.....................cgacaucuccgacuuuucgu...	43	0	0G2
.....................cgacaucuccgacuuuucguA..	15	1	0G2
.....................cgacaucuccgacuuuucgucA...	3	1	0G2
......................Aacaucuccgacuuuucg..	3	1	0G2

```
                                 Mature                                    Star
gaagggagaaaaucccugccccgacaucuccgacuuuucgucuuuagcauauggaugaaaaccgagaagucggaguugaaaaaguucgagucuuugcuccgacaucucca
......................gacaucuccgacuuuucgu.....................................................................    13    0    0G2
......................Aacaucuccgacuuuucgu.....................................................................    22    1    0G2
......................gacaucuccgacuuuucguUuua.................................................................    3     1    0G2
.......................acaucuccgacuuuucguA....................................................................    2     1    0G2
.......................acaucuccgacuuuucguUuua.................................................................    9     1    0G2
........................................................................................agucuuugcuccgacaucucA.    2     1    0G2
.........................................................................................Aucuuugcuccgacaucucc.    4     1    0G2

...................cccgacaucucUgacuuuucg......................................................................    1     1    0A2
...................cccgacaucuccgacuuuucg......................................................................    7     0    0A2
...................cccgacaucuccgacuuuucA......................................................................    4     1    0A2
...................ccGgacaucuccgacuuuucg......................................................................    7     1    0A2
...................cccgacaucuccgacuuuucguA....................................................................    3     1    0A2
....................ccgacaucuccgacuuuucg......................................................................    5     0    0A2
....................ccgacaucuccgacuuuucgu.....................................................................    4     0    0A2
....................ccgacaucuccgacuuuucguA....................................................................    1     1    0A2
.....................cAacaucuccgacuuuucg......................................................................    3     1    0A2
.....................Ugacaucuccgacuuuucg......................................................................    1     1    0A2
.....................cgacaucuccgacuuuucgu.....................................................................    72    0    0A2
.....................cAacaucuccgacuuuucgu.....................................................................    60    1    0A2
.....................cgacaucuccgacuuuucguA....................................................................    20    1    0A2
.....................cgacaucuccgacuuuucguAuu..................................................................    3     1    0A2
......................gacaucuccgacuuuucA......................................................................    1     1    0A2
......................Aacaucuccgacuuuucgu.....................................................................    3     1    0A2
......................gacaucuccgacuuuucgu.....................................................................    4     0    0A2
......................gacaucuccgacuuuucguA....................................................................    2     1    0A2
```

Provisional ID	:	scaffold204_29914
Score total	:	153038.8
Score for star read(s)	:	3.9
Score for read counts	:	153029.7
Score for mfe	:	0.5
Score for randfold	:	1.6
Score for cons. seed	:	3
Total read count	:	300173
Mature read count	:	299327
Loop read count	:	0
Star read count	:	846

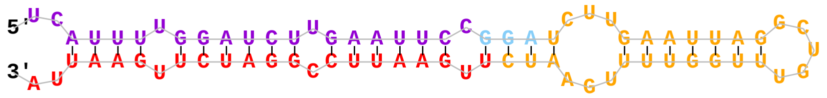

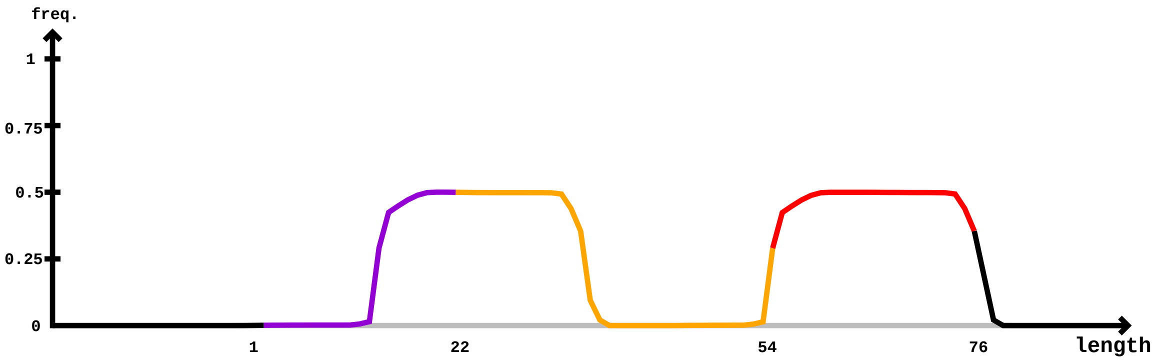

Star **Mature**

5'-	ggaucuugaauuaggcuguuucauuuuggaucuugaauuccggaucuugaauuaggcuguuugguuuugaaucuugaauuccggaucuugaauuaggcuguuugguuuug	-3'	obs	
	ggaucuugaauuaggcuguuucauuuuggaucuugaauuccggaucuugaauuaggcuguuugguuuugaaucuugaauuccggaucuugaauuaggcuguuugguuuug		exp	
	.......(((((((((.((((.((((.((((((.((((((.((((...(((((((.....)))))))...)))).)))))).)))))).)))).)))).)))))))))..	reads	mm	sample
	................uguuucauuCuggaucuugaa...	2	1	0A2
	..................uuucauuCuggaucuugaauu...	1	1	0A2
	..................uuucGuuuuggaucuugaauuc..	7	1	0A2
	..................uuucauuCuggaucuugaauuc..	11	1	0A2
	...................uucauuuuggaucuugaauuA..	2	1	0A2
	...................uucauuCuggaucuugaauuc..	29	1	0A2
	...................uucauuuuggaucuugaauucA...	4	1	0A2
	...................uucauuCuggaucuugaauucc...	14	1	0A2
	....................ucauuCuggaucuugaauuc..	30	1	0A2
	....................ucauuGuggaucuugaauuc..	1	1	0A2
	....................ucauuuuggaucuugaauucA...	23	1	0A2
	....................ucauuCuggaucuugaauucc...	65	1	0A2
	....................ucauuuuggaucuugaauucG...	3	1	0A2
	....................ucauuuuggaucuCgaauuccg..	4	1	0A2
	....................ucauuuuggaucuugaauuccg..	7	0	0A2
	.....................cauuCuggaucuugaauuc..	6	1	0A2
	.....................cauuuuggGucuugaauucc...	3	1	0A2
	.....................cauuuuggaucuugaauucc...	1	0	0A2
	.....................cauuCuggaucuugaauucc...	23	1	0A2
	.....................cauuuuggaucuugaauucA...	3	1	0A2
	......................auuuuggaucuugaauucA...	2	1	0A2
	......................auuCuggaucuugaauucc...	17	1	0A2
	......................auuuuggGucuugaauuccg..	3	1	0A2
	......................aCuuuggaucuugaauuccg..	2	1	0A2
	......................auuCuggaucuugaauuccg..	4	1	0A2
	......................auuuuggaucuugaauuccg..	2	0	0A2
	......................auuuugUaucuugaauuccgg...	1	1	0A2
	.......................uuuuggaucuugaauucUg..	3	1	0A2
	.......................uuuuggaucuugaauuccg..	5	0	0A2
	.......................uuuuggGucuugaauuccg..	13	1	0A2
	.......................uuuuggaucuugaGuuccgg...	1	1	0A2
	.......................uuuuggGucuugaauuccgg...	2	1	0A2
	.......................uuuuggGucuugaauuccgga..	20	1	0A2

```
                    Star                                                 Mature
ggaucuugaauuaggcuguuucauuuuggaucuugaauuccggaucuugaauuaggcuguuugguuuugaaucuugaauuccggaucuugaauuaggcuguuugguuuug
.......................uuuuggaucuugaauuccgga..................................................................    15      0     0A2
........................uuuggGucuugaauuccgga..................................................................    2       1     0A2
.........................uuggaucuugaauuccggaucA...............................................................    3       1     0A2
..........................uggGucuugaauuccggauc................................................................    2       1     0A2
.............................aucuugaauuccggaucuu..............................................................    12      0     0A2
.............................aucuugaauuccggaucuug.............................................................    6       0     0A2
.............................aucGugaauuccggaucuug.............................................................    6       1     0A2
.............................aucuugaauuccggaucuugaauAa........................................................    4       1     0A2
.............................aucuugaauuccggaucuugUauua........................................................    6       1     0A2
.............................aucuugaauuccggaucuugaauuagg......................................................    14      0     0A2
..............................ucuugaauuccggaucuA..............................................................    2       1     0A2
..............................ucuugaauuccggaucuug.............................................................    12      0     0A2
..............................ucuugaauuccggaucuAga............................................................    80      1     0A2
..............................ucuugaauuccggaucuuga............................................................    14      0     0A2
..............................ucuugaauuccggaucuAgaa...........................................................    110     1     0A2
..............................ucuugaauuccggaucuugaa...........................................................    44      0     0A2
..............................Ccuugaauuccggaucuugaa...........................................................    10      1     0A2
..............................ucGugaauuccggaucuugaa...........................................................    52      1     0A2
..............................ucuugaauuccggaucuCgaa...........................................................    2       1     0A2
..............................ucuugaauuccggaucuAgaau..........................................................    24      1     0A2
..............................ucuugaauuccggaucuugaau..........................................................    368     0     0A2
..............................Ccuugaauuccggaucuugaau..........................................................    2       1     0A2
..............................ucGugaauuccggaucuugaau..........................................................    68      1     0A2
..............................ucuugaauuccggaucuugaauu.........................................................    46      0     0A2
..............................uUuugaauuccggaucuugaauu.........................................................    2       1     0A2
..............................ucGugaauuccggaucuugaauu.........................................................    6       1     0A2
..............................uGuugaauuccggaucuugaauu.........................................................    14      1     0A2
..............................ucuugaauuccggaucuAgaauua........................................................    44      1     0A2
..............................ucuugaauuccggaucuugaaCua........................................................    2       1     0A2
..............................uUuugaauuccggaucuugaauua........................................................    6       1     0A2
..............................ucuugaauuccggaucuCgaauua........................................................    2       1     0A2
..............................uGuugaauuccggaucuugaauua........................................................    4       1     0A2
..............................ucuugaauuccggaucuugaauua........................................................    226     0     0A2
..............................ucuugaauuccggaucuugaauAa........................................................    464     1     0A2
..............................ucuugaGuuccggaucuugaauua........................................................    2       1     0A2
..............................ucuugaauuccggaucuAgaauuag.......................................................    38      1     0A2
..............................ucuugaauuccggaucuugaauuag.......................................................    48      0     0A2
..............................ucuugaauuccggaucuugaauuagg......................................................    36      0     0A2
..............................ucuugaauuccggaucuugaaCuagg......................................................    14      1     0A2
..............................ucuugaauuccggaucuugaauuaggc.....................................................    18      0     0A2
...............................cuugaauuccggaucuAga............................................................    34      1     0A2
...............................cGugaauuccggaucuugaa...........................................................    4       1     0A2
...............................cuugaauuccggaucuAgaa...........................................................    582     1     0A2
...............................Nuugaauuccggaucuugaa...........................................................    2       1     0A2
...............................cuugaGuuccggaucuugaa...........................................................    2       1     0A2
...............................cuugaauuccggaucuugaa...........................................................    190     0     0A2
...............................cuugaauuccggaucuAgaau..........................................................    84      1     0A2
...............................cGugaauuccggaucuugaau..........................................................    58      1     0A2
...............................cuugaauuccggaucuugaau..........................................................    304     0     0A2
...............................cuugaauuccggaucuAgaauu.........................................................    114     1     0A2
...............................cuugaauuccggaucuugaauu.........................................................    458     0     0A2
...............................cGugaauuccggaucuugaauu.........................................................    12      1     0A2
...............................cuugaaGuccggaucuugaauu.........................................................    2       1     0A2
...............................cuugaauuccggaucuugaauua........................................................    1160    0     0A2
...............................cuugaauuccggaucuugaauCa........................................................    48      1     0A2
...............................cuugaauuccggCucuugaauua........................................................    2       1     0A2
...............................Nuugaauuccggaucuugaauua........................................................    2       1     0A2
...............................cuugaauuccggaucuugGauua........................................................    6       1     0A2
...............................cGugaauuccggaucuugaauua........................................................    10      1     0A2
...............................cuugaauuccggaucuAgaauua........................................................    244     1     0A2
...............................cuugaGuuccggaucuugaauua........................................................    2       1     0A2
...............................cuugaauuccggaGcuugaauua........................................................    2       1     0A2
...............................cuugaaGuccggaucuugaauua........................................................    4       1     0A2
...............................cuugaauuccggaucuugaauAa........................................................    268     1     0A2
...............................cuugaauuccggaucuAgaauuag.......................................................    2       1     0A2
...............................cuugaauuccggaucuugaaCuag.......................................................    4       1     0A2
...............................cuugaauuccggaucuugaauuag.......................................................    12      0     0A2
...............................cuugaauuccggaucuugaauuagg......................................................    6       0     0A2
................................uugaauuccggaucuAga............................................................    12      1     0A2
................................uugaauuccggaucuugaa...........................................................    98      0     0A2
```

```
                    Star                                                        Mature
ggaucuugaauuaggcuguuucauuuuggaucuugaauuccggaucuugaauuaggcuguugguuuugaaucuugaauuccggaucuugaauuaggcuguugguuuug
................................uugaauuccggaucuAgaa.........................................................	384	1	0A2
................................uNgaauuccggaucuugaau........................................................	6	1	0A2
................................uugaauuccggaucuAgaau........................................................	3154	1	0A2
................................uugaauuccggauAuugaau........................................................	4	1	0A2
................................uugaauuccggaGcuugaau........................................................	14	1	0A2
................................uugaauuccggaCcuugaau........................................................	8	1	0A2
................................uugaauuccggaucuugaaG........................................................	2	1	0A2
................................uugaauuccggaucuGgaau........................................................	8	1	0A2
................................Nugaauuccggaucuugaau........................................................	30	1	0A2
................................Augaauuccggaucuugaau........................................................	4	1	0A2
................................uugaauuAcggaucuugaau........................................................	6	1	0A2
................................uugaauuccggaucAugaau........................................................	2	1	0A2
................................Cugaauuccggaucuugaau........................................................	6	1	0A2
................................uugaauAccggaucuugaau........................................................	10	1	0A2
................................uugaauuccggCucuugaau........................................................	2	1	0A2
................................uugaauuccggaucuugaaC........................................................	4	1	0A2
................................uugaauuccggaucuCgaau........................................................	4	1	0A2
................................uugaGuuccggaucuugaau........................................................	162	1	0A2
................................Gugaauuccggaucuugaau........................................................	36	1	0A2
................................uugaauuccggaucCugaau........................................................	6	1	0A2
................................uugaauuccggGucuugaau........................................................	26	1	0A2
................................uugaauuccggaucuugUau........................................................	2	1	0A2
................................uugaauuccggaucuugaau........................................................	14644	0	0A2
................................uugaaGuccggaucuugaau........................................................	16	1	0A2
................................uugaauuccggaucuCgaauu.......................................................	46	1	0A2
................................uNgaauuccggaucuugaauu.......................................................	2	1	0A2
................................uugaauuccggCucuugaauu.......................................................	6	1	0A2
................................uGgaauuccggaucuugaauu.......................................................	10	1	0A2
................................uugaauuccggaucCugaauu.......................................................	4	1	0A2
................................uugaauuccggaGcuugaauu.......................................................	18	1	0A2
................................uugaauuccggaucuugaauu.......................................................	18324	0	0A2
................................uugaauuccggaCcuugaauu.......................................................	8	1	0A2
................................uugaauuccggaucuuUaauu.......................................................	14	1	0A2
................................uugaauuccggauGuugaauu.......................................................	2	1	0A2
................................uugaauAccggaucuugaauu.......................................................	14	1	0A2
................................uuNaauuccggaucuugaauu.......................................................	2	1	0A2
................................uugaauuccggaucuugaaGu.......................................................	2	1	0A2
................................uugaGuuccggaucuugaauu.......................................................	310	1	0A2
................................Augaauuccggaucuugaauu.......................................................	2	1	0A2
................................uugaauuccggGucuugaauu.......................................................	6	1	0A2
................................Cugaauuccggaucuugaauu.......................................................	2	1	0A2
................................Gugaauuccggaucuugaauu.......................................................	14	1	0A2
................................uugaaGuccggaucuugaauu.......................................................	2	1	0A2
................................uugaauuccggauAuugaauu.......................................................	2	1	0A2
................................uugaauuccggaucuAgaauu.......................................................	4628	1	0A2
................................uugaauuccggaucuugaaCu.......................................................	10	1	0A2
................................uugaauuAcggaucuugaauu.......................................................	10	1	0A2
................................Nugaauuccggaucuugaauu.......................................................	56	1	0A2
................................uugaauuccggaucuugGauu.......................................................	6	1	0A2
................................uugaauuccggaucuGgaauu.......................................................	6	1	0A2
................................uugaauuccggaucAugaauu.......................................................	6	1	0A2
................................uugaauuccggauAuugaauua......................................................	22	1	0A2
................................Nugaauuccggaucuugaauua......................................................	98	1	0A2
................................uugaauuGcggaucuugaauua......................................................	6	1	0A2
................................uugaauuccggaCcuugaauua......................................................	14	1	0A2
................................uGgaauuccggaucuugaauua......................................................	14	1	0A2
................................uugaauuccggaucuugaauAa......................................................	7444	1	0A2
................................uugaaGuccggaucuugaauua......................................................	18	1	0A2
................................Cugaauuccggaucuugaauua......................................................	32	1	0A2
................................uugaauuccggauGuugaauua......................................................	4	1	0A2
................................uugaauuccggGucuugaauua......................................................	10	1	0A2
................................uugaauuAcggaucuugaauua......................................................	40	1	0A2
................................uugaauuccggaucuugaauCa......................................................	3958	1	0A2
................................Augaauuccggaucuugaauua......................................................	18	1	0A2
................................uuNaauuccggaucuugaauua......................................................	4	1	0A2
................................uugaauuccggaucAugaauua......................................................	6	1	0A2
................................uugaauuccggaucuugUauua......................................................	2	1	0A2
................................uAgaauuccggaucuugaauua......................................................	8	1	0A2
................................uugaauuccggaGcuugaauua......................................................	38	1	0A2
................................uugaauuccggaucuugGauua......................................................	40	1	0A2
```

Star Mature

ggaucuugaauuaggcuguuucauuuuggaucuugaauuccggaucuugaauuaggcuguuugguuuugaaucuugaauuccggaucuugaauuaggcuguuugguuuug			
................................uugaauuccggaucCugaauua..	8	1	0A2
................................uugaauuccggaucuugCauua..	2	1	0A2
................................uNgaauuccggaucuugaauua..	10	1	0A2
................................uugaauuccgCaucuugaauua..	2	1	0A2
................................uugaauuccggaucuCgaauua..	22	1	0A2
................................uugaauuccggaucuugaauua..	46990	0	0A2
................................Gugaauuccggaucuugaauua..	74	1	0A2
................................uugaauuccggaucuGgaauua..	8	1	0A2
................................uugaauuccCgaucuugaauua..	2	1	0A2
................................uugaGuuccggaucuugaauua..	1250	1	0A2
................................uugaauuccggaucuugaaGua..	8	1	0A2
................................uugaauuccggaucuuUaauua..	30	1	0A2
................................uugaauuccggaucuAgaauua..	10386	1	0A2
................................uugaauAccggaucuugaauua..	20	1	0A2
................................uugaauuccggCucuugaauua..	12	1	0A2
................................uugaauuccggaucuugaaCua..	94	1	0A2
................................uugaauuccggaucuGgaauuag...	2	1	0A2
................................uugaauuccggaucuugaauCag...	8	1	0A2
................................uugaGuuccggaucuugaauuag...	26	1	0A2
................................uugaauuccggaucuugaaGuag...	4	1	0A2
................................uugaauuccggaucuAgaauuag...	2540	1	0A2
................................uugaauuccggaucuuUaauuag...	4	1	0A2
................................uugaauuccggaucuugaauuag...	568	0	0A2
................................uugaauuccggaucuugaaCuag...	40	1	0A2
................................uugaauuccggaucuugaauAag...	76	1	0A2
................................uugaauuccggaucuugaauuagA..	14	1	0A2
................................uugaauuccggaucuugaauuagg..	22	0	0A2
.................................ugaauuccggaucuAgaa...	98	1	0A2
.................................ugaauuccggaucuugaaG..	2	1	0A2
.................................ugaauuccggGucuugaau..	6	1	0A2
.................................ugaauuccggaucuAgaau..	172	1	0A2
.................................ugaGuuccggaucuugaau..	12	1	0A2
.................................ugaauuccggaucuugaau..	634	0	0A2
.................................ugaauuccggaGcuugaau..	2	1	0A2
.................................Agaauuccggaucuugaau..	2	1	0A2
.................................ugaauuccggaucuugaaGu...	2	1	0A2
.................................ugaauuccggaucuugaaCu...	14	1	0A2
.................................ugaauuccgCaucuugaauu...	2	1	0A2
.................................ugaauuccggaucuCgaauu...	6	1	0A2
.................................ugaauuccggaCcuugaauu...	2	1	0A2
.................................ugaauuccggaucCugaauu...	2	1	0A2
.................................ugaGuuccggaucuugaauu...	86	1	0A2
.................................uNaauuccggaucuugaauu...	4	1	0A2
.................................ugaauuccggaucuAgaauu...	1502	1	0A2
.................................ugaCuuccggaucuugaauu...	2	1	0A2
.................................ugaauuccggaGcuugaauu...	6	1	0A2
.................................ugaauuccggaucuugGauu...	2	1	0A2
.................................ugaauuccggaucuuUaauu...	4	1	0A2
.................................Ggaauuccggaucuugaauu...	4	1	0A2
.................................ugaauuAcggaucuugaauu...	4	1	0A2
.................................ugaauuccggGucuugaauu...	4	1	0A2
.................................ugaauuccggaucuugaauu...	4500	0	0A2
.................................Ngaauuccggaucuugaauu...	6	1	0A2
.................................ugaaGuccggaucuugaauu...	8	1	0A2
.................................ugaauuccggaucuCgaauua..	12	1	0A2
.................................Agaauuccggaucuugaauua..	12	1	0A2
.................................ugaauuccggaucuuUaauua..	22	1	0A2
.................................ugaauuGcggaucuugaauua..	22	1	0A2
.................................ugaauuccggauAuugaauua..	12	1	0A2
.................................ugaauuccggaucuAgaauua..	9536	1	0A2
.................................ugaauuccggaucuugaaCua..	40	1	0A2
.................................Ngaauuccggaucuugaauua..	52	1	0A2
.................................ugaauuccggCucuugaauua..	16	1	0A2
.................................ugaauuccggaCcuugaauua..	12	1	0A2
.................................ugaauuccggaucuugaaGua..	4	1	0A2
.................................Ggaauuccggaucuugaauua..	8	1	0A2
.................................uNaauuccggaucuugaauua..	6	1	0A2
.................................ugaGuuccggaucuugaauua..	512	1	0A2
.................................ugaauuccggaucuugGauua..	20	1	0A2
.................................ugaauuccggaucuugaauAa..	542	1	0A2

```
                    Star                                                   Mature
ggaucuugaauuaggcuguuucauuuuggaucuugaauuccggaucuugaauuaggcuguuugguuuugaaucuugaauuccggaucuugaauuaggcuguuugguuuug
.................................ugaauuccggaucuugaauCa........................................................   634     1   0A2
.................................ugaauuAcggaucuugaauua........................................................   22      1   0A2
.................................ugaauuccggaucuugaauua........................................................   26330   0   0A2
.................................ugaauuccggaucCugaauua........................................................   6       1   0A2
.................................ugaauuccggaGcuugaauua........................................................   16      1   0A2
.................................ugaauuccggaucuugCauua........................................................   4       1   0A2
.................................ugaauuccggaucuGgaauua........................................................   4       1   0A2
.................................ugaaGuccggaucuugaauua........................................................   42      1   0A2
.................................ugaauuccggGucuugaauua........................................................   20      1   0A2
.................................ugaauuccggaucuugaauAag.......................................................   20      1   0A2
.................................ugaauuccggaucuugaaGuag.......................................................   14      1   0A2
.................................ugaauuccggaucuugaauCag.......................................................   24      1   0A2
.................................ugaauuccggaucuAgaauuag.......................................................   7582    1   0A2
.................................ugaauuccggaucuugaauuag.......................................................   1894    0   0A2
.................................ugaauuccggaucCugaauuag.......................................................   4       1   0A2
.................................ugaaGuccggaucuugaauuag.......................................................   4       1   0A2
.................................Agaauuccggaucuugaauuag.......................................................   2       1   0A2
.................................ugaGuuccggaucuugaauuag.......................................................   36      1   0A2
.................................ugaauuccggGucuugaauuag.......................................................   2       1   0A2
.................................ugaauuccggaucuugaauuagg......................................................   58      0   0A2
.................................ugaauuccggaucuugaaCuagg......................................................   4       1   0A2
.................................ugaauuccggaucuAgaauuagg......................................................   8       1   0A2
.................................ugaauuccggauAuugaauuagg......................................................   2       1   0A2
.................................ugaauuAcggaucuugaauuagg......................................................   4       1   0A2
.................................ugaauuccggaucuugaauuagC......................................................   8       1   0A2
..................................gaauuccggaucuAgaau..........................................................   14      1   0A2
..................................gaauuccggaucuugaau..........................................................   42      0   0A2
..................................gaauuccggGucuugaauu.........................................................   2       1   0A2
..................................gaauuccggaucuAgaauu.........................................................   92      1   0A2
..................................gaGuuccggaucuugaauu.........................................................   4       1   0A2
..................................gaauuccggaucuugaauu.........................................................   214     0   0A2
..................................gaauuccggaCcuugaauua........................................................   6       1   0A2
..................................gaauuccggaucuugaaCua........................................................   4       1   0A2
..................................gaauuccggaucuAgaauua........................................................   926     1   0A2
..................................Naauuccggaucuugaauua........................................................   4       1   0A2
..................................gaauuccggaucuugaauCa........................................................   168     1   0A2
..................................gaauuccggaucuuUaauua........................................................   2       1   0A2
..................................gaauuccggaucuugaauAa........................................................   6       1   0A2
..................................gaaGuccggaucuugaauua........................................................   2       1   0A2
..................................gaGuuccggaucuugaauua........................................................   46      1   0A2
..................................gaauuccggaucuugaaGua........................................................   2       1   0A2
..................................gaauuccggaucuugaauua........................................................   1670    0   0A2
..................................gaauuccggaucuugGauua........................................................   2       1   0A2
..................................gaauuccggaucuAgaauuag.......................................................   5374    1   0A2
..................................gaauuccggaucuugaauuag.......................................................   1086    0   0A2
..................................gaauuccggGucuugaauuag.......................................................   2       1   0A2
..................................gaauuccggauAuugaauuag.......................................................   2       1   0A2
..................................Naauuccggaucuugaauuag.......................................................   4       1   0A2
..................................gaauuccggaCcuugaauuag.......................................................   2       1   0A2
..................................gaauuccggaucuAgaauuagg......................................................   10      1   0A2
..................................gaauuccggaucuugaauuagg......................................................   48      0   0A2
...................................aauuccggaucuugaauu.........................................................   20      0   0A2
...................................aGuuccggaucuugaauua........................................................   14      1   0A2
...................................aauuccggaucuugaauAa........................................................   32      1   0A2
...................................Nauuccggaucuugaauua........................................................   2       1   0A2
...................................aauuccggaucuugaauCa........................................................   34      1   0A2
...................................aauuccggCucuugaauua........................................................   2       1   0A2
...................................aauuAcggaucuugaauua........................................................   2       1   0A2
...................................aauuGcggaucuugaauua........................................................   2       1   0A2
...................................aauuccggaucuugaauua........................................................   1052    0   0A2
...................................Nauuccggaucuugaauuag.......................................................   24      1   0A2
...................................aauuccggaucuugCauuag.......................................................   2       1   0A2
...................................aauuccggaucAugaauuag.......................................................   12      1   0A2
...................................aauuccggaCcuugaauuag.......................................................   2       1   0A2
...................................aauuccggaucuugaauAag.......................................................   2       1   0A2
...................................aauuccggaucuuUaauuag.......................................................   12      1   0A2
...................................aauuccggaucuGgaauuag.......................................................   4       1   0A2
...................................aGuuccggaucuugaauuag.......................................................   138     1   0A2
...................................aauuccggaucuCgaauuag.......................................................   6       1   0A2
...................................aauuccCgaucuugaauuag.......................................................   8       1   0A2
```

```
                    Star                                                   Mature
ggaucuugaauuaggcuguuucauuuuggaucuugaauuccggaucuugaauuaggcuguuugguuuugaaucuugaauuccggaucuugaauuaggcuguuugguuuug
...................................aauuccggGucuugaauuag.......................................................  2     1  0A2
...................................aauuccggaGcuugaauuag.......................................................  2     1  0A2
...................................aauuAcggaucuugaauuag.......................................................  2     1  0A2
...................................aNuuccggaucuugaauuag.......................................................  2     1  0A2
...................................aauuccggCucuugaauuag.......................................................  4     1  0A2
...................................aauuccggaucuugaaCuag.......................................................  2     1  0A2
...................................aCuuccggaucuugaauuag.......................................................  4     1  0A2
...................................aauuccggaucuugGauuag.......................................................  12    1  0A2
...................................aauuccggaucuugaauuag.......................................................  7498  0  0A2
...................................aauuccggaucuugaaGuag.......................................................  2     1  0A2
...................................aauuccggaucuugaauCag.......................................................  30    1  0A2
...................................aauuccggaucuugaauuagA......................................................  38    1  0A2
...................................aauuccggaucuGgaauuagg......................................................  2     1  0A2
...................................aauuccggaucuugaaCuagg......................................................  2     1  0A2
...................................aauuccggaucuCgaauuagg......................................................  6     1  0A2
...................................aauuccggGucuugaauuagg......................................................  2     1  0A2
...................................Nauuccggaucuugaauuagg......................................................  2     1  0A2
...................................aauuccggaucuugaauuagg......................................................  540   0  0A2
...................................aauuccggaucuAgaauuagg......................................................  22    1  0A2
....................................auuccggaucuugaauua........................................................  366   0  0A2
....................................auuccggaucuugaaCua........................................................  4     1  0A2
....................................Nuuccggaucuugaauua........................................................  4     1  0A2
....................................auuccggaucuugaauCa........................................................  20    1  0A2
....................................Guuccggaucuugaauua........................................................  8     1  0A2
....................................auuccggaucuugaauAa........................................................  8     1  0A2
....................................auuccggaucuugaauCag.......................................................  18    1  0A2
....................................auuccgCaucuugaauuag.......................................................  2     1  0A2
....................................Guuccggaucuugaauuag.......................................................  24    1  0A2
....................................Cuuccggaucuugaauuag.......................................................  10    1  0A2
....................................auuccggaucuugaauAag.......................................................  6     1  0A2
....................................auuccggGucuugaauuag.......................................................  4     1  0A2
....................................auuccggaucuuUaauuag.......................................................  6     1  0A2
....................................auuccggaucuCgaauuag.......................................................  4     1  0A2
....................................Nuuccggaucuugaauuag.......................................................  8     1  0A2
....................................auuccggaucuGgaauuag.......................................................  4     1  0A2
....................................auuccggaucAugaauuag.......................................................  24    1  0A2
....................................auuccggaGcuugaauuag.......................................................  4     1  0A2
....................................auuccggaucuugaauuag.......................................................  2084  0  0A2
....................................auuccggCucuugaauuag.......................................................  4     1  0A2
....................................auuccggaucAugaauuagg......................................................  44    1  0A2
....................................Guuccggaucuugaauuagg......................................................  82    1  0A2
....................................auuccggaucuugaauuagA......................................................  24    1  0A2
....................................auuccggaucuAgaauuagg......................................................  58    1  0A2
....................................auuccggaucuugaauGagg......................................................  4     1  0A2
....................................auuccggaucuugaaCuagg......................................................  8     1  0A2
....................................auuccggaucuugGauuagg......................................................  6     1  0A2
....................................auuccggaucuugaauuCgg......................................................  2     1  0A2
....................................auuccggaucuCgaauuagg......................................................  6     1  0A2
....................................auuccggaucuugaauuagg......................................................  4064  0  0A2
....................................auuccggaucuGgaauuagg......................................................  2     1  0A2
....................................auuccggGucuugaauuagg......................................................  2     1  0A2
....................................auuccggaucuuUaauuagg......................................................  6     1  0A2
....................................auuccggaucuugaauuagC......................................................  8     1  0A2
....................................Cuuccggaucuugaauuagg......................................................  8     1  0A2
....................................auuAcggaucuugaauuagg......................................................  16    1  0A2
....................................auuccggaucuugaauuaUg......................................................  28    1  0A2
....................................Nuuccggaucuugaauuagg......................................................  8     1  0A2
.....................................Nuccggaucuugaauuag.......................................................  4     1  0A2
.....................................uuccggCucuugaauuag.......................................................  4     1  0A2
.....................................uuccggauAuugaauuag.......................................................  2     1  0A2
.....................................uuccggaucuCgaauuag.......................................................  2     1  0A2
.....................................uuccggaucAugaauuag.......................................................  6     1  0A2
.....................................uuccggaucuugaauuag.......................................................  1958  0  0A2
.....................................uuccggaucuugGauuag.......................................................  6     1  0A2
.....................................uuccggaCcuugaauuag.......................................................  2     1  0A2
.....................................uuccggGucuugaauuag.......................................................  2     1  0A2
.....................................uuccggaucuugaauCag.......................................................  58    1  0A2
.....................................uuccggaucuGgaauuagg......................................................  8     1  0A2
.....................................uuccggaucuAgaauuagg......................................................  20    1  0A2
.....................................uuccggaucuugaaGuagg......................................................  2     1  0A2
```

```
                     Star                                                      Mature
ggaucuugaauuaggcuguuucauuuuggaucuugaauuccggaucuugaauuaggcuguuugguuuugaaucuugaauuccggaucuugaauuaggcuguuugguuuug
.....................................uuccggaucuugGauuagg......................................................    2       1    0A2
.....................................Nuccggaucuugaauuagg......................................................    4       1    0A2
.....................................uuccggaucuugaauuaUg......................................................    4       1    0A2
.....................................uuccggaucuugaaCuagg......................................................    8       1    0A2
.....................................uuccggCucuugaauuagg......................................................    2       1    0A2
.....................................uuccggaucuugCauuagg......................................................    4       1    0A2
.....................................uuAcggaucuugaauuagg......................................................    2       1    0A2
.....................................uuccggaucuuUaauuagg......................................................    4       1    0A2
.....................................uuccggaucuugaauuagA......................................................    22      1    0A2
.....................................uuccggaucAugaauuagg......................................................    42      1    0A2
.....................................uucGggaucuugaauuagg......................................................    12      1    0A2
.....................................uuccggGucuugaauuagg......................................................    6       1    0A2
.....................................uuccggaucuugaauuagg......................................................    2276    0    0A2
.....................................uuccggaGcuugaauuagg......................................................    4       1    0A2
.....................................uuccggaucuugaauuagUcu....................................................    6       1    0A2
.....................................uuccggaucuugaauuaggcuC...................................................    8       1    0A2
......................................Nccggaucuugaauuagg......................................................    4       1    0A2
......................................uccggaucuAgaauuagg......................................................    6       1    0A2
......................................uccggaucAugaauuagg......................................................    10      1    0A2
......................................uccggaucuGgaauuagg......................................................    6       1    0A2
......................................uccggaCcuugaauuagg......................................................    2       1    0A2
......................................uccggaucuugaauuagA......................................................    2       1    0A2
......................................uccggaucuugaauuagg......................................................    632     0    0A2
......................................uAcggaucuugaauuagg......................................................    4       1    0A2
......................................uccggaucuugaauuaggcuA...................................................    2       1    0A2
...............................................................Cuuuugaaucuugaauuccgga.........................    2       1    0A2
................................................................uuuugaaucuugaauuccgg..........................    71      0    0A2
................................................................uuuugaauUuugaauuccgg..........................    5       1    0A2
................................................................uuuugaGucuugaauuccgga.........................    9       1    0A2
................................................................uuuugaaucuugaauuccgga.........................    14      0    0A2
................................................................uuuugaaucuugaauuccggG.........................    4       1    0A2
................................................................uuuugaaucGugaauuccgga.........................    5       1    0A2
................................................................uuuugaaucuugaauuccggaG........................    1       1    0A2
................................................................uuuugaaucuugaauuccggau........................    7       0    0A2
.................................................................uuugaaucuugaauuccgg..........................    7       0    0A2
.................................................................uuugaaucuugaauuccgga.........................    7       0    0A2
.................................................................uuugaCucuugaauuccgga.........................    3       1    0A2
..................................................................uugaaucuugaauuccggauA.......................    14      1    0A2
..................................................................uugaaucuugaauuccggauc.......................    3       0    0A2
..................................................................uugaGucuugaauuccggauc.......................    3       1    0A2
..................................................................uugaaucuugaauuccggaucA......................    7       1    0A2
..................................................................uugaaucuugaauuccggaucuugaau.................    21      0    0A2
...................................................................ugaaucuugaauuccggau........................    3       0    0A2
...................................................................ugaaucuugaauuccggauA.......................    12      1    0A2
...................................................................ugaaucuugaauuccggauc.......................    3       0    0A2
...................................................................ugaGucuugaauuccggaucu......................    2       1    0A2
...................................................................ugaCucuugaauuccggaucuug....................    1       1    0A2
...................................................................ugaaucuugaauuccggaucuAgaa..................    11      1    0A2
...................................................................ugaaucuugaauuccggaucuAgaau.................    3       1    0A2
...................................................................ugaaucuugaauuccggaucuugaau.................    3       0    0A2
...................................................................ugaaucuugaauucCgaucuugaauu................    1       1    0A2
...................................................................ugaaucuugaauuccggaucuAgaauu................    1       1    0A2
...................................................................ugaaucuugaauuccggaucuugaauu................    19      0    0A2
.....................................................................aaucuugaauuccggaucuA.....................    1       1    0A2
.....................................................................aaucuugaauuccggaucuug....................    11      0    0A2
.....................................................................aaucuugaauuccggaucuAg....................    2       1    0A2
.....................................................................aaucuugaauuccggaucuAgaauua...............    3       1    0A2
.....................................................................aaucuugaauuccggaucuAgaauuag..............    1       1    0A2
......................................................................aucuugaauuccggaucuu.....................    12      0    0A2
......................................................................aucGugaauuccggaucuug....................    6       1    0A2
......................................................................aucuugaauuccggaucuug....................    6       0    0A2
......................................................................aucuugaauuccggaucuugUauua...............    6       1    0A2
......................................................................aucuugaauuccggaucuugaauAa...............    4       1    0A2
......................................................................aucuugaauuccggaucuugaauuagg.............    14      0    0A2
.......................................................................ucuugaauuccggaucuA.....................    2       1    0A2
.......................................................................ucuugaauuccggaucuug....................    12      0    0A2
.......................................................................ucuugaauuccggaucuuga...................    14      0    0A2
.......................................................................ucuugaauuccggaucuAga...................    80      1    0A2
.......................................................................ucuugaauuccggaucuAgaa..................    110     1    0A2
.......................................................................Ccuugaauuccggaucuugaa..................    10      1    0A2
```

Star Mature

ggaucuugaauuaggcuguuucauuuuggaucuugaauuccggaucuugaauuaggcuguuugguuuugaaucuugaauuccggaucuugaauuaggcuguuugguuuug

....ucGugaauuccggaucuugaa..................	52	1	0A2
....ucuugaauuccggaucuCgaa..................	2	1	0A2
....ucuugaauuccggaucuugaa..................	44	0	0A2
....ucuugaauuccggaucuugaau.................	368	0	0A2
....Ccuugaauuccggaucuugaau.................	2	1	0A2
....ucuugaauuccggaucuAgaau.................	24	1	0A2
....ucGugaauuccggaucuugaau.................	68	1	0A2
....uGuugaauuccggaucuugaauu................	14	1	0A2
....ucuugaauuccggaucuugaauu................	46	0	0A2
....uUuugaauuccggaucuugaauu................	2	1	0A2
....ucGugaauuccggaucuugaauu................	6	1	0A2
....ucuugaauuccggaucuugaaCua...............	2	1	0A2
....uGuugaauuccggaucuugaauua...............	4	1	0A2
....ucuugaauuccggaucuAgaauua...............	44	1	0A2
....ucuugaauuccggaucuugaauua...............	226	0	0A2
....uUuugaauuccggaucuugaauua...............	6	1	0A2
....ucuugaauuccggaucuugaauAa...............	464	1	0A2
....ucuugaauuccggaucuCgaauua...............	2	1	0A2
....ucuugaGuuccggaucuugaauua...............	2	1	0A2
....ucuugaauuccggaucuAgaauuag..............	38	1	0A2
....ucuugaauuccggaucuugaauuag..............	48	0	0A2
....ucuugaauuccggaucuugaauuagg.............	36	0	0A2
....ucuugaauuccggaucuugaaCuagg.............	14	1	0A2
....ucuugaauuccggaucuugaauuaggc............	18	0	0A2
....cuugaauuccggaucuAga...................	34	1	0A2
....cuugaGuuccggaucuugaa..................	2	1	0A2
....cuugaauuccggaucuugaa..................	190	0	0A2
....cuugaauuccggaucuAgaa..................	582	1	0A2
....cGugaauuccggaucuugaa..................	4	1	0A2
....Nuugaauuccggaucuugaa..................	2	1	0A2
....cGugaauuccggaucuugaau.................	58	1	0A2
....cuugaauuccggaucuAgaau.................	84	1	0A2
....cuugaauuccggaucuugaau.................	304	0	0A2
....cuugaaGuccggaucuugaauu................	2	1	0A2
....cuugaauuccggaucuugaauu................	458	0	0A2
....cuugaauuccggaucuAgaauu................	114	1	0A2
....cGugaauuccggaucuugaauu................	12	1	0A2
....cGugaauuccggaucuugaauua...............	10	1	0A2
....Nuugaauuccggaucuugaauua...............	2	1	0A2
....cuugaauuccggaucuugaauAa...............	268	1	0A2
....cuugaaGuccggaucuugaauua...............	4	1	0A2
....cuugaauuccggCucuugaauua...............	2	1	0A2
....cuugaGuuccggaucuugaauua...............	2	1	0A2
....cuugaauuccggaucuugaauua...............	1160	0	0A2
....cuugaauuccggaucuugGauua...............	6	1	0A2
....cuugaauuccggaucuAgaauua...............	244	1	0A2
....cuugaauuccggaGcuugaauua...............	2	1	0A2
....cuugaauuccggaucuugaauCa...............	48	1	0A2
....cuugaauuccggaucuugaauuag..............	12	0	0A2
....cuugaauuccggaucuAgaauuag..............	2	1	0A2
....cuugaauuccggaucuugaaCuag..............	4	1	0A2
....cuugaauuccggaucuugaauuagg.............	6	0	0A2
....uugaauuccggaucuAga...................	12	1	0A2
....uugaauuccggaucuugaa..................	98	0	0A2
....uugaauuccggaucuAgaa..................	384	1	0A2
....uugaauuccggaucuugaaC.................	4	1	0A2
....uugaauAccggaucuugaau.................	10	1	0A2
....uugaaGuccggaucuugaau.................	16	1	0A2
....uugaauuccggaucAugaau.................	2	1	0A2
....uugaauuccggaucuGgaau.................	8	1	0A2
....uugaauuccggaGcuugaau.................	14	1	0A2
....uugaauuccggaucuugaaG.................	2	1	0A2
....uugaauuAcggaucuugaau.................	6	1	0A2
....Gugaauuccggaucuugaau.................	36	1	0A2
....Cugaauuccggaucuugaau.................	6	1	0A2
....uugaauuccggGucuugaau.................	26	1	0A2
....uugaauuccggaucuCgaau.................	4	1	0A2
....uugaGuuccggaucuugaau.................	162	1	0A2
....uugaauuccggCucuugaau.................	2	1	0A2
....Augaauuccggaucuugaau.................	4	1	0A2

```
                    Star                                              Mature
ggaucuugaauuaggcuguuucauuuuggaucuugaauuccggaucuugaauuaggcuguuugguuuugaaucuugaauuccggaucuugaauuaggcuguuugguuuug
.........................................................................uugaauuccggaucuAgaau.................  3154   1  0A2
.........................................................................Nugaauuccggaucuugaau.................  30     1  0A2
.........................................................................uugaauuccggaucuugaau.................  14644  0  0A2
.........................................................................uNgaauuccggaucuugaau.................  6      1  0A2
.........................................................................uugaauuccggaCcuugaau.................  8      1  0A2
.........................................................................uugaauuccggauAuugaau.................  4      1  0A2
.........................................................................uugaauuccggaucCugaau.................  6      1  0A2
.........................................................................uugaauuccggaucuugUau.................  2      1  0A2
.........................................................................uugaauuccggaucuugaauu................  18324  0  0A2
.........................................................................Augaauuccggaucuugaauu................  2      1  0A2
.........................................................................uGgaauuccggaucuugaauu................  10     1  0A2
.........................................................................uugaGuuccggaucuugaauu................  310    1  0A2
.........................................................................uugaauuccggaucAugaauu................  6      1  0A2
.........................................................................uugaauuccggaucCugaauu................  4      1  0A2
.........................................................................uugaauuccggaucuuUaauu................  14     1  0A2
.........................................................................uugaauuccggaucuCgaauu................  46     1  0A2
.........................................................................uugaauuccggaucuugGauu................  6      1  0A2
.........................................................................uugaauuccggaucuugaaGu................  2      1  0A2
.........................................................................uugaauuccggaCcuugaauu................  8      1  0A2
.........................................................................uugaauuccggauAuugaauu................  2      1  0A2
.........................................................................uugaauuccggaucuugaaCu................  10     1  0A2
.........................................................................uugaaGuccggaucuugaauu................  2      1  0A2
.........................................................................uugaauuccggCucuugaauu................  6      1  0A2
.........................................................................uugaauuccggaGcuugaauu................  18     1  0A2
.........................................................................Cugaauuccggaucuugaauu................  2      1  0A2
.........................................................................uugaauuccggaucuAgaauu................  4628   1  0A2
.........................................................................Nugaauuccggaucuugaauu................  56     1  0A2
.........................................................................uugaauuccggGucuugaauu................  6      1  0A2
.........................................................................uugaauuAcggaucuugaauu................  10     1  0A2
.........................................................................uugaauAccggaucuugaauu................  14     1  0A2
.........................................................................Gugaauuccggaucuugaauu................  14     1  0A2
.........................................................................uugaauuccggauGuugaauu................  2      1  0A2
.........................................................................uugaauuccggaucuGgaauu................  6      1  0A2
.........................................................................uuNaauuccggaucuugaauu................  2      1  0A2
.........................................................................uNgaauuccggaucuugaauu................  2      1  0A2
.........................................................................uAgaauuccggaucuugaauua...............  8      1  0A2
.........................................................................Nugaauuccggaucuugaauua...............  98     1  0A2
.........................................................................Cugaauuccggaucuugaauua...............  32     1  0A2
.........................................................................uugaauuccggaucuugaauua...............  46990  0  0A2
.........................................................................uugaaGuccggaucuugaauua...............  18     1  0A2
.........................................................................uugaauuccggaucuugaaCua...............  94     1  0A2
.........................................................................uugaGuuccggaucuugaauua...............  1250   1  0A2
.........................................................................Augaauuccggaucuugaauua...............  18     1  0A2
.........................................................................uGgaauuccggaucuugaauua...............  14     1  0A2
.........................................................................uugaauuccggaucuAgaauua...............  10386  1  0A2
.........................................................................uugaauuccggaucAugaauua...............  6      1  0A2
.........................................................................uugaauAccggaucuugaauua...............  20     1  0A2
.........................................................................uugaauuccCgaucuugaauua...............  2      1  0A2
.........................................................................uugaauuccggaucuCgaauua...............  22     1  0A2
.........................................................................uugaauuccggaucuugCauua...............  2      1  0A2
.........................................................................uugaauuccggaucCugaauua...............  8      1  0A2
.........................................................................uNgaauuccggaucuugaauua...............  10     1  0A2
.........................................................................uugaauuccgCaucuugaauua...............  2      1  0A2
.........................................................................uuNaauuccggaucuugaauua...............  4      1  0A2
.........................................................................uugaauuccggauGuugaauua...............  4      1  0A2
.........................................................................uugaauuccggaCcuugaauua...............  14     1  0A2
.........................................................................uugaauuGcggaucuugaauua...............  6      1  0A2
.........................................................................uugaauuccggaucuugaauCa...............  3958   1  0A2
.........................................................................Gugaauuccggaucuugaauua...............  74     1  0A2
.........................................................................uugaauuccggaucuugaauAa...............  7444   1  0A2
.........................................................................uugaauuAcggaucuugaauua...............  40     1  0A2
.........................................................................uugaauuccggGucuugaauua...............  10     1  0A2
.........................................................................uugaauuccggaucuugGauua...............  40     1  0A2
.........................................................................uugaauuccggCucuugaauua...............  12     1  0A2
.........................................................................uugaauuccggauAuugaauua...............  22     1  0A2
.........................................................................uugaauuccggaucuugaaGua...............  8      1  0A2
.........................................................................uugaauuccggaucuuUaauua...............  30     1  0A2
.........................................................................uugaauuccggaGcuugaauua...............  38     1  0A2
.........................................................................uugaauuccggaucuGgaauua...............  8      1  0A2
.........................................................................uugaauuccggaucuugUauua...............  2      1  0A2
```

Star Mature

ggaucuugaauuaggcuguuucauuuuggaucuugaauuccggaucuugaauuaggcuguuugguuuugaaucuugaauuccggaucuugaauuaggcuguuugguuuug			
....uugaauuccggaucuugaauuag..............	568	0	0A2
....uugaauuccggaucuugaaCuag..............	40	1	0A2
....uugaauuccggaucuugaauAag..............	76	1	0A2
....uugaauuccggaucuAgaauuag..............	2540	1	0A2
....uugaauuccggaucuugaaGuag..............	4	1	0A2
....uugaauuccggaucuuUaauuag..............	4	1	0A2
....uugaGuuccggaucuugaauuag..............	26	1	0A2
....uugaauuccggaucuGgaauuag..............	2	1	0A2
....uugaauuccggaucuugaauCag..............	8	1	0A2
....uugaauuccggaucuugaauuagg.............	22	0	0A2
....uugaauuccggaucuugaauuagA.............	14	1	0A2
....ugaauuccggaucuAgaa..................	98	1	0A2
....ugaauuccggaucuAgaau.................	172	1	0A2
....ugaauuccggaGcuugaau.................	2	1	0A2
....ugaauuccggaucuugaaG.................	2	1	0A2
....ugaGuuccggaucuugaau.................	12	1	0A2
....ugaauuccggaucuugaau.................	634	0	0A2
....Agaauuccggaucuugaau.................	2	1	0A2
....ugaauuccggGucuugaau.................	6	1	0A2
....ugaauuccggaucuAgaauu................	1502	1	0A2
....ugaauuccgCaucuugaauu................	2	1	0A2
....Ggaauuccggaucuugaauu................	4	1	0A2
....ugaaGuccggaucuugaauu................	8	1	0A2
....ugaauuccggaucuuUaauu................	4	1	0A2
....uNaauuccggaucuugaauu................	4	1	0A2
....ugaCuuccggaucuugaauu................	2	1	0A2
....ugaauuccggaucuugaauu................	4500	0	0A2
....Ngaauuccggaucuugaauu................	6	1	0A2
....ugaauuccggaCcuugaauu................	2	1	0A2
....ugaauuccggaucCugaauu................	2	1	0A2
....ugaauuccggaucuugGauu................	2	1	0A2
....ugaauuccggaucuugaaCu................	14	1	0A2
....ugaauuccggaucuugaaGu................	2	1	0A2
....ugaauuAcggaucuugaauu................	4	1	0A2
....ugaauuccggaGcuugaauu................	6	1	0A2
....ugaGuuccggaucuugaauu................	86	1	0A2
....ugaauuccggaucuCgaauu................	6	1	0A2
....ugaauuccggGucuugaauu................	4	1	0A2
....Agaauuccggaucuugaauua...............	12	1	0A2
....ugaauuccggaucuGgaauua...............	4	1	0A2
....ugaauuccggaucuugaaCua...............	40	1	0A2
....ugaauuccggaucuCgaauua...............	12	1	0A2
....Ngaauuccggaucuugaauua...............	52	1	0A2
....Ggaauuccggaucuugaauua...............	8	1	0A2
....ugaauuccggGucuugaauua...............	20	1	0A2
....ugaauuccggaucuugaauua...............	26330	0	0A2
....ugaGuuccggaucuugaauua...............	512	1	0A2
....ugaauuccggCucuugaauua...............	16	1	0A2
....ugaauuccggaucuuUaauua...............	22	1	0A2
....ugaaGuccggaucuugaauua...............	42	1	0A2
....ugaauuccggaucuugaauAa...............	542	1	0A2
....ugaauuccggaCcuugaauua...............	12	1	0A2
....ugaauuccggaucuugGauua...............	20	1	0A2
....ugaauuccggaucCugaauua...............	6	1	0A2
....ugaauuAcggaucuugaauua...............	22	1	0A2
....ugaauuGcggaucuugaauua...............	22	1	0A2
....uNaauuccggaucuugaauua...............	6	1	0A2
....ugaauuccggauAuugaauua...............	12	1	0A2
....ugaauuccggaGcuugaauua...............	16	1	0A2
....ugaauuccggaucuugaaGua...............	4	1	0A2
....ugaauuccggaucuugCauua...............	4	1	0A2
....ugaauuccggaucuAgaauua...............	9536	1	0A2
....ugaauuccggaucuugaauCa...............	634	1	0A2
....ugaauuccggaucuugaauuag..............	1894	0	0A2
....ugaauuccggaucuugaaGuag..............	14	1	0A2
....ugaauuccggaucuAgaauuag..............	7582	1	0A2
....ugaauuccggGucuugaauuag..............	2	1	0A2
....ugaauuccggaucuugaauAag..............	20	1	0A2
....ugaaGuccggaucuugaauuag..............	4	1	0A2
....ugaauuccggaucuugaauCag..............	24	1	0A2

Star **Mature**

ggaucuugaauuaggcuguuucauuuuggaucuugaauuccggaucuugaauuaggcuguuugguuuugaaucuugaauuccggaucuugaauuaggcuguuugguuuug

....ugaauuccggaucCugaauuag..............	4	1	0A2
....ugaGuuccggaucuugaauuag..............	36	1	0A2
....Agaauuccggaucuugaauuag..............	2	1	0A2
....ugaauuAcggaucuugaauuagg.............	4	1	0A2
....ugaauuccggaucuAgaauuagg.............	8	1	0A2
....ugaauuccggaucuugaaCuagg.............	4	1	0A2
....ugaauuccggauAuugaauuagg.............	2	1	0A2
....ugaauuccggaucuugaauuagC.............	8	1	0A2
....ugaauuccggaucuugaauuagg.............	58	0	0A2
....gaauuccggaucuAgaau.................	14	1	0A2
....gaauuccggaucuugaau.................	42	0	0A2
....gaauuccggGucuugaauu................	2	1	0A2
....gaauuccggaucuugaauu................	214	0	0A2
....gaauuccggaucuAgaauu................	92	1	0A2
....gaGuuccggaucuugaauu................	4	1	0A2
....gaauuccggaucuugaaCua...............	4	1	0A2
....gaauuccggaucuugaauAa...............	6	1	0A2
....gaauuccggaucuAgaauua...............	926	1	0A2
....gaauuccggaCcuugaauua...............	6	1	0A2
....gaauuccggaucuuUaauua...............	2	1	0A2
....gaauuccggaucuugaauCa...............	168	1	0A2
....gaauuccggaucuugGauua...............	2	1	0A2
....gaauuccggaucuugaauua...............	1670	0	0A2
....gaaGuccggaucuugaauua...............	2	1	0A2
....Naauuccggaucuugaauua...............	4	1	0A2
....gaGuuccggaucuugaauua...............	46	1	0A2
....gaauuccggaucuugaaGua...............	2	1	0A2
....gaauuccggaCcuugaauuag..............	2	1	0A2
....gaauuccggaucuugaauuag..............	1086	0	0A2
....gaauuccggGucuugaauuag..............	2	1	0A2
....Naauuccggaucuugaauuag..............	4	1	0A2
....gaauuccggauAuugaauuag..............	2	1	0A2
....gaauuccggaucuAgaauuag..............	5374	1	0A2
....gaauuccggaucuugaauuagg.............	48	0	0A2
....gaauuccggaucuAgaauuagg.............	10	1	0A2
....aauuccggaucuugaauu................	20	0	0A2
....aauuccggaucuugaauAa...............	32	1	0A2
....Nauuccggaucuugaauua...............	2	1	0A2
....aauuccggCucuugaauua...............	2	1	0A2
....aGuuccggaucuugaauua...............	14	1	0A2
....aauuAcggaucuugaauua...............	2	1	0A2
....aauuccggaucuugaauCa...............	34	1	0A2
....aauuccggaucuugaauua...............	1052	0	0A2
....aauuGcggaucuugaauua...............	2	1	0A2
....aNuuccggaucuugaauuag..............	2	1	0A2
....aGuuccggaucuugaauuag..............	138	1	0A2
....aauuccggaucAugaauuag..............	12	1	0A2
....aauuccggaucuCgaauuag..............	6	1	0A2
....aauuccggCucuugaauuag..............	4	1	0A2
....aauuccggaucuuUaauuag..............	12	1	0A2
....aauuccCgaucuugaauuag..............	8	1	0A2
....aauuccggaucuugCauuag..............	2	1	0A2
....aauuccggaucuugaauCag..............	30	1	0A2
....aauuccggaucuugaaCuag..............	2	1	0A2
....aauuccggaucuugGauuag..............	12	1	0A2
....aauuAcggaucuugaauuag..............	2	1	0A2
....aCuuccggaucuugaauuag..............	4	1	0A2
....Nauuccggaucuugaauuag..............	24	1	0A2
....aauuccggaGcuugaauuag..............	2	1	0A2
....aauuccggaucuugaauuag..............	7498	0	0A2
....aauuccggaucuGgaauuag..............	4	1	0A2
....aauuccggGucuugaauuag..............	2	1	0A2
....aauuccggaCcuugaauuag..............	2	1	0A2
....aauuccggaucuugaauAag..............	2	1	0A2
....aauuccggaucuugaaGuag..............	2	1	0A2
....aauuccggaucuugaaCuagg.............	2	1	0A2
....aauuccggaucuugaauuagA.............	38	1	0A2
....aauuccggaucuGgaauuagg.............	2	1	0A2
....Nauuccggaucuugaauuagg.............	2	1	0A2
....aauuccggaucuugaauuagg.............	540	0	0A2

Star **Mature**

ggaucuugaauuaggcuguuucauuuuggaucuugaauuccggaucuugaauuaggcuguuugguuuugaaucuugaauuccggaucuugaauuaggcuguuugguuuug			
....aauuccggaucuCgaauuagg.............	6	1	0A2
....aauuccggaucuAgaauuagg.............	22	1	0A2
....aauuccggGucuugaauuagg.............	2	1	0A2
....auuccggaucuugaaCua...............	4	1	0A2
....Guuccggaucuugaauua...............	8	1	0A2
....auuccggaucuugaauua...............	366	0	0A2
....auuccggaucuugaauAa...............	8	1	0A2
....Nuuccggaucuugaauua...............	4	1	0A2
....auuccggaucuugaauCa...............	20	1	0A2
....auuccggGucuugaauuag..............	4	1	0A2
....auuccgCaucuugaauuag..............	2	1	0A2
....auuccggaucuugaauAag..............	6	1	0A2
....auuccggCucuugaauuag..............	4	1	0A2
....auuccggaucuugaauuag..............	2084	0	0A2
....auuccggaucuCgaauuag..............	4	1	0A2
....Cuuccggaucuugaauuag..............	10	1	0A2
....auuccggaucuuUaauuag..............	6	1	0A2
....auuccggaucuGgaauuag..............	4	1	0A2
....Nuuccggaucuugaauuag..............	8	1	0A2
....Guuccggaucuugaauuag..............	24	1	0A2
....auuccggaGcuugaauuag..............	4	1	0A2
....auuccggaucAugaauuag..............	24	1	0A2
....auuccggaucuugaauCag..............	18	1	0A2
....auuccggaucuGgaauuagg.............	2	1	0A2
....auuccggaucuugaauuaUg.............	28	1	0A2
....auuccggaucuAgaauuagg.............	58	1	0A2
....auuccggaucuuUaauuagg.............	6	1	0A2
....auuccggaucuugaauuCgg.............	2	1	0A2
....auuccggaucuugaauuagC.............	8	1	0A2
....auuccggaucuugaaCuagg.............	8	1	0A2
....Nuuccggaucuugaauuagg.............	8	1	0A2
....auuccggaucuugaauuagA.............	24	1	0A2
....auuccggaucAugaauuagg.............	44	1	0A2
....Guuccggaucuugaauuagg.............	82	1	0A2
....auuAcggaucuugaauuagg.............	16	1	0A2
....auuccggGucuugaauuagg.............	2	1	0A2
....Cuuccggaucuugaauuagg.............	8	1	0A2
....auuccggaucuugaauuagg.............	4064	0	0A2
....auuccggaucuugaauGagg.............	4	1	0A2
....auuccggaucuCgaauuagg.............	6	1	0A2
....auuccggaucuugGauuagg.............	6	1	0A2
....uuccggaucuugaauCag..............	58	1	0A2
....uuccggaucuCgaauuag..............	2	1	0A2
....uuccggGucuugaauuag..............	2	1	0A2
....Nuccggaucuugaauuag..............	4	1	0A2
....uuccggCucuugaauuag..............	4	1	0A2
....uuccggaucuugaauuag..............	1958	0	0A2
....uuccggaCcuugaauuag..............	2	1	0A2
....uuccggaucuugGauuag..............	6	1	0A2
....uuccggaucAugaauuag..............	6	1	0A2
....uuccggauAuugaauuag..............	2	1	0A2
....uuccggaucuugGauuagg.............	2	1	0A2
....uuccggaucuugaauuaUg.............	4	1	0A2
....uuccggaucuugaauuagA.............	22	1	0A2
....uuAcggaucuugaauuagg.............	2	1	0A2
....uuccggaucuAgaauuagg.............	20	1	0A2
....uuccggGucuugaauuagg.............	6	1	0A2
....Nuccggaucuugaauuagg.............	4	1	0A2
....uuccggaucuugaaCuagg.............	8	1	0A2
....uuccggaGcuugaauuagg.............	4	1	0A2
....uuccggCucuugaauuagg.............	2	1	0A2
....uuccggaucuugCauuagg.............	4	1	0A2
....uuccggaucAugaauuagg.............	42	1	0A2
....uuccggaucuugaauuagg.............	2276	0	0A2
....uuccggaucuugaaGuagg.............	2	1	0A2
....uuccggaucuGgaauuagg.............	8	1	0A2
....uucGggaucuugaauuagg.............	12	1	0A2
....uuccggaucuuUaauuagg.............	4	1	0A2
....uuccggaucuugaauuagUcu...........	6	1	0A2
....uuccggaucuugaauuaggcuC..........	8	1	0A2

Star Mature

```
ggaucuugaauuaggcuguuucauuuuggaucuugaauuccggaucuugaauuaggcuguuugguuuugaaucuugaauuccggaucuugaauuaggcuguuugguuuug
...............................................................................uAcggaucuugaauuagg.............   4     1   0A2
...............................................................................uccggaucAugaauuagg.............   10    1   0A2
...............................................................................uccggaucuAgaauuagg.............   6     1   0A2
...............................................................................uccggaCcuugaauuagg.............   2     1   0A2
...............................................................................uccggaucuGgaauuagg.............   6     1   0A2
...............................................................................Nccggaucuugaauuagg.............   4     1   0A2
...............................................................................uccggaucuugaauuagA.............   2     1   0A2
...............................................................................uccggaucuugaauuagg.............   632   0   0A2
...............................................................................uccggaucuugaauuaggcuA..........   2     1   0A2

................uguuucauuGuggaucuuga..........................................................................   2     1   0B2
................uguGucauuuuggaucuugaa.........................................................................   6     1   0B2
..................uuucauuCuggaucuugaau........................................................................   2     1   0B2
..................uuucauuGuggaucuugaauu.......................................................................   1     1   0B2
..................uuucauuCuggaucuugaauu.......................................................................   7     1   0B2
..................uuucauuuuggaucuugaauA.......................................................................   4     1   0B2
..................uuucauuCuggaucuugaauuc......................................................................   18    1   0B2
..................uuucauuCuggaucuugaauucc.....................................................................   10    1   0B2
...................uucauuCuggaucuugaauu.......................................................................   7     1   0B2
...................uucauuGuggaucuugaauu.......................................................................   4     1   0B2
...................uucauuGuggaucuugaauuc......................................................................   1     1   0B2
...................uucauuCuggaucuugaauuc......................................................................   48    1   0B2
...................uucGuuuuggaucuugaauucc.....................................................................   2     1   0B2
...................uucauuCuggaucuugaauucc.....................................................................   11    1   0B2
....................ucauuCuggaucuugaauuc......................................................................   44    1   0B2
....................ucauuuuggaucuugaauuA......................................................................   3     1   0B2
....................ucauuuuggaucuugaauucU.....................................................................   2     1   0B2
....................ucauuuuggaucuugaauucA.....................................................................   19    1   0B2
....................ucauuCuggaucuugaauucc.....................................................................   49    1   0B2
.....................cauuCuggaucuugaauuc......................................................................   9     1   0B2
.....................cauuuuggaucuugaauucA.....................................................................   2     1   0B2
.....................cGuuuuggaucuugaauucc.....................................................................   2     1   0B2
.....................cauuCuggaucuugaauucc.....................................................................   41    1   0B2
.....................cauuuuggaucuugaauuccg....................................................................   1     0   0B2
.....................cauuCuggaucuugaauuccg....................................................................   2     1   0B2
......................auuCuggaucuugaauucc.....................................................................   25    1   0B2
......................auuCuggaucuugaauuccg....................................................................   8     1   0B2
......................auuuuggaucuugaauucUg....................................................................   2     1   0B2
......................aCuuuggaucuugaauuccg....................................................................   5     1   0B2
......................auuuuggaucuugaauuccg....................................................................   2     0   0B2
......................auuuuggGucuugaauuccg....................................................................   4     1   0B2
......................auuuuggaucuugaauuccgg...................................................................   5     0   0B2
.......................uuCuggaucuugaauuccg....................................................................   5     1   0B2
.......................uuuuggaucuugaauuccg....................................................................   1     0   0B2
.......................uuuuggaucuugaauucUg....................................................................   3     1   0B2
.......................uuuuggGucuugaauuccg....................................................................   3     1   0B2
.......................uuuuggaucuugaauucUgga..................................................................   1     1   0B2
.......................uuuuggGucuugaauuccgga..................................................................   17    1   0B2
.......................uuuuggaucuugaauuccgga..................................................................   4     0   0B2
........................uuuggUucuugaauuccgg...................................................................   3     1   0B2
........................uuuggGucuugaauuccgga..................................................................   1     1   0B2
........................uuuggaucuugaauuccgga..................................................................   1     0   0B2
.........................uuggGucuugaauuccgga..................................................................   2     1   0B2
.........................uuggGucuugaauuccggau.................................................................   2     1   0B2
.........................uuggGucuugaauuccggauc................................................................   2     1   0B2
.........................uuggaucuugaauuccggaucA...............................................................   3     1   0B2
..........................uggaucuugaauuUcgga..................................................................   1     1   0B2
..........................uggaucuugaauuccCgaucuugaauu.........................................................   1     1   0B2
.............................aucuugaauuccggaucu...............................................................   8     0   0B2
.............................aucuugaauuccggaucuA..............................................................   4     1   0B2
.............................aucuugaauuccggaucuu..............................................................   12    0   0B2
.............................aucGugaauuccggaucuuga............................................................   14    1   0B2
.............................aucuugaauuccggaucuuga............................................................   10    0   0B2
.............................Cucuugaauuccggaucuugaa...........................................................   4     1   0B2
.............................aucuugaauuccggaucuugaau..........................................................   22    0   0B2
.............................aucuugaauuccggaucuugaauua........................................................   4     0   0B2
..............................ucuugaauuccggaucuA..............................................................   2     1   0B2
..............................ucuugaauuccggaucuu..............................................................   2     0   0B2
..............................ucuugaauuccggaucuug.............................................................   10    0   0B2
..............................ucuugaauuccggaucuAg.............................................................   22    1   0B2
```

Star **Mature**

ggaucuugaauuaggcuguuucauuuuggaucuugaauuccggaucuugaauuaggcuguuugguuuugaaucuugaauuccggaucuugaauuaggcuguuugguuuug			
..............................ucuugaauuccggaucuuga..	30	0	0B2
..............................ucuugaauuccggaucuAga..	88	1	0B2
..............................ucGugaauuccggaucuugaa...	52	1	0B2
..............................ucuugaauuccggaucuGgaa...	8	1	0B2
..............................ucuugaauuccggaucuugaa...	40	0	0B2
..............................ucuugaauuccggaucuAgaa...	88	1	0B2
..............................ucuugaauuccggaucuAgaau..	32	1	0B2
..............................ucuugaauuccggaucuugaaG..	10	1	0B2
..............................ucGugaauuccggaucuugaau..	36	1	0B2
..............................Ccuugaauuccggaucuugaau..	2	1	0B2
..............................Ncuugaauuccggaucuugaau..	6	1	0B2
..............................ucuugaauuccggaucuCgaau..	4	1	0B2
..............................ucuugaauuccggaucuugaau..	524	0	0B2
..............................ucuugaauuccggaucuAgaauu...	20	1	0B2
..............................uUuugaauuccggaucuugaauu...	6	1	0B2
..............................ucuugaauuccggaucuugaauu...	84	0	0B2
..............................Ncuugaauuccggaucuugaauu...	2	1	0B2
..............................uGuugaauuccggaucuugaauu...	14	1	0B2
..............................ucuugaauuccggaucuugaauua..	126	0	0B2
..............................uGuugaauuccggaucuugaauua..	12	1	0B2
..............................uUuugaauuccggaucuugaauua..	18	1	0B2
..............................ucuugaauuccggaucuAgaauua..	16	1	0B2
..............................ucuugaauuccggaucuugaauAa..	132	1	0B2
..............................ucuugaauuccggaucuugaauCa..	4	1	0B2
..............................ucuugaauuccggaucuugaauuag...	2	0	0B2
..............................ucuugaauuccggaucuugaauAag...	6	1	0B2
..............................ucuugaauuccggaucuAgaauuag...	22	1	0B2
..............................ucuugaauuccggaucuugaauuagg..	4	0	0B2
..............................ucuugaauuccggaucuugaaCuagg..	4	1	0B2
...............................cuugaauuccggaucuug...	4	0	0B2
...............................cuugaauuccggaucuAg...	2	1	0B2
...............................cuugaauuccggaucuuga..	10	0	0B2
...............................cuugaauuccggaucuAga..	22	1	0B2
...............................cGugaauuccggaucuugaa...	8	1	0B2
...............................cuugaauuccggaucuAgaa...	732	1	0B2
...............................Nuugaauuccggaucuugaa...	2	1	0B2
...............................cuugaauuccggaucuugaa...	180	0	0B2
...............................cuugaauuccggaucuAgaau..	30	1	0B2
...............................cGugaauuccggaucuugaau..	56	1	0B2
...............................Nuugaauuccggaucuugaau..	2	1	0B2
...............................cuugaauuccggaucuugaau..	408	0	0B2
...............................cuugaauuccggaucuugaaC..	2	1	0B2
...............................cuugaauuccggaGcuugaau..	2	1	0B2
...............................cuugaauuccggaucuugaauu...	468	0	0B2
...............................cuugaauuccggaucuAgaauu...	148	1	0B2
...............................Nuugaauuccggaucuugaauu...	2	1	0B2
...............................cuugaauuccggaucuAgaauua..	74	1	0B2
...............................Guugaauuccggaucuugaauua..	2	1	0B2
...............................cuugaauuccggauGuugaauua..	14	1	0B2
...............................cuugaauuccggaucuugaauCa..	54	1	0B2
...............................Nuugaauuccggaucuugaauua..	4	1	0B2
...............................cuugaauuccggaucuugaauAa..	76	1	0B2
...............................cuugaauuccggaucuugaauua..	478	0	0B2
...............................cuugaauuccggaucuugGauua..	6	1	0B2
...............................cuugaauuccggaucuAgaauuag...	16	1	0B2
...............................cuugaauuccggaucuugaauuag...	4	0	0B2
...............................cuugaauuccggaucuugaauuagg..	2	0	0B2
................................uugaauuccggaucuAga..	28	1	0B2
................................uugaauuccggaucuugaa...	106	0	0B2
................................uugaauuccggaucuAgaa...	568	1	0B2
................................uugaauuccggaucuGgaa...	2	1	0B2
................................uugaauuccggCucuugaa...	2	1	0B2
................................uugaauuccggauAuugaau..	2	1	0B2
................................uugaGuuccggaucuugaau..	182	1	0B2
................................Nugaauuccggaucuugaau..	146	1	0B2
................................uugaauuccggaucuGgaau..	18	1	0B2
................................uugaCuuccggaucuugaau..	2	1	0B2
................................uugaauuccggaucuAgaau..	3882	1	0B2
................................uGgaauuccggaucuugaau..	2	1	0B2
................................uugaauuccggaucuCgaau..	4	1	0B2

```
                     Star                                              Mature
ggaucuugaauuaggcuguuucauuuuggaucuugaauuccggaucuugaauuaggcuguuugguuuugaaucuugaauuccggaucuugaauuaggcuguuugguuuug
................................uugaauuccggaGcuugaau..........................................................    8       1    0B2
................................uugaauuccggCucuugaau..........................................................    2       1    0B2
................................uugaauuccgCaucuugaau..........................................................    8       1    0B2
................................Gugaauuccggaucuugaau..........................................................    14      1    0B2
................................uugaauuccggGucuugaau..........................................................    16      1    0B2
................................uugaauuccggaCcuugaau..........................................................    6       1    0B2
................................uugaauuccggaucuugaaC..........................................................    20      1    0B2
................................Cugaauuccggaucuugaau..........................................................    16      1    0B2
................................uugaauuccggaucAugaau..........................................................    8       1    0B2
................................uugaauuccggaucuugaaG..........................................................    2       1    0B2
................................uugaauuccggaucuugaau..........................................................    18978   0    0B2
................................uugaauuccggauNuugaauu.........................................................    2       1    0B2
................................uugaGuuccggaucuugaauu.........................................................    332     1    0B2
................................uugaauuccggauAuugaauu.........................................................    2       1    0B2
................................uugaauuccggaucuugaaGu.........................................................    6       1    0B2
................................Gugaauuccggaucuugaauu.........................................................    18      1    0B2
................................uugaauuccggaucuCgaauu.........................................................    18      1    0B2
................................Nugaauuccggaucuugaauu.........................................................    154     1    0B2
................................uugaauuccggaucCugaauu.........................................................    8       1    0B2
................................Augaauuccggaucuugaauu.........................................................    6       1    0B2
................................uugaauuccggaucuugGauu.........................................................    12      1    0B2
................................uugaauAccggaucuugaauu.........................................................    2       1    0B2
................................uugaaGuccggaucuugaauu.........................................................    8       1    0B2
................................uugaauuAcggaucuugaauu.........................................................    6       1    0B2
................................uugaauuccggaucuugaauu.........................................................    19318   0    0B2
................................uugaauuccggaucuugaaCu.........................................................    66      1    0B2
................................uugaauuccggaucuAgaauu.........................................................    5504    1    0B2
................................uugaauuccggGucuugaauu.........................................................    2       1    0B2
................................uugaauuccggaCcuugaauu.........................................................    4       1    0B2
................................Cugaauuccggaucuugaauu.........................................................    4       1    0B2
................................uugaauuccggaucuGgaauu.........................................................    4       1    0B2
................................uugaauuccggaGcuugaauu.........................................................    6       1    0B2
................................uugaauuccggCucuugaauu.........................................................    4       1    0B2
................................uugaauuccggaCcuugaauua........................................................    6       1    0B2
................................uugaauNccggaucuugaauua........................................................    2       1    0B2
................................uugaauuccggaucuugaauCa........................................................    1868    1    0B2
................................uugaauuccggaucuGgaauua........................................................    4       1    0B2
................................uugaauuccggNucuugaauua........................................................    2       1    0B2
................................Gugaauuccggaucuugaauua........................................................    8       1    0B2
................................uGgaauuccggaucuugaauua........................................................    4       1    0B2
................................uugaauuccggCucuugaauua........................................................    18      1    0B2
................................Cugaauuccggaucuugaauua........................................................    18      1    0B2
................................Augaauuccggaucuugaauua........................................................    6       1    0B2
................................uugaauuGcggaucuugaauua........................................................    4       1    0B2
................................uugaauuccggaucuugaauua........................................................    21770   0    0B2
................................uugaauuccggaucuCgaauua........................................................    30      1    0B2
................................uugaaGuccggaucuugaauua........................................................    24      1    0B2
................................uugaauuAcggaucuugaauua........................................................    2       1    0B2
................................uugaauuccggauAuugaauua........................................................    2       1    0B2
................................uugaauuccggaGcuugaauua........................................................    8       1    0B2
................................uugaauuccggaucuugaauAa........................................................    1638    1    0B2
................................Nugaauuccggaucuugaauua........................................................    182     1    0B2
................................uugaauuccggaucuugUauua........................................................    2       1    0B2
................................uugaauuccggaucuugGauua........................................................    36      1    0B2
................................uugaGuuccggaucuugaauua........................................................    526     1    0B2
................................uugaauuccggaucuuUaauua........................................................    2       1    0B2
................................uugaauuccggGucuugaauua........................................................    4       1    0B2
................................uugaCuuccggaucuugaauua........................................................    4       1    0B2
................................uugaauuccggaucuAgaauua........................................................    5186    1    0B2
................................uugaauuccggaucuugaaCua........................................................    32      1    0B2
................................Nugaauuccggaucuugaauuag.......................................................    10      1    0B2
................................uugaauuccggaucuAgaauuag.......................................................    1514    1    0B2
................................uugaauuccggaucuugaauAag.......................................................    8       1    0B2
................................uugaauuccggaucuugaaCuag.......................................................    8       1    0B2
................................uugaauuccggaucuugaauuag.......................................................    486     0    0B2
................................uugaaGuccggaucuugaauuag.......................................................    4       1    0B2
................................uugaauuccggauGuugaauuag.......................................................    2       1    0B2
................................Cugaauuccggaucuugaauuag.......................................................    18      1    0B2
................................uugaauuccCgaucuugaauuag.......................................................    2       1    0B2
................................uugaGuuccggaucuugaauuag.......................................................    28      1    0B2
```

Star Mature

```
ggaucuugaauuaggcuguuucauuuuggaucuugaauuccggaucuugaauuaggcuguuugguuuugaaucuugaauuccggaucuugaauuaggcuguuugguuuug
................................uugaauuccggaucuugaaCuagg......................................................   4      1   0B2
................................uugaauuccggaucuugaauuagA......................................................   6      1   0B2
................................uugaGuuccggaucuugaauuagg......................................................   6      1   0B2
.................................ugaauuccggaucuugaa...........................................................   10     0   0B2
.................................ugaauuccggaucuAgaa...........................................................   140    1   0B2
.................................ugaauuccggaucuAgaau..........................................................   298    1   0B2
.................................ugaGuuccggaucuugaau..........................................................   14     1   0B2
.................................Ngaauuccggaucuugaau..........................................................   4      1   0B2
.................................ugaauuccggaucuugaau..........................................................   996    0   0B2
.................................ugaauuccggaGcuugaau..........................................................   4      1   0B2
.................................ugaaGuccggaucuugaauu.........................................................   10     1   0B2
.................................Ngaauuccggaucuugaauu.........................................................   28     1   0B2
.................................ugaGuuccggaucuugaauu.........................................................   132    1   0B2
.................................ugaauuccggaucuCgaauu.........................................................   4      1   0B2
.................................ugaauuccggaucuugaauu.........................................................   6110   0   0B2
.................................ugaauuccggaucuuUaauu.........................................................   2      1   0B2
.................................ugaauuccggaGcuugaauu.........................................................   2      1   0B2
.................................ugaauuccggaucuAgaauu.........................................................   2800   1   0B2
.................................ugaauuccggaucuugaaGu.........................................................   2      1   0B2
.................................ugaauuccggGucuugaauu.........................................................   4      1   0B2
.................................ugaauuccggauAuugaauu.........................................................   2      1   0B2
.................................ugaauuccggaucCugaauu.........................................................   4      1   0B2
.................................Ngaauuccggaucuugaauua........................................................   112    1   0B2
.................................ugaauuccggaucuugaaCua........................................................   12     1   0B2
.................................ugaauuAcggaucuugaauua........................................................   2      1   0B2
.................................ugaauuccggGucuugaauua........................................................   2      1   0B2
.................................ugaauuccggaucuugaauCa........................................................   560    1   0B2
.................................ugaGuuccggaucuugaauua........................................................   292    1   0B2
.................................ugaauuccggaucuugGauua........................................................   20     1   0B2
.................................ugaauuccggauAuugaauua........................................................   8      1   0B2
.................................Ggaauuccggaucuugaauua........................................................   8      1   0B2
.................................ugaauuccgCaucuugaauua........................................................   2      1   0B2
.................................ugaauuccggaucCugaauua........................................................   6      1   0B2
.................................ugaauuccggaucuugaauua........................................................   15826  0   0B2
.................................ugaauuccggaCcuugaauua........................................................   4      1   0B2
.................................ugaauuccggaucuugaauAa........................................................   116    1   0B2
.................................ugaauuccggaucuAgaauua........................................................   6298   1   0B2
.................................ugaauuccggaucuCgaauua........................................................   6      1   0B2
.................................uNaauuccggaucuugaauua........................................................   2      1   0B2
.................................ugaaGuccggaucuugaauua........................................................   44     1   0B2
.................................ugaauuccggaucuGgaauua........................................................   2      1   0B2
.................................ugaauuccCgaucuugaauua........................................................   2      1   0B2
.................................ugaauuccggaucAugaauua........................................................   8      1   0B2
.................................ugaauuccggaGcuugaauua........................................................   10     1   0B2
.................................ugaauuccggaucuugUauua........................................................   2      1   0B2
.................................ugaauuccggaucuugGauuag.......................................................   2      1   0B2
.................................ugaauuccggaucuugaauuag.......................................................   1450   0   0B2
.................................ugaauuccggaucuAgaauuag.......................................................   5118   1   0B2
.................................Ngaauuccggaucuugaauuag.......................................................   12     1   0B2
.................................ugaauuccggaucuGgaauuag.......................................................   2      1   0B2
.................................ugaauuccggGucuugaauuag.......................................................   2      1   0B2
.................................ugaauuccggaucuugaauCag.......................................................   26     1   0B2
.................................ugaauuccggaucuugaauAag.......................................................   18     1   0B2
.................................ugaGuuccggaucuugaauuag.......................................................   26     1   0B2
.................................ugaauuccggaucuugaauuagg......................................................   122    0   0B2
.................................ugaaGuccggaucuugaauuagg......................................................   2      1   0B2
.................................ugaauuccggaucuugaauuaUg......................................................   2      1   0B2
.................................ugaauuccggaucuugaaCuagg......................................................   2      1   0B2
.................................ugaauuccggaucuugaauuagA......................................................   2      1   0B2
..................................gaauuccggaucuugaau..........................................................   42     0   0B2
..................................gaauuccggaucuAgaau..........................................................   2      1   0B2
..................................gaauuccggaucuugaauu.........................................................   262    0   0B2
..................................gaauuccggaucuAgaauu.........................................................   172    1   0B2
..................................gaauuccggaucuAgaauua........................................................   862    1   0B2
..................................gaGuuccggaucuugaauua........................................................   14     1   0B2
..................................gaauuccggaucAugaauua........................................................   4      1   0B2
..................................gaauuccggaucuugCauua........................................................   2      1   0B2
..................................gaauuccggaucuugaauua........................................................   1374   0   0B2
..................................gaaGuccggaucuugaauua........................................................   6      1   0B2
..................................gaauuccggaucuugaauCa........................................................   118    1   0B2
```

Star Mature

ggaucuugaauuaggcuguuucauuuuggaucuugaauuccggaucuugaauuaggcuguuugguuuugaaucuugaauuccggaucuugaauuaggcuguuugguuuug			
..................................gaauuccggaucuugaauAa..	4	1	0B2
..................................Naauuccggaucuugaauua..	20	1	0B2
..................................gaGuuccggaucuugaauuag...	2	1	0B2
..................................gaauuccggaucuAgaauuag...	4018	1	0B2
..................................gaauuccggaucCugaauuag...	2	1	0B2
..................................gaauuccggaucuugaauCag...	6	1	0B2
..................................gaauuccggaucuugaaCuag...	6	1	0B2
..................................gaauuccggaucuCgaauuag...	2	1	0B2
..................................Naauuccggaucuugaauuag...	10	1	0B2
..................................gaauuccggaucuugaauuag...	984	0	0B2
..................................Aaauuccggaucuugaauuagg..	16	1	0B2
..................................gaauuccggaucuugaauuagA..	2	1	0B2
..................................gaauuccggaucuugaauGagg..	2	1	0B2
..................................gaauuccggaucuugaauuagg..	22	0	0B2
...................................aauuccggaucuugaauu...	14	0	0B2
...................................aauuccggaucuugaauAa..	8	1	0B2
...................................aauuccggaucuugaauua..	626	0	0B2
...................................aauuccggaucuCgaauua..	2	1	0B2
...................................aCuuccggaucuugaauua..	2	1	0B2
...................................aauuccggaCcuugaauua..	2	1	0B2
...................................aauuccggaucuugaauCa..	16	1	0B2
...................................aGuuccggaucuugaauuag...	70	1	0B2
...................................aauuccggaucuugaauCag...	26	1	0B2
...................................aauuccggaGcuugaauuag...	2	1	0B2
...................................aauuccggaucuugGauuag...	22	1	0B2
...................................aauuccggaucuugaauAag...	2	1	0B2
...................................aauuccggaucAugaauuag...	2	1	0B2
...................................aauuccggaucuugaaGuag...	2	1	0B2
...................................aauuccggaucuCgaauuag...	4	1	0B2
...................................aauAccggaucuugaauuag...	4	1	0B2
...................................aaGuccggaucuugaauuag...	14	1	0B2
...................................aauuccggaucuugaauuag...	6030	0	0B2
...................................Nauuccggaucuugaauuag...	24	1	0B2
...................................aauuccggaucuuUaauuag...	14	1	0B2
...................................aauuccggaCcuugaauuag...	2	1	0B2
...................................aauuccggaucuugaauuagA..	18	1	0B2
...................................aauuccggaucuAgaauuagg..	20	1	0B2
...................................aauuccggaucuugaauuaUg..	8	1	0B2
...................................Nauuccggaucuugaauuagg..	4	1	0B2
...................................aauuccggaucuugGauuagg..	2	1	0B2
...................................aauuccggaucuCgaauuagg..	2	1	0B2
...................................aauuccggaCcuugaauuagg..	2	1	0B2
...................................aauuccggaucuugaauuagg..	486	0	0B2
....................................auuccggaucuugaauCa..	8	1	0B2
....................................Nuuccggaucuugaauua..	4	1	0B2
....................................auuccggaGcuugaauua..	2	1	0B2
....................................auuccggaucuugaauAa..	2	1	0B2
....................................auuccggaucuugaauua..	220	0	0B2
....................................auuccggaucAugaauuag...	10	1	0B2
....................................auuccggaucuugaauCag...	16	1	0B2
....................................Guuccggaucuugaauuag...	18	1	0B2
....................................auuccggaucuugaauuag...	1402	0	0B2
....................................auuccggGucuugaauuag...	2	1	0B2
....................................auuccggauAuugaauuag...	2	1	0B2
....................................auuccggaGcuugaauuag...	4	1	0B2
....................................Nuuccggaucuugaauuag...	6	1	0B2
....................................auuccggaGcuugaauuagg..	4	1	0B2
....................................auuccggaucuCgaauuagg..	6	1	0B2
....................................auuccggaucuugaaGuagg..	2	1	0B2
....................................auuccggaucuugaauAagg..	2	1	0B2
....................................auuccggaucAugaauuagg..	56	1	0B2
....................................auuccggaucuugaauuagA..	88	1	0B2
....................................auucGggaucuugaauuagg..	12	1	0B2
....................................auuccggGucuugaauuagg..	12	1	0B2
....................................auuccgCaucuugaauuagg..	2	1	0B2
....................................Guuccggaucuugaauuagg..	66	1	0B2
....................................auuccggaucuugaauuagg..	3760	0	0B2
....................................auuccggaucuAgaauuagg..	38	1	0B2
....................................auuccggaucuugGauuagg..	4	1	0B2
....................................auuccggaucuugaauGagg..	4	1	0B2

Star Mature

ggaucuugaauuaggcuguuucauuuuggaucuugaauuccggaucuugaauuaggcuguuugguuuugaaucuugaauuccggaucuugaauuaggcuguuugguuuug

....................................Nuuccggaucuugaauuagg..	30	1	0B2
....................................auuccggCucuugaauuagg..	2	1	0B2
....................................auuccggaucuugaauuaUg..	18	1	0B2
....................................auuccggaucuugaaCuagg..	2	1	0B2
....................................auuccggaCcuugaauuagg..	2	1	0B2
....................................auuccggaucuugaauuaggcA..	10	1	0B2
.....................................uuccggaucuugGauuag...	2	1	0B2
.....................................uuccggaucuugaauuag...	1006	0	0B2
.....................................uuccggaucuugaauCag...	10	1	0B2
.....................................Nuccggaucuugaauuag...	12	1	0B2
.....................................uuccggaucuGgaauuag...	4	1	0B2
.....................................uuccggaGcuugaauuag...	2	1	0B2
.....................................uuccggaucuugaauuagg..	1688	0	0B2
.....................................uuccggaucuugGauuagg..	4	1	0B2
.....................................uucGggaucuugaauuagg..	4	1	0B2
.....................................uuccggaGcuugaauuagg..	2	1	0B2
.....................................uuccggaucuGgaauuagg..	8	1	0B2
.....................................uuccggauAuugaauuagg..	2	1	0B2
.....................................uuccggaucuuUaauuagg..	4	1	0B2
.....................................uuccggaucuAgaauuagg..	16	1	0B2
.....................................uuAcggaucuugaauuagg..	4	1	0B2
.....................................Nuccggaucuugaauuagg..	8	1	0B2
.....................................uuccggaucuugaauuagA..	24	1	0B2
.....................................uucAggaucuugaauuagg..	16	1	0B2
.....................................uuccggaucAugaauuagg..	56	1	0B2
.....................................uuccggaucuugaaCuagg..	10	1	0B2
.....................................uuccggaucuugaauuaggcA..	4	1	0B2
......................................Nccggaucuugaauuagg..	2	1	0B2
......................................uccggaucuAgaauuagg..	8	1	0B2
......................................uccggaucAugaauuagg..	2	1	0B2
......................................uccggaucuugaauuagg..	384	0	0B2
......................................uccggGucuugaauuagg..	2	1	0B2
......................................uccggaCcuugaauuagg..	6	1	0B2
....cggaucuugaauuaggcuA...	6	1	0B2
....uuuugaCucuugaauuccg...........................	4	1	0B2
....uuuugaCucuugaauuccgg..........................	9	1	0B2
....uuuugaaucuugaauuccgg..........................	37	0	0B2
....uuuuAaaucuugaauuccgg..........................	2	1	0B2
....uuuugaGucuugaauuccgga.........................	7	1	0B2
....uuuugaaucuugaauuccgga.........................	23	0	0B2
....uuuugaCucuugaauuccgga.........................	10	1	0B2
....Nuuugaaucuugaauuccgga.........................	1	1	0B2
....uuuugaaucGugaauuccgga.........................	2	1	0B2
....uuuugaaucuugaauuccggaucA......................	4	1	0B2
....uuuugaaucuugaauuccggaucuuU....................	2	1	0B2
....uuugaGucuugaauuccgga.........................	3	1	0B2
....uuugaCucuugaauuccgga.........................	1	1	0B2
....uuugaaucuugaauuccgga.........................	13	0	0B2
....uugaaucuugaauuccgga.........................	2	0	0B2
....uugaaucuugaauuccggau........................	4	0	0B2
....uugaGucuugaauuccggau........................	3	1	0B2
....uugaaucuugaauuccggauc.......................	17	0	0B2
....uugaaucuugaauuccggauA.......................	5	1	0B2
....uugaaucuugaauuccggaucA......................	7	1	0B2
....uugaGucuugaauuccggaucu......................	3	1	0B2
....uugaaucuugaauuccggaucuugaau.................	8	0	0B2
....ugaaucuugaauuccggauc.......................	5	0	0B2
....ugaaucuugaauuccggaucA......................	3	1	0B2
....ugaaucuugaauuccggaucuugaau.................	3	0	0B2
....ugaaucuugaauuccggaucuAgaau.................	6	1	0B2
....ugaaucuugaauuccggaucuAgaauu................	7	1	0B2
....ugaaucuugaauuccggaucuugaauu................	3	0	0B2
....gaaucuugaauuccggaucuugaauu................	1	0	0B2
....gaaucuugaauuccggaucuAgaauua...............	3	1	0B2
....aaucuugaauuccggaucuug....................	2	0	0B2
....aaucuugaauuccggaucuAg....................	6	1	0B2
....aucuugaauuccggaucu......................	8	0	0B2
....aucuugaauuccggaucuA.....................	4	1	0B2
....aucuugaauuccggaucuu.....................	12	0	0B2
....aucuugaauuccggaucuuga...................	10	0	0B2

```
                    Star                                                 Mature
ggaucuugaauuaggcuguuucauuuuggaucuugaauuccggaucuugaauuaggcuguuugguuuugaaucuugaauuccggaucuugaauuaggcuguuugguuuug
......................................................................aucGugaauuccggaucuuga...................    14    1    0B2
......................................................................Cucuugaauuccggaucuugaa..................    4     1    0B2
......................................................................aucuugaauuccggaucuugaau.................    22    0    0B2
......................................................................aucuugaauuccggaucuugaauua...............    4     0    0B2
.......................................................................ucuugaauuccggaucuu.....................    2     0    0B2
.......................................................................ucuugaauuccggaucuA.....................    2     1    0B2
.......................................................................ucuugaauuccggaucuAg....................    22    1    0B2
.......................................................................ucuugaauuccggaucuug....................    10    0    0B2
.......................................................................ucuugaauuccggaucuAga...................    88    1    0B2
.......................................................................ucuugaauuccggaucuuga...................    30    0    0B2
.......................................................................ucuugaauuccggaucuAgaa..................    88    1    0B2
.......................................................................ucGugaauuccggaucuugaa..................    52    1    0B2
.......................................................................ucuugaauuccggaucuGgaa..................    8     1    0B2
.......................................................................ucuugaauuccggaucuugaa..................    40    0    0B2
.......................................................................ucGugaauuccggaucuugaau.................    36    1    0B2
.......................................................................Ncuugaauuccggaucuugaau.................    6     1    0B2
.......................................................................ucuugaauuccggaucuugaau.................    524   0    0B2
.......................................................................ucuugaauuccggaucuAgaau.................    32    1    0B2
.......................................................................Ccuugaauuccggaucuugaau.................    2     1    0B2
.......................................................................ucuugaauuccggaucuCgaau.................    4     1    0B2
.......................................................................ucuugaauuccggaucuugaaG.................    10    1    0B2
.......................................................................uGuugaauuccggaucuugaauu................    14    1    0B2
.......................................................................ucuugaauuccggaucuAgaauu................    20    1    0B2
.......................................................................uUuugaauuccggaucuugaauu................    6     1    0B2
.......................................................................ucuugaauuccggaucuugaauu................    84    0    0B2
.......................................................................Ncuugaauuccggaucuugaauu................    2     1    0B2
.......................................................................ucuugaauuccggaucuAgaauua...............    16    1    0B2
.......................................................................uUuugaauuccggaucuugaauua...............    18    1    0B2
.......................................................................ucuugaauuccggaucuugaauAa...............    132   1    0B2
.......................................................................ucuugaauuccggaucuugaauua...............    126   0    0B2
.......................................................................ucuugaauuccggaucuugaauCa...............    4     1    0B2
.......................................................................uGuugaauuccggaucuugaauua...............    12    1    0B2
.......................................................................ucuugaauuccggaucuAgaauuag..............    22    1    0B2
.......................................................................ucuugaauuccggaucuugaauAag..............    6     1    0B2
.......................................................................ucuugaauuccggaucuugaauuag..............    2     0    0B2
.......................................................................ucuugaauuccggaucuugaaCuagg.............    4     1    0B2
.......................................................................ucuugaauuccggaucuugaauuagg.............    4     0    0B2
........................................................................cuugaauuccggaucuAg....................    2     1    0B2
........................................................................cuugaauuccggaucuug....................    4     0    0B2
........................................................................cuugaauuccggaucuAga...................    22    1    0B2
........................................................................cuugaauuccggaucuuga...................    10    0    0B2
........................................................................cuugaauuccggaucuugaa..................    180   0    0B2
........................................................................Nuugaauuccggaucuugaa..................    2     1    0B2
........................................................................cGugaauuccggaucuugaa..................    8     1    0B2
........................................................................cuugaauuccggaucuAgaa..................    732   1    0B2
........................................................................cuugaauuccggaucuugaau.................    408   0    0B2
........................................................................cuugaauuccggaucuAgaau.................    30    1    0B2
........................................................................Nuugaauuccggaucuugaau.................    2     1    0B2
........................................................................cuugaauuccggaucuugaaC.................    2     1    0B2
........................................................................cuugaauuccggaGcuugaau.................    2     1    0B2
........................................................................cGugaauuccggaucuugaau.................    56    1    0B2
........................................................................cuugaauuccggaucuAgaauu................    148   1    0B2
........................................................................cuugaauuccggaucuugaauu................    468   0    0B2
........................................................................Nuugaauuccggaucuugaauu................    2     1    0B2
........................................................................Nuugaauuccggaucuugaauua...............    4     1    0B2
........................................................................cuugaauuccggaucuugaauCa...............    54    1    0B2
........................................................................cuugaauuccggauGuugaauua...............    14    1    0B2
........................................................................cuugaauuccggaucuugGauua...............    6     1    0B2
........................................................................cuugaauuccggaucuAgaauua...............    74    1    0B2
........................................................................cuugaauuccggaucuugaauAa...............    76    1    0B2
........................................................................cuugaauuccggaucuugaauua...............    478   0    0B2
........................................................................Guugaauuccggaucuugaauua...............    2     1    0B2
........................................................................cuugaauuccggaucuugaauuag..............    4     0    0B2
........................................................................cuugaauuccggaucuAgaauuag..............    16    1    0B2
........................................................................cuugaauuccggaucuugaauuagg.............    2     0    0B2
.........................................................................uugaauuccggaucuAga...................    28    1    0B2
.........................................................................uugaauuccggaucuugaa..................    106   0    0B2
.........................................................................uugaauuccggaucuGgaa..................    2     1    0B2
.........................................................................uugaauuccggCucuugaa..................    2     1    0B2
.........................................................................uugaauuccggaucuAgaa..................    568   1    0B2
```

```
                    Star                                                  Mature
ggaucuugaauuaggcuguuucauuuuggaucuugaauuccggaucuugaauuaggcuguuugguuuugaaucuugaauuccggaucuugaauuaggcuguuugguuuug
.........................................................................Nugaauuccggaucuugaau.................   146     1   0B2
.........................................................................uugaauuccggauAuugaau.................   2       1   0B2
.........................................................................uugaauuccgCaucuugaau.................   8       1   0B2
.........................................................................Cugaauuccggaucuugaau.................   16      1   0B2
.........................................................................uugaauuccggaucAugaau.................   8       1   0B2
.........................................................................uugaauuccggaCcuugaau.................   6       1   0B2
.........................................................................uugaauuccggaucuugaaC.................   20      1   0B2
.........................................................................uugaCuuccggaucuugaau.................   2       1   0B2
.........................................................................uugaauuccggaGcuugaau.................   8       1   0B2
.........................................................................Gugaauuccggaucuugaau.................   14      1   0B2
.........................................................................uugaauuccggaucuAgaau.................   3882    1   0B2
.........................................................................uGgaauuccggaucuugaau.................   2       1   0B2
.........................................................................uugaauuccggaucuugaau.................   18978   0   0B2
.........................................................................uugaauuccggaucuugaaG.................   2       1   0B2
.........................................................................uugaauuccggGucuugaau.................   16      1   0B2
.........................................................................uugaauuccggaucuGgaau.................   18      1   0B2
.........................................................................uugaGuuccggaucuugaau.................   182     1   0B2
.........................................................................uugaauuccggaucuCgaau.................   4       1   0B2
.........................................................................uugaauuccggCucuugaau.................   2       1   0B2
.........................................................................Gugaauuccggaucuugaauu................   18      1   0B2
.........................................................................uugaaGuccggaucuugaauu................   8       1   0B2
.........................................................................uugaauuccggaGcuugaauu................   6       1   0B2
.........................................................................uugaauuccggauAuugaauu................   2       1   0B2
.........................................................................uugaauuccggaucuGgaauu................   4       1   0B2
.........................................................................uugaauuAcggaucuugaauu................   6       1   0B2
.........................................................................Nugaauuccggaucuugaauu................   154     1   0B2
.........................................................................uugaauuccggaucuCgaauu................   18      1   0B2
.........................................................................uugaauAccggaucuugaauu................   2       1   0B2
.........................................................................uugaauuccggGucuugaauu................   2       1   0B2
.........................................................................uugaGuuccggaucuugaauu................   332     1   0B2
.........................................................................uugaauuccggCucuugaauu................   4       1   0B2
.........................................................................uugaauuccggaucuugaaCu................   66      1   0B2
.........................................................................uugaauuccggaucuugGauu................   12      1   0B2
.........................................................................uugaauuccggauNuugaauu................   2       1   0B2
.........................................................................uugaauuccggaucCugaauu................   8       1   0B2
.........................................................................uugaauuccggaCcuugaauu................   4       1   0B2
.........................................................................uugaauuccggaucuugaaGu................   6       1   0B2
.........................................................................Augaauuccggaucuugaauu................   6       1   0B2
.........................................................................uugaauuccggaucuugaauu................   19318   0   0B2
.........................................................................uugaauuccggaucuAgaauu................   5504    1   0B2
.........................................................................Cugaauuccggaucuugaauu................   4       1   0B2
.........................................................................uugaauuGcggaucuugaauua...............   4       1   0B2
.........................................................................uugaGuuccggaucuugaauua...............   526     1   0B2
.........................................................................uugaauuccggaucuuUaauua...............   2       1   0B2
.........................................................................Gugaauuccggaucuugaauua...............   8       1   0B2
.........................................................................uugaauuccggaucuugGauua...............   36      1   0B2
.........................................................................Augaauuccggaucuugaauua...............   6       1   0B2
.........................................................................uugaauuccggaucuGgaauua...............   4       1   0B2
.........................................................................uugaauNccggaucuugaauua...............   2       1   0B2
.........................................................................uugaauuAcggaucuugaauua...............   2       1   0B2
.........................................................................uugaauuccggNucuugaauua...............   2       1   0B2
.........................................................................uugaaGuccggaucuugaauua...............   24      1   0B2
.........................................................................uugaauuccggaCcuugaauua...............   6       1   0B2
.........................................................................uugaauuccggaGcuugaauua...............   8       1   0B2
.........................................................................uugaauuccggaucuCgaauua...............   30      1   0B2
.........................................................................uugaauuccggCucuugaauua...............   18      1   0B2
.........................................................................uugaauuccggauAuugaauua...............   2       1   0B2
.........................................................................uugaauuccggGucuugaauua...............   4       1   0B2
.........................................................................Cugaauuccggaucuugaauua...............   18      1   0B2
.........................................................................uugaauuccggaucuugaauua...............   21770   0   0B2
.........................................................................uugaauuccggaucuugaauCa...............   1868    1   0B2
.........................................................................Nugaauuccggaucuugaauua...............   182     1   0B2
.........................................................................uugaauuccggaucuugaauAa...............   1638    1   0B2
.........................................................................uugaauuccggaucuugaaCua...............   32      1   0B2
.........................................................................uugaCuuccggaucuugaauua...............   4       1   0B2
.........................................................................uugaauuccggaucuAgaauua...............   5186    1   0B2
.........................................................................uugaauuccggaucuugUauua...............   2       1   0B2
.........................................................................uGgaauuccggaucuugaauua...............   4       1   0B2
.........................................................................uugaauuccggaucuugaaCuag..............   8       1   0B2
.........................................................................uugaauuccggaucuAgaauuag..............   1514    1   0B2
```

Star Mature

```
ggaucuugaauuaggcuguuucauuuuggaucuugaauuccggaucuugaauuaggcuguuugguuuugaaucuugaauuccggaucuugaauuaggcuguuugguuuug
.........................................................................uugaauuccCgaucuugaauuag..............   2       1   0B2
.........................................................................uugaauuccggauGuugaauuag..............   2       1   0B2
.........................................................................uugaauuccggaucuugaauuag..............   486     0   0B2
.........................................................................Cugaauuccggaucuugaauuag..............   18      1   0B2
.........................................................................Nugaauuccggaucuugaauuag..............   10      1   0B2
.........................................................................uugaauuccggaucuugaauAag..............   8       1   0B2
.........................................................................uugaaGuccggaucuugaauuag..............   4       1   0B2
.........................................................................uugaGuuccggaucuugaauuag..............   28      1   0B2
.........................................................................uugaGuuccggaucuugaauuagg.............   6       1   0B2
.........................................................................uugaauuccggaucuugaaCuagg.............   4       1   0B2
.........................................................................uugaauuccggaucuugaauuagA.............   6       1   0B2
..........................................................................ugaauuccggaucuAgaa..................   140     1   0B2
..........................................................................ugaauuccggaucuugaa..................   10      0   0B2
..........................................................................ugaauuccggaucuugaau.................   996     0   0B2
..........................................................................ugaauuccggaucuAgaau.................   298     1   0B2
..........................................................................Ngaauuccggaucuugaau.................   4       1   0B2
..........................................................................ugaauuccggaGcuugaau.................   4       1   0B2
..........................................................................ugaGuuccggaucuugaau.................   14      1   0B2
..........................................................................ugaauuccggaucuAgaauu................   2800    1   0B2
..........................................................................ugaauuccggauAuugaauu................   2       1   0B2
..........................................................................ugaauuccggaucuuUaauu................   2       1   0B2
..........................................................................ugaauuccggaGcuugaauu................   2       1   0B2
..........................................................................ugaauuccggGucuugaauu................   4       1   0B2
..........................................................................ugaauuccggaucuugaauu................   6110    0   0B2
..........................................................................Ngaauuccggaucuugaauu................   28      1   0B2
..........................................................................ugaauuccggaucuCgaauu................   4       1   0B2
..........................................................................ugaauuccggaucuugaaGu................   2       1   0B2
..........................................................................ugaaGuccggaucuugaauu................   10      1   0B2
..........................................................................ugaauuccggaucCugaauu................   4       1   0B2
..........................................................................ugaGuuccggaucuugaauu................   132     1   0B2
..........................................................................ugaauuccggaucuGgaauua...............   2       1   0B2
..........................................................................ugaauuccggaucuugaauua...............   15826   0   0B2
..........................................................................ugaauuccggGucuugaauua...............   2       1   0B2
..........................................................................ugaauuccggaCcuugaauua...............   4       1   0B2
..........................................................................ugaauuccggaucuugaaCua...............   12      1   0B2
..........................................................................ugaaGuccggaucuugaauua...............   44      1   0B2
..........................................................................ugaauuccggaucCugaauua...............   6       1   0B2
..........................................................................ugaauuccggaucuugaauCa...............   560     1   0B2
..........................................................................ugaauuAcggaucuugaauua...............   2       1   0B2
..........................................................................ugaauuccggaucuCgaauua...............   6       1   0B2
..........................................................................Ngaauuccggaucuugaauua...............   112     1   0B2
..........................................................................ugaauuccggaucuugaauAa...............   116     1   0B2
..........................................................................ugaauuccggaucuugGauua...............   20      1   0B2
..........................................................................ugaauuccggaucAugaauua...............   8       1   0B2
..........................................................................ugaauuccggaucuAgaauua...............   6298    1   0B2
..........................................................................ugaGuuccggaucuugaauua...............   292     1   0B2
..........................................................................uNaauuccggaucuugaauua...............   2       1   0B2
..........................................................................ugaauuccggaGcuugaauua...............   10      1   0B2
..........................................................................ugaauuccggaucuugUauua...............   2       1   0B2
..........................................................................ugaauuccgCaucuugaauua...............   2       1   0B2
..........................................................................Ggaauuccggaucuugaauua...............   8       1   0B2
..........................................................................ugaauuccggauAuugaauua...............   8       1   0B2
..........................................................................ugaauuccCgaucuugaauua...............   2       1   0B2
..........................................................................ugaauuccggaucuAgaauuag..............   5118    1   0B2
..........................................................................ugaGuuccggaucuugaauuag..............   26      1   0B2
..........................................................................ugaauuccggaucuugaauuag..............   1450    0   0B2
..........................................................................ugaauuccggGucuugaauuag..............   2       1   0B2
..........................................................................ugaauuccggaucuugGauuag..............   2       1   0B2
..........................................................................ugaauuccggaucuGgaauuag..............   2       1   0B2
..........................................................................ugaauuccggaucuugaauAag..............   18      1   0B2
..........................................................................ugaauuccggaucuugaauCag..............   26      1   0B2
..........................................................................Ngaauuccggaucuugaauuag..............   12      1   0B2
..........................................................................ugaauuccggaucuugaauuaUg.............   2       1   0B2
..........................................................................ugaauuccggaucuugaauuagA.............   2       1   0B2
..........................................................................ugaauuccggaucuugaauuagg.............   122     0   0B2
..........................................................................ugaauuccggaucuugaaCuagg.............   2       1   0B2
..........................................................................ugaaGuccggaucuugaauuagg.............   2       1   0B2
...........................................................................gaauuccggaucuAgaau.................   2       1   0B2
...........................................................................gaauuccggaucuugaau.................   42      0   0B2
...........................................................................gaauuccggaucuugaauu................   262     0   0B2
```

```
                    Star                                                   Mature
ggaucuugaauuaggcuguuucauuuuggaucuugaauuccggaucuugaauuaggcuguuugguuuugaaucuugaauuccggaucuugaauuaggcuguuugguuuug
...........................................................................gaauuccggaucuAgaauu................   172   1   0B2
...........................................................................gaauuccggaucuugCauua...............   2     1   0B2
...........................................................................gaauuccggaucuugaauCa...............   118   1   0B2
...........................................................................gaauuccggaucuugaauAa...............   4     1   0B2
...........................................................................gaauuccggaucuAgaauua...............   862   1   0B2
...........................................................................gaauuccggaucuugaauua...............   1374  0   0B2
...........................................................................gaGuuccggaucuugaauua...............   14    1   0B2
...........................................................................gaaGuccggaucuugaauua...............   6     1   0B2
...........................................................................Naauuccggaucuugaauua...............   20    1   0B2
...........................................................................gaauuccggaucAugaauua...............   4     1   0B2
...........................................................................Naauuccggaucuugaauuag..............   10    1   0B2
...........................................................................gaauuccggaucuCgaauuag..............   2     1   0B2
...........................................................................gaauuccggaucuugaaCuag..............   6     1   0B2
...........................................................................gaauuccggaucCugaauuag..............   2     1   0B2
...........................................................................gaauuccggaucuugaauCag..............   6     1   0B2
...........................................................................gaauuccggaucuAgaauuag..............   4018  1   0B2
...........................................................................gaGuuccggaucuugaauuag..............   2     1   0B2
...........................................................................gaauuccggaucuugaauuag..............   984   0   0B2
...........................................................................gaauuccggaucuugaauGagg.............   2     1   0B2
...........................................................................Aaauuccggaucuugaauuagg.............   16    1   0B2
...........................................................................gaauuccggaucuugaauuagg.............   22    0   0B2
...........................................................................gaauuccggaucuugaauuagA.............   2     1   0B2
............................................................................aauuccggaucuugaauu................   14    0   0B2
............................................................................aauuccggaucuCgaauua...............   2     1   0B2
............................................................................aauuccggaucuugaauCa...............   16    1   0B2
............................................................................aCuuccggaucuugaauua...............   2     1   0B2
............................................................................aauuccggaCcuugaauua...............   2     1   0B2
............................................................................aauuccggaucuugaauua...............   626   0   0B2
............................................................................aauuccggaucuugaauAa...............   8     1   0B2
............................................................................aaGuccggaucuugaauuag..............   14    1   0B2
............................................................................aauAccggaucuugaauuag..............   4     1   0B2
............................................................................Nauuccggaucuugaauuag..............   24    1   0B2
............................................................................aauuccggaucuugaauCag..............   26    1   0B2
............................................................................aauuccggaucuugGauuag..............   22    1   0B2
............................................................................aauuccggaucAugaauuag..............   2     1   0B2
............................................................................aGuuccggaucuugaauuag..............   70    1   0B2
............................................................................aauuccggaucuugaauuag..............   6030  0   0B2
............................................................................aauuccggaGcuugaauuag..............   2     1   0B2
............................................................................aauuccggaucuugaauAag..............   2     1   0B2
............................................................................aauuccggaucuuUaauuag..............   14    1   0B2
............................................................................aauuccggaucuCgaauuag..............   4     1   0B2
............................................................................aauuccggaucuugaaGuag..............   2     1   0B2
............................................................................aauuccggaCcuugaauuag..............   2     1   0B2
............................................................................aauuccggaucuugaauuaUg.............   8     1   0B2
............................................................................Nauuccggaucuugaauuagg.............   4     1   0B2
............................................................................aauuccggaucuAgaauuagg.............   20    1   0B2
............................................................................aauuccggaucuugaauuagA.............   18    1   0B2
............................................................................aauuccggaucuugaauuagg.............   486   0   0B2
............................................................................aauuccggaucuCgaauuagg.............   2     1   0B2
............................................................................aauuccggaucuugGauuagg.............   2     1   0B2
............................................................................aauuccggaCcuugaauuagg.............   2     1   0B2
.............................................................................auuccggaucuugaauCa...............   8     1   0B2
.............................................................................Nuuccggaucuugaauua...............   4     1   0B2
.............................................................................auuccggaucuugaauua...............   220   0   0B2
.............................................................................auuccggaucuugaauAa...............   2     1   0B2
.............................................................................auuccggaGcuugaauua...............   2     1   0B2
.............................................................................auuccggGucuugaauuag..............   2     1   0B2
.............................................................................Guuccggaucuugaauuag..............   18    1   0B2
.............................................................................Nuuccggaucuugaauuag..............   6     1   0B2
.............................................................................auuccggaGcuugaauuag..............   4     1   0B2
.............................................................................auuccggaucAugaauuag..............   10    1   0B2
.............................................................................auuccggaucuugaauCag..............   16    1   0B2
.............................................................................auuccggauAuugaauuag..............   2     1   0B2
.............................................................................auuccggaucuugaauuag..............   1402  0   0B2
.............................................................................Guuccggaucuugaauuagg.............   66    1   0B2
.............................................................................auuccggaucuugaauuagA.............   88    1   0B2
.............................................................................auucGggaucuugaauuagg.............   12    1   0B2
.............................................................................auuccggaucuugGauuagg.............   4     1   0B2
.............................................................................auuccggaucuAgaauuagg.............   38    1   0B2
.............................................................................auuccggaucuugaauuaUg.............   18    1   0B2
```

Star **Mature**

ggaucuugaauuaggcuguuucauuuuggaucuugaauuccggaucuugaauuaggcuguuugguuuugaaucuugaauuccggaucuugaauuaggcuguuugguuuug			
....auuccggaCcuugaauuagg.............	2	1	0B2
....auuccggaucuugaauuagg.............	3760	0	0B2
....auuccggGucuugaauuagg.............	12	1	0B2
....auuccggaucuugaaCuagg.............	2	1	0B2
....auuccggaucuCgaauuagg.............	6	1	0B2
....Nuuccggaucuugaauuagg.............	30	1	0B2
....auuccggaucuugaauGagg.............	4	1	0B2
....auuccggaucuugaaGuagg.............	2	1	0B2
....auuccggaucAugaauuagg.............	56	1	0B2
....auuccggaGcuugaauuagg.............	4	1	0B2
....auuccggaucuugaauAagg.............	2	1	0B2
....auuccgCaucuugaauuagg.............	2	1	0B2
....auuccggCucuugaauuagg.............	2	1	0B2
....auuccggaucuugaauuaggcA...........	10	1	0B2
....uuccggaucuugaauCag..............	10	1	0B2
....uuccggaucuugGauuag..............	2	1	0B2
....uuccggaucuugaauuag..............	1006	0	0B2
....uuccggaGcuugaauuag..............	2	1	0B2
....uuccggaucuGgaauuag..............	4	1	0B2
....Nuccggaucuugaauuag..............	12	1	0B2
....uuccggaucuuUaauuagg.............	4	1	0B2
....uuccggaucuugaaCuagg.............	10	1	0B2
....Nuccggaucuugaauuagg.............	8	1	0B2
....uuAcggaucuugaauuagg.............	4	1	0B2
....uuccggaGcuugaauuagg.............	2	1	0B2
....uucAggaucuugaauuagg.............	16	1	0B2
....uuccggaucuAgaauuagg.............	16	1	0B2
....uucGggaucuugaauuagg.............	4	1	0B2
....uuccggaucAugaauuagg.............	56	1	0B2
....uuccggaucuugaauuagg.............	1688	0	0B2
....uuccggaucuugGauuagg.............	4	1	0B2
....uuccggaucuugaauuagA.............	24	1	0B2
....uuccggauAuugaauuagg.............	2	1	0B2
....uuccggaucuGgaauuagg.............	8	1	0B2
....uuccggaucuugaauuaggcA...........	4	1	0B2
....uccggGucuugaauuagg.............	2	1	0B2
....uccggaucAugaauuagg.............	2	1	0B2
....Nccggaucuugaauuagg.............	2	1	0B2
....uccggaCcuugaauuagg.............	6	1	0B2
....uccggaucuugaauuagg.............	384	0	0B2
....uccggaucuAgaauuagg.............	8	1	0B2
....cggaucuugaauuaggcuA..........	6	1	0B2
..................uuucauuCuggaucuugaauu...	5	1	0G2
..................uuucauuCuggaucuugaauuc..	2	1	0G2
..................uuucauuCuggaucuugaauucc...	5	1	0G2
...................uucauuuuggGucuugaauu...	6	1	0G2
...................uucauuCuggaucuugaauuc..	56	1	0G2
...................uucauuCuggaucuugaauucc...	5	1	0G2
...................uucauuuuggaucuugaauucA...	8	1	0G2
....................ucauuGuggaucuugaauu...	4	1	0G2
....................ucauuuuggaucuugaauuA..	8	1	0G2
....................ucauuCuggaucuugaauuc..	14	1	0G2
....................ucauuuuggaucuugaauucc...	4	0	0G2
....................ucauuCuggaucuugaauucc...	39	1	0G2
....................ucauuuuggaucuugaauucA...	19	1	0G2
....................ucauuuuggaucuugaauuccg..	2	0	0G2
....................ucauuCuggaucuugaauuccg..	9	1	0G2
.....................cauuCuggaucuugaauuc..	11	1	0G2
.....................cauuCuggaucuugaauucc...	57	1	0G2
.....................cauuuuggaucuugaauucA...	21	1	0G2
.....................cGuuuuggaucuugaauuccg..	1	1	0G2
.....................cauuuuggaucuugaauuccgga..	5	0	0G2
......................auuCuggaucuugaauucc...	7	1	0G2
......................auuuuggaucuugaauucUg..	3	1	0G2
......................auuCuggaucuugaauuccg..	13	1	0G2
......................auuuuggaucuugaauuccg..	7	0	0G2
.......................uuCuggaucuugaauucc...	6	1	0G2
.......................uuuuggGucuugaauuccg..	2	1	0G2
.......................Cuuuggaucuugaauuccgg...	2	1	0G2

Star **Mature**

```
ggaucuugaauuaggcuguuucauuuuggaucuugaauuccggaucuugaauuaggcuguuugguuuugaaucuugaauuccggaucuugaauuaggcuguuugguuuug
.......................uuuuggGucuugaauuccgg...................................................................  1     1  0G2
.......................uuuuggGucuugaauuccgga..................................................................  23    1  0G2
.......................uuuuggaucuugaauuccgga..................................................................  10    0  0G2
........................uuuggGucuugaauuccgga..................................................................  4     1  0G2
.........................uuggGucuugaauuccgg...................................................................  5     1  0G2
.........................uuggaucuugaauuccgga..................................................................  8     0  0G2
.........................uuggaucuugaauuccggau.................................................................  1     0  0G2
.........................uuggaucuugaauuccggauc................................................................  5     0  0G2
.........................uuggGucuugaauuccggauc................................................................  11    1  0G2
.........................uuggaucuugaauuccggauA................................................................  1     1  0G2
............................gGucuugaauuccggaucuu..............................................................  3     1  0G2
.............................aucuugaauuccggaucuug.............................................................  6     0  0G2
.............................aucuugaauuccggaucuAga............................................................  4     1  0G2
.............................aucuugaauuccggaucuugaau..........................................................  16    0  0G2
.............................aucuugaauuccggaucuugaauua........................................................  26    0  0G2
.............................aucuugaauuccggaucuugaauuag.......................................................  2     0  0G2
..............................ucuugaauuccggaucuA..............................................................  4     1  0G2
..............................ucuugaauuccggaucuug.............................................................  2     0  0G2
..............................ucuugaauuccggaucuuga............................................................  38    0  0G2
..............................ucuugaauuccggaucuAga............................................................  102   1  0G2
..............................ucGugaauuccggaucuugaa...........................................................  32    1  0G2
..............................ucuugaauuccggaucuugaa...........................................................  52    0  0G2
..............................ucuugaauuccggaucuAgaa...........................................................  148   1  0G2
..............................ucuugaauuAcggaucuugaau..........................................................  2     1  0G2
..............................ucuugaauuccggaucuAgaau..........................................................  20    1  0G2
..............................ucGugaauuccggaucuugaau..........................................................  94    1  0G2
..............................ucuugaauuccggaucuugaaG..........................................................  6     1  0G2
..............................ucuugaauuccggaCcuugaau..........................................................  2     1  0G2
..............................ucuugaauuccggaucuugaau..........................................................  454   0  0G2
..............................Ccuugaauuccggaucuugaau..........................................................  2     1  0G2
..............................ucuugaauuccggaucuugaauu.........................................................  104   0  0G2
..............................uUuugaauuccggaucuugaauu.........................................................  22    1  0G2
..............................uGuugaauuccggaucuugaauu.........................................................  8     1  0G2
..............................ucuugaaGuccggaucuugaauu.........................................................  20    1  0G2
..............................ucuugaauuccggaucuGgaauua........................................................  6     1  0G2
..............................ucuugaauuccggaucuugaauua........................................................  200   0  0G2
..............................uUuugaauuccggaucuugaauua........................................................  12    1  0G2
..............................ucuugaauuccggaucuAgaauua........................................................  14    1  0G2
..............................ucuugaauuccggaucuugaauAa........................................................  330   1  0G2
..............................ucuugaauuccggaucuugaauuag.......................................................  24    0  0G2
..............................ucuugaauuccggaucuAgaauuag.......................................................  14    1  0G2
..............................ucuugaauuccggaucuugaauAag.......................................................  6     1  0G2
..............................ucuugaauuccggaucuugaauuagg......................................................  20    0  0G2
...............................cuugaauuccggaucuug.............................................................  14    0  0G2
...............................cuugaauuccggaucuuga............................................................  4     0  0G2
...............................cuugaauuccggaucuAga............................................................  108   1  0G2
...............................Nuugaauuccggaucuugaa...........................................................  4     1  0G2
...............................cuugaauuccggaucuugaa...........................................................  192   0  0G2
...............................cuugaauuccggaucuAgaa...........................................................  726   1  0G2
...............................cGugaauuccggaucuugaa...........................................................  2     1  0G2
...............................Nuugaauuccggaucuugaau..........................................................  4     1  0G2
...............................cGugaauuccggaucuugaau..........................................................  30    1  0G2
...............................cuugaauuccggaucuAgaau..........................................................  42    1  0G2
...............................cuugaauuccggaucuugaau..........................................................  454   0  0G2
...............................cNugaauuccggaucuugaauu.........................................................  2     1  0G2
...............................cuugaauuccggaucuugaauu.........................................................  452   0  0G2
...............................cAugaauuccggaucuugaauu.........................................................  2     1  0G2
...............................cuugaauuccggaucuAgaauu.........................................................  148   1  0G2
...............................cuugaauuccggaucuugaauCa........................................................  70    1  0G2
...............................Nuugaauuccggaucuugaauua........................................................  2     1  0G2
...............................cuugaauuccggaucuugaaGua........................................................  2     1  0G2
...............................cuugaauuccggaucuugaauAa........................................................  136   1  0G2
...............................cuugaauuccggaucuAgaauua........................................................  320   1  0G2
...............................cuugaauuccggaucuugaauua........................................................  1032  0  0G2
...............................cuugaauuccggaucuugaaCua........................................................  10    1  0G2
...............................cuugaauuccggaucuugaauuag.......................................................  8     0  0G2
...............................cuugaauuccggaucuAgaauuag.......................................................  4     1  0G2
................................uugaauuccggaucuAga............................................................  52    1  0G2
................................uugaauuccggaucuugaa...........................................................  206   0  0G2
................................uugaauuccggaucuAgaa...........................................................  476   1  0G2
```

```
                    Star                                                  Mature
ggaucuugaauuaggcuguuucauuuuggaucuugaauuccggaucuugaauuaggcuguuugguuuugaaucuugaauuccggaucuugaauuaggcuguuugguuuug
................................uugaauuccggaGcuugaau..........................................................   4       1   0G2
................................uugaauuccggaucuAgaau..........................................................   2996    1   0G2
................................uugaauuccggauAuugaau..........................................................   10      1   0G2
................................Gugaauuccggaucuugaau..........................................................   24      1   0G2
................................uugaauuccggaucuugaaC..........................................................   6       1   0G2
................................uugaauuccggGucuugaau..........................................................   14      1   0G2
................................uGgaauuccggaucuugaau..........................................................   4       1   0G2
................................uAgaauuccggaucuugaau..........................................................   4       1   0G2
................................uugaauuccggaCcuugaau..........................................................   2       1   0G2
................................uugaauuccggaucCugaau..........................................................   2       1   0G2
................................uNgaauuccggaucuugaau..........................................................   12      1   0G2
................................uugaaGuccggaucuugaau..........................................................   2       1   0G2
................................uugaauuAcggaucuugaau..........................................................   6       1   0G2
................................uugaauuccggaucuGgaau..........................................................   6       1   0G2
................................uugaGuuccggaucuugaau..........................................................   114     1   0G2
................................uugaauuccggCucuugaau..........................................................   2       1   0G2
................................Cugaauuccggaucuugaau..........................................................   4       1   0G2
................................uugaauuccggaucuugaaG..........................................................   14      1   0G2
................................uugaauuccggaucuugaau..........................................................   14612   0   0G2
................................Augaauuccggaucuugaau..........................................................   12      1   0G2
................................Nugaauuccggaucuugaau..........................................................   56      1   0G2
................................uugaauuccggaucuGgaauu.........................................................   6       1   0G2
................................uugaauuccggaucuAgaauu.........................................................   5240    1   0G2
................................uugaGuuccggaucuugaauu.........................................................   260     1   0G2
................................uugaauuccggaucuuUaauu.........................................................   2       1   0G2
................................uugaauuccggaucuCgaauu.........................................................   10      1   0G2
................................uugaauuccggaucuugaaGu.........................................................   8       1   0G2
................................Cugaauuccggaucuugaauu.........................................................   6       1   0G2
................................uugaauuccggaGcuugaauu.........................................................   4       1   0G2
................................Gugaauuccggaucuugaauu.........................................................   10      1   0G2
................................uNgaauuccggaucuugaauu.........................................................   8       1   0G2
................................uugaauuccggaCcuugaauu.........................................................   10      1   0G2
................................uugaauuccggGucuugaauu.........................................................   8       1   0G2
................................uugaauuccggCucuugaauu.........................................................   8       1   0G2
................................uugaauuccggaucCugaauu.........................................................   4       1   0G2
................................uugaauuccggaucuugGauu.........................................................   14      1   0G2
................................Augaauuccggaucuugaauu.........................................................   4       1   0G2
................................uugaauuAcggaucuugaauu.........................................................   4       1   0G2
................................uugaauuccggaucuugaaCu.........................................................   38      1   0G2
................................uGgaauuccggaucuugaauu.........................................................   2       1   0G2
................................Nugaauuccggaucuugaauu.........................................................   60      1   0G2
................................uugaauuccggaucuugaauu.........................................................   21266   0   0G2
................................uugaaGuccggaucuugaauu.........................................................   4       1   0G2
................................Gugaauuccggaucuugaauua........................................................   24      1   0G2
................................uAgaauuccggaucuugaauua........................................................   4       1   0G2
................................uugaauuccggaucuugaauCa........................................................   4852    1   0G2
................................uugaauuccggaucuugaaGua........................................................   6       1   0G2
................................uugaauuccggaucuugCauua........................................................   2       1   0G2
................................uugaauuccggaucAugaauua........................................................   2       1   0G2
................................uGgaauuccggaucuugaauua........................................................   2       1   0G2
................................uugaauuccggauGuugaauua........................................................   6       1   0G2
................................uugaauuccggaucuuUaauua........................................................   6       1   0G2
................................uugaauuAcggaucuugaauua........................................................   28      1   0G2
................................uugaCuuccggaucuugaauua........................................................   2       1   0G2
................................uugaauuccggaucuAgaauua........................................................   13424   1   0G2
................................uugaauuccggaucuugaauAa........................................................   5360    1   0G2
................................uNgaauuccggaucuugaauua........................................................   24      1   0G2
................................Augaauuccggaucuugaauua........................................................   14      1   0G2
................................uugaauuccggaucCugaauua........................................................   18      1   0G2
................................uugaauuccggaucuugaauua........................................................   58180   0   0G2
................................uugaauuccggaucuCgaauua........................................................   10      1   0G2
................................uugaGuuccggaucuugaauua........................................................   1278    1   0G2
................................uugaauuccggaucuugGauua........................................................   74      1   0G2
................................uugaauuccggaucuGgaauua........................................................   12      1   0G2
................................Cugaauuccggaucuugaauua........................................................   34      1   0G2
................................uugaauuGcggaucuugaauua........................................................   2       1   0G2
................................uugaauuccgCaucuugaauua........................................................   2       1   0G2
................................uugaauuccCgaucuugaauua........................................................   16      1   0G2
................................uugaauuccggaucuugaaCua........................................................   64      1   0G2
................................uugaauuccggauAuugaauua........................................................   14      1   0G2
```

```
                    Star                                                  Mature
ggaucuugaauuaggcuguuucauuuuggaucuugaauuccggaucuugaauuaggcuguuugguuuugaaucuugaauuccggaucuugaauuaggcuguuugguuuug
................................uugaauuccggGucuugaauua........................................................    16      1    0G2
................................uugaaGuccggaucuugaauua........................................................    36      1    0G2
................................uugaauuccggaGcuugaauua........................................................    14      1    0G2
................................uugaauuccggaCcuugaauua........................................................    8       1    0G2
................................Nugaauuccggaucuugaauua........................................................    164     1    0G2
................................uugaauAccggaucuugaauua........................................................    22      1    0G2
................................uugaauuccggaucuCgaauuag.......................................................    8       1    0G2
................................uugaauuccggaucuAgaauuag.......................................................    2626    1    0G2
................................uugaauuccggaucuugaauCag.......................................................    16      1    0G2
................................uugaauuccggaucuugaaCuag.......................................................    30      1    0G2
................................uugaauuccggaucuugaauuag.......................................................    606     0    0G2
................................uugaauuccggaucuugaauAag.......................................................    18      1    0G2
................................uugaauuccggaucuugGauuag.......................................................    2       1    0G2
................................uNgaauuccggaucuugaauuag.......................................................    2       1    0G2
................................Cugaauuccggaucuugaauuag.......................................................    2       1    0G2
................................uugaauuccggaucuGgaauuag.......................................................    2       1    0G2
................................uugaaGuccggaucuugaauuag.......................................................    14      1    0G2
................................uugaGuuccggaucuugaauuag.......................................................    14      1    0G2
................................Nugaauuccggaucuugaauuag.......................................................    2       1    0G2
................................uAgaauuccggaucuugaauuag.......................................................    6       1    0G2
................................uugaauuccggaucAugaauuagg......................................................    22      1    0G2
................................uugaauuccggaucuugaauuagg......................................................    2       0    0G2
................................uugaauuccggaucuugaauuagA......................................................    6       1    0G2
................................uugaauuccggaucuugaauuaUg......................................................    2       1    0G2
................................uugaauuccggaucuugaauuaggcuA...................................................    2       1    0G2
.................................ugaauuccggaucuAgaa...........................................................    132     1    0G2
.................................ugaauuccggaucuGgaa...........................................................    2       1    0G2
.................................ugaauuccggaucuugaa...........................................................    4       0    0G2
.................................ugaauuAcggaucuugaau..........................................................    2       1    0G2
.................................ugaauuccggaucuAgaau..........................................................    136     1    0G2
.................................ugaauuccggaucuugaau..........................................................    480     0    0G2
.................................ugaauuccggaucuCgaauu.........................................................    2       1    0G2
.................................ugaauuccggaucuAgaauu.........................................................    1700    1    0G2
.................................Ngaauuccggaucuugaauu.........................................................    14      1    0G2
.................................ugaauuccggaucuugaaCu.........................................................    8       1    0G2
.................................ugaauuccggaucuugaauu.........................................................    5018    0    0G2
.................................Agaauuccggaucuugaauu.........................................................    14      1    0G2
.................................ugaauuccggCucuugaauu.........................................................    2       1    0G2
.................................ugaaGuccggaucuugaauu.........................................................    2       1    0G2
.................................uNaauuccggaucuugaauu.........................................................    2       1    0G2
.................................ugaGuuccggaucuugaauu.........................................................    64      1    0G2
.................................ugaauuAcggaucuugaauu.........................................................    2       1    0G2
.................................ugaauuAcggaucuugaauua........................................................    32      1    0G2
.................................ugaauuccggaucuugaauAa........................................................    294     1    0G2
.................................ugaauuccggaucuuUaauua........................................................    10      1    0G2
.................................ugaauuccggaucAugaauua........................................................    6       1    0G2
.................................Agaauuccggaucuugaauua........................................................    6       1    0G2
.................................ugaauuccggaucuAgaauua........................................................    12002   1    0G2
.................................ugaauuccggaCcuugaauua........................................................    8       1    0G2
.................................ugaauuccggaGcuugaauua........................................................    14      1    0G2
.................................ugaauuccggaucuugaaCua........................................................    36      1    0G2
.................................ugaauuccggGucuugaauua........................................................    2       1    0G2
.................................ugaGuuccggaucuugaauua........................................................    612     1    0G2
.................................Ggaauuccggaucuugaauua........................................................    14      1    0G2
.................................ugaauuccggaucuCgaauua........................................................    20      1    0G2
.................................ugaauuccggauAuugaauua........................................................    10      1    0G2
.................................ugaauuccggaucuugaauCa........................................................    948     1    0G2
.................................ugaauuccggaucuugUauua........................................................    4       1    0G2
.................................ugaauuccggaucuugaauua........................................................    31676   0    0G2
.................................ugaauuccggaucuGgaauua........................................................    2       1    0G2
.................................Ngaauuccggaucuugaauua........................................................    106     1    0G2
.................................uNaauuccggaucuugaauua........................................................    22      1    0G2
.................................ugaauuccCgaucuugaauua........................................................    2       1    0G2
.................................ugaauuccggaucuugGauua........................................................    42      1    0G2
.................................ugaauuccggaucCugaauua........................................................    6       1    0G2
.................................ugaauuccggCucuugaauua........................................................    6       1    0G2
.................................ugaaGuccggaucuugaauua........................................................    52      1    0G2
.................................ugaauuccggaGcuugaauuag.......................................................    2       1    0G2
.................................ugaauuccggaucuAgaauuag.......................................................    9070    1    0G2
.................................Ngaauuccggaucuugaauuag.......................................................    14      1    0G2
```

```
                    Star                                                   Mature
ggaucuugaauuaggcuguuucauuuuggaucuugaauuccggaucuugaauuaggcuguuugguuuugaaucuugaauuccggaucuugaauuaggcuguuugguuuug
.................................Agaauuccggaucuugaauuag.......................................................   2     1   0G2
.................................ugaauuccggGucuugaauuag.......................................................   2     1   0G2
.................................ugaauuccggaucuugGauuag.......................................................   2     1   0G2
.................................ugaauuccggaucuugaauCag.......................................................   16    1   0G2
.................................ugaauuccggaucuugaauAag.......................................................   2     1   0G2
.................................ugaauuccggaucuGgaauuag.......................................................   4     1   0G2
.................................ugaauuccggaucuugaauuag.......................................................   2092  0   0G2
.................................ugaGuuccggaucuugaauuag.......................................................   24    1   0G2
.................................ugaaGuccggaucuugaauuagg......................................................   10    1   0G2
.................................ugaauuccggaucuugaauuagA......................................................   4     1   0G2
.................................ugaauuccggaucuugaauuagg......................................................   146   0   0G2
..................................gaauuccggaucuugaau..........................................................   20    0   0G2
..................................gaauuccggaucuAgaau..........................................................   30    1   0G2
..................................gaauuccggaucuuUaauu.........................................................   2     1   0G2
..................................gaauuccggaucuCgaauu.........................................................   4     1   0G2
..................................gaauuccggaucuAgaauu.........................................................   70    1   0G2
..................................gaauuccggauAuugaauu.........................................................   2     1   0G2
..................................gaauuccggaucuugaauu.........................................................   286   0   0G2
..................................gaauuccggaucuugaauCa........................................................   206   1   0G2
..................................gaauuccggaucuugaauua........................................................   2166  0   0G2
..................................gaauuccggaucuAgaauua........................................................   1214  1   0G2
..................................Naauuccggaucuugaauua........................................................   10    1   0G2
..................................gNauuccggaucuugaauua........................................................   2     1   0G2
..................................gaauuccggaucCugaauua........................................................   2     1   0G2
..................................gaauuccggaucuugaauAa........................................................   8     1   0G2
..................................gaauuccggauAuugaauua........................................................   2     1   0G2
..................................gaauuccggaGcuugaauua........................................................   2     1   0G2
..................................gaGuuccggaucuugaauua........................................................   26    1   0G2
..................................gaauuccggaucuuUaauua........................................................   2     1   0G2
..................................gaauuccggaucuugaaCua........................................................   8     1   0G2
..................................gaGuuccggaucuugaauuag.......................................................   12    1   0G2
..................................gaauuccggaucuugaauuag.......................................................   1122  0   0G2
..................................gaauuccggaucuAgaauuag.......................................................   6458  1   0G2
..................................gaauuccggaucuugaauuagA......................................................   16    1   0G2
..................................gaauuccggaucuugaauuagg......................................................   10    0   0G2
..................................gaauuccggaucuAgaauuagg......................................................   6     1   0G2
...................................aauuccggaucuugaauu.........................................................   8     0   0G2
...................................aauuccggaucuugaauua........................................................   992   0   0G2
...................................aauuccggaucuugaauAa........................................................   22    1   0G2
...................................aNuuccggaucuugaauua........................................................   2     1   0G2
...................................aauuccggaucuugaauCa........................................................   38    1   0G2
...................................aauuccggaucAugaauuag.......................................................   4     1   0G2
...................................aauuccggaucuGgaauuag.......................................................   12    1   0G2
...................................aauuccggGucuugaauuag.......................................................   14    1   0G2
...................................aauuccggaucuugaauCag.......................................................   40    1   0G2
...................................aNuuccggaucuugaauuag.......................................................   2     1   0G2
...................................aGuuccggaucuugaauuag.......................................................   112   1   0G2
...................................aauuccggaGcuugaauuag.......................................................   2     1   0G2
...................................aauuccggaCcuugaauuag.......................................................   4     1   0G2
...................................aauuccggaucuugaauuag.......................................................   8186  0   0G2
...................................aauuccggaucuCgaauuag.......................................................   30    1   0G2
...................................aauuccggauAuugaauuag.......................................................   4     1   0G2
...................................aauAccggaucuugaauuag.......................................................   2     1   0G2
...................................Nauuccggaucuugaauuag.......................................................   12    1   0G2
...................................aauuccggaucuugaaCuag.......................................................   4     1   0G2
...................................aauuccggaucuuUaauuag.......................................................   18    1   0G2
...................................Nauuccggaucuugaauuagg......................................................   2     1   0G2
...................................aauuccggaucuugaauuaUg......................................................   2     1   0G2
...................................aGuuccggaucuugaauuagg......................................................   14    1   0G2
...................................aauuccggaucuugaauuagg......................................................   558   0   0G2
...................................aauuccggaucuugaauuagA......................................................   8     1   0G2
...................................aauuccggaucuAgaauuagg......................................................   14    1   0G2
...................................aNuuccggaucuugaauuagg......................................................   2     1   0G2
...................................aauuccggaucAugaauuagg......................................................   8     1   0G2
....................................auuccggaucuugaauua........................................................   358   0   0G2
....................................aNuccggaucuugaauua........................................................   2     1   0G2
....................................auuccggaucuGgaauua........................................................   2     1   0G2
....................................Guuccggaucuugaauua........................................................   8     1   0G2
....................................auuccggaucuugaauAa........................................................   20    1   0G2
....................................auuccggaucuugaauCa........................................................   24    1   0G2
```

```
                    Star                                                   Mature
ggaucuugaauuaggcuguuucauuuuggaucuugaauuccggaucuugaauuaggcuguuugguuuugaaucuugaauuccggaucuugaauuaggcuguuugguuuug
....................................auuccggaGcuugaauua........................................................   2      1   0G2
....................................Nuuccggaucuugaauuag.......................................................   4      1   0G2
....................................auuccggauAuugaauuag.......................................................   2      1   0G2
....................................auuAcggaucuugaauuag.......................................................   2      1   0G2
....................................Guuccggaucuugaauuag.......................................................   4      1   0G2
....................................auuccggaucuGgaauuag.......................................................   24     1   0G2
....................................auuccggaucAugaauuag.......................................................   28     1   0G2
....................................auuccggaucuugaauuag.......................................................   2374   0   0G2
....................................auuccggaucuugaauCag.......................................................   22     1   0G2
....................................auuccggaucuugaaGuagg......................................................   2      1   0G2
....................................auuccggaucuugaauuagA......................................................   90     1   0G2
....................................auuccggGucuugaauuagg......................................................   2      1   0G2
....................................auuccggaucuugaauuagg......................................................   4754   0   0G2
....................................auuccggaucuGgaauuagg......................................................   4      1   0G2
....................................auuccggaucuugGauuagg......................................................   2      1   0G2
....................................auucGggaucuugaauuagg......................................................   18     1   0G2
....................................auuccggaucuAgaauuagg......................................................   38     1   0G2
....................................Nuuccggaucuugaauuagg......................................................   16     1   0G2
....................................aNuccggaucuugaauuagg......................................................   2      1   0G2
....................................auuccggaucuCgaauuagg......................................................   18     1   0G2
....................................auuccggCucuugaauuagg......................................................   18     1   0G2
....................................auuccggaCcuugaauuagg......................................................   2      1   0G2
....................................auuccggaucuugaauuaUg......................................................   26     1   0G2
....................................auuAcggaucuugaauuagg......................................................   6      1   0G2
....................................auuccggaucuugaauCagg......................................................   2      1   0G2
....................................auuccggaucAugaauuagg......................................................   118    1   0G2
....................................auuccggaucuugaaCuagg......................................................   32     1   0G2
....................................Guuccggaucuugaauuagg......................................................   64     1   0G2
.....................................uuccggGucuugaauuag.......................................................   8      1   0G2
.....................................Nuccggaucuugaauuag.......................................................   6      1   0G2
.....................................uuccggaucuugaauCag.......................................................   10     1   0G2
.....................................uuccggaucuGgaauuag.......................................................   8      1   0G2
.....................................uuccggaucuugGauuag.......................................................   2      1   0G2
.....................................uuccggaucuuUaauuag.......................................................   8      1   0G2
.....................................uuccggaucuugaauuag.......................................................   1866   0   0G2
.....................................uuccggaucuugaauuagg......................................................   2582   0   0G2
.....................................uuccggaucuAgaauuagg......................................................   22     1   0G2
.....................................uuccggaucuugaaCuagg......................................................   26     1   0G2
.....................................uuccggaucuugaauuagA......................................................   6      1   0G2
.....................................uuccggaucuugaauuaUg......................................................   8      1   0G2
.....................................uucGggaucuugaauuagg......................................................   20     1   0G2
.....................................Nuccggaucuugaauuagg......................................................   8      1   0G2
.....................................uuccggaucuGgaauuagg......................................................   8      1   0G2
.....................................uuccggaucAugaauuagg......................................................   28     1   0G2
.....................................uuccggCucuugaauuagg......................................................   2      1   0G2
.....................................uNccggaucuugaauuagg......................................................   4      1   0G2
.....................................uuccggaCcuugaauuagg......................................................   4      1   0G2
.....................................uuccggGucuugaauuagg......................................................   4      1   0G2
.....................................uuccggauGuugaauuagg......................................................   6      1   0G2
......................................uccggaucuGgaauuagg......................................................   6      1   0G2
......................................ucGggaucuugaauuagg......................................................   8      1   0G2
......................................uccggaucuugaauuagg......................................................   888    0   0G2
......................................uccggaucAugaauuagg......................................................   10     1   0G2
......................................uccggaucuugaauuaUg......................................................   2      1   0G2
......................................Nccggaucuugaauuagg......................................................   6      1   0G2
......................................uccggaucuugaauuagA......................................................   6      1   0G2
......................................uccggaucuCgaauuagg......................................................   20     1   0G2
......................................uccggaucuAgaauuagg......................................................   10     1   0G2
......................................uccggGucuugaauuagg......................................................   2      1   0G2
.......................................ccggaucuugaauuaggcuA...................................................   6      1   0G2
................................................................uuuuAaaucuugaauuccgg..........................   3      1   0G2
................................................................uuuugaaucuugaauuccgg..........................   62     0   0G2
................................................................uuuugaGucuugaauuccgg..........................   3      1   0G2
................................................................uuCugaaucuugaauuccgg..........................   11     1   0G2
................................................................uuuugaCucuugaauuccgg..........................   3      1   0G2
................................................................uuuugaaucGugaauuccgg..........................   4      1   0G2
................................................................uuuugaaucuugaauuccgga.........................   23     0   0G2
................................................................uuuugaCucuugaauuccgga.........................   10     1   0G2
................................................................uNuugaaucuugaauuccgga.........................   1      1   0G2
................................................................Nuuugaaucuugaauuccgga.........................   1      1   0G2
```

Star Mature

ggaucuugaauuaggcuguuucauuuuggaucuugaauuccggaucuugaauuaggcuguuugguuuugaaucuugaauuccggaucuugaauuaggcuguuugguuuug			
....uuuugaaucuugaauuccggauA.......................	18	1	0G2
....uuugaaucuugaauuccgg..........................	1	0	0G2
....uuugaaucuugaauuccgga.........................	2	0	0G2
....uuugaaucuugaauuccggauA.......................	14	1	0G2
....uuugaaucuugaauuccggauc.......................	5	0	0G2
....uugaaucuugaauuccggau........................	2	0	0G2
....Nugaaucuugaauuccggau........................	1	1	0G2
....uugaaucuugaauuccggauA.......................	27	1	0G2
....uugaaucGugaauuccggauc.......................	1	1	0G2
....uugaaucuugaauuccggaucA......................	12	1	0G2
....uugaaucuugaauuccggaucuugaau.................	13	0	0G2
....ugaGucuugaauuccggauc.......................	3	1	0G2
....ugaCucuugaauuccggauc.......................	4	1	0G2
....ugaaucuugaauuccggauc.......................	3	0	0G2
....ugaaucuugaauuccggauA.......................	10	1	0G2
....ugaaucuugaauuccggaucu......................	20	0	0G2
....ugaaucuugaauuccggaucA......................	1	1	0G2
....ugaaucuugaauuccggaucuAgaauu................	3	1	0G2
....ugaaucuugaauuccggaucuugaauu................	22	0	0G2
....ugaaucuugaauuccCgaucuugaauu................	3	1	0G2
....gaaucuugaauuccggauc.......................	6	0	0G2
....gaaucuugaauuccggaucuAgaauua...............	2	1	0G2
....aaucuugaauuccggaucA......................	5	1	0G2
....aaucuugaauuccggaucu......................	5	0	0G2
....aaucuugaauuccggaucuA.....................	5	1	0G2
....aaucuugaauuccggaucuAg....................	18	1	0G2
....aGucuugaauuccggaucuug....................	10	1	0G2
....aaucuugaauuccggaucuugUa..................	5	1	0G2
....aaucuugaauuccggaucuAgaauuag..............	5	1	0G2
....aucuugaauuccggaucuug....................	6	0	0G2
....aucuugaauuccggaucuAga...................	4	1	0G2
....aucuugaauuccggaucuugaau.................	16	0	0G2
....aucuugaauuccggaucuugaauua...............	26	0	0G2
....aucuugaauuccggaucuugaauuag..............	2	0	0G2
....ucuugaauuccggaucuA.....................	4	1	0G2
....ucuugaauuccggaucuug....................	2	0	0G2
....ucuugaauuccggaucuAga...................	102	1	0G2
....ucuugaauuccggaucuuga...................	38	0	0G2
....ucuugaauuccggaucuugaa..................	52	0	0G2
....ucGugaauuccggaucuugaa..................	32	1	0G2
....ucuugaauuccggaucuAgaa..................	148	1	0G2
....ucGugaauuccggaucuugaau.................	94	1	0G2
....ucuugaauuccggaucuAgaau.................	20	1	0G2
....ucuugaauuAcggaucuugaau.................	2	1	0G2
....ucuugaauuccggaucuugaau.................	454	0	0G2
....ucuugaauuccggaCcuugaau.................	2	1	0G2
....Ccuugaauuccggaucuugaau.................	2	1	0G2
....ucuugaauuccggaucuugaaG.................	6	1	0G2
....uGuugaauuccggaucuugaauu................	8	1	0G2
....uUuugaauuccggaucuugaauu................	22	1	0G2
....ucuugaauuccggaucuugaauu................	104	0	0G2
....ucuugaaGuccggaucuugaauu................	20	1	0G2
....ucuugaauuccggaucuGgaauua...............	6	1	0G2
....ucuugaauuccggaucuAgaauua...............	14	1	0G2
....ucuugaauuccggaucuugaauAa...............	330	1	0G2
....uUuugaauuccggaucuugaauua...............	12	1	0G2
....ucuugaauuccggaucuugaauua...............	200	0	0G2
....ucuugaauuccggaucuugaauuag..............	24	0	0G2
....ucuugaauuccggaucuAgaauuag..............	14	1	0G2
....ucuugaauuccggaucuugaauAag..............	6	1	0G2
....ucuugaauuccggaucuugaauuagg.............	20	0	0G2
....cuugaauuccggaucuug....................	14	0	0G2
....cuugaauuccggaucuAga...................	108	1	0G2
....cuugaauuccggaucuuga...................	4	0	0G2
....cuugaauuccggaucuAgaa..................	726	1	0G2
....Nuugaauuccggaucuugaa..................	4	1	0G2
....cGugaauuccggaucuugaa..................	2	1	0G2
....cuugaauuccggaucuugaa..................	192	0	0G2
....cuugaauuccggaucuAgaau.................	42	1	0G2
....cuugaauuccggaucuugaau.................	454	0	0G2

```
                    Star                                                      Mature
ggaucuugaauuaggcuguuucauuuuggaucuugaauuccggaucuugaauuaggcuguuugguuuugaaucuugaauuccggaucuugaauuaggcuguuugguuuug
........................................................................cGugaauuccggaucuugaau.................    30       1    0G2
........................................................................Nuugaauuccggaucuugaau.................    4        1    0G2
........................................................................cuugaauuccggaucuAgaauu................    148      1    0G2
........................................................................cuugaauuccggaucuugaauu................    452      0    0G2
........................................................................cAugaauuccggaucuugaauu................    2        1    0G2
........................................................................cNugaauuccggaucuugaauu................    2        1    0G2
........................................................................cuugaauuccggaucuugaaGua...............    2        1    0G2
........................................................................cuugaauuccggaucuugaaCua...............    10       1    0G2
........................................................................cuugaauuccggaucuugaauAa...............    136      1    0G2
........................................................................cuugaauuccggaucuAgaauua...............    320      1    0G2
........................................................................cuugaauuccggaucuugaauCa...............    70       1    0G2
........................................................................cuugaauuccggaucuugaauua...............    1032     0    0G2
........................................................................Nuugaauuccggaucuugaauua...............    2        1    0G2
........................................................................cuugaauuccggaucuugaauuag..............    8        0    0G2
........................................................................cuugaauuccggaucuAgaauuag..............    4        1    0G2
.........................................................................uugaauuccggaucuAga...................    52       1    0G2
.........................................................................uugaauuccggaucuAgaa..................    476      1    0G2
.........................................................................uugaauuccggaucuugaa..................    206      0    0G2
.........................................................................uAgaauuccggaucuugaau.................    4        1    0G2
.........................................................................uugaauuccggaGcuugaau.................    4        1    0G2
.........................................................................uugaGuuccggaucuugaau.................    114      1    0G2
.........................................................................Nugaauuccggaucuugaau.................    56       1    0G2
.........................................................................uugaauuAcggaucuugaau.................    6        1    0G2
.........................................................................Augaauuccggaucuugaau.................    12       1    0G2
.........................................................................uugaauuccggaucuugaau.................    14612    0    0G2
.........................................................................uGgaauuccggaucuugaau.................    4        1    0G2
.........................................................................Gugaauuccggaucuugaau.................    24       1    0G2
.........................................................................uugaauuccggauAuugaau.................    10       1    0G2
.........................................................................uugaauuccggaucuAgaau.................    2996     1    0G2
.........................................................................uugaaGuccggaucuugaau.................    2        1    0G2
.........................................................................uugaauuccggaucuugaaG.................    14       1    0G2
.........................................................................uugaauuccggaCcuugaau.................    2        1    0G2
.........................................................................uNgaauuccggaucuugaau.................    12       1    0G2
.........................................................................Cugaauuccggaucuugaau.................    4        1    0G2
.........................................................................uugaauuccggaucCugaau.................    2        1    0G2
.........................................................................uugaauuccggaucuGgaau.................    6        1    0G2
.........................................................................uugaauuccggCucuugaau.................    2        1    0G2
.........................................................................uugaauuccggGucuugaau.................    14       1    0G2
.........................................................................uugaauuccggaucuugaaC.................    6        1    0G2
.........................................................................uugaauuccggaucuGgaauu................    6        1    0G2
.........................................................................uugaauuccggaCcuugaauu................    10       1    0G2
.........................................................................Gugaauuccggaucuugaauu................    10       1    0G2
.........................................................................uugaaGuccggaucuugaauu................    4        1    0G2
.........................................................................uugaGuuccggaucuugaauu................    260      1    0G2
.........................................................................uNgaauuccggaucuugaauu................    8        1    0G2
.........................................................................Nugaauuccggaucuugaauu................    60       1    0G2
.........................................................................uugaauuccggaucCugaauu................    4        1    0G2
.........................................................................uugaauuccggaucuugaaGu................    8        1    0G2
.........................................................................uugaauuccggGucuugaauu................    8        1    0G2
.........................................................................uugaauuccggaucuAgaauu................    5240     1    0G2
.........................................................................uugaauuccggaGcuugaauu................    4        1    0G2
.........................................................................uugaauuccggaucuCgaauu................    10       1    0G2
.........................................................................uugaauuccggaucuuUaauu................    2        1    0G2
.........................................................................Cugaauuccggaucuugaauu................    6        1    0G2
.........................................................................Augaauuccggaucuugaauu................    4        1    0G2
.........................................................................uugaauuccggaucuugGauu................    14       1    0G2
.........................................................................uugaauuccggaucuugaaCu................    38       1    0G2
.........................................................................uugaauuccggaucuugaauu................    21266    0    0G2
.........................................................................uGgaauuccggaucuugaauu................    2        1    0G2
.........................................................................uugaauuAcggaucuugaauu................    4        1    0G2
.........................................................................uugaauuccggCucuugaauu................    8        1    0G2
.........................................................................uugaauuccggaucuugaaGua...............    6        1    0G2
.........................................................................uugaauuccggaucAugaauua...............    2        1    0G2
.........................................................................uugaauuccggaucuugCauua...............    2        1    0G2
.........................................................................uugaauuccggGucuugaauua...............    16       1    0G2
.........................................................................uugaauuccCgaucuugaauua...............    16       1    0G2
.........................................................................uugaauAccggaucuugaauua...............    22       1    0G2
.........................................................................uugaauuccggauAuugaauua...............    14       1    0G2
.........................................................................uugaaGuccggaucuugaauua...............    36       1    0G2
.........................................................................uugaauuccggaucuugaaCua...............    64       1    0G2
```

Star Mature

ggaucuugaauuaggcuguuucauuuuggaucuugaauuccggaucuugaauuaggcuguuugguuuugaaucuugaauuccggaucuugaauuaggcuguuugguuuug

....uugaauuccggaucCugaauua...............	18	1	0G2
....uugaauuccgCaucuugaauua...............	2	1	0G2
....uugaauuccggaucuuUaauua...............	6	1	0G2
....uNgaauuccggaucuugaauua...............	24	1	0G2
....uugaauuccggaucuugaauua...............	58180	0	0G2
....Augaauuccggaucuugaauua...............	14	1	0G2
....uugaGuuccggaucuugaauua...............	1278	1	0G2
....Cugaauuccggaucuugaauua...............	34	1	0G2
....uugaauuccggaucuugGauua...............	74	1	0G2
....uugaauuccggaucuCgaauua...............	10	1	0G2
....uugaauuAcggaucuugaauua...............	28	1	0G2
....uugaauuccggaGcuugaauua...............	14	1	0G2
....uugaauuccggaCcuugaauua...............	8	1	0G2
....Nugaauuccggaucuugaauua...............	164	1	0G2
....uugaCuuccggaucuugaauua...............	2	1	0G2
....uugaauuGcggaucuugaauua...............	2	1	0G2
....uugaauuccggaucuugaauCa...............	4852	1	0G2
....uugaauuccggauGuugaauua...............	6	1	0G2
....uugaauuccggaucuugaauAa...............	5360	1	0G2
....uugaauuccggaucuAgaauua...............	13424	1	0G2
....uAgaauuccggaucuugaauua...............	4	1	0G2
....uGgaauuccggaucuugaauua...............	2	1	0G2
....uugaauuccggaucuGgaauua...............	12	1	0G2
....Gugaauuccggaucuugaauua...............	24	1	0G2
....Nugaauuccggaucuugaauuag..............	2	1	0G2
....uugaauuccggaucuCgaauuag..............	8	1	0G2
....uugaauuccggaucuugaauCag..............	16	1	0G2
....uugaauuccggaucuugaauuag..............	606	0	0G2
....uugaauuccggaucuGgaauuag..............	2	1	0G2
....uugaauuccggaucuugaauAag..............	18	1	0G2
....uAgaauuccggaucuugaauuag..............	6	1	0G2
....uNgaauuccggaucuugaauuag..............	2	1	0G2
....Cugaauuccggaucuugaauuag..............	2	1	0G2
....uugaaGuccggaucuugaauuag..............	14	1	0G2
....uugaauuccggaucuAgaauuag..............	2626	1	0G2
....uugaGuuccggaucuugaauuag..............	14	1	0G2
....uugaauuccggaucuugGauuag..............	2	1	0G2
....uugaauuccggaucuugaaCuag..............	30	1	0G2
....uugaauuccggaucuugaauuagA.............	6	1	0G2
....uugaauuccggaucuugaauuagg.............	2	0	0G2
....uugaauuccggaucuugaauuaUg.............	2	1	0G2
....uugaauuccggaucAugaauuagg.............	22	1	0G2
....uugaauuccggaucuugaauuaggcuA..........	2	1	0G2
....ugaauuccggaucuAgaa..................	132	1	0G2
....ugaauuccggaucuGgaa..................	2	1	0G2
....ugaauuccggaucuugaa..................	4	0	0G2
....ugaauuccggaucuugaau.................	480	0	0G2
....ugaauuAcggaucuugaau.................	2	1	0G2
....ugaauuccggaucuAgaau.................	136	1	0G2
....ugaGuuccggaucuugaauu................	64	1	0G2
....ugaauuAcggaucuugaauu................	2	1	0G2
....uNaauuccggaucuugaauu................	2	1	0G2
....ugaauuccggaucuugaauu................	5018	0	0G2
....ugaauuccggaucuCgaauu................	2	1	0G2
....ugaauuccggaucuugaaCu................	8	1	0G2
....ugaauuccggCucuugaauu................	2	1	0G2
....Ngaauuccggaucuugaauu................	14	1	0G2
....Agaauuccggaucuugaauu................	14	1	0G2
....ugaauuccggaucuAgaauu................	1700	1	0G2
....ugaaGuccggaucuugaauu................	2	1	0G2
....ugaauuccggGucuugaauua...............	2	1	0G2
....ugaauuccggaGcuugaauua...............	14	1	0G2
....ugaauuccggaucuuUaauua...............	10	1	0G2
....ugaauuccggaucuugaauua...............	31676	0	0G2
....Ggaauuccggaucuugaauua...............	14	1	0G2
....Agaauuccggaucuugaauua...............	6	1	0G2
....ugaauuccggaCcuugaauua...............	8	1	0G2
....ugaauuccggaucCugaauua...............	6	1	0G2
....ugaauuccggaucuugaauCa...............	948	1	0G2
....ugaauuccggaucuugaauAa...............	294	1	0G2

Star   Mature

```
ggaucuugaauuaggcuguuucauuuuggaucuugaauuccggaucuugaauuaggcuguuugguuuugaaucuugaauuccggaucuugaauuaggcuguuugguuuug
..........................................................................ugaauuccggauAuugaauua...............    10      1    0G2
..........................................................................ugaGuuccggaucuugaauua...............    612     1    0G2
..........................................................................ugaauuccggaucuCgaauua...............    20      1    0G2
..........................................................................ugaauuccggCucuugaauua...............    6       1    0G2
..........................................................................ugaauuccggaucAugaauua...............    6       1    0G2
..........................................................................ugaaGuccggaucuugaauua...............    52      1    0G2
..........................................................................ugaauuccggaucuAgaauua...............    12002   1    0G2
..........................................................................ugaauuccCgaucuugaauua...............    2       1    0G2
..........................................................................ugaauuccggaucuugGauua...............    42      1    0G2
..........................................................................ugaauuccggaucuGgaauua...............    2       1    0G2
..........................................................................ugaauuccggaucuugaaCua...............    36      1    0G2
..........................................................................ugaauuAcggaucuugaauua...............    32      1    0G2
..........................................................................Ngaauuccggaucuugaauua...............    106     1    0G2
..........................................................................ugaauuccggaucuugUauua...............    4       1    0G2
..........................................................................uNaauuccggaucuugaauua...............    22      1    0G2
..........................................................................Ngaauuccggaucuugaauuag..............    14      1    0G2
..........................................................................ugaauuccggGucuugaauuag..............    2       1    0G2
..........................................................................Agaauuccggaucuugaauuag..............    2       1    0G2
..........................................................................ugaGuuccggaucuugaauuag..............    24      1    0G2
..........................................................................ugaauuccggaucuugGauuag..............    2       1    0G2
..........................................................................ugaauuccggaucuAgaauuag..............    9070    1    0G2
..........................................................................ugaauuccggaucuugaauCag..............    16      1    0G2
..........................................................................ugaauuccggaGcuugaauuag..............    2       1    0G2
..........................................................................ugaauuccggaucuugaauuag..............    2092    0    0G2
..........................................................................ugaauuccggaucuGgaauuag..............    4       1    0G2
..........................................................................ugaauuccggaucuugaauAag..............    2       1    0G2
..........................................................................ugaauuccggaucuugaauuagg.............    146     0    0G2
..........................................................................ugaauuccggaucuugaauuagA.............    4       1    0G2
..........................................................................ugaaGuccggaucuugaauuagg.............    10      1    0G2
...........................................................................gaauuccggaucuugaau.................    20      0    0G2
...........................................................................gaauuccggaucuAgaau.................    30      1    0G2
...........................................................................gaauuccggaucuuUaauu................    2       1    0G2
...........................................................................gaauuccggaucuCgaauu................    4       1    0G2
...........................................................................gaauuccggauAuugaauu................    2       1    0G2
...........................................................................gaauuccggaucuugaauu................    286     0    0G2
...........................................................................gaauuccggaucuAgaauu................    70      1    0G2
...........................................................................gaauuccggaucuugaauCa...............    206     1    0G2
...........................................................................gaauuccggaucuugaauua...............    2166    0    0G2
...........................................................................Naauuccggaucuugaauua...............    10      1    0G2
...........................................................................gaauuccggaucCugaauua...............    2       1    0G2
...........................................................................gaauuccggaucuuUaauua...............    2       1    0G2
...........................................................................gaauuccggaucuugaaCua...............    8       1    0G2
...........................................................................gaauuccggaucuAgaauua...............    1214    1    0G2
...........................................................................gaauuccggauAuugaauua...............    2       1    0G2
...........................................................................gaauuccggaucuugaauAa...............    8       1    0G2
...........................................................................gaGuuccggaucuugaauua...............    26      1    0G2
...........................................................................gaauuccggaGcuugaauua...............    2       1    0G2
...........................................................................gNauuccggaucuugaauua...............    2       1    0G2
...........................................................................gaauuccggaucuugaauuag..............    1122    0    0G2
...........................................................................gaGuuccggaucuugaauuag..............    12      1    0G2
...........................................................................gaauuccggaucuAgaauuag..............    6458    1    0G2
...........................................................................gaauuccggaucuugaauuagg.............    10      0    0G2
...........................................................................gaauuccggaucuugaauuagA.............    16      1    0G2
...........................................................................gaauuccggaucuAgaauuagg.............    6       1    0G2
............................................................................aauuccggaucuugaauu................    8       0    0G2
............................................................................aNuuccggaucuugaauua...............    2       1    0G2
............................................................................aauuccggaucuugaauAa...............    22      1    0G2
............................................................................aauuccggaucuugaauua...............    992     0    0G2
............................................................................aauuccggaucuugaauCa...............    38      1    0G2
............................................................................aauuccggaucuugaaCuag..............    4       1    0G2
............................................................................aauuccggaucAugaauuag..............    4       1    0G2
............................................................................aauuccggaucuGgaauuag..............    12      1    0G2
............................................................................aauuccggaucuugaauuag..............    8186    0    0G2
............................................................................aauuccggaCcuugaauuag..............    4       1    0G2
............................................................................aauuccggaucuugaauCag..............    40      1    0G2
............................................................................aauuccggaucuuUaauuag..............    18      1    0G2
............................................................................aNuuccggaucuugaauuag..............    2       1    0G2
............................................................................aauuccggaGcuugaauuag..............    2       1    0G2
............................................................................aauAccggaucuugaauuag..............    2       1    0G2
............................................................................aauuccggGucuugaauuag..............    14      1    0G2
```

```
                    Star                                             Mature
ggaucuugaauuaggcuguuucauuuuggaucuugaauuccggaucuugaauuaggcuguuugguuuugaaucuugaauuccggaucuugaauuaggcuguuugguuuug
............................................................................aauuccggaucuCgaauuag..............   30     1   0G2
............................................................................aauuccggauAuugaauuag..............   4      1   0G2
............................................................................aGuuccggaucuugaauuag..............   112    1   0G2
............................................................................Nauuccggaucuugaauuag..............   12     1   0G2
............................................................................aauuccggaucuugaauuaUg.............   2      1   0G2
............................................................................aGuuccggaucuugaauuagg.............   14     1   0G2
............................................................................aauuccggaucAugaauuagg.............   8      1   0G2
............................................................................aNuuccggaucuugaauuagg.............   2      1   0G2
............................................................................aauuccggaucuugaauuagA.............   8      1   0G2
............................................................................aauuccggaucuugaauuagg.............   558    0   0G2
............................................................................aauuccggaucuAgaauuagg.............   14     1   0G2
............................................................................Nauuccggaucuugaauuagg.............   2      1   0G2
.............................................................................auuccggaucuugaauAa...............   20     1   0G2
.............................................................................auuccggaucuugaauCa...............   24     1   0G2
.............................................................................auuccggaucuGgaauua...............   2      1   0G2
.............................................................................Guuccggaucuugaauua...............   8      1   0G2
.............................................................................aNuccggaucuugaauua...............   2      1   0G2
.............................................................................auuccggaGcuugaauua...............   2      1   0G2
.............................................................................auuccggaucuugaauua...............   358    0   0G2
.............................................................................auuccggaucuugaauuag..............   2374   0   0G2
.............................................................................Guuccggaucuugaauuag..............   4      1   0G2
.............................................................................auuAcggaucuugaauuag..............   2      1   0G2
.............................................................................Nuuccggaucuugaauuag..............   4      1   0G2
.............................................................................auuccggaucAugaauuag..............   28     1   0G2
.............................................................................auuccggaucuugaauCag..............   22     1   0G2
.............................................................................auuccggaucuGgaauuag..............   24     1   0G2
.............................................................................auuccggauAuugaauuag..............   2      1   0G2
.............................................................................auuccggGucuugaauuagg.............   2      1   0G2
.............................................................................auuccggaucuugaauCagg.............   2      1   0G2
.............................................................................auuccggaCcuugaauuagg.............   2      1   0G2
.............................................................................auuccggaucuugaaCuagg.............   32     1   0G2
.............................................................................auucGggaucuugaauuagg.............   18     1   0G2
.............................................................................auuccggaucuugaauuagg.............   4754   0   0G2
.............................................................................auuccggaucuugaaGuagg.............   2      1   0G2
.............................................................................auuccggaucuugGauuagg.............   2      1   0G2
.............................................................................auuccggaucuAgaauuagg.............   38     1   0G2
.............................................................................Guuccggaucuugaauuagg.............   64     1   0G2
.............................................................................Nuuccggaucuugaauuagg.............   16     1   0G2
.............................................................................auuccggaucuugaauuagA.............   90     1   0G2
.............................................................................aNuccggaucuugaauuagg.............   2      1   0G2
.............................................................................auuccggaucuGgaauuagg.............   4      1   0G2
.............................................................................auuccggaucuugaauuaUg.............   26     1   0G2
.............................................................................auuAcggaucuugaauuagg.............   6      1   0G2
.............................................................................auuccggaucuCgaauuagg.............   18     1   0G2
.............................................................................auuccggaucAugaauuagg.............   118    1   0G2
.............................................................................auuccggCucuugaauuagg.............   18     1   0G2
..............................................................................uuccggaucuugGauuag..............   2      1   0G2
..............................................................................uuccggaucuugaauuag..............   1866   0   0G2
..............................................................................uuccggaucuuUaauuag..............   8      1   0G2
..............................................................................uuccggaucuugaauCag..............   10     1   0G2
..............................................................................uuccggGucuugaauuag..............   8      1   0G2
..............................................................................uuccggaucuGgaauuag..............   8      1   0G2
..............................................................................Nuccggaucuugaauuag..............   6      1   0G2
..............................................................................uuccggauGuugaauuagg.............   6      1   0G2
..............................................................................uuccggaucuugaauuagA.............   6      1   0G2
..............................................................................uuccggaucAugaauuagg.............   28     1   0G2
..............................................................................uNccggaucuugaauuagg.............   4      1   0G2
..............................................................................uuccggaucuGgaauuagg.............   8      1   0G2
..............................................................................uuccggaucuugaauuagg.............   2582   0   0G2
..............................................................................uuccggaCcuugaauuagg.............   4      1   0G2
..............................................................................uucGggaucuugaauuagg.............   20     1   0G2
..............................................................................uuccggCucuugaauuagg.............   2      1   0G2
..............................................................................uuccggaucuugaauuaUg.............   8      1   0G2
..............................................................................uuccggaucuAgaauuagg.............   22     1   0G2
..............................................................................Nuccggaucuugaauuagg.............   8      1   0G2
..............................................................................uuccggGucuugaauuagg.............   4      1   0G2
..............................................................................uuccggaucuugaaCuagg.............   26     1   0G2
...............................................................................uccggGucuugaauuagg.............   2      1   0G2
...............................................................................uccggaucuCgaauuagg.............   20     1   0G2
...............................................................................uccggaucuAgaauuagg.............   10     1   0G2
```

```
                    Star                                                   Mature
ggaucuugaauuaggcuguuucauuuuggaucuugaauuccggaucuugaauuaggcuguuugguuuugaaucuugaauuccggaucuugaauuaggcuguuugguuuug
...............................................................................ucGggaucuugaauuagg.............   8     1     0G2
...............................................................................Nccggaucuugaauuagg.............   6     1     0G2
...............................................................................uccggaucuugaauuaUg.............   2     1     0G2
...............................................................................uccggaucAugaauuagg.............   10    1     0G2
...............................................................................uccggaucuGgaauuagg.............   6     1     0G2
...............................................................................uccggaucuugaauuagg.............   888   0     0G2
...............................................................................uccggaucuugaauuagA.............   6     1     0G2
................................................................................ccggaucuugaauuaggcuA..........   6     1     0G2
```

Provisional ID	:	scaffold216_30740
Score total	:	5.2
Score for star read(s)	:	-1.3
Score for read counts	:	0
Score for mfe	:	1.9
Score for randfold	:	1.6
Score for cons. seed	:	3
Total read count	:	26918
Mature read count	:	26918
Loop read count	:	0
Star read count	:	0

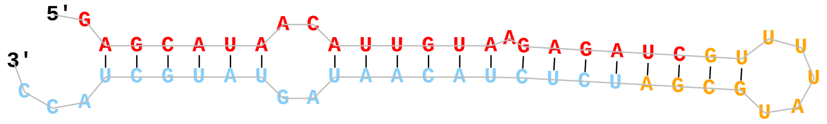

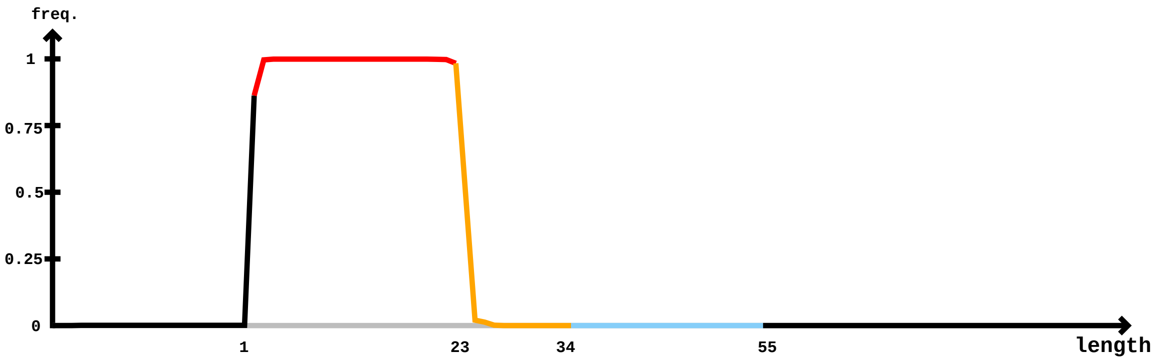

Mature   Star

```
5'- aguauguuggaauuccggggagcauaacauuguaagagaucguuuuaugcgaucucuacaauaguaugcuaccuggcauauccuaugcaauaugcaguuucuacguugga  -3'   exp
    .((((((((((((.(((((.((((((..((((((.((((((((.....))))))))))))))..)))))).))))).)).))).....)))))))...............   reads   mm   sample
    .guauguuggaauuccggg...........................................................................................   15      0    0B2
    ...................Uagcauaacauuguaagaga.......................................................................   19      1    0B2
    ...................Uagcauaacauuguaagagau......................................................................   129     1    0B2
    ...................Aagcauaacauuguaagagauc.....................................................................   1       1    0B2
    ...................gagcauaacauuguaagagauc.....................................................................   1       0    0B2
    ...................Nagcauaacauuguaagagauc.....................................................................   84      1    0B2
    ...................Cagcauaacauuguaagagauc.....................................................................   1       1    0B2
    ...................Uagcauaacauuguaagagauc.....................................................................   10480   1    0B2
    ...................Nagcauaacauuguaagagaucg....................................................................   29      1    0B2
    ...................Uagcauaacauuguaagagaucg....................................................................   2715    1    0B2
    ...................Uagcauaacauuguaagagaucgu...................................................................   83      1    0B2
    ...................Uagcauaacauuguaagagaucguu..................................................................   115     1    0B2
    ...................Nagcauaacauuguaagagaucguu..................................................................   1       1    0B2
    ...................Uagcauaacauuguaagagaucguuu.................................................................   5       1    0B2
    ....................agcauaacauuguaagagau......................................................................   8       0    0B2
    ....................agcauaacauGguaagagauc.....................................................................   1       1    0B2
    ....................agcaCaacauuguaagagauc.....................................................................   1       1    0B2
    ....................agcauaacauuguaagagauA.....................................................................   26      1    0B2
    ....................Ngcauaacauuguaagagauc.....................................................................   12      1    0B2
    ....................agcauaacauuguaagagauc.....................................................................   1230    0    0B2
    ....................agcauaGcauuguaagagauc.....................................................................   1       1    0B2
    ....................agcauaacauuguaagagaCc.....................................................................   4       1    0B2
    ....................Ggcauaacauuguaagagauc.....................................................................   2       1    0B2
    ....................agcauaacaGuguaagagauc.....................................................................   1       1    0B2
    ....................agcauaacauuguaagGgauc.....................................................................   1       1    0B2
    ....................agcauaacauuguaagagaucA....................................................................   104     1    0B2
    ....................agcauaacauugGaagagaucg....................................................................   2       1    0B2
    ....................agcauaacauuguaagagaucg....................................................................   453     0    0B2
    ....................agcauaacauuguaagagaucU....................................................................   33      1    0B2
    ....................Ngcauaacauuguaagagaucg....................................................................   3       1    0B2
    ....................agcauaacauuguaagagaGcg....................................................................   1       1    0B2
    ....................agcauaGcauuguaagagaucg....................................................................   1       1    0B2
    ....................agcauaacauuguaagaUaucg....................................................................   1       1    0B2
    ....................agcauaacauuguaagagaucgA...................................................................   4       1    0B2
```

```
                    Mature                          Star
aguauguuggaauuccggggagcauaacauuguaagagaucguuuuaugcgaucucuacaauaguaugcuaccuggcauauccuaugcaauaugcaguuucuacguugga
....................agcauaacauuguaagagaucgu...................................................................  2     0  0B2
....................agcauaacauuguaagagaucguu..................................................................  4     0  0B2
....................agcauaacauuguaagagaucguG..................................................................  8     1  0B2
.....................gcauaacauuguaagagauc.....................................................................  32    0  0B2
.....................gcauaacauuguaagagauA.....................................................................  6     1  0B2
.....................gcauaacauuguaagagaucg....................................................................  5     0  0B2

aguauguuggaauuccggg...........................................................................................  3     0  0G2
...................Uagcauaacauuguaagag........................................................................  3     1  0G2
...................Uagcauaacauuguaagaga.......................................................................  2     1  0G2
...................Uagcauaacauuguaagagau......................................................................  95    1  0G2
...................Uagcauaacauuguaagagauc.....................................................................  2186  1  0G2
...................Cagcauaacauuguaagagauc.....................................................................  1     1  0G2
...................Aagcauaacauuguaagagauc.....................................................................  1     1  0G2
...................Nagcauaacauuguaagagauc.....................................................................  5     1  0G2
...................Nagcauaacauuguaagagaucg....................................................................  1     1  0G2
...................Uagcauaacauuguaagagaucg....................................................................  892   1  0G2
...................Uagcauaacauuguaagagaucgu...................................................................  22    1  0G2
...................Uagcauaacauuguaagagaucguu..................................................................  19    1  0G2
...................Uagcauaacauuguaagagaucguuu.................................................................  4     1  0G2
....................agcauaacauuguaagagau......................................................................  1     0  0G2
....................agcaCaacauuguaagagauc.....................................................................  1     1  0G2
....................aAcauaacauuguaagagauc.....................................................................  3     1  0G2
....................agcauaacauuguaagagauA.....................................................................  20    1  0G2
....................Ngcauaacauuguaagagauc.....................................................................  1     1  0G2
....................agcauaacauuguaagagauc.....................................................................  331   0  0G2
....................agcauaacauuguaagagaGcg....................................................................  1     1  0G2
....................agcauaacauuguaagagaucg....................................................................  142   0  0G2
....................agcauaGcauuguaagagaucg....................................................................  4     1  0G2
....................agcauaacauuguaaAagaucg....................................................................  1     1  0G2
....................agcauaacauuguaagagaucU....................................................................  12    1  0G2
....................agcauaacauuguaagagaucA....................................................................  126   1  0G2
....................agcaCaacauuguaagagaucg....................................................................  1     1  0G2
....................agcauaacauuguaagagaucgA...................................................................  10    1  0G2
....................agcauaacauuguaagagaucAuu..................................................................  14    1  0G2
....................agcauaacauuguaagagaucgAuu.................................................................  9     1  0G2
.....................gcauaacauuguaagagauc.....................................................................  11    0  0G2
.....................gcauaacauuguaagagaucA....................................................................  4     1  0G2

.guauguuggaauuccggg...........................................................................................  7     0  0A2
...................Uagcauaacauuguaagag........................................................................  12    1  0A2
...................Uagcauaacauuguaagaga.......................................................................  4     1  0A2
...................Nagcauaacauuguaagagau......................................................................  3     1  0A2
...................Uagcauaacauuguaagagau......................................................................  135   1  0A2
...................Uagcauaacauuguaagagauc.....................................................................  4273  1  0A2
...................Nagcauaacauuguaagagauc.....................................................................  6     1  0A2
...................Nagcauaacauuguaagagaucg....................................................................  3     1  0A2
...................Uagcauaacauuguaagagaucg....................................................................  1684  1  0A2
...................Aagcauaacauuguaagagaucg....................................................................  1     1  0A2
...................Uagcauaacauuguaagagaucgu...................................................................  69    1  0A2
...................Uagcauaacauuguaagagaucguu..................................................................  133   1  0A2
...................Uagcauaacauuguaagagaucguuu.................................................................  19    1  0A2
....................agcauaacauuguaagag........................................................................  3     0  0A2
....................agcauaacauuguaagagau......................................................................  9     0  0A2
....................agcauaacauuguaaUagauc.....................................................................  1     1  0A2
....................agcauaacGuuguaagagauc.....................................................................  1     1  0A2
....................agcauaacauuguaagagauc.....................................................................  496   0  0A2
....................Ngcauaacauuguaagagauc.....................................................................  1     1  0A2
....................agcauaacauuguaagagauA.....................................................................  9     1  0A2
....................agcauaacauuguaagagCuc.....................................................................  1     1  0A2
....................agcauaacaGuguaagagauc.....................................................................  1     1  0A2
....................agcauaacauuguaagagaucA....................................................................  128   1  0A2
....................agcauaacauuguaagagaucU....................................................................  15    1  0A2
....................agcauaacauuguaagagaucg....................................................................  329   0  0A2
....................agcauaaAauuguaagagaucg....................................................................  1     1  0A2
....................agcauaacGuuguaagagaucg....................................................................  2     1  0A2
....................agUauaacauuguaagagaucg....................................................................  1     1  0A2
....................agcauaacauuguaagagauUg....................................................................  1     1  0A2
....................agcauaacauuguaagagaucgu...................................................................  10    0  0A2
....................agcauaacauuguaagagaucguu..................................................................  3     0  0A2
```

Mature **Star**

```
aguauguuggaauuccggggagcauaacauuguaagagaucguuuuaugcgaucucuacaauaguaugcuaccuggcauauccuaugcaauaugcaguuucuacguugga
....................agcauaacauuguaagagaucAuu..................................................................   14   1   0A2
.....................gcauaacauuguaagagauc.....................................................................   5    0   0A2
.....................gcauaacauuguaagagauA.....................................................................   3    1   0A2
.....................gcauaacauuguaagagaucg....................................................................   4    0   0A2
.....................gcauaacauuguaagagaucU....................................................................   6    1   0A2
```

Provisional ID	:	scaffold216_30742
Score total	:	5.2
Score for star read(s)	:	-1.3
Score for read counts	:	0
Score for mfe	:	1.9
Score for randfold	:	1.6
Score for cons. seed	:	3
Total read count	:	26918
Mature read count	:	26918
Loop read count	:	0
Star read count	:	0

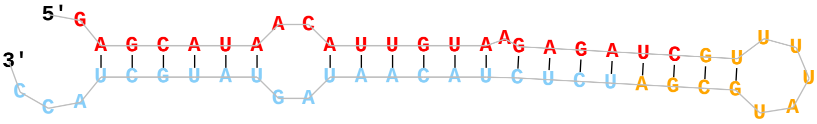

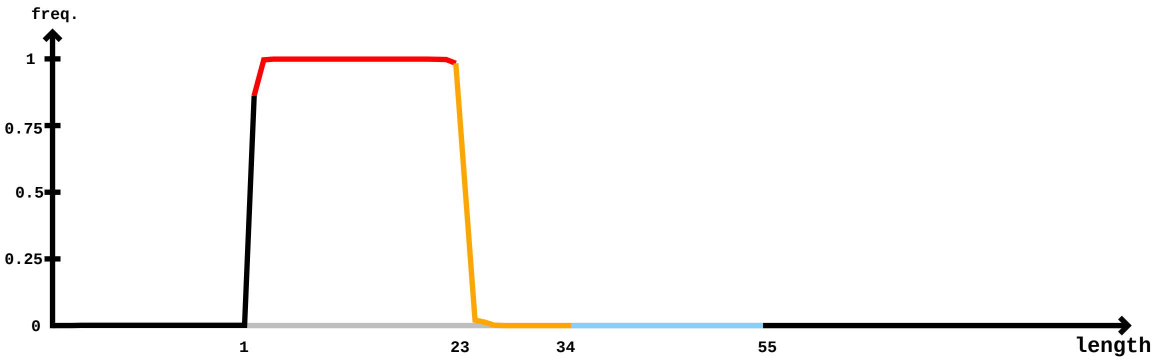

Mature   Star

```
5'- aguauguuggaauuccggggagcauaacauuguaagagaucguuuuaugcgaucucuacaauaguaugcuaccuggcauauccuaugcaauaugcaguuucuacguugga  -3'  exp
    .((((((((((((.(((((.((((((..((((((.((((((((.....))))))))))))))..)))))).))))).)).))).....)))))))...............   reads   mm   sample
    .guauguuggaauuccggg...........................................................................................   7       0    0A2
    ...................Uagcauaacauuguaagag........................................................................   12      1    0A2
    ...................Uagcauaacauuguaagaga.......................................................................   4       1    0A2
    ...................Nagcauaacauuguaagagau......................................................................   3       1    0A2
    ...................Uagcauaacauuguaagagau......................................................................   135     1    0A2
    ...................Nagcauaacauuguaagagauc.....................................................................   6       1    0A2
    ...................Uagcauaacauuguaagagauc.....................................................................   4273    1    0A2
    ...................Aagcauaacauuguaagagaucg....................................................................   1       1    0A2
    ...................Nagcauaacauuguaagagaucg....................................................................   3       1    0A2
    ...................Uagcauaacauuguaagagaucg....................................................................   1684    1    0A2
    ...................Uagcauaacauuguaagagaucgu...................................................................   69      1    0A2
    ...................Uagcauaacauuguaagagaucguu..................................................................   133     1    0A2
    ...................Uagcauaacauuguaagagaucguuu.................................................................   19      1    0A2
    ....................agcauaacauuguaagag........................................................................   3       0    0A2
    ....................agcauaacauuguaagagau......................................................................   9       0    0A2
    ....................agcauaacauuguaaUagauc.....................................................................   1       1    0A2
    ....................Ngcauaacauuguaagagauc.....................................................................   1       1    0A2
    ....................agcauaacauuguaagagauA.....................................................................   9       1    0A2
    ....................agcauaacauuguaagagauc.....................................................................   496     0    0A2
    ....................agcauaacauuguaagagCuc.....................................................................   1       1    0A2
    ....................agcauaacaGuguaagagauc.....................................................................   1       1    0A2
    ....................agcauaacGuuguaagagauc.....................................................................   1       1    0A2
    ....................agcauaacGuuguaagagaucg....................................................................   2       1    0A2
    ....................agcauaacauuguaagagaucg....................................................................   329     0    0A2
    ....................agUauaacauuguaagagaucg....................................................................   1       1    0A2
    ....................agcauaacauuguaagagaucU....................................................................   15      1    0A2
    ....................agcauaacauuguaagagaucA....................................................................   128     1    0A2
    ....................agcauaacauuguaagagauUg....................................................................   1       1    0A2
    ....................agcauaaAauuguaagagaucg....................................................................   1       1    0A2
    ....................agcauaacauuguaagagaucgu...................................................................   10      0    0A2
    ....................agcauaacauuguaagagaucguu..................................................................   3       0    0A2
    ....................agcauaacauuguaagagaucAuu..................................................................   14      1    0A2
    .....................gcauaacauuguaagagauA.....................................................................   3       1    0A2
    .....................gcauaacauuguaagagauc.....................................................................   5       0    0A2
```

Mature **Star**

```
aguauguuggaauuccggggagcauaacauuguaagagaucguuuuaugcgaucucuacaauaguaugcuaccuggcauauccuaugcaauaugcaguuucuacguugga
.....................gcauaacauuguaagagaucU....................................................................    6      1  0A2
.....................gcauaacauuguaagagaucg....................................................................    4      0  0A2

.guauguuggaauuccggg...........................................................................................    15     0  0B2
...................Uagcauaacauuguaagaga.......................................................................    19     1  0B2
...................Uagcauaacauuguaagagau......................................................................    129    1  0B2
...................gagcauaacauuguaagagauc.....................................................................    1      0  0B2
...................Uagcauaacauuguaagagauc.....................................................................    10480  1  0B2
...................Nagcauaacauuguaagagauc.....................................................................    84     1  0B2
...................Cagcauaacauuguaagagauc.....................................................................    1      1  0B2
...................Aagcauaacauuguaagagauc.....................................................................    1      1  0B2
...................Uagcauaacauuguaagagaucg....................................................................    2715   1  0B2
...................Nagcauaacauuguaagagaucg....................................................................    29     1  0B2
...................Uagcauaacauuguaagagaucgu...................................................................    83     1  0B2
...................Nagcauaacauuguaagagaucguu..................................................................    1      1  0B2
...................Uagcauaacauuguaagagaucguu..................................................................    115    1  0B2
...................Uagcauaacauuguaagagaucguuu.................................................................    5      1  0B2
....................agcauaacauuguaagagau......................................................................    8      0  0B2
....................agcauaacauGguaagagauc.....................................................................    1      1  0B2
....................agcauaacauuguaagagauc.....................................................................    1230   0  0B2
....................Ngcauaacauuguaagagauc.....................................................................    12     1  0B2
....................agcaCaacauuguaagagauc.....................................................................    1      1  0B2
....................agcauaacauuguaagGgauc.....................................................................    1      1  0B2
....................agcauaGcauuguaagagauc.....................................................................    1      1  0B2
....................Ggcauaacauuguaagagauc.....................................................................    2      1  0B2
....................agcauaacauuguaagagauA.....................................................................    26     1  0B2
....................agcauaacaGuguaagagauc.....................................................................    1      1  0B2
....................agcauaacauuguaagagaCc.....................................................................    4      1  0B2
....................agcauaacauuguaagagaucA....................................................................    104    1  0B2
....................agcauaacauuguaagaUaucg....................................................................    1      1  0B2
....................agcauaacauuguaagagaucU....................................................................    33     1  0B2
....................Ngcauaacauuguaagagaucg....................................................................    3      1  0B2
....................agcauaGcauuguaagagaucg....................................................................    1      1  0B2
....................agcauaacauuguaagagaucg....................................................................    453    0  0B2
....................agcauaacauugGaagagaucg....................................................................    2      1  0B2
....................agcauaacauuguaagagaGcg....................................................................    1      1  0B2
....................agcauaacauuguaagagaucgA...................................................................    4      1  0B2
....................agcauaacauuguaagagaucgu...................................................................    2      0  0B2
....................agcauaacauuguaagagaucguG..................................................................    8      1  0B2
....................agcauaacauuguaagagaucguu..................................................................    4      0  0B2
.....................gcauaacauuguaagagauA.....................................................................    6      1  0B2
.....................gcauaacauuguaagagauc.....................................................................    32     0  0B2
.....................gcauaacauuguaagagaucg....................................................................    5      0  0B2

aguauguuggaauuccggg...........................................................................................    3      0  0G2
...................Uagcauaacauuguaagag........................................................................    3      1  0G2
...................Uagcauaacauuguaagaga.......................................................................    2      1  0G2
...................Uagcauaacauuguaagagau......................................................................    95     1  0G2
...................Cagcauaacauuguaagagauc.....................................................................    1      1  0G2
...................Aagcauaacauuguaagagauc.....................................................................    1      1  0G2
...................Uagcauaacauuguaagagauc.....................................................................    2186   1  0G2
...................Nagcauaacauuguaagagauc.....................................................................    5      1  0G2
...................Uagcauaacauuguaagagaucg....................................................................    892    1  0G2
...................Nagcauaacauuguaagagaucg....................................................................    1      1  0G2
...................Uagcauaacauuguaagagaucgu...................................................................    22     1  0G2
...................Uagcauaacauuguaagagaucguu..................................................................    19     1  0G2
...................Uagcauaacauuguaagagaucguuu.................................................................    4      1  0G2
....................agcauaacauuguaagagau......................................................................    1      0  0G2
....................agcauaacauuguaagagauc.....................................................................    331    0  0G2
....................Ngcauaacauuguaagagauc.....................................................................    1      1  0G2
....................aAcauaacauuguaagagauc.....................................................................    3      1  0G2
....................agcauaacauuguaagagauA.....................................................................    20     1  0G2
....................agcaCaacauuguaagagauc.....................................................................    1      1  0G2
....................agcauaacauuguaaAagaucg....................................................................    1      1  0G2
....................agcauaacauuguaagagaucA....................................................................    126    1  0G2
....................agcauaGcauuguaagagaucg....................................................................    4      1  0G2
....................agcauaacauuguaagagaucU....................................................................    12     1  0G2
....................agcaCaacauuguaagagaucg....................................................................    1      1  0G2
....................agcauaacauuguaagagaucg....................................................................    142    0  0G2
....................agcauaacauuguaagagaGcg....................................................................    1      1  0G2
```

Mature **Star**

```
aguauguuggaauuccggggagcauaacauuguaagagaucguuuuaugcgaucucuacaauaguaugcuaccuggcauauccuaugcaauaugcaguuucuacguugga
....................agcauaacauuguaagagaucgA...................................................................	10	1	0G2
....................agcauaacauuguaagagaucAuu..................................................................	14	1	0G2
....................agcauaacauuguaagagaucgAuu.................................................................	9	1	0G2
.....................gcauaacauuguaagagauc.....................................................................	11	0	0G2
.....................gcauaacauuguaagagaucA....................................................................	4	1	0G2
```

Provisional ID	:	scaffold265_34282
Score total	:	3087.2
Score for star read(s)	:	3.9
Score for read counts	:	3076.4
Score for mfe	:	2.3
Score for randfold	:	1.6
Score for cons. seed	:	3
Total read count	:	6046
Mature read count	:	5614
Loop read count	:	0
Star read count	:	432

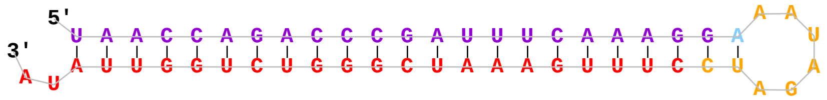

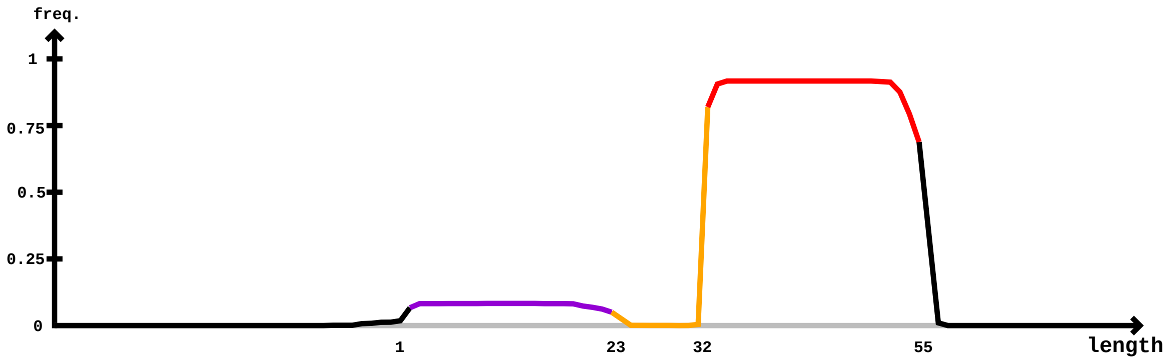

Star **Mature**

5'-	caauauacgccccugccuccuacgccuuagccugauaaccagacccgauuucaaaggaaauagaucuuugaaaucgggucugguuauaaggcuaccuacgccugugaacg	-3'	obs		
	caauauacgccccugccuccuacgccuuagccugauaaccagacccgauuucaaaggaaauagaucuuugaaaucgggucugguuauaaggcuaccuacgccugugaacg		exp		
	...((((.((....((.......))..((((((.((((((((((((((((((((((((......)))))))))))))))))))))))).)))))).....)).))))....		reads	mm	sample
	...........................uagccugauaaccagacccgauuuA...		2	1	0A2
	..............................cAugauaaccagacccgauuuca..		20	1	0A2
	...............................cugauaaccaCacccgauuuca..		1	1	0A2
	...............................Augauaaccagacccgauuuca..		7	1	0A2
	................................ugauaaccagaAccgauuucaa...		10	1	0A2
	................................ugauaaccagacccgauuucaa...		2	0	0A2
	................................ugauaaccagaAccgauuucaaa..		5	1	0A2
	..................................auaaccagacccgauuucaa...		4	0	0A2
	..................................auaaccagacccgauuucaaa..		1	0	0A2
	..................................auaaccagaAccgauuucaaa..		6	1	0A2
	...................................uaaccagaAccgauuucaaa..		1	1	0A2
	...................................uaaccagacccgauuucaaa..		1	0	0A2
	...................................Caaccagacccgauuucaaa..		3	1	0A2
	...................................Caaccagacccgauuucaaag...		10	1	0A2
	...................................uaaccagacccgauuucaaag...		8	0	0A2
	...................................uaGccagacccgauuucaaagg..		4	1	0A2
	...................................Caaccagacccgauuucaaagg..		99	1	0A2
	...................................uaaccagacccgauuucaaaAg..		4	1	0A2
	...................................Caaccagacccgauuucaaagga...		4	1	0A2
	....................................aaccagacccgauuucaa...		8	0	0A2
	....................................aaccagacccgauuucaaaA...		2	1	0A2
	....................................aaccagacccgauuucaaag...		3	0	0A2
	....................................aaccagacccgauuucaaagg..		3	0	0A2
	....................................aaccagacccgauuucaaaAga...		23	1	0A2
	....cagacccgauuucaaaAgaa..		1	1	0A2
	....cagacccgauuucaaaAgaaa...		1	1	0A2
	....ucUuuugaaaucgggucugguua........................		1	1	0A2
	....ucUuuugaaaucgggucugguuau.......................		1	1	0A2
	....ccuuugaaaucgggucugguua........................		2	0	0A2
	....ccuuugaaaucgggucugUuua........................		6	1	0A2
	....cUuuugaaaucgggucugguuaua......................		1	1	0A2
	....cUuuugaaaucgggucugguuauaa.....................		4	1	0A2
	....cuuugaaaucgggucugg...........................		1	0	0A2

Star **Mature**

caauauacgccccugccuccuacgccuuagccugauaaccagacccgauuucaaaggaaauagauccuuugaaaucgggucugguuauaaggcuaccuacgccugugaacg			
....cuuugaaaucgggucuggu..........................	2	0	0A2
....cuuugaaaucgggucugguu.........................	68	0	0A2
....cuuugaGaucgggucugguua........................	1	1	0A2
....cuuugaaaucgggucugguuG........................	10	1	0A2
....cuuugaaaucgggucugguuC........................	5	1	0A2
....cuuugaaauGgggucugguua........................	1	1	0A2
....Nuuugaaaucgggucugguua........................	1	1	0A2
....cuuugaaaucgggucugguua........................	186	0	0A2
....cuuugaaaucgggCcugguuau.......................	1	1	0A2
....cuuugaaaucgggucugguuau.......................	150	0	0A2
....cuuugaCaucgggucugguuau.......................	1	1	0A2
....cuuugaaaucggguUugguuau.......................	1	1	0A2
....cuuugaGaucgggucugguuau.......................	1	1	0A2
....cuuugaaaucgggucugguuaC.......................	7	1	0A2
....cuuugaaaucgggucugguuUu.......................	33	1	0A2
....Nuuugaaaucgggucugguuau.......................	1	1	0A2
....cuuugaaaucgggucugguuGu.......................	40	1	0A2
....Uuuugaaaucgggucugguuaua......................	53	1	0A2
....cuuugaCaucgggucugguuaua......................	1	1	0A2
....cuuugaaaucgggucugguuGua......................	2	1	0A2
....cuuugaaaucgggucugguuaAa......................	341	1	0A2
....cuuugaaauUgggucugguuaua......................	24	1	0A2
....cuuugaaaucgggucugguuUua......................	161	1	0A2
....cuuugaaaucgggucugguuaua......................	840	0	0A2
....cuuugaaaGcgggucugguuaua......................	1	1	0A2
....cuuugaaaucgggGcugguuaua......................	1	1	0A2
....cuuugaaaucgggucugUuuaua......................	1	1	0A2
....cuuugaaaucggAucugguuaua......................	1	1	0A2
....cuuugaaaucggUucugguuaua......................	1	1	0A2
....cuuugaaaucgggucugguuaCa......................	31	1	0A2
....cuuugaaaucAggucugguuaua......................	1	1	0A2
....cuuugaaaucgggucuAguuaua......................	2	1	0A2
....cuuugaGaucgggucugguuaua......................	3	1	0A2
....cuuugaaaucgggCcugguuaua......................	1	1	0A2
....Nuuugaaaucgggucugguuaua......................	4	1	0A2
....cuuugaaaucUggucugguuauaa.....................	1	1	0A2
....cuuugaaaucgggucugguuGuaa.....................	2	1	0A2
....cuuugaaaucgggucugguuauaU.....................	25	1	0A2
....cuuugaaaucgggucugguuaCaa.....................	16	1	0A2
....cuuugaaaucgggucugguuUuaa.....................	8	1	0A2
....Uuuugaaaucgggucugguuauaa.....................	4	1	0A2
....cuuugaaaucgggucugguuaAaa.....................	98	1	0A2
....cCuugaaaucgggucugguuauaa.....................	5	1	0A2
....cuuugaaaucgggucuggGuauaa.....................	1	1	0A2
....cuuugaaaucgggucugguuauaG.....................	11	1	0A2
....Nuuugaaaucgggucugguuauaa.....................	1	1	0A2
....cuuugaaauUgggucugguuauaa.....................	3	1	0A2
....cuuugaaaucgggucugguuauaa.....................	131	0	0A2
....cuuugaaaucgggucugguuauaaU....................	10	1	0A2
....cuuugaaaucgggucugguuauaaA....................	21	1	0A2
....uuugaaaucgggGcugguu.........................	1	1	0A2
....uuugaaaucgggucugguu.........................	11	0	0A2
....uuugaaaucgggucugguua........................	13	0	0A2
....uuugaaaucgggucugguuUu.......................	1	1	0A2
....uuugaaaucgggucugguuau.......................	35	0	0A2
....uuugaaaucgggGcugguuau.......................	1	1	0A2
....uuugaaaucgggucugguGau.......................	1	1	0A2
....uuuAaaaucgggucugguuau.......................	6	1	0A2
....uuugaaaucUggucugguuau.......................	1	1	0A2
....uuugaaauUgggucugguuaua......................	6	1	0A2
....uuugaaaucgggucugguuUua......................	46	1	0A2
....uuuAaaaucgggucugguuaua......................	1	1	0A2
....uuugaaaucgggucugguuaAa......................	33	1	0A2
....uuugaaaucggAucugguuaua......................	19	1	0A2
....uuugaaaucgggucugguuaua......................	65	0	0A2
....uuugaaaucgggucugguuauaU.....................	3	1	0A2
....uuugaaaucgggucugguuauaa.....................	1	0	0A2
....uuugaaaucgggucugguuaAaa.....................	10	1	0A2
....uuugaaaucggAucugguuauaa.....................	3	1	0A2
....uuugaaaucgggucugguuauaaA....................	5	1	0A2

Star **Mature**

```
caauauacgccccugccuccuacgccuuagccugauaaccagacccgauuucaaaggaaauagauccuuugaaaucgggucugguuauaaggcuaccuacgccugugaacg
....................................................................uugaaaucgggucugguuaua......................   29   0   0A2
....................................................................uugaaaucggAucugguuaua......................   2    1   0A2
....................................................................uugaaaucggguGugguuaua......................   1    1   0A2

..............................cAugauaaccagacccgauuuc...........................................................   2    1   0G2
..............................cAugauaaccagacccgauuuca..........................................................   15   1   0G2
................................ugauaaccagacccgauuucaa.........................................................   3    0   0G2
................................ugauaaccagaAccgauuucaaa........................................................   2    1   0G2
.................................gauaaccagacccgauuuca..........................................................   2    0   0G2
..................................auaaccagacccgauuuca..........................................................   2    0   0G2
..................................auaaccagacccgauuucaa.........................................................   1    0   0G2
..................................auaaccagacccgauuucaaa........................................................   2    0   0G2
..................................Nuaaccagacccgauuucaaa........................................................   1    1   0G2
...................................uaaccGgacccgauuucaaa........................................................   4    1   0G2
...................................Caaccagacccgauuucaaag.......................................................   2    1   0G2
...................................uaaccagacccgauuucaaaAg......................................................   1    1   0G2
...................................Caaccagacccgauuucaaagg......................................................   10   1   0G2
...................................uaaccagacccgauuucaaagg......................................................   7    0   0G2
....................................aaccagacccgauuucaaag.......................................................   9    0   0G2
....................................aaccagacccgauuucaaaAga.....................................................   1    1   0G2
.................................................................ccuuugaaaucgggucugguua........................   3    0   0G2
.................................................................cUuuugaaaucgggucugguuaua......................   8    1   0G2
..................................................................cuuugaaaucgggucugg...........................   6    0   0G2
..................................................................cuuugaaGucgggucuggu..........................   1    1   0G2
..................................................................cuuugaaaucgggucuggu..........................   4    0   0G2
..................................................................cuuugaaaucgggucugguu.........................   67   0   0G2
..................................................................cuuugaaaucgggucugguuG........................   3    1   0G2
..................................................................cuuugaaaucgggucugguua........................   111  0   0G2
..................................................................Nuuugaaaucgggucugguua........................   1    1   0G2
..................................................................cuuugaaaucgggucugguuUu.......................   12   1   0G2
..................................................................Nuuugaaaucgggucugguuau.......................   1    1   0G2
..................................................................cuuugaaaucgggucugguuGu.......................   27   1   0G2
..................................................................cAuugaaaucgggucugguuau.......................   1    1   0G2
..................................................................cuuugaaaucgggucugguuau.......................   163  0   0G2
..................................................................cuuugaaaucgggucugguuaG.......................   6    1   0G2
..................................................................cuuugaaaucgggucugguuUua......................   57   1   0G2
..................................................................cuCugaaaucgggucugguuaua......................   3    1   0G2
..................................................................cuuugaaauUgggucugguuaua......................   25   1   0G2
..................................................................Uuuugaaaucgggucugguuaua......................   79   1   0G2
..................................................................cuuugaaaucgggucugguuaAa......................   327  1   0G2
..................................................................cuuugaaaucgggucugguuaua......................   423  0   0G2
..................................................................cNuugaaaucgggucugguuaua......................   1    1   0G2
..................................................................cuuugaaaucgggucugguuaCa......................   6    1   0G2
..................................................................cuuugaaaucgggucugguuauaa.....................   80   0   0G2
..................................................................cuuugaaaucgggucugguuUuaa.....................   4    1   0G2
..................................................................cuuugaaaucgggucugguuaAaa.....................   37   1   0G2
..................................................................cuuugaaaucgggucugguuauaU.....................   13   1   0G2
..................................................................cuuugaaaucgggucugguuauaG.....................   2    1   0G2
..................................................................cuuugaaaucgggucugguuGuaa.....................   2    1   0G2
..................................................................cuuugaaaucgggucugguuauaC.....................   1    1   0G2
..................................................................Uuuugaaaucgggucugguuauaa.....................   7    1   0G2
..................................................................cuuugaaaucgggucugguuaCaa.....................   9    1   0G2
..................................................................cuuugaaaucgggucugguuauaaA....................   14   1   0G2
..................................................................cuuugaaaucgggucugguuauaGg....................   4    1   0G2
...................................................................uuugaaaucgggucugguuG........................   7    1   0G2
...................................................................uuugaaaucgggucugguua........................   23   0   0G2
...................................................................uuugaaaucgggucugguuau.......................   2    0   0G2
...................................................................uuugaaaucggAucugguuaua......................   13   1   0G2
...................................................................Cuugaaaucgggucugguuaua......................   2    1   0G2
...................................................................uuugaaaucgggucugguuaCa......................   4    1   0G2
...................................................................uuugaaaucgggucugguuaua......................   52   0   0G2
...................................................................uuugaaaucgggucugguuUua......................   12   1   0G2
...................................................................uuugaaaucgggucugguuaAa......................   24   1   0G2
...................................................................uuugaaauUgggucugguuaua......................   8    1   0G2
...................................................................uuugaaaucgggucugguuauaaU....................   1    1   0G2
....................................................................uugaaaucgggGcugguua........................   1    1   0G2
....................................................................uugaaaucgggucugguua........................   2    0   0G2
....................................................................uugaaaCcgggucugguuau.......................   1    1   0G2
....................................................................uugaaaucgggucugguuau.......................   2    0   0G2
....................................................................uugaaaucgggucugguuaua......................   19   0   0G2
```

```
                                   Star                          Mature
caauauacgccccugccuccuacgccuuagccugauaaccagacccgauuucaaaggaaauagauccuuugaaaucgggucugguuauaaggcuaccuacgccugugaacg
....................................................................uugaGaucgggucugguuaua......................    2    1    0G2
....................................................................uugaaauUgggucugguuaua......................    4    1    0G2
....................................................................uugaaaucgggucugguuauaG.....................    1    1    0G2

...........................uagccugauaacUagacccgau..............................................................    5    1    0B2
................................ugauaaccagaAccgauuucaa.........................................................    2    1    0B2
..................................auaaccagacccgauuucaaa........................................................   11    0    0B2
..................................aCaaccagacccgauuucaaag.......................................................    5    1    0B2
...................................Caaccagacccgauuuca..........................................................    3    1    0B2
...................................Caaccagacccgauuucaaa........................................................    3    1    0B2
...................................Caaccagacccgauuucaaag.......................................................   26    1    0B2
...................................uaaccagacccgauuucaaaAg......................................................    2    1    0B2
...................................Caaccagacccgauuucaaagg......................................................   98    1    0B2
...................................uaGccagacccgauuucaaagg......................................................    1    1    0B2
...................................uaaccagacccgauuucaaagga.....................................................    7    0    0B2
....................................aaccagacccgauuucaaag.......................................................    7    0    0B2
....................................aaccGgacccgauuucaaagg......................................................    5    1    0B2
....................................aaccagacGcgauuucaaagg......................................................    1    1    0B2
....................................aaccagacccgauuucaaagg......................................................   32    0    0B2
...........................................cccgauuucaaagAaaauag................................................    3    1    0B2
.................................................................ccuuugaaaucgggucugguu.........................    1    0    0B2
..................................................................cuuugaaaucgggucugg...........................    6    0    0B2
..................................................................cuuugaaaucgggucuggu..........................    6    0    0B2
..................................................................cuuugaaaucgggucugguC.........................    1    1    0B2
..................................................................cuuugaaaucgggucugguu.........................   72    0    0B2
..................................................................cuuugaaaucgggucugguua........................  101    0    0B2
..................................................................cuuugaaaucgggucugguuC........................    5    1    0B2
..................................................................cuuugaaaucgggucugguuG........................    7    1    0B2
..................................................................Nuuugaaaucgggucugguua........................    2    1    0B2
..................................................................cuuugaaaCcgggucugguuau.......................    1    1    0B2
..................................................................cuuugaaaucgggucuggGuau.......................    1    1    0B2
..................................................................cuuugaaaucgggucugguuGu.......................   14    1    0B2
..................................................................cuuugaaaucgggucugguuau.......................   83    0    0B2
..................................................................cuuuAaaaucgggucugguuau.......................    2    1    0B2
..................................................................cAuugaaaucgggucugguuau.......................    1    1    0B2
..................................................................cuuugaaaucgggucugguuUu.......................   14    1    0B2
..................................................................Nuuugaaaucgggucugguuau.......................    2    1    0B2
..................................................................cuuugaaaGcgggucugguuaua......................    1    1    0B2
..................................................................Nuuugaaaucgggucugguuaua......................    3    1    0B2
..................................................................cuuugaaaucgggucugguuaua......................  348    0    0B2
..................................................................cuuugaaaucgggucugguuauG......................    2    1    0B2
..................................................................cuuugaaaucgggucugguuaCa......................   17    1    0B2
..................................................................cuuugaaaucgggucCgguuaua......................    1    1    0B2
..................................................................Uuuugaaaucgggucugguuaua......................   32    1    0B2
..................................................................cuuugaaaucgggucugguuGua......................    7    1    0B2
..................................................................cuuugaaaucgggucugguuaAa......................  273    1    0B2
..................................................................cuuugaaaucgggucuggCuaua......................    1    1    0B2
..................................................................cuuugaaaucgggucugguuUua......................   68    1    0B2
..................................................................cuuugaaauUgggucugguuaua......................    8    1    0B2
..................................................................cuuugaaaucgggucugguuauaG.....................    1    1    0B2
..................................................................cuuugaaaucgggucugguuauaU.....................   16    1    0B2
..................................................................cuuugaaaucgggucugguuaAaa.....................   35    1    0B2
..................................................................cuuugaaaucgggucugguuaCaa.....................   14    1    0B2
..................................................................cuuugaaaucgggucugguuauaC.....................    8    1    0B2
..................................................................cuuugaaaucgggucugguuauaa.....................   18    0    0B2
..................................................................Nuuugaaaucgggucugguuauaa.....................    1    1    0B2
..................................................................cuuugaaaucgggucugguuauaaU....................    3    1    0B2
..................................................................cuuugaaaucgggucugguuauaaA....................    1    1    0B2
...................................................................uuugaaaucgggucugguua........................   17    0    0B2
...................................................................uuugaaaucgggucugguuau.......................   23    0    0B2
...................................................................uuugaaaucgggucugguuGu.......................    3    1    0B2
...................................................................Nuugaaaucgggucugguuaua......................    1    1    0B2
...................................................................uuugaaaucgggucuUguuaua......................    3    1    0B2
...................................................................uuugaaaucggAucugguuaua......................    5    1    0B2
...................................................................uuugaaaucgggucugguuaAa......................   26    1    0B2
...................................................................uuugaaaucgggucugguuaua......................   27    0    0B2
...................................................................uuugaaaucgggucugguuGua......................    1    1    0B2
...................................................................uuugaaaucgggucugguuUua......................    6    1    0B2
...................................................................uuugaaaucgggucugguuUaa.....................    3    1    0B2
...................................................................uuugaaaucgggucugguuauaaA....................    2    1    0B2
```

Star **Mature**

```
caauauacgccccugccuccuacgccuuagccugauaaccagacccgauuucaaaggaaauagauccuuugaaaucgggucugguuauaaggcuaccuacgccugugaacg
```

```
...................................................................uuugaaaucgggucugguuauaaC.....................   1   1   0B2
...................................................................uugaaaucgggucugguA..........................   6   1   0B2
```

Provisional ID	:	scaffold275_34783
Score total	:	5.5
Score for star read(s)	:	-1.3
Score for read counts	:	0
Score for mfe	:	2.2
Score for randfold	:	1.6
Score for cons. seed	:	3
Total read count	:	331
Mature read count	:	331
Loop read count	:	0
Star read count	:	0

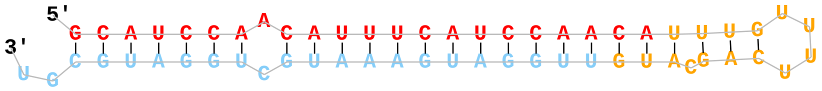

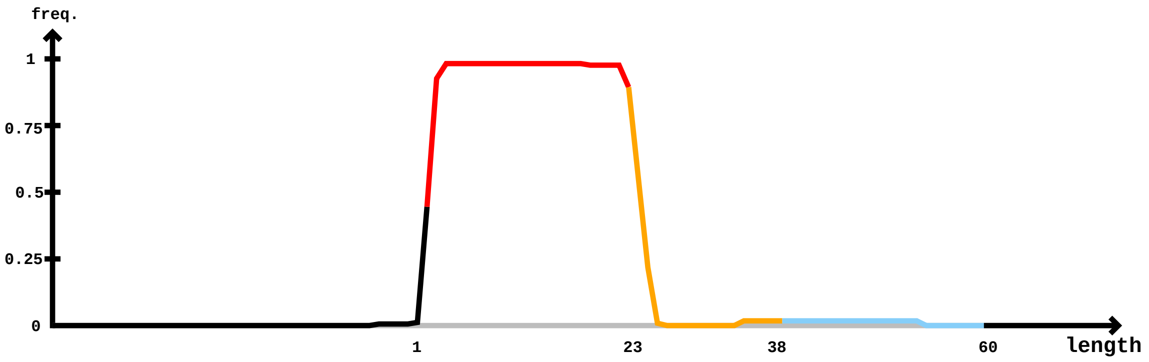

Mature Star

```
5'- guaaaaauccuuuuuuaagccuugagggaaaucaaacgcauccaacauuucauccaacauuuguuuucagcauguuggaugaaaugcuggaugcguuugaccacucaug -3'   exp
    .((((((....)))))).....((((((...(((((((((((((.((((((((((((((((((....))).))))))))))))))).))))))))))))))).))))..    reads   mm   sample
    .....................................Ccauccaacauuucauccaac...................................................    8       1    0A2
    .....................................gcauccaacauuucauccaaca..................................................    3       0    0A2
    .....................................Ccauccaacauuucauccaaca..................................................    18      1    0A2
    ......................................cauccaacauuucauccaaca..................................................    4       0    0A2
    ......................................cauccaCcauuucauccaacau.................................................    1       1    0A2
    ......................................caCccaacauuucauccaacau.................................................    4       1    0A2
    ......................................cauccaacauuucauccaacau.................................................    16      0    0A2
    ......................................cauccaacauuucauccaacauu................................................    2       0    0A2
    ......................................cauccaacauuucauccaacauA................................................    18      1    0A2
    .......................................auccaacauuucauccaaca..................................................    4       0    0A2
    .......................................auccaacauuucauccaacauA................................................    4       1    0A2

    ....................................cgcauccaacauuucauccaac...................................................    2       0    0G2
    .....................................Ccauccaacauuucauccaac...................................................    6       1    0G2
    .....................................Ccauccaacauuucauccaaca..................................................    57      1    0G2
    ......................................cauccaacauuucauccaaca..................................................    10      0    0G2
    ......................................cauccaacauuucauccaacau.................................................    8       0    0G2
    ......................................cauccaAauuucauccaacau.................................................    1       1    0G2
    ......................................cauccaacauuucauccaacauA................................................    26      1    0G2
    ......................................caucc Uacauuucauccaacauu................................................    12      1    0G2
    .......................................auccaacauuucauccaaca..................................................    5       0    0G2
    ......................................................................cauguuggaugaaaugcug....................    6       0    0G2

    ................................caaacgcauccaacauuucauA.......................................................    2       1    0B2
    .....................................Ccauccaacauuucauccaac...................................................    8       1    0B2
    .....................................gcauccaacauuucauccaac...................................................    4       0    0B2
    .....................................Ccauccaacauuucauccaaca..................................................    43      1    0B2
    ......................................cauccaacauuucauccaaca..................................................    16      0    0B2
    ......................................caCccaacauuucauccaacau.................................................    5       1    0B2
    ......................................cauccaacauuucauccaacau.................................................    28      0    0B2
    ......................................cauccaacauuucauccaacauA................................................    8       1    0B2
    ......................................cauccaacauuucauccaacauu................................................    1       0    0B2
    ......................................cauccaacauuucauccaacauAu...............................................    3       1    0B2
    .......................................auccaacauuucauccaacau.................................................    6       0    0B2
```

Mature Star

guaaaaauccuuuuuuaagccuugagggaaaucaaacgcauccaacauuucauccaacauuuguuuucagcauguuggaugaaaugcuggaugcguuugaccacucaug

Provisional ID	:	scaffold291_35518
Score total	:	5.9
Score for star read(s)	:	-1.3
Score for read counts	:	0
Score for mfe	:	2.6
Score for randfold	:	1.6
Score for cons. seed	:	3
Total read count	:	202
Mature read count	:	142
Loop read count	:	0
Star read count	:	60

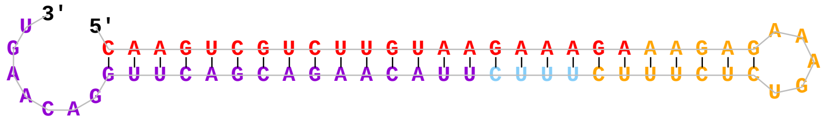

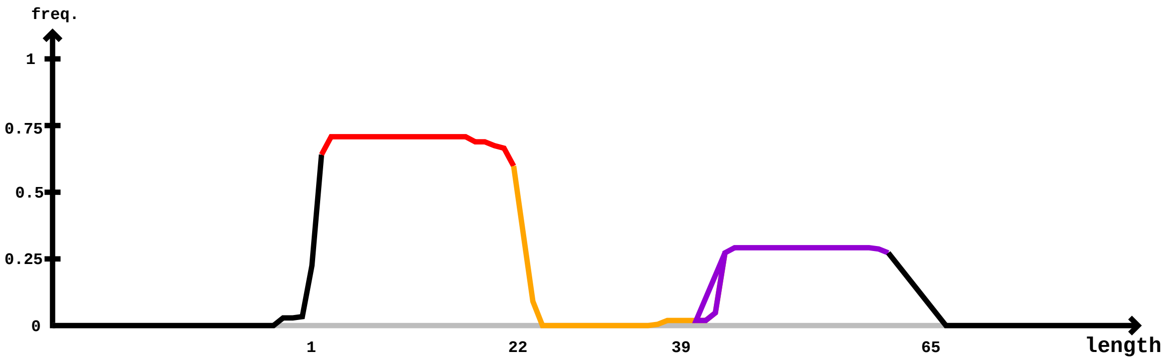

Mature **Star**

```
5'- auaaguagcaaacuacuggacuugcccaagucgucuuguaagaaagaaagagaaagucucuuucuuucuuacaagacgacuuggacaaguccagcaaacuacuggcuuuga  -3'   obs
    auaaguagcaaacuacuggacuugcccaagucgucuuguaagaaagaaagagaaagucucuuucuuucuuacaagacgacuuggacaaguccagcaaacuacuggcuuuga        exp
    ...(((((.......((((((((.((((((((((((((((((((((((((((.....)))))))))))))))))))))))))))).)))))))))....)))))........   reads   mm   sample
    ........................ccUagucgucuuguaagaaa...................................................................   1       1    0A2
    ........................ccaagucgucuuguaagaaag..................................................................   6       0    0A2
    ........................ccUagucgucuuguaagaaag..................................................................   1       1    0A2
    ........................ccaagucgucuuguaagaaaga.................................................................   16      0    0A2
    ..........................caagucgucuuguaagaG...................................................................   1       1    0A2
    ..........................caagucgucuuguaagaaaga................................................................   19      0    0A2
    ..........................Uaagucgucuuguaagaaaga................................................................   5       1    0A2
    ..........................caagucgucuuguaagaaagaa...............................................................   1       0    0A2
    ..........................caagucgucuuguaagaaagaaa..............................................................   8       0    0A2
    ...........................aagucgucuuguaagaaagaaa..............................................................   2       0    0A2
    ..............................................................ucuuCcuuacaagacgacuugga..........................   3       1    0A2
    ....................................................................uuacaagacgacuuggacaagA.....................   4       1    0A2
    ....................................................................uuacaagacgacuuggacaagu.....................   8       0    0A2

    ......................ugccUaagucgucuuguaag.....................................................................   4       1    0B2
    .......................cccUagucgucuuguaagaaaga.................................................................   1       1    0B2
    ........................ccaagucgucuuguaagaaag..................................................................   3       0    0B2
    ........................ccaagucgucuuguaagaaaga.................................................................   5       0    0B2
    ........................ccaagucgucuuguaagaaagaa................................................................   2       0    0B2
    ..........................caagucgucuuguaagaaa..................................................................   1       0    0B2
    ..........................caagucgucuuguaagaaaga................................................................   15      0    0B2
    ..........................Uaagucgucuuguaagaaaga................................................................   3       1    0B2
    ..........................caagucgucuuguaagaaagaa...............................................................   11      0    0B2
    ..........................caagucgucuuguaagaaagaaG..............................................................   2       1    0B2
    ...........................aagucgucuuguaagaaaga................................................................   5       0    0B2
    ...........................aagucgucuuguaagaaagaaa..............................................................   2       0    0B2

    ......................ugccUaagucgucuuguaagaa...................................................................   2       1    0G2
    ........................ccaagucgucuuguaagaaag..................................................................   4       0    0G2
    ........................ccaagucgucuuguaagaaaga.................................................................   2       0    0G2
    ..........................Uaagucgucuuguaagaaaga................................................................   9       1    0G2
    ..........................caagucgucuuguaagaaaga................................................................   12      0    0G2
    ...........................aagucgucuuguaagaaagaaa..............................................................   5       0    0G2
```

```
                          Mature                                    Star
auaaguagcaaacuacuggacuugcccaagucgucuuguaagaaagaaagagaaagucucuuucuuucuuacaagacgacuuggacaaguccagcaaacuacuggcuuuga
.............................................................uucuuucuuacaagacgaUuugg...........................   1    1    0G2
...................................................................cuuacaagacgacuuggacaa.......................   3    0    0G2
...................................................................cuuacaagacgacuuggacaaA......................   3    1    0G2
....................................................................uuacaagacgacuuggacaa.......................   9    0    0G2
....................................................................uuacaagacgacuuggacaag......................   12   0    0G2
....................................................................uuacaagacgacuuggacaagu.....................   14   0    0G2
.....................................................................uacaagacgacuuggacaagu.....................   4    0    0G2
```

Provisional ID	:	scaffold336_37608
Score total	:	166.2
Score for star read(s)	:	3.9
Score for read counts	:	156.1
Score for mfe	:	1.5
Score for randfold	:	1.6
Score for cons. seed	:	3
Total read count	:	318
Mature read count	:	302
Loop read count	:	0
Star read count	:	16

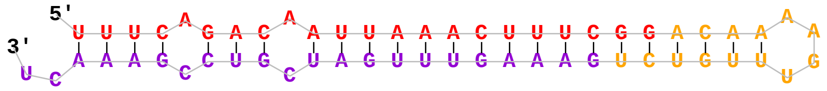

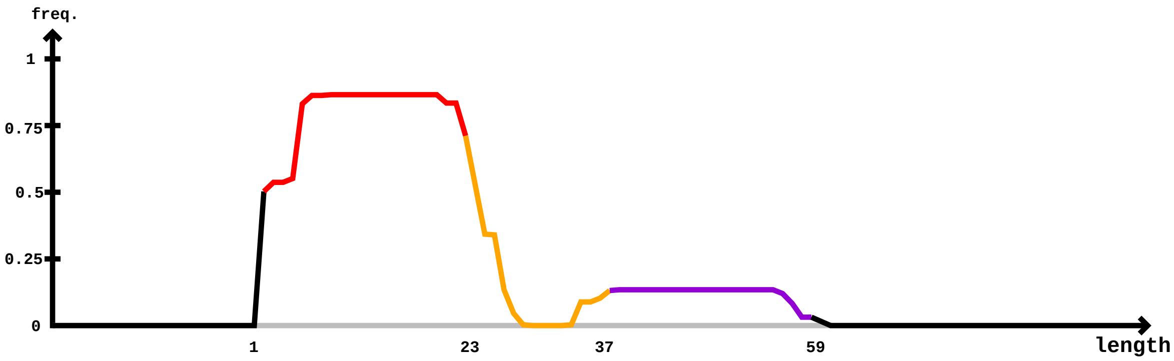

Mature Star

```
5'- aacuuucggacaaaaccagauuucagacaauuaaacuuucggacaaaaguuugucugaaaguuugaucguccgaaacuuuuugguuuucauacauacaaaugaaagcaaauu   -3'   obs
    aacuuucggacaaaaccagauuucagacaauuaaacuuucggacaaaaguuugucugaaaguuugaucguccgaaacuuuuugguuuucauacauacaaaugaaagcaaauu         exp
    ..(((((...((((((((((((.(((.((((((((((((((((....)))))))))))))))).))).))))...)))))))))...........))))))......   reads   mm   sample
    ....................uuucagacaauuaaacuuucg.......................................................................   3   0   0B2
    ....................uuucUgacaauuaaacuuucg.......................................................................   5   1   0B2
    ....................uuucagacaauuaaacuuucgg......................................................................   29   0   0B2
    ....................uuucagacaauuaaacuuucgga.....................................................................   1   0   0B2
    ....................uuucagacaauuaaacuuucggaca...................................................................   5   0   0B2
    .....................uucagacaauuaaacuuucg.......................................................................   4   0   0B2
    .......................cagacaauuaaacuuucAgac....................................................................   1   1   0B2
    ........................agacaauuaaacuuucggaca...................................................................   13   0   0B2
    ........................agacaauuaGacuuucggaca...................................................................   1   1   0B2
    ........................Ngacaauuaaacuuucggaca...................................................................   1   1   0B2
    ........................agacaauAaaacuuucggacaa..................................................................   1   1   0B2
    ........................agacaauuaaacuuucggacaa..................................................................   14   0   0B2
    ........................agacaauuaaacuuucggacaaU.................................................................   4   1   0B2
    ........................Ugacaauuaaacuuucggacaaa.................................................................   1   1   0B2
    .....................................................ucCgaaaguuugaucguccgaa.....................................   8   1   0B2
    .....................................................ucCgaaaguuugaucguccgaaa....................................   1   1   0B2
    ........................................................gaaaguuugaucguccgaaaGu..................................   1   1   0B2
    .........................................................aaaguuugaucguccgaaaGu..................................   1   1   0B2

    ....................uuucagacaauuaaacuuu.........................................................................   11   0   0G2
    ....................uuucagacaauuaaacuuucg.......................................................................   15   0   0G2
    ....................uuucagacaauuaaacuuucgg......................................................................   43   0   0G2
    .......................cagacaauuaaacuuucggaca...................................................................   4   0   0G2
    ........................agacGauuaaacuuucggaca...................................................................   1   1   0G2
    ........................agacaauuaaacuuucggaca...................................................................   25   0   0G2
    ........................agacaauuaaacuuucggacaaa.................................................................   10   0   0G2
    .........................gacaauuaaacuuucggacaa..................................................................   8   0   0G2
    ...........................caauuaaacuuucggacaaaa................................................................   1   0   0G2
    ....................................................gucCgaaaguuugaucguccga......................................   1   1   0G2
    .....................................................ucugaaaguuugauUguccgaa.....................................   3   1   0G2
    ........................................................gaaaguuugaucguccgaaaGu..................................   9   1   0G2

    ....................uuucUgacaauuaaacuuucg.......................................................................   4   1   0A2
```

```
                    Mature                            Star
aacuuucggacaaaaccagauuucagacaauuaaacuuucggacaaaaguuugucugaaaguuugaucguccgaaacuuuuugguuuucauacauacaaaugaaagcaaauu
....................uuucCgacaauuaaacuuucg.......................................................................    1     1     0A2
....................uuucagacaauuaaacuuucg.......................................................................    8     0     0A2
....................uuucagacaauuaaacuuucgg......................................................................    50    0     0A2
....................uuucUgacaauuaaacuuucgg......................................................................    1     1     0A2
.....................uucagacaauuaaacuuucA.......................................................................    3     1     0A2
.....................uucagacaauuaaacuuucgga.....................................................................    5     0     0A2
........................agacaauuaaacuuucggaca...................................................................    16    0     0A2
........................Ugacaauuaaacuuucggaca...................................................................    2     1     0A2
........................agacaauCaaacuuucggaca...................................................................    1     1     0A2
........................agacaauuaaUcuuucggacaa..................................................................    4     1     0A2
........................agacaauuaaacuuucggacaa..................................................................    4     0     0A2
.........................gacaauuaaacuuucggaca...................................................................    3     0     0A2
.....................................................ucCgaaaguuugaucguccga......................................    4     1     0A2
.....................................................ucCgaaaguuugaucguccgaa.....................................    2     1     0A2
.....................................................ucCgaaaguuugaucguccgaaa....................................    12    1     0A2
.......................................................Cgaaaguuugaucguccgaaa....................................    5     1     0A2
```

Provisional ID	: scaffold336_37640
Score total	: 1.2
Score for star read(s)	: -1.3
Score for read counts	: 0
Score for mfe	: 1.5
Score for randfold	: 1.6
Score for cons. seed	: -0.6
Total read count	: 3243
Mature read count	: 3009
Loop read count	: 0
Star read count	: 234

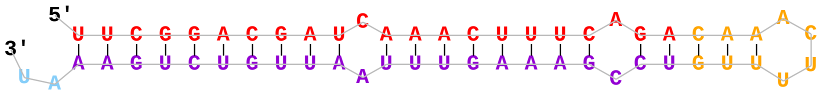

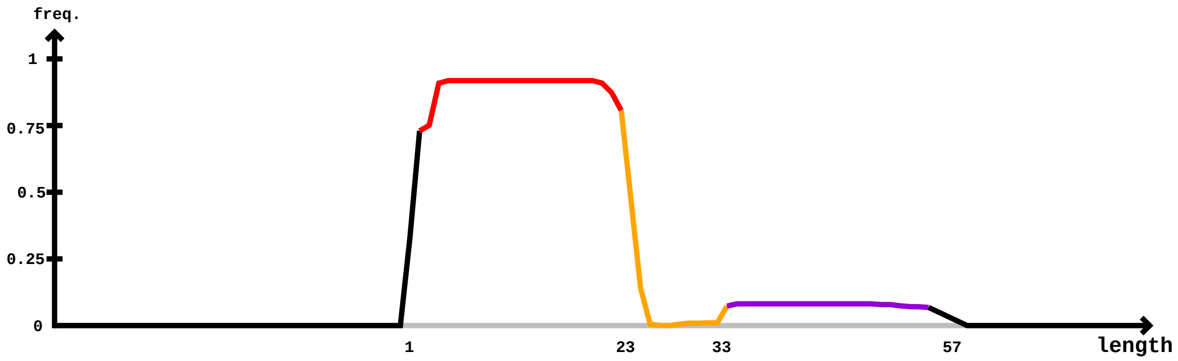

Mature Star

5'-	auuugcuuucauuuguauguaugaaaaccaaaaaguuucggacgaucaaacuuucagacaaacuuuuguccgaaaguuuaauugucugaaaucugguuuuguccgaaaguuu	-3'	obs	
	auuugcuuucauuuguauguaugaaaaccaaaaaguuucggacgaucaaacuuucagacaaacuuuuguccgaaaguuuaauugucugaaaucugguuuuguccgaaaguuu		exp	
	....((((((....((....)).(((((((....((((((((((((.((((((((.(((((....))))).)))))))).)))))))))))).)))))))....))))))..	reads	mm	sample
	...................................uuucggacgaucaaacuuCc...	4	1	0A2
	...................................uuucggacgaucaaacuuuc...	3	0	0A2
	...................................uuucggacgaucaaacuuuca..	9	0	0A2
	...................................uuucggaGgaucaaacuuuca..	27	1	0A2
	...................................uuucggacgaucaaacuuucag...	8	0	0A2
	...................................uuucggaGgaucaaacuuucag...	41	1	0A2
	...................................uuucggacgaucaaacuuucUg...	1	1	0A2
	...................................uuucggaGgaucaaacuuucaga..	244	1	0A2
	...................................uuucggacgaucaaacuuucaga..	17	0	0A2
	...................................uuucggacgaucaaacuuucagU..	2	1	0A2
	...................................uuucggacgaucaaacuuucagaA...	3	1	0A2
	...................................uuucggaGgaucaaacuuucagaca..	17	1	0A2
	...................................uuucggaGgaucaaacuuucagacaaa..	1	1	0A2
	....................................uucggacgaucaaacuuuc...	5	0	0A2
	....................................uucggacgaucaaacuuucaga..	3	0	0A2
	....................................uucggaGgaucaaacuuucaga..	355	1	0A2
	....................................uucggacgauUaaacuuucaga..	2	1	0A2
	....................................uucggaGgaucaaacuuucagaca..	4	1	0A2
	.....................................ucggaGgaucaaacuuucaga..	16	1	0A2
	......................................Nggacgaucaaacuuuca..	1	1	0A2
	......................................cggaUgaucaaacuuucaga..	65	1	0A2
	......................................Nggacgaucaaacuuucagaca..	3	1	0A2
	......................................cggaGgaucaaacuuucagaca..	114	1	0A2
	......................................cggacUaucaaacuuucagaca..	1	1	0A2
	......................................cgAacgaucaaacuuucagaca..	1	1	0A2
	......................................cgUacgaucaaacuuucagaca..	1	1	0A2
	......................................cggacgGucaaacuuucagaca..	2	1	0A2
	......................................Uggacgaucaaacuuucagaca..	1	1	0A2
	......................................cggGcgaucaaacuuucagaca..	1	1	0A2
	......................................Nggacgaucaaacuuucagacaa...	2	1	0A2
	......................................cggaGgaucaaacuuucagacaa...	9	1	0A2
	....ggaGgaucaaacuuucagaca..	4	1	0A2
	....uuuuguccgaaaguuuaauuA............................	7	1	0A2

```
                                   Mature                Star
auuugcuuucauuuguauguaugaaaaccaaaaaguuucggacgaucaaacuuucagacaaacuuuuguccgaaaguuuaauugucugaaaucugguuuuguccgaaaguuu
...............................................................uuuuguccgaaaguuuaauuguc..........................    3    0    0A2
................................................................uuuguccgaaaguuuaauug............................    2    0    0A2
................................................................uuuguccgaaaguuuaauugucG.........................    8    1    0A2
..................................................................uguccgaaaguuuaauuAucug........................    2    1    0A2
....................................................................uccgaaaguuuaauugucAga.......................    2    1    0A2
....................................................................uccgaaaguuuaauugucGgaa......................    2    1    0A2
....................................................................uccgaaaguuuGauugucugaa......................    1    1    0A2
....................................................................uccgaaaguuuaauugucAgaa......................    3    1    0A2
....................................................................uccgaaaguuuaauugucugaa......................    20   0    0A2
....................................................................uccgaaaguuuaauugucugaaa.....................    13   0    0A2
....................................................................uccgaaaguuuaauugucugaaUu....................    2    1    0A2
.....................................................................ccgaaaguuuaauugucugaa......................    6    0    0A2
.....................................................................ccgaaaguuuaauugucAgaa......................    3    1    0A2
.....................................................................ccgaaaguuuaauugucugaaauU...................    1    1    0A2

...................................uuucggacgaucaaacuuuc.........................................................    3    0    0B2
...................................uuucggaGgaucaaacuuuca........................................................    21   1    0B2
...................................uuucggacgaucaaacuuuca........................................................    6    0    0B2
...................................uuucggacgaucaaacuuucag.......................................................    7    0    0B2
...................................uuucggaGgaucaaacuuucag.......................................................    97   1    0B2
...................................uuucggaGgaucaaacuuucaga......................................................    250  1    0B2
...................................uuucggacgaucaaacuuucaga......................................................    14   0    0B2
...................................uuucggaGgaucaaacuuucagac.....................................................    4    1    0B2
...................................uuucggaGgaucaaacuuucagaca....................................................    3    1    0B2
...................................uuucggacgaucaaacuuucagaca....................................................    2    0    0B2
....................................uucggacgaucaaacuuuca........................................................    1    0    0B2
....................................uucggacgaucaaacuuucag.......................................................    1    0    0B2
....................................uucggaGgaucaaacuuucag.......................................................    4    1    0B2
....................................uucggaUgaucaaacuuucaga......................................................    1    1    0B2
....................................uucggaGgaucaaacuuucaga......................................................    423  1    0B2
....................................uucggacgaucaaacuuucaga......................................................    18   0    0B2
.....................................ucggaGgaucaaacuuucaga......................................................    23   1    0B2
......................................cggacAaucaaacuuuca........................................................    4    1    0B2
......................................cggGcgaucaaacuuuca........................................................    1    1    0B2
......................................cggaGgaucaaacuuucaga......................................................    2    1    0B2
......................................cggGcgaucaaacuuucaga......................................................    2    1    0B2
......................................cggGcgaucaaacuuucagac.....................................................    3    1    0B2
......................................cgUacgaucaaacuuucagaca....................................................    1    1    0B2
......................................Uggacgaucaaacuuucagaca....................................................    1    1    0B2
......................................cggaGgaucaaacuuucagaca....................................................    98   1    0B2
......................................Nggacgaucaaacuuucagaca....................................................    13   1    0B2
.......................................gUacgaucaaacuuucagac.....................................................    1    1    0B2
.......................................ggaGgaucaaacuuucagaca....................................................    10   1    0B2
...............................................................uuuuguccgaaaguuuaauuguc..........................    2    0    0B2
...............................................................uuuuguccgaaaguuuaauugucA.........................    2    1    0B2
................................................................uuuguccgaaaguuuaauuguc..........................    3    0    0B2
..................................................................uguccgaaaguuuaauuAucuga.......................    2    1    0B2
....................................................................uccgaaaguuuaauuguA..........................    5    1    0B2
....................................................................uccgaaaguuuaauugucuga.......................    4    0    0B2
....................................................................Nccgaaaguuuaauugucugaa......................    1    1    0B2
....................................................................uccgaaaguuuaaAugucugaa......................    1    1    0B2
....................................................................uccgaaaguuuaauugucugaa......................    37   0    0B2
....................................................................uccgaaaguuuaauugucAgaa......................    29   1    0B2
....................................................................uccgaaaguuuaauugucugaaU.....................    4    1    0B2
....................................................................uccgaaaguuuaauugucugaaa.....................    8    0    0B2
....................................................................uccgaaaguuuaauugucugaaUu....................    3    1    0B2
.....................................................................ccgaaaguuuaauugucugaa......................    3    0    0B2
......................................................................cgaaaguuuaauugucugaa......................    1    0    0B2

...................................uuucggacgaucaaacuuuc.........................................................    16   0    0G2
...................................uuucggacAaucaaacuuuca........................................................    2    1    0G2
...................................uuucggaGgaucaaacuuuca........................................................    32   1    0G2
...................................uuucggacgaucaaacuuuca........................................................    14   0    0G2
...................................uuucggaGgaucaaacuuucag.......................................................    61   1    0G2
...................................uuucggaGgaucaaacuuucaga......................................................    134  1    0G2
...................................uuucggacgaucaaacuuucaga......................................................    12   0    0G2
...................................uuucggacgaucaaacuuucagaUa....................................................    2    1    0G2
...................................uuucggaGgaucaaacuuucagaca....................................................    30   1    0G2
....................................uucggacgaucaaacuuucaga......................................................    4    0    0G2
....................................uucggaGgaucaaacuuucaga......................................................    474  1    0G2
```

```
                                   Mature                   Star
auuugcuuucauuuguauguaugaaaaccaaaaaguuucggacgaucaaacuuucagacaaacuuuuguccgaaaguuuaauugucugaaaucugguuuuguccgaaaguuu
....................................uucggaGgaucaaacuuucagaca....................................................    8     1    0G2
....................................uucggacgaucaaacuuucagaca....................................................    2     0    0G2
.....................................ucggaGgaucaaacuuucaga......................................................    25    1    0G2
.....................................ucggaGgaucaaacuuucagaca....................................................    3     1    0G2
......................................cggaUgaucaaacuuucaga......................................................    68    1    0G2
......................................cggaGgaucaaacuuucaga......................................................    4     1    0G2
......................................cggGcgaucaaacuuucagaca....................................................    2     1    0G2
......................................Uggacgaucaaacuuucagaca....................................................    1     1    0G2
......................................Nggacgaucaaacuuucagaca....................................................    1     1    0G2
......................................cggaGgaucaaacuuucagaca....................................................    111   1    0G2
......................................cggaGgaucaaacuuucagacaa...................................................    5     1    0G2
.......................................ggaUgaucaaacuuucaga......................................................    6     1    0G2
.......................................gUacgaucaaacuuucaga......................................................    1     1    0G2
.......................................ggaGgaucaaacuuucagaca....................................................    10    1    0G2
................................................................uuuguccgaaaguuuaauuguA..........................    2     1    0G2
....................................................................uccgaaaguuuaauugucuga.......................    1     0    0G2
....................................................................uAcgaaaguuuaauugucugaa......................    3     1    0G2
....................................................................uccgaaaguuuaauugucAgaa......................    6     1    0G2
....................................................................uccgaaaguuuaauugucugaa......................    32    0    0G2
....................................................................uccgaaaguuuaauugucugaaU.....................    2     1    0G2
....................................................................uccgaaaguuuaauugucugaaa.....................    19    0    0G2
....................................................................uccgaaaguuuaauugucugaaUu....................    2     1    0G2
....................................................................uccgaaaguuuaauugucugaaauA...................    6     1    0G2
.....................................................................ccgaaaguuuaauugucugaa......................    9     0    0G2
.....................................................................ccgaaaguuuaauugucugaaa.....................    5     0    0G2
```

Provisional ID	:	scaffold355_38279
Score total	:	5.2
Score for star read(s)	:	-1.3
Score for read counts	:	0
Score for mfe	:	1.9
Score for randfold	:	1.6
Score for cons. seed	:	3
Total read count	:	851
Mature read count	:	851
Loop read count	:	0
Star read count	:	0

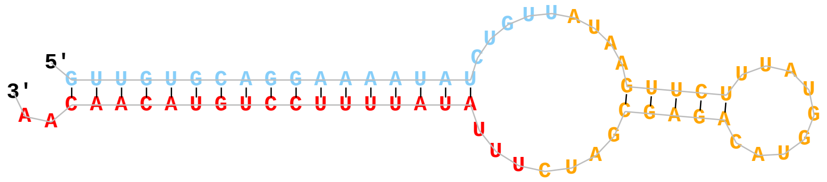

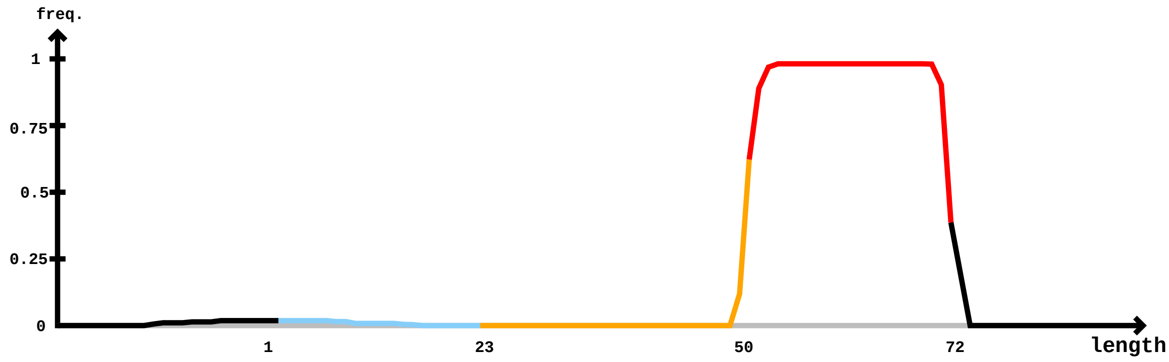

Star **Mature**

```
5'- gcgcauacuaaccgguuuguuguugugcaggaaaauaucuguuauaaguucuuuaugguacagagcgaucuuuauauuuuccuguacaacaaugauaccgguaccgguuag   -3'    exp
    .......((((((((((..(((((((((((((((((((.........(((((.........))))).......)))))))))))))))))))..).......)))))))))    reads    mm    sample
    ........uaaccgguuuguAguugug....................................................................................    3        1     0G2
    ............cgguuuguAguugugcaggaaa.............................................................................    3        1     0G2
    .....................................................................cuuuauauuuuccuguacaac.....................    6        0     0G2
    .....................................................................cuuuauauCuuccuguacaac.....................    2        1     0G2
    .....................................................................cuuuauauGuuccuguacaaca....................    6        1     0G2
    .....................................................................cuuuauauuuuUcuguacaaca....................    1        1     0G2
    .....................................................................cuuuauauuuuccuguacaaca....................    32       0     0G2
    ......................................................................uuuauauuuuccuguacaac.....................    9        0     0G2
    ......................................................................uuuauauuuuccuguacaacU....................    29       1     0G2
    ......................................................................uuuauauuuuccuguacaaca....................    33       0     0G2
    ......................................................................uuuauauuuuccuguacaacC....................    6        1     0G2
    ......................................................................uuuauauuuuccuguacaacCa...................    1        1     0G2
    ......................................................................uuuauauuuuccuguacaacUa...................    1        1     0G2
    .......................................................................uuauauuuCccuguacaaca....................    4        1     0G2
    .......................................................................uuauauuuuccuguacaaca....................    11       0     0G2
    .......................................................................uuauauuuuccuguacaacU....................    10       1     0G2
    .......................................................................uuauauuuuccuguacaacUa...................    13       1     0G2
    .......................................................................uuauauuuuccuguacaacCa...................    10       1     0G2
    .......................................................................uuauauuuuccuguacaacaCu..................    8        1     0G2
    ........................................................................uauauuuuccuguacaaca....................    5        0     0G2
    ........................................................................uauauuuuccuguacaacCa...................    6        1     0G2
    ........................................................................uauauuuuccuguacaacUa...................    1        1     0G2
    ........................................................................uauauuuuccuguacaacaCu..................    12       1     0G2

    .........aaccgguuuguAguugugca..................................................................................    4        1     0B2
    ...............uuuguAguugugcaggaaaa............................................................................    1        1     0B2
    ...............uuuguuguugugcGgaaaau............................................................................    3        1     0B2
    .....................................................................cuuuauauCuuccuguacaac.....................    1        1     0B2
    .....................................................................cuuuauauuuuccuguacaac.....................    2        0     0B2
    .....................................................................cuuuauauuuuccuguacaacU....................    5        1     0B2
    .....................................................................cuuuauauuuuccuguacaaca....................    5        0     0B2
    ......................................................................uuuauauuuuccuguacaac.....................    5        0     0B2
    ......................................................................uuuauauCuuccuguacaac.....................    6        1     0B2
    ......................................................................uuuauauuGuccuguacaac.....................    2        1     0B2
```

```
                     Star                                                 Mature
gcgcauacuaaccgguuuguuguugugcaggaaaauaucuguuauaaguucuuuaugguacagagcgaucuuuauauuuuccuguacaacaaugauaccgguaccgguuag
......................................................................uuuauauuuuccuguacaacU....................    44     1     0B2
......................................................................uuuauauCuuccuguacaaca....................    3      1     0B2
......................................................................uuuauauuuuccuguacaaca....................    10     0     0B2
......................................................................uuuauauuuuccuguacaacUa...................    2      1     0B2
......................................................................uuuauauuuuccuguacaacUau..................    4      1     0B2
.......................................................................uuauauuuuccuguacaac.....................    3      0     0B2
.......................................................................uuauauuuuccuguacaaca....................    1      0     0B2
.......................................................................uuauauuuuccuguacaacU....................    9      1     0B2
.......................................................................uuauauuuuccuguacaacUa...................    3      1     0B2
.......................................................................uuauauuuuccuguacaacaCu..................    13     1     0B2
........................................................................uauauuuuccuguacaacUau..................    2      1     0B2
........................................................................uauauuuuccuguacaacaCu..................    11     1     0B2
.........................................................................auauuuuccuguacaacaCu..................    6      1     0B2

........uaaccgguuuguAguugugca..................................................................................    2      1     0A2
.....................................................................cuuuauauuuuccuguacaac.....................    6      0     0A2
.....................................................................cuuuauauCuuccuguacaac.....................    9      1     0A2
.....................................................................cuuuauauuuCccuguacaaca....................    2      1     0A2
.....................................................................cuuuauauuuuccuguacaaca....................    24     0     0A2
.....................................................................cuuuauCuuuuccuguacaaca....................    1      1     0A2
.....................................................................cuuuauauuuuccuAuacaaca....................    1      1     0A2
......................................................................uuuauauuuuccuguacaa......................    1      0     0A2
......................................................................uuuauauuuuccuguacaac.....................    14     0     0A2
......................................................................uuuauauuuuccuguacaaca....................    69     0     0A2
......................................................................uuuauauuuuccuguacaacC....................    9      1     0A2
......................................................................uuuauauuuuccuguacaacU....................    70     1     0A2
......................................................................uuuauauuuuccuguacaacUa...................    101    1     0A2
......................................................................uuuauauuuuccuguacaacCa...................    4      1     0A2
......................................................................uuuauauuuuccuguacaacUau..................    14     1     0A2
.......................................................................uuauauuuuccuguacaac.....................    2      0     0A2
.......................................................................uuauauuuuccuguacaacC....................    4      1     0A2
.......................................................................uuauauuuuccuguacaaca....................    16     0     0A2
.......................................................................uuauauuuuccuguacaacU....................    26     1     0A2
.......................................................................uuauauuCuccuguacaaca....................    1      1     0A2
.......................................................................uuauauuuuccuguacaacCa...................    13     1     0A2
.......................................................................uuauauuuuccuguacaacUa...................    48     1     0A2
.......................................................................uuauauuuuccuguacaacaCu..................    36     1     0A2
........................................................................uauauuuuccuguacaacU....................    6      1     0A2
........................................................................uauauuuuccuguacaaca....................    5      0     0A2
........................................................................uauauuuuccuguacaacUa...................    8      1     0A2
........................................................................uauauuuuccuguacaacCa...................    5      1     0A2
........................................................................uauauuuuccuguacaacUau..................    1      1     0A2
........................................................................uauauuuuccuguacaacaCu..................    7      1     0A2
.........................................................................auauuuuccuguacaacUa...................    2      1     0A2
.........................................................................auauuuuccuguacaacaCu..................    3      1     0A2
```

Provisional ID	:	scaffold360_38565
Score total	:	5.1
Score for star read(s)	:	-1.3
Score for read counts	:	0
Score for mfe	:	1.8
Score for randfold	:	1.6
Score for cons. seed	:	3
Total read count	:	2027
Mature read count	:	2026
Loop read count	:	0
Star read count	:	1

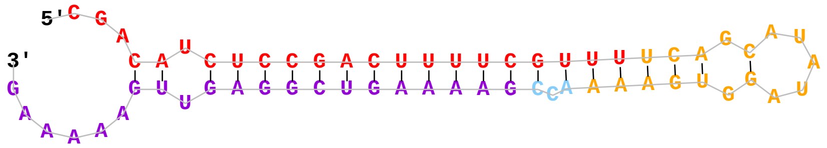

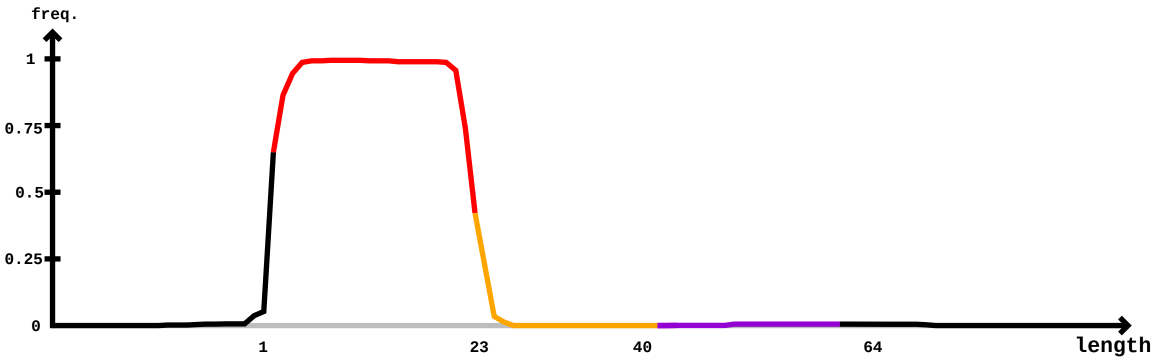

Mature **Star**

```
5'- gaagggagaaaaucccugccccgacaucuccgacuuuucguuuucagcauauaggugaaaaccgaaaagucggaguugaaaaaguuugaguccuugcuccgagaucuccg  -3'   obs
    gaagggagaaaaucccugccccgacaucuccgacuuuucguuuucagcauauaggugaaaaccgaaaagucggaguugaaaaaguuugaguccuugcuccgagaucuccg         exp
    ..(((((.....))))).......((.((((((((((((((((((.(.....).)))))).))))))))))))).))...........(((..((((...))))..)))..   reads    mm      sample
    ...................ccGgacaucuccgacuuuucg......................................................................   7        1       0A2
    ...................cccgacaucuccgacuuuucA......................................................................   4        1       0A2
    ...................cccgacaucucUgacuuuucg......................................................................   1        1       0A2
    ...................cccgacaucuccgacuuuucg......................................................................   7        0       0A2
    ...................cccgacaucuccgacuuuucguA....................................................................   3        1       0A2
    ...................ccGgacaucuccgacuuuucguu....................................................................   1        1       0A2
    ....................ccgacaucuccgacuuuucg......................................................................   5        0       0A2
    ....................ccgacaucuccgacuuuucgu.....................................................................   4        0       0A2
    ....................ccgacaucuccgacuuuucguA....................................................................   1        1       0A2
    ....................ccgacaucuccgacuuuucguuA...................................................................   3        1       0A2
    .....................cAacaucuccgacuuuucg......................................................................   3        1       0A2
    .....................Ugacaucuccgacuuuucg......................................................................   1        1       0A2
    .....................cgacaucuccgacuuuucgu.....................................................................   72       0       0A2
    .....................cAacaucuccgacuuuucgu.....................................................................   60       1       0A2
    .....................Ugacaucuccgacuuuucguu....................................................................   16       1       0A2
    .....................cgacaucCccgacuuuucguu....................................................................   1        1       0A2
    .....................cgacaucuccgacuuuucUuu....................................................................   1        1       0A2
    .....................cAacaucuccgacuuuucguu....................................................................   32       1       0A2
    .....................cgacaucuccgacuuuucguu....................................................................   93       0       0A2
    .....................cgacaucuccgacuuuucguA....................................................................   20       1       0A2
    .....................Agacaucuccgacuuuucguu....................................................................   1        1       0A2
    .....................cgacGucuccgacuuuucguu....................................................................   3        1       0A2
    .....................cgacaucuccgacuuuucguuA...................................................................   123      1       0A2
    .....................cAacaucuccgacuuuucguuu...................................................................   10       1       0A2
    .....................cgacaucuccgacuuuucguuu...................................................................   15       0       0A2
    .....................Ugacaucuccgacuuuucguuu...................................................................   7        1       0A2
    .....................Ugacaucuccgacuuuucguuuu..................................................................   5        1       0A2
    .....................cgacaucuccgacuuuucguuuu..................................................................   3        0       0A2
    .....................cgacaucuccgacuuuucguAuu..................................................................   3        1       0A2
    .....................cgacaucuccgacuuuucguuAu..................................................................   9        1       0A2
    .....................Ngacaucuccgacuuuucguuuu..................................................................   1        1       0A2
    .....................cgacaucuccgacuuuucguuuuA.................................................................   3        1       0A2
    ......................gacaucuccgacuuuucA......................................................................   1        1       0A2
```

```
                      Mature                                    Star
gaagggagaaaaucccugccccgacaucuccgacuuuucguuuucagcauauaggugaaaaccgaaaagucggaguugaaaaaguuugaguccuugcuccgagaucuccg
......................gacaucuccgacuuuucgu.....................................................................    4    0    0A2
......................Aacaucuccgacuuuucgu.....................................................................    3    1    0A2
......................gacaucuccgacuuuucguu....................................................................    35   0    0A2
......................gaAaucuccgacuuuucguu....................................................................    1    1    0A2
......................Aacaucuccgacuuuucguu....................................................................    17   1    0A2
......................gacaucuccgacuuuucguA....................................................................    2    1    0A2
......................Uacaucuccgacuuuucguu....................................................................    3    1    0A2
......................Aacaucuccgacuuuucguuu...................................................................    13   1    0A2
......................gacaucuccgacuuuucguuu...................................................................    18   0    0A2
......................gacaucuccgacuuuucguuA...................................................................    11   1    0A2
......................gacaucuccgacuuuucguuuu..................................................................    1    0    0A2
......................gacaucuccgacuuuucguuuuA.................................................................    7    1    0A2
......................gacaucuccgacuuuucguuuuAa................................................................    1    1    0A2
.......................acaucuccgacuuuucguu....................................................................    7    0    0A2
.......................acaucuccgacuuuucgGuu...................................................................    1    1    0A2
.......................acaucuccgacuuuucguuu...................................................................    16   0    0A2
.......................acaucuccgacuuuucguuA...................................................................    20   1    0A2
.......................acaucuccgacuuuucguuuuA.................................................................    8    1    0A2
........................caucuccgacuuuucguuu...................................................................    2    0    0A2
........................caucuccgacuCuucguuuu..................................................................    3    1    0A2
........................caucuccgacuuuucguuuu..................................................................    14   0    0A2
........................caucuccgacuuuucguuuuA.................................................................    11   1    0A2
.........................Gucuccgacuuuucguuuu..................................................................    2    1    0A2
...............................................................gaaaagucggagAugaaaaag..........................    1    1    0A2
.....................................................................ucggagAugaaaaaguuuga.....................    4    1    0A2
.....................................................................ucggagAugaaaaaguuugag....................    6    1    0A2

................ugccccgacaucuccgacuuuucgA.....................................................................    2    1    0G2
...................cccgacaucuccgacuuuucA......................................................................    2    1    0G2
...................Nccgacaucuccgacuuuucg......................................................................    1    1    0G2
...................cccgacaucuccgacuuuucg......................................................................    13   0    0G2
...................ccGgacaucuccgacuuuucgu.....................................................................    1    1    0G2
...................ccGgacaucuccgacuuuucguu....................................................................    8    1    0G2
....................ccgacaucuccgacuuuucA......................................................................    1    1    0G2
....................ccgacaucuccgacuuuucgu.....................................................................    1    0    0G2
....................cUgacaucuccgacuuuucguu....................................................................    1    1    0G2
....................ccAacaucuccgacuuuucguu....................................................................    2    1    0G2
.....................cAacaucuccgacuuuucg......................................................................    2    1    0G2
.....................cAacaucuccgacuuuucgu.....................................................................    64   1    0G2
.....................cgacaucuccgGcuuuucgu.....................................................................    2    1    0G2
.....................cgacaucuccgacuuuucgu.....................................................................    43   0    0G2
.....................cgacaucuccgacuuuucAuu....................................................................    6    1    0G2
.....................cAacaucuccgacuuuucguu....................................................................    37   1    0G2
.....................cgacaucuccgacuuuucguA....................................................................    15   1    0G2
.....................cgacaucuccgacuuuucguu....................................................................    26   0    0G2
.....................cgacaucuccgacuuuucgGu....................................................................    1    1    0G2
.....................cgacaucuccgacuuuucCuu....................................................................    3    1    0G2
.....................cgacaucuccgacuuuuGguuu...................................................................    1    1    0G2
.....................cgacaucuccgacuuuucguuA...................................................................    116  1    0G2
.....................cgacaucuccgacuuuucguuu...................................................................    2    0    0G2
.....................cgacaucuccgacuuuucguuAu..................................................................    1    1    0G2
.....................cgacaucuccgacuuuucguuuuA.................................................................    2    1    0G2
......................Aacaucuccgacuuuucg......................................................................    3    1    0G2
......................Aacaucuccgacuuuucgu.....................................................................    22   1    0G2
......................gacaucuccgacuuuucgu.....................................................................    13   0    0G2
......................gacaucuccgacuuuucguu....................................................................    42   0    0G2
......................Aacaucuccgacuuuucguu....................................................................    32   1    0G2
......................Aacaucuccgacuuuucguuu...................................................................    11   1    0G2
......................gacaucuccgacuuuucguuA...................................................................    15   1    0G2
......................gacaucuccgacuuuucguuu...................................................................    24   0    0G2
......................gacaucuccgacuuuucguuuuUa................................................................    3    1    0G2
.......................acaucuccgacuuuucguu....................................................................    6    0    0G2
.......................acaucuccgacuuuucguA....................................................................    2    1    0G2
.......................acaucuccgGcuuuucguuu...................................................................    2    1    0G2
.......................acaucuccgacuuuucguuA...................................................................    18   1    0G2
.......................acaucuccgacuuuucguuu...................................................................    29   0    0G2
.......................acaucuccgacuuuucguuuG..................................................................    4    1    0G2
.......................acaucuccgacuuuucguuuu..................................................................    4    0    0G2
.......................acaucuccgacuuuucguuuuUa................................................................    9    1    0G2
........................caucuccgacuuuucguuA...................................................................    1    1    0G2
```

Mature **Star**

```
gaagggagaaaaucccugccccgacaucuccgacuuuucguuuucagcauauaggugaaaaccgaaaagucggaguugaaaaaguuugaguccuugcuccgagaucuccg
........................caucuccgacuuuucguuuG..................................................................   4   1   0G2
........................caucuccgacuuuuUguuuu..................................................................   5   1   0G2
........................caucuccgacuuuucguuuu..................................................................   10  0   0G2
........................caucuccgacuuuucguuuuAa................................................................   4   1   0G2
.........................aucuccgacuuuucguuuuA.................................................................   5   1   0G2
.........................aucuccgacuuuucguuuuAa................................................................   5   1   0G2

..........aaucccugcccGgacaucucc...............................................................................   4   1   0B2
.............cccugcccGgacaucuccgac............................................................................   4   1   0B2
..............ccugcccGgacaucuccgac............................................................................   3   1   0B2
...................cccgacaucuccgacuuuuc.......................................................................   4   0   0B2
...................cccgacaucucUgacuuuucg......................................................................   1   1   0B2
...................cccgacaucuccgacuuuucA......................................................................   1   1   0B2
...................cccgacaucuccgacuuuucgu.....................................................................   1   0   0B2
...................cccgacaucuccgacuuuucguuA...................................................................   7   1   0B2
...................cccgacaucuccgacuuuucguuuA..................................................................   1   1   0B2
....................ccgacaucuccgacuuuuc.......................................................................   1   0   0B2
....................ccgacaucuccgacuuuucg......................................................................   7   0   0B2
....................ccAacaucuccgacuuuucg......................................................................   2   1   0B2
....................ccAacaucuccgacuuuucgu.....................................................................   1   1   0B2
....................ccgacaucuccgacuuuucguu....................................................................   1   0   0B2
....................cUgacaucuccgacuuuucguu....................................................................   2   1   0B2
.....................cAacaucuccgacuuuucgu.....................................................................   77  1   0B2
.....................cgacaucuccgacuuuucAu.....................................................................   2   1   0B2
.....................cgacaucuccgacuuuucgu.....................................................................   54  0   0B2
.....................cAacaucuccgacuuuucguu....................................................................   41  1   0B2
.....................Ngacaucuccgacuuuucguu....................................................................   2   1   0B2
.....................cgacaucuccgGcuuuucguu....................................................................   2   1   0B2
.....................cgacaucuccgacuuuucguu....................................................................   95  0   0B2
.....................cgaUaucuccgacuuuucguu....................................................................   2   1   0B2
.....................cgacaucuccgacuuuucguA....................................................................   20  1   0B2
.....................Ugacaucuccgacuuuucguu....................................................................   1   1   0B2
.....................cgacaucuccgacuuuucguuu...................................................................   25  0   0B2
.....................Ugacaucuccgacuuuucguuu...................................................................   2   1   0B2
.....................Ngacaucuccgacuuuucguuu...................................................................   1   1   0B2
.....................cAacaucuccgacuuuucguuu...................................................................   3   1   0B2
.....................cgacaucuccgacuuCucguuu...................................................................   7   1   0B2
.....................cgacaucuccgacuuuucguuA...................................................................   69  1   0B2
.....................cgacaucuccgacuuuucguuuu..................................................................   11  0   0B2
.....................cgacaucuccgacuuuucguuAu..................................................................   5   1   0B2
.....................cgacaucuccgacuuuucguuuuU.................................................................   2   1   0B2
......................Aacaucuccgacuuuucgu.....................................................................   9   1   0B2
......................gacaucuccgacuuuucgu.....................................................................   12  0   0B2
......................Aacaucuccgacuuuucguu....................................................................   32  1   0B2
......................gacaucuccgacuuuucguu....................................................................   31  0   0B2
......................gacauAuccgacuuuucguu....................................................................   1   1   0B2
......................Nacaucuccgacuuuucguu....................................................................   1   1   0B2
......................gacaucuccgacuuuucguuu...................................................................   21  0   0B2
......................gacaucuccgacuuuucguuA...................................................................   24  1   0B2
......................Aacaucuccgacuuuucguuu...................................................................   21  1   0B2
......................gacaucuccgacuuuucguuAu..................................................................   4   1   0B2
.......................acaucuccgacuuuucgGuu...................................................................   1   1   0B2
.......................Ncaucuccgacuuuucguuu...................................................................   1   1   0B2
.......................acaucuccgacuuuucguuA...................................................................   10  1   0B2
.......................acaucuccgacuuuucguuu...................................................................   16  0   0B2
.......................acaucuUcgacuuuucguuuu..................................................................   5   1   0B2
.......................acaucuccgacuuuucguuuu..................................................................   9   0   0B2
........................caucuccgacuuuucguuu...................................................................   11  0   0B2
........................caucuccgacuuuucguuuu..................................................................   13  0   0B2
........................caucuccgacuGuucguuuu..................................................................   1   1   0B2
........................caucuccgacuuuucguuuuA.................................................................   4   1   0B2
........................caucuccgacuuuucguuuuAa................................................................   2   1   0B2
...........................cuccgacuuuucguuuucC................................................................   4   1   0B2
```

Provisional ID	:	scaffold368_38885
Score total	:	310
Score for star read(s)	:	3.9
Score for read counts	:	299.9
Score for mfe	:	1.6
Score for randfold	:	1.6
Score for cons. seed	:	3
Total read count	:	600
Mature read count	:	599
Loop read count	:	0
Star read count	:	1

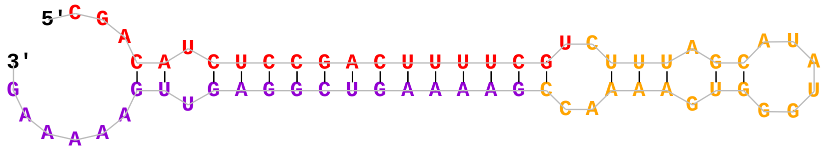

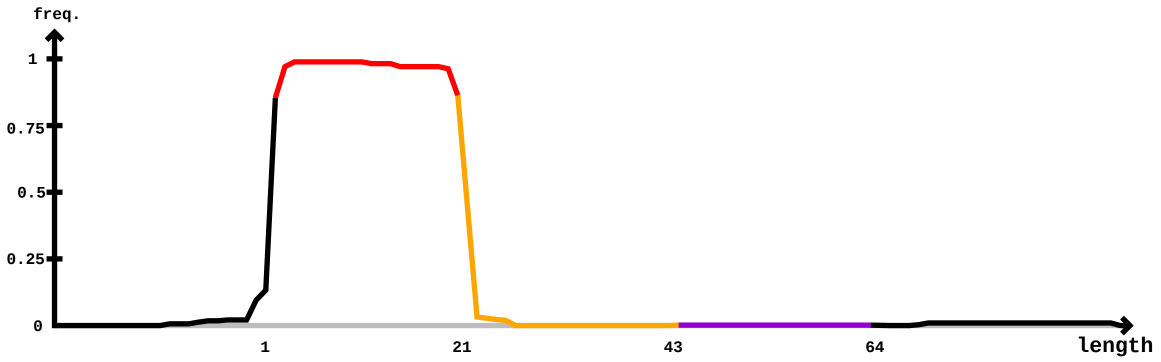

Mature   Star

```
5'- gaagggagaaaaucccugccccgacaucuccgacuuuucgucuuuagcauaugggugaaaaccgaaaagucggaguugaaaaaguucgagucuuugcuccgacaucucca  -3'   obs
    gaagggagaaaaucccugccccgacaucuccgacuuuucgucuuuagcauaugggugaaaaccgaaaagucggaguugaaaaaguucgagucuuugcuccgacaucucca        exp
    ....(((((...............((.(((((((((((((..(((.((......)).)))..))))))))))))).)).....(((.((((....)))).))).))))).   reads   mm   sample
    ...................cccgacaucucUgacuuuucg......................................................................   1       1    0A2
    ...................ccGgacaucuccgacuuuucg......................................................................   7       1    0A2
    ...................cccgacaucuccgacuuuucg......................................................................   7       0    0A2
    ...................cccgacaucuccgacuuuucA......................................................................   4       1    0A2
    ...................cccgacaucuccgacuuuucguA....................................................................   3       1    0A2
    ....................ccgacaucuccgacuuuucg......................................................................   5       0    0A2
    ....................ccgacaucuccgacuuuucgu.....................................................................   4       0    0A2
    ....................ccgacaucuccgacuuuucguA....................................................................   1       1    0A2
    .....................Ugacaucuccgacuuuucg......................................................................   1       1    0A2
    .....................cAacaucuccgacuuuucg......................................................................   3       1    0A2
    .....................cgacaucuccgacuuuucgu.....................................................................   72      0    0A2
    .....................cAacaucuccgacuuuucgu.....................................................................   60      1    0A2
    .....................cgacaucuccgacuuuucguA....................................................................   20      1    0A2
    .....................cgacaucuccgacuuuucguAuu..................................................................   3       1    0A2
    ......................gacaucuccgacuuuucA......................................................................   1       1    0A2
    ......................Aacaucuccgacuuuucgu.....................................................................   3       1    0A2
    ......................gacaucuccgacuuuucgu.....................................................................   4       0    0A2
    ......................gacaucuccgacuuuucguA....................................................................   2       1    0A2
    ...............................................................gaaaagucggagAugaaaaag..........................   1       1    0A2

    ................ugccccgacaucuccgacuuuucgA.....................................................................   2       1    0G2
    ...................cccgacaucuccgacuuuucA......................................................................   2       1    0G2
    ...................cccgacaucuccgacuuuucg......................................................................   13      0    0G2
    ...................Nccgacaucuccgacuuuucg......................................................................   1       1    0G2
    ...................ccGgacaucuccgacuuuucgu.....................................................................   1       1    0G2
    ....................ccgacaucuccgacuuuucA......................................................................   1       1    0G2
    ....................ccgacaucuccgacuuuucgu.....................................................................   1       0    0G2
    .....................cAacaucuccgacuuuucg......................................................................   2       1    0G2
    .....................cgacaucuccgacuuuucgu.....................................................................   43      0    0G2
    .....................cAacaucuccgacuuuucgu.....................................................................   64      1    0G2
    .....................cgacaucuccgGcuuuucgu.....................................................................   2       1    0G2
    .....................cgacaucuccgacuuuucguA....................................................................   15      1    0G2
    .....................cgacaucuccgacuuuucgucA...................................................................   3       1    0G2
```

Mature **Star**

```
gaagggagaaaaucccugccccgacaucuccgacuuuucgucuuuagcauaugggugaaaaccgaaaagucggaguugaaaaaguucgagucuuugcuccgacaucucca
......................Aacaucuccgacuuuucg......................................................................   3    1    0G2
......................gacaucuccgacuuuucgu.....................................................................   13   0    0G2
......................Aacaucuccgacuuuucgu.....................................................................   22   1    0G2
......................gacaucuccgacuuuucguUuuua................................................................   3    1    0G2
.......................acaucuccgacuuuucguA....................................................................   2    1    0G2
.......................acaucuccgacuuuucguUuuua................................................................   9    1    0G2
........................................................................................agucuuugcuccgacaucucA.   2    1    0G2
.........................................................................................Aucuuugcuccgacaucucc.   4    1    0G2

..........aaucccugcccGgacaucucc...............................................................................   4    1    0B2
.............cccugcccGgacaucuccgac............................................................................   4    1    0B2
..............ccugcccGgacaucuccgac............................................................................   3    1    0B2
...................cccgacaucuccgacuuuuc.......................................................................   4    0    0B2
...................cccgacaucucUgacuuuucg......................................................................   1    1    0B2
...................cccgacaucuccgacuuuucA......................................................................   1    1    0B2
...................cccgacaucuccgacuuuucgu.....................................................................   1    0    0B2
....................ccgacaucuccgacuuuuc.......................................................................   1    0    0B2
....................ccAacaucuccgacuuuucg......................................................................   2    1    0B2
....................ccgacaucuccgacuuuucg......................................................................   7    0    0B2
....................ccAacaucuccgacuuuucgu.....................................................................   1    1    0B2
.....................cAacaucuccgacuuuucgu.....................................................................   77   1    0B2
.....................cgacaucuccgacuuuucgu.....................................................................   54   0    0B2
.....................cgacaucuccgacuuuucAu.....................................................................   2    1    0B2
.....................cgacaucuccgacuuuucguA....................................................................   20   1    0B2
.....................cgacaucuccgacuuuucguc....................................................................   4    0    0B2
.....................cgacaucuccgacuuuucguUuuu.................................................................   2    1    0B2
......................gacaucuccgacuuuucgu.....................................................................   12   0    0B2
......................Aacaucuccgacuuuucgu.....................................................................   9    1    0B2
```

Provisional ID	:	scaffold395_39926
Score total	:	2.1
Score for star read(s)	:	-1.3
Score for read counts	:	0
Score for mfe	:	2.4
Score for randfold	:	1.6
Score for cons. seed	:	-0.6
Total read count	:	2255
Mature read count	:	2255
Loop read count	:	0
Star read count	:	0

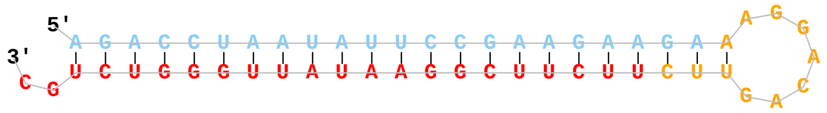

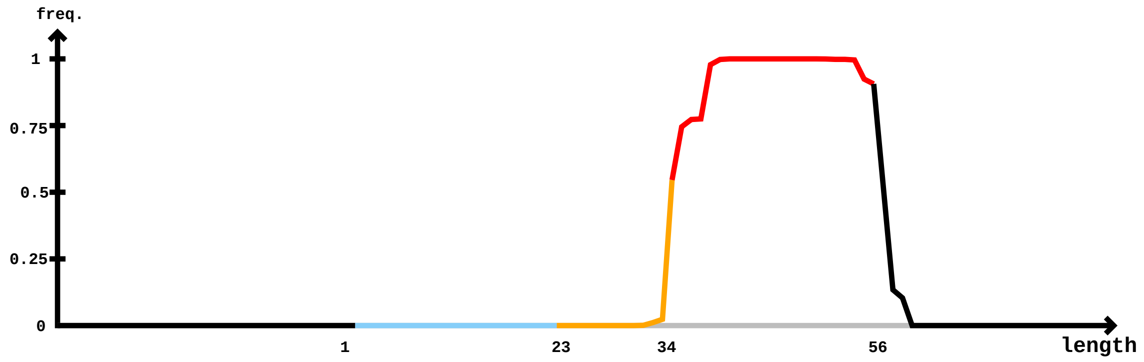

Star   Mature

```
5'- caaugcugccuaaccauuagccugcgccuagaccuaauauuccgaagaagaaaggacaguucuucuucggaauauugggucugcgugcaggcuaccuaaccauuaguu   -3'    exp
    ....((((.........((((((((((.((((((((((((((((((((((((.......)))))))))))))))))))))))).)))))))))).........)))).    reads    mm    sample
    ...........................................................uucuucuucggaauauuggg.............................    1    0    0A2
    ............................................................ucuucuucggaGuauuggg.............................    2    1    0A2
    ............................................................ucuucuucggaauauuggUuc...........................    3    1    0A2
    ............................................................ucuucuucggaauauuggUucu..........................    7    1    0A2
    .............................................................cuucuucggaauauugggucu..........................    6    0    0A2
    .............................................................cuucuucggaauauuggUucu..........................    6    1    0A2
    ..............................................................uucuucggaauauugggucu..........................    14    0    0A2
    ..............................................................uucuucggaGuauugggucu..........................    5    1    0A2
    ..............................................................uucuucggaauauuggUucu..........................    20    1    0A2
    ..............................................................uucuucggaauauuggUucug.........................    4    1    0A2
    ..............................................................uucuucggaauauugggucuU.........................    2    1    0A2
    ..............................................................uucuucggaauauugggucug.........................    3    0    0A2
    ..............................................................uucuucggaauauuggUucugc........................    279    1    0A2
    ..............................................................uucuucggaauauugggucugU........................    1    1    0A2
    ..............................................................uucuucAgaauauugggucugc........................    2    1    0A2
    ..............................................................uucuucggaauauugggucugc........................    8    0    0A2
    ..............................................................uucuucggaauauugggucugcU.......................    1    1    0A2
    ...............................................................ucuucggaauauugggucu..........................    3    0    0A2
    ...............................................................ucuucggaauauugggucug.........................    1    0    0A2
    ...............................................................ucuucggaauaGugggucugc........................    1    1    0A2
    ...............................................................ucuucggaauauugggucugc........................    39    0    0A2
    ...............................................................ucuucAgaauauugggucugc........................    4    1    0A2
    ...............................................................ucuucggaauauugggucugU........................    1    1    0A2
    ...............................................................ucuucggaauauuggUucugc........................    59    1    0A2
    ...............................................................ucuucggaauauugggucugcA.......................    1    1    0A2
    ...............................................................ucuucgCaauauugggucugcg.......................    1    1    0A2
    ...............................................................ucuucggaauauugggucugcU.......................    2    1    0A2
    ...............................................................ucuucggaauauugggucugcg.......................    18    0    0A2
    ...............................................................ucuucggaauauugggucugcgA......................    6    1    0A2
    ...............................................................ucuucggaauauugggucugcguU.....................    2    1    0A2
    ................................................................cuucggaauauuggUucu..........................    9    1    0A2
    ................................................................cuucggaauauuggUucugc........................    4    1    0A2
    ................................................................cuucggaauauugggucugc........................    17    0    0A2
    ................................................................cuucggaauauugggucugcA.......................    6    1    0A2
```

Star Mature

caaugcugccuaaccauuagccugcgccuagaccuaauauuccgaagaagaaaggacaguucuucuucggaauauugggucugcgugcaggcuaccuaaccauuaguu			
....cuucggaauauugggucugcgu......................	2	0	0A2
....ucggaauauugggucugc........................	19	0	0A2
....ucAgaauauugggucugc........................	1	1	0A2
....ucggaauauugggucugcg.......................	27	0	0A2
....ucggaauauugggucugcA.......................	6	1	0A2
....ucggaauauugggucugcgu......................	20	0	0A2
....ucggaauauugggucugcguA.....................	13	1	0A2
....ucggaauauugggucugcguU.....................	46	1	0A2
....cggaauauugggucugcA.......................	3	1	0A2
....cggaauauugggucugcguU.....................	9	1	0A2
....ggaauauugggucugcgu......................	3	0	0A2
....ucuucuucggaauauuggUucu..........................	3	1	0G2
....cuucuucggaauauuggUucu..........................	6	1	0G2
....uucuucggaauauugggucu..........................	2	0	0G2
....uucuucggaauauuggUucu..........................	27	1	0G2
....uucuucggaauauuggUucug.........................	9	1	0G2
....uucuucggaGuauugggucugc........................	1	1	0G2
....uucuucggaauauugggucugc........................	3	0	0G2
....uucuucggaauauuggUucugc........................	404	1	0G2
....ucuucggaauauugggucug.........................	7	0	0G2
....ucuucAgaauauugggucugc........................	1	1	0G2
....ucuucggaauauugggucugc........................	22	0	0G2
....ucuucggaauauuggUucugc........................	40	1	0G2
....ucuucggaauauugggucugU........................	8	1	0G2
....ucuucggaauauugggucugcA.......................	16	1	0G2
....ucuucggaauauugggucugcg.......................	21	0	0G2
....ucuucggaauauugggucugcU.......................	1	1	0G2
....ucuucggaauauugggucugcgA......................	2	1	0G2
....ucuucggaauauugggucugcgu......................	4	0	0G2
....cuucggaauauugggucu..........................	1	0	0G2
....cuucggaauauugggucugc........................	5	0	0G2
....uucggaauauugggucugc........................	4	0	0G2
....ucggaauauugggucugc........................	56	0	0G2
....ucggGauauugggucugc........................	1	1	0G2
....ucggaauauugggucugcA.......................	2	1	0G2
....ucggaauauugggucugcg.......................	15	0	0G2
....ucggaauauugggucugcgu......................	12	0	0G2
....ucggaauauugggucugcguA.....................	13	1	0G2
....ucggaauauugggucugcguU.....................	45	1	0G2
....ucggaauauugggucugcguC.....................	3	1	0G2
....cggaauauugggucugcA.......................	7	1	0G2
....cggaauauugggucugcguU.....................	11	1	0G2
....ggaauauugggucugcgu......................	1	0	0G2
....uucGucuucggaauauugg..............................	1	1	0B2
....ucuucuucggaauauugggucu..........................	5	0	0B2
....ucuucuucggaauauuggUucu..........................	3	1	0B2
....cAucuucggaauauugggucu..........................	4	1	0B2
....cAucuucggaauauugggucugc........................	6	1	0B2
....Gucuucggaauauuggguc...........................	2	1	0B2
....uucuucggaauauuggUucu..........................	26	1	0B2
....uucuucggaauauugggucu..........................	7	0	0B2
....uucuucggaauauuggUucug.........................	6	1	0B2
....uucuucggaauauuggUucugc........................	314	1	0B2
....uucuucggaauauugggucugc........................	38	0	0B2
....uucuucggaauauugggucugcA.......................	1	1	0B2
....Ncuucggaauauugggucu..........................	2	1	0B2
....ucuucggaauauugggucu..........................	6	0	0B2
....ucuucggaauauugggucug.........................	6	0	0B2
....Ncuucggaauauugggucug.........................	1	1	0B2
....ucuucggaauauugggucuU.........................	2	1	0B2
....ucuucggaauauugggucugU........................	6	1	0B2
....ucuucggaauauuggUucugc........................	64	1	0B2
....ucuucggaauauugggucugc........................	83	0	0B2
....ucuucggaauauugggucugcU.......................	2	1	0B2
....ucuucggaauauugggucugcA.......................	5	1	0B2
....ucuucggaauauugggucugcg.......................	2	0	0B2
....ucuucggaauauugggucugcC.......................	2	1	0B2
....ucuucggaauauugggucugcgu......................	4	0	0B2

```
                                  Star                          Mature
caaugcugccuaaccauuagccugcgccuagaccuaauauuccgaagaagaaaggacaguucuucuucggaauauugggucugcgugcaggcuaccuaaccauuaguu
...............................................................ucuucggaauauugggucugcUu......................   2   1   0B2
...............................................................ucuucggaauauugggucugcgA......................   3   1   0B2
................................................................cuucggaGuauugggucugc........................   4   1   0B2
................................................................cuucggaauauugggucugc........................   2   0   0B2
................................................................cuucggaauauuggUucugc........................   3   1   0B2
................................................................cuucggaauauugggucugcA.......................   2   1   0B2
................................................................cuucggaauauugggucugcU.......................   7   1   0B2
.................................................................uucggaauauugggucugc........................   1   0   0B2
..................................................................ucggaauauugggucugc........................  39   0   0B2
..................................................................ucggaauaGugggucugc........................   1   1   0B2
..................................................................Ncggaauauugggucugc........................   1   1   0B2
..................................................................ucggaauauugggucugcg.......................  47   0   0B2
..................................................................ucggaauauugggucugcA.......................   3   1   0B2
..................................................................ucggaauauugggucugcgu......................   6   0   0B2
..................................................................ucggaauauugggucugcUu......................   3   1   0B2
..................................................................Ncggaauauugggucugcgu......................   1   1   0B2
..................................................................ucggaauauugggucugcguA.....................  10   1   0B2
..................................................................ucggaauauugggucugcguU.....................  66   1   0B2
..................................................................ucggaauauugggucugcguC.....................   2   1   0B2
..................................................................ucggaauauugggucugcgug.....................   1   0   0B2
...................................................................cggaauauugggucugcg.......................   2   0   0B2
...................................................................cggaauauugggucugcguU.....................  12   1   0B2
....................................................................ggaauauugggucugcguU.....................   1   1   0B2
```

Provisional ID	:	scaffold411_40327
Score total	:	5.7
Score for star read(s)	:	-1.3
Score for read counts	:	0
Score for mfe	:	2.4
Score for randfold	:	1.6
Score for cons. seed	:	3
Total read count	:	1744
Mature read count	:	1744
Loop read count	:	0
Star read count	:	0

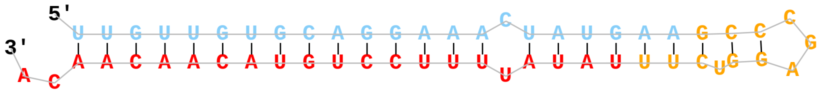

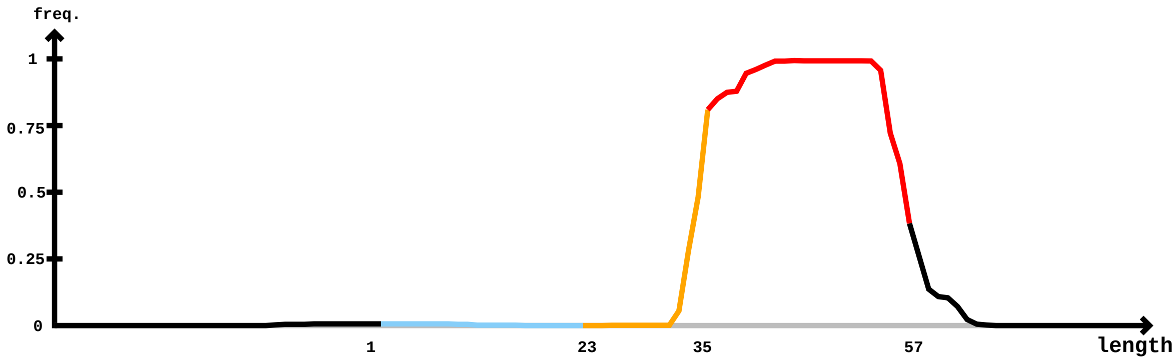

Star Mature

```
5'- caacaugacauucgcuuuuacuaaccgguuuguuguugugcaggaaacuaugaagcccgaggucuuuauauuuuccuguacaacaacaaaaccgguugguaugagcuuuauu   -3'    exp
    .............((((.(((((((((((((((((((((((((((((.(((((((((...)).))))))).)))))))))))))))).))))))))))))).)))).......    reads    mm    sample
    .....................uaaccgguuuguAguugugca......................................................................    2        1     0A2
    ...............................................................cuuuauauuuuccuguacaac............................    6        0     0A2
    ...............................................................cuuuauauCuuccuguacaac............................    9        1     0A2
    ...............................................................cuuuauauuuCccuguacaaca...........................    2        1     0A2
    ...............................................................cuuuauauuuuccuAuacaaca...........................    1        1     0A2
    ...............................................................cuuuauauuuuccuguacaaca...........................    24       0     0A2
    ...............................................................cuuuauCuuuuccuguacaaca...........................    1        1     0A2
    ................................................................uuuauauuuuccuguacaa.............................    1        0     0A2
    ................................................................uuuauauuuuccuguacaac............................    14       0     0A2
    ................................................................uuuauauuuuccuguacaaca...........................    69       0     0A2
    ................................................................uuuauauuuuccuguacaacU...........................    70       1     0A2
    ................................................................uuuauauuuuccuguacaacC...........................    9        1     0A2
    ................................................................uuuauauuuuccuguacaacUa..........................    101      1     0A2
    ................................................................uuuauauuuuccuguacaacCa..........................    4        1     0A2
    ................................................................uuuauauuuuccuguacaacUac.........................    7        1     0A2
    ................................................................uuuauauuuuccuguacaacUaca........................    2        1     0A2
    .................................................................uuauauuuuccuguacaac............................    2        0     0A2
    .................................................................uuauauuuuccuguacaaca...........................    16       0     0A2
    .................................................................uuauauuuuccuguacaacU...........................    26       1     0A2
    .................................................................uuauauuuuccuguacaacC...........................    4        1     0A2
    .................................................................uuauauuCuccuguacaaca...........................    1        1     0A2
    .................................................................uuauauuuuccuguacaacCa..........................    13       1     0A2
    .................................................................uuauauuuuccuguacaacUa..........................    48       1     0A2
    .................................................................uuauauuuuccuguacaacUac.........................    38       1     0A2
    .................................................................uuauauuuuccuguacaacUaca........................    85       1     0A2
    .................................................................uuauauuuCccuguacaacaaca........................    1        1     0A2
    .................................................................uuauauuuuccuguacaacUacaaa......................    7        1     0A2
    ..................................................................uauauuuuccuguacaacU...........................    6        1     0A2
    ..................................................................uauauuuuccuguacaaca...........................    5        0     0A2
    ..................................................................uauauuuuccuguacaacCa..........................    5        1     0A2
    ..................................................................uauauuuuccuguacaacUa..........................    8        1     0A2
    ..................................................................uauauuuCccuguacaacaac.........................    2        1     0A2
    ..................................................................uauauuuuccuguacaacaac.........................    2        0     0A2
    ..................................................................uauauCuuccuguacaacaac.........................    6        1     0A2
```

Star Mature

caacaugacauucgcuuuuacuaaccgguuuguuguugugcaggaaacuaugaagcccgaggucuuuauauuuuccuguacaacaacaaaaccgguugguaugagcuuuauu			
....uauauuuuccuguacaacUac.........................	143	1	0A2
....uauauuuuccuguacaacaaca........................	2	0	0A2
....uauauuuuccuguacaacUaca........................	192	1	0A2
....uauauuuuccuguacaacUacaaa......................	1	1	0A2
....auauuuuccuguacaacUa..........................	2	1	0A2
....auauCuuccuguacaacaac.........................	3	1	0A2
....auauuuuccuguacaacUac.........................	15	1	0A2
....auauuuuccuguacaacUaca........................	23	1	0A2
....auauuuuccuguacaacUacaa.......................	3	1	0A2
....auauuuuccuguacaacUacaaa......................	1	1	0A2
....uauuuuccuguacaacUaca........................	7	1	0A2
....uauuuuccuguacaacUacaaa......................	11	1	0A2
....uuuuccuguacaacUacaaa......................	9	1	0A2
....uuuuccuguacaacUacaaaa.....................	1	1	0A2
....uuuuccuguacaacaacaaaac....................	12	0	0A2
....uuuuccuguacaacUacaaaac....................	3	1	0A2
....uuuuccuguacaacUacaaaacc...................	1	1	0A2
....uuuuccuguacaacaacaaaacA...................	34	1	0A2
....uuuccuguacaacaacaaaac....................	2	0	0A2
....uuuccuguacaacUacaaaac....................	5	1	0A2
....uuuccuguacaacaacaaaacA...................	13	1	0A2
....uuccuguacaacaacaaaac....................	1	0	0A2
....uuccuguacaacUacaaaac....................	4	1	0A2
....uuccuguacaacUacaaaacc...................	3	1	0A2
....uuccuguacaacaacaaaaccA..................	8	1	0A2
....uuccuguacaacUacaaaaccgg.................	2	1	0A2
....cuguacaacUacaaaaccggu................	4	1	0A2
.....................uaaccgguuuguAguugug..	3	1	0G2
.........................cgguuuguAguugugcaggaaa...	3	1	0G2
....cuuuauauCuuccuguacaac............................	2	1	0G2
....cuuuauauuuuccuguacaac............................	6	0	0G2
....cuuuauauuuuccuguacaaca...........................	32	0	0G2
....cuuuauauuuuUcuguacaaca...........................	1	1	0G2
....cuuuauauGuuccuguacaaca...........................	6	1	0G2
....uuuauauuuuccuguacaac............................	9	0	0G2
....uuuauauuuuccuguacaacC...........................	6	1	0G2
....uuuauauuuuccuguacaacU...........................	29	1	0G2
....uuuauauuuuccuguacaaca...........................	33	0	0G2
....uuuauauuuuccuguacaacUa..........................	1	1	0G2
....uuuauauuuuccuguacaacCa..........................	1	1	0G2
....uuuauauuuuccuguacaacUac.........................	2	1	0G2
....uuauauuuuccuguacaacU...........................	10	1	0G2
....uuauauuuCccuguacaaca...........................	4	1	0G2
....uuauauuuuccuguacaaca...........................	11	0	0G2
....uuauauuuuccuguacaacCa..........................	10	1	0G2
....uuauauuuuccuguacaacUa..........................	13	1	0G2
....uuauauuuuccuguacaacUac.........................	22	1	0G2
....uuauauCuuccuguacaacaac.........................	2	1	0G2
....uuauauuuuccuguacaacUaca........................	19	1	0G2
....uauauuuuccuguacaaca...........................	5	0	0G2
....uauauuuuccuguacaacCa..........................	6	1	0G2
....uauauuuuccuguacaacUa..........................	1	1	0G2
....uauauuuuccuguacaacUac.........................	93	1	0G2
....uauauCuuccuguacaacaac.........................	3	1	0G2
....uauauuuuccuguacaacaac.........................	3	0	0G2
....uauauuuuccuguacaacUaca........................	64	1	0G2
....auauuuuccuguacaacUac.........................	13	1	0G2
....auauCuuccuguacaacaac.........................	4	1	0G2
....auauuuuccuguacaacUaca........................	12	1	0G2
....uauuuuccuguacaacUaca........................	9	1	0G2
....uauuuuccuguacaacUacaaa......................	5	1	0G2
....uauuuuccuguacaacUacaaaa.....................	2	1	0G2
....auuuuccuguacaacUacaaaa.....................	4	1	0G2
....uuuuccuguacaacUacaaa......................	3	1	0G2
....uuuuccuguacaacUacaaaa.....................	2	1	0G2
....uuuuccuguacaacaacaaaac....................	6	0	0G2
....uuuuccuguacaacUacaaaac....................	12	1	0G2
....uuuuccuguacaacaacaaaacA...................	17	1	0G2
....uuuccuguacaacaacaaaacA...................	4	1	0G2

Star Mature

caacaugacauucgcuuuuacuaaccgguuuguuguugugcaggaaacuaugaagcccgaggucuuuauauuuuccuguacaacaacaaaaccgguugguaugagcuuuauu

....uuccuguacaacaacaaaac....................	1	0	0G2
....uuccuguacaacaacaaaaccA..................	6	1	0G2
....uccuguacaacUacaaaaccg..................	12	1	0G2
....uccuguacaacUacaaaaccgg.................	4	1	0G2
......................aaccgguuuguAguugugca..	4	1	0B2
....ccgaggucuuuauauuuucA....................................	2	1	0B2
....cuuuauauuuuccuguacaac............................	2	0	0B2
....cuuuauauCuuccuguacaac............................	1	1	0B2
....cuuuauauuuuccuguacaaca...........................	5	0	0B2
....cuuuauauuuuccuguacaacU...........................	5	1	0B2
....uuuauauuuuccuguacaac............................	5	0	0B2
....uuuauauCuuccuguacaac............................	6	1	0B2
....uuuauauuGuccuguacaac............................	2	1	0B2
....uuuauauCuuccuguacaaca...........................	3	1	0B2
....uuuauauuuuccuguacaacU...........................	44	1	0B2
....uuuauauuuuccuguacaaca...........................	10	0	0B2
....uuuauauuuuccuguacaacUa..........................	2	1	0B2
....uuuauauuuuccuguacaacUac.........................	2	1	0B2
....uuauauuuuccuguacaac............................	3	0	0B2
....uuauauuuuccuguacaaca...........................	1	0	0B2
....uuauauuuuccuguacaacU...........................	9	1	0B2
....uuauauuuuccuguacaacUa..........................	3	1	0B2
....uuauauuuuccuguacaacUac.........................	25	1	0B2
....uuauauuuCccuguacaacaaca........................	3	1	0B2
....uuauauuuuccuguacaacUaca........................	5	1	0B2
....uauauuuuccuguacaacUac.........................	39	1	0B2
....uauauuuuccuguGcaacaac.........................	2	1	0B2
....uauauCuuccuguacaacaac.........................	1	1	0B2
....uauauuuuccuguacaacUaca........................	34	1	0B2
....auauuuuccuguacaacUaca........................	2	1	0B2
....uauuuuccuguacaacUaca........................	5	1	0B2
....uauuuuccuguacaacUacaaa......................	7	1	0B2
....auuuuccuguacaacGaca........................	1	1	0B2
....auuuuccuguacaacUacaaa......................	3	1	0B2
....uuuuccuguacaacUacaaa......................	6	1	0B2
....uuuuccuguacaacaacaaaac....................	13	0	0B2
....uuuuccuguacaacaacaaaacA...................	9	1	0B2
....uuuccuguacaacUacaaaac....................	1	1	0B2
....uuuccuguacaacaacaaaac....................	2	0	0B2
....uuccuguacaacUacaaaacc...................	6	1	0B2
....uccuguacaacUacaaaacc...................	6	1	0B2
....uccuguacaacUacaaaaccg..................	7	1	0B2

Provisional ID	:	scaffold437_41122
Score total	:	0.4
Score for star read(s)	:	-1.3
Score for read counts	:	0
Score for mfe	:	0.7
Score for randfold	:	1.6
Score for cons. seed	:	-0.6
Total read count	:	96
Mature read count	:	96
Loop read count	:	0
Star read count	:	0

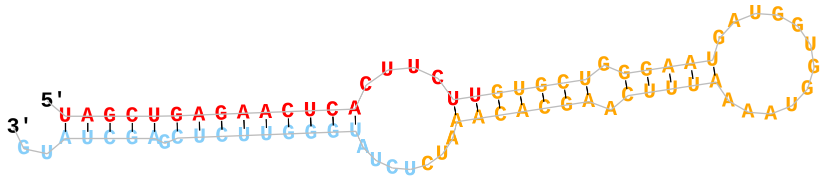

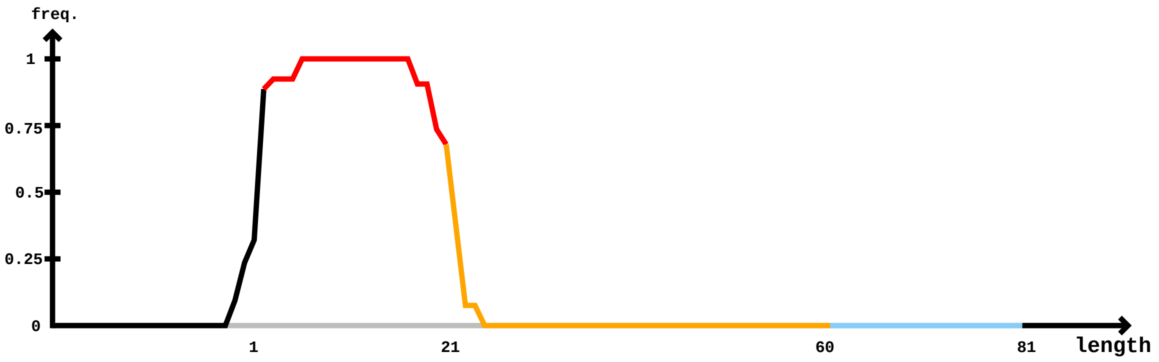

Mature **Star**

```
5'- aacgcccauuucgacagaaauagcugagaacucacuucuugugcugggaaugauggugguaaaauuucaagcacaaaucucuauggguucucgagcuaugaguuauuuuu   -3'    exp
    ............(((....(((((((((((((((....(((((((.(((((...........))))).))))))).......))))))))).))))))..)))......   reads    mm    sample
    ..................aGauagcugagaacucacu.........................................................................   3        1     0A2
    ...................Gauagcugagaacucacuuc.......................................................................   7        1     0A2
    ...................Gauagcugagaacucacuucu......................................................................   2        1     0A2
    ....................uagcugagaacucacuucuu......................................................................   2        0     0A2
    ....................uGgcugagaacucacuucuu......................................................................   6        1     0A2

    ...................auagcugagaacucacuuc........................................................................   5        0     0B2
    ...................auagcugagaacucacuucu.......................................................................   4        0     0B2
    ....................uagcugagaacucGcuucuu......................................................................   4        1     0B2
    ....................uagcugagaacucacuucuu......................................................................   12       0     0B2
    ....................uagcugagaacucacuucuug.....................................................................   2        0     0B2
    .....................agcugagaacucacuucuu......................................................................   4        0     0B2
    ........................ugagaacucacuucuugug...................................................................   4        0     0B2

    ..................aGauagcugagaacucacu.........................................................................   7        1     0G2
    ...................Gauagcugagaacucacuuc.......................................................................   6        1     0G2
    ....................uagcugagaacucacuucuu......................................................................   10       0     0G2
    ....................uagcugagaGcucacuucuug.....................................................................   4        1     0G2
    ....................uagcugagaacucacuucuuA.....................................................................   10       1     0G2
    ....................uagcugagaacucacuucuug.....................................................................   10       0     0G2
    ........................ugagaacucacuucuugug...................................................................   4        0     0G2
```

Provisional ID	:	scaffold445_41442
Score total	:	2.1
Score for star read(s)	:	-1.3
Score for read counts	:	0
Score for mfe	:	2.4
Score for randfold	:	1.6
Score for cons. seed	:	-0.6
Total read count	:	2119
Mature read count	:	2119
Loop read count	:	0
Star read count	:	0

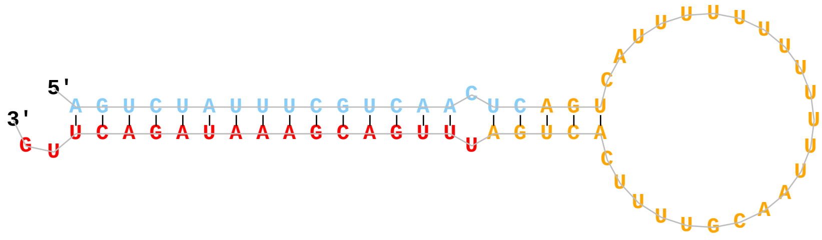

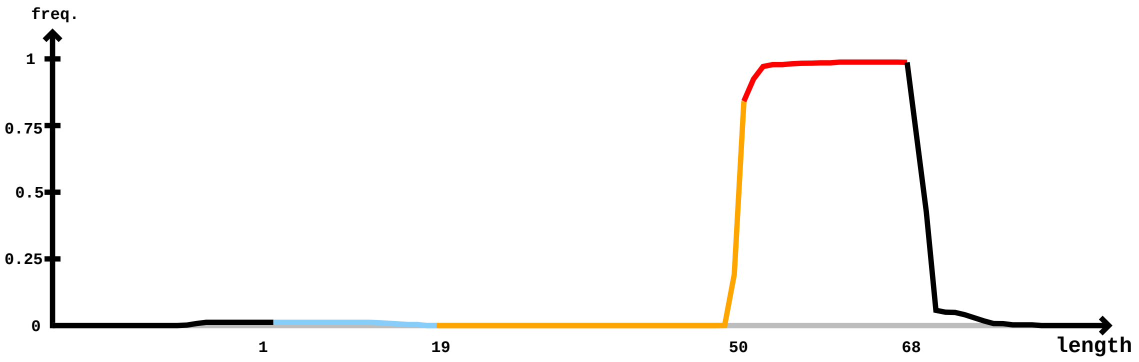

Star Mature

```
5'- agaaaaacuagacuugccccaagucuauuucgucaacucagucauuuuuuuuuuuuaacguuuucacugauuugacgaaauagacuuggggcaagucuagagaaagac   -3'    exp
    .......((((((((((((((((((((((((((((.(((((........................))))).)))))))))))))))))))))))))))).........   reads   mm    sample
    .............uugccccaagucuauuucgu...........................................................................   4       0     0A2
    .............uugccccaagucuGuuucguca.........................................................................   1       1     0A2
    .............uugccccaagucuauuucguca.........................................................................   4       0     0A2
    ..............ugccccaagucuauuucgucaaU.......................................................................   6       1     0A2
    .....................................................................Uuuugacgaaauagacuug....................   70      1     0A2
    .....................................................................Cuuugacgaaauagacuug....................   76      1     0A2
    .....................................................................auuugacgaaauagacuug....................   4       0     0A2
    .....................................................................Uuuugacgaaauagacuugg...................   6       1     0A2
    .....................................................................Cuuugacgaaauagacuuggg..................   1       1     0A2
    .....................................................................Uuuugacgaaauagacuuggg..................   5       1     0A2
    .....................................................................Uuuugacgaaauagacuuggggcaa..............   4       1     0A2
    ......................................................................uuugacgaaauagacuug....................   187     0     0A2
    ......................................................................uuugacgaaauagacCug....................   2       1     0A2
    ......................................................................Nuugacgaaauagacuug....................   1       1     0A2
    ......................................................................uuugacgaaUuagacuug....................   5       1     0A2
    ......................................................................uuugacgaGauagacuug....................   1       1     0A2
    ......................................................................uuugacAaaauagacuug....................   36      1     0A2
    ......................................................................uuugacgaaauagacuGg....................   1       1     0A2
    ......................................................................uuugacAaaauagacuugg...................   12      1     0A2
    ......................................................................uuugacgaaauagacuugg...................   51      0     0A2
    ......................................................................uuugacAaaauagacuuggg..................   88      1     0A2
    ......................................................................uuugacgaaauagacuuggg..................   96      0     0A2
    ......................................................................uuugacgaGauagacuuggg..................   4       1     0A2
    ......................................................................uuugacAaaauagacuugggg.................   4       1     0A2
    ......................................................................uuugacgaaauagacuugUggca...............   6       1     0A2
    .......................................................................uugacgaaauagacuugg...................   19      0     0A2
    .......................................................................uugacgaaauagacuuAgg..................   2       1     0A2
    .......................................................................uugacgaaauagacuugUg..................   8       1     0A2
    .......................................................................uugacgaaauagacuuggg..................   23      0     0A2
    .......................................................................Cugacgaaauagacuuggg..................   3       1     0A2
    ........................................................................uNacgaaauagacuuggg..................   1       1     0A2
    ........................................................................ugacgaaauagacuuggg..................   3       0     0A2
    ........................................................................ugacgaaauagacuugUg..................   2       1     0A2
    ........................................................................ugacgaaauagacuugggU.................   5       1     0A2
```

Star Mature

agaaaaacuagacuugccccaagucuauuucgucaacucagucauuuuuuuuuuuuaacguuuucacugauuugacgaaauagacuuggggcaagucuagagaaagac			
....ugacgaaauagacuugUggcaa..............	6	1	0A2
....ugacgaaauagacuugUggcaag.............	3	1	0A2
....ugacgaaauGgacuuggggcaagucu..........	4	1	0A2
....gacgaaauagacuugUggcaa..............	6	1	0A2
....cgaaauagacuuggggcaaUu............	1	1	0A2
....Gauagacuuggggcaagucu..........	3	1	0A2
....uagacuuggggcaagucuaCa.......	1	1	0A2
....uGgacuuggggcaagucuaga.......	5	1	0A2
............cuugccccaagucuGuuucg..	2	1	0B2
.............uugccccaagucuauuucguc..	4	0	0B2
....Uuuugacgaaauagacuug....................	32	1	0B2
....Cuuugacgaaauagacuug....................	74	1	0B2
....Cuuugacgaaauagacuugg...................	3	1	0B2
....Uuuugacgaaauagacuugg...................	13	1	0B2
....Cuuugacgaaauagacuuggg..................	5	1	0B2
....Uuuugacgaaauagacuuggg..................	7	1	0B2
....uuugacgaaauagacCug....................	2	1	0B2
....uuugacAaaauagacuug....................	28	1	0B2
....Auugacgaaauagacuug....................	4	1	0B2
....uuugacgaaauagacuug....................	109	0	0B2
....Nuugacgaaauagacuug....................	1	1	0B2
....uuugacgaaauagacuugg...................	44	0	0B2
....uuugacAaaauagacuugg...................	19	1	0B2
....uuugacgaaauagacuuggg..................	105	0	0B2
....uuugacAaaauagacuuggg..................	65	1	0B2
....uuugacgaaauagacuuggA..................	4	1	0B2
....uugaUgaaauagacuugg...................	5	1	0B2
....uugacgaaauagacuugg...................	10	0	0B2
....uugaUgaaauagacuuggg..................	4	1	0B2
....uugacgaaauagacuuggg..................	37	0	0B2
....uugacgaaauagacuugAgg.................	4	1	0B2
....ugacgaaauagacuuggg..................	19	0	0B2
....ugacgaaauagacuugUg..................	1	1	0B2
....ugacgaaauagacuugUggca...............	5	1	0B2
....ugacgaaauagacuugggAca...............	4	1	0B2
....ugacgaaauagacuugUggcaa..............	1	1	0B2
....ugacgaaauagacuugUggcaag.............	7	1	0B2
....ugacgaaauagacuuggggcaaUu............	4	1	0B2
....gacgaaauagacuugggU.................	1	1	0B2
....gacgaaauagacuugUggcaa..............	2	1	0B2
....cgaaauagacuuggggcaaUu............	2	1	0B2
....cgaaauagacuuggggcaagu............	2	0	0B2
....cgaaauagacuuggggcaaguUu..........	2	1	0B2
....gaGauagacuuggggcaaguc...........	1	1	0B2
....aGauagacuuggggcaagucu..........	1	1	0B2
............cuugccccaaguAuauuucg..	1	1	0G2
............cuugccccaagucuauuucgu...	1	0	0G2
..............ugccccaagucuauuucguA..	1	1	0G2
..............ugccccaagucuauuucgucaaU...	3	1	0G2
....gUuuugacgaaauagacuugg...................	1	1	0G2
....Cuuugacgaaauagacuu.....................	2	1	0G2
....Guuugacgaaauagacuug....................	2	1	0G2
....Cuuugacgaaauagacuug....................	34	1	0G2
....Uuuugacgaaauagacuug....................	63	1	0G2
....Uuuugacgaaauagacuugg...................	6	1	0G2
....Uuuugacgaaauagacuuggg..................	11	1	0G2
....Uuuugacgaaauagacuuggggcaagu............	10	1	0G2
....uuugacgaaUuagacuug....................	8	1	0G2
....uuugacAaaauagacuug....................	42	1	0G2
....uuugacgaaauagacuug....................	208	0	0G2
....Nuugacgaaauagacuugg...................	1	1	0G2
....uuugacgaaauagacuugU...................	4	1	0G2
....uuugacgaaauagacuugg...................	37	0	0G2
....uuugacAaaauagacuugg...................	16	1	0G2
....Cuugacgaaauagacuugg...................	1	1	0G2
....uuugacgaaauagacCuggg..................	5	1	0G2
....uuugacgaaauagacuugAg..................	3	1	0G2
....uuugacAaaauagacuuggg..................	65	1	0G2

```
                            Star                                          Mature
agaaaaacuagacuugccccaagucuauuucgucaacucagucauuuuuuuuuuuuaacguuuucacugauuugacgaaauagacuuggggcaagucuagagaaagac
......................................................................uuugacgaGauagacuuggg..................	1	1	0G2
......................................................................uuugacgaaauagacuuggg..................	180	0	0G2
......................................................................Nuugacgaaauagacuuggg..................	2	1	0G2
......................................................................uuugacgaaauagacuugggg.................	2	0	0G2
......................................................................uuugacgaaauagacuuggggcaagu............	3	0	0G2
.......................................................................uugacgaaauagacuugg...................	16	0	0G2
.......................................................................uugacgaaauagacuuggg..................	52	0	0G2
.......................................................................uugacgaaauagacuuggggc................	2	0	0G2
........................................................................ugacgaaauagacuuAgg..................	4	1	0G2
........................................................................uAacgaaauagacuuggg..................	2	1	0G2
........................................................................ugacgaaauagacuuggg..................	14	0	0G2
........................................................................ugacUaaauagacuuggg..................	4	1	0G2
........................................................................ugacgaaauagacuugggAca...............	3	1	0G2
........................................................................ugacgaaauagacuugUggcaa..............	7	1	0G2
........................................................................ugacgaaauagacuugUggcaag.............	7	1	0G2
.........................................................................gacgaaauagacuugggAca...............	1	1	0G2
.........................................................................gacgaaauagacuugUggcaag.............	6	1	0G2
............................................................................gaaauagacuuggggcaaU.............	3	1	0G2
```

Provisional ID	:	scaffold483_42298
Score total	:	5.8
Score for star read(s)	:	-1.3
Score for read counts	:	0
Score for mfe	:	2.5
Score for randfold	:	1.6
Score for cons. seed	:	3
Total read count	:	3541
Mature read count	:	3525
Loop read count	:	11
Star read count	:	5

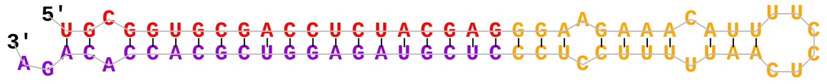

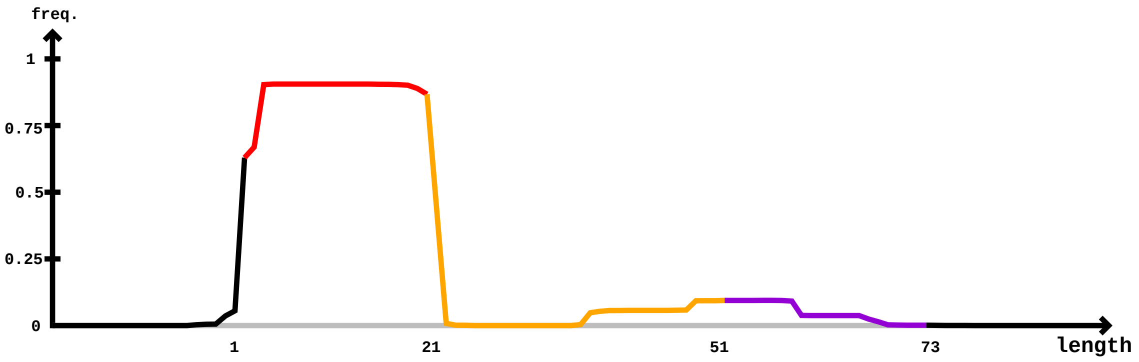

Mature Star

```
5'- acagagucuaugauagucugcggugcgaccucuacgagggaagaaacauuuuccucaauuuuuccucccucguagaggucgcaccacagacuaagucuaugaguucau  -3'   obs
    acagagucuaugauagucugcggugcgaccucuacgagggaagaaacauuuuccucaauuuuuccucccucguagaggucgcaccacagacuaagucuaugaguucau         exp
    ...(((..(((((((((((.(((((((((((((((((((.((((.(((......))).)))).))))))))))))))))))).))))))..))).)))..)))..    reads      mm       sample
    ..............agucugcggugcgaccucuaU.........................................................................    3          1        0G2
    ................ucugcggugcgaccucuacg........................................................................    5          0        0G2
    ................ucugcggugcgaccucuacgag......................................................................    17         0        0G2
    .................cugcggugcgaccucuacg........................................................................    2          0        0G2
    .................cugcggugcgaccucuacgag......................................................................    19         0        0G2
    ..................ugcggugcgaccucuacg........................................................................    1          0        0G2
    ..................ugcggugcgaccucuacAa.......................................................................    5          1        0G2
    ..................ugcggugcgaccucuacga.......................................................................    10         0        0G2
    ..................Ngcggugcgaccucuacgag......................................................................    1          1        0G2
    ..................ugcggugcgGccucuacgag......................................................................    1          1        0G2
    ..................ugcggGgcgaccucuacgag......................................................................    1          1        0G2
    ..................ugcggugcgaccucuacgag......................................................................    130        0        0G2
    ..................ugcggugcgaccucAacgag......................................................................    2          1        0G2
    ..................ugcggugcgaccucuacgaAg.....................................................................    3          1        0G2
    ..................ugcggugcgaccucuacgagg.....................................................................    9          0        0G2
    ...................gcggugcgaccucuacgag......................................................................    7          0        0G2
    ....................cggugcgaccucAacgag......................................................................    1          1        0G2
    ....................cggugcgaccucuacAag......................................................................    7          1        0G2
    ....................cUgugcgaccucuacgag......................................................................    1          1        0G2
    ....................cggugcgaccucuGcgag......................................................................    2          1        0G2
    ....................cggugcgaccucuacgag......................................................................    139        0        0G2
    ....................cggugcgaccucuacgagg.....................................................................    13         0        0G2
    ....................cggugcgaccucuacgaggg....................................................................    2          0        0G2
    .....................................................Uucaauuuuuccucccucguag.................................    3          1        0G2
    ......................................................ucaauuuuuccucccucguaga................................    41         0        0G2
    .......................................................caauuuuuccucccucguaga................................    4          0        0G2
    ..........................................................uuuuuccucccucguagag...............................    2          0        0G2
    ...............................................................ccucccucguagaggucgcacU.......................    2          1        0G2
    .................................................................ucccuUguagaggucgca.........................    10         1        0G2
    .................................................................ucccuUguagaggucgcac........................    11         1        0G2
    .................................................................ucccucguagaggucgcacU.......................    3          1        0G2
    .................................................................ucccuUguagaggucgcacc.......................    4          1        0G2

    .............uagucugcggugcgaccucua..........................................................................    1          0        0B2
```

Mature **Star**

```
acagagucuaugauagucugcggugcgaccucuacgagggaagaaacauuuuccucaauuuuuccucccucguagaggucgcaccacagacuaagucuaugaguucau
.............uagucugcgguAcgaccucua.......................................................................... 3 1 0B2
..............agucugcggugcgaccuc............................................................................ 3 0 0B2
................ucugcggugcgaccucuac......................................................................... 5 0 0B2
................ucugcggugcgaccucuacg........................................................................ 11 0 0B2
................Ncugcggugcgaccucuacgag...................................................................... 1 1 0B2
................ucugcggugcgaccCcuacgag...................................................................... 1 1 0B2
................ucugcggugcgaccucuacgag...................................................................... 19 0 0B2
................ucugcggugcgaccucuacgagU..................................................................... 2 1 0B2
.................cugcggugcgaccucuacg........................................................................ 3 0 0B2
.................cugcggugcgaccucuacga....................................................................... 5 0 0B2
.................cugcggugcgaccucuacgag...................................................................... 25 0 0B2
..................ugcggugcgaccucuacg........................................................................ 5 0 0B2
..................ugcggugcgaccucuacga....................................................................... 35 0 0B2
..................ugcggugcgaccucuacgag...................................................................... 972 0 0B2
..................ugcUgugcgaccucuacgag...................................................................... 1 1 0B2
..................ugcggGgcgaccucuacgag...................................................................... 1 1 0B2
..................ugcgAugcgaccucuacgag...................................................................... 1 1 0B2
..................Cgcggugcgaccucuacgag...................................................................... 1 1 0B2
..................Ngcggugcgaccucuacgag...................................................................... 8 1 0B2
..................ugcggugcgaccucuGcgag...................................................................... 5 1 0B2
..................ugcgguUcgaccucuacgag...................................................................... 2 1 0B2
..................ugcggugcgaccucuacgagg..................................................................... 27 0 0B2
..................ugcggugcgaccucuacgagA..................................................................... 2 1 0B2
..................ugcggugcgaccucuacgagU..................................................................... 9 1 0B2
..................Ngcggugcgaccucuacgagg..................................................................... 1 1 0B2
..................ugcggugcgaccucuacgaAg..................................................................... 7 1 0B2
..................ugcggugcgaccucuacgagAg.................................................................... 2 1 0B2
...................gcggugcgaccucuacgag...................................................................... 72 0 0B2
...................Ncggugcgaccucuacgag...................................................................... 1 1 0B2
....................cggugcgaccucuacAag...................................................................... 7 1 0B2
....................cggugcgaccucAacgag...................................................................... 5 1 0B2
....................cggugcgaccucuacgaC...................................................................... 4 1 0B2
....................cggugcgaccucuacgag...................................................................... 301 0 0B2
....................cggugcgGccucuacgag...................................................................... 1 1 0B2
....................Nggugcgaccucuacgag...................................................................... 2 1 0B2
....................cggugcgaccucuacgaU...................................................................... 11 1 0B2
....................Nggugcgaccucuacgagg..................................................................... 1 1 0B2
....................cggugcgaccucuacgagg..................................................................... 40 0 0B2
....................cggugcgaccucuacgaggg.................................................................... 10 0 0B2
....................cggugcgaccucuacgagggaU.................................................................. 4 1 0B2
.....................ggugcgaccucuacgagg..................................................................... 2 0 0B2
.....................ggugcgaccucuacgaggg.................................................................... 3 0 0B2
.....................................................Uucaauuuuuccucccucguag................................. 5 1 0B2
......................................................ucaauuuuuccucccucguaga................................ 45 0 0B2
......................................................ucGauuuuuccucccucguaga................................ 2 1 0B2
.......................................................caauuuuuccucccucguaga................................ 5 0 0B2
........................................................aauuuuuccucccucguaga................................ 5 0 0B2
................................................................cucccucguagaggucgcacca...................... 1 0 0B2
.................................................................ucccuUguagaggucgca......................... 22 1 0B2
.................................................................ucccuUguagaggucgcac........................ 28 1 0B2
.................................................................ucccucguagaggucgcacU....................... 6 1 0B2
.................................................................ucccuUguagaggucgcacc....................... 17 1 0B2
....................................................................cucguagaggucgcaccacagU.................. 4 1 0B2
........................................................................uagaggucgcaccGcagacuaa.............. 1 1 0B2

.............uagucugcggugcgaccucA........................................................................... 1 1 0A2
.............uagucugcggugcgaccucuacA........................................................................ 7 1 0A2
..............agucugcggugcgaccucuacg........................................................................ 1 0 0A2
...............gucugcggugcgaccucuac......................................................................... 1 0 0A2
...............gucugcggugcgaccucuacA........................................................................ 1 1 0A2
................ucugcggugcgaccucuacA........................................................................ 1 1 0A2
................ucugcggugcgaccucuacg........................................................................ 3 0 0A2
................ucugcggugcgaccucuacga....................................................................... 1 0 0A2
................ucugcggugcgaccucuacgag...................................................................... 52 0 0A2
................ucugcggugcUaccucuacgag...................................................................... 1 1 0A2
................ucugcggugcgaccucuacgagU..................................................................... 1 1 0A2
.................cugcggugcgaccucuacg........................................................................ 7 0 0A2
.................cugcggugcgaccucuacA........................................................................ 1 1 0A2
.................cugcggugcgaccucuacga....................................................................... 2 0 0A2
.................cugcggugcgaccucuacgag...................................................................... 12 0 0A2
```

Mature **Star**

```
acagagucuaugauagucugcggugcgaccucuacgagggaagaaacauuuuccucaauuuuuccucccucguagaggucgcaccacagacuaagucuaugaguucau
..................ugcggugcgaccucuacga.......................................................................    23     0     0A2
..................ugcggugcgGccucuacga.......................................................................    3      1     0A2
..................ugcggugAgaccucuacgag......................................................................    1      1     0A2
..................ugcggugcgaccucuaUgag......................................................................    1      1     0A2
..................Cgcggugcgaccucuacgag......................................................................    1      1     0A2
..................ugcggugcgGccucuacgag......................................................................    1      1     0A2
..................ugcggugcgaccucuGcgag......................................................................    1      1     0A2
..................ugcgguAcgaccucuacgag......................................................................    2      1     0A2
..................ugcggugcgaccucuacgaU......................................................................    2      1     0A2
..................Ngcggugcgaccucuacgag......................................................................    4      1     0A2
..................ugcgguUcgaccucuacgag......................................................................    1      1     0A2
..................ugcUgugcgaccucuacgag......................................................................    1      1     0A2
..................ugcggugcgaccucuacgag......................................................................    942    0     0A2
..................Ggcggugcgaccucuacgag......................................................................    2      1     0A2
..................ugcggugcgaccCcuacgag......................................................................    3      1     0A2
..................ugcggugcgaccucuacgagU.....................................................................    1      1     0A2
..................ugcggugcgaccucuacgagg.....................................................................    17     0     0A2
...................gcggugcgaccucuacgag......................................................................    69     0     0A2
...................gcggugcgaccucuGcgag......................................................................    2      1     0A2
...................gcggugcgaccucuacgagg.....................................................................    2      0     0A2
...................gcggugcgaccucuacgaggg....................................................................    2      0     0A2
....................cggugcgaccucuacgag......................................................................    332    0     0A2
....................cggugcgaccucuacAag......................................................................    2      1     0A2
....................cggugcgaGcucuacgag......................................................................    1      1     0A2
....................cCgugcgaccucuacgag......................................................................    1      1     0A2
....................cNgugcgaccucuacgag......................................................................    1      1     0A2
....................cggugcgaccucAacgag......................................................................    1      1     0A2
....................cgAugcgaccucuacgag......................................................................    1      1     0A2
....................cggugcgaccucuacgaU......................................................................    1      1     0A2
....................cggugcgaccucuacgagg.....................................................................    15     0     0A2
....................cggugcgaccucuacgagU.....................................................................    6      1     0A2
....................cggugcgaccucuacgaggg....................................................................    5      0     0A2
....................cggugcgaccucuacgaggga...................................................................    1      0     0A2
.....................ggugcgaccucuacgaggg....................................................................    3      0     0A2
.....................................................Uucaauuuuuccucccucguag.................................    1      1     0A2
.....................................................Uucaauuuuuccucccucguaga................................    6      1     0A2
......................................................ucaauuuuuccucccucgua..................................    2      0     0A2
......................................................ucaauuuCuccucccucguaga................................    1      1     0A2
......................................................ucaauCuuuccucccucguaga................................    1      1     0A2
......................................................ucaauuuuuccucccucguaga................................    81     0     0A2
.......................................................caauuuuuccucccucguaga................................    11     0     0A2
.......................................................caGuuuuuccucccucguaga................................    1      1     0A2
........................................................aauuuuuccucccucguaga................................    7      0     0A2
................................................................cucccucguagaggucgcacA.......................    1      1     0A2
................................................................cucccucguagaggucgcacca......................    1      0     0A2
.................................................................ucccuUguagaggucgca.........................    20     1     0A2
.................................................................ucccuUguagaggucgcacc.......................    3      1     0A2
.................................................................ucccucguagaggucgcacU.......................    9      1     0A2
.................................................................ucccucguagaggucgcaccaA.....................    3      1     0A2
..................................................................cccucguagaggucgcacca......................    1      0     0A2
```

Provisional ID : scaffold550_43911
Score total : 0.1
Score for star read(s) : -1.3
Score for read counts : 0
Score for mfe : 0.4
Score for randfold : 1.6
Score for cons. seed : -0.6
Total read count : 3278
Mature read count : 3230
Loop read count : 0
Star read count : 48

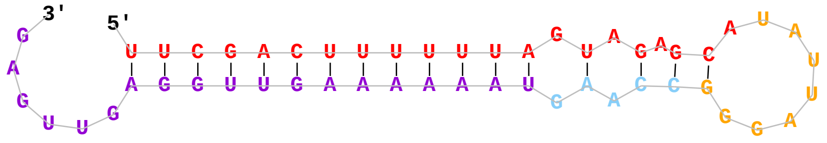

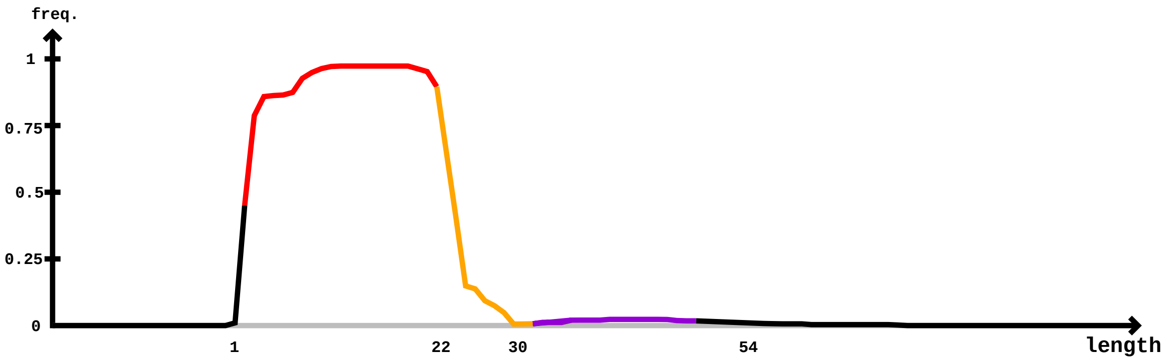

Mature **Star**

```
5'- cuaagcagaucaauuagcuucgacuuuuuuuaguagagcauauuagggccaaguaaaaaaguuggaguugagaaauguuggagaaucuugccaagacaucgcugauaucucu  -3'   obs
    cuaagcagaucaauuagcuucgacuuuuuuuaguagagcauauuagggccaaguaaaaaaguuggaguugagaaauguuggagaaucuugccaagacaucgcugauaucucu        exp
    ...((((..((..((((((((((((((((((.(.(.((........))).).)))))))))))))))))))..)))).(((((((..((.........)).))).)))).   reads   mm   sample
    ................cuCcgacuuuuuuuaguaga............................................................................   16      1    0A2
    ..................uCcgacuuuuuuuaguaga...........................................................................   4       1    0A2
    ..................uCcgacuuuuuuuaguagagc.........................................................................   41      1    0A2
    ..................uAcgacuuuuuuuaguagagca........................................................................   1       1    0A2
    ..................uCcgacuuuuuuuaguagagca........................................................................   304     1    0A2
    ..................uCcgacuuuuuuuaguagagcau.......................................................................   26      1    0A2
    ..................uCcgacuuuuuuuaguagagcaua......................................................................   82      1    0A2
    ..................uCcgacuuuuuuuaguagagcauau.....................................................................   4       1    0A2
    ..................uCcgacuuuuuuuaguagagcauauua...................................................................   6       1    0A2
    ...................Ccgacuuuuuuuaguagagc.........................................................................   6       1    0A2
    ...................Ccgacuuuuuuuaguagagca........................................................................   176     1    0A2
    ...................Ccgacuuuuuuuaguagagcaua......................................................................   194     1    0A2
    ...................Ccgacuuuuuuuaguagagcauau.....................................................................   4       1    0A2
    ...................Ccgacuuuuuuuaguagagcauauu....................................................................   7       1    0A2
    ...................Ccgacuuuuuuuaguagagcauauua...................................................................   8       1    0A2
    ....................cgacuuuuuuuaguagagca........................................................................   5       0    0A2
    ....................cgacuuuuuuuaguagagcaA.......................................................................   11      1    0A2
    ....................Ugacuuuuuuuaguagagcau.......................................................................   1       1    0A2
    ....................cgacuuuuuuuagCagagcaua......................................................................   1       1    0A2
    ....................cgacuuuuuuuaguagagcaua......................................................................   43      0    0A2
    ....................cgacuuuuuuuaguagagcauau.....................................................................   2       0    0A2
    ....................cgacuuuuuuuaguagagcauUu.....................................................................   7       1    0A2
    ....................cgacuuuuuuuaguagagcauauA....................................................................   3       1    0A2
    ....................cgacuuuuuuuaguagagcauauu....................................................................   3       0    0A2
    ....................cgacuuuuuuuaguagagcauauua...................................................................   1       0    0A2
    .....................gacuuuuuuuaguagagcaua......................................................................   6       0    0A2
    .....................gacuuuuuuuaguagagcauU......................................................................   8       1    0A2
    ......................acuuuuuuuaguagagcauauA....................................................................   4       1    0A2
    .......................cuuuuuuuaguagagcauauA....................................................................   15      1    0A2
    ........................uuuuuuuaguagagcauauA....................................................................   10      1    0A2
    ........................uuuuuuuaguagagcauauAa...................................................................   8       1    0A2
    ........................uuuuuuuaguagagcauauAag..................................................................   15      1    0A2
    .........................uuuuuuaguagagcauauA....................................................................   4       1    0A2
```

Mature Star

```
cuaagcagaucaauuagcuucgacuuuuuuaguagagcauauuagggccaaguaaaaaaguuggaguugagaaauguuggagaaucuugccaagacaucgcugauaucucu
.........................uuuuuaguagagcauauAa...................................................................    2     1    0A2
.........................uuuuuaguagagcauauAag..................................................................    10    1    0A2
.........................uuuuuaguagagcauauAagg.................................................................    21    1    0A2
..........................uuuuaguagagcauauAagg.................................................................    23    1    0A2
...........................uuuaguagagcauauAagg.................................................................    2     1    0A2
..........................................uagggccaaguaGaaaaguug................................................    2     1    0A2
..........................................uagggccaaguaaaCaaguugg...............................................    4     1    0A2
.................................................aaguaaaCaaguuggaguug..........................................    3     1    0A2
.................................................aaguaaaaaaguCggaguug..........................................    6     1    0A2
....................................................uaaaaaaguCggaguuga.........................................    2     1    0A2
....................................................uaaaaaaguCggaguugaga.......................................    2     1    0A2
....................................................uaaaaaaguCggaguugagaa......................................    2     1    0A2
....................................................uaaaaaaguCggaguugagaaa.....................................    2     1    0A2
........................................................aaaguuggaguugagaaaCgu..................................    2     1    0A2

.................cuCcgacuuuuuuaguaga...........................................................................    10    1    0G2
.................cuCcgacuuuuuuaguagagca........................................................................    1     1    0G2
..................uCcgacuuuuuuaguagag..........................................................................    16    1    0G2
..................uCcgacuuuuuuaguagagc.........................................................................    40    1    0G2
..................uNcgacuuuuuuaguagagca........................................................................    1     1    0G2
..................uCcgacuuuuuuaguagagca........................................................................    399   1    0G2
..................uCcgacuuuuuuaguagagcau.......................................................................    13    1    0G2
..................uCcgacuuuuuuaguagagcaua......................................................................    66    1    0G2
..................uCcgacuuuuuuaguagagcauau.....................................................................    3     1    0G2
..................uCcgacuuuuuuaguagagcauauu....................................................................    7     1    0G2
...................Ccgacuuuuuuaguagag..........................................................................    3     1    0G2
...................Ccgacuuuuuuaguagagc.........................................................................    3     1    0G2
...................Ncgacuuuuuuaguagagca........................................................................    2     1    0G2
...................Acgacuuuuuuaguagagca........................................................................    1     1    0G2
...................Ccgacuuuuuuaguagagca........................................................................    173   1    0G2
...................Ccgacuuuuuuaguagagcau.......................................................................    9     1    0G2
...................ucgacuuuuCuaguagagcaua......................................................................    4     1    0G2
...................Ncgacuuuuuuaguagagcaua......................................................................    1     1    0G2
...................Ccgacuuuuuuaguagagcaua......................................................................    259   1    0G2
...................Ccgacuuuuuuaguagagcauau.....................................................................    11    1    0G2
...................Ccgacuuuuuuaguagagcauauu....................................................................    11    1    0G2
...................Ccgacuuuuuuaguagagcauauua...................................................................    6     1    0G2
....................Ugacuuuuuuaguagagca........................................................................    11    1    0G2
....................cgacuuuuuuaguagagca........................................................................    5     0    0G2
....................cgacuuuuuuaguagagcaA.......................................................................    9     1    0G2
....................cgacuuuuuuaguagagcaua......................................................................    44    0    0G2
....................cgacuuuuuuaguagagcauU......................................................................    6     1    0G2
....................cgacuuuuuuaguagagcauaA.....................................................................    3     1    0G2
....................cgacCuuuuuaguagagcauauu....................................................................    4     1    0G2
....................cgacuuuuuuaguagagcauauu....................................................................    5     0    0G2
......................acuuGuuuaguagagcauau.....................................................................    3     1    0G2
.......................cuuuuuuaguagagcauauA....................................................................    11    1    0G2
.......................cCuuuuuaguagagcauauu....................................................................    5     1    0G2
........................uuuuuuaguagagcauauA....................................................................    37    1    0G2
........................uuuuuuaguagagcauauAa...................................................................    22    1    0G2
........................uuuuuuaguagagcauauAag..................................................................    32    1    0G2
........................uuuuuuaguagagcauauAagg.................................................................    27    1    0G2
.........................uuuuuaguagagcauauA....................................................................    1     1    0G2
.........................uuuuuaguagagcauauAa...................................................................    2     1    0G2
.........................uuuuuaguagagcauauAag..................................................................    16    1    0G2
.........................uuuuuaguagagcauauAagg.................................................................    7     1    0G2
..........................uuuuaguagagcauauAagg.................................................................    21    1    0G2
...........................uuuaguagagcauauAag..................................................................    8     1    0G2
...........................uuuaguagagcauauAagg.................................................................    4     1    0G2
............................uuaguagagcauauAagg.................................................................    6     1    0G2
..........................................uagggccaaguaaaaaaguCg................................................    8     1    0G2
.................................................aaguaaaaaaguCggaguug..........................................    3     1    0G2
.................................................aGguaaaaaaguuggaguug..........................................    1     1    0G2
....................................................uaaaaaaguuggaguugag........................................    7     0    0G2
....................................................uaaaaaaguCggaguugag........................................    12    1    0G2
.....................................................aaaaaUguuggaguugag........................................    1     1    0G2
........................................................aaaguuggaguugagaaaCgu..................................    8     1    0G2
..................................................................uugagaaaCguuggagaauc.........................    4     1    0G2
..................................................................uugagaaaCguuggagaaucu........................    7     1    0G2

.................cuCcgacuuuuuuaguaga...........................................................................    7     1    0B2
```

Mature **Star**

cuaagcagaucaauuagcuucgacuuuuuuaguagagcauauuagggccaaguaaaaaaguuggaguugagaaauguuggagaaucuugccaagacaucgcugauaucucu			
.................cuCcgacuuuuuuaguagagc...	1	1	0B2
.................cuCcgacuuuuuuaguagagca..	1	1	0B2
..................uCcgacuuuuuuaguagag..	17	1	0B2
..................uCcgacuuuuuuaguagagc...	101	1	0B2
..................uCcgacuuuuuuaguagagca..	358	1	0B2
..................uCcgacuuuuuuaguagagcau...	31	1	0B2
..................uCcgacuuuuuuaguagagcaua..	43	1	0B2
..................uCcgacuuuuuuaguagagcauau...	10	1	0B2
...................Ccgacuuuuuuaguagag..	1	1	0B2
...................Ccgacuuuuuuaguagagc...	10	1	0B2
...................Ncgacuuuuuuaguagagca..	2	1	0B2
...................Ccgacuuuuuuaguagagca..	174	1	0B2
...................Ccgacuuuuuuaguagagcau...	7	1	0B2
...................Ccgacuuuuuuaguagagcaua..	120	1	0B2
...................Ccgacuuuuuuaguagagcauau...	9	1	0B2
...................Ccgacuuuuuuaguagagcauauu..	2	1	0B2
....................cgacCuuuuuaguagagc...	1	1	0B2
....................cgacCuuuuuaguagagca..	1	1	0B2
....................cgacuuuuuuaguagagca..	24	0	0B2
....................cgacuuuuuuaguagagcau...	7	0	0B2
....................cgacuuuuuuaguagagcaA...	5	1	0B2
....................cgacuuuuuuaguagagcaua..	27	0	0B2
....................cgacCuuuuuaguagagcaua..	6	1	0B2
....................cgacuuuuuuaguagagcauaA...	1	1	0B2
....................cgacuuuuuuaguagagcauau...	3	0	0B2
....................cgacuuuuuuaguagagcauauu..	6	0	0B2
....................cgacuuuuuuaguagagcauauA..	5	1	0B2
....................cgacuuuuuuaguagagcauauua...	5	0	0B2
.......................cuuuuuuaguagagcauauA..	2	1	0B2
.......................cuuuuuuaguagagcauauC..	2	1	0B2
........................uuuuuuaguagagcauauA..	14	1	0B2
........................uuuuuuaguagagcauauAa...	7	1	0B2
........................uuuuuuaguagagcauauAag..	12	1	0B2
........................uuuuuuaguagagcauauAagg...	4	1	0B2
.........................uuuuuaguagagcauauAagg...	16	1	0B2
..........................uuuuaguagagcauauAagg...	8	1	0B2
...........................uuuaguagagcauauAagg...	14	1	0B2
....uagggccaaguaaaaaaguC...	2	1	0B2
....uagggccaaguaaaCaaguug..	4	1	0B2
....ccaaguaaaaaaguCggaguug..	1	1	0B2
....caaguaaaaaaguCggaguug..	3	1	0B2
....aaguaaaaaaguCggaguug..	5	1	0B2
....uaaaaaaguCggaguugaga.......................................	2	1	0B2
....uaaaaaaguCggaguugagaa......................................	1	1	0B2
....uugagaaaCguuggagaauc.........................	2	1	0B2

Provisional ID	:	scaffold635_45073
Score total	:	152613.4
Score for star read(s)	:	3.9
Score for read counts	:	152605.1
Score for mfe	:	-0.1
Score for randfold	:	1.6
Score for cons. seed	:	3
Total read count	:	299340
Mature read count	:	299327
Loop read count	:	0
Star read count	:	13

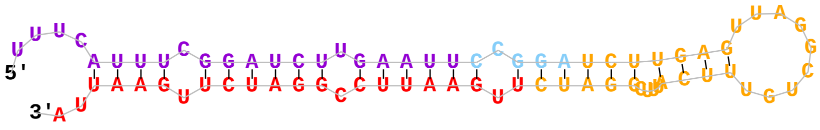

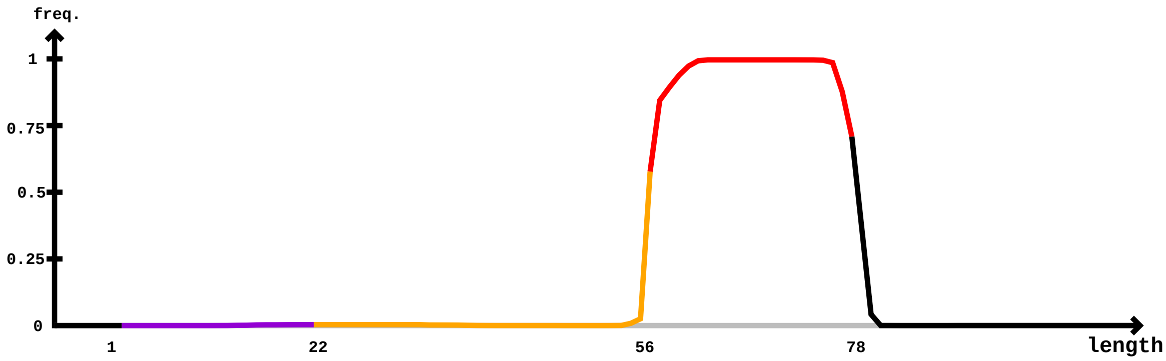

Star **Mature**

```
5'- ggcuguuucauuucggaucuugaauuccggaucuugaguuaggcuguuucauuucggaucuugaauuccggaucuugaauuaggcuguuugguuuugaaucuugaauucug  -3'  obs
    ggcuguuucauuucggaucuugaauuccggaucuugaguuaggcuguuucauuucggaucuugaauuccggaucuugaauuaggcuguuugguuuugaaucuugaauucug       exp
    ((((..((((....((((((.((((((.((((((((((.........))))....)))))).)))))).))))))))))...)))).........((((.....))))..  reads  mm  sample
    ................aucuugaauuccggaucuu............................................................................  12  0  0A2
    ................aucuugaauuccggaucuug...........................................................................  6   0  0A2
    ................aucGugaauuccggaucuug...........................................................................  6   1  0A2
    .................ucuugaauuccggaucuA............................................................................  2   1  0A2
    .................ucuugaauuccggaucuug...........................................................................  12  0  0A2
    .................ucuugaauuccggaucuuga..........................................................................  14  0  0A2
    .................ucuugaauuccggaucuAga..........................................................................  80  1  0A2
    .................ucGugaauuccggaucuugag.........................................................................  4   1  0A2
    ..................cuugaauuccggaucuAga..........................................................................  34  1  0A2
    ...................uugaauuccggaucuAga..........................................................................  12  1  0A2
    ...................uugaauuccggaucuugag.........................................................................  1   0  0A2
    ...................uugaGuuccggaucuugagu........................................................................  9   1  0A2
    ...................uugaauuccggaucuAgagu........................................................................  9   1  0A2
    ...................uugaauuccggaucuugagu........................................................................  5   0  0A2
    ...................uugaauuccggaucuugaguu.......................................................................  14  0  0A2
    ...................uugaauuccggaucuugaguA.......................................................................  5   1  0A2
    ...................uugaauuccggaucuugaguC.......................................................................  1   1  0A2
    ...................uugaauuccggaucuugaguua......................................................................  17  0  0A2
    ...................uugaauuccggaucuAgaguua......................................................................  13  1  0A2
    ...................uugaauuccggaucuugGguua......................................................................  15  1  0A2
    ...................uugaauuccggaucuugaguuG......................................................................  4   1  0A2
    ...................uugaGuuccggaucuugaguua......................................................................  3   1  0A2
    ...................uugaauuccggGucuugaguua......................................................................  2   1  0A2
    ...................uugaauuccggaucuugaguAa......................................................................  3   1  0A2
    ...................uugaauuccggaucuugaguuaU.....................................................................  9   1  0A2
    ...................uugaauuccggaucuugaguuaA.....................................................................  3   1  0A2
    ...................uugaauuccggaucuugaguuaC.....................................................................  1   1  0A2
    ...................uugaauuccggaucuAgaguuag.....................................................................  6   1  0A2
    ...................uugaauuccggaucuugaguuag.....................................................................  2   0  0A2
    ....................ugaauuccggaucuAgaguu.......................................................................  4   1  0A2
    ....................ugaauuccggaucuugaguu.......................................................................  1   0  0A2
    ....................Agaauuccggaucuugaguua......................................................................  1   1  0A2
    ....................ugaauuccggaucuugaguua......................................................................  14  0  0A2
```

Star **Mature**

```
ggcuguuucauuucggaucuugaauuccggaucuugaguuaggcuguuucauuucggaucuugaauuccggaucuugaauuaggcuguuugguuuugaaucuugaauucug
....................ugaauucUggaucuugaguua......................................................................   1     1     0A2
....................ugaauuccggaucuugGguua......................................................................   2     1     0A2
....................ugaauuccggaucuAgaguua......................................................................   4     1     0A2
....................ugaauuccggaucuugaguuGg.....................................................................   2     1     0A2
....................ugaauuccggaucuGgaguuag.....................................................................   1     1     0A2
....................ugaauuccggaucuAgaguuag.....................................................................   8     1     0A2
......................aauuccggaucuugaguua......................................................................   2     0     0A2
......................aauuccggaucuugaguuGg.....................................................................   2     1     0A2
......................aauuccggaucuugaguuag.....................................................................   1     0     0A2
......................aauuccggaucuAgaguuag.....................................................................   13    1     0A2
......................aauuccggaucuugGguuag.....................................................................   5     1     0A2
......................aauuccggaucuugGguuagg....................................................................   2     1     0A2
.......................auccggaucuugaguuaA......................................................................   2     1     0A2
.......................auccggaucuugaguuag......................................................................   4     0     0A2
.......................auccggaucuugaguuagg.....................................................................   4     0     0A2
.......................auccggaucuugaguuaggG....................................................................   6     1     0A2
........................uuccggUucuugaguuagg....................................................................   3     1     0A2
........................uuccggaucuugaguuagg....................................................................   4     0     0A2
........................uuccggaucuugaguuaggGug.................................................................   2     1     0A2
.........................uccggaucuugaguuagg....................................................................   1     0     0A2
.........................uccggaucuugaguuaggGuguuuca............................................................   7     1     0A2
..........................ccggaucuugaguuaggGu..................................................................   1     1     0A2
..........................ccggaucuugaguuaggGug.................................................................   2     1     0A2
.........................................................aucuugaauuccggaucuu...................................   12    0     0A2
.........................................................aucuugaauuccggaucuug..................................   6     0     0A2
.........................................................aucGugaauuccggaucuug..................................   6     1     0A2
.........................................................aucuugaauuccggaucuugUauua.............................   3     1     0A2
.........................................................aucuugaauuccggaucuugaauAa.............................   2     1     0A2
.........................................................aucuugaauuccggaucuugaauuagg...........................   7     0     0A2
..........................................................ucuugaauuccggaucuA...................................   2     1     0A2
..........................................................ucuugaauuccggaucuug..................................   12    0     0A2
..........................................................ucuugaauuccggaucuuga.................................   14    0     0A2
..........................................................ucuugaauuccggaucuAga.................................   80    1     0A2
..........................................................ucuugaauuccggaucuCgaa................................   1     1     0A2
..........................................................ucuugaauuccggaucuAgaa................................   55    1     0A2
..........................................................ucGugaauuccggaucuugaa................................   26    1     0A2
..........................................................Ccuugaauuccggaucuugaa................................   5     1     0A2
..........................................................ucuugaauuccggaucuugaa................................   22    0     0A2
..........................................................ucuugaauuccggaucuAgaau...............................   12    1     0A2
..........................................................ucuugaauuccggaucuugaau...............................   184   0     0A2
..........................................................Ccuugaauuccggaucuugaau...............................   1     1     0A2
..........................................................ucGugaauuccggaucuugaau...............................   34    1     0A2
..........................................................uGuugaauuccggaucuugaauu..............................   7     1     0A2
..........................................................uUuugaauuccggaucuugaauu..............................   1     1     0A2
..........................................................ucGugaauuccggaucuugaauu..............................   3     1     0A2
..........................................................ucuugaauuccggaucuugaauu..............................   23    0     0A2
..........................................................ucuugaauuccggaucuCgaauua.............................   1     1     0A2
..........................................................ucuugaGuuccggaucuugaauua.............................   1     1     0A2
..........................................................uUuugaauuccggaucuugaauua.............................   3     1     0A2
..........................................................ucuugaauuccggaucuugaauua.............................   113   0     0A2
..........................................................ucuugaauuccggaucuugaauAa.............................   232   1     0A2
..........................................................ucuugaauuccggaucuAgaauua.............................   22    1     0A2
..........................................................ucuugaauuccggaucuugaaCua.............................   1     1     0A2
..........................................................uGuugaauuccggaucuugaauua.............................   2     1     0A2
..........................................................ucuugaauuccggaucuugaauuag............................   24    0     0A2
..........................................................ucuugaauuccggaucuAgaauuag............................   19    1     0A2
..........................................................ucuugaauuccggaucuugaaCuagg...........................   7     1     0A2
..........................................................ucuugaauuccggaucuugaauuagg...........................   18    0     0A2
..........................................................ucuugaauuccggaucuugaauuaggc..........................   9     0     0A2
...........................................................cuugaauuccggaucuAga.................................   34    1     0A2
...........................................................cGugaauuccggaucuugaa................................   2     1     0A2
...........................................................cuugaauuccggaucuugaa................................   95    0     0A2
...........................................................Nuugaauuccggaucuugaa................................   1     1     0A2
...........................................................cuugaauuccggaucuAgaa................................   291   1     0A2
...........................................................cuugaGuuccggaucuugaa................................   1     1     0A2
...........................................................cuugaauuccggaucuugaau...............................   152   0     0A2
...........................................................cuugaauuccggaucuAgaau...............................   42    1     0A2
...........................................................cGugaauuccggaucuugaau...............................   29    1     0A2
...........................................................cuugaauuccggaucuAgaauu..............................   57    1     0A2
...........................................................cuugaaGuccggaucuugaauu..............................   1     1     0A2
```

Star **Mature**

ggcuguuucauuucggaucuugaauuccggaucuugaguuaggcuguuucauuucggaucuugaauuccggaucuugaauuaggcuguuugguuuugaaucuugaauucug			
....cGugaauuccggaucuugaauu.............................	6	1	0A2
....cuugaauuccggaucuugaauu.............................	229	0	0A2
....cGugaauuccggaucuugaauua............................	5	1	0A2
....cuugaaGuccggaucuugaauua............................	2	1	0A2
....cuugaauuccggCucuugaauua............................	1	1	0A2
....Nuugaauuccggaucuugaauua............................	1	1	0A2
....cuugaauuccggaucuugaauua............................	580	0	0A2
....cuugaGuuccggaucuugaauua............................	1	1	0A2
....cuugaauuccggaucuAgaauua............................	122	1	0A2
....cuugaauuccggaucuugaauAa............................	134	1	0A2
....cuugaauuccggaucuugaauCa............................	24	1	0A2
....cuugaauuccggaGcuugaauua............................	1	1	0A2
....cuugaauuccggaucuugGauua............................	3	1	0A2
....cuugaauuccggaucuAgaauuag...........................	1	1	0A2
....cuugaauuccggaucuugaaCuag...........................	2	1	0A2
....cuugaauuccggaucuugaauuag...........................	6	0	0A2
....cuugaauuccggaucuugaauuagg..........................	3	0	0A2
....uugaauuccggaucuAga................................	12	1	0A2
....uugaauuccggaucuugaa...............................	49	0	0A2
....uugaauuccggaucuAgaa...............................	192	1	0A2
....uugaaGuccggaucuugaau..............................	8	1	0A2
....uugaauuccggauAuugaau..............................	2	1	0A2
....uugaauuccggaucAugaau..............................	1	1	0A2
....uugaauAccggaucuugaau..............................	5	1	0A2
....uugaauuccggCucuugaau..............................	1	1	0A2
....Gugaauuccggaucuugaau..............................	18	1	0A2
....uugaauuccggaCcuugaau..............................	4	1	0A2
....uugaauuccggaucuugaaG..............................	1	1	0A2
....Cugaauuccggaucuugaau..............................	3	1	0A2
....uugaauuccggaucuAgaau..............................	1577	1	0A2
....uugaauuccggGucuugaau..............................	13	1	0A2
....uugaauuccggaucuugaaC..............................	2	1	0A2
....uugaauuccggaucuugaau..............................	7322	0	0A2
....uugaauuccggaGcuugaau..............................	7	1	0A2
....uugaGuuccggaucuugaau..............................	81	1	0A2
....Augaauuccggaucuugaau..............................	2	1	0A2
....uugaauuccggaucuGgaau..............................	4	1	0A2
....uugaauuAcggaucuugaau..............................	3	1	0A2
....uugaauuccggaucuugUau..............................	1	1	0A2
....uNgaauuccggaucuugaau..............................	3	1	0A2
....uugaauuccggaucCugaau..............................	3	1	0A2
....uugaauuccggaucuCgaau..............................	2	1	0A2
....Nugaauuccggaucuugaau..............................	15	1	0A2
....Gugaauuccggaucuugaauu.............................	7	1	0A2
....uugaauuccggauAuugaauu.............................	1	1	0A2
....uugaaGuccggaucuugaauu.............................	1	1	0A2
....uugaauuccggCucuugaauu.............................	3	1	0A2
....uugaauuccggauGuugaauu.............................	1	1	0A2
....uugaauuAcggaucuugaauu.............................	5	1	0A2
....uugaauuccggaucuGgaauu.............................	3	1	0A2
....uugaauuccggaucCugaauu.............................	2	1	0A2
....uugaauuccggaucuugGauu.............................	3	1	0A2
....uugaauuccggaucuCgaauu.............................	23	1	0A2
....uugaauuccggaGcuugaauu.............................	9	1	0A2
....uugaauuccggaucuuUaauu.............................	7	1	0A2
....uNgaauuccggaucuugaauu.............................	1	1	0A2
....uuNaauuccggaucuugaauu.............................	1	1	0A2
....uugaauuccggaucuugaaCu.............................	5	1	0A2
....uGgaauuccggaucuugaauu.............................	5	1	0A2
....uugaauuccggaucuugaauu.............................	9162	0	0A2
....uugaGuuccggaucuugaauu.............................	155	1	0A2
....uugaauuccggaucuugaaGu.............................	1	1	0A2
....uugaauAccggaucuugaauu.............................	7	1	0A2
....Augaauuccggaucuugaauu.............................	1	1	0A2
....Cugaauuccggaucuugaauu.............................	1	1	0A2
....uugaauuccggGucuugaauu.............................	3	1	0A2
....Nugaauuccggaucuugaauu.............................	28	1	0A2
....uugaauuccggaucAugaauu.............................	3	1	0A2
....uugaauuccggaucuAgaauu.............................	2314	1	0A2
....uugaauuccggaCcuugaauu.............................	4	1	0A2

```
   Star                                               Mature
ggcuguuucauuucggaucuugaauuccggaucuugaguuaggcuguuucauuucggaucuugaauuccggaucuugaauuaggcuguuugguuuugaaucuugaauucug
............................................................uugaauAccggaucuugaauua.............................   10      1    0A2
............................................................uugaauuccggaucuugaauAa.............................   3722    1    0A2
............................................................uugaauuccggaucuAgaauua.............................   5193    1    0A2
............................................................Nugaauuccggaucuugaauua.............................   49      1    0A2
............................................................uugaauuccggauGuugaauua.............................   2       1    0A2
............................................................uAgaauuccggaucuugaauua.............................   4       1    0A2
............................................................uugaauuccggaucuCgaauua.............................   11      1    0A2
............................................................Gugaauuccggaucuugaauua.............................   37      1    0A2
............................................................uGgaauuccggaucuugaauua.............................   7       1    0A2
............................................................Cugaauuccggaucuugaauua.............................   16      1    0A2
............................................................uugaauuccggaucuGgaauua.............................   4       1    0A2
............................................................uugaauuAcggaucuugaauua.............................   20      1    0A2
............................................................uugaauuGcggaucuugaauua.............................   3       1    0A2
............................................................uuNaauuccggaucuugaauua.............................   2       1    0A2
............................................................uugaauuccCgaucuugaauua.............................   1       1    0A2
............................................................uugaauuccggaucAugaauua.............................   3       1    0A2
............................................................uugaauuccggaucuugGauua.............................   20      1    0A2
............................................................uugaauuccgCaucuugaauua.............................   1       1    0A2
............................................................uugaaGuccggaucuugaauua.............................   9       1    0A2
............................................................uugaauuccggaCcuugaauua.............................   7       1    0A2
............................................................uugaauuccggGucuugaauua.............................   5       1    0A2
............................................................uugaauuccggaucuugCauua.............................   1       1    0A2
............................................................uugaauuccggaucCugaauua.............................   4       1    0A2
............................................................uugaauuccggaucuugUauua.............................   1       1    0A2
............................................................uugaauuccggauAuugaauua.............................   11      1    0A2
............................................................Augaauuccggaucuugaauua.............................   9       1    0A2
............................................................uugaauuccggaucuugaaCua.............................   47      1    0A2
............................................................uugaauuccggaucuugaauCa.............................   1979    1    0A2
............................................................uugaGuuccggaucuugaauua.............................   625     1    0A2
............................................................uugaauuccggaucuugaaGua.............................   4       1    0A2
............................................................uugaauuccggaucuugaauua.............................   23495   0    0A2
............................................................uugaauuccggaGcuugaauua.............................   19      1    0A2
............................................................uNgaauuccggaucuugaauua.............................   5       1    0A2
............................................................uugaauuccggCucuugaauua.............................   6       1    0A2
............................................................uugaauuccggaucuuUaauua.............................   15      1    0A2
............................................................uugaauuccggaucuuUaauuag............................   2       1    0A2
............................................................uugaauuccggaucuGgaauuag............................   1       1    0A2
............................................................uugaauuccggaucuAgaauuag............................   1270    1    0A2
............................................................uugaauuccggaucuugaauCag............................   4       1    0A2
............................................................uugaauuccggaucuugaaCuag............................   20      1    0A2
............................................................uugaauuccggaucuugaauuag............................   284     0    0A2
............................................................uugaauuccggaucuugaauAag............................   38      1    0A2
............................................................uugaauuccggaucuugaaGuag............................   2       1    0A2
............................................................uugaGuuccggaucuugaauuag............................   13      1    0A2
............................................................uugaauuccggaucuugaauuagg...........................   11      0    0A2
............................................................uugaauuccggaucuugaauuagA...........................   7       1    0A2
.............................................................ugaauuccggaucuAgaa................................   49      1    0A2
.............................................................ugaauuccggaucuAgaau...............................   86      1    0A2
.............................................................Agaauuccggaucuugaau...............................   1       1    0A2
.............................................................ugaauuccggGucuugaau...............................   3       1    0A2
.............................................................ugaauuccggaGcuugaau...............................   1       1    0A2
.............................................................ugaGuuccggaucuugaau...............................   6       1    0A2
.............................................................ugaauuccggaucuugaaG...............................   1       1    0A2
.............................................................ugaauuccggaucuugaau...............................   317     0    0A2
.............................................................ugaauuccggaucuAgaauu..............................   751     1    0A2
.............................................................uNaauuccggaucuugaauu..............................   2       1    0A2
.............................................................ugaauuccgCaucuugaauu..............................   1       1    0A2
.............................................................ugaGuuccggaucuugaauu..............................   43      1    0A2
.............................................................ugaauuccggaucuugaaGu..............................   1       1    0A2
.............................................................Ngaauuccggaucuugaauu..............................   3       1    0A2
.............................................................ugaaGuccggaucuugaauu..............................   4       1    0A2
.............................................................ugaauuccggaucCugaauu..............................   1       1    0A2
.............................................................ugaauuccggaucuuUaauu..............................   2       1    0A2
.............................................................ugaauuAcggaucuugaauu..............................   2       1    0A2
.............................................................Ggaauuccggaucuugaauu..............................   2       1    0A2
.............................................................ugaauuccggaucuugGauu..............................   1       1    0A2
.............................................................ugaauuccggaucuugaauu..............................   2250    0    0A2
.............................................................ugaauuccggaucuCgaauu..............................   3       1    0A2
.............................................................ugaCuuccggaucuugaauu..............................   1       1    0A2
.............................................................ugaauuccggaCcuugaauu..............................   1       1    0A2
```

Star Mature

```
ggcuguuucauuucggaucuugaauuccggaucuugaguuaggcuguuucauuucggaucuugaauuccggaucuugaauuaggcuguuugguuuugaaucuugaauucug
.............................................................ugaauuccggaucuugaaCu..............................   7      1   0A2
.............................................................ugaauuccggGucuugaauu..............................   2      1   0A2
.............................................................ugaauuccggaGcuugaauu..............................   3      1   0A2
.............................................................ugaauuccggGucuugaauua.............................   10     1   0A2
.............................................................ugaauuGcggaucuugaauua.............................   11     1   0A2
.............................................................ugaGuuccggaucuugaauua.............................   256    1   0A2
.............................................................ugaauuAcggaucuugaauua.............................   11     1   0A2
.............................................................ugaauuccggaucuugaauAa.............................   271    1   0A2
.............................................................Agaauuccggaucuugaauua.............................   6      1   0A2
.............................................................ugaauuccggaCcuugaauua.............................   6      1   0A2
.............................................................ugaauuccggaucuAgaauua.............................   4768   1   0A2
.............................................................ugaauuccggaucuugGauua.............................   10     1   0A2
.............................................................ugaaGuccggaucuugaauua.............................   21     1   0A2
.............................................................Ngaauuccggaucuugaauua.............................   26     1   0A2
.............................................................ugaauuccggaucuugaaCua.............................   20     1   0A2
.............................................................ugaauuccggaucuuUaauua.............................   11     1   0A2
.............................................................ugaauuccggaucuugaaGua.............................   2      1   0A2
.............................................................ugaauuccggaucCugaauua.............................   3      1   0A2
.............................................................ugaauuccggaucuugCauua.............................   2      1   0A2
.............................................................ugaauuccggaucuugaauCa.............................   317    1   0A2
.............................................................ugaauuccggaucuGgaauua.............................   2      1   0A2
.............................................................ugaauuccggCucuugaauua.............................   8      1   0A2
.............................................................ugaauuccggaGcuugaauua.............................   8      1   0A2
.............................................................ugaauuccggaucuCgaauua.............................   6      1   0A2
.............................................................Ggaauuccggaucuugaauua.............................   4      1   0A2
.............................................................ugaauuccggaucuugaauua.............................   13165  0   0A2
.............................................................ugaauuccggauAuugaauua.............................   6      1   0A2
.............................................................uNaauuccggaucuugaauua.............................   3      1   0A2
.............................................................ugaauuccggGucuugaauuag............................   1      1   0A2
.............................................................ugaauuccggaucuugaauAag............................   10     1   0A2
.............................................................ugaauuccggaucuAgaauuag............................   3791   1   0A2
.............................................................ugaauuccggaucuugaaGuag............................   7      1   0A2
.............................................................ugaauuccggaucCugaauuag............................   2      1   0A2
.............................................................ugaGuuccggaucuugaauuag............................   18     1   0A2
.............................................................ugaaGuccggaucuugaauuag............................   2      1   0A2
.............................................................ugaauuccggaucuugaauCag............................   12     1   0A2
.............................................................ugaauuccggaucuugaauuag............................   947    0   0A2
.............................................................Agaauuccggaucuugaauuag............................   1      1   0A2
.............................................................ugaauuccggaucuugaaCuagg...........................   2      1   0A2
.............................................................ugaauuccggauAuugaauuagg...........................   1      1   0A2
.............................................................ugaauuAcggaucuugaauuagg...........................   2      1   0A2
.............................................................ugaauuccggaucuugaauuagC...........................   4      1   0A2
.............................................................ugaauuccggaucuugaauuagg...........................   29     0   0A2
.............................................................ugaauuccggaucuAgaauuagg...........................   4      1   0A2
..............................................................gaauuccggaucuAgaau...............................   7      1   0A2
..............................................................gaauuccggaucuugaau...............................   21     0   0A2
..............................................................gaauuccggaucuAgaauu..............................   46     1   0A2
..............................................................gaauuccggGucuugaauu..............................   1      1   0A2
..............................................................gaauuccggaucuugaauu..............................   107    0   0A2
..............................................................gaGuuccggaucuugaauu..............................   2      1   0A2
..............................................................gaauuccggaucuugGauua.............................   1      1   0A2
..............................................................gaaGuccggaucuugaauua.............................   1      1   0A2
..............................................................Naauuccggaucuugaauua.............................   2      1   0A2
..............................................................gaauuccggaucuugaauAa.............................   3      1   0A2
..............................................................gaauuccggaucuugaaCua.............................   2      1   0A2
..............................................................gaauuccggaucuugaaGua.............................   1      1   0A2
..............................................................gaauuccggaCcuugaauua.............................   3      1   0A2
..............................................................gaauuccggaucuugaauua.............................   835    0   0A2
..............................................................gaGuuccggaucuugaauua.............................   23     1   0A2
..............................................................gaauuccggaucuuUaauua.............................   1      1   0A2
..............................................................gaauuccggaucuAgaauua.............................   463    1   0A2
..............................................................gaauuccggaucuugaauCa.............................   84     1   0A2
..............................................................gaauuccggaCcuugaauuag............................   1      1   0A2
..............................................................gaauuccggaucuAgaauuag............................   2687   1   0A2
..............................................................Naauuccggaucuugaauuag............................   2      1   0A2
..............................................................gaauuccggauAuugaauuag............................   1      1   0A2
..............................................................gaauuccggGucuugaauuag............................   1      1   0A2
..............................................................gaauuccggaucuugaauuag............................   543    0   0A2
..............................................................gaauuccggaucuugaauuagg...........................   24     0   0A2
..............................................................gaauuccggaucuAgaauuagg...........................   5      1   0A2
```

```
       Star                                                   Mature
ggcuguuucauuucggaucuugaauuccggaucuugaguuaggcuguuucauuucggaucuugaauuccggaucuugaauuaggcuguuugguuuugaaucuugaauucug
...............................................................aauuccggaucuugaauu..............................    10     0    0A2
...............................................................aauuccggCucuugaauua.............................    1      1    0A2
...............................................................aauuGcggaucuugaauua.............................    1      1    0A2
...............................................................aauuAcggaucuugaauua.............................    1      1    0A2
...............................................................aauuccggaucuugaauCa.............................    17     1    0A2
...............................................................aGuuccggaucuugaauua.............................    7      1    0A2
...............................................................aauuccggaucuugaauAa.............................    16     1    0A2
...............................................................aauuccggaucuugaauua.............................    526    0    0A2
...............................................................Nauuccggaucuugaauua.............................    1      1    0A2
...............................................................aauuccggaucuugaauCag............................    15     1    0A2
...............................................................aauuccggaucuCgaauuag............................    3      1    0A2
...............................................................aauuccggCucuugaauuag............................    2      1    0A2
...............................................................aauuccggaucuGgaauuag............................    2      1    0A2
...............................................................aauuccggaucuugCauuag............................    1      1    0A2
...............................................................aauuccCgaucuugaauuag............................    4      1    0A2
...............................................................aauuccggaCcuugaauuag............................    1      1    0A2
...............................................................aauuAcggaucuugaauuag............................    1      1    0A2
...............................................................aauuccggGucuugaauuag............................    1      1    0A2
...............................................................aauuccggaucuugaauuag............................    3749   0    0A2
...............................................................aauuccggaucuuUaauuag............................    6      1    0A2
...............................................................aauuccggaucAugaauuag............................    6      1    0A2
...............................................................aNuuccggaucuugaauuag............................    1      1    0A2
...............................................................aauuccggaucuugaaCuag............................    1      1    0A2
...............................................................aCuuccggaucuugaauuag............................    2      1    0A2
...............................................................aauuccggaucuugGauuag............................    6      1    0A2
...............................................................aGuuccggaucuugaauuag............................    69     1    0A2
...............................................................aauuccggaucuugaauAag............................    1      1    0A2
...............................................................Nauuccggaucuugaauuag............................    12     1    0A2
...............................................................aauuccggaucuugaaGuag............................    1      1    0A2
...............................................................aauuccggaGcuugaauuag............................    1      1    0A2
...............................................................aauuccggaucuGgaauuagg...........................    1      1    0A2
...............................................................aauuccggaucuugaaCuagg...........................    1      1    0A2
...............................................................aauuccggaucuAgaauuagg...........................    11     1    0A2
...............................................................aauuccggaucuugaauuagg...........................    270    0    0A2
...............................................................aauuccggaucuCgaauuagg...........................    3      1    0A2
...............................................................aauuccggaucuugaauuagA...........................    19     1    0A2
...............................................................Nauuccggaucuugaauuagg...........................    1      1    0A2
...............................................................aauuccggGucuugaauuagg...........................    1      1    0A2
................................................................Nuuccggaucuugaauua.............................    2      1    0A2
................................................................Guuccggaucuugaauua.............................    4      1    0A2
................................................................auuccggaucuugaauAa.............................    4      1    0A2
................................................................auuccggaucuugaauua.............................    183    0    0A2
................................................................auuccggaucuugaaCua.............................    2      1    0A2
................................................................auuccggaucuugaauCa.............................    10     1    0A2
................................................................auuccggCucuugaauuag............................    2      1    0A2
................................................................auuccggaucuuUaauuag............................    3      1    0A2
................................................................auuccggaucuGgaauuag............................    2      1    0A2
................................................................auuccggaucAugaauuag............................    12     1    0A2
................................................................auuccggaucuugaauAag............................    3      1    0A2
................................................................auuccggaucuugaauuag............................    1042   0    0A2
................................................................Nuuccggaucuugaauuag............................    4      1    0A2
................................................................Cuuccggaucuugaauuag............................    5      1    0A2
................................................................auuccggaucuugaauCag............................    9      1    0A2
................................................................auuccggaGcuugaauuag............................    2      1    0A2
................................................................auuccggaucuCgaauuag............................    2      1    0A2
................................................................auuccgCaucuugaauuag............................    1      1    0A2
................................................................Guuccggaucuugaauuag............................    12     1    0A2
................................................................auuccggGucuugaauuag............................    2      1    0A2
................................................................auuccggaucuugaaCuagg...........................    4      1    0A2
................................................................auuccggaucuugaauuCgg...........................    1      1    0A2
................................................................auuccggaucuugaauuagA...........................    12     1    0A2
................................................................auuccggaucuGgaauuagg...........................    1      1    0A2
................................................................auuccggaucuugaauuagC...........................    4      1    0A2
................................................................Guuccggaucuugaauuagg...........................    41     1    0A2
................................................................auuccggaucuAgaauuagg...........................    29     1    0A2
................................................................Nuuccggaucuugaauuagg...........................    4      1    0A2
................................................................auuccggGucuugaauuagg...........................    1      1    0A2
................................................................Cuuccggaucuugaauuagg...........................    4      1    0A2
................................................................auuccggaucuugaauuaUg...........................    14     1    0A2
................................................................auuccggaucAugaauuagg...........................    22     1    0A2
```

Star **Mature**

ggcuguuucauuucggaucuugaauuccggaucuugaguuaggcuguuucauuucggaucuugaauuccggaucuugaauuaggcuguuugguuuugaaucuugaauucug			
....auuccggaucuuUaauuagg...........................	3	1	0A2
....auuAcggaucuugaauuagg...........................	8	1	0A2
....auuccggaucuugaauGagg...........................	2	1	0A2
....auuccggaucuCgaauuagg...........................	3	1	0A2
....auuccggaucuugGauuagg...........................	3	1	0A2
....auuccggaucuugaauuagg...........................	2032	0	0A2
....uuccggaucuugGauuag............................	3	1	0A2
....uuccggaucuCgaauuag............................	1	1	0A2
....uuccggaucuugaauuag............................	979	0	0A2
....uuccggaucAugaauuag............................	3	1	0A2
....uuccggGucuugaauuag............................	1	1	0A2
....Nuccggaucuugaauuag............................	2	1	0A2
....uuccggauAuugaauuag............................	1	1	0A2
....uuccggaCcuugaauuag............................	1	1	0A2
....uuccggaucuugaauCag............................	29	1	0A2
....uuccggCucuugaauuag............................	2	1	0A2
....uuccggaucuugaauuaUg...........................	2	1	0A2
....uuccggaucuugGauuagg...........................	1	1	0A2
....uucGggaucuugaauuagg...........................	6	1	0A2
....uuccggaucuugaaGuagg...........................	1	1	0A2
....uuccggaucuAgaauuagg...........................	10	1	0A2
....uuccggaucuuUaauuagg...........................	2	1	0A2
....uuccggaucuugaauuagA...........................	11	1	0A2
....uuccggGucuugaauuagg...........................	3	1	0A2
....uuccggaucuGgaauuagg...........................	4	1	0A2
....uuAcggaucuugaauuagg...........................	1	1	0A2
....Nuccggaucuugaauuagg...........................	2	1	0A2
....uuccggaucuugaaCuagg...........................	4	1	0A2
....uuccggaucuugCauuagg...........................	2	1	0A2
....uuccggCucuugaauuagg...........................	1	1	0A2
....uuccggaucuugaauuagg...........................	1138	0	0A2
....uuccggaGcuugaauuagg...........................	2	1	0A2
....uuccggaucAugaauuagg...........................	21	1	0A2
....uuccggaucuugaauuagUcu.........................	3	1	0A2
....uuccggaucuugaauuaggcuC........................	4	1	0A2
....uccggaucAugaauuagg...........................	5	1	0A2
....uAcggaucuugaauuagg...........................	2	1	0A2
....uccggaCcuugaauuagg...........................	1	1	0A2
....uccggaucuugaauuagA...........................	1	1	0A2
....uccggaucuugaauuagg...........................	316	0	0A2
....Nccggaucuugaauuagg...........................	2	1	0A2
....uccggaucuGgaauuagg...........................	3	1	0A2
....uccggaucuAgaauuagg...........................	3	1	0A2
....uccggaucuugaauuaggcuA........................	1	1	0A2
....gCuuugaaucuugaauucug	1	1	0A2
....Cuuugaaucuugaauucug	3	1	0A2
....uuuugaaucuugaauucug	5	0	0A2
....uuuugGaucuugaauucug	3	1	0A2
.....uuGcauuucggaucuugaauu...	14	1	0B2
................aucuugaauuccggaucu...	8	0	0B2
................aucuugaauuccggaucuu..	12	0	0B2
................aucuugaauuccggaucuA..	4	1	0B2
................aucGugaauuccggaucuuga..	14	1	0B2
................aucuugaauuccggaucuuga..	10	0	0B2
.................ucuugaauuccggaucuA..	2	1	0B2
.................ucuugaauuccggaucuu..	2	0	0B2
.................ucuugaauuccggaucuAg...	22	1	0B2
.................ucuugaauuccggaucuug...	10	0	0B2
.................ucuugaauuccggaucuuga..	30	0	0B2
.................ucuugaauuccggaucuAga..	88	1	0B2
..................cuugaauuccggaucuAg...	2	1	0B2
..................cuugaauuccggaucuug...	4	0	0B2
..................cuugaauuccggaucuAga..	22	1	0B2
..................cuugaauuccggaucuuga..	10	0	0B2
..................cuugaauuccggaucuAgag...	1	1	0B2
...................uugaauuccggaucuAga..	28	1	0B2
...................uugaauuccggaucuAgag...	5	1	0B2
...................uugaauuccggaucuAgagu..	11	1	0B2
...................uugGauuccggaucuugagu..	1	1	0B2

Star **Mature**

```
ggcuguuucauuucggaucuugaauuccggaucuugaguuaggcuguuucauuucggaucuugaauuccggaucuugaauuaggcuguuugguuuugaaucuugaauucug
...................uugaauuccggaucuugagu........................................................................ 1 0 0B2
...................uugaauuccggaucuugGguu....................................................................... 8 1 0B2
...................uugaauuccggaucuAgaguu....................................................................... 6 1 0B2
...................uugaauuccggaucuugaguA....................................................................... 3 1 0B2
...................uugaauuccggaucuugaguC....................................................................... 1 1 0B2
...................uugaauuccggaucuugaguua...................................................................... 15 0 0B2
...................uugaauuccggaucuugaguCa...................................................................... 3 1 0B2
...................uugaauuccggaucuAgaguua...................................................................... 6 1 0B2
...................uugaGuuccggaucuugaguua...................................................................... 2 1 0B2
...................uugaauuccggaucuugGguua...................................................................... 17 1 0B2
...................uugaauuccggaucuAgaguuag..................................................................... 5 1 0B2
...................uugaauuccggaucuugaguuaA..................................................................... 2 1 0B2
...................uugaauuccggaucuugaguuag..................................................................... 2 0 0B2
....................ugaauuccggaucuGgagu........................................................................ 2 1 0B2
....................ugaauuccggaucuAgaguu....................................................................... 2 1 0B2
....................ugaauuccggaucuugaguua...................................................................... 7 0 0B2
....................ugaauuccggaucuAgaguua...................................................................... 6 1 0B2
....................ugaauuccggaucuugaguuag..................................................................... 4 0 0B2
....................ugaauuccggaucuugaguuagg.................................................................... 1 0 0B2
.....................gaauuccggaucuAgaguua...................................................................... 2 1 0B2
.....................gaauuccggaucuAgaguuag..................................................................... 1 1 0B2
......................aauuccggUucuugaguuag..................................................................... 2 1 0B2
......................aauuccggaucuugaguuag..................................................................... 7 0 0B2
......................aauuccggaucuGgaguuag..................................................................... 2 1 0B2
......................aauuccggaucuugGguuag..................................................................... 2 1 0B2
......................aauuccggaucuAgaguuag..................................................................... 3 1 0B2
.......................auuccggaucuugaguuag..................................................................... 1 0 0B2
.......................Guuccggaucuugaguuagg.................................................................... 1 1 0B2
.......................auuccggaucuugaguuagU.................................................................... 1 1 0B2
.......................auuccggaucuugaguuagg.................................................................... 3 0 0B2
.......................auuccggaucuugaguuaggG................................................................... 5 1 0B2
........................uuccggUucuugaguuag..................................................................... 1 1 0B2
........................uuccggaucuugaguuaA..................................................................... 2 1 0B2
........................uuccggUucuugaguuagg.................................................................... 10 1 0B2
.........................uccggaucuugaguuagg.................................................................... 5 0 0B2
.........................uccggaucuugaguuaggGug................................................................. 6 1 0B2
..........................ccggaucuugaguuaggGug................................................................. 9 1 0B2
..........................ccggaucuugaguuaggGugu................................................................ 1 1 0B2
..........................ccggaucuugaguuaggAugu................................................................ 2 1 0B2
..............................................uuGcauuucggaucuugaauu............................................ 14 1 0B2
.........................................................aucuugaauuccggaucu.................................... 8 0 0B2
.........................................................aucuugaauuccggaucuu................................... 12 0 0B2
.........................................................aucuugaauuccggaucuA................................... 4 1 0B2
.........................................................aucGugaauuccggaucuuga................................. 14 1 0B2
.........................................................aucuugaauuccggaucuuga................................. 10 0 0B2
.........................................................Cucuugaauuccggaucuugaa................................ 2 1 0B2
.........................................................aucuugaauuccggaucuugaau............................... 11 0 0B2
.........................................................aucuugaauuccggaucuugaauua............................. 2 0 0B2
..........................................................ucuugaauuccggaucuu................................... 2 0 0B2
..........................................................ucuugaauuccggaucuA................................... 2 1 0B2
..........................................................ucuugaauuccggaucuAg.................................. 22 1 0B2
..........................................................ucuugaauuccggaucuug.................................. 10 0 0B2
..........................................................ucuugaauuccggaucuuga................................. 30 0 0B2
..........................................................ucuugaauuccggaucuAga................................. 88 1 0B2
..........................................................ucGugaauuccggaucuugaa................................ 26 1 0B2
..........................................................ucuugaauuccggaucuugaa................................ 20 0 0B2
..........................................................ucuugaauuccggaucuGgaa................................ 4 1 0B2
..........................................................ucuugaauuccggaucuAgaa................................ 44 1 0B2
..........................................................ucGugaauuccggaucuugaau............................... 18 1 0B2
..........................................................ucuugaauuccggaucuugaaG............................... 5 1 0B2
..........................................................ucuugaauuccggaucuugaau............................... 262 0 0B2
..........................................................ucuugaauuccggaucuAgaau............................... 16 1 0B2
..........................................................Ccuugaauuccggaucuugaau............................... 1 1 0B2
..........................................................ucuugaauuccggaucuCgaau............................... 2 1 0B2
..........................................................Ncuugaauuccggaucuugaau............................... 3 1 0B2
..........................................................ucuugaauuccggaucuugaauu.............................. 42 0 0B2
..........................................................uUuugaauuccggaucuugaauu.............................. 3 1 0B2
..........................................................ucuugaauuccggaucuAgaauu.............................. 10 1 0B2
..........................................................Ncuugaauuccggaucuugaauu.............................. 1 1 0B2
..........................................................uGuugaauuccggaucuugaauu.............................. 7 1 0B2
```

Star Mature

```
ggcuguuucauuucggaucuugaauuccggaucuugaguuaggcuguuucauuucggaucuugaauuccggaucuugaauuaggcuguuugguuuugaaucuugaauucug
..........................................................ucuugaauuccggaucuugaauua.............................    63    0    0B2
..........................................................ucuugaauuccggaucuugaauCa.............................    2     1    0B2
..........................................................uUuugaauuccggaucuugaauua.............................    9     1    0B2
..........................................................ucuugaauuccggaucuAgaauua.............................    8     1    0B2
..........................................................uGuugaauuccggaucuugaauua.............................    6     1    0B2
..........................................................ucuugaauuccggaucuugaauAa.............................    66    1    0B2
..........................................................ucuugaauuccggaucuAgaauuag............................    11    1    0B2
..........................................................ucuugaauuccggaucuugaauuag............................    1     0    0B2
..........................................................ucuugaauuccggaucuugaauAag............................    3     1    0B2
..........................................................ucuugaauuccggaucuugaaCuagg...........................    2     1    0B2
..........................................................ucuugaauuccggaucuugaauuagg...........................    2     0    0B2
...........................................................cuugaauuccggaucuug..................................    4     0    0B2
...........................................................cuugaauuccggaucuAg..................................    2     1    0B2
...........................................................cuugaauuccggaucuuga.................................    10    0    0B2
...........................................................cuugaauuccggaucuAga.................................    22    1    0B2
...........................................................cuugaauuccggaucuugaa................................    90    0    0B2
...........................................................cGugaauuccggaucuugaa................................    4     1    0B2
...........................................................cuugaauuccggaucuAgaa................................    366   1    0B2
...........................................................Nuugaauuccggaucuugaa................................    1     1    0B2
...........................................................cuugaauuccggaucuugaau...............................    204   0    0B2
...........................................................cuugaauuccggaucuAgaau...............................    15    1    0B2
...........................................................Nuugaauuccggaucuugaau...............................    1     1    0B2
...........................................................cGugaauuccggaucuugaau...............................    28    1    0B2
...........................................................cuugaauuccggaucuugaaC...............................    1     1    0B2
...........................................................cuugaauuccggaGcuugaau...............................    1     1    0B2
...........................................................cuugaauuccggaucuAgaauu..............................    74    1    0B2
...........................................................Nuugaauuccggaucuugaauu..............................    1     1    0B2
...........................................................cuugaauuccggaucuugaauu..............................    234   0    0B2
...........................................................cuugaauuccggaucuugaauua.............................    239   0    0B2
...........................................................cuugaauuccggauGuugaauua.............................    7     1    0B2
...........................................................Guugaauuccggaucuugaauua.............................    1     1    0B2
...........................................................cuugaauuccggaucuugaauCa.............................    27    1    0B2
...........................................................Nuugaauuccggaucuugaauua.............................    2     1    0B2
...........................................................cuugaauuccggaucuugGauua.............................    3     1    0B2
...........................................................cuugaauuccggaucuugaauAa.............................    38    1    0B2
...........................................................cuugaauuccggaucuAgaauua.............................    37    1    0B2
...........................................................cuugaauuccggaucuugaauuag............................    2     0    0B2
...........................................................cuugaauuccggaucuAgaauuag............................    8     1    0B2
...........................................................cuugaauuccggaucuugaauuagg...........................    1     0    0B2
............................................................uugaauuccggaucuAga.................................    28    1    0B2
............................................................uugaauuccggaucuGgaa................................    1     1    0B2
............................................................uugaauuccggaucuAgaa................................    284   1    0B2
............................................................uugaauuccggCucuugaa................................    1     1    0B2
............................................................uugaauuccggaucuugaa................................    53    0    0B2
............................................................uugaauuccggaucuugaau...............................    9489  0    0B2
............................................................uugaGuuccggaucuugaau...............................    91    1    0B2
............................................................uugaauuccggCucuugaau...............................    1     1    0B2
............................................................uGgaauuccggaucuugaau...............................    1     1    0B2
............................................................uugaauuccggGucuugaau...............................    8     1    0B2
............................................................uugaauuccggaucuugaaG...............................    1     1    0B2
............................................................Cugaauuccggaucuugaau...............................    8     1    0B2
............................................................uugaauuccggaucuCgaau...............................    2     1    0B2
............................................................uugaauuccggaucuGgaau...............................    9     1    0B2
............................................................uugaauuccgCaucuugaau...............................    4     1    0B2
............................................................Nugaauuccggaucuugaau...............................    73    1    0B2
............................................................uugaauuccggaucuAgaau...............................    1941  1    0B2
............................................................Gugaauuccggaucuugaau...............................    7     1    0B2
............................................................uugaauuccggauAuugaau...............................    1     1    0B2
............................................................uugaauuccggaucAugaau...............................    4     1    0B2
............................................................uugaauuccggaucuugaaC...............................    10    1    0B2
............................................................uugaauuccggaCcuugaau...............................    3     1    0B2
............................................................uugaCuuccggaucuugaau...............................    1     1    0B2
............................................................uugaauuccggaGcuugaau...............................    4     1    0B2
............................................................uugaauuccggauNuugaauu..............................    1     1    0B2
............................................................uugaaGuccggaucuugaauu..............................    4     1    0B2
............................................................uugaauuccggaucuCgaauu..............................    9     1    0B2
............................................................uugaauuccggaucuGgaauu..............................    2     1    0B2
............................................................Augaauuccggaucuugaauu..............................    3     1    0B2
............................................................uugaauuccggaucuugaaGu..............................    3     1    0B2
............................................................uugaauuccggaucuugGauu..............................    6     1    0B2
```

Star Mature

```
ggcuguuucauuucggaucuugaauuccggaucuugaguuaggcuguuucauuucggaucuugaauuccggaucuugaauuaggcuguuugguuuugaaucuugaauucug
............................................................uugaGuuccggaucuugaauu..............................	166	1	0B2
............................................................uugaauuccggaucuugaauu..............................	9659	0	0B2
............................................................Nugaauuccggaucuugaauu..............................	77	1	0B2
............................................................uugaauuccggCucuugaauu..............................	2	1	0B2
............................................................uugaauuccggaucuAgaauu..............................	2752	1	0B2
............................................................uugaauAccggaucuugaauu..............................	1	1	0B2
............................................................uugaauuccggaucuugaaCu..............................	33	1	0B2
............................................................Gugaauuccggaucuugaauu..............................	9	1	0B2
............................................................uugaauuAcggaucuugaauu..............................	3	1	0B2
............................................................uugaauuccggaucCugaauu..............................	4	1	0B2
............................................................uugaauuccggGucuugaauu..............................	1	1	0B2
............................................................uugaauuccggauAuugaauu..............................	1	1	0B2
............................................................uugaauuccggaCcuugaauu..............................	2	1	0B2
............................................................uugaauuccggaGcuugaauu..............................	3	1	0B2
............................................................Cugaauuccggaucuugaauu..............................	2	1	0B2
............................................................uugaauuccggGucuugaauua.............................	2	1	0B2
............................................................uugaauuccggaucuugUauua.............................	1	1	0B2
............................................................uugaauuccggaucuugaauAa.............................	819	1	0B2
............................................................uugaauuccggaucuAgaauua.............................	2593	1	0B2
............................................................uugaauuGcggaucuugaauua.............................	2	1	0B2
............................................................uugaGuuccggaucuugaauua.............................	263	1	0B2
............................................................uugaauuAcggaucuugaauua.............................	1	1	0B2
............................................................Augaauuccggaucuugaauua.............................	3	1	0B2
............................................................uugaauuccggaucuugaauua.............................	10885	0	0B2
............................................................uugaauuccggauAuugaauua.............................	1	1	0B2
............................................................uugaauuccggaucuugaauCa.............................	934	1	0B2
............................................................uugaauuccggCucuugaauua.............................	9	1	0B2
............................................................Nugaauuccggaucuugaauua.............................	91	1	0B2
............................................................Gugaauuccggaucuugaauua.............................	4	1	0B2
............................................................uugaauuccggaucuGgaauua.............................	2	1	0B2
............................................................uugaauNccggaucuugaauua.............................	1	1	0B2
............................................................uGgaauuccggaucuugaauua.............................	2	1	0B2
............................................................Cugaauuccggaucuugaauua.............................	9	1	0B2
............................................................uugaauuccggaucuuUaauua.............................	1	1	0B2
```
[truncated: 293,821 more chars]
