# Supplementary material for: Jellyfish genomes reveal distinct homeobox gene clusters and conservation of small RNA processing
Source: Nat Commun. 2020 Jun 19;11:3051. doi: 10.1038/s41467-020-16801-9 (PMC7305137; doi:10.1038/s41467-020-16801-9)
Supplement: Supplementary file 5 — Supplementary Data 1 [file 41467_2020_16801_MOESM5_ESM.pdf]

Provisional ID	:	ScYm1zk_139_2193
Score total	:	4303.1
Score for star read(s)	:	3.9
Score for read counts	:	4292.3
Score for mfe	:	2.2
Score for randfold	:	1.6
Score for cons. seed	:	3
Total read count	:	8431
Mature read count	:	8425
Loop read count	:	0
Star read count	:	6

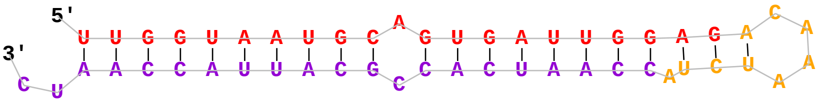

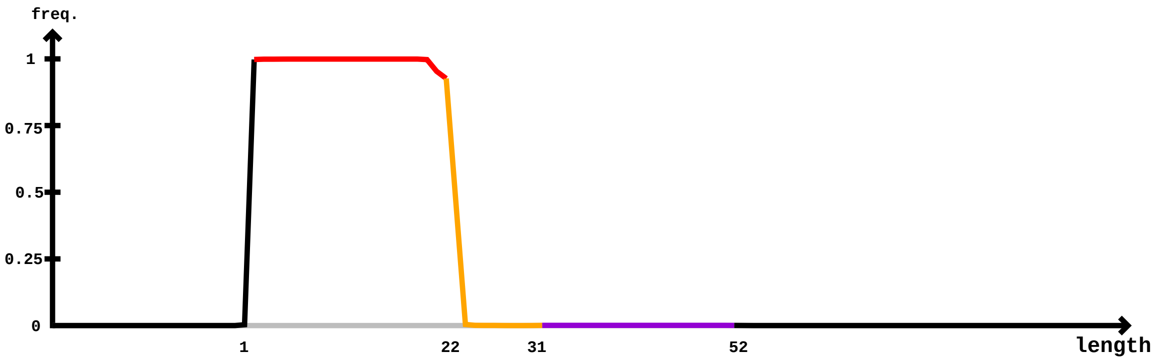

Mature Star

```
5'- uggagacaaaucuacuugauugguaaugcagugauuggagacaaaucuaccaaucaccgcauuaccaaucaaguuucuaugcuaugcacaguaguggaugggcaaagaga   -3'   obs
    uggagacaaaucuacuugauugguaaugcagugauuggagacaaaucuaccaaucaccgcauuaccaaucaaguuucuaugcuaugcacaguaguggaugggcaaagaga         exp
    ((....)).....(((((((((((((((.((((((((((....))).)))))))).))))))))))))))))((((.((((((.(((....))).)).)))).)))).    reads   mm   sample
    ...................uugguaaugcagugauugg........................................................................    12      0    st1
    ...................uugguaaugcagugauugga.......................................................................    3       0    st1
    ...................uugguaaugcagugauuggUg......................................................................    1       1    st1
    ...................uugguaaugcGgugauuggag......................................................................    2       1    st1
    ...................uugguaaugcagugauugAag......................................................................    1       1    st1
    ...................uugguaaugcagugauuggaA......................................................................    17      1    st1
    ...................uugguaaugcagugauuggag......................................................................    193     0    st1
    ...................uAgguaaugcagugauuggag......................................................................    1       1    st1
    ...................uGgguaaugcagugauuggag......................................................................    1       1    st1
    ...................uugguaaugcagAgauuggag......................................................................    1       1    st1
    ...................uugguaaugcagugGuuggag......................................................................    1       1    st1
    ...................uugguaaugcagugauuggaga.....................................................................    6       0    st1
    ....................uUguaaugcagugauuggag......................................................................    1       1    st1
    ....................ugguaaugcagugauuggagaU....................................................................    1       1    st1

    ..................Cuugguaaugcagugauugg........................................................................    4       1    sr1
    ..................Cuugguaaugcagugauugga.......................................................................    1       1    sr1
    ..................Cuugguaaugcagugauuggag......................................................................    20      1    sr1
    ..................Cuugguaaugcagugauuggaga.....................................................................    1       1    sr1
    ...................Gugguaaugcagugauug.........................................................................    2       1    sr1
    ...................uugguaaugcagugauug.........................................................................    14      0    sr1
    ...................uugguaaugcagugauuA.........................................................................    1       1    sr1
    ...................uugguaaugcagugauugg........................................................................    247     0    sr1
    ...................uugguaaugcagugauCgg........................................................................    1       1    sr1
    ...................uugguaaugcagugGuugg........................................................................    1       1    sr1
    ...................Gugguaaugcagugauugg........................................................................    46      1    sr1
    ...................uugguaaugcagugauugU........................................................................    1       1    sr1
    ...................uugguaaugcagugauugC........................................................................    1       1    sr1
    ...................uugguaaugcagugauugA........................................................................    26      1    sr1
    ...................Cugguaaugcagugauugg........................................................................    2       1    sr1
    ...................uCgguaaugcagugauugg........................................................................    1       1    sr1
    ...................uugguaaugcagugauugga.......................................................................    171     0    sr1
    ...................uugguaGugcagugauugga.......................................................................    1       1    sr1
```

```
                   Mature              Star
uggagacaaaucuacuugauugguaaugcagugauuggagacaaaucuaccaaucaccgcauuaccaaucaaguuucuaugcuaugcacaguaguggaugggcaaagaga
...................uugguaaugcagugauuggC.......................................................................   1      1    sr1
...................Augguaaugcagugauugga.......................................................................   1      1    sr1
...................uugguaaugcagugauCgga.......................................................................   1      1    sr1
...................Cugguaaugcagugauugga.......................................................................   1      1    sr1
...................uugguaaugcagugauuggG.......................................................................   3      1    sr1
...................Gugguaaugcagugauugga.......................................................................   20     1    sr1
...................uugguGaugcagugauugga.......................................................................   2      1    sr1
...................uugguaaugcGgugauugga.......................................................................   2      1    sr1
...................uugguaaugcagugauuAgag......................................................................   3      1    sr1
...................uCgguaaugcagugauuggag......................................................................   12     1    sr1
...................Augguaaugcagugauuggag......................................................................   2      1    sr1
...................uugguaaugcagugGuuggag......................................................................   13     1    sr1
...................uugguaaugcagugauugAag......................................................................   2      1    sr1
...................uugguaaugcagugauAggag......................................................................   4      1    sr1
...................uugguaaGgcagugauuggag......................................................................   1      1    sr1
...................uugguaaugcagugauGggag......................................................................   3      1    sr1
...................uugguaaugcagCgauuggag......................................................................   5      1    sr1
...................uugguaaCgcagugauuggag......................................................................   13     1    sr1
...................uugguaauCcagugauuggag......................................................................   1      1    sr1
...................uugguaaugcagugaCuggag......................................................................   7      1    sr1
...................uugguaaugcUgugauuggag......................................................................   6      1    sr1
...................uugguaaugcGgugauuggag......................................................................   63     1    sr1
...................uAgguaaugcagugauuggag......................................................................   4      1    sr1
...................uugguaaugcagGgauuggag......................................................................   1      1    sr1
...................uugguaauUcagugauuggag......................................................................   1      1    sr1
...................uugguaaugcagugauuggaA......................................................................   330    1    sr1
...................uuAguaaugcagugauuggag......................................................................   5      1    sr1
...................uugguaaugcagAgauuggag......................................................................   5      1    sr1
...................uuggAaaugcagugauuggag......................................................................   3      1    sr1
...................uugguaaugcagugauuggUg......................................................................   6      1    sr1
...................uugguaaugcagugauCggag......................................................................   13     1    sr1
...................uugguaaugcaCugauuggag......................................................................   2      1    sr1
...................uugUuaaugcagugauuggag......................................................................   4      1    sr1
...................uugguaauAcagugauuggag......................................................................   6      1    sr1
...................uugguaaugcaUugauuggag......................................................................   4      1    sr1
...................uugAuaaugcagugauuggag......................................................................   2      1    sr1
...................uugguaaugcagugCuuggag......................................................................   2      1    sr1
...................uuUguaaugcagugauuggag......................................................................   6      1    sr1
...................uugguaaugcagugaGuggag......................................................................   4      1    sr1
...................uugguaaugcaAugauuggag......................................................................   6      1    sr1
...................uugguaaugcagugauuggag......................................................................   5133   0    sr1
...................uugguGaugcagugauuggag......................................................................   11     1    sr1
...................uugguaaugcCgugauuggag......................................................................   3      1    sr1
...................Gugguaaugcagugauuggag......................................................................   948    1    sr1
...................Cugguaaugcagugauuggag......................................................................   15     1    sr1
...................uugguaaugUagugauuggag......................................................................   4      1    sr1
...................uugguaGugcagugauuggag......................................................................   10     1    sr1
...................uugguaaugcagugauuggGg......................................................................   27     1    sr1
...................uugguaaugcagugauuggaC......................................................................   6      1    sr1
...................uugguaaugcagugauugCag......................................................................   1      1    sr1
...................uuggGaaugcagugauuggag......................................................................   1      1    sr1
...................uugguaCugcagugauuggag......................................................................   2      1    sr1
...................uugguaaugcaguAauuggag......................................................................   4      1    sr1
...................uugguUaugcagugauuggag......................................................................   2      1    sr1
...................uuggCaaugcagugauuggag......................................................................   8      1    sr1
...................uugguaaugcagugauuggCg......................................................................   3      1    sr1
...................uugguaaugcagugauuggaU......................................................................   25     1    sr1
...................uugguaaugcagugauuggaUa.....................................................................   3      1    sr1
...................uugguaaugcagugauuggagU.....................................................................   2      1    sr1
...................uugguaaugcagugauuggaga.....................................................................   22     0    sr1
...................Gugguaaugcagugauuggaga.....................................................................   2      1    sr1
...................uugguaaugcagugauuggagG.....................................................................   1      1    sr1
...................uugguaaugcagugauuggaAa.....................................................................   7      1    sr1
...................uugguaaugcGgugauuggaga.....................................................................   1      1    sr1
...................uugguaaugcagugauuggCga.....................................................................   1      1    sr1
...................uugguaaugcagugauuggagaA....................................................................   22     1    sr1
...................uugguaaugcagugauuggagaAa...................................................................   2      1    sr1
...................uugguaaugcagugauuggagaUa...................................................................   1      1    sr1
...................uugguaaugcagugauuggagacaaau................................................................   1      0    sr1
....................uUguaaugcagugauuggag......................................................................   1      1    sr1
```

```
                   Mature                        Star
uggagacaaaucuacuugauugguaaugcagugauuggagacaaaucuaccaaucaccgcauuaccaaucaaguuucuaugcuaugcacaguaguggaugggcaaagaga
....................ugguaaugcagugauuggag......................................................................    4      0      sr1
....................ugguaaugcagugUuuggag......................................................................    1      1      sr1
....................ugguaaugcagugauuggagaA....................................................................    2      1      sr1
......................guaaugcagugauuggag......................................................................    2      0      sr1
.......................uaaugcagugauuggagacaaa.................................................................    1      0      sr1
................................................accaaucaccgcauuaccaau.........................................    2      0      sr1
.................................................ccaaucaccgcauuaccaauc........................................    2      0      sr1

..................Cuugguaaugcagugauuggag......................................................................    1      1      so1
...................uugguaaugcagugGuugg........................................................................    1      1      so1
...................uugguaaugcagugauugg........................................................................    18     0      so1
...................uugguaaugcagugauugC........................................................................    1      1      so1
...................uugguaaugcagugauugA........................................................................    5      1      so1
...................uugguaaugcagugauugga.......................................................................    12     0      so1
...................uugguaaugUagugauuggag......................................................................    1      1      so1
...................uugguaaugcagugGuuggag......................................................................    3      1      so1
...................uugguaaugcagugauuggaU......................................................................    5      1      so1
...................uugguaaugcGgugauuggag......................................................................    5      1      so1
...................uugguaaugcagugauGggag......................................................................    1      1      so1
...................uugguaaugcagugauuggag......................................................................    495    0      so1
...................uugguaaugcagugauuggaA......................................................................    45     1      so1
...................Augguaaugcagugauuggag......................................................................    1      1      so1
...................uugguaaCgcagugauuggag......................................................................    1      1      so1
...................Cugguaaugcagugauuggag......................................................................    4      1      so1
...................uugguaaugcagugauuggUg......................................................................    3      1      so1
...................uugguGaugcagugauuggag......................................................................    3      1      so1
...................uugguaaugcaAugauuggag......................................................................    1      1      so1
...................uCgguaaugcagugauuggag......................................................................    1      1      so1
...................uugguaaugcagugauuggGg......................................................................    5      1      so1
...................uugguaauAcagugauuggag......................................................................    1      1      so1
...................uugguaaugcagugauuggagU.....................................................................    2      1      so1
...................uugguaaugcagugauuggaga.....................................................................    9      0      so1
...................uugguaaugcagugauuggaUa.....................................................................    1      1      so1
...................uugguaaugcGgugauuggaga.....................................................................    2      1      so1
...................uugguaaugcagugauuggagC.....................................................................    1      1      so1
...................uugguaaugcagugauuggagaA....................................................................    2      1      so1
...................uugguaaugcagugauuggagac....................................................................    1      0      so1
...................uugguaaugcagugauuggagacaaa.................................................................    2      0      so1
.................................................ccaaucaccgcauuaccaauc........................................    2      0      so1

...................uugguaaugcagugauugg........................................................................    3      0      sg1
...................uugguaaugcagugauugga.......................................................................    8      0      sg1
...................uugguaaugcagugauGggag......................................................................    1      1      sg1
...................uugguaaugcagugauuggaU......................................................................    1      1      sg1
...................uugguaaugcagugauuggCg......................................................................    1      1      sg1
...................Cugguaaugcagugauuggag......................................................................    1      1      sg1
...................uugguaaugcagugauuggaA......................................................................    6      1      sg1
...................uugguaaugcagugauuggag......................................................................    139    0      sg1
...................uugguaaugcGgugauuggag......................................................................    1      1      sg1
...................uugguaaugcagugauuggaga.....................................................................    1      0      sg1
```

Provisional ID : ScYm1zk_191_2366
Score total : 6558.3
Score for star read(s) : 3.9
Score for read counts : 6551.4
Score for mfe : 2
Score for randfold : 1.6
Score for cons. seed : -0.6
Total read count : 12862
Mature read count : 6525
Loop read count : 0
Star read count : 6337

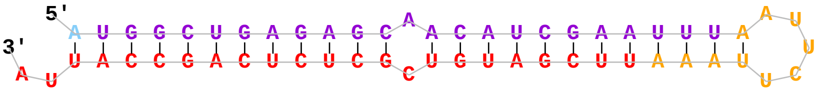

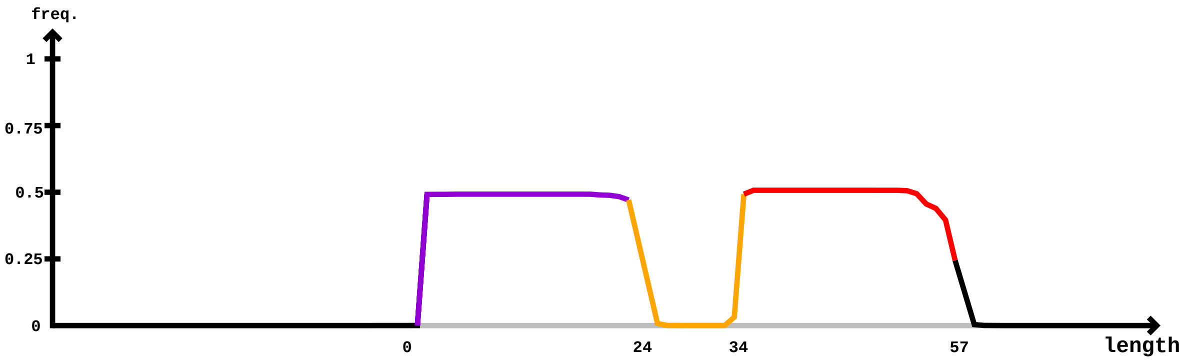

Star Mature

```
5'- auuuguuugccuauuuuuguacagauuagaauuauaauggcugagagcaacaucgaauuuaauucuuaaauucgaugucgcucucagccauuauaagaaacuuuaaacuaucu  -3'   obs
    auuuguuugccuauuuuuguacagauuagaauuauaauggcugagagcaacaucgaauuuaauucuuaaauucgaugucgcucucagccauuauaagaaacuuuaaacuaucu         exp
    ....(((((.(((.(((.....))).)))..(((((((((((((((((.((((((((((((.....)))))))))))).))))))))))))))))).......)))))..... reads  mm  sample
    .....................................uggcugagagcaacaucg.......................................................... 2      0   sg1
    .....................................uggcugagagcaacaucga......................................................... 2      0   sg1
    .....................................uggcugagagcaacaucgaa........................................................ 8      0   sg1
    .....................................uggcugagagcaGcaucgaa........................................................ 1      1   sg1
    .....................................uggcugagagcGacaucgaau....................................................... 1      1   sg1
    .....................................uggcugagagcaacaucgaau....................................................... 12     0   sg1
    .....................................uggcugagagcaacaucgaUu....................................................... 1      1   sg1
    .....................................uggcugagagcaacaucgaaC....................................................... 1      1   sg1
    .....................................uggcugagagcaacaucgaGuu...................................................... 1      1   sg1
    .....................................uggcugagagcaacaCcgaauu...................................................... 1      1   sg1
    .....................................Aggcugagagcaacaucgaauu...................................................... 1      1   sg1
    .....................................uggcugagagcGacaucgaauu...................................................... 5      1   sg1
    .....................................uggcugagagcaacaucgaauA...................................................... 15     1   sg1
    .....................................uggcugagagcaacaucgaauC...................................................... 8      1   sg1
    .....................................uggcugagGgcaacaucgaauu...................................................... 2      1   sg1
    .....................................uggcugagagcaacaucgaauu...................................................... 130    0   sg1
    .....................................uggcugaAagcaacaucgaauu...................................................... 1      1   sg1
    .....................................Cggcugagagcaacaucgaauuu..................................................... 2      1   sg1
    .....................................uggcugagagcaacaucgaauuu..................................................... 223    0   sg1
    .....................................uggcugagagcaacaucgaGuuu..................................................... 7      1   sg1
    .....................................uggcugagagcaacGucgaauuu..................................................... 1      1   sg1
    .....................................uggcugagagcaacaucgaauuC..................................................... 14     1   sg1
    .....................................uggcugagagcaacauAgaauuu..................................................... 1      1   sg1
    .....................................uggcugagagcaacaucUaauuu..................................................... 1      1   sg1
    .....................................uAgcugagagcaacaucgaauuu..................................................... 1      1   sg1
    .....................................uggcugagagcaacaucgaaCuu..................................................... 1      1   sg1
    .....................................uggcugagagcaacaucgaauuA..................................................... 96     1   sg1
    .....................................uggcugagagcaacaucgGauuu..................................................... 1      1   sg1
    .....................................uggcugagagcGacaucgaauuu..................................................... 2      1   sg1
    .....................................uggcugagagcaGcaucgaauuu..................................................... 2      1   sg1
    .....................................uggcugagagcaacaCcgaauuu..................................................... 3      1   sg1
    .....................................uggcugagagcaacaucgaauuG..................................................... 1      1   sg1
    .....................................uggcugagagcaacaucgaGuuua.................................................... 19     1   sg1
```

Star Mature

```
auuuguuugccuauuuuuguacagauuagaauuauaauggcugagagcaacaucgaauuuaauucuuaaauucgaugucgcucucagccauuauaagaaacuuuaaacuaucu
.....................................uggcugagagcaacaucgaaCuua....................................................   1     1   sg1
.....................................ugAcugagagcaacaucgaauuua....................................................   1     1   sg1
.....................................uggcugagagcaacaucgaauuuG....................................................   12    1   sg1
.....................................uggcugagaAcaacaucgaauuua....................................................   1     1   sg1
.....................................uggcugagagcaacGucgaauuua....................................................   2     1   sg1
.....................................uggcugagagcGacaucgaauuua....................................................   6     1   sg1
.....................................Aggcugagagcaacaucgaauuua....................................................   1     1   sg1
.....................................uggcugagagcaacaucgaauuua....................................................   274   0   sg1
.....................................uggcugagagcaacaucgGauuua....................................................   2     1   sg1
.....................................uggcCgagagcaacaucgaauuua....................................................   1     1   sg1
.....................................uggcugagagcaacaucgaauuAa....................................................   2     1   sg1
.....................................uggcuAagagcaacaucgaauuua....................................................   3     1   sg1
.....................................uggcugGgagcaacaucgaauuua....................................................   1     1   sg1
.....................................uggcugagagcaacaucgaauuuU....................................................   10    1   sg1
.....................................uggcugagagcaacaucgaauCua....................................................   2     1   sg1
.....................................uggcugagagcaacaucgaauuuaG...................................................   1     1   sg1
.....................................uggcugagagcaacaucgaauuuaU...................................................   1     1   sg1
.....................................uggcugagagcaacaucgaauuuaa...................................................   16    0   sg1
.....................................uggcugagagcaacaucgaauuuUa...................................................   8     1   sg1
........................................cugagagcaacaucgaauuU.....................................................   1     1   sg1
........................................cugagagcaacaucgaauuua....................................................   1     0   sg1
...............................................................ucuuaaauucgaugucgcucu.............................   1     0   sg1
.....................................................................auucgaugucgcucucag..........................   1     0   sg1
.....................................................................auucgaugucgcucucagc.........................   4     0   sg1
.....................................................................auucgaugucgcucucagcU........................   2     1   sg1
.....................................................................auucgaugucgcucucagcc........................   3     0   sg1
.....................................................................auucgaugucgcucucagcG........................   1     1   sg1
.....................................................................auucgaugucgcucucagcA........................   1     1   sg1
.....................................................................auucgaugucgcucucagcca.......................   14    0   sg1
.....................................................................auucgaugucgcucucagccCu......................   1     1   sg1
.....................................................................auucgaugucgcucucagccau......................   5     0   sg1
.....................................................................auucgaugucgcucucagccaC......................   2     1   sg1
.....................................................................auucgaugucgcucucagccaA......................   2     1   sg1
.....................................................................auucgaugucgcucucagccauA.....................   2     1   sg1
.....................................................................auucgaugucgcucucagccauC.....................   1     1   sg1
.....................................................................auucgaugucgcucucagccaCu.....................   1     1   sg1
.....................................................................auucgaugucUcucucagccauu.....................   1     1   sg1
.....................................................................auucgaugucgcucucagccauu.....................   9     0   sg1
.....................................................................auucgaugucgcucucagccauua....................   3     0   sg1
.....................................................................auucgaugucgcucucagccauuU....................   3     1   sg1
......................................................................uCcgaugucgcucucagc.........................   1     1   sg1
......................................................................uucgaugucgcucucagA.........................   1     1   sg1
......................................................................uucgGugucgcucucagc.........................   1     1   sg1
......................................................................uucgaugucgcucucagU.........................   1     1   sg1
......................................................................uucgaugucgcucucagc.........................   20    0   sg1
......................................................................uucgaugucgcucucagcc........................   43    0   sg1
......................................................................uucgaugucgcucucagcA........................   4     1   sg1
......................................................................uucgaugucgcucucagcU........................   7     1   sg1
......................................................................Aucgaugucgcucucagcc........................   1     1   sg1
......................................................................uucgaugucgcCcucagcc........................   1     1   sg1
......................................................................uucgaugucgcucucagcca.......................   41    0   sg1
......................................................................uucgaugucgcucucagccU.......................   3     1   sg1
......................................................................uucgaugucgcucucagccG.......................   1     1   sg1
......................................................................Cucgaugucgcucucagcca.......................   1     1   sg1
......................................................................uucgaugucgcucuUagccau......................   1     1   sg1
......................................................................uucgaugAcgcucucagccau......................   1     1   sg1
......................................................................uucgaugCcgcucucagccau......................   2     1   sg1
......................................................................uucgaugucgcucucagccaC......................   5     1   sg1
......................................................................uucgaugucgcucucaAccau......................   1     1   sg1
......................................................................uucgaugucgcucucagccau......................   56    0   sg1
......................................................................uucgaugucgcucucagccaA......................   4     1   sg1
......................................................................uucgaugucgcucucagccaAu.....................   4     1   sg1
......................................................................uucgaugucgcucucagccauA.....................   44    1   sg1
......................................................................uucgaugucgcucucGgccauu.....................   1     1   sg1
......................................................................uucgaugucgcucucagccGuu.....................   2     1   sg1
......................................................................uucgaugucgcucucagccauC.....................   2     1   sg1
......................................................................uucgaugucgcucAcagccauu.....................   2     1   sg1
......................................................................uucgaCgucgcucucagccauu.....................   1     1   sg1
......................................................................uucgaugucgcucucagccauG.....................   4     1   sg1
......................................................................uucgaugucgcucucagccauu.....................   216   0   sg1
```

Star **Mature**

```
auuuguuugccuauuuuuguacagauuagaauuauaauggcugagagcaacaucgaauuuaauucuuaaauucgaugucgcucucagccauuauaagaaacuuuaaacuaucu
......................................................................uucgGugucgcucucagccauu.....................  5    1  sg1
......................................................................uucgauguUgcucucagccauu.....................  1    1  sg1
......................................................................uucgaugucgcucucagccaCu.....................  1    1  sg1
......................................................................Cucgaugucgcucucagccauu.....................  1    1  sg1
......................................................................uucgauguAgcucucagccauu.....................  2    1  sg1
......................................................................uucgaugucAcucucagccauua....................  1    1  sg1
......................................................................uucgaugucgcucucagccauua....................  609  0  sg1
......................................................................uucgaugucgcucucGgccauua....................  1    1  sg1
......................................................................uCcgaugucgcucucagccauua....................  3    1  sg1
......................................................................uucgaugucgcucucagcUauua....................  2    1  sg1
......................................................................uucgaugucgcucucagUcauua....................  1    1  sg1
......................................................................uucgGugucgcucucagccauua....................  2    1  sg1
......................................................................uucgaGgucgcucucagccauua....................  1    1  sg1
......................................................................uAcgaugucgcucucagccauua....................  1    1  sg1
......................................................................uucgaugucgUucucagccauua....................  1    1  sg1
......................................................................uucgaCgucgcucucagccauua....................  1    1  sg1
......................................................................uucgaugucgcCcucagccauua....................  2    1  sg1
......................................................................uucgaugucgcucucagccauuU....................  53   1  sg1
......................................................................uuUgaugucgcucucagccauua....................  1    1  sg1
......................................................................uucgaugucgcucucagccauuG....................  4    1  sg1
......................................................................uucgaugucgcucucagccauAa....................  1    1  sg1
......................................................................uucgaugucgcucucagccUuua....................  1    1  sg1
......................................................................uucgaugucgcucucagccGuua....................  1    1  sg1
......................................................................uucgaugucgcucucagccaCua....................  1    1  sg1
......................................................................Cucgaugucgcucucagccauua....................  2    1  sg1
......................................................................uucgaugCcgcucucagccauua....................  2    1  sg1
......................................................................uucgaugucgcucucagccauuC....................  2    1  sg1
......................................................................uucgaugucgcucCcagccauua....................  1    1  sg1
......................................................................uucgaugucgcucucagccauuaA...................  5    1  sg1
......................................................................uucgaugucgcucucagccauuau...................  2    0  sg1
......................................................................uucgaugucgcucucagccauuUu...................  1    1  sg1
......................................................................uucgaugucgcucucagccauuCu...................  1    1  sg1
......................................................................uucgaugucgcucucagccauuauG..................  1    1  sg1
......................................................................uucgaugucgcucucagccauuaua..................  7    0  sg1
......................................................................uucgaugucgcucucagccauuUua..................  11   1  sg1
......................................................................uucgaugucgcucucagccauuauUa.................  1    1  sg1
.......................................................................ucgaugucgcucucagcc........................  2    0  sg1
.......................................................................ucgaugucgcucucagccau......................  2    0  sg1
.......................................................................ucgaugucgcucucagccaA......................  1    1  sg1
.......................................................................ucgaugucgcucucagccaC......................  1    1  sg1
.......................................................................ucgaugucgcucucagccauu.....................  6    0  sg1
.......................................................................ucgaugucgcucucagccaCu.....................  1    1  sg1
.......................................................................ucgaugucgcucucagccauU.....................  1    1  sg1
.......................................................................ucgaugucgcucucagccauC.....................  2    1  sg1
.......................................................................ucgaugucgcucucagccauua....................  12   0  sg1
.......................................................................ucgaugucgcucucagccaGua....................  1    1  sg1
.......................................................................ucgaugucgcucucagccauuA....................  1    1  sg1
.......................................................................ucgaugucgcucucagccauuaua..................  1    0  sg1

....................................Cuggcugagagcaacaucgaauu......................................................  1    1  so1
.....................................uggcugagagcaacaucg..........................................................  2    0  so1
.....................................uggcugagagcGacaucg..........................................................  2    1  so1
.....................................uggcugagagcaacaucga.........................................................  3    0  so1
.....................................uggcugagagcaacaucgaa........................................................  11   0  so1
.....................................uggcugagagcaacaucgaG........................................................  2    1  so1
.....................................uggcugagagcaGcaucgaau.......................................................  3    1  so1
.....................................uggcugagagcaacaucgaGu.......................................................  1    1  so1
.....................................uggcugagagcaacaucgaau.......................................................  29   0  so1
.....................................uggcugagagcaacaucgaUu.......................................................  2    1  so1
.....................................uggcugagagcGacaucgaau.......................................................  1    1  so1
.....................................uggcugagagcaacaucgaaC.......................................................  2    1  so1
.....................................uggcugagagcaacaucgGauu......................................................  1    1  so1
.....................................Cggcugagagcaacaucgaauu......................................................  2    1  so1
.....................................uggcugagagcaGcaucgaauu......................................................  1    1  so1
.....................................uggcugaAagcaacaucgaauu......................................................  2    1  so1
.....................................uggcugagagcaacaucgaUuu......................................................  1    1  so1
.....................................uggcugagagcaacaucgaauG......................................................  4    1  so1
.....................................uggcugagagcaacaucgaauA......................................................  16   1  so1
.....................................uggcugagagcaacaucgaauu......................................................  222  0  so1
.....................................uggcugagagcaacGucgaauu......................................................  2    1  so1
```

```
                                     Star                            Mature
auuuguuugccuauuuuuguacagauuagaauuauaauggcugagagcaacaucgaauuuaauucuuaaauucgaugucgcucucagccauuauaagaaacuuuaaacuaucu
.....................................uggcugagagcaacaucgaauC......................................................    24     1     so1
.....................................uggcugagagcaacaucgaGuu......................................................    16     1     so1
.....................................Aggcugagagcaacaucgaauu......................................................    1      1     so1
.....................................uggcugagagcGacaucgaauu......................................................    7      1     so1
.....................................uggcugGgagcaacaucgaauu......................................................    1      1     so1
.....................................Aggcugagagcaacaucgaauuu.....................................................    1      1     so1
.....................................ugAcugagagcaacaucgaauuu.....................................................    2      1     so1
.....................................uggcugagagcaacaucgaauuG.....................................................    5      1     so1
.....................................uggcugagGgcaacaucgaauuu.....................................................    4      1     so1
.....................................uggcugagagcaacaucgaauuu.....................................................    488    0     so1
.....................................uggcugagagcaacaucAaauuu.....................................................    1      1     so1
.....................................uggcugagagcGacaucgaauuu.....................................................    5      1     so1
.....................................uggcugagagcaacaucgaauuC.....................................................    33     1     so1
.....................................uggcCgagagcaacaucgaauuu.....................................................    1      1     so1
.....................................uggcugagagcaacaucgaGuuu.....................................................    17     1     so1
.....................................uggcugagagcaGcaucgaauuu.....................................................    8      1     so1
.....................................uggcugagagcaacaucgaauuA.....................................................    108    1     so1
.....................................Cggcugagagcaacaucgaauuu.....................................................    1      1     so1
.....................................Gggcugagagcaacaucgaauuu.....................................................    1      1     so1
.....................................uggcugagagcaacaucgGauuu.....................................................    3      1     so1
.....................................uggcugGgagcaacaucgaauuu.....................................................    1      1     so1
.....................................uggcugagagcaacaCcgaauuu.....................................................    1      1     so1
.....................................uggUugagagcaacaucgaauuu.....................................................    1      1     so1
.....................................uggcugagagcaacaucgaaGuu.....................................................    1      1     so1
.....................................uAgcugagagcaacaucgaauuua....................................................    7      1     so1
.....................................uggcugagagcaacaucgaGuuua....................................................    40     1     so1
.....................................uggcugagagcaacaucgaauuAa....................................................    1      1     so1
.....................................uggcugagagcaacaCcgaauuua....................................................    1      1     so1
.....................................Cggcugagagcaacaucgaauuua....................................................    1      1     so1
.....................................Aggcugagagcaacaucgaauuua....................................................    1      1     so1
.....................................uggcugGgagcaacaucgaauuua....................................................    2      1     so1
.....................................uggcugagagcGacaucgaauuua....................................................    7      1     so1
.....................................uggcugagagcaacaucgaauuuU....................................................    20     1     so1
.....................................uggcugagagcaacaucgaauuua....................................................    418    0     so1
.....................................uggcugUgagcaacaucgaauuua....................................................    1      1     so1
.....................................uggcugagagcaacaucgaauCua....................................................    2      1     so1
.....................................uggcCgagagcaacaucgaauuua....................................................    5      1     so1
.....................................uggcugagagcaGcaucgaauuua....................................................    2      1     so1
.....................................uggcugagagcaacaucgaauuuG....................................................    20     1     so1
.....................................uggcugagagcaacaucgaauuuC....................................................    1      1     so1
.....................................uggcugagagcaacaucgaaCuua....................................................    1      1     so1
.....................................uggAugagagcaacaucgaauuua....................................................    1      1     so1
.....................................uggcugagagcaacaucgGauuua....................................................    2      1     so1
.....................................uggcugagagcaacaucgaauuuUa...................................................    16     1     so1
.....................................uggcugagagcaacaucgaauuuaG...................................................    1      1     so1
.....................................uggcugagagcaacaucgaauuuaa...................................................    4      0     so1
.....................................uggcugagagcaacaucgaauuuaU...................................................    4      1     so1
.....................................uggcugagagcaacaucgaauuuaUu..................................................    1      1     so1
.....................................uggcugagagcaacaucgaauuuaUuu.................................................    1      1     so1
.....................................uggcugagagcaacaucgaauuuaauu.................................................    1      0     so1
......................................Ugcugagagcaacaucgaauu......................................................    1      1     so1
......................................ggcugagagcaacaucgaauuuaU...................................................    1      1     so1
........................................cugagagcaacaucgaauu......................................................    1      0     so1
........................................cugagagcaacaucgaauuua....................................................    2      0     so1
.....................................................................auucgaugucgcucucagc.........................    3      0     so1
.....................................................................auucgaugucgcucucagcU........................    3      1     so1
.....................................................................auucgaugucgcucucagcc........................    7      0     so1
.....................................................................auucgaugucgcucucagccU.......................    1      1     so1
.....................................................................auucgaugucgcucucagcca.......................    1      0     so1
.....................................................................auucgaugucgcucucagccau......................    11     0     so1
.....................................................................auucgaugucgcucCcagccau......................    1      1     so1
.....................................................................auucgaugucgcucucagccaA......................    1      1     so1
.....................................................................auucgaugucgcuAucagccauu.....................    1      1     so1
.....................................................................auucgaugucgcucucagccauu.....................    18     0     so1
.....................................................................auucgaugucgcucucagccaAu.....................    1      1     so1
.....................................................................auucgaugucgcucucagccauC.....................    5      1     so1
.....................................................................auCcgaugucgcucucagccauu.....................    1      1     so1
.....................................................................auucgaugucgcucucagccauA.....................    4      1     so1
.....................................................................auucgaugucgcucucagccauua....................    11     0     so1
.....................................................................auucgaugucgAucucagccauua....................    1      1     so1
```

Star Mature

```
auuuguuugccuauuuuuguacagauuagaauuauaauggcugagagcaacaucgaauuuaauucuuaaauucgaugucgcucucagccauuauaagaaacuuuaaacuaucu
.....................................................................auucgaugucgUucucagccauua....................    1    1    so1
.....................................................................auucgaugucgcucucagccauuU....................    10   1    so1
.....................................................................auucgaugucgcucucagccauuauaU.................    1    1    so1
......................................................................uucgaugucgcucucagU.........................    1    1    so1
......................................................................uucgaugucgcucucagA.........................    2    1    so1
......................................................................uucgaugucgcucucagc.........................    9    0    so1
......................................................................uucgaugucgcucucagcG........................    1    1    so1
......................................................................uucgaugucgcucucagcU........................    16   1    so1
......................................................................uucgaugucgcucucagcc........................    35   0    so1
......................................................................uucgaugucgcucucagcA........................    3    1    so1
......................................................................uucgaugucgcucCcagcca.......................    1    1    so1
......................................................................uucgaugucgcucucagcca.......................    25   0    so1
......................................................................uucgaugucgcucucagccU.......................    1    1    so1
......................................................................uucgaugucgcucucagccG.......................    1    1    so1
......................................................................uucgaugucgcucucagcUau......................    1    1    so1
......................................................................uucgaugucgcucucagccau......................    103  0    so1
......................................................................uucgaugucgcucucagccaA......................    4    1    so1
......................................................................uucgaugucgcucucagccaC......................    19   1    so1
......................................................................uucgaugucgcucucagccUu......................    1    1    so1
......................................................................uCcgaugucgcucucagccau......................    1    1    so1
......................................................................uucgaugucgcucucagccGu......................    2    1    so1
......................................................................uucgaugucgcucucagccaG......................    3    1    so1
......................................................................uucgGugucgcucucagccauu.....................    3    1    so1
......................................................................uucgaCgucgcucucagccauu.....................    1    1    so1
......................................................................uucgaugucgcucucGgccauu.....................    1    1    so1
......................................................................uucgaAgucgcucucagccauu.....................    1    1    so1
......................................................................uucgaugucgcAcucagccauu.....................    1    1    so1
......................................................................Cucgaugucgcucucagccauu.....................    2    1    so1
......................................................................uucgaugucgcucucagccauu.....................    366  0    so1
......................................................................uucgaugucgcucucagccauC.....................    42   1    so1
......................................................................uucgaugAcgcucucagccauu.....................    2    1    so1
......................................................................uucgaugucgcucAcagccauu.....................    1    1    so1
......................................................................uucgaugucgcucucagcAauu.....................    1    1    so1
......................................................................uucgaugucgcucucagUcauu.....................    1    1    so1
......................................................................uucgaugucgcucucagccauA.....................    41   1    so1
......................................................................uucgaugucgcucucagccauG.....................    7    1    so1
......................................................................uucgaugucgcucucagccaGu.....................    2    1    so1
......................................................................uucgaugCcgcucucagccauu.....................    1    1    so1
......................................................................uucgaugucgcucCcagccauu.....................    2    1    so1
......................................................................uucgaugucgcucucagccUuu.....................    2    1    so1
......................................................................uucgaugucgcucucagccCuu.....................    3    1    so1
......................................................................uucgaugAcgcucucagccauua....................    1    1    so1
......................................................................uucgaugucgcucucagccauAa....................    1    1    so1
......................................................................uucgaugucgcucucagcUauua....................    1    1    so1
......................................................................uucgaugucgcucucagccauuC....................    12   1    so1
......................................................................uucgaugucgcucucagccauua....................    474  0    so1
......................................................................uucgaugucgcucucagccauuU....................    119  1    so1
......................................................................uucgaugucUcucucagccauua....................    1    1    so1
......................................................................uucgaugCcgcucucagccauua....................    2    1    so1
......................................................................uucgaugucgcucucagccaCua....................    1    1    so1
......................................................................uucgaugucgcCcucagccauua....................    2    1    so1
......................................................................uucgaugucgcucucagccaAua....................    2    1    so1
......................................................................uucgaugucgcucCcagccauua....................    1    1    so1
......................................................................Cucgaugucgcucucagccauua....................    3    1    so1
......................................................................uucgaAgucgcucucagccauua....................    1    1    so1
......................................................................uucgaugucgcucucagccauuG....................    2    1    so1
......................................................................uucgaugucgcucucagccauuaA...................    4    1    so1
......................................................................uucgaugucgcucucagccauuau...................    3    0    so1
......................................................................uucgaugucgcucucagccauuUu...................    4    1    so1
......................................................................uucgaugucgcucucagccauuUua..................    7    1    so1
......................................................................uucgaugucgcucucagccauuaua..................    1    0    so1
......................................................................uucgaugucgcucucagccauuGua..................    1    1    so1
.......................................................................ucgaugucgcucucagccaC......................    1    1    so1
.......................................................................ucgaugucgcuAucagccau......................    1    1    so1
.......................................................................ucgaGgucgcucucagccauu.....................    1    1    so1
.......................................................................ucgaugucgcucucagccaAu.....................    2    1    so1
.......................................................................ucgaugucgcucucagccauu.....................    9    0    so1
.......................................................................ucgaugucgcucucagccaCu.....................    5    1    so1
.......................................................................ucgaugucgcucucagccauuU....................    8    1    so1
.......................................................................ucgaugucgcucucagccauua....................    15   0    so1
```

Star **Mature**

auuuguuugccuauuuuuguacagauuagaauuauaauggcugagagcaacaucgaauuuaauucuuaaauucgaugucgcucucagccauuauaagaaacuuuaaacuaucu

....................................Cuggcugagagcaacaucgaauuua..	1	1	st1
.....................................uggcugagagcaacaucg..	14	0	st1
.....................................uggcugagagcaacaucA..	2	1	st1
.....................................uggcugagagcaacaucga...	4	0	st1
.....................................uggcugagagcaGcaucga...	2	1	st1
.....................................uggcugagagcaacaucgaG..	1	1	st1
.....................................uggcugagagcGacaucgaa..	1	1	st1
.....................................uggcugagagcaacaucgaa..	9	0	st1
.....................................uggcugagagcaacaucgaGu...	2	1	st1
.....................................uggcugagagcaacaucgaUu...	1	1	st1
.....................................uggcugagagcaacaucgaau...	45	0	st1
.....................................uggcugagagcaacaucgaaC...	2	1	st1
.....................................uggcugagagcGacaucgaau...	1	1	st1
.....................................Aggcugagagcaacaucgaauu..	1	1	st1
.....................................uggcugagagcaacaCcgaauu..	2	1	st1
.....................................uggcugagagcaacaucAaauu..	1	1	st1
.....................................uggcugagCgcaacaucgaauu..	3	1	st1
.....................................uggUugagagcaacaucgaauu..	1	1	st1
.....................................uggcCgagagcaacaucgaauu..	2	1	st1
.....................................uggcugagagcaacaucgaauA..	9	1	st1
.....................................Cggcugagagcaacaucgaauu..	6	1	st1
.....................................uggcugagagcaacaucgaauC..	29	1	st1
.....................................uggcugagagcaGcaucgaauu..	1	1	st1
.....................................uggcugagagcaacaucgaauu..	379	0	st1
.....................................uggcuAagagcaacaucgaauu..	1	1	st1
.....................................uggcugagGgcaacaucgaauu..	3	1	st1
.....................................uggcugagagcaacGucgaauu..	2	1	st1
.....................................uggcugagaAcaacaucgaauu..	1	1	st1
.....................................uggcugUgagcaacaucgaauu..	1	1	st1
.....................................uggcugagagcaacaucgaauuu...	817	0	st1
.....................................uggcCgagagcaacaucgaauuu...	2	1	st1
.....................................uggcugagGgcaacaucgaauuu...	2	1	st1
.....................................Cggcugagagcaacaucgaauuu...	1	1	st1
.....................................uggcugagagcaacaucgaGuuu...	17	1	st1
.....................................uggcugagagcaacauUgaauuu...	2	1	st1
.....................................uggcugagagcaacaucgaauCu...	3	1	st1
.....................................uggcugagagcaUcaucgaauuu...	1	1	st1
.....................................uggcugagagcaacaucgGauuu...	8	1	st1
.....................................uggcugagagcaaUaucgaauuu...	1	1	st1
.....................................uggcugagagcGacaucgaauuu...	2	1	st1
.....................................uggcugGgagcaacaucgaauuu...	3	1	st1
.....................................uggcugaAagcaacaucgaauuu...	3	1	st1
.....................................uAgcugagagcaacaucgaauuu...	2	1	st1
.....................................uggcugagagcaGcaucgaauuu...	5	1	st1
.....................................uggcugagagcaacaucgaauuA...	42	1	st1
.....................................uggcugagagcaacaCcgaauuu...	3	1	st1
.....................................uggcugagagcaacaucUaauuu...	1	1	st1
.....................................uggcugagagcaacaucgaauuG...	4	1	st1
.....................................uggcugagCgcaacaucgaauuu...	3	1	st1
.....................................uggcugagagcaacGucgaauuu...	2	1	st1
.....................................uggcugagagcaacCucgaauuu...	1	1	st1
.....................................Aggcugagagcaacaucgaauuu...	3	1	st1
.....................................Gggcugagagcaacaucgaauuu...	1	1	st1
.....................................uggcugagagcaacaucgaauuC...	64	1	st1
.....................................uggcugCgagcaacaucgaauuu...	1	1	st1
.....................................uggcugagagcaacaucgaaCuu...	3	1	st1
.....................................uggcugagagcaacaucgaauuuC..	6	1	st1
.....................................uggcugagagcaacGucgaauuua..	1	1	st1
.....................................uggcugagagcaacaucgaaCuua..	1	1	st1
.....................................uAgcugagagcaacaucgaauuua..	1	1	st1
.....................................uggcugagagcaacaucgaauuuU..	33	1	st1
.....................................uggcugagagcGacaucgaauuua..	1	1	st1
.....................................uggcugagagcaacaucgaauuuG..	6	1	st1
.....................................uggcugagagcaacaucgaGuuua..	3	1	st1
.....................................uggcugagCgcaacaucgaauuua..	1	1	st1
.....................................uggcugagagcaacaucgaauuua..	166	0	st1
.....................................uggcugagagcaacaucgaauuuaa...	1	0	st1
.....................................uggcugagagcaacaucgaauuuUa...	11	1	st1
.....................................uggcugagagcaacaucgaauuuaG...	1	1	st1

```
                                    Star                             Mature
auuuguuugccuauuuuuguacagauuagaauuauaauggcugagagcaacaucgaauuuaauucuuaaauucgaugucgcucucagccauuauaagaaacuuuaaacuaucu
.......................................gcugagagcaacaucgaauu......................................................   1     0    st1
.....................................................................auucgaugucgcucucagc.........................   2     0    st1
.....................................................................auucgaugucgcucucagU.........................   1     1    st1
.....................................................................auucgaugucgcucucagcc........................   4     0    st1
.....................................................................auucgaugucgcucucagcU........................   1     1    st1
.....................................................................auucgaugucgcucucagcca.......................   11    0    st1
.....................................................................auucgaugucgcucucagccaC......................   3     1    st1
.....................................................................auucgaugucgcucucagccau......................   15    0    st1
.....................................................................auucgauguUgcucucagccau......................   1     1    st1
.....................................................................auucgaugucgcucucagccauu.....................   15    0    st1
.....................................................................auucgaugucgcucucagccauA.....................   5     1    st1
.....................................................................auucgaugucgcucucagccaAu.....................   1     1    st1
.....................................................................auucgaugucgcucucagccauC.....................   3     1    st1
.....................................................................auucgaugucgcucucagccauuU....................   6     1    st1
.....................................................................auucgaugucgcucucagccauuC....................   1     1    st1
.....................................................................auucgaugucgcucucagccauua....................   1     0    st1
......................................................................uucgaugucgcucucagU.........................   3     1    st1
......................................................................uucgaugucgcucucagc.........................   13    0    st1
......................................................................uucgaugucgcucucGgc.........................   1     1    st1
......................................................................uucgaugucgcucucagcA........................   4     1    st1
......................................................................uucgaugucgcucucagcc........................   43    0    st1
......................................................................uCcgaugucgcucucagcc........................   1     1    st1
......................................................................uucgaugucgcucucagcU........................   11    1    st1
......................................................................uucgaugucgcucucagcca.......................   10    0    st1
......................................................................uucgaugucgcucucagccaA......................   1     1    st1
......................................................................uucgaugucgcucucagccaC......................   5     1    st1
......................................................................uucgaugucgcCcucagccau......................   1     1    st1
......................................................................uucgaugucgcucucagccCu......................   1     1    st1
......................................................................uucgaugucgcucucagccaG......................   2     1    st1
......................................................................uucgaugucgcucucagccau......................   46    0    st1
......................................................................uucgaugucgcucucagccaAu.....................   1     1    st1
......................................................................uucgaugucgcucucagccauC.....................   21    1    st1
......................................................................uucgaugucgcCcucagccauu.....................   1     1    st1
......................................................................uucgaugucgcucucagccauu.....................   256   0    st1
......................................................................uucgaugucgcucuGagccauu.....................   1     1    st1
......................................................................uucgaugucgcucucagccCuu.....................   2     1    st1
......................................................................uucAaugucgcucucagccauu.....................   1     1    st1
......................................................................uucgaugucgcucGcagccauu.....................   1     1    st1
......................................................................uucgGugucgcucucagccauu.....................   3     1    st1
......................................................................uucgaugAcgcucucagccauu.....................   3     1    st1
......................................................................uucgaugucgcucucagccauA.....................   10    1    st1
......................................................................Cucgaugucgcucucagccauu.....................   1     1    st1
......................................................................uucgaugucgcuUucagccauu.....................   1     1    st1
......................................................................uucgUugucgcucucagccauu.....................   1     1    st1
......................................................................uucgaugucgcucucagccaCu.....................   2     1    st1
......................................................................uucgaugucgcucCcagccauua....................   1     1    st1
......................................................................uucgaugucgcCcucagccauua....................   1     1    st1
......................................................................uucgaugucgcucucagccauCa....................   1     1    st1
......................................................................uucgaugucgcucucagccauua....................   102   0    st1
......................................................................uucgaugucgcucucagccauuU....................   30    1    st1
......................................................................uucgaCgucgcucucagccauua....................   2     1    st1
......................................................................uucgaugucgcucucagccauuG....................   2     1    st1
......................................................................uucgaugucgUucucagccauua....................   1     1    st1
......................................................................uucgaugucgcucucagccauuC....................   1     1    st1
......................................................................uucgaugucgcucucagccauuUu...................   3     1    st1
.......................................................................ucgaugucgcucucagcc........................   2     0    st1
.......................................................................ucgaGgucgcucucagccauu.....................   1     1    st1
.......................................................................ucgaugucgcucucagccauu.....................   7     0    st1
.......................................................................ucgaugucgcucucagccauuU....................   4     1    st1
.......................................................................ucgaugucgcucucagccauua....................   6     0    st1

................................uauaauggcugagagcGacauc...........................................................   1     1    sr1
.....................................uggcugagagcaacaucg..........................................................   10    0    sr1
.....................................Gggcugagagcaacaucg..........................................................   2     1    sr1
.....................................uggcugagagcaacaucA..........................................................   2     1    sr1
.....................................uggcugagagcaacaucga.........................................................   4     0    sr1
.....................................Gggcugagagcaacaucgaa........................................................   4     1    sr1
.....................................uggcugagagcaacaucgaU........................................................   3     1    sr1
.....................................uggcugagagcGacaucgaa........................................................   2     1    sr1
.....................................Cggcugagagcaacaucgaa........................................................   1     1    sr1
```

```
                                     Star                               Mature
auuuguuugccuauuuuuguacagauuagaauuauaauggcugagagcaacaucgaauuuaauucuuaaauucgaugucgcucucagccauuauaagaaacuuuaaacuaucu
.....................................uggcugagagcaacaucgaa........................................................	22	0	sr1
.....................................uggcugagagcaacaucgaaC.......................................................	5	1	sr1
.....................................uggcugagagcaacaucgaaG.......................................................	3	1	sr1
.....................................uggcugagagcGacaucgaau.......................................................	1	1	sr1
.....................................uggcugagagcaUcaucgaau.......................................................	1	1	sr1
.....................................uggcugagagcaacaucgaUu.......................................................	3	1	sr1
.....................................uggcugagagcaacaucgaaA.......................................................	2	1	sr1
.....................................Gggcugagagcaacaucgaau.......................................................	6	1	sr1
.....................................uggcugagagcaacaucgaau.......................................................	36	0	sr1
.....................................uggcugagagcaacaucgaauG......................................................	5	1	sr1
.....................................uggcugagagcaacaucgaauC......................................................	23	1	sr1
.....................................uggcugagagcGacaucgaauu......................................................	6	1	sr1
.....................................uggcugagagcaacaucgGauu......................................................	1	1	sr1
.....................................uggcugagGgcaacaucgaauu......................................................	2	1	sr1
.....................................uggcGgagagcaacaucgaauu......................................................	1	1	sr1
.....................................uggcugagagcaacaucgaGuu......................................................	4	1	sr1
.....................................uggcAgagagcaacaucgaauu......................................................	1	1	sr1
.....................................uggcugagagcaGcaucgaauu......................................................	2	1	sr1
.....................................uggcugagagcaacaucgaauA......................................................	46	1	sr1
.....................................uggcCgagagcaacaucgaauu......................................................	2	1	sr1
.....................................uggcugagagcaacaucgaauu......................................................	295	0	sr1
.....................................uggcugagagcaacGucgaauu......................................................	4	1	sr1
.....................................uggcugGgagcaacaucgaauu......................................................	1	1	sr1
.....................................Gggcugagagcaacaucgaauu......................................................	58	1	sr1
.....................................uggUugagagcaacaucgaauu......................................................	2	1	sr1
.....................................uggcugagagcaaUaucgaauuu.....................................................	3	1	sr1
.....................................uggcugagagcaacaucgaauuC.....................................................	29	1	sr1
.....................................uggcugagagcaacaucgaauuu.....................................................	364	0	sr1
.....................................uggcugagagcaaAaucgaauuu.....................................................	1	1	sr1
.....................................uggcuAagagcaacaucgaauuu.....................................................	3	1	sr1
.....................................uggcugagagcaacaAcgaauuu.....................................................	3	1	sr1
.....................................Gggcugagagcaacaucgaauuu.....................................................	86	1	sr1
.....................................uggcugagagcaacaucgaauuG.....................................................	17	1	sr1
.....................................uggcugagagcaacaucgaauCu.....................................................	2	1	sr1
.....................................uggUugagagcaacaucgaauuu.....................................................	1	1	sr1
.....................................uggcugagagcGacaucgaauuu.....................................................	2	1	sr1
.....................................uggcugagGgcaacaucgaauuu.....................................................	1	1	sr1
.....................................Cggcugagagcaacaucgaauuu.....................................................	2	1	sr1
.....................................uggcugagagcUacaucgaauuu.....................................................	1	1	sr1
.....................................uggcugagagcaacaucgaGuuu.....................................................	11	1	sr1
.....................................uggcugagagcaacaucgaauuA.....................................................	187	1	sr1
.....................................uAgcugagagcaacaucgaauuu.....................................................	1	1	sr1
.....................................uggcugagagcaacGucgaauuu.....................................................	2	1	sr1
.....................................uggcugagagcaGcaucgaauuu.....................................................	2	1	sr1
.....................................Aggcugagagcaacaucgaauuu.....................................................	1	1	sr1
.....................................uggcAgagagcaacaucgaauuu.....................................................	1	1	sr1
.....................................uggcCgagagcaacaucgaauuua....................................................	3	1	sr1
.....................................uggcugagagcGacaucgaauuua....................................................	3	1	sr1
.....................................Cggcugagagcaacaucgaauuua....................................................	2	1	sr1
.....................................Gggcugagagcaacaucgaauuua....................................................	135	1	sr1
.....................................uggcugagagcaacaucgaauuAa....................................................	4	1	sr1
.....................................uggcugGgagcaacaucgaauuua....................................................	3	1	sr1
.....................................uggcugagagcaacaucgGauuua....................................................	3	1	sr1
.....................................uggcugagagcaacaCcgaauuua....................................................	1	1	sr1
.....................................uggcugagagcaacaucgaauuuG....................................................	33	1	sr1
.....................................uggcugagagcaacaucgaauuua....................................................	507	0	sr1
.....................................uggcugagagcaacGucgaauuua....................................................	1	1	sr1
.....................................uggcugagagcaaAaucgaauuua....................................................	1	1	sr1
.....................................uggcugagGgcaacaucgaauuua....................................................	4	1	sr1
.....................................uggcugagagcaacaucgaauuuU....................................................	7	1	sr1
.....................................uggcugagagcaacaucgaGuuua....................................................	25	1	sr1
.....................................uggcugagagcaacaucgaauuCa....................................................	1	1	sr1
.....................................uggcugagagcaGcaucgaauuua....................................................	2	1	sr1
.....................................uggcugagagcaacaucgaauuuC....................................................	3	1	sr1
.....................................uggcugagagcaacaucgaaGuua....................................................	1	1	sr1
.....................................uggcugagagcaacaucgaauuuaa...................................................	17	0	sr1
.....................................uggcugagagcaacaucgaauuuaU...................................................	3	1	sr1
.....................................uggcugagagcaacaucgaauuuaG...................................................	1	1	sr1
.....................................uUgcugagagcaacaucgaauuuaa...................................................	1	1	sr1
.....................................uggcugagagcaacaucgaauuuUa...................................................	7	1	sr1
```

Star **Mature**

auuuguuugccuauuuuuguacagauuagaauuauaauggcugagagcaacaucgaauuuaauucuuaaauucgaugucgcucucagccauuauaagaaacuuuaaacuaucu			
.....................................uggcugagagcaacaucgaGuuuaa...	3	1	sr1
.....................................Gggcugagagcaacaucgaauuuaa...	3	1	sr1
.....................................uggcugagagcaacaucgaauuuaaA..	1	1	sr1
.....................................Gggcugagagcaacaucgaauuuaauu...	1	1	sr1
....gcugagagcaacaucgaauu..	1	0	sr1
....cugagagcaacaucgaauuua..	2	0	sr1
....cuuaaauucgaugucgcucu.............................	1	0	sr1
....auucgaugucgcucucGg..........................	1	1	sr1
....auucgaugucgcucucag..........................	17	0	sr1
....auucgaugucgcucucagc.........................	19	0	sr1
....Guucgaugucgcucucagc.........................	1	1	sr1
....auucgaugucgcucucagA.........................	1	1	sr1
....auucgaugucgcucucagcG........................	1	1	sr1
....Cuucgaugucgcucucagcc........................	1	1	sr1
....auucgaugucgcucucagcc........................	23	0	sr1
....auucgaugucgcucucagcU........................	11	1	sr1
....auucgaugucgcucucagcA........................	9	1	sr1
....auucgaugucgcucucagccC.......................	1	1	sr1
....auucgaugucgcucucagccG.......................	1	1	sr1
....auucgaugucgcucucagccU.......................	2	1	sr1
....auucgaugucgcucucagcca.......................	14	0	sr1
....auucgaugucgcucucagccau......................	18	0	sr1
....auucgaugucgcucucagccaG......................	2	1	sr1
....auucgaugucgcucucagccUu......................	1	1	sr1
....auucgaugucgcucucagccaA......................	2	1	sr1
....auucgaugucgcucucagccaC......................	2	1	sr1
....auucgaugucgcucucagccUuu.....................	1	1	sr1
....auucgaugucgcucucagccauA.....................	23	1	sr1
....auucgaugCcgcucucagccauu.....................	1	1	sr1
....Guucgaugucgcucucagccauu.....................	1	1	sr1
....auucgaugucgcucucagccauC.....................	2	1	sr1
....auucgaugucgcucucagccauu.....................	19	0	sr1
....auucgaugucgcucucagccauG.....................	1	1	sr1
....Cuucgaugucgcucucagccauu.....................	1	1	sr1
....auucgaugucgcucucagccauua....................	14	0	sr1
....auucgaugucgcucucagccUuua....................	2	1	sr1
....auucgaugucgcucucagccauuU....................	5	1	sr1
....auucgaugucgcucucagccauuUua..................	1	1	sr1
....uucgaugucgcucucagU.........................	4	1	sr1
....Gucgaugucgcucucagc.........................	1	1	sr1
....uucgaugucAcucucagc.........................	1	1	sr1
....uucgaugucgcucucUgc.........................	1	1	sr1
....uucgaugucgcucCcagc.........................	1	1	sr1
....uucgaugucgcucucagG.........................	1	1	sr1
....uucgaugucgcucucagc.........................	46	0	sr1
....uucgaugucgcucucagA.........................	6	1	sr1
....Cucgaugucgcucucagcc........................	1	1	sr1
....Gucgaugucgcucucagcc........................	10	1	sr1
....uucgaugucgcucucagcA........................	27	1	sr1
....uucgUugucgcucucagcc........................	1	1	sr1
....uucgaugucgcucucagcG........................	2	1	sr1
....uucgaugucgcucucagcU........................	51	1	sr1
....uucgGugucgcucucagcc........................	1	1	sr1
....uucgaugCcgcucucagcc........................	2	1	sr1
....uucgaCgucgcucucagcc........................	1	1	sr1
....uucgaugucgcucCcagcc........................	1	1	sr1
....uucgaugucgcucucagcc........................	153	0	sr1
....Gucgaugucgcucucagcca.......................	1	1	sr1
....uucgaugucgcucucagccG.......................	2	1	sr1
....uucgaugucgcucCcagcca.......................	1	1	sr1
....uucgaugucgcucucagcca.......................	74	0	sr1
....uucgaugucgcucucagccU.......................	1	1	sr1
....uCcgaugucgcucucagcca.......................	1	1	sr1
....uucgaugucgcucucagAca.......................	1	1	sr1
....uucgaugucgcucuAagccau......................	1	1	sr1
....uucgaugucgcucucGgccau......................	2	1	sr1
....uucgaugucgcucucagccCu......................	2	1	sr1
....uucgaugucgcucucagccaG......................	8	1	sr1
....uucgauguUgcucucagccau......................	1	1	sr1
....uucgaugucgcucucagccaA......................	8	1	sr1

```
                                    Star                             Mature
auuuguuugccuauuuuuguacagauuagaauuauaauggcugagagcaacaucgaauuuaauucuuaaauucgaugucgcucucagccauuauaagaaacuuuaaacuaucu
......................................................................Gucgaugucgcucucagccau......................    1       1       sr1
......................................................................uucgaugucgcucucagccau......................    174     0       sr1
......................................................................uucgaugucgcucucagccaC......................    20      1       sr1
......................................................................uucgaugucgcucCcagccau......................    2       1       sr1
......................................................................uucgaugucgcucucagccCuu.....................    6       1       sr1
......................................................................uucgaugucgcucucagccauu.....................    447     0       sr1
......................................................................uucgaugucgcuUucagccauu.....................    1       1       sr1
......................................................................Gucgaugucgcucucagccauu.....................    8       1       sr1
......................................................................uucAaugucgcucucagccauu.....................    1       1       sr1
......................................................................uucgaugucgcAcucagccauu.....................    1       1       sr1
......................................................................uucgaugucgcucCcagccauu.....................    2       1       sr1
......................................................................uucgaugucgcucucagccauA.....................    155     1       sr1
......................................................................uCcgaugucgcucucagccauu.....................    1       1       sr1
......................................................................uuUgaugucgcucucagccauu.....................    1       1       sr1
......................................................................uucgaCgucgcucucagccauu.....................    1       1       sr1
......................................................................uucgaugCcgcucucagccauu.....................    1       1       sr1
......................................................................uucgaugucgcucucagccaGu.....................    1       1       sr1
......................................................................uucgaugucgcucucagccaCu.....................    4       1       sr1
......................................................................uucgGugucgcucucagccauu.....................    3       1       sr1
......................................................................uucgaugucgcucucagccauG.....................    26      1       sr1
......................................................................uucgaugucgcCcucagccauu.....................    3       1       sr1
......................................................................uucgaugucgcucucagccaAu.....................    4       1       sr1
......................................................................uucgaugucgcucucGgccauu.....................    1       1       sr1
......................................................................uucgaugucgcucucagccUuu.....................    2       1       sr1
......................................................................uucgaugucgcucucagccauC.....................    42      1       sr1
......................................................................uucgGugucgcucucagccauua....................    5       1       sr1
......................................................................uucgaugucgcucucagccauCa....................    1       1       sr1
......................................................................uucgaugucgcucucagccauua....................    1265    0       sr1
......................................................................uucgaugucgcucucGgccauua....................    4       1       sr1
......................................................................Gucgaugucgcucucagccauua....................    17      1       sr1
......................................................................uucgaugucgcucucagccauuU....................    55      1       sr1
......................................................................uucgaugucgcucucaAccauua....................    1       1       sr1
......................................................................uucgaugucgcucucagUcauua....................    1       1       sr1
......................................................................Cucgaugucgcucucagccauua....................    2       1       sr1
......................................................................uucgaugucgcucucagccaCua....................    1       1       sr1
......................................................................uucgaugucgcucucagccauAa....................    3       1       sr1
......................................................................uucgaugucgcucucagccUuua....................    2       1       sr1
......................................................................uucgaugucgcucuUagccauua....................    3       1       sr1
......................................................................uucgaAgucgcucucagccauua....................    1       1       sr1
......................................................................uucgaGgucgcucucagccauua....................    1       1       sr1
......................................................................uucgaugCcgcucucagccauua....................    6       1       sr1
......................................................................Aucgaugucgcucucagccauua....................    2       1       sr1
......................................................................uucgaCgucgcucucagccauua....................    3       1       sr1
......................................................................uucgaugucgcucucagccCuua....................    1       1       sr1
......................................................................uucgaugucgcucCcagccauua....................    2       1       sr1
......................................................................uuUgaugucgcucucagccauua....................    2       1       sr1
......................................................................uucgaugucgcucucUgccauua....................    1       1       sr1
......................................................................uucgaugucgcucucagccauuG....................    26      1       sr1
......................................................................uucgaugucgcucucagccauuC....................    13      1       sr1
......................................................................uCcgaugucgcucucagccauua....................    1       1       sr1
......................................................................uucgauguAgcucucagccauua....................    1       1       sr1
......................................................................uAcgaugucgcucucagccauua....................    2       1       sr1
......................................................................uucgaugucgcucucagccauuUu...................    5       1       sr1
......................................................................uucgaugCcgcucucagccauuau...................    1       1       sr1
......................................................................uucgaugucgcucucagccauuau...................    2       0       sr1
......................................................................uucgaugucgcucucagccauuaA...................    10      1       sr1
......................................................................uucgaugucgcucucagccauuaC...................    4       1       sr1
......................................................................uucgaugucgcucucagccauuUua..................    5       1       sr1
......................................................................uucgaugucgcucucagccauuaAa..................    2       1       sr1
......................................................................uucgaugucgcucucagccauuaua..................    5       0       sr1
......................................................................uucgaugucgcucucagccauuauaU.................    1       1       sr1
......................................................................uucgaugucgcucucagccauuauUa.................    2       1       sr1
......................................................................uucgaugucgcucucagccauuauaUg................    2       1       sr1
.......................................................................ucgaugucgcucucagcc........................    5       0       sr1
.......................................................................ucgaugucgcucucagcU........................    1       1       sr1
.......................................................................ucgaugucAcucucagcc........................    1       1       sr1
.......................................................................ucgaugucgcucucagcca.......................    1       0       sr1
.......................................................................ucgaugucgcucucagccCu......................    2       1       sr1
.......................................................................ucgaugucgcCcucagccau......................    1       1       sr1
.......................................................................ucgaugucgcucucagccau......................    4       0       sr1
```

Star **Mature**

```
auuuguuugccuauuuuuguacagauuagaauuauaauggcugagagcaacaucgaauuuaauucuuaaauucgaugucgcucucagccauuauaagaaacuuuaaacuaucu
.......................................................................ucgaugucgcucucagccUuu......................	2	1	sr1
.......................................................................ucgaugucgcucucagccauu......................	6	0	sr1
.......................................................................ucgaugucgcucucagccauG......................	4	1	sr1
.......................................................................ucgaugucgcucucagccauA......................	6	1	sr1
.......................................................................ucgaugucgcucucagccaCu......................	3	1	sr1
.......................................................................ucgaugucgcucucagccCuu......................	1	1	sr1
.......................................................................Gcgaugucgcucucagccauua.....................	1	1	sr1
.......................................................................ucgaugucgcucucagccUuua.....................	1	1	sr1
.......................................................................ucgaugucgcucucagccauua.....................	51	0	sr1
.......................................................................ucgaugucgcucucagccauuU.....................	3	1	sr1
.......................................................................ucgaugucgcucucagccauuC.....................	1	1	sr1
.......................................................................ucgaugucgcucucagccCuuau....................	1	1	sr1
```

Provisional ID	:	ScYm1zk_198_2402
Score total	:	11508.7
Score for star read(s)	:	3.9
Score for read counts	:	11498.2
Score for mfe	:	2
Score for randfold	:	1.6
Score for cons. seed	:	3
Total read count	:	22565
Mature read count	:	22397
Loop read count	:	0
Star read count	:	168

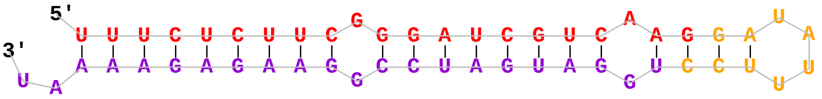

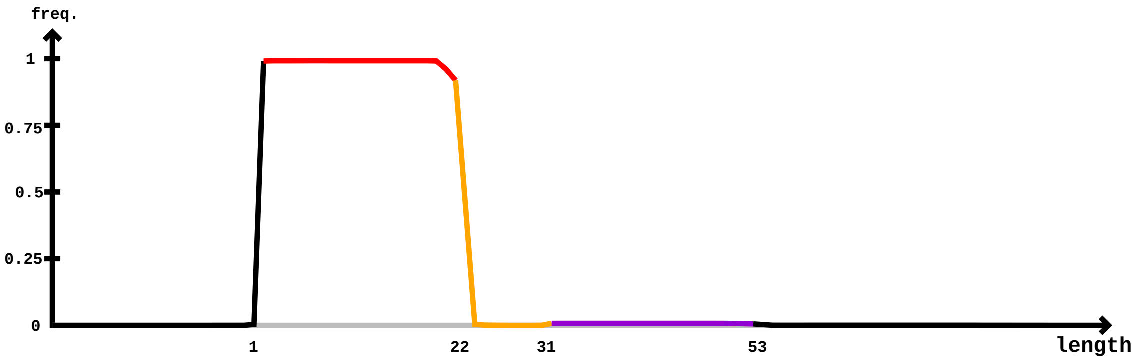

Mature Star

```
5'- aauaguucgucacgaaauauuuucucuucgggaucgucaaggauauuuccuggaugauccggaagagaaaauauucgaaaaugucgaauuuggcgcucaaaauugaaa  -3'   obs
    aauaguucgucacgaaauauuuucucuucgggaucgucaaggauauuuccuggaugauccggaagagaaaauauucgaaaaugucgaauuuggcgcucaaaauugaaa         exp
    ...((((((.((((((.((((((((((((.((((((((.((((....)))).)))))))).))))))))))))))))....)).))))))..................   reads   mm   sample
    .......cgucacgaaauauuuucucu.................................................................................   1       0    sr1
    ...............aauauuuucucuucgggaucg........................................................................   1       0    sr1
    ................auauuuucucuucgggaucguca.....................................................................   1       0    sr1
    ..................auuuucucuucgggaucguca.....................................................................   1       0    sr1
    ...................uuuucucuucCggaucguc......................................................................   1       1    sr1
    ...................uuuucucuucgggaucguca.....................................................................   11      0    sr1
    ...................Guuucucuucgggaucgucaag...................................................................   1       1    sr1
    ...................uuCucucuucgggaucgucaag...................................................................   2       1    sr1
    ...................uuuucucuucgggaucgucaag...................................................................   23      0    sr1
    ...................Auuucucuucgggaucgucaagg..................................................................   1       1    sr1
    ....................uuucucuucgggaucguU......................................................................   1       1    sr1
    ....................uuucucuucgggaucguc......................................................................   7       0    sr1
    ....................Guucucuucgggaucguc......................................................................   1       1    sr1
    ....................uuucCcuucgggaucguca.....................................................................   1       1    sr1
    ....................uuucucuucgggaucguAa.....................................................................   2       1    sr1
    ....................uuucucuucgggaucguca.....................................................................   293     0    sr1
    ....................uuucucuucgggaucguUa.....................................................................   1       1    sr1
    ....................uuucucuucgggauAguca.....................................................................   1       1    sr1
    ....................uuucucCucgggaucguca.....................................................................   2       1    sr1
    ....................uCucucuucgggaucguca.....................................................................   1       1    sr1
    ....................uuucucuucgggaucgucG.....................................................................   14      1    sr1
    ....................uuucucuucgggaucgucC.....................................................................   1       1    sr1
    ....................Guucucuucgggaucguca.....................................................................   13      1    sr1
    ....................Auucucuucgggaucguca.....................................................................   1       1    sr1
    ....................uuucCcuucgggaucgucaa....................................................................   1       1    sr1
    ....................uuucucuucgggaucguUaa....................................................................   1       1    sr1
    ....................uuucucuucgggaucgCcaa....................................................................   2       1    sr1
    ....................uuucucuucgggaucgucaG....................................................................   16      1    sr1
    ....................Cuucucuucgggaucgucaa....................................................................   3       1    sr1
    ....................uuucucuucgggaucgucaU....................................................................   1       1    sr1
    ....................uuucucuucgggaCcgucaa....................................................................   1       1    sr1
    ....................uuucucuucAggaucgucaa....................................................................   1       1    sr1
    ....................uuucucuucgggaucgucGa....................................................................   1       1    sr1
```

```
                    Mature                       Star
aauaguucgucacgaaauauuuucucuucgggaucgucaaggauauuuccuggaugauccggaagagaaaauauucgaaaaugucgaauuuggcgcucaaaauugaaa
....................Guucucuucgggaucgucaa....................................................................   20     1    sr1
....................uCucucuucgggaucgucaa....................................................................   2      1    sr1
....................uuucucuucgggaucguAaa....................................................................   1      1    sr1
....................uuucucuucgggGucgucaa....................................................................   1      1    sr1
....................uuucucuucgggaucgucaa....................................................................   308    0    sr1
....................uuCcucuucgggaucgucaa....................................................................   1      1    sr1
....................uuucucuucgggaucgucaC....................................................................   2      1    sr1
....................uuucucGucgggaucgucaag...................................................................   2      1    sr1
....................uuucucuucgggaucgucaag...................................................................   5470   0    sr1
....................uuucuAuucgggaucgucaag...................................................................   1      1    sr1
....................uCucucuucgggaucgucaag...................................................................   15     1    sr1
....................uuucucuucCggaucgucaag...................................................................   1      1    sr1
....................uuuAucuucgggaucgucaag...................................................................   4      1    sr1
....................uuucuGuucgggaucgucaag...................................................................   1      1    sr1
....................uuucucuucgggaucguUaag...................................................................   6      1    sr1
....................uuucucuucgggaucgucUag...................................................................   5      1    sr1
....................uuAcucuucgggaucgucaag...................................................................   1      1    sr1
....................uuucuUuucgggaucgucaag...................................................................   2      1    sr1
....................uuucucuucgggauUgucaag...................................................................   6      1    sr1
....................Guucucuucgggaucgucaag...................................................................   226    1    sr1
....................Cuucucuucgggaucgucaag...................................................................   26     1    sr1
....................uuucucuucgggaucgucaCg...................................................................   3      1    sr1
....................uuucucuucgAgaucgucaag...................................................................   4      1    sr1
....................uuucucuucgggaucUucaag...................................................................   1      1    sr1
....................uuucAcuucgggaucgucaag...................................................................   3      1    sr1
....................uuucucuucgggGucgucaag...................................................................   8      1    sr1
....................uuuUucuucgggaucgucaag...................................................................   2      1    sr1
....................uuucucuucgggaucgucaUg...................................................................   5      1    sr1
....................uuucucuucgggaucguAaag...................................................................   2      1    sr1
....................uuucucuucgggaCcgucaag...................................................................   12     1    sr1
....................uuucucuucgggaucgucaaU...................................................................   26     1    sr1
....................Auucucuucgggaucgucaag...................................................................   2      1    sr1
....................uuucucCucgggaucgucaag...................................................................   14     1    sr1
....................uuucucuucgggaucgucaaA...................................................................   307    1    sr1
....................uAucucuucgggaucgucaag...................................................................   2      1    sr1
....................uuucucuucgggaGcgucaag...................................................................   2      1    sr1
....................uuucucuucgggaucgucaaC...................................................................   7      1    sr1
....................uuucucAucgggaucgucaag...................................................................   1      1    sr1
....................uuucucuucgggaucgucGag...................................................................   19     1    sr1
....................uuucucuucUggaucgucaag...................................................................   2      1    sr1
....................uuucucuucgUgaucgucaag...................................................................   3      1    sr1
....................uuucucuucgggaAcgucaag...................................................................   4      1    sr1
....................uuucucuucgggaucgucaGg...................................................................   36     1    sr1
....................uuucCcuucgggaucgucaag...................................................................   13     1    sr1
....................uuucucuucgggaucgCcaag...................................................................   31     1    sr1
....................uuCcucuucgggaucgucaag...................................................................   25     1    sr1
....................uuucucuCcgggaucgucaag...................................................................   9      1    sr1
....................uuucucuucgggaucgAcaag...................................................................   1      1    sr1
....................uuucucuucgggaucAucaag...................................................................   12     1    sr1
....................uuucucuucAggaucgucaag...................................................................   4      1    sr1
....................uuucucuuUgggaucgucaag...................................................................   4      1    sr1
....................uuucucuucgggaAcgucaagg..................................................................   2      1    sr1
....................uuucCcuucgggaucgucaagg..................................................................   8      1    sr1
....................Guucucuucgggaucgucaagg..................................................................   133    1    sr1
....................uuucucuucgggaucgucaaCg..................................................................   3      1    sr1
....................uuucucuucgggaucgucaagC..................................................................   19     1    sr1
....................uuucucuucgggaucgCcaagg..................................................................   8      1    sr1
....................Cuucucuucgggaucgucaagg..................................................................   15     1    sr1
....................uuuUucuucgggaucgucaagg..................................................................   2      1    sr1
....................uuucucuucgggaucgucaaAg..................................................................   3      1    sr1
....................uuGcucuucgggaucgucaagg..................................................................   1      1    sr1
....................uuucucuucgAgaucgucaagg..................................................................   2      1    sr1
....................uuucuAuucgggaucgucaagg..................................................................   1      1    sr1
....................uuucucuucgggGucgucaagg..................................................................   12     1    sr1
....................uuucucuuUgggaucgucaagg..................................................................   1      1    sr1
....................uCucucuucgggaucgucaagg..................................................................   14     1    sr1
....................uuucucuucgggaucgucaCgg..................................................................   1      1    sr1
....................uuucucuucgggaucgucaagU..................................................................   22     1    sr1
....................uuucucuucgggaucgucaagA..................................................................   179    1    sr1
....................uuuAucuucgggaucgucaagg..................................................................   1      1    sr1
```

Mature **Star**

```
aauaguucgucacgaaauauuuucucuucgggaucgucaaggauauuuccuggaugauccggaagagaaaauauucgaaaaugucgaauuuggcgcucaaaauugaaa
....................uuucucuucgggaucAucaagg..................................................................  3     1  sr1
....................uuucucuucgggaCcgucaagg..................................................................  5     1  sr1
....................uuucucuucgggUucgucaagg..................................................................  1     1  sr1
....................uuucucuucgggaucgucUagg..................................................................  2     1  sr1
....................uuucucCucgggaucgucaagg..................................................................  4     1  sr1
....................uAucucuucgggaucgucaagg..................................................................  1     1  sr1
....................uuCcucuucgggaucgucaagg..................................................................  7     1  sr1
....................uuucucuucgggaucgucaUgg..................................................................  3     1  sr1
....................uuucucuCcgggaucgucaagg..................................................................  4     1  sr1
....................uuucucuucgggaucgucCagg..................................................................  3     1  sr1
....................uuucucuucgggaucgucGagg..................................................................  16    1  sr1
....................uuAcucuucgggaucgucaagg..................................................................  2     1  sr1
....................uuucucuucgggauUgucaagg..................................................................  1     1  sr1
....................Auucucuucgggaucgucaagg..................................................................  6     1  sr1
....................uuucucuucgggaucgucaGgg..................................................................  25    1  sr1
....................uuucucuucAggaucgucaagg..................................................................  3     1  sr1
....................uuucucuucgggaucgucaagg..................................................................  2573  0  sr1
....................uuucucuucgggaucgucaagAa.................................................................  1     1  sr1
....................uuucucuucgggaucgucaaggU.................................................................  1     1  sr1
....................uuucucuucgggaucgucaagUa.................................................................  3     1  sr1
....................Guucucuucgggaucgucaagga.................................................................  1     1  sr1
....................uuucucuucgggaucgucaagga.................................................................  18    0  sr1
....................uuucucuucgggaucgucaaggau................................................................  1     0  sr1
....................uuucucuucgggaucgucaaggaA................................................................  5     1  sr1
....................uuucucuucgggaucgucaaggUua...............................................................  1     1  sr1
....................uuucucuucgggaucgucaagAaua...............................................................  1     1  sr1
.....................uucucuucgggaucgucaa....................................................................  1     0  sr1
.....................uuUucuucgggaucgucaag...................................................................  1     1  sr1
.....................uucucuucgggaucgucaag...................................................................  1     0  sr1
.....................uucucuucgggaucgucaagg..................................................................  8     0  sr1
.....................uuUucuucgggaucgucaagg..................................................................  1     1  sr1
.....................uucucuucgggaucgucaaggauC...............................................................  1     1  sr1
......................ucucuucgggaucgucaagg..................................................................  1     0  sr1
.........................cuucgggaucgucaaggauauA.............................................................  1     1  sr1
..................................................uggaugauccggaagagaa.......................................  2     0  sr1
..................................................Gggaugauccggaagagaa.......................................  1     1  sr1
..................................................uggaugauccggaagagaaa......................................  8     0  sr1
..................................................uggaugauccggaagGgaaa......................................  1     1  sr1
..................................................uggaugauccggaagagaaaa.....................................  2     0  sr1
..................................................Gggaugauccggaagagaaaa.....................................  2     1  sr1
..................................................uggaugauccggaGgagaaaau....................................  1     1  sr1
..................................................uggaugauccggaagagaaaUu....................................  1     1  sr1
..................................................uggaugaCccggaagagaaaau....................................  1     1  sr1
..................................................Gggaugauccggaagagaaaau....................................  2     1  sr1
..................................................uggaugauccggaagagaaaau....................................  17    0  sr1
..................................................uggaCgauccggaagagaaaau....................................  1     1  sr1
..................................................uggaugauccggaagagaaaaC....................................  1     1  sr1
..................................................uggaugauccggaagagaaaauU...................................  2     1  sr1
..................................................uggaugauccggaagagaaaauG...................................  1     1  sr1
..................................................uggaugauccggaagagaaaaua...................................  13    0  sr1
..................................................uggaugauccggaagagaaaauau..................................  1     0  sr1
..................................................uggaugauccggaagagaaaauUu..................................  2     1  sr1
.........................................................................uucgaaaaugucgaauuugg...............  2     0  sr1
.........................................................................uucgaaaaugucgaauuuggcg.............  1     0  sr1
.........................................................................uucgaaaaugucgaauuuggcgc............  1     0  sr1
..........................................................................ucgaaaaugucgaauuuggcg.............  1     0  sr1
................................................................................augucgaauuuggcgcucU.........  1     1  sr1

..................auuuucucuucgggaucgucaaA...................................................................  1     1  st1
...................Cuuucucuucgggaucgucaa....................................................................  1     1  st1
...................uuuucucuucgggaucgucaag...................................................................  12    0  st1
...................Cuuucucuucgggaucgucaag...................................................................  3     1  st1
....................uuucucuucgggaucguc......................................................................  3     0  st1
....................uuucucuucgggaucguU......................................................................  2     1  st1
....................uuucucuCcgggaucguca.....................................................................  1     1  st1
....................uuucucuucgggaucgucG.....................................................................  5     1  st1
....................Auucucuucgggaucguca.....................................................................  1     1  st1
....................uuucucuucgggaucguca.....................................................................  170   0  st1
....................uuucucuucgggaucgucU.....................................................................  4     1  st1
....................uuCcucuucgggaucguca.....................................................................  1     1  st1
```

Mature Star

```
aauaguucgucacgaaauauuuucucuucgggaucgucaaggauauuuccuggaugauccggaagagaaaauauucgaaaaugucgaauuggcgcucaaaauugaaa
....................uuucucuucgggaucguAa....................................................................  3     1  st1
....................uuucucuCcgggaucgucaa...................................................................  1     1  st1
....................uuucucuucgggaucgucaU...................................................................  1     1  st1
....................uuucucuucgggaucgucaC...................................................................  1     1  st1
....................uuucucuucgggaucCucaa...................................................................  1     1  st1
....................uuucucuucgggaucgucGa...................................................................  1     1  st1
....................uuucucuucAggaucgucaa...................................................................  1     1  st1
....................uuucucuucgggaCcgucaa...................................................................  3     1  st1
....................uuCcucuucgggaucgucaa...................................................................  1     1  st1
....................uuucucuucgggaucAucaa...................................................................  1     1  st1
....................uuucucuucgggaucgucaG...................................................................  4     1  st1
....................uuucucuucgggaucgucaa...................................................................  277   0  st1
....................uuucucuucgggauGgucaa...................................................................  1     1  st1
....................Cuucucuucgggaucgucaa...................................................................  2     1  st1
....................uuucucuucgggGucgucaag..................................................................  13    1  st1
....................uuucucuucgggaCcgucaag..................................................................  5     1  st1
....................uuucucGucgggaucgucaag..................................................................  1     1  st1
....................uuucucuucgggaucguUaag..................................................................  1     1  st1
....................uuucucuucgggaAcgucaag..................................................................  3     1  st1
....................uuucucuucUggaucgucaag..................................................................  1     1  st1
....................uuucucuucAggaucgucaag..................................................................  4     1  st1
....................uuucucuAcgggaucgucaag..................................................................  3     1  st1
....................Cuucucuucgggaucgucaag..................................................................  7     1  st1
....................uuucuAuucgggaucgucaag..................................................................  1     1  st1
....................uuucucuucgggaucgAcaag..................................................................  2     1  st1
....................uuucucuucgggaucgucUag..................................................................  6     1  st1
....................uuucucuucgggaucAucaag..................................................................  1     1  st1
....................Auucucuucgggaucgucaag..................................................................  3     1  st1
....................uuucucuucgUgaucgucaag..................................................................  1     1  st1
....................uuucucuucgggaucgucaGg..................................................................  5     1  st1
....................uuCcucuucgggaucgucaag..................................................................  9     1  st1
....................uuucucuucgggaucgCcaag..................................................................  8     1  st1
....................uuucucuucgggaucgucGag..................................................................  8     1  st1
....................uuucucuucgggaucgucaaA..................................................................  140   1  st1
....................uuucucuucgggaucgucaaC..................................................................  9     1  st1
....................uuucucCucgggaucgucaag..................................................................  5     1  st1
....................uuucucuucggAaucgucaag..................................................................  2     1  st1
....................uuucucuuUgggaucgucaag..................................................................  1     1  st1
....................uuucCcuucgggaucgucaag..................................................................  3     1  st1
....................uuucucuucgggaGcgucaag..................................................................  1     1  st1
....................uuucucuCcgggaucgucaag..................................................................  1     1  st1
....................uuucucuucgggaucgucaag..................................................................  2712  0  st1
....................uCucucuucgggaucgucaag..................................................................  8     1  st1
....................uuuUucuucgggaucgucaag..................................................................  4     1  st1
....................uuucucuucgggauUgucaag..................................................................  8     1  st1
....................uuucucuucgggauAgucaag..................................................................  2     1  st1
....................uuucucuucgggaucgucaaU..................................................................  14    1  st1
....................uAucucuucgggaucgucaag..................................................................  1     1  st1
....................uuucucuucgggauGgucaag..................................................................  4     1  st1
....................uuucucuucCggaucgucaag..................................................................  1     1  st1
....................uuucAcuucgggaucgucaag..................................................................  1     1  st1
....................uuucucuucgggaAcgucaagg.................................................................  3     1  st1
....................uuucucuucgggGucgucaagg.................................................................  8     1  st1
....................uuucucuucgggaucgucaaAg.................................................................  1     1  st1
....................uuucucuucgUgaucgucaagg.................................................................  1     1  st1
....................Cuucucuucgggaucgucaagg.................................................................  3     1  st1
....................uuucucuucgggauUgucaagg.................................................................  5     1  st1
....................uuCcucuucgggaucgucaagg.................................................................  13    1  st1
....................uuucucuucgggaucgucGagg.................................................................  10    1  st1
....................uuucucCucgggaucgucaagg.................................................................  9     1  st1
....................uuucucuucgggaucgucaagg.................................................................  2051  0  st1
....................uuucucuucgggaucgucaUgg.................................................................  2     1  st1
....................uuucucuucggAaucgucaagg.................................................................  4     1  st1
....................uuucAcuucgggaucgucaagg.................................................................  1     1  st1
....................uuucucuucgggauGgucaagg.................................................................  2     1  st1
....................uuucucuAcgggaucgucaagg.................................................................  1     1  st1
....................uuucucAucgggaucgucaagg.................................................................  1     1  st1
....................uuucucuucgggaucgucaagC.................................................................  12    1  st1
....................uuuUucuucgggaucgucaagg.................................................................  1     1  st1
....................uuucucuucgggaucgucaagU.................................................................  19    1  st1
```

```
                    Mature                        Star
aauaguucgucacgaaauauuuucucuucgggaucgucaaggauauuuccuggaugauccggaagagaaaauauucgaaaaugucgaauuuggcgcucaaaauugaaa
....................uuucucuucgggaucgucaGgg..................................................................   8    1   st1
....................uuucucuCcgggaucgucaagg..................................................................   3    1   st1
....................uuucucuucgCgaucgucaagg..................................................................   1    1   st1
....................uuucuUuucgggaucgucaagg..................................................................   1    1   st1
....................uCucucuucgggaucgucaagg..................................................................   5    1   st1
....................uuAcucuucgggaucgucaagg..................................................................   1    1   st1
....................uuucucuucgggaCcgucaagg..................................................................   2    1   st1
....................uuucucuucgggaucgCcaagg..................................................................   9    1   st1
....................uuucCcuucgggaucgucaagg..................................................................   3    1   st1
....................uuucucuucgggauAgucaagg..................................................................   3    1   st1
....................uuucucuucgggaucgucaagA..................................................................   147  1   st1
....................uuucucuucgggaucgucaagUa.................................................................   2    1   st1
....................uuucucuucgggaucgucaagga.................................................................   5    0   st1
....................uuucucuucgggaucgucaaggU.................................................................   3    1   st1
....................uuucucuucgggaucgucaaggau................................................................   1    0   st1
....................uuucucuucgggaucgucaaggUua...............................................................   1    1   st1
.....................uucucuucgggaucgucaagg..................................................................   1    0   st1
.....................uucucuucgggaucgucaGgg..................................................................   1    1   st1
.......................cucuucgggaucgucaagg..................................................................   1    0   st1
........................ucuucgggaucgucaagg..................................................................   1    0   st1
........................ucuucgggaucgucaGggaua...............................................................   1    1   st1
...............................................uccuggaugauccggaagagaaaau....................................   1    0   st1
.................................................cuggaugauccggaagagaaaau....................................   1    0   st1
..................................................uggaugauccggaagagaa.......................................   1    0   st1
..................................................uggaugauccggaagagaaa......................................   1    0   st1
..................................................uggaugauccggaagagaaaa.....................................   4    0   st1
..................................................uggaugauccggaagagaaaaC....................................   1    1   st1
..................................................uggaugauccggaagagaaaau....................................   22   0   st1
...............................................................................aaugucgaauuuggcgcuca.........   1    0   st1

...................uuuucucuucgggaucguca.....................................................................   2    0   so1
...................uuuucucuucgggaucgucaa....................................................................   1    0   so1
...................Cuuucucuucgggaucgucaag...................................................................   1    1   so1
...................uuuucucuucgggaucgucaag...................................................................   3    0   so1
...................Cuuucucuucgggaucgucaagg..................................................................   1    1   so1
....................uuucucuucgggaucgucG.....................................................................   1    1   so1
....................uuCcucuucgggaucguca.....................................................................   1    1   so1
....................uuucucuucgggaucguca.....................................................................   48   0   so1
....................uuucucuucgggaucgucaG....................................................................   3    1   so1
....................uuucucuucgggaucgCcaa....................................................................   1    1   so1
....................uuucucuucgggaucgucaa....................................................................   94   0   so1
....................uuucucuucgggaucgucaU....................................................................   1    1   so1
....................uuucucuucgggaCcgucaag...................................................................   1    1   so1
....................Cuucucuucgggaucgucaag...................................................................   3    1   so1
....................uuucucuucgggaucgucCag...................................................................   1    1   so1
....................uuucCcuucgggaucgucaag...................................................................   3    1   so1
....................uuucucuucgggaucgucaUg...................................................................   1    1   so1
....................uuCcucuucgggaucgucaag...................................................................   6    1   so1
....................uuucucuucgggaucgucGag...................................................................   7    1   so1
....................uuucucuucgggaucgucaaA...................................................................   60   1   so1
....................uuucucuucgggaucgucaaU...................................................................   11   1   so1
....................uuucucuucgggaucgCcaag...................................................................   5    1   so1
....................uuucucuCcgggaucgucaag...................................................................   3    1   so1
....................uuucucCucgggaucgucaag...................................................................   1    1   so1
....................uuucucuucgggaucgucaag...................................................................   967  0   so1
....................uuucucAucgggaucgucaag...................................................................   2    1   so1
....................uuucucuucgggaucgucaGg...................................................................   11   1   so1
....................Auucucuucgggaucgucaag...................................................................   1    1   so1
....................uuucucuucgggaucgAcaag...................................................................   1    1   so1
....................uuucucuucgggauAgucaag...................................................................   1    1   so1
....................uuucucuucgggaucgucaagg..................................................................   626  0   so1
....................uuucucCucgggaucgucaagg..................................................................   4    1   so1
....................uuucucuucgggaucgucaagU..................................................................   20   1   so1
....................uuucucuucgggaucgucGagg..................................................................   4    1   so1
....................uuucucAucgggaucgucaagg..................................................................   3    1   so1
....................uuucucuucgggaucgucaaUg..................................................................   1    1   so1
....................uuucucuucgggaucgucaGgg..................................................................   17   1   so1
....................uuucucuucgggaucgucaagC..................................................................   4    1   so1
....................uuucucuucgggaCcgucaagg..................................................................   1    1   so1
....................uuucucuucgggGucgucaagg..................................................................   3    1   so1
```

Mature **Star**

```
aauaguucgucacgaaauauuuucucuucgggaucgucaaggauauuuccuggaugauccggaagagaaaauauucgaaaaugucgaauuuggcgcucaaaauugaaa
....................uuucucuucgAgaucgucaagg..................................................................  3     1   so1
....................uCucucuucgggaucgucaagg..................................................................  1     1   so1
....................uuucucuucgggaucgCcaagg..................................................................  5     1   so1
....................uuucucuucgggaucgucaagA..................................................................  75    1   so1
....................Cuucucuucgggaucgucaagg..................................................................  3     1   so1
....................uuucucuucgggaucgucaaggU.................................................................  2     1   so1
....................uuucucuucgggaucgucaagUa.................................................................  2     1   so1
....................uuucucuucgggaucgucaagga.................................................................  4     0   so1
....................uuucucuucgggaucgucaGggau................................................................  1     1   so1
....................uuucucuucgggaucgucaaggaA................................................................  2     1   so1
..................................................uggaugauccggaagaga........................................  1     0   so1
..................................................uggaugauccggaagagaa.......................................  2     0   so1
..................................................uggaugauccggaagagaaU......................................  1     1   so1
..................................................uggaugauccggaagagaaa......................................  6     0   so1
..................................................uggaugauccggaagagaaaa.....................................  1     0   so1
..................................................uggaugauccggaagagaaaau....................................  9     0   so1
..................................................uggaugauccggaagagaaaaC....................................  2     1   so1
..................................................uggaugauccggaGgagaaaau....................................  1     1   so1
..................................................uggaugauccggaagagaaaaua...................................  4     0   so1
..................................................uggaugaucAggaagagaaaaua...................................  1     1   so1
..................................................uggaugauccggaagagaaaauUu..................................  2     1   so1

...................uuuucucuucgggaucguca.....................................................................  1     0   sg1
...................uuuucucuucgggaucgucG.....................................................................  1     1   sg1
...................Cuuucucuucgggaucgucaa....................................................................  1     1   sg1
...................uuuucucuucgggaucgucaa....................................................................  2     0   sg1
...................uuuucucuucgggaucgucaGg...................................................................  1     1   sg1
...................uuuucucuucgggaucgucaag...................................................................  3     0   sg1
....................uuucucuucgggaucguc......................................................................  1     0   sg1
....................uuucucuucgggaucguca.....................................................................  105   0   sg1
....................Cuucucuucgggaucguca.....................................................................  1     1   sg1
....................uuucucuucAggaucguca.....................................................................  1     1   sg1
....................uuucucuucgggaucgCca.....................................................................  1     1   sg1
....................uuucucuucgggaucguAa.....................................................................  1     1   sg1
....................uuucucuucgggaucgucU.....................................................................  2     1   sg1
....................uuucucuucgggaucgucaU....................................................................  2     1   sg1
....................uuCcucuucgggaucgucaa....................................................................  1     1   sg1
....................uuucucuucgggaucguAaa....................................................................  1     1   sg1
....................uuucucuucgggaucgucGa....................................................................  2     1   sg1
....................uuucucuCcgggaucgucaa....................................................................  1     1   sg1
....................uuucucuucgggaucAucaa....................................................................  1     1   sg1
....................uuucucuucgggaucgucaG....................................................................  1     1   sg1
....................uuucucuucgggaucgucaa....................................................................  175   0   sg1
....................uuucucuucgAgaucgucaa....................................................................  1     1   sg1
....................uuucucAucgggaucgucaa....................................................................  1     1   sg1
....................uuucucuucgggaucgucaag...................................................................  2111  0   sg1
....................Cuucucuucgggaucgucaag...................................................................  10    1   sg1
....................uuucucuucgggaucgucaGg...................................................................  20    1   sg1
....................uuucucuucgggaucgucGag...................................................................  10    1   sg1
....................uuucucuucgggaucgucUag...................................................................  3     1   sg1
....................uuucucuucgggaucgCcaag...................................................................  8     1   sg1
....................uuucucCucgggaucgucaag...................................................................  7     1   sg1
....................uuucAcuucgggaucgucaag...................................................................  2     1   sg1
....................uuucucuucgggaucgucaaC...................................................................  3     1   sg1
....................uuucucuCcgggaucgucaag...................................................................  1     1   sg1
....................uuucucuucgggaucgucaaA...................................................................  44    1   sg1
....................uCucucuucgggaucgucaag...................................................................  1     1   sg1
....................uuucucuucgggaCcgucaag...................................................................  5     1   sg1
....................uuucucuucgggaucgucaCg...................................................................  1     1   sg1
....................uuucucuucgggaucAucaag...................................................................  1     1   sg1
....................uuucucuuUgggaucgucaag...................................................................  5     1   sg1
....................uuucucuucCggaucgucaag...................................................................  2     1   sg1
....................uuucucuucgggaucguUaag...................................................................  3     1   sg1
....................uuucucuucAggaucgucaag...................................................................  1     1   sg1
....................uuucCcuucgggaucgucaag...................................................................  5     1   sg1
....................uuucucuucgggaucgucaaU...................................................................  7     1   sg1
....................uuCcucuucgggaucgucaag...................................................................  6     1   sg1
....................uuuUucuucgggaucgucaag...................................................................  2     1   sg1
....................uuucucuucgggGucgucaag...................................................................  8     1   sg1
....................uAucucuucgggaucgucaag...................................................................  1     1   sg1
```

Mature Star

```
aauaguucgucacgaaauauuuucucuucgggaucgucaaggauauuuccuggaugauccggaagagaaaauauucgaaaaugucgaauuuggcgcucaaaauugaaa
....................uuucucuucgggauUgucaag...................................................................  2     1  sg1
....................uuucucuucgggaucgucaUg...................................................................  1     1  sg1
....................Auucucuucgggaucgucaag...................................................................  2     1  sg1
....................Cuucucuucgggaucgucaagg..................................................................  6     1  sg1
....................uuucCcuucgggaucgucaagg..................................................................  2     1  sg1
....................uuucAcuucgggaucgucaagg..................................................................  1     1  sg1
....................uuucucuucgggaucgucaaAg..................................................................  3     1  sg1
....................uuucucuuUgggaucgucaagg..................................................................  3     1  sg1
....................uCucucuucgggaucgucaagg..................................................................  6     1  sg1
....................uuucucuucgAgaucgucaagg..................................................................  3     1  sg1
....................uuucucuucgggaucgucGagg..................................................................  6     1  sg1
....................uuuUucuucgggaucgucaagg..................................................................  1     1  sg1
....................uuucucuucgggaucgucaagU..................................................................  18    1  sg1
....................uuucucCucgggaucgucaagg..................................................................  2     1  sg1
....................Auucucuucgggaucgucaagg..................................................................  1     1  sg1
....................uuucucuucgggaucAucaagg..................................................................  2     1  sg1
....................uuucucuucgggaCcgucaagg..................................................................  5     1  sg1
....................uuucucuucgggauAgucaagg..................................................................  3     1  sg1
....................uuucucuucAggaucgucaagg..................................................................  1     1  sg1
....................uuucucuucgggaucgucaagg..................................................................  1495  0  sg1
....................uuucucuucgggaucgucaGgg..................................................................  30    1  sg1
....................uuucucuCcgggaucgucaagg..................................................................  4     1  sg1
....................uuucucuucgggaucguAaagg..................................................................  1     1  sg1
....................uuAcucuucgggaucgucaagg..................................................................  2     1  sg1
....................uuucucuucgggaucgucaagC..................................................................  14    1  sg1
....................uuucucuucgggaucgucaagA..................................................................  92    1  sg1
....................uuucucuucgCgaucgucaagg..................................................................  1     1  sg1
....................uuucucuucgggGucgucaagg..................................................................  9     1  sg1
....................uGucucuucgggaucgucaagg..................................................................  1     1  sg1
....................uuCcucuucgggaucgucaagg..................................................................  9     1  sg1
....................uuucucAucgggaucgucaagg..................................................................  1     1  sg1
....................uuucucuucgggaucgCcaagg..................................................................  4     1  sg1
....................uuucucuucgggaucgucaagUa.................................................................  1     1  sg1
....................uuucucuucgggaucgucaagga.................................................................  7     0  sg1
....................uuucucuucgggaucgucaGgga.................................................................  4     1  sg1
....................uuucucuucgggaucgucaaggaA................................................................  2     1  sg1
....................uuucucuucgggaucgucaaggauG...............................................................  1     1  sg1
....................uuucucuucgggaucgucaaggauaA..............................................................  1     1  sg1
.....................uucucuucgggaucgucaagg..................................................................  2     0  sg1
.................................................cuggaugauccggaagagaaaa.....................................  4     0  sg1
..................................................uggaugauccggaagagaa.......................................  4     0  sg1
..................................................uggaugauccggaagagaaa......................................  1     0  sg1
..................................................uggaugauccggaagagaaaa.....................................  6     0  sg1
..................................................uggaugauccggaagagGaaau....................................  1     1  sg1
..................................................uggaugauccggaagagaaaau....................................  24    0  sg1
..................................................uggaugauccggaagagaaaaua...................................  5     0  sg1
..................................................uggaugauccggaagagaaaauU...................................  1     1  sg1
..................................................uggaugauccggaGgagaaaaua...................................  1     1  sg1
..................................................uggaugauccggaagagaaaauauA.................................  1     1  sg1
...................................................ggaugauccggaagagaaaau....................................  1     0  sg1
..........................................................................ucgaaaaugucgaauuuggcg.............  1     0  sg1
.............................................................................aaaaugucgaauuuggcgcucaaaa......  2     0  sg1
```

Provisional ID	:	ScYm1zk_320_3230
Score total	:	1336399.5
Score for star read(s)	:	3.9
Score for read counts	:	1336388.9
Score for mfe	:	2
Score for randfold	:	1.6
Score for cons. seed	:	3
Total read count	:	2621281
Mature read count	:	2607680
Loop read count	:	0
Star read count	:	13601

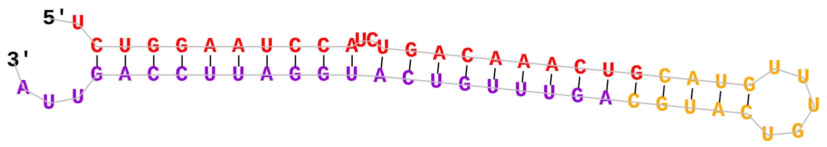

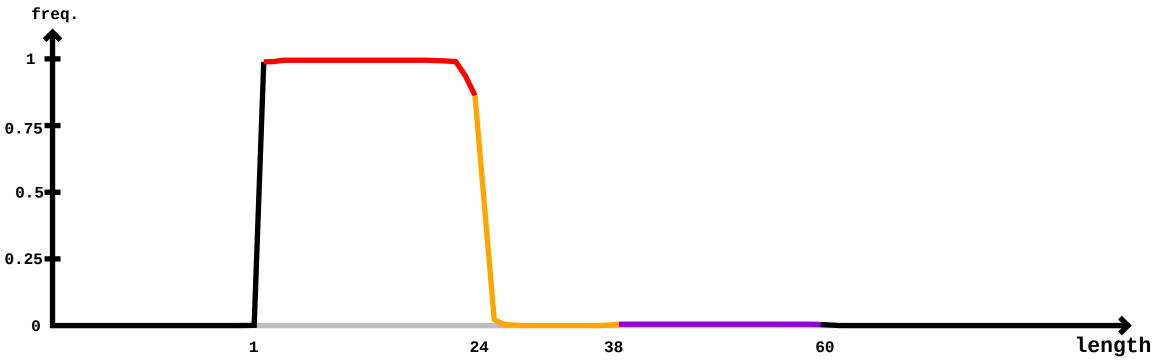

Mature **Star**

```
5'- acuuuauucuuuuuuuuuuaucuggaauccaucugacaaacugcauguuugucaugcaguuugucauggauuccaguuaaaaaaaagcaugcaaucguacgucaaggaga  -3'   obs
    acuuuauucuuuuuuuuuuaucuggaauccaucugacaaacugcauguuugucaugcaguuugucauggauuccaguuaaaaaaaagcaugcaaucguacgucaaggaga         exp
    ......(((((((((((((.((((((((((..((((((((((((((.....))))))))))))))))))))))))).)))))))).((.(((....))).)).)))))).    reads     mm      sample
    .........uuuuuuuuuuaucuggaauA.................................................................................    1         1       sr1
    ..................Caucuggaauccaucugacaa.......................................................................    1         1       sr1
    ..................uaucuggaauccaucugacaa.......................................................................    5         0       sr1
    ..................uaucuggGauccaucugacaaa......................................................................    2         1       sr1
    ..................Caucuggaauccaucugacaaa......................................................................    1         1       sr1
    ..................uaucuggaauccaucugacaaa......................................................................    9         0       sr1
    ..................uaucuggaauccaucugacaaac.....................................................................    7         0       sr1
    ..................uaucuggaauccaucugacaaaU.....................................................................    3         1       sr1
    ..................uaucuggaauccaucugacaaaA.....................................................................    2         1       sr1
    ..................uaucuggaGuccaucugacaaac.....................................................................    2         1       sr1
    ..................uaucAggaauccaucugacaaacu....................................................................    1         1       sr1
    ..................uaucuggaauccaucugacaaacG....................................................................    2         1       sr1
    ..................uaucuggaauccaucugacaaacC....................................................................    3         1       sr1
    ..................uaucuggaauccaucugacaaacA....................................................................    2         1       sr1
    ..................uaucuggaGuccaucugacaaacu....................................................................    1         1       sr1
    ..................uaucuggaauccaucugacaaacu....................................................................    6         0       sr1
    ..................Gaucuggaauccaucugacaaacu....................................................................    1         1       sr1
    ..................uaucuggaauccaucugacaaacCg...................................................................    1         1       sr1
    ..................uaucuggaauccaucugacaaacug...................................................................    33        0       sr1
    ..................Aaucuggaauccaucugacaaacug...................................................................    3         1       sr1
    ..................uaucuggaGuccaucugacaaacug...................................................................    3         1       sr1
    ..................uaucuggaauccaucugacaGacug...................................................................    1         1       sr1
    ..................Gaucuggaauccaucugacaaacug...................................................................    2         1       sr1
    ..................uaucuggaauccaucugacaaacuA...................................................................    5         1       sr1
    ..................uCucuggaauccaucugacaaacug...................................................................    17        1       sr1
    ..................uaucuggaGuccaucugacaaacugc..................................................................    1         1       sr1
    ...................Cucuggaauccaucugac.........................................................................    1         1       sr1
    ...................Cucuggaauccaucugaca........................................................................    2         1       sr1
    ...................Cucuggaauccaucugacaa.......................................................................    3         1       sr1
    ...................aucuggaaCccaucugacaaa......................................................................    2         1       sr1
    ...................Cucuggaauccaucugacaaa......................................................................    4         1       sr1
    ...................Cucuggaauccaucugacaaac.....................................................................    55        1       sr1
    ...................aucuggaauccaucugacaaacu....................................................................    1         0       sr1
```

```
                    Mature                         Star
acuuuauucuuuuuuuuuuaucuggaauccaucugacaaacugcauguuugucaugcaguuugucauggauuccaguuaaaaaaaagcaugcaaucguacgucaaggaga
...................Cucuggaauccaucugacaaacu....................................................................   67     1   sr1
...................Cucuggaauccaucugacaaacug...................................................................   1123   1   sr1
...................Uucuggaauccaucugacaaacug...................................................................   4      1   sr1
...................aucuggaauccaucugacaaacug...................................................................   8      0   sr1
...................Gucuggaauccaucugacaaacug...................................................................   22     1   sr1
...................Cucuggaauccaucugacaaacugc..................................................................   16     1   sr1
...................Gucuggaauccaucugacaaacugca.................................................................   1      1   sr1
...................Cucuggaauccaucugacaaacugca.................................................................   27     1   sr1
....................ucuggaCuccaucugaca........................................................................   1      1   sr1
....................ucuggaGuccaucugaca........................................................................   18     1   sr1
....................ucCggaauccaucugaca........................................................................   5      1   sr1
....................Gcuggaauccaucugaca........................................................................   94     1   sr1
....................ucuggGauccaucugaca........................................................................   12     1   sr1
....................ucuggaauccaucugacC........................................................................   4      1   sr1
....................ucuggaauccaucCgaca........................................................................   8      1   sr1
....................ucuggaauccaucugUca........................................................................   3      1   sr1
....................ucuggaauccaGcugaca........................................................................   1      1   sr1
....................ucuggaauccaucGgaca........................................................................   2      1   sr1
....................ucuCgaauccaucugaca........................................................................   1      1   sr1
....................ucuggaauccaucuAaca........................................................................   2      1   sr1
....................ucuggaauccGucugaca........................................................................   5      1   sr1
....................ucuggaaCccaucugaca........................................................................   6      1   sr1
....................ucuggaaucAaucugaca........................................................................   2      1   sr1
....................ucuggaauccaCcugaca........................................................................   7      1   sr1
....................ucAggaauccaucugaca........................................................................   1      1   sr1
....................ucuggaauccaucugacG........................................................................   132    1   sr1
....................ucuggaauccaucugaUa........................................................................   1      1   sr1
....................ucuggaauccaucugacU........................................................................   18     1   sr1
....................ucuggaauccaucugaca........................................................................   1950   0   sr1
....................ucuggaauccaucugGca........................................................................   21     1   sr1
....................ucuggaauccaucugaAa........................................................................   4      1   sr1
....................ucuggaauccCucugaca........................................................................   1      1   sr1
....................ucugAaauccaucugaca........................................................................   3      1   sr1
....................ucuggaauccauUugaca........................................................................   3      1   sr1
....................ucuAgaauccaucugaca........................................................................   1      1   sr1
....................ucuggaauccauGugaca........................................................................   2      1   sr1
....................ucGggaauccaucugaca........................................................................   2      1   sr1
....................ucuggaauccaucAgaca........................................................................   1      1   sr1
....................ucuggaauUcaucugaca........................................................................   4      1   sr1
....................ucuggGauccaucugacaa.......................................................................   8      1   sr1
....................ucuggaauccaucCgacaa.......................................................................   2      1   sr1
....................ucuggaauccaucugCcaa.......................................................................   1      1   sr1
....................ucuggaaCccaucugacaa.......................................................................   3      1   sr1
....................ucuggaauccGucugacaa.......................................................................   7      1   sr1
....................ucuggUauccaucugacaa.......................................................................   1      1   sr1
....................ucuggaauccaAcugacaa.......................................................................   1      1   sr1
....................uAuggaauccaucugacaa.......................................................................   1      1   sr1
....................ucuggaauccaucugaAaa.......................................................................   1      1   sr1
....................ucuggaauccaucugUcaa.......................................................................   1      1   sr1
....................ucuggaauccaucugacUa.......................................................................   2      1   sr1
....................ucCggaauccaucugacaa.......................................................................   3      1   sr1
....................Acuggaauccaucugacaa.......................................................................   1      1   sr1
....................ucuggaauccaucugacaU.......................................................................   17     1   sr1
....................ucugAaauccaucugacaa.......................................................................   1      1   sr1
....................ucGggaauccaucugacaa.......................................................................   1      1   sr1
....................ucuggaGuccaucugacaa.......................................................................   25     1   sr1
....................ucuggaauccauUugacaa.......................................................................   1      1   sr1
....................ucuggaauccaucugGcaa.......................................................................   28     1   sr1
....................ucuggaauccaucugacaC.......................................................................   1      1   sr1
....................ucuAgaauccaucugacaa.......................................................................   2      1   sr1
....................ucuggaauccaucAgacaa.......................................................................   1      1   sr1
....................ucuggaauccaucugaUaa.......................................................................   1      1   sr1
....................ucuggaauccaucugacCa.......................................................................   1      1   sr1
....................ucuggaauccaCcugacaa.......................................................................   4      1   sr1
....................ucuggaauccaucuUacaa.......................................................................   2      1   sr1
....................ucuggaauccaucugacGa.......................................................................   7      1   sr1
....................ucuggaauccUucugacaa.......................................................................   1      1   sr1
....................ucuggaauccaGcugacaa.......................................................................   1      1   sr1
....................ucuggaauccaucugacaa.......................................................................   2033   0   sr1
....................ucuggaauccaucugacaG.......................................................................   95     1   sr1
```

```
                    Mature                               Star
acuuuauucuuuuuuuuuuaucuggaauccaucugacaaacugcauguuugucaugcaguuugucauggauuccaguuaaaaaaaagcaugcaaucguacgucaaggaga
....................Gcuggaauccaucugacaa.......................................................................	40	1	sr1
....................ucuggaauccaucuAacaa.......................................................................	3	1	sr1
....................ucuggaauccaucugacaaC......................................................................	14	1	sr1
....................ucuggaauccaucugacCaa......................................................................	2	1	sr1
....................ucuggaauccaucugacaaa......................................................................	4111	0	sr1
....................ucuggaauccCucugacaaa......................................................................	1	1	sr1
....................ucuUgaauccaucugacaaa......................................................................	2	1	sr1
....................ucuggaauUcaucugacaaa......................................................................	7	1	sr1
....................ucuggaauccaucugaUaaa......................................................................	2	1	sr1
....................uUuggaauccaucugacaaa......................................................................	2	1	sr1
....................Acuggaauccaucugacaaa......................................................................	4	1	sr1
....................ucuggaauccaucugacaUa......................................................................	1	1	sr1
....................ucCggaauccaucugacaaa......................................................................	6	1	sr1
....................ucuggaauccaucugaAaaa......................................................................	6	1	sr1
....................ucuggaauccaAcugacaaa......................................................................	1	1	sr1
....................ucuggaGuccaucugacaaa......................................................................	45	1	sr1
....................ucuggaauccaucugacUaa......................................................................	9	1	sr1
....................ucuggaauccaucAgacaaa......................................................................	2	1	sr1
....................Gcuggaauccaucugacaaa......................................................................	57	1	sr1
....................ucuggaaCccaucugacaaa......................................................................	7	1	sr1
....................ucuggaaAccaucugacaaa......................................................................	3	1	sr1
....................ucuggaaucAaucugacaaa......................................................................	2	1	sr1
....................ucuggaauccaucugacaGa......................................................................	94	1	sr1
....................ucuggaauccGucugacaaa......................................................................	7	1	sr1
....................ucuggaauccauUugacaaa......................................................................	3	1	sr1
....................ucuggaauccaucugGcaaa......................................................................	45	1	sr1
....................ucuggaauccaucuAacaaa......................................................................	2	1	sr1
....................ucugAaauccaucugacaaa......................................................................	4	1	sr1
....................ucAggaauccaucugacaaa......................................................................	1	1	sr1
....................ucuggaauccaucCgacaaa......................................................................	12	1	sr1
....................ucuggaauccaCcugacaaa......................................................................	10	1	sr1
....................ucuggaauccaucugacGaa......................................................................	14	1	sr1
....................ucuggaauccaucugacaaG......................................................................	94	1	sr1
....................ucuggaauccaGcugacaaa......................................................................	1	1	sr1
....................ucuggUauccaucugacaaa......................................................................	1	1	sr1
....................ucuggaauccaucugacaaU......................................................................	7	1	sr1
....................ucuggaaucUaucugacaaa......................................................................	2	1	sr1
....................ucuggGauccaucugacaaa......................................................................	15	1	sr1
....................uGuggaauccaucugacaaa......................................................................	1	1	sr1
....................ucuggaauccaucugUcaaa......................................................................	2	1	sr1
....................ucuggaauccaucugacaaGc.....................................................................	439	1	sr1
....................Acuggaauccaucugacaaac.....................................................................	66	1	sr1
....................ucuggaauccaucugacaaaG.....................................................................	62	1	sr1
....................ucuggaauccaucugacaUac.....................................................................	18	1	sr1
....................ucuggaauccaAcugacaaac.....................................................................	25	1	sr1
....................ucuggaauccauAugacaaac.....................................................................	8	1	sr1
....................ucuggaauccaucugCcaaac.....................................................................	16	1	sr1
....................ucuggaauccCucugacaaac.....................................................................	3	1	sr1
....................ucuggaauccaucugacaaUc.....................................................................	28	1	sr1
....................ucuggUauccaucugacaaac.....................................................................	33	1	sr1
....................ucuggaauccaCcugacaaac.....................................................................	198	1	sr1
....................ucuggaauccaucugacCaac.....................................................................	10	1	sr1
....................ucuCgaauccaucugacaaac.....................................................................	5	1	sr1
....................ucuggaauccaucuCacaaac.....................................................................	2	1	sr1
....................ucuggaauccaGcugacaaac.....................................................................	15	1	sr1
....................ucuggCauccaucugacaaac.....................................................................	5	1	sr1
....................ucuggaUuccaucugacaaac.....................................................................	10	1	sr1
....................ucuggaauccaucugacaGac.....................................................................	1401	1	sr1
....................ucugCaauccaucugacaaac.....................................................................	2	1	sr1
....................ucuggaauccaucugacaaaU.....................................................................	5082	1	sr1
....................ucuggaauccaucuUacaaac.....................................................................	1	1	sr1
....................ucuggaauGcaucugacaaac.....................................................................	2	1	sr1
....................ucuggaauccaucGgacaaac.....................................................................	17	1	sr1
....................ucGggaauccaucugacaaac.....................................................................	11	1	sr1
....................ucuAgaauccaucugacaaac.....................................................................	58	1	sr1
....................ucuggaauccaucugUcaaac.....................................................................	43	1	sr1
....................ucuggaauccaucugGcaaac.....................................................................	1159	1	sr1
....................ucuggaGuccaucugacaaac.....................................................................	526	1	sr1
....................ucuggGauccaucugacaaac.....................................................................	334	1	sr1
....................ucuggaaGccaucugacaaac.....................................................................	8	1	sr1
```

```
                    Mature                                      Star
acuuuauucuuuuuuuuuuaucuggaauccaucugacaaacugcauguuugucaugcaguuugucauggauuccaguuaaaaaaaagcaugcaaucguacgucaaggaga
....................ucuggaCuccaucugacaaac.....................................................................    6      1    sr1
....................ucugAaauccaucugacaaac.....................................................................    34     1    sr1
....................ucCggaauccaucugacaaac.....................................................................    206    1    sr1
....................ucuggaauccUucugacaaac.....................................................................    24     1    sr1
....................ucuUgaauccaucugacaaac.....................................................................    8      1    sr1
....................ucugUaauccaucugacaaac.....................................................................    10     1    sr1
....................ucAggaauccaucugacaaac.....................................................................    45     1    sr1
....................ucuggaauAcaucugacaaac.....................................................................    6      1    sr1
....................ucuggaauccaucugacGaac.....................................................................    271    1    sr1
....................uAuggaauccaucugacaaac.....................................................................    12     1    sr1
....................ucuggaauccaucuAacaaac.....................................................................    26     1    sr1
....................ucuggaauccaucugacaaCc.....................................................................    47     1    sr1
....................uUuggaauccaucugacaaac.....................................................................    51     1    sr1
....................ucuggaauccGucugacaaac.....................................................................    151    1    sr1
....................ucuggaauccaucugacaaaA.....................................................................    952    1    sr1
....................ucuggaauccaucAgacaaac.....................................................................    58     1    sr1
....................ucuggaauUcaucugacaaac.....................................................................    53     1    sr1
....................ucuggaaucAaucugacaaac.....................................................................    10     1    sr1
....................ucuggaauccaucugacaaac.....................................................................    68771  0    sr1
....................uGuggaauccaucugacaaac.....................................................................    1      1    sr1
....................ucuggaaCccaucugacaaac.....................................................................    187    1    sr1
....................ucuggaaucUaucugacaaac.....................................................................    51     1    sr1
....................ucuggaauccaucugaAaaac.....................................................................    14     1    sr1
....................ucuggaauccaucCgacaaac.....................................................................    254    1    sr1
....................Gcuggaauccaucugacaaac.....................................................................    1982   1    sr1
....................ucuggaauccauUugacaaac.....................................................................    36     1    sr1
....................ucuggaauccaucugacUaac.....................................................................    48     1    sr1
....................ucuggaauccaucugacaCac.....................................................................    6      1    sr1
....................ucuggaauccaucugaUaaac.....................................................................    37     1    sr1
....................ucuggaAccaucugacaaac......................................................................    16     1    sr1
....................ucuggaauccaucugaUaaacu....................................................................    53     1    sr1
....................Gcuggaauccaucugacaaacu....................................................................    3889   1    sr1
....................uGuggaauccaucugacaaacu....................................................................    2      1    sr1
....................ucuggGauccaucugacaaacu....................................................................    389    1    sr1
....................ucuggaaucUaucugacaaacu....................................................................    77     1    sr1
....................ucuggaauccaucugacaaacG....................................................................    3555   1    sr1
....................ucuggaauccaucugacGaacu....................................................................    352    1    sr1
....................ucuggaauccauUugacaaacu....................................................................    32     1    sr1
....................ucGggaauccaucugacaaacu....................................................................    16     1    sr1
....................ucuggaauccaucugacaaaUu....................................................................    61     1    sr1
....................ucuggaauccaucugacaUacu....................................................................    19     1    sr1
....................ucCggaauccaucugacaaacu....................................................................    203    1    sr1
....................ucuggaauccaucugacUaacu....................................................................    87     1    sr1
....................ucuggaaucAaucugacaaacu....................................................................    10     1    sr1
....................ucuggaauccaucAgacaaacu....................................................................    61     1    sr1
....................uAuggaauccaucugacaaacu....................................................................    16     1    sr1
....................ucuggaauccaucugacaaCcu....................................................................    28     1    sr1
....................ucuggaauccauGugacaaacu....................................................................    2      1    sr1
....................ucuggaauccaucugacCaacu....................................................................    15     1    sr1
....................ucuggaauccGucugacaaacu....................................................................    183    1    sr1
....................ucugAaauccaucugacaaacu....................................................................    63     1    sr1
....................ucuggaauccaucugacaaaAu....................................................................    22     1    sr1
....................ucuggaGuccaucugacaaacu....................................................................    772    1    sr1
....................ucuggaauGcaucugacaaacu....................................................................    2      1    sr1
....................ucuggaauccCucugacaaacu....................................................................    10     1    sr1
....................ucuggaaGccaucugacaaacu....................................................................    6      1    sr1
....................ucuggaUuccaucugacaaacu....................................................................    12     1    sr1
....................ucuggaauccaucugaAaaacu....................................................................    10     1    sr1
....................ucugCaauccaucugacaaacu....................................................................    1      1    sr1
....................ucuUgaauccaucugacaaacu....................................................................    15     1    sr1
....................ucuggaauccaucugUcaaacu....................................................................    52     1    sr1
....................ucuggaauccaucugacaaacu....................................................................    79488  0    sr1
....................ucuggaauccaucugacaaaGu....................................................................    2      1    sr1
....................ucuggaaucGaucugacaaacu....................................................................    2      1    sr1
....................ucuAgaauccaucugacaaacu....................................................................    72     1    sr1
....................ucuggCauccaucugacaaacu....................................................................    9      1    sr1
....................ucuggaauccaucugacaaUcu....................................................................    23     1    sr1
....................ucuggaauccUucugacaaacu....................................................................    16     1    sr1
....................ucuggaaCccaucugacaaacu....................................................................    167    1    sr1
....................ucuggaauAcaucugacaaacu....................................................................    11     1    sr1
```

Mature Star

```
acuuuauucuuuuuuuuuuaucuggaauccaucugacaaacugcauguuugucaugcaguuugucauggauuccaguuaaaaaaaagcaugcaaucguacgucaaggaga
....................ucuggaauccaucugGcaaacu....................................................................   1009    1   sr1
....................ucuggaauccaucCgacaaacu....................................................................   276     1   sr1
....................ucuggaauUcaucugacaaacu....................................................................   52      1   sr1
....................ucuggaauccaucuAacaaacu....................................................................   50      1   sr1
....................ucuggaauccaucugacaGacu....................................................................   1607    1   sr1
....................ucuggaauccauAugacaaacu....................................................................   9       1   sr1
....................ucuggaauccaucuUacaaacu....................................................................   3       1   sr1
....................ucuggaauccaucugacaaacC....................................................................   10125   1   sr1
....................ucuggaauccaucugacaaacA....................................................................   3793    1   sr1
....................ucuggaauccaucGgacaaacu....................................................................   20      1   sr1
....................Acuggaauccaucugacaaacu....................................................................   55      1   sr1
....................ucuggUauccaucugacaaacu....................................................................   26      1   sr1
....................ucuggaauccaucuCacaaacu....................................................................   5       1   sr1
....................ucugUaauccaucugacaaacu....................................................................   11      1   sr1
....................ucuggaauccaGcugacaaacu....................................................................   13      1   sr1
....................ucuggaCuccaucugacaaacu....................................................................   7       1   sr1
....................ucuggaauccaAcugacaaacu....................................................................   29      1   sr1
....................uUuggaauccaucugacaaacu....................................................................   60      1   sr1
....................ucAggaauccaucugacaaacu....................................................................   53      1   sr1
....................ucuggaaAccaucugacaaacu....................................................................   15      1   sr1
....................ucuggaauccaucugacaaGcu....................................................................   468     1   sr1
....................ucuCgaauccaucugacaaacu....................................................................   6       1   sr1
....................ucuggaauccaCcugacaaacu....................................................................   217     1   sr1
....................ucuggaauccaucugCcaaacu....................................................................   19      1   sr1
....................ucuggaauccaucugacaCacu....................................................................   8       1   sr1
....................ucuggaauccaucugacaaaAug...................................................................   180     1   sr1
....................ucCggaauccaucugacaaacug...................................................................   2617    1   sr1
....................ucuggaauccaucugacaGacug...................................................................   37614   1   sr1
....................ucuggaaCccaucugacaaacug...................................................................   2646    1   sr1
....................uAuggaauccaucugacaaacug...................................................................   192     1   sr1
....................ucuggaauccaucCgacaaacug...................................................................   3354    1   sr1
....................ucuggaaucGaucugacaaacug...................................................................   42      1   sr1
....................ucugUaauccaucugacaaacug...................................................................   129     1   sr1
....................ucuggaauccauGugacaaacug...................................................................   24      1   sr1
....................ucuggaauccaucugUcaaacug...................................................................   588     1   sr1
....................ucuggaauccaucugacaaacuU...................................................................   7056    1   sr1
....................ucuggaauccaucugacaaacGg...................................................................   1381    1   sr1
....................ucuggaauccaucugacaaaUug...................................................................   595     1   sr1
....................ucuggaauccaAcugacaaacug...................................................................   285     1   sr1
....................ucuggaauccaucGgacaaacug...................................................................   213     1   sr1
....................Gcuggaauccaucugacaaacug...................................................................   51599   1   sr1
....................ucuggaauccaucugacaaacAg...................................................................   907     1   sr1
....................ucuggaauccaucugacaaacuC...................................................................   4634    1   sr1
....................ucuggaaAccaucugacaaacug...................................................................   167     1   sr1
....................ucuggaauccUucugacaaacug...................................................................   278     1   sr1
....................ucuggaauccaucugacaaacug...................................................................   992950  0   sr1
....................ucuggaauccaucugacaaaGug...................................................................   23      1   sr1
....................ucuggaauAcaucugacaaacug...................................................................   134     1   sr1
....................uGuggaauccaucugacaaacug...................................................................   37      1   sr1
....................ucuggUauccaucugacaaacug...................................................................   435     1   sr1
....................ucuggaauccaucugacaaacuA...................................................................   93644   1   sr1
....................ucuggaauccaucuCacaaacug...................................................................   28      1   sr1
....................ucuggaCuccaucugacaaacug...................................................................   100     1   sr1
....................ucuggaauccaucugacGaacug...................................................................   4017    1   sr1
....................Acuggaauccaucugacaaacug...................................................................   889     1   sr1
....................ucuggaauccaucugacaaCcug...................................................................   385     1   sr1
....................ucuggaauccaucugacaaGcug...................................................................   4366    1   sr1
....................ucuggaaGccaucugacaaacug...................................................................   122     1   sr1
....................ucugCaauccaucugacaaacug...................................................................   39      1   sr1
....................ucuggaauccaucuAacaaacug...................................................................   552     1   sr1
....................ucuggaauccaGcugacaaacug...................................................................   113     1   sr1
....................ucuggaauccaucugacaaUcug...................................................................   257     1   sr1
....................ucuCgaauccaucugacaaacug...................................................................   64      1   sr1
....................ucuggaauccaucugacaUacug...................................................................   345     1   sr1
....................ucuggaUuccaucugacaaacug...................................................................   99      1   sr1
....................ucuggaauccCucugacaaacug...................................................................   91      1   sr1
....................ucuggaaucAaucugacaaacug...................................................................   157     1   sr1
....................ucuggaauccaucugaUaaacug...................................................................   646     1   sr1
....................ucuggCauccaucugacaaacug...................................................................   96      1   sr1
....................ucuggaauccaucugacaCacug...................................................................   112     1   sr1
```

```
                    Mature                 Star
acuuuauucuuuuuuuuuuaucuggaauccaucugacaaacugcauguuugucaugcaguuugucauggauuccaguuaaaaaaaagcaugcaaucguacgucaaggaga
....................ucuggaauccaucugacaaacCg...................................................................   12730   1   sr1
....................ucuggaauccGucugacaaacug...................................................................   2373    1   sr1
....................ucuggaauccaucAgacaaacug...................................................................   809     1   sr1
....................ucuggaauccaucugacCaacug...................................................................   207     1   sr1
....................ucuggaauccaucuUacaaacug...................................................................   50      1   sr1
....................uUuggaauccaucugacaaacug...................................................................   698     1   sr1
....................ucuggaauccaucugaAaaacug...................................................................   264     1   sr1
....................ucuggaauccaucugGcaaacug...................................................................   10976   1   sr1
....................ucuUgaauccaucugacaaacug...................................................................   209     1   sr1
....................ucuggaauUcaucugacaaacug...................................................................   619     1   sr1
....................ucuAgaauccaucugacaaacug...................................................................   721     1   sr1
....................ucuggaauccauUugacaaacug...................................................................   451     1   sr1
....................ucuggaaucUaucugacaaacug...................................................................   751     1   sr1
....................ucuggGauccaucugacaaacug...................................................................   4862    1   sr1
....................ucuggaauccaucugaGaaacug...................................................................   15      1   sr1
....................ucuggaauccaCcugacaaacug...................................................................   2581    1   sr1
....................ucuggaGuccaucugacaaacug...................................................................   13834   1   sr1
....................ucuggaauGcaucugacaaacug...................................................................   32      1   sr1
....................ucAggaauccaucugacaaacug...................................................................   688     1   sr1
....................ucuggaauccauAugacaaacug...................................................................   100     1   sr1
....................ucuggaauccaucugCcaaacug...................................................................   280     1   sr1
....................ucugAaauccaucugacaaacug...................................................................   613     1   sr1
....................ucuggaauccaucugacUaacug...................................................................   1018    1   sr1
....................ucGggaauccaucugacaaacug...................................................................   134     1   sr1
....................ucuggaauccaucugacaaacugA..................................................................   3312    1   sr1
....................ucuggaauccaucugacaaUcugc..................................................................   6       1   sr1
....................ucuggaGuccaucugacaaacugc..................................................................   225     1   sr1
....................ucuggaaucGaucugacaaacugc..................................................................   1       1   sr1
....................ucCggaauccaucugacaaacugc..................................................................   39      1   sr1
....................ucuggaauccaucugacaGacugc..................................................................   230     1   sr1
....................ucuggaauccaucugacaaacuAc..................................................................   124     1   sr1
....................ucuggaauccaucugacaaCcugc..................................................................   3       1   sr1
....................ucuggaauccaucCgacaaacugc..................................................................   48      1   sr1
....................ucugAaauccaucugacaaacugc..................................................................   11      1   sr1
....................ucuggaauccaucugacaaacugc..................................................................   14529   0   sr1
....................ucAggaauccaucugacaaacugc..................................................................   7       1   sr1
....................ucuggaauccaAcugacaaacugc..................................................................   4       1   sr1
....................ucuggUauccaucugacaaacugc..................................................................   3       1   sr1
....................ucuggaauAcaucugacaaacugc..................................................................   3       1   sr1
....................ucuggaauccaucugacaaacugG..................................................................   302     1   sr1
....................ucuggaauccUucugacaaacugc..................................................................   2       1   sr1
....................ucuggaauccaucugacaaaAugc..................................................................   1       1   sr1
....................Gcuggaauccaucugacaaacugc..................................................................   752     1   sr1
....................ucuggaauccaucugaAaaacugc..................................................................   4       1   sr1
....................ucuggaauccaucugacaaacuUc..................................................................   21      1   sr1
....................ucGggaauccaucugacaaacugc..................................................................   1       1   sr1
....................ucuggaauccaucugacaaacCgc..................................................................   28      1   sr1
....................ucuggaauccaucugacaaacGgc..................................................................   1       1   sr1
....................ucuggaauccauAugacaaacugc..................................................................   2       1   sr1
....................ucuggaauccaucugacaaaUugc..................................................................   7       1   sr1
....................ucugCaauccaucugacaaacugc..................................................................   1       1   sr1
....................ucuggCauccaucugacaaacugc..................................................................   4       1   sr1
....................ucuggaauccaucugacaCacugc..................................................................   1       1   sr1
....................ucuggaauccaCcugacaaacugc..................................................................   38      1   sr1
....................ucuggaaAccaucugacaaacugc..................................................................   3       1   sr1
....................ucuggaauccaucugacaUacugc..................................................................   9       1   sr1
....................ucuggaCuccaucugacaaacugc..................................................................   3       1   sr1
....................ucuggaauccaucugacaaGcugc..................................................................   52      1   sr1
....................ucuggaauccaucAgacaaacugc..................................................................   23      1   sr1
....................ucuggaauccaucugaUaaacugc..................................................................   11      1   sr1
....................ucuggaauccGucugacaaacugc..................................................................   34      1   sr1
....................ucuggaauccaucGgacaaacugc..................................................................   3       1   sr1
....................ucuggaaucUaucugacaaacugc..................................................................   8       1   sr1
....................ucuggaauccaucugacaaacugU..................................................................   2762    1   sr1
....................uUuggaauccaucugacaaacugc..................................................................   12      1   sr1
....................ucuggaauUcaucugacaaacugc..................................................................   9       1   sr1
....................ucuggaauccauUugacaaacugc..................................................................   12      1   sr1
....................ucuggaaucAaucugacaaacugc..................................................................   2       1   sr1
....................ucuggaauccaucugGcaaacugc..................................................................   173     1   sr1
....................ucuggaauccaucugCcaaacugc..................................................................   2       1   sr1
```

```
                    Mature                              Star
acuuuauucuuuuuuuuuuaucuggaauccaucugacaaacugcauguuugucaugcaguuugucauggauuccaguuaaaaaaaagcaugcaaucguacgucaaggaga
....................ucuggaauccaucugacaaacAgc..................................................................	7	1	sr1
....................ucuggaauccaucugUcaaacugc..................................................................	6	1	sr1
....................ucuggaaCccaucugacaaacugc..................................................................	44	1	sr1
....................Acuggaauccaucugacaaacugc..................................................................	16	1	sr1
....................ucuUgaauccaucugacaaacugc..................................................................	4	1	sr1
....................ucuggaauccaucugacaaacuCc..................................................................	38	1	sr1
....................ucuggaaGccaucugacaaacugc..................................................................	1	1	sr1
....................ucuggaauccaucugacGaacugc..................................................................	58	1	sr1
....................ucuggaUuccaucugacaaacugc..................................................................	2	1	sr1
....................ucuggaauccaucuAacaaacugc..................................................................	3	1	sr1
....................ucugUaauccaucugacaaacugc..................................................................	1	1	sr1
....................ucuggaauccaucugaGaaacugc..................................................................	1	1	sr1
....................ucuggGauccaucugacaaacugc..................................................................	70	1	sr1
....................ucuAgaauccaucugacaaacugc..................................................................	10	1	sr1
....................ucuggaauccaucugacUaacugc..................................................................	14	1	sr1
....................ucuggaauccGucugacaaacugca.................................................................	31	1	sr1
....................ucGggaauccaucugacaaacugca.................................................................	2	1	sr1
....................Gcuggaauccaucugacaaacugca.................................................................	340	1	sr1
....................ucuggaauccaucugacaaacuUca.................................................................	6	1	sr1
....................ucuggaauccaucugacaaCcugca.................................................................	3	1	sr1
....................ucuggaauccaCcugacaaacugca.................................................................	37	1	sr1
....................ucuggaUuccaucugacaaacugca.................................................................	4	1	sr1
....................ucuggaaAccaucugacaaacugca.................................................................	3	1	sr1
....................ucuAgaauccaucugacaaacugca.................................................................	20	1	sr1
....................ucugAaauccaucugacaaacugca.................................................................	10	1	sr1
....................ucuggaauccaucugacaaacugcU.................................................................	534	1	sr1
....................ucuggaauccaucugacaaGcugca.................................................................	36	1	sr1
....................ucuggUauccaucugacaaacugca.................................................................	8	1	sr1
....................ucuggaauccaucugacaaacugca.................................................................	15137	0	sr1
....................ucuggaGuccaucugacaaacugca.................................................................	99	1	sr1
....................ucuggaauAcaucugacaaacugca.................................................................	4	1	sr1
....................ucuggaauccaucugacaaacuAca.................................................................	62	1	sr1
....................ucuggaauccaucuAacaaacugca.................................................................	11	1	sr1
....................Acuggaauccaucugacaaacugca.................................................................	6	1	sr1
....................ucuggaauccaucugacaaacCgca.................................................................	42	1	sr1
....................ucuggaauUcaucugacaaacugca.................................................................	13	1	sr1
....................ucuggaauccaucugacaaacAgca.................................................................	1	1	sr1
....................ucuggaauccaucugacaaacuCca.................................................................	34	1	sr1
....................ucuUgaauccaucugacaaacugca.................................................................	3	1	sr1
....................ucuggaauccCucugacaaacugca.................................................................	1	1	sr1
....................ucuggaauccauAugacaaacugca.................................................................	1	1	sr1
....................ucuggaauccaucugacaUacugca.................................................................	5	1	sr1
....................ucuggaauccUucugacaaacugca.................................................................	11	1	sr1
....................ucuggaauccaucugUcaaacugca.................................................................	9	1	sr1
....................ucuggaaGccaucugacaaacugca.................................................................	3	1	sr1
....................ucuggaauccaucugacaaacugcG.................................................................	859	1	sr1
....................ucuggaauccaucugacaaUcugca.................................................................	1	1	sr1
....................ucuggaaucAaucugacaaacugca.................................................................	4	1	sr1
....................ucuggaaucUaucugacaaacugca.................................................................	19	1	sr1
....................ucuggaauccaucuCacaaacugca.................................................................	1	1	sr1
....................uAuggaauccaucugacaaacugca.................................................................	2	1	sr1
....................ucugUaauccaucugacaaacugca.................................................................	2	1	sr1
....................ucuggaauccaucugaAaaacugca.................................................................	3	1	sr1
....................ucuggaauccaucugCcaaacugca.................................................................	5	1	sr1
....................ucuggaCuccaucugacaaacugca.................................................................	1	1	sr1
....................uGuggaauccaucugacaaacugca.................................................................	1	1	sr1
....................ucuggGauccaucugacaaacugca.................................................................	34	1	sr1
....................ucuggaaCccaucugacaaacugca.................................................................	47	1	sr1
....................ucuggaauccaucCgacaaacugca.................................................................	52	1	sr1
....................ucCggaauccaucugacaaacugca.................................................................	40	1	sr1
....................ucuggaauccauUugacaaacugca.................................................................	8	1	sr1
....................ucuggaauccaGcugacaaacugca.................................................................	3	1	sr1
....................ucuggaauccaucugacaaaAugca.................................................................	7	1	sr1
....................ucuggCauccaucugacaaacugca.................................................................	3	1	sr1
....................ucuggaauccaucugacaaacugAa.................................................................	31	1	sr1
....................ucuggaauccaucugacaGacugca.................................................................	192	1	sr1
....................ucuggaauccaucugacaCacugca.................................................................	2	1	sr1
....................ucuggaauccaucugaUaaacugca.................................................................	12	1	sr1
....................ucAggaauccaucugacaaacugca.................................................................	7	1	sr1
....................ucuggaauccaucugacGaacugca.................................................................	69	1	sr1
```

```
                    Mature                                     Star
acuuuauucuuuuuuuuuuaucuggaauccaucugacaaacugcauguuugucaugcaguuugucauggauuccaguuaaaaaaaagcaugcaaucguacgucaaggaga
....................ucuggaauccaucugacaaacugcC.................................................................    103    1    sr1
....................ucuggaauccaucAgacaaacugca.................................................................    12     1    sr1
....................ucuggaauccaucGgacaaacugca.................................................................    2      1    sr1
....................ucuggaauccaucugacaaaUugca.................................................................    14     1    sr1
....................ucuggaauccaucugacaaacugGa.................................................................    8      1    sr1
....................uUuggaauccaucugacaaacugca.................................................................    6      1    sr1
....................ucuggaauccaucugacUaacugca.................................................................    8      1    sr1
....................ucuggaauccaucugacaaacugUa.................................................................    3314   1    sr1
....................ucuggaauccaucugGcaaacugca.................................................................    217    1    sr1
....................ucuggaauccaAcugacaaacugca.................................................................    5      1    sr1
....................ucuggaauccaucugacaaacugcGu................................................................    4      1    sr1
....................ucuggaauccaucugacaGacugcau................................................................    9      1    sr1
....................ucuggaauccaucugacaaacugcCu................................................................    1      1    sr1
....................ucuggaGuccaucugacaaacugcau................................................................    2      1    sr1
....................ucuggaauccaucugacaaacGgcau................................................................    1      1    sr1
....................uUuggaauccaucugacaaacugcau................................................................    2      1    sr1
....................ucuggaauccaucugacaaacuCcau................................................................    2      1    sr1
....................ucCggaauccaucugacaaacugcau................................................................    3      1    sr1
....................ucuggaauccaucugacaaacCgcau................................................................    1      1    sr1
....................ucuggaauccaucugacaaacugcaG................................................................    761    1    sr1
....................ucuggaauccaucugacaaacugcUu................................................................    78     1    sr1
....................ucuggaauccaucugacaaacugcaC................................................................    38     1    sr1
....................ucuggaauccaucugacaaacugcaA................................................................    413    1    sr1
....................ucuggaauccaucugacaaacuUcau................................................................    1      1    sr1
....................ucuggaauccaucugacaaacugUau................................................................    51     1    sr1
....................ucuggaauccaucugaUaaacugcau................................................................    1      1    sr1
....................ucuggaauccaCcugacaaacugcau................................................................    4      1    sr1
....................Gcuggaauccaucugacaaacugcau................................................................    10     1    sr1
....................ucuggaauccaucugacaaacuAcau................................................................    2      1    sr1
....................ucuggaauccaucugGcaaacugcau................................................................    23     1    sr1
....................ucuggaauccaucugacaaacugGau................................................................    2      1    sr1
....................ucuggaauccaucugacaaacugcau................................................................    305    0    sr1
....................ucuggGauccaucugacaaacugcau................................................................    1      1    sr1
....................ucuggaauccaucugacaaGcugcau................................................................    1      1    sr1
....................ucuggaauccaucugacaaacugcUug...............................................................    12     1    sr1
....................ucuggaGuccaucugacaaacugcaug...............................................................    2      1    sr1
....................uUuggaauccaucugacaaacugcaug...............................................................    1      1    sr1
....................Gcuggaauccaucugacaaacugcaug...............................................................    7      1    sr1
....................ucuggaauccaucugacaaacugcauA...............................................................    1355   1    sr1
....................ucuggaauccaucugacaaacugcaug...............................................................    165    0    sr1
....................ucuggaauccaucugacaaacugcaAg...............................................................    17     1    sr1
....................Acuggaauccaucugacaaacugcaug...............................................................    1      1    sr1
....................ucuggaauccaucugacaaacugcGug...............................................................    1      1    sr1
....................ucuggaauUcaucugacaaacugcaug...............................................................    1      1    sr1
....................ucuggaauccaucugacaaacugcaGg...............................................................    1      1    sr1
....................ucuggaauccaucugacaaacugcaCg...............................................................    3      1    sr1
....................ucuggaauccaucugGcaaacugcaug...............................................................    5      1    sr1
....................ucCggaauccaucugacaaacugcaug...............................................................    2      1    sr1
....................ucuggaaCccaucugacaaacugcaug...............................................................    1      1    sr1
....................ucuggaauccaucugacaaacugUaug...............................................................    9      1    sr1
....................ucuggaauccaucugacaaacugcauU...............................................................    174    1    sr1
....................ucuggaauccaucugacaaacugAaug...............................................................    1      1    sr1
....................ucuggaauccaucugacaaacugcauC...............................................................    26     1    sr1
....................ucuggaaGccaucugacaaacugcaug...............................................................    1      1    sr1
....................ucAggaauccaucugacaaacugcaug...............................................................    1      1    sr1
....................ucuggaauccaCcugacaaacugcaug...............................................................    1      1    sr1
....................ucuggaauccaucCgacaaacugcaug...............................................................    2      1    sr1
.....................cuggaauccaucugacaa.......................................................................    1      0    sr1
.....................Guggaauccaucugacaaa......................................................................    1      1    sr1
.....................cuggaauccaucugacaaa......................................................................    5      0    sr1
.....................Guggaauccaucugacaaac.....................................................................    2      1    sr1
.....................cuggaauccaucugacaaUc.....................................................................    1      1    sr1
.....................cuggaauccaucugacaaGc.....................................................................    1      1    sr1
.....................cuggaauccaucugacaaac.....................................................................    33     0    sr1
.....................cuggaauccaucugacGaac.....................................................................    1      1    sr1
.....................cuggaauccaucugacaaaU.....................................................................    8      1    sr1
.....................cuggGauccaucugacaaac.....................................................................    1      1    sr1
.....................cuggaauccaucugacaaaA.....................................................................    2      1    sr1
.....................Uuggaauccaucugacaaac.....................................................................    4      1    sr1
.....................cuggaauccaucugacaGacu....................................................................    4      1    sr1
```

Mature **Star**

```
acuuuauucuuuuuuuuuuaucuggaauccaucugacaaacugcauguuugucaugcaguuugucauggauuccaguuaaaaaaaagcaugcaaucguacgucaaggaga
.....................cuggaauccaucugacaaacu....................................................................  78    0  sr1
.....................Guggaauccaucugacaaacu....................................................................  2     1  sr1
.....................cuggaauccaucugacaaacC....................................................................  11    1  sr1
.....................cuggaauccaucugacaaacA....................................................................  3     1  sr1
.....................cuggaauccaucugacaaacG....................................................................  5     1  sr1
.....................cuggaaCccaucugacaaacu....................................................................  1     1  sr1
.....................Uuggaauccaucugacaaacu....................................................................  1     1  sr1
.....................cuggaauccaucugacUaacug...................................................................  1     1  sr1
.....................cuggaaucAaucugacaaacug...................................................................  1     1  sr1
.....................cuggaauccGucugacaaacug...................................................................  5     1  sr1
.....................cuggaauccaucugacaaacCg...................................................................  11    1  sr1
.....................cuggaaucUaucugacaaacug...................................................................  2     1  sr1
.....................cugUaauccaucugacaaacug...................................................................  1     1  sr1
.....................cuggaauccaucugCcaaacug...................................................................  1     1  sr1
.....................cuggaauccaucCgacaaacug...................................................................  4     1  sr1
.....................cuggaauccaucGgacaaacug...................................................................  1     1  sr1
.....................cuggaauUcaucugacaaacug...................................................................  1     1  sr1
.....................cuggaauccaucugacaCacug...................................................................  2     1  sr1
.....................cuUgaauccaucugacaaacug...................................................................  1     1  sr1
.....................cuggaauccaucugacaaacug...................................................................  1065  0  sr1
.....................cuggaauccaucugacaGacug...................................................................  32    1  sr1
.....................cuggaauccaucugacaaUcug...................................................................  1     1  sr1
.....................Guggaauccaucugacaaacug...................................................................  14    1  sr1
.....................cuggaauccaCcugacaaacug...................................................................  3     1  sr1
.....................cuggaauccaucuAacaaacug...................................................................  1     1  sr1
.....................cuggaauccaucAgacaaacug...................................................................  2     1  sr1
.....................cuggaauccaucugacaaacuA...................................................................  103   1  sr1
.....................cuggGauccaucugacaaacug...................................................................  6     1  sr1
.....................cuggaauccaucugacaUacug...................................................................  1     1  sr1
.....................cuggaaGccaucugacaaacug...................................................................  1     1  sr1
.....................cuggaauccaucugacaaacGg...................................................................  1     1  sr1
.....................cCggaauccaucugacaaacug...................................................................  11    1  sr1
.....................cuggaauccaAcugacaaacug...................................................................  1     1  sr1
.....................Uuggaauccaucugacaaacug...................................................................  20    1  sr1
.....................cugAaauccaucugacaaacug...................................................................  1     1  sr1
.....................cuggaauccaucugacaaacuC...................................................................  5     1  sr1
.....................Auggaauccaucugacaaacug...................................................................  2     1  sr1
.....................cuggaauccaucugGcaaacug...................................................................  2     1  sr1
.....................cuggaaAccaucugacaaacug...................................................................  3     1  sr1
.....................cuggaauccaucugacaaGcug...................................................................  8     1  sr1
.....................cuggaauccaucugacaaacuU...................................................................  9     1  sr1
.....................cuggaGuccaucugacaaacug...................................................................  9     1  sr1
.....................cuggaauccauUugacaaacug...................................................................  1     1  sr1
.....................cuggaaCccaucugacaaacug...................................................................  2     1  sr1
.....................cuggaauccaucugUcaaacug...................................................................  3     1  sr1
.....................cAggaauccaucugacaaacug...................................................................  1     1  sr1
.....................cuggaauccaucugacGaacug...................................................................  4     1  sr1
.....................cuggaauccaucugGcaaacugc..................................................................  3     1  sr1
.....................cuggaauccaucugacaaacugA..................................................................  1     1  sr1
.....................cuggaauccCucugacaaacugc..................................................................  1     1  sr1
.....................cuggaauccaucugacaaacugU..................................................................  3     1  sr1
.....................cuggaauccaucugacaGacugc..................................................................  1     1  sr1
.....................cuggaauccaucugacaaacugc..................................................................  17    0  sr1
.....................cCggaauccaucugacaaacugca.................................................................  1     1  sr1
.....................cuggaauccaucugacaaacugUa.................................................................  12    1  sr1
.....................cuggaauccaucugacaaacugca.................................................................  12    0  sr1
.....................cuggaauccaucugacaaacugcaG................................................................  1     1  sr1
.....................cuggaauccaucugacaaacugcauA...............................................................  3     1  sr1
......................Gggaauccaucugacaaa......................................................................  1     1  sr1
......................uggaauccaucugacaaa......................................................................  9     0  sr1
......................uggGauccaucugacaaac.....................................................................  2     1  sr1
......................uggaauccaucugacaaaU.....................................................................  38    1  sr1
......................uAgaauccaucugacaaac.....................................................................  1     1  sr1
......................uggaauccaucugacaaUc.....................................................................  1     1  sr1
......................uggaauccGucugacaaac.....................................................................  1     1  sr1
......................uggaauccaucugacaGac.....................................................................  3     1  sr1
......................Aggaauccaucugacaaac.....................................................................  1     1  sr1
......................uggaauccaucugGcaaac.....................................................................  67    1  sr1
......................uggaauccaucCgacaaac.....................................................................  3     1  sr1
......................uggaauccaucugacaaGc.....................................................................  1     1  sr1
```

```
                    Mature                                   Star
acuuuauucuuuuuuuuuuaucuggaauccaucugacaaacugcauguuugucaugcaguuugucauggauuccaguuaaaaaaaagcaugcaaucguacgucaaggaga
......................Gggaauccaucugacaaac.....................................................................    20     1    sr1
......................uggaauccaucugacaaaA.....................................................................    6      1    sr1
......................uggaauccaCcugacaaac.....................................................................    1      1    sr1
......................Cggaauccaucugacaaac.....................................................................    1      1    sr1
......................uggaGuccaucugacaaac.....................................................................    3      1    sr1
......................uggaauccaucugacaaac.....................................................................    390    0    sr1
......................uggaauccaucugacGaac.....................................................................    1      1    sr1
......................uggaaCccaucugacaaac.....................................................................    2      1    sr1
......................uggaauccaucugGcaaacu....................................................................    38     1    sr1
......................uggaauccaucugacGaacu....................................................................    3      1    sr1
......................uggaGuccaucugacaaacu....................................................................    3      1    sr1
......................uggaauccaucugacaaacC....................................................................    42     1    sr1
......................uggGauccaucugacaaacu....................................................................    3      1    sr1
......................Aggaauccaucugacaaacu....................................................................    1      1    sr1
......................uggaauccaucugacUaacu....................................................................    1      1    sr1
......................uggaauccaucCgacaaacu....................................................................    2      1    sr1
......................uggaauccaucugacaaacG....................................................................    18     1    sr1
......................uggaauccUucugacaaacu....................................................................    1      1    sr1
......................uggaaucAaucugacaaacu....................................................................    1      1    sr1
......................uggaauccaucugacaGacu....................................................................    7      1    sr1
......................uggaauccaucugacaaacu....................................................................    372    0    sr1
......................uggaauccaucugacaaacA....................................................................    20     1    sr1
......................Gggaauccaucugacaaacu....................................................................    27     1    sr1
......................uggaauccaucugacaaUcug...................................................................    1      1    sr1
......................uggaauUcaucugacaaacug...................................................................    5      1    sr1
......................uggaauccaucugacCaacug...................................................................    1      1    sr1
......................uggaaAccaucugacaaacug...................................................................    1      1    sr1
......................uggaCuccaucugacaaacug...................................................................    4      1    sr1
......................uggaauccaucugacaaaAug...................................................................    6      1    sr1
......................uggaauccaucGgacaaacug...................................................................    4      1    sr1
......................uggaauccaucugacaaCcug...................................................................    1      1    sr1
......................uCgaauccaucugacaaacug...................................................................    1      1    sr1
......................uggaauccaucugaGaaacug...................................................................    1      1    sr1
......................uggaauccaucugUcaaacug...................................................................    5      1    sr1
......................uggaauccaucugacaaacuU...................................................................    38     1    sr1
......................uggaaucAaucugacaaacug...................................................................    2      1    sr1
......................uggaauccaucugaUaaacug...................................................................    6      1    sr1
......................uggaauccaucugacaGacug...................................................................    152    1    sr1
......................uggUauccaucugacaaacug...................................................................    5      1    sr1
......................uggaauccUucugacaaacug...................................................................    2      1    sr1
......................uggaaucUaucugacaaacug...................................................................    5      1    sr1
......................uAgaauccaucugacaaacug...................................................................    1      1    sr1
......................uggaauccaucugacaaacug...................................................................    5784   0    sr1
......................uggaGuccaucugacaaacug...................................................................    40     1    sr1
......................uggaauccaucugacaaacAg...................................................................    9      1    sr1
......................Cggaauccaucugacaaacug...................................................................    18     1    sr1
......................uggaauccauUugacaaacug...................................................................    5      1    sr1
......................uggaaCccaucugacaaacug...................................................................    8      1    sr1
......................uggaauccaucugacaUacug...................................................................    2      1    sr1
......................Gggaauccaucugacaaacug...................................................................    402    1    sr1
......................uggaauccaCcugacaaacug...................................................................    19     1    sr1
......................uggaauccaucugacaaGcug...................................................................    29     1    sr1
......................Aggaauccaucugacaaacug...................................................................    5      1    sr1
......................uggaauccaucugacaCacug...................................................................    1      1    sr1
......................uggaauccaucugacaaacuC...................................................................    14     1    sr1
......................uggaauccaucugacaaacGg...................................................................    5      1    sr1
......................uggaauccaucugacaaaUug...................................................................    12     1    sr1
......................uggaauccaucugacGaacug...................................................................    17     1    sr1
......................uggaauccaucugaAaaacug...................................................................    1      1    sr1
......................uggaauccaucugGcaaacug...................................................................    345    1    sr1
......................uggaauccaucAgacaaacug...................................................................    5      1    sr1
......................uUgaauccaucugacaaacug...................................................................    4      1    sr1
......................uggaUuccaucugacaaacug...................................................................    1      1    sr1
......................uggGauccaucugacaaacug...................................................................    35     1    sr1
......................uggaauccaucugacaaacuA...................................................................    445    1    sr1
......................uggaauccaucCgacaaacug...................................................................    19     1    sr1
......................uggaauccaucugacaaacCg...................................................................    64     1    sr1
......................uggaauccaucuAacaaacug...................................................................    1      1    sr1
......................ugAaauccaucugacaaacug...................................................................    2      1    sr1
......................uggaauccaucugacUaacug...................................................................    6      1    sr1
```

Mature Star

acuuuauucuuuuuuuuuuaucuggaauccaucugacaaacugcauguuugucaugcaguuugucauggauuccaguuaaaaaaaagcaugcaaucguacgucaaggaga			
......................uggaauccaucuUacaaacug...	1	1	sr1
......................uggaauccaAcugacaaacug...	4	1	sr1
......................uggaauccGucugacaaacug...	10	1	sr1
......................uggaaGccaucugacaaacug...	1	1	sr1
......................uggaauccaucugGcaaacugc..	17	1	sr1
......................uggaauccaucugacaaacuCc..	1	1	sr1
......................uggaauccaucugacGaacugc..	1	1	sr1
......................uggaauccaucugacaaCcugc..	1	1	sr1
......................uggaauccaCcugacaaacugc..	1	1	sr1
......................uggaauccaucugacaaacugU..	52	1	sr1
......................uggaauccaucugacaaacugG..	1	1	sr1
......................uggaGuccaucugacaaacugc..	3	1	sr1
......................uggaauccaucugacaaacugA..	15	1	sr1
......................uggaauccaucugacaaacugc..	262	0	sr1
......................Gggaauccaucugacaaacugc..	13	1	sr1
......................uggaauccaucugacaaacuAc..	1	1	sr1
......................uggaauccaucugaUaaacugc..	2	1	sr1
......................uggGauccaucugacaaacugc..	3	1	sr1
......................uggaauccaucugacaGacugc..	5	1	sr1
......................Cggaauccaucugacaaacugc..	1	1	sr1
......................uggaauccaucugacaaacCgca...	1	1	sr1
......................uggaauccaucugacaaacugcU...	9	1	sr1
......................uggaauccaucugacaGacugca...	2	1	sr1
......................uggaauccaucugacaaacuAca...	1	1	sr1
......................uggaauccaucugacaaacugcG...	21	1	sr1
......................uggaauccaucugacGaacugca...	3	1	sr1
......................uggaauccaucugacaaacugcC...	4	1	sr1
......................uggaauccaucugacaaacugUa...	25	1	sr1
......................uggaauccaucugUcaaacugca...	1	1	sr1
......................uggaauccaucugacaaaUugca...	1	1	sr1
......................Gggaauccaucugacaaacugca...	26	1	sr1
......................uggaauccaucugacaaacugca...	282	0	sr1
......................uggaGuccaucugacaaacugca...	1	1	sr1
......................Aggaauccaucugacaaacugca...	1	1	sr1
......................uggaauccaucCgacaaacugca...	1	1	sr1
......................uggaauccaucugGcaaacugca...	24	1	sr1
......................uggaauccaucugacaaacugcaC..	12	1	sr1
......................uggaauccaucCgacaaacugcau..	1	1	sr1
......................uggaauccaucugacaaacugcaA..	69	1	sr1
......................uggaauccaucugacaaacugcau..	129	0	sr1
......................uggaauccaucugUcaaacugcau..	1	1	sr1
......................uggaauccaucugGcaaacugcau..	13	1	sr1
......................uggaauccaucugacaGacugcau..	2	1	sr1
......................uggaauccaucugacaaacugcaG..	69	1	sr1
......................uggaauccaucugacaaacugcUu..	7	1	sr1
......................uggaaAccaucugacaaacugcau..	1	1	sr1
......................uggaauccaucugacaaacugcGu..	2	1	sr1
......................Gggaauccaucugacaaacugcau..	15	1	sr1
......................uggaauccaucugacaaGcugcau..	1	1	sr1
......................uggaauccaucuUacaaacugcau..	1	1	sr1
......................uggaauccaucugacaaacugcCu..	1	1	sr1
......................Gggaauccaucugacaaacugcaug...	2	1	sr1
......................uggaauccaucugacaaacugcaug...	24	0	sr1
......................uggaauccaucugacaaacugcauU...	62	1	sr1
......................uggaauccaucugacaaacugcauA...	577	1	sr1
......................uggaauccaucugGcaaacugcaug...	3	1	sr1
......................uggaauccaucugacaaacugcaCg...	1	1	sr1
......................uggaauccaucugacaaacugcaAg...	2	1	sr1
......................uggaauccaucugacaaacugcauC...	10	1	sr1
......................uggaauccaucugacaaGcugcaug...	1	1	sr1
......................uggaauccaucugacaaacugUaug...	1	1	sr1
......................Aggaauccaucugacaaacugcaug...	1	1	sr1
......................uggaauccaucugacaaacugcauUu..	11	1	sr1
......................uggaauccaucugacaaacugcauAu..	36	1	sr1
......................uggaauccaucugacaaacugcauUuu...	4	1	sr1
......................uggaauccaucugacaaacugcauAuu...	6	1	sr1
.......................ggaauccaucugacaaac...	4	0	sr1
.......................ggaauccaucugacaaacA..	1	1	sr1
.......................ggaauccaucugacaaacu..	1	0	sr1
.......................ggaauccaucugacaaacug...	45	0	sr1

Mature   Star

```
acuuuauucuuuuuuuuuuaucuggaauccaucugacaaacugcauguuugucaugcaguuugucauggauuccaguuaaaaaaaagcaugcaaucguacgucaaggaga
.......................ggGauccaucugacaaacug...................................................................   1    1    sr1
.......................ggaauccaucugacaaacuC...................................................................   1    1    sr1
.......................ggaauccaucugacaGacug...................................................................   2    1    sr1
.......................ggaauccaucugacaaacuA...................................................................   3    1    sr1
.......................ggaauccaucCgacaaacug...................................................................   1    1    sr1
.......................ggaauccaucugacaaGcug...................................................................   2    1    sr1
.......................Ugaauccaucugacaaacug...................................................................   2    1    sr1
.......................ggaauccaucugacaaacuU...................................................................   2    1    sr1
.......................ggaauccaucugacaaacugc..................................................................   2    0    sr1
.......................ggaauccaucugacaGacugc..................................................................   1    1    sr1
.......................ggaauccaucugacaaacugU..................................................................   1    1    sr1
.......................ggaauccaucugacaaacugca.................................................................   4    0    sr1
.......................ggaauccaucugacaaacugUa.................................................................   1    1    sr1
.......................ggaauUcaucugacaaacugca.................................................................   1    1    sr1
........................gaauccaucCgacaaacug...................................................................   1    1    sr1
........................gaauccaucugacaaacug...................................................................   3    0    sr1
........................gaauccaucugacaaacugca.................................................................   2    0    sr1
........................gaauccaucugGcaaacugca.................................................................   1    1    sr1
.........................aauccaucugacaaacug...................................................................   1    0    sr1
.........................aauccaucugacaaacuA...................................................................   2    1    sr1
.........................aauccaucugacaaacugUa.................................................................   2    1    sr1
...........................uccaucugacaaacugca.................................................................   1    0    sr1
.............................caucugacaaacugcauA...............................................................   1    1    sr1
......................................................ugcaguuugucauggauuc.....................................   1    0    sr1
......................................................ugcaguuugucauggauucU....................................   1    1    sr1
........................................................caguuugucauggauuUc....................................   1    1    sr1
........................................................caguuugucauggauucc....................................   45   0    sr1
........................................................caguuugucauggauucA....................................   4    1    sr1
........................................................Gaguuugucauggauucc....................................   1    1    sr1
........................................................caguuugucauggauucU....................................   11   1    sr1
........................................................caguuugucauggauucca...................................   7    0    sr1
........................................................cUguuugucauggauuccag..................................   1    1    sr1
........................................................cagGuugucauggauuccag..................................   2    1    sr1
........................................................caguuugucauggauCccag..................................   1    1    sr1
........................................................caguuugucauggauuccGg..................................   1    1    sr1
........................................................Gaguuugucauggauuccag..................................   2    1    sr1
........................................................caguuugucauggauuccaA..................................   9    1    sr1
........................................................caguuugucauggauuccag..................................   101  0    sr1
........................................................caguuugucauggauuccagC.................................   5    1    sr1
........................................................caguuugucaugAauuccagu.................................   1    1    sr1
........................................................caguuugucauggauuccagA.................................   10   1    sr1
........................................................caguuugucauggauuccagG.................................   1    1    sr1
........................................................caguuugucauggauuccaUu.................................   1    1    sr1
........................................................caguuugucauggauuccagu.................................   44   0    sr1
........................................................caguuugCcauggauuccaguu................................   1    1    sr1
........................................................caguuugucauggauuccaguC................................   59   1    sr1
........................................................Gaguuugucauggauuccaguu................................   9    1    sr1
........................................................caguuugucauggauuccGguu................................   2    1    sr1
........................................................caguuCgucauggauuccaguu................................   4    1    sr1
........................................................caguuugucauggauuccaguu................................   457  0    sr1
........................................................caguuugucGuggauuccaguu................................   1    1    sr1
........................................................caguuugucaCggauuccaguu................................   1    1    sr1
........................................................caguuugucauggaCuccaguu................................   2    1    sr1
........................................................caguuugucauggauuccaguA................................   37   1    sr1
........................................................caguuugucUuggauuccaguu................................   2    1    sr1
........................................................cUguuugucauggauuccaguu................................   2    1    sr1
........................................................cGguuugucauggauuccaguu................................   2    1    sr1
........................................................caguuugucauggauuccaguG................................   16   1    sr1
........................................................caguuugucauggauuUcaguu................................   1    1    sr1
........................................................caguuugucauggauuccaguuC...............................   11   1    sr1
........................................................caguuugucauggauuccaguuG...............................   30   1    sr1
........................................................Uaguuugucauggauuccaguua...............................   1    1    sr1
........................................................Gaguuugucauggauuccaguua...............................   13   1    sr1
........................................................caguuugucauggaCuccaguua...............................   2    1    sr1
........................................................caguuugucauggauuccGguua...............................   3    1    sr1
........................................................caguuugucauggGuuccaguua...............................   1    1    sr1
........................................................caguuugucauggUuuccaguua...............................   2    1    sr1
........................................................caguuugucGuggauuccaguua...............................   5    1    sr1
........................................................caguuugucauggauuccaguuU...............................   63   1    sr1
........................................................caguuugucauggauucUaguua...............................   2    1    sr1
```

```
                    Mature                               Star
acuuuauucuuuuuuuuuuaucuggaauccaucugacaaacugcauguuugucaugcaguuugucauggauuccaguuaaaaaaaagcaugcaaucguacgucaaggaga
........................................................caguuugucaCggauuccaguua...............................   1     1   sr1
........................................................caguuugucaugUauuccaguua...............................   1     1   sr1
........................................................cGguuugucauggauuccaguua...............................   10    1   sr1
........................................................caguuugucauggauucAaguua...............................   1     1   sr1
........................................................caguuugucauggauuccaguCa...............................   4     1   sr1
........................................................caguuugucauggauuccaguAa...............................   2     1   sr1
........................................................caguuugucauggauuccagCua...............................   1     1   sr1
........................................................cUguuugucauggauuccaguua...............................   2     1   sr1
........................................................caguuugucauggauuUcaguua...............................   1     1   sr1
........................................................caguuCgucauggauuccaguua...............................   6     1   sr1
........................................................caguuugucauggauuccaguGa...............................   1     1   sr1
........................................................caguCugucauggauuccaguua...............................   4     1   sr1
........................................................Aaguuugucauggauuccaguua...............................   1     1   sr1
........................................................caguuugucauggauCccaguua...............................   3     1   sr1
........................................................caguuugCcauggauuccaguua...............................   1     1   sr1
........................................................cagGuugucauggauuccaguua...............................   1     1   sr1
........................................................caguuugucauggauuccaguua...............................   1233  0   sr1
........................................................cagCuugucauggauuccaguua...............................   6     1   sr1
........................................................caguuugucauggauuccaguuaG..............................   3     1   sr1
........................................................caguuugucauggauuccaguuCa..............................   1     1   sr1
........................................................caguuugucauggauuccaguuaC..............................   2     1   sr1
........................................................caguuugucauggauuccaguuUa..............................   33    1   sr1
........................................................caguuugucauggauuccaguuaU..............................   29    1   sr1
........................................................caguuugucauggauuccaguuaa..............................   29    0   sr1
........................................................caguuugucauggauuccaguuaGa.............................   1     1   sr1
........................................................caguuugucauggauuccaguuUaa.............................   1     1   sr1
........................................................caguuugucauggauuccaguuaaa.............................   1     0   sr1
........................................................caguuugucauggauuccaguuaUa.............................   7     1   sr1
.........................................................aguuugucauggauucca...................................   5     0   sr1
.........................................................aguuugucauggauuccG...................................   1     1   sr1
.........................................................aguuugucauggauuccag..................................   4     0   sr1
.........................................................aguuugucauggauCccag..................................   1     1   sr1
.........................................................Gguuugucauggauuccag..................................   1     1   sr1
.........................................................aguuugucauggauuccagA.................................   8     1   sr1
.........................................................aguuugucaCggauuccagu.................................   1     1   sr1
.........................................................aguuugucauggGuuccagu.................................   1     1   sr1
.........................................................Gguuugucauggauuccagu.................................   1     1   sr1
.........................................................aguuugucauggauuccagu.................................   54    0   sr1
.........................................................aguuugucauggauuccagC.................................   3     1   sr1
.........................................................aguuugucauggauuccagG.................................   7     1   sr1
.........................................................aguAugucauggauuccagu.................................   1     1   sr1
.........................................................aguuugCcauggauuccaguu................................   3     1   sr1
.........................................................aguuugucauggGuuccaguu................................   1     1   sr1
.........................................................aguuugucaugAauuccaguu................................   3     1   sr1
.........................................................aguuugucauggauucUaguu................................   1     1   sr1
.........................................................aguuugucauggauuccagGu................................   1     1   sr1
.........................................................aguuugucauggauuccaguu................................   753   0   sr1
.........................................................Uguuugucauggauuccaguu................................   4     1   sr1
.........................................................aguuugucauggauuccaguG................................   25    1   sr1
.........................................................aguuugucauggauuccaguA................................   41    1   sr1
.........................................................Gguuugucauggauuccaguu................................   22    1   sr1
.........................................................aguuugucauggauuccGguu................................   5     1   sr1
.........................................................aguuugucauggauuccagCu................................   3     1   sr1
.........................................................aguuugucGuggauuccaguu................................   5     1   sr1
.........................................................aguuugGcauggauuccaguu................................   1     1   sr1
.........................................................aguuugucauggauuccaguC................................   82    1   sr1
.........................................................aguuugucauggauuccUguu................................   1     1   sr1
.........................................................aguuCgucauggauuccaguu................................   1     1   sr1
.........................................................aguuugucauggaGuccaguu................................   2     1   sr1
.........................................................aguuugucauggauCccaguu................................   3     1   sr1
.........................................................aguuugucauggaCuccaguu................................   2     1   sr1
.........................................................agCuugucauggauuccaguu................................   1     1   sr1
.........................................................aguCugucauggauuccaguu................................   3     1   sr1
.........................................................aguCugucauggauuccaguua...............................   4     1   sr1
.........................................................aguuugucauggauuccaguAa...............................   3     1   sr1
.........................................................Gguuugucauggauuccaguua...............................   58    1   sr1
.........................................................aguuugucauggauuAcaguua...............................   1     1   sr1
.........................................................aguuugucauggauCccaguua...............................   2     1   sr1
.........................................................aguuugucauggauuccUguua...............................   1     1   sr1
.........................................................aguuugucauggauuccaguCa...............................   6     1   sr1
```

Mature **Star**

```
acuuuauucuuuuuuuuuuaucuggaauccaucugacaaacugcauguuugucaugcaguuugucauggauuccaguuaaaaaaaagcaugcaaucguacgucaaggaga
.........................................................aguuuguUauggauuccaguua...............................	3	1	sr1
.........................................................aguuugucauAgauuccaguua...............................	2	1	sr1
.........................................................aguuugucaugAauuccaguua...............................	1	1	sr1
.........................................................aguuugCcauggauuccaguua...............................	8	1	sr1
.........................................................aguuugucauggauuccaguua...............................	1843	0	sr1
.........................................................aguuugucaCggauuccaguua...............................	7	1	sr1
.........................................................aguuugGcauggauuccaguua...............................	1	1	sr1
.........................................................aguuugucauggauuccaguuU...............................	188	1	sr1
.........................................................aguuugucauggGuuccaguua...............................	4	1	sr1
.........................................................aguuugucauggauuccagCua...............................	1	1	sr1
.........................................................aguuugucauggauuccGguua...............................	2	1	sr1
.........................................................aguuugucauggauuccaguuC...............................	36	1	sr1
.........................................................aguuCgucauggauuccaguua...............................	6	1	sr1
.........................................................aguuuAucauggauuccaguua...............................	1	1	sr1
.........................................................aguAugucauggauuccaguua...............................	1	1	sr1
.........................................................agCuugucauggauuccaguua...............................	4	1	sr1
.........................................................aguuugucGuggauuccaguua...............................	3	1	sr1
.........................................................aguuugucauggauuUcaguua...............................	1	1	sr1
.........................................................aguuugucauggaCuccaguua...............................	8	1	sr1
.........................................................aguuugucauggauuccaguuG...............................	27	1	sr1
.........................................................aguuugucauggGuuccaguuaa..............................	1	1	sr1
.........................................................aguuugucGuggauuccaguuaa..............................	1	1	sr1
.........................................................aguuugucauggauuccaguuaC..............................	5	1	sr1
.........................................................aguuugucauggauuccaguuaa..............................	124	0	sr1
.........................................................aguuugucauggauuccaguAaa..............................	1	1	sr1
.........................................................aguuugucauggauuUcaguuaa..............................	1	1	sr1
.........................................................Gguuugucauggauuccaguuaa..............................	1	1	sr1
.........................................................aguuugucauggauuccaguuCa..............................	1	1	sr1
.........................................................aguuugucauggauCccaguuaa..............................	1	1	sr1
.........................................................aguuugucauggauuccaguuaU..............................	68	1	sr1
.........................................................aguuugucauggauuccaguuUa..............................	179	1	sr1
.........................................................Cguuugucauggauuccaguuaa..............................	1	1	sr1
.........................................................aguuugucauggauucAaguuaa..............................	1	1	sr1
.........................................................agGuugucauggauuccaguuaa..............................	1	1	sr1
.........................................................aguuugucauggauuccaguuaG..............................	20	1	sr1
.........................................................aguuugucauggauuccaguuaUa.............................	31	1	sr1
.........................................................aguuugucauggauuccaguuaGa.............................	2	1	sr1
.........................................................aguuugucauggauuccaguuUaa.............................	8	1	sr1
.........................................................aguuugucauggauuccaguuaaU.............................	3	1	sr1
.........................................................aguuugucauggauuccaguuaaa.............................	1	0	sr1
.........................................................aguuugucauggauuccaguuaaUa............................	1	1	sr1
.........................................................aguuugucauggauuccaguuaUaa............................	1	1	sr1
..........................................................guuugucauggauuccagC.................................	1	1	sr1
..........................................................guuugucauggauuccaguu................................	3	0	sr1
..........................................................guuugucauggauuccaguua...............................	1	0	sr1
...........................................................uuugucauggauuccaguG................................	1	1	sr1
...........................................................Guugucauggauuccaguu................................	1	1	sr1
...........................................................uuugucauggauuccaguC................................	2	1	sr1
...........................................................uuugucauggauuccaguA................................	1	1	sr1
...........................................................uuugucauggauuccaguu................................	14	0	sr1
...........................................................uuugucauggauuccagCua...............................	1	1	sr1
...........................................................uuugucauggauuccaguua...............................	99	0	sr1
...........................................................uuugucauggauuccaguuU...............................	9	1	sr1
...........................................................uuugucauggauCccaguua...............................	1	1	sr1
...........................................................uuugucauggauuccaguuG...............................	1	1	sr1
...........................................................uuugucauAgauuccaguua...............................	2	1	sr1
...........................................................uuugAcauggauuccaguua...............................	1	1	sr1
...........................................................Guugucauggauuccaguua...............................	13	1	sr1
...........................................................uuugucauggaCuccaguua...............................	1	1	sr1
...........................................................uuugucauggGuuccaguua...............................	1	1	sr1
...........................................................uuugucauggauuccGguua...............................	1	1	sr1
...........................................................uuugucauggauuccaguuaa..............................	24	0	sr1
...........................................................uuugucauggauuccaguuaG..............................	2	1	sr1
...........................................................uuAgucauggauuccaguuaa..............................	1	1	sr1
...........................................................uuugucauggauuccaguuUa..............................	12	1	sr1
...........................................................uuugucauggauuccaguuaU..............................	7	1	sr1
...........................................................Guugucauggauuccaguuaa..............................	3	1	sr1
...........................................................uuugucauggauuccaguuaC..............................	1	1	sr1
...........................................................uuugucauggauuccaguuaUa.............................	7	1	sr1
...........................................................uuugucauggauuccUguuaaa.............................	1	1	sr1
```

Mature Star

```
acuuuauucuuuuuuuuuuaucuggaauccaucugacaaacugcauguuugucaugcaguuugucauggauuccaguuaaaaaaaagcaugcaaucguacgucaaggaga
...........................................................uuugucauggauuccaguuUaa.............................    1    1    sr1
...........................................................uuugucauggauuccaguuaaUa............................    2    1    sr1
...........................................................uuugucauggauuccaguuaaaUa...........................    1    1    sr1
............................................................uugucauggauuccaguua...............................    1    0    sr1
............................................................uugucauggUuuccaguua...............................    1    1    sr1
............................................................uugucauggauuccaguuaU..............................    2    1    sr1
............................................................uugucauggauuccaguuaUa.............................    1    1    sr1
............................................................uugucauggauuccaguuaaUa............................    1    1    sr1
......................................................................................gAaugcaaucguacgucaagga..    1    1    sr1
........................................................................................augcaaucguacgucaaggag.    1    0    sr1

................uuuaucAggaauccaucugacaaa......................................................................    1    1    st1
................uuuaucuggaauccaucugacaaacuA...................................................................    1    1    st1
.................uuaucuggaauccaucugacaaac.....................................................................    1    0    st1
..................uaucuggaauccaucugacaa.......................................................................    1    0    st1
..................uaucuggaauccaucugacaaU......................................................................    1    1    st1
..................uaucuggaauccaucugacaaa......................................................................    4    0    st1
..................uaucuggaauccaucugacaaac.....................................................................    4    0    st1
..................uaucuggaauccaucugacaaaU.....................................................................    1    1    st1
..................uaucuggaauccaucugacaaacu....................................................................    5    0    st1
..................uaucuggaauccaucugacaaacC....................................................................    1    1    st1
..................uCucuggaauccaucugacaaacu....................................................................    1    1    st1
..................uaucuggaauccaucugacaaaGu....................................................................    1    1    st1
..................uaucuggaauccaucugacaaacuA...................................................................    1    1    st1
..................uCucuggaauccaucugacaaacug...................................................................    17   1    st1
..................uaucuggaauccaucugacaaacCg...................................................................    1    1    st1
..................uaucuggaGuccaucugacaaacug...................................................................    1    1    st1
..................uaucuggaauccaucugGcaaacug...................................................................    1    1    st1
..................Aaucuggaauccaucugacaaacug...................................................................    1    1    st1
..................uaucuggaauccaucugacaaacug...................................................................    12   0    st1
..................uaucuggaauccaucugacaGacug...................................................................    2    1    st1
..................uCucuggaauccaucugacaaacugc..................................................................    1    1    st1
..................uaucuggaauccaucugacaaacugA..................................................................    1    1    st1
...................Cucuggaauccaucugacaaac.....................................................................    29   1    st1
...................Cucuggaauccaucugacaaacu....................................................................    47   1    st1
...................Uucuggaauccaucugacaaacu....................................................................    1    1    st1
...................Cucuggaauccaucugacaaacug...................................................................    641  1    st1
...................aucuggaauccaucugacaaacug...................................................................    2    0    st1
...................Cucuggaauccaucugacaaacugc..................................................................    13   1    st1
...................Cucuggaauccaucugacaaacugca.................................................................    7    1    st1
....................ucuggaaCccaucugaca........................................................................    1    1    st1
....................ucuggaauccCucugaca........................................................................    1    1    st1
....................ucuggaauccaucCgaca........................................................................    2    1    st1
....................ucuggaauccaAcugaca........................................................................    1    1    st1
....................ucuggaauccaucugaca........................................................................    736  0    st1
....................Acuggaauccaucugaca........................................................................    2    1    st1
....................ucuggaauccGucugaca........................................................................    1    1    st1
....................ucuggaauccaucugGca........................................................................    11   1    st1
....................ucuggaGuccaucugaca........................................................................    7    1    st1
....................ucuggaauccaucugacU........................................................................    5    1    st1
....................ucuggaauccaucugUca........................................................................    1    1    st1
....................ucuggaauccaCcugaca........................................................................    6    1    st1
....................ucuggaauccaucuAaca........................................................................    1    1    st1
....................ucuggaauccaucAgaca........................................................................    2    1    st1
....................ucuggaaGccaucugaca........................................................................    1    1    st1
....................uAuggaauccaucugaca........................................................................    1    1    st1
....................ucuggaauccaucugacG........................................................................    25   1    st1
....................ucuggGauccaucugaca........................................................................    1    1    st1
....................ucuggaGuccaucugacaa.......................................................................    4    1    st1
....................ucuggaauccaucugaUaa.......................................................................    1    1    st1
....................uUuggaauccaucugacaa.......................................................................    2    1    st1
....................ucuggaauccaucugacGa.......................................................................    2    1    st1
....................ucuggaauccaucuCacaa.......................................................................    2    1    st1
....................ucuggaauccaGcugacaa.......................................................................    1    1    st1
....................ucuggaauccUucugacaa.......................................................................    2    1    st1
....................ucuggaauccaucCgacaa.......................................................................    4    1    st1
....................ucAggaauccaucugacaa.......................................................................    2    1    st1
....................ucuggaauccaCcugacaa.......................................................................    3    1    st1
....................Gcuggaauccaucugacaa.......................................................................    5    1    st1
....................ucuggaauccaucugGcaa.......................................................................    8    1    st1
```

```
                   Mature                                Star
acuuuauucuuuuuuuuuuaucuggaauccaucugacaaacugcauguuugucaugcaguuugucauggauuccaguuaaaaaaaagcaugcaaucguacgucaaggaga
....................ucCggaauccaucugacaa.......................................................................  5    1  st1
....................ucuggaauccaucugacUa.......................................................................  1    1  st1
....................ucuggaauccaucugacCa.......................................................................  1    1  st1
....................ucuggaauccaucugacaa.......................................................................  755  0  st1
....................ucuggGauccaucugacaa.......................................................................  4    1  st1
....................ucuggaauccaucugacaG.......................................................................  15   1  st1
....................ucuggaauccaAcugacaa.......................................................................  1    1  st1
....................ucuggaauccaucugacaU.......................................................................  6    1  st1
....................ucuggaauccaucAgacaa.......................................................................  1    1  st1
....................ucuggaauccaucuAacaa.......................................................................  2    1  st1
....................ucuggaaucUaucugacaaa......................................................................  1    1  st1
....................ucugAaauccaucugacaaa......................................................................  1    1  st1
....................ucuggaauccaucCgacaaa......................................................................  5    1  st1
....................ucuggGauccaucugacaaa......................................................................  5    1  st1
....................ucuggaaAccaucugacaaa......................................................................  1    1  st1
....................ucuggaauccaucugGcaaa......................................................................  7    1  st1
....................ucuggaauccaucugacGaa......................................................................  4    1  st1
....................ucuggaauccaGcugacaaa......................................................................  2    1  st1
....................ucCggaauccaucugacaaa......................................................................  1    1  st1
....................ucuggaauccaucugacaaC......................................................................  2    1  st1
....................ucuggaauccGucugacaaa......................................................................  4    1  st1
....................ucuggaauccaucugacaaG......................................................................  2    1  st1
....................ucuggaauccaucugacaGa......................................................................  16   1  st1
....................ucuggaaCccaucugacaaa......................................................................  2    1  st1
....................ucuggaauccaucuAacaaa......................................................................  1    1  st1
....................ucuggaGuccaucugacaaa......................................................................  4    1  st1
....................ucuggaauccaucugacaaa......................................................................  691  0  st1
....................ucuggaUuccaucugacaaac.....................................................................  3    1  st1
....................ucuUgaauccaucugacaaac.....................................................................  9    1  st1
....................ucuCgaauccaucugacaaac.....................................................................  1    1  st1
....................ucuggaauccaucugacaaaG.....................................................................  9    1  st1
....................ucuggaauccUucugacaaac.....................................................................  14   1  st1
....................ucuggaauAcaucugacaaac.....................................................................  5    1  st1
....................ucuggaGuccaucugacaaac.....................................................................  157  1  st1
....................Acuggaauccaucugacaaac.....................................................................  30   1  st1
....................ucuggCauccaucugacaaac.....................................................................  8    1  st1
....................ucuggaauccGucugacaaac.....................................................................  80   1  st1
....................ucuggaauccaucuAacaaac.....................................................................  47   1  st1
....................ucuggaaAccaucugacaaac.....................................................................  2    1  st1
....................ucuggaauUcaucugacaaac.....................................................................  25   1  st1
....................ucuggaauccaucugacaaCc.....................................................................  37   1  st1
....................ucuggaauccaucugaAaaac.....................................................................  7    1  st1
....................ucuAgaauccaucugacaaac.....................................................................  35   1  st1
....................ucuggGauccaucugacaaac.....................................................................  127  1  st1
....................uAuggaauccaucugacaaac.....................................................................  12   1  st1
....................ucuggaauccaucugacCaac.....................................................................  16   1  st1
....................ucuggaauccaucugGcaaac.....................................................................  612  1  st1
....................ucuggaCuccaucugacaaac.....................................................................  3    1  st1
....................ucugUaauccaucugacaaac.....................................................................  9    1  st1
....................ucuggaauccauUugacaaac.....................................................................  21   1  st1
....................ucuggaauccCucugacaaac.....................................................................  9    1  st1
....................Gcuggaauccaucugacaaac.....................................................................  9    1  st1
....................ucuggaauccaucugacaUac.....................................................................  14   1  st1
....................ucuggaauccaAcugacaaac.....................................................................  13   1  st1
....................ucuggaauccaGcugacaaac.....................................................................  3    1  st1
....................ucuggaaGccaucugacaaac.....................................................................  4    1  st1
....................ucuggaauccaucCgacaaac.....................................................................  139  1  st1
....................ucuggaaCccaucugacaaac.....................................................................  115  1  st1
....................ucuggaauccauAugacaaac.....................................................................  6    1  st1
....................ucuggaauccaucugacaaUc.....................................................................  12   1  st1
....................ucCggaauccaucugacaaac.....................................................................  104  1  st1
....................ucuggUauccaucugacaaac.....................................................................  15   1  st1
....................ucuggaauccaCcugacaaac.....................................................................  106  1  st1
....................ucuggaauccaucugacaaaA.....................................................................  592  1  st1
....................ucuggaaucAaucugacaaac.....................................................................  4    1  st1
....................ucuggaauccaucugacaGac.....................................................................  449  1  st1
....................ucuggaauccaucugacUaac.....................................................................  35   1  st1
....................ucuggaauccaucGgacaaac.....................................................................  9    1  st1
....................ucuggaauccaucugaUaaac.....................................................................  21   1  st1
....................ucuggaaucUaucugacaaac.....................................................................  31   1  st1
```

```
                    Mature                                     Star
acuuuauucuuuuuuuuuuaucuggaauccaucugacaaacugcauguuugucaugcaguuugucauggauuccaguuaaaaaaaagcaugcaaucguacgucaaggaga
....................ucuggaauccaucugacaCac.....................................................................    3       1    st1
....................ucuggaauccaucugacaaGc.....................................................................    186     1    st1
....................ucuggaauccaucugacGaac.....................................................................    125     1    st1
....................ucuggaauccaucugUcaaac.....................................................................    15      1    st1
....................ucuggaauccauGugacaaac.....................................................................    3       1    st1
....................ucuggaauccaucugCcaaac.....................................................................    4       1    st1
....................ucGggaauccaucugacaaac.....................................................................    7       1    st1
....................ucuggaauccaucugacaaac.....................................................................    37478   0    st1
....................ucuggaauccaucugacaaaU.....................................................................    3044    1    st1
....................ucuggaauccaucuCacaaac.....................................................................    26      1    st1
....................ucuggaauccaucAgacaaac.....................................................................    31      1    st1
....................uUuggaauccaucugacaaac.....................................................................    18      1    st1
....................ucugAaauccaucugacaaac.....................................................................    22      1    st1
....................ucAggaauccaucugacaaac.....................................................................    25      1    st1
....................uGuggaauccaucugacaaac.....................................................................    5       1    st1
....................ucuggaauccaucuUacaaac.....................................................................    3       1    st1
....................ucuggaauccaucAgacaaacu....................................................................    40      1    st1
....................uAuggaauccaucugacaaacu....................................................................    5       1    st1
....................ucugCaauccaucugacaaacu....................................................................    1       1    st1
....................ucuggaauccaucugacaaacG....................................................................    465     1    st1
....................ucuggaaCccaucugacaaacu....................................................................    127     1    st1
....................ucuggaGuccaucugacaaacu....................................................................    275     1    st1
....................ucuggaauccaucugaGaaacu....................................................................    1       1    st1
....................uGuggaauccaucugacaaacu....................................................................    3       1    st1
....................ucuggaauccaucuAacaaacu....................................................................    66      1    st1
....................ucuggaauccaucugacaUacu....................................................................    6       1    st1
....................ucuggCauccaucugacaaacu....................................................................    3       1    st1
....................ucuggUauccaucugacaaacu....................................................................    22      1    st1
....................ucuggaauccaucugacaaGcu....................................................................    200     1    st1
....................ucuggaauccCucugacaaacu....................................................................    8       1    st1
....................ucuggaaucGaucugacaaacu....................................................................    1       1    st1
....................ucuggaauccaucugacaaCcu....................................................................    27      1    st1
....................ucuggaaAccaucugacaaacu....................................................................    7       1    st1
....................ucuggaauccaucugacaCacu....................................................................    6       1    st1
....................ucugUaauccaucugacaaacu....................................................................    10      1    st1
....................ucuggaauccaAcugacaaacu....................................................................    16      1    st1
....................ucuggaauccaCcugacaaacu....................................................................    129     1    st1
....................ucuggaauccauAugacaaacu....................................................................    8       1    st1
....................ucuggaauccaGcugacaaacu....................................................................    7       1    st1
....................uUuggaauccaucugacaaacu....................................................................    30      1    st1
....................ucuggaauccaucugacaaUcu....................................................................    15      1    st1
....................ucuggaaucAaucugacaaacu....................................................................    7       1    st1
....................ucuggaauccaucGgacaaacu....................................................................    13      1    st1
....................ucuggaauAcaucugacaaacu....................................................................    9       1    st1
....................ucuggaUuccaucugacaaacu....................................................................    9       1    st1
....................ucuggaauccaucugacaaacC....................................................................    4927    1    st1
....................ucuggaauGcaucugacaaacu....................................................................    3       1    st1
....................ucuggGauccaucugacaaacu....................................................................    208     1    st1
....................ucuggaaGccaucugacaaacu....................................................................    7       1    st1
....................ucuggaCuccaucugacaaacu....................................................................    3       1    st1
....................ucuggaauccGucugacaaacu....................................................................    101     1    st1
....................ucGggaauccaucugacaaacu....................................................................    5       1    st1
....................ucuggaauccaucugacCaacu....................................................................    4       1    st1
....................ucugAaauccaucugacaaacu....................................................................    28      1    st1
....................ucuggaauccaucugGcaaacu....................................................................    529     1    st1
....................ucuCgaauccaucugacaaacu....................................................................    4       1    st1
....................ucCggaauccaucugacaaacu....................................................................    108     1    st1
....................ucuggaauccauGugacaaacu....................................................................    3       1    st1
....................ucuUgaauccaucugacaaacu....................................................................    5       1    st1
....................ucuggaaucUaucugacaaacu....................................................................    43      1    st1
....................ucuggaauccaucugacaGacu....................................................................    725     1    st1
....................ucuggaauccaucuUacaaacu....................................................................    8       1    st1
....................ucuggaauccaucugacGaacu....................................................................    177     1    st1
....................ucuggaauccaucugacaaaUu....................................................................    35      1    st1
....................ucuggaauccaucugUcaaacu....................................................................    16      1    st1
....................Acuggaauccaucugacaaacu....................................................................    31      1    st1
....................ucuggaauccaucugacUaacu....................................................................    46      1    st1
....................ucuggaauccaucugacaaacu....................................................................    46047   0    st1
....................ucuggaauccaucugacaaaAu....................................................................    11      1    st1
....................ucuggaauccaucugacaaacA....................................................................    1808    1    st1
```

Mature **Star**

```
acuuuauucuuuuuuuuuuaucuggaauccaucugacaaacugcauguuugucaugcaguuugucauggauuccaguuaaaaaaaagcaugcaaucguacgucaaggaga
....................ucuAgaauccaucugacaaacu....................................................................   24      1   st1
....................ucuggaauUcaucugacaaacu....................................................................   40      1   st1
....................ucuggaauccUucugacaaacu....................................................................   14      1   st1
....................ucuggaauccaucugCcaaacu....................................................................   4       1   st1
....................ucuggaauccaucCgacaaacu....................................................................   170     1   st1
....................ucuggaauccauUugacaaacu....................................................................   20      1   st1
....................ucuggaauccaucugaAaaacu....................................................................   4       1   st1
....................ucAggaauccaucugacaaacu....................................................................   27      1   st1
....................ucuggaauccaucugaUaaacu....................................................................   19      1   st1
....................Gcuggaauccaucugacaaacu....................................................................   10      1   st1
....................ucuggaauccaucuCacaaacu....................................................................   38      1   st1
....................ucuggaauccauAugacaaacug...................................................................   57      1   st1
....................ucuggaauccaucugCcaaacug...................................................................   58      1   st1
....................ucuggaauccaucCgacaaacug...................................................................   1883    1   st1
....................ucuggaaucUaucugacaaacug...................................................................   443     1   st1
....................ucuggaauccaucugacaaGcug...................................................................   2052    1   st1
....................ucuggaauccaucugGcaaacug...................................................................   5249    1   st1
....................ucuggCauccaucugacaaacug...................................................................   72      1   st1
....................uGuggaauccaucugacaaacug...................................................................   39      1   st1
....................ucuggaauccauUugacaaacug...................................................................   245     1   st1
....................Gcuggaauccaucugacaaacug...................................................................   162     1   st1
....................ucuggaauccaucugacaaacCg...................................................................   3120    1   st1
....................ucuggaauccaucugacaUacug...................................................................   198     1   st1
....................ucCggaauccaucugacaaacug...................................................................   1409    1   st1
....................ucuggaauccaAcugacaaacug...................................................................   211     1   st1
....................ucuggaauccaGcugacaaacug...................................................................   52      1   st1
....................ucugUaauccaucugacaaacug...................................................................   55      1   st1
....................uUuggaauccaucugacaaacug...................................................................   404     1   st1
....................ucuggaauccaucugacaaaGug...................................................................   8       1   st1
....................ucuggaauccaucugacUaacug...................................................................   553     1   st1
....................ucuggaaucGaucugacaaacug...................................................................   16      1   st1
....................ucuggUauccaucugacaaacug...................................................................   256     1   st1
....................ucuggaaucAaucugacaaacug...................................................................   90      1   st1
....................ucuggaauccGucugacaaacug...................................................................   1283    1   st1
....................ucuggaauUcaucugacaaacug...................................................................   322     1   st1
....................ucuggaauAcaucugacaaacug...................................................................   61      1   st1
....................ucuggaauccauGugacaaacug...................................................................   9       1   st1
....................ucuggaauGcaucugacaaacug...................................................................   13      1   st1
....................ucuggaauccaucAgacaaacug...................................................................   484     1   st1
....................uAuggaauccaucugacaaacug...................................................................   135     1   st1
....................ucuggaauccaucugacaaacGg...................................................................   681     1   st1
....................ucuggaauccaucugacaaacuC...................................................................   2595    1   st1
....................ucugCaauccaucugacaaacug...................................................................   29      1   st1
....................ucuggaauccaucugacaaacuA...................................................................   40746   1   st1
....................ucuggaaAccaucugacaaacug...................................................................   92      1   st1
....................ucuggaaGccaucugacaaacug...................................................................   42      1   st1
....................ucuggaauccaucugacGaacug...................................................................   2252    1   st1
....................ucuggaauccaucuAacaaacug...................................................................   822     1   st1
....................ucuUgaauccaucugacaaacug...................................................................   94      1   st1
....................ucuggaaCccaucugacaaacug...................................................................   1385    1   st1
....................ucAggaauccaucugacaaacug...................................................................   378     1   st1
....................ucuggaauccaucugaAaaacug...................................................................   138     1   st1
....................ucuggaGuccaucugacaaacug...................................................................   4535    1   st1
....................ucuggaauccaucugaUaaacug...................................................................   300     1   st1
....................ucuggaauccaucugacCaacug...................................................................   94      1   st1
....................ucuCgaauccaucugacaaacug...................................................................   14      1   st1
....................ucuggaauccUucugacaaacug...................................................................   187     1   st1
....................ucuggaauccCucugacaaacug...................................................................   86      1   st1
....................ucuggaauccaucugacaaacAg...................................................................   404     1   st1
....................ucuggaauccaucugacaaCcug...................................................................   273     1   st1
....................ucugAaauccaucugacaaacug...................................................................   335     1   st1
....................ucuggGauccaucugacaaacug...................................................................   2365    1   st1
....................ucuggaauccaucuCacaaacug...................................................................   441     1   st1
....................ucuggaCuccaucugacaaacug...................................................................   75      1   st1
....................ucuggaauccaucugacaGacug...................................................................   12583   1   st1
....................Acuggaauccaucugacaaacug...................................................................   362     1   st1
....................ucuggaauccaucugacaaaUug...................................................................   288     1   st1
....................ucuggaauccaucugacaaacuU...................................................................   4320    1   st1
....................ucuggaauccaucugaGaaacug...................................................................   10      1   st1
....................ucGggaauccaucugacaaacug...................................................................   72      1   st1
```

```
                    Mature                     Star
acuuuauucuuuuuuuuuuaucuggaauccaucugacaaacugcauguuugucaugcaguuugucauggauuccaguuaaaaaaaagcaugcaaucguacgucaaggaga
....................ucuggaauccaucugacaaaAug...................................................................   67      1   st1
....................ucuggaauccaucugUcaaacug...................................................................   294     1   st1
....................ucuggaauccaucuUacaaacug...................................................................   50      1   st1
....................ucuggaauccaucugacaCacug...................................................................   65      1   st1
....................ucuggaauccaucGgacaaacug...................................................................   127     1   st1
....................ucuggaUuccaucugacaaacug...................................................................   61      1   st1
....................ucuggaauccaucugacaaUcug...................................................................   121     1   st1
....................ucuAgaauccaucugacaaacug...................................................................   366     1   st1
....................ucuggaauccaucugacaaacug...................................................................   550369  0   st1
....................ucuggaauccaCcugacaaacug...................................................................   1449    1   st1
....................ucuggaauccaucugacaaacuCc..................................................................   39      1   st1
....................ucuggUauccaucugacaaacugc..................................................................   8       1   st1
....................ucuggaauccaucGgacaaacugc..................................................................   3       1   st1
....................ucuggaauccaucugacaaaUugc..................................................................   19      1   st1
....................ucuggaauccaucugacUaacugc..................................................................   17      1   st1
....................ucuggaauccCucugacaaacugc..................................................................   4       1   st1
....................ucuggaauccaGcugacaaacugc..................................................................   3       1   st1
....................ucuggaauccaucugacaUacugc..................................................................   10      1   st1
....................ucuggaauccGucugacaaacugc..................................................................   25      1   st1
....................ucuggaauccaCcugacaaacugc..................................................................   34      1   st1
....................ucuggaauccaucugacaaacugA..................................................................   1819    1   st1
....................uAuggaauccaucugacaaacugc..................................................................   7       1   st1
....................ucugAaauccaucugacaaacugc..................................................................   8       1   st1
....................ucuggaauccaucCgacaaacugc..................................................................   44      1   st1
....................ucuggaauccaucugacaaacugG..................................................................   105     1   st1
....................ucuggaauccaucugacaaCcugc..................................................................   6       1   st1
....................ucuggaauccaucugacaGacugc..................................................................   154     1   st1
....................ucuggaauccaucugacaaacuUc..................................................................   23      1   st1
....................ucuggaauccaucugacCaacugc..................................................................   4       1   st1
....................ucuggaauAcaucugacaaacugc..................................................................   1       1   st1
....................ucuggaaAccaucugacaaacugc..................................................................   5       1   st1
....................ucuggaauccUucugacaaacugc..................................................................   2       1   st1
....................ucuggaauccaucugacaaGcugc..................................................................   45      1   st1
....................ucuggaauccaAcugacaaacugc..................................................................   4       1   st1
....................ucuggaauccaucugacaaaGugc..................................................................   1       1   st1
....................ucuggaaCccaucugacaaacugc..................................................................   47      1   st1
....................ucuggaaucUaucugacaaacugc..................................................................   13      1   st1
....................ucuggaauccauAugacaaacugc..................................................................   2       1   st1
....................ucuggaaUcaucugacaaacugc...................................................................   9       1   st1
....................ucuggGauccaucugacaaacugc..................................................................   45      1   st1
....................ucuAgaauccaucugacaaacugc..................................................................   5       1   st1
....................ucuggaauccaucugacaaacGgc..................................................................   1       1   st1
....................ucuggaGuccaucugacaaacugc..................................................................   177     1   st1
....................ucuggaauccaucugacaaacugc..................................................................   14207   0   st1
....................ucuggaauccaucugacaaacAgc..................................................................   11      1   st1
....................ucAggaauccaucugacaaacugc..................................................................   12      1   st1
....................ucuggaauccaucugacaaUcugc..................................................................   2       1   st1
....................ucuggaauccauUugacaaacugc..................................................................   9       1   st1
....................ucuggaauccaucugacaCacugc..................................................................   1       1   st1
....................ucuggaauccaucuAacaaacugc..................................................................   28      1   st1
....................ucugUaauccaucugacaaacugc..................................................................   2       1   st1
....................ucuggaauccaucugacGaacugc..................................................................   68      1   st1
....................ucuggaauccaucugUcaaacugc..................................................................   8       1   st1
....................ucCggaauccaucugacaaacugc..................................................................   38      1   st1
....................ucuggaauccaucugacaaacuAc..................................................................   40      1   st1
....................ucuggaauccaucuCacaaacugc..................................................................   12      1   st1
....................Acuggaauccaucugacaaacugc..................................................................   16      1   st1
....................ucuggaaucGaucugacaaacugc..................................................................   1       1   st1
....................ucuggaauccaucugacaaacCgc..................................................................   31      1   st1
....................ucuggaauccaucugGcaaacugc..................................................................   164     1   st1
....................ucuggaauccaucugacaaacugU..................................................................   2667    1   st1
....................ucuggaauccaucAgacaaacugc..................................................................   11      1   st1
....................ucuggaaucAaucugacaaacugc..................................................................   2       1   st1
....................ucuggaauccaucugaUaaacugc..................................................................   4       1   st1
....................ucuggaauccaucuUacaaacugc..................................................................   2       1   st1
....................ucuUgaauccaucugacaaacugc..................................................................   5       1   st1
....................uUuggaauccaucugacaaacugc..................................................................   9       1   st1
....................Gcuggaauccaucugacaaacugc..................................................................   8       1   st1
....................ucuggaCuccaucugacaaacugc..................................................................   3       1   st1
....................ucuggaauccauGugacaaacugc..................................................................   1       1   st1
```

```
                    Mature                              Star
acuuuauucuuuuuuuuuuaucuggaauccaucugacaaacugcauguuugucaugcaguuugucauggauuccaguuaaaaaaaagcaugcaaucguacgucaaggaga
....................ucuggaauccaucugacaaacGgca.................................................................    1       1    st1
....................ucuggaauccGucugacaaacugca.................................................................    35      1    st1
....................ucuggaauccaucugacaaacAgca.................................................................    5       1    st1
....................ucuggaauccaucugCcaaacugca.................................................................    1       1    st1
....................ucuggaauccCucugacaaacugca.................................................................    2       1    st1
....................ucuggUauccaucugacaaacugca.................................................................    3       1    st1
....................uAuggaauccaucugacaaacugca.................................................................    6       1    st1
....................uGuggaauccaucugacaaacugca.................................................................    1       1    st1
....................Acuggaauccaucugacaaacugca.................................................................    9       1    st1
....................ucuggaauccaucugacaaGcugca.................................................................    35      1    st1
....................ucuggaauAcaucugacaaacugca.................................................................    2       1    st1
....................ucuggaauccaucugacaaaUugca.................................................................    4       1    st1
....................ucuggaaGccaucugacaaacugca.................................................................    2       1    st1
....................ucuggaauccaucAgacaaacugca.................................................................    15      1    st1
....................ucugAaauccaucugacaaacugca.................................................................    11      1    st1
....................ucuggaauccaucugacaaacuUca.................................................................    11      1    st1
....................ucuggaauccaucugacaaCcugca.................................................................    4       1    st1
....................ucuggaauccaucuAacaaacugca.................................................................    9       1    st1
....................ucuggaUuccaucugacaaacugca.................................................................    1       1    st1
....................ucuggaGuccaucugacaaacugca.................................................................    80      1    st1
....................ucuggaauccaCcugacaaacugca.................................................................    27      1    st1
....................ucuAgaauccaucugacaaacugca.................................................................    10      1    st1
....................ucuggaauccaucugacaaacugca.................................................................    13371   0    st1
....................ucuggaauccaucugacaaacuAca.................................................................    34      1    st1
....................ucuggaauccaucugacaaacugcG.................................................................    267     1    st1
....................ucAggaauccaucugacaaacugca.................................................................    11      1    st1
....................ucuggaauccaucCgacaaacugca.................................................................    45      1    st1
....................ucuggaaucAaucugacaaacugca.................................................................    3       1    st1
....................ucuggaauccaucugacaaacugUa.................................................................    307     1    st1
....................ucuggaauccaucuUacaaacugca.................................................................    1       1    st1
....................ucuggaauccaucugaUaaacugca.................................................................    11      1    st1
....................ucuggaauccaucugGcaaacugca.................................................................    202     1    st1
....................ucuggaaAccaucugacaaacugca.................................................................    2       1    st1
....................ucuggaauccaucugacaaacugcU.................................................................    212     1    st1
....................ucuggaaCccaucugacaaacugca.................................................................    26      1    st1
....................ucuggaauccaucugaAaaacugca.................................................................    2       1    st1
....................ucuggaauccaucugacUaacugca.................................................................    13      1    st1
....................ucuggaauccaucugacaaacCgca.................................................................    34      1    st1
....................ucuggaauccauUugacaaacugca.................................................................    3       1    st1
....................uUuggaauccaucugacaaacugca.................................................................    8       1    st1
....................ucuggaauccauAugacaaacugca.................................................................    3       1    st1
....................Gcuggaauccaucugacaaacugca.................................................................    1       1    st1
....................ucuggaauccaucGgacaaacugca.................................................................    8       1    st1
....................ucuggaauccaucugacaCacugca.................................................................    1       1    st1
....................ucuggaauccauGugacaaacugca.................................................................    1       1    st1
....................ucuggaauccUucugacaaacugca.................................................................    1       1    st1
....................ucuggCauccaucugacaaacugca.................................................................    3       1    st1
....................ucuggaauUcaucugacaaacugca.................................................................    9       1    st1
....................ucuggaauccaGcugacaaacugca.................................................................    1       1    st1
....................ucuggGauccaucugacaaacugca.................................................................    37      1    st1
....................ucuggaauccaucuCacaaacugca.................................................................    8       1    st1
....................ucuggaaucUaucugacaaacugca.................................................................    8       1    st1
....................ucuggaauccaucugacCaacugca.................................................................    4       1    st1
....................ucuggaauccaucugacaaacuCca.................................................................    32      1    st1
....................ucuggaauccaucugacaUacugca.................................................................    6       1    st1
....................ucuggaauccaucugacaaacugGa.................................................................    4       1    st1
....................ucuggaauccaucugacaaacugcC.................................................................    48      1    st1
....................ucuggaauccaucugUcaaacugca.................................................................    6       1    st1
....................ucuggaauccaucugacaaacugAa.................................................................    12      1    st1
....................ucugUaauccaucugacaaacugca.................................................................    1       1    st1
....................ucuggaauccaucugacGaacugca.................................................................    64      1    st1
....................ucuggaauccaucugacaGacugca.................................................................    109     1    st1
....................ucCggaauccaucugacaaacugca.................................................................    26      1    st1
....................ucuggaauccaAcugacaaacugca.................................................................    5       1    st1
....................ucuggaauccaucugacUaacugcau................................................................    1       1    st1
....................Acuggaauccaucugacaaacugcau................................................................    1       1    st1
....................ucuggaauccaAcugacaaacugcau................................................................    2       1    st1
....................ucuggaauccaucugacaaacugcUu................................................................    71      1    st1
....................ucuggaauUcaucugacaaacugcau................................................................    2       1    st1
....................ucuggaauccaucugacaaacuCcau................................................................    1       1    st1
```

```
                    Mature                               Star
acuuuauucuuuuuuuuuuaucuggaauccaucugacaaacugcauguuugucaugcaguuugucauggauuccaguuaaaaaaaagcaugcaaucguacgucaaggaga
....................ucuggaauccaucugacaaGcugcau................................................................    2     1    st1
....................ucuggaGuccaucugacaaacugcau................................................................    4     1    st1
....................ucuggaauccaucugacaaacugcCu................................................................    3     1    st1
....................ucuggaauccaucugaUaaacugcau................................................................    1     1    st1
....................ucuggaauccaucCgacaaacugcau................................................................    2     1    st1
....................ucuggGauccaucugacaaacugcau................................................................    2     1    st1
....................ucuggaauccaucugacaaacugUau................................................................    10    1    st1
....................ucuggaauccaucugacaaacGgcau................................................................    1     1    st1
....................ucuggaauccaucugacaaacugcaA................................................................    551   1    st1
....................ucuggaauccGucugacaaacugcau................................................................    1     1    st1
....................ucuggaauccaucugacaaacuUcau................................................................    1     1    st1
....................ucuggaauccaucugGcaaacugcau................................................................    27    1    st1
....................ucuggaauccaucugacaaacugcaG................................................................    1472  1    st1
....................ucuggaauccaucugacaaacugcaC................................................................    51    1    st1
....................ucuggaauccaucugacaaacugcau................................................................    431   0    st1
....................ucuggaaAccaucugacaaacugcau................................................................    1     1    st1
....................ucuAgaauccaucugacaaacugcau................................................................    1     1    st1
....................ucuggaauccaucugacaaacugAau................................................................    2     1    st1
....................ucuggaauccaucugacaaacCgcau................................................................    2     1    st1
....................ucCggaauccaucugacaaacugcau................................................................    4     1    st1
....................ucuggaauccaucugacaGacugcau................................................................    7     1    st1
....................ucuggaauccaucugacaaaUugcau................................................................    1     1    st1
....................ucuggaauccaucugacaaacuAcau................................................................    1     1    st1
....................uUuggaauccaucugacaaacugcau................................................................    1     1    st1
....................ucuggaaCccaucugacaaacugcau................................................................    1     1    st1
....................ucuggaauccaucugacaaacugcaAg...............................................................    63    1    st1
....................ucuggaauccaucugacaaacugcaGg...............................................................    4     1    st1
....................ucuggaauccaucAgacaaacugcaug...............................................................    1     1    st1
....................ucuggaauccaucugacaaacugcUug...............................................................    3     1    st1
....................ucuggaauccaCcugacaaacugcaug...............................................................    1     1    st1
....................ucuggaauccaucugacaaacugcauA...............................................................    575   1    st1
....................ucuggaauccaucugacaaacugcauU...............................................................    671   1    st1
....................ucuggaauccaucugacaaacugcauC...............................................................    67    1    st1
....................ucuggaauccaucugacaaGcugcaug...............................................................    1     1    st1
....................ucuggaauccaucuAacaaacugcaug...............................................................    1     1    st1
....................ucuggGauccaucugacaaacugcaug...............................................................    1     1    st1
....................ucuggaaCccaucugacaaacugcaug...............................................................    1     1    st1
....................ucuggaauccaucugacaaacugcaCg...............................................................    1     1    st1
....................ucuggaauccaucugacaaacugcaug...............................................................    82    0    st1
.....................cuggaauccaucugacaa.......................................................................    1     0    st1
.....................Uuggaauccaucugacaaac.....................................................................    1     1    st1
.....................cuggaauccaucugacaaac.....................................................................    21    0    st1
.....................cuggaauccaucugacaaaU.....................................................................    1     1    st1
.....................cuggaauccaucugacaaacA....................................................................    1     1    st1
.....................cuggaauccaucugacaaacC....................................................................    1     1    st1
.....................cuggGauccaucugacaaacu....................................................................    1     1    st1
.....................Uuggaauccaucugacaaacu....................................................................    1     1    st1
.....................cuggaauccaucugacaaacu....................................................................    26    0    st1
.....................cuggaGuccaucugacaaacu....................................................................    1     1    st1
.....................cuggaauccaucugacaaacug...................................................................    432   0    st1
.....................cuggaauccaucugacGaacug...................................................................    3     1    st1
.....................cGggaauccaucugacaaacug...................................................................    2     1    st1
.....................cuggaauccaucugacaaacuC...................................................................    3     1    st1
.....................cuggaauccaucugacaGacug...................................................................    7     1    st1
.....................cuggaGuccaucugacaaacug...................................................................    6     1    st1
.....................cuggaauccaCcugacaaacug...................................................................    1     1    st1
.....................cuggaauccaucugaUaaacug...................................................................    1     1    st1
.....................cuggaaCccaucugacaaacug...................................................................    1     1    st1
.....................cuggaauccaucugacaaacuA...................................................................    31    1    st1
.....................cCggaauccaucugacaaacug...................................................................    1     1    st1
.....................cuggaauccaAcugacaaacug...................................................................    1     1    st1
.....................cuggaauccaucugacaaacuU...................................................................    5     1    st1
.....................cuggGauccaucugacaaacug...................................................................    2     1    st1
.....................cuggaauccaucCgacaaacug...................................................................    1     1    st1
.....................cuggaauccaucugUcaaacug...................................................................    1     1    st1
.....................cugAaauccaucugacaaacug...................................................................    1     1    st1
.....................cuggaauccaucugGcaaacug...................................................................    2     1    st1
.....................cuggaauccaucugacaaacCg...................................................................    1     1    st1
.....................cuggaauccaucugacaaGcug...................................................................    3     1    st1
.....................Auggaauccaucugacaaacug...................................................................    1     1    st1
```

```
                    Mature                              Star
acuuuauucuuuuuuuuuuaucuggaauccaucugacaaacugcauguuugucaugcaguuugucauggauuccaguuaaaaaaaagcaugcaaucguacgucaaggaga
.....................cuAgaauccaucugacaaacug...................................................................    1    1    st1
.....................Uuggaauccaucugacaaacug...................................................................    14   1    st1
.....................cuggaauccGucugacaaacug...................................................................    2    1    st1
.....................cuggaauccaucugacaaacugc..................................................................    13   0    st1
.....................cuAgaauccaucugacaaacugc..................................................................    1    1    st1
.....................Uuggaauccaucugacaaacugc..................................................................    1    1    st1
.....................cuggaauccaucugacaaacugU..................................................................    2    1    st1
.....................cuggaauccaucugacaaacugcC.................................................................    3    1    st1
.....................cuggaauccaucugacaaacugUa.................................................................    1    1    st1
.....................cuggaauccaucugacaaacugca.................................................................    10   0    st1
.....................cuggaauccaucugacaaacugcaG................................................................    1    1    st1
.....................cuggaauccaucugacaaacugcCu................................................................    1    1    st1
.....................cuggaauccaucugacaaacugcauU...............................................................    1    1    st1
......................uggaauccaucugacaaa......................................................................    1    0    st1
......................uAgaauccaucugacaaac.....................................................................    1    1    st1
......................uggaauccaucugacaaac.....................................................................    41   0    st1
......................uggaauccaucugacaGac.....................................................................    2    1    st1
......................uggaauccaucugacaaaU.....................................................................    5    1    st1
......................uggaauccaucugacaaaA.....................................................................    1    1    st1
......................uggaauccaucugGcaaac.....................................................................    4    1    st1
......................uggGauccaucugacaaac.....................................................................    1    1    st1
......................uggGauccaucugacaaacu....................................................................    1    1    st1
......................uggaauccaucugGcaaacu....................................................................    3    1    st1
......................uggaaAccaucugacaaacu....................................................................    2    1    st1
......................Cggaauccaucugacaaacu....................................................................    1    1    st1
......................uggaaCccaucugacaaacu....................................................................    1    1    st1
......................uggaauccaucugacaaacC....................................................................    3    1    st1
......................uggaauccaucugacaaacu....................................................................    45   0    st1
......................uggaauccaucugacaaacuU...................................................................    3    1    st1
......................uggaauccaucugacGaacug...................................................................    2    1    st1
......................ugAaauccaucugacaaacug...................................................................    1    1    st1
......................uggaauccaucugaUaaacug...................................................................    1    1    st1
......................uggaauccaucugacaaacuA...................................................................    45   1    st1
......................uggaauccaucugacaaGcug...................................................................    1    1    st1
......................uggaauccaucugacaaacug...................................................................    605  0    st1
......................uggaGuccaucugacaaacug...................................................................    5    1    st1
......................uggaauccaucAgacaaacug...................................................................    1    1    st1
......................uggaauccaucugacaaacuC...................................................................    1    1    st1
......................uAgaauccaucugacaaacug...................................................................    1    1    st1
......................uggaauccaucCgacaaacug...................................................................    3    1    st1
......................uggaauccaucugGcaaacug...................................................................    23   1    st1
......................uggaaucAaucugacaaacug...................................................................    1    1    st1
......................uggGauccaucugacaaacug...................................................................    5    1    st1
......................Aggaauccaucugacaaacug...................................................................    3    1    st1
......................uggaauccaucugacaaacGg...................................................................    1    1    st1
......................uggaauccaucugacaaacCg...................................................................    3    1    st1
......................uggaauccCucugacaaacug...................................................................    9    1    st1
......................uggaaCccaucugacaaacug...................................................................    2    1    st1
......................uggaauGcaucugacaaacug...................................................................    1    1    st1
......................Cggaauccaucugacaaacug...................................................................    5    1    st1
......................uggaauccaucugacaGacug...................................................................    9    1    st1
......................uggaCuccaucugacaaacug...................................................................    1    1    st1
......................uUgaauccaucugacaaacug...................................................................    2    1    st1
......................uggaauccauUugacaaacug...................................................................    1    1    st1
......................uggaauccaucugacaaacugA..................................................................    2    1    st1
......................uggaauccaucugacaaacugc..................................................................    32   0    st1
......................uggaauccCucugacaaacugc..................................................................    1    1    st1
......................uggaauccaucugacaaacugU..................................................................    11   1    st1
......................uggaauccaucugGcaaacugc..................................................................    2    1    st1
......................uggaauccaucugacaaacugcU.................................................................    1    1    st1
......................uggaauccaucugGcaaacugca.................................................................    2    1    st1
......................uggaauccaucugacaaacugca.................................................................    60   0    st1
......................uAgaauccaucugacaaacugca.................................................................    2    1    st1
......................uggaauccaucugacaaacugUa.................................................................    1    1    st1
......................uggaauccGucugacaaacugca.................................................................    1    1    st1
......................uggaauccaucugacaaacugcau................................................................    32   0    st1
......................uggaauccaucugGcaaacugcau................................................................    2    1    st1
......................uggaauccaucugacaaacugcUu................................................................    2    1    st1
......................uggaauccaucugacaaacugcaC................................................................    8    1    st1
......................uggaauccaucugacaaacugcGu................................................................    4    1    st1
```

```
                    Mature                               Star
acuuuauucuuuuuuuuuuaucuggaauccaucugacaaacugcauguuugucaugcaguuugucauggauuccaguuaaaaaaaagcaugcaaucguacgucaaggaga
......................uggaauccaucugacaaacugcaA................................................................	8	1	st1
......................uggaauccaucugacaaacugcaG................................................................	7	1	st1
......................uggaauccaucugacaaacugcauU...............................................................	17	1	st1
......................uggaauccaucugacaaacugcaAg...............................................................	3	1	st1
......................uggaauccaucugacaaacugcauA...............................................................	32	1	st1
......................uggaauccaucugacaaacugcaug...............................................................	2	0	st1
......................uggaauccaucugacaaacugcauC...............................................................	2	1	st1
......................uggaauccaucugacaaacugcauUu..............................................................	9	1	st1
......................uggaauccaucugacaaacugcauAu..............................................................	6	1	st1
......................uggaauccaucugacaaacugcauUuu.............................................................	4	1	st1
.......................ggaauccaucugacaaac.....................................................................	1	0	st1
.......................ggaauccaucugacaaaU.....................................................................	1	1	st1
.......................ggaauccaucugacaaacu....................................................................	2	0	st1
.......................ggaauccaucugacaaaAug...................................................................	1	1	st1
.......................ggaauccaucugacaaacug...................................................................	21	0	st1
.......................ggaauccaucugacaaacuA...................................................................	2	1	st1
.......................ggGauccaucugacaaacug...................................................................	1	1	st1
.......................ggaGuccaucugacaaacug...................................................................	1	1	st1
.......................ggaauccaucugacaaacugc..................................................................	1	0	st1
........................gaauccaucugacaaacug...................................................................	1	0	st1
.........................aauccaucugacaaacug...................................................................	1	0	st1
.........................aauccaucugacaaacugcauUu..............................................................	1	1	st1
........................................................caguuugucauggauucc....................................	36	0	st1
........................................................caguuugucauggauucU....................................	13	1	st1
........................................................caguuugucauggauucA....................................	1	1	st1
........................................................caguuugucauggauucca...................................	1	0	st1
........................................................caguuugucauggauuccag..................................	14	0	st1
........................................................caguuuguUauggauuccagu.................................	1	1	st1
........................................................caguuugucauggauuccagu.................................	21	0	st1
........................................................cagCuugucauggauuccagu.................................	2	1	st1
........................................................caguuugucauggauuccaCu.................................	1	1	st1
........................................................caguuugucauggauuccagA.................................	3	1	st1
........................................................caguuugucauggauuccagGu................................	1	1	st1
........................................................cagCuugucauggauuccaguu................................	1	1	st1
........................................................caguuugucauggaGuccaguu................................	1	1	st1
........................................................caguuugucauggauuccGguu................................	2	1	st1
........................................................caguuugucauggGuuccaguu................................	1	1	st1
........................................................cagAuugucauggauuccaguu................................	1	1	st1
........................................................caguuugucauggauuccaguA................................	4	1	st1
........................................................caguuugucauggauuccagCu................................	2	1	st1
........................................................caguuugucauggaCuccaguu................................	4	1	st1
........................................................caguuCgucauggauuccaguu................................	2	1	st1
........................................................caguuugucGuggauuccaguu................................	2	1	st1
........................................................cGguuugucauggauuccaguu................................	1	1	st1
........................................................caguuugucauggauuccaguu................................	377	0	st1
........................................................caguuugucauggauuccaguG................................	2	1	st1
........................................................caguuugucauggauuccaguC................................	27	1	st1
........................................................caguCugucauggauuccaguu................................	3	1	st1
........................................................caguuugCcauggauuccaguu................................	1	1	st1
........................................................Gaguuugucauggauuccaguu................................	1	1	st1
........................................................Aaguuugucauggauuccaguua...............................	1	1	st1
........................................................caguuugucauggauuccaguGa...............................	3	1	st1
........................................................caguuugucaCggauuccaguua...............................	4	1	st1
........................................................caguuugucauggauuccGguua...............................	3	1	st1
........................................................caguuugucauggaCuccaguua...............................	4	1	st1
........................................................caguuugucaugAauuccaguua...............................	1	1	st1
........................................................caguuugucauggauuccaguCa...............................	2	1	st1
........................................................caguuugCcauggauuccaguua...............................	2	1	st1
........................................................caguuCgucauggauuccaguua...............................	8	1	st1
........................................................caguuugucauggauuccaguuC...............................	14	1	st1
........................................................caguuugucauggauCccaguua...............................	4	1	st1
........................................................caguCugucauggauuccaguua...............................	5	1	st1
........................................................cUguuugucauggauuccaguua...............................	4	1	st1
........................................................caguuuAucauggauuccaguua...............................	1	1	st1
........................................................caguuugucauggauuccaguuU...............................	48	1	st1
........................................................cagCuugucauggauuccaguua...............................	3	1	st1
........................................................caguuugucauAgauuccaguua...............................	1	1	st1
........................................................caguuugucGuggauuccaguua...............................	4	1	st1
........................................................caguuGgucauggauuccaguua...............................	1	1	st1
........................................................caguuugucauggaGuccaguua...............................	6	1	st1
```

```
                    Mature                                   Star
acuuuauucuuuuuuuuuuaucuggaauccaucugacaaacugcauguuugucaugcaguuugucauggauuccaguuaaaaaaaagcaugcaaucguacgucaaggaga
........................................................caguGugucauggauuccaguua...............................   1     1   st1
........................................................caguuugucauggauAccaguua...............................   1     1   st1
........................................................caguuugucauggauuccaguuG...............................   9     1   st1
........................................................caguuugucauggauuccaguua...............................   1837  0   st1
........................................................caguuugucaAggauuccaguua...............................   1     1   st1
........................................................caguuugucauggauuUcaguua...............................   2     1   st1
........................................................caguuuguUauggauuccaguua...............................   1     1   st1
........................................................caguuugucauggGuuccaguua...............................   1     1   st1
........................................................cagAuugucauggauuccaguua...............................   1     1   st1
........................................................cCguuugucauggauuccaguua...............................   2     1   st1
........................................................caguuugucauggauuccagCua...............................   3     1   st1
........................................................Uaguuugucauggauuccaguua...............................   1     1   st1
........................................................caguuugucaugUauuccaguua...............................   1     1   st1
........................................................caguuugucauggauucAaguua...............................   1     1   st1
........................................................caguuugucauggauuccaguuaa..............................   12    0   st1
........................................................caguuugucauggauuccaguuaC..............................   4     1   st1
........................................................caguuugucauggauuccaguuUa..............................   10    1   st1
........................................................caguuugucauggauuccaguuaU..............................   24    1   st1
........................................................caguuugucauggauuccaguuaG..............................   5     1   st1
........................................................caguuugucauggauuccaguuaUa.............................   3     1   st1
........................................................caguuugucauggauuccaguuaaaG............................   1     1   st1
.........................................................aguuugucauggauuccC...................................   1     1   st1
.........................................................aguuugucauggauuccag..................................   1     0   st1
.........................................................aguuugucaCggauuccagu.................................   1     1   st1
.........................................................aguuugucauggauuccagG.................................   1     1   st1
.........................................................Gguuugucauggauuccagu.................................   1     1   st1
.........................................................aguuugucauggauuccagu.................................   17    0   st1
.........................................................aguuugucauggauuccagA.................................   1     1   st1
.........................................................aguuugucauggauuccagC.................................   1     1   st1
.........................................................aguuugucauggauuccaguA................................   3     1   st1
.........................................................Gguuugucauggauuccaguu................................   3     1   st1
.........................................................aguuCgucauggauuccaguu................................   1     1   st1
.........................................................aguuugucCuggauuccaguu................................   2     1   st1
.........................................................agAuugucauggauuccaguu................................   2     1   st1
.........................................................Uguuugucauggauuccaguu................................   1     1   st1
.........................................................aguuugucGuggauuccaguu................................   4     1   st1
.........................................................aguuugAcauggauuccaguu................................   1     1   st1
.........................................................aguuugucauggauuccaguC................................   31    1   st1
.........................................................aguuugucauggGuuccaguu................................   2     1   st1
.........................................................aguCugucauggauuccaguu................................   3     1   st1
.........................................................aguuugucauggauuccaguG................................   2     1   st1
.........................................................aguuugucauggaCuccaguu................................   1     1   st1
.........................................................aguuugucauggauuccaguu................................   279   0   st1
.........................................................aguuugucauggauCccaguu................................   2     1   st1
.........................................................aguuugucauggauuAcaguua...............................   1     1   st1
.........................................................aguuugucauggauucAaguua...............................   1     1   st1
.........................................................Uguuugucauggauuccaguua...............................   2     1   st1
.........................................................aguuugucauggauuccagCua...............................   2     1   st1
.........................................................Gguuugucauggauuccaguua...............................   5     1   st1
.........................................................aguuugucGuggauuccaguua...............................   4     1   st1
.........................................................aguuugucaCggauuccaguua...............................   2     1   st1
.........................................................aguuugucaugAauuccaguua...............................   1     1   st1
.........................................................aguuugucauggauuccaguuC...............................   6     1   st1
.........................................................aguuugucauggauuccaguuU...............................   65    1   st1
.........................................................aguuugucauggGuuccaguua...............................   7     1   st1
.........................................................aguuugucauggauGccaguua...............................   3     1   st1
.........................................................aguuugucUuggauuccaguua...............................   4     1   st1
.........................................................aguuugAcauggauuccaguua...............................   1     1   st1
.........................................................aguuAgucauggauuccaguua...............................   1     1   st1
.........................................................aguuugucauggauuccUguua...............................   1     1   st1
.........................................................aguuugucauggauuccaguuG...............................   15    1   st1
.........................................................aguuugucauggauCccaguua...............................   5     1   st1
.........................................................aguuugucCuggauuccaguua...............................   11    1   st1
.........................................................aguuugucaAggauuccaguua...............................   1     1   st1
.........................................................aguuugCcauggauuccaguua...............................   3     1   st1
.........................................................aguuugucauggauucUaguua...............................   7     1   st1
.........................................................aguuugucauggauuUcaguua...............................   2     1   st1
.........................................................aguuugucauggauuccaguua...............................   1478  0   st1
.........................................................aguuugucauggauAccaguua...............................   3     1   st1
.........................................................aguuugucauggauuccaguCa...............................   3     1   st1
```

```
                    Mature                               Star
acuuuauucuuuuuuuuuuaucuggaauccaucugacaaacugcauguuugucaugcaguuugucauggauuccaguuaaaaaaaagcaugcaaucguacgucaaggaga
.........................................................aguuugucauggauuccGguua...............................  4    1    st1
.........................................................aguuugucauAgauuccaguua...............................  2    1    st1
.........................................................aguuugucauggaCuccaguua...............................  6    1    st1
.........................................................aguuugucauUgauuccaguua...............................  1    1    st1
.........................................................aguuCgucauggauuccaguua...............................  4    1    st1
.........................................................agCuugucauggauuccaguua...............................  3    1    st1
.........................................................aguuugucauggauuccaguuaG..............................  2    1    st1
.........................................................aguuugucauggauuccGguuaa..............................  1    1    st1
.........................................................aguuugucauggauuccaguuUa..............................  18   1    st1
.........................................................agCuugucauggauuccaguuaa..............................  2    1    st1
.........................................................Gguuugucauggauuccaguuaa..............................  1    1    st1
.........................................................aguuugucCuggauuccaguuaa..............................  2    1    st1
.........................................................aguuugucauggauuccaguuaU..............................  42   1    st1
.........................................................aguuugucauggauuccaguuaC..............................  3    1    st1
.........................................................aguuugucauggauuccaguuaa..............................  29   0    st1
.........................................................aguuugucauggauuccaguuaGa.............................  2    1    st1
.........................................................aguuugucauggauuccaguuaaU.............................  1    1    st1
.........................................................aguuugucauggauuccaguuaUa.............................  6    1    st1
.........................................................aguuugucauggauuccaguuaaG.............................  4    1    st1
.........................................................aguuugucauggauuccaguuaaGa............................  1    1    st1
..........................................................guuugucauggauuccaguuaa..............................  1    0    st1
...........................................................uuugucauggauuccaguua...............................  4    0    st1
...........................................................uuugucauggauuccaguuC...............................  1    1    st1
...........................................................uuugucauggauuccaguuU...............................  1    1    st1
...........................................................uuugucauggauuccaguuG...............................  2    1    st1
...........................................................uuugucauggauuccaguuaa..............................  1    0    st1
...........................................................uuugucauggauuccaguuUa..............................  2    1    st1
...........................................................uuugucauggauuccaguuaU..............................  3    1    st1
............................................................uugucauggauuccaguua...............................  1    0    st1
.............................................................ugucauggauuccaguua...............................  1    0    st1

..................uaucuggaauccaucugacaaacug...................................................................  2    0    sg1
..................uCucuggaauccaucugacaaacug...................................................................  3    1    sg1
..................uaucuggaGuccaucugacaaacug...................................................................  1    1    sg1
..................uaucuggaauccaucugacaaacuA...................................................................  1    1    sg1
..................uCucuggaauccaucugacaaacugca.................................................................  1    1    sg1
...................Cucuggaauccaucugacaaac.....................................................................  5    1    sg1
...................Cucuggaauccaucugacaaacu....................................................................  8    1    sg1
...................Cucuggaauccaucugacaaacug...................................................................  102  1    sg1
...................Cucuggaauccaucugacaaacugc..................................................................  5    1    sg1
...................Cucuggaauccaucugacaaacugca.................................................................  4    1    sg1
...................Cucuggaauccaucugacaaacugcau................................................................  1    1    sg1
....................ucuggaauccaucugacG........................................................................  6    1    sg1
....................ucuggaauccaucugGca........................................................................  2    1    sg1
....................ucAggaauccaucugaca........................................................................  1    1    sg1
....................ucuggaauccaucugaca........................................................................  157  0    sg1
....................ucuggaauccaucAgaca........................................................................  1    1    sg1
....................ucuggaauccaucugacaG.......................................................................  8    1    sg1
....................ucuggaauccaucugacaC.......................................................................  1    1    sg1
....................ucuggaauccaucugGcaa.......................................................................  1    1    sg1
....................ucuggaauccaucugacaU.......................................................................  3    1    sg1
....................ucuggGauccaucugacaa.......................................................................  1    1    sg1
....................ucCggaauccaucugacaa.......................................................................  1    1    sg1
....................ucuggaauccaucugacaa.......................................................................  263  0    sg1
....................ucuggaaCccaucugacaa.......................................................................  1    1    sg1
....................ucuggaauccaucugacGa.......................................................................  1    1    sg1
....................ucuggaauccaCcugacaa.......................................................................  2    1    sg1
....................ucuggaauccaucuAacaa.......................................................................  1    1    sg1
....................ucuggaaCccaucugacaaa......................................................................  1    1    sg1
....................ucuggaauccaucugGcaaa......................................................................  1    1    sg1
....................uUuggaauccaucugacaaa......................................................................  1    1    sg1
....................ucuggaauccaucugUcaaa......................................................................  1    1    sg1
....................ucuggaCuccaucugacaaa......................................................................  3    1    sg1
....................ucuAgaauccaucugacaaa......................................................................  1    1    sg1
....................ucuggaauccaucugacaaG......................................................................  5    1    sg1
....................ucuggaauccaucuAacaaa......................................................................  1    1    sg1
....................ucuggaauccaucCgacaaa......................................................................  2    1    sg1
....................ucuggaauccaucugaUaaa......................................................................  1    1    sg1
....................ucuggGauccaucugacaaa......................................................................  5    1    sg1
....................ucuggaauccGucugacaaa......................................................................  3    1    sg1
```

Mature **Star**

```
acuuuauucuuuuuuuuuuaucuggaauccaucugacaaacugcauguuugucaugcaguuugucauggauuccaguuaaaaaaaagcaugcaaucguacgucaaggaga
....................ucuggaauccaucugacGaa......................................................................   4     1  sg1
....................ucuggaauccaucugacaaa......................................................................   899   0  sg1
....................ucuggaaAccaucugacaaa......................................................................   1     1  sg1
....................ucCggaauccaucugacaaa......................................................................   4     1  sg1
....................ucuggaauccaucugacUaa......................................................................   1     1  sg1
....................ucuggaauccauUugacaaa......................................................................   1     1  sg1
....................ucuggaauccaucugacaGa......................................................................   12    1  sg1
....................Acuggaauccaucugacaaa......................................................................   1     1  sg1
....................ucuggaauUcaucugacaaa......................................................................   1     1  sg1
....................ucuggaGuccaucugacaaa......................................................................   1     1  sg1
....................ucuggaauccaucugacaaUc.....................................................................   2     1  sg1
....................ucuggaauccaucugGcaaac.....................................................................   89    1  sg1
....................ucuggaauccaucugacUaac.....................................................................   5     1  sg1
....................ucuggaauccaucugaUaaac.....................................................................   8     1  sg1
....................ucuggaauccaucugacaaaA.....................................................................   32    1  sg1
....................ucuggaauccaucugacaaGc.....................................................................   52    1  sg1
....................ucuggaauccaucAgacaaac.....................................................................   4     1  sg1
....................ucuggaauccaucugacaaCc.....................................................................   3     1  sg1
....................ucuggaauccaucCgacaaac.....................................................................   25    1  sg1
....................ucAggaauccaucugacaaac.....................................................................   10    1  sg1
....................ucCggaauccaucugacaaac.....................................................................   11    1  sg1
....................ucuggaauccaucugacaUac.....................................................................   1     1  sg1
....................ucuggaauccaucugacCaac.....................................................................   4     1  sg1
....................ucugAaauccaucugacaaac.....................................................................   1     1  sg1
....................ucuggaauccaucuAacaaac.....................................................................   3     1  sg1
....................ucuggaaGccaucugacaaac.....................................................................   1     1  sg1
....................ucuggUauccaucugacaaac.....................................................................   2     1  sg1
....................ucuggaauccaucugacaGac.....................................................................   134   1  sg1
....................ucuggaCuccaucugacaaac.....................................................................   2     1  sg1
....................ucuUgaauccaucugacaaac.....................................................................   2     1  sg1
....................ucuggaauAcaucugacaaac.....................................................................   1     1  sg1
....................ucuggaauccCucugacaaac.....................................................................   1     1  sg1
....................ucuggaauccaAcugacaaac.....................................................................   1     1  sg1
....................ucuggaaucAaucugacaaac.....................................................................   1     1  sg1
....................ucuggaaucUaucugacaaac.....................................................................   3     1  sg1
....................uUuggaauccaucugacaaac.....................................................................   5     1  sg1
....................ucuggaauccaucGgacaaac.....................................................................   1     1  sg1
....................ucuggaGuccaucugacaaac.....................................................................   55    1  sg1
....................Acuggaauccaucugacaaac.....................................................................   6     1  sg1
....................ucuggaauccaucugUcaaac.....................................................................   3     1  sg1
....................ucuggaauccauAugacaaac.....................................................................   2     1  sg1
....................ucuggaauccaCcugacaaac.....................................................................   13    1  sg1
....................ucuggaauccaucugaAaaac.....................................................................   2     1  sg1
....................ucuggGauccaucugacaaac.....................................................................   26    1  sg1
....................ucuggaauccaucugacaaaU.....................................................................   209   1  sg1
....................ucuggaauUcaucugacaaac.....................................................................   6     1  sg1
....................ucuggaauccaucugacaaaG.....................................................................   5     1  sg1
....................ucuggaauccaucugacGaac.....................................................................   23    1  sg1
....................ucuAgaauccaucugacaaac.....................................................................   6     1  sg1
....................ucuggaauccGucugacaaac.....................................................................   14    1  sg1
....................ucuggaauccUucugacaaac.....................................................................   6     1  sg1
....................ucuggaauccaucugacaaac.....................................................................   6305  0  sg1
....................ucuggaaCccaucugacaaac.....................................................................   17    1  sg1
....................ucuggaauccaucuUacaaac.....................................................................   1     1  sg1
....................ucuggaauccaucugaGaaac.....................................................................   1     1  sg1
....................Gcuggaauccaucugacaaac.....................................................................   4     1  sg1
....................ucGggaauccaucugacaaac.....................................................................   1     1  sg1
....................uAuggaauccaucugacaaac.....................................................................   2     1  sg1
....................ucuggaauccaucugGcaaacu....................................................................   169   1  sg1
....................ucuggaCuccaucugacaaacu....................................................................   4     1  sg1
....................ucuggaauccaucugUcaaacu....................................................................   7     1  sg1
....................ucuggaauccaucugacaUacu....................................................................   6     1  sg1
....................uUuggaauccaucugacaaacu....................................................................   11    1  sg1
....................ucuggaauccaucugaAaaacu....................................................................   3     1  sg1
....................ucuggaauccaucugaUaaacu....................................................................   13    1  sg1
....................ucuggaauccaucAgacaaacu....................................................................   10    1  sg1
....................ucuggaauccaucugacaaaGu....................................................................   1     1  sg1
....................ucuAgaauccaucugacaaacu....................................................................   3     1  sg1
....................uGuggaauccaucugacaaacu....................................................................   1     1  sg1
....................ucuggaauccGucugacaaacu....................................................................   28    1  sg1
```

```
                    Mature                                Star
acuuuauucuuuuuuuuuuaucuggaauccaucugacaaacugcauguuugucaugcaguuugucauggauuccaguuaaaaaaaagcaugcaaucguacgucaaggaga
....................ucuggaauAcaucugacaaacu....................................................................    2      1    sg1
....................ucuggaauccaucugacaaacG....................................................................    130    1    sg1
....................ucuggaauccaucugacUaacu....................................................................    19     1    sg1
....................ucuggUauccaucugacaaacu....................................................................    3      1    sg1
....................ucuggaaCccaucugacaaacu....................................................................    36     1    sg1
....................ucuggaauccaucCgacaaacu....................................................................    43     1    sg1
....................ucuggaauccaucugacaaacu....................................................................    12127  0    sg1
....................ucuggaauccaucugacaaaAu....................................................................    2      1    sg1
....................ucuggaauccaucugCcaaacu....................................................................    1      1    sg1
....................ucuggaauccaCcugacaaacu....................................................................    25     1    sg1
....................ucuggaauccaucugacaaacA....................................................................    275    1    sg1
....................ucuggaauccaucugacaCacu....................................................................    2      1    sg1
....................ucuggaauccaGcugacaaacu....................................................................    2      1    sg1
....................ucuggaauccauUugacaaacu....................................................................    6      1    sg1
....................ucuggaauccaAcugacaaacu....................................................................    6      1    sg1
....................ucuggaauccaucGgacaaacu....................................................................    2      1    sg1
....................ucuggaaucUaucugacaaacu....................................................................    11     1    sg1
....................ucuggaauccaucugacaGacu....................................................................    267    1    sg1
....................Acuggaauccaucugacaaacu....................................................................    14     1    sg1
....................ucuggaauUcaucugacaaacu....................................................................    8      1    sg1
....................ucuggaauccaucugacCaacu....................................................................    3      1    sg1
....................Gcuggaauccaucugacaaacu....................................................................    5      1    sg1
....................ucuUgaauccaucugacaaacu....................................................................    2      1    sg1
....................ucuggaauccaucuAacaaacu....................................................................    8      1    sg1
....................ucuggGauccaucugacaaacu....................................................................    77     1    sg1
....................ucuggaauccauAugacaaacu....................................................................    5      1    sg1
....................ucuggaaAccaucugacaaacu....................................................................    2      1    sg1
....................ucuggaauccaucugacaaaUu....................................................................    16     1    sg1
....................uAuggaauccaucugacaaacu....................................................................    1      1    sg1
....................ucuggaauccaucugacGaacu....................................................................    38     1    sg1
....................ucuggaauccaucugacaaCcu....................................................................    4      1    sg1
....................ucCggaauccaucugacaaacu....................................................................    30     1    sg1
....................ucuggaauccaucugacaaUcu....................................................................    6      1    sg1
....................ucuggaauccaucugacaaacC....................................................................    1037   1    sg1
....................ucugAaauccaucugacaaacu....................................................................    2      1    sg1
....................ucuggaauccaucugacaaGcu....................................................................    72     1    sg1
....................ucAggaauccaucugacaaacu....................................................................    10     1    sg1
....................ucuggaGuccaucugacaaacu....................................................................    92     1    sg1
....................ucugUaauccaucugacaaacu....................................................................    1      1    sg1
....................ucuggCauccaucugacaaacu....................................................................    2      1    sg1
....................ucuggaGuccaucugacaaacug...................................................................    1324   1    sg1
....................ucuggaauccaucugacaaacAg...................................................................    79     1    sg1
....................ucuggaauccaucugacaaGcug...................................................................    487    1    sg1
....................ucuggUauccaucugacaaacug...................................................................    42     1    sg1
....................ucuggaauccaGcugacaaacug...................................................................    6      1    sg1
....................ucuggaauccaucugCcaaacug...................................................................    16     1    sg1
....................Acuggaauccaucugacaaacug...................................................................    71     1    sg1
....................ucuggaauccaucugacaaaGug...................................................................    3      1    sg1
....................ucAggaauccaucugacaaacug...................................................................    63     1    sg1
....................ucuggaauccaucugGcaaacug...................................................................    1298   1    sg1
....................ucuggaauccaucugacaaacuC...................................................................    145    1    sg1
....................ucuggaauccaucugacaaacCg...................................................................    431    1    sg1
....................ucuggaauccaucugaUaaacug...................................................................    55     1    sg1
....................uAuggaauccaucugacaaacug...................................................................    20     1    sg1
....................ucuCgaauccaucugacaaacug...................................................................    2      1    sg1
....................ucuggaUuccaucugacaaacug...................................................................    3      1    sg1
....................ucuggaaGccaucugacaaacug...................................................................    14     1    sg1
....................ucuggaaucGaucugacaaacug...................................................................    1      1    sg1
....................ucuggaauccaucugacaGacug...................................................................    3707   1    sg1
....................ucuggaauccaucugacaUacug...................................................................    37     1    sg1
....................ucuggaauccaAcugacaaacug...................................................................    36     1    sg1
....................ucuggCauccaucugacaaacug...................................................................    11     1    sg1
....................ucuUgaauccaucugacaaacug...................................................................    12     1    sg1
....................ucuggaauUcaucugacaaacug...................................................................    70     1    sg1
....................ucuggaauccCucugacaaacug...................................................................    15     1    sg1
....................ucuggaaAccaucugacaaacug...................................................................    14     1    sg1
....................ucuggaauccauGugacaaacug...................................................................    1      1    sg1
....................ucugAaauccaucugacaaacug...................................................................    57     1    sg1
....................ucuggaauccaucugacaaaUug...................................................................    50     1    sg1
....................ucuggaauccGucugacaaacug...................................................................    232    1    sg1
```

```
                    Mature                                     Star
acuuuauucuuuuuuuuuuaucuggaauccaucugacaaacugcauguuugucaugcaguuugucauggauuccaguuaaaaaaaagcaugcaaucguacgucaaggaga
....................ucuggaauGcaucugacaaacug...................................................................    2      1    sg1
....................ucuggaauccaucugacaaacuU...................................................................    377    1    sg1
....................ucuggaauccaucugacaCacug...................................................................    11     1    sg1
....................ucuggaauccaucugacaaaAug...................................................................    20     1    sg1
....................ucuggaaucAaucugacaaacug...................................................................    10     1    sg1
....................ucuggaauccauUugacaaacug...................................................................    36     1    sg1
....................ucuggaaucUaucugacaaacug...................................................................    81     1    sg1
....................ucuggaauAcaucugacaaacug...................................................................    7      1    sg1
....................ucuggaauccaucuUacaaacug...................................................................    5      1    sg1
....................ucuggaauccaucugacaaCcug...................................................................    36     1    sg1
....................ucuggaauccUucugacaaacug...................................................................    23     1    sg1
....................ucuggaauccaucugacUaacug...................................................................    77     1    sg1
....................ucuggaauccaucugaAaaacug...................................................................    30     1    sg1
....................ucuggaauccaCcugacaaacug...................................................................    242    1    sg1
....................ucuggaaCccaucugacaaacug...................................................................    253    1    sg1
....................ucugUaauccaucugacaaacug...................................................................    9      1    sg1
....................uUuggaauccaucugacaaacug...................................................................    85     1    sg1
....................ucuAgaauccaucugacaaacug...................................................................    51     1    sg1
....................ucuggaauccaucugacGaacug...................................................................    435    1    sg1
....................ucuggaauccaucugacCaacug...................................................................    19     1    sg1
....................Gcuggaauccaucugacaaacug...................................................................    21     1    sg1
....................ucuggaauccaucugUcaaacug...................................................................    33     1    sg1
....................ucuggaauccaucugacaaacug...................................................................    96196  0    sg1
....................ucuggaauccaucugaGaaacug...................................................................    4      1    sg1
....................ucuggaauccaucAgacaaacug...................................................................    60     1    sg1
....................ucuggaauccaucCgacaaacug...................................................................    307    1    sg1
....................ucuggaCuccaucugacaaacug...................................................................    6      1    sg1
....................ucuggaauccaucGgacaaacug...................................................................    19     1    sg1
....................ucuggGauccaucugacaaacug...................................................................    494    1    sg1
....................ucuggaauccaucuAacaaacug...................................................................    41     1    sg1
....................ucuggaauccaucugacaaUcug...................................................................    27     1    sg1
....................uGuggaauccaucugacaaacug...................................................................    7      1    sg1
....................ucuggaauccaucuCacaaacug...................................................................    1      1    sg1
....................ucGggaauccaucugacaaacug...................................................................    12     1    sg1
....................ucugCaauccaucugacaaacug...................................................................    3      1    sg1
....................ucuggaauccaucugacaaacGg...................................................................    172    1    sg1
....................ucCggaauccaucugacaaacug...................................................................    263    1    sg1
....................ucuggaauccaucugacaaacuA...................................................................    6852   1    sg1
....................ucuggaauccauAugacaaacug...................................................................    8      1    sg1
....................ucuggaauccaucugacaaacugA..................................................................    474    1    sg1
....................ucuggaauccaucugacaCacugc..................................................................    2      1    sg1
....................Acuggaauccaucugacaaacugc..................................................................    11     1    sg1
....................ucuggaauccauUugacaaacugc..................................................................    2      1    sg1
....................ucuggaauccaucugacaUacugc..................................................................    2      1    sg1
....................ucuggaauccGucugacaaacugc..................................................................    22     1    sg1
....................ucuggaauccaucugacaaacuAc..................................................................    16     1    sg1
....................ucuggaauccaucGgacaaacugc..................................................................    2      1    sg1
....................Gcuggaauccaucugacaaacugc..................................................................    1      1    sg1
....................ucuggaGuccaucugacaaacugc..................................................................    62     1    sg1
....................ucuggaauUcaucugacaaacugc..................................................................    3      1    sg1
....................ucuggaauccaucugacaaacugc..................................................................    6835   0    sg1
....................ucuggGauccaucugacaaacugc..................................................................    32     1    sg1
....................ucuggaauccaucugacaaUcugc..................................................................    2      1    sg1
....................ucuggaauccaucugacaaGcugc..................................................................    23     1    sg1
....................ucuggaauccaucuAacaaacugc..................................................................    3      1    sg1
....................ucuggaauccaucugGcaaacugc..................................................................    81     1    sg1
....................ucuggaauccaucugacaaaUugc..................................................................    2      1    sg1
....................ucuggaauccaucugacaaacuUc..................................................................    1      1    sg1
....................ucuggCauccaucugacaaacugc..................................................................    1      1    sg1
....................ucuggaauccaucugacaaacuCc..................................................................    2      1    sg1
....................ucuggaauccaAcugacaaacugc..................................................................    2      1    sg1
....................ucuAgaauccaucugacaaacugc..................................................................    2      1    sg1
....................ucuggaauccaucuUacaaacugc..................................................................    1      1    sg1
....................ucCggaauccaucugacaaacugc..................................................................    10     1    sg1
....................ucugAaauccaucugacaaacugc..................................................................    3      1    sg1
....................ucuggaauccaucugacaGacugc..................................................................    124    1    sg1
....................ucuggaauccaucAgacaaacugc..................................................................    5      1    sg1
....................ucuggaauccaucugUcaaacugc..................................................................    1      1    sg1
....................ucuggaaucUaucugacaaacugc..................................................................    8      1    sg1
....................uAuggaauccaucugacaaacugc..................................................................    1      1    sg1
```

```
                    Mature                                Star
acuuuauucuuuuuuuuuuaucuggaauccaucugacaaacugcauguuugucaugcaguuugucauggauuccaguuaaaaaaaagcaugcaaucguacgucaaggaga
....................ucuggaauccaucugacaaacugU..................................................................   694    1    sg1
....................ucuggaauccaucugacGaacugc..................................................................   30     1    sg1
....................ucuggaaGccaucugacaaacugc..................................................................   1      1    sg1
....................ucAggaauccaucugacaaacugc..................................................................   7      1    sg1
....................ucuggaauccaCcugacaaacugc..................................................................   21     1    sg1
....................ucGggaauccaucugacaaacugc..................................................................   4      1    sg1
....................ucuggaauccaucugacUaacugc..................................................................   4      1    sg1
....................ucuggUauccaucugacaaacugc..................................................................   1      1    sg1
....................uUuggaauccaucugacaaacugc..................................................................   8      1    sg1
....................ucuggaaAccaucugacaaacugc..................................................................   2      1    sg1
....................ucuggaauccaucugacaaacCgc..................................................................   10     1    sg1
....................ucuggaaCccaucugacaaacugc..................................................................   15     1    sg1
....................ucuggaauccaucCgacaaacugc..................................................................   20     1    sg1
....................ucuggaauccaucugacaaacugG..................................................................   27     1    sg1
....................ucuggaauccUucugacaaacugc..................................................................   2      1    sg1
....................ucugUaauccaucugacaaacugc..................................................................   1      1    sg1
....................ucuggaauccaucugacaaCcugc..................................................................   3      1    sg1
....................ucuggaauccaucugacaaacugcU.................................................................   324    1    sg1
....................uAuggaauccaucugacaaacugca.................................................................   5      1    sg1
....................ucuggaauccaucugUcaaacugca.................................................................   3      1    sg1
....................ucuggaauccaucCgacaaacugca.................................................................   18     1    sg1
....................ucuggaauccaucugGcaaacugca.................................................................   104    1    sg1
....................ucuggaauccaucugacaaUcugca.................................................................   1      1    sg1
....................ucuggaauccaucugacaaCcugca.................................................................   2      1    sg1
....................ucuggaauccaucugacCaacugca.................................................................   1      1    sg1
....................ucuggaaAccaucugacaaacugca.................................................................   1      1    sg1
....................ucuggaauccaucugacGaacugca.................................................................   20     1    sg1
....................ucuggaauccaGcugacaaacugca.................................................................   1      1    sg1
....................ucuggaaCccaucugacaaacugca.................................................................   8      1    sg1
....................ucuggaauccaAcugacaaacugca.................................................................   2      1    sg1
....................ucuggaaucAaucugacaaacugca.................................................................   3      1    sg1
....................ucugAaauccaucugacaaacugca.................................................................   1      1    sg1
....................uUuggaauccaucugacaaacugca.................................................................   2      1    sg1
....................ucuggaaucUaucugacaaacugca.................................................................   6      1    sg1
....................ucCggaauccaucugacaaacugca.................................................................   21     1    sg1
....................ucuggaaGccaucugacaaacugca.................................................................   1      1    sg1
....................ucuggaauccaCcugacaaacugca.................................................................   11     1    sg1
....................ucuggaauccaucAgacaaacugca.................................................................   3      1    sg1
....................ucuggaauccaucugaAaaacugca.................................................................   1      1    sg1
....................ucuggaauAcaucugacaaacugca.................................................................   2      1    sg1
....................ucuggaauccaucugaUaaacugca.................................................................   2      1    sg1
....................ucGggaauccaucugacaaacugca.................................................................   2      1    sg1
....................ucuggaauccaucugacaaacugcG.................................................................   88     1    sg1
....................ucuggaauccGucugacaaacugca.................................................................   13     1    sg1
....................ucuggaauccaucugacaaacugcC.................................................................   47     1    sg1
....................ucuggaauccaucugacaaacCgca.................................................................   11     1    sg1
....................ucuggaauccaucugacaaacugGa.................................................................   1      1    sg1
....................ucuggaGuccaucugacaaacugca.................................................................   37     1    sg1
....................ucuggaauccaucugacaaacugUa.................................................................   379    1    sg1
....................ucuggaauccaucugacaaacugAa.................................................................   11     1    sg1
....................ucuggaauccaucugacaaaGugca.................................................................   1      1    sg1
....................ucuggaauccaucugacaaaUugca.................................................................   3      1    sg1
....................ucAggaauccaucugacaaacugca.................................................................   5      1    sg1
....................ucuggaauccaucugacaaGcugca.................................................................   17     1    sg1
....................Acuggaauccaucugacaaacugca.................................................................   3      1    sg1
....................ucuggaauccaucugacaGacugca.................................................................   75     1    sg1
....................ucuggaauccaucugacaCacugca.................................................................   3      1    sg1
....................ucuggaauccaucugaGaaacugca.................................................................   1      1    sg1
....................uGuggaauccaucugacaaacugca.................................................................   1      1    sg1
....................ucuggaauGcaucugacaaacugca.................................................................   1      1    sg1
....................ucuggUauccaucugacaaacugca.................................................................   2      1    sg1
....................ucuggaauccaucGgacaaacugca.................................................................   3      1    sg1
....................ucuggaauUcaucugacaaacugca.................................................................   4      1    sg1
....................ucuggaauccaucugacUaacugca.................................................................   5      1    sg1
....................ucuggaauccaucugacaaacGgca.................................................................   2      1    sg1
....................ucuggGauccaucugacaaacugca.................................................................   15     1    sg1
....................ucuggaauccaucugacaaacAgca.................................................................   3      1    sg1
....................ucuggaauccaucuAacaaacugca.................................................................   3      1    sg1
....................ucuggaauccauUugacaaacugca.................................................................   1      1    sg1
....................ucuggaauccaucugacaaacugca.................................................................   4632   0    sg1
```

Mature Star

acuuuauucuuuuuuuuuuaucuggaauccaucugacaaacugcauguuugucaugcaguuugucauggauuccaguuaaaaaaaagcaugcaaucguacgucaaggaga			
....................ucuggaauccauAugacaaacugca...	1	1	sg1
....................ucuggaauccaucugacaaacuAca...	5	1	sg1
....................ucuggaauccGucugacaaacugcau..	1	1	sg1
....................ucuggaauccaucugacaaacugcCu..	2	1	sg1
....................ucuggaauccaucugacaaacuCcau..	1	1	sg1
....................ucuggaauccaucugacaaacugUau..	13	1	sg1
....................ucuggaauccaucugGcaaacugcau..	4	1	sg1
....................ucuggaauccaucugaAaaacugcau..	1	1	sg1
....................ucuggaauccaucugacaaacugcUu..	39	1	sg1
....................ucuggaauccaucugacaaacugAau..	1	1	sg1
....................ucuggaauccaucugacaaacugcaA..	111	1	sg1
....................ucuggaauccaucugacGaacugcau..	1	1	sg1
....................ucuggaauccaucugacaaacugcaC..	8	1	sg1
....................ucuggaauccaucCgacaaacugcau..	1	1	sg1
....................ucuggaauccaucugacaaacugcaG..	194	1	sg1
....................ucuggaauccaucugacaaacugcau..	135	0	sg1
....................ucuggaauccaCcugacaaacugcau..	2	1	sg1
....................ucuggaauccaucugacaGacugcau..	7	1	sg1
....................ucuggaaucUaucugacaaacugcau..	1	1	sg1
....................ucuggaauUcaucugacaaacugcau..	1	1	sg1
....................ucuggaauccaucugacaaacugcaAg...	3	1	sg1
....................ucuggaauccaucugacaaacugcaGg...	1	1	sg1
....................ucuggaauccaucugacaUacugcaug...	1	1	sg1
....................ucuggaauccaucugGcaaacugcaug...	1	1	sg1
....................ucuggaauccaucugacaaacugcUug...	1	1	sg1
....................ucuggaauccaucugacaaacugcauC...	21	1	sg1
....................ucuggaauccaucugacaaacugcauU...	214	1	sg1
....................ucuggaauccaucugacaaacugUaug...	2	1	sg1
....................ucuggaauccaucugacaGacugcaug...	1	1	sg1
....................ucuggaauccaucugacaaacugcaug...	22	0	sg1
....................ucuggaauccaucugacaaacugcauA...	297	1	sg1
.....................cuggaauccaucugacaaac...	6	0	sg1
.....................cuggaauccaucugacaaacC..	1	1	sg1
.....................cuggaauccaucugacaaacu..	9	0	sg1
.....................cuggaauccaucugacaGacu..	1	1	sg1
.....................cCggaauccaucugacaaacu..	1	1	sg1
.....................cuggaauccaucugacaaacCg...	2	1	sg1
.....................cuggaauccaucugacaGacug...	5	1	sg1
.....................cuggUauccaucugacaaacug...	1	1	sg1
.....................cuggaauccaucugacaaacuA...	10	1	sg1
.....................cuggaauccaCcugacaaacug...	1	1	sg1
.....................cuggaauccaucugacGaacug...	1	1	sg1
.....................cuggaauccaucugacaaacug...	75	0	sg1
.....................Uuggaauccaucugacaaacug...	1	1	sg1
.....................cuggaauccaucugGcaaacugc..	1	1	sg1
.....................cuggaauccaucugacaaacugc..	7	0	sg1
.....................cuggaauccaucugacaaacugca...	2	0	sg1
.....................cuggaauccaucugacaaacugcauA...	1	1	sg1
......................uggaauccaucugacaaa..	1	0	sg1
......................uggaauccaucugacaaac...	17	0	sg1
......................uggaauccaucugGcaaac...	4	1	sg1
......................uggaauccaucugacaGac...	2	1	sg1
......................uggaauccaucugacaaacu..	44	0	sg1
......................uggaauccaucugacaGacu..	3	1	sg1
......................uggaauccaucugGcaaacu..	2	1	sg1
......................uggaauccaucugacaaacA..	1	1	sg1
......................uggaauccaucugacGaacu..	1	1	sg1
......................uggaaCccaucugacaaacu..	1	1	sg1
......................uggaauccaucugacaGacug...	12	1	sg1
......................Cggaauccaucugacaaacug...	4	1	sg1
......................uggGauccaucugacaaacug...	3	1	sg1
......................uggaauccaucugacaaacuU...	2	1	sg1
......................uggaGuccaucugacaaacug...	1	1	sg1
......................uggaauccaucugacGaacug...	2	1	sg1
......................uAgaauccaucugacaaacug...	1	1	sg1
......................uggaauccaucugacaaacug...	332	0	sg1
......................Aggaauccaucugacaaacug...	1	1	sg1
......................uggaauccaucugacaaacCg...	1	1	sg1
......................uggaauUcaucugacaaacug...	1	1	sg1
......................uggaaucUaucugacaaacug...	3	1	sg1

```
                    Mature                                 Star
acuuuauucuuuuuuuuuuaucuggaauccaucugacaaacugcauguuugucaugcaguuugucauggauuccaguuaaaaaaaagcaugcaaucguacgucaaggaga
......................uggaauccaucugacaaacuA...................................................................  18   1  sg1
......................uggaauccaucugGcaaacug...................................................................  38   1  sg1
......................uggaauccaucugacaaacuC...................................................................  2    1  sg1
......................uggaauccaucugacaaacugc..................................................................  66   0  sg1
......................uggaauccaucugacaaacugA..................................................................  4    1  sg1
......................uggaauccaucugGcaaacugc..................................................................  2    1  sg1
......................uggaauccaucugacaaacugU..................................................................  7    1  sg1
......................Cggaauccaucugacaaacugc..................................................................  1    1  sg1
......................uggaauccaucugacaaacAgc..................................................................  1    1  sg1
......................uggaauccaucugacaGacugc..................................................................  1    1  sg1
......................uggaauccaucugacaaacugUa.................................................................  1    1  sg1
......................uggaauccaucugacaaacugcU.................................................................  4    1  sg1
......................uggaauccaucugacaGacugca.................................................................  4    1  sg1
......................uggaauccaucugGcaaacugca.................................................................  13   1  sg1
......................uggaauccaucugacaaacugca.................................................................  65   0  sg1
......................uggaauccaucugacGaacugca.................................................................  3    1  sg1
......................uggaauccaucugacaaacugcUu................................................................  1    1  sg1
......................uggaauccaucugGcaaacugcau................................................................  2    1  sg1
......................uggaauccaucugacaaacugcaA................................................................  8    1  sg1
......................uggGauccaucugacaaacugcau................................................................  1    1  sg1
......................uggaauccaucugacaaacugcaC................................................................  1    1  sg1
......................uggaauccaucugacaaacugcaG................................................................  16   1  sg1
......................uggaauccaucugacaaacugcau................................................................  17   0  sg1
......................uggaauccaucugacaaacugcaAg...............................................................  1    1  sg1
......................uggaauccaucugacaGacugcaug...............................................................  1    1  sg1
......................uggaauccaucugacaaacugcauA...............................................................  85   1  sg1
......................uggaauccaucugacaaacugcauU...............................................................  12   1  sg1
......................uggaauccaucugacaaacugcaug...............................................................  1    0  sg1
......................uggaauccaucugacaaacugcauUu..............................................................  1    1  sg1
......................uggaauccaucugacaaacugcauAu..............................................................  8    1  sg1
......................uggaauccaucugacaaacugcauAuu.............................................................  1    1  sg1
.......................ggaauccaucugacaaacug...................................................................  3    0  sg1
.......................ggaauccaucugacaaacugc..................................................................  2    0  sg1
........................gaauccaucugacaaacu....................................................................  1    0  sg1
.........................aauccaucugacaaacugcauUu..............................................................  1    1  sg1
..........................auccaucugacaaacugca.................................................................  2    0  sg1
........................................................caguuugucauggauucc....................................  4    0  sg1
........................................................caguuugucauggauuccag..................................  9    0  sg1
........................................................caguuugucauggauuccagu.................................  3    0  sg1
........................................................caguuugucauggauuccagA.................................  1    1  sg1
........................................................caguuugucauggauuccagGu................................  1    1  sg1
........................................................caguuugucauggauuccaguA................................  3    1  sg1
........................................................cUguuugucauggauuccaguu................................  2    1  sg1
........................................................caguuugucauggauCccaguu................................  1    1  sg1
........................................................caguuugucauggauuccaguu................................  68   0  sg1
........................................................caguuugucauggauuccaguC................................  7    1  sg1
........................................................cGguuugucauggauuccaguua...............................  2    1  sg1
........................................................caguuugucaugUauuccaguua...............................  1    1  sg1
........................................................caguuugucGuggauuccaguua...............................  1    1  sg1
........................................................cagCuugucauggauuccaguua...............................  1    1  sg1
........................................................caguuugucauggGuuccaguua...............................  1    1  sg1
........................................................caguuCgucauggauuccaguua...............................  2    1  sg1
........................................................caguuugucauggauuccaguuC...............................  1    1  sg1
........................................................caguuugucauggauuccaguuU...............................  5    1  sg1
........................................................caguuugucauggauuccaguua...............................  215  0  sg1
........................................................caguuugucauggauuccaguuUa..............................  5    1  sg1
........................................................caguuugucauggauuccaguuaa..............................  9    0  sg1
........................................................caguuugucauggauuccaguuaU..............................  4    1  sg1
........................................................caguuugucauggauuccaguuaG..............................  1    1  sg1
........................................................caguuugucauggauuccaguuaUa.............................  2    1  sg1
........................................................caguuugucauggauuccaguuaaUa............................  1    1  sg1
.........................................................aguuugucauggauucca...................................  2    0  sg1
.........................................................aguuugucauggauuccagu.................................  7    0  sg1
.........................................................aguuugucauggauuccaguA................................  1    1  sg1
.........................................................aguuugucauggauuccagGu................................  1    1  sg1
.........................................................aguuugucGuggauuccaguu................................  1    1  sg1
.........................................................aguuugucaCggauuccaguu................................  1    1  sg1
.........................................................aguuugucauggauuccaguu................................  95   0  sg1
.........................................................aguuugucauggauuccaguC................................  7    1  sg1
.........................................................aguuugucauggauuUcaguua...............................  1    1  sg1
```

Mature Star

```
acuuuauucuuuuuuuuuuaucuggaauccaucugacaaacugcauguuugucaugcaguuugucauggauuccaguuaaaaaaaagcaugcaaucguacgucaaggaga
.........................................................aguuugCcauggauuccaguua...............................   3    1    sg1
.........................................................aguuugucauggauuccagAua...............................   1    1    sg1
.........................................................aguuugucaugAauuccaguua...............................   1    1    sg1
.........................................................aguuugucauggauuccaguuU...............................   28   1    sg1
.........................................................aguuugucauggauuccaguua...............................   286  0    sg1
.........................................................aguuugucauggaCuccaguua...............................   3    1    sg1
.........................................................aguuugucauggauuccCguua...............................   1    1    sg1
.........................................................aguuugucauggGuuccaguua...............................   2    1    sg1
.........................................................Uguuugucauggauuccaguua...............................   2    1    sg1
.........................................................aguuugucauggauuccaguuC...............................   2    1    sg1
.........................................................aguuugucauggauuccaguuaa..............................   22   0    sg1
.........................................................aguuugucauggauuccaguuaG..............................   2    1    sg1
.........................................................aguuugucauggauuccaguuUa..............................   43   1    sg1
.........................................................aguuugucauggauuccaguuaU..............................   6    1    sg1
.........................................................aguuugucauggauuccaguuaGa.............................   1    1    sg1
.........................................................aguuugucauggauuccaguuaUa.............................   18   1    sg1
.........................................................aguuugucauggauuccaguuaUaa............................   1    1    sg1
.........................................................aguuugucauggauuccaguuaUaaa...........................   1    1    sg1
...........................................................uuugucauggauuccaguuU...............................   1    1    sg1
...........................................................uuugucauggauuccaguua...............................   4    0    sg1
...........................................................uuugucauggauuccaguuUa..............................   2    1    sg1
...........................................................uuugucauggauuccaguuaUa.............................   1    1    sg1

..................uaucuggaauccaucugacaaacu....................................................................   3    0    so1
..................uaucuggaauccaucugacaaacuA...................................................................   1    1    so1
..................uCucuggaauccaucugacaaacug...................................................................   4    1    so1
..................uaucuggaauccaucugacaaacug...................................................................   7    0    so1
...................Cucuggaauccaucugacaaac.....................................................................   5    1    so1
...................Cucuggaauccaucugacaaacu....................................................................   10   1    so1
...................Cucuggaauccaucugacaaacug...................................................................   92   1    so1
...................aucuggaauccaucugacaaacuA...................................................................   2    1    so1
...................Cucuggaauccaucugacaaacugc..................................................................   4    1    so1
...................Cucuggaauccaucugacaaacugca.................................................................   2    1    so1
....................ucuggGauccaucugaca........................................................................   1    1    so1
....................ucuggaauccaucugaca........................................................................   144  0    so1
....................ucuggaGuccaucugaca........................................................................   2    1    so1
....................ucuggaauccaucugacG........................................................................   5    1    so1
....................ucuggaauccaucugGca........................................................................   2    1    so1
....................ucuggaauccaucugacU........................................................................   2    1    so1
....................ucuggaauccauUugaca........................................................................   1    1    so1
....................ucuggaauccaCcugacaa.......................................................................   3    1    so1
....................ucuggaauccaucugacaa.......................................................................   128  0    so1
....................ucuggaauccaucugacaG.......................................................................   6    1    so1
....................ucuggaauccaucugacUa.......................................................................   1    1    so1
....................ucuggaaCccaucugacaa.......................................................................   2    1    so1
....................ucuggaauccaAcugacaa.......................................................................   1    1    so1
....................ucuggaauccaucugGcaa.......................................................................   1    1    so1
....................ucuggaauccaucugacCa.......................................................................   2    1    so1
....................ucuggaGuccaucugacaa.......................................................................   1    1    so1
....................ucuggaauccaucugacaaa......................................................................   452  0    so1
....................ucuggaauccaucugacaaG......................................................................   1    1    so1
....................ucuggGauccaucugacaaa......................................................................   1    1    so1
....................ucuggaaucUaucugacaaa......................................................................   1    1    so1
....................ucuggaauccaCcugacaaa......................................................................   2    1    so1
....................ucuAgaauccaucugacaaa......................................................................   1    1    so1
....................ucuggaaAccaucugacaaa......................................................................   1    1    so1
....................ucCggaauccaucugacaaa......................................................................   4    1    so1
....................ucGggaauccaucugacaaa......................................................................   2    1    so1
....................ucuggaauccaucugacaaC......................................................................   4    1    so1
....................ucuggaGuccaucugacaaa......................................................................   2    1    so1
....................ucuggaauccaucugacaaU......................................................................   1    1    so1
....................ucuggaauccaucugGcaaa......................................................................   4    1    so1
....................ucuggaauccaucugacaGa......................................................................   9    1    so1
....................ucuggaauUcaucugacaaa......................................................................   2    1    so1
....................uUuggaauccaucugacaaa......................................................................   1    1    so1
....................ucuggaauccaucCgacaaa......................................................................   5    1    so1
....................ucuggaauccaucugaUaaa......................................................................   1    1    so1
....................ucuAgaauccaucugacaaac.....................................................................   2    1    so1
....................ucuggaauccaucugacUaac.....................................................................   3    1    so1
....................ucuggaaAccaucugacaaac.....................................................................   1    1    so1
```

```
                    Mature                                    Star
acuuuauucuuuuuuuuuuaucuggaauccaucugacaaacugcauguuugucaugcaguuugucauggauuccaguuaaaaaaaagcaugcaaucguacgucaaggaga
....................ucuggaauccaucugCcaaac.....................................................................    3       1   so1
....................ucuggaauccGucugacaaac.....................................................................    16      1   so1
....................ucuggaauccaucugacaUac.....................................................................    2       1   so1
....................ucugAaauccaucugacaaac.....................................................................    2       1   so1
....................ucuggUauccaucugacaaac.....................................................................    1       1   so1
....................ucuggaauccaucugacaaaA.....................................................................    57      1   so1
....................ucuggaauccaucugacaGac.....................................................................    82      1   so1
....................ucuggaauccaucugacaaac.....................................................................    4095    0   so1
....................ucuggaauccaCcugacaaac.....................................................................    9       1   so1
....................ucuggaauAcaucugacaaac.....................................................................    2       1   so1
....................ucCggaauccaucugacaaac.....................................................................    6       1   so1
....................ucuggaauccaucAgacaaac.....................................................................    2       1   so1
....................ucuggaauUcaucugacaaac.....................................................................    5       1   so1
....................ucuggaauccaucugUcaaac.....................................................................    1       1   so1
....................ucuggaauccaAcugacaaac.....................................................................    2       1   so1
....................ucuggaauccaucuUacaaac.....................................................................    2       1   so1
....................ucuggGauccaucugacaaac.....................................................................    27      1   so1
....................ucuggaGuccaucugacaaac.....................................................................    27      1   so1
....................ucuggaauccaucCgacaaac.....................................................................    17      1   so1
....................ucuggaauccaucugacCaac.....................................................................    1       1   so1
....................Acuggaauccaucugacaaac.....................................................................    7       1   so1
....................Gcuggaauccaucugacaaac.....................................................................    1       1   so1
....................ucuggaaCccaucugacaaac.....................................................................    14      1   so1
....................ucuggaauccaucugacaaaU.....................................................................    290     1   so1
....................uAuggaauccaucugacaaac.....................................................................    1       1   so1
....................ucuggaauccaucugaUaaac.....................................................................    2       1   so1
....................ucuggaauccaucugacGaac.....................................................................    22      1   so1
....................ucuggaauccaucugGcaaac.....................................................................    83      1   so1
....................ucuggaauccaucuAacaaac.....................................................................    4       1   so1
....................ucuggaauccaucugacaaGc.....................................................................    32      1   so1
....................uUuggaauccaucugacaaac.....................................................................    1       1   so1
....................ucuggaauccaucugacaaCc.....................................................................    7       1   so1
....................ucuggaauccaucugacUaacu....................................................................    9       1   so1
....................ucuggaauccUucugacaaacu....................................................................    4       1   so1
....................ucuggaauccaucugGcaaacu....................................................................    129     1   so1
....................ucuggaGuccaucugacaaacu....................................................................    83      1   so1
....................ucuggaauccaucugaGaaacu....................................................................    1       1   so1
....................ucuggaauccaucugacaaacu....................................................................    10866   0   so1
....................ucugCaauccaucugacaaacu....................................................................    1       1   so1
....................ucuggaUuccaucugacaaacu....................................................................    1       1   so1
....................Gcuggaauccaucugacaaacu....................................................................    4       1   so1
....................ucuggaauccaucugacaaaAu....................................................................    3       1   so1
....................ucuggaauccaucugacaaaUu....................................................................    8       1   so1
....................ucuggaauccaucugacGaacu....................................................................    42      1   so1
....................ucuggaauccaucugacaaaGu....................................................................    1       1   so1
....................ucuggaauccauUugacaaacu....................................................................    3       1   so1
....................ucuggaaucUaucugacaaacu....................................................................    10      1   so1
....................uUuggaauccaucugacaaacu....................................................................    4       1   so1
....................ucuggaauccaucugUcaaacu....................................................................    4       1   so1
....................ucuggaauccaucugacaUacu....................................................................    3       1   so1
....................uAuggaauccaucugacaaacu....................................................................    4       1   so1
....................ucuggaauccaucugacaaacA....................................................................    453     1   so1
....................ucuAgaauccaucugacaaacu....................................................................    10      1   so1
....................ucuggaauccGucugacaaacu....................................................................    21      1   so1
....................ucuggaauccCucugacaaacu....................................................................    2       1   so1
....................ucugUaauccaucugacaaacu....................................................................    2       1   so1
....................ucuggaauccaucGgacaaacu....................................................................    1       1   so1
....................ucuggaauccaucugacaGacu....................................................................    238     1   so1
....................ucGggaauccaucugacaaacu....................................................................    1       1   so1
....................ucuggaaCccaucugacaaacu....................................................................    24      1   so1
....................ucuggaauccaucCgacaaacu....................................................................    27      1   so1
....................ucugAaauccaucugacaaacu....................................................................    5       1   so1
....................ucuggaauccaucugacaaGcu....................................................................    64      1   so1
....................ucuggaauccaucugaUaaacu....................................................................    3       1   so1
....................ucuUgaauccaucugacaaacu....................................................................    3       1   so1
....................ucuCgaauccaucugacaaacu....................................................................    1       1   so1
....................ucuggaauccaucugCcaaacu....................................................................    2       1   so1
....................ucuggaauccaCcugacaaacu....................................................................    37      1   so1
....................ucuggaauccauAugacaaacu....................................................................    4       1   so1
....................ucuggaaAccaucugacaaacu....................................................................    1       1   so1
```

```
                    Mature                                    Star
acuuuauucuuuuuuuuuuaucuggaauccaucugacaaacugcauguuugucaugcaguuugucauggauuccaguuaaaaaaaagcaugcaaucguacgucaaggaga
....................ucuggaauccaAcugacaaacu....................................................................	4	1	so1
....................ucuggaauccaucugaAaaacu....................................................................	4	1	so1
....................ucAggaauccaucugacaaacu....................................................................	7	1	so1
....................ucuggaauccaucugacaaacG....................................................................	241	1	so1
....................ucuggaauccaucugacaaCcu....................................................................	8	1	so1
....................ucuggaauccauGugacaaacu....................................................................	1	1	so1
....................ucuggaauccaucugacaaacC....................................................................	1595	1	so1
....................uGuggaauccaucugacaaacu....................................................................	3	1	so1
....................ucuggaauUcaucugacaaacu....................................................................	10	1	so1
....................ucCggaauccaucugacaaacu....................................................................	33	1	so1
....................ucuggaauccaucugacCaacu....................................................................	2	1	so1
....................ucuggGauccaucugacaaacu....................................................................	66	1	so1
....................ucuggaauccaucuAacaaacu....................................................................	5	1	so1
....................ucuggUauccaucugacaaacu....................................................................	5	1	so1
....................ucuggaauccaucugacaaUcu....................................................................	3	1	so1
....................ucuggaauccaucAgacaaacu....................................................................	6	1	so1
....................ucuggaaucAaucugacaaacu....................................................................	2	1	so1
....................Acuggaauccaucugacaaacu....................................................................	15	1	so1
....................ucuggaauccaucugacaaacug...................................................................	77873	0	so1
....................ucugCaauccaucugacaaacug...................................................................	3	1	so1
....................ucuggaauccaucugacaaaUug...................................................................	50	1	so1
....................ucuggaaucAaucugacaaacug...................................................................	31	1	so1
....................ucugUaauccaucugacaaacug...................................................................	17	1	so1
....................ucuggaauccUucugacaaacug...................................................................	25	1	so1
....................ucuggaauccauAugacaaacug...................................................................	6	1	so1
....................ucuggaauccaucugaAaaacug...................................................................	22	1	so1
....................ucuggaaAccaucugacaaacug...................................................................	10	1	so1
....................Gcuggaauccaucugacaaacug...................................................................	17	1	so1
....................ucuggaauccaucugaUaaacug...................................................................	35	1	so1
....................ucuggaCuccaucugacaaacug...................................................................	4	1	so1
....................ucuUgaauccaucugacaaacug...................................................................	8	1	so1
....................ucuggaaGccaucugacaaacug...................................................................	4	1	so1
....................ucCggaauccaucugacaaacug...................................................................	207	1	so1
....................uUuggaauccaucugacaaacug...................................................................	60	1	so1
....................ucuggaauccaucugaGaaacug...................................................................	2	1	so1
....................ucuggaauccaucugacaaacCg...................................................................	835	1	so1
....................ucuggaauccauGugacaaacug...................................................................	1	1	so1
....................ucuAgaauccaucugacaaacug...................................................................	33	1	so1
....................ucuggaauccaucuUacaaacug...................................................................	4	1	so1
....................ucuggaauccaucugacaaCcug...................................................................	43	1	so1
....................uAuggaauccaucugacaaacug...................................................................	26	1	so1
....................ucuggaauccaCcugacaaacug...................................................................	237	1	so1
....................ucuggaauccaucugacGaacug...................................................................	329	1	so1
....................ucuggUauccaucugacaaacug...................................................................	34	1	so1
....................ucuggaauAcaucugacaaacug...................................................................	11	1	so1
....................ucuggaauccaAcugacaaacug...................................................................	24	1	so1
....................ucuggaauccaucugGcaaacug...................................................................	1221	1	so1
....................ucuggaauccaucugacaaGcug...................................................................	450	1	so1
....................ucuggaauccaucugUcaaacug...................................................................	34	1	so1
....................ucuggaauccaucugacaUacug...................................................................	21	1	so1
....................ucuggaauccCucugacaaacug...................................................................	6	1	so1
....................ucuggaauUcaucugacaaacug...................................................................	32	1	so1
....................ucuggaaucUaucugacaaacug...................................................................	66	1	so1
....................ucuggaaCccaucugacaaacug...................................................................	210	1	so1
....................Acuggaauccaucugacaaacug...................................................................	67	1	so1
....................ucuggCauccaucugacaaacug...................................................................	5	1	so1
....................ucuggaauccaucCgacaaacug...................................................................	273	1	so1
....................ucuggaauccaGcugacaaacug...................................................................	6	1	so1
....................ucuggaauccaucugacaGacug...................................................................	2834	1	so1
....................ucuggaauccaucAgacaaacug...................................................................	73	1	so1
....................ucuggaauccaucugacaaaAug...................................................................	17	1	so1
....................ucGggaauccaucugacaaacug...................................................................	9	1	so1
....................ucuggaauccaucuCacaaacug...................................................................	3	1	so1
....................ucuggaauccaucugacaaacuU...................................................................	876	1	so1
....................ucuggaauccaucugacaaacuC...................................................................	294	1	so1
....................ucugAaauccaucugacaaacug...................................................................	40	1	so1
....................ucuCgaauccaucugacaaacug...................................................................	3	1	so1
....................ucuggaauccaucugacUaacug...................................................................	96	1	so1
....................ucuggaGuccaucugacaaacug...................................................................	946	1	so1
....................ucuggGauccaucugacaaacug...................................................................	428	1	so1
```

Mature / Star			
acuuuauucuuuuuuuuuuaucuggaauccaucugacaaacugcauguuugucaugcaguuugucauggauuccaguuaaaaaaaagcaugcaaucguacgucaaggaga			
....................ucuggaauccaucugacaaUcug...	11	1	so1
....................ucuggaauccaucugCcaaacug...	9	1	so1
....................ucuggaauccaucugacaCacug...	15	1	so1
....................ucuggaauccaucugacaaacGg...	231	1	so1
....................ucuggaauccaucuAacaaacug...	29	1	so1
....................ucuggaauccauUugacaaacug...	34	1	so1
....................ucuggaauGcaucugacaaacug...	3	1	so1
....................ucuggaauccaucugacaaacuA...	9582	1	so1
....................uGuggaauccaucugacaaacug...	8	1	so1
....................ucuggaUuccaucugacaaacug...	5	1	so1
....................ucuggaauccaucugacCaacug...	19	1	so1
....................ucuggaauccaucGgacaaacug...	12	1	so1
....................ucuggaauccaucugacaaaGug...	3	1	so1
....................ucuggaauccaucugacaaacAg...	66	1	so1
....................ucuggaauccGucugacaaacug...	209	1	so1
....................ucAggaauccaucugacaaacug...	54	1	so1
....................ucuggaauccauAugacaaacugc..	1	1	so1
....................uGuggaauccaucugacaaacugc..	1	1	so1
....................ucuggaauccaucugacaaacugU..	754	1	so1
....................Acuggaauccaucugacaaacugc..	1	1	so1
....................ucuggaauccaucugacaUacugc..	1	1	so1
....................ucuggaauccaucugacaaacugG..	22	1	so1
....................ucuggaauccaucugacaaacugc..	3546	0	so1
....................ucuggaauccaucugacaaacugA..	540	1	so1
....................ucuggaauccaucugaAaaacugc..	3	1	so1
....................ucuAgaauccaucugacaaacugc..	4	1	so1
....................ucuggaauccaucugacaaacGgc..	1	1	so1
....................ucuggaauccaucugacaCacugc..	1	1	so1
....................ucuggaauccaCcugacaaacugc..	5	1	so1
....................ucuggaGuccaucugacaaacugc..	37	1	so1
....................ucuggaaCccaucugacaaacugc..	9	1	so1
....................ucuggaauccaucugUcaaacugc..	1	1	so1
....................ucuggaauccaucugaUaaacugc..	4	1	so1
....................ucuggaauccaucugacaaaUugc..	2	1	so1
....................ucuggaauccaucugacaaCcugc..	3	1	so1
....................uUuggaauccaucugacaaacugc..	1	1	so1
....................ucuggaCuccaucugacaaacugc..	1	1	so1
....................ucuggaauUcaucugacaaacugc..	3	1	so1
....................ucuggaauccaucugacGaacugc..	14	1	so1
....................ucuggaauccaucugacaaacuUc..	3	1	so1
....................ucuggaauccaucugCcaaacugc..	1	1	so1
....................ucuggaauccaucugacaGacugc..	55	1	so1
....................ucuggaauccaucugacaaacAgc..	1	1	so1
....................ucuggaauccaucugGcaaacugc..	47	1	so1
....................ucuggaauccaucugacUaacugc..	5	1	so1
....................ucuggCauccaucugacaaacugc..	2	1	so1
....................ucuggaauccGucugacaaacugc..	12	1	so1
....................ucuggaauccaucCgacaaacugc..	15	1	so1
....................ucuggaauccaucugacaaGcugc..	11	1	so1
....................ucuggaaucUaucugacaaacugc..	1	1	so1
....................ucuggaauccaucugacaaacCgc..	7	1	so1
....................uAuggaauccaucugacaaacugc..	1	1	so1
....................ucuggGauccaucugacaaacugc..	24	1	so1
....................ucCggaauccaucugacaaacugc..	8	1	so1
....................ucugUaauccaucugacaaacugc..	2	1	so1
....................ucuggaauccaucuUacaaacugc..	2	1	so1
....................ucuggaauccaucuAacaaacugc..	1	1	so1
....................ucuggaauccaucugacaaacuAc..	16	1	so1
....................ucuggaauccUucugacaaacugc..	1	1	so1
....................ucuggaauUcaucugacaaacugca...	2	1	so1
....................ucuggaauccaucugacUaacugca...	3	1	so1
....................ucuggaauccaCcugacaaacugca...	11	1	so1
....................ucuggaauccaucugGcaaacugca...	64	1	so1
....................ucuggaaucUaucugacaaacugca...	1	1	so1
....................ucuggaUuccaucugacaaacugca...	1	1	so1
....................ucugAaauccaucugacaaacugca...	1	1	so1
....................ucuggaauccaucCgacaaacugca...	12	1	so1
....................ucuggaauccaucGgacaaacugca...	5	1	so1
....................ucuggaauccaucAgacaaacugca...	1	1	so1
....................ucuggaauccUucugacaaacugca...	3	1	so1

```
                    Mature                               Star
acuuuauucuuuuuuuuuuaucuggaauccaucugacaaacugcauguuugucaugcaguuugucauggauuccaguuaaaaaaaagcaugcaaucguacgucaaggaga
....................ucuggaauccaucugacaaacugca.................................................................    3315  0  so1
....................ucugUaauccaucugacaaacugca.................................................................    2     1  so1
....................ucuggaauccaucugacaaacugcU.................................................................    131   1  so1
....................uUuggaauccaucugacaaacugca.................................................................    3     1  so1
....................ucuggaaGccaucugacaaacugca.................................................................    2     1  so1
....................ucuggaauccaucugacaGacugca.................................................................    40    1  so1
....................ucuggaauccaucugacaaCcugca.................................................................    3     1  so1
....................Gcuggaauccaucugacaaacugca.................................................................    3     1  so1
....................ucAggaauccaucugacaaacugca.................................................................    3     1  so1
....................ucuggaauccaucugacaaacugcC.................................................................    42    1  so1
....................ucuggaGuccaucugacaaacugca.................................................................    34    1  so1
....................ucuggaauccaucugacaaaUugca.................................................................    2     1  so1
....................ucuggaauccaucugaUaaacugca.................................................................    4     1  so1
....................ucuggaauccaucugacaaacugAa.................................................................    2     1  so1
....................ucuggaauccaucugacaaacAgca.................................................................    2     1  so1
....................ucuggaauccaucugacaaacuAca.................................................................    1     1  so1
....................ucuggaaCccaucugacaaacugca.................................................................    16    1  so1
....................ucuggaauccaucugacGaacugca.................................................................    13    1  so1
....................ucuggaauccaucugacaaacugUa.................................................................    406   1  so1
....................ucuggGauccaucugacaaacugca.................................................................    11    1  so1
....................ucuggaauccGucugacaaacugca.................................................................    8     1  so1
....................ucuUgaauccaucugacaaacugca.................................................................    1     1  so1
....................ucuggaauccaucugUcaaacugca.................................................................    1     1  so1
....................ucuggaauccauUugacaaacugca.................................................................    1     1  so1
....................ucuggaauccaucugacaaacugcG.................................................................    114   1  so1
....................ucuggaauccaucugacaaGcugca.................................................................    10    1  so1
....................ucCggaauccaucugacaaacugca.................................................................    10    1  so1
....................ucuggaauccaucugacaaacCgca.................................................................    14    1  so1
....................ucuggaauccaucugacaUacugca.................................................................    1     1  so1
....................ucuggaGuccaucugacaaacugcau................................................................    2     1  so1
....................ucuggaauccaucCgacaaacugcau................................................................    1     1  so1
....................ucuggaauccaucugacaaacugcUu................................................................    21    1  so1
....................ucuggaauccaucugacaaacugUau................................................................    11    1  so1
....................ucuggaauccaucugacaaacugcaG................................................................    168   1  so1
....................ucuggaauccaucugacaaacugcaA................................................................    121   1  so1
....................ucuggaauccaucugacaaacugcau................................................................    112   0  so1
....................ucuggaauccaucugacaaacugcaC................................................................    11    1  so1
....................ucuggaauccaucugacaGacugcau................................................................    10    1  so1
....................ucuggGauccaucugacaaacugcau................................................................    1     1  so1
....................ucuggaauccaucugGcaaacugcau................................................................    4     1  so1
....................ucuggaauccaucugacaaacugcaAg...............................................................    5     1  so1
....................ucuggaauccaucugacaaacugUaug...............................................................    2     1  so1
....................ucuggaauccaucugacaaacuAcaug...............................................................    1     1  so1
....................ucuggaauccaucugacaaacugcauU...............................................................    71    1  so1
....................ucuggaauccaucugGcaaacugcaug...............................................................    1     1  so1
....................ucuggaauccaucugacaaacugcauA...............................................................    278   1  so1
....................ucuggaauccaucugacaaacugcaug...............................................................    36    0  so1
....................ucuggaGuccaucugacaaacugcaug...............................................................    1     1  so1
....................ucuggaauccaucugacaaacugcUug...............................................................    1     1  so1
....................ucuggaauccaucugacaaacugcauC...............................................................    7     1  so1
.....................cuggaauccaucugacaaa......................................................................    1     0  so1
.....................cuggaauccaucugacaaac.....................................................................    3     0  so1
.....................cuggaauccaucugacaaacA....................................................................    1     1  so1
.....................cuggaauccaucugGcaaacu....................................................................    1     1  so1
.....................cuggaauccaucugacaaacu....................................................................    15    0  so1
.....................cuggaauccaucugacaaacuU...................................................................    1     1  so1
.....................cuggaauccaucugGcaaacug...................................................................    1     1  so1
.....................cuggaauccaucugacaaacuC...................................................................    1     1  so1
.....................Auggaauccaucugacaaacug...................................................................    1     1  so1
.....................cuggaauccaucugacaaGcug...................................................................    1     1  so1
.....................cuggaauccaucugacGaacug...................................................................    1     1  so1
.....................cuggaauccaucugacaaacug...................................................................    78    0  so1
.....................Uuggaauccaucugacaaacug...................................................................    3     1  so1
.....................cuggaauccaucugacaGacug...................................................................    4     1  so1
.....................cuggaauccaucugacaaacuA...................................................................    7     1  so1
.....................cuggaauccaucugacaaacugc..................................................................    2     0  so1
.....................cCggaauccaucugacaaacugc..................................................................    1     1  so1
.....................cuggaauccaucugacaaacugU..................................................................    1     1  so1
.....................cuggaauccaucugacaaacugca.................................................................    1     0  so1
.....................cuggaauccaucugacaaacugcUu................................................................    1     1  so1
```

Mature   Star

```
acuuuauucuuuuuuuuuuaucuggaauccaucugacaaacugcauguuugucaugcaguuugucauggauuccaguuaaaaaaaagcaugcaaucguacgucaaggaga
.....................cuggaauccaucugacaaacugcauA...............................................................    2    1    so1
......................uggaauccaucugacaaaA.....................................................................    3    1    so1
......................uggaauccaucugacaaaU.....................................................................    2    1    so1
......................uggaauccaucugacaaac.....................................................................    11   0    so1
......................uggaauccaucugGcaaac.....................................................................    1    1    so1
......................uggaauccaucAgacaaacu....................................................................    1    1    so1
......................uggaauccaucugacaaacC....................................................................    5    1    so1
......................uggaauccaucugGcaaacu....................................................................    3    1    so1
......................uggaauccaucugacaaacu....................................................................    27   0    so1
......................uggaauccaucCgacaaacu....................................................................    1    1    so1
......................uggaauccaAcugacaaacu....................................................................    1    1    so1
......................uggaauccaucugacaaacA....................................................................    7    1    so1
......................uggaaCccaucugacaaacu....................................................................    1    1    so1
......................uggaauccaucugUcaaacu....................................................................    2    1    so1
......................uggaauccaucugacaaacGg...................................................................    1    1    so1
......................uggaaCccaucugacaaacug...................................................................    3    1    so1
......................uggaauccaucugacaaGcug...................................................................    1    1    so1
......................uggaGuccaucugacaaacug...................................................................    1    1    so1
......................uggaauccaucugGcaaacug...................................................................    28   1    so1
......................uggaauccaucugacaaCcug...................................................................    1    1    so1
......................uggaaucUaucugacaaacug...................................................................    1    1    so1
......................uggGauccaucugacaaacug...................................................................    3    1    so1
......................uggaauccaucugacaGacug...................................................................    8    1    so1
......................uggaauAcaucugacaaacug...................................................................    1    1    so1
......................uAgaauccaucugacaaacug...................................................................    3    1    so1
......................uggaauccaucugacaaacuA...................................................................    36   1    so1
......................uggaauccaCcugacaaacug...................................................................    2    1    so1
......................uggaauccaucugacaaacCg...................................................................    1    1    so1
......................uggaauccaucugacaaaUug...................................................................    2    1    so1
......................uggaauUcaucugacaaacug...................................................................    1    1    so1
......................uggaauccaucugacGaacug...................................................................    1    1    so1
......................uggaauccaucCgacaaacug...................................................................    1    1    so1
......................uggaauccGucugacaaacug...................................................................    1    1    so1
......................uggaauccaucugacaaacuU...................................................................    2    1    so1
......................uggaauccaucugacaaacug...................................................................    365  0    so1
......................uggaauccaucugacaaaAug...................................................................    1    1    so1
......................uggaauccaucugacaaacugA..................................................................    5    1    so1
......................uggaauccaucugacaaacugc..................................................................    40   0    so1
......................uggaauccaucugGcaaacugc..................................................................    2    1    so1
......................uggaauccaucugacaaacGgc..................................................................    1    1    so1
......................uggaauccaucugacaaacugU..................................................................    8    1    so1
......................uggaGuccaucugacaaacugca.................................................................    1    1    so1
......................uggaauccaucugacaGacugca.................................................................    3    1    so1
......................uggaauccaucugacaaacugca.................................................................    44   0    so1
......................uggaauccaucugacaaacugcU.................................................................    1    1    so1
......................uggaauccaucugGcaaacugca.................................................................    3    1    so1
......................uggaauccaucugacaaacugcG.................................................................    1    1    so1
......................uggaauccaucugacaaacugcaG................................................................    7    1    so1
......................uggaauccaucugacaaacugcUu................................................................    3    1    so1
......................uggGauccaucugacaaacugcau................................................................    1    1    so1
......................uggaauccaucugacaaacugcCu................................................................    1    1    so1
......................uggaauccaucugacaaacugcaA................................................................    3    1    so1
......................uggaauccaucugacaaacugcau................................................................    30   0    so1
......................uggaauccaucugacaaacugcaC................................................................    1    1    so1
......................uggaauccaucugacGaacugcau................................................................    2    1    so1
......................uggaauccaucugGcaaacugcau................................................................    3    1    so1
......................uggaauccaucugacaaacugcaug...............................................................    4    0    so1
......................uggaauccaucugacaaacugcauA...............................................................    71   1    so1
......................uggGauccaucugacaaacugcaug...............................................................    1    1    so1
......................uggaauccaucugacaaacugcauU...............................................................    11   1    so1
......................uggaauccaucugacaaacugcauUu..............................................................    1    1    so1
......................uggaauccaucugacaaacugcauAu..............................................................    12   1    so1
......................uggaauccaucugacaaacugcauAuu.............................................................    2    1    so1
......................uggaauccaucugacaaacugcauUuu.............................................................    1    1    so1
.......................ggaauccaucugacaaacu.....................................................................    1    0    so1
.......................ggaauccaucugacaaacug....................................................................    5    0    so1
.......................ggaauccaucugGcaaacug....................................................................    1    1    so1
.......................ggaauccaucugacaaacuA....................................................................    1    1    so1
.......................ggaauccaucugacaaacugcaG.................................................................    1    1    so1
.......................ggaauccaucugacaaacugcauUu...............................................................    1    1    so1
```

Mature **Star**

```
acuuuauucuuuuuuuuuuaucuggaauccaucugacaaacugcauguuugucaugcaguuugucauggauuccaguuaaaaaaaagcaugcaaucguacgucaaggaga
........................................................caguuugucauggauucc....................................    4     0     so1
........................................................caguuugucauggauucA....................................    1     1     so1
........................................................caAuuugucauggauuccag..................................    1     1     so1
........................................................caguuugucauggauuccag..................................    10    0     so1
........................................................caguuugucauggauuccGg..................................    1     1     so1
........................................................caguuugucauggauuccagu.................................    11    0     so1
........................................................caguuugucauggauuccagC.................................    2     1     so1
........................................................caguuugucauggauuccagGu................................    5     1     so1
........................................................cagCuugucauggauuccaguu................................    2     1     so1
........................................................caguuugucauggauuccaguA................................    10    1     so1
........................................................caguuugucauggauuccagCu................................    1     1     so1
........................................................caguuugucauggauuccaguu................................    133   0     so1
........................................................caguuugucauggauuccaguC................................    17    1     so1
........................................................caguuugucauggauuccaguG................................    2     1     so1
........................................................caguuugucauggCuuccaguua...............................    1     1     so1
........................................................caguuugucauggauuccaguuC...............................    3     1     so1
........................................................caguuugucauggauuccaguuU...............................    23    1     so1
........................................................caguuugucauggauuccaguuG...............................    6     1     so1
........................................................caguuugucauggauuccaAuua...............................    1     1     so1
........................................................cGguuugucauggauuccaguua...............................    2     1     so1
........................................................caguuugucauggGuuccaguua...............................    2     1     so1
........................................................caguuugucauggauuccaguGa...............................    1     1     so1
........................................................caguuugucauggauuccagGua...............................    2     1     so1
........................................................caguuugucauggauuccaguCa...............................    1     1     so1
........................................................Uaguuugucauggauuccaguua...............................    1     1     so1
........................................................caguuugucauggauuccaguua...............................    314   0     so1
........................................................caguuugucauggauuccagCua...............................    1     1     so1
........................................................caguuCgucauggauuccaguua...............................    3     1     so1
........................................................caguuugCcauggauuccaguua...............................    2     1     so1
........................................................caguuugucauggauuccGguua...............................    5     1     so1
........................................................caguuuAucauggauuccaguua...............................    1     1     so1
........................................................caguuugucauggaCuccaguua...............................    3     1     so1
........................................................caguuugucauggauuccaguuaG..............................    3     1     so1
........................................................caguuugucauggauuccaguuUa..............................    19    1     so1
........................................................caguuugucauggauuccaguuaa..............................    6     0     so1
........................................................caguuugucauggauuccaguuaC..............................    5     1     so1
........................................................caguuugucauggauuccGguuaa..............................    1     1     so1
........................................................caguuugucauggauuccaguuaU..............................    8     1     so1
........................................................caguuugucauggauuccaguuaUa.............................    6     1     so1
........................................................caguuugucauggauuccaguuaaG.............................    1     1     so1
........................................................caguuugucauggauuccaguuaUaa............................    2     1     so1
.........................................................aguuugucauggauuccagC.................................    3     1     so1
.........................................................aguuugucauggauuccagu.................................    18    0     so1
.........................................................aguuugucauggauuccagG.................................    1     1     so1
.........................................................agCuugucauggauuccaguu................................    1     1     so1
.........................................................aguuugucauggauCccaguu................................    1     1     so1
.........................................................aguuugucauggauuccaguA................................    5     1     so1
.........................................................Cguuugucauggauuccaguu................................    1     1     so1
.........................................................aguuugucGuggauuccaguu................................    1     1     so1
.........................................................aguuugCcauggauuccaguu................................    1     1     so1
.........................................................aguuugucauggauuccaguu................................    211   0     so1
.........................................................aguuugucauggauuccaguC................................    32    1     so1
.........................................................aguuugucauggauucUaguua...............................    1     1     so1
.........................................................Cguuugucauggauuccaguua...............................    1     1     so1
.........................................................aguuugucauggGuuccaguua...............................    1     1     so1
.........................................................aguuugucauggaCuccaguua...............................    4     1     so1
.........................................................aguuugucGuggauuccaguua...............................    6     1     so1
.........................................................aguAugucauggauuccaguua...............................    1     1     so1
.........................................................aguuugucauggauucAaguua...............................    1     1     so1
.........................................................Gguuugucauggauuccaguua...............................    4     1     so1
.........................................................aguuugucauggauuccGguua...............................    2     1     so1
.........................................................aguuugucauggauuccaguuU...............................    97    1     so1
.........................................................aguuugucauggauuccaguuC...............................    8     1     so1
.........................................................aguuCgucauggauuccaguua...............................    2     1     so1
.........................................................aguuugucauggauuccaguua...............................    474   0     so1
.........................................................aguuugCcauggauuccaguua...............................    2     1     so1
.........................................................aguuugucauggauuAcaguua...............................    1     1     so1
.........................................................aguCugucauggauuccaguua...............................    1     1     so1
.........................................................Uguuugucauggauuccaguua...............................    1     1     so1
.........................................................aguuugucauggauuccUguua...............................    1     1     so1
```

Mature   Star

acuuuauucuuuuuuuuuuaucuggaauccaucugacaaacugcauguuugucaugcaguuugucauggauuccaguuaaaaaaaagcaugcaaucguacgucaaggaga

....aguuugucauggauCccaguua...............................	1	1	so1
....aguuugucauggauuccaguuG...............................	4	1	so1
....aguuugucauUgauuccaguua...............................	1	1	so1
....aguuugucaAggauuccaguua...............................	1	1	so1
....aguuugucUuggauuccaguua...............................	1	1	so1
....aguuugucauggauuccaguGa...............................	1	1	so1
....aguuugucauggauuccagCua...............................	1	1	so1
....agGuugucauggauuccaguua...............................	1	1	so1
....aguuugucauggauuccaguuaC..............................	2	1	so1
....aguuugucauggauuccaguuaU..............................	15	1	so1
....aguuugucauggauuccaguuaG..............................	5	1	so1
....aguuugucauggauuccaguuUa..............................	89	1	so1
....aguuugucauggauuccaguuaa..............................	17	0	so1
....aguuugucauggauuccaguuaUa.............................	20	1	so1
....aguuugucauggauuccaguuaaG.............................	2	1	so1
....aguuugucauggauuccaguuaUaa............................	1	1	so1
....guuugucauggauuccaguua...............................	1	0	so1
....uuugucauggauuccaguua...............................	5	0	so1
....uuugucauggauuccaguuU...............................	3	1	so1
....uuugucauggauuccaguuUa..............................	4	1	so1

Provisional ID	:	ScYm1zk_320_3242
Score total	:	354105.1
Score for star read(s)	:	3.9
Score for read counts	:	354094.6
Score for mfe	:	2
Score for randfold	:	1.6
Score for cons. seed	:	3
Total read count	:	694553
Mature read count	:	680952
Loop read count	:	0
Star read count	:	13601

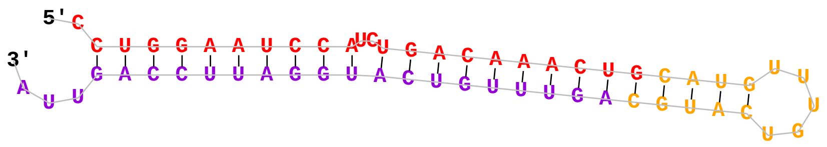

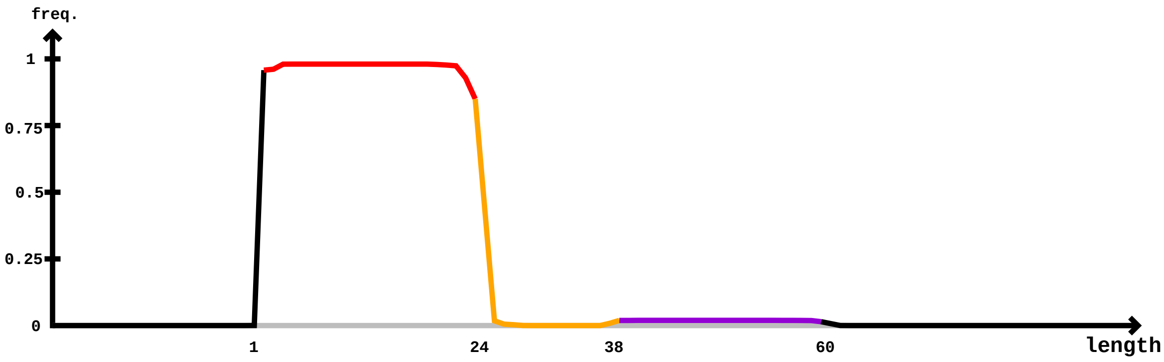

Mature Star

```
5'- acuuuauucuuuuuuuuuuaccuggaauccaucugacaaacugcauguuugucaugcaguuugucauggauuccaguuaaaaaaaagcaugcaaucguacgucaaggagaa  -3'  obs
    acuuuauucuuuuuuuuuuaccuggaauccaucugacaaacugcauguuugucaugcaguuugucauggauuccaguuaaaaaaaagcaugcaaucguacgucaaggagaa       exp
    ......((((((((((((.((((((((((..((((((((((((((.....))))))))))))))))))))))))).)))))))).((.(((....))).)).))))))..  reads  mm  sample
    .................Guaccuggaauccaucugacaaa.......................................................................  1  1  sr1
    ..................uaccuggaauccaucugacaaac......................................................................  1  0  sr1
    ..................uaccuggaauccaucugGcaaacu.....................................................................  1  1  sr1
    ..................uaccuggaauccaucugacaaacug....................................................................  1  0  sr1
    ..................uaccuggaGuccaucugacaaacug....................................................................  1  1  sr1
    ..................uCccuggaauccaucugacaaacug....................................................................  8  1  sr1
    ...................Cccuggaauccaucugacaaac......................................................................  1  1  sr1
    ...................Cccuggaauccaucugacaaacu.....................................................................  7  1  sr1
    ...................accuggaauccaucugacaaacC.....................................................................  1  1  sr1
    ...................accuggaauccaucugacaaacug....................................................................  20  0  sr1
    ...................Uccuggaauccaucugacaaacug....................................................................  5  1  sr1
    ...................accuggaauccaucugacaaacGg....................................................................  1  1  sr1
    ...................accuggaauccaucugacaaacuA....................................................................  1  1  sr1
    ...................Cccuggaauccaucugacaaacug....................................................................  49  1  sr1
    ...................accuggaauccaucugacaaacugU...................................................................  2  1  sr1
    ...................accuggaauccaucugacaaacugc...................................................................  5  0  sr1
    ...................accuggaauccaucugacaaacugA...................................................................  1  1  sr1
    ...................accuggaauUcaucugacaaacugca..................................................................  1  1  sr1
    ...................accuggaauccaucugacaaacugcU..................................................................  1  1  sr1
    ...................accuggaauccaucugacaaacugUa..................................................................  1  1  sr1
    ....................ccuggGauccaucugaca.........................................................................  1  1  sr1
    ....................ccuggaauccaucugacU.........................................................................  3  1  sr1
    ....................ccuggaauccGucugaca.........................................................................  5  1  sr1
    ....................ccuggaaCccaucugaca.........................................................................  1  1  sr1
    ....................ccuggaauccaucugaAa.........................................................................  2  1  sr1
    ....................ccuggaauccaucugGca.........................................................................  3  1  sr1
    ....................ccuggaauUcaucugaca.........................................................................  1  1  sr1
    ....................ccuggaauccaucugacG.........................................................................  40  1  sr1
    ....................ccuggaauccaucCgaca.........................................................................  3  1  sr1
    ....................ccuggaGuccaucugaca.........................................................................  6  1  sr1
    ....................ccuggaaucUaucugaca.........................................................................  1  1  sr1
    ....................ccuggaauccaucugaca.........................................................................  590  0  sr1
    ....................ccuggaauccauAugaca.........................................................................  1  1  sr1
```

```
                    Mature                              Star
acuuuauucuuuuuuuuuuaccuggaauccaucugacaaacugcauguuugucaugcaguuugucauggauuccaguuaaaaaaaagcaugcaaucguacgucaaggagaa
....................ccuggaauccaAcugaca.........................................................................	1	1	sr1
....................cAuggaauccaucugaca.........................................................................	1	1	sr1
....................Gcuggaauccaucugaca.........................................................................	94	1	sr1
....................ccuggaauccaucugaUa.........................................................................	1	1	sr1
....................ccuggaauccaucugCca.........................................................................	2	1	sr1
....................ccuggaauccaCcugaca.........................................................................	2	1	sr1
....................ccCggaauccaucugaca.........................................................................	1	1	sr1
....................ccuggaauccaAcugacaa........................................................................	2	1	sr1
....................Acuggaauccaucugacaa........................................................................	1	1	sr1
....................ccuggaauccaCcugacaa........................................................................	2	1	sr1
....................ccuggaauccaucugacGa........................................................................	2	1	sr1
....................ccuggaauccaucugacaU........................................................................	7	1	sr1
....................ccuggaaucAaucugacaa........................................................................	1	1	sr1
....................ccuggaaucUaucugacaa........................................................................	1	1	sr1
....................ccuggaauccGucugacaa........................................................................	1	1	sr1
....................ccCggaauccaucugacaa........................................................................	1	1	sr1
....................ccuggaauccaucAgacaa........................................................................	1	1	sr1
....................ccuggaauccaucuAacaa........................................................................	1	1	sr1
....................ccuggaauccaucCgacaa........................................................................	5	1	sr1
....................ccuggaaCccaucugacaa........................................................................	3	1	sr1
....................Gcuggaauccaucugacaa........................................................................	40	1	sr1
....................ccugAaauccaucugacaa........................................................................	1	1	sr1
....................ccuggaauccaucugacaa........................................................................	849	0	sr1
....................ccuggaauccaucugaUaa........................................................................	1	1	sr1
....................ccuggUauccaucugacaa........................................................................	3	1	sr1
....................ccuggaauccaucugacaG........................................................................	50	1	sr1
....................ccuAgaauccaucugacaa........................................................................	2	1	sr1
....................ccuUgaauccaucugacaa........................................................................	1	1	sr1
....................ccuggGauccaucugacaa........................................................................	1	1	sr1
....................ccuggaGuccaucugacaa........................................................................	9	1	sr1
....................cUuggaauccaucugacaa........................................................................	2	1	sr1
....................ccuggaauccaucugGcaa........................................................................	2	1	sr1
....................ccuggaGuccaucugacaaa.......................................................................	8	1	sr1
....................ccAggaauccaucugacaaa.......................................................................	2	1	sr1
....................ccuggaauccaucugaUaaa.......................................................................	3	1	sr1
....................ccuggaauccauAugacaaa.......................................................................	1	1	sr1
....................cAuggaauccaucugacaaa.......................................................................	1	1	sr1
....................ccuggaauccaucAgacaaa.......................................................................	2	1	sr1
....................ccuggaauccaucugUcaaa.......................................................................	1	1	sr1
....................Gcuggaauccaucugacaaa.......................................................................	57	1	sr1
....................ccuggaauccaucugacUaa.......................................................................	1	1	sr1
....................ccuggUauccaucugacaaa.......................................................................	1	1	sr1
....................ccuUgaauccaucugacaaa.......................................................................	1	1	sr1
....................ccuggaauccaucugGcaaa.......................................................................	5	1	sr1
....................ccuggaaAccaucugacaaa.......................................................................	1	1	sr1
....................ccugUaauccaucugacaaa.......................................................................	1	1	sr1
....................ccuggaauccaucugaAaaa.......................................................................	1	1	sr1
....................ccuggaauccGucugacaaa.......................................................................	2	1	sr1
....................ccuggGauccaucugacaaa.......................................................................	7	1	sr1
....................ccuggaauccaGcugacaaa.......................................................................	1	1	sr1
....................ccuggaauccaucuAacaaa.......................................................................	1	1	sr1
....................ccuAgaauccaucugacaaa.......................................................................	1	1	sr1
....................ccuggaauccaucugacaaG.......................................................................	34	1	sr1
....................ccuggaauccaucugacaaa.......................................................................	1308	0	sr1
....................ccuggaauccaucugacaGa.......................................................................	22	1	sr1
....................ccuggaaCccaucugacaaa.......................................................................	4	1	sr1
....................ccuggaauccaCcugacaaa.......................................................................	1	1	sr1
....................Acuggaauccaucugacaaa.......................................................................	4	1	sr1
....................ccuggaaucUaucugacaaa.......................................................................	1	1	sr1
....................ccuggaauccaucugacGaa.......................................................................	10	1	sr1
....................ccCggaauccaucugacaaa.......................................................................	2	1	sr1
....................ccuggaauccaucCgacaaa.......................................................................	7	1	sr1
....................ccuggaauccaucugacaaC.......................................................................	4	1	sr1
....................ccuggaauccaucugacaaU.......................................................................	1	1	sr1
....................ccuggaauccaucugacaaUc......................................................................	7	1	sr1
....................ccuggaauccauAugacaaac......................................................................	2	1	sr1
....................ccuggaauccaucugacaaaU......................................................................	1236	1	sr1
....................ccuggaauccaCcugacaaac......................................................................	52	1	sr1
....................ccAggaauccaucugacaaac......................................................................	16	1	sr1
....................ccuggaaAccaucugacaaac......................................................................	2	1	sr1
```

Mature Star

```
acuuuauucuuuuuuuuuuaccuggaauccaucugacaaacugcauguuugucaugcaguuugucauggauuccaguuaaaaaaaagcaugcaaucguacgucaaggagaa
....................ccuggaaucAaucugacaaac......................................................................   3      1   sr1
....................ccuggaauccaucugacUaac......................................................................   16     1   sr1
....................ccuggaauccaucugaAaaac......................................................................   5      1   sr1
....................ccuggaCuccaucugacaaac......................................................................   1      1   sr1
....................ccuggaauccaucugacaaaA......................................................................   309    1   sr1
....................ccuggGauccaucugacaaac......................................................................   77     1   sr1
....................ccuggaauccaucugacaGac......................................................................   273    1   sr1
....................ccuggaauccaucAgacaaac......................................................................   18     1   sr1
....................ccuggaauccaucugacaUac......................................................................   9      1   sr1
....................ccGggaauccaucugacaaac......................................................................   6      1   sr1
....................ccuggaauccUucugacaaac......................................................................   5      1   sr1
....................ccuggaauccGucugacaaac......................................................................   37     1   sr1
....................ccuggaauccaucugacaaCc......................................................................   12     1   sr1
....................ccuggaauccaucugacaaac......................................................................   16573  0   sr1
....................ccuggaaucUaucugacaaac......................................................................   13     1   sr1
....................ccuggaaCccaucugacaaac......................................................................   42     1   sr1
....................ccuggaauccaucugCcaaac......................................................................   9      1   sr1
....................Acuggaauccaucugacaaac......................................................................   66     1   sr1
....................ccuggaauccaucugUcaaac......................................................................   7      1   sr1
....................ccuggaauccaucCgacaaac......................................................................   68     1   sr1
....................ccuggaauccauUugacaaac......................................................................   15     1   sr1
....................ccuggCauccaucugacaaac......................................................................   2      1   sr1
....................ccuggaauccaucugGcaaac......................................................................   143    1   sr1
....................ccugCaauccaucugacaaac......................................................................   1      1   sr1
....................ccuggaauccaucugaUaaac......................................................................   7      1   sr1
....................ccuggaauUcaucugacaaac......................................................................   14     1   sr1
....................ccuggaauccaucugacaaGc......................................................................   79     1   sr1
....................ccuggaUuccaucugacaaac......................................................................   1      1   sr1
....................ccuggaauccaucGgacaaac......................................................................   3      1   sr1
....................ccuggaauccaucugacGaac......................................................................   63     1   sr1
....................ccugUaauccaucugacaaac......................................................................   2      1   sr1
....................ccuggaauccaAcugacaaac......................................................................   5      1   sr1
....................ccCggaauccaucugacaaac......................................................................   55     1   sr1
....................ccuggaauccaucuAacaaac......................................................................   9      1   sr1
....................ccuggaGuccaucugacaaac......................................................................   84     1   sr1
....................ccuggUauccaucugacaaac......................................................................   4      1   sr1
....................ccuggaaGccaucugacaaac......................................................................   3      1   sr1
....................cUuggaauccaucugacaaac......................................................................   15     1   sr1
....................cAuggaauccaucugacaaac......................................................................   2      1   sr1
....................ccuAgaauccaucugacaaac......................................................................   12     1   sr1
....................ccugAaauccaucugacaaac......................................................................   8      1   sr1
....................ccuggaauccaucugacaaaG......................................................................   19     1   sr1
....................Gcuggaauccaucugacaaac......................................................................   1982   1   sr1
....................ccuggaauccaucugacCaac......................................................................   4      1   sr1
....................ccuggaauccCucugacaaac......................................................................   2      1   sr1
....................ccuUgaauccaucugacaaac......................................................................   3      1   sr1
....................ccuggaauccaGcugacaaac......................................................................   3      1   sr1
....................ccuAgaauccaucugacaaacu.....................................................................   21     1   sr1
....................ccuggaaucAaucugacaaacu.....................................................................   3      1   sr1
....................ccuggaauccaCcugacaaacu.....................................................................   75     1   sr1
....................cAuggaauccaucugacaaacu.....................................................................   8      1   sr1
....................ccuggaauccauGugacaaacu.....................................................................   1      1   sr1
....................Gcuggaauccaucugacaaacu.....................................................................   3889   1   sr1
....................ccGggaauccaucugacaaacu.....................................................................   2      1   sr1
....................ccuggCauccaucugacaaacu.....................................................................   3      1   sr1
....................ccuggaauccaucugacaaacC.....................................................................   2994   1   sr1
....................ccugUaauccaucugacaaacu.....................................................................   5      1   sr1
....................ccuggaauccaucugacGaacu.....................................................................   115    1   sr1
....................ccuggaauccaucugacaUacu.....................................................................   8      1   sr1
....................ccuggaauccaucugaUaaacu.....................................................................   16     1   sr1
....................ccuggaauccaucugacaaacu.....................................................................   24073  0   sr1
....................ccuggaauccaucGgacaaacu.....................................................................   4      1   sr1
....................ccuggaauccaGcugacaaacu.....................................................................   1      1   sr1
....................cUuggaauccaucugacaaacu.....................................................................   17     1   sr1
....................ccuggaauccaucugacaaacG.....................................................................   1174   1   sr1
....................ccuggaauccaucugacaaCcu.....................................................................   11     1   sr1
....................ccuggaauccaucugacaCacu.....................................................................   3      1   sr1
....................ccuggaaucUaucugacaaacu.....................................................................   29     1   sr1
....................ccuggaaAccaucugacaaacu.....................................................................   7      1   sr1
....................ccuggaauccaucugacUaacu.....................................................................   19     1   sr1
```

```
                    Mature                                    Star
acuuuauucuuuuuuuuuuaccuggaauccaucugacaaacugcauguuugucaugcaguuugucauggauuccaguuaaaaaaaagcaugcaaucguacgucaaggagaa
....................ccuggaauccauAugacaaacu.....................................................................  1      1  sr1
....................ccuggaauccGucugacaaacu.....................................................................  57     1  sr1
....................ccuggaaGccaucugacaaacu.....................................................................  1      1  sr1
....................ccuggGauccaucugacaaacu.....................................................................  114    1  sr1
....................ccuggaauccaucugacaaaUu.....................................................................  28     1  sr1
....................ccuggaaCccaucugacaaacu.....................................................................  54     1  sr1
....................ccuggaauccaucugacaaUcu.....................................................................  5      1  sr1
....................ccuggaauccaucugUcaaacu.....................................................................  9      1  sr1
....................ccuggaauccaucuCacaaacu.....................................................................  1      1  sr1
....................ccuggaauccaucugCcaaacu.....................................................................  9      1  sr1
....................ccuggaauccCucugacaaacu.....................................................................  4      1  sr1
....................ccuggUauccaucugacaaacu.....................................................................  12     1  sr1
....................ccugAaauccaucugacaaacu.....................................................................  17     1  sr1
....................ccuggaauccaAcugacaaacu.....................................................................  9      1  sr1
....................ccuggaauccaucAgacaaacu.....................................................................  28     1  sr1
....................ccCggaauccaucugacaaacu.....................................................................  54     1  sr1
....................Acuggaauccaucugacaaacu.....................................................................  55     1  sr1
....................ccuggaauccaucugGcaaacu.....................................................................  169    1  sr1
....................ccuggaauUcaucugacaaacu.....................................................................  22     1  sr1
....................ccuggaauAcaucugacaaacu.....................................................................  1      1  sr1
....................ccuggaauccaucugacaaacA.....................................................................  1113   1  sr1
....................ccuggaauccaucugacaaaGu.....................................................................  1      1  sr1
....................ccugCaauccaucugacaaacu.....................................................................  2      1  sr1
....................ccAggaauccaucugacaaacu.....................................................................  24     1  sr1
....................ccuggaGuccaucugacaaacu.....................................................................  189    1  sr1
....................ccuggaauccaucugaAaaacu.....................................................................  2      1  sr1
....................ccuggaaucGaucugacaaacu.....................................................................  1      1  sr1
....................ccuggaauccaucugacaaaAu.....................................................................  13     1  sr1
....................ccuggaauccaucugacaGacu.....................................................................  415    1  sr1
....................ccuggaauccaucCgacaaacu.....................................................................  92     1  sr1
....................ccuggaauccauUugacaaacu.....................................................................  17     1  sr1
....................ccuggaUuccaucugacaaacu.....................................................................  5      1  sr1
....................ccuggaauccaucuAacaaacu.....................................................................  17     1  sr1
....................ccuggaauccaucugaGaaacu.....................................................................  1      1  sr1
....................ccuggaauccUucugacaaacu.....................................................................  13     1  sr1
....................ccuggaauccaucugacaaGcu.....................................................................  89     1  sr1
....................ccuUgaauccaucugacaaacu.....................................................................  5      1  sr1
....................ccuCgaauccaucugacaaacug....................................................................  29     1  sr1
....................ccuggCauccaucugacaaacug....................................................................  23     1  sr1
....................ccuggaauccaGcugacaaacug....................................................................  31     1  sr1
....................ccuggaauccauAugacaaacug....................................................................  31     1  sr1
....................ccuggaauccaucugacaaGcug....................................................................  1074   1  sr1
....................ccuggaauccaucGgacaaacug....................................................................  64     1  sr1
....................ccuggaauccaAcugacaaacug....................................................................  93     1  sr1
....................ccuggaauccaucAgacaaacug....................................................................  243    1  sr1
....................ccuggaauccaucugacaCacug....................................................................  23     1  sr1
....................ccuggaauccaucugacaaaAug....................................................................  37     1  sr1
....................ccuggaaucAaucugacaaacug....................................................................  28     1  sr1
....................ccuggaauccaucugacaGacug....................................................................  9112   1  sr1
....................ccuggaauUcaucugacaaacug....................................................................  191    1  sr1
....................cUuggaauccaucugacaaacug....................................................................  187    1  sr1
....................ccuggaauccaucuUacaaacug....................................................................  13     1  sr1
....................ccuggaauccCucugacaaacug....................................................................  32     1  sr1
....................ccuggGauccaucugacaaacug....................................................................  1410   1  sr1
....................ccuggaauccaucugacaaacAg....................................................................  243    1  sr1
....................ccuggaaGccaucugacaaacug....................................................................  26     1  sr1
....................ccuggaauccaucugaAaaacug....................................................................  65     1  sr1
....................ccugAaauccaucugacaaacug....................................................................  154    1  sr1
....................ccuggaauccauGugacaaacug....................................................................  4      1  sr1
....................ccuggaauccaucugacaaacuA....................................................................  24832  1  sr1
....................ccuggaauccaucugacaaaGug....................................................................  9      1  sr1
....................ccugCaauccaucugacaaacug....................................................................  9      1  sr1
....................ccuggaauccGucugacaaacug....................................................................  662    1  sr1
....................ccuggaauGcaucugacaaacug....................................................................  11     1  sr1
....................Gcuggaauccaucugacaaacug....................................................................  51599  1  sr1
....................ccuggaauccaucuCacaaacug....................................................................  14     1  sr1
....................ccuggaaAccaucugacaaacug....................................................................  43     1  sr1
....................ccAggaauccaucugacaaacug....................................................................  177    1  sr1
....................ccuggaauAcaucugacaaacug....................................................................  38     1  sr1
....................ccuggaUuccaucugacaaacug....................................................................  43     1  sr1
```

```
                    Mature                                Star
acuuuauucuuuuuuuuuuaccuggaauccaucugacaaacugcauguuugucaugcaguuugucauggauuccaguuaaaaaaaagcaugcaaucguacgucaaggagaa
....................ccuggaaucGaucugacaaacug....................................................................    8       1    sr1
....................ccugUaauccaucugacaaacug....................................................................    39      1    sr1
....................ccuggaauccaucugCcaaacug....................................................................    97      1    sr1
....................ccuggaauccaucugacaaacuC....................................................................    1327    1    sr1
....................Acuggaauccaucugacaaacug....................................................................    889     1    sr1
....................ccuUgaauccaucugacaaacug....................................................................    49      1    sr1
....................ccuggaauccaucugacaaCcug....................................................................    99      1    sr1
....................ccuggaauccauUugacaaacug....................................................................    99      1    sr1
....................ccuggaauccaucugacGaacug....................................................................    1226    1    sr1
....................ccuggaaucUaucugacaaacug....................................................................    257     1    sr1
....................ccuggaauccaucugacaUacug....................................................................    87      1    sr1
....................cAuggaauccaucugacaaacug....................................................................    62      1    sr1
....................cGuggaauccaucugacaaacug....................................................................    15      1    sr1
....................ccuggaauccaucugacUaacug....................................................................    249     1    sr1
....................ccuggaauccaucugaUaaacug....................................................................    125     1    sr1
....................ccuggaauccaucugUcaaacug....................................................................    155     1    sr1
....................ccuggaauccaucugacaaacuU....................................................................    2019    1    sr1
....................ccuAgaauccaucugacaaacug....................................................................    197     1    sr1
....................ccuggaauccUucugacaaacug....................................................................    90      1    sr1
....................ccuggaauccaucugacaaacGg....................................................................    361     1    sr1
....................ccuggaauccaucugGcaaacug....................................................................    1848    1    sr1
....................ccuggaauccaucugacaaUcug....................................................................    53      1    sr1
....................ccGggaauccaucugacaaacug....................................................................    54      1    sr1
....................ccuggaauccaucugacaaaUug....................................................................    160     1    sr1
....................ccuggaauccaucugacaaacCg....................................................................    3615    1    sr1
....................ccuggaauccaucuAacaaacug....................................................................    136     1    sr1
....................ccuggaauccaucCgacaaacug....................................................................    996     1    sr1
....................ccuggaGuccaucugacaaacug....................................................................    2868    1    sr1
....................ccuggaauccaucugacCaacug....................................................................    45      1    sr1
....................ccuggaauccaCcugacaaacug....................................................................    685     1    sr1
....................ccuggaauccaucugacaaacug....................................................................    275242  0    sr1
....................ccuggUauccaucugacaaacug....................................................................    115     1    sr1
....................ccuggaaCccaucugacaaacug....................................................................    705     1    sr1
....................ccCggaauccaucugacaaacug....................................................................    684     1    sr1
....................ccuggaCuccaucugacaaacug....................................................................    45      1    sr1
....................cUuggaauccaucugacaaacugc...................................................................    4       1    sr1
....................ccuggaauccaucuAacaaacugc...................................................................    5       1    sr1
....................ccuggaauccaucugacaaacCgc...................................................................    9       1    sr1
....................ccuggaaGccaucugacaaacugc...................................................................    3       1    sr1
....................ccuggaauccaucugacaaacuAc...................................................................    39      1    sr1
....................ccuggaauccaucugacaaacAgc...................................................................    3       1    sr1
....................ccugAaauccaucugacaaacugc...................................................................    1       1    sr1
....................ccuggaauccaAcugacaaacugc...................................................................    1       1    sr1
....................ccuggaauccaucugacaaacuCc...................................................................    19      1    sr1
....................Acuggaauccaucugacaaacugc...................................................................    16      1    sr1
....................ccuggaaCccaucugacaaacugc...................................................................    9       1    sr1
....................ccuggaauccaucugUcaaacugc...................................................................    2       1    sr1
....................ccuggaauccaucCgacaaacugc...................................................................    11      1    sr1
....................ccuggaauccaucugacaaCcugc...................................................................    5       1    sr1
....................ccuggaauccaucugacaUacugc...................................................................    2       1    sr1
....................ccuggaauccaucugCcaaacugc...................................................................    3       1    sr1
....................ccuggaauccaucugacaaacugG...................................................................    48      1    sr1
....................Gcuggaauccaucugacaaacugc...................................................................    752     1    sr1
....................ccuggaauccaucugacaaacugU...................................................................    917     1    sr1
....................ccuggaauccaucugacGaacugc...................................................................    20      1    sr1
....................ccuggaauccaucugGcaaacugc...................................................................    37      1    sr1
....................ccuggaauccaucugacaGacugc...................................................................    45      1    sr1
....................ccuggaauccaucugacaaacugA...................................................................    636     1    sr1
....................ccuggaauccaucugacaaaUugc...................................................................    3       1    sr1
....................ccCggaauccaucugacaaacugc...................................................................    6       1    sr1
....................ccuggaauccGucugacaaacugc...................................................................    9       1    sr1
....................ccuggaauccaucugacaaGcugc...................................................................    10      1    sr1
....................ccuggaauccaucugacaaacugc...................................................................    4015    0    sr1
....................ccuggaauccaucuUacaaacugc...................................................................    1       1    sr1
....................ccuggaauccaucugaAaaacugc...................................................................    1       1    sr1
....................ccuggaGuccaucugacaaacugc...................................................................    57      1    sr1
....................ccuggaauccaCcugacaaacugc...................................................................    10      1    sr1
....................ccuggaauccaucugacaaacuUc...................................................................    7       1    sr1
....................ccuggaAccaucugacaaacugc....................................................................    1       1    sr1
....................ccuggaauccaucugaUaaacugc...................................................................    1       1    sr1
```

```
                    Mature                                     Star
acuuuauucuuuuuuuuuuaccuggaauccaucugacaaacugcauguuugucaugcaguuugucauggauuccaguuaaaaaaaagcaugcaaucguacgucaaggagaa
....................ccuggaaucUaucugacaaacugc...................................................................   2      1   sr1
....................ccuggaauccaucAgacaaacugc...................................................................   1      1   sr1
....................ccuggaauAcaucugacaaacugc...................................................................   1      1   sr1
....................ccuggaauUcaucugacaaacugc...................................................................   4      1   sr1
....................ccuggaauccauUugacaaacugc...................................................................   3      1   sr1
....................ccuggaCuccaucugacaaacugc...................................................................   1      1   sr1
....................ccuggUauccaucugacaaacugc...................................................................   1      1   sr1
....................ccuCgaauccaucugacaaacugc...................................................................   1      1   sr1
....................ccAggaauccaucugacaaacugc...................................................................   1      1   sr1
....................ccuggaauccaucugacUaacugc...................................................................   4      1   sr1
....................ccuggGauccaucugacaaacugc...................................................................   18     1   sr1
....................ccuggaauccaucugacCaacugca..................................................................   2      1   sr1
....................ccuggaauccaucugacUaacugca..................................................................   2      1   sr1
....................ccuggaauUcaucugacaaacugca..................................................................   1      1   sr1
....................ccuggaauccauAugacaaacugca..................................................................   2      1   sr1
....................ccuggaauccaucugacaaacugcU..................................................................   217    1   sr1
....................ccuggaauccaucugacaaacCgca..................................................................   4      1   sr1
....................ccuggaauccaucugacaaacugUa..................................................................   1737   1   sr1
....................ccuggaauccaucCgacaaacugca..................................................................   4      1   sr1
....................ccuggUauccaucugacaaacugca..................................................................   2      1   sr1
....................ccuggaauccaucugacaaacugGa..................................................................   2      1   sr1
....................ccuggaGuccaucugacaaacugca..................................................................   11     1   sr1
....................ccuggaauccaucugacaaacugcG..................................................................   123    1   sr1
....................ccuggaauccaucugacaaacuUca..................................................................   1      1   sr1
....................Acuggaauccaucugacaaacugca..................................................................   6      1   sr1
....................ccuggaauccaucugacaaacugAa..................................................................   12     1   sr1
....................ccuggaauccaCcugacaaacugca..................................................................   7      1   sr1
....................ccuggaauccaucugaAaaacugca..................................................................   3      1   sr1
....................Gcuggaauccaucugacaaacugca..................................................................   340    1   sr1
....................ccuggaauccaucugacGaacugca..................................................................   11     1   sr1
....................ccuggaaucUaucugacaaacugca..................................................................   4      1   sr1
....................ccuggaauccGucugacaaacugca..................................................................   9      1   sr1
....................ccuggaauccaucugacaaacAgca..................................................................   2      1   sr1
....................cUuggaauccaucugacaaacugca..................................................................   1      1   sr1
....................ccuggaauccaucugacaaacuCca..................................................................   3      1   sr1
....................ccuggaauccaucugUcaaacugca..................................................................   1      1   sr1
....................ccuggaauccaucugacaaacugcC..................................................................   49     1   sr1
....................ccuCgaauccaucugacaaacugca..................................................................   1      1   sr1
....................ccuggaauccUucugacaaacugca..................................................................   1      1   sr1
....................ccugAaauccaucugacaaacugca..................................................................   2      1   sr1
....................ccuggaauccaucugacaUacugca..................................................................   1      1   sr1
....................ccuggaaucAaucugacaaacugca..................................................................   1      1   sr1
....................ccuggaauccaucugacaaacuAca..................................................................   4      1   sr1
....................ccCggaauccaucugacaaacugca..................................................................   10     1   sr1
....................ccuggaauccaucugGcaaacugca..................................................................   31     1   sr1
....................ccuggaaAccaucugacaaacugca..................................................................   1      1   sr1
....................ccuggaauccaucugacaaGcugca..................................................................   5      1   sr1
....................ccuggaauccauUugacaaacugca..................................................................   1      1   sr1
....................ccuggaaCccaucugacaaacugca..................................................................   8      1   sr1
....................ccuggaauccaucAgacaaacugca..................................................................   1      1   sr1
....................ccuggCauccaucugacaaacugca..................................................................   1      1   sr1
....................ccuggaauccaucugacaaacugca..................................................................   2021   0   sr1
....................ccuggaauccaucugacaGacugca..................................................................   33     1   sr1
....................ccuggGauccaucugacaaacugca..................................................................   5      1   sr1
....................ccuggaauccaucugacaGacugcau.................................................................   5      1   sr1
....................ccuggaauccaucugacaaacugUau.................................................................   43     1   sr1
....................ccuggaauccaucugaUaaacugcau.................................................................   2      1   sr1
....................ccuggaauccaucugacaaacugcaC.................................................................   8      1   sr1
....................ccuggaauccaucugacaaacugcCu.................................................................   2      1   sr1
....................ccuggaauccaucugacaaacugcUu.................................................................   47     1   sr1
....................ccuggaaCccaucugacaaacugcau.................................................................   2      1   sr1
....................ccuggaauccaucugacaaacugcau.................................................................   103    0   sr1
....................ccuggaauccaucugacaaacugcaA.................................................................   63     1   sr1
....................ccuggaauccaucugGcaaacugcau.................................................................   1      1   sr1
....................ccuggaauccaucugacaaacuAcau.................................................................   1      1   sr1
....................ccuggaauccaucugacaaacugcaG.................................................................   120    1   sr1
....................Gcuggaauccaucugacaaacugcau.................................................................   10     1   sr1
....................Gcuggaauccaucugacaaacugcaug................................................................   7      1   sr1
....................ccuggaauccaucugacaaacugcauC................................................................   4      1   sr1
....................ccuggaauccaCcugacaaacugcaug................................................................   1      1   sr1
```

Mature **Star**

acuuuauucuuuuuuuuuuaccuggaauccaucugacaaacugcauguuugucaugcaguuugucauggauuccaguuaaaaaaaagcaugcaaucguacgucaaggagaa			
....................ccuggaauccaucugacaaacugcaug..	65	0	sr1
....................ccuggaauccaucugacaaacugcauA..	326	1	sr1
....................Acuggaauccaucugacaaacugcaug..	1	1	sr1
....................ccuggaauccaucugacaaacugUaug..	12	1	sr1
....................ccuggaauccaucugacaaacugcUug..	3	1	sr1
....................ccuggaauccaucugacaaacugcaAg..	7	1	sr1
....................ccuggaauccaucugacaaacugcauU..	64	1	sr1
.....................cuggaauccaucugacaa..	1	0	sr1
.....................Guggaauccaucugacaaa...	1	1	sr1
.....................cuggaauccaucugacaaa...	5	0	sr1
.....................cuggGauccaucugacaaac..	1	1	sr1
.....................cuggaauccaucugacGaac..	1	1	sr1
.....................cuggaauccaucugacaaaU..	8	1	sr1
.....................cuggaauccaucugacaaGc..	1	1	sr1
.....................Guggaauccaucugacaaac..	2	1	sr1
.....................cuggaauccaucugacaaaA..	2	1	sr1
.....................cuggaauccaucugacaaac..	33	0	sr1
.....................Uuggaauccaucugacaaac..	4	1	sr1
.....................cuggaauccaucugacaaUc..	1	1	sr1
.....................cuggaauccaucugacaaacA...	3	1	sr1
.....................cuggaaCccaucugacaaacu...	1	1	sr1
.....................cuggaauccaucugacaaacu...	78	0	sr1
.....................cuggaauccaucugacaaacC...	11	1	sr1
.....................cuggaauccaucugacaaacG...	5	1	sr1
.....................Guggaauccaucugacaaacu...	2	1	sr1
.....................cuggaauccaucugacaGacu...	4	1	sr1
.....................Uuggaauccaucugacaaacu...	1	1	sr1
.....................cuggGauccaucugacaaacug..	6	1	sr1
.....................cuggaauccaucugacaGacug..	32	1	sr1
.....................cuggaauccaucugUcaaacug..	3	1	sr1
.....................Guggaauccaucugacaaacug..	14	1	sr1
.....................cuggaauccaucugacGaacug..	4	1	sr1
.....................cuggaGuccaucugacaaacug..	9	1	sr1
.....................cuggaauccaucugacaaacCg..	11	1	sr1
.....................cuggaauccaucugacaaacug..	1065	0	sr1
.....................cuggaaucAaucugacaaacug..	1	1	sr1
.....................cuggaauccaucugacUaacug..	1	1	sr1
.....................cuggaauccauUugacaaacug..	1	1	sr1
.....................Uuggaauccaucugacaaacug..	20	1	sr1
.....................cuggaauccaucugCcaaacug..	1	1	sr1
.....................cugAaauccaucugacaaacug..	1	1	sr1
.....................Auggaauccaucugacaaacug..	2	1	sr1
.....................cCggaauccaucugacaaacug..	11	1	sr1
.....................cugUaauccaucugacaaacug..	1	1	sr1
.....................cuggaauccaucugacaaGcug..	8	1	sr1
.....................cuggaauccaucugacaaacuC..	5	1	sr1
.....................cuggaauccaAcugacaaacug..	1	1	sr1
.....................cuggaauccaucugacaaacuU..	9	1	sr1
.....................cuggaauccaucugacaUacug..	1	1	sr1
.....................cuggaaCccaucugacaaacug..	2	1	sr1
.....................cuggaauUcaucugacaaacug..	1	1	sr1
.....................cuggaauccaucugacaaacuA..	103	1	sr1
.....................cuggaauccaCcugacaaacug..	3	1	sr1
.....................cuggaaGccaucugacaaacug..	1	1	sr1
.....................cuggaaAccaucugacaaacug..	3	1	sr1
.....................cuggaauccaucGgacaaacug..	1	1	sr1
.....................cuggaauccaucAgacaaacug..	2	1	sr1
.....................cuggaauccGucugacaaacug..	5	1	sr1
.....................cuggaauccaucuAacaaacug..	1	1	sr1
.....................cAggaauccaucugacaaacug..	1	1	sr1
.....................cuggaauccaucugGcaaacug..	2	1	sr1
.....................cuggaauccaucugacaaUcug..	1	1	sr1
.....................cuggaaucUaucugacaaacug..	2	1	sr1
.....................cuggaauccaucugacaCacug..	2	1	sr1
.....................cuUgaauccaucugacaaacug..	1	1	sr1
.....................cuggaauccaucCgacaaacug..	4	1	sr1
.....................cuggaauccaucugacaaacGg..	1	1	sr1
.....................cuggaauccaucugacaaacugU...	3	1	sr1
.....................cuggaauccaucugacaaacugA...	1	1	sr1
.....................cuggaauccaucugacaGacugc...	1	1	sr1

Mature Star			
acuuuauucuuuuuuuuuuaccuggaauccaucugacaaacugcauguuugucaugcaguuugucauggauuccaguuaaaaaaaagcaugcaaucguacgucaaggagaa			
.....................cuggaauccaucugGcaaacugc...	3	1	sr1
.....................cuggaauccCucugacaaacugc...	1	1	sr1
.....................cuggaauccaucugacaaacugc...	17	0	sr1
.....................cCggaauccaucugacaaacugca..	1	1	sr1
.....................cuggaauccaucugacaaacugca..	12	0	sr1
.....................cuggaauccaucugacaaacugUa..	12	1	sr1
.....................cuggaauccaucugacaaacugcaG...	1	1	sr1
.....................cuggaauccaucugacaaacugcauA..	3	1	sr1
......................Gggaauccaucugacaaa...	1	1	sr1
......................uggaauccaucugacaaa...	9	0	sr1
......................uggaauccGucugacaaac..	1	1	sr1
......................uggaaCccaucugacaaac..	2	1	sr1
......................uggaGuccaucugacaaac..	3	1	sr1
......................uggaauccaucugacaGac..	3	1	sr1
......................uggaauccaCcugacaaac..	1	1	sr1
......................uggaauccaucugacGaac..	1	1	sr1
......................uggGauccaucugacaaac..	2	1	sr1
......................uggaauccaucugacaaaU..	38	1	sr1
......................uggaauccaucugacaaUc..	1	1	sr1
......................Aggaauccaucugacaaac..	1	1	sr1
......................uAgaauccaucugacaaac..	1	1	sr1
......................uggaauccaucugGcaaac..	67	1	sr1
......................Gggaauccaucugacaaac..	20	1	sr1
......................Cggaauccaucugacaaac..	1	1	sr1
......................uggaauccaucugacaaac..	390	0	sr1
......................uggaauccaucCgacaaac..	3	1	sr1
......................uggaauccaucugacaaaA..	6	1	sr1
......................uggaauccaucugacaaGc..	1	1	sr1
......................uggaauccaucugacaaacC...	42	1	sr1
......................uggaGuccaucugacaaacu...	3	1	sr1
......................uggaauccaucugacaaacG...	18	1	sr1
......................uggGauccaucugacaaacu...	3	1	sr1
......................uggaaucAaucugacaaacu...	1	1	sr1
......................uggaauccaucCgacaaacu...	2	1	sr1
......................Gggaauccaucugacaaacu...	27	1	sr1
......................uggaauccaucugacaaacA...	20	1	sr1
......................Aggaauccaucugacaaacu...	1	1	sr1
......................uggaauccaucugacaGacu...	7	1	sr1
......................uggaauccaucugacaaacu...	372	0	sr1
......................uggaauccUucugacaaacu...	1	1	sr1
......................uggaauccaucugGcaaacu...	38	1	sr1
......................uggaauccaucugacGaacu...	3	1	sr1
......................uggaauccaucugacUaacu...	1	1	sr1
......................uggaauccaAcugacaaacug..	4	1	sr1
......................uggaauccaucugacaaaUug..	12	1	sr1
......................uggaauccaucuUacaaacug..	1	1	sr1
......................Aggaauccaucugacaaacug..	5	1	sr1
......................uggaauccGucugacaaacug..	10	1	sr1
......................uggaauccaucugacGaacug..	17	1	sr1
......................uggaauccaucugacaaGcug..	29	1	sr1
......................uggaauUcaucugacaaacug..	5	1	sr1
......................uggaaAccaucugacaaacug..	1	1	sr1
......................uggaauccaucugGcaaacug..	345	1	sr1
......................uggaaCccaucugacaaacug..	8	1	sr1
......................uggaauccaucugacUaacug..	6	1	sr1
......................uggaUuccaucugacaaacug..	1	1	sr1
......................uggaGuccaucugacaaacug..	40	1	sr1
......................uggaauccaucugaGaaacug..	1	1	sr1
......................uggaaucUaucugacaaacug..	5	1	sr1
......................uggaauccaucugacaaacuA..	445	1	sr1
......................uggaauccaucugacaaUcug..	1	1	sr1
......................uggaauccaucugacaCacug..	1	1	sr1
......................uggaauccaucugacaaaAug..	6	1	sr1
......................uggaauccaucugacaaacGg..	5	1	sr1
......................uggaauccaucugacaaacug..	5784	0	sr1
......................Gggaauccaucugacaaacug..	402	1	sr1
......................uggaauccauUugacaaacug..	5	1	sr1
......................uggaauccaucugacaGacug..	152	1	sr1
......................uggaauccUucugacaaacug..	2	1	sr1
......................uggaauccaucugaAaaacug..	1	1	sr1

Mature **Star**

```
acuuuauucuuuuuuuuuuaccuggaauccaucugacaaacugcauguuugucaugcaguuugucauggauuccaguuaaaaaaaagcaugcaaucguacgucaaggagaa
......................uCgaauccaucugacaaacug....................................................................  1    1  sr1
......................uggaauccaucGgacaaacug....................................................................  4    1  sr1
......................uAgaauccaucugacaaacug....................................................................  1    1  sr1
......................uggaauccaucugacCaacug....................................................................  1    1  sr1
......................Cggaauccaucugacaaacug....................................................................  18   1  sr1
......................uggaauccaucugaUaaacug....................................................................  6    1  sr1
......................ugAaauccaucugacaaacug....................................................................  2    1  sr1
......................uggaCuccaucugacaaacug....................................................................  4    1  sr1
......................uggaauccaucugUcaaacug....................................................................  5    1  sr1
......................uggaauccaucugacaUacug....................................................................  2    1  sr1
......................uggaauccaucugacaaacCg....................................................................  64   1  sr1
......................uggaauccaCcugacaaacug....................................................................  19   1  sr1
......................uggUauccaucugacaaacug....................................................................  5    1  sr1
......................uggaauccaucugacaaCcug....................................................................  1    1  sr1
......................uggaaucAaucugacaaacug....................................................................  2    1  sr1
......................uggaaGccaucugacaaacug....................................................................  1    1  sr1
......................uggGauccaucugacaaacug....................................................................  35   1  sr1
......................uggaauccaucuAacaaacug....................................................................  1    1  sr1
......................uUgaauccaucugacaaacug....................................................................  4    1  sr1
......................uggaauccaucugacaaacAg....................................................................  9    1  sr1
......................uggaauccaucugacaaacuC....................................................................  14   1  sr1
......................uggaauccaucugacaaacuU....................................................................  38   1  sr1
......................uggaauccaucCgacaaacug....................................................................  19   1  sr1
......................uggaauccaucAgacaaacug....................................................................  5    1  sr1
......................uggaauccaucugaUaaacugc...................................................................  2    1  sr1
......................uggaauccaucugacaaacugG...................................................................  1    1  sr1
......................uggaauccaCcugacaaacugc...................................................................  1    1  sr1
......................Gggaauccaucugacaaacugc...................................................................  13   1  sr1
......................uggGauccaucugacaaacugc...................................................................  3    1  sr1
......................uggaauccaucugacaaacugA...................................................................  15   1  sr1
......................uggaauccaucugacGaacugc...................................................................  1    1  sr1
......................uggaauccaucugacaaCcugc...................................................................  1    1  sr1
......................uggaauccaucugGcaaacugc...................................................................  17   1  sr1
......................uggaauccaucugacaGacugc...................................................................  5    1  sr1
......................Cggaauccaucugacaaacugc...................................................................  1    1  sr1
......................uggaauccaucugacaaacugU...................................................................  52   1  sr1
......................uggaauccaucugacaaacugc...................................................................  262  0  sr1
......................uggaGuccaucugacaaacugc...................................................................  3    1  sr1
......................uggaauccaucugacaaacuCc...................................................................  1    1  sr1
......................uggaauccaucugacaaacuAc...................................................................  1    1  sr1
......................uggaauccaucugacaaaUugca..................................................................  1    1  sr1
......................uggaauccaucugacaGacugca..................................................................  2    1  sr1
......................uggaauccaucugacaaacugcG..................................................................  21   1  sr1
......................uggaauccaucugGcaaacugca..................................................................  24   1  sr1
......................uggaGuccaucugacaaacugca..................................................................  1    1  sr1
......................uggaauccaucugacaaacugcU..................................................................  9    1  sr1
......................uggaauccaucugUcaaacugca..................................................................  1    1  sr1
......................uggaauccaucugacaaacCgca..................................................................  1    1  sr1
......................uggaauccaucugacaaacuAca..................................................................  1    1  sr1
......................uggaauccaucCgacaaacugca..................................................................  1    1  sr1
......................uggaauccaucugacGaacugca..................................................................  3    1  sr1
......................Aggaauccaucugacaaacugca..................................................................  1    1  sr1
......................uggaauccaucugacaaacugcC..................................................................  4    1  sr1
......................Gggaauccaucugacaaacugca..................................................................  26   1  sr1
......................uggaauccaucugacaaacugUa..................................................................  25   1  sr1
......................uggaauccaucugacaaacugca..................................................................  282  0  sr1
......................uggaauccaucuUacaaacugcau.................................................................  1    1  sr1
......................Gggaauccaucugacaaacugcau.................................................................  15   1  sr1
......................uggaauccaucugacaaacugcGu.................................................................  2    1  sr1
......................uggaauccaucugUcaaacugcau.................................................................  1    1  sr1
......................uggaauccaucugGcaaacugcau.................................................................  13   1  sr1
......................uggaauccaucCgacaaacugcau.................................................................  1    1  sr1
......................uggaauccaucugacaaacugcaC.................................................................  12   1  sr1
......................uggaauccaucugacaaGcugcau.................................................................  1    1  sr1
......................uggaauccaucugacaaacugcUu.................................................................  7    1  sr1
......................uggaaAccaucugacaaacugcau.................................................................  1    1  sr1
......................uggaauccaucugacaaacugcaA.................................................................  69   1  sr1
......................uggaauccaucugacaGacugcau.................................................................  2    1  sr1
......................uggaauccaucugacaaacugcCu.................................................................  1    1  sr1
......................uggaauccaucugacaaacugcaG.................................................................  69   1  sr1
```

Mature Star

```
acuuuauucuuuuuuuuuuaccuggaauccaucugacaaacugcauguuugucaugcaguuugucauggauuccaguuaaaaaaaagcaugcaaucguacgucaaggagaa
......................uggaauccaucugacaaacugcau.................................................................	129	0	sr1
......................uggaauccaucugacaaacugcaCg................................................................	1	1	sr1
......................uggaauccaucugacaaacugcaug................................................................	24	0	sr1
......................uggaauccaucugacaaGcugcaug................................................................	1	1	sr1
......................uggaauccaucugGcaaacugcaug................................................................	3	1	sr1
......................uggaauccaucugacaaacugcauC................................................................	10	1	sr1
......................uggaauccaucugacaaacugUaug................................................................	1	1	sr1
......................Aggaauccaucugacaaacugcaug................................................................	1	1	sr1
......................uggaauccaucugacaaacugcaAg................................................................	2	1	sr1
......................Gggaauccaucugacaaacugcaug................................................................	2	1	sr1
......................uggaauccaucugacaaacugcauA................................................................	577	1	sr1
......................uggaauccaucugacaaacugcauU................................................................	62	1	sr1
......................uggaauccaucugacaaacugcauAu...............................................................	36	1	sr1
......................uggaauccaucugacaaacugcauUu...............................................................	11	1	sr1
......................uggaauccaucugacaaacugcauUuu..............................................................	4	1	sr1
......................uggaauccaucugacaaacugcauAuu..............................................................	6	1	sr1
.......................ggaauccaucugacaaac......................................................................	4	0	sr1
.......................ggaauccaucugacaaacu.....................................................................	1	0	sr1
.......................ggaauccaucugacaaacA.....................................................................	1	1	sr1
.......................ggaauccaucugacaaacuC....................................................................	1	1	sr1
.......................ggaauccaucugacaGacug....................................................................	2	1	sr1
.......................ggaauccaucugacaaacuU....................................................................	2	1	sr1
.......................ggGauccaucugacaaacug....................................................................	1	1	sr1
.......................ggaauccaucugacaaacug....................................................................	45	0	sr1
.......................Ugaauccaucugacaaacug....................................................................	2	1	sr1
.......................ggaauccaucugacaaGcug....................................................................	2	1	sr1
.......................ggaauccaucugacaaacuA....................................................................	3	1	sr1
.......................ggaauccaucCgacaaacug....................................................................	1	1	sr1
.......................ggaauccaucugacaaacugU...................................................................	1	1	sr1
.......................ggaauccaucugacaGacugc...................................................................	1	1	sr1
.......................ggaauccaucugacaaacugc...................................................................	2	0	sr1
.......................ggaauccaucugacaaacugca..................................................................	4	0	sr1
.......................ggaauccaucugacaaacugUa..................................................................	1	1	sr1
.......................ggaauUcaucugacaaacugca..................................................................	1	1	sr1
........................gaauccaucugacaaacug....................................................................	3	0	sr1
........................gaauccaucCgacaaacug....................................................................	1	1	sr1
........................gaauccaucugacaaacugca..................................................................	2	0	sr1
........................gaauccaucugGcaaacugca..................................................................	1	1	sr1
.........................aauccaucugacaaacug....................................................................	1	0	sr1
.........................aauccaucugacaaacuA....................................................................	2	1	sr1
.........................aauccaucugacaaacugUa..................................................................	2	1	sr1
...........................uccaucugacaaacugca..................................................................	1	0	sr1
.............................caucugacaaacugcauA................................................................	1	1	sr1
......................................................ugcaguuugucauggauuc......................................	1	0	sr1
......................................................ugcaguuugucauggauucU.....................................	1	1	sr1
........................................................caguuugucauggauuUc.....................................	1	1	sr1
........................................................caguuugucauggauucc.....................................	45	0	sr1
........................................................caguuugucauggauucU.....................................	11	1	sr1
........................................................Gaguuugucauggauucc.....................................	1	1	sr1
........................................................caguuugucauggauucA.....................................	4	1	sr1
........................................................caguuugucauggauucca....................................	7	0	sr1
........................................................caguuugucauggauuccag...................................	101	0	sr1
........................................................caguuugucauggauuccaA...................................	9	1	sr1
........................................................caguuugucauggauCccag...................................	1	1	sr1
........................................................cUguuugucauggauuccag...................................	1	1	sr1
........................................................caguuugucauggauuccGg...................................	1	1	sr1
........................................................cagGuugucauggauuccag...................................	2	1	sr1
........................................................Gaguuugucauggauuccag...................................	2	1	sr1
........................................................caguuugucaugAauuccagu..................................	1	1	sr1
........................................................caguuugucauggauuccagA..................................	10	1	sr1
........................................................caguuugucauggauuccaUu..................................	1	1	sr1
........................................................caguuugucauggauuccagG..................................	1	1	sr1
........................................................caguuugucauggauuccagu..................................	44	0	sr1
........................................................caguuugucauggauuccagC..................................	5	1	sr1
........................................................caguuugucauggauuccaguu.................................	457	0	sr1
........................................................caguuugucauggauuUcaguu.................................	1	1	sr1
........................................................caguuugucauggauuccaguC.................................	59	1	sr1
........................................................caguuugucauggauuccaguG.................................	16	1	sr1
........................................................caguuugucaCggauuccaguu.................................	1	1	sr1
........................................................cGguuugucauggauuccaguu.................................	2	1	sr1
```

```
                    Mature                                Star
acuuuauucuuuuuuuuuuaccuggaauccaucugacaaacugcauguuugucaugcaguuugucauggauuccaguuaaaaaaaagcaugcaaucguacgucaaggagaa
........................................................caguuugucauggauuccGguu.................................   2      1     sr1
........................................................caguuugucauggaCuccaguu.................................   2      1     sr1
........................................................caguuugCcauggauuccaguu.................................   1      1     sr1
........................................................cUguuugucauggauuccaguu.................................   2      1     sr1
........................................................caguuCgucauggauuccaguu.................................   4      1     sr1
........................................................caguuugucauggauuccaguA.................................   37     1     sr1
........................................................Gaguuugucauggauuccaguu.................................   9      1     sr1
........................................................caguuugucUuggauuccaguu.................................   2      1     sr1
........................................................caguuugucGuggauuccaguu.................................   1      1     sr1
........................................................caguuugucauggaCuccaguua................................   2      1     sr1
........................................................caguuugucauggauuccaguCa................................   4      1     sr1
........................................................caguuugucaugUauuccaguua................................   1      1     sr1
........................................................caguuCgucauggauuccaguua................................   6      1     sr1
........................................................cagCuugucauggauuccaguua................................   6      1     sr1
........................................................cGguuugucauggauuccaguua................................   10     1     sr1
........................................................caguuugCcauggauuccaguua................................   1      1     sr1
........................................................caguuugucauggauuccaguAa................................   2      1     sr1
........................................................caguuugucauggauuccGguua................................   3      1     sr1
........................................................caguuugucauggauucUaguua................................   2      1     sr1
........................................................caguuugucauggauuccaguuG................................   30     1     sr1
........................................................caguuugucauggauuUcaguua................................   1      1     sr1
........................................................caguuugucauggauuccaguGa................................   1      1     sr1
........................................................caguuugucauggauuccagCua................................   1      1     sr1
........................................................caguuugucauggauuccaguuC................................   11     1     sr1
........................................................caguuugucaCggauuccaguua................................   1      1     sr1
........................................................caguuugucauggGuuccaguua................................   1      1     sr1
........................................................Aaguuugucauggauuccaguua................................   1      1     sr1
........................................................Gaguuugucauggauuccaguua................................   13     1     sr1
........................................................cUguuugucauggauuccaguua................................   2      1     sr1
........................................................Uaguuugucauggauuccaguua................................   1      1     sr1
........................................................caguuugucauggauCccaguua................................   3      1     sr1
........................................................cagGuugucauggauuccaguua................................   1      1     sr1
........................................................caguuugucauggUuuccaguua................................   2      1     sr1
........................................................caguuugucauggauucAaguua................................   1      1     sr1
........................................................caguuugucauggauuccaguuU................................   63     1     sr1
........................................................caguuugucGuggauuccaguua................................   5      1     sr1
........................................................caguCugucauggauuccaguua................................   4      1     sr1
........................................................caguuugucauggauuccaguua................................   1233   0     sr1
........................................................caguuugucauggauuccaguuUa...............................   33     1     sr1
........................................................caguuugucauggauuccaguuaC...............................   2      1     sr1
........................................................caguuugucauggauuccaguuaU...............................   29     1     sr1
........................................................caguuugucauggauuccaguuaG...............................   3      1     sr1
........................................................caguuugucauggauuccaguuaa...............................   29     0     sr1
........................................................caguuugucauggauuccaguuCa...............................   1      1     sr1
........................................................caguuugucauggauuccaguuaaa..............................   1      0     sr1
........................................................caguuugucauggauuccaguuaUa..............................   7      1     sr1
........................................................caguuugucauggauuccaguuUaa..............................   1      1     sr1
........................................................caguuugucauggauuccaguuaGa..............................   1      1     sr1
.........................................................aguuugucauggauuccG....................................   1      1     sr1
.........................................................aguuugucauggauucca....................................   5      0     sr1
.........................................................Gguuugucauggauuccag...................................   1      1     sr1
.........................................................aguuugucauggauuccag...................................   4      0     sr1
.........................................................aguuugucauggauCccag...................................   1      1     sr1
.........................................................aguuugucauggauuccagu..................................   54     0     sr1
.........................................................aguuugucauggauuccagG..................................   7      1     sr1
.........................................................aguAugucauggauuccagu..................................   1      1     sr1
.........................................................aguuugucauggauuccagC..................................   3      1     sr1
.........................................................Gguuugucauggauuccagu..................................   1      1     sr1
.........................................................aguuugucauggauuccagA..................................   8      1     sr1
.........................................................aguuugucaCggauuccagu..................................   1      1     sr1
.........................................................aguuugucauggGuuccagu..................................   1      1     sr1
.........................................................agCuugucauggauuccaguu.................................   1      1     sr1
.........................................................aguuugucauggauuccaguu.................................   753    0     sr1
.........................................................aguuugucGuggauuccaguu.................................   5      1     sr1
.........................................................aguuugucauggauuccaguC.................................   82     1     sr1
.........................................................aguuugCcauggauuccaguu.................................   3      1     sr1
.........................................................aguuugGcauggauuccaguu.................................   1      1     sr1
.........................................................aguuugucauggauucUaguu.................................   1      1     sr1
.........................................................aguuugucauggaCuccaguu.................................   2      1     sr1
.........................................................aguuugucauggauuccGguu.................................   5      1     sr1
```

```
                    Mature                                    Star
acuuuauucuuuuuuuuuuaccuggaauccaucugacaaacugcauguuugucaugcaguuugucauggauuccaguuaaaaaaaagcaugcaaucguacgucaaggagaa
.........................................................Uguuugucauggauuccaguu.................................    4     1    sr1
.........................................................aguCugucauggauuccaguu.................................    3     1    sr1
.........................................................aguuugucauggGuuccaguu.................................    1     1    sr1
.........................................................aguuugucauggauuccagGu.................................    1     1    sr1
.........................................................aguuugucaugAauuccaguu.................................    3     1    sr1
.........................................................aguuugucauggauuccaguG.................................    25    1    sr1
.........................................................aguuugucauggauCccaguu.................................    3     1    sr1
.........................................................aguuugucauggauuccaguA.................................    41    1    sr1
.........................................................aguuugucauggauuccagCu.................................    3     1    sr1
.........................................................aguuugucauggauuccUguu.................................    1     1    sr1
.........................................................aguuCgucauggauuccaguu.................................    1     1    sr1
.........................................................aguuugucauggaGuccaguu.................................    2     1    sr1
.........................................................Gguuugucauggauuccaguu.................................    22    1    sr1
.........................................................aguuugucauggauuAcaguua................................    1     1    sr1
.........................................................aguuugucaugAauuccaguua................................    1     1    sr1
.........................................................aguuugucauggauuccaguua................................    1843  0    sr1
.........................................................aguuCgucauggauuccaguua................................    6     1    sr1
.........................................................aguuugGcauggauuccaguua................................    1     1    sr1
.........................................................aguuugucauggauuccUguua................................    1     1    sr1
.........................................................aguuugucauggauuccaguAa................................    3     1    sr1
.........................................................aguuugucauAgauuccaguua................................    2     1    sr1
.........................................................agCuugucauggauuccaguua................................    4     1    sr1
.........................................................aguuugCcauggauuccaguua................................    8     1    sr1
.........................................................aguuugucauggaCuccaguua................................    8     1    sr1
.........................................................aguCugucauggauuccaguua................................    4     1    sr1
.........................................................aguuugucGuggauuccaguua................................    3     1    sr1
.........................................................aguuuAucauggauuccaguua................................    1     1    sr1
.........................................................aguuugucauggauuccaguuC................................    36    1    sr1
.........................................................aguuugucauggauCccaguua................................    2     1    sr1
.........................................................aguuugucauggauuccagCua................................    1     1    sr1
.........................................................aguuugucauggGuuccaguua................................    4     1    sr1
.........................................................aguuugucauggauuccaguuG................................    27    1    sr1
.........................................................aguuugucaCggauuccaguua................................    7     1    sr1
.........................................................aguuugucauggauuccGguua................................    2     1    sr1
.........................................................aguuuguUauggauuccaguua................................    3     1    sr1
.........................................................aguuugucauggauuccaguCa................................    6     1    sr1
.........................................................aguuugucauggauuUcaguua................................    1     1    sr1
.........................................................aguAugucauggauuccaguua................................    1     1    sr1
.........................................................Gguuugucauggauuccaguua................................    58    1    sr1
.........................................................aguuugucauggauuccaguuU................................    188   1    sr1
.........................................................aguuugucauggauuccaguuUa...............................    179   1    sr1
.........................................................aguuugucauggauuUcaguuaa...............................    1     1    sr1
.........................................................aguuugucauggauuccaguAaa...............................    1     1    sr1
.........................................................aguuugucauggauuccaguuaa...............................    124   0    sr1
.........................................................Gguuugucauggauuccaguuaa...............................    1     1    sr1
.........................................................aguuugucauggauCccaguuaa...............................    1     1    sr1
.........................................................aguuugucauggauuccaguuaG...............................    20    1    sr1
.........................................................Cguuugucauggauuccaguuaa...............................    1     1    sr1
.........................................................aguuugucauggauucAaguuaa...............................    1     1    sr1
.........................................................aguuugucGuggauuccaguuaa...............................    1     1    sr1
.........................................................aguuugucauggauuccaguuaC...............................    5     1    sr1
.........................................................aguuugucauggauuccaguuCa...............................    1     1    sr1
.........................................................aguuugucauggauuccaguuaU...............................    68    1    sr1
.........................................................aguuugucauggGuuccaguuaa...............................    1     1    sr1
.........................................................agGuugucauggauuccaguuaa...............................    1     1    sr1
.........................................................aguuugucauggauuccaguuaGa..............................    2     1    sr1
.........................................................aguuugucauggauuccaguuUaa..............................    8     1    sr1
.........................................................aguuugucauggauuccaguuaaa..............................    1     0    sr1
.........................................................aguuugucauggauuccaguuaUa..............................    31    1    sr1
.........................................................aguuugucauggauuccaguuaaU..............................    3     1    sr1
.........................................................aguuugucauggauuccaguuaUaa.............................    1     1    sr1
.........................................................aguuugucauggauuccaguuaaUa.............................    1     1    sr1
..........................................................guuugucauggauuccagC..................................    1     1    sr1
..........................................................guuugucauggauuccaguu.................................    3     0    sr1
..........................................................guuugucauggauuccaguua................................    1     0    sr1
...........................................................uuugucauggauuccaguu.................................    14    0    sr1
...........................................................uuugucauggauuccaguG.................................    1     1    sr1
...........................................................uuugucauggauuccaguC.................................    2     1    sr1
...........................................................uuugucauggauuccaguA.................................    1     1    sr1
...........................................................Guugucauggauuccaguu.................................    1     1    sr1
```

```
                    Mature                                   Star
acuuuauucuuuuuuuuuuaccuggaauccaucugacaaacugcauguuugucaugcaguuugucauggauuccaguuaaaaaaaagcaugcaaucguacgucaaggagaa
...........................................................Guugucauggauuccaguua................................ 13   1  sr1
...........................................................uuugucauggauuccGguua................................ 1    1  sr1
...........................................................uuugucauggauuccaguuU................................ 9    1  sr1
...........................................................uuugucauggauuccagCua................................ 1    1  sr1
...........................................................uuugucauggauCccaguua................................ 1    1  sr1
...........................................................uuugAcauggauuccaguua................................ 1    1  sr1
...........................................................uuugucauggauuccaguuG................................ 1    1  sr1
...........................................................uuugucauggGuuccaguua................................ 1    1  sr1
...........................................................uuugucauggaCuccaguua................................ 1    1  sr1
...........................................................uuugucauggauuccaguua................................ 99   0  sr1
...........................................................uuugucauAgauuccaguua................................ 2    1  sr1
...........................................................uuAgucauggauuccaguuaa............................... 1    1  sr1
...........................................................uuugucauggauuccaguuaG............................... 2    1  sr1
...........................................................Guugucauggauuccaguuaa............................... 3    1  sr1
...........................................................uuugucauggauuccaguuaa............................... 24   0  sr1
...........................................................uuugucauggauuccaguuUa............................... 12   1  sr1
...........................................................uuugucauggauuccaguuaU............................... 7    1  sr1
...........................................................uuugucauggauuccaguuaC............................... 1    1  sr1
...........................................................uuugucauggauuccUguuaaa.............................. 1    1  sr1
...........................................................uuugucauggauuccaguuUaa.............................. 1    1  sr1
...........................................................uuugucauggauuccaguuaUa.............................. 7    1  sr1
...........................................................uuugucauggauuccaguuaaUa............................. 2    1  sr1
...........................................................uuugucauggauuccaguuaaaUa............................ 1    1  sr1
............................................................uugucauggauuccaguua................................ 1    0  sr1
............................................................uugucauggUuuccaguua................................ 1    1  sr1
............................................................uugucauggauuccaguuaU............................... 2    1  sr1
............................................................uugucauggauuccaguuaUa.............................. 1    1  sr1
............................................................uugucauggauuccaguuaaUa............................. 1    1  sr1
......................................................................................gAaugcaaucguacgucaagga... 1    1  sr1
........................................................................................augcaaucguacgucaaggag.. 1    0  sr1

..................uaccuggaauccaucugacaaa....................................................................... 1    0  st1
..................uaccuggaauccaucugacaaacug.................................................................... 1    0  st1
..................uCccuggaauccaucugacaaacug.................................................................... 1    1  st1
...................Uccuggaauccaucugacaaacug.................................................................... 2    1  st1
...................accuggaauccaucCgacaaacug.................................................................... 1    1  st1
...................Cccuggaauccaucugacaaacug.................................................................... 13   1  st1
....................ccuggaauccaCcugaca......................................................................... 1    1  st1
....................ccuggaauUcaucugaca......................................................................... 2    1  st1
....................ccuggaauccaucugGca......................................................................... 1    1  st1
....................ccuggGauccaucugaca......................................................................... 2    1  st1
....................Acuggaauccaucugaca......................................................................... 2    1  st1
....................ccuggaauccUucugaca......................................................................... 1    1  st1
....................ccuggaauccaucugaca......................................................................... 120  0  st1
....................ccuggaauccaucugacG......................................................................... 4    1  st1
....................ccuggaauccaucugacU......................................................................... 2    1  st1
....................ccuggaauccaucAgaca......................................................................... 2    1  st1
....................ccCggaauccaucugaca......................................................................... 1    1  st1
....................ccuAgaauccaucugacaa........................................................................ 1    1  st1
....................ccuggaauccaucugacGa........................................................................ 1    1  st1
....................ccuggaauccaucugacaG........................................................................ 4    1  st1
....................Gcuggaauccaucugacaa........................................................................ 5    1  st1
....................ccuggaauccaucugGcaa........................................................................ 2    1  st1
....................ccuggaauccaucugacaa........................................................................ 230  0  st1
....................ccuggaGuccaucugacaa........................................................................ 3    1  st1
....................ccuggGauccaucugacaa........................................................................ 1    1  st1
....................ccuggaauccaucCgacaa........................................................................ 5    1  st1
....................ccuggaauccaucugacaaa....................................................................... 140  0  st1
....................ccuggaGuccaucugacaaa....................................................................... 1    1  st1
....................ccuggaauccaucugacaaC....................................................................... 3    1  st1
....................ccuggaauccaucugacaaG....................................................................... 3    1  st1
....................ccuggaauccaucugGcaaa....................................................................... 4    1  st1
....................ccuggaaCccaucugacaaac...................................................................... 11   1  st1
....................ccuggaauccauAugacaaac...................................................................... 1    1  st1
....................ccuggaauccaucugacGaac...................................................................... 16   1  st1
....................cGuggaauccaucugacaaac...................................................................... 1    1  st1
....................ccuggaauUcaucugacaaac...................................................................... 2    1  st1
....................ccAggaauccaucugacaaac...................................................................... 5    1  st1
....................ccuggaauccaucugacaGac...................................................................... 61   1  st1
....................ccuggaaucAaucugacaaac...................................................................... 3    1  st1
```

```
                   Mature                                Star
acuuuauucuuuuuuuuuuaccuggaauccaucugacaaacugcauguuugucaugcaguuugucauggauuccaguuaaaaaaaagcaugcaaucguacgucaaggagaa
....................ccuggaaAccaucugacaaac......................................................................    5     1    st1
....................ccuggaauAcaucugacaaac......................................................................    1     1    st1
....................ccCggaauccaucugacaaac......................................................................    14    1    st1
....................ccuggaaucUaucugacaaac......................................................................    1     1    st1
....................ccuUgaauccaucugacaaac......................................................................    2     1    st1
....................ccuggaauccaucGgacaaac......................................................................    2     1    st1
....................ccuggaauccaucugacaaGc......................................................................    33    1    st1
....................ccuggaauGcaucugacaaac......................................................................    1     1    st1
....................ccuggaauccaucugCcaaac......................................................................    1     1    st1
....................ccugAaauccaucugacaaac......................................................................    3     1    st1
....................ccuggaauccaucuCacaaac......................................................................    5     1    st1
....................ccuggaauccaCcugacaaac......................................................................    14    1    st1
....................ccuggaauccaucugacUaac......................................................................    5     1    st1
....................cUuggaauccaucugacaaac......................................................................    3     1    st1
....................ccuAgaauccaucugacaaac......................................................................    5     1    st1
....................ccuggaauccaucugGcaaac......................................................................    53    1    st1
....................ccuggUauccaucugacaaac......................................................................    1     1    st1
....................Acuggaauccaucugacaaac......................................................................    30    1    st1
....................ccuggaauccaucugacaaCc......................................................................    7     1    st1
....................ccuggaauccaucugUcaaac......................................................................    2     1    st1
....................ccuggaauccaucugaAaaac......................................................................    1     1    st1
....................ccuggaauccaucugacaaaU......................................................................    501   1    st1
....................ccuggaauccaucugacaaaG......................................................................    3     1    st1
....................ccuggaauccaucugaUaaac......................................................................    1     1    st1
....................ccuggaauccaucugacCaac......................................................................    1     1    st1
....................ccuggaauccaucAgacaaac......................................................................    8     1    st1
....................ccuggaauccaucuAacaaac......................................................................    9     1    st1
....................ccuggaauccaucugacaaaA......................................................................    116   1    st1
....................cAuggaauccaucugacaaac......................................................................    3     1    st1
....................ccuggaauccaAcugacaaac......................................................................    1     1    st1
....................Gcuggaauccaucugacaaac......................................................................    9     1    st1
....................ccugUaauccaucugacaaac......................................................................    1     1    st1
....................ccuggaauccaucugacaUac......................................................................    2     1    st1
....................ccuggaauccaucugacaaac......................................................................    5592  0    st1
....................ccuggaauccCucugacaaac......................................................................    1     1    st1
....................ccuggGauccaucugacaaac......................................................................    27    1    st1
....................ccuggaauccGucugacaaac......................................................................    11    1    st1
....................ccuggaGuccaucugacaaac......................................................................    26    1    st1
....................ccuggaauccaucCgacaaac......................................................................    16    1    st1
....................ccCggaauccaucugacaaacu.....................................................................    18    1    st1
....................Gcuggaauccaucugacaaacu.....................................................................    10    1    st1
....................ccuggaauccaGcugacaaacu.....................................................................    2     1    st1
....................ccuggaauccaucugacaaCcu.....................................................................    4     1    st1
....................ccuggaauccaucugacaaacu.....................................................................    8351  0    st1
....................ccuggaauccaucugacaUacu.....................................................................    1     1    st1
....................ccuggaauccaucAgacaaacu.....................................................................    7     1    st1
....................ccuggaauccaucGgacaaacu.....................................................................    2     1    st1
....................ccuggaauccaucugacaaaUu.....................................................................    7     1    st1
....................ccGggaauccaucugacaaacu.....................................................................    2     1    st1
....................ccuggaaAccaucugacaaacu.....................................................................    3     1    st1
....................ccuggaauccaucCgacaaacu.....................................................................    24    1    st1
....................ccuggaauccaucugacaaaGu.....................................................................    1     1    st1
....................ccuggaauUcaucugacaaacu.....................................................................    6     1    st1
....................ccuggaauccaucugacaaGcu.....................................................................    30    1    st1
....................ccuggUauccaucugacaaacu.....................................................................    3     1    st1
....................cUuggaauccaucugacaaacu.....................................................................    3     1    st1
....................ccuggaauccGucugacaaacu.....................................................................    18    1    st1
....................ccAggaauccaucugacaaacu.....................................................................    3     1    st1
....................ccuggaauccaAcugacaaacu.....................................................................    8     1    st1
....................ccuUgaauccaucugacaaacu.....................................................................    2     1    st1
....................ccuggaauccaucugaUaaacu.....................................................................    1     1    st1
....................ccuggGauccaucugacaaacu.....................................................................    33    1    st1
....................ccuggaauAcaucugacaaacu.....................................................................    2     1    st1
....................ccuggaauccaucugacaaUcu.....................................................................    1     1    st1
....................ccuggaauccaCcugacaaacu.....................................................................    33    1    st1
....................ccuggaauccaucugacUaacu.....................................................................    6     1    st1
....................ccuggaauccaucuCacaaacu.....................................................................    8     1    st1
....................ccuggaauccaucugacaaacA.....................................................................    307   1    st1
....................ccugAaauccaucugacaaacu.....................................................................    5     1    st1
....................ccuggaauccauUugacaaacu.....................................................................    3     1    st1
```

```
                    Mature                                 Star
acuuuauucuuuuuuuuuuaccuggaauccaucugacaaacugcauguuugucaugcaguuugucauggauuccaguuaaaaaaaagcaugcaaucguacgucaaggagaa
....................ccuggaauccaucugUcaaacu.....................................................................    2     1    st1
....................ccuggaauccaucugacaCacu.....................................................................    1     1    st1
....................ccuggaaCccaucugacaaacu.....................................................................    15    1    st1
....................ccuggaauccaucugacaGacu.....................................................................    89    1    st1
....................ccuggaauccaucugacaaacG.....................................................................    71    1    st1
....................ccuggaCuccaucugacaaacu.....................................................................    2     1    st1
....................ccuggaauccaucugacaaaAu.....................................................................    1     1    st1
....................ccuggaauccauAugacaaacu.....................................................................    1     1    st1
....................ccuggaGuccaucugacaaacu.....................................................................    41    1    st1
....................ccuggaauccaucugGcaaacu.....................................................................    65    1    st1
....................ccuAgaauccaucugacaaacu.....................................................................    10    1    st1
....................ccuggaauccaucugacaaacC.....................................................................    917   1    st1
....................cAuggaauccaucugacaaacu.....................................................................    1     1    st1
....................ccuggaauccaucugacGaacu.....................................................................    39    1    st1
....................ccuggaaucUaucugacaaacu.....................................................................    10    1    st1
....................ccuggaauccaucuAacaaacu.....................................................................    13    1    st1
....................Acuggaauccaucugacaaacu.....................................................................    31    1    st1
....................ccuggaUuccaucugacaaacu.....................................................................    2     1    st1
....................ccuggaauccaucugacUaacug....................................................................    109   1    st1
....................ccuggaauccaCcugacaaacug....................................................................    238   1    st1
....................ccuggaauccaucugacaGacug....................................................................    1814  1    st1
....................ccugUaauccaucugacaaacug....................................................................    12    1    st1
....................ccuggaauccaGcugacaaacug....................................................................    8     1    st1
....................ccuggaauccaucugacaaacuC....................................................................    480   1    st1
....................ccuggCauccaucugacaaacug....................................................................    13    1    st1
....................ccGggaauccaucugacaaacug....................................................................    8     1    st1
....................ccuggaauGcaucugacaaacug....................................................................    4     1    st1
....................ccAggaauccaucugacaaacug....................................................................    61    1    st1
....................ccuggaaGccaucugacaaacug....................................................................    9     1    st1
....................cGuggaauccaucugacaaacug....................................................................    6     1    st1
....................ccuggaauccaucugGcaaacug....................................................................    550   1    st1
....................ccuggaauccaucugacaaUcug....................................................................    16    1    st1
....................ccuggaGuccaucugacaaacug....................................................................    600   1    st1
....................ccuggaauccaucuCacaaacug....................................................................    72    1    st1
....................ccugCaauccaucugacaaacug....................................................................    4     1    st1
....................cUuggaauccaucugacaaacug....................................................................    63    1    st1
....................ccuggaauccaucugacaaGcug....................................................................    355   1    st1
....................ccuggaauccauGugacaaacug....................................................................    1     1    st1
....................ccuggaauccaucuAacaaacug....................................................................    130   1    st1
....................ccuggaaucUaucugacaaacug....................................................................    66    1    st1
....................ccuggaauccaucugacaaacGg....................................................................    107   1    st1
....................Gcuggaauccaucugacaaacug....................................................................    162   1    st1
....................ccuCgaauccaucugacaaacug....................................................................    2     1    st1
....................cAuggaauccaucugacaaacug....................................................................    27    1    st1
....................ccuAgaauccaucugacaaacug....................................................................    69    1    st1
....................ccCggaauccaucugacaaacug....................................................................    237   1    st1
....................ccuggaauccaucugacaaaGug....................................................................    1     1    st1
....................ccuggaauccaucGgacaaacug....................................................................    20    1    st1
....................ccuggaauccaucCgacaaacug....................................................................    326   1    st1
....................ccuggaCuccaucugacaaacug....................................................................    11    1    st1
....................ccuggaauccaucugacaaCcug....................................................................    41    1    st1
....................ccuggaauccaucugacaUacug....................................................................    37    1    st1
....................ccuggUauccaucugacaaacug....................................................................    42    1    st1
....................ccuggaauccaucugacaCacug....................................................................    14    1    st1
....................ccuggaauccaucugacCaacug....................................................................    16    1    st1
....................ccuggaauccaucugacaaaAug....................................................................    11    1    st1
....................ccuggGauccaucugacaaacug....................................................................    390   1    st1
....................ccuggaauccauUugacaaacug....................................................................    51    1    st1
....................ccuggaauccaucugacaaacuA....................................................................    7166  1    st1
....................ccuggaauccaucugCcaaacug....................................................................    15    1    st1
....................ccuggaauccaucugaUaaacug....................................................................    52    1    st1
....................ccuggaauccCucugacaaacug....................................................................    13    1    st1
....................ccuggaauccaucugacaaacAg....................................................................    92    1    st1
....................ccuggaauccUucugacaaacug....................................................................    22    1    st1
....................ccuggaauccaucugaAaaacug....................................................................    6     1    st1
....................ccuggaaucAaucugacaaacug....................................................................    11    1    st1
....................ccuggaUuccaucugacaaacug....................................................................    14    1    st1
....................ccuggaauccaucuUacaaacug....................................................................    13    1    st1
....................Acuggaauccaucugacaaacug....................................................................    362   1    st1
....................ccugAaauccaucugacaaacug....................................................................    64    1    st1
```

```
                    Mature                                  Star
acuuuauucuuuuuuuuuuaccuggaauccaucugacaaacugcauguuugucaugcaguuugucauggauuccaguuaaaaaaaagcaugcaaucguacgucaaggagaa
....................ccuggaAccaucugacaaacug....................................................................    20     1   st1
....................ccuUgaauccaucugacaaacug....................................................................    12     1   st1
....................ccuggaauccaucugacaaacuU....................................................................    715    1   st1
....................ccuggaaucGaucugacaaacug....................................................................    7      1   st1
....................ccuggaauccaucugacaaacCg....................................................................    542    1   st1
....................ccuggaauUcaucugacaaacug....................................................................    77     1   st1
....................ccuggaauccaucAgacaaacug....................................................................    63     1   st1
....................ccuggaauccaucugacaaacug....................................................................    93712  0   st1
....................ccuggaauccauAugacaaacug....................................................................    13     1   st1
....................ccuggaauccaAcugacaaacug....................................................................    37     1   st1
....................ccuggaauAcaucugacaaacug....................................................................    12     1   st1
....................ccuggaauccaucugacGaacug....................................................................    390    1   st1
....................ccuggaaCccaucugacaaacug....................................................................    234    1   st1
....................ccuggaauccaucugUcaaacug....................................................................    43     1   st1
....................ccuggaauccaucugacaaaUug....................................................................    52     1   st1
....................ccuggaauccGucugacaaacug....................................................................    229    1   st1
....................ccuggaauccaucugacaaacugG...................................................................    7      1   st1
....................ccuggaauccaucuAacaaacugc...................................................................    3      1   st1
....................ccuggaauccaucCgacaaacugc...................................................................    7      1   st1
....................ccuggaauccGucugacaaacugc...................................................................    4      1   st1
....................ccuUgaauccaucugacaaacugc...................................................................    2      1   st1
....................ccuggGauccaucugacaaacugc...................................................................    14     1   st1
....................ccuggaauccaucugacaaacuCc...................................................................    7      1   st1
....................ccuggaauccauAugacaaacugc...................................................................    1      1   st1
....................ccuggaauccaucugacaaCcugc...................................................................    1      1   st1
....................ccuggaauccaCcugacaaacugc...................................................................    9      1   st1
....................ccuggaauccaucugacaaacuAc...................................................................    10     1   st1
....................Gcuggaauccaucugacaaacugc...................................................................    8      1   st1
....................ccuggaauccaucugacGaacugc...................................................................    19     1   st1
....................ccuggaaCccaucugacaaacugc...................................................................    4      1   st1
....................ccuggaauccaucAgacaaacugc...................................................................    1      1   st1
....................ccuggaAccaucugacaaacugc...................................................................    1      1   st1
....................ccugUaauccaucugacaaacugc...................................................................    1      1   st1
....................ccuggaauUcaucugacaaacugc...................................................................    2      1   st1
....................ccuggaaucUaucugacaaacugc...................................................................    1      1   st1
....................ccuggaauccaucugacaaacugc...................................................................    2649   0   st1
....................ccugAaauccaucugacaaacugc...................................................................    2      1   st1
....................ccuggaauccaucugUcaaacugc...................................................................    1      1   st1
....................ccuggaauccaucugacaaacCgc...................................................................    7      1   st1
....................ccuggaauccaucugCcaaacugc...................................................................    1      1   st1
....................Acuggaauccaucugacaaacugc...................................................................    16     1   st1
....................ccuggaauccauUugacaaacugc...................................................................    2      1   st1
....................ccuggaauccaucugacaaaUugc...................................................................    3      1   st1
....................cUuggaauccaucugacaaacugc...................................................................    2      1   st1
....................ccuggaauccaucugacaaacuUc...................................................................    3      1   st1
....................ccAggaauccaucugacaaacugc...................................................................    2      1   st1
....................ccuggaauccaucGgacaaacugc...................................................................    3      1   st1
....................ccuggaGuccaucugacaaacugc...................................................................    17     1   st1
....................ccuggaauccaucugacaaacugU...................................................................    478    1   st1
....................ccCggaauccaucugacaaacugc...................................................................    11     1   st1
....................ccuggaauccaucugacaGacugc...................................................................    21     1   st1
....................ccuggaauccaucugGcaaacugc...................................................................    23     1   st1
....................ccuggaauAcaucugacaaacugc...................................................................    1      1   st1
....................ccuggaauccaucuCacaaacugc...................................................................    4      1   st1
....................ccuggaauccaucugacaaGcugc...................................................................    9      1   st1
....................ccuggaauccaucugacUaacugc...................................................................    3      1   st1
....................ccuggaauccaucugacaaacugA...................................................................    271    1   st1
....................ccuggaauccaucugacaaacAgc...................................................................    2      1   st1
....................ccuggaauccaucugacaaGcugca..................................................................    4      1   st1
....................ccuggGauccaucugacaaacugca..................................................................    4      1   st1
....................ccuggaauccaucugGcaaacugca..................................................................    17     1   st1
....................ccuggaauccaucugacGaacugca..................................................................    4      1   st1
....................ccuggaauccaucCgacaaacugca..................................................................    4      1   st1
....................ccuggaauccauUugacaaacugca..................................................................    1      1   st1
....................ccuAgaauccaucugacaaacugca..................................................................    3      1   st1
....................ccuggaaCccaucugacaaacugca..................................................................    2      1   st1
....................ccuggaauccaucugacaaacugcC..................................................................    8      1   st1
....................ccuggaauccaucugacaaacugUa..................................................................    65     1   st1
....................ccuggaauccaucugacaaacCgca..................................................................    2      1   st1
....................ccuggaauccaucugaUaaacugca..................................................................    4      1   st1
```

Mature Star			
acuuuauucuuuuuuuuuuaccuggaauccaucugacaaacugcauguuugucaugcaguuugucauggauuccaguuaaaaaaaagcaugcaaucguacgucaaggagaa			
....................ccugCaauccaucugacaaacugca..	1	1	st1
....................cUuggaauccaucugacaaacugca..	3	1	st1
....................ccCggaauccaucugacaaacugca..	4	1	st1
....................Acuggaauccaucugacaaacugca..	9	1	st1
....................ccuggaauccaucugacaaacugGa..	2	1	st1
....................ccuggaGuccaucugacaaacugca..	7	1	st1
....................ccuggaauccaucugacaGacugca..	16	1	st1
....................ccuggaauUcaucugacaaacugca..	1	1	st1
....................ccuggaauccaucugacaaacugcU..	65	1	st1
....................ccuggaauccaCcugacaaacugca..	2	1	st1
....................Gcuggaauccaucugacaaacugca..	1	1	st1
....................ccuggaauccGucugacaaacugca..	6	1	st1
....................ccuggaauccaucugacaaacuAca..	6	1	st1
....................ccuggaauccaucugacaaCcugca..	2	1	st1
....................ccuggaauccaucAgacaaacugca..	2	1	st1
....................ccuggaaAccaucugacaaacugca..	1	1	st1
....................ccuggaaucUaucugacaaacugca..	1	1	st1
....................ccuggaauccaucugacaaacugcG..	31	1	st1
....................ccuggUauccaucugacaaacugca..	1	1	st1
....................ccuggaauccaucuAacaaacugca..	1	1	st1
....................ccuggaauccaucugacaaacuCca..	2	1	st1
....................ccuggaauccaucugacaaacugca..	1259	0	st1
....................Acuggaauccaucugacaaacugcau...	1	1	st1
....................ccuggaauccaucugacaGacugcau...	1	1	st1
....................ccCggaauccaucugacaaacugcau...	2	1	st1
....................ccuggaauccaucugacaaacuAcau...	1	1	st1
....................ccuggaauccaucugacaaacugcUu...	31	1	st1
....................ccuggaauccaucugacGaacugcau...	1	1	st1
....................ccuggaauccaCcugacaaacugcau...	1	1	st1
....................ccuggaauccaucugacaaacugcaC...	15	1	st1
....................ccuggaauccaucugacaaacugUau...	4	1	st1
....................ccuggaauccaucugacaaacugcaG...	181	1	st1
....................ccuggaauccaucugacaaacugcau...	129	0	st1
....................ccuggaauccaucugacaaaAugcau...	1	1	st1
....................ccuggaaCccaucugacaaacugcau...	2	1	st1
....................ccuggaauccaucugGcaaacugcau...	2	1	st1
....................ccuggaauccaucugacaaacugcaA...	60	1	st1
....................ccuggaauccaucCgacaaacugcau...	1	1	st1
....................ccuggaauccaucugacaaacugcaug..	30	0	st1
....................ccuggaauccaucugacaaacugcaAg..	15	1	st1
....................ccuggaauccaucugacaaacugcUug..	2	1	st1
....................ccuggaauccaucugacaaacugcauC..	21	1	st1
....................ccuggaauccaucugacaaacugcauU..	187	1	st1
....................ccuggaauccaucugacaaacugcaCg..	1	1	st1
....................ccuggaauccaucugacaaacugcauA..	136	1	st1
.....................cuggaauccaucugacaa..	1	0	st1
.....................cuggaauccaucugacaaac..	21	0	st1
.....................cuggaauccaucugacaaaU..	1	1	st1
.....................Uuggaauccaucugacaaac..	1	1	st1
.....................cuggaGuccaucugacaaacu...	1	1	st1
.....................cuggaauccaucugacaaacu...	26	0	st1
.....................cuggaauccaucugacaaacA...	1	1	st1
.....................Uuggaauccaucugacaaacu...	1	1	st1
.....................cuggGauccaucugacaaacu...	1	1	st1
.....................cuggaauccaucugacaaacC...	1	1	st1
.....................cuggaauccaucugacaaacuA..	31	1	st1
.....................cuggaauccaCcugacaaacug..	1	1	st1
.....................cuggaauccaucugacaaGcug..	3	1	st1
.....................cugAaauccaucugacaaacug..	1	1	st1
.....................cuggaauccaucugacaGacug..	7	1	st1
.....................cuggaauccaucCgacaaacug..	1	1	st1
.....................Auggaauccaucugacaaacug..	1	1	st1
.....................cuggaauccGucugacaaacug..	2	1	st1
.....................cuggaauccaucugGcaaacug..	2	1	st1
.....................cCggaauccaucugacaaacug..	1	1	st1
.....................cuAgaauccaucugacaaacug..	1	1	st1
.....................cuggaauccaucugUcaaacug..	1	1	st1
.....................cuggaauccaucugacGaacug..	3	1	st1
.....................cuggaauccaucugacaaacuC..	3	1	st1
.....................cuggaauccaucugaUaaacug..	1	1	st1

```
                    Mature                               Star
acuuuauucuuuuuuuuuuaccuggaauccaucugacaaacugcauguuugucaugcaguuugucauggauuccaguuaaaaaaaagcaugcaaucguacgucaaggagaa
.....................cuggaaCccaucugacaaacug....................................................................    1    1    st1
.....................cuggaauccaucugacaaacCg....................................................................    1    1    st1
.....................Uuggaauccaucugacaaacug....................................................................    14   1    st1
.....................cGggaauccaucugacaaacug....................................................................    2    1    st1
.....................cuggGauccaucugacaaacug....................................................................    2    1    st1
.....................cuggaauccaAcugacaaacug....................................................................    1    1    st1
.....................cuggaGuccaucugacaaacug....................................................................    6    1    st1
.....................cuggaauccaucugacaaacug....................................................................    432  0    st1
.....................cuggaauccaucugacaaacuU....................................................................    5    1    st1
.....................cuggaauccaucugacaaacugU...................................................................    2    1    st1
.....................cuAgaauccaucugacaaacugc...................................................................    1    1    st1
.....................cuggaauccaucugacaaacugc...................................................................    13   0    st1
.....................Uuggaauccaucugacaaacugc...................................................................    1    1    st1
.....................cuggaauccaucugacaaacugUa..................................................................    1    1    st1
.....................cuggaauccaucugacaaacugcC..................................................................    3    1    st1
.....................cuggaauccaucugacaaacugca..................................................................    10   0    st1
.....................cuggaauccaucugacaaacugcCu.................................................................    1    1    st1
.....................cuggaauccaucugacaaacugcaG.................................................................    1    1    st1
.....................cuggaauccaucugacaaacugcauU................................................................    1    1    st1
......................uggaauccaucugacaaa.......................................................................    1    0    st1
......................uggaauccaucugacaGac......................................................................    2    1    st1
......................uAgaauccaucugacaaac......................................................................    1    1    st1
......................uggaauccaucugacaaac......................................................................    41   0    st1
......................uggaauccaucugacaaaA......................................................................    1    1    st1
......................uggaauccaucugacaaaU......................................................................    5    1    st1
......................uggaauccaucugGcaaac......................................................................    4    1    st1
......................uggGauccaucugacaaac......................................................................    1    1    st1
......................uggaauccaucugacaaacu.....................................................................    45   0    st1
......................uggaaAccaucugacaaacu.....................................................................    2    1    st1
......................uggaauccaucugGcaaacu.....................................................................    3    1    st1
......................uggGauccaucugacaaacu.....................................................................    1    1    st1
......................uggaauccaucugacaaacC.....................................................................    3    1    st1
......................Cggaauccaucugacaaacu.....................................................................    1    1    st1
......................uggaaCccaucugacaaacu.....................................................................    1    1    st1
......................Cggaauccaucugacaaacug....................................................................    5    1    st1
......................uggaauccCucugacaaacug....................................................................    9    1    st1
......................uggaaCccaucugacaaacug....................................................................    2    1    st1
......................uggaauccaucugacaaacuU....................................................................    3    1    st1
......................uggaauccaucugacaGacug....................................................................    9    1    st1
......................uggaauccaucugaUaaacug....................................................................    1    1    st1
......................uggaauccauUugacaaacug....................................................................    1    1    st1
......................uggaauccaucugacaaacCg....................................................................    3    1    st1
......................uggGauccaucugacaaacug....................................................................    5    1    st1
......................ugAaauccaucugacaaacug....................................................................    1    1    st1
......................Aggaauccaucugacaaacug....................................................................    3    1    st1
......................uggaauccaucAgacaaacug....................................................................    1    1    st1
......................uggaauGcaucugacaaacug....................................................................    1    1    st1
......................uggaGuccaucugacaaacug....................................................................    5    1    st1
......................uggaaucAaucugacaaacug....................................................................    1    1    st1
......................uggaauccaucugacaaacuC....................................................................    1    1    st1
......................uggaauccaucugacaaGcug....................................................................    1    1    st1
......................uggaauccaucugGcaaacug....................................................................    23   1    st1
......................uggaauccaucCgacaaacug....................................................................    3    1    st1
......................uggaauccaucugacaaacuA....................................................................    45   1    st1
......................uUgaauccaucugacaaacug....................................................................    2    1    st1
......................uggaauccaucugacaaacGg....................................................................    1    1    st1
......................uggaCuccaucugacaaacug....................................................................    1    1    st1
......................uggaauccaucugacaaacug....................................................................    605  0    st1
......................uggaauccaucugacGaacug....................................................................    2    1    st1
......................uAgaauccaucugacaaacug....................................................................    1    1    st1
......................uggaauccCucugacaaacugc...................................................................    1    1    st1
......................uggaauccaucugacaaacugA...................................................................    2    1    st1
......................uggaauccaucugGcaaacugc...................................................................    2    1    st1
......................uggaauccaucugacaaacugU...................................................................    11   1    st1
......................uggaauccaucugacaaacugc...................................................................    32   0    st1
......................uggaauccaucugacaaacugUa..................................................................    1    1    st1
......................uggaauccaucugGcaaacugca..................................................................    2    1    st1
......................uAgaauccaucugacaaacugca..................................................................    2    1    st1
......................uggaauccaucugacaaacugcU..................................................................    1    1    st1
......................uggaauccaucugacaaacugca..................................................................    60   0    st1
```

```
                    Mature                              Star
acuuuauucuuuuuuuuuuaccuggaauccaucugacaaacugcauguuugucaugcaguuugucauggauuccaguuaaaaaaaagcaugcaaucguacgucaaggagaa
......................uggaauccGucugacaaacugca..................................................................  1     1  st1
......................uggaauccaucugacaaacugcUu.................................................................  2     1  st1
......................uggaauccaucugacaaacugcaC.................................................................  8     1  st1
......................uggaauccaucugacaaacugcaG.................................................................  7     1  st1
......................uggaauccaucugGcaaacugcau.................................................................  2     1  st1
......................uggaauccaucugacaaacugcGu.................................................................  4     1  st1
......................uggaauccaucugacaaacugcaA.................................................................  8     1  st1
......................uggaauccaucugacaaacugcau.................................................................  32    0  st1
......................uggaauccaucugacaaacugcauA................................................................  32    1  st1
......................uggaauccaucugacaaacugcaug................................................................  2     0  st1
......................uggaauccaucugacaaacugcauU................................................................  17    1  st1
......................uggaauccaucugacaaacugcauC................................................................  2     1  st1
......................uggaauccaucugacaaacugcaAg................................................................  3     1  st1
......................uggaauccaucugacaaacugcauUu...............................................................  9     1  st1
......................uggaauccaucugacaaacugcauAu...............................................................  6     1  st1
......................uggaauccaucugacaaacugcauUuu..............................................................  4     1  st1
.......................ggaauccaucugacaaac......................................................................  1     0  st1
.......................ggaauccaucugacaaaU......................................................................  1     1  st1
.......................ggaauccaucugacaaacu.....................................................................  2     0  st1
.......................ggaauccaucugacaaacuA....................................................................  2     1  st1
.......................ggaGuccaucugacaaacug....................................................................  1     1  st1
.......................ggGauccaucugacaaacug....................................................................  1     1  st1
.......................ggaauccaucugacaaaAug....................................................................  1     1  st1
.......................ggaauccaucugacaaacug....................................................................  21    0  st1
.......................ggaauccaucugacaaacugc...................................................................  1     0  st1
........................gaauccaucugacaaacug....................................................................  1     0  st1
.........................aauccaucugacaaacug....................................................................  1     0  st1
.........................aauccaucugacaaacugcauUu...............................................................  1     1  st1
........................................................caguuugucauggauucA.....................................  1     1  st1
........................................................caguuugucauggauucc.....................................  36    0  st1
........................................................caguuugucauggauucU.....................................  13    1  st1
........................................................caguuugucauggauucca....................................  1     0  st1
........................................................caguuugucauggauuccag...................................  14    0  st1
........................................................caguuuguUauggauuccagu..................................  1     1  st1
........................................................caguuugucauggauuccagA..................................  3     1  st1
........................................................caguuugucauggauuccaCu..................................  1     1  st1
........................................................caguuugucauggauuccagu..................................  21    0  st1
........................................................cagCuugucauggauuccagu..................................  2     1  st1
........................................................cagAuugucauggauuccaguu.................................  1     1  st1
........................................................caguuugucauggauuccagGu.................................  1     1  st1
........................................................caguuugucauggaCuccaguu.................................  4     1  st1
........................................................cGguuugucauggauuccaguu.................................  1     1  st1
........................................................caguuugCcauggauuccaguu.................................  1     1  st1
........................................................caguuugucauggauuccaguC.................................  27    1  st1
........................................................caguuugucauggauuccagCu.................................  2     1  st1
........................................................caguuugucauggauuccGguu.................................  2     1  st1
........................................................Gaguuugucauggauuccaguu.................................  1     1  st1
........................................................caguuugucauggaGuccaguu.................................  1     1  st1
........................................................caguuCgucauggauuccaguu.................................  2     1  st1
........................................................caguuugucauggauuccaguG.................................  2     1  st1
........................................................caguuugucGuggauuccaguu.................................  2     1  st1
........................................................caguuugucauggGuuccaguu.................................  1     1  st1
........................................................cagCuugucauggauuccaguu.................................  1     1  st1
........................................................caguuugucauggauuccaguu.................................  377   0  st1
........................................................caguCugucauggauuccaguu.................................  3     1  st1
........................................................caguuugucauggauuccaguA.................................  4     1  st1
........................................................caguuugucauggGuuccaguua................................  1     1  st1
........................................................cCguuugucauggauuccaguua................................  2     1  st1
........................................................caguuugucauggauucAaguua................................  1     1  st1
........................................................caguCugucauggauuccaguua................................  5     1  st1
........................................................cagCuugucauggauuccaguua................................  3     1  st1
........................................................caguuugucaCggauuccaguua................................  4     1  st1
........................................................caguuugucauggauuccaguua................................  1837  0  st1
........................................................caguuugucauggaGuccaguua................................  6     1  st1
........................................................Uaguuugucauggauuccaguua................................  1     1  st1
........................................................caguuugucaugAauuccaguua................................  1     1  st1
........................................................caguuugucGuggauuccaguua................................  4     1  st1
........................................................caguuuAucauggauuccaguua................................  1     1  st1
........................................................caguuugucauggauuccaguuU................................  48    1  st1
........................................................caguuuguUauggauuccaguua................................  1     1  st1
```

```
                    Mature                                Star
acuuuauucuuuuuuuuuuaccuggaauccaucugacaaacugcauguuugucaugcaguuugucauggauuccaguuaaaaaaaagcaugcaaucguacgucaaggagaa
........................................................caguuugucauggauuUcaguua................................  2     1    st1
........................................................cUguuugucauggauuccaguua................................  4     1    st1
........................................................caguuugucauggauCccaguua................................  4     1    st1
........................................................Aaguuugucauggauuccaguua................................  1     1    st1
........................................................caguuugucauggauuccaguuC................................  14    1    st1
........................................................caguuGgucauggauuccaguua................................  1     1    st1
........................................................caguuugucauggauuccGguua................................  3     1    st1
........................................................caguuugucaugUauuccaguua................................  1     1    st1
........................................................cagAuugucauggauuccaguua................................  1     1    st1
........................................................caguuCgucauggauuccaguua................................  8     1    st1
........................................................caguGugucauggauuccaguua................................  1     1    st1
........................................................caguuugucauggauuccaguGa................................  3     1    st1
........................................................caguuugucauAgauuccaguua................................  1     1    st1
........................................................caguuugucauggaCuccaguua................................  4     1    st1
........................................................caguuugucauggauuccagCua................................  3     1    st1
........................................................caguuugucaAggauuccaguua................................  1     1    st1
........................................................caguuugucauggauuccaguuG................................  9     1    st1
........................................................caguuugCcauggauuccaguua................................  2     1    st1
........................................................caguuugucauggauuccaguCa................................  2     1    st1
........................................................caguuugucauggauAccaguua................................  1     1    st1
........................................................caguuugucauggauuccaguuaa...............................  12    0    st1
........................................................caguuugucauggauuccaguuaU...............................  24    1    st1
........................................................caguuugucauggauuccaguuUa...............................  10    1    st1
........................................................caguuugucauggauuccaguuaG...............................  5     1    st1
........................................................caguuugucauggauuccaguuaC...............................  4     1    st1
........................................................caguuugucauggauuccaguuaUa..............................  3     1    st1
........................................................caguuugucauggauuccaguuaaaG.............................  1     1    st1
.........................................................aguuugucauggauuccC....................................  1     1    st1
.........................................................aguuugucauggauuccag...................................  1     0    st1
.........................................................aguuugucauggauuccagC..................................  1     1    st1
.........................................................aguuugucauggauuccagG..................................  1     1    st1
.........................................................aguuugucauggauuccagu..................................  17    0    st1
.........................................................aguuugucaCggauuccagu..................................  1     1    st1
.........................................................aguuugucauggauuccagA..................................  1     1    st1
.........................................................Gguuugucauggauuccagu..................................  1     1    st1
.........................................................aguuCgucauggauuccaguu.................................  1     1    st1
.........................................................aguuugucCuggauuccaguu.................................  2     1    st1
.........................................................Uguuugucauggauuccaguu.................................  1     1    st1
.........................................................aguuugucauggauuccaguu.................................  279   0    st1
.........................................................Gguuugucauggauuccaguu.................................  3     1    st1
.........................................................aguuugucauggauuccaguA.................................  3     1    st1
.........................................................aguuugucauggauCccaguu.................................  2     1    st1
.........................................................aguuugucauggauuccaguG.................................  2     1    st1
.........................................................aguCugucauggauuccaguu.................................  3     1    st1
.........................................................agAuugucauggauuccaguu.................................  2     1    st1
.........................................................aguuugucGuggauuccaguu.................................  4     1    st1
.........................................................aguuugAcauggauuccaguu.................................  1     1    st1
.........................................................aguuugucauggGuuccaguu.................................  2     1    st1
.........................................................aguuugucauggaCuccaguu.................................  1     1    st1
.........................................................aguuugucauggauuccaguC.................................  31    1    st1
.........................................................aguuugucauUgauuccaguua................................  1     1    st1
.........................................................aguuAgucauggauuccaguua................................  1     1    st1
.........................................................aguuugucauggauuccaguua................................  1478  0    st1
.........................................................aguuugucauggGuuccaguua................................  7     1    st1
.........................................................aguuugucaCggauuccaguua................................  2     1    st1
.........................................................aguuugAcauggauuccaguua................................  1     1    st1
.........................................................aguuugucauggauucUaguua................................  7     1    st1
.........................................................aguuugucauggauucAaguua................................  1     1    st1
.........................................................aguuugucaugAauuccaguua................................  1     1    st1
.........................................................aguuugucauAgauuccaguua................................  2     1    st1
.........................................................aguuugucCuggauuccaguua................................  11    1    st1
.........................................................aguuCgucauggauuccaguua................................  4     1    st1
.........................................................aguuugucauggauuccagCua................................  2     1    st1
.........................................................agCuugucauggauuccaguua................................  3     1    st1
.........................................................aguuugucauggauuccUguua................................  1     1    st1
.........................................................aguuugCcauggauuccaguua................................  3     1    st1
.........................................................Gguuugucauggauuccaguua................................  5     1    st1
.........................................................aguuugucauggauuccaguCa................................  3     1    st1
.........................................................aguuugucauggauAccaguua................................  3     1    st1
.........................................................Uguuugucauggauuccaguua................................  2     1    st1
```

```
                    Mature                                 Star
acuuuauucuuuuuuuuuuaccuggaauccaucugacaaacugcauguuugucaugcaguuugucauggauuccaguuaaaaaaaagcaugcaaucguacgucaaggagaa
.........................................................aguuugucauggauuAcaguua................................   1     1    st1
.........................................................aguuugucauggaCuccaguua................................   6     1    st1
.........................................................aguuugucauggauCccaguua................................   5     1    st1
.........................................................aguuugucauggauuccaguuC................................   6     1    st1
.........................................................aguuugucaAggauuccaguua................................   1     1    st1
.........................................................aguuugucGuggauuccaguua................................   4     1    st1
.........................................................aguuugucauggauuccaguuU................................   65    1    st1
.........................................................aguuugucUuggauuccaguua................................   4     1    st1
.........................................................aguuugucauggauuccGguua................................   4     1    st1
.........................................................aguuugucauggauuccaguuG................................   15    1    st1
.........................................................aguuugucauggauuUcaguua................................   2     1    st1
.........................................................aguuugucauggauGccaguua................................   3     1    st1
.........................................................Gguuugucauggauuccaguuaa...............................   1     1    st1
.........................................................aguuugucauggauuccaguuaC...............................   3     1    st1
.........................................................aguuugucauggauuccaguuaa...............................   29    0    st1
.........................................................aguuugucauggauuccaguuaU...............................   42    1    st1
.........................................................aguuugucauggauuccaguuaG...............................   2     1    st1
.........................................................aguuugucCuggauuccaguuaa...............................   2     1    st1
.........................................................aguuugucauggauuccaguuUa...............................   18    1    st1
.........................................................agCuugucauggauuccaguuaa...............................   2     1    st1
.........................................................aguuugucauggauuccGguuaa...............................   1     1    st1
.........................................................aguuugucauggauuccaguuaaG..............................   4     1    st1
.........................................................aguuugucauggauuccaguuaUa..............................   6     1    st1
.........................................................aguuugucauggauuccaguuaGa..............................   2     1    st1
.........................................................aguuugucauggauuccaguuaaU..............................   1     1    st1
.........................................................aguuugucauggauuccaguuaaGa.............................   1     1    st1
..........................................................guuugucauggauuccaguuaa...............................   1     0    st1
...........................................................uuugucauggauuccaguua................................   4     0    st1
...........................................................uuugucauggauuccaguuU................................   1     1    st1
...........................................................uuugucauggauuccaguuC................................   1     1    st1
...........................................................uuugucauggauuccaguuG................................   2     1    st1
...........................................................uuugucauggauuccaguuaa...............................   1     0    st1
...........................................................uuugucauggauuccaguuUa...............................   2     1    st1
...........................................................uuugucauggauuccaguuaU...............................   3     1    st1
............................................................uugucauggauuccaguua................................   1     0    st1
.............................................................ugucauggauuccaguua................................   1     0    st1

...................accuggaauccaucugacaaacug....................................................................   2     0    so1
...................Cccuggaauccaucugacaaacug....................................................................   5     1    so1
...................Cccuggaauccaucugacaaacugc...................................................................   1     1    so1
....................ccuggaauccaucugaca.........................................................................   48    0    so1
....................ccuggaauccaucugacG.........................................................................   3     1    so1
....................ccuggaaCccaucugaca.........................................................................   1     1    so1
....................ccuggaGuccaucugaca.........................................................................   1     1    so1
....................ccuggaauccaucugacaG........................................................................   5     1    so1
....................ccuggaauccaucugacaa........................................................................   77    0    so1
....................ccuggaauccaucugCcaa........................................................................   1     1    so1
....................ccuggaauccaucugacaU........................................................................   1     1    so1
....................ccuggGauccaucugacaa........................................................................   1     1    so1
....................ccuggaaCccaucugacaaa.......................................................................   1     1    so1
....................ccuggaauccaucugacaaa.......................................................................   149   0    so1
....................ccuggaauccaucugacaGa.......................................................................   5     1    so1
....................ccuggaauccaGcugacaaa.......................................................................   1     1    so1
....................ccuggaauccaucugacaaG.......................................................................   3     1    so1
....................ccuggaauccGucugacaaa.......................................................................   1     1    so1
....................ccuUgaauccaucugacaaac......................................................................   3     1    so1
....................ccCggaauccaucugacaaac......................................................................   4     1    so1
....................ccuggaauccaucugacaaaA......................................................................   14    1    so1
....................ccuggaaucUaucugacaaac......................................................................   1     1    so1
....................ccuggaaAccaucugacaaac......................................................................   1     1    so1
....................ccuggaauccaucugacaaac......................................................................   1083  0    so1
....................ccuggGauccaucugacaaac......................................................................   10    1    so1
....................ccuggaauccaucugUcaaac......................................................................   2     1    so1
....................ccuggaauccaucugacaaCc......................................................................   2     1    so1
....................ccuggaauccaucCgacaaac......................................................................   4     1    so1
....................ccuggaauccaucugacaGac......................................................................   16    1    so1
....................ccuCgaauccaucugacaaac......................................................................   1     1    so1
....................ccuggaauccaCcugacaaac......................................................................   3     1    so1
....................ccuggaauccaucugacaaUc......................................................................   2     1    so1
....................ccuggaauccaucugaUaaac......................................................................   1     1    so1
```

Mature Star

```
acuuuauucuuuuuuuuuuaccuggaauccaucugacaaacugcauguuugucaugcaguuugucauggauuccaguuaaaaaaaagcaugcaaucguacgucaaggagaa
....................ccuggaauccGucugacaaac......................................................................   5     1   so1
....................ccuggaauccaucugacaaGc......................................................................   5     1   so1
....................ccuggaauccaucugacaaaU......................................................................   77    1   so1
....................ccuggaauccauUugacaaac......................................................................   3     1   so1
....................ccuggaauccaucugGcaaac......................................................................   11    1   so1
....................ccuggaauccaucugacUaac......................................................................   3     1   so1
....................Gcuggaauccaucugacaaac......................................................................   1     1   so1
....................cAuggaauccaucugacaaac......................................................................   1     1   so1
....................Acuggaauccaucugacaaac......................................................................   7     1   so1
....................ccuggaauUcaucugacaaac......................................................................   1     1   so1
....................ccuggaauccaucugacGaac......................................................................   2     1   so1
....................ccuggaaCccaucugacaaac......................................................................   4     1   so1
....................ccuggaGuccaucugacaaac......................................................................   8     1   so1
....................ccCggaauccaucugacaaacu.....................................................................   8     1   so1
....................ccuggaauccaucugGcaaacu.....................................................................   45    1   so1
....................ccuggaauccaucugacGaacu.....................................................................   16    1   so1
....................ccuggaauccauUugacaaacu.....................................................................   1     1   so1
....................ccuggaGuccaucugacaaacu.....................................................................   22    1   so1
....................ccGggaauccaucugacaaacu.....................................................................   2     1   so1
....................ccuggaauUcaucugacaaacu.....................................................................   4     1   so1
....................ccuggaauccaucugUcaaacu.....................................................................   2     1   so1
....................ccuggGauccaucugacaaacu.....................................................................   18    1   so1
....................ccuggaauccaucugacCaacu.....................................................................   1     1   so1
....................ccuggaauccaucCgacaaacu.....................................................................   21    1   so1
....................ccugAaauccaucugacaaacu.....................................................................   1     1   so1
....................ccuggaauccaucugacaCacu.....................................................................   3     1   so1
....................ccuggaauccaucugacaGacu.....................................................................   74    1   so1
....................Acuggaauccaucugacaaacu.....................................................................   15    1   so1
....................ccuggaauccGucugacaaacu.....................................................................   13    1   so1
....................ccuggaauccUucugacaaacu.....................................................................   1     1   so1
....................ccuggaauccaucugacaaacu.....................................................................   3865  0   so1
....................ccuggaauccaCcugacaaacu.....................................................................   13    1   so1
....................ccuggaauccaucugacaaaUu.....................................................................   2     1   so1
....................ccuggaaucUaucugacaaacu.....................................................................   2     1   so1
....................ccuggaauccaucugacaaacG.....................................................................   70    1   so1
....................ccuggaauccaucuAacaaacu.....................................................................   4     1   so1
....................ccuggaauccaucugacaaacA.....................................................................   167   1   so1
....................cUuggaauccaucugacaaacu.....................................................................   4     1   so1
....................ccuggaCuccaucugacaaacu.....................................................................   1     1   so1
....................ccuggaauccaucugaUaaacu.....................................................................   1     1   so1
....................ccugUaauccaucugacaaacu.....................................................................   1     1   so1
....................ccAggaauccaucugacaaacu.....................................................................   1     1   so1
....................ccuggaauccaucugacaaGcu.....................................................................   17    1   so1
....................Gcuggaauccaucugacaaacu.....................................................................   4     1   so1
....................ccuAgaauccaucugacaaacu.....................................................................   1     1   so1
....................ccuggaaCccaucugacaaacu.....................................................................   15    1   so1
....................ccuggaauccaucugaAaaacu.....................................................................   1     1   so1
....................ccuggaauccaucAgacaaacu.....................................................................   5     1   so1
....................ccuggaauccaucugacUaacu.....................................................................   1     1   so1
....................ccuggaauccaucugacaaCcu.....................................................................   3     1   so1
....................ccuggaauccaucugacaUacu.....................................................................   1     1   so1
....................ccuggaauccaucugacaaacC.....................................................................   540   1   so1
....................cUuggaauccaucugacaaacug....................................................................   11    1   so1
....................ccuggaaGccaucugacaaacug....................................................................   2     1   so1
....................ccuggaauccaucugGcaaacug....................................................................   279   1   so1
....................ccuggaauccaGcugacaaacug....................................................................   2     1   so1
....................ccuggaauccaucugacaaacAg....................................................................   15    1   so1
....................ccuggaauccaucugacaaaGug....................................................................   2     1   so1
....................ccuggaUuccaucugacaaacug....................................................................   1     1   so1
....................Acuggaauccaucugacaaacug....................................................................   67    1   so1
....................ccuggaauAcaucugacaaacug....................................................................   3     1   so1
....................ccuggaauccaucugacaaGcug....................................................................   134   1   so1
....................ccuggaauccaucugacaaacuA....................................................................   3067  1   so1
....................ccuggaauccaucugacaaaUug....................................................................   17    1   so1
....................ccuggaauccaucugacaaacGg....................................................................   78    1   so1
....................ccuggaGuccaucugacaaacug....................................................................   268   1   so1
....................ccuggaauccaucugacaGacug....................................................................   748   1   so1
....................ccAggaauccaucugacaaacug....................................................................   13    1   so1
....................ccuggaauccaucCgacaaacug....................................................................   84    1   so1
....................ccuggaaucUaucugacaaacug....................................................................   25    1   so1
```

Mature Star

acuuuauucuuuuuuuuuuaccuggaauccaucugacaaacugcauguuugucaugcaguuugucauggauuccaguuaaaaaaaagcaugcaaucguacgucaaggagaa			
....................ccuggaauccaAcugacaaacug..	6	1	so1
....................ccuggaauccaucugCcaaacug..	3	1	so1
....................Gcuggaauccaucugacaaacug..	17	1	so1
....................ccuggaauccaucugUcaaacug..	11	1	so1
....................ccuggaauccaucugacaaacuC..	90	1	so1
....................ccuggaauccaucuUacaaacug..	1	1	so1
....................ccuggaauccCucugacaaacug..	1	1	so1
....................ccuggaauccaucugacaaacCg..	240	1	so1
....................ccuUgaauccaucugacaaacug..	8	1	so1
....................ccuggaauccaucugacaaaAug..	3	1	so1
....................ccuggaCuccaucugacaaacug..	2	1	so1
....................ccuggGauccaucugacaaacug..	145	1	so1
....................ccuggaauccaucGgacaaacug..	10	1	so1
....................ccuggaauccaucugacaaacug..	25061	0	so1
....................ccuggaaAccaucugacaaacug..	2	1	so1
....................ccuggaauccaucugacaaCcug..	10	1	so1
....................ccuAgaauccaucugacaaacug..	15	1	so1
....................ccugAaauccaucugacaaacug..	14	1	so1
....................ccuggaauccaucugaAaaacug..	3	1	so1
....................ccuCgaauccaucugacaaacug..	1	1	so1
....................cGuggaauccaucugacaaacug..	1	1	so1
....................ccugUaauccaucugacaaacug..	2	1	so1
....................ccuggaauUcaucugacaaacug..	19	1	so1
....................ccuggCauccaucugacaaacug..	2	1	so1
....................ccuggaauccaCcugacaaacug..	68	1	so1
....................ccuggaauccauUugacaaacug..	16	1	so1
....................ccuggaaCccaucugacaaacug..	74	1	so1
....................ccuggaauccaucuAacaaacug..	8	1	so1
....................ccuggaauccaucugacGaacug..	113	1	so1
....................ccuggaauccaucuCacaaacug..	1	1	so1
....................ccuggaauccUucugacaaacug..	6	1	so1
....................ccuggaauccaucugacaUacug..	6	1	so1
....................cAuggaauccaucugacaaacug..	13	1	so1
....................ccuggaauccaucugacaCacug..	3	1	so1
....................ccuggaaucGaucugacaaacug..	1	1	so1
....................ccuggaauccaucugacCaacug..	7	1	so1
....................ccCggaauccaucugacaaacug..	67	1	so1
....................ccuggaauccaucugacUaacug..	16	1	so1
....................ccuggaauccauGugacaaacug..	2	1	so1
....................ccuggaaucAaucugacaaacug..	7	1	so1
....................ccuggaauccGucugacaaacug..	76	1	so1
....................ccuggUauccaucugacaaacug..	3	1	so1
....................ccuggaauccauAugacaaacug..	2	1	so1
....................ccuggaauccaucugacaaacuU..	302	1	so1
....................ccuggaauccaucAgacaaacug..	15	1	so1
....................ccuggaauccaucugacaaUcug..	9	1	so1
....................ccuggaauccaucugaUaaacug..	8	1	so1
....................ccuggaauccaucuAacaaacugc...	1	1	so1
....................ccuggaauccauUugacaaacugc...	1	1	so1
....................ccuggaauccaucAgacaaacugc...	6	1	so1
....................ccuggaauccaucugacaUacugc...	1	1	so1
....................ccuggaauccaucugacaaacugU...	331	1	so1
....................ccuggaauccaucugacaaacugA...	171	1	so1
....................ccCggaauccaucugacaaacugc...	5	1	so1
....................cUuggaauccaucugacaaacugc...	2	1	so1
....................ccugAaauccaucugacaaacugc...	2	1	so1
....................ccuggaauccaucCgacaaacugc...	7	1	so1
....................ccuggaauccGucugacaaacugc...	3	1	so1
....................ccuggaauccaucugacaaaUugc...	1	1	so1
....................ccuggGauccaucugacaaacugc...	8	1	so1
....................ccuggaauccaucugGcaaacugc...	15	1	so1
....................ccuggaGuccaucugacaaacugc...	5	1	so1
....................ccuggaauccaucugacaaacuAc...	9	1	so1
....................ccuggaauccUucugacaaacugc...	2	1	so1
....................ccuggaauccaCcugacaaacugc...	1	1	so1
....................ccuggaauccaucugacaaacGgc...	1	1	so1
....................Acuggaauccaucugacaaacugc...	1	1	so1
....................ccuggaauccaucugacaaGcugc...	1	1	so1
....................ccGggaauccaucugacaaacugc...	1	1	so1
....................ccuggaauUcaucugacaaacugc...	1	1	so1

Mature Star

```
acuuuauucuuuuuuuuuuaccuggaauccaucugacaaacugcauguuugucaugcaguuugucauggauuccaguuaaaaaaaagcaugcaaucguacgucaaggagaa
....................ccuAgaauccaucugacaaacugc................................................................... 1     1   so1
....................ccuggaauccaucugacaaacugG................................................................... 8     1   so1
....................ccuggaauccaucugacaGacugc................................................................... 12    1   so1
....................ccuggaauccaucugacaaacugc................................................................... 1170  0   so1
....................ccuggaauccaucugacaaacuCc................................................................... 4     1   so1
....................ccuggaaCccaucugacaaacugc................................................................... 3     1   so1
....................ccuggCauccaucugacaaacugc................................................................... 1     1   so1
....................ccuggaauccaucugacGaacugc................................................................... 7     1   so1
....................ccCggaauccaucugacaaacugca.................................................................. 3     1   so1
....................ccuggaauccaucugacGaacugca.................................................................. 3     1   so1
....................ccuggaGuccaucugacaaacugca.................................................................. 2     1   so1
....................ccuggaauccaucugGcaaacugca.................................................................. 18    1   so1
....................ccuggaauccaucugacaaacugGa.................................................................. 1     1   so1
....................ccuggaauccaucugacaaacugcU.................................................................. 62    1   so1
....................ccuggaauccaucugacaGacugca.................................................................. 16    1   so1
....................ccuggaauccaucugacaaacCgca.................................................................. 2     1   so1
....................ccuggaauccaucCgacaaacugca.................................................................. 3     1   so1
....................ccuggaauccaucugacaaacugcC.................................................................. 11    1   so1
....................ccugAaauccaucugacaaacugca.................................................................. 1     1   so1
....................ccuUgaauccaucugacaaacugca.................................................................. 1     1   so1
....................Gcuggaauccaucugacaaacugca.................................................................. 3     1   so1
....................ccuggaauccaucugacaaGcugca.................................................................. 2     1   so1
....................ccuggaauUcaucugacaaacugca.................................................................. 1     1   so1
....................ccuggaauccaucugacaaacugUa.................................................................. 248   1   so1
....................ccuggaauccaucugacaaacugcG.................................................................. 26    1   so1
....................ccuggaauccaucugacaaCcugca.................................................................. 1     1   so1
....................ccuggaauccauUugacaaacugca.................................................................. 1     1   so1
....................ccuggaauccaucugacaaacuAca.................................................................. 2     1   so1
....................ccuggaauccaucugacaaacugca.................................................................. 560   0   so1
....................ccuggaaCccaucugacaaacugca.................................................................. 1     1   so1
....................ccuggGauccaucugacaaacugca.................................................................. 1     1   so1
....................ccuggaaucUaucugacaaacugca.................................................................. 2     1   so1
....................ccuggaauccaucugacUaacugca.................................................................. 1     1   so1
....................ccuggaauccauAugacaaacugca.................................................................. 1     1   so1
....................ccuggaauccaucugacaaacugcaA................................................................. 32    1   so1
....................ccuggaauccaucugacaaacugcUu................................................................. 17    1   so1
....................ccuggaauccaucugacaaacugcaG................................................................. 24    1   so1
....................ccuggaauccaucugacaaacugUau................................................................. 16    1   so1
....................ccuggaauccaucugacaGacugcau................................................................. 2     1   so1
....................ccuggaauccaucugacaaacugcau................................................................. 28    0   so1
....................ccuggaauccaucugGcaaacugcau................................................................. 1     1   so1
....................ccuggaauccaucugacaaacugcaC................................................................. 8     1   so1
....................ccuggaauccaucugacaaacugcaAg................................................................ 2     1   so1
....................ccuggaauccaucugacaaacugcaug................................................................ 4     0   so1
....................ccuggaauccaucugacaaacugcUug................................................................ 1     1   so1
....................ccuggaauccaucugacaaacugcauU................................................................ 18    1   so1
....................ccuggaauccaucugacaaacugcauC................................................................ 2     1   so1
....................ccuggaauccaucugacaaacugcauA................................................................ 78    1   so1
.....................cuggaauccaucugacaaa....................................................................... 1     0   so1
.....................cuggaauccaucugacaaac...................................................................... 3     0   so1
.....................cuggaauccaucugacaaacA..................................................................... 1     1   so1
.....................cuggaauccaucugGcaaacu..................................................................... 1     1   so1
.....................cuggaauccaucugacaaacu..................................................................... 15    0   so1
.....................cuggaauccaucugacaGacug.................................................................... 4     1   so1
.....................Auggaauccaucugacaaacug.................................................................... 1     1   so1
.....................cuggaauccaucugGcaaacug.................................................................... 1     1   so1
.....................cuggaauccaucugacaaGcug.................................................................... 1     1   so1
.....................cuggaauccaucugacGaacug.................................................................... 1     1   so1
.....................Uuggaauccaucugacaaacug.................................................................... 3     1   so1
.....................cuggaauccaucugacaaacug.................................................................... 78    0   so1
.....................cuggaauccaucugacaaacuA.................................................................... 7     1   so1
.....................cuggaauccaucugacaaacuU.................................................................... 1     1   so1
.....................cuggaauccaucugacaaacuC.................................................................... 1     1   so1
.....................cuggaauccaucugacaaacugU................................................................... 1     1   so1
.....................cCggaauccaucugacaaacugc................................................................... 1     1   so1
.....................cuggaauccaucugacaaacugc................................................................... 2     0   so1
.....................cuggaauccaucugacaaacugca.................................................................. 1     0   so1
.....................cuggaauccaucugacaaacugcUu................................................................. 1     1   so1
.....................cuggaauccaucugacaaacugcauA................................................................ 2     1   so1
......................uggaauccaucugGcaaac...................................................................... 1     1   so1
```

```
                    Mature                              Star
acuuuauucuuuuuuuuuuaccuggaauccaucugacaaacugcauguuugucaugcaguuugucauggauuccaguuaaaaaaaagcaugcaaucguacgucaaggagaa
......................uggaauccaucugacaaaA......................................................................   3    1    so1
......................uggaauccaucugacaaaU......................................................................   2    1    so1
......................uggaauccaucugacaaac......................................................................   11   0    so1
......................uggaauccaucCgacaaacu.....................................................................   1    1    so1
......................uggaauccaucugacaaacC.....................................................................   5    1    so1
......................uggaauccaucugacaaacA.....................................................................   7    1    so1
......................uggaauccaAcugacaaacu.....................................................................   1    1    so1
......................uggaauccaucugUcaaacu.....................................................................   2    1    so1
......................uggaauccaucugGcaaacu.....................................................................   3    1    so1
......................uggaaCccaucugacaaacu.....................................................................   1    1    so1
......................uggaauccaucugacaaacu.....................................................................   27   0    so1
......................uggaauccaucAgacaaacu.....................................................................   1    1    so1
......................uggaauccaucugacaaaUug....................................................................   2    1    so1
......................uggaauccaucugacaaacGg....................................................................   1    1    so1
......................uggaauccaucCgacaaacug....................................................................   1    1    so1
......................uggaauccaucugGcaaacug....................................................................   28   1    so1
......................uggaauccaucugacaaCcug....................................................................   1    1    so1
......................uggaGuccaucugacaaacug....................................................................   1    1    so1
......................uggaauccaucugacaaacug....................................................................   365  0    so1
......................uggaauccaucugacGaacug....................................................................   1    1    so1
......................uggaauccaCcugacaaacug....................................................................   2    1    so1
......................uggaauccaucugacaaaAug....................................................................   1    1    so1
......................uggaauAcaucugacaaacug....................................................................   1    1    so1
......................uggaauccaucugacaaacuU....................................................................   2    1    so1
......................uggaauUcaucugacaaacug....................................................................   1    1    so1
......................uggaaucUaucugacaaacug....................................................................   1    1    so1
......................uggaauccaucugacaaacuA....................................................................   36   1    so1
......................uggaauccaucugacaaacCg....................................................................   1    1    so1
......................uggaauccGucugacaaacug....................................................................   1    1    so1
......................uggaaCccaucugacaaacug....................................................................   3    1    so1
......................uAgaauccaucugacaaacug....................................................................   3    1    so1
......................uggaauccaucugacaGacug....................................................................   8    1    so1
......................uggaauccaucugacaaGcug....................................................................   1    1    so1
......................uggGauccaucugacaaacug....................................................................   3    1    so1
......................uggaauccaucugacaaacugc...................................................................   40   0    so1
......................uggaauccaucugacaaacugA...................................................................   5    1    so1
......................uggaauccaucugacaaacGgc...................................................................   1    1    so1
......................uggaauccaucugGcaaacugc...................................................................   2    1    so1
......................uggaauccaucugacaaacugU...................................................................   8    1    so1
......................uggaauccaucugacaGacugca..................................................................   3    1    so1
......................uggaauccaucugacaaacugca..................................................................   44   0    so1
......................uggaGuccaucugacaaacugca..................................................................   1    1    so1
......................uggaauccaucugacaaacugcG..................................................................   1    1    so1
......................uggaauccaucugGcaaacugca..................................................................   3    1    so1
......................uggaauccaucugacaaacugcU..................................................................   1    1    so1
......................uggaauccaucugGcaaacugcau.................................................................   3    1    so1
......................uggaauccaucugacGaacugcau.................................................................   2    1    so1
......................uggaauccaucugacaaacugcUu.................................................................   3    1    so1
......................uggaauccaucugacaaacugcCu.................................................................   1    1    so1
......................uggaauccaucugacaaacugcaA.................................................................   3    1    so1
......................uggaauccaucugacaaacugcaG.................................................................   7    1    so1
......................uggGauccaucugacaaacugcau.................................................................   1    1    so1
......................uggaauccaucugacaaacugcaC.................................................................   1    1    so1
......................uggaauccaucugacaaacugcau.................................................................   30   0    so1
......................uggaauccaucugacaaacugcaug................................................................   4    0    so1
......................uggaauccaucugacaaacugcauA................................................................   71   1    so1
......................uggGauccaucugacaaacugcaug................................................................   1    1    so1
......................uggaauccaucugacaaacugcauU................................................................   11   1    so1
......................uggaauccaucugacaaacugcauUu...............................................................   1    1    so1
......................uggaauccaucugacaaacugcauAu...............................................................   12   1    so1
......................uggaauccaucugacaaacugcauAuu..............................................................   2    1    so1
......................uggaauccaucugacaaacugcauUuu..............................................................   1    1    so1
.......................ggaauccaucugacaaacu.....................................................................   1    0    so1
.......................ggaauccaucugacaaacug....................................................................   5    0    so1
.......................ggaauccaucugacaaacuA....................................................................   1    1    so1
.......................ggaauccaucugGcaaacug....................................................................   1    1    so1
.......................ggaauccaucugacaaacugcaG.................................................................   1    1    so1
.......................ggaauccaucugacaaacugcauUu...............................................................   1    1    so1
........................................................caguuugucauggauucc.....................................   4    0    so1
........................................................caguuugucauggauucA.....................................   1    1    so1
```

Mature **Star**

```
acuuuauucuuuuuuuuuuaccuggaauccaucugacaaacugcauguuugucaugcaguuugucauggauuccaguuaaaaaaaagcaugcaaucguacgucaaggagaa
........................................................caguuugucauggauuccGg...................................  1    1  so1
........................................................caguuugucauggauuccag...................................  10   0  so1
........................................................caAuuugucauggauuccag...................................  1    1  so1
........................................................caguuugucauggauuccagu..................................  11   0  so1
........................................................caguuugucauggauuccagC..................................  2    1  so1
........................................................caguuugucauggauuccaguu.................................  133  0  so1
........................................................caguuugucauggauuccagCu.................................  1    1  so1
........................................................caguuugucauggauuccaguC.................................  17   1  so1
........................................................cagCuugucauggauuccaguu.................................  2    1  so1
........................................................caguuugucauggauuccagGu.................................  5    1  so1
........................................................caguuugucauggauuccaguA.................................  10   1  so1
........................................................caguuugucauggauuccaguG.................................  2    1  so1
........................................................caguuugucauggCuuccaguua................................  1    1  so1
........................................................caguuugucauggauuccaguuU................................  23   1  so1
........................................................caguuugucauggauuccagGua................................  2    1  so1
........................................................caguuuAucauggauuccaguua................................  1    1  so1
........................................................caguuugucauggauuccaguuC................................  3    1  so1
........................................................caguuCgucauggauuccaguua................................  3    1  so1
........................................................caguuugucauggauuccagCua................................  1    1  so1
........................................................caguuugCcauggauuccaguua................................  2    1  so1
........................................................caguuugucauggauuccGguua................................  5    1  so1
........................................................caguuugucauggauuccaguua................................  314  0  so1
........................................................Uaguuugucauggauuccaguua................................  1    1  so1
........................................................caguuugucauggauuccaguGa................................  1    1  so1
........................................................caguuugucauggaCuccaguua................................  3    1  so1
........................................................caguuugucauggauuccaguuG................................  6    1  so1
........................................................caguuugucauggGuuccaguua................................  2    1  so1
........................................................cGguuugucauggauuccaguua................................  2    1  so1
........................................................caguuugucauggauuccaguCa................................  1    1  so1
........................................................caguuugucauggauuccaAuua................................  1    1  so1
........................................................caguuugucauggauuccaguuaU...............................  8    1  so1
........................................................caguuugucauggauuccGguuaa...............................  1    1  so1
........................................................caguuugucauggauuccaguuaC...............................  5    1  so1
........................................................caguuugucauggauuccaguuaa...............................  6    0  so1
........................................................caguuugucauggauuccaguuUa...............................  19   1  so1
........................................................caguuugucauggauuccaguuaG...............................  3    1  so1
........................................................caguuugucauggauuccaguuaaG..............................  1    1  so1
........................................................caguuugucauggauuccaguuaUa..............................  6    1  so1
........................................................caguuugucauggauuccaguuaUaa.............................  2    1  so1
.........................................................aguuugucauggauuccagC..................................  3    1  so1
.........................................................aguuugucauggauuccagu..................................  18   0  so1
.........................................................aguuugucauggauuccagG..................................  1    1  so1
.........................................................aguuugucauggauuccaguC.................................  32   1  so1
.........................................................Cguuugucauggauuccaguu.................................  1    1  so1
.........................................................aguuugucGuggauuccaguu.................................  1    1  so1
.........................................................agCuugucauggauuccaguu.................................  1    1  so1
.........................................................aguuugCcauggauuccaguu.................................  1    1  so1
.........................................................aguuugucauggauCccaguu.................................  1    1  so1
.........................................................aguuugucauggauuccaguu.................................  211  0  so1
.........................................................aguuugucauggauuccaguA.................................  5    1  so1
.........................................................aguuugucGuggauuccaguua................................  6    1  so1
.........................................................agGuugucauggauuccaguua................................  1    1  so1
.........................................................aguAugucauggauuccaguua................................  1    1  so1
.........................................................aguCugucauggauuccaguua................................  1    1  so1
.........................................................aguuugucauggauuccUguua................................  1    1  so1
.........................................................Gguuugucauggauuccaguua................................  4    1  so1
.........................................................aguuugucauggauucUaguua................................  1    1  so1
.........................................................aguuugucauggaCuccaguua................................  4    1  so1
.........................................................aguuugucauggauucAaguua................................  1    1  so1
.........................................................aguuCgucauggauuccaguua................................  2    1  so1
.........................................................aguuugucauggauuAcaguua................................  1    1  so1
.........................................................aguuugCcauggauuccaguua................................  2    1  so1
.........................................................Cguuugucauggauuccaguua................................  1    1  so1
.........................................................aguuugucauggauuccGguua................................  2    1  so1
.........................................................aguuugucUuggauuccaguua................................  1    1  so1
.........................................................aguuugucauggauCccaguua................................  1    1  so1
.........................................................aguuugucauggGuuccaguua................................  1    1  so1
.........................................................aguuugucauggauuccaguuG................................  4    1  so1
.........................................................aguuugucauUgauuccaguua................................  1    1  so1
.........................................................aguuugucauggauuccaguGa................................  1    1  so1
```

```
                    Mature                                Star
acuuuauucuuuuuuuuuuaccuggaauccaucugacaaacugcauguuugucaugcaguuugucauggauuccaguuaaaaaaaagcaugcaaucguacgucaaggagaa
.........................................................aguuugucaAggauuccaguua................................   1     1   so1
.........................................................aguuugucauggauuccaguuU................................   97    1   so1
.........................................................aguuugucauggauuccaguuC................................   8     1   so1
.........................................................Uguuugucauggauuccaguua................................   1     1   so1
.........................................................aguuugucauggauuccaguua................................   474   0   so1
.........................................................aguuugucauggauuccagCua................................   1     1   so1
.........................................................aguuugucauggauuccaguuUa...............................   89    1   so1
.........................................................aguuugucauggauuccaguuaU...............................   15    1   so1
.........................................................aguuugucauggauuccaguuaa...............................   17    0   so1
.........................................................aguuugucauggauuccaguuaC...............................   2     1   so1
.........................................................aguuugucauggauuccaguuaG...............................   5     1   so1
.........................................................aguuugucauggauuccaguuaaG..............................   2     1   so1
.........................................................aguuugucauggauuccaguuaUa..............................   20    1   so1
.........................................................aguuugucauggauuccaguuaUaa.............................   1     1   so1
..........................................................guuugucauggauuccaguua................................   1     0   so1
...........................................................uuugucauggauuccaguua................................   5     0   so1
...........................................................uuugucauggauuccaguuU................................   3     1   so1
...........................................................uuugucauggauuccaguuUa...............................   4     1   so1

...................Cccuggaauccaucugacaaacug....................................................................   2     1   sg1
...................accuggaauccaucugacaaacug....................................................................   1     0   sg1
...................accuggaauccaucCgacaaacugc...................................................................   1     1   sg1
....................ccuggaauccaucugacG.........................................................................   1     1   sg1
....................ccuggaauccaucugaca.........................................................................   52    0   sg1
....................ccuggGauccaucugaca.........................................................................   1     1   sg1
....................ccuggaGuccaucugaca.........................................................................   1     1   sg1
....................ccuggaauccaucugGca.........................................................................   1     1   sg1
....................ccuggaauccaucugacaa........................................................................   74    0   sg1
....................ccuggaauccaucugacGa........................................................................   1     1   sg1
....................ccuggGauccaucugacaa........................................................................   1     1   sg1
....................ccuggaauccaucugGcaa........................................................................   1     1   sg1
....................ccuggaauccaucugacaG........................................................................   2     1   sg1
....................cAuggaauccaucugacaaa.......................................................................   1     1   sg1
....................ccuggaauccaucCgacaaa.......................................................................   2     1   sg1
....................ccuggaauccaucugacGaa.......................................................................   2     1   sg1
....................ccuggaauccaucugacaaG.......................................................................   4     1   sg1
....................ccuggaauccaucugacaGa.......................................................................   2     1   sg1
....................ccuggGauccaucugacaaa.......................................................................   1     1   sg1
....................ccuggaauccaucugGcaaa.......................................................................   3     1   sg1
....................ccuggaauccaCcugacaaa.......................................................................   1     1   sg1
....................ccuggaauccGucugacaaa.......................................................................   3     1   sg1
....................Acuggaauccaucugacaaa.......................................................................   1     1   sg1
....................ccuggaauccaucugacaaa.......................................................................   223   0   sg1
....................ccuggaauccaucugacGaac......................................................................   10    1   sg1
....................ccuggaauccCucugacaaac......................................................................   1     1   sg1
....................ccuAgaauccaucugacaaac......................................................................   1     1   sg1
....................ccuggaauccaucugacaGac......................................................................   21    1   sg1
....................ccuggaauccaucugGcaaac......................................................................   16    1   sg1
....................ccuggaaAccaucugacaaac......................................................................   1     1   sg1
....................ccuggaauccaucuAacaaac......................................................................   4     1   sg1
....................ccuggaauccaucugacaaaA......................................................................   14    1   sg1
....................ccuggaaucUaucugacaaac......................................................................   2     1   sg1
....................ccuggaauccaucugacaaac......................................................................   1330  0   sg1
....................ccCggaauccaucugacaaac......................................................................   3     1   sg1
....................cUuggaauccaucugacaaac......................................................................   3     1   sg1
....................Acuggaauccaucugacaaac......................................................................   6     1   sg1
....................ccuggGauccaucugacaaac......................................................................   7     1   sg1
....................ccuggaauccaucugacUaac......................................................................   1     1   sg1
....................ccuggaauccaucugacaaaU......................................................................   37    1   sg1
....................ccAggaauccaucugacaaac......................................................................   1     1   sg1
....................ccuggaauccaucugacaaUc......................................................................   2     1   sg1
....................ccugAaauccaucugacaaac......................................................................   1     1   sg1
....................ccuggaaCccaucugacaaac......................................................................   4     1   sg1
....................ccuggaauccGucugacaaac......................................................................   3     1   sg1
....................Gcuggaauccaucugacaaac......................................................................   4     1   sg1
....................ccuggaauccaucugacaaGc......................................................................   10    1   sg1
....................ccuggaauccaucAgacaaac......................................................................   2     1   sg1
....................ccuggaaucAaucugacaaac......................................................................   1     1   sg1
....................ccuggaauccaucCgacaaac......................................................................   3     1   sg1
....................ccuggaauccaCcugacaaac......................................................................   4     1   sg1
```

```
                     Mature                 Star
acuuuauucuuuuuuuuuuuaccuggaauccaucugacaaacugcauguuugucaugcaguuugucauggauuccaguuaaaaaaaagcaugcaaucguacgucaaggagaa
.....................ccuggaGuccaucugacaaac......................................................................   7       1   sg1
.....................ccuggaauccaucGgacaaac......................................................................   1       1   sg1
.....................ccuggaauccaucugacaaacu.....................................................................   3006    0   sg1
.....................ccCggaauccaucugacaaacu.....................................................................   5       1   sg1
.....................ccuggaauccaucugacaaGcu.....................................................................   20      1   sg1
.....................ccuggGauccaucugacaaacu.....................................................................   15      1   sg1
.....................ccuggUauccaucugacaaacu.....................................................................   1       1   sg1
.....................cUuggaauccaucugacaaacu.....................................................................   1       1   sg1
.....................ccuggaauccaucugGcaaacu.....................................................................   29      1   sg1
.....................Acuggaauccaucugacaaacu.....................................................................   14      1   sg1
.....................ccuggaaucUaucugacaaacu.....................................................................   1       1   sg1
.....................ccuAgaauccaucugacaaacu.....................................................................   3       1   sg1
.....................ccuggaauccaucugacaGacu.....................................................................   56      1   sg1
.....................ccuggaauccaucugacaaaUu.....................................................................   2       1   sg1
.....................ccuggaauccaucugacaaaAu.....................................................................   1       1   sg1
.....................ccuggaauccaCcugacaaacu.....................................................................   17      1   sg1
.....................ccuggaauccGucugacaaacu.....................................................................   8       1   sg1
.....................ccuggaauccaucAgacaaacu.....................................................................   2       1   sg1
.....................ccuggaauccaucugacaaacG.....................................................................   27      1   sg1
.....................ccuggaauccaucugCcaaacu.....................................................................   3       1   sg1
.....................ccuggaauccaucugacGaacu.....................................................................   11      1   sg1
.....................ccuggaauccaucugacCaacu.....................................................................   1       1   sg1
.....................Gcuggaauccaucugacaaacu.....................................................................   5       1   sg1
.....................ccuggaauccaucugacaaacA.....................................................................   49      1   sg1
.....................ccuggaaCccaucugacaaacu.....................................................................   2       1   sg1
.....................ccuggaauccaucugacaUacu.....................................................................   2       1   sg1
.....................ccugAaauccaucugacaaacu.....................................................................   3       1   sg1
.....................ccuggaGuccaucugacaaacu.....................................................................   13      1   sg1
.....................ccAggaauccaucugacaaacu.....................................................................   1       1   sg1
.....................ccuggaauccaucugacUaacu.....................................................................   2       1   sg1
.....................ccuggaauUcaucugacaaacu.....................................................................   1       1   sg1
.....................ccuggaaucAaucugacaaacu.....................................................................   2       1   sg1
.....................ccuggaauccaucugacaaCcu.....................................................................   3       1   sg1
.....................ccuggaauccauUugacaaacu.....................................................................   1       1   sg1
.....................ccuggaauccaucugacaaacC.....................................................................   251     1   sg1
.....................ccuggaauccaucCgacaaacu.....................................................................   11      1   sg1
.....................ccuggaauccaCcugacaaacug....................................................................   51      1   sg1
.....................ccuggaauccCucugacaaacug....................................................................   6       1   sg1
.....................ccuggaauGcaucugacaaacug....................................................................   2       1   sg1
.....................Gcuggaauccaucugacaaacug....................................................................   21      1   sg1
.....................ccuggaauccaucugaAaaacug....................................................................   2       1   sg1
.....................ccuggaauccaucugacCaacug....................................................................   4       1   sg1
.....................ccugAaauccaucugacaaacug....................................................................   18      1   sg1
.....................ccuggaauccaucugacaaUcug....................................................................   3       1   sg1
.....................ccuggaauccaucugacaaacuA....................................................................   1576    1   sg1
.....................ccuggaauccaucugCcaaacug....................................................................   4       1   sg1
.....................ccuggaauccGucugacaaacug....................................................................   37      1   sg1
.....................ccuggaauccaAcugacaaacug....................................................................   10      1   sg1
.....................ccuggaauccaucugacaaaUug....................................................................   8       1   sg1
.....................cGuggaauccaucugacaaacug....................................................................   1       1   sg1
.....................Acuggaauccaucugacaaacug....................................................................   71      1   sg1
.....................ccuggaauUcaucugacaaacug....................................................................   17      1   sg1
.....................ccuggaaCccaucugacaaacug....................................................................   57      1   sg1
.....................ccuggaauccaucCgacaaacug....................................................................   73      1   sg1
.....................ccuggaauccaucugacGaacug....................................................................   90      1   sg1
.....................ccuggGauccaucugacaaacug....................................................................   129     1   sg1
.....................ccuggaauccaucGgacaaacug....................................................................   4       1   sg1
.....................ccuggaauccaucugacaaacCg....................................................................   95      1   sg1
.....................ccuggUauccaucugacaaacug....................................................................   7       1   sg1
.....................ccuggaauccaucugacaaacuU....................................................................   95      1   sg1
.....................ccuggaauccaucugacaaGcug....................................................................   114     1   sg1
.....................ccuggaauccaucugacaaacuC....................................................................   47      1   sg1
.....................ccuggaauccaucugacaaacug....................................................................   21246   0   sg1
.....................ccuggaaGccaucugacaaacug....................................................................   1       1   sg1
.....................ccuggaauccaucugacaGacug....................................................................   690     1   sg1
.....................ccuggaauccaucugacUaacug....................................................................   13      1   sg1
.....................ccugUaauccaucugacaaacug....................................................................   2       1   sg1
.....................ccuggaauccaucAgacaaacug....................................................................   9       1   sg1
.....................ccCggaauccaucugacaaacug....................................................................   63      1   sg1
.....................ccuggaauccauAugacaaacug....................................................................   1       1   sg1
```

```
                    Mature                Star
acuuuauucuuuuuuuuuuaccuggaauccaucugacaaacugcauguuugucaugcaguuugucauggauuccaguuaaaaaaaagcaugcaaucguacgucaaggagaa
....................ccGggaauccaucugacaaacug....................................................................   7      1   sg1
....................ccuggaauccaucugaUaaacug....................................................................   12     1   sg1
....................cAuggaauccaucugacaaacug....................................................................   2      1   sg1
....................ccuggaauccaucugGcaaacug....................................................................   194    1   sg1
....................ccuggaauccaucugacaaCcug....................................................................   8      1   sg1
....................ccuggaauccaucugacaaacAg....................................................................   15     1   sg1
....................ccuUgaauccaucugacaaacug....................................................................   3      1   sg1
....................ccuggaauccaucugUcaaacug....................................................................   6      1   sg1
....................ccuggaauccaucugacaaaAug....................................................................   4      1   sg1
....................ccuggCauccaucugacaaacug....................................................................   2      1   sg1
....................ccuggaUuccaucugacaaacug....................................................................   1      1   sg1
....................ccuggaauccaucugacaaacGg....................................................................   29     1   sg1
....................ccuggaauccauUugacaaacug....................................................................   7      1   sg1
....................ccuggaauAcaucugacaaacug....................................................................   2      1   sg1
....................ccuggaaucUaucugacaaacug....................................................................   21     1   sg1
....................ccuggaauccaucugacaUacug....................................................................   2      1   sg1
....................cUuggaauccaucugacaaacug....................................................................   12     1   sg1
....................ccuggaaucAaucugacaaacug....................................................................   5      1   sg1
....................ccuggaGuccaucugacaaacug....................................................................   233    1   sg1
....................ccuggaaAccaucugacaaacug....................................................................   4      1   sg1
....................ccuAgaauccaucugacaaacug....................................................................   12     1   sg1
....................ccuggaauccUucugacaaacug....................................................................   7      1   sg1
....................ccAggaauccaucugacaaacug....................................................................   19     1   sg1
....................ccuggaauccaucuAacaaacug....................................................................   14     1   sg1
....................cUuggaauccaucugacaaacugc...................................................................   2      1   sg1
....................ccuggaauccaucugacaaacugU...................................................................   161    1   sg1
....................ccuggaauccaucugacaaacGgc...................................................................   1      1   sg1
....................ccuggaauccaucugGcaaacugc...................................................................   22     1   sg1
....................ccuggaaCccaucugacaaacugc...................................................................   8      1   sg1
....................ccuggaauccaucugacaaacuAc...................................................................   4      1   sg1
....................Acuggaauccaucugacaaacugc...................................................................   11     1   sg1
....................ccuggaauccaucugacaaGcugc...................................................................   7      1   sg1
....................ccuggaauccaucugacGaacugc...................................................................   8      1   sg1
....................ccuggGauccaucugacaaacugc...................................................................   3      1   sg1
....................ccuggaGuccaucugacaaacugc...................................................................   17     1   sg1
....................ccuggaauccaucCgacaaacugc...................................................................   6      1   sg1
....................ccuggaauccaucugacaaCcugc...................................................................   1      1   sg1
....................ccuggaauccaCcugacaaacugc...................................................................   4      1   sg1
....................ccuggaauccGucugacaaacugc...................................................................   3      1   sg1
....................ccuggaaucUaucugacaaacugc...................................................................   2      1   sg1
....................ccuggaauccaucugacaaacuUc...................................................................   1      1   sg1
....................ccuggaauccaucugacaaacCgc...................................................................   6      1   sg1
....................ccuggaauccaucugacaaacAgc...................................................................   1      1   sg1
....................ccuggaauccaucugacaGacugc...................................................................   24     1   sg1
....................ccuggaauccaucugacaaacugG...................................................................   7      1   sg1
....................ccAggaauccaucugacaaacugc...................................................................   1      1   sg1
....................Gcuggaauccaucugacaaacugc...................................................................   1      1   sg1
....................ccuggaauccCucugacaaacugc...................................................................   1      1   sg1
....................ccuggaauccaucugaGaaacugc...................................................................   1      1   sg1
....................cAuggaauccaucugacaaacugc...................................................................   1      1   sg1
....................ccuggaauccaucugacaaacugc...................................................................   1315   0   sg1
....................ccuggaauccaucugacaaacugA...................................................................   84     1   sg1
....................ccCggaauccaucugacaaacugc...................................................................   5      1   sg1
....................ccuggaauccaucugacGaacugca..................................................................   3      1   sg1
....................ccuggaauccaucugacaaacCgca..................................................................   2      1   sg1
....................ccuggaauccaucugacaaacugUa..................................................................   159    1   sg1
....................ccuggaauccaucugGcaaacugca..................................................................   13     1   sg1
....................ccuggaaAccaucugacaaacugca..................................................................   1      1   sg1
....................ccuggaauccaucugacaGacugca..................................................................   7      1   sg1
....................ccuggaauccaucCgacaaacugca..................................................................   1      1   sg1
....................ccuggGauccaucugacaaacugca..................................................................   1      1   sg1
....................ccuggaauccaucugacaaacugca..................................................................   507    0   sg1
....................ccuggaGuccaucugacaaacugca..................................................................   2      1   sg1
....................ccuggaaCccaucugacaaacugca..................................................................   3      1   sg1
....................ccuggaauccaucugacaaacugAa..................................................................   5      1   sg1
....................Acuggaauccaucugacaaacugca..................................................................   3      1   sg1
....................ccuggaauccaucugacaaacugcU..................................................................   68     1   sg1
....................ccuggaauccaucugacaaacuAca..................................................................   1      1   sg1
....................ccuggaauccaucugacUaacugca..................................................................   1      1   sg1
....................ccuggaauccaucugacaaacugcG..................................................................   9      1   sg1
```

```
                    Mature                               Star
acuuuauucuuuuuuuuuuaccuggaauccaucugacaaacugcauguuugucaugcaguuugucauggauuccaguuaaaaaaaagcaugcaaucguacgucaaggagaa
....................ccuggaauccaucugacaaacugcC..................................................................   12    1   sg1
....................ccuggaauccaucugacaaGcugca..................................................................   2     1   sg1
....................ccuggaauccaucugacaaacugcaC.................................................................   2     1   sg1
....................ccuggaauccaucugacaaacugcau.................................................................   20    0   sg1
....................ccuggaauccaucugacaaacugUau.................................................................   2     1   sg1
....................ccuggaauccaucugacaaacugcUu.................................................................   8     1   sg1
....................ccuggaauccaucugacaaacugcaG.................................................................   34    1   sg1
....................ccuggaaCccaucugacaaacugcau.................................................................   1     1   sg1
....................ccuggaauccaucugacaaacugcaA.................................................................   9     1   sg1
....................ccuggaauccaucugGcaaacugcau.................................................................   4     1   sg1
....................ccuggaauccaucugacaGacugcau.................................................................   5     1   sg1
....................ccuggaauccaucugacaaacugcauA................................................................   47    1   sg1
....................ccuggaauccaucugacaaacugcaug................................................................   5     0   sg1
....................ccuggaauccaucugacaaacugcauC................................................................   1     1   sg1
....................ccuggaauccaucugacaaacugcaGg................................................................   1     1   sg1
....................ccuggaauccaucugacaaacugUaug................................................................   2     1   sg1
....................ccuggaauccaucugacaaacugcauU................................................................   41    1   sg1
.....................cuggaauccaucugacaaac......................................................................   6     0   sg1
.....................cuggaauccaucugacaaacC.....................................................................   1     1   sg1
.....................cuggaauccaucugacaGacu.....................................................................   1     1   sg1
.....................cCggaauccaucugacaaacu.....................................................................   1     1   sg1
.....................cuggaauccaucugacaaacu.....................................................................   9     0   sg1
.....................cuggaauccaCcugacaaacug....................................................................   1     1   sg1
.....................cuggUauccaucugacaaacug....................................................................   1     1   sg1
.....................cuggaauccaucugacGaacug....................................................................   1     1   sg1
.....................Uuggaauccaucugacaaacug....................................................................   1     1   sg1
.....................cuggaauccaucugacaGacug....................................................................   5     1   sg1
.....................cuggaauccaucugacaaacuA....................................................................   10    1   sg1
.....................cuggaauccaucugacaaacug....................................................................   75    0   sg1
.....................cuggaauccaucugacaaacCg....................................................................   2     1   sg1
.....................cuggaauccaucugGcaaacugc...................................................................   1     1   sg1
.....................cuggaauccaucugacaaacugc...................................................................   7     0   sg1
.....................cuggaauccaucugacaaacugca..................................................................   2     0   sg1
.....................cuggaauccaucugacaaacugcauA................................................................   1     1   sg1
......................uggaauccaucugacaaa.......................................................................   1     0   sg1
......................uggaauccaucugGcaaac......................................................................   4     1   sg1
......................uggaauccaucugacaaac......................................................................   17    0   sg1
......................uggaauccaucugacaGac......................................................................   2     1   sg1
......................uggaauccaucugacaGacu.....................................................................   3     1   sg1
......................uggaauccaucugacaaacA.....................................................................   1     1   sg1
......................uggaauccaucugGcaaacu.....................................................................   2     1   sg1
......................uggaauccaucugacaaacu.....................................................................   44    0   sg1
......................uggaauccaucugacGaacu.....................................................................   1     1   sg1
......................uggaaCccaucugacaaacu.....................................................................   1     1   sg1
......................Aggaauccaucugacaaacug....................................................................   1     1   sg1
......................uggaauccaucugacaaacCg....................................................................   1     1   sg1
......................uggaauccaucugacaaacug....................................................................   332   0   sg1
......................Cggaauccaucugacaaacug....................................................................   4     1   sg1
......................uggaauccaucugacaGacug....................................................................   12    1   sg1
......................uggaauccaucugacGaacug....................................................................   2     1   sg1
......................uggaauccaucugGcaaacug....................................................................   38    1   sg1
......................uggaGuccaucugacaaacug....................................................................   1     1   sg1
......................uAgaauccaucugacaaacug....................................................................   1     1   sg1
......................uggGauccaucugacaaacug....................................................................   3     1   sg1
......................uggaauccaucugacaaacuA....................................................................   18    1   sg1
......................uggaauUcaucugacaaacug....................................................................   1     1   sg1
......................uggaauccaucugacaaacuU....................................................................   2     1   sg1
......................uggaaucUaucugacaaacug....................................................................   3     1   sg1
......................uggaauccaucugacaaacuC....................................................................   2     1   sg1
......................uggaauccaucugacaaacAgc...................................................................   1     1   sg1
......................uggaauccaucugacaaacugU...................................................................   7     1   sg1
......................Cggaauccaucugacaaacugc...................................................................   1     1   sg1
......................uggaauccaucugGcaaacugc...................................................................   2     1   sg1
......................uggaauccaucugacaaacugA...................................................................   4     1   sg1
......................uggaauccaucugacaGacugc...................................................................   1     1   sg1
......................uggaauccaucugacaaacugc...................................................................   66    0   sg1
......................uggaauccaucugacaaacugUa..................................................................   1     1   sg1
......................uggaauccaucugacaaacugca..................................................................   65    0   sg1
......................uggaauccaucugGcaaacugca..................................................................   13    1   sg1
......................uggaauccaucugacaaacugcU..................................................................   4     1   sg1
```

Mature **Star**

acuuuauucuuuuuuuuuuaccuggaauccaucugacaaacugcauguuugucaugcaguuugucauggauuccaguuaaaaaaaagcaugcaaucguacgucaaggagaa			
......................uggaauccaucugacaGacugca..	4	1	sg1
......................uggaauccaucugacGaacugca..	3	1	sg1
......................uggGauccaucugacaaacugcau...	1	1	sg1
......................uggaauccaucugacaaacugcaG...	16	1	sg1
......................uggaauccaucugacaaacugcaA...	8	1	sg1
......................uggaauccaucugGcaaacugcau...	2	1	sg1
......................uggaauccaucugacaaacugcUu...	1	1	sg1
......................uggaauccaucugacaaacugcau...	17	0	sg1
......................uggaauccaucugacaaacugcaC...	1	1	sg1
......................uggaauccaucugacaaacugcaAg..	1	1	sg1
......................uggaauccaucugacaaacugcauA..	85	1	sg1
......................uggaauccaucugacaaacugcaug..	1	0	sg1
......................uggaauccaucugacaaacugcauU..	12	1	sg1
......................uggaauccaucugacaGacugcaug..	1	1	sg1
......................uggaauccaucugacaaacugcauAu...	8	1	sg1
......................uggaauccaucugacaaacugcauUu...	1	1	sg1
......................uggaauccaucugacaaacugcauAuu..	1	1	sg1
.......................ggaauccaucugacaaacug..	3	0	sg1
.......................ggaauccaucugacaaacugc...	2	0	sg1
........................gaauccaucugacaaacu...	1	0	sg1
.........................aauccaucugacaaacugcauUu...	1	1	sg1
..........................auccaucugacaaacugca..	2	0	sg1
....caguuugucauggauucc.....................................	4	0	sg1
....caguuugucauggauuccag...................................	9	0	sg1
....caguuugucauggauuccagu..................................	3	0	sg1
....caguuugucauggauuccagA..................................	1	1	sg1
....caguuugucauggauuccaguu.................................	68	0	sg1
....caguuugucauggauCccaguu.................................	1	1	sg1
....cUguuugucauggauuccaguu.................................	2	1	sg1
....caguuugucauggauuccagGu.................................	1	1	sg1
....caguuugucauggauuccaguC.................................	7	1	sg1
....caguuugucauggauuccaguA.................................	3	1	sg1
....caguuCgucauggauuccaguua................................	2	1	sg1
....caguuugucauggauuccaguuU................................	5	1	sg1
....caguuugucauggauuccaguua................................	215	0	sg1
....cagCuugucauggauuccaguua................................	1	1	sg1
....caguuugucaugUauuccaguua................................	1	1	sg1
....caguuugucGuggauuccaguua................................	1	1	sg1
....cGguuugucauggauuccaguua................................	2	1	sg1
....caguuugucauggGuuccaguua................................	1	1	sg1
....caguuugucauggauuccaguuC................................	1	1	sg1
....caguuugucauggauuccaguuaG...............................	1	1	sg1
....caguuugucauggauuccaguuUa...............................	5	1	sg1
....caguuugucauggauuccaguuaU...............................	4	1	sg1
....caguuugucauggauuccaguuaa...............................	9	0	sg1
....caguuugucauggauuccaguuaUa..............................	2	1	sg1
....caguuugucauggauuccaguuaaUa.............................	1	1	sg1
....aguuugucauggauucca....................................	2	0	sg1
....aguuugucauggauuccagu..................................	7	0	sg1
....aguuugucauggauuccaguu.................................	95	0	sg1
....aguuugucaCggauuccaguu.................................	1	1	sg1
....aguuugucauggauuccaguC.................................	7	1	sg1
....aguuugucauggauuccagGu.................................	1	1	sg1
....aguuugucauggauuccaguA.................................	1	1	sg1
....aguuugucGuggauuccaguu.................................	1	1	sg1
....aguuugucauggaCuccaguua................................	3	1	sg1
....aguuugucauggauuccCguua................................	1	1	sg1
....aguuugucauggauuccaguua................................	286	0	sg1
....Uguuugucauggauuccaguua................................	2	1	sg1
....aguuugucauggauuccaguuU................................	28	1	sg1
....aguuugucauggauuUcaguua................................	1	1	sg1
....aguuugucauggauuccaguuC................................	2	1	sg1
....aguuugucauggauuccagAua................................	1	1	sg1
....aguuugucaugAauuccaguua................................	1	1	sg1
....aguuugucauggGuuccaguua................................	2	1	sg1
....aguuugCcauggauuccaguua................................	3	1	sg1
....aguuugucauggauuccaguuUa...............................	43	1	sg1
....aguuugucauggauuccaguuaa...............................	22	0	sg1
....aguuugucauggauuccaguuaG...............................	2	1	sg1
....aguuugucauggauuccaguuaU...............................	6	1	sg1

Mature Star

acuuuauucuuuuuuuuuuuaccuggaauccaucugacaaacugcauguuugucaugcaguuugucauggauuccaguuaaaaaaaagcaugcaaucguacgucaaggagaa			
....aguuugucauggauuccaguuaUa..............................	18	1	sg1
....aguuugucauggauuccaguuaGa..............................	1	1	sg1
....aguuugucauggauuccaguuaUaa.............................	1	1	sg1
....aguuugucauggauuccaguuaUaaa............................	1	1	sg1
....uuugucauggauuccaguua................................	4	0	sg1
....uuugucauggauuccaguuU................................	1	1	sg1
....uuugucauggauuccaguuUa...............................	2	1	sg1
....uuugucauggauuccaguuaUa..............................	1	1	sg1

Provisional ID	:	ScYm1zk_361_3658
Score total	:	1295.1
Score for star read(s)	:	3.9
Score for read counts	:	1285.4
Score for mfe	:	1.2
Score for randfold	:	1.6
Score for cons. seed	:	3
Total read count	:	2533
Mature read count	:	2513
Loop read count	:	0
Star read count	:	20

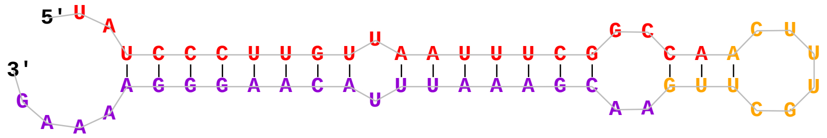

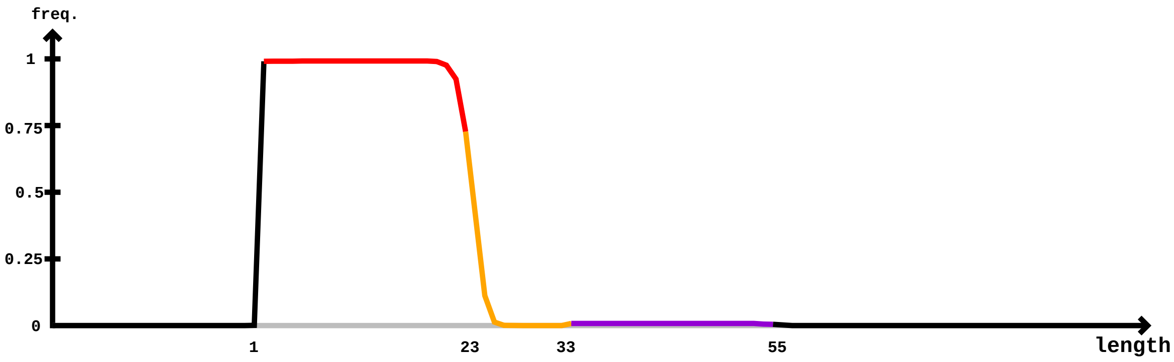

Mature Star

```
5'- uggucaucacauuccccucuuaucccuuguuaauuucggccaacuuugcuugaacgaaauuuacaagggaaaagaggcgccugccagaaguacuacuacaucaugcucgucg  -3'  obs
    uggucaucacauuccccucuuaucccuuguuaauuucggccaacuuugcuugaacgaaauuuacaagggaaaagaggcgccugccagaaguacuacuacaucaugcucgucg        exp
    ..............((((((.((((((((.(((((((..(((......)))..))))))).)))))))).)))))).((..((..((.(((....))).))..))..))..   reads   mm   sample
    ...................Cuaucccuuguuaauuucggcca......................................................................   2       1    sr1
    ....................uaucccuuguuaauuucg..........................................................................   5       0    sr1
    ....................uaucccuuguuaauuucgA.........................................................................   1       1    sr1
    ....................uaucccuuguuaGuuucgg.........................................................................   1       1    sr1
    ....................uaucccuuguuaauuucgg.........................................................................   23      0    sr1
    ....................uaucccuuguuaauCucgg.........................................................................   1       1    sr1
    ....................Gaucccuuguuaauuucggc........................................................................   14      1    sr1
    ....................Aaucccuuguuaauuucggc........................................................................   1       1    sr1
    ....................uaucccuuguuaauuucggU........................................................................   10      1    sr1
    ....................uGucccuuguuaauuucggc........................................................................   1       1    sr1
    ....................uaucccuuguuaauuucggc........................................................................   53      0    sr1
    ....................uaucccuuguuaauuucggA........................................................................   3       1    sr1
    ....................uaucccuuguuaauuucggcc.......................................................................   95      0    sr1
    ....................uaucccuCguuaauuucggcc.......................................................................   1       1    sr1
    ....................uaucccuuguuaauuucggcA.......................................................................   118     1    sr1
    ....................Gaucccuuguuaauuucggcc.......................................................................   11      1    sr1
    ....................uaucccuuguuaauuCcggcc.......................................................................   1       1    sr1
    ....................uaucccCuguuaauuucggcc.......................................................................   2       1    sr1
    ....................uaucccuuguuaauuucggcG.......................................................................   6       1    sr1
    ....................uaucccuuguuaauuucggcU.......................................................................   58      1    sr1
    ....................uaucccuugCuaauuucggcca......................................................................   2       1    sr1
    ....................uaucccuugAuaauuucggcca......................................................................   1       1    sr1
    ....................Gaucccuuguuaauuucggcca......................................................................   90      1    sr1
    ....................uaucccuuguuaauCucggcca......................................................................   1       1    sr1
    ....................uaucccuuguuaauuucggccU......................................................................   12      1    sr1
    ....................uaucccuuguuGauuucggcca......................................................................   1       1    sr1
    ....................uaucccuuguuaauuucCgcca......................................................................   1       1    sr1
    ....................uaucccuuguuaGuuucggcca......................................................................   2       1    sr1
    ....................uaucccuCguuaauuucggcca......................................................................   1       1    sr1
    ....................uaucccuuguuaauuucggccG......................................................................   30      1    sr1
    ....................uaucccuuguuaauuCcggcca......................................................................   1       1    sr1
    ....................uaucccuuguuaauuucggcUa......................................................................   4       1    sr1
    ....................uauUccuuguuaauuucggcca......................................................................   2       1    sr1
```

Mature **Star**

```
uggucaucacauuccccucuuaucccuuguuaauuucggccaacuuugcuugaacgaaauuuacaagggaaaagaggcgccugccagaaguacuacuacaucaugcucgucg
....................uaucccuGguuaauuucggcca......................................................................   1     1   sr1
....................uaucccuuguuaauuucggcca......................................................................   350   0   sr1
....................uaucccuuguuaauuucggccC......................................................................   14    1   sr1
....................uaucccuuguuaauuucggcAa......................................................................   1     1   sr1
....................uaucccCuguuaauuucggcca......................................................................   1     1   sr1
....................uaCcccuuguuaauuucggcca......................................................................   1     1   sr1
....................uaucccuuguuaauuucggccaU.....................................................................   140   1   sr1
....................uaCcccuuguuaauuucggccaa.....................................................................   1     1   sr1
....................uaucccuuguuaauCucggccaa.....................................................................   1     1   sr1
....................uaucccuuguuaauuucggccaa.....................................................................   29    0   sr1
....................uaucccuuguuaauuucggccaG.....................................................................   10    1   sr1
....................Gaucccuuguuaauuucggccaa.....................................................................   6     1   sr1
....................uaucccuuguuaauuucggcUaa.....................................................................   5     1   sr1
....................uaucccuuguuaauuucggccaC.....................................................................   22    1   sr1
....................uaucccuuguuaauuucggccaaU....................................................................   37    1   sr1
....................uaucccuuguuaauuucggccaac....................................................................   8     0   sr1
....................Gaucccuuguuaauuucggccaac....................................................................   1     1   sr1
....................uaucccuuguuaauuucggccaUc....................................................................   17    1   sr1
....................uaucccuuguuaauuucggccaaA....................................................................   25    1   sr1
....................uaucccuuguuaauuucggccaaG....................................................................   14    1   sr1
....................uaucccuuguuaauuucggccaaUu...................................................................   5     1   sr1
....................uaucccuuguuaauuucggccaacA...................................................................   1     1   sr1
....................uaucccuuguuaauuucggccaaAu...................................................................   1     1   sr1
....................uaucccuuguuaauuucggccaUcu...................................................................   1     1   sr1
........................ccuuguuaauuucggccaa.....................................................................   1     0   sr1
....................................................aacgaaauuuacaagggaaa........................................   4     0   sr1
....................................................aacgaaauuuGcaagggaaa........................................   1     1   sr1
....................................................Gacgaaauuuacaagggaaaa.......................................   1     1   sr1
....................................................aacgaaauuuacaagggaaaa.......................................   1     0   sr1
....................................................aacgaaauuuacaagggaaaag......................................   9     0   sr1

....................uaucccuuguuaauuucgg.........................................................................   4     0   st1
....................uaucccuuguuaaCuucgg.........................................................................   1     1   st1
....................uaucccuuguuaauuucggc........................................................................   10    0   st1
....................uaucccuuguuaauuucggU........................................................................   4     1   st1
....................uaucccuuguuaauuucggA........................................................................   1     1   st1
....................uaucccuuguuaGuuucggcc.......................................................................   1     1   st1
....................uaucccuuguuaauuucggcA.......................................................................   5     1   st1
....................uaucccuuguuaauuucggcc.......................................................................   40    0   st1
....................uaucccuuguuaauuucggcU.......................................................................   21    1   st1
....................uaucccuugCuaauuucggcca......................................................................   2     1   st1
....................uaucccuuguuaauCucggcca......................................................................   1     1   st1
....................Caucccuuguuaauuucggcca......................................................................   1     1   st1
....................uaucccuuguuaauuucggccG......................................................................   7     1   st1
....................uaucccuugGuaauuucggcca......................................................................   2     1   st1
....................uaucccuCguuaauuucggcca......................................................................   2     1   st1
....................uaucccuuguuaGuuucggcca......................................................................   1     1   st1
....................uaucccuuguuaauuucggccC......................................................................   7     1   st1
....................uaucccuuguuaaCuucggcca......................................................................   1     1   st1
....................uaucccuuguuaauuucggccU......................................................................   10    1   st1
....................uaucccCuguuaauuucggcca......................................................................   1     1   st1
....................uaucccuuguuCauuucggcca......................................................................   1     1   st1
....................uaucccuuguuaauuCcggcca......................................................................   3     1   st1
....................uaucccuuguCaauuucggcca......................................................................   2     1   st1
....................uaucccuuguuaauuucggcca......................................................................   257   0   st1
....................uaucccuuguuaauuucggccaG.....................................................................   2     1   st1
....................uaucccuuguuaauuucggccGa.....................................................................   1     1   st1
....................uaucccuuguuaauuucggccaU.....................................................................   67    1   st1
....................uaucccuuguuaauuucggccaC.....................................................................   12    1   st1
....................uaucccuuguuaauuucggccaa.....................................................................   18    0   st1
....................uaucccuuguuaauuucggccaaG....................................................................   5     1   st1
....................uaucccuuguuaauuucggccaGc....................................................................   1     1   st1
....................uaucccuuguuaauuucggccaaA....................................................................   14    1   st1
....................uaucccuuguuaauuucggccaaU....................................................................   23    1   st1
....................uaucccuuguuaauuucggccaUc....................................................................   16    1   st1
....................uaucccuuguuaauuucggccaac....................................................................   1     0   st1
....................uaucccuuguuaauuucggccaaUu...................................................................   1     1   st1
....................uaucccuuguuaauuucggccaaAu...................................................................   2     1   st1
....................uaucccuuguuaauuucggccaUcu...................................................................   2     1   st1
....................uaucccuuguuaauuucggccaacuA..................................................................   1     1   st1
```

Mature Star

```
uggucaucacauuccccucuuaucccuuguuaauuucggccaacuuugcuugaacgaaauuuacaagggaaaagaggcgccugccagaaguacuacuacaucaugcucgucg
........................ccuuguuaauuucggccaU......................................................................   1    1   st1
....................................................aacgaaauuuacaagggaaaag......................................   3    0   st1

....................uaucccuuguuaauuucggc........................................................................   6    0   sg1
....................uaucccuuguuaauuucggU........................................................................   1    1   sg1
....................uaucccuuguuaauuuUggcc.......................................................................   1    1   sg1
....................uaucccuuguuaauuucggcU.......................................................................   3    1   sg1
....................uaucccCuguuaauuucggcc.......................................................................   1    1   sg1
....................uaucccuuguuaauuucggcA.......................................................................   5    1   sg1
....................uaucccuuguuaauuucggcc.......................................................................   24   0   sg1
....................uaucUcuuguuaauuucggcca......................................................................   1    1   sg1
....................uaucccuuguuaauuucggcca......................................................................   78   0   sg1
....................uaucccuuguuaauuucggccC......................................................................   1    1   sg1
....................uaucccuuguuGauuucggcca......................................................................   2    1   sg1
....................uaucccuuguuaauuucggccU......................................................................   6    1   sg1
....................uaucccuuguuaauuucggccG......................................................................   4    1   sg1
....................uaucccuCguuaauuucggcca......................................................................   1    1   sg1
....................uaucccuuguuaauuucggccaC.....................................................................   9    1   sg1
....................uaucccuuguuaauuucggccaG.....................................................................   3    1   sg1
....................uaucccuuguuaauuucggccaU.....................................................................   32   1   sg1
....................uaucccuuguuaauuucggccaa.....................................................................   7    0   sg1
....................uaucccuuguuaauuucggccaUc....................................................................   9    1   sg1
....................uaucccuuguuaauuucggccaac....................................................................   1    0   sg1
....................uaucccuuguuaauuucggccaaA....................................................................   3    1   sg1
....................uaucccuuguuaauuucggccaaU....................................................................   12   1   sg1
....................uaucccuuguuaauuucggccaUcu...................................................................   4    1   sg1
....................uaucccuuguuaauuucggccaaUu...................................................................   1    1   sg1

....................uaucccuuguuaauuucgg.........................................................................   2    0   so1
....................uaucccuuguuaauuucgA.........................................................................   2    1   so1
....................uaucccuuguuaauuucggA........................................................................   2    1   so1
....................uaucccuuguuaauuucggU........................................................................   3    1   so1
....................uaucccuuguuaauuucggc........................................................................   23   0   so1
....................uaucccuuguuaauuucggcU.......................................................................   30   1   so1
....................uaCcccuuguuaauuucggcc.......................................................................   1    1   so1
....................uaucccuuguuaauuucggcA.......................................................................   22   1   so1
....................uaucccCuguuaauuucggcc.......................................................................   1    1   so1
....................uaucccuuguuaauuucggcc.......................................................................   52   0   so1
....................uauccAuuguuaauuucggcca......................................................................   1    1   so1
....................uaucccuuguuaauCucggcca......................................................................   1    1   so1
....................Caucccuuguuaauuucggcca......................................................................   2    1   so1
....................uaucccuuguuaauuucggccC......................................................................   6    1   so1
....................uaucccuuguuaauuucggccU......................................................................   8    1   so1
....................uaucccuuguuaauuucggcca......................................................................   150  0   so1
....................Aaucccuuguuaauuucggcca......................................................................   1    1   so1
....................uaucccuuguuaauuucggccG......................................................................   2    1   so1
....................uaucccAuguuaauuucggcca......................................................................   1    1   so1
....................uaucccuuguuaauuucggccaU.....................................................................   78   1   so1
....................uaucccuuguuaauuucggcUaa.....................................................................   1    1   so1
....................uaucccuuguuaauuucggccaG.....................................................................   3    1   so1
....................uaucccuuguuaauuucggccaC.....................................................................   11   1   so1
....................uaucccuuguuaauuucggccaa.....................................................................   16   0   so1
....................uaucccuuguuaauuucggccaaG....................................................................   1    1   so1
....................uaucccuuguuaauuucggccaUc....................................................................   17   1   so1
....................uaucccuuguuaauuucggccaaU....................................................................   32   1   so1
....................uaucccuuguuaauuucggccaaA....................................................................   12   1   so1
....................uaucccuuguuaauuucggccaac....................................................................   2    0   so1
....................uaucccuuguuaauuucggccaaUu...................................................................   7    1   so1
....................uaucccuuguuaauuucggccaUcu...................................................................   2    1   so1
....................uaucccuuguuaauuucggccaaAu...................................................................   4    1   so1
....................uaucccuuguuaauuucggccaacuuu.................................................................   1    0   so1
.....................aucccuuguuaauuucggcca......................................................................   1    0   so1
....................................................aacgaaauuuacaagggaaa........................................   1    0   so1
```

Provisional ID	:	ScYm1zk_361_3660
Score total	:	1.6
Score for star read(s)	:	-1.3
Score for read counts	:	0
Score for mfe	:	1.9
Score for randfold	:	1.6
Score for cons. seed	:	-0.6
Total read count	:	29819
Mature read count	:	29495
Loop read count	:	0
Star read count	:	324

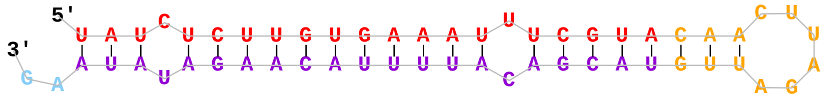

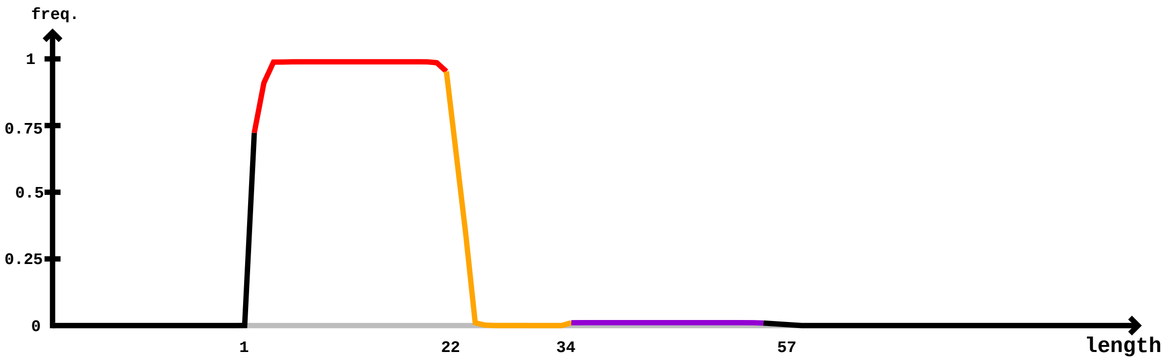

Mature Star

```
5'- gucgucaucacauuccucuuaucucuugugaaauuucguacaacuuagauuguacgacauuuuacaagauauaagaggugccaaaagugggauucgaacccacgcaugcuu  -3'   obs
    gucgucaucacauuccucuuaucucuugugaaauuucguacaacuuagauuguacgacauuuuacaagauauaagaggugccaaaagugggauucgaacccacgcaugcuu         exp
    ..............((((((((.(((((((((((.((((((((......)))))))).))))))))))).)))))))).((.....(((((.......)))))....))..   reads   mm   sample
    ...................uaucucuugugaaauuuc..........................................................................   1       0    so1
    ...................uaucucuugugaaauuucg.........................................................................   3       0    so1
    ...................uaucucuugugaaauuucgG........................................................................   1       1    so1
    ...................uaucucuugugaaauuucgC........................................................................   8       1    so1
    ...................uaucucuugCgaaauuucgu........................................................................   1       1    so1
    ...................uaucucuCgugaaauuucgu........................................................................   1       1    so1
    ...................uaucucuugugaaauuucgu........................................................................   52      0    so1
    ...................uaucucuCgugaaauuucgua.......................................................................   3       1    so1
    ...................uaucucuugugaaauCucgua.......................................................................   1       1    so1
    ...................uUucucuugugaaauuucgua.......................................................................   2       1    so1
    ...................Caucucuugugaaauuucgua.......................................................................   1       1    so1
    ...................uaucucuugugaaauuCcgua.......................................................................   1       1    so1
    ...................uaucucuugugaaauuAcgua.......................................................................   1       1    so1
    ...................uGucucuugugaaauuucgua.......................................................................   1       1    so1
    ...................uaucucuugugaaauuucguG.......................................................................   6       1    so1
    ...................uaucucuugugaaauuucgua.......................................................................   532     0    so1
    ...................uaucucuugugaaauuucguU.......................................................................   2       1    so1
    ...................uaucucuugugaGauuucgua.......................................................................   4       1    so1
    ...................uaucucuugugGaauuucgua.......................................................................   3       1    so1
    ...................uaucucuugugaaauuucgGac......................................................................   1       1    so1
    ...................uaucucuugCgaaauuucguac......................................................................   3       1    so1
    ...................uaucucuCgugaaauuucguac......................................................................   2       1    so1
    ...................uaucucCugugaaauuucguac......................................................................   1       1    so1
    ...................uaucucuugugGaauuucguac......................................................................   5       1    so1
    ...................uaucucuugugaaauuucguGc......................................................................   2       1    so1
    ...................uaucucuugugaaauuucguaA......................................................................   19      1    so1
    ...................uaucucuuguAaaauuucguac......................................................................   1       1    so1
    ...................uaucGcuugugaaauuucguac......................................................................   2       1    so1
    ...................uaucucuugugaaauuucguac......................................................................   451     0    so1
    ...................uaucucuugugaaauuucguaG......................................................................   5       1    so1
    ...................uGucucuugugaaauuucguac......................................................................   7       1    so1
    ...................uauAucuugugaaauuucguac......................................................................   1       1    so1
    ...................uaucucuugugaaauGucguac......................................................................   2       1    so1
```

```
                   Mature                          Star
gucgucaucacauuccucuuaucucuugugaaauuucguacaacuuagauuguacgacauuuuacaagauauaagaggugccaaaagugggauucgaacccacgcaugcuu
...................uaCcucuugugaaauuucguac......................................................................   2     1   so1
...................uaucucuugugaGauuucguac......................................................................   2     1   so1
...................uaucucuugugaaauuucguaU......................................................................   78    1   so1
...................uaucCcuugugaaauuucguac......................................................................   3     1   so1
...................uaucucuugugaaauuucAuac......................................................................   1     1   so1
...................uaucucuugugaaauCucguac......................................................................   1     1   so1
...................Caucucuugugaaauuucguac......................................................................   1     1   so1
...................uaucucuugugaaauuucguaAa.....................................................................   8     1   so1
...................uaucucuCgugaaauuucguaca.....................................................................   1     1   so1
...................uaucucuugugaaauuucguaUa.....................................................................   25    1   so1
...................uaucucuugugaaauuAcguaca.....................................................................   1     1   so1
...................uaucuAuugugaaauuucguaca.....................................................................   1     1   so1
...................uaucuUuugugaaauuucguaca.....................................................................   1     1   so1
...................uaucucuugCgaaauuucguaca.....................................................................   1     1   so1
...................uaucucuugugaGauuucguaca.....................................................................   1     1   so1
...................uaucucuugugaaauuucguacU.....................................................................   7     1   so1
...................uaucucuugugaaauuucguaca.....................................................................   436   0   so1
...................uaucucuugugaaauuucguacC.....................................................................   3     1   so1
...................uaucucuugugGaauuucguaca.....................................................................   2     1   so1
...................uaucCcuugugaaauuucguaca.....................................................................   2     1   so1
...................uaucucuugugaaauuucguacG.....................................................................   23    1   so1
...................uaucucCugugaaauuucguaca.....................................................................   1     1   so1
...................uaucucuugugaaauuucguGca.....................................................................   2     1   so1
...................uGucucuugugaaauuucguaca.....................................................................   1     1   so1
...................uaucucuugugaaauuucguacaU....................................................................   5     1   so1
...................uaucucuugugaaauuucguaUaa....................................................................   2     1   so1
...................uaucucuugugaaauuucguacaC....................................................................   1     1   so1
...................uaucucuugugaaauuucguacaa....................................................................   2     0   so1
...................uaucucuugugaaauuucguacCa....................................................................   1     1   so1
...................uaucucuugugaaauuucguacUa....................................................................   7     1   so1
...................uaucucuugugaaauuucguacaac...................................................................   1     0   so1
...................uaucucuugugaaauuucguacaCc...................................................................   2     1   so1
...................uaucucuugugaaauuucguacaaA...................................................................   6     1   so1
...................uaucucuugugaaauuucguacaaAu..................................................................   2     1   so1
....................aucucuugugaaauuucgA........................................................................   1     1   so1
....................aucucuugugaaauuucgC........................................................................   1     1   so1
....................aucucuugugaaauuucgu........................................................................   4     0   so1
....................Gucucuugugaaauuucgua.......................................................................   1     1   so1
....................aucucuAgugaaauuucgua.......................................................................   1     1   so1
....................aCcucuugugaaauuucgua.......................................................................   1     1   so1
....................aucucuugugaaauuucgua.......................................................................   41    0   so1
....................aucucuugugaaauCucgua.......................................................................   4     1   so1
....................aucucuugugaaauuucguaA......................................................................   2     1   so1
....................aucucuugugaaauuucguGc......................................................................   1     1   so1
....................aucucuuguAaaauuucguac......................................................................   1     1   so1
....................aucucuugugaaauuucguaU......................................................................   26    1   so1
....................aucucuugugaaauuCcguac......................................................................   1     1   so1
....................aucucuugugaaauuucguac......................................................................   122   0   so1
....................aucucuugugaaauCucguac......................................................................   1     1   so1
....................aucucuugugaaauuucguaUa.....................................................................   17    1   so1
....................aucucuugugaaauuucguGca.....................................................................   1     1   so1
....................aucucuugugaaauuCcguaca.....................................................................   1     1   so1
....................aucucuugugaaauuucguaca.....................................................................   62    0   so1
....................aucucuugugaaauuucguacG.....................................................................   5     1   so1
....................aucucuugugaaauuucguacC.....................................................................   1     1   so1
....................aucucuugugaaauuucguacaa....................................................................   1     0   so1
....................aucucuugugaaauuucguacUa....................................................................   1     1   so1
....................aucucuugugaaauuucguacaCc...................................................................   1     1   so1
.....................ucucuugugaaauuucgua.......................................................................   4     0   so1
.....................ucucuugugaaauuucguU.......................................................................   2     1   so1
.....................ucucuugugaaauuucguaU......................................................................   10    1   so1
.....................ucucuugugaaauuucguac......................................................................   64    0   so1
.....................Ccucuugugaaauuucguac......................................................................   1     1   so1
.....................ucCcuugugaaauuucguac......................................................................   1     1   so1
.....................ucucCugugaaauuucguac......................................................................   1     1   so1
.....................ucucuugugaaauuucgCaca.....................................................................   1     1   so1
.....................ucucuugugaaauuucguacU.....................................................................   1     1   so1
.....................ucucuuAugaaauuucguaca.....................................................................   1     1   so1
.....................ucucuugugaGauuucguaca.....................................................................   1     1   so1
.....................ucucuugugaaauuucguaUa.....................................................................   2     1   so1
```

Mature **Star**

```
gucgucaucacauuccucuuaucucuugugaaauuucguacaacuuagauuguacgacauuuuacaagauauaagaggugccaaaagugggauucgaacccacgcaugcuu
.....................ucCcuugugaaauuucguaca.....................................................................  1    1  so1
.....................ucucuugugaaauuucguacC.....................................................................  1    1  so1
.....................ucucuugugaaauuucguaca.....................................................................  56   0  so1
.....................ucucuugugaaauuucguacG.....................................................................  1    1  so1
.....................ucucuugugaaauuucguacaC....................................................................  2    1  so1
.....................ucucuugugaaauuucguacaU....................................................................  2    1  so1
.....................ucucuugugaaauuucguacaacuuag...............................................................  2    0  so1
......................cucuugugaaauuucguaca.....................................................................  1    0  so1
....................................................uacgacauuuuacaagau.........................................  1    0  so1
....................................................uacgacauuuuacaagauau.......................................  2    0  so1
....................................................uacgacauuuuacaagauaua......................................  7    0  so1
....................................................uacgacauuuuacaagauauaa.....................................  5    0  so1
....................................................uacgacauuuuacaagauauaag....................................  6    0  so1

...................uaucucuugugaaauuucg.........................................................................  6    0  sg1
...................uaucucuugugaaauuucgu........................................................................  23   0  sg1
...................uaucucuugugaaauuucgC........................................................................  1    1  sg1
...................uaucucuugugaaauuucgA........................................................................  1    1  sg1
...................uaucucuugugaaauuucgG........................................................................  1    1  sg1
...................uaucucuuguAaaauuucgua.......................................................................  1    1  sg1
...................uGucucuugugaaauuucgua.......................................................................  1    1  sg1
...................uaucucuugugaaauGucgua.......................................................................  1    1  sg1
...................uaucucuugugaaauuucguG.......................................................................  4    1  sg1
...................uaucucuugugaaauuucgGa.......................................................................  2    1  sg1
...................uaucucuugugaaauuCcgua.......................................................................  3    1  sg1
...................uaucucuugugGaauuucgua.......................................................................  1    1  sg1
...................uaucucCugugaaauuucgua.......................................................................  1    1  sg1
...................uaucucuAgugaaauuucgua.......................................................................  1    1  sg1
...................uaucucuugugaaauuucgua.......................................................................  244  0  sg1
...................uaucucuugCgaaauuucgua.......................................................................  1    1  sg1
...................uaucucuugugaaauuucguac......................................................................  342  0  sg1
...................uaCcucuugugaaauuucguac......................................................................  4    1  sg1
...................uaucucuugugaaauCucguac......................................................................  1    1  sg1
...................uaucucuugugaaauuucguGc......................................................................  3    1  sg1
...................uaucucCugugaaauuucguac......................................................................  1    1  sg1
...................uaucucuugugaaauuucguaG......................................................................  2    1  sg1
...................uaucucuugugaaaCuucguac......................................................................  1    1  sg1
...................uaucucuugugaGauuucguac......................................................................  1    1  sg1
...................uaucucuugugaaauuucguaU......................................................................  26   1  sg1
...................uaucucuugugGaauuucguac......................................................................  3    1  sg1
...................uaucucuuguAaaauuucguac......................................................................  1    1  sg1
...................uaucucuugugaaauuucguaA......................................................................  6    1  sg1
...................uGucucuugugaaauuucguaca.....................................................................  2    1  sg1
...................uaucucuugugaaauuucguUca.....................................................................  1    1  sg1
...................uaucucuugugaaauuucguaca.....................................................................  574  0  sg1
...................uaucucuugugaaauuucguGca.....................................................................  6    1  sg1
...................uaucucuugugaaauuucguaUa.....................................................................  22   1  sg1
...................uaucucuugugaGauuucguaca.....................................................................  1    1  sg1
...................uaucCcuugugaaauuucguaca.....................................................................  2    1  sg1
...................Caucucuugugaaauuucguaca.....................................................................  2    1  sg1
...................uaucucuAgugaaauuucguaca.....................................................................  1    1  sg1
...................uaucucuCgugaaauuucguaca.....................................................................  1    1  sg1
...................uaucucuugugaaauuucguacU.....................................................................  11   1  sg1
...................uaucucuugugaaaCuucguaca.....................................................................  2    1  sg1
...................uaucucuugugaaauCucguaca.....................................................................  1    1  sg1
...................uaucucCugugaaauuucguaca.....................................................................  1    1  sg1
...................uaucucuugCgaaauuucguaca.....................................................................  2    1  sg1
...................uaucucuugugaaauuucguaAa.....................................................................  4    1  sg1
...................uaucucuugugaaauuucguacC.....................................................................  6    1  sg1
...................uaucucuugugaaauuucguacG.....................................................................  8    1  sg1
...................uaucucuugugaCauuucguaca.....................................................................  1    1  sg1
...................uaucucuugugaaauuucguacaa....................................................................  4    0  sg1
...................uaucucuugugaaauuucguacaU....................................................................  2    1  sg1
...................uaucucuugugaaauuucguacUa....................................................................  2    1  sg1
...................uaucucuugugaaauuucguacaaA...................................................................  3    1  sg1
....................aucucuugugaaauuucgu........................................................................  5    0  sg1
....................aucucuugugaaauuucgua.......................................................................  16   0  sg1
....................aucucuugugaaauuCcgua.......................................................................  1    1  sg1
....................aucucuugugaaauuucguG.......................................................................  1    1  sg1
....................aucucuugugaGauuucguac......................................................................  1    1  sg1
```

Mature Star

```
gucgucaucacauuccucuuaucucuugugaaauuucguacaacuuagauuguacgacauuuuacaagauauaagaggugccaaaagugggauucgaacccacgcaugcuu
....................aucucCugugaaauuucguac......................................................................  1     1  sg1
....................aucucuugugaaauuucguac......................................................................  41    0  sg1
....................aucucuugugaaauuucguaG......................................................................  1     1  sg1
....................aucucuugugaaauuucguaU......................................................................  7     1  sg1
....................aucucuugCgaaauuucguac......................................................................  1     1  sg1
....................aucucuugugaaaCuucguac......................................................................  2     1  sg1
....................aucucuCgugaaauuucguaca.....................................................................  1     1  sg1
....................Gucucuugugaaauuucguaca.....................................................................  1     1  sg1
....................aucucuugugaaauuucguaAa.....................................................................  3     1  sg1
....................aucucuugugaaauCucguaca.....................................................................  2     1  sg1
....................aucucuugugaaauuucguaca.....................................................................  62    0  sg1
....................aucucuugugaaauuucguacU.....................................................................  5     1  sg1
....................aucucuugugaaauuucgCaca.....................................................................  1     1  sg1
....................aucucuugugaaauuucguaUa.....................................................................  17    1  sg1
....................aucucuugugaaauuucguaUaa....................................................................  1     1  sg1
....................aucucuugugaaauuucguacUa....................................................................  1     1  sg1
.....................ucucuugugaaauuucgu........................................................................  1     0  sg1
.....................ucucuugugaaauuucgua.......................................................................  4     0  sg1
.....................ucucuugugaaGuuucguac......................................................................  3     1  sg1
.....................ucucuugugaaauuucguac......................................................................  28    0  sg1
.....................ucucuugugaaauuucguacG.....................................................................  2     1  sg1
.....................ucucuugugaaauuucguaca.....................................................................  63    0  sg1
.....................ucucCugugaaauuucguaca.....................................................................  1     1  sg1
.....................ucucuugugaaauuucguacCa....................................................................  1     1  sg1
.....................ucucuugugaaauuucguacaC....................................................................  1     1  sg1
.....................ucucuugugaaauuucguaUaa....................................................................  1     1  sg1
.....................ucucuugugaaauuucguacaCc...................................................................  1     1  sg1
.......................ucuugugaaauuucguac......................................................................  1     0  sg1
........................................caacuuagauuguacgacauuu.................................................  1     0  sg1
....................................................uacgacauuuuacaagauaC.......................................  1     1  sg1
....................................................uacgacauuuuacaagauaua......................................  3     0  sg1
....................................................uacgacauuuuacaagauauaa.....................................  10    0  sg1
....................................................uacgacauuuuacaagauauaag....................................  4     0  sg1
.....................................................acgacauuuuacaagauaua......................................  1     0  sg1

..................Cuaucucuugugaaauuucgua.......................................................................  1     1  sr1
..................Cuaucucuugugaaauuucguac......................................................................  3     1  sr1
...................uaucucuugugaaauuuc..........................................................................  2     0  sr1
...................uaucucuugugaaauCucg.........................................................................  2     1  sr1
...................uaucucuugugaaauuucA.........................................................................  4     1  sr1
...................uaucucuugugaGauuucg.........................................................................  4     1  sr1
...................uaucucuugCgaaauuucg.........................................................................  1     1  sr1
...................uaucucuugugaaauuucC.........................................................................  1     1  sr1
...................uaucucuugugaaauuucg.........................................................................  63    0  sr1
...................uaucucuugugaaauGucg.........................................................................  1     1  sr1
...................uaucucCugugaaauuucg.........................................................................  1     1  sr1
...................uaucucuugugaaauuucgA........................................................................  2     1  sr1
...................uaucucCugugaaauuucgu........................................................................  1     1  sr1
...................uaucCcuugugaaauuucgu........................................................................  1     1  sr1
...................uaucucuCgugaaauuucgu........................................................................  2     1  sr1
...................uaucucuugugaaauuucgC........................................................................  43    1  sr1
...................uaCcucuugugaaauuucgu........................................................................  2     1  sr1
...................uaucucuugugaaauuucgu........................................................................  356   0  sr1
...................uaucucuugugaaGuuucgu........................................................................  1     1  sr1
...................uaucucuugugaGauuucgu........................................................................  1     1  sr1
...................Gaucucuugugaaauuucgu........................................................................  21    1  sr1
...................uaucucuugugaaauCucgu........................................................................  2     1  sr1
...................uGucucuugugaaauuucgu........................................................................  3     1  sr1
...................uaucucuugugaaauuCcgu........................................................................  1     1  sr1
...................uaucucuugugaaauuucgG........................................................................  9     1  sr1
...................Caucucuugugaaauuucgua.......................................................................  10    1  sr1
...................uaucucuugugaaauuCcgua.......................................................................  14    1  sr1
...................uaCcucuugugaaauuucgua.......................................................................  23    1  sr1
...................uaucucuuguAaaauuucgua.......................................................................  3     1  sr1
...................uaucucuugugaaauuucgua.......................................................................  4253  0  sr1
...................uaucucuuAugaaauuucgua.......................................................................  4     1  sr1
...................uaucucuugCgaaauuucgua.......................................................................  10    1  sr1
...................uaucucuugAgaaauuucgua.......................................................................  4     1  sr1
...................uaucucuugGgaaauuucgua.......................................................................  1     1  sr1
...................uaucucuugugaaauuuAgua.......................................................................  3     1  sr1
```

```
                   Mature           Star
gucgucaucacauuccucuuaucucuugugaaauuucguacaacuuagauuguacgacauuuuacaagauauaagaggugccaaaagugggauucgaacccacgcaugcuu
...................uaucucuugugaaauuucgCa.......................................................................	11	1	sr1
...................uaucAcuugugaaauuucgua.......................................................................	1	1	sr1
...................uaucucuugugaaauuucgGa.......................................................................	2	1	sr1
...................uaucucuCgugaaauuucgua.......................................................................	14	1	sr1
...................uUucucuugugaaauuucgua.......................................................................	1	1	sr1
...................uaucucuugugaGauuucgua.......................................................................	15	1	sr1
...................uaucucuugugaaauuucguU.......................................................................	9	1	sr1
...................uaucucuAgugaaauuucgua.......................................................................	4	1	sr1
...................uaucucuugugUaauuucgua.......................................................................	3	1	sr1
...................uaGcucuugugaaauuucgua.......................................................................	1	1	sr1
...................uaucucGugugaaauuucgua.......................................................................	1	1	sr1
...................uaucucuugugaaauuucguG.......................................................................	113	1	sr1
...................uaucucuugugaaGuuucgua.......................................................................	5	1	sr1
...................uaucucuuUugaaauuucgua.......................................................................	3	1	sr1
...................uaucuUuugugaaauuucgua.......................................................................	2	1	sr1
...................uaucucuugugaaauuuGgua.......................................................................	1	1	sr1
...................uaucCcuugugaaauuucgua.......................................................................	13	1	sr1
...................uauUucuugugaaauuucgua.......................................................................	3	1	sr1
...................uaAcucuugugaaauuucgua.......................................................................	1	1	sr1
...................uaucucCugugaaauuucgua.......................................................................	7	1	sr1
...................uaucucuugugaaaCuucgua.......................................................................	13	1	sr1
...................uaucucuugugaaauuAcgua.......................................................................	1	1	sr1
...................Aaucucuugugaaauuucgua.......................................................................	4	1	sr1
...................uaucucuugugaaauAucgua.......................................................................	3	1	sr1
...................uGucucuugugaaauuucgua.......................................................................	33	1	sr1
...................uaucucuugugaaauCucgua.......................................................................	7	1	sr1
...................uaucucuugugaaauuucAua.......................................................................	1	1	sr1
...................Gaucucuugugaaauuucgua.......................................................................	287	1	sr1
...................uaucuAuugugaaauuucgua.......................................................................	1	1	sr1
...................uaucucuugugaaaAuucgua.......................................................................	3	1	sr1
...................uaucucuugugGaauuucgua.......................................................................	20	1	sr1
...................uaucucuugugaaauGucgua.......................................................................	5	1	sr1
...................uaCcucuugugaaauuucguac......................................................................	3	1	sr1
...................uaucucuuAugaaauuucguac......................................................................	1	1	sr1
...................uaucucuugugGaauuucguac......................................................................	11	1	sr1
...................uaucucuugugaaauuucguUc......................................................................	2	1	sr1
...................uaucucuugugaaauuAcguac......................................................................	3	1	sr1
...................uaucGcuugugaaauuucguac......................................................................	1	1	sr1
...................uaucucuugugCaauuucguac......................................................................	1	1	sr1
...................Gaucucuugugaaauuucguac......................................................................	212	1	sr1
...................uaucucuugugaaauuucguGc......................................................................	14	1	sr1
...................uaucucuugugaaauuucgCac......................................................................	1	1	sr1
...................uaucucuugugaaaCuucguac......................................................................	6	1	sr1
...................uaucucuugCgaaauuucguac......................................................................	11	1	sr1
...................uaucucuuguAaaauuucguac......................................................................	2	1	sr1
...................uaucucuugugaaauuucgGac......................................................................	1	1	sr1
...................uauUucuugugaaauuucguac......................................................................	2	1	sr1
...................uaucucuugugaaauCucguac......................................................................	2	1	sr1
...................uaucucuugugaaauuuAguac......................................................................	2	1	sr1
...................uaucuUuugugaaauuucguac......................................................................	1	1	sr1
...................uaucucuugugaaauuCcguac......................................................................	3	1	sr1
...................uaucucuugugaaauuucguaU......................................................................	345	1	sr1
...................uaucucCugugaaauuucguac......................................................................	3	1	sr1
...................uaucucAugugaaauuucguac......................................................................	1	1	sr1
...................uaucucuugAgaaauuucguac......................................................................	1	1	sr1
...................uaGcucuugugaaauuucguac......................................................................	1	1	sr1
...................uGucucuugugaaauuucguac......................................................................	12	1	sr1
...................uaucucuugugUaauuucguac......................................................................	1	1	sr1
...................uaucucuugugaaauGucguac......................................................................	6	1	sr1
...................Aaucucuugugaaauuucguac......................................................................	1	1	sr1
...................uaucucuugugaaauuucguac......................................................................	2270	0	sr1
...................uauAucuugugaaauuucguac......................................................................	2	1	sr1
...................uaucCcuugugaaauuucguac......................................................................	9	1	sr1
...................uaucucuCgugaaauuucguac......................................................................	3	1	sr1
...................uaucucuAgugaaauuucguac......................................................................	3	1	sr1
...................uaucucuugugaGauuucguac......................................................................	5	1	sr1
...................uaucucuugugaaauuucguaA......................................................................	163	1	sr1
...................Caucucuugugaaauuucguac......................................................................	7	1	sr1
...................uaucucuugugaaGuuucguac......................................................................	8	1	sr1
...................uaucucuugugaaauuucAuac......................................................................	5	1	sr1
```

```
                   Mature              Star
gucgucaucacauuccucuuaucucuugugaaauuucguacaacuuagauuguacgacauuuuacaagauauaagaggugccaaaagugggauucgaacccacgcaugcuu
...................uaucucuugugaaaGuucguac......................................................................  1     1  sr1
...................uaucucuugugaaauuucguaG......................................................................  35    1  sr1
...................uaucucuGgugaaauuucguac......................................................................  1     1  sr1
...................uaucucuugugaaauuucgAaca.....................................................................  1     1  sr1
...................uaucucuugCgaaauuucguaca.....................................................................  8     1  sr1
...................uaucucuugugaaauuucgCaca.....................................................................  1     1  sr1
...................uaucucAugugaaauuucguaca.....................................................................  1     1  sr1
...................uaucucuugugGaauuucguaca.....................................................................  19    1  sr1
...................uaucucuugugaaauuucguaca.....................................................................  2501  0  sr1
...................uaucucuugugaaauuucguUca.....................................................................  2     1  sr1
...................uaucucuugugaaauGucguaca.....................................................................  6     1  sr1
...................uGucucuugugaaauuucguaca.....................................................................  26    1  sr1
...................Aaucucuugugaaauuucguaca.....................................................................  1     1  sr1
...................uaucucuugugaaaAuucguaca.....................................................................  1     1  sr1
...................uaucucuugugaaauuucguaGa.....................................................................  2     1  sr1
...................uaucucuugugaaauuAcguaca.....................................................................  1     1  sr1
...................uaucucuugugaGauuucguaca.....................................................................  7     1  sr1
...................uaucucuugugaaauuucguacC.....................................................................  14    1  sr1
...................Caucucuugugaaauuucguaca.....................................................................  9     1  sr1
...................uaucucuugugaaauuCcguaca.....................................................................  5     1  sr1
...................uaucucuugGgaaauuucguaca.....................................................................  1     1  sr1
...................uaucCcuugugaaauuucguaca.....................................................................  3     1  sr1
...................uaucucuuAugaaauuucguaca.....................................................................  1     1  sr1
...................uaucucuugugaaaCuucguaca.....................................................................  8     1  sr1
...................uaucucuugugaaauuucguacU.....................................................................  55    1  sr1
...................uaucucuugugaaauAucguaca.....................................................................  1     1  sr1
...................uaucucuugugaaauCucguaca.....................................................................  6     1  sr1
...................uaucucuugugaaauuucAuaca.....................................................................  4     1  sr1
...................uaucucuCgugaaauuucguaca.....................................................................  5     1  sr1
...................uaucucuugugaaauuucguGca.....................................................................  29    1  sr1
...................uaucucuuguAaaauuucguaca.....................................................................  1     1  sr1
...................uaucucuugugaaauuuAguaca.....................................................................  3     1  sr1
...................uUucucuugugaaauuucguaca.....................................................................  1     1  sr1
...................uaCcucuugugaaauuucguaca.....................................................................  6     1  sr1
...................uaucucuugugaaauuucguacG.....................................................................  208   1  sr1
...................uaucucuugugaaauuucguaUa.....................................................................  311   1  sr1
...................uaAcucuugugaaauuucguaca.....................................................................  1     1  sr1
...................uauAucuugugaaauuucguaca.....................................................................  1     1  sr1
...................uaucucGugugaaauuucguaca.....................................................................  1     1  sr1
...................uaucucuugugUaauuucguaca.....................................................................  1     1  sr1
...................uaucucuugugaaGuuucguaca.....................................................................  11    1  sr1
...................Gaucucuugugaaauuucguaca.....................................................................  375   1  sr1
...................uaucucCugugaaauuucguaca.....................................................................  9     1  sr1
...................uaucucuugugaaauuucguaAa.....................................................................  48    1  sr1
...................uaucucuAgugaaauuucguaca.....................................................................  5     1  sr1
...................uaucucuugugaaauuucguacUa...................................................................  22    1  sr1
...................uaucucuugugaaauuucguacaU...................................................................  19    1  sr1
...................uaucucuugugaaauuucguGcaa...................................................................  2     1  sr1
...................Gaucucuugugaaauuucguacaa...................................................................  2     1  sr1
...................uaucucuugugaaauuucguacaC...................................................................  2     1  sr1
...................uaucucuugugaaauuucguacCa...................................................................  4     1  sr1
...................uaucucuugugaaauuucguaUaa...................................................................  13    1  sr1
...................uaucucuugugaaGuuucguacaa...................................................................  1     1  sr1
...................uaucucuugugaaauuucguacaa...................................................................  29    0  sr1
...................uaucucuugugaaauuucguacaG...................................................................  8     1  sr1
...................uaucucuugugaaauuucguacaaG..................................................................  1     1  sr1
...................uaucucuugugaaauuucguacaCc..................................................................  5     1  sr1
...................uaucucuugugaaauuucguacaaA..................................................................  15    1  sr1
....................aucucuugugaaauuucg.........................................................................  2     0  sr1
....................Gucucuugugaaauuucgu........................................................................  4     1  sr1
....................aucucuugugaaGuuucgu........................................................................  1     1  sr1
....................aucucuugugaaauuucgC........................................................................  10    1  sr1
....................aucucuugugaaauuucgG........................................................................  4     1  sr1
....................aucucuuguAaaauuucgu........................................................................  1     1  sr1
....................aucucuugugaaauuucgA........................................................................  3     1  sr1
....................aucucuAgugaaauuucgu........................................................................  1     1  sr1
....................aucucuugCgaaauuucgu........................................................................  1     1  sr1
....................aucucuugugaaauuucgu........................................................................  129   0  sr1
....................aucCcuugugaaauuucgu........................................................................  1     1  sr1
....................aucucuugugaaGuuucgua.......................................................................  1     1  sr1
```

Mature Star

gucgucaucacauuccucuuaucucuugugaaauuucguacaacuuagauuguacgacauuuuacaagauauaagaggugccaaaagugggauucgaacccacgcaugcuu			
....................aucucuugugaaauuAcgua...	1	1	sr1
....................aucucuugugaaauuucAua...	1	1	sr1
....................aucucuugugGaauuucgua...	7	1	sr1
....................Gucucuugugaaauuucgua...	26	1	sr1
....................aucucuugugaaauuucguG...	19	1	sr1
....................aucucuugugaGauuucgua...	1	1	sr1
....................aucucuugugaaauuucgCa...	7	1	sr1
....................aucucuugugaaaCuucgua...	1	1	sr1
....................aucucuugugaaaAuucgua...	1	1	sr1
....................aucucuCgugaaauuucgua...	8	1	sr1
....................aCcucuugugaaauuucgua...	2	1	sr1
....................aucCcuugugaaauuucgua...	3	1	sr1
....................aucucCugugaaauuucgua...	6	1	sr1
....................aucAcuugugaaauuucgua...	1	1	sr1
....................aucucuugCgaaauuucgua...	3	1	sr1
....................aucucuugugaaauuCcgua...	2	1	sr1
....................aucucuugugaaauuGcgua...	1	1	sr1
....................aucucuugugaaauCucgua...	2	1	sr1
....................aucucuugugaaauuucgua...	766	0	sr1
....................aucucuugugaaaGuucgua...	1	1	sr1
....................aucucAugugaaauuucgua...	1	1	sr1
....................aucucuAgugaaauuucgua...	2	1	sr1
....................aucucuAgugaaauuucguac..	3	1	sr1
....................aucucuugugaaauuAcguac..	2	1	sr1
....................aucucuugugaaauuucguaU..	211	1	sr1
....................aucAcuugugaaauuucguac..	2	1	sr1
....................aucCcuugugaaauuucguac..	6	1	sr1
....................aucucuugugaaauuucAuac..	4	1	sr1
....................aucucuugugaaauuucguaG..	7	1	sr1
....................aucucuugugaaauuCcguac..	3	1	sr1
....................aucucuugugUaauuucguac..	2	1	sr1
....................auUucuugugaaauuucguac..	1	1	sr1
....................aucucuuAugaaauuucguac..	1	1	sr1
....................aCcucuugugaaauuucguac..	2	1	sr1
....................aucucuugugaaauGucguac..	1	1	sr1
....................aucucuugugaaGuuucguac..	2	1	sr1
....................aucucuugugGaauuucguac..	8	1	sr1
....................Gucucuugugaaauuucguac..	46	1	sr1
....................aucucuugugaGauuucguac..	4	1	sr1
....................aucuUuugugaaauuucguac..	3	1	sr1
....................aucucuugugaaauuucguaA..	57	1	sr1
....................aucucuugugaaauuucgAac..	1	1	sr1
....................aucucCugugaaauuucguac..	3	1	sr1
....................aucucuugCgaaauuucguac..	5	1	sr1
....................aucucuugugaaauCucguac..	1	1	sr1
....................aucucuugugaaauuucguGc..	4	1	sr1
....................aucucuCgugaaauuucguac..	1	1	sr1
....................aucucuugugaaauuucguUc..	1	1	sr1
....................aucucuugugaaaCuucguac..	6	1	sr1
....................aucucuugugaaauuucguac..	1379	0	sr1
....................aucucGugugaaauuucguac..	1	1	sr1
....................aucucuugugaaauuucguacC...	18	1	sr1
....................aucucuugugGaauuucguaca...	1	1	sr1
....................aucCcuugugaaauuucguaca...	7	1	sr1
....................aucucuugugaaauuucguaAa...	5	1	sr1
....................aucucuugugaaauuucgCaca...	1	1	sr1
....................aucucuugugaaauuucAuaca...	3	1	sr1
....................aucucuugugaaauuucguaUa...	282	1	sr1
....................aucucuugugaaauuucguaca...	707	0	sr1
....................aucucuCgugaaauuucguaca...	4	1	sr1
....................aucucuugugaaauuucguacG...	44	1	sr1
....................aucucuugugaaauuAcguaca...	1	1	sr1
....................aucucuugugaaauuCcguaca...	1	1	sr1
....................aucucuugugaaaAuucguaca...	1	1	sr1
....................aucucuugugaGauuucguaca...	1	1	sr1
....................auAucuugugaaauuucguaca...	1	1	sr1
....................aucucuuguAaaauuucguaca...	1	1	sr1
....................aucucuugugaaGuuucguaca...	1	1	sr1
....................Gucucuugugaaauuucguaca...	14	1	sr1
....................aucucuAgugaaauuucguaca...	2	1	sr1

Mature **Star**

gucgucaucacauuccucuuaucucuugugaaauuucguacaacuuagauuguacgacauuuuacaagauauaagaggugccaaaagugggauucgaacccacgcaugcuu

....................aucucuugugaaauuucguGca...	3	1	sr1
....................aCcucuugugaaauuucguaca...	1	1	sr1
....................aucucuugugaaauuucguacU...	27	1	sr1
....................aucucuuAugaaauuucguaca...	1	1	sr1
....................aucucuugugaaauCucguaca...	1	1	sr1
....................aucucuugCgaaauuucguaca...	1	1	sr1
....................aucucuugugaaauuucguacCa..	5	1	sr1
....................aucucuugugaaauuucguacUa..	7	1	sr1
....................aucucuugugaaauuucguaAaa..	1	1	sr1
....................aucucuugugaaauuucguacaU..	6	1	sr1
....................aucucuugugaaauuucguacaa..	7	0	sr1
....................aucucuugugaaauuucguaUaa..	11	1	sr1
....................aucucuugugaaauuucguacaC..	1	1	sr1
....................aucucuugugaaauuucguacaCc...	1	1	sr1
....................aucucuugugaaauuucguacaaA...	3	1	sr1
.....................ucucuugugaaauuucgC..	4	1	sr1
.....................ucucuugugaaauuucgG..	2	1	sr1
.....................ucucuugugaaauuucgu..	23	0	sr1
.....................ucucuugugaaauuucgA..	3	1	sr1
.....................ucucuugugGaauuucgu..	1	1	sr1
.....................Gcucuugugaaauuucgua...	1	1	sr1
.....................Ccucuugugaaauuucgua...	1	1	sr1
.....................ucucuCgugaaauuucgua...	1	1	sr1
.....................ucucuugugGaauuucgua...	1	1	sr1
.....................ucucuugugaaauuucgua...	108	0	sr1
.....................ucucuugugaaauuucguG...	4	1	sr1
.....................ucucuugugUaauuucgua...	1	1	sr1
.....................Gcucuugugaaauuucguac..	24	1	sr1
.....................ucucuugugaaauuucguac..	378	0	sr1
.....................Ccucuugugaaauuucguac..	4	1	sr1
.....................ucucuugugaaauuucguaA..	11	1	sr1
.....................ucucCugugaaauuucguac..	1	1	sr1
.....................uAucuugugaaauuucguac..	2	1	sr1
.....................ucucuugugaaauGucguac..	1	1	sr1
.....................ucucuCgugaaauuucguac..	1	1	sr1
.....................ucucuugugaaauuucCuac..	1	1	sr1
.....................ucucuugugaaauuucguGc..	2	1	sr1
.....................ucucuugugaGauuucguac..	3	1	sr1
.....................ucucuugugaaauuucguaG..	2	1	sr1
.....................ucucuugugaaauuucguaU..	31	1	sr1
.....................ucucuugugaaGuuucguac..	1	1	sr1
.....................ucucuugugaaauuucgCac..	1	1	sr1
.....................ucucuugugaaauuucguacU...	14	1	sr1
.....................ucucuugugaaauuucguaca...	862	0	sr1
.....................ucucuugCgaaauuucguaca...	6	1	sr1
.....................ucucCugugaaauuucguaca...	3	1	sr1
.....................ucucuugugaaauuucgAaca...	1	1	sr1
.....................ucucuugugaaauuucguaUa...	7	1	sr1
.....................ucucuugAgaaauuucguaca...	1	1	sr1
.....................Gcucuugugaaauuucguaca...	8	1	sr1
.....................Acucuugugaaauuucguaca...	2	1	sr1
.....................ucucuugugaaauCucguaca...	1	1	sr1
.....................Ccucuugugaaauuucguaca...	2	1	sr1
.....................ucucuugugaaauuucguacG...	53	1	sr1
.....................ucucuugugaaauuucguacC...	7	1	sr1
.....................ucucuugugaGauuucguaca...	3	1	sr1
.....................ucucuugugaaUuuucguaca...	3	1	sr1
.....................ucucuugugGaauuucguaca...	6	1	sr1
.....................ucucuugugaaaCuucguaca...	1	1	sr1
.....................ucucuugugaaauuucguUca...	1	1	sr1
.....................ucucuugugUaauuucguaca...	1	1	sr1
.....................ucCcuugugaaauuucguaca...	1	1	sr1
.....................uUcucuugugaaauuucguaca...	1	1	sr1
.....................ucucuugugaaGuuucguaca...	5	1	sr1
.....................ucucuugGgaaauuucguaca...	1	1	sr1
.....................ucucuugugaaauuucguGca...	6	1	sr1
.....................ucucuugugaaauuucguacaG..	2	1	sr1
.....................ucucuugugaaauuucguacaa..	2	0	sr1
.....................ucucuugugaaauuucguacaC..	4	1	sr1
.....................ucucuugugaaauuucguacCa..	4	1	sr1

Mature **Star**

```
gucgucaucacauuccucuuaucucuugugaaauuucguacaacuuagauuguacgacauuuuacaagauauaagaggugccaaaagugggauucgaacccacgcaugcuu
.....................ucucuugugaaauuucguacaU....................................................................	6	1	sr1
.....................ucucuugugaaauuucguacUa....................................................................	7	1	sr1
.....................ucucuugugaaauuucguacaCc...................................................................	1	1	sr1
.....................ucucuugugaaauuucguacaacuuag...............................................................	1	0	sr1
......................cucuugugaaauuucgua.......................................................................	1	0	sr1
......................cucuugugaaauuucguaU......................................................................	1	1	sr1
......................cucuugugaaauuucguac......................................................................	1	0	sr1
......................cucuugugaaauuucguaca.....................................................................	2	0	sr1
......................cucuugugaaauuucguacaG....................................................................	1	1	sr1
.......................ucuugugaaauuucguac......................................................................	6	0	sr1
.......................ucuugugGaauuucguac......................................................................	1	1	sr1
.......................ucuugugaaauuucguaca.....................................................................	6	0	sr1
.......................Gcuugugaaauuucguaca.....................................................................	1	1	sr1
.......................ucuugugaaauuucguaUa.....................................................................	3	1	sr1
.......................ucuugugaaauuucguacaC....................................................................	1	1	sr1
.......................ucuugugaaauuucguacUa....................................................................	1	1	sr1
.......................ucuugugaaauuucguacaUc...................................................................	1	1	sr1
........................cuugugaaauuucguacG.....................................................................	1	1	sr1
....................................................uacgacauuuuacaagauU........................................	1	1	sr1
....................................................uacgacauuuuacaagaua........................................	3	0	sr1
....................................................Gacgacauuuuacaagauau.......................................	1	1	sr1
....................................................uacgacauuuuacaagauau.......................................	19	0	sr1
....................................................uacgacauuuuacaagauaG.......................................	2	1	sr1
....................................................uacgacauuuuacaagauaA.......................................	1	1	sr1
....................................................Gacgacauuuuacaagauaua......................................	7	1	sr1
....................................................uacgacauuuuacaagauaua......................................	85	0	sr1
....................................................uGcgacauuuuacaagauaua......................................	1	1	sr1
....................................................uacgacaCuuuacaagauaua......................................	1	1	sr1
....................................................uacgacauuuuacaagauauaG.....................................	1	1	sr1
....................................................uacgacauuuuacaagauauaU.....................................	4	1	sr1
....................................................uacgacGuuuuacaagauauaa.....................................	3	1	sr1
....................................................Gacgacauuuuacaagauauaa.....................................	2	1	sr1
....................................................uaUgacauuuuacaagauauaa.....................................	1	1	sr1
....................................................uacgacauuuuacaaCauauaa.....................................	1	1	sr1
....................................................uacgacauuuuacaagauauaa.....................................	65	0	sr1
....................................................uacgacGuuuuacaagauauaag....................................	5	1	sr1
....................................................uacgacauuuuacaagauauaag....................................	27	0	sr1
....................................................uacgacauuuuacaagauauaaA....................................	4	1	sr1
....................................................uacgacauuuuacaagauauaGg....................................	1	1	sr1
.....................................................acgacauuuuacaagauaua......................................	1	0	sr1
.....................................................acgacauuuuacaagauauaa.....................................	2	0	sr1
.....................................................acgacauuuuacaagauauaag....................................	1	0	sr1
......................................................cgacauuuuacaagauau.......................................	1	0	sr1
......................................................cgacauuuuacaagauaua......................................	1	0	sr1
...........................................................................aggugccaaaagugggauA.................	1	1	sr1

..................Cuaucucuugugaaauuucguac......................................................................	1	1	st1
..................Cuaucucuugugaaauuucguaca.....................................................................	2	1	st1
...................uaucucuugugaaauuuc..........................................................................	1	0	st1
...................uaucucuugugaaauuucg.........................................................................	11	0	st1
...................uaucucuugugaaauuucA.........................................................................	1	1	st1
...................uaucucuugugaaaAuucgu........................................................................	2	1	st1
...................uaucucuugugCaauuucgu........................................................................	1	1	st1
...................uaucCcuugugaaauuucgu........................................................................	1	1	st1
...................uaucucuugugaGauuucgu........................................................................	1	1	st1
...................uaucucuugugGaauuucgu........................................................................	2	1	st1
...................uaucucuugugaaauGucgu........................................................................	3	1	st1
...................uaucucuugugaaaGuucgu........................................................................	1	1	st1
...................uaucucuugugaaauuucgG........................................................................	4	1	st1
...................uaucucuugugaaauuucgC........................................................................	25	1	st1
...................uaucucuugugaaauuucgA........................................................................	3	1	st1
...................uaucucuugugaaauuucgu........................................................................	148	0	st1
...................uaucucuugugCaauuucgua.......................................................................	1	1	st1
...................uaucucuugugaaauGucgua.......................................................................	3	1	st1
...................uaucucuugugaGauuucgua.......................................................................	6	1	st1
...................Aaucucuugugaaauuucgua.......................................................................	1	1	st1
...................uaucucuugugaaGuuucgua.......................................................................	5	1	st1
...................uaucucCugugaaauuucgua.......................................................................	4	1	st1
...................uaucucuugugaaauCucgua.......................................................................	4	1	st1
...................uaucucuCgugaaauuucgua.......................................................................	4	1	st1
```

```
                   Mature             Star
gucgucaucacauuccucuuaucucuugugaaauuucguacaacuuagauuguacgacauuuuacaagauauaagaggugccaaaagugggauucgaacccacgcaugcuu
...................uaucucuugGgaaauuucgua.......................................................................    1     1    st1
...................uaucucuAgugaaauuucgua.......................................................................    3     1    st1
...................uaucucuugugaaauuucguG.......................................................................    8     1    st1
...................uCucucuugugaaauuucgua.......................................................................    1     1    st1
...................uaucucuugAgaaauuucgua.......................................................................    4     1    st1
...................Caucucuugugaaauuucgua.......................................................................    3     1    st1
...................uaucCcuugugaaauuucgua.......................................................................    2     1    st1
...................uaucucuugugaaUuuucgua.......................................................................    2     1    st1
...................uaucucuugCgaaauuucgua.......................................................................    3     1    st1
...................uaucucuugugaaauuucgua.......................................................................    859   0    st1
...................uaucucuugugaaauuCcgua.......................................................................    2     1    st1
...................uaucucuugugaaaGuucgua.......................................................................    2     1    st1
...................uaucucuuAugaaauuucgua.......................................................................    1     1    st1
...................uaucuUuugugaaauuucgua.......................................................................    1     1    st1
...................uaCcucuugugaaauuucgua.......................................................................    2     1    st1
...................uGucucuugugaaauuucgua.......................................................................    8     1    st1
...................uaucucuugugaaauuucgGa.......................................................................    1     1    st1
...................uaucucuugugaaauuucAua.......................................................................    1     1    st1
...................uaucucuugugGaauuucgua.......................................................................    7     1    st1
...................uaucucuugugaaauuucgCa.......................................................................    2     1    st1
...................uaucucuugugUaauuucgua.......................................................................    2     1    st1
...................uauUucuugugaaauuucgua.......................................................................    2     1    st1
...................Aaucucuugugaaauuucguac......................................................................    2     1    st1
...................uaucucuugugaaauuucguac......................................................................    2157  0    st1
...................uaucAcuugugaaauuucguac......................................................................    3     1    st1
...................uaucucuugugGaauuucguac......................................................................    9     1    st1
...................uaucucuugugaaauuucAuac......................................................................    4     1    st1
...................uaucucuugCgaaauuucguac......................................................................    6     1    st1
...................uGucucuugugaaauuucguac......................................................................    8     1    st1
...................uaGcucuugugaaauuucguac......................................................................    1     1    st1
...................uaucucuugugaaauuucguaA......................................................................    49    1    st1
...................uaucucuugugCaauuucguac......................................................................    4     1    st1
...................uaucucuugugaGauuucguac......................................................................    10    1    st1
...................uaucucuCgugaaauuucguac......................................................................    3     1    st1
...................Gaucucuugugaaauuucguac......................................................................    1     1    st1
...................uaucucuugAgaaauuucguac......................................................................    1     1    st1
...................uaucucuAgugaaauuucguac......................................................................    2     1    st1
...................uaucucuugugaaaGuucguac......................................................................    2     1    st1
...................uaucCcuugugaaauuucguac......................................................................    6     1    st1
...................uaucucCugugaaauuucguac......................................................................    1     1    st1
...................uaucucuugugaaaCuucguac......................................................................    10    1    st1
...................uaucucuugugaaauuucgGac......................................................................    1     1    st1
...................uaucucuugugaaauCucguac......................................................................    7     1    st1
...................uaucucuugugaaauuCcguac......................................................................    8     1    st1
...................uaucucuugugaaauuucguGc......................................................................    2     1    st1
...................uaucucuugugaaauGucguac......................................................................    15    1    st1
...................uaCcucuugugaaauuucguac......................................................................    5     1    st1
...................Caucucuugugaaauuucguac......................................................................    5     1    st1
...................uaucucuugugaaauuucguaU......................................................................    283   1    st1
...................uaucucuugugaaauuucguaG......................................................................    11    1    st1
...................uaucucuugugaaGuuucguac......................................................................    9     1    st1
...................uaAcucuugugaaauuucguac......................................................................    3     1    st1
...................uaucucuuguAaaauuucguac......................................................................    2     1    st1
...................uaucucuugugaaauuucgCac......................................................................    1     1    st1
...................uaucucuugugaUauuucguac......................................................................    2     1    st1
...................uUucucuugugaaauuucguac......................................................................    2     1    st1
...................uaucucuugugaaauuucguUc......................................................................    2     1    st1
...................uaucucuuAugaaauuucguac......................................................................    1     1    st1
...................uGucucuugugaaauuucguaca.....................................................................    13    1    st1
...................uaAcucuugugaaauuucguaca.....................................................................    1     1    st1
...................uaucucuugugaaauuGcguaca.....................................................................    2     1    st1
...................uaucucuugugaaauCucguaca.....................................................................    7     1    st1
...................uaucucGugugaaauuucguaca.....................................................................    1     1    st1
...................uaucucuugCgaaauuucguaca.....................................................................    2     1    st1
...................Caucucuugugaaauuucguaca.....................................................................    4     1    st1
...................uaucucuugugaaauuucguacC.....................................................................    30    1    st1
...................uaucucuugugaaauGucguaca.....................................................................    5     1    st1
...................uaucucuuAugaaauuucguaca.....................................................................    3     1    st1
...................uaucucuugugaaauuucguUca.....................................................................    2     1    st1
...................uaucucuugugaaauuCcguaca.....................................................................    4     1    st1
```

```
                   Mature                Star
gucgucaucacauuccucuuaucucuugugaaauuucguacaacuuagauuguacgacauuuuacaagauauaagaggugccaaaagugggauucgaacccacgcaugcuu
...................uCucucuugugaaauuucguaca.....................................................................  1     1  st1
...................uaucucuugugaGauuucguaca.....................................................................  8     1  st1
...................uaucucuugugaaCuuucguaca.....................................................................  1     1  st1
...................uaucucuugugaaauuucguaUa.....................................................................  33    1  st1
...................uaucucuugugGaauuucguaca.....................................................................  10    1  st1
...................uaCcucuugugaaauuucguaca.....................................................................  5     1  st1
...................uaucucuugugaaauuucAuaca.....................................................................  4     1  st1
...................uaucucuugugaaauuucgCaca.....................................................................  3     1  st1
...................uaucucAugugaaauuucguaca.....................................................................  1     1  st1
...................uaucucuugugaaauuucguGca.....................................................................  2     1  st1
...................uaucucuAgugaaauuucguaca.....................................................................  2     1  st1
...................Gaucucuugugaaauuucguaca.....................................................................  1     1  st1
...................uaucucuugugaaauuucguacG.....................................................................  48    1  st1
...................uaucucuugugaaaCuucguaca.....................................................................  6     1  st1
...................uaucucuugugaaauuucguaca.....................................................................  1872  0  st1
...................uaucucuugugCaauuucguaca.....................................................................  6     1  st1
...................uaucuAuugugaaauuucguaca.....................................................................  1     1  st1
...................uaucucuugugaaGuuucguaca.....................................................................  4     1  st1
...................uaucCcuugugaaauuucguaca.....................................................................  5     1  st1
...................uUucucuugugaaauuucguaca.....................................................................  1     1  st1
...................uaucucuugugaaaGuucguaca.....................................................................  5     1  st1
...................uaucucuCgugaaauuucguaca.....................................................................  4     1  st1
...................uaucucuugugaaauuucguacU.....................................................................  96    1  st1
...................uaucucuugugaaauuucguaAa.....................................................................  3     1  st1
...................uaucucCugugaaauuucguaca.....................................................................  6     1  st1
...................uaucucuugugaaauuucCuaca.....................................................................  1     1  st1
...................uaucucuugugaaauuucguacUa....................................................................  9     1  st1
...................uaucucuugugaaauuucguacCa....................................................................  1     1  st1
...................uaucucuugugaaauuucguacaU....................................................................  14    1  st1
...................uaucucuugugaaauuucguacaa....................................................................  4     0  st1
...................uaucucuugugaaauuucguaUaa....................................................................  1     1  st1
...................uaucucuugugaaauuucguacaaA...................................................................  4     1  st1
....................aucucuugugaaauuucgC........................................................................  4     1  st1
....................aucucuugugaaauuucgu........................................................................  36    0  st1
....................aucucuugugaaauGucgu........................................................................  1     1  st1
....................aucucuugugaaauuucgG........................................................................  1     1  st1
....................aucucuugugaCauuucgu........................................................................  1     1  st1
....................aucucuugugaCauuucgua.......................................................................  1     1  st1
....................aucucuugugaaauuucgCa.......................................................................  2     1  st1
....................aucCcuugugaaauuucgua.......................................................................  1     1  st1
....................aucucuugugaaGuuucgua.......................................................................  1     1  st1
....................aucucuugugaaauuCcgua.......................................................................  1     1  st1
....................aucucuugugaaauuucgua.......................................................................  89    0  st1
....................aucucuugugaaauuAcguac......................................................................  1     1  st1
....................aucucuugugGaauuucguac......................................................................  2     1  st1
....................aCcucuugugaaauuucguac......................................................................  3     1  st1
....................aucucuugugaaGuuucguac......................................................................  4     1  st1
....................aucGcuugugaaauuucguac......................................................................  1     1  st1
....................aucucuugugaaauuucguUc......................................................................  1     1  st1
....................aucucuugugaGauuucguac......................................................................  3     1  st1
....................aucucuugugaaauuucguaA......................................................................  7     1  st1
....................aucucuugugaaauCucguac......................................................................  1     1  st1
....................Uucucuugugaaauuucguac......................................................................  1     1  st1
....................aucucuugugaaaCuucguac......................................................................  2     1  st1
....................aucucuugugaaauuucAuac......................................................................  1     1  st1
....................aucucuugugaUauuucguac......................................................................  1     1  st1
....................Gucucuugugaaauuucguac......................................................................  2     1  st1
....................aucucuCgugaaauuucguac......................................................................  2     1  st1
....................aucucuugugaCauuucguac......................................................................  7     1  st1
....................aucucuugugaaauuucguGc......................................................................  3     1  st1
....................aucCcuugugaaauuucguac......................................................................  2     1  st1
....................aucucuugugaaauuucguaU......................................................................  87    1  st1
....................aucucuugugaaauuCcguac......................................................................  4     1  st1
....................aucucuugugaaauuucguac......................................................................  642   0  st1
....................aucucuugugaaauuucguGca.....................................................................  1     1  st1
....................aucucuugugaCauuucguaca.....................................................................  1     1  st1
....................aucucuugugaaauuucguacG.....................................................................  3     1  st1
....................aucucuugugaaauuCcguaca.....................................................................  1     1  st1
....................aucucuugugaaauuAcguaca.....................................................................  1     1  st1
....................aucCcuugugaaauuucguaca.....................................................................  1     1  st1
```

```
                   Mature                         Star
gucgucaucacauuccucuuaucucuugugaaauuucguacaacuuagauuguacgacauuuuacaagauauaagaggugccaaaagugggauucgaacccacgcaugcuu
....................aucucuugugaaauuucguaAa.....................................................................	1	1	st1
....................aucucuugCgaaauuucguaca.....................................................................	1	1	st1
....................aucucuugugaaauuucguaca.....................................................................	177	0	st1
....................aucucuugugaaauuucAuaca.....................................................................	1	1	st1
....................aucucuugugaaauuucguacC.....................................................................	1	1	st1
....................aucucuugugaaauuucguaUa.....................................................................	21	1	st1
....................aucucuugugaGauuucguaca.....................................................................	2	1	st1
....................aucucuugugaaauuucguacU.....................................................................	23	1	st1
....................aucucuugugaaauuucguacUa....................................................................	3	1	st1
....................aucucuugugaaauuucguacaa....................................................................	1	0	st1
.....................ucucuugugaaauuucgG........................................................................	1	1	st1
.....................ucucuugugaaauuucgC........................................................................	1	1	st1
.....................ucucuugugaaauuucgu........................................................................	7	0	st1
.....................ucucuugugaaauuucgua.......................................................................	13	0	st1
.....................ucucuCgugaaauuucguac......................................................................	1	1	st1
.....................ucucuugugaaGuuucguac......................................................................	1	1	st1
.....................uUucuugugaaauuucguac......................................................................	1	1	st1
.....................ucucuAgugaaauuucguac......................................................................	1	1	st1
.....................ucucuugugaaauuucguaA......................................................................	1	1	st1
.....................ucucuugugaaauuucguaU......................................................................	9	1	st1
.....................ucucuugugGaauuucguac......................................................................	2	1	st1
.....................ucucuugugaGauuucguac......................................................................	1	1	st1
.....................ucucuugugaaauuucUuac......................................................................	1	1	st1
.....................ucucuugugaaauuucguac......................................................................	84	0	st1
.....................ucucuugugaaauuucguacU.....................................................................	2	1	st1
.....................ucucuugugaaauuucguacC.....................................................................	5	1	st1
.....................ucucuugugaaauuucguaca.....................................................................	267	0	st1
.....................ucucuugugaaCuuucguaca.....................................................................	1	1	st1
.....................Ccucuugugaaauuucguaca.....................................................................	2	1	st1
.....................ucCcuugugaaauuucguaca.....................................................................	1	1	st1
.....................ucucuugugaaGuuucguaca.....................................................................	3	1	st1
.....................ucucuugugaaauuucguacG.....................................................................	3	1	st1
.....................ucucuugugaaauGucguaca.....................................................................	4	1	st1
.....................ucucuugugGaauuucguaca.....................................................................	1	1	st1
.....................ucucuugugaaauuucgCaca.....................................................................	1	1	st1
.....................ucucuugugaaauuucguaUa.....................................................................	2	1	st1
.....................ucucuugugaaauuGcguaca.....................................................................	1	1	st1
.....................ucucuuAugaaauuucguaca.....................................................................	1	1	st1
.....................ucucuugugaaauuucguacaa....................................................................	1	0	st1
.....................ucucuugugaaauuucguacaU....................................................................	6	1	st1
.....................ucucuugugaaauuucguacCa....................................................................	2	1	st1
.....................ucucuugugaaauuucguacaC....................................................................	2	1	st1
.....................ucucuugugaaauuucguacaaA...................................................................	2	1	st1
.....................ucucuugugaaauuucguacaCc...................................................................	2	1	st1
......................cCcuugugaaauuucguac......................................................................	1	1	st1
......................cucuugugaaauuucguaca.....................................................................	1	0	st1
.......................ucuugugaaauuucguac......................................................................	1	0	st1
.......................ucuugugaaauuucguaca.....................................................................	2	0	st1
........................cuugugaaauuucguGca.....................................................................	1	1	st1
....................................................uacgacauuuuacaagaua........................................	2	0	st1
....................................................uacgacauuuuacaagauau.......................................	7	0	st1
....................................................uacgacauuuuacaagauaC.......................................	2	1	st1
....................................................uacgacauuuuacaagauaua......................................	9	0	st1
....................................................uacgacauuuuacaagauauaa.....................................	8	0	st1
....................................................uacgacaCuuuacaagauauaa.....................................	1	1	st1
....................................................uacgacauuuuacaagauauaU.....................................	1	1	st1
....................................................uacgacauuuuacaagauauUag....................................	1	1	st1
....................................................uacgacauuuuacaagauauaag....................................	9	0	st1
....................................................uacgacauuuuacaagauauaaga...................................	1	0	st1
.....................................................acgacauuuuacaagauauaa.....................................	1	0	st1
.....................................................acgacauuuuacaagauauaag....................................	1	0	st1
```

Provisional ID	: ScYm1zk_361_3661
Score total	: 17301.6
Score for star read(s)	: 3.9
Score for read counts	: 17292.4
Score for mfe	: 0.7
Score for randfold	: 1.6
Score for cons. seed	: 3
Total read count	: 33930
Mature read count	: 19411
Loop read count	: 0
Star read count	: 14519

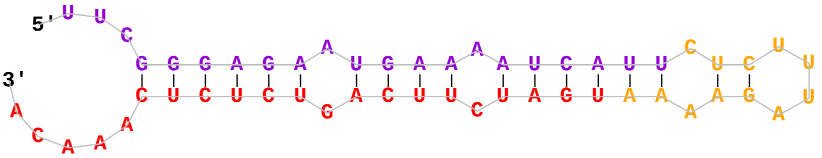

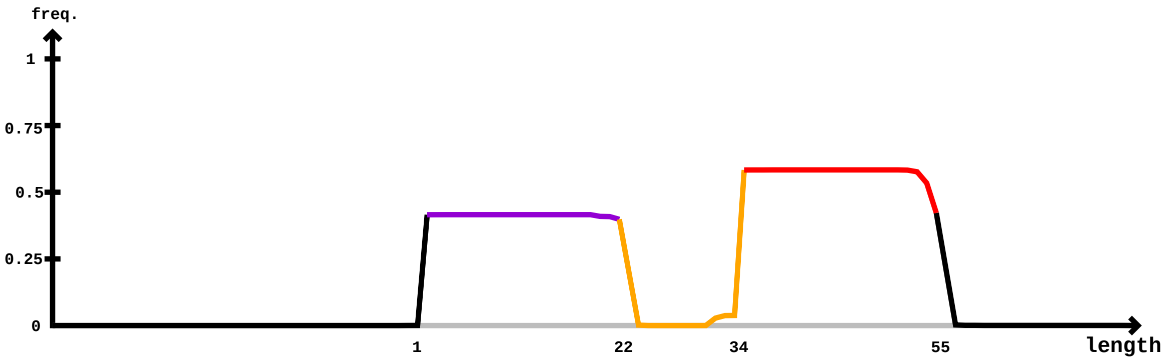

Star Mature

5'- cgcacagaagacgaauaucgcaacaccauccuaccuguucgggagaaugaaaaucauucucuuuagaaaaugaucuucagucucucaaacaaguagcaacacugaucgucg -3'	obs		
cgcacagaagacgaauaucgcaacaccauccuaccuguucgggagaaugaaaaucauucucuuuagaaaaugaucuucagucucucaaacaaguagcaacacugaucgucg	exp		
.........(((((................((((.((((.((((((.((((.((((((.((....)).)))))).)))).)))))).)))).))))((....)).))))).	reads	mm	sample
....................................Cuucgggagaaugaaaaucauuc..	2	1	so1
.....................................uucgggagaaugaaaauc..	4	0	so1
.....................................uucgggagaaugaaaauca...	5	0	so1
.....................................uucgggagaaugaaaaucaC..	10	1	so1
.....................................uucgggagaaugaaaaucaA..	1	1	so1
.....................................uucgggagaaugaaaaucau..	32	0	so1
.....................................uucgggagaaugaaaaAcauu...	1	1	so1
.....................................uucgggagaaugGaaaucauu...	6	1	so1
.....................................uucgggagaaugaaGaucauu...	10	1	so1
.....................................uucgggUgaaugaaaaucauu...	1	1	so1
.....................................uucgggagaGugaaaaucauu...	6	1	so1
.....................................uucgggagaaugaaaaucaCu...	1	1	so1
.....................................uucgggagaaugaaaaucaGu...	2	1	so1
.....................................Cucgggagaaugaaaaucauu...	1	1	so1
.....................................uucgggagaaugaGaaucauu...	1	1	so1
.....................................uucgggagaaugaaaaucauu...	1027	0	so1
.....................................uucgggagaaugaaaaucauG...	3	1	so1
.....................................uucgggGgaaugaaaaucauu...	5	1	so1
.....................................Aucgggagaaugaaaaucauu...	2	1	so1
.....................................uucgggagaaugaaaaucauA...	13	1	so1
.....................................uucgggagaaugaaaauGauu...	1	1	so1
.....................................uucgggagaaugaaCaucauu...	2	1	so1
.....................................uucgUgagaaugaaaaucauu...	1	1	so1
.....................................uucgggagaaugaaaaucUuu...	2	1	so1
.....................................uucgggagGaugaaaaucauu...	5	1	so1
.....................................uucgAgagaaugaaaaucauu...	2	1	so1
.....................................uucgggagaaugaaaGucauu...	2	1	so1
.....................................uucgggagaaugaaaaucauC...	117	1	so1
.....................................uucAggagaaugaaaaucauu...	1	1	so1
.....................................uucgggagaaugaaaaCcauu...	1	1	so1
.....................................uucgggagaaugaaaauAauu...	1	1	so1
.....................................uCcgggagaaugaaaaucauu...	3	1	so1
.....................................uucgggagaaCgaaaaucauu...	1	1	so1

```
                                     Star                          Mature
cgcacagaagacgaauaucgcaacaccauccuaccuguucgggagaaugaaaaucauucucuuuagaaaaugaucuucagucucucaaacaaguagcaacacugaucgucg
.....................................uucgggagaaugaaaaucauAc....................................................    1     1    so1
.....................................uucgggagaaugaaaaucauuU....................................................    21    1    so1
.....................................uucgggagaaugaaaaucGuuc....................................................    1     1    so1
.....................................uucgggagaaugaaaaucauuA....................................................    515   1    so1
.....................................uucgggagaaugaaGaucauuc....................................................    1     1    so1
.....................................uucggUagaaugaaaaucauuc....................................................    1     1    so1
.....................................uucgggagaaugGaaaucauuc....................................................    1     1    so1
.....................................uucgggagaaugaaaaucauuc....................................................    73    0    so1
.....................................uucgggagaaugaaaaucauuG....................................................    2     1    so1
.....................................uucgggagaaugaaaaucauuUu...................................................    1     1    so1
.....................................uucgggagaaugaaaaucauucA...................................................    2     1    so1
.....................................uucgggagaaugaaaaucauucu...................................................    2     0    so1
.....................................uucgggagaaugaaaaucauucuUu.................................................    1     1    so1
...................................................................aaaugaucuucagucucuca........................    3     0    so1
...................................................................aaaugaucuucagucucucaG.......................    2     1    so1
...................................................................aaaugaucuucagucucucaa.......................    20    0    so1
...................................................................aaaugaucuCcagucucucaaa......................    2     1    so1
...................................................................aaaugaucuucagucucucaaa......................    58    0    so1
...................................................................aaaugaucCucagucucucaaa......................    1     1    so1
...................................................................aaaugaucuucagAcucucaaa......................    1     1    so1
...................................................................aaaugaucuucagucucucaaU......................    7     1    so1
...................................................................aaaugaucuucagucucucaaaA.....................    2     1    so1
...................................................................aaaugaucuucagucucucaaac.....................    3     0    so1
...................................................................aaaugaucuucagucucucaaaca....................    3     0    so1
...................................................................aaaugaucuucagucucucaaaUa....................    2     1    so1
...................................................................aaaugaucuucagucucucaaacUa...................    1     1    so1
...................................................................aaaugaucuucagucucucaaaUaa...................    2     1    so1
....................................................................aaugaucuucagucucuc.........................    1     0    so1
....................................................................aaugaucuucagucucucaa.......................    1     0    so1
....................................................................aaugaucuucagucucucaaa......................    7     0    so1
....................................................................aaugaucuucagucucucaGa......................    1     1    so1
....................................................................aaugaucuucagucucucaaaU.....................    1     1    so1
....................................................................aaugaucuucagucucucaaaca....................    8     0    so1
....................................................................aaugaucuucagucucucaaacaa...................    1     0    so1
.....................................................................augaucuucagucucucaaaca....................    1     0    so1
.....................................................................Cugaucuucagucucucaaaca....................    2     1    so1
......................................................................ugaucuucagucucucaaa......................    8     0    so1
......................................................................ugaucuucagucucucaaU......................    2     1    so1
......................................................................ugaucuucagucucucaaaU.....................    16    1    so1
......................................................................ugaucuucagucucucaaac.....................    124   0    so1
......................................................................ugaucuucagucCcucaaac.....................    1     1    so1
......................................................................ugaucuucagucucucaaaA.....................    1     1    so1
......................................................................ugaucAucagucucucaaaca....................    1     1    so1
......................................................................ugaucuucagucucucaaacC....................    1     1    so1
......................................................................ugaucuucagucucCcaaaca....................    1     1    so1
......................................................................ugaucuucagucucucGaaca....................    4     1    so1
......................................................................ugaucuucagucucucaaaca....................    577   0    so1
......................................................................ugaucuucagAcucucaaaca....................    1     1    so1
......................................................................ugaCcuucagucucucaaaca....................    1     1    so1
......................................................................ugaucuucagucCcucaaaca....................    1     1    so1
......................................................................uAaucuucagucucucaaaca....................    1     1    so1
......................................................................ugaucuucagucucucaaacG....................    12    1    so1
......................................................................ugaucCucagucucucaaaca....................    5     1    so1
......................................................................ugaucuucGgucucucaaaca....................    3     1    so1
......................................................................ugaucuucagucucucaaacU....................    5     1    so1
......................................................................ugaucuucagucucucaaaUa....................    2     1    so1
......................................................................ugaucuucagCcucucaaaca....................    1     1    so1
......................................................................ugaucGucagucucucaaaca....................    1     1    so1
......................................................................ugaucuCcagucucucaaaca....................    2     1    so1
......................................................................ugaucuucagucucucaaacaU...................    20    1    so1
......................................................................ugaucuucagucucucaaacaa...................    33    0    so1
......................................................................ugaucuucagucucucaaacUa...................    3     1    so1
......................................................................ugaucuucagucucucaaacaC...................    1     1    so1
......................................................................ugaucuucagucucucaaacaaA..................    2     1    so1
......................................................................ugaucuucagucucucaaacaagua................    1     0    so1
...........................................................................................aguagcaacacugaucgucA    1     1    so1

.....................................uucgggagaaugaaaauc........................................................    5     0    sg1
.....................................uucgggagaaugaaaauca.......................................................    2     0    sg1
.....................................uucgggagaaugaaaaucau......................................................    17    0    sg1
```

```
                                     Star                             Mature
cgcacagaagacgaauaucgcaacaccauccuaccuguucgggagaaugaaaaucauucucuuuagaaaaugaucuucagucucucaaacaaguagcaacacugaucgucg
.....................................uucgggagaaugaaaaucaCu.....................................................   3     1   sg1
.....................................uucgggagaaugaaaaucauC.....................................................   21    1   sg1
.....................................uucgggagaaugaaaUucauu.....................................................   1     1   sg1
.....................................uucgggagGaugaaaaucauu.....................................................   1     1   sg1
.....................................uucgggagaaugaaaGucauu.....................................................   2     1   sg1
.....................................uucgggagaaCgaaaaucauu.....................................................   2     1   sg1
.....................................uucgggagaaugaGaaucauu.....................................................   2     1   sg1
.....................................uucgggagaaugaaaaucaAu.....................................................   1     1   sg1
.....................................uucgggagaaugaaaaucUuu.....................................................   1     1   sg1
.....................................uucgggagaaugGaaaucauu.....................................................   2     1   sg1
.....................................uucgggagaGugaaaaucauu.....................................................   3     1   sg1
.....................................uucgggagaaugaaaaucauu.....................................................   427   0   sg1
.....................................uucgggGgaaugaaaaucauu.....................................................   3     1   sg1
.....................................uucAggagaaugaaaaucauu.....................................................   4     1   sg1
.....................................uucgggagaaugaaaaCcauu.....................................................   1     1   sg1
.....................................uucgggagaaugaaGaucauu.....................................................   3     1   sg1
.....................................uucgggagaaugaaaaucauA.....................................................   3     1   sg1
.....................................uucgggagaaugaaaaucauG.....................................................   2     1   sg1
.....................................uucgggagaaugaaaaucauuU....................................................   8     1   sg1
.....................................uucgggagaaugaaaaucauuc....................................................   23    0   sg1
.....................................uucgggagaaCgaaaaucauuc....................................................   1     1   sg1
.....................................uucgggagaaugaaaaucauuA....................................................   237   1   sg1
.....................................uucgggagaaugaaaaucauuG....................................................   3     1   sg1
.....................................uuUgggagaaugaaaaucauuc....................................................   2     1   sg1
.....................................uucgggagaaugaaaaucauucA...................................................   1     1   sg1
.....................................uucgggagaaugaaaaucauuUu...................................................   1     1   sg1
.....................................uucgggagaaugaaaaucauucu...................................................   2     0   sg1
.....................................uucgggagaaugaaaaucauucuA..................................................   3     1   sg1
...............................................................uagaaaaugaucuucagucucucaaac.....................   1     0   sg1
...................................................................aaaugaucuucagucucucaa.......................   9     0   sg1
...................................................................aaaugaucuucagCcucucaaa......................   1     1   sg1
...................................................................aaaugaucuucagucucucaaU......................   5     1   sg1
...................................................................aaaugaucuucagucucucaaa......................   43    0   sg1
...................................................................aGaugaucuucagucucucaaa......................   1     1   sg1
...................................................................aaaugaucuCcagucucucaaa......................   1     1   sg1
...................................................................aaaugaucuucagucucucGaac.....................   1     1   sg1
...................................................................aaaugaucuucagucucucaaaA.....................   3     1   sg1
...................................................................aaaugaucuucagucucucaaac.....................   5     0   sg1
...................................................................aaaugaucuucagucucucaaaUa....................   1     1   sg1
...................................................................aaaugaucuucagucucucaaaca....................   1     0   sg1
...................................................................aaaugaucuucagucucucaaaUaa...................   1     1   sg1
...................................................................aaaugaucuucagucucucaaacUa...................   1     1   sg1
....................................................................aaugaucuucagucucucaaa......................   7     0   sg1
....................................................................aaugaucuucagCcucucaaa......................   3     1   sg1
....................................................................aaugaucuucagucucucaaaca....................   6     0   sg1
....................................................................aaugaucuucagucucucaaacaa...................   2     0   sg1
.....................................................................augaucuucagucucucaa.......................   1     0   sg1
......................................................................ugaucuucagucucucaa.......................   1     0   sg1
......................................................................ugaucuucagucucucaaU......................   3     1   sg1
......................................................................ugaucCucagucucucaaa......................   1     1   sg1
......................................................................ugaucuucagucucucaaa......................   15    0   sg1
......................................................................ugaucuucagucucucaaac.....................   178   0   sg1
......................................................................ugaucuCcagucucucaaac.....................   1     1   sg1
......................................................................ugaucCucagucucucaaac.....................   1     1   sg1
......................................................................ugaucuucGgucucucaaac.....................   1     1   sg1
......................................................................ugaucuucagucucucaaaU.....................   4     1   sg1
......................................................................ugaucuucagucucucaaaA.....................   3     1   sg1
......................................................................ugaucuucagucucCcaaac.....................   1     1   sg1
......................................................................ugaucuucagucucucUaaca....................   2     1   sg1
......................................................................ugaucuuUagucucucaaaca....................   1     1   sg1
......................................................................ugaucCucagucucucaaaca....................   7     1   sg1
......................................................................ugaucuucagucucucaaacU....................   4     1   sg1
......................................................................ugaucuucagCcucucaaaca....................   5     1   sg1
......................................................................ugaucuucagucucucGaaca....................   7     1   sg1
......................................................................ugaucuucagucucucaaaca....................   786   0   sg1
......................................................................ugaCcuucagucucucaaaca....................   2     1   sg1
......................................................................ugaucuucagucucucaGaca....................   5     1   sg1
......................................................................ugUucuucagucucucaaaca....................   2     1   sg1
......................................................................ugaucuuAagucucucaaaca....................   1     1   sg1
......................................................................ugaucuCcagucucucaaaca....................   2     1   sg1
```

```
                                     Star                            Mature
cgcacagaagacgaauaucgcaacaccauccuaccuguucgggagaaugaaaaucauucucuuuagaaaaugaucuucagucucucaaacaaguagcaacacugaucgucg
......................................................................ugaucuucagucucucaaacG....................   8     1    sg1
......................................................................ugaucuucagucCcucaaaca....................   4     1    sg1
......................................................................ugaucuucagucucucaaaUa....................   2     1    sg1
......................................................................ugauUuucagucucucaaaca....................   2     1    sg1
......................................................................Agaucuucagucucucaaaca....................   1     1    sg1
......................................................................ugaAcuucagucucucaaaca....................   1     1    sg1
......................................................................ugaucuucagucucCcaaaca....................   1     1    sg1
......................................................................ugGucuucagucucucaaaca....................   6     1    sg1
......................................................................ugaucuucagucucucaaGca....................   2     1    sg1
......................................................................ugaucuucagucucucaaCca....................   2     1    sg1
......................................................................ugaucuucagucucucaaacaU...................   13    1    sg1
......................................................................ugaucuucagucucucaGacaa...................   1     1    sg1
......................................................................ugaucuucGgucucucaaacaa...................   1     1    sg1
......................................................................ugaucuucagucucucaaacaG...................   2     1    sg1
......................................................................ugaucCucagucucucaaacaa...................   2     1    sg1
......................................................................ugaucuucagucucucaaacUa...................   10    1    sg1
......................................................................ugaucuucagucucucaaacaa...................   73    0    sg1
......................................................................ugaucuucagucucucaaacaaA..................   2     1    sg1
......................................................................ugaucuucagucucucaaacaUg..................   1     1    sg1
......................................................................ugaucuucagucucucaaacaaU..................   1     1    sg1

...................................uAuucgggagaaugaaaauc........................................................   8     1    sr1
....................................Cuucgggagaaugaaaaucauu.....................................................   3     1    sr1
.....................................Cucgggagaaugaaaauc........................................................   2     1    sr1
.....................................uucgggagaaugaaaauc........................................................   130   0    sr1
.....................................uucgggagaaugaGaauc........................................................   1     1    sr1
.....................................uucgggagaaugaaaauG........................................................   1     1    sr1
.....................................Gucgggagaaugaaaauc........................................................   9     1    sr1
.....................................uucgggagaGugaaaauc........................................................   1     1    sr1
.....................................uucgAgagaaugaaaauc........................................................   2     1    sr1
.....................................uucgggagGaugaaaauc........................................................   3     1    sr1
.....................................uuUgggagaaugaaaauc........................................................   1     1    sr1
.....................................uucgggagaaugaaGauc........................................................   1     1    sr1
.....................................uucgggagaaugaaaauA........................................................   5     1    sr1
.....................................uucgggagaaugaaaauca.......................................................   18    0    sr1
.....................................Gucgggagaaugaaaauca.......................................................   1     1    sr1
.....................................uucgggGgaaugaaaaucau......................................................   4     1    sr1
.....................................uucgggagaaugaaaauAau......................................................   1     1    sr1
.....................................uucgUgagaaugaaaaucau......................................................   1     1    sr1
.....................................uucgggagaaugaaaaucaA......................................................   4     1    sr1
.....................................uucgggagaaugaaaauUau......................................................   2     1    sr1
.....................................uucgggagaaugUaaaucau......................................................   1     1    sr1
.....................................uucgggagaaugaaaaucaC......................................................   27    1    sr1
.....................................uucAggagaaugaaaaucau......................................................   1     1    sr1
.....................................Gucgggagaaugaaaaucau......................................................   7     1    sr1
.....................................uucgggagaaugGaaaucau......................................................   1     1    sr1
.....................................uucgggagaaugaaaaucGu......................................................   3     1    sr1
.....................................uucgggagaaugaaaaucaG......................................................   9     1    sr1
.....................................uucgggagaaugaaaaucau......................................................   161   0    sr1
.....................................uucgggagaaugUaaaucauu.....................................................   1     1    sr1
.....................................Aucgggagaaugaaaaucauu.....................................................   3     1    sr1
.....................................uucgggagaaugaaCaucauu.....................................................   2     1    sr1
.....................................uucgggagaaugaaaaAcauu.....................................................   2     1    sr1
.....................................uucgggagaaugaaaauUauu.....................................................   2     1    sr1
.....................................uCcgggagaaugaaaaucauu.....................................................   15    1    sr1
.....................................Cucgggagaaugaaaaucauu.....................................................   18    1    sr1
.....................................uucgggagaaugaaaaucaCu.....................................................   4     1    sr1
.....................................uucgggagaaugCaaaucauu.....................................................   1     1    sr1
.....................................uucgggagGaugaaaaucauu.....................................................   18    1    sr1
.....................................uucgggUgaaugaaaaucauu.....................................................   2     1    sr1
.....................................uucgggagUaugaaaaucauu.....................................................   3     1    sr1
.....................................uucAggagaaugaaaaucauu.....................................................   4     1    sr1
.....................................uucgggagaaugaaaaucaAu.....................................................   2     1    sr1
.....................................uucgggagaaugaaaGucauu.....................................................   7     1    sr1
.....................................uucgggagaaugaaaaucaGu.....................................................   4     1    sr1
.....................................uucgggagaaugaaaaCcauu.....................................................   10    1    sr1
.....................................uucgggagaaugGaaaucauu.....................................................   21    1    sr1
.....................................uucgggagaaugaaGaucauu.....................................................   33    1    sr1
.....................................uucgggGgaaugaaaaucauu.....................................................   10    1    sr1
.....................................uucgggagaaugaGaaucauu.....................................................   15    1    sr1
```

```
                                     Star                            Mature
cgcacagaagacgaauaucgcaacaccauccuaccuguucgggagaaugaaaaucauucucuuuagaaaaugaucuucagucucucaaacaaguagcaacacugaucgucg
.....................................uucgggagaaugaaaaucUuu.....................................................   1     1   sr1
.....................................uucgggagaGugaaaaucauu.....................................................   15    1   sr1
.....................................uAcgggagaaugaaaaucauu.....................................................   3     1   sr1
.....................................uucgggagaaugaaaaucauu.....................................................   4188  0   sr1
.....................................uucgggagaaAgaaaaucauu.....................................................   2     1   sr1
.....................................uucgggagaaugaaaaucGuu.....................................................   12    1   sr1
.....................................Gucgggagaaugaaaaucauu.....................................................   257   1   sr1
.....................................uucgggagaaugaaaaucauC.....................................................   379   1   sr1
.....................................uucgggagaauAaaaaucauu.....................................................   2     1   sr1
.....................................uucgggagaaugaaaaucauA.....................................................   52    1   sr1
.....................................uucggAagaaugaaaaucauu.....................................................   1     1   sr1
.....................................uucgAgagaaugaaaaucauu.....................................................   2     1   sr1
.....................................uucgggagaaugaaaaGcauu.....................................................   1     1   sr1
.....................................uucgggagaaugaUaaucauu.....................................................   3     1   sr1
.....................................uucgggagaaugaCaaucauu.....................................................   1     1   sr1
.....................................uucgggagaaugaaaauAauu.....................................................   1     1   sr1
.....................................uucgggagaaugaaaUucauu.....................................................   1     1   sr1
.....................................uucgggagaCugaaaaucauu.....................................................   1     1   sr1
.....................................uucgggagaaugaaUaucauu.....................................................   3     1   sr1
.....................................uucgggagaaCgaaaaucauu.....................................................   4     1   sr1
.....................................uucgggagaaugaaaaucauG.....................................................   63    1   sr1
.....................................uuUgggagaaugaaaaucauu.....................................................   4     1   sr1
.....................................uucgggagaaugaaaaucauuA....................................................   1762  1   sr1
.....................................uuAgggagaaugaaaaucauuc....................................................   1     1   sr1
.....................................uucgggagaaugaUaaucauuc....................................................   1     1   sr1
.....................................uucgggagaGugaaaaucauuc....................................................   1     1   sr1
.....................................uucgggagaaugaaaaucauuc....................................................   260   0   sr1
.....................................uucgggGgaaugaaaaucauuc....................................................   1     1   sr1
.....................................uucgggagaaugaaaaucauuU....................................................   52    1   sr1
.....................................uucgggagaaugaaaaucauuG....................................................   34    1   sr1
.....................................uucgggagaaugGaaaucauuc....................................................   3     1   sr1
.....................................uucgggagaaugaaaaCcauuc....................................................   2     1   sr1
.....................................uucgggagaaugaaGaucauuc....................................................   12    1   sr1
.....................................uucgggagaaugaaaaucaCuc....................................................   3     1   sr1
.....................................Cucgggagaaugaaaaucauuc....................................................   2     1   sr1
.....................................Gucgggagaaugaaaaucauuc....................................................   14    1   sr1
.....................................uucgggagaaugaaaaucGuuc....................................................   2     1   sr1
.....................................uucgggagaaugaaaGucauuc....................................................   1     1   sr1
.....................................uucgggagaaugaaaaucauucA...................................................   4     1   sr1
.....................................uucgggagaaugaaaaucauucu...................................................   7     0   sr1
.....................................uucgggagaaugaaaaucauuUu...................................................   10    1   sr1
.....................................uucgggagaaugaaaaucauucG...................................................   1     1   sr1
.....................................uucgggagaaugaaaaucauuAu...................................................   5     1   sr1
.....................................uucgggagaaugaaaaucauucuA..................................................   1     1   sr1
......................................ucgggagaaugaaaaucauu.....................................................   1     0   sr1
.......................................cgggagaaugaaaaucauuc....................................................   1     0   sr1
..................................................................aaaaugaucuucagucucucaaaca....................   1     0   sr1
...................................................................aaaugaucuucagucucuca........................   15    0   sr1
...................................................................Gaaugaucuucagucucucaa.......................   1     1   sr1
...................................................................aaaugaucuucagucucucaa.......................   77    0   sr1
...................................................................aaaugGucuucagucucucaa.......................   1     1   sr1
...................................................................aaaugaucuucagucucCcaa.......................   1     1   sr1
...................................................................aaaugaucuucaguUucucaa.......................   1     1   sr1
...................................................................aaaAgaucuucagucucucaa.......................   1     1   sr1
...................................................................aaaugaucuucagucucucaG.......................   2     1   sr1
...................................................................aaaugaucuucagucucucaaa......................   362   0   sr1
...................................................................aaaugaucuucagucucucaaC......................   3     1   sr1
...................................................................aaaugaucuucagucucucaaU......................   30    1   sr1
...................................................................aaaugaAcuucagucucucaaa......................   1     1   sr1
...................................................................aaGugaucuucagucucucaaa......................   1     1   sr1
...................................................................aaaugaucuucaAucucucaaa......................   2     1   sr1
...................................................................Uaaugaucuucagucucucaaa......................   3     1   sr1
...................................................................aaaugaucuucagucucucaaG......................   14    1   sr1
...................................................................aaaugaucCucagucucucaaa......................   2     1   sr1
...................................................................aaaugaucuucagucucucaUa......................   1     1   sr1
...................................................................Gaaugaucuucagucucucaaa......................   18    1   sr1
...................................................................aaauCaucuucagucucucaaa......................   1     1   sr1
...................................................................aaaugaucuCcagucucucaaa......................   5     1   sr1
...................................................................aaaugaucuucagucucucaGa......................   1     1   sr1
...................................................................aaaugaucuucagucCcucaaa......................   1     1   sr1
```

Star **Mature**

cgcacagaagacgaauaucgcaacaccauccuaccuguucgggagaaugaaaaucauucucuuuagaaaaugaucuucagucucucaaacaaguagcaacacugaucgucg

....aaaCgaucuucagucucucaaa......................	1	1	sr1
....aaaugGucuucagucucucaaa......................	3	1	sr1
....aaaugaucuucagucucucaaaG.....................	1	1	sr1
....aaaugaucuucagCcucucaaac.....................	1	1	sr1
....aaaugaucuucagucucucaaac.....................	25	0	sr1
....aaaugaucuucagucucucaaaA.....................	34	1	sr1
....aaaugaucuucagucucucaaUc.....................	2	1	sr1
....aaaugaucuucagucucucaaaU.....................	15	1	sr1
....Gaaugaucuucagucucucaaaca....................	1	1	sr1
....aaaugaucuucagCcucucaaaca....................	1	1	sr1
....aaaugaucuucagucucucaaaUa....................	8	1	sr1
....aaaugaucuucagucucucaaaca....................	14	0	sr1
....aaaugaucuucagucucucaaacG....................	3	1	sr1
....aaaugaucuucagucucucaaacC....................	1	1	sr1
....aaaugaucuucagucucucaaacU....................	7	1	sr1
....aaaugaucuucagucucucaaacaa...................	1	0	sr1
....aaaugaucuucagucucucaaacUa...................	13	1	sr1
....aaugaucuucagucucucG........................	1	1	sr1
....aaugaucuucagucucuca........................	7	0	sr1
....aaugaucuucagucucucaa.......................	28	0	sr1
....aaugGucuucagucucucaa.......................	1	1	sr1
....aGugaucuucagucucucaaa......................	4	1	sr1
....Gaugaucuucagucucucaaa......................	6	1	sr1
....aaugaucuucagucucucaaU......................	7	1	sr1
....aaugaucuucagCcucucaaa......................	1	1	sr1
....aaugaucuCcagucucucaaa......................	2	1	sr1
....aaugaucuucagucucucaaa......................	129	0	sr1
....aaugGucuucagucucucaaa......................	1	1	sr1
....aaugaucCucagucucucaaa......................	1	1	sr1
....aaugaucuucagucucucaaG......................	9	1	sr1
....aaugaucuucagucucucaUa......................	1	1	sr1
....aaugaucuucagucucucaaaA.....................	6	1	sr1
....aaugaucuucagucucucaaaG.....................	1	1	sr1
....aGugaucuucagucucucaaac.....................	3	1	sr1
....aaugaucuucagucucucaaac.....................	19	0	sr1
....aaugaucuucagCcucucaaaca....................	1	1	sr1
....Gaugaucuucagucucucaaaca....................	1	1	sr1
....aaugGucuucagucucucaaaca....................	1	1	sr1
....aaugaucuucagucucucaaacG....................	2	1	sr1
....aaugaucuucagucucucaaaUa....................	1	1	sr1
....aaugaucuucagucucucaaaca....................	25	0	sr1
....aaugaucuucagucucucaaacU....................	1	1	sr1
....aaugaucuucagucucucaaacaa...................	4	0	sr1
....aaugaucuucagucucucaaacUa...................	2	1	sr1
....augaucuucagucucucaaU......................	3	1	sr1
....augaucuucagucucucaaaU.....................	1	1	sr1
....augaucuucagucucucaaac.....................	3	0	sr1
....Cugaucuucagucucucaaac.....................	1	1	sr1
....augaucuucagucucucaaaca....................	3	0	sr1
....Cugaucuucagucucucaaaca....................	1	1	sr1
....Cugaucuucagucucucaaacaa...................	1	1	sr1
....ugaucuucagucucucGa.......................	1	1	sr1
....ugGucuucagucucucaa.......................	1	1	sr1
....ugaucuucagucucucaU.......................	2	1	sr1
....ugaucuucagucucuAaa.......................	2	1	sr1
....Ggaucuucagucucucaa.......................	1	1	sr1
....ugaucuucagucucucaa.......................	57	0	sr1
....ugaucuucagucucucaaU......................	48	1	sr1
....ugaucuCcagucucucaaa......................	1	1	sr1
....ugUucuucagucucucaaa......................	2	1	sr1
....ugaucuucagucucucaaC......................	5	1	sr1
....ugaAcuucagucucucaaa......................	1	1	sr1
....ugaucuucagucucucaUa......................	7	1	sr1
....ugaucuucagucucucaaa......................	504	0	sr1
....ugaucuucagCcucucaaa......................	1	1	sr1
....ugaucuucagucucucaaG......................	17	1	sr1
....ugGucuucagucucucaaa......................	5	1	sr1
....ugaucuucagucCcucaaa......................	2	1	sr1
....Ggaucuucagucucucaaa......................	12	1	sr1
....ugaucuucaguUucucaaa......................	1	1	sr1

Star **Mature**

```
cgcacagaagacgaauaucgcaacaccauccuaccuguucgggagaaugaaaaucauucucuuuagaaaaugaucuucagucucucaaacaaguagcaacacugaucgucg
......................................................................ugaucuucagucucCcaaa......................	2	1	sr1
......................................................................ugaCcuucagucucucaaa......................	2	1	sr1
......................................................................ugaucCucagucucucaaa......................	3	1	sr1
......................................................................ugaucuucagucucucaGa......................	1	1	sr1
......................................................................ugaucCucagucucucaaac.....................	18	1	sr1
......................................................................ugaucuucagAcucucaaac.....................	2	1	sr1
......................................................................ugaucuucagucucucaaCc.....................	1	1	sr1
......................................................................ugaucuucagucucucaaUc.....................	2	1	sr1
......................................................................ugUucuucagucucucaaac.....................	1	1	sr1
......................................................................ugaucuucagucucuUaaac.....................	1	1	sr1
......................................................................ugauUuucagucucucaaac.....................	2	1	sr1
......................................................................ugaucuucagucucucaaaU.....................	180	1	sr1
......................................................................ugaucuAcagucucucaaac.....................	3	1	sr1
......................................................................ugaucuucagucuUucaaac.....................	2	1	sr1
......................................................................ugaucuucagCcucucaaac.....................	8	1	sr1
......................................................................Agaucuucagucucucaaac.....................	2	1	sr1
......................................................................ugaGcuucagucucucaaac.....................	1	1	sr1
......................................................................ugaucuuUagucucucaaac.....................	3	1	sr1
......................................................................ugaucuucagucucCcaaac.....................	9	1	sr1
......................................................................ugaucuucagucucucaaaG.....................	3	1	sr1
......................................................................ugaucuucagucucucGaac.....................	7	1	sr1
......................................................................ugaucuucGgucucucaaac.....................	12	1	sr1
......................................................................ugaAcuucagucucucaaac.....................	4	1	sr1
......................................................................Ggaucuucagucucucaaac.....................	66	1	sr1
......................................................................ugaucuucagucucAcaaac.....................	1	1	sr1
......................................................................ugaucuucagucCcucaaac.....................	9	1	sr1
......................................................................ugaucuucagucuAucaaac.....................	1	1	sr1
......................................................................ugaucuucagucucucaaac.....................	2713	0	sr1
......................................................................Cgaucuucagucucucaaac.....................	8	1	sr1
......................................................................ugaCcuucagucucucaaac.....................	4	1	sr1
......................................................................uAaucuucagucucucaaac.....................	3	1	sr1
......................................................................ugaucuCcagucucucaaac.....................	3	1	sr1
......................................................................ugaucuucagucucucaaGc.....................	6	1	sr1
......................................................................ugaucuucagucucucaaaA.....................	48	1	sr1
......................................................................ugaucuucagucucucaGac.....................	9	1	sr1
......................................................................ugaucuucCgucucucaaac.....................	1	1	sr1
......................................................................ugGucuucagucucucaaac.....................	10	1	sr1
......................................................................ugaucuucUgucucucaaac.....................	1	1	sr1
......................................................................ugaucuucagucucCcaaaca....................	33	1	sr1
......................................................................ugaucCucagucucucaaaca....................	26	1	sr1
......................................................................ugaucuucagucucuGaaaca....................	1	1	sr1
......................................................................ugaucuucaguGucucaaaca....................	2	1	sr1
......................................................................ugCucuucagucucucaaaca....................	2	1	sr1
......................................................................ugaucuucagucucucaaCca....................	1	1	sr1
......................................................................ugaucuucagucucAcaaaca....................	7	1	sr1
......................................................................ugaucGucagucucucaaaca....................	2	1	sr1
......................................................................ugaucuucaAucucucaaaca....................	3	1	sr1
......................................................................ugaucuucagucucucaaaUa....................	13	1	sr1
......................................................................ugaucuuGagucucucaaaca....................	1	1	sr1
......................................................................ugaCcuucagucucucaaaca....................	37	1	sr1
......................................................................ugaucuucagCcucucaaaca....................	24	1	sr1
......................................................................ugaucAucagucucucaaaca....................	1	1	sr1
......................................................................ugaucuAcagucucucaaaca....................	4	1	sr1
......................................................................ugaucuucagucucucaaacG....................	369	1	sr1
......................................................................ugaucuucagucucucaGaca....................	20	1	sr1
......................................................................ugauGuucagucucucaaaca....................	1	1	sr1
......................................................................ugaGcuucagucucucaaaca....................	1	1	sr1
......................................................................ugaAcuucagucucucaaaca....................	7	1	sr1
......................................................................Agaucuucagucucucaaaca....................	17	1	sr1
......................................................................uAaucuucagucucucaaaca....................	6	1	sr1
......................................................................ugaucuGcagucucucaaaca....................	3	1	sr1
......................................................................ugGucuucagucucucaaaca....................	68	1	sr1
......................................................................ugaucuCcagucucucaaaca....................	27	1	sr1
......................................................................ugaucuucagucucucaaaAa....................	2	1	sr1
......................................................................ugaucuucUgucucucaaaca....................	3	1	sr1
......................................................................ugaucuucagucucucaaacC....................	11	1	sr1
......................................................................ugaucuucagucAcucaaaca....................	3	1	sr1
......................................................................ugaucuucagucucucaaacU....................	86	1	sr1
......................................................................ugaucuucagucucucCaaca....................	3	1	sr1
......................................................................ugaucuucGgucucucaaaca....................	22	1	sr1
```

Star Mature

```
cgcacagaagacgaauaucgcaacaccauccuaccuguucgggagaaugaaaaucauucucuuuagaaaaugaucuucagucucucaaacaaguagcaacacugaucgucg
......................................................................ugauUuucagucucucaaaca....................  1  1  sr1
......................................................................ugaucuuUagucucucaaaca....................  12  1  sr1
......................................................................ugaucuucagucucucaUaca....................  1  1  sr1
......................................................................ugaucuucagucuUucaaaca....................  7  1  sr1
......................................................................ugaucuucagucuAucaaaca....................  1  1  sr1
......................................................................ugaucuucagucucucaaaca....................  8915  0  sr1
......................................................................uUaucuucagucucucaaaca....................  2  1  sr1
......................................................................ugaucuucagucucucaaUca....................  2  1  sr1
......................................................................Cgaucuucagucucucaaaca....................  26  1  sr1
......................................................................ugaucuucagucucuUaaaca....................  7  1  sr1
......................................................................ugaucuucagucucucGaaca....................  31  1  sr1
......................................................................ugaucuuAagucucucaaaca....................  2  1  sr1
......................................................................ugauAuucagucucucaaaca....................  1  1  sr1
......................................................................ugaucuucagucucucaaGca....................  23  1  sr1
......................................................................ugaucuucagucCcucaaaca....................  28  1  sr1
......................................................................Ggaucuucagucucucaaaca....................  490  1  sr1
......................................................................ugaucuucagucucuAaaaca....................  1  1  sr1
......................................................................ugaucuucaUucucucaaaca....................  1  1  sr1
......................................................................ugaucuucagucucucUaaca....................  7  1  sr1
......................................................................ugUucuucagucucucaaacaa...................  4  1  sr1
......................................................................ugaucuucagucucucaaacCa...................  1  1  sr1
......................................................................Cgaucuucagucucucaaacaa...................  2  1  sr1
......................................................................ugaucGucagucucucaaacaa...................  2  1  sr1
......................................................................ugaucuucagucucucaaacUa...................  107  1  sr1
......................................................................ugaucuucGgucucucaaacaa...................  2  1  sr1
......................................................................ugaucuucagucucucaCacaa...................  1  1  sr1
......................................................................ugaucuucagucucucaaacGa...................  1  1  sr1
......................................................................ugaucuucagucucCcaaacaa...................  3  1  sr1
......................................................................ugaucuucagucucucaaacaG...................  30  1  sr1
......................................................................ugaucuucagucucucaaacaa...................  708  0  sr1
......................................................................ugauUuucagucucucaaacaa...................  1  1  sr1
......................................................................ugaucuucagucucucaaacaC...................  17  1  sr1
......................................................................Ggaucuucagucucucaaacaa...................  11  1  sr1
......................................................................ugaucuucagucucucaaaAaa...................  1  1  sr1
......................................................................ugaucuCcagucucucaaacaa...................  1  1  sr1
......................................................................ugaucuucagucucucaGacaa...................  6  1  sr1
......................................................................ugaucuucaAucucucaaacaa...................  2  1  sr1
......................................................................ugaucuuUagucucucaaacaa...................  4  1  sr1
......................................................................ugaucuucagucucucaaaUaa...................  1  1  sr1
......................................................................ugaucuucagAcucucaaacaa...................  1  1  sr1
......................................................................ugaAcuucagucucucaaacaa...................  3  1  sr1
......................................................................ugaucuucagucucucaaCcaa...................  2  1  sr1
......................................................................ugaucuucagucucucaaGcaa...................  1  1  sr1
......................................................................ugaucuucagucucucaaacaU...................  113  1  sr1
......................................................................ugaCcuucagucucucaaacaa...................  2  1  sr1
......................................................................ugaucuucagucCcucaaacaa...................  5  1  sr1
......................................................................ugaucuucagCcucucaaacaa...................  1  1  sr1
......................................................................ugaucCucagucucucaaacaa...................  4  1  sr1
......................................................................ugaucuucagucucucGaacaa...................  1  1  sr1
......................................................................ugaucuucagucucucaaacaUg..................  15  1  sr1
......................................................................ugaucuucagucucucaaacaaA..................  27  1  sr1
......................................................................ugaucuucagucucucaaacUag..................  2  1  sr1
......................................................................ugaucuucagucucucaaacGag..................  1  1  sr1
......................................................................ugaucuucagucucucaaacaaC..................  2  1  sr1
......................................................................ugaucuucagucucucaaacaaU..................  3  1  sr1
......................................................................ugaucuucagucucucaaacaaAu.................  1  1  sr1
......................................................................ugaucuucagucucucaaacaaUua................  5  1  sr1
......................................................................ugaucuucagucucucaaacaaAua................  3  1  sr1
.......................................................................gaucuucagucucucaaa......................  1  0  sr1
.......................................................................gaucuucagucucucaaac.....................  2  0  sr1
.......................................................................gaucuucagucucucaaaU.....................  1  1  sr1
.......................................................................gaucuucagucucucaaaca....................  1  0  sr1
........................................................................aucuucagucucucaaac.....................  1  0  sr1
.........................................................................ucuucagucucucaaacG....................  2  1  sr1
.........................................................................ucuucagucucucaaaca....................  2  0  sr1
.........................................................................Gcuucagucucucaaaca....................  1  1  sr1
..........................................................................cuucagucucucaaacaU...................  1  1  sr1
...........................................................................................aguagcaacacugaucguG.  1  1  sr1
...........................................................................................aguagcaacacugaucguc.  1  0  sr1
...........................................................................................aguagcaacacugaucguA.  3  1  sr1
```

Star Mature

cgcacagaagacgaauaucgcaacaccauccuaccuguucgggagaaugaaaaucauucucuuuagaaaaugaucuucagucucucaaacaaguagcaacacugaucgucg			
....aguagcaacacugaucgucg	6	0	sr1
....aguagcaacacugaucguAg	1	1	sr1
...................................uAuucgggagaaugaaaauc..	1	1	st1
....................................Cuucgggagaaugaaaaucauu...	1	1	st1
.....................................uucgggagaaugaaaauc..	45	0	st1
.....................................uCcgggagaaugaaaauc..	1	1	st1
.....................................uucgggagaaugaaaaCc..	2	1	st1
.....................................uucgggagaaugaaaauca...	3	0	st1
.....................................uCcgggagaaugaaaaucau..	1	1	st1
.....................................uucgggagCaugaaaaucau..	1	1	st1
.....................................uucgggagaaugaaaaucaC..	6	1	st1
.....................................uucgggagaaugaGaaucau..	1	1	st1
.....................................uAcgggagaaugaaaaucau..	1	1	st1
.....................................uucgggagaaugaaaaucaG..	1	1	st1
.....................................uucgggagaaugaaUaucau..	1	1	st1
.....................................uucgggagaaugaaaaucau..	57	0	st1
.....................................uucgggagaaugaaaaucaA..	2	1	st1
.....................................uucgggagaaugaaGaucau..	1	1	st1
.....................................uucgggagaaugaaaaucauu...	2995	0	st1
.....................................uucggAagaaugaaaaucauu...	1	1	st1
.....................................uucgggGgaaugaaaaucauu...	12	1	st1
.....................................uucgggagaaAgaaaaucauu...	1	1	st1
.....................................uucgggagaaugaaaaucauA...	31	1	st1
.....................................uucgggagaaugaaaaucauC...	249	1	st1
.....................................uAcgggagaaugaaaaucauu...	4	1	st1
.....................................uucgggagaauAaaaaucauu...	2	1	st1
.....................................uGcgggagaaugaaaaucauu...	2	1	st1
.....................................uucgggagaaugGaaaucauu...	7	1	st1
.....................................uucgggagCaugaaaaucauu...	19	1	st1
.....................................uucggUagaaugaaaaucauu...	1	1	st1
.....................................uucgggagaaCgaaaaucauu...	4	1	st1
.....................................uucgggagaaugaaaCucauu...	2	1	st1
.....................................uucgggagaaugaaCaucauu...	2	1	st1
.....................................uucgggaAaaugaaaaucauu...	2	1	st1
.....................................Cucgggagaaugaaaaucauu...	10	1	st1
.....................................uucgggagaaugUaaaucauu...	2	1	st1
.....................................uucgggCgaaugaaaaucauu...	2	1	st1
.....................................uucAggagaaugaaaaucauu...	1	1	st1
.....................................uucgggagaaugaaGaucauu...	17	1	st1
.....................................uucUggagaaugaaaaucauu...	2	1	st1
.....................................uucgggagaaugaGaaucauu...	13	1	st1
.....................................uucgggagaaugaaaGucauu...	10	1	st1
.....................................uuAgggagaaugaaaaucauu...	1	1	st1
.....................................uucgggagaaugaaaaucUuu...	1	1	st1
.....................................uucgggagaaugaaaaucGuu...	9	1	st1
.....................................uucgggagUaugaaaaucauu...	1	1	st1
.....................................uucgggagaaugaaaaucaGu...	1	1	st1
.....................................uucgggagaaugaaaaucaCu...	7	1	st1
.....................................uucgggagaaugaaaaCcauu...	9	1	st1
.....................................uuUgggagaaugaaaaucauu...	2	1	st1
.....................................uucgggagGaugaaaaucauu...	5	1	st1
.....................................uucgggagaGugaaaaucauu...	8	1	st1
.....................................uCcgggagaaugaaaaucauu...	9	1	st1
.....................................uucgggagaaugaaaaucauG...	16	1	st1
.....................................uucgggagaaugaaaaucaAu...	1	1	st1
.....................................uucgggagaaugaGaaucauuc..	1	1	st1
.....................................uucgggagaaugaaaaucauuc..	66	0	st1
.....................................uAcgggagaaugaaaaucauuc..	2	1	st1
.....................................uucgggagaaugaaaaucauuG..	1	1	st1
.....................................uucgggagaaugaaaaucauuA..	340	1	st1
.....................................uucgggagaaugaaaaCcauuc..	1	1	st1
.....................................uucgggagaaugaaGaucauuc..	1	1	st1
.....................................uucgggagaaugaaaaucauCc..	2	1	st1
.....................................uucgggagaaugaaaaucGuuc..	1	1	st1
.....................................uCcgggagaaugaaaaucauuc..	1	1	st1
.....................................uucgggagaaugaaaaucauuU..	47	1	st1
.....................................uucgggGgaaugaaaaucauuc..	2	1	st1
.....................................uucgggagaaugaaaaucauucA...	1	1	st1
.....................................uucgggagaaugaaaaucauuAu...	1	1	st1

Star **Mature**

```
cgcacagaagacgaauaucgcaacaccauccuaccuguucgggagaaugaaaaucauucucuuuagaaaaugaucuucagucucucaaacaaguagcaacacugaucgucg
.....................................uucgggagaaugaaaaucauucu...................................................	1	0	st1
.....................................uucgggagaaugaaaaucauuUu...................................................	18	1	st1
.....................................uucgggagaaugaaaaucauuGu...................................................	1	1	st1
......................................ucgggagaaugaaaaucauu.....................................................	1	0	st1
......................................ucgggagaaugaaaaucauC.....................................................	1	1	st1
......................................ucgggagaaugaaaaucauuA....................................................	1	1	st1
...................................................................aaaugaucuucagucucuca........................	1	0	st1
...................................................................aaaugaucuucagucucucaa.......................	5	0	st1
...................................................................aaaugaucCucagucucucaaa......................	1	1	st1
...................................................................aaaugaucuucagucucucaaa......................	48	0	st1
...................................................................aaaugaucuucagucucucaaC......................	1	1	st1
...................................................................aaaugaucuucagucucucaaU......................	13	1	st1
...................................................................aaaugaucuucagucucucaaac.....................	11	0	st1
...................................................................aaaugaucuucagucucucaaGc.....................	1	1	st1
...................................................................aaaugaucuucagucucucaaaA.....................	4	1	st1
...................................................................aaauAaucuucagucucucaaac.....................	1	1	st1
...................................................................aaaugaucuucagucucucaaaG.....................	1	1	st1
...................................................................aaaugaucuucagucucucaaaU.....................	11	1	st1
...................................................................aaaugaucuucagucucucaaacU....................	6	1	st1
...................................................................aaaugaucuucagucucucaaacC....................	1	1	st1
...................................................................aaaugaucuucagucucucaaaca....................	10	0	st1
...................................................................aaaugaucuucagucucucaaacUa...................	1	1	st1
....................................................................aaugaucuucagucucucaa.......................	2	0	st1
....................................................................aaugaucuucagucucucaaa......................	6	0	st1
....................................................................aaugaucuucagucucucaaac.....................	10	0	st1
....................................................................aaugaucuucagucucucaaacG....................	1	1	st1
....................................................................aaugaucuucagucucucaaaca....................	7	0	st1
....................................................................aaugaucuucagucucucaaaUa....................	1	1	st1
....................................................................aaugaucuucagucucucaaacU....................	1	1	st1
....................................................................aaugaucuucagucucucaaacaU...................	1	1	st1
.....................................................................augaucuucagucucucaaac.....................	1	0	st1
.....................................................................augaucuucagucucucaaaca....................	1	0	st1
.....................................................................Cugaucuucagucucucaaaca....................	1	1	st1
......................................................................ugaucuucagucucucaa.......................	2	0	st1
......................................................................ugaucuucagucucucaaa......................	14	0	st1
......................................................................ugaucuucagucucucaaU......................	1	1	st1
......................................................................Agaucuucagucucucaaac.....................	1	1	st1
......................................................................ugaucuucagucucucaaaU.....................	13	1	st1
......................................................................ugaucuucagucucucaaGc.....................	1	1	st1
......................................................................ugaGcuucagucucucaaac.....................	1	1	st1
......................................................................ugaucCucagucucucaaac.....................	2	1	st1
......................................................................ugaucuucCgucucucaaac.....................	2	1	st1
......................................................................Cgaucuucagucucucaaac.....................	2	1	st1
......................................................................ugaucuucagucCcucaaac.....................	1	1	st1
......................................................................ugaucuucagucucucUaac.....................	1	1	st1
......................................................................ugaucuucagucucucaGac.....................	1	1	st1
......................................................................ugaucuucagucucCcaaac.....................	4	1	st1
......................................................................ugaucuucagucucucaaac.....................	240	0	st1
......................................................................ugaucuucGgucucucaaac.....................	1	1	st1
......................................................................ugGucuucagucucucaaac.....................	1	1	st1
......................................................................uAaucuucagucucucaaac.....................	1	1	st1
......................................................................ugaucuucaUucucucaaac.....................	1	1	st1
......................................................................ugaucuucagucucucGaaca....................	4	1	st1
......................................................................ugaucuucagucucCcaaaca....................	1	1	st1
......................................................................ugaucuucagucucucaaUca....................	1	1	st1
......................................................................ugaucuucagucucucaaaca....................	1244	0	st1
......................................................................ugaucuucagucCcucaaaca....................	3	1	st1
......................................................................ugaucuucaUucucucaaaca....................	1	1	st1
......................................................................ugaucuucGgucucucaaaca....................	4	1	st1
......................................................................ugaucuAcagucucucaaaca....................	2	1	st1
......................................................................ugaucuucagucucucaaacU....................	7	1	st1
......................................................................ugaucuucagucucucaaGca....................	2	1	st1
......................................................................ugaucuCcagucucucaaaca....................	5	1	st1
......................................................................ugaucuucagCcucucaaaca....................	7	1	st1
......................................................................ugaucGucagucucucaaaca....................	1	1	st1
......................................................................ugaucuucagucuUucaaaca....................	1	1	st1
......................................................................ugaucuucagucucucaGaca....................	2	1	st1
......................................................................ugauUuucagucucucaaaca....................	1	1	st1
......................................................................ugaucuucCgucucucaaaca....................	13	1	st1
......................................................................uUaucuucagucucucaaaca....................	1	1	st1
```

Star Mature

```
cgcacagaagacgaauaucgcaacaccauccuaccuguucgggagaaugaaaaucauucucuuuagaaaaugaucuucagucucucaaacaaguagcaacacugaucgucg
......................................................................ugaucuucagucucucaaacC....................    1    1    st1
......................................................................ugGucuucagucucucaaaca....................    8    1    st1
......................................................................ugaucuucagucucucaaaUa....................    1    1    st1
......................................................................ugaucuucagucucucaaCca....................    1    1    st1
......................................................................uAaucuucagucucucaaaca....................    4    1    st1
......................................................................ugaCcuucagucucucaaaca....................    3    1    st1
......................................................................ugaucuucUgucucucaaaca....................    1    1    st1
......................................................................ugaucuucagucucucaaacG....................    29   1    st1
......................................................................ugaucCucagucucucaaaca....................    1    1    st1
......................................................................ugaucuucagucAcucaaaca....................    2    1    st1
......................................................................ugaucuuUagucucucaaaca....................    1    1    st1
......................................................................ugaucAucagucucucaaaca....................    3    1    st1
......................................................................ugaucuucagucucGcaaaca....................    1    1    st1
......................................................................ugaucuucagAcucucaaaca....................    1    1    st1
......................................................................Agaucuucagucucucaaaca....................    2    1    st1
......................................................................Cgaucuucagucucucaaaca....................    3    1    st1
......................................................................ugaucuucagucucAcaaacaa...................    1    1    st1
......................................................................ugaucuucagucucucaaacUa...................    3    1    st1
......................................................................ugaucuucagucucucaaacaU...................    48   1    st1
......................................................................ugaucuucagucucucaaacaC...................    2    1    st1
......................................................................ugaucuucCgucucucaaacaa...................    1    1    st1
......................................................................ugaucuucagucucucaaacaa...................    53   0    st1
......................................................................ugaucuucagucucucaaacaaU..................    1    1    st1
......................................................................ugaucuucagucucucaaacaaAu.................    1    1    st1
......................................................................ugaucuucagucucucaaacaaUua................    3    1    st1
...........................................................................................aguagcaacacugaucgA..    1    1    st1
...........................................................................................aguagcaacacugaucguc.    1    0    st1
...........................................................................................Uguagcaacacugaucgucg    1    1    st1
...........................................................................................aguagcaacacugaucgucg    2    0    st1
```

Provisional ID	:	ScYm1zk_361_3669
Score total	:	2982.8
Score for star read(s)	:	3.9
Score for read counts	:	2977
Score for mfe	:	0.9
Score for randfold	:	1.6
Score for cons. seed	:	-0.6
Total read count	:	5851
Mature read count	:	5837
Loop read count	:	0
Star read count	:	14

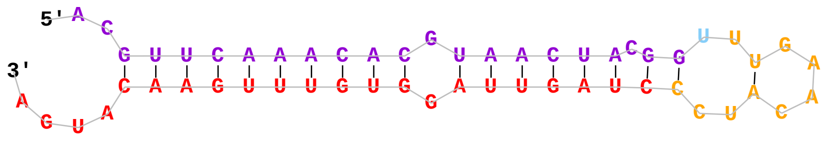

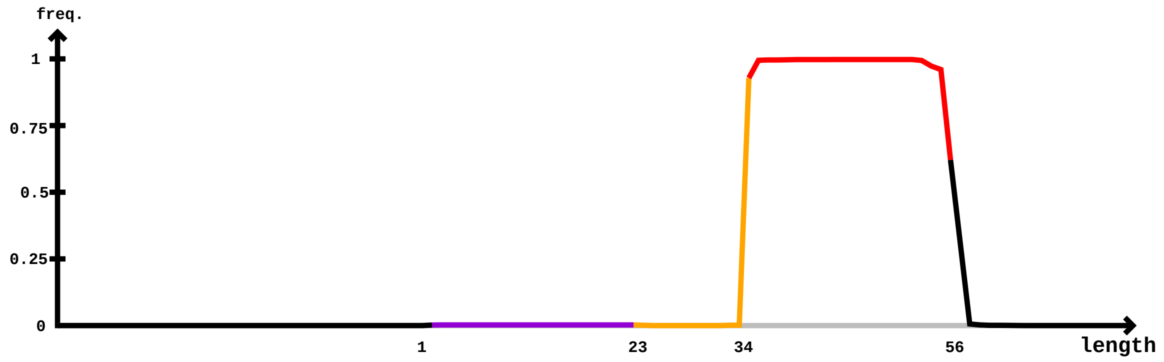

Star **Mature**

5'- guucaagcgucaacaaugaaacaucgcaugacaauucacguucaaacacguaacuacgguuugaacaucccuaguuagguguuugaacaugaauucgaaaaaguguauua -3'	obs		
guucaagcgucaacaaugaaacaucgcaugacaauucacguucaaacacguaacuacgguuugaacaucccuaguuagguguuugaacaugaauucgaaaaaguguauua	exp		
..(((.((((((....))).....))).))).((((((.((((((((((.((((((.((..(....)..)))))))).)))))))))).)))))))..............	reads	mm	sample
.....................................acguucaaacacguaacuacgg...	3	0	sg1
....cuaguuagguguuugaac......................	1	0	sg1
....Auaguuagguguuugaacaug...................	4	1	sg1
....cuaguuagguguuugaacaug...................	20	0	sg1
....cuaguuagguguuugaacGug...................	1	1	sg1
....Auaguuagguguuugaacauga..................	13	1	sg1
....Guaguuagguguuugaacauga..................	4	1	sg1
....cuaguuagAuguuugaacauga..................	27	1	sg1
....cuaguuagguguuugaacGuga..................	3	1	sg1
....cuaguuagguguuugaacauga..................	48	0	sg1
....cuaguuGgguguuugaacauga..................	1	1	sg1
....cuaguuagguguuugaacaugaa.................	4	0	sg1
....cuaguuagAuguuugaacaugaa.................	1	1	sg1
....cuaguuaggCguuugaacaugaa.................	1	1	sg1
....cuaguuagguguuugaacaugaaA................	3	1	sg1
....cuaguuagguguuugaacaugaauu...............	1	0	sg1
....uaguuagguguuugaacaug...................	5	0	sg1
....uaguuagAuguuugaacauga..................	2	1	sg1
....uaguuagguguuugaacauga..................	17	0	sg1
....uaguuGgguguuugaacaugaa.................	1	1	sg1
....uaguuagguguuugaacaugaa.................	2	0	sg1
....uaguuagguguuugaacaugUa.................	3	1	sg1
....uaguuagguguuugaacaugaaA................	2	1	sg1
.....................................acguucaaacacguaacuacgg...	1	0	so1
.....................................Ucguucaaacacguaacuacgg...	1	1	so1
....cccuaguuagguguuugaacaug...................	1	0	so1
....cuaguuagguguuugaac......................	3	0	so1
....cuaguuagAuguuugaaca.....................	1	1	so1
....cuaguuagguguuugaacau....................	1	0	so1
....cuaguuagAuguuugaacaug...................	10	1	so1
....Guaguuagguguuugaacaug...................	2	1	so1
....cuaguuagguguuugaacauU...................	1	1	so1

Star **Mature**

```
guucaagcgucaacaaugaaacaucgcaugacaauucacguucaaacacguaacuacgguuugaacaucccuaguuagguguuugaacaugaauucgaaaaaguguauua
......................................................................cuaguuagguguCugaacaug...................   2     1   so1
......................................................................cuaguuGgguguuugaacaug...................   2     1   so1
......................................................................cuaguuagguguuugaacaug...................   63    0   so1
......................................................................cuaguuagguguuugaacauA...................   5     1   so1
......................................................................cuaguAagguguuugaacaug...................   1     1   so1
......................................................................cuaguuagguguuugaacGug...................   1     1   so1
......................................................................Auaguuagguguuugaacaug...................   1     1   so1
......................................................................cuGguuagguguuugaacauga..................   4     1   so1
......................................................................cuaguuagguguuGgaacauga..................   2     1   so1
......................................................................cuaguuagguguuugaacauga..................   70    0   so1
......................................................................cuaguuaggugAuugaacauga..................   1     1   so1
......................................................................Auaguuagguguuugaacauga..................   9     1   so1
......................................................................cuaguuagguguuugaacaGga..................   1     1   so1
......................................................................cuaguuaAguguuugaacauga..................   1     1   so1
......................................................................cuaguuagguguCugaacauga..................   2     1   so1
......................................................................cuaguuagguguuugaacaugG..................   1     1   so1
......................................................................cuaguuagguguuugaacauAa..................   1     1   so1
......................................................................Guaguuagguguuugaacauga..................   4     1   so1
......................................................................cuaguuagguguuugaacaugU..................   1     1   so1
......................................................................cuaguuagAuguuugaacauga..................   29    1   so1
......................................................................cuaguuagguguuugaacaugaa.................   3     0   so1
......................................................................cuaguuagguguuugaacaugaC.................   1     1   so1
......................................................................cuaguuagAuguuugaacaugaa.................   3     1   so1
......................................................................cuaguuagguguuugaacaugaaA................   2     1   so1
.......................................................................uaguuagguguuugaacau....................   1     0   so1
.......................................................................uaguuagguguuugaacauA...................   3     1   so1
.......................................................................uaguuagguguuugaacaCg...................   1     1   so1
.......................................................................uaguuagguguuugaacaug...................   12    0   so1
.......................................................................uaguuUgguguuugaacauga..................   1     1   so1
.......................................................................uaguuagAuguuugaacauga..................   2     1   so1
.......................................................................uaguuagguguuugaacauga..................   27    0   so1
.......................................................................uaguuagguguuugaacaugUa.................   1     1   so1
.......................................................................uaguuagguguuugaacaugaauA...............   1     1   so1
..........................................................................uuagAuguuugaacauga..................   1     1   so1
............................................................................uagguguuugaacaugaauuc.............   1     0   so1

.....................................acguucaaacacguaacuacgg...................................................   3     0   sr1
.....................................acguucGaacacguaacuacgg...................................................   1     1   sr1
......................................cguucaaacacguaacuacgg...................................................   1     0   sr1
......................................cguucaaacacguaacuacgA...................................................   1     1   sr1
......................................cguucaaacacguaacuacggA..................................................   1     1   sr1
...................................................................Gccuaguuagguguuugaacaug...................   1     1   sr1
...................................................................cccuaguuagAuguuugaacaug...................   1     1   sr1
...................................................................cccuaguuagguguuugaacaug...................   2     0   sr1
....................................................................ccuaguuagguguuugaacaug...................   2     0   sr1
......................................................................Guaguuagguguuugaac......................   12    1   sr1
......................................................................cuaguuagguguuugaac......................   4     0   sr1
......................................................................cuaguuagAuguuugaaca.....................   3     1   sr1
......................................................................cuaguuagguguuugaaca.....................   20    0   sr1
......................................................................Guaguuagguguuugaaca.....................   90    1   sr1
......................................................................Guaguuagguguuugaacau....................   57    1   sr1
......................................................................cuaguuagguguuugaacaC....................   3     1   sr1
......................................................................cuaguuagAuguuugaacau....................   1     1   sr1
......................................................................cuaguuagguguuugaacau....................   8     0   sr1
......................................................................cuaguuagguguuugaacUug...................   1     1   sr1
......................................................................cuaguuagguguuugGacaug...................   2     1   sr1
......................................................................cuaguuagguguuugaacaug...................   305   0   sr1
......................................................................cuaguuagguguuGgaacaug...................   1     1   sr1
......................................................................Guaguuagguguuugaacaug...................   1210  1   sr1
......................................................................cuaguuagguguuugaacaCg...................   9     1   sr1
......................................................................cuagCuagguguuugaacaug...................   1     1   sr1
......................................................................cuaguuGgguguuugaacaug...................   7     1   sr1
......................................................................cuaguuagguguuugaacauA...................   27    1   sr1
......................................................................cuaguuaggAguuugaacaug...................   1     1   sr1
......................................................................cuaguCagguguuugaacaug...................   1     1   sr1
......................................................................cuaguuagAuguuugaacaug...................   25    1   sr1
......................................................................cuaguuagguguCugaacaug...................   1     1   sr1
......................................................................cuaguuagguguuugaacauU...................   3     1   sr1
......................................................................cuaguuaggCguuugaacaug...................   1     1   sr1
......................................................................Auaguuagguguuugaacaug...................   5     1   sr1
```

Star   Mature

```
guucaagcgucaacaaugaaacaucgcaugacaauucacguucaaacacguaacuacgguuugaacaucccuaguuagguguuugaacaugaauucgaaaaaguguauua
......................................................................cuaguuagguguuugaaUaug................... 2     1  sr1
......................................................................cuaguuagguguuugaGcaug................... 1     1  sr1
......................................................................cuaguuagguguuugaacaugG.................. 7     1  sr1
......................................................................cuaguuagguAuuugaacauga.................. 1     1  sr1
......................................................................cuaguuagguguuugCacauga.................. 1     1  sr1
......................................................................cuaguuagguguuugaacauga.................. 233   0  sr1
......................................................................cuaguuagguguuugaacauAa.................. 1     1  sr1
......................................................................Guaguuagguguuugaacauga.................. 2296  1  sr1
......................................................................cuaguuGgguguuugaacauga.................. 7     1  sr1
......................................................................Auaguuagguguuugaacauga.................. 14    1  sr1
......................................................................cuaguuaggCguuugaacauga.................. 2     1  sr1
......................................................................cuaguuagAuguuugaacauga.................. 81    1  sr1
......................................................................cuaguuagguguuugGacauga.................. 2     1  sr1
......................................................................cuaguuagguguuugaaUauga.................. 1     1  sr1
......................................................................cuaguuagguguuugaacaugU.................. 4     1  sr1
......................................................................cuaguuagguguuugaGcauga.................. 1     1  sr1
......................................................................Guaguuagguguuugaacaugaa................. 126   1  sr1
......................................................................cuaguuagAuguuugaacaugaa................. 10    1  sr1
......................................................................cuaguuagguguuugaacaugUa................. 1     1  sr1
......................................................................Auaguuagguguuugaacaugaa................. 2     1  sr1
......................................................................cuaguuagguguuugaacaugaa................. 14    0  sr1
......................................................................cuaguuagguguuugaacaugaG................. 1     1  sr1
......................................................................cuaguuagguguuugaacaugaC................. 1     1  sr1
......................................................................cuaguuagguguuugaacaugaaA................ 8     1  sr1
......................................................................cuaguuagguguuugaacaugaUuu............... 1     1  sr1
......................................................................cuaguuagguguuugaacaugaauA............... 2     1  sr1
......................................................................Guaguuagguguuugaacaugaauu............... 2     1  sr1
.......................................................................uaguuagguguuugaacG..................... 1     1  sr1
.......................................................................uaguuagguguuugaaca..................... 1     0  sr1
.......................................................................uaguuagguguuugaacauA................... 3     1  sr1
.......................................................................uaguuagguguuugaacaug................... 27    0  sr1
.......................................................................uaguuagguguuugaacaGg................... 1     1  sr1
.......................................................................uaguuagguguuugaacaCg................... 1     1  sr1
.......................................................................uaguuagguguuugaacauC................... 1     1  sr1
.......................................................................uaguuaggugCuugaacaug................... 1     1  sr1
.......................................................................Gaguuagguguuugaacaug................... 8     1  sr1
.......................................................................uaguuGgguguuugaacaug................... 1     1  sr1
.......................................................................uaguuagguguuugaacaugU.................. 1     1  sr1
.......................................................................uaguuagAuguuugaacauga.................. 10    1  sr1
.......................................................................uaguuaggugCuugaacauga.................. 1     1  sr1
.......................................................................uaguuGgguguuugaacauga.................. 3     1  sr1
.......................................................................uaguuaAguguuugaacauga.................. 1     1  sr1
.......................................................................uaguuUgguguuugaacauga.................. 1     1  sr1
.......................................................................Gaguuagguguuugaacauga.................. 21    1  sr1
.......................................................................uaguuagguguuugaacaugG.................. 9     1  sr1
.......................................................................uaguuagguguuugaacauga.................. 109   0  sr1
.......................................................................Gaguuagguguuugaacaugaa................. 4     1  sr1
.......................................................................uaguuagguguuugaacaugaa................. 5     0  sr1
.......................................................................uaguuagAuguuugaacaugaa................. 2     1  sr1
........................................................................aguuagAuguuugaacauga.................. 3     1  sr1
..........................................................................uuagAuguuugaacauga.................. 2     1  sr1
...........................................................................uagguguuugaacaugaauucA............. 1     1  sr1
...........................................................................uagAuguuugaacaugaauucga............ 1     1  sr1
...............................................................................uguuugaacaugaauucgaa........... 1     0  sr1

.....................................acguucaGacacguaacuacgg................................................... 1     1  st1
.....................................acguucaaacacguaacuacgg................................................... 1     0  st1
....................................................................cccuaguuagAuguuugaacaug................... 2     1  st1
......................................................................cuaguuagguguuugaac...................... 1     0  st1
......................................................................cuaguuagAuguuugaaca..................... 4     1  st1
......................................................................cuaguuagguguuugaaca..................... 3     0  st1
......................................................................cuaguuagAuguuugaacau.................... 1     1  st1
......................................................................cuaguuagguguuugaacaC.................... 1     1  st1
......................................................................cuaguuagguguuugaacau.................... 3     0  st1
......................................................................Guaguuagguguuugaacaug................... 2     1  st1
......................................................................Auaguuagguguuugaacaug................... 1     1  st1
......................................................................cuaguuagguguuugaacauA................... 13    1  st1
......................................................................cuaguuagguguuugaacauU................... 6     1  st1
......................................................................cuaguuagguguuugaacaug................... 153   0  st1
......................................................................cuGguuagguguuugaacaug................... 2     1  st1
```

Star Mature

```
guucaagcgucaacaaugaaacaucgcaugacaauucacguucaaacacguaacuacgguuugaacaucccuaguuagguguuugaacaugaauucgaaaaaguguauua
......................................................................cuaguuagAuguuugaacaug...................  7    1  st1
......................................................................cuagCuagguguuugaacaug...................  1    1  st1
......................................................................cuaguuagguguuugGacaug...................  1    1  st1
......................................................................cuaguuagguguuugaacaCg...................  1    1  st1
......................................................................cuaguuagguguuugaacauga..................  158  0  st1
......................................................................Guaguuagguguuugaacauga..................  8    1  st1
......................................................................cuaguuagAuguuugaacauga..................  49   1  st1
......................................................................cuaguuagguguuugaacGuga..................  1    1  st1
......................................................................cuaguuagguguuCgaacauga..................  1    1  st1
......................................................................cuaguuagguguuuAaacauga..................  1    1  st1
......................................................................cuaguuagguguuugaacaugG..................  1    1  st1
......................................................................cuaguuagguguCugaacauga..................  1    1  st1
......................................................................cuaguuagguguuugaacaCga..................  2    1  st1
......................................................................cuaguuaggugCuugaacauga..................  2    1  st1
......................................................................Auaguuagguguuugaacauga..................  9    1  st1
......................................................................cuaguuagAuguuugaacaugaa.................  4    1  st1
......................................................................cuaguuagguguuugaacaugaa.................  3    0  st1
......................................................................cuaguuagguguuugaacaugaG.................  1    1  st1
......................................................................cuaguuagguguuugaacaugaaA................  4    1  st1
......................................................................cuaguuagguguuugaacaugaaC................  1    1  st1
......................................................................cuaguuagguguuugaacaugaauA...............  1    1  st1
.......................................................................uaguuagguguuugCacaug...................  1    1  st1
.......................................................................uaguuagguguuugaacaug...................  8    0  st1
.......................................................................uaguuagAuguuugaacauga..................  10   1  st1
.......................................................................uaguuagguguCugaacauga..................  1    1  st1
.......................................................................uaguuagguguuugaacaugU..................  4    1  st1
.......................................................................uagAuagguguuugaacauga..................  1    1  st1
.......................................................................uaguuagguguuugaacauga..................  68   0  st1
.......................................................................uaguCagguguuugaacauga..................  1    1  st1
.......................................................................uaguuagguguuugaacaugaauA...............  1    1  st1
........................................................................aguuagAuguuugaacauga..................  3    1  st1
........................................................................aguuagguguuugaacaugU..................  1    1  st1
..........................................................................uuagAuguuugaacauga..................  1    1  st1
...........................................................................uagAuguuugaacaugaauucga............  2    1  st1
```

Provisional ID	: ScYm1zk_361_3678
Score total	: 1295.1
Score for star read(s)	: 3.9
Score for read counts	: 1285.4
Score for mfe	: 1.2
Score for randfold	: 1.6
Score for cons. seed	: 3
Total read count	: 2533
Mature read count	: 2513
Loop read count	: 0
Star read count	: 20

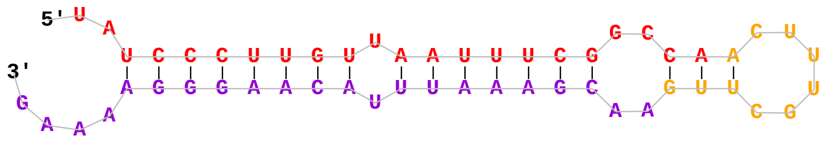

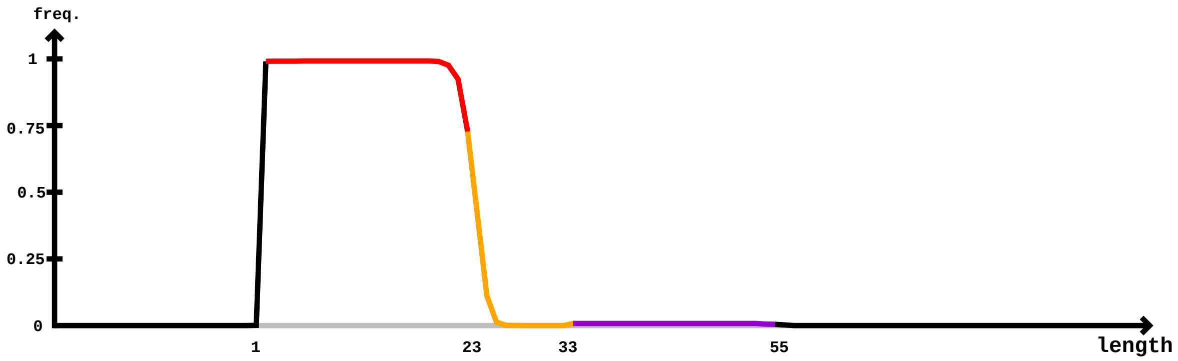

Mature Star

5'-	ucgucaucacauuccccucuuaucccuuguuaauuucggccaacuuugcuugaacgaaauuuacaagggaaaagaggcgccugccagaaguacuacaucaugcucgucg	-3'	obs	
	ucgucaucacauuccccucuuaucccuuguuaauuucggccaacuuugcuugaacgaaauuuacaagggaaaagaggcgccugccagaaguacuacaucaugcucgucg		exp	
	.((.(..........((((((.((((((((.(((((((..(((......)))..))))))).)))))))).)))))).....((..((.(((....))).))..))..).))	reads	mm	sample
	....................uaucccuuguuaauuucgA..	2	1	so1
	....................uaucccuuguuaauuucgg..	2	0	so1
	....................uaucccuuguuaauuucggU...	3	1	so1
	....................uaucccuuguuaauuucggA...	2	1	so1
	....................uaucccuuguuaauuucggc...	23	0	so1
	....................uaucccCuguuaauuucggcc..	1	1	so1
	....................uaucccuuguuaauuucggcc..	52	0	so1
	....................uaCcccuuguuaauuucggcc..	1	1	so1
	....................uaucccuuguuaauuucggcA..	22	1	so1
	....................uaucccuuguuaauuucggcU..	30	1	so1
	....................Caucccuuguuaauuucggcca...	2	1	so1
	....................uaucccuuguuaauuucggccC...	6	1	so1
	....................Aaucccuuguuaauuucggcca...	1	1	so1
	....................uaucccuuguuaauCucggcca...	1	1	so1
	....................uaucccuuguuaauuucggcca...	150	0	so1
	....................uaucccuuguuaauuucggccG...	2	1	so1
	....................uauccAuuguuaauuucggcca...	1	1	so1
	....................uaucccAuguuaauuucggcca...	1	1	so1
	....................uaucccuuguuaauuucggccU...	8	1	so1
	....................uaucccuuguuaauuucggccaG..	3	1	so1
	....................uaucccuuguuaauuucggccaU..	78	1	so1
	....................uaucccuuguuaauuucggccaa..	16	0	so1
	....................uaucccuuguuaauuucggcUaa..	1	1	so1
	....................uaucccuuguuaauuucggccaC..	11	1	so1
	....................uaucccuuguuaauuucggccaaG...	1	1	so1
	....................uaucccuuguuaauuucggccaaU...	32	1	so1
	....................uaucccuuguuaauuucggccaac...	2	0	so1
	....................uaucccuuguuaauuucggccaaA...	12	1	so1
	....................uaucccuuguuaauuucggccaUc...	17	1	so1
	....................uaucccuuguuaauuucggccaaUu..	7	1	so1
	....................uaucccuuguuaauuucggccaaAu..	4	1	so1
	....................uaucccuuguuaauuucggccaUcu..	2	1	so1
	....................uaucccuuguuaauuucggccaacuuu..	1	0	so1

```
                    Mature                          Star
ucgucaucacauuccccucuuaucccuuguuaauuucggccaacuuugcuugaacgaaauuuacaagggaaaagaggcgccugccagaaguacuacaucaugcucgucg
.....................aucccuuguuaauuucggcca...................................................................  1    0    so1
...................................................aacgaaauuuacaagggaaa......................................  1    0    so1

....................uaucccuuguuaauuucggU.....................................................................  1    1    sg1
....................uaucccuuguuaauuucggc.....................................................................  6    0    sg1
....................uaucccCuguuaauuucggcc....................................................................  1    1    sg1
....................uaucccuuguuaauuucggcA....................................................................  5    1    sg1
....................uaucccuuguuaauuucggcU....................................................................  3    1    sg1
....................uaucccuuguuaauuucggcc....................................................................  24   0    sg1
....................uaucccuuguuaauuuUggcc....................................................................  1    1    sg1
....................uaucccuCguuaauuucggcca...................................................................  1    1    sg1
....................uaucccuuguuGauuucggcca...................................................................  2    1    sg1
....................uaucccuuguuaauuucggccU...................................................................  6    1    sg1
....................uaucccuuguuaauuucggccC...................................................................  1    1    sg1
....................uaucccuuguuaauuucggccG...................................................................  4    1    sg1
....................uaucccuuguuaauuucggcca...................................................................  78   0    sg1
....................uaucUcuuguuaauuucggcca...................................................................  1    1    sg1
....................uaucccuuguuaauuucggccaG..................................................................  3    1    sg1
....................uaucccuuguuaauuucggccaa..................................................................  7    0    sg1
....................uaucccuuguuaauuucggccaC..................................................................  9    1    sg1
....................uaucccuuguuaauuucggccaU..................................................................  32   1    sg1
....................uaucccuuguuaauuucggccaac.................................................................  1    0    sg1
....................uaucccuuguuaauuucggccaaA.................................................................  3    1    sg1
....................uaucccuuguuaauuucggccaaU.................................................................  12   1    sg1
....................uaucccuuguuaauuucggccaUc.................................................................  9    1    sg1
....................uaucccuuguuaauuucggccaaUu................................................................  1    1    sg1
....................uaucccuuguuaauuucggccaUcu................................................................  4    1    sg1

....................uaucccuuguuaauuucgg......................................................................  4    0    st1
....................uaucccuuguuaaCuucgg......................................................................  1    1    st1
....................uaucccuuguuaauuucggU.....................................................................  4    1    st1
....................uaucccuuguuaauuucggc.....................................................................  10   0    st1
....................uaucccuuguuaauuucggA.....................................................................  1    1    st1
....................uaucccuuguuaauuucggcA....................................................................  5    1    st1
....................uaucccuuguuaGuuucggcc....................................................................  1    1    st1
....................uaucccuuguuaauuucggcc....................................................................  40   0    st1
....................uaucccuuguuaauuucggcU....................................................................  21   1    st1
....................uaucccCuguuaauuucggcca...................................................................  1    1    st1
....................uaucccuCguuaauuucggcca...................................................................  2    1    st1
....................uaucccuuguCaauuucggcca...................................................................  2    1    st1
....................uaucccuuguuaGuuucggcca...................................................................  1    1    st1
....................uaucccuuguuaauuucggccC...................................................................  7    1    st1
....................uaucccuuguuCauuucggcca...................................................................  1    1    st1
....................uaucccuuguuaaCuucggcca...................................................................  1    1    st1
....................uaucccuugCuaauuucggcca...................................................................  2    1    st1
....................uaucccuuguuaauCucggcca...................................................................  1    1    st1
....................uaucccuuguuaauuucggccU...................................................................  10   1    st1
....................Caucccuuguuaauuucggcca...................................................................  1    1    st1
....................uaucccuuguuaauuucggccG...................................................................  7    1    st1
....................uaucccuuguuaauuCcggcca...................................................................  3    1    st1
....................uaucccuugGuaauuucggcca...................................................................  2    1    st1
....................uaucccuuguuaauuucggcca...................................................................  257  0    st1
....................uaucccuuguuaauuucggccaC..................................................................  12   1    st1
....................uaucccuuguuaauuucggccaU..................................................................  67   1    st1
....................uaucccuuguuaauuucggccaa..................................................................  18   0    st1
....................uaucccuuguuaauuucggccGa..................................................................  1    1    st1
....................uaucccuuguuaauuucggccaG..................................................................  2    1    st1
....................uaucccuuguuaauuucggccaaG.................................................................  5    1    st1
....................uaucccuuguuaauuucggccaaA.................................................................  14   1    st1
....................uaucccuuguuaauuucggccaaU.................................................................  23   1    st1
....................uaucccuuguuaauuucggccaUc.................................................................  16   1    st1
....................uaucccuuguuaauuucggccaGc.................................................................  1    1    st1
....................uaucccuuguuaauuucggccaac.................................................................  1    0    st1
....................uaucccuuguuaauuucggccaaAu................................................................  2    1    st1
....................uaucccuuguuaauuucggccaaUu................................................................  1    1    st1
....................uaucccuuguuaauuucggccaUcu................................................................  2    1    st1
....................uaucccuuguuaauuucggccaacuA...............................................................  1    1    st1
........................ccuuguuaauuucggccaU..................................................................  1    1    st1
...................................................aacgaaauuuacaagggaaaag....................................  3    0    st1

...................Cuaucccuuguuaauuucggcca...................................................................  2    1    sr1
```

Mature **Star**

```
ucgucaucacauuccccucuuaucccuuguuaauuucggccaacuuugcuugaacgaaauuuacaagggaaaagaggcgccugccagaaguacuacuacaucaugcucgucg
....................uaucccuuguuaauuucg..........................................................................	5	0	sr1
....................uaucccuuguuaauuucgg.........................................................................	23	0	sr1
....................uaucccuuguuaauCucgg.........................................................................	1	1	sr1
....................uaucccuuguuaauuucgA.........................................................................	1	1	sr1
....................uaucccuuguuaGuuucgg.........................................................................	1	1	sr1
....................uaucccuuguuaauuucggc........................................................................	53	0	sr1
....................uaucccuuguuaauuucggA........................................................................	3	1	sr1
....................Gaucccuuguuaauuucggc........................................................................	14	1	sr1
....................uaucccuuguuaauuucggU........................................................................	10	1	sr1
....................uGucccuuguuaauuucggc........................................................................	1	1	sr1
....................Aaucccuuguuaauuucggc........................................................................	1	1	sr1
....................uaucccuCguuaauuucggcc.......................................................................	1	1	sr1
....................uaucccuuguuaauuucggcc.......................................................................	95	0	sr1
....................uaucccCuguuaauuucggcc.......................................................................	2	1	sr1
....................uaucccuuguuaauuucggcG.......................................................................	6	1	sr1
....................uaucccuuguuaauuucggcU.......................................................................	58	1	sr1
....................Gaucccuuguuaauuucggcc.......................................................................	11	1	sr1
....................uaucccuuguuaauuucggcA.......................................................................	118	1	sr1
....................uaucccuuguuaauuCcggcc.......................................................................	1	1	sr1
....................uaCcccuuguuaauuucggcca......................................................................	1	1	sr1
....................uaucccuCguuaauuucggcca......................................................................	1	1	sr1
....................uaucccuugAuaauuucggcca......................................................................	1	1	sr1
....................uaucccuuguuaauCucggcca......................................................................	1	1	sr1
....................uaucccuuguuaauuucggccG......................................................................	30	1	sr1
....................uaucccuuguuaauuCcggcca......................................................................	1	1	sr1
....................uaucccuuguuaauuucggccC......................................................................	14	1	sr1
....................uaucccuuguuaGuuucggcca......................................................................	2	1	sr1
....................uaucccuGguuaauuucggcca......................................................................	1	1	sr1
....................uaucccuuguuaauuucggcUa......................................................................	4	1	sr1
....................uaucccuuguuGauuucggcca......................................................................	1	1	sr1
....................uaucccuuguuaauuucCgcca......................................................................	1	1	sr1
....................uaucccuugCuaauuucggcca......................................................................	2	1	sr1
....................uaucccuuguuaauuucggcca......................................................................	350	0	sr1
....................uaucccCuguuaauuucggcca......................................................................	1	1	sr1
....................Gaucccuuguuaauuucggcca......................................................................	90	1	sr1
....................uaucccuuguuaauuucggcAa......................................................................	1	1	sr1
....................uauUccuuguuaauuucggcca......................................................................	2	1	sr1
....................uaucccuuguuaauuucggccU......................................................................	12	1	sr1
....................uaucccuuguuaauuucggcUaa.....................................................................	5	1	sr1
....................uaucccuuguuaauuucggccaa.....................................................................	29	0	sr1
....................Gaucccuuguuaauuucggccaa.....................................................................	6	1	sr1
....................uaucccuuguuaauuucggccaG.....................................................................	10	1	sr1
....................uaucccuuguuaauuucggccaC.....................................................................	22	1	sr1
....................uaucccuuguuaauCucggccaa.....................................................................	1	1	sr1
....................uaCcccuuguuaauuucggccaa.....................................................................	1	1	sr1
....................uaucccuuguuaauuucggccaU.....................................................................	140	1	sr1
....................uaucccuuguuaauuucggccaac....................................................................	8	0	sr1
....................uaucccuuguuaauuucggccaaU....................................................................	37	1	sr1
....................uaucccuuguuaauuucggccaUc....................................................................	17	1	sr1
....................uaucccuuguuaauuucggccaaG....................................................................	14	1	sr1
....................Gaucccuuguuaauuucggccaac....................................................................	1	1	sr1
....................uaucccuuguuaauuucggccaaA....................................................................	25	1	sr1
....................uaucccuuguuaauuucggccaacA...................................................................	1	1	sr1
....................uaucccuuguuaauuucggccaUcu...................................................................	1	1	sr1
....................uaucccuuguuaauuucggccaaUu...................................................................	5	1	sr1
....................uaucccuuguuaauuucggccaaAu...................................................................	1	1	sr1
........................ccuuguuaauuucggccaa.....................................................................	1	0	sr1
....................................................aacgaaauuuGcaagggaaa........................................	1	1	sr1
....................................................aacgaaauuuacaagggaaa........................................	4	0	sr1
....................................................Gacgaaauuuacaagggaaaa.......................................	1	1	sr1
....................................................aacgaaauuuacaagggaaaa.......................................	1	0	sr1
....................................................aacgaaauuuacaagggaaaag......................................	9	0	sr1
```

Provisional ID	: ScYm1zk_361_3679
Score total	: 9914.3
Score for star read(s)	: 3.9
Score for read counts	: 9905
Score for mfe	: 0.8
Score for randfold	: 1.6
Score for cons. seed	: 3
Total read count	: 19440
Mature read count	: 19411
Loop read count	: 0
Star read count	: 29

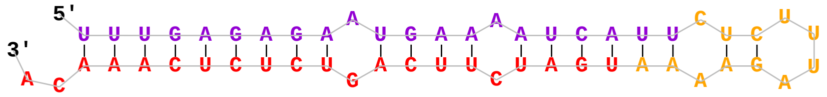

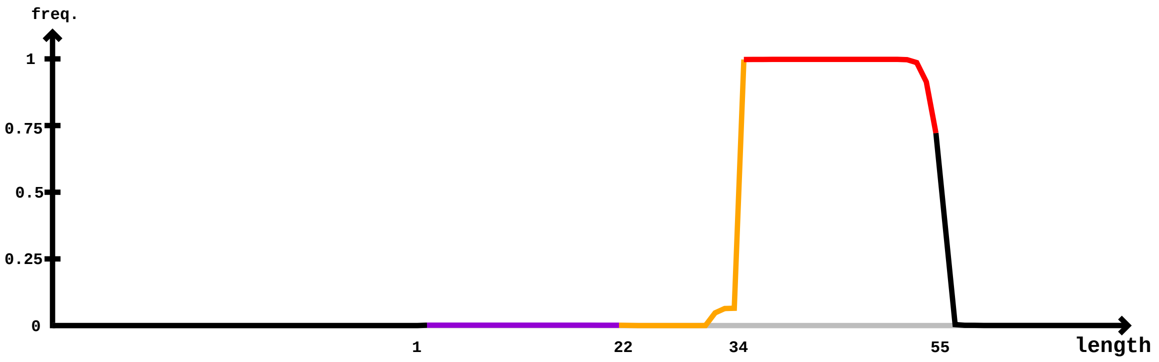

Star Mature

```
5'- ccuuaaccacucggccauucuaacaccauccuaccuguuugagagaaugaaaaucauucucuuuagaaaaugaucuucagucucucaaacaaguagcaacacugaucguc  -3'   obs
    ccuuaaccacucggccauucuaacaccauccuaccuguuugagagaaugaaaaucauucucuuuagaaaaugaucuucagucucucaaacaaguagcaacacugaucguc         exp
    ......((....))................((((.(((((((((((.((((.((((((.((....)).)))))).)))).))))))))))).))))..............    reads     mm       sample
    .....................................uuugagagaaugaaaauc.......................................................    1         0        st1
    .....................................uuugagagaaugaaaaucGuu....................................................    1         1        st1
    .....................................uuugGgagaaugaaaaucauu....................................................    2         1        st1
    .....................................uuugagagaaugaaaaucauu....................................................    6         0        st1
    .....................................uuugagagaaugaaaaucauucu..................................................    1         0        st1
    ...................................................................aaaugaucuucagucucuca.......................    1         0        st1
    ...................................................................aaaugaucuucagucucucaa......................    5         0        st1
    ...................................................................aaaugaucCucagucucucaaa.....................    1         1        st1
    ...................................................................aaaugaucuucagucucucaaC.....................    1         1        st1
    ...................................................................aaaugaucuucagucucucaaa.....................    48        0        st1
    ...................................................................aaaugaucuucagucucucaaU.....................    13        1        st1
    ...................................................................aaaugaucuucagucucucaaac....................    11        0        st1
    ...................................................................aaaugaucuucagucucucaaaA....................    4         1        st1
    ...................................................................aaaugaucuucagucucucaaaG....................    1         1        st1
    ...................................................................aaaugaucuucagucucucaaaU....................    11        1        st1
    ...................................................................aaauAaucuucagucucucaaac....................    1         1        st1
    ...................................................................aaaugaucuucagucucucaaGc....................    1         1        st1
    ...................................................................aaaugaucuucagucucucaaaca...................    10        0        st1
    ...................................................................aaaugaucuucagucucucaaacU...................    6         1        st1
    ...................................................................aaaugaucuucagucucucaaacC...................    1         1        st1
    ...................................................................aaaugaucuucagucucucaaacUa..................    1         1        st1
    ....................................................................aaugaucuucagucucucaa......................    2         0        st1
    ....................................................................aaugaucuucagucucucaaa.....................    6         0        st1
    ....................................................................aaugaucuucagucucucaaac....................    10        0        st1
    ....................................................................aaugaucuucagucucucaaaUa...................    1         1        st1
    ....................................................................aaugaucuucagucucucaaacU...................    1         1        st1
    ....................................................................aaugaucuucagucucucaaaca...................    7         0        st1
    ....................................................................aaugaucuucagucucucaaacG...................    1         1        st1
    ....................................................................aaugaucuucagucucucaaacaU..................    1         1        st1
    .....................................................................augaucuucagucucucaaac....................    1         0        st1
    .....................................................................augaucuucagucucucaaaca...................    1         0        st1
    .....................................................................Cugaucuucagucucucaaaca...................    1         1        st1
    ......................................................................ugaucuucagucucucaa......................    2         0        st1
```

```
                                     Star                 Mature
ccuuaaccacucggccauucuaacaccauccuaccuguuugagagaaugaaaaucauucucuuuagaaaaugaucuucagucucucaaacaaguagcaacacugaucguc
......................................................................ugaucuucagucucucaaa.....................   14    0  st1
......................................................................ugaucuucagucucucaaU.....................   1     1  st1
......................................................................ugaucuucagucucucaaaU....................   13    1  st1
......................................................................ugGucuucagucucucaaac....................   1     1  st1
......................................................................ugaucuucagucucucUaac....................   1     1  st1
......................................................................ugaucuucagucucucaGac....................   1     1  st1
......................................................................uAaucuucagucucucaaac....................   1     1  st1
......................................................................Agaucuucagucucucaaac....................   1     1  st1
......................................................................ugaucuucagucCcucaaac....................   1     1  st1
......................................................................ugaGcuucagucucucaaac....................   1     1  st1
......................................................................ugaucuucagucucucaaGc....................   1     1  st1
......................................................................ugaucuucCgucucucaaac....................   2     1  st1
......................................................................ugaucuucaUucucucaaac....................   1     1  st1
......................................................................ugaucuucGgucucucaaac....................   1     1  st1
......................................................................Cgaucuucagucucucaaac....................   2     1  st1
......................................................................ugaucCucagucucucaaac....................   2     1  st1
......................................................................ugaucuucagucucCcaaac....................   4     1  st1
......................................................................ugaucuucagucucucaaac....................   240   0  st1
......................................................................ugaucuucagucucucaaacC...................   1     1  st1
......................................................................ugaucuucagucAcucaaaca...................   2     1  st1
......................................................................ugaucuucagucucucaaacU...................   7     1  st1
......................................................................ugaucuucagucucucaaacG...................   29    1  st1
......................................................................ugaucCucagucucucaaaca...................   1     1  st1
......................................................................ugaucAucagucucucaaaca...................   3     1  st1
......................................................................ugauUuucagucucucaaaca...................   1     1  st1
......................................................................uAaucuucagucucucaaaca...................   4     1  st1
......................................................................ugaucuAcagucucucaaaca...................   2     1  st1
......................................................................ugaucuucGgucucucaaaca...................   4     1  st1
......................................................................Agaucuucagucucucaaaca...................   2     1  st1
......................................................................ugaucuCcagucucucaaaca...................   5     1  st1
......................................................................ugaucuucagucucucGaaca...................   4     1  st1
......................................................................uUaucuucagucucucaaaca...................   1     1  st1
......................................................................ugaucuucagucCcucaaaca...................   3     1  st1
......................................................................ugaucuucagucucucaaCca...................   1     1  st1
......................................................................ugaucuucagucucucaaUca...................   1     1  st1
......................................................................ugaucuucagucucucaaaUa...................   1     1  st1
......................................................................ugaucuucagucucucaaaca...................   1244  0  st1
......................................................................ugaucuucagucucGcaaaca...................   1     1  st1
......................................................................Cgaucuucagucucucaaaca...................   3     1  st1
......................................................................ugaCcuucagucucucaaaca...................   3     1  st1
......................................................................ugaucuucUgucucucaaaca...................   1     1  st1
......................................................................ugaucuucagucucCcaaaca...................   1     1  st1
......................................................................ugaucuucagCcucucaaaca...................   7     1  st1
......................................................................ugGucuucagucucucaaaca...................   8     1  st1
......................................................................ugaucuucCgucucucaaaca...................   13    1  st1
......................................................................ugaucuucaUucucucaaaca...................   1     1  st1
......................................................................ugaucuucagucuUucaaaca...................   1     1  st1
......................................................................ugaucuucagAcucucaaaca...................   1     1  st1
......................................................................ugaucuucagucucucaGaca...................   2     1  st1
......................................................................ugaucuucagucucucaaGca...................   2     1  st1
......................................................................ugaucGucagucucucaaaca...................   1     1  st1
......................................................................ugaucuuUagucucucaaaca...................   1     1  st1
......................................................................ugaucuucagucucAcaaacaa..................   1     1  st1
......................................................................ugaucuucCgucucucaaacaa..................   1     1  st1
......................................................................ugaucuucagucucucaaacUa..................   3     1  st1
......................................................................ugaucuucagucucucaaacaU..................   48    1  st1
......................................................................ugaucuucagucucucaaacaa..................   53    0  st1
......................................................................ugaucuucagucucucaaacaC..................   2     1  st1
......................................................................ugaucuucagucucucaaacaaU.................   1     1  st1
......................................................................ugaucuucagucucucaaacaaAu................   1     1  st1
......................................................................ugaucuucagucucucaaacaaUua...............   3     1  st1
...........................................................................................aguagcaacacugaucgA.   1     1  st1
...........................................................................................aguagcaacacugaucguc   1     0  st1

.....................................uuugGgagaaugaaaauc.......................................................   1     1  sr1
.....................................uuCgagagaaugaaaauc.......................................................   2     1  sr1
.....................................uuCgagagaaugaaaaucauu....................................................   2     1  sr1
.....................................uuugagagaaugGaaaucauu....................................................   1     1  sr1
.....................................uuugagagaaugaaaaucauu....................................................   1     0  sr1
.....................................uuAgagagaaugaaaaucauu....................................................   1     1  sr1
```

Star **Mature**

```
ccuuaaccacucggccauucuaacaccauccuaccuguuugagagaaugaaaaucauucucuuuagaaaaugaucuucagucucucaaacaaguagcaacacugaucguc
.....................................uuugGgagaaugaaaaucauu....................................................  4    1  sr1
.....................................uuugagagaaugaaaaucauuA...................................................  1    1  sr1
..................................................................aaaaugaucuucagucucucaaaca...................  1    0  sr1
...................................................................aaaugaucuucagucucuca.......................  15   0  sr1
...................................................................aaaugaucuucagucucucaG......................  2    1  sr1
...................................................................aaaugaucuucagucucucaa......................  77   0  sr1
...................................................................aaaugaucuucaguUucucaa......................  1    1  sr1
...................................................................aaaugaucuucagucucCcaa......................  1    1  sr1
...................................................................aaaugGucuucagucucucaa......................  1    1  sr1
...................................................................aaaAgaucuucagucucucaa......................  1    1  sr1
...................................................................Gaaugaucuucagucucucaa......................  1    1  sr1
...................................................................Uaaugaucuucagucucucaaa.....................  3    1  sr1
...................................................................aaaugaucuucagucucucaaU.....................  30   1  sr1
...................................................................aaaugaucuucagucucucaUa.....................  1    1  sr1
...................................................................aaaugaucuucaAucucucaaa.....................  2    1  sr1
...................................................................aaaugaucuCcagucucucaaa.....................  5    1  sr1
...................................................................aaauCaucuucagucucucaaa.....................  1    1  sr1
...................................................................aaaugGucuucagucucucaaa.....................  3    1  sr1
...................................................................aaaugaucuucagucucucaaa.....................  362  0  sr1
...................................................................aaaugaAcuucagucucucaaa.....................  1    1  sr1
...................................................................aaaugaucuucagucucucaGa.....................  1    1  sr1
...................................................................Gaaugaucuucagucucucaaa.....................  18   1  sr1
...................................................................aaaugaucCucagucucucaaa.....................  2    1  sr1
...................................................................aaaugaucuucagucucucaaG.....................  14   1  sr1
...................................................................aaaCgaucuucagucucucaaa.....................  1    1  sr1
...................................................................aaaugaucuucagucucucaaC.....................  3    1  sr1
...................................................................aaaugaucuucagucCcucaaa.....................  1    1  sr1
...................................................................aaGugaucuucagucucucaaa.....................  1    1  sr1
...................................................................aaaugaucuucagucucucaaaG....................  1    1  sr1
...................................................................aaaugaucuucagCcucucaaac....................  1    1  sr1
...................................................................aaaugaucuucagucucucaaaA....................  34   1  sr1
...................................................................aaaugaucuucagucucucaaaU....................  15   1  sr1
...................................................................aaaugaucuucagucucucaaUc....................  2    1  sr1
...................................................................aaaugaucuucagucucucaaac....................  25   0  sr1
...................................................................aaaugaucuucagucucucaaaca...................  14   0  sr1
...................................................................Gaaugaucuucagucucucaaaca...................  1    1  sr1
...................................................................aaaugaucuucagCcucucaaaca...................  1    1  sr1
...................................................................aaaugaucuucagucucucaaacG...................  3    1  sr1
...................................................................aaaugaucuucagucucucaaacC...................  1    1  sr1
...................................................................aaaugaucuucagucucucaaaUa...................  8    1  sr1
...................................................................aaaugaucuucagucucucaaacU...................  7    1  sr1
...................................................................aaaugaucuucagucucucaaacaa..................  1    0  sr1
...................................................................aaaugaucuucagucucucaaacUa..................  13   1  sr1
....................................................................aaugaucuucagucucuca.......................  7    0  sr1
....................................................................aaugaucuucagucucucG.......................  1    1  sr1
....................................................................aaugGucuucagucucucaa......................  1    1  sr1
....................................................................aaugaucuucagucucucaa......................  28   0  sr1
....................................................................aaugaucuucagucucucaaU.....................  7    1  sr1
....................................................................aaugaucuucagucucucaaG.....................  9    1  sr1
....................................................................aaugaucCucagucucucaaa.....................  1    1  sr1
....................................................................aaugaucuucagucucucaUa.....................  1    1  sr1
....................................................................aGugaucuucagucucucaaa.....................  4    1  sr1
....................................................................aaugaucuucagucucucaaa.....................  129  0  sr1
....................................................................Gaugaucuucagucucucaaa.....................  6    1  sr1
....................................................................aaugaucuucagCcucucaaa.....................  1    1  sr1
....................................................................aaugGucuucagucucucaaa.....................  1    1  sr1
....................................................................aaugaucuCcagucucucaaa.....................  2    1  sr1
....................................................................aGugaucuucagucucucaaac....................  3    1  sr1
....................................................................aaugaucuucagucucucaaaA....................  6    1  sr1
....................................................................aaugaucuucagucucucaaac....................  19   0  sr1
....................................................................aaugaucuucagucucucaaaG....................  1    1  sr1
....................................................................Gaugaucuucagucucucaaaca...................  1    1  sr1
....................................................................aaugaucuucagucucucaaaUa...................  1    1  sr1
....................................................................aaugaucuucagucucucaaacU...................  1    1  sr1
....................................................................aaugaucuucagCcucucaaaca...................  1    1  sr1
....................................................................aaugaucuucagucucucaaacG...................  2    1  sr1
....................................................................aaugGucuucagucucucaaaca...................  1    1  sr1
....................................................................aaugaucuucagucucucaaaca...................  25   0  sr1
....................................................................aaugaucuucagucucucaaacaa..................  4    0  sr1
....................................................................aaugaucuucagucucucaaacUa..................  2    1  sr1
```

Star Mature

ccuuaaccacucggccauucuaacaccauccuaccuguuugagagaaugaaaaucauucucuuuagaaaaugaucuucagucucucaaacaaguagcaacacugaucguc			
....augaucuucagucucucaaU.....................	3	1	sr1
....augaucuucagucucucaaaU....................	1	1	sr1
....Cugaucuucagucucucaaac....................	1	1	sr1
....augaucuucagucucucaaac....................	3	0	sr1
....augaucuucagucucucaaaca...................	3	0	sr1
....Cugaucuucagucucucaaaca...................	1	1	sr1
....Cugaucuucagucucucaaacaa..................	1	1	sr1
....ugaucuucagucucucaU......................	2	1	sr1
....ugGucuucagucucucaa......................	1	1	sr1
....ugaucuucagucucucaa......................	57	0	sr1
....ugaucuucagucucucGa......................	1	1	sr1
....Ggaucuucagucucucaa......................	1	1	sr1
....ugaucuucagucucuAaa......................	2	1	sr1
....ugaucuucagCcucucaaa.....................	1	1	sr1
....ugaucCucagucucucaaa.....................	3	1	sr1
....ugaCcuucagucucucaaa.....................	2	1	sr1
....ugaucuucagucucucaGa.....................	1	1	sr1
....ugaucuucagucucCcaaa.....................	2	1	sr1
....ugaucuucagucucucaUa.....................	7	1	sr1
....ugaucuucagucucucaaC.....................	5	1	sr1
....ugaucuCcagucucucaaa.....................	1	1	sr1
....ugGucuucagucucucaaa.....................	5	1	sr1
....ugaucuucagucucucaaa.....................	504	0	sr1
....Ggaucuucagucucucaaa.....................	12	1	sr1
....ugUucuucagucucucaaa.....................	2	1	sr1
....ugaucuucagucucucaaU.....................	48	1	sr1
....ugaucuucagucucucaaG.....................	17	1	sr1
....ugaAcuucagucucucaaa.....................	1	1	sr1
....ugaucuucagucCcucaaa.....................	2	1	sr1
....ugaucuucaguUucucaaa.....................	1	1	sr1
....ugauUuucagucucucaaac....................	2	1	sr1
....ugaucuucagucCcucaaac....................	9	1	sr1
....ugaucuucGgucucucaaac....................	12	1	sr1
....Ggaucuucagucucucaaac....................	66	1	sr1
....ugaucuucagucucucaGac....................	9	1	sr1
....ugaucCucagucucucaaac....................	18	1	sr1
....ugaucuucagCcucucaaac....................	8	1	sr1
....ugaCcuucagucucucaaac....................	4	1	sr1
....ugaucuucagucucucaaUc....................	2	1	sr1
....ugGucuucagucucucaaac....................	10	1	sr1
....ugaucuucagucucucaaac....................	2713	0	sr1
....ugaucuucagAcucucaaac....................	2	1	sr1
....ugaucuucagucucucGaac....................	7	1	sr1
....ugaucuucagucuAucaaac....................	1	1	sr1
....ugaucuucagucucAcaaac....................	1	1	sr1
....ugaucuCcagucucucaaac....................	3	1	sr1
....ugaucuucagucucucaaaA....................	48	1	sr1
....ugaucuAcagucucucaaac....................	3	1	sr1
....ugUucuucagucucucaaac....................	1	1	sr1
....ugaucuucCgucucucaaac....................	1	1	sr1
....Agaucuucagucucucaaac....................	2	1	sr1
....ugaucuucagucucucaaaU....................	180	1	sr1
....uAaucuucagucucucaaac....................	3	1	sr1
....ugaAcuucagucucucaaac....................	4	1	sr1
....ugaucuucagucucucaaaG....................	3	1	sr1
....ugaGcuucagucucucaaac....................	1	1	sr1
....Cgaucuucagucucucaaac....................	8	1	sr1
....ugaucuucagucucCcaaac....................	9	1	sr1
....ugaucuucagucucucaaGc....................	6	1	sr1
....ugaucuuUagucucucaaac....................	3	1	sr1
....ugaucuucagucuUucaaac....................	2	1	sr1
....ugaucuucUgucucucaaac....................	1	1	sr1
....ugaucuucagucucucaaCc....................	1	1	sr1
....ugaucuucagucucuUaaac....................	1	1	sr1
....ugaucuucagucucucaaaca...................	8915	0	sr1
....ugaucuucaUucucucaaaca...................	1	1	sr1
....ugaucuucagucucucUaaca...................	7	1	sr1
....ugaucuucagucucAcaaaca...................	7	1	sr1
....ugaucuucagucucucaUaca...................	1	1	sr1
....ugaucuucagucucucGaaca...................	31	1	sr1

```
                                                         Star                              Mature
ccuuaaccacucggccauucuaacaccauccuaccuguuugagagaaugaaaaucauucucuuuagaaaaugaucuucagucucucaaacaaguagcaacacugaucguc
......................................................................ugaucGucagucucucaaaca...................    2      1     sr1
......................................................................ugaucuucagucucuUaaaca...................    7      1     sr1
......................................................................ugGucuucagucucucaaaca...................    68     1     sr1
......................................................................ugauAuucagucucucaaaca...................    1      1     sr1
......................................................................uUaucuucagucucucaaaca...................    2      1     sr1
......................................................................ugaucuucagCcucucaaaca...................    24     1     sr1
......................................................................ugaucuucaAucucucaaaca...................    3      1     sr1
......................................................................ugaucuCcagucucucaaaca...................    27     1     sr1
......................................................................uAaucuucagucucucaaaca...................    6      1     sr1
......................................................................ugaucuGcagucucucaaaca...................    3      1     sr1
......................................................................ugaucuucagucCcucaaaca...................    28     1     sr1
......................................................................ugaucuuUagucucucaaaca...................    12     1     sr1
......................................................................Cgaucuucagucucucaaaca...................    26     1     sr1
......................................................................ugaucuucagucuUucaaaca...................    7      1     sr1
......................................................................ugaucuucUgucucucaaaca...................    3      1     sr1
......................................................................ugaucuucagucAcucaaaca...................    3      1     sr1
......................................................................ugaucuuAagucucucaaaca...................    2      1     sr1
......................................................................ugaucuucagucucucaaacC...................    11     1     sr1
......................................................................ugaucuucGgucucucaaaca...................    22     1     sr1
......................................................................ugaucuAcagucucucaaaca...................    4      1     sr1
......................................................................ugaucuucagucucuGaaaca...................    1      1     sr1
......................................................................ugaucuucagucucuAaaaca...................    1      1     sr1
......................................................................ugauUuucagucucucaaaca...................    1      1     sr1
......................................................................ugaucuucaguGucucaaaca...................    2      1     sr1
......................................................................ugaucuucagucucucaaaUa...................    13     1     sr1
......................................................................ugaucuucagucucucaaacU...................    86     1     sr1
......................................................................ugaucuuGagucucucaaaca...................    1      1     sr1
......................................................................Ggaucuucagucucucaaaca...................    490    1     sr1
......................................................................ugaucuucagucucucaaacG...................    369    1     sr1
......................................................................ugaucuucagucucucaaaAa...................    2      1     sr1
......................................................................ugauGuucagucucucaaaca...................    1      1     sr1
......................................................................ugaucuucagucucCcaaaca...................    33     1     sr1
......................................................................ugaucAucagucucucaaaca...................    1      1     sr1
......................................................................ugaucuucagucucucCaaca...................    3      1     sr1
......................................................................ugaucuucagucuAucaaaca...................    1      1     sr1
......................................................................Agaucuucagucucucaaaca...................    17     1     sr1
......................................................................ugaGcuucagucucucaaaca...................    1      1     sr1
......................................................................ugaAcuucagucucucaaaca...................    7      1     sr1
......................................................................ugaucuucagucucucaaGca...................    23     1     sr1
......................................................................ugaucuucagucucucaaUca...................    2      1     sr1
......................................................................ugaCcuucagucucucaaaca...................    37     1     sr1
......................................................................ugCucuucagucucucaaaca...................    2      1     sr1
......................................................................ugaucuucagucucucaGaca...................    20     1     sr1
......................................................................ugaucCucagucucucaaaca...................    26     1     sr1
......................................................................ugaucuucagucucucaaCca...................    1      1     sr1
......................................................................ugaucuucagucucucaaacGa..................    1      1     sr1
......................................................................ugaucuucagucucucaaacaU..................    113    1     sr1
......................................................................ugaucuucagucucucaaaAaa..................    1      1     sr1
......................................................................ugaucuucagucucucaaacaa..................    708    0     sr1
......................................................................ugaucuucagucucucaaacaC..................    17     1     sr1
......................................................................ugaucuucaAucucucaaacaa..................    2      1     sr1
......................................................................ugaucuucGgucucucaaacaa..................    2      1     sr1
......................................................................ugaucuucagCcucucaaacaa..................    1      1     sr1
......................................................................ugaucuucagucucucaGacaa..................    6      1     sr1
......................................................................ugUucuucagucucucaaacaa..................    4      1     sr1
......................................................................ugaucuuUagucucucaaacaa..................    4      1     sr1
......................................................................Ggaucuucagucucucaaacaa..................    11     1     sr1
......................................................................ugaucuucagucucCcaaacaa..................    3      1     sr1
......................................................................ugaucuucagucucucaaaUaa..................    1      1     sr1
......................................................................ugaucuucagucucucaaCcaa..................    2      1     sr1
......................................................................ugaucuucagAcucucaaacaa..................    1      1     sr1
......................................................................ugaucuucagucucucaCacaa..................    1      1     sr1
......................................................................ugaucuucagucucucaaacUa..................    107    1     sr1
......................................................................ugaucuucagucucucaaacCa..................    1      1     sr1
......................................................................ugaAcuucagucucucaaacaa..................    3      1     sr1
......................................................................ugaucuCcagucucucaaacaa..................    1      1     sr1
......................................................................ugaCcuucagucucucaaacaa..................    2      1     sr1
......................................................................ugauUuucagucucucaaacaa..................    1      1     sr1
......................................................................ugaucuucagucCcucaaacaa..................    5      1     sr1
......................................................................ugaucuucagucucucGaacaa..................    1      1     sr1
```

Star Mature

```
ccuuaaccacucggccauucuaacaccauccuaccuguuugagagaaugaaaaucauucucuuuagaaaaugaucuucagucucucaaacaaguagcaacacugaucguc
......................................................................ugaucuucagucucucaaacaG..................  30  1  sr1
......................................................................Cgaucuucagucucucaaacaa..................  2   1  sr1
......................................................................ugaucCucagucucucaaacaa..................  4   1  sr1
......................................................................ugaucuucagucucucaaGcaa..................  1   1  sr1
......................................................................ugaucGucagucucucaaacaa..................  2   1  sr1
......................................................................ugaucuucagucucucaaacUag.................  2   1  sr1
......................................................................ugaucuucagucucucaaacaaU.................  3   1  sr1
......................................................................ugaucuucagucucucaaacaaC.................  2   1  sr1
......................................................................ugaucuucagucucucaaacaaA.................  27  1  sr1
......................................................................ugaucuucagucucucaaacaUg.................  15  1  sr1
......................................................................ugaucuucagucucucaaacGag.................  1   1  sr1
......................................................................ugaucuucagucucucaaacaaAu................  1   1  sr1
......................................................................ugaucuucagucucucaaacaaUua...............  5   1  sr1
......................................................................ugaucuucagucucucaaacaaAua...............  3   1  sr1
.......................................................................gaucuucagucucucaaa.....................  1   0  sr1
.......................................................................gaucuucagucucucaaac....................  2   0  sr1
.......................................................................gaucuucagucucucaaaU....................  1   1  sr1
.......................................................................gaucuucagucucucaaaca...................  1   0  sr1
........................................................................aucuucagucucucaaac....................  1   0  sr1
.........................................................................Gcuucagucucucaaaca...................  1   1  sr1
.........................................................................ucuucagucucucaaaca...................  2   0  sr1
.........................................................................ucuucagucucucaaacG...................  2   1  sr1
..........................................................................cuucagucucucaaacaU..................  1   1  sr1
...........................................................................................aguagcaacacugaucguG  1   1  sr1
...........................................................................................aguagcaacacugaucguc  1   0  sr1
...........................................................................................aguagcaacacugaucguA  3   1  sr1

.....................................uuAgagagaaugaaaaucauu....................................................  1   1  so1
.....................................uuCgagagaaugaaaaucauu....................................................  2   1  so1
...................................................................aaaugaucuucagucucuca.......................  3   0  so1
...................................................................aaaugaucuucagucucucaa......................  20  0  so1
...................................................................aaaugaucuucagucucucaG......................  2   1  so1
...................................................................aaaugaucuCcagucucucaaa.....................  2   1  so1
...................................................................aaaugaucuucagucucucaaU.....................  7   1  so1
...................................................................aaaugaucuucagucucucaaa.....................  58  0  so1
...................................................................aaaugaucuucagAcucucaaa.....................  1   1  so1
...................................................................aaaugaucCucagucucucaaa.....................  1   1  so1
...................................................................aaaugaucuucagucucucaaac....................  3   0  so1
...................................................................aaaugaucuucagucucucaaaA....................  2   1  so1
...................................................................aaaugaucuucagucucucaaaUa...................  2   1  so1
...................................................................aaaugaucuucagucucucaaaca...................  3   0  so1
...................................................................aaaugaucuucagucucucaaaUaa..................  2   1  so1
...................................................................aaaugaucuucagucucucaaacUa..................  1   1  so1
....................................................................aaugaucuucagucucuc........................  1   0  so1
....................................................................aaugaucuucagucucucaa......................  1   0  so1
....................................................................aaugaucuucagucucucaaa.....................  7   0  so1
....................................................................aaugaucuucagucucucaGa.....................  1   1  so1
....................................................................aaugaucuucagucucucaaaU....................  1   1  so1
....................................................................aaugaucuucagucucucaaaca...................  8   0  so1
....................................................................aaugaucuucagucucucaaacaa..................  1   0  so1
.....................................................................Cugaucuucagucucucaaaca...................  2   1  so1
.....................................................................augaucuucagucucucaaaca...................  1   0  so1
......................................................................ugaucuucagucucucaaa.....................  8   0  so1
......................................................................ugaucuucagucucucaaU.....................  2   1  so1
......................................................................ugaucuucagucucucaaac....................  124 0  so1
......................................................................ugaucuucagucucucaaaA....................  1   1  so1
......................................................................ugaucuucagucCcucaaac....................  1   1  so1
......................................................................ugaucuucagucucucaaaU....................  16  1  so1
......................................................................ugaucuucagCcucucaaaca...................  1   1  so1
......................................................................ugaucuucagucucucGaaca...................  4   1  so1
......................................................................ugaucGucagucucucaaaca...................  1   1  so1
......................................................................ugaucuucagAcucucaaaca...................  1   1  so1
......................................................................ugaucuucagucucucaaacU...................  5   1  so1
......................................................................ugaucCucagucucucaaaca...................  5   1  so1
......................................................................ugaucuucagucucucaaaUa...................  2   1  so1
......................................................................ugaucuucagucucucaaacG...................  12  1  so1
......................................................................uAaucuucagucucucaaaca...................  1   1  so1
......................................................................ugaucuucagucCcucaaaca...................  1   1  so1
......................................................................ugaucuucGgucucucaaaca...................  3   1  so1
......................................................................ugaucuucagucucCcaaaca...................  1   1  so1
```

Star Mature

```
ccuuaaccacucggccauucuaacaccauccuaccuguuugagagaaugaaaaucauucucuuuagaaaaugaucuucagucucucaaacaaguagcaacacugaucguc
......................................................................ugaucuucagucucucaaacC...................  1    1   so1
......................................................................ugaCcuucagucucucaaaca...................  1    1   so1
......................................................................ugaucuCcagucucucaaaca...................  2    1   so1
......................................................................ugaucAucagucucucaaaca...................  1    1   so1
......................................................................ugaucuucagucucucaaaca...................  577  0   so1
......................................................................ugaucuucagucucucaaacaC..................  1    1   so1
......................................................................ugaucuucagucucucaaacUa..................  3    1   so1
......................................................................ugaucuucagucucucaaacaU..................  20   1   so1
......................................................................ugaucuucagucucucaaacaa..................  33   0   so1
......................................................................ugaucuucagucucucaaacaaA.................  2    1   so1
......................................................................ugaucuucagucucucaaacaagua...............  1    0   so1

.....................................uuugGgagaaugaaaaucauuc...................................................  2    1   sg1
...............................................................uagaaaaugaucuucagucucucaaac....................  1    0   sg1
...................................................................aaaugaucuucagucucucaa......................  9    0   sg1
...................................................................aaaugaucuucagucucucaaU.....................  5    1   sg1
...................................................................aaaugaucuucagCcucucaaa.....................  1    1   sg1
...................................................................aaaugaucuCcagucucucaaa.....................  1    1   sg1
...................................................................aGaugaucuucagucucucaaa.....................  1    1   sg1
...................................................................aaaugaucuucagucucucaaa.....................  43   0   sg1
...................................................................aaaugaucuucagucucucaaaA....................  3    1   sg1
...................................................................aaaugaucuucagucucucaaac....................  5    0   sg1
...................................................................aaaugaucuucagucucucGaac....................  1    1   sg1
...................................................................aaaugaucuucagucucucaaaca...................  1    0   sg1
...................................................................aaaugaucuucagucucucaaaUa...................  1    1   sg1
...................................................................aaaugaucuucagucucucaaaUaa..................  1    1   sg1
...................................................................aaaugaucuucagucucucaaacUa..................  1    1   sg1
....................................................................aaugaucuucagCcucucaaa.....................  3    1   sg1
....................................................................aaugaucuucagucucucaaa.....................  7    0   sg1
....................................................................aaugaucuucagucucucaaaca...................  6    0   sg1
....................................................................aaugaucuucagucucucaaacaa..................  2    0   sg1
.....................................................................augaucuucagucucucaa......................  1    0   sg1
......................................................................ugaucuucagucucucaa......................  1    0   sg1
......................................................................ugaucCucagucucucaaa.....................  1    1   sg1
......................................................................ugaucuucagucucucaaU.....................  3    1   sg1
......................................................................ugaucuucagucucucaaa.....................  15   0   sg1
......................................................................ugaucuucagucucucaaaU....................  4    1   sg1
......................................................................ugaucuucagucucucaaaA....................  3    1   sg1
......................................................................ugaucuucagucucCcaaac....................  1    1   sg1
......................................................................ugaucuucGgucucucaaac....................  1    1   sg1
......................................................................ugaucuCcagucucucaaac....................  1    1   sg1
......................................................................ugaucuucagucucucaaac....................  178  0   sg1
......................................................................ugaucCucagucucucaaac....................  1    1   sg1
......................................................................ugaucCucagucucucaaaca...................  7    1   sg1
......................................................................ugaucuucagucucucaaCca...................  2    1   sg1
......................................................................ugaucuuUagucucucaaaca...................  1    1   sg1
......................................................................ugaucuCcagucucucaaaca...................  2    1   sg1
......................................................................ugaucuucagucucucaaacU...................  4    1   sg1
......................................................................ugaCcuucagucucucaaaca...................  2    1   sg1
......................................................................ugaucuucagucucucaaacG...................  8    1   sg1
......................................................................ugaucuucagucucucaaGca...................  2    1   sg1
......................................................................ugaucuucagucucucGaaca...................  7    1   sg1
......................................................................ugaucuucagucucucaaaUa...................  2    1   sg1
......................................................................ugaucuucagCcucucaaaca...................  5    1   sg1
......................................................................ugaucuucagucucCcaaaca...................  1    1   sg1
......................................................................ugaAcuucagucucucaaaca...................  1    1   sg1
......................................................................ugaucuucagucCcucaaaca...................  4    1   sg1
......................................................................ugaucuucagucucucaaaca...................  786  0   sg1
......................................................................ugUucuucagucucucaaaca...................  2    1   sg1
......................................................................ugaucuucagucucucaGaca...................  5    1   sg1
......................................................................ugGucuucagucucucaaaca...................  6    1   sg1
......................................................................ugauUuucagucucucaaaca...................  2    1   sg1
......................................................................ugaucuucagucucucUaaca...................  2    1   sg1
......................................................................ugaucuuAagucucucaaaca...................  1    1   sg1
......................................................................Agaucuucagucucucaaaca...................  1    1   sg1
......................................................................ugaucuucGgucucucaaacaa..................  1    1   sg1
......................................................................ugaucuucagucucucaaacaG..................  2    1   sg1
......................................................................ugaucuucagucucucaGacaa..................  1    1   sg1
......................................................................ugaucCucagucucucaaacaa..................  2    1   sg1
......................................................................ugaucuucagucucucaaacaU..................  13   1   sg1
```

```
                                                      Star                                Mature
ccuuaaccacucggccauucuaacaccauccuaccuguuugagagaaugaaaaucauucucuuuagaaaaugaucuucagucucucaaacaaguagcaacacugaucguc
......................................................................ugaucuucagucucucaaacaa..................    73    0    sg1
......................................................................ugaucuucagucucucaaacUa..................    10    1    sg1
......................................................................ugaucuucagucucucaaacaaA.................    2     1    sg1
......................................................................ugaucuucagucucucaaacaaU.................    1     1    sg1
......................................................................ugaucuucagucucucaaacaUg.................    1     1    sg1
```

Provisional ID	:	ScYm1zk_361_3722
Score total	:	9914.6
Score for star read(s)	:	3.9
Score for read counts	:	9905
Score for mfe	:	1.1
Score for randfold	:	1.6
Score for cons. seed	:	3
Total read count	:	19440
Mature read count	:	19411
Loop read count	:	0
Star read count	:	29

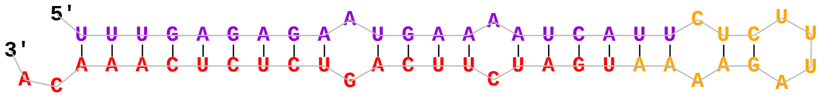

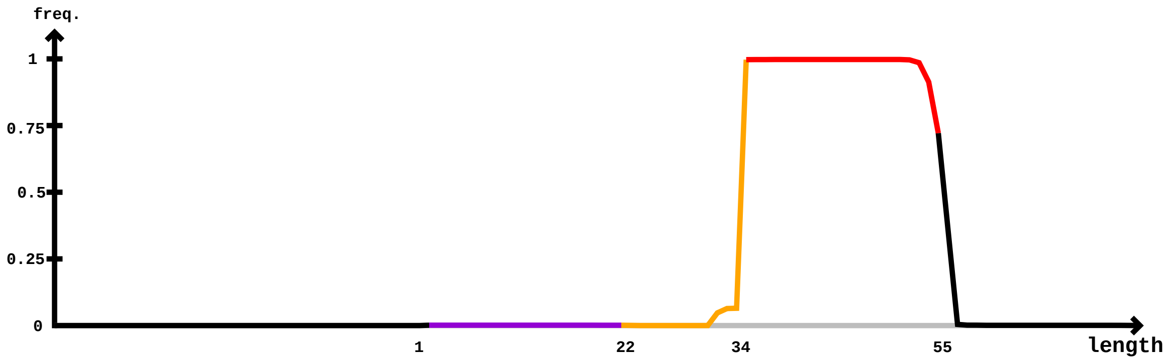

Star Mature

```
5'- ccuuaaccacucggccauucuaacaccauccuaccuguuugagagaaugaaaaucauucucuuuagaaaaugaucuucagucucucaaacaaguagcaacacugaucgucg   -3'   obs
    ccuuaaccacucggccauucuaacaccauccuaccuguuugagagaaugaaaaucauucucuuuagaaaaugaucuucagucucucaaacaaguagcaacacugaucgucg         exp
    ...........((((...((..........((((.(((((((((((.((((.((((((.((....)).)))))).)))).))))))))))).)))).......))..))))   reads   mm   sample
    .....................................uuAgagagaaugaaaaucauu.....................................................   1       1    so1
    .....................................uuCgagagaaugaaaaucauu.....................................................   2       1    so1
    ...................................................................aaaugaucuucagucucuca........................   3       0    so1
    ...................................................................aaaugaucuucagucucucaa.......................   20      0    so1
    ...................................................................aaaugaucuucagucucucaG.......................   2       1    so1
    ...................................................................aaaugaucuucagucucucaaa......................   58      0    so1
    ...................................................................aaaugaucuucagAcucucaaa......................   1       1    so1
    ...................................................................aaaugaucuCcagucucucaaa......................   2       1    so1
    ...................................................................aaaugaucCucagucucucaaa......................   1       1    so1
    ...................................................................aaaugaucuucagucucucaaU......................   7       1    so1
    ...................................................................aaaugaucuucagucucucaaaA.....................   2       1    so1
    ...................................................................aaaugaucuucagucucucaaac.....................   3       0    so1
    ...................................................................aaaugaucuucagucucucaaaca....................   3       0    so1
    ...................................................................aaaugaucuucagucucucaaaUa....................   2       1    so1
    ...................................................................aaaugaucuucagucucucaaacUa...................   1       1    so1
    ...................................................................aaaugaucuucagucucucaaaUaa...................   2       1    so1
    ....................................................................aaugaucuucagucucuc.........................   1       0    so1
    ....................................................................aaugaucuucagucucucaa.......................   1       0    so1
    ....................................................................aaugaucuucagucucucaaa......................   7       0    so1
    ....................................................................aaugaucuucagucucucaGa......................   1       1    so1
    ....................................................................aaugaucuucagucucucaaaU.....................   1       1    so1
    ....................................................................aaugaucuucagucucucaaaca....................   8       0    so1
    ....................................................................aaugaucuucagucucucaaacaa...................   1       0    so1
    .....................................................................augaucuucagucucucaaaca....................   1       0    so1
    .....................................................................Cugaucuucagucucucaaaca....................   2       1    so1
    ......................................................................ugaucuucagucucucaaa......................   8       0    so1
    ......................................................................ugaucuucagucucucaaU......................   2       1    so1
    ......................................................................ugaucuucagucucucaaac.....................   124     0    so1
    ......................................................................ugaucuucagucCcucaaac.....................   1       1    so1
    ......................................................................ugaucuucagucucucaaaA.....................   1       1    so1
    ......................................................................ugaucuucagucucucaaaU.....................   16      1    so1
    ......................................................................ugaucuucagucucucaaaUa....................   2       1    so1
    ......................................................................ugaucuucagucucucaaacG....................   12      1    so1
```

```
                                     Star                            Mature
ccuuaaccacucggccauucuaacaccauccuaccuguuugagagaaugaaaaucauucucuuuagaaaaugaucuucagucucucaaacaaguagcaacacugaucgucg
......................................................................ugaucuucagucucucaaaca....................   577   0   so1
......................................................................ugaucuucagucucCcaaaca....................   1     1   so1
......................................................................ugaucuucGgucucucaaaca....................   3     1   so1
......................................................................ugaucuucagCcucucaaaca....................   1     1   so1
......................................................................ugaCcuucagucucucaaaca....................   1     1   so1
......................................................................ugaucuCcagucucucaaaca....................   2     1   so1
......................................................................ugaucuucagucucucGaaca....................   4     1   so1
......................................................................uAaucuucagucucucaaaca....................   1     1   so1
......................................................................ugaucuucagucucucaaacC....................   1     1   so1
......................................................................ugaucuucagucucucaaacU....................   5     1   so1
......................................................................ugaucuucagAcucucaaaca....................   1     1   so1
......................................................................ugaucuucagucCcucaaaca....................   1     1   so1
......................................................................ugaucAucagucucucaaaca....................   1     1   so1
......................................................................ugaucGucagucucucaaaca....................   1     1   so1
......................................................................ugaucCucagucucucaaaca....................   5     1   so1
......................................................................ugaucuucagucucucaaacaa...................   33    0   so1
......................................................................ugaucuucagucucucaaacUa...................   3     1   so1
......................................................................ugaucuucagucucucaaacaC...................   1     1   so1
......................................................................ugaucuucagucucucaaacaU...................   20    1   so1
......................................................................ugaucuucagucucucaaacaaA..................   2     1   so1
......................................................................ugaucuucagucucucaaacaagua................   1     0   so1
...........................................................................................aguagcaacacugaucgucA   1     1   so1

.....................................uuugGgagaaugaaaaucauuc....................................................   2     1   sg1
...............................................................uagaaaaugaucuucagucucucaaac.....................   1     0   sg1
...................................................................aaaugaucuucagucucucaa.......................   9     0   sg1
...................................................................aaaugaucuucagucucucaaa......................   43    0   sg1
...................................................................aaaugaucuucagucucucaaU......................   5     1   sg1
...................................................................aaaugaucuucagCcucucaaa......................   1     1   sg1
...................................................................aGaugaucuucagucucucaaa......................   1     1   sg1
...................................................................aaaugaucuCcagucucucaaa......................   1     1   sg1
...................................................................aaaugaucuucagucucucaaac.....................   5     0   sg1
...................................................................aaaugaucuucagucucucGaac.....................   1     1   sg1
...................................................................aaaugaucuucagucucucaaaA.....................   3     1   sg1
...................................................................aaaugaucuucagucucucaaaUa....................   1     1   sg1
...................................................................aaaugaucuucagucucucaaaca....................   1     0   sg1
...................................................................aaaugaucuucagucucucaaacUa...................   1     1   sg1
...................................................................aaaugaucuucagucucucaaaUaa...................   1     1   sg1
....................................................................aaugaucuucagCcucucaaa......................   3     1   sg1
....................................................................aaugaucuucagucucucaaa......................   7     0   sg1
....................................................................aaugaucuucagucucucaaaca....................   6     0   sg1
....................................................................aaugaucuucagucucucaaacaa...................   2     0   sg1
.....................................................................augaucuucagucucucaa.......................   1     0   sg1
......................................................................ugaucuucagucucucaa.......................   1     0   sg1
......................................................................ugaucuucagucucucaaU......................   3     1   sg1
......................................................................ugaucCucagucucucaaa......................   1     1   sg1
......................................................................ugaucuucagucucucaaa......................   15    0   sg1
......................................................................ugaucuucagucucucaaac.....................   178   0   sg1
......................................................................ugaucuucagucucucaaaU.....................   4     1   sg1
......................................................................ugaucuCcagucucucaaac.....................   1     1   sg1
......................................................................ugaucuucagucucucaaaA.....................   3     1   sg1
......................................................................ugaucuucGgucucucaaac.....................   1     1   sg1
......................................................................ugaucuucagucucCcaaac.....................   1     1   sg1
......................................................................ugaucCucagucucucaaac.....................   1     1   sg1
......................................................................ugaucuucagucucucUaaca....................   2     1   sg1
......................................................................ugaCcuucagucucucaaaca....................   2     1   sg1
......................................................................Agaucuucagucucucaaaca....................   1     1   sg1
......................................................................ugaucuucagucucucaaGca....................   2     1   sg1
......................................................................ugGucuucagucucucaaaca....................   6     1   sg1
......................................................................ugaucuucagucucCcaaaca....................   1     1   sg1
......................................................................ugaucuuUagucucucaaaca....................   1     1   sg1
......................................................................ugaucuCcagucucucaaaca....................   2     1   sg1
......................................................................ugaucuucagucucucaaacU....................   4     1   sg1
......................................................................ugaAcuucagucucucaaaca....................   1     1   sg1
......................................................................ugaucuucagCcucucaaaca....................   5     1   sg1
......................................................................ugaucuucagucucucaaacG....................   8     1   sg1
......................................................................ugaucuucagucucucaGaca....................   5     1   sg1
......................................................................ugUucuucagucucucaaaca....................   2     1   sg1
......................................................................ugaucuucagucucucaaaca....................   786   0   sg1
......................................................................ugaucCucagucucucaaaca....................   7     1   sg1
```

Star Mature

ccuuaaccacucggccauucuaacaccauccuaccuguuugagagaaugaaaaucauucucuuuagaaaaugaucuucagucucucaaacaaguagcaacacugaucgucg			
....ugaucuucagucCcucaaaca....................	4	1	sg1
....ugaucuucagucucucaaCca....................	2	1	sg1
....ugaucuuAagucucucaaaca....................	1	1	sg1
....ugaucuucagucucucaaaUa....................	2	1	sg1
....ugaucuucagucucucGaaca....................	7	1	sg1
....ugauUuucagucucucaaaca....................	2	1	sg1
....ugaucuucGgucucucaaacaa...................	1	1	sg1
....ugaucuucagucucucaaacaU...................	13	1	sg1
....ugaucuucagucucucaaacaG...................	2	1	sg1
....ugaucCucagucucucaaacaa...................	2	1	sg1
....ugaucuucagucucucaaacaa...................	73	0	sg1
....ugaucuucagucucucaGacaa...................	1	1	sg1
....ugaucuucagucucucaaacUa...................	10	1	sg1
....ugaucuucagucucucaaacaaU..................	1	1	sg1
....ugaucuucagucucucaaacaaA..................	2	1	sg1
....ugaucuucagucucucaaacaUg..................	1	1	sg1
.....................................uuugagagaaugaaaauc..	1	0	st1
.....................................uuugGgagaaugaaaaucauu...	2	1	st1
.....................................uuugagagaaugaaaaucauu...	6	0	st1
.....................................uuugagagaaugaaaaucGuu...	1	1	st1
.....................................uuugagagaaugaaaaucauucu...	1	0	st1
....aaaugaucuucagucucuca........................	1	0	st1
....aaaugaucuucagucucucaa.......................	5	0	st1
....aaaugaucuucagucucucaaU......................	13	1	st1
....aaaugaucCucagucucucaaa......................	1	1	st1
....aaaugaucuucagucucucaaC......................	1	1	st1
....aaaugaucuucagucucucaaa......................	48	0	st1
....aaaugaucuucagucucucaaGc.....................	1	1	st1
....aaaugaucuucagucucucaaaA.....................	4	1	st1
....aaaugaucuucagucucucaaaG.....................	1	1	st1
....aaauAaucuucagucucucaaac.....................	1	1	st1
....aaaugaucuucagucucucaaac.....................	11	0	st1
....aaaugaucuucagucucucaaaU.....................	11	1	st1
....aaaugaucuucagucucucaaacC....................	1	1	st1
....aaaugaucuucagucucucaaaca....................	10	0	st1
....aaaugaucuucagucucucaaacU....................	6	1	st1
....aaaugaucuucagucucucaaacUa...................	1	1	st1
....aaugaucuucagucucucaa.......................	2	0	st1
....aaugaucuucagucucucaaa......................	6	0	st1
....aaugaucuucagucucucaaac.....................	10	0	st1
....aaugaucuucagucucucaaaUa....................	1	1	st1
....aaugaucuucagucucucaaaca....................	7	0	st1
....aaugaucuucagucucucaaacU....................	1	1	st1
....aaugaucuucagucucucaaacG....................	1	1	st1
....aaugaucuucagucucucaaacaU...................	1	1	st1
....augaucuucagucucucaaac.....................	1	0	st1
....augaucuucagucucucaaaca....................	1	0	st1
....Cugaucuucagucucucaaaca....................	1	1	st1
....ugaucuucagucucucaa.......................	2	0	st1
....ugaucuucagucucucaaa......................	14	0	st1
....ugaucuucagucucucaaU......................	1	1	st1
....ugaucuucagucCcucaaac.....................	1	1	st1
....uAaucuucagucucucaaac.....................	1	1	st1
....ugaucuucaUucucucaaac.....................	1	1	st1
....ugaucCucagucucucaaac.....................	2	1	st1
....ugaucuucagucucucaaGc.....................	1	1	st1
....ugaucuucGgucucucaaac.....................	1	1	st1
....ugaucuucagucucCcaaac.....................	4	1	st1
....ugaucuucCgucucucaaac.....................	2	1	st1
....ugaGcuucagucucucaaac.....................	1	1	st1
....ugaucuucagucucucUaac.....................	1	1	st1
....Agaucuucagucucucaaac.....................	1	1	st1
....ugaucuucagucucucaaaU.....................	13	1	st1
....Cgaucuucagucucucaaac.....................	2	1	st1
....ugGucuucagucucucaaac.....................	1	1	st1
....ugaucuucagucucucaaac.....................	240	0	st1
....ugaucuucagucucucaGac.....................	1	1	st1
....ugaucuucagucuUucaaaca....................	1	1	st1
....ugaucGucagucucucaaaca....................	1	1	st1

```
                                      Star                              Mature
ccuuaaccacucggccauucuaaccaccauccuaccuguuugagagaaugaaaaucauucucuuuagaaaaugaucuucagucucucaaacaaguagcaacacugaucgucg
.......................................................................ugaucuucagucAcucaaaca....................   2      1     st1
.......................................................................ugaucuCcagucucucaaaca....................   5      1     st1
.......................................................................uAaucuucagucucucaaaca....................   4      1     st1
.......................................................................ugaucuucagucucucaaaUa....................   1      1     st1
.......................................................................ugaucuucagucucucaaacU....................   7      1     st1
.......................................................................ugaucuucUgucucucaaaca....................   1      1     st1
.......................................................................ugGucuucagucucucaaaca....................   8      1     st1
.......................................................................ugaucuucagucucucaaCca....................   1      1     st1
.......................................................................ugaucuucGgucucucaaaca....................   4      1     st1
.......................................................................ugaucuucagucucucaaUca....................   1      1     st1
.......................................................................ugaucuucagucucucGaaca....................   4      1     st1
.......................................................................ugaucuucagucucCcaaaca....................   1      1     st1
.......................................................................ugaucuucagucCcucaaaca....................   3      1     st1
.......................................................................ugaucuucagucucucaaGca....................   2      1     st1
.......................................................................ugauUuucagucucucaaaca....................   1      1     st1
.......................................................................ugaucuucaUucucucaaaca....................   1      1     st1
.......................................................................Cgaucuucagucucucaaaca....................   3      1     st1
.......................................................................ugaucuucCgucucucaaaca....................   13     1     st1
.......................................................................ugaucuAcagucucucaaaca....................   2      1     st1
.......................................................................ugaCcuucagucucucaaaca....................   3      1     st1
.......................................................................Agaucuucagucucucaaaca....................   2      1     st1
.......................................................................ugaucuucagucucucaaaca....................   1244   0     st1
.......................................................................uUaucuucagucucucaaaca....................   1      1     st1
.......................................................................ugaucuucagAcucucaaaca....................   1      1     st1
.......................................................................ugaucuucagucucucaaacG....................   29     1     st1
.......................................................................ugaucuuUagucucucaaaca....................   1      1     st1
.......................................................................ugaucuucagCcucucaaaca....................   7      1     st1
.......................................................................ugaucCucagucucucaaaca....................   1      1     st1
.......................................................................ugaucuucagucucucaGaca....................   2      1     st1
.......................................................................ugaucuucagucucucaaacC....................   1      1     st1
.......................................................................ugaucuucagucucGcaaaca....................   1      1     st1
.......................................................................ugaucAucagucucucaaaca....................   3      1     st1
.......................................................................ugaucuucagucucucaaacUa...................   3      1     st1
.......................................................................ugaucuucagucucucaaacaU...................   48     1     st1
.......................................................................ugaucuucCgucucucaaacaa...................   1      1     st1
.......................................................................ugaucuucagucucucaaacaa...................   53     0     st1
.......................................................................ugaucuucagucucAcaaacaa...................   1      1     st1
.......................................................................ugaucuucagucucucaaacaC...................   2      1     st1
.......................................................................ugaucuucagucucucaaacaaU..................   1      1     st1
.......................................................................ugaucuucagucucucaaacaaAu.................   1      1     st1
.......................................................................ugaucuucagucucucaaacaaUa.................   3      1     st1
............................................................................................aguagcaacacugaucgA..   1      1     st1
............................................................................................aguagcaacacugaucguc.   1      0     st1
............................................................................................aguagcaacacugaucgucg   2      0     st1
............................................................................................Uguagcaacacugaucgucg   1      1     st1

......................................uuCgagagaaugaaaauc........................................................   2      1     sr1
......................................uuugGgagaaugaaaauc........................................................   1      1     sr1
......................................uuCgagagaaugaaaaucauu.....................................................   2      1     sr1
......................................uuugGgagaaugaaaaucauu.....................................................   4      1     sr1
......................................uuAgagagaaugaaaaucauu.....................................................   1      1     sr1
......................................uuugagagaaugaaaaucauu.....................................................   1      0     sr1
......................................uuugagagaaugGaaaucauu.....................................................   1      1     sr1
......................................uuugagagaaugaaaaucauuA....................................................   1      1     sr1
...................................................................aaaaugaucuucagucucucaaaca....................   1      0     sr1
....................................................................aaaugaucuucagucucuca........................   15     0     sr1
....................................................................aaaAgaucuucagucucucaa.......................   1      1     sr1
....................................................................aaaugGucuucagucucucaa.......................   1      1     sr1
....................................................................aaaugaucuucagucucucaG.......................   2      1     sr1
....................................................................aaaugaucuucagucucucaa.......................   77     0     sr1
....................................................................aaaugaucuucagucucCcaa.......................   1      1     sr1
....................................................................Gaaugaucuucagucucucaa.......................   1      1     sr1
....................................................................aaaugaucuucaguUucucaa.......................   1      1     sr1
....................................................................aaaCgaucuucagucucucaaa......................   1      1     sr1
....................................................................aaaugaucuucagucucucaUa......................   1      1     sr1
....................................................................aaaugaucuucagucucucaaG......................   14     1     sr1
....................................................................aaauCaucuucagucucucaaa......................   1      1     sr1
....................................................................aaGugaucuucagucucucaaa......................   1      1     sr1
....................................................................aaaugaucCucagucucucaaa......................   2      1     sr1
....................................................................aaaugaAcuucagucucucaaa......................   1      1     sr1
```

Star Mature

```
ccuuaaccacucggccauucuaacaccauccuaccuguuugagagaaugaaaaucauucucuuuagaaaaugaucuucagucucucaaacaaguagcaacacugaucgucg
...................................................................aaaugaucuCcagucucucaaa......................	5	1	sr1
...................................................................aaaugaucuucagucucucaaC......................	3	1	sr1
...................................................................aaaugaucuucaAucucucaaa......................	2	1	sr1
...................................................................aaaugaucuucagucCcucaaa......................	1	1	sr1
...................................................................Uaaugaucuucagucucucaaa......................	3	1	sr1
...................................................................aaaugaucuucagucucucaaa......................	362	0	sr1
...................................................................aaaugaucuucagucucucaGa......................	1	1	sr1
...................................................................aaaugGucuucagucucucaaa......................	3	1	sr1
...................................................................Gaaugaucuucagucucucaaa......................	18	1	sr1
...................................................................aaaugaucuucagucucucaaU......................	30	1	sr1
...................................................................aaaugaucuucagucucucaaaA.....................	34	1	sr1
...................................................................aaaugaucuucagucucucaaaG.....................	1	1	sr1
...................................................................aaaugaucuucagCcucucaaac.....................	1	1	sr1
...................................................................aaaugaucuucagucucucaaaU.....................	15	1	sr1
...................................................................aaaugaucuucagucucucaaac.....................	25	0	sr1
...................................................................aaaugaucuucagucucucaaUc.....................	2	1	sr1
...................................................................aaaugaucuucagucucucaaaca....................	14	0	sr1
...................................................................Gaaugaucuucagucucucaaaca....................	1	1	sr1
...................................................................aaaugaucuucagCcucucaaaca....................	1	1	sr1
...................................................................aaaugaucuucagucucucaaaUa....................	8	1	sr1
...................................................................aaaugaucuucagucucucaaacU....................	7	1	sr1
...................................................................aaaugaucuucagucucucaaacC....................	1	1	sr1
...................................................................aaaugaucuucagucucucaaacG....................	3	1	sr1
...................................................................aaaugaucuucagucucucaaacaa...................	1	0	sr1
...................................................................aaaugaucuucagucucucaaacUa...................	13	1	sr1
....................................................................aaugaucuucagucucucG........................	1	1	sr1
....................................................................aaugaucuucagucucuca........................	7	0	sr1
....................................................................aaugGucuucagucucucaa.......................	1	1	sr1
....................................................................aaugaucuucagucucucaa.......................	28	0	sr1
....................................................................aaugaucuucagCcucucaa.......................	1	1	sr1
....................................................................aaugaucuucagucucucaaa......................	129	0	sr1
....................................................................aaugaucuucagucucucaaU......................	7	1	sr1
....................................................................aaugGucuucagucucucaaa......................	1	1	sr1
....................................................................aaugaucuucagucucucaUa......................	1	1	sr1
....................................................................aaugaucuCcagucucucaaa......................	2	1	sr1
....................................................................aGugaucuucagucucucaaa......................	4	1	sr1
....................................................................Gaugaucuucagucucucaaa......................	6	1	sr1
....................................................................aaugaucCucagucucucaaa......................	1	1	sr1
....................................................................aaugaucuucagucucucaaG......................	9	1	sr1
....................................................................aGugaucuucagucucucaaac.....................	3	1	sr1
....................................................................aaugaucuucagucucucaaaG.....................	1	1	sr1
....................................................................aaugaucuucagucucucaaaA.....................	6	1	sr1
....................................................................aaugaucuucagucucucaaac.....................	19	0	sr1
....................................................................aaugaucuucagucucucaaacG....................	2	1	sr1
....................................................................aaugaucuucagucucucaaaca....................	25	0	sr1
....................................................................aaugGucuucagucucucaaaca....................	1	1	sr1
....................................................................aaugaucuucagCcucucaaaca....................	1	1	sr1
....................................................................aaugaucuucagucucucaaaUa....................	1	1	sr1
....................................................................aaugaucuucagucucucaaacU....................	1	1	sr1
....................................................................Gaugaucuucagucucucaaaca....................	1	1	sr1
....................................................................aaugaucuucagucucucaaacaa...................	4	0	sr1
....................................................................aaugaucuucagucucucaaacUa...................	2	1	sr1
.....................................................................augaucuucagucucucaaU......................	3	1	sr1
.....................................................................augaucuucagucucucaaaU.....................	1	1	sr1
.....................................................................augaucuucagucucucaaac.....................	3	0	sr1
.....................................................................Cugaucuucagucucucaaac.....................	1	1	sr1
.....................................................................Cugaucuucagucucucaaaca....................	1	1	sr1
.....................................................................augaucuucagucucucaaaca....................	3	0	sr1
.....................................................................Cugaucuucagucucucaaacaa...................	1	1	sr1
......................................................................ugaucuucagucucuAaa.......................	2	1	sr1
......................................................................Ggaucuucagucucucaa.......................	1	1	sr1
......................................................................ugaucuucagucucucGa.......................	1	1	sr1
......................................................................ugaucuucagucucucaa.......................	57	0	sr1
......................................................................ugaucuucagucucucaU.......................	2	1	sr1
......................................................................ugGucuucagucucucaa.......................	1	1	sr1
......................................................................ugaucuucagucucucaaC......................	5	1	sr1
......................................................................ugaucuucagucucucaUa......................	7	1	sr1
......................................................................ugaucuucaguUucucaaa......................	1	1	sr1
......................................................................ugaucuucagucucCcaaa......................	2	1	sr1
......................................................................ugGucuucagucucucaaa......................	5	1	sr1
```

```
                                     Star                           Mature
ccuuaaccacucggccauucuaacaccauccuaccuguuugagagaaugaaaaucauucucuuuagaaaaugaucuucagucucucaaacaaguagcaacacugaucgucg
......................................................................ugaCcuucagucucucaaa......................    2     1    sr1
......................................................................ugaucuucagucucucaaU......................    48    1    sr1
......................................................................Ggaucuucagucucucaaa......................    12    1    sr1
......................................................................ugaucuCcagucucucaaa......................    1     1    sr1
......................................................................ugaucuucagucucucaaa......................    504   0    sr1
......................................................................ugaucuucagucucucaaG......................    17    1    sr1
......................................................................ugaucuucagucCcucaaa......................    2     1    sr1
......................................................................ugaAcuucagucucucaaa......................    1     1    sr1
......................................................................ugaucuucagCcucucaaa......................    1     1    sr1
......................................................................ugaucCucagucucucaaa......................    3     1    sr1
......................................................................ugaucuucagucucucaGa......................    1     1    sr1
......................................................................ugUucuucagucucucaaa......................    2     1    sr1
......................................................................ugaCcuucagucucucaaac.....................    4     1    sr1
......................................................................Cgaucuucagucucucaaac.....................    8     1    sr1
......................................................................ugaucuucagucucucaaaU.....................    180   1    sr1
......................................................................ugaucuucagucucucaaGc.....................    6     1    sr1
......................................................................ugauUuucagucucucaaac.....................    2     1    sr1
......................................................................ugaucuuUagucucucaaac.....................    3     1    sr1
......................................................................ugaucuucagAcucucaaac.....................    2     1    sr1
......................................................................ugaucuucagucucucaGac.....................    9     1    sr1
......................................................................ugaucCucagucucucaaac.....................    18    1    sr1
......................................................................ugaucuucagucCcucaaac.....................    9     1    sr1
......................................................................Agaucuucagucucucaaac.....................    2     1    sr1
......................................................................ugaucuucagucuUucaaac.....................    2     1    sr1
......................................................................ugGucuucagucucucaaac.....................    10    1    sr1
......................................................................ugaucuucagucucAcaaac.....................    1     1    sr1
......................................................................ugaucuuCgucucucaaac......................    1     1    sr1
......................................................................ugaucuucagucucucaaaA.....................    48    1    sr1
......................................................................ugaAcuucagucucucaaac.....................    4     1    sr1
......................................................................ugaucuucUgucucucaaac.....................    1     1    sr1
......................................................................ugaucuucagucucCcaaac.....................    9     1    sr1
......................................................................ugaucuucagucucucaaaG.....................    3     1    sr1
......................................................................ugaucuucagucucuUaaac.....................    1     1    sr1
......................................................................ugaucuAcagucucucaaac.....................    3     1    sr1
......................................................................ugaucuucagucucucaaUc.....................    2     1    sr1
......................................................................ugaucuucGgucucucaaac.....................    12    1    sr1
......................................................................ugaucuucagucuAucaaac.....................    1     1    sr1
......................................................................ugaucuucagucucucGaac.....................    7     1    sr1
......................................................................ugaucuucagucucucaaCc.....................    1     1    sr1
......................................................................Ggaucuucagucucucaaac.....................    66    1    sr1
......................................................................ugaucuCcagucucucaaac.....................    3     1    sr1
......................................................................ugUucuucagucucucaaac.....................    1     1    sr1
......................................................................ugaucuucagucucucaaac.....................    2713  0    sr1
......................................................................ugaGcuucagucucucaaac.....................    1     1    sr1
......................................................................uAaucuucagucucucaaac.....................    3     1    sr1
......................................................................ugaucuucagCcucucaaac.....................    8     1    sr1
......................................................................ugGucuucagucucucaaaca....................    68    1    sr1
......................................................................ugaucuucagucucucGaaca....................    31    1    sr1
......................................................................ugaucuucagucucucaaaAa....................    2     1    sr1
......................................................................ugaGcuucagucucucaaaca....................    1     1    sr1
......................................................................Cgaucuucagucucucaaaca....................    26    1    sr1
......................................................................ugaucuucUgucucucaaaca....................    3     1    sr1
......................................................................ugaucuucagucucucaaGca....................    23    1    sr1
......................................................................ugaucuucagucucucaGaca....................    20    1    sr1
......................................................................ugaucuAcagucucucaaaca....................    4     1    sr1
......................................................................ugaucuCcagucucucaaaca....................    27    1    sr1
......................................................................ugaucuucaAucucucaaaca....................    3     1    sr1
......................................................................ugaucuucagucucucaaUca....................    2     1    sr1
......................................................................ugaucuucagucucucaUaca....................    1     1    sr1
......................................................................ugauGuucagucucucaaaca....................    1     1    sr1
......................................................................Ggaucuucagucucucaaaca....................    490   1    sr1
......................................................................ugaucAucagucucucaaaca....................    1     1    sr1
......................................................................ugaucuucagucuAucaaaca....................    1     1    sr1
......................................................................ugaCcuucagucucucaaaca....................    37    1    sr1
......................................................................ugaucuucagucucucaaacC....................    11    1    sr1
......................................................................ugaAcuucagucucucaaaca....................    7     1    sr1
......................................................................ugaucuucagucAcucaaaca....................    3     1    sr1
......................................................................ugaucuucagCcucucaaaca....................    24    1    sr1
......................................................................ugauUuucagucucucaaaca....................    1     1    sr1
......................................................................ugaucuucagucuUucaaaca....................    7     1    sr1
```

Star **Mature**

```
ccuuaaccacucggccauucuaacaccauccuaccuguuugagagaaugaaaaucauucucuuuagaaaaugaucuucagucucucaaacaaguagcaacacugaucgucg
......................................................................ugCucuucagucucucaaaca....................  2     1  sr1
......................................................................ugaucuucagucucucCaaca....................  3     1  sr1
......................................................................ugaucuucagucucucaaCca....................  1     1  sr1
......................................................................ugaucuucagucucucaaaca....................  8915  0  sr1
......................................................................ugaucuucagucucucaaacU....................  86    1  sr1
......................................................................ugaucuGcagucucucaaaca....................  3     1  sr1
......................................................................ugaucuucagucCcucaaaca....................  28    1  sr1
......................................................................ugauAuucagucucucaaaca....................  1     1  sr1
......................................................................uAaucuucagucucucaaaca....................  6     1  sr1
......................................................................Agaucuucagucucucaaaca....................  17    1  sr1
......................................................................ugaucGucagucucucaaaca....................  2     1  sr1
......................................................................ugaucuucagucucuUaaaca....................  7     1  sr1
......................................................................ugaucuucagucucuGaaaca....................  1     1  sr1
......................................................................ugaucuucagucucuAaaaca....................  1     1  sr1
......................................................................ugaucuucagucucAcaaaca....................  7     1  sr1
......................................................................ugaucCucagucucucaaaca....................  26    1  sr1
......................................................................ugaucuucagucucucaaacG....................  369   1  sr1
......................................................................ugaucuuUagucucucaaaca....................  12    1  sr1
......................................................................ugaucuuGagucucucaaaca....................  1     1  sr1
......................................................................ugaucuucaguGucucaaaca....................  2     1  sr1
......................................................................ugaucuuAagucucucaaaca....................  2     1  sr1
......................................................................ugaucuucagucucCcaaaca....................  33    1  sr1
......................................................................ugaucuucagucucucaaaUa....................  13    1  sr1
......................................................................ugaucuucagucucucUaaca....................  7     1  sr1
......................................................................ugaucuucGgucucucaaaca....................  22    1  sr1
......................................................................uUaucuucagucucucaaaca....................  2     1  sr1
......................................................................ugaucuucaUucucucaaaca....................  1     1  sr1
......................................................................ugaucuuUagucucucaaacaa...................  4     1  sr1
......................................................................ugaucuucagucucucaaacUa...................  107   1  sr1
......................................................................ugaAcuucagucucucaaacaa...................  3     1  sr1
......................................................................ugaucuucagucucucaaGcaa...................  1     1  sr1
......................................................................ugaucuucagucucucaGacaa...................  6     1  sr1
......................................................................ugaucuucGgucucucaaacaa...................  2     1  sr1
......................................................................ugauUuucagucucucaaacaa...................  1     1  sr1
......................................................................Ggaucuucagucucucaaacaa...................  11    1  sr1
......................................................................ugaucuucagucucucaaacaU...................  113   1  sr1
......................................................................ugUucuucagucucucaaacaa...................  4     1  sr1
......................................................................ugaucuucaAucucucaaacaa...................  2     1  sr1
......................................................................ugaCcuucagucucucaaacaa...................  2     1  sr1
......................................................................ugaucuucagucucucGaacaa...................  1     1  sr1
......................................................................ugaucuucagucucucaaCcaa...................  2     1  sr1
......................................................................Cgaucuucagucucucaaacaa...................  2     1  sr1
......................................................................ugaucuucagucCcucaaacaa...................  5     1  sr1
......................................................................ugaucuucagucucucaaacaG...................  30    1  sr1
......................................................................ugaucuucagucucucaaacCa...................  1     1  sr1
......................................................................ugaucCucagucucucaaacaa...................  4     1  sr1
......................................................................ugaucuucagucucucaaacaa...................  708   0  sr1
......................................................................ugaucuucagAcucucaaacaa...................  1     1  sr1
......................................................................ugaucuucagCcucucaaacaa...................  1     1  sr1
......................................................................ugaucGucagucucucaaacaa...................  2     1  sr1
......................................................................ugaucuucagucucucaaaUaa...................  1     1  sr1
......................................................................ugaucuucagucucucaaacaC...................  17    1  sr1
......................................................................ugaucuCcagucucucaaacaa...................  1     1  sr1
......................................................................ugaucuucagucucucaaaAaa...................  1     1  sr1
......................................................................ugaucuucagucucucaaacGa...................  1     1  sr1
......................................................................ugaucuucagucucucaCacaa...................  1     1  sr1
......................................................................ugaucuucagucucCcaaacaa...................  3     1  sr1
......................................................................ugaucuucagucucucaaacaaU..................  3     1  sr1
......................................................................ugaucuucagucucucaaacUag..................  2     1  sr1
......................................................................ugaucuucagucucucaaacaUg..................  15    1  sr1
......................................................................ugaucuucagucucucaaacaaA..................  27    1  sr1
......................................................................ugaucuucagucucucaaacGag..................  1     1  sr1
......................................................................ugaucuucagucucucaaacaaC..................  2     1  sr1
......................................................................ugaucuucagucucucaaacaaAu.................  1     1  sr1
......................................................................ugaucuucagucucucaaacaaAua................  3     1  sr1
......................................................................ugaucuucagucucucaaacaaUua................  5     1  sr1
.......................................................................gaucuucagucucucaaa......................  1     0  sr1
.......................................................................gaucuucagucucucaaac.....................  2     0  sr1
.......................................................................gaucuucagucucucaaaU.....................  1     1  sr1
.......................................................................gaucuucagucucucaaaca....................  1     0  sr1
```

```
                                     Star                              Mature
ccuuaaccacucggccauucuaacaccauccuaccuguuugagagaaugaaaaucauucucuuuagaaaaugaucuucagucucucaaacaaguagcaacacugaucgucg
........................................................................aucuucagucucucaaac.....................    1    0    sr1
.........................................................................Gcuucagucucucaaaca....................    1    1    sr1
.........................................................................ucuucagucucucaaacG....................    2    1    sr1
.........................................................................ucuucagucucucaaaca....................    2    0    sr1
..........................................................................cuucagucucucaaacaU...................    1    1    sr1
...........................................................................................aguagcaacacugaucguA.    3    1    sr1
...........................................................................................aguagcaacacugaucguc.    1    0    sr1
...........................................................................................aguagcaacacugaucguG.    1    1    sr1
...........................................................................................aguagcaacacugaucgucg    6    0    sr1
...........................................................................................aguagcaacacugaucguAg    1    1    sr1
```

Provisional ID : ScYm1zk_361_3723
Score total : 1.8
Score for star read(s) : -1.3
Score for read counts : 0
Score for mfe : 2.1
Score for randfold : 1.6
Score for cons. seed : -0.6
Total read count : 29501
Mature read count : 29495
Loop read count : 0
Star read count : 6

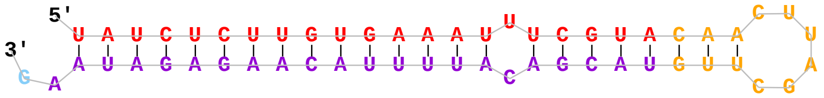

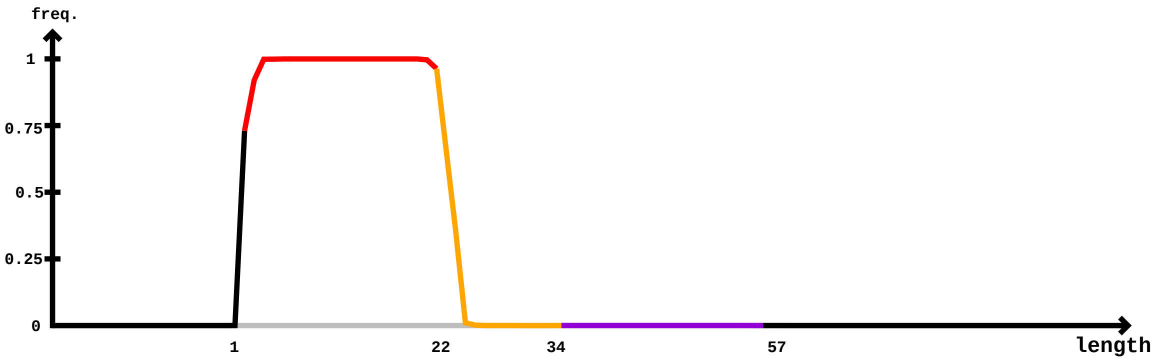

Mature **Star**

Sequence			
5'- ucgucgucacauuccucuuaucucuugugaaauuucguacaacuuagcuuguacgacauuuuacaagagauaagaggugccagaagugggauucgaacccacgcaugcuu -3'	obs		
ucgucgucacauuccucuuaucucuugugaaauuucguacaacuuagcuuguacgacauuuuacaagagauaagaggugccagaagugggauucgaacccacgcaugcuu	exp		
............((((((((((((((((((.((((((((......)))))))).)))))))))))))))))).((.....(((((.......)))))....))..	reads	mm	sample
..................uaucucuugugaaauuuc..	1	0	so1
..................uaucucuugugaaauuucg...	3	0	so1
..................uaucucuugugaaauuucgu..	52	0	so1
..................uaucucuugugaaauuucgG..	1	1	so1
..................uaucucuugugaaauuucgC..	8	1	so1
..................uaucucuCgugaaauuucgu..	1	1	so1
..................uaucucuugCgaaauuucgu..	1	1	so1
..................uaucucuCgugaaauuucgua...	3	1	so1
..................uaucucuugugaaauuCcgua...	1	1	so1
..................uUucucuugugaaauuucgua...	2	1	so1
..................uGucucuugugaaauuucgua...	1	1	so1
..................uaucucuugugaaauuAcgua...	1	1	so1
..................uaucucuugugaaauuucguU...	2	1	so1
..................uaucucuugugGaauuucgua...	3	1	so1
..................uaucucuugugaaauuucgua...	532	0	so1
..................Caucucuugugaaauuucgua...	1	1	so1
..................uaucucuugugaaauuucguG...	6	1	so1
..................uaucucuugugaGauuucgua...	4	1	so1
..................uaucucuugugaaauCucgua...	1	1	so1
..................uaucucuugugaaauCucguac..	1	1	so1
..................uaucucuugugaaauuucguac..	451	0	so1
..................uaucCcuugugaaauuucguac..	3	1	so1
..................uaucGcuugugaaauuucguac..	2	1	so1
..................uaucucuugugaaauuucguGc..	2	1	so1
..................uauAucuugugaaauuucguac..	1	1	so1
..................uGucucuugugaaauuucguac..	7	1	so1
..................uaucucuugugaaauuucguaU..	78	1	so1
..................uaucucuugugaaauuucgGac..	1	1	so1
..................uaucucuugugaaauuucguaA..	19	1	so1
..................uaucucuugCgaaauuucguac..	3	1	so1
..................Caucucuugugaaauuucguac..	1	1	so1
..................uaucucuugugaaauGucguac..	2	1	so1
..................uaCcucuugugaaauuucguac..	2	1	so1

Mature **Star**

ucgucgucacauuccucuuaucucuugugaaauuucguacaacuuagcuuguacgacauuuuacaagagauaagaggugccagaagugggauucgaacccacgcaugcuu			
..................uaucucuugugGaauuucguac..	5	1	so1
..................uaucucuugugaaauuucguaG..	5	1	so1
..................uaucucuCgugaaauuucguac..	2	1	so1
..................uaucucuugugaGauuucguac..	2	1	so1
..................uaucucuugugaaauuucAuac..	1	1	so1
..................uaucucuuguAaaauuucguac..	1	1	so1
..................uaucucCugugaaauuucguac..	1	1	so1
..................uaucucuugugaGauuucguaca...	1	1	so1
..................uaucucuugugaaauuucguaca...	436	0	so1
..................uaucucuugugaaauuucguGca...	2	1	so1
..................uaucucuCgugaaauuucguaca...	1	1	so1
..................uaucucuugugaaauuucguacC...	3	1	so1
..................uaucucuugugaaauuucguaUa...	25	1	so1
..................uaucucuugugaaauuucguaAa...	8	1	so1
..................uaucucuugugGaauuucguaca...	2	1	so1
..................uaucucCugugaaauuucguaca...	1	1	so1
..................uGucucuugugaaauuucguaca...	1	1	so1
..................uaucuAuugugaaauuucguaca...	1	1	so1
..................uaucuUuugugaaauuucguaca...	1	1	so1
..................uaucucuugugaaauuucguacG...	23	1	so1
..................uaucucuugugaaauuucguacU...	7	1	so1
..................uaucCcuugugaaauuucguaca...	2	1	so1
..................uaucucuugugaaauuAcguaca...	1	1	so1
..................uaucucuugCgaaauuucguaca...	1	1	so1
..................uaucucuugugaaauuucguacCa..	1	1	so1
..................uaucucuugugaaauuucguacaa..	2	0	so1
..................uaucucuugugaaauuucguaUaa..	2	1	so1
..................uaucucuugugaaauuucguacaU..	5	1	so1
..................uaucucuugugaaauuucguacaC..	1	1	so1
..................uaucucuugugaaauuucguacUa..	7	1	so1
..................uaucucuugugaaauuucguacaaA...	6	1	so1
..................uaucucuugugaaauuucguacaCc...	2	1	so1
..................uaucucuugugaaauuucguacaac...	1	0	so1
..................uaucucuugugaaauuucguacaaAu..	2	1	so1
...................aucucuugugaaauuucgA..	1	1	so1
...................aucucuugugaaauuucgu..	4	0	so1
...................aucucuugugaaauuucgC..	1	1	so1
...................aCcucuugugaaauuucgua...	1	1	so1
...................aucucuugugaaauCucgua...	4	1	so1
...................Gucucuugugaaauuucgua...	1	1	so1
...................aucucuugugaaauuucgua...	41	0	so1
...................aucucuAgugaaauuucgua...	1	1	so1
...................aucucuugugaaauuucguaA..	2	1	so1
...................aucucuuguAaaauuucguac..	1	1	so1
...................aucucuugugaaauCucguac..	1	1	so1
...................aucucuugugaaauuucguaU..	26	1	so1
...................aucucuugugaaauuCcguac..	1	1	so1
...................aucucuugugaaauuucguGc..	1	1	so1
...................aucucuugugaaauuucguac..	122	0	so1
...................aucucuugugaaauuucguacG...	5	1	so1
...................aucucuugugaaauuCcguaca...	1	1	so1
...................aucucuugugaaauuucguGca...	1	1	so1
...................aucucuugugaaauuucguaUa...	17	1	so1
...................aucucuugugaaauuucguaca...	62	0	so1
...................aucucuugugaaauuucguacC...	1	1	so1
...................aucucuugugaaauuucguacaa..	1	0	so1
...................aucucuugugaaauuucguacUa..	1	1	so1
...................aucucuugugaaauuucguacaCc...	1	1	so1
....................ucucuugugaaauuucgua...	4	0	so1
....................ucucuugugaaauuucguU...	2	1	so1
....................ucucuugugaaauuucguaU..	10	1	so1
....................Ccucuugugaaauuucguac..	1	1	so1
....................ucucCugugaaauuucguac..	1	1	so1
....................ucCcuugugaaauuucguac..	1	1	so1
....................ucucuugugaaauuucguac..	64	0	so1
....................ucCcuugugaaauuucguaca...	1	1	so1
....................ucucuugugaaauuucguaUa...	2	1	so1
....................ucucuugugaaauuucguaca...	56	0	so1
....................ucucuugugaaauuucguacU...	1	1	so1
....................ucucuugugaaauuucgCaca...	1	1	so1

```
                  Mature                          Star
ucgucgucacauuccucuuaucucuugugaaauuucguacaacuuagcuuguacgacauuuuacaagagauaagaggugccagaagugggauucgaacccacgcaugcuu
....................ucucuugugaGauuucguaca.....................................................................   1     1     so1
....................ucucuuAugaaauuucguaca.....................................................................   1     1     so1
....................ucucuugugaaauuucguacG.....................................................................   1     1     so1
....................ucucuugugaaauuucguacC.....................................................................   1     1     so1
....................ucucuugugaaauuucguacaC....................................................................   2     1     so1
....................ucucuugugaaauuucguacaU....................................................................   2     1     so1
....................ucucuugugaaauuucguacaacuuag...............................................................   2     0     so1
.....................cucuugugaaauuucguaca.....................................................................   1     0     so1

..................uaucucuugugaaauuucg.........................................................................   6     0     sg1
..................uaucucuugugaaauuucgA........................................................................   1     1     sg1
..................uaucucuugugaaauuucgC........................................................................   1     1     sg1
..................uaucucuugugaaauuucgG........................................................................   1     1     sg1
..................uaucucuugugaaauuucgu........................................................................   23    0     sg1
..................uaucucuugugGaauuucgua.......................................................................   1     1     sg1
..................uaucucuugugaaauuCcgua.......................................................................   3     1     sg1
..................uaucucuugugaaauGucgua.......................................................................   1     1     sg1
..................uGucucuugugaaauuucgua.......................................................................   1     1     sg1
..................uaucucuuguAaaauuucgua.......................................................................   1     1     sg1
..................uaucucCugugaaauuucgua.......................................................................   1     1     sg1
..................uaucucuugugaaauuucgGa.......................................................................   2     1     sg1
..................uaucucuAgugaaauuucgua.......................................................................   1     1     sg1
..................uaucucuugugaaauuucgua.......................................................................   244   0     sg1
..................uaucucuugugaaauuucguG.......................................................................   4     1     sg1
..................uaucucuugCgaaauuucgua.......................................................................   1     1     sg1
..................uaucucuugugaaauuucguaA......................................................................   6     1     sg1
..................uaucucuugugaaaCuucguac......................................................................   1     1     sg1
..................uaucucuugugaGauuucguac......................................................................   1     1     sg1
..................uaCcucuugugaaauuucguac......................................................................   4     1     sg1
..................uaucucCugugaaauuucguac......................................................................   1     1     sg1
..................uaucucuugugaaauuucguac......................................................................   342   0     sg1
..................uaucucuugugaaauuucguaG......................................................................   2     1     sg1
..................uaucucuugugaaauuucguGc......................................................................   3     1     sg1
..................uaucucuugugaaauuucguaU......................................................................   26    1     sg1
..................uaucucuugugGaauuucguac......................................................................   3     1     sg1
..................uaucucuuguAaaauuucguac......................................................................   1     1     sg1
..................uaucucuugugaaauCucguac......................................................................   1     1     sg1
..................uaucucuugugaaauuucguacC.....................................................................   6     1     sg1
..................uaucucuugugaaauuucguUca.....................................................................   1     1     sg1
..................uaucucCugugaaauuucguaca.....................................................................   1     1     sg1
..................uGucucuugugaaauuucguaca.....................................................................   2     1     sg1
..................Caucucuugugaaauuucguaca.....................................................................   2     1     sg1
..................uaucucuugugaaauuucguacG.....................................................................   8     1     sg1
..................uaucucuugugaaauCucguaca.....................................................................   1     1     sg1
..................uaucucuugugaaaCuucguaca.....................................................................   2     1     sg1
..................uaucucuugugaaauuucguGca.....................................................................   6     1     sg1
..................uaucucuugugaGauuucguaca.....................................................................   1     1     sg1
..................uaucucuCgugaaauuucguaca.....................................................................   1     1     sg1
..................uaucucuugugaaauuucguaUa.....................................................................   22    1     sg1
..................uaucucuugCgaaauuucguaca.....................................................................   2     1     sg1
..................uaucucuAgugaaauuucguaca.....................................................................   1     1     sg1
..................uaucucuugugaaauuucguaca.....................................................................   574   0     sg1
..................uaucCcuugugaaauuucguaca.....................................................................   2     1     sg1
..................uaucucuugugaCauuucguaca.....................................................................   1     1     sg1
..................uaucucuugugaaauuucguaAa.....................................................................   4     1     sg1
..................uaucucuugugaaauuucguacU.....................................................................   11    1     sg1
..................uaucucuugugaaauuucguacaU....................................................................   2     1     sg1
..................uaucucuugugaaauuucguacaa....................................................................   4     0     sg1
..................uaucucuugugaaauuucguacUa....................................................................   2     1     sg1
..................uaucucuugugaaauuucguacaaA...................................................................   3     1     sg1
...................aucucuugugaaauuucgu........................................................................   5     0     sg1
...................aucucuugugaaauuucguG.......................................................................   1     1     sg1
...................aucucuugugaaauuCcgua.......................................................................   1     1     sg1
...................aucucuugugaaauuucgua.......................................................................   16    0     sg1
...................aucucuugugaGauuucguac......................................................................   1     1     sg1
...................aucucuugugaaauuucguaU......................................................................   7     1     sg1
...................aucucCugugaaauuucguac......................................................................   1     1     sg1
...................aucucuugugaaaCuucguac......................................................................   2     1     sg1
...................aucucuugugaaauuucguaG......................................................................   1     1     sg1
...................aucucuugugaaauuucguac......................................................................   41    0     sg1
```

```
                  Mature                           Star
ucgucgucacauuccucuuaucucuugugaaauuucguacaacuuagcuuguacgacauuuuacaagagauaagaggugccagaagugggauucgaacccacgcaugcuu
...................aucucuugCgaaauuucguac......................................................................   1    1    sg1
...................aucucuugugaaauuucguacU.....................................................................   5    1    sg1
...................aucucuCgugaaauuucguaca.....................................................................   1    1    sg1
...................aucucuugugaaauuucguaUa.....................................................................   17   1    sg1
...................Gucucuugugaaauuucguaca.....................................................................   1    1    sg1
...................aucucuugugaaauuucguaAa.....................................................................   3    1    sg1
...................aucucuugugaaauCucguaca.....................................................................   2    1    sg1
...................aucucuugugaaauuucgCaca.....................................................................   1    1    sg1
...................aucucuugugaaauuucguaca.....................................................................   62   0    sg1
...................aucucuugugaaauuucguaUaa....................................................................   1    1    sg1
...................aucucuugugaaauuucguacUa....................................................................   1    1    sg1
....................ucucuugugaaauuucgu........................................................................   1    0    sg1
....................ucucuugugaaauuucgua.......................................................................   4    0    sg1
....................ucucuugugaaGuuucguac......................................................................   3    1    sg1
....................ucucuugugaaauuucguac......................................................................   28   0    sg1
....................ucucuugugaaauuucguaca.....................................................................   63   0    sg1
....................ucucuugugaaauuucguacG.....................................................................   2    1    sg1
....................ucucCugugaaauuucguaca.....................................................................   1    1    sg1
....................ucucuugugaaauuucguacaC....................................................................   1    1    sg1
....................ucucuugugaaauuucguacCa....................................................................   1    1    sg1
....................ucucuugugaaauuucguaUaa....................................................................   1    1    sg1
....................ucucuugugaaauuucguacaCc...................................................................   1    1    sg1
......................ucuugugaaauuucguac......................................................................   1    0    sg1
...................................................uacgacauuuuacaagagau.......................................   1    0    sg1
...................................................uacgacauuuuacaagagaua......................................   1    0    sg1
...................................................uacgacauuuuacaagagauaa.....................................   1    0    sg1

.................Cuaucucuugugaaauuucgua.......................................................................   1    1    sr1
.................Cuaucucuugugaaauuucguac......................................................................   3    1    sr1
..................uaucucuugugaaauuuc..........................................................................   2    0    sr1
..................uaucucuugugaaauuucg.........................................................................   63   0    sr1
..................uaucucuugCgaaauuucg.........................................................................   1    1    sr1
..................uaucucuugugaaauuucA.........................................................................   4    1    sr1
..................uaucucuugugaaauGucg.........................................................................   1    1    sr1
..................uaucucuugugaGauuucg.........................................................................   4    1    sr1
..................uaucucuugugaaauuucC.........................................................................   1    1    sr1
..................uaucucuugugaaauCucg.........................................................................   2    1    sr1
..................uaucucCugugaaauuucg.........................................................................   1    1    sr1
..................uaucucuugugaaauuucgA........................................................................   2    1    sr1
..................uaucucuugugaaGuuucgu........................................................................   1    1    sr1
..................uaucucCugugaaauuucgu........................................................................   1    1    sr1
..................uaCcucuugugaaauuucgu........................................................................   2    1    sr1
..................uaucucuugugaGauuucgu........................................................................   1    1    sr1
..................uaucucuugugaaauuucgC........................................................................   43   1    sr1
..................uaucCcuugugaaauuucgu........................................................................   1    1    sr1
..................uaucucuCgugaaauuucgu........................................................................   2    1    sr1
..................uaucucuugugaaauuCcgu........................................................................   1    1    sr1
..................uaucucuugugaaauuucgG........................................................................   9    1    sr1
..................uaucucuugugaaauCucgu........................................................................   2    1    sr1
..................uGucucuugugaaauuucgu........................................................................   3    1    sr1
..................Gaucucuugugaaauuucgu........................................................................   21   1    sr1
..................uaucucuugugaaauuucgu........................................................................   356  0    sr1
..................uaucucuuAugaaauuucgua.......................................................................   4    1    sr1
..................uaGcucuugugaaauuucgua.......................................................................   1    1    sr1
..................uaucucuugugUaauuucgua.......................................................................   3    1    sr1
..................uaCcucuugugaaauuucgua.......................................................................   23   1    sr1
..................uaucucuAgugaaauuucgua.......................................................................   4    1    sr1
..................Aaucucuugugaaauuucgua.......................................................................   4    1    sr1
..................uaucucuugugaaGuuucgua.......................................................................   5    1    sr1
..................Caucucuugugaaauuucgua.......................................................................   10   1    sr1
..................uaucucGugugaaauuucgua.......................................................................   1    1    sr1
..................uaucucuugugaaauuuAgua.......................................................................   3    1    sr1
..................uaucuUuugugaaauuucgua.......................................................................   2    1    sr1
..................uaucucuugugaaauGucgua.......................................................................   5    1    sr1
..................uaucucuugugaGauuucgua.......................................................................   15   1    sr1
..................uaucucuugugaaauuCcgua.......................................................................   14   1    sr1
..................uaucucuugugaaauuucgCa.......................................................................   11   1    sr1
..................uaucucuugugaaauuAcgua.......................................................................   1    1    sr1
..................uaucucuugugaaauuucgGa.......................................................................   2    1    sr1
..................uaucCcuugugaaauuucgua.......................................................................   13   1    sr1
```

```
                  Mature                          Star
ucgucgucacauuccucuuaucucuugugaaauuucguacaacuuagcuuguacgacauuuuacaagagauaagaggugccagaagugggauucgaacccacgcaugcuu
..................uaucucuugugaaaAuucgua.......................................................................  3     1  sr1
..................uaucucuuguAaaauuucgua.......................................................................  3     1  sr1
..................uaucucuugGgaaauuucgua.......................................................................  1     1  sr1
..................uaucucuCgugaaauuucgua.......................................................................  14    1  sr1
..................uaucucuugugaaauAucgua.......................................................................  3     1  sr1
..................uaucuAuugugaaauuucgua.......................................................................  1     1  sr1
..................uaucAcuugugaaauuucgua.......................................................................  1     1  sr1
..................uGucucuugugaaauuucgua.......................................................................  33    1  sr1
..................uaucucuugugaaauCucgua.......................................................................  7     1  sr1
..................uaucucuugugaaaCuucgua.......................................................................  13    1  sr1
..................uaucucuugAgaaauuucgua.......................................................................  4     1  sr1
..................uaucucuugugaaauuucgua.......................................................................  4253  0  sr1
..................uaucucuugugaaauuucguG.......................................................................  113   1  sr1
..................uaucucuugugGaauuucgua.......................................................................  20    1  sr1
..................Gaucucuugugaaauuucgua.......................................................................  287   1  sr1
..................uUucucuugugaaauuucgua.......................................................................  1     1  sr1
..................uaucucCugugaaauuucgua.......................................................................  7     1  sr1
..................uaucucuugugaaauuucAua.......................................................................  1     1  sr1
..................uaucucuugugaaauuucguU.......................................................................  9     1  sr1
..................uauUucuugugaaauuucgua.......................................................................  3     1  sr1
..................uaucucuugCgaaauuucgua.......................................................................  10    1  sr1
..................uaAcucuugugaaauuucgua.......................................................................  1     1  sr1
..................uaucucuuUugaaauuucgua.......................................................................  3     1  sr1
..................uaucucuugugaaauuuGgua.......................................................................  1     1  sr1
..................uaucucuugugaaauuucguaU......................................................................  345   1  sr1
..................uaucucuuAugaaauuucguac......................................................................  1     1  sr1
..................uaucucuugugaaauuuAguac......................................................................  2     1  sr1
..................Caucucuugugaaauuucguac......................................................................  7     1  sr1
..................uaucucuugugaaGuuucguac......................................................................  8     1  sr1
..................Gaucucuugugaaauuucguac......................................................................  212   1  sr1
..................uaucucuugugaaauuucguUc......................................................................  2     1  sr1
..................uaucucuugugaaauuucguaA......................................................................  163   1  sr1
..................uaucucuuguAaaauuucguac......................................................................  2     1  sr1
..................uauAucuugugaaauuucguac......................................................................  2     1  sr1
..................uaucucAugugaaauuucguac......................................................................  1     1  sr1
..................uaucucuugugaaaCuucguac......................................................................  6     1  sr1
..................uaucucuugugaaauCucguac......................................................................  2     1  sr1
..................Aaucucuugugaaauuucguac......................................................................  1     1  sr1
..................uaucucuugugCaauuucguac......................................................................  1     1  sr1
..................uaGcucuugugaaauuucguac......................................................................  1     1  sr1
..................uaucucuugugaaauuucAuac......................................................................  5     1  sr1
..................uaucCcuugugaaauuucguac......................................................................  9     1  sr1
..................uaucucuugCgaaauuucguac......................................................................  11    1  sr1
..................uaucucuugugaaauuucguac......................................................................  2270  0  sr1
..................uaucucuugugaaaGuucguac......................................................................  1     1  sr1
..................uGucucuugugaaauuucguac......................................................................  12    1  sr1
..................uaucuUuugugaaauuucguac......................................................................  1     1  sr1
..................uaucucuugugUaauuucguac......................................................................  1     1  sr1
..................uaucucuugugaaauuucguaG......................................................................  35    1  sr1
..................uaucucuCgugaaauuucguac......................................................................  3     1  sr1
..................uaucucuAgugaaauuucguac......................................................................  3     1  sr1
..................uaucucuugugaaauuucgCac......................................................................  1     1  sr1
..................uaucucCugugaaauuucguac......................................................................  3     1  sr1
..................uaucucuugugaaauuucguGc......................................................................  14    1  sr1
..................uaucucuugugaaauuAcguac......................................................................  3     1  sr1
..................uaucucuugugaaauGucguac......................................................................  6     1  sr1
..................uaucucuGgugaaauuucguac......................................................................  1     1  sr1
..................uauUucuugugaaauuucguac......................................................................  2     1  sr1
..................uaucucuugugaGauuucguac......................................................................  5     1  sr1
..................uaucucuugAgaaauuucguac......................................................................  1     1  sr1
..................uaucGcuugugaaauuucguac......................................................................  1     1  sr1
..................uaucucuugugaaauuucgGac......................................................................  1     1  sr1
..................uaucucuugugaaauuCcguac......................................................................  3     1  sr1
..................uaucucuugugGaauuucguac......................................................................  11    1  sr1
..................uaCcucuugugaaauuucguac......................................................................  3     1  sr1
..................uaucucuCgugaaauuucguaca.....................................................................  5     1  sr1
..................uaucucuugugaaauuucguaAa.....................................................................  48    1  sr1
..................uaucucuugugaaauuuAguaca.....................................................................  3     1  sr1
..................uaucucuugugaaauuucguUca.....................................................................  2     1  sr1
..................uaucucuugugaaauuucAuaca.....................................................................  4     1  sr1
```

```
                  Mature                           Star
ucgucgucacauuccucuuaucucuugugaaauuucguacaacuuagcuuguacgacauuuuacaagagauaagaggugccagaagugggauucgaacccacgcaugcuu
..................uUucucuugugaaauuucguaca.....................................................................    1      1    sr1
..................uaucCcuugugaaauuucguaca.....................................................................    3      1    sr1
..................uaucucuuAugaaauuucguaca.....................................................................    1      1    sr1
..................uaucucuugugaaauuucguaGa.....................................................................    2      1    sr1
..................uauAucuugugaaauuucguaca.....................................................................    1      1    sr1
..................uaucucuugugaaauuucguaca.....................................................................    2501   0    sr1
..................uaucucuugugaaauuucguGca.....................................................................    29     1    sr1
..................uaucucAugugaaauuucguaca.....................................................................    1      1    sr1
..................Gaucucuugugaaauuucguaca.....................................................................    375    1    sr1
..................uaucucuugugaaauuAcguaca.....................................................................    1      1    sr1
..................uaucucuugugGaauuucguaca.....................................................................    19     1    sr1
..................Caucucuugugaaauuucguaca.....................................................................    9      1    sr1
..................uaucucuugugaaauAucguaca.....................................................................    1      1    sr1
..................uaucucuugugaaGuuucguaca.....................................................................    11     1    sr1
..................uaucucuuguAaaauuucguaca.....................................................................    1      1    sr1
..................uaucucuugCgaaauuucguaca.....................................................................    8      1    sr1
..................uaucucGugugaaauuucguaca.....................................................................    1      1    sr1
..................uaucucuugugUaauuucguaca.....................................................................    1      1    sr1
..................uaucucuugugaaauuucgAaca.....................................................................    1      1    sr1
..................uaucucuugugaaauCucguaca.....................................................................    6      1    sr1
..................uaucucuugugaaaCuucguaca.....................................................................    8      1    sr1
..................uaucucuugugaaauGucguaca.....................................................................    6      1    sr1
..................uaucucuugugaaauuCcguaca.....................................................................    5      1    sr1
..................uaucucuugugaaaAuucguaca.....................................................................    1      1    sr1
..................uaucucuAgugaaauuucguaca.....................................................................    5      1    sr1
..................uaucucuugGgaaauuucguaca.....................................................................    1      1    sr1
..................uaucucuugugaaauuucgCaca.....................................................................    1      1    sr1
..................uaucucCugugaaauuucguaca.....................................................................    9      1    sr1
..................uaucucuugugaaauuucguacU.....................................................................    55     1    sr1
..................uaucucuugugaaauuucguacC.....................................................................    14     1    sr1
..................uGucucuugugaaauuucguaca.....................................................................    26     1    sr1
..................Aaucucuugugaaauuucguaca.....................................................................    1      1    sr1
..................uaucucuugugaGauuucguaca.....................................................................    7      1    sr1
..................uaucucuugugaaauuucguaUa.....................................................................    311    1    sr1
..................uaucucuugugaaauuucguacG.....................................................................    208    1    sr1
..................uaCcucuugugaaauuucguaca.....................................................................    6      1    sr1
..................uaAcucuugugaaauuucguaca.....................................................................    1      1    sr1
..................uaucucuugugaaGuuucguacaa....................................................................    1      1    sr1
..................uaucucuugugaaauuucguacCa....................................................................    4      1    sr1
..................uaucucuugugaaauuucguacaC....................................................................    2      1    sr1
..................uaucucuugugaaauuucguacaa....................................................................    29     0    sr1
..................uaucucuugugaaauuucguGcaa....................................................................    2      1    sr1
..................uaucucuugugaaauuucguaUaa....................................................................    13     1    sr1
..................uaucucuugugaaauuucguacUa....................................................................    22     1    sr1
..................Gaucucuugugaaauuucguacaa....................................................................    2      1    sr1
..................uaucucuugugaaauuucguacaG....................................................................    8      1    sr1
..................uaucucuugugaaauuucguacaU....................................................................    19     1    sr1
..................uaucucuugugaaauuucguacaaG...................................................................    1      1    sr1
..................uaucucuugugaaauuucguacaCc...................................................................    5      1    sr1
..................uaucucuugugaaauuucguacaaA...................................................................    15     1    sr1
...................aucucuugugaaauuucg.........................................................................    2      0    sr1
...................aucucuugugaaauuucgA........................................................................    3      1    sr1
...................aucucuugugaaauuucgC........................................................................    10     1    sr1
...................aucCcuugugaaauuucgu........................................................................    1      1    sr1
...................aucucuugugaaauuucgG........................................................................    4      1    sr1
...................aucucuuguAaaauuucgu........................................................................    1      1    sr1
...................Gucucuugugaaauuucgu........................................................................    4      1    sr1
...................aucucuugCgaaauuucgu........................................................................    1      1    sr1
...................aucucuugugaaGuuucgu........................................................................    1      1    sr1
...................aucucuAgugaaauuucgu........................................................................    1      1    sr1
...................aucucuugugaaauuucgu........................................................................    129    0    sr1
...................aucucuugugaaauuucgCa.......................................................................    7      1    sr1
...................aucucuugugaGauuucgua.......................................................................    1      1    sr1
...................aucucuugugaaGuuucgua.......................................................................    1      1    sr1
...................aucucuugugaaauuucgua.......................................................................    766    0    sr1
...................aucucCugugaaauuucgua.......................................................................    6      1    sr1
...................aucCcuugugaaauuucgua.......................................................................    3      1    sr1
...................aucucuCgugaaauuucgua.......................................................................    8      1    sr1
...................aucucuugCgaaauuucgua.......................................................................    3      1    sr1
...................aucucuugugaaauuCcgua.......................................................................    2      1    sr1
```

Mature **Star**

```
ucgucgucacauuccucuuaucucuugugaaauuucguacaacuuagcuuguacgacauuuuacaagagauaagaggugccagaagugggauucgaacccacgcaugcuu
...................aCcucuugugaaauuucgua.......................................................................   2     1   sr1
...................aucucuugugaaauCucgua.......................................................................   2     1   sr1
...................aucucuugugaaauuucAua.......................................................................   1     1   sr1
...................aucucuugugaaaCuucgua.......................................................................   1     1   sr1
...................aucucuAgugaaauuucgua.......................................................................   2     1   sr1
...................aucucuugugaaaAuucgua.......................................................................   1     1   sr1
...................Gucucuugugaaauuucgua.......................................................................   26    1   sr1
...................aucucuugugaaauuGcgua.......................................................................   1     1   sr1
...................aucucuugugaaaGuucgua.......................................................................   1     1   sr1
...................aucAcuugugaaauuucgua.......................................................................   1     1   sr1
...................aucucuugugaaauuucguG.......................................................................   19    1   sr1
...................aucucuugugaaauuAcgua.......................................................................   1     1   sr1
...................aucucAugugaaauuucgua.......................................................................   1     1   sr1
...................aucucuugugGaauuucgua.......................................................................   7     1   sr1
...................auUucuugugaaauuucguac......................................................................   1     1   sr1
...................aucucuugugGaauuucguac......................................................................   8     1   sr1
...................aucuUuugugaaauuucguac......................................................................   3     1   sr1
...................aucucuugugaaaCuucguac......................................................................   6     1   sr1
...................aucucGugugaaauuucguac......................................................................   1     1   sr1
...................aucucuugugaaauCucguac......................................................................   1     1   sr1
...................aucucuugugaaGuuucguac......................................................................   2     1   sr1
...................aucucuugugUaauuucguac......................................................................   2     1   sr1
...................aucucuugugaaauuucAuac......................................................................   4     1   sr1
...................aucucCugugaaauuucguac......................................................................   3     1   sr1
...................aucucuugCgaaauuucguac......................................................................   5     1   sr1
...................aucucuuAugaaauuucguac......................................................................   1     1   sr1
...................aucucuCgugaaauuucguac......................................................................   1     1   sr1
...................aucucuugugaaauuucgAac......................................................................   1     1   sr1
...................aucucuugugaGauuucguac......................................................................   4     1   sr1
...................aucucuugugaaauuucguaU......................................................................   211   1   sr1
...................aCcucuugugaaauuucguac......................................................................   2     1   sr1
...................aucucuAgugaaauuucguac......................................................................   3     1   sr1
...................aucucuugugaaauuAcguac......................................................................   2     1   sr1
...................aucucuugugaaauGucguac......................................................................   1     1   sr1
...................aucucuugugaaauuCcguac......................................................................   3     1   sr1
...................aucucuugugaaauuucguaA......................................................................   57    1   sr1
...................aucucuugugaaauuucguGc......................................................................   4     1   sr1
...................Gucucuugugaaauuucguac......................................................................   46    1   sr1
...................aucucuugugaaauuucguUc......................................................................   1     1   sr1
...................aucAcuugugaaauuucguac......................................................................   2     1   sr1
...................aucCcuugugaaauuucguac......................................................................   6     1   sr1
...................aucucuugugaaauuucguac......................................................................   1379  0   sr1
...................aucucuugugaaauuucguaG......................................................................   7     1   sr1
...................aucucuuguAaaauuucguaca.....................................................................   1     1   sr1
...................aucucuugCgaaauuucguaca.....................................................................   1     1   sr1
...................aucucuugugaaaAuucguaca.....................................................................   1     1   sr1
...................aucCcuugugaaauuucguaca.....................................................................   7     1   sr1
...................Gucucuugugaaauuucguaca.....................................................................   14    1   sr1
...................aucucuugugaaauuucguacG.....................................................................   44    1   sr1
...................aucucuugugaaauuucguGca.....................................................................   3     1   sr1
...................aucucuAgugaaauuucguaca.....................................................................   2     1   sr1
...................aucucuugugaaauCucguaca.....................................................................   1     1   sr1
...................aucucuugugaaGuuucguaca.....................................................................   1     1   sr1
...................aucucuugugaGauuucguaca.....................................................................   1     1   sr1
...................aucucuugugaaauuCcguaca.....................................................................   1     1   sr1
...................aucucuugugaaauuAcguaca.....................................................................   1     1   sr1
...................aucucuugugaaauuucguaAa.....................................................................   5     1   sr1
...................aCcucuugugaaauuucguaca.....................................................................   1     1   sr1
...................aucucuugugaaauuucguacU.....................................................................   27    1   sr1
...................aucucuugugaaauuucAuaca.....................................................................   3     1   sr1
...................aucucuugugaaauuucgCaca.....................................................................   1     1   sr1
...................aucucuugugaaauuucguaUa.....................................................................   282   1   sr1
...................aucucuuAugaaauuucguaca.....................................................................   1     1   sr1
...................aucucuugugGaauuucguaca.....................................................................   1     1   sr1
...................auAucuugugaaauuucguaca.....................................................................   1     1   sr1
...................aucucuugugaaauuucguacC.....................................................................   18    1   sr1
...................aucucuugugaaauuucguaca.....................................................................   707   0   sr1
...................aucucuCgugaaauuucguaca.....................................................................   4     1   sr1
...................aucucuugugaaauuucguaAaa....................................................................   1     1   sr1
...................aucucuugugaaauuucguacCa....................................................................   5     1   sr1
```

Mature Star

```
ucgucgucacauuccucuuaucucuugugaaauuucguacaacuuagcuuguacgacauuuuacaagagauaagaggugccagaagugggauucgaacccacgcaugcuu
...................aucucuugugaaauuucguacUa....................................................................  7    1  sr1
...................aucucuugugaaauuucguacaa....................................................................  7    0  sr1
...................aucucuugugaaauuucguacaU....................................................................  6    1  sr1
...................aucucuugugaaauuucguaUaa....................................................................  11   1  sr1
...................aucucuugugaaauuucguacaC....................................................................  1    1  sr1
...................aucucuugugaaauuucguacaCc...................................................................  1    1  sr1
...................aucucuugugaaauuucguacaaA...................................................................  3    1  sr1
....................ucucuugugaaauuucgA........................................................................  3    1  sr1
....................ucucuugugaaauuucgC........................................................................  4    1  sr1
....................ucucuugugaaauuucgG........................................................................  2    1  sr1
....................ucucuugugGaauuucgu........................................................................  1    1  sr1
....................ucucuugugaaauuucgu........................................................................  23   0  sr1
....................ucucuCgugaaauuucgua.......................................................................  1    1  sr1
....................ucucuugugaaauuucgua.......................................................................  108  0  sr1
....................Gcucuugugaaauuucgua.......................................................................  1    1  sr1
....................ucucuugugaaauuucguG.......................................................................  4    1  sr1
....................Ccucuugugaaauuucgua.......................................................................  1    1  sr1
....................ucucuugugUaauuucgua.......................................................................  1    1  sr1
....................ucucuugugGaauuucgua.......................................................................  1    1  sr1
....................ucucuugugaaauuucgCac......................................................................  1    1  sr1
....................ucucuugugaaauuucguaG......................................................................  2    1  sr1
....................ucucuugugaaauuucguaU......................................................................  31   1  sr1
....................Gcucuugugaaauuucguac......................................................................  24   1  sr1
....................ucucuugugaaauGucguac......................................................................  1    1  sr1
....................Ccucuugugaaauuucguac......................................................................  4    1  sr1
....................uAucuugugaaauuucguac......................................................................  2    1  sr1
....................ucucuCgugaaauuucguac......................................................................  1    1  sr1
....................ucucuugugaaauuucguaA......................................................................  11   1  sr1
....................ucucuugugaGauuucguac......................................................................  3    1  sr1
....................ucucuugugaaauuucCuac......................................................................  1    1  sr1
....................ucucuugugaaauuucguac......................................................................  378  0  sr1
....................ucucuugugaaGuuucguac......................................................................  1    1  sr1
....................ucucuugugaaauuucguGc......................................................................  2    1  sr1
....................ucucCugugaaauuucguac......................................................................  1    1  sr1
....................Gcucuugugaaauuucguaca.....................................................................  8    1  sr1
....................ucucuugugUaauuucguaca.....................................................................  1    1  sr1
....................ucucuugugGaauuucguaca.....................................................................  6    1  sr1
....................ucucuugugaaauuucguUca.....................................................................  1    1  sr1
....................ucucuugugaaauuucguaca.....................................................................  862  0  sr1
....................ucucCugugaaauuucguaca.....................................................................  3    1  sr1
....................Acucuugugaaauuucguaca.....................................................................  2    1  sr1
....................ucCcuugugaaauuucguaca.....................................................................  1    1  sr1
....................ucucuugugaaauCucguaca.....................................................................  1    1  sr1
....................ucucuugugaaauuucguacC.....................................................................  7    1  sr1
....................Ccucuugugaaauuucguaca.....................................................................  2    1  sr1
....................ucucuugugaaauuucgAaca.....................................................................  1    1  sr1
....................ucucuugugaaGuuucguaca.....................................................................  5    1  sr1
....................ucucuugugaaUuuucguaca.....................................................................  3    1  sr1
....................uUucuugugaaauuucguaca.....................................................................  1    1  sr1
....................ucucuugugaaauuucguaUa.....................................................................  7    1  sr1
....................ucucuugugaGauuucguaca.....................................................................  3    1  sr1
....................ucucuugCgaaauuucguaca.....................................................................  6    1  sr1
....................ucucuugugaaauuucguacU.....................................................................  14   1  sr1
....................ucucuugugaaaCuucguaca.....................................................................  1    1  sr1
....................ucucuugGgaaauuucguaca.....................................................................  1    1  sr1
....................ucucuugugaaauuucguacG.....................................................................  53   1  sr1
....................ucucuugugaaauuucguGca.....................................................................  6    1  sr1
....................ucucuugAgaaauuucguaca.....................................................................  1    1  sr1
....................ucucuugugaaauuucguacaa....................................................................  2    0  sr1
....................ucucuugugaaauuucguacaC....................................................................  4    1  sr1
....................ucucuugugaaauuucguacCa....................................................................  4    1  sr1
....................ucucuugugaaauuucguacaU....................................................................  6    1  sr1
....................ucucuugugaaauuucguacaG....................................................................  2    1  sr1
....................ucucuugugaaauuucguacUa....................................................................  7    1  sr1
....................ucucuugugaaauuucguacaCc...................................................................  1    1  sr1
....................ucucuugugaaauuucguacaacuuag...............................................................  1    0  sr1
.....................cucuugugaaauuucgua.......................................................................  1    0  sr1
.....................cucuugugaaauuucguaU......................................................................  1    1  sr1
.....................cucuugugaaauuucguac......................................................................  1    0  sr1
.....................cucuugugaaauuucguaca.....................................................................  2    0  sr1
```

Mature **Star**

```
ucgucgucacauuccucuuaucucuugugaaauuucguacaacuuagcuuguacgacauuuuacaagagauaagaggugccagaagugggauucgaacccacgcaugcuu
.....................cucuugugaaauuucguacaG....................................................................    1    1    sr1
......................ucuugugaaauuucguac......................................................................    6    0    sr1
......................ucuugugGaauuucguac......................................................................    1    1    sr1
......................Gcuugugaaauuucguaca.....................................................................    1    1    sr1
......................ucuugugaaauuucguaUa.....................................................................    3    1    sr1
......................ucuugugaaauuucguaca.....................................................................    6    0    sr1
......................ucuugugaaauuucguacUa....................................................................    1    1    sr1
......................ucuugugaaauuucguacaC....................................................................    1    1    sr1
......................ucuugugaaauuucguacaUc...................................................................    1    1    sr1
.......................cuugugaaauuucguacG.....................................................................    1    1    sr1
...................................................uacgacGuuuuacaagagauaa.....................................    2    1    sr1

.................Cuaucucuugugaaauuucguac......................................................................    1    1    st1
.................Cuaucucuugugaaauuucguaca.....................................................................    2    1    st1
..................uaucucuugugaaauuuc..........................................................................    1    0    st1
..................uaucucuugugaaauuucg.........................................................................    11   0    st1
..................uaucucuugugaaauuucA.........................................................................    1    1    st1
..................uaucucuugugaaauuucgA........................................................................    3    1    st1
..................uaucucuugugCaauuucgu........................................................................    1    1    st1
..................uaucucuugugGaauuucgu........................................................................    2    1    st1
..................uaucucuugugaaaAuucgu........................................................................    2    1    st1
..................uaucucuugugaaauuucgG........................................................................    4    1    st1
..................uaucucuugugaaauuucgC........................................................................    25   1    st1
..................uaucCcuugugaaauuucgu........................................................................    1    1    st1
..................uaucucuugugaaauGucgu........................................................................    3    1    st1
..................uaucucuugugaGauuucgu........................................................................    1    1    st1
..................uaucucuugugaaaGuucgu........................................................................    1    1    st1
..................uaucucuugugaaauuucgu........................................................................    148  0    st1
..................uaucucCugugaaauuucgua.......................................................................    4    1    st1
..................uaucucuuAugaaauuucgua.......................................................................    1    1    st1
..................uaucucuugugaaauuucguG.......................................................................    8    1    st1
..................uaucucuugugaaGuuucgua.......................................................................    5    1    st1
..................uaucucuugAgaaauuucgua.......................................................................    4    1    st1
..................uaucucuugugaaauuucgGa.......................................................................    1    1    st1
..................uaCcucuugugaaauuucgua.......................................................................    2    1    st1
..................uaucucuugugUaauuucgua.......................................................................    2    1    st1
..................uGucucuugugaaauuucgua.......................................................................    8    1    st1
..................uaucucuCgugaaauuucgua.......................................................................    4    1    st1
..................uaucucuugugCaauuucgua.......................................................................    1    1    st1
..................uaucucuugugaaauuucgua.......................................................................    859  0    st1
..................uaucCcuugugaaauuucgua.......................................................................    2    1    st1
..................uaucucuAgugaaauuucgua.......................................................................    3    1    st1
..................uaucucuugugaaaGuucgua.......................................................................    2    1    st1
..................uCucucuugugaaauuucgua.......................................................................    1    1    st1
..................uaucucuugCgaaauuucgua.......................................................................    3    1    st1
..................uaucucuugugaaauGucgua.......................................................................    3    1    st1
..................uaucucuugGgaaauuucgua.......................................................................    1    1    st1
..................uauUucuugugaaauuucgua.......................................................................    2    1    st1
..................uaucucuugugaaauuCcgua.......................................................................    2    1    st1
..................uaucucuugugGaauuucgua.......................................................................    7    1    st1
..................uaucuUuugugaaauuucgua.......................................................................    1    1    st1
..................uaucucuugugaGauuucgua.......................................................................    6    1    st1
..................uaucucuugugaaUuuucgua.......................................................................    2    1    st1
..................Caucucuugugaaauuucgua.......................................................................    3    1    st1
..................Aaucucuugugaaauuucgua.......................................................................    1    1    st1
..................uaucucuugugaaauuucgCa.......................................................................    2    1    st1
..................uaucucuugugaaauuucAua.......................................................................    1    1    st1
..................uaucucuugugaaauCucgua.......................................................................    4    1    st1
..................uGucucuugugaaauuucguac......................................................................    8    1    st1
..................uaucucuugCgaaauuucguac......................................................................    6    1    st1
..................Gaucucuugugaaauuucguac......................................................................    1    1    st1
..................uaucucuugugaaauuucguaA......................................................................    49   1    st1
..................uaucucuugugaUauuucguac......................................................................    2    1    st1
..................uaucucuugAgaaauuucguac......................................................................    1    1    st1
..................uaucucuugugaaaGuucguac......................................................................    2    1    st1
..................uaucucuugugaaauuucgCac......................................................................    1    1    st1
..................uaucucuugugCaauuucguac......................................................................    4    1    st1
..................uaucucuugugaaauCucguac......................................................................    7    1    st1
..................uaucucuAgugaaauuucguac......................................................................    2    1    st1
..................uaucucuugugaaGuuucguac......................................................................    9    1    st1
```

```
                  Mature                          Star
ucgucgucacauuccucuuaucucuugugaaauuucguacaacuuagcuuguacgacauuuuacaagagauaagaggugccagaaguggauucgaacccacgcaugcuu
..................uaucucuugugaaauuucguaU......................................................................    283    1    st1
..................uaucucuugugaGauuucguac......................................................................    10     1    st1
..................uaucucuugugaaauGucguac......................................................................    15     1    st1
..................uaCcucuugugaaauuucguac......................................................................    5      1    st1
..................Aaucucuugugaaauuucguac......................................................................    2      1    st1
..................uaucucuugugaaauuucAuac......................................................................    4      1    st1
..................uaucucCugugaaauuucguac......................................................................    1      1    st1
..................uaucucuCgugaaauuucguac......................................................................    3      1    st1
..................uaucucuugugaaaCuucguac......................................................................    10     1    st1
..................Caucucuugugaaauuucguac......................................................................    5      1    st1
..................uUucucuugugaaauuucguac......................................................................    2      1    st1
..................uaucCcuugugaaauuucguac......................................................................    6      1    st1
..................uaucAcuugugaaauuucguac......................................................................    3      1    st1
..................uaucucuuAugaaauuucguac......................................................................    1      1    st1
..................uaucucuugugaaauuucgGac......................................................................    1      1    st1
..................uaucucuugugaaauuucguac......................................................................    2157   0    st1
..................uaucucuugugaaauuucguaG......................................................................    11     1    st1
..................uaucucuugugaaauuCcguac......................................................................    8      1    st1
..................uaAcucuugugaaauuucguac......................................................................    3      1    st1
..................uaGcucuugugaaauuucguac......................................................................    1      1    st1
..................uaucucuuguAaaauuucguac......................................................................    2      1    st1
..................uaucucuugugGaauuucguac......................................................................    9      1    st1
..................uaucucuugugaaauuucguUc......................................................................    2      1    st1
..................uaucucuugugaaauuucguGc......................................................................    2      1    st1
..................uaucucuugugaaauuucgCaca.....................................................................    3      1    st1
..................uaucucuugCgaaauuucguaca.....................................................................    2      1    st1
..................uaucucuugugaaauCucguaca.....................................................................    7      1    st1
..................uaucucuugugaaCuuucguaca.....................................................................    1      1    st1
..................uCucucuugugaaauuucguaca.....................................................................    1      1    st1
..................uaucucGugugaaauuucguaca.....................................................................    1      1    st1
..................uaucucAugugaaauuucguaca.....................................................................    1      1    st1
..................uaucucuugugaaauuucguGca.....................................................................    2      1    st1
..................uaucucCugugaaauuucguaca.....................................................................    6      1    st1
..................uaucucuugugaaauGucguaca.....................................................................    5      1    st1
..................Caucucuugugaaauuucguaca.....................................................................    4      1    st1
..................uaucucuugugCaauuucguaca.....................................................................    6      1    st1
..................uaucucuuAugaaauuucguaca.....................................................................    3      1    st1
..................uaucCcuugugaaauuucguaca.....................................................................    5      1    st1
..................uaucucuugugaaaGuucguaca.....................................................................    5      1    st1
..................uaucucuugugGaauuucguaca.....................................................................    10     1    st1
..................uaucucuugugaaauuucCuaca.....................................................................    1      1    st1
..................uaucucuugugaaauuucguaca.....................................................................    1872   0    st1
..................uaAcucuugugaaauuucguaca.....................................................................    1      1    st1
..................uaucucuugugaaauuucAuaca.....................................................................    4      1    st1
..................uaucucuugugaaauuucguaAa.....................................................................    3      1    st1
..................uaucucuCgugaaauuucguaca.....................................................................    4      1    st1
..................uaucucuugugaaauuCcguaca.....................................................................    4      1    st1
..................uaucucuugugaGauuucguaca.....................................................................    8      1    st1
..................uaCcucuugugaaauuucguaca.....................................................................    5      1    st1
..................Gaucucuugugaaauuucguaca.....................................................................    1      1    st1
..................uUucucuugugaaauuucguaca.....................................................................    1      1    st1
..................uaucucuugugaaauuGcguaca.....................................................................    2      1    st1
..................uaucucuugugaaauuucguacU.....................................................................    96     1    st1
..................uaucucuAgugaaauuucguaca.....................................................................    2      1    st1
..................uaucucuugugaaauuucguaUa.....................................................................    33     1    st1
..................uaucucuugugaaauuucguacC.....................................................................    30     1    st1
..................uaucuAuugugaaauuucguaca.....................................................................    1      1    st1
..................uaucucuugugaaaCuucguaca.....................................................................    6      1    st1
..................uGucucuugugaaauuucguaca.....................................................................    13     1    st1
..................uaucucuugugaaauuucguacG.....................................................................    48     1    st1
..................uaucucuugugaaauuucguUca.....................................................................    2      1    st1
..................uaucucuugugaaGuuucguaca.....................................................................    4      1    st1
..................uaucucuugugaaauuucguacaa....................................................................    4      0    st1
..................uaucucuugugaaauuucguacaU....................................................................    14     1    st1
..................uaucucuugugaaauuucguacCa....................................................................    1      1    st1
..................uaucucuugugaaauuucguaUaa....................................................................    1      1    st1
..................uaucucuugugaaauuucguacUa....................................................................    9      1    st1
..................uaucucuugugaaauuucguacaaA...................................................................    4      1    st1
...................aucucuugugaCauuucgu........................................................................    1      1    st1
...................aucucuugugaaauGucgu........................................................................    1      1    st1
```

```
                  Mature              Star
ucgucgucacauuccucuuaucucuugugaaauuucguacaacuuagcuuguacgacauuuuacaagagauaagaggugccagaagugggauucgaacccacgcaugcuu
...................aucucuugugaaauuucgu........................................................................	36	0	st1
...................aucucuugugaaauuucgC........................................................................	4	1	st1
...................aucucuugugaaauuucgG........................................................................	1	1	st1
...................aucucuugugaCauuucgua.......................................................................	1	1	st1
...................aucucuugugaaauuucgCa.......................................................................	2	1	st1
...................aucucuugugaaGuuucgua.......................................................................	1	1	st1
...................aucCcuugugaaauuucgua.......................................................................	1	1	st1
...................aucucuugugaaauuCcgua.......................................................................	1	1	st1
...................aucucuugugaaauuucgua.......................................................................	89	0	st1
...................aucucuugugaaauuAcguac......................................................................	1	1	st1
...................aucucuugugaaauuucguaU......................................................................	87	1	st1
...................Uucucuugugaaauuucguac......................................................................	1	1	st1
...................aucucuugugaCauuucguac......................................................................	7	1	st1
...................aucGcuugugaaauuucguac......................................................................	1	1	st1
...................aCcucuugugaaauuucguac......................................................................	3	1	st1
...................Gucucuugugaaauuucguac......................................................................	2	1	st1
...................aucucuugugGaauuucguac......................................................................	2	1	st1
...................aucucuugugaaauuucguGc......................................................................	3	1	st1
...................aucucuugugaaauuCcguac......................................................................	4	1	st1
...................aucucuCgugaaauuucguac......................................................................	2	1	st1
...................aucucuugugaaauuucguaA......................................................................	7	1	st1
...................aucucuugugaaauuucguUc......................................................................	1	1	st1
...................aucucuugugaaauuucAuac......................................................................	1	1	st1
...................aucucuugugaaGuuucguac......................................................................	4	1	st1
...................aucucuugugaaauCucguac......................................................................	1	1	st1
...................aucucuugugaGauuucguac......................................................................	3	1	st1
...................aucucuugugaaauuucguac......................................................................	642	0	st1
...................aucucuugugaaaCuucguac......................................................................	2	1	st1
...................aucCcuugugaaauuucguac......................................................................	2	1	st1
...................aucucuugugaUauuucguac......................................................................	1	1	st1
...................aucucuugugaaauuucguaAa.....................................................................	1	1	st1
...................aucucuugugaaauuucguGca.....................................................................	1	1	st1
...................aucucuugugaaauuucAuaca.....................................................................	1	1	st1
...................aucucuugCgaaauuucguaca.....................................................................	1	1	st1
...................aucucuugugaaauuucguacC.....................................................................	1	1	st1
...................aucucuugugaaauuCcguaca.....................................................................	1	1	st1
...................aucucuugugaaauuucguacG.....................................................................	3	1	st1
...................aucucuugugaaauuAcguaca.....................................................................	1	1	st1
...................aucucuugugaaauuucguaca.....................................................................	177	0	st1
...................aucucuugugaaauuucguacU.....................................................................	23	1	st1
...................aucucuugugaCauuucguaca.....................................................................	1	1	st1
...................aucucuugugaaauuucguaUa.....................................................................	21	1	st1
...................aucucuugugaGauuucguaca.....................................................................	2	1	st1
...................aucCcuugugaaauuucguaca.....................................................................	1	1	st1
...................aucucuugugaaauuucguacaa....................................................................	1	0	st1
...................aucucuugugaaauuucguacUa....................................................................	3	1	st1
....................ucucuugugaaauuucgC........................................................................	1	1	st1
....................ucucuugugaaauuucgG........................................................................	1	1	st1
....................ucucuugugaaauuucgu........................................................................	7	0	st1
....................ucucuugugaaauuucgua.......................................................................	13	0	st1
....................ucucuugugGaauuucguac......................................................................	2	1	st1
....................ucucuugugaGauuucguac......................................................................	1	1	st1
....................ucucuugugaaauuucguaU......................................................................	9	1	st1
....................ucucuugugaaauuucguaA......................................................................	1	1	st1
....................ucucuCgugaaauuucguac......................................................................	1	1	st1
....................ucucuugugaaGuuucguac......................................................................	1	1	st1
....................ucucuugugaaauuucUuac......................................................................	1	1	st1
....................ucucuugugaaauuucguac......................................................................	84	0	st1
....................uUucuugugaaauuucguac......................................................................	1	1	st1
....................ucucuAgugaaauuucguac......................................................................	1	1	st1
....................ucucuugugaaauuucguaUa.....................................................................	2	1	st1
....................ucucuuAugaaauuucguaca.....................................................................	1	1	st1
....................ucucuugugaaauuGcguaca.....................................................................	1	1	st1
....................ucucuugugaaauuucgCaca.....................................................................	1	1	st1
....................ucucuugugaaCuuucguaca.....................................................................	1	1	st1
....................Ccucuugugaaauuucguaca.....................................................................	2	1	st1
....................ucucuugugaaGuuucguaca.....................................................................	3	1	st1
....................ucucuugugaaauGucguaca.....................................................................	4	1	st1
....................ucucuugugGaauuucguaca.....................................................................	1	1	st1
....................ucucuugugaaauuucguacG.....................................................................	3	1	st1
```

```
                  Mature                          Star
ucgucgucacauuccucuuaucucuugugaaauuucguacaacuuagcuuguacgacauuuuacaagagauaagaggugccagaagugggauucgaacccacgcaugcuu
....................ucCcuugugaaauuucguaca.....................................................................   1     1   st1
....................ucucuugugaaauuucguaca.....................................................................   267   0   st1
....................ucucuugugaaauuucguacC.....................................................................   5     1   st1
....................ucucuugugaaauuucguacU.....................................................................   2     1   st1
....................ucucuugugaaauuucguacaC....................................................................   2     1   st1
....................ucucuugugaaauuucguacCa....................................................................   2     1   st1
....................ucucuugugaaauuucguacaU....................................................................   6     1   st1
....................ucucuugugaaauuucguacaa....................................................................   1     0   st1
....................ucucuugugaaauuucguacaCc...................................................................   2     1   st1
....................ucucuugugaaauuucguacaaA...................................................................   2     1   st1
.....................cCcuugugaaauuucguac......................................................................   1     1   st1
.....................cucuugugaaauuucguaca.....................................................................   1     0   st1
......................ucuugugaaauuucguac......................................................................   1     0   st1
......................ucuugugaaauuucguaca.....................................................................   2     0   st1
.......................cuugugaaauuucguGca.....................................................................   1     1   st1
...................................................uacgacauuuuacaagagaC.......................................   1     1   st1
```

Provisional ID	:	ScYm1zk_361_3726
Score total	:	5
Score for star read(s)	:	-1.3
Score for read counts	:	0
Score for mfe	:	1.7
Score for randfold	:	1.6
Score for cons. seed	:	3
Total read count	:	2291
Mature read count	:	1226
Loop read count	:	0
Star read count	:	1065

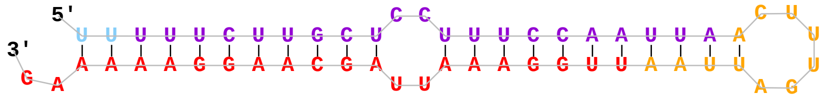

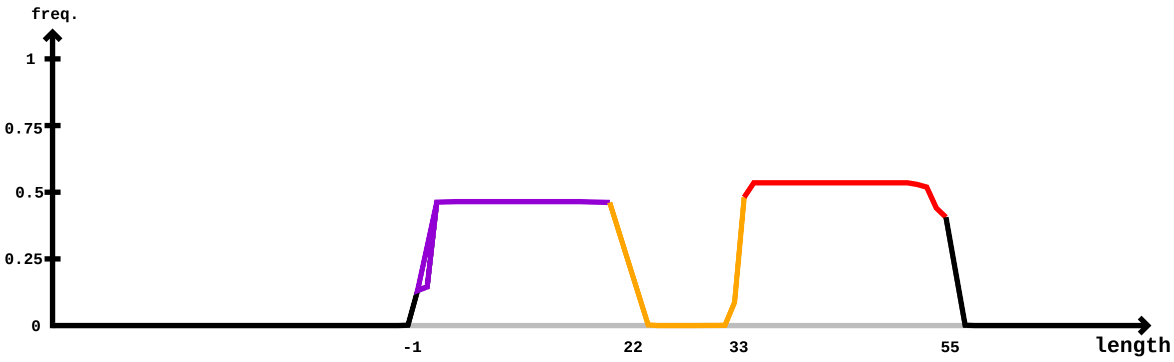


```
                                                   Star                              Mature
5'- ccucgggacuuucccucggcacaucgaucguccuccuuuuucuugcuccuuuccaauuaacuuugauuaauuggaaauuagcaaggaaaaagaggcgccaacagaaguacug  -3'   obs
    ccucgggacuuucccucggcacaucgaucguccuccuuuuucuugcuccuuuccaauuaacuuugauuaauuggaaauuagcaaggaaaaagaggcgccaacagaaguacug        exp
    ....(((.....))).(((.((.((...((.((((.(((((((((((..(((((((((((......)))))))))))..))))))))))).))))))......)).)).)))   reads    mm     sample
    ....................................uuuuucuugcuccuuuccaauu......................................................   9        0      sg1
    ....................................uAuuucuugcuccuuuccaauu......................................................   2        1      sg1
    ....................................uuuuucuugUuccuuuccaauu......................................................   1        1      sg1
    ....................................uuuuucuugcuccuuuccaauua.....................................................   6        0      sg1
    ....................................uAuuucuugcuccuuuccaauua.....................................................   1        1      sg1
    ....................................uuuuucuugcuccuuuccaauuaU....................................................   1        1      sg1
    ....................................uuuuucuugcuccuuuccaauuaa....................................................   1        0      sg1
    ....................................uuuuucuugcuccuuuccaauuaaG...................................................   1        1      sg1
    .....................................uuuucuugcuccuuuccaauua.....................................................   3        0      sg1
    ......................................uuucuugcuccuuuccaauu......................................................   4        0      sg1
    ......................................uuucuugcuccCuuccaauua.....................................................   1        1      sg1
    ......................................uuucuugcuccuuuccaauua.....................................................   10       0      sg1
    ......................................uuucuugcuccuuuccaauuaa....................................................   1        0      sg1
    ......................................uuucuugcuccuuuccaauuaU....................................................   1        1      sg1
    ......................................uuucuugcuUcuuuccaauuaa....................................................   2        1      sg1
    ......................................uuucuugcuccuuuccaauuaaA...................................................   4        1      sg1
    .....................................................................auuggaaauuagcaaggaa........................   1        0      sg1
    .....................................................................auuggaaauuagcaaggaaaa......................   1        0      sg1
    .....................................................................auuggaaauuagcaaggaaaaa.....................   3        0      sg1
    .....................................................................auuggaaauuagcaaggaaaaag....................   5        0      sg1
    .....................................................................auuggaaauuagcaaggaaaaaC....................   1        1      sg1
    ......................................................................uuggaaauuagcaaggaa........................   1        0      sg1
    ......................................................................uuggaaauuagcaaggaaa.......................   1        0      sg1
    ......................................................................uuggaaauuGgcaaggaaaa......................   1        1      sg1
    ......................................................................uuggaaauuagcaaggaaaa......................   6        0      sg1
    ......................................................................uuggaaauuagcaaggaaGa......................   1        1      sg1
    ......................................................................uuggaaauuagcaaggaaaUa.....................   1        1      sg1
    ......................................................................uuggaaauuagcaaggaaaaa.....................   2        0      sg1
    ......................................................................uuggaGauuagcaaggaaaaag....................   1        1      sg1
    ......................................................................uuggaaauuagcaaggaaaaGg....................   1        1      sg1
    ......................................................................uuggaaauuagcaaggaaaaag....................   43       0      sg1
    ......................................................................uuggaaauuagcaGggaaaaag....................   1        1      sg1
    ......................................................................uuggaaauuagcaaggaaaaaU....................   1        1      sg1
```

Star **Mature**

```
ccucgggacuuucccucggcacaucgaucguccuccuuuuucuugcuccuuuccaauuaacuuugauuaauuggaaauuagcaaggaaaaagaggcgccaacagaaguacug
......................................................................uuggGaauuagcaaggaaaaag....................  1    1  sg1
......................................................................uuggaaauuagcaaggaaaaaA....................  3    1  sg1
......................................................................uuggaaauuagcaaggaaaaaga...................  1    0  sg1
.......................................................................uggaaauuagcaaggaaaaaA....................  1    1  sg1
.......................................................................uggaaauuagcaaggaaaaag....................  1    0  sg1

...................................Uuuuuucuugcuccuuuccaauu......................................................  1    1  so1
....................................uuuuucuugcuccuuCccaau.......................................................  1    1  so1
....................................uuuuucuugcuccuuuccaau.......................................................  3    0  so1
....................................uuuuucuugcuccuuuccaaC.......................................................  1    1  so1
....................................uAuuucuugcuccuuuccaauu......................................................  6    1  so1
....................................Cuuuucuugcuccuuuccaauu......................................................  1    1  so1
....................................uuuuucuugcuccuuuccaauu......................................................  24   0  so1
....................................uuuuucuugcuccuuuccaauA......................................................  1    1  so1
....................................uuuuucuugcuccuuuccaauC......................................................  3    1  so1
....................................uuuuucuugcuccCuuccaauua.....................................................  2    1  so1
....................................uuuuucuugcuccuuuccaauua.....................................................  20   0  so1
....................................uuuuucuugcuccuuuccaauuU.....................................................  1    1  so1
....................................uAuuucuugcuccuuuccaauua.....................................................  1    1  so1
....................................uuuuucCugcuccuuuccaauua.....................................................  1    1  so1
....................................uuuuucuugcuccuuuccaauuUa....................................................  1    1  so1
....................................uuuuucuugcuccuuuccaauuaa....................................................  2    0  so1
....................................uuuuucuugcuccuuuccaauuaU....................................................  5    1  so1
.....................................uuuucuugcuccuuuccaauu......................................................  3    0  so1
.....................................uuuucuugcuccuuuccaauua.....................................................  4    0  so1
......................................uuucuugcuccuuuccaau.......................................................  2    0  so1
......................................uuucuugcuccuuuccaauu......................................................  22   0  so1
......................................uuucuugcuccuuuccaauC......................................................  4    1  so1
......................................uuucuugcuccuuuccaauua.....................................................  85   0  so1
......................................uuucuugcuccuuuccaauuU.....................................................  1    1  so1
......................................uuucuugcuccuuuccaauuaa....................................................  5    0  so1
.......................................uucuugcuccuuuccaauuaa....................................................  2    0  so1
.....................................................................auuggaaauuagcaaggaa........................  1    0  so1
.....................................................................auuggaaauuagcaaggaaa.......................  1    0  so1
.....................................................................auuggaaauuagcaaggaaaa......................  4    0  so1
.....................................................................auuggaaauuagcaaggaaaGa.....................  1    1  so1
.....................................................................auuggaaauuagcaaggaaaaag....................  22   0  so1
.....................................................................auuggaaauuagcaaggaaaaagU...................  1    1  so1
......................................................................uuggaaauuagcaaggaa........................  1    0  so1
......................................................................uuggaaauuagcaaggaaa.......................  2    0  so1
......................................................................uuggaaauuagcaaggaaaa......................  17   0  so1
......................................................................uuggaaauuagcaaggaaUa......................  1    1  so1
......................................................................uuggaaauuagcaaggGaaa......................  1    1  so1
......................................................................uuggaaauuagcaaggaaaaa.....................  7    0  so1
......................................................................uuggaaauuagcaaggaaGaag....................  1    1  so1
......................................................................uuggaaauuagcaaggaaaaaA....................  4    1  so1
......................................................................uuggaaauuagcaaggaaaaag....................  102  0  so1
......................................................................uuggaaauuagcaaggaaaaGg....................  2    1  so1
......................................................................uuggaGauuagcaaggaaaaag....................  1    1  so1
......................................................................uuggaaauuagcaaggaaaaCg....................  1    1  so1
......................................................................uuggaaauuagcaGggaaaaag....................  1    1  so1
......................................................................uuggaaauuagcaaggGaaaag....................  1    1  so1
......................................................................uCggaaauuagcaaggaaaaag....................  1    1  so1
.......................................................................uggaaauuagcaaggaaaa......................  2    0  so1
.......................................................................uggaaauuagcaaggaaGaag....................  1    1  so1
.......................................................................uggaaauuagcaaggaaaaag....................  19   0  so1
.......................................................................uggaaauuagcaaggaaaaaA....................  1    1  so1

....................................uuuuucuugcuccuuucc..........................................................  1    0  st1
....................................uAuuucuugcuccuuuccaau.......................................................  1    1  st1
....................................uuuuucuugcuccuCuccaau.......................................................  1    1  st1
....................................uuuuucuugcuccuuuccaaC.......................................................  2    1  st1
....................................uuuuucuugcuccuuuccaau.......................................................  17   0  st1
....................................uuuuucuugcuccuuuccaauu......................................................  47   0  st1
....................................uuuuucuugcuccuuuccaauG......................................................  1    1  st1
....................................uuuuucuugcuccuuuccaGuu......................................................  1    1  st1
....................................uuuuucuugcuccuuuccaauA......................................................  1    1  st1
....................................uuuuucuugcuccuuuccaauC......................................................  1    1  st1
....................................uuuuucuugcuccuuuccGauu......................................................  1    1  st1
....................................uuuuucuugcuccuuuccaauua.....................................................  12   0  st1
```

Star **Mature**

ccucgggacuuucccucggcacaucgaucguccuccuuuuucuugcuccuuuccaauuaacuuugauuaauuggaaauuagcaaggaaaaagaggcgccaacagaaguacug

....................................uuuuucuugcuccuuuccaauuU...	2	1	st1
....................................uuuuucuugcuccuuuccaauuaa..	2	0	st1
....................................uuuuucuugcuccuuuccaauuUa..	1	1	st1
.....................................uuuucuugcuccuuucca...	1	0	st1
.....................................uuuucuugcuccuuuccaauu..	3	0	st1
.....................................uuuucuugcuccuuuccaauua...	3	0	st1
......................................uuucuugcuccuuuUcaau...	2	1	st1
......................................uuucuugcuccuuuccaUu...	1	1	st1
......................................uuucuugcuccuuuccaauu..	26	0	st1
......................................uuucuugcCccuuuccaauu..	1	1	st1
......................................uuucuCgcuccuuuccaauu..	1	1	st1
......................................uuucuugcuccuuCccaauu..	1	1	st1
......................................uuucuugcuccuuuccaauC..	1	1	st1
......................................uuucuugcuccuuuAcaauua...	1	1	st1
......................................uuucuugcuccuuuccaauua...	102	0	st1
......................................uuucuugcuccuuuccaauuU...	2	1	st1
......................................uuucuugcuccuCuccaauua...	2	1	st1
......................................uuucuugcuccuuuGcaauua...	1	1	st1
......................................uuCcuugcuccuuuccaauua...	1	1	st1
......................................uuucuugcuccuuuccaauuaa..	5	0	st1
......................................uuucuugcuccuuuccaauuaG..	1	1	st1
....uucuugcuccuuuccaauua...	1	0	st1
....auuggaaauuagcaaggaaa.......................	1	0	st1
....auuggaaauuCgcaaggaaaa......................	1	1	st1
....auuggaaauuagcaaggaaaa......................	18	0	st1
....auuggaaauuagcaagAaaaa......................	1	1	st1
....auuggaaauuagcaaggaaaaa.....................	3	0	st1
....auuggaaauuagcaaggaaaaG.....................	2	1	st1
....auuggaaauuagcaaggaaaaaA....................	1	1	st1
....auuggaaauuagcaaggaaaCag....................	2	1	st1
....auuggaaauuagcaaggaaaaag....................	41	0	st1
....auuggaaauuagcaaggGaaaag....................	1	1	st1
....uuggaaauuagcaaggaa........................	2	0	st1
....uuggaaauuagcaaggaaa.......................	4	0	st1
....uuggaaauuagcaaggaaaa......................	16	0	st1
....uuggaaauuUgcaaggaaaaa.....................	1	1	st1
....uuggaaauuagcaaggaaaaa.....................	6	0	st1
....uuggaaauuagcaaggUaaaag....................	1	1	st1
....uuggaaauuagcGaggaaaaag....................	2	1	st1
....uuggaaauuaUcaaggaaaaag....................	1	1	st1
....uuggaGauuagcaaggaaaaag....................	1	1	st1
....uuggaaGuuagcaaggaaaaag....................	1	1	st1
....Cuggaaauuagcaaggaaaaag....................	1	1	st1
....uuggaaauuagcaaggaaaaaA....................	9	1	st1
....uuggaaauuagcaaggaGaaag....................	1	1	st1
....uuggaaauCagcaaggaaaaag....................	1	1	st1
....uuggaaauuagcaaggaaaaag....................	105	0	st1
....uuggaaauuagcaaggaaaaaga...................	2	0	st1
....uggaaauuagcaaggaaaa......................	8	0	st1
....uggaaauuagcaaggaaaaa.....................	4	0	st1
....uggaaauuagcaaggaaaaag....................	16	0	st1
....uggGaauuagcaaggaaaaag....................	1	1	st1
....uggaaauuagcaaggaaaaaA....................	1	1	st1
....uggaaauuagcaaggaGaaag....................	1	1	st1
....uggaaauuagcaagCaaaaaga...................	1	1	st1
...................................Uuuuuucuugcuccuuuccaauua...	2	1	sr1
....................................uuuuucuugcuccuuucc..	1	0	sr1
....................................uuuAucuugcuccuuucc..	1	1	sr1
....................................uuuuucuugcuccuuucca...	1	0	sr1
....................................uuuuucuugcuccuuuccaa..	2	0	sr1
....................................uuuuucuugcuccuuuccaau...	11	0	sr1
....................................uuuuucuugcuccuuuccaaC...	1	1	sr1
....................................uuuuucuugcuccuuuccaauu..	33	0	sr1
....................................uAuuucuugcuccuuuccaauu..	3	1	sr1
....................................uuuuucuugcuccuuuccaauA..	2	1	sr1
....................................uuuuucuugcuccuuuccaauC..	8	1	sr1
....................................uuuuucuugcuccuuuccGauua...	1	1	sr1
....................................uAuuucuugcuccuuuccaauua...	2	1	sr1
....................................uuuuucuugcuccuuuccaauuG...	4	1	sr1

Star Mature

```
ccucgggacuuucccucggcacaucgaucguccuccuuuuucuugcuccuuuccaauuaacuuugauuaauuggaaauuagcaaggaaaaagaggcgccaacagaaguacug
....................................uuCuucuugcuccuuuccaauua.....................................................	1	1	sr1
....................................uuuuucuugcuccuuuccaauuU.....................................................	1	1	sr1
....................................uuuuucuugcuccuuuccaauua.....................................................	30	0	sr1
....................................uuuuucuugcuccuuuccaauuaU....................................................	1	1	sr1
....................................Guuuucuugcuccuuuccaauuaa....................................................	1	1	sr1
....................................uuuuucuugcuccuuuccaauuGa....................................................	1	1	sr1
....................................uuuuucuugcuccuuuccaauuaa....................................................	4	0	sr1
....................................uuuuucuugcuccuuuccaauuUa....................................................	3	1	sr1
.....................................uuuucuugcuccuuuccaauu......................................................	6	0	sr1
.....................................uuuucuugcuccuuuccaauC......................................................	1	1	sr1
.....................................uuuucuugcuccuuuccaauua.....................................................	8	0	sr1
......................................uuucuugcuccuuuccaaG.......................................................	1	1	sr1
......................................uuucuugcuccuuuccaau.......................................................	4	0	sr1
......................................Guucuugcuccuuuccaauu......................................................	6	1	sr1
......................................uuucuugcuccuuuccaauu......................................................	76	0	sr1
......................................uuucuAgcuccuuuccaauu......................................................	2	1	sr1
......................................uuucuugcuccuuuUcaauu......................................................	3	1	sr1
......................................uuucuugcuccuuuccaauG......................................................	2	1	sr1
......................................uuucuugUuccuuuccaauu......................................................	1	1	sr1
......................................uuucuugcuccuuuccaauC......................................................	4	1	sr1
......................................uuucuugcuccuuuccaauuC.....................................................	1	1	sr1
......................................uuucuugcuccuuuccaauuU.....................................................	4	1	sr1
......................................uuucuugUuccuuuccaauua.....................................................	4	1	sr1
......................................uuucuCgcuccuuuccaauua.....................................................	2	1	sr1
......................................uuucCugcuccuuuccaauua.....................................................	1	1	sr1
......................................uuucuugcuccuuuccaauua.....................................................	267	0	sr1
......................................Auucuugcuccuuuccaauua.....................................................	2	1	sr1
......................................uuucuugcuccuuuccGauua.....................................................	1	1	sr1
......................................Cuucuugcuccuuuccaauua.....................................................	3	1	sr1
......................................Guucuugcuccuuuccaauua.....................................................	24	1	sr1
......................................uuucuugcuAcuuuccaauua.....................................................	1	1	sr1
......................................uuucuugcuccuuuccaauuG.....................................................	3	1	sr1
......................................Cuucuugcuccuuuccaauuaa....................................................	1	1	sr1
......................................uuucuugcuccuuuccaauuaa....................................................	17	0	sr1
......................................Guucuugcuccuuuccaauuaa....................................................	3	1	sr1
......................................uuucuugcuccuuuccaauuaU....................................................	3	1	sr1
......................................uuucuugcuccuuuccaauuaUc...................................................	1	1	sr1
........................................ucuugcuccuuuccaauua.....................................................	2	0	sr1
..................................................................uuaauuggaaauuagcaaggaaaaag....................	1	0	sr1
....................................................................aauuggaaauuagcaaggaaaa......................	1	0	sr1
.....................................................................auuggaaauuagcaaggaaa.......................	4	0	sr1
.....................................................................auuggaaauuagcaaggaaaG......................	2	1	sr1
.....................................................................Guuggaaauuagcaaggaaaa......................	1	1	sr1
.....................................................................auuggaaauuagcaaggaaaa......................	14	0	sr1
.....................................................................auuggaaauuagcaaggaaaaa.....................	4	0	sr1
.....................................................................Guuggaaauuagcaaggaaaaa.....................	1	1	sr1
.....................................................................auuggaaauuagcaaggaGaaa.....................	1	1	sr1
.....................................................................auuggaaauuagcaagAaaaaa.....................	1	1	sr1
.....................................................................auuggaaauuagcaaggaaaaGg....................	1	1	sr1
.....................................................................auuggaaauuagcaaggaaaaaA....................	4	1	sr1
.....................................................................auuggaaauuagcaaggaaaaag....................	46	0	sr1
.....................................................................auuggaaauuagcaaggaaGaag....................	1	1	sr1
.....................................................................auuggaaauuagcaaggaaaaaC....................	2	1	sr1
.....................................................................Guuggaaauuagcaaggaaaaag....................	3	1	sr1
.....................................................................auuggGaauuagcaaggaaaaag....................	1	1	sr1
......................................................................Guggaaauuagcaaggaa........................	2	1	sr1
......................................................................uuggaaauuagcaaggaa........................	5	0	sr1
......................................................................uuggaaauuagcaaggaU........................	1	1	sr1
......................................................................Guggaaauuagcaaggaaa.......................	6	1	sr1
......................................................................uuggaaauuagcaaggaaa.......................	4	0	sr1
......................................................................uuggaaauCagcaaggaaaa......................	2	1	sr1
......................................................................uuggaaauuagcaaggaaaa......................	59	0	sr1
......................................................................uuggaaauuagcaaggaaaG......................	1	1	sr1
......................................................................Guggaaauuagcaaggaaaa......................	14	1	sr1
......................................................................uuggaaauuagcaaggaaaaa.....................	25	0	sr1
......................................................................uuggaaGuuagcaaggaaaaa.....................	1	1	sr1
......................................................................Guggaaauuagcaaggaaaaa.....................	9	1	sr1
......................................................................uuggaaauuagcaaggaaaaG.....................	1	1	sr1
......................................................................uuggaaauuagcGaggaaaaag....................	2	1	sr1
......................................................................uuggaaauuagUaaggaaaaag....................	3	1	sr1
```

Star Mature

```
ccucgggacuuucccucggcacaucgaucguccuccuuuuucuugcuccuuuccaauuaacuuugauuaauuggaaauuagcaaggaaaaagaggcgccaacagaaguacug
......................................................................Guggaaauuagcaaggaaaaag....................   77    1   sr1
......................................................................uuggGaauuagcaaggaaaaag....................   2     1   sr1
......................................................................uuAgaaauuagcaaggaaaaag....................   1     1   sr1
......................................................................uuggaaauuagcaaggaaaGag....................   2     1   sr1
......................................................................uuggaaauuGgcaaggaaaaag....................   5     1   sr1
......................................................................uuggaaauuagcaaggaaaaUg....................   2     1   sr1
......................................................................uuggaaauCagcaaggaaaaag....................   1     1   sr1
......................................................................uuggaaauuagcaaggaaaaaA....................   3     1   sr1
......................................................................uuggaaauuagcaaggaaaaGg....................   8     1   sr1
......................................................................uuggaCauuagcaaggaaaaag....................   1     1   sr1
......................................................................uuggaaGuuagcaaggaaaaag....................   2     1   sr1
......................................................................uuggaGauuagcaaggaaaaag....................   2     1   sr1
......................................................................uuggaaauuagcaagUaaaaag....................   1     1   sr1
......................................................................uuggaaauuagcaaggaaaaag....................   288   0   sr1
......................................................................uuggaaauuagcaaggaaUaag....................   2     1   sr1
......................................................................uuggaaauuagcaaggaaaaaga...................   3     0   sr1
......................................................................Guggaaauuagcaaggaaaaaga...................   1     1   sr1
......................................................................uuggaaauuagcaaggaaaaagC...................   1     1   sr1
......................................................................uuggaaauuagcaaggaaaaagU...................   1     1   sr1
......................................................................uuggaaauuagcaaggaaaaagaA..................   3     1   sr1
.......................................................................uggaaauuagcaaggaaaa......................   6     0   sr1
.......................................................................Gggaaauuagcaaggaaaa......................   2     1   sr1
.......................................................................uggaaauuagcaaggaaaaa.....................   5     0   sr1
.......................................................................uggaaauuagcaagAaaaaa.....................   1     1   sr1
.......................................................................uggaaauuagcaaggaaUaag....................   1     1   sr1
.......................................................................uggaaauuagcaaggaaaaag....................   39    0   sr1
.......................................................................Gggaaauuagcaaggaaaaag....................   9     1   sr1
.......................................................................uggaaauuagcaaggaaaaGg....................   3     1   sr1
.......................................................................uggaaauuagcaaggaaaaaA....................   1     1   sr1
```

Provisional ID	:	ScYm1zk_374_7192
Score total	:	575
Score for star read(s)	:	3.9
Score for read counts	:	568.6
Score for mfe	:	1.5
Score for randfold	:	1.6
Score for cons. seed	:	-0.6
Total read count	:	1127
Mature read count	:	1123
Loop read count	:	0
Star read count	:	4

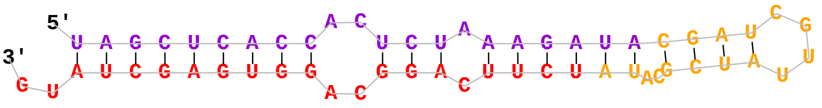

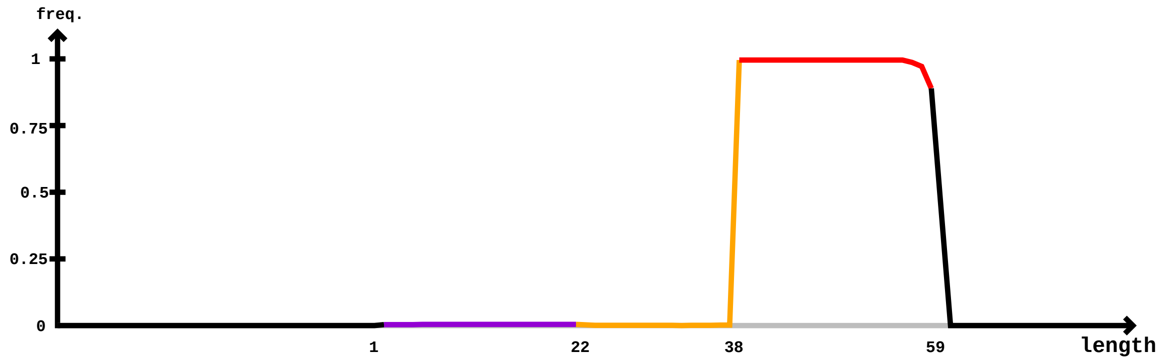

Star Mature

```
5'- agauucaaagauacagaagcauauaucagccauagcucaccacucuaaagauacgaucguuaucgcauaucuucaggcaggugagcuaugagugauucuuugauuucaaa   -3'   obs
    agauucaaagauacagaagcauauaucagccauagcucaccacucuaaagauacgaucguuaucgcauaucuucaggcaggugagcuaugagugauucuuugauuucaaa         exp
    .((.(((((((.............((((..(((((((((((..(((.((((((((((....))))..)))))).)))..)))))))))))..)))))))))))..))...   reads   mm   sample
    .....................................................................ucuucaggcaggugagcu.......................   1   0   so1
    .....................................................................ucuucaggcaggugagcC.......................   2   1   so1
    .....................................................................ucuucaggcaggugagcua......................   2   0   so1
    .....................................................................ucuucaggcaggugagcuaC.....................   7   1   so1
    .....................................................................ucuucaggcaggugagcuau.....................   13   0   so1
    .....................................................................ucCucaggcaggugagcuaug....................   1   1   so1
    .....................................................................ucuucaggcaggugagcuauC....................   2   1   so1
    .....................................................................ucuucaggcaggugagcuaug....................   99   0   so1
    .....................................................................ucuucaggcaggugagcuaCg....................   2   1   so1
    .....................................................................ucuucaggcaggugagcuauU....................   7   1   so1
    .....................................................................ucuAcaggcaggugagcuaug....................   1   1   so1
    .....................................................................ucuucGggcaggugagcuaug....................   2   1   so1
    .....................................................................ucuucaggcaggugagcuauA....................   6   1   so1
    .....................................................................ucuucaggcaggugagcuauUa...................   5   1   so1
    .....................................................................ucuucaggcaggugagcuauAa...................   1   1   so1
    .....................................................................ucuucaggcaggugagcuauga...................   3   0   so1

    ................................uagcucaccacucuaaagaua.........................................................   1   0   sg1
    ....................................ucaccacucuaaagauacgaucguuau...............................................   1   0   sg1
    ................................................................gcauaucuucaggcaggugagcuauga...................   1   0   sg1
    ...................................................................uaucuucaggcaggugagcuauU....................   1   1   sg1
    .....................................................................ucuucaggcaggugagcu.......................   4   0   sg1
    .....................................................................ucuucaggcaggugagcua......................   5   0   sg1
    .....................................................................ucuucaggcaggugagcuau.....................   34   0   sg1
    .....................................................................ucuucaggcaggugagcuaC.....................   4   1   sg1
    .....................................................................ucuucaggcGggugagcuau.....................   1   1   sg1
    .....................................................................ucuucaggcaggugagcuaA.....................   1   1   sg1
    .....................................................................Ccuucaggcaggugagcuaug....................   1   1   sg1
    .....................................................................ucCucaggcaggugagcuaug....................   2   1   sg1
    .....................................................................ucuucaggcaggugagcAaug....................   1   1   sg1
    .....................................................................ucuucaggcaggugagcuaug....................   355   0   sg1
    .....................................................................ucuucaggcagUugagcuaug....................   1   1   sg1
    .....................................................................ucuucaggcaggugagcuauA....................   18   1   sg1
```

Star / Mature

```
                                Star                                   Mature
agauucaaagauacagaagcauauaucagccauagcucaccacucuaaagauacgaucguuaucgcauaucuucaggcaggugagcuaugagugauucuuugauuucaaa
.....................................................................ucuucaggcaggugagcuauU....................    22    1    sg1
.....................................................................uAuucaggcaggugagcuaug....................    1     1    sg1
.....................................................................ucuucaggcaggCgagcuaug....................    1     1    sg1
.....................................................................ucuCcaggcaggugagcuaug....................    2     1    sg1
.....................................................................ucuucaggcaggugagcuaGg....................    1     1    sg1
.....................................................................ucuucaggcaggugagcCaug....................    1     1    sg1
.....................................................................ucuucGggcaggugagcuaug....................    1     1    sg1
.....................................................................ucuucaggcaggugagUuaug....................    1     1    sg1
.....................................................................ucuucaggcaggugagcuaCg....................    2     1    sg1
.....................................................................ucuucaggcaAgugagcuaug....................    1     1    sg1
.....................................................................uUuucaggcaggugagcuaug....................    1     1    sg1
.....................................................................ucuucaggcaggugagcuaugU...................    4     1    sg1
.....................................................................ucuucaggcaggugagcuauUa...................    7     1    sg1
.....................................................................ucuucaggcaggugagcuauAa...................    1     1    sg1
.....................................................................ucuucaggcaggugagcuauga...................    8     0    sg1

................................Gagcucaccacucuaaagaua.........................................................    2     1    sr1
................................uagcucaccacucuaaagaua.........................................................    1     0    sr1
.....................................................................ucuucaggcaggugagcua......................    5     0    sr1
.....................................................................Gcuucaggcaggugagcuau.....................    1     1    sr1
.....................................................................ucuucaggcaggugagcuaC.....................    1     1    sr1
.....................................................................ucuucaggcaggugagcuau.....................    11    0    sr1
.....................................................................uUuucaggcaggugagcuaug....................    1     1    sr1
.....................................................................ucuucaggcaggugGgcuaug....................    2     1    sr1
.....................................................................ucuucaggcaggugagcCaug....................    1     1    sr1
.....................................................................ucuucaggcaggugagcuauA....................    28    1    sr1
.....................................................................ucuucaggcaggugagcuaCg....................    2     1    sr1
.....................................................................ucuucagAcaggugagcuaug....................    1     1    sr1
.....................................................................ucuucaggcaggCgagcuaug....................    1     1    sr1
.....................................................................ucuCcaggcaggugagcuaug....................    2     1    sr1
.....................................................................ucuucaggcaggugagcuauU....................    12    1    sr1
.....................................................................Gcuucaggcaggugagcuaug....................    9     1    sr1
.....................................................................ucuucaggcaggugagcuauC....................    1     1    sr1
.....................................................................ucuucaggcaggugagcuaug....................    168   0    sr1
.....................................................................ucuucaggcaggugaAcuaug....................    1     1    sr1
.....................................................................ucuucaggcaggugagcuauUa...................    15    1    sr1
.....................................................................ucuucaggcaggugagcuauAa...................    1     1    sr1

.....................................................................ucuucaggcaggugagcu.......................    2     0    st1
.....................................................................ucuucaggcaggugagcG.......................    1     1    st1
.....................................................................ucuucaggcaggugagcuG......................    1     1    st1
.....................................................................ucuucaggcaggugagcua......................    4     0    st1
.....................................................................ucuucaggcaggugagcuau.....................    19    0    st1
.....................................................................ucuucaggcaggugagcuaC.....................    1     1    st1
.....................................................................ucuucaggcaggugGgcuaug....................    2     1    st1
.....................................................................ucuucaggcaggugagcuauA....................    9     1    st1
.....................................................................ucuucagAcaggugagcuaug....................    2     1    st1
.....................................................................ucuucGggcaggugagcuaug....................    1     1    st1
.....................................................................ucuCcaggcaggugagcuaug....................    1     1    st1
.....................................................................ucuucaggcaggugagcuauU....................    9     1    st1
.....................................................................ucuucaggcaggugagcuauC....................    1     1    st1
.....................................................................ucuucaggcaggugagcuaug....................    168   0    st1
.....................................................................ucuucaggcaggugagcuauUa...................    1     1    st1
.....................................................................ucuucaggcaggugagcuaugU...................    1     1    st1
.....................................................................ucuucaggcaggugagcuauga...................    1     0    st1
```

Provisional ID : ScYm1zk_397_10438
Score total : 1002.1
Score for star read(s) : 3.9
Score for read counts : 991.7
Score for mfe : 1.8
Score for randfold : 1.6
Score for cons. seed : 3
Total read count : 1957
Mature read count : 1863
Loop read count : 0
Star read count : 94

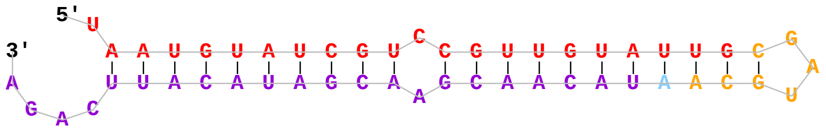

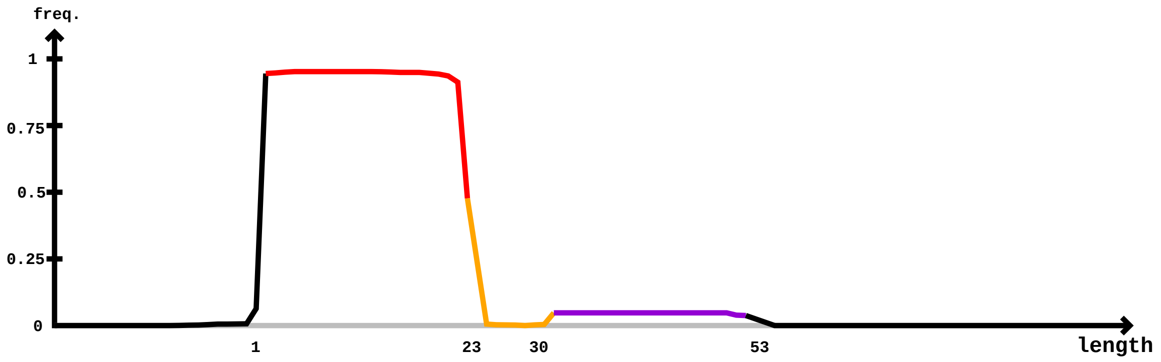

Mature Star

```
5'- aauuguucaaaauauugucuuaauguaucguccguuguauugcgaugcaauacaacgaacgauacauucagacaauacauucagacaauauaagacacgcgccgaauaau  -3'   obs
    aauuguucaaaauauugucuuaauguaucguccguuguauugcgaugcaauacaacgaacgauacauucagacaauacauucagacaauauaagacacgcgccgaauaau         exp
    .((((((.....((((((((.((((((((((.(((((((((((...))))))))))).)))))))))).))))))))......)))))).....................   reads   mm   sample
    .............auugucuuaauguaucguccA............................................................................   1       1    sr1
    ..............uugucuuaauguaucguccg............................................................................   2       0    sr1
    ..............uugucuuaauguaucguccguug.........................................................................   1       0    sr1
    ...............ugucuuaauguaucguccguug.........................................................................   2       0    sr1
    .................ucuuaauguaucguccguug.........................................................................   1       0    sr1
    ..................cuuaauguaucguccguuguauA.....................................................................   1       1    sr1
    ...................uuaauguaucguUcguugua.......................................................................   1       1    sr1
    ...................uuaauguaucguccguuguauu.....................................................................   1       0    sr1
    ...................uuaauguaucguccguuguauA.....................................................................   2       1    sr1
    ...................uGaauguaucguccguuguauu.....................................................................   3       1    sr1
    ...................uGaauguaucguccguuguauug....................................................................   1       1    sr1
    ...................uuaauguaucguccguuguauuA....................................................................   18      1    sr1
    ....................uaauguaucguccguugu........................................................................   1       0    sr1
    ....................uaauguaucguccguugua.......................................................................   8       0    sr1
    ....................uaauguauUguccguugua.......................................................................   1       1    sr1
    ....................uaauguaucguccguuguau......................................................................   12      0    sr1
    ....................uaauguaucguccguuguUu......................................................................   1       1    sr1
    ....................uaauguaucguccguuguaG......................................................................   1       1    sr1
    ....................uaauguaucguccguuguaC......................................................................   1       1    sr1
    ....................Gaauguaucguccguuguau......................................................................   1       1    sr1
    ....................Caauguaucguccguuguauu.....................................................................   1       1    sr1
    ....................uaauguaucguccguuguauu.....................................................................   98      0    sr1
    ....................uaauguaucguccguuguauC.....................................................................   2       1    sr1
    ....................uaauguaucguccguuguauA.....................................................................   74      1    sr1
    ....................Gaauguaucguccguuguauu.....................................................................   21      1    sr1
    ....................uaauguaCcguccguuguauu.....................................................................   1       1    sr1
    ....................uaauguaucguccguuguauG.....................................................................   8       1    sr1
    ....................uaauguaucguccgCuguauu.....................................................................   1       1    sr1
    ....................Gaauguaucguccguuguauug....................................................................   4       1    sr1
    ....................uaauguaucguccguuguaCug....................................................................   1       1    sr1
    ....................uaauguaucguccguuguauug....................................................................   30      0    sr1
    ....................uaauguaucguccguuguauCg....................................................................   1       1    sr1
    ....................uaauguaucguccguuguauuU....................................................................   1       1    sr1
```

```
                    Mature                       Star
aauuguucaaaauauugucuuaauguaucguccguuguauugcgaugcaauacaacgaacgauacauucagacaauacauucagacaauauaagacacgcgccgaauaau
....................uaaAguaucguccguuguauug....................................................................    1     1    sr1
....................uaauguaucguccguuguauuA....................................................................    408   1    sr1
....................uaauguaucguccguuguauugc...................................................................    1     0    sr1
....................Gaauguaucguccguuguauugc...................................................................    1     1    sr1
....................Gaauguaucguccguuguauugcga.................................................................    1     1    sr1
....................uaauguaucguccguuguauugcgaug...............................................................    2     0    sr1
.....................aauguaucguccguuguauug....................................................................    1     0    sr1
......................auguaucguccguuguauu.....................................................................    2     0    sr1
......................auguaucguccguugCauu.....................................................................    1     1    sr1
......................auguaucguccguuguauuA....................................................................    1     1    sr1
......................auguaucguccguuguauug....................................................................    1     0    sr1
.......................uguaucguccguuguauugc...................................................................    1     0    sr1
.......................uguaucguccguuguauugcgaA................................................................    1     1    sr1
................................................aauacaacgaacgauacauuca........................................    2     0    sr1
................................................aauacaacgaacgauacauucag.......................................    1     0    sr1
.................................................auacaacgaacgauacauuAag.......................................    1     1    sr1
..................................................uacaacgaacgauacauuA.........................................    2     1    sr1
..................................................uacaacgaacgauacauuAag.......................................    2     1    sr1
..................................................uacaacgaacgauacauuAaga......................................    13    1    sr1
..................................................uacaacgGacgauacauucaga......................................    2     1    sr1

...................uuaauguaucguccguuguauA.....................................................................    2     1    st1
...................uuaauguaucguccguuguauu.....................................................................    3     0    st1
...................uuaauguaucguUcguuguauu.....................................................................    1     1    st1
...................uGaauguaucguccguuguauu.....................................................................    1     1    st1
...................uuaauguaucguccguuguauuA....................................................................    1     1    st1
....................uaauguaucguccguuguaG......................................................................    1     1    st1
....................uaauguaucguccguuguau......................................................................    2     0    st1
....................uaauguaucguccguuguaC......................................................................    1     1    st1
....................uaauguaucguccguuguauu.....................................................................    63    0    st1
....................uaauguaucguccguuAuauu.....................................................................    1     1    st1
....................uaauguaucguccguCguauu.....................................................................    1     1    st1
....................uaauguaucgCccguuguauu.....................................................................    1     1    st1
....................uaauguaucguccguuguauC.....................................................................    3     1    st1
....................uaauguaucguccguuguauA.....................................................................    18    1    st1
....................uaauguaucguccguuguGuu.....................................................................    1     1    st1
....................uaauguaucguccguuguauug....................................................................    4     0    st1
....................uaauguaucguccguCguauug....................................................................    1     1    st1
....................uaauguaucguccguuguauuA....................................................................    96    1    st1
....................uaauguaucguccguuguauuU....................................................................    3     1    st1
....................uaauguaucguccguuguauugcg..................................................................    1     0    st1
.....................Uauguaucguccguuguauu.....................................................................    1     1    st1
.......................uguaucguccguuguauugcg..................................................................    1     0    st1
..................................................uacaacgGacgauacauuc.........................................    2     1    st1
..................................................uacaacgaacgauacauuA.........................................    1     1    st1
..................................................uacaacgaacgauacauuAaga......................................    2     1    st1

...........auauugucuuaauguaucgucc.............................................................................    1     0    sg1
............uauugucuuaauguaucgucc.............................................................................    1     0    sg1
...............ugucuuaauguaucguccguug.........................................................................    1     0    sg1
...................uGaauguaucguccguugu........................................................................    1     1    sg1
...................uGaauguaucguccguuguau......................................................................    3     1    sg1
...................uuaauguaucguUcguuguau......................................................................    1     1    sg1
...................uuaauguaucguccguuguauC.....................................................................    1     1    sg1
...................uuaauguaucguccguuguauA.....................................................................    1     1    sg1
...................uuaauguaucguccguuguauu.....................................................................    13    0    sg1
...................uGaauguaucguccguuguauu.....................................................................    35    1    sg1
...................uuaauguaucguccguuguauug....................................................................    1     0    sg1
...................uGaauguaucguccguuguauug....................................................................    2     1    sg1
...................uuaauguaucguccguuguauuA....................................................................    2     1    sg1
....................Caauguaucguccguugu........................................................................    1     1    sg1
....................uaauguaucguccguugC........................................................................    1     1    sg1
....................uaauguaucguccguugu........................................................................    1     0    sg1
....................uaauguaucguccguugua.......................................................................    4     0    sg1
....................uaauguaCcguccguuguau......................................................................    1     1    sg1
....................uaauguaucguccguuguaC......................................................................    1     1    sg1
....................uaauguaucguccguuguau......................................................................    11    0    sg1
....................uaaugCaucguccguuguauu.....................................................................    2     1    sg1
....................uaauguaUguccguuguauu......................................................................    1     1    sg1
....................uaauguaucguccguuguGuu.....................................................................    5     1    sg1
```

```
                    Mature                        Star
aauuguucaaaauauugucuuaauguaucguccguuguauugcgaugcaauacaacgaacgauacauucagacaauacauucagacaauauaagacacgcgccgaauaau
....................uaauguaucguccguuguauG.....................................................................   2     1     sg1
....................Gaauguaucguccguuguauu.....................................................................   2     1     sg1
....................uaaCguaucguccguuguauu.....................................................................   2     1     sg1
....................uaauguaucguccguuguauC.....................................................................   15    1     sg1
....................uaauguaucguccguuguauA.....................................................................   16    1     sg1
....................uaauguaucguccguugCauu.....................................................................   1     1     sg1
....................uaauguGucguccguuguauu.....................................................................   3     1     sg1
....................uaauguaucguccguuguauu.....................................................................   378   0     sg1
....................uaGuguaucguccguuguauu.....................................................................   1     1     sg1
....................uaauguaCcguccguuguauu.....................................................................   2     1     sg1
....................uaauguauAguccguuguauu.....................................................................   1     1     sg1
....................uaauguaucguccguCguauu.....................................................................   1     1     sg1
....................uGauguaucguccguuguauu.....................................................................   2     1     sg1
....................uaauguaucgCccguuguauu.....................................................................   1     1     sg1
....................uaauguaucguccguuguGuug....................................................................   1     1     sg1
....................uaauguaucguccguuguauuA....................................................................   106   1     sg1
....................uaauguaucguccguuguauuU....................................................................   9     1     sg1
....................uaauguaucguccguuguauug....................................................................   13    0     sg1
......................auguaucguUcguuguauugcg..................................................................   1     1     sg1
.......................uguaucguccguuguauugcg..................................................................   1     0     sg1
................................................aauacaacgaacgauacauucag.......................................   1     0     sg1
.................................................auacaacgaacgauacauuAag.......................................   1     1     sg1
..................................................uacaacgGacgauacauuc.........................................   2     1     sg1
..................................................uacaacgaacgauacauuA.........................................   6     1     sg1
..................................................uacaacgaacgauacauuAag.......................................   10    1     sg1
..................................................uacaacgaacgauacauucag.......................................   4     0     sg1
..................................................uacaacgGacgauacauucag.......................................   5     1     sg1
..................................................uacaacgaacgauacauuAaga......................................   19    1     sg1
..................................................uacaacgGacgauacauucaga......................................   3     1     sg1

............uauugucuuaauguaucguc..............................................................................   1     0     so1
...............ugucuuaauguaucguccguug.........................................................................   1     0     so1
...................uuaauguaucguccguuguaG......................................................................   1     1     so1
...................uGaauguaucguccguuguauu.....................................................................   2     1     so1
...................uuaauguaucguccguuguGuu.....................................................................   2     1     so1
...................uuaauguaucguccguuguauuA....................................................................   13    1     so1
....................uaauguaucguccguugC........................................................................   1     1     so1
....................uaauguaucguccguuguaC......................................................................   2     1     so1
....................uaauguaucguccguuguau......................................................................   4     0     so1
....................uaauAuaucguccguuguau......................................................................   2     1     so1
....................uaauguaucguccguuguauC.....................................................................   4     1     so1
....................uaauguaucguccguuguauG.....................................................................   1     1     so1
....................uaauguaucguccguuguauA.....................................................................   25    1     so1
....................uaauguaucguccguuguauu.....................................................................   25    0     so1
....................uaauguaucguccguuguauuC....................................................................   1     1     so1
....................uaauguaucguccguuguauug....................................................................   14    0     so1
....................uaauguaucguccguuguauuA....................................................................   188   1     so1
....................uaauguaucguccguuguauuU....................................................................   2     1     so1
....................uaauguaucguccguuguauugcg..................................................................   1     0     so1
....................uaaugCaucguccguuguauugcgaug...............................................................   1     1     so1
....................uaauguaucguccguuguauugcgaug...............................................................   1     0     so1
.....................aauguaucguccAuuguauu.....................................................................   2     1     so1
................................................aauacaacgGacgauacauuc.........................................   1     1     so1
.................................................auacaacgaacgauacauucag.......................................   1     0     so1
..................................................uacaacgGacgauacauuc.........................................   3     1     so1
..................................................uacaacgaacgauacauuAa........................................   1     1     so1
..................................................uacaacgaacgauacauuAag.......................................   5     1     so1
..................................................uacaacgaacgauacauuAaga......................................   4     1     so1
```

Provisional ID	:	ScYm1zk_397_10462
Score total	:	82945.5
Score for star read(s)	:	3.9
Score for read counts	:	82934.9
Score for mfe	:	2.1
Score for randfold	:	1.6
Score for cons. seed	:	3
Total read count	:	162685
Mature read count	:	123497
Loop read count	:	0
Star read count	:	39188

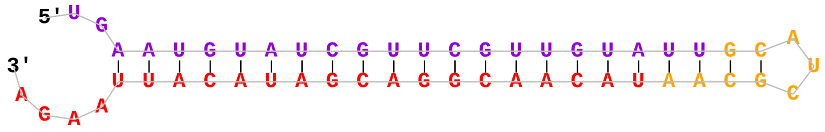

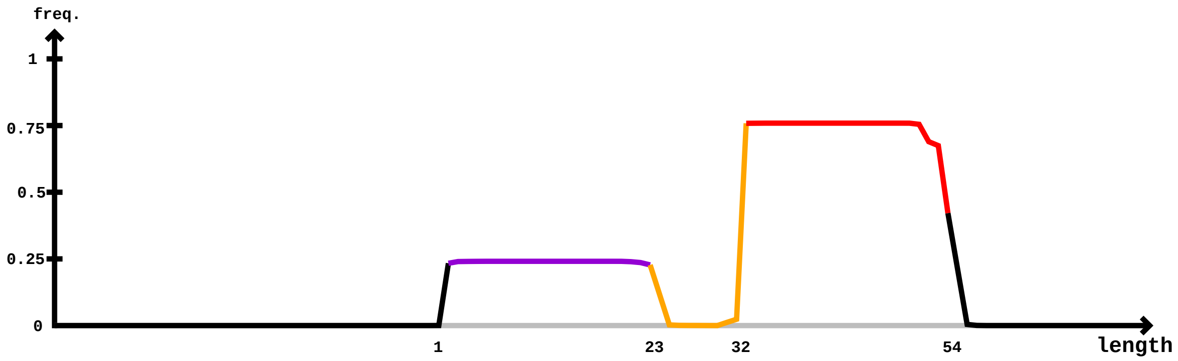


```
                                           Star                         Mature
5'- uuauucggcgcgugucuuauauugucugaauguauugucugaauguaucguucguuguauugcaucgcaauacaacggacgauacauuaagacaauauuuugaacaauuugg  -3'  obs
    uuauucggcgcgugucuuauauugucugaauguauugucugaauguaucguucguuguauugcaucgcaauacaacggacgauacauuaagacaauauuuugaacaauuugg       exp
    ......(((....)))....(((((..(((.(((((((((.((((((((((((((((((((((...))))))))))))))))))))))).))))))))))))..)))))....   reads   mm   sample
    .......................................ugaauguaucguucguug.......................................................   1       0    so1
    .......................................ugaauguaucguucguugC......................................................   1       1    so1
    .......................................ugaauguaucguucguugG......................................................   1       1    so1
    .......................................ugaauguaucguucguugu......................................................   16      0    so1
    .......................................ugaauguaucguucguugA......................................................   1       1    so1
    .......................................ugaGuguaucguucguugua.....................................................   1       1    so1
    .......................................ugaauguaucguucguuguG.....................................................   1       1    so1
    .......................................ugaauguauUguucguugua.....................................................   3       1    so1
    .......................................ugaauguaucguucguugua.....................................................   85      0    so1
    .......................................ugaauguaucUuucguugua.....................................................   1       1    so1
    .......................................ugaaCguaucguucguugua.....................................................   1       1    so1
    .......................................ugaauguaCcguucguugua.....................................................   1       1    so1
    .......................................ugaauguaucguucguCguau....................................................   1       1    so1
    .......................................ugaauguaucguucguuguGu....................................................   1       1    so1
    .......................................Cgaauguaucguucguuguau....................................................   2       1    so1
    .......................................ugaauguaucguucguuguaA....................................................   7       1    so1
    .......................................ugaauguaucguucguuguau....................................................   197     0    so1
    .......................................ugaaCguaucguucguuguau....................................................   2       1    so1
    .......................................ugaauguaucguucguuguaC....................................................   19      1    so1
    .......................................ugaauguaucguucguuguaG....................................................   3       1    so1
    .......................................ugaauguaucguucguuguauG...................................................   24      1    so1
    .......................................ugaauguaucguucguugCauu...................................................   1       1    so1
    .......................................ugaauguaucAuucguuguauu...................................................   1       1    so1
    .......................................ugaauguaucguucguuUuauu...................................................   1       1    so1
    .......................................ugaauguaucguucguuguGuu...................................................   1       1    so1
    .......................................ugaauguaucguucguuguauC...................................................   67      1    so1
    .......................................Cgaauguaucguucguuguauu...................................................   1       1    so1
    .......................................ugaauguaucguucguuguaCu...................................................   1       1    so1
    .......................................ugaCuguaucguucguuguauu...................................................   1       1    so1
    .......................................ugaauguaucgCucguuguauu...................................................   2       1    so1
    .......................................ugaauguaucguCcguuguauu...................................................   2       1    so1
    .......................................ugaauguaucguucguugAauu...................................................   2       1    so1
    .......................................ugGauguaucguucguuguauu...................................................   3       1    so1
```

```
                                       Star                          Mature
uuauucggcgcgugucuuauauugucugaauguauugucugaauguaucguucguuguauugcaucgcaauacaacggacgauacauuaagacaauauuugaacaauuugg
.......................................ugaaugCaucguucguuguauu..................................................  3     1  so1
.......................................ugaauguaucguucguuguauu..................................................  526   0  so1
.......................................ugaaCguaucguucguuguauu..................................................  5     1  so1
.......................................ugaauguaucguucgCuguauu..................................................  1     1  so1
.......................................ugaauguaucguucguCguauu..................................................  1     1  so1
.......................................ugaauguaucguucguuguauA..................................................  1450  1  so1
.......................................ugaauguaucguucguuguauCg.................................................  1     1  so1
.......................................ugaauguaucguucguuguauug.................................................  271   0  so1
.......................................ugaauguaucguucguugCauug.................................................  1     1  so1
.......................................ugaauAuaucguucguuguauug.................................................  1     1  so1
.......................................ugaauguaucguuUguuguauug.................................................  1     1  so1
.......................................ugaauguaucguucguuguauuA.................................................  1254  1  so1
.......................................ugaauguaucguucguuguauAg.................................................  3     1  so1
.......................................ugaauguaucAuucguuguauug.................................................  4     1  so1
.......................................ugaauguaucguucguuguauuC.................................................  8     1  so1
.......................................ugaauguaucguucguuguGuug.................................................  2     1  so1
.......................................ugaauguaucguucguuguauuU.................................................  42    1  so1
.......................................ugaauguaucguucUuuguauug.................................................  1     1  so1
.......................................ugaauguaucguucguCguauug.................................................  3     1  so1
.......................................ugaauguGucguucguuguauug.................................................  2     1  so1
.......................................ugaauguaucguucgCuguauugc................................................  1     1  so1
.......................................ugaauguaucguucguuguauuAc................................................  1     1  so1
.......................................ugaauguaucguucguuguauugA................................................  1     1  so1
.......................................ugaauguaucguucguuguauugU................................................  2     1  so1
.......................................ugaauguaucguucguuguauugc................................................  7     0  so1
.......................................ugaauguaucguucguuguauuUc................................................  1     1  so1
.......................................ugaauguaucguucguuguauugUa...............................................  2     1  so1
.......................................ugaauguaucguucguuguauugcau..............................................  2     0  so1
.......................................ugaauguaucguucguuguauugcauc.............................................  4     0  so1
........................................gaauguaucguucguuguau...................................................  5     0  so1
........................................gaauguaucguucguuguauu..................................................  26    0  so1
........................................gaauguaucguucguuguauA..................................................  18    1  so1
........................................gaauguaucguucguuguauC..................................................  3     1  so1
........................................gaauguaCcguucguuguauu..................................................  1     1  so1
........................................gaauguaucguucguuAuauug.................................................  1     1  so1
........................................gaauguaucguucguuguauug.................................................  30    0  so1
........................................gGauguaucguucguuguauug.................................................  2     1  so1
........................................gaauguaucguucguuguaCug.................................................  2     1  so1
........................................gaauguaucguucguuguauuA.................................................  35    1  so1
........................................gaauguaucguucguuguauuAcauc.............................................  1     1  so1
........................................gaauguaucguucguuguauugcauA.............................................  1     1  so1
........................................gaauguaucguucguuguauugcaucg............................................  5     0  so1
.........................................aauguaucguucguuguaC...................................................  1     1  so1
.........................................aauguaucguucguuguauug.................................................  3     0  so1
..........................................auguaucguucguuguauug.................................................  2     0  so1
..........................................auguaucguucguuguauuA.................................................  3     1  so1
...........................................uguaucguucguuguauug.................................................  3     0  so1
...........................................uguaucguucguuguauugUa...............................................  1     1  so1
.............................................uaucguucguuguauugcauA.............................................  1     1  so1
....................................................................aauacaacggacgauacauC.......................  5     1  so1
....................................................................aauacaacggacgauacauA.......................  1     1  so1
....................................................................aauacaacggacgauacauu.......................  14    0  so1
....................................................................aauacaacggacgGuacauua......................  2     1  so1
....................................................................aauacaacggacgauacauua......................  63    0  so1
....................................................................aauacaacggGcgauacauua......................  1     1  so1
....................................................................aauacUacggacgauacauua......................  1     1  so1
....................................................................aauacaacggacgauacauuU......................  3     1  so1
....................................................................aauacaacggacgauacauuC......................  1     1  so1
....................................................................Gauacaacggacgauacauua......................  1     1  so1
....................................................................aauacaacggacgauacauuaU.....................  3     1  so1
....................................................................aauacaacggGcgauacauuaa.....................  2     1  so1
....................................................................aauacaacggacgauacauuaC.....................  2     1  so1
....................................................................aauacaacggacgauacGuuaa.....................  1     1  so1
....................................................................aauacaacggacgauacauuaa.....................  37    0  so1
....................................................................aauacaacggacgauacauuUa.....................  2     1  so1
....................................................................aauacaacggacgauacauuaaC....................  3     1  so1
....................................................................Gauacaacggacgauacauuaag....................  1     1  so1
....................................................................aauacaacggacgauacauuaag....................  86    0  so1
....................................................................aauacaaUggacgauacauuaag....................  1     1  so1
....................................................................aauacaacggacgauacauuaaU....................  4     1  so1
```

Star **Mature**

```
uuauucggcgcgugucuuauauugucugaauguauugucugaauguaucguucguuguauugcaucgcaauacaacggacgauacauuaagacaauauuuugaacaauuugg
....................................................................aauacaacggacgauacauuaaA.....................	9	1	so1
.....................................................................auacaacggacgauacauu........................	7	0	so1
.....................................................................auacaacggacgaCacauua.......................	1	1	so1
.....................................................................auacaacggacgauacauuU.......................	1	1	so1
.....................................................................Cuacaacggacgauacauua.......................	2	1	so1
.....................................................................auacaacggacgauacauua.......................	29	0	so1
.....................................................................auacaacggacgauacauuaU......................	5	1	so1
.....................................................................auacaacggacgauacauuUa......................	1	1	so1
.....................................................................auacaacggacgauacauuaC......................	1	1	so1
.....................................................................auacaacggacgaCacauuaa......................	1	1	so1
.....................................................................auacaacggacgauacauuaa......................	41	0	so1
.....................................................................Cuacaacggacgauacauuaag.....................	8	1	so1
.....................................................................Guacaacggacgauacauuaag.....................	2	1	so1
.....................................................................auacaacggacgauacauuaaA.....................	3	1	so1
.....................................................................auacaacggacgauacauuaag.....................	152	0	so1
.....................................................................auacaacggacgauacauuaaU.....................	3	1	so1
.....................................................................auacaGcggacgauacauuaag.....................	1	1	so1
.....................................................................auacaacggacgGuacauuaag.....................	1	1	so1
.....................................................................auacaacggacgauacaCuaag.....................	2	1	so1
.....................................................................aAacaacggacgauacauuaag.....................	2	1	so1
.....................................................................auacaacggacgauacauuaGg.....................	1	1	so1
.....................................................................Cuacaacggacgauacauuaaga....................	8	1	so1
.....................................................................auacaacggacgauacauuaaga....................	8	0	so1
......................................................................uacaacggacgauacauA........................	1	1	so1
......................................................................uacaacggacgauacauu........................	70	0	so1
......................................................................uacaacggacgauaUauu........................	1	1	so1
......................................................................uacaacggacgauGcauu........................	1	1	so1
......................................................................uacaacggacgauacauG........................	1	1	so1
......................................................................uacaacggacgauacauC........................	10	1	so1
......................................................................uacGacggacgauacauua.......................	3	1	so1
......................................................................uCcaacggacgauacauua.......................	1	1	so1
......................................................................uacaacggacgauacauCa.......................	2	1	so1
......................................................................uacaacggGcgauacauua.......................	4	1	so1
......................................................................uacaacggaUgauacauua.......................	1	1	so1
......................................................................uacaacggacgUuacauua.......................	1	1	so1
......................................................................uacaGcggacgauacauua.......................	3	1	so1
......................................................................Cacaacggacgauacauua.......................	4	1	so1
......................................................................uacaacggacAauacauua.......................	1	1	so1
......................................................................uacaacggacgauacauua.......................	1209	0	so1
......................................................................uacaacggacgauacaAua.......................	1	1	so1
......................................................................uacUacggacgauacauua.......................	1	1	so1
......................................................................uacaacggacgauacauuG.......................	5	1	so1
......................................................................uacaacggacgauacauuU.......................	8	1	so1
......................................................................uacaacggacgauacaGua.......................	1	1	so1
......................................................................uacaacggacgauGcauua.......................	2	1	so1
......................................................................uacaacggacgGuacauua.......................	5	1	so1
......................................................................uacaacggacgauacauuC.......................	3	1	so1
......................................................................uacaacggacgauUcauua.......................	1	1	so1
......................................................................Aacaacggacgauacauua.......................	1	1	so1
......................................................................uacaaAggacgauacauua.......................	2	1	so1
......................................................................uacaacggacgauacauAa.......................	1	1	so1
......................................................................uacaacggacgCuacauua.......................	1	1	so1
......................................................................uacaacggacgauaUauua.......................	2	1	so1
......................................................................uacaacggacgauacauGa.......................	1	1	so1
......................................................................uacaacggacgauacauuaC......................	1	1	so1
......................................................................uacaacggacgauacGuuaa......................	4	1	so1
......................................................................uacaGcggacgauacauuaa......................	1	1	so1
......................................................................uacaUcggacgauacauuaa......................	2	1	so1
......................................................................uacaacggGcgauacauuaa......................	1	1	so1
......................................................................uacaacggacgauacauuaU......................	1	1	so1
......................................................................uacaacggacgCuacauuaa......................	1	1	so1
......................................................................uacaacggacgaCacauuaa......................	1	1	so1
......................................................................Cacaacggacgauacauuaa......................	1	1	so1
......................................................................uacCacggacgauacauuaa......................	1	1	so1
......................................................................uacaacggacgGuacauuaa......................	4	1	so1
......................................................................uacaacggacgauacauuaa......................	437	0	so1
......................................................................uacaaUggacgauacauuaa......................	1	1	so1
......................................................................uacGacggacgauacauuaa......................	1	1	so1
......................................................................uacaacgAacgauacauuaa......................	1	1	so1
......................................................................uacaacggacgauacaCuaa......................	1	1	so1
```

```
                                                        Star                             Mature
uuauucggcgcgugucuuauauuugucugaauguauugucugaauguaucguucguuguauugcaucgcaauacaacggacgauacauuaagacaauauuuugaacaauuugg
.......................................................................uacaacAgacgauacauuaa......................	4	1	so1
.......................................................................uacaacggacgUuacauuaag.....................	6	1	so1
.......................................................................uacaacAgacgauacauuaag.....................	4	1	so1
.......................................................................uacaacggacgauacauuaGg.....................	23	1	so1
.......................................................................uacaacggacgauacauuUag.....................	6	1	so1
.......................................................................uacaacggacAauacauuaag.....................	9	1	so1
.......................................................................uacaacUgacgauacauuaag.....................	1	1	so1
.......................................................................uacaacggacgauacauuaaA.....................	273	1	so1
.......................................................................uacaacggacgauacauuGag.....................	15	1	so1
.......................................................................uacaacgAacgauacauuaag.....................	5	1	so1
.......................................................................uacaacggacgauacauAaag.....................	1	1	so1
.......................................................................uaUaacggacgauacauuaag.....................	10	1	so1
.......................................................................uacaGcggacgauacauuaag.....................	14	1	so1
.......................................................................uacaacggacgauacauuaaU.....................	13	1	so1
.......................................................................uGcaacggacgauacauuaag.....................	19	1	so1
.......................................................................uacaacggacUauacauuaag.....................	2	1	so1
.......................................................................uacaacggacgaCacauuaag.....................	11	1	so1
.......................................................................uacaacggacgauacauGaag.....................	1	1	so1
.......................................................................uacaacggacgaGacauuaag.....................	2	1	so1
.......................................................................uacaacggacgauacauCaag.....................	7	1	so1
.......................................................................uUcaacggacgauacauuaag.....................	6	1	so1
.......................................................................Cacaacggacgauacauuaag.....................	18	1	so1
.......................................................................uCcaacggacgauacauuaag.....................	2	1	so1
.......................................................................uacaacggacgauacCuuaag.....................	2	1	so1
.......................................................................uacaacggacgauGcauuaag.....................	21	1	so1
.......................................................................uacaaUggacgauacauuaag.....................	6	1	so1
.......................................................................uacaacggacgauacGuuaag.....................	33	1	so1
.......................................................................uacUacggacgauacauuaag.....................	2	1	so1
.......................................................................uacCacggacgauacauuaag.....................	2	1	so1
.......................................................................uacaacggUcgauacauuaag.....................	1	1	so1
.......................................................................uacaCcggacgauacauuaag.....................	1	1	so1
.......................................................................uacaacggaUgauacauuaag.....................	11	1	so1
.......................................................................uacaacggacgauacauuaaC.....................	8	1	so1
.......................................................................uacaaAggacgauacauuaag.....................	3	1	so1
.......................................................................uacGacggacgauacauuaag.....................	10	1	so1
.......................................................................uacaacggacgauacauuaag.....................	7370	0	so1
.......................................................................Gacaacggacgauacauuaag.....................	2	1	so1
.......................................................................uacaacggacgauacUuuaag.....................	2	1	so1
.......................................................................Aacaacggacgauacauuaag.....................	6	1	so1
.......................................................................uacaacggacgauacaCuaag.....................	12	1	so1
.......................................................................uacaacggGcgauacauuaag.....................	32	1	so1
.......................................................................uacaacggacgauUcauuaag.....................	1	1	so1
.......................................................................uacaacggacgGuacauuaag.....................	32	1	so1
.......................................................................uacaacggacgauaUauuaag.....................	10	1	so1
.......................................................................uacaUcggacgauacauuaag.....................	2	1	so1
.......................................................................uacaacggacgauGcauuaaga....................	20	1	so1
.......................................................................uacaacgCacgauacauuaaga....................	1	1	so1
.......................................................................uacaacggacgauacaCuaaga....................	12	1	so1
.......................................................................uacaacggUcgauacauuaaga....................	1	1	so1
.......................................................................uacaacggacgauacauuaagU....................	68	1	so1
.......................................................................uacaacggacgauacauCaaga....................	7	1	so1
.......................................................................uacaacggacgauacauuaaUa....................	10	1	so1
.......................................................................uacaacggacgaCacauuaaga....................	10	1	so1
.......................................................................uacaUcggacgauacauuaaga....................	1	1	so1
.......................................................................uacaacAgacgauacauuaaga....................	12	1	so1
.......................................................................uacaacggacUauacauuaaga....................	4	1	so1
.......................................................................uacaacggacgaAacauuaaga....................	2	1	so1
.......................................................................uacaacUgacgauacauuaaga....................	2	1	so1
.......................................................................Cacaacggacgauacauuaaga....................	24	1	so1
.......................................................................Aacaacggacgauacauuaaga....................	2	1	so1
.......................................................................uacaacggacgauacauuaGga....................	23	1	so1
.......................................................................uCcaacggacgauacauuaaga....................	2	1	so1
.......................................................................uacaacggacgGuacauuaaga....................	30	1	so1
.......................................................................uacaacggacgauacauAaaga....................	2	1	so1
.......................................................................uacaacggacgauacGuuaaga....................	20	1	so1
.......................................................................uacaGcggacgauacauuaaga....................	21	1	so1
.......................................................................uacaacggacgauacauuaagC....................	9	1	so1
.......................................................................uaUaacggacgauacauuaaga....................	11	1	so1
.......................................................................uacaacggaAgauacauuaaga....................	2	1	so1
.......................................................................uacaacggacAauacauuaaga....................	5	1	so1
```

```
                                             Star                      Mature
uuauucggcgcgugucuuauauugucugaaguauugucugaauguaucguucguuguauugcaucgcaauacaacggacgauacauuaagacaauauuuugaacaauuugg
.....................................................................uacaaUggacgauacauuaaga....................  4     1  so1
.....................................................................uacaacggaUgauacauuaaga....................  5     1  so1
.....................................................................uacaaAggacgauacauuaaga....................  1     1  so1
.....................................................................uacaacggacgauaUauuaaga....................  8     1  so1
.....................................................................uacaacggacgauacauuaaCa....................  1     1  so1
.....................................................................uacaacgAacgauacauuaaga....................  4     1  so1
.....................................................................uacaacggacgauacauuGaga....................  13    1  so1
.....................................................................uacaacggacgauacauuaaAa....................  1     1  so1
.....................................................................uacaacggacgauacauuaUga....................  1     1  so1
.....................................................................uacaacggacgauacUuuaaga....................  2     1  so1
.....................................................................uacaacggacgUuacauuaaga....................  2     1  so1
.....................................................................uacaacggacgauacauuaagG....................  93    1  so1
.....................................................................uacUacggacgauacauuaaga....................  1     1  so1
.....................................................................uacGacggacgauacauuaaga....................  32    1  so1
.....................................................................uacaacggacgauacauuaaga....................  6911  0  so1
.....................................................................uacaacggacgauacaAuaaga....................  1     1  so1
.....................................................................uUcaacggacgauacauuaaga....................  5     1  so1
.....................................................................uacaacggacgauacCuuaaga....................  1     1  so1
.....................................................................uacaacggacgauUcauuaaga....................  1     1  so1
.....................................................................uacaCcggacgauacauuaaga....................  2     1  so1
.....................................................................uGcaacggacgauacauuaaga....................  22    1  so1
.....................................................................uacaacggGcgauacauuaaga....................  21    1  so1
.....................................................................uacaacggacgauacauuaagaU...................  131   1  so1
.....................................................................uacaacggacgauacauuaagac...................  10    0  so1
.....................................................................uacaacggacgauacauuaagaA...................  1170  1  so1
.....................................................................uacaacggacgauacauuaagaG...................  93    1  so1
.....................................................................uacaacggacgauacauuaagaca..................  2     0  so1
.....................................................................uacaacggacgauacauuaagacC..................  3     1  so1
.....................................................................uacaacggacgauacauuaagaAa..................  31    1  so1
.....................................................................uacaacggacgauacauuaagaUa..................  143   1  so1
.....................................................................uacaacggacgauacauuaagacU..................  1     1  so1
.....................................................................uacaacggacgauacauuaagacaa.................  1     0  so1
.....................................................................uacaacggacgauacauuaagaAaa.................  5     1  so1
.....................................................................uacaacggacgauacauuaagaUaa.................  13    1  so1
.....................................................................uacaacggacgauacauuaagaAaaua...............  1     1  so1
.....................................................................uacaacggacgauacauuaagaUaaua...............  1     1  so1
......................................................................acaacggacgauacauuaa......................  1     0  so1
......................................................................acaacggacgauacauuaag.....................  1     0  so1
......................................................................acaacggacgauacauuaaga....................  1     0  so1
.......................................................................caacggacgauacauuaag.....................  2     0  so1

.........gcgugucuuauauugucugaaugua.............................................................................  4     0  sg1
.....................................cugaauguaucguucguuguau....................................................  1     0  sg1
.....................................cugGauguaucguucguuguauu...................................................  1     1  sg1
.....................................cugaauguaucguucguuguauC...................................................  2     1  sg1
.....................................Uugaauguaucguucguuguauu...................................................  1     1  sg1
.....................................cugaauguaucguucguuguauu...................................................  18    0  sg1
.....................................cugaauguaucguucguuguauuA..................................................  1     1  sg1
.....................................cugaauguaucguucguuguauugU.................................................  1     1  sg1
......................................ugaauguaucguucguug.......................................................  2     0  sg1
......................................ugaauguaucguucguugG......................................................  4     1  sg1
......................................ugaauguaucguCcguugu......................................................  1     1  sg1
......................................ugaaugCaucguucguugu......................................................  1     1  sg1
......................................ugaauguaucguucguugu......................................................  131   0  sg1
......................................ugGauguaucguucguugu......................................................  4     1  sg1
......................................ugaauguaucguucguugA......................................................  1     1  sg1
......................................Ggaauguaucguucguugu......................................................  1     1  sg1
......................................ugaauguaucguucguugC......................................................  16    1  sg1
......................................ugaauguaucguucguuguU.....................................................  2     1  sg1
......................................ugaauguaucguucguuguG.....................................................  2     1  sg1
......................................ugaauguaucguucguugua.....................................................  209   0  sg1
......................................ugGauguaucguucguugua.....................................................  3     1  sg1
......................................ugaauguaucguucguCgua.....................................................  2     1  sg1
......................................ugaauguaucgCucguugua.....................................................  1     1  sg1
......................................uAaauguaucguucguugua.....................................................  1     1  sg1
......................................ugaauguaAcguucguugua.....................................................  1     1  sg1
......................................ugaaCguaucguucguugua.....................................................  1     1  sg1
......................................ugaauguaucguucguuAuau....................................................  3     1  sg1
......................................ugaauguaCcguucguuguau....................................................  1     1  sg1
......................................ugaauguaucguucguCguau....................................................  1     1  sg1
```

Star **Mature**

uuauucggcgcgugucuuauauugucugaauguauugucugaauguaucguucguuguauugcaucgcaauacaacggacgauacauuaagacaauauuuugaacaauuugg			
....ugaauguaucguCcguuguau..	3	1	sg1
....ugaauguaucguucgCuguau..	3	1	sg1
....ugaauguaucguuUguuguau..	1	1	sg1
....ugaaugCaucguucguuguau..	1	1	sg1
....uAaauguaucguucguuguau..	1	1	sg1
....ugaauguaucgCucguuguau..	2	1	sg1
....ugaGuguaucguucguuguau..	2	1	sg1
....ugaauguaucguucguuguaA..	7	1	sg1
....ugaauguaucguucguuguau..	694	0	sg1
....ugaaCguaucguucguuguau..	1	1	sg1
....ugaauguaucguucguuguaG..	7	1	sg1
....ugaauguaucguucguuguaC..	47	1	sg1
....uUaauguaucguucguuguau..	1	1	sg1
....ugaauguaucAuucguuguau..	1	1	sg1
....ugaauguaAcguucguuguau..	1	1	sg1
....ugaaugAaucguucguuguau..	1	1	sg1
....ugaauguaucguucguuguGu..	5	1	sg1
....Cgaauguaucguucguuguau..	3	1	sg1
....ugGauguaucguucguuguau..	4	1	sg1
....ugaauguaucguucguuguauG...	69	1	sg1
....ugaCuguaucguucguuguauu...	2	1	sg1
....ugaauguGucguucguuguauu...	39	1	sg1
....ugaGuguaucguucguuguauu...	62	1	sg1
....ugaauguCucguucguuguauu...	1	1	sg1
....ugaauguaucguucguuguaGu...	12	1	sg1
....ugaauguaucguucguCguauu...	52	1	sg1
....ugaauguaucguucAuuguauu...	7	1	sg1
....Agaauguaucguucguuguauu...	25	1	sg1
....ugaauCuaucguucguuguauu...	2	1	sg1
....ugaauguaucguucguuguGuu...	33	1	sg1
....ugaauguaucguucguuguauC...	1088	1	sg1
....ugaaGguaucguucguuguauu...	1	1	sg1
....ugaauguUucguucguuguauu...	6	1	sg1
....ugaauguauAguucguuguauu...	6	1	sg1
....ugaaCguaucguucguuguauu...	60	1	sg1
....ugaaugGaucguucguuguauu...	2	1	sg1
....ugUauguaucguucguuguauu...	14	1	sg1
....ugaauguaucguucguuguauu...	17637	0	sg1
....ugaauguaCcguucguuguauu...	69	1	sg1
....ugaauguaucguucguugCauu...	40	1	sg1
....ugaauguaucguAcguuguauu...	6	1	sg1
....ugaauguauGguucguuguauu...	1	1	sg1
....ugaauguaucguucguGguauu...	4	1	sg1
....ugaauguaucguucgGuguauu...	2	1	sg1
....ugaauguaucguucguuCuauu...	3	1	sg1
....ugaauguaucguucguuguaCu...	20	1	sg1
....Cgaauguaucguucguuguauu...	51	1	sg1
....ugaauUuaucguucguuguauu...	1	1	sg1
....ugaauguaucguGcguuguauu...	1	1	sg1
....ugaauguaucUuucguuguauu...	3	1	sg1
....ugaauguaucguucguugGauu...	4	1	sg1
....ugaaugCaucguucguuguauu...	35	1	sg1
....ugaauguaGcguucguuguauu...	2	1	sg1
....ugaauguaucguucguuguauA...	978	1	sg1
....ugaaAguaucguucguuguauu...	5	1	sg1
....ugaauguaucguucguAguauu...	13	1	sg1
....ugaauguaucguucguuguaAu...	4	1	sg1
....ugaauguaucguucgCuguauu...	41	1	sg1
....ugaauAuaucguucguuguauu...	12	1	sg1
....ugaauguauUguucguuguauu...	19	1	sg1
....ugaauguaucAuucguuguauu...	17	1	sg1
....ugaauguaucguucguuAuauu...	15	1	sg1
....ugaauguaucgGucguuguauu...	3	1	sg1
....ugaauguaucguucguugAauu...	6	1	sg1
....ugaauguaucguuAguuguauu...	2	1	sg1
....ugaUuguaucguucguuguauu...	2	1	sg1
....ugaauguaucguCcguuguauu...	35	1	sg1
....ugaauguaucgCucguuguauu...	79	1	sg1
....ugGauguaucguucguuguauu...	52	1	sg1
....ugaaugAaucguucguuguauu...	6	1	sg1

```
                                       Star                Mature
uuauucggcgcgugucuuauauugucugaauguauugucugaauguaucguucguuguauugcaucgcaauacaacggacgauacauuaagacaauauuuugaacaauuugg
.......................................ugaauguaucguucguuguCuu...................................................	3	1	sg1
.......................................ugaauguaucguucguuguUuu...................................................	3	1	sg1
.......................................uCaauguaucguucguuguauu...................................................	1	1	sg1
.......................................Ggaauguaucguucguuguauu...................................................	5	1	sg1
.......................................ugCauguaucguucguuguauu...................................................	2	1	sg1
.......................................ugaauguaAcguucguuguauu...................................................	7	1	sg1
.......................................ugaauguaucguucgAuguauu...................................................	10	1	sg1
.......................................uAaauguaucguucguuguauu...................................................	17	1	sg1
.......................................ugaauguaucgAucguuguauu...................................................	9	1	sg1
.......................................ugaauguaucguuUguuguauu...................................................	8	1	sg1
.......................................ugaauguaucguucguCguauug..................................................	5	1	sg1
.......................................ugaauguauUguucguuguauug..................................................	1	1	sg1
.......................................ugUauguaucguucguuguauug..................................................	1	1	sg1
.......................................ugaauguaucguCcguuguauug..................................................	2	1	sg1
.......................................ugaauguaucguucguuguauuU..................................................	416	1	sg1
.......................................ugaauguaucguucguuguauAg..................................................	3	1	sg1
.......................................ugaaCguaucguucguuguauug..................................................	4	1	sg1
.......................................ugaauguaucguucAuuguauug..................................................	2	1	sg1
.......................................ugaauguaucgCucguuguauug..................................................	1	1	sg1
.......................................ugaauguaucguucguuguauug..................................................	951	0	sg1
.......................................Ggaauguaucguucguuguauug..................................................	1	1	sg1
.......................................ugaauguaucguucguuguauCg..................................................	1	1	sg1
.......................................ugaauguaucguucgCuguauug..................................................	3	1	sg1
.......................................ugaauguaucguucguuguauuC..................................................	13	1	sg1
.......................................ugaauguaucguucguuguGuug..................................................	3	1	sg1
.......................................ugaauguaucguucguuguauuA..................................................	1954	1	sg1
.......................................ugGauguaucguucguuguauug..................................................	5	1	sg1
.......................................ugaauguaucguucguugAauug..................................................	1	1	sg1
.......................................ugaaugCaucguucguuguauug..................................................	1	1	sg1
.......................................ugaauguaucguucguuguauGg..................................................	1	1	sg1
.......................................ugaauguaucguucguuguaCug..................................................	2	1	sg1
.......................................ugaGuguaucguucguuguauug..................................................	1	1	sg1
.......................................ugaauguaCcguucguuguauug..................................................	1	1	sg1
.......................................Cgaauguaucguucguuguauug..................................................	1	1	sg1
.......................................ugaauguGucguucguuguauug..................................................	2	1	sg1
.......................................ugaauguaucguuUguuguauugc.................................................	1	1	sg1
.......................................ugaauguauUguucguuguauugc.................................................	1	1	sg1
.......................................ugaauguaucguucguuguGuugc.................................................	1	1	sg1
.......................................ugaauguaucguucguuguauugc.................................................	178	0	sg1
.......................................ugaauguaucguucguuguauuUc.................................................	1	1	sg1
.......................................ugaauguaucguucguuguauGgc.................................................	1	1	sg1
.......................................ugGauguaucguucguuguauugc.................................................	1	1	sg1
.......................................ugaUuguaucguucguuguauugc.................................................	1	1	sg1
.......................................ugaaCguaucguucguuguauugc.................................................	1	1	sg1
.......................................ugaauguaucguucguuguauuAc.................................................	1	1	sg1
.......................................ugaauguaucguucgGuguauugc.................................................	1	1	sg1
.......................................ugaauguaucguucgCuguauugc.................................................	1	1	sg1
.......................................ugaaGguaucguucguuguauugc.................................................	1	1	sg1
.......................................ugaauguaucguucguuguauugG.................................................	1	1	sg1
.......................................Cgaauguaucguucguuguauugc.................................................	2	1	sg1
.......................................ugaauguaucguucguuguauugU.................................................	23	1	sg1
.......................................ugaauguaucguucguuguauugA.................................................	8	1	sg1
.......................................ugaauguaucguucguuguauugcU................................................	15	1	sg1
.......................................ugaauguaucguucguuguauugcC................................................	4	1	sg1
.......................................ugaauguaucguucguuguauugcG................................................	1	1	sg1
.......................................ugaauguaucguucguuguauugUa................................................	1	1	sg1
.......................................ugaauguaucguucguuguauugca................................................	7	0	sg1
.......................................ugaauguaucguucguuguauugcau...............................................	2	0	sg1
.......................................ugaauguaucguucguuguauugcaA...............................................	1	1	sg1
.......................................Cgaauguaucguucguuguauugcau...............................................	1	1	sg1
.......................................ugaauguaucguucguuguauugcauc..............................................	1	0	sg1
.......................................ugaauguaucguucguuguauugcauA..............................................	2	1	sg1
.......................................ugaauguaucguucguuguauugcauU..............................................	4	1	sg1
........................................gaauguaucguucguugu......................................................	5	0	sg1
........................................gaauguaucguucguuguaC....................................................	1	1	sg1
........................................gaauguaucguucguuguau....................................................	8	0	sg1
........................................gaauguaucguucguuguaCu...................................................	5	1	sg1
........................................gaauguaucgCucguuguauu...................................................	3	1	sg1
........................................gaauguaucguucguuguauG...................................................	2	1	sg1
........................................gaGuguaucguucguuguauu...................................................	3	1	sg1
```

Star **Mature**

```
uuauucggcgcgugucuuauauugucugaauguauugucugaauguaucguucguuguauugcaucgcaauacaacggacgauacauuaagacaauauuuugaacaauuugg
........................................gaauguaCcguucguuguauu...................................................	2	1	sg1
........................................gaauguaucguCcguuguauu...................................................	2	1	sg1
........................................gaauguGucguucguuguauu...................................................	1	1	sg1
........................................gaauguaucguucgCuguauu...................................................	2	1	sg1
........................................gaauguaucguucguuguauu...................................................	329	0	sg1
........................................gaauguaucguucguugCauu...................................................	2	1	sg1
........................................gaauguaucguucguuguauA...................................................	9	1	sg1
........................................gaauguaucguucguuguauC...................................................	15	1	sg1
........................................gaauguaucguucguuAuauu...................................................	1	1	sg1
........................................gaauguaucguucguCguauu...................................................	1	1	sg1
........................................gaauguaucguucguAguauug..................................................	1	1	sg1
........................................gaauguaucguucguuguaCug..................................................	1	1	sg1
........................................gaauguaucguucguuguGuug..................................................	1	1	sg1
........................................gaGuguaucguucguuguauug..................................................	1	1	sg1
........................................gaauguaucguucguuguauug..................................................	48	0	sg1
........................................gaauguaucguucguuguauuA..................................................	56	1	sg1
........................................gaauguaucguucguugCauug..................................................	1	1	sg1
........................................gaauguaucguucguuguauuU..................................................	2	1	sg1
........................................gaauguaucguucguuguauugU.................................................	1	1	sg1
........................................gaauguaucguucguuguauugc.................................................	15	0	sg1
........................................gGauguaucguucguuguauugc.................................................	2	1	sg1
........................................gaauguaucguucguuguauugca................................................	2	0	sg1
........................................gaauguaucguucguuguauugcU................................................	2	1	sg1
........................................gaauguaucguucguuguauugcauc..............................................	3	0	sg1
........................................gaauguaucguucguuguauugcauA..............................................	1	1	sg1
........................................gaauguaucguucguCguauugcaucg.............................................	1	1	sg1
........................................gaauguaucguucguuguauugcaucg.............................................	4	0	sg1
.........................................aauguaucguucguuguauug..................................................	12	0	sg1
.........................................aauguaucguucguuguauugc.................................................	3	0	sg1
..........................................auguaucguucguuguauu...................................................	1	0	sg1
..........................................auguaucguucguCguauug..................................................	1	1	sg1
..........................................auguaucguucguuguauug..................................................	2	0	sg1
..........................................auguaucguucguuguauugc.................................................	15	0	sg1
..........................................auguaucguucguuguauugU.................................................	3	1	sg1
..........................................auguaucguucguuguauugcG................................................	1	1	sg1
..........................................auguaucguucguuguauugcU................................................	2	1	sg1
..........................................auguaucguucguuguauugcauU..............................................	2	1	sg1
...........................................uguaucguucguuguauu...................................................	1	0	sg1
...........................................uguaucguucguuguauug..................................................	3	0	sg1
...........................................uguaucguucguuguauugcauA..............................................	2	1	sg1
...........................................uguaucguucguuguauugcauU..............................................	1	1	sg1
...................................................................caauacaacggacgauacauua.......................	1	0	sg1
....................................................................aauacaacggacgauaca..........................	1	0	sg1
....................................................................aauacaacggacgauacau.........................	3	0	sg1
....................................................................aauacaacggacgauacauC........................	1	1	sg1
....................................................................aauacaacggacgauacauu........................	29	0	sg1
....................................................................aauacaacggacgauacUuu........................	1	1	sg1
....................................................................aauacaacggacgauacauuU.......................	2	1	sg1
....................................................................aGuacaacggacgauacauua.......................	1	1	sg1
....................................................................aauacGacggacgauacauua.......................	2	1	sg1
....................................................................aauacaacggacgauacauua.......................	178	0	sg1
....................................................................aauacaGcggacgauacauua.......................	2	1	sg1
....................................................................aauacaacggacAauacauua.......................	1	1	sg1
....................................................................aauacaacggacgauacauuG.......................	1	1	sg1
....................................................................aauacaacggacgauacauCa.......................	2	1	sg1
....................................................................aauacaacggGcgauacauua.......................	1	1	sg1
....................................................................Uauacaacggacgauacauuaa......................	1	1	sg1
....................................................................aauacaacggacgauGcauuaa......................	2	1	sg1
....................................................................aauacaacggacgauacauuaU......................	4	1	sg1
....................................................................aCuacaacggacgauacauuaa......................	1	1	sg1
....................................................................aauacaacggacgauacauuUa......................	1	1	sg1
....................................................................aauacaacggacgauacauuaG......................	1	1	sg1
....................................................................aauacaacggacgauacauuaa......................	159	0	sg1
....................................................................aauacaacggGcgauacauuaa......................	2	1	sg1
....................................................................aauacaacggacgUuacauuaag.....................	2	1	sg1
....................................................................aCuacaacggacgauacauuaag.....................	1	1	sg1
....................................................................aauacaacggacgauacauuaag.....................	732	0	sg1
....................................................................aauacaacggacgauacaCuaag.....................	1	1	sg1
....................................................................aauacaacggacgauacauuaaC.....................	1	1	sg1
....................................................................aauacaacggacgauacauuaaA.....................	14	1	sg1
```

Star Mature

uuauucggcgcgugucuuauauugucugaauguauugucugaauguaucguucguuguauugcaucgcaauacaacggacgauacauuaagacaauauuuugaacaauuugg			
....aauacaacggacgauacGuuaag.....................	5	1	sg1
....aauacaacggacgauacauuaaU.....................	13	1	sg1
....aauacaacggacgauaUauuaag.....................	1	1	sg1
....aauacaacggacgauacCuuaag.....................	1	1	sg1
....Cauacaacggacgauacauuaag.....................	2	1	sg1
....aauGcaacggacgauacauuaag.....................	3	1	sg1
....aauacaacggacgGuacauuaag.....................	1	1	sg1
....aauacaacggacgauacUuuaag.....................	2	1	sg1
....aauacGacggacgauacauuaag.....................	3	1	sg1
....aauacCacggacgauacauuaag.....................	1	1	sg1
....aGuacaacggacgauacauuaag.....................	1	1	sg1
....aauacaacggacgauacauuGag.....................	2	1	sg1
....aaCacaacggacgauacauuaag.....................	1	1	sg1
....aauacaacggacgauGcauuaag.....................	1	1	sg1
....aauacaacggacgauacauCaag.....................	1	1	sg1
....aauacaacggacgaCacauuaag.....................	2	1	sg1
....aauacaacggacgauacauuaGg.....................	1	1	sg1
....Uauacaacggacgauacauuaag.....................	4	1	sg1
....aauacaacggGcgauacauuaag.....................	1	1	sg1
....aauacaaUggacgauacauuaag.....................	1	1	sg1
....Gauacaacggacgauacauuaag.....................	3	1	sg1
....aauacaacggacgauacauuaagU....................	6	1	sg1
....aauacaacggacgauacauuaaUa....................	1	1	sg1
....auacaacggacgauacau.........................	1	0	sg1
....Cuacaacggacgauacauu........................	1	1	sg1
....auacaacggacgauacauu........................	11	0	sg1
....Cuacaacggacgauacauua.......................	4	1	sg1
....auacaacggacgauacauua.......................	87	0	sg1
....auacaacggacgauacaCua.......................	1	1	sg1
....auacaacggacgauacauuU.......................	1	1	sg1
....auacaacggacgauacauuaa......................	96	0	sg1
....auacaacggacgauacauuUa......................	1	1	sg1
....auacaacggacgauacauuaC......................	1	1	sg1
....auacaacggacgauacauuaU......................	1	1	sg1
....auacaacggacgauacauuaGg.....................	2	1	sg1
....auacaacggacgauacauuaaU.....................	10	1	sg1
....auacaaUggacgauacauuaag.....................	2	1	sg1
....auacaGcggacgauacauuaag.....................	5	1	sg1
....auacaacgAacgauacauuaag.....................	1	1	sg1
....auGcaacggacgauacauuaag.....................	1	1	sg1
....auacaacggacgGuacauuaag.....................	6	1	sg1
....auacaacggacgauacauuaag.....................	581	0	sg1
....auacaacggacgauacaCuaag.....................	1	1	sg1
....aCacaacggacgauacauuaag.....................	2	1	sg1
....auacaacggacgauacauuaaC.....................	2	1	sg1
....Guacaacggacgauacauuaag.....................	3	1	sg1
....auacaacggacgauacauuaaA.....................	10	1	sg1
....auacaacggacgauaAauuaag.....................	1	1	sg1
....auacaacggaUgauacauuaag.....................	2	1	sg1
....auacaacggacgauacGuuaag.....................	1	1	sg1
....aAacaacggacgauacauuaag.....................	1	1	sg1
....auacaacggacgauacauAaag.....................	1	1	sg1
....auacaacggacgUuacauuaag.....................	1	1	sg1
....Cuacaacggacgauacauuaag.....................	13	1	sg1
....Cuacaacggacgauacauuaaga....................	28	1	sg1
....auacaacggacgauacauuaagU....................	3	1	sg1
....auacaacggacgauacauuaaga....................	49	0	sg1
....auacaacggacgauacauuaagG....................	1	1	sg1
....auacaacggacgauacauuaagaA...................	2	1	sg1
....uaUaacggacgauacauu........................	1	1	sg1
....uacaacggacgauacauG........................	4	1	sg1
....uacaacggacgauacaCu........................	1	1	sg1
....uacaacggacgauacauu........................	228	0	sg1
....Cacaacggacgauacauu........................	2	1	sg1
....uacaacggacgGuacauu........................	1	1	sg1
....uacaacggacgauacauC........................	22	1	sg1
....uacaacggGcgauacauu........................	1	1	sg1
....uacaacggacgauacauA........................	1	1	sg1
....uacaacgUacgauacauua.......................	3	1	sg1
....uacaacggacAauacauua.......................	6	1	sg1

Star **Mature**

uuauucggcgcgugucuuauauugucugaauguauugucugaauguaucguucguuguauugcaucgcaauacaacggacgauacauuaagacaauauuuugaacaauuugg

....uacaacggacUauacauua.......................	1	1	sg1
....uacaacggacgauCcauua.......................	1	1	sg1
....uacUacggacgauacauua.......................	2	1	sg1
....uacaacggacgauacCuua.......................	1	1	sg1
....uacaCcggacgauacauua.......................	1	1	sg1
....uaUaacggacgauacauua.......................	7	1	sg1
....uacaacggacgUuacauua.......................	1	1	sg1
....uacaacgAacgauacauua.......................	6	1	sg1
....uacaacggacgauacauCa.......................	7	1	sg1
....uacaaUggacgauacauua.......................	5	1	sg1
....uacaacggacgauaUauua.......................	6	1	sg1
....uacaacggGcgauacauua.......................	10	1	sg1
....Aacaacggacgauacauua.......................	2	1	sg1
....uacaacggacgauacauGa.......................	1	1	sg1
....uacaacggUcgauacauua.......................	5	1	sg1
....uacaacggacgauacauuU.......................	6	1	sg1
....uGcaacggacgauacauua.......................	13	1	sg1
....uacaacggacgaAacauua.......................	1	1	sg1
....uCcaacggacgauacauua.......................	4	1	sg1
....uacaacggacgauacaAua.......................	1	1	sg1
....uacaacggacgauUcauua.......................	2	1	sg1
....uacaacggacgCuacauua.......................	2	1	sg1
....uacaacggaUgauacauua.......................	2	1	sg1
....uacaacggacgauacaCua.......................	10	1	sg1
....uacaacAgacgauacauua.......................	6	1	sg1
....uacaacggacgauacaGua.......................	3	1	sg1
....uacaacggacgauacGuua.......................	9	1	sg1
....uacaGcggacgauacauua.......................	19	1	sg1
....uacGacggacgauacauua.......................	17	1	sg1
....uacaacggacgaCacauua.......................	5	1	sg1
....uacaacggCcgauacauua.......................	1	1	sg1
....uacaacggacgGuacauua.......................	13	1	sg1
....uUcaacggacgauacauua.......................	4	1	sg1
....uacaacUgacgauacauua.......................	1	1	sg1
....uacaacggacgauacauuG.......................	12	1	sg1
....Cacaacggacgauacauua.......................	11	1	sg1
....uacaacggacgauGcauua.......................	10	1	sg1
....uacaacggacgauacauAa.......................	2	1	sg1
....uacaacggacgauacauuC.......................	2	1	sg1
....uacaacggacgauacauua.......................	4674	0	sg1
....Gacaacggacgauacauua.......................	3	1	sg1
....uacaacggaGgauacauua.......................	1	1	sg1
....uacaacggacgauacauuGa......................	1	1	sg1
....uacaacggGcgauacauuaa......................	1	1	sg1
....uUcaacggacgauacauuaa......................	1	1	sg1
....uacaacggaUgauacauuaa......................	2	1	sg1
....uacaacggacgauacauuaG......................	2	1	sg1
....uacaacggacgauacCuuaa......................	1	1	sg1
....uacaacggacgauacauuaU......................	2	1	sg1
....uacaacggacgauacauuUa......................	1	1	sg1
....uacaacggacAauacauuaa......................	1	1	sg1
....uacaacggacgGuacauuaa......................	1	1	sg1
....uacaacggacgauacauAaa......................	1	1	sg1
....uacaacggacgauacGuuaa......................	2	1	sg1
....uacaaUggacgauacauuaa......................	1	1	sg1
....uacaGcggacgauacauuaa......................	4	1	sg1
....Cacaacggacgauacauuaa......................	2	1	sg1
....uacaacggacgauacauuaa......................	706	0	sg1
....uGcaacggacgauacauuaa......................	3	1	sg1
....uacaacggacgauGcauuaa......................	1	1	sg1
....uacaacUgacgauacauuaag.....................	2	1	sg1
....uacaacggacgauacauuGag.....................	25	1	sg1
....Aacaacggacgauacauuaag.....................	13	1	sg1
....uacaacggacgauacauuaaC.....................	17	1	sg1
....uacaacggacgauacaCuaag.....................	29	1	sg1
....uacaCcggacgauacauuaag.....................	2	1	sg1
....uUcaacggacgauacauuaag.....................	6	1	sg1
....uacaacggacgaAacauuaag.....................	3	1	sg1
....uaUaacggacgauacauuaag.....................	10	1	sg1
....uacaacggacgauGcauuaag.....................	23	1	sg1

```
                                       Star                          Mature
uuauucggcgcgugucuuauauugucugaauguauugucugaauguaucguucguuguauugcaucgcaauacaacggacgauacauuaagacaauauuuugaacaauuugg
......................................................................uacaacggacgaCacauuaag.....................	25	1	sg1
......................................................................uacaacggacAauacauuaag.....................	10	1	sg1
......................................................................uacaacggacgauacauuaaA.....................	323	1	sg1
......................................................................uacaacggaUgauacauuaag.....................	11	1	sg1
......................................................................uacaacggaAgauacauuaag.....................	1	1	sg1
......................................................................uacaacggacgGuacauuaag.....................	40	1	sg1
......................................................................uacaacggGcgauacauuaag.....................	47	1	sg1
......................................................................uacaacggacgUuacauuaag.....................	5	1	sg1
......................................................................uacaacggacgauacauuaag.....................	16484	0	sg1
......................................................................uacaacggacgauacauuaUg.....................	4	1	sg1
......................................................................uaAaacggacgauacauuaag.....................	3	1	sg1
......................................................................uaGaacggacgauacauuaag.....................	1	1	sg1
......................................................................uacaacggaGgauacauuaag.....................	1	1	sg1
......................................................................uacaacggacgauacGuuaag.....................	55	1	sg1
......................................................................Gacaacggacgauacauuaag.....................	3	1	sg1
......................................................................uacaacggacgauUcauuaag.....................	3	1	sg1
......................................................................uacUacggacgauacauuaag.....................	10	1	sg1
......................................................................uacaGcggacgauacauuaag.....................	63	1	sg1
......................................................................uacaacggacgauaUauuaag.....................	16	1	sg1
......................................................................uacaacggacgaGacauuaag.....................	3	1	sg1
......................................................................uacaacggacgauacUuuaag.....................	8	1	sg1
......................................................................uacaacggacgauacauuaaU.....................	24	1	sg1
......................................................................uacCacggacgauacauuaag.....................	2	1	sg1
......................................................................uacaacggacgauacauGaag.....................	1	1	sg1
......................................................................uacaacggacgauacauCaag.....................	16	1	sg1
......................................................................uacaacgAacgauacauuaag.....................	10	1	sg1
......................................................................uacaacggacgauacCuuaag.....................	2	1	sg1
......................................................................uacaacggacgauacauAaag.....................	1	1	sg1
......................................................................uacaacggacgauacauuaGg.....................	48	1	sg1
......................................................................uacaacggacUauacauuaag.....................	4	1	sg1
......................................................................uacGacggacgauacauuaag.....................	41	1	sg1
......................................................................uacaacggUcgauacauuaag.....................	7	1	sg1
......................................................................uacaacAgacgauacauuaag.....................	20	1	sg1
......................................................................uacaacggCcgauacauuaag.....................	2	1	sg1
......................................................................uacaacggacgauacaAuaag.....................	1	1	sg1
......................................................................uacaaUggacgauacauuaag.....................	10	1	sg1
......................................................................uacaacggacgCuacauuaag.....................	4	1	sg1
......................................................................uacaacggacgauacauuaCg.....................	1	1	sg1
......................................................................uCcaacggacgauacauuaag.....................	3	1	sg1
......................................................................uacaacggacgauacaGuaag.....................	4	1	sg1
......................................................................uacaUcggacgauacauuaag.....................	1	1	sg1
......................................................................uGcaacggacgauacauuaag.....................	39	1	sg1
......................................................................Cacaacggacgauacauuaag.....................	44	1	sg1
......................................................................uacaaAggacgauacauuaag.....................	1	1	sg1
......................................................................uacaacggacgauCcauuaag.....................	4	1	sg1
......................................................................uacaacggacgauacauuCag.....................	5	1	sg1
......................................................................uacaacgUacgauacauuaag.....................	1	1	sg1
......................................................................uacaacggacgauacauuUag.....................	3	1	sg1
......................................................................uacaacggacgauaAauuaag.....................	2	1	sg1
......................................................................uacaacCgacgauacauuaaga....................	3	1	sg1
......................................................................uacaacggacgauacUuuaaga....................	16	1	sg1
......................................................................uacaaGggacgauacauuaaga....................	1	1	sg1
......................................................................Cacaacggacgauacauuaaga....................	98	1	sg1
......................................................................uacaacggacgauUcauuaaga....................	9	1	sg1
......................................................................uacaacggacgauacauuaagU....................	236	1	sg1
......................................................................uacaacggacgauacauCaaga....................	59	1	sg1
......................................................................uacaacggacgauacauuaagC....................	20	1	sg1
......................................................................uacaacggacUauacauuaaga....................	4	1	sg1
......................................................................uacaacggaGgauacauuaaga....................	4	1	sg1
......................................................................uacaacggacgauacauuaaUa....................	11	1	sg1
......................................................................uacaacggacgauacGuuaaga....................	106	1	sg1
......................................................................uacaacggacgauacauuaCga....................	9	1	sg1
......................................................................uacaacggacgauacaCuaaga....................	44	1	sg1
......................................................................uacaacggacgauacauuUaga....................	5	1	sg1
......................................................................uacaUcggacgauacauuaaga....................	10	1	sg1
......................................................................uacaacggacgauacauAaaga....................	4	1	sg1
......................................................................uacaacggacAauacauuaaga....................	27	1	sg1
......................................................................uacaacggaUgauacauuaaga....................	40	1	sg1
......................................................................uacaacgAacgauacauuaaga....................	19	1	sg1
......................................................................uacaacggacgauGcauuaaga....................	111	1	sg1
```

Star Mature

uuauucggcgcgugucuuauauugucugaauguauugucugaauguaucguucguuguauugcaucgcaauacaacggacgauacauuaagacaauauuuugaacaauuugg			
....uaGaacggacgauacauuaaga....................	1	1	sg1
....uacaacggacgauacauGaaga....................	6	1	sg1
....uacaacggacgaCacauuaaga....................	59	1	sg1
....uacaacggacgauacauuaagG....................	307	1	sg1
....uacaacggacgauacCuuaaga....................	2	1	sg1
....uacaaUggacgauacauuaaga....................	25	1	sg1
....uacaacggacgauacauuaUga....................	11	1	sg1
....uacaacggacgaGacauuaaga....................	1	1	sg1
....uacaacUgacgauacauuaaga....................	2	1	sg1
....uacaaAggacgauacauuaaga....................	5	1	sg1
....uacaacggacCauacauuaaga....................	3	1	sg1
....uacaacggUcgauacauuaaga....................	14	1	sg1
....uaUaacggacgauacauuaaga....................	28	1	sg1
....uacaacggacgauCcauuaaga....................	4	1	sg1
....uacaacggacgUuacauuaaga....................	20	1	sg1
....uacaacggacgauaUauuaaga....................	33	1	sg1
....uacaacggacgauacauuGaga....................	61	1	sg1
....uacaacggacgauaAauuaaga....................	5	1	sg1
....uacaacggacgauacauuaaAa....................	4	1	sg1
....Aacaacggacgauacauuaaga....................	40	1	sg1
....uacaacggGcgauacauuaaga....................	104	1	sg1
....uaAaacggacgauacauuaaga....................	4	1	sg1
....uacaGcggacgauacauuaaga....................	123	1	sg1
....uGcaacggacgauacauuaaga....................	99	1	sg1
....uacaacggacgauacauuaGga....................	64	1	sg1
....uacaacggacgCuacauuaaga....................	3	1	sg1
....uacGacggacgauacauuaaga....................	107	1	sg1
....uacaCcggacgauacauuaaga....................	4	1	sg1
....uacaacgCacgauacauuaaga....................	1	1	sg1
....uacaacggacgGuacauuaaga....................	124	1	sg1
....Gacaacggacgauacauuaaga....................	13	1	sg1
....uacaacAgacgauacauuaaga....................	41	1	sg1
....uacaacgUacgauacauuaaga....................	3	1	sg1
....uacUacggacgauacauuaaga....................	14	1	sg1
....uacaacggacgauacauuaaga....................	36645	0	sg1
....uacaacggacgaAacauuaaga....................	8	1	sg1
....uacaacggCcgauacauuaaga....................	6	1	sg1
....uacCacggacgauacauuaaga....................	5	1	sg1
....uacaacggaAgauacauuaaga....................	2	1	sg1
....uacaacggacgauacauuCaga....................	3	1	sg1
....uacaacggacgauacauuaaCa....................	7	1	sg1
....uCcaacggacgauacauuaaga....................	11	1	sg1
....uacaacggacgauacaGuaaga....................	3	1	sg1
....uUcaacggacgauacauuaaga....................	21	1	sg1
....uacaacggacgauacaAuaaga....................	8	1	sg1
....uacaacggacgauacauuaagaU...................	504	1	sg1
....uacaacggacgauacauuaagac...................	53	0	sg1
....uacaacggacgauacauuaagaA...................	1471	1	sg1
....uacaacggacgauacauuaagaG...................	179	1	sg1
....uacaacggacgauaUauuaagac...................	1	1	sg1
....uacaacggaUgauacauuaagac...................	1	1	sg1
....uacaacggacgauacauuGagac...................	1	1	sg1
....uacaacggacgauacauuaagUc...................	1	1	sg1
....uacaacggacgGuacauuaagac...................	1	1	sg1
....uacaacggacgauacauuaagacU..................	1	1	sg1
....uacaacggacgauacauuaagacG..................	1	1	sg1
....uacaacggacgauacauuaagaAa..................	21	1	sg1
....uacaacggacgauacauuaagacC..................	1	1	sg1
....uacaacggacgauacauuaagaUa..................	119	1	sg1
....uacaacggacgauacauuaagaca..................	2	0	sg1
....uacaacggacgauacauuaagacGa.................	1	1	sg1
....uacaacggacgauacauuaagaAaa.................	6	1	sg1
....uacaacggacgauacauuaagacaU.................	1	1	sg1
....uacaacggacgauacauuaagacaG.................	1	1	sg1
....uacaacggacgauacauuaagacaa.................	1	0	sg1
....uacaacggacgauacauuaagaUaa.................	17	1	sg1
....uacaacggacgauacauuaagacUa.................	1	1	sg1
....uacaacggacgauacauuaagacUau................	1	1	sg1
....acaacggacgauacauuaag.....................	8	0	sg1
....acaacggacgauacauuGaga....................	2	1	sg1

Star **Mature**

```
uuauucggcgcgugucuuauauugucugaauguauugucugaauguaucguucguuguauugcaucgcaauacaacggacgauacauuaagacaauauuuugaacaauuugg
.......................................................................acGacggacgauacauuaaga....................  2    1   sg1
.......................................................................acaacggacgauacauuaaga....................  12   0   sg1
........................................................................caacggacgauacauuaag.....................  13   0   sg1
........................................................................caacggacgauacauuaaga....................  20   0   sg1
........................................................................caacggacgauacauuaagU....................  1    1   sg1
.........................................................................aacggacgauacauuaaga....................  2    0   sg1
.........................................................................aacggacgauacauuaagaA...................  1    1   sg1

......................................cugaauguaucguucguuguau....................................................  2    0   st1
.......................................ugaauguaucguucguugu......................................................  9    0   st1
.......................................ugaaCguaucguucguugu......................................................  2    1   st1
.......................................ugaauguaucguucguugua.....................................................  24   0   st1
.......................................ugaauguaucguucguuguaC....................................................  12   1   st1
.......................................Ggaauguaucguucguuguau....................................................  1    1   st1
.......................................ugaauguaucguucguuguaA....................................................  2    1   st1
.......................................ugaauguaucguucAuuguau....................................................  1    1   st1
.......................................ugaauguaucguucguuguau....................................................  89   0   st1
.......................................ugaauguaucguucguuguauC...................................................  68   1   st1
.......................................ugaauguaucguucguuAuauu...................................................  1    1   st1
.......................................ugaauguaucguucguCguauu...................................................  1    1   st1
.......................................uAaauguaucguucguuguauu...................................................  1    1   st1
.......................................ugaauguaucguucguuguauA...................................................  320  1   st1
.......................................ugaauguaucguucAuuguauu...................................................  1    1   st1
.......................................ugaauguaucguucgCuguauu...................................................  1    1   st1
.......................................ugaGuguaucguucguuguauu...................................................  2    1   st1
.......................................ugaauguaucguucguuguauu...................................................  714  0   st1
.......................................ugaaugGaucguucguuguauu...................................................  1    1   st1
.......................................ugaauguaucguucgGuguauu...................................................  1    1   st1
.......................................Cgaauguaucguucguuguauu...................................................  1    1   st1
.......................................ugaauguaucguucguuguGuu...................................................  2    1   st1
.......................................ugaauguGucguucguuguauu...................................................  1    1   st1
.......................................ugaauguaucguucguugCauu...................................................  1    1   st1
.......................................ugaauguaucguucUuuguauu...................................................  1    1   st1
.......................................ugaauguaucguucguuguauG...................................................  16   1   st1
.......................................ugGauguaucguucguuguauu...................................................  2    1   st1
.......................................ugaauCuaucguucguuguauu...................................................  1    1   st1
.......................................ugaauguaucAuucguuguauu...................................................  2    1   st1
.......................................ugaaAguaucguucguuguauu...................................................  1    1   st1
.......................................ugaauguaCcguucguuguauu...................................................  6    1   st1
.......................................ugaauguaAcguucguuguauu...................................................  2    1   st1
.......................................ugaauguaucguucguuguaCu...................................................  3    1   st1
.......................................ugaauguaucgCucguuguauu...................................................  2    1   st1
.......................................uUaauguaucguucguuguauu...................................................  1    1   st1
.......................................ugaauguaucguCcguuguauu...................................................  1    1   st1
.......................................ugaaugCaucguucguuguauu...................................................  1    1   st1
.......................................ugaauguaucguucguuguauuU..................................................  10   1   st1
.......................................ugaauguaucguucguuguauuA..................................................  304  1   st1
.......................................ugaauguaucguucguuguauug..................................................  98   0   st1
.......................................uAaauguaucguucguuguauug..................................................  2    1   st1
.......................................ugaauguaucgCucguuguauug..................................................  1    1   st1
.......................................ugGauguaucguucguuguauug..................................................  1    1   st1
.......................................ugaauguaucguucguuguauuC..................................................  5    1   st1
.......................................ugaGuguaucguucguuguauug..................................................  1    1   st1
.......................................ugaauguaucguucguuguauugU.................................................  2    1   st1
.......................................ugaauguaucguucguuguauugc.................................................  17   0   st1
.......................................ugaauguaucguucguuguauugcauA..............................................  2    1   st1
........................................gaauguaucguucguugC......................................................  1    1   st1
........................................gaauguaucguucguugu......................................................  1    0   st1
........................................gaauguaucguucguuguauA...................................................  15   1   st1
........................................gaauguaucguucguuguauu...................................................  35   0   st1
........................................gaauguaucguucguuguauC...................................................  4    1   st1
........................................gaGuguaucguucguuguauu...................................................  1    1   st1
........................................gaauguaucguucguuguauug..................................................  13   0   st1
........................................gaauguaucguucguuguauuA..................................................  10   1   st1
........................................gaauguaucguucguuguauuU..................................................  2    1   st1
........................................gaauguaucguucguuguauugcau...............................................  1    0   st1
........................................gaauguaucguucgCuguauugcaucg.............................................  1    1   st1
........................................gaauguaucguucguuguauugcaucg.............................................  2    0   st1
.........................................aauguaucguucguuguau....................................................  1    0   st1
.........................................aauguaucguucguuguauuA..................................................  1    1   st1
```

```
                                       Star                 Mature
uuauucggcgcgugucuuauauugucugaauguauugucugaauguaucguucguuguauugcaucgcaauacaacggacgauacauuaagacaauauuuugaacaauuugg
.........................................aauguaucguucguuguauug..................................................    3    0    st1
..........................................auguaucguucguuguauuA..................................................    1    1    st1
..........................................auguaucguucguuguauug..................................................    2    0    st1
...........................................uguaucguucguuguauugcaA...............................................    1    1    st1
...........................................uguaucguucguuguauugcauA..............................................    2    1    st1
....................................................................aauacaacggacgauacauu........................    5    0    st1
....................................................................aauacaacggacgauacauuU.......................    3    1    st1
....................................................................aauacaacggacgauacauua.......................    25   0    st1
....................................................................aauacaacggacgauacauuaU......................    3    1    st1
....................................................................aauacaacggacgauacauuaa......................    19   0    st1
....................................................................aauacaacggacAauacauuaa......................    1    1    st1
....................................................................Gauacaacggacgauacauuaa......................    1    1    st1
....................................................................aauacaacggacgauacauuaaC.....................    2    1    st1
....................................................................aauGcaacggacgauacauuaag.....................    1    1    st1
....................................................................aauacaacggGcgauacauuaag.....................    1    1    st1
....................................................................aauacaacggacgauacauuaaA.....................    2    1    st1
....................................................................aauacaacggacgauacaCuaag.....................    2    1    st1
....................................................................aauacaaUggacgauacauuaag.....................    1    1    st1
....................................................................aauacaacggacgauacGuuaag.....................    1    1    st1
....................................................................aauacaacggacgauacauuaaU.....................    2    1    st1
....................................................................aauacaacggacgauacauuaag.....................    87   0    st1
.....................................................................auacaacggacgauacauu........................    4    0    st1
.....................................................................auacaacggacgauacauua.......................    14   0    st1
.....................................................................Cuacaacggacgauacauua.......................    1    1    st1
.....................................................................auacaacggacgauacauuaU......................    2    1    st1
.....................................................................auacaacggacgauacauuaa......................    13   0    st1
.....................................................................auacaacggacgauacauuaGg.....................    1    1    st1
.....................................................................auacaacggacgauacauuaaA.....................    3    1    st1
.....................................................................Cuacaacggacgauacauuaag.....................    2    1    st1
.....................................................................auacaacggacgauacauuaag.....................    90   0    st1
.....................................................................auacaacggacgauacGuuaag.....................    2    1    st1
.....................................................................Cuacaacggacgauacauuaaga....................    4    1    st1
.....................................................................auacaacggacgauacauuaaga....................    5    0    st1
......................................................................uacaacggacgauacauu........................    32   0    st1
......................................................................uacaacggacgauacauG........................    1    1    st1
......................................................................uacaacggacgauacauA........................    2    1    st1
......................................................................uacaacggacgauacauuG.......................    5    1    st1
......................................................................uacaacggacgauaGauua.......................    1    1    st1
......................................................................uacaacggacgauacGuua.......................    3    1    st1
......................................................................uacaacggacgGuacauua.......................    3    1    st1
......................................................................uacGacggacgauacauua.......................    1    1    st1
......................................................................uacaacgAacgauacauua.......................    1    1    st1
......................................................................Gacaacggacgauacauua.......................    1    1    st1
......................................................................uaUaacggacgauacauua.......................    1    1    st1
......................................................................uacaacggaUgauacauua.......................    1    1    st1
......................................................................uacaacggacgCuacauua.......................    3    1    st1
......................................................................uacaacggacgauacauuU.......................    6    1    st1
......................................................................uacaaUggacgauacauua.......................    1    1    st1
......................................................................uacaacggacgaCacauua.......................    1    1    st1
......................................................................uacaacggacgauacauCa.......................    1    1    st1
......................................................................uacaacAgacgauacauua.......................    1    1    st1
......................................................................uGcaacggacgauacauua.......................    4    1    st1
......................................................................uacaacggacgUuacauua.......................    1    1    st1
......................................................................uacaacggacAauacauua.......................    1    1    st1
......................................................................uacaacggacgauacauuC.......................    2    1    st1
......................................................................uacaacggacgauacauua.......................    955  0    st1
......................................................................uacaacggacgauGcauua.......................    1    1    st1
......................................................................uacaGcggacgauacauua.......................    4    1    st1
......................................................................Cacaacggacgauacauua.......................    1    1    st1
......................................................................uacaacggCcgauacauua.......................    9    1    st1
......................................................................uacaacggGcgauacauua.......................    1    1    st1
......................................................................uacaacggacgauacUuua.......................    1    1    st1
......................................................................uacUacggacgauacauuaa......................    2    1    st1
......................................................................uacaGcggacgauacauuaa......................    2    1    st1
......................................................................uGcaacggacgauacauuaa......................    1    1    st1
......................................................................Aacaacggacgauacauuaa......................    1    1    st1
......................................................................uacaacggacgauaGauuaa......................    1    1    st1
......................................................................uacaacggacgauacauuaG......................    1    1    st1
......................................................................uacaacggacgauaUauuaa......................    1    1    st1
......................................................................Cacaacggacgauacauuaa......................    1    1    st1
```

Star **Mature**

```
uuauucggcgcgugucuuauauuugucugaauguauugucugaauguaucguucguuguauugcaucgcaauacaacggacgauacauuaagacaauauuuugaacaauuugg
.......................................................................uacaacggacgauacauuaa......................  91    0  st1
.......................................................................uacaacggacgauacauuaaU.....................  6     1  st1
.......................................................................uacaacggacgaAacauuaag.....................  1     1  st1
.......................................................................uacaacggacgauaAauuaag.....................  1     1  st1
.......................................................................uacaacUgacgauacauuaag.....................  1     1  st1
.......................................................................uGcaacggacgauacauuaag.....................  4     1  st1
.......................................................................uacaUcggacgauacauuaag.....................  2     1  st1
.......................................................................Cacaacggacgauacauuaag.....................  5     1  st1
.......................................................................uacaacggacgauUcauuaag.....................  2     1  st1
.......................................................................uacaacggUcgauacauuaag.....................  1     1  st1
.......................................................................uacUacggacgauacauuaag.....................  1     1  st1
.......................................................................uacaacggCcgauacauuaag.....................  16    1  st1
.......................................................................uacaacAgacgauacauuaag.....................  3     1  st1
.......................................................................uacaacggacgauaUauuaag.....................  3     1  st1
.......................................................................uacaacggacgauGcauuaag.....................  5     1  st1
.......................................................................uacaGcggacgauacauuaag.....................  8     1  st1
.......................................................................uacaacggacgauacauuaaC.....................  3     1  st1
.......................................................................uacaacggacgauacGuuaag.....................  7     1  st1
.......................................................................uacaacggacgauacaCuaag.....................  7     1  st1
.......................................................................uacaacggacgUuacauuaag.....................  2     1  st1
.......................................................................uacaacggacgGuacauuaag.....................  1     1  st1
.......................................................................uacaacggacgCuacauuaag.....................  8     1  st1
.......................................................................uacaacggacgauacauuaUg.....................  2     1  st1
.......................................................................uacaacggacgauaGauuaag.....................  2     1  st1
.......................................................................Gacaacggacgauacauuaag.....................  1     1  st1
.......................................................................uacaacggacAauacauuaag.....................  3     1  st1
.......................................................................uCcaacggacgauacauuaag.....................  1     1  st1
.......................................................................uacaacggGcgauacauuaag.....................  7     1  st1
.......................................................................uacGacggacgauacauuaag.....................  8     1  st1
.......................................................................uacaacggacgauacauuaag.....................  2464  0  st1
.......................................................................uacaaUggacgauacauuaag.....................  1     1  st1
.......................................................................uacaacggaUgauacauuaag.....................  4     1  st1
.......................................................................uacaacggacgauacauuaGg.....................  10    1  st1
.......................................................................uUcaacggacgauacauuaag.....................  3     1  st1
.......................................................................uacaacggacgauacUuuaag.....................  1     1  st1
.......................................................................uaUaacggacgauacauuaag.....................  1     1  st1
.......................................................................uacaacggacgaCacauuaag.....................  5     1  st1
.......................................................................Aacaacggacgauacauuaag.....................  1     1  st1
.......................................................................uacaacggacgauacauuaaA.....................  128   1  st1
.......................................................................uacaacggacgauacauuGag.....................  3     1  st1
.......................................................................uacaacggacgCuacauuaaga....................  6     1  st1
.......................................................................uacaaUggacgauacauuaaga....................  3     1  st1
.......................................................................uacaUcggacgauacauuaaga....................  1     1  st1
.......................................................................uacaacggacgauacauuaaAa....................  4     1  st1
.......................................................................uacaacggacgauGcauuaaga....................  7     1  st1
.......................................................................uacaacggacgaAacauuaaga....................  1     1  st1
.......................................................................uacaacggCcgauacauuaaga....................  20    1  st1
.......................................................................uGcaacggacgauacauuaaga....................  9     1  st1
.......................................................................uacaCcggacgauacauuaaga....................  1     1  st1
.......................................................................uacaacggacgaGacauuaaga....................  2     1  st1
.......................................................................uacaacggacgauaGauuaaga....................  4     1  st1
.......................................................................uacaacggacgauacauuaGga....................  11    1  st1
.......................................................................uacaacAgacgauacauuaaga....................  3     1  st1
.......................................................................uacaacggacgauaAauuaaga....................  2     1  st1
.......................................................................uacaGcggacgauacauuaaga....................  18    1  st1
.......................................................................uacaacggacgauacauuaaUa....................  1     1  st1
.......................................................................uCcaacggacgauacauuaaga....................  1     1  st1
.......................................................................uacaacgAacgauacauuaaga....................  2     1  st1
.......................................................................uacaacggacgauacauuaUga....................  1     1  st1
.......................................................................uacaacggacgauacauuaagU....................  27    1  st1
.......................................................................Cacaacggacgauacauuaaga....................  13    1  st1
.......................................................................uacaacggacgauacauuaagC....................  4     1  st1
.......................................................................uacaacggacgauacGuuaaga....................  15    1  st1
.......................................................................uacUacggacgauacauuaaga....................  1     1  st1
.......................................................................uacGacggacgauacauuaaga....................  9     1  st1
.......................................................................uacaacggacgauacauuaagG....................  44    1  st1
.......................................................................uacaacggaUgauacauuaaga....................  6     1  st1
.......................................................................uacaacggacgauacCuuaaga....................  1     1  st1
.......................................................................uacaacggaGgauacauuaaga....................  1     1  st1
.......................................................................uacaacggacgauaUauuaaga....................  8     1  st1
```

Star **Mature**

```
uuauucggcgcgugucuuauauugucugaauguauugucugaauguaucguucguuguauugcaucgcaauacaacggacgauacauuaagacaauauuuugaacaauuugg
......................................................................uacaacggacgauacauuGaga....................    6     1    st1
......................................................................uacaacggacAauacauuaaga....................    2     1    st1
......................................................................uacaacggacgauacauuUaga....................    1     1    st1
......................................................................uacaacggacgauacaCuaaga....................    10    1    st1
......................................................................Aacaacggacgauacauuaaga....................    3     1    st1
......................................................................uacaacggacgauUcauuaaga....................    2     1    st1
......................................................................uacaacggacgaCacauuaaga....................    7     1    st1
......................................................................uUcaacggacgauacauuaaga....................    3     1    st1
......................................................................uacaacggacgauacauCaaga....................    3     1    st1
......................................................................uacaacggacgauacUuuaaga....................    1     1    st1
......................................................................uacaacggacgGuacauuaaga....................    9     1    st1
......................................................................uaUaacggacgauacauuaaga....................    4     1    st1
......................................................................Gacaacggacgauacauuaaga....................    1     1    st1
......................................................................uacaacggacgauacauuaaga....................    3708  0    st1
......................................................................uacaacggGcgauacauuaaga....................    7     1    st1
......................................................................uacaaGggacgauacauuaaga....................    1     1    st1
......................................................................uacaacggacgauacauuaagaA...................    197   1    st1
......................................................................uacaacggacgauacauuaagac...................    5     0    st1
......................................................................uacaacggacgauacauuaagaU...................    73    1    st1
......................................................................uacaacggacgauacauuaagaG...................    10    1    st1
......................................................................uacaacggacgauacauuaagacU..................    1     1    st1
......................................................................uacaacggacgauacauuaagaUa..................    27    1    st1
......................................................................uacaacggacgauacauuaagaAa..................    10    1    st1
......................................................................uacaacggacgauacauuaagaca..................    1     0    st1
......................................................................uacaacggacgauacauuaagaUaa.................    3     1    st1
.......................................................................acaacggacgauacauuaa......................    1     0    st1
.......................................................................acaacggacgauacauuaag.....................    1     0    st1
.......................................................................acaacggacgauacauuGaga....................    1     1    st1
.......................................................................acaacggacgauacauuaaga....................    2     0    st1
........................................................................caacggacgauacauuaag.....................    1     0    st1

...................uauugucugaauguauuguc.........................................................................    1     0    sr1
.....................uugucugaauguauuguc.........................................................................    1     0    sr1
..........................Ggaauguauugucugaauguauc...............................................................    1     1    sr1
..................................uugucugaauguaucguucguug.......................................................    1     0    sr1
......................................cugaauguaucguucguuguauA...................................................    1     1    sr1
......................................cugaauguaucguucguuguauuA..................................................    2     1    sr1
......................................cugaauguaucguucguuguauug..................................................    3     0    sr1
.......................................ugaauguaucguucguuA.......................................................    4     1    sr1
.......................................ugaauguaucguucguug.......................................................    1     0    sr1
.......................................ugaGuguaucguucguugu......................................................    1     1    sr1
.......................................ugaauguaucguuUguugu......................................................    1     1    sr1
.......................................ugaauguaucguucguugG......................................................    1     1    sr1
.......................................ugaauguaucguucguugu......................................................    31    0    sr1
.......................................ugaauguaucgCucguugu......................................................    1     1    sr1
.......................................ugaauguaucguucguugC......................................................    4     1    sr1
.......................................ugaauguaucguucguuguU.....................................................    1     1    sr1
.......................................ugaauguaucguucguugua.....................................................    172   0    sr1
.......................................ugaauguaCcguucguugua.....................................................    1     1    sr1
.......................................ugaauguauUguucguugua.....................................................    1     1    sr1
.......................................ugaauguaucguucguCgua.....................................................    2     1    sr1
.......................................ugaauguaucguucgAugua.....................................................    1     1    sr1
.......................................ugaauguaAcguucguugua.....................................................    1     1    sr1
.......................................uUaauguaucguucguugua.....................................................    1     1    sr1
.......................................ugaaugCaucguucguugua.....................................................    1     1    sr1
.......................................ugaauguaucguuUguugua.....................................................    1     1    sr1
.......................................ugaauguaucguucguuguG.....................................................    7     1    sr1
.......................................ugaauguGucguucguugua.....................................................    1     1    sr1
.......................................Ggaauguaucguucguugua.....................................................    5     1    sr1
.......................................ugaauguaucAuucguuguau....................................................    1     1    sr1
.......................................ugaauguaucgCucguuguau....................................................    2     1    sr1
.......................................ugaauguaucUuucguuguau....................................................    1     1    sr1
.......................................ugaauguaucguucguuguaC....................................................    22    1    sr1
.......................................ugaauUuaucguucguuguau....................................................    1     1    sr1
.......................................ugaauguaucguucAuuguau....................................................    1     1    sr1
.......................................ugaauguaucguucguuguaG....................................................    8     1    sr1
.......................................ugGauguaucguucguuguau....................................................    2     1    sr1
.......................................ugaauguaucguucguuguGu....................................................    1     1    sr1
.......................................ugaauguaCcguucguuguau....................................................    1     1    sr1
.......................................Ggaauguaucguucguuguau....................................................    9     1    sr1
```

Star **Mature**

```
uuauucggcgcgugucuuauauugucugaauguauugucugaauguaucguucguuguauugcaucgcaauacaacggacgauacauuaagacaauauuuugaacaauuugg
.......................................ugaCuguaucguucguuguau.....................................................  1     1  sr1
.......................................ugaauguaucguucgCuguau.....................................................  2     1  sr1
.......................................ugaauguaucguucguuguau.....................................................  212   0  sr1
.......................................ugaauguaucguucguuguaA....................................................  8     1  sr1
.......................................ugGauguaucguucguuguauu...................................................  3     1  sr1
.......................................ugCauguaucguucguuguauu...................................................  1     1  sr1
.......................................ugaauguaucguucguuguauA...................................................  1825  1  sr1
.......................................ugaauguaucguAcguuguauu...................................................  1     1  sr1
.......................................ugaauguaucguucguuguGuu...................................................  1     1  sr1
.......................................Cgaauguaucguucguuguauu...................................................  1     1  sr1
.......................................ugaaugAaucguucguuguauu...................................................  2     1  sr1
.......................................ugaaCguaucguucguuguauu...................................................  2     1  sr1
.......................................ugaauguaAcguucguuguauu...................................................  3     1  sr1
.......................................ugaauguaucguucguCguauu...................................................  3     1  sr1
.......................................ugaauguaucguucguuguaGu...................................................  1     1  sr1
.......................................ugaauguaucguucguugCauu...................................................  1     1  sr1
.......................................ugaauguaucguucguuguauG...................................................  58    1  sr1
.......................................ugaauguaucguucgCuguauu...................................................  1     1  sr1
.......................................ugaauguaucguucguuguUuu...................................................  1     1  sr1
.......................................ugaauguaucguucguuguauu...................................................  651   0  sr1
.......................................ugaauguaucguucguuAuauu...................................................  2     1  sr1
.......................................ugaGuguaucguucguuguauu...................................................  1     1  sr1
.......................................Ggaauguaucguucguuguauu...................................................  27    1  sr1
.......................................ugaauguauUguucguuguauu...................................................  1     1  sr1
.......................................ugaauguGucguucguuguauu...................................................  2     1  sr1
.......................................uCaauguaucguucguuguauu...................................................  1     1  sr1
.......................................ugaauguaucguCcguuguauu...................................................  3     1  sr1
.......................................ugaauguaucgCucguuguauu...................................................  2     1  sr1
.......................................ugaauguaucguucguuguauC...................................................  87    1  sr1
.......................................ugaauguaucguucgAuguauug..................................................  1     1  sr1
.......................................ugaauguaucguucguuguauuC..................................................  10    1  sr1
.......................................ugaaugAaucguucguuguauug..................................................  1     1  sr1
.......................................ugaauguaCcguucguuguauug..................................................  2     1  sr1
.......................................ugaGuguaucguucguuguauug..................................................  2     1  sr1
.......................................Ggaauguaucguucguuguauug..................................................  22    1  sr1
.......................................ugaauguaucguucguCguauug..................................................  3     1  sr1
.......................................ugaauguaucguucguuguauAg..................................................  2     1  sr1
.......................................ugGauguaucguucguuguauug..................................................  1     1  sr1
.......................................ugaauguaucgCucguuguauug..................................................  2     1  sr1
.......................................ugaauguaucgAucguuguauug..................................................  1     1  sr1
.......................................ugaauguaucguCcguuguauug..................................................  1     1  sr1
.......................................Cgaauguaucguucguuguauug..................................................  4     1  sr1
.......................................ugaaCguaucguucguuguauug..................................................  2     1  sr1
.......................................ugaauguaucguucguuguauug..................................................  577   0  sr1
.......................................ugaauguaucguucguuguGuug..................................................  7     1  sr1
.......................................ugaauguaucguucguuguauCg..................................................  8     1  sr1
.......................................ugaauguaucguucguGguauug..................................................  1     1  sr1
.......................................ugaauguaucguucguuguauuU..................................................  34    1  sr1
.......................................ugaauguaucguucguuguauuA..................................................  2762  1  sr1
.......................................ugaauguaCcguucguuguauugc.................................................  1     1  sr1
.......................................Ggaauguaucguucguuguauugc.................................................  1     1  sr1
.......................................ugaauguaucguucguuguauugA.................................................  2     1  sr1
.......................................ugaauguaucguucguuguauugU.................................................  2     1  sr1
.......................................ugaauguaucguucguuguauugc.................................................  15    0  sr1
.......................................ugaauguaucguucguuguauugca................................................  1     0  sr1
.......................................ugaauguaucguucguuguauugUa................................................  2     1  sr1
.......................................ugaauguaucguucguuguauugcau...............................................  2     0  sr1
.......................................ugaauguaucguucguuguauugcauc..............................................  2     0  sr1
.......................................ugaauguaucguucguuguauugcauA..............................................  1     1  sr1
........................................gaauguaucguucguugu......................................................  1     0  sr1
........................................gaauguaucguucguugua.....................................................  2     0  sr1
........................................gaauguaucguCcguuguau....................................................  1     1  sr1
........................................gaauguaucguucguuguaG....................................................  1     1  sr1
........................................gaauguaucguucguuguau....................................................  5     0  sr1
........................................gaauguaucguucguuguaC....................................................  1     1  sr1
........................................gaauguaucguucguugCauu...................................................  1     1  sr1
........................................gaauguaucguucguuguauG...................................................  1     1  sr1
........................................gaauguaucguCcguuguauu...................................................  21    1  sr1
........................................gaauguaucguucguuguauC...................................................  1     1  sr1
........................................gaauguaucguucguuguauu...................................................  29    0  sr1
```

```
                                        Star                          Mature
uuauucggcgcgugucuuauauugucugaauguauugucugaauguaucguucguuguauugcaucgcaauacaacggacgauacauuaagacaauauuuugaacaauuugg
........................................gaauguaucguucguuguauA...................................................  33   1  sr1
........................................gaauguauUguucguuguauug..................................................  1    1  sr1
........................................gaauguaucguCcguuguauug..................................................  4    1  sr1
........................................gaauguaucguucguuguauCg..................................................  3    1  sr1
........................................gGauguaucguucguuguauug..................................................  1    1  sr1
........................................gaauguaucguucguuguauuA..................................................  79   1  sr1
........................................gaauguaucguucguuguauug..................................................  88   0  sr1
........................................gaaCguaucguucguuguauug..................................................  2    1  sr1
........................................gaauguaucguucguCguauug..................................................  1    1  sr1
........................................gaauguaucguucguuguauuU..................................................  3    1  sr1
........................................gaauguaucguucguuguauugU.................................................  2    1  sr1
........................................gaauguaucguCcguuguauugc.................................................  1    1  sr1
........................................gaauguaucguucguuguauugcauA..............................................  1    1  sr1
........................................gaauguaucguucguuguauugcaucA.............................................  1    1  sr1
........................................gaauguaucguucguuguauugcaucg.............................................  6    0  sr1
.........................................aauguaucguucguuguau....................................................  1    0  sr1
.........................................aauguaucguucguuguauu...................................................  1    0  sr1
.........................................aauguaucguucguuguauA...................................................  1    1  sr1
.........................................aauguaucguucguuguauuA..................................................  3    1  sr1
.........................................aauguaucguucguuguauCg..................................................  1    1  sr1
.........................................aauguaucguucguuguauug..................................................  39   0  sr1
.........................................Gauguaucguucguuguauug..................................................  1    1  sr1
..........................................auguaucguucguuguau....................................................  1    0  sr1
..........................................auguaucguucguuguauu...................................................  1    0  sr1
..........................................auguaucguuUguuguauug..................................................  2    1  sr1
..........................................auguaucguucguuguauuA..................................................  6    1  sr1
..........................................auguaucguucguuguauuC..................................................  1    1  sr1
..........................................auguaucguucguuguauug..................................................  9    0  sr1
...........................................uguaucguucguuguauug..................................................  7    0  sr1
...........................................Gguaucguucguuguauug..................................................  1    1  sr1
...........................................uguaucguucguuguauuA..................................................  1    1  sr1
...........................................uguaucguucguuguauugUa................................................  3    1  sr1
...........................................uguaucguucguuguauugcauA..............................................  2    1  sr1
.............................................uaucguucguuguauugcau...............................................  1    0  sr1
.............................................uaucguucguuguauugcaucgcaau.........................................  1    0  sr1
....................................................................aauacaacggacgauacau.........................  4    0  sr1
....................................................................aCuacaacggacgauacau.........................  1    1  sr1
....................................................................Gauacaacggacgauacau.........................  1    1  sr1
....................................................................Gauacaacggacgauacauu........................  1    1  sr1
....................................................................aauacaacggacgauacauu........................  30   0  sr1
....................................................................aauacaacggacgauacauA........................  2    1  sr1
....................................................................aauacaacggacgauacauC........................  6    1  sr1
....................................................................Gauacaacggacgauacauua.......................  3    1  sr1
....................................................................aauacaacggacgauacauAa.......................  1    1  sr1
....................................................................aauacaacggacgauacGuua.......................  1    1  sr1
....................................................................aauacaGcggacgauacauua.......................  1    1  sr1
....................................................................aauacaacggacgauacauuG.......................  3    1  sr1
....................................................................aauacaacggacgauacauCa.......................  2    1  sr1
....................................................................aauacaaUggacgauacauua.......................  1    1  sr1
....................................................................aauacaacggacgauacauua.......................  98   0  sr1
....................................................................aauacGacggacgauacauua.......................  1    1  sr1
....................................................................aauacaacggacgauacauuU.......................  1    1  sr1
....................................................................aauacaacggacgauacauuUa......................  1    1  sr1
....................................................................aauacaacggacgauacauuaa......................  55   0  sr1
....................................................................aauUcaacggacgauacauuaa......................  1    1  sr1
....................................................................aauacaacggacgauacauuaC......................  1    1  sr1
....................................................................aauacaacggacgauacauuaU......................  2    1  sr1
....................................................................aauacaacggacgauacauuaaU.....................  6    1  sr1
....................................................................Gauacaacggacgauacauuaag.....................  1    1  sr1
....................................................................aauacaacggacgauacauuaGg.....................  1    1  sr1
....................................................................aauacaacggacgauacaCuaag.....................  1    1  sr1
....................................................................aauacaacggacgauacauuaaC.....................  4    1  sr1
....................................................................aauacaacggacgauacGuuaag.....................  2    1  sr1
....................................................................aauacaacggacgUuacauuaag.....................  1    1  sr1
....................................................................aauacaacggacgauacauuaag.....................  105  0  sr1
....................................................................aauacaacggacgauacauuaaA.....................  5    1  sr1
.....................................................................auacaacggacgauacaC.........................  2    1  sr1
.....................................................................auacaacggacgauacauC........................  3    1  sr1
.....................................................................auacaacggacgauacauA........................  1    1  sr1
.....................................................................auacaacggacgauacauu........................  17   0  sr1
```

Star **Mature**

uuauucggcgcgugucuuauauuugucugaauguauugucugaauguaucguucguuguauugcaucgcaauacaacggacgauacauuaagacaauauuuugaacaauuugg			
....auacaacggacgauacauua.......................	79	0	sr1
....auacaacggacgaCacauua.......................	1	1	sr1
....auacaacUgacgauacauua.......................	1	1	sr1
....Guacaacggacgauacauua.......................	1	1	sr1
....auacaacggacgauacGuua.......................	1	1	sr1
....auacaacggacgauacauuU.......................	2	1	sr1
....Cuacaacggacgauacauua.......................	5	1	sr1
....auacaacggacgauUcauua.......................	1	1	sr1
....auacaacggacgauacaCua.......................	1	1	sr1
....auacaacggacgauacauuaU......................	5	1	sr1
....Guacaacggacgauacauuaa......................	3	1	sr1
....auacaacggacgauacaCuaa......................	1	1	sr1
....auacaacggacgauacauuaG......................	3	1	sr1
....auacaacggacgauacGuuaa......................	1	1	sr1
....auacaacggacgauacauuaa......................	108	0	sr1
....Cuacaacggacgauacauuaa......................	2	1	sr1
....auacUacggacgauacauuaa......................	1	1	sr1
....Guacaacggacgauacauuaag.....................	5	1	sr1
....auacaacggacgauacauuaaC.....................	1	1	sr1
....auacaacggacgaCacauuaag.....................	1	1	sr1
....auacaacggacgauacauAaag.....................	1	1	sr1
....auacaacggacgauacauuaaU.....................	1	1	sr1
....auacaacggacgauacaCuaag.....................	1	1	sr1
....auacaacggacgauacauuaaA.....................	12	1	sr1
....auGcaacggacgauacauuaag.....................	1	1	sr1
....auacaacgAacgauacauuaag.....................	1	1	sr1
....auaGaacggacgauacauuaag.....................	1	1	sr1
....Cuacaacggacgauacauuaag.....................	8	1	sr1
....auacaacggUcgauacauuaag.....................	1	1	sr1
....auacaacggacgauacauuaGg.....................	1	1	sr1
....auacaacggacgauacauuaag.....................	241	0	sr1
....auacaacggacgauUcauuaag.....................	2	1	sr1
....auacaacggacgauacauuaaga....................	12	0	sr1
....Guacaacggacgauacauuaaga....................	1	1	sr1
....Cuacaacggacgauacauuaaga....................	3	1	sr1
....auacaacggacgauacauuaagU....................	2	1	sr1
....auacaacggacgauacauuaagaA...................	1	1	sr1
....uacaacggacgauacauC........................	16	1	sr1
....uacaacggCcgauacauu........................	1	1	sr1
....uacaacggGcgauacauu........................	2	1	sr1
....uacaacggacgauacauA........................	4	1	sr1
....uacaacggacgauacauu........................	136	0	sr1
....uGcaacggacgauacauu........................	3	1	sr1
....Gacaacggacgauacauu........................	13	1	sr1
....uacaacAgacgauacauua.......................	2	1	sr1
....uacaacggacgauacCuua.......................	2	1	sr1
....uacaacggacgGuacauua.......................	11	1	sr1
....uacaacggCcgauacauua.......................	2	1	sr1
....uacaacggacgauacauua.......................	2396	0	sr1
....Aacaacggacgauacauua.......................	4	1	sr1
....uacaacggacgauacGuua.......................	7	1	sr1
....uacGacggacgauacauua.......................	12	1	sr1
....uacaacggacgauaUauua.......................	3	1	sr1
....uacaacggacgauacauuU.......................	7	1	sr1
....uacaacggacgaCacauua.......................	3	1	sr1
....uacaacggacgauacauuG.......................	29	1	sr1
....uaUaacggacgauacauua.......................	2	1	sr1
....uacaacggacgauacUuua.......................	1	1	sr1
....uacaacgAacgauacauua.......................	2	1	sr1
....uacCacggacgauacauua.......................	2	1	sr1
....uacaacggaUgauacauua.......................	3	1	sr1
....uacaacggacgauUcauua.......................	4	1	sr1
....uacaacggacgUuacauua.......................	2	1	sr1
....uacaacggacgauGcauua.......................	8	1	sr1
....uacaacggGcgauacauua.......................	6	1	sr1
....Gacaacggacgauacauua.......................	221	1	sr1
....uacaacggacgauacauCa.......................	12	1	sr1
....uacaacggacgaAacauua.......................	1	1	sr1
....uacaGcggacgauacauua.......................	8	1	sr1
....uGcaacggacgauacauua.......................	8	1	sr1

Star **Mature**

```
uuauucggcgcgugucuuauauugucugaauguauugucugaauguaucguucguuguauugcaucgcaauacaacggacgauacauuaagacaauauuuugaacaauuugg
......................................................................Cacaacggacgauacauua.......................   5     1   sr1
......................................................................uacaacggacgauacaAua.......................   1     1   sr1
......................................................................uUcaacggacgauacauua.......................   3     1   sr1
......................................................................uacaacggacAauacauua.......................   3     1   sr1
......................................................................uacaaUggacgauacauua.......................   2     1   sr1
......................................................................uacaacggUcgauacauuaa......................   1     1   sr1
......................................................................uacaacggacUauacauuaa......................   1     1   sr1
......................................................................uacaacggacgaCacauuaa......................   1     1   sr1
......................................................................uaUaacggacgauacauuaa......................   1     1   sr1
......................................................................uacaacggacgauacauuaa......................   470   0   sr1
......................................................................uacaaUggacgauacauuaa......................   1     1   sr1
......................................................................uacaacAgacgauacauuaa......................   4     1   sr1
......................................................................uacaacggacgauacauuaC......................   1     1   sr1
......................................................................uacaacggGcgauacauuaa......................   2     1   sr1
......................................................................uacaacggacgauacGuuaa......................   2     1   sr1
......................................................................uacaacggaUgauacauuaa......................   1     1   sr1
......................................................................uacaacggacgauacauuaU......................   5     1   sr1
......................................................................uacaacggacgauGcauuaa......................   1     1   sr1
......................................................................Gacaacggacgauacauuaa......................   36    1   sr1
......................................................................uacaGcggacgauacauuaa......................   1     1   sr1
......................................................................uacaacggacgauacUuuaa......................   1     1   sr1
......................................................................uacaacggacgauacauuaG......................   11    1   sr1
......................................................................uGcaacggacgauacauuaa......................   2     1   sr1
......................................................................uacaacggacgauacUuuaag.....................   9     1   sr1
......................................................................uacaacggacgauacauuaGg.....................   41    1   sr1
......................................................................uacaacggacgaCacauuaag.....................   15    1   sr1
......................................................................uacaacggacgauacauuaaC.....................   9     1   sr1
......................................................................Aacaacggacgauacauuaag.....................   7     1   sr1
......................................................................uacaacggacgauacauuaaU.....................   18    1   sr1
......................................................................uacaacggacgauacauuaag.....................   9022  0   sr1
......................................................................uacaacggacgauGcauuaag.....................   18    1   sr1
......................................................................uacaacggaUgauacauuaag.....................   6     1   sr1
......................................................................uacaacggGcgauacauuaag.....................   35    1   sr1
......................................................................uacaacggacgauaUauuaag.....................   4     1   sr1
......................................................................uacaacggacgauacauCaag.....................   7     1   sr1
......................................................................uacaCcggacgauacauuaag.....................   4     1   sr1
......................................................................uacaacggacgauacauUag.....................   3     1   sr1
......................................................................uacaacggUcgauacauuaag.....................   2     1   sr1
......................................................................uacCacggacgauacauuaag.....................   2     1   sr1
......................................................................uacaacggacgauacauuaUg.....................   6     1   sr1
......................................................................uacaacggacgaGacauuaag.....................   1     1   sr1
......................................................................uacaacgAacgauacauuaag.....................   2     1   sr1
......................................................................uacaacggacgauacauuaaA.....................   388   1   sr1
......................................................................uacaacggacgauacaCuaag.....................   20    1   sr1
......................................................................uacaacggacgauacauAaag.....................   1     1   sr1
......................................................................uacUacggacgauacauuaag.....................   4     1   sr1
......................................................................uacaacggacgauacauuGag.....................   22    1   sr1
......................................................................uacaacggacgauacauuaCg.....................   2     1   sr1
......................................................................uacaacAgacgauacauuaag.....................   14    1   sr1
......................................................................uacaacggacgauacaAuaag.....................   1     1   sr1
......................................................................uacaaGggacgauacauuaag.....................   1     1   sr1
......................................................................uaUaacggacgauacauuaag.....................   8     1   sr1
......................................................................uacaacggacUauacauuaag.....................   3     1   sr1
......................................................................uacaacggacgaAacauuaag.....................   2     1   sr1
......................................................................uacaacggacgCuacauuaag.....................   2     1   sr1
......................................................................uacaaAggacgauacauuaag.....................   3     1   sr1
......................................................................Cacaacggacgauacauuaag.....................   40    1   sr1
......................................................................uacaacggacgauUcauuaag.....................   3     1   sr1
......................................................................uacaGcggacgauacauuaag.....................   27    1   sr1
......................................................................uacaaUggacgauacauuaag.....................   5     1   sr1
......................................................................uacaacggacgauacaGuaag.....................   2     1   sr1
......................................................................uacaacggacgauaGauuaag.....................   1     1   sr1
......................................................................uacaacggacgauacGuuaag.....................   24    1   sr1
......................................................................uUcaacggacgauacauuaag.....................   2     1   sr1
......................................................................uacaacggacAauacauuaag.....................   8     1   sr1
......................................................................uacaacggacgGuacauuaag.....................   33    1   sr1
......................................................................uacaacggacgUuacauuaag.....................   3     1   sr1
......................................................................uacaUcggacgauacauuaag.....................   2     1   sr1
......................................................................uacGacggacgauacauuaag.....................   40    1   sr1
......................................................................uacaacggacgauacCuuaag.....................   3     1   sr1
```

```
                                        Star                            Mature
uuauucggcgcgugucuuauauugucugaauguauugucugaauguaucguucguuguauugcaucgcaauacaacggacgauacauuaagacaauauuuugaacaauuugg
......................................................................Gacaacggacgauacauuaag.....................  647   1  sr1
......................................................................uGcaacggacgauacauuaag.....................  18    1  sr1
......................................................................uacaacggacgauacauuCaga....................  2     1  sr1
......................................................................uacaacgCacgauacauuaaga....................  1     1  sr1
......................................................................uacaGcggacgauacauuaaga....................  33    1  sr1
......................................................................uacaacggacgauacauuUaga....................  3     1  sr1
......................................................................uacaacggacgauacauuaaCa....................  1     1  sr1
......................................................................uacaacggacgauacauuaagC....................  8     1  sr1
......................................................................uacaacggacgauacauuaagG....................  254   1  sr1
......................................................................uUcaacggacgauacauuaaga....................  5     1  sr1
......................................................................uacaacggaAgauacauuaaga....................  3     1  sr1
......................................................................uacaacgAacgauacauuaaga....................  13    1  sr1
......................................................................uacaacggacgauaUauuaaga....................  4     1  sr1
......................................................................Cacaacggacgauacauuaaga....................  22    1  sr1
......................................................................uacaacggacgauacCuuaaga....................  2     1  sr1
......................................................................uacaacggacgauaGauuaaga....................  1     1  sr1
......................................................................uGcaacggacgauacauuaaga....................  21    1  sr1
......................................................................uacaUcggacgauacauuaaga....................  2     1  sr1
......................................................................uacaacggacgauacGuuaaga....................  34    1  sr1
......................................................................uacaacggacgauUcauuaaga....................  8     1  sr1
......................................................................Gacaacggacgauacauuaaga....................  820   1  sr1
......................................................................uacaacggacgauacauuaaAa....................  3     1  sr1
......................................................................uacaacggacUauacauuaaga....................  3     1  sr1
......................................................................uacaacggacgGuacauuaaga....................  29    1  sr1
......................................................................uaUaacggacgauacauuaaga....................  3     1  sr1
......................................................................uacaacggacgauacauuaagU....................  61    1  sr1
......................................................................uacaacggacgCuacauuaaga....................  1     1  sr1
......................................................................uacaaAggacgauacauuaaga....................  2     1  sr1
......................................................................uacaaUggacgauacauuaaga....................  5     1  sr1
......................................................................uacaacggacAauacauuaaga....................  9     1  sr1
......................................................................uaAaacggacgauacauuaaga....................  1     1  sr1
......................................................................uacaacggacgauacauuGaga....................  15    1  sr1
......................................................................uacaacggUcgauacauuaaga....................  5     1  sr1
......................................................................uacaacggacgauacaGuaaga....................  2     1  sr1
......................................................................uacaacggacgauacauGaaga....................  1     1  sr1
......................................................................uacaacggacgauacauuaaga....................  9103  0  sr1
......................................................................uacUacggacgauacauuaaga....................  5     1  sr1
......................................................................uacaacggacgauacauuaUga....................  7     1  sr1
......................................................................uacaacAgacgauacauuaaga....................  7     1  sr1
......................................................................uacaacggacgaCacauuaaga....................  18    1  sr1
......................................................................uacaacggacgaGacauuaaga....................  1     1  sr1
......................................................................uacaacggacgauacauuaaUa....................  18    1  sr1
......................................................................uacaacggaUgauacauuaaga....................  11    1  sr1
......................................................................uCcaacggacgauacauuaaga....................  2     1  sr1
......................................................................uacaacggGcgauacauuaaga....................  27    1  sr1
......................................................................uacaacggacgaAacauuaaga....................  2     1  sr1
......................................................................uacaacggacgUuacauuaaga....................  2     1  sr1
......................................................................uacaacggacgauacauuaGga....................  24    1  sr1
......................................................................uacGacggacgauacauuaaga....................  50    1  sr1
......................................................................uacaacggacgauacUuuaaga....................  8     1  sr1
......................................................................uacaacggacgauacauCaaga....................  21    1  sr1
......................................................................uacaacggacCauacauuaaga....................  1     1  sr1
......................................................................Aacaacggacgauacauuaaga....................  9     1  sr1
......................................................................uacaacggacgauGcauuaaga....................  28    1  sr1
......................................................................uacaacUgacgauacauuaaga....................  2     1  sr1
......................................................................uacaacggacgauacaCuaaga....................  9     1  sr1
......................................................................uacaacggacgauacauuaagaA...................  2489  1  sr1
......................................................................uacaacggacgauacauuaagaG...................  148   1  sr1
......................................................................Gacaacggacgauacauuaagac...................  1     1  sr1
......................................................................uacaacggacgauacauuaagac...................  17    0  sr1
......................................................................uacaacggacgauacauuaagGc...................  1     1  sr1
......................................................................uacaacggacgauacauuaagaU...................  77    1  sr1
......................................................................uacaacggacgauacauuGagaca..................  1     1  sr1
......................................................................uacaacggacgauacauuaagacC..................  3     1  sr1
......................................................................uacaacggacgauacauuaagacU..................  1     1  sr1
......................................................................uacaacggacgauacauuaagaAa..................  101   1  sr1
......................................................................uacaacggacgauacauuaagaUa..................  160   1  sr1
......................................................................uacaacggacgauacauuaagacCa.................  1     1  sr1
......................................................................uacaacggacgauacauuaagaAaa.................  14    1  sr1
......................................................................uacaacggacgauacauuaagaUaa.................  16    1  sr1
```

Star Mature

```
uuauucggcgcgugucuuauauugucugaauguauugucugaauguaucguucguuguauugcaucgcaauacaacggacgauacauuaagacaauauuuugaacaauuugg
.......................................................................acaacggacgauacauua.......................     1     0     sr1
.......................................................................Gcaacggacgauacauuaa......................     1     1     sr1
.......................................................................acaCcggacgauacauuaag.....................     1     1     sr1
.......................................................................acaacggGcgauacauuaag.....................     1     1     sr1
.......................................................................acaacggacgauacauuaag.....................     2     0     sr1
.......................................................................acaacggacgauacauuaaga....................     4     0     sr1
........................................................................caacggacgauacauuaag.....................     2     0     sr1
........................................................................caacggacgauacauuaaga....................     2     0     sr1
........................................................................caGcggacgauacauuaaga....................     1     1     sr1
........................................................................caacggacgauacauuaagaA...................     1     1     sr1
```

Provisional ID : ScYm1zk_406_10488
Score total : 416.4
Score for star read(s) : 3.9
Score for read counts : 405.9
Score for mfe : 1.9
Score for randfold : 1.6
Score for cons. seed : 3
Total read count : 808
Mature read count : 640
Loop read count : 0
Star read count : 168

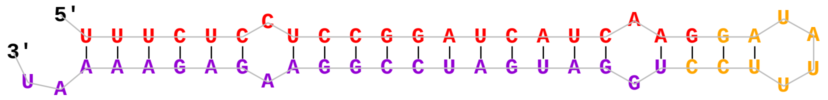

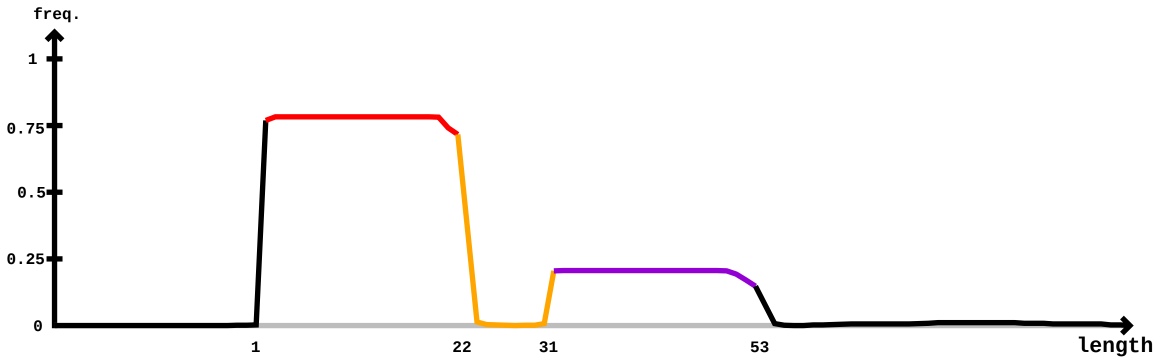


```
                     Mature                Star
5'- aauaguucgucacgaaauauuuucuccuccggaucaucaagggauauuuccuggaugauccggaagagaaaauauucaaaaaugucgaauuuggcgcucaaaauuugaaag  -3'  obs
    aauaguucgucacgaaauauuuucuccuccggaucaucaagggauauuuccuggaugauccggaagagaaaauauucaaaaaugucgaauuuggcgcucaaaauuugaaag       exp
    .............(((.(((((((((.(((((((((((.((((....)))).))))))))))).)))))))))))).......((((((((........))))))))...   reads   mm   sample
    .................uauuuucucUuccggaucauc........................................................................   1       1    sr1
    ....................uuucuccuccggaucGuca.......................................................................   2       1    sr1
    ....................uuucuccuccggaucauca.......................................................................   12      0    sr1
    ....................uuucuccuccggaucaucaa......................................................................   7       0    sr1
    ....................uuucuccuccggaucaucaag.....................................................................   130     0    sr1
    ....................uuucuccuccggaucGucaag.....................................................................   17      1    sr1
    ....................uuucuccuccggauGaucaag.....................................................................   1       1    sr1
    ....................uuucuccuccggaucaucaaA.....................................................................   8       1    sr1
    ....................Cuucuccuccggaucaucaag.....................................................................   1       1    sr1
    ....................uuucuccuccggaCcaucaag.....................................................................   1       1    sr1
    ....................uuAcuccuccggaucaucaag.....................................................................   1       1    sr1
    ....................Guucuccuccggaucaucaag.....................................................................   5       1    sr1
    ....................uuucuccuccggaucGucaagg....................................................................   4       1    sr1
    ....................uuucuccCccggaucaucaagg....................................................................   1       1    sr1
    ....................uuucuccuccggaucaucaagC....................................................................   1       1    sr1
    ....................uuucuccuccggaucaucaagA....................................................................   11      1    sr1
    ....................uuucuccuccggaucaucaagg....................................................................   50      0    sr1
    ....................uuucuccuccggaucaucaGgg....................................................................   1       1    sr1
    ....................Guucuccuccggaucaucaagg....................................................................   3       1    sr1
    ....................uuucuccuccggaucaucaagga...................................................................   3       0    sr1
    ....................uuucuccuccggaucaucaGggaua.................................................................   1       1    sr1
    .....................uucuccuccggaucaucaagg....................................................................   4       0    sr1
    .....................uucuccuccggaucaucaGgga...................................................................   1       1    sr1
    ..................................................uggaugauccggaagagaa.........................................   2       0    sr1
    ..................................................Gggaugauccggaagagaa.........................................   1       1    sr1
    ..................................................uggaugauccggaagagaaa........................................   8       0    sr1
    ..................................................uggaugauccggaagGgaaa........................................   1       1    sr1
    ..................................................Gggaugauccggaagagaaaa.......................................   2       1    sr1
    ..................................................uggaugauccggaagagaaaa.......................................   2       0    sr1
    ..................................................uggaugauccggaagagaaaUu......................................   1       1    sr1
    ..................................................uggaugauccggaGgagaaaau......................................   1       1    sr1
    ..................................................Gggaugauccggaagagaaaau......................................   2       1    sr1
    ..................................................uggaugauccggaagagaaaaC......................................   1       1    sr1
```

Mature **Star**

```
aauaguucgucacgaaauauuuucuccuccggaucaucaaggauauuuccuggaugauccggaagagaaaauauucaaaaaugucgaauuggcgcucaaaauuugaaag
..................................................uggaCgauccggaagagaaaau.....................................	1	1	sr1
..................................................uggaugaCccggaagagaaaau.....................................	1	1	sr1
..................................................uggaugauccggaagagaaaau.....................................	17	0	sr1
..................................................uggaugauccggaagagaaaauG....................................	1	1	sr1
..................................................uggaugauccggaagagaaaaua....................................	13	0	sr1
..................................................uggaugauccggaagagaaaauU....................................	2	1	sr1
..................................................uggaugauccggaagagaaaauUu...................................	2	1	sr1
..................................................uggaugauccggaagagaaaauau...................................	1	0	sr1
................................................................................augucgaauuggcgcucU...........	1	1	sr1
.........................................................................................uggcgcucaaaauuugaaag	2	0	sr1

....................uuucuccuccggaucauca......................................................................	10	0	st1
....................uuucuccuccggaucaucaa.....................................................................	2	0	st1
....................uuucuccuccggaucGucaag....................................................................	2	1	st1
....................uuucuccuccggaucaucaaU....................................................................	1	1	st1
....................uuucuccuccAgaucaucaag....................................................................	1	1	st1
....................uuucuccuccggauAaucaag....................................................................	1	1	st1
....................uuucuccuccggaucaucaag....................................................................	60	0	st1
....................uuucuccuccggaucaucaaA....................................................................	4	1	st1
....................uuucuccuccggaucaucaagU...................................................................	2	1	st1
....................uuucCcuccggaucaucaagg...................................................................	1	1	st1
....................uuucuccuccggaucaucaagC...................................................................	1	1	st1
....................uuucuccuccggaucaucaagA...................................................................	3	1	st1
....................uuucuccuccggaucaucaagg...................................................................	34	0	st1
....................uuucuccuccggaucaucaGgg...................................................................	1	1	st1
....................uuCcuccuccggaucaucaagg...................................................................	1	1	st1
....................uuucuccuccggGucaucaagg...................................................................	1	1	st1
....................uuucuccuccggaucaucaGgga..................................................................	1	1	st1
....................uuucuccuccggaucaucaagga..................................................................	1	0	st1
....................uuucuccuccggaucaucaaggauUu...............................................................	1	1	st1
.....................uucuccuccggaucaucaagga..................................................................	1	0	st1
...............................................uccuggaugauccggaagagaaaau.....................................	1	0	st1
.................................................cuggaugauccggaagagaaaau.....................................	1	0	st1
..................................................uggaugauccggaagagaa........................................	1	0	st1
..................................................uggaugauccggaagagaaa.......................................	1	0	st1
..................................................uggaugauccggaagagaaaa......................................	4	0	st1
..................................................uggaugauccggaagagaaaau.....................................	22	0	st1
..................................................uggaugauccggaagagaaaaC.....................................	1	1	st1
...............................................................................aaugucgaauuggcgcuca...........	1	0	st1

....................uuucuccuccggaucauca......................................................................	4	0	so1
....................uuucuccuccggaucGuca......................................................................	1	1	so1
....................uuucuccuccggaucaucaa.....................................................................	2	0	so1
....................uuucuccuccggaucGucaa.....................................................................	1	1	so1
....................uuucuccuccggaucaucaag....................................................................	35	0	so1
....................uuucuccuccggaucGucaag....................................................................	2	1	so1
....................uAucuccuccggaucaucaag....................................................................	2	1	so1
....................uuucuccuccggaucaucaagU...................................................................	3	1	so1
....................uuucuccuccggaucGucaagg...................................................................	2	1	so1
....................uuCcuccuccggaucaucaagg...................................................................	1	1	so1
....................uuucuccuccggaucaucaagg...................................................................	22	0	so1
....................uuucuccuccggaucaucaagga..................................................................	1	0	so1
..................................................uggaugauccggaagaga.........................................	1	0	so1
..................................................uggaugauccggaagagaa........................................	2	0	so1
..................................................uggaugauccggaagagaaa.......................................	6	0	so1
..................................................uggaugauccggaagagaaU.......................................	1	1	so1
..................................................uggaugauccggaagagaaaa......................................	1	0	so1
..................................................uggaugauccggaGgagaaaau.....................................	1	1	so1
..................................................uggaugauccggaagagaaaaC.....................................	2	1	so1
..................................................uggaugauccggaagagaaaau.....................................	9	0	so1
..................................................uggaugaucAggaagagaaaaua....................................	1	1	so1
..................................................uggaugauccggaagagaaaaua....................................	4	0	so1
..................................................uggaugauccggaagagaaaauUu...................................	2	1	so1
.......................................................................................uuuggcgcucaaaauuugaa..	1	0	so1
........................................................................................uuggcgcucaaaauuugaa..	1	0	so1

...................Cuuucuccuccggaucaucaag....................................................................	1	1	sg1
....................uuucuccuccggaucauca......................................................................	4	0	sg1
....................uuucuccuccggaucaCcaa.....................................................................	1	1	sg1
....................uuucuccuccggaucGucaa.....................................................................	1	1	sg1
```

```
                    Mature                        Star
aauaguucgucacgaaauauuuucuccuccggaucaucaaggauauuuccuggaugauccggaagagaaaauauucaaaaaugucgaauuuggcgcucaaaauuugaaag
....................uuucuccuccggaucaucaa......................................................................  6   0  sg1
....................uuucCccuccggaucaucaag.....................................................................  2   1  sg1
....................uuucuccuccggaucaucaaA.....................................................................  2   1  sg1
....................uuucuccuccggaucaCcaag.....................................................................  1   1  sg1
....................uuucuccuccAgaucaucaag.....................................................................  1   1  sg1
....................uuucuccuccggaucGucaag..................................................................... 12   1  sg1
....................uuucuccuccggaucaucaag..................................................................... 83   0  sg1
....................uCucuccuccggaucaucaag.....................................................................  2   1  sg1
....................uuucuccuccggGucaucaagg....................................................................  1   1  sg1
....................uuucuccuccggaucaucaagA....................................................................  6   1  sg1
....................uuucuccuccggaucGucaagg....................................................................  5   1  sg1
....................uuucuccuccggaucaucaagg.................................................................... 36   0  sg1
....................uuucuccuccggaucaucaaggCu..................................................................  1   1  sg1
.....................uucuccuccggaucaucaag.....................................................................  2   0  sg1
.....................uucuccuccggaucaucaagg....................................................................  3   0  sg1
.................................................cuggaugauccggaagagaaaa.......................................  4   0  sg1
..................................................uggaugauccggaagagaa.........................................  4   0  sg1
..................................................uggaugauccggaagagaaa........................................  1   0  sg1
..................................................uggaugauccggaagagaaaa.......................................  6   0  sg1
..................................................uggaugauccggaagagGaaau......................................  1   1  sg1
..................................................uggaugauccggaagagaaaau...................................... 24   0  sg1
..................................................uggaugauccggaagagaaaaua.....................................  5   0  sg1
..................................................uggaugauccggaGgagaaaaua.....................................  1   1  sg1
..................................................uggaugauccggaagagaaaauU.....................................  1   1  sg1
..................................................uggaugauccggaagagaaaauauA...................................  1   1  sg1
...................................................ggaugauccggaagagaaaau......................................  1   0  sg1
.............................................................................aaaaugucgaauuuggcgcucaaaa........  2   0  sg1
.................................................................................ugucgaauuuggcgcucaaaauuugaa..  1   0  sg1
```

Provisional ID	:	ScYm1zk_434_10966
Score total	:	1049
Score for star read(s)	:	3.9
Score for read counts	:	1041.7
Score for mfe	:	2.4
Score for randfold	:	1.6
Score for cons. seed	:	-0.6
Total read count	:	2055
Mature read count	:	2026
Loop read count	:	0
Star read count	:	29

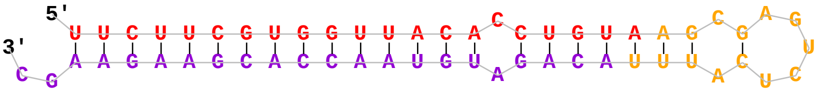

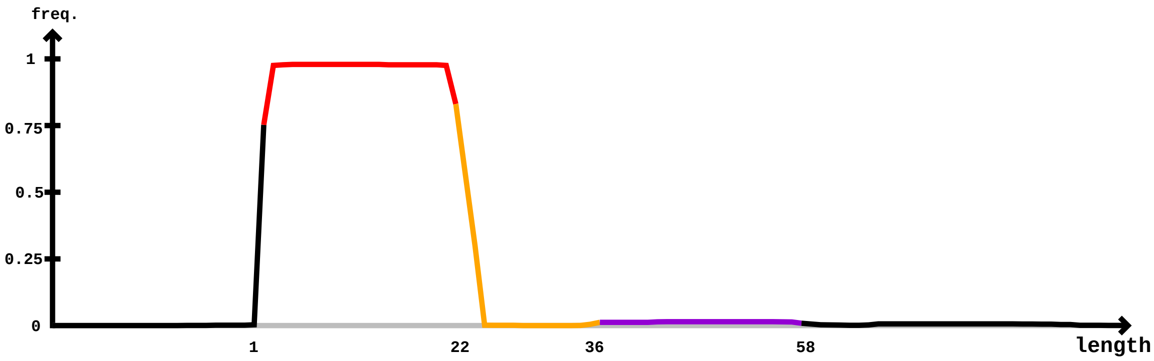

Mature **Star**

```
5'- aaagcaaaacgggugucggcuucuucgugguuacaccuguaagcgagucucauuuacagauguaaccacgaagaagccgacugauguuuugguucugcuugugugggcuu  -3'   obs
    aaagcaaaacgggugucggcuucuucgugguuacaccuguaagcgagucucauuuacagauguaaccacgaagaagccgacugauguuuugguucugcuugugugggcuu         exp
    .((.(((((((...(((((((((((((((((((((.(((((((.(.....).))))))).)))))))))))))))))))))...))))))).))..((((....))))..   reads   mm   sample
    ............gugucggcuucuucgugguuG.............................................................................   1       1    sr1
    ...............ucggcuucuucgugguAa.............................................................................   1       1    sr1
    ...............ucggcuucuucgugguuG.............................................................................   1       1    sr1
    ...................cuucuucgugguuacaccugua.....................................................................   1       0    sr1
    ...................cuucuucgugguuacaccuguaU....................................................................   1       1    sr1
    ....................uucuucgugguuacaccug.......................................................................   2       0    sr1
    ....................uucuucgugguuacaccAg.......................................................................   1       1    sr1
    ....................Cucuucgugguuacaccugu......................................................................   2       1    sr1
    ....................uucuucgugguuacaccugC......................................................................   11      1    sr1
    ....................uucuucgugguCacaccugu......................................................................   3       1    sr1
    ....................uucuucgugguuacaAcugu......................................................................   16      1    sr1
    ....................uucuucgugguuacGccugu......................................................................   1       1    sr1
    ....................uucuucgugguuacaGcugu......................................................................   18      1    sr1
    ....................uucuucgugguuacaccugA......................................................................   1       1    sr1
    ....................Gucuucgugguuacaccugu......................................................................   8       1    sr1
    ....................uucuucguggCuacaccugu......................................................................   1       1    sr1
    ....................uucuucgugguuacaccugG......................................................................   2       1    sr1
    ....................uucuucgugguuacaccugu......................................................................   115     0    sr1
    ....................uucuucgugguuacaAcugua.....................................................................   48      1    sr1
    ....................uucuucgugguuacaccuUua.....................................................................   4       1    sr1
    ....................uucuucUugguuacaccugua.....................................................................   1       1    sr1
    ....................uucuucgugguuacaccugua.....................................................................   356     0    sr1
    ....................uCcuucgugguuacaccugua.....................................................................   2       1    sr1
    ....................uucuucgugguuacaccugGa.....................................................................   3       1    sr1
    ....................Gucuucgugguuacaccugua.....................................................................   5       1    sr1
    ....................Aucuucgugguuacaccugua.....................................................................   1       1    sr1
    ....................uucuucgugguCacaccugua.....................................................................   2       1    sr1
    ....................uucuucgugguuacaccuguG.....................................................................   12      1    sr1
    ....................uucuucgugguuacaGcugua.....................................................................   39      1    sr1
    ....................uucuucgugguuacGccugua.....................................................................   1       1    sr1
    ....................uucuucgugguuacaccuguU.....................................................................   4       1    sr1
    ....................uucuucgugguuaUaccugua.....................................................................   2       1    sr1
    ....................uuUuucgugguuacaccugua.....................................................................   2       1    sr1
```

```
                    Mature                            Star
aaagcaaaacgggugucggcuucuucgugguuacaccuguaagcgagucucauuuacagauguaaccacgaagaagccgacugauguuuugguucugcuugugugggcuu
....................uucCucgugguuacaccugua.....................................................................   3     1   sr1
....................uucuucgugguuacaccuguaa....................................................................   120   0   sr1
....................uucuucgugguCacaccuguaa....................................................................   1     1   sr1
....................uucuucgugguuGcaccuguaa....................................................................   1     1   sr1
....................uucuucgugguuacaccugCaa....................................................................   1     1   sr1
....................uucuucgugguuacaccuguGa....................................................................   1     1   sr1
....................uucuucgugguuacaccuguaG....................................................................   1     1   sr1
....................uucuucgugguuacaccuguaU....................................................................   11    1   sr1
....................uucuucgugguuacaAcuguaa....................................................................   19    1   sr1
....................uucuucgugguuacaGcuguaa....................................................................   9     1   sr1
....................uucuucgCgguuacaccuguaa....................................................................   1     1   sr1
....................uucuucgugguuacaUcuguaag...................................................................   164   1   sr1
....................uucuucgugguAacaccuguaag...................................................................   1     1   sr1
....................uucuucgugguuacaccuguaUg...................................................................   1     1   sr1
....................uucuucgugguuacaccuguaaA...................................................................   4     1   sr1
....................uucuucgugguuacaccuguaag...................................................................   2     0   sr1
....................uucuucgugguuacaUcuguaagcgag...............................................................   2     1   sr1
.....................ucuucgugguuacaccugu......................................................................   12    0   sr1
.....................ucuucgugguuacaAcugu......................................................................   1     1   sr1
.....................ucuucgugguuacaccugua.....................................................................   20    0   sr1
.....................ucuucgugguuacaAcugua.....................................................................   3     1   sr1
.....................Gcuucgugguuacaccugua.....................................................................   2     1   sr1
.....................ucuGcgugguuacaccuguaa....................................................................   1     1   sr1
.....................ucuucgugguuacaccuguaa....................................................................   26    0   sr1
.....................uUuucgugguuacaccuguaa....................................................................   1     1   sr1
.....................ucuucgugguuGcaccuguaa....................................................................   1     1   sr1
.....................ucuucgugguuacaccuguaG....................................................................   4     1   sr1
.....................ucuucgugguuacaGcuguaa....................................................................   2     1   sr1
.....................Gcuucgugguuacaccuguaag...................................................................   1     1   sr1
.....................ucuucgugguuacaUcuguaag...................................................................   245   1   sr1
.....................ucuucgugguuacaAcuguaag...................................................................   1     1   sr1
.....................ucuucgugguuacaccuguaaA...................................................................   27    1   sr1
......................cuucgugguuacaccuguaU....................................................................   1     1   sr1
......................cuucgCgguuacaccuguaa....................................................................   1     1   sr1
.......................uucgugguuacaUcuguaag...................................................................   2     1   sr1
......................................................uacagauguaaccacgaaga....................................   1     0   sr1
......................................................uacagauguaaccacgaagaag..................................   2     0   sr1
......................................................uacagauguaaccacgaagaA...................................   1     1   sr1
.......................................................acagauguaaccacgaagaagc.................................   3     0   sr1
..............................................................................gacugauguuuugguucugcG...........   1     1   sr1
................................................................................cugGuguuuugguucugcuug.........   1     1   sr1
...................................................................................auguuuuCguucugcuugug.......   1     1   sr1
...................................................................................auguuuugguucugcuugug.......   2     0   sr1
....................................................................................uguuuugguucugcuugugug.....   1     0   sr1
....................................................................................uguuuuCguucugcuugugug.....   3     1   sr1

....................uucuucgugguuacaccugC......................................................................   1     1   st1
....................uucuucgugguuacaAcugu......................................................................   1     1   st1
....................uucuucgugguuacaccugA......................................................................   1     1   st1
....................uucuucgugguuacaccugu......................................................................   40    0   st1
....................uucuucgugguuGcaccugu......................................................................   1     1   st1
....................uucuucgugguuacaccugua.....................................................................   71    0   st1
....................uucuucgugguuacaGcugua.....................................................................   1     1   st1
....................uucuucgugguuacaAcugua.....................................................................   8     1   st1
....................uucuucgugguuacaccuguaU....................................................................   1     1   st1
....................uucuucgugguuacaccuguaa....................................................................   34    0   st1
....................uucuucgugguuacaAcuguaa....................................................................   3     1   st1
....................uucuucgugAuuacaccuguaa....................................................................   1     1   st1
....................uucuucgugguuacaUcuguaag...................................................................   34    1   st1
....................uucuucgugguuacaccuguaaA...................................................................   12    1   st1
.....................ucuucgugguuacaccugu......................................................................   5     0   st1
.....................ucuucgugguuacaccugua.....................................................................   3     0   st1
.....................ucuucgugguuacaGcuguaa....................................................................   1     1   st1
.....................ucuucgugguuacaAcuguaa....................................................................   1     1   st1
.....................ucuucgugguuacaccuguaa....................................................................   4     0   st1
.....................uUuucgugguuacaccuguaa....................................................................   1     1   st1
.....................ucuucgugguuacaccuguaaA...................................................................   12    1   st1
.....................ucuucgugguuacaccuguaaU...................................................................   1     1   st1
.....................ucuucgugguuacaUcuguaag...................................................................   20    1   st1
......................Uuucgugguuacaccugua.....................................................................   2     1   st1
```

```
                    Mature                          Star
aaagcaaaacgggugucggcuucuucgugguuacaccuguaagcgagucucauuuacagauguaaccacgaagaagccgacugauguuuugguucugcuugugugggcuu
......................cuucgugguuacaccuguaa....................................................................    1    0    st1
.......................uucgugguuacaccuguaa....................................................................    1    0    st1
.....................................................uuacagauguaaccacgaagaa...................................    1    0    st1
.......................................................acagauguaaccacgaagaagc.................................    4    0    st1
.......................................................acagauguaaccacgaagaagA.................................    1    1    st1
.............................................................guaaccacgaagaagcUg...............................    1    1    st1
.............................................................guaaccacgaagaagcUga..............................    2    1    st1
.............................................................guaaccacgaagaagcUgac.............................    1    1    st1
..............................................................uaaccacgaagaagcUgac.............................    1    1    st1
....................................................................................uguuuugguucugcuuguguggAc..    1    1    st1

...................cuucuucgugguuacaAcugua.....................................................................    1    1    sg1
....................uucuucgugguuacaccugA......................................................................    1    1    sg1
....................uucuucgugguuacaccugC......................................................................    1    1    sg1
....................uucuucgugguuacaccugu......................................................................    19   0    sg1
....................uCcuucgugguuacaccugu......................................................................    1    1    sg1
....................uucuucgugguuacaAcugu......................................................................    1    1    sg1
....................uucuucgugguuacaGcugu......................................................................    2    1    sg1
....................uucuucgugguuacaccuAu......................................................................    2    1    sg1
....................uucuucgugguuacaAcugua.....................................................................    1    1    sg1
....................uucuucgugguuacaccugua.....................................................................    25   0    sg1
....................uucuucgugguuacaccugCa.....................................................................    1    1    sg1
....................uucuucgugguuacaGcugua.....................................................................    2    1    sg1
....................uucuucgugguuacaccuguU.....................................................................    2    1    sg1
....................uucuucgugguuacaccuguaU....................................................................    1    1    sg1
....................uucuucgugguuacaAcuguaa....................................................................    1    1    sg1
....................uucuucgugguuacaGcuguaa....................................................................    3    1    sg1
....................uucuucgugguuacaccCguaa....................................................................    1    1    sg1
....................uucuucgugguuacaccuguaa....................................................................    23   0    sg1
....................uucuucgugguuacaUcuguaag...................................................................    16   1    sg1
....................uucuucgugguuacaccuguaaA...................................................................    2    1    sg1
....................uucuucgugguuacaUcuguaagc..................................................................    1    1    sg1
.....................ucuucgugguuGcaccugua.....................................................................    1    1    sg1
.....................ucuucgugguuacaccugua.....................................................................    1    0    sg1
.....................ucuucgugguuacaccuguaa....................................................................    4    0    sg1
.....................ucuucgugguuacaccuguaaA...................................................................    1    1    sg1
.....................ucuucgugguuacaUcuguaag...................................................................    8    1    sg1
....................................................................................uguuuugguucugcuuguguggAcuu    1    1    sg1

....................uucuucgugguuacaccug.......................................................................    2    0    so1
....................uucuucgugguuacaccugu......................................................................    28   0    so1
....................uucuucgugguuacaGcugu......................................................................    1    1    so1
....................uucuucgugguuacaccugC......................................................................    2    1    so1
....................uucuucAugguuacaccugu......................................................................    1    1    so1
....................uucuucgugguuacaccugCa.....................................................................    1    1    so1
....................uucuucgugguuacaccuguC.....................................................................    1    1    so1
....................uucuucgugguuacaAcugua.....................................................................    6    1    so1
....................uucuucgugguuacaGcugua.....................................................................    1    1    so1
....................uucuucgugguuacGccugua.....................................................................    2    1    so1
....................uucuucgugguuacaccuguG.....................................................................    2    1    so1
....................uucuucgugguuacaccugua.....................................................................    84   0    so1
....................uucuucguAguuacaccugua.....................................................................    1    1    so1
....................uucuucgCgguuacaccuguaa....................................................................    1    1    so1
....................uucuucgugguuacaccuguaG....................................................................    2    1    so1
....................uucuucgugguuacaGcuguaa....................................................................    4    1    so1
....................uucuucgugguuacaccuguaU....................................................................    6    1    so1
....................uucuucgugguuacaAcuguaa....................................................................    4    1    so1
....................Cucuucgugguuacaccuguaa....................................................................    2    1    so1
....................uucuucgugguuacaccuguaa....................................................................    47   0    so1
....................uucuucgugguuacaUcuguaag...................................................................    34   1    so1
....................uucuucgugguuacaccuguaaA...................................................................    2    1    so1
....................uucuucgugguuacaccuguaaU...................................................................    1    1    so1
.....................ucuucgugguuacaccugu......................................................................    1    0    so1
.....................ucuucgugguuacaccugua.....................................................................    8    0    so1
.....................ucuucgugguuacaccuguC.....................................................................    1    1    so1
.....................uUuucgugguuacaccugua.....................................................................    1    1    so1
.....................ucuucgCgguuacaccuguaa....................................................................    1    1    so1
.....................ucuCcgugguuacaccuguaa....................................................................    2    1    so1
.....................ucuucgugguuacaccuguaa....................................................................    11   0    so1
.....................ucuucgugguuacaUcuguaag...................................................................    14   1    so1
```

Mature Star

```
aaagcaaaacggguguc ggcuucuucgugguuacaccuguaagcgagucucauuuacagauguaaccacgaagaagccgacugauguuuugguucugcuugugugggcuu
.....................ucuucgugguuacaccuguaaA...................................................................   8   1   so1
.....................ucuucgugguuacaccuguaag...................................................................   5   0   so1
......................................................uacagauguaaccacgaagaag..................................   5   0   so1
.......................................................acagauguaaccacgaagaag..................................   2   0   so1
.......................................................acagauguaaccacgaagaagc.................................   5   0   so1
....................................................................................uguuuugguucugcuugugug.....   1   0   so1
....................................................................................uguuuuCguucugcuugugug.....   1   1   so1
```

Provisional ID	:	ScYm1zk_434_10971
Score total	:	242750.5
Score for star read(s)	:	3.9
Score for read counts	:	242743.2
Score for mfe	:	2.4
Score for randfold	:	1.6
Score for cons. seed	:	-0.6
Total read count	:	476142
Mature read count	:	475146
Loop read count	:	0
Star read count	:	996

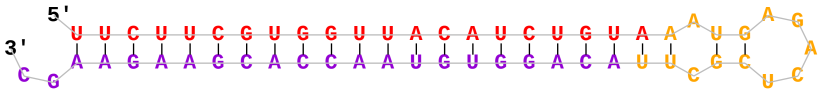

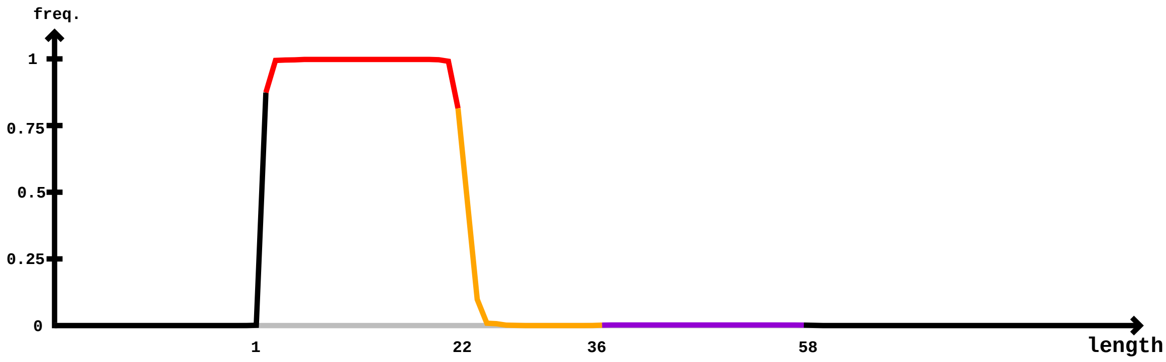

Mature **Star**

```
5'- gaaccaaaacaucagucggcuucuucgugguuacaucuguaaaugagacucgcuuacagguguaaccacgaagaagccgacacccguuuugcuuugcaugcaucucuugaa  -3'  obs
    gaaccaaaacaucagucggcuucuucgugguuacaucuguaaaugagacucgcuuacagguguaaccacgaagaagccgacacccguuuugcuuugcaugcaucucuugaa       exp
    (((.((((((....((((((((((((((((((((((((((((.((.....)).))))))))))))))))))))))))))))....)))))).)))................  reads  mm  sample
    ...........uUagucggcuucuucgug..................................................................................  1  1  sr1
    ...........ucagucggcuucuucgugg.................................................................................  1  0  sr1
    ...........ucagucggcuucuucgugA.................................................................................  1  1  sr1
    ...........ucagucggcuucuucgugguua..............................................................................  1  0  sr1
    ...............ucggcuucuucgugguuG..............................................................................  1  1  sr1
    ...............ucggcuucuucgugguAa..............................................................................  1  1  sr1
    ...............ucggcuucuucgugguuacau...........................................................................  2  0  sr1
    ..................Ucuucuucgugguuacaucugu.......................................................................  2  1  sr1
    ..................Ucuucuucgugguuacaucugua......................................................................  11  1  sr1
    ..................gcuucuucgugguuacaucuguaa.....................................................................  1  0  sr1
    ..................Ucuucuucgugguuacaucuguaa.....................................................................  2  1  sr1
    ...................cuucuucgugguuacaucu.........................................................................  1  0  sr1
    ...................cuucuucgugguuacaucug........................................................................  1  0  sr1
    ...................cuucuucgugguuacaucuA........................................................................  1  1  sr1
    ...................cuucuucgugguuacaucugC.......................................................................  10  1  sr1
    ...................cuucuucgugguuacaucugG.......................................................................  1  1  sr1
    ...................cuucuucgugguCacaucugu.......................................................................  1  1  sr1
    ...................cuucuucgugguuacaucugu.......................................................................  57  0  sr1
    ...................cuucuAcgugguuacaucugu.......................................................................  1  1  sr1
    ...................cuucuucgugguuaUaucugua......................................................................  1  1  sr1
    ...................cuCcuucgugguuacaucugua......................................................................  1  1  sr1
    ...................cuucuucgugguuacaucugua......................................................................  156  0  sr1
    ...................Guucuucgugguuacaucugua......................................................................  1  1  sr1
    ...................cuucCucgugguuacaucugua......................................................................  1  1  sr1
    ...................cuucuucgugguuacaucuguG......................................................................  11  1  sr1
    ...................cuucCucgugguuacaucuguaa.....................................................................  1  1  sr1
    ...................cuucuucgugguuacaucuguaa.....................................................................  44  0  sr1
    ...................cuucuucgugguCacaucuguaa.....................................................................  1  1  sr1
    ...................cuucuucgugguuacaucuguGa.....................................................................  1  1  sr1
    ...................cuucuucgugguuacaucuguaU.....................................................................  3  1  sr1
    ...................cuucuucgugguuacaucuguaG.....................................................................  1  1  sr1
    ...................cuucuucgCgguuacaucuguaa.....................................................................  1  1  sr1
    ...................Guucuucgugguuacaucuguaa.....................................................................  2  1  sr1
```

```
                    Mature                         Star
gaaccaaaacaucagucggcuucuucgugguuacaucuguaaaugagacucgcuuacagguguaaccacgaagaagccgacacccguuuugcuuugcaugcaucucuugaa
...................cuucuucgugguuacaucuguaUa.................................................................... 5      1  sr1
...................cuucuucgugguuacaucuguaaa.................................................................... 3      0  sr1
....................uucuucguggAuacaucu......................................................................... 1      1  sr1
....................uCcuucgugguuacaucu......................................................................... 1      1  sr1
....................uucuucgAgguuacaucu......................................................................... 1      1  sr1
....................uucuucgugguuacaucA......................................................................... 20     1  sr1
....................uucuucgugguuacaucC......................................................................... 34     1  sr1
....................uucuucgugguCacaucu......................................................................... 1      1  sr1
....................uucuCcgugguuacaucu......................................................................... 4      1  sr1
....................Aucuucgugguuacaucu......................................................................... 1      1  sr1
....................uucuucgugguuacaucG......................................................................... 13     1  sr1
....................uucCucgugguuacaucu......................................................................... 1      1  sr1
....................Gucuucgugguuacaucu......................................................................... 15     1  sr1
....................uucuucgugguuacaucu......................................................................... 302    0  sr1
....................Cucuucgugguuacaucu......................................................................... 1      1  sr1
....................uuUuucgugguuacaucug........................................................................ 3      1  sr1
....................uucuucgugguuacaucAg........................................................................ 40     1  sr1
....................uucuucgugguuacaucCg........................................................................ 3      1  sr1
....................uucCucgugguuacaucug........................................................................ 8      1  sr1
....................uucuucguAguuacaucug........................................................................ 2      1  sr1
....................uucuucgugguuacaucug........................................................................ 1588   0  sr1
....................uuAuucgugguuacaucug........................................................................ 1      1  sr1
....................uCcuucgugguuacaucug........................................................................ 3      1  sr1
....................Cucuucgugguuacaucug........................................................................ 2      1  sr1
....................uucuucguggCuacaucug........................................................................ 5      1  sr1
....................uucuCcgugguuacaucug........................................................................ 1      1  sr1
....................uucuucgugguuacaucuU........................................................................ 11     1  sr1
....................uucuuUgugguuacaucug........................................................................ 1      1  sr1
....................uucuucgugguCacaucug........................................................................ 1      1  sr1
....................uucuucgugguuacGucug........................................................................ 5      1  sr1
....................uucuucgugguuGcaucug........................................................................ 2      1  sr1
....................uucuucgCgguuacaucug........................................................................ 3      1  sr1
....................uucuucguUguuacaucug........................................................................ 1      1  sr1
....................Gucuucgugguuacaucug........................................................................ 44     1  sr1
....................Aucuucgugguuacaucug........................................................................ 1      1  sr1
....................uucuucgugguuacauGug........................................................................ 2      1  sr1
....................uucuucgugguuacauAug........................................................................ 1      1  sr1
....................uucuuAgugguuacaucug........................................................................ 1      1  sr1
....................uucuucgugguuacaucuA........................................................................ 86     1  sr1
....................uucuucgugguuacaucuC........................................................................ 2      1  sr1
....................uucuucgugguuacaucGg........................................................................ 2      1  sr1
....................uAcuucgugguuacaucug........................................................................ 1      1  sr1
....................uucuucgugguGacaucugu....................................................................... 9      1  sr1
....................uucuucgugguuacCucugu....................................................................... 1      1  sr1
....................uucuucgugUuacaucugu........................................................................ 2      1  sr1
....................uucuucgugguuacaucGgu....................................................................... 11     1  sr1
....................uAcuucgugguuacaucugu....................................................................... 15     1  sr1
....................uucuucgugguuacaucugC....................................................................... 4945   1  sr1
....................uucuucguggCuacaucugu....................................................................... 81     1  sr1
....................uucuuUgugguuacaucugu....................................................................... 21     1  sr1
....................uucuCcgugguuacaucugu....................................................................... 122    1  sr1
....................uucuucgugguuacaucugA....................................................................... 915    1  sr1
....................Cucuucgugguuacaucugu....................................................................... 134    1  sr1
....................uucuucgugguuCcaucugu....................................................................... 8      1  sr1
....................uucuucgugguuacaucuCu....................................................................... 22     1  sr1
....................uucuucgugguuacUucugu....................................................................... 5      1  sr1
....................uucuucgugguuUcaucugu....................................................................... 12     1  sr1
....................Aucuucgugguuacaucugu....................................................................... 31     1  sr1
....................uucuucgugguuacaucugu....................................................................... 41939  0  sr1
....................uucuucgugguuacauUugu....................................................................... 26     1  sr1
....................uucuGcgugguuacaucugu....................................................................... 1      1  sr1
....................uucuucgCgguuacaucugu....................................................................... 79     1  sr1
....................uucAucgugguuacaucugu....................................................................... 18     1  sr1
....................uucuucAugguuacaucugu....................................................................... 25     1  sr1
....................uucuucgugguuaUaucugu....................................................................... 36     1  sr1
....................uucuucgugguuaAaucugu....................................................................... 2      1  sr1
....................uucCucgugguuacaucugu....................................................................... 125    1  sr1
....................uucuucguUguuacaucugu....................................................................... 5      1  sr1
....................uucuucgugguuacGucugu....................................................................... 144    1  sr1
....................uucuucguAguuacaucugu....................................................................... 33     1  sr1
```

```
                    Mature                            Star
gaaccaaaacaucagucggcuucuucgugguuacaucuguaaaugagacucgcuuacagguguaaccacgaagaagccgacacccguuuugcuuugcaugcaucucuugaa
....................Gucuucgugguuacaucugu.......................................................................   1068    1   sr1
....................uucuucgugguuGcaucugu.......................................................................   107     1   sr1
....................uucuucguggAuacaucugu.......................................................................   11      1   sr1
....................uucuucgugguuacaucuUu.......................................................................   4       1   sr1
....................uucuucCugguuacaucugu.......................................................................   2       1   sr1
....................uucuucgugguAacaucugu.......................................................................   15      1   sr1
....................uucuuGgugguuacaucugu.......................................................................   1       1   sr1
....................uucuucgugguuacauAugu.......................................................................   16      1   sr1
....................uucuucgugAuuacaucugu.......................................................................   30      1   sr1
....................uuUuucgugguuacaucugu.......................................................................   24      1   sr1
....................uuAuucgugguuacaucugu.......................................................................   10      1   sr1
....................uucuucguggGuacaucugu.......................................................................   6       1   sr1
....................uucuucgugguuacaucCgu.......................................................................   98      1   sr1
....................uucuucgugguCacaucugu.......................................................................   106     1   sr1
....................uCcuucgugguuacaucugu.......................................................................   103     1   sr1
....................uucGucgugguuacaucugu.......................................................................   15      1   sr1
....................uucuucgugguuacaucAgu.......................................................................   27      1   sr1
....................uucuucgugguuacauGugu.......................................................................   5       1   sr1
....................uucuucgGgguuacaucugu.......................................................................   12      1   sr1
....................uucuucgugguuacaAcugu.......................................................................   16      1   sr1
....................uucuucguCguuacaucugu.......................................................................   1       1   sr1
....................uucuuAgugguuacaucugu.......................................................................   10      1   sr1
....................uucuucgAgguuacaucugu.......................................................................   20      1   sr1
....................uucuAcgugguuacaucugu.......................................................................   26      1   sr1
....................uGcuucgugguuacaucugu.......................................................................   2       1   sr1
....................uucuucgugguuacaGcugu.......................................................................   18      1   sr1
....................uucuucgugguuacaucugG.......................................................................   1854    1   sr1
....................uucuucUugguuacaucugu.......................................................................   2       1   sr1
....................uucuucgugguuacaucuAu.......................................................................   22      1   sr1
....................uucuucCugguuacaucugua......................................................................   14      1   sr1
....................uucuucUugguuacaucugua......................................................................   15      1   sr1
....................uucuucgugguuacaucuUua......................................................................   16      1   sr1
....................Gucuucgugguuacaucugua......................................................................   3874    1   sr1
....................uucuucgugguuCcaucugua......................................................................   13      1   sr1
....................uucuucgugguuaGaucugua......................................................................   1       1   sr1
....................uucuucgugguuaAaucugua......................................................................   6       1   sr1
....................uucuucgugguuacauAugua......................................................................   33      1   sr1
....................uucuucguAguuacaucugua......................................................................   89      1   sr1
....................uucuucgugAuuacaucugua......................................................................   71      1   sr1
....................uucuGcgugguuacaucugua......................................................................   7       1   sr1
....................uucuucgAgguuacaucugua......................................................................   26      1   sr1
....................uucuucgugguAacaucugua......................................................................   50      1   sr1
....................uucuucgugguuacaucugua......................................................................   133942  0   sr1
....................uucuucgCgguuacaucugua......................................................................   209     1   sr1
....................uucuucgugguuacaucAgua......................................................................   63      1   sr1
....................uucuucguCguuacaucugua......................................................................   4       1   sr1
....................uucGucgugguuacaucugua......................................................................   50      1   sr1
....................uucuucgugguuacGucugua......................................................................   439     1   sr1
....................uucuucgugguuacCucugua......................................................................   13      1   sr1
....................uucCucgugguuacaucugua......................................................................   426     1   sr1
....................uucuucgugguuacaucuCua......................................................................   3       1   sr1
....................Cucuucgugguuacaucugua......................................................................   467     1   sr1
....................uucuucgugguuacaucGgua......................................................................   19      1   sr1
....................uucuucgugguuaUaucugua......................................................................   113     1   sr1
....................uucuucgugguuacaucuguG.....................................................................   3729    1   sr1
....................uucuucguggCuacaucugua......................................................................   324     1   sr1
....................uucuucgugguuacUucugua......................................................................   41      1   sr1
....................uAcuucgugguuacaucugua......................................................................   42      1   sr1
....................uucuucAugguuacaucugua......................................................................   82      1   sr1
....................uucuucguUguuacaucugua......................................................................   27      1   sr1
....................uucuucgugguuacaucugCa......................................................................   404     1   sr1
....................uuGuucgugguuacaucugua......................................................................   2       1   sr1
....................uucuucgugguuacaucuguU.....................................................................   512     1   sr1
....................uucuucgugguuacaucuguC.....................................................................   78      1   sr1
....................uucuCcgugguuacaucugua......................................................................   367     1   sr1
....................uucuucgugguuacaucuAua......................................................................   73      1   sr1
....................Aucuucgugguuacaucugua......................................................................   82      1   sr1
....................uucuucgugguuUcaucugua......................................................................   26      1   sr1
....................uucuucgugguuacauGugua......................................................................   4       1   sr1
....................uucuucgugguuacaGcugua......................................................................   39      1   sr1
```

```
                    Mature                Star
gaaccaaaacaucagucggcuucuucgugguuacaucuguaaaugagacucgcuuacagguguaaccacgaagaagccgacacccguuuugcuuugcaugcaucucuugaa
....................uGcuucgugguuacaucugua......................................................................    13      1   sr1
....................uucuucgugguuacaucugAa......................................................................    46      1   sr1
....................uucuuAgugguuacaucugua......................................................................    30      1   sr1
....................uucuucgugCuuacaucugua......................................................................    2       1   sr1
....................uucuucgGgguuacaucugua......................................................................    33      1   sr1
....................uucuucgugguuacauUugua......................................................................    71      1   sr1
....................uucuuUgugguuacaucugua......................................................................    96      1   sr1
....................uuAuucgugguuacaucugua......................................................................    19      1   sr1
....................uucuucgugUuuacaucugua......................................................................    20      1   sr1
....................uucuAcgugguuacaucugua......................................................................    74      1   sr1
....................uCcuucgugguuacaucugua......................................................................    375     1   sr1
....................uucuucguggGuacaucugua......................................................................    24      1   sr1
....................uucAucgugguuacaucugua......................................................................    62      1   sr1
....................uucuucguggAuacaucugua......................................................................    53      1   sr1
....................uucuucgugguuacaAcugua......................................................................    48      1   sr1
....................uucuucgugguuacaucugGa......................................................................    72      1   sr1
....................uucuucgugguuacaucCgua......................................................................    373     1   sr1
....................uucuucgugguuGcaucugua......................................................................    297     1   sr1
....................uucuucgugguCacaucugua......................................................................    272     1   sr1
....................uucuucgugguGacaucugua......................................................................    9       1   sr1
....................uuUuucgugguuacaucugua......................................................................    63      1   sr1
....................uucuucguCguuacaucuguaa.....................................................................    2       1   sr1
....................uucuucgugguuacaucCguaa.....................................................................    176     1   sr1
....................uucuCcgugguuacaucuguaa.....................................................................    153     1   sr1
....................uucuucgugguuacaucAguaa.....................................................................    37      1   sr1
....................uucuucgugguuaAaucuguaa.....................................................................    2       1   sr1
....................uucuucgugguuUcaucuguaa.....................................................................    12      1   sr1
....................uucuucgugguuacauUuguaa.....................................................................    32      1   sr1
....................uucCucgugguuacaucuguaa.....................................................................    143     1   sr1
....................uGcuucgugguuacaucuguaa.....................................................................    5       1   sr1
....................uucuucguggCuacaucuguaa.....................................................................    126     1   sr1
....................uucuucgugguuacaucuguaG.....................................................................    1713    1   sr1
....................uucuucgugguuacaucugAaa.....................................................................    8       1   sr1
....................uucuuUgugguuacaucuguaa.....................................................................    43      1   sr1
....................uucuucgugguuacaucuguaC.....................................................................    522     1   sr1
....................uucuucgugguuGcaucuguaa.....................................................................    287     1   sr1
....................Aucuucgugguuacaucuguaa.....................................................................    28      1   sr1
....................uucuucgugguuacaGcuguaa.....................................................................    9       1   sr1
....................uucuucgugguuacaucuCuaa.....................................................................    4       1   sr1
....................uucuucgugguuacaucGguaa.....................................................................    15      1   sr1
....................uucuucgugguuacaucugGaa.....................................................................    3       1   sr1
....................uucuucgugguuacCucuguaa.....................................................................    5       1   sr1
....................uucuucgugguuacaucugCaa.....................................................................    70      1   sr1
....................uucGucgugguuacaucuguaa.....................................................................    10      1   sr1
....................uucuucgugguuacaAcuguaa.....................................................................    19      1   sr1
....................uucuucgugguGacaucuguaa.....................................................................    2       1   sr1
....................uucuucgugguuacaucuguCa.....................................................................    9       1   sr1
....................uucuucgugguCacaucuguaa.....................................................................    110     1   sr1
....................Gucuucgugguuacaucuguaa.....................................................................    1385    1   sr1
....................uucuucgugguuacaucuguaU.....................................................................    3307    1   sr1
....................uucuucUugguuacaucuguaa.....................................................................    4       1   sr1
....................Cucuucgugguuacaucuguaa.....................................................................    160     1   sr1
....................uucuucgugguuacaucuguaa.....................................................................    48638   0   sr1
....................uucuucgugguuacauAuguaa.....................................................................    15      1   sr1
....................uucuucgAgguuacaucuguaa.....................................................................    12      1   sr1
....................uucuucgugguuacUucuguaa.....................................................................    13      1   sr1
....................uuAuucgugguuacaucuguaa.....................................................................    10      1   sr1
....................uCcuucgugguuacaucuguaa.....................................................................    112     1   sr1
....................uuGuucgugguuacaucuguaa.....................................................................    2       1   sr1
....................uucAucgugguuacaucuguaa.....................................................................    25      1   sr1
....................uucuGcgugguuacaucuguaa.....................................................................    3       1   sr1
....................uucuucgGgguuacaucuguaa.....................................................................    11      1   sr1
....................uucuucguggGuacaucuguaa.....................................................................    5       1   sr1
....................uucuucgugAuuacaucuguaa.....................................................................    24      1   sr1
....................uucuucguggAuacaucuguaa.....................................................................    13      1   sr1
....................uucuuAgugguuacaucuguaa.....................................................................    13      1   sr1
....................uAcuucgugguuacaucuguaa.....................................................................    22      1   sr1
....................uucuucgugguuacaucuUuaa.....................................................................    2       1   sr1
....................uucuAcgugguuacaucuguaa.....................................................................    22      1   sr1
....................uucuucgugguuacaucuguUa.....................................................................    64      1   sr1
```

```
                    Mature                                      Star
gaaccaaaacaucagucggcuucuucgugguuacaucuguaaaugagacucgcuuacagguguaaccacgaagaagccgacacccguuuugcuuugcaugcaucucuugaa
....................uucuucgugguuaUaucuguaa.....................................................................    54     1    sr1
....................uucuucgCgguuacaucuguaa.....................................................................    89     1    sr1
....................uucuucguAguuacaucuguaa.....................................................................    31     1    sr1
....................uucuucgugUuuacaucuguaa.....................................................................    7      1    sr1
....................uucuucgugguuCcaucuguaa.....................................................................    4      1    sr1
....................uucuucgugguuacaucuguGa.....................................................................    125    1    sr1
....................uuUuucgugguuacaucuguaa.....................................................................    17     1    sr1
....................uucuucCugguuacaucuguaa.....................................................................    3      1    sr1
....................uucuucgugguAacaucuguaa.....................................................................    20     1    sr1
....................uucuucAugguuacaucuguaa.....................................................................    42     1    sr1
....................uucuucguUguuacaucuguaa.....................................................................    10     1    sr1
....................uucuucgugguuacaucuAuaa.....................................................................    14     1    sr1
....................uucuucgugguuacGucuguaa.....................................................................    166    1    sr1
....................uucuucgugguCacaucuguaaa....................................................................    15     1    sr1
....................uucuucguggCuacaucuguaaa....................................................................    10     1    sr1
....................uucuucgugguuacaucuguaaG....................................................................    164    1    sr1
....................uucuuUgugguuacaucuguaaa....................................................................    4      1    sr1
....................uucuucgugguuacauGuguaaa....................................................................    1      1    sr1
....................uCcuucgugguuacaucuguaaa....................................................................    16     1    sr1
....................uucuucgugguuacaucuguaCa....................................................................    106    1    sr1
....................uucuucgugguuacaucGguaaa....................................................................    2      1    sr1
....................uucuucgugguuGcaucuguaaa....................................................................    15     1    sr1
....................uucuucgugguuCcaucuguaaa....................................................................    1      1    sr1
....................Aucuucgugguuacaucuguaaa....................................................................    4      1    sr1
....................uGcuucgugguuacaucuguaaa....................................................................    1      1    sr1
....................uucuucgugguuaUaucuguaaa....................................................................    8      1    sr1
....................uucuucguUguuacaucuguaaa....................................................................    2      1    sr1
....................uucuucgCgguuacaucuguaaa....................................................................    11     1    sr1
....................uucuucgugguuacaucuguGaa....................................................................    7      1    sr1
....................uAcuucgugguuacaucuguaaa....................................................................    7      1    sr1
....................uucuucgugguuacaucAguaaa....................................................................    3      1    sr1
....................uucuucgugguuacaucuAuaaa....................................................................    2      1    sr1
....................uucuucCugguuacaucuguaaa....................................................................    1      1    sr1
....................uucuucgugguuacGucuguaaa....................................................................    22     1    sr1
....................uuAuucgugguuacaucuguaaa....................................................................    1      1    sr1
....................uucuucgugguuacaucuguaaa....................................................................    4755   0    sr1
....................uucuucgugguuacaucuguaaU....................................................................    146    1    sr1
....................uucuucguggGuacaucuguaaa....................................................................    1      1    sr1
....................uucuucAugguuacaucuguaaa....................................................................    1      1    sr1
....................uucuucgugguAacaucuguaaa....................................................................    1      1    sr1
....................uucuucgugguuacaucuguUaa....................................................................    17     1    sr1
....................uucuAcgugguuacaucuguaaa....................................................................    1      1    sr1
....................uucCucgugguuacaucuguaaa....................................................................    9      1    sr1
....................uucuCcgugguuacaucuguaaa....................................................................    14     1    sr1
....................uucAucgugguuacaucuguaaa....................................................................    2      1    sr1
....................uucuucgugguuacaucCguaaa....................................................................    13     1    sr1
....................Cucuucgugguuacaucuguaaa....................................................................    26     1    sr1
....................uucuucgugguuacaucugCaaa....................................................................    5      1    sr1
....................uucuucgugguuacaGcuguaaa....................................................................    1      1    sr1
....................uucuucgugguuacCucuguaaa....................................................................    1      1    sr1
....................uucuucgugguuacaucuguaUa....................................................................    8156   1    sr1
....................uucuucgugguuacauUuguaaa....................................................................    3      1    sr1
....................Gucuucgugguuacaucuguaaa....................................................................    127    1    sr1
....................uucuucgugguuacaucuguaGa....................................................................    24     1    sr1
....................uucuucgugguuacaucuguaaC....................................................................    34     1    sr1
....................uucuucgugguuacaAcuguaaa....................................................................    1      1    sr1
....................uucuucgugguuacaCcuguaaa....................................................................    4      1    sr1
....................uucuucgugAuuacaucuguaaa....................................................................    1      1    sr1
....................uucuuAgugguuacaucuguaaa....................................................................    1      1    sr1
....................uucuucgAgguuacaucuguaaa....................................................................    2      1    sr1
....................uucGucgugguuacaucuguaaa....................................................................    2      1    sr1
....................uucuucguAguuacaucuguaaa....................................................................    5      1    sr1
....................uucuucgugguuacaucuguCaa....................................................................    2      1    sr1
....................uucuucgugguuacaucuguGaau...................................................................    1      1    sr1
....................uucuucgugguuacaucuguaaGu...................................................................    2      1    sr1
....................uucuucgugguuacaucuguaaaG...................................................................    12     1    sr1
....................uucuucgugguuacaucuguaaUu...................................................................    15     1    sr1
....................uucuucgugguuacaucuguaaaA...................................................................    44     1    sr1
....................uucuucgugguuacaucuguaaaC...................................................................    14     1    sr1
....................uucuucgCgguuacaucuguaaau...................................................................    1      1    sr1
```

```
                    Mature                              Star
gaaccaaaacaucagucggcuucuucgugguuacaucuguaaaugagacucgcuuacagguguaaccacgaagaagccgacacccguuuugcuuugcaugcaucucuugaa
....................uucuucgugguuacaucuguaUau...................................................................    38     1     sr1
....................uuUuucgugguuacaucuguaaau...................................................................    1      1     sr1
....................uucuucgugguuacaucuguaaCu...................................................................    2      1     sr1
....................Gucuucgugguuacaucuguaaau...................................................................    3      1     sr1
....................uucuucgugguuacaucuguaaau...................................................................    80     0     sr1
....................uucuucguggCuacaucuguaaau...................................................................    1      1     sr1
....................uucuucgugguuaUaucuguaaaug..................................................................    1      1     sr1
....................uucuucgugguuacaucuguaaauA..................................................................    215    1     sr1
....................uucuucgugguuacGucuguaaaug..................................................................    1      1     sr1
....................uucuucgugguuacaucuguaaGug..................................................................    2      1     sr1
....................uucuucgugguuacaucuguGaaug..................................................................    1      1     sr1
....................uucuucgugguuacaucuguaGaug..................................................................    1      1     sr1
....................uucuucgugguuacaucuguaaaAg..................................................................    1      1     sr1
....................uucuucgugguCacaucuguaaaug..................................................................    2      1     sr1
....................uCcuucgugguuacaucuguaaaug..................................................................    1      1     sr1
....................uucuucgugguuacaucugCaaaug..................................................................    1      1     sr1
....................uucuucguAguuacaucuguaaaug..................................................................    1      1     sr1
....................ucAucgugguuacaucuguaaaug..................................................................    1      1     sr1
....................uucuucgugguuacaucuguaaauC..................................................................    1      1     sr1
....................uucuucgugguuacaucCguaaaug..................................................................    2      1     sr1
....................uucuucgugguuacaucuguaUaug..................................................................    7      1     sr1
....................Cucuucgugguuacaucuguaaaug..................................................................    2      1     sr1
....................uucuucgugguuacaucuguaaaCg..................................................................    4      1     sr1
....................Gucuucgugguuacaucuguaaaug..................................................................    23     1     sr1
....................uucuucgugguuGcaucuguaaaug..................................................................    2      1     sr1
....................uucuCcgugguuacaucuguaaaug..................................................................    1      1     sr1
....................uucCucgugguuacaucuguaaaug..................................................................    3      1     sr1
....................uucuucgugguuacaucuguaCaug..................................................................    1      1     sr1
....................uucuucgugguuacaCcuguaaaug..................................................................    5      1     sr1
....................uucuucgugguuacaucuguaaaug..................................................................    634    0     sr1
....................uucuucguggCuacaucuguaaaug..................................................................    1      1     sr1
....................uucuAcgugguuacaucuguaaaug..................................................................    2      1     sr1
....................uucuucgugguuacaucugAaaaug..................................................................    1      1     sr1
....................uucuucgCgguuacaucuguaaaug..................................................................    2      1     sr1
....................uucuucAugguuacaucuguaaaug..................................................................    2      1     sr1
....................uucuucgugguuacaucuguaaaGg..................................................................    1      1     sr1
....................uucuucgugguuacaucuguaaCug..................................................................    1      1     sr1
....................uucuucgugguuacaucuguaaauU..................................................................    13     1     sr1
....................uucuucgugguuacaucuguaaauAa.................................................................    2      1     sr1
....................uucuucguggCuacaucuguaaauga.................................................................    1      1     sr1
....................Gucuucgugguuacaucuguaaauga.................................................................    6      1     sr1
....................uucuucgugguuacaCcuguaaauga.................................................................    1      1     sr1
....................Cucuucgugguuacaucuguaaauga.................................................................    1      1     sr1
....................uucuucgugguuacaucuguaaauga.................................................................    173    0     sr1
....................uucuucgugguCacaucuguaaauga.................................................................    1      1     sr1
....................uucuucgugguuacaucuguaaaugG.................................................................    8      1     sr1
....................uucuucgugAuuacaucuguaaauga.................................................................    1      1     sr1
....................uucuucgugguuGcaucuguaaauga.................................................................    2      1     sr1
....................uucuucgugguuacaucuguaaauUa.................................................................    5      1     sr1
....................uucuucgugguuacaucuguaaaugU.................................................................    5      1     sr1
....................uucuucgugguuacaucuguaaUuga.................................................................    1      1     sr1
....................Gucuucgugguuacaucuguaaaugag................................................................    4      1     sr1
....................uucuucgugguuacaucuguaaaugag................................................................    115    0     sr1
....................uucuucgugguuacGucuguaaaugag................................................................    1      1     sr1
....................uucuucgugguuacaucuguGaaugag................................................................    1      1     sr1
....................Cucuucgugguuacaucuguaaaugag................................................................    2      1     sr1
....................uCcuucgugguuacaucuguaaaugag................................................................    1      1     sr1
....................uucuucgugguuacaucuguaaaugaA................................................................    16     1     sr1
....................uucuucgugguuacaucuguaaaugaC................................................................    2      1     sr1
....................uucuucgugguuacaucuguaaaugaU................................................................    1      1     sr1
.....................ucuucgugguuacaucuA........................................................................    7      1     sr1
.....................ucuucgugguuacaucAg........................................................................    4      1     sr1
.....................ucuucgugguuacaucug........................................................................    50     0     sr1
.....................ucuucgugguuacaucuC........................................................................    1      1     sr1
.....................Gcuucgugguuacaucug........................................................................    2      1     sr1
.....................ucuucgugguuacaucugG.......................................................................    206    1     sr1
.....................ucuucguggCuacaucugu.......................................................................    16     1     sr1
.....................ucuucgugguuacauAugu.......................................................................    3      1     sr1
.....................ucuucgugguuacaAcugu.......................................................................    1      1     sr1
.....................Acuucgugguuacaucugu.......................................................................    3      1     sr1
```

Mature   Star

```
gaaccaaaacaucagucggcuucuucgugguuacaucuguaaaugagacucgcuuacagguguaaccacgaagaagccgacacccguuuugcuuugcaugcaucucuugaa
.....................ucuucgugguuacaucugu.......................................................................	4136	0	sr1
.....................uUuucgugguuacaucugu.......................................................................	2	1	sr1
.....................ucuucgugguuacauUugu.......................................................................	4	1	sr1
.....................ucuucgugguCacaucugu.......................................................................	10	1	sr1
.....................ucuucgugguuacaucCgu.......................................................................	10	1	sr1
.....................ucuucgugguAacaucugu.......................................................................	1	1	sr1
.....................ucuucgugguuCcaucugu.......................................................................	2	1	sr1
.....................ucuucgGgguuacaucugu.......................................................................	2	1	sr1
.....................ucuucgugguuacaucugC.......................................................................	517	1	sr1
.....................ucuuAgugguuacaucugu.......................................................................	1	1	sr1
.....................ucuGcgugguuacaucugu.......................................................................	1	1	sr1
.....................ucuucgugguuGcaucugu.......................................................................	11	1	sr1
.....................ucuucgCgguuacaucugu.......................................................................	10	1	sr1
.....................ucuucguAguuacaucugu.......................................................................	2	1	sr1
.....................ucuucgugguuaUaucugu.......................................................................	3	1	sr1
.....................ucuucgAgguuacaucugu.......................................................................	2	1	sr1
.....................ucuAcgugguuacaucugu.......................................................................	3	1	sr1
.....................ucCucgugguuacaucugu.......................................................................	16	1	sr1
.....................ucuucgugguuacaucGgu.......................................................................	4	1	sr1
.....................ucuucgugguuacGucugu.......................................................................	12	1	sr1
.....................ucuuUgugguuacaucugu.......................................................................	2	1	sr1
.....................Ccuucgugguuacaucugu.......................................................................	7	1	sr1
.....................ucuucgugguuacaucuCu.......................................................................	5	1	sr1
.....................ucGucgugguuacaucugu.......................................................................	1	1	sr1
.....................Gcuucgugguuacaucugu.......................................................................	160	1	sr1
.....................ucuucgugguuacaucugA.......................................................................	105	1	sr1
.....................ucuucgugguuacaucuAu.......................................................................	1	1	sr1
.....................uAuucgugguuacaucugu.......................................................................	1	1	sr1
.....................ucAucgugguuacaucugu.......................................................................	1	1	sr1
.....................ucuCcgugguuacaucugu.......................................................................	18	1	sr1
.....................ucuucgugAuuacaucugu.......................................................................	1	1	sr1
.....................ucuucAugguuacaucugu.......................................................................	1	1	sr1
.....................ucuucgugguuacaucugua......................................................................	7647	0	sr1
.....................ucuucgugguuGcaucugua......................................................................	23	1	sr1
.....................ucuucgugguuacaucugGa......................................................................	6	1	sr1
.....................ucuucgugguuacaucuguG......................................................................	200	1	sr1
.....................ucuucgugguuacauGugua......................................................................	3	1	sr1
.....................ucuucgugguAacaucugua......................................................................	3	1	sr1
.....................Ccuucgugguuacaucugua......................................................................	14	1	sr1
.....................ucuucguAguuacaucugua......................................................................	11	1	sr1
.....................ucuucgugguuacaucuAua......................................................................	9	1	sr1
.....................ucuuUgugguuacaucugua......................................................................	6	1	sr1
.....................ucCucgugguuacaucugua......................................................................	32	1	sr1
.....................ucuucgugCuuacaucugua......................................................................	1	1	sr1
.....................ucuucgugguuacaucugCa......................................................................	24	1	sr1
.....................uAuucgugguuacaucugua......................................................................	1	1	sr1
.....................ucuucguggGuacaucugua......................................................................	2	1	sr1
.....................ucuucgugguuacaucuguC......................................................................	6	1	sr1
.....................ucuGcgugguuacaucugua......................................................................	1	1	sr1
.....................ucuucgugguCacaucugua......................................................................	17	1	sr1
.....................ucuAcgugguuacaucugua......................................................................	4	1	sr1
.....................ucuucgugAuuacaucugua......................................................................	2	1	sr1
.....................ucuucgugguuacauUugua......................................................................	3	1	sr1
.....................ucuucguggCuacaucugua......................................................................	19	1	sr1
.....................ucuucgugguuaAaucugua......................................................................	1	1	sr1
.....................ucuucgugguuacaucuguU......................................................................	42	1	sr1
.....................ucuucguggAuacaucugua......................................................................	2	1	sr1
.....................ucuucAugguuacaucugua......................................................................	5	1	sr1
.....................ucuucgugguuacUucugua......................................................................	5	1	sr1
.....................uUuucgugguuacaucugua......................................................................	10	1	sr1
.....................Gcuucgugguuacaucugua......................................................................	329	1	sr1
.....................ucuucgugguuacaucAgua......................................................................	4	1	sr1
.....................ucuucgugguuacaucuCua......................................................................	1	1	sr1
.....................ucuucgugguuaUaucugua......................................................................	3	1	sr1
.....................Acuucgugguuacaucugua......................................................................	10	1	sr1
.....................ucuucgugguuacaucCgua......................................................................	22	1	sr1
.....................ucuucgugguuacauAugua......................................................................	6	1	sr1
.....................ucuucgugguuUcaucugua......................................................................	2	1	sr1
.....................ucuucgugguuacaucGgua......................................................................	1	1	sr1
.....................ucuucgugguuacaAcugua......................................................................	3	1	sr1
```

Mature **Star**

```
gaaccaaaacaucagucggcuucuucgugguuacaucuguaaaugagacucgcuuacagguguaaccacgaagaagccgacacccguuuugcuuugcaugcaucucuugaa
.....................ucuucgCgguuacaucugua......................................................................  16     1  sr1
.....................ucuCcgugguuacaucugua......................................................................  25     1  sr1
.....................ucuucgugguuacGucugua......................................................................  29     1  sr1
.....................ucuucgugguuCcaucugua......................................................................  1      1  sr1
.....................uUuucgugguuacaucuguaa.....................................................................  14     1  sr1
.....................ucuucgugguuGcaucuguaa.....................................................................  164    1  sr1
.....................ucuucguggCuacaucuguaa.....................................................................  32     1  sr1
.....................ucuucguUguuacaucuguaa.....................................................................  2      1  sr1
.....................ucuucguAguuacaucuguaa.....................................................................  13     1  sr1
.....................ucuucgugguuacauGuguaa.....................................................................  1      1  sr1
.....................ucuucgugguuacaucuguaG.....................................................................  356    1  sr1
.....................ucuucgugguuacaucuAuaa.....................................................................  3      1  sr1
.....................ucuCcgugguuacaucuguaa.....................................................................  26     1  sr1
.....................ucuucgugguCacaucuguaa.....................................................................  17     1  sr1
.....................ucuucgugguuacaucCguaa.....................................................................  34     1  sr1
.....................ucuucgugguuacaGcuguaa.....................................................................  2      1  sr1
.....................Acuucgugguuacaucuguaa.....................................................................  7      1  sr1
.....................ucuucgugguGacaucuguaa.....................................................................  3      1  sr1
.....................ucuucgugguuacaucGguaa.....................................................................  2      1  sr1
.....................ucuAcgugguuacaucuguaa.....................................................................  6      1  sr1
.....................ucuucgugguuacauUuguaa.....................................................................  5      1  sr1
.....................ucuucgugguuacaucuUuaa.....................................................................  1      1  sr1
.....................ucuucgugguuacGucuguaa.....................................................................  36     1  sr1
.....................ucuuGgugguuacaucuguaa.....................................................................  1      1  sr1
.....................ucuucgugguuacaucuguaa.....................................................................  10306  0  sr1
.....................ucuucgGgguuacaucuguaa.....................................................................  1      1  sr1
.....................ucuucgugguuacaucAguaa.....................................................................  11     1  sr1
.....................ucuucgugguuacaucugCaa.....................................................................  12     1  sr1
.....................ucuucgugAuuacaucuguaa.....................................................................  7      1  sr1
.....................ucuucgugUuuacaucuguaa.....................................................................  2      1  sr1
.....................uAuucgugguuacaucuguaa.....................................................................  1      1  sr1
.....................ucuucgugguAacaucuguaa.....................................................................  3      1  sr1
.....................ucuucCugguuacaucuguaa.....................................................................  1      1  sr1
.....................ucuucAugguuacaucuguaa.....................................................................  5      1  sr1
.....................ucuuAgugguuacaucuguaa.....................................................................  1      1  sr1
.....................ucAucgugguuacaucuguaa.....................................................................  6      1  sr1
.....................ucuucgAgguuacaucuguaa.....................................................................  2      1  sr1
.....................ucuucgugguuacaucuguaU.....................................................................  735    1  sr1
.....................ucuucgugguuacaucuguUa.....................................................................  6      1  sr1
.....................Ccuucgugguuacaucuguaa.....................................................................  21     1  sr1
.....................ucuucgugguuacaucuguGa.....................................................................  24     1  sr1
.....................ucuucgugguuaUaucuguaa.....................................................................  12     1  sr1
.....................ucuucgugguuacauAuguaa.....................................................................  4      1  sr1
.....................Gcuucgugguuacaucuguaa.....................................................................  401    1  sr1
.....................ucCucgugguuacaucuguaa.....................................................................  34     1  sr1
.....................ucuucUugguuacaucuguaa.....................................................................  2      1  sr1
.....................ucGucgugguuacaucuguaa.....................................................................  2      1  sr1
.....................ucuucgugguuacaucuguCa.....................................................................  3      1  sr1
.....................ucuucgugguuacaucugAaa.....................................................................  3      1  sr1
.....................ucuuUgugguuacaucuguaa.....................................................................  7      1  sr1
.....................ucuucguggAuacaucuguaa.....................................................................  4      1  sr1
.....................ucuucgCgguuacaucuguaa.....................................................................  19     1  sr1
.....................ucuucgugCuuacaucuguaa.....................................................................  1      1  sr1
.....................ucuucgugguuUcaucuguaa.....................................................................  1      1  sr1
.....................ucuucgugguuacUucuguaa.....................................................................  5      1  sr1
.....................ucuucgugguuacaucuguaC.....................................................................  395    1  sr1
.....................ucuucgCgguuacaucuguaaa....................................................................  16     1  sr1
.....................ucuCcgugguuacaucuguaaa....................................................................  31     1  sr1
.....................ucuucguUguuacaucuguaaa....................................................................  2      1  sr1
.....................ucuucguCguuacaucuguaaa....................................................................  2      1  sr1
.....................ucuucgugguuacaucuguaaG....................................................................  245    1  sr1
.....................Gcuucgugguuacaucuguaaa....................................................................  387    1  sr1
.....................ucCucgugguuacaucuguaaa....................................................................  28     1  sr1
.....................ucuucgugguuaUaucuguaaa....................................................................  9      1  sr1
.....................ucuucgugguGacaucuguaaa....................................................................  2      1  sr1
.....................ucuGcgugguuacaucuguaaa....................................................................  2      1  sr1
.....................ucuucgugguuacauGuguaaa....................................................................  2      1  sr1
.....................ucuucgugguuacGucuguaaa....................................................................  44     1  sr1
.....................ucuucgugguuacUucuguaaa....................................................................  2      1  sr1
.....................ucuucgugguuacaucuCuaaa....................................................................  1      1  sr1
```

Mature Star

```
gaaccaaaacaucagucggcuucuucgugguuacaucuguaaaugagacucgcuuacagguguaaccacgaagaagccgacacccguuuugcuuugcaugcaucucuugaa
.....................ucuucgugguCacaucuguaaa....................................................................  29  1  sr1
.....................ucuucguAguuacaucuguaaa....................................................................  13  1  sr1
.....................ucAucgugguuacaucuguaaa....................................................................  4  1  sr1
.....................ucuucgugguuacaucuguaaa....................................................................  10104  0  sr1
.....................ucuucguggAuacaucuguaaa....................................................................  6  1  sr1
.....................ucuucUugguuacaucuguaaa....................................................................  2  1  sr1
.....................ucuuUgugguuacaucuguaaa....................................................................  7  1  sr1
.....................ucuucgugguuacauAuguaaa....................................................................  3  1  sr1
.....................ucuucgugguuacaucugGaaa....................................................................  2  1  sr1
.....................ucuucgugguuGcaucuguaaa....................................................................  47  1  sr1
.....................ucuucgugguuacaucAguaaa....................................................................  8  1  sr1
.....................ucuucgugguuacaucuguaaC....................................................................  29  1  sr1
.....................ucuAcgugguuacaucuguaaa....................................................................  9  1  sr1
.....................ucuucgugguuacaucuguaUa....................................................................  2098  1  sr1
.....................ucuucgugguuacaucuguaGa....................................................................  35  1  sr1
.....................ucuucgGgguuacaucuguaaa....................................................................  2  1  sr1
.....................ucuucgugguuacCucuguaaa....................................................................  2  1  sr1
.....................ucuucgugguuacaucuUuaaa....................................................................  2  1  sr1
.....................uUuucgugguuacaucuguaaa....................................................................  7  1  sr1
.....................Acuucgugguuacaucuguaaa....................................................................  10  1  sr1
.....................uGuucgugguuacaucuguaaa....................................................................  1  1  sr1
.....................ucuucCugguuacaucuguaaa....................................................................  2  1  sr1
.....................Ccuucgugguuacaucuguaaa....................................................................  24  1  sr1
.....................ucuucgugguuacaucCguaaa....................................................................  31  1  sr1
.....................ucuucgugguuacaucuguGaa....................................................................  24  1  sr1
.....................ucuucgAgguuacaucuguaaa....................................................................  5  1  sr1
.....................ucuucgugguuacaucuguaaU....................................................................  113  1  sr1
.....................ucuucgugguuacaAcuguaaa....................................................................  3  1  sr1
.....................ucuucgugguuacaucuguUaa....................................................................  1  1  sr1
.....................ucuucgugguuacaucugCaaa....................................................................  10  1  sr1
.....................ucuucgugguuacauUuguaaa....................................................................  11  1  sr1
.....................ucuucguggCuacaucuguaaa....................................................................  24  1  sr1
.....................ucuucgugguuacaCcuguaaa....................................................................  27  1  sr1
.....................ucuucgugAuuacaucuguaaa....................................................................  3  1  sr1
.....................ucuucgugguAacaucuguaaa....................................................................  7  1  sr1
.....................ucuucgugguuacaucuAuaaa....................................................................  6  1  sr1
.....................ucuucgugguuacaucGguaaa....................................................................  5  1  sr1
.....................ucuuAgugguuacaucuguaaa....................................................................  1  1  sr1
.....................ucuucAugguuacaucuguaaa....................................................................  8  1  sr1
.....................ucuucgugCuuacaucuguaaa....................................................................  1  1  sr1
.....................ucGucgugguuacaucuguaaa....................................................................  1  1  sr1
.....................ucuucgugguuacaucuguaCa....................................................................  234  1  sr1
.....................ucuucgugguuUcaucuguaaa....................................................................  2  1  sr1
.....................ucuucgugguuacaucuguGaau...................................................................  1  1  sr1
.....................ucuucguggCuacaucuguaaau...................................................................  1  1  sr1
.....................ucuucgugguuacaucuguaUau...................................................................  5  1  sr1
.....................ucuucgugguuacaucuguaaau...................................................................  74  0  sr1
.....................ucuucgugguuacaucuguaaUu...................................................................  6  1  sr1
.....................ucuucgugguuacaucuguaaCu...................................................................  2  1  sr1
.....................ucuucgugguuacaucuguaaaA...................................................................  60  1  sr1
.....................ucuucgugguuacaucCguaaau...................................................................  1  1  sr1
.....................ucuucgugguuacaucuguaaaG...................................................................  15  1  sr1
.....................ucuucgugguuacaucuguaaaC...................................................................  18  1  sr1
.....................Gcuucgugguuacaucuguaaau...................................................................  2  1  sr1
.....................ucuucgugguuacaucuguaaaAg..................................................................  1  1  sr1
.....................ucuucgugguuacaucuguaaauA..................................................................  94  1  sr1
.....................ucuucgugguuacaucuguaaaug..................................................................  4  0  sr1
.....................ucuucgugguuacaucuguaaauU..................................................................  3  1  sr1
.....................ucuucgugguuacaucuguaaauUa.................................................................  1  1  sr1
......................cuucgugguuacaucugC.......................................................................  8  1  sr1
......................cuucgugguuacaucugu.......................................................................  53  0  sr1
......................Uuucgugguuacaucugu.......................................................................  2  1  sr1
......................Guucgugguuacaucugu.......................................................................  1  1  sr1
......................cuucgugguuacaucugA.......................................................................  3  1  sr1
......................cuucgugguuacaucugG.......................................................................  2  1  sr1
......................cuucgugguuacaucugua......................................................................  107  0  sr1
......................Uuucgugguuacaucugua......................................................................  17  1  sr1
......................cuucgugguuacaucuguG......................................................................  3  1  sr1
......................cuucguggCuacaucugua......................................................................  2  1  sr1
......................cuucgugguuacaucuguaG.....................................................................  13  1  sr1
```

```
                    Mature                Star
gaaccaaaacaucagucggcuucuucgugguuacaucuguaaaugagacucgcuuacagguguaaccacgaagaagccgacacccguuuugcuuugcaugcaucucuugaa
......................cuucgugguuGcaucuguaa.....................................................................   3     1     sr1
......................cuucgugguuacaucuguaa.....................................................................   172   0     sr1
......................Uuucgugguuacaucuguaa.....................................................................   4     1     sr1
......................Guucgugguuacaucuguaa.....................................................................   1     1     sr1
......................cuucguggAuacaucuguaa.....................................................................   1     1     sr1
......................cuucgugguuacaucGguaa.....................................................................   1     1     sr1
......................cuucgugguuaUaucuguaa.....................................................................   1     1     sr1
......................cuucgugguuacaucAguaa.....................................................................   1     1     sr1
......................cuucgugguuacaucuguaU.....................................................................   6     1     sr1
......................cuucgugguuacaucuguaaa....................................................................   41    0     sr1
......................cuucgugguuacaucuguaCa....................................................................   5     1     sr1
......................Guucgugguuacaucuguaaa....................................................................   4     1     sr1
......................cuucgugguuacaucAguaaa....................................................................   2     1     sr1
......................cuucgugguuGcaucuguaaa....................................................................   1     1     sr1
......................cuucgugguuacaucuguaUa....................................................................   47    1     sr1
......................cuucgugguuacaucuguaGa....................................................................   1     1     sr1
......................cuucgugguCacaucuguaaa....................................................................   1     1     sr1
......................cuucgugguuacaucuguCaa....................................................................   1     1     sr1
......................cuucgugguuacaucuguaaau...................................................................   1     0     sr1
......................cuucgugguuacaucuguaaaA...................................................................   1     1     sr1
......................cuucgugguuacaucuguaaauA..................................................................   2     1     sr1
......................cuucgugguuacaucuguaaauU..................................................................   1     1     sr1
.......................Gucgugguuacaucugua......................................................................   1     1     sr1
.......................uucgugguuacaucugua......................................................................   34    0     sr1
.......................uucgCgguuacaucugua......................................................................   1     1     sr1
.......................uucgugguuacaucuguaG.....................................................................   3     1     sr1
.......................uucgugAuuacaucuguaa.....................................................................   1     1     sr1
.......................Gucgugguuacaucuguaa.....................................................................   1     1     sr1
.......................uucgugguuacaucuguaa.....................................................................   50    0     sr1
.......................uucgugguuacGucuguaa.....................................................................   2     1     sr1
.......................uucgugguuacaucuguGa.....................................................................   1     1     sr1
.......................uucgugguuacaucuguaC.....................................................................   3     1     sr1
.......................uucgugguuacaucAguaa.....................................................................   1     1     sr1
.......................uucgugguuacaucuguaU.....................................................................   3     1     sr1
.......................Gucgugguuacaucuguaaa....................................................................   3     1     sr1
.......................uucgugguuacaucuguaaa....................................................................   38    0     sr1
.......................uucgugguuacaucuguaCa....................................................................   7     1     sr1
.......................uucgugguuacaucuguaaU....................................................................   2     1     sr1
.......................uucgugguuacaucuguaUa....................................................................   30    1     sr1
.......................uucgugguuacaucuguaaG....................................................................   2     1     sr1
.......................uucgugguuacaucuguaaaC...................................................................   3     1     sr1
.......................uucgugguuacaucCguaaau...................................................................   1     1     sr1
.......................uucgugguuacaucuguaaau...................................................................   2     0     sr1
.......................Gucgugguuacaucuguaaau...................................................................   2     1     sr1
.......................uucgugguuacaucuguaaauA..................................................................   11    1     sr1
.......................uucgugguuacaucuguaaaug..................................................................   24    0     sr1
.......................Gucgugguuacaucuguaaaug..................................................................   4     1     sr1
.......................uucgugguuacaucuguaaauU..................................................................   1     1     sr1
.......................uCcgugguuacaucuguaaaug..................................................................   1     1     sr1
.......................Cucgugguuacaucuguaaaug..................................................................   1     1     sr1
.......................uucgugguuacaucuguaaaCg..................................................................   1     1     sr1
.......................uucgugguuacaucuguaaaugag................................................................   1     0     sr1
.......................uucgugguuacaucuguaaaugagaA..............................................................   1     1     sr1
.......................uucgugguuacaucuguaaaugagacC.............................................................   1     1     sr1
.......................uucgugguuacaucuguaaaugagacu.............................................................   1     0     sr1
........................ucAugguuacaucuguaa.....................................................................   1     1     sr1
........................ucgugguuacaucuguaa.....................................................................   20    0     sr1
........................ucgugguuacaucuguaC.....................................................................   1     1     sr1
........................Gcgugguuacaucuguaa.....................................................................   2     1     sr1
........................ucgugguuacaucuguaaa....................................................................   28    0     sr1
........................ucgugguuacaucuAuaaa....................................................................   1     1     sr1
........................ucgCgguuacaucuguaaa....................................................................   1     1     sr1
........................ucgugguuacaucuguaCa....................................................................   1     1     sr1
........................Gcgugguuacaucuguaaa....................................................................   2     1     sr1
........................ucgugguuacaucuguaUa....................................................................   9     1     sr1
........................ucgugguuacaucuguaUau...................................................................   1     1     sr1
........................ucgugguuacaucuguaaaC...................................................................   1     1     sr1
........................ucgugguuacaucuguaaau...................................................................   22    0     sr1
........................uUgugguuacaucuguaaaug..................................................................   1     1     sr1
........................ucgugguuacaucuguaaaGg..................................................................   1     1     sr1
```

```
                    Mature                              Star
gaaccaaaacaucagucggcuucuucgugguuacaucuguaaaugagacucgcuuacagguguaaccacgaagaagccgacacccguuuugcuuugcaugcaucucuugaa
........................ucgugguCacaucuguaaaug..................................................................    3     1     sr1
........................ucguAguuacaucuguaaaug..................................................................    3     1     sr1
........................Gcgugguuacaucuguaaaug..................................................................    22    1     sr1
........................ucgugguuacaucuguaaaug..................................................................    334   0     sr1
........................ucgugguuacaCcuguaaaug..................................................................    2     1     sr1
........................ucgugguuacaucuguaaUug..................................................................    1     1     sr1
........................ucgugguuacaucuguaaauA..................................................................    76    1     sr1
........................ucgugguuacaucCguaaaug..................................................................    1     1     sr1
........................ucguggCuacaucuguaaaug..................................................................    1     1     sr1
........................ucgugguuacGucuguaaaug..................................................................    2     1     sr1
........................ucgugguuacaucuguaaauga.................................................................    3     0     sr1
........................Gcgugguuacaucuguaaauga.................................................................    1     1     sr1
........................ucgugguuacaucuguaaauAa.................................................................    1     1     sr1
........................ucgugguuacaucuguaaaugag................................................................    2     0     sr1
........................ucgugguuacaucuguaaaugaga...............................................................    1     0     sr1
.........................cgugguuacaucuguaaaug..................................................................    1     0     sr1
.........................cgugguuacaucuguaaaugag................................................................    1     0     sr1
..........................gugguuacaucuguaaaug..................................................................    1     0     sr1
..........................gugguuacaucuguaaaugaga...............................................................    1     0     sr1
...........................ugguuacaucuguaaaug..................................................................    1     0     sr1
...........................ugguuacaucuguaaaugag................................................................    2     0     sr1
...........................ugguuacaucuguaaaugaA................................................................    1     1     sr1
...........................Ggguuacaucuguaaaugaga...............................................................    1     1     sr1
...........................ugguuacaucuguaaaugaga...............................................................    6     0     sr1
...........................ugguuacaucuguaaaugagaU..............................................................    1     1     sr1
...........................ugguuacaucuguaaaugagaA..............................................................    2     1     sr1
.................................................ucgcuuacagguguaaccacgUa.......................................    1     1     sr1
...................................................gcuuacagguguaaccacga........................................    1     0     sr1
...................................................gcuuacagguguaaccacgaa.......................................    1     0     sr1
....................................................cuuacagguguaaccacgaagaa....................................    1     0     sr1
.....................................................uuacagguguaaccacgaaga.....................................    1     0     sr1
.....................................................uuacagguguaaccacgaagaagc..................................    1     0     sr1
......................................................uacagguguaaccacgaaga.....................................    3     0     sr1
......................................................uacagguguaaccacgaagaa....................................    1     0     sr1
......................................................uacagguguaaccacgaagaag...................................    31    0     sr1
......................................................uGcagguguaaccacgaagaag...................................    2     1     sr1
......................................................uacagguguaaccacgaagGag...................................    1     1     sr1
......................................................uacagguguaaccacgaagaaA...................................    2     1     sr1
......................................................uacagguguaaccacgaagaGg...................................    1     1     sr1
......................................................uGcagguguaaccacgaagaagc..................................    1     1     sr1
......................................................uacagguguaaccacgaagaagU..................................    1     1     sr1
.......................................................acagguAuaaccacgaaga.....................................    3     1     sr1
.......................................................Gcagguguaaccacgaaga.....................................    1     1     sr1
.......................................................acagguguaaccacgaaga.....................................    13    0     sr1
.......................................................acagguguaaccacgaagaa....................................    3     0     sr1
.......................................................acagguguaaccacgaagaGg...................................    1     1     sr1
.......................................................acagguguaaccUcgaagaag...................................    1     1     sr1
.......................................................acagguguaaccacAaagaag...................................    1     1     sr1
.......................................................acagguguaaccacgaagaag...................................    33    0     sr1
.......................................................acaggugCaaccacgaagaag...................................    1     1     sr1
.......................................................acagguguaaccacAaagaagc..................................    4     1     sr1
.......................................................acGgguguaaccacgaagaagc..................................    1     1     sr1
.......................................................acagguguaaccacgaagaagU..................................    46    1     sr1
.......................................................acagguguaaccacgaagaagc..................................    278   0     sr1
.......................................................acagguguaaccacgGagaagc..................................    1     1     sr1
.......................................................Gcagguguaaccacgaagaagc..................................    10    1     sr1
.......................................................acagguguaGccacgaagaagc..................................    1     1     sr1
.......................................................acagguguaaccacgaagaGgc..................................    1     1     sr1
.......................................................acaggugCaaccacgaagaagc..................................    1     1     sr1
.......................................................acaggCguaaccacgaagaagc..................................    2     1     sr1
.......................................................acagguguaaccacgaagaagA..................................    4     1     sr1
.......................................................acagguguaaccUcgaagaagc..................................    1     1     sr1
.......................................................acagguguaaUcacgaagaagc..................................    1     1     sr1
.......................................................acaggGguaaccacgaagaagc..................................    1     1     sr1
.......................................................acagguguaaccacgaGgaagc..................................    1     1     sr1
.......................................................acagguguaaccacgaagaagcA.................................    1     1     sr1
.......................................................acagguguaaccacgaagaagcU.................................    1     1     sr1
.......................................................acagguguaaccacgaagaagcUga...............................    1     1     sr1
........................................................cagguguaaccacgaaga.....................................    4     0     sr1
........................................................cagguguaaccacgGagaag...................................    1     1     sr1
```

Mature Star

```
gaaccaaaacaucagucggcuucuucgugguuacaucuguaaaugagacucgcuuacagguguaaccacgaagaagccgacacccguuuugcuuugcaugcaucucuugaa
........................................................Gagguguaaccacgaagaag...................................	1	1	sr1
........................................................cagguguaaccacgaagaag...................................	2	0	sr1
........................................................cagguguaaccacgaagaagA..................................	2	1	sr1
........................................................cagguguaacGacgaagaagc..................................	1	1	sr1
........................................................caggugua Gccacgaagaagc..................................	1	1	sr1
........................................................cagguguaaccacgaGgaagc..................................	1	1	sr1
........................................................Gagguguaaccacgaagaagc..................................	3	1	sr1
........................................................cagguguaaccacgaagaagc..................................	106	0	sr1
........................................................cagguguaaccacgaagaagU..................................	14	1	sr1
........................................................cagguguaaccacgaagaCgc..................................	1	1	sr1
.........................................................agguguaaccacgaagaaC...................................	1	1	sr1
.........................................................agguguaaccGcgaagaag...................................	1	1	sr1
.............................................................guaaccacgaagaagcUgaca.............................	1	1	sr1

..................Ucuucuucgugguuacaucugua......................................................................	1	1	st1
...................cuucuucgugguuacaucug........................................................................	2	0	st1
...................cuucuucgugguuacaucugC.......................................................................	5	1	st1
...................Uuucuucgugguuacaucugu.......................................................................	1	1	st1
...................cuucuucgugguuacaucugu.......................................................................	18	0	st1
...................cuucuucguAguuacaucugua......................................................................	2	1	st1
...................cuucuucgugguuacaucugua......................................................................	26	0	st1
...................cuucuGcgugguuacaucuguaa.....................................................................	1	1	st1
...................cuucuucgugguuacaucuguaa.....................................................................	16	0	st1
...................cuucuucgugguuacaucuguaaa....................................................................	2	0	st1
....................uucuAcgugguuacaucu.........................................................................	1	1	st1
....................uucuucgugguuacaucu.........................................................................	82	0	st1
....................uucuucgugguCacaucu.........................................................................	2	1	st1
....................uucuucgugguuacaucC.........................................................................	13	1	st1
....................uucuucgugguuacaucA.........................................................................	2	1	st1
....................uucuucgugguuacaucG.........................................................................	6	1	st1
....................uucuucgugguuacaucAg........................................................................	5	1	st1
....................Cucuucgugguuacaucug........................................................................	1	1	st1
....................uucAucgugguuacaucug........................................................................	1	1	st1
....................uucuucgugguuacaucuA........................................................................	13	1	st1
....................uucuucAugguuacaucug........................................................................	1	1	st1
....................uucuucgugguuacaucug........................................................................	241	0	st1
....................uucuucgugguuacaucuU........................................................................	1	1	st1
....................uucuucguggCuacaucug........................................................................	2	1	st1
....................uucuucgugguCacaucug........................................................................	1	1	st1
....................uucuCcgugguuacaucug........................................................................	1	1	st1
....................uucuucgugguuacaucugA.......................................................................	250	1	st1
....................uucuucguggCuacaucugu.......................................................................	22	1	st1
....................uucuucAugguuacaucugu.......................................................................	10	1	st1
....................uAcuucgugguuacaucugu.......................................................................	5	1	st1
....................uucuucgugguuacCucugu.......................................................................	5	1	st1
....................uucuucgugguuacaucugC.......................................................................	1222	1	st1
....................uucuucgugAuuacaucugu.......................................................................	7	1	st1
....................uucuucgugguuacGucugu.......................................................................	57	1	st1
....................Gucuucgugguuacaucugu.......................................................................	2	1	st1
....................uucuuUgugguuacaucugu.......................................................................	8	1	st1
....................uucuuAgugguuacaucugu.......................................................................	1	1	st1
....................Aucuucgugguuacaucugu.......................................................................	7	1	st1
....................uuAuucgugguuacaucugu.......................................................................	4	1	st1
....................uucGucgugguuacaucugu.......................................................................	4	1	st1
....................uucuucgCgguuacaucugu.......................................................................	17	1	st1
....................uucAucgugguuacaucugu.......................................................................	6	1	st1
....................uucuucguggGuacaucugu.......................................................................	3	1	st1
....................uGcuucgugguuacaucugu.......................................................................	1	1	st1
....................uucuucgugguuacaucAgu.......................................................................	5	1	st1
....................uucuucgugguuacaucuAu.......................................................................	10	1	st1
....................uucuucguUguuacaucugu.......................................................................	2	1	st1
....................uucuucgugguuacaucGgu.......................................................................	4	1	st1
....................uucuucguggAuacaucugu.......................................................................	4	1	st1
....................uucuucgugguuUcaucugu.......................................................................	1	1	st1
....................uuUuucgugguuacaucugu.......................................................................	8	1	st1
....................uucuucgugguGacaucugu.......................................................................	1	1	st1
....................uucuGcgugguuacaucugu.......................................................................	2	1	st1
....................Cucuucgugguuacaucugu.......................................................................	57	1	st1
....................uucuucgugguuacauUugu.......................................................................	7	1	st1
....................uucuucgugguuacaucCgu.......................................................................	31	1	st1
```

```
                    Mature                              Star
gaaccaaaacaucagucggcuucuucgugguuacaucuguaaaugagacucgcuuacagguguaaccacgaagaagccgacacccguuuugcuuugcaugcaucucuugaa
....................uucuCcgugguuacaucugu.......................................................................   35     1   st1
....................uucuucgugguuacUucugu.......................................................................   14     1   st1
....................uucuucgugUuuacaucugu.......................................................................   3      1   st1
....................uucuucgugguuacaucuCu.......................................................................   7      1   st1
....................uucuAcgugguuacaucugu.......................................................................   5      1   st1
....................uucuucgugguCacaucugu.......................................................................   26     1   st1
....................uucuucgugguAacaucugu.......................................................................   2      1   st1
....................uucuucgugguuaAaucugu.......................................................................   4      1   st1
....................uCcuucgugguuacaucugu.......................................................................   34     1   st1
....................uucuucgugguuaGaucugu.......................................................................   1      1   st1
....................uucuucgugguuGcaucugu.......................................................................   28     1   st1
....................uucuucgugguuacauAugu.......................................................................   1      1   st1
....................uuGuucgugguuacaucugu.......................................................................   1      1   st1
....................uucuucgAgguuacaucugu.......................................................................   3      1   st1
....................uucuucgugguuacaucugu.......................................................................   11673  0   st1
....................uucuucguAguuacaucugu.......................................................................   8      1   st1
....................uucuucgugguuaUaucugu.......................................................................   9      1   st1
....................uucCucgugguuacaucugu.......................................................................   31     1   st1
....................uucuucgugguuacaAcugu.......................................................................   1      1   st1
....................uucuucgugguuCcaucugu.......................................................................   1      1   st1
....................uucuucgGgguuacaucugu.......................................................................   2      1   st1
....................uucuucgugguuacaucugG.......................................................................   169    1   st1
....................uucuucgugguuacaucuUu.......................................................................   3      1   st1
....................uucuuUgugguuacaucugua......................................................................   24     1   st1
....................uucuucguggGuacaucugua......................................................................   2      1   st1
....................uucuucgugguuacaucugua......................................................................   25349  0   st1
....................uucuuGgugguuacaucugua......................................................................   1      1   st1
....................uucuucguUguuacaucugua......................................................................   2      1   st1
....................uucuucgugguAacaucugua......................................................................   7      1   st1
....................uucuCcgugguuacaucugua......................................................................   72     1   st1
....................uucuuAgugguuacaucugua......................................................................   5      1   st1
....................uucuucgugguuacaucAgua......................................................................   6      1   st1
....................uucuucgugguCacaucugua......................................................................   50     1   st1
....................uGcuucgugguuacaucugua......................................................................   1      1   st1
....................uucuucgugguuCcaucugua......................................................................   2      1   st1
....................uucuucguggCuacaucugua......................................................................   80     1   st1
....................uucuucgugguuacGucugua......................................................................   105    1   st1
....................Aucuucgugguuacaucugua......................................................................   20     1   st1
....................uucuAcgugguuacaucugua......................................................................   6      1   st1
....................uucuucgGgguuacaucugua......................................................................   3      1   st1
....................uucuucguAguuacaucugua......................................................................   13     1   st1
....................uucuucgugguuaAaucugua......................................................................   3      1   st1
....................uucuucgugguuacauUugua......................................................................   12     1   st1
....................Cucuucgugguuacaucugua......................................................................   81     1   st1
....................Gucuucgugguuacaucugua......................................................................   5      1   st1
....................uucuucgugguuacauAugua......................................................................   4      1   st1
....................uucuucgugguuacaGcugua......................................................................   1      1   st1
....................uucuucgugguuacaAcugua......................................................................   8      1   st1
....................uucuucgugguuacaucCgua......................................................................   45     1   st1
....................uucuucgugguuacaucuguC......................................................................   20     1   st1
....................uucuucgugguuacaucuguU......................................................................   78     1   st1
....................uucuucgugguuacaucuAua......................................................................   14     1   st1
....................uucuucgCgguuacaucugua......................................................................   40     1   st1
....................uucuucgugguUcaucugua.......................................................................   5      1   st1
....................uucuucgugguuacaucugAa......................................................................   4      1   st1
....................uucuucgugguuacaucuCua......................................................................   1      1   st1
....................uucuucgugguuaGaucugua......................................................................   1      1   st1
....................uuUuucgugguuacaucugua......................................................................   13     1   st1
....................uucuucguggu Gacaucugua.....................................................................   1      1   st1
....................uucuucgugguuGcaucugua......................................................................   53     1   st1
....................uucuucAugguuacaucugua......................................................................   17     1   st1
....................uucAucgugguuacaucugua......................................................................   9      1   st1
....................uucuucgugguuaUaucugua......................................................................   13     1   st1
....................uucuucgugguuacUucugua......................................................................   35     1   st1
....................uucGucgugguuacaucugua......................................................................   3      1   st1
....................uucuucgugguuacaucugCa......................................................................   52     1   st1
....................uCcuucgugguuacaucugua......................................................................   72     1   st1
....................uucCucgugguuacaucugua......................................................................   76     1   st1
....................uucuucgugguuacCucugua......................................................................   4      1   st1
....................uucuucgugguuacaucGgua......................................................................   3      1   st1
```

Mature Star

```
gaaccaaaacaucagucggcuucuucgugguuacaucuguaaaugagacucgcuuacagguguaaccacgaagaagccgacacccguuuugcuuugcaugcaucucuugaa
....................uucuucgugguuacaucugGa......................................................................  4      1  st1
....................uucuucgAgguuacaucugua......................................................................  5      1  st1
....................uucuucgugUuuacaucugua......................................................................  5      1  st1
....................uucuucgugAuuacaucugua......................................................................  14     1  st1
....................uAcuucgugguuacaucugua......................................................................  10     1  st1
....................uucuGcgugguuacaucugua......................................................................  2      1  st1
....................uucuucguggAuacaucugua......................................................................  7      1  st1
....................uucuucgugguuacaucuguG......................................................................  241    1  st1
....................uucuucUugguuacaucugua......................................................................  1      1  st1
....................uucuucgugAuuacaucuguaa.....................................................................  13     1  st1
....................uucuucgugguuacaucuguaa.....................................................................  13223  0  st1
....................uucuucgugguuacaucuUuaa.....................................................................  1      1  st1
....................uucuucgugguuGcaucuguaa.....................................................................  57     1  st1
....................uucuucgugguuacaucugAaa.....................................................................  1      1  st1
....................uucuucgugguuacaucuAuaa.....................................................................  8      1  st1
....................uucuucgugguuacGucuguaa.....................................................................  59     1  st1
....................uucuuAgugguuacaucuguaa.....................................................................  1      1  st1
....................uucuucguUguuacaucuguaa.....................................................................  2      1  st1
....................uucuucgugguuacauAuguaa.....................................................................  12     1  st1
....................Cucuucgugguuacaucuguaa.....................................................................  50     1  st1
....................uucuucgugguCacaucuguaa.....................................................................  27     1  st1
....................uucuucgugguuacaucCguaa.....................................................................  33     1  st1
....................uucuucgCgguuacaucuguaa.....................................................................  21     1  st1
....................uucuucgugguuacauUuguaa.....................................................................  8      1  st1
....................uucuucgugguuacaucuCuaa.....................................................................  2      1  st1
....................uCcuucgugguuacaucuguaa.....................................................................  40     1  st1
....................uucuucguggAuacaucuguaa.....................................................................  3      1  st1
....................uucuucgugguuacaucGguaa.....................................................................  2      1  st1
....................uucuucgugguuacaucugCaa.....................................................................  11     1  st1
....................uucuucgugguuacaucuguaG.....................................................................  201    1  st1
....................uucuucgugguuUcaucuguaa.....................................................................  3      1  st1
....................uucuucAugguuacaucuguaa.....................................................................  10     1  st1
....................uucuucgugUuuacaucuguaa.....................................................................  4      1  st1
....................uucCucgugguuacaucuguaa.....................................................................  51     1  st1
....................uucuucgugguuacaAcuguaa.....................................................................  3      1  st1
....................uucuucgugguuaUaucuguaa.....................................................................  9      1  st1
....................uucuucgugguuaGaucuguaa.....................................................................  1      1  st1
....................uuUuucgugguuacaucuguaa.....................................................................  8      1  st1
....................uucAucgugguuacaucuguaa.....................................................................  4      1  st1
....................uucuucgAgguuacaucuguaa.....................................................................  3      1  st1
....................uucuucgugguAacaucuguaa.....................................................................  3      1  st1
....................Aucuucgugguuacaucuguaa.....................................................................  3      1  st1
....................uucuAcgugguuacaucuguaa.....................................................................  11     1  st1
....................uucuucgugguuacCucuguaa.....................................................................  2      1  st1
....................uucuucgugguuacaucugGaa.....................................................................  3      1  st1
....................uucuucguggCuacaucuguaa.....................................................................  17     1  st1
....................uucuucgugguuacaucuguaU.....................................................................  580    1  st1
....................uucuucgugguuacaucAguaa.....................................................................  14     1  st1
....................uucuucUugguuacaucuguaa.....................................................................  1      1  st1
....................uucuucgugguuaAaucuguaa.....................................................................  2      1  st1
....................uucuucgugguuacaucuguGa.....................................................................  31     1  st1
....................uucuCcgugguuacaucuguaa.....................................................................  29     1  st1
....................uucuucguAguuacaucuguaa.....................................................................  9      1  st1
....................Gucuucgugguuacaucuguaa.....................................................................  1      1  st1
....................uucuucgugguuacaucuguUa.....................................................................  7      1  st1
....................uucGucgugguuacaucuguaa.....................................................................  3      1  st1
....................uucuuUgugguuacaucuguaa.....................................................................  17     1  st1
....................uAcuucgugguuacaucuguaa.....................................................................  5      1  st1
....................uucuucgugguuacUucuguaa.....................................................................  20     1  st1
....................uucuucgugguuacaucuguaC.....................................................................  76     1  st1
....................uucuucgugguuacaucuguaUa....................................................................  590    1  st1
....................uucuucgugguuGcaucuguaaa....................................................................  5      1  st1
....................uucuuUgugguuacaucuguaaa....................................................................  1      1  st1
....................uucuucgugguuUcaucuguaaa....................................................................  2      1  st1
....................uucuucgugguuacGucuguaaa....................................................................  11     1  st1
....................uucuCcgugguuacaucuguaaa....................................................................  3      1  st1
....................uucuucguggCuacaucuguaaa....................................................................  3      1  st1
....................uucuAcgugguuacaucuguaaa....................................................................  1      1  st1
....................uucuuAgugguuacaucuguaaa....................................................................  1      1  st1
....................uucCucgugguuacaucuguaaa....................................................................  2      1  st1
```

```
                    Mature                 Star
gaaccaaaacaucagucggcuucuucgugguuacaucuguaaaugagacucgcuuacagguguaaccacgaagaagccgacacccguuuugcuuugcaugcaucucuugaa
....................Cucuucgugguuacaucuguaaa....................................................................   13    1  st1
....................uucuucgugguuacaCcuguaaa....................................................................   12    1  st1
....................uucuucgugguuacaucuguaCa....................................................................   7     1  st1
....................uucuucgugguGacaucuguaaa....................................................................   1     1  st1
....................uucuucgugguuacaucuguGaa....................................................................   2     1  st1
....................uucuucgugguuacaucuguaaa....................................................................   1928  0  st1
....................uucuucgugguuacaucuguaGa....................................................................   10    1  st1
....................uucuucgugguuacaucuguaaU....................................................................   41    1  st1
....................uucuucguAguuacaucuguaaa....................................................................   3     1  st1
....................uAcuucgugguuacaucuguaaa....................................................................   3     1  st1
....................uucuucgugguuacUucuguaaa....................................................................   3     1  st1
....................uucuucgCgguuacaucuguaaa....................................................................   2     1  st1
....................uCcuucgugguuacaucuguaaa....................................................................   3     1  st1
....................uucuucgugguuacaucGguaaa....................................................................   1     1  st1
....................uucAucgugguuacaucuguaaa....................................................................   1     1  st1
....................uucuucgugguuaAaucuguaaa....................................................................   1     1  st1
....................uucuucgugguuacaucuguaaC....................................................................   7     1  st1
....................uucuucgugguuacaucuguaaG....................................................................   34    1  st1
....................uucuucgugguuaUaucuguaaa....................................................................   3     1  st1
....................uucuucgugguCacaucuguaaa....................................................................   3     1  st1
....................uucuucgugguuacaucCguaaa....................................................................   2     1  st1
....................uucuuUgugguuacaucuguaaau...................................................................   1     1  st1
....................uucuucgugguuacaucuguaaaG...................................................................   8     1  st1
....................uucuucgugguuacaucuguaaaA...................................................................   8     1  st1
....................uucuucgugguuacaucuguaaaC...................................................................   5     1  st1
....................uucuucgugguuacaucuguaaCu...................................................................   2     1  st1
....................uucuucgugguuacaucuguaUau...................................................................   4     1  st1
....................uucuucgugguuacaucuguaaau...................................................................   24    0  st1
....................uucuucgugguuacaucuguaaUu...................................................................   6     1  st1
....................uucuucgugguuacaucuguaUaug..................................................................   1     1  st1
....................uucuucgugguuacGucuguaaaug..................................................................   3     1  st1
....................uucuucgugAuuacaucuguaaaug..................................................................   1     1  st1
....................uucuucgugguuacaCcuguaaaug..................................................................   2     1  st1
....................uucuuUgugguuacaucuguaaaug..................................................................   1     1  st1
....................uucuucgugguuacaucuguaaCug..................................................................   1     1  st1
....................uucuucgugguuacaucuguaaauU..................................................................   4     1  st1
....................uucuucgugguuacaucuguaaauA..................................................................   17    1  st1
....................uucuucgugguuacaucuguaaaug..................................................................   54    0  st1
....................uucuucgugguuacaucuguaaauga.................................................................   33    0  st1
....................uucuucgugguuacaucuguaaaugU.................................................................   1     1  st1
....................uucuucgugguuacaCcuguaaauga.................................................................   1     1  st1
....................uucuucgugguuacaucuguaaaugaU................................................................   1     1  st1
....................uucuucgugguuacaucuguaaaugaA................................................................   1     1  st1
....................uucuucgugguuacaucuguaaaugag................................................................   22    0  st1
....................uucuucgugguuacaucuguaaGugag................................................................   1     1  st1
.....................ucuucgugguuacaucug........................................................................   8     0  st1
.....................ucuucgugguuGcaucug........................................................................   1     1  st1
.....................Ccuucgugguuacaucugu.......................................................................   5     1  st1
.....................ucuucgugguuacaucugG.......................................................................   8     1  st1
.....................ucuucgugguuaUaucugu.......................................................................   2     1  st1
.....................ucuCcgugguuacaucugu.......................................................................   1     1  st1
.....................ucuucguggCuacaucugu.......................................................................   2     1  st1
.....................ucuucgugUuuacaucugu.......................................................................   1     1  st1
.....................ucuucgugguuacGucugu.......................................................................   4     1  st1
.....................ucuucguAguuacaucugu.......................................................................   1     1  st1
.....................ucuucgugguuacaucugC.......................................................................   58    1  st1
.....................ucuAcgugguuacaucugu.......................................................................   1     1  st1
.....................ucuucgCgguuacaucugu.......................................................................   1     1  st1
.....................ucuucguUguuacaucugu.......................................................................   1     1  st1
.....................ucuucgugguuacaucugA.......................................................................   7     1  st1
.....................ucuucgugguuCcaucugu.......................................................................   2     1  st1
.....................ucCucgugguuacaucugu.......................................................................   3     1  st1
.....................ucuucgugguuUcaucugu.......................................................................   1     1  st1
.....................ucuucgugguCacaucugu.......................................................................   2     1  st1
.....................ucuucgugguuacaucugu.......................................................................   567   0  st1
.....................Acuucgugguuacaucugua......................................................................   1     1  st1
.....................ucuucgugguuacGucugua......................................................................   2     1  st1
.....................ucuucgCgguuacaucugua......................................................................   1     1  st1
.....................ucuucgugguuacaucuguG......................................................................   5     1  st1
.....................ucuucgugguuacaucugCa......................................................................   3     1  st1
```

Mature **Star**

gaaccaaaacaucagucggcuucuucgugguuacaucuguaaaugagacucgcuuacagguguaaccacgaagaagccgacacccguuuugcuuugcaugcaucucuugaa			
.....................ucuucguggCuacaucugua..	2	1	st1
.....................ucuucgugAuuacaucugua..	1	1	st1
.....................ucuucguggAuacaucugua..	1	1	st1
.....................ucuucUugguuacaucugua..	1	1	st1
.....................ucuucgugguuacaucCgua..	2	1	st1
.....................ucuucgugguAacaucugua..	2	1	st1
.....................ucCucgugguuacaucugua..	3	1	st1
.....................Ccuucgugguuacaucugua..	1	1	st1
.....................uUuucgugguuacaucugua..	1	1	st1
.....................ucuucgugguuacaucuguU..	5	1	st1
.....................ucuCcgugguuacaucugua..	1	1	st1
.....................ucuucgugguuacaucugua..	752	0	st1
.....................ucuucgugguCacaucugua..	1	1	st1
.....................ucuucgugguuaUaucugua..	1	1	st1
.....................ucGucgugguuacaucugua..	1	1	st1
.....................ucuAcgugguuacaucugua..	1	1	st1
.....................ucuucgugguuCcaucugua..	1	1	st1
.....................ucuucgCgguuacaucuguaa...	2	1	st1
.....................ucuucgugguuacaucuguaa...	1330	0	st1
.....................ucuucgugguuacaucuAuaa...	1	1	st1
.....................ucuucgugguuacGucuguaa...	7	1	st1
.....................ucGucgugguuacaucuguaa...	1	1	st1
.....................ucuucgugguuacaucuguaU...	50	1	st1
.....................ucuucgugguuaUaucuguaa...	2	1	st1
.....................ucuucgugguuacaucugCaa...	1	1	st1
.....................ucuucgugguuacaAcuguaa...	1	1	st1
.....................ucuucgugguuacauUuguaa...	1	1	st1
.....................ucuucgugguuacaucuguaG...	22	1	st1
.....................Acuucgugguuacaucuguaa...	3	1	st1
.....................ucuucgugguuacaucCguaa...	3	1	st1
.....................ucuuUgugguuacaucuguaa...	2	1	st1
.....................ucuucgugguCacaucuguaa...	2	1	st1
.....................uAuucgugguuacaucuguaa...	1	1	st1
.....................ucuucgugguuacauAuguaa...	2	1	st1
.....................ucuucgugguuGcaucuguaa...	16	1	st1
.....................ucuucgugguuacaucuguaC...	28	1	st1
.....................ucuucgugguuacaucAguaa...	2	1	st1
.....................Ccuucgugguuacaucuguaa...	3	1	st1
.....................ucuucgugguuacCucuguaa...	2	1	st1
.....................ucuCcgugguuacaucuguaa...	3	1	st1
.....................ucuucgugguuacaGcuguaa...	1	1	st1
.....................ucuucgugguuacaucuguGa...	4	1	st1
.....................ucuucguUguuacaucuguaa...	1	1	st1
.....................ucuucguggCuacaucuguaa...	1	1	st1
.....................ucuucgugguuCcaucuguaa...	8	1	st1
.....................ucuAcgugguuacaucuguaa...	1	1	st1
.....................ucCucgugguuacaucuguaa...	6	1	st1
.....................ucuucgugguuacaucugAaa...	1	1	st1
.....................ucuucgugguuacaucuUuaaa..	1	1	st1
.....................ucuucgugguuacaucuguaaC..	4	1	st1
.....................ucuucgugguuacaucuguaaa..	3032	0	st1
.....................ucuucgugguuacaucugCaaa..	2	1	st1
.....................ucuucguggGuacaucuguaaa..	1	1	st1
.....................ucuucgugguuacaucuguGaa..	3	1	st1
.....................ucuucgugguuacaCcuguaaa..	12	1	st1
.....................uAuucgugguuacaucuguaaa..	2	1	st1
.....................ucuucgugguuacaucuAuaaa..	1	1	st1
.....................ucuucgCgguuacaucuguaaa..	5	1	st1
.....................ucuucgugguCacaucuguaaa..	2	1	st1
.....................ucuucgugguuacaGcuguaaa..	1	1	st1
.....................ucuucgugguuacaucuguaaG..	20	1	st1
.....................ucuucgugguuacauAuguaaa..	2	1	st1
.....................ucuAcgugguuacaucuguaaa..	5	1	st1
.....................ucuucgugguuacGucuguaaa..	12	1	st1
.....................ucuucguggCuacaucuguaaa..	5	1	st1
.....................ucuucgugguuCcaucuguaaa..	4	1	st1
.....................ucuCcgugguuacaucuguaaa..	9	1	st1
.....................ucuucgugguuacaucCguaaa..	11	1	st1
.....................ucuuUgugguuacaucuguaaa..	2	1	st1
.....................ucuucgugguuacaucuguaCa..	6	1	st1

Mature **Star**

```
gaaccaaaacaucagucggcuucuucgugguuacaucuguaaaugagacucgcuuacagguguaaccacgaagaagccgacacccguuuugcuuugcaugcaucucuugaa
.....................Acuucgugguuacaucuguaaa....................................................................  2   1   st1
.....................ucuucguggAuacaucuguaaa....................................................................  1   1   st1
.....................ucCucgugguuacaucuguaaa....................................................................  7   1   st1
.....................Ccuucgugguuacaucuguaaa....................................................................  6   1   st1
.....................ucuucgugguuacaucuguaUa....................................................................  61  1   st1
.....................ucuucgugguuacaAcuguaaa....................................................................  3   1   st1
.....................ucuucgugguuacaucuguaGa....................................................................  8   1   st1
.....................ucuucgugguuaUaucuguaaa....................................................................  2   1   st1
.....................ucuucgugguuacaucAguaaa....................................................................  5   1   st1
.....................ucAucgugguuacaucuguaaa....................................................................  1   1   st1
.....................ucuucgugguuacaucuguaaU....................................................................  14  1   st1
.....................ucuucgugguuGcaucuguaaa....................................................................  15  1   st1
.....................uGuucgugguuacaucuguaaa....................................................................  1   1   st1
.....................uUuucgugguuacaucuguaaa....................................................................  4   1   st1
.....................ucuucUugguuacaucuguaaa....................................................................  1   1   st1
.....................ucuucgugguuacaucuguaUau...................................................................  1   1   st1
.....................ucuucgugguuGcaucuguaaau...................................................................  1   1   st1
.....................ucuucgugguuacaucuguaaaA...................................................................  20  1   st1
.....................ucuucgugguuacaucuguaaau...................................................................  25  0   st1
.....................ucuucgugguuacaucuguaaaC...................................................................  3   1   st1
.....................ucuucgugguuacaucuguaaaG...................................................................  3   1   st1
.....................ucuucgugguuacaucuguaaUu...................................................................  5   1   st1
.....................ucuucgugguuacaucuguaaauA..................................................................  12  1   st1
.....................ucuucgugguuacaucuguaaaugaga...............................................................  1   0   st1
......................cuucgugguuacaucugu.......................................................................  8   0   st1
......................cuucgugguuacaucugua......................................................................  14  0   st1
......................cuucgugguuacauGugua......................................................................  1   1   st1
......................Uuucgugguuacaucuguaa.....................................................................  2   1   st1
......................cuucgugguuacaucuguaU.....................................................................  4   1   st1
......................cuucgugguuacaucuguaa.....................................................................  25  0   st1
......................cuucgugguCacaucuguaaa....................................................................  1   1   st1
......................cuucgugguuacaucuguaaa....................................................................  10  0   st1
......................cuucgugguuacaucuguaUa....................................................................  3   1   st1
......................Uuucgugguuacaucuguaaa....................................................................  1   1   st1
......................cuucgugguuacaucuguaaU....................................................................  1   1   st1
......................cuucgugguuacaucuguaaaug..................................................................  1   0   st1
.......................uucgugguuacaucugua......................................................................  2   0   st1
.......................uucgugguuacaucuguaa.....................................................................  3   0   st1
.......................uucgugguuacaucuguaaa....................................................................  6   0   st1
.......................uucgugguuacaucuguaaaC...................................................................  1   1   st1
.......................uucgugguuacaucuguaaaug..................................................................  3   0   st1
.......................uucgugguuacaucuguaaauA..................................................................  2   1   st1
.......................uucgugguuacaucuguaaaugaga...............................................................  1   0   st1
........................ucgugguuacaucuguaU.....................................................................  2   1   st1
........................ucgugguuacaucuguaa.....................................................................  2   0   st1
........................ucgugguCacaucuguaaa....................................................................  3   1   st1
........................ucgugguuacaucuguaaa....................................................................  2   0   st1
........................ucgugguuacaucuguaaaug..................................................................  4   0   st1
........................ucgugguuacaucuguaaauA..................................................................  1   1   st1
......................................................uacagguguaaccacgaaga.....................................  2   0   st1
......................................................uGcagguguaaccacgaaga.....................................  1   1   st1
......................................................uacagguguaaccacgaagaa....................................  1   0   st1
......................................................uacagguguaaccacgaagaaA...................................  1   1   st1
......................................................uacagguguaaccacgaagaag...................................  10  0   st1
......................................................uGcagguguaaccacgaagaagc..................................  2   1   st1
.......................................................acagguguaaccacgaaga.....................................  3   0   st1
.......................................................acagguguaaccacgaagaag...................................  3   0   st1
.......................................................acagguguaaccacgaagaagc..................................  72  0   st1
.......................................................acagguguaaccacgaagaGgc..................................  1   1   st1
.......................................................acagguguaaccacgaagaagU..................................  14  1   st1
.......................................................acagguguaaccacgGagaagc..................................  1   1   st1
.......................................................acagguguaaccacgaagaagA..................................  2   1   st1
.......................................................Gcagguguaaccacgaagaagc..................................  1   1   st1
........................................................cagguguaaccacgaagaagU..................................  3   1   st1
........................................................cagguguaaccacgaagaagA..................................  1   1   st1
........................................................Uagguguaaccacgaagaagc..................................  1   1   st1
........................................................cagguguaaccacgaagaagc..................................  39  0   st1
.............................................................guaaccacgaagaagcUg................................  1   1   st1
.............................................................guaaccacgaagaagcUga...............................  2   1   st1
.............................................................guaaccacgaagaagcUgac..............................  1   1   st1
```

```
                    Mature                              Star
gaaccaaaacaucagucggcuucuucgugguuacaucuguaaaugagacucgcuuacagguguaaccacgaagaagccgacacccguuuugcuuugcaugcaucucuugaa
.............................................................guaaccacgaagaagcUgaca.............................	1	1	st1
..............................................................uaaccacgaagaagcUgac..............................	1	1	st1

.................Agcuucuucgugguuacaucugu.......................................................................	1	1	so1
.................Agcuucuucgugguuacaucuguaa.....................................................................	2	1	so1
..................Ucuucuucgugguuacaucugua......................................................................	1	1	so1
...................cuucuucgugguuacaucu.........................................................................	1	0	so1
...................cuucuucgugguuacaucug........................................................................	1	0	so1
...................cuucuucgugguuacaucugu.......................................................................	5	0	so1
...................cuucuucgugguuacaucugC.......................................................................	3	1	so1
...................Uuucuucgugguuacaucugua......................................................................	1	1	so1
...................cuucuucgugguuacaucugCa......................................................................	1	1	so1
...................cuucuucgugguuacaucuguU......................................................................	1	1	so1
...................cuucuucgugguuacaucugua......................................................................	27	0	so1
...................cuucuucgugguuCcaucugua......................................................................	1	1	so1
...................cuucuucgugAuuacaucugua......................................................................	1	1	so1
...................cuucuucgugguuacaucuguaa.....................................................................	6	0	so1
...................cuucuucgugguuacaucuguaG.....................................................................	2	1	so1
...................cuucuucgugguuacaucuguaU.....................................................................	1	1	so1
...................cuucuucgugguuacaucuguaUa....................................................................	3	1	so1
...................cuucuucgugguuacaucuguaaa....................................................................	1	0	so1
...................cuucuucgugguuacaucuguaaaug..................................................................	1	0	so1
....................uucuucgugguGacaucu.........................................................................	1	1	so1
....................uucuucgugguuacaucu.........................................................................	38	0	so1
....................uucuucgugguuacaucC.........................................................................	8	1	so1
....................uCcuucgugguuacaucu.........................................................................	1	1	so1
....................uucuCcgugguuacaucu.........................................................................	1	1	so1
....................uucuucgAgguuacaucug........................................................................	1	1	so1
....................uucuucgugguuacaucAg........................................................................	4	1	so1
....................uucuucgGgguuacaucug........................................................................	1	1	so1
....................Cucuucgugguuacaucug........................................................................	2	1	so1
....................uucuucgugguuaUaucug........................................................................	1	1	so1
....................uucuucgugguuacaucuA........................................................................	38	1	so1
....................uucuucgugguuacaucug........................................................................	335	0	so1
....................uucGucgugguuacaucug........................................................................	1	1	so1
....................uucuucguggCuacaucug........................................................................	2	1	so1
....................uucuucgugguuacaucuU........................................................................	4	1	so1
....................uucuucgugguuacaucuC........................................................................	2	1	so1
....................Aucuucgugguuacaucug........................................................................	2	1	so1
....................uucuucgugguuacaucCg........................................................................	1	1	so1
....................uucuCcgugguuacaucug........................................................................	2	1	so1
....................uucuucgGgguuacaucugu.......................................................................	4	1	so1
....................uucuucgugguuacaucuUu.......................................................................	2	1	so1
....................uucuucgugguCacaucugu.......................................................................	5	1	so1
....................uucuCcgugguuacaucugu.......................................................................	9	1	so1
....................uucuuUgugguuacaucugu.......................................................................	4	1	so1
....................uucuucgugguuacaucuCu.......................................................................	2	1	so1
....................uucuucgugUuuacaucugu.......................................................................	1	1	so1
....................uCcuucgugguuacaucugu.......................................................................	13	1	so1
....................Cucuucgugguuacaucugu.......................................................................	20	1	so1
....................uAcuucgugguuacaucugu.......................................................................	3	1	so1
....................uucuuAgugguuacaucugu.......................................................................	1	1	so1
....................Aucuucgugguuacaucugu.......................................................................	6	1	so1
....................uucuucgugguuGcaucugu.......................................................................	8	1	so1
....................uucuucgugguuacaucugG.......................................................................	103	1	so1
....................uucuucgugAuuacaucugu.......................................................................	7	1	so1
....................uucuucgugguAacaucugu.......................................................................	1	1	so1
....................uucuucgugguuacaucugA.......................................................................	99	1	so1
....................uucuucgugguuacaGcugu.......................................................................	1	1	so1
....................uucCucgugguuacaucugu.......................................................................	20	1	so1
....................uucuucguAguuacaucugu.......................................................................	2	1	so1
....................uucAucgugguuacaucugu.......................................................................	5	1	so1
....................uucuucgugguuacaucugu.......................................................................	5506	0	so1
....................uucuucgugguuUcaucugu.......................................................................	1	1	so1
....................uucuucguggCuacaucugu.......................................................................	18	1	so1
....................uucuucgugguuaUaucugu.......................................................................	10	1	so1
....................uucuucgugCuuacaucugu.......................................................................	1	1	so1
....................uucuucgAgguuacaucugu.......................................................................	3	1	so1
....................uucuucgugguuacaucCgu.......................................................................	11	1	so1
....................uucuucgCgguuacaucugu.......................................................................	13	1	so1
```

```
                    Mature                          Star
gaaccaaaacaucagucggcuucuucgugguuacaucuguaaaugagacucgcuuacagguguaaccacgaagaagccgacacccguuuugcuuugcaugcaucucuugaa
....................uuUuucgugguuacaucugu.......................................................................    6        1    so1
....................uucuucgugguuacGucugu.......................................................................    18       1    so1
....................uucuucgugguuacaucGgu.......................................................................    7        1    so1
....................uucuucAugguuacaucugu.......................................................................    6        1    so1
....................uucuucgugguuacaucAgu.......................................................................    6        1    so1
....................uucuucgugguuacauUugu.......................................................................    3        1    so1
....................uucuucgugguGacaucugu.......................................................................    1        1    so1
....................uucuucgugguuacaucugC.......................................................................    828      1    so1
....................uucuucgugguuacUucugu.......................................................................    3        1    so1
....................uucuAcgugguuacaucugu.......................................................................    3        1    so1
....................uucuucgugguuacaucuAu.......................................................................    4        1    so1
....................uucuucCugguuacaucugua......................................................................    1        1    so1
....................uucuGcgugguuacaucugua......................................................................    4        1    so1
....................uucuucgGgguuacaucugua......................................................................    9        1    so1
....................uucuucgugguuacaucAgua......................................................................    9        1    so1
....................uucuucgugguAacaucugua......................................................................    12       1    so1
....................uucuucgugguuacaucGgua......................................................................    5        1    so1
....................uucuucgugguuaUaucugua......................................................................    17       1    so1
....................uucuucgugguCacaucugua......................................................................    72       1    so1
....................uCcuucgugguuacaucugua......................................................................    50       1    so1
....................uucuCcgugguuacaucugua......................................................................    57       1    so1
....................uucuucAugguuacaucugua......................................................................    22       1    so1
....................uucuuUgugguuacaucugua......................................................................    18       1    so1
....................uucuucgugguuacauGugua......................................................................    2        1    so1
....................uucuucgugguuacaGcugua......................................................................    1        1    so1
....................uucuucgugguuacaucugua......................................................................    22537    0    so1
....................uucuucgugguuacUucugua......................................................................    6        1    so1
....................uucuucgugguuacaucuAua......................................................................    13       1    so1
....................uucuucgugguuacaucuguU......................................................................    144      1    so1
....................uucuucgugguuacaucuUua......................................................................    2        1    so1
....................uucuucgugguuacauUugua......................................................................    7        1    so1
....................uucuucUugguuacaucugua......................................................................    3        1    so1
....................uucuucgugAuuacaucugua......................................................................    11       1    so1
....................uuGuucgugguuacaucugua......................................................................    1        1    so1
....................Cucuucgugguuacaucugua......................................................................    70       1    so1
....................uucuucgugguGacaucugua......................................................................    1        1    so1
....................uucuAcgugguuacaucugua......................................................................    8        1    so1
....................uucuucgugguuacCucugua......................................................................    3        1    so1
....................uuUuucgugguuacaucugua......................................................................    8        1    so1
....................uucuucguggAuacaucugua......................................................................    11       1    so1
....................uucuucgugguuacauAugua......................................................................    2        1    so1
....................uucuucgugguuCcaucugua......................................................................    1        1    so1
....................uucuucgugguuacaucugGa......................................................................    25       1    so1
....................uucuucgugguuUcaucugua......................................................................    2        1    so1
....................uucuucguggGuacaucugua......................................................................    4        1    so1
....................uucuucguAguuacaucugua......................................................................    24       1    so1
....................uucuucgugguuacaucuguC......................................................................    18       1    so1
....................uucuucguggCuacaucugua......................................................................    56       1    so1
....................uucuucgugguuacaucuguG......................................................................    251      1    so1
....................uucuucgugguuGcaucugua......................................................................    61       1    so1
....................uucuucgAgguuacaucugua......................................................................    11       1    so1
....................uucuucgugguuaGaucugua......................................................................    2        1    so1
....................uucuucgugguuacaucugAa......................................................................    6        1    so1
....................uucCucgugguuacaucugua......................................................................    61       1    so1
....................uucuucgugguuacaucuCua......................................................................    1        1    so1
....................uucuucgugguuacaAcugua......................................................................    6        1    so1
....................uAcuucgugguuacaucugua......................................................................    12       1    so1
....................uuAuucgugguuacaucugua......................................................................    1        1    so1
....................uucAucgugguuacaucugua......................................................................    7        1    so1
....................Aucuucgugguuacaucugua......................................................................    8        1    so1
....................uucuucgugUuuacaucugua......................................................................    1        1    so1
....................uucuucgugguuacGucugua......................................................................    80       1    so1
....................uucuuAgugguuacaucugua......................................................................    4        1    so1
....................uucuucgugguuacaucugCa......................................................................    93       1    so1
....................uucuucgugguuacaucCgua......................................................................    67       1    so1
....................uucuucgCgguuacaucugua......................................................................    40       1    so1
....................uucuucguUguuacaucugua......................................................................    5        1    so1
....................uucuucgugguuaAaucugua......................................................................    2        1    so1
....................Gucuucgugguuacaucugua......................................................................    2        1    so1
....................uucuCcgugguuacaucuguaa.....................................................................    56       1    so1
```

Mature Star

gaaccaaaacaucagucggcuucuucgugguuacaucuguaaaugagacucgcuuacagguguaaccacgaagaagccgacacccguuuugcuuugcaugcaucucuugaa			
....................Aucuucgugguuacaucuguaa...	10	1	so1
....................uucuucgugguuacaucuUuaa...	1	1	so1
....................uucuucgCgguuacaucuguaa...	23	1	so1
....................uucuucguggGuacaucuguaa...	4	1	so1
....................uucuucgugguuacaucuguUa...	22	1	so1
....................uucuucgugguuCcaucuguaa...	2	1	so1
....................uucuuAgugguuacaucuguaa...	1	1	so1
....................Gucuucgugguuacaucuguaa...	3	1	so1
....................uucuucgugguCacaucuguaa...	24	1	so1
....................uuUuucgugguuacaucuguaa...	8	1	so1
....................uucuucgugguuacaucugCaa...	18	1	so1
....................uucuucgugguuacaucugAaa...	1	1	so1
....................uucuGcgugguuacaucuguaa...	4	1	so1
....................uucuucgugguuacCucuguaa...	2	1	so1
....................uucuucguAguuacaucuguaa...	6	1	so1
....................uGcuucgugguuacaucuguaa...	3	1	so1
....................uucuucgugguuaGaucuguaa...	1	1	so1
....................uucuucgugguuacaucuguaC...	247	1	so1
....................uucuucguUguuacaucuguaa...	2	1	so1
....................uucuucgugguuacUucuguaa...	5	1	so1
....................uucuucgugguuacaucCguaa...	49	1	so1
....................uucuucgugguuacaucuguaU...	1259	1	so1
....................uucuucgugguuacaAcuguaa...	4	1	so1
....................uucuucgugUuuacaucuguaa...	4	1	so1
....................uucuucgGgguuacaucuguaa...	8	1	so1
....................uAcuucgugguuacaucuguaa...	1	1	so1
....................uucuucguggAuacaucuguaa...	4	1	so1
....................uucuucgugguuacaucuguaG...	177	1	so1
....................uucuucgugguGacaucuguaa...	2	1	so1
....................uucuucgugguuGcaucuguaa...	74	1	so1
....................uucuucguggCuacaucuguaa...	32	1	so1
....................uucuAcgugguuacaucuguaa...	7	1	so1
....................uucuucgugguuacauAuguaa...	4	1	so1
....................uucuucgugguAacaucuguaa...	6	1	so1
....................uucuucgugAuuacaucuguaa...	2	1	so1
....................uucuucAugguuacaucuguaa...	9	1	so1
....................uuAuucgugguuacaucuguaa...	3	1	so1
....................uucuucgugguuacaucugGaa...	2	1	so1
....................Cucuucgugguuacaucuguaa...	52	1	so1
....................uucuucgAgguuacaucuguaa...	9	1	so1
....................uucuucgugguuacauUuguaa...	4	1	so1
....................uucuucgugguuacGucuguaa...	66	1	so1
....................uucAucgugguuacaucuguaa...	3	1	so1
....................uucuucgugguuacaucuguaa...	14392	0	so1
....................uCcuucgugguuacaucuguaa...	33	1	so1
....................uucCucgugguuacaucuguaa...	26	1	so1
....................uucuucgugguuaUaucuguaa...	14	1	so1
....................uucuucgugguuacaucuAuaa...	7	1	so1
....................uucuucgugguuacaucuguGa...	41	1	so1
....................uucuucgugguuUcaucuguaa...	6	1	so1
....................uucuucgugguuacaucAguaa...	19	1	so1
....................uucuucgugguuacaucGguaa...	3	1	so1
....................uucuucgugguuacaGcuguaa...	4	1	so1
....................uucuuUgugguuacaucuguaa...	12	1	so1
....................uucCucgugguuacaucuguaaa..	2	1	so1
....................uucuucgugguuacaucuguaUa..	1951	1	so1
....................uucuucgugguuGcaucuguaaa..	4	1	so1
....................uucuGcgugguuacaucuguaaa..	1	1	so1
....................uucuucgugguuacaGcuguaaa..	1	1	so1
....................uucuucgCgguuacaucuguaaa..	2	1	so1
....................uucuucgugguuacaucuguaaU..	86	1	so1
....................uucuucgAgguuacaucuguaaa..	1	1	so1
....................uucuucgugguuacUucuguaaa..	1	1	so1
....................uucuucAugguuacaucuguaaa..	1	1	so1
....................Cucuucgugguuacaucuguaaa..	5	1	so1
....................uucuucgugguuacaucuguaaa..	970	0	so1
....................uucuucgugguuacaucuguaGa..	4	1	so1
....................uucuucgugguuacaCcuguaaa..	2	1	so1
....................uucuucgugguuacaucCguaaa..	3	1	so1
....................uucuucgugguCacaucuguaaa..	4	1	so1

Mature Star

gaaccaaaacaucagucggcuucuucgugguuacaucuguaaaugagacucgcuuacagguguaaccacgaagaagccgacacccguuuugcuuugcaugcaucucuugaa			
....................uucuucgugguuacaucuguaaC..	8	1	so1
....................uucuucgugguuacaucuguaaG..	34	1	so1
....................uucuucgugguuacGucuguaaa..	1	1	so1
....................uucuucguggCuacaucuguaaa..	2	1	so1
....................uucuucgugguuacaucuguGaa..	2	1	so1
....................uucuucgugguuacaucuguaCa..	8	1	so1
....................uucuucgugguuacaucuguaaau...	74	0	so1
....................uucuucgugguuacaucuguaUau...	56	1	so1
....................uucCucgugguuacaucuguaaau...	1	1	so1
....................uucuucgugguuacaucuguaaUu...	10	1	so1
....................uucuucguggCuacaucuguaaau...	1	1	so1
....................uucuucgugguuacaucuguaaCu...	1	1	so1
....................uucuucgugguuacaucuguaaaC...	12	1	so1
....................uucuucgugguCacaucuguaaau...	1	1	so1
....................uucuucgugguuacaucuguaaaA...	17	1	so1
....................uucuucgugguuacaucuguaaaG...	4	1	so1
....................uucuucgugguuacaucuguaaaug..	446	0	so1
....................uucuucgugguuacaucuguaaauU..	12	1	so1
....................uucuCcgugguuacaucuguaaaug..	3	1	so1
....................uCcuucgugguuacaucuguaaaug..	1	1	so1
....................uucuucgugguCacaucuguaaaug..	4	1	so1
....................uucuucgugguuacaucuguaCaug..	1	1	so1
....................uucuucgugguuacaCcuguaaaug..	1	1	so1
....................uucuucgugguuGcaucuguaaaug..	3	1	so1
....................uucuucgugguuacaucCguaaaug..	1	1	so1
....................uucuucgugguuacaucAguaaaug..	3	1	so1
....................uucuucguAguuacaucuguaaaug..	1	1	so1
....................uucuucgugguuacaucuguaaaCg..	4	1	so1
....................uucuucgugguuacaucuguaaauC..	4	1	so1
....................uucuucgugguuUcaucuguaaaug..	1	1	so1
....................uucuucgugguuacaucuguaaaGg..	2	1	so1
....................uucuucgugguuacaucuguaaGug..	1	1	so1
....................uucuucgugguuacaucuguaUaug..	1	1	so1
....................uucuucgugguuacaucuguGaaug..	1	1	so1
....................uucuucgugguuacaucuguaaauA..	85	1	so1
....................uucuucgugguuacaucuguGaauga...	1	1	so1
....................uucuucgugguuacaucuguaaaugG...	3	1	so1
....................uucuucgugguuacaucuguaaaugU...	1	1	so1
....................uucuucgugguuacaucuguaaauUa...	4	1	so1
....................Cucuucgugguuacaucuguaaauga...	1	1	so1
....................uucuucgugguuacaucuguaaauga...	80	0	so1
....................uucuucgugguAacaucuguaaauga...	1	1	so1
....................uucuucgugguuacaucuguaaaugaA..	7	1	so1
....................uucuucgugguuacaucuguaaaugag..	97	0	so1
....................uucuucgugguuacaucuguaGaugag..	3	1	so1
....................uucuucgugguuacaucuguaaaugaU..	1	1	so1
....................uucuCcgugguuacaucuguaaaugag..	1	1	so1
....................uucuucgugguuacaucuguaaaugGg..	2	1	so1
....................uucuucgugguuacaucuguaaaugaC..	2	1	so1
.....................ucuucgugguuacaucug..	1	0	so1
.....................ucuucgugguuacaucugA...	14	1	so1
.....................ucuucgugguAacaucugu...	1	1	so1
.....................ucuucgugguuacaucugG...	11	1	so1
.....................ucuucgugguuacaucAgu...	1	1	so1
.....................ucuucgugguuaUaucugu...	1	1	so1
.....................ucuucgugAuuacaucugu...	1	1	so1
.....................ucuucguUguuacaucugu...	1	1	so1
.....................ucuucgugguuacGucugu...	1	1	so1
.....................ucuucgCgguuacaucugu...	3	1	so1
.....................ucCucgugguuacaucugu...	1	1	so1
.....................ucuucgugguuacaucugC...	72	1	so1
.....................ucuucgugguCacaucugu...	1	1	so1
.....................ucuucgugguuacaucugu...	430	0	so1
.....................Ccuucgugguuacaucugu...	5	1	so1
.....................uUuucgugguuacaucugu...	2	1	so1
.....................ucuucgugguuGcaucugu...	4	1	so1
.....................ucuucgAgguuacaucugu...	1	1	so1
.....................Ccuucgugguuacaucugua..	1	1	so1
.....................ucuucgCgguuacaucugua..	2	1	so1
.....................ucuucgugguuacaucugCa..	5	1	so1

Mature **Star**

```
gaaccaaaacaucagucggcuucuucgugguuacaucuguaaaugagacucgcuuacagguguaaccacgaagaagccgacacccguuuugcuuugcaugcaucucuugaa
.....................ucuucgugguuacaucuguC......................................................................  2     1  so1
.....................ucCucgugguuacaucugua......................................................................  2     1  so1
.....................Acuucgugguuacaucugua......................................................................  1     1  so1
.....................ucuucgugguCacaucugua......................................................................  2     1  so1
.....................ucuucAugguuacaucugua......................................................................  2     1  so1
.....................ucuucgugguGacaucugua......................................................................  1     1  so1
.....................ucuucgugguuacaucugAa......................................................................  1     1  so1
.....................ucuucguggCuacaucugua......................................................................  6     1  so1
.....................ucuucgAgguuacaucugua......................................................................  1     1  so1
.....................ucuucgugguuacaucugua......................................................................  1320  0  so1
.....................ucuucgugguuacaucuguU......................................................................  13    1  so1
.....................ucuucgugguuacaucuguG......................................................................  19    1  so1
.....................ucAucgugguuacaucugua......................................................................  1     1  so1
.....................ucuucgugguuacGucugua......................................................................  7     1  so1
.....................ucuucgugguuacUucugua......................................................................  1     1  so1
.....................ucuucgugguuacauUugua......................................................................  2     1  so1
.....................ucuucgugguuacaucGgua......................................................................  1     1  so1
.....................ucuucguAguuacaucugua......................................................................  1     1  so1
.....................ucuuUgugguuacaucugua......................................................................  1     1  so1
.....................ucuucgugguuGcaucugua......................................................................  9     1  so1
.....................ucuCcgugguuacaucugua......................................................................  7     1  so1
.....................ucuucgugguuacaucCgua......................................................................  6     1  so1
.....................ucuucgugguuacaucugGa......................................................................  2     1  so1
.....................ucuucgugguuacaucAgua......................................................................  4     1  so1
.....................ucuucgugguuacaucuguGa.....................................................................  5     1  so1
.....................Acuucgugguuacaucuguaa.....................................................................  1     1  so1
.....................ucuuUgugguuacaucuguaa.....................................................................  5     1  so1
.....................ucCucgugguuacaucuguaa.....................................................................  7     1  so1
.....................ucuucgAgguuacaucuguaa.....................................................................  1     1  so1
.....................ucuucgugguuacGucuguaa.....................................................................  1     1  so1
.....................ucuucgugguuacaucuguUa.....................................................................  3     1  so1
.....................ucuucgugguuacaucuguaG.....................................................................  22    1  so1
.....................ucuucguAguuacaucuguaa.....................................................................  5     1  so1
.....................ucuucgugguuacaucuguaa.....................................................................  1911  0  so1
.....................ucuucgugguuacaucuguaC.....................................................................  85    1  so1
.....................ucuucgugguuacaucuguCa.....................................................................  1     1  so1
.....................ucuucgugguuGcaucuguaa.....................................................................  50    1  so1
.....................ucuucgugguuacaucCguaa.....................................................................  5     1  so1
.....................ucuucgCgguuacaucuguaa.....................................................................  4     1  so1
.....................Ccuucgugguuacaucuguaa.....................................................................  6     1  so1
.....................ucuCcgugguuacaucuguaa.....................................................................  4     1  so1
.....................ucuucgugguuacaucugCaa.....................................................................  1     1  so1
.....................uGuucgugguuacaucuguaa.....................................................................  1     1  so1
.....................uUuucgugguuacaucuguaa.....................................................................  2     1  so1
.....................ucuucgugguuacCucuguaa.....................................................................  1     1  so1
.....................ucuucgugguCacaucuguaa.....................................................................  3     1  so1
.....................ucuucAugguuacaucuguaa.....................................................................  1     1  so1
.....................ucuucgugguuacaucAguaa.....................................................................  2     1  so1
.....................ucuAcgugguuacaucuguaa.....................................................................  2     1  so1
.....................ucuucgugguuacaucuguaU.....................................................................  150   1  so1
.....................ucuucguggCuacaucuguaa.....................................................................  2     1  so1
.....................ucuucgugguCacaucuguaaa....................................................................  7     1  so1
.....................ucuucgugguuacaucuguGaa....................................................................  2     1  so1
.....................ucuucgugguuacaucuguaaG....................................................................  14    1  so1
.....................ucuucgugguuacaucAguaaa....................................................................  1     1  so1
.....................ucuucgugguuacaucuguaaa....................................................................  1464  0  so1
.....................ucuucgugguuacaucugCaaa....................................................................  2     1  so1
.....................ucuucgCgguuacaucuguaaa....................................................................  4     1  so1
.....................ucuucgugguuacauUuguaaa....................................................................  2     1  so1
.....................ucuucgugguuacaucuguaUa....................................................................  305   1  so1
.....................ucuCcgugguuacaucuguaaa....................................................................  3     1  so1
.....................ucCucgugguuacaucuguaaa....................................................................  7     1  so1
.....................ucuucgugguuacaucCguaaa....................................................................  4     1  so1
.....................ucuucgugguuacaucuguaCa....................................................................  12    1  so1
.....................ucuucgugguAacaucuguaaa....................................................................  1     1  so1
.....................ucuucgugguuaUaucuguaaa....................................................................  1     1  so1
.....................ucuucgugguuacaCcuguaaa....................................................................  8     1  so1
.....................ucuucgugguuacaucuguaGa....................................................................  5     1  so1
.....................ucuucguggCuacaucuguaaa....................................................................  3     1  so1
.....................ucuucguggGuacaucuguaaa....................................................................  1     1  so1
```

Mature **Star**

gaaccaaaacaucagucggcuucuucgugguuacaucuguaaaugagacucgcuuacagguguaaccacgaagaagccgacacccguuuugcuuugcaugcaucucuugaa			
.....................ucuucgugguuacaucuAuaaa..	2	1	so1
.....................Ccuucgugguuacaucuguaaa..	4	1	so1
.....................ucuucgugguuacaucugAaaa..	1	1	so1
.....................ucuucgugguuacGucuguaaa..	4	1	so1
.....................ucuucAugguuacaucuguaaa..	2	1	so1
.....................ucuucgugguuacaucuguaaC..	5	1	so1
.....................ucuucgugguuacauAuguaaa..	1	1	so1
.....................ucuucgugguuacaucuguaaU..	30	1	so1
.....................ucuucgugguuGcaucuguaaa..	7	1	so1
.....................ucuucgugguuUcaucuguaaa..	1	1	so1
.....................ucuucgugguuacaucuguaaaC...	8	1	so1
.....................ucuucgugguuacaucuguaaaA...	7	1	so1
.....................ucuucgugguuacaucuguaaUu...	5	1	so1
.....................ucuucgugguuacaucuguaaaG...	3	1	so1
.....................ucuucgugguuacaucuguaGau...	1	1	so1
.....................ucuucgugguuacaucuguaUau...	2	1	so1
.....................ucuucgugguuacaucuguaaau...	22	0	so1
.....................ucuucgugguuacaucuguaCau...	1	1	so1
.....................ucuucgugguuacaucuguaaaug..	5	0	so1
.....................ucuucgugguuacaucuguaaauA..	18	1	so1
.....................ucuucgugguuacaucuguaaauU..	1	1	so1
.....................ucuucgugguuacaucuguaaauUa...	1	1	so1
......................Uuucgugguuacaucugu...	1	1	so1
......................cuucgugguuacaucugu...	5	0	so1
......................cuucgugguuacaucugua..	13	0	so1
......................cuucgugguuacaucuguaC...	1	1	so1
......................cuucgugguuacaucuguaU...	1	1	so1
......................Uuucgugguuacaucuguaa...	5	1	so1
......................cuucgugguuacaucuguaa...	30	0	so1
......................cuucgugguuacaucuguaaa..	7	0	so1
......................Uuucgugguuacaucuguaaa..	2	1	so1
......................cuucgugguuacaucuguaUa..	3	1	so1
......................cuucgugguuacaucuguaaaug..	1	0	so1
.......................uucgugguuacaucugua..	7	0	so1
.......................uucgugguuacaucuguaa...	6	0	so1
.......................uucgugguuacaucuguaaa..	4	0	so1
.......................uucgugguuacaucuguaUa..	4	1	so1
.......................uucgugguuacaucuguaaau...	1	0	so1
.......................uucgugguuacaucAguaaaug..	1	1	so1
.......................uucgugguuacaucuguaaaug..	15	0	so1
.......................uucgugguuacaucuguaaauA..	1	1	so1
.......................uucgugguuacaucuguaaauga...	4	0	so1
.......................uucgugguuacaucuguaaauUa...	1	1	so1
.......................uucgugguuacaucuguaaaugaga...	1	0	so1
........................ucgugguuacaucuguaa...	5	0	so1
........................ucgugguuacaucuguaaa..	1	0	so1
........................ucgugguuacaucuguaUa..	1	1	so1
........................ucgugguuacaucuguaaau...	5	0	so1
........................ucgugguuacaucuguaaaC...	3	1	so1
........................Ccgugguuacaucuguaaaug..	1	1	so1
........................ucgugguuacaucuguaaaug..	86	0	so1
........................ucgugguuacaucuguaaauC..	2	1	so1
........................ucgugguuacaucAguaaaug..	1	1	so1
........................ucgugguuacaucuguaaauA..	21	1	so1
........................ucgugguuacaucuguaaaCg..	1	1	so1
........................ucgugguuacaucuguaaauga...	1	0	so1
....uuacagguguaaccacgaagaa....................................	1	0	so1
....uacagguguaaccacgaaga.....................................	1	0	so1
....uacagguguaaccacgaagaa....................................	3	0	so1
....uacagguguaaccacgaagaag...................................	7	0	so1
....uacGgguguaaccacgaagaag...................................	1	1	so1
....uacagguguaaccacgaagaagc..................................	1	0	so1
....acagguguaaccacgaaga.....................................	4	0	so1
....acagguguaaccacgaagaa....................................	2	0	so1
....acagguguaaccacgaagaag...................................	5	0	so1
....acagguguaaccacgaagaagc..................................	69	0	so1
....acagguguaaccacgGagaagc..................................	3	1	so1
....acagguguaaccacgaagaagA..................................	1	1	so1
....acagguguaaccacgaagaagU..................................	12	1	so1
....cagguguaaccacgaaga.....................................	1	0	so1

```
                    Mature                               Star
gaaccaaaacaucagucggcuucuucgugguuacaucuguaaaugagacucgcuuacagguguaaccacgaagaagccgacacccguuuugcuuugcaugcaucucuugaa
........................................................cagguguaaccacgaagaag...................................	1	0	so1
........................................................cagguguaaccacgaagaagc..................................	18	0	so1
........................................................cUgguguaaccacgaagaagc..................................	1	1	so1
........................................................cagguguaaccacgaagaCgc..................................	1	1	so1
........................................................cagguguaaccacgaagaagA..................................	2	1	so1
........................................................cagguguaaccacgaagaagU..................................	4	1	so1
........................................................cagguguaaccacgaagaGgc..................................	1	1	so1
.........................................................agguguaaccacgaagaaCc..................................	1	1	so1
.............................................................................cgacacccguuuugcuuug...............	1	0	so1

..................Ucuucuucgugguuacaucuguaa.....................................................................	2	1	sg1
...................cuucuucgugguuacGucugu.......................................................................	1	1	sg1
...................cuCcuucgugguuacaucugu.......................................................................	2	1	sg1
...................cuucuucgugguuacaucugu.......................................................................	3	0	sg1
...................cuucuucCugguuacaucugu.......................................................................	1	1	sg1
...................cuucuucgugguuacaucugC.......................................................................	1	1	sg1
...................cuucuucgugguuacaucugua......................................................................	22	0	sg1
...................cuucuucgugguuacaAcugua......................................................................	1	1	sg1
...................Auucuucgugguuacaucugua......................................................................	1	1	sg1
...................cuucuucgugguuacaucuguG......................................................................	1	1	sg1
...................cuucuucgugguuacaucuguaa.....................................................................	4	0	sg1
...................cuucuCcgugguuacaucuguaa.....................................................................	1	1	sg1
...................cuucuucgugguuacaucuguaaa....................................................................	1	0	sg1
...................cuucuucgugguuacaucuguaUa....................................................................	1	1	sg1
....................uucuucgugguuacaucG.........................................................................	2	1	sg1
....................uucuucgugguuacaucC.........................................................................	3	1	sg1
....................uucuucgugguuacaucu.........................................................................	23	0	sg1
....................uucuucgugguuacaucAg........................................................................	1	1	sg1
....................uucuucgugguuacaucCg........................................................................	1	1	sg1
....................uucuucgugguAacaucug........................................................................	1	1	sg1
....................uucuucgugguuacaucuA........................................................................	7	1	sg1
....................uucuucgugguuacaucug........................................................................	126	0	sg1
....................uuAuucgugguuacaucugu.......................................................................	1	1	sg1
....................uucuucgugguuacaucGgu.......................................................................	3	1	sg1
....................uuUuucgugguuacaucugu.......................................................................	2	1	sg1
....................uucuucgGgguuacaucugu.......................................................................	1	1	sg1
....................uucuucgugguuGcaucugu.......................................................................	9	1	sg1
....................uucuucguggAuacaucugu.......................................................................	2	1	sg1
....................uucuucgugguuacauUugu.......................................................................	6	1	sg1
....................uucAucgugguuacaucugu.......................................................................	1	1	sg1
....................uucuucgugguuacaGcugu.......................................................................	2	1	sg1
....................uucuuUgugguuacaucugu.......................................................................	3	1	sg1
....................uucuucgugguGacaucugu.......................................................................	1	1	sg1
....................uucuucgugguAacaucugu.......................................................................	3	1	sg1
....................uucCucgugguuacaucugu.......................................................................	3	1	sg1
....................uAcuucgugguuacaucugu.......................................................................	4	1	sg1
....................uucuucguggCuacaucugu.......................................................................	4	1	sg1
....................uCcuucgugguuacaucugu.......................................................................	12	1	sg1
....................uucuucguggGuacaucugu.......................................................................	2	1	sg1
....................uucuucgCgguuacaucugu.......................................................................	3	1	sg1
....................uucuucAugguuacaucugu.......................................................................	3	1	sg1
....................Cucuucgugguuacaucugu.......................................................................	7	1	sg1
....................uucuucgugguuacaucugG.......................................................................	42	1	sg1
....................uucGucgugguuacaucugu.......................................................................	2	1	sg1
....................uucuucgAgguuacaucugu.......................................................................	3	1	sg1
....................uucuucgugguuacaucuAu.......................................................................	4	1	sg1
....................uucuucgugguuacaAcugu.......................................................................	1	1	sg1
....................uucuucgugguuacaucugu.......................................................................	3513	0	sg1
....................uucuucgugguuUcaucugu.......................................................................	1	1	sg1
....................uucuCcgugguuacaucugu.......................................................................	8	1	sg1
....................uucuucgugguuacGucugu.......................................................................	10	1	sg1
....................uucuucgugguuacaucAgu.......................................................................	2	1	sg1
....................uucuucgugguuacCucugu.......................................................................	1	1	sg1
....................uucuuAgugguuacaucugu.......................................................................	4	1	sg1
....................uucuucgugguuacaucuUu.......................................................................	1	1	sg1
....................uucuucgugguuacaucugA.......................................................................	53	1	sg1
....................uucuucguAguuacaucugu.......................................................................	6	1	sg1
....................uucuucgugguuacaucCgu.......................................................................	11	1	sg1
....................uucuucgugguuacaucugC.......................................................................	351	1	sg1
....................uucuucgugguuacaucuCu.......................................................................	1	1	sg1
```

Mature **Star**

```
gaaccaaaacaucagucggcuucuucgugguuacaucuguaaaugagacucgcuuacagguguaaccacgaagaagccgacacccguuuugcuuugcaugcaucucuugaa
....................uucuucgugguCacaucugu.......................................................................    5      1    sg1
....................uucuucgugguuaUaucugu.......................................................................    5      1    sg1
....................Aucuucgugguuacaucugua......................................................................    8      1    sg1
....................uGcuucgugguuacaucugua......................................................................    2      1    sg1
....................uucGucgugguuacaucugua......................................................................    2      1    sg1
....................uucuucgugguAacaucugua......................................................................    4      1    sg1
....................uucuucgugguuacaucuguG......................................................................    100    1    sg1
....................uucuucguggAuacaucugua......................................................................    1      1    sg1
....................uucuGcgugguuacaucugua......................................................................    1      1    sg1
....................uucuucgAgguuacaucugua......................................................................    9      1    sg1
....................uuUuucgugguuacaucugua......................................................................    3      1    sg1
....................uucuucgugguuacaAcugua......................................................................    1      1    sg1
....................uucuucgugguuacaucugGa......................................................................    12     1    sg1
....................uucCucgugguuacaucugua......................................................................    40     1    sg1
....................uucuucgugguuacaucugua......................................................................    12796  0    sg1
....................uucuucguAguuacaucugua......................................................................    4      1    sg1
....................uucuuAgugguuacaucugua......................................................................    4      1    sg1
....................uucuCcgugguuacaucugua......................................................................    34     1    sg1
....................uucuAcgugguuacaucugua......................................................................    7      1    sg1
....................uucuucgugguuacaucuguU......................................................................    45     1    sg1
....................uucuucgugguuacaGcugua......................................................................    2      1    sg1
....................uucuucgugguuUcaucugua......................................................................    1      1    sg1
....................uucuucgugguuacGucugua......................................................................    31     1    sg1
....................uucuucgugguuacauAugua......................................................................    3      1    sg1
....................uucuucgugguuaUaucugua......................................................................    5      1    sg1
....................uucuucgugUuuacaucugua......................................................................    1      1    sg1
....................uucuucgugguuacaucuUua......................................................................    4      1    sg1
....................uucuucgugguuacCucugua......................................................................    1      1    sg1
....................uAcuucgugguuacaucugua......................................................................    7      1    sg1
....................uucuucgugguuacUucugua......................................................................    3      1    sg1
....................uCcuucgugguuacaucugua......................................................................    21     1    sg1
....................Gucuucgugguuacaucugua......................................................................    2      1    sg1
....................uucAucgugguuacaucugua......................................................................    4      1    sg1
....................uucuuUgugguuacaucugua......................................................................    11     1    sg1
....................uucuucAugguuacaucugua......................................................................    10     1    sg1
....................uuAuucgugguuacaucugua......................................................................    2      1    sg1
....................uucuucgCgguuacaucugua......................................................................    39     1    sg1
....................uucuucguggGuacaucugua......................................................................    3      1    sg1
....................uucuucgugguuacauUugua......................................................................    8      1    sg1
....................uucuucgugguuacaucCgua......................................................................    36     1    sg1
....................uucuucgugguuaAaucugua......................................................................    1      1    sg1
....................uucuucgugguuacaucuguC......................................................................    7      1    sg1
....................uucuucgugguCacaucugua......................................................................    30     1    sg1
....................uucuucgugguuGcaucugua......................................................................    41     1    sg1
....................uucuucgugguuacaucugAa......................................................................    6      1    sg1
....................uucuucgugAuuacaucugua......................................................................    9      1    sg1
....................Cucuucgugguuacaucugua......................................................................    35     1    sg1
....................uucuucgugguuCcaucugua......................................................................    4      1    sg1
....................uucuucgugguuacaucGgua......................................................................    9      1    sg1
....................uucuucgugguuacaucAgua......................................................................    4      1    sg1
....................uucuucguUguuacaucugua......................................................................    1      1    sg1
....................uucuucgGgguuacaucugua......................................................................    10     1    sg1
....................uucuucguggCuacaucugua......................................................................    22     1    sg1
....................uucuucgugguuacaucugCa......................................................................    38     1    sg1
....................uucuucgugguuacauGugua......................................................................    1      1    sg1
....................uucuucgugguuacaucuAua......................................................................    10     1    sg1
....................uAcuucgugguuacaucuguaa.....................................................................    4      1    sg1
....................uucuucgugguuacaucuguaa.....................................................................    8262   0    sg1
....................uucuucgAgguuacaucuguaa.....................................................................    1      1    sg1
....................uucuucgugguuaUaucuguaa.....................................................................    12     1    sg1
....................uucuucgugguuacaucAguaa.....................................................................    2      1    sg1
....................uucuucgCgguuacaucuguaa.....................................................................    13     1    sg1
....................uucuucgugguuacaucuguUa.....................................................................    4      1    sg1
....................Aucuucgugguuacaucuguaa.....................................................................    4      1    sg1
....................uucuucgugAuuacaucuguaa.....................................................................    7      1    sg1
....................uucuucguggGuacaucuguaa.....................................................................    3      1    sg1
....................uucuucgugguuacaucuAuaa.....................................................................    4      1    sg1
....................uucuucgugguuacaucugCaa.....................................................................    13     1    sg1
....................uCcuucgugguuacaucuguaa.....................................................................    15     1    sg1
....................uucuucgugguuacaucGguaa.....................................................................    2      1    sg1
```

Mature Star

```
gaaccaaaacaucagucggcuucuucgugguuacaucuguaaaugagacucgcuuacagguguaaccacgaagaagccgacacccguuuugcuuugcaugcaucucuugaa
....................uucuucgGgguuacaucuguaa.....................................................................  1     1  sg1
....................uucuucgugguuacaGcuguaa.....................................................................  3     1  sg1
....................uucuucguggCuacaucuguaa.....................................................................  23    1  sg1
....................uucuucCugguuacaucuguaa.....................................................................  1     1  sg1
....................uucuucgugguuacaucuguaU.....................................................................  482   1  sg1
....................uucuucgugguAacaucuguaa.....................................................................  2     1  sg1
....................Cucuucgugguuacaucuguaa.....................................................................  23    1  sg1
....................uucuucgugguuacaucuguaC.....................................................................  54    1  sg1
....................uucuucgugguuacaucuguGa.....................................................................  19    1  sg1
....................uucuucgugguuGcaucuguaa.....................................................................  41    1  sg1
....................uucuucgugguCacaucuguaa.....................................................................  20    1  sg1
....................uucuucgugguuUcaucuguaa.....................................................................  1     1  sg1
....................uucuucgugguuacaucugAaa.....................................................................  1     1  sg1
....................uucuucgugguuacauUuguaa.....................................................................  7     1  sg1
....................uucuucgugguuacCucuguaa.....................................................................  3     1  sg1
....................uucuucgugUuuacaucuguaa.....................................................................  1     1  sg1
....................uucuucgugguuacaucuCuaa.....................................................................  1     1  sg1
....................uucAucgugguuacaucuguaa.....................................................................  2     1  sg1
....................uucuucAugguuacaucuguaa.....................................................................  7     1  sg1
....................uucuucUugguuacaucuguaa.....................................................................  2     1  sg1
....................uucuuUgugguuacaucuguaa.....................................................................  6     1  sg1
....................uucuucguggAuacaucuguaa.....................................................................  3     1  sg1
....................uucGucgugguuacaucuguaa.....................................................................  1     1  sg1
....................uucuucgugguuacaucuguaG.....................................................................  104   1  sg1
....................uucuucgugguuacUucuguaa.....................................................................  4     1  sg1
....................uucCucgugguuacaucuguaa.....................................................................  24    1  sg1
....................uucuCcgugguuacaucuguaa.....................................................................  19    1  sg1
....................uucuucgugguuacGucuguaa.....................................................................  25    1  sg1
....................uucuucguAguuacaucuguaa.....................................................................  5     1  sg1
....................uucuuAgugguuacaucuguaa.....................................................................  2     1  sg1
....................uucuucgugguuacaAcuguaa.....................................................................  1     1  sg1
....................uucuAcgugguuacaucuguaa.....................................................................  4     1  sg1
....................uucuucgugguuacauAuguaa.....................................................................  1     1  sg1
....................uucuucgugguuacaucCguaa.....................................................................  23    1  sg1
....................uucuucgugguuacaucuguaGa....................................................................  6     1  sg1
....................uucuucgugguuacaucuguaaG....................................................................  16    1  sg1
....................Cucuucgugguuacaucuguaaa....................................................................  1     1  sg1
....................uucuucguUguuacaucuguaaa....................................................................  1     1  sg1
....................uucuucgugguuacaucuguaaC....................................................................  3     1  sg1
....................uAcuucgugguuacaucuguaaa....................................................................  1     1  sg1
....................uucuucgugguuacaucCguaaa....................................................................  5     1  sg1
....................uucuucguggCuacaucuguaaa....................................................................  2     1  sg1
....................uucuucgugguuacaucugCaaa....................................................................  2     1  sg1
....................uucuucgugguuacGucuguaaa....................................................................  6     1  sg1
....................uucuucgugguCacaucuguaaa....................................................................  3     1  sg1
....................uucuucguggGuacaucuguaaa....................................................................  1     1  sg1
....................uucuucgugguGacaucuguaaa....................................................................  1     1  sg1
....................uucuucgugAuuacaucuguaaa....................................................................  2     1  sg1
....................uucuuUgugguuacaucuguaaa....................................................................  1     1  sg1
....................uucuucgugguuacaucuguaUa....................................................................  1238  1  sg1
....................uucuucgugguuGcaucuguaaa....................................................................  4     1  sg1
....................uCcuucgugguuacaucuguaaa....................................................................  1     1  sg1
....................uucuucgugguuacaucuguaCa....................................................................  23    1  sg1
....................uucuucgCgguuacaucuguaaa....................................................................  1     1  sg1
....................uucuucgugguuacaCcuguaaa....................................................................  2     1  sg1
....................uucuucUugguuacaucuguaaa....................................................................  1     1  sg1
....................uucuCcgugguuacaucuguaaa....................................................................  2     1  sg1
....................uucuucgugguuacaucuguaaU....................................................................  34    1  sg1
....................uucuucgugguuacaucuguaaa....................................................................  946   0  sg1
....................uucuucgugguuacaucuguGaa....................................................................  1     1  sg1
....................uucuucgugguuacaucuguUaa....................................................................  3     1  sg1
....................uucuucgugguuacaAcuguaaa....................................................................  1     1  sg1
....................uucuucgugguuacaucuguaUau...................................................................  10    1  sg1
....................uucuucgugguuacaucuguaaau...................................................................  30    0  sg1
....................uucuucgugguuacaucuguaCau...................................................................  2     1  sg1
....................uucuucgugguuacaucuguaaUu...................................................................  4     1  sg1
....................uucuucgugguuacaucuguaaaA...................................................................  4     1  sg1
....................uucuucgugguuacaucuguaaaG...................................................................  3     1  sg1
....................uucuucgugguuacaucuguaaUug..................................................................  1     1  sg1
....................uucuucgugguuacaCcuguaaaug..................................................................  1     1  sg1
```

```
                    Mature                Star
gaaccaaaacaucagucggcuucuucgugguuacaucuguaaaugagacucgcuuacagguguaaccacgaagaagccgacacccguuuugcuuugcaugcaucucuugaa
....................uucuucgugguuacaucuguaaaug..................................................................    123     0    sg1
....................uucuucgugguuacaucuguaaaCg..................................................................    1       1    sg1
....................uuAuucgugguuacaucuguaaaug..................................................................    1       1    sg1
....................Gucuucgugguuacaucuguaaaug..................................................................    1       1    sg1
....................Cucuucgugguuacaucuguaaaug..................................................................    1       1    sg1
....................uucuucgugguAacaucuguaaaug..................................................................    1       1    sg1
....................uucuucgugguuacaucuguaaauU..................................................................    2       1    sg1
....................uCcuucgugguuacaucuguaaaug..................................................................    1       1    sg1
....................uucuucgugguuacaucuguaaaAg..................................................................    2       1    sg1
....................uucuucgugguuacaucuguaGaug..................................................................    1       1    sg1
....................uucuucgugguuacaucuguaaauA..................................................................    29      1    sg1
....................uucuucgugguuacaucuguaaauga.................................................................    45      0    sg1
....................uucuucgugguuacaucuguaaauUa.................................................................    1       1    sg1
....................Aucuucgugguuacaucuguaaauga.................................................................    1       1    sg1
....................uucuCcgugguuacaucuguaaauga.................................................................    1       1    sg1
....................uucuucgugguuacaucuguaaaCga.................................................................    1       1    sg1
....................uucuucgugguuacaucuguaaaugaU................................................................    2       1    sg1
....................uucuucgugguuacaucuguaaaugaA................................................................    1       1    sg1
....................uucuucgugguuacaucuguaaaugag................................................................    36      0    sg1
.....................ucuucgugguuacaucug........................................................................    7       0    sg1
.....................ucuucgugguuacaucCgu.......................................................................    1       1    sg1
.....................ucuucgugguuacaucugC.......................................................................    33      1    sg1
.....................ucuucgugguuGcaucugu.......................................................................    1       1    sg1
.....................ucuucgugguuacaucugu.......................................................................    319     0    sg1
.....................Ccuucgugguuacaucugu.......................................................................    2       1    sg1
.....................ucuucgugguuaAaucugu.......................................................................    1       1    sg1
.....................ucuucgugguCacaucugu.......................................................................    1       1    sg1
.....................ucuucgugguuacaucugG.......................................................................    2       1    sg1
.....................ucuucgCgguuacaucugu.......................................................................    3       1    sg1
.....................ucuucguAguuacaucugu.......................................................................    2       1    sg1
.....................ucuucgugguuacaucugA.......................................................................    3       1    sg1
.....................ucuucgugguuacGucugu.......................................................................    1       1    sg1
.....................ucuucgugguuacUucugua......................................................................    1       1    sg1
.....................ucGucgugguuacaucugua......................................................................    1       1    sg1
.....................ucuucgugguuacaucuguG......................................................................    5       1    sg1
.....................ucuucgugguuacaucugCa......................................................................    1       1    sg1
.....................ucuucguggCuacaucugua......................................................................    5       1    sg1
.....................ucuucgugguuacaucAgua......................................................................    1       1    sg1
.....................ucuucguAguuacaucugua......................................................................    1       1    sg1
.....................ucuucgugguuacGucugua......................................................................    1       1    sg1
.....................ucuucgugguuacaucuguU......................................................................    1       1    sg1
.....................ucuucgugguCacaucugua......................................................................    1       1    sg1
.....................ucuucgugguuacaucugAa......................................................................    1       1    sg1
.....................ucuCcgugguuacaucugua......................................................................    4       1    sg1
.....................uAuucgugguuacaucugua......................................................................    1       1    sg1
.....................ucuucgugguuGcaucugua......................................................................    3       1    sg1
.....................ucuucgugguuacaucCgua......................................................................    8       1    sg1
.....................ucuucgugguuacaucuAua......................................................................    1       1    sg1
.....................ucuucgugguuacaucGgua......................................................................    1       1    sg1
.....................ucuucgugguuacaucugua......................................................................    812     0    sg1
.....................ucCucgugguuacaucugua......................................................................    1       1    sg1
.....................ucuucAugguuacaucuguaa.....................................................................    1       1    sg1
.....................ucuuUgugguuacaucuguaa.....................................................................    1       1    sg1
.....................ucuucgugguuacauUuguaa.....................................................................    1       1    sg1
.....................Acuucgugguuacaucuguaa.....................................................................    1       1    sg1
.....................ucuucgugguCacaucuguaa.....................................................................    2       1    sg1
.....................ucuucgugguuacauAuguaa.....................................................................    1       1    sg1
.....................Ccuucgugguuacaucuguaa.....................................................................    1       1    sg1
.....................ucAucgugguuacaucuguaa.....................................................................    1       1    sg1
.....................ucuucgugguuGcaucuguaa.....................................................................    19      1    sg1
.....................ucuucgugguuacaucuguaG.....................................................................    11      1    sg1
.....................ucuucgugguAacaucuguaa.....................................................................    2       1    sg1
.....................ucuucgugguuacaucugCaa.....................................................................    1       1    sg1
.....................ucuucgugguuacaucuguaU.....................................................................    58      1    sg1
.....................ucuucgugAuuacaucuguaa.....................................................................    1       1    sg1
.....................ucuucgugguuacaucuguUa.....................................................................    2       1    sg1
.....................ucuucgugguuacaucuguGa.....................................................................    2       1    sg1
.....................ucuucgugguuacaucuguaa.....................................................................    1244    0    sg1
.....................ucuucgugguuacaucuguaC.....................................................................    27      1    sg1
.....................ucuucgugguuacaucCguaa.....................................................................    3       1    sg1
```

Mature Star

gaaccaaaacaucagucggcuucuucgugguuacaucuguaaaugagacucgcuuacagguguaaccacgaagaagccgacacccguuuugcuuugcaugcaucucuugaa

.....................ucuCcgugguuacaucuguaa...	8	1	sg1
.....................ucuucguggCuacaucuguaa...	3	1	sg1
.....................ucCucgugguuacaucuguaa...	10	1	sg1
.....................uAuucgugguuacaucuguaa...	1	1	sg1
.....................ucuucguAguuacaucuguaa...	1	1	sg1
.....................ucuucgugguuacGucuguaa...	4	1	sg1
.....................ucuucgugguuacaucuguaGa..	2	1	sg1
.....................ucuucgugAuuacaucuguaaa..	1	1	sg1
.....................ucuucgugguCacaucuguaaa..	2	1	sg1
.....................ucuucgugguuacaucuguaaa..	1187	0	sg1
.....................ucuucgugguuacaCcuguaaa..	1	1	sg1
.....................ucuucgugguuacaucuguaaU..	12	1	sg1
.....................ucuucgugguuacaucGguaaa..	1	1	sg1
.....................ucuucgugguuacaucugAaaa..	1	1	sg1
.....................ucuucgugguuacaucuguaaG..	8	1	sg1
.....................ucuucgugguuaUaucuguaaa..	2	1	sg1
.....................ucuucgugguuacaucuguGaa..	2	1	sg1
.....................ucuucgugguuacaucuguaaC..	4	1	sg1
.....................ucuucgugguuUcaucuguaaa..	1	1	sg1
.....................ucuucAugguuacaucuguaaa..	1	1	sg1
.....................ucuucgugguuacaucuguaUa..	171	1	sg1
.....................ucuucgugguuacaucuguaCa..	35	1	sg1
.....................ucuucgugguuGcaucuguaaa..	5	1	sg1
.....................ucCucgugguuacaucuguaaa..	2	1	sg1
.....................ucuucguggCuacaucuguaaa..	1	1	sg1
.....................Ccuucgugguuacaucuguaaa..	4	1	sg1
.....................ucuucgugguuacaucAguaaa..	5	1	sg1
.....................ucuucgugguuacGucuguaaa..	2	1	sg1
.....................ucuucgugguAacaucuguaaa..	1	1	sg1
.....................ucuucgCgguuacaucuguaaa..	3	1	sg1
.....................ucuucgugguuacaucuguaaau...	24	0	sg1
.....................ucuucgugguuacaucuguaUau...	3	1	sg1
.....................ucuucgugguuacaucuguaaaC...	2	1	sg1
.....................ucuucgugguuacaucuguaCau...	1	1	sg1
.....................ucuucgugguuacaucuguaaaG...	3	1	sg1
.....................ucuucgugguuacaucuguaaaA...	8	1	sg1
.....................ucuucgugguuacaucuguaaauA..	22	1	sg1
.....................ucuucgugguuacaucuguaaaug..	3	0	sg1
.....................ucuucgugguuacaucuguaaauU..	1	1	sg1
.....................ucuucgugguuacaucuguaaauAa...	1	1	sg1
......................cuucgugguuGcaucugu...	1	1	sg1
......................cuucgugguuacaucugu...	6	0	sg1
......................cuucgugguuacaucugua..	12	0	sg1
......................cuucgugguuacaucuguG..	1	1	sg1
......................Uuucgugguuacaucuguaa...	2	1	sg1
......................cuucgugguuacauUuguaa...	1	1	sg1
......................cuucgugguuacaucuguaa...	19	0	sg1
......................cuucgugguuacaucuguaU...	2	1	sg1
......................cuucgugguuacaucuguaUa..	7	1	sg1
......................cuucgugguuacaucuguaaa..	8	0	sg1
.......................uucgugguuacaucuguaa...	10	0	sg1
.......................uucgugguuacaucuguaaa..	1	0	sg1
.......................uucgugguuacaucuguaCa..	2	1	sg1
.......................uucgugguuacaucuguaUa..	1	1	sg1
.......................uucgugguuacaucuguaaau...	2	0	sg1
........................ucgugguuacaucuguaa...	4	0	sg1
........................ucgugguuacaucuguaaa..	2	0	sg1
........................ucgugguuacaucuguaUa..	3	1	sg1
........................ucgugguuacaCcuguaaaug..	1	1	sg1
........................ucgugguuacaucuguaaauA..	8	1	sg1
........................ucgCgguuacaucuguaaaug..	1	1	sg1
........................ucgugguuacaucuguaaaug..	17	0	sg1
........................ucgugguuacaucuguaaauga...	1	0	sg1
........................ucgugguuacaucuguaaaugag..	1	0	sg1
...........................ugguuacaucuguaaaugag..	1	0	sg1
....uacagguguaaccacgaagaa....................................	2	0	sg1
....uacaggugu Gaccacgaagaag...................................	2	1	sg1
....uacagguguaGccacgaagaag...................................	1	1	sg1
....uacagguguaaccacgaagaag...................................	9	0	sg1
....uGcagguguaaccacgaagaag...................................	4	1	sg1

Mature **Star**

```
gaaccaaaacaucagucggcuucuucgugguuacaucuguaaaugagacucgcuuacagguguaaccacgaagaagccgacacccguuuugcuuugcaugcaucucuugaa
.......................................................acagguguaaccacgaaga.....................................	2	0	sg1
.......................................................acagguguaaccacgaagaa....................................	1	0	sg1
.......................................................acagguguaaccacgaagaag...................................	5	0	sg1
.......................................................acUgguguaaccacgaagaagc..................................	1	1	sg1
.......................................................acagguguaaccacgaagaagU..................................	4	1	sg1
.......................................................acagguguGaccacgaagaagc..................................	1	1	sg1
.......................................................acagguguaaccacgaagaagc..................................	40	0	sg1
........................................................cagguguaaccacgaaga.....................................	4	0	sg1
........................................................cagguguaaccacgaagaag...................................	1	0	sg1
........................................................cGgguguaaccacgaagaagc..................................	3	1	sg1
........................................................cagguguaaccacgaagaagU..................................	1	1	sg1
........................................................cagguguaaccacgaagaagc..................................	15	0	sg1
........................................................cagguguaaccacgaagaagcA.................................	1	1	sg1
..............................................................uaaccacgaagaagcUgacac............................	1	1	sg1
```

Provisional ID	:	ScYm1zk_454_11699
Score total	:	6699.2
Score for star read(s)	:	3.9
Score for read counts	:	6692.6
Score for mfe	:	1.7
Score for randfold	:	1.6
Score for cons. seed	:	-0.6
Total read count	:	13139
Mature read count	:	12976
Loop read count	:	0
Star read count	:	163

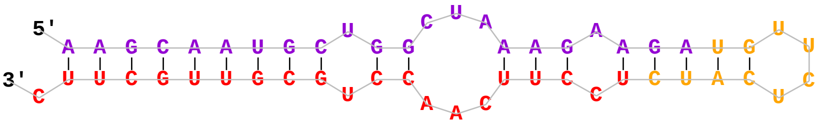

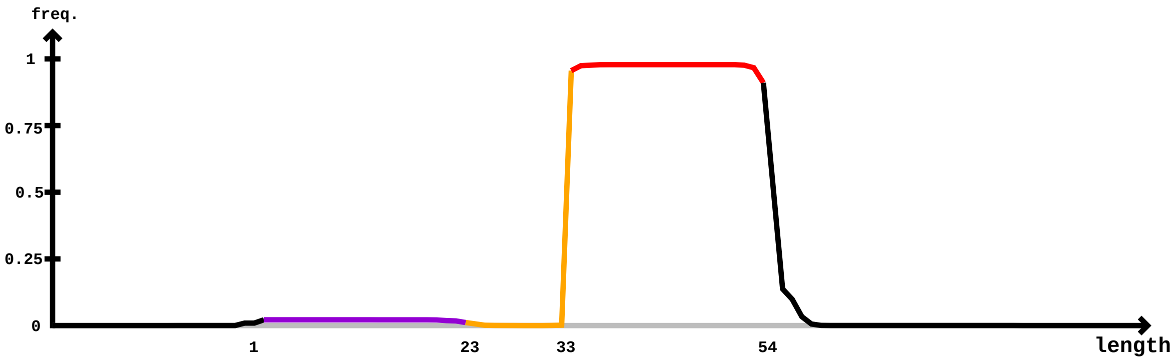


```
                          Star                            Mature
5'- uggagaagcugauugaaaugaagcaaugcuggcuaaagaagauguucucaucuccuucaaccugcguugcuucauuucauuguuaaauguauguccauuugugaauccugauu   -3'   obs
    uggagaagcugauugaaaugaagcaaugcuggcuaaagaagauguucucaucuccuucaaccugcguugcuucauuucauuguuaaauguauguccauuugugaauccugauu         exp
    ((((.(.((((((((((((((((((((((.((...(((.(((((....))))).)))...)).))))))))))))))))..))))..)).).)))).................   reads   mm   sample
    ...........auugaaaugaagcaaugcugg.................................................................................   1       0    st1
    ..................ugaagcaaugcuggcuaaag...........................................................................   2       0    st1
    ..................ugaagcaaugcuggcuaaaga..........................................................................   3       0    st1
    ..................ugaagcaaugcuggcuaaagaa.........................................................................   5       0    st1
    ..................ugaagcaaugcuggcuaaagaag........................................................................   15      0    st1
    ..................ugaagcaaugcuggcuaaagaaA........................................................................   1       1    st1
    ..................ugaagcaaugcuggcuaaagaaU........................................................................   2       1    st1
    ..................ugaagcaaugcuggcuaaagaagUu......................................................................   1       1    st1
    ...................gaagcaaugcuggcuaaagaagau......................................................................   1       0    st1
    ....................aagcaaugcuggcuaaaga..........................................................................   2       0    st1
    ....................aagcGaugcuggcuaaagaaga.......................................................................   1       1    st1
    ....................aagcaaugcuggcuaaagaaga.......................................................................   39      0    st1
    ....................aagcaaugcuggcuCaagaaga.......................................................................   1       1    st1
    ....................aagcaGugcuggcuaaagaaga.......................................................................   2       1    st1
    ....................aagcaaugcuggcuaaagaagau......................................................................   5       0    st1
    ....................aagcaaugcuggcuaaagaagauU.....................................................................   4       1    st1
    ....................aagcaaugcuggcuaaagaagauC.....................................................................   3       1    st1
    ....................aagcaaugcuggcuaaagaGgauguuc..................................................................   1       1    st1
    ...................................................cuccuucaaccugcguugcuucUu......................................   1       1    st1
    ....................................................uccuucaaccugcguugc...........................................   2       0    st1
    ....................................................uccuucaaccugcguugU...........................................   1       1    st1
    ....................................................uccuucaaccugcguugcu..........................................   7       0    st1
    ....................................................uccuucaaccugcguugGuu.........................................   1       1    st1
    ....................................................uccuucaaccugcguugcuC.........................................   4       1    st1
    ....................................................uccuucaaccuAcguugcuu.........................................   3       1    st1
    ....................................................uccuucaacUugcguugcuu.........................................   1       1    st1
    ....................................................uccuucaaccugcguugcuu.........................................   50      0    st1
    ....................................................uccuucaGccugcguugcuu.........................................   2       1    st1
    ....................................................uccuucaaccugcguugcuA.........................................   3       1    st1
    ....................................................uccuucaaccugcguugcCuc........................................   2       1    st1
    ....................................................uccuucaGccugcguugcuuc........................................   9       1    st1
    ....................................................uccuucaaccugcUuugcuuc........................................   1       1    st1
    ....................................................uccuucaaccugcguCgcuuc........................................   1       1    st1
```

Star **Mature**

```
uggagaagcugauugaaaugaagcaaugcuggcuaaagaagauguucucaucuccuucaaccugcguugcuucauuucauuguuaauguauguccauuugugaauccugauu
....................................................uccuucaaccugcguugcuuA.......................................   85    1    st1
....................................................uccuucaaccugcguugcuuU.......................................   54    1    st1
....................................................uccuucaaccugGguugcuuc.......................................   1     1    st1
....................................................uccuucaaccugcAuugcuuc.......................................   1     1    st1
....................................................uccuucaaccugUguugcuuc.......................................   3     1    st1
....................................................uccuucaacUugcguugcuuc.......................................   1     1    st1
....................................................uccuucaaccugcguugcuuG.......................................   1     1    st1
....................................................uccuucaaAcugcguugcuuc.......................................   1     1    st1
....................................................ucGuucaaccugcguugcuuc.......................................   1     1    st1
....................................................uccuAcaaccugcguugcuuc.......................................   1     1    st1
....................................................uccuucGaccugcguugcuuc.......................................   1     1    st1
....................................................uccuCcaaccugcguugcuuc.......................................   1     1    st1
....................................................uccuucaaccugcguugcuCc.......................................   3     1    st1
....................................................uccuucaaccCgcguugcuuc.......................................   2     1    st1
....................................................uccuucaaccugcguugUuuc.......................................   1     1    st1
....................................................uccuucaaccugcguugcuuc.......................................   610   0    st1
....................................................uccuucaaccugcgCugcuuc.......................................   3     1    st1
....................................................ucUuucaaccugcguugcuuca......................................   1     1    st1
....................................................Gccuucaaccugcguugcuuca......................................   2     1    st1
....................................................uccuucaaccugcguugcuuAa......................................   4     1    st1
....................................................uccuucaaccugcguugcCuca......................................   1     1    st1
....................................................uUcuucaaccugcguugcuuca......................................   1     1    st1
....................................................uccuucaaccugcguugcuucU......................................   110   1    st1
....................................................uccAucaaccugcguugcuuca......................................   1     1    st1
....................................................uccuucaaccugcgCugcuuca......................................   1     1    st1
....................................................uccuucaUccugcguugcuuca......................................   1     1    st1
....................................................uccuucaaccugcguugcuucC......................................   12    1    st1
....................................................uccuucaGccugcguugcuuca......................................   6     1    st1
....................................................uccuucaaccugcguugcuuca......................................   676   0    st1
....................................................uccuucGaccugcguugcuuca......................................   1     1    st1
....................................................uccuucaaccugcgAugcuuca......................................   1     1    st1
....................................................uccuucaaccCgcguugcuuca......................................   2     1    st1
....................................................uccuCcaaccugcguugcuuca......................................   1     1    st1
....................................................uccuucaaccugcguugcuCca......................................   4     1    st1
....................................................uccuucaaccugcguugGuuca......................................   1     1    st1
....................................................uccuucaaccugcCuugcuuca......................................   2     1    st1
....................................................uccuucaaAcugcguugcuuca......................................   1     1    st1
....................................................uccuucaaccugcAuugcuuca......................................   2     1    st1
....................................................uccuucaaccugcguugcuucG......................................   12    1    st1
....................................................uccuucaaccugcguCgcuuca......................................   1     1    st1
....................................................uccuucaaccugcguugcuuUa......................................   4     1    st1
....................................................uccuucaaccugcguugcuucUu.....................................   5     1    st1
....................................................uccuucaaccugcguugcuucau.....................................   68    0    st1
....................................................uccuucaaccugcguugcuucaG.....................................   1     1    st1
....................................................uccuucaaccugcguugcuCcau.....................................   1     1    st1
....................................................uccuucaaccugcguugcuucaC.....................................   8     1    st1
....................................................uccuucaaccugcguCgcuucau.....................................   2     1    st1
....................................................uccuucaaccugcguugcuucaA.....................................   62    1    st1
....................................................Cccuucaaccugcguugcuucau.....................................   1     1    st1
....................................................uccuucaaccugcguugcuucUuu....................................   2     1    st1
....................................................uccuucaGccugcguugcuucauu....................................   2     1    st1
....................................................uccuucaaccugcguugcuuUauu....................................   1     1    st1
....................................................uccuucaaccugcguuAcuucauu....................................   2     1    st1
....................................................uccuucaaccugcguugcuucauA....................................   29    1    st1
....................................................uccuucaaccugcguugcuucaGu....................................   1     1    st1
....................................................uccuucaaccugcguugcuucaCu....................................   1     1    st1
....................................................uccuucaaccugcguugcuCcauu....................................   1     1    st1
....................................................uccuucaaccugcguugcuucaAu....................................   29    1    st1
....................................................uccuucaaccugcguugGuucauu....................................   1     1    st1
....................................................uccuucaaccugcguugcuucauu....................................   90    0    st1
....................................................uccuucaaccugcguugcuucauC....................................   16    1    st1
....................................................uccuucaaccugcguugcuucauG....................................   1     1    st1
....................................................uccuucaaccugcgCugcuucauu....................................   1     1    st1
....................................................uccuucaaccugcguugcuucauuC...................................   3     1    st1
....................................................uccuucaaccugcguugcuucauuu...................................   12    0    st1
....................................................uccuucaaccugcguugcuucaAuu...................................   15    1    st1
....................................................uccuucaaccugcguugcuucauuA...................................   5     1    st1
....................................................uccuuUaaccugcguugcuucauuu...................................   1     1    st1
....................................................uccuucaaccugcguugcuucauCu...................................   1     1    st1
....................................................uccuucaaccugcguugcuucUuuu...................................   5     1    st1
```

Star Mature

```
uggagaagcugauugaaaugaagcaaugcuggcuaaagaagauguucucaucuccuucaaccugcguugcuucauuucauuguuaauguauguccauuugugaauccugauu
....................................................uccuucaaccugcguugcuucauAu...................................   5   1   st1
....................................................uccuucaaccugcguugcuucauuuA..................................   4   1   st1
....................................................uccuucaaccugcguugcuucauuuAa.................................   1   1   st1
.....................................................ccuucaaccugcguugcuu........................................   1   0   st1
.....................................................ccuucaaccugcguugcuuc.......................................   6   0   st1
.....................................................ccuucaGccugcguugcuuc.......................................   4   1   st1
.....................................................ccuucaaccugcguugcuucG......................................   1   1   st1
.....................................................ccuucaGccugcguugcuuca......................................   8   1   st1
.....................................................ccuucaaccugcguugcuuca......................................   3   0   st1
.....................................................ccuucaaccugcguugcuucau.....................................   2   0   st1
.....................................................ccuucaaccugcguugcuucauu....................................   21  0   st1
.....................................................ccuucaaccugcguugcuucauC....................................   3   1   st1
.....................................................ccuucaaccugcguuAcuucauu....................................   1   1   st1
.....................................................ccuucaaccugcgCugcuucauu....................................   1   1   st1
.....................................................ccuucaGccugcguugcuucauu....................................   4   1   st1
.....................................................ccuucaaccugcguugcuucauuu...................................   3   0   st1
.....................................................ccuucaaccugcguugcuucauuC...................................   4   1   st1
.....................................................ccuucaaccugcguugcuucauuA...................................   3   1   st1
.....................................................ccuucaaccugcguugcuucauuuA..................................   2   1   st1
......................................................cuucaaccugUguugcuuc.......................................   1   1   st1
......................................................cuucaaccugcguugcuuca......................................   1   0   st1
......................................................cuucaaccugcguugcuucauu....................................   2   0   st1
.......................................................uucGaccugcguugcuucauu....................................   1   1   st1
.......................................................uucaaccugcguugcuucauu....................................   2   0   st1
.......................................................uucaaccugcguugcuucauuuA..................................   1   1   st1
.......................................................uucaaccugcguugcuucauuuAa.................................   1   1   st1

..................Ggaagcaaugcuggcuaaag..........................................................................   1   1   sr1
..................ugaagcaaugcuggcuaaag..........................................................................   1   0   sr1
..................Cgaagcaaugcuggcuaaaga.........................................................................   2   1   sr1
..................ugaagcaaugcuggcuaaaga.........................................................................   6   0   sr1
..................Ggaagcaaugcuggcuaaaga.........................................................................   5   1   sr1
..................ugaagcaaugcuggcuaaagaa........................................................................   3   0   sr1
..................ugaagcaaugcuggcuaaagaag.......................................................................   11  0   sr1
..................ugaagcaaugcuggcCaaagaag.......................................................................   3   1   sr1
..................Ggaagcaaugcuggcuaaagaag.......................................................................   21  1   sr1
....................aagcaaugcuggcuaaaga.........................................................................   5   0   sr1
....................aagcaaugcuggcuaaagaag.......................................................................   2   0   sr1
....................aagcaaugcuggcuaaagGaga......................................................................   1   1   sr1
....................aagcaaugcuggcuaaagaaga......................................................................   43  0   sr1
....................aagcaaugcCggcuaaagaaga......................................................................   1   1   sr1
....................aagcaaugcuggcuaaagaagau.....................................................................   4   0   sr1
....................aagcaaugcuggcuaaagaagaA.....................................................................   1   1   sr1
....................aagcaaugcuggcuaaagaagaug....................................................................   1   0   sr1
....................aagcaaugcuggcuaaagaagauA....................................................................   1   1   sr1
....................aagcaaugcuggcuaaagaagauguC..................................................................   1   1   sr1
..................................................ucuccuucaaccugcguugcuu........................................   1   0   sr1
..................................................ucuccuucaaccugcguugcuuA.......................................   2   1   sr1
...................................................cuccuucaaccugcguugcuuA.......................................   3   1   sr1
...................................................cuccuucGaccugcguugcuuc.......................................   1   1   sr1
...................................................cuccuucaaccugcguugcuuc.......................................   2   0   sr1
....................................................Gccuucaaccugcguugc..........................................   1   1   sr1
....................................................uccuucaaccugcguugc..........................................   16  0   sr1
....................................................uccuucaaccugcguugU..........................................   2   1   sr1
....................................................uccuucaaccugcguugA..........................................   1   1   sr1
....................................................Gccuucaaccugcguugcu.........................................   3   1   sr1
....................................................Cccuucaaccugcguugcu.........................................   1   1   sr1
....................................................uccuucaaccugcguugcC.........................................   6   1   sr1
....................................................uccuucaaccugcguugUu.........................................   2   1   sr1
....................................................uccuucaaccugcguugcG.........................................   5   1   sr1
....................................................uccuucaaccugcguugcu.........................................   42  0   sr1
....................................................uccuucaaccugcguugcA.........................................   3   1   sr1
....................................................uccuCcaaccugcguugcu.........................................   1   1   sr1
....................................................Gccuucaaccugcguugcuu........................................   14  1   sr1
....................................................uccuucaaccugcguugcuC........................................   22  1   sr1
....................................................uccuucaaccugcguugcuu........................................   210 0   sr1
....................................................uccuucaGccugcguugcuu........................................   3   1   sr1
....................................................uccuucaaccugcguugUuu........................................   1   1   sr1
....................................................uccuucaaccugAguugcuu........................................   1   1   sr1
....................................................uccuucaaccugcguugcCu........................................   2   1   sr1
```

```
                    Star                           Mature
uggagaagcugauugaaaugaagcaaugcuggcuaaagaagauguucucaucuccuucaaccugcguugcuucauuucauuguuaauguauguccauuugugaauccugauu
....................................................uccuucaaccugcguugcuA........................................ 4     1   sr1
....................................................uccuucaaccugcguuAcuu........................................ 1     1   sr1
....................................................uccuucGaccugcguugcuu........................................ 1     1   sr1
....................................................uccuucaaccugcguugcuG........................................ 11    1   sr1
....................................................uccuucaaccugcguugcuuc....................................... 1345  0   sr1
....................................................uccuucaaccugcguugcuGc....................................... 2     1   sr1
....................................................uccuucaaccugcguugcuuU....................................... 182   1   sr1
....................................................uccuucaaccugcAuugcuuc....................................... 2     1   sr1
....................................................uccuucaaccAgcguugcuuc....................................... 1     1   sr1
....................................................uUcuucaaccugcguugcuuc....................................... 1     1   sr1
....................................................ucUuucaaccugcguugcuuc....................................... 1     1   sr1
....................................................uccuucaaccugcguugcuuA....................................... 1107  1   sr1
....................................................uccuCcaaccugcguugcuuc....................................... 8     1   sr1
....................................................uccuucaaccugcguAgcuuc....................................... 3     1   sr1
....................................................uccuucaaccugcguugUuuc....................................... 1     1   sr1
....................................................Cccuucaaccugcguugcuuc....................................... 1     1   sr1
....................................................uccuucaaccugcguCgcuuc....................................... 4     1   sr1
....................................................uccuucaaccugcguugcGuc....................................... 1     1   sr1
....................................................uccuucaaccugcguugcAuc....................................... 2     1   sr1
....................................................uccuucaaccugUguugcuuc....................................... 1     1   sr1
....................................................uccuucGaccugcguugcuuc....................................... 4     1   sr1
....................................................uccuucaacGugcguugcuuc....................................... 1     1   sr1
....................................................uccAucaaccugcguugcuuc....................................... 3     1   sr1
....................................................uccuucaaccugAguugcuuc....................................... 1     1   sr1
....................................................uccuucaaccugcgCugcuuc....................................... 2     1   sr1
....................................................uccuucaaccCgcguugcuuc....................................... 10    1   sr1
....................................................Gccuucaaccugcguugcuuc....................................... 106   1   sr1
....................................................uccuucaaccugcguugcuuG....................................... 19    1   sr1
....................................................uccuucaaccugcguuCcuuc....................................... 1     1   sr1
....................................................uccuAcaaccugcguugcuuc....................................... 4     1   sr1
....................................................uccuucaaccuAcguugcuuc....................................... 1     1   sr1
....................................................uccuucaGccugcguugcuuc....................................... 10    1   sr1
....................................................uccuucaaccugcguugcuCc....................................... 9     1   sr1
....................................................uccuucaaccugcguugcCuc....................................... 6     1   sr1
....................................................uccuucaaccAgcguugcuuca...................................... 1     1   sr1
....................................................uccuucaaccugcguuAcuuca...................................... 1     1   sr1
....................................................Cccuucaaccugcguugcuuca...................................... 3     1   sr1
....................................................uccuucaaccugUguugcuuca...................................... 2     1   sr1
....................................................Accuucaaccugcguugcuuca...................................... 1     1   sr1
....................................................uccuucaaccugcguugcuAca...................................... 2     1   sr1
....................................................uccuucaaccugcguugcuuca...................................... 635   0   sr1
....................................................uccuucaaccugcguugcCuca...................................... 4     1   sr1
....................................................uccuuUaaccugcguugcuuca...................................... 1     1   sr1
....................................................uccuucaaccugcguugcuuUa...................................... 54    1   sr1
....................................................uccuucaaccugcguAgcuuca...................................... 1     1   sr1
....................................................uccuucaaccugcguugAuuca...................................... 1     1   sr1
....................................................uccuucaaccugcgAugcuuca...................................... 1     1   sr1
....................................................uccuucaaccugcguugcuuAa...................................... 52    1   sr1
....................................................uccuucaaccugcguugGuuca...................................... 1     1   sr1
....................................................uccuCcaaccugcguugcuuca...................................... 4     1   sr1
....................................................Gccuucaaccugcguugcuuca...................................... 38    1   sr1
....................................................uccuucaaccCgcguugcuuca...................................... 5     1   sr1
....................................................uccuucGaccugcguugcuuca...................................... 1     1   sr1
....................................................uccuGcaaccugcguugcuuca...................................... 1     1   sr1
....................................................uccuucaaUcugcguugcuuca...................................... 1     1   sr1
....................................................uccuucaaccugcguugcuucU...................................... 153   1   sr1
....................................................uccuucaaccugcgCugcuuca...................................... 3     1   sr1
....................................................uccuucaaccugcAuugcuuca...................................... 1     1   sr1
....................................................uccuucaGccugcguugcuuca...................................... 10    1   sr1
....................................................uccuucaaccugcguugcuucG...................................... 55    1   sr1
....................................................uccuucaaccugcguugcuucC...................................... 13    1   sr1
....................................................uccuucaaccugcguuAcuucau..................................... 1     1   sr1
....................................................uccuucaaccugcguugcuucaC..................................... 4     1   sr1
....................................................uccuucaaccugcguugcuucUu..................................... 5     1   sr1
....................................................Gccuucaaccugcguugcuucau..................................... 4     1   sr1
....................................................uccuCcaaccugcguugcuucau..................................... 2     1   sr1
....................................................uccuucaaccugcguugcuucaG..................................... 6     1   sr1
....................................................uccuucaaccugcAuugcuucau..................................... 1     1   sr1
....................................................uccuucaaccugcguugcuucau..................................... 62    0   sr1
....................................................uccuucaaccugcguugcuuAau..................................... 1     1   sr1
```

```
                    Star                            Mature
uggagaagcugauugaaaugaagcaaugcuggcuaaagaagauguucucaucuccuucaaccugcguugcuucauuucauuguuaaauguauguccauuugugaauccugauu
....................................................uccuucaaccugcguugcuucaA.......................................  41   1  sr1
....................................................uUcuucaaccugcguugcuucau......................................  1    1  sr1
....................................................uccuucaaccugcguugcuucauG.....................................  9    1  sr1
....................................................uccuucaaccugcguugcuucauA.....................................  80   1  sr1
....................................................uccuucaaccugcguugcuucauu.....................................  61   0  sr1
....................................................uccuucaaccugcguugcuucauC.....................................  5    1  sr1
....................................................uccuucaaccugcguugcuucaAu.....................................  29   1  sr1
....................................................Gccuucaaccugcguugcuucauu.....................................  2    1  sr1
....................................................uccuucaaccugcguugcuucUuu.....................................  6    1  sr1
....................................................uccuucaaccugcguugcuucaCu.....................................  1    1  sr1
....................................................uccuucaaccugcguugcuucauuu....................................  22   0  sr1
....................................................uccuucaaccugcguugcuucauuG....................................  1    1  sr1
....................................................uccuucaaccugcAuugcuucauuu....................................  1    1  sr1
....................................................uccuucaaccugcguugcuucaAuu....................................  12   1  sr1
....................................................uccuucaaccugcguugcuucauuA....................................  43   1  sr1
....................................................uccuucaaccugcguugcuucUuuu....................................  4    1  sr1
....................................................uccuucaaccugcguugcuucauAu....................................  10   1  sr1
....................................................uccuucaaccugcguugcuucauuuA...................................  17   1  sr1
....................................................uccuucaaccugcguugcuucaAuuc...................................  2    1  sr1
.....................................................ccuucaaccugcguugcuu.........................................  1    0  sr1
.....................................................ccuucaaccugcguugcuuA........................................  5    1  sr1
.....................................................cAuucaaccugcguugcuuc........................................  1    1  sr1
.....................................................ccuucaaccugcguugcuuc........................................  17   0  sr1
.....................................................ccuucaaccugcguugcuuU........................................  1    1  sr1
.....................................................ccuucaGccugcguugcuuc........................................  5    1  sr1
.....................................................ccuucaaccuAcguugcuuc........................................  1    1  sr1
.....................................................ccuucaaccuAcguugcuuca.......................................  1    1  sr1
.....................................................ccuucaGccugcguugcuuca.......................................  14   1  sr1
.....................................................ccuucaaccugcguugcuuca.......................................  7    0  sr1
.....................................................ccuucaaccugcguugcuucaA......................................  1    1  sr1
.....................................................ccuucaaccugcguugcuucaG......................................  1    1  sr1
.....................................................ccuucaaccugcguugcuucau......................................  1    0  sr1
.....................................................ccuucaaccugcguugcuucauA.....................................  4    1  sr1
.....................................................ccuucaGccugcguugcuucauu.....................................  1    1  sr1
.....................................................ccuucaaccugcguugcuucauu.....................................  11   0  sr1
.....................................................ccuucaaccugcguugcuucauC.....................................  5    1  sr1
.....................................................ccuucaaccugcguugcuucauG.....................................  1    1  sr1
.....................................................ccuucaaccugcguugcuucauuC....................................  1    1  sr1
.....................................................ccuucaaccugcguugcuucauuA....................................  7    1  sr1
.....................................................ccuucaaccugcguugcuucauuuA...................................  1    1  sr1
.....................................................ccuucaaccugcguugcuucauuAc...................................  1    1  sr1
.....................................................ccuucaaccugcguugcuucauuuAa..................................  1    1  sr1
......................................................cuucaaccugcguugcuuU........................................  2    1  sr1
......................................................cuucaaccugcguugcuuc........................................  2    0  sr1
......................................................cuucaaccugcguugcuuca.......................................  2    0  sr1
......................................................cuucaaccugcguugcuucauu.....................................  1    0  sr1
.......................................................uucaaccugcguugcuucauu.....................................  2    0  sr1
.......................................................uucaaccugcguugcuucauuA....................................  3    1  sr1
.......................................................uucaaccugcguugcuucGuuu....................................  1    1  sr1
.......................................................uucaaccugcguugcuucauuuA...................................  1    1  sr1
........................................................ucaaccugcguugcuucauuuA...................................  2    1  sr1
..........................................................................uuucauuguuaaauguauguccauuu.............  2    0  sr1

..................ugaagcaaugcuggcuaaag...........................................................................  1    0  sg1
..................ugaagcaaugcuggcuaaaga..........................................................................  3    0  sg1
..................ugaagcaauUcuggcuaaaga..........................................................................  1    1  sg1
..................ugaagcaaugcuggcuaaagaa.........................................................................  5    0  sg1
..................ugaagcaaugcuggcuaaagaaA........................................................................  1    1  sg1
..................ugaagcaaugcuggcuUaagaag........................................................................  1    1  sg1
..................ugaagcaaugcuggcuaaagaag........................................................................  5    0  sg1
....................aagcaaugcuggcuaaagaag........................................................................  1    0  sg1
....................aagcaaugcuggcuaaagaaga.......................................................................  12   0  sg1
....................aagcaaugcuggcuaaagaagauU.....................................................................  1    1  sg1
....................................................uccuucaaccugcguugcu..........................................  1    0  sg1
....................................................uccuucaaccugcguugcCu.........................................  1    1  sg1
....................................................uccuucaaccugcguugcuu.........................................  26   0  sg1
....................................................uccuucaaccugcguugcuC.........................................  3    1  sg1
....................................................Cccuucaaccugcguugcuuc........................................  2    1  sg1
....................................................uccuucaaccugcguugcuuA........................................  198  1  sg1
....................................................ucUuucaaccugcguugcuuc........................................  1    1  sg1
```

Star Mature

```
uggagaagcugauugaaaugaagcaaugcuggcuaaagaagauguucucaucuccuucaaccugcguugcuucauuucauuguuaaauguauguccauuugugaauccugauu
....................................................uccuucaaUcugcguugcuuc........................................ 1 1 sg1
....................................................uccuucaaccugcguugcuuG........................................ 2 1 sg1
....................................................uccuucaaccugcguCgcuuc........................................ 3 1 sg1
....................................................uccuucaaccugcguugcuuc........................................ 273 0 sg1
....................................................uccuucaaccugcguugcuCc........................................ 3 1 sg1
....................................................uccuucaaccugcguugcCuc........................................ 1 1 sg1
....................................................uccuucaaccugcAuugcuuc........................................ 1 1 sg1
....................................................Accuucaaccugcguugcuuc........................................ 2 1 sg1
....................................................uccuucaaccuAcguugcuuc........................................ 1 1 sg1
....................................................uccuucaaccugcguugcuuU........................................ 31 1 sg1
....................................................uccuucaGccugcguugcuuc........................................ 5 1 sg1
....................................................uccuCcaaccugcguugcuuc........................................ 1 1 sg1
....................................................uccuucaaccugcgCugcuuc........................................ 2 1 sg1
....................................................uccuucaaccCgcguugcuuc........................................ 1 1 sg1
....................................................uccuucaaccugcguugcuucC....................................... 3 1 sg1
....................................................uccuucaaccugcguuAcuuca....................................... 1 1 sg1
....................................................uccuucaGccugcguugcuuca....................................... 5 1 sg1
....................................................uccuucaaccugcguugcuuca....................................... 293 0 sg1
....................................................uccuucaaccugcguugcuuUa....................................... 13 1 sg1
....................................................uccuucaaccugcguugcuuAa....................................... 8 1 sg1
....................................................uccuucaaccugcguugcuucU....................................... 63 1 sg1
....................................................uccuCcaaccugcguugcuuca....................................... 2 1 sg1
....................................................uccuucaaccugcguugUuuca....................................... 1 1 sg1
....................................................uccuucaaccCgcguugcuuca....................................... 2 1 sg1
....................................................uccuucGaccugcguugcuuca....................................... 1 1 sg1
....................................................uccuucaaccugcgCugcuuca....................................... 1 1 sg1
....................................................uccuucaaccugcguugcuucG....................................... 6 1 sg1
....................................................uccuucaaccugcguugcuucUu...................................... 1 1 sg1
....................................................uccuucaaccugcguugcuuUau...................................... 2 1 sg1
....................................................uccuucaaccugcguugcuucaC...................................... 1 1 sg1
....................................................uccuucaGccugcguugcuucau...................................... 3 1 sg1
....................................................uccuucaaccugcguugcuucaG...................................... 1 1 sg1
....................................................uccuucaaccugcguugcuuAau...................................... 1 1 sg1
....................................................uccuucaaccugcguugcuucaA...................................... 25 1 sg1
....................................................uccuucaaccugcguugcuucau...................................... 25 0 sg1
....................................................uccuucaaccugcguugcuucauG..................................... 1 1 sg1
....................................................uccuucaaccugcguugcuucauA..................................... 30 1 sg1
....................................................uccuucaacUugcguugcuucauu..................................... 1 1 sg1
....................................................uccuucaaccugcguugcuucaAu..................................... 13 1 sg1
....................................................uccuucaaccugcguugcuucUuu..................................... 4 1 sg1
....................................................uccuucaaccugcguuUcuucauu..................................... 1 1 sg1
....................................................uccuucaaccugcguugcuucauC..................................... 3 1 sg1
....................................................uccuucaaccugcguugcuucauu..................................... 41 0 sg1
....................................................uccuucaaccugcguugcuucauuu.................................... 12 0 sg1
....................................................uccuucaaccugcguugcuucauuA.................................... 16 1 sg1
....................................................uccuucaaccugcguCgcuucauuu.................................... 1 1 sg1
....................................................uccuucaaccugcguugcuucauAu.................................... 6 1 sg1
....................................................uccuucaaccugcguugcuucaAuu.................................... 2 1 sg1
....................................................uccuucaaccugcguugcuucauuuA................................... 6 1 sg1
.....................................................ccuucaGccugcguugcuuc........................................ 3 1 sg1
.....................................................ccuucaaccugcguugcuuc........................................ 5 0 sg1
.....................................................ccuucaaccugcguugcuuca....................................... 2 0 sg1
.....................................................ccuucaGccugcguugcuuca....................................... 1 1 sg1
.....................................................ccuucaaccugcguugcuucU....................................... 1 1 sg1
.....................................................ccuucaaccugcguugcuucau...................................... 1 0 sg1
.....................................................ccuucaaccugcguugcuucaA...................................... 1 1 sg1
.....................................................ccuucaGccugcguugcuucauu..................................... 2 1 sg1
.....................................................ccuucaaccugcguugcuucauu..................................... 1 0 sg1
.....................................................ccuucaaccugcguugcuucauuA.................................... 2 1 sg1
.....................................................ccuucaaccugcguugcuucauuu.................................... 2 0 sg1
......................................................cuucaaccugcguugcuucauu..................................... 2 0 sg1

..................ugaagcaaugcuggcuaaaga.......................................................................... 5 0 so1
..................ugaagcaaugcuggcuaaagaa......................................................................... 2 0 so1
..................ugaagcGaugcuggcuaaagaa......................................................................... 1 1 so1
..................ugaagcaaugcuggcuaaagaaA........................................................................ 1 1 so1
..................ugaagcaaugcuggcuaaagaag........................................................................ 11 0 so1
..................ugaagcaaugcuggcuaaagaaU........................................................................ 2 1 so1
..................ugaagcaaugcuggcuaaGgaag........................................................................ 1 1 so1
....................aaUcaaugcuggcuaaaga.......................................................................... 1 1 so1
```

```
                    Star                          Mature
uggagaagcugauugaaaugaagcaaugcuggcuaaagaagauguucucaucuccuucaaccugcguugcuucauuucauuguuaauguauguccauuugugaauccugauu
....................aagcaaugcuggcuaaagaag.......................................................................	1	0	so1
....................aagcaaugcuggcuaaagGag.......................................................................	1	1	so1
....................aagcaaugcuggcuaaagaagU......................................................................	2	1	so1
....................aagcaaugcuggcuaaagaaCa......................................................................	3	1	so1
....................aGgcaaugcuggcuaaagaaga......................................................................	1	1	so1
....................aagcaaugcuggcuaaagaaga......................................................................	15	0	so1
....................aagcaaugcuggcuaaagaagau.....................................................................	2	0	so1
....................aagcaaugcuggcuaaagaagaugC...................................................................	2	1	so1
....................aagcaaugcuggcuaaagaagauguu..................................................................	1	0	so1
....................aagcaaugcuggcuaaagaagauguuc.................................................................	1	0	so1
..................................................ucuccuucaaccugcguugcuuU.......................................	2	1	so1
..................................................ucuccuucaaccugcguugcuuc.......................................	1	0	so1
...................................................cuccuucaaccugcguugcuu........................................	1	0	so1
...................................................cuccuucaaccugcguugcuuA.......................................	2	1	so1
...................................................cuccuucaaccugcguugcuuc.......................................	2	0	so1
...................................................cuccuucaaccugcguugcuucUu.....................................	1	1	so1
...................................................cuccuucaaccugcguugcuucaAu....................................	1	1	so1
....................................................uccuucaaccugcguugc..........................................	4	0	so1
....................................................uccuCcaaccugcguugcu.........................................	1	1	so1
....................................................uccuucaaccugcguugcC.........................................	7	1	so1
....................................................uccuucaaccugcguugcu.........................................	45	0	so1
....................................................uccuucaaccugcguugcA.........................................	1	1	so1
....................................................uccuucaaccugcguugcuG........................................	5	1	so1
....................................................uccuucaGccugcguugcuu........................................	3	1	so1
....................................................uccuucGaccugcguugcuu........................................	1	1	so1
....................................................uccuucaaccugcguugcuu........................................	300	0	so1
....................................................uccuucaaccugcguugAuu........................................	1	1	so1
....................................................uccuucaaUcugcguugcuu........................................	1	1	so1
....................................................Cccuucaaccugcguugcuu........................................	1	1	so1
....................................................uccuCcaaccugcguugcuu........................................	1	1	so1
....................................................uccuucaaccugcguugcuA........................................	13	1	so1
....................................................uccuucaaccugcgCugcuu........................................	2	1	so1
....................................................uccuucaaccugcguugcuC........................................	41	1	so1
....................................................uccuucaaccugAguugcuu........................................	1	1	so1
....................................................uccuucaaccugcguCgcuu........................................	1	1	so1
....................................................uccuucaaccugcguugcCu........................................	1	1	so1
....................................................uccuucaaccCgcguugcuu........................................	4	1	so1
....................................................uccuucaaccugcguugcGu........................................	3	1	so1
....................................................Cccuucaaccugcguugcuuc.......................................	3	1	so1
....................................................uccuucaaccugcguugcuuc.......................................	1192	0	so1
....................................................uccuucaaccugcguCgcuuc.......................................	3	1	so1
....................................................Accuucaaccugcguugcuuc.......................................	2	1	so1
....................................................uccuucaaccugcguugcuAc.......................................	1	1	so1
....................................................uccuAcaaccugcguugcuuc.......................................	1	1	so1
....................................................ucAuucaaccugcguugcuuc.......................................	1	1	so1
....................................................uccuCcaaccugcguugcuuc.......................................	4	1	so1
....................................................uccuucaaccugcguAgcuuc.......................................	1	1	so1
....................................................uccuucaaccugcgCugcuuc.......................................	7	1	so1
....................................................uccuucaaccugcguugcuuA.......................................	983	1	so1
....................................................uccuucaaccCgcguugcuuc.......................................	4	1	so1
....................................................uccuucaaccugcguugcCuc.......................................	5	1	so1
....................................................uccuucGaccugcguugcuuc.......................................	4	1	so1
....................................................uccuucaaccugcguugcuuG.......................................	10	1	so1
....................................................uccuucaaccugcguugcuCc.......................................	7	1	so1
....................................................uccuucaaccugcguugUuuc.......................................	2	1	so1
....................................................uccuucaaccugcguugcuGc.......................................	5	1	so1
....................................................uccuucaGccugcguugcuuc.......................................	15	1	so1
....................................................uccuucaaccugUguugcuuc.......................................	1	1	so1
....................................................uccuucaaccugcguugcuuU.......................................	243	1	so1
....................................................uccuucaaccugcguugGuuc.......................................	1	1	so1
....................................................ucAuucaaccugcguugcuuca......................................	1	1	so1
....................................................Accuucaaccugcguugcuuca......................................	3	1	so1
....................................................uccuucaaccugcguugcuuUa......................................	87	1	so1
....................................................uccuucaaccugcguugcuucC......................................	25	1	so1
....................................................uccuucaaccugcguugcuCca......................................	3	1	so1
....................................................uccuucaaccugcAuugcuuca......................................	3	1	so1
....................................................uccuucaaccugcguugcuucG......................................	29	1	so1
....................................................uccuucaaccAgcguugcuuca......................................	1	1	so1
....................................................uccuucaaccuAcguugcuuca......................................	1	1	so1
....................................................uccuucaaccugcguugcuuAa......................................	22	1	so1
```

Star **Mature**

```
uggagaagcugauugaaaugaagcaaugcuggcuaaagaagauguucucaucuccuucaaccugcguugcuucauuucauuguuaauguaugccauuugugaauccugauu
....................................................uccuucaaccugcguCgcuuca.....................................    1      1      so1
....................................................ucUuucaaccugcguugcuuca.....................................    2      1      so1
....................................................uccuucaaccugcguugcAuca.....................................    1      1      so1
....................................................uccuCcaaccugcguugcuuca.....................................    2      1      so1
....................................................uccuucaaccugcgCugcuuca.....................................    1      1      so1
....................................................uccuucaGccugcguugcuuca.....................................    4      1      so1
....................................................uGcuucaaccugcguugcuuca.....................................    2      1      so1
....................................................uccuucaaccugcguugcuucU.....................................    170    1      so1
....................................................uccuucGaccugcguugcuuca.....................................    3      1      so1
....................................................uAcuucaaccugcguugcuuca.....................................    1      1      so1
....................................................uccuucaaccCgcguugcuuca.....................................    2      1      so1
....................................................Cccuucaaccugcguugcuuca.....................................    1      1      so1
....................................................uccuucaaccugcguugcuuca.....................................    817    0      so1
....................................................uccuCcaaccugcguugcuucau....................................    1      1      so1
....................................................uccuucaaccugcguugcuucaC....................................    18     1      so1
....................................................uccuucaaccugcguugcuuUau....................................    8      1      so1
....................................................uccuucaaccugcguugcuucau....................................    73     0      so1
....................................................uccuucaaccugcguugcuuAau....................................    1      1      so1
....................................................uccuucaaccugcguugcuucaA....................................    46     1      so1
....................................................uccuucaGccugcguugcuucau....................................    1      1      so1
....................................................uccuucaaccugcguugcuucUu....................................    11     1      so1
....................................................uccuucaaccugcguugcuucaG....................................    3      1      so1
....................................................uccuucaaccugcguugcCucau....................................    3      1      so1
....................................................uccuucGaccugcguugcuucau....................................    1      1      so1
....................................................uccuucaaccugcguugcuCcau....................................    1      1      so1
....................................................uccuucaaccugcguugcuucaAu...................................    32     1      so1
....................................................uccuucaaccugcguugcCucauu...................................    3      1      so1
....................................................uccuucaaccugcguugcuucUuu...................................    18     1      so1
....................................................uccuucaaccugcguugcuucauu...................................    168    0      so1
....................................................uccuucaaccugUguugcuucauu...................................    1      1      so1
....................................................uccuucaaccCgcguugcuucauu...................................    2      1      so1
....................................................uccuucaaccugcguugcuucauG...................................    1      1      so1
....................................................uccuucaaccugcguugcuucauA...................................    48     1      so1
....................................................uccuCcaaccugcguugcuucauu...................................    1      1      so1
....................................................uccuucaGccugcguugcuucauu...................................    1      1      so1
....................................................uccuucaaccugcguugcuuUauu...................................    1      1      so1
....................................................uccuucaaccugcguugcuucauC...................................    21     1      so1
....................................................uccuucaaccugcguugcuuAauu...................................    2      1      so1
....................................................uUcuucaaccugcguugcuucauu...................................    1      1      so1
....................................................Cccuucaaccugcguugcuucauuu..................................    1      1      so1
....................................................uccuucaaccugcguugcuucaAuu..................................    14     1      so1
....................................................uccuucaaccugcguugcuucauAu..................................    16     1      so1
....................................................uccuucaaccugcguugcuucUuuu..................................    6      1      so1
....................................................uccuucaaccugcguugcuucauuu..................................    60     0      so1
....................................................uccuucaaccugcguugcuucCuuu..................................    1      1      so1
....................................................uccuucaaccugcguugcCucauuu..................................    1      1      so1
....................................................uccuucaaccugcguugcuucauuC..................................    4      1      so1
....................................................uccuucaaccugcguugcuucauuA..................................    44     1      so1
....................................................uccuucaaccugcguugcuucauAuc.................................    2      1      so1
....................................................uccuucaaccugcguugcuucauuuA.................................    23     1      so1
....................................................uccuucaaccugcguugcuucauuuc.................................    2      0      so1
....................................................uccuucaaccugcguugcuucaCuuc.................................    1      1      so1
....................................................uccuucaaccugcguugcuucauuuAa................................    3      1      so1
.....................................................ccuucaaccugcguugcuC.......................................    1      1      so1
.....................................................ccuucaGccugcguugcuu.......................................    1      1      so1
.....................................................ccuucaaccugcguugcuuU......................................    1      1      so1
.....................................................ccuucaGccugcguugcuuc......................................    5      1      so1
.....................................................ccuucaaccugcguugcuuA......................................    4      1      so1
.....................................................ccuucaaccugcguugcuuc......................................    12     0      so1
.....................................................ccuucaGccugcguugcuuca.....................................    3      1      so1
.....................................................ccuucaaccugcguugcuucC.....................................    1      1      so1
.....................................................ccuucaaccugcguugcuuca.....................................    3      0      so1
.....................................................ccuucaaccugcguugcuucau....................................    2      0      so1
.....................................................ccuucaGccugcguugcuucau....................................    2      1      so1
.....................................................ccuucaaccugcguugcuucauC...................................    2      1      so1
.....................................................ccuucaGccugcguugcuucauu...................................    2      1      so1
.....................................................ccuucaaccugcguugcuucauu...................................    20     0      so1
.....................................................ccuucaaccugcguugcuucauuA..................................    2      1      so1
.....................................................ccuucaaccugcguugcuucauuu..................................    6      0      so1
.....................................................ccuucaaccugcguugcuucauuuA.................................    3      1      so1
```

Star Mature

```
uggagaagcugauugaaaugaagcaaugcuggcuaaagaagauguucucaucuccuucaaccugcguugcuucauuucauuguuaaauguauguccauuugugaauccugauu
......................................................cuucaaccugcguugcuu.........................................	1	0	so1
......................................................cuucaaccugcguugcuuA........................................	2	1	so1
......................................................cuucaaccugcguugcuuca.......................................	1	0	so1
......................................................cuucaaccugcguugcuucauu.....................................	2	0	so1
......................................................cuucaaccugcguugcuucauC.....................................	2	1	so1
......................................................cuucaaccugcguugcuucauuA....................................	2	1	so1
......................................................cuucaaccugcguugcuucauuu....................................	1	0	so1
.......................................................uucaaccugcguugcuucauA.....................................	3	1	so1
.......................................................uucaaccugcguugcuucauC.....................................	1	1	so1
.......................................................uucaaccugcguugcuucauu.....................................	1	0	so1
.......................................................uucaaccugcguugcuucauuA....................................	1	1	so1
.......................................................uucaaccugcguugcuucauuu....................................	4	0	so1
.......................................................uucaaccugcguugcuucauuuA...................................	1	1	so1
........................................................ucaaccugcguugcuucauuA....................................	1	1	so1
```

Provisional ID	:	ScYm1zk_454_11705
Score total	:	1.9
Score for star read(s)	:	-1.3
Score for read counts	:	0
Score for mfe	:	2.2
Score for randfold	:	1.6
Score for cons. seed	:	-0.6
Total read count	:	69580
Mature read count	:	62529
Loop read count	:	0
Star read count	:	7051

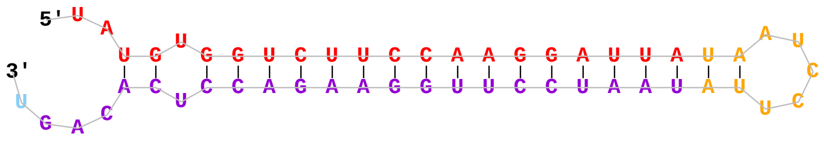

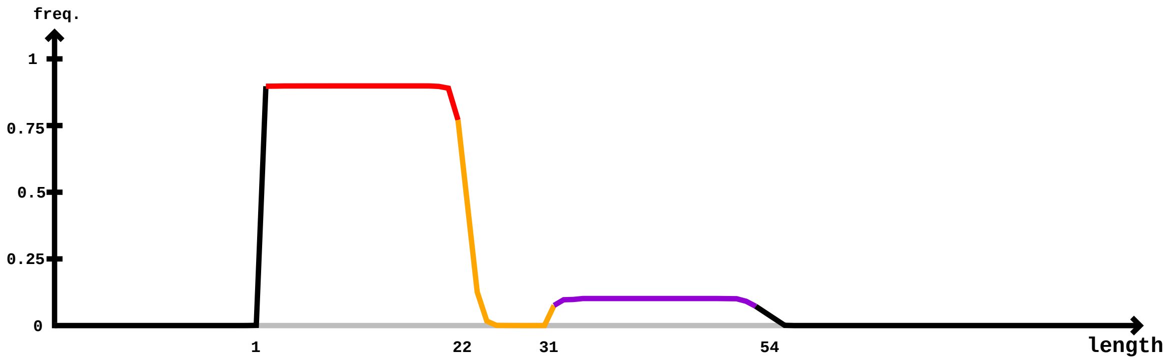

Mature Star

```
5'- cuccgcaucaaaguuuugacuauguggucuuccaaggauuauaauccuuauaauccuuggaagaccucacagucaacacuuuuuugaugcaaccaaagagagguagcaaca  -3'   obs
    cuccgcaucaaaguuuugacuauguggucuuccaaggauuauaauccuuauaauccuuggaagaccucacagucaacacuuuuuugaugcaaccaaagagagguagcaaca         exp
    ....(((((((((.((((((.((.((((((((((((((((((.....)))))))))))))))))).)).)))))).)))...))))))).(((.......)))....... reads   mm   sample
    .uccgcaucaaaguuuugac...........................................................................................   1      0    sr1
    ..................aAuauguggucuuccaaggauua......................................................................   1      1    sr1
    ..................Ucuauguggucuuccaaggauua......................................................................   1      1    sr1
    ...................cuauguggucuuccaaggauua......................................................................   6      0    sr1
    ...................cuauguggucuuccaaggauuaA.....................................................................   2      1    sr1
    ...................cuauguggucuuccaaggauuaC.....................................................................   1      1    sr1
    ...................cuauguggucuuccaaggauuaua....................................................................   5      0    sr1
    ....................uauguggucuuccaagAa.........................................................................   3      1    sr1
    ....................uauguggucuuccaaggG.........................................................................   4      1    sr1
    ....................Gauguggucuuccaagga.........................................................................   8      1    sr1
    ....................uauguggucuuccaagga.........................................................................   45     0    sr1
    ....................uauguggucuGccaagga.........................................................................   1      1    sr1
    ....................uauguggucuuccaGgga.........................................................................   7      1    sr1
    ....................uauguggucuuccaGggau........................................................................   9      1    sr1
    ....................uauguggucuuccaaggUu........................................................................   1      1    sr1
    ....................Gauguggucuuccaaggau........................................................................   14     1    sr1
    ....................uauguggCcuuccaaggau........................................................................   1      1    sr1
    ....................uauguggucuuccaaggaC........................................................................   14     1    sr1
    ....................uauguggucuuccaaggaG........................................................................   1      1    sr1
    ....................uauguggucuuccaaggGu........................................................................   1      1    sr1
    ....................uauguggucuAccaaggau........................................................................   1      1    sr1
    ....................uauguggucuuccaaggaA........................................................................   4      1    sr1
    ....................uauguggucuuccaaggCu........................................................................   1      1    sr1
    ....................uauguggucuuccaaggau........................................................................   139    0    sr1
    ....................Cauguggucuuccaaggau........................................................................   1      1    sr1
    ....................uauguggucuuUcaaggau........................................................................   1      1    sr1
    ....................uauguggucCuccaaggau........................................................................   1      1    sr1
    ....................uaugugguUuuccaaggauu.......................................................................   2      1    sr1
    ....................uauguggucuuccaGggauu.......................................................................   122    1    sr1
    ....................uauguggucuuccaaUgauu.......................................................................   1      1    sr1
    ....................uauguggucuuccUaggauu.......................................................................   1      1    sr1
    ....................uauguggGcuuccaaggauu.......................................................................   1      1    sr1
    ....................uauguggucuuccGaggauu.......................................................................   5      1    sr1
```

```
                    Mature                   Star
cuccgcaucaaaguuuugacuauguggucuuccaaggauuauaaauccuuauaauccuuggaagaccucacagucaacacuuuuuugaugcaaccaaagagagguagcaaca
....................uauguggucuuccaaggaCu........................................................................    2       1       sr1
....................uaugCggucuuccaaggauu........................................................................    2       1       sr1
....................uauguggucuuccaaggauC........................................................................    212     1       sr1
....................uaugAggucuuccaaggauu........................................................................    4       1       sr1
....................uauguggucuCccaaggauu........................................................................    9       1       sr1
....................uauguggCcuuccaaggauu........................................................................    7       1       sr1
....................uauguggucuuccaUggauu........................................................................    1       1       sr1
....................uCuguggucuuccaaggauu........................................................................    1       1       sr1
....................uauguggucuuccaaggGuu........................................................................    9       1       sr1
....................Gauguggucuuccaaggauu........................................................................    249     1       sr1
....................Aauguggucuuccaaggauu........................................................................    1       1       sr1
....................uauguggucuuccaaCgauu........................................................................    2       1       sr1
....................uUuguggucuuccaaggauu........................................................................    1       1       sr1
....................Cauguggucuuccaaggauu........................................................................    1       1       sr1
....................uauguggucuuccaCggauu........................................................................    1       1       sr1
....................uauguAgucuuccaaggauu........................................................................    1       1       sr1
....................uauguggucuuccaaggaGu........................................................................    1       1       sr1
....................uaCguggucuuccaaggauu........................................................................    9       1       sr1
....................uauguggucuuccaaggauA........................................................................    79      1       sr1
....................uauguggucuuccaaggauu........................................................................    2072    0       sr1
....................uauguggucuuccaagAauu........................................................................    2       1       sr1
....................uauguggucuuccaaggauG........................................................................    44      1       sr1
....................uauguggucCuccaaggauu........................................................................    2       1       sr1
....................uauguggucuGccaaggauu........................................................................    24      1       sr1
....................uauguggucuAccaaggauu........................................................................    1       1       sr1
....................uauguggucuuccaaggUuu........................................................................    4       1       sr1
....................uauguggucuuccCaggauu........................................................................    1       1       sr1
....................uauguggucuucGaaggauu........................................................................    1       1       sr1
....................uauguggucuucUaaggauu........................................................................    2       1       sr1
....................uauguggucuuccaaggaAua.......................................................................    2       1       sr1
....................uaugugAucuuccaaggauua.......................................................................    7       1       sr1
....................uauguggucuCccaaggauua.......................................................................    33      1       sr1
....................uauguggucCuccaaggauua.......................................................................    24      1       sr1
....................uauguggucuuccaaggauCa.......................................................................    22      1       sr1
....................uaugugguGuccaaggauua.......................................................................    1       1       sr1
....................uauguggucuuccaagAauua.......................................................................    6       1       sr1
....................Cauguggucuuccaaggauua.......................................................................    22      1       sr1
....................uauguggGcuuccaaggauua.......................................................................    1       1       sr1
....................uaAguggucuuccaaggauua.......................................................................    5       1       sr1
....................uauguggucuucUaaggauua.......................................................................    16      1       sr1
....................uauguggucuuccaaggauuC.......................................................................    6       1       sr1
....................uauguggucuuccUaggauua.......................................................................    6       1       sr1
....................uaugugguUuccaaggauua.......................................................................    3       1       sr1
....................uaugAggucuuccaaggauua.......................................................................    9       1       sr1
....................uauguggucuuccaCggauua.......................................................................    1       1       sr1
....................uauguggucuuccaaAgauua.......................................................................    4       1       sr1
....................uUuguggucuuccaaggauua.......................................................................    5       1       sr1
....................uauguggCcuuccaaggauua.......................................................................    28      1       sr1
....................Gauguggucuuccaaggauua.......................................................................    1135    1       sr1
....................uauguggucuGccaaggauua.......................................................................    100     1       sr1
....................uauguggucuuccaaggauuG.......................................................................    413     1       sr1
....................uauguggucuuccaaggauuU.......................................................................    96      1       sr1
....................uauguAgucuuccaaggauua.......................................................................    9       1       sr1
....................uauguggucuuccaaUgauua.......................................................................    2       1       sr1
....................uauguggucuuccGaggauua.......................................................................    22      1       sr1
....................uauguggucuuccaaggauAa.......................................................................    23      1       sr1
....................Aauguggucuuccaaggauua.......................................................................    10      1       sr1
....................uauguggucuuUcaaggauua.......................................................................    3       1       sr1
....................uauguggucuuccaaggauua.......................................................................    9235    0       sr1
....................uauguggucuuccCaggauua.......................................................................    2       1       sr1
....................uauguggucuucAaaggauua.......................................................................    2       1       sr1
....................uauguggAcuuccaaggauua.......................................................................    3       1       sr1
....................uauAuggucuuccaaggauua.......................................................................    5       1       sr1
....................uauguggucuuccaaggaCua.......................................................................    13      1       sr1
....................uauguUgucuuccaaggauua.......................................................................    1       1       sr1
....................uauguggucuuccaUggauua.......................................................................    4       1       sr1
....................uaCguggucuuccaaggauua.......................................................................    32      1       sr1
....................uaugCggucuuccaaggauua.......................................................................    27      1       sr1
....................uauguggucuuccaaggGuua.......................................................................    28      1       sr1
....................uauUuggucuuccaaggauua.......................................................................    2       1       sr1
```

```
                    Mature               Star
cuccgcaucaaaguuuugacuauguggucuuccaaggauuauaauccuuauaauccuuggaagaccucacagucaacacuuuuuugaugcaaccaaagagagguagcaaca
....................uauguggucuuccaaggUuua......................................................................    2       1    sr1
....................uauguggucuuccaGggauua......................................................................    466     1    sr1
....................uauguggucuuccaaggaGua......................................................................    2       1    sr1
....................uauguggucAuccaaggauua......................................................................    6       1    sr1
....................uauguggucuAccaaggauua......................................................................    2       1    sr1
....................uCuguggucuuccaaggauua......................................................................    9       1    sr1
....................uauguggucuuccaaggauCau.....................................................................    4       1    sr1
....................uauguggGcuuccaaggauuau.....................................................................    1       1    sr1
....................uauguggucuuccaaCgauuau.....................................................................    1       1    sr1
....................uauguggucuuccGaggauuau.....................................................................    4       1    sr1
....................uauguggucuuccaaggaCuau.....................................................................    7       1    sr1
....................uauguggucuuccaaggauuaC.....................................................................    262     1    sr1
....................uauguAgucuuccaaggauuau.....................................................................    2       1    sr1
....................uauguggucuuUcaaggauuau.....................................................................    1       1    sr1
....................uaAguggucuuccaaggauuau.....................................................................    1       1    sr1
....................uauguggucCuccaaggauuau.....................................................................    8       1    sr1
....................Gauguggucuuccaaggauuau.....................................................................    265     1    sr1
....................uauguggCcuuccaaggauuau.....................................................................    11      1    sr1
....................uauguggucuuccaaggauuUu.....................................................................    11      1    sr1
....................uaugugguUuuccaaggauuau.....................................................................    1       1    sr1
....................uauguggucuucAaaggauuau.....................................................................    2       1    sr1
....................uauguggucuuccaaAgauuau.....................................................................    1       1    sr1
....................uauguggucuuccaaggGuuau.....................................................................    12      1    sr1
....................uauguggucuuccaaggauuGu.....................................................................    24      1    sr1
....................uaCguggucuuccaaggauuau.....................................................................    4       1    sr1
....................uauguggucuCccaaggauuau.....................................................................    3       1    sr1
....................uauguggucuuccaaggauuaA.....................................................................    1767    1    sr1
....................uauguggucuuccaUggauuau.....................................................................    1       1    sr1
....................uauguggucuuccaaggauuau.....................................................................    2479    0    sr1
....................Cauguggucuuccaaggauuau.....................................................................    3       1    sr1
....................uaugCggucuuccaaggauuau.....................................................................    12      1    sr1
....................uauguggucuuccaaggauuCu.....................................................................    2       1    sr1
....................uauguggucuuccaGggauuau.....................................................................    213     1    sr1
....................uauUggucuuccaaggauuau.....................................................................    1       1    sr1
....................uauguggucuuccCaggauuau.....................................................................    1       1    sr1
....................uauguggAcuuccaaggauuau.....................................................................    1       1    sr1
....................uauguggucuucUaaggauuau.....................................................................    1       1    sr1
....................uauguggucuuccaaggauGau.....................................................................    1       1    sr1
....................uauguggucuuccaaggauuaG.....................................................................    126     1    sr1
....................uUguggucuuccaaggauuau.....................................................................    2       1    sr1
....................uaugAggucuuccaaggauuau.....................................................................    3       1    sr1
....................uauguggucuuccaaggCuuau.....................................................................    1       1    sr1
....................uaugugAucuuccaaggauuau.....................................................................    2       1    sr1
....................uauguggucuGccaaggauuau.....................................................................    50      1    sr1
....................uauAuggucuuccaaggauuau.....................................................................    3       1    sr1
....................uauguggucAuccaaggauuau.....................................................................    1       1    sr1
....................uauguggucuuccUaggauuau.....................................................................    3       1    sr1
....................uauguggucuuccaaggGuuaua....................................................................    6       1    sr1
....................uauguggucAuccaaggauuaua....................................................................    1       1    sr1
....................uaugAggucuuccaaggauuaua....................................................................    6       1    sr1
....................uauguggucuuAcaaggauuaua....................................................................    1       1    sr1
....................uauguggCcuuccaaggauuaua....................................................................    11      1    sr1
....................uauguggucuuccaaggauuauU....................................................................    114     1    sr1
....................uauguUgucuuccaaggauuaua....................................................................    1       1    sr1
....................uauguggucuuccaaggaAuaua....................................................................    3       1    sr1
....................uauAuggucuuccaaggauuaua....................................................................    2       1    sr1
....................uaugugguUuuccaaggauuaua....................................................................    3       1    sr1
....................uauguggAcuuccaaggauuaua....................................................................    1       1    sr1
....................uauguggucuuccaaAgauuaua....................................................................    2       1    sr1
....................uauguggucuucUaaggauuaua....................................................................    1       1    sr1
....................Aauguggucuuccaaggauuaua....................................................................    3       1    sr1
....................Cauguggucuuccaaggauuaua....................................................................    5       1    sr1
....................uauguggucuuccUaggauuaua....................................................................    1       1    sr1
....................uauguggucuuccaaggauuGua....................................................................    120     1    sr1
....................uauguggucuuccaaggauuauC....................................................................    15      1    sr1
....................uauguggucuuccaGggauuaua....................................................................    149     1    sr1
....................uauguggucuuccaaggauuaCa....................................................................    4       1    sr1
....................uauguggucCuccaaggauuaua....................................................................    6       1    sr1
....................uauguggucuuccaaggauuUua....................................................................    20      1    sr1
....................uUguggucuuccaaggauuaua....................................................................    2       1    sr1
```

```
                    Mature            Star
cuccgcaucaaaguuuugacuauguggucuuccaaggauuauaauccuuauaauccuuggaagaccucacagucaacacuuuuuugaugcaaccaaagagagguagcaaca
....................uauguggucuuccaaggauuaAa....................................................................	60	1	sr1
....................uaCguggucuuccaaggauuaua....................................................................	6	1	sr1
....................uauguggucuuccaaggauuCua....................................................................	2	1	sr1
....................uauguggucuGccaaggauuaua....................................................................	39	1	sr1
....................uauguggucuuccGaggauuaua....................................................................	11	1	sr1
....................uauUuggucuuccaaggauuaua....................................................................	1	1	sr1
....................uauguggucuuccaaggauuaua....................................................................	2323	0	sr1
....................uauguggucuuccaaggauGaua....................................................................	1	1	sr1
....................uaAguggucuuccaaggauuaua....................................................................	1	1	sr1
....................uauguggucuAccaaggauuaua....................................................................	2	1	sr1
....................uaGguggucuuccaaggauuaua....................................................................	1	1	sr1
....................Gauguggucuuccaaggauuaua....................................................................	279	1	sr1
....................uauguggucuuccaaggauuauG....................................................................	370	1	sr1
....................uaugugAucuuccaaggauuaua....................................................................	1	1	sr1
....................uaugCggucuuccaaggauuaua....................................................................	8	1	sr1
....................uauguggucuuccaaggaCuaua....................................................................	1	1	sr1
....................uauguggucuuccaaggauuaGa....................................................................	1	1	sr1
....................uauguggucuuUcaaggauuaua....................................................................	1	1	sr1
....................uauguggucuCccaaggauuaua....................................................................	11	1	sr1
....................uauguggucuuccaUggauuaua....................................................................	1	1	sr1
....................uaCguggucuuccaaggauuauaa...................................................................	2	1	sr1
....................uauguggucuuccaGggauuauaa...................................................................	32	1	sr1
....................uauguggucuuccaaggauuaGaa...................................................................	1	1	sr1
....................uauguggucuuccaaggauuauGa...................................................................	10	1	sr1
....................uauguggucuGccaaggauuauaa...................................................................	3	1	sr1
....................uauguggucuuccaaggauuGuaa...................................................................	57	1	sr1
....................uauguggucuuccaaggauCauaa...................................................................	1	1	sr1
....................uauguggucuuccaaggauuauaG...................................................................	1	1	sr1
....................uauguggucAuccaaggauuauaa...................................................................	1	1	sr1
....................uauguggucuuccaaggauuauCa...................................................................	2	1	sr1
....................uauguggucuuccaaggauuUuaa...................................................................	8	1	sr1
....................Gauguggucuuccaaggauuauaa...................................................................	23	1	sr1
....................uauguggucuuccaaggauuauaa...................................................................	283	0	sr1
....................uauguggucuuccaaggGuuauaa...................................................................	2	1	sr1
....................uauguggucuuccaaggauuauaU...................................................................	8	1	sr1
....................uauguggucuuccaaggauuauUa...................................................................	128	1	sr1
....................uauguggucuuccaagUauuauaa...................................................................	1	1	sr1
....................uauguggucuuccaaggauuaAaa...................................................................	13	1	sr1
....................uauguggucuAccaaggauuauaa...................................................................	1	1	sr1
....................uauguggucuuccGaggauuauaa...................................................................	1	1	sr1
....................uauguggucuuccaaggauuauaaG..................................................................	2	1	sr1
....................uauguggucuuccaaggauuaAaau..................................................................	3	1	sr1
....................uauguggucuuccaaggauuaGaau..................................................................	2	1	sr1
....................uauguggucuuccaaggauuauaaA..................................................................	1	1	sr1
....................uauguggucuuccaaggauuauaaC..................................................................	2	1	sr1
....................uauguggucuuccaaggauuauaau..................................................................	2	0	sr1
....................uauguggucuuccaaggauuauaauA.................................................................	1	1	sr1
....................uauguggucuuccaaggauuGuaauc.................................................................	1	1	sr1
....................uauguggucuuccaaggauuauaauU.................................................................	2	1	sr1
.....................auguggucuuccaaggauu.......................................................................	2	0	sr1
.....................auguggucuuccaaggauua......................................................................	1	0	sr1
.....................auguggucuuccaGggauua......................................................................	1	1	sr1
.....................auguggucuuccaaggauuau.....................................................................	1	0	sr1
.....................auguggucuuccaaggauuaua....................................................................	3	0	sr1
......................Gguggucuuccaaggauua......................................................................	1	1	sr1
......................uguggucuuccaaggauua......................................................................	2	0	sr1
......................uguggucuuccaaggauuG......................................................................	1	1	sr1
......................uguggucuAccaaggauuau.....................................................................	1	1	sr1
......................uguggucuuccaaggauuaC.....................................................................	1	1	sr1
......................uAuggucuuccaaggauuau.....................................................................	3	1	sr1
......................uguggucuuccaaggauuaA.....................................................................	2	1	sr1
......................uguggucuuccaaggauuau.....................................................................	1	0	sr1
......................uAuggucuuccaaggauuaua....................................................................	1	1	sr1
......................uguggucuuccaaggauuauU....................................................................	1	1	sr1
......................uguggucuuccaaggauuaua....................................................................	2	0	sr1
......................uguggucuuccaaggauuauaa...................................................................	2	0	sr1
........................uggucuuccaaggauuaua....................................................................	1	0	sr1
........................uggucuuccaaggauuauaU...................................................................	1	1	sr1
...........................ucuuccaaggauuauaaucU................................................................	4	1	sr1
...........................................auUcuuauaauccuugga..................................................	1	1	sr1
```

```
                    Mature                Star
cuccgcaucaaaguuuugacuaugugguucuuccaaggauuauaauccuuauaauccuuggaagaccucacagucaacacuuuuuugaugcaaccaaagagagguagcaaca
................................................uauaauccuuggaagaccuc...........................................   1     0   sr1
................................................uauaauccuuggaagaccucac.........................................   1     0   sr1
................................................uauaauccuuggaagaccucaca........................................   1     0   sr1
.................................................auaauccuuggaagaccuca..........................................   3     0   sr1
..................................................Gaauccuuggaagaccuc...........................................   1     1   sr1
..................................................uaauccuuggaagaccuU...........................................   1     1   sr1
..................................................Aaauccuuggaagaccuc...........................................   1     1   sr1
..................................................uaauccuuggaagaccuc...........................................   9     0   sr1
..................................................uaauccuuggaagaccuA...........................................   1     1   sr1
..................................................uaauccuuggaagaccuca..........................................   11    0   sr1
..................................................uaGuccuuggaagaccuca..........................................   1     1   sr1
..................................................uGauccuuggaagaccuca..........................................   1     1   sr1
..................................................uaauccuuggaagaccCcac.........................................   2     1   sr1
..................................................uaauccuuggaagaccucaG.........................................   1     1   sr1
..................................................uGauccuuggaagaccucac.........................................   10    1   sr1
..................................................Gaauccuuggaagaccucac.........................................   8     1   sr1
..................................................uaGuccuuggaagaccucac.........................................   3     1   sr1
..................................................uaauccuuggaagaccucGc.........................................   1     1   sr1
..................................................uaauccuuggaagaccucaA.........................................   4     1   sr1
..................................................uaauccCuggaagaccucac.........................................   1     1   sr1
..................................................uaauccuuggaGgaccucac.........................................   2     1   sr1
..................................................uaauccuuggaagaccucac.........................................   148   0   sr1
..................................................Caauccuuggaagaccucac.........................................   1     1   sr1
..................................................uaauccuuggaagaccucaU.........................................   10    1   sr1
..................................................uaauccuuggaagaccucaUa........................................   4     1   sr1
..................................................uaauccuuggaagGccucaca........................................   1     1   sr1
..................................................uaauccuuggaagaccucacU........................................   7     1   sr1
..................................................uaauccuCggaagaccucaca........................................   1     1   sr1
..................................................uaGuccuuggaagaccucaca........................................   5     1   sr1
..................................................uaauccuuggaagaccucacG........................................   23    1   sr1
..................................................uGauccuuggaagaccucaca........................................   26    1   sr1
..................................................uaauccAuggaagaccucaca........................................   1     1   sr1
..................................................uCauccuuggaagaccucaca........................................   1     1   sr1
..................................................uaauccuuAgaagaccucaca........................................   1     1   sr1
..................................................Gaauccuuggaagaccucaca........................................   19    1   sr1
..................................................uaauccuuggaagaccucaAa........................................   2     1   sr1
..................................................uaaCccuuggaagaccucaca........................................   1     1   sr1
..................................................uaauccuuggGagaccucaca........................................   1     1   sr1
..................................................uaauccuuggaagaccucaca........................................   409   0   sr1
..................................................uaauccuuggaagacGucaca........................................   1     1   sr1
..................................................uGauccuuggaagaccucacag.......................................   90    1   sr1
..................................................uaauccuuggaGgaccucacag.......................................   3     1   sr1
..................................................uaauccuuggaagaccuUacag.......................................   1     1   sr1
..................................................uaauccuuggaagaccucCcag.......................................   1     1   sr1
..................................................uaaCccuuggaagaccucacag.......................................   2     1   sr1
..................................................uaGuccuuggaagaccucacag.......................................   10    1   sr1
..................................................uUauccuuggaagaccucacag.......................................   1     1   sr1
..................................................uaUuccuuggaagaccucacag.......................................   3     1   sr1
..................................................uaauccCuggaagaccucacag.......................................   2     1   sr1
..................................................uCauccuuggaagaccucacag.......................................   1     1   sr1
..................................................uaauccuuggaagaccCcacag.......................................   1     1   sr1
..................................................uaauccuuggaagaccucacaC.......................................   3     1   sr1
..................................................Caauccuuggaagaccucacag.......................................   1     1   sr1
..................................................uaauccuuggaagaccucacag.......................................   462   0   sr1
..................................................uaauccuuggGagaccucacag.......................................   2     1   sr1
..................................................uaauccuuggaagaccucacaA.......................................   42    1   sr1
..................................................uaauccuCggaagaccucacag.......................................   1     1   sr1
..................................................uaauccuuggaagaccucacaU.......................................   5     1   sr1
..................................................Aaauccuuggaagaccucacag.......................................   1     1   sr1
..................................................Gaauccuuggaagaccucacag.......................................   14    1   sr1
..................................................Gaauccuuggaagaccucacagu......................................   4     1   sr1
..................................................uGauccuuggaagaccucacagu......................................   5     1   sr1
..................................................uaauccuuggaagaccucacagC......................................   13    1   sr1
..................................................uaauccuuggaagaccucacagu......................................   61    0   sr1
..................................................uaGuccuuggaagaccucacagu......................................   1     1   sr1
..................................................uaauccuuggaagaccucacagA......................................   12    1   sr1
..................................................uaauccuuggaagaccucacaguG.....................................   12    1   sr1
..................................................uaauccuuggaagaccucacaguU.....................................   12    1   sr1
..................................................uaauccuuggaagaccucacaguA.....................................   249   1   sr1
..................................................uaauccuuggaagaccucacaguUa....................................   4     1   sr1
```

```
                    Mature              Star
cuccgcaucaaaguuuugacuauguggucuuccaaggauuauaauccuuauaauccuuggaagaccucacagucaacacuuuuuugaugcaaccaaagagagguagcaaca
..................................................uaauccuuggaagaccucacaguAa....................................    5    1    sr1
...................................................aauccuuggaagaccuca..........................................    2    0    sr1
...................................................aauccuuggaagaccucac.........................................    47   0    sr1
...................................................aauccAuggaagaccucac.........................................    1    1    sr1
...................................................aauccuuggaagaUcucac.........................................    2    1    sr1
...................................................aauccuuggaGgaccucac.........................................    3    1    sr1
...................................................Gauccuuggaagaccucac.........................................    2    1    sr1
...................................................aauccuuggaagaccucaU.........................................    1    1    sr1
...................................................aGuccuuggaagaccucac.........................................    1    1    sr1
...................................................aauccuuggaagaccucacC........................................    2    1    sr1
...................................................aauccuuggaagaccucaca........................................    67   0    sr1
...................................................aauccuuggaagaccucacU........................................    1    1    sr1
...................................................aauccuuggaagaccCcaca........................................    3    1    sr1
...................................................Gauccuuggaagaccucacag.......................................    3    1    sr1
...................................................aGuccuuggaagaccucacag.......................................    1    1    sr1
...................................................aauccuuggaagaccucacag.......................................    166  0    sr1
...................................................aauccuuggaagaccucacaA.......................................    14   1    sr1
...................................................aauccuuggGagaccucacag.......................................    3    1    sr1
...................................................aauccuuggaagaccucGcag.......................................    1    1    sr1
...................................................aauccuuggaagaccCcacag.......................................    1    1    sr1
...................................................aauccCuggaagaccucacag.......................................    1    1    sr1
...................................................aauccuuggaagaccucacGg.......................................    1    1    sr1
...................................................aauccuuggaagaccucacagu......................................    69   0    sr1
...................................................aUuccuuggaagaccucacagu......................................    1    1    sr1
...................................................aauccuuggaagaccucacagG......................................    3    1    sr1
...................................................Gauccuuggaagaccucacagu......................................    3    1    sr1
...................................................aGuccuuggaagaccucacagu......................................    4    1    sr1
...................................................aauccuuggaagaccucacagA......................................    4    1    sr1
...................................................aauccuuggaagaccucacagC......................................    12   1    sr1
...................................................aauccuuggaagaccucacaguA.....................................    38   1    sr1
...................................................aauccuuggaagaccucacaguG.....................................    1    1    sr1
....................................................auccuuggaagaccucac.........................................    2    0    sr1
....................................................auccuuggaagaccucaA.........................................    1    1    sr1
....................................................auccuuggaagaccucaca........................................    4    0    sr1
....................................................auccuuggaagaccucacag.......................................    1    0    sr1
....................................................auccuuggaagaccucacaA.......................................    1    1    sr1
....................................................auccuuggaagaccucacagu......................................    6    0    sr1
....................................................auccCuggaagaccucacagu......................................    2    1    sr1
....................................................auccuuggaagGccucacagu......................................    1    1    sr1
....................................................auccuuggaagaccucacaguG.....................................    1    1    sr1
....................................................auccuuggaagaccucacaguA.....................................    8    1    sr1
....................................................auccuuggaagaccucacaguUa....................................    1    1    sr1
.....................................................uccuuggaagaccucacag.......................................    3    0    sr1
.....................................................uccuuggaagaccucGcagu......................................    1    1    sr1
.....................................................uccuuggUagaccucacagu......................................    1    1    sr1
.....................................................uccuuggaagaccucacagu......................................    13   0    sr1
.....................................................Gccuuggaagaccucacagu......................................    1    1    sr1
.....................................................uccuuggaagaccucacagC......................................    1    1    sr1
.....................................................uccuuggaagacAucacagu......................................    2    1    sr1
.....................................................uccuuggaagaccucacaguU.....................................    3    1    sr1
.....................................................uccuuggaagaccucacaguG.....................................    3    1    sr1
.....................................................uccuuggaagaccucacaguA.....................................    108  1    sr1
.....................................................uccuuggaagaccucacaguAa....................................    4    1    sr1
.....................................................uccuuggaagaccucacaguUa....................................    7    1    sr1

..................Ucuauguggucuuccaaggauua......................................................................    1    1    st1
..................aAuauguggucuuccaaggauuau.....................................................................    1    1    st1
...................cuauguggucuuccaaggauu.......................................................................    6    0    st1
...................cuauguggucuuccaGggauu.......................................................................    1    1    st1
...................cuauguggucuuccaaggauua......................................................................    5    0    st1
...................cuauguggucuuccaGggauua......................................................................    1    1    st1
...................Auauguggucuuccaaggauuau.....................................................................    2    1    st1
...................cuauguggucuuccaaggauuaC.....................................................................    1    1    st1
...................cuauguggucCuccaaggauuau.....................................................................    2    1    st1
...................cuauguggucuuccaaggauuau.....................................................................    5    0    st1
...................cuauguggucuGccaaggauuau.....................................................................    2    1    st1
...................cuauguggucuuccaaggauuGu.....................................................................    1    1    st1
...................cuauguggucuuccaaggauuauU....................................................................    3    1    st1
....................uauguggucuuccGagga.........................................................................    1    1    st1
....................uauguggucuuccaGgga.........................................................................    4    1    st1
```

```
                    Mature              Star
cuccgcaucaaaguuuugacuauguggucuuccaaggauuauaauccuuauaauccuuggaagaccucacagucaacacuuuuuugaugcaaccaaagagagguagcaaca
....................uauguggucuuccaaggU.........................................................................    1     1   st1
....................uauguggucuGccaagga.........................................................................    3     1   st1
....................uauguggucuuccaagga.........................................................................    29    0   st1
....................uauguggucuuccaaggaG........................................................................    5     1   st1
....................uauguggucuuccaaggaC........................................................................    10    1   st1
....................uauguggucuuccaaggau........................................................................    104   0   st1
....................uauguggucuGccaaggau........................................................................    2     1   st1
....................uauguggucuuccaGggau........................................................................    5     1   st1
....................uauguggucuuccaaggaA........................................................................    6     1   st1
....................uaugAggucuuccaaggauu.......................................................................    6     1   st1
....................uaugCggucuuccaaggauu.......................................................................    6     1   st1
....................uUguggucuuccaaggauu.......................................................................    1     1   st1
....................uauguggucuuccaCggauu.......................................................................    1     1   st1
....................uauguggucuAccaaggauu.......................................................................    1     1   st1
....................uaugugguCuuccaaggauu.......................................................................    4     1   st1
....................uauguggucuuccUaggauu.......................................................................    4     1   st1
....................uauguggucuuccaaggauC.......................................................................    188   1   st1
....................uauguggucuGccaaggauu.......................................................................    38    1   st1
....................uaugugguAuuccaaggauu.......................................................................    2     1   st1
....................uauguggucuuccaaggauG.......................................................................    16    1   st1
....................uCuguggucuuccaaggauu.......................................................................    2     1   st1
....................uauguggucuCccaaggauu.......................................................................    10    1   st1
....................uaugugAucuuccaaggauu.......................................................................    2     1   st1
....................uauguggucuuccGaggauu.......................................................................    4     1   st1
....................uaugugguUuuccaaggauu.......................................................................    1     1   st1
....................Cauguggucuuccaaggauu.......................................................................    3     1   st1
....................uauguggucuuccaaggaGu.......................................................................    1     1   st1
....................uauguggCcuuccaaggauu.......................................................................    6     1   st1
....................uauguggucuuccaGggauu.......................................................................    67    1   st1
....................uauguggucuuccaaggGuu.......................................................................    6     1   st1
....................Gauguggucuuccaaggauu.......................................................................    1     1   st1
....................uauguggucuucUaaggauu.......................................................................    2     1   st1
....................uauguggucuuccaUggauu.......................................................................    4     1   st1
....................Aauguggucuuccaaggauu.......................................................................    1     1   st1
....................uauguggucuuccaaggauA.......................................................................    44    1   st1
....................uauguggucuuccaaggauu.......................................................................    2355  0   st1
....................uaCguggucuuccaaggauu.......................................................................    2     1   st1
....................uauguggucuuUcaaggauu.......................................................................    1     1   st1
....................uauguggucuuccaaggUuu.......................................................................    6     1   st1
....................uauguggucuuccaaggaCu.......................................................................    4     1   st1
....................uauguggucuuccaagAauua......................................................................    1     1   st1
....................uauguggucuuccaaggauuC......................................................................    6     1   st1
....................uauguggucuCccaaggauua......................................................................    17    1   st1
....................uaugugUucuuccaaggauua......................................................................    1     1   st1
....................uauguggucuAccaaggauua......................................................................    8     1   st1
....................uaugugguUuuccaaggauua......................................................................    3     1   st1
....................Gauguggucuuccaaggauua......................................................................    5     1   st1
....................uauguggucuuccaaggaCua......................................................................    10    1   st1
....................uauguggucuuccaaggaAua......................................................................    1     1   st1
....................uauAuggucuuccaaggauua......................................................................    6     1   st1
....................Cauguggucuuccaaggauua......................................................................    6     1   st1
....................uauguggucuucGaaggauua......................................................................    1     1   st1
....................uauguggucuuccaaggauAa......................................................................    3     1   st1
....................uCuguggucuuccaaggauua......................................................................    2     1   st1
....................uauguggucuuccaaggauuG......................................................................    78    1   st1
....................uaugCggucuuccaaggauua......................................................................    17    1   st1
....................uaugugAucuuccaaggauua......................................................................    3     1   st1
....................uauguggucuucUaaggauua......................................................................    5     1   st1
....................uauguggucuuUcaaggauua......................................................................    7     1   st1
....................uauguggucuuccaaggauua......................................................................    5161  0   st1
....................uaGguggucuuccaaggauua......................................................................    2     1   st1
....................uaugAggucuuccaaggauua......................................................................    8     1   st1
....................uauguggucuuccGaggauua......................................................................    15    1   st1
....................uauguggucuucAaaggauua......................................................................    4     1   st1
....................uauguggucuuccaaggauCa......................................................................    8     1   st1
....................uauguggucuuccaaggGuua......................................................................    13    1   st1
....................uaugugguAuuccaaggauua......................................................................    2     1   st1
....................uaCguggucuuccaaggauua......................................................................    12    1   st1
....................uauguggucuuccaCggauua......................................................................    3     1   st1
....................uauguAgucuuccaaggauua......................................................................    4     1   st1
```

Mature **Star**

```
cuccgcaucaaaguuuugacuauguggucuuccaaggauuauaauccuuauaauccuuggaagaccucacagucaacacuuuuuugaugcaaccaaagagagguagcaaca
....................uauUuggucuuccaaggauua......................................................................	1	1	st1
....................Aauguggucuuccaaggauua......................................................................	4	1	st1
....................uauguggAcuuccaaggauua......................................................................	3	1	st1
....................uauguggucuGccaaggauua......................................................................	51	1	st1
....................uauguggucuuccaGggauua......................................................................	128	1	st1
....................uauguggucuuccaaUgauua......................................................................	1	1	st1
....................uUuguggucuuccaaggauua......................................................................	1	1	st1
....................uauguggucuuccaaggCuua......................................................................	1	1	st1
....................uauguggCcuuccaaggauua......................................................................	13	1	st1
....................uauguggucuuccaaggauGa......................................................................	2	1	st1
....................uauguggucuuccaUggauua......................................................................	3	1	st1
....................uauguggucuuccaaggauuU......................................................................	85	1	st1
....................uauguggucuuccUaggauua......................................................................	3	1	st1
....................uaAguggucuuccaaggauua......................................................................	1	1	st1
....................uauguggucCuccaaggauua......................................................................	15	1	st1
....................uaugAggucuuccaaggauuau.....................................................................	13	1	st1
....................uauguggCcuuccaaggauuau.....................................................................	21	1	st1
....................uauguggucuuccaaAgauuau.....................................................................	4	1	st1
....................uauguggucuuccaaggauuGu.....................................................................	33	1	st1
....................uauguggucuuccGaggauuau.....................................................................	15	1	st1
....................uauguAgucuuccaaggauuau.....................................................................	5	1	st1
....................Aauguggucuuccaaggauuau.....................................................................	3	1	st1
....................uaugugUucuuccaaggauuau.....................................................................	1	1	st1
....................uauguggucuuccaagAauuau.....................................................................	2	1	st1
....................uaCguggucuuccaaggauuau.....................................................................	16	1	st1
....................uauguggucuuccUaggauuau.....................................................................	5	1	st1
....................uauguggucuuccaaggauuaG.....................................................................	81	1	st1
....................uauguggucuuccaaggaCuau.....................................................................	9	1	st1
....................uauguggucuucUaaggauuau.....................................................................	4	1	st1
....................uauguggucuuccaUggauuau.....................................................................	5	1	st1
....................uauguggucuuccaaggauuaA.....................................................................	588	1	st1
....................uauguggucuuccaaggauuaC.....................................................................	535	1	st1
....................uauguggucuuccaaggGuuau.....................................................................	23	1	st1
....................uauguggucuuUcaaggauuau.....................................................................	4	1	st1
....................uauguggucuuccaaggauuUu.....................................................................	10	1	st1
....................uauguggucuuccaaggauCau.....................................................................	4	1	st1
....................uauguggucCuccaaggauuau.....................................................................	19	1	st1
....................uaugCggucuuccaaggauuau.....................................................................	34	1	st1
....................uauguggucuuccaaggauAau.....................................................................	1	1	st1
....................uaugugAucuuccaaggauuau.....................................................................	4	1	st1
....................uauguggucuuccaagUauuau.....................................................................	1	1	st1
....................uauguggucuuccaaggauuau.....................................................................	5699	0	st1
....................Cauguggucuuccaaggauuau.....................................................................	8	1	st1
....................uCuguggucuuccaaggauuau.....................................................................	10	1	st1
....................uauguggucuuccaaggaGuau.....................................................................	1	1	st1
....................uauguggucuGccaaggauuau.....................................................................	213	1	st1
....................uaGguggucuuccaaggauuau.....................................................................	2	1	st1
....................uauguggucuuccaaggUuuau.....................................................................	2	1	st1
....................uaugGggucuuccaaggauuau.....................................................................	1	1	st1
....................uauguggucuuAcaaggauuau.....................................................................	1	1	st1
....................uauguggucuuGcaaggauuau.....................................................................	1	1	st1
....................uauguggucuCccaaggauuau.....................................................................	16	1	st1
....................uauguggucuuccaaggauuCu.....................................................................	2	1	st1
....................uauguggAcuuccaaggauuau.....................................................................	2	1	st1
....................uauguggucuAccaaggauuau.....................................................................	2	1	st1
....................uauguggucuuccaCggauuau.....................................................................	2	1	st1
....................uaugugguUuuccaaggauuau.....................................................................	5	1	st1
....................uauguggucuuccCaggauuau.....................................................................	2	1	st1
....................uauAuggucuuccaaggauuau.....................................................................	3	1	st1
....................uUuguggucuuccaaggauuau.....................................................................	6	1	st1
....................uauguggucuuccaGggauuau.....................................................................	172	1	st1
....................uaAguggucuuccaaggauuau.....................................................................	6	1	st1
....................uauguggucuucAaaggauuaua....................................................................	1	1	st1
....................uauguggucCuccaaggauuaua....................................................................	2	1	st1
....................uaugugguUuuccaaggauuaua....................................................................	2	1	st1
....................uauguggucuuccaaggauuaAa....................................................................	45	1	st1
....................uauguggucuuccaaggauuGua....................................................................	23	1	st1
....................uauguggucuuccaaggauuauU....................................................................	443	1	st1
....................uauguggucuuccaGggauuaua....................................................................	23	1	st1
....................uauguggucuAccaaggauuaua....................................................................	1	1	st1
```

Mature **Star**

cuccgcaucaaaguuuugacuauguggucuuccaaggauuauaauccuuauaauccuuggaagaccucacagucaacacuuuuuugaugcaaccaaagagagguagcaaca			
....................uauguggucuuccaaggauuauC..	33	1	st1
....................Gauguggucuuccaaggauuaua..	1	1	st1
....................uauguggucuuccaaggauCaua..	1	1	st1
....................uauguggucuGccaaggauuaua..	21	1	st1
....................uaugugAucuuccaaggauuaua..	2	1	st1
....................uauguggucuuccaaggauuaGa..	1	1	st1
....................uauguggucuuccaaggauuaua..	1008	0	st1
....................uaugCggucuuccaaggauuaua..	2	1	st1
....................uauguggucuuccaaggauuaCa..	5	1	st1
....................uauguggucuuccaaggauuauG..	130	1	st1
....................uauguggucuuccaaggaCuaua..	6	1	st1
....................uauguggucAuccaaggauuaua..	1	1	st1
....................uauguggucuuccaaggGuuaua..	2	1	st1
....................Cauguggucuuccaaggauuaua..	1	1	st1
....................uauguggucuucGaaggauuaua..	1	1	st1
....................uauguggucuuccGaggauuaua..	4	1	st1
....................uauguggucuuccaaggauuUua..	4	1	st1
....................uauguggCcuuccaaggauuaua..	4	1	st1
....................uauguggucuCccaaggauuaua..	6	1	st1
....................uauguggucuuccUaggauuaua..	2	1	st1
....................uauguggucuuccaaAgauuaua..	1	1	st1
....................uaCguggucuuccaaggauuaua..	3	1	st1
....................uauguAgucuuccaaggauuaua..	1	1	st1
....................uauguggucuuccaaggauuauGa...	2	1	st1
....................uauguggucuuccaaggGuuauaa...	1	1	st1
....................uauguggucuuUcaaggauuauaa...	2	1	st1
....................uauguggucuuccaaggauuauaa...	59	0	st1
....................uauguggucuuccaaggauuauaC...	2	1	st1
....................uauguggucuuccaaggauuauaU...	3	1	st1
....................uauguggucuuccaaggauuaAaa...	4	1	st1
....................uaugCggucuuccaaggauuauaa...	1	1	st1
....................uauguggucuuccaaggauuGuaa...	6	1	st1
....................uauguggucuuccaaggauuauUa...	49	1	st1
....................uauguggucuGccaaggauuauaa...	4	1	st1
....................uauguggucuuccaaggauuaGaa...	1	1	st1
....................uauguggucuuccaaggauuaAaau..	1	1	st1
....................uauguggucuuccaaggauuauaaA..	1	1	st1
....................uauguggucuuccaaggauuauaau..	1	0	st1
....................uauguggucuuccaaggauuauGauc...	2	1	st1
....................uauguggucuuccaaggauuauaauUc..	1	1	st1
....................uauguggucuuccaaggauuauaaucc..	1	0	st1
....................uauguggucuuccaaggauuauaCucc..	3	1	st1
.....................auguggucuuccaaggauu...	1	0	st1
.....................auguggucuuccaaggauua..	1	0	st1
.....................auguggucuuccaaggauuau...	2	0	st1
.....................auguggucuuccaaggauuauU..	2	1	st1
.....................auguggucuuccaaggauuaua..	2	0	st1
......................uguggucuuccaaggauu...	1	0	st1
......................uguggucuuccaaggauua..	3	0	st1
......................uguggucuuccaaggauuau...	2	0	st1
......................uguggucuuccaaggauuauU..	7	1	st1
......................uguggucuuccaaggauuauC..	1	1	st1
.......................guggucuuccaaggauuaua..	1	0	st1
.......................guggucuuccaaggauuauaa...	1	0	st1
........................uggucuuccaaggauuau...	1	0	st1
........................uggucuuccaaggauuaua..	1	0	st1
....uaauccuuggaagaccuc...	5	0	st1
....uaauccAuggaagaccuc...	1	1	st1
....uaauccuuggaagaccuca..	1	0	st1
....uaauccuuggaagaccAcac...	1	1	st1
....uaauccuuggaagaccucaU...	15	1	st1
....uaauccuuggaagaccucac...	108	0	st1
....uaauccuuggaCgaccucac...	2	1	st1
....uaauccuuggaaAaccucac...	1	1	st1
....uaGuccuuggaagaccucac...	1	1	st1
....uaauccuuggaagaccucaA...	4	1	st1
....uaauccuuggaagaGcucaca..	1	1	st1
....uaauccuuggGagaccucaca..	3	1	st1
....uaGuccuuggaagaccucaca..	2	1	st1
....uaauccuuggaagaccucacG..	2	1	st1

```
                     Mature                   Star
cuccgcaucaaaguuuugacuauguggucuuccaaggauuauaauccuuauaauccuuggaagaccucacagucaacacuuuuuugaugcaaccaaagagagguagcaaca
..................................................uaauccuuAgaagaccucaca........................................    1    1    st1
..................................................uaauccCuggaagaccucaca........................................    1    1    st1
..................................................uaauccuGggaagaccucaca........................................    1    1    st1
..................................................uaauccuuggaGgaccucaca........................................    1    1    st1
..................................................uGauccuuggaagaccucaca........................................    3    1    st1
..................................................uaauccuuggaagaccucacU........................................    7    1    st1
..................................................uaauccuuggaagGccucaca........................................    2    1    st1
..................................................uaauccuCggaagaccucaca........................................    1    1    st1
..................................................uaaCccuuggaagaccucaca........................................    4    1    st1
..................................................uaauccuuggaagaccucaca........................................    146  0    st1
..................................................uaauccuuggaagaccucacaA.......................................    22   1    st1
..................................................uaauccuuggGagaccucacag.......................................    2    1    st1
..................................................uaauccuuggaagaccucacaU.......................................    6    1    st1
..................................................uaauccuuggaagaccucacCg.......................................    1    1    st1
..................................................uaauccuuggaagaccucacag.......................................    242  0    st1
..................................................uaGuccuuggaagaccucacag.......................................    1    1    st1
..................................................uaauccuuggaagaccAcacag.......................................    1    1    st1
..................................................Aaauccuuggaagaccucacag.......................................    2    1    st1
..................................................uaauccuuggaagaccucacaC.......................................    5    1    st1
..................................................uaauccuuggaCgaccucacag.......................................    2    1    st1
..................................................uaauccuuggaGgaccucacag.......................................    1    1    st1
..................................................uaauccCuggaagaccucacag.......................................    2    1    st1
..................................................Caauccuuggaagaccucacag.......................................    1    1    st1
..................................................uGauccuuggaagaccucacag.......................................    9    1    st1
..................................................uaauccuAggaagaccucacag.......................................    1    1    st1
..................................................uaauccuuggaagaccucacagG......................................    3    1    st1
..................................................uaauccuCggaagaccucacagu......................................    1    1    st1
..................................................uaauccuuggaagaccucacagC......................................    27   1    st1
..................................................uaauccuuggaagaccucacaUu......................................    1    1    st1
..................................................uaauccuuggaGgaccucacagu......................................    2    1    st1
..................................................uaauccuGggaagaccucacagu......................................    1    1    st1
..................................................uaauccCuggaagaccucacagu......................................    2    1    st1
..................................................uaauccuuggaagaccucGcagu......................................    3    1    st1
..................................................uaauccuuggaagaccucacagu......................................    329  0    st1
..................................................uaauccuuggUagaccucacagu......................................    2    1    st1
..................................................uGauccuuggaagaccucacagu......................................    7    1    st1
..................................................uaauccuuggGagaccucacagu......................................    1    1    st1
..................................................uaauccuuggaagaccucacGgu......................................    1    1    st1
..................................................uaauccuuggaagaccucacagA......................................    10   1    st1
..................................................uaauccuuggaagaccuUacagu......................................    1    1    st1
..................................................uaauccuuggaagaccucacaguA.....................................    155  1    st1
..................................................uaauccuuggaagaccucacaguU.....................................    12   1    st1
..................................................uaauccuuggaagaccucacaguG.....................................    2    1    st1
..................................................uaauccuuggaagaccucacaguc.....................................    4    0    st1
..................................................uaauccuuggaagaccucacaguUa....................................    4    1    st1
..................................................uaauccuuggaagaccucacaguAa....................................    3    1    st1
...................................................aauccuuggaagaccucaA.........................................    1    1    st1
...................................................aauccuuggaagaccucaU.........................................    6    1    st1
...................................................aauccuuggaagaccucac.........................................    47   0    st1
...................................................aauccuuggaagGccucac.........................................    3    1    st1
...................................................aauccuuggaagaUcucac.........................................    1    1    st1
...................................................aauccuuggaagaccAcac.........................................    1    1    st1
...................................................aauccCuggaagaccucaca........................................    2    1    st1
...................................................aauccuuggaagGccucaca........................................    2    1    st1
...................................................aauccuuggaagaccucacU........................................    1    1    st1
...................................................aauccGuggaagaccucaca........................................    1    1    st1
...................................................aauccuuggaagaccucaca........................................    74   0    st1
...................................................aauccuuggaGgaccucacag.......................................    1    1    st1
...................................................aauccuuggaagaccucGcag.......................................    1    1    st1
...................................................aauccuuggaagaccucacaA.......................................    12   1    st1
...................................................aauccuuggGagaccucacag.......................................    1    1    st1
...................................................aauccuuggaagaccucacGg.......................................    1    1    st1
...................................................aauccuuggaagaccucacaU.......................................    2    1    st1
...................................................aauccCuggaagaccucacag.......................................    2    1    st1
...................................................aauccuuggaagaccucacag.......................................    110  0    st1
...................................................aaCccuuggaagaccucacagu......................................    1    1    st1
...................................................aCuccuuggaagaccucacagu......................................    1    1    st1
...................................................aauccuuggaagaccCcacagu......................................    1    1    st1
...................................................aauccuuggaagacAucacagu......................................    1    1    st1
...................................................aauccuuUgaagaccucacagu......................................    1    1    st1
```

Mature **Star**

cuccgcaucaaaguuuugacuauguggucuuccaaggauuauaauccuuauaauccuuggaagaccucacagucaacacuuuuuugaugcaaccaaagagagguagcaaca			
....aauccuuggaagaccucacCgu......................................	2	1	st1
....aauccuuggaagGccucacagu......................................	2	1	st1
....aauccuuggGagaccucacagu......................................	1	1	st1
....aauccCuggaagaccucacagu......................................	2	1	st1
....aauccuuggaagaccucacagu......................................	182	0	st1
....aauccuuggaagaccucacagC......................................	15	1	st1
....aauccuuggaagaccuGacagu......................................	2	1	st1
....aauccuuggaagaccucacUgu......................................	1	1	st1
....aauccuugUaagaccucacagu......................................	1	1	st1
....aauccuuggaGgaccucacagu......................................	1	1	st1
....aauccuuggaagaccucacagA......................................	4	1	st1
....aauccuuggaagaccucacaguA.....................................	47	1	st1
....aauccuuggaagaccucacaguG.....................................	2	1	st1
....aauccuuggaagaccucacaguU.....................................	2	1	st1
....aauccuuggaagaccucacaguUa....................................	2	1	st1
....aauccuuggaagaccucacaguAa....................................	1	1	st1
....auccuuggaagaccucac...	1	0	st1
....auccuuggaagaccucaca..	3	0	st1
....auccuuggaagaccucacag.......................................	2	0	st1
....auccuuggaagaccucacagu......................................	9	0	st1
....auccuuggaagaccCcacagu......................................	2	1	st1
....auccuuggaagaccucacaguA.....................................	2	1	st1
....uccuuggaagaccucacagu......................................	1	0	st1
....uccuuggaagacGucacagu......................................	1	1	st1
....uccuuggaagaccucacaguA.....................................	3	1	st1
....uccuuggaagaccucacaguU.....................................	2	1	st1
....uccuuggaagaccucacaguUa....................................	2	1	st1
....cuuggaagaccucacagu......................................	2	0	st1
....................uauguggucuuccaagga...	1	0	sg1
....................uauguggucuuccaaggau..	34	0	sg1
....................uauguggucuuccaaggaA..	2	1	sg1
....................uauguggucuuccaGggau..	2	1	sg1
....................uauAuggucuuccaaggau..	1	1	sg1
....................uauguggucuuccaaggaC..	3	1	sg1
....................uauguAgucuuccaaggauu...	2	1	sg1
....................uaugCggucuuccaaggauu...	2	1	sg1
....................uauguggucCuccaaggauu...	5	1	sg1
....................uauguggucuucUaaggauu...	2	1	sg1
....................uauguggucuuccaaggauG...	1	1	sg1
....................uauguggucuCccaaggauu...	1	1	sg1
....................uauguggucuuccaaggauC...	44	1	sg1
....................uauguggCcuuccaaggauu...	1	1	sg1
....................uauguggucuuccaaggauA...	19	1	sg1
....................uauguggucuuccaaggGuu...	2	1	sg1
....................uauAuggucuuccaaggauu...	3	1	sg1
....................uauguggucuuccaaggauu...	587	0	sg1
....................uauguggucuuccaGggauu...	28	1	sg1
....................Cauguggucuuccaaggauu...	1	1	sg1
....................uauguggucuuccaCggauu...	1	1	sg1
....................uaCguggucuuccaaggauu...	1	1	sg1
....................uauguggucuuccaaggUuu...	1	1	sg1
....................uauguggucuuccGaggauu...	1	1	sg1
....................uaugAggucuuccaaggauu...	1	1	sg1
....................uauguggucuuccaaggaGu...	1	1	sg1
....................uauguggucuGccaaggauu...	9	1	sg1
....................uauguggucuucAaaggauu...	1	1	sg1
....................uUuguggucuuccaaggauu...	1	1	sg1
....................uaCguggucuuccaaggauua..	5	1	sg1
....................uauguggucuuccaaggauua..	2174	0	sg1
....................uaugCggucuuccaaggauua..	13	1	sg1
....................uauguggucGuccaaggauua..	1	1	sg1
....................uauguggucAuccaaggauua..	1	1	sg1
....................uauguggucuucAaaggauua..	2	1	sg1
....................uauguggCcuuccaaggauua..	6	1	sg1
....................uaugugguUuuccaaggauua..	1	1	sg1
....................uauguggucuAccaaggauua..	2	1	sg1
....................uauguUgucuuccaaggauua..	1	1	sg1
....................uauguggucuuccUaggauua..	2	1	sg1
....................uauguggucuuccaaggauuU..	36	1	sg1

```
                    Mature                        Star
cuccgcaucaaaguuuugacuauguggucuuccaaggauuauaaauccuuauaauccuuggaagaccucacagucaacacuuuuuugaugcaaccaaagagagguagcaaca
....................uauguggucuuccaaggauGa.......................................................................   1     1   sg1
....................uauguggucCuccaaggauua.......................................................................   7     1   sg1
....................uauguggucuuUcaaggauua.......................................................................   1     1   sg1
....................uauguggucuuccaaggauAa.......................................................................   1     1   sg1
....................Cauguggucuuccaaggauua.......................................................................   2     1   sg1
....................uauAuggucuuccaaggauua.......................................................................   2     1   sg1
....................uauguggucuuAcaaggauua.......................................................................   1     1   sg1
....................Gauguggucuuccaaggauua.......................................................................   1     1   sg1
....................uauguggucuuccaaggGuua.......................................................................   12    1   sg1
....................uauguggucuGccaaggauua.......................................................................   36    1   sg1
....................uauguggucuuccaaggaCua.......................................................................   2     1   sg1
....................uauguggucuucUaaggauua.......................................................................   2     1   sg1
....................uauguggucuCccaaggauua.......................................................................   12    1   sg1
....................uauguggucuuccaGggauua.......................................................................   142   1   sg1
....................uauguggucuuccaaggauuG.......................................................................   63    1   sg1
....................uauguggucuuccaaggauCa.......................................................................   5     1   sg1
....................uauguAgucuuccaaggauua.......................................................................   2     1   sg1
....................uaugAggucuuccaaggauua.......................................................................   3     1   sg1
....................uauguggucuuccaagAauua.......................................................................   1     1   sg1
....................uauguggucuuccGaggauua.......................................................................   10    1   sg1
....................uUuguggucuuccaaggauua.......................................................................   2     1   sg1
....................uauguggucuuccaaggauuC.......................................................................   2     1   sg1
....................Aauguggucuuccaaggauua.......................................................................   2     1   sg1
....................uauguggCcuuccaaggauuau......................................................................   3     1   sg1
....................uauguggucuuccaaggauuGu......................................................................   12    1   sg1
....................uauguggAcuuccaaggauuau......................................................................   1     1   sg1
....................uauguggucuuccaaggauuau......................................................................   991   0   sg1
....................uaugugguUuuccaaggauuau......................................................................   1     1   sg1
....................uauUuggucuuccaaggauuau......................................................................   1     1   sg1
....................uauguggucuuccaaggauuUu......................................................................   3     1   sg1
....................uauguggucuuccaaggauuaG......................................................................   22    1   sg1
....................uauAuggucuuccaaggauuau......................................................................   1     1   sg1
....................uauguggucuGccaaggauuau......................................................................   33    1   sg1
....................uauguggucuuccaaggauuaA......................................................................   385   1   sg1
....................uauguggucuuccaaggGuuau......................................................................   6     1   sg1
....................uauguggucuucUaaggauuau......................................................................   1     1   sg1
....................Gauguggucuuccaaggauuau......................................................................   2     1   sg1
....................uaugCggucuuccaaggauuau......................................................................   11    1   sg1
....................uauguggucCuccaaggauuau......................................................................   3     1   sg1
....................uauguggucuuccaUggauuau......................................................................   1     1   sg1
....................uUuguggucuuccaaggauuau......................................................................   2     1   sg1
....................uauguggucuuccaaggauuaC......................................................................   66    1   sg1
....................uauguAgucuuccaaggauuau......................................................................   1     1   sg1
....................uaugAggucuuccaaggauuau......................................................................   4     1   sg1
....................uauguggucuuccaGggauuau......................................................................   90    1   sg1
....................uauguggucuuccGaggauuau......................................................................   5     1   sg1
....................uaCguggucuuccaaggauuau......................................................................   1     1   sg1
....................uauguggGcuuccaaggauuau......................................................................   1     1   sg1
....................uauguggucuCccaaggauuau......................................................................   3     1   sg1
....................uaugugAucuuccaaggauuau......................................................................   1     1   sg1
....................uaCguggucuuccaaggauuaua.....................................................................   3     1   sg1
....................uauguggucuCccaaggauuaua.....................................................................   5     1   sg1
....................uauguggucuGccaaggauuaua.....................................................................   13    1   sg1
....................uauguggucuuccaaggauuGua.....................................................................   29    1   sg1
....................uauguggucuuccaaggGuuaua.....................................................................   4     1   sg1
....................uauguggucuuccCaggauuaua.....................................................................   1     1   sg1
....................uauguggCcuuccaaggauuaua.....................................................................   2     1   sg1
....................uaugugguUuuccaaggauuaua.....................................................................   1     1   sg1
....................Gauguggucuuccaaggauuaua.....................................................................   1     1   sg1
....................uauguggucuuccGaggauuaua.....................................................................   2     1   sg1
....................uauguggucuuccaaggauAaua.....................................................................   1     1   sg1
....................uauguggucuuccaGggauuaua.....................................................................   47    1   sg1
....................uauguggucuuccaaggauuaua.....................................................................   644   0   sg1
....................Cauguggucuuccaaggauuaua.....................................................................   1     1   sg1
....................uaugCggucuuccaaggauuaua.....................................................................   1     1   sg1
....................uCuguggucuuccaaggauuaua.....................................................................   1     1   sg1
....................uauguggucuuccaaggauuUua.....................................................................   3     1   sg1
....................uauguggucuAccaaggauuaua.....................................................................   1     1   sg1
....................uauguggucuucGaaggauuaua.....................................................................   1     1   sg1
....................uauguggucuuccaaggauuaAa.....................................................................   15    1   sg1
```

Mature Star

```
cuccgcaucaaaguuuugacuauguggucuuccaaggauuauaauccuuauaauccuuggaagaccucacagucaacacuuuuuugaugcaaccaaagagagguagcaaca
....................uUuguggucuuccaaggauuaua....................................................................  2    1  sg1
....................uauguggucuuccaaggauuauC....................................................................  5    1  sg1
....................uaugAggucuuccaaggauuaua....................................................................  1    1  sg1
....................uauguggucuuccaaggauuauU....................................................................  59   1  sg1
....................uauguggucuuccaaggauuauG....................................................................  90   1  sg1
....................uauguggucCuccaaggauuaua....................................................................  4    1  sg1
....................uauguggucuuccaaggauuaCa....................................................................  1    1  sg1
....................uauguAgucuuccaaggauuaua....................................................................  1    1  sg1
....................uauAuggucuuccaaggauuaua....................................................................  1    1  sg1
....................uauguggucuuUcaaggauuaua....................................................................  1    1  sg1
....................uauguggucuuccaaggaCuaua....................................................................  1    1  sg1
....................uauguggucuuccaaggCuuaua....................................................................  1    1  sg1
....................uauguggucuuccaaggauuUuaa...................................................................  4    1  sg1
....................uauguggucuuccaaggauuauaG...................................................................  2    1  sg1
....................uauguggucuuccaaggauuGuaa...................................................................  12   1  sg1
....................uauguggucuuccaaggauuauaU...................................................................  1    1  sg1
....................uauguggucuGccaaggauuauaa...................................................................  1    1  sg1
```
[truncated: 987,866 more chars]
